# Systems Biology Modeling of the Complement System Under Immune Susceptible Pathogens

**DOI:** 10.3389/fphy.2021.603704

**Published:** 2021-04-29

**Authors:** Nehemiah T. Zewde, Rohaine V. Hsu, Dimitrios Morikis, Giulia Palermo

**Affiliations:** 1 Department of Bioengineering, University of California, Riverside, Riverside, CA, United States; 2 Department of Chemistry, University of California, Riverside, Riverside, CA, United States

**Keywords:** predictive modeling, computational systems biology, protein network, predictive network biology, complement system, *Neisseria meningitidis*

## Abstract

The complement system is assembled from a network of proteins that function to bring about the first line of defense of the body against invading pathogens. However, complement deficiencies or invasive pathogens can hijack complement to subsequently increase susceptibility of the body to infections. Moreover, invasive pathogens are increasingly becoming resistant to the currently available therapies. Hence, it is important to gain insights into the highly dynamic interaction between complement and invading microbes in the frontlines of immunity. Here, we developed a mathematical model of the complement system composed of 670 ordinary differential equations with 328 kinetic parameters, which describes all three complement pathways (alternative, classical, and lectin) and includes description of mannose-binding lectin, collectins, ficolins, factor H-related proteins, immunoglobulin M, and pentraxins. Additionally, we incorporate two pathogens: (type 1) complement susceptible pathogen and (type 2) *Neisseria meningitidis* located in either nasopharynx or bloodstream. In both cases, we generate time profiles of the pathogen surface occupied by complement components and the membrane attack complex (MAC). Our model shows both pathogen types in bloodstream are saturated by complement proteins, whereas MACs occupy <<1.0% of the pathogen surface. Conversely, the MAC production in nasopharynx occupies about 1.5–10% of the total *N. meningitidis* surface, thus making nasal MAC levels at least about eight orders of magnitude higher. Altogether, we predict complement-imbalance, favoring overactivation, is associated with nasopharynx homeostasis. Conversely, orientating toward complement-balance may cause disruption to the nasopharynx homeostasis. Thus, for sporadic meningococcal disease, our model predicts rising nasal levels of complement regulators as early infection biomarkers.

## INTRODUCTION

The human respiratory tract is a complex system whose primary function is to exchange oxygen and carbon dioxide to meet the metabolic requirements of an organism. However, this physiological need also creates vulnerability through constant exposure to toxic particles and pathogens [1–3]. To counteract this susceptibility, defense mechanism is deployed in the respiratory tract to clear entrapped particles and target invading pathogens [4]. Part of the innate immunity, the complement system also forms a defensive unit in the respiratory tract to identify and remove infectious agents [4–7]. The complement system is composed of three pathways known as alternative (AP), classical (CP), and lectin (LP) that coordinate an immune response to directly engage invading pathogens. Once activated, the complement system achieves host immunity by generating anaphylatoxins, opsonins, and the membrane attack complex (MAC) [8–19]. Additionally, complement forms a bridge between innate and adaptive immunity. Activation of the complement system assists in lymphocyte stimulation, regulation, and antigen presentation. For instance, complement protein C3d, which tags the pathogen surface, has acted as a molecular adjuvant by lowering the threshold for B-cell activation within the lymphoid follicle. Complement-tagged immune complexes also promote the development of germinal center, which subsequently is essential for maintenance of memory B-cell repertoires.

To counteract the deadly effects of complement activation, microorganisms evolved several strategies to evade the immune system and potentially cause infections [20–24]. These counterstrategies include recruitment of complement system regulators to the surface of pathogen, secretion of proteins that inhibit complement activation, and proteases that cleave complement effector molecules. For instance, human-specific *Neisseria meningitidis* is an important cause of meningitis and septicemia; it is associated with mortality rates ranging from 10 to 40% [25, 26]. Of the many counterstrategies used by *N. meningitidis*, one method involves evading the complement system by expressing a surface lipoprotein known as factor H binding protein (fHbp) [27–30]. Presence of this lipoprotein promotes survival of *N. meningitidis* by recruiting the complement regulators factor H (FH) and factor H-like protein 1 (FHL-1) to its surface. In addition to recruiting AP regulators (FH and FHL-1), *N. meningitidis* uses additional membrane proteins such as porin A (porA) and Meningococcal surface fibril (Msf) to recruit complement regulators C4b-binding protein (C4BP) and vitronectin (Vn), respectively [20]. By utilizing these strategies, *N. meningitidis* inhibits all three pathways of the complement system (AP, CP, and LP) and effectively interferes with the bactericidal function of complement.

The importance of the complement system against invading microbes is reflected in studies that show how genetic deficiencies in complement lead to recurrent infections [31]. For instance, deficiencies in the classical pathway are associated with *Streptococcus pneumoniae* bacteremia, and *Haemophilus influenzae* meningitis [31–33]. In addition to the classical pathway, deficiencies in the lectin pathway are associated with an increased risk of meningococcal and pneumococcal infections [31, 32, 34]. Lastly, deficiencies in the alternative pathway are also associated with invasive diseases. Individuals with inherited and acquired deficiency in C3 develop recurrent and severe infections, such as meningitis, bacteremia, pneumonia, and otitis media [31, 32, 35, 36].

Deficiencies in the MAC components are also associated with an increased predisposition to invasive meningococcal disease and disseminated gonococcal infection [32]. Individuals with defective MAC components have 7,000- to 10,000-fold higher risk of developing meningococcal disease [37]. Furthermore, 40–50% of MAC-deficient individuals will suffer from recurrent infections at a rate ~100–150 times greater than the normal population. Moreover, using complement inhibitors to interfere with MAC assembly might also increase the susceptibility of acquiring bacterial infections [38–40].

Altogether, a competent complement system that spans all three pathways forms a pivotal junction in the fight against pathogens. Knowing this importance, computational models have been assembled to shed light on the dynamics of complement activation and propagation [41–46]. However, to our knowledge, a comprehensive model that covers all three pathways of the complement system has not been developed. Here, we have implemented a computational predictive model of the complement system that contains all three pathways: alternative, classical, and lectin. Building on the frameworks of our previous models [47, 48], the presented approach contains immunoglobulin M (IgM), mannose-binding lectin (MBL), collectins, and ficolins, factor H-related proteins 1–5 (FHR1-5), and pentraxins (C-reactive protein, CRP; serum-amyloid P component, SAP; and long pentraxin 3, PTX3).

**FIGURE 1 |:**
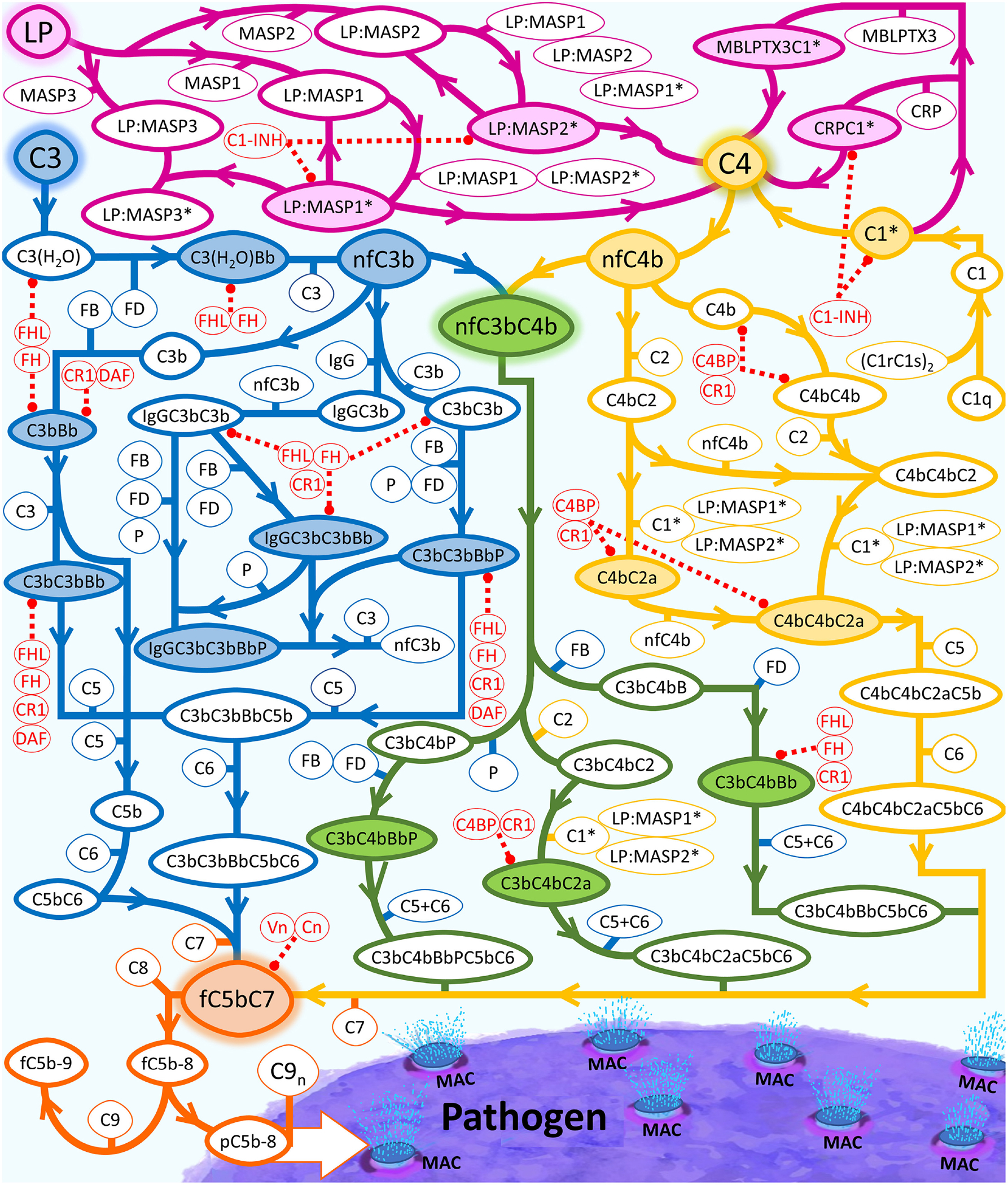
Summary of the complement system biochemical network. Activation and propagation of the alternative pathway are shown in blue, the classical pathway is shown in yellow, and the lectin pathway in conjunction with different segments of humoral immunity is shown in magenta. The interconnection between all pathways is shown in green. The continued propagation terminates in the formation of either fC5b-9 (soluble MAC) or surface-bound MAC. LP is used as an umbrella term that equates to pattern recognition molecules, such as MBL trimers and tetramers, collectin liver 1 (CL-L1), collectin kidney 1 (CL-K1), heteromeric CL-L1/K1 known as CL-LK, ficolin-1 (M-ficolin), ficolin-2 (L-ficolin), and ficolin-3 (H-ficolin), MBL-associated serine proteases (MASPs)-1, MASP-2, MASP-3, MBL-associated proteins (MAp19 and MAp44). Complement proteins that make up the regulatory checkpoints such FH, FHL-1, and Vn are shown in red. Activated C1 (C1*) will use C1s* to cleave C4 into C4a and nascent C4b (nC4b). Similar to nC3b, nC4b can also covalently attach to nearby cells and initiate complement.

Additionally, since complement activation leads to indirect killing through opsonophagocytosis and direct killing through MAC production, we used our model to determine the complement response in the presence of two types of pathogens: (type 1) complement-susceptible pathogen incapable of immune evasion, (type 2) model of *N. meningitidis* that recruits complement regulators (C4BP, Vn, FH, and FHL-1), and also an activator (FHR-3). Since bactericidal kinetics of MAC may take 3 h [19], our calculations were performed within a 3-h timespan. A summary of the complement cascade used in generating our model is shown in Figure 1. By comparing both pathogen types, we can use the model to examine how the complement activation varies in response to invasion by different pathogen groups. Moreover, comparing the immune response on different pathogen types may also identify potential strategies to boost bactericidal activities against invasive- or therapy-resistant pathogens. These insights can also be used to gain an understanding of which strategies to evade the complement system that can lead to infections. Lastly, incorporating both pathogen types can be used to elucidate how environmental changes, such as the bloodstream or nasal cavity, may alter the response of complement on the level of opsonization or MAC deposition.

## RESULTS

**FIGURE 2 |:**
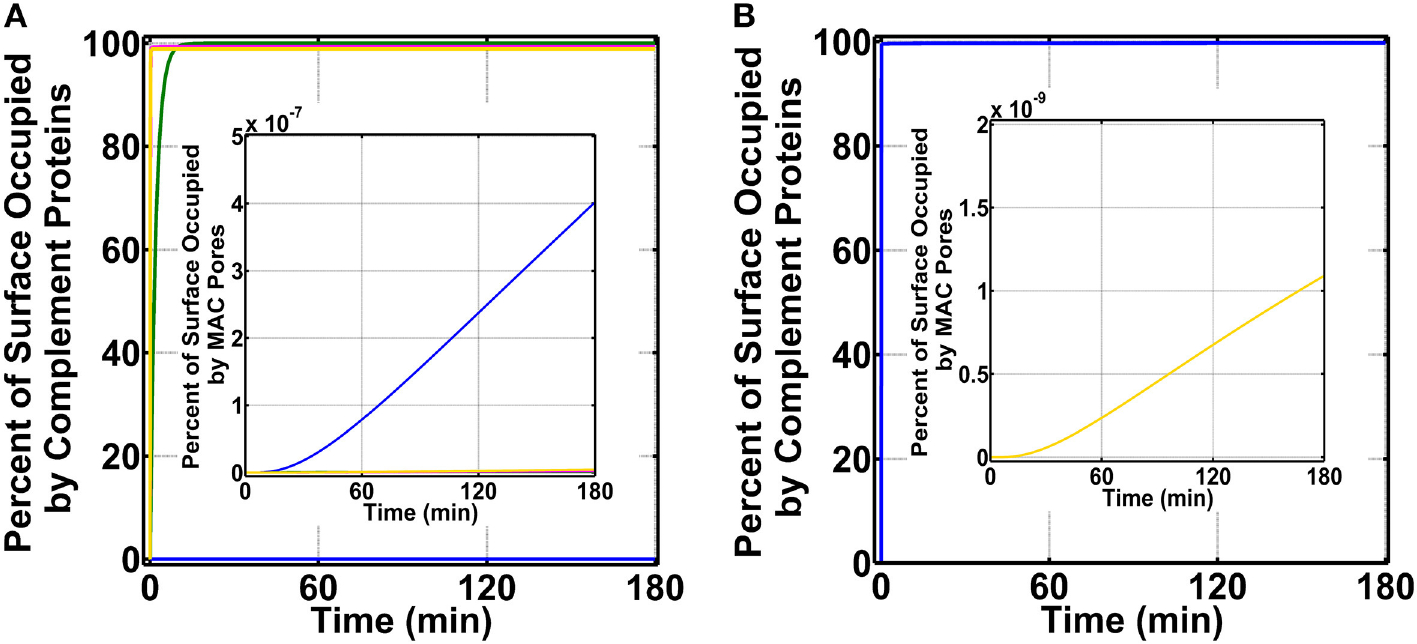
Percent of surface occupation (type 1 pathogens) under the different modes of complement activation of (i) FP in blue (ii) CP in green, (iii) LP in magenta, and (iv) FHR1-5 in yellow, and complement system with pentraxins (CSP). **(A)** FP occupied <1.0% of the pathogen surface, whereas all other modes of complement activation rapidly saturate the pathogen surface. The inset shows membrane attack complex (MAC) production is the highest under FP, but all four modes occupied <<1.0% of the pathogen surface. **(B)** Pathogen surface is rapidly saturated by CSP components as shown in blue. The inset shows MAC production is similar to **(A)** inset where MACs occupied <<1.0% of the pathogen surface.

To initiate our modeling efforts, we focused on complement targeting of type 1 pathogens. We compared the different modes of complement activations: (i) fluid phase (FP) of alternative and classical pathways, (ii) CP, (iii) LP, and (iv) FHR1-5 in generating complement components, such as C3b, proconvertases, C3/C5 convertases, and MAC pores that occupy the pathogen surface within 180 min (Figure 2A). Under FP, about 99.0% of the total pathogen surface remains unoccupied (Figure 2A blue), whereas, under CP (Figure 2A green), the pathogen surface is fully occupied as shown in Figure 2A and Supplementary Material 1 (green). Moreover, under LP (Figure 2A and Supplementary Material 1, magenta) and FHR1-5 (Figure 2A and Supplementary Material 1, yellow), complement components nearly saturate the pathogen surface. The CP-based surface occupation however took longer (~10 min) compared to LP/FHR-based surface occupation (<1 min) as shown in Figure 2A and Supplementary Material 1. Next, we generated time profiles of MAC by using the same modes of the above-mentioned complement activation. As shown in the insets of Figure 2A and Supplementary Material 1, MAC production under all four modes occupied <<1.0% of the total pathogen surface. However, MAC production was the highest under FP activation (Figure 2A inset) and subsequently followed in decreasing order by FHR1-5 (Supplementary Figure 1 inset, yellow), LP (Supplementary Figure 1 inset, magenta), and CP (Supplementary Figure 1 inset, green). Overall, these results show under CP, LP, and FHR1-5 complement-mediated surface occupation is the primary response against type 1 pathogens. And despite occupying <1.0% of the total pathogen surface, FP produced the highest MAC level in comparison to the different modes of complement activation.

We then combined FP, CP, LP, FHR1-5, and pentraxins (PTX3, SAP, and CRP) to generate a combined mode containing both the complement system and pentraxins (CSP). We compared the effects of CSP on pathogen surface occupation and MAC production. As shown in Figure 2B, type 1 pathogens (blue) are rapidly occupied by CSP in <1 min. Furthermore, as shown in Figure 2B inset (yellow), CSP produce MAC levels that occupy <<1.0% of the pathogen surface in 180 min.

After generating complement profiles on type 1 pathogens, we continued our modeling efforts of the complement system against *N. meningitidis* in the bloodstream and nasopharynx. *N. meningitidis* evades the complement system by recruiting complement regulators C4BP, Vn, FH, and FHL-1 to its surface. C4BP and Vn are recruited by using membrane proteins known as porA and Msf, respectively. Additionally, alternative pathways regulators (FH and FHL-1) are also recruited to the surface using a membrane lipoprotein known as fHbp. However, fHbp also recruits a promotor of complement activation known as FHR-3. Hence survival of *N. meningitidis* may depend on the dynamics between complement regulators (C4BP, Vn, FH, and FHL-1) and promotor (FHR-3).

To better understand this dynamic, we first picked the complement activation mode that favored MAC production (not favoring opsonophagocytosis) since individuals with MAC deficiency have 7,000- to 10,000-fold higher risk of developing meningococcal disease [37] and complement-based opsonophagocytosis is still achievable in the absence of MAC deficiency. And then we generated three conditions: (i) FP activation and recruitment of FHR-3 on *N. meningitidis* (ii) FP activation and recruitment of C4BP, Vn, FH, and FHL-1 on *N. meningitidis*, and (iii) FP activation and recruitment of C4BP, Vn, FH, FHL-1, and FHR-3 on *N. meningitidis.*

**FIGURE 3 |:**
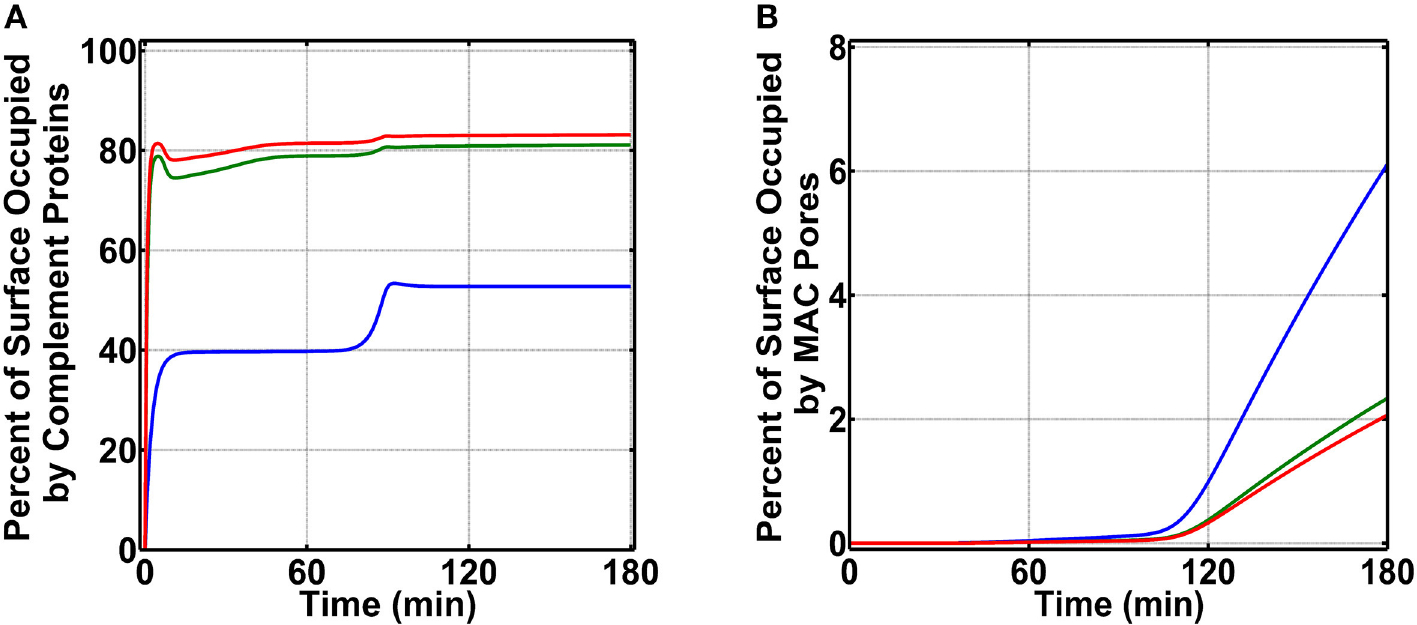
Nasal complement profiles on *N. meningitidis* that occupy the pathogen surface under three conditions: (i) FP and FHR-3 recruitment in blue; (ii) FP and C4BP, Vn, FH, and FHL-1 recruitment in green; and (iii) FP and FHR-3, C4BP, Vn, FH, and FHL-1 recruitment in red. **(A)** Conditions (ii) and (iii) rapidly occupy the surface of *N. meningitidis* and account for 81.1 and 83.1% of its surface, respectively. Condition (i) occupies a lower percent of the bacterial surface (52.8%). **(B)** Conversely, condition (i) produces the highest membrane attack complex (MAC) level, occupying 6.1% *N. meningitidis* surface, whereas, conditions (ii) and (iii) led to lower MAC production that occupied 2.3 and 2.1% of the bacterial surface, respectively.

To generate percent surface occupation levels and MAC production on *N. meningitidis*, we started our studies by using nasal complement levels (Supplementary Table 1). As shown in Figure 3A (blue) for condition (i), we observe a surface occupation of about 40.0% within 15 min. This level of surface occupation is maintained until about 80 min, and there after there is another increase in the occupation level to reach a final value of 52.8% in 180 min. Under condition (ii) as shown in Figure 3A (green), we observe a more rapid occupation of *N. meningitidis* (<5 min). However, this phase of rapid occupation is followed by a transient increase in the available surface and then a subsequent decrease to reach a final occupation level of 81.1%. Lastly, under condition (iii) as shown in Figure 3A (red), we generate a time profile similar to condition (ii). However, under condition (iii), the final occupation level settles to 83.1% in 180 min. After generating complement occupation levels on *N. meningitidis*, we continued our calculations for MAC levels (Figure 3B). Condition (i) generates the highest MAC level (Figure 3B, blue) with 6.1% of the pathogen surface being occupied by MAC pores. Condition (ii) generates the second highest MAC level (Figure 3B, green) with 2.3% of the pathogen surface being occupied by MAC pores. And lastly, the lowest level of MAC pores is generated under condition (iii) where MACs occupy (Figure 3B, red) 2.1% of the pathogen surface. Altogether, condition (i) produced the highest MAC level, followed in order by conditions (ii) and (iii).

Our model shows a majority of the recruited proteins are not involved in biochemical cascades that propagate/inhibit complement but as entities in complex with the pathogen surface (e.g., pathogen: FHR-3) as shown in Supplementary Figures 2A–C for conditions (i), (ii), and (iii), respectively. For instance, for condition (ii) shown as a green time profile in Supplementary Figure 2B, out of the total 81.1% surface occupation by complement proteins (180 min), Vn (magenta) in complex with just the pathogen surface accounts for 41.1 of 81.1%, FH and FHL-1 (cyan) account for 21.8%, and C4BP (brown) accounts for 9.8%. Thus, a total of 72.7% out of 81.1% of *N. meningitidis* surface is occupied by just recruiting complement proteins (C4BP, Vn, FH, and FHL-1) as entities with pathogen surface. Moreover, for condition (iii) shown as a red time profile in Supplementary Figure 2C, out of the total pathogen surface occupation of 83.1%, Vn (magenta) in complex with just the pathogen surface accounts for 36.6% of 83.1%, FH and FHL-1 (cyan) account for 19.4%, FHR-3 (yellow) accounts for 11.0%, and C4BP (brown) accounts for 8.7%. Thus, a total of 75.7% out of 83.1% of *N. meningitidis* surface is occupied by just recruiting complement proteins in complex with pathogen surface.

**FIGURE 4 |:**
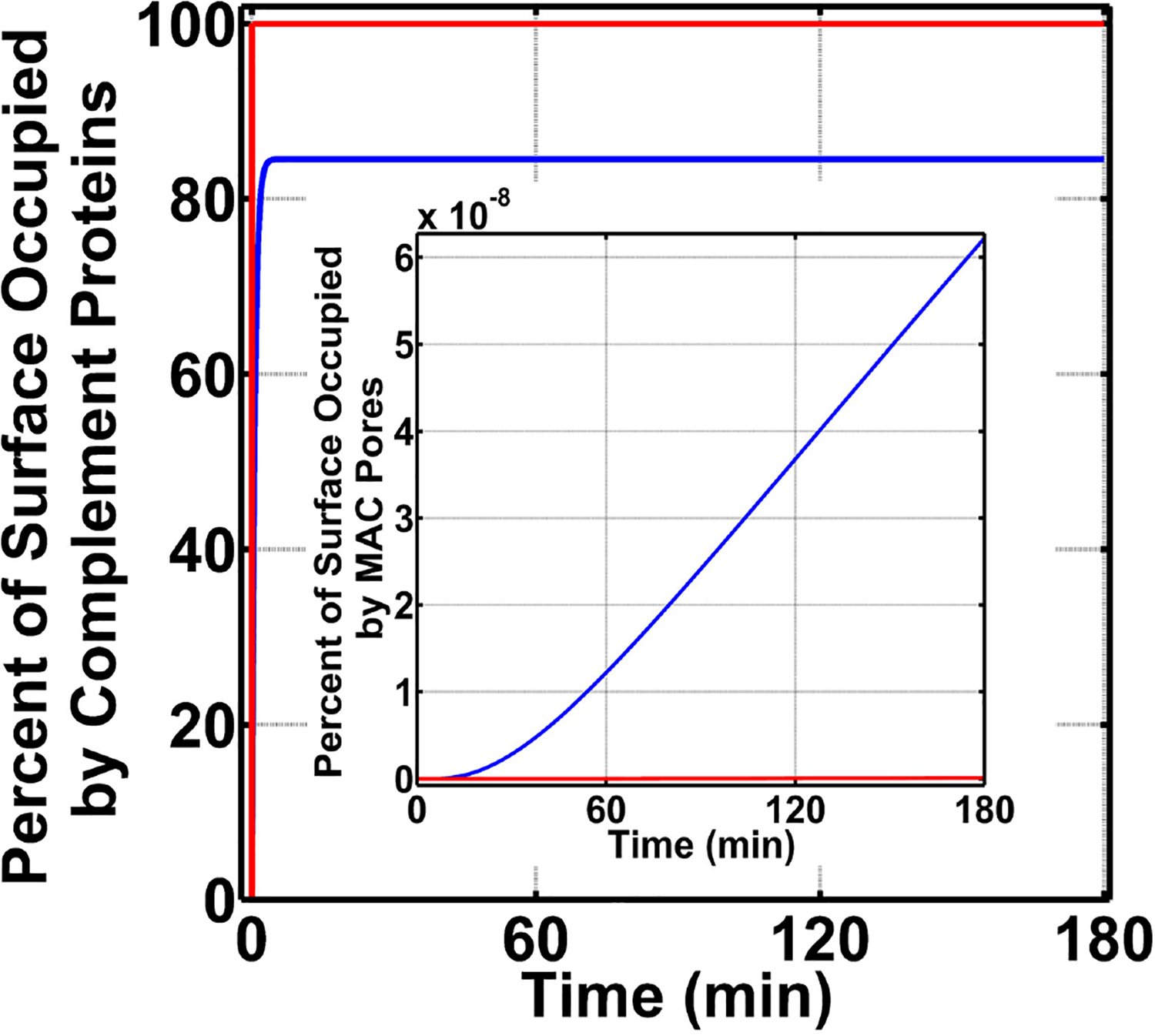
Bloodstream time profiles on *N. meningitidis* under three conditions: (i) FP and FHR-3 recruitment in blue; (ii) FP and C4BP, Vn, FH, and FHL-1 recruitment in green; and (iii) FP and FHR-3, C4BP, Vn, FH, and FHL-1 recruitment in red. All three conditions rapidly occupy the bacterial surface. However, condition (i) occupied a lower amount of 84.5% of the bacterial surface. Time profile of condition (ii) is under the profile of condition (iii). Moreover, membrane attack complex (MAC) profiles in the inset show condition (i) produced the highest amount, whereas under conditions (ii) and (iii) MAC levels are lower and overlapping.

We next generated time profiles for surface occupation and MAC production in the bloodstream by using the same three above-mentioned conditions. As shown in Figure 4, the surface of *N. meningitidis* is rapidly occupied under all three conditions. However, under condition (i) shown in blue, complement components occupy 84.5% of the pathogen surface and do not fully occupy the surface as conditions (ii) and (iii), shown in green and red, respectively. The result for condition (ii) is under the time profile of condition (iii). Later, we generated the MAC production profiles under the same conditions. The MAC production under all the three conditions occupied <<1.0% of the pathogen surface as shown in Figure 4 inset. Condition (i), however, generated the highest MACs followed by conditions (ii) and (iii). MAC levels for conditions (ii) and (iii) are overlapping as shown in Figure 4 inset. Overall, these results show MAC levels on *N. meningitidis* are similar to type 1 pathogens in the bloodstream where MAC levels in both pathogen types occupy <<1.0% of the total surface. However, the MAC production under condition (i) in nasopharynx is about eight orders of magnitude higher to that generated under condition (i) in the bloodstream. Moreover, under conditions (ii) and (iii) in the nasopharynx, MAC levels are about 10 orders of magnitude higher compared to those generated under conditions (ii) and (iii) in the bloodstream.

Similar to the above-mentioned time profiles, we also generated profiles for recruited complement proteins on the surface of the pathogen to determine if these proteins are mostly propagating/inhibiting complement cascade or bound to the pathogen surface as free entities. The results are shown in Supplementary Figures 3A–C for conditions (i), (ii), and (iii), respectively. In the bloodstream, our model shows almost all of the occupied surface on the *N. meningitidis* is due to the complement proteins in complex with the pathogen surface as entities, such as pathogen: FHR-3 or pathogen:Vn. For instance, for conditions (ii) shown as a green time profile in Supplementary Figure 3B, out of the total 99.9736% surface occupation by complement proteins (180 min), Vn (magenta) in complex with just the pathogen surface accounts for 57.32%, FH and FHL-1 (cyan) account for 30.44%, and C4BP (brown) accounts for 12.21%. In total, almost all of 99.9736% of *N. meningitidis* surface is occupied by just recruiting complement proteins C4BP, Vn, FH, and FHL-1, and subsequently leaving little room for other complement components, such as MACs. Moreover, although a similar response is also seen for condition (iii) shown as a red time profile in Supplementary Figure 3C, FHR-3 (yellow) accounts for only 0.14% of the occupied surface, whereas complement regulators (recruited) account for almost all of the remaining percent of surface occupation on *N. meningitidis.*

**FIGURE 5 |:**
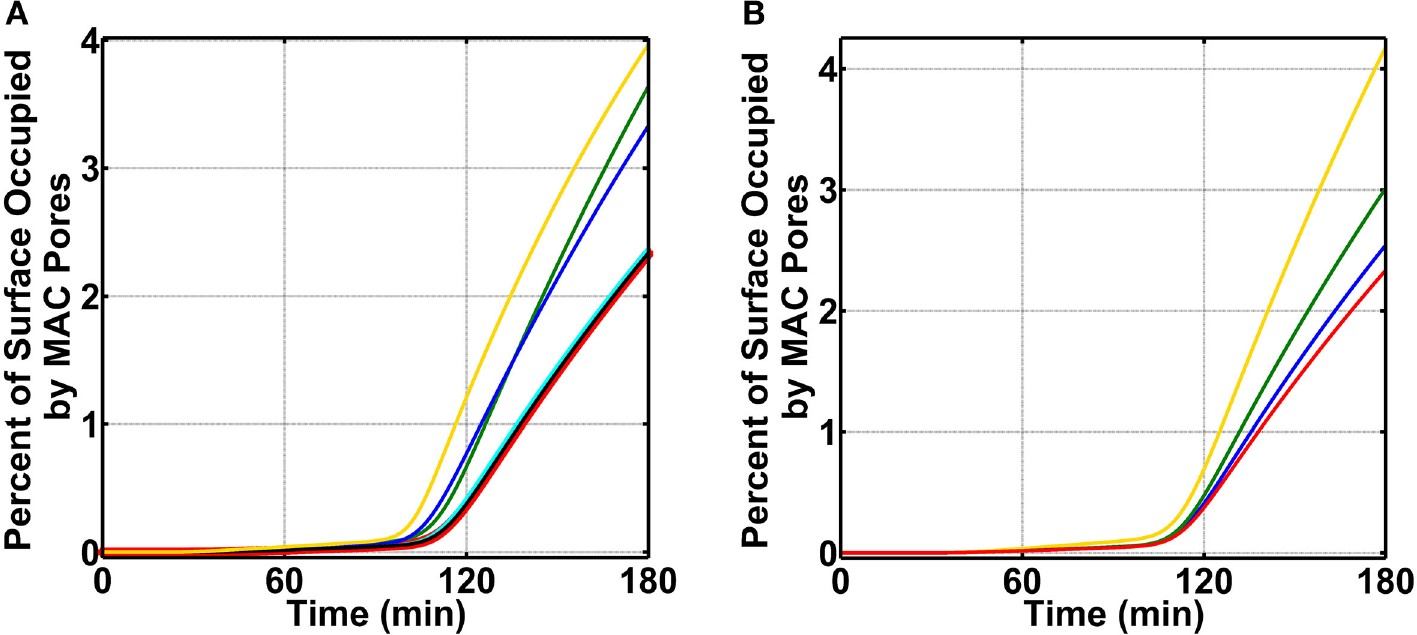
Nasal complement profiles on *N. meningitidis* under complement enhancement, varying recruitment capabilities, and absence of FHR-3. **(A)** Complement proteins are increased to 20.0% of their serum values. Compared to the membrane attack complex (MAC) deposition under *N. meningitidis* that recruits C4BP, Vn, FH, and FHL-1 (red, 2.3% occupation), the terminal module enhancement of proteins C5 (green, 3.3% occupation), C6 (blue, 3.6% occupation), and C9 (yellow, 4.0% occupation) led to higher MAC levels. However, enhancing the concentrations of C7 (cyan) and C8 (black) had minor effects. Time profile for *N. meningitidis* that recruits C4BP, Vn, FH, and FHL-1 (red) was increased to show the source of comparison. **(B)** Compared to the MAC deposition under *N. meningitidis* that recruits C4BP, Vn, FH, and FHL-1 (red), removing the ability of *N. meningitidis* to recruit Vn (yellow) had the largest effect with MAC level increasing by 2-fold. This was followed by an increase in the MAC deposition through the removal of FH and FHL-1 (green) recruitment capabilities. Lastly, removing the ability of *N. meningitidis* to recruit C4BP (blue) also increased the MAC deposition, but this had the smallest effect in comparison to Vn, FH, and FHL-1.

We continued our modeling efforts where we examined if FHR-3 was absent in the nasopharynx can enhance complement activation (FP) on *N. meningitidis* offset the protective effects of recruiting C4BP, Vn, FH, and FHL-1. For this, we first divide our initial concentration into three modules: AP module [C3, factor B (FB), factor D (FD), and properdin], CP module [C1, C1q, (C1rC1s)_2_, C2, and C4], and terminal module (C5, C6, C7, C8, and C9). We then increased the concentration of a single protein to 20.0% of its serum level, while keeping the concentration of others constant at 12.0% of their serum levels (Supplementary Table 1). This choice is based on the assumption that nasal complement proteins, like that of measured nasal C3 and immunoglobulin G (IgG) [49], can be as high as 20.0% of their serum levels. All modules were compared to the MAC production on *N. meningitidis* that recruits C4BP, Vn, FH, and FHL-1 and has no complement enhancement in the nasopharynx as mentioned above in Figure 3B for condition (ii). In Figure 5A, we present our results for the terminal module, whereas AP and CP modules are shown in Supplementary Figures 4A,B, respectively. Compared to MAC levels on *N. meningitidis* without terminal module enhancement (2.3% surface occupation, red), increasing the concentration of C9 had the largest effect in the MAC production with MAC levels occupying 4.0% of the pathogen surface in 180 min (yellow). Furthermore, increasing the concentration of C5 or C6 also led to a higher MAC level. As shown in Figure 5A, increasing the level of C5 increased the MAC production to occupy 3.6% (green) and increasing C6 lead to a MAC level occupying 3.3% of the *N. meningitidis* surface in 180 min. Conversely, increasing the concentration of C7 (cyan) or C8 (black) had minor effects on the MAC production. Lastly, increasing AP or CP modules also had minor effects on the MAC production (Supplementary Figures 4A,B). Overall, our results show increasing the terminal components C5, C6, and C9 to 20.0% of their serum levels led to an increase in MAC levels despite the absence of FHR-3 and the presence of the protective effects in recruiting C4BP, Vn, FH, and FHL-1.

Since enhancing terminal components (C5, C6, and C9) led to an increase in the MAC deposition on *N. meningitidis*, we next examined which of the recruited complement regulators (C4BP, Vn, FH, and FHL-1) had a greater effect on the MAC modulation (absent FHR-3). For this, we first carried out calculations by using nasal complement levels without enhancement and removing (making association rate constant zero) the ability of *N. meningitidis* to recruit C4BP, while still having the ability to recruit Vn, FH, and FHL-1. Subsequently, we performed a similar calculation by removing the ability of *N. meningitidis* to recruit Vn (making association rate constant zero), while still having the ability to recruit C4BP, FH, and FHL-1. We then completed our calculations by performing the same procedures for the recruitment of FH and FHL-1. All permutations are compared to *N. meningitidis* that recruits all four regulators and have no enhancement in complement activation as shown in Figure 5B (red, 2.3% surface occupation). Our model shows removing the ability of *N. meningitidis* to recruit Vn led to the largest effect with MAC level occupying 4.2% of the pathogen surface in 180 min (Figure 5B, yellow). This equates to about a 2-fold increase in the MAC deposition compared to *N. meningitidis* that recruits all four regulators and has no enhancement (Figure 5B, red). This is followed by the loss in the ability to recruit FH and FHL-1, where MACs occupy 3.0% of the surface in 180 min (Figure 5B, green). Lastly, removing the ability of *N. meningitidis* to recruit C4BP led to the smallest increase in the MAC occupation (2.5%) in 180 min (Figure 5B, blue). Altogether, our model shows removing the ability to recruit Vn had the largest effect on the MAC production, followed by FH and FHL-1, and then C4BP.

**FIGURE 6 |:**
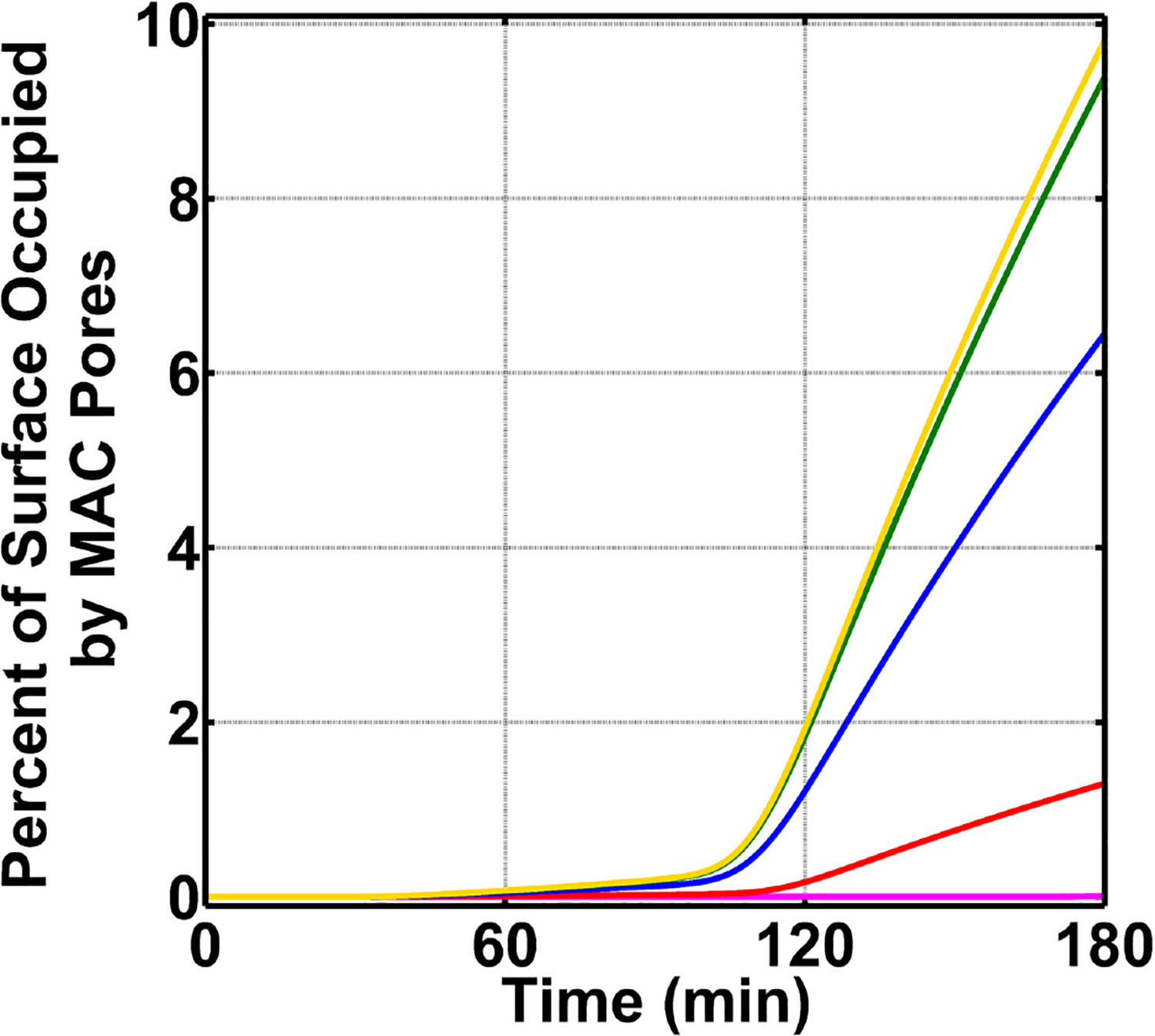
Nasal complement profiles on *N. meningitidis* with varying regulatory levels and absence of FHR-3. Concentrations of C1-INH, C4BP, Vn, Cn, FH, and FHL-1 are varied between 2.0 and 0.0002% of their respective serum levels. The highest membrane attack complex (MAC) levels with the surface occupation of about 10.0% are produced when the concentration of the complement regulators is reduced to either 0.002% (green) or 0.0002% (yellow) of their serum levels. This is followed by 0.02% (blue) of serum levels where MACs occupied 6.4% of the bacterial surface. Lastly, the MAC production under 0.2% (red) of serum occupies 1.3% of the bacterial surface, and subsequently increasing the complement regulators to 2.0% of their serum levels led to MAC pores occupying 0.008% of the bacterial surface.

We concluded our studies by exploring how the board range in nasal regulatory levels (0.2–0.0002% of their serum levels) affects MAC production on *N. meningitidis* (absent FHR-3). This range is based on the assumption that the nasal complement regulatory levels can vary similarly to that of mucosal FH [50]. For this, we reduced the concentrations of C1 inhibitor (C1-INH), C4BP, Vn, clusterin (Cn), FH, and FHL-1 between 0.2 and 0.0002% of their respective serum levels (Supplementary Table 1). As shown in Figure 6, as we reduce the concentrations of complement regulators from 0.2 to 0.0002% of their serum levels, we see an increase in the MAC deposition. The MAC production increases from occupying 1.3% of the surface under 0.2% (red) to an occupation level of 6.4% under nasal regulatory levels of 0.02% of their serum levels (blue). Further reducing the nasal regulatory levels to 0.002% (green) or 0.0002% (yellow) of their serum levels has increased the MAC occupation level to about 10% of the pathogen surface. In contrast, increasing the concentration of complement regulators to 2.0% of their serum levels reduced the MAC production to occupy 0.008% of the pathogen surface as shown in Figure 6 in magenta. From this, our model predicts if complement proteins are maintained at 12.0% of their serum levels, and nasal C1-INH, C4BP, Vn, Cn, FH, and FHL-1 are maintained at 0.02% of their serum levels or lower, MAC pores can go from occupying 6.4% (0.02%) to about 10% (under 0.002% or 0.0002%) of the total *N. meningitidis* surface. Conversely, increasing the concentration of complement regulators to 0.2% led to a decrease in the MAC occupation of 1.3%. Moreover, further increasing the concentration of regulators to 2.0% led to reduced MAC occupation levels of 0.008%. Altogether, our model shows mounting higher levels of MAC may require a concentration barrier between complement proteins and regulators (C1-INH, C4BP, Vn, Cn, FH, and FHL-1). However, as this concentration barrier is reduced, the MAC deposition is potently regulated on the surface of *N. meningitidis*.

## DISCUSSION

In this study, we developed a computational model of the complement system that includes all three pathways and the crosstalk with different subsets of the immune system. Our comprehensive model includes the alternative, classical, and lectin pathways of the complement system and components of the humoral immunity such as IgG, IgM, CRP, SAP, and PTX3. Biomolecular details and kinetic parameters of the complement system are assembled from the available literature data, whereas unknown parameters are estimated/assumed as mentioned in section “Methods.” Subsequently, we compare the different phases of complement activation by generating time profiles of the pathogen surface that is occupied by the complement components and MAC pores (C5b-9_18_).

Our computational model shows that pathogens (type 1 and type 2) are rapidly occupied by complement components in the bloodstream. However, in both pathogen types, our model shows MACs occupied <<1.0% of the total pathogen surface. Thus, our model predicts if a pathogen enters the bloodstream, phagocytosis, following opsonization, may serve as the primary mechanism of eliminating pathogens. To highlight this point, in the absence of MACs, specific antibodies in conjunction with complement activation can elicit bacterial killing of *N. meningitidis* through opsonophagocytosis [51–53]. Furthermore, opsonophagocytosis may also compensate for the MAC deficiency as impairment in assembling MACs increases the risk for *Neisseria* and disseminated gonococcal infections, whereas not predisposing individuals for contracting infections caused by other Gram-negative pathogens [54]. Altogether, pathogen elimination through opsonization followed by phagocytosis may form a potent immune response in the absence of MAC-based directed response.

In contrast to the terminal cascade in the bloodstream, MAC levels in the nasopharynx were much higher. Production of MAC under condition (i) occupied 6.1% of the total pathogen surface (*N. meningitidis*), followed by condition (ii) with 2.3% surface occupation, and then condition (iii) with 2.1% surface occupation, as shown in Figure 3B. This difference in nasal MAC production equates to about eight orders of magnitude higher in the nasopharynx, compared to MAC levels on pathogens in the bloodstream. And this elevated MAC production in nasopharynx stems mainly from the differences between levels of complement activators and regulators. Nasopharyngeal secretions of C3 are estimated to be between 4.2 and 20.2% of serum C3 levels [49], whereas levels of mucosal FH are below 0.2% (in a range between 0.2 and 0.0002%) of plasma FH levels [50]. These concentration differences in nasopharynx will favor overactivation of the complement system as nasal C3 levels are 50- to 233-fold greater than nasal FH levels. Conversely, complement activation in the bloodstream is regulated as the mean plasma C3 (7.1 μM) is about 2-fold higher than plasma FH (3.2 μM). It should be noted, unlike other regulators of the alternative pathway, FH is the main regulator of AP that targets complement proteins such as C3(H_2_O) and C3b or complement complexes such as C3/C5 convertases C3bBb.

Altogether, *N. meningitidis* may be susceptible to MAC-mediated lysis due to overactivation of the complement system in the nasopharynx. Our model shows that the MAC deposition on *N. meningitidis* is dependent on the recruitment of complement regulators (Figure 5B). Removing the ability of *N. meningitidis* to recruit complement regulators led to enhanced MAC deposition. The role of complement regulators is shown in recent genome-wide association studies where variants on the complement regulator factor H gene are associated with susceptibility to meningococcal disease, caused by *N. meningitidis* [55, 56]. It should be noted that FHL-1 is an alternative splice variant of FH and that the earlier study [56] did also show genetic variation within FHR-3. Although these studies did not find any association with genes of other complement regulators to the susceptibility of meningococcal disease, our model predicts recruitment of Vn as an important factor used to evade the complement system. And this may explain why the majority of the sequenced meningococcal isolates contain Msf (used to recruit Vn) [57].

Increasing the concentration of complement proteins to 20% of their serum values showed the terminal module (C5, C6, and C9) enhanced MAC production and subsequently counteracted the effects of recruiting complement regulators by porA, Msf, and fHbp (Figure 5A). In contrast, increasing the concentrations AP and CP modules had minor effects on the MAC production as shown in Supplementary Figures 4A,B, respectively. To our surprise, increasing the concentration of properdin in the AP module did not enhance the MAC deposition. In our current model, properdin is taken as a stabilizer of the convertases and not as a recognition molecule. This choice in properdin function comes from recent studies showing properdin binding to zymosan, *Escherichia coli*, human umbilical vein endothelial cells, and *N. meningitidis* is solely dependent on the initial C3b deposition [58, 59]. Although past studies have shown properdin as a recognition molecule that initiates AP [60–64], caution needs to be taken when interpreting these results depending on the serum conditions of intact C3 and molecular structure of properdin [58, 65]. However, a recent study showed generating a highly polymerized artificial form of properdin by recombinant techniques was able to directly bind to *N. meningitidis* and elicit an enhanced complement activation [66]. High-order oligomers of properdin have additional functions that include direct binding to pathogens and serving as a platform for the complement activation. And this function may potentially be manipulated for therapeutic interventions to target invasive pathogens, such as *N. meningitidis.*

**FIGURE 7 |:**
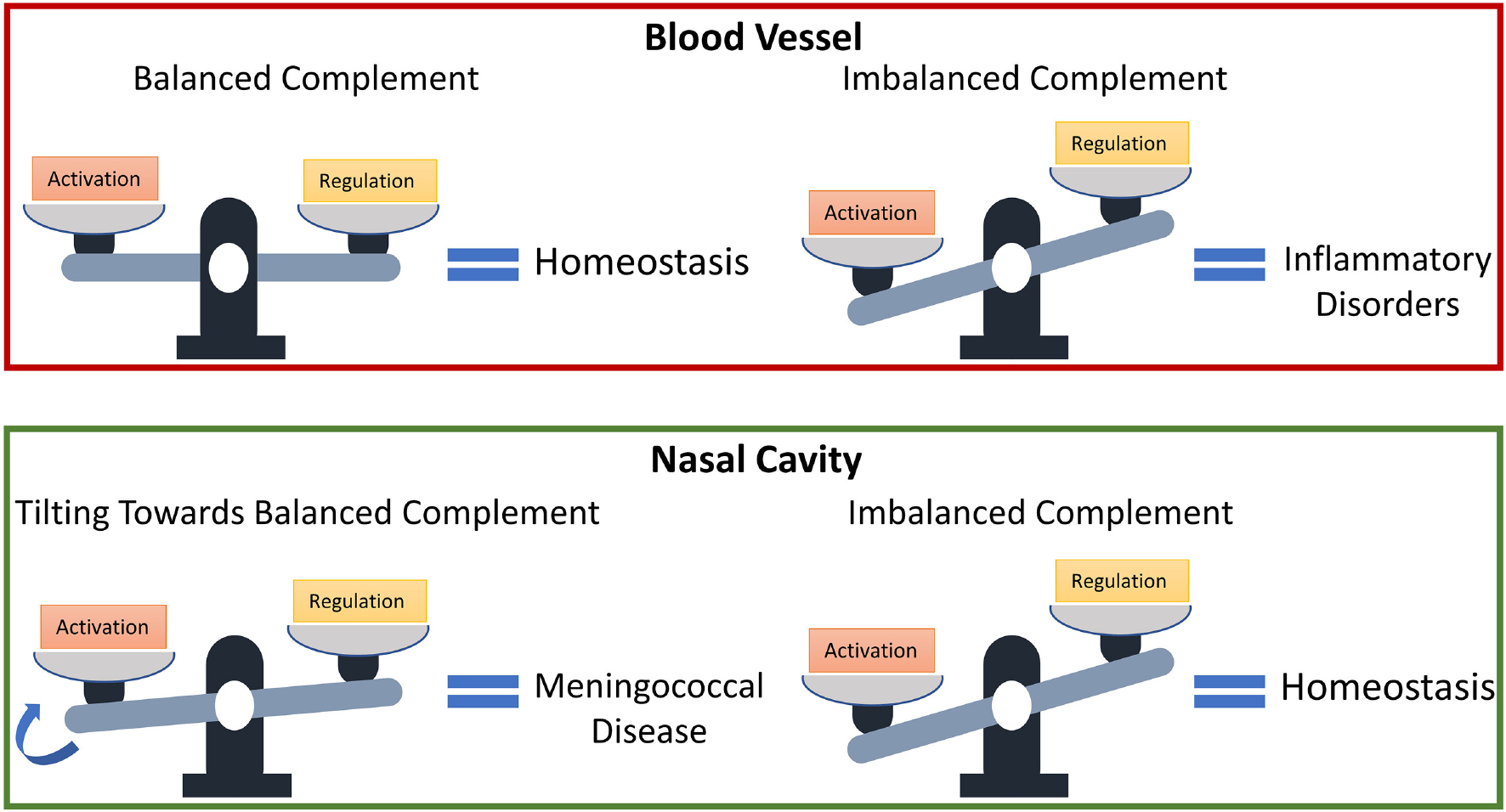
Model predictions for the susceptibility of developing meningococcal disease. States associated with a dysfunctional complement system due to an overactivation may have protective effects in the nasal cavity against invasive pathogens.

Our findings show that levels of complement regulators C1-INH, C4BP, Vn, Cn, FH, and FHL-1 may have to be reassessed in conjunction with their local host microenvironments to determine if they function as protective or not. Balanced complement activation and regulation are essential to avoid inflammatory disorders; however, an imbalanced complement system (favoring overactivation) may signify complement homeostasis in regions, such as the nasopharynx that experiences constant insults from toxins and pathogens [67–69]. And if genetic and environmental factors, which affect complement regulatory (FH) plasma levels [70], also affect nasal levels, i.e., increasing their levels, pathogenic or commensal strains may use the elevated levels of complement regulatory proteins to modulate the MAC deposition and potentially develop into invasive disease. Altogether, if the complement activation is balanced in the nasopharynx as shown in the bloodstream under normal conditions, this new state may promote increased survival of pathogens in the nasopharynx. And this subsequently makes elevated nasal levels of C1-INH, C4BP, Vn, Cn, FH, and FHL-1 as early biomarkers for an individual potentially developing meningococcal disease. A summary of our model prediction is shown in Figure 7.

A major limitation of this study is that the findings are exclusively computational. Experimental studies were not conducted to either support or refute the findings. Future studies will need to be conducted to examine the predictions of the model. Additionally, our model does not account for the flow-mediated transport of complement proteins and complexes. This will not account for multiscale dynamics of complement reactions on the pathogen surface that occurs in a different time and spatial scales. Moreover, the presence of flow-mediated transport will affect both the assembly and deposition of MAC pores on the pathogen surface. Subsequently, altering the lysis rate of pathogens. Thus, by not including the transport of MAC proteins, further modeling efforts should be taken to better compare the dynamics of MAC (and complement activation) in the nasal cavity.

## METHODS

### Computational Model

Our model is based on the manual construction of biochemical reactions of the complement system. Figure 1 summarizes the biochemical reactions that form a network of proteins, fragments, and complexes that either activate or regulate the complement system. This network of interactions is also coupled with other segments of the humoral immunity that includes IgM, CRP, SAP, and PTX3. The formulated biochemical model entails the different phases of complement activation, propagation, and regulation in both fluid and cellular surfaces. Human erythrocytes were used as a host cell model, whereas *Escherichia coli* was used as a pathogen cell model [47]. We incorporated two types of pathogens: (type 1) complement susceptible pathogen, and (type 2) model of *N. meningitidis* located in either nasopharynx or bloodstream. Moreover, using our biochemical network, we assembled a system of 670 ordinary differential equations (ODEs) with 328 kinetic parameters. Our system was organized into modules of equations that describe the biochemical cascade for initiation and propagation, host cell, pathogen, regulation, proteins, and fragments as shown in Supplementary Equation 1. The equations describe the reaction rates of complement proteins, fragments, and complexes in conjunctions with immunoglobins and pentraxins. Enzymatic reactions are based on Michaelis–Menten kinetics with substrate competitions taken into account for the complement complexes, such as C3/C5 convertases [71]. Model parameters of concentration and kinetic parameters, when available, are acquired from the published literature data or experimental measurements (Supplementary Table 2). We used the ode15s solver of MATLAB (MathWorks, Natick, MA, USA) to solve our ODEs.

For unknown kinetic rate constants in our model, we assumed those parameters to be the same for proteins that are structurally or functionally homologous with experimentally determined parameter values. For instance, the decay rate of C3bBb by FH is known [72], whereas the decay rate of C3(H_2_O)Bb by the regulatory function of FH is not known. However, we assumed that these two rates to be the same for the following reasons. First, C3(H_2_O) is known as a C3b-like molecule that has a homologous function as C3b by forming a proconvertase with FB. Second, proconvertase C3(H_2_O)B, just like C3bB, is activated by the serine protease factor D to form convertases [C3(H_2_O)Bb and C3bBb]. Lastly, without the FH involvement, both convertases of C3(H_2_O)Bb and C3bBb have very similar decay rates of 9.0 × 10^−3^ s^−1^ and 7.7 × 10^−3^ s^−1^, respectively [73]. Similarly, we assumed complement regulators: complement receptor 1 (CR1), decay-accelerating factor (DAF), and C4BP accelerate the decay of C3/C5 convertases with the same kinetic rate constant as FH. We made this assumption because all three regulators are functionally homologous to FH by either inhibiting or deactivating C3/C5 convertases.

We performed global sensitivity analysis by using multiparametric sensitivity analysis (MPSA) as described in our previous paper [47] to identify complement parameters for MAC production under FP activation and recruitment of C4BP, Vn, FH, FHL-1, and FHR-3 on *N. meningitidis.* We emphasized in the nasopharynx as MAC production was more pronounced unlike the MAC production in bloodstream that occupied <<1% of the pathogen surface. Briefly, we first selected all the kinetic parameters in our biochemical network of the complement system. We then set broad ranges for each parameter value to cover all feasible variations and subsequently used the Latin hypercube sampling method to generate our random parameter vectors. We simulated our model for each parameter set to calculate the objective function value for 1,585 kinetic variations. The objective function is the sum of squared errors between the observed and perturbed system output values. Subsequently, the given set of parameters were determined to be acceptable or unacceptable by comparing the objective function value to a threshold of 50% divisions of the 1,585 sorted objective functions. Lastly, we calculated the cumulative frequency for each parameter with both acceptable and unacceptable cases. The sensitivity of each parameter was evaluated using Kolmogorov–Smirnov statistic. The parameter ranges used in MPSA can be found in Supplementary Table 3 of Supporting Information and the result is shown in Supplementary Figure 5.

### Biochemical Model

#### Alternative Pathway

The alternative pathway auto-activates in the fluid phase by the spontaneous hydrolysis of the complement protein C3. Once activated, C3 will generate a C3b-like molecule known as C3(H_2_O) [74]. This activated molecule is targeted by the complement protease FB to form a proconvertase known as C3(H_2_O)B [75]. Subsequently, this complex is targeted by the serine protease FD that cleaves the bound FB complex into Ba and Bb, whereas Ba is released into the fluid, and Bb remains attached to C3(H_2_O) to form the initial C3 convertase of the alternative pathway, C3(H_2_O)Bb [73, 74]. This short-lived enzyme catalyzes the cleavage of C3 into a smaller fragment C3a and a larger molecule called nascent C3b (nC3b) [73, 74]. The first product, C3a plays a major role in mediating inflammation by inducing histamine production by mast and basophil cells, resulting in vasodilatation [13, 76, 77], whereas in other cases, C3a plays an anti-inflammatory role by preventing neutrophil migration and degranulation [13, 78]. Nascent fluid C3b (nfC3b) is highly reactive due to its exposed internal thioester bond and is capable of indiscriminately attaching to cell surfaces *via* covalent linkage [79–81]. However, the newly formed nfC3b has a short half-life of 60 μs before it loses the ability to covalently attach to nearby cell surfaces and remain solely in the fluid phase to form fluid C3b (fC3b) [82]. Similar to the cascade of reactions initiated by C3(H_2_O), fC3b or surface-bound nC3b can associate with FB, followed by FD cleavage to form the C3/C5 convertase C3bBb [73, 83]. This reaction scheme is also followed by nC3b if it interacts with IgG already in complex with nC3b to form IgGC3bC3b and subsequently interacts with FB and FD to form the C3 convertase IgGC3bC3bBb [84].

Formation of surface-bound convertases will then recruit the complement protein properdin to form the C3/C5 convertase, such as C3bBbP [85, 86]. The recruitment of properdin stabilizes C3/C5 convertases by extending their half-lives by 10-fold and hence initiating the amplification loop. The presence of stabilized convertases will cleave more C3 (into C3a and nC3b) and C5 (into C5a and C5b) than convertases without properdin. The smaller fragments C3a and C5a are mediators of inflammation, but C5a is more potent [13]. However, the larger fragments, nC3b, will initiate the same cascade of reaction by interacting with FB and FD to form C3bBb, whereas C5b initiates the terminal step by first interacting with C6 to form C5b6. This step in complex formation between C5b and C6 is affected by a 5-min short half-life of C5b before irreversibly losing its binding ability to C6 [87]. However, if C5b6 complex is formed, C7 and C8 will sequentially interact C5b6 to form C5b-8. This complex (C5b-8) forms a small pore that later expands by the insertion of multiple C9s to form the MAC [16, 88, 89].

#### Classical Pathway

Similar to the spontaneous activation of C3 in the alternative pathway, the classical pathway is also spontaneously activated in the fluid phase through its first component of complement, C1 [9–11]. C1 is composed of two complexes of C1q and the heterotetramer (C1rC1s) containing two copies of C1r and C1s serine proteases. Activation of C1 proceeds by the autocatalytic cleavage of C1r, which subsequently activates/cleaves C1s subunits (C1s*). However, nC4b has a short half-life for its exposed thioester and hence becomes rapidly hydrolyzed in solution to generate the fluid C4b (fC4b) [90]. This molecule cannot covalently attach to cellular surfaces but can associate with complement C2 to form the classical pathway proconvertase C4bC2. Soon after, C1* will cleave C2 to form the C3/C5 convertase of the classical pathway, C4b2a. The same cascade of reactions is followed by surface-bound C4b to form C4b2a. On the one hand, the formation of C4b2a leads to cleavage of C3 into C3a and nC3b to propagate the alternative pathway. On the other hand, nC3b or nC4b molecules can also form covalent linkage with each other to form homo- or heterodimers, such as C3bC3b/C4bC4b and C3bC4b that can interact with FB or C2 to form C3/C5 convertases (C3bC3bBb/C4bC4bC2a and C3bC4bBb/C3bC4bC2a) [91–93].

In addition to fluid phase activation, the classical pathway can directly act on cells through the presence or absence of antigen–antibody complexes [94]. In the presence of antigen–antibody immune complexes, C1q (as part of C1) will bind to fragment crystallizable (Fc) portion region of IgG clusters and IgM [94–98]. However, in the absence of antigen–antibody immune complexes, C1q recognizes different targets, such as the short pentraxin CRP and the long pentraxin PTX3. Once C1 binds to its different targets, it follows the same cascade of reaction as in the fluid phase by the auto-activation of C1r, which subsequently activates C1s (C1*). This will lead to cleavage of C4 and C2 to form the convertase C4b2a. Overall, the classical pathway can recognize various targets through the recognition molecule C1q. But once bound, C1 goes through the same cascade of reactions to generate C1*.

#### Lectin Pathway

Unlike the alternative and classical pathways, the lectin pathway contains numerous pattern recognition molecules (PRMs) in complex with either three types of serine proteases or with two types of non-enzymatic protein molecules. The PRMs of the lectin pathway include MBL, CL-L1, CL-K1, CL-LK, M-ficolin, L-ficolin, and H-ficolin [99, 100]. These molecules have a common feature where they can associate with serine proteases known as MASP-1, MASP-2, and MASP-3. In addition to associating with MASPs, the pattern recognition molecules can also interact with MAp19 and MAp44 that do not contain a serine protease domain [101, 102]. The serine proteases (MASP-1,-2, and -3) are in their zymogen forms and follow subsequent mechanisms to become activated with various cross-activation steps. For instance, MASP-1 in complex with PRM auto-activates and cleaves zymogen MASP-2 and MASP-3 [103, 104]. Furthermore, activated MASP-1 cleaves zymogen MASP-1, whereas activated MASP-2 cross-activates zymogen MASP-1 [103, 105]. However, once PRM–MASP-1 or PRM–MASP-2 are activated, they both can propagate the lectin pathway by cleaving complement proteins C2 and C4 to generate the C3/C5 convertase C4b2a [106, 107]. Once this convertase is formed, it can generate C3a and nC3b or C5a and C5b. And through this mechanism, the lectin pathway can initiate either the alternative pathway or the terminal step to form MACs.

#### Factor H-Related Proteins

Factor H is the main regulator of an alternative pathway that deactivates C3b and accelerates the decay of C3/C5 convertases. However, factor H belongs to a family protein that includes factor H-like protein 1 (FHL-1) and five factor H-related proteins (FHR-1, FHR-2, FHR-3, FHR-4, and FHR-5) [108–110]. The genes of FHRs are located downstream of the FH gene and each gene codes for complement control proteins (CCP) that are homologous to CCP domains 6–9 and 18–20 of FH [108–110]. However, FHRs lack the regulatory domains of CCP1-4 of FH/FHL-1 and hence implies FHRs may not inhibit complement. Recent evidence suggests that factor H-related proteins may function as positive regulators of the complement system that compete against FH and further enhance complement activation and propagation. For instance, FHR-1 was shown to enhance complement in several ways, such as binding to C3b and allowing convertase formation, not accelerating the decay of convertases, and not inhibiting convertase decay activity of FH [111]. Furthermore, FHR-4 in complex with CRP was able to activate complement and result in C3 and C4 deposition [112]. Lastly, FHR-5 was shown to generate the C3/C5 convertase when incubated with C3b, FB, FD, and properdin [113].

#### Regulation

Due to the potency of complement activation and propagation, multiple regulatory checkpoints are present to ensure host cell protection. In the alternative pathway, FH, followed by FHL-1, are the two main regulators present in both fluid and cellular surfaces. Both FH and FHL-1 bind to C3b and act as cofactors for Factor I (FI) cleavage of C3b into inactivated C3b (iC3b) [114–118]. Additionally, FH and FHL-1 also target convertases, such as C3bBb and accelerate the rate of decay into its respective components of C3b and Bb [114–116]. Similarly, the classical and lectin pathways also contain two main regulatory proteins known as C1-INH and C4BP that act in both fluid and surface phases. Activated C1, MASP-1, and MASP-2 in complex with PRMs are all regulated by C1-INH [119, 120], whereas C4b is targeted by C4BP and subsequently deactivated into C4d with the help of the serum protease FI [121]. Furthermore, C4BP targets convertases, such as C4b2a and accelerates their decay into their respective components [121].

As a secondary checkpoint, host cells also contain membrane-bound regulators, such as CR1 that regulates activation of the complement system. CR1 regulates in a similar manner as FH/C4BP by binding to either C3b/C4b and using the protease FI to deactivate the complement proteins into iC3b/C4d [122]. Furthermore, iC3b is further cleaved into C3dg by the action of CR1 in collaboration with FI. CR1 can also induce decay acceleration of C3/C5 convertases into their respective parts [123]. In addition, another membrane-bound regulator, DAF, also targets C3/C5 convertases and rapidly dissociates the components [124, 125], whereas DAF only targets convertases, another cell-bound regulator, membrane cofactor protein (MCP), deactivates C3b or C4b with the aid of FI. Since our system uses erythrocytes as a cellular model [47], MCP is not included because it is not expressed on erythrocytes. Lastly, the terminal cascade is regulated through the action of vitronectin or clusterin that inhibits surface binding of C5b-7, whereas CD59 (protectin) inhibits C9 polymerization to regulate MAC pore formation [116, 126].

## Supplementary Material

Data_Sheet_1_Systems Biology Modeling of the Complement System Under Immune Susceptible Pathogens

Table_1_Systems Biology Modeling of the Complement System Under Immune Susceptible Pathogens

Table_2_Systems Biology Modeling of the Complement System Under Immune Susceptible Pathogens

Table_3_Systems Biology Modeling of the Complement System Under Immune Susceptible Pathogens

Data_Sheet_2_Systems Biology Modeling of the Complement System Under Immune Susceptible Pathogens

## Data Availability

The original contributions presented in the study are included in the article/Supplementary Material, further inquiries can be directed to the corresponding author/s.
