# Supplementary material for: Systems Biology Modeling of the Complement System Under Immune Susceptible Pathogens
Source: Front Phys. Author manuscript; Available in PMC 2022 Feb 9. (PMC8827490; doi:10.3389/fphy.2021.603704)
Supplement: Data_Sheet_1_Systems Biology Modeling of the Complement System Under Immune Susceptible Pathogens [file NIHMS1768816-supplement-Data_Sheet_1_Systems_Biology_Modeling_of_the_Complement_System_Under_Immune_Susceptible_Pathogens.pdf]

Supporting Information

Computational Modeling of the Complement System Under Immune Susceptible Pathogens

Nehemiah T. Zewde,[1] Rohaine V. Hsu,[1] Dimitrios Morikis[1] and Giulia Palermo[1,2*]

1. Department of Bioengineering and 2. Department of Chemistry, University of California, Riverside, Riverside, California, United States of America.

Table of Contents

Supplementary Figures S1-S5.

Table S1. (A) Complement molecular masses and concentrations. (B) Surface concentrations. (C) Nasal complement concentrations. (D) Nasal concentration of complement regulators. (E) Nasal complement enhancement. (F) Nasal range of complement regulators. *Provided as a separate file.*

Table S2. Kinetic rate constants. *Provided as a separate file.*

Table S3. Range of kinetic rate constants implemented in sensitivity analysis. *Provided as a separate file.*

Supplementary Equations. Theoretical model of complement system. System of 670 ordinary differential equations. *Provided as a separate file.*

*Corresponding author:
giulia.palermo@ucr.edu

Supplementary Figures

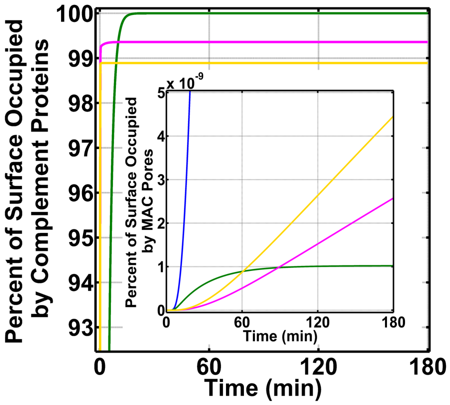

Figure S1. Zoom-in panel of Fig 2A. Percent surface occupation on type 1 pathogens under: (i) FP in blue (ii) CP in green, (iii) LP in magenta, and (iv) FHR1-5 in yellow. Complement components under LP and FHR1-5 nearly saturate the pathogen surface, whereas CP-based surface occupation covered the entire pathogen surface. Inset shows FP produced highest MAC level, and then followed in order by FHR1-5, LP, and CP.

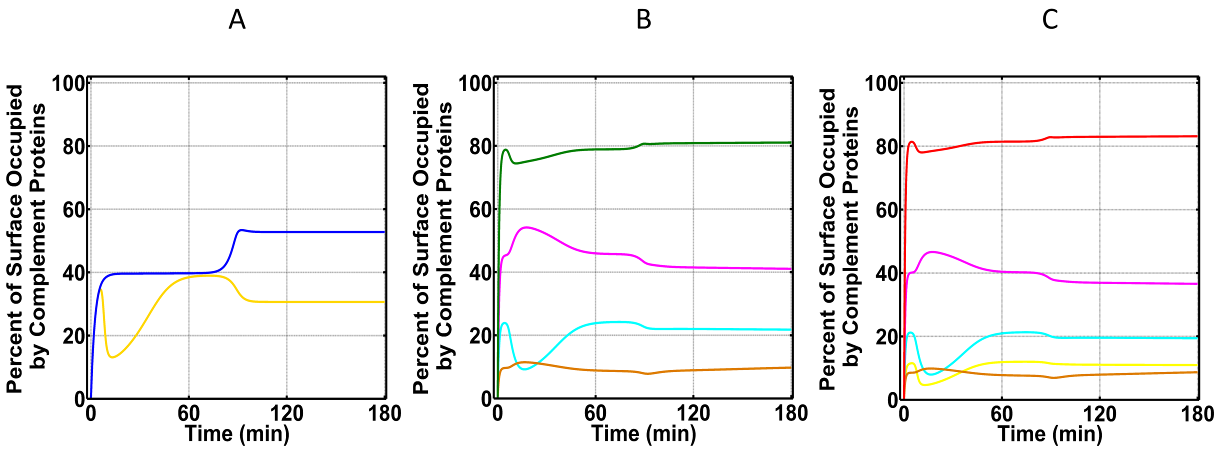

Figure S2. Nasal complement profiles on *N. meningitides* that occupy the pathogen surface under three conditions: (i) FP and FHR-3 recruitment in panel A (ii) FP and C4BP, Vn, FH, and FHL-1 recruitment in panel B, (iii) FP and FHR-3, C4BP, Vn, FH, and FHL-1 recruitment in panel C. (A) Total surface occupation under condition (i) accounts for 52.8% in 180 minutes (blue), whereas FHR-3 (yellow) complex with just the pathogen surface accounts for 30.6% of the 52.8%. (B) Out of the total 81.1% surface occupation by complement proteins under conditions (ii) shown in green (180 minutes), Vn (magenta) in complex with pathogen alone accounts for 41.1%, FH and FHL-1 (cyan) account for 21.8%, and C4BP (brown) accounts for 9.8%. (C) Total surface occupation under condition (iii) accounts for 83.1% in 180 minutes (red), whereas Vn (magenta) in complex with pathogen alone accounts for 36.6%, FH and FHL-1 (cyan) account for 19.4%, FHR-3 (yellow) accounts for 11.0%, and C4BP (brown) accounts for 8.7%.

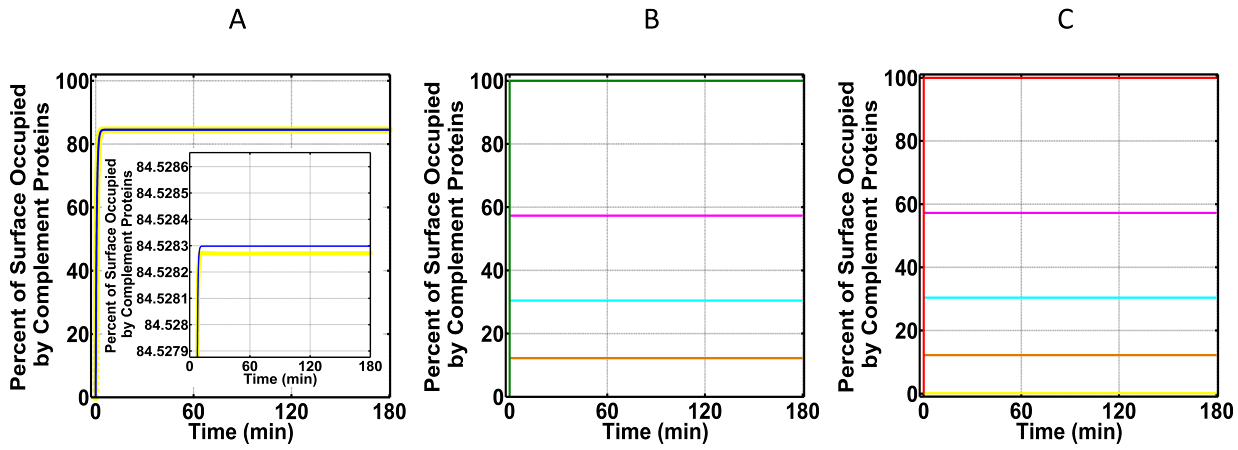

Figure S3. Bloodstream time profiles on *N. meningitides* under three conditions: (i) FP and FHR-3 recruitment in panel A (ii) FP and C4BP, Vn, FH, and FHL-1 recruitment in panel B, (iii) FP and FHR-3, C4BP, Vn, FH, and FHL-1 recruitment in panel C. (A) FHR-3 (yellow) in complex with just the pathogen surface accounts for almost all of the 84.5% of the pathogen surface occupied under condition (i) shown in blue. Time profile for pathogen:FHR-3 (yellow) complex was increased to show better comparison as shown in inset. (B) Similarly, almost all of the pathogen surface occupation (99.9736%) under conditions (ii) shown in green is due to Vn (magenta) in complex with pathogen surface accounting for 57.32%, FH and FHL-1 (cyan) accounting for 30.44%, and C4BP (brown) accounting for 12.21%. (C) Lastly, similar response is also seen under condition (iii) shown in red, where almost all of the pathogen surface occupied (99.9736%) is due to Vn (magenta) in complex with pathogen surface accounting for 57.23%, FH and FHL-1 (cyan) accounting for 30.40%, FHR-3 (yellow) accounting for 0.14%, and C4BP (brown) accounting for 12.20%.

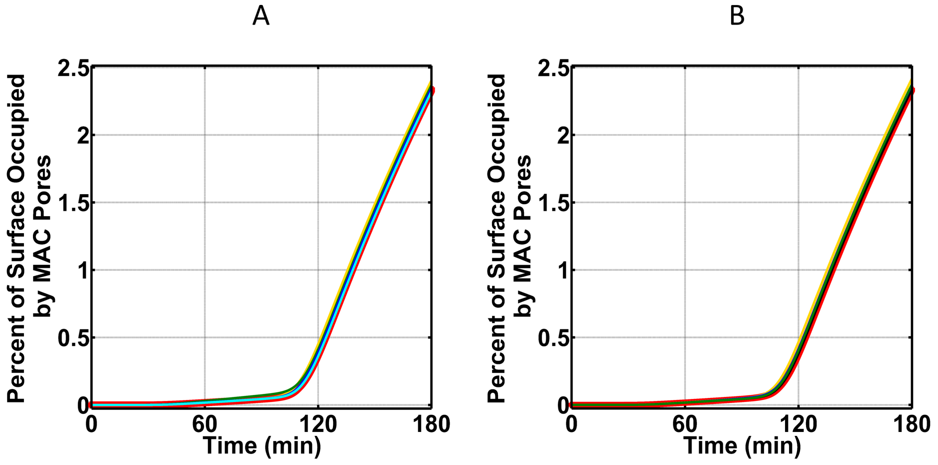

Figure S4. Nasal complement profiles on *N. meningitides* under AP and CP module enhancement and absence of FHR-3. Complement proteins are increased to 20.0% of their serum values. Increasing AP or CP modules had minor effects in MAC production. Time profile for *N. meningitidis* that recruits C4BP, Vn, FH, FHL-1 (red) was increased to show the source of comparison. AP modules include C3 (yellow), factor B (green), factor D (blue), properdin (cyan). Factor B and factor D profiles are under the profiles of C3 and *N. meningitides* that recruits C4BP, Vn, FH, and FHL-1. CP modules include C1 (yellow), C1q (cyan), $(C1rC1s)_2$ (black), C2 (blue), and C4 (green). C1q and C2 profiles are under the profiles of C1, C4, and $(C1rC1s)_2$.

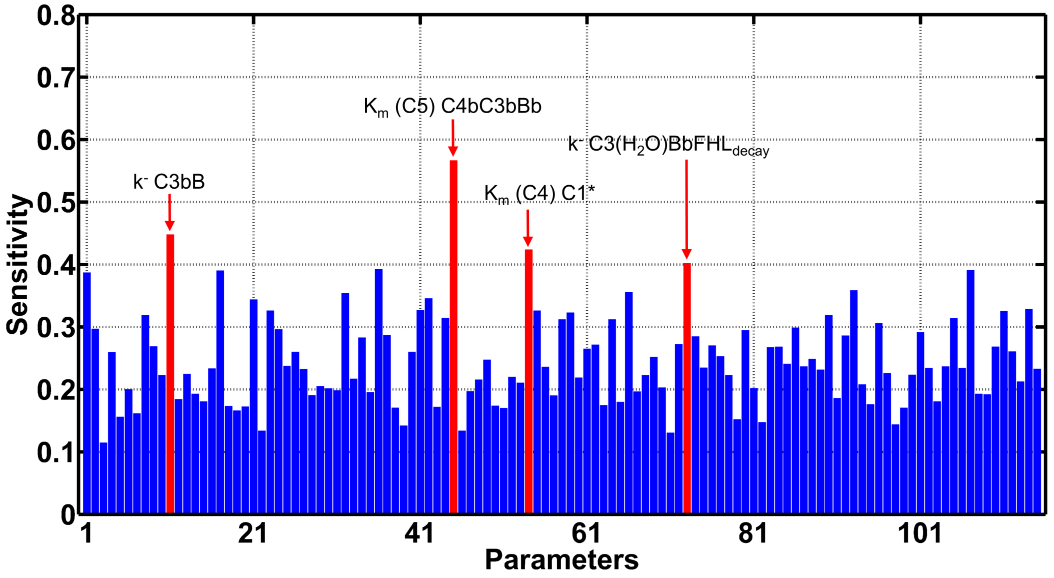

Figure S5 Global sensitivity analysis. MPSA was implemented to identify parameters that affect the biochemical cascade of MAC formation on *N. meningitides* under FP activation and recruitment of C4BP, Vn, FH, FHL-1, and FHR-3 in the nasopharynx. The topmost sensitive parameters, shown in red, are as follows in descending order: K_m (C5) C4bC3bBb, k^- C3bB, K_m (C4) C1*, and k^- C3(H_2O)BbFHL$_{decay}$.
