# Supplementary material for: Systems Biology Modeling of the Complement System Under Immune Susceptible Pathogens
Source: Front Phys. Author manuscript; Available in PMC 2022 Feb 9. (PMC8827490; doi:10.3389/fphy.2021.603704)
Supplement: Table_1_Systems Biology Modeling of the Complement System Under Immune Susceptible Pathogens [file NIHMS1768816-supplement-Table_1_Systems_Biology_Modeling_of_the_Complement_System_Under_Immune_Susceptible_Pathogens.pdf]

Table S1. **(A)** Complement molecular masses and concentrations. **(B)** Surface concentrations. **(C)** Nasal complement concentrations. **(D)** Nasal concentration of complement regulators. **(E)** Nasal complement enhancement. **(F)** Nasal range of complement regulators.

A. Complement molecular masses and concentrations.

Complement protein	Molecular mass (kDa)	Concentration (µM)	Source
C1	632	0.14	[1]
C2	102	0.20	[1]
C3	183	7.1	[2]
C4	205	2.9	[1]
C5	191	0.37	[3]
C1q	459	0.030	[1]
$(C1rC1s)_2$	173	0.060	[1]
C4BP	560	1.3	[1]
C1-INH	110	1.8	[1]
C6	120	0.53	[3]
C7	110	0.51	[3]
C8	151	0.36	[3]
C9	71	0.83	[3]
FB	93	2.2	[2]
FD	24	0.083	[2]
FI	88	0.40	[2]
Properdin	53	0.47	[2]
FH	155	3.2	[2]
FHL-1	43	0.04	[4,5]
FHR1 Homodimers	79	0.19	[6]
FHR2 Homodimers	55	0.012	[6]
FHR5 Homodimers	130	0.013	[6]
FHR3	43.5	0.016	[7]
FHR4	86	0.03	[8]
Carboxypeptidase N	280	0.12	[9]
CR1	190	0.024	[2,10]
DAF	70	0.027	[2,10]
Vn	83	6.1	[3]
Cn	80	0.88	[11]
CD59	18	0.21	[12]
IgG	150	81.0	[13,14]
IgG that recognizes pathogens. 0.5% of serum IgG	150	0.41	Assumption
IgM	970	1.5	[13,15]

IgM that recognizes pathogens. 0.5% of serum IgM	970	0.0075	Assumption

Complement protein	Molecular mass (kDa)	Concentration (µM)	Source
MBL Trimer	216	0.0039	[16,17]
MBL Tetramer	288	0.0029	[16,17]
H-ficolin	372	0.11	[16,17]
L-ficolin	372	0.0074	[16,17]
M-ficolin	384	0.0063	[16,17]
MASP-1	154	0.07	[16,17]
MASP-2	146	0.0028	[16,17]
MASP-3	158	0.042	[16,17]
MAp19	36	0.011	[16,17]
MAp44	82	0.027	[16,17]
CL-L1	444	0.0011	[16,18]
CL-K1	396	0.0012	[16,19]
CL-LK	430	0.00073	[19–21]
PTX3	340	0.0000059	[22,23]
CRP	115	0.007	[24,25]
SAP	115	0.24	[26,27]

B. Surface concentrations.

Host cell	Concentration (M)	Source
Red blood cell	1.2×10^{-5}	[28]
Pathogen	5.2×10^{-15}	[28]

C. Nasal complement concentrations. Values are assumed to be 12.0% (median) of their serum levels like that of measured C3 and IgG in nasopharyngeal secretions (range 4.0 – 20.0% of serum) [29].

Complement protein	Molecular mass (kDa)	Concentration (µM)
C1	632	0.017
C2	102	0.024
C3	183	0.85
C4	205	0.35
C5	191	0.044
C1q	459	0.0036
$(C1rC1s)_2$	173	0.0072
C6	120	0.064
C7	110	0.061

C8	151	0.043
C9	71	0.10
FB	93	0.26
FD	24	0.01
FI	88	0.048
Properdin	53	0.056
Carboxypeptidase N	280	0.014
IgG	150	9.7
FHR3	43.5	0.0019

D. Nasal concentration of complement regulators. Values are assumed to be 0.1% (median) of their serum levels as measured for mucosal factor H (range 0.0002% – 0.2% of serum) [30].

Complement protein	Molecular mass (kDa)	Concentration (μM)
FH	155	0.0032
FHL-1	43	0.00004
C4BP	560	0.0013
C1-INH	110	0.0018
Vn	83	0.0061
Cn	80	0.00088

E. Nasal complement enhancement. Values are increased to 20.0% of their serum levels.

Complement protein	Molecular mass (kDa)	Concentration (μM)
C1	632	0.028
C2	102	0.04
C3	183	1.4
C4	205	0.58
C5	191	0.074
C1q	459	0.006
$(C1rC1s)_2$	173	0.012
C6	120	0.11
C7	110	0.1
C8	151	0.072
C9	71	0.17
FB	93	0.44
FD	24	0.017
Properdin	53	0.094

		Concentration (M)				
**Complement protein**	**Molecular mass (kDa)**	**2.0% of serum**	**0.2% of serum**	**0.02% of serum**	**0.002% of serum**	**0.0002% of serum**
FH	155	6.4×10^{-8}	6.4×10^{-9}	6.4×10^{-10}	6.4×10^{-11}	6.4×10^{-12}
FHL-1	43	8.0×10^{-10}	8.0×10^{-11}	8.0×10^{-12}	8.0×10^{-13}	8.0×10^{-14}
C4BP	560	2.6×10^{-8}	2.6×10^{-9}	2.6×10^{-10}	2.6×10^{-11}	2.6×10^{-12}
C1-INH	110	3.6×10^{-8}	3.6×10^{-9}	3.6×10^{-10}	3.6×10^{-11}	3.6×10^{-12}
Vn	83	1.2×10^{-7}	1.2×10^{-8}	1.2×10^{-9}	1.2×10^{-10}	1.2×10^{-11}
Cn	80	1.8×10^{-8}	1.8×10^{-9}	1.8×10^{-10}	1.8×10^{-11}	1.8×10^{-12}

F. Nasal range of complement regulators. Values are varied between 0.0002% – 2.0% of serum.

References

1. Arlaud GJ, Colomb MG. Complement: Classical Pathway. eLS. John Wiley & Sons, Ltd; 2001. doi:10.1038/npg.els.0000510

2. Zipfel PF. Complement: Alternative Pathway. eLS. John Wiley & Sons, Ltd; 2001. Available: http://onlinelibrary.wiley.com/doi/10.1038/npg.els.0000509/abstract

3. Muller-Eberhard HJ. The Membrane Attack Complex of Complement. Annu Rev Immunol. 1986;4: 503–528. doi:10.1146/annurev.iy.04.040186.002443

4. Dopler A, Guntau L, Harder MJ, Palmer A, Höchsmann B, Schrezenmeier H, et al. Self versus Nonself Discrimination by the Soluble Complement Regulators Factor H and FHL-1. J Immunol Baltim Md 1950. 2019;202: 2082–2094. doi:10.4049/jimmunol.1801545

5. Schwaeble W, Zwirner J, Schulz TF, Linke RP, Dierich MP, Weiss EH. Human complement factor H: expression of an additional truncated gene product of 43 kDa in human liver. Eur J Immunol. 1987;17: 1485–1489. doi:10.1002/eji.1830171015

6. van Beek AE, Pouw RB, Brouwer MC, van Mierlo G, Geissler J, Ooijevaar-de Heer P, et al. Factor H-Related (FHR)-1 and FHR-2 Form Homo- and Heterodimers, while FHR-5 Circulates Only As Homodimer in Human Plasma. Front Immunol. 2017;8: 1328. doi:10.3389/fimmu.2017.01328

7. Pouw RB, Brouwer MC, Geissler J, van Herpen LV, Zeerleder SS, Wuillemin WA, et al. Complement Factor H-Related Protein 3 Serum Levels Are Low Compared to Factor H and Mainly Determined by Gene Copy Number Variation in CFHR3. PLoS ONE. 2016;11. doi:10.1371/journal.pone.0152164

8. Pouw RB, Brouwer MC, van Beek AE, Józsi M, Wouters D, Kuijpers TW. Complement Factor H-Related Protein 4A Is the Dominant Circulating Splice Variant of CFHR4. Front Immunol. 2018;9: 729. doi:10.3389/fimmu.2018.00729

9. Matthews KW, Mueller-Ortiz SL, Wetsel RA. Carboxypeptidase N: a pleiotropic regulator of inflammation. Mol Immunol. 2004;40: 785–793. doi:10.1016/j.molimm.2003.10.002

10. Seya T. Human Regulator of Complement Activation (RCA) Gene Family Proteins and Their Relationship to Microbial Infection. Microbiol Immunol. 1995;39: 295–305. doi:10.1111/j.1348-0421.1995.tb02205.x

11. Murphy BF, Kirszbaum L, Walker ID, d'Apice AJ. SP-40,40, a newly identified normal human serum protein found in the SC5b-9 complex of complement and in the immune deposits in glomerulonephritis. J Clin Invest. 1988;81: 1858–1864.

12. Meri S, Morgan BP, Davies A, Daniels RH, Olavesen MG, Waldmann H, et al. Human protectin (CD59), an 18,000-20,000 MW complement lysis restricting factor, inhibits C5b-8 catalysed insertion of C9 into lipid bilayers. Immunology. 1990;71: 1–9.

13. Gonzalez-Quintela A, Alende R, Gude F, Campos J, Rey J, Meijide LM, et al. Serum levels of immunoglobulins (IgG, IgA, IgM) in a general adult population and their relationship with alcohol consumption, smoking and common metabolic abnormalities. Clin Exp Immunol. 2008;151: 42–50. doi:10.1111/j.1365-2249.2007.03545.x

14. Charles A Janeway J, Travers P, Walport M, Shlomchik MJ. The structure of a typical antibody molecule. Immunobiol Immune Syst Health Dis 5th Ed. 2001 [cited 25 May 2019]. Available: https://www.ncbi.nlm.nih.gov/books/NBK27144/

15. Casali P. IgM. In: Delves PJ, editor. Encyclopedia of Immunology (Second Edition). Oxford: Elsevier; 1998. pp. 1212–1217. doi:10.1006/rwei.1999.0314

16. Troldborg A, Hansen A, Hansen SWK, Jensenius JC, Stengaard-Pedersen K, Thiel S. Lectin complement pathway proteins in healthy individuals. Clin Exp Immunol. 2017;188: 138–147. doi:10.1111/cei.12909

17. Thiel S, Jensen L, Degn SE, Nielsen HJ, Gál P, Dobó J, et al. Mannan-binding lectin (MBL)-associated serine protease-1 (MASP-1), a serine protease associated with humoral pattern-recognition molecules: normal and acute-phase levels in serum and stoichiometry of lectin pathway components. Clin Exp Immunol. 2012;169: 38–48. doi:10.1111/j.1365-2249.2012.04584.x

18. Axelgaard E, Jensen L, Dyrlund TF, Nielsen HJ, Enghild JJ, Thiel S, et al. Investigations on collectin liver 1. J Biol Chem. 2013;288: 23407–23420. doi:10.1074/jbc.M113.492603

19. Henriksen ML, Brandt J, Andrieu J-P, Nielsen C, Jensen PH, Holmskov U, et al. Heteromeric complexes of native collectin kidney 1 and collectin liver 1 are found in the circulation with MASPs and activate the complement system. J Immunol Baltim Md 1950. 2013;191: 6117–6127. doi:10.4049/jimmunol.1302121

20. Yoshizaki T, Ohtani K, Motomura W, Jang S-J, Mori K, Kitamoto N, et al. Comparison of human blood concentrations of collectin kidney 1 and mannan-binding lectin. J Biochem (Tokyo). 2012;151: 57–64. doi:10.1093/jb/mvr114

21. Selman L, Henriksen ML, Brandt J, Palarasah Y, Waters A, Beales PL, et al. An enzyme-linked immunosorbent assay (ELISA) for quantification of human collectin 11 (CL-11, CL-K1). J Immunol Methods. 2012;375: 182–188. doi:10.1016/j.jim.2011.10.010

22. Yamasaki K, Kurimura M, Kasai T, Sagara M, Kodama T, Inoue K. Determination of physiological plasma pentraxin 3 (PTX3) levels in healthy populations. Clin Chem Lab Med. 2009;47: 471–477. doi:10.1515/CCLM.2009.110

23. Inforzato A, Rivieccio V, Morreale AP, Bastone A, Salustri A, Scarchilli L, et al. Structural characterization of PTX3 disulfide bond network and its multimeric status in cumulus matrix organization. J Biol Chem. 2008;283: 10147–10161. doi:10.1074/jbc.M708535200

24. Shine B, de Beer FC, Pepys MB. Solid phase radioimmunoassays for human C-reactive protein. Clin Chim Acta Int J Clin Chem. 1981;117: 13–23.

25. Shrive AK, Cheetham GM, Holden D, Myles DA, Turnell WG, Volanakis JE, et al. Three dimensional structure of human C-reactive protein. Nat Struct Biol. 1996;3: 346–354.

26. Nelson SR, Tennent GA, Sethi D, Gower PE, Ballardie FW, Amatayakul-Chantler S, et al. Serum amyloid P component in chronic renal failure and dialysis. Clin Chim Acta Int J Clin Chem. 1991;200: 191–199.

27. Emsley J, White HE, O'Hara BP, Oliva G, Srinivasan N, Tickle IJ, et al. Structure of pentameric human serum amyloid P component. Nature. 1994;367: 338–345. doi:10.1038/367338a0

28. Zewde N, Jr RDG, Dorado A, Morikis D. Quantitative Modeling of the Alternative Pathway of the Complement System. PLOS ONE. 2016;11: e0152337. doi:10.1371/journal.pone.0152337

29. Prellner K, Stjernquist-Desatnik A. A simple method for collecting nasopharyngeal secretions, using cross-linked dextran. Clin Otolaryngol Allied Sci. 1985;10: 85–87. doi:10.1111/j.1365-2273.1985.tb01172.x

30. Glennie S, Gritzfeld JF, Pennington SH, Garner-Jones M, Coombes N, Hopkins MJ, et al. Modulation of nasopharyngeal innate defenses by viral coinfection predisposes individuals to experimental pneumococcal carriage. Mucosal Immunol. 2016;9: 56–67. doi:10.1038/mi.2015.35
