# Supplementary material for: Systems Biology Modeling of the Complement System Under Immune Susceptible Pathogens
Source: Front Phys. Author manuscript; Available in PMC 2022 Feb 9. (PMC8827490; doi:10.3389/fphy.2021.603704)
Supplement: Table_2_Systems Biology Modeling of the Complement System Under Immune Susceptible Pathogens [file NIHMS1768816-supplement-Table_2_Systems_Biology_Modeling_of_the_Complement_System_Under_Immune_Susceptible_Pathogens.pdf]

S2 Table. Kinetic rate constants.

Biochemical reaction	Rate constant	Value	Source
Hydrolysis of $C3(H_2O)$	$k^{+}_{C3(H_2O)}$	4.5×10^{-6} s^{-1}	[1]
Association of Factor B to $C3(H_2O)$	$k^{+}_{C3(H_2O)B}$	1.1×10^{4} $M^{-1}s^{-1}$	[2]
Dissociation of complex $C3(H_2O)B$	$k^{-}_{C3(H_2O)B}$	1.4×10^{-3} s^{-1}	[2]
Association of Factor H to $C3(H_2O)$	$k^{+}_{C3(H_2O)H}$	1.1×10^{6} $M^{-1}s^{-1}$	Assumption based on the interaction of functionally homologous protein C3b and FH
Dissociation of complex $C3(H_2O)H$	$k^{-}_{C3(H_2O)H}$	6.0×10^{-2} s^{-1}	Assumption based on the interaction of functionally homologous protein C3b and FH
Association of Factor H-like protein 1 to $C3(H_2O)$	$k^{+}_{C3(H_2O)FHL1}$	1.8×10^{4} $M^{-1}s^{-1}$	Assumption based on the interaction of functionally homologous protein C3b and FHL
Dissociation of complex $C3(H_2O)FHL1$	$k^{-}_{C3(H_2O)FHL1}$	1.9×10^{-2} s^{-1}	Assumption based on the interaction of functionally homologous protein C3b and FHL
Dissociation of complex $C3(H_2O)Bb$	$k^{-}_{C3(H_2O)Bb}$	9.0×10^{-3} s^{-1}	[3]
Association of Factor B to C3b	k^{+}_{C3bB}	21.3×10^{4} $M^{-1}s^{-1}$	[4]
Dissociation of complex C3bB	k^{-}_{C3bB}	15.5×10^{-2} s^{-1}	[4]
Dissociation of complex C3bBb	k^{-}_{C3bBb}	7.7×10^{-3} s^{-1}	[3]
Dissociation of complex C3bBbP	k^{-}_{C3bBbP}	7.7×10^{-4} s^{-1}	[3,5]
Dissociation of complex C4bC2a	k^{-}_{C4bC2a}	4.0×10^{-3} s^{-1}	[6]

Association of Properdin to C3b	k^{+}_{C3bP}	1.5×10^{5} M^{-1}s^{-1}	[7]
Dissociation of complex C3bP	k^{-}_{C3bP}	15.3×10^{-5} s^{-1}	[7]
Attachment of nfC3b to host cell or pathogen	k^{+}_{hC3b} or k^{+}_{pC3b}	4.2×10^{8} M^{-1}s^{-1}	[8] Calculated based on rates of diffusion in blood
Association of nfC3b to water	k^{+}_{fC3b}	4.2×10^{8} M^{-1}s^{-1}	[8] Calculated based on rates of diffusion in blood
Association of nfC3b to C3b	k^{+}_{C3bC3b}	4.2×10^{8} M^{-1}s^{-1}	[8] Calculated based on rates of diffusion in blood
Association of nfC3b to IgG	k^{+}_{IgGC3b}	4.2×10^{8} M^{-1}s^{-1}	[8] Calculated based on rates of diffusion in blood
Association of nfC3b/ nfC4b to C4b/C3b	k^{+}_{C3bC4b}	4.2×10^{8} M^{-1}s^{-1}	[8] Calculated based on rates of diffusion in blood
Attachment of nfC4b to host cell or pathogen	k^{+}_{hC4b} or k^{+}_{pC4b}	4.2×10^{8} M^{-1}s^{-1}	[8] Calculated based on rates of diffusion in blood
Association of nfC4b to water	k^{+}_{fC4b}	4.2×10^{8} M^{-1}s^{-1}	[8] Calculated based on rates of diffusion in blood
Association of nfC4b to C4b	k^{+}_{C4bC4b}	4.2×10^{8} M^{-1}s^{-1}	[8] Calculated based on rates of diffusion in blood
Association of Factor H to C3b	k^{+}_{C3bH}	1.1×10^{6} M^{-1}s^{-1}	[9]
Dissociation of complex C3bH	k^{-}_{C3bH}	6.0×10^{-2} s^{-1}	[9]
Association of Factor H-like protein 1 to C3b	k^{+}_{C3bFHL1}	1.8×10^{4} M^{-1}s^{-1}	[10]
Dissociation of complex C3bFHL1	k^{-}_{C3bFHL1}	1.9×10^{-2} s^{-1}	[10]
Association of CR1 to C3b	k^{+}_{C3bCR1}	4.4×10^{6} M^{-1}s^{-1}	[9]
Dissociation of complex C3bCR1	k^{-}_{C3bCR1}	5.7×10^{-2} s^{-1}	[9]

Association of DAF to convertase on host cell	$k^{+}_{\mathrm{C3bBbDAF}}$	1.4×10^{3} $M^{-1}s^{-1}$	[11]
Dissociation of complex C3bBbDAF	$k^{-}_{\mathrm{C3bBbDAF}}$	1.2×10^{-3} s^{-1}	[11]
Decay of convertase by inhibitor DAF on host cell	$k^{-}_{\mathrm{C3bBbDAF}_{\mathrm{decay}}}$	1.7×10^{-2} s^{-1}	Assumption based on the decay rate of functionally homologous protein FH on C3bBb
Decay of convertase by inhibitor CR1	$k^{-}_{\mathrm{C3bBbCR1}_{\mathrm{decay}}}$	1.7×10^{-2} s^{-1}	Assumption based on the decay rate of functionally homologous protein FH on C3bBb
Decay of convertase by inhibitor CR1	$k^{-}_{\mathrm{C4bC2aCR1}_{\mathrm{decay}}}$	1.7×10^{-2} s^{-1}	Assumption based on the decay rate of functionally homologous protein FH on C3bBb
Decay of convertase by inhibitor C4BP	$k^{-}_{\mathrm{C4bC2aC4BP}_{\mathrm{decay}}}$	1.7×10^{-2} s^{-1}	Assumption based on the decay rate of functionally homologous protein FH on C3bBb
Decay of C3 convertase by inhibitor Factor H	$k^{-}_{\mathrm{C3bBbH}_{\mathrm{decay}}}$	1.7×10^{-2} s^{-1}	[12]
Decay of convertase by inhibitor Factor H-like protein 1	$k^{-}_{\mathrm{C3bBbFHL1}_{\mathrm{decay}}}$	3.3×10^{-2} s^{-1}	[12]
Decay of convertase by inhibitor Factor H	$k^{-}_{\mathrm{C3(H_2O)BbH}_{\mathrm{decay}}}$	1.7×10^{-2} s^{-1}	Assumption based on the decay rate of functionally homologous protein FH on C3bBb
Decay of convertase by inhibitor Factor H-like protein 1	$k^{-}_{\mathrm{C3(H_2O)BbFHL1}_{\mathrm{decay}}}$	3.3×10^{-2} s^{-1}	Assumption based on the decay rate of functionally homologous protein FHL on C3bBb
Association of CR1 to iC3b	k^{+}_{iC3bCR1}	1.8×10^{4} $M^{-1}s^{-1}$	[12]
Dissociation of complex iC3bCR1	k^{-}_{iC3bCR1}	1.0×10^{-2} s^{-1}	[12]
Association of CR1 to C3bC3b	$k^{+}_{\mathrm{C3bC3bCR1}}$	9.8×10^{4} $M^{-1}s^{-1}$	[13]

Dissociation of complex C3bC3bCR1	$k^{-}_{\mathrm{C3bC3bCR1}}$	2.1×10^{-3} s^{-1}	[13]
Association of CR1 to C3biC3b	$k^{+}_{\mathrm{C3biC3bCR1}}$	9.8×10^{4} M^{-1}s^{-1}	Assumption based on the interaction of dimerized C3b and CR1
Dissociation of complex C3biC3bCR1	$k^{-}_{\mathrm{C3biC3bCR1}}$	2.1×10^{-3} s^{-1}	Assumption based on the interaction of dimerized C3b and CR1
Association of CR1 to iC3biC3b	$k^{+}_{\mathrm{iC3biC3bCR1}}$	9.8×10^{4} M^{-1}s^{-1}	Assumption based on the interaction of dimerized C3b and CR1
Dissociation of complex iC3biC3bCR1	$k^{-}_{\mathrm{iC3biC3bCR1}}$	2.1×10^{-3} s^{-1}	Assumption based on the interaction of dimerized C3b and CR1
Association of CR1 to iC3bC3dg	$k^{+}_{\mathrm{iC3bC3dgCR1}}$	9.8×10^{4} M^{-1}s^{-1}	Assumption based on the interaction of dimerized C3b and CR1
Dissociation of complex iC3bC3dgCR1	$k^{-}_{\mathrm{iC3bC3dgCR1}}$	2.1×10^{-3} s^{-1}	Assumption based on the interaction of dimerized C3b and CR1
Association of CR1 to C3bC4b	$k^{+}_{\mathrm{C3bC4bCR1}}$	9.8×10^{4} M^{-1}s^{-1}	Assumption based on the interaction of dimerized C3b and CR1
Dissociation of complex C3bC4bCR1	$k^{-}_{\mathrm{C3bC4bCR1}}$	2.1×10^{-3} s^{-1}	Assumption based on the interaction of dimerized C3b and CR1
Association of CR1 to C3bC4d	$k^{+}_{\mathrm{C3bC4dCR1}}$	9.8×10^{4} M^{-1}s^{-1}	Assumption based on the interaction of dimerized C3b and CR1
Dissociation of complex C3bC4dCR1	$k^{-}_{\mathrm{C3bC4dCR1}}$	2.1×10^{-3} s^{-1}	Assumption based on the interaction of dimerized C3b and CR1
Association of CR1 to iC3bC4b	$k^{+}_{\mathrm{iC3bC4bCR1}}$	9.8×10^{4} M^{-1}s^{-1}	Assumption based on the interaction of dimerized C3b and CR1
Dissociation of complex iC3bC4bCR1	$k^{-}_{\mathrm{iC3bC4bCR1}}$	2.1×10^{-3} s^{-1}	Assumption based on the interaction of dimerized C3b and CR1
Association of CR1 to iC3bC4d	$k^{+}_{\mathrm{iC3bC4dCR1}}$	9.8×10^{4} M^{-1}s^{-1}	Assumption based on the interaction dimerized C3b and CR1

Dissociation of complex iC3bC4dCR1	$k^{-}_{\mathrm{iC3bC4dCR1}}$	2.1×10^{-3} s^{-1}	Assumption based on the interaction of dimerized C3b and CR1
Association of CR1 to C3dgC4b	$k^{+}_{\mathrm{C3dgC4bCR1}}$	9.8×10^{4} $M^{-1}s^{-1}$	Assumption based on the interaction of dimerized C3b and CR1
Dissociation of complex C3dgC4bCR1	$k^{-}_{\mathrm{C3dgC4bCR1}}$	2.1×10^{-3} s^{-1}	Assumption based on the interaction of dimerized C3b and CR1
Association of CR1 to C4b	k^{+}_{C4bCR1}	3.8×10^{6} $M^{-1}s^{-1}$	[9]
Dissociation of complex C4bCR1	k^{-}_{C4bCR1}	4.2×10^{-2} s^{-1}	[9]
Association of C4BP to C4b	k^{+}_{C4bC4BP}	2.0×10^{5} $M^{-1}s^{-1}$	[14,15]
Dissociation of complex C4bC4BP	k^{-}_{C4bC4BP}	1.6×10^{-2} s^{-1}	[14–16]
Association of C2 to C4b	k^{+}_{C4bC2}	1.6×10^{6} $M^{-1}s^{-1}$	[2]
Dissociation of complex C4bC2	k^{-}_{C4bC2}	4.2×10^{-3} s^{-1}	[2]
Association of CR1 to C4bC4b	$k^{+}_{\mathrm{C4bC4bCR1}}$	9.8×10^{4} $M^{-1}s^{-1}$	Assumption based on the interaction of structurally and functionally homologous protein C3b and CR1
Dissociation of complex C4bC4bCR1	$k^{-}_{\mathrm{C4bC4bCR1}}$	2.1×10^{-3} s^{-1}	Assumption based on the interaction of structurally and functionally homologous protein C3b and CR1
Association of CR1 to C4bC4d	$k^{+}_{\mathrm{C4bC4dCR1}}$	9.8×10^{4} $M^{-1}s^{-1}$	Assumption based on the interaction of structurally and functionally homologous protein C3b and CR1
Dissociation of complex C4bC4dCR1	$k^{-}_{\mathrm{C4bC4dCR1}}$	2.1×10^{-3} s^{-1}	Assumption based on the interaction of structurally and functionally homologous protein C3b and CR1
Association of C1q to $(C1rC1s)_2$	k^{+}_{C1}	0.8×10^{6} $M^{-1}s^{-1}$	[17]

Dissociation of complex C1	k^{-}_{C1}	1.2×10^{-3} s^{-1}	[17]
Activation of C1	$k^{+}_{\mathrm{activation}}$	2.1×10^{-5} s^{-1}	[18]
Association of C1-INH to C1*	$k^{+}_{\mathrm{C1*C1INH}}$	4.3×10^{5} $M^{-1}s^{-1}$	[19]
Dissociation of complex C3bC3bBb/ IgGC3bC3bBb	$k^{-}_{\mathrm{C3bC3bBb}}$	5.7×10^{-3} s^{-1}	[20]
Dissociation of complex C3bC3bBbP	$k^{-}_{\mathrm{C3bC3bBbP}}$	5.7×10^{-4} s^{-1}	[5,20]
Dissociation of complex C3bC4bBb	$k^{-}_{\mathrm{C3bC4bBb}}$	5.7×10^{-3} s^{-1}	Assumption based on the interaction of structurally and functionally homologous protein C3bC3bBb
Dissociation of complex C3bC4bBbP	$k^{-}_{\mathrm{C3bC4bBbP}}$	5.7×10^{-4} s^{-1}	Assumption based on the interaction of structurally and functionally homologous protein C3bC3bBbP
Dissociation of complex C3bC4bC2a	$k^{-}_{\mathrm{C3bC4bC2a}}$	5.0×10^{-3} s^{-1}	[6]
Dissociation of complex C4bC4bC2a	$k^{-}_{\mathrm{C4bC4bC2a}}$	6.0×10^{-3} s^{-1}	[6]
Dissociation from complex C5b	$k^{-}_{\mathrm{C5b*}}$	5.0×10^{-3} s^{-1}	[20]
Association of C6 to C5b	k^{+}_{C5bC6}	6.0×10^{4} $M^{-1}s^{-1}$	[14,21]
Dissociation of complex C5bC6	k^{-}_{C5bC6}	9.0×10^{-8} s^{-1}	[14,21]
Association of C7 to C5bC6	k^{+}_{C5b7}	7.3×10^{5} $M^{-1}s^{-1}$	[14,21,22]
Dissociation of complex C5bC6C7	k^{-}_{C5b7}	1.5×10^{-7} s^{-1}	[14,21,22]
Attachment of C5b7 to host cell or pathogen	$k^{+}_{\mathrm{C5b7_{surface}}}$	4.2×10^{8} $M^{-1}s^{-1}$	[8]

Formation of C5b7 micelle in fluid	k^{+}_{micelle}	69.3 s^{-1}	[20]
Association of C8 to C5b7	k^{+}_{C5b8}	1.1×10^{6} $M^{-1}s^{-1}$	[14,21,22]
Dissociation of complex C5b8	k^{-}_{C5b8}	9.8×10^{-7} s^{-1}	[14,21,22]
Association of C9 to C5b8	k^{+}_{C5b9}	2.8×10^{6} $M^{-1}s^{-1}$	[14,21,22]
Dissociation of complex C5b9	k^{-}_{C5b9}	2.8×10^{-7} s^{-1}	[14,21,22]
Association of $C9_1$ to surface C5b8	$k^{+}_{\text{C5b9}_1}$	7.8×10^{2} $M^{-1}s^{-1}$	[23]
Association of $C9_{n>=2}$ to surface $C5b9_1$	$k^{+}_{\text{C5b9}_{\text{poly}}}$	1.1×10^{5} $M^{-1}s^{-1}$	[23]
Association of Cn to C5b7	k^{+}_{CnC5b7}	2.4×10^{5} $M^{-1}s^{-1}$	[14,24]
Dissociation of complex CnC5b7	k^{-}_{CnC5b7}	4.0×10^{-3} s^{-1}	Assumption based on the interaction of structurally and functionally homologous protein Cn and C5b8
Association of Cn to C5b8	k^{+}_{CnC5b8}	4.2×10^{5} $M^{-1}s^{-1}$	[14]
Dissociation of complex CnC5b8	k^{-}_{CnC5b8}	4.0×10^{-3} s^{-1}	[14]
Association of Cn to C5b9	k^{+}_{CnC5b9}	4.2×10^{5} $M^{-1}s^{-1}$	Assumption based on the interaction of structurally and functionally homologous protein Cn and C5b8
Dissociation of complex CnC5b9	k^{-}_{CnC5b9}	4.0×10^{-3} s^{-1}	Assumption based on the interaction of structurally and functionally homologous protein Cn and C5b8
Association of Vn to C5b7	k^{+}_{VnC5b7}	2.4×10^{5} $M^{-1}s^{-1}$	Assumption based on the interaction of structurally and functionally homologous protein Cn and C5b7
Dissociation of complex VnC5b7	k^{-}_{VnC5b7}	4.0×10^{-3} s^{-1}	Assumption based on the interaction of structurally and

			functionally homologous protein Cn and C5b8
Association of Vn to C5b8	$k^{+}_{\mathtt{VnC5b8}}$	4.2×10^{5} $M^{-1}s^{-1}$	Assumption based on the interaction of structurally and functionally homologous protein Cn and C5b8
Dissociation of complex VnC5b8	$k^{-}_{\mathtt{VnC5b8}}$	4.0×10^{-3} s^{-1}	Assumption based on the interaction of structurally and functionally homologous protein Cn and C5b8
Association of Vn to C5b9	$k^{+}_{\mathtt{VnC5b9}}$	4.2×10^{5} $M^{-1}s^{-1}$	Assumption based on the interaction of structurally and functionally homologous protein Cn and C5b8
Dissociation of complex VnC5b9	$k^{-}_{\mathtt{VnC5b9}}$	4.0×10^{-3} s^{-1}	Assumption based on the interaction of structurally and functionally homologous protein Cn and C5b8
Association of CD59 to C5b8	$k^{+}_{\mathtt{C5b8CD59}}$	1.0×10^{6} $M^{-1}s^{-1}$	[8]<br>Assumption
Dissociation of complex C5b8CD59	$k^{-}_{\mathtt{C5b8CD59}}$	2.0×10^{-4} s^{-1}	[8]<br>Assumption
Association of CD59 to C5b9	$k^{+}_{\mathtt{C5b9CD59}}$	1.0×10^{6} $M^{-1}s^{-1}$	[8]<br>Assumption
Dissociation of complex C5b9CD59	$k^{-}_{\mathtt{C5b9CD59}}$	2.0×10^{-4} s^{-1}	[8]<br>Assumption
Cleavage of C3 by C3 convertase, $C3(H_2O)Bb$	k_{cat} $C3(H_2O)Bb$<br><br>K_m $C3(H_2O)Bb$	1.8 s^{-1}<br><br>10.6×10^{-6} M	[3]<br>Assumption based on the increasing the K_m of C3bBb
Cleavage of C3 by C3 convertase, C3bBb	k_{cat} C3bBb<br><br>K_m C3bBb	1.8 s^{-1}<br><br>5.9×10^{-6} M	[3]
Cleavage of C3 by convertase, IgGC3bC3bBb	k_{cat} C3bBb<br><br>K_m C3bBb	1.8 s^{-1}<br><br>5.9×10^{-6} M	Assumption based on the interaction of functionally homologous protein C3bBb
Cleavage of C5 by C3 convertase, C3bBb	k_{cat} C3bBb<br><br>K_m C3bBb	1.1×10^{-2} s^{-1}<br><br>24.0×10^{-6} M	[25]

Cleavage of C3 by C3 convertase, C4bC2a	k_{cat} C4bC2a<br><br>K_m C4bC2a	3.2 s^{-1}<br><br>1.8×10^{-6} M	[14,26]
Cleavage of C5 by the C3 convertase, C4bC2a	k_{cat} C4bC2a<br><br>K_m C4bC2a	2.2×10^{-2} s^{-1}<br><br>8.9×10^{-6} M	[6]
Cleavage of C4 by activated C1, C1*	k_{cat} C1*<br><br>K_m C1*	5.4 s^{-1}<br><br>6100×10^{-9} M	[27]
Cleavage of C2 by activated C1, C1*	k_{cat} C1*<br><br>K_m C1*	5.1 s^{-1}<br><br>6.1×10^{-6} M	[27]
Activation of complex C3bB by enzyme factor D	k_{cat} C3bB<br><br>K_m C3bB	5.0 s^{-1}<br><br>2.5×10^{-6} M	Assumption based on the FD activation of functionally homologous protein $C3(H_2O)B$
Activation of complex $C3(H_2O)B$ by enzyme factor D	k_{cat} $C3(H_2O)B$<br><br>K_m $C3(H_2O)B$	5.0 s^{-1}<br><br>2.5×10^{-6} M	[28]
Cleavage of C3b by inhibitor factor I	k_{cat} C3bH<br><br>K_m C3bH	1.3 s^{-1}<br><br>2.5×10^{-7} M	[29]
Cleavage of C5 by the C5 convertase, C3bC3bBb	k_{cat} C3bC3bBb<br><br>K_m C3bC3bBb	3.0×10^{-3} s^{-1}<br><br>1.7×10^{-6} M	[30]
Cleavage of C5 by the C5 convertase, C3bC4bBb	k_{cat} C3bC4bBb<br><br>K_m C3bC4bBb	3.0×10^{-3} s^{-1}<br><br>1.7×10^{-6} M	Assumption based on the interaction of structurally and functionally homologous protein C3bC3bBb
Cleavage of C5 by the C5 convertase, C3bC4bC2a	k_{cat} C3bC4bC2a<br><br>K_m C3bC4bC2a	1.8×10^{-2} s^{-1}<br><br>5.1×10^{-9} M	[6]
Cleavage of C5 by the C5 convertase, C4bC4bC2a	k_{cat} C4bC4bC2a<br><br>K_m C4bC4bC2a	3.0×10^{-2} s^{-1}<br><br>5.6×10^{-6} M	[6]
Cleavage of C3a by Carboxypeptidase N,	k_{cat} CPN	57.9 s^{-1}	[31]

CPN	K_m CPN	77.1×10^{-6} M	
Cleavage of C5a by Carboxypeptidase N, CPN	k_{cat} CPN<br><br>K_m CPN	$9.3\ s^{-1}$<br><br>602.2×10^{-6} M	[31]
Association of complement Factor H-related protein 1 to C3b	$k^{+}_{\mathrm{C3bCFHR1}}$	$1.9\times10^{5}\ M^{-1}s^{-1}$	[32]
Dissociation of complex C3bCFHR1	$k^{-}_{\mathrm{C3bCFHR1}}$	$5.0\times10^{-3}\ s^{-1}$	[32]
Association of complement Factor H-related protein 2 to C3b	$k^{+}_{\mathrm{C3bCFHR2}}$	$6.7\times10^{3}\ M^{-1}s^{-1}$	[33]
Dissociation of complex C3bCFHR2	$k^{-}_{\mathrm{C3bCFHR2}}$	$2.8\times10^{-2}\ s^{-1}$	[33]
Association of complement Factor H-related protein 3 to C3b	$k^{+}_{\mathrm{C3bCFHR3}}$	$1.7\times10^{4}\ M^{-1}s^{-1}$	[34]
Dissociation of complex C3bCFHR3	$k^{-}_{\mathrm{C3bCFHR3}}$	$1.4\times10^{-3}\ s^{-1}$	[34]
Association of complement Factor H-related protein 4 to C3b	$k^{+}_{\mathrm{C3bCFHR4}}$	$2.3\times10^{4}\ M^{-1}s^{-1}$	[34]
Dissociation of complex C3bCFHR4	$k^{-}_{\mathrm{C3bCFHR4}}$	$5.6\times10^{-3}\ s^{-1}$	[34]
Association of complement Factor H-related protein 5 to C3b	$k^{+}_{\mathrm{C3bCFHR5}}$	$4.0\times10^{4}\ M^{-1}s^{-1}$	[35]
Dissociation of complex C3bCFHR5	$k^{-}_{\mathrm{C3bCFHR5}}$	$3.8\times10^{-2}\ s^{-1}$	[35]
Association of C1q to IgG	k^{+}_{C1qIgG}	$1.1\times10^{4}\ M^{-1}s^{-1}$	[36]
Dissociation of complex C1qIgG	k^{-}_{C1qIgG}	$2.3\times10^{-3}\ s^{-1}$	[36]

Association of C1q to IgM	k^{+}_{C1qIgM}	1.1×10^{4} $M^{-1}s^{-1}$	Assumption based on the interaction of functionally homologous protein C1qIgG
Dissociation of complex C1qIgM	k^{-}_{C1qIgM}	2.3×10^{-3} s^{-1}	Assumption based on the interaction of functionally homologous protein C1qIgG
Association of $IgG_{n=1-6}$ to pathogen surface (Proteins A and G)	k^{+}_{pIgG}	2.0×10^{4} $M^{-1}s^{-1}$	[37]
Dissociation of Pathogen:$IgG_{n=1-6}$	k^{-}_{pIgG}	2.8×10^{-6} s^{-1}	[37]<br>Reduced the disassociation constant to account for higher avidity with IgG clusters with multiple binding sites
Association of IgM to pathogen surface (Proteins A and G)	k^{+}_{pIgM}	2.0×10^{4} $M^{-1}s^{-1}$	Assumption based on the interaction of functionally homologous protein Pathogen:IgG
Dissociation of Pathogen:IgM	k^{-}_{pIgM}	2.8×10^{-6} s^{-1}	Assumption based on the interaction of functionally homologous protein Pathogen:IgG
MASP1 zymogen activation of MASP1 zymogen	k_{cat} $MASP1_{zym}$<br>K_m $MASP1_{zym}$	2.3×10^{-3} s^{-1}<br>5.7×10^{-6} M	[38]<br>Calculated k_{cat} by averaging experimental K_M values for complement activators
MASP1* activation of MASP1 zymogen	k_{cat} MASP1*<br>K_m MASP1*	5.4×10^{-1} s^{-1}<br>5.7×10^{-6} M	[38]<br>Calculated k_{cat} by averaging experimental K_M values for complement activators
MASP2 zymogen activation of MASP2 zymogen	k_{cat} $MASP2_{zym}$<br>K_m $MASP2_{zym}$	6.3×10^{-7} s^{-1}<br>5.7×10^{-6} M	[38]<br>Calculated k_{cat} by averaging experimental K_M values for complement activators
MASP1 zymogen activation of MASP2 zymogen	k_{cat} $MASP1_{zym}$<br>K_m $MASP1_{zym}$	1.7×10^{-3} s^{-1}<br>5.7×10^{-6} M	[38]<br>Calculated k_{cat} by averaging experimental K_M values for complement activators
MASP2* activation of MASP1 zymogen	k_{cat} MASP2*<br>K_m MASP2*	2.7×10^{-2} s^{-1}<br>5.7×10^{-6} M	[38]<br>Calculated k_{cat} by averaging experimental K_M values for complement activators
MASP1* activation of MASP3 zymogen	k_{cat} MASP1*<br>K_m MASP1*	6.3×10^{-3} s^{-1}<br>5.7×10^{-6} M	[38]

			Calculated k_{cat} by averaging experimental K_M values for complement activators
MASP1* activation of MASP2 zymogen	k_{cat} MASP1*<br><br>K_m MASP1*	6.8×10^{-2} s^{-1}<br><br>5.7×10^{-6} M	[39]<br>Calculated k_{cat} by averaging experimental K_M values for complement activators
MASP2* activation of MASP2 zymogen	k_{cat} MASP2*<br><br>K_m MASP2*	3.4×10^{-3} s^{-1}<br><br>5.7×10^{-6} M	[39]<br>Calculated k_{cat} by averaging experimental K_M values for complement activators
Cleavage of C2 by MASP1*	k_{cat} MASP1*<br><br>K_m MASP1*	0.1 s^{-1}<br><br>4.8×10^{-6} M	[40]
Cleavage of C2 by MASP2*	k_{cat} MASP2*<br><br>K_m MASP2*	5.6 s^{-1}<br><br>5.2×10^{-6} M	[27]
Cleavage of C4 by MASP1*	k_{cat} MASP1*<br><br>K_m MASP1*	2.0×10^{-3} s^{-1}<br><br>2.7×10^{-6} M	[40]
Cleavage of C4 by MASP2*	k_{cat} MASP2*<br><br>K_m MASP2*	1.9 s^{-1}<br><br>85.0×10^{-9} M	[27]
Association of MBL trimer to pathogen surface	$k^{+}_{\mathrm{pMBL}_3}$	5.2×10^{6} $M^{-1}s^{-1}$	[41]
Dissociation of PathogenMBL$_3$	$k^{-}_{\mathrm{pMBL}_3}$	7.7×10^{-3} s^{-1}	[41]
Association of MBL tetramer to pathogen surface	$k^{+}_{\mathrm{pMBL}_4}$	2.0×10^{6} $M^{-1}s^{-1}$	[41]
Dissociation of PathogenMBL$_4$	$k^{-}_{\mathrm{pMBL}_4}$	1.7×10^{-3} s^{-1}	[41]
Association of L-Ficolin to pathogen surface	k^{+}_{pLF}	2.0×10^{6} $M^{-1}s^{-1}$	Assumption based on the interaction of structurally and functionally homologous protein PathogenMBL$_4$

Dissociation of PathogenLF	k_{pLF}^{-}	1.7×10^{-3} s^{-1}	Assumption based on the interaction of structurally and functionally homologous protein $PathogenMBL_4$
Association of M-Ficolin to pathogen surface	k_{pMF}^{+}	2.0×10^{6} $M^{-1}s^{-1}$	Assumption based on the interaction of structurally and functionally homologous protein $PathogenMBL_4$
Dissociation of PathogenMF	k_{pMF}^{-}	1.7×10^{-3} s^{-1}	Assumption based on the interaction of structurally and functionally homologous protein $PathogenMBL_4$
Association of H-Ficolin to pathogen surface	k_{pHF}^{+}	2.0×10^{6} $M^{-1}s^{-1}$	Assumption based on the interaction of structurally and functionally homologous protein $PathogenMBL_4$
Dissociation of PathogenHF	k_{pHF}^{-}	1.7×10^{-3} s^{-1}	Assumption based on the interaction of structurally and functionally homologous protein $PathogenMBL_4$
Association of CL-LK to pathogen surface	k_{pCLLK}^{+}	2.0×10^{6} $M^{-1}s^{-1}$	Assumption based on the interaction of structurally and functionally homologous protein $PathogenMBL_4$
Dissociation of PathogenCLLK	k_{pCLLK}^{-}	1.7×10^{-3} s^{-1}	Assumption based on the interaction of structurally and functionally homologous protein $PathogenMBL_4$
Association of CL-L1 to pathogen surface	k_{pCLL1}^{+}	2.0×10^{6} $M^{-1}s^{-1}$	Assumption based on the interaction of structurally and functionally homologous protein $PathogenMBL_4$
Dissociation of PathogenCLL1	k_{pCLL1}^{-}	1.7×10^{-3} s^{-1}	Assumption based on the interaction of structurally and functionally homologous protein $PathogenMBL_4$
Association of CL-K1 to pathogen surface	k_{pCLK1}^{+}	2.0×10^{6} $M^{-1}s^{-1}$	Assumption based on the interaction of structurally and functionally homologous protein $PathogenMBL_4$

Dissociation of PathogenCLK1	k^{-}_{pCLK1}	1.7×10^{-3} s^{-1}	Assumption based on the interaction of structurally and functionally homologous protein $\mathrm{PathogenMBL_4}$
Association of MBL trimer to MASP1	$k^{+}_{\mathrm{MBL_3MASP1}}$	2.1×10^{5} $M^{-1}s^{-1}$	[41]
Dissociation of $\mathrm{MBL_3MASP1}$	$k^{-}_{\mathrm{MBL_3MASP1}}$	6.8×10^{-4} s^{-1}	[41]
Association of MBL trimer to MASP2	$k^{+}_{\mathrm{MBL_3MASP2}}$	2.3×10^{5} $M^{-1}s^{-1}$	[41]
Dissociation of $\mathrm{MBL_3MASP2}$	$k^{-}_{\mathrm{MBL_3MASP2}}$	5.5×10^{-4} s^{-1}	[41]
Association of MBL trimer to MASP3	$k^{+}_{\mathrm{MBL_3MASP3}}$	2.6×10^{5} $M^{-1}s^{-1}$	[42]
Dissociation of $\mathrm{MBL_3MASP3}$	$k^{-}_{\mathrm{MBL_3MASP3}}$	6.8×10^{-4} s^{-1}	[42]
Association of MBL trimer to MAp44	$k^{+}_{\mathrm{MBL_3MAp44}}$	1.9×10^{5} $M^{-1}s^{-1}$	[43]
Dissociation of $\mathrm{MBL_3MAp44}$	$k^{-}_{\mathrm{MBL_3MAp44}}$	1.3×10^{-3} s^{-1}	[43]
Association of MBL trimer to MAp19	$k^{+}_{\mathrm{MBL_3MAp19}}$	7.1×10^{5} $M^{-1}s^{-1}$	[41]
Dissociation of $\mathrm{MBL_3MAp19}$	$k^{-}_{\mathrm{MBL_3MAp19}}$	7.2×10^{-3} s^{-1}	[41]
Association of MBL tetramer to MASP1	$k^{+}_{\mathrm{MBL_4MASP1}}$	1.6×10^{5} $M^{-1}s^{-1}$	[41]
Dissociation of $\mathrm{MBL_4MASP1}$	$k^{-}_{\mathrm{MBL_4MASP1}}$	5.4×10^{-4} s^{-1}	[41]
Association of MBL tetramer to MASP2	$k^{+}_{\mathrm{MBL_4MASP2}}$	1.9×10^{5} $M^{-1}s^{-1}$	[41]

Dissociation of MBL_4MASP2	$k^{-}_{\mathrm{MBL_4MASP2}}$	4.6×10^{-4} s^{-1}	[41]
Association of MBL tetramer to MASP3	$k^{+}_{\mathrm{MBL_4MASP3}}$	2.1×10^{5} $M^{-1}s^{-1}$	[41]
Dissociation of MBL_4MASP3	$k^{-}_{\mathrm{MBL_4MASP3}}$	5.3×10^{-4} s^{-1}	[41]
Association of MBL tetramer to MAp44	$k^{+}_{\mathrm{MBL_4MAp44}}$	1.9×10^{5} $M^{-1}s^{-1}$	[43]
Dissociation of MBL_4MAp44	$k^{-}_{\mathrm{MBL_4MAp44}}$	1.3×10^{-3} s^{-1}	[43]
Association of MBL tetramer to MAp19	$k^{+}_{\mathrm{MBL_4MAp19}}$	6.0×10^{5} $M^{-1}s^{-1}$	[41]
Dissociation of MBL_4MAp19	$k^{-}_{\mathrm{MBL_4MAp19}}$	5.8×10^{-3} s^{-1}	[41]
Association of L-Ficolin to MASP1	k^{+}_{LFMASP1}	1.3×10^{5} $M^{-1}s^{-1}$	[43]
Dissociation of LFMASP1	k^{-}_{LFMASP1}	1.2×10^{-3} s^{-1}	[43]
Association of L-Ficolin to MASP2	k^{+}_{LFMASP2}	2.6×10^{5} $M^{-1}s^{-1}$	[43]
Dissociation of LFMASP2	k^{-}_{LFMASP2}	1.2×10^{-3} s^{-1}	[43]
Association of L-Ficolin to MASP3	k^{+}_{LFMASP3}	1.8×10^{5} $M^{-1}s^{-1}$	[42]
Dissociation of LFMASP3	k^{-}_{LFMASP3}	1.3×10^{-3} s^{-1}	[42]
Association of L-Ficolin to MAp44	k^{+}_{LFMAp44}	1.1×10^{5} $M^{-1}s^{-1}$	[43]
Dissociation of LFMAp44	k^{-}_{LFMAp44}	1.1×10^{-3} s^{-1}	[43]
Association of L-Ficolin to MAp19	k^{+}_{LFMAp19}	2.7×10^{5} $M^{-1}s^{-1}$	[43]
Dissociation of LFMAp19	k^{-}_{LFMAp19}	3.9×10^{-3} s^{-1}	[43]

Association of M-Ficolin to MASP1	k^{+}_{MFMASP1}	1.3×10^{5} M^{-1}s^{-1}	Assumption based on the interaction of structurally and functionally homologous protein LFMASP1
Dissociation of MFMASP1	k^{-}_{MFMASP1}	1.2×10^{-3} s^{-1}	Assumption based on the interaction of structurally and functionally homologous protein LFMASP1
Association of M-Ficolin to MASP2	k^{+}_{MFMASP2}	2.6×10^{5} M^{-1}s^{-1}	Assumption based on the interaction of structurally and functionally homologous protein LFMASP2
Dissociation of MFMASP2	k^{-}_{MFMASP2}	1.2×10^{-3} s^{-1}	Assumption based on the interaction of structurally and functionally homologous protein LFMASP2
Association of M-Ficolin to MASP3	k^{+}_{MFMASP3}	1.8×10^{5} M^{-1}s^{-1}	Assumption based on the interaction of structurally and functionally homologous protein LFMASP3
Dissociation of MFMASP3	k^{-}_{MFMASP3}	1.3×10^{-3} s^{-1}	Assumption based on the interaction of structurally and functionally homologous protein LFMASP3
Association of M-Ficolin to MAp44	k^{+}_{MFMAp44}	1.1×10^{5} M^{-1}s^{-1}	Assumption based on the interaction of structurally and functionally homologous protein LFMAp44
Dissociation of MFMAp44	k^{-}_{MFMAp44}	1.1×10^{-3} s^{-1}	Assumption based on the interaction of structurally and functionally homologous protein LFMAp44
Association of M-Ficolin to MAp19	k^{+}_{MFMAp19}	2.7×10^{5} M^{-1}s^{-1}	Assumption based on the interaction of structurally and functionally homologous protein LFMAp19
Dissociation of MFMAp19	k^{-}_{MFMAp19}	3.9×10^{-3} s^{-1}	Assumption based on the interaction of structurally and functionally homologous protein LFMAp19
Association of H-Ficolin to MASP1	k^{+}_{HFMASP1}	1.3×10^{5} M^{-1}s^{-1}	Assumption based on the interaction of structurally and functionally homologous protein LFMASP1

Dissociation of HFMASP1	k^{-}_{HFMASP1}	1.2×10^{-3} s^{-1}	Assumption based on the interaction of structurally and functionally homologous protein LFMASP1
Association of H-Ficolin to MASP2	k^{+}_{HFMASP2}	2.6×10^{5} M^{-1}s^{-1}	Assumption based on the interaction of structurally and functionally homologous protein LFMASP2
Dissociation of HFMASP2	k^{-}_{HFMASP2}	1.2×10^{-3} s^{-1}	Assumption based on the interaction of structurally and functionally homologous protein LFMASP2
Association of H-Ficolin to MASP3	k^{+}_{HFMASP3}	1.8×10^{5} M^{-1}s^{-1}	Assumption based on the interaction of structurally and functionally homologous protein LFMASP3
Dissociation of HFMASP3	k^{-}_{HFMASP3}	1.3×10^{-3} s^{-1}	Assumption based on the interaction of structurally and functionally homologous protein LFMASP3
Association of H-Ficolin to MAp44	k^{+}_{HFMAp44}	1.1×10^{5} M^{-1}s^{-1}	Assumption based on the interaction of structurally and functionally homologous protein LFMAp44
Dissociation of HFMAp44	k^{-}_{HFMAp44}	1.1×10^{-3} s^{-1}	Assumption based on the interaction of structurally and functionally homologous protein LFMAp44
Association of H-Ficolin to MAp19	k^{+}_{HFMAp19}	2.7×10^{5} M^{-1}s^{-1}	Assumption based on the interaction of structurally and functionally homologous protein LFMAp19
Dissociation of HFMAp19	k^{-}_{HFMAp19}	3.9×10^{-3} s^{-1}	Assumption based on the interaction of structurally and functionally homologous protein LFMAp19
Association of CL-LK to MASP1	$k^{+}_{\mathrm{CLLKMASP1}}$	9.9×10^{5} M^{-1}s^{-1}	Assumption based on the interaction of structurally and functionally homologous protein CL-LKMASP3
Dissociation of CL-LKMASP1	$k^{-}_{\mathrm{CLLKMASP1}}$	1.7×10^{-3} s^{-1}	Assumption based on the interaction of structurally and functionally homologous protein CL-LKMASP3
Association of CL-LK to MASP2	$k^{+}_{\mathrm{CLLKMASP2}}$	3.8×10^{5} M^{-1}s^{-1}	[44]

Dissociation of CL-LKMASP2	$k^{-}_{\mathrm{CLLKMASP2}}$	2.1×10^{-3} s^{-1}	[44]
Association of CL-LK to MASP3	$k^{+}_{\mathrm{CLLKMASP3}}$	9.9×10^{5} M^{-1}s^{-1}	[44]
Dissociation of CL-LKMASP3	$k^{-}_{\mathrm{CLLKMASP3}}$	1.7×10^{-3} s^{-1}	[44]
Association of CL-LK to MAp44	$k^{+}_{\mathrm{CLLKMAp44}}$	5.0×10^{5} M^{-1}s^{-1}	[44]
Dissociation of CL-LKMAp44	$k^{-}_{\mathrm{CLLKMAp44}}$	1.2×10^{-3} s^{-1}	[44]
Association of CL-LK to MAp19	$k^{+}_{\mathrm{CLLKMAp19}}$	3.8×10^{5} M^{-1}s^{-1}	Assumption based on the interaction of structurally and functionally homologous protein CL-LKMASP2
Dissociation of CL-LKMAp19	$k^{-}_{\mathrm{CLLKMAp19}}$	2.1×10^{-3} s^{-1}	Assumption based on the interaction of structurally and functionally homologous protein CL-LKMASP2
Association of CL-L1 to MASP1	$k^{+}_{\mathrm{CLL1MASP1}}$	9.9×10^{5} M^{-1}s^{-1}	Assumption based on the interaction of structurally and functionally homologous protein CL-LKMASP3
Dissociation of CL-L1MASP1	$k^{-}_{\mathrm{CLL1MASP1}}$	1.7×10^{-3} s^{-1}	Assumption based on the interaction of structurally and functionally homologous protein CL-LKMASP3
Association of CL-L1 to MASP2	$k^{+}_{\mathrm{CLL1MASP2}}$	3.8×10^{5} M^{-1}s^{-1}	Assumption based on the interaction of structurally and functionally homologous protein CL-LKMASP2
Dissociation of CL-L1MASP2	$k^{-}_{\mathrm{CLL1MASP2}}$	2.1×10^{-3} s^{-1}	Assumption based on the interaction of structurally and functionally homologous protein CL-LKMASP2
Association of CL-L1 to MASP3	$k^{+}_{\mathrm{CLL1MASP3}}$	9.9×10^{5} M^{-1}s^{-1}	Assumption based on the interaction of structurally and functionally homologous protein CL-LKMASP3
Dissociation of CL-L1MASP3	$k^{-}_{\mathrm{CLL1MASP3}}$	1.7×10^{-3} s^{-1}	Assumption based on the interaction of structurally and

			functionally homologous protein CL-LKMASP3
Association of CL-L1 to MAp44	$k^{+}_{\mathrm{CLL1MAp44}}$	5.0×10^{5} $M^{-1}s^{-1}$	Assumption based on the interaction of structurally and functionally homologous protein CL-LKMAp44
Dissociation of CL-L1MAp44	$k^{-}_{\mathrm{CLL1MAp44}}$	1.2×10^{-3} s^{-1}	Assumption based on the interaction of structurally and functionally homologous protein CL-LKMAp44
Association of CL-L1 to MAp19	$k^{+}_{\mathrm{CLL1MAp19}}$	3.8×10^{5} $M^{-1}s^{-1}$	Assumption based on the interaction of structurally and functionally homologous protein CL-LKMASP2
Dissociation of CL-L1MAp19	$k^{-}_{\mathrm{CLL1MAp19}}$	2.1×10^{-3} s^{-1}	Assumption based on the interaction of structurally and functionally homologous protein CL-LKMASP2
Association of CL-K1 to MASP1	$k^{+}_{\mathrm{CLK1MASP1}}$	1.7×10^{6} $M^{-1}s^{-1}$	Assumption based on the interaction of structurally and functionally homologous protein CL-K1MASP3
Dissociation of CL-K1MASP1	$k^{-}_{\mathrm{CLK1MASP1}}$	4.7×10^{-3} s^{-1}	Assumption based on the interaction of structurally and functionally homologous protein CL-K1MASP3
Association of CL-K1 to MASP2	$k^{+}_{\mathrm{CLK1MASP2}}$	2.2×10^{5} $M^{-1}s^{-1}$	[44]
Dissociation of CL-K1MASP2	$k^{-}_{\mathrm{CLK1MASP2}}$	2.6×10^{-3} s^{-1}	[44]
Association of CL-K1 to MASP3	$k^{+}_{\mathrm{CLK1MASP3}}$	1.7×10^{6} $M^{-1}s^{-1}$	[44]
Dissociation of CL-K1MASP3	$k^{-}_{\mathrm{CLK1MASP3}}$	4.7×10^{-3} s^{-1}	[44]
Association of CL-K1 to MAp44	$k^{+}_{\mathrm{CLK1MAp44}}$	5.0×10^{5} $M^{-1}s^{-1}$	Assumption based on the interaction of structurally and functionally homologous protein CL-LKMAp44
Dissociation of CL-K1MAp44	$k^{-}_{\mathrm{CLK1MAp44}}$	1.2×10^{-3} s^{-1}	Assumption based on the interaction of structurally and functionally homologous protein CL-LKMAp44

Association of CL-K1 to MAp19	$k^{+}_{\mathrm{CLK1MAp19}}$	2.2×10^{5} $M^{-1}s^{-1}$	Assumption based on the interaction of structurally and functionally homologous protein CL-K1MASP2
Dissociation of CL-K1MAp19	$k^{-}_{\mathrm{CLK1MAp19}}$	2.6×10^{-3} s^{-1}	Assumption based on the interaction of structurally and functionally homologous protein CL-K1MASP2
Association of C1-INH to MASP1	$k^{+}_{\mathrm{C1INHMASP1}}$	6.2×10^{3} $M^{-1}s^{-1}$	[45]
Association of C1-INH to MASP2	$k^{+}_{\mathrm{C1INHMASP2}}$	2.2×10^{7} $M^{-1}s^{-1}$	[19]
Association of CFHR1 to pathogen surface	k^{+}_{pCFHR1}	1.4×10^{6} $M^{-1}s^{-1}$	Assumption based on the interaction of structurally and functionally homologous protein PathogenCFHR3
Dissociation of PathogenCFHR1	k^{-}_{pCFHR1}	4.1×10^{-3} s^{-1}	Assumption based on the interaction of structurally and functionally homologous protein PathogenCFHR3
Association of CFHR2 to pathogen surface	k^{+}_{pCFHR2}	1.4×10^{6} $M^{-1}s^{-1}$	Assumption based on the interaction of structurally and functionally homologous protein PathogenCFHR3
Dissociation of PathogenCFHR2	k^{-}_{pCFHR2}	4.1×10^{-3} s^{-1}	Assumption based on the interaction of structurally and functionally homologous protein PathogenCFHR3
Association of CFHR3 to pathogen surface (Fhbp V3)	k^{+}_{pCFHR3}	1.4×10^{6} $M^{-1}s^{-1}$	[46]
Dissociation of PathogenCFHR3	k^{-}_{pCFHR3}	4.1×10^{-3} s^{-1}	[46]
Association of CFHR4 to pathogen surface	k^{+}_{pCFHR4}	1.4×10^{6} $M^{-1}s^{-1}$	Assumption based on the interaction of structurally and functionally homologous protein PathogenCFHR3

Dissociation of PathogenCFHR4	k^-_{pCFHR4}	4.1×10^{-3} s^{-1}	Assumption based on the interaction of structurally and functionally homologous protein PathogenCFHR3
Association of CFHR5 to pathogen surface	k^+_{pCFHR5}	1.4×10^{6} M^{-1}s^{-1}	Assumption based on the interaction of structurally and functionally homologous protein PathogenCFHR3
Dissociation of PathogenCFHR5	k^-_{pCFHR5}	4.1×10^{-3} s^{-1}	Assumption based on the interaction of structurally and functionally homologous protein PathogenCFHR3
Association of PTX3 to pathogen surface	k^+_{pPTX3}	2.0×10^{6} M^{-1}s^{-1}	Calculated by averaging experimental values of $k^+_{\mathrm{pMBL_3}}$, $k^+_{\mathrm{pMBL_4}}$, k^+_{pCFHR3}, k^+_{pFH}, k^+_{pFHL1}, and k^+_{pC4BP}
Dissociation of PathogenPTX3	k^-_{pPTX3}	6.3×10^{-3} s^{-1}	Calculated by averaging experimental values of $k^-_{\mathrm{pMBL_3}}$, $k^-_{\mathrm{pMBL_4}}$, k^-_{pCFHR3}, k^-_{pFH}, k^-_{pFHL1}, and k^-_{pC4BP}
Association of PTX3 to MBL$_3$	$k^+_{\mathrm{MBL_3PTX3}}$	3.0×10^{5} M^{-1}s^{-1}	Calculated by averaging experimental values of k^+_{FHPTX3}, k^+_{C1qPTX3}, k^+_{LFPTX3}, k^+_{CFHR4CRP}, and k^+_{C1qCRP}
Dissociation of MBL$_3$PTX3	$k^-_{\mathrm{MBL_3PTX3}}$	3.0×10^{-3} s^{-1}	Calculated by averaging experimental values of k^-_{FHPTX3}, k^-_{C1qPTX3}, k^-_{LFCRP}, k^-_{CFRH4CRP}, and k^-_{C1qCRP}
Association of PTX3 to MBL$_4$	$k^+_{\mathrm{MBL_4PTX3}}$	3.0×10^{5} M^{-1}s^{-1}	Calculated by averaging experimental values of k^+_{FHPTX3}, k^+_{C1qPTX3}, k^+_{LFPTX3}, k^+_{CFHR4CRP}, and k^+_{C1qCRP}
Dissociation of MBL$_4$PTX3	$k^-_{\mathrm{MBL_4PTX3}}$	3.0×10^{-3} s^{-1}	Calculated by averaging experimental values of $k^-_{\mathrm{FH:PTX3}}$, $k^-_{\mathrm{C1q:PTX3}}$, $k^-_{\mathrm{LF:CRP}}$, $k^-_{\mathrm{CFRH4:CRP}}$, and $k^-_{\mathrm{C1q:CRP}}$

Association of PTX3 to C4BP	$k^{+}_{\mathrm{C4BPPTX3}}$	5.0×10^{4} $M^{-1}s^{-1}$	Assumption based on the interaction of functionally homologous protein FHPTX3
Dissociation of C4BPPTX3	$\mathrm{k}^{-}_{\mathrm{C4BPPTX3}}$	5.5×10^{-3} s^{-1}	Assumption based on the interaction of functionally homologous protein FHPTX3
Association of PTX3 to FH	k^{+}_{FHPTX3}	5.0×10^{4} $M^{-1}s^{-1}$	[47]
Dissociation of FHPTX3	$\mathrm{k}^{-}_{\mathrm{FHPTX3}}$	5.5×10^{-3} s^{-1}	[47]
Association of PTX3 to FHL1	$k^{+}_{\mathrm{FHL1PTX3}}$	5.0×10^{4} $M^{-1}s^{-1}$	Assumption based on the interaction of structurally and functionally homologous protein FHPTX3
Dissociation of FHL1PTX3	$\mathrm{k}^{-}_{\mathrm{FHL1PTX3}}$	5.5×10^{-3} s^{-1}	Assumption based on the interaction of structurally and functionally homologous protein FHPTX3
Association of PTX3 to C1q	k^{+}_{C1qPTX3}	3.9×10^{4} $M^{-1}s^{-1}$	[48]
Dissociation of C1qPTX3	$\mathrm{k}^{-}_{\mathrm{C1qPTX3}}$	6.7×10^{-4} s^{-1}	[48]
Association of PTX3 to LF	k^{+}_{LFPTX3}	6.0×10^{5} $M^{-1}s^{-1}$	[49]
Dissociation of LFPTX3	$\mathrm{k}^{-}_{\mathrm{LFPTX3}}$	3.9×10^{-3} s^{-1}	[49]
Association of PTX3 to CFHR1	$k^{+}_{\mathrm{CFHR1PTX3}}$	5.0×10^{4} $M^{-1}s^{-1}$	Assumption based on the interaction of structurally and functionally homologous protein FHPTX3

Dissociation of CFHR1PTX3	$k^{-}_{\mathrm{CFHR1PTX3}}$	$5.5\times10^{-3}\ s^{-1}$	Assumption based on the interaction of structurally and functionally homologous protein FHPTX3
Association of PTX3 to CFHR5	$k^{+}_{\mathrm{CFHR5PTX3}}$	$5.0\times10^{4}\ M^{-1}s^{-1}$	Assumption based on the interaction of structurally and functionally homologous protein FHPTX3
Dissociation of CFHR5PTX3	$k^{-}_{\mathrm{CFHR5PTX3}}$	$5.5\times10^{-3}\ s^{-1}$	Assumption based on the interaction of structurally and functionally homologous protein FHPTX3
Association of SAP to pathogen surface	k^{+}_{pSAP}	$2.0\times10^{6}\ M^{-1}s^{-1}$	Calculated by averaging experimental values of $k^{+}_{\mathrm{pMBL_3}}$, $k^{+}_{\mathrm{pMBL_4}}$, k^{+}_{pCFHR3}, k^{+}_{pFH}, k^{+}_{pFHL1}, and k^{+}_{pC4BP}
Dissociation of PathogenSAP	k^{-}_{pSAP}	$6.3\times10^{-3}\ s^{-1}$	Calculated by averaging experimental values of $k^{-}_{\mathrm{pMBL_3}}$, $k^{-}_{\mathrm{pMBL_4}}$, k^{-}_{pCFHR3}, k^{-}_{pFH}, k^{-}_{pFHL1}, and k^{-}_{pC4BP}
Association of SAP to MBL_3	$k^{+}_{\mathrm{MBL_3\,SAP}}$	$3.0\times10^{5}\ M^{-1}s^{-1}$	Calculated by averaging experimental values of k^{+}_{FHPTX3}, k^{+}_{C1qPTX3}, k^{+}_{LFPTX3}, $k^{+}_{\mathrm{CFHR4CRP}}$, and k^{+}_{C1qCRP}
Dissociation of MBL_3SAP	$k^{-}_{\mathrm{MBL_3\,SAP}}$	$3.0\times10^{-3}\ s^{-1}$	Calculated by averaging experimental values of k^{-}_{FHPTX3}, k^{-}_{C1qPTX3}, k^{-}_{LFCRP}, $k^{-}_{\mathrm{CFRH4CRP}}$, and k^{-}_{C1qCRP}
Association of SAP to MBL_4	$k^{+}_{\mathrm{MBL_4\,SAP}}$	$3.0\times10^{5}\ M^{-1}s^{-1}$	Calculated by averaging experimental values of k^{+}_{FHPTX3}, k^{+}_{C1qPTX3}, k^{+}_{LFPTX3}, $k^{+}_{\mathrm{CFHR4CRP}}$, and k^{+}_{C1qCRP}
Dissociation of MBL_4SAP	$k^{-}_{\mathrm{MBL_4\,SAP}}$	$3.0\times10^{-3}\ s^{-1}$	Calculated by averaging experimental values of k^{-}_{FHPTX3}, k^{-}_{C1qPTX3}, k^{-}_{LFCRP}, $k^{-}_{\mathrm{CFRH4CRP}}$, and k^{-}_{C1qCRP}

Association of CRP to pathogen surface	k^{+}_{pCRP}	2.0×10^{6} M^{-1}s^{-1}	Calculated by averaging experimental values of $k^{+}_{\mathrm{pMBL}_3}$, $k^{+}_{\mathrm{pMBL}_4}$, k^{+}_{pCFHR3}, k^{+}_{pFH}, k^{+}_{pFHL1}, and k^{+}_{pC4BP}
Dissociation of PathogenCRP	k^{-}_{pCRP}	6.3×10^{-3} s^{-1}	Calculated by averaging experimental values of $k^{-}_{\mathrm{pMBL}_3}$, $k^{-}_{\mathrm{pMBL}_4}$, k^{-}_{pCFHR3}, k^{-}_{pFH}, k^{-}_{pFHL1}, and k^{-}_{pC4BP}
Association of CRP to CFHR4	$k^{+}_{\mathrm{CFHR4CRP}}$	8.3×10^{4} M^{-1}s^{-1}	[50]
Dissociation of CFHR4CRP	$k^{-}_{\mathrm{CFHR4CRP}}$	3.6×10^{-3} s^{-1}	[50]
Association of CRP to C1q	k^{+}_{C1qCRP}	7.4×10^{4} M^{-1}s^{-1}	[51]
Dissociation of C1qCRP	k^{-}_{C1qCRP}	1.1×10^{-3} s^{-1}	[51]
Association of FH to pathogen surface (fHbp V3)	k^{+}_{pFH}	1.6×10^{6} M^{-1}s^{-1}	[52]
Dissociation of fHbpFH	$\mathrm{k}^{-}_{\mathrm{pFH}}$	4.5×10^{-3} s^{-1}	[52]
Association of FHL-1 to pathogen surface (fHbp V3)	$k^{+}_{\mathrm{FhbpFHL1}}$	1.6×10^{6} M^{-1}s^{-1}	[52]
Dissociation of fHbpFHL1	$\mathrm{k}^{-}_{\mathrm{FhbpFHL1}}$	4.5×10^{-3} s^{-1}	[52]

Association of C4BP to pathogen surface (porA)	k_{pC4BP}^{+}	1.6×10^{6} M^{-1}s^{-1}	Assumption based on the interaction of structurally and functionally homologous protein fHbpFH
Dissociation of porAC4BP	$\mathrm{k}_{\mathrm{pC4BP}}^{-}$	4.5×10^{-3} s^{-1}	Assumption based on the interaction of structurally and functionally homologous protein fHbpFH
Association of Vn to pathogen surface (Msf)	k_{pVn}^{+}	1.6×10^{6} M^{-1}s^{-1}	Assumption based on the interaction of structurally and functionally homologous protein fHbpFH
Dissociation of MsfVn	$\mathrm{k}_{\mathrm{pVn}}^{-}$	4.5×10^{-3} s^{-1}	Assumption based on the interaction of structurally and functionally homologous protein fHbpFH

References

1. Pangburn MK, Schreiber RD, Müller-Eberhard HJ. Formation of the initial C3 convertase of the alternative complement pathway. Acquisition of C3b-like activities by spontaneous hydrolysis of the putative thioester in native C3. J Exp Med. 1981;154: 856–867.

2. Laich A, Sim RB. Complement C4bC2 complex formation: an investigation by surface plasmon resonance. Biochim Biophys Acta. 2001;1544: 96–112.

3. Pangburn MK, Müller-Eberhard HJ. The C3 convertase of the alternative pathway of human complement. Enzymic properties of the bimolecular proteinase. Biochem J. 1986;235: 723–730.

4. Chen H, Ricklin D, Hammel M, Garcia BL, McWhorter WJ, Sfyroera G, et al. Allosteric inhibition of complement function by a staphylococcal immune evasion protein. Proc Natl Acad Sci. 2010;107: 17621–17626. doi:10.1073/pnas.1003750107

5. Hourcade DE. The Role of Properdin in the Assembly of the Alternative Pathway C3 Convertases of Complement. J Biol Chem. 2006;281: 2128–2132. doi:10.1074/jbc.M508928200

6. Rawal N, Pangburn MK. Formation of High Affinity C5 Convertase of the Classical Pathway of Complement. J Biol Chem. 2003;278: 38476–38483. doi:10.1074/jbc.M307017200

7. Pedersen DV, Roumenina L, Jensen RK, Gadeberg TA, Marinozzi C, Picard C, et al. Functional and structural insight into properdin control of complement alternative pathway amplification. EMBO J. 2017;36: 1084–1099. doi:10.15252/embj.201696173

8. Zewde N, Jr RDG, Dorado A, Morikis D. Quantitative Modeling of the Alternative Pathway of the Complement System. PLOS ONE. 2016;11: e0152337. doi:10.1371/journal.pone.0152337

9. Bernet J, Mullick J, Panse Y, Parab PB, Sahu A. Kinetic Analysis of the Interactions between Vaccinia Virus Complement Control Protein and Human Complement Proteins C3b and C4b. J Virol. 2004;78: 9446–9457. doi:10.1128/JVI.78.17.9446-9457.2004

10. Dopler A, Guntau L, Harder MJ, Palmer A, Höchsmann B, Schrezenmeier H, et al. Self versus Nonself Discrimination by the Soluble Complement Regulators Factor H and FHL-1. J Immunol Baltim Md 1950. 2019;202: 2082–2094. doi:10.4049/jimmunol.1801545

11. Harris CL, Abbott RJM, Smith RA, Morgan BP, Lea SM. Molecular Dissection of Interactions between Components of the Alternative Pathway of Complement and Decay Accelerating Factor (CD55). J Biol Chem. 2005;280: 2569–2578. doi:10.1074/jbc.M410179200

12. Harder MJ, Anliker M, Höchsmann B, Simmet T, Huber-Lang M, Schrezenmeier H, et al. Comparative analysis of novel complement-targeted inhibitors, miniFH, and the natural

regulators Factor H and Factor H-like protein 1 reveal functional determinants of complement regulation. J Immunol Baltim Md 1950. 2016;196: 866–876. doi:10.4049/jimmunol.1501919

13. Klickstein LB, Barbashov SF, Liu T, Jack RM, Nicholson-Weller A. Complement receptor type 1 (CR1, CD35) is a receptor for C1q. Immunity. 1997;7: 345–355.

14. Korotaevskiy AA, Hanin LG, Khanin MA. Non-linear dynamics of the complement system activation. Math Biosci. 2009;222: 127–143. doi:10.1016/j.mbs.2009.10.003

15. Fujita T, Tamura N. Interaction of C4-binding protein with cell-bound C4b. A quantitative analysis of binding and the role of C4-binding protein in proteolysis of cell-bound C4b. J Exp Med. 1983;157: 1239–1251.

16. Ziccardi RJ, Dahlback B, Müller-Eberhard HJ. Characterization of the interaction of human C4b-binding protein with physiological ligands. J Biol Chem. 1984;259: 13674–13679.

17. Bally I, Rossi V, Lunardi T, Thielens NM, Gaboriaud C, Arlaud GJ. Identification of the C1q-binding Sites of Human C1r and C1s A REFINED THREE-DIMENSIONAL MODEL OF THE C1 COMPLEX OF COMPLEMENT. J Biol Chem. 2009;284: 19340–19348. doi:10.1074/jbc.M109.004473

18. Bianchino AC, Poon PH, Schumaker VN. A mechanism for the spontaneous activation of the first component of complement, C1, and its regulation by C1-inhibitor. J Immunol. 1988;141: 3930–3936.

19. Kerr FK, Thomas AR, Wijeyewickrema LC, Whisstock JC, Boyd SE, Kaiserman D, et al. Elucidation of the substrate specificity of the MASP-2 protease of the lectin complement pathway and identification of the enzyme as a major physiological target of the serpin, C1-inhibitor. Mol Immunol. 2008;45: 670–677. doi:10.1016/j.molimm.2007.07.008

20. Muller-Eberhard HJ. The Membrane Attack Complex of Complement. Annu Rev Immunol. 1986;4: 503–528. doi:10.1146/annurev.iy.04.040186.002443

21. Li CKN, Levine RP. Rate process in the final stage of complement hemolysis. Immunochemistry. 1977;14: 421–428. doi:10.1016/0019-2791(77)90167-7

22. Podack ER, Biesecker G, Kolb WP, Müller-Eberhard HJ. The C5b-6 complex: reaction with C7, C8, C9. J Immunol Baltim Md 1950. 1978;121: 484–490.

23. Parsons ES, Stanley GJ, Pyne ALB, Hodel AW, Nievergelt AP, Menny A, et al. Single-molecule kinetics of pore assembly by the membrane attack complex. Nat Commun. 2019;10: 2066. doi:10.1038/s41467-019-10058-7

24. McDonald JF, Nelsestuen GL. Potent inhibition of terminal complement assembly by clusterin: characterization of its impact on C9 polymerization. Biochemistry (Mosc). 1997;36: 7464–7473. doi:10.1021/bi962895r

25. Rawal N, Pangburn MK. C5 convertase of the alternative pathway of complement. Kinetic analysis of the free and surface-bound forms of the enzyme. J Biol Chem. 1998;273: 16828–16835.

26. Cooper NR. Enzymatic activity of the second component of complement. Biochemistry (Mosc). 1975;14: 4245–4251.

27. Rossi V, Teillet F, Thielens NM, Bally I, Arlaud GJ. Functional Characterization of Complement Proteases C1s/Mannan-binding Lectin-associated Serine Protease-2 (MASP-2) Chimeras Reveals the Higher C4 Recognition Efficacy of the MASP-2 Complement Control Protein Modules. J Biol Chem. 2005;280: 41811–41818. doi:10.1074/jbc.M503813200

28. Taylor FR, Bixler SA, Budman JI, Wen D, Karpusas M, Ryan ST, et al. Induced Fit Activation Mechanism of the Exceptionally Specific Serine Protease, Complement Factor D†. Biochemistry (Mosc). 1999;38: 2849–2859. doi:10.1021/bi982140f

29. Pangburn MK, Mueller-Eberhard HJ. Kinetic and thermodynamic analysis of the control of C3b by the complement regulatory proteins factors H and I. Biochemistry (Mosc). 1983;22: 178–185. doi:10.1021/bi00270a026

30. Rawal N, Pangburn M. Formation of high-affinity C5 convertases of the alternative pathway of complement. J Immunol Baltim Md 1950. 2001;166: 2635–2642.

31. Du X-Y, Zabel BA, Myles T, Allen SJ, Handel TM, Lee PP, et al. Regulation of chemerin bioactivity by plasma carboxypeptidase N, carboxypeptidase B (activated thrombin-activable fibrinolysis inhibitor), and platelets. J Biol Chem. 2009;284: 751–758. doi:10.1074/jbc.M805000200

32. Heinen S, Hartmann A, Lauer N, Wiehl U, Dahse H-M, Schirmer S, et al. Factor H-related protein 1 (CFHR-1) inhibits complement C5 convertase activity and terminal complex formation. Blood. 2009;114: 2439–2447. doi:10.1182/blood-2009-02-205641

33. Eberhardt HU, Buhlmann D, Hortschansky P, Chen Q, Böhm S, Kemper MJ, et al. Human factor H-related protein 2 (CFHR2) regulates complement activation. PloS One. 2013;8: e78617. doi:10.1371/journal.pone.0078617

34. Hellwage J, Jokiranta TS, Koistinen V, Vaarala O, Meri S, Zipfel PF. Functional properties of complement factor H-related proteins FHR-3 and FHR-4: binding to the C3d region of C3b and differential regulation by heparin. FEBS Lett. 1999;462: 345–352.

35. Goicoechea de Jorge E, Caesar JJE, Malik TH, Patel M, Colledge M, Johnson S, et al. Dimerization of complement factor H-related proteins modulates complement activation in vivo. Proc Natl Acad Sci U S A. 2013;110: 4685–4690. doi:10.1073/pnas.1219260110

36. Bally I, Ancelet S, Moriscot C, Gonnet F, Mantovani A, Daniel R, et al. Expression of recombinant human complement C1q allows identification of the C1r/C1s-binding sites. Proc Natl Acad Sci U S A. 2013;110: 8650–8655. doi:10.1073/pnas.1304894110

37. Saha K, Bender F, Gizeli E. Comparative study of IgG binding to proteins G and A: nonequilibrium kinetic and binding constant determination with the acoustic waveguide device. Anal Chem. 2003;75: 835–842.

38. Megyeri M, Harmat V, Major B, Végh Á, Balczer J, Héja D, et al. Quantitative characterization of the activation steps of mannan-binding lectin (MBL)-associated serine proteases (MASPs) points to the central role of MASP-1 in the initiation of the complement lectin pathway. J Biol Chem. 2013;288: 8922–8934. doi:10.1074/jbc.M112.446500

39. Héja D, Kocsis A, Dobó J, Szilágyi K, Szász R, Závodszky P, et al. Revised mechanism of complement lectin-pathway activation revealing the role of serine protease MASP-1 as the exclusive activator of MASP-2. Proc Natl Acad Sci U S A. 2012;109: 10498–10503. doi:10.1073/pnas.1202588109

40. Ambrus G, Gál P, Kojima M, Szilágyi K, Balczer J, Antal J, et al. Natural Substrates and Inhibitors of Mannan-Binding Lectin-Associated Serine Protease-1 and -2: A Study on Recombinant Catalytic Fragments. J Immunol. 2003;170: 1374–1382. doi:10.4049/jimmunol.170.3.1374

41. Teillet F, Dublet B, Andrieu J-P, Gaboriaud C, Arlaud GJ, Thielens NM. The two major oligomeric forms of human mannan-binding lectin: chemical characterization, carbohydrate-binding properties, and interaction with MBL-associated serine proteases. J Immunol Baltim Md 1950. 2005;174: 2870–2877. doi:10.4049/jimmunol.174.5.2870

42. Zundel S, Cseh S, Lacroix M, Dahl MR, Matsushita M, Andrieu J-P, et al. Characterization of recombinant mannan-binding lectin-associated serine protease (MASP)-3 suggests an activation mechanism different from that of MASP-1 and MASP-2. J Immunol Baltim Md 1950. 2004;172: 4342–4350. doi:10.4049/jimmunol.172.7.4342

43. Cseh S, Vera L, Matsushita M, Fujita T, Arlaud GJ, Thielens NM. Characterization of the interaction between L-ficolin/p35 and mannan-binding lectin-associated serine proteases-1 and -2. J Immunol Baltim Md 1950. 2002;169: 5735–5743. doi:10.4049/jimmunol.169.10.5735

44. Henriksen ML, Brandt J, Andrieu J-P, Nielsen C, Jensen PH, Holmskov U, et al. Heteromeric complexes of native collectin kidney 1 and collectin liver 1 are found in the circulation with MASPs and activate the complement system. J Immunol Baltim Md 1950. 2013;191: 6117–6127. doi:10.4049/jimmunol.1302121

45. Dobó J, Harmat V, Beinrohr L, Sebestyén E, Závodszky P, Gál P. MASP-1, a promiscuous complement protease: structure of its catalytic region reveals the basis of its broad specificity. J Immunol Baltim Md 1950. 2009;183: 1207–1214. doi:10.4049/jimmunol.0901141

46. Caesar JJE, Lavender H, Ward PN, Exley RM, Eaton J, Chittock E, et al. Competition between antagonistic complement factors for a single protein on N. meningitidis rules disease susceptibility. eLife. 2014;3. doi:10.7554/eLife.04008

47. Deban L, Jarva H, Lehtinen MJ, Bottazzi B, Bastone A, Doni A, et al. Binding of the long pentraxin PTX3 to factor H: interacting domains and function in the regulation of complement activation. J Immunol Baltim Md 1950. 2008;181: 8433–8440. doi:10.4049/jimmunol.181.12.8433

48. Moreau C, Bally I, Chouquet A, Bottazzi B, Ghebrehiwet B, Gaboriaud C, et al. Structural and Functional Characterization of a Single-Chain Form of the Recognition Domain of Complement Protein C1q. Front Immunol. 2016;7: 79. doi:10.3389/fimmu.2016.00079

49. Ma YJ, Doni A, Hummelshøj T, Honoré C, Bastone A, Mantovani A, et al. Synergy between ficolin-2 and pentraxin 3 boosts innate immune recognition and complement deposition. J Biol Chem. 2009;284: 28263–28275. doi:10.1074/jbc.M109.009225

50. Mihlan M, Hebecker M, Dahse H-M, Hälbich S, Huber-Lang M, Dahse R, et al. Human complement factor H-related protein 4 binds and recruits native pentameric C-reactive protein to necrotic cells. Mol Immunol. 2009;46: 335–344. doi:10.1016/j.molimm.2008.10.029

51. Bíró A, Rovó Z, Papp D, Cervenak L, Varga L, Füst G, et al. Studies on the interactions between C-reactive protein and complement proteins. Immunology. 2007;121: 40–50. doi:10.1111/j.1365-2567.2007.02535.x

52. Johnson S, Tan L, van der Veen S, Caesar J, Goicoechea De Jorge E, Harding RJ, et al. Design and evaluation of meningococcal vaccines through structure-based modification of host and pathogen molecules. PLoS Pathog. 2012;8: e1002981. doi:10.1371/journal.ppat.1002981
