# Supplementary material for: Systems Biology Modeling of the Complement System Under Immune Susceptible Pathogens
Source: Front Phys. Author manuscript; Available in PMC 2022 Feb 9. (PMC8827490; doi:10.3389/fphy.2021.603704)
Supplement: Table_3_Systems Biology Modeling of the Complement System Under Immune Susceptible Pathogens [file NIHMS1768816-supplement-Table_3_Systems_Biology_Modeling_of_the_Complement_System_Under_Immune_Susceptible_Pathogens.pdf]

S3 Table. Range of kinetic rate constants implemented in sensitivity analysis.

Biochemical reaction	Rate constant	Value	Source
Hydrolysis of $C3(H_2O)$	$k^{+}_{\mathrm{C3(H_2O)}}$	4.5×10^{-6} s^{-1}	$4.5\times10^{-7} - 4.5\times10^{-5}$ s^{-1}
Association of Factor B to $C3(H_2O)$	$k^{+}_{\mathrm{C3(H_2O)B}}$	1.1×10^{4} $M^{-1}s^{-1}$	$1.1\times10^{3} - 1.1\times10^{5}$ $M^{-1}s^{-1}$
Dissociation of complex $C3(H_2O)B$	$k^{-}_{\mathrm{C3(H_2O)B}}$	1.4×10^{-3} s^{-1}	$1.4\times10^{-4} - 1.4\times10^{-2}$ s^{-1}
Association of Factor H to $C3(H_2O)$	$k^{+}_{\mathrm{C3(H_2O)H}}$	1.1×10^{6} $M^{-1}s^{-1}$	$1.1\times10^{5} - 1.1\times10^{7}$ $M^{-1}s^{-1}$
Dissociation of complex $C3(H_2O)H$	$k^{-}_{\mathrm{C3(H_2O)H}}$	6.0×10^{-2} s^{-1}	$6.0\times10^{-3} - 6.0\times10^{-1}$ s^{-1}
Association of Factor H-like protein 1 to $C3(H_2O)$	$k^{+}_{\mathrm{C3(H_2O)FHL1}}$	1.8×10^{4} $M^{-1}s^{-1}$	$1.8\times10^{3} - 1.8\times10^{5}$ $M^{-1}s^{-1}$
Dissociation of complex $C3(H_2O)FHL1$	$k^{-}_{\mathrm{C3(H_2O)FHL1}}$	1.9×10^{-2} s^{-1}	$1.9\times10^{-3} - 1.9\times10^{-1}$ s^{-1}
Dissociation of complex $C3(H_2O)Bb$	$k^{-}_{\mathrm{C3(H_2O)Bb}}$	9.0×10^{-3} s^{-1}	$9.0\times10^{-4} - 9.0\times10^{-2}$ s^{-1}
Association of Factor B to C3b	k^{+}_{C3bB}	21.3×10^{4} $M^{-1}s^{-1}$	$21.3\times10^{3} - 21.3\times10^{5}$ $M^{-1}s^{-1}$
Dissociation of complex C3bB	k^{-}_{C3bB}	15.5×10^{-2} s^{-1}	$15.5\times10^{-3} - 15.5\times10^{-1}$ s^{-1}
Dissociation of complex C3bBb	k^{-}_{C3bBb}	7.7×10^{-3} s^{-1}	$7.7\times10^{-4} - 7.7\times10^{-2}$ s^{-1}
Dissociation of complex C3bBbP	k^{-}_{C3bBbP}	7.7×10^{-4} s^{-1}	$7.7\times10^{-5} - 7.7\times10^{-3}$ s^{-1}
Dissociation of complex C4bC2a	k^{-}_{C4bC2a}	4.0×10^{-3} s^{-1}	$4.0\times10^{-4} - 4.0\times10^{-2}$ s^{-1}
Association of Properdin to C3b	k^{+}_{C3bP}	1.5×10^{5} $M^{-1}s^{-1}$	$1.5\times10^{4} - 1.5\times10^{6}$ $M^{-1}s^{-1}$

Dissociation of complex C3bP	k^{-}_{C3bP}	15.3×10^{-5} s^{-1}	$15.3\times10^{-6} - 15.3\times10^{-4}$ s^{-1}
Attachment of nfC3b to host cell or pathogen	$\mathrm{k}^{+}_{\mathrm{hC3b}}$ or $\mathrm{k}^{+}_{\mathrm{pC3b}}$	4.2×10^{8} $M^{-1}s^{-1}$	$4.2\times10^{7} - 4.2\times10^{9}$ $M^{-1}s^{-1}$
Association of nfC3b to water	k^{+}_{fC3b}	4.2×10^{8} $M^{-1}s^{-1}$	$4.2\times10^{7} - 4.2\times10^{9}$ $M^{-1}s^{-1}$
Association of nfC3b to C3b	k^{+}_{C3bC3b}	4.2×10^{8} $M^{-1}s^{-1}$	$4.2\times10^{7} - 4.2\times10^{9}$ $M^{-1}s^{-1}$
Association of nfC3b to IgG	k^{+}_{IgGC3b}	4.2×10^{8} $M^{-1}s^{-1}$	$4.2\times10^{7} - 4.2\times10^{9}$ $M^{-1}s^{-1}$
Association of nfC3b/ nfC4b to C4b/C3b	k^{+}_{C3bC4b}	4.2×10^{8} $M^{-1}s^{-1}$	$4.2\times10^{7} - 4.2\times10^{9}$ $M^{-1}s^{-1}$
Attachment of nfC4b to host cell or pathogen	k^{+}_{hC4b} or k^{+}_{pC4b}	4.2×10^{8} $M^{-1}s^{-1}$	$4.2\times10^{7} - 4.2\times10^{9}$ $M^{-1}s^{-1}$
Association of nfC4b to water	k^{+}_{fC4b}	4.2×10^{8} $M^{-1}s^{-1}$	$4.2\times10^{7} - 4.2\times10^{9}$ $M^{-1}s^{-1}$
Association of nfC4b to C4b	k^{+}_{C4bC4b}	4.2×10^{8} $M^{-1}s^{-1}$	$4.2\times10^{7} - 4.2\times10^{9}$ $M^{-1}s^{-1}$
Association of Factor H to C3b	k^{+}_{C3bH}	1.1×10^{6} $M^{-1}s^{-1}$	$1.1\times10^{5} - 1.1\times10^{7}$ $M^{-1}s^{-1}$
Dissociation of complex C3bH	k^{-}_{C3bH}	6.0×10^{-2} s^{-1}	$6.0\times10^{-3} - 6.0\times10^{-1}$ s^{-1}
Association of Factor H-like protein 1 to C3b	k^{+}_{C3bFHL1}	1.8×10^{4} $M^{-1}s^{-1}$	$1.8\times10^{3} - 1.8\times10^{5}$ $M^{-1}s^{-1}$
Dissociation of complex C3bFHL1	k^{-}_{C3bFHL1}	1.9×10^{-2} s^{-1}	$1.9\times10^{-3} - 1.9\times10^{-1}$ s^{-1}
Decay of convertase by inhibitor C4BP	$k^{-}_{\mathrm{C4bC2aC4BP_{decay}}}$	1.7×10^{-2} s^{-1}	$1.7\times10^{-3} - 1.7\times10^{-1}$ s^{-1}

Decay of C3 convertase by inhibitor Factor H	$k^{-}_{\mathrm{C3bBbH}_{\mathrm{decay}}}$	1.7×10^{-2} s^{-1}	1.7×10^{-3} – 1.7×10^{-1} s^{-1}
Decay of convertase by inhibitor Factor H-like protein 1	$k^{-}_{\mathrm{C3bBbFHL1}_{\mathrm{decay}}}$	3.3×10^{-2} s^{-1}	3.3×10^{-3} – 3.3×10^{-1} s^{-1}
Decay of convertase by inhibitor Factor H	$k^{-}_{\mathrm{C3(H_2O)BbH}_{\mathrm{decay}}}$	1.7×10^{-2} s^{-1}	1.7×10^{-3} – 1.7×10^{-1} s^{-1}
Decay of convertase by inhibitor Factor H-like protein 1	$k^{-}_{\mathrm{C3(H_2O)BbFHL1}_{\mathrm{decay}}}$	3.3×10^{-2} s^{-1}	3.3×10^{-3} – 3.3×10^{-1} s^{-1}
Association of C4BP to C4b	k^{+}_{C4bC4BP}	2.0×10^{5} M^{-1}s^{-1}	2.0×10^{4} – 2.0×10^{6} M^{-1}s^{-1}
Dissociation of complex C4bC4BP	k^{-}_{C4bC4BP}	1.6×10^{-2} s^{-1}	1.6×10^{-3} – 1.6×10^{-1} s^{-1}
Association of C2 to C4b	k^{+}_{C4bC2}	1.6×10^{6} M^{-1}s^{-1}	1.6×10^{5} – 1.6×10^{7} M^{-1}s^{-1}
Dissociation of complex C4bC2	k^{-}_{C4bC2}	4.2×10^{-3} s^{-1}	4.2×10^{-4} – 4.2×10^{-2} s^{-1}
Association of C1q to $(\mathrm{C1rC1s})_2$	k^{+}_{C1}	0.82×10^{6} M^{-1}s^{-1}	0.82×10^{5} – 0.82×10^{7} M^{-1}s^{-1}
Dissociation of complex C1	k^{-}_{C1}	1.2×10^{-3} s^{-1}	1.2×10^{-4} – 1.2×10^{-2} s^{-1}
Activation of C1	$k^{+}_{\mathrm{activation}}$	2.1×10^{-5} s^{-1}	2.1×10^{-6} – 2.1×10^{-4} s^{-1}
Association of C1-INH to C1*	$k^{+}_{\mathrm{C1*C1INH}}$	4.3×10^{5} M^{-1}s^{-1}	4.3×10^{4} – 4.3×10^{6} M^{-1}s^{-1}
Dissociation of complex C3bC3bBb/IgGC3bC3bBb	$k^{-}_{\mathrm{C3bC3bBb}}$	5.7×10^{-3} s^{-1}	5.7×10^{-4} – 5.7×10^{-2} s^{-1}
Dissociation of complex C3bC3bBbP	$k^{-}_{\mathrm{C3bC3bBbP}}$	5.7×10^{-4} s^{-1}	5.7×10^{-5} – 5.7×10^{-3} s^{-1}

Dissociation of complex C3bC4bBb	$k^{-}_{\mathrm{C3bC4bBb}}$	5.7×10^{-3} s^{-1}	5.7×10^{-4} – 5.7×10^{-2} s^{-1}
Dissociation of complex C3bC4bBbP	$k^{-}_{\mathrm{C3bC4bBbP}}$	5.7×10^{-4} s^{-1}	5.7×10^{-5} – 5.7×10^{-3} s^{-1}
Dissociation of complex C3bC4bC2a	$k^{-}_{\mathrm{C3bC4bC2a}}$	5.0×10^{-3} s^{-1}	5.0×10^{-4} – 5.0×10^{-2} s^{-1}
Dissociation of complex C4bC4bC2a	$k^{-}_{\mathrm{C4bC4bC2a}}$	6.0×10^{-3} s^{-1}	6.0×10^{-4} – 6.0×10^{-2} s^{-1}
Dissociation from complex C5b	$k^{-}_{\mathrm{C5b*}}$	5.0×10^{-3} s^{-1}	5.0×10^{-4} – 5.0×10^{-2} s^{-1}
Association of C6 to C5b	k^{+}_{C5bC6}	6.0×10^{4} M^{-1}s^{-1}	6.0×10^{3} – 6.0×10^{5} M^{-1}s^{-1}
Dissociation of complex C5bC6	k^{-}_{C5bC6}	9.0×10^{-8} s^{-1}	9.0×10^{-9} – 9.0×10^{-7} s^{-1}
Association of C7 to C5bC6	k^{+}_{C5b7}	7.3×10^{5} M^{-1}s^{-1}	7.3×10^{4} – 7.3×10^{6} M^{-1}s^{-1}
Dissociation of complex C5bC6C7	k^{-}_{C5b7}	1.5×10^{-7} s^{-1}	1.5×10^{-8} – 1.5×10^{-6} s^{-1}
Attachment of C5b7 to host cell or pathogen	$k^{+}_{\mathrm{C5b7_{surface}}}$	4.2×10^{8} M^{-1}s^{-1}	4.2×10^{7} – 4.2×10^{9} M^{-1}s^{-1}
Formation of C5b7 micelle in fluid	k^{+}_{micelle}	69.3 s^{-1}	6.93 – 693 s^{-1}
Association of C8 to C5b7	k^{+}_{C5b8}	1.1×10^{6} M^{-1}s^{-1}	1.1×10^{5} – 1.1×10^{7} M^{-1}s^{-1}
Dissociation of complex C5b8	k^{-}_{C5b8}	9.8×10^{-7} s^{-1}	9.8×10^{-8} – 9.8×10^{-6} M^{-1}s^{-1}
Association of C9 to C5b8	k^{+}_{C5b9}	2.8×10^{6} M^{-1}s^{-1}	2.8×10^{5} – 2.8×10^{7} M^{-1}s^{-1}
Dissociation of complex C5b9	k^{-}_{C5b9}	2.8×10^{-7} s^{-1}	2.8×10^{-8} – 2.8×10^{-6} M^{-1}s^{-1}

Association of $C9_1$ to surface C5b8	$k^+_{\mathrm{C5b9}_1}$	7.8×10^2 $M^{-1}s^{-1}$	$7.8\times10^1 - 7.8\times10^3$ $M^{-1}s^{-1}$
Association of $C9_{n>=2}$ to surface $C5b9_1$	$k^+_{\mathrm{C5b9}_{\mathrm{poly}}}$	1.1×10^5 $M^{-1}s^{-1}$	$1.1\times10^4 - 1.1\times10^6$ $M^{-1}s^{-1}$
Association of Cn to C5b7	k^+_{CnC5b7}	2.4×10^5 $M^{-1}s^{-1}$	$2.4\times10^4 - 2.4\times10^6$ $M^{-1}s^{-1}$
Dissociation of complex CnC5b7	k^-_{CnC5b7}	4.0×10^{-3} s^{-1}	$4.0\times10^{-4} - 4.0\times10^{-2}$ s^{-1}
Association of Cn to C5b8	k^+_{CnC5b8}	4.2×10^5 $M^{-1}s^{-1}$	$4.2\times10^4 - 4.2\times10^6$ $M^{-1}s^{-1}$
Dissociation of complex CnC5b8	k^-_{CnC5b8}	4.0×10^{-3} s^{-1}	$4.0\times10^{-4} - 4.0\times10^{-2}$ s^{-1}
Association of Cn to C5b9	k^+_{CnC5b9}	4.2×10^5 $M^{-1}s^{-1}$	$4.2\times10^4 - 4.2\times10^6$ $M^{-1}s^{-1}$
Dissociation of complex CnC5b9	k^-_{CnC5b9}	4.0×10^{-3} s^{-1}	$4.0\times10^{-4} - 4.0\times10^{-2}$ s^{-1}
Association of Vn to C5b7	k^+_{VnC5b7}	2.4×10^5 $M^{-1}s^{-1}$	$2.4\times10^4 - 2.4\times10^6$ $M^{-1}s^{-1}$
Dissociation of complex VnC5b7	k^-_{VnC5b7}	4.0×10^{-3} s^{-1}	$4.0\times10^{-4} - 4.0\times10^{-2}$ s^{-1}
Association of Vn to C5b8	k^+_{VnC5b8}	4.2×10^5 $M^{-1}s^{-1}$	$4.2\times10^4 - 4.2\times10^6$ $M^{-1}s^{-1}$
Dissociation of complex VnC5b8	k^-_{VnC5b8}	4.0×10^{-3} s^{-1}	$4.0\times10^{-4} - 4.0\times10^{-2}$ s^{-1}
Association of Vn to C5b9	k^+_{VnC5b9}	4.2×10^5 $M^{-1}s^{-1}$	$4.2\times10^4 - 4.2\times10^6$ $M^{-1}s^{-1}$
Dissociation of complex VnC5b9	k^-_{VnC5b9}	4.0×10^{-3} s^{-1}	$4.0\times10^{-4} - 4.0\times10^{-2}$ s^{-1}

Association of complement Factor H-related protein 3 to C3b	$k^{+}_{\mathrm{C3bCFHR3}}$	1.7×10^{4} $M^{-1}s^{-1}$	$1.7\times10^{3} - 1.7\times10^{5}$ $M^{-1}s^{-1}$
Dissociation of complex C3bCFHR3	$k^{-}_{\mathrm{C3bCFHR3}}$	1.4×10^{-3} s^{-1}	$1.4\times10^{-4} - 1.4\times10^{-2}$ s^{-1}
Association of CFHR3 to pathogen surface	k^{+}_{pCFHR3}	1.4×10^{6} $M^{-1}s^{-1}$	$1.4\times10^{5} - 1.4\times10^{7}$ $M^{-1}s^{-1}$
Dissociation of PathogenCFHR3	k^{-}_{pCFHR3}	4.1×10^{-3} s^{-1}	$4.1\times10^{-4} - 4.1\times10^{-2}$ s^{-1}
Association of FH to pathogen surface	k^{+}_{pFH}	1.6×10^{6} $M^{-1}s^{-1}$	$1.6\times10^{5} - 1.6\times10^{7}$ $M^{-1}s^{-1}$
Dissociation of fHbpFH	$\mathrm{k}^{-}_{\mathrm{pFH}}$	4.5×10^{-3} s^{-1}	$4.5\times10^{-4} - 4.5\times10^{-2}$ s^{-1}
Association of FHL-1 to pathogen surface	$k^{+}_{\mathrm{FhbpFHL1}}$	1.6×10^{6} $M^{-1}s^{-1}$	$1.6\times10^{5} - 1.6\times10^{7}$ $M^{-1}s^{-1}$
Dissociation of fHbpFHL1	$\mathrm{k}^{-}_{\mathrm{FhbpFHL1}}$	4.5×10^{-3} s^{-1}	$4.5\times10^{-4} - 4.5\times10^{-2}$ s^{-1}
Association of C4BP to pathogen surface	k^{+}_{pC4BP}	1.6×10^{6} $M^{-1}s^{-1}$	$1.6\times10^{5} - 1.6\times10^{7}$ $M^{-1}s^{-1}$
Dissociation of porAC4BP	$\mathrm{k}^{-}_{\mathrm{pC4BP}}$	4.5×10^{-3} s^{-1}	$4.5\times10^{-4} - 4.5\times10^{-2}$ s^{-1}
Association of Vn to pathogen surface	k^{+}_{pVn}	1.6×10^{6} $M^{-1}s^{-1}$	$1.6\times10^{5} - 1.6\times10^{7}$ $M^{-1}s^{-1}$
Dissociation of MsfVn	$\mathrm{k}^{-}_{\mathrm{pVn}}$	4.5×10^{-3} s^{-1}	$4.5\times10^{-4} - 4.5\times10^{-2}$ s^{-1}

Cleavage of C3 by C3 convertase, $C3(H_2O)Bb$	k_{cat} $C3(H_2O)Bb$<br><br>K_m $C3(H_2O)Bb$	$1.8\ s^{-1}$<br><br>10.6×10^{-6} M	$0.18 - 18\ s^{-1}$<br><br>$10.6\times10^{-7} - 10.6\times10^{-5}$ M
Cleavage of C3 by C3 convertase, C3bBb	k_{cat} C3bBb<br><br>K_m C3bBb	$1.8\ s^{-1}$<br><br>5.9×10^{-6} M	$0.18 - 18\ s^{-1}$<br><br>$5.9\times10^{-7} - 5.9\times10^{-5}$ M
Cleavage of C3 by convertase, IgGC3bC3bBb	k_{cat} C3bBb<br><br>K_m C3bBb	$1.8\ s^{-1}$<br><br>5.9×10^{-6} M	$0.18 - 18\ s^{-1}$<br><br>$5.9\times10^{-7} - 5.9\times10^{-5}$ M
Cleavage of C5 by C3 convertase, C3bBb	k_{cat} C3bBb<br><br>K_m C3bBb	$1.1\times10^{-2}\ s^{-1}$<br><br>24.0×10^{-6} M	$1.1\times10^{-3} - 1.1\times10^{-1}\ s^{-1}$<br><br>$24.0\times10^{-7} - 24.0\times10^{-5}$ M
Cleavage of C3 by C3 convertase, C4bC2a	k_{cat} C4bC2a<br><br>K_m C4bC2a	$3.2\ s^{-1}$<br><br>1.8×10^{-6} M	$0.32 - 32\ s^{-1}$<br><br>$1.8\times10^{-7} - 1.8\times10^{-5}$ M
Cleavage of C5 by the C3 convertase, C4bC2a	k_{cat} C4bC2a<br><br>K_m C4bC2a	$2.2\times10^{-2}\ s^{-1}$<br><br>8.9×10^{-6} M	$2.2\times10^{-3} - 2.2\times10^{-1}\ s^{-1}$<br><br>$8.9\times10^{-7} - 8.9\times10^{-5}$ M
Cleavage of C4 by activated C1, C1*	k_{cat} C1*<br><br>K_m C1*	$5.4\ s^{-1}$<br><br>6100×10^{-9} M	$0.54 - 54\ s^{-1}$<br><br>$6100\times10^{-10} - 6100\times10^{-8}$ M
Cleavage of C2 by activated C1, C1*	k_{cat} C1*<br><br>K_m C1*	$5.1\ s^{-1}$<br><br>6.1×10^{-6} M	$0.51 - 51\ s^{-1}$<br><br>$6.1\times10^{-7} - 6.1\times10^{-5}$ M
Activation of complex $C3bB/C3(H_2O)B$ by enzyme Factor D	k_{cat} C3bB<br><br>K_m C3bB	$5.0\ s^{-1}$<br><br>2.5×10^{-6} M	$0.50 - 50\ s^{-1}$<br><br>$2.5\times10^{-7} - 2.5\times10^{-5}$ M
Cleavage of C3b by inhibitor Factor I	k_{cat} C3bH<br><br>K_m C3bH	$1.3\ s^{-1}$<br><br>2.5×10^{-7} M	$0.13 - 13\ s^{-1}$<br><br>$2.5\times10^{-8} - 2.5\times10^{-6}$ M
Cleavage of C5 by the C5 convertase, C3bC3bBb	k_{cat} C3bC3bBb<br><br>K_m C3bC3bBb	$3.0\times10^{-3}\ s^{-1}$<br><br>1.7×10^{-6} M	$3.0\times10^{-4} - 3.0\times10^{-2}\ s^{-1}$<br><br>$1.7\times10^{-7} - 1.7\times10^{-5}$ M

Cleavage of C5 by the C5 convertase, C3bC4bBb	k_{cat} C3bC4bBb<br><br>K_m C3bC4bBb	3.0×10^{-3} s^{-1}<br><br>1.7×10^{-6} M	$3.0\times10^{-4} - 3.0\times10^{-2}$ s^{-1}<br><br>$1.7\times10^{-7} - 1.7\times10^{-5}$ M
Cleavage of C5 by the C5 convertase, C3bC4bC2a	k_{cat} C3bC4bC2a<br><br>K_m C3bC4bC2a	1.8×10^{-2} s^{-1}<br><br>5.1×10^{-9} M	$1.8\times10^{-3} - 1.8\times10^{-1}$ s^{-1}<br><br>$5.1\times10^{-10} - 5.1\times10^{-8}$ M
Cleavage of C5 by the C5 convertase, C4bC4bC2a	k_{cat} C4bC4bC2a<br><br>K_m C4bC4bC2a	3.0×10^{-2} s^{-1}<br><br>5.6×10^{-6} M	$3.0\times10^{-3} - 3.0\times10^{-1}$ s^{-1}<br><br>$5.6\times10^{-7} - 5.6\times10^{-5}$ M
Cleavage of C3a by Carboxypeptidase N, CPN	k_{cat} CPN<br><br>K_m CPN	57.9 s^{-1}<br><br>77.1×10^{-6} M	5.79 – 579 s^{-1}<br><br>$77.1\times10^{-7} - 77.1\times10^{-5}$ M
Cleavage of C5a by Carboxypeptidase N, CPN	k_{cat} CPN<br><br>K_m CPN	9.3 s^{-1}<br><br>602.2×10^{-6} M	0.93 – 93 s^{-1}<br><br>$602.2\times10^{-7} - 602.2\times10^{-5}$ M
