# Supplementary material for: Systems Biology Modeling of the Complement System Under Immune Susceptible Pathogens
Source: Front Phys. Author manuscript; Available in PMC 2022 Feb 9. (PMC8827490; doi:10.3389/fphy.2021.603704)
Supplement: Data_Sheet_2_Systems Biology Modeling of the Complement System Under Immune Susceptible Pathogens [file NIHMS1768816-supplement-Data_Sheet_2_Systems_Biology_Modeling_of_the_Complement_System_Under_Immune_Susceptible_Pathogens.pdf]

Initiation and Propagation (Fluid)

1

$$\frac{d\left[\mathrm{C3}\left(\mathrm{H_2O}\right)\right]}{dt}=k^{+}_{\mathrm{C3(H_2O)}}\left[\mathrm{C3}\right]-k^{+}_{\mathrm{C3(H_2O)B}}\left[\mathrm{C3}\left(\mathrm{H_2O}\right)\right]\left[\mathrm{FB}\right]+k^{-}_{\mathrm{C3(H_2O)B}}\left[\mathrm{C3}\left(\mathrm{H_2O}\right)\mathrm{B}\right]+k^{-}_{\mathrm{C3(H_2O)Bb}}\left[\mathrm{C3}\left(\mathrm{H_2O}\right)\mathrm{Bb}\right]$$
$$-k^{+}_{\mathrm{C3(H_2O)H}}\left[\mathrm{C3}\left(\mathrm{H_2O}\right)\right]\left[\mathrm{FH}\right]+k^{-}_{\mathrm{C3(H_2O)H}}\left[\mathrm{C3}\left(\mathrm{H_2O}\right)\mathrm{FH}\right]+k^{-}_{\mathrm{C3(H_2O)BbH_{decay}}}\left[\mathrm{C3}\left(\mathrm{H_2O}\right)\mathrm{BbFH}\right]-k^{+}_{\mathrm{C3(H_2O)FHL1}}\left[\mathrm{C3}\left(\mathrm{H_2O}\right)\right]\left[\mathrm{FHL1}\right]$$
$$+k^{-}_{\mathrm{C3(H_2O)FHL1}}\left[\mathrm{C3}\left(\mathrm{H_2O}\right)\mathrm{FHL1}\right]+k^{-}_{\mathrm{C3(H_2O)BbFHL1_{decay}}}\left[\mathrm{C3}\left(\mathrm{H_2O}\right)\mathrm{BbFHL1}\right]$$

2

$$\frac{d\left[\mathrm{C3}\left(\mathrm{H_2O}\right)\mathrm{B}\right]}{dt}=k^{+}_{\mathrm{C3(H_2O)B}}\left[\mathrm{C3}\left(\mathrm{H_2O}\right)\right]\left[\mathrm{FB}\right]-k^{-}_{\mathrm{C3(H_2O)B}}\left[\mathrm{C3}\left(\mathrm{H_2O}\right)\mathrm{B}\right]$$
$$-\frac{k^{\mathrm{FD}}_{\mathrm{cat\ C3(H_2O)B}}\left[\mathrm{FD}\right]\left[\mathrm{C3}\left(\mathrm{H_2O}\right)\mathrm{B}\right]}{\left(\begin{array}{l}K^{\mathrm{D}}_{\mathrm{m\ C3(H_2O)B}}+\left[\mathrm{C3}\left(\mathrm{H_2O}\right)\mathrm{B}\right]+\left[\mathrm{fC3bB}\right]+\left[\mathrm{fC3bC3bB}\right]+\left[\mathrm{fC3bC3bBP}\right]+\left[\mathrm{IgGC3bC3bB}\right]+\left[\mathrm{IgGC3bC3bBP}\right]\\+\left[\mathrm{fC4bC3bB}\right]+\left[\mathrm{fC4bC3bBP}\right]+\left[\mathrm{pC3bBCFHR1}\right]+\left[\mathrm{pC3bBCFHR2}\right]+\left[\mathrm{pC3bBCFHR3}\right]+\left[\mathrm{pC3bBCFHR4}\right]\\+\left[\mathrm{pC3bBCFHR5}\right]+\left[\mathrm{hC4bC3bBP}\right]+\left[\mathrm{hC4bC3bB}\right]+\left[\mathrm{hC3bC3bB}\right]+\left[\mathrm{hC3bC3bBP}\right]+\left[\mathrm{hC3bB}\right]+\left[\mathrm{pC4bC3bB}\right]\\+\left[\mathrm{pC4bC3bBP}\right]+\left[\mathrm{pC3bC3bB}\right]+\left[\mathrm{pC3bC3bBP}\right]+\left[\mathrm{pC3bB}\right]\end{array}\right)}$$

3

$$\frac{d\left[\mathrm{C3}\left(\mathrm{H_2O}\right)\mathrm{Bb}\right]}{dt}=$$
$$\frac{k^{\mathrm{FD}}_{\mathrm{cat\ C3(H_2O)B}}\left[\mathrm{FD}\right]\left[\mathrm{C3}\left(\mathrm{H_2O}\right)\mathrm{B}\right]}{\left(\begin{array}{l}K^{\mathrm{D}}_{\mathrm{m\ C3(H_2O)B}}+\left[\mathrm{C3}\left(\mathrm{H_2O}\right)\mathrm{B}\right]+\left[\mathrm{fC3bB}\right]+\left[\mathrm{fC3bC3bB}\right]+\left[\mathrm{fC3bC3bBP}\right]+\left[\mathrm{IgGC3bC3bB}\right]+\left[\mathrm{IgGC3bC3bBP}\right]\\+\left[\mathrm{fC4bC3bB}\right]+\left[\mathrm{fC4bC3bBP}\right]+\left[\mathrm{pC3bBCFHR1}\right]+\left[\mathrm{pC3bBCFHR2}\right]+\left[\mathrm{pC3bBCFHR3}\right]+\left[\mathrm{pC3bBCFHR4}\right]\\+\left[\mathrm{pC3bBCFHR5}\right]+\left[\mathrm{hC4bC3bBP}\right]+\left[\mathrm{hC4bC3bB}\right]+\left[\mathrm{hC3bC3bB}\right]+\left[\mathrm{hC3bC3bBP}\right]+\left[\mathrm{hC3bB}\right]+\left[\mathrm{pC4bC3bB}\right]\\+\left[\mathrm{pC4bC3bBP}\right]+\left[\mathrm{pC3bC3bB}\right]+\left[\mathrm{pC3bC3bBP}\right]+\left[\mathrm{pC3bB}\right]\end{array}\right)}$$
$$-k^{-}_{\mathrm{C3(H_2O)Bb}}\left[\mathrm{C3}\left(\mathrm{H_2O}\right)\mathrm{Bb}\right]-k^{+}_{\mathrm{C3(H_2O)H}}\left[\mathrm{C3}\left(\mathrm{H_2O}\right)\mathrm{Bb}\right]\left[\mathrm{FH}\right]+k^{-}_{\mathrm{C3(H_2O)H}}\left[\mathrm{C3}\left(\mathrm{H_2O}\right)\mathrm{BbFH}\right]$$
$$-k^{+}_{\mathrm{C3(H_2O)FHL1}}\left[\mathrm{C3}\left(\mathrm{H_2O}\right)\mathrm{Bb}\right]\left[\mathrm{FHL1}\right]+k^{-}_{\mathrm{C3(H_2O)FHL1}}\left[\mathrm{C3}\left(\mathrm{H_2O}\right)\mathrm{BbFHL1}\right]$$

4

$$\frac{d[\mathrm{fC3b}]}{dt}=k^{+}_{\mathrm{fC3b}}[\mathrm{nfC3b}][\mathrm{H_2O}]-k^{+}_{\mathrm{C3bB}}[\mathrm{fC3b}][\mathrm{FB}]+k^{-}_{\mathrm{C3bB}}[\mathrm{fC3bB}]+k^{-}_{\mathrm{C3bBb}}[\mathrm{fC3bBb}]-k^{+}_{\mathrm{C3bC3b}}[\mathrm{nfC3b}][\mathrm{fC3b}]$$
$$-k^{+}_{\mathrm{C3bC4b}}[\mathrm{nfC4b}][\mathrm{fC3b}]-k^{+}_{\mathrm{C3bH}}[\mathrm{fC3b}][\mathrm{FH}]+k^{-}_{\mathrm{C3bH}}[\mathrm{fC3bFH}]+k^{-}_{\mathrm{C3bBbH_{decay}}}[\mathrm{fC3bBbFH}]-k^{+}_{\mathrm{C3bFHL1}}[\mathrm{fC3b}][\mathrm{FHL1}]$$
$$+k^{-}_{\mathrm{C3bFHL1}}[\mathrm{fC3bFHL1}]+k^{-}_{\mathrm{C3bBbFHL1_{decay}}}[\mathrm{fC3bBbFHL1}]-k^{+}_{\mathrm{C3bCR1}}[\mathrm{fC3b}][\mathrm{CR1}]+k^{-}_{\mathrm{C3bCR1}}[\mathrm{fC3bCR1}]$$
$$+k^{-}_{\mathrm{C3bBbCR1_{decay}}}[\mathrm{fC3bBbCR1}]-k^{+}_{\mathrm{C3bCFHR1}}[\mathrm{fC3b}][\mathrm{pCFHR1}]+k^{-}_{\mathrm{C3bCFHR1}}[\mathrm{pC3bCFHR1}]+k^{-}_{\mathrm{pCFHR1}}[\mathrm{pC3bCFHR1}]$$
$$+k^{-}_{\mathrm{pCFHR1}}[\mathrm{pC3bBCFHR1}]+k^{-}_{\mathrm{pCFHR1}}[\mathrm{pC3bBbCFHR1}]+k^{-}_{\mathrm{pCFHR1}}[\mathrm{pC3bBbPCFHR1}]-k^{+}_{\mathrm{C3bCFHR2}}[\mathrm{fC3b}][\mathrm{pCFHR2}]$$
$$+k^{-}_{\mathrm{C3bCFHR2}}[\mathrm{pC3bCFHR2}]+k^{-}_{\mathrm{pCFHR2}}[\mathrm{pC3bCFHR2}]+k^{-}_{\mathrm{pCFHR2}}[\mathrm{pC3bBCFHR2}]+k^{-}_{\mathrm{pCFHR2}}[\mathrm{pC3bBbCFHR2}]$$
$$+k^{-}_{\mathrm{pCFHR2}}[\mathrm{pC3bBbPCFHR2}]-k^{+}_{\mathrm{C3bCFHR3}}[\mathrm{fC3b}][\mathrm{pCFHR3}]+k^{-}_{\mathrm{C3bCFHR3}}[\mathrm{pC3bCFHR3}]+k^{-}_{\mathrm{pCFHR3}}[\mathrm{pC3bCFHR3}]$$
$$+k^{-}_{\mathrm{pCFHR3}}[\mathrm{pC3bBCFHR3}]+k^{-}_{\mathrm{pCFHR3}}[\mathrm{pC3bBbCFHR3}]+k^{-}_{\mathrm{pCFHR3}}[\mathrm{pC3bBbPCFHR3}]-k^{+}_{\mathrm{C3bCFHR4}}[\mathrm{fC3b}][\mathrm{pCFHR4}]$$
$$+k^{-}_{\mathrm{C3bCFHR4}}[\mathrm{pC3bCFHR4}]+k^{-}_{\mathrm{pCFHR4}}[\mathrm{pC3bCFHR4}]+k^{-}_{\mathrm{pCFHR4}}[\mathrm{pC3bBCFHR4}]+k^{-}_{\mathrm{pCFHR4}}[\mathrm{pC3bBbCFHR4}]$$
$$+k^{-}_{\mathrm{pCFHR4}}[\mathrm{pC3bBbPCFHR4}]-k^{+}_{\mathrm{C3bCFHR5}}[\mathrm{fC3b}][\mathrm{pCFHR5}]+k^{-}_{\mathrm{C3bCFHR5}}[\mathrm{pC3bCFHR5}]+k^{-}_{\mathrm{pCFHR5}}[\mathrm{pC3bCFHR5}]$$
$$+k^{-}_{\mathrm{pCFHR5}}[\mathrm{pC3bBCFHR5}]+k^{-}_{\mathrm{pCFHR5}}[\mathrm{pC3bBbCFHR5}]+k^{-}_{\mathrm{pCFHR5}}[\mathrm{pC3bBbPCFHR5}]$$

5

$$\frac{d[\mathrm{fC3bB}]}{dt}=k^{+}_{\mathrm{C3bB}}[\mathrm{fC3b}][\mathrm{FB}]-k^{-}_{\mathrm{C3bB}}[\mathrm{fC3bB}]$$
$$-\frac{k^{\mathrm{FD}}_{\mathrm{cat\ C3(H_2O)B}}[\mathrm{FD}][\mathrm{fC3bB}]}{\left(\begin{array}{l}K^{\mathrm{D}}_{\mathrm{m\ C3(H_2O)B}}+[\mathrm{C3(H_2O)B}]+[\mathrm{fC3bB}]+[\mathrm{fC3bC3bB}]+[\mathrm{fC3bC3bBP}]+[\mathrm{IgGC3bC3bB}]+[\mathrm{IgGC3bC3bBP}]\\+[\mathrm{fC4bC3bB}]+[\mathrm{fC4bC3bBP}]+[\mathrm{pC3bBCFHR1}]+[\mathrm{pC3bBCFHR2}]+[\mathrm{pC3bBCFHR3}]+[\mathrm{pC3bBCFHR4}]\\+[\mathrm{pC3bBCFHR5}]+[\mathrm{hC4bC3bBP}]+[\mathrm{hC4bC3bB}]+[\mathrm{hC3bC3bB}]+[\mathrm{hC3bC3bBP}]+[\mathrm{hC3bB}]+[\mathrm{pC4bC3bB}]\\+[\mathrm{pC4bC3bBP}]+[\mathrm{pC3bC3bB}]+[\mathrm{pC3bC3bBP}]+[\mathrm{pC3bB}]\end{array}\right)}$$

6

$$\frac{d[\mathrm{fC3bBb}]}{dt}=$$
$$\frac{k^{\mathrm{FD}}_{\mathrm{cat\ C3(H_2O)B}}[\mathrm{FD}][\mathrm{fC3bB}]}{\left(\begin{array}{l}K^{\mathrm{D}}_{\mathrm{m\ C3(H_2O)B}}+[\mathrm{C3(H_2O)B}]+[\mathrm{fC3bB}]+[\mathrm{fC3bC3bB}]+[\mathrm{fC3bC3bBP}]+[\mathrm{IgGC3bC3bB}]+[\mathrm{IgGC3bC3bBP}]\\+[\mathrm{fC4bC3bB}]+[\mathrm{fC4bC3bBP}]+[\mathrm{pC3bBCFHR1}]+[\mathrm{pC3bBCFHR2}]+[\mathrm{pC3bBCFHR3}]+[\mathrm{pC3bBCFHR4}]\\+[\mathrm{pC3bBCFHR5}]+[\mathrm{hC4bC3bBP}]+[\mathrm{hC4bC3bB}]+[\mathrm{hC3bC3bB}]+[\mathrm{hC3bC3bBP}]+[\mathrm{hC3bB}]+[\mathrm{pC4bC3bB}]\\+[\mathrm{pC4bC3bBP}]+[\mathrm{pC3bC3bB}]+[\mathrm{pC3bC3bBP}]+[\mathrm{pC3bB}]\end{array}\right)}$$
$$-k^{-}_{\mathrm{C3bBb}}[\mathrm{fC3bBb}]-k^{+}_{\mathrm{C3bC3b}}[\mathrm{fC3bBb}][\mathrm{nfC3b}]-k^{+}_{\mathrm{C3bC4b}}[\mathrm{fC3bBb}][\mathrm{nfC4b}]-k^{+}_{\mathrm{C3bH}}[\mathrm{fC3bBb}][\mathrm{FH}]+k^{-}_{\mathrm{C3bH}}[\mathrm{fC3bBbFH}]$$
$$-k^{+}_{\mathrm{C3bFHL1}}[\mathrm{fC3bBb}][\mathrm{FHL1}]+k^{-}_{\mathrm{C3bFHL1}}[\mathrm{fC3bBbFHL1}]-k^{+}_{\mathrm{C3bCR1}}[\mathrm{fC3bBb}][\mathrm{CR1}]+k^{-}_{\mathrm{C3bCR1}}[\mathrm{fC3bBbCR1}]$$

7

$$\frac{d[\mathrm{nfC3b}]}{dt}=\frac{k^{\mathrm{C3}}_{\mathrm{cat\ C3(H_2O)Bb}}[\mathrm{C3}][\mathrm{C3(H_2O)Bb}]}{K^{\mathrm{C3}}_{\mathrm{m\ C3(H_2O)Bb}}+[\mathrm{C3}]}+\frac{k^{\mathrm{C3}}_{\mathrm{cat\ C3bBb}}[\mathrm{C3}][\mathrm{fC3bBb}]}{K^{\mathrm{C3}}_{\mathrm{m\ C3bBb}}\left(1+\frac{[\mathrm{C3}]}{K^{\mathrm{C3}}_{\mathrm{m\ C3bBb}}}+\frac{[\mathrm{C5}]}{K^{\mathrm{C5}}_{\mathrm{m\ C3bBb}}}\right)}+\frac{k^{\mathrm{C3}}_{\mathrm{cat\ IgGC3bC3bBb}}[\mathrm{C3}][\mathrm{IgGC3bC3bBb}]}{K^{\mathrm{C3}}_{\mathrm{m\ IgGC3bC3bBb}}+[\mathrm{C3}]}$$

$$+\frac{k^{\mathrm{C3}}_{\mathrm{cat\ IgGC3bC3bBb}}[\mathrm{C3}][\mathrm{IgGC3bC3bBbP}]}{K^{\mathrm{C3}}_{\mathrm{m\ IgGC3bC3bBb}}+[\mathrm{C3}]}+\frac{k^{\mathrm{C3}}_{\mathrm{cat\ C4bC2a}}[\mathrm{C3}][\mathrm{fC4bC2a}]}{K^{\mathrm{C3}}_{\mathrm{m\ C4bC2a}}\left(1+\frac{[\mathrm{C3}]}{K^{\mathrm{C3}}_{\mathrm{m\ C4bC2a}}}+\frac{[\mathrm{C5}]}{K^{\mathrm{C5}}_{\mathrm{m\ C4bC2a}}}\right)}+\frac{k^{\mathrm{C3}}_{\mathrm{cat\ C3bBb}}[\mathrm{C3}][\mathrm{hC3bBb}]}{K^{\mathrm{C3}}_{\mathrm{m\ C3bBb}}\left(1+\frac{[\mathrm{C3}]}{K^{\mathrm{C3}}_{\mathrm{m\ C3bBb}}}+\frac{[\mathrm{C5}]}{K^{\mathrm{C5}}_{\mathrm{m\ C3bBb}}}\right)}$$

$$+\frac{k^{\mathrm{C3}}_{\mathrm{cat\ C3bBb}}[\mathrm{C3}][\mathrm{hC3bBbP}]}{K^{\mathrm{C3}}_{\mathrm{m\ C3bBb}}\left(1+\frac{[\mathrm{C3}]}{K^{\mathrm{C3}}_{\mathrm{m\ C3bBb}}}+\frac{[\mathrm{C5}]}{K^{\mathrm{C5}}_{\mathrm{m\ C3bBb}}}\right)}+\frac{k^{\mathrm{C3}}_{\mathrm{cat\ C3bBb}}[\mathrm{C3}][\mathrm{fC3bC3bBb}]}{K^{\mathrm{C3}}_{\mathrm{m\ C3bBb}}\left(1+\frac{[\mathrm{C3}]}{K^{\mathrm{C3}}_{\mathrm{m\ C3bBb}}}+\frac{[\mathrm{C5}]}{K^{\mathrm{C5}}_{\mathrm{m\ C3bC3bBb}}}\right)}+\frac{k^{\mathrm{C3}}_{\mathrm{cat\ C3bBb}}[\mathrm{C3}][\mathrm{fC3bC3bBbP}]}{K^{\mathrm{C3}}_{\mathrm{m\ C3bBb}}\left(1+\frac{[\mathrm{C3}]}{K^{\mathrm{C3}}_{\mathrm{m\ C3bBb}}}+\frac{[\mathrm{C5}]}{K^{\mathrm{C5}}_{\mathrm{m\ C3bC3bBb}}}\right)}$$

$$+\frac{k^{\mathrm{C3}}_{\mathrm{cat\ C3bBb}}[\mathrm{C3}][\mathrm{fC4bC3bBb}]}{K^{\mathrm{C3}}_{\mathrm{m\ C3bBb}}\left(1+\frac{[\mathrm{C3}]}{K^{\mathrm{C3}}_{\mathrm{m\ C3bBb}}}+\frac{[\mathrm{C5}]}{K^{\mathrm{C5}}_{\mathrm{m\ C3bC4bBb}}}\right)}+\frac{k^{\mathrm{C3}}_{\mathrm{cat\ C3bBb}}[\mathrm{C3}][\mathrm{fC4bC3bBbP}]}{K^{\mathrm{C3}}_{\mathrm{m\ C3bBb}}\left(1+\frac{[\mathrm{C3}]}{K^{\mathrm{C3}}_{\mathrm{m\ C3bBb}}}+\frac{[\mathrm{C5}]}{K^{\mathrm{C5}}_{\mathrm{m\ C3bC4bBb}}}\right)}+\frac{k^{\mathrm{C3}}_{\mathrm{cat\ C4bC2a}}[\mathrm{C3}][\mathrm{fC4bC4bC2a}]}{K^{\mathrm{C3}}_{\mathrm{m\ C4bC2a}}\left(1+\frac{[\mathrm{C3}]}{K^{\mathrm{C3}}_{\mathrm{m\ C4bC2a}}}+\frac{[\mathrm{C5}]}{K^{\mathrm{C5}}_{\mathrm{m\ C4bC4bC2a}}}\right)}$$

$$+\frac{k^{\mathrm{C3}}_{\mathrm{cat\ C4bC2a}}[\mathrm{C3}][\mathrm{fC3bC4bC2a}]}{K^{\mathrm{C3}}_{\mathrm{m\ C4bC2a}}\left(1+\frac{[\mathrm{C3}]}{K^{\mathrm{C3}}_{\mathrm{m\ C4bC2a}}}+\frac{[\mathrm{C5}]}{K^{\mathrm{C5}}_{\mathrm{m\ C3bC4bC2a}}}\right)}+\frac{k^{\mathrm{C3}}_{\mathrm{cat\ C3bBb}}[\mathrm{C3}][\mathrm{hC4bC3bBbP}]}{K^{\mathrm{C3}}_{\mathrm{m\ C3bBb}}\left(1+\frac{[\mathrm{C3}]}{K^{\mathrm{C3}}_{\mathrm{m\ C3bBb}}}+\frac{[\mathrm{C5}]}{K^{\mathrm{C5}}_{\mathrm{m\ C3bC4bBb}}}\right)}+\frac{k^{\mathrm{C3}}_{\mathrm{cat\ C3bBb}}[\mathrm{C3}][\mathrm{hC4bC3bBb}]}{K^{\mathrm{C3}}_{\mathrm{m\ C3bBb}}\left(1+\frac{[\mathrm{C3}]}{K^{\mathrm{C3}}_{\mathrm{m\ C3bBb}}}+\frac{[\mathrm{C5}]}{K^{\mathrm{C5}}_{\mathrm{m\ C3bC4bBb}}}\right)}$$

$$+\frac{k^{\mathrm{C3}}_{\mathrm{cat\ C3bBb}}[\mathrm{C3}][\mathrm{hC3bC3bBb}]}{K^{\mathrm{C3}}_{\mathrm{m\ C3bBb}}\left(1+\frac{[\mathrm{C3}]}{K^{\mathrm{C3}}_{\mathrm{m\ C3bBb}}}+\frac{[\mathrm{C5}]}{K^{\mathrm{C5}}_{\mathrm{m\ C3bC3bBb}}}\right)}+\frac{k^{\mathrm{C3}}_{\mathrm{cat\ C3bBb}}[\mathrm{C3}][\mathrm{hC3bC3bBbP}]}{K^{\mathrm{C3}}_{\mathrm{m\ C3bBb}}\left(1+\frac{[\mathrm{C3}]}{K^{\mathrm{C3}}_{\mathrm{m\ C3bBb}}}+\frac{[\mathrm{C5}]}{K^{\mathrm{C5}}_{\mathrm{m\ C3bC3bBb}}}\right)}+\frac{k^{\mathrm{C3}}_{\mathrm{cat\ C4bC2a}}[\mathrm{C3}][\mathrm{pC4bC2a}]}{K^{\mathrm{C3}}_{\mathrm{m\ C4bC2a}}\left(1+\frac{[\mathrm{C3}]}{K^{\mathrm{C3}}_{\mathrm{m\ C4bC2a}}}+\frac{[\mathrm{C5}]}{K^{\mathrm{C5}}_{\mathrm{m\ C4bC2a}}}\right)}$$

$$+\frac{k^{\mathrm{C3}}_{\mathrm{cat\ C4bC2a}}[\mathrm{C3}][\mathrm{pC4bC4bC2a}]}{K^{\mathrm{C3}}_{\mathrm{m\ C4bC2a}}\left(1+\frac{[\mathrm{C3}]}{K^{\mathrm{C3}}_{\mathrm{m\ C4bC2a}}}+\frac{[\mathrm{C5}]}{K^{\mathrm{C5}}_{\mathrm{m\ C4bC4bC2a}}}\right)}+\frac{k^{\mathrm{C3}}_{\mathrm{cat\ C4bC2a}}[\mathrm{C3}][\mathrm{pC3bC4bC2a}]}{K^{\mathrm{C3}}_{\mathrm{m\ C4bC2a}}\left(1+\frac{[\mathrm{C3}]}{K^{\mathrm{C3}}_{\mathrm{m\ C4bC2a}}}+\frac{[\mathrm{C5}]}{K^{\mathrm{C5}}_{\mathrm{m\ C3bC4bC2a}}}\right)}+\frac{k^{\mathrm{C3}}_{\mathrm{cat\ C3bBb}}[\mathrm{C3}][\mathrm{pC4bC3bBb}]}{K^{\mathrm{C3}}_{\mathrm{m\ C3bBb}}\left(1+\frac{[\mathrm{C3}]}{K^{\mathrm{C3}}_{\mathrm{m\ C3bBb}}}+\frac{[\mathrm{C5}]}{K^{\mathrm{C5}}_{\mathrm{m\ C3bC4bBb}}}\right)}$$

$$+\frac{k^{\mathrm{C3}}_{\mathrm{cat\ C3bBb}}[\mathrm{C3}][\mathrm{pC4bC3bBbP}]}{K^{\mathrm{C3}}_{\mathrm{m\ C3bBb}}\left(1+\frac{[\mathrm{C3}]}{K^{\mathrm{C3}}_{\mathrm{m\ C3bBb}}}+\frac{[\mathrm{C5}]}{K^{\mathrm{C5}}_{\mathrm{m\ C3bC4bBb}}}\right)}+\frac{k^{\mathrm{C3}}_{\mathrm{cat\ C3bBb}}[\mathrm{C3}][\mathrm{pC3bC3bBb}]}{K^{\mathrm{C3}}_{\mathrm{m\ C3bBb}}\left(1+\frac{[\mathrm{C3}]}{K^{\mathrm{C3}}_{\mathrm{m\ C3bBb}}}+\frac{[\mathrm{C5}]}{K^{\mathrm{C5}}_{\mathrm{m\ C3bC3bBb}}}\right)}+\frac{k^{\mathrm{C3}}_{\mathrm{cat\ C3bBb}}[\mathrm{C3}][\mathrm{pC3bC3bBbP}]}{K^{\mathrm{C3}}_{\mathrm{m\ C3bBb}}\left(1+\frac{[\mathrm{C3}]}{K^{\mathrm{C3}}_{\mathrm{m\ C3bBb}}}+\frac{[\mathrm{C5}]}{K^{\mathrm{C5}}_{\mathrm{m\ C3bC3bBb}}}\right)}$$

$$+\frac{k^{\mathrm{C3}}_{\mathrm{cat\ C3bBb}}[\mathrm{C3}][\mathrm{pC3bBb}]}{K^{\mathrm{C3}}_{\mathrm{m\ C3bBb}}\left(1+\frac{[\mathrm{C3}]}{K^{\mathrm{C3}}_{\mathrm{m\ C3bBb}}}+\frac{[\mathrm{C5}]}{K^{\mathrm{C5}}_{\mathrm{m\ C3bBb}}}\right)}+\frac{k^{\mathrm{C3}}_{\mathrm{cat\ C3bBb}}[\mathrm{C3}][\mathrm{pC3bBbP}]}{K^{\mathrm{C3}}_{\mathrm{m\ C3bBb}}\left(1+\frac{[\mathrm{C3}]}{K^{\mathrm{C3}}_{\mathrm{m\ C3bBb}}}+\frac{[\mathrm{C5}]}{K^{\mathrm{C5}}_{\mathrm{m\ C3bBb}}}\right)}-k^{+}_{\mathrm{IgGC3b}}[\mathrm{IgG}][\mathrm{nfC3b}]$$

$$-k^{+}_{\mathrm{C3bC3b}}[\mathrm{IgGfC3b}][\mathrm{nfC3b}]-k^{+}_{\mathrm{C3bC3b}}[\mathrm{nfC3b}][\mathrm{nfC3b}]-k^{+}_{\mathrm{C3bC4b}}[\mathrm{nfC3b}][\mathrm{nfC4b}]-k^{+}_{\mathrm{fC3b}}[\mathrm{nfC3b}][\mathrm{H_2O}]$$

$$-k^{+}_{\mathrm{C3bC3b}}[\mathrm{IgGnfC3b}][\mathrm{nfC3b}]-k^{+}_{\mathrm{C3bC3b}}[\mathrm{fC3bBb}][\mathrm{nfC3b}]-k^{+}_{\mathrm{C3bC4b}}[\mathrm{nfC3b}][\mathrm{fC4b}]-k^{+}_{\mathrm{C3bC3b}}[\mathrm{nfC3b}][\mathrm{fC3b}]$$

$$-k^{+}_{\mathrm{C4bC3b}}[\mathrm{fC4bC2a}][\mathrm{nfC3b}]-k^{+}_{\mathrm{C4bC3b}}[\mathrm{hC4b}][\mathrm{nfC3b}]-k^{+}_{\mathrm{hC3b}}[\mathrm{Host\ Surface}][\mathrm{nfC3b}]-k^{+}_{\mathrm{C3bC3b}}[\mathrm{hC3b}][\mathrm{nfC3b}]$$

$$-k^{+}_{\mathrm{C3bC3b}}[\mathrm{hC3bBb}][\mathrm{nfC3b}]-k^{+}_{\mathrm{C3bC3b}}[\mathrm{hC3bBbP}][\mathrm{nfC3b}]-k^{+}_{\mathrm{C4bC3b}}[\mathrm{pC4b}][\mathrm{nfC3b}]-k^{+}_{\mathrm{C4bC3b}}[\mathrm{pC4bC2a}][\mathrm{nfC3b}]$$

$$-k^{+}_{\mathrm{pC3b}}[\mathrm{Pathogen\ Surface}][\mathrm{nfC3b}]-k^{+}_{\mathrm{C3bC3b}}[\mathrm{pC3b}][\mathrm{nfC3b}]-k^{+}_{\mathrm{C3bC3b}}[\mathrm{pC3bBb}][\mathrm{nfC3b}]$$

$$-k^{+}_{\mathrm{C3bC3b}}[\mathrm{pC3bBbP}][\mathrm{nfC3b}]+\frac{k^{\mathrm{C3}}_{\mathrm{cat\ C3bBb}}[\mathrm{C3}][\mathrm{pC3bBbCFHR1}]}{K^{\mathrm{C3}}_{\mathrm{m\ C3bBb}}\left(1+\frac{[\mathrm{C3}]}{K^{\mathrm{C3}}_{\mathrm{m\ C3bBb}}}+\frac{[\mathrm{C5}]}{K^{\mathrm{C5}}_{\mathrm{m\ C3bBb}}}\right)}+\frac{k^{\mathrm{C3}}_{\mathrm{cat\ C3bBb}}[\mathrm{C3}][\mathrm{pC3bBbPCFHR1}]}{K^{\mathrm{C3}}_{\mathrm{m\ C3bBb}}\left(1+\frac{[\mathrm{C3}]}{K^{\mathrm{C3}}_{\mathrm{m\ C3bBb}}}+\frac{[\mathrm{C5}]}{K^{\mathrm{C5}}_{\mathrm{m\ C3bBb}}}\right)}$$

$$+\frac{k_{\text{cat C3bBb}}^{\text{C3}}[\text{C3}][\text{pC3bBbCFHR2}]}{K_{\text{m C3bBb}}^{\text{C3}}\left(1+\frac{[\text{C3}]}{K_{\text{m C3bBb}}^{\text{C3}}}+\frac{[\text{C5}]}{K_{\text{m C3bBb}}^{\text{C5}}}\right)}+\frac{k_{\text{cat C3bBb}}^{\text{C3}}[\text{C3}][\text{pC3bBbPCFHR2}]}{K_{\text{m C3bBb}}^{\text{C3}}\left(1+\frac{[\text{C3}]}{K_{\text{m C3bBb}}^{\text{C3}}}+\frac{[\text{C5}]}{K_{\text{m C3bBb}}^{\text{C5}}}\right)}+\frac{k_{\text{cat C3bBb}}^{\text{C3}}[\text{C3}][\text{pC3bBbCFHR3}]}{K_{\text{m C3bBb}}^{\text{C3}}\left(1+\frac{[\text{C3}]}{K_{\text{m C3bBb}}^{\text{C3}}}+\frac{[\text{C5}]}{K_{\text{m C3bBb}}^{\text{C5}}}\right)}$$

$$+\frac{k_{\text{cat C3bBb}}^{\text{C3}}[\text{C3}][\text{pC3bBbPCFHR3}]}{K_{\text{m C3bBb}}^{\text{C3}}\left(1+\frac{[\text{C3}]}{K_{\text{m C3bBb}}^{\text{C3}}}+\frac{[\text{C5}]}{K_{\text{m C3bBb}}^{\text{C5}}}\right)}+\frac{k_{\text{cat C3bBb}}^{\text{C3}}[\text{C3}][\text{pC3bBbCFHR4}]}{K_{\text{m C3bBb}}^{\text{C3}}\left(1+\frac{[\text{C3}]}{K_{\text{m C3bBb}}^{\text{C3}}}+\frac{[\text{C5}]}{K_{\text{m C3bBb}}^{\text{C5}}}\right)}+\frac{k_{\text{cat C3bBb}}^{\text{C3}}[\text{C3}][\text{pC3bBbPCFHR4}]}{K_{\text{m C3bBb}}^{\text{C3}}\left(1+\frac{[\text{C3}]}{K_{\text{m C3bBb}}^{\text{C3}}}+\frac{[\text{C5}]}{K_{\text{m C3bBb}}^{\text{C5}}}\right)}$$

$$+\frac{k_{\text{cat C3bBb}}^{\text{C3}}[\text{C3}][\text{pC3bBbCFHR5}]}{K_{\text{m C3bBb}}^{\text{C3}}\left(1+\frac{[\text{C3}]}{K_{\text{m C3bBb}}^{\text{C3}}}+\frac{[\text{C5}]}{K_{\text{m C3bBb}}^{\text{C5}}}\right)}+\frac{k_{\text{cat C3bBb}}^{\text{C3}}[\text{C3}][\text{pC3bBbPCFHR5}]}{K_{\text{m C3bBb}}^{\text{C3}}\left(1+\frac{[\text{C3}]}{K_{\text{m C3bBb}}^{\text{C3}}}+\frac{[\text{C5}]}{K_{\text{m C3bBb}}^{\text{C5}}}\right)}$$

8

$$\frac{d[\text{H}_2\text{O}]}{dt}=-k_{\text{fC3b}}^{+}[\text{nfC3b}][\text{H}_2\text{O}]-k_{\text{fC3b}}^{+}[\text{nfC3bC3b}][\text{H}_2\text{O}]-k_{\text{fC3b}}^{+}[\text{IgGnC3b}][\text{H}_2\text{O}]-k_{\text{fC3b}}^{+}[\text{IgGnC3bnC3b}][\text{H}_2\text{O}]$$
$$-k_{\text{fC3b}}^{+}[\text{nC3bnC4b}][\text{H}_2\text{O}]-k_{\text{fC4b}}^{+}[\text{nC4b}][\text{H}_2\text{O}]-k_{\text{fC4b}}^{+}[\text{nC4bnC4b}][\text{H}_2\text{O}]$$

9

$$\frac{d[\text{nfC3bC3b}]}{dt}=k_{\text{C3bC3b}}^{+}[\text{nfC3b}][\text{nfC3b}]-k_{\text{fC3b}}^{+}[\text{nfC3bC3b}][\text{H}_2\text{O}]$$

10

$$\frac{d[\text{fC3bC3b}]}{dt}=k_{\text{C3bC3b}}^{+}[\text{nfC3b}][\text{fC3b}]+k_{\text{fC3b}}^{+}[\text{nfC3bC3b}][\text{H}_2\text{O}]-k_{\text{C3bB}}^{+}[\text{fC3bC3b}][\text{FB}]+k_{\text{C3bB}}^{-}[\text{fC3bC3bB}]$$
$$-k_{\text{C3bP}}^{+}[\text{fC3bC3b}][\text{P}]+k_{\text{C3bP}}^{-}[\text{fC3bC3bP}]+k_{\text{C3bC3bBb}}^{-}[\text{fC3bC3bBb}]+k_{\text{C3bC3bBbP}}^{-}[\text{fC3bC3bBbP}]$$
$$-k_{\text{C3bH}}^{+}[\text{fC3bC3b}][\text{FH}]+k_{\text{C3bH}}^{-}[\text{fC3bC3bFH}]+k_{\text{C3bBbH}_{\text{decay}}}^{-}[\text{fC3bC3bBbFH}]-k_{\text{C3bFHL1}}^{+}[\text{fC3bC3b}][\text{FHL1}]$$
$$+k_{\text{C3bFHL1}}^{-}[\text{fC3bC3bFHL1}]+k_{\text{C3bBbFHL1}_{\text{decay}}}^{-}[\text{fC3bC3bBbFHL1}]-k_{\text{C3bCR1}}^{+}[\text{fC3bC3b}][\text{CR1}]+k_{\text{C3bCR1}}^{-}[\text{fC3bC3bCR1}]$$
$$+k_{\text{C3bBbH}_{\text{decay}}}^{-}[\text{fC3bC3bBbCR1}]$$

11

$$\frac{d[\text{fC3bC3bB}]}{dt}=k_{\text{C3bB}}^{+}[\text{fC3bC3b}][\text{FB}]-k_{\text{C3bB}}^{-}[\text{fC3bC3bB}]$$
$$-\frac{k_{\text{cat C3(H}_2\text{O)B}}^{\text{FD}}[\text{FD}][\text{fC3bC3bB}]}{\left(\begin{array}{l}K_{\text{m C3(H}_2\text{O)B}}^{\text{D}}+[\text{C3(H}_2\text{O)B}]+[\text{fC3bB}]+[\text{fC3bC3bB}]+[\text{fC3bC3bBP}]+[\text{IgGC3bC3bB}]+[\text{IgGC3bC3bBP}]\\+[\text{fC4bC3bB}]+[\text{fC4bC3bBP}]+[\text{pC3bBCFHR1}]+[\text{pC3bBCFHR2}]+[\text{pC3bBCFHR3}]+[\text{pC3bBCFHR4}]\\+[\text{pC3bBCFHR5}]+[\text{hC4bC3bBP}]+[\text{hC4bC3bB}]+[\text{hC3bC3bB}]+[\text{hC3bC3bBP}]+[\text{hC3bB}]+[\text{pC4bC3bB}]\\+[\text{pC4bC3bBP}]+[\text{pC3bC3bB}]+[\text{pC3bC3bBP}]+[\text{pC3bB}]\end{array}\right)}$$

12

$$\frac{d\left[\mathrm{fC3bC3bBb}\right]}{dt}=$$

$$\frac{k^{\mathrm{FD}}_{\mathrm{cat\ C3(H_2O)B}}\left[\mathrm{FD}\right]\left[\mathrm{fC3bC3bB}\right]}{\left(\begin{array}{l} K^{\mathrm{D}}_{\mathrm{m\ C3(H_2O)B}}+\left[\mathrm{C3(H_2O)B}\right]+\left[\mathrm{fC3bB}\right]+\left[\mathrm{fC3bC3bB}\right]+\left[\mathrm{fC3bC3bBP}\right]+\left[\mathrm{IgGC3bC3bB}\right]+\left[\mathrm{IgGC3bC3bBP}\right]\\ +\left[\mathrm{fC4bC3bB}\right]+\left[\mathrm{fC4bC3bBP}\right]+\left[\mathrm{pC3bBCFHR1}\right]+\left[\mathrm{pC3bBCFHR2}\right]+\left[\mathrm{pC3bBCFHR3}\right]+\left[\mathrm{pC3bBCFHR4}\right]\\ +\left[\mathrm{pC3bBCFHR5}\right]+\left[\mathrm{hC4bC3bBP}\right]+\left[\mathrm{hC4bC3bB}\right]+\left[\mathrm{hC3bC3bB}\right]+\left[\mathrm{hC3bC3bBP}\right]+\left[\mathrm{hC3bB}\right]+\left[\mathrm{pC4bC3bB}\right]\\ +\left[\mathrm{pC4bC3bBP}\right]+\left[\mathrm{pC3bC3bB}\right]+\left[\mathrm{pC3bC3bBP}\right]+\left[\mathrm{pC3bB}\right]\end{array}\right)}$$

$$+k^{+}_{\mathrm{C3bC3b}}\left[\mathrm{fC3bBb}\right]\left[\mathrm{nfC3b}\right]-k^{-}_{\mathrm{C3bC3bBb}}\left[\mathrm{fC3bC3bBb}\right]-k^{+}_{\mathrm{C3bP}}\left[\mathrm{fC3bC3bBb}\right]\left[\mathrm{P}\right]+k^{-}_{\mathrm{C3bP}}\left[\mathrm{fC3bC3bBbP}\right]$$

$$+k^{+}_{\mathrm{C5b7}}\left[\mathrm{fC3bC3bBbC5b6}\right]\left[\mathrm{C7}\right]+k^{-}_{\mathrm{C5b^*}}\left[\mathrm{fC3bC3bBbC5b}\right]-k^{+}_{\mathrm{C3bH}}\left[\mathrm{fC3bC3bBb}\right]\left[\mathrm{FH}\right]+k^{-}_{\mathrm{C3bH}}\left[\mathrm{fC3bC3bBbFH}\right]$$

$$-k^{+}_{\mathrm{C3bFHL1}}\left[\mathrm{fC3bC3bBb}\right]\left[\mathrm{FHL1}\right]+k^{-}_{\mathrm{C3bFHL1}}\left[\mathrm{fC3bC3bBbFHL1}\right]-k^{+}_{\mathrm{C3bCR1}}\left[\mathrm{fC3bC3bBb}\right]\left[\mathrm{CR1}\right]$$

$$+k^{-}_{\mathrm{C3bCR1}}\left[\mathrm{fC3bC3bBbCR1}\right]$$

13

$$\frac{d\left[\mathrm{fC3bC3bP}\right]}{dt}=k^{+}_{\mathrm{C3bP}}\left[\mathrm{fC3bC3b}\right]\left[\mathrm{P}\right]-k^{-}_{\mathrm{C3bP}}\left[\mathrm{fC3bC3bP}\right]-k^{+}_{\mathrm{C3bB}}\left[\mathrm{fC3bC3bP}\right]\left[\mathrm{FB}\right]+k^{-}_{\mathrm{C3bB}}\left[\mathrm{fC3bC3bBP}\right]$$

14

$$\frac{d\left[\mathrm{fC3bC3bBP}\right]}{dt}=k^{+}_{\mathrm{C3bB}}\left[\mathrm{fC3bC3bP}\right]\left[\mathrm{FB}\right]-k^{-}_{\mathrm{C3bB}}\left[\mathrm{fC3bC3bBP}\right]$$

$$-\frac{k^{\mathrm{FD}}_{\mathrm{cat\ C3(H_2O)B}}\left[\mathrm{FD}\right]\left[\mathrm{fC3bC3bBP}\right]}{\left(\begin{array}{l} K^{\mathrm{D}}_{\mathrm{m\ C3(H_2O)B}}+\left[\mathrm{C3(H_2O)B}\right]+\left[\mathrm{fC3bB}\right]+\left[\mathrm{fC3bC3bB}\right]+\left[\mathrm{fC3bC3bBP}\right]+\left[\mathrm{IgGC3bC3bB}\right]+\left[\mathrm{IgGC3bC3bBP}\right]\\ +\left[\mathrm{fC4bC3bB}\right]+\left[\mathrm{fC4bC3bBP}\right]+\left[\mathrm{pC3bBCFHR1}\right]+\left[\mathrm{pC3bBCFHR2}\right]+\left[\mathrm{pC3bBCFHR3}\right]+\left[\mathrm{pC3bBCFHR4}\right]\\ +\left[\mathrm{pC3bBCFHR5}\right]+\left[\mathrm{hC4bC3bBP}\right]+\left[\mathrm{hC4bC3bB}\right]+\left[\mathrm{hC3bC3bB}\right]+\left[\mathrm{hC3bC3bBP}\right]+\left[\mathrm{hC3bB}\right]+\left[\mathrm{pC4bC3bB}\right]\\ +\left[\mathrm{pC4bC3bBP}\right]+\left[\mathrm{pC3bC3bB}\right]+\left[\mathrm{pC3bC3bBP}\right]+\left[\mathrm{pC3bB}\right]\end{array}\right)}$$

15

$$\frac{d\left[\mathrm{fC3bC3bBbP}\right]}{dt}=$$

$$\frac{k_{\mathrm{cat\ C3(H_2O)B}}^{\mathrm{FD}}\left[\mathrm{FD}\right]\left[\mathrm{fC3bC3bBP}\right]}{\begin{pmatrix} K_{\mathrm{m\ C3(H_2O)B}}^{\mathrm{D}}+\left[\mathrm{C3}\left(\mathrm{H_2O}\right)\mathrm{B}\right]+\left[\mathrm{fC3bB}\right]+\left[\mathrm{fC3bC3bB}\right]+\left[\mathrm{fC3bC3bBP}\right]+\left[\mathrm{IgGC3bC3bB}\right]+\left[\mathrm{IgGC3bC3bBP}\right] \\ +\left[\mathrm{fC4bC3bB}\right]+\left[\mathrm{fC4bC3bBP}\right]+\left[\mathrm{pC3bBCFHR1}\right]+\left[\mathrm{pC3bBCFHR2}\right]+\left[\mathrm{pC3bBCFHR3}\right]+\left[\mathrm{pC3bBCFHR4}\right] \\ +\left[\mathrm{pC3bBCFHR5}\right]+\left[\mathrm{hC4bC3bBP}\right]+\left[\mathrm{hC4bC3bB}\right]+\left[\mathrm{hC3bC3bB}\right]+\left[\mathrm{hC3bC3bBP}\right]+\left[\mathrm{hC3bB}\right]+\left[\mathrm{pC4bC3bB}\right] \\ +\left[\mathrm{pC4bC3bBP}\right]+\left[\mathrm{pC3bC3bB}\right]+\left[\mathrm{pC3bC3bBP}\right]+\left[\mathrm{pC3bB}\right] \end{pmatrix}}$$

$$+k_{\mathrm{C3bP}}^{+}\left[\mathrm{fC3bC3bBb}\right]\left[\mathrm{P}\right]-k_{\mathrm{C3bP}}^{-}\left[\mathrm{fC3bC3bBbP}\right]-k_{\mathrm{C3bC3bBbP}}^{-}\left[\mathrm{fC3bC3bBbP}\right]+k_{\mathrm{C5b7}}^{+}\left[\mathrm{fC3bC3bBbPC5b6}\right]\left[\mathrm{C7}\right]$$
$$+k_{\mathrm{C5b^*}}^{-}\left[\mathrm{fC3bC3bBbPC5b}\right]$$

16

$$\frac{d\left[\mathrm{IgGnfC3b}\right]}{dt}=k_{\mathrm{IgGC3b}}^{+}\left[\mathrm{IgG}\right]\left[\mathrm{nfC3b}\right]-k_{\mathrm{C3bC3b}}^{+}\left[\mathrm{IgGnfC3b}\right]\left[\mathrm{nfC3b}\right]-k_{\mathrm{fC3b}}^{+}\left[\mathrm{IgGnfC3b}\right]\left[\mathrm{H_2O}\right]$$

17

$$\frac{d\left[\mathrm{IgGfC3b}\right]}{dt}=k_{\mathrm{fC3b}}^{+}\left[\mathrm{IgGnfC3b}\right]\left[\mathrm{H_2O}\right]-k_{\mathrm{C3bC3b}}^{+}\left[\mathrm{IgGfC3b}\right]\left[\mathrm{nfC3b}\right]$$

18

$$\frac{d\left[\mathrm{IgGnfC3bC3b}\right]}{dt}=k_{\mathrm{C3bC3b}}^{+}\left[\mathrm{IgGnfC3b}\right]\left[\mathrm{nfC3b}\right]-k_{\mathrm{fC3b}}^{+}\left[\mathrm{IgGnfC3bC3b}\right]\left[\mathrm{H_2O}\right]$$

19

$$\frac{d\left[\mathrm{IgGC3bC3b}\right]}{dt}=k_{\mathrm{fC3b}}^{+}\left[\mathrm{IgGnfC3bC3b}\right]\left[\mathrm{H_2O}\right]+k_{\mathrm{C3bC3b}}^{+}\left[\mathrm{IgGfC3b}\right]\left[\mathrm{nfC3b}\right]-k_{\mathrm{C3bB}}^{+}\left[\mathrm{IgGC3bC3b}\right]\left[\mathrm{FB}\right]$$
$$+k_{\mathrm{C3bB}}^{-}\left[\mathrm{IgGC3bC3bB}\right]-k_{\mathrm{C3bP}}^{+}\left[\mathrm{IgGC3bC3b}\right]\left[\mathrm{P}\right]+k_{\mathrm{C3bP}}^{-}\left[\mathrm{IgGC3bC3bP}\right]+k_{\mathrm{C3bC3bBb}}^{-}\left[\mathrm{IgGC3bC3bBb}\right]$$
$$+k_{\mathrm{C3bC3bBbP}}^{-}\left[\mathrm{IgGC3bC3bBbP}\right]-k_{\mathrm{C3bH}}^{+}\left[\mathrm{IgGC3bC3b}\right]\left[\mathrm{FH}\right]+k_{\mathrm{C3bH}}^{-}\left[\mathrm{IgGC3bC3bFH}\right]+k_{\mathrm{C3bBbH_{decay}}}^{-}\left[\mathrm{IgGC3bC3bBbFH}\right]$$
$$-k_{\mathrm{C3bFHL1}}^{+}\left[\mathrm{IgGC3bC3b}\right]\left[\mathrm{FHL1}\right]+k_{\mathrm{C3bFHL1}}^{-}\left[\mathrm{IgGC3bC3bFHL1}\right]+k_{\mathrm{C3bBbFHL1_{decay}}}^{-}\left[\mathrm{IgGC3bC3bBbFHL1}\right]$$
$$-k_{\mathrm{C3bC3bCR1}}^{+}\left[\mathrm{IgGC3bC3b}\right]\left[\mathrm{CR1}\right]+k_{\mathrm{C3bC3bCR1}}^{-}\left[\mathrm{IgGC3bC3bCR1}\right]+k_{\mathrm{C3bBbCR1_{decay}}}^{-}\left[\mathrm{IgGC3bC3bBbCR1}\right]$$

20

$$\frac{d[\mathrm{IgGC3bC3bB}]}{dt} = k^{+}_{\mathrm{C3bB}}[\mathrm{IgGC3bC3b}][\mathrm{FB}] - k^{-}_{\mathrm{C3bB}}[\mathrm{IgGC3bC3bB}]$$

$$-\frac{k^{\mathrm{FD}}_{\mathrm{cat\,C3(H_2O)B}}[\mathrm{FD}][\mathrm{IgGC3bC3bB}]}{\left(\begin{array}{l} K^{\mathrm{D}}_{\mathrm{m\,C3(H_2O)B}} + [\mathrm{C3(H_2O)B}] + [\mathrm{fC3bB}] + [\mathrm{fC3bC3bB}] + [\mathrm{fC3bC3bBP}] + [\mathrm{IgGC3bC3bB}] + [\mathrm{IgGC3bC3bBP}] \\ + [\mathrm{fC4bC3bB}] + [\mathrm{fC4bC3bBP}] + [\mathrm{pC3bBCFHR1}] + [\mathrm{pC3bBCFHR2}] + [\mathrm{pC3bBCFHR3}] + [\mathrm{pC3bBCFHR4}] \\ + [\mathrm{pC3bBCFHR5}] + [\mathrm{hC4bC3bBP}] + [\mathrm{hC4bC3bB}] + [\mathrm{hC3bC3bB}] + [\mathrm{hC3bC3bBP}] + [\mathrm{hC3bB}] + [\mathrm{pC4bC3bB}] \\ + [\mathrm{pC4bC3bBP}] + [\mathrm{pC3bC3bB}] + [\mathrm{pC3bC3bBP}] + [\mathrm{pC3bB}] \end{array}\right)}$$

21

$$\frac{d[\mathrm{IgGC3bC3bBb}]}{dt} =$$

$$\frac{k^{\mathrm{FD}}_{\mathrm{cat\,C3(H_2O)B}}[\mathrm{FD}][\mathrm{IgGC3bC3bB}]}{\left(\begin{array}{l} K^{\mathrm{D}}_{\mathrm{m\,C3(H_2O)B}} + [\mathrm{C3(H_2O)B}] + [\mathrm{fC3bB}] + [\mathrm{fC3bC3bB}] + [\mathrm{fC3bC3bBP}] + [\mathrm{IgGC3bC3bB}] + [\mathrm{IgGC3bC3bBP}] \\ + [\mathrm{fC4bC3bB}] + [\mathrm{fC4bC3bBP}] + [\mathrm{pC3bBCFHR1}] + [\mathrm{pC3bBCFHR2}] + [\mathrm{pC3bBCFHR3}] + [\mathrm{pC3bBCFHR4}] \\ + [\mathrm{pC3bBCFHR5}] + [\mathrm{hC4bC3bBP}] + [\mathrm{hC4bC3bB}] + [\mathrm{hC3bC3bB}] + [\mathrm{hC3bC3bBP}] + [\mathrm{hC3bB}] + [\mathrm{pC4bC3bB}] \\ + [\mathrm{pC4bC3bBP}] + [\mathrm{pC3bC3bB}] + [\mathrm{pC3bC3bBP}] + [\mathrm{pC3bB}] \end{array}\right)}$$

$$-k^{-}_{\mathrm{C3bC3bBb}}[\mathrm{IgGC3bC3bBb}] - k^{+}_{\mathrm{C3bP}}[\mathrm{IgGC3bC3bBb}][\mathrm{P}] + k^{-}_{\mathrm{C3bP}}[\mathrm{IgGC3bC3bBbP}] - k^{+}_{\mathrm{C3bH}}[\mathrm{IgGC3bC3bBb}][\mathrm{FH}]$$
$$+k^{-}_{\mathrm{C3bH}}[\mathrm{IgGC3bC3bBbFH}] - k^{+}_{\mathrm{C3bFHL1}}[\mathrm{IgGC3bC3bBb}][\mathrm{FHL1}] + k^{-}_{\mathrm{C3bFHL1}}[\mathrm{IgGC3bC3bBbFHL1}]$$
$$-k^{+}_{\mathrm{C3bC3bCR1}}[\mathrm{IgGC3bC3bBb}][\mathrm{CR1}] + k^{-}_{\mathrm{C3bC3bCR1}}[\mathrm{IgGC3bC3bBbCR1}]$$

22

$$\frac{d[\mathrm{IgGC3bC3bP}]}{dt} = k^{+}_{\mathrm{C3bP}}[\mathrm{IgGC3bC3bBb}][\mathrm{P}] - k^{-}_{\mathrm{C3bP}}[\mathrm{IgGC3bC3bBbP}] - k^{+}_{\mathrm{C3bB}}[\mathrm{IgGC3bC3bP}][\mathrm{FB}]$$
$$+k^{-}_{\mathrm{C3bB}}[\mathrm{IgGC3bC3bBP}]$$

23

$$\frac{d[\mathrm{IgGC3bC3bBP}]}{dt} = k^{+}_{\mathrm{C3bB}}[\mathrm{IgGC3bC3bP}][\mathrm{FB}] - k^{-}_{\mathrm{C3bB}}[\mathrm{IgGC3bC3bBP}]$$

$$-\frac{k^{\mathrm{FD}}_{\mathrm{cat\,C3(H_2O)B}}[\mathrm{FD}][\mathrm{IgGC3bC3bBP}]}{\left(\begin{array}{l} K^{\mathrm{D}}_{\mathrm{m\,C3(H_2O)B}} + [\mathrm{C3(H_2O)B}] + [\mathrm{fC3bB}] + [\mathrm{fC3bC3bB}] + [\mathrm{fC3bC3bBP}] + [\mathrm{IgGC3bC3bB}] + [\mathrm{IgGC3bC3bBP}] \\ + [\mathrm{fC4bC3bB}] + [\mathrm{fC4bC3bBP}] + [\mathrm{pC3bBCFHR1}] + [\mathrm{pC3bBCFHR2}] + [\mathrm{pC3bBCFHR3}] + [\mathrm{pC3bBCFHR4}] \\ + [\mathrm{pC3bBCFHR5}] + [\mathrm{hC4bC3bBP}] + [\mathrm{hC4bC3bB}] + [\mathrm{hC3bC3bB}] + [\mathrm{hC3bC3bBP}] + [\mathrm{hC3bB}] + [\mathrm{pC4bC3bB}] \\ + [\mathrm{pC4bC3bBP}] + [\mathrm{pC3bC3bB}] + [\mathrm{pC3bC3bBP}] + [\mathrm{pC3bB}] \end{array}\right)}$$

24

$$\frac{d\left[\mathrm{IgGC3bC3bBbP}\right]}{dt}=$$

$$\frac{k_{\mathrm{cat\,C3(H_2O)B}}^{\mathrm{FD}}\left[\mathrm{FD}\right]\left[\mathrm{IgGC3bC3bBP}\right]}{\left(\begin{array}{l}K_{\mathrm{m\,C3(H_2O)B}}^{\mathrm{D}}+\left[\mathrm{C3(H_2O)B}\right]+\left[\mathrm{fC3bB}\right]+\left[\mathrm{fC3bC3bB}\right]+\left[\mathrm{fC3bC3bBP}\right]+\left[\mathrm{IgGC3bC3bB}\right]+\left[\mathrm{IgGC3bC3bBP}\right]\\+\left[\mathrm{fC4bC3bB}\right]+\left[\mathrm{fC4bC3bBP}\right]+\left[\mathrm{pC3bBCFHR1}\right]+\left[\mathrm{pC3bBCFHR2}\right]+\left[\mathrm{pC3bBCFHR3}\right]+\left[\mathrm{pC3bBCFHR4}\right]\\+\left[\mathrm{pC3bBCFHR5}\right]+\left[\mathrm{hC4bC3bBP}\right]+\left[\mathrm{hC4bC3bB}\right]+\left[\mathrm{hC3bC3bB}\right]+\left[\mathrm{hC3bC3bBP}\right]+\left[\mathrm{hC3bB}\right]+\left[\mathrm{pC4bC3bB}\right]\\+\left[\mathrm{pC4bC3bBP}\right]+\left[\mathrm{pC3bC3bB}\right]+\left[\mathrm{pC3bC3bBP}\right]+\left[\mathrm{pC3bB}\right]\end{array}\right)}$$

$$+k_{\mathrm{C3bP}}^{+}\left[\mathrm{IgGC3bC3bBb}\right]\left[\mathrm{P}\right]-k_{\mathrm{C3bP}}^{-}\left[\mathrm{IgGC3bC3bBbP}\right]-k_{\mathrm{C3bC3bBbP}}^{-}\left[\mathrm{IgGC3bC3bBbP}\right]$$

25

$$\frac{d\left[\mathrm{nfC3bC4b}\right]}{dt}=k_{\mathrm{C3bC4b}}^{+}\left[\mathrm{nfC3b}\right]\left[\mathrm{nfC4b}\right]-k_{\mathrm{fC3b}}^{+}\left[\mathrm{nfC3bC4b}\right]\left[\mathrm{H_2O}\right]$$

26

$$\frac{d\left[\mathrm{fC3bC4b}\right]}{dt}=k_{\mathrm{fC3b}}^{+}\left[\mathrm{nfC3bC4b}\right]\left[\mathrm{H_2O}\right]+k_{\mathrm{C3bC4b}}^{+}\left[\mathrm{nfC3b}\right]\left[\mathrm{fC4b}\right]+k_{\mathrm{C3bC4b}}^{+}\left[\mathrm{nfC4b}\right]\left[\mathrm{fC3b}\right]-k_{\mathrm{C3bB}}^{+}\left[\mathrm{fC3bC4b}\right]\left[\mathrm{FB}\right]$$

$$+k_{\mathrm{C3bB}}^{-}\left[\mathrm{fC4bC3bB}\right]+k_{\mathrm{C3bC4bBb}}^{-}\left[\mathrm{fC4bC3bBb}\right]-k_{\mathrm{C4bC2}}^{+}\left[\mathrm{fC3bC4b}\right]\left[\mathrm{C2}\right]+k_{\mathrm{C4bC2}}^{-}\left[\mathrm{fC3bC4bC2}\right]$$

$$+k_{\mathrm{C3bC4bC2a}}^{-}\left[\mathrm{fC3bC4bC2a}\right]-k_{\mathrm{C3bH}}^{+}\left[\mathrm{fC3bC4b}\right]\left[\mathrm{FH}\right]+k_{\mathrm{C3bH}}^{-}\left[\mathrm{fC3bC4bFH}\right]+k_{\mathrm{C3bBbH_{decay}}}^{-}\left[\mathrm{fC4bC3bBbFH}\right]$$

$$-k_{\mathrm{C3bFHL1}}^{+}\left[\mathrm{fC3bC4b}\right]\left[\mathrm{FHL1}\right]+k_{\mathrm{C3bFHL1}}^{-}\left[\mathrm{fC3bC4bFHL1}\right]+k_{\mathrm{C3bBbFHL1_{decay}}}^{-}\left[\mathrm{fC4bC3bBbFHL1}\right]$$

$$-k_{\mathrm{C3bC4bCR1}}^{+}\left[\mathrm{fC3bC4b}\right]\left[\mathrm{CR1}\right]+k_{\mathrm{C3bC4bCR1}}^{-}\left[\mathrm{fC3bC4bCR1}\right]+k_{\mathrm{C3bBbCR1_{decay}}}^{-}\left[\mathrm{fC4bC3bBbCR1}\right]-k_{\mathrm{C4bC4BP}}^{+}\left[\mathrm{fC3bC4b}\right]\left[\mathrm{C4BP}\right]$$

$$+k_{\mathrm{C4bC4BP}}^{-}\left[\mathrm{fC3bC4bC4BP}\right]+k_{\mathrm{C4bC2aC4BP_{decay}}}^{-}\left[\mathrm{fC3bC4bC2aC4BP}\right]+k_{\mathrm{C3bBbCR1_{decay}}}^{-}\left[\mathrm{fC3bC4bC2aCR1}\right]$$

$$-k_{\mathrm{C3bP}}^{+}\left[\mathrm{fC3bC4b}\right]\left[\mathrm{P}\right]+k_{\mathrm{C3bP}}^{-}\left[\mathrm{fC3bC4bP}\right]+k_{\mathrm{C3bC4bBbP}}^{-}\left[\mathrm{fC4bC3bBbP}\right]$$

27

$$\frac{d[\mathrm{fC4bC3bB}]}{dt} = k^{+}_{\mathrm{C3bB}}[\mathrm{fC3bC4b}][\mathrm{FB}] - k^{-}_{\mathrm{C3bB}}[\mathrm{fC4bC3bB}]$$
$$- \frac{k^{\mathrm{FD}}_{\mathrm{cat\,C3(H_2O)B}}[\mathrm{FD}][\mathrm{fC4bC3bB}]}{\begin{pmatrix} K^{\mathrm{D}}_{\mathrm{m\,C3(H_2O)B}} + [\mathrm{C3(H_2O)B}] + [\mathrm{fC3bB}] + [\mathrm{fC3bC3bB}] + [\mathrm{fC3bC3bBP}] + [\mathrm{IgGC3bC3bB}] + [\mathrm{IgGC3bC3bBP}] \\ + [\mathrm{fC4bC3bB}] + [\mathrm{fC4bC3bBP}] + [\mathrm{pC3bBCFHR1}] + [\mathrm{pC3bBCFHR2}] + [\mathrm{pC3bBCFHR3}] + [\mathrm{pC3bBCFHR4}] \\ + [\mathrm{pC3bBCFHR5}] + [\mathrm{hC4bC3bBP}] + [\mathrm{hC4bC3bB}] + [\mathrm{hC3bC3bB}] + [\mathrm{hC3bC3bBP}] + [\mathrm{hC3bB}] + [\mathrm{pC4bC3bB}] \\ + [\mathrm{pC4bC3bBP}] + [\mathrm{pC3bC3bB}] + [\mathrm{pC3bC3bBP}] + [\mathrm{pC3bB}] \end{pmatrix}}$$

28

$$\frac{d[\mathrm{fC4bC3bBb}]}{dt} =$$
$$\frac{k^{\mathrm{FD}}_{\mathrm{cat\,C3(H_2O)B}}[\mathrm{FD}][\mathrm{fC4bC3bB}]}{\begin{pmatrix} K^{\mathrm{D}}_{\mathrm{m\,C3(H_2O)B}} + [\mathrm{C3(H_2O)B}] + [\mathrm{fC3bB}] + [\mathrm{fC3bC3bB}] + [\mathrm{fC3bC3bBP}] + [\mathrm{IgGC3bC3bB}] + [\mathrm{IgGC3bC3bBP}] \\ + [\mathrm{fC4bC3bB}] + [\mathrm{fC4bC3bBP}] + [\mathrm{pC3bBCFHR1}] + [\mathrm{pC3bBCFHR2}] + [\mathrm{pC3bBCFHR3}] + [\mathrm{pC3bBCFHR4}] \\ + [\mathrm{pC3bBCFHR5}] + [\mathrm{hC4bC3bBP}] + [\mathrm{hC4bC3bB}] + [\mathrm{hC3bC3bB}] + [\mathrm{hC3bC3bBP}] + [\mathrm{hC3bB}] + [\mathrm{pC4bC3bB}] \\ + [\mathrm{pC4bC3bBP}] + [\mathrm{pC3bC3bB}] + [\mathrm{pC3bC3bBP}] + [\mathrm{pC3bB}] \end{pmatrix}}$$
$$+ k^{+}_{\mathrm{C3bC4b}}[\mathrm{fC3bBb}][\mathrm{nfC4b}] - k^{-}_{\mathrm{C3bC4bBb}}[\mathrm{fC4bC3bBb}] + k^{+}_{\mathrm{C5b7}}[\mathrm{fC4bC3bBbC5b6}][\mathrm{C7}] + k^{-}_{\mathrm{C5b^*}}[\mathrm{fC4bC3bBbC5b}]$$
$$- k^{+}_{\mathrm{C3bH}}[\mathrm{fC4bC3bBb}][\mathrm{FH}] + k^{-}_{\mathrm{C3bH}}[\mathrm{fC4bC3bBbFH}] - k^{+}_{\mathrm{C3bFHL1}}[\mathrm{fC4bC3bBb}][\mathrm{FHL1}] + k^{-}_{\mathrm{C3bFHL1}}[\mathrm{fC4bC3bBbFHL1}]$$
$$- k^{+}_{\mathrm{C3bC4bCR1}}[\mathrm{fC4bC3bBb}][\mathrm{CR1}] + k^{-}_{\mathrm{C3bC4bCR1}}[\mathrm{fC4bC3bBbCR1}] - k^{+}_{\mathrm{C3bP}}[\mathrm{fC4bC3bBb}][\mathrm{P}] + k^{-}_{\mathrm{C3bP}}[\mathrm{fC4bC3bBbP}]$$

29

$$\frac{d[\mathrm{fC3bC4bP}]}{dt} = k^{+}_{\mathrm{C3bP}}[\mathrm{fC3bC4b}][\mathrm{P}] - k^{-}_{\mathrm{C3bP}}[\mathrm{fC3bC4bP}] - k^{+}_{\mathrm{C3bB}}[\mathrm{fC3bC4bP}][\mathrm{FB}] + k^{-}_{\mathrm{C3bB}}[\mathrm{fC4bC3bBP}]$$

30

$$\frac{d[\mathrm{fC4bC3bBP}]}{dt} = k^{+}_{\mathrm{C3bB}}[\mathrm{fC3bC4bP}][\mathrm{FB}] - k^{-}_{\mathrm{C3bB}}[\mathrm{fC4bC3bBP}]$$
$$- \frac{k^{\mathrm{FD}}_{\mathrm{cat\,C3(H_2O)B}}[\mathrm{FD}][\mathrm{fC4bC3bBP}]}{\begin{pmatrix} K^{\mathrm{D}}_{\mathrm{m\,C3(H_2O)B}} + [\mathrm{C3(H_2O)B}] + [\mathrm{fC3bB}] + [\mathrm{fC3bC3bB}] + [\mathrm{fC3bC3bBP}] + [\mathrm{IgGC3bC3bB}] + [\mathrm{IgGC3bC3bBP}] \\ + [\mathrm{fC4bC3bB}] + [\mathrm{fC4bC3bBP}] + [\mathrm{pC3bBCFHR1}] + [\mathrm{pC3bBCFHR2}] + [\mathrm{pC3bBCFHR3}] + [\mathrm{pC3bBCFHR4}] \\ + [\mathrm{pC3bBCFHR5}] + [\mathrm{hC4bC3bBP}] + [\mathrm{hC4bC3bB}] + [\mathrm{hC3bC3bB}] + [\mathrm{hC3bC3bBP}] + [\mathrm{hC3bB}] + [\mathrm{pC4bC3bB}] \\ + [\mathrm{pC4bC3bBP}] + [\mathrm{pC3bC3bB}] + [\mathrm{pC3bC3bBP}] + [\mathrm{pC3bB}] \end{pmatrix}}$$

31

$$\frac{d\left[\mathrm{fC4bC3bBbP}\right]}{dt}=$$

$$\frac{k_{\mathrm{cat\ C3(H_2O)B}}^{\mathrm{FD}}\left[\mathrm{FD}\right]\left[\mathrm{fC4bC3bBP}\right]}{\left(\begin{array}{l}K_{\mathrm{m\ C3(H_2O)B}}^{\mathrm{D}}+\left[\mathrm{C3}\left(\mathrm{H_2O}\right)\mathrm{B}\right]+\left[\mathrm{fC3bB}\right]+\left[\mathrm{fC3bC3bB}\right]+\left[\mathrm{fC3bC3bBP}\right]+\left[\mathrm{IgGC3bC3bB}\right]+\left[\mathrm{IgGC3bC3bBP}\right]\\+\left[\mathrm{fC4bC3bB}\right]+\left[\mathrm{fC4bC3bBP}\right]+\left[\mathrm{pC3bBCFHR1}\right]+\left[\mathrm{pC3bBCFHR2}\right]+\left[\mathrm{pC3bBCFHR3}\right]+\left[\mathrm{pC3bBCFHR4}\right]\\+\left[\mathrm{pC3bBCFHR5}\right]+\left[\mathrm{hC4bC3bBP}\right]+\left[\mathrm{hC4bC3bB}\right]+\left[\mathrm{hC3bC3bB}\right]+\left[\mathrm{hC3bC3bBP}\right]+\left[\mathrm{hC3bB}\right]+\left[\mathrm{pC4bC3bB}\right]\\+\left[\mathrm{pC4bC3bBP}\right]+\left[\mathrm{pC3bC3bB}\right]+\left[\mathrm{pC3bC3bBP}\right]+\left[\mathrm{pC3bB}\right]\end{array}\right)}$$

$$-k_{\mathrm{C3bC4bBbP}}^{-}\left[\mathrm{fC4bC3bBbP}\right]+k_{\mathrm{C3bP}}^{+}\left[\mathrm{fC4bC3bBb}\right]\left[\mathrm{P}\right]-k_{\mathrm{C3bP}}^{-}\left[\mathrm{fC4bC3bBbP}\right]+k_{\mathrm{C5b7}}^{+}\left[\mathrm{fC4bC3bBbPC5b6}\right]\left[\mathrm{C7}\right]$$
$$+k_{\mathrm{C5b^*}}^{-}\left[\mathrm{fC4bC3bBbPC5b}\right]$$

32

$$\frac{d\left[\mathrm{fC3bC4bC2}\right]}{dt}=k_{\mathrm{C4bC2}}^{+}\left[\mathrm{fC3bC4b}\right]\left[\mathrm{C2}\right]-k_{\mathrm{C4bC2}}^{-}\left[\mathrm{fC3bC4bC2}\right]$$

$$-\frac{k_{\mathrm{cat\ C1^*}}^{\mathrm{C2}}\left[\mathrm{C1^*}\right]\left[\mathrm{fC3bC4bC2}\right]}{K_{\mathrm{m\ C1^*}}^{\mathrm{C2}}\left(1+\frac{\left[\mathrm{C4}\right]}{K_{\mathrm{m\ C1^*}}^{\mathrm{C4}}}+\frac{\left[\mathrm{fC4bC2}\right]}{K_{\mathrm{m\ C1^*}}^{\mathrm{C2}}}+\frac{\left[\mathrm{fC4bC4bC2}\right]}{K_{\mathrm{m\ C1^*}}^{\mathrm{C2}}}+\frac{\left[\mathrm{fC3bC4bC2}\right]}{K_{\mathrm{m\ C1^*}}^{\mathrm{C2}}}+\frac{\left[\mathrm{pC4bC2}\right]}{K_{\mathrm{m\ C1^*}}^{\mathrm{C2}}}+\frac{\left[\mathrm{pC4bC4bC2}\right]}{K_{\mathrm{m\ C1^*}}^{\mathrm{C2}}}+\frac{\left[\mathrm{pC3bC4bC2}\right]}{K_{\mathrm{m\ C1^*}}^{\mathrm{C2}}}\right)}$$

$$-\frac{k_{\mathrm{cat\ C1^*}}^{\mathrm{C2}}\left[\mathrm{pIgG_6C1^*}\right]\left[\mathrm{fC3bC4bC2}\right]}{K_{\mathrm{m\ C1^*}}^{\mathrm{C2}}\left(1+\frac{\left[\mathrm{C4}\right]}{K_{\mathrm{m\ C1^*}}^{\mathrm{C4}}}+\frac{\left[\mathrm{fC4bC2}\right]}{K_{\mathrm{m\ C1^*}}^{\mathrm{C2}}}+\frac{\left[\mathrm{fC4bC4bC2}\right]}{K_{\mathrm{m\ C1^*}}^{\mathrm{C2}}}+\frac{\left[\mathrm{fC3bC4bC2}\right]}{K_{\mathrm{m\ C1^*}}^{\mathrm{C2}}}+\frac{\left[\mathrm{pC4bC2}\right]}{K_{\mathrm{m\ C1^*}}^{\mathrm{C2}}}+\frac{\left[\mathrm{pC4bC4bC2}\right]}{K_{\mathrm{m\ C1^*}}^{\mathrm{C2}}}+\frac{\left[\mathrm{pC3bC4bC2}\right]}{K_{\mathrm{m\ C1^*}}^{\mathrm{C2}}}\right)}$$

$$-\frac{k_{\mathrm{cat\ C1^*}}^{\mathrm{C2}}\left[\mathrm{pIgMC1^*}\right]\left[\mathrm{fC3bC4bC2}\right]}{K_{\mathrm{m\ C1^*}}^{\mathrm{C2}}\left(1+\frac{\left[\mathrm{C4}\right]}{K_{\mathrm{m\ C1^*}}^{\mathrm{C4}}}+\frac{\left[\mathrm{fC4bC2}\right]}{K_{\mathrm{m\ C1^*}}^{\mathrm{C2}}}+\frac{\left[\mathrm{fC4bC4bC2}\right]}{K_{\mathrm{m\ C1^*}}^{\mathrm{C2}}}+\frac{\left[\mathrm{fC3bC4bC2}\right]}{K_{\mathrm{m\ C1^*}}^{\mathrm{C2}}}+\frac{\left[\mathrm{pC4bC2}\right]}{K_{\mathrm{m\ C1^*}}^{\mathrm{C2}}}+\frac{\left[\mathrm{pC4bC4bC2}\right]}{K_{\mathrm{m\ C1^*}}^{\mathrm{C2}}}+\frac{\left[\mathrm{pC3bC4bC2}\right]}{K_{\mathrm{m\ C1^*}}^{\mathrm{C2}}}\right)}$$

$$
-\frac{k_{\mathrm{cat\,MASP1^*}}^{\mathrm{C2}}\left[\mathrm{pMBL_3MASP1^*}\right]\left[\mathrm{fC3bC4bC2}\right]}{K_{\mathrm{m\,MASP1^*}}^{\mathrm{C2}}\left(\begin{array}{l}
1+\frac{[\mathrm{C4}]}{K_{\mathrm{m\,MASP1^*}}^{\mathrm{C4}}}+\frac{[\mathrm{fC4bC2}]}{K_{\mathrm{m\,MASP1^*}}^{\mathrm{C2}}}+\frac{[\mathrm{fC4bC4bC2}]}{K_{\mathrm{m\,MASP1^*}}^{\mathrm{C2}}}+\frac{[\mathrm{fC3bC4bC2}]}{K_{\mathrm{m\,MASP1^*}}^{\mathrm{C2}}}+\frac{[\mathrm{pC4bC2}]}{K_{\mathrm{m\,MASP1^*}}^{\mathrm{C2}}}+\frac{[\mathrm{pC4bC4bC2}]}{K_{\mathrm{m\,MASP1^*}}^{\mathrm{C2}}}\\
+\frac{[\mathrm{pC3bC4bC2}]}{K_{\mathrm{m\,MASP1^*}}^{\mathrm{C2}}}+\frac{\left[\mathrm{pMBL_3MASP1_{zym}}\right]}{K_{\mathrm{m\,MASP1^*}}^{\mathrm{MASP1_{zym}}}}+\frac{\left[\mathrm{pMBL_3MASP2_{zym}}\right]}{K_{\mathrm{m\,MASP1^*}}^{\mathrm{MASP2_{zym}}}}+\frac{\left[\mathrm{pMBL_3MASP3_{zym}}\right]}{K_{\mathrm{m\,MASP1^*}}^{\mathrm{MASP3_{zym}}}}\\
+\frac{\left[\mathrm{pMBL_4MASP1_{zym}}\right]}{K_{\mathrm{m\,MASP1^*}}^{\mathrm{MASP1_{zym}}}}+\frac{\left[\mathrm{pMBL_4MASP2_{zym}}\right]}{K_{\mathrm{m\,MASP1^*}}^{\mathrm{MASP2_{zym}}}}+\frac{\left[\mathrm{pMBL_4MASP3_{zym}}\right]}{K_{\mathrm{m\,MASP1^*}}^{\mathrm{MASP3_{zym}}}}+\frac{\left[\mathrm{pCLLKMASP1_{zym}}\right]}{K_{\mathrm{m\,MASP1^*}}^{\mathrm{MASP1_{zym}}}}\\
+\frac{\left[\mathrm{pCLLKMASP2_{zym}}\right]}{K_{\mathrm{m\,MASP1^*}}^{\mathrm{MASP2_{zym}}}}+\frac{\left[\mathrm{pCLLKMASP3_{zym}}\right]}{K_{\mathrm{m\,MASP1^*}}^{\mathrm{MASP3_{zym}}}}+\frac{\left[\mathrm{pCLK1MASP1_{zym}}\right]}{K_{\mathrm{m\,MASP1^*}}^{\mathrm{MASP1_{zym}}}}+\frac{\left[\mathrm{pCLK1MASP2_{zym}}\right]}{K_{\mathrm{m\,MASP1^*}}^{\mathrm{MASP2_{zym}}}}\\
+\frac{\left[\mathrm{pCLK1MASP3_{zym}}\right]}{K_{\mathrm{m\,MASP1^*}}^{\mathrm{MASP3_{zym}}}}+\frac{\left[\mathrm{pCLL1MASP1_{zym}}\right]}{K_{\mathrm{m\,MASP1^*}}^{\mathrm{MASP1_{zym}}}}+\frac{\left[\mathrm{pCLL1MASP2_{zym}}\right]}{K_{\mathrm{m\,MASP1^*}}^{\mathrm{MASP2_{zym}}}}+\frac{\left[\mathrm{pCLL1MASP3_{zym}}\right]}{K_{\mathrm{m\,MASP1^*}}^{\mathrm{MASP3_{zym}}}}\\
+\frac{\left[\mathrm{pLFMASP1_{zym}}\right]}{K_{\mathrm{m\,MASP1^*}}^{\mathrm{MASP1_{zym}}}}+\frac{\left[\mathrm{pLFMASP2_{zym}}\right]}{K_{\mathrm{m\,MASP1^*}}^{\mathrm{MASP2_{zym}}}}+\frac{\left[\mathrm{pLFMASP3_{zym}}\right]}{K_{\mathrm{m\,MASP1^*}}^{\mathrm{MASP3_{zym}}}}+\frac{\left[\mathrm{pHFMASP1_{zym}}\right]}{K_{\mathrm{m\,MASP1^*}}^{\mathrm{MASP1_{zym}}}}+\frac{\left[\mathrm{pHFMASP2_{zym}}\right]}{K_{\mathrm{m\,MASP1^*}}^{\mathrm{MASP2_{zym}}}}\\
+\frac{\left[\mathrm{pHFMASP3_{zym}}\right]}{K_{\mathrm{m\,MASP1^*}}^{\mathrm{MASP3_{zym}}}}+\frac{\left[\mathrm{pMFMASP1_{zym}}\right]}{K_{\mathrm{m\,MASP1^*}}^{\mathrm{MASP1_{zym}}}}+\frac{\left[\mathrm{pMFMASP2_{zym}}\right]}{K_{\mathrm{m\,MASP1^*}}^{\mathrm{MASP2_{zym}}}}+\frac{\left[\mathrm{pMFMASP3_{zym}}\right]}{K_{\mathrm{m\,MASP1^*}}^{\mathrm{MASP3_{zym}}}}
\end{array}\right)}
$$

$$
-\frac{k_{\mathrm{cat\,MASP1^*}}^{\mathrm{C2}}\left[\mathrm{pMBL_4MASP1^*}\right]\left[\mathrm{fC3bC4bC2}\right]}{K_{\mathrm{m\,MASP1^*}}^{\mathrm{C2}}\left(\begin{array}{l}
1+\frac{[\mathrm{C4}]}{K_{\mathrm{m\,MASP1^*}}^{\mathrm{C4}}}+\frac{[\mathrm{fC4bC2}]}{K_{\mathrm{m\,MASP1^*}}^{\mathrm{C2}}}+\frac{[\mathrm{fC4bC4bC2}]}{K_{\mathrm{m\,MASP1^*}}^{\mathrm{C2}}}+\frac{[\mathrm{fC3bC4bC2}]}{K_{\mathrm{m\,MASP1^*}}^{\mathrm{C2}}}+\frac{[\mathrm{pC4bC2}]}{K_{\mathrm{m\,MASP1^*}}^{\mathrm{C2}}}+\frac{[\mathrm{pC4bC4bC2}]}{K_{\mathrm{m\,MASP1^*}}^{\mathrm{C2}}}\\
+\frac{[\mathrm{pC3bC4bC2}]}{K_{\mathrm{m\,MASP1^*}}^{\mathrm{C2}}}+\frac{\left[\mathrm{pMBL_3MASP1_{zym}}\right]}{K_{\mathrm{m\,MASP1^*}}^{\mathrm{MASP1_{zym}}}}+\frac{\left[\mathrm{pMBL_3MASP2_{zym}}\right]}{K_{\mathrm{m\,MASP1^*}}^{\mathrm{MASP2_{zym}}}}+\frac{\left[\mathrm{pMBL_3MASP3_{zym}}\right]}{K_{\mathrm{m\,MASP1^*}}^{\mathrm{MASP3_{zym}}}}\\
+\frac{\left[\mathrm{pMBL_4MASP1_{zym}}\right]}{K_{\mathrm{m\,MASP1^*}}^{\mathrm{MASP1_{zym}}}}+\frac{\left[\mathrm{pMBL_4MASP2_{zym}}\right]}{K_{\mathrm{m\,MASP1^*}}^{\mathrm{MASP2_{zym}}}}+\frac{\left[\mathrm{pMBL_4MASP3_{zym}}\right]}{K_{\mathrm{m\,MASP1^*}}^{\mathrm{MASP3_{zym}}}}+\frac{\left[\mathrm{pCLLKMASP1_{zym}}\right]}{K_{\mathrm{m\,MASP1^*}}^{\mathrm{MASP1_{zym}}}}\\
+\frac{\left[\mathrm{pCLLKMASP2_{zym}}\right]}{K_{\mathrm{m\,MASP1^*}}^{\mathrm{MASP2_{zym}}}}+\frac{\left[\mathrm{pCLLKMASP3_{zym}}\right]}{K_{\mathrm{m\,MASP1^*}}^{\mathrm{MASP3_{zym}}}}+\frac{\left[\mathrm{pCLK1MASP1_{zym}}\right]}{K_{\mathrm{m\,MASP1^*}}^{\mathrm{MASP1_{zym}}}}+\frac{\left[\mathrm{pCLK1MASP2_{zym}}\right]}{K_{\mathrm{m\,MASP1^*}}^{\mathrm{MASP2_{zym}}}}\\
+\frac{\left[\mathrm{pCLK1MASP3_{zym}}\right]}{K_{\mathrm{m\,MASP1^*}}^{\mathrm{MASP3_{zym}}}}+\frac{\left[\mathrm{pCLL1MASP1_{zym}}\right]}{K_{\mathrm{m\,MASP1^*}}^{\mathrm{MASP1_{zym}}}}+\frac{\left[\mathrm{pCLL1MASP2_{zym}}\right]}{K_{\mathrm{m\,MASP1^*}}^{\mathrm{MASP2_{zym}}}}+\frac{\left[\mathrm{pCLL1MASP3_{zym}}\right]}{K_{\mathrm{m\,MASP1^*}}^{\mathrm{MASP3_{zym}}}}\\
+\frac{\left[\mathrm{pLFMASP1_{zym}}\right]}{K_{\mathrm{m\,MASP1^*}}^{\mathrm{MASP1_{zym}}}}+\frac{\left[\mathrm{pLFMASP2_{zym}}\right]}{K_{\mathrm{m\,MASP1^*}}^{\mathrm{MASP2_{zym}}}}+\frac{\left[\mathrm{pLFMASP3_{zym}}\right]}{K_{\mathrm{m\,MASP1^*}}^{\mathrm{MASP3_{zym}}}}+\frac{\left[\mathrm{pHFMASP1_{zym}}\right]}{K_{\mathrm{m\,MASP1^*}}^{\mathrm{MASP1_{zym}}}}+\frac{\left[\mathrm{pHFMASP2_{zym}}\right]}{K_{\mathrm{m\,MASP1^*}}^{\mathrm{MASP2_{zym}}}}\\
+\frac{\left[\mathrm{pHFMASP3_{zym}}\right]}{K_{\mathrm{m\,MASP1^*}}^{\mathrm{MASP3_{zym}}}}+\frac{\left[\mathrm{pMFMASP1_{zym}}\right]}{K_{\mathrm{m\,MASP1^*}}^{\mathrm{MASP1_{zym}}}}+\frac{\left[\mathrm{pMFMASP2_{zym}}\right]}{K_{\mathrm{m\,MASP1^*}}^{\mathrm{MASP2_{zym}}}}+\frac{\left[\mathrm{pMFMASP3_{zym}}\right]}{K_{\mathrm{m\,MASP1^*}}^{\mathrm{MASP3_{zym}}}}
\end{array}\right)}
$$

$$-\frac{k_{\mathrm{cat\ MASP1^*}}^{\mathrm{C2}}\left[\mathrm{pCLLKMASP1^*}\right]\left[\mathrm{fC3bC4bC2}\right]}{K_{\mathrm{m\ MASP1^*}}^{\mathrm{C2}}\left(\begin{array}{l}1+\frac{[\mathrm{C4}]}{K_{\mathrm{m\ MASP1^*}}^{\mathrm{C4}}}+\frac{[\mathrm{fC4bC2}]}{K_{\mathrm{m\ MASP1^*}}^{\mathrm{C2}}}+\frac{[\mathrm{fC4bC4bC2}]}{K_{\mathrm{m\ MASP1^*}}^{\mathrm{C2}}}+\frac{[\mathrm{fC3bC4bC2}]}{K_{\mathrm{m\ MASP1^*}}^{\mathrm{C2}}}+\frac{[\mathrm{pC4bC2}]}{K_{\mathrm{m\ MASP1^*}}^{\mathrm{C2}}}+\frac{[\mathrm{pC4bC4bC2}]}{K_{\mathrm{m\ MASP1^*}}^{\mathrm{C2}}}\\
+\frac{[\mathrm{pC3bC4bC2}]}{K_{\mathrm{m\ MASP1^*}}^{\mathrm{C2}}}+\frac{[\mathrm{pMBL_3MASP1_{zym}}]}{K_{\mathrm{m\ MASP1^*}}^{\mathrm{MASP1_{zym}}}}+\frac{[\mathrm{pMBL_3MASP2_{zym}}]}{K_{\mathrm{m\ MASP1^*}}^{\mathrm{MASP2_{zym}}}}+\frac{[\mathrm{pMBL_3MASP3_{zym}}]}{K_{\mathrm{m\ MASP1^*}}^{\mathrm{MASP3_{zym}}}}\\
+\frac{[\mathrm{pMBL_4MASP1_{zym}}]}{K_{\mathrm{m\ MASP1^*}}^{\mathrm{MASP1_{zym}}}}+\frac{[\mathrm{pMBL_4MASP2_{zym}}]}{K_{\mathrm{m\ MASP1^*}}^{\mathrm{MASP2_{zym}}}}+\frac{[\mathrm{pMBL_4MASP3_{zym}}]}{K_{\mathrm{m\ MASP1^*}}^{\mathrm{MASP3_{zym}}}}+\frac{[\mathrm{pCLLKMASP1_{zym}}]}{K_{\mathrm{m\ MASP1^*}}^{\mathrm{MASP1_{zym}}}}\\
+\frac{[\mathrm{pCLLKMASP2_{zym}}]}{K_{\mathrm{m\ MASP1^*}}^{\mathrm{MASP2_{zym}}}}+\frac{[\mathrm{pCLLKMASP3_{zym}}]}{K_{\mathrm{m\ MASP1^*}}^{\mathrm{MASP3_{zym}}}}+\frac{[\mathrm{pCLK1MASP1_{zym}}]}{K_{\mathrm{m\ MASP1^*}}^{\mathrm{MASP1_{zym}}}}+\frac{[\mathrm{pCLK1MASP2_{zym}}]}{K_{\mathrm{m\ MASP1^*}}^{\mathrm{MASP2_{zym}}}}\\
+\frac{[\mathrm{pCLK1MASP3_{zym}}]}{K_{\mathrm{m\ MASP1^*}}^{\mathrm{MASP3_{zym}}}}+\frac{[\mathrm{pCLL1MASP1_{zym}}]}{K_{\mathrm{m\ MASP1^*}}^{\mathrm{MASP1_{zym}}}}+\frac{[\mathrm{pCLL1MASP2_{zym}}]}{K_{\mathrm{m\ MASP1^*}}^{\mathrm{MASP2_{zym}}}}+\frac{[\mathrm{pCLL1MASP3_{zym}}]}{K_{\mathrm{m\ MASP1^*}}^{\mathrm{MASP3_{zym}}}}\\
+\frac{[\mathrm{pLFMASP1_{zym}}]}{K_{\mathrm{m\ MASP1^*}}^{\mathrm{MASP1_{zym}}}}+\frac{[\mathrm{pLFMASP2_{zym}}]}{K_{\mathrm{m\ MASP1^*}}^{\mathrm{MASP2_{zym}}}}+\frac{[\mathrm{pLFMASP3_{zym}}]}{K_{\mathrm{m\ MASP1^*}}^{\mathrm{MASP3_{zym}}}}+\frac{[\mathrm{pHFMASP1_{zym}}]}{K_{\mathrm{m\ MASP1^*}}^{\mathrm{MASP1_{zym}}}}+\frac{[\mathrm{pHFMASP2_{zym}}]}{K_{\mathrm{m\ MASP1^*}}^{\mathrm{MASP2_{zym}}}}\\
+\frac{[\mathrm{pHFMASP3_{zym}}]}{K_{\mathrm{m\ MASP1^*}}^{\mathrm{MASP3_{zym}}}}+\frac{[\mathrm{pMFMASP1_{zym}}]}{K_{\mathrm{m\ MASP1^*}}^{\mathrm{MASP1_{zym}}}}+\frac{[\mathrm{pMFMASP2_{zym}}]}{K_{\mathrm{m\ MASP1^*}}^{\mathrm{MASP2_{zym}}}}+\frac{[\mathrm{pMFMASP3_{zym}}]}{K_{\mathrm{m\ MASP1^*}}^{\mathrm{MASP3_{zym}}}}\end{array}\right)}$$

$$-\frac{k_{\mathrm{cat\ MASP1^*}}^{\mathrm{C2}}\left[\mathrm{pCLK1MASP1^*}\right]\left[\mathrm{fC3bC4bC2}\right]}{K_{\mathrm{m\ MASP1^*}}^{\mathrm{C2}}\left(\begin{array}{l}1+\frac{[\mathrm{C4}]}{K_{\mathrm{m\ MASP1^*}}^{\mathrm{C4}}}+\frac{[\mathrm{fC4bC2}]}{K_{\mathrm{m\ MASP1^*}}^{\mathrm{C2}}}+\frac{[\mathrm{fC4bC4bC2}]}{K_{\mathrm{m\ MASP1^*}}^{\mathrm{C2}}}+\frac{[\mathrm{fC3bC4bC2}]}{K_{\mathrm{m\ MASP1^*}}^{\mathrm{C2}}}+\frac{[\mathrm{pC4bC2}]}{K_{\mathrm{m\ MASP1^*}}^{\mathrm{C2}}}+\frac{[\mathrm{pC4bC4bC2}]}{K_{\mathrm{m\ MASP1^*}}^{\mathrm{C2}}}\\
+\frac{[\mathrm{pC3bC4bC2}]}{K_{\mathrm{m\ MASP1^*}}^{\mathrm{C2}}}+\frac{[\mathrm{pMBL_3MASP1_{zym}}]}{K_{\mathrm{m\ MASP1^*}}^{\mathrm{MASP1_{zym}}}}+\frac{[\mathrm{pMBL_3MASP2_{zym}}]}{K_{\mathrm{m\ MASP1^*}}^{\mathrm{MASP2_{zym}}}}+\frac{[\mathrm{pMBL_3MASP3_{zym}}]}{K_{\mathrm{m\ MASP1^*}}^{\mathrm{MASP3_{zym}}}}\\
+\frac{[\mathrm{pMBL_4MASP1_{zym}}]}{K_{\mathrm{m\ MASP1^*}}^{\mathrm{MASP1_{zym}}}}+\frac{[\mathrm{pMBL_4MASP2_{zym}}]}{K_{\mathrm{m\ MASP1^*}}^{\mathrm{MASP2_{zym}}}}+\frac{[\mathrm{pMBL_4MASP3_{zym}}]}{K_{\mathrm{m\ MASP1^*}}^{\mathrm{MASP3_{zym}}}}+\frac{[\mathrm{pCLLKMASP1_{zym}}]}{K_{\mathrm{m\ MASP1^*}}^{\mathrm{MASP1_{zym}}}}\\
+\frac{[\mathrm{pCLLKMASP2_{zym}}]}{K_{\mathrm{m\ MASP1^*}}^{\mathrm{MASP2_{zym}}}}+\frac{[\mathrm{pCLLKMASP3_{zym}}]}{K_{\mathrm{m\ MASP1^*}}^{\mathrm{MASP3_{zym}}}}+\frac{[\mathrm{pCLK1MASP1_{zym}}]}{K_{\mathrm{m\ MASP1^*}}^{\mathrm{MASP1_{zym}}}}+\frac{[\mathrm{pCLK1MASP2_{zym}}]}{K_{\mathrm{m\ MASP1^*}}^{\mathrm{MASP2_{zym}}}}\\
+\frac{[\mathrm{pCLK1MASP3_{zym}}]}{K_{\mathrm{m\ MASP1^*}}^{\mathrm{MASP3_{zym}}}}+\frac{[\mathrm{pCLL1MASP1_{zym}}]}{K_{\mathrm{m\ MASP1^*}}^{\mathrm{MASP1_{zym}}}}+\frac{[\mathrm{pCLL1MASP2_{zym}}]}{K_{\mathrm{m\ MASP1^*}}^{\mathrm{MASP2_{zym}}}}+\frac{[\mathrm{pCLL1MASP3_{zym}}]}{K_{\mathrm{m\ MASP1^*}}^{\mathrm{MASP3_{zym}}}}\\
+\frac{[\mathrm{pLFMASP1_{zym}}]}{K_{\mathrm{m\ MASP1^*}}^{\mathrm{MASP1_{zym}}}}+\frac{[\mathrm{pLFMASP2_{zym}}]}{K_{\mathrm{m\ MASP1^*}}^{\mathrm{MASP2_{zym}}}}+\frac{[\mathrm{pLFMASP3_{zym}}]}{K_{\mathrm{m\ MASP1^*}}^{\mathrm{MASP3_{zym}}}}+\frac{[\mathrm{pHFMASP1_{zym}}]}{K_{\mathrm{m\ MASP1^*}}^{\mathrm{MASP1_{zym}}}}+\frac{[\mathrm{pHFMASP2_{zym}}]}{K_{\mathrm{m\ MASP1^*}}^{\mathrm{MASP2_{zym}}}}\\
+\frac{[\mathrm{pHFMASP3_{zym}}]}{K_{\mathrm{m\ MASP1^*}}^{\mathrm{MASP3_{zym}}}}+\frac{[\mathrm{pMFMASP1_{zym}}]}{K_{\mathrm{m\ MASP1^*}}^{\mathrm{MASP1_{zym}}}}+\frac{[\mathrm{pMFMASP2_{zym}}]}{K_{\mathrm{m\ MASP1^*}}^{\mathrm{MASP2_{zym}}}}+\frac{[\mathrm{pMFMASP3_{zym}}]}{K_{\mathrm{m\ MASP1^*}}^{\mathrm{MASP3_{zym}}}}\end{array}\right)}$$

$$-\frac{k_{\text{cat MASP1}^*}^{\text{C2}}\left[\text{pCLL1MASP1}^*\right]\left[\text{fC3bC4bC2}\right]}{K_{\text{m MASP1}^*}^{\text{C2}}\left(\begin{array}{l}1+\frac{[\text{C4}]}{K_{\text{m MASP1}^*}^{\text{C4}}}+\frac{[\text{fC4bC2}]}{K_{\text{m MASP1}^*}^{\text{C2}}}+\frac{[\text{fC4bC4bC2}]}{K_{\text{m MASP1}^*}^{\text{C2}}}+\frac{[\text{fC3bC4bC2}]}{K_{\text{m MASP1}^*}^{\text{C2}}}+\frac{[\text{pC4bC2}]}{K_{\text{m MASP1}^*}^{\text{C2}}}+\frac{[\text{pC4bC4bC2}]}{K_{\text{m MASP1}^*}^{\text{C2}}}\\+\frac{[\text{pC3bC4bC2}]}{K_{\text{m MASP1}^*}^{\text{C2}}}+\frac{[\text{pMBL}_3\text{MASP1}_{\text{zym}}]}{K_{\text{m MASP1}^*}^{\text{MASP1}_{\text{zym}}}}+\frac{[\text{pMBL}_3\text{MASP2}_{\text{zym}}]}{K_{\text{m MASP1}^*}^{\text{MASP2}_{\text{zym}}}}+\frac{[\text{pMBL}_3\text{MASP3}_{\text{zym}}]}{K_{\text{m MASP1}^*}^{\text{MASP3}_{\text{zym}}}}\\+\frac{[\text{pMBL}_4\text{MASP1}_{\text{zym}}]}{K_{\text{m MASP1}^*}^{\text{MASP1}_{\text{zym}}}}+\frac{[\text{pMBL}_4\text{MASP2}_{\text{zym}}]}{K_{\text{m MASP1}^*}^{\text{MASP2}_{\text{zym}}}}+\frac{[\text{pMBL}_4\text{MASP3}_{\text{zym}}]}{K_{\text{m MASP1}^*}^{\text{MASP3}_{\text{zym}}}}+\frac{[\text{pCLLKMASP1}_{\text{zym}}]}{K_{\text{m MASP1}^*}^{\text{MASP1}_{\text{zym}}}}\\+\frac{[\text{pCLLKMASP2}_{\text{zym}}]}{K_{\text{m MASP1}^*}^{\text{MASP2}_{\text{zym}}}}+\frac{[\text{pCLLKMASP3}_{\text{zym}}]}{K_{\text{m MASP1}^*}^{\text{MASP3}_{\text{zym}}}}+\frac{[\text{pCLK1MASP1}_{\text{zym}}]}{K_{\text{m MASP1}^*}^{\text{MASP1}_{\text{zym}}}}+\frac{[\text{pCLK1MASP2}_{\text{zym}}]}{K_{\text{m MASP1}^*}^{\text{MASP2}_{\text{zym}}}}\\+\frac{[\text{pCLK1MASP3}_{\text{zym}}]}{K_{\text{m MASP1}^*}^{\text{MASP3}_{\text{zym}}}}+\frac{[\text{pCLL1MASP1}_{\text{zym}}]}{K_{\text{m MASP1}^*}^{\text{MASP1}_{\text{zym}}}}+\frac{[\text{pCLL1MASP2}_{\text{zym}}]}{K_{\text{m MASP1}^*}^{\text{MASP2}_{\text{zym}}}}+\frac{[\text{pCLL1MASP3}_{\text{zym}}]}{K_{\text{m MASP1}^*}^{\text{MASP3}_{\text{zym}}}}\\+\frac{[\text{pLFMASP1}_{\text{zym}}]}{K_{\text{m MASP1}^*}^{\text{MASP1}_{\text{zym}}}}+\frac{[\text{pLFMASP2}_{\text{zym}}]}{K_{\text{m MASP1}^*}^{\text{MASP2}_{\text{zym}}}}+\frac{[\text{pLFMASP3}_{\text{zym}}]}{K_{\text{m MASP1}^*}^{\text{MASP3}_{\text{zym}}}}+\frac{[\text{pHFMASP1}_{\text{zym}}]}{K_{\text{m MASP1}^*}^{\text{MASP1}_{\text{zym}}}}+\frac{[\text{pHFMASP2}_{\text{zym}}]}{K_{\text{m MASP1}^*}^{\text{MASP2}_{\text{zym}}}}\\+\frac{[\text{pHFMASP3}_{\text{zym}}]}{K_{\text{m MASP1}^*}^{\text{MASP3}_{\text{zym}}}}+\frac{[\text{pMFMASP1}_{\text{zym}}]}{K_{\text{m MASP1}^*}^{\text{MASP1}_{\text{zym}}}}+\frac{[\text{pMFMASP2}_{\text{zym}}]}{K_{\text{m MASP1}^*}^{\text{MASP2}_{\text{zym}}}}+\frac{[\text{pMFMASP3}_{\text{zym}}]}{K_{\text{m MASP1}^*}^{\text{MASP3}_{\text{zym}}}}\end{array}\right)}$$

$$-\frac{k_{\text{cat MASP1}^*}^{\text{C2}}\left[\text{pLFMASP1}^*\right]\left[\text{fC3bC4bC2}\right]}{K_{\text{m MASP1}^*}^{\text{C2}}\left(\begin{array}{l}1+\frac{[\text{C4}]}{K_{\text{m MASP1}^*}^{\text{C4}}}+\frac{[\text{fC4bC2}]}{K_{\text{m MASP1}^*}^{\text{C2}}}+\frac{[\text{fC4bC4bC2}]}{K_{\text{m MASP1}^*}^{\text{C2}}}+\frac{[\text{fC3bC4bC2}]}{K_{\text{m MASP1}^*}^{\text{C2}}}+\frac{[\text{pC4bC2}]}{K_{\text{m MASP1}^*}^{\text{C2}}}+\frac{[\text{pC4bC4bC2}]}{K_{\text{m MASP1}^*}^{\text{C2}}}\\+\frac{[\text{pC3bC4bC2}]}{K_{\text{m MASP1}^*}^{\text{C2}}}+\frac{[\text{pMBL}_3\text{MASP1}_{\text{zym}}]}{K_{\text{m MASP1}^*}^{\text{MASP1}_{\text{zym}}}}+\frac{[\text{pMBL}_3\text{MASP2}_{\text{zym}}]}{K_{\text{m MASP1}^*}^{\text{MASP2}_{\text{zym}}}}+\frac{[\text{pMBL}_3\text{MASP3}_{\text{zym}}]}{K_{\text{m MASP1}^*}^{\text{MASP3}_{\text{zym}}}}\\+\frac{[\text{pMBL}_4\text{MASP1}_{\text{zym}}]}{K_{\text{m MASP1}^*}^{\text{MASP1}_{\text{zym}}}}+\frac{[\text{pMBL}_4\text{MASP2}_{\text{zym}}]}{K_{\text{m MASP1}^*}^{\text{MASP2}_{\text{zym}}}}+\frac{[\text{pMBL}_4\text{MASP3}_{\text{zym}}]}{K_{\text{m MASP1}^*}^{\text{MASP3}_{\text{zym}}}}+\frac{[\text{pCLLKMASP1}_{\text{zym}}]}{K_{\text{m MASP1}^*}^{\text{MASP1}_{\text{zym}}}}\\+\frac{[\text{pCLLKMASP2}_{\text{zym}}]}{K_{\text{m MASP1}^*}^{\text{MASP2}_{\text{zym}}}}+\frac{[\text{pCLLKMASP3}_{\text{zym}}]}{K_{\text{m MASP1}^*}^{\text{MASP3}_{\text{zym}}}}+\frac{[\text{pCLK1MASP1}_{\text{zym}}]}{K_{\text{m MASP1}^*}^{\text{MASP1}_{\text{zym}}}}+\frac{[\text{pCLK1MASP2}_{\text{zym}}]}{K_{\text{m MASP1}^*}^{\text{MASP2}_{\text{zym}}}}\\+\frac{[\text{pCLK1MASP3}_{\text{zym}}]}{K_{\text{m MASP1}^*}^{\text{MASP3}_{\text{zym}}}}+\frac{[\text{pCLL1MASP1}_{\text{zym}}]}{K_{\text{m MASP1}^*}^{\text{MASP1}_{\text{zym}}}}+\frac{[\text{pCLL1MASP2}_{\text{zym}}]}{K_{\text{m MASP1}^*}^{\text{MASP2}_{\text{zym}}}}+\frac{[\text{pCLL1MASP3}_{\text{zym}}]}{K_{\text{m MASP1}^*}^{\text{MASP3}_{\text{zym}}}}\\+\frac{[\text{pLFMASP1}_{\text{zym}}]}{K_{\text{m MASP1}^*}^{\text{MASP1}_{\text{zym}}}}+\frac{[\text{pLFMASP2}_{\text{zym}}]}{K_{\text{m MASP1}^*}^{\text{MASP2}_{\text{zym}}}}+\frac{[\text{pLFMASP3}_{\text{zym}}]}{K_{\text{m MASP1}^*}^{\text{MASP3}_{\text{zym}}}}+\frac{[\text{pHFMASP1}_{\text{zym}}]}{K_{\text{m MASP1}^*}^{\text{MASP1}_{\text{zym}}}}+\frac{[\text{pHFMASP2}_{\text{zym}}]}{K_{\text{m MASP1}^*}^{\text{MASP2}_{\text{zym}}}}\\+\frac{[\text{pHFMASP3}_{\text{zym}}]}{K_{\text{m MASP1}^*}^{\text{MASP3}_{\text{zym}}}}+\frac{[\text{pMFMASP1}_{\text{zym}}]}{K_{\text{m MASP1}^*}^{\text{MASP1}_{\text{zym}}}}+\frac{[\text{pMFMASP2}_{\text{zym}}]}{K_{\text{m MASP1}^*}^{\text{MASP2}_{\text{zym}}}}+\frac{[\text{pMFMASP3}_{\text{zym}}]}{K_{\text{m MASP1}^*}^{\text{MASP3}_{\text{zym}}}}\end{array}\right)}$$

$$-\frac{k^{\mathrm{C2}}_{\mathrm{cat\,MASP1^*}}\left[\mathrm{pHFMASP1^*}\right]\left[\mathrm{fC3bC4bC2}\right]}{K^{\mathrm{C2}}_{\mathrm{m\,MASP1^*}}\left(\begin{array}{l}1+\frac{[\mathrm{C4}]}{K^{\mathrm{C4}}_{\mathrm{m\,MASP1^*}}}+\frac{[\mathrm{fC4bC2}]}{K^{\mathrm{C2}}_{\mathrm{m\,MASP1^*}}}+\frac{[\mathrm{fC4bC4bC2}]}{K^{\mathrm{C2}}_{\mathrm{m\,MASP1^*}}}+\frac{[\mathrm{fC3bC4bC2}]}{K^{\mathrm{C2}}_{\mathrm{m\,MASP1^*}}}+\frac{[\mathrm{pC4bC2}]}{K^{\mathrm{C2}}_{\mathrm{m\,MASP1^*}}}+\frac{[\mathrm{pC4bC4bC2}]}{K^{\mathrm{C2}}_{\mathrm{m\,MASP1^*}}}\\
+\frac{[\mathrm{pC3bC4bC2}]}{K^{\mathrm{C2}}_{\mathrm{m\,MASP1^*}}}+\frac{[\mathrm{pMBL_3MASP1_{zym}}]}{K^{\mathrm{MASP1_{zym}}}_{\mathrm{m\,MASP1^*}}}+\frac{[\mathrm{pMBL_3MASP2_{zym}}]}{K^{\mathrm{MASP2_{zym}}}_{\mathrm{m\,MASP1^*}}}+\frac{[\mathrm{pMBL_3MASP3_{zym}}]}{K^{\mathrm{MASP3_{zym}}}_{\mathrm{m\,MASP1^*}}}\\
+\frac{[\mathrm{pMBL_4MASP1_{zym}}]}{K^{\mathrm{MASP1_{zym}}}_{\mathrm{m\,MASP1^*}}}+\frac{[\mathrm{pMBL_4MASP2_{zym}}]}{K^{\mathrm{MASP2_{zym}}}_{\mathrm{m\,MASP1^*}}}+\frac{[\mathrm{pMBL_4MASP3_{zym}}]}{K^{\mathrm{MASP3_{zym}}}_{\mathrm{m\,MASP1^*}}}+\frac{[\mathrm{pCLLKMASP1_{zym}}]}{K^{\mathrm{MASP1_{zym}}}_{\mathrm{m\,MASP1^*}}}\\
+\frac{[\mathrm{pCLLKMASP2_{zym}}]}{K^{\mathrm{MASP2_{zym}}}_{\mathrm{m\,MASP1^*}}}+\frac{[\mathrm{pCLLKMASP3_{zym}}]}{K^{\mathrm{MASP3_{zym}}}_{\mathrm{m\,MASP1^*}}}+\frac{[\mathrm{pCLK1MASP1_{zym}}]}{K^{\mathrm{MASP1_{zym}}}_{\mathrm{m\,MASP1^*}}}+\frac{[\mathrm{pCLK1MASP2_{zym}}]}{K^{\mathrm{MASP2_{zym}}}_{\mathrm{m\,MASP1^*}}}\\
+\frac{[\mathrm{pCLK1MASP3_{zym}}]}{K^{\mathrm{MASP3_{zym}}}_{\mathrm{m\,MASP1^*}}}+\frac{[\mathrm{pCLL1MASP1_{zym}}]}{K^{\mathrm{MASP1_{zym}}}_{\mathrm{m\,MASP1^*}}}+\frac{[\mathrm{pCLL1MASP2_{zym}}]}{K^{\mathrm{MASP2_{zym}}}_{\mathrm{m\,MASP1^*}}}+\frac{[\mathrm{pCLL1MASP3_{zym}}]}{K^{\mathrm{MASP3_{zym}}}_{\mathrm{m\,MASP1^*}}}\\
+\frac{[\mathrm{pLFMASP1_{zym}}]}{K^{\mathrm{MASP1_{zym}}}_{\mathrm{m\,MASP1^*}}}+\frac{[\mathrm{pLFMASP2_{zym}}]}{K^{\mathrm{MASP2_{zym}}}_{\mathrm{m\,MASP1^*}}}+\frac{[\mathrm{pLFMASP3_{zym}}]}{K^{\mathrm{MASP3_{zym}}}_{\mathrm{m\,MASP1^*}}}+\frac{[\mathrm{pHFMASP1_{zym}}]}{K^{\mathrm{MASP1_{zym}}}_{\mathrm{m\,MASP1^*}}}+\frac{[\mathrm{pHFMASP2_{zym}}]}{K^{\mathrm{MASP2_{zym}}}_{\mathrm{m\,MASP1^*}}}\\
+\frac{[\mathrm{pHFMASP3_{zym}}]}{K^{\mathrm{MASP3_{zym}}}_{\mathrm{m\,MASP1^*}}}+\frac{[\mathrm{pMFMASP1_{zym}}]}{K^{\mathrm{MASP1_{zym}}}_{\mathrm{m\,MASP1^*}}}+\frac{[\mathrm{pMFMASP2_{zym}}]}{K^{\mathrm{MASP2_{zym}}}_{\mathrm{m\,MASP1^*}}}+\frac{[\mathrm{pMFMASP3_{zym}}]}{K^{\mathrm{MASP3_{zym}}}_{\mathrm{m\,MASP1^*}}}\end{array}\right)}$$

$$-\frac{k^{\mathrm{C2}}_{\mathrm{cat\,MASP1^*}}\left[\mathrm{pMFMASP1^*}\right]\left[\mathrm{fC3bC4bC2}\right]}{K^{\mathrm{C2}}_{\mathrm{m\,MASP1^*}}\left(\begin{array}{l}1+\frac{[\mathrm{C4}]}{K^{\mathrm{C4}}_{\mathrm{m\,MASP1^*}}}+\frac{[\mathrm{fC4bC2}]}{K^{\mathrm{C2}}_{\mathrm{m\,MASP1^*}}}+\frac{[\mathrm{fC4bC4bC2}]}{K^{\mathrm{C2}}_{\mathrm{m\,MASP1^*}}}+\frac{[\mathrm{fC3bC4bC2}]}{K^{\mathrm{C2}}_{\mathrm{m\,MASP1^*}}}+\frac{[\mathrm{pC4bC2}]}{K^{\mathrm{C2}}_{\mathrm{m\,MASP1^*}}}+\frac{[\mathrm{pC4bC4bC2}]}{K^{\mathrm{C2}}_{\mathrm{m\,MASP1^*}}}\\
+\frac{[\mathrm{pC3bC4bC2}]}{K^{\mathrm{C2}}_{\mathrm{m\,MASP1^*}}}+\frac{[\mathrm{pMBL_3MASP1_{zym}}]}{K^{\mathrm{MASP1_{zym}}}_{\mathrm{m\,MASP1^*}}}+\frac{[\mathrm{pMBL_3MASP2_{zym}}]}{K^{\mathrm{MASP2_{zym}}}_{\mathrm{m\,MASP1^*}}}+\frac{[\mathrm{pMBL_3MASP3_{zym}}]}{K^{\mathrm{MASP3_{zym}}}_{\mathrm{m\,MASP1^*}}}\\
+\frac{[\mathrm{pMBL_4MASP1_{zym}}]}{K^{\mathrm{MASP1_{zym}}}_{\mathrm{m\,MASP1^*}}}+\frac{[\mathrm{pMBL_4MASP2_{zym}}]}{K^{\mathrm{MASP2_{zym}}}_{\mathrm{m\,MASP1^*}}}+\frac{[\mathrm{pMBL_4MASP3_{zym}}]}{K^{\mathrm{MASP3_{zym}}}_{\mathrm{m\,MASP1^*}}}+\frac{[\mathrm{pCLLKMASP1_{zym}}]}{K^{\mathrm{MASP1_{zym}}}_{\mathrm{m\,MASP1^*}}}\\
+\frac{[\mathrm{pCLLKMASP2_{zym}}]}{K^{\mathrm{MASP2_{zym}}}_{\mathrm{m\,MASP1^*}}}+\frac{[\mathrm{pCLLKMASP3_{zym}}]}{K^{\mathrm{MASP3_{zym}}}_{\mathrm{m\,MASP1^*}}}+\frac{[\mathrm{pCLK1MASP1_{zym}}]}{K^{\mathrm{MASP1_{zym}}}_{\mathrm{m\,MASP1^*}}}+\frac{[\mathrm{pCLK1MASP2_{zym}}]}{K^{\mathrm{MASP2_{zym}}}_{\mathrm{m\,MASP1^*}}}\\
+\frac{[\mathrm{pCLK1MASP3_{zym}}]}{K^{\mathrm{MASP3_{zym}}}_{\mathrm{m\,MASP1^*}}}+\frac{[\mathrm{pCLL1MASP1_{zym}}]}{K^{\mathrm{MASP1_{zym}}}_{\mathrm{m\,MASP1^*}}}+\frac{[\mathrm{pCLL1MASP2_{zym}}]}{K^{\mathrm{MASP2_{zym}}}_{\mathrm{m\,MASP1^*}}}+\frac{[\mathrm{pCLL1MASP3_{zym}}]}{K^{\mathrm{MASP3_{zym}}}_{\mathrm{m\,MASP1^*}}}\\
+\frac{[\mathrm{pLFMASP1_{zym}}]}{K^{\mathrm{MASP1_{zym}}}_{\mathrm{m\,MASP1^*}}}+\frac{[\mathrm{pLFMASP2_{zym}}]}{K^{\mathrm{MASP2_{zym}}}_{\mathrm{m\,MASP1^*}}}+\frac{[\mathrm{pLFMASP3_{zym}}]}{K^{\mathrm{MASP3_{zym}}}_{\mathrm{m\,MASP1^*}}}+\frac{[\mathrm{pHFMASP1_{zym}}]}{K^{\mathrm{MASP1_{zym}}}_{\mathrm{m\,MASP1^*}}}+\frac{[\mathrm{pHFMASP2_{zym}}]}{K^{\mathrm{MASP2_{zym}}}_{\mathrm{m\,MASP1^*}}}\\
+\frac{[\mathrm{pHFMASP3_{zym}}]}{K^{\mathrm{MASP3_{zym}}}_{\mathrm{m\,MASP1^*}}}+\frac{[\mathrm{pMFMASP1_{zym}}]}{K^{\mathrm{MASP1_{zym}}}_{\mathrm{m\,MASP1^*}}}+\frac{[\mathrm{pMFMASP2_{zym}}]}{K^{\mathrm{MASP2_{zym}}}_{\mathrm{m\,MASP1^*}}}+\frac{[\mathrm{pMFMASP3_{zym}}]}{K^{\mathrm{MASP3_{zym}}}_{\mathrm{m\,MASP1^*}}}\end{array}\right)}$$

$$
-\frac{k_{\mathrm{cat\ MASP2^*}}^{\mathrm{C2}}\left[\mathrm{pMBL_3MASP2^*}\right]\left[\mathrm{fC3bC4bC2}\right]}{K_{\mathrm{m\ MASP2^*}}^{\mathrm{C2}}\left(\begin{array}{l}1+\frac{[\mathrm{C4}]}{K_{\mathrm{m\ MASP2^*}}^{\mathrm{C4}}}+\frac{[\mathrm{fC4bC2}]}{K_{\mathrm{m\ MASP2^*}}^{\mathrm{C2}}}+\frac{[\mathrm{fC4bC4bC2}]}{K_{\mathrm{m\ MASP2^*}}^{\mathrm{C2}}}+\frac{[\mathrm{fC3bC4bC2}]}{K_{\mathrm{m\ MASP2^*}}^{\mathrm{C2}}}+\frac{[\mathrm{pC4bC2}]}{K_{\mathrm{m\ MASP2^*}}^{\mathrm{C2}}}+\frac{[\mathrm{pC4bC4bC2}]}{K_{\mathrm{m\ MASP2^*}}^{\mathrm{C2}}}\\ +\frac{[\mathrm{pC3bC4bC2}]}{K_{\mathrm{m\ MASP2^*}}^{\mathrm{C2}}}+\frac{[\mathrm{pMBL_3MASP1_{zym}}]}{K_{\mathrm{m\ MASP2^*}}^{\mathrm{MASP1_{zym}}}}+\frac{[\mathrm{pMBL_3MASP2_{zym}}]}{K_{\mathrm{m\ MASP2^*}}^{\mathrm{MASP2_{zym}}}}+\frac{[\mathrm{pMBL_4MASP1_{zym}}]}{K_{\mathrm{m\ MASP2^*}}^{\mathrm{MASP1_{zym}}}}\\ +\frac{[\mathrm{pMBL_4MASP2_{zym}}]}{K_{\mathrm{m\ MASP2^*}}^{\mathrm{MASP2_{zym}}}}+\frac{[\mathrm{pCLLKMASP1_{zym}}]}{K_{\mathrm{m\ MASP2^*}}^{\mathrm{MASP1_{zym}}}}+\frac{[\mathrm{pCLLKMASP2_{zym}}]}{K_{\mathrm{m\ MASP2^*}}^{\mathrm{MASP2_{zym}}}}+\frac{[\mathrm{pCLK1MASP1_{zym}}]}{K_{\mathrm{m\ MASP2^*}}^{\mathrm{MASP1_{zym}}}}\\ +\frac{[\mathrm{pCLK1MASP2_{zym}}]}{K_{\mathrm{m\ MASP2^*}}^{\mathrm{MASP2_{zym}}}}+\frac{[\mathrm{pCLL1MASP1_{zym}}]}{K_{\mathrm{m\ MASP2^*}}^{\mathrm{MASP1_{zym}}}}+\frac{[\mathrm{pCLL1MASP2_{zym}}]}{K_{\mathrm{m\ MASP2^*}}^{\mathrm{MASP2_{zym}}}}+\frac{[\mathrm{pLFMASP1_{zym}}]}{K_{\mathrm{m\ MASP2^*}}^{\mathrm{MASP1_{zym}}}}\\ +\frac{[\mathrm{pLFMASP2_{zym}}]}{K_{\mathrm{m\ MASP2^*}}^{\mathrm{MASP2_{zym}}}}+\frac{[\mathrm{pHFMASP1_{zym}}]}{K_{\mathrm{m\ MASP2^*}}^{\mathrm{MASP1_{zym}}}}+\frac{[\mathrm{pHFMASP2_{zym}}]}{K_{\mathrm{m\ MASP2^*}}^{\mathrm{MASP2_{zym}}}}+\frac{[\mathrm{pMFMASP1_{zym}}]}{K_{\mathrm{m\ MASP2^*}}^{\mathrm{MASP1_{zym}}}}+\frac{[\mathrm{pMFMASP2_{zym}}]}{K_{\mathrm{m\ MASP2^*}}^{\mathrm{MASP2_{zym}}}}\end{array}\right)}
$$

$$
-\frac{k_{\mathrm{cat\ MASP2^*}}^{\mathrm{C2}}\left[\mathrm{pMBL_4MASP2^*}\right]\left[\mathrm{fC3bC4bC2}\right]}{K_{\mathrm{m\ MASP2^*}}^{\mathrm{C2}}\left(\begin{array}{l}1+\frac{[\mathrm{C4}]}{K_{\mathrm{m\ MASP2^*}}^{\mathrm{C4}}}+\frac{[\mathrm{fC4bC2}]}{K_{\mathrm{m\ MASP2^*}}^{\mathrm{C2}}}+\frac{[\mathrm{fC4bC4bC2}]}{K_{\mathrm{m\ MASP2^*}}^{\mathrm{C2}}}+\frac{[\mathrm{fC3bC4bC2}]}{K_{\mathrm{m\ MASP2^*}}^{\mathrm{C2}}}+\frac{[\mathrm{pC4bC2}]}{K_{\mathrm{m\ MASP2^*}}^{\mathrm{C2}}}+\frac{[\mathrm{pC4bC4bC2}]}{K_{\mathrm{m\ MASP2^*}}^{\mathrm{C2}}}\\ +\frac{[\mathrm{pC3bC4bC2}]}{K_{\mathrm{m\ MASP2^*}}^{\mathrm{C2}}}+\frac{[\mathrm{pMBL_3MASP1_{zym}}]}{K_{\mathrm{m\ MASP2^*}}^{\mathrm{MASP1_{zym}}}}+\frac{[\mathrm{pMBL_3MASP2_{zym}}]}{K_{\mathrm{m\ MASP2^*}}^{\mathrm{MASP2_{zym}}}}+\frac{[\mathrm{pMBL_4MASP1_{zym}}]}{K_{\mathrm{m\ MASP2^*}}^{\mathrm{MASP1_{zym}}}}\\ +\frac{[\mathrm{pMBL_4MASP2_{zym}}]}{K_{\mathrm{m\ MASP2^*}}^{\mathrm{MASP2_{zym}}}}+\frac{[\mathrm{pCLLKMASP1_{zym}}]}{K_{\mathrm{m\ MASP2^*}}^{\mathrm{MASP1_{zym}}}}+\frac{[\mathrm{pCLLKMASP2_{zym}}]}{K_{\mathrm{m\ MASP2^*}}^{\mathrm{MASP2_{zym}}}}+\frac{[\mathrm{pCLK1MASP1_{zym}}]}{K_{\mathrm{m\ MASP2^*}}^{\mathrm{MASP1_{zym}}}}\\ +\frac{[\mathrm{pCLK1MASP2_{zym}}]}{K_{\mathrm{m\ MASP2^*}}^{\mathrm{MASP2_{zym}}}}+\frac{[\mathrm{pCLL1MASP1_{zym}}]}{K_{\mathrm{m\ MASP2^*}}^{\mathrm{MASP1_{zym}}}}+\frac{[\mathrm{pCLL1MASP2_{zym}}]}{K_{\mathrm{m\ MASP2^*}}^{\mathrm{MASP2_{zym}}}}+\frac{[\mathrm{pLFMASP1_{zym}}]}{K_{\mathrm{m\ MASP2^*}}^{\mathrm{MASP1_{zym}}}}\\ +\frac{[\mathrm{pLFMASP2_{zym}}]}{K_{\mathrm{m\ MASP2^*}}^{\mathrm{MASP2_{zym}}}}+\frac{[\mathrm{pHFMASP1_{zym}}]}{K_{\mathrm{m\ MASP2^*}}^{\mathrm{MASP1_{zym}}}}+\frac{[\mathrm{pHFMASP2_{zym}}]}{K_{\mathrm{m\ MASP2^*}}^{\mathrm{MASP2_{zym}}}}+\frac{[\mathrm{pMFMASP1_{zym}}]}{K_{\mathrm{m\ MASP2^*}}^{\mathrm{MASP1_{zym}}}}+\frac{[\mathrm{pMFMASP2_{zym}}]}{K_{\mathrm{m\ MASP2^*}}^{\mathrm{MASP2_{zym}}}}\end{array}\right)}
$$

$$
-\frac{k_{\mathrm{cat\ MASP2^*}}^{\mathrm{C2}}\left[\mathrm{pCLLKMASP2^*}\right]\left[\mathrm{fC3bC4bC2}\right]}{K_{\mathrm{m\ MASP2^*}}^{\mathrm{C2}}\left(\begin{array}{l}1+\frac{[\mathrm{C4}]}{K_{\mathrm{m\ MASP2^*}}^{\mathrm{C4}}}+\frac{[\mathrm{fC4bC2}]}{K_{\mathrm{m\ MASP2^*}}^{\mathrm{C2}}}+\frac{[\mathrm{fC4bC4bC2}]}{K_{\mathrm{m\ MASP2^*}}^{\mathrm{C2}}}+\frac{[\mathrm{fC3bC4bC2}]}{K_{\mathrm{m\ MASP2^*}}^{\mathrm{C2}}}+\frac{[\mathrm{pC4bC2}]}{K_{\mathrm{m\ MASP2^*}}^{\mathrm{C2}}}+\frac{[\mathrm{pC4bC4bC2}]}{K_{\mathrm{m\ MASP2^*}}^{\mathrm{C2}}}\\ +\frac{[\mathrm{pC3bC4bC2}]}{K_{\mathrm{m\ MASP2^*}}^{\mathrm{C2}}}+\frac{[\mathrm{pMBL_3MASP1_{zym}}]}{K_{\mathrm{m\ MASP2^*}}^{\mathrm{MASP1_{zym}}}}+\frac{[\mathrm{pMBL_3MASP2_{zym}}]}{K_{\mathrm{m\ MASP2^*}}^{\mathrm{MASP2_{zym}}}}+\frac{[\mathrm{pMBL_4MASP1_{zym}}]}{K_{\mathrm{m\ MASP2^*}}^{\mathrm{MASP1_{zym}}}}\\ +\frac{[\mathrm{pMBL_4MASP2_{zym}}]}{K_{\mathrm{m\ MASP2^*}}^{\mathrm{MASP2_{zym}}}}+\frac{[\mathrm{pCLLKMASP1_{zym}}]}{K_{\mathrm{m\ MASP2^*}}^{\mathrm{MASP1_{zym}}}}+\frac{[\mathrm{pCLLKMASP2_{zym}}]}{K_{\mathrm{m\ MASP2^*}}^{\mathrm{MASP2_{zym}}}}+\frac{[\mathrm{pCLK1MASP1_{zym}}]}{K_{\mathrm{m\ MASP2^*}}^{\mathrm{MASP1_{zym}}}}\\ +\frac{[\mathrm{pCLK1MASP2_{zym}}]}{K_{\mathrm{m\ MASP2^*}}^{\mathrm{MASP2_{zym}}}}+\frac{[\mathrm{pCLL1MASP1_{zym}}]}{K_{\mathrm{m\ MASP2^*}}^{\mathrm{MASP1_{zym}}}}+\frac{[\mathrm{pCLL1MASP2_{zym}}]}{K_{\mathrm{m\ MASP2^*}}^{\mathrm{MASP2_{zym}}}}+\frac{[\mathrm{pLFMASP1_{zym}}]}{K_{\mathrm{m\ MASP2^*}}^{\mathrm{MASP1_{zym}}}}\\ +\frac{[\mathrm{pLFMASP2_{zym}}]}{K_{\mathrm{m\ MASP2^*}}^{\mathrm{MASP2_{zym}}}}+\frac{[\mathrm{pHFMASP1_{zym}}]}{K_{\mathrm{m\ MASP2^*}}^{\mathrm{MASP1_{zym}}}}+\frac{[\mathrm{pHFMASP2_{zym}}]}{K_{\mathrm{m\ MASP2^*}}^{\mathrm{MASP2_{zym}}}}+\frac{[\mathrm{pMFMASP1_{zym}}]}{K_{\mathrm{m\ MASP2^*}}^{\mathrm{MASP1_{zym}}}}+\frac{[\mathrm{pMFMASP2_{zym}}]}{K_{\mathrm{m\ MASP2^*}}^{\mathrm{MASP2_{zym}}}}\end{array}\right)}
$$

$$-\frac{k_{\mathrm{cat\ MASP2^*}}^{\mathrm{C2}}\left[\mathrm{pCLK1MASP2^*}\right]\left[\mathrm{fC3bC4bC2}\right]}{K_{\mathrm{m\ MASP2^*}}^{\mathrm{C2}}\left(\begin{array}{l}1+\frac{[\mathrm{C4}]}{K_{\mathrm{m\ MASP2^*}}^{\mathrm{C4}}}+\frac{[\mathrm{fC4bC2}]}{K_{\mathrm{m\ MASP2^*}}^{\mathrm{C2}}}+\frac{[\mathrm{fC4bC4bC2}]}{K_{\mathrm{m\ MASP2^*}}^{\mathrm{C2}}}+\frac{[\mathrm{fC3bC4bC2}]}{K_{\mathrm{m\ MASP2^*}}^{\mathrm{C2}}}+\frac{[\mathrm{pC4bC2}]}{K_{\mathrm{m\ MASP2^*}}^{\mathrm{C2}}}+\frac{[\mathrm{pC4bC4bC2}]}{K_{\mathrm{m\ MASP2^*}}^{\mathrm{C2}}}\\+\frac{[\mathrm{pC3bC4bC2}]}{K_{\mathrm{m\ MASP2^*}}^{\mathrm{C2}}}+\frac{[\mathrm{pMBL_3MASP1_{zym}}]}{K_{\mathrm{m\ MASP2^*}}^{\mathrm{MASP1_{zym}}}}+\frac{[\mathrm{pMBL_3MASP2_{zym}}]}{K_{\mathrm{m\ MASP2^*}}^{\mathrm{MASP2_{zym}}}}+\frac{[\mathrm{pMBL_4MASP1_{zym}}]}{K_{\mathrm{m\ MASP2^*}}^{\mathrm{MASP1_{zym}}}}\\+\frac{[\mathrm{pMBL_4MASP2_{zym}}]}{K_{\mathrm{m\ MASP2^*}}^{\mathrm{MASP2_{zym}}}}+\frac{[\mathrm{pCLLKMASP1_{zym}}]}{K_{\mathrm{m\ MASP2^*}}^{\mathrm{MASP1_{zym}}}}+\frac{[\mathrm{pCLLKMASP2_{zym}}]}{K_{\mathrm{m\ MASP2^*}}^{\mathrm{MASP2_{zym}}}}+\frac{[\mathrm{pCLK1MASP1_{zym}}]}{K_{\mathrm{m\ MASP2^*}}^{\mathrm{MASP1_{zym}}}}\\+\frac{[\mathrm{pCLK1MASP2_{zym}}]}{K_{\mathrm{m\ MASP2^*}}^{\mathrm{MASP2_{zym}}}}+\frac{[\mathrm{pCLL1MASP1_{zym}}]}{K_{\mathrm{m\ MASP2^*}}^{\mathrm{MASP1_{zym}}}}+\frac{[\mathrm{pCLL1MASP2_{zym}}]}{K_{\mathrm{m\ MASP2^*}}^{\mathrm{MASP2_{zym}}}}+\frac{[\mathrm{pLFMASP1_{zym}}]}{K_{\mathrm{m\ MASP2^*}}^{\mathrm{MASP1_{zym}}}}\\+\frac{[\mathrm{pLFMASP2_{zym}}]}{K_{\mathrm{m\ MASP2^*}}^{\mathrm{MASP2_{zym}}}}+\frac{[\mathrm{pHFMASP1_{zym}}]}{K_{\mathrm{m\ MASP2^*}}^{\mathrm{MASP1_{zym}}}}+\frac{[\mathrm{pHFMASP2_{zym}}]}{K_{\mathrm{m\ MASP2^*}}^{\mathrm{MASP2_{zym}}}}+\frac{[\mathrm{pMFMASP1_{zym}}]}{K_{\mathrm{m\ MASP2^*}}^{\mathrm{MASP1_{zym}}}}+\frac{[\mathrm{pMFMASP2_{zym}}]}{K_{\mathrm{m\ MASP2^*}}^{\mathrm{MASP2_{zym}}}}\end{array}\right)}$$

$$-\frac{k_{\mathrm{cat\ MASP2^*}}^{\mathrm{C2}}\left[\mathrm{pCLL1MASP2^*}\right]\left[\mathrm{fC3bC4bC2}\right]}{K_{\mathrm{m\ MASP2^*}}^{\mathrm{C2}}\left(\begin{array}{l}1+\frac{[\mathrm{C4}]}{K_{\mathrm{m\ MASP2^*}}^{\mathrm{C4}}}+\frac{[\mathrm{fC4bC2}]}{K_{\mathrm{m\ MASP2^*}}^{\mathrm{C2}}}+\frac{[\mathrm{fC4bC4bC2}]}{K_{\mathrm{m\ MASP2^*}}^{\mathrm{C2}}}+\frac{[\mathrm{fC3bC4bC2}]}{K_{\mathrm{m\ MASP2^*}}^{\mathrm{C2}}}+\frac{[\mathrm{pC4bC2}]}{K_{\mathrm{m\ MASP2^*}}^{\mathrm{C2}}}+\frac{[\mathrm{pC4bC4bC2}]}{K_{\mathrm{m\ MASP2^*}}^{\mathrm{C2}}}\\+\frac{[\mathrm{pC3bC4bC2}]}{K_{\mathrm{m\ MASP2^*}}^{\mathrm{C2}}}+\frac{[\mathrm{pMBL_3MASP1_{zym}}]}{K_{\mathrm{m\ MASP2^*}}^{\mathrm{MASP1_{zym}}}}+\frac{[\mathrm{pMBL_3MASP2_{zym}}]}{K_{\mathrm{m\ MASP2^*}}^{\mathrm{MASP2_{zym}}}}+\frac{[\mathrm{pMBL_4MASP1_{zym}}]}{K_{\mathrm{m\ MASP2^*}}^{\mathrm{MASP1_{zym}}}}\\+\frac{[\mathrm{pMBL_4MASP2_{zym}}]}{K_{\mathrm{m\ MASP2^*}}^{\mathrm{MASP2_{zym}}}}+\frac{[\mathrm{pCLLKMASP1_{zym}}]}{K_{\mathrm{m\ MASP2^*}}^{\mathrm{MASP1_{zym}}}}+\frac{[\mathrm{pCLLKMASP2_{zym}}]}{K_{\mathrm{m\ MASP2^*}}^{\mathrm{MASP2_{zym}}}}+\frac{[\mathrm{pCLK1MASP1_{zym}}]}{K_{\mathrm{m\ MASP2^*}}^{\mathrm{MASP1_{zym}}}}\\+\frac{[\mathrm{pCLK1MASP2_{zym}}]}{K_{\mathrm{m\ MASP2^*}}^{\mathrm{MASP2_{zym}}}}+\frac{[\mathrm{pCLL1MASP1_{zym}}]}{K_{\mathrm{m\ MASP2^*}}^{\mathrm{MASP1_{zym}}}}+\frac{[\mathrm{pCLL1MASP2_{zym}}]}{K_{\mathrm{m\ MASP2^*}}^{\mathrm{MASP2_{zym}}}}+\frac{[\mathrm{pLFMASP1_{zym}}]}{K_{\mathrm{m\ MASP2^*}}^{\mathrm{MASP1_{zym}}}}\\+\frac{[\mathrm{pLFMASP2_{zym}}]}{K_{\mathrm{m\ MASP2^*}}^{\mathrm{MASP2_{zym}}}}+\frac{[\mathrm{pHFMASP1_{zym}}]}{K_{\mathrm{m\ MASP2^*}}^{\mathrm{MASP1_{zym}}}}+\frac{[\mathrm{pHFMASP2_{zym}}]}{K_{\mathrm{m\ MASP2^*}}^{\mathrm{MASP2_{zym}}}}+\frac{[\mathrm{pMFMASP1_{zym}}]}{K_{\mathrm{m\ MASP2^*}}^{\mathrm{MASP1_{zym}}}}+\frac{[\mathrm{pMFMASP2_{zym}}]}{K_{\mathrm{m\ MASP2^*}}^{\mathrm{MASP2_{zym}}}}\end{array}\right)}$$

$$-\frac{k_{\mathrm{cat\ MASP2^*}}^{\mathrm{C2}}\left[\mathrm{pLFMASP2^*}\right]\left[\mathrm{fC3bC4bC2}\right]}{K_{\mathrm{m\ MASP2^*}}^{\mathrm{C2}}\left(\begin{array}{l}1+\frac{[\mathrm{C4}]}{K_{\mathrm{m\ MASP2^*}}^{\mathrm{C4}}}+\frac{[\mathrm{fC4bC2}]}{K_{\mathrm{m\ MASP2^*}}^{\mathrm{C2}}}+\frac{[\mathrm{fC4bC4bC2}]}{K_{\mathrm{m\ MASP2^*}}^{\mathrm{C2}}}+\frac{[\mathrm{fC3bC4bC2}]}{K_{\mathrm{m\ MASP2^*}}^{\mathrm{C2}}}+\frac{[\mathrm{pC4bC2}]}{K_{\mathrm{m\ MASP2^*}}^{\mathrm{C2}}}+\frac{[\mathrm{pC4bC4bC2}]}{K_{\mathrm{m\ MASP2^*}}^{\mathrm{C2}}}\\+\frac{[\mathrm{pC3bC4bC2}]}{K_{\mathrm{m\ MASP2^*}}^{\mathrm{C2}}}+\frac{[\mathrm{pMBL_3MASP1_{zym}}]}{K_{\mathrm{m\ MASP2^*}}^{\mathrm{MASP1_{zym}}}}+\frac{[\mathrm{pMBL_3MASP2_{zym}}]}{K_{\mathrm{m\ MASP2^*}}^{\mathrm{MASP2_{zym}}}}+\frac{[\mathrm{pMBL_4MASP1_{zym}}]}{K_{\mathrm{m\ MASP2^*}}^{\mathrm{MASP1_{zym}}}}\\+\frac{[\mathrm{pMBL_4MASP2_{zym}}]}{K_{\mathrm{m\ MASP2^*}}^{\mathrm{MASP2_{zym}}}}+\frac{[\mathrm{pCLLKMASP1_{zym}}]}{K_{\mathrm{m\ MASP2^*}}^{\mathrm{MASP1_{zym}}}}+\frac{[\mathrm{pCLLKMASP2_{zym}}]}{K_{\mathrm{m\ MASP2^*}}^{\mathrm{MASP2_{zym}}}}+\frac{[\mathrm{pCLK1MASP1_{zym}}]}{K_{\mathrm{m\ MASP2^*}}^{\mathrm{MASP1_{zym}}}}\\+\frac{[\mathrm{pCLK1MASP2_{zym}}]}{K_{\mathrm{m\ MASP2^*}}^{\mathrm{MASP2_{zym}}}}+\frac{[\mathrm{pCLL1MASP1_{zym}}]}{K_{\mathrm{m\ MASP2^*}}^{\mathrm{MASP1_{zym}}}}+\frac{[\mathrm{pCLL1MASP2_{zym}}]}{K_{\mathrm{m\ MASP2^*}}^{\mathrm{MASP2_{zym}}}}+\frac{[\mathrm{pLFMASP1_{zym}}]}{K_{\mathrm{m\ MASP2^*}}^{\mathrm{MASP1_{zym}}}}\\+\frac{[\mathrm{pLFMASP2_{zym}}]}{K_{\mathrm{m\ MASP2^*}}^{\mathrm{MASP2_{zym}}}}+\frac{[\mathrm{pHFMASP1_{zym}}]}{K_{\mathrm{m\ MASP2^*}}^{\mathrm{MASP1_{zym}}}}+\frac{[\mathrm{pHFMASP2_{zym}}]}{K_{\mathrm{m\ MASP2^*}}^{\mathrm{MASP2_{zym}}}}+\frac{[\mathrm{pMFMASP1_{zym}}]}{K_{\mathrm{m\ MASP2^*}}^{\mathrm{MASP1_{zym}}}}+\frac{[\mathrm{pMFMASP2_{zym}}]}{K_{\mathrm{m\ MASP2^*}}^{\mathrm{MASP2_{zym}}}}\end{array}\right)}$$

$$
-\frac{k_{\mathrm{cat\,MASP2^*}}^{\mathrm{C2}}\left[\mathrm{pHFMASP2^*}\right]\left[\mathrm{fC3bC4bC2}\right]}{K_{\mathrm{m\,MASP2^*}}^{\mathrm{C2}}\left(\begin{array}{l}1+\frac{[\mathrm{C4}]}{K_{\mathrm{m\,MASP2^*}}^{\mathrm{C4}}}+\frac{[\mathrm{fC4bC2}]}{K_{\mathrm{m\,MASP2^*}}^{\mathrm{C2}}}+\frac{[\mathrm{fC4bC4bC2}]}{K_{\mathrm{m\,MASP2^*}}^{\mathrm{C2}}}+\frac{[\mathrm{fC3bC4bC2}]}{K_{\mathrm{m\,MASP2^*}}^{\mathrm{C2}}}+\frac{[\mathrm{pC4bC2}]}{K_{\mathrm{m\,MASP2^*}}^{\mathrm{C2}}}+\frac{[\mathrm{pC4bC4bC2}]}{K_{\mathrm{m\,MASP2^*}}^{\mathrm{C2}}}\\
+\frac{[\mathrm{pC3bC4bC2}]}{K_{\mathrm{m\,MASP2^*}}^{\mathrm{C2}}}+\frac{[\mathrm{pMBL_3MASP1_{zym}}]}{K_{\mathrm{m\,MASP2^*}}^{\mathrm{MASP1_{zym}}}}+\frac{[\mathrm{pMBL_3MASP2_{zym}}]}{K_{\mathrm{m\,MASP2^*}}^{\mathrm{MASP2_{zym}}}}+\frac{[\mathrm{pMBL_4MASP1_{zym}}]}{K_{\mathrm{m\,MASP2^*}}^{\mathrm{MASP1_{zym}}}}\\
+\frac{[\mathrm{pMBL_4MASP2_{zym}}]}{K_{\mathrm{m\,MASP2^*}}^{\mathrm{MASP2_{zym}}}}+\frac{[\mathrm{pCLLKMASP1_{zym}}]}{K_{\mathrm{m\,MASP2^*}}^{\mathrm{MASP1_{zym}}}}+\frac{[\mathrm{pCLLKMASP2_{zym}}]}{K_{\mathrm{m\,MASP2^*}}^{\mathrm{MASP2_{zym}}}}+\frac{[\mathrm{pCLK1MASP1_{zym}}]}{K_{\mathrm{m\,MASP2^*}}^{\mathrm{MASP1_{zym}}}}\\
+\frac{[\mathrm{pCLK1MASP2_{zym}}]}{K_{\mathrm{m\,MASP2^*}}^{\mathrm{MASP2_{zym}}}}+\frac{[\mathrm{pCLL1MASP1_{zym}}]}{K_{\mathrm{m\,MASP2^*}}^{\mathrm{MASP1_{zym}}}}+\frac{[\mathrm{pCLL1MASP2_{zym}}]}{K_{\mathrm{m\,MASP2^*}}^{\mathrm{MASP2_{zym}}}}+\frac{[\mathrm{pLFMASP1_{zym}}]}{K_{\mathrm{m\,MASP2^*}}^{\mathrm{MASP1_{zym}}}}\\
+\frac{[\mathrm{pLFMASP2_{zym}}]}{K_{\mathrm{m\,MASP2^*}}^{\mathrm{MASP2_{zym}}}}+\frac{[\mathrm{pHFMASP1_{zym}}]}{K_{\mathrm{m\,MASP2^*}}^{\mathrm{MASP1_{zym}}}}+\frac{[\mathrm{pHFMASP2_{zym}}]}{K_{\mathrm{m\,MASP2^*}}^{\mathrm{MASP2_{zym}}}}+\frac{[\mathrm{pMFMASP1_{zym}}]}{K_{\mathrm{m\,MASP2^*}}^{\mathrm{MASP1_{zym}}}}+\frac{[\mathrm{pMFMASP2_{zym}}]}{K_{\mathrm{m\,MASP2^*}}^{\mathrm{MASP2_{zym}}}}\end{array}\right)}
$$

$$
-\frac{k_{\mathrm{cat\,MASP2^*}}^{\mathrm{C2}}\left[\mathrm{pMFMASP2^*}\right]\left[\mathrm{fC3bC4bC2}\right]}{K_{\mathrm{m\,MASP2^*}}^{\mathrm{C2}}\left(\begin{array}{l}1+\frac{[\mathrm{C4}]}{K_{\mathrm{m\,MASP2^*}}^{\mathrm{C4}}}+\frac{[\mathrm{fC4bC2}]}{K_{\mathrm{m\,MASP2^*}}^{\mathrm{C2}}}+\frac{[\mathrm{fC4bC4bC2}]}{K_{\mathrm{m\,MASP2^*}}^{\mathrm{C2}}}+\frac{[\mathrm{fC3bC4bC2}]}{K_{\mathrm{m\,MASP2^*}}^{\mathrm{C2}}}+\frac{[\mathrm{pC4bC2}]}{K_{\mathrm{m\,MASP2^*}}^{\mathrm{C2}}}+\frac{[\mathrm{pC4bC4bC2}]}{K_{\mathrm{m\,MASP2^*}}^{\mathrm{C2}}}\\
+\frac{[\mathrm{pC3bC4bC2}]}{K_{\mathrm{m\,MASP2^*}}^{\mathrm{C2}}}+\frac{[\mathrm{pMBL_3MASP1_{zym}}]}{K_{\mathrm{m\,MASP2^*}}^{\mathrm{MASP1_{zym}}}}+\frac{[\mathrm{pMBL_3MASP2_{zym}}]}{K_{\mathrm{m\,MASP2^*}}^{\mathrm{MASP2_{zym}}}}+\frac{[\mathrm{pMBL_4MASP1_{zym}}]}{K_{\mathrm{m\,MASP2^*}}^{\mathrm{MASP1_{zym}}}}\\
+\frac{[\mathrm{pMBL_4MASP2_{zym}}]}{K_{\mathrm{m\,MASP2^*}}^{\mathrm{MASP2_{zym}}}}+\frac{[\mathrm{pCLLKMASP1_{zym}}]}{K_{\mathrm{m\,MASP2^*}}^{\mathrm{MASP1_{zym}}}}+\frac{[\mathrm{pCLLKMASP2_{zym}}]}{K_{\mathrm{m\,MASP2^*}}^{\mathrm{MASP2_{zym}}}}+\frac{[\mathrm{pCLK1MASP1_{zym}}]}{K_{\mathrm{m\,MASP2^*}}^{\mathrm{MASP1_{zym}}}}\\
+\frac{[\mathrm{pCLK1MASP2_{zym}}]}{K_{\mathrm{m\,MASP2^*}}^{\mathrm{MASP2_{zym}}}}+\frac{[\mathrm{pCLL1MASP1_{zym}}]}{K_{\mathrm{m\,MASP2^*}}^{\mathrm{MASP1_{zym}}}}+\frac{[\mathrm{pCLL1MASP2_{zym}}]}{K_{\mathrm{m\,MASP2^*}}^{\mathrm{MASP2_{zym}}}}+\frac{[\mathrm{pLFMASP1_{zym}}]}{K_{\mathrm{m\,MASP2^*}}^{\mathrm{MASP1_{zym}}}}\\
+\frac{[\mathrm{pLFMASP2_{zym}}]}{K_{\mathrm{m\,MASP2^*}}^{\mathrm{MASP2_{zym}}}}+\frac{[\mathrm{pHFMASP1_{zym}}]}{K_{\mathrm{m\,MASP2^*}}^{\mathrm{MASP1_{zym}}}}+\frac{[\mathrm{pHFMASP2_{zym}}]}{K_{\mathrm{m\,MASP2^*}}^{\mathrm{MASP2_{zym}}}}+\frac{[\mathrm{pMFMASP1_{zym}}]}{K_{\mathrm{m\,MASP2^*}}^{\mathrm{MASP1_{zym}}}}+\frac{[\mathrm{pMFMASP2_{zym}}]}{K_{\mathrm{m\,MASP2^*}}^{\mathrm{MASP2_{zym}}}}\end{array}\right)}
$$

$$
-\frac{k_{\mathrm{cat\,C1^*}}^{\mathrm{C2}}\left[\mathrm{pMBL_3PTX3C1^*}\right]\left[\mathrm{fC3bC4bC2}\right]}{K_{\mathrm{m\,C1^*}}^{\mathrm{C2}}\left(1+\frac{[\mathrm{C4}]}{K_{\mathrm{m\,C1^*}}^{\mathrm{C4}}}+\frac{[\mathrm{fC4bC2}]}{K_{\mathrm{m\,C1^*}}^{\mathrm{C2}}}+\frac{[\mathrm{fC4bC4bC2}]}{K_{\mathrm{m\,C1^*}}^{\mathrm{C2}}}+\frac{[\mathrm{fC3bC4bC2}]}{K_{\mathrm{m\,C1^*}}^{\mathrm{C2}}}+\frac{[\mathrm{pC4bC2}]}{K_{\mathrm{m\,C1^*}}^{\mathrm{C2}}}+\frac{[\mathrm{pC4bC4bC2}]}{K_{\mathrm{m\,C1^*}}^{\mathrm{C2}}}+\frac{[\mathrm{pC3bC4bC2}]}{K_{\mathrm{m\,C1^*}}^{\mathrm{C2}}}\right)}
$$

$$
-\frac{k_{\mathrm{cat\,C1^*}}^{\mathrm{C2}}\left[\mathrm{pMBL_4PTX3C1^*}\right]\left[\mathrm{fC3bC4bC2}\right]}{K_{\mathrm{m\,C1^*}}^{\mathrm{C2}}\left(1+\frac{[\mathrm{C4}]}{K_{\mathrm{m\,C1^*}}^{\mathrm{C4}}}+\frac{[\mathrm{fC4bC2}]}{K_{\mathrm{m\,C1^*}}^{\mathrm{C2}}}+\frac{[\mathrm{fC4bC4bC2}]}{K_{\mathrm{m\,C1^*}}^{\mathrm{C2}}}+\frac{[\mathrm{fC3bC4bC2}]}{K_{\mathrm{m\,C1^*}}^{\mathrm{C2}}}+\frac{[\mathrm{pC4bC2}]}{K_{\mathrm{m\,C1^*}}^{\mathrm{C2}}}+\frac{[\mathrm{pC4bC4bC2}]}{K_{\mathrm{m\,C1^*}}^{\mathrm{C2}}}+\frac{[\mathrm{pC3bC4bC2}]}{K_{\mathrm{m\,C1^*}}^{\mathrm{C2}}}\right)}
$$

$$
-\frac{k_{\mathrm{cat\,C1^*}}^{\mathrm{C2}}\left[\mathrm{CRPC1^*}\right]\left[\mathrm{fC3bC4bC2}\right]}{K_{\mathrm{m\,C1^*}}^{\mathrm{C2}}\left(1+\frac{[\mathrm{C4}]}{K_{\mathrm{m\,C1^*}}^{\mathrm{C4}}}+\frac{[\mathrm{fC4bC2}]}{K_{\mathrm{m\,C1^*}}^{\mathrm{C2}}}+\frac{[\mathrm{fC4bC4bC2}]}{K_{\mathrm{m\,C1^*}}^{\mathrm{C2}}}+\frac{[\mathrm{fC3bC4bC2}]}{K_{\mathrm{m\,C1^*}}^{\mathrm{C2}}}\right)}
$$

33

$$\frac{d\left[\mathrm{fC3bC4bC2a}\right]}{dt}=k_{\mathrm{C3bC4b}}^{+}\left[\mathrm{fC4bC2a}\right]\left[\mathrm{nfC3b}\right]$$

$$+\frac{k_{\mathrm{cat\ C1^*}}^{\mathrm{C2}}\left[\mathrm{C1^*}\right]\left[\mathrm{fC3bC4bC2}\right]}{K_{\mathrm{m\ C1^*}}^{\mathrm{C2}}\left(1+\frac{\left[\mathrm{C4}\right]}{K_{\mathrm{m\ C1^*}}^{\mathrm{C4}}}+\frac{\left[\mathrm{fC4bC2}\right]}{K_{\mathrm{m\ C1^*}}^{\mathrm{C2}}}+\frac{\left[\mathrm{fC4bC4bC2}\right]}{K_{\mathrm{m\ C1^*}}^{\mathrm{C2}}}+\frac{\left[\mathrm{fC3bC4bC2}\right]}{K_{\mathrm{m\ C1^*}}^{\mathrm{C2}}}+\frac{\left[\mathrm{pC4bC2}\right]}{K_{\mathrm{m\ C1^*}}^{\mathrm{C2}}}+\frac{\left[\mathrm{pC4bC4bC2}\right]}{K_{\mathrm{m\ C1^*}}^{\mathrm{C2}}}+\frac{\left[\mathrm{pC3bC4bC2}\right]}{K_{\mathrm{m\ C1^*}}^{\mathrm{C2}}}\right)}$$

$$-k_{\mathrm{C3bC4bC2a}}^{-}\left[\mathrm{fC3bC4bC2a}\right]+k_{\mathrm{C5b^*}}^{-}\left[\mathrm{fC3bC4bC2aC5b}\right]-k_{\mathrm{C4bC4BP}}^{+}\left[\mathrm{fC3bC4bC2a}\right]\left[\mathrm{C4BP}\right]+k_{\mathrm{C4bC4BP}}^{-}\left[\mathrm{fC3bC4bC2aC4BP}\right]$$

$$-k_{\mathrm{C3bC4bCR1}}^{+}\left[\mathrm{fC3bC4bC2a}\right]\left[\mathrm{CR1}\right]+k_{\mathrm{C3bC4bCR1}}^{-}\left[\mathrm{fC3bC4bC2aCR1}\right]+k_{\mathrm{C5b7}}^{+}\left[\mathrm{fC3bC4bC2aC5b}\right]\left[\mathrm{C7}\right]$$

$$+\frac{k_{\mathrm{cat\ C1^*}}^{\mathrm{C2}}\left[\mathrm{pIgG_6C1^*}\right]\left[\mathrm{fC3bC4bC2}\right]}{K_{\mathrm{m\ C1^*}}^{\mathrm{C2}}\left(1+\frac{\left[\mathrm{C4}\right]}{K_{\mathrm{m\ C1^*}}^{\mathrm{C4}}}+\frac{\left[\mathrm{fC4bC2}\right]}{K_{\mathrm{m\ C1^*}}^{\mathrm{C2}}}+\frac{\left[\mathrm{fC4bC4bC2}\right]}{K_{\mathrm{m\ C1^*}}^{\mathrm{C2}}}+\frac{\left[\mathrm{fC3bC4bC2}\right]}{K_{\mathrm{m\ C1^*}}^{\mathrm{C2}}}+\frac{\left[\mathrm{pC4bC2}\right]}{K_{\mathrm{m\ C1^*}}^{\mathrm{C2}}}+\frac{\left[\mathrm{pC4bC4bC2}\right]}{K_{\mathrm{m\ C1^*}}^{\mathrm{C2}}}+\frac{\left[\mathrm{pC3bC4bC2}\right]}{K_{\mathrm{m\ C1^*}}^{\mathrm{C2}}}\right)}$$

$$+\frac{k_{\mathrm{cat\ C1^*}}^{\mathrm{C2}}\left[\mathrm{pIgMC1^*}\right]\left[\mathrm{fC3bC4bC2}\right]}{K_{\mathrm{m\ C1^*}}^{\mathrm{C2}}\left(1+\frac{\left[\mathrm{C4}\right]}{K_{\mathrm{m\ C1^*}}^{\mathrm{C4}}}+\frac{\left[\mathrm{fC4bC2}\right]}{K_{\mathrm{m\ C1^*}}^{\mathrm{C2}}}+\frac{\left[\mathrm{fC4bC4bC2}\right]}{K_{\mathrm{m\ C1^*}}^{\mathrm{C2}}}+\frac{\left[\mathrm{fC3bC4bC2}\right]}{K_{\mathrm{m\ C1^*}}^{\mathrm{C2}}}+\frac{\left[\mathrm{pC4bC2}\right]}{K_{\mathrm{m\ C1^*}}^{\mathrm{C2}}}+\frac{\left[\mathrm{pC4bC4bC2}\right]}{K_{\mathrm{m\ C1^*}}^{\mathrm{C2}}}+\frac{\left[\mathrm{pC3bC4bC2}\right]}{K_{\mathrm{m\ C1^*}}^{\mathrm{C2}}}\right)}$$

$$+\frac{k_{\mathrm{cat\ MASP1^*}}^{\mathrm{C2}}\left[\mathrm{pMBL_3MASP1^*}\right]\left[\mathrm{fC3bC4bC2}\right]}{K_{\mathrm{m\ MASP1^*}}^{\mathrm{C2}}\left(\begin{array}{l}1+\frac{\left[\mathrm{C4}\right]}{K_{\mathrm{m\ MASP1^*}}^{\mathrm{C4}}}+\frac{\left[\mathrm{fC4bC2}\right]}{K_{\mathrm{m\ MASP1^*}}^{\mathrm{C2}}}+\frac{\left[\mathrm{fC4bC4bC2}\right]}{K_{\mathrm{m\ MASP1^*}}^{\mathrm{C2}}}+\frac{\left[\mathrm{fC3bC4bC2}\right]}{K_{\mathrm{m\ MASP1^*}}^{\mathrm{C2}}}+\frac{\left[\mathrm{pC4bC2}\right]}{K_{\mathrm{m\ MASP1^*}}^{\mathrm{C2}}}+\frac{\left[\mathrm{pC4bC4bC2}\right]}{K_{\mathrm{m\ MASP1^*}}^{\mathrm{C2}}}\\+\frac{\left[\mathrm{pC3bC4bC2}\right]}{K_{\mathrm{m\ MASP1^*}}^{\mathrm{C2}}}+\frac{\left[\mathrm{pMBL_3MASP1_{zym}}\right]}{K_{\mathrm{m\ MASP1^*}}^{\mathrm{MASP1_{zym}}}}+\frac{\left[\mathrm{pMBL_3MASP2_{zym}}\right]}{K_{\mathrm{m\ MASP1^*}}^{\mathrm{MASP2_{zym}}}}+\frac{\left[\mathrm{pMBL_3MASP3_{zym}}\right]}{K_{\mathrm{m\ MASP1^*}}^{\mathrm{MASP3_{zym}}}}\\+\frac{\left[\mathrm{pMBL_4MASP1_{zym}}\right]}{K_{\mathrm{m\ MASP1^*}}^{\mathrm{MASP1_{zym}}}}+\frac{\left[\mathrm{pMBL_4MASP2_{zym}}\right]}{K_{\mathrm{m\ MASP1^*}}^{\mathrm{MASP2_{zym}}}}+\frac{\left[\mathrm{pMBL_4MASP3_{zym}}\right]}{K_{\mathrm{m\ MASP1^*}}^{\mathrm{MASP3_{zym}}}}+\frac{\left[\mathrm{pCLLKMASP1_{zym}}\right]}{K_{\mathrm{m\ MASP1^*}}^{\mathrm{MASP1_{zym}}}}\\+\frac{\left[\mathrm{pCLLKMASP2_{zym}}\right]}{K_{\mathrm{m\ MASP1^*}}^{\mathrm{MASP2_{zym}}}}+\frac{\left[\mathrm{pCLLKMASP3_{zym}}\right]}{K_{\mathrm{m\ MASP1^*}}^{\mathrm{MASP3_{zym}}}}+\frac{\left[\mathrm{pCLK1MASP1_{zym}}\right]}{K_{\mathrm{m\ MASP1^*}}^{\mathrm{MASP1_{zym}}}}+\frac{\left[\mathrm{pCLK1MASP2_{zym}}\right]}{K_{\mathrm{m\ MASP1^*}}^{\mathrm{MASP2_{zym}}}}\\+\frac{\left[\mathrm{pCLK1MASP3_{zym}}\right]}{K_{\mathrm{m\ MASP1^*}}^{\mathrm{MASP3_{zym}}}}+\frac{\left[\mathrm{pCLL1MASP1_{zym}}\right]}{K_{\mathrm{m\ MASP1^*}}^{\mathrm{MASP1_{zym}}}}+\frac{\left[\mathrm{pCLL1MASP2_{zym}}\right]}{K_{\mathrm{m\ MASP1^*}}^{\mathrm{MASP2_{zym}}}}+\frac{\left[\mathrm{pCLL1MASP3_{zym}}\right]}{K_{\mathrm{m\ MASP1^*}}^{\mathrm{MASP3_{zym}}}}\\+\frac{\left[\mathrm{pLFMASP1_{zym}}\right]}{K_{\mathrm{m\ MASP1^*}}^{\mathrm{MASP1_{zym}}}}+\frac{\left[\mathrm{pLFMASP2_{zym}}\right]}{K_{\mathrm{m\ MASP1^*}}^{\mathrm{MASP2_{zym}}}}+\frac{\left[\mathrm{pLFMASP3_{zym}}\right]}{K_{\mathrm{m\ MASP1^*}}^{\mathrm{MASP3_{zym}}}}+\frac{\left[\mathrm{pHFMASP1_{zym}}\right]}{K_{\mathrm{m\ MASP1^*}}^{\mathrm{MASP1_{zym}}}}+\frac{\left[\mathrm{pHFMASP2_{zym}}\right]}{K_{\mathrm{m\ MASP1^*}}^{\mathrm{MASP2_{zym}}}}\\+\frac{\left[\mathrm{pHFMASP3_{zym}}\right]}{K_{\mathrm{m\ MASP1^*}}^{\mathrm{MASP3_{zym}}}}+\frac{\left[\mathrm{pMFMASP1_{zym}}\right]}{K_{\mathrm{m\ MASP1^*}}^{\mathrm{MASP1_{zym}}}}+\frac{\left[\mathrm{pMFMASP2_{zym}}\right]}{K_{\mathrm{m\ MASP1^*}}^{\mathrm{MASP2_{zym}}}}+\frac{\left[\mathrm{pMFMASP3_{zym}}\right]}{K_{\mathrm{m\ MASP1^*}}^{\mathrm{MASP3_{zym}}}}\end{array}\right)}$$

$$
+\frac{k^{\mathrm{C2}}_{\mathrm{cat\,MASP1^*}}\left[\mathrm{pMBL_4MASP1^*}\right]\left[\mathrm{fC3bC4bC2}\right]}{K^{\mathrm{C2}}_{\mathrm{m\,MASP1^*}}\left(\begin{aligned}
&1+\frac{[\mathrm{C4}]}{K^{\mathrm{C4}}_{\mathrm{m\,MASP1^*}}}+\frac{[\mathrm{fC4bC2}]}{K^{\mathrm{C2}}_{\mathrm{m\,MASP1^*}}}+\frac{[\mathrm{fC4bC4bC2}]}{K^{\mathrm{C2}}_{\mathrm{m\,MASP1^*}}}+\frac{[\mathrm{fC3bC4bC2}]}{K^{\mathrm{C2}}_{\mathrm{m\,MASP1^*}}}+\frac{[\mathrm{pC4bC2}]}{K^{\mathrm{C2}}_{\mathrm{m\,MASP1^*}}}+\frac{[\mathrm{pC4bC4bC2}]}{K^{\mathrm{C2}}_{\mathrm{m\,MASP1^*}}}\\
&+\frac{[\mathrm{pC3bC4bC2}]}{K^{\mathrm{C2}}_{\mathrm{m\,MASP1^*}}}+\frac{\left[\mathrm{pMBL_3MASP1_{zym}}\right]}{K^{\mathrm{MASP1_{zym}}}_{\mathrm{m\,MASP1^*}}}+\frac{\left[\mathrm{pMBL_3MASP2_{zym}}\right]}{K^{\mathrm{MASP2_{zym}}}_{\mathrm{m\,MASP1^*}}}+\frac{\left[\mathrm{pMBL_3MASP3_{zym}}\right]}{K^{\mathrm{MASP3_{zym}}}_{\mathrm{m\,MASP1^*}}}\\
&+\frac{\left[\mathrm{pMBL_4MASP1_{zym}}\right]}{K^{\mathrm{MASP1_{zym}}}_{\mathrm{m\,MASP1^*}}}+\frac{\left[\mathrm{pMBL_4MASP2_{zym}}\right]}{K^{\mathrm{MASP2_{zym}}}_{\mathrm{m\,MASP1^*}}}+\frac{\left[\mathrm{pMBL_4MASP3_{zym}}\right]}{K^{\mathrm{MASP3_{zym}}}_{\mathrm{m\,MASP1^*}}}+\frac{\left[\mathrm{pCLLKMASP1_{zym}}\right]}{K^{\mathrm{MASP1_{zym}}}_{\mathrm{m\,MASP1^*}}}\\
&+\frac{\left[\mathrm{pCLLKMASP2_{zym}}\right]}{K^{\mathrm{MASP2_{zym}}}_{\mathrm{m\,MASP1^*}}}+\frac{\left[\mathrm{pCLLKMASP3_{zym}}\right]}{K^{\mathrm{MASP3_{zym}}}_{\mathrm{m\,MASP1^*}}}+\frac{\left[\mathrm{pCLK1MASP1_{zym}}\right]}{K^{\mathrm{MASP1_{zym}}}_{\mathrm{m\,MASP1^*}}}+\frac{\left[\mathrm{pCLK1MASP2_{zym}}\right]}{K^{\mathrm{MASP2_{zym}}}_{\mathrm{m\,MASP1^*}}}\\
&+\frac{\left[\mathrm{pCLK1MASP3_{zym}}\right]}{K^{\mathrm{MASP3_{zym}}}_{\mathrm{m\,MASP1^*}}}+\frac{\left[\mathrm{pCLL1MASP1_{zym}}\right]}{K^{\mathrm{MASP1_{zym}}}_{\mathrm{m\,MASP1^*}}}+\frac{\left[\mathrm{pCLL1MASP2_{zym}}\right]}{K^{\mathrm{MASP2_{zym}}}_{\mathrm{m\,MASP1^*}}}+\frac{\left[\mathrm{pCLL1MASP3_{zym}}\right]}{K^{\mathrm{MASP3_{zym}}}_{\mathrm{m\,MASP1^*}}}\\
&+\frac{\left[\mathrm{pLFMASP1_{zym}}\right]}{K^{\mathrm{MASP1_{zym}}}_{\mathrm{m\,MASP1^*}}}+\frac{\left[\mathrm{pLFMASP2_{zym}}\right]}{K^{\mathrm{MASP2_{zym}}}_{\mathrm{m\,MASP1^*}}}+\frac{\left[\mathrm{pLFMASP3_{zym}}\right]}{K^{\mathrm{MASP3_{zym}}}_{\mathrm{m\,MASP1^*}}}+\frac{\left[\mathrm{pHFMASP1_{zym}}\right]}{K^{\mathrm{MASP1_{zym}}}_{\mathrm{m\,MASP1^*}}}+\frac{\left[\mathrm{pHFMASP2_{zym}}\right]}{K^{\mathrm{MASP2_{zym}}}_{\mathrm{m\,MASP1^*}}}\\
&+\frac{\left[\mathrm{pHFMASP3_{zym}}\right]}{K^{\mathrm{MASP3_{zym}}}_{\mathrm{m\,MASP1^*}}}+\frac{\left[\mathrm{pMFMASP1_{zym}}\right]}{K^{\mathrm{MASP1_{zym}}}_{\mathrm{m\,MASP1^*}}}+\frac{\left[\mathrm{pMFMASP2_{zym}}\right]}{K^{\mathrm{MASP2_{zym}}}_{\mathrm{m\,MASP1^*}}}+\frac{\left[\mathrm{pMFMASP3_{zym}}\right]}{K^{\mathrm{MASP3_{zym}}}_{\mathrm{m\,MASP1^*}}}
\end{aligned}\right)}
$$

$$
+\frac{k^{\mathrm{C2}}_{\mathrm{cat\,MASP1^*}}\left[\mathrm{pCLLKMASP1^*}\right]\left[\mathrm{fC3bC4bC2}\right]}{K^{\mathrm{C2}}_{\mathrm{m\,MASP1^*}}\left(\begin{aligned}
&1+\frac{[\mathrm{C4}]}{K^{\mathrm{C4}}_{\mathrm{m\,MASP1^*}}}+\frac{[\mathrm{fC4bC2}]}{K^{\mathrm{C2}}_{\mathrm{m\,MASP1^*}}}+\frac{[\mathrm{fC4bC4bC2}]}{K^{\mathrm{C2}}_{\mathrm{m\,MASP1^*}}}+\frac{[\mathrm{fC3bC4bC2}]}{K^{\mathrm{C2}}_{\mathrm{m\,MASP1^*}}}+\frac{[\mathrm{pC4bC2}]}{K^{\mathrm{C2}}_{\mathrm{m\,MASP1^*}}}+\frac{[\mathrm{pC4bC4bC2}]}{K^{\mathrm{C2}}_{\mathrm{m\,MASP1^*}}}\\
&+\frac{[\mathrm{pC3bC4bC2}]}{K^{\mathrm{C2}}_{\mathrm{m\,MASP1^*}}}+\frac{\left[\mathrm{pMBL_3MASP1_{zym}}\right]}{K^{\mathrm{MASP1_{zym}}}_{\mathrm{m\,MASP1^*}}}+\frac{\left[\mathrm{pMBL_3MASP2_{zym}}\right]}{K^{\mathrm{MASP2_{zym}}}_{\mathrm{m\,MASP1^*}}}+\frac{\left[\mathrm{pMBL_3MASP3_{zym}}\right]}{K^{\mathrm{MASP3_{zym}}}_{\mathrm{m\,MASP1^*}}}\\
&+\frac{\left[\mathrm{pMBL_4MASP1_{zym}}\right]}{K^{\mathrm{MASP1_{zym}}}_{\mathrm{m\,MASP1^*}}}+\frac{\left[\mathrm{pMBL_4MASP2_{zym}}\right]}{K^{\mathrm{MASP2_{zym}}}_{\mathrm{m\,MASP1^*}}}+\frac{\left[\mathrm{pMBL_4MASP3_{zym}}\right]}{K^{\mathrm{MASP3_{zym}}}_{\mathrm{m\,MASP1^*}}}+\frac{\left[\mathrm{pCLLKMASP1_{zym}}\right]}{K^{\mathrm{MASP1_{zym}}}_{\mathrm{m\,MASP1^*}}}\\
&+\frac{\left[\mathrm{pCLLKMASP2_{zym}}\right]}{K^{\mathrm{MASP2_{zym}}}_{\mathrm{m\,MASP1^*}}}+\frac{\left[\mathrm{pCLLKMASP3_{zym}}\right]}{K^{\mathrm{MASP3_{zym}}}_{\mathrm{m\,MASP1^*}}}+\frac{\left[\mathrm{pCLK1MASP1_{zym}}\right]}{K^{\mathrm{MASP1_{zym}}}_{\mathrm{m\,MASP1^*}}}+\frac{\left[\mathrm{pCLK1MASP2_{zym}}\right]}{K^{\mathrm{MASP2_{zym}}}_{\mathrm{m\,MASP1^*}}}\\
&+\frac{\left[\mathrm{pCLK1MASP3_{zym}}\right]}{K^{\mathrm{MASP3_{zym}}}_{\mathrm{m\,MASP1^*}}}+\frac{\left[\mathrm{pCLL1MASP1_{zym}}\right]}{K^{\mathrm{MASP1_{zym}}}_{\mathrm{m\,MASP1^*}}}+\frac{\left[\mathrm{pCLL1MASP2_{zym}}\right]}{K^{\mathrm{MASP2_{zym}}}_{\mathrm{m\,MASP1^*}}}+\frac{\left[\mathrm{pCLL1MASP3_{zym}}\right]}{K^{\mathrm{MASP3_{zym}}}_{\mathrm{m\,MASP1^*}}}\\
&+\frac{\left[\mathrm{pLFMASP1_{zym}}\right]}{K^{\mathrm{MASP1_{zym}}}_{\mathrm{m\,MASP1^*}}}+\frac{\left[\mathrm{pLFMASP2_{zym}}\right]}{K^{\mathrm{MASP2_{zym}}}_{\mathrm{m\,MASP1^*}}}+\frac{\left[\mathrm{pLFMASP3_{zym}}\right]}{K^{\mathrm{MASP3_{zym}}}_{\mathrm{m\,MASP1^*}}}+\frac{\left[\mathrm{pHFMASP1_{zym}}\right]}{K^{\mathrm{MASP1_{zym}}}_{\mathrm{m\,MASP1^*}}}+\frac{\left[\mathrm{pHFMASP2_{zym}}\right]}{K^{\mathrm{MASP2_{zym}}}_{\mathrm{m\,MASP1^*}}}\\
&+\frac{\left[\mathrm{pHFMASP3_{zym}}\right]}{K^{\mathrm{MASP3_{zym}}}_{\mathrm{m\,MASP1^*}}}+\frac{\left[\mathrm{pMFMASP1_{zym}}\right]}{K^{\mathrm{MASP1_{zym}}}_{\mathrm{m\,MASP1^*}}}+\frac{\left[\mathrm{pMFMASP2_{zym}}\right]}{K^{\mathrm{MASP2_{zym}}}_{\mathrm{m\,MASP1^*}}}+\frac{\left[\mathrm{pMFMASP3_{zym}}\right]}{K^{\mathrm{MASP3_{zym}}}_{\mathrm{m\,MASP1^*}}}
\end{aligned}\right)}
$$

$$+\frac{k_{\mathrm{cat\,MASP1^*}}^{\mathrm{C2}}\left[\mathrm{pCLK1MASP1^*}\right]\left[\mathrm{fC3bC4bC2}\right]}{K_{\mathrm{m\,MASP1^*}}^{\mathrm{C2}}\left(\begin{array}{l} 1+\frac{[\mathrm{C4}]}{K_{\mathrm{m\,MASP1^*}}^{\mathrm{C4}}}+\frac{[\mathrm{fC4bC2}]}{K_{\mathrm{m\,MASP1^*}}^{\mathrm{C2}}}+\frac{[\mathrm{fC4bC4bC2}]}{K_{\mathrm{m\,MASP1^*}}^{\mathrm{C2}}}+\frac{[\mathrm{fC3bC4bC2}]}{K_{\mathrm{m\,MASP1^*}}^{\mathrm{C2}}}+\frac{[\mathrm{pC4bC2}]}{K_{\mathrm{m\,MASP1^*}}^{\mathrm{C2}}}+\frac{[\mathrm{pC4bC4bC2}]}{K_{\mathrm{m\,MASP1^*}}^{\mathrm{C2}}} \\ +\frac{[\mathrm{pC3bC4bC2}]}{K_{\mathrm{m\,MASP1^*}}^{\mathrm{C2}}}+\frac{[\mathrm{pMBL_3MASP1_{zym}}]}{K_{\mathrm{m\,MASP1^*}}^{\mathrm{MASP1_{zym}}}}+\frac{[\mathrm{pMBL_3MASP2_{zym}}]}{K_{\mathrm{m\,MASP1^*}}^{\mathrm{MASP2_{zym}}}}+\frac{[\mathrm{pMBL_3MASP3_{zym}}]}{K_{\mathrm{m\,MASP1^*}}^{\mathrm{MASP3_{zym}}}} \\ +\frac{[\mathrm{pMBL_4MASP1_{zym}}]}{K_{\mathrm{m\,MASP1^*}}^{\mathrm{MASP1_{zym}}}}+\frac{[\mathrm{pMBL_4MASP2_{zym}}]}{K_{\mathrm{m\,MASP1^*}}^{\mathrm{MASP2_{zym}}}}+\frac{[\mathrm{pMBL_4MASP3_{zym}}]}{K_{\mathrm{m\,MASP1^*}}^{\mathrm{MASP3_{zym}}}}+\frac{[\mathrm{pCLLKMASP1_{zym}}]}{K_{\mathrm{m\,MASP1^*}}^{\mathrm{MASP1_{zym}}}} \\ +\frac{[\mathrm{pCLLKMASP2_{zym}}]}{K_{\mathrm{m\,MASP1^*}}^{\mathrm{MASP2_{zym}}}}+\frac{[\mathrm{pCLLKMASP3_{zym}}]}{K_{\mathrm{m\,MASP1^*}}^{\mathrm{MASP3_{zym}}}}+\frac{[\mathrm{pCLK1MASP1_{zym}}]}{K_{\mathrm{m\,MASP1^*}}^{\mathrm{MASP1_{zym}}}}+\frac{[\mathrm{pCLK1MASP2_{zym}}]}{K_{\mathrm{m\,MASP1^*}}^{\mathrm{MASP2_{zym}}}} \\ +\frac{[\mathrm{pCLK1MASP3_{zym}}]}{K_{\mathrm{m\,MASP1^*}}^{\mathrm{MASP3_{zym}}}}+\frac{[\mathrm{pCLL1MASP1_{zym}}]}{K_{\mathrm{m\,MASP1^*}}^{\mathrm{MASP1_{zym}}}}+\frac{[\mathrm{pCLL1MASP2_{zym}}]}{K_{\mathrm{m\,MASP1^*}}^{\mathrm{MASP2_{zym}}}}+\frac{[\mathrm{pCLL1MASP3_{zym}}]}{K_{\mathrm{m\,MASP1^*}}^{\mathrm{MASP3_{zym}}}} \\ +\frac{[\mathrm{pLFMASP1_{zym}}]}{K_{\mathrm{m\,MASP1^*}}^{\mathrm{MASP1_{zym}}}}+\frac{[\mathrm{pLFMASP2_{zym}}]}{K_{\mathrm{m\,MASP1^*}}^{\mathrm{MASP2_{zym}}}}+\frac{[\mathrm{pLFMASP3_{zym}}]}{K_{\mathrm{m\,MASP1^*}}^{\mathrm{MASP3_{zym}}}}+\frac{[\mathrm{pHFMASP1_{zym}}]}{K_{\mathrm{m\,MASP1^*}}^{\mathrm{MASP1_{zym}}}}+\frac{[\mathrm{pHFMASP2_{zym}}]}{K_{\mathrm{m\,MASP1^*}}^{\mathrm{MASP2_{zym}}}} \\ +\frac{[\mathrm{pHFMASP3_{zym}}]}{K_{\mathrm{m\,MASP1^*}}^{\mathrm{MASP3_{zym}}}}+\frac{[\mathrm{pMFMASP1_{zym}}]}{K_{\mathrm{m\,MASP1^*}}^{\mathrm{MASP1_{zym}}}}+\frac{[\mathrm{pMFMASP2_{zym}}]}{K_{\mathrm{m\,MASP1^*}}^{\mathrm{MASP2_{zym}}}}+\frac{[\mathrm{pMFMASP3_{zym}}]}{K_{\mathrm{m\,MASP1^*}}^{\mathrm{MASP3_{zym}}}} \end{array}\right)}$$

$$+\frac{k_{\mathrm{cat\,MASP1^*}}^{\mathrm{C2}}\left[\mathrm{pCLL1MASP1^*}\right]\left[\mathrm{fC3bC4bC2}\right]}{K_{\mathrm{m\,MASP1^*}}^{\mathrm{C2}}\left(\begin{array}{l} 1+\frac{[\mathrm{C4}]}{K_{\mathrm{m\,MASP1^*}}^{\mathrm{C4}}}+\frac{[\mathrm{fC4bC2}]}{K_{\mathrm{m\,MASP1^*}}^{\mathrm{C2}}}+\frac{[\mathrm{fC4bC4bC2}]}{K_{\mathrm{m\,MASP1^*}}^{\mathrm{C2}}}+\frac{[\mathrm{fC3bC4bC2}]}{K_{\mathrm{m\,MASP1^*}}^{\mathrm{C2}}}+\frac{[\mathrm{pC4bC2}]}{K_{\mathrm{m\,MASP1^*}}^{\mathrm{C2}}}+\frac{[\mathrm{pC4bC4bC2}]}{K_{\mathrm{m\,MASP1^*}}^{\mathrm{C2}}} \\ +\frac{[\mathrm{pC3bC4bC2}]}{K_{\mathrm{m\,MASP1^*}}^{\mathrm{C2}}}+\frac{[\mathrm{pMBL_3MASP1_{zym}}]}{K_{\mathrm{m\,MASP1^*}}^{\mathrm{MASP1_{zym}}}}+\frac{[\mathrm{pMBL_3MASP2_{zym}}]}{K_{\mathrm{m\,MASP1^*}}^{\mathrm{MASP2_{zym}}}}+\frac{[\mathrm{pMBL_3MASP3_{zym}}]}{K_{\mathrm{m\,MASP1^*}}^{\mathrm{MASP3_{zym}}}} \\ +\frac{[\mathrm{pMBL_4MASP1_{zym}}]}{K_{\mathrm{m\,MASP1^*}}^{\mathrm{MASP1_{zym}}}}+\frac{[\mathrm{pMBL_4MASP2_{zym}}]}{K_{\mathrm{m\,MASP1^*}}^{\mathrm{MASP2_{zym}}}}+\frac{[\mathrm{pMBL_4MASP3_{zym}}]}{K_{\mathrm{m\,MASP1^*}}^{\mathrm{MASP3_{zym}}}}+\frac{[\mathrm{pCLLKMASP1_{zym}}]}{K_{\mathrm{m\,MASP1^*}}^{\mathrm{MASP1_{zym}}}} \\ +\frac{[\mathrm{pCLLKMASP2_{zym}}]}{K_{\mathrm{m\,MASP1^*}}^{\mathrm{MASP2_{zym}}}}+\frac{[\mathrm{pCLLKMASP3_{zym}}]}{K_{\mathrm{m\,MASP1^*}}^{\mathrm{MASP3_{zym}}}}+\frac{[\mathrm{pCLK1MASP1_{zym}}]}{K_{\mathrm{m\,MASP1^*}}^{\mathrm{MASP1_{zym}}}}+\frac{[\mathrm{pCLK1MASP2_{zym}}]}{K_{\mathrm{m\,MASP1^*}}^{\mathrm{MASP2_{zym}}}} \\ +\frac{[\mathrm{pCLK1MASP3_{zym}}]}{K_{\mathrm{m\,MASP1^*}}^{\mathrm{MASP3_{zym}}}}+\frac{[\mathrm{pCLL1MASP1_{zym}}]}{K_{\mathrm{m\,MASP1^*}}^{\mathrm{MASP1_{zym}}}}+\frac{[\mathrm{pCLL1MASP2_{zym}}]}{K_{\mathrm{m\,MASP1^*}}^{\mathrm{MASP2_{zym}}}}+\frac{[\mathrm{pCLL1MASP3_{zym}}]}{K_{\mathrm{m\,MASP1^*}}^{\mathrm{MASP3_{zym}}}} \\ +\frac{[\mathrm{pLFMASP1_{zym}}]}{K_{\mathrm{m\,MASP1^*}}^{\mathrm{MASP1_{zym}}}}+\frac{[\mathrm{pLFMASP2_{zym}}]}{K_{\mathrm{m\,MASP1^*}}^{\mathrm{MASP2_{zym}}}}+\frac{[\mathrm{pLFMASP3_{zym}}]}{K_{\mathrm{m\,MASP1^*}}^{\mathrm{MASP3_{zym}}}}+\frac{[\mathrm{pHFMASP1_{zym}}]}{K_{\mathrm{m\,MASP1^*}}^{\mathrm{MASP1_{zym}}}}+\frac{[\mathrm{pHFMASP2_{zym}}]}{K_{\mathrm{m\,MASP1^*}}^{\mathrm{MASP2_{zym}}}} \\ +\frac{[\mathrm{pHFMASP3_{zym}}]}{K_{\mathrm{m\,MASP1^*}}^{\mathrm{MASP3_{zym}}}}+\frac{[\mathrm{pMFMASP1_{zym}}]}{K_{\mathrm{m\,MASP1^*}}^{\mathrm{MASP1_{zym}}}}+\frac{[\mathrm{pMFMASP2_{zym}}]}{K_{\mathrm{m\,MASP1^*}}^{\mathrm{MASP2_{zym}}}}+\frac{[\mathrm{pMFMASP3_{zym}}]}{K_{\mathrm{m\,MASP1^*}}^{\mathrm{MASP3_{zym}}}} \end{array}\right)}$$

$$
+\frac{k_{\mathrm{cat\,MASP1^*}}^{\mathrm{C2}}\left[\mathrm{pLFMASP1^*}\right]\left[\mathrm{fC3bC4bC2}\right]}{K_{\mathrm{m\,MASP1^*}}^{\mathrm{C2}}\left(\begin{array}{l}
1+\frac{[\mathrm{C4}]}{K_{\mathrm{m\,MASP1^*}}^{\mathrm{C4}}}+\frac{[\mathrm{fC4bC2}]}{K_{\mathrm{m\,MASP1^*}}^{\mathrm{C2}}}+\frac{[\mathrm{fC4bC4bC2}]}{K_{\mathrm{m\,MASP1^*}}^{\mathrm{C2}}}+\frac{[\mathrm{fC3bC4bC2}]}{K_{\mathrm{m\,MASP1^*}}^{\mathrm{C2}}}+\frac{[\mathrm{pC4bC2}]}{K_{\mathrm{m\,MASP1^*}}^{\mathrm{C2}}}+\frac{[\mathrm{pC4bC4bC2}]}{K_{\mathrm{m\,MASP1^*}}^{\mathrm{C2}}}\\
+\frac{[\mathrm{pC3bC4bC2}]}{K_{\mathrm{m\,MASP1^*}}^{\mathrm{C2}}}+\frac{[\mathrm{pMBL_3MASP1_{zym}}]}{K_{\mathrm{m\,MASP1^*}}^{\mathrm{MASP1_{zym}}}}+\frac{[\mathrm{pMBL_3MASP2_{zym}}]}{K_{\mathrm{m\,MASP1^*}}^{\mathrm{MASP2_{zym}}}}+\frac{[\mathrm{pMBL_3MASP3_{zym}}]}{K_{\mathrm{m\,MASP1^*}}^{\mathrm{MASP3_{zym}}}}\\
+\frac{[\mathrm{pMBL_4MASP1_{zym}}]}{K_{\mathrm{m\,MASP1^*}}^{\mathrm{MASP1_{zym}}}}+\frac{[\mathrm{pMBL_4MASP2_{zym}}]}{K_{\mathrm{m\,MASP1^*}}^{\mathrm{MASP2_{zym}}}}+\frac{[\mathrm{pMBL_4MASP3_{zym}}]}{K_{\mathrm{m\,MASP1^*}}^{\mathrm{MASP3_{zym}}}}+\frac{[\mathrm{pCLLKMASP1_{zym}}]}{K_{\mathrm{m\,MASP1^*}}^{\mathrm{MASP1_{zym}}}}\\
+\frac{[\mathrm{pCLLKMASP2_{zym}}]}{K_{\mathrm{m\,MASP1^*}}^{\mathrm{MASP2_{zym}}}}+\frac{[\mathrm{pCLLKMASP3_{zym}}]}{K_{\mathrm{m\,MASP1^*}}^{\mathrm{MASP3_{zym}}}}+\frac{[\mathrm{pCLK1MASP1_{zym}}]}{K_{\mathrm{m\,MASP1^*}}^{\mathrm{MASP1_{zym}}}}+\frac{[\mathrm{pCLK1MASP2_{zym}}]}{K_{\mathrm{m\,MASP1^*}}^{\mathrm{MASP2_{zym}}}}\\
+\frac{[\mathrm{pCLK1MASP3_{zym}}]}{K_{\mathrm{m\,MASP1^*}}^{\mathrm{MASP3_{zym}}}}+\frac{[\mathrm{pCLL1MASP1_{zym}}]}{K_{\mathrm{m\,MASP1^*}}^{\mathrm{MASP1_{zym}}}}+\frac{[\mathrm{pCLL1MASP2_{zym}}]}{K_{\mathrm{m\,MASP1^*}}^{\mathrm{MASP2_{zym}}}}+\frac{[\mathrm{pCLL1MASP3_{zym}}]}{K_{\mathrm{m\,MASP1^*}}^{\mathrm{MASP3_{zym}}}}\\
+\frac{[\mathrm{pLFMASP1_{zym}}]}{K_{\mathrm{m\,MASP1^*}}^{\mathrm{MASP1_{zym}}}}+\frac{[\mathrm{pLFMASP2_{zym}}]}{K_{\mathrm{m\,MASP1^*}}^{\mathrm{MASP2_{zym}}}}+\frac{[\mathrm{pLFMASP3_{zym}}]}{K_{\mathrm{m\,MASP1^*}}^{\mathrm{MASP3_{zym}}}}+\frac{[\mathrm{pHFMASP1_{zym}}]}{K_{\mathrm{m\,MASP1^*}}^{\mathrm{MASP1_{zym}}}}+\frac{[\mathrm{pHFMASP2_{zym}}]}{K_{\mathrm{m\,MASP1^*}}^{\mathrm{MASP2_{zym}}}}\\
+\frac{[\mathrm{pHFMASP3_{zym}}]}{K_{\mathrm{m\,MASP1^*}}^{\mathrm{MASP3_{zym}}}}+\frac{[\mathrm{pMFMASP1_{zym}}]}{K_{\mathrm{m\,MASP1^*}}^{\mathrm{MASP1_{zym}}}}+\frac{[\mathrm{pMFMASP2_{zym}}]}{K_{\mathrm{m\,MASP1^*}}^{\mathrm{MASP2_{zym}}}}+\frac{[\mathrm{pMFMASP3_{zym}}]}{K_{\mathrm{m\,MASP1^*}}^{\mathrm{MASP3_{zym}}}}
\end{array}\right)}
$$

$$
+\frac{k_{\mathrm{cat\,MASP1^*}}^{\mathrm{C2}}\left[\mathrm{pHFMASP1^*}\right]\left[\mathrm{fC3bC4bC2}\right]}{K_{\mathrm{m\,MASP1^*}}^{\mathrm{C2}}\left(\begin{array}{l}
1+\frac{[\mathrm{C4}]}{K_{\mathrm{m\,MASP1^*}}^{\mathrm{C4}}}+\frac{[\mathrm{fC4bC2}]}{K_{\mathrm{m\,MASP1^*}}^{\mathrm{C2}}}+\frac{[\mathrm{fC4bC4bC2}]}{K_{\mathrm{m\,MASP1^*}}^{\mathrm{C2}}}+\frac{[\mathrm{fC3bC4bC2}]}{K_{\mathrm{m\,MASP1^*}}^{\mathrm{C2}}}+\frac{[\mathrm{pC4bC2}]}{K_{\mathrm{m\,MASP1^*}}^{\mathrm{C2}}}+\frac{[\mathrm{pC4bC4bC2}]}{K_{\mathrm{m\,MASP1^*}}^{\mathrm{C2}}}\\
+\frac{[\mathrm{pC3bC4bC2}]}{K_{\mathrm{m\,MASP1^*}}^{\mathrm{C2}}}+\frac{[\mathrm{pMBL_3MASP1_{zym}}]}{K_{\mathrm{m\,MASP1^*}}^{\mathrm{MASP1_{zym}}}}+\frac{[\mathrm{pMBL_3MASP2_{zym}}]}{K_{\mathrm{m\,MASP1^*}}^{\mathrm{MASP2_{zym}}}}+\frac{[\mathrm{pMBL_3MASP3_{zym}}]}{K_{\mathrm{m\,MASP1^*}}^{\mathrm{MASP3_{zym}}}}\\
+\frac{[\mathrm{pMBL_4MASP1_{zym}}]}{K_{\mathrm{m\,MASP1^*}}^{\mathrm{MASP1_{zym}}}}+\frac{[\mathrm{pMBL_4MASP2_{zym}}]}{K_{\mathrm{m\,MASP1^*}}^{\mathrm{MASP2_{zym}}}}+\frac{[\mathrm{pMBL_4MASP3_{zym}}]}{K_{\mathrm{m\,MASP1^*}}^{\mathrm{MASP3_{zym}}}}+\frac{[\mathrm{pCLLKMASP1_{zym}}]}{K_{\mathrm{m\,MASP1^*}}^{\mathrm{MASP1_{zym}}}}\\
+\frac{[\mathrm{pCLLKMASP2_{zym}}]}{K_{\mathrm{m\,MASP1^*}}^{\mathrm{MASP2_{zym}}}}+\frac{[\mathrm{pCLLKMASP3_{zym}}]}{K_{\mathrm{m\,MASP1^*}}^{\mathrm{MASP3_{zym}}}}+\frac{[\mathrm{pCLK1MASP1_{zym}}]}{K_{\mathrm{m\,MASP1^*}}^{\mathrm{MASP1_{zym}}}}+\frac{[\mathrm{pCLK1MASP2_{zym}}]}{K_{\mathrm{m\,MASP1^*}}^{\mathrm{MASP2_{zym}}}}\\
+\frac{[\mathrm{pCLK1MASP3_{zym}}]}{K_{\mathrm{m\,MASP1^*}}^{\mathrm{MASP3_{zym}}}}+\frac{[\mathrm{pCLL1MASP1_{zym}}]}{K_{\mathrm{m\,MASP1^*}}^{\mathrm{MASP1_{zym}}}}+\frac{[\mathrm{pCLL1MASP2_{zym}}]}{K_{\mathrm{m\,MASP1^*}}^{\mathrm{MASP2_{zym}}}}+\frac{[\mathrm{pCLL1MASP3_{zym}}]}{K_{\mathrm{m\,MASP1^*}}^{\mathrm{MASP3_{zym}}}}\\
+\frac{[\mathrm{pLFMASP1_{zym}}]}{K_{\mathrm{m\,MASP1^*}}^{\mathrm{MASP1_{zym}}}}+\frac{[\mathrm{pLFMASP2_{zym}}]}{K_{\mathrm{m\,MASP1^*}}^{\mathrm{MASP2_{zym}}}}+\frac{[\mathrm{pLFMASP3_{zym}}]}{K_{\mathrm{m\,MASP1^*}}^{\mathrm{MASP3_{zym}}}}+\frac{[\mathrm{pHFMASP1_{zym}}]}{K_{\mathrm{m\,MASP1^*}}^{\mathrm{MASP1_{zym}}}}+\frac{[\mathrm{pHFMASP2_{zym}}]}{K_{\mathrm{m\,MASP1^*}}^{\mathrm{MASP2_{zym}}}}\\
+\frac{[\mathrm{pHFMASP3_{zym}}]}{K_{\mathrm{m\,MASP1^*}}^{\mathrm{MASP3_{zym}}}}+\frac{[\mathrm{pMFMASP1_{zym}}]}{K_{\mathrm{m\,MASP1^*}}^{\mathrm{MASP1_{zym}}}}+\frac{[\mathrm{pMFMASP2_{zym}}]}{K_{\mathrm{m\,MASP1^*}}^{\mathrm{MASP2_{zym}}}}+\frac{[\mathrm{pMFMASP3_{zym}}]}{K_{\mathrm{m\,MASP1^*}}^{\mathrm{MASP3_{zym}}}}
\end{array}\right)}
$$

$$
+\frac{k^{\mathrm{C2}}_{\mathrm{cat\,MASP1^*}}\left[\mathrm{pMFMASP1^*}\right]\left[\mathrm{fC3bC4bC2}\right]}{K^{\mathrm{C2}}_{\mathrm{m\,MASP1^*}}\left(\begin{aligned}
&1+\frac{[\mathrm{C4}]}{K^{\mathrm{C4}}_{\mathrm{m\,MASP1^*}}}+\frac{[\mathrm{fC4bC2}]}{K^{\mathrm{C2}}_{\mathrm{m\,MASP1^*}}}+\frac{[\mathrm{fC4bC4bC2}]}{K^{\mathrm{C2}}_{\mathrm{m\,MASP1^*}}}+\frac{[\mathrm{fC3bC4bC2}]}{K^{\mathrm{C2}}_{\mathrm{m\,MASP1^*}}}+\frac{[\mathrm{pC4bC2}]}{K^{\mathrm{C2}}_{\mathrm{m\,MASP1^*}}}+\frac{[\mathrm{pC4bC4bC2}]}{K^{\mathrm{C2}}_{\mathrm{m\,MASP1^*}}}\\
&+\frac{[\mathrm{pC3bC4bC2}]}{K^{\mathrm{C2}}_{\mathrm{m\,MASP1^*}}}+\frac{[\mathrm{pMBL_3MASP1_{zym}}]}{K^{\mathrm{MASP1_{zym}}}_{\mathrm{m\,MASP1^*}}}+\frac{[\mathrm{pMBL_3MASP2_{zym}}]}{K^{\mathrm{MASP2_{zym}}}_{\mathrm{m\,MASP1^*}}}+\frac{[\mathrm{pMBL_3MASP3_{zym}}]}{K^{\mathrm{MASP3_{zym}}}_{\mathrm{m\,MASP1^*}}}\\
&+\frac{[\mathrm{pMBL_4MASP1_{zym}}]}{K^{\mathrm{MASP1_{zym}}}_{\mathrm{m\,MASP1^*}}}+\frac{[\mathrm{pMBL_4MASP2_{zym}}]}{K^{\mathrm{MASP2_{zym}}}_{\mathrm{m\,MASP1^*}}}+\frac{[\mathrm{pMBL_4MASP3_{zym}}]}{K^{\mathrm{MASP3_{zym}}}_{\mathrm{m\,MASP1^*}}}+\frac{[\mathrm{pCLLKMASP1_{zym}}]}{K^{\mathrm{MASP1_{zym}}}_{\mathrm{m\,MASP1^*}}}\\
&+\frac{[\mathrm{pCLLKMASP2_{zym}}]}{K^{\mathrm{MASP2_{zym}}}_{\mathrm{m\,MASP1^*}}}+\frac{[\mathrm{pCLLKMASP3_{zym}}]}{K^{\mathrm{MASP3_{zym}}}_{\mathrm{m\,MASP1^*}}}+\frac{[\mathrm{pCLK1MASP1_{zym}}]}{K^{\mathrm{MASP1_{zym}}}_{\mathrm{m\,MASP1^*}}}+\frac{[\mathrm{pCLK1MASP2_{zym}}]}{K^{\mathrm{MASP2_{zym}}}_{\mathrm{m\,MASP1^*}}}\\
&+\frac{[\mathrm{pCLK1MASP3_{zym}}]}{K^{\mathrm{MASP3_{zym}}}_{\mathrm{m\,MASP1^*}}}+\frac{[\mathrm{pCLL1MASP1_{zym}}]}{K^{\mathrm{MASP1_{zym}}}_{\mathrm{m\,MASP1^*}}}+\frac{[\mathrm{pCLL1MASP2_{zym}}]}{K^{\mathrm{MASP2_{zym}}}_{\mathrm{m\,MASP1^*}}}+\frac{[\mathrm{pCLL1MASP3_{zym}}]}{K^{\mathrm{MASP3_{zym}}}_{\mathrm{m\,MASP1^*}}}\\
&+\frac{[\mathrm{pLFMASP1_{zym}}]}{K^{\mathrm{MASP1_{zym}}}_{\mathrm{m\,MASP1^*}}}+\frac{[\mathrm{pLFMASP2_{zym}}]}{K^{\mathrm{MASP2_{zym}}}_{\mathrm{m\,MASP1^*}}}+\frac{[\mathrm{pLFMASP3_{zym}}]}{K^{\mathrm{MASP3_{zym}}}_{\mathrm{m\,MASP1^*}}}+\frac{[\mathrm{pHFMASP1_{zym}}]}{K^{\mathrm{MASP1_{zym}}}_{\mathrm{m\,MASP1^*}}}+\frac{[\mathrm{pHFMASP2_{zym}}]}{K^{\mathrm{MASP2_{zym}}}_{\mathrm{m\,MASP1^*}}}\\
&+\frac{[\mathrm{pHFMASP3_{zym}}]}{K^{\mathrm{MASP3_{zym}}}_{\mathrm{m\,MASP1^*}}}+\frac{[\mathrm{pMFMASP1_{zym}}]}{K^{\mathrm{MASP1_{zym}}}_{\mathrm{m\,MASP1^*}}}+\frac{[\mathrm{pMFMASP2_{zym}}]}{K^{\mathrm{MASP2_{zym}}}_{\mathrm{m\,MASP1^*}}}+\frac{[\mathrm{pMFMASP3_{zym}}]}{K^{\mathrm{MASP3_{zym}}}_{\mathrm{m\,MASP1^*}}}
\end{aligned}\right)}
$$

$$
+\frac{k^{\mathrm{C2}}_{\mathrm{cat\,MASP2^*}}\left[\mathrm{pMBL_3MASP2^*}\right]\left[\mathrm{fC3bC4bC2}\right]}{K^{\mathrm{C2}}_{\mathrm{m\,MASP2^*}}\left(\begin{aligned}
&1+\frac{[\mathrm{C4}]}{K^{\mathrm{C4}}_{\mathrm{m\,MASP2^*}}}+\frac{[\mathrm{fC4bC2}]}{K^{\mathrm{C2}}_{\mathrm{m\,MASP2^*}}}+\frac{[\mathrm{fC4bC4bC2}]}{K^{\mathrm{C2}}_{\mathrm{m\,MASP2^*}}}+\frac{[\mathrm{fC3bC4bC2}]}{K^{\mathrm{C2}}_{\mathrm{m\,MASP2^*}}}+\frac{[\mathrm{pC4bC2}]}{K^{\mathrm{C2}}_{\mathrm{m\,MASP2^*}}}+\frac{[\mathrm{pC4bC4bC2}]}{K^{\mathrm{C2}}_{\mathrm{m\,MASP2^*}}}\\
&+\frac{[\mathrm{pC3bC4bC2}]}{K^{\mathrm{C2}}_{\mathrm{m\,MASP2^*}}}+\frac{[\mathrm{pMBL_3MASP1_{zym}}]}{K^{\mathrm{MASP1_{zym}}}_{\mathrm{m\,MASP2^*}}}+\frac{[\mathrm{pMBL_3MASP2_{zym}}]}{K^{\mathrm{MASP2_{zym}}}_{\mathrm{m\,MASP2^*}}}+\frac{[\mathrm{pMBL_4MASP1_{zym}}]}{K^{\mathrm{MASP1_{zym}}}_{\mathrm{m\,MASP2^*}}}\\
&+\frac{[\mathrm{pMBL_4MASP2_{zym}}]}{K^{\mathrm{MASP2_{zym}}}_{\mathrm{m\,MASP2^*}}}+\frac{[\mathrm{pCLLKMASP1_{zym}}]}{K^{\mathrm{MASP1_{zym}}}_{\mathrm{m\,MASP2^*}}}+\frac{[\mathrm{pCLLKMASP2_{zym}}]}{K^{\mathrm{MASP2_{zym}}}_{\mathrm{m\,MASP2^*}}}+\frac{[\mathrm{pCLK1MASP1_{zym}}]}{K^{\mathrm{MASP1_{zym}}}_{\mathrm{m\,MASP2^*}}}\\
&+\frac{[\mathrm{pCLK1MASP2_{zym}}]}{K^{\mathrm{MASP2_{zym}}}_{\mathrm{m\,MASP2^*}}}+\frac{[\mathrm{pCLL1MASP1_{zym}}]}{K^{\mathrm{MASP1_{zym}}}_{\mathrm{m\,MASP2^*}}}+\frac{[\mathrm{pCLL1MASP2_{zym}}]}{K^{\mathrm{MASP2_{zym}}}_{\mathrm{m\,MASP2^*}}}+\frac{[\mathrm{pLFMASP1_{zym}}]}{K^{\mathrm{MASP1_{zym}}}_{\mathrm{m\,MASP2^*}}}\\
&+\frac{[\mathrm{pLFMASP2_{zym}}]}{K^{\mathrm{MASP2_{zym}}}_{\mathrm{m\,MASP2^*}}}+\frac{[\mathrm{pHFMASP1_{zym}}]}{K^{\mathrm{MASP1_{zym}}}_{\mathrm{m\,MASP2^*}}}+\frac{[\mathrm{pHFMASP2_{zym}}]}{K^{\mathrm{MASP2_{zym}}}_{\mathrm{m\,MASP2^*}}}+\frac{[\mathrm{pMFMASP1_{zym}}]}{K^{\mathrm{MASP1_{zym}}}_{\mathrm{m\,MASP2^*}}}+\frac{[\mathrm{pMFMASP2_{zym}}]}{K^{\mathrm{MASP2_{zym}}}_{\mathrm{m\,MASP2^*}}}
\end{aligned}\right)}
$$

$$+\frac{k^{\mathrm{C2}}_{\mathrm{cat\,MASP2^*}}\left[\mathrm{pMBL_4MASP2^*}\right]\left[\mathrm{fC3bC4bC2}\right]}{K^{\mathrm{C2}}_{\mathrm{m\,MASP2^*}}\left(\begin{aligned}&1+\frac{[\mathrm{C4}]}{K^{\mathrm{C4}}_{\mathrm{m\,MASP2^*}}}+\frac{[\mathrm{fC4bC2}]}{K^{\mathrm{C2}}_{\mathrm{m\,MASP2^*}}}+\frac{[\mathrm{fC4bC4bC2}]}{K^{\mathrm{C2}}_{\mathrm{m\,MASP2^*}}}+\frac{[\mathrm{fC3bC4bC2}]}{K^{\mathrm{C2}}_{\mathrm{m\,MASP2^*}}}+\frac{[\mathrm{pC4bC2}]}{K^{\mathrm{C2}}_{\mathrm{m\,MASP2^*}}}+\frac{[\mathrm{pC4bC4bC2}]}{K^{\mathrm{C2}}_{\mathrm{m\,MASP2^*}}}\\&+\frac{[\mathrm{pC3bC4bC2}]}{K^{\mathrm{C2}}_{\mathrm{m\,MASP2^*}}}+\frac{[\mathrm{pMBL_3MASP1_{zym}}]}{K^{\mathrm{MASP1_{zym}}}_{\mathrm{m\,MASP2^*}}}+\frac{[\mathrm{pMBL_3MASP2_{zym}}]}{K^{\mathrm{MASP2_{zym}}}_{\mathrm{m\,MASP2^*}}}+\frac{[\mathrm{pMBL_4MASP1_{zym}}]}{K^{\mathrm{MASP1_{zym}}}_{\mathrm{m\,MASP2^*}}}\\&+\frac{[\mathrm{pMBL_4MASP2_{zym}}]}{K^{\mathrm{MASP2_{zym}}}_{\mathrm{m\,MASP2^*}}}+\frac{[\mathrm{pCLLKMASP1_{zym}}]}{K^{\mathrm{MASP1_{zym}}}_{\mathrm{m\,MASP2^*}}}+\frac{[\mathrm{pCLLKMASP2_{zym}}]}{K^{\mathrm{MASP2_{zym}}}_{\mathrm{m\,MASP2^*}}}+\frac{[\mathrm{pCLK1MASP1_{zym}}]}{K^{\mathrm{MASP1_{zym}}}_{\mathrm{m\,MASP2^*}}}\\&+\frac{[\mathrm{pCLK1MASP2_{zym}}]}{K^{\mathrm{MASP2_{zym}}}_{\mathrm{m\,MASP2^*}}}+\frac{[\mathrm{pCLL1MASP1_{zym}}]}{K^{\mathrm{MASP1_{zym}}}_{\mathrm{m\,MASP2^*}}}+\frac{[\mathrm{pCLL1MASP2_{zym}}]}{K^{\mathrm{MASP2_{zym}}}_{\mathrm{m\,MASP2^*}}}+\frac{[\mathrm{pLFMASP1_{zym}}]}{K^{\mathrm{MASP1_{zym}}}_{\mathrm{m\,MASP2^*}}}\\&+\frac{[\mathrm{pLFMASP2_{zym}}]}{K^{\mathrm{MASP2_{zym}}}_{\mathrm{m\,MASP2^*}}}+\frac{[\mathrm{pHFMASP1_{zym}}]}{K^{\mathrm{MASP1_{zym}}}_{\mathrm{m\,MASP2^*}}}+\frac{[\mathrm{pHFMASP2_{zym}}]}{K^{\mathrm{MASP2_{zym}}}_{\mathrm{m\,MASP2^*}}}+\frac{[\mathrm{pMFMASP1_{zym}}]}{K^{\mathrm{MASP1_{zym}}}_{\mathrm{m\,MASP2^*}}}+\frac{[\mathrm{pMFMASP2_{zym}}]}{K^{\mathrm{MASP2_{zym}}}_{\mathrm{m\,MASP2^*}}}\end{aligned}\right)}$$

$$+\frac{k^{\mathrm{C2}}_{\mathrm{cat\,MASP2^*}}\left[\mathrm{pCLLKMASP2^*}\right]\left[\mathrm{fC3bC4bC2}\right]}{K^{\mathrm{C2}}_{\mathrm{m\,MASP2^*}}\left(\begin{aligned}&1+\frac{[\mathrm{C4}]}{K^{\mathrm{C4}}_{\mathrm{m\,MASP2^*}}}+\frac{[\mathrm{fC4bC2}]}{K^{\mathrm{C2}}_{\mathrm{m\,MASP2^*}}}+\frac{[\mathrm{fC4bC4bC2}]}{K^{\mathrm{C2}}_{\mathrm{m\,MASP2^*}}}+\frac{[\mathrm{fC3bC4bC2}]}{K^{\mathrm{C2}}_{\mathrm{m\,MASP2^*}}}+\frac{[\mathrm{pC4bC2}]}{K^{\mathrm{C2}}_{\mathrm{m\,MASP2^*}}}+\frac{[\mathrm{pC4bC4bC2}]}{K^{\mathrm{C2}}_{\mathrm{m\,MASP2^*}}}\\&+\frac{[\mathrm{pC3bC4bC2}]}{K^{\mathrm{C2}}_{\mathrm{m\,MASP2^*}}}+\frac{[\mathrm{pMBL_3MASP1_{zym}}]}{K^{\mathrm{MASP1_{zym}}}_{\mathrm{m\,MASP2^*}}}+\frac{[\mathrm{pMBL_3MASP2_{zym}}]}{K^{\mathrm{MASP2_{zym}}}_{\mathrm{m\,MASP2^*}}}+\frac{[\mathrm{pMBL_4MASP1_{zym}}]}{K^{\mathrm{MASP1_{zym}}}_{\mathrm{m\,MASP2^*}}}\\&+\frac{[\mathrm{pMBL_4MASP2_{zym}}]}{K^{\mathrm{MASP2_{zym}}}_{\mathrm{m\,MASP2^*}}}+\frac{[\mathrm{pCLLKMASP1_{zym}}]}{K^{\mathrm{MASP1_{zym}}}_{\mathrm{m\,MASP2^*}}}+\frac{[\mathrm{pCLLKMASP2_{zym}}]}{K^{\mathrm{MASP2_{zym}}}_{\mathrm{m\,MASP2^*}}}+\frac{[\mathrm{pCLK1MASP1_{zym}}]}{K^{\mathrm{MASP1_{zym}}}_{\mathrm{m\,MASP2^*}}}\\&+\frac{[\mathrm{pCLK1MASP2_{zym}}]}{K^{\mathrm{MASP2_{zym}}}_{\mathrm{m\,MASP2^*}}}+\frac{[\mathrm{pCLL1MASP1_{zym}}]}{K^{\mathrm{MASP1_{zym}}}_{\mathrm{m\,MASP2^*}}}+\frac{[\mathrm{pCLL1MASP2_{zym}}]}{K^{\mathrm{MASP2_{zym}}}_{\mathrm{m\,MASP2^*}}}+\frac{[\mathrm{pLFMASP1_{zym}}]}{K^{\mathrm{MASP1_{zym}}}_{\mathrm{m\,MASP2^*}}}\\&+\frac{[\mathrm{pLFMASP2_{zym}}]}{K^{\mathrm{MASP2_{zym}}}_{\mathrm{m\,MASP2^*}}}+\frac{[\mathrm{pHFMASP1_{zym}}]}{K^{\mathrm{MASP1_{zym}}}_{\mathrm{m\,MASP2^*}}}+\frac{[\mathrm{pHFMASP2_{zym}}]}{K^{\mathrm{MASP2_{zym}}}_{\mathrm{m\,MASP2^*}}}+\frac{[\mathrm{pMFMASP1_{zym}}]}{K^{\mathrm{MASP1_{zym}}}_{\mathrm{m\,MASP2^*}}}+\frac{[\mathrm{pMFMASP2_{zym}}]}{K^{\mathrm{MASP2_{zym}}}_{\mathrm{m\,MASP2^*}}}\end{aligned}\right)}$$

$$+\frac{k^{\mathrm{C2}}_{\mathrm{cat\,MASP2^*}}\left[\mathrm{pCLK1MASP2^*}\right]\left[\mathrm{fC3bC4bC2}\right]}{K^{\mathrm{C2}}_{\mathrm{m\,MASP2^*}}\left(\begin{aligned}&1+\frac{[\mathrm{C4}]}{K^{\mathrm{C4}}_{\mathrm{m\,MASP2^*}}}+\frac{[\mathrm{fC4bC2}]}{K^{\mathrm{C2}}_{\mathrm{m\,MASP2^*}}}+\frac{[\mathrm{fC4bC4bC2}]}{K^{\mathrm{C2}}_{\mathrm{m\,MASP2^*}}}+\frac{[\mathrm{fC3bC4bC2}]}{K^{\mathrm{C2}}_{\mathrm{m\,MASP2^*}}}+\frac{[\mathrm{pC4bC2}]}{K^{\mathrm{C2}}_{\mathrm{m\,MASP2^*}}}+\frac{[\mathrm{pC4bC4bC2}]}{K^{\mathrm{C2}}_{\mathrm{m\,MASP2^*}}}\\&+\frac{[\mathrm{pC3bC4bC2}]}{K^{\mathrm{C2}}_{\mathrm{m\,MASP2^*}}}+\frac{[\mathrm{pMBL_3MASP1_{zym}}]}{K^{\mathrm{MASP1_{zym}}}_{\mathrm{m\,MASP2^*}}}+\frac{[\mathrm{pMBL_3MASP2_{zym}}]}{K^{\mathrm{MASP2_{zym}}}_{\mathrm{m\,MASP2^*}}}+\frac{[\mathrm{pMBL_4MASP1_{zym}}]}{K^{\mathrm{MASP1_{zym}}}_{\mathrm{m\,MASP2^*}}}\\&+\frac{[\mathrm{pMBL_4MASP2_{zym}}]}{K^{\mathrm{MASP2_{zym}}}_{\mathrm{m\,MASP2^*}}}+\frac{[\mathrm{pCLLKMASP1_{zym}}]}{K^{\mathrm{MASP1_{zym}}}_{\mathrm{m\,MASP2^*}}}+\frac{[\mathrm{pCLLKMASP2_{zym}}]}{K^{\mathrm{MASP2_{zym}}}_{\mathrm{m\,MASP2^*}}}+\frac{[\mathrm{pCLK1MASP1_{zym}}]}{K^{\mathrm{MASP1_{zym}}}_{\mathrm{m\,MASP2^*}}}\\&+\frac{[\mathrm{pCLK1MASP2_{zym}}]}{K^{\mathrm{MASP2_{zym}}}_{\mathrm{m\,MASP2^*}}}+\frac{[\mathrm{pCLL1MASP1_{zym}}]}{K^{\mathrm{MASP1_{zym}}}_{\mathrm{m\,MASP2^*}}}+\frac{[\mathrm{pCLL1MASP2_{zym}}]}{K^{\mathrm{MASP2_{zym}}}_{\mathrm{m\,MASP2^*}}}+\frac{[\mathrm{pLFMASP1_{zym}}]}{K^{\mathrm{MASP1_{zym}}}_{\mathrm{m\,MASP2^*}}}\\&+\frac{[\mathrm{pLFMASP2_{zym}}]}{K^{\mathrm{MASP2_{zym}}}_{\mathrm{m\,MASP2^*}}}+\frac{[\mathrm{pHFMASP1_{zym}}]}{K^{\mathrm{MASP1_{zym}}}_{\mathrm{m\,MASP2^*}}}+\frac{[\mathrm{pHFMASP2_{zym}}]}{K^{\mathrm{MASP2_{zym}}}_{\mathrm{m\,MASP2^*}}}+\frac{[\mathrm{pMFMASP1_{zym}}]}{K^{\mathrm{MASP1_{zym}}}_{\mathrm{m\,MASP2^*}}}+\frac{[\mathrm{pMFMASP2_{zym}}]}{K^{\mathrm{MASP2_{zym}}}_{\mathrm{m\,MASP2^*}}}\end{aligned}\right)}$$

$$
+\frac{k^{\mathrm{C2}}_{\mathrm{cat\ MASP2^*}}\left[\mathrm{pCLL1MASP2^*}\right]\left[\mathrm{fC3bC4bC2}\right]}{K^{\mathrm{C2}}_{\mathrm{m\ MASP2^*}}\left(\begin{array}{l}1+\frac{\left[\mathrm{C4}\right]}{K^{\mathrm{C4}}_{\mathrm{m\ MASP2^*}}}+\frac{\left[\mathrm{fC4bC2}\right]}{K^{\mathrm{C2}}_{\mathrm{m\ MASP2^*}}}+\frac{\left[\mathrm{fC4bC4bC2}\right]}{K^{\mathrm{C2}}_{\mathrm{m\ MASP2^*}}}+\frac{\left[\mathrm{fC3bC4bC2}\right]}{K^{\mathrm{C2}}_{\mathrm{m\ MASP2^*}}}+\frac{\left[\mathrm{pC4bC2}\right]}{K^{\mathrm{C2}}_{\mathrm{m\ MASP2^*}}}+\frac{\left[\mathrm{pC4bC4bC2}\right]}{K^{\mathrm{C2}}_{\mathrm{m\ MASP2^*}}}\\
+\frac{\left[\mathrm{pC3bC4bC2}\right]}{K^{\mathrm{C2}}_{\mathrm{m\ MASP2^*}}}+\frac{\left[\mathrm{pMBL_3MASP1_{zym}}\right]}{K^{\mathrm{MASP1_{zym}}}_{\mathrm{m\ MASP2^*}}}+\frac{\left[\mathrm{pMBL_3MASP2_{zym}}\right]}{K^{\mathrm{MASP2_{zym}}}_{\mathrm{m\ MASP2^*}}}+\frac{\left[\mathrm{pMBL_4MASP1_{zym}}\right]}{K^{\mathrm{MASP1_{zym}}}_{\mathrm{m\ MASP2^*}}}\\
+\frac{\left[\mathrm{pMBL_4MASP2_{zym}}\right]}{K^{\mathrm{MASP2_{zym}}}_{\mathrm{m\ MASP2^*}}}+\frac{\left[\mathrm{pCLLKMASP1_{zym}}\right]}{K^{\mathrm{MASP1_{zym}}}_{\mathrm{m\ MASP2^*}}}+\frac{\left[\mathrm{pCLLKMASP2_{zym}}\right]}{K^{\mathrm{MASP2_{zym}}}_{\mathrm{m\ MASP2^*}}}+\frac{\left[\mathrm{pCLK1MASP1_{zym}}\right]}{K^{\mathrm{MASP1_{zym}}}_{\mathrm{m\ MASP2^*}}}\\
+\frac{\left[\mathrm{pCLK1MASP2_{zym}}\right]}{K^{\mathrm{MASP2_{zym}}}_{\mathrm{m\ MASP2^*}}}+\frac{\left[\mathrm{pCLL1MASP1_{zym}}\right]}{K^{\mathrm{MASP1_{zym}}}_{\mathrm{m\ MASP2^*}}}+\frac{\left[\mathrm{pCLL1MASP2_{zym}}\right]}{K^{\mathrm{MASP2_{zym}}}_{\mathrm{m\ MASP2^*}}}+\frac{\left[\mathrm{pLFMASP1_{zym}}\right]}{K^{\mathrm{MASP1_{zym}}}_{\mathrm{m\ MASP2^*}}}\\
+\frac{\left[\mathrm{pLFMASP2_{zym}}\right]}{K^{\mathrm{MASP2_{zym}}}_{\mathrm{m\ MASP2^*}}}+\frac{\left[\mathrm{pHFMASP1_{zym}}\right]}{K^{\mathrm{MASP1_{zym}}}_{\mathrm{m\ MASP2^*}}}+\frac{\left[\mathrm{pHFMASP2_{zym}}\right]}{K^{\mathrm{MASP2_{zym}}}_{\mathrm{m\ MASP2^*}}}+\frac{\left[\mathrm{pMFMASP1_{zym}}\right]}{K^{\mathrm{MASP1_{zym}}}_{\mathrm{m\ MASP2^*}}}+\frac{\left[\mathrm{pMFMASP2_{zym}}\right]}{K^{\mathrm{MASP2_{zym}}}_{\mathrm{m\ MASP2^*}}}\end{array}\right)}
$$

$$
+\frac{k^{\mathrm{C2}}_{\mathrm{cat\ MASP2^*}}\left[\mathrm{pLFMASP2^*}\right]\left[\mathrm{fC3bC4bC2}\right]}{K^{\mathrm{C2}}_{\mathrm{m\ MASP2^*}}\left(\begin{array}{l}1+\frac{\left[\mathrm{C4}\right]}{K^{\mathrm{C4}}_{\mathrm{m\ MASP2^*}}}+\frac{\left[\mathrm{fC4bC2}\right]}{K^{\mathrm{C2}}_{\mathrm{m\ MASP2^*}}}+\frac{\left[\mathrm{fC4bC4bC2}\right]}{K^{\mathrm{C2}}_{\mathrm{m\ MASP2^*}}}+\frac{\left[\mathrm{fC3bC4bC2}\right]}{K^{\mathrm{C2}}_{\mathrm{m\ MASP2^*}}}+\frac{\left[\mathrm{pC4bC2}\right]}{K^{\mathrm{C2}}_{\mathrm{m\ MASP2^*}}}+\frac{\left[\mathrm{pC4bC4bC2}\right]}{K^{\mathrm{C2}}_{\mathrm{m\ MASP2^*}}}\\
+\frac{\left[\mathrm{pC3bC4bC2}\right]}{K^{\mathrm{C2}}_{\mathrm{m\ MASP2^*}}}+\frac{\left[\mathrm{pMBL_3MASP1_{zym}}\right]}{K^{\mathrm{MASP1_{zym}}}_{\mathrm{m\ MASP2^*}}}+\frac{\left[\mathrm{pMBL_3MASP2_{zym}}\right]}{K^{\mathrm{MASP2_{zym}}}_{\mathrm{m\ MASP2^*}}}+\frac{\left[\mathrm{pMBL_4MASP1_{zym}}\right]}{K^{\mathrm{MASP1_{zym}}}_{\mathrm{m\ MASP2^*}}}\\
+\frac{\left[\mathrm{pMBL_4MASP2_{zym}}\right]}{K^{\mathrm{MASP2_{zym}}}_{\mathrm{m\ MASP2^*}}}+\frac{\left[\mathrm{pCLLKMASP1_{zym}}\right]}{K^{\mathrm{MASP1_{zym}}}_{\mathrm{m\ MASP2^*}}}+\frac{\left[\mathrm{pCLLKMASP2_{zym}}\right]}{K^{\mathrm{MASP2_{zym}}}_{\mathrm{m\ MASP2^*}}}+\frac{\left[\mathrm{pCLK1MASP1_{zym}}\right]}{K^{\mathrm{MASP1_{zym}}}_{\mathrm{m\ MASP2^*}}}\\
+\frac{\left[\mathrm{pCLK1MASP2_{zym}}\right]}{K^{\mathrm{MASP2_{zym}}}_{\mathrm{m\ MASP2^*}}}+\frac{\left[\mathrm{pCLL1MASP1_{zym}}\right]}{K^{\mathrm{MASP1_{zym}}}_{\mathrm{m\ MASP2^*}}}+\frac{\left[\mathrm{pCLL1MASP2_{zym}}\right]}{K^{\mathrm{MASP2_{zym}}}_{\mathrm{m\ MASP2^*}}}+\frac{\left[\mathrm{pLFMASP1_{zym}}\right]}{K^{\mathrm{MASP1_{zym}}}_{\mathrm{m\ MASP2^*}}}\\
+\frac{\left[\mathrm{pLFMASP2_{zym}}\right]}{K^{\mathrm{MASP2_{zym}}}_{\mathrm{m\ MASP2^*}}}+\frac{\left[\mathrm{pHFMASP1_{zym}}\right]}{K^{\mathrm{MASP1_{zym}}}_{\mathrm{m\ MASP2^*}}}+\frac{\left[\mathrm{pHFMASP2_{zym}}\right]}{K^{\mathrm{MASP2_{zym}}}_{\mathrm{m\ MASP2^*}}}+\frac{\left[\mathrm{pMFMASP1_{zym}}\right]}{K^{\mathrm{MASP1_{zym}}}_{\mathrm{m\ MASP2^*}}}+\frac{\left[\mathrm{pMFMASP2_{zym}}\right]}{K^{\mathrm{MASP2_{zym}}}_{\mathrm{m\ MASP2^*}}}\end{array}\right)}
$$

$$
+\frac{k^{\mathrm{C2}}_{\mathrm{cat\ MASP2^*}}\left[\mathrm{pHFMASP2^*}\right]\left[\mathrm{fC3bC4bC2}\right]}{K^{\mathrm{C2}}_{\mathrm{m\ MASP2^*}}\left(\begin{array}{l}1+\frac{\left[\mathrm{C4}\right]}{K^{\mathrm{C4}}_{\mathrm{m\ MASP2^*}}}+\frac{\left[\mathrm{fC4bC2}\right]}{K^{\mathrm{C2}}_{\mathrm{m\ MASP2^*}}}+\frac{\left[\mathrm{fC4bC4bC2}\right]}{K^{\mathrm{C2}}_{\mathrm{m\ MASP2^*}}}+\frac{\left[\mathrm{fC3bC4bC2}\right]}{K^{\mathrm{C2}}_{\mathrm{m\ MASP2^*}}}+\frac{\left[\mathrm{pC4bC2}\right]}{K^{\mathrm{C2}}_{\mathrm{m\ MASP2^*}}}+\frac{\left[\mathrm{pC4bC4bC2}\right]}{K^{\mathrm{C2}}_{\mathrm{m\ MASP2^*}}}\\
+\frac{\left[\mathrm{pC3bC4bC2}\right]}{K^{\mathrm{C2}}_{\mathrm{m\ MASP2^*}}}+\frac{\left[\mathrm{pMBL_3MASP1_{zym}}\right]}{K^{\mathrm{MASP1_{zym}}}_{\mathrm{m\ MASP2^*}}}+\frac{\left[\mathrm{pMBL_3MASP2_{zym}}\right]}{K^{\mathrm{MASP2_{zym}}}_{\mathrm{m\ MASP2^*}}}+\frac{\left[\mathrm{pMBL_4MASP1_{zym}}\right]}{K^{\mathrm{MASP1_{zym}}}_{\mathrm{m\ MASP2^*}}}\\
+\frac{\left[\mathrm{pMBL_4MASP2_{zym}}\right]}{K^{\mathrm{MASP2_{zym}}}_{\mathrm{m\ MASP2^*}}}+\frac{\left[\mathrm{pCLLKMASP1_{zym}}\right]}{K^{\mathrm{MASP1_{zym}}}_{\mathrm{m\ MASP2^*}}}+\frac{\left[\mathrm{pCLLKMASP2_{zym}}\right]}{K^{\mathrm{MASP2_{zym}}}_{\mathrm{m\ MASP2^*}}}+\frac{\left[\mathrm{pCLK1MASP1_{zym}}\right]}{K^{\mathrm{MASP1_{zym}}}_{\mathrm{m\ MASP2^*}}}\\
+\frac{\left[\mathrm{pCLK1MASP2_{zym}}\right]}{K^{\mathrm{MASP2_{zym}}}_{\mathrm{m\ MASP2^*}}}+\frac{\left[\mathrm{pCLL1MASP1_{zym}}\right]}{K^{\mathrm{MASP1_{zym}}}_{\mathrm{m\ MASP2^*}}}+\frac{\left[\mathrm{pCLL1MASP2_{zym}}\right]}{K^{\mathrm{MASP2_{zym}}}_{\mathrm{m\ MASP2^*}}}+\frac{\left[\mathrm{pLFMASP1_{zym}}\right]}{K^{\mathrm{MASP1_{zym}}}_{\mathrm{m\ MASP2^*}}}\\
+\frac{\left[\mathrm{pLFMASP2_{zym}}\right]}{K^{\mathrm{MASP2_{zym}}}_{\mathrm{m\ MASP2^*}}}+\frac{\left[\mathrm{pHFMASP1_{zym}}\right]}{K^{\mathrm{MASP1_{zym}}}_{\mathrm{m\ MASP2^*}}}+\frac{\left[\mathrm{pHFMASP2_{zym}}\right]}{K^{\mathrm{MASP2_{zym}}}_{\mathrm{m\ MASP2^*}}}+\frac{\left[\mathrm{pMFMASP1_{zym}}\right]}{K^{\mathrm{MASP1_{zym}}}_{\mathrm{m\ MASP2^*}}}+\frac{\left[\mathrm{pMFMASP2_{zym}}\right]}{K^{\mathrm{MASP2_{zym}}}_{\mathrm{m\ MASP2^*}}}\end{array}\right)}
$$

$$+\frac{k_{\text{cat MASP2}^*}^{\text{C2}}\left[\text{pMFMASP2}^*\right]\left[\text{fC3bC4bC2}\right]}{K_{\text{m MASP2}^*}^{\text{C2}}\left(\begin{aligned}&1+\frac{[\text{C4}]}{K_{\text{m MASP2}^*}^{\text{C4}}}+\frac{[\text{fC4bC2}]}{K_{\text{m MASP2}^*}^{\text{C2}}}+\frac{[\text{fC4bC4bC2}]}{K_{\text{m MASP2}^*}^{\text{C2}}}+\frac{[\text{fC3bC4bC2}]}{K_{\text{m MASP2}^*}^{\text{C2}}}+\frac{[\text{pC4bC2}]}{K_{\text{m MASP2}^*}^{\text{C2}}}+\frac{[\text{pC4bC4bC2}]}{K_{\text{m MASP2}^*}^{\text{C2}}}\\&+\frac{[\text{pC3bC4bC2}]}{K_{\text{m MASP2}^*}^{\text{C2}}}+\frac{[\text{pMBL}_3\text{MASP1}_{\text{zym}}]}{K_{\text{m MASP2}^*}^{\text{MASP1}_{\text{zym}}}}+\frac{[\text{pMBL}_3\text{MASP2}_{\text{zym}}]}{K_{\text{m MASP2}^*}^{\text{MASP2}_{\text{zym}}}}+\frac{[\text{pMBL}_4\text{MASP1}_{\text{zym}}]}{K_{\text{m MASP2}^*}^{\text{MASP1}_{\text{zym}}}}\\&+\frac{[\text{pMBL}_4\text{MASP2}_{\text{zym}}]}{K_{\text{m MASP2}^*}^{\text{MASP2}_{\text{zym}}}}+\frac{[\text{pCLLKMASP1}_{\text{zym}}]}{K_{\text{m MASP2}^*}^{\text{MASP1}_{\text{zym}}}}+\frac{[\text{pCLLKMASP2}_{\text{zym}}]}{K_{\text{m MASP2}^*}^{\text{MASP2}_{\text{zym}}}}+\frac{[\text{pCLK1MASP1}_{\text{zym}}]}{K_{\text{m MASP2}^*}^{\text{MASP1}_{\text{zym}}}}\\&+\frac{[\text{pCLK1MASP2}_{\text{zym}}]}{K_{\text{m MASP2}^*}^{\text{MASP2}_{\text{zym}}}}+\frac{[\text{pCLL1MASP1}_{\text{zym}}]}{K_{\text{m MASP2}^*}^{\text{MASP1}_{\text{zym}}}}+\frac{[\text{pCLL1MASP2}_{\text{zym}}]}{K_{\text{m MASP2}^*}^{\text{MASP2}_{\text{zym}}}}+\frac{[\text{pLFMASP1}_{\text{zym}}]}{K_{\text{m MASP2}^*}^{\text{MASP1}_{\text{zym}}}}\\&+\frac{[\text{pLFMASP2}_{\text{zym}}]}{K_{\text{m MASP2}^*}^{\text{MASP2}_{\text{zym}}}}+\frac{[\text{pHFMASP1}_{\text{zym}}]}{K_{\text{m MASP2}^*}^{\text{MASP1}_{\text{zym}}}}+\frac{[\text{pHFMASP2}_{\text{zym}}]}{K_{\text{m MASP2}^*}^{\text{MASP2}_{\text{zym}}}}+\frac{[\text{pMFMASP1}_{\text{zym}}]}{K_{\text{m MASP2}^*}^{\text{MASP1}_{\text{zym}}}}+\frac{[\text{pMFMASP2}_{\text{zym}}]}{K_{\text{m MASP2}^*}^{\text{MASP2}_{\text{zym}}}}\end{aligned}\right)}$$

$$+\frac{k_{\text{cat C1}^*}^{\text{C2}}\left[\text{pMBL}_3\text{PTX3C1}^*\right]\left[\text{fC3bC4bC2}\right]}{K_{\text{m C1}^*}^{\text{C2}}\left(1+\frac{[\text{C4}]}{K_{\text{m C1}^*}^{\text{C4}}}+\frac{[\text{fC4bC2}]}{K_{\text{m C1}^*}^{\text{C2}}}+\frac{[\text{fC4bC4bC2}]}{K_{\text{m C1}^*}^{\text{C2}}}+\frac{[\text{fC3bC4bC2}]}{K_{\text{m C1}^*}^{\text{C2}}}+\frac{[\text{pC4bC2}]}{K_{\text{m C1}^*}^{\text{C2}}}+\frac{[\text{pC4bC4bC2}]}{K_{\text{m C1}^*}^{\text{C2}}}+\frac{[\text{pC3bC4bC2}]}{K_{\text{m C1}^*}^{\text{C2}}}\right)}$$

$$+\frac{k_{\text{cat C1}^*}^{\text{C2}}\left[\text{pMBL}_4\text{PTX3C1}^*\right]\left[\text{fC3bC4bC2}\right]}{K_{\text{m C1}^*}^{\text{C2}}\left(1+\frac{[\text{C4}]}{K_{\text{m C1}^*}^{\text{C4}}}+\frac{[\text{fC4bC2}]}{K_{\text{m C1}^*}^{\text{C2}}}+\frac{[\text{fC4bC4bC2}]}{K_{\text{m C1}^*}^{\text{C2}}}+\frac{[\text{fC3bC4bC2}]}{K_{\text{m C1}^*}^{\text{C2}}}+\frac{[\text{pC4bC2}]}{K_{\text{m C1}^*}^{\text{C2}}}+\frac{[\text{pC4bC4bC2}]}{K_{\text{m C1}^*}^{\text{C2}}}+\frac{[\text{pC3bC4bC2}]}{K_{\text{m C1}^*}^{\text{C2}}}\right)}$$

$$+\frac{k_{\text{cat C1}^*}^{\text{C2}}\left[\text{CRPC1}^*\right]\left[\text{fC3bC4bC2}\right]}{K_{\text{m C1}^*}^{\text{C2}}\left(1+\frac{[\text{C4}]}{K_{\text{m C1}^*}^{\text{C4}}}+\frac{[\text{fC4bC2}]}{K_{\text{m C1}^*}^{\text{C2}}}+\frac{[\text{fC4bC4bC2}]}{K_{\text{m C1}^*}^{\text{C2}}}+\frac{[\text{fC3bC4bC2}]}{K_{\text{m C1}^*}^{\text{C2}}}\right)}$$

34

$$\frac{d[\text{nfC4b}]}{dt}=$$

$$\frac{k_{\text{cat C1}^*}^{\text{C4}}\left[\text{C1}^*\right]\left[\text{C4}\right]}{K_{\text{m C1}^*}^{\text{C4}}\left(1+\frac{[\text{C4}]}{K_{\text{m C1}^*}^{\text{C4}}}+\frac{[\text{fC4bC2}]}{K_{\text{m C1}^*}^{\text{C2}}}+\frac{[\text{fC4bC4bC2}]}{K_{\text{m C1}^*}^{\text{C2}}}+\frac{[\text{fC3bC4bC2}]}{K_{\text{m C1}^*}^{\text{C2}}}+\frac{[\text{pC4bC2}]}{K_{\text{m C1}^*}^{\text{C2}}}+\frac{[\text{pC4bC4bC2}]}{K_{\text{m C1}^*}^{\text{C2}}}+\frac{[\text{pC3bC4bC2}]}{K_{\text{m C1}^*}^{\text{C2}}}\right)}$$

$$+\frac{k_{\text{cat C1}^*}^{\text{C4}}\left[\text{pIgG}_6\text{C1}^*\right]\left[\text{C4}\right]}{K_{\text{m C1}^*}^{\text{C4}}\left(1+\frac{[\text{C4}]}{K_{\text{m C1}^*}^{\text{C4}}}+\frac{[\text{fC4bC2}]}{K_{\text{m C1}^*}^{\text{C2}}}+\frac{[\text{fC4bC4bC2}]}{K_{\text{m C1}^*}^{\text{C2}}}+\frac{[\text{fC3bC4bC2}]}{K_{\text{m C1}^*}^{\text{C2}}}+\frac{[\text{pC4bC2}]}{K_{\text{m C1}^*}^{\text{C2}}}+\frac{[\text{pC4bC4bC2}]}{K_{\text{m C1}^*}^{\text{C2}}}+\frac{[\text{pC3bC4bC2}]}{K_{\text{m C1}^*}^{\text{C2}}}\right)}$$

$$+\frac{k_{\text{cat C1}^*}^{\text{C4}}\left[\text{pIgMC1}^*\right]\left[\text{C4}\right]}{K_{\text{m C1}^*}^{\text{C4}}\left(1+\frac{[\text{C4}]}{K_{\text{m C1}^*}^{\text{C4}}}+\frac{[\text{fC4bC2}]}{K_{\text{m C1}^*}^{\text{C2}}}+\frac{[\text{fC4bC4bC2}]}{K_{\text{m C1}^*}^{\text{C2}}}+\frac{[\text{fC3bC4bC2}]}{K_{\text{m C1}^*}^{\text{C2}}}+\frac{[\text{pC4bC2}]}{K_{\text{m C1}^*}^{\text{C2}}}+\frac{[\text{pC4bC4bC2}]}{K_{\text{m C1}^*}^{\text{C2}}}+\frac{[\text{pC3bC4bC2}]}{K_{\text{m C1}^*}^{\text{C2}}}\right)}$$

$$-k^{+}_{\mathrm{fC4b}}[\mathrm{nfC4b}][\mathrm{H_2O}]-k^{+}_{\mathrm{C4bC4b}}[\mathrm{nfC4b}][\mathrm{nfC4b}]-k^{+}_{\mathrm{C4bC4b}}[\mathrm{nfC4b}][\mathrm{fC4b}]-k^{+}_{\mathrm{C3bC4b}}[\mathrm{nfC4b}][\mathrm{nfC3b}]$$

$$-k^{+}_{\mathrm{C3bC4b}}[\mathrm{nfC4b}][\mathrm{fC3b}]-k^{+}_{\mathrm{C3bC4b}}[\mathrm{fC3bBb}][\mathrm{nfC4b}]-k^{+}_{\mathrm{C4bC4b}}[\mathrm{fC4bC2a}][\mathrm{nfC4b}]-k^{+}_{\mathrm{C4bC4BP}}[\mathrm{nfC4b}][\mathrm{C4BP}]$$

$$-k^{+}_{\mathrm{hC4b}}[\mathrm{Host\ Surface}][\mathrm{nfC4b}]-k^{+}_{\mathrm{C4bC4b}}[\mathrm{hC4b}][\mathrm{nfC4b}]-k^{+}_{\mathrm{C3bC4b}}[\mathrm{hC3bBb}][\mathrm{nfC4b}]-k^{+}_{\mathrm{C3bC4b}}[\mathrm{hC3bBbP}][\mathrm{nfC4b}]$$

$$-k^{+}_{\mathrm{C3bC4b}}[\mathrm{hC3b}][\mathrm{nfC4b}]-k^{+}_{\mathrm{pC4b}}[\mathrm{Pathogen\ surface}][\mathrm{nfC4b}]-k^{+}_{\mathrm{C4bC4b}}[\mathrm{pC4b}][\mathrm{nfC4b}]-k^{+}_{\mathrm{C4bC4b}}[\mathrm{pC4bC2a}][\mathrm{nfC4b}]$$

$$-k^{+}_{\mathrm{C4bC3b}}[\mathrm{pC3b}][\mathrm{nfC4b}]-k^{+}_{\mathrm{C4bC3b}}[\mathrm{pC3bBb}][\mathrm{nfC4b}]-k^{+}_{\mathrm{C4bC3b}}[\mathrm{pC3bBbP}][\mathrm{nfC4b}]$$

$$+\frac{k^{\mathrm{C4}}_{\mathrm{cat\ MASP1^*}}\left[\mathrm{pMBL_3MASP1^*}\right][\mathrm{C4}]}{K^{\mathrm{C4}}_{\mathrm{m\ MASP1^*}}\left(\begin{array}{l}1+\frac{[\mathrm{C4}]}{K^{\mathrm{C4}}_{\mathrm{m\ MASP1^*}}}+\frac{[\mathrm{fC4bC2}]}{K^{\mathrm{C2}}_{\mathrm{m\ MASP1^*}}}+\frac{[\mathrm{fC4bC4bC2}]}{K^{\mathrm{C2}}_{\mathrm{m\ MASP1^*}}}+\frac{[\mathrm{fC3bC4bC2}]}{K^{\mathrm{C2}}_{\mathrm{m\ MASP1^*}}}+\frac{[\mathrm{pC4bC2}]}{K^{\mathrm{C2}}_{\mathrm{m\ MASP1^*}}}+\frac{[\mathrm{pC4bC4bC2}]}{K^{\mathrm{C2}}_{\mathrm{m\ MASP1^*}}}\\
+\frac{[\mathrm{pC3bC4bC2}]}{K^{\mathrm{C2}}_{\mathrm{m\ MASP1^*}}}+\frac{[\mathrm{pMBL_3MASP1_{zym}}]}{K^{\mathrm{MASP1_{zym}}}_{\mathrm{m\ MASP1^*}}}+\frac{[\mathrm{pMBL_3MASP2_{zym}}]}{K^{\mathrm{MASP2_{zym}}}_{\mathrm{m\ MASP1^*}}}+\frac{[\mathrm{pMBL_3MASP3_{zym}}]}{K^{\mathrm{MASP3_{zym}}}_{\mathrm{m\ MASP1^*}}}\\
+\frac{[\mathrm{pMBL_4MASP1_{zym}}]}{K^{\mathrm{MASP1_{zym}}}_{\mathrm{m\ MASP1^*}}}+\frac{[\mathrm{pMBL_4MASP2_{zym}}]}{K^{\mathrm{MASP2_{zym}}}_{\mathrm{m\ MASP1^*}}}+\frac{[\mathrm{pMBL_4MASP3_{zym}}]}{K^{\mathrm{MASP3_{zym}}}_{\mathrm{m\ MASP1^*}}}+\frac{[\mathrm{pCLLKMASP1_{zym}}]}{K^{\mathrm{MASP1_{zym}}}_{\mathrm{m\ MASP1^*}}}\\
+\frac{[\mathrm{pCLLKMASP2_{zym}}]}{K^{\mathrm{MASP2_{zym}}}_{\mathrm{m\ MASP1^*}}}+\frac{[\mathrm{pCLLKMASP3_{zym}}]}{K^{\mathrm{MASP3_{zym}}}_{\mathrm{m\ MASP1^*}}}+\frac{[\mathrm{pCLK1MASP1_{zym}}]}{K^{\mathrm{MASP1_{zym}}}_{\mathrm{m\ MASP1^*}}}+\frac{[\mathrm{pCLK1MASP2_{zym}}]}{K^{\mathrm{MASP2_{zym}}}_{\mathrm{m\ MASP1^*}}}\\
+\frac{[\mathrm{pCLK1MASP3_{zym}}]}{K^{\mathrm{MASP3_{zym}}}_{\mathrm{m\ MASP1^*}}}+\frac{[\mathrm{pCLL1MASP1_{zym}}]}{K^{\mathrm{MASP1_{zym}}}_{\mathrm{m\ MASP1^*}}}+\frac{[\mathrm{pCLL1MASP2_{zym}}]}{K^{\mathrm{MASP2_{zym}}}_{\mathrm{m\ MASP1^*}}}+\frac{[\mathrm{pCLL1MASP3_{zym}}]}{K^{\mathrm{MASP3_{zym}}}_{\mathrm{m\ MASP1^*}}}\\
+\frac{[\mathrm{pLFMASP1_{zym}}]}{K^{\mathrm{MASP1_{zym}}}_{\mathrm{m\ MASP1^*}}}+\frac{[\mathrm{pLFMASP2_{zym}}]}{K^{\mathrm{MASP2_{zym}}}_{\mathrm{m\ MASP1^*}}}+\frac{[\mathrm{pLFMASP3_{zym}}]}{K^{\mathrm{MASP3_{zym}}}_{\mathrm{m\ MASP1^*}}}+\frac{[\mathrm{pHFMASP1_{zym}}]}{K^{\mathrm{MASP1_{zym}}}_{\mathrm{m\ MASP1^*}}}+\frac{[\mathrm{pHFMASP2_{zym}}]}{K^{\mathrm{MASP2_{zym}}}_{\mathrm{m\ MASP1^*}}}\\
+\frac{[\mathrm{pHFMASP3_{zym}}]}{K^{\mathrm{MASP3_{zym}}}_{\mathrm{m\ MASP1^*}}}+\frac{[\mathrm{pMFMASP1_{zym}}]}{K^{\mathrm{MASP1_{zym}}}_{\mathrm{m\ MASP1^*}}}+\frac{[\mathrm{pMFMASP2_{zym}}]}{K^{\mathrm{MASP2_{zym}}}_{\mathrm{m\ MASP1^*}}}+\frac{[\mathrm{pMFMASP3_{zym}}]}{K^{\mathrm{MASP3_{zym}}}_{\mathrm{m\ MASP1^*}}}\end{array}\right)}$$

$$
+\frac{k^{\mathrm{C4}}_{\mathrm{cat\ MASP1^*}}\left[\mathrm{pMBL_4MASP1^*}\right]\left[\mathrm{C4}\right]}{K^{\mathrm{C4}}_{\mathrm{m\ MASP1^*}}\left(\begin{array}{l}1+\frac{[\mathrm{C4}]}{K^{\mathrm{C4}}_{\mathrm{m\ MASP1^*}}}+\frac{[\mathrm{fC4bC2}]}{K^{\mathrm{C2}}_{\mathrm{m\ MASP1^*}}}+\frac{[\mathrm{fC4bC4bC2}]}{K^{\mathrm{C2}}_{\mathrm{m\ MASP1^*}}}+\frac{[\mathrm{fC3bC4bC2}]}{K^{\mathrm{C2}}_{\mathrm{m\ MASP1^*}}}+\frac{[\mathrm{pC4bC2}]}{K^{\mathrm{C2}}_{\mathrm{m\ MASP1^*}}}+\frac{[\mathrm{pC4bC4bC2}]}{K^{\mathrm{C2}}_{\mathrm{m\ MASP1^*}}}\\
+\frac{[\mathrm{pC3bC4bC2}]}{K^{\mathrm{C2}}_{\mathrm{m\ MASP1^*}}}+\frac{[\mathrm{pMBL_3MASP1_{zym}}]}{K^{\mathrm{MASP1_{zym}}}_{\mathrm{m\ MASP1^*}}}+\frac{[\mathrm{pMBL_3MASP2_{zym}}]}{K^{\mathrm{MASP2_{zym}}}_{\mathrm{m\ MASP1^*}}}+\frac{[\mathrm{pMBL_3MASP3_{zym}}]}{K^{\mathrm{MASP3_{zym}}}_{\mathrm{m\ MASP1^*}}}\\
+\frac{[\mathrm{pMBL_4MASP1_{zym}}]}{K^{\mathrm{MASP1_{zym}}}_{\mathrm{m\ MASP1^*}}}+\frac{[\mathrm{pMBL_4MASP2_{zym}}]}{K^{\mathrm{MASP2_{zym}}}_{\mathrm{m\ MASP1^*}}}+\frac{[\mathrm{pMBL_4MASP3_{zym}}]}{K^{\mathrm{MASP3_{zym}}}_{\mathrm{m\ MASP1^*}}}+\frac{[\mathrm{pCLLKMASP1_{zym}}]}{K^{\mathrm{MASP1_{zym}}}_{\mathrm{m\ MASP1^*}}}\\
+\frac{[\mathrm{pCLLKMASP2_{zym}}]}{K^{\mathrm{MASP2_{zym}}}_{\mathrm{m\ MASP1^*}}}+\frac{[\mathrm{pCLLKMASP3_{zym}}]}{K^{\mathrm{MASP3_{zym}}}_{\mathrm{m\ MASP1^*}}}+\frac{[\mathrm{pCLK1MASP1_{zym}}]}{K^{\mathrm{MASP1_{zym}}}_{\mathrm{m\ MASP1^*}}}+\frac{[\mathrm{pCLK1MASP2_{zym}}]}{K^{\mathrm{MASP2_{zym}}}_{\mathrm{m\ MASP1^*}}}\\
+\frac{[\mathrm{pCLK1MASP3_{zym}}]}{K^{\mathrm{MASP3_{zym}}}_{\mathrm{m\ MASP1^*}}}+\frac{[\mathrm{pCLL1MASP1_{zym}}]}{K^{\mathrm{MASP1_{zym}}}_{\mathrm{m\ MASP1^*}}}+\frac{[\mathrm{pCLL1MASP2_{zym}}]}{K^{\mathrm{MASP2_{zym}}}_{\mathrm{m\ MASP1^*}}}+\frac{[\mathrm{pCLL1MASP3_{zym}}]}{K^{\mathrm{MASP3_{zym}}}_{\mathrm{m\ MASP1^*}}}\\
+\frac{[\mathrm{pLFMASP1_{zym}}]}{K^{\mathrm{MASP1_{zym}}}_{\mathrm{m\ MASP1^*}}}+\frac{[\mathrm{pLFMASP2_{zym}}]}{K^{\mathrm{MASP2_{zym}}}_{\mathrm{m\ MASP1^*}}}+\frac{[\mathrm{pLFMASP3_{zym}}]}{K^{\mathrm{MASP3_{zym}}}_{\mathrm{m\ MASP1^*}}}+\frac{[\mathrm{pHFMASP1_{zym}}]}{K^{\mathrm{MASP1_{zym}}}_{\mathrm{m\ MASP1^*}}}+\frac{[\mathrm{pHFMASP2_{zym}}]}{K^{\mathrm{MASP2_{zym}}}_{\mathrm{m\ MASP1^*}}}\\
+\frac{[\mathrm{pHFMASP3_{zym}}]}{K^{\mathrm{MASP3_{zym}}}_{\mathrm{m\ MASP1^*}}}+\frac{[\mathrm{pMFMASP1_{zym}}]}{K^{\mathrm{MASP1_{zym}}}_{\mathrm{m\ MASP1^*}}}+\frac{[\mathrm{pMFMASP2_{zym}}]}{K^{\mathrm{MASP2_{zym}}}_{\mathrm{m\ MASP1^*}}}+\frac{[\mathrm{pMFMASP3_{zym}}]}{K^{\mathrm{MASP3_{zym}}}_{\mathrm{m\ MASP1^*}}}\end{array}\right)}
$$

$$
+\frac{k^{\mathrm{C4}}_{\mathrm{cat\ MASP1^*}}\left[\mathrm{pCLLKMASP1^*}\right]\left[\mathrm{C4}\right]}{K^{\mathrm{C4}}_{\mathrm{m\ MASP1^*}}\left(\begin{array}{l}1+\frac{[\mathrm{C4}]}{K^{\mathrm{C4}}_{\mathrm{m\ MASP1^*}}}+\frac{[\mathrm{fC4bC2}]}{K^{\mathrm{C2}}_{\mathrm{m\ MASP1^*}}}+\frac{[\mathrm{fC4bC4bC2}]}{K^{\mathrm{C2}}_{\mathrm{m\ MASP1^*}}}+\frac{[\mathrm{fC3bC4bC2}]}{K^{\mathrm{C2}}_{\mathrm{m\ MASP1^*}}}+\frac{[\mathrm{pC4bC2}]}{K^{\mathrm{C2}}_{\mathrm{m\ MASP1^*}}}+\frac{[\mathrm{pC4bC4bC2}]}{K^{\mathrm{C2}}_{\mathrm{m\ MASP1^*}}}\\
+\frac{[\mathrm{pC3bC4bC2}]}{K^{\mathrm{C2}}_{\mathrm{m\ MASP1^*}}}+\frac{[\mathrm{pMBL_3MASP1_{zym}}]}{K^{\mathrm{MASP1_{zym}}}_{\mathrm{m\ MASP1^*}}}+\frac{[\mathrm{pMBL_3MASP2_{zym}}]}{K^{\mathrm{MASP2_{zym}}}_{\mathrm{m\ MASP1^*}}}+\frac{[\mathrm{pMBL_3MASP3_{zym}}]}{K^{\mathrm{MASP3_{zym}}}_{\mathrm{m\ MASP1^*}}}\\
+\frac{[\mathrm{pMBL_4MASP1_{zym}}]}{K^{\mathrm{MASP1_{zym}}}_{\mathrm{m\ MASP1^*}}}+\frac{[\mathrm{pMBL_4MASP2_{zym}}]}{K^{\mathrm{MASP2_{zym}}}_{\mathrm{m\ MASP1^*}}}+\frac{[\mathrm{pMBL_4MASP3_{zym}}]}{K^{\mathrm{MASP3_{zym}}}_{\mathrm{m\ MASP1^*}}}+\frac{[\mathrm{pCLLKMASP1_{zym}}]}{K^{\mathrm{MASP1_{zym}}}_{\mathrm{m\ MASP1^*}}}\\
+\frac{[\mathrm{pCLLKMASP2_{zym}}]}{K^{\mathrm{MASP2_{zym}}}_{\mathrm{m\ MASP1^*}}}+\frac{[\mathrm{pCLLKMASP3_{zym}}]}{K^{\mathrm{MASP3_{zym}}}_{\mathrm{m\ MASP1^*}}}+\frac{[\mathrm{pCLK1MASP1_{zym}}]}{K^{\mathrm{MASP1_{zym}}}_{\mathrm{m\ MASP1^*}}}+\frac{[\mathrm{pCLK1MASP2_{zym}}]}{K^{\mathrm{MASP2_{zym}}}_{\mathrm{m\ MASP1^*}}}\\
+\frac{[\mathrm{pCLK1MASP3_{zym}}]}{K^{\mathrm{MASP3_{zym}}}_{\mathrm{m\ MASP1^*}}}+\frac{[\mathrm{pCLL1MASP1_{zym}}]}{K^{\mathrm{MASP1_{zym}}}_{\mathrm{m\ MASP1^*}}}+\frac{[\mathrm{pCLL1MASP2_{zym}}]}{K^{\mathrm{MASP2_{zym}}}_{\mathrm{m\ MASP1^*}}}+\frac{[\mathrm{pCLL1MASP3_{zym}}]}{K^{\mathrm{MASP3_{zym}}}_{\mathrm{m\ MASP1^*}}}\\
+\frac{[\mathrm{pLFMASP1_{zym}}]}{K^{\mathrm{MASP1_{zym}}}_{\mathrm{m\ MASP1^*}}}+\frac{[\mathrm{pLFMASP2_{zym}}]}{K^{\mathrm{MASP2_{zym}}}_{\mathrm{m\ MASP1^*}}}+\frac{[\mathrm{pLFMASP3_{zym}}]}{K^{\mathrm{MASP3_{zym}}}_{\mathrm{m\ MASP1^*}}}+\frac{[\mathrm{pHFMASP1_{zym}}]}{K^{\mathrm{MASP1_{zym}}}_{\mathrm{m\ MASP1^*}}}+\frac{[\mathrm{pHFMASP2_{zym}}]}{K^{\mathrm{MASP2_{zym}}}_{\mathrm{m\ MASP1^*}}}\\
+\frac{[\mathrm{pHFMASP3_{zym}}]}{K^{\mathrm{MASP3_{zym}}}_{\mathrm{m\ MASP1^*}}}+\frac{[\mathrm{pMFMASP1_{zym}}]}{K^{\mathrm{MASP1_{zym}}}_{\mathrm{m\ MASP1^*}}}+\frac{[\mathrm{pMFMASP2_{zym}}]}{K^{\mathrm{MASP2_{zym}}}_{\mathrm{m\ MASP1^*}}}+\frac{[\mathrm{pMFMASP3_{zym}}]}{K^{\mathrm{MASP3_{zym}}}_{\mathrm{m\ MASP1^*}}}\end{array}\right)}
$$

$$+\frac{k^{\mathrm{C4}}_{\mathrm{cat\,MASP1^*}}\left[\mathrm{pCLK1MASP1^*}\right]\left[\mathrm{C4}\right]}{K^{\mathrm{C4}}_{\mathrm{m\,MASP1^*}}\left(\begin{array}{l}1+\frac{[\mathrm{C4}]}{K^{\mathrm{C4}}_{\mathrm{m\,MASP1^*}}}+\frac{[\mathrm{fC4bC2}]}{K^{\mathrm{C2}}_{\mathrm{m\,MASP1^*}}}+\frac{[\mathrm{fC4bC4bC2}]}{K^{\mathrm{C2}}_{\mathrm{m\,MASP1^*}}}+\frac{[\mathrm{fC3bC4bC2}]}{K^{\mathrm{C2}}_{\mathrm{m\,MASP1^*}}}+\frac{[\mathrm{pC4bC2}]}{K^{\mathrm{C2}}_{\mathrm{m\,MASP1^*}}}+\frac{[\mathrm{pC4bC4bC2}]}{K^{\mathrm{C2}}_{\mathrm{m\,MASP1^*}}}\\
+\frac{[\mathrm{pC3bC4bC2}]}{K^{\mathrm{C2}}_{\mathrm{m\,MASP1^*}}}+\frac{[\mathrm{pMBL_3MASP1_{zym}}]}{K^{\mathrm{MASP1_{zym}}}_{\mathrm{m\,MASP1^*}}}+\frac{[\mathrm{pMBL_3MASP2_{zym}}]}{K^{\mathrm{MASP2_{zym}}}_{\mathrm{m\,MASP1^*}}}+\frac{[\mathrm{pMBL_3MASP3_{zym}}]}{K^{\mathrm{MASP3_{zym}}}_{\mathrm{m\,MASP1^*}}}\\
+\frac{[\mathrm{pMBL_4MASP1_{zym}}]}{K^{\mathrm{MASP1_{zym}}}_{\mathrm{m\,MASP1^*}}}+\frac{[\mathrm{pMBL_4MASP2_{zym}}]}{K^{\mathrm{MASP2_{zym}}}_{\mathrm{m\,MASP1^*}}}+\frac{[\mathrm{pMBL_4MASP3_{zym}}]}{K^{\mathrm{MASP3_{zym}}}_{\mathrm{m\,MASP1^*}}}+\frac{[\mathrm{pCLLKMASP1_{zym}}]}{K^{\mathrm{MASP1_{zym}}}_{\mathrm{m\,MASP1^*}}}\\
+\frac{[\mathrm{pCLLKMASP2_{zym}}]}{K^{\mathrm{MASP2_{zym}}}_{\mathrm{m\,MASP1^*}}}+\frac{[\mathrm{pCLLKMASP3_{zym}}]}{K^{\mathrm{MASP3_{zym}}}_{\mathrm{m\,MASP1^*}}}+\frac{[\mathrm{pCLK1MASP1_{zym}}]}{K^{\mathrm{MASP1_{zym}}}_{\mathrm{m\,MASP1^*}}}+\frac{[\mathrm{pCLK1MASP2_{zym}}]}{K^{\mathrm{MASP2_{zym}}}_{\mathrm{m\,MASP1^*}}}\\
+\frac{[\mathrm{pCLK1MASP3_{zym}}]}{K^{\mathrm{MASP3_{zym}}}_{\mathrm{m\,MASP1^*}}}+\frac{[\mathrm{pCLL1MASP1_{zym}}]}{K^{\mathrm{MASP1_{zym}}}_{\mathrm{m\,MASP1^*}}}+\frac{[\mathrm{pCLL1MASP2_{zym}}]}{K^{\mathrm{MASP2_{zym}}}_{\mathrm{m\,MASP1^*}}}+\frac{[\mathrm{pCLL1MASP3_{zym}}]}{K^{\mathrm{MASP3_{zym}}}_{\mathrm{m\,MASP1^*}}}\\
+\frac{[\mathrm{pLFMASP1_{zym}}]}{K^{\mathrm{MASP1_{zym}}}_{\mathrm{m\,MASP1^*}}}+\frac{[\mathrm{pLFMASP2_{zym}}]}{K^{\mathrm{MASP2_{zym}}}_{\mathrm{m\,MASP1^*}}}+\frac{[\mathrm{pLFMASP3_{zym}}]}{K^{\mathrm{MASP3_{zym}}}_{\mathrm{m\,MASP1^*}}}+\frac{[\mathrm{pHFMASP1_{zym}}]}{K^{\mathrm{MASP1_{zym}}}_{\mathrm{m\,MASP1^*}}}+\frac{[\mathrm{pHFMASP2_{zym}}]}{K^{\mathrm{MASP2_{zym}}}_{\mathrm{m\,MASP1^*}}}\\
+\frac{[\mathrm{pHFMASP3_{zym}}]}{K^{\mathrm{MASP3_{zym}}}_{\mathrm{m\,MASP1^*}}}+\frac{[\mathrm{pMFMASP1_{zym}}]}{K^{\mathrm{MASP1_{zym}}}_{\mathrm{m\,MASP1^*}}}+\frac{[\mathrm{pMFMASP2_{zym}}]}{K^{\mathrm{MASP2_{zym}}}_{\mathrm{m\,MASP1^*}}}+\frac{[\mathrm{pMFMASP3_{zym}}]}{K^{\mathrm{MASP3_{zym}}}_{\mathrm{m\,MASP1^*}}}\end{array}\right)}$$

$$+\frac{k^{\mathrm{C4}}_{\mathrm{cat\,MASP1^*}}\left[\mathrm{pCLL1MASP1^*}\right]\left[\mathrm{C4}\right]}{K^{\mathrm{C4}}_{\mathrm{m\,MASP1^*}}\left(\begin{array}{l}1+\frac{[\mathrm{C4}]}{K^{\mathrm{C4}}_{\mathrm{m\,MASP1^*}}}+\frac{[\mathrm{fC4bC2}]}{K^{\mathrm{C2}}_{\mathrm{m\,MASP1^*}}}+\frac{[\mathrm{fC4bC4bC2}]}{K^{\mathrm{C2}}_{\mathrm{m\,MASP1^*}}}+\frac{[\mathrm{fC3bC4bC2}]}{K^{\mathrm{C2}}_{\mathrm{m\,MASP1^*}}}+\frac{[\mathrm{pC4bC2}]}{K^{\mathrm{C2}}_{\mathrm{m\,MASP1^*}}}+\frac{[\mathrm{pC4bC4bC2}]}{K^{\mathrm{C2}}_{\mathrm{m\,MASP1^*}}}\\
+\frac{[\mathrm{pC3bC4bC2}]}{K^{\mathrm{C2}}_{\mathrm{m\,MASP1^*}}}+\frac{[\mathrm{pMBL_3MASP1_{zym}}]}{K^{\mathrm{MASP1_{zym}}}_{\mathrm{m\,MASP1^*}}}+\frac{[\mathrm{pMBL_3MASP2_{zym}}]}{K^{\mathrm{MASP2_{zym}}}_{\mathrm{m\,MASP1^*}}}+\frac{[\mathrm{pMBL_3MASP3_{zym}}]}{K^{\mathrm{MASP3_{zym}}}_{\mathrm{m\,MASP1^*}}}\\
+\frac{[\mathrm{pMBL_4MASP1_{zym}}]}{K^{\mathrm{MASP1_{zym}}}_{\mathrm{m\,MASP1^*}}}+\frac{[\mathrm{pMBL_4MASP2_{zym}}]}{K^{\mathrm{MASP2_{zym}}}_{\mathrm{m\,MASP1^*}}}+\frac{[\mathrm{pMBL_4MASP3_{zym}}]}{K^{\mathrm{MASP3_{zym}}}_{\mathrm{m\,MASP1^*}}}+\frac{[\mathrm{pCLLKMASP1_{zym}}]}{K^{\mathrm{MASP1_{zym}}}_{\mathrm{m\,MASP1^*}}}\\
+\frac{[\mathrm{pCLLKMASP2_{zym}}]}{K^{\mathrm{MASP2_{zym}}}_{\mathrm{m\,MASP1^*}}}+\frac{[\mathrm{pCLLKMASP3_{zym}}]}{K^{\mathrm{MASP3_{zym}}}_{\mathrm{m\,MASP1^*}}}+\frac{[\mathrm{pCLK1MASP1_{zym}}]}{K^{\mathrm{MASP1_{zym}}}_{\mathrm{m\,MASP1^*}}}+\frac{[\mathrm{pCLK1MASP2_{zym}}]}{K^{\mathrm{MASP2_{zym}}}_{\mathrm{m\,MASP1^*}}}\\
+\frac{[\mathrm{pCLK1MASP3_{zym}}]}{K^{\mathrm{MASP3_{zym}}}_{\mathrm{m\,MASP1^*}}}+\frac{[\mathrm{pCLL1MASP1_{zym}}]}{K^{\mathrm{MASP1_{zym}}}_{\mathrm{m\,MASP1^*}}}+\frac{[\mathrm{pCLL1MASP2_{zym}}]}{K^{\mathrm{MASP2_{zym}}}_{\mathrm{m\,MASP1^*}}}+\frac{[\mathrm{pCLL1MASP3_{zym}}]}{K^{\mathrm{MASP3_{zym}}}_{\mathrm{m\,MASP1^*}}}\\
+\frac{[\mathrm{pLFMASP1_{zym}}]}{K^{\mathrm{MASP1_{zym}}}_{\mathrm{m\,MASP1^*}}}+\frac{[\mathrm{pLFMASP2_{zym}}]}{K^{\mathrm{MASP2_{zym}}}_{\mathrm{m\,MASP1^*}}}+\frac{[\mathrm{pLFMASP3_{zym}}]}{K^{\mathrm{MASP3_{zym}}}_{\mathrm{m\,MASP1^*}}}+\frac{[\mathrm{pHFMASP1_{zym}}]}{K^{\mathrm{MASP1_{zym}}}_{\mathrm{m\,MASP1^*}}}+\frac{[\mathrm{pHFMASP2_{zym}}]}{K^{\mathrm{MASP2_{zym}}}_{\mathrm{m\,MASP1^*}}}\\
+\frac{[\mathrm{pHFMASP3_{zym}}]}{K^{\mathrm{MASP3_{zym}}}_{\mathrm{m\,MASP1^*}}}+\frac{[\mathrm{pMFMASP1_{zym}}]}{K^{\mathrm{MASP1_{zym}}}_{\mathrm{m\,MASP1^*}}}+\frac{[\mathrm{pMFMASP2_{zym}}]}{K^{\mathrm{MASP2_{zym}}}_{\mathrm{m\,MASP1^*}}}+\frac{[\mathrm{pMFMASP3_{zym}}]}{K^{\mathrm{MASP3_{zym}}}_{\mathrm{m\,MASP1^*}}}\end{array}\right)}$$

$$
+\frac{k_{\mathrm{cat\,MASP1^*}}^{\mathrm{C4}}\left[\mathrm{pLFMASP1^*}\right]\left[\mathrm{C4}\right]}{K_{\mathrm{m\,MASP1^*}}^{\mathrm{C4}}\left(\begin{array}{l}
1+\frac{[\mathrm{C4}]}{K_{\mathrm{m\,MASP1^*}}^{\mathrm{C4}}}+\frac{[\mathrm{fC4bC2}]}{K_{\mathrm{m\,MASP1^*}}^{\mathrm{C2}}}+\frac{[\mathrm{fC4bC4bC2}]}{K_{\mathrm{m\,MASP1^*}}^{\mathrm{C2}}}+\frac{[\mathrm{fC3bC4bC2}]}{K_{\mathrm{m\,MASP1^*}}^{\mathrm{C2}}}+\frac{[\mathrm{pC4bC2}]}{K_{\mathrm{m\,MASP1^*}}^{\mathrm{C2}}}+\frac{[\mathrm{pC4bC4bC2}]}{K_{\mathrm{m\,MASP1^*}}^{\mathrm{C2}}} \\
+\frac{[\mathrm{pC3bC4bC2}]}{K_{\mathrm{m\,MASP1^*}}^{\mathrm{C2}}}+\frac{\left[\mathrm{pMBL_3MASP1_{zym}}\right]}{K_{\mathrm{m\,MASP1^*}}^{\mathrm{MASP1_{zym}}}}+\frac{\left[\mathrm{pMBL_3MASP2_{zym}}\right]}{K_{\mathrm{m\,MASP1^*}}^{\mathrm{MASP2_{zym}}}}+\frac{\left[\mathrm{pMBL_3MASP3_{zym}}\right]}{K_{\mathrm{m\,MASP1^*}}^{\mathrm{MASP3_{zym}}}} \\
+\frac{\left[\mathrm{pMBL_4MASP1_{zym}}\right]}{K_{\mathrm{m\,MASP1^*}}^{\mathrm{MASP1_{zym}}}}+\frac{\left[\mathrm{pMBL_4MASP2_{zym}}\right]}{K_{\mathrm{m\,MASP1^*}}^{\mathrm{MASP2_{zym}}}}+\frac{\left[\mathrm{pMBL_4MASP3_{zym}}\right]}{K_{\mathrm{m\,MASP1^*}}^{\mathrm{MASP3_{zym}}}}+\frac{\left[\mathrm{pCLLKMASP1_{zym}}\right]}{K_{\mathrm{m\,MASP1^*}}^{\mathrm{MASP1_{zym}}}} \\
+\frac{\left[\mathrm{pCLLKMASP2_{zym}}\right]}{K_{\mathrm{m\,MASP1^*}}^{\mathrm{MASP2_{zym}}}}+\frac{\left[\mathrm{pCLLKMASP3_{zym}}\right]}{K_{\mathrm{m\,MASP1^*}}^{\mathrm{MASP3_{zym}}}}+\frac{\left[\mathrm{pCLK1MASP1_{zym}}\right]}{K_{\mathrm{m\,MASP1^*}}^{\mathrm{MASP1_{zym}}}}+\frac{\left[\mathrm{pCLK1MASP2_{zym}}\right]}{K_{\mathrm{m\,MASP1^*}}^{\mathrm{MASP2_{zym}}}} \\
+\frac{\left[\mathrm{pCLK1MASP3_{zym}}\right]}{K_{\mathrm{m\,MASP1^*}}^{\mathrm{MASP3_{zym}}}}+\frac{\left[\mathrm{pCLL1MASP1_{zym}}\right]}{K_{\mathrm{m\,MASP1^*}}^{\mathrm{MASP1_{zym}}}}+\frac{\left[\mathrm{pCLL1MASP2_{zym}}\right]}{K_{\mathrm{m\,MASP1^*}}^{\mathrm{MASP2_{zym}}}}+\frac{\left[\mathrm{pCLL1MASP3_{zym}}\right]}{K_{\mathrm{m\,MASP1^*}}^{\mathrm{MASP3_{zym}}}} \\
+\frac{\left[\mathrm{pLFMASP1_{zym}}\right]}{K_{\mathrm{m\,MASP1^*}}^{\mathrm{MASP1_{zym}}}}+\frac{\left[\mathrm{pLFMASP2_{zym}}\right]}{K_{\mathrm{m\,MASP1^*}}^{\mathrm{MASP2_{zym}}}}+\frac{\left[\mathrm{pLFMASP3_{zym}}\right]}{K_{\mathrm{m\,MASP1^*}}^{\mathrm{MASP3_{zym}}}}+\frac{\left[\mathrm{pHFMASP1_{zym}}\right]}{K_{\mathrm{m\,MASP1^*}}^{\mathrm{MASP1_{zym}}}}+\frac{\left[\mathrm{pHFMASP2_{zym}}\right]}{K_{\mathrm{m\,MASP1^*}}^{\mathrm{MASP2_{zym}}}} \\
+\frac{\left[\mathrm{pHFMASP3_{zym}}\right]}{K_{\mathrm{m\,MASP1^*}}^{\mathrm{MASP3_{zym}}}}+\frac{\left[\mathrm{pMFMASP1_{zym}}\right]}{K_{\mathrm{m\,MASP1^*}}^{\mathrm{MASP1_{zym}}}}+\frac{\left[\mathrm{pMFMASP2_{zym}}\right]}{K_{\mathrm{m\,MASP1^*}}^{\mathrm{MASP2_{zym}}}}+\frac{\left[\mathrm{pMFMASP3_{zym}}\right]}{K_{\mathrm{m\,MASP1^*}}^{\mathrm{MASP3_{zym}}}}
\end{array}\right)}
$$

$$
+\frac{k_{\mathrm{cat\,MASP1^*}}^{\mathrm{C4}}\left[\mathrm{pHFMASP1^*}\right]\left[\mathrm{C4}\right]}{K_{\mathrm{m\,MASP1^*}}^{\mathrm{C4}}\left(\begin{array}{l}
1+\frac{[\mathrm{C4}]}{K_{\mathrm{m\,MASP1^*}}^{\mathrm{C4}}}+\frac{[\mathrm{fC4bC2}]}{K_{\mathrm{m\,MASP1^*}}^{\mathrm{C2}}}+\frac{[\mathrm{fC4bC4bC2}]}{K_{\mathrm{m\,MASP1^*}}^{\mathrm{C2}}}+\frac{[\mathrm{fC3bC4bC2}]}{K_{\mathrm{m\,MASP1^*}}^{\mathrm{C2}}}+\frac{[\mathrm{pC4bC2}]}{K_{\mathrm{m\,MASP1^*}}^{\mathrm{C2}}}+\frac{[\mathrm{pC4bC4bC2}]}{K_{\mathrm{m\,MASP1^*}}^{\mathrm{C2}}} \\
+\frac{[\mathrm{pC3bC4bC2}]}{K_{\mathrm{m\,MASP1^*}}^{\mathrm{C2}}}+\frac{\left[\mathrm{pMBL_3MASP1_{zym}}\right]}{K_{\mathrm{m\,MASP1^*}}^{\mathrm{MASP1_{zym}}}}+\frac{\left[\mathrm{pMBL_3MASP2_{zym}}\right]}{K_{\mathrm{m\,MASP1^*}}^{\mathrm{MASP2_{zym}}}}+\frac{\left[\mathrm{pMBL_3MASP3_{zym}}\right]}{K_{\mathrm{m\,MASP1^*}}^{\mathrm{MASP3_{zym}}}} \\
+\frac{\left[\mathrm{pMBL_4MASP1_{zym}}\right]}{K_{\mathrm{m\,MASP1^*}}^{\mathrm{MASP1_{zym}}}}+\frac{\left[\mathrm{pMBL_4MASP2_{zym}}\right]}{K_{\mathrm{m\,MASP1^*}}^{\mathrm{MASP2_{zym}}}}+\frac{\left[\mathrm{pMBL_4MASP3_{zym}}\right]}{K_{\mathrm{m\,MASP1^*}}^{\mathrm{MASP3_{zym}}}}+\frac{\left[\mathrm{pCLLKMASP1_{zym}}\right]}{K_{\mathrm{m\,MASP1^*}}^{\mathrm{MASP1_{zym}}}} \\
+\frac{\left[\mathrm{pCLLKMASP2_{zym}}\right]}{K_{\mathrm{m\,MASP1^*}}^{\mathrm{MASP2_{zym}}}}+\frac{\left[\mathrm{pCLLKMASP3_{zym}}\right]}{K_{\mathrm{m\,MASP1^*}}^{\mathrm{MASP3_{zym}}}}+\frac{\left[\mathrm{pCLK1MASP1_{zym}}\right]}{K_{\mathrm{m\,MASP1^*}}^{\mathrm{MASP1_{zym}}}}+\frac{\left[\mathrm{pCLK1MASP2_{zym}}\right]}{K_{\mathrm{m\,MASP1^*}}^{\mathrm{MASP2_{zym}}}} \\
+\frac{\left[\mathrm{pCLK1MASP3_{zym}}\right]}{K_{\mathrm{m\,MASP1^*}}^{\mathrm{MASP3_{zym}}}}+\frac{\left[\mathrm{pCLL1MASP1_{zym}}\right]}{K_{\mathrm{m\,MASP1^*}}^{\mathrm{MASP1_{zym}}}}+\frac{\left[\mathrm{pCLL1MASP2_{zym}}\right]}{K_{\mathrm{m\,MASP1^*}}^{\mathrm{MASP2_{zym}}}}+\frac{\left[\mathrm{pCLL1MASP3_{zym}}\right]}{K_{\mathrm{m\,MASP1^*}}^{\mathrm{MASP3_{zym}}}} \\
+\frac{\left[\mathrm{pLFMASP1_{zym}}\right]}{K_{\mathrm{m\,MASP1^*}}^{\mathrm{MASP1_{zym}}}}+\frac{\left[\mathrm{pLFMASP2_{zym}}\right]}{K_{\mathrm{m\,MASP1^*}}^{\mathrm{MASP2_{zym}}}}+\frac{\left[\mathrm{pLFMASP3_{zym}}\right]}{K_{\mathrm{m\,MASP1^*}}^{\mathrm{MASP3_{zym}}}}+\frac{\left[\mathrm{pHFMASP1_{zym}}\right]}{K_{\mathrm{m\,MASP1^*}}^{\mathrm{MASP1_{zym}}}}+\frac{\left[\mathrm{pHFMASP2_{zym}}\right]}{K_{\mathrm{m\,MASP1^*}}^{\mathrm{MASP2_{zym}}}} \\
+\frac{\left[\mathrm{pHFMASP3_{zym}}\right]}{K_{\mathrm{m\,MASP1^*}}^{\mathrm{MASP3_{zym}}}}+\frac{\left[\mathrm{pMFMASP1_{zym}}\right]}{K_{\mathrm{m\,MASP1^*}}^{\mathrm{MASP1_{zym}}}}+\frac{\left[\mathrm{pMFMASP2_{zym}}\right]}{K_{\mathrm{m\,MASP1^*}}^{\mathrm{MASP2_{zym}}}}+\frac{\left[\mathrm{pMFMASP3_{zym}}\right]}{K_{\mathrm{m\,MASP1^*}}^{\mathrm{MASP3_{zym}}}}
\end{array}\right)}
$$

$$
+\frac{k^{\mathrm{C4}}_{\mathrm{cat\,MASP1^*}}\left[\mathrm{pMFMASP1^*}\right]\left[\mathrm{C4}\right]}{K^{\mathrm{C4}}_{\mathrm{m\,MASP1^*}}\left(\begin{array}{l}
1+\frac{[\mathrm{C4}]}{K^{\mathrm{C4}}_{\mathrm{m\,MASP1^*}}}+\frac{[\mathrm{fC4bC2}]}{K^{\mathrm{C2}}_{\mathrm{m\,MASP1^*}}}+\frac{[\mathrm{fC4bC4bC2}]}{K^{\mathrm{C2}}_{\mathrm{m\,MASP1^*}}}+\frac{[\mathrm{fC3bC4bC2}]}{K^{\mathrm{C2}}_{\mathrm{m\,MASP1^*}}}+\frac{[\mathrm{pC4bC2}]}{K^{\mathrm{C2}}_{\mathrm{m\,MASP1^*}}}+\frac{[\mathrm{pC4bC4bC2}]}{K^{\mathrm{C2}}_{\mathrm{m\,MASP1^*}}}\\
+\frac{[\mathrm{pC3bC4bC2}]}{K^{\mathrm{C2}}_{\mathrm{m\,MASP1^*}}}+\frac{[\mathrm{pMBL_3MASP1_{zym}}]}{K^{\mathrm{MASP1_{zym}}}_{\mathrm{m\,MASP1^*}}}+\frac{[\mathrm{pMBL_3MASP2_{zym}}]}{K^{\mathrm{MASP2_{zym}}}_{\mathrm{m\,MASP1^*}}}+\frac{[\mathrm{pMBL_3MASP3_{zym}}]}{K^{\mathrm{MASP3_{zym}}}_{\mathrm{m\,MASP1^*}}}\\
+\frac{[\mathrm{pMBL_4MASP1_{zym}}]}{K^{\mathrm{MASP1_{zym}}}_{\mathrm{m\,MASP1^*}}}+\frac{[\mathrm{pMBL_4MASP2_{zym}}]}{K^{\mathrm{MASP2_{zym}}}_{\mathrm{m\,MASP1^*}}}+\frac{[\mathrm{pMBL_4MASP3_{zym}}]}{K^{\mathrm{MASP3_{zym}}}_{\mathrm{m\,MASP1^*}}}+\frac{[\mathrm{pCLLKMASP1_{zym}}]}{K^{\mathrm{MASP1_{zym}}}_{\mathrm{m\,MASP1^*}}}\\
+\frac{[\mathrm{pCLLKMASP2_{zym}}]}{K^{\mathrm{MASP2_{zym}}}_{\mathrm{m\,MASP1^*}}}+\frac{[\mathrm{pCLLKMASP3_{zym}}]}{K^{\mathrm{MASP3_{zym}}}_{\mathrm{m\,MASP1^*}}}+\frac{[\mathrm{pCLK1MASP1_{zym}}]}{K^{\mathrm{MASP1_{zym}}}_{\mathrm{m\,MASP1^*}}}+\frac{[\mathrm{pCLK1MASP2_{zym}}]}{K^{\mathrm{MASP2_{zym}}}_{\mathrm{m\,MASP1^*}}}\\
+\frac{[\mathrm{pCLK1MASP3_{zym}}]}{K^{\mathrm{MASP3_{zym}}}_{\mathrm{m\,MASP1^*}}}+\frac{[\mathrm{pCLL1MASP1_{zym}}]}{K^{\mathrm{MASP1_{zym}}}_{\mathrm{m\,MASP1^*}}}+\frac{[\mathrm{pCLL1MASP2_{zym}}]}{K^{\mathrm{MASP2_{zym}}}_{\mathrm{m\,MASP1^*}}}+\frac{[\mathrm{pCLL1MASP3_{zym}}]}{K^{\mathrm{MASP3_{zym}}}_{\mathrm{m\,MASP1^*}}}\\
+\frac{[\mathrm{pLFMASP1_{zym}}]}{K^{\mathrm{MASP1_{zym}}}_{\mathrm{m\,MASP1^*}}}+\frac{[\mathrm{pLFMASP2_{zym}}]}{K^{\mathrm{MASP2_{zym}}}_{\mathrm{m\,MASP1^*}}}+\frac{[\mathrm{pLFMASP3_{zym}}]}{K^{\mathrm{MASP3_{zym}}}_{\mathrm{m\,MASP1^*}}}+\frac{[\mathrm{pHFMASP1_{zym}}]}{K^{\mathrm{MASP1_{zym}}}_{\mathrm{m\,MASP1^*}}}+\frac{[\mathrm{pHFMASP2_{zym}}]}{K^{\mathrm{MASP2_{zym}}}_{\mathrm{m\,MASP1^*}}}\\
+\frac{[\mathrm{pHFMASP3_{zym}}]}{K^{\mathrm{MASP3_{zym}}}_{\mathrm{m\,MASP1^*}}}+\frac{[\mathrm{pMFMASP1_{zym}}]}{K^{\mathrm{MASP1_{zym}}}_{\mathrm{m\,MASP1^*}}}+\frac{[\mathrm{pMFMASP2_{zym}}]}{K^{\mathrm{MASP2_{zym}}}_{\mathrm{m\,MASP1^*}}}+\frac{[\mathrm{pMFMASP3_{zym}}]}{K^{\mathrm{MASP3_{zym}}}_{\mathrm{m\,MASP1^*}}}
\end{array}\right)}
$$

$$
+\frac{k^{\mathrm{C4}}_{\mathrm{cat\,MASP2^*}}\left[\mathrm{pMBL_3MASP2^*}\right]\left[\mathrm{C4}\right]}{K^{\mathrm{C4}}_{\mathrm{m\,MASP2^*}}\left(\begin{array}{l}
1+\frac{[\mathrm{C4}]}{K^{\mathrm{C4}}_{\mathrm{m\,MASP2^*}}}+\frac{[\mathrm{fC4bC2}]}{K^{\mathrm{C2}}_{\mathrm{m\,MASP2^*}}}+\frac{[\mathrm{fC4bC4bC2}]}{K^{\mathrm{C2}}_{\mathrm{m\,MASP2^*}}}+\frac{[\mathrm{fC3bC4bC2}]}{K^{\mathrm{C2}}_{\mathrm{m\,MASP2^*}}}+\frac{[\mathrm{pC4bC2}]}{K^{\mathrm{C2}}_{\mathrm{m\,MASP2^*}}}+\frac{[\mathrm{pC4bC4bC2}]}{K^{\mathrm{C2}}_{\mathrm{m\,MASP2^*}}}\\
+\frac{[\mathrm{pC3bC4bC2}]}{K^{\mathrm{C2}}_{\mathrm{m\,MASP2^*}}}+\frac{[\mathrm{pMBL_3MASP1_{zym}}]}{K^{\mathrm{MASP1_{zym}}}_{\mathrm{m\,MASP2^*}}}+\frac{[\mathrm{pMBL_3MASP2_{zym}}]}{K^{\mathrm{MASP2_{zym}}}_{\mathrm{m\,MASP2^*}}}+\frac{[\mathrm{pMBL_4MASP1_{zym}}]}{K^{\mathrm{MASP1_{zym}}}_{\mathrm{m\,MASP2^*}}}\\
+\frac{[\mathrm{pMBL_4MASP2_{zym}}]}{K^{\mathrm{MASP2_{zym}}}_{\mathrm{m\,MASP2^*}}}+\frac{[\mathrm{pCLLKMASP1_{zym}}]}{K^{\mathrm{MASP1_{zym}}}_{\mathrm{m\,MASP2^*}}}+\frac{[\mathrm{pCLLKMASP2_{zym}}]}{K^{\mathrm{MASP2_{zym}}}_{\mathrm{m\,MASP2^*}}}+\frac{[\mathrm{pCLK1MASP1_{zym}}]}{K^{\mathrm{MASP1_{zym}}}_{\mathrm{m\,MASP2^*}}}\\
+\frac{[\mathrm{pCLK1MASP2_{zym}}]}{K^{\mathrm{MASP2_{zym}}}_{\mathrm{m\,MASP2^*}}}+\frac{[\mathrm{pCLL1MASP1_{zym}}]}{K^{\mathrm{MASP1_{zym}}}_{\mathrm{m\,MASP2^*}}}+\frac{[\mathrm{pCLL1MASP2_{zym}}]}{K^{\mathrm{MASP2_{zym}}}_{\mathrm{m\,MASP2^*}}}+\frac{[\mathrm{pLFMASP1_{zym}}]}{K^{\mathrm{MASP1_{zym}}}_{\mathrm{m\,MASP2^*}}}\\
+\frac{[\mathrm{pLFMASP2_{zym}}]}{K^{\mathrm{MASP2_{zym}}}_{\mathrm{m\,MASP2^*}}}+\frac{[\mathrm{pHFMASP1_{zym}}]}{K^{\mathrm{MASP1_{zym}}}_{\mathrm{m\,MASP2^*}}}+\frac{[\mathrm{pHFMASP2_{zym}}]}{K^{\mathrm{MASP2_{zym}}}_{\mathrm{m\,MASP2^*}}}+\frac{[\mathrm{pMFMASP1_{zym}}]}{K^{\mathrm{MASP1_{zym}}}_{\mathrm{m\,MASP2^*}}}+\frac{[\mathrm{pMFMASP2_{zym}}]}{K^{\mathrm{MASP2_{zym}}}_{\mathrm{m\,MASP2^*}}}
\end{array}\right)}
$$

$$
+\frac{k_{\mathrm{cat\ MASP2^*}}^{\mathrm{C4}}\left[\mathrm{pMBL_4MASP2^*}\right]\left[\mathrm{C4}\right]}{K_{\mathrm{m\ MASP2^*}}^{\mathrm{C4}}\left(\begin{array}{l}1+\frac{[\mathrm{C4}]}{K_{\mathrm{m\ MASP2^*}}^{\mathrm{C4}}}+\frac{[\mathrm{fC4bC2}]}{K_{\mathrm{m\ MASP2^*}}^{\mathrm{C2}}}+\frac{[\mathrm{fC4bC4bC2}]}{K_{\mathrm{m\ MASP2^*}}^{\mathrm{C2}}}+\frac{[\mathrm{fC3bC4bC2}]}{K_{\mathrm{m\ MASP2^*}}^{\mathrm{C2}}}+\frac{[\mathrm{pC4bC2}]}{K_{\mathrm{m\ MASP2^*}}^{\mathrm{C2}}}+\frac{[\mathrm{pC4bC4bC2}]}{K_{\mathrm{m\ MASP2^*}}^{\mathrm{C2}}}\\ +\frac{[\mathrm{pC3bC4bC2}]}{K_{\mathrm{m\ MASP2^*}}^{\mathrm{C2}}}+\frac{[\mathrm{pMBL_3MASP1_{zym}}]}{K_{\mathrm{m\ MASP2^*}}^{\mathrm{MASP1_{zym}}}}+\frac{[\mathrm{pMBL_3MASP2_{zym}}]}{K_{\mathrm{m\ MASP2^*}}^{\mathrm{MASP2_{zym}}}}+\frac{[\mathrm{pMBL_4MASP1_{zym}}]}{K_{\mathrm{m\ MASP2^*}}^{\mathrm{MASP1_{zym}}}}\\ +\frac{[\mathrm{pMBL_4MASP2_{zym}}]}{K_{\mathrm{m\ MASP2^*}}^{\mathrm{MASP2_{zym}}}}+\frac{[\mathrm{pCLLKMASP1_{zym}}]}{K_{\mathrm{m\ MASP2^*}}^{\mathrm{MASP1_{zym}}}}+\frac{[\mathrm{pCLLKMASP2_{zym}}]}{K_{\mathrm{m\ MASP2^*}}^{\mathrm{MASP2_{zym}}}}+\frac{[\mathrm{pCLK1MASP1_{zym}}]}{K_{\mathrm{m\ MASP2^*}}^{\mathrm{MASP1_{zym}}}}\\ +\frac{[\mathrm{pCLK1MASP2_{zym}}]}{K_{\mathrm{m\ MASP2^*}}^{\mathrm{MASP2_{zym}}}}+\frac{[\mathrm{pCLL1MASP1_{zym}}]}{K_{\mathrm{m\ MASP2^*}}^{\mathrm{MASP1_{zym}}}}+\frac{[\mathrm{pCLL1MASP2_{zym}}]}{K_{\mathrm{m\ MASP2^*}}^{\mathrm{MASP2_{zym}}}}+\frac{[\mathrm{pLFMASP1_{zym}}]}{K_{\mathrm{m\ MASP2^*}}^{\mathrm{MASP1_{zym}}}}\\ +\frac{[\mathrm{pLFMASP2_{zym}}]}{K_{\mathrm{m\ MASP2^*}}^{\mathrm{MASP2_{zym}}}}+\frac{[\mathrm{pHFMASP1_{zym}}]}{K_{\mathrm{m\ MASP2^*}}^{\mathrm{MASP1_{zym}}}}+\frac{[\mathrm{pHFMASP2_{zym}}]}{K_{\mathrm{m\ MASP2^*}}^{\mathrm{MASP2_{zym}}}}+\frac{[\mathrm{pMFMASP1_{zym}}]}{K_{\mathrm{m\ MASP2^*}}^{\mathrm{MASP1_{zym}}}}+\frac{[\mathrm{pMFMASP2_{zym}}]}{K_{\mathrm{m\ MASP2^*}}^{\mathrm{MASP2_{zym}}}}\end{array}\right)}
$$

$$
+\frac{k_{\mathrm{cat\ MASP2^*}}^{\mathrm{C4}}\left[\mathrm{pCLLKMASP2^*}\right]\left[\mathrm{C4}\right]}{K_{\mathrm{m\ MASP2^*}}^{\mathrm{C4}}\left(\begin{array}{l}1+\frac{[\mathrm{C4}]}{K_{\mathrm{m\ MASP2^*}}^{\mathrm{C4}}}+\frac{[\mathrm{fC4bC2}]}{K_{\mathrm{m\ MASP2^*}}^{\mathrm{C2}}}+\frac{[\mathrm{fC4bC4bC2}]}{K_{\mathrm{m\ MASP2^*}}^{\mathrm{C2}}}+\frac{[\mathrm{fC3bC4bC2}]}{K_{\mathrm{m\ MASP2^*}}^{\mathrm{C2}}}+\frac{[\mathrm{pC4bC2}]}{K_{\mathrm{m\ MASP2^*}}^{\mathrm{C2}}}+\frac{[\mathrm{pC4bC4bC2}]}{K_{\mathrm{m\ MASP2^*}}^{\mathrm{C2}}}\\ +\frac{[\mathrm{pC3bC4bC2}]}{K_{\mathrm{m\ MASP2^*}}^{\mathrm{C2}}}+\frac{[\mathrm{pMBL_3MASP1_{zym}}]}{K_{\mathrm{m\ MASP2^*}}^{\mathrm{MASP1_{zym}}}}+\frac{[\mathrm{pMBL_3MASP2_{zym}}]}{K_{\mathrm{m\ MASP2^*}}^{\mathrm{MASP2_{zym}}}}+\frac{[\mathrm{pMBL_4MASP1_{zym}}]}{K_{\mathrm{m\ MASP2^*}}^{\mathrm{MASP1_{zym}}}}\\ +\frac{[\mathrm{pMBL_4MASP2_{zym}}]}{K_{\mathrm{m\ MASP2^*}}^{\mathrm{MASP2_{zym}}}}+\frac{[\mathrm{pCLLKMASP1_{zym}}]}{K_{\mathrm{m\ MASP2^*}}^{\mathrm{MASP1_{zym}}}}+\frac{[\mathrm{pCLLKMASP2_{zym}}]}{K_{\mathrm{m\ MASP2^*}}^{\mathrm{MASP2_{zym}}}}+\frac{[\mathrm{pCLK1MASP1_{zym}}]}{K_{\mathrm{m\ MASP2^*}}^{\mathrm{MASP1_{zym}}}}\\ +\frac{[\mathrm{pCLK1MASP2_{zym}}]}{K_{\mathrm{m\ MASP2^*}}^{\mathrm{MASP2_{zym}}}}+\frac{[\mathrm{pCLL1MASP1_{zym}}]}{K_{\mathrm{m\ MASP2^*}}^{\mathrm{MASP1_{zym}}}}+\frac{[\mathrm{pCLL1MASP2_{zym}}]}{K_{\mathrm{m\ MASP2^*}}^{\mathrm{MASP2_{zym}}}}+\frac{[\mathrm{pLFMASP1_{zym}}]}{K_{\mathrm{m\ MASP2^*}}^{\mathrm{MASP1_{zym}}}}\\ +\frac{[\mathrm{pLFMASP2_{zym}}]}{K_{\mathrm{m\ MASP2^*}}^{\mathrm{MASP2_{zym}}}}+\frac{[\mathrm{pHFMASP1_{zym}}]}{K_{\mathrm{m\ MASP2^*}}^{\mathrm{MASP1_{zym}}}}+\frac{[\mathrm{pHFMASP2_{zym}}]}{K_{\mathrm{m\ MASP2^*}}^{\mathrm{MASP2_{zym}}}}+\frac{[\mathrm{pMFMASP1_{zym}}]}{K_{\mathrm{m\ MASP2^*}}^{\mathrm{MASP1_{zym}}}}+\frac{[\mathrm{pMFMASP2_{zym}}]}{K_{\mathrm{m\ MASP2^*}}^{\mathrm{MASP2_{zym}}}}\end{array}\right)}
$$

$$
+\frac{k_{\mathrm{cat\ MASP2^*}}^{\mathrm{C4}}\left[\mathrm{pCLK1MASP2^*}\right]\left[\mathrm{C4}\right]}{K_{\mathrm{m\ MASP2^*}}^{\mathrm{C4}}\left(\begin{array}{l}1+\frac{[\mathrm{C4}]}{K_{\mathrm{m\ MASP2^*}}^{\mathrm{C4}}}+\frac{[\mathrm{fC4bC2}]}{K_{\mathrm{m\ MASP2^*}}^{\mathrm{C2}}}+\frac{[\mathrm{fC4bC4bC2}]}{K_{\mathrm{m\ MASP2^*}}^{\mathrm{C2}}}+\frac{[\mathrm{fC3bC4bC2}]}{K_{\mathrm{m\ MASP2^*}}^{\mathrm{C2}}}+\frac{[\mathrm{pC4bC2}]}{K_{\mathrm{m\ MASP2^*}}^{\mathrm{C2}}}+\frac{[\mathrm{pC4bC4bC2}]}{K_{\mathrm{m\ MASP2^*}}^{\mathrm{C2}}}\\ +\frac{[\mathrm{pC3bC4bC2}]}{K_{\mathrm{m\ MASP2^*}}^{\mathrm{C2}}}+\frac{[\mathrm{pMBL_3MASP1_{zym}}]}{K_{\mathrm{m\ MASP2^*}}^{\mathrm{MASP1_{zym}}}}+\frac{[\mathrm{pMBL_3MASP2_{zym}}]}{K_{\mathrm{m\ MASP2^*}}^{\mathrm{MASP2_{zym}}}}+\frac{[\mathrm{pMBL_4MASP1_{zym}}]}{K_{\mathrm{m\ MASP2^*}}^{\mathrm{MASP1_{zym}}}}\\ +\frac{[\mathrm{pMBL_4MASP2_{zym}}]}{K_{\mathrm{m\ MASP2^*}}^{\mathrm{MASP2_{zym}}}}+\frac{[\mathrm{pCLLKMASP1_{zym}}]}{K_{\mathrm{m\ MASP2^*}}^{\mathrm{MASP1_{zym}}}}+\frac{[\mathrm{pCLLKMASP2_{zym}}]}{K_{\mathrm{m\ MASP2^*}}^{\mathrm{MASP2_{zym}}}}+\frac{[\mathrm{pCLK1MASP1_{zym}}]}{K_{\mathrm{m\ MASP2^*}}^{\mathrm{MASP1_{zym}}}}\\ +\frac{[\mathrm{pCLK1MASP2_{zym}}]}{K_{\mathrm{m\ MASP2^*}}^{\mathrm{MASP2_{zym}}}}+\frac{[\mathrm{pCLL1MASP1_{zym}}]}{K_{\mathrm{m\ MASP2^*}}^{\mathrm{MASP1_{zym}}}}+\frac{[\mathrm{pCLL1MASP2_{zym}}]}{K_{\mathrm{m\ MASP2^*}}^{\mathrm{MASP2_{zym}}}}+\frac{[\mathrm{pLFMASP1_{zym}}]}{K_{\mathrm{m\ MASP2^*}}^{\mathrm{MASP1_{zym}}}}\\ +\frac{[\mathrm{pLFMASP2_{zym}}]}{K_{\mathrm{m\ MASP2^*}}^{\mathrm{MASP2_{zym}}}}+\frac{[\mathrm{pHFMASP1_{zym}}]}{K_{\mathrm{m\ MASP2^*}}^{\mathrm{MASP1_{zym}}}}+\frac{[\mathrm{pHFMASP2_{zym}}]}{K_{\mathrm{m\ MASP2^*}}^{\mathrm{MASP2_{zym}}}}+\frac{[\mathrm{pMFMASP1_{zym}}]}{K_{\mathrm{m\ MASP2^*}}^{\mathrm{MASP1_{zym}}}}+\frac{[\mathrm{pMFMASP2_{zym}}]}{K_{\mathrm{m\ MASP2^*}}^{\mathrm{MASP2_{zym}}}}\end{array}\right)}
$$

$$+\frac{k^{\mathrm{C4}}_{\mathrm{cat\,MASP2^*}}\left[\mathrm{pCLL1MASP2^*}\right]\left[\mathrm{C4}\right]}{K^{\mathrm{C4}}_{\mathrm{m\,MASP2^*}}\left(1+\frac{[\mathrm{C4}]}{K^{\mathrm{C4}}_{\mathrm{m\,MASP2^*}}}+\frac{[\mathrm{fC4bC2}]}{K^{\mathrm{C2}}_{\mathrm{m\,MASP2^*}}}+\frac{[\mathrm{fC4bC4bC2}]}{K^{\mathrm{C2}}_{\mathrm{m\,MASP2^*}}}+\frac{[\mathrm{fC3bC4bC2}]}{K^{\mathrm{C2}}_{\mathrm{m\,MASP2^*}}}+\frac{[\mathrm{pC4bC2}]}{K^{\mathrm{C2}}_{\mathrm{m\,MASP2^*}}}+\frac{[\mathrm{pC4bC4bC2}]}{K^{\mathrm{C2}}_{\mathrm{m\,MASP2^*}}}+\frac{[\mathrm{pC3bC4bC2}]}{K^{\mathrm{C2}}_{\mathrm{m\,MASP2^*}}}+\frac{[\mathrm{pMBL_3MASP1_{zym}}]}{K^{\mathrm{MASP1_{zym}}}_{\mathrm{m\,MASP2^*}}}+\frac{[\mathrm{pMBL_3MASP2_{zym}}]}{K^{\mathrm{MASP2_{zym}}}_{\mathrm{m\,MASP2^*}}}+\frac{[\mathrm{pMBL_4MASP1_{zym}}]}{K^{\mathrm{MASP1_{zym}}}_{\mathrm{m\,MASP2^*}}}+\frac{[\mathrm{pMBL_4MASP2_{zym}}]}{K^{\mathrm{MASP2_{zym}}}_{\mathrm{m\,MASP2^*}}}+\frac{[\mathrm{pCLLKMASP1_{zym}}]}{K^{\mathrm{MASP1_{zym}}}_{\mathrm{m\,MASP2^*}}}+\frac{[\mathrm{pCLLKMASP2_{zym}}]}{K^{\mathrm{MASP2_{zym}}}_{\mathrm{m\,MASP2^*}}}+\frac{[\mathrm{pCLK1MASP1_{zym}}]}{K^{\mathrm{MASP1_{zym}}}_{\mathrm{m\,MASP2^*}}}+\frac{[\mathrm{pCLK1MASP2_{zym}}]}{K^{\mathrm{MASP2_{zym}}}_{\mathrm{m\,MASP2^*}}}+\frac{[\mathrm{pCLL1MASP1_{zym}}]}{K^{\mathrm{MASP1_{zym}}}_{\mathrm{m\,MASP2^*}}}+\frac{[\mathrm{pCLL1MASP2_{zym}}]}{K^{\mathrm{MASP2_{zym}}}_{\mathrm{m\,MASP2^*}}}+\frac{[\mathrm{pLFMASP1_{zym}}]}{K^{\mathrm{MASP1_{zym}}}_{\mathrm{m\,MASP2^*}}}+\frac{[\mathrm{pLFMASP2_{zym}}]}{K^{\mathrm{MASP2_{zym}}}_{\mathrm{m\,MASP2^*}}}+\frac{[\mathrm{pHFMASP1_{zym}}]}{K^{\mathrm{MASP1_{zym}}}_{\mathrm{m\,MASP2^*}}}+\frac{[\mathrm{pHFMASP2_{zym}}]}{K^{\mathrm{MASP2_{zym}}}_{\mathrm{m\,MASP2^*}}}+\frac{[\mathrm{pMFMASP1_{zym}}]}{K^{\mathrm{MASP1_{zym}}}_{\mathrm{m\,MASP2^*}}}+\frac{[\mathrm{pMFMASP2_{zym}}]}{K^{\mathrm{MASP2_{zym}}}_{\mathrm{m\,MASP2^*}}}\right)}$$

$$+\frac{k^{\mathrm{C4}}_{\mathrm{cat\,MASP2^*}}\left[\mathrm{pLFMASP2^*}\right]\left[\mathrm{C4}\right]}{K^{\mathrm{C4}}_{\mathrm{m\,MASP2^*}}\left(1+\frac{[\mathrm{C4}]}{K^{\mathrm{C4}}_{\mathrm{m\,MASP2^*}}}+\frac{[\mathrm{fC4bC2}]}{K^{\mathrm{C2}}_{\mathrm{m\,MASP2^*}}}+\frac{[\mathrm{fC4bC4bC2}]}{K^{\mathrm{C2}}_{\mathrm{m\,MASP2^*}}}+\frac{[\mathrm{fC3bC4bC2}]}{K^{\mathrm{C2}}_{\mathrm{m\,MASP2^*}}}+\frac{[\mathrm{pC4bC2}]}{K^{\mathrm{C2}}_{\mathrm{m\,MASP2^*}}}+\frac{[\mathrm{pC4bC4bC2}]}{K^{\mathrm{C2}}_{\mathrm{m\,MASP2^*}}}+\frac{[\mathrm{pC3bC4bC2}]}{K^{\mathrm{C2}}_{\mathrm{m\,MASP2^*}}}+\frac{[\mathrm{pMBL_3MASP1_{zym}}]}{K^{\mathrm{MASP1_{zym}}}_{\mathrm{m\,MASP2^*}}}+\frac{[\mathrm{pMBL_3MASP2_{zym}}]}{K^{\mathrm{MASP2_{zym}}}_{\mathrm{m\,MASP2^*}}}+\frac{[\mathrm{pMBL_4MASP1_{zym}}]}{K^{\mathrm{MASP1_{zym}}}_{\mathrm{m\,MASP2^*}}}+\frac{[\mathrm{pMBL_4MASP2_{zym}}]}{K^{\mathrm{MASP2_{zym}}}_{\mathrm{m\,MASP2^*}}}+\frac{[\mathrm{pCLLKMASP1_{zym}}]}{K^{\mathrm{MASP1_{zym}}}_{\mathrm{m\,MASP2^*}}}+\frac{[\mathrm{pCLLKMASP2_{zym}}]}{K^{\mathrm{MASP2_{zym}}}_{\mathrm{m\,MASP2^*}}}+\frac{[\mathrm{pCLK1MASP1_{zym}}]}{K^{\mathrm{MASP1_{zym}}}_{\mathrm{m\,MASP2^*}}}+\frac{[\mathrm{pCLK1MASP2_{zym}}]}{K^{\mathrm{MASP2_{zym}}}_{\mathrm{m\,MASP2^*}}}+\frac{[\mathrm{pCLL1MASP1_{zym}}]}{K^{\mathrm{MASP1_{zym}}}_{\mathrm{m\,MASP2^*}}}+\frac{[\mathrm{pCLL1MASP2_{zym}}]}{K^{\mathrm{MASP2_{zym}}}_{\mathrm{m\,MASP2^*}}}+\frac{[\mathrm{pLFMASP1_{zym}}]}{K^{\mathrm{MASP1_{zym}}}_{\mathrm{m\,MASP2^*}}}+\frac{[\mathrm{pLFMASP2_{zym}}]}{K^{\mathrm{MASP2_{zym}}}_{\mathrm{m\,MASP2^*}}}+\frac{[\mathrm{pHFMASP1_{zym}}]}{K^{\mathrm{MASP1_{zym}}}_{\mathrm{m\,MASP2^*}}}+\frac{[\mathrm{pHFMASP2_{zym}}]}{K^{\mathrm{MASP2_{zym}}}_{\mathrm{m\,MASP2^*}}}+\frac{[\mathrm{pMFMASP1_{zym}}]}{K^{\mathrm{MASP1_{zym}}}_{\mathrm{m\,MASP2^*}}}+\frac{[\mathrm{pMFMASP2_{zym}}]}{K^{\mathrm{MASP2_{zym}}}_{\mathrm{m\,MASP2^*}}}\right)}$$

$$+\frac{k^{\mathrm{C4}}_{\mathrm{cat\,MASP2^*}}\left[\mathrm{pHFMASP2^*}\right]\left[\mathrm{C4}\right]}{K^{\mathrm{C4}}_{\mathrm{m\,MASP2^*}}\left(1+\frac{[\mathrm{C4}]}{K^{\mathrm{C4}}_{\mathrm{m\,MASP2^*}}}+\frac{[\mathrm{fC4bC2}]}{K^{\mathrm{C2}}_{\mathrm{m\,MASP2^*}}}+\frac{[\mathrm{fC4bC4bC2}]}{K^{\mathrm{C2}}_{\mathrm{m\,MASP2^*}}}+\frac{[\mathrm{fC3bC4bC2}]}{K^{\mathrm{C2}}_{\mathrm{m\,MASP2^*}}}+\frac{[\mathrm{pC4bC2}]}{K^{\mathrm{C2}}_{\mathrm{m\,MASP2^*}}}+\frac{[\mathrm{pC4bC4bC2}]}{K^{\mathrm{C2}}_{\mathrm{m\,MASP2^*}}}+\frac{[\mathrm{pC3bC4bC2}]}{K^{\mathrm{C2}}_{\mathrm{m\,MASP2^*}}}+\frac{[\mathrm{pMBL_3MASP1_{zym}}]}{K^{\mathrm{MASP1_{zym}}}_{\mathrm{m\,MASP2^*}}}+\frac{[\mathrm{pMBL_3MASP2_{zym}}]}{K^{\mathrm{MASP2_{zym}}}_{\mathrm{m\,MASP2^*}}}+\frac{[\mathrm{pMBL_4MASP1_{zym}}]}{K^{\mathrm{MASP1_{zym}}}_{\mathrm{m\,MASP2^*}}}+\frac{[\mathrm{pMBL_4MASP2_{zym}}]}{K^{\mathrm{MASP2_{zym}}}_{\mathrm{m\,MASP2^*}}}+\frac{[\mathrm{pCLLKMASP1_{zym}}]}{K^{\mathrm{MASP1_{zym}}}_{\mathrm{m\,MASP2^*}}}+\frac{[\mathrm{pCLLKMASP2_{zym}}]}{K^{\mathrm{MASP2_{zym}}}_{\mathrm{m\,MASP2^*}}}+\frac{[\mathrm{pCLK1MASP1_{zym}}]}{K^{\mathrm{MASP1_{zym}}}_{\mathrm{m\,MASP2^*}}}+\frac{[\mathrm{pCLK1MASP2_{zym}}]}{K^{\mathrm{MASP2_{zym}}}_{\mathrm{m\,MASP2^*}}}+\frac{[\mathrm{pCLL1MASP1_{zym}}]}{K^{\mathrm{MASP1_{zym}}}_{\mathrm{m\,MASP2^*}}}+\frac{[\mathrm{pCLL1MASP2_{zym}}]}{K^{\mathrm{MASP2_{zym}}}_{\mathrm{m\,MASP2^*}}}+\frac{[\mathrm{pLFMASP1_{zym}}]}{K^{\mathrm{MASP1_{zym}}}_{\mathrm{m\,MASP2^*}}}+\frac{[\mathrm{pLFMASP2_{zym}}]}{K^{\mathrm{MASP2_{zym}}}_{\mathrm{m\,MASP2^*}}}+\frac{[\mathrm{pHFMASP1_{zym}}]}{K^{\mathrm{MASP1_{zym}}}_{\mathrm{m\,MASP2^*}}}+\frac{[\mathrm{pHFMASP2_{zym}}]}{K^{\mathrm{MASP2_{zym}}}_{\mathrm{m\,MASP2^*}}}+\frac{[\mathrm{pMFMASP1_{zym}}]}{K^{\mathrm{MASP1_{zym}}}_{\mathrm{m\,MASP2^*}}}+\frac{[\mathrm{pMFMASP2_{zym}}]}{K^{\mathrm{MASP2_{zym}}}_{\mathrm{m\,MASP2^*}}}\right)}$$

$$+\frac{k^{\mathrm{C4}}_{\mathrm{cat\,MASP2^*}}\left[\mathrm{pMFMASP2^*}\right]\left[\mathrm{C4}\right]}{K^{\mathrm{C4}}_{\mathrm{m\,MASP2^*}}\left(\begin{array}{l}1+\frac{[\mathrm{C4}]}{K^{\mathrm{C4}}_{\mathrm{m\,MASP2^*}}}+\frac{[\mathrm{fC4bC2}]}{K^{\mathrm{C2}}_{\mathrm{m\,MASP2^*}}}+\frac{[\mathrm{fC4bC4bC2}]}{K^{\mathrm{C2}}_{\mathrm{m\,MASP2^*}}}+\frac{[\mathrm{fC3bC4bC2}]}{K^{\mathrm{C2}}_{\mathrm{m\,MASP2^*}}}+\frac{[\mathrm{pC4bC2}]}{K^{\mathrm{C2}}_{\mathrm{m\,MASP2^*}}}+\frac{[\mathrm{pC4bC4bC2}]}{K^{\mathrm{C2}}_{\mathrm{m\,MASP2^*}}}\\+\frac{[\mathrm{pC3bC4bC2}]}{K^{\mathrm{C2}}_{\mathrm{m\,MASP2^*}}}+\frac{[\mathrm{pMBL_3MASP1_{zym}}]}{K^{\mathrm{MASP1_{zym}}}_{\mathrm{m\,MASP2^*}}}+\frac{[\mathrm{pMBL_3MASP2_{zym}}]}{K^{\mathrm{MASP2_{zym}}}_{\mathrm{m\,MASP2^*}}}+\frac{[\mathrm{pMBL_4MASP1_{zym}}]}{K^{\mathrm{MASP1_{zym}}}_{\mathrm{m\,MASP2^*}}}\\+\frac{[\mathrm{pMBL_4MASP2_{zym}}]}{K^{\mathrm{MASP2_{zym}}}_{\mathrm{m\,MASP2^*}}}+\frac{[\mathrm{pCLLKMASP1_{zym}}]}{K^{\mathrm{MASP1_{zym}}}_{\mathrm{m\,MASP2^*}}}+\frac{[\mathrm{pCLLKMASP2_{zym}}]}{K^{\mathrm{MASP2_{zym}}}_{\mathrm{m\,MASP2^*}}}+\frac{[\mathrm{pCLK1MASP1_{zym}}]}{K^{\mathrm{MASP1_{zym}}}_{\mathrm{m\,MASP2^*}}}\\+\frac{[\mathrm{pCLK1MASP2_{zym}}]}{K^{\mathrm{MASP2_{zym}}}_{\mathrm{m\,MASP2^*}}}+\frac{[\mathrm{pCLL1MASP1_{zym}}]}{K^{\mathrm{MASP1_{zym}}}_{\mathrm{m\,MASP2^*}}}+\frac{[\mathrm{pCLL1MASP2_{zym}}]}{K^{\mathrm{MASP2_{zym}}}_{\mathrm{m\,MASP2^*}}}+\frac{[\mathrm{pLFMASP1_{zym}}]}{K^{\mathrm{MASP1_{zym}}}_{\mathrm{m\,MASP2^*}}}\\+\frac{[\mathrm{pLFMASP2_{zym}}]}{K^{\mathrm{MASP2_{zym}}}_{\mathrm{m\,MASP2^*}}}+\frac{[\mathrm{pHFMASP1_{zym}}]}{K^{\mathrm{MASP1_{zym}}}_{\mathrm{m\,MASP2^*}}}+\frac{[\mathrm{pHFMASP2_{zym}}]}{K^{\mathrm{MASP2_{zym}}}_{\mathrm{m\,MASP2^*}}}+\frac{[\mathrm{pMFMASP1_{zym}}]}{K^{\mathrm{MASP1_{zym}}}_{\mathrm{m\,MASP2^*}}}+\frac{[\mathrm{pMFMASP2_{zym}}]}{K^{\mathrm{MASP2_{zym}}}_{\mathrm{m\,MASP2^*}}}\end{array}\right)}$$

$$+\frac{k^{\mathrm{C4}}_{\mathrm{cat\,C1^*}}\left[\mathrm{pMBL_3PTX3C1^*}\right]\left[\mathrm{C4}\right]}{K^{\mathrm{C4}}_{\mathrm{m\,C1^*}}\left(1+\frac{[\mathrm{C4}]}{K^{\mathrm{C4}}_{\mathrm{m\,C1^*}}}+\frac{[\mathrm{fC4bC2}]}{K^{\mathrm{C2}}_{\mathrm{m\,C1^*}}}+\frac{[\mathrm{fC4bC4bC2}]}{K^{\mathrm{C2}}_{\mathrm{m\,C1^*}}}+\frac{[\mathrm{fC3bC4bC2}]}{K^{\mathrm{C2}}_{\mathrm{m\,C1^*}}}+\frac{[\mathrm{pC4bC2}]}{K^{\mathrm{C2}}_{\mathrm{m\,C1^*}}}+\frac{[\mathrm{pC4bC4bC2}]}{K^{\mathrm{C2}}_{\mathrm{m\,C1^*}}}+\frac{[\mathrm{pC3bC4bC2}]}{K^{\mathrm{C2}}_{\mathrm{m\,C1^*}}}\right)}$$

$$+\frac{k^{\mathrm{C4}}_{\mathrm{cat\,C1^*}}\left[\mathrm{pMBL_4PTX3C1^*}\right]\left[\mathrm{C4}\right]}{K^{\mathrm{C4}}_{\mathrm{m\,C1^*}}\left(1+\frac{[\mathrm{C4}]}{K^{\mathrm{C4}}_{\mathrm{m\,C1^*}}}+\frac{[\mathrm{fC4bC2}]}{K^{\mathrm{C2}}_{\mathrm{m\,C1^*}}}+\frac{[\mathrm{fC4bC4bC2}]}{K^{\mathrm{C2}}_{\mathrm{m\,C1^*}}}+\frac{[\mathrm{fC3bC4bC2}]}{K^{\mathrm{C2}}_{\mathrm{m\,C1^*}}}+\frac{[\mathrm{pC4bC2}]}{K^{\mathrm{C2}}_{\mathrm{m\,C1^*}}}+\frac{[\mathrm{pC4bC4bC2}]}{K^{\mathrm{C2}}_{\mathrm{m\,C1^*}}}+\frac{[\mathrm{pC3bC4bC2}]}{K^{\mathrm{C2}}_{\mathrm{m\,C1^*}}}\right)}$$

$$+\frac{k^{\mathrm{C4}}_{\mathrm{cat\,C1^*}}\left[\mathrm{CRPC1^*}\right]\left[\mathrm{C4}\right]}{K^{\mathrm{C4}}_{\mathrm{m\,C1^*}}\left(1+\frac{[\mathrm{C4}]}{K^{\mathrm{C4}}_{\mathrm{m\,C1^*}}}+\frac{[\mathrm{fC4bC2}]}{K^{\mathrm{C2}}_{\mathrm{m\,C1^*}}}+\frac{[\mathrm{fC4bC4bC2}]}{K^{\mathrm{C2}}_{\mathrm{m\,C1^*}}}+\frac{[\mathrm{fC3bC4bC2}]}{K^{\mathrm{C2}}_{\mathrm{m\,C1^*}}}\right)}$$

35

$$\frac{d[\mathrm{fC4b}]}{dt}=k^{+}_{\mathrm{fC4b}}[\mathrm{nfC4b}][\mathrm{H_2O}]-k^{+}_{\mathrm{C4bC2}}[\mathrm{fC4b}][\mathrm{C2}]+k^{-}_{\mathrm{C4bC2}}[\mathrm{fC4bC2}]+k^{-}_{\mathrm{C4bC2a}}[\mathrm{fC4bC2a}]-k^{+}_{\mathrm{C4bC4b}}[\mathrm{nfC4b}][\mathrm{fC4b}]$$
$$-k^{+}_{\mathrm{C4bC4BP}}[\mathrm{fC4b}][\mathrm{C4BP}]+k^{-}_{\mathrm{C4bC4BP}}[\mathrm{fC4bC4BP}]+k^{-}_{\mathrm{C4bC2aC4BP_{decay}}}[\mathrm{fC4bC2aC4BP}]-k^{+}_{\mathrm{C4bCR1}}[\mathrm{fC4b}][\mathrm{CR1}]$$
$$+k^{-}_{\mathrm{C4bCR1}}[\mathrm{fC4bCR1}]+k^{-}_{\mathrm{C4bC2aCR1_{decay}}}[\mathrm{fC4bC2aCR1}]-k^{+}_{\mathrm{C3bC4b}}[\mathrm{nfC3b}][\mathrm{fC4b}]$$

36

$$\frac{d[\mathrm{fC4bC2}]}{dt} = k^{+}_{\mathrm{C4bC2}}[\mathrm{fC4b}][\mathrm{C2}] - k^{-}_{\mathrm{C4bC2}}[\mathrm{fC4bC2}]$$

$$-\frac{k^{\mathrm{C2}}_{\mathrm{cat\ C1^*}}\left[\mathrm{C1^*}\right][\mathrm{fC4bC2}]}{K^{\mathrm{C2}}_{\mathrm{m\ C1^*}}\left(1+\frac{[\mathrm{C4}]}{K^{\mathrm{C4}}_{\mathrm{m\ C1^*}}}+\frac{[\mathrm{fC4bC2}]}{K^{\mathrm{C2}}_{\mathrm{m\ C1^*}}}+\frac{[\mathrm{fC4bC4bC2}]}{K^{\mathrm{C2}}_{\mathrm{m\ C1^*}}}+\frac{[\mathrm{fC3bC4bC2}]}{K^{\mathrm{C2}}_{\mathrm{m\ C1^*}}}+\frac{[\mathrm{pC4bC2}]}{K^{\mathrm{C2}}_{\mathrm{m\ C1^*}}}+\frac{[\mathrm{pC4bC4bC2}]}{K^{\mathrm{C2}}_{\mathrm{m\ C1^*}}}+\frac{[\mathrm{pC3bC4bC2}]}{K^{\mathrm{C2}}_{\mathrm{m\ C1^*}}}\right)}$$

$$-\frac{k^{\mathrm{C2}}_{\mathrm{cat\ C1^*}}\left[\mathrm{pIgG_6C1^*}\right][\mathrm{fC4bC2}]}{K^{\mathrm{C2}}_{\mathrm{m\ C1^*}}\left(1+\frac{[\mathrm{C4}]}{K^{\mathrm{C4}}_{\mathrm{m\ C1^*}}}+\frac{[\mathrm{fC4bC2}]}{K^{\mathrm{C2}}_{\mathrm{m\ C1^*}}}+\frac{[\mathrm{fC4bC4bC2}]}{K^{\mathrm{C2}}_{\mathrm{m\ C1^*}}}+\frac{[\mathrm{fC3bC4bC2}]}{K^{\mathrm{C2}}_{\mathrm{m\ C1^*}}}+\frac{[\mathrm{pC4bC2}]}{K^{\mathrm{C2}}_{\mathrm{m\ C1^*}}}+\frac{[\mathrm{pC4bC4bC2}]}{K^{\mathrm{C2}}_{\mathrm{m\ C1^*}}}+\frac{[\mathrm{pC3bC4bC2}]}{K^{\mathrm{C2}}_{\mathrm{m\ C1^*}}}\right)}$$

$$-\frac{k^{\mathrm{C2}}_{\mathrm{cat\ C1^*}}\left[\mathrm{pIgMC1^*}\right][\mathrm{fC4bC2}]}{K^{\mathrm{C2}}_{\mathrm{m\ C1^*}}\left(1+\frac{[\mathrm{C4}]}{K^{\mathrm{C4}}_{\mathrm{m\ C1^*}}}+\frac{[\mathrm{fC4bC2}]}{K^{\mathrm{C2}}_{\mathrm{m\ C1^*}}}+\frac{[\mathrm{fC4bC4bC2}]}{K^{\mathrm{C2}}_{\mathrm{m\ C1^*}}}+\frac{[\mathrm{fC3bC4bC2}]}{K^{\mathrm{C2}}_{\mathrm{m\ C1^*}}}+\frac{[\mathrm{pC4bC2}]}{K^{\mathrm{C2}}_{\mathrm{m\ C1^*}}}+\frac{[\mathrm{pC4bC4bC2}]}{K^{\mathrm{C2}}_{\mathrm{m\ C1^*}}}+\frac{[\mathrm{pC3bC4bC2}]}{K^{\mathrm{C2}}_{\mathrm{m\ C1^*}}}\right)}$$

$$-\frac{k^{\mathrm{C2}}_{\mathrm{cat\ MASP1^*}}\left[\mathrm{pMBL_3MASP1^*}\right][\mathrm{fC4bC2}]}{K^{\mathrm{C2}}_{\mathrm{m\ MASP1^*}}\left(\begin{array}{l}1+\frac{[\mathrm{C4}]}{K^{\mathrm{C4}}_{\mathrm{m\ MASP1^*}}}+\frac{[\mathrm{fC4bC2}]}{K^{\mathrm{C2}}_{\mathrm{m\ MASP1^*}}}+\frac{[\mathrm{fC4bC4bC2}]}{K^{\mathrm{C2}}_{\mathrm{m\ MASP1^*}}}+\frac{[\mathrm{fC3bC4bC2}]}{K^{\mathrm{C2}}_{\mathrm{m\ MASP1^*}}}+\frac{[\mathrm{pC4bC2}]}{K^{\mathrm{C2}}_{\mathrm{m\ MASP1^*}}}+\frac{[\mathrm{pC4bC4bC2}]}{K^{\mathrm{C2}}_{\mathrm{m\ MASP1^*}}}\\
+\frac{[\mathrm{pC3bC4bC2}]}{K^{\mathrm{C2}}_{\mathrm{m\ MASP1^*}}}+\frac{[\mathrm{pMBL_3MASP1_{zym}}]}{K^{\mathrm{MASP1_{zym}}}_{\mathrm{m\ MASP1^*}}}+\frac{[\mathrm{pMBL_3MASP2_{zym}}]}{K^{\mathrm{MASP2_{zym}}}_{\mathrm{m\ MASP1^*}}}+\frac{[\mathrm{pMBL_3MASP3_{zym}}]}{K^{\mathrm{MASP3_{zym}}}_{\mathrm{m\ MASP1^*}}}\\
+\frac{[\mathrm{pMBL_4MASP1_{zym}}]}{K^{\mathrm{MASP1_{zym}}}_{\mathrm{m\ MASP1^*}}}+\frac{[\mathrm{pMBL_4MASP2_{zym}}]}{K^{\mathrm{MASP2_{zym}}}_{\mathrm{m\ MASP1^*}}}+\frac{[\mathrm{pMBL_4MASP3_{zym}}]}{K^{\mathrm{MASP3_{zym}}}_{\mathrm{m\ MASP1^*}}}+\frac{[\mathrm{pCLLKMASP1_{zym}}]}{K^{\mathrm{MASP1_{zym}}}_{\mathrm{m\ MASP1^*}}}\\
+\frac{[\mathrm{pCLLKMASP2_{zym}}]}{K^{\mathrm{MASP2_{zym}}}_{\mathrm{m\ MASP1^*}}}+\frac{[\mathrm{pCLLKMASP3_{zym}}]}{K^{\mathrm{MASP3_{zym}}}_{\mathrm{m\ MASP1^*}}}+\frac{[\mathrm{pCLK1MASP1_{zym}}]}{K^{\mathrm{MASP1_{zym}}}_{\mathrm{m\ MASP1^*}}}+\frac{[\mathrm{pCLK1MASP2_{zym}}]}{K^{\mathrm{MASP2_{zym}}}_{\mathrm{m\ MASP1^*}}}\\
+\frac{[\mathrm{pCLK1MASP3_{zym}}]}{K^{\mathrm{MASP3_{zym}}}_{\mathrm{m\ MASP1^*}}}+\frac{[\mathrm{pCLL1MASP1_{zym}}]}{K^{\mathrm{MASP1_{zym}}}_{\mathrm{m\ MASP1^*}}}+\frac{[\mathrm{pCLL1MASP2_{zym}}]}{K^{\mathrm{MASP2_{zym}}}_{\mathrm{m\ MASP1^*}}}+\frac{[\mathrm{pCLL1MASP3_{zym}}]}{K^{\mathrm{MASP3_{zym}}}_{\mathrm{m\ MASP1^*}}}\\
+\frac{[\mathrm{pLFMASP1_{zym}}]}{K^{\mathrm{MASP1_{zym}}}_{\mathrm{m\ MASP1^*}}}+\frac{[\mathrm{pLFMASP2_{zym}}]}{K^{\mathrm{MASP2_{zym}}}_{\mathrm{m\ MASP1^*}}}+\frac{[\mathrm{pLFMASP3_{zym}}]}{K^{\mathrm{MASP3_{zym}}}_{\mathrm{m\ MASP1^*}}}+\frac{[\mathrm{pHFMASP1_{zym}}]}{K^{\mathrm{MASP1_{zym}}}_{\mathrm{m\ MASP1^*}}}+\frac{[\mathrm{pHFMASP2_{zym}}]}{K^{\mathrm{MASP2_{zym}}}_{\mathrm{m\ MASP1^*}}}\\
+\frac{[\mathrm{pHFMASP3_{zym}}]}{K^{\mathrm{MASP3_{zym}}}_{\mathrm{m\ MASP1^*}}}+\frac{[\mathrm{pMFMASP1_{zym}}]}{K^{\mathrm{MASP1_{zym}}}_{\mathrm{m\ MASP1^*}}}+\frac{[\mathrm{pMFMASP2_{zym}}]}{K^{\mathrm{MASP2_{zym}}}_{\mathrm{m\ MASP1^*}}}+\frac{[\mathrm{pMFMASP3_{zym}}]}{K^{\mathrm{MASP3_{zym}}}_{\mathrm{m\ MASP1^*}}}\end{array}\right)}$$

$$
-\frac{k^{\mathrm{C2}}_{\mathrm{cat\,MASP1^*}}\left[\mathrm{pMBL_4MASP1^*}\right]\left[\mathrm{fC4bC2}\right]}{K^{\mathrm{C2}}_{\mathrm{m\,MASP1^*}}\left(\begin{array}{l}1+\frac{[\mathrm{C4}]}{K^{\mathrm{C4}}_{\mathrm{m\,MASP1^*}}}+\frac{[\mathrm{fC4bC2}]}{K^{\mathrm{C2}}_{\mathrm{m\,MASP1^*}}}+\frac{[\mathrm{fC4bC4bC2}]}{K^{\mathrm{C2}}_{\mathrm{m\,MASP1^*}}}+\frac{[\mathrm{fC3bC4bC2}]}{K^{\mathrm{C2}}_{\mathrm{m\,MASP1^*}}}+\frac{[\mathrm{pC4bC2}]}{K^{\mathrm{C2}}_{\mathrm{m\,MASP1^*}}}+\frac{[\mathrm{pC4bC4bC2}]}{K^{\mathrm{C2}}_{\mathrm{m\,MASP1^*}}}\\
+\frac{[\mathrm{pC3bC4bC2}]}{K^{\mathrm{C2}}_{\mathrm{m\,MASP1^*}}}+\frac{\left[\mathrm{pMBL_3MASP1_{zym}}\right]}{K^{\mathrm{MASP1_{zym}}}_{\mathrm{m\,MASP1^*}}}+\frac{\left[\mathrm{pMBL_3MASP2_{zym}}\right]}{K^{\mathrm{MASP2_{zym}}}_{\mathrm{m\,MASP1^*}}}+\frac{\left[\mathrm{pMBL_3MASP3_{zym}}\right]}{K^{\mathrm{MASP3_{zym}}}_{\mathrm{m\,MASP1^*}}}\\
+\frac{\left[\mathrm{pMBL_4MASP1_{zym}}\right]}{K^{\mathrm{MASP1_{zym}}}_{\mathrm{m\,MASP1^*}}}+\frac{\left[\mathrm{pMBL_4MASP2_{zym}}\right]}{K^{\mathrm{MASP2_{zym}}}_{\mathrm{m\,MASP1^*}}}+\frac{\left[\mathrm{pMBL_4MASP3_{zym}}\right]}{K^{\mathrm{MASP3_{zym}}}_{\mathrm{m\,MASP1^*}}}+\frac{\left[\mathrm{pCLLKMASP1_{zym}}\right]}{K^{\mathrm{MASP1_{zym}}}_{\mathrm{m\,MASP1^*}}}\\
+\frac{\left[\mathrm{pCLLKMASP2_{zym}}\right]}{K^{\mathrm{MASP2_{zym}}}_{\mathrm{m\,MASP1^*}}}+\frac{\left[\mathrm{pCLLKMASP3_{zym}}\right]}{K^{\mathrm{MASP3_{zym}}}_{\mathrm{m\,MASP1^*}}}+\frac{\left[\mathrm{pCLK1MASP1_{zym}}\right]}{K^{\mathrm{MASP1_{zym}}}_{\mathrm{m\,MASP1^*}}}+\frac{\left[\mathrm{pCLK1MASP2_{zym}}\right]}{K^{\mathrm{MASP2_{zym}}}_{\mathrm{m\,MASP1^*}}}\\
+\frac{\left[\mathrm{pCLK1MASP3_{zym}}\right]}{K^{\mathrm{MASP3_{zym}}}_{\mathrm{m\,MASP1^*}}}+\frac{\left[\mathrm{pCLL1MASP1_{zym}}\right]}{K^{\mathrm{MASP1_{zym}}}_{\mathrm{m\,MASP1^*}}}+\frac{\left[\mathrm{pCLL1MASP2_{zym}}\right]}{K^{\mathrm{MASP2_{zym}}}_{\mathrm{m\,MASP1^*}}}+\frac{\left[\mathrm{pCLL1MASP3_{zym}}\right]}{K^{\mathrm{MASP3_{zym}}}_{\mathrm{m\,MASP1^*}}}\\
+\frac{\left[\mathrm{pLFMASP1_{zym}}\right]}{K^{\mathrm{MASP1_{zym}}}_{\mathrm{m\,MASP1^*}}}+\frac{\left[\mathrm{pLFMASP2_{zym}}\right]}{K^{\mathrm{MASP2_{zym}}}_{\mathrm{m\,MASP1^*}}}+\frac{\left[\mathrm{pLFMASP3_{zym}}\right]}{K^{\mathrm{MASP3_{zym}}}_{\mathrm{m\,MASP1^*}}}+\frac{\left[\mathrm{pHFMASP1_{zym}}\right]}{K^{\mathrm{MASP1_{zym}}}_{\mathrm{m\,MASP1^*}}}+\frac{\left[\mathrm{pHFMASP2_{zym}}\right]}{K^{\mathrm{MASP2_{zym}}}_{\mathrm{m\,MASP1^*}}}\\
+\frac{\left[\mathrm{pHFMASP3_{zym}}\right]}{K^{\mathrm{MASP3_{zym}}}_{\mathrm{m\,MASP1^*}}}+\frac{\left[\mathrm{pMFMASP1_{zym}}\right]}{K^{\mathrm{MASP1_{zym}}}_{\mathrm{m\,MASP1^*}}}+\frac{\left[\mathrm{pMFMASP2_{zym}}\right]}{K^{\mathrm{MASP2_{zym}}}_{\mathrm{m\,MASP1^*}}}+\frac{\left[\mathrm{pMFMASP3_{zym}}\right]}{K^{\mathrm{MASP3_{zym}}}_{\mathrm{m\,MASP1^*}}}\end{array}\right)}
$$

$$
-\frac{k^{\mathrm{C2}}_{\mathrm{cat\,MASP1^*}}\left[\mathrm{pCLLKMASP1^*}\right]\left[\mathrm{fC4bC2}\right]}{K^{\mathrm{C2}}_{\mathrm{m\,MASP1^*}}\left(\begin{array}{l}1+\frac{[\mathrm{C4}]}{K^{\mathrm{C4}}_{\mathrm{m\,MASP1^*}}}+\frac{[\mathrm{fC4bC2}]}{K^{\mathrm{C2}}_{\mathrm{m\,MASP1^*}}}+\frac{[\mathrm{fC4bC4bC2}]}{K^{\mathrm{C2}}_{\mathrm{m\,MASP1^*}}}+\frac{[\mathrm{fC3bC4bC2}]}{K^{\mathrm{C2}}_{\mathrm{m\,MASP1^*}}}+\frac{[\mathrm{pC4bC2}]}{K^{\mathrm{C2}}_{\mathrm{m\,MASP1^*}}}+\frac{[\mathrm{pC4bC4bC2}]}{K^{\mathrm{C2}}_{\mathrm{m\,MASP1^*}}}\\
+\frac{[\mathrm{pC3bC4bC2}]}{K^{\mathrm{C2}}_{\mathrm{m\,MASP1^*}}}+\frac{\left[\mathrm{pMBL_3MASP1_{zym}}\right]}{K^{\mathrm{MASP1_{zym}}}_{\mathrm{m\,MASP1^*}}}+\frac{\left[\mathrm{pMBL_3MASP2_{zym}}\right]}{K^{\mathrm{MASP2_{zym}}}_{\mathrm{m\,MASP1^*}}}+\frac{\left[\mathrm{pMBL_3MASP3_{zym}}\right]}{K^{\mathrm{MASP3_{zym}}}_{\mathrm{m\,MASP1^*}}}\\
+\frac{\left[\mathrm{pMBL_4MASP1_{zym}}\right]}{K^{\mathrm{MASP1_{zym}}}_{\mathrm{m\,MASP1^*}}}+\frac{\left[\mathrm{pMBL_4MASP2_{zym}}\right]}{K^{\mathrm{MASP2_{zym}}}_{\mathrm{m\,MASP1^*}}}+\frac{\left[\mathrm{pMBL_4MASP3_{zym}}\right]}{K^{\mathrm{MASP3_{zym}}}_{\mathrm{m\,MASP1^*}}}+\frac{\left[\mathrm{pCLLKMASP1_{zym}}\right]}{K^{\mathrm{MASP1_{zym}}}_{\mathrm{m\,MASP1^*}}}\\
+\frac{\left[\mathrm{pCLLKMASP2_{zym}}\right]}{K^{\mathrm{MASP2_{zym}}}_{\mathrm{m\,MASP1^*}}}+\frac{\left[\mathrm{pCLLKMASP3_{zym}}\right]}{K^{\mathrm{MASP3_{zym}}}_{\mathrm{m\,MASP1^*}}}+\frac{\left[\mathrm{pCLK1MASP1_{zym}}\right]}{K^{\mathrm{MASP1_{zym}}}_{\mathrm{m\,MASP1^*}}}+\frac{\left[\mathrm{pCLK1MASP2_{zym}}\right]}{K^{\mathrm{MASP2_{zym}}}_{\mathrm{m\,MASP1^*}}}\\
+\frac{\left[\mathrm{pCLK1MASP3_{zym}}\right]}{K^{\mathrm{MASP3_{zym}}}_{\mathrm{m\,MASP1^*}}}+\frac{\left[\mathrm{pCLL1MASP1_{zym}}\right]}{K^{\mathrm{MASP1_{zym}}}_{\mathrm{m\,MASP1^*}}}+\frac{\left[\mathrm{pCLL1MASP2_{zym}}\right]}{K^{\mathrm{MASP2_{zym}}}_{\mathrm{m\,MASP1^*}}}+\frac{\left[\mathrm{pCLL1MASP3_{zym}}\right]}{K^{\mathrm{MASP3_{zym}}}_{\mathrm{m\,MASP1^*}}}\\
+\frac{\left[\mathrm{pLFMASP1_{zym}}\right]}{K^{\mathrm{MASP1_{zym}}}_{\mathrm{m\,MASP1^*}}}+\frac{\left[\mathrm{pLFMASP2_{zym}}\right]}{K^{\mathrm{MASP2_{zym}}}_{\mathrm{m\,MASP1^*}}}+\frac{\left[\mathrm{pLFMASP3_{zym}}\right]}{K^{\mathrm{MASP3_{zym}}}_{\mathrm{m\,MASP1^*}}}+\frac{\left[\mathrm{pHFMASP1_{zym}}\right]}{K^{\mathrm{MASP1_{zym}}}_{\mathrm{m\,MASP1^*}}}+\frac{\left[\mathrm{pHFMASP2_{zym}}\right]}{K^{\mathrm{MASP2_{zym}}}_{\mathrm{m\,MASP1^*}}}\\
+\frac{\left[\mathrm{pHFMASP3_{zym}}\right]}{K^{\mathrm{MASP3_{zym}}}_{\mathrm{m\,MASP1^*}}}+\frac{\left[\mathrm{pMFMASP1_{zym}}\right]}{K^{\mathrm{MASP1_{zym}}}_{\mathrm{m\,MASP1^*}}}+\frac{\left[\mathrm{pMFMASP2_{zym}}\right]}{K^{\mathrm{MASP2_{zym}}}_{\mathrm{m\,MASP1^*}}}+\frac{\left[\mathrm{pMFMASP3_{zym}}\right]}{K^{\mathrm{MASP3_{zym}}}_{\mathrm{m\,MASP1^*}}}\end{array}\right)}
$$

$$
-\frac{k_{\mathrm{cat\,MASP1^*}}^{\mathrm{C2}}\left[\mathrm{pCLK1MASP1^*}\right]\left[\mathrm{fC4bC2}\right]}{K_{\mathrm{m\,MASP1^*}}^{\mathrm{C2}}\left(\begin{aligned}&1+\frac{[\mathrm{C4}]}{K_{\mathrm{m\,MASP1^*}}^{\mathrm{C4}}}+\frac{[\mathrm{fC4bC2}]}{K_{\mathrm{m\,MASP1^*}}^{\mathrm{C2}}}+\frac{[\mathrm{fC4bC4bC2}]}{K_{\mathrm{m\,MASP1^*}}^{\mathrm{C2}}}+\frac{[\mathrm{fC3bC4bC2}]}{K_{\mathrm{m\,MASP1^*}}^{\mathrm{C2}}}+\frac{[\mathrm{pC4bC2}]}{K_{\mathrm{m\,MASP1^*}}^{\mathrm{C2}}}+\frac{[\mathrm{pC4bC4bC2}]}{K_{\mathrm{m\,MASP1^*}}^{\mathrm{C2}}}\\
&+\frac{[\mathrm{pC3bC4bC2}]}{K_{\mathrm{m\,MASP1^*}}^{\mathrm{C2}}}+\frac{[\mathrm{pMBL_3MASP1_{zym}}]}{K_{\mathrm{m\,MASP1^*}}^{\mathrm{MASP1_{zym}}}}+\frac{[\mathrm{pMBL_3MASP2_{zym}}]}{K_{\mathrm{m\,MASP1^*}}^{\mathrm{MASP2_{zym}}}}+\frac{[\mathrm{pMBL_3MASP3_{zym}}]}{K_{\mathrm{m\,MASP1^*}}^{\mathrm{MASP3_{zym}}}}\\
&+\frac{[\mathrm{pMBL_4MASP1_{zym}}]}{K_{\mathrm{m\,MASP1^*}}^{\mathrm{MASP1_{zym}}}}+\frac{[\mathrm{pMBL_4MASP2_{zym}}]}{K_{\mathrm{m\,MASP1^*}}^{\mathrm{MASP2_{zym}}}}+\frac{[\mathrm{pMBL_4MASP3_{zym}}]}{K_{\mathrm{m\,MASP1^*}}^{\mathrm{MASP3_{zym}}}}+\frac{[\mathrm{pCLLKMASP1_{zym}}]}{K_{\mathrm{m\,MASP1^*}}^{\mathrm{MASP1_{zym}}}}\\
&+\frac{[\mathrm{pCLLKMASP2_{zym}}]}{K_{\mathrm{m\,MASP1^*}}^{\mathrm{MASP2_{zym}}}}+\frac{[\mathrm{pCLLKMASP3_{zym}}]}{K_{\mathrm{m\,MASP1^*}}^{\mathrm{MASP3_{zym}}}}+\frac{[\mathrm{pCLK1MASP1_{zym}}]}{K_{\mathrm{m\,MASP1^*}}^{\mathrm{MASP1_{zym}}}}+\frac{[\mathrm{pCLK1MASP2_{zym}}]}{K_{\mathrm{m\,MASP1^*}}^{\mathrm{MASP2_{zym}}}}\\
&+\frac{[\mathrm{pCLK1MASP3_{zym}}]}{K_{\mathrm{m\,MASP1^*}}^{\mathrm{MASP3_{zym}}}}+\frac{[\mathrm{pCLL1MASP1_{zym}}]}{K_{\mathrm{m\,MASP1^*}}^{\mathrm{MASP1_{zym}}}}+\frac{[\mathrm{pCLL1MASP2_{zym}}]}{K_{\mathrm{m\,MASP1^*}}^{\mathrm{MASP2_{zym}}}}+\frac{[\mathrm{pCLL1MASP3_{zym}}]}{K_{\mathrm{m\,MASP1^*}}^{\mathrm{MASP3_{zym}}}}\\
&+\frac{[\mathrm{pLFMASP1_{zym}}]}{K_{\mathrm{m\,MASP1^*}}^{\mathrm{MASP1_{zym}}}}+\frac{[\mathrm{pLFMASP2_{zym}}]}{K_{\mathrm{m\,MASP1^*}}^{\mathrm{MASP2_{zym}}}}+\frac{[\mathrm{pLFMASP3_{zym}}]}{K_{\mathrm{m\,MASP1^*}}^{\mathrm{MASP3_{zym}}}}+\frac{[\mathrm{pHFMASP1_{zym}}]}{K_{\mathrm{m\,MASP1^*}}^{\mathrm{MASP1_{zym}}}}+\frac{[\mathrm{pHFMASP2_{zym}}]}{K_{\mathrm{m\,MASP1^*}}^{\mathrm{MASP2_{zym}}}}\\
&+\frac{[\mathrm{pHFMASP3_{zym}}]}{K_{\mathrm{m\,MASP1^*}}^{\mathrm{MASP3_{zym}}}}+\frac{[\mathrm{pMFMASP1_{zym}}]}{K_{\mathrm{m\,MASP1^*}}^{\mathrm{MASP1_{zym}}}}+\frac{[\mathrm{pMFMASP2_{zym}}]}{K_{\mathrm{m\,MASP1^*}}^{\mathrm{MASP2_{zym}}}}+\frac{[\mathrm{pMFMASP3_{zym}}]}{K_{\mathrm{m\,MASP1^*}}^{\mathrm{MASP3_{zym}}}}\end{aligned}\right)}
$$

$$
-\frac{k_{\mathrm{cat\,MASP1^*}}^{\mathrm{C2}}\left[\mathrm{pCLL1MASP1^*}\right]\left[\mathrm{fC4bC2}\right]}{K_{\mathrm{m\,MASP1^*}}^{\mathrm{C2}}\left(\begin{aligned}&1+\frac{[\mathrm{C4}]}{K_{\mathrm{m\,MASP1^*}}^{\mathrm{C4}}}+\frac{[\mathrm{fC4bC2}]}{K_{\mathrm{m\,MASP1^*}}^{\mathrm{C2}}}+\frac{[\mathrm{fC4bC4bC2}]}{K_{\mathrm{m\,MASP1^*}}^{\mathrm{C2}}}+\frac{[\mathrm{fC3bC4bC2}]}{K_{\mathrm{m\,MASP1^*}}^{\mathrm{C2}}}+\frac{[\mathrm{pC4bC2}]}{K_{\mathrm{m\,MASP1^*}}^{\mathrm{C2}}}+\frac{[\mathrm{pC4bC4bC2}]}{K_{\mathrm{m\,MASP1^*}}^{\mathrm{C2}}}\\
&+\frac{[\mathrm{pC3bC4bC2}]}{K_{\mathrm{m\,MASP1^*}}^{\mathrm{C2}}}+\frac{[\mathrm{pMBL_3MASP1_{zym}}]}{K_{\mathrm{m\,MASP1^*}}^{\mathrm{MASP1_{zym}}}}+\frac{[\mathrm{pMBL_3MASP2_{zym}}]}{K_{\mathrm{m\,MASP1^*}}^{\mathrm{MASP2_{zym}}}}+\frac{[\mathrm{pMBL_3MASP3_{zym}}]}{K_{\mathrm{m\,MASP1^*}}^{\mathrm{MASP3_{zym}}}}\\
&+\frac{[\mathrm{pMBL_4MASP1_{zym}}]}{K_{\mathrm{m\,MASP1^*}}^{\mathrm{MASP1_{zym}}}}+\frac{[\mathrm{pMBL_4MASP2_{zym}}]}{K_{\mathrm{m\,MASP1^*}}^{\mathrm{MASP2_{zym}}}}+\frac{[\mathrm{pMBL_4MASP3_{zym}}]}{K_{\mathrm{m\,MASP1^*}}^{\mathrm{MASP3_{zym}}}}+\frac{[\mathrm{pCLLKMASP1_{zym}}]}{K_{\mathrm{m\,MASP1^*}}^{\mathrm{MASP1_{zym}}}}\\
&+\frac{[\mathrm{pCLLKMASP2_{zym}}]}{K_{\mathrm{m\,MASP1^*}}^{\mathrm{MASP2_{zym}}}}+\frac{[\mathrm{pCLLKMASP3_{zym}}]}{K_{\mathrm{m\,MASP1^*}}^{\mathrm{MASP3_{zym}}}}+\frac{[\mathrm{pCLK1MASP1_{zym}}]}{K_{\mathrm{m\,MASP1^*}}^{\mathrm{MASP1_{zym}}}}+\frac{[\mathrm{pCLK1MASP2_{zym}}]}{K_{\mathrm{m\,MASP1^*}}^{\mathrm{MASP2_{zym}}}}\\
&+\frac{[\mathrm{pCLK1MASP3_{zym}}]}{K_{\mathrm{m\,MASP1^*}}^{\mathrm{MASP3_{zym}}}}+\frac{[\mathrm{pCLL1MASP1_{zym}}]}{K_{\mathrm{m\,MASP1^*}}^{\mathrm{MASP1_{zym}}}}+\frac{[\mathrm{pCLL1MASP2_{zym}}]}{K_{\mathrm{m\,MASP1^*}}^{\mathrm{MASP2_{zym}}}}+\frac{[\mathrm{pCLL1MASP3_{zym}}]}{K_{\mathrm{m\,MASP1^*}}^{\mathrm{MASP3_{zym}}}}\\
&+\frac{[\mathrm{pLFMASP1_{zym}}]}{K_{\mathrm{m\,MASP1^*}}^{\mathrm{MASP1_{zym}}}}+\frac{[\mathrm{pLFMASP2_{zym}}]}{K_{\mathrm{m\,MASP1^*}}^{\mathrm{MASP2_{zym}}}}+\frac{[\mathrm{pLFMASP3_{zym}}]}{K_{\mathrm{m\,MASP1^*}}^{\mathrm{MASP3_{zym}}}}+\frac{[\mathrm{pHFMASP1_{zym}}]}{K_{\mathrm{m\,MASP1^*}}^{\mathrm{MASP1_{zym}}}}+\frac{[\mathrm{pHFMASP2_{zym}}]}{K_{\mathrm{m\,MASP1^*}}^{\mathrm{MASP2_{zym}}}}\\
&+\frac{[\mathrm{pHFMASP3_{zym}}]}{K_{\mathrm{m\,MASP1^*}}^{\mathrm{MASP3_{zym}}}}+\frac{[\mathrm{pMFMASP1_{zym}}]}{K_{\mathrm{m\,MASP1^*}}^{\mathrm{MASP1_{zym}}}}+\frac{[\mathrm{pMFMASP2_{zym}}]}{K_{\mathrm{m\,MASP1^*}}^{\mathrm{MASP2_{zym}}}}+\frac{[\mathrm{pMFMASP3_{zym}}]}{K_{\mathrm{m\,MASP1^*}}^{\mathrm{MASP3_{zym}}}}\end{aligned}\right)}
$$

$$-\frac{k_{\text{cat MASP1}^*}^{\text{C2}}\left[\text{pLFMASP1}^*\right]\left[\text{fC4bC2}\right]}{K_{\text{m MASP1}^*}^{\text{C2}}\left(\begin{aligned}&1+\frac{[\text{C4}]}{K_{\text{m MASP1}^*}^{\text{C4}}}+\frac{[\text{fC4bC2}]}{K_{\text{m MASP1}^*}^{\text{C2}}}+\frac{[\text{fC4bC4bC2}]}{K_{\text{m MASP1}^*}^{\text{C2}}}+\frac{[\text{fC3bC4bC2}]}{K_{\text{m MASP1}^*}^{\text{C2}}}+\frac{[\text{pC4bC2}]}{K_{\text{m MASP1}^*}^{\text{C2}}}+\frac{[\text{pC4bC4bC2}]}{K_{\text{m MASP1}^*}^{\text{C2}}}\\&+\frac{[\text{pC3bC4bC2}]}{K_{\text{m MASP1}^*}^{\text{C2}}}+\frac{[\text{pMBL}_3\text{MASP1}_{\text{zym}}]}{K_{\text{m MASP1}^*}^{\text{MASP1}_{\text{zym}}}}+\frac{[\text{pMBL}_3\text{MASP2}_{\text{zym}}]}{K_{\text{m MASP1}^*}^{\text{MASP2}_{\text{zym}}}}+\frac{[\text{pMBL}_3\text{MASP3}_{\text{zym}}]}{K_{\text{m MASP1}^*}^{\text{MASP3}_{\text{zym}}}}\\&+\frac{[\text{pMBL}_4\text{MASP1}_{\text{zym}}]}{K_{\text{m MASP1}^*}^{\text{MASP1}_{\text{zym}}}}+\frac{[\text{pMBL}_4\text{MASP2}_{\text{zym}}]}{K_{\text{m MASP1}^*}^{\text{MASP2}_{\text{zym}}}}+\frac{[\text{pMBL}_4\text{MASP3}_{\text{zym}}]}{K_{\text{m MASP1}^*}^{\text{MASP3}_{\text{zym}}}}+\frac{[\text{pCLLKMASP1}_{\text{zym}}]}{K_{\text{m MASP1}^*}^{\text{MASP1}_{\text{zym}}}}\\&+\frac{[\text{pCLLKMASP2}_{\text{zym}}]}{K_{\text{m MASP1}^*}^{\text{MASP2}_{\text{zym}}}}+\frac{[\text{pCLLKMASP3}_{\text{zym}}]}{K_{\text{m MASP1}^*}^{\text{MASP3}_{\text{zym}}}}+\frac{[\text{pCLK1MASP1}_{\text{zym}}]}{K_{\text{m MASP1}^*}^{\text{MASP1}_{\text{zym}}}}+\frac{[\text{pCLK1MASP2}_{\text{zym}}]}{K_{\text{m MASP1}^*}^{\text{MASP2}_{\text{zym}}}}\\&+\frac{[\text{pCLK1MASP3}_{\text{zym}}]}{K_{\text{m MASP1}^*}^{\text{MASP3}_{\text{zym}}}}+\frac{[\text{pCLL1MASP1}_{\text{zym}}]}{K_{\text{m MASP1}^*}^{\text{MASP1}_{\text{zym}}}}+\frac{[\text{pCLL1MASP2}_{\text{zym}}]}{K_{\text{m MASP1}^*}^{\text{MASP2}_{\text{zym}}}}+\frac{[\text{pCLL1MASP3}_{\text{zym}}]}{K_{\text{m MASP1}^*}^{\text{MASP3}_{\text{zym}}}}\\&+\frac{[\text{pLFMASP1}_{\text{zym}}]}{K_{\text{m MASP1}^*}^{\text{MASP1}_{\text{zym}}}}+\frac{[\text{pLFMASP2}_{\text{zym}}]}{K_{\text{m MASP1}^*}^{\text{MASP2}_{\text{zym}}}}+\frac{[\text{pLFMASP3}_{\text{zym}}]}{K_{\text{m MASP1}^*}^{\text{MASP3}_{\text{zym}}}}+\frac{[\text{pHFMASP1}_{\text{zym}}]}{K_{\text{m MASP1}^*}^{\text{MASP1}_{\text{zym}}}}+\frac{[\text{pHFMASP2}_{\text{zym}}]}{K_{\text{m MASP1}^*}^{\text{MASP2}_{\text{zym}}}}\\&+\frac{[\text{pHFMASP3}_{\text{zym}}]}{K_{\text{m MASP1}^*}^{\text{MASP3}_{\text{zym}}}}+\frac{[\text{pMFMASP1}_{\text{zym}}]}{K_{\text{m MASP1}^*}^{\text{MASP1}_{\text{zym}}}}+\frac{[\text{pMFMASP2}_{\text{zym}}]}{K_{\text{m MASP1}^*}^{\text{MASP2}_{\text{zym}}}}+\frac{[\text{pMFMASP3}_{\text{zym}}]}{K_{\text{m MASP1}^*}^{\text{MASP3}_{\text{zym}}}}\end{aligned}\right)}$$

$$-\frac{k_{\text{cat MASP1}^*}^{\text{C2}}\left[\text{pHFMASP1}^*\right]\left[\text{fC4bC2}\right]}{K_{\text{m MASP1}^*}^{\text{C2}}\left(\begin{aligned}&1+\frac{[\text{C4}]}{K_{\text{m MASP1}^*}^{\text{C4}}}+\frac{[\text{fC4bC2}]}{K_{\text{m MASP1}^*}^{\text{C2}}}+\frac{[\text{fC4bC4bC2}]}{K_{\text{m MASP1}^*}^{\text{C2}}}+\frac{[\text{fC3bC4bC2}]}{K_{\text{m MASP1}^*}^{\text{C2}}}+\frac{[\text{pC4bC2}]}{K_{\text{m MASP1}^*}^{\text{C2}}}+\frac{[\text{pC4bC4bC2}]}{K_{\text{m MASP1}^*}^{\text{C2}}}\\&+\frac{[\text{pC3bC4bC2}]}{K_{\text{m MASP1}^*}^{\text{C2}}}+\frac{[\text{pMBL}_3\text{MASP1}_{\text{zym}}]}{K_{\text{m MASP1}^*}^{\text{MASP1}_{\text{zym}}}}+\frac{[\text{pMBL}_3\text{MASP2}_{\text{zym}}]}{K_{\text{m MASP1}^*}^{\text{MASP2}_{\text{zym}}}}+\frac{[\text{pMBL}_3\text{MASP3}_{\text{zym}}]}{K_{\text{m MASP1}^*}^{\text{MASP3}_{\text{zym}}}}\\&+\frac{[\text{pMBL}_4\text{MASP1}_{\text{zym}}]}{K_{\text{m MASP1}^*}^{\text{MASP1}_{\text{zym}}}}+\frac{[\text{pMBL}_4\text{MASP2}_{\text{zym}}]}{K_{\text{m MASP1}^*}^{\text{MASP2}_{\text{zym}}}}+\frac{[\text{pMBL}_4\text{MASP3}_{\text{zym}}]}{K_{\text{m MASP1}^*}^{\text{MASP3}_{\text{zym}}}}+\frac{[\text{pCLLKMASP1}_{\text{zym}}]}{K_{\text{m MASP1}^*}^{\text{MASP1}_{\text{zym}}}}\\&+\frac{[\text{pCLLKMASP2}_{\text{zym}}]}{K_{\text{m MASP1}^*}^{\text{MASP2}_{\text{zym}}}}+\frac{[\text{pCLLKMASP3}_{\text{zym}}]}{K_{\text{m MASP1}^*}^{\text{MASP3}_{\text{zym}}}}+\frac{[\text{pCLK1MASP1}_{\text{zym}}]}{K_{\text{m MASP1}^*}^{\text{MASP1}_{\text{zym}}}}+\frac{[\text{pCLK1MASP2}_{\text{zym}}]}{K_{\text{m MASP1}^*}^{\text{MASP2}_{\text{zym}}}}\\&+\frac{[\text{pCLK1MASP3}_{\text{zym}}]}{K_{\text{m MASP1}^*}^{\text{MASP3}_{\text{zym}}}}+\frac{[\text{pCLL1MASP1}_{\text{zym}}]}{K_{\text{m MASP1}^*}^{\text{MASP1}_{\text{zym}}}}+\frac{[\text{pCLL1MASP2}_{\text{zym}}]}{K_{\text{m MASP1}^*}^{\text{MASP2}_{\text{zym}}}}+\frac{[\text{pCLL1MASP3}_{\text{zym}}]}{K_{\text{m MASP1}^*}^{\text{MASP3}_{\text{zym}}}}\\&+\frac{[\text{pLFMASP1}_{\text{zym}}]}{K_{\text{m MASP1}^*}^{\text{MASP1}_{\text{zym}}}}+\frac{[\text{pLFMASP2}_{\text{zym}}]}{K_{\text{m MASP1}^*}^{\text{MASP2}_{\text{zym}}}}+\frac{[\text{pLFMASP3}_{\text{zym}}]}{K_{\text{m MASP1}^*}^{\text{MASP3}_{\text{zym}}}}+\frac{[\text{pHFMASP1}_{\text{zym}}]}{K_{\text{m MASP1}^*}^{\text{MASP1}_{\text{zym}}}}+\frac{[\text{pHFMASP2}_{\text{zym}}]}{K_{\text{m MASP1}^*}^{\text{MASP2}_{\text{zym}}}}\\&+\frac{[\text{pHFMASP3}_{\text{zym}}]}{K_{\text{m MASP1}^*}^{\text{MASP3}_{\text{zym}}}}+\frac{[\text{pMFMASP1}_{\text{zym}}]}{K_{\text{m MASP1}^*}^{\text{MASP1}_{\text{zym}}}}+\frac{[\text{pMFMASP2}_{\text{zym}}]}{K_{\text{m MASP1}^*}^{\text{MASP2}_{\text{zym}}}}+\frac{[\text{pMFMASP3}_{\text{zym}}]}{K_{\text{m MASP1}^*}^{\text{MASP3}_{\text{zym}}}}\end{aligned}\right)}$$

$$-\frac{k^{\mathrm{C2}}_{\mathrm{cat\,MASP1^*}}\left[\mathrm{pMFMASP1^*}\right]\left[\mathrm{fC4bC2}\right]}{K^{\mathrm{C2}}_{\mathrm{m\,MASP1^*}}\left(\begin{array}{l}1+\frac{[\mathrm{C4}]}{K^{\mathrm{C4}}_{\mathrm{m\,MASP1^*}}}+\frac{[\mathrm{fC4bC2}]}{K^{\mathrm{C2}}_{\mathrm{m\,MASP1^*}}}+\frac{[\mathrm{fC4bC4bC2}]}{K^{\mathrm{C2}}_{\mathrm{m\,MASP1^*}}}+\frac{[\mathrm{fC3bC4bC2}]}{K^{\mathrm{C2}}_{\mathrm{m\,MASP1^*}}}+\frac{[\mathrm{pC4bC2}]}{K^{\mathrm{C2}}_{\mathrm{m\,MASP1^*}}}+\frac{[\mathrm{pC4bC4bC2}]}{K^{\mathrm{C2}}_{\mathrm{m\,MASP1^*}}}\\
+\frac{[\mathrm{pC3bC4bC2}]}{K^{\mathrm{C2}}_{\mathrm{m\,MASP1^*}}}+\frac{[\mathrm{pMBL_3MASP1_{zym}}]}{K^{\mathrm{MASP1_{zym}}}_{\mathrm{m\,MASP1^*}}}+\frac{[\mathrm{pMBL_3MASP2_{zym}}]}{K^{\mathrm{MASP2_{zym}}}_{\mathrm{m\,MASP1^*}}}+\frac{[\mathrm{pMBL_3MASP3_{zym}}]}{K^{\mathrm{MASP3_{zym}}}_{\mathrm{m\,MASP1^*}}}\\
+\frac{[\mathrm{pMBL_4MASP1_{zym}}]}{K^{\mathrm{MASP1_{zym}}}_{\mathrm{m\,MASP1^*}}}+\frac{[\mathrm{pMBL_4MASP2_{zym}}]}{K^{\mathrm{MASP2_{zym}}}_{\mathrm{m\,MASP1^*}}}+\frac{[\mathrm{pMBL_4MASP3_{zym}}]}{K^{\mathrm{MASP3_{zym}}}_{\mathrm{m\,MASP1^*}}}+\frac{[\mathrm{pCLLKMASP1_{zym}}]}{K^{\mathrm{MASP1_{zym}}}_{\mathrm{m\,MASP1^*}}}\\
+\frac{[\mathrm{pCLLKMASP2_{zym}}]}{K^{\mathrm{MASP2_{zym}}}_{\mathrm{m\,MASP1^*}}}+\frac{[\mathrm{pCLLKMASP3_{zym}}]}{K^{\mathrm{MASP3_{zym}}}_{\mathrm{m\,MASP1^*}}}+\frac{[\mathrm{pCLK1MASP1_{zym}}]}{K^{\mathrm{MASP1_{zym}}}_{\mathrm{m\,MASP1^*}}}+\frac{[\mathrm{pCLK1MASP2_{zym}}]}{K^{\mathrm{MASP2_{zym}}}_{\mathrm{m\,MASP1^*}}}\\
+\frac{[\mathrm{pCLK1MASP3_{zym}}]}{K^{\mathrm{MASP3_{zym}}}_{\mathrm{m\,MASP1^*}}}+\frac{[\mathrm{pCLL1MASP1_{zym}}]}{K^{\mathrm{MASP1_{zym}}}_{\mathrm{m\,MASP1^*}}}+\frac{[\mathrm{pCLL1MASP2_{zym}}]}{K^{\mathrm{MASP2_{zym}}}_{\mathrm{m\,MASP1^*}}}+\frac{[\mathrm{pCLL1MASP3_{zym}}]}{K^{\mathrm{MASP3_{zym}}}_{\mathrm{m\,MASP1^*}}}\\
+\frac{[\mathrm{pLFMASP1_{zym}}]}{K^{\mathrm{MASP1_{zym}}}_{\mathrm{m\,MASP1^*}}}+\frac{[\mathrm{pLFMASP2_{zym}}]}{K^{\mathrm{MASP2_{zym}}}_{\mathrm{m\,MASP1^*}}}+\frac{[\mathrm{pLFMASP3_{zym}}]}{K^{\mathrm{MASP3_{zym}}}_{\mathrm{m\,MASP1^*}}}+\frac{[\mathrm{pHFMASP1_{zym}}]}{K^{\mathrm{MASP1_{zym}}}_{\mathrm{m\,MASP1^*}}}+\frac{[\mathrm{pHFMASP2_{zym}}]}{K^{\mathrm{MASP2_{zym}}}_{\mathrm{m\,MASP1^*}}}\\
+\frac{[\mathrm{pHFMASP3_{zym}}]}{K^{\mathrm{MASP3_{zym}}}_{\mathrm{m\,MASP1^*}}}+\frac{[\mathrm{pMFMASP1_{zym}}]}{K^{\mathrm{MASP1_{zym}}}_{\mathrm{m\,MASP1^*}}}+\frac{[\mathrm{pMFMASP2_{zym}}]}{K^{\mathrm{MASP2_{zym}}}_{\mathrm{m\,MASP1^*}}}+\frac{[\mathrm{pMFMASP3_{zym}}]}{K^{\mathrm{MASP3_{zym}}}_{\mathrm{m\,MASP1^*}}}\end{array}\right)}$$

$$-\frac{k^{\mathrm{C2}}_{\mathrm{cat\,MASP2^*}}\left[\mathrm{pMBL_3MASP2^*}\right]\left[\mathrm{fC4bC2}\right]}{K^{\mathrm{C2}}_{\mathrm{m\,MASP2^*}}\left(\begin{array}{l}1+\frac{[\mathrm{C4}]}{K^{\mathrm{C4}}_{\mathrm{m\,MASP2^*}}}+\frac{[\mathrm{fC4bC2}]}{K^{\mathrm{C2}}_{\mathrm{m\,MASP2^*}}}+\frac{[\mathrm{fC4bC4bC2}]}{K^{\mathrm{C2}}_{\mathrm{m\,MASP2^*}}}+\frac{[\mathrm{fC3bC4bC2}]}{K^{\mathrm{C2}}_{\mathrm{m\,MASP2^*}}}+\frac{[\mathrm{pC4bC2}]}{K^{\mathrm{C2}}_{\mathrm{m\,MASP2^*}}}+\frac{[\mathrm{pC4bC4bC2}]}{K^{\mathrm{C2}}_{\mathrm{m\,MASP2^*}}}\\
+\frac{[\mathrm{pC3bC4bC2}]}{K^{\mathrm{C2}}_{\mathrm{m\,MASP2^*}}}+\frac{[\mathrm{pMBL_3MASP1_{zym}}]}{K^{\mathrm{MASP1_{zym}}}_{\mathrm{m\,MASP2^*}}}+\frac{[\mathrm{pMBL_3MASP2_{zym}}]}{K^{\mathrm{MASP2_{zym}}}_{\mathrm{m\,MASP2^*}}}+\frac{[\mathrm{pMBL_4MASP1_{zym}}]}{K^{\mathrm{MASP1_{zym}}}_{\mathrm{m\,MASP2^*}}}\\
+\frac{[\mathrm{pMBL_4MASP2_{zym}}]}{K^{\mathrm{MASP2_{zym}}}_{\mathrm{m\,MASP2^*}}}+\frac{[\mathrm{pCLLKMASP1_{zym}}]}{K^{\mathrm{MASP1_{zym}}}_{\mathrm{m\,MASP2^*}}}+\frac{[\mathrm{pCLLKMASP2_{zym}}]}{K^{\mathrm{MASP2_{zym}}}_{\mathrm{m\,MASP2^*}}}+\frac{[\mathrm{pCLK1MASP1_{zym}}]}{K^{\mathrm{MASP1_{zym}}}_{\mathrm{m\,MASP2^*}}}\\
+\frac{[\mathrm{pCLK1MASP2_{zym}}]}{K^{\mathrm{MASP2_{zym}}}_{\mathrm{m\,MASP2^*}}}+\frac{[\mathrm{pCLL1MASP1_{zym}}]}{K^{\mathrm{MASP1_{zym}}}_{\mathrm{m\,MASP2^*}}}+\frac{[\mathrm{pCLL1MASP2_{zym}}]}{K^{\mathrm{MASP2_{zym}}}_{\mathrm{m\,MASP2^*}}}+\frac{[\mathrm{pLFMASP1_{zym}}]}{K^{\mathrm{MASP1_{zym}}}_{\mathrm{m\,MASP2^*}}}\\
+\frac{[\mathrm{pLFMASP2_{zym}}]}{K^{\mathrm{MASP2_{zym}}}_{\mathrm{m\,MASP2^*}}}+\frac{[\mathrm{pHFMASP1_{zym}}]}{K^{\mathrm{MASP1_{zym}}}_{\mathrm{m\,MASP2^*}}}+\frac{[\mathrm{pHFMASP2_{zym}}]}{K^{\mathrm{MASP2_{zym}}}_{\mathrm{m\,MASP2^*}}}+\frac{[\mathrm{pMFMASP1_{zym}}]}{K^{\mathrm{MASP1_{zym}}}_{\mathrm{m\,MASP2^*}}}+\frac{[\mathrm{pMFMASP2_{zym}}]}{K^{\mathrm{MASP2_{zym}}}_{\mathrm{m\,MASP2^*}}}\end{array}\right)}$$

$$-\frac{k^{\mathrm{C2}}_{\mathrm{cat\ MASP2^*}}\left[\mathrm{pMBL_4MASP2^*}\right]\left[\mathrm{fC4bC2}\right]}{K^{\mathrm{C2}}_{\mathrm{m\ MASP2^*}}\left(\begin{aligned}&1+\frac{[\mathrm{C4}]}{K^{\mathrm{C4}}_{\mathrm{m\ MASP2^*}}}+\frac{[\mathrm{fC4bC2}]}{K^{\mathrm{C2}}_{\mathrm{m\ MASP2^*}}}+\frac{[\mathrm{fC4bC4bC2}]}{K^{\mathrm{C2}}_{\mathrm{m\ MASP2^*}}}+\frac{[\mathrm{fC3bC4bC2}]}{K^{\mathrm{C2}}_{\mathrm{m\ MASP2^*}}}+\frac{[\mathrm{pC4bC2}]}{K^{\mathrm{C2}}_{\mathrm{m\ MASP2^*}}}+\frac{[\mathrm{pC4bC4bC2}]}{K^{\mathrm{C2}}_{\mathrm{m\ MASP2^*}}}\\&+\frac{[\mathrm{pC3bC4bC2}]}{K^{\mathrm{C2}}_{\mathrm{m\ MASP2^*}}}+\frac{[\mathrm{pMBL_3MASP1_{zym}}]}{K^{\mathrm{MASP1_{zym}}}_{\mathrm{m\ MASP2^*}}}+\frac{[\mathrm{pMBL_3MASP2_{zym}}]}{K^{\mathrm{MASP2_{zym}}}_{\mathrm{m\ MASP2^*}}}+\frac{[\mathrm{pMBL_4MASP1_{zym}}]}{K^{\mathrm{MASP1_{zym}}}_{\mathrm{m\ MASP2^*}}}\\&+\frac{[\mathrm{pMBL_4MASP2_{zym}}]}{K^{\mathrm{MASP2_{zym}}}_{\mathrm{m\ MASP2^*}}}+\frac{[\mathrm{pCLLKMASP1_{zym}}]}{K^{\mathrm{MASP1_{zym}}}_{\mathrm{m\ MASP2^*}}}+\frac{[\mathrm{pCLLKMASP2_{zym}}]}{K^{\mathrm{MASP2_{zym}}}_{\mathrm{m\ MASP2^*}}}+\frac{[\mathrm{pCLK1MASP1_{zym}}]}{K^{\mathrm{MASP1_{zym}}}_{\mathrm{m\ MASP2^*}}}\\&+\frac{[\mathrm{pCLK1MASP2_{zym}}]}{K^{\mathrm{MASP2_{zym}}}_{\mathrm{m\ MASP2^*}}}+\frac{[\mathrm{pCLL1MASP1_{zym}}]}{K^{\mathrm{MASP1_{zym}}}_{\mathrm{m\ MASP2^*}}}+\frac{[\mathrm{pCLL1MASP2_{zym}}]}{K^{\mathrm{MASP2_{zym}}}_{\mathrm{m\ MASP2^*}}}+\frac{[\mathrm{pLFMASP1_{zym}}]}{K^{\mathrm{MASP1_{zym}}}_{\mathrm{m\ MASP2^*}}}\\&+\frac{[\mathrm{pLFMASP2_{zym}}]}{K^{\mathrm{MASP2_{zym}}}_{\mathrm{m\ MASP2^*}}}+\frac{[\mathrm{pHFMASP1_{zym}}]}{K^{\mathrm{MASP1_{zym}}}_{\mathrm{m\ MASP2^*}}}+\frac{[\mathrm{pHFMASP2_{zym}}]}{K^{\mathrm{MASP2_{zym}}}_{\mathrm{m\ MASP2^*}}}+\frac{[\mathrm{pMFMASP1_{zym}}]}{K^{\mathrm{MASP1_{zym}}}_{\mathrm{m\ MASP2^*}}}+\frac{[\mathrm{pMFMASP2_{zym}}]}{K^{\mathrm{MASP2_{zym}}}_{\mathrm{m\ MASP2^*}}}\end{aligned}\right)}$$

$$-\frac{k^{\mathrm{C2}}_{\mathrm{cat\ MASP2^*}}\left[\mathrm{pCLLKMASP2^*}\right]\left[\mathrm{fC4bC2}\right]}{K^{\mathrm{C2}}_{\mathrm{m\ MASP2^*}}\left(\begin{aligned}&1+\frac{[\mathrm{C4}]}{K^{\mathrm{C4}}_{\mathrm{m\ MASP2^*}}}+\frac{[\mathrm{fC4bC2}]}{K^{\mathrm{C2}}_{\mathrm{m\ MASP2^*}}}+\frac{[\mathrm{fC4bC4bC2}]}{K^{\mathrm{C2}}_{\mathrm{m\ MASP2^*}}}+\frac{[\mathrm{fC3bC4bC2}]}{K^{\mathrm{C2}}_{\mathrm{m\ MASP2^*}}}+\frac{[\mathrm{pC4bC2}]}{K^{\mathrm{C2}}_{\mathrm{m\ MASP2^*}}}+\frac{[\mathrm{pC4bC4bC2}]}{K^{\mathrm{C2}}_{\mathrm{m\ MASP2^*}}}\\&+\frac{[\mathrm{pC3bC4bC2}]}{K^{\mathrm{C2}}_{\mathrm{m\ MASP2^*}}}+\frac{[\mathrm{pMBL_3MASP1_{zym}}]}{K^{\mathrm{MASP1_{zym}}}_{\mathrm{m\ MASP2^*}}}+\frac{[\mathrm{pMBL_3MASP2_{zym}}]}{K^{\mathrm{MASP2_{zym}}}_{\mathrm{m\ MASP2^*}}}+\frac{[\mathrm{pMBL_4MASP1_{zym}}]}{K^{\mathrm{MASP1_{zym}}}_{\mathrm{m\ MASP2^*}}}\\&+\frac{[\mathrm{pMBL_4MASP2_{zym}}]}{K^{\mathrm{MASP2_{zym}}}_{\mathrm{m\ MASP2^*}}}+\frac{[\mathrm{pCLLKMASP1_{zym}}]}{K^{\mathrm{MASP1_{zym}}}_{\mathrm{m\ MASP2^*}}}+\frac{[\mathrm{pCLLKMASP2_{zym}}]}{K^{\mathrm{MASP2_{zym}}}_{\mathrm{m\ MASP2^*}}}+\frac{[\mathrm{pCLK1MASP1_{zym}}]}{K^{\mathrm{MASP1_{zym}}}_{\mathrm{m\ MASP2^*}}}\\&+\frac{[\mathrm{pCLK1MASP2_{zym}}]}{K^{\mathrm{MASP2_{zym}}}_{\mathrm{m\ MASP2^*}}}+\frac{[\mathrm{pCLL1MASP1_{zym}}]}{K^{\mathrm{MASP1_{zym}}}_{\mathrm{m\ MASP2^*}}}+\frac{[\mathrm{pCLL1MASP2_{zym}}]}{K^{\mathrm{MASP2_{zym}}}_{\mathrm{m\ MASP2^*}}}+\frac{[\mathrm{pLFMASP1_{zym}}]}{K^{\mathrm{MASP1_{zym}}}_{\mathrm{m\ MASP2^*}}}\\&+\frac{[\mathrm{pLFMASP2_{zym}}]}{K^{\mathrm{MASP2_{zym}}}_{\mathrm{m\ MASP2^*}}}+\frac{[\mathrm{pHFMASP1_{zym}}]}{K^{\mathrm{MASP1_{zym}}}_{\mathrm{m\ MASP2^*}}}+\frac{[\mathrm{pHFMASP2_{zym}}]}{K^{\mathrm{MASP2_{zym}}}_{\mathrm{m\ MASP2^*}}}+\frac{[\mathrm{pMFMASP1_{zym}}]}{K^{\mathrm{MASP1_{zym}}}_{\mathrm{m\ MASP2^*}}}+\frac{[\mathrm{pMFMASP2_{zym}}]}{K^{\mathrm{MASP2_{zym}}}_{\mathrm{m\ MASP2^*}}}\end{aligned}\right)}$$

$$-\frac{k^{\mathrm{C2}}_{\mathrm{cat\ MASP2^*}}\left[\mathrm{pCLK1MASP2^*}\right]\left[\mathrm{fC4bC2}\right]}{K^{\mathrm{C2}}_{\mathrm{m\ MASP2^*}}\left(\begin{aligned}&1+\frac{[\mathrm{C4}]}{K^{\mathrm{C4}}_{\mathrm{m\ MASP2^*}}}+\frac{[\mathrm{fC4bC2}]}{K^{\mathrm{C2}}_{\mathrm{m\ MASP2^*}}}+\frac{[\mathrm{fC4bC4bC2}]}{K^{\mathrm{C2}}_{\mathrm{m\ MASP2^*}}}+\frac{[\mathrm{fC3bC4bC2}]}{K^{\mathrm{C2}}_{\mathrm{m\ MASP2^*}}}+\frac{[\mathrm{pC4bC2}]}{K^{\mathrm{C2}}_{\mathrm{m\ MASP2^*}}}+\frac{[\mathrm{pC4bC4bC2}]}{K^{\mathrm{C2}}_{\mathrm{m\ MASP2^*}}}\\&+\frac{[\mathrm{pC3bC4bC2}]}{K^{\mathrm{C2}}_{\mathrm{m\ MASP2^*}}}+\frac{[\mathrm{pMBL_3MASP1_{zym}}]}{K^{\mathrm{MASP1_{zym}}}_{\mathrm{m\ MASP2^*}}}+\frac{[\mathrm{pMBL_3MASP2_{zym}}]}{K^{\mathrm{MASP2_{zym}}}_{\mathrm{m\ MASP2^*}}}+\frac{[\mathrm{pMBL_4MASP1_{zym}}]}{K^{\mathrm{MASP1_{zym}}}_{\mathrm{m\ MASP2^*}}}\\&+\frac{[\mathrm{pMBL_4MASP2_{zym}}]}{K^{\mathrm{MASP2_{zym}}}_{\mathrm{m\ MASP2^*}}}+\frac{[\mathrm{pCLLKMASP1_{zym}}]}{K^{\mathrm{MASP1_{zym}}}_{\mathrm{m\ MASP2^*}}}+\frac{[\mathrm{pCLLKMASP2_{zym}}]}{K^{\mathrm{MASP2_{zym}}}_{\mathrm{m\ MASP2^*}}}+\frac{[\mathrm{pCLK1MASP1_{zym}}]}{K^{\mathrm{MASP1_{zym}}}_{\mathrm{m\ MASP2^*}}}\\&+\frac{[\mathrm{pCLK1MASP2_{zym}}]}{K^{\mathrm{MASP2_{zym}}}_{\mathrm{m\ MASP2^*}}}+\frac{[\mathrm{pCLL1MASP1_{zym}}]}{K^{\mathrm{MASP1_{zym}}}_{\mathrm{m\ MASP2^*}}}+\frac{[\mathrm{pCLL1MASP2_{zym}}]}{K^{\mathrm{MASP2_{zym}}}_{\mathrm{m\ MASP2^*}}}+\frac{[\mathrm{pLFMASP1_{zym}}]}{K^{\mathrm{MASP1_{zym}}}_{\mathrm{m\ MASP2^*}}}\\&+\frac{[\mathrm{pLFMASP2_{zym}}]}{K^{\mathrm{MASP2_{zym}}}_{\mathrm{m\ MASP2^*}}}+\frac{[\mathrm{pHFMASP1_{zym}}]}{K^{\mathrm{MASP1_{zym}}}_{\mathrm{m\ MASP2^*}}}+\frac{[\mathrm{pHFMASP2_{zym}}]}{K^{\mathrm{MASP2_{zym}}}_{\mathrm{m\ MASP2^*}}}+\frac{[\mathrm{pMFMASP1_{zym}}]}{K^{\mathrm{MASP1_{zym}}}_{\mathrm{m\ MASP2^*}}}+\frac{[\mathrm{pMFMASP2_{zym}}]}{K^{\mathrm{MASP2_{zym}}}_{\mathrm{m\ MASP2^*}}}\end{aligned}\right)}$$

$$-\frac{k_{\mathrm{cat\ MASP2^*}}^{\mathrm{C2}}\left[\mathrm{pCLL1MASP2^*}\right]\left[\mathrm{fC4bC2}\right]}{K_{\mathrm{m\ MASP2^*}}^{\mathrm{C2}}\left(1+\frac{[\mathrm{C4}]}{K_{\mathrm{m\ MASP2^*}}^{\mathrm{C4}}}+\frac{[\mathrm{fC4bC2}]}{K_{\mathrm{m\ MASP2^*}}^{\mathrm{C2}}}+\frac{[\mathrm{fC4bC4bC2}]}{K_{\mathrm{m\ MASP2^*}}^{\mathrm{C2}}}+\frac{[\mathrm{fC3bC4bC2}]}{K_{\mathrm{m\ MASP2^*}}^{\mathrm{C2}}}+\frac{[\mathrm{pC4bC2}]}{K_{\mathrm{m\ MASP2^*}}^{\mathrm{C2}}}+\frac{[\mathrm{pC4bC4bC2}]}{K_{\mathrm{m\ MASP2^*}}^{\mathrm{C2}}}+\frac{[\mathrm{pC3bC4bC2}]}{K_{\mathrm{m\ MASP2^*}}^{\mathrm{C2}}}+\frac{[\mathrm{pMBL_3MASP1_{zym}}]}{K_{\mathrm{m\ MASP2^*}}^{\mathrm{MASP1_{zym}}}}+\frac{[\mathrm{pMBL_3MASP2_{zym}}]}{K_{\mathrm{m\ MASP2^*}}^{\mathrm{MASP2_{zym}}}}+\frac{[\mathrm{pMBL_4MASP1_{zym}}]}{K_{\mathrm{m\ MASP2^*}}^{\mathrm{MASP1_{zym}}}}+\frac{[\mathrm{pMBL_4MASP2_{zym}}]}{K_{\mathrm{m\ MASP2^*}}^{\mathrm{MASP2_{zym}}}}+\frac{[\mathrm{pCLLKMASP1_{zym}}]}{K_{\mathrm{m\ MASP2^*}}^{\mathrm{MASP1_{zym}}}}+\frac{[\mathrm{pCLLKMASP2_{zym}}]}{K_{\mathrm{m\ MASP2^*}}^{\mathrm{MASP2_{zym}}}}+\frac{[\mathrm{pCLK1MASP1_{zym}}]}{K_{\mathrm{m\ MASP2^*}}^{\mathrm{MASP1_{zym}}}}+\frac{[\mathrm{pCLK1MASP2_{zym}}]}{K_{\mathrm{m\ MASP2^*}}^{\mathrm{MASP2_{zym}}}}+\frac{[\mathrm{pCLL1MASP1_{zym}}]}{K_{\mathrm{m\ MASP2^*}}^{\mathrm{MASP1_{zym}}}}+\frac{[\mathrm{pCLL1MASP2_{zym}}]}{K_{\mathrm{m\ MASP2^*}}^{\mathrm{MASP2_{zym}}}}+\frac{[\mathrm{pLFMASP1_{zym}}]}{K_{\mathrm{m\ MASP2^*}}^{\mathrm{MASP1_{zym}}}}+\frac{[\mathrm{pLFMASP2_{zym}}]}{K_{\mathrm{m\ MASP2^*}}^{\mathrm{MASP2_{zym}}}}+\frac{[\mathrm{pHFMASP1_{zym}}]}{K_{\mathrm{m\ MASP2^*}}^{\mathrm{MASP1_{zym}}}}+\frac{[\mathrm{pHFMASP2_{zym}}]}{K_{\mathrm{m\ MASP2^*}}^{\mathrm{MASP2_{zym}}}}+\frac{[\mathrm{pMFMASP1_{zym}}]}{K_{\mathrm{m\ MASP2^*}}^{\mathrm{MASP1_{zym}}}}+\frac{[\mathrm{pMFMASP2_{zym}}]}{K_{\mathrm{m\ MASP2^*}}^{\mathrm{MASP2_{zym}}}}\right)}$$

$$-\frac{k_{\mathrm{cat\ MASP2^*}}^{\mathrm{C2}}\left[\mathrm{pLFMASP2^*}\right]\left[\mathrm{fC4bC2}\right]}{K_{\mathrm{m\ MASP2^*}}^{\mathrm{C2}}\left(1+\frac{[\mathrm{C4}]}{K_{\mathrm{m\ MASP2^*}}^{\mathrm{C4}}}+\frac{[\mathrm{fC4bC2}]}{K_{\mathrm{m\ MASP2^*}}^{\mathrm{C2}}}+\frac{[\mathrm{fC4bC4bC2}]}{K_{\mathrm{m\ MASP2^*}}^{\mathrm{C2}}}+\frac{[\mathrm{fC3bC4bC2}]}{K_{\mathrm{m\ MASP2^*}}^{\mathrm{C2}}}+\frac{[\mathrm{pC4bC2}]}{K_{\mathrm{m\ MASP2^*}}^{\mathrm{C2}}}+\frac{[\mathrm{pC4bC4bC2}]}{K_{\mathrm{m\ MASP2^*}}^{\mathrm{C2}}}+\frac{[\mathrm{pC3bC4bC2}]}{K_{\mathrm{m\ MASP2^*}}^{\mathrm{C2}}}+\frac{[\mathrm{pMBL_3MASP1_{zym}}]}{K_{\mathrm{m\ MASP2^*}}^{\mathrm{MASP1_{zym}}}}+\frac{[\mathrm{pMBL_3MASP2_{zym}}]}{K_{\mathrm{m\ MASP2^*}}^{\mathrm{MASP2_{zym}}}}+\frac{[\mathrm{pMBL_4MASP1_{zym}}]}{K_{\mathrm{m\ MASP2^*}}^{\mathrm{MASP1_{zym}}}}+\frac{[\mathrm{pMBL_4MASP2_{zym}}]}{K_{\mathrm{m\ MASP2^*}}^{\mathrm{MASP2_{zym}}}}+\frac{[\mathrm{pCLLKMASP1_{zym}}]}{K_{\mathrm{m\ MASP2^*}}^{\mathrm{MASP1_{zym}}}}+\frac{[\mathrm{pCLLKMASP2_{zym}}]}{K_{\mathrm{m\ MASP2^*}}^{\mathrm{MASP2_{zym}}}}+\frac{[\mathrm{pCLK1MASP1_{zym}}]}{K_{\mathrm{m\ MASP2^*}}^{\mathrm{MASP1_{zym}}}}+\frac{[\mathrm{pCLK1MASP2_{zym}}]}{K_{\mathrm{m\ MASP2^*}}^{\mathrm{MASP2_{zym}}}}+\frac{[\mathrm{pCLL1MASP1_{zym}}]}{K_{\mathrm{m\ MASP2^*}}^{\mathrm{MASP1_{zym}}}}+\frac{[\mathrm{pCLL1MASP2_{zym}}]}{K_{\mathrm{m\ MASP2^*}}^{\mathrm{MASP2_{zym}}}}+\frac{[\mathrm{pLFMASP1_{zym}}]}{K_{\mathrm{m\ MASP2^*}}^{\mathrm{MASP1_{zym}}}}+\frac{[\mathrm{pLFMASP2_{zym}}]}{K_{\mathrm{m\ MASP2^*}}^{\mathrm{MASP2_{zym}}}}+\frac{[\mathrm{pHFMASP1_{zym}}]}{K_{\mathrm{m\ MASP2^*}}^{\mathrm{MASP1_{zym}}}}+\frac{[\mathrm{pHFMASP2_{zym}}]}{K_{\mathrm{m\ MASP2^*}}^{\mathrm{MASP2_{zym}}}}+\frac{[\mathrm{pMFMASP1_{zym}}]}{K_{\mathrm{m\ MASP2^*}}^{\mathrm{MASP1_{zym}}}}+\frac{[\mathrm{pMFMASP2_{zym}}]}{K_{\mathrm{m\ MASP2^*}}^{\mathrm{MASP2_{zym}}}}\right)}$$

$$-\frac{k_{\mathrm{cat\ MASP2^*}}^{\mathrm{C2}}\left[\mathrm{pHFMASP2^*}\right]\left[\mathrm{fC4bC2}\right]}{K_{\mathrm{m\ MASP2^*}}^{\mathrm{C2}}\left(1+\frac{[\mathrm{C4}]}{K_{\mathrm{m\ MASP2^*}}^{\mathrm{C4}}}+\frac{[\mathrm{fC4bC2}]}{K_{\mathrm{m\ MASP2^*}}^{\mathrm{C2}}}+\frac{[\mathrm{fC4bC4bC2}]}{K_{\mathrm{m\ MASP2^*}}^{\mathrm{C2}}}+\frac{[\mathrm{fC3bC4bC2}]}{K_{\mathrm{m\ MASP2^*}}^{\mathrm{C2}}}+\frac{[\mathrm{pC4bC2}]}{K_{\mathrm{m\ MASP2^*}}^{\mathrm{C2}}}+\frac{[\mathrm{pC4bC4bC2}]}{K_{\mathrm{m\ MASP2^*}}^{\mathrm{C2}}}+\frac{[\mathrm{pC3bC4bC2}]}{K_{\mathrm{m\ MASP2^*}}^{\mathrm{C2}}}+\frac{[\mathrm{pMBL_3MASP1_{zym}}]}{K_{\mathrm{m\ MASP2^*}}^{\mathrm{MASP1_{zym}}}}+\frac{[\mathrm{pMBL_3MASP2_{zym}}]}{K_{\mathrm{m\ MASP2^*}}^{\mathrm{MASP2_{zym}}}}+\frac{[\mathrm{pMBL_4MASP1_{zym}}]}{K_{\mathrm{m\ MASP2^*}}^{\mathrm{MASP1_{zym}}}}+\frac{[\mathrm{pMBL_4MASP2_{zym}}]}{K_{\mathrm{m\ MASP2^*}}^{\mathrm{MASP2_{zym}}}}+\frac{[\mathrm{pCLLKMASP1_{zym}}]}{K_{\mathrm{m\ MASP2^*}}^{\mathrm{MASP1_{zym}}}}+\frac{[\mathrm{pCLLKMASP2_{zym}}]}{K_{\mathrm{m\ MASP2^*}}^{\mathrm{MASP2_{zym}}}}+\frac{[\mathrm{pCLK1MASP1_{zym}}]}{K_{\mathrm{m\ MASP2^*}}^{\mathrm{MASP1_{zym}}}}+\frac{[\mathrm{pCLK1MASP2_{zym}}]}{K_{\mathrm{m\ MASP2^*}}^{\mathrm{MASP2_{zym}}}}+\frac{[\mathrm{pCLL1MASP1_{zym}}]}{K_{\mathrm{m\ MASP2^*}}^{\mathrm{MASP1_{zym}}}}+\frac{[\mathrm{pCLL1MASP2_{zym}}]}{K_{\mathrm{m\ MASP2^*}}^{\mathrm{MASP2_{zym}}}}+\frac{[\mathrm{pLFMASP1_{zym}}]}{K_{\mathrm{m\ MASP2^*}}^{\mathrm{MASP1_{zym}}}}+\frac{[\mathrm{pLFMASP2_{zym}}]}{K_{\mathrm{m\ MASP2^*}}^{\mathrm{MASP2_{zym}}}}+\frac{[\mathrm{pHFMASP1_{zym}}]}{K_{\mathrm{m\ MASP2^*}}^{\mathrm{MASP1_{zym}}}}+\frac{[\mathrm{pHFMASP2_{zym}}]}{K_{\mathrm{m\ MASP2^*}}^{\mathrm{MASP2_{zym}}}}+\frac{[\mathrm{pMFMASP1_{zym}}]}{K_{\mathrm{m\ MASP2^*}}^{\mathrm{MASP1_{zym}}}}+\frac{[\mathrm{pMFMASP2_{zym}}]}{K_{\mathrm{m\ MASP2^*}}^{\mathrm{MASP2_{zym}}}}\right)}$$

$$-\frac{k_{\text{cat MASP2}^*}^{\text{C2}}\left[\text{pMFMASP2}^*\right]\left[\text{fC4bC2}\right]}{K_{\text{m MASP2}^*}^{\text{C2}}\left(\begin{array}{l}1+\frac{[\text{C4}]}{K_{\text{m MASP2}^*}^{\text{C4}}}+\frac{[\text{fC4bC2}]}{K_{\text{m MASP2}^*}^{\text{C2}}}+\frac{[\text{fC4bC4bC2}]}{K_{\text{m MASP2}^*}^{\text{C2}}}+\frac{[\text{fC3bC4bC2}]}{K_{\text{m MASP2}^*}^{\text{C2}}}+\frac{[\text{pC4bC2}]}{K_{\text{m MASP2}^*}^{\text{C2}}}+\frac{[\text{pC4bC4bC2}]}{K_{\text{m MASP2}^*}^{\text{C2}}}\\+\frac{[\text{pC3bC4bC2}]}{K_{\text{m MASP2}^*}^{\text{C2}}}+\frac{[\text{pMBL}_3\text{MASP1}_{\text{zym}}]}{K_{\text{m MASP2}^*}^{\text{MASP1}_{\text{zym}}}}+\frac{[\text{pMBL}_3\text{MASP2}_{\text{zym}}]}{K_{\text{m MASP2}^*}^{\text{MASP2}_{\text{zym}}}}+\frac{[\text{pMBL}_4\text{MASP1}_{\text{zym}}]}{K_{\text{m MASP2}^*}^{\text{MASP1}_{\text{zym}}}}\\+\frac{[\text{pMBL}_4\text{MASP2}_{\text{zym}}]}{K_{\text{m MASP2}^*}^{\text{MASP2}_{\text{zym}}}}+\frac{[\text{pCLLKMASP1}_{\text{zym}}]}{K_{\text{m MASP2}^*}^{\text{MASP1}_{\text{zym}}}}+\frac{[\text{pCLLKMASP2}_{\text{zym}}]}{K_{\text{m MASP2}^*}^{\text{MASP2}_{\text{zym}}}}+\frac{[\text{pCLK1MASP1}_{\text{zym}}]}{K_{\text{m MASP2}^*}^{\text{MASP1}_{\text{zym}}}}\\+\frac{[\text{pCLK1MASP2}_{\text{zym}}]}{K_{\text{m MASP2}^*}^{\text{MASP2}_{\text{zym}}}}+\frac{[\text{pCLL1MASP1}_{\text{zym}}]}{K_{\text{m MASP2}^*}^{\text{MASP1}_{\text{zym}}}}+\frac{[\text{pCLL1MASP2}_{\text{zym}}]}{K_{\text{m MASP2}^*}^{\text{MASP2}_{\text{zym}}}}+\frac{[\text{pLFMASP1}_{\text{zym}}]}{K_{\text{m MASP2}^*}^{\text{MASP1}_{\text{zym}}}}\\+\frac{[\text{pLFMASP2}_{\text{zym}}]}{K_{\text{m MASP2}^*}^{\text{MASP2}_{\text{zym}}}}+\frac{[\text{pHFMASP1}_{\text{zym}}]}{K_{\text{m MASP2}^*}^{\text{MASP1}_{\text{zym}}}}+\frac{[\text{pHFMASP2}_{\text{zym}}]}{K_{\text{m MASP2}^*}^{\text{MASP2}_{\text{zym}}}}+\frac{[\text{pMFMASP1}_{\text{zym}}]}{K_{\text{m MASP2}^*}^{\text{MASP1}_{\text{zym}}}}+\frac{[\text{pMFMASP2}_{\text{zym}}]}{K_{\text{m MASP2}^*}^{\text{MASP2}_{\text{zym}}}}\end{array}\right)}$$

$$-\frac{k_{\text{cat C1}^*}^{\text{C2}}\left[\text{pMBL}_3\text{PTX3C1}^*\right]\left[\text{fC4bC2}\right]}{K_{\text{m C1}^*}^{\text{C2}}\left(1+\frac{[\text{C4}]}{K_{\text{m C1}^*}^{\text{C4}}}+\frac{[\text{fC4bC2}]}{K_{\text{m C1}^*}^{\text{C2}}}+\frac{[\text{fC4bC4bC2}]}{K_{\text{m C1}^*}^{\text{C2}}}+\frac{[\text{fC3bC4bC2}]}{K_{\text{m C1}^*}^{\text{C2}}}+\frac{[\text{pC4bC2}]}{K_{\text{m C1}^*}^{\text{C2}}}+\frac{[\text{pC4bC4bC2}]}{K_{\text{m C1}^*}^{\text{C2}}}+\frac{[\text{pC3bC4bC2}]}{K_{\text{m C1}^*}^{\text{C2}}}\right)}$$

$$-\frac{k_{\text{cat C1}^*}^{\text{C2}}\left[\text{pMBL}_4\text{PTX3C1}^*\right]\left[\text{fC4bC2}\right]}{K_{\text{m C1}^*}^{\text{C2}}\left(1+\frac{[\text{C4}]}{K_{\text{m C1}^*}^{\text{C4}}}+\frac{[\text{fC4bC2}]}{K_{\text{m C1}^*}^{\text{C2}}}+\frac{[\text{fC4bC4bC2}]}{K_{\text{m C1}^*}^{\text{C2}}}+\frac{[\text{fC3bC4bC2}]}{K_{\text{m C1}^*}^{\text{C2}}}+\frac{[\text{pC4bC2}]}{K_{\text{m C1}^*}^{\text{C2}}}+\frac{[\text{pC4bC4bC2}]}{K_{\text{m C1}^*}^{\text{C2}}}+\frac{[\text{pC3bC4bC2}]}{K_{\text{m C1}^*}^{\text{C2}}}\right)}$$

$$-\frac{k_{\text{cat C1}^*}^{\text{C2}}\left[\text{CRPC1}^*\right]\left[\text{fC4bC2}\right]}{K_{\text{m C1}^*}^{\text{C2}}\left(1+\frac{[\text{C4}]}{K_{\text{m C1}^*}^{\text{C4}}}+\frac{[\text{fC4bC2}]}{K_{\text{m C1}^*}^{\text{C2}}}+\frac{[\text{fC4bC4bC2}]}{K_{\text{m C1}^*}^{\text{C2}}}+\frac{[\text{fC3bC4bC2}]}{K_{\text{m C1}^*}^{\text{C2}}}\right)}$$

37

$$\frac{d\left[\mathrm{fC4bC2a}\right]}{dt}=$$

$$\frac{k_{\mathrm{cat\,C1^*}}^{\mathrm{C2}}\left[\mathrm{C1^*}\right]\left[\mathrm{fC4bC2}\right]}{K_{\mathrm{m\,C1^*}}^{\mathrm{C2}}\left(1+\frac{[\mathrm{C4}]}{K_{\mathrm{m\,C1^*}}^{\mathrm{C4}}}+\frac{[\mathrm{fC4bC2}]}{K_{\mathrm{m\,C1^*}}^{\mathrm{C2}}}+\frac{[\mathrm{fC4bC4bC2}]}{K_{\mathrm{m\,C1^*}}^{\mathrm{C2}}}+\frac{[\mathrm{fC3bC4bC2}]}{K_{\mathrm{m\,C1^*}}^{\mathrm{C2}}}+\frac{[\mathrm{pC4bC2}]}{K_{\mathrm{m\,C1^*}}^{\mathrm{C2}}}+\frac{[\mathrm{pC4bC4bC2}]}{K_{\mathrm{m\,C1^*}}^{\mathrm{C2}}}+\frac{[\mathrm{pC3bC4bC2}]}{K_{\mathrm{m\,C1^*}}^{\mathrm{C2}}}\right)}$$

$$-k_{\mathrm{C4bC4b}}^{+}\left[\mathrm{fC4bC2a}\right]\left[\mathrm{nfC4b}\right]-k_{\mathrm{C3bC4b}}^{+}\left[\mathrm{fC4bC2a}\right]\left[\mathrm{nfC3b}\right]-k_{\mathrm{C4bC2a}}^{-}\left[\mathrm{fC4bC2a}\right]-k_{\mathrm{C4bC4BP}}^{+}\left[\mathrm{fC4bC2a}\right]\left[\mathrm{C4BP}\right]$$

$$+k_{\mathrm{C4bC4BP}}^{-}\left[\mathrm{fC4bC2aC4BP}\right]-k_{\mathrm{C4bCR1}}^{+}\left[\mathrm{fC4bC2a}\right]\left[\mathrm{CR1}\right]+k_{\mathrm{C4bCR1}}^{-}\left[\mathrm{fC4bC2aCR1}\right]$$

$$+\frac{k_{\mathrm{cat\,C1^*}}^{\mathrm{C2}}\left[\mathrm{pIgG_6C1^*}\right]\left[\mathrm{fC4bC2}\right]}{K_{\mathrm{m\,C1^*}}^{\mathrm{C2}}\left(1+\frac{[\mathrm{C4}]}{K_{\mathrm{m\,C1^*}}^{\mathrm{C4}}}+\frac{[\mathrm{fC4bC2}]}{K_{\mathrm{m\,C1^*}}^{\mathrm{C2}}}+\frac{[\mathrm{fC4bC4bC2}]}{K_{\mathrm{m\,C1^*}}^{\mathrm{C2}}}+\frac{[\mathrm{fC3bC4bC2}]}{K_{\mathrm{m\,C1^*}}^{\mathrm{C2}}}+\frac{[\mathrm{pC4bC2}]}{K_{\mathrm{m\,C1^*}}^{\mathrm{C2}}}+\frac{[\mathrm{pC4bC4bC2}]}{K_{\mathrm{m\,C1^*}}^{\mathrm{C2}}}+\frac{[\mathrm{pC3bC4bC2}]}{K_{\mathrm{m\,C1^*}}^{\mathrm{C2}}}\right)}$$

$$+\frac{k_{\mathrm{cat\,C1^*}}^{\mathrm{C2}}\left[\mathrm{pIgMC1^*}\right]\left[\mathrm{fC4bC2}\right]}{K_{\mathrm{m\,C1^*}}^{\mathrm{C2}}\left(1+\frac{[\mathrm{C4}]}{K_{\mathrm{m\,C1^*}}^{\mathrm{C4}}}+\frac{[\mathrm{fC4bC2}]}{K_{\mathrm{m\,C1^*}}^{\mathrm{C2}}}+\frac{[\mathrm{fC4bC4bC2}]}{K_{\mathrm{m\,C1^*}}^{\mathrm{C2}}}+\frac{[\mathrm{fC3bC4bC2}]}{K_{\mathrm{m\,C1^*}}^{\mathrm{C2}}}+\frac{[\mathrm{pC4bC2}]}{K_{\mathrm{m\,C1^*}}^{\mathrm{C2}}}+\frac{[\mathrm{pC4bC4bC2}]}{K_{\mathrm{m\,C1^*}}^{\mathrm{C2}}}+\frac{[\mathrm{pC3bC4bC2}]}{K_{\mathrm{m\,C1^*}}^{\mathrm{C2}}}\right)}$$

$$+\frac{k_{\mathrm{cat\,MASP1^*}}^{\mathrm{C2}}\left[\mathrm{pMBL_3MASP1^*}\right]\left[\mathrm{fC4bC2}\right]}{K_{\mathrm{m\,MASP1^*}}^{\mathrm{C2}}\left(\begin{aligned}&1+\frac{[\mathrm{C4}]}{K_{\mathrm{m\,MASP1^*}}^{\mathrm{C4}}}+\frac{[\mathrm{fC4bC2}]}{K_{\mathrm{m\,MASP1^*}}^{\mathrm{C2}}}+\frac{[\mathrm{fC4bC4bC2}]}{K_{\mathrm{m\,MASP1^*}}^{\mathrm{C2}}}+\frac{[\mathrm{fC3bC4bC2}]}{K_{\mathrm{m\,MASP1^*}}^{\mathrm{C2}}}+\frac{[\mathrm{pC4bC2}]}{K_{\mathrm{m\,MASP1^*}}^{\mathrm{C2}}}+\frac{[\mathrm{pC4bC4bC2}]}{K_{\mathrm{m\,MASP1^*}}^{\mathrm{C2}}}\\&+\frac{[\mathrm{pC3bC4bC2}]}{K_{\mathrm{m\,MASP1^*}}^{\mathrm{C2}}}+\frac{[\mathrm{pMBL_3MASP1_{zym}}]}{K_{\mathrm{m\,MASP1^*}}^{\mathrm{MASP1_{zym}}}}+\frac{[\mathrm{pMBL_3MASP2_{zym}}]}{K_{\mathrm{m\,MASP1^*}}^{\mathrm{MASP2_{zym}}}}+\frac{[\mathrm{pMBL_3MASP3_{zym}}]}{K_{\mathrm{m\,MASP1^*}}^{\mathrm{MASP3_{zym}}}}\\&+\frac{[\mathrm{pMBL_4MASP1_{zym}}]}{K_{\mathrm{m\,MASP1^*}}^{\mathrm{MASP1_{zym}}}}+\frac{[\mathrm{pMBL_4MASP2_{zym}}]}{K_{\mathrm{m\,MASP1^*}}^{\mathrm{MASP2_{zym}}}}+\frac{[\mathrm{pMBL_4MASP3_{zym}}]}{K_{\mathrm{m\,MASP1^*}}^{\mathrm{MASP3_{zym}}}}+\frac{[\mathrm{pCLLKMASP1_{zym}}]}{K_{\mathrm{m\,MASP1^*}}^{\mathrm{MASP1_{zym}}}}\\&+\frac{[\mathrm{pCLLKMASP2_{zym}}]}{K_{\mathrm{m\,MASP1^*}}^{\mathrm{MASP2_{zym}}}}+\frac{[\mathrm{pCLLKMASP3_{zym}}]}{K_{\mathrm{m\,MASP1^*}}^{\mathrm{MASP3_{zym}}}}+\frac{[\mathrm{pCLK1MASP1_{zym}}]}{K_{\mathrm{m\,MASP1^*}}^{\mathrm{MASP1_{zym}}}}+\frac{[\mathrm{pCLK1MASP2_{zym}}]}{K_{\mathrm{m\,MASP1^*}}^{\mathrm{MASP2_{zym}}}}\\&+\frac{[\mathrm{pCLK1MASP3_{zym}}]}{K_{\mathrm{m\,MASP1^*}}^{\mathrm{MASP3_{zym}}}}+\frac{[\mathrm{pCLL1MASP1_{zym}}]}{K_{\mathrm{m\,MASP1^*}}^{\mathrm{MASP1_{zym}}}}+\frac{[\mathrm{pCLL1MASP2_{zym}}]}{K_{\mathrm{m\,MASP1^*}}^{\mathrm{MASP2_{zym}}}}+\frac{[\mathrm{pCLL1MASP3_{zym}}]}{K_{\mathrm{m\,MASP1^*}}^{\mathrm{MASP3_{zym}}}}\\&+\frac{[\mathrm{pLFMASP1_{zym}}]}{K_{\mathrm{m\,MASP1^*}}^{\mathrm{MASP1_{zym}}}}+\frac{[\mathrm{pLFMASP2_{zym}}]}{K_{\mathrm{m\,MASP1^*}}^{\mathrm{MASP2_{zym}}}}+\frac{[\mathrm{pLFMASP3_{zym}}]}{K_{\mathrm{m\,MASP1^*}}^{\mathrm{MASP3_{zym}}}}+\frac{[\mathrm{pHFMASP1_{zym}}]}{K_{\mathrm{m\,MASP1^*}}^{\mathrm{MASP1_{zym}}}}+\frac{[\mathrm{pHFMASP2_{zym}}]}{K_{\mathrm{m\,MASP1^*}}^{\mathrm{MASP2_{zym}}}}\\&+\frac{[\mathrm{pHFMASP3_{zym}}]}{K_{\mathrm{m\,MASP1^*}}^{\mathrm{MASP3_{zym}}}}+\frac{[\mathrm{pMFMASP1_{zym}}]}{K_{\mathrm{m\,MASP1^*}}^{\mathrm{MASP1_{zym}}}}+\frac{[\mathrm{pMFMASP2_{zym}}]}{K_{\mathrm{m\,MASP1^*}}^{\mathrm{MASP2_{zym}}}}+\frac{[\mathrm{pMFMASP3_{zym}}]}{K_{\mathrm{m\,MASP1^*}}^{\mathrm{MASP3_{zym}}}}\end{aligned}\right)}$$

$$+\frac{k^{\mathrm{C2}}_{\mathrm{cat\,MASP1^*}}\left[\mathrm{pMBL_4MASP1^*}\right]\left[\mathrm{fC4bC2}\right]}{K^{\mathrm{C2}}_{\mathrm{m\,MASP1^*}}\left(\begin{array}{l}1+\frac{[\mathrm{C4}]}{K^{\mathrm{C4}}_{\mathrm{m\,MASP1^*}}}+\frac{[\mathrm{fC4bC2}]}{K^{\mathrm{C2}}_{\mathrm{m\,MASP1^*}}}+\frac{[\mathrm{fC4bC4bC2}]}{K^{\mathrm{C2}}_{\mathrm{m\,MASP1^*}}}+\frac{[\mathrm{fC3bC4bC2}]}{K^{\mathrm{C2}}_{\mathrm{m\,MASP1^*}}}+\frac{[\mathrm{pC4bC2}]}{K^{\mathrm{C2}}_{\mathrm{m\,MASP1^*}}}+\frac{[\mathrm{pC4bC4bC2}]}{K^{\mathrm{C2}}_{\mathrm{m\,MASP1^*}}}\\ +\frac{[\mathrm{pC3bC4bC2}]}{K^{\mathrm{C2}}_{\mathrm{m\,MASP1^*}}}+\frac{[\mathrm{pMBL_3MASP1_{zym}}]}{K^{\mathrm{MASP1_{zym}}}_{\mathrm{m\,MASP1^*}}}+\frac{[\mathrm{pMBL_3MASP2_{zym}}]}{K^{\mathrm{MASP2_{zym}}}_{\mathrm{m\,MASP1^*}}}+\frac{[\mathrm{pMBL_3MASP3_{zym}}]}{K^{\mathrm{MASP3_{zym}}}_{\mathrm{m\,MASP1^*}}}\\ +\frac{[\mathrm{pMBL_4MASP1_{zym}}]}{K^{\mathrm{MASP1_{zym}}}_{\mathrm{m\,MASP1^*}}}+\frac{[\mathrm{pMBL_4MASP2_{zym}}]}{K^{\mathrm{MASP2_{zym}}}_{\mathrm{m\,MASP1^*}}}+\frac{[\mathrm{pMBL_4MASP3_{zym}}]}{K^{\mathrm{MASP3_{zym}}}_{\mathrm{m\,MASP1^*}}}+\frac{[\mathrm{pCLLKMASP1_{zym}}]}{K^{\mathrm{MASP1_{zym}}}_{\mathrm{m\,MASP1^*}}}\\ +\frac{[\mathrm{pCLLKMASP2_{zym}}]}{K^{\mathrm{MASP2_{zym}}}_{\mathrm{m\,MASP1^*}}}+\frac{[\mathrm{pCLLKMASP3_{zym}}]}{K^{\mathrm{MASP3_{zym}}}_{\mathrm{m\,MASP1^*}}}+\frac{[\mathrm{pCLK1MASP1_{zym}}]}{K^{\mathrm{MASP1_{zym}}}_{\mathrm{m\,MASP1^*}}}+\frac{[\mathrm{pCLK1MASP2_{zym}}]}{K^{\mathrm{MASP2_{zym}}}_{\mathrm{m\,MASP1^*}}}\\ +\frac{[\mathrm{pCLK1MASP3_{zym}}]}{K^{\mathrm{MASP3_{zym}}}_{\mathrm{m\,MASP1^*}}}+\frac{[\mathrm{pCLL1MASP1_{zym}}]}{K^{\mathrm{MASP1_{zym}}}_{\mathrm{m\,MASP1^*}}}+\frac{[\mathrm{pCLL1MASP2_{zym}}]}{K^{\mathrm{MASP2_{zym}}}_{\mathrm{m\,MASP1^*}}}+\frac{[\mathrm{pCLL1MASP3_{zym}}]}{K^{\mathrm{MASP3_{zym}}}_{\mathrm{m\,MASP1^*}}}\\ +\frac{[\mathrm{pLFMASP1_{zym}}]}{K^{\mathrm{MASP1_{zym}}}_{\mathrm{m\,MASP1^*}}}+\frac{[\mathrm{pLFMASP2_{zym}}]}{K^{\mathrm{MASP2_{zym}}}_{\mathrm{m\,MASP1^*}}}+\frac{[\mathrm{pLFMASP3_{zym}}]}{K^{\mathrm{MASP3_{zym}}}_{\mathrm{m\,MASP1^*}}}+\frac{[\mathrm{pHFMASP1_{zym}}]}{K^{\mathrm{MASP1_{zym}}}_{\mathrm{m\,MASP1^*}}}+\frac{[\mathrm{pHFMASP2_{zym}}]}{K^{\mathrm{MASP2_{zym}}}_{\mathrm{m\,MASP1^*}}}\\ +\frac{[\mathrm{pHFMASP3_{zym}}]}{K^{\mathrm{MASP3_{zym}}}_{\mathrm{m\,MASP1^*}}}+\frac{[\mathrm{pMFMASP1_{zym}}]}{K^{\mathrm{MASP1_{zym}}}_{\mathrm{m\,MASP1^*}}}+\frac{[\mathrm{pMFMASP2_{zym}}]}{K^{\mathrm{MASP2_{zym}}}_{\mathrm{m\,MASP1^*}}}+\frac{[\mathrm{pMFMASP3_{zym}}]}{K^{\mathrm{MASP3_{zym}}}_{\mathrm{m\,MASP1^*}}}\end{array}\right)}$$

$$+\frac{k^{\mathrm{C2}}_{\mathrm{cat\,MASP1^*}}\left[\mathrm{pCLLKMASP1^*}\right]\left[\mathrm{fC4bC2}\right]}{K^{\mathrm{C2}}_{\mathrm{m\,MASP1^*}}\left(\begin{array}{l}1+\frac{[\mathrm{C4}]}{K^{\mathrm{C4}}_{\mathrm{m\,MASP1^*}}}+\frac{[\mathrm{fC4bC2}]}{K^{\mathrm{C2}}_{\mathrm{m\,MASP1^*}}}+\frac{[\mathrm{fC4bC4bC2}]}{K^{\mathrm{C2}}_{\mathrm{m\,MASP1^*}}}+\frac{[\mathrm{fC3bC4bC2}]}{K^{\mathrm{C2}}_{\mathrm{m\,MASP1^*}}}+\frac{[\mathrm{pC4bC2}]}{K^{\mathrm{C2}}_{\mathrm{m\,MASP1^*}}}+\frac{[\mathrm{pC4bC4bC2}]}{K^{\mathrm{C2}}_{\mathrm{m\,MASP1^*}}}\\ +\frac{[\mathrm{pC3bC4bC2}]}{K^{\mathrm{C2}}_{\mathrm{m\,MASP1^*}}}+\frac{[\mathrm{pMBL_3MASP1_{zym}}]}{K^{\mathrm{MASP1_{zym}}}_{\mathrm{m\,MASP1^*}}}+\frac{[\mathrm{pMBL_3MASP2_{zym}}]}{K^{\mathrm{MASP2_{zym}}}_{\mathrm{m\,MASP1^*}}}+\frac{[\mathrm{pMBL_3MASP3_{zym}}]}{K^{\mathrm{MASP3_{zym}}}_{\mathrm{m\,MASP1^*}}}\\ +\frac{[\mathrm{pMBL_4MASP1_{zym}}]}{K^{\mathrm{MASP1_{zym}}}_{\mathrm{m\,MASP1^*}}}+\frac{[\mathrm{pMBL_4MASP2_{zym}}]}{K^{\mathrm{MASP2_{zym}}}_{\mathrm{m\,MASP1^*}}}+\frac{[\mathrm{pMBL_4MASP3_{zym}}]}{K^{\mathrm{MASP3_{zym}}}_{\mathrm{m\,MASP1^*}}}+\frac{[\mathrm{pCLLKMASP1_{zym}}]}{K^{\mathrm{MASP1_{zym}}}_{\mathrm{m\,MASP1^*}}}\\ +\frac{[\mathrm{pCLLKMASP2_{zym}}]}{K^{\mathrm{MASP2_{zym}}}_{\mathrm{m\,MASP1^*}}}+\frac{[\mathrm{pCLLKMASP3_{zym}}]}{K^{\mathrm{MASP3_{zym}}}_{\mathrm{m\,MASP1^*}}}+\frac{[\mathrm{pCLK1MASP1_{zym}}]}{K^{\mathrm{MASP1_{zym}}}_{\mathrm{m\,MASP1^*}}}+\frac{[\mathrm{pCLK1MASP2_{zym}}]}{K^{\mathrm{MASP2_{zym}}}_{\mathrm{m\,MASP1^*}}}\\ +\frac{[\mathrm{pCLK1MASP3_{zym}}]}{K^{\mathrm{MASP3_{zym}}}_{\mathrm{m\,MASP1^*}}}+\frac{[\mathrm{pCLL1MASP1_{zym}}]}{K^{\mathrm{MASP1_{zym}}}_{\mathrm{m\,MASP1^*}}}+\frac{[\mathrm{pCLL1MASP2_{zym}}]}{K^{\mathrm{MASP2_{zym}}}_{\mathrm{m\,MASP1^*}}}+\frac{[\mathrm{pCLL1MASP3_{zym}}]}{K^{\mathrm{MASP3_{zym}}}_{\mathrm{m\,MASP1^*}}}\\ +\frac{[\mathrm{pLFMASP1_{zym}}]}{K^{\mathrm{MASP1_{zym}}}_{\mathrm{m\,MASP1^*}}}+\frac{[\mathrm{pLFMASP2_{zym}}]}{K^{\mathrm{MASP2_{zym}}}_{\mathrm{m\,MASP1^*}}}+\frac{[\mathrm{pLFMASP3_{zym}}]}{K^{\mathrm{MASP3_{zym}}}_{\mathrm{m\,MASP1^*}}}+\frac{[\mathrm{pHFMASP1_{zym}}]}{K^{\mathrm{MASP1_{zym}}}_{\mathrm{m\,MASP1^*}}}+\frac{[\mathrm{pHFMASP2_{zym}}]}{K^{\mathrm{MASP2_{zym}}}_{\mathrm{m\,MASP1^*}}}\\ +\frac{[\mathrm{pHFMASP3_{zym}}]}{K^{\mathrm{MASP3_{zym}}}_{\mathrm{m\,MASP1^*}}}+\frac{[\mathrm{pMFMASP1_{zym}}]}{K^{\mathrm{MASP1_{zym}}}_{\mathrm{m\,MASP1^*}}}+\frac{[\mathrm{pMFMASP2_{zym}}]}{K^{\mathrm{MASP2_{zym}}}_{\mathrm{m\,MASP1^*}}}+\frac{[\mathrm{pMFMASP3_{zym}}]}{K^{\mathrm{MASP3_{zym}}}_{\mathrm{m\,MASP1^*}}}\end{array}\right)}$$

$$+\frac{k_{\mathrm{cat\ MASP1^*}}^{\mathrm{C2}}\left[\mathrm{pCLK1MASP1^*}\right]\left[\mathrm{fC4bC2}\right]}{K_{\mathrm{m\ MASP1^*}}^{\mathrm{C2}}\left(\begin{array}{l}1+\frac{[\mathrm{C4}]}{K_{\mathrm{m\ MASP1^*}}^{\mathrm{C4}}}+\frac{[\mathrm{fC4bC2}]}{K_{\mathrm{m\ MASP1^*}}^{\mathrm{C2}}}+\frac{[\mathrm{fC4bC4bC2}]}{K_{\mathrm{m\ MASP1^*}}^{\mathrm{C2}}}+\frac{[\mathrm{fC3bC4bC2}]}{K_{\mathrm{m\ MASP1^*}}^{\mathrm{C2}}}+\frac{[\mathrm{pC4bC2}]}{K_{\mathrm{m\ MASP1^*}}^{\mathrm{C2}}}+\frac{[\mathrm{pC4bC4bC2}]}{K_{\mathrm{m\ MASP1^*}}^{\mathrm{C2}}}\\+\frac{[\mathrm{pC3bC4bC2}]}{K_{\mathrm{m\ MASP1^*}}^{\mathrm{C2}}}+\frac{[\mathrm{pMBL_3MASP1_{zym}}]}{K_{\mathrm{m\ MASP1^*}}^{\mathrm{MASP1_{zym}}}}+\frac{[\mathrm{pMBL_3MASP2_{zym}}]}{K_{\mathrm{m\ MASP1^*}}^{\mathrm{MASP2_{zym}}}}+\frac{[\mathrm{pMBL_3MASP3_{zym}}]}{K_{\mathrm{m\ MASP1^*}}^{\mathrm{MASP3_{zym}}}}\\+\frac{[\mathrm{pMBL_4MASP1_{zym}}]}{K_{\mathrm{m\ MASP1^*}}^{\mathrm{MASP1_{zym}}}}+\frac{[\mathrm{pMBL_4MASP2_{zym}}]}{K_{\mathrm{m\ MASP1^*}}^{\mathrm{MASP2_{zym}}}}+\frac{[\mathrm{pMBL_4MASP3_{zym}}]}{K_{\mathrm{m\ MASP1^*}}^{\mathrm{MASP3_{zym}}}}+\frac{[\mathrm{pCLLKMASP1_{zym}}]}{K_{\mathrm{m\ MASP1^*}}^{\mathrm{MASP1_{zym}}}}\\+\frac{[\mathrm{pCLLKMASP2_{zym}}]}{K_{\mathrm{m\ MASP1^*}}^{\mathrm{MASP2_{zym}}}}+\frac{[\mathrm{pCLLKMASP3_{zym}}]}{K_{\mathrm{m\ MASP1^*}}^{\mathrm{MASP3_{zym}}}}+\frac{[\mathrm{pCLK1MASP1_{zym}}]}{K_{\mathrm{m\ MASP1^*}}^{\mathrm{MASP1_{zym}}}}+\frac{[\mathrm{pCLK1MASP2_{zym}}]}{K_{\mathrm{m\ MASP1^*}}^{\mathrm{MASP2_{zym}}}}\\+\frac{[\mathrm{pCLK1MASP3_{zym}}]}{K_{\mathrm{m\ MASP1^*}}^{\mathrm{MASP3_{zym}}}}+\frac{[\mathrm{pCLL1MASP1_{zym}}]}{K_{\mathrm{m\ MASP1^*}}^{\mathrm{MASP1_{zym}}}}+\frac{[\mathrm{pCLL1MASP2_{zym}}]}{K_{\mathrm{m\ MASP1^*}}^{\mathrm{MASP2_{zym}}}}+\frac{[\mathrm{pCLL1MASP3_{zym}}]}{K_{\mathrm{m\ MASP1^*}}^{\mathrm{MASP3_{zym}}}}\\+\frac{[\mathrm{pLFMASP1_{zym}}]}{K_{\mathrm{m\ MASP1^*}}^{\mathrm{MASP1_{zym}}}}+\frac{[\mathrm{pLFMASP2_{zym}}]}{K_{\mathrm{m\ MASP1^*}}^{\mathrm{MASP2_{zym}}}}+\frac{[\mathrm{pLFMASP3_{zym}}]}{K_{\mathrm{m\ MASP1^*}}^{\mathrm{MASP3_{zym}}}}+\frac{[\mathrm{pHFMASP1_{zym}}]}{K_{\mathrm{m\ MASP1^*}}^{\mathrm{MASP1_{zym}}}}+\frac{[\mathrm{pHFMASP2_{zym}}]}{K_{\mathrm{m\ MASP1^*}}^{\mathrm{MASP2_{zym}}}}\\+\frac{[\mathrm{pHFMASP3_{zym}}]}{K_{\mathrm{m\ MASP1^*}}^{\mathrm{MASP3_{zym}}}}+\frac{[\mathrm{pMFMASP1_{zym}}]}{K_{\mathrm{m\ MASP1^*}}^{\mathrm{MASP1_{zym}}}}+\frac{[\mathrm{pMFMASP2_{zym}}]}{K_{\mathrm{m\ MASP1^*}}^{\mathrm{MASP2_{zym}}}}+\frac{[\mathrm{pMFMASP3_{zym}}]}{K_{\mathrm{m\ MASP1^*}}^{\mathrm{MASP3_{zym}}}}\end{array}\right)}$$

$$+\frac{k_{\mathrm{cat\ MASP1^*}}^{\mathrm{C2}}\left[\mathrm{pCLL1MASP1^*}\right]\left[\mathrm{fC4bC2}\right]}{K_{\mathrm{m\ MASP1^*}}^{\mathrm{C2}}\left(\begin{array}{l}1+\frac{[\mathrm{C4}]}{K_{\mathrm{m\ MASP1^*}}^{\mathrm{C4}}}+\frac{[\mathrm{fC4bC2}]}{K_{\mathrm{m\ MASP1^*}}^{\mathrm{C2}}}+\frac{[\mathrm{fC4bC4bC2}]}{K_{\mathrm{m\ MASP1^*}}^{\mathrm{C2}}}+\frac{[\mathrm{fC3bC4bC2}]}{K_{\mathrm{m\ MASP1^*}}^{\mathrm{C2}}}+\frac{[\mathrm{pC4bC2}]}{K_{\mathrm{m\ MASP1^*}}^{\mathrm{C2}}}+\frac{[\mathrm{pC4bC4bC2}]}{K_{\mathrm{m\ MASP1^*}}^{\mathrm{C2}}}\\+\frac{[\mathrm{pC3bC4bC2}]}{K_{\mathrm{m\ MASP1^*}}^{\mathrm{C2}}}+\frac{[\mathrm{pMBL_3MASP1_{zym}}]}{K_{\mathrm{m\ MASP1^*}}^{\mathrm{MASP1_{zym}}}}+\frac{[\mathrm{pMBL_3MASP2_{zym}}]}{K_{\mathrm{m\ MASP1^*}}^{\mathrm{MASP2_{zym}}}}+\frac{[\mathrm{pMBL_3MASP3_{zym}}]}{K_{\mathrm{m\ MASP1^*}}^{\mathrm{MASP3_{zym}}}}\\+\frac{[\mathrm{pMBL_4MASP1_{zym}}]}{K_{\mathrm{m\ MASP1^*}}^{\mathrm{MASP1_{zym}}}}+\frac{[\mathrm{pMBL_4MASP2_{zym}}]}{K_{\mathrm{m\ MASP1^*}}^{\mathrm{MASP2_{zym}}}}+\frac{[\mathrm{pMBL_4MASP3_{zym}}]}{K_{\mathrm{m\ MASP1^*}}^{\mathrm{MASP3_{zym}}}}+\frac{[\mathrm{pCLLKMASP1_{zym}}]}{K_{\mathrm{m\ MASP1^*}}^{\mathrm{MASP1_{zym}}}}\\+\frac{[\mathrm{pCLLKMASP2_{zym}}]}{K_{\mathrm{m\ MASP1^*}}^{\mathrm{MASP2_{zym}}}}+\frac{[\mathrm{pCLLKMASP3_{zym}}]}{K_{\mathrm{m\ MASP1^*}}^{\mathrm{MASP3_{zym}}}}+\frac{[\mathrm{pCLK1MASP1_{zym}}]}{K_{\mathrm{m\ MASP1^*}}^{\mathrm{MASP1_{zym}}}}+\frac{[\mathrm{pCLK1MASP2_{zym}}]}{K_{\mathrm{m\ MASP1^*}}^{\mathrm{MASP2_{zym}}}}\\+\frac{[\mathrm{pCLK1MASP3_{zym}}]}{K_{\mathrm{m\ MASP1^*}}^{\mathrm{MASP3_{zym}}}}+\frac{[\mathrm{pCLL1MASP1_{zym}}]}{K_{\mathrm{m\ MASP1^*}}^{\mathrm{MASP1_{zym}}}}+\frac{[\mathrm{pCLL1MASP2_{zym}}]}{K_{\mathrm{m\ MASP1^*}}^{\mathrm{MASP2_{zym}}}}+\frac{[\mathrm{pCLL1MASP3_{zym}}]}{K_{\mathrm{m\ MASP1^*}}^{\mathrm{MASP3_{zym}}}}\\+\frac{[\mathrm{pLFMASP1_{zym}}]}{K_{\mathrm{m\ MASP1^*}}^{\mathrm{MASP1_{zym}}}}+\frac{[\mathrm{pLFMASP2_{zym}}]}{K_{\mathrm{m\ MASP1^*}}^{\mathrm{MASP2_{zym}}}}+\frac{[\mathrm{pLFMASP3_{zym}}]}{K_{\mathrm{m\ MASP1^*}}^{\mathrm{MASP3_{zym}}}}+\frac{[\mathrm{pHFMASP1_{zym}}]}{K_{\mathrm{m\ MASP1^*}}^{\mathrm{MASP1_{zym}}}}+\frac{[\mathrm{pHFMASP2_{zym}}]}{K_{\mathrm{m\ MASP1^*}}^{\mathrm{MASP2_{zym}}}}\\+\frac{[\mathrm{pHFMASP3_{zym}}]}{K_{\mathrm{m\ MASP1^*}}^{\mathrm{MASP3_{zym}}}}+\frac{[\mathrm{pMFMASP1_{zym}}]}{K_{\mathrm{m\ MASP1^*}}^{\mathrm{MASP1_{zym}}}}+\frac{[\mathrm{pMFMASP2_{zym}}]}{K_{\mathrm{m\ MASP1^*}}^{\mathrm{MASP2_{zym}}}}+\frac{[\mathrm{pMFMASP3_{zym}}]}{K_{\mathrm{m\ MASP1^*}}^{\mathrm{MASP3_{zym}}}}\end{array}\right)}$$

$$+\frac{k_{\text{cat MASP1}^*}^{\text{C2}}\left[\text{pLFMASP1}^*\right]\left[\text{fC4bC2}\right]}{K_{\text{m MASP1}^*}^{\text{C2}}\left(\begin{array}{l}1+\frac{[\text{C4}]}{K_{\text{m MASP1}^*}^{\text{C4}}}+\frac{[\text{fC4bC2}]}{K_{\text{m MASP1}^*}^{\text{C2}}}+\frac{[\text{fC4bC4bC2}]}{K_{\text{m MASP1}^*}^{\text{C2}}}+\frac{[\text{fC3bC4bC2}]}{K_{\text{m MASP1}^*}^{\text{C2}}}+\frac{[\text{pC4bC2}]}{K_{\text{m MASP1}^*}^{\text{C2}}}+\frac{[\text{pC4bC4bC2}]}{K_{\text{m MASP1}^*}^{\text{C2}}}\\+\frac{[\text{pC3bC4bC2}]}{K_{\text{m MASP1}^*}^{\text{C2}}}+\frac{[\text{pMBL}_3\text{MASP1}_{\text{zym}}]}{K_{\text{m MASP1}^*}^{\text{MASP1}_{\text{zym}}}}+\frac{[\text{pMBL}_3\text{MASP2}_{\text{zym}}]}{K_{\text{m MASP1}^*}^{\text{MASP2}_{\text{zym}}}}+\frac{[\text{pMBL}_3\text{MASP3}_{\text{zym}}]}{K_{\text{m MASP1}^*}^{\text{MASP3}_{\text{zym}}}}\\+\frac{[\text{pMBL}_4\text{MASP1}_{\text{zym}}]}{K_{\text{m MASP1}^*}^{\text{MASP1}_{\text{zym}}}}+\frac{[\text{pMBL}_4\text{MASP2}_{\text{zym}}]}{K_{\text{m MASP1}^*}^{\text{MASP2}_{\text{zym}}}}+\frac{[\text{pMBL}_4\text{MASP3}_{\text{zym}}]}{K_{\text{m MASP1}^*}^{\text{MASP3}_{\text{zym}}}}+\frac{[\text{pCLLKMASP1}_{\text{zym}}]}{K_{\text{m MASP1}^*}^{\text{MASP1}_{\text{zym}}}}\\+\frac{[\text{pCLLKMASP2}_{\text{zym}}]}{K_{\text{m MASP1}^*}^{\text{MASP2}_{\text{zym}}}}+\frac{[\text{pCLLKMASP3}_{\text{zym}}]}{K_{\text{m MASP1}^*}^{\text{MASP3}_{\text{zym}}}}+\frac{[\text{pCLK1MASP1}_{\text{zym}}]}{K_{\text{m MASP1}^*}^{\text{MASP1}_{\text{zym}}}}+\frac{[\text{pCLK1MASP2}_{\text{zym}}]}{K_{\text{m MASP1}^*}^{\text{MASP2}_{\text{zym}}}}\\+\frac{[\text{pCLK1MASP3}_{\text{zym}}]}{K_{\text{m MASP1}^*}^{\text{MASP3}_{\text{zym}}}}+\frac{[\text{pCLL1MASP1}_{\text{zym}}]}{K_{\text{m MASP1}^*}^{\text{MASP1}_{\text{zym}}}}+\frac{[\text{pCLL1MASP2}_{\text{zym}}]}{K_{\text{m MASP1}^*}^{\text{MASP2}_{\text{zym}}}}+\frac{[\text{pCLL1MASP3}_{\text{zym}}]}{K_{\text{m MASP1}^*}^{\text{MASP3}_{\text{zym}}}}\\+\frac{[\text{pLFMASP1}_{\text{zym}}]}{K_{\text{m MASP1}^*}^{\text{MASP1}_{\text{zym}}}}+\frac{[\text{pLFMASP2}_{\text{zym}}]}{K_{\text{m MASP1}^*}^{\text{MASP2}_{\text{zym}}}}+\frac{[\text{pLFMASP3}_{\text{zym}}]}{K_{\text{m MASP1}^*}^{\text{MASP3}_{\text{zym}}}}+\frac{[\text{pHFMASP1}_{\text{zym}}]}{K_{\text{m MASP1}^*}^{\text{MASP1}_{\text{zym}}}}+\frac{[\text{pHFMASP2}_{\text{zym}}]}{K_{\text{m MASP1}^*}^{\text{MASP2}_{\text{zym}}}}\\+\frac{[\text{pHFMASP3}_{\text{zym}}]}{K_{\text{m MASP1}^*}^{\text{MASP3}_{\text{zym}}}}+\frac{[\text{pMFMASP1}_{\text{zym}}]}{K_{\text{m MASP1}^*}^{\text{MASP1}_{\text{zym}}}}+\frac{[\text{pMFMASP2}_{\text{zym}}]}{K_{\text{m MASP1}^*}^{\text{MASP2}_{\text{zym}}}}+\frac{[\text{pMFMASP3}_{\text{zym}}]}{K_{\text{m MASP1}^*}^{\text{MASP3}_{\text{zym}}}}\end{array}\right)}$$

$$+\frac{k_{\text{cat MASP1}^*}^{\text{C2}}\left[\text{pHFMASP1}^*\right]\left[\text{fC4bC2}\right]}{K_{\text{m MASP1}^*}^{\text{C2}}\left(\begin{array}{l}1+\frac{[\text{C4}]}{K_{\text{m MASP1}^*}^{\text{C4}}}+\frac{[\text{fC4bC2}]}{K_{\text{m MASP1}^*}^{\text{C2}}}+\frac{[\text{fC4bC4bC2}]}{K_{\text{m MASP1}^*}^{\text{C2}}}+\frac{[\text{fC3bC4bC2}]}{K_{\text{m MASP1}^*}^{\text{C2}}}+\frac{[\text{pC4bC2}]}{K_{\text{m MASP1}^*}^{\text{C2}}}+\frac{[\text{pC4bC4bC2}]}{K_{\text{m MASP1}^*}^{\text{C2}}}\\+\frac{[\text{pC3bC4bC2}]}{K_{\text{m MASP1}^*}^{\text{C2}}}+\frac{[\text{pMBL}_3\text{MASP1}_{\text{zym}}]}{K_{\text{m MASP1}^*}^{\text{MASP1}_{\text{zym}}}}+\frac{[\text{pMBL}_3\text{MASP2}_{\text{zym}}]}{K_{\text{m MASP1}^*}^{\text{MASP2}_{\text{zym}}}}+\frac{[\text{pMBL}_3\text{MASP3}_{\text{zym}}]}{K_{\text{m MASP1}^*}^{\text{MASP3}_{\text{zym}}}}\\+\frac{[\text{pMBL}_4\text{MASP1}_{\text{zym}}]}{K_{\text{m MASP1}^*}^{\text{MASP1}_{\text{zym}}}}+\frac{[\text{pMBL}_4\text{MASP2}_{\text{zym}}]}{K_{\text{m MASP1}^*}^{\text{MASP2}_{\text{zym}}}}+\frac{[\text{pMBL}_4\text{MASP3}_{\text{zym}}]}{K_{\text{m MASP1}^*}^{\text{MASP3}_{\text{zym}}}}+\frac{[\text{pCLLKMASP1}_{\text{zym}}]}{K_{\text{m MASP1}^*}^{\text{MASP1}_{\text{zym}}}}\\+\frac{[\text{pCLLKMASP2}_{\text{zym}}]}{K_{\text{m MASP1}^*}^{\text{MASP2}_{\text{zym}}}}+\frac{[\text{pCLLKMASP3}_{\text{zym}}]}{K_{\text{m MASP1}^*}^{\text{MASP3}_{\text{zym}}}}+\frac{[\text{pCLK1MASP1}_{\text{zym}}]}{K_{\text{m MASP1}^*}^{\text{MASP1}_{\text{zym}}}}+\frac{[\text{pCLK1MASP2}_{\text{zym}}]}{K_{\text{m MASP1}^*}^{\text{MASP2}_{\text{zym}}}}\\+\frac{[\text{pCLK1MASP3}_{\text{zym}}]}{K_{\text{m MASP1}^*}^{\text{MASP3}_{\text{zym}}}}+\frac{[\text{pCLL1MASP1}_{\text{zym}}]}{K_{\text{m MASP1}^*}^{\text{MASP1}_{\text{zym}}}}+\frac{[\text{pCLL1MASP2}_{\text{zym}}]}{K_{\text{m MASP1}^*}^{\text{MASP2}_{\text{zym}}}}+\frac{[\text{pCLL1MASP3}_{\text{zym}}]}{K_{\text{m MASP1}^*}^{\text{MASP3}_{\text{zym}}}}\\+\frac{[\text{pLFMASP1}_{\text{zym}}]}{K_{\text{m MASP1}^*}^{\text{MASP1}_{\text{zym}}}}+\frac{[\text{pLFMASP2}_{\text{zym}}]}{K_{\text{m MASP1}^*}^{\text{MASP2}_{\text{zym}}}}+\frac{[\text{pLFMASP3}_{\text{zym}}]}{K_{\text{m MASP1}^*}^{\text{MASP3}_{\text{zym}}}}+\frac{[\text{pHFMASP1}_{\text{zym}}]}{K_{\text{m MASP1}^*}^{\text{MASP1}_{\text{zym}}}}+\frac{[\text{pHFMASP2}_{\text{zym}}]}{K_{\text{m MASP1}^*}^{\text{MASP2}_{\text{zym}}}}\\+\frac{[\text{pHFMASP3}_{\text{zym}}]}{K_{\text{m MASP1}^*}^{\text{MASP3}_{\text{zym}}}}+\frac{[\text{pMFMASP1}_{\text{zym}}]}{K_{\text{m MASP1}^*}^{\text{MASP1}_{\text{zym}}}}+\frac{[\text{pMFMASP2}_{\text{zym}}]}{K_{\text{m MASP1}^*}^{\text{MASP2}_{\text{zym}}}}+\frac{[\text{pMFMASP3}_{\text{zym}}]}{K_{\text{m MASP1}^*}^{\text{MASP3}_{\text{zym}}}}\end{array}\right)}$$

$$
+\frac{k_{\mathrm{cat\ MASP1^*}}^{\mathrm{C2}}\left[\mathrm{pMFMASP1^*}\right]\left[\mathrm{fC4bC2}\right]}{K_{\mathrm{m\ MASP1^*}}^{\mathrm{C2}}\left(\begin{aligned}&1+\frac{[\mathrm{C4}]}{K_{\mathrm{m\ MASP1^*}}^{\mathrm{C4}}}+\frac{[\mathrm{fC4bC2}]}{K_{\mathrm{m\ MASP1^*}}^{\mathrm{C2}}}+\frac{[\mathrm{fC4bC4bC2}]}{K_{\mathrm{m\ MASP1^*}}^{\mathrm{C2}}}+\frac{[\mathrm{fC3bC4bC2}]}{K_{\mathrm{m\ MASP1^*}}^{\mathrm{C2}}}+\frac{[\mathrm{pC4bC2}]}{K_{\mathrm{m\ MASP1^*}}^{\mathrm{C2}}}+\frac{[\mathrm{pC4bC4bC2}]}{K_{\mathrm{m\ MASP1^*}}^{\mathrm{C2}}}\\
&+\frac{[\mathrm{pC3bC4bC2}]}{K_{\mathrm{m\ MASP1^*}}^{\mathrm{C2}}}+\frac{\left[\mathrm{pMBL_3MASP1_{zym}}\right]}{K_{\mathrm{m\ MASP1^*}}^{\mathrm{MASP1_{zym}}}}+\frac{\left[\mathrm{pMBL_3MASP2_{zym}}\right]}{K_{\mathrm{m\ MASP1^*}}^{\mathrm{MASP2_{zym}}}}+\frac{\left[\mathrm{pMBL_3MASP3_{zym}}\right]}{K_{\mathrm{m\ MASP1^*}}^{\mathrm{MASP3_{zym}}}}\\
&+\frac{\left[\mathrm{pMBL_4MASP1_{zym}}\right]}{K_{\mathrm{m\ MASP1^*}}^{\mathrm{MASP1_{zym}}}}+\frac{\left[\mathrm{pMBL_4MASP2_{zym}}\right]}{K_{\mathrm{m\ MASP1^*}}^{\mathrm{MASP2_{zym}}}}+\frac{\left[\mathrm{pMBL_4MASP3_{zym}}\right]}{K_{\mathrm{m\ MASP1^*}}^{\mathrm{MASP3_{zym}}}}+\frac{\left[\mathrm{pCLLKMASP1_{zym}}\right]}{K_{\mathrm{m\ MASP1^*}}^{\mathrm{MASP1_{zym}}}}\\
&+\frac{\left[\mathrm{pCLLKMASP2_{zym}}\right]}{K_{\mathrm{m\ MASP1^*}}^{\mathrm{MASP2_{zym}}}}+\frac{\left[\mathrm{pCLLKMASP3_{zym}}\right]}{K_{\mathrm{m\ MASP1^*}}^{\mathrm{MASP3_{zym}}}}+\frac{\left[\mathrm{pCLK1MASP1_{zym}}\right]}{K_{\mathrm{m\ MASP1^*}}^{\mathrm{MASP1_{zym}}}}+\frac{\left[\mathrm{pCLK1MASP2_{zym}}\right]}{K_{\mathrm{m\ MASP1^*}}^{\mathrm{MASP2_{zym}}}}\\
&+\frac{\left[\mathrm{pCLK1MASP3_{zym}}\right]}{K_{\mathrm{m\ MASP1^*}}^{\mathrm{MASP3_{zym}}}}+\frac{\left[\mathrm{pCLL1MASP1_{zym}}\right]}{K_{\mathrm{m\ MASP1^*}}^{\mathrm{MASP1_{zym}}}}+\frac{\left[\mathrm{pCLL1MASP2_{zym}}\right]}{K_{\mathrm{m\ MASP1^*}}^{\mathrm{MASP2_{zym}}}}+\frac{\left[\mathrm{pCLL1MASP3_{zym}}\right]}{K_{\mathrm{m\ MASP1^*}}^{\mathrm{MASP3_{zym}}}}\\
&+\frac{\left[\mathrm{pLFMASP1_{zym}}\right]}{K_{\mathrm{m\ MASP1^*}}^{\mathrm{MASP1_{zym}}}}+\frac{\left[\mathrm{pLFMASP2_{zym}}\right]}{K_{\mathrm{m\ MASP1^*}}^{\mathrm{MASP2_{zym}}}}+\frac{\left[\mathrm{pLFMASP3_{zym}}\right]}{K_{\mathrm{m\ MASP1^*}}^{\mathrm{MASP3_{zym}}}}+\frac{\left[\mathrm{pHFMASP1_{zym}}\right]}{K_{\mathrm{m\ MASP1^*}}^{\mathrm{MASP1_{zym}}}}+\frac{\left[\mathrm{pHFMASP2_{zym}}\right]}{K_{\mathrm{m\ MASP1^*}}^{\mathrm{MASP2_{zym}}}}\\
&+\frac{\left[\mathrm{pHFMASP3_{zym}}\right]}{K_{\mathrm{m\ MASP1^*}}^{\mathrm{MASP3_{zym}}}}+\frac{\left[\mathrm{pMFMASP1_{zym}}\right]}{K_{\mathrm{m\ MASP1^*}}^{\mathrm{MASP1_{zym}}}}+\frac{\left[\mathrm{pMFMASP2_{zym}}\right]}{K_{\mathrm{m\ MASP1^*}}^{\mathrm{MASP2_{zym}}}}+\frac{\left[\mathrm{pMFMASP3_{zym}}\right]}{K_{\mathrm{m\ MASP1^*}}^{\mathrm{MASP3_{zym}}}}\end{aligned}\right)}
$$

$$
+\frac{k_{\mathrm{cat\ MASP2^*}}^{\mathrm{C2}}\left[\mathrm{pMBL_3MASP2^*}\right]\left[\mathrm{fC4bC2}\right]}{K_{\mathrm{m\ MASP2^*}}^{\mathrm{C2}}\left(\begin{aligned}&1+\frac{[\mathrm{C4}]}{K_{\mathrm{m\ MASP2^*}}^{\mathrm{C4}}}+\frac{[\mathrm{fC4bC2}]}{K_{\mathrm{m\ MASP2^*}}^{\mathrm{C2}}}+\frac{[\mathrm{fC4bC4bC2}]}{K_{\mathrm{m\ MASP2^*}}^{\mathrm{C2}}}+\frac{[\mathrm{fC3bC4bC2}]}{K_{\mathrm{m\ MASP2^*}}^{\mathrm{C2}}}+\frac{[\mathrm{pC4bC2}]}{K_{\mathrm{m\ MASP2^*}}^{\mathrm{C2}}}+\frac{[\mathrm{pC4bC4bC2}]}{K_{\mathrm{m\ MASP2^*}}^{\mathrm{C2}}}\\
&+\frac{[\mathrm{pC3bC4bC2}]}{K_{\mathrm{m\ MASP2^*}}^{\mathrm{C2}}}+\frac{\left[\mathrm{pMBL_3MASP1_{zym}}\right]}{K_{\mathrm{m\ MASP2^*}}^{\mathrm{MASP1_{zym}}}}+\frac{\left[\mathrm{pMBL_3MASP2_{zym}}\right]}{K_{\mathrm{m\ MASP2^*}}^{\mathrm{MASP2_{zym}}}}+\frac{\left[\mathrm{pMBL_4MASP1_{zym}}\right]}{K_{\mathrm{m\ MASP2^*}}^{\mathrm{MASP1_{zym}}}}\\
&+\frac{\left[\mathrm{pMBL_4MASP2_{zym}}\right]}{K_{\mathrm{m\ MASP2^*}}^{\mathrm{MASP2_{zym}}}}+\frac{\left[\mathrm{pCLLKMASP1_{zym}}\right]}{K_{\mathrm{m\ MASP2^*}}^{\mathrm{MASP1_{zym}}}}+\frac{\left[\mathrm{pCLLKMASP2_{zym}}\right]}{K_{\mathrm{m\ MASP2^*}}^{\mathrm{MASP2_{zym}}}}+\frac{\left[\mathrm{pCLK1MASP1_{zym}}\right]}{K_{\mathrm{m\ MASP2^*}}^{\mathrm{MASP1_{zym}}}}\\
&+\frac{\left[\mathrm{pCLK1MASP2_{zym}}\right]}{K_{\mathrm{m\ MASP2^*}}^{\mathrm{MASP2_{zym}}}}+\frac{\left[\mathrm{pCLL1MASP1_{zym}}\right]}{K_{\mathrm{m\ MASP2^*}}^{\mathrm{MASP1_{zym}}}}+\frac{\left[\mathrm{pCLL1MASP2_{zym}}\right]}{K_{\mathrm{m\ MASP2^*}}^{\mathrm{MASP2_{zym}}}}+\frac{\left[\mathrm{pLFMASP1_{zym}}\right]}{K_{\mathrm{m\ MASP2^*}}^{\mathrm{MASP1_{zym}}}}\\
&+\frac{\left[\mathrm{pLFMASP2_{zym}}\right]}{K_{\mathrm{m\ MASP2^*}}^{\mathrm{MASP2_{zym}}}}+\frac{\left[\mathrm{pHFMASP1_{zym}}\right]}{K_{\mathrm{m\ MASP2^*}}^{\mathrm{MASP1_{zym}}}}+\frac{\left[\mathrm{pHFMASP2_{zym}}\right]}{K_{\mathrm{m\ MASP2^*}}^{\mathrm{MASP2_{zym}}}}+\frac{\left[\mathrm{pMFMASP1_{zym}}\right]}{K_{\mathrm{m\ MASP2^*}}^{\mathrm{MASP1_{zym}}}}+\frac{\left[\mathrm{pMFMASP2_{zym}}\right]}{K_{\mathrm{m\ MASP2^*}}^{\mathrm{MASP2_{zym}}}}\end{aligned}\right)}
$$

$$+\frac{k^{\mathrm{C2}}_{\mathrm{cat\ MASP2^*}}\left[\mathrm{pMBL_4MASP2^*}\right]\left[\mathrm{fC4bC2}\right]}{K^{\mathrm{C2}}_{\mathrm{m\ MASP2^*}}\left(\begin{array}{l}1+\frac{[\mathrm{C4}]}{K^{\mathrm{C4}}_{\mathrm{m\ MASP2^*}}}+\frac{[\mathrm{fC4bC2}]}{K^{\mathrm{C2}}_{\mathrm{m\ MASP2^*}}}+\frac{[\mathrm{fC4bC4bC2}]}{K^{\mathrm{C2}}_{\mathrm{m\ MASP2^*}}}+\frac{[\mathrm{fC3bC4bC2}]}{K^{\mathrm{C2}}_{\mathrm{m\ MASP2^*}}}+\frac{[\mathrm{pC4bC2}]}{K^{\mathrm{C2}}_{\mathrm{m\ MASP2^*}}}+\frac{[\mathrm{pC4bC4bC2}]}{K^{\mathrm{C2}}_{\mathrm{m\ MASP2^*}}}\\+\frac{[\mathrm{pC3bC4bC2}]}{K^{\mathrm{C2}}_{\mathrm{m\ MASP2^*}}}+\frac{[\mathrm{pMBL_3MASP1_{zym}}]}{K^{\mathrm{MASP1_{zym}}}_{\mathrm{m\ MASP2^*}}}+\frac{[\mathrm{pMBL_3MASP2_{zym}}]}{K^{\mathrm{MASP2_{zym}}}_{\mathrm{m\ MASP2^*}}}+\frac{[\mathrm{pMBL_4MASP1_{zym}}]}{K^{\mathrm{MASP1_{zym}}}_{\mathrm{m\ MASP2^*}}}\\+\frac{[\mathrm{pMBL_4MASP2_{zym}}]}{K^{\mathrm{MASP2_{zym}}}_{\mathrm{m\ MASP2^*}}}+\frac{[\mathrm{pCLLKMASP1_{zym}}]}{K^{\mathrm{MASP1_{zym}}}_{\mathrm{m\ MASP2^*}}}+\frac{[\mathrm{pCLLKMASP2_{zym}}]}{K^{\mathrm{MASP2_{zym}}}_{\mathrm{m\ MASP2^*}}}+\frac{[\mathrm{pCLK1MASP1_{zym}}]}{K^{\mathrm{MASP1_{zym}}}_{\mathrm{m\ MASP2^*}}}\\+\frac{[\mathrm{pCLK1MASP2_{zym}}]}{K^{\mathrm{MASP2_{zym}}}_{\mathrm{m\ MASP2^*}}}+\frac{[\mathrm{pCLL1MASP1_{zym}}]}{K^{\mathrm{MASP1_{zym}}}_{\mathrm{m\ MASP2^*}}}+\frac{[\mathrm{pCLL1MASP2_{zym}}]}{K^{\mathrm{MASP2_{zym}}}_{\mathrm{m\ MASP2^*}}}+\frac{[\mathrm{pLFMASP1_{zym}}]}{K^{\mathrm{MASP1_{zym}}}_{\mathrm{m\ MASP2^*}}}\\+\frac{[\mathrm{pLFMASP2_{zym}}]}{K^{\mathrm{MASP2_{zym}}}_{\mathrm{m\ MASP2^*}}}+\frac{[\mathrm{pHFMASP1_{zym}}]}{K^{\mathrm{MASP1_{zym}}}_{\mathrm{m\ MASP2^*}}}+\frac{[\mathrm{pHFMASP2_{zym}}]}{K^{\mathrm{MASP2_{zym}}}_{\mathrm{m\ MASP2^*}}}+\frac{[\mathrm{pMFMASP1_{zym}}]}{K^{\mathrm{MASP1_{zym}}}_{\mathrm{m\ MASP2^*}}}+\frac{[\mathrm{pMFMASP2_{zym}}]}{K^{\mathrm{MASP2_{zym}}}_{\mathrm{m\ MASP2^*}}}\end{array}\right)}$$

$$+\frac{k^{\mathrm{C2}}_{\mathrm{cat\ MASP2^*}}\left[\mathrm{pCLLKMASP2^*}\right]\left[\mathrm{fC4bC2}\right]}{K^{\mathrm{C2}}_{\mathrm{m\ MASP2^*}}\left(\begin{array}{l}1+\frac{[\mathrm{C4}]}{K^{\mathrm{C4}}_{\mathrm{m\ MASP2^*}}}+\frac{[\mathrm{fC4bC2}]}{K^{\mathrm{C2}}_{\mathrm{m\ MASP2^*}}}+\frac{[\mathrm{fC4bC4bC2}]}{K^{\mathrm{C2}}_{\mathrm{m\ MASP2^*}}}+\frac{[\mathrm{fC3bC4bC2}]}{K^{\mathrm{C2}}_{\mathrm{m\ MASP2^*}}}+\frac{[\mathrm{pC4bC2}]}{K^{\mathrm{C2}}_{\mathrm{m\ MASP2^*}}}+\frac{[\mathrm{pC4bC4bC2}]}{K^{\mathrm{C2}}_{\mathrm{m\ MASP2^*}}}\\+\frac{[\mathrm{pC3bC4bC2}]}{K^{\mathrm{C2}}_{\mathrm{m\ MASP2^*}}}+\frac{[\mathrm{pMBL_3MASP1_{zym}}]}{K^{\mathrm{MASP1_{zym}}}_{\mathrm{m\ MASP2^*}}}+\frac{[\mathrm{pMBL_3MASP2_{zym}}]}{K^{\mathrm{MASP2_{zym}}}_{\mathrm{m\ MASP2^*}}}+\frac{[\mathrm{pMBL_4MASP1_{zym}}]}{K^{\mathrm{MASP1_{zym}}}_{\mathrm{m\ MASP2^*}}}\\+\frac{[\mathrm{pMBL_4MASP2_{zym}}]}{K^{\mathrm{MASP2_{zym}}}_{\mathrm{m\ MASP2^*}}}+\frac{[\mathrm{pCLLKMASP1_{zym}}]}{K^{\mathrm{MASP1_{zym}}}_{\mathrm{m\ MASP2^*}}}+\frac{[\mathrm{pCLLKMASP2_{zym}}]}{K^{\mathrm{MASP2_{zym}}}_{\mathrm{m\ MASP2^*}}}+\frac{[\mathrm{pCLK1MASP1_{zym}}]}{K^{\mathrm{MASP1_{zym}}}_{\mathrm{m\ MASP2^*}}}\\+\frac{[\mathrm{pCLK1MASP2_{zym}}]}{K^{\mathrm{MASP2_{zym}}}_{\mathrm{m\ MASP2^*}}}+\frac{[\mathrm{pCLL1MASP1_{zym}}]}{K^{\mathrm{MASP1_{zym}}}_{\mathrm{m\ MASP2^*}}}+\frac{[\mathrm{pCLL1MASP2_{zym}}]}{K^{\mathrm{MASP2_{zym}}}_{\mathrm{m\ MASP2^*}}}+\frac{[\mathrm{pLFMASP1_{zym}}]}{K^{\mathrm{MASP1_{zym}}}_{\mathrm{m\ MASP2^*}}}\\+\frac{[\mathrm{pLFMASP2_{zym}}]}{K^{\mathrm{MASP2_{zym}}}_{\mathrm{m\ MASP2^*}}}+\frac{[\mathrm{pHFMASP1_{zym}}]}{K^{\mathrm{MASP1_{zym}}}_{\mathrm{m\ MASP2^*}}}+\frac{[\mathrm{pHFMASP2_{zym}}]}{K^{\mathrm{MASP2_{zym}}}_{\mathrm{m\ MASP2^*}}}+\frac{[\mathrm{pMFMASP1_{zym}}]}{K^{\mathrm{MASP1_{zym}}}_{\mathrm{m\ MASP2^*}}}+\frac{[\mathrm{pMFMASP2_{zym}}]}{K^{\mathrm{MASP2_{zym}}}_{\mathrm{m\ MASP2^*}}}\end{array}\right)}$$

$$+\frac{k^{\mathrm{C2}}_{\mathrm{cat\ MASP2^*}}\left[\mathrm{pCLK1MASP2^*}\right]\left[\mathrm{fC4bC2}\right]}{K^{\mathrm{C2}}_{\mathrm{m\ MASP2^*}}\left(\begin{array}{l}1+\frac{[\mathrm{C4}]}{K^{\mathrm{C4}}_{\mathrm{m\ MASP2^*}}}+\frac{[\mathrm{fC4bC2}]}{K^{\mathrm{C2}}_{\mathrm{m\ MASP2^*}}}+\frac{[\mathrm{fC4bC4bC2}]}{K^{\mathrm{C2}}_{\mathrm{m\ MASP2^*}}}+\frac{[\mathrm{fC3bC4bC2}]}{K^{\mathrm{C2}}_{\mathrm{m\ MASP2^*}}}+\frac{[\mathrm{pC4bC2}]}{K^{\mathrm{C2}}_{\mathrm{m\ MASP2^*}}}+\frac{[\mathrm{pC4bC4bC2}]}{K^{\mathrm{C2}}_{\mathrm{m\ MASP2^*}}}\\+\frac{[\mathrm{pC3bC4bC2}]}{K^{\mathrm{C2}}_{\mathrm{m\ MASP2^*}}}+\frac{[\mathrm{pMBL_3MASP1_{zym}}]}{K^{\mathrm{MASP1_{zym}}}_{\mathrm{m\ MASP2^*}}}+\frac{[\mathrm{pMBL_3MASP2_{zym}}]}{K^{\mathrm{MASP2_{zym}}}_{\mathrm{m\ MASP2^*}}}+\frac{[\mathrm{pMBL_4MASP1_{zym}}]}{K^{\mathrm{MASP1_{zym}}}_{\mathrm{m\ MASP2^*}}}\\+\frac{[\mathrm{pMBL_4MASP2_{zym}}]}{K^{\mathrm{MASP2_{zym}}}_{\mathrm{m\ MASP2^*}}}+\frac{[\mathrm{pCLLKMASP1_{zym}}]}{K^{\mathrm{MASP1_{zym}}}_{\mathrm{m\ MASP2^*}}}+\frac{[\mathrm{pCLLKMASP2_{zym}}]}{K^{\mathrm{MASP2_{zym}}}_{\mathrm{m\ MASP2^*}}}+\frac{[\mathrm{pCLK1MASP1_{zym}}]}{K^{\mathrm{MASP1_{zym}}}_{\mathrm{m\ MASP2^*}}}\\+\frac{[\mathrm{pCLK1MASP2_{zym}}]}{K^{\mathrm{MASP2_{zym}}}_{\mathrm{m\ MASP2^*}}}+\frac{[\mathrm{pCLL1MASP1_{zym}}]}{K^{\mathrm{MASP1_{zym}}}_{\mathrm{m\ MASP2^*}}}+\frac{[\mathrm{pCLL1MASP2_{zym}}]}{K^{\mathrm{MASP2_{zym}}}_{\mathrm{m\ MASP2^*}}}+\frac{[\mathrm{pLFMASP1_{zym}}]}{K^{\mathrm{MASP1_{zym}}}_{\mathrm{m\ MASP2^*}}}\\+\frac{[\mathrm{pLFMASP2_{zym}}]}{K^{\mathrm{MASP2_{zym}}}_{\mathrm{m\ MASP2^*}}}+\frac{[\mathrm{pHFMASP1_{zym}}]}{K^{\mathrm{MASP1_{zym}}}_{\mathrm{m\ MASP2^*}}}+\frac{[\mathrm{pHFMASP2_{zym}}]}{K^{\mathrm{MASP2_{zym}}}_{\mathrm{m\ MASP2^*}}}+\frac{[\mathrm{pMFMASP1_{zym}}]}{K^{\mathrm{MASP1_{zym}}}_{\mathrm{m\ MASP2^*}}}+\frac{[\mathrm{pMFMASP2_{zym}}]}{K^{\mathrm{MASP2_{zym}}}_{\mathrm{m\ MASP2^*}}}\end{array}\right)}$$

$$
+\frac{k^{\mathrm{C2}}_{\mathrm{cat\ MASP2^*}}\left[\mathrm{pCLL1MASP2^*}\right]\left[\mathrm{fC4bC2}\right]}{K^{\mathrm{C2}}_{\mathrm{m\ MASP2^*}}\left(\begin{array}{l}1+\frac{[\mathrm{C4}]}{K^{\mathrm{C4}}_{\mathrm{m\ MASP2^*}}}+\frac{[\mathrm{fC4bC2}]}{K^{\mathrm{C2}}_{\mathrm{m\ MASP2^*}}}+\frac{[\mathrm{fC4bC4bC2}]}{K^{\mathrm{C2}}_{\mathrm{m\ MASP2^*}}}+\frac{[\mathrm{fC3bC4bC2}]}{K^{\mathrm{C2}}_{\mathrm{m\ MASP2^*}}}+\frac{[\mathrm{pC4bC2}]}{K^{\mathrm{C2}}_{\mathrm{m\ MASP2^*}}}+\frac{[\mathrm{pC4bC4bC2}]}{K^{\mathrm{C2}}_{\mathrm{m\ MASP2^*}}}\\
+\frac{[\mathrm{pC3bC4bC2}]}{K^{\mathrm{C2}}_{\mathrm{m\ MASP2^*}}}+\frac{[\mathrm{pMBL_3MASP1_{zym}}]}{K^{\mathrm{MASP1_{zym}}}_{\mathrm{m\ MASP2^*}}}+\frac{[\mathrm{pMBL_3MASP2_{zym}}]}{K^{\mathrm{MASP2_{zym}}}_{\mathrm{m\ MASP2^*}}}+\frac{[\mathrm{pMBL_4MASP1_{zym}}]}{K^{\mathrm{MASP1_{zym}}}_{\mathrm{m\ MASP2^*}}}\\
+\frac{[\mathrm{pMBL_4MASP2_{zym}}]}{K^{\mathrm{MASP2_{zym}}}_{\mathrm{m\ MASP2^*}}}+\frac{[\mathrm{pCLLKMASP1_{zym}}]}{K^{\mathrm{MASP1_{zym}}}_{\mathrm{m\ MASP2^*}}}+\frac{[\mathrm{pCLLKMASP2_{zym}}]}{K^{\mathrm{MASP2_{zym}}}_{\mathrm{m\ MASP2^*}}}+\frac{[\mathrm{pCLK1MASP1_{zym}}]}{K^{\mathrm{MASP1_{zym}}}_{\mathrm{m\ MASP2^*}}}\\
+\frac{[\mathrm{pCLK1MASP2_{zym}}]}{K^{\mathrm{MASP2_{zym}}}_{\mathrm{m\ MASP2^*}}}+\frac{[\mathrm{pCLL1MASP1_{zym}}]}{K^{\mathrm{MASP1_{zym}}}_{\mathrm{m\ MASP2^*}}}+\frac{[\mathrm{pCLL1MASP2_{zym}}]}{K^{\mathrm{MASP2_{zym}}}_{\mathrm{m\ MASP2^*}}}+\frac{[\mathrm{pLFMASP1_{zym}}]}{K^{\mathrm{MASP1_{zym}}}_{\mathrm{m\ MASP2^*}}}\\
+\frac{[\mathrm{pLFMASP2_{zym}}]}{K^{\mathrm{MASP2_{zym}}}_{\mathrm{m\ MASP2^*}}}+\frac{[\mathrm{pHFMASP1_{zym}}]}{K^{\mathrm{MASP1_{zym}}}_{\mathrm{m\ MASP2^*}}}+\frac{[\mathrm{pHFMASP2_{zym}}]}{K^{\mathrm{MASP2_{zym}}}_{\mathrm{m\ MASP2^*}}}+\frac{[\mathrm{pMFMASP1_{zym}}]}{K^{\mathrm{MASP1_{zym}}}_{\mathrm{m\ MASP2^*}}}+\frac{[\mathrm{pMFMASP2_{zym}}]}{K^{\mathrm{MASP2_{zym}}}_{\mathrm{m\ MASP2^*}}}\end{array}\right)}
$$

$$
+\frac{k^{\mathrm{C2}}_{\mathrm{cat\ MASP2^*}}\left[\mathrm{pLFMASP2^*}\right]\left[\mathrm{fC4bC2}\right]}{K^{\mathrm{C2}}_{\mathrm{m\ MASP2^*}}\left(\begin{array}{l}1+\frac{[\mathrm{C4}]}{K^{\mathrm{C4}}_{\mathrm{m\ MASP2^*}}}+\frac{[\mathrm{fC4bC2}]}{K^{\mathrm{C2}}_{\mathrm{m\ MASP2^*}}}+\frac{[\mathrm{fC4bC4bC2}]}{K^{\mathrm{C2}}_{\mathrm{m\ MASP2^*}}}+\frac{[\mathrm{fC3bC4bC2}]}{K^{\mathrm{C2}}_{\mathrm{m\ MASP2^*}}}+\frac{[\mathrm{pC4bC2}]}{K^{\mathrm{C2}}_{\mathrm{m\ MASP2^*}}}+\frac{[\mathrm{pC4bC4bC2}]}{K^{\mathrm{C2}}_{\mathrm{m\ MASP2^*}}}\\
+\frac{[\mathrm{pC3bC4bC2}]}{K^{\mathrm{C2}}_{\mathrm{m\ MASP2^*}}}+\frac{[\mathrm{pMBL_3MASP1_{zym}}]}{K^{\mathrm{MASP1_{zym}}}_{\mathrm{m\ MASP2^*}}}+\frac{[\mathrm{pMBL_3MASP2_{zym}}]}{K^{\mathrm{MASP2_{zym}}}_{\mathrm{m\ MASP2^*}}}+\frac{[\mathrm{pMBL_4MASP1_{zym}}]}{K^{\mathrm{MASP1_{zym}}}_{\mathrm{m\ MASP2^*}}}\\
+\frac{[\mathrm{pMBL_4MASP2_{zym}}]}{K^{\mathrm{MASP2_{zym}}}_{\mathrm{m\ MASP2^*}}}+\frac{[\mathrm{pCLLKMASP1_{zym}}]}{K^{\mathrm{MASP1_{zym}}}_{\mathrm{m\ MASP2^*}}}+\frac{[\mathrm{pCLLKMASP2_{zym}}]}{K^{\mathrm{MASP2_{zym}}}_{\mathrm{m\ MASP2^*}}}+\frac{[\mathrm{pCLK1MASP1_{zym}}]}{K^{\mathrm{MASP1_{zym}}}_{\mathrm{m\ MASP2^*}}}\\
+\frac{[\mathrm{pCLK1MASP2_{zym}}]}{K^{\mathrm{MASP2_{zym}}}_{\mathrm{m\ MASP2^*}}}+\frac{[\mathrm{pCLL1MASP1_{zym}}]}{K^{\mathrm{MASP1_{zym}}}_{\mathrm{m\ MASP2^*}}}+\frac{[\mathrm{pCLL1MASP2_{zym}}]}{K^{\mathrm{MASP2_{zym}}}_{\mathrm{m\ MASP2^*}}}+\frac{[\mathrm{pLFMASP1_{zym}}]}{K^{\mathrm{MASP1_{zym}}}_{\mathrm{m\ MASP2^*}}}\\
+\frac{[\mathrm{pLFMASP2_{zym}}]}{K^{\mathrm{MASP2_{zym}}}_{\mathrm{m\ MASP2^*}}}+\frac{[\mathrm{pHFMASP1_{zym}}]}{K^{\mathrm{MASP1_{zym}}}_{\mathrm{m\ MASP2^*}}}+\frac{[\mathrm{pHFMASP2_{zym}}]}{K^{\mathrm{MASP2_{zym}}}_{\mathrm{m\ MASP2^*}}}+\frac{[\mathrm{pMFMASP1_{zym}}]}{K^{\mathrm{MASP1_{zym}}}_{\mathrm{m\ MASP2^*}}}+\frac{[\mathrm{pMFMASP2_{zym}}]}{K^{\mathrm{MASP2_{zym}}}_{\mathrm{m\ MASP2^*}}}\end{array}\right)}
$$

$$
+\frac{k^{\mathrm{C2}}_{\mathrm{cat\ MASP2^*}}\left[\mathrm{pHFMASP2^*}\right]\left[\mathrm{fC4bC2}\right]}{K^{\mathrm{C2}}_{\mathrm{m\ MASP2^*}}\left(\begin{array}{l}1+\frac{[\mathrm{C4}]}{K^{\mathrm{C4}}_{\mathrm{m\ MASP2^*}}}+\frac{[\mathrm{fC4bC2}]}{K^{\mathrm{C2}}_{\mathrm{m\ MASP2^*}}}+\frac{[\mathrm{fC4bC4bC2}]}{K^{\mathrm{C2}}_{\mathrm{m\ MASP2^*}}}+\frac{[\mathrm{fC3bC4bC2}]}{K^{\mathrm{C2}}_{\mathrm{m\ MASP2^*}}}+\frac{[\mathrm{pC4bC2}]}{K^{\mathrm{C2}}_{\mathrm{m\ MASP2^*}}}+\frac{[\mathrm{pC4bC4bC2}]}{K^{\mathrm{C2}}_{\mathrm{m\ MASP2^*}}}\\
+\frac{[\mathrm{pC3bC4bC2}]}{K^{\mathrm{C2}}_{\mathrm{m\ MASP2^*}}}+\frac{[\mathrm{pMBL_3MASP1_{zym}}]}{K^{\mathrm{MASP1_{zym}}}_{\mathrm{m\ MASP2^*}}}+\frac{[\mathrm{pMBL_3MASP2_{zym}}]}{K^{\mathrm{MASP2_{zym}}}_{\mathrm{m\ MASP2^*}}}+\frac{[\mathrm{pMBL_4MASP1_{zym}}]}{K^{\mathrm{MASP1_{zym}}}_{\mathrm{m\ MASP2^*}}}\\
+\frac{[\mathrm{pMBL_4MASP2_{zym}}]}{K^{\mathrm{MASP2_{zym}}}_{\mathrm{m\ MASP2^*}}}+\frac{[\mathrm{pCLLKMASP1_{zym}}]}{K^{\mathrm{MASP1_{zym}}}_{\mathrm{m\ MASP2^*}}}+\frac{[\mathrm{pCLLKMASP2_{zym}}]}{K^{\mathrm{MASP2_{zym}}}_{\mathrm{m\ MASP2^*}}}+\frac{[\mathrm{pCLK1MASP1_{zym}}]}{K^{\mathrm{MASP1_{zym}}}_{\mathrm{m\ MASP2^*}}}\\
+\frac{[\mathrm{pCLK1MASP2_{zym}}]}{K^{\mathrm{MASP2_{zym}}}_{\mathrm{m\ MASP2^*}}}+\frac{[\mathrm{pCLL1MASP1_{zym}}]}{K^{\mathrm{MASP1_{zym}}}_{\mathrm{m\ MASP2^*}}}+\frac{[\mathrm{pCLL1MASP2_{zym}}]}{K^{\mathrm{MASP2_{zym}}}_{\mathrm{m\ MASP2^*}}}+\frac{[\mathrm{pLFMASP1_{zym}}]}{K^{\mathrm{MASP1_{zym}}}_{\mathrm{m\ MASP2^*}}}\\
+\frac{[\mathrm{pLFMASP2_{zym}}]}{K^{\mathrm{MASP2_{zym}}}_{\mathrm{m\ MASP2^*}}}+\frac{[\mathrm{pHFMASP1_{zym}}]}{K^{\mathrm{MASP1_{zym}}}_{\mathrm{m\ MASP2^*}}}+\frac{[\mathrm{pHFMASP2_{zym}}]}{K^{\mathrm{MASP2_{zym}}}_{\mathrm{m\ MASP2^*}}}+\frac{[\mathrm{pMFMASP1_{zym}}]}{K^{\mathrm{MASP1_{zym}}}_{\mathrm{m\ MASP2^*}}}+\frac{[\mathrm{pMFMASP2_{zym}}]}{K^{\mathrm{MASP2_{zym}}}_{\mathrm{m\ MASP2^*}}}\end{array}\right)}
$$

$$+\frac{k_{\text{cat MASP2}^*}^{\text{C2}}\left[\text{pMFMASP2}^*\right]\left[\text{fC4bC2}\right]}{K_{\text{m MASP2}^*}^{\text{C2}}\left(\begin{aligned}&1+\frac{[\text{C4}]}{K_{\text{m MASP2}^*}^{\text{C4}}}+\frac{[\text{fC4bC2}]}{K_{\text{m MASP2}^*}^{\text{C2}}}+\frac{[\text{fC4bC4bC2}]}{K_{\text{m MASP2}^*}^{\text{C2}}}+\frac{[\text{fC3bC4bC2}]}{K_{\text{m MASP2}^*}^{\text{C2}}}+\frac{[\text{pC4bC2}]}{K_{\text{m MASP2}^*}^{\text{C2}}}+\frac{[\text{pC4bC4bC2}]}{K_{\text{m MASP2}^*}^{\text{C2}}}\\&+\frac{[\text{pC3bC4bC2}]}{K_{\text{m MASP2}^*}^{\text{C2}}}+\frac{[\text{pMBL}_3\text{MASP1}_{\text{zym}}]}{K_{\text{m MASP2}^*}^{\text{MASP1}_{\text{zym}}}}+\frac{[\text{pMBL}_3\text{MASP2}_{\text{zym}}]}{K_{\text{m MASP2}^*}^{\text{MASP2}_{\text{zym}}}}+\frac{[\text{pMBL}_4\text{MASP1}_{\text{zym}}]}{K_{\text{m MASP2}^*}^{\text{MASP1}_{\text{zym}}}}\\&+\frac{[\text{pMBL}_4\text{MASP2}_{\text{zym}}]}{K_{\text{m MASP2}^*}^{\text{MASP2}_{\text{zym}}}}+\frac{[\text{pCLLKMASP1}_{\text{zym}}]}{K_{\text{m MASP2}^*}^{\text{MASP1}_{\text{zym}}}}+\frac{[\text{pCLLKMASP2}_{\text{zym}}]}{K_{\text{m MASP2}^*}^{\text{MASP2}_{\text{zym}}}}+\frac{[\text{pCLK1MASP1}_{\text{zym}}]}{K_{\text{m MASP2}^*}^{\text{MASP1}_{\text{zym}}}}\\&+\frac{[\text{pCLK1MASP2}_{\text{zym}}]}{K_{\text{m MASP2}^*}^{\text{MASP2}_{\text{zym}}}}+\frac{[\text{pCLL1MASP1}_{\text{zym}}]}{K_{\text{m MASP2}^*}^{\text{MASP1}_{\text{zym}}}}+\frac{[\text{pCLL1MASP2}_{\text{zym}}]}{K_{\text{m MASP2}^*}^{\text{MASP2}_{\text{zym}}}}+\frac{[\text{pLFMASP1}_{\text{zym}}]}{K_{\text{m MASP2}^*}^{\text{MASP1}_{\text{zym}}}}\\&+\frac{[\text{pLFMASP2}_{\text{zym}}]}{K_{\text{m MASP2}^*}^{\text{MASP2}_{\text{zym}}}}+\frac{[\text{pHFMASP1}_{\text{zym}}]}{K_{\text{m MASP2}^*}^{\text{MASP1}_{\text{zym}}}}+\frac{[\text{pHFMASP2}_{\text{zym}}]}{K_{\text{m MASP2}^*}^{\text{MASP2}_{\text{zym}}}}+\frac{[\text{pMFMASP1}_{\text{zym}}]}{K_{\text{m MASP2}^*}^{\text{MASP1}_{\text{zym}}}}+\frac{[\text{pMFMASP2}_{\text{zym}}]}{K_{\text{m MASP2}^*}^{\text{MASP2}_{\text{zym}}}}\end{aligned}\right)}$$

$$+\frac{k_{\text{cat C1}^*}^{\text{C2}}\left[\text{pMBL}_3\text{PTX3C1}^*\right]\left[\text{fC4bC2}\right]}{K_{\text{m C1}^*}^{\text{C2}}\left(1+\frac{[\text{C4}]}{K_{\text{m C1}^*}^{\text{C4}}}+\frac{[\text{fC4bC2}]}{K_{\text{m C1}^*}^{\text{C2}}}+\frac{[\text{fC4bC4bC2}]}{K_{\text{m C1}^*}^{\text{C2}}}+\frac{[\text{fC3bC4bC2}]}{K_{\text{m C1}^*}^{\text{C2}}}+\frac{[\text{pC4bC2}]}{K_{\text{m C1}^*}^{\text{C2}}}+\frac{[\text{pC4bC4bC2}]}{K_{\text{m C1}^*}^{\text{C2}}}+\frac{[\text{pC3bC4bC2}]}{K_{\text{m C1}^*}^{\text{C2}}}\right)}$$

$$+\frac{k_{\text{cat C1}^*}^{\text{C2}}\left[\text{pMBL}_4\text{PTX3C1}^*\right]\left[\text{fC4bC2}\right]}{K_{\text{m C1}^*}^{\text{C2}}\left(1+\frac{[\text{C4}]}{K_{\text{m C1}^*}^{\text{C4}}}+\frac{[\text{fC4bC2}]}{K_{\text{m C1}^*}^{\text{C2}}}+\frac{[\text{fC4bC4bC2}]}{K_{\text{m C1}^*}^{\text{C2}}}+\frac{[\text{fC3bC4bC2}]}{K_{\text{m C1}^*}^{\text{C2}}}+\frac{[\text{pC4bC2}]}{K_{\text{m C1}^*}^{\text{C2}}}+\frac{[\text{pC4bC4bC2}]}{K_{\text{m C1}^*}^{\text{C2}}}+\frac{[\text{pC3bC4bC2}]}{K_{\text{m C1}^*}^{\text{C2}}}\right)}$$

$$+\frac{k_{\text{cat C1}^*}^{\text{C2}}\left[\text{CRPC1}^*\right]\left[\text{fC4bC2}\right]}{K_{\text{m C1}^*}^{\text{C2}}\left(1+\frac{[\text{C4}]}{K_{\text{m C1}^*}^{\text{C4}}}+\frac{[\text{fC4bC2}]}{K_{\text{m C1}^*}^{\text{C2}}}+\frac{[\text{fC4bC4bC2}]}{K_{\text{m C1}^*}^{\text{C2}}}+\frac{[\text{fC3bC4bC2}]}{K_{\text{m C1}^*}^{\text{C2}}}\right)}$$

38

$$\frac{d[\text{nfC4bC4b}]}{dt}=k_{\text{C4bC4b}}^{+}[\text{nfC4b}][\text{nfC4b}]-k_{\text{fC4b}}^{+}[\text{nfC4bC4b}][\text{H}_2\text{O}]$$

39

$$\begin{aligned}\frac{d[\text{fC4bC4b}]}{dt}=&\,k_{\text{fC4b}}^{+}[\text{nfC4bC4b}][\text{H}_2\text{O}]-k_{\text{C4bC2}}^{+}[\text{fC4bC4b}][\text{C2}]+k_{\text{C4bC2}}^{-}[\text{fC4bC4bC2}]+k_{\text{C4bC4bC2a}}^{-}[\text{fC4bC4bC2a}]\\&+k_{\text{C4bC4b}}^{+}[\text{nfC4b}][\text{fC4b}]-k_{\text{C4bC4BP}}^{+}[\text{fC4bC4b}][\text{C4BP}]+k_{\text{C4bC4BP}}^{-}[\text{fC4bC4bC4BP}]+k_{\text{C4bC2aC4BP}_{\text{decay}}}^{-}[\text{fC4bC4bC2aC4BP}]\\&-k_{\text{C4bC4bCR1}}^{+}[\text{fC4bC4b}][\text{CR1}]+k_{\text{C4bC4bCR1}}^{-}[\text{fC4bC4bCR1}]+k_{\text{C4bC2aCR1}_{\text{decay}}}^{-}[\text{fC4bC4bCR1}]\end{aligned}$$

40

$$\frac{d\left[\mathrm{fC4bC4bC2}\right]}{dt}=k^{+}_{\mathrm{C4bC2}}\left[\mathrm{fC4bC4b}\right]\left[\mathrm{C2}\right]-k^{-}_{\mathrm{C4bC2}}\left[\mathrm{fC4bC4bC2}\right]$$

$$-\frac{k^{\mathrm{C2}}_{\mathrm{cat\ C1^*}}\left[\mathrm{C1^*}\right]\left[\mathrm{fC4bC4bC2}\right]}{K^{\mathrm{C2}}_{\mathrm{m\ C1^*}}\left(1+\frac{[\mathrm{C4}]}{K^{\mathrm{C4}}_{\mathrm{m\ C1^*}}}+\frac{[\mathrm{fC4bC2}]}{K^{\mathrm{C2}}_{\mathrm{m\ C1^*}}}+\frac{[\mathrm{fC4bC4bC2}]}{K^{\mathrm{C2}}_{\mathrm{m\ C1^*}}}+\frac{[\mathrm{fC3bC4bC2}]}{K^{\mathrm{C2}}_{\mathrm{m\ C1^*}}}+\frac{[\mathrm{pC4bC2}]}{K^{\mathrm{C2}}_{\mathrm{m\ C1^*}}}+\frac{[\mathrm{pC4bC4bC2}]}{K^{\mathrm{C2}}_{\mathrm{m\ C1^*}}}+\frac{[\mathrm{pC3bC4bC2}]}{K^{\mathrm{C2}}_{\mathrm{m\ C1^*}}}\right)}$$

$$-\frac{k^{\mathrm{C2}}_{\mathrm{cat\ C1^*}}\left[\mathrm{pIgG_6C1^*}\right]\left[\mathrm{fC4bC4bC2}\right]}{K^{\mathrm{C2}}_{\mathrm{m\ C1^*}}\left(1+\frac{[\mathrm{C4}]}{K^{\mathrm{C4}}_{\mathrm{m\ C1^*}}}+\frac{[\mathrm{fC4bC2}]}{K^{\mathrm{C2}}_{\mathrm{m\ C1^*}}}+\frac{[\mathrm{fC4bC4bC2}]}{K^{\mathrm{C2}}_{\mathrm{m\ C1^*}}}+\frac{[\mathrm{fC3bC4bC2}]}{K^{\mathrm{C2}}_{\mathrm{m\ C1^*}}}+\frac{[\mathrm{pC4bC2}]}{K^{\mathrm{C2}}_{\mathrm{m\ C1^*}}}+\frac{[\mathrm{pC4bC4bC2}]}{K^{\mathrm{C2}}_{\mathrm{m\ C1^*}}}+\frac{[\mathrm{pC3bC4bC2}]}{K^{\mathrm{C2}}_{\mathrm{m\ C1^*}}}\right)}$$

$$-\frac{k^{\mathrm{C2}}_{\mathrm{cat\ C1^*}}\left[\mathrm{pIgMC1^*}\right]\left[\mathrm{fC4bC4bC2}\right]}{K^{\mathrm{C2}}_{\mathrm{m\ C1^*}}\left(1+\frac{[\mathrm{C4}]}{K^{\mathrm{C4}}_{\mathrm{m\ C1^*}}}+\frac{[\mathrm{fC4bC2}]}{K^{\mathrm{C2}}_{\mathrm{m\ C1^*}}}+\frac{[\mathrm{fC4bC4bC2}]}{K^{\mathrm{C2}}_{\mathrm{m\ C1^*}}}+\frac{[\mathrm{fC3bC4bC2}]}{K^{\mathrm{C2}}_{\mathrm{m\ C1^*}}}+\frac{[\mathrm{pC4bC2}]}{K^{\mathrm{C2}}_{\mathrm{m\ C1^*}}}+\frac{[\mathrm{pC4bC4bC2}]}{K^{\mathrm{C2}}_{\mathrm{m\ C1^*}}}+\frac{[\mathrm{pC3bC4bC2}]}{K^{\mathrm{C2}}_{\mathrm{m\ C1^*}}}\right)}$$

$$-\frac{k^{\mathrm{C2}}_{\mathrm{cat\ MASP1^*}}\left[\mathrm{pMBL_3MASP1^*}\right]\left[\mathrm{fC4bC4bC2}\right]}{K^{\mathrm{C2}}_{\mathrm{m\ MASP1^*}}\left(\begin{aligned}&1+\frac{[\mathrm{C4}]}{K^{\mathrm{C4}}_{\mathrm{m\ MASP1^*}}}+\frac{[\mathrm{fC4bC2}]}{K^{\mathrm{C2}}_{\mathrm{m\ MASP1^*}}}+\frac{[\mathrm{fC4bC4bC2}]}{K^{\mathrm{C2}}_{\mathrm{m\ MASP1^*}}}+\frac{[\mathrm{fC3bC4bC2}]}{K^{\mathrm{C2}}_{\mathrm{m\ MASP1^*}}}+\frac{[\mathrm{pC4bC2}]}{K^{\mathrm{C2}}_{\mathrm{m\ MASP1^*}}}+\frac{[\mathrm{pC4bC4bC2}]}{K^{\mathrm{C2}}_{\mathrm{m\ MASP1^*}}}\\&+\frac{[\mathrm{pC3bC4bC2}]}{K^{\mathrm{C2}}_{\mathrm{m\ MASP1^*}}}+\frac{[\mathrm{pMBL_3MASP1_{zym}}]}{K^{\mathrm{MASP1_{zym}}}_{\mathrm{m\ MASP1^*}}}+\frac{[\mathrm{pMBL_3MASP2_{zym}}]}{K^{\mathrm{MASP2_{zym}}}_{\mathrm{m\ MASP1^*}}}+\frac{[\mathrm{pMBL_3MASP3_{zym}}]}{K^{\mathrm{MASP3_{zym}}}_{\mathrm{m\ MASP1^*}}}\\&+\frac{[\mathrm{pMBL_4MASP1_{zym}}]}{K^{\mathrm{MASP1_{zym}}}_{\mathrm{m\ MASP1^*}}}+\frac{[\mathrm{pMBL_4MASP2_{zym}}]}{K^{\mathrm{MASP2_{zym}}}_{\mathrm{m\ MASP1^*}}}+\frac{[\mathrm{pMBL_4MASP3_{zym}}]}{K^{\mathrm{MASP3_{zym}}}_{\mathrm{m\ MASP1^*}}}+\frac{[\mathrm{pCLLKMASP1_{zym}}]}{K^{\mathrm{MASP1_{zym}}}_{\mathrm{m\ MASP1^*}}}\\&+\frac{[\mathrm{pCLLKMASP2_{zym}}]}{K^{\mathrm{MASP2_{zym}}}_{\mathrm{m\ MASP1^*}}}+\frac{[\mathrm{pCLLKMASP3_{zym}}]}{K^{\mathrm{MASP3_{zym}}}_{\mathrm{m\ MASP1^*}}}+\frac{[\mathrm{pCLK1MASP1_{zym}}]}{K^{\mathrm{MASP1_{zym}}}_{\mathrm{m\ MASP1^*}}}+\frac{[\mathrm{pCLK1MASP2_{zym}}]}{K^{\mathrm{MASP2_{zym}}}_{\mathrm{m\ MASP1^*}}}\\&+\frac{[\mathrm{pCLK1MASP3_{zym}}]}{K^{\mathrm{MASP3_{zym}}}_{\mathrm{m\ MASP1^*}}}+\frac{[\mathrm{pCLL1MASP1_{zym}}]}{K^{\mathrm{MASP1_{zym}}}_{\mathrm{m\ MASP1^*}}}+\frac{[\mathrm{pCLL1MASP2_{zym}}]}{K^{\mathrm{MASP2_{zym}}}_{\mathrm{m\ MASP1^*}}}+\frac{[\mathrm{pCLL1MASP3_{zym}}]}{K^{\mathrm{MASP3_{zym}}}_{\mathrm{m\ MASP1^*}}}\\&+\frac{[\mathrm{pLFMASP1_{zym}}]}{K^{\mathrm{MASP1_{zym}}}_{\mathrm{m\ MASP1^*}}}+\frac{[\mathrm{pLFMASP2_{zym}}]}{K^{\mathrm{MASP2_{zym}}}_{\mathrm{m\ MASP1^*}}}+\frac{[\mathrm{pLFMASP3_{zym}}]}{K^{\mathrm{MASP3_{zym}}}_{\mathrm{m\ MASP1^*}}}+\frac{[\mathrm{pHFMASP1_{zym}}]}{K^{\mathrm{MASP1_{zym}}}_{\mathrm{m\ MASP1^*}}}+\frac{[\mathrm{pHFMASP2_{zym}}]}{K^{\mathrm{MASP2_{zym}}}_{\mathrm{m\ MASP1^*}}}\\&+\frac{[\mathrm{pHFMASP3_{zym}}]}{K^{\mathrm{MASP3_{zym}}}_{\mathrm{m\ MASP1^*}}}+\frac{[\mathrm{pMFMASP1_{zym}}]}{K^{\mathrm{MASP1_{zym}}}_{\mathrm{m\ MASP1^*}}}+\frac{[\mathrm{pMFMASP2_{zym}}]}{K^{\mathrm{MASP2_{zym}}}_{\mathrm{m\ MASP1^*}}}+\frac{[\mathrm{pMFMASP3_{zym}}]}{K^{\mathrm{MASP3_{zym}}}_{\mathrm{m\ MASP1^*}}}\end{aligned}\right)}$$

$$
-\frac{k_{\text{cat MASP1}^*}^{\text{C2}}\left[\text{pMBL}_4\text{MASP1}^*\right]\left[\text{fC4bC4bC2}\right]}{K_{\text{m MASP1}^*}^{\text{C2}}\left(\begin{aligned}&1+\frac{[\text{C4}]}{K_{\text{m MASP1}^*}^{\text{C4}}}+\frac{[\text{fC4bC2}]}{K_{\text{m MASP1}^*}^{\text{C2}}}+\frac{[\text{fC4bC4bC2}]}{K_{\text{m MASP1}^*}^{\text{C2}}}+\frac{[\text{fC3bC4bC2}]}{K_{\text{m MASP1}^*}^{\text{C2}}}+\frac{[\text{pC4bC2}]}{K_{\text{m MASP1}^*}^{\text{C2}}}+\frac{[\text{pC4bC4bC2}]}{K_{\text{m MASP1}^*}^{\text{C2}}}\\&+\frac{[\text{pC3bC4bC2}]}{K_{\text{m MASP1}^*}^{\text{C2}}}+\frac{[\text{pMBL}_3\text{MASP1}_{\text{zym}}]}{K_{\text{m MASP1}^*}^{\text{MASP1}_{\text{zym}}}}+\frac{[\text{pMBL}_3\text{MASP2}_{\text{zym}}]}{K_{\text{m MASP1}^*}^{\text{MASP2}_{\text{zym}}}}+\frac{[\text{pMBL}_3\text{MASP3}_{\text{zym}}]}{K_{\text{m MASP1}^*}^{\text{MASP3}_{\text{zym}}}}\\&+\frac{[\text{pMBL}_4\text{MASP1}_{\text{zym}}]}{K_{\text{m MASP1}^*}^{\text{MASP1}_{\text{zym}}}}+\frac{[\text{pMBL}_4\text{MASP2}_{\text{zym}}]}{K_{\text{m MASP1}^*}^{\text{MASP2}_{\text{zym}}}}+\frac{[\text{pMBL}_4\text{MASP3}_{\text{zym}}]}{K_{\text{m MASP1}^*}^{\text{MASP3}_{\text{zym}}}}+\frac{[\text{pCLLKMASP1}_{\text{zym}}]}{K_{\text{m MASP1}^*}^{\text{MASP1}_{\text{zym}}}}\\&+\frac{[\text{pCLLKMASP2}_{\text{zym}}]}{K_{\text{m MASP1}^*}^{\text{MASP2}_{\text{zym}}}}+\frac{[\text{pCLLKMASP3}_{\text{zym}}]}{K_{\text{m MASP1}^*}^{\text{MASP3}_{\text{zym}}}}+\frac{[\text{pCLK1MASP1}_{\text{zym}}]}{K_{\text{m MASP1}^*}^{\text{MASP1}_{\text{zym}}}}+\frac{[\text{pCLK1MASP2}_{\text{zym}}]}{K_{\text{m MASP1}^*}^{\text{MASP2}_{\text{zym}}}}\\&+\frac{[\text{pCLK1MASP3}_{\text{zym}}]}{K_{\text{m MASP1}^*}^{\text{MASP3}_{\text{zym}}}}+\frac{[\text{pCLL1MASP1}_{\text{zym}}]}{K_{\text{m MASP1}^*}^{\text{MASP1}_{\text{zym}}}}+\frac{[\text{pCLL1MASP2}_{\text{zym}}]}{K_{\text{m MASP1}^*}^{\text{MASP2}_{\text{zym}}}}+\frac{[\text{pCLL1MASP3}_{\text{zym}}]}{K_{\text{m MASP1}^*}^{\text{MASP3}_{\text{zym}}}}\\&+\frac{[\text{pLFMASP1}_{\text{zym}}]}{K_{\text{m MASP1}^*}^{\text{MASP1}_{\text{zym}}}}+\frac{[\text{pLFMASP2}_{\text{zym}}]}{K_{\text{m MASP1}^*}^{\text{MASP2}_{\text{zym}}}}+\frac{[\text{pLFMASP3}_{\text{zym}}]}{K_{\text{m MASP1}^*}^{\text{MASP3}_{\text{zym}}}}+\frac{[\text{pHFMASP1}_{\text{zym}}]}{K_{\text{m MASP1}^*}^{\text{MASP1}_{\text{zym}}}}+\frac{[\text{pHFMASP2}_{\text{zym}}]}{K_{\text{m MASP1}^*}^{\text{MASP2}_{\text{zym}}}}\\&+\frac{[\text{pHFMASP3}_{\text{zym}}]}{K_{\text{m MASP1}^*}^{\text{MASP3}_{\text{zym}}}}+\frac{[\text{pMFMASP1}_{\text{zym}}]}{K_{\text{m MASP1}^*}^{\text{MASP1}_{\text{zym}}}}+\frac{[\text{pMFMASP2}_{\text{zym}}]}{K_{\text{m MASP1}^*}^{\text{MASP2}_{\text{zym}}}}+\frac{[\text{pMFMASP3}_{\text{zym}}]}{K_{\text{m MASP1}^*}^{\text{MASP3}_{\text{zym}}}}\end{aligned}\right)}
$$

$$
-\frac{k_{\text{cat MASP1}^*}^{\text{C2}}\left[\text{pCLLKMASP1}^*\right]\left[\text{fC4bC4bC2}\right]}{K_{\text{m MASP1}^*}^{\text{C2}}\left(\begin{aligned}&1+\frac{[\text{C4}]}{K_{\text{m MASP1}^*}^{\text{C4}}}+\frac{[\text{fC4bC2}]}{K_{\text{m MASP1}^*}^{\text{C2}}}+\frac{[\text{fC4bC4bC2}]}{K_{\text{m MASP1}^*}^{\text{C2}}}+\frac{[\text{fC3bC4bC2}]}{K_{\text{m MASP1}^*}^{\text{C2}}}+\frac{[\text{pC4bC2}]}{K_{\text{m MASP1}^*}^{\text{C2}}}+\frac{[\text{pC4bC4bC2}]}{K_{\text{m MASP1}^*}^{\text{C2}}}\\&+\frac{[\text{pC3bC4bC2}]}{K_{\text{m MASP1}^*}^{\text{C2}}}+\frac{[\text{pMBL}_3\text{MASP1}_{\text{zym}}]}{K_{\text{m MASP1}^*}^{\text{MASP1}_{\text{zym}}}}+\frac{[\text{pMBL}_3\text{MASP2}_{\text{zym}}]}{K_{\text{m MASP1}^*}^{\text{MASP2}_{\text{zym}}}}+\frac{[\text{pMBL}_3\text{MASP3}_{\text{zym}}]}{K_{\text{m MASP1}^*}^{\text{MASP3}_{\text{zym}}}}\\&+\frac{[\text{pMBL}_4\text{MASP1}_{\text{zym}}]}{K_{\text{m MASP1}^*}^{\text{MASP1}_{\text{zym}}}}+\frac{[\text{pMBL}_4\text{MASP2}_{\text{zym}}]}{K_{\text{m MASP1}^*}^{\text{MASP2}_{\text{zym}}}}+\frac{[\text{pMBL}_4\text{MASP3}_{\text{zym}}]}{K_{\text{m MASP1}^*}^{\text{MASP3}_{\text{zym}}}}+\frac{[\text{pCLLKMASP1}_{\text{zym}}]}{K_{\text{m MASP1}^*}^{\text{MASP1}_{\text{zym}}}}\\&+\frac{[\text{pCLLKMASP2}_{\text{zym}}]}{K_{\text{m MASP1}^*}^{\text{MASP2}_{\text{zym}}}}+\frac{[\text{pCLLKMASP3}_{\text{zym}}]}{K_{\text{m MASP1}^*}^{\text{MASP3}_{\text{zym}}}}+\frac{[\text{pCLK1MASP1}_{\text{zym}}]}{K_{\text{m MASP1}^*}^{\text{MASP1}_{\text{zym}}}}+\frac{[\text{pCLK1MASP2}_{\text{zym}}]}{K_{\text{m MASP1}^*}^{\text{MASP2}_{\text{zym}}}}\\&+\frac{[\text{pCLK1MASP3}_{\text{zym}}]}{K_{\text{m MASP1}^*}^{\text{MASP3}_{\text{zym}}}}+\frac{[\text{pCLL1MASP1}_{\text{zym}}]}{K_{\text{m MASP1}^*}^{\text{MASP1}_{\text{zym}}}}+\frac{[\text{pCLL1MASP2}_{\text{zym}}]}{K_{\text{m MASP1}^*}^{\text{MASP2}_{\text{zym}}}}+\frac{[\text{pCLL1MASP3}_{\text{zym}}]}{K_{\text{m MASP1}^*}^{\text{MASP3}_{\text{zym}}}}\\&+\frac{[\text{pLFMASP1}_{\text{zym}}]}{K_{\text{m MASP1}^*}^{\text{MASP1}_{\text{zym}}}}+\frac{[\text{pLFMASP2}_{\text{zym}}]}{K_{\text{m MASP1}^*}^{\text{MASP2}_{\text{zym}}}}+\frac{[\text{pLFMASP3}_{\text{zym}}]}{K_{\text{m MASP1}^*}^{\text{MASP3}_{\text{zym}}}}+\frac{[\text{pHFMASP1}_{\text{zym}}]}{K_{\text{m MASP1}^*}^{\text{MASP1}_{\text{zym}}}}+\frac{[\text{pHFMASP2}_{\text{zym}}]}{K_{\text{m MASP1}^*}^{\text{MASP2}_{\text{zym}}}}\\&+\frac{[\text{pHFMASP3}_{\text{zym}}]}{K_{\text{m MASP1}^*}^{\text{MASP3}_{\text{zym}}}}+\frac{[\text{pMFMASP1}_{\text{zym}}]}{K_{\text{m MASP1}^*}^{\text{MASP1}_{\text{zym}}}}+\frac{[\text{pMFMASP2}_{\text{zym}}]}{K_{\text{m MASP1}^*}^{\text{MASP2}_{\text{zym}}}}+\frac{[\text{pMFMASP3}_{\text{zym}}]}{K_{\text{m MASP1}^*}^{\text{MASP3}_{\text{zym}}}}\end{aligned}\right)}
$$

$$-\frac{k_{\text{cat MASP1}^*}^{\text{C2}}\left[\text{pCLK1MASP1}^*\right]\left[\text{fC4bC4bC2}\right]}{K_{\text{m MASP1}^*}^{\text{C2}}\left(\begin{aligned}&1+\frac{[\text{C4}]}{K_{\text{m MASP1}^*}^{\text{C4}}}+\frac{[\text{fC4bC2}]}{K_{\text{m MASP1}^*}^{\text{C2}}}+\frac{[\text{fC4bC4bC2}]}{K_{\text{m MASP1}^*}^{\text{C2}}}+\frac{[\text{fC3bC4bC2}]}{K_{\text{m MASP1}^*}^{\text{C2}}}+\frac{[\text{pC4bC2}]}{K_{\text{m MASP1}^*}^{\text{C2}}}+\frac{[\text{pC4bC4bC2}]}{K_{\text{m MASP1}^*}^{\text{C2}}}\\&+\frac{[\text{pC3bC4bC2}]}{K_{\text{m MASP1}^*}^{\text{C2}}}+\frac{\left[\text{pMBL}_3\text{MASP1}_{\text{zym}}\right]}{K_{\text{m MASP1}^*}^{\text{MASP1}_{\text{zym}}}}+\frac{\left[\text{pMBL}_3\text{MASP2}_{\text{zym}}\right]}{K_{\text{m MASP1}^*}^{\text{MASP2}_{\text{zym}}}}+\frac{\left[\text{pMBL}_3\text{MASP3}_{\text{zym}}\right]}{K_{\text{m MASP1}^*}^{\text{MASP3}_{\text{zym}}}}\\&+\frac{\left[\text{pMBL}_4\text{MASP1}_{\text{zym}}\right]}{K_{\text{m MASP1}^*}^{\text{MASP1}_{\text{zym}}}}+\frac{\left[\text{pMBL}_4\text{MASP2}_{\text{zym}}\right]}{K_{\text{m MASP1}^*}^{\text{MASP2}_{\text{zym}}}}+\frac{\left[\text{pMBL}_4\text{MASP3}_{\text{zym}}\right]}{K_{\text{m MASP1}^*}^{\text{MASP3}_{\text{zym}}}}+\frac{\left[\text{pCLLKMASP1}_{\text{zym}}\right]}{K_{\text{m MASP1}^*}^{\text{MASP1}_{\text{zym}}}}\\&+\frac{\left[\text{pCLLKMASP2}_{\text{zym}}\right]}{K_{\text{m MASP1}^*}^{\text{MASP2}_{\text{zym}}}}+\frac{\left[\text{pCLLKMASP3}_{\text{zym}}\right]}{K_{\text{m MASP1}^*}^{\text{MASP3}_{\text{zym}}}}+\frac{\left[\text{pCLK1MASP1}_{\text{zym}}\right]}{K_{\text{m MASP1}^*}^{\text{MASP1}_{\text{zym}}}}+\frac{\left[\text{pCLK1MASP2}_{\text{zym}}\right]}{K_{\text{m MASP1}^*}^{\text{MASP2}_{\text{zym}}}}\\&+\frac{\left[\text{pCLK1MASP3}_{\text{zym}}\right]}{K_{\text{m MASP1}^*}^{\text{MASP3}_{\text{zym}}}}+\frac{\left[\text{pCLL1MASP1}_{\text{zym}}\right]}{K_{\text{m MASP1}^*}^{\text{MASP1}_{\text{zym}}}}+\frac{\left[\text{pCLL1MASP2}_{\text{zym}}\right]}{K_{\text{m MASP1}^*}^{\text{MASP2}_{\text{zym}}}}+\frac{\left[\text{pCLL1MASP3}_{\text{zym}}\right]}{K_{\text{m MASP1}^*}^{\text{MASP3}_{\text{zym}}}}\\&+\frac{\left[\text{pLFMASP1}_{\text{zym}}\right]}{K_{\text{m MASP1}^*}^{\text{MASP1}_{\text{zym}}}}+\frac{\left[\text{pLFMASP2}_{\text{zym}}\right]}{K_{\text{m MASP1}^*}^{\text{MASP2}_{\text{zym}}}}+\frac{\left[\text{pLFMASP3}_{\text{zym}}\right]}{K_{\text{m MASP1}^*}^{\text{MASP3}_{\text{zym}}}}+\frac{\left[\text{pHFMASP1}_{\text{zym}}\right]}{K_{\text{m MASP1}^*}^{\text{MASP1}_{\text{zym}}}}+\frac{\left[\text{pHFMASP2}_{\text{zym}}\right]}{K_{\text{m MASP1}^*}^{\text{MASP2}_{\text{zym}}}}\\&+\frac{\left[\text{pHFMASP3}_{\text{zym}}\right]}{K_{\text{m MASP1}^*}^{\text{MASP3}_{\text{zym}}}}+\frac{\left[\text{pMFMASP1}_{\text{zym}}\right]}{K_{\text{m MASP1}^*}^{\text{MASP1}_{\text{zym}}}}+\frac{\left[\text{pMFMASP2}_{\text{zym}}\right]}{K_{\text{m MASP1}^*}^{\text{MASP2}_{\text{zym}}}}+\frac{\left[\text{pMFMASP3}_{\text{zym}}\right]}{K_{\text{m MASP1}^*}^{\text{MASP3}_{\text{zym}}}}\end{aligned}\right)}$$

$$-\frac{k_{\text{cat MASP1}^*}^{\text{C2}}\left[\text{pCLL1MASP1}^*\right]\left[\text{fC4bC4bC2}\right]}{K_{\text{m MASP1}^*}^{\text{C2}}\left(\begin{aligned}&1+\frac{[\text{C4}]}{K_{\text{m MASP1}^*}^{\text{C4}}}+\frac{[\text{fC4bC2}]}{K_{\text{m MASP1}^*}^{\text{C2}}}+\frac{[\text{fC4bC4bC2}]}{K_{\text{m MASP1}^*}^{\text{C2}}}+\frac{[\text{fC3bC4bC2}]}{K_{\text{m MASP1}^*}^{\text{C2}}}+\frac{[\text{pC4bC2}]}{K_{\text{m MASP1}^*}^{\text{C2}}}+\frac{[\text{pC4bC4bC2}]}{K_{\text{m MASP1}^*}^{\text{C2}}}\\&+\frac{[\text{pC3bC4bC2}]}{K_{\text{m MASP1}^*}^{\text{C2}}}+\frac{\left[\text{pMBL}_3\text{MASP1}_{\text{zym}}\right]}{K_{\text{m MASP1}^*}^{\text{MASP1}_{\text{zym}}}}+\frac{\left[\text{pMBL}_3\text{MASP2}_{\text{zym}}\right]}{K_{\text{m MASP1}^*}^{\text{MASP2}_{\text{zym}}}}+\frac{\left[\text{pMBL}_3\text{MASP3}_{\text{zym}}\right]}{K_{\text{m MASP1}^*}^{\text{MASP3}_{\text{zym}}}}\\&+\frac{\left[\text{pMBL}_4\text{MASP1}_{\text{zym}}\right]}{K_{\text{m MASP1}^*}^{\text{MASP1}_{\text{zym}}}}+\frac{\left[\text{pMBL}_4\text{MASP2}_{\text{zym}}\right]}{K_{\text{m MASP1}^*}^{\text{MASP2}_{\text{zym}}}}+\frac{\left[\text{pMBL}_4\text{MASP3}_{\text{zym}}\right]}{K_{\text{m MASP1}^*}^{\text{MASP3}_{\text{zym}}}}+\frac{\left[\text{pCLLKMASP1}_{\text{zym}}\right]}{K_{\text{m MASP1}^*}^{\text{MASP1}_{\text{zym}}}}\\&+\frac{\left[\text{pCLLKMASP2}_{\text{zym}}\right]}{K_{\text{m MASP1}^*}^{\text{MASP2}_{\text{zym}}}}+\frac{\left[\text{pCLLKMASP3}_{\text{zym}}\right]}{K_{\text{m MASP1}^*}^{\text{MASP3}_{\text{zym}}}}+\frac{\left[\text{pCLK1MASP1}_{\text{zym}}\right]}{K_{\text{m MASP1}^*}^{\text{MASP1}_{\text{zym}}}}+\frac{\left[\text{pCLK1MASP2}_{\text{zym}}\right]}{K_{\text{m MASP1}^*}^{\text{MASP2}_{\text{zym}}}}\\&+\frac{\left[\text{pCLK1MASP3}_{\text{zym}}\right]}{K_{\text{m MASP1}^*}^{\text{MASP3}_{\text{zym}}}}+\frac{\left[\text{pCLL1MASP1}_{\text{zym}}\right]}{K_{\text{m MASP1}^*}^{\text{MASP1}_{\text{zym}}}}+\frac{\left[\text{pCLL1MASP2}_{\text{zym}}\right]}{K_{\text{m MASP1}^*}^{\text{MASP2}_{\text{zym}}}}+\frac{\left[\text{pCLL1MASP3}_{\text{zym}}\right]}{K_{\text{m MASP1}^*}^{\text{MASP3}_{\text{zym}}}}\\&+\frac{\left[\text{pLFMASP1}_{\text{zym}}\right]}{K_{\text{m MASP1}^*}^{\text{MASP1}_{\text{zym}}}}+\frac{\left[\text{pLFMASP2}_{\text{zym}}\right]}{K_{\text{m MASP1}^*}^{\text{MASP2}_{\text{zym}}}}+\frac{\left[\text{pLFMASP3}_{\text{zym}}\right]}{K_{\text{m MASP1}^*}^{\text{MASP3}_{\text{zym}}}}+\frac{\left[\text{pHFMASP1}_{\text{zym}}\right]}{K_{\text{m MASP1}^*}^{\text{MASP1}_{\text{zym}}}}+\frac{\left[\text{pHFMASP2}_{\text{zym}}\right]}{K_{\text{m MASP1}^*}^{\text{MASP2}_{\text{zym}}}}\\&+\frac{\left[\text{pHFMASP3}_{\text{zym}}\right]}{K_{\text{m MASP1}^*}^{\text{MASP3}_{\text{zym}}}}+\frac{\left[\text{pMFMASP1}_{\text{zym}}\right]}{K_{\text{m MASP1}^*}^{\text{MASP1}_{\text{zym}}}}+\frac{\left[\text{pMFMASP2}_{\text{zym}}\right]}{K_{\text{m MASP1}^*}^{\text{MASP2}_{\text{zym}}}}+\frac{\left[\text{pMFMASP3}_{\text{zym}}\right]}{K_{\text{m MASP1}^*}^{\text{MASP3}_{\text{zym}}}}\end{aligned}\right)}$$

$$
-\frac{k_{\mathrm{cat\,MASP1^*}}^{\mathrm{C2}}\left[\mathrm{pLFMASP1^*}\right]\left[\mathrm{fC4bC4bC2}\right]}{K_{\mathrm{m\,MASP1^*}}^{\mathrm{C2}}\left(\begin{array}{l}
1+\frac{[\mathrm{C4}]}{K_{\mathrm{m\,MASP1^*}}^{\mathrm{C4}}}+\frac{[\mathrm{fC4bC2}]}{K_{\mathrm{m\,MASP1^*}}^{\mathrm{C2}}}+\frac{[\mathrm{fC4bC4bC2}]}{K_{\mathrm{m\,MASP1^*}}^{\mathrm{C2}}}+\frac{[\mathrm{fC3bC4bC2}]}{K_{\mathrm{m\,MASP1^*}}^{\mathrm{C2}}}+\frac{[\mathrm{pC4bC2}]}{K_{\mathrm{m\,MASP1^*}}^{\mathrm{C2}}}+\frac{[\mathrm{pC4bC4bC2}]}{K_{\mathrm{m\,MASP1^*}}^{\mathrm{C2}}}\\
+\frac{[\mathrm{pC3bC4bC2}]}{K_{\mathrm{m\,MASP1^*}}^{\mathrm{C2}}}+\frac{[\mathrm{pMBL_3MASP1_{zym}}]}{K_{\mathrm{m\,MASP1^*}}^{\mathrm{MASP1_{zym}}}}+\frac{[\mathrm{pMBL_3MASP2_{zym}}]}{K_{\mathrm{m\,MASP1^*}}^{\mathrm{MASP2_{zym}}}}+\frac{[\mathrm{pMBL_3MASP3_{zym}}]}{K_{\mathrm{m\,MASP1^*}}^{\mathrm{MASP3_{zym}}}}\\
+\frac{[\mathrm{pMBL_4MASP1_{zym}}]}{K_{\mathrm{m\,MASP1^*}}^{\mathrm{MASP1_{zym}}}}+\frac{[\mathrm{pMBL_4MASP2_{zym}}]}{K_{\mathrm{m\,MASP1^*}}^{\mathrm{MASP2_{zym}}}}+\frac{[\mathrm{pMBL_4MASP3_{zym}}]}{K_{\mathrm{m\,MASP1^*}}^{\mathrm{MASP3_{zym}}}}+\frac{[\mathrm{pCLLKMASP1_{zym}}]}{K_{\mathrm{m\,MASP1^*}}^{\mathrm{MASP1_{zym}}}}\\
+\frac{[\mathrm{pCLLKMASP2_{zym}}]}{K_{\mathrm{m\,MASP1^*}}^{\mathrm{MASP2_{zym}}}}+\frac{[\mathrm{pCLLKMASP3_{zym}}]}{K_{\mathrm{m\,MASP1^*}}^{\mathrm{MASP3_{zym}}}}+\frac{[\mathrm{pCLK1MASP1_{zym}}]}{K_{\mathrm{m\,MASP1^*}}^{\mathrm{MASP1_{zym}}}}+\frac{[\mathrm{pCLK1MASP2_{zym}}]}{K_{\mathrm{m\,MASP1^*}}^{\mathrm{MASP2_{zym}}}}\\
+\frac{[\mathrm{pCLK1MASP3_{zym}}]}{K_{\mathrm{m\,MASP1^*}}^{\mathrm{MASP3_{zym}}}}+\frac{[\mathrm{pCLL1MASP1_{zym}}]}{K_{\mathrm{m\,MASP1^*}}^{\mathrm{MASP1_{zym}}}}+\frac{[\mathrm{pCLL1MASP2_{zym}}]}{K_{\mathrm{m\,MASP1^*}}^{\mathrm{MASP2_{zym}}}}+\frac{[\mathrm{pCLL1MASP3_{zym}}]}{K_{\mathrm{m\,MASP1^*}}^{\mathrm{MASP3_{zym}}}}\\
+\frac{[\mathrm{pLFMASP1_{zym}}]}{K_{\mathrm{m\,MASP1^*}}^{\mathrm{MASP1_{zym}}}}+\frac{[\mathrm{pLFMASP2_{zym}}]}{K_{\mathrm{m\,MASP1^*}}^{\mathrm{MASP2_{zym}}}}+\frac{[\mathrm{pLFMASP3_{zym}}]}{K_{\mathrm{m\,MASP1^*}}^{\mathrm{MASP3_{zym}}}}+\frac{[\mathrm{pHFMASP1_{zym}}]}{K_{\mathrm{m\,MASP1^*}}^{\mathrm{MASP1_{zym}}}}+\frac{[\mathrm{pHFMASP2_{zym}}]}{K_{\mathrm{m\,MASP1^*}}^{\mathrm{MASP2_{zym}}}}\\
+\frac{[\mathrm{pHFMASP3_{zym}}]}{K_{\mathrm{m\,MASP1^*}}^{\mathrm{MASP3_{zym}}}}+\frac{[\mathrm{pMFMASP1_{zym}}]}{K_{\mathrm{m\,MASP1^*}}^{\mathrm{MASP1_{zym}}}}+\frac{[\mathrm{pMFMASP2_{zym}}]}{K_{\mathrm{m\,MASP1^*}}^{\mathrm{MASP2_{zym}}}}+\frac{[\mathrm{pMFMASP3_{zym}}]}{K_{\mathrm{m\,MASP1^*}}^{\mathrm{MASP3_{zym}}}}
\end{array}\right)}
$$

$$
-\frac{k_{\mathrm{cat\,MASP1^*}}^{\mathrm{C2}}\left[\mathrm{pHFMASP1^*}\right]\left[\mathrm{fC4bC4bC2}\right]}{K_{\mathrm{m\,MASP1^*}}^{\mathrm{C2}}\left(\begin{array}{l}
1+\frac{[\mathrm{C4}]}{K_{\mathrm{m\,MASP1^*}}^{\mathrm{C4}}}+\frac{[\mathrm{fC4bC2}]}{K_{\mathrm{m\,MASP1^*}}^{\mathrm{C2}}}+\frac{[\mathrm{fC4bC4bC2}]}{K_{\mathrm{m\,MASP1^*}}^{\mathrm{C2}}}+\frac{[\mathrm{fC3bC4bC2}]}{K_{\mathrm{m\,MASP1^*}}^{\mathrm{C2}}}+\frac{[\mathrm{pC4bC2}]}{K_{\mathrm{m\,MASP1^*}}^{\mathrm{C2}}}+\frac{[\mathrm{pC4bC4bC2}]}{K_{\mathrm{m\,MASP1^*}}^{\mathrm{C2}}}\\
+\frac{[\mathrm{pC3bC4bC2}]}{K_{\mathrm{m\,MASP1^*}}^{\mathrm{C2}}}+\frac{[\mathrm{pMBL_3MASP1_{zym}}]}{K_{\mathrm{m\,MASP1^*}}^{\mathrm{MASP1_{zym}}}}+\frac{[\mathrm{pMBL_3MASP2_{zym}}]}{K_{\mathrm{m\,MASP1^*}}^{\mathrm{MASP2_{zym}}}}+\frac{[\mathrm{pMBL_3MASP3_{zym}}]}{K_{\mathrm{m\,MASP1^*}}^{\mathrm{MASP3_{zym}}}}\\
+\frac{[\mathrm{pMBL_4MASP1_{zym}}]}{K_{\mathrm{m\,MASP1^*}}^{\mathrm{MASP1_{zym}}}}+\frac{[\mathrm{pMBL_4MASP2_{zym}}]}{K_{\mathrm{m\,MASP1^*}}^{\mathrm{MASP2_{zym}}}}+\frac{[\mathrm{pMBL_4MASP3_{zym}}]}{K_{\mathrm{m\,MASP1^*}}^{\mathrm{MASP3_{zym}}}}+\frac{[\mathrm{pCLLKMASP1_{zym}}]}{K_{\mathrm{m\,MASP1^*}}^{\mathrm{MASP1_{zym}}}}\\
+\frac{[\mathrm{pCLLKMASP2_{zym}}]}{K_{\mathrm{m\,MASP1^*}}^{\mathrm{MASP2_{zym}}}}+\frac{[\mathrm{pCLLKMASP3_{zym}}]}{K_{\mathrm{m\,MASP1^*}}^{\mathrm{MASP3_{zym}}}}+\frac{[\mathrm{pCLK1MASP1_{zym}}]}{K_{\mathrm{m\,MASP1^*}}^{\mathrm{MASP1_{zym}}}}+\frac{[\mathrm{pCLK1MASP2_{zym}}]}{K_{\mathrm{m\,MASP1^*}}^{\mathrm{MASP2_{zym}}}}\\
+\frac{[\mathrm{pCLK1MASP3_{zym}}]}{K_{\mathrm{m\,MASP1^*}}^{\mathrm{MASP3_{zym}}}}+\frac{[\mathrm{pCLL1MASP1_{zym}}]}{K_{\mathrm{m\,MASP1^*}}^{\mathrm{MASP1_{zym}}}}+\frac{[\mathrm{pCLL1MASP2_{zym}}]}{K_{\mathrm{m\,MASP1^*}}^{\mathrm{MASP2_{zym}}}}+\frac{[\mathrm{pCLL1MASP3_{zym}}]}{K_{\mathrm{m\,MASP1^*}}^{\mathrm{MASP3_{zym}}}}\\
+\frac{[\mathrm{pLFMASP1_{zym}}]}{K_{\mathrm{m\,MASP1^*}}^{\mathrm{MASP1_{zym}}}}+\frac{[\mathrm{pLFMASP2_{zym}}]}{K_{\mathrm{m\,MASP1^*}}^{\mathrm{MASP2_{zym}}}}+\frac{[\mathrm{pLFMASP3_{zym}}]}{K_{\mathrm{m\,MASP1^*}}^{\mathrm{MASP3_{zym}}}}+\frac{[\mathrm{pHFMASP1_{zym}}]}{K_{\mathrm{m\,MASP1^*}}^{\mathrm{MASP1_{zym}}}}+\frac{[\mathrm{pHFMASP2_{zym}}]}{K_{\mathrm{m\,MASP1^*}}^{\mathrm{MASP2_{zym}}}}\\
+\frac{[\mathrm{pHFMASP3_{zym}}]}{K_{\mathrm{m\,MASP1^*}}^{\mathrm{MASP3_{zym}}}}+\frac{[\mathrm{pMFMASP1_{zym}}]}{K_{\mathrm{m\,MASP1^*}}^{\mathrm{MASP1_{zym}}}}+\frac{[\mathrm{pMFMASP2_{zym}}]}{K_{\mathrm{m\,MASP1^*}}^{\mathrm{MASP2_{zym}}}}+\frac{[\mathrm{pMFMASP3_{zym}}]}{K_{\mathrm{m\,MASP1^*}}^{\mathrm{MASP3_{zym}}}}
\end{array}\right)}
$$

$$-\frac{k_{\text{cat MASP1}^*}^{\text{C2}}\left[\text{pMFMASP1}^*\right]\left[\text{fC4bC4bC2}\right]}{K_{\text{m MASP1}^*}^{\text{C2}}\left(\begin{array}{l}1+\frac{[\text{C4}]}{K_{\text{m MASP1}^*}^{\text{C4}}}+\frac{[\text{fC4bC2}]}{K_{\text{m MASP1}^*}^{\text{C2}}}+\frac{[\text{fC4bC4bC2}]}{K_{\text{m MASP1}^*}^{\text{C2}}}+\frac{[\text{fC3bC4bC2}]}{K_{\text{m MASP1}^*}^{\text{C2}}}+\frac{[\text{pC4bC2}]}{K_{\text{m MASP1}^*}^{\text{C2}}}+\frac{[\text{pC4bC4bC2}]}{K_{\text{m MASP1}^*}^{\text{C2}}}\\
+\frac{[\text{pC3bC4bC2}]}{K_{\text{m MASP1}^*}^{\text{C2}}}+\frac{[\text{pMBL}_3\text{MASP1}_{\text{zym}}]}{K_{\text{m MASP1}^*}^{\text{MASP1}_{\text{zym}}}}+\frac{[\text{pMBL}_3\text{MASP2}_{\text{zym}}]}{K_{\text{m MASP1}^*}^{\text{MASP2}_{\text{zym}}}}+\frac{[\text{pMBL}_3\text{MASP3}_{\text{zym}}]}{K_{\text{m MASP1}^*}^{\text{MASP3}_{\text{zym}}}}\\
+\frac{[\text{pMBL}_4\text{MASP1}_{\text{zym}}]}{K_{\text{m MASP1}^*}^{\text{MASP1}_{\text{zym}}}}+\frac{[\text{pMBL}_4\text{MASP2}_{\text{zym}}]}{K_{\text{m MASP1}^*}^{\text{MASP2}_{\text{zym}}}}+\frac{[\text{pMBL}_4\text{MASP3}_{\text{zym}}]}{K_{\text{m MASP1}^*}^{\text{MASP3}_{\text{zym}}}}+\frac{[\text{pCLLKMASP1}_{\text{zym}}]}{K_{\text{m MASP1}^*}^{\text{MASP1}_{\text{zym}}}}\\
+\frac{[\text{pCLLKMASP2}_{\text{zym}}]}{K_{\text{m MASP1}^*}^{\text{MASP2}_{\text{zym}}}}+\frac{[\text{pCLLKMASP3}_{\text{zym}}]}{K_{\text{m MASP1}^*}^{\text{MASP3}_{\text{zym}}}}+\frac{[\text{pCLK1MASP1}_{\text{zym}}]}{K_{\text{m MASP1}^*}^{\text{MASP1}_{\text{zym}}}}+\frac{[\text{pCLK1MASP2}_{\text{zym}}]}{K_{\text{m MASP1}^*}^{\text{MASP2}_{\text{zym}}}}\\
+\frac{[\text{pCLK1MASP3}_{\text{zym}}]}{K_{\text{m MASP1}^*}^{\text{MASP3}_{\text{zym}}}}+\frac{[\text{pCLL1MASP1}_{\text{zym}}]}{K_{\text{m MASP1}^*}^{\text{MASP1}_{\text{zym}}}}+\frac{[\text{pCLL1MASP2}_{\text{zym}}]}{K_{\text{m MASP1}^*}^{\text{MASP2}_{\text{zym}}}}+\frac{[\text{pCLL1MASP3}_{\text{zym}}]}{K_{\text{m MASP1}^*}^{\text{MASP3}_{\text{zym}}}}\\
+\frac{[\text{pLFMASP1}_{\text{zym}}]}{K_{\text{m MASP1}^*}^{\text{MASP1}_{\text{zym}}}}+\frac{[\text{pLFMASP2}_{\text{zym}}]}{K_{\text{m MASP1}^*}^{\text{MASP2}_{\text{zym}}}}+\frac{[\text{pLFMASP3}_{\text{zym}}]}{K_{\text{m MASP1}^*}^{\text{MASP3}_{\text{zym}}}}+\frac{[\text{pHFMASP1}_{\text{zym}}]}{K_{\text{m MASP1}^*}^{\text{MASP1}_{\text{zym}}}}+\frac{[\text{pHFMASP2}_{\text{zym}}]}{K_{\text{m MASP1}^*}^{\text{MASP2}_{\text{zym}}}}\\
+\frac{[\text{pHFMASP3}_{\text{zym}}]}{K_{\text{m MASP1}^*}^{\text{MASP3}_{\text{zym}}}}+\frac{[\text{pMFMASP1}_{\text{zym}}]}{K_{\text{m MASP1}^*}^{\text{MASP1}_{\text{zym}}}}+\frac{[\text{pMFMASP2}_{\text{zym}}]}{K_{\text{m MASP1}^*}^{\text{MASP2}_{\text{zym}}}}+\frac{[\text{pMFMASP3}_{\text{zym}}]}{K_{\text{m MASP1}^*}^{\text{MASP3}_{\text{zym}}}}\end{array}\right)}$$

$$-\frac{k_{\text{cat MASP2}^*}^{\text{C2}}\left[\text{pMBL}_3\text{MASP2}^*\right]\left[\text{fC4bC4bC2}\right]}{K_{\text{m MASP2}^*}^{\text{C2}}\left(\begin{array}{l}1+\frac{[\text{C4}]}{K_{\text{m MASP2}^*}^{\text{C4}}}+\frac{[\text{fC4bC2}]}{K_{\text{m MASP2}^*}^{\text{C2}}}+\frac{[\text{fC4bC4bC2}]}{K_{\text{m MASP2}^*}^{\text{C2}}}+\frac{[\text{fC3bC4bC2}]}{K_{\text{m MASP2}^*}^{\text{C2}}}+\frac{[\text{pC4bC2}]}{K_{\text{m MASP2}^*}^{\text{C2}}}+\frac{[\text{pC4bC4bC2}]}{K_{\text{m MASP2}^*}^{\text{C2}}}\\
+\frac{[\text{pC3bC4bC2}]}{K_{\text{m MASP2}^*}^{\text{C2}}}+\frac{[\text{pMBL}_3\text{MASP1}_{\text{zym}}]}{K_{\text{m MASP2}^*}^{\text{MASP1}_{\text{zym}}}}+\frac{[\text{pMBL}_3\text{MASP2}_{\text{zym}}]}{K_{\text{m MASP2}^*}^{\text{MASP2}_{\text{zym}}}}+\frac{[\text{pMBL}_4\text{MASP1}_{\text{zym}}]}{K_{\text{m MASP2}^*}^{\text{MASP1}_{\text{zym}}}}\\
+\frac{[\text{pMBL}_4\text{MASP2}_{\text{zym}}]}{K_{\text{m MASP2}^*}^{\text{MASP2}_{\text{zym}}}}+\frac{[\text{pCLLKMASP1}_{\text{zym}}]}{K_{\text{m MASP2}^*}^{\text{MASP1}_{\text{zym}}}}+\frac{[\text{pCLLKMASP2}_{\text{zym}}]}{K_{\text{m MASP2}^*}^{\text{MASP2}_{\text{zym}}}}+\frac{[\text{pCLK1MASP1}_{\text{zym}}]}{K_{\text{m MASP2}^*}^{\text{MASP1}_{\text{zym}}}}\\
+\frac{[\text{pCLK1MASP2}_{\text{zym}}]}{K_{\text{m MASP2}^*}^{\text{MASP2}_{\text{zym}}}}+\frac{[\text{pCLL1MASP1}_{\text{zym}}]}{K_{\text{m MASP2}^*}^{\text{MASP1}_{\text{zym}}}}+\frac{[\text{pCLL1MASP2}_{\text{zym}}]}{K_{\text{m MASP2}^*}^{\text{MASP2}_{\text{zym}}}}+\frac{[\text{pLFMASP1}_{\text{zym}}]}{K_{\text{m MASP2}^*}^{\text{MASP1}_{\text{zym}}}}\\
+\frac{[\text{pLFMASP2}_{\text{zym}}]}{K_{\text{m MASP2}^*}^{\text{MASP2}_{\text{zym}}}}+\frac{[\text{pHFMASP1}_{\text{zym}}]}{K_{\text{m MASP2}^*}^{\text{MASP1}_{\text{zym}}}}+\frac{[\text{pHFMASP2}_{\text{zym}}]}{K_{\text{m MASP2}^*}^{\text{MASP2}_{\text{zym}}}}+\frac{[\text{pMFMASP1}_{\text{zym}}]}{K_{\text{m MASP2}^*}^{\text{MASP1}_{\text{zym}}}}+\frac{[\text{pMFMASP2}_{\text{zym}}]}{K_{\text{m MASP2}^*}^{\text{MASP2}_{\text{zym}}}}\end{array}\right)}$$

$$-\frac{k_{\mathrm{cat\,MASP2^*}}^{\mathrm{C2}}\left[\mathrm{pMBL_4MASP2^*}\right]\left[\mathrm{fC4bC4bC2}\right]}{K_{\mathrm{m\,MASP2^*}}^{\mathrm{C2}}\left(\begin{array}{l}1+\frac{[\mathrm{C4}]}{K_{\mathrm{m\,MASP2^*}}^{\mathrm{C4}}}+\frac{[\mathrm{fC4bC2}]}{K_{\mathrm{m\,MASP2^*}}^{\mathrm{C2}}}+\frac{[\mathrm{fC4bC4bC2}]}{K_{\mathrm{m\,MASP2^*}}^{\mathrm{C2}}}+\frac{[\mathrm{fC3bC4bC2}]}{K_{\mathrm{m\,MASP2^*}}^{\mathrm{C2}}}+\frac{[\mathrm{pC4bC2}]}{K_{\mathrm{m\,MASP2^*}}^{\mathrm{C2}}}+\frac{[\mathrm{pC4bC4bC2}]}{K_{\mathrm{m\,MASP2^*}}^{\mathrm{C2}}}\\+\frac{[\mathrm{pC3bC4bC2}]}{K_{\mathrm{m\,MASP2^*}}^{\mathrm{C2}}}+\frac{[\mathrm{pMBL_3MASP1_{zym}}]}{K_{\mathrm{m\,MASP2^*}}^{\mathrm{MASP1_{zym}}}}+\frac{[\mathrm{pMBL_3MASP2_{zym}}]}{K_{\mathrm{m\,MASP2^*}}^{\mathrm{MASP2_{zym}}}}+\frac{[\mathrm{pMBL_4MASP1_{zym}}]}{K_{\mathrm{m\,MASP2^*}}^{\mathrm{MASP1_{zym}}}}\\+\frac{[\mathrm{pMBL_4MASP2_{zym}}]}{K_{\mathrm{m\,MASP2^*}}^{\mathrm{MASP2_{zym}}}}+\frac{[\mathrm{pCLLKMASP1_{zym}}]}{K_{\mathrm{m\,MASP2^*}}^{\mathrm{MASP1_{zym}}}}+\frac{[\mathrm{pCLLKMASP2_{zym}}]}{K_{\mathrm{m\,MASP2^*}}^{\mathrm{MASP2_{zym}}}}+\frac{[\mathrm{pCLK1MASP1_{zym}}]}{K_{\mathrm{m\,MASP2^*}}^{\mathrm{MASP1_{zym}}}}\\+\frac{[\mathrm{pCLK1MASP2_{zym}}]}{K_{\mathrm{m\,MASP2^*}}^{\mathrm{MASP2_{zym}}}}+\frac{[\mathrm{pCLL1MASP1_{zym}}]}{K_{\mathrm{m\,MASP2^*}}^{\mathrm{MASP1_{zym}}}}+\frac{[\mathrm{pCLL1MASP2_{zym}}]}{K_{\mathrm{m\,MASP2^*}}^{\mathrm{MASP2_{zym}}}}+\frac{[\mathrm{pLFMASP1_{zym}}]}{K_{\mathrm{m\,MASP2^*}}^{\mathrm{MASP1_{zym}}}}\\+\frac{[\mathrm{pLFMASP2_{zym}}]}{K_{\mathrm{m\,MASP2^*}}^{\mathrm{MASP2_{zym}}}}+\frac{[\mathrm{pHFMASP1_{zym}}]}{K_{\mathrm{m\,MASP2^*}}^{\mathrm{MASP1_{zym}}}}+\frac{[\mathrm{pHFMASP2_{zym}}]}{K_{\mathrm{m\,MASP2^*}}^{\mathrm{MASP2_{zym}}}}+\frac{[\mathrm{pMFMASP1_{zym}}]}{K_{\mathrm{m\,MASP2^*}}^{\mathrm{MASP1_{zym}}}}+\frac{[\mathrm{pMFMASP2_{zym}}]}{K_{\mathrm{m\,MASP2^*}}^{\mathrm{MASP2_{zym}}}}\end{array}\right)}$$

$$-\frac{k_{\mathrm{cat\,MASP2^*}}^{\mathrm{C2}}\left[\mathrm{pCLLKMASP2^*}\right]\left[\mathrm{fC4bC4bC2}\right]}{K_{\mathrm{m\,MASP2^*}}^{\mathrm{C2}}\left(\begin{array}{l}1+\frac{[\mathrm{C4}]}{K_{\mathrm{m\,MASP2^*}}^{\mathrm{C4}}}+\frac{[\mathrm{fC4bC2}]}{K_{\mathrm{m\,MASP2^*}}^{\mathrm{C2}}}+\frac{[\mathrm{fC4bC4bC2}]}{K_{\mathrm{m\,MASP2^*}}^{\mathrm{C2}}}+\frac{[\mathrm{fC3bC4bC2}]}{K_{\mathrm{m\,MASP2^*}}^{\mathrm{C2}}}+\frac{[\mathrm{pC4bC2}]}{K_{\mathrm{m\,MASP2^*}}^{\mathrm{C2}}}+\frac{[\mathrm{pC4bC4bC2}]}{K_{\mathrm{m\,MASP2^*}}^{\mathrm{C2}}}\\+\frac{[\mathrm{pC3bC4bC2}]}{K_{\mathrm{m\,MASP2^*}}^{\mathrm{C2}}}+\frac{[\mathrm{pMBL_3MASP1_{zym}}]}{K_{\mathrm{m\,MASP2^*}}^{\mathrm{MASP1_{zym}}}}+\frac{[\mathrm{pMBL_3MASP2_{zym}}]}{K_{\mathrm{m\,MASP2^*}}^{\mathrm{MASP2_{zym}}}}+\frac{[\mathrm{pMBL_4MASP1_{zym}}]}{K_{\mathrm{m\,MASP2^*}}^{\mathrm{MASP1_{zym}}}}\\+\frac{[\mathrm{pMBL_4MASP2_{zym}}]}{K_{\mathrm{m\,MASP2^*}}^{\mathrm{MASP2_{zym}}}}+\frac{[\mathrm{pCLLKMASP1_{zym}}]}{K_{\mathrm{m\,MASP2^*}}^{\mathrm{MASP1_{zym}}}}+\frac{[\mathrm{pCLLKMASP2_{zym}}]}{K_{\mathrm{m\,MASP2^*}}^{\mathrm{MASP2_{zym}}}}+\frac{[\mathrm{pCLK1MASP1_{zym}}]}{K_{\mathrm{m\,MASP2^*}}^{\mathrm{MASP1_{zym}}}}\\+\frac{[\mathrm{pCLK1MASP2_{zym}}]}{K_{\mathrm{m\,MASP2^*}}^{\mathrm{MASP2_{zym}}}}+\frac{[\mathrm{pCLL1MASP1_{zym}}]}{K_{\mathrm{m\,MASP2^*}}^{\mathrm{MASP1_{zym}}}}+\frac{[\mathrm{pCLL1MASP2_{zym}}]}{K_{\mathrm{m\,MASP2^*}}^{\mathrm{MASP2_{zym}}}}+\frac{[\mathrm{pLFMASP1_{zym}}]}{K_{\mathrm{m\,MASP2^*}}^{\mathrm{MASP1_{zym}}}}\\+\frac{[\mathrm{pLFMASP2_{zym}}]}{K_{\mathrm{m\,MASP2^*}}^{\mathrm{MASP2_{zym}}}}+\frac{[\mathrm{pHFMASP1_{zym}}]}{K_{\mathrm{m\,MASP2^*}}^{\mathrm{MASP1_{zym}}}}+\frac{[\mathrm{pHFMASP2_{zym}}]}{K_{\mathrm{m\,MASP2^*}}^{\mathrm{MASP2_{zym}}}}+\frac{[\mathrm{pMFMASP1_{zym}}]}{K_{\mathrm{m\,MASP2^*}}^{\mathrm{MASP1_{zym}}}}+\frac{[\mathrm{pMFMASP2_{zym}}]}{K_{\mathrm{m\,MASP2^*}}^{\mathrm{MASP2_{zym}}}}\end{array}\right)}$$

$$-\frac{k_{\mathrm{cat\,MASP2^*}}^{\mathrm{C2}}\left[\mathrm{pCLK1MASP2^*}\right]\left[\mathrm{fC4bC4bC2}\right]}{K_{\mathrm{m\,MASP2^*}}^{\mathrm{C2}}\left(\begin{array}{l}1+\frac{[\mathrm{C4}]}{K_{\mathrm{m\,MASP2^*}}^{\mathrm{C4}}}+\frac{[\mathrm{fC4bC2}]}{K_{\mathrm{m\,MASP2^*}}^{\mathrm{C2}}}+\frac{[\mathrm{fC4bC4bC2}]}{K_{\mathrm{m\,MASP2^*}}^{\mathrm{C2}}}+\frac{[\mathrm{fC3bC4bC2}]}{K_{\mathrm{m\,MASP2^*}}^{\mathrm{C2}}}+\frac{[\mathrm{pC4bC2}]}{K_{\mathrm{m\,MASP2^*}}^{\mathrm{C2}}}+\frac{[\mathrm{pC4bC4bC2}]}{K_{\mathrm{m\,MASP2^*}}^{\mathrm{C2}}}\\+\frac{[\mathrm{pC3bC4bC2}]}{K_{\mathrm{m\,MASP2^*}}^{\mathrm{C2}}}+\frac{[\mathrm{pMBL_3MASP1_{zym}}]}{K_{\mathrm{m\,MASP2^*}}^{\mathrm{MASP1_{zym}}}}+\frac{[\mathrm{pMBL_3MASP2_{zym}}]}{K_{\mathrm{m\,MASP2^*}}^{\mathrm{MASP2_{zym}}}}+\frac{[\mathrm{pMBL_4MASP1_{zym}}]}{K_{\mathrm{m\,MASP2^*}}^{\mathrm{MASP1_{zym}}}}\\+\frac{[\mathrm{pMBL_4MASP2_{zym}}]}{K_{\mathrm{m\,MASP2^*}}^{\mathrm{MASP2_{zym}}}}+\frac{[\mathrm{pCLLKMASP1_{zym}}]}{K_{\mathrm{m\,MASP2^*}}^{\mathrm{MASP1_{zym}}}}+\frac{[\mathrm{pCLLKMASP2_{zym}}]}{K_{\mathrm{m\,MASP2^*}}^{\mathrm{MASP2_{zym}}}}+\frac{[\mathrm{pCLK1MASP1_{zym}}]}{K_{\mathrm{m\,MASP2^*}}^{\mathrm{MASP1_{zym}}}}\\+\frac{[\mathrm{pCLK1MASP2_{zym}}]}{K_{\mathrm{m\,MASP2^*}}^{\mathrm{MASP2_{zym}}}}+\frac{[\mathrm{pCLL1MASP1_{zym}}]}{K_{\mathrm{m\,MASP2^*}}^{\mathrm{MASP1_{zym}}}}+\frac{[\mathrm{pCLL1MASP2_{zym}}]}{K_{\mathrm{m\,MASP2^*}}^{\mathrm{MASP2_{zym}}}}+\frac{[\mathrm{pLFMASP1_{zym}}]}{K_{\mathrm{m\,MASP2^*}}^{\mathrm{MASP1_{zym}}}}\\+\frac{[\mathrm{pLFMASP2_{zym}}]}{K_{\mathrm{m\,MASP2^*}}^{\mathrm{MASP2_{zym}}}}+\frac{[\mathrm{pHFMASP1_{zym}}]}{K_{\mathrm{m\,MASP2^*}}^{\mathrm{MASP1_{zym}}}}+\frac{[\mathrm{pHFMASP2_{zym}}]}{K_{\mathrm{m\,MASP2^*}}^{\mathrm{MASP2_{zym}}}}+\frac{[\mathrm{pMFMASP1_{zym}}]}{K_{\mathrm{m\,MASP2^*}}^{\mathrm{MASP1_{zym}}}}+\frac{[\mathrm{pMFMASP2_{zym}}]}{K_{\mathrm{m\,MASP2^*}}^{\mathrm{MASP2_{zym}}}}\end{array}\right)}$$

$$
-\frac{k^{\mathrm{C2}}_{\mathrm{cat\,MASP2^*}}\left[\mathrm{pCLL1MASP2^*}\right]\left[\mathrm{fC4bC4bC2}\right]}{K^{\mathrm{C2}}_{\mathrm{m\,MASP2^*}}\left(\begin{aligned}&1+\frac{[\mathrm{C4}]}{K^{\mathrm{C4}}_{\mathrm{m\,MASP2^*}}}+\frac{[\mathrm{fC4bC2}]}{K^{\mathrm{C2}}_{\mathrm{m\,MASP2^*}}}+\frac{[\mathrm{fC4bC4bC2}]}{K^{\mathrm{C2}}_{\mathrm{m\,MASP2^*}}}+\frac{[\mathrm{fC3bC4bC2}]}{K^{\mathrm{C2}}_{\mathrm{m\,MASP2^*}}}+\frac{[\mathrm{pC4bC2}]}{K^{\mathrm{C2}}_{\mathrm{m\,MASP2^*}}}+\frac{[\mathrm{pC4bC4bC2}]}{K^{\mathrm{C2}}_{\mathrm{m\,MASP2^*}}}\\&+\frac{[\mathrm{pC3bC4bC2}]}{K^{\mathrm{C2}}_{\mathrm{m\,MASP2^*}}}+\frac{[\mathrm{pMBL_3MASP1_{zym}}]}{K^{\mathrm{MASP1_{zym}}}_{\mathrm{m\,MASP2^*}}}+\frac{[\mathrm{pMBL_3MASP2_{zym}}]}{K^{\mathrm{MASP2_{zym}}}_{\mathrm{m\,MASP2^*}}}+\frac{[\mathrm{pMBL_4MASP1_{zym}}]}{K^{\mathrm{MASP1_{zym}}}_{\mathrm{m\,MASP2^*}}}\\&+\frac{[\mathrm{pMBL_4MASP2_{zym}}]}{K^{\mathrm{MASP2_{zym}}}_{\mathrm{m\,MASP2^*}}}+\frac{[\mathrm{pCLLKMASP1_{zym}}]}{K^{\mathrm{MASP1_{zym}}}_{\mathrm{m\,MASP2^*}}}+\frac{[\mathrm{pCLLKMASP2_{zym}}]}{K^{\mathrm{MASP2_{zym}}}_{\mathrm{m\,MASP2^*}}}+\frac{[\mathrm{pCLK1MASP1_{zym}}]}{K^{\mathrm{MASP1_{zym}}}_{\mathrm{m\,MASP2^*}}}\\&+\frac{[\mathrm{pCLK1MASP2_{zym}}]}{K^{\mathrm{MASP2_{zym}}}_{\mathrm{m\,MASP2^*}}}+\frac{[\mathrm{pCLL1MASP1_{zym}}]}{K^{\mathrm{MASP1_{zym}}}_{\mathrm{m\,MASP2^*}}}+\frac{[\mathrm{pCLL1MASP2_{zym}}]}{K^{\mathrm{MASP2_{zym}}}_{\mathrm{m\,MASP2^*}}}+\frac{[\mathrm{pLFMASP1_{zym}}]}{K^{\mathrm{MASP1_{zym}}}_{\mathrm{m\,MASP2^*}}}\\&+\frac{[\mathrm{pLFMASP2_{zym}}]}{K^{\mathrm{MASP2_{zym}}}_{\mathrm{m\,MASP2^*}}}+\frac{[\mathrm{pHFMASP1_{zym}}]}{K^{\mathrm{MASP1_{zym}}}_{\mathrm{m\,MASP2^*}}}+\frac{[\mathrm{pHFMASP2_{zym}}]}{K^{\mathrm{MASP2_{zym}}}_{\mathrm{m\,MASP2^*}}}+\frac{[\mathrm{pMFMASP1_{zym}}]}{K^{\mathrm{MASP1_{zym}}}_{\mathrm{m\,MASP2^*}}}+\frac{[\mathrm{pMFMASP2_{zym}}]}{K^{\mathrm{MASP2_{zym}}}_{\mathrm{m\,MASP2^*}}}\end{aligned}\right)}
$$

$$
-\frac{k^{\mathrm{C2}}_{\mathrm{cat\,MASP2^*}}\left[\mathrm{pLFMASP2^*}\right]\left[\mathrm{fC4bC4bC2}\right]}{K^{\mathrm{C2}}_{\mathrm{m\,MASP2^*}}\left(\begin{aligned}&1+\frac{[\mathrm{C4}]}{K^{\mathrm{C4}}_{\mathrm{m\,MASP2^*}}}+\frac{[\mathrm{fC4bC2}]}{K^{\mathrm{C2}}_{\mathrm{m\,MASP2^*}}}+\frac{[\mathrm{fC4bC4bC2}]}{K^{\mathrm{C2}}_{\mathrm{m\,MASP2^*}}}+\frac{[\mathrm{fC3bC4bC2}]}{K^{\mathrm{C2}}_{\mathrm{m\,MASP2^*}}}+\frac{[\mathrm{pC4bC2}]}{K^{\mathrm{C2}}_{\mathrm{m\,MASP2^*}}}+\frac{[\mathrm{pC4bC4bC2}]}{K^{\mathrm{C2}}_{\mathrm{m\,MASP2^*}}}\\&+\frac{[\mathrm{pC3bC4bC2}]}{K^{\mathrm{C2}}_{\mathrm{m\,MASP2^*}}}+\frac{[\mathrm{pMBL_3MASP1_{zym}}]}{K^{\mathrm{MASP1_{zym}}}_{\mathrm{m\,MASP2^*}}}+\frac{[\mathrm{pMBL_3MASP2_{zym}}]}{K^{\mathrm{MASP2_{zym}}}_{\mathrm{m\,MASP2^*}}}+\frac{[\mathrm{pMBL_4MASP1_{zym}}]}{K^{\mathrm{MASP1_{zym}}}_{\mathrm{m\,MASP2^*}}}\\&+\frac{[\mathrm{pMBL_4MASP2_{zym}}]}{K^{\mathrm{MASP2_{zym}}}_{\mathrm{m\,MASP2^*}}}+\frac{[\mathrm{pCLLKMASP1_{zym}}]}{K^{\mathrm{MASP1_{zym}}}_{\mathrm{m\,MASP2^*}}}+\frac{[\mathrm{pCLLKMASP2_{zym}}]}{K^{\mathrm{MASP2_{zym}}}_{\mathrm{m\,MASP2^*}}}+\frac{[\mathrm{pCLK1MASP1_{zym}}]}{K^{\mathrm{MASP1_{zym}}}_{\mathrm{m\,MASP2^*}}}\\&+\frac{[\mathrm{pCLK1MASP2_{zym}}]}{K^{\mathrm{MASP2_{zym}}}_{\mathrm{m\,MASP2^*}}}+\frac{[\mathrm{pCLL1MASP1_{zym}}]}{K^{\mathrm{MASP1_{zym}}}_{\mathrm{m\,MASP2^*}}}+\frac{[\mathrm{pCLL1MASP2_{zym}}]}{K^{\mathrm{MASP2_{zym}}}_{\mathrm{m\,MASP2^*}}}+\frac{[\mathrm{pLFMASP1_{zym}}]}{K^{\mathrm{MASP1_{zym}}}_{\mathrm{m\,MASP2^*}}}\\&+\frac{[\mathrm{pLFMASP2_{zym}}]}{K^{\mathrm{MASP2_{zym}}}_{\mathrm{m\,MASP2^*}}}+\frac{[\mathrm{pHFMASP1_{zym}}]}{K^{\mathrm{MASP1_{zym}}}_{\mathrm{m\,MASP2^*}}}+\frac{[\mathrm{pHFMASP2_{zym}}]}{K^{\mathrm{MASP2_{zym}}}_{\mathrm{m\,MASP2^*}}}+\frac{[\mathrm{pMFMASP1_{zym}}]}{K^{\mathrm{MASP1_{zym}}}_{\mathrm{m\,MASP2^*}}}+\frac{[\mathrm{pMFMASP2_{zym}}]}{K^{\mathrm{MASP2_{zym}}}_{\mathrm{m\,MASP2^*}}}\end{aligned}\right)}
$$

$$
-\frac{k^{\mathrm{C2}}_{\mathrm{cat\,MASP2^*}}\left[\mathrm{pHFMASP2^*}\right]\left[\mathrm{fC4bC4bC2}\right]}{K^{\mathrm{C2}}_{\mathrm{m\,MASP2^*}}\left(\begin{aligned}&1+\frac{[\mathrm{C4}]}{K^{\mathrm{C4}}_{\mathrm{m\,MASP2^*}}}+\frac{[\mathrm{fC4bC2}]}{K^{\mathrm{C2}}_{\mathrm{m\,MASP2^*}}}+\frac{[\mathrm{fC4bC4bC2}]}{K^{\mathrm{C2}}_{\mathrm{m\,MASP2^*}}}+\frac{[\mathrm{fC3bC4bC2}]}{K^{\mathrm{C2}}_{\mathrm{m\,MASP2^*}}}+\frac{[\mathrm{pC4bC2}]}{K^{\mathrm{C2}}_{\mathrm{m\,MASP2^*}}}+\frac{[\mathrm{pC4bC4bC2}]}{K^{\mathrm{C2}}_{\mathrm{m\,MASP2^*}}}\\&+\frac{[\mathrm{pC3bC4bC2}]}{K^{\mathrm{C2}}_{\mathrm{m\,MASP2^*}}}+\frac{[\mathrm{pMBL_3MASP1_{zym}}]}{K^{\mathrm{MASP1_{zym}}}_{\mathrm{m\,MASP2^*}}}+\frac{[\mathrm{pMBL_3MASP2_{zym}}]}{K^{\mathrm{MASP2_{zym}}}_{\mathrm{m\,MASP2^*}}}+\frac{[\mathrm{pMBL_4MASP1_{zym}}]}{K^{\mathrm{MASP1_{zym}}}_{\mathrm{m\,MASP2^*}}}\\&+\frac{[\mathrm{pMBL_4MASP2_{zym}}]}{K^{\mathrm{MASP2_{zym}}}_{\mathrm{m\,MASP2^*}}}+\frac{[\mathrm{pCLLKMASP1_{zym}}]}{K^{\mathrm{MASP1_{zym}}}_{\mathrm{m\,MASP2^*}}}+\frac{[\mathrm{pCLLKMASP2_{zym}}]}{K^{\mathrm{MASP2_{zym}}}_{\mathrm{m\,MASP2^*}}}+\frac{[\mathrm{pCLK1MASP1_{zym}}]}{K^{\mathrm{MASP1_{zym}}}_{\mathrm{m\,MASP2^*}}}\\&+\frac{[\mathrm{pCLK1MASP2_{zym}}]}{K^{\mathrm{MASP2_{zym}}}_{\mathrm{m\,MASP2^*}}}+\frac{[\mathrm{pCLL1MASP1_{zym}}]}{K^{\mathrm{MASP1_{zym}}}_{\mathrm{m\,MASP2^*}}}+\frac{[\mathrm{pCLL1MASP2_{zym}}]}{K^{\mathrm{MASP2_{zym}}}_{\mathrm{m\,MASP2^*}}}+\frac{[\mathrm{pLFMASP1_{zym}}]}{K^{\mathrm{MASP1_{zym}}}_{\mathrm{m\,MASP2^*}}}\\&+\frac{[\mathrm{pLFMASP2_{zym}}]}{K^{\mathrm{MASP2_{zym}}}_{\mathrm{m\,MASP2^*}}}+\frac{[\mathrm{pHFMASP1_{zym}}]}{K^{\mathrm{MASP1_{zym}}}_{\mathrm{m\,MASP2^*}}}+\frac{[\mathrm{pHFMASP2_{zym}}]}{K^{\mathrm{MASP2_{zym}}}_{\mathrm{m\,MASP2^*}}}+\frac{[\mathrm{pMFMASP1_{zym}}]}{K^{\mathrm{MASP1_{zym}}}_{\mathrm{m\,MASP2^*}}}+\frac{[\mathrm{pMFMASP2_{zym}}]}{K^{\mathrm{MASP2_{zym}}}_{\mathrm{m\,MASP2^*}}}\end{aligned}\right)}
$$

$$-\frac{k_{\text{cat MASP2}^*}^{\text{C2}}\left[\text{pMFMASP2}^*\right]\left[\text{fC4bC4bC2}\right]}{K_{\text{m MASP2}^*}^{\text{C2}}\left(\begin{array}{l}1+\frac{[\text{C4}]}{K_{\text{m MASP2}^*}^{\text{C4}}}+\frac{[\text{fC4bC2}]}{K_{\text{m MASP2}^*}^{\text{C2}}}+\frac{[\text{fC4bC4bC2}]}{K_{\text{m MASP2}^*}^{\text{C2}}}+\frac{[\text{fC3bC4bC2}]}{K_{\text{m MASP2}^*}^{\text{C2}}}+\frac{[\text{pC4bC2}]}{K_{\text{m MASP2}^*}^{\text{C2}}}+\frac{[\text{pC4bC4bC2}]}{K_{\text{m MASP2}^*}^{\text{C2}}}\\+\frac{[\text{pC3bC4bC2}]}{K_{\text{m MASP2}^*}^{\text{C2}}}+\frac{[\text{pMBL}_3\text{MASP1}_{\text{zym}}]}{K_{\text{m MASP2}^*}^{\text{MASP1}_{\text{zym}}}}+\frac{[\text{pMBL}_3\text{MASP2}_{\text{zym}}]}{K_{\text{m MASP2}^*}^{\text{MASP2}_{\text{zym}}}}+\frac{[\text{pMBL}_4\text{MASP1}_{\text{zym}}]}{K_{\text{m MASP2}^*}^{\text{MASP1}_{\text{zym}}}}\\+\frac{[\text{pMBL}_4\text{MASP2}_{\text{zym}}]}{K_{\text{m MASP2}^*}^{\text{MASP2}_{\text{zym}}}}+\frac{[\text{pCLLKMASP1}_{\text{zym}}]}{K_{\text{m MASP2}^*}^{\text{MASP1}_{\text{zym}}}}+\frac{[\text{pCLLKMASP2}_{\text{zym}}]}{K_{\text{m MASP2}^*}^{\text{MASP2}_{\text{zym}}}}+\frac{[\text{pCLK1MASP1}_{\text{zym}}]}{K_{\text{m MASP2}^*}^{\text{MASP1}_{\text{zym}}}}\\+\frac{[\text{pCLK1MASP2}_{\text{zym}}]}{K_{\text{m MASP2}^*}^{\text{MASP2}_{\text{zym}}}}+\frac{[\text{pCLL1MASP1}_{\text{zym}}]}{K_{\text{m MASP2}^*}^{\text{MASP1}_{\text{zym}}}}+\frac{[\text{pCLL1MASP2}_{\text{zym}}]}{K_{\text{m MASP2}^*}^{\text{MASP2}_{\text{zym}}}}+\frac{[\text{pLFMASP1}_{\text{zym}}]}{K_{\text{m MASP2}^*}^{\text{MASP1}_{\text{zym}}}}\\+\frac{[\text{pLFMASP2}_{\text{zym}}]}{K_{\text{m MASP2}^*}^{\text{MASP2}_{\text{zym}}}}+\frac{[\text{pHFMASP1}_{\text{zym}}]}{K_{\text{m MASP2}^*}^{\text{MASP1}_{\text{zym}}}}+\frac{[\text{pHFMASP2}_{\text{zym}}]}{K_{\text{m MASP2}^*}^{\text{MASP2}_{\text{zym}}}}+\frac{[\text{pMFMASP1}_{\text{zym}}]}{K_{\text{m MASP2}^*}^{\text{MASP1}_{\text{zym}}}}+\frac{[\text{pMFMASP2}_{\text{zym}}]}{K_{\text{m MASP2}^*}^{\text{MASP2}_{\text{zym}}}}\end{array}\right)}$$

$$-\frac{k_{\text{cat C1}^*}^{\text{C2}}\left[\text{pMBL}_3\text{PTX3C1}^*\right]\left[\text{fC4bC4bC2}\right]}{K_{\text{m C1}^*}^{\text{C2}}\left(1+\frac{[\text{C4}]}{K_{\text{m C1}^*}^{\text{C4}}}+\frac{[\text{fC4bC2}]}{K_{\text{m C1}^*}^{\text{C2}}}+\frac{[\text{fC4bC4bC2}]}{K_{\text{m C1}^*}^{\text{C2}}}+\frac{[\text{fC3bC4bC2}]}{K_{\text{m C1}^*}^{\text{C2}}}+\frac{[\text{pC4bC2}]}{K_{\text{m C1}^*}^{\text{C2}}}+\frac{[\text{pC4bC4bC2}]}{K_{\text{m C1}^*}^{\text{C2}}}+\frac{[\text{pC3bC4bC2}]}{K_{\text{m C1}^*}^{\text{C2}}}\right)}$$

$$-\frac{k_{\text{cat C1}^*}^{\text{C2}}\left[\text{pMBL}_4\text{PTX3C1}^*\right]\left[\text{fC4bC4bC2}\right]}{K_{\text{m C1}^*}^{\text{C2}}\left(1+\frac{[\text{C4}]}{K_{\text{m C1}^*}^{\text{C4}}}+\frac{[\text{fC4bC2}]}{K_{\text{m C1}^*}^{\text{C2}}}+\frac{[\text{fC4bC4bC2}]}{K_{\text{m C1}^*}^{\text{C2}}}+\frac{[\text{fC3bC4bC2}]}{K_{\text{m C1}^*}^{\text{C2}}}+\frac{[\text{pC4bC2}]}{K_{\text{m C1}^*}^{\text{C2}}}+\frac{[\text{pC4bC4bC2}]}{K_{\text{m C1}^*}^{\text{C2}}}+\frac{[\text{pC3bC4bC2}]}{K_{\text{m C1}^*}^{\text{C2}}}\right)}$$

$$-\frac{k_{\text{cat C1}^*}^{\text{C2}}\left[\text{CRPC1}^*\right]\left[\text{fC4bC4bC2}\right]}{K_{\text{m C1}^*}^{\text{C2}}\left(1+\frac{[\text{C4}]}{K_{\text{m C1}^*}^{\text{C4}}}+\frac{[\text{fC4bC2}]}{K_{\text{m C1}^*}^{\text{C2}}}+\frac{[\text{fC4bC4bC2}]}{K_{\text{m C1}^*}^{\text{C2}}}+\frac{[\text{fC3bC4bC2}]}{K_{\text{m C1}^*}^{\text{C2}}}\right)}$$

41

$$
\begin{aligned}
\frac{d[\mathrm{fC4bC4bC2a}]}{dt} &= k^{+}_{\mathrm{C4bC4b}}[\mathrm{fC4bC2a}][\mathrm{nfC4b}] \\
&+ \frac{k^{\mathrm{C2}}_{\mathrm{cat\ C1^*}}[\mathrm{C1^*}][\mathrm{fC4bC4bC2}]}{K^{\mathrm{C2}}_{\mathrm{m\ C1^*}}\left(1+\frac{[\mathrm{C4}]}{K^{\mathrm{C4}}_{\mathrm{m\ C1^*}}}+\frac{[\mathrm{fC4bC2}]}{K^{\mathrm{C2}}_{\mathrm{m\ C1^*}}}+\frac{[\mathrm{fC4bC4bC2}]}{K^{\mathrm{C2}}_{\mathrm{m\ C1^*}}}+\frac{[\mathrm{fC3bC4bC2}]}{K^{\mathrm{C2}}_{\mathrm{m\ C1^*}}}+\frac{[\mathrm{pC4bC2}]}{K^{\mathrm{C2}}_{\mathrm{m\ C1^*}}}+\frac{[\mathrm{pC4bC4bC2}]}{K^{\mathrm{C2}}_{\mathrm{m\ C1^*}}}+\frac{[\mathrm{pC3bC4bC2}]}{K^{\mathrm{C2}}_{\mathrm{m\ C1^*}}}\right)} \\
&- k^{-}_{\mathrm{C4bC4bC2a}}[\mathrm{fC4bC4bC2a}] + k^{-}_{\mathrm{C5b^*}}[\mathrm{fC4bC4bC2aC5b}] + k^{+}_{\mathrm{C5b7}}[\mathrm{fC4bC4bC2aC5b}][\mathrm{C7}] - k^{+}_{\mathrm{C4bC4BP}}[\mathrm{fC4bC4bC2a}][\mathrm{C4BP}] \\
&+ k^{-}_{\mathrm{C4bC4BP}}[\mathrm{fC4bC4bC2aC4BP}] - k^{+}_{\mathrm{C4bC4bCR1}}[\mathrm{fC4bC4bC2a}][\mathrm{CR1}] + k^{-}_{\mathrm{C4bC4bCR1}}[\mathrm{fC4bC4bC2aCR1}] \\
&+ \frac{k^{\mathrm{C2}}_{\mathrm{cat\ C1^*}}[\mathrm{pIgG_6C1^*}][\mathrm{fC4bC4bC2}]}{K^{\mathrm{C2}}_{\mathrm{m\ C1^*}}\left(1+\frac{[\mathrm{C4}]}{K^{\mathrm{C4}}_{\mathrm{m\ C1^*}}}+\frac{[\mathrm{fC4bC2}]}{K^{\mathrm{C2}}_{\mathrm{m\ C1^*}}}+\frac{[\mathrm{fC4bC4bC2}]}{K^{\mathrm{C2}}_{\mathrm{m\ C1^*}}}+\frac{[\mathrm{fC3bC4bC2}]}{K^{\mathrm{C2}}_{\mathrm{m\ C1^*}}}+\frac{[\mathrm{pC4bC2}]}{K^{\mathrm{C2}}_{\mathrm{m\ C1^*}}}+\frac{[\mathrm{pC4bC4bC2}]}{K^{\mathrm{C2}}_{\mathrm{m\ C1^*}}}+\frac{[\mathrm{pC3bC4bC2}]}{K^{\mathrm{C2}}_{\mathrm{m\ C1^*}}}\right)} \\
&+ \frac{k^{\mathrm{C2}}_{\mathrm{cat\ C1^*}}[\mathrm{pIgMC1^*}][\mathrm{fC4bC4bC2}]}{K^{\mathrm{C2}}_{\mathrm{m\ C1^*}}\left(1+\frac{[\mathrm{C4}]}{K^{\mathrm{C4}}_{\mathrm{m\ C1^*}}}+\frac{[\mathrm{fC4bC2}]}{K^{\mathrm{C2}}_{\mathrm{m\ C1^*}}}+\frac{[\mathrm{fC4bC4bC2}]}{K^{\mathrm{C2}}_{\mathrm{m\ C1^*}}}+\frac{[\mathrm{fC3bC4bC2}]}{K^{\mathrm{C2}}_{\mathrm{m\ C1^*}}}+\frac{[\mathrm{pC4bC2}]}{K^{\mathrm{C2}}_{\mathrm{m\ C1^*}}}+\frac{[\mathrm{pC4bC4bC2}]}{K^{\mathrm{C2}}_{\mathrm{m\ C1^*}}}+\frac{[\mathrm{pC3bC4bC2}]}{K^{\mathrm{C2}}_{\mathrm{m\ C1^*}}}\right)} \\
&+ \frac{k^{\mathrm{C2}}_{\mathrm{cat\ MASP1^*}}[\mathrm{pMBL_3MASP1^*}][\mathrm{fC4bC4bC2}]}{K^{\mathrm{C2}}_{\mathrm{m\ MASP1^*}}\left(\begin{aligned}&1+\frac{[\mathrm{C4}]}{K^{\mathrm{C4}}_{\mathrm{m\ MASP1^*}}}+\frac{[\mathrm{fC4bC2}]}{K^{\mathrm{C2}}_{\mathrm{m\ MASP1^*}}}+\frac{[\mathrm{fC4bC4bC2}]}{K^{\mathrm{C2}}_{\mathrm{m\ MASP1^*}}}+\frac{[\mathrm{fC3bC4bC2}]}{K^{\mathrm{C2}}_{\mathrm{m\ MASP1^*}}}+\frac{[\mathrm{pC4bC2}]}{K^{\mathrm{C2}}_{\mathrm{m\ MASP1^*}}}+\frac{[\mathrm{pC4bC4bC2}]}{K^{\mathrm{C2}}_{\mathrm{m\ MASP1^*}}}\\
&+\frac{[\mathrm{pC3bC4bC2}]}{K^{\mathrm{C2}}_{\mathrm{m\ MASP1^*}}}+\frac{[\mathrm{pMBL_3MASP1_{zym}}]}{K^{\mathrm{MASP1_{zym}}}_{\mathrm{m\ MASP1^*}}}+\frac{[\mathrm{pMBL_3MASP2_{zym}}]}{K^{\mathrm{MASP2_{zym}}}_{\mathrm{m\ MASP1^*}}}+\frac{[\mathrm{pMBL_3MASP3_{zym}}]}{K^{\mathrm{MASP3_{zym}}}_{\mathrm{m\ MASP1^*}}}\\
&+\frac{[\mathrm{pMBL_4MASP1_{zym}}]}{K^{\mathrm{MASP1_{zym}}}_{\mathrm{m\ MASP1^*}}}+\frac{[\mathrm{pMBL_4MASP2_{zym}}]}{K^{\mathrm{MASP2_{zym}}}_{\mathrm{m\ MASP1^*}}}+\frac{[\mathrm{pMBL_4MASP3_{zym}}]}{K^{\mathrm{MASP3_{zym}}}_{\mathrm{m\ MASP1^*}}}+\frac{[\mathrm{pCLLKMASP1_{zym}}]}{K^{\mathrm{MASP1_{zym}}}_{\mathrm{m\ MASP1^*}}}\\
&+\frac{[\mathrm{pCLLKMASP2_{zym}}]}{K^{\mathrm{MASP2_{zym}}}_{\mathrm{m\ MASP1^*}}}+\frac{[\mathrm{pCLLKMASP3_{zym}}]}{K^{\mathrm{MASP3_{zym}}}_{\mathrm{m\ MASP1^*}}}+\frac{[\mathrm{pCLK1MASP1_{zym}}]}{K^{\mathrm{MASP1_{zym}}}_{\mathrm{m\ MASP1^*}}}+\frac{[\mathrm{pCLK1MASP2_{zym}}]}{K^{\mathrm{MASP2_{zym}}}_{\mathrm{m\ MASP1^*}}}\\
&+\frac{[\mathrm{pCLK1MASP3_{zym}}]}{K^{\mathrm{MASP3_{zym}}}_{\mathrm{m\ MASP1^*}}}+\frac{[\mathrm{pCLL1MASP1_{zym}}]}{K^{\mathrm{MASP1_{zym}}}_{\mathrm{m\ MASP1^*}}}+\frac{[\mathrm{pCLL1MASP2_{zym}}]}{K^{\mathrm{MASP2_{zym}}}_{\mathrm{m\ MASP1^*}}}+\frac{[\mathrm{pCLL1MASP3_{zym}}]}{K^{\mathrm{MASP3_{zym}}}_{\mathrm{m\ MASP1^*}}}\\
&+\frac{[\mathrm{pLFMASP1_{zym}}]}{K^{\mathrm{MASP1_{zym}}}_{\mathrm{m\ MASP1^*}}}+\frac{[\mathrm{pLFMASP2_{zym}}]}{K^{\mathrm{MASP2_{zym}}}_{\mathrm{m\ MASP1^*}}}+\frac{[\mathrm{pLFMASP3_{zym}}]}{K^{\mathrm{MASP3_{zym}}}_{\mathrm{m\ MASP1^*}}}+\frac{[\mathrm{pHFMASP1_{zym}}]}{K^{\mathrm{MASP1_{zym}}}_{\mathrm{m\ MASP1^*}}}+\frac{[\mathrm{pHFMASP2_{zym}}]}{K^{\mathrm{MASP2_{zym}}}_{\mathrm{m\ MASP1^*}}}\\
&+\frac{[\mathrm{pHFMASP3_{zym}}]}{K^{\mathrm{MASP3_{zym}}}_{\mathrm{m\ MASP1^*}}}+\frac{[\mathrm{pMFMASP1_{zym}}]}{K^{\mathrm{MASP1_{zym}}}_{\mathrm{m\ MASP1^*}}}+\frac{[\mathrm{pMFMASP2_{zym}}]}{K^{\mathrm{MASP2_{zym}}}_{\mathrm{m\ MASP1^*}}}+\frac{[\mathrm{pMFMASP3_{zym}}]}{K^{\mathrm{MASP3_{zym}}}_{\mathrm{m\ MASP1^*}}}\end{aligned}\right)}
\end{aligned}
$$

$$
+\frac{k_{\mathrm{cat\,MASP1^*}}^{\mathrm{C2}}\left[\mathrm{pMBL_4MASP1^*}\right]\left[\mathrm{fC4bC4bC2}\right]}{K_{\mathrm{m\,MASP1^*}}^{\mathrm{C2}}\left(\begin{aligned}&1+\frac{[\mathrm{C4}]}{K_{\mathrm{m\,MASP1^*}}^{\mathrm{C4}}}+\frac{[\mathrm{fC4bC2}]}{K_{\mathrm{m\,MASP1^*}}^{\mathrm{C2}}}+\frac{[\mathrm{fC4bC4bC2}]}{K_{\mathrm{m\,MASP1^*}}^{\mathrm{C2}}}+\frac{[\mathrm{fC3bC4bC2}]}{K_{\mathrm{m\,MASP1^*}}^{\mathrm{C2}}}+\frac{[\mathrm{pC4bC2}]}{K_{\mathrm{m\,MASP1^*}}^{\mathrm{C2}}}+\frac{[\mathrm{pC4bC4bC2}]}{K_{\mathrm{m\,MASP1^*}}^{\mathrm{C2}}}\\
&+\frac{[\mathrm{pC3bC4bC2}]}{K_{\mathrm{m\,MASP1^*}}^{\mathrm{C2}}}+\frac{\left[\mathrm{pMBL_3MASP1_{zym}}\right]}{K_{\mathrm{m\,MASP1^*}}^{\mathrm{MASP1_{zym}}}}+\frac{\left[\mathrm{pMBL_3MASP2_{zym}}\right]}{K_{\mathrm{m\,MASP1^*}}^{\mathrm{MASP2_{zym}}}}+\frac{\left[\mathrm{pMBL_3MASP3_{zym}}\right]}{K_{\mathrm{m\,MASP1^*}}^{\mathrm{MASP3_{zym}}}}\\
&+\frac{\left[\mathrm{pMBL_4MASP1_{zym}}\right]}{K_{\mathrm{m\,MASP1^*}}^{\mathrm{MASP1_{zym}}}}+\frac{\left[\mathrm{pMBL_4MASP2_{zym}}\right]}{K_{\mathrm{m\,MASP1^*}}^{\mathrm{MASP2_{zym}}}}+\frac{\left[\mathrm{pMBL_4MASP3_{zym}}\right]}{K_{\mathrm{m\,MASP1^*}}^{\mathrm{MASP3_{zym}}}}+\frac{\left[\mathrm{pCLLKMASP1_{zym}}\right]}{K_{\mathrm{m\,MASP1^*}}^{\mathrm{MASP1_{zym}}}}\\
&+\frac{\left[\mathrm{pCLLKMASP2_{zym}}\right]}{K_{\mathrm{m\,MASP1^*}}^{\mathrm{MASP2_{zym}}}}+\frac{\left[\mathrm{pCLLKMASP3_{zym}}\right]}{K_{\mathrm{m\,MASP1^*}}^{\mathrm{MASP3_{zym}}}}+\frac{\left[\mathrm{pCLK1MASP1_{zym}}\right]}{K_{\mathrm{m\,MASP1^*}}^{\mathrm{MASP1_{zym}}}}+\frac{\left[\mathrm{pCLK1MASP2_{zym}}\right]}{K_{\mathrm{m\,MASP1^*}}^{\mathrm{MASP2_{zym}}}}\\
&+\frac{\left[\mathrm{pCLK1MASP3_{zym}}\right]}{K_{\mathrm{m\,MASP1^*}}^{\mathrm{MASP3_{zym}}}}+\frac{\left[\mathrm{pCLL1MASP1_{zym}}\right]}{K_{\mathrm{m\,MASP1^*}}^{\mathrm{MASP1_{zym}}}}+\frac{\left[\mathrm{pCLL1MASP2_{zym}}\right]}{K_{\mathrm{m\,MASP1^*}}^{\mathrm{MASP2_{zym}}}}+\frac{\left[\mathrm{pCLL1MASP3_{zym}}\right]}{K_{\mathrm{m\,MASP1^*}}^{\mathrm{MASP3_{zym}}}}\\
&+\frac{\left[\mathrm{pLFMASP1_{zym}}\right]}{K_{\mathrm{m\,MASP1^*}}^{\mathrm{MASP1_{zym}}}}+\frac{\left[\mathrm{pLFMASP2_{zym}}\right]}{K_{\mathrm{m\,MASP1^*}}^{\mathrm{MASP2_{zym}}}}+\frac{\left[\mathrm{pLFMASP3_{zym}}\right]}{K_{\mathrm{m\,MASP1^*}}^{\mathrm{MASP3_{zym}}}}+\frac{\left[\mathrm{pHFMASP1_{zym}}\right]}{K_{\mathrm{m\,MASP1^*}}^{\mathrm{MASP1_{zym}}}}+\frac{\left[\mathrm{pHFMASP2_{zym}}\right]}{K_{\mathrm{m\,MASP1^*}}^{\mathrm{MASP2_{zym}}}}\\
&+\frac{\left[\mathrm{pHFMASP3_{zym}}\right]}{K_{\mathrm{m\,MASP1^*}}^{\mathrm{MASP3_{zym}}}}+\frac{\left[\mathrm{pMFMASP1_{zym}}\right]}{K_{\mathrm{m\,MASP1^*}}^{\mathrm{MASP1_{zym}}}}+\frac{\left[\mathrm{pMFMASP2_{zym}}\right]}{K_{\mathrm{m\,MASP1^*}}^{\mathrm{MASP2_{zym}}}}+\frac{\left[\mathrm{pMFMASP3_{zym}}\right]}{K_{\mathrm{m\,MASP1^*}}^{\mathrm{MASP3_{zym}}}}\end{aligned}\right)}
$$

$$
+\frac{k_{\mathrm{cat\,MASP1^*}}^{\mathrm{C2}}\left[\mathrm{pCLLKMASP1^*}\right]\left[\mathrm{fC4bC4bC2}\right]}{K_{\mathrm{m\,MASP1^*}}^{\mathrm{C2}}\left(\begin{aligned}&1+\frac{[\mathrm{C4}]}{K_{\mathrm{m\,MASP1^*}}^{\mathrm{C4}}}+\frac{[\mathrm{fC4bC2}]}{K_{\mathrm{m\,MASP1^*}}^{\mathrm{C2}}}+\frac{[\mathrm{fC4bC4bC2}]}{K_{\mathrm{m\,MASP1^*}}^{\mathrm{C2}}}+\frac{[\mathrm{fC3bC4bC2}]}{K_{\mathrm{m\,MASP1^*}}^{\mathrm{C2}}}+\frac{[\mathrm{pC4bC2}]}{K_{\mathrm{m\,MASP1^*}}^{\mathrm{C2}}}+\frac{[\mathrm{pC4bC4bC2}]}{K_{\mathrm{m\,MASP1^*}}^{\mathrm{C2}}}\\
&+\frac{[\mathrm{pC3bC4bC2}]}{K_{\mathrm{m\,MASP1^*}}^{\mathrm{C2}}}+\frac{\left[\mathrm{pMBL_3MASP1_{zym}}\right]}{K_{\mathrm{m\,MASP1^*}}^{\mathrm{MASP1_{zym}}}}+\frac{\left[\mathrm{pMBL_3MASP2_{zym}}\right]}{K_{\mathrm{m\,MASP1^*}}^{\mathrm{MASP2_{zym}}}}+\frac{\left[\mathrm{pMBL_3MASP3_{zym}}\right]}{K_{\mathrm{m\,MASP1^*}}^{\mathrm{MASP3_{zym}}}}\\
&+\frac{\left[\mathrm{pMBL_4MASP1_{zym}}\right]}{K_{\mathrm{m\,MASP1^*}}^{\mathrm{MASP1_{zym}}}}+\frac{\left[\mathrm{pMBL_4MASP2_{zym}}\right]}{K_{\mathrm{m\,MASP1^*}}^{\mathrm{MASP2_{zym}}}}+\frac{\left[\mathrm{pMBL_4MASP3_{zym}}\right]}{K_{\mathrm{m\,MASP1^*}}^{\mathrm{MASP3_{zym}}}}+\frac{\left[\mathrm{pCLLKMASP1_{zym}}\right]}{K_{\mathrm{m\,MASP1^*}}^{\mathrm{MASP1_{zym}}}}\\
&+\frac{\left[\mathrm{pCLLKMASP2_{zym}}\right]}{K_{\mathrm{m\,MASP1^*}}^{\mathrm{MASP2_{zym}}}}+\frac{\left[\mathrm{pCLLKMASP3_{zym}}\right]}{K_{\mathrm{m\,MASP1^*}}^{\mathrm{MASP3_{zym}}}}+\frac{\left[\mathrm{pCLK1MASP1_{zym}}\right]}{K_{\mathrm{m\,MASP1^*}}^{\mathrm{MASP1_{zym}}}}+\frac{\left[\mathrm{pCLK1MASP2_{zym}}\right]}{K_{\mathrm{m\,MASP1^*}}^{\mathrm{MASP2_{zym}}}}\\
&+\frac{\left[\mathrm{pCLK1MASP3_{zym}}\right]}{K_{\mathrm{m\,MASP1^*}}^{\mathrm{MASP3_{zym}}}}+\frac{\left[\mathrm{pCLL1MASP1_{zym}}\right]}{K_{\mathrm{m\,MASP1^*}}^{\mathrm{MASP1_{zym}}}}+\frac{\left[\mathrm{pCLL1MASP2_{zym}}\right]}{K_{\mathrm{m\,MASP1^*}}^{\mathrm{MASP2_{zym}}}}+\frac{\left[\mathrm{pCLL1MASP3_{zym}}\right]}{K_{\mathrm{m\,MASP1^*}}^{\mathrm{MASP3_{zym}}}}\\
&+\frac{\left[\mathrm{pLFMASP1_{zym}}\right]}{K_{\mathrm{m\,MASP1^*}}^{\mathrm{MASP1_{zym}}}}+\frac{\left[\mathrm{pLFMASP2_{zym}}\right]}{K_{\mathrm{m\,MASP1^*}}^{\mathrm{MASP2_{zym}}}}+\frac{\left[\mathrm{pLFMASP3_{zym}}\right]}{K_{\mathrm{m\,MASP1^*}}^{\mathrm{MASP3_{zym}}}}+\frac{\left[\mathrm{pHFMASP1_{zym}}\right]}{K_{\mathrm{m\,MASP1^*}}^{\mathrm{MASP1_{zym}}}}+\frac{\left[\mathrm{pHFMASP2_{zym}}\right]}{K_{\mathrm{m\,MASP1^*}}^{\mathrm{MASP2_{zym}}}}\\
&+\frac{\left[\mathrm{pHFMASP3_{zym}}\right]}{K_{\mathrm{m\,MASP1^*}}^{\mathrm{MASP3_{zym}}}}+\frac{\left[\mathrm{pMFMASP1_{zym}}\right]}{K_{\mathrm{m\,MASP1^*}}^{\mathrm{MASP1_{zym}}}}+\frac{\left[\mathrm{pMFMASP2_{zym}}\right]}{K_{\mathrm{m\,MASP1^*}}^{\mathrm{MASP2_{zym}}}}+\frac{\left[\mathrm{pMFMASP3_{zym}}\right]}{K_{\mathrm{m\,MASP1^*}}^{\mathrm{MASP3_{zym}}}}\end{aligned}\right)}
$$

$$+\frac{k^{\mathrm{C2}}_{\mathrm{cat\,MASP1^*}}\left[\mathrm{pCLK1MASP1^*}\right]\left[\mathrm{fC4bC4bC2}\right]}{K^{\mathrm{C2}}_{\mathrm{m\,MASP1^*}}\left(\begin{array}{l}1+\frac{[\mathrm{C4}]}{K^{\mathrm{C4}}_{\mathrm{m\,MASP1^*}}}+\frac{[\mathrm{fC4bC2}]}{K^{\mathrm{C2}}_{\mathrm{m\,MASP1^*}}}+\frac{[\mathrm{fC4bC4bC2}]}{K^{\mathrm{C2}}_{\mathrm{m\,MASP1^*}}}+\frac{[\mathrm{fC3bC4bC2}]}{K^{\mathrm{C2}}_{\mathrm{m\,MASP1^*}}}+\frac{[\mathrm{pC4bC2}]}{K^{\mathrm{C2}}_{\mathrm{m\,MASP1^*}}}+\frac{[\mathrm{pC4bC4bC2}]}{K^{\mathrm{C2}}_{\mathrm{m\,MASP1^*}}}\\
+\frac{[\mathrm{pC3bC4bC2}]}{K^{\mathrm{C2}}_{\mathrm{m\,MASP1^*}}}+\frac{[\mathrm{pMBL_3MASP1_{zym}}]}{K^{\mathrm{MASP1_{zym}}}_{\mathrm{m\,MASP1^*}}}+\frac{[\mathrm{pMBL_3MASP2_{zym}}]}{K^{\mathrm{MASP2_{zym}}}_{\mathrm{m\,MASP1^*}}}+\frac{[\mathrm{pMBL_3MASP3_{zym}}]}{K^{\mathrm{MASP3_{zym}}}_{\mathrm{m\,MASP1^*}}}\\
+\frac{[\mathrm{pMBL_4MASP1_{zym}}]}{K^{\mathrm{MASP1_{zym}}}_{\mathrm{m\,MASP1^*}}}+\frac{[\mathrm{pMBL_4MASP2_{zym}}]}{K^{\mathrm{MASP2_{zym}}}_{\mathrm{m\,MASP1^*}}}+\frac{[\mathrm{pMBL_4MASP3_{zym}}]}{K^{\mathrm{MASP3_{zym}}}_{\mathrm{m\,MASP1^*}}}+\frac{[\mathrm{pCLLKMASP1_{zym}}]}{K^{\mathrm{MASP1_{zym}}}_{\mathrm{m\,MASP1^*}}}\\
+\frac{[\mathrm{pCLLKMASP2_{zym}}]}{K^{\mathrm{MASP2_{zym}}}_{\mathrm{m\,MASP1^*}}}+\frac{[\mathrm{pCLLKMASP3_{zym}}]}{K^{\mathrm{MASP3_{zym}}}_{\mathrm{m\,MASP1^*}}}+\frac{[\mathrm{pCLK1MASP1_{zym}}]}{K^{\mathrm{MASP1_{zym}}}_{\mathrm{m\,MASP1^*}}}+\frac{[\mathrm{pCLK1MASP2_{zym}}]}{K^{\mathrm{MASP2_{zym}}}_{\mathrm{m\,MASP1^*}}}\\
+\frac{[\mathrm{pCLK1MASP3_{zym}}]}{K^{\mathrm{MASP3_{zym}}}_{\mathrm{m\,MASP1^*}}}+\frac{[\mathrm{pCLL1MASP1_{zym}}]}{K^{\mathrm{MASP1_{zym}}}_{\mathrm{m\,MASP1^*}}}+\frac{[\mathrm{pCLL1MASP2_{zym}}]}{K^{\mathrm{MASP2_{zym}}}_{\mathrm{m\,MASP1^*}}}+\frac{[\mathrm{pCLL1MASP3_{zym}}]}{K^{\mathrm{MASP3_{zym}}}_{\mathrm{m\,MASP1^*}}}\\
+\frac{[\mathrm{pLFMASP1_{zym}}]}{K^{\mathrm{MASP1_{zym}}}_{\mathrm{m\,MASP1^*}}}+\frac{[\mathrm{pLFMASP2_{zym}}]}{K^{\mathrm{MASP2_{zym}}}_{\mathrm{m\,MASP1^*}}}+\frac{[\mathrm{pLFMASP3_{zym}}]}{K^{\mathrm{MASP3_{zym}}}_{\mathrm{m\,MASP1^*}}}+\frac{[\mathrm{pHFMASP1_{zym}}]}{K^{\mathrm{MASP1_{zym}}}_{\mathrm{m\,MASP1^*}}}+\frac{[\mathrm{pHFMASP2_{zym}}]}{K^{\mathrm{MASP2_{zym}}}_{\mathrm{m\,MASP1^*}}}\\
+\frac{[\mathrm{pHFMASP3_{zym}}]}{K^{\mathrm{MASP3_{zym}}}_{\mathrm{m\,MASP1^*}}}+\frac{[\mathrm{pMFMASP1_{zym}}]}{K^{\mathrm{MASP1_{zym}}}_{\mathrm{m\,MASP1^*}}}+\frac{[\mathrm{pMFMASP2_{zym}}]}{K^{\mathrm{MASP2_{zym}}}_{\mathrm{m\,MASP1^*}}}+\frac{[\mathrm{pMFMASP3_{zym}}]}{K^{\mathrm{MASP3_{zym}}}_{\mathrm{m\,MASP1^*}}}\end{array}\right)}$$

$$+\frac{k^{\mathrm{C2}}_{\mathrm{cat\,MASP1^*}}\left[\mathrm{pCLL1MASP1^*}\right]\left[\mathrm{fC4bC4bC2}\right]}{K^{\mathrm{C2}}_{\mathrm{m\,MASP1^*}}\left(\begin{array}{l}1+\frac{[\mathrm{C4}]}{K^{\mathrm{C4}}_{\mathrm{m\,MASP1^*}}}+\frac{[\mathrm{fC4bC2}]}{K^{\mathrm{C2}}_{\mathrm{m\,MASP1^*}}}+\frac{[\mathrm{fC4bC4bC2}]}{K^{\mathrm{C2}}_{\mathrm{m\,MASP1^*}}}+\frac{[\mathrm{fC3bC4bC2}]}{K^{\mathrm{C2}}_{\mathrm{m\,MASP1^*}}}+\frac{[\mathrm{pC4bC2}]}{K^{\mathrm{C2}}_{\mathrm{m\,MASP1^*}}}+\frac{[\mathrm{pC4bC4bC2}]}{K^{\mathrm{C2}}_{\mathrm{m\,MASP1^*}}}\\
+\frac{[\mathrm{pC3bC4bC2}]}{K^{\mathrm{C2}}_{\mathrm{m\,MASP1^*}}}+\frac{[\mathrm{pMBL_3MASP1_{zym}}]}{K^{\mathrm{MASP1_{zym}}}_{\mathrm{m\,MASP1^*}}}+\frac{[\mathrm{pMBL_3MASP2_{zym}}]}{K^{\mathrm{MASP2_{zym}}}_{\mathrm{m\,MASP1^*}}}+\frac{[\mathrm{pMBL_3MASP3_{zym}}]}{K^{\mathrm{MASP3_{zym}}}_{\mathrm{m\,MASP1^*}}}\\
+\frac{[\mathrm{pMBL_4MASP1_{zym}}]}{K^{\mathrm{MASP1_{zym}}}_{\mathrm{m\,MASP1^*}}}+\frac{[\mathrm{pMBL_4MASP2_{zym}}]}{K^{\mathrm{MASP2_{zym}}}_{\mathrm{m\,MASP1^*}}}+\frac{[\mathrm{pMBL_4MASP3_{zym}}]}{K^{\mathrm{MASP3_{zym}}}_{\mathrm{m\,MASP1^*}}}+\frac{[\mathrm{pCLLKMASP1_{zym}}]}{K^{\mathrm{MASP1_{zym}}}_{\mathrm{m\,MASP1^*}}}\\
+\frac{[\mathrm{pCLLKMASP2_{zym}}]}{K^{\mathrm{MASP2_{zym}}}_{\mathrm{m\,MASP1^*}}}+\frac{[\mathrm{pCLLKMASP3_{zym}}]}{K^{\mathrm{MASP3_{zym}}}_{\mathrm{m\,MASP1^*}}}+\frac{[\mathrm{pCLK1MASP1_{zym}}]}{K^{\mathrm{MASP1_{zym}}}_{\mathrm{m\,MASP1^*}}}+\frac{[\mathrm{pCLK1MASP2_{zym}}]}{K^{\mathrm{MASP2_{zym}}}_{\mathrm{m\,MASP1^*}}}\\
+\frac{[\mathrm{pCLK1MASP3_{zym}}]}{K^{\mathrm{MASP3_{zym}}}_{\mathrm{m\,MASP1^*}}}+\frac{[\mathrm{pCLL1MASP1_{zym}}]}{K^{\mathrm{MASP1_{zym}}}_{\mathrm{m\,MASP1^*}}}+\frac{[\mathrm{pCLL1MASP2_{zym}}]}{K^{\mathrm{MASP2_{zym}}}_{\mathrm{m\,MASP1^*}}}+\frac{[\mathrm{pCLL1MASP3_{zym}}]}{K^{\mathrm{MASP3_{zym}}}_{\mathrm{m\,MASP1^*}}}\\
+\frac{[\mathrm{pLFMASP1_{zym}}]}{K^{\mathrm{MASP1_{zym}}}_{\mathrm{m\,MASP1^*}}}+\frac{[\mathrm{pLFMASP2_{zym}}]}{K^{\mathrm{MASP2_{zym}}}_{\mathrm{m\,MASP1^*}}}+\frac{[\mathrm{pLFMASP3_{zym}}]}{K^{\mathrm{MASP3_{zym}}}_{\mathrm{m\,MASP1^*}}}+\frac{[\mathrm{pHFMASP1_{zym}}]}{K^{\mathrm{MASP1_{zym}}}_{\mathrm{m\,MASP1^*}}}+\frac{[\mathrm{pHFMASP2_{zym}}]}{K^{\mathrm{MASP2_{zym}}}_{\mathrm{m\,MASP1^*}}}\\
+\frac{[\mathrm{pHFMASP3_{zym}}]}{K^{\mathrm{MASP3_{zym}}}_{\mathrm{m\,MASP1^*}}}+\frac{[\mathrm{pMFMASP1_{zym}}]}{K^{\mathrm{MASP1_{zym}}}_{\mathrm{m\,MASP1^*}}}+\frac{[\mathrm{pMFMASP2_{zym}}]}{K^{\mathrm{MASP2_{zym}}}_{\mathrm{m\,MASP1^*}}}+\frac{[\mathrm{pMFMASP3_{zym}}]}{K^{\mathrm{MASP3_{zym}}}_{\mathrm{m\,MASP1^*}}}\end{array}\right)}$$

$$
+\frac{k_{\mathrm{cat\,MASP1^*}}^{\mathrm{C2}}\left[\mathrm{pLFMASP1^*}\right]\left[\mathrm{fC4bC4bC2}\right]}{K_{\mathrm{m\,MASP1^*}}^{\mathrm{C2}}\left(\begin{array}{l}
1+\frac{[\mathrm{C4}]}{K_{\mathrm{m\,MASP1^*}}^{\mathrm{C4}}}+\frac{[\mathrm{fC4bC2}]}{K_{\mathrm{m\,MASP1^*}}^{\mathrm{C2}}}+\frac{[\mathrm{fC4bC4bC2}]}{K_{\mathrm{m\,MASP1^*}}^{\mathrm{C2}}}+\frac{[\mathrm{fC3bC4bC2}]}{K_{\mathrm{m\,MASP1^*}}^{\mathrm{C2}}}+\frac{[\mathrm{pC4bC2}]}{K_{\mathrm{m\,MASP1^*}}^{\mathrm{C2}}}+\frac{[\mathrm{pC4bC4bC2}]}{K_{\mathrm{m\,MASP1^*}}^{\mathrm{C2}}}\\
+\frac{[\mathrm{pC3bC4bC2}]}{K_{\mathrm{m\,MASP1^*}}^{\mathrm{C2}}}+\frac{\left[\mathrm{pMBL_3MASP1_{zym}}\right]}{K_{\mathrm{m\,MASP1^*}}^{\mathrm{MASP1_{zym}}}}+\frac{\left[\mathrm{pMBL_3MASP2_{zym}}\right]}{K_{\mathrm{m\,MASP1^*}}^{\mathrm{MASP2_{zym}}}}+\frac{\left[\mathrm{pMBL_3MASP3_{zym}}\right]}{K_{\mathrm{m\,MASP1^*}}^{\mathrm{MASP3_{zym}}}}\\
+\frac{\left[\mathrm{pMBL_4MASP1_{zym}}\right]}{K_{\mathrm{m\,MASP1^*}}^{\mathrm{MASP1_{zym}}}}+\frac{\left[\mathrm{pMBL_4MASP2_{zym}}\right]}{K_{\mathrm{m\,MASP1^*}}^{\mathrm{MASP2_{zym}}}}+\frac{\left[\mathrm{pMBL_4MASP3_{zym}}\right]}{K_{\mathrm{m\,MASP1^*}}^{\mathrm{MASP3_{zym}}}}+\frac{\left[\mathrm{pCLLKMASP1_{zym}}\right]}{K_{\mathrm{m\,MASP1^*}}^{\mathrm{MASP1_{zym}}}}\\
+\frac{\left[\mathrm{pCLLKMASP2_{zym}}\right]}{K_{\mathrm{m\,MASP1^*}}^{\mathrm{MASP2_{zym}}}}+\frac{\left[\mathrm{pCLLKMASP3_{zym}}\right]}{K_{\mathrm{m\,MASP1^*}}^{\mathrm{MASP3_{zym}}}}+\frac{\left[\mathrm{pCLK1MASP1_{zym}}\right]}{K_{\mathrm{m\,MASP1^*}}^{\mathrm{MASP1_{zym}}}}+\frac{\left[\mathrm{pCLK1MASP2_{zym}}\right]}{K_{\mathrm{m\,MASP1^*}}^{\mathrm{MASP2_{zym}}}}\\
+\frac{\left[\mathrm{pCLK1MASP3_{zym}}\right]}{K_{\mathrm{m\,MASP1^*}}^{\mathrm{MASP3_{zym}}}}+\frac{\left[\mathrm{pCLL1MASP1_{zym}}\right]}{K_{\mathrm{m\,MASP1^*}}^{\mathrm{MASP1_{zym}}}}+\frac{\left[\mathrm{pCLL1MASP2_{zym}}\right]}{K_{\mathrm{m\,MASP1^*}}^{\mathrm{MASP2_{zym}}}}+\frac{\left[\mathrm{pCLL1MASP3_{zym}}\right]}{K_{\mathrm{m\,MASP1^*}}^{\mathrm{MASP3_{zym}}}}\\
+\frac{\left[\mathrm{pLFMASP1_{zym}}\right]}{K_{\mathrm{m\,MASP1^*}}^{\mathrm{MASP1_{zym}}}}+\frac{\left[\mathrm{pLFMASP2_{zym}}\right]}{K_{\mathrm{m\,MASP1^*}}^{\mathrm{MASP2_{zym}}}}+\frac{\left[\mathrm{pLFMASP3_{zym}}\right]}{K_{\mathrm{m\,MASP1^*}}^{\mathrm{MASP3_{zym}}}}+\frac{\left[\mathrm{pHFMASP1_{zym}}\right]}{K_{\mathrm{m\,MASP1^*}}^{\mathrm{MASP1_{zym}}}}+\frac{\left[\mathrm{pHFMASP2_{zym}}\right]}{K_{\mathrm{m\,MASP1^*}}^{\mathrm{MASP2_{zym}}}}\\
+\frac{\left[\mathrm{pHFMASP3_{zym}}\right]}{K_{\mathrm{m\,MASP1^*}}^{\mathrm{MASP3_{zym}}}}+\frac{\left[\mathrm{pMFMASP1_{zym}}\right]}{K_{\mathrm{m\,MASP1^*}}^{\mathrm{MASP1_{zym}}}}+\frac{\left[\mathrm{pMFMASP2_{zym}}\right]}{K_{\mathrm{m\,MASP1^*}}^{\mathrm{MASP2_{zym}}}}+\frac{\left[\mathrm{pMFMASP3_{zym}}\right]}{K_{\mathrm{m\,MASP1^*}}^{\mathrm{MASP3_{zym}}}}
\end{array}\right)}
$$

$$
+\frac{k_{\mathrm{cat\,MASP1^*}}^{\mathrm{C2}}\left[\mathrm{pHFMASP1^*}\right]\left[\mathrm{fC4bC4bC2}\right]}{K_{\mathrm{m\,MASP1^*}}^{\mathrm{C2}}\left(\begin{array}{l}
1+\frac{[\mathrm{C4}]}{K_{\mathrm{m\,MASP1^*}}^{\mathrm{C4}}}+\frac{[\mathrm{fC4bC2}]}{K_{\mathrm{m\,MASP1^*}}^{\mathrm{C2}}}+\frac{[\mathrm{fC4bC4bC2}]}{K_{\mathrm{m\,MASP1^*}}^{\mathrm{C2}}}+\frac{[\mathrm{fC3bC4bC2}]}{K_{\mathrm{m\,MASP1^*}}^{\mathrm{C2}}}+\frac{[\mathrm{pC4bC2}]}{K_{\mathrm{m\,MASP1^*}}^{\mathrm{C2}}}+\frac{[\mathrm{pC4bC4bC2}]}{K_{\mathrm{m\,MASP1^*}}^{\mathrm{C2}}}\\
+\frac{[\mathrm{pC3bC4bC2}]}{K_{\mathrm{m\,MASP1^*}}^{\mathrm{C2}}}+\frac{\left[\mathrm{pMBL_3MASP1_{zym}}\right]}{K_{\mathrm{m\,MASP1^*}}^{\mathrm{MASP1_{zym}}}}+\frac{\left[\mathrm{pMBL_3MASP2_{zym}}\right]}{K_{\mathrm{m\,MASP1^*}}^{\mathrm{MASP2_{zym}}}}+\frac{\left[\mathrm{pMBL_3MASP3_{zym}}\right]}{K_{\mathrm{m\,MASP1^*}}^{\mathrm{MASP3_{zym}}}}\\
+\frac{\left[\mathrm{pMBL_4MASP1_{zym}}\right]}{K_{\mathrm{m\,MASP1^*}}^{\mathrm{MASP1_{zym}}}}+\frac{\left[\mathrm{pMBL_4MASP2_{zym}}\right]}{K_{\mathrm{m\,MASP1^*}}^{\mathrm{MASP2_{zym}}}}+\frac{\left[\mathrm{pMBL_4MASP3_{zym}}\right]}{K_{\mathrm{m\,MASP1^*}}^{\mathrm{MASP3_{zym}}}}+\frac{\left[\mathrm{pCLLKMASP1_{zym}}\right]}{K_{\mathrm{m\,MASP1^*}}^{\mathrm{MASP1_{zym}}}}\\
+\frac{\left[\mathrm{pCLLKMASP2_{zym}}\right]}{K_{\mathrm{m\,MASP1^*}}^{\mathrm{MASP2_{zym}}}}+\frac{\left[\mathrm{pCLLKMASP3_{zym}}\right]}{K_{\mathrm{m\,MASP1^*}}^{\mathrm{MASP3_{zym}}}}+\frac{\left[\mathrm{pCLK1MASP1_{zym}}\right]}{K_{\mathrm{m\,MASP1^*}}^{\mathrm{MASP1_{zym}}}}+\frac{\left[\mathrm{pCLK1MASP2_{zym}}\right]}{K_{\mathrm{m\,MASP1^*}}^{\mathrm{MASP2_{zym}}}}\\
+\frac{\left[\mathrm{pCLK1MASP3_{zym}}\right]}{K_{\mathrm{m\,MASP1^*}}^{\mathrm{MASP3_{zym}}}}+\frac{\left[\mathrm{pCLL1MASP1_{zym}}\right]}{K_{\mathrm{m\,MASP1^*}}^{\mathrm{MASP1_{zym}}}}+\frac{\left[\mathrm{pCLL1MASP2_{zym}}\right]}{K_{\mathrm{m\,MASP1^*}}^{\mathrm{MASP2_{zym}}}}+\frac{\left[\mathrm{pCLL1MASP3_{zym}}\right]}{K_{\mathrm{m\,MASP1^*}}^{\mathrm{MASP3_{zym}}}}\\
+\frac{\left[\mathrm{pLFMASP1_{zym}}\right]}{K_{\mathrm{m\,MASP1^*}}^{\mathrm{MASP1_{zym}}}}+\frac{\left[\mathrm{pLFMASP2_{zym}}\right]}{K_{\mathrm{m\,MASP1^*}}^{\mathrm{MASP2_{zym}}}}+\frac{\left[\mathrm{pLFMASP3_{zym}}\right]}{K_{\mathrm{m\,MASP1^*}}^{\mathrm{MASP3_{zym}}}}+\frac{\left[\mathrm{pHFMASP1_{zym}}\right]}{K_{\mathrm{m\,MASP1^*}}^{\mathrm{MASP1_{zym}}}}+\frac{\left[\mathrm{pHFMASP2_{zym}}\right]}{K_{\mathrm{m\,MASP1^*}}^{\mathrm{MASP2_{zym}}}}\\
+\frac{\left[\mathrm{pHFMASP3_{zym}}\right]}{K_{\mathrm{m\,MASP1^*}}^{\mathrm{MASP3_{zym}}}}+\frac{\left[\mathrm{pMFMASP1_{zym}}\right]}{K_{\mathrm{m\,MASP1^*}}^{\mathrm{MASP1_{zym}}}}+\frac{\left[\mathrm{pMFMASP2_{zym}}\right]}{K_{\mathrm{m\,MASP1^*}}^{\mathrm{MASP2_{zym}}}}+\frac{\left[\mathrm{pMFMASP3_{zym}}\right]}{K_{\mathrm{m\,MASP1^*}}^{\mathrm{MASP3_{zym}}}}
\end{array}\right)}
$$

$$+\frac{k_{\mathrm{cat\ MASP1^*}}^{\mathrm{C2}}\left[\mathrm{pMFMASP1^*}\right]\left[\mathrm{fC4bC4bC2}\right]}{K_{\mathrm{m\ MASP1^*}}^{\mathrm{C2}}\left(\begin{aligned}&1+\frac{[\mathrm{C4}]}{K_{\mathrm{m\ MASP1^*}}^{\mathrm{C4}}}+\frac{[\mathrm{fC4bC2}]}{K_{\mathrm{m\ MASP1^*}}^{\mathrm{C2}}}+\frac{[\mathrm{fC4bC4bC2}]}{K_{\mathrm{m\ MASP1^*}}^{\mathrm{C2}}}+\frac{[\mathrm{fC3bC4bC2}]}{K_{\mathrm{m\ MASP1^*}}^{\mathrm{C2}}}+\frac{[\mathrm{pC4bC2}]}{K_{\mathrm{m\ MASP1^*}}^{\mathrm{C2}}}+\frac{[\mathrm{pC4bC4bC2}]}{K_{\mathrm{m\ MASP1^*}}^{\mathrm{C2}}}\\&+\frac{[\mathrm{pC3bC4bC2}]}{K_{\mathrm{m\ MASP1^*}}^{\mathrm{C2}}}+\frac{[\mathrm{pMBL_3MASP1_{zym}}]}{K_{\mathrm{m\ MASP1^*}}^{\mathrm{MASP1_{zym}}}}+\frac{[\mathrm{pMBL_3MASP2_{zym}}]}{K_{\mathrm{m\ MASP1^*}}^{\mathrm{MASP2_{zym}}}}+\frac{[\mathrm{pMBL_3MASP3_{zym}}]}{K_{\mathrm{m\ MASP1^*}}^{\mathrm{MASP3_{zym}}}}\\&+\frac{[\mathrm{pMBL_4MASP1_{zym}}]}{K_{\mathrm{m\ MASP1^*}}^{\mathrm{MASP1_{zym}}}}+\frac{[\mathrm{pMBL_4MASP2_{zym}}]}{K_{\mathrm{m\ MASP1^*}}^{\mathrm{MASP2_{zym}}}}+\frac{[\mathrm{pMBL_4MASP3_{zym}}]}{K_{\mathrm{m\ MASP1^*}}^{\mathrm{MASP3_{zym}}}}+\frac{[\mathrm{pCLLKMASP1_{zym}}]}{K_{\mathrm{m\ MASP1^*}}^{\mathrm{MASP1_{zym}}}}\\&+\frac{[\mathrm{pCLLKMASP2_{zym}}]}{K_{\mathrm{m\ MASP1^*}}^{\mathrm{MASP2_{zym}}}}+\frac{[\mathrm{pCLLKMASP3_{zym}}]}{K_{\mathrm{m\ MASP1^*}}^{\mathrm{MASP3_{zym}}}}+\frac{[\mathrm{pCLK1MASP1_{zym}}]}{K_{\mathrm{m\ MASP1^*}}^{\mathrm{MASP1_{zym}}}}+\frac{[\mathrm{pCLK1MASP2_{zym}}]}{K_{\mathrm{m\ MASP1^*}}^{\mathrm{MASP2_{zym}}}}\\&+\frac{[\mathrm{pCLK1MASP3_{zym}}]}{K_{\mathrm{m\ MASP1^*}}^{\mathrm{MASP3_{zym}}}}+\frac{[\mathrm{pCLL1MASP1_{zym}}]}{K_{\mathrm{m\ MASP1^*}}^{\mathrm{MASP1_{zym}}}}+\frac{[\mathrm{pCLL1MASP2_{zym}}]}{K_{\mathrm{m\ MASP1^*}}^{\mathrm{MASP2_{zym}}}}+\frac{[\mathrm{pCLL1MASP3_{zym}}]}{K_{\mathrm{m\ MASP1^*}}^{\mathrm{MASP3_{zym}}}}\\&+\frac{[\mathrm{pLFMASP1_{zym}}]}{K_{\mathrm{m\ MASP1^*}}^{\mathrm{MASP1_{zym}}}}+\frac{[\mathrm{pLFMASP2_{zym}}]}{K_{\mathrm{m\ MASP1^*}}^{\mathrm{MASP2_{zym}}}}+\frac{[\mathrm{pLFMASP3_{zym}}]}{K_{\mathrm{m\ MASP1^*}}^{\mathrm{MASP3_{zym}}}}+\frac{[\mathrm{pHFMASP1_{zym}}]}{K_{\mathrm{m\ MASP1^*}}^{\mathrm{MASP1_{zym}}}}+\frac{[\mathrm{pHFMASP2_{zym}}]}{K_{\mathrm{m\ MASP1^*}}^{\mathrm{MASP2_{zym}}}}\\&+\frac{[\mathrm{pHFMASP3_{zym}}]}{K_{\mathrm{m\ MASP1^*}}^{\mathrm{MASP3_{zym}}}}+\frac{[\mathrm{pMFMASP1_{zym}}]}{K_{\mathrm{m\ MASP1^*}}^{\mathrm{MASP1_{zym}}}}+\frac{[\mathrm{pMFMASP2_{zym}}]}{K_{\mathrm{m\ MASP1^*}}^{\mathrm{MASP2_{zym}}}}+\frac{[\mathrm{pMFMASP3_{zym}}]}{K_{\mathrm{m\ MASP1^*}}^{\mathrm{MASP3_{zym}}}}\end{aligned}\right)}$$

$$+\frac{k_{\mathrm{cat\ MASP2^*}}^{\mathrm{C2}}\left[\mathrm{pMBL_3MASP2^*}\right]\left[\mathrm{fC4bC4bC2}\right]}{K_{\mathrm{m\ MASP2^*}}^{\mathrm{C2}}\left(\begin{aligned}&1+\frac{[\mathrm{C4}]}{K_{\mathrm{m\ MASP2^*}}^{\mathrm{C4}}}+\frac{[\mathrm{fC4bC2}]}{K_{\mathrm{m\ MASP2^*}}^{\mathrm{C2}}}+\frac{[\mathrm{fC4bC4bC2}]}{K_{\mathrm{m\ MASP2^*}}^{\mathrm{C2}}}+\frac{[\mathrm{fC3bC4bC2}]}{K_{\mathrm{m\ MASP2^*}}^{\mathrm{C2}}}+\frac{[\mathrm{pC4bC2}]}{K_{\mathrm{m\ MASP2^*}}^{\mathrm{C2}}}+\frac{[\mathrm{pC4bC4bC2}]}{K_{\mathrm{m\ MASP2^*}}^{\mathrm{C2}}}\\&+\frac{[\mathrm{pC3bC4bC2}]}{K_{\mathrm{m\ MASP2^*}}^{\mathrm{C2}}}+\frac{[\mathrm{pMBL_3MASP1_{zym}}]}{K_{\mathrm{m\ MASP2^*}}^{\mathrm{MASP1_{zym}}}}+\frac{[\mathrm{pMBL_3MASP2_{zym}}]}{K_{\mathrm{m\ MASP2^*}}^{\mathrm{MASP2_{zym}}}}+\frac{[\mathrm{pMBL_4MASP1_{zym}}]}{K_{\mathrm{m\ MASP2^*}}^{\mathrm{MASP1_{zym}}}}\\&+\frac{[\mathrm{pMBL_4MASP2_{zym}}]}{K_{\mathrm{m\ MASP2^*}}^{\mathrm{MASP2_{zym}}}}+\frac{[\mathrm{pCLLKMASP1_{zym}}]}{K_{\mathrm{m\ MASP2^*}}^{\mathrm{MASP1_{zym}}}}+\frac{[\mathrm{pCLLKMASP2_{zym}}]}{K_{\mathrm{m\ MASP2^*}}^{\mathrm{MASP2_{zym}}}}+\frac{[\mathrm{pCLK1MASP1_{zym}}]}{K_{\mathrm{m\ MASP2^*}}^{\mathrm{MASP1_{zym}}}}\\&+\frac{[\mathrm{pCLK1MASP2_{zym}}]}{K_{\mathrm{m\ MASP2^*}}^{\mathrm{MASP2_{zym}}}}+\frac{[\mathrm{pCLL1MASP1_{zym}}]}{K_{\mathrm{m\ MASP2^*}}^{\mathrm{MASP1_{zym}}}}+\frac{[\mathrm{pCLL1MASP2_{zym}}]}{K_{\mathrm{m\ MASP2^*}}^{\mathrm{MASP2_{zym}}}}+\frac{[\mathrm{pLFMASP1_{zym}}]}{K_{\mathrm{m\ MASP2^*}}^{\mathrm{MASP1_{zym}}}}\\&+\frac{[\mathrm{pLFMASP2_{zym}}]}{K_{\mathrm{m\ MASP2^*}}^{\mathrm{MASP2_{zym}}}}+\frac{[\mathrm{pHFMASP1_{zym}}]}{K_{\mathrm{m\ MASP2^*}}^{\mathrm{MASP1_{zym}}}}+\frac{[\mathrm{pHFMASP2_{zym}}]}{K_{\mathrm{m\ MASP2^*}}^{\mathrm{MASP2_{zym}}}}+\frac{[\mathrm{pMFMASP1_{zym}}]}{K_{\mathrm{m\ MASP2^*}}^{\mathrm{MASP1_{zym}}}}+\frac{[\mathrm{pMFMASP2_{zym}}]}{K_{\mathrm{m\ MASP2^*}}^{\mathrm{MASP2_{zym}}}}\end{aligned}\right)}$$

$$
+\frac{k_{\mathrm{cat\,MASP2^*}}^{\mathrm{C2}}\left[\mathrm{pMBL_4MASP2^*}\right]\left[\mathrm{fC4bC4bC2}\right]}{K_{\mathrm{m\,MASP2^*}}^{\mathrm{C2}}\left(\begin{aligned}&1+\frac{[\mathrm{C4}]}{K_{\mathrm{m\,MASP2^*}}^{\mathrm{C4}}}+\frac{[\mathrm{fC4bC2}]}{K_{\mathrm{m\,MASP2^*}}^{\mathrm{C2}}}+\frac{[\mathrm{fC4bC4bC2}]}{K_{\mathrm{m\,MASP2^*}}^{\mathrm{C2}}}+\frac{[\mathrm{fC3bC4bC2}]}{K_{\mathrm{m\,MASP2^*}}^{\mathrm{C2}}}+\frac{[\mathrm{pC4bC2}]}{K_{\mathrm{m\,MASP2^*}}^{\mathrm{C2}}}+\frac{[\mathrm{pC4bC4bC2}]}{K_{\mathrm{m\,MASP2^*}}^{\mathrm{C2}}}\\&+\frac{[\mathrm{pC3bC4bC2}]}{K_{\mathrm{m\,MASP2^*}}^{\mathrm{C2}}}+\frac{[\mathrm{pMBL_3MASP1_{zym}}]}{K_{\mathrm{m\,MASP2^*}}^{\mathrm{MASP1_{zym}}}}+\frac{[\mathrm{pMBL_3MASP2_{zym}}]}{K_{\mathrm{m\,MASP2^*}}^{\mathrm{MASP2_{zym}}}}+\frac{[\mathrm{pMBL_4MASP1_{zym}}]}{K_{\mathrm{m\,MASP2^*}}^{\mathrm{MASP1_{zym}}}}\\&+\frac{[\mathrm{pMBL_4MASP2_{zym}}]}{K_{\mathrm{m\,MASP2^*}}^{\mathrm{MASP2_{zym}}}}+\frac{[\mathrm{pCLLKMASP1_{zym}}]}{K_{\mathrm{m\,MASP2^*}}^{\mathrm{MASP1_{zym}}}}+\frac{[\mathrm{pCLLKMASP2_{zym}}]}{K_{\mathrm{m\,MASP2^*}}^{\mathrm{MASP2_{zym}}}}+\frac{[\mathrm{pCLK1MASP1_{zym}}]}{K_{\mathrm{m\,MASP2^*}}^{\mathrm{MASP1_{zym}}}}\\&+\frac{[\mathrm{pCLK1MASP2_{zym}}]}{K_{\mathrm{m\,MASP2^*}}^{\mathrm{MASP2_{zym}}}}+\frac{[\mathrm{pCLL1MASP1_{zym}}]}{K_{\mathrm{m\,MASP2^*}}^{\mathrm{MASP1_{zym}}}}+\frac{[\mathrm{pCLL1MASP2_{zym}}]}{K_{\mathrm{m\,MASP2^*}}^{\mathrm{MASP2_{zym}}}}+\frac{[\mathrm{pLFMASP1_{zym}}]}{K_{\mathrm{m\,MASP2^*}}^{\mathrm{MASP1_{zym}}}}\\&+\frac{[\mathrm{pLFMASP2_{zym}}]}{K_{\mathrm{m\,MASP2^*}}^{\mathrm{MASP2_{zym}}}}+\frac{[\mathrm{pHFMASP1_{zym}}]}{K_{\mathrm{m\,MASP2^*}}^{\mathrm{MASP1_{zym}}}}+\frac{[\mathrm{pHFMASP2_{zym}}]}{K_{\mathrm{m\,MASP2^*}}^{\mathrm{MASP2_{zym}}}}+\frac{[\mathrm{pMFMASP1_{zym}}]}{K_{\mathrm{m\,MASP2^*}}^{\mathrm{MASP1_{zym}}}}+\frac{[\mathrm{pMFMASP2_{zym}}]}{K_{\mathrm{m\,MASP2^*}}^{\mathrm{MASP2_{zym}}}}\end{aligned}\right)}
$$

$$
+\frac{k_{\mathrm{cat\,MASP2^*}}^{\mathrm{C2}}\left[\mathrm{pCLLKMASP2^*}\right]\left[\mathrm{fC4bC4bC2}\right]}{K_{\mathrm{m\,MASP2^*}}^{\mathrm{C2}}\left(\begin{aligned}&1+\frac{[\mathrm{C4}]}{K_{\mathrm{m\,MASP2^*}}^{\mathrm{C4}}}+\frac{[\mathrm{fC4bC2}]}{K_{\mathrm{m\,MASP2^*}}^{\mathrm{C2}}}+\frac{[\mathrm{fC4bC4bC2}]}{K_{\mathrm{m\,MASP2^*}}^{\mathrm{C2}}}+\frac{[\mathrm{fC3bC4bC2}]}{K_{\mathrm{m\,MASP2^*}}^{\mathrm{C2}}}+\frac{[\mathrm{pC4bC2}]}{K_{\mathrm{m\,MASP2^*}}^{\mathrm{C2}}}+\frac{[\mathrm{pC4bC4bC2}]}{K_{\mathrm{m\,MASP2^*}}^{\mathrm{C2}}}\\&+\frac{[\mathrm{pC3bC4bC2}]}{K_{\mathrm{m\,MASP2^*}}^{\mathrm{C2}}}+\frac{[\mathrm{pMBL_3MASP1_{zym}}]}{K_{\mathrm{m\,MASP2^*}}^{\mathrm{MASP1_{zym}}}}+\frac{[\mathrm{pMBL_3MASP2_{zym}}]}{K_{\mathrm{m\,MASP2^*}}^{\mathrm{MASP2_{zym}}}}+\frac{[\mathrm{pMBL_4MASP1_{zym}}]}{K_{\mathrm{m\,MASP2^*}}^{\mathrm{MASP1_{zym}}}}\\&+\frac{[\mathrm{pMBL_4MASP2_{zym}}]}{K_{\mathrm{m\,MASP2^*}}^{\mathrm{MASP2_{zym}}}}+\frac{[\mathrm{pCLLKMASP1_{zym}}]}{K_{\mathrm{m\,MASP2^*}}^{\mathrm{MASP1_{zym}}}}+\frac{[\mathrm{pCLLKMASP2_{zym}}]}{K_{\mathrm{m\,MASP2^*}}^{\mathrm{MASP2_{zym}}}}+\frac{[\mathrm{pCLK1MASP1_{zym}}]}{K_{\mathrm{m\,MASP2^*}}^{\mathrm{MASP1_{zym}}}}\\&+\frac{[\mathrm{pCLK1MASP2_{zym}}]}{K_{\mathrm{m\,MASP2^*}}^{\mathrm{MASP2_{zym}}}}+\frac{[\mathrm{pCLL1MASP1_{zym}}]}{K_{\mathrm{m\,MASP2^*}}^{\mathrm{MASP1_{zym}}}}+\frac{[\mathrm{pCLL1MASP2_{zym}}]}{K_{\mathrm{m\,MASP2^*}}^{\mathrm{MASP2_{zym}}}}+\frac{[\mathrm{pLFMASP1_{zym}}]}{K_{\mathrm{m\,MASP2^*}}^{\mathrm{MASP1_{zym}}}}\\&+\frac{[\mathrm{pLFMASP2_{zym}}]}{K_{\mathrm{m\,MASP2^*}}^{\mathrm{MASP2_{zym}}}}+\frac{[\mathrm{pHFMASP1_{zym}}]}{K_{\mathrm{m\,MASP2^*}}^{\mathrm{MASP1_{zym}}}}+\frac{[\mathrm{pHFMASP2_{zym}}]}{K_{\mathrm{m\,MASP2^*}}^{\mathrm{MASP2_{zym}}}}+\frac{[\mathrm{pMFMASP1_{zym}}]}{K_{\mathrm{m\,MASP2^*}}^{\mathrm{MASP1_{zym}}}}+\frac{[\mathrm{pMFMASP2_{zym}}]}{K_{\mathrm{m\,MASP2^*}}^{\mathrm{MASP2_{zym}}}}\end{aligned}\right)}
$$

$$
+\frac{k_{\mathrm{cat\,MASP2^*}}^{\mathrm{C2}}\left[\mathrm{pCLK1MASP2^*}\right]\left[\mathrm{fC4bC4bC2}\right]}{K_{\mathrm{m\,MASP2^*}}^{\mathrm{C2}}\left(\begin{aligned}&1+\frac{[\mathrm{C4}]}{K_{\mathrm{m\,MASP2^*}}^{\mathrm{C4}}}+\frac{[\mathrm{fC4bC2}]}{K_{\mathrm{m\,MASP2^*}}^{\mathrm{C2}}}+\frac{[\mathrm{fC4bC4bC2}]}{K_{\mathrm{m\,MASP2^*}}^{\mathrm{C2}}}+\frac{[\mathrm{fC3bC4bC2}]}{K_{\mathrm{m\,MASP2^*}}^{\mathrm{C2}}}+\frac{[\mathrm{pC4bC2}]}{K_{\mathrm{m\,MASP2^*}}^{\mathrm{C2}}}+\frac{[\mathrm{pC4bC4bC2}]}{K_{\mathrm{m\,MASP2^*}}^{\mathrm{C2}}}\\&+\frac{[\mathrm{pC3bC4bC2}]}{K_{\mathrm{m\,MASP2^*}}^{\mathrm{C2}}}+\frac{[\mathrm{pMBL_3MASP1_{zym}}]}{K_{\mathrm{m\,MASP2^*}}^{\mathrm{MASP1_{zym}}}}+\frac{[\mathrm{pMBL_3MASP2_{zym}}]}{K_{\mathrm{m\,MASP2^*}}^{\mathrm{MASP2_{zym}}}}+\frac{[\mathrm{pMBL_4MASP1_{zym}}]}{K_{\mathrm{m\,MASP2^*}}^{\mathrm{MASP1_{zym}}}}\\&+\frac{[\mathrm{pMBL_4MASP2_{zym}}]}{K_{\mathrm{m\,MASP2^*}}^{\mathrm{MASP2_{zym}}}}+\frac{[\mathrm{pCLLKMASP1_{zym}}]}{K_{\mathrm{m\,MASP2^*}}^{\mathrm{MASP1_{zym}}}}+\frac{[\mathrm{pCLLKMASP2_{zym}}]}{K_{\mathrm{m\,MASP2^*}}^{\mathrm{MASP2_{zym}}}}+\frac{[\mathrm{pCLK1MASP1_{zym}}]}{K_{\mathrm{m\,MASP2^*}}^{\mathrm{MASP1_{zym}}}}\\&+\frac{[\mathrm{pCLK1MASP2_{zym}}]}{K_{\mathrm{m\,MASP2^*}}^{\mathrm{MASP2_{zym}}}}+\frac{[\mathrm{pCLL1MASP1_{zym}}]}{K_{\mathrm{m\,MASP2^*}}^{\mathrm{MASP1_{zym}}}}+\frac{[\mathrm{pCLL1MASP2_{zym}}]}{K_{\mathrm{m\,MASP2^*}}^{\mathrm{MASP2_{zym}}}}+\frac{[\mathrm{pLFMASP1_{zym}}]}{K_{\mathrm{m\,MASP2^*}}^{\mathrm{MASP1_{zym}}}}\\&+\frac{[\mathrm{pLFMASP2_{zym}}]}{K_{\mathrm{m\,MASP2^*}}^{\mathrm{MASP2_{zym}}}}+\frac{[\mathrm{pHFMASP1_{zym}}]}{K_{\mathrm{m\,MASP2^*}}^{\mathrm{MASP1_{zym}}}}+\frac{[\mathrm{pHFMASP2_{zym}}]}{K_{\mathrm{m\,MASP2^*}}^{\mathrm{MASP2_{zym}}}}+\frac{[\mathrm{pMFMASP1_{zym}}]}{K_{\mathrm{m\,MASP2^*}}^{\mathrm{MASP1_{zym}}}}+\frac{[\mathrm{pMFMASP2_{zym}}]}{K_{\mathrm{m\,MASP2^*}}^{\mathrm{MASP2_{zym}}}}\end{aligned}\right)}
$$

$$+\frac{k^{\mathrm{C2}}_{\mathrm{cat\,MASP2^*}}\left[\mathrm{pCLL1MASP2^*}\right]\left[\mathrm{fC4bC4bC2}\right]}{K^{\mathrm{C2}}_{\mathrm{m\,MASP2^*}}\left(\begin{aligned}&1+\frac{[\mathrm{C4}]}{K^{\mathrm{C4}}_{\mathrm{m\,MASP2^*}}}+\frac{[\mathrm{fC4bC2}]}{K^{\mathrm{C2}}_{\mathrm{m\,MASP2^*}}}+\frac{[\mathrm{fC4bC4bC2}]}{K^{\mathrm{C2}}_{\mathrm{m\,MASP2^*}}}+\frac{[\mathrm{fC3bC4bC2}]}{K^{\mathrm{C2}}_{\mathrm{m\,MASP2^*}}}+\frac{[\mathrm{pC4bC2}]}{K^{\mathrm{C2}}_{\mathrm{m\,MASP2^*}}}+\frac{[\mathrm{pC4bC4bC2}]}{K^{\mathrm{C2}}_{\mathrm{m\,MASP2^*}}}\\&+\frac{[\mathrm{pC3bC4bC2}]}{K^{\mathrm{C2}}_{\mathrm{m\,MASP2^*}}}+\frac{[\mathrm{pMBL_3MASP1_{zym}}]}{K^{\mathrm{MASP1_{zym}}}_{\mathrm{m\,MASP2^*}}}+\frac{[\mathrm{pMBL_3MASP2_{zym}}]}{K^{\mathrm{MASP2_{zym}}}_{\mathrm{m\,MASP2^*}}}+\frac{[\mathrm{pMBL_4MASP1_{zym}}]}{K^{\mathrm{MASP1_{zym}}}_{\mathrm{m\,MASP2^*}}}\\&+\frac{[\mathrm{pMBL_4MASP2_{zym}}]}{K^{\mathrm{MASP2_{zym}}}_{\mathrm{m\,MASP2^*}}}+\frac{[\mathrm{pCLLKMASP1_{zym}}]}{K^{\mathrm{MASP1_{zym}}}_{\mathrm{m\,MASP2^*}}}+\frac{[\mathrm{pCLLKMASP2_{zym}}]}{K^{\mathrm{MASP2_{zym}}}_{\mathrm{m\,MASP2^*}}}+\frac{[\mathrm{pCLK1MASP1_{zym}}]}{K^{\mathrm{MASP1_{zym}}}_{\mathrm{m\,MASP2^*}}}\\&+\frac{[\mathrm{pCLK1MASP2_{zym}}]}{K^{\mathrm{MASP2_{zym}}}_{\mathrm{m\,MASP2^*}}}+\frac{[\mathrm{pCLL1MASP1_{zym}}]}{K^{\mathrm{MASP1_{zym}}}_{\mathrm{m\,MASP2^*}}}+\frac{[\mathrm{pCLL1MASP2_{zym}}]}{K^{\mathrm{MASP2_{zym}}}_{\mathrm{m\,MASP2^*}}}+\frac{[\mathrm{pLFMASP1_{zym}}]}{K^{\mathrm{MASP1_{zym}}}_{\mathrm{m\,MASP2^*}}}\\&+\frac{[\mathrm{pLFMASP2_{zym}}]}{K^{\mathrm{MASP2_{zym}}}_{\mathrm{m\,MASP2^*}}}+\frac{[\mathrm{pHFMASP1_{zym}}]}{K^{\mathrm{MASP1_{zym}}}_{\mathrm{m\,MASP2^*}}}+\frac{[\mathrm{pHFMASP2_{zym}}]}{K^{\mathrm{MASP2_{zym}}}_{\mathrm{m\,MASP2^*}}}+\frac{[\mathrm{pMFMASP1_{zym}}]}{K^{\mathrm{MASP1_{zym}}}_{\mathrm{m\,MASP2^*}}}+\frac{[\mathrm{pMFMASP2_{zym}}]}{K^{\mathrm{MASP2_{zym}}}_{\mathrm{m\,MASP2^*}}}\end{aligned}\right)}$$

$$+\frac{k^{\mathrm{C2}}_{\mathrm{cat\,MASP2^*}}\left[\mathrm{pLFMASP2^*}\right]\left[\mathrm{fC4bC4bC2}\right]}{K^{\mathrm{C2}}_{\mathrm{m\,MASP2^*}}\left(\begin{aligned}&1+\frac{[\mathrm{C4}]}{K^{\mathrm{C4}}_{\mathrm{m\,MASP2^*}}}+\frac{[\mathrm{fC4bC2}]}{K^{\mathrm{C2}}_{\mathrm{m\,MASP2^*}}}+\frac{[\mathrm{fC4bC4bC2}]}{K^{\mathrm{C2}}_{\mathrm{m\,MASP2^*}}}+\frac{[\mathrm{fC3bC4bC2}]}{K^{\mathrm{C2}}_{\mathrm{m\,MASP2^*}}}+\frac{[\mathrm{pC4bC2}]}{K^{\mathrm{C2}}_{\mathrm{m\,MASP2^*}}}+\frac{[\mathrm{pC4bC4bC2}]}{K^{\mathrm{C2}}_{\mathrm{m\,MASP2^*}}}\\&+\frac{[\mathrm{pC3bC4bC2}]}{K^{\mathrm{C2}}_{\mathrm{m\,MASP2^*}}}+\frac{[\mathrm{pMBL_3MASP1_{zym}}]}{K^{\mathrm{MASP1_{zym}}}_{\mathrm{m\,MASP2^*}}}+\frac{[\mathrm{pMBL_3MASP2_{zym}}]}{K^{\mathrm{MASP2_{zym}}}_{\mathrm{m\,MASP2^*}}}+\frac{[\mathrm{pMBL_4MASP1_{zym}}]}{K^{\mathrm{MASP1_{zym}}}_{\mathrm{m\,MASP2^*}}}\\&+\frac{[\mathrm{pMBL_4MASP2_{zym}}]}{K^{\mathrm{MASP2_{zym}}}_{\mathrm{m\,MASP2^*}}}+\frac{[\mathrm{pCLLKMASP1_{zym}}]}{K^{\mathrm{MASP1_{zym}}}_{\mathrm{m\,MASP2^*}}}+\frac{[\mathrm{pCLLKMASP2_{zym}}]}{K^{\mathrm{MASP2_{zym}}}_{\mathrm{m\,MASP2^*}}}+\frac{[\mathrm{pCLK1MASP1_{zym}}]}{K^{\mathrm{MASP1_{zym}}}_{\mathrm{m\,MASP2^*}}}\\&+\frac{[\mathrm{pCLK1MASP2_{zym}}]}{K^{\mathrm{MASP2_{zym}}}_{\mathrm{m\,MASP2^*}}}+\frac{[\mathrm{pCLL1MASP1_{zym}}]}{K^{\mathrm{MASP1_{zym}}}_{\mathrm{m\,MASP2^*}}}+\frac{[\mathrm{pCLL1MASP2_{zym}}]}{K^{\mathrm{MASP2_{zym}}}_{\mathrm{m\,MASP2^*}}}+\frac{[\mathrm{pLFMASP1_{zym}}]}{K^{\mathrm{MASP1_{zym}}}_{\mathrm{m\,MASP2^*}}}\\&+\frac{[\mathrm{pLFMASP2_{zym}}]}{K^{\mathrm{MASP2_{zym}}}_{\mathrm{m\,MASP2^*}}}+\frac{[\mathrm{pHFMASP1_{zym}}]}{K^{\mathrm{MASP1_{zym}}}_{\mathrm{m\,MASP2^*}}}+\frac{[\mathrm{pHFMASP2_{zym}}]}{K^{\mathrm{MASP2_{zym}}}_{\mathrm{m\,MASP2^*}}}+\frac{[\mathrm{pMFMASP1_{zym}}]}{K^{\mathrm{MASP1_{zym}}}_{\mathrm{m\,MASP2^*}}}+\frac{[\mathrm{pMFMASP2_{zym}}]}{K^{\mathrm{MASP2_{zym}}}_{\mathrm{m\,MASP2^*}}}\end{aligned}\right)}$$

$$+\frac{k^{\mathrm{C2}}_{\mathrm{cat\,MASP2^*}}\left[\mathrm{pHFMASP2^*}\right]\left[\mathrm{fC4bC4bC2}\right]}{K^{\mathrm{C2}}_{\mathrm{m\,MASP2^*}}\left(\begin{aligned}&1+\frac{[\mathrm{C4}]}{K^{\mathrm{C4}}_{\mathrm{m\,MASP2^*}}}+\frac{[\mathrm{fC4bC2}]}{K^{\mathrm{C2}}_{\mathrm{m\,MASP2^*}}}+\frac{[\mathrm{fC4bC4bC2}]}{K^{\mathrm{C2}}_{\mathrm{m\,MASP2^*}}}+\frac{[\mathrm{fC3bC4bC2}]}{K^{\mathrm{C2}}_{\mathrm{m\,MASP2^*}}}+\frac{[\mathrm{pC4bC2}]}{K^{\mathrm{C2}}_{\mathrm{m\,MASP2^*}}}+\frac{[\mathrm{pC4bC4bC2}]}{K^{\mathrm{C2}}_{\mathrm{m\,MASP2^*}}}\\&+\frac{[\mathrm{pC3bC4bC2}]}{K^{\mathrm{C2}}_{\mathrm{m\,MASP2^*}}}+\frac{[\mathrm{pMBL_3MASP1_{zym}}]}{K^{\mathrm{MASP1_{zym}}}_{\mathrm{m\,MASP2^*}}}+\frac{[\mathrm{pMBL_3MASP2_{zym}}]}{K^{\mathrm{MASP2_{zym}}}_{\mathrm{m\,MASP2^*}}}+\frac{[\mathrm{pMBL_4MASP1_{zym}}]}{K^{\mathrm{MASP1_{zym}}}_{\mathrm{m\,MASP2^*}}}\\&+\frac{[\mathrm{pMBL_4MASP2_{zym}}]}{K^{\mathrm{MASP2_{zym}}}_{\mathrm{m\,MASP2^*}}}+\frac{[\mathrm{pCLLKMASP1_{zym}}]}{K^{\mathrm{MASP1_{zym}}}_{\mathrm{m\,MASP2^*}}}+\frac{[\mathrm{pCLLKMASP2_{zym}}]}{K^{\mathrm{MASP2_{zym}}}_{\mathrm{m\,MASP2^*}}}+\frac{[\mathrm{pCLK1MASP1_{zym}}]}{K^{\mathrm{MASP1_{zym}}}_{\mathrm{m\,MASP2^*}}}\\&+\frac{[\mathrm{pCLK1MASP2_{zym}}]}{K^{\mathrm{MASP2_{zym}}}_{\mathrm{m\,MASP2^*}}}+\frac{[\mathrm{pCLL1MASP1_{zym}}]}{K^{\mathrm{MASP1_{zym}}}_{\mathrm{m\,MASP2^*}}}+\frac{[\mathrm{pCLL1MASP2_{zym}}]}{K^{\mathrm{MASP2_{zym}}}_{\mathrm{m\,MASP2^*}}}+\frac{[\mathrm{pLFMASP1_{zym}}]}{K^{\mathrm{MASP1_{zym}}}_{\mathrm{m\,MASP2^*}}}\\&+\frac{[\mathrm{pLFMASP2_{zym}}]}{K^{\mathrm{MASP2_{zym}}}_{\mathrm{m\,MASP2^*}}}+\frac{[\mathrm{pHFMASP1_{zym}}]}{K^{\mathrm{MASP1_{zym}}}_{\mathrm{m\,MASP2^*}}}+\frac{[\mathrm{pHFMASP2_{zym}}]}{K^{\mathrm{MASP2_{zym}}}_{\mathrm{m\,MASP2^*}}}+\frac{[\mathrm{pMFMASP1_{zym}}]}{K^{\mathrm{MASP1_{zym}}}_{\mathrm{m\,MASP2^*}}}+\frac{[\mathrm{pMFMASP2_{zym}}]}{K^{\mathrm{MASP2_{zym}}}_{\mathrm{m\,MASP2^*}}}\end{aligned}\right)}$$

$$+\frac{k_{\text{cat MASP2}^*}^{\text{C2}}\left[\text{pMFMASP2}^*\right]\left[\text{fC4bC4bC2}\right]}{K_{\text{m MASP2}^*}^{\text{C2}}\left(\begin{array}{l}1+\frac{[\text{C4}]}{K_{\text{m MASP2}^*}^{\text{C4}}}+\frac{[\text{fC4bC2}]}{K_{\text{m MASP2}^*}^{\text{C2}}}+\frac{[\text{fC4bC4bC2}]}{K_{\text{m MASP2}^*}^{\text{C2}}}+\frac{[\text{fC3bC4bC2}]}{K_{\text{m MASP2}^*}^{\text{C2}}}+\frac{[\text{pC4bC2}]}{K_{\text{m MASP2}^*}^{\text{C2}}}+\frac{[\text{pC4bC4bC2}]}{K_{\text{m MASP2}^*}^{\text{C2}}}\\ +\frac{[\text{pC3bC4bC2}]}{K_{\text{m MASP2}^*}^{\text{C2}}}+\frac{[\text{pMBL}_3\text{MASP1}_{\text{zym}}]}{K_{\text{m MASP2}^*}^{\text{MASP1}_{\text{zym}}}}+\frac{[\text{pMBL}_3\text{MASP2}_{\text{zym}}]}{K_{\text{m MASP2}^*}^{\text{MASP2}_{\text{zym}}}}+\frac{[\text{pMBL}_4\text{MASP1}_{\text{zym}}]}{K_{\text{m MASP2}^*}^{\text{MASP1}_{\text{zym}}}}\\ +\frac{[\text{pMBL}_4\text{MASP2}_{\text{zym}}]}{K_{\text{m MASP2}^*}^{\text{MASP2}_{\text{zym}}}}+\frac{[\text{pCLLKMASP1}_{\text{zym}}]}{K_{\text{m MASP2}^*}^{\text{MASP1}_{\text{zym}}}}+\frac{[\text{pCLLKMASP2}_{\text{zym}}]}{K_{\text{m MASP2}^*}^{\text{MASP2}_{\text{zym}}}}+\frac{[\text{pCLK1MASP1}_{\text{zym}}]}{K_{\text{m MASP2}^*}^{\text{MASP1}_{\text{zym}}}}\\ +\frac{[\text{pCLK1MASP2}_{\text{zym}}]}{K_{\text{m MASP2}^*}^{\text{MASP2}_{\text{zym}}}}+\frac{[\text{pCLL1MASP1}_{\text{zym}}]}{K_{\text{m MASP2}^*}^{\text{MASP1}_{\text{zym}}}}+\frac{[\text{pCLL1MASP2}_{\text{zym}}]}{K_{\text{m MASP2}^*}^{\text{MASP2}_{\text{zym}}}}+\frac{[\text{pLFMASP1}_{\text{zym}}]}{K_{\text{m MASP2}^*}^{\text{MASP1}_{\text{zym}}}}\\ +\frac{[\text{pLFMASP2}_{\text{zym}}]}{K_{\text{m MASP2}^*}^{\text{MASP2}_{\text{zym}}}}+\frac{[\text{pHFMASP1}_{\text{zym}}]}{K_{\text{m MASP2}^*}^{\text{MASP1}_{\text{zym}}}}+\frac{[\text{pHFMASP2}_{\text{zym}}]}{K_{\text{m MASP2}^*}^{\text{MASP2}_{\text{zym}}}}+\frac{[\text{pMFMASP1}_{\text{zym}}]}{K_{\text{m MASP2}^*}^{\text{MASP1}_{\text{zym}}}}+\frac{[\text{pMFMASP2}_{\text{zym}}]}{K_{\text{m MASP2}^*}^{\text{MASP2}_{\text{zym}}}}\end{array}\right)}$$

$$+\frac{k_{\text{cat C1}^*}^{\text{C2}}\left[\text{pMBL}_3\text{PTX3C1}^*\right]\left[\text{fC4bC4bC2}\right]}{K_{\text{m C1}^*}^{\text{C2}}\left(1+\frac{[\text{C4}]}{K_{\text{m C1}^*}^{\text{C4}}}+\frac{[\text{fC4bC2}]}{K_{\text{m C1}^*}^{\text{C2}}}+\frac{[\text{fC4bC4bC2}]}{K_{\text{m C1}^*}^{\text{C2}}}+\frac{[\text{fC3bC4bC2}]}{K_{\text{m C1}^*}^{\text{C2}}}+\frac{[\text{pC4bC2}]}{K_{\text{m C1}^*}^{\text{C2}}}+\frac{[\text{pC4bC4bC2}]}{K_{\text{m C1}^*}^{\text{C2}}}+\frac{[\text{pC3bC4bC2}]}{K_{\text{m C1}^*}^{\text{C2}}}\right)}$$

$$+\frac{k_{\text{cat C1}^*}^{\text{C2}}\left[\text{pMBL}_4\text{PTX3C1}^*\right]\left[\text{fC4bC4bC2}\right]}{K_{\text{m C1}^*}^{\text{C2}}\left(1+\frac{[\text{C4}]}{K_{\text{m C1}^*}^{\text{C4}}}+\frac{[\text{fC4bC2}]}{K_{\text{m C1}^*}^{\text{C2}}}+\frac{[\text{fC4bC4bC2}]}{K_{\text{m C1}^*}^{\text{C2}}}+\frac{[\text{fC3bC4bC2}]}{K_{\text{m C1}^*}^{\text{C2}}}+\frac{[\text{pC4bC2}]}{K_{\text{m C1}^*}^{\text{C2}}}+\frac{[\text{pC4bC4bC2}]}{K_{\text{m C1}^*}^{\text{C2}}}+\frac{[\text{pC3bC4bC2}]}{K_{\text{m C1}^*}^{\text{C2}}}\right)}$$

$$+\frac{k_{\text{cat C1}^*}^{\text{C2}}\left[\text{CRPC1}^*\right]\left[\text{fC4bC4bC2}\right]}{K_{\text{m C1}^*}^{\text{C2}}\left(1+\frac{[\text{C4}]}{K_{\text{m C1}^*}^{\text{C4}}}+\frac{[\text{fC4bC2}]}{K_{\text{m C1}^*}^{\text{C2}}}+\frac{[\text{fC4bC4bC2}]}{K_{\text{m C1}^*}^{\text{C2}}}+\frac{[\text{fC3bC4bC2}]}{K_{\text{m C1}^*}^{\text{C2}}}\right)}$$

42

$$\frac{d[\mathrm{fC5b}]}{dt}=\frac{k_{\mathrm{cat\ C3bBb}}^{\mathrm{C5}}[\mathrm{fC3bBb}]}{K_{\mathrm{m\ C3bBb}}^{\mathrm{C5}}\left(1+\frac{[\mathrm{C3}]}{K_{\mathrm{m\ C3bBb}}^{\mathrm{C3}}}+\frac{[\mathrm{C5}]}{K_{\mathrm{m\ C3bBb}}^{\mathrm{C5}}}\right)}+\frac{k_{\mathrm{cat\ C4bC2a}}^{\mathrm{C5}}[\mathrm{fC4bC2a}]}{K_{\mathrm{m\ C4bC2a}}^{\mathrm{C5}}\left(1+\frac{[\mathrm{C3}]}{K_{\mathrm{m\ C4bC2a}}^{\mathrm{C3}}}+\frac{[\mathrm{C5}]}{K_{\mathrm{m\ C4bC2a}}^{\mathrm{C5}}}\right)}+\frac{k_{\mathrm{cat\ C3bBb}}^{\mathrm{C5}}[\mathrm{hC3bBb}]}{K_{\mathrm{m\ C3bBb}}^{\mathrm{C5}}\left(1+\frac{[\mathrm{C3}]}{K_{\mathrm{m\ C3bBb}}^{\mathrm{C3}}}+\frac{[\mathrm{C5}]}{K_{\mathrm{m\ C3bBb}}^{\mathrm{C5}}}\right)}$$

$$+\frac{k_{\mathrm{cat\ C3bBb}}^{\mathrm{C5}}[\mathrm{hC3bBbP}]}{K_{\mathrm{m\ C3bBb}}^{\mathrm{C5}}\left(1+\frac{[\mathrm{C3}]}{K_{\mathrm{m\ C3bBb}}^{\mathrm{C3}}}+\frac{[\mathrm{C5}]}{K_{\mathrm{m\ C3bBb}}^{\mathrm{C5}}}\right)}-k_{\mathrm{C5b^*}}^{-}[\mathrm{fC5b}]-k_{\mathrm{C5b6}}^{+}[\mathrm{fC5b}][\mathrm{C6}]+k_{\mathrm{C5b6}}^{-}[\mathrm{fC5b6}]$$

$$+\frac{k_{\mathrm{cat\ C4bC2a}}^{\mathrm{C5}}[\mathrm{pC4bC2a}]}{K_{\mathrm{m\ C4bC2a}}^{\mathrm{C5}}\left(1+\frac{[\mathrm{C3}]}{K_{\mathrm{m\ C4bC2a}}^{\mathrm{C3}}}+\frac{[\mathrm{C5}]}{K_{\mathrm{m\ C4bC2a}}^{\mathrm{C5}}}\right)}+\frac{k_{\mathrm{cat\ C3bBb}}^{\mathrm{C5}}[\mathrm{pC3bBb}]}{K_{\mathrm{m\ C3bBb}}^{\mathrm{C5}}\left(1+\frac{[\mathrm{C3}]}{K_{\mathrm{m\ C3bBb}}^{\mathrm{C3}}}+\frac{[\mathrm{C5}]}{K_{\mathrm{m\ C3bBb}}^{\mathrm{C5}}}\right)}+\frac{k_{\mathrm{cat\ C3bBb}}^{\mathrm{C5}}[\mathrm{pC3bBbP}]}{K_{\mathrm{m\ C3bBb}}^{\mathrm{C5}}\left(1+\frac{[\mathrm{C3}]}{K_{\mathrm{m\ C3bBb}}^{\mathrm{C3}}}+\frac{[\mathrm{C5}]}{K_{\mathrm{m\ C3bBb}}^{\mathrm{C5}}}\right)}$$

$$+\frac{k_{\mathrm{cat\ C3bBb}}^{\mathrm{C5}}[\mathrm{pC3bBbCFHR1}]}{K_{\mathrm{m\ C3bBb}}^{\mathrm{C5}}\left(1+\frac{[\mathrm{C3}]}{K_{\mathrm{m\ C3bBb}}^{\mathrm{C3}}}+\frac{[\mathrm{C5}]}{K_{\mathrm{m\ C3bBb}}^{\mathrm{C5}}}\right)}+\frac{k_{\mathrm{cat\ C3bBb}}^{\mathrm{C5}}[\mathrm{pC3bBbPCFHR1}]}{K_{\mathrm{m\ C3bBb}}^{\mathrm{C5}}\left(1+\frac{[\mathrm{C3}]}{K_{\mathrm{m\ C3bBb}}^{\mathrm{C3}}}+\frac{[\mathrm{C5}]}{K_{\mathrm{m\ C3bBb}}^{\mathrm{C5}}}\right)}+\frac{k_{\mathrm{cat\ C3bBb}}^{\mathrm{C5}}[\mathrm{pC3bBbCFHR2}]}{K_{\mathrm{m\ C3bBb}}^{\mathrm{C5}}\left(1+\frac{[\mathrm{C3}]}{K_{\mathrm{m\ C3bBb}}^{\mathrm{C3}}}+\frac{[\mathrm{C5}]}{K_{\mathrm{m\ C3bBb}}^{\mathrm{C5}}}\right)}$$

$$+\frac{k_{\mathrm{cat\ C3bBb}}^{\mathrm{C5}}[\mathrm{pC3bBbPCFHR2}]}{K_{\mathrm{m\ C3bBb}}^{\mathrm{C5}}\left(1+\frac{[\mathrm{C3}]}{K_{\mathrm{m\ C3bBb}}^{\mathrm{C3}}}+\frac{[\mathrm{C5}]}{K_{\mathrm{m\ C3bBb}}^{\mathrm{C5}}}\right)}+\frac{k_{\mathrm{cat\ C3bBb}}^{\mathrm{C5}}[\mathrm{pC3bBbCFHR3}]}{K_{\mathrm{m\ C3bBb}}^{\mathrm{C5}}\left(1+\frac{[\mathrm{C3}]}{K_{\mathrm{m\ C3bBb}}^{\mathrm{C3}}}+\frac{[\mathrm{C5}]}{K_{\mathrm{m\ C3bBb}}^{\mathrm{C5}}}\right)}+\frac{k_{\mathrm{cat\ C3bBb}}^{\mathrm{C5}}[\mathrm{pC3bBbPCFHR3}]}{K_{\mathrm{m\ C3bBb}}^{\mathrm{C5}}\left(1+\frac{[\mathrm{C3}]}{K_{\mathrm{m\ C3bBb}}^{\mathrm{C3}}}+\frac{[\mathrm{C5}]}{K_{\mathrm{m\ C3bBb}}^{\mathrm{C5}}}\right)}$$

$$+\frac{k_{\mathrm{cat\ C3bBb}}^{\mathrm{C5}}[\mathrm{pC3bBbCFHR4}]}{K_{\mathrm{m\ C3bBb}}^{\mathrm{C5}}\left(1+\frac{[\mathrm{C3}]}{K_{\mathrm{m\ C3bBb}}^{\mathrm{C3}}}+\frac{[\mathrm{C5}]}{K_{\mathrm{m\ C3bBb}}^{\mathrm{C5}}}\right)}+\frac{k_{\mathrm{cat\ C3bBb}}^{\mathrm{C5}}[\mathrm{pC3bBbPCFHR4}]}{K_{\mathrm{m\ C3bBb}}^{\mathrm{C5}}\left(1+\frac{[\mathrm{C3}]}{K_{\mathrm{m\ C3bBb}}^{\mathrm{C3}}}+\frac{[\mathrm{C5}]}{K_{\mathrm{m\ C3bBb}}^{\mathrm{C5}}}\right)}+\frac{k_{\mathrm{cat\ C3bBb}}^{\mathrm{C5}}[\mathrm{pC3bBbCFHR5}]}{K_{\mathrm{m\ C3bBb}}^{\mathrm{C5}}\left(1+\frac{[\mathrm{C3}]}{K_{\mathrm{m\ C3bBb}}^{\mathrm{C3}}}+\frac{[\mathrm{C5}]}{K_{\mathrm{m\ C3bBb}}^{\mathrm{C5}}}\right)}$$

$$+\frac{k_{\mathrm{cat\ C3bBb}}^{\mathrm{C5}}[\mathrm{pC3bBbPCFHR5}]}{K_{\mathrm{m\ C3bBb}}^{\mathrm{C5}}\left(1+\frac{[\mathrm{C3}]}{K_{\mathrm{m\ C3bBb}}^{\mathrm{C3}}}+\frac{[\mathrm{C5}]}{K_{\mathrm{m\ C3bBb}}^{\mathrm{C5}}}\right)}$$

43

$$\frac{d[\mathrm{fC5b6}]}{dt}=k_{\mathrm{C5b6}}^{+}[\mathrm{fC5b}][\mathrm{C6}]-k_{\mathrm{C5b6}}^{-}[\mathrm{fC5b6}]-k_{\mathrm{C5b7}}^{+}[\mathrm{fC5b6}][\mathrm{C7}]+k_{\mathrm{C5b7}}^{-}[\mathrm{fC5b7}]$$

44

$$\frac{d[\mathrm{fC3bC3bBbC5b}]}{dt}=\frac{k_{\mathrm{cat\ C3bC3bBb}}^{\mathrm{C5}}[\mathrm{C5}][\mathrm{fC3bC3bBb}]}{K_{\mathrm{m\ C3bC3bBb}}^{\mathrm{C5}}\left(1+\frac{[\mathrm{C3}]}{K_{\mathrm{m\ C3bBb}}^{\mathrm{C3}}}+\frac{[\mathrm{C5}]}{K_{\mathrm{m\ C3bC3bBb}}^{\mathrm{C5}}}\right)}-k_{\mathrm{C5b^*}}^{-}[\mathrm{fC3bC3bBbC5b}]-k_{\mathrm{C5b6}}^{+}[\mathrm{fC3bC3bBbC5b}][\mathrm{C6}]$$

$$+k_{\mathrm{C5b6}}^{-}[\mathrm{fC3bC3bBbC5b6}]$$

45

$$\frac{d[\mathrm{fC3bC3bBbC5b6}]}{dt}=k_{\mathrm{C5b6}}^{+}[\mathrm{fC3bC3bBbC5b}][\mathrm{C6}]-k_{\mathrm{C5b6}}^{-}[\mathrm{fC3bC3bBbC5b6}]-k_{\mathrm{C5b7}}^{+}[\mathrm{fC3bC3bBbC5b6}][\mathrm{C7}]$$

46

$$\frac{d\left[\mathrm{fC3bC3bBbPC5b}\right]}{dt}=\frac{k_{\mathrm{cat\ C3bC3bBb}}^{\mathrm{C5}}\left[\mathrm{C5}\right]\left[\mathrm{fC3bC3bBbP}\right]}{K_{\mathrm{m\ C3bC3bBb}}^{\mathrm{C5}}\left(1+\frac{\left[\mathrm{C3}\right]}{K_{\mathrm{m\ C3bBb}}^{\mathrm{C3}}}+\frac{\left[\mathrm{C5}\right]}{K_{\mathrm{m\ C3bC3bBb}}^{\mathrm{C5}}}\right)}-k_{\mathrm{C5b^*}}^{-}\left[\mathrm{fC3bC3bBbPC5b}\right]$$

$$-k_{\mathrm{C5b6}}^{+}\left[\mathrm{fC3bC3bBbPC5b}\right]\left[\mathrm{C6}\right]+k_{\mathrm{C5b6}}^{-}\left[\mathrm{fC3bC3bBbPC5b6}\right]$$

47

$$\frac{d\left[\mathrm{fC3bC3bBbPC5b6}\right]}{dt}=k_{\mathrm{C5b6}}^{+}\left[\mathrm{fC3bC3bBbPC5b}\right]\left[\mathrm{C6}\right]-k_{\mathrm{C5b6}}^{-}\left[\mathrm{fC3bC3bBbPC5b6}\right]-k_{\mathrm{C5b7}}^{+}\left[\mathrm{fC3bC3bBbPC5b6}\right]\left[\mathrm{C7}\right]$$

48

$$\frac{d\left[\mathrm{fC4bC3bBbC5b}\right]}{dt}=\frac{k_{\mathrm{cat\ C3bC4bBb}}^{\mathrm{C5}}\left[\mathrm{C5}\right]\left[\mathrm{fC4bC3bBb}\right]}{K_{\mathrm{m\ C3bC4bBb}}^{\mathrm{C5}}\left(1+\frac{\left[\mathrm{C3}\right]}{K_{\mathrm{m\ C3bBb}}^{\mathrm{C3}}}+\frac{\left[\mathrm{C5}\right]}{K_{\mathrm{m\ C3bC4bBb}}^{\mathrm{C5}}}\right)}-k_{\mathrm{C5b^*}}^{-}\left[\mathrm{fC4bC3bBbC5b}\right]$$

$$-k_{\mathrm{C5b6}}^{+}\left[\mathrm{fC4bC3bBbC5b}\right]\left[\mathrm{C6}\right]+k_{\mathrm{C5b6}}^{-}\left[\mathrm{fC4bC3bBbC5b6}\right]$$

49

$$\frac{d\left[\mathrm{fC4bC3bBbC5b6}\right]}{dt}=k_{\mathrm{C5b6}}^{+}\left[\mathrm{fC4bC3bBbC5b}\right]\left[\mathrm{C6}\right]-k_{\mathrm{C5b6}}^{-}\left[\mathrm{fC4bC3bBbC5b6}\right]-k_{\mathrm{C5b7}}^{+}\left[\mathrm{fC4bC3bBbC5b6}\right]\left[\mathrm{C7}\right]$$

50

$$\frac{d\left[\mathrm{fC4bC3bBbPC5b}\right]}{dt}=\frac{k_{\mathrm{cat\ C3bC4bBb}}^{\mathrm{C5}}\left[\mathrm{C5}\right]\left[\mathrm{fC4bC3bBbP}\right]}{K_{\mathrm{m\ C3bC4bBb}}^{\mathrm{C5}}\left(1+\frac{\left[\mathrm{C3}\right]}{K_{\mathrm{m\ C3bBb}}^{\mathrm{C3}}}+\frac{\left[\mathrm{C5}\right]}{K_{\mathrm{m\ C3bC4bBb}}^{\mathrm{C5}}}\right)}-k_{\mathrm{C5b^*}}^{-}\left[\mathrm{fC4bC3bBbPC5b}\right]$$

$$-k_{\mathrm{C5b6}}^{+}\left[\mathrm{fC4bC3bBbPC5b}\right]\left[\mathrm{C6}\right]+k_{\mathrm{C5b6}}^{-}\left[\mathrm{fC4bC3bBbPC5b6}\right]$$

51

$$\frac{d\left[\mathrm{fC4bC3bBbPC5b6}\right]}{dt}=k_{\mathrm{C5b6}}^{+}\left[\mathrm{fC4bC3bBbPC5b}\right]\left[\mathrm{C6}\right]-k_{\mathrm{C5b6}}^{-}\left[\mathrm{fC4bC3bBbPC5b6}\right]-k_{\mathrm{C5b7}}^{+}\left[\mathrm{fC4bC3bBbPC5b6}\right]\left[\mathrm{C7}\right]$$

52

$$\frac{d\left[\mathrm{fC3bC4bC2aC5b}\right]}{dt}=\frac{k_{\mathrm{cat\ C3bC4bC2a}}^{\mathrm{C5}}\left[\mathrm{C5}\right]\left[\mathrm{fC3bC4bC2a}\right]}{K_{\mathrm{m\ C3bC4bC2a}}^{\mathrm{C5}}\left(1+\frac{\left[\mathrm{C3}\right]}{K_{\mathrm{m\ C3bBb}}^{\mathrm{C3}}}+\frac{\left[\mathrm{C5}\right]}{K_{\mathrm{m\ C3bC4bC2a}}^{\mathrm{C5}}}\right)}-k_{\mathrm{C5b^*}}^{-}\left[\mathrm{fC3bC4bC2aC5b}\right]$$

$$-k_{\mathrm{C5b6}}^{+}\left[\mathrm{fC3bC4bC2aC5b}\right]\left[\mathrm{C6}\right]+k_{\mathrm{C5b6}}^{-}\left[\mathrm{fC3bC4bC2aC5b6}\right]$$

53

$$\frac{d\left[\mathrm{fC3bC4bC2aC5b6}\right]}{dt}=k_{\mathrm{C5b6}}^{+}\left[\mathrm{fC3bC4bC2aC5b}\right]\left[\mathrm{C6}\right]-k_{\mathrm{C5b6}}^{-}\left[\mathrm{fC3bC4bC2aC5b6}\right]-k_{\mathrm{C5b7}}^{+}\left[\mathrm{fC3bC4bC2aC5b6}\right]\left[\mathrm{C7}\right]$$

54

$$\frac{d\left[\text{fC4bC4bC2aC5b}\right]}{dt}=\frac{k_{\text{cat C4bC4bC2a}}^{\text{C5}}\left[\text{C5}\right]\left[\text{fC4bC4bC2a}\right]}{K_{\text{m C4bC4bC2a}}^{\text{C5}}\left(1+\frac{\left[\text{C3}\right]}{K_{\text{m C3bBb}}^{\text{C3}}}+\frac{\left[\text{C5}\right]}{K_{\text{m C4bC4bC2a}}^{\text{C5}}}\right)}-k_{\text{C5b}^*}^{-}\left[\text{fC4bC4bC2aC5b}\right]$$
$$-k_{\text{C5b6}}^{+}\left[\text{fC4bC4bC2aC5b}\right]\left[\text{C6}\right]+k_{\text{C5b6}}^{-}\left[\text{fC4bC4bC2aC5b6}\right]$$

55

$$\frac{d\left[\text{fC4bC4bC2aC5b6}\right]}{dt}=k_{\text{C5b6}}^{+}\left[\text{fC4bC4bC2aC5b}\right]\left[\text{C6}\right]-k_{\text{C5b6}}^{-}\left[\text{fC4bC4bC2aC5b6}\right]-k_{\text{C5b7}}^{+}\left[\text{fC4bC4bC2aC5b6}\right]\left[\text{C7}\right]$$

Host Cell (Fluid and Surface)

56

$$\frac{d\left[\text{Host Surface}\right]}{dt}=-k_{\text{hC4b}}^{+}\left[\text{Host Surface}\right]\left[\text{nfC4b}\right]-k_{\text{hC3b}}^{+}\left[\text{Host Surface}\right]\left[\text{nfC3b}\right]$$
$$-k_{\text{C5b7}_{\text{surface}}}^{+}\left[\text{fC5b7}\right]\left[\text{Host Surface}\right]\times 4$$

57

$$\frac{d\left[\text{hC3b}\right]}{dt}=k_{\text{hC3b}}^{+}\left[\text{Host Surface}\right]\left[\text{nfC3b}\right]-k_{\text{C3bC4b}}^{+}\left[\text{hC3b}\right]\left[\text{nfC4b}\right]-k_{\text{C3bC3b}}^{+}\left[\text{hC3b}\right]\left[\text{nfC3b}\right]-k_{\text{C3bB}}^{+}\left[\text{hC3b}\right]\left[\text{FB}\right]$$
$$+k_{\text{C3bB}}^{-}\left[\text{hC3bB}\right]+k_{\text{C3bBb}}^{-}\left[\text{hC3bBb}\right]+k_{\text{C3bBbP}}^{-}\left[\text{hC3bBbP}\right]-k_{\text{C3bH}}^{+}\left[\text{hC3b}\right]\left[\text{FH}\right]+k_{\text{C3bH}}^{-}\left[\text{hC3bFH}\right]$$
$$+k_{\text{C3bBbH}_{\text{decay}}}^{-}\left[\text{hC3bBbFH}\right]-k_{\text{C3bFHL1}}^{+}\left[\text{hC3b}\right]\left[\text{FHL1}\right]+k_{\text{C3bFHL1}}^{-}\left[\text{hC3bFHL1}\right]+k_{\text{C3bBbFHL1}_{\text{decay}}}^{-}\left[\text{hC3bBbFHL1}\right]$$
$$-k_{\text{C3bCR1}}^{+}\left[\text{hC3b}\right]\left[\text{CR1}\right]+k_{\text{C3bCR1}}^{-}\left[\text{hC3bCR1}\right]+k_{\text{C3bBbCR1}_{\text{decay}}}^{-}\left[\text{hC3bBbCR1}\right]+k_{\text{C3bBbDAF}_{\text{decay}}}^{-}\left[\text{hC3bBbDAF}\right]$$

58

$$\frac{d\left[\text{hC3bB}\right]}{dt}=k_{\text{C3bB}}^{+}\left[\text{hC3b}\right]\left[\text{FB}\right]-k_{\text{C3bB}}^{-}\left[\text{hC3bB}\right]$$
$$-\frac{k_{\text{cat C3}(\text{H}_2\text{O})\text{B}}^{\text{FD}}\left[\text{FD}\right]\left[\text{hC3bB}\right]}{\left(\begin{array}{l}K_{\text{m C3}(\text{H}_2\text{O})\text{B}}^{\text{D}}+\left[\text{C3}\left(\text{H}_2\text{O}\right)\text{B}\right]+\left[\text{fC3bB}\right]+\left[\text{fC3bC3bB}\right]+\left[\text{fC3bC3bBP}\right]+\left[\text{IgGC3bC3bB}\right]+\left[\text{IgGC3bC3bBP}\right]\\+\left[\text{fC4bC3bB}\right]+\left[\text{fC4bC3bBP}\right]+\left[\text{pC3bBCFHR1}\right]+\left[\text{pC3bBCFHR2}\right]+\left[\text{pC3bBCFHR3}\right]+\left[\text{pC3bBCFHR4}\right]\\+\left[\text{pC3bBCFHR5}\right]+\left[\text{hC4bC3bBP}\right]+\left[\text{hC4bC3bB}\right]+\left[\text{hC3bC3bB}\right]+\left[\text{hC3bC3bBP}\right]+\left[\text{hC3bB}\right]+\left[\text{pC4bC3bB}\right]\\+\left[\text{pC4bC3bBP}\right]+\left[\text{pC3bC3bB}\right]+\left[\text{pC3bC3bBP}\right]+\left[\text{pC3bB}\right]\end{array}\right)}$$

59

$$\frac{d[\mathrm{hC3bBb}]}{dt}=$$

$$\frac{k^{\mathrm{FD}}_{\mathrm{cat\,C3(H_2O)B}}[\mathrm{FD}][\mathrm{hC3bB}]}{\left(\begin{array}{l}K^{\mathrm{D}}_{\mathrm{m\,C3(H_2O)B}}+[\mathrm{C3(H_2O)B}]+[\mathrm{fC3bB}]+[\mathrm{fC3bC3bB}]+[\mathrm{fC3bC3bBP}]+[\mathrm{IgGC3bC3bB}]+[\mathrm{IgGC3bC3bBP}]\\+[\mathrm{fC4bC3bB}]+[\mathrm{fC4bC3bBP}]+[\mathrm{pC3bBCFHR1}]+[\mathrm{pC3bBCFHR2}]+[\mathrm{pC3bBCFHR3}]+[\mathrm{pC3bBCFHR4}]\\+[\mathrm{pC3bBCFHR5}]+[\mathrm{hC4bC3bBP}]+[\mathrm{hC4bC3bB}]+[\mathrm{hC3bC3bB}]+[\mathrm{hC3bC3bBP}]+[\mathrm{hC3bB}]+[\mathrm{pC4bC3bB}]\\+[\mathrm{pC4bC3bBP}]+[\mathrm{pC3bC3bB}]+[\mathrm{pC3bC3bBP}]+[\mathrm{pC3bB}]\end{array}\right)}$$

$$-k^{+}_{\mathrm{C3bC4b}}[\mathrm{nfC4b}][\mathrm{hC3bBb}]-k^{+}_{\mathrm{C3bC3b}}[\mathrm{nfC3b}][\mathrm{hC3bBb}]-k^{-}_{\mathrm{C3bBb}}[\mathrm{hC3bBb}]-k^{+}_{\mathrm{C3bP}}[\mathrm{hC3bBb}][\mathrm{P}]+k^{-}_{\mathrm{C3bP}}[\mathrm{hC3bBbP}]$$
$$-k^{+}_{\mathrm{C3bH}}[\mathrm{hC3bBb}][\mathrm{FH}]+k^{-}_{\mathrm{C3bH}}[\mathrm{hC3bBbFH}]-k^{+}_{\mathrm{C3bFHL1}}[\mathrm{hC3bBb}][\mathrm{FHL1}]+k^{-}_{\mathrm{C3bFHL1}}[\mathrm{hC3bBbFHL1}]$$
$$-k^{+}_{\mathrm{C3bCR1}}[\mathrm{hC3bBb}][\mathrm{CR1}]+k^{-}_{\mathrm{C3bCR1}}[\mathrm{hC3bBbCR1}]-k^{+}_{\mathrm{C3bBbDAF}}[\mathrm{hC3bBb}][\mathrm{DAF}]+k^{-}_{\mathrm{C3bBbDAF}}[\mathrm{hC3bBbDAF}]$$

60

$$\frac{d[\mathrm{hC3bBbP}]}{dt}=k^{+}_{\mathrm{C3bP}}[\mathrm{hC3bBb}][\mathrm{P}]-k^{-}_{\mathrm{C3bP}}[\mathrm{hC3bBbP}]-k^{-}_{\mathrm{C3bBbP}}[\mathrm{hC3bBbP}]-k^{+}_{\mathrm{C3bC3b}}[\mathrm{nfC3b}][\mathrm{hC3bBbP}]$$
$$-k^{+}_{\mathrm{C3bC4b}}[\mathrm{nfC4b}][\mathrm{hC3bBbP}]$$

61

$$\frac{d[\mathrm{hC3bC3b}]}{dt}=k^{+}_{\mathrm{C3bC3b}}[\mathrm{hC3b}][\mathrm{nfC3b}]-k^{+}_{\mathrm{C3bB}}[\mathrm{hC3bC3b}][\mathrm{FB}]+k^{-}_{\mathrm{C3bB}}[\mathrm{hC3bC3bB}]-k^{+}_{\mathrm{C3bP}}[\mathrm{hC3bC3b}][\mathrm{P}]$$
$$+k^{-}_{\mathrm{C3bP}}[\mathrm{hC3bC3bP}]+k^{-}_{\mathrm{C3bC3bBb}}[\mathrm{hC3bC3bBb}]+k^{-}_{\mathrm{C3bC3bBbP}}[\mathrm{hC3bC3bBbP}]-k^{+}_{\mathrm{C3bH}}[\mathrm{hC3bC3b}][\mathrm{FH}]$$
$$+k^{-}_{\mathrm{C3bH}}[\mathrm{hC3bC3bFH}]+k^{-}_{\mathrm{C3bBbH_{decay}}}[\mathrm{hC3bC3bBbFH}]-k^{+}_{\mathrm{C3bFHL1}}[\mathrm{hC3bC3b}][\mathrm{FHL1}]+k^{-}_{\mathrm{C3bFHL1}}[\mathrm{hC3bC3bFHL1}]$$
$$+k^{-}_{\mathrm{C3bBbFHL1_{decay}}}[\mathrm{hC3bC3bBbFHL1}]-k^{+}_{\mathrm{C3bC3bCR1}}[\mathrm{hC3bC3b}][\mathrm{CR1}]+k^{-}_{\mathrm{C3bC3bCR1}}[\mathrm{hC3bC3bCR1}]$$
$$+k^{-}_{\mathrm{C3bBbCR1_{decay}}}[\mathrm{hC3bC3bBbCR1}]+k^{-}_{\mathrm{C3bBbDAF_{decay}}}[\mathrm{hC3bC3bBbDAF}]$$

62

$$\frac{d[\mathrm{hC3bC3bB}]}{dt}=k^{+}_{\mathrm{C3bB}}[\mathrm{hC3bC3b}][\mathrm{FB}]-k^{-}_{\mathrm{C3bB}}[\mathrm{hC3bC3bB}]$$
$$-\frac{k^{\mathrm{FD}}_{\mathrm{cat\,C3(H_2O)B}}[\mathrm{FD}][\mathrm{hC3bC3bB}]}{\left(\begin{array}{l}K^{\mathrm{D}}_{\mathrm{m\,C3(H_2O)B}}+[\mathrm{C3(H_2O)B}]+[\mathrm{fC3bB}]+[\mathrm{fC3bC3bB}]+[\mathrm{fC3bC3bBP}]+[\mathrm{IgGC3bC3bB}]+[\mathrm{IgGC3bC3bBP}]\\+[\mathrm{fC4bC3bB}]+[\mathrm{fC4bC3bBP}]+[\mathrm{pC3bBCFHR1}]+[\mathrm{pC3bBCFHR2}]+[\mathrm{pC3bBCFHR3}]+[\mathrm{pC3bBCFHR4}]\\+[\mathrm{pC3bBCFHR5}]+[\mathrm{hC4bC3bBP}]+[\mathrm{hC4bC3bB}]+[\mathrm{hC3bC3bB}]+[\mathrm{hC3bC3bBP}]+[\mathrm{hC3bB}]+[\mathrm{pC4bC3bB}]\\+[\mathrm{pC4bC3bBP}]+[\mathrm{pC3bC3bB}]+[\mathrm{pC3bC3bBP}]+[\mathrm{pC3bB}]\end{array}\right)}$$

63

$$\frac{d[\mathrm{hC3bC3bBb}]}{dt}=$$

$$\frac{k^{\mathrm{FD}}_{\mathrm{cat\ C3(H_2O)B}}[\mathrm{FD}][\mathrm{hC3bC3bB}]}{\begin{pmatrix}K^{\mathrm{D}}_{\mathrm{m\ C3(H_2O)B}}+[\mathrm{C3(H_2O)B}]+[\mathrm{fC3bB}]+[\mathrm{fC3bC3bB}]+[\mathrm{fC3bC3bBP}]+[\mathrm{IgGC3bC3bB}]+[\mathrm{IgGC3bC3bBP}]\\+[\mathrm{fC4bC3bB}]+[\mathrm{fC4bC3bBP}]+[\mathrm{pC3bBCFHR1}]+[\mathrm{pC3bBCFHR2}]+[\mathrm{pC3bBCFHR3}]+[\mathrm{pC3bBCFHR4}]\\+[\mathrm{pC3bBCFHR5}]+[\mathrm{hC4bC3bBP}]+[\mathrm{hC4bC3bB}]+[\mathrm{hC3bC3bB}]+[\mathrm{hC3bC3bBP}]+[\mathrm{hC3bB}]+[\mathrm{pC4bC3bB}]\\+[\mathrm{pC4bC3bBP}]+[\mathrm{pC3bC3bB}]+[\mathrm{pC3bC3bBP}]+[\mathrm{pC3bB}]\end{pmatrix}}$$

$$+k^{+}_{\mathrm{C3bC3b}}[\mathrm{nfC3b}][\mathrm{hC3bBb}]-k^{-}_{\mathrm{C3bC3bBb}}[\mathrm{hC3bC3bBb}]-k^{+}_{\mathrm{C3bP}}[\mathrm{hC3bC3bBb}][\mathrm{P}]+k^{-}_{\mathrm{C3bP}}[\mathrm{hC3bC3bBbP}]$$
$$+k^{-}_{\mathrm{C5b^*}}[\mathrm{hC3bC3bBbC5b}]+k^{+}_{\mathrm{C5b7}}[\mathrm{hC3bC3bBbC5b6}][\mathrm{C7}]-k^{+}_{\mathrm{C3bH}}[\mathrm{hC3bC3bBb}][\mathrm{FH}]+k^{-}_{\mathrm{C3bH}}[\mathrm{hC3bC3bBbFH}]$$
$$-k^{+}_{\mathrm{C3bFHL1}}[\mathrm{hC3bC3bBb}][\mathrm{FHL1}]+k^{-}_{\mathrm{C3bFHL1}}[\mathrm{hC3bC3bBbFHL1}]-k^{+}_{\mathrm{C3bC3bCR1}}[\mathrm{hC3bC3bBb}][\mathrm{CR1}]$$
$$+k^{-}_{\mathrm{C3bC3bCR1}}[\mathrm{hC3bC3bBbCR1}]-k^{+}_{\mathrm{C3bBbDAF}}[\mathrm{hC3bC3bBb}][\mathrm{DAF}]+k^{-}_{\mathrm{C3bBbDAF}}[\mathrm{hC3bC3bBbDAF}]$$

64

$$\frac{d[\mathrm{hC3bC3bP}]}{dt}=k^{+}_{\mathrm{C3bP}}[\mathrm{hC3bC3b}][\mathrm{P}]-k^{-}_{\mathrm{C3bP}}[\mathrm{hC3bC3bP}]-k^{+}_{\mathrm{C3bB}}[\mathrm{hC3bC3bP}][\mathrm{FB}]+k^{-}_{\mathrm{C3bB}}[\mathrm{hC3bC3bBP}]$$

65

$$\frac{d[\mathrm{hC3bC3bBP}]}{dt}=k^{+}_{\mathrm{C3bB}}[\mathrm{hC3bC3bP}][\mathrm{FB}]-k^{-}_{\mathrm{C3bB}}[\mathrm{hC3bC3bBP}]$$

$$-\frac{k^{\mathrm{FD}}_{\mathrm{cat\ C3(H_2O)B}}[\mathrm{FD}][\mathrm{hC3bC3bBP}]}{\begin{pmatrix}K^{\mathrm{D}}_{\mathrm{m\ C3(H_2O)B}}+[\mathrm{C3(H_2O)B}]+[\mathrm{fC3bB}]+[\mathrm{fC3bC3bB}]+[\mathrm{fC3bC3bBP}]+[\mathrm{IgGC3bC3bB}]+[\mathrm{IgGC3bC3bBP}]\\+[\mathrm{fC4bC3bB}]+[\mathrm{fC4bC3bBP}]+[\mathrm{pC3bBCFHR1}]+[\mathrm{pC3bBCFHR2}]+[\mathrm{pC3bBCFHR3}]+[\mathrm{pC3bBCFHR4}]\\+[\mathrm{pC3bBCFHR5}]+[\mathrm{hC4bC3bBP}]+[\mathrm{hC4bC3bB}]+[\mathrm{hC3bC3bB}]+[\mathrm{hC3bC3bBP}]+[\mathrm{hC3bB}]+[\mathrm{pC4bC3bB}]\\+[\mathrm{pC4bC3bBP}]+[\mathrm{pC3bC3bB}]+[\mathrm{pC3bC3bBP}]+[\mathrm{pC3bB}]\end{pmatrix}}$$

66

$$\frac{d\left[\mathrm{hC3bC3bBbP}\right]}{dt}=$$

$$\frac{k_{\mathrm{cat\ C3(H_2O)B}}^{\mathrm{FD}}\left[\mathrm{FD}\right]\left[\mathrm{hC3bC3bBP}\right]}{\left(\begin{array}{l}K_{\mathrm{m\ C3(H_2O)B}}^{\mathrm{D}}+\left[\mathrm{C3}\left(\mathrm{H_2O}\right)\mathrm{B}\right]+\left[\mathrm{fC3bB}\right]+\left[\mathrm{fC3bC3bB}\right]+\left[\mathrm{fC3bC3bBP}\right]+\left[\mathrm{IgGC3bC3bB}\right]+\left[\mathrm{IgGC3bC3bBP}\right]\\+\left[\mathrm{fC4bC3bB}\right]+\left[\mathrm{fC4bC3bBP}\right]+\left[\mathrm{pC3bBCFHR1}\right]+\left[\mathrm{pC3bBCFHR2}\right]+\left[\mathrm{pC3bBCFHR3}\right]+\left[\mathrm{pC3bBCFHR4}\right]\\+\left[\mathrm{pC3bBCFHR5}\right]+\left[\mathrm{hC4bC3bBP}\right]+\left[\mathrm{hC4bC3bB}\right]+\left[\mathrm{hC3bC3bB}\right]+\left[\mathrm{hC3bC3bBP}\right]+\left[\mathrm{hC3bB}\right]+\left[\mathrm{pC4bC3bB}\right]\\+\left[\mathrm{pC4bC3bBP}\right]+\left[\mathrm{pC3bC3bB}\right]+\left[\mathrm{pC3bC3bBP}\right]+\left[\mathrm{pC3bB}\right]\end{array}\right)}$$

$$+k_{\mathrm{C3bC3b}}^{+}\left[\mathrm{nfC3b}\right]\left[\mathrm{hC3bBbP}\right]+k_{\mathrm{C3bP}}^{+}\left[\mathrm{hC3bC3bBb}\right]\left[\mathrm{P}\right]-k_{\mathrm{C3bP}}^{-}\left[\mathrm{hC3bC3bBbP}\right]-k_{\mathrm{C3bC3bBbP}}^{-}\left[\mathrm{hC3bC3bBbP}\right]$$

$$+k_{\mathrm{C5b^*}}^{-}\left[\mathrm{hC3bC3bBbPC5b}\right]+k_{\mathrm{C5b7}}^{+}\left[\mathrm{hC3bC3bBbPC5b6}\right]\left[\mathrm{C7}\right]$$

67

$$\frac{d\left[\mathrm{hC4b}\right]}{dt}=k_{\mathrm{hC4b}}^{+}\left[\mathrm{Host\ Surface}\right]\left[\mathrm{nfC4b}\right]-k_{\mathrm{C4bC4b}}^{+}\left[\mathrm{hC4b}\right]\left[\mathrm{nfC4b}\right]-k_{\mathrm{C4bC2}}^{+}\left[\mathrm{hC4b}\right]\left[\mathrm{C2}\right]+k_{\mathrm{C4bC2}}^{-}\left[\mathrm{hC4bC2}\right]$$

$$-k_{\mathrm{C3bC4b}}^{+}\left[\mathrm{hC4b}\right]\left[\mathrm{nfC3b}\right]-k_{\mathrm{C4bCR1}}^{+}\left[\mathrm{hC4b}\right]\left[\mathrm{CR1}\right]+k_{\mathrm{C4bCR1}}^{-}\left[\mathrm{hC4bCR1}\right]-k_{\mathrm{C4bC4BP}}^{+}\left[\mathrm{hC4b}\right]\left[\mathrm{C4BP}\right]+k_{\mathrm{C4bC4BP}}^{-}\left[\mathrm{hC4bC4BP}\right]$$

68

$$\frac{d\left[\mathrm{hC4bC2}\right]}{dt}=k_{\mathrm{C4bC2}}^{+}\left[\mathrm{hC4b}\right]\left[\mathrm{C2}\right]-k_{\mathrm{C4bC2}}^{-}\left[\mathrm{hC4bC2}\right]$$

69

$$\frac{d\left[\mathrm{hC4bC4b}\right]}{dt}=k_{\mathrm{C4bC4b}}^{+}\left[\mathrm{hC4b}\right]\left[\mathrm{nfC4b}\right]-k_{\mathrm{C4bC2}}^{+}\left[\mathrm{hC4bC4b}\right]\left[\mathrm{C2}\right]+k_{\mathrm{C4bC2}}^{-}\left[\mathrm{hC4bC4bC2}\right]$$

$$-k_{\mathrm{C4bC4bCR1}}^{+}\left[\mathrm{hC4bC4b}\right]\left[\mathrm{CR1}\right]+k_{\mathrm{C4bC4bCR1}}^{-}\left[\mathrm{hC4bC4bCR1}\right]-k_{\mathrm{C4bC4BP}}^{+}\left[\mathrm{hC4bC4b}\right]\left[\mathrm{C4BP}\right]+k_{\mathrm{C4bC4BP}}^{-}\left[\mathrm{hC4bC4bC4BP}\right]$$

70

$$\frac{d\left[\mathrm{hC4bC4bC2}\right]}{dt}=k_{\mathrm{C4bC2}}^{+}\left[\mathrm{hC4bC4b}\right]\left[\mathrm{C2}\right]-k_{\mathrm{C4bC2}}^{-}\left[\mathrm{hC4bC4bC2}\right]$$

71

$$\frac{d\left[\mathrm{hC3bC4b}\right]}{dt}=k_{\mathrm{C3bC4b}}^{+}\left[\mathrm{hC4b}\right]\left[\mathrm{nfC3b}\right]-k_{\mathrm{C4bC2}}^{+}\left[\mathrm{hC3bC4b}\right]\left[\mathrm{C2}\right]+k_{\mathrm{C4bC2}}^{-}\left[\mathrm{hC3bC4bC2}\right]-k_{\mathrm{C3bB}}^{+}\left[\mathrm{hC3bC4b}\right]\left[\mathrm{FB}\right]$$

$$+k_{\mathrm{C3bB}}^{-}\left[\mathrm{hC4bC3bB}\right]+k_{\mathrm{C3bC4b}}^{+}\left[\mathrm{hC3b}\right]\left[\mathrm{nfC4b}\right]+k_{\mathrm{C3bC4bBb}}^{-}\left[\mathrm{hC4bC3bBb}\right]-k_{\mathrm{C3bH}}^{+}\left[\mathrm{hC3bC4b}\right]\left[\mathrm{FH}\right]+k_{\mathrm{C3bH}}^{-}\left[\mathrm{hC3bC4bFH}\right]$$

$$-k_{\mathrm{C3bFHL1}}^{+}\left[\mathrm{hC3bC4b}\right]\left[\mathrm{FHL1}\right]+k_{\mathrm{C3bFHL1}}^{-}\left[\mathrm{hC3bC4bFHL1}\right]-k_{\mathrm{C3bC4bCR1}}^{+}\left[\mathrm{hC3bC4b}\right]\left[\mathrm{CR1}\right]+k_{\mathrm{C3bC4bCR1}}^{-}\left[\mathrm{hC3bC4bCR1}\right]$$

$$-k_{\mathrm{C4bC4BP}}^{+}\left[\mathrm{hC3bC4b}\right]\left[\mathrm{C4BP}\right]+k_{\mathrm{C4bC4BP}}^{-}\left[\mathrm{hC3bC4bC4BP}\right]+k_{\mathrm{C3bBbH_{decay}}}^{-}\left[\mathrm{hC4bC3bBbFH}\right]$$

$$+k_{\mathrm{C3bBbFHL1_{decay}}}^{-}\left[\mathrm{hC4bC3bBbFHL1}\right]+k_{\mathrm{C3bBbCR1_{decay}}}^{-}\left[\mathrm{hC4bC3bBbCR1}\right]+k_{\mathrm{C3bBbDAF_{decay}}}^{-}\left[\mathrm{hC4bC3bBbDAF}\right]$$

$$-k_{\mathrm{C3bP}}^{+}\left[\mathrm{hC3bC4b}\right]\left[\mathrm{P}\right]+k_{\mathrm{C3bP}}^{-}\left[\mathrm{hC3bC4bP}\right]+k_{\mathrm{C3bC4bBbP}}^{-}\left[\mathrm{hC4bC3bBbP}\right]$$

72

$$\frac{d[\text{hC3bC4bC2}]}{dt}=k^{+}_{\text{C4bC2}}[\text{hC3bC4b}][\text{C2}]-k^{-}_{\text{C4bC2}}[\text{hC3bC4bC2}]$$

73

$$\frac{d[\text{hC4bC3bB}]}{dt}=k^{+}_{\text{C3bB}}[\text{hC3bC4b}][\text{FB}]-k^{-}_{\text{C3bB}}[\text{hC4bC3bB}]$$
$$-\frac{k^{\text{FD}}_{\text{cat C3(H}_2\text{O)B}}[\text{FD}][\text{hC4bC3bB}]}{\left(\begin{array}{l}K^{\text{D}}_{\text{m C3(H}_2\text{O)B}}+[\text{C3(H}_2\text{O)B}]+[\text{fC3bB}]+[\text{fC3bC3bB}]+[\text{fC3bC3bBP}]+[\text{IgGC3bC3bB}]+[\text{IgGC3bC3bBP}]\\+[\text{fC4bC3bB}]+[\text{fC4bC3bBP}]+[\text{pC3bBCFHR1}]+[\text{pC3bBCFHR2}]+[\text{pC3bBCFHR3}]+[\text{pC3bBCFHR4}]\\+[\text{pC3bBCFHR5}]+[\text{hC4bC3bBP}]+[\text{hC4bC3bB}]+[\text{hC3bC3bB}]+[\text{hC3bC3bBP}]+[\text{hC3bB}]+[\text{pC4bC3bB}]\\+[\text{pC4bC3bBP}]+[\text{pC3bC3bB}]+[\text{pC3bC3bBP}]+[\text{pC3bB}]\end{array}\right)}$$

74

$$\frac{d[\text{hC4bC3bBb}]}{dt}=$$
$$\frac{k^{\text{FD}}_{\text{cat C3(H}_2\text{O)B}}[\text{FD}][\text{hC4bC3bB}]}{\left(\begin{array}{l}K^{\text{D}}_{\text{m C3(H}_2\text{O)B}}+[\text{C3(H}_2\text{O)B}]+[\text{fC3bB}]+[\text{fC3bC3bB}]+[\text{fC3bC3bBP}]+[\text{IgGC3bC3bB}]+[\text{IgGC3bC3bBP}]\\+[\text{fC4bC3bB}]+[\text{fC4bC3bBP}]+[\text{pC3bBCFHR1}]+[\text{pC3bBCFHR2}]+[\text{pC3bBCFHR3}]+[\text{pC3bBCFHR4}]\\+[\text{pC3bBCFHR5}]+[\text{hC4bC3bBP}]+[\text{hC4bC3bB}]+[\text{hC3bC3bB}]+[\text{hC3bC3bBP}]+[\text{hC3bB}]+[\text{pC4bC3bB}]\\+[\text{pC4bC3bBP}]+[\text{pC3bC3bB}]+[\text{pC3bC3bBP}]+[\text{pC3bB}]\end{array}\right)}$$
$$+k^{+}_{\text{C3bC4b}}[\text{nfC4b}][\text{hC3bBb}]-k^{-}_{\text{C3bC4bBb}}[\text{hC4bC3bBb}]+k^{-}_{\text{C5b}^*}[\text{hC4bC3bBbC5b}]+k^{+}_{\text{C5b7}}[\text{hC4bC3bBbC5b6}][\text{C7}]$$
$$-k^{+}_{\text{C3bH}}[\text{hC4bC3bBb}][\text{FH}]+k^{-}_{\text{C3bH}}[\text{hC4bC3bBbFH}]-k^{+}_{\text{C3bFHL1}}[\text{hC4bC3bBb}][\text{FHL1}]+k^{-}_{\text{C3bFHL1}}[\text{hC4bC3bBbFHL1}]$$
$$-k^{+}_{\text{C3bC4bCR1}}[\text{hC4bC3bBb}][\text{CR1}]+k^{-}_{\text{C3bC4bCR1}}[\text{hC4bC3bBbCR1}]-k^{+}_{\text{C3bBbDAF}}[\text{hC4bC3bBb}][\text{DAF}]$$
$$+k^{-}_{\text{C3bBbDAF}}[\text{hC4bC3bBbDAF}]-k^{+}_{\text{C3bP}}[\text{hC4bC3bBb}][\text{P}]+k^{-}_{\text{C3bP}}[\text{hC4bC3bBbP}]$$

75

$$\frac{d[\text{hC3bC4bP}]}{dt}=k^{+}_{\text{C3bP}}[\text{hC3bC4b}][\text{P}]-k^{-}_{\text{C3bP}}[\text{hC3bC4bP}]-k^{+}_{\text{C3bB}}[\text{hC3bC4bP}][\text{FB}]+k^{-}_{\text{C3bB}}[\text{hC4bC3bBP}]$$

76

$$\frac{d\left[\mathrm{hC4bC3bBP}\right]}{dt}=k_{\mathrm{C3bB}}^{+}\left[\mathrm{hC3bC4bP}\right]\left[\mathrm{FB}\right]-k_{\mathrm{C3bB}}^{-}\left[\mathrm{hC4bC3bBP}\right]$$
$$-\frac{k_{\mathrm{cat\ C3(H_2O)B}}^{\mathrm{FD}}\left[\mathrm{FD}\right]\left[\mathrm{hC4bC3bBP}\right]}{\left(\begin{array}{l}K_{\mathrm{m\ C3(H_2O)B}}^{\mathrm{D}}+\left[\mathrm{C3(H_2O)B}\right]+\left[\mathrm{fC3bB}\right]+\left[\mathrm{fC3bC3bB}\right]+\left[\mathrm{fC3bC3bBP}\right]+\left[\mathrm{IgGC3bC3bB}\right]+\left[\mathrm{IgGC3bC3bBP}\right]\\+\left[\mathrm{fC4bC3bB}\right]+\left[\mathrm{fC4bC3bBP}\right]+\left[\mathrm{pC3bBCFHR1}\right]+\left[\mathrm{pC3bBCFHR2}\right]+\left[\mathrm{pC3bBCFHR3}\right]+\left[\mathrm{pC3bBCFHR4}\right]\\+\left[\mathrm{pC3bBCFHR5}\right]+\left[\mathrm{hC4bC3bBP}\right]+\left[\mathrm{hC4bC3bB}\right]+\left[\mathrm{hC3bC3bB}\right]+\left[\mathrm{hC3bC3bBP}\right]+\left[\mathrm{hC3bB}\right]+\left[\mathrm{pC4bC3bB}\right]\\+\left[\mathrm{pC4bC3bBP}\right]+\left[\mathrm{pC3bC3bB}\right]+\left[\mathrm{pC3bC3bBP}\right]+\left[\mathrm{pC3bB}\right]\end{array}\right)}$$

77

$$\frac{d\left[\mathrm{hC4bC3bBbP}\right]}{dt}=$$
$$\frac{k_{\mathrm{cat\ C3(H_2O)B}}^{\mathrm{FD}}\left[\mathrm{FD}\right]\left[\mathrm{hC4bC3bBP}\right]}{\left(\begin{array}{l}K_{\mathrm{m\ C3(H_2O)B}}^{\mathrm{D}}+\left[\mathrm{C3(H_2O)B}\right]+\left[\mathrm{fC3bB}\right]+\left[\mathrm{fC3bC3bB}\right]+\left[\mathrm{fC3bC3bBP}\right]+\left[\mathrm{IgGC3bC3bB}\right]+\left[\mathrm{IgGC3bC3bBP}\right]\\+\left[\mathrm{fC4bC3bB}\right]+\left[\mathrm{fC4bC3bBP}\right]+\left[\mathrm{pC3bBCFHR1}\right]+\left[\mathrm{pC3bBCFHR2}\right]+\left[\mathrm{pC3bBCFHR3}\right]+\left[\mathrm{pC3bBCFHR4}\right]\\+\left[\mathrm{pC3bBCFHR5}\right]+\left[\mathrm{hC4bC3bBP}\right]+\left[\mathrm{hC4bC3bB}\right]+\left[\mathrm{hC3bC3bB}\right]+\left[\mathrm{hC3bC3bBP}\right]+\left[\mathrm{hC3bB}\right]+\left[\mathrm{pC4bC3bB}\right]\\+\left[\mathrm{pC4bC3bBP}\right]+\left[\mathrm{pC3bC3bB}\right]+\left[\mathrm{pC3bC3bBP}\right]+\left[\mathrm{pC3bB}\right]\end{array}\right)}$$
$$+k_{\mathrm{C3bC4b}}^{+}\left[\mathrm{nfC4b}\right]\left[\mathrm{hC3bBbP}\right]+k_{\mathrm{C3bP}}^{+}\left[\mathrm{hC4bC3bBb}\right]\left[\mathrm{P}\right]-k_{\mathrm{C3bP}}^{-}\left[\mathrm{hC4bC3bBbP}\right]-k_{\mathrm{C3bC4bBbP}}^{-}\left[\mathrm{hC4bC3bBbP}\right]$$
$$+k_{\mathrm{C5b^*}}^{-}\left[\mathrm{hC4bC3bBbPC5b}\right]+k_{\mathrm{C5b7}}^{+}\left[\mathrm{hC4bC3bBbPC5b6}\right]\left[\mathrm{C7}\right]$$

78

$$\frac{d\left[\mathrm{hC4bC3bBbC5b}\right]}{dt}=\frac{k_{\mathrm{cat\ C3bC4bBb}}^{\mathrm{C5}}\left[\mathrm{C5}\right]\left[\mathrm{hC4bC3bBb}\right]}{K_{\mathrm{m\ C3bC4bBb}}^{\mathrm{C5}}\left(1+\frac{\left[\mathrm{C3}\right]}{K_{\mathrm{m\ C3bBb}}^{\mathrm{C3}}}+\frac{\left[\mathrm{C5}\right]}{K_{\mathrm{m\ C3bC4bBb}}^{\mathrm{C5}}}\right)}-k_{\mathrm{C5b^*}}^{-}\left[\mathrm{hC4bC3bBbC5b}\right]$$
$$-k_{\mathrm{C5b6}}^{+}\left[\mathrm{hC4bC3bBbC5b}\right]\left[\mathrm{C6}\right]+k_{\mathrm{C5b6}}^{-}\left[\mathrm{hC4bC3bBbC5b6}\right]$$

79

$$\frac{d\left[\mathrm{hC4bC3bBbC5b6}\right]}{dt}=k_{\mathrm{C5b6}}^{+}\left[\mathrm{hC4bC3bBbC5b}\right]\left[\mathrm{C6}\right]-k_{\mathrm{C5b6}}^{-}\left[\mathrm{hC4bC3bBbC5b6}\right]-k_{\mathrm{C5b7}}^{+}\left[\mathrm{hC4bC3bBbC5b6}\right]\left[\mathrm{C7}\right]$$

80

$$\frac{d\left[\mathrm{hC4bC3bBbPC5b}\right]}{dt}=\frac{k_{\mathrm{cat\ C3bC4bBb}}^{\mathrm{C5}}\left[\mathrm{C5}\right]\left[\mathrm{hC4bC3bBbP}\right]}{K_{\mathrm{m\ C3bC4bBb}}^{\mathrm{C5}}\left(1+\frac{\left[\mathrm{C3}\right]}{K_{\mathrm{m\ C3bBb}}^{\mathrm{C3}}}+\frac{\left[\mathrm{C5}\right]}{K_{\mathrm{m\ C3bC4bBb}}^{\mathrm{C5}}}\right)}-k_{\mathrm{C5b^*}}^{-}\left[\mathrm{hC4bC3bBbPC5b}\right]$$
$$-k_{\mathrm{C5b6}}^{+}\left[\mathrm{hC4bC3bBbPC5b}\right]\left[\mathrm{C6}\right]+k_{\mathrm{C5b6}}^{-}\left[\mathrm{hC4bC3bBbPC5b6}\right]$$

81

$$\frac{d[\text{hC4bC3bBbPC5b6}]}{dt}=k_{\text{C5b6}}^{+}[\text{hC4bC3bBbPC5b}][\text{C6}]-k_{\text{C5b6}}^{-}[\text{hC4bC3bBbPC5b6}]$$
$$-k_{\text{C5b7}}^{+}[\text{hC4bC3bBbPC5b6}][\text{C7}]$$

82

$$\frac{d[\text{hC3bC3bBbC5b}]}{dt}=\frac{k_{\text{cat C3bC3bBb}}^{\text{C5}}[\text{C5}][\text{hC3bC3bBb}]}{K_{\text{m C3bC3bBb}}^{\text{C5}}\left(1+\frac{[\text{C3}]}{K_{\text{m C3bBb}}^{\text{C3}}}+\frac{[\text{C5}]}{K_{\text{m C3bC3bBb}}^{\text{C5}}}\right)}-k_{\text{C5b}^*}^{-}[\text{hC3bC3bBbC5b}]$$
$$-k_{\text{C5b6}}^{+}[\text{hC3bC3bBbC5b}][\text{C6}]+k_{\text{C5b6}}^{-}[\text{hC3bC3bBbC5b6}]$$

83

$$\frac{d[\text{hC3bC3bBbC5b6}]}{dt}=k_{\text{C5b6}}^{+}[\text{hC3bC3bBbC5b}][\text{C6}]-k_{\text{C5b6}}^{-}[\text{hC3bC3bBbC5b6}]-k_{\text{C5b7}}^{+}[\text{hC3bC3bBbC5b6}][\text{C7}]$$

84

$$\frac{d[\text{hC3bC3bBbPC5b}]}{dt}=\frac{k_{\text{cat C3bC3bBb}}^{\text{C5}}[\text{C5}][\text{hC3bC3bBbP}]}{K_{\text{m C3bC3bBb}}^{\text{C5}}\left(1+\frac{[\text{C3}]}{K_{\text{m C3bBb}}^{\text{C3}}}+\frac{[\text{C5}]}{K_{\text{m C3bC3bBb}}^{\text{C5}}}\right)}-k_{\text{C5b}^*}^{-}[\text{hC3bC3bBbPC5b}]$$
$$-k_{\text{C5b6}}^{+}[\text{hC3bC3bBbPC5b}][\text{C6}]+k_{\text{C5b6}}^{-}[\text{hC3bC3bBbPC5b6}]$$

85

$$\frac{d[\text{hC3bC3bBbPC5b6}]}{dt}=k_{\text{C5b6}}^{+}[\text{hC3bC3bBbPC5b}][\text{C6}]-k_{\text{C5b6}}^{-}[\text{hC3bC3bBbPC5b6}]$$
$$-k_{\text{C5b7}}^{+}[\text{hC3bC3bBbPC5b6}][\text{C7}]$$

86

$$\frac{d[\text{fC5b7}]}{dt}=k_{\text{C5b7}}^{+}[\text{fC5b6}][\text{C7}]-k_{\text{C5b7}}^{-}[\text{fC5b7}]+k_{\text{C5b7}}^{+}[\text{fC3bC3bBbC5b6}][\text{C7}]+k_{\text{C5b7}}^{+}[\text{fC3bC3bBbPC5b6}][\text{C7}]$$
$$+k_{\text{C5b7}}^{+}[\text{fC4bC3bBbC5b6}][\text{C7}]+k_{\text{C5b7}}^{+}[\text{fC3bC4bC2aC5b6}][\text{C7}]+k_{\text{C5b7}}^{+}[\text{fC4bC4bC2aC5b6}][\text{C7}]$$
$$+k_{\text{C5b7}}^{+}[\text{fC4bC3bBbPC5b6}][\text{C7}]-k_{\text{C5b8}}^{+}[\text{fC5b7}][\text{C8}]+k_{\text{C5b8}}^{-}[\text{fC5b8}]-k_{\text{C5b7}_{\text{micelle}}}^{+}[\text{fC5b7}]-k_{\text{VnC5b7}}^{+}[\text{fC5b7}][\text{Vn}]$$
$$+k_{\text{VnC5b7}}^{-}[\text{VnC5b7}]-k_{\text{CnC5b7}}^{+}[\text{fC5b7}][\text{Cn}]+k_{\text{CnC5b7}}^{-}[\text{CnC5b7}]-k_{\text{C5b7}_{\text{surface}}}^{+}[\text{fC5b7}][\text{Host Surface}]\times 4$$
$$+k_{\text{C5b7}}^{+}[\text{hC4bC3bBbC5b6}][\text{C7}]+k_{\text{C5b7}}^{+}[\text{hC4bC3bBbPC5b6}][\text{C7}]+k_{\text{C5b7}}^{+}[\text{hC3bC3bBbC5b6}][\text{C7}]$$
$$+k_{\text{C5b7}}^{+}[\text{hC3bC3bBbPC5b6}][\text{C7}]+k_{\text{C5b7}}^{+}[\text{pC4bC4bC2aC5b6}][\text{C7}]+k_{\text{C5b7}}^{+}[\text{pC4bC3bC2aC5b6}][\text{C7}]$$
$$+k_{\text{C5b7}}^{+}[\text{pC4bC3bBbC5b6}][\text{C7}]+k_{\text{C5b7}}^{+}[\text{pC4bC3bBbPC5b6}][\text{C7}]+k_{\text{C5b7}}^{+}[\text{pC3bC3bBbC5b6}][\text{C7}]$$
$$+k_{\text{C5b7}}^{+}[\text{pC3bC3bBbPC5b6}][\text{C7}]-k_{\text{C5b7}_{\text{surface}}}^{+}[\text{fC5b7}][\text{Pathogen Surface}]\times 4$$

87

$$\frac{d\left[\text{fC5b}^*_{\text{inactive}}\right]}{dt}=k^-_{\text{C5b}^*}\left[\text{fC5b}^*\right]+k^-_{\text{C5b}^*}\left[\text{fC3bC3bBbC5b}\right]+k^-_{\text{C5b}^*}\left[\text{fC3bC3bBbPC5b}\right]+k^-_{\text{C5b}^*}\left[\text{fC4bC3bBbC5b}\right]$$
$$+k^-_{\text{C5b}^*}\left[\text{fC4bC3bBbPC5b}\right]+k^-_{\text{C5b}^*}\left[\text{fC4bC4bC2aC5b}\right]+k^-_{\text{C5b}^*}\left[\text{fC3bC4bC2aC5b}\right]+k^-_{\text{C5b}^*}\left[\text{hC4bC3bBbC5b}\right]$$
$$+k^-_{\text{C5b}^*}\left[\text{hC4bC3bBbPC5b}\right]+k^-_{\text{C5b}^*}\left[\text{hC3bC3bBbC5b}\right]+k^-_{\text{C5b}^*}\left[\text{hC3bC3bBbPC5b}\right]+k^-_{\text{C5b}^*}\left[\text{pC4bC4bC2aC5b}\right]$$
$$+k^-_{\text{C5b}^*}\left[\text{pC3bC4bC2aC5b}\right]+k^-_{\text{C5b}^*}\left[\text{pC4bC3bBbC5b}\right]+k^-_{\text{C5b}^*}\left[\text{pC4bC3bBbPC5b}\right]+k^-_{\text{C5b}^*}\left[\text{pC3bC3bBbC5b}\right]$$
$$+k^-_{\text{C5b}^*}\left[\text{pC3bC3bBbPC5b}\right]$$

88

$$\frac{d\left[\text{fC5b7}_{\text{micelle}}\right]}{dt}=k^+_{\text{micelle}}\left[\text{fC5b7}\right]$$

89

$$\frac{d\left[\text{fC5b8}\right]}{dt}=k^+_{\text{C5b8}}\left[\text{fC5b7}\right]\left[\text{C8}\right]-k^-_{\text{C5b8}}\left[\text{fC5b8}\right]-k^+_{\text{C5b9}}\left[\text{fC5b8}\right]\left[\text{C9}_1\right]+k^-_{\text{C5b9}}\left[\text{fC5b9}_1\right]-k^+_{\text{VnC5b8}}\left[\text{fC5b8}\right]\left[\text{Vn}\right]$$
$$+k^-_{\text{VnC5b8}}\left[\text{VnC5b8}\right]-k^+_{\text{CnC5b8}}\left[\text{fC5b8}\right]\left[\text{Cn}\right]+k^-_{\text{CnC5b8}}\left[\text{CnC5b8}\right]$$

90

$$\frac{d\left[\text{fC5b9}_1\right]}{dt}=k^+_{\text{C5b9}}\left[\text{fC5b8}\right]\left[\text{C9}_1\right]-k^-_{\text{C5b9}}\left[\text{fC5b9}_1\right]-k^+_{\text{VnC5b9}}\left[\text{fC5b9}\right]\left[\text{Vn}\right]+k^-_{\text{VnC5b9}}\left[\text{VnC5b9}\right]-k^+_{\text{CnC5b9}}\left[\text{fC5b9}\right]\left[\text{Cn}\right]$$
$$+k^-_{\text{CnC5b9}}\left[\text{CnC5b9}\right]$$

91

$$\frac{d\left[\text{hC5b7}\right]}{dt}=k^+_{\text{C5b7}_{\text{surface}}}\left[\text{fC5b7}\right]\left[\text{Host Surface}\right]\times 4-k^+_{\text{C5b8}}\left[\text{hC5b7}\right]\left[\text{C8}\right]$$

92

$$\frac{d\left[\text{hC5b8}\right]}{dt}=k^+_{\text{C5b8}}\left[\text{hC5b7}\right]\left[\text{C8}\right]-k^+_{\text{C5b9}_1}\left[\text{hC5b8}\right]\left[\text{C9}_1\right]-k^+_{\text{C5b8CD59}}\left[\text{hC5b8}\right]\left[\text{CD59}\right]+k^-_{\text{C5b8CD59}}\left[\text{hC5b8CD59}\right]$$

93

$$\frac{d\left[\text{hC5b9}_1\right]}{dt}=k^+_{\text{C5b9}_1}\left[\text{hC5b8}\right]\left[\text{C9}_1\right]-k^+_{\text{C5b9}_{\text{poly}}}\left[\text{hC5b9}_1\right]\left[\text{C9}_2\right]-k^+_{\text{C5b9CD59}}\left[\text{hC5b9}_1\right]\left[\text{CD59}\right]+k^-_{\text{C5b9CD59}}\left[\text{hC5b9}_1\text{CD59}\right]$$

94

$$\frac{d\left[\text{hC5b9}_2\right]}{dt}=k^+_{\text{C5b9}_{\text{poly}}}\left[\text{hC5b9}_1\right]\left[\text{C9}_2\right]-k^+_{\text{C5b9}_{\text{poly}}}\left[\text{hC5b9}_2\right]\left[\text{C9}_3\right]$$

95

$$\frac{d\left[\text{hC5b9}_3\right]}{dt}=k^+_{\text{C5b9}_{\text{poly}}}\left[\text{hC5b9}_2\right]\left[\text{C9}_3\right]-k^+_{\text{C5b9}_{\text{poly}}}\left[\text{hC5b9}_3\right]\left[\text{C9}_4\right]$$

96
$$\frac{d\left[\mathrm{hC5b9}_{4}\right]}{dt}=k^{+}_{\mathrm{C5b9}_{\mathrm{poly}}}\left[\mathrm{hC5b9}_{3}\right]\left[\mathrm{C9}_{4}\right]-k^{+}_{\mathrm{C5b9}_{\mathrm{poly}}}\left[\mathrm{hC5b9}_{4}\right]\left[\mathrm{C9}_{5}\right]$$

97
$$\frac{d\left[\mathrm{hC5b9}_{5}\right]}{dt}=k^{+}_{\mathrm{C5b9}_{\mathrm{poly}}}\left[\mathrm{hC5b9}_{4}\right]\left[\mathrm{C9}_{5}\right]-k^{+}_{\mathrm{C5b9}_{\mathrm{poly}}}\left[\mathrm{hC5b9}_{5}\right]\left[\mathrm{C9}_{6}\right]$$

98
$$\frac{d\left[\mathrm{hC5b9}_{6}\right]}{dt}=k^{+}_{\mathrm{C5b9}_{\mathrm{poly}}}\left[\mathrm{hC5b9}_{5}\right]\left[\mathrm{C9}_{6}\right]-k^{+}_{\mathrm{C5b9}_{\mathrm{poly}}}\left[\mathrm{hC5b9}_{6}\right]\left[\mathrm{C9}_{7}\right]$$

99
$$\frac{d\left[\mathrm{hC5b9}_{7}\right]}{dt}=k^{+}_{\mathrm{C5b9}_{\mathrm{poly}}}\left[\mathrm{hC5b9}_{6}\right]\left[\mathrm{C9}_{7}\right]-k^{+}_{\mathrm{C5b9}_{\mathrm{poly}}}\left[\mathrm{hC5b9}_{7}\right]\left[\mathrm{C9}_{8}\right]$$

100
$$\frac{d\left[\mathrm{hC5b9}_{8}\right]}{dt}=k^{+}_{\mathrm{C5b9}_{\mathrm{poly}}}\left[\mathrm{hC5b9}_{7}\right]\left[\mathrm{C9}_{8}\right]-k^{+}_{\mathrm{C5b9}_{\mathrm{poly}}}\left[\mathrm{hC5b9}_{8}\right]\left[\mathrm{C9}_{9}\right]$$

101
$$\frac{d\left[\mathrm{hC5b9}_{9}\right]}{dt}=k^{+}_{\mathrm{C5b9}_{\mathrm{poly}}}\left[\mathrm{hC5b9}_{8}\right]\left[\mathrm{C9}_{9}\right]-k^{+}_{\mathrm{C5b9}_{\mathrm{poly}}}\left[\mathrm{hC5b9}_{9}\right]\left[\mathrm{C9}_{10}\right]$$

102
$$\frac{d\left[\mathrm{hC5b9}_{10}\right]}{dt}=k^{+}_{\mathrm{C5b9}_{\mathrm{poly}}}\left[\mathrm{hC5b9}_{9}\right]\left[\mathrm{C9}_{10}\right]-k^{+}_{\mathrm{C5b9}_{\mathrm{poly}}}\left[\mathrm{hC5b9}_{10}\right]\left[\mathrm{C9}_{11}\right]$$

103
$$\frac{d\left[\mathrm{hC5b9}_{11}\right]}{dt}=k^{+}_{\mathrm{C5b9}_{\mathrm{poly}}}\left[\mathrm{hC5b9}_{10}\right]\left[\mathrm{C9}_{11}\right]-k^{+}_{\mathrm{C5b9}_{\mathrm{poly}}}\left[\mathrm{hC5b9}_{11}\right]\left[\mathrm{C9}_{12}\right]$$

104
$$\frac{d\left[\mathrm{hC5b9}_{12}\right]}{dt}=k^{+}_{\mathrm{C5b9}_{\mathrm{poly}}}\left[\mathrm{hC5b9}_{11}\right]\left[\mathrm{C9}_{12}\right]-k^{+}_{\mathrm{C5b9}_{\mathrm{poly}}}\left[\mathrm{hC5b9}_{12}\right]\left[\mathrm{C9}_{13}\right]$$

105
$$\frac{d\left[\mathrm{hC5b9}_{13}\right]}{dt}=k^{+}_{\mathrm{C5b9}_{\mathrm{poly}}}\left[\mathrm{hC5b9}_{12}\right]\left[\mathrm{C9}_{13}\right]-k^{+}_{\mathrm{C5b9}_{\mathrm{poly}}}\left[\mathrm{hC5b9}_{13}\right]\left[\mathrm{C9}_{14}\right]$$

106
$$\frac{d\left[\mathrm{hC5b9}_{14}\right]}{dt}=k^{+}_{\mathrm{C5b9}_{\mathrm{poly}}}\left[\mathrm{hC5b9}_{13}\right]\left[\mathrm{C9}_{14}\right]-k^{+}_{\mathrm{C5b9}_{\mathrm{poly}}}\left[\mathrm{hC5b9}_{14}\right]\left[\mathrm{C9}_{15}\right]$$

107

$$\frac{d\left[\mathrm{hC5b9}_{15}\right]}{dt}=k^{+}_{\mathrm{C5b9_{poly}}}\left[\mathrm{hC5b9}_{14}\right]\left[\mathrm{C9}_{15}\right]-k^{+}_{\mathrm{C5b9_{poly}}}\left[\mathrm{hC5b9}_{15}\right]\left[\mathrm{C9}_{16}\right]$$

108

$$\frac{d\left[\mathrm{hC5b9}_{16}\right]}{dt}=k^{+}_{\mathrm{C5b9_{poly}}}\left[\mathrm{hC5b9}_{15}\right]\left[\mathrm{C9}_{16}\right]-k^{+}_{\mathrm{C5b9_{poly}}}\left[\mathrm{hC5b9}_{16}\right]\left[\mathrm{C9}_{17}\right]$$

109

$$\frac{d\left[\mathrm{hC5b9}_{17}\right]}{dt}=k^{+}_{\mathrm{C5b9_{poly}}}\left[\mathrm{hC5b9}_{16}\right]\left[\mathrm{C9}_{17}\right]-k^{+}_{\mathrm{C5b9_{poly}}}\left[\mathrm{hC5b9}_{17}\right]\left[\mathrm{C9}_{18}\right]$$

110

$$\frac{d\left[\mathrm{hC5b9}_{18}\right]}{dt}=k^{+}_{\mathrm{C5b9_{poly}}}\left[\mathrm{hC5b9}_{17}\right]\left[\mathrm{C9}_{18}\right]$$

Pathogen (Fluid and Surface)

111

$$\frac{d\left[\mathrm{pC3bCFHR1}\right]}{dt}=k^{+}_{\mathrm{C3bCFHR1}}\left[\mathrm{fC3b}\right]\left[\mathrm{pCFHR1}\right]-k^{-}_{\mathrm{C3bCFHR1}}\left[\mathrm{pC3bCFHR1}\right]-k^{+}_{\mathrm{C3bB}}\left[\mathrm{pC3bCFHR1}\right]\left[\mathrm{FB}\right]$$
$$+k^{-}_{\mathrm{C3bB}}\left[\mathrm{pC3bBCFHR1}\right]+k^{-}_{\mathrm{C3bBb}}\left[\mathrm{pC3bBbCFHR1}\right]+k^{-}_{\mathrm{C3bBbP}}\left[\mathrm{pC3bBbPCFHR1}\right]-k^{-}_{\mathrm{pCFHR1}}\left[\mathrm{pC3bCFHR1}\right]$$

112

$$\frac{d\left[\mathrm{pC3bBCFHR1}\right]}{dt}=k^{+}_{\mathrm{C3bB}}\left[\mathrm{pC3bCFHR1}\right]\left[\mathrm{FB}\right]-k^{-}_{\mathrm{C3bB}}\left[\mathrm{pC3bBCFHR1}\right]$$
$$-\frac{k^{\mathrm{FD}}_{\mathrm{cat\ C3(H_2O)B}}\left[\mathrm{FD}\right]\left[\mathrm{pC3bBCFHR1}\right]}{\left(\begin{array}{l}K^{\mathrm{D}}_{\mathrm{m\ C3(H_2O)B}}+\left[\mathrm{C3(H_2O)B}\right]+\left[\mathrm{fC3bB}\right]+\left[\mathrm{fC3bC3bB}\right]+\left[\mathrm{fC3bC3bBP}\right]+\left[\mathrm{IgGC3bC3bB}\right]+\left[\mathrm{IgGC3bC3bBP}\right]\\+\left[\mathrm{fC4bC3bB}\right]+\left[\mathrm{fC4bC3bBP}\right]+\left[\mathrm{pC3bBCFHR1}\right]+\left[\mathrm{pC3bBCFHR2}\right]+\left[\mathrm{pC3bBCFHR3}\right]+\left[\mathrm{pC3bBCFHR4}\right]\\+\left[\mathrm{pC3bBCFHR5}\right]+\left[\mathrm{hC4bC3bBP}\right]+\left[\mathrm{hC4bC3bB}\right]+\left[\mathrm{hC3bC3bB}\right]+\left[\mathrm{hC3bC3bBP}\right]+\left[\mathrm{hC3bB}\right]+\left[\mathrm{pC4bC3bB}\right]\\+\left[\mathrm{pC4bC3bBP}\right]+\left[\mathrm{pC3bC3bB}\right]+\left[\mathrm{pC3bC3bBP}\right]+\left[\mathrm{pC3bB}\right]\end{array}\right)}$$
$$-k^{-}_{\mathrm{pCFHR1}}\left[\mathrm{pC3bBCFHR1}\right]$$

113

$$\frac{d[\text{pC3bBbCFHR1}]}{dt}=$$

$$\frac{k^{\text{FD}}_{\text{cat C3(H}_2\text{O)B}}[\text{FD}][\text{pC3bBCFHR1}]}{\begin{pmatrix} K^{\text{D}}_{\text{m C3(H}_2\text{O)B}}+[\text{C3(H}_2\text{O)B}]+[\text{fC3bB}]+[\text{fC3bC3bB}]+[\text{fC3bC3bBP}]+[\text{IgGC3bC3bB}]+[\text{IgGC3bC3bBP}] \\ +[\text{fC4bC3bB}]+[\text{fC4bC3bBP}]+[\text{pC3bBCFHR1}]+[\text{pC3bBCFHR2}]+[\text{pC3bBCFHR3}]+[\text{pC3bBCFHR4}] \\ +[\text{pC3bBCFHR5}]+[\text{hC4bC3bBP}]+[\text{hC4bC3bB}]+[\text{hC3bC3bB}]+[\text{hC3bC3bBP}]+[\text{hC3bB}]+[\text{pC4bC3bB}] \\ +[\text{pC4bC3bBP}]+[\text{pC3bC3bB}]+[\text{pC3bC3bBP}]+[\text{pC3bB}] \end{pmatrix}}$$

$$-k^{-}_{\text{C3bBb}}[\text{pC3bBbCFHR1}]-k^{+}_{\text{C3bP}}[\text{pC3bBbCFHR1}][\text{P}]+k^{-}_{\text{C3bP}}[\text{pC3bBbPCFHR1}]-k^{-}_{\text{pCFHR1}}[\text{pC3bBbCFHR1}]$$

114

$$\frac{d[\text{pC3bBbPCFHR1}]}{dt}=k^{+}_{\text{C3bP}}[\text{pC3bBbCFHR1}][\text{P}]-k^{-}_{\text{C3bP}}[\text{pC3bBbPCFHR1}]-k^{-}_{\text{C3bBbP}}[\text{pC3bBbPCFHR1}]$$

$$-k^{-}_{\text{pCFHR1}}[\text{pC3bBbPCFHR1}]$$

115

$$\frac{d[\text{pC3bCFHR2}]}{dt}=k^{+}_{\text{C3bCFHR2}}[\text{fC3b}][\text{pCFHR2}]-k^{-}_{\text{C3bCFHR2}}[\text{pC3bCFHR2}]-k^{+}_{\text{C3bB}}[\text{pC3bCFHR2}][\text{FB}]$$

$$+k^{-}_{\text{C3bB}}[\text{pC3bBCFHR2}]+k^{-}_{\text{C3bBb}}[\text{pC3bBbCFHR2}]+k^{-}_{\text{C3bBbP}}[\text{pC3bBbPCFHR2}]-k^{-}_{\text{pCFHR2}}[\text{pC3bCFHR2}]$$

116

$$\frac{d[\text{pC3bBCFHR2}]}{dt}=k^{+}_{\text{C3bB}}[\text{pC3bCFHR2}][\text{FB}]-k^{-}_{\text{C3bB}}[\text{pC3bBCFHR2}]$$

$$-\frac{k^{\text{FD}}_{\text{cat C3(H}_2\text{O)B}}[\text{FD}][\text{pC3bBCFHR2}]}{\begin{pmatrix} K^{\text{D}}_{\text{m C3(H}_2\text{O)B}}+[\text{C3(H}_2\text{O)B}]+[\text{fC3bB}]+[\text{fC3bC3bB}]+[\text{fC3bC3bBP}]+[\text{IgGC3bC3bB}]+[\text{IgGC3bC3bBP}] \\ +[\text{fC4bC3bB}]+[\text{fC4bC3bBP}]+[\text{pC3bBCFHR1}]+[\text{pC3bBCFHR2}]+[\text{pC3bBCFHR3}]+[\text{pC3bBCFHR4}] \\ +[\text{pC3bBCFHR5}]+[\text{hC4bC3bBP}]+[\text{hC4bC3bB}]+[\text{hC3bC3bB}]+[\text{hC3bC3bBP}]+[\text{hC3bB}]+[\text{pC4bC3bB}] \\ +[\text{pC4bC3bBP}]+[\text{pC3bC3bB}]+[\text{pC3bC3bBP}]+[\text{pC3bB}] \end{pmatrix}}$$

$$-k^{-}_{\text{pCFHR2}}[\text{pC3bBCFHR2}]$$

117

$$\frac{d\left[\mathrm{pC3bBbCFHR2}\right]}{dt}=$$

$$\frac{k_{\mathrm{cat\ C3(H_2O)B}}^{\mathrm{FD}}\left[\mathrm{FD}\right]\left[\mathrm{pC3bBCFHR2}\right]}{\left(\begin{array}{l}K_{\mathrm{m\ C3(H_2O)B}}^{\mathrm{D}}+\left[\mathrm{C3(H_2O)B}\right]+\left[\mathrm{fC3bB}\right]+\left[\mathrm{fC3bC3bB}\right]+\left[\mathrm{fC3bC3bBP}\right]+\left[\mathrm{IgGC3bC3bB}\right]+\left[\mathrm{IgGC3bC3bBP}\right]\\+\left[\mathrm{fC4bC3bB}\right]+\left[\mathrm{fC4bC3bBP}\right]+\left[\mathrm{pC3bBCFHR1}\right]+\left[\mathrm{pC3bBCFHR2}\right]+\left[\mathrm{pC3bBCFHR3}\right]+\left[\mathrm{pC3bBCFHR4}\right]\\+\left[\mathrm{pC3bBCFHR5}\right]+\left[\mathrm{hC4bC3bBP}\right]+\left[\mathrm{hC4bC3bB}\right]+\left[\mathrm{hC3bC3bB}\right]+\left[\mathrm{hC3bC3bBP}\right]+\left[\mathrm{hC3bB}\right]+\left[\mathrm{pC4bC3bB}\right]\\+\left[\mathrm{pC4bC3bBP}\right]+\left[\mathrm{pC3bC3bB}\right]+\left[\mathrm{pC3bC3bBP}\right]+\left[\mathrm{pC3bB}\right]\end{array}\right)}$$

$$-k_{\mathrm{C3bBb}}^{-}\left[\mathrm{pC3bBbCFHR2}\right]-k_{\mathrm{C3bP}}^{+}\left[\mathrm{pC3bBbCFHR2}\right]\left[\mathrm{P}\right]+k_{\mathrm{C3bP}}^{-}\left[\mathrm{pC3bBbPCFHR2}\right]-k_{\mathrm{pCFHR2}}^{-}\left[\mathrm{pC3bBbCFHR2}\right]$$

118

$$\frac{d\left[\mathrm{pC3bBbPCFHR2}\right]}{dt}=k_{\mathrm{C3bP}}^{+}\left[\mathrm{pC3bBbCFHR2}\right]\left[\mathrm{P}\right]-k_{\mathrm{C3bP}}^{-}\left[\mathrm{pC3bBbPCFHR2}\right]-k_{\mathrm{C3bBbP}}^{-}\left[\mathrm{pC3bBbPCFHR2}\right]$$

$$-k_{\mathrm{pCFHR2}}^{-}\left[\mathrm{pC3bBbPCFHR2}\right]$$

119

$$\frac{d\left[\mathrm{pC3bCFHR3}\right]}{dt}=k_{\mathrm{C3bCFHR3}}^{+}\left[\mathrm{fC3b}\right]\left[\mathrm{pCFHR3}\right]-k_{\mathrm{C3bCFHR3}}^{-}\left[\mathrm{pC3bCFHR3}\right]-k_{\mathrm{C3bB}}^{+}\left[\mathrm{pC3bCFHR3}\right]\left[\mathrm{FB}\right]$$

$$+k_{\mathrm{C3bB}}^{-}\left[\mathrm{pC3bBCFHR3}\right]+k_{\mathrm{C3bBb}}^{-}\left[\mathrm{pC3bBbCFHR3}\right]+k_{\mathrm{C3bBbP}}^{-}\left[\mathrm{pC3bBbPCFHR3}\right]-k_{\mathrm{pCFHR3}}^{-}\left[\mathrm{pC3bCFHR3}\right]$$

120

$$\frac{d\left[\mathrm{pC3bBCFHR3}\right]}{dt}=k_{\mathrm{C3bB}}^{+}\left[\mathrm{pC3bCFHR3}\right]\left[\mathrm{FB}\right]-k_{\mathrm{C3bB}}^{-}\left[\mathrm{pC3bBCFHR3}\right]$$

$$-\frac{k_{\mathrm{cat\ C3(H_2O)B}}^{\mathrm{FD}}\left[\mathrm{FD}\right]\left[\mathrm{pC3bBCFHR3}\right]}{\left(\begin{array}{l}K_{\mathrm{m\ C3(H_2O)B}}^{\mathrm{D}}+\left[\mathrm{C3(H_2O)B}\right]+\left[\mathrm{fC3bB}\right]+\left[\mathrm{fC3bC3bB}\right]+\left[\mathrm{fC3bC3bBP}\right]+\left[\mathrm{IgGC3bC3bB}\right]+\left[\mathrm{IgGC3bC3bBP}\right]\\+\left[\mathrm{fC4bC3bB}\right]+\left[\mathrm{fC4bC3bBP}\right]+\left[\mathrm{pC3bBCFHR1}\right]+\left[\mathrm{pC3bBCFHR2}\right]+\left[\mathrm{pC3bBCFHR3}\right]+\left[\mathrm{pC3bBCFHR4}\right]\\+\left[\mathrm{pC3bBCFHR5}\right]+\left[\mathrm{hC4bC3bBP}\right]+\left[\mathrm{hC4bC3bB}\right]+\left[\mathrm{hC3bC3bB}\right]+\left[\mathrm{hC3bC3bBP}\right]+\left[\mathrm{hC3bB}\right]+\left[\mathrm{pC4bC3bB}\right]\\+\left[\mathrm{pC4bC3bBP}\right]+\left[\mathrm{pC3bC3bB}\right]+\left[\mathrm{pC3bC3bBP}\right]+\left[\mathrm{pC3bB}\right]\end{array}\right)}$$

$$-k_{\mathrm{pCFHR3}}^{-}\left[\mathrm{pC3bBCFHR3}\right]$$

121

$$\frac{d\left[\mathrm{pC3bBbCFHR3}\right]}{dt} =$$

$$\frac{k^{\mathrm{FD}}_{\mathrm{cat\ C3(H_2O)B}}\left[\mathrm{FD}\right]\left[\mathrm{pC3bBCFHR3}\right]}{\begin{pmatrix} K^{\mathrm{D}}_{\mathrm{m\ C3(H_2O)B}}+\left[\mathrm{C3}\left(\mathrm{H_2O}\right)\mathrm{B}\right]+\left[\mathrm{fC3bB}\right]+\left[\mathrm{fC3bC3bB}\right]+\left[\mathrm{fC3bC3bBP}\right]+\left[\mathrm{IgGC3bC3bB}\right]+\left[\mathrm{IgGC3bC3bBP}\right] \\ +\left[\mathrm{fC4bC3bB}\right]+\left[\mathrm{fC4bC3bBP}\right]+\left[\mathrm{pC3bBCFHR1}\right]+\left[\mathrm{pC3bBCFHR2}\right]+\left[\mathrm{pC3bBCFHR3}\right]+\left[\mathrm{pC3bBCFHR4}\right] \\ +\left[\mathrm{pC3bBCFHR5}\right]+\left[\mathrm{hC4bC3bBP}\right]+\left[\mathrm{hC4bC3bB}\right]+\left[\mathrm{hC3bC3bB}\right]+\left[\mathrm{hC3bC3bBP}\right]+\left[\mathrm{hC3bB}\right]+\left[\mathrm{pC4bC3bB}\right] \\ +\left[\mathrm{pC4bC3bBP}\right]+\left[\mathrm{pC3bC3bB}\right]+\left[\mathrm{pC3bC3bBP}\right]+\left[\mathrm{pC3bB}\right] \end{pmatrix}}$$

$$-k^{-}_{\mathrm{C3bBb}}\left[\mathrm{pC3bBbCFHR3}\right]-k^{+}_{\mathrm{C3bP}}\left[\mathrm{pC3bBbCFHR3}\right]\left[\mathrm{P}\right]+k^{-}_{\mathrm{C3bP}}\left[\mathrm{pC3bBbPCFHR3}\right]-k^{-}_{\mathrm{pCFHR3}}\left[\mathrm{pC3bBbCFHR3}\right]$$

122

$$\frac{d\left[\mathrm{pC3bBbPCFHR3}\right]}{dt} = k^{+}_{\mathrm{C3bP}}\left[\mathrm{pC3bBbCFHR3}\right]\left[\mathrm{P}\right]-k^{-}_{\mathrm{C3bP}}\left[\mathrm{pC3bBbPCFHR3}\right]-k^{-}_{\mathrm{C3bBbP}}\left[\mathrm{pC3bBbPCFHR3}\right]$$

$$-k^{-}_{\mathrm{pCFHR3}}\left[\mathrm{pC3bBbPCFHR3}\right]$$

123

$$\frac{d\left[\mathrm{pC3bCFHR4}\right]}{dt} = k^{+}_{\mathrm{C3bCFHR4}}\left[\mathrm{fC3b}\right]\left[\mathrm{pCFHR4}\right]-k^{-}_{\mathrm{C3bCFHR4}}\left[\mathrm{pC3bCFHR4}\right]-k^{+}_{\mathrm{C3bB}}\left[\mathrm{pC3bCFHR4}\right]\left[\mathrm{FB}\right]$$

$$+k^{-}_{\mathrm{C3bB}}\left[\mathrm{pC3bBCFHR4}\right]+k^{-}_{\mathrm{C3bBb}}\left[\mathrm{pC3bBbCFHR4}\right]+k^{-}_{\mathrm{C3bBbP}}\left[\mathrm{pC3bBbPCFHR4}\right]-k^{-}_{\mathrm{pCFHR4}}\left[\mathrm{pC3bCFHR4}\right]$$

124

$$\frac{d\left[\mathrm{pC3bBCFHR4}\right]}{dt} = k^{+}_{\mathrm{C3bB}}\left[\mathrm{pC3bCFHR4}\right]\left[\mathrm{FB}\right]-k^{-}_{\mathrm{C3bB}}\left[\mathrm{pC3bBCFHR4}\right]$$

$$-\frac{k^{\mathrm{FD}}_{\mathrm{cat\ C3(H_2O)B}}\left[\mathrm{FD}\right]\left[\mathrm{pC3bBCFHR4}\right]}{\begin{pmatrix} K^{\mathrm{D}}_{\mathrm{m\ C3(H_2O)B}}+\left[\mathrm{C3}\left(\mathrm{H_2O}\right)\mathrm{B}\right]+\left[\mathrm{fC3bB}\right]+\left[\mathrm{fC3bC3bB}\right]+\left[\mathrm{fC3bC3bBP}\right]+\left[\mathrm{IgGC3bC3bB}\right]+\left[\mathrm{IgGC3bC3bBP}\right] \\ +\left[\mathrm{fC4bC3bB}\right]+\left[\mathrm{fC4bC3bBP}\right]+\left[\mathrm{pC3bBCFHR1}\right]+\left[\mathrm{pC3bBCFHR2}\right]+\left[\mathrm{pC3bBCFHR3}\right]+\left[\mathrm{pC3bBCFHR4}\right] \\ +\left[\mathrm{pC3bBCFHR5}\right]+\left[\mathrm{hC4bC3bBP}\right]+\left[\mathrm{hC4bC3bB}\right]+\left[\mathrm{hC3bC3bB}\right]+\left[\mathrm{hC3bC3bBP}\right]+\left[\mathrm{hC3bB}\right]+\left[\mathrm{pC4bC3bB}\right] \\ +\left[\mathrm{pC4bC3bBP}\right]+\left[\mathrm{pC3bC3bB}\right]+\left[\mathrm{pC3bC3bBP}\right]+\left[\mathrm{pC3bB}\right] \end{pmatrix}}$$

$$-k^{-}_{\mathrm{pCFHR4}}\left[\mathrm{pC3bBCFHR4}\right]$$

125

$$\frac{d\left[\text{pC3bBbCFHR4}\right]}{dt}=$$

$$\frac{k_{\text{cat C3}(\text{H}_2\text{O})\text{B}}^{\text{FD}}\left[\text{FD}\right]\left[\text{pC3bBCFHR4}\right]}{\left(\begin{array}{l}K_{\text{m C3}(\text{H}_2\text{O})\text{B}}^{\text{D}}+\left[\text{C3}\left(\text{H}_2\text{O}\right)\text{B}\right]+\left[\text{fC3bB}\right]+\left[\text{fC3bC3bB}\right]+\left[\text{fC3bC3bBP}\right]+\left[\text{IgGC3bC3bB}\right]+\left[\text{IgGC3bC3bBP}\right]\\+\left[\text{fC4bC3bB}\right]+\left[\text{fC4bC3bBP}\right]+\left[\text{pC3bBCFHR1}\right]+\left[\text{pC3bBCFHR2}\right]+\left[\text{pC3bBCFHR3}\right]+\left[\text{pC3bBCFHR4}\right]\\+\left[\text{pC3bBCFHR5}\right]+\left[\text{hC4bC3bBP}\right]+\left[\text{hC4bC3bB}\right]+\left[\text{hC3bC3bB}\right]+\left[\text{hC3bC3bBP}\right]+\left[\text{hC3bB}\right]+\left[\text{pC4bC3bB}\right]\\+\left[\text{pC4bC3bBP}\right]+\left[\text{pC3bC3bB}\right]+\left[\text{pC3bC3bBP}\right]+\left[\text{pC3bB}\right]\end{array}\right)}$$

$$-k_{\text{C3bBb}}^{-}\left[\text{pC3bBbCFHR4}\right]-k_{\text{C3bP}}^{+}\left[\text{pC3bBbCFHR4}\right]\left[\text{P}\right]+k_{\text{C3bP}}^{-}\left[\text{pC3bBbPCFHR4}\right]-k_{\text{pCFHR4}}^{-}\left[\text{pC3bBbCFHR4}\right]$$

126

$$\frac{d\left[\text{pC3bBbPCFHR4}\right]}{dt}=k_{\text{C3bP}}^{+}\left[\text{pC3bBbCFHR4}\right]\left[\text{P}\right]-k_{\text{C3bP}}^{-}\left[\text{pC3bBbPCFHR4}\right]-k_{\text{C3bBbP}}^{-}\left[\text{pC3bBbPCFHR4}\right]$$
$$-k_{\text{pCFHR4}}^{-}\left[\text{pC3bBbPCFHR4}\right]$$

127

$$\frac{d\left[\text{pC3bCFHR5}\right]}{dt}=k_{\text{C3bCFHR5}}^{+}\left[\text{fC3b}\right]\left[\text{pCFHR5}\right]-k_{\text{C3bCFHR5}}^{-}\left[\text{pC3bCFHR5}\right]-k_{\text{C3bB}}^{+}\left[\text{pC3bCFHR5}\right]\left[\text{FB}\right]$$
$$+k_{\text{C3bB}}^{-}\left[\text{pC3bBCFHR5}\right]+k_{\text{C3bBb}}^{-}\left[\text{pC3bBbCFHR5}\right]+k_{\text{C3bBbP}}^{-}\left[\text{pC3bBbPCFHR5}\right]-k_{\text{pCFHR5}}^{-}\left[\text{pC3bCFHR5}\right]$$

128

$$\frac{d\left[\text{pC3bBCFHR5}\right]}{dt}=k_{\text{C3bB}}^{+}\left[\text{pC3bCFHR5}\right]\left[\text{FB}\right]-k_{\text{C3bB}}^{-}\left[\text{pC3bBCFHR5}\right]$$

$$-\frac{k_{\text{cat C3}(\text{H}_2\text{O})\text{B}}^{\text{FD}}\left[\text{FD}\right]\left[\text{pC3bBCFHR5}\right]}{\left(\begin{array}{l}K_{\text{m C3}(\text{H}_2\text{O})\text{B}}^{\text{D}}+\left[\text{C3}\left(\text{H}_2\text{O}\right)\text{B}\right]+\left[\text{fC3bB}\right]+\left[\text{fC3bC3bB}\right]+\left[\text{fC3bC3bBP}\right]+\left[\text{IgGC3bC3bB}\right]+\left[\text{IgGC3bC3bBP}\right]\\+\left[\text{fC4bC3bB}\right]+\left[\text{fC4bC3bBP}\right]+\left[\text{pC3bBCFHR1}\right]+\left[\text{pC3bBCFHR2}\right]+\left[\text{pC3bBCFHR3}\right]+\left[\text{pC3bBCFHR4}\right]\\+\left[\text{pC3bBCFHR5}\right]+\left[\text{hC4bC3bBP}\right]+\left[\text{hC4bC3bB}\right]+\left[\text{hC3bC3bB}\right]+\left[\text{hC3bC3bBP}\right]+\left[\text{hC3bB}\right]+\left[\text{pC4bC3bB}\right]\\+\left[\text{pC4bC3bBP}\right]+\left[\text{pC3bC3bB}\right]+\left[\text{pC3bC3bBP}\right]+\left[\text{pC3bB}\right]\end{array}\right)}$$

$$-k_{\text{pCFHR5}}^{-}\left[\text{pC3bBCFHR5}\right]$$

129

$$\frac{d\left[\text{pC3bBbCFHR5}\right]}{dt}=$$

$$\frac{k_{\text{cat C3}\left(\text{H}_2\text{O}\right)\text{B}}^{\text{FD}}\left[\text{FD}\right]\left[\text{pC3bBCFHR5}\right]}{\left(\begin{array}{l}K_{\text{m C3}\left(\text{H}_2\text{O}\right)\text{B}}^{\text{D}}+\left[\text{C3}\left(\text{H}_2\text{O}\right)\text{B}\right]+\left[\text{fC3bB}\right]+\left[\text{fC3bC3bB}\right]+\left[\text{fC3bC3bBP}\right]+\left[\text{IgGC3bC3bB}\right]+\left[\text{IgGC3bC3bBP}\right]\\+\left[\text{fC4bC3bB}\right]+\left[\text{fC4bC3bBP}\right]+\left[\text{pC3bBCFHR1}\right]+\left[\text{pC3bBCFHR2}\right]+\left[\text{pC3bBCFHR3}\right]+\left[\text{pC3bBCFHR4}\right]\\+\left[\text{pC3bBCFHR5}\right]+\left[\text{hC4bC3bBP}\right]+\left[\text{hC4bC3bB}\right]+\left[\text{hC3bC3bB}\right]+\left[\text{hC3bC3bBP}\right]+\left[\text{hC3bB}\right]+\left[\text{pC4bC3bB}\right]\\+\left[\text{pC4bC3bBP}\right]+\left[\text{pC3bC3bB}\right]+\left[\text{pC3bC3bBP}\right]+\left[\text{pC3bB}\right]\end{array}\right)}$$

$$-k_{\text{C3bBb}}^{-}\left[\text{pC3bBbCFHR5}\right]-k_{\text{C3bP}}^{+}\left[\text{pC3bBbCFHR5}\right]\left[\text{P}\right]+k_{\text{C3bP}}^{-}\left[\text{pC3bBbPCFHR5}\right]-k_{\text{pCFHR5}}^{-}\left[\text{pC3bBbCFHR5}\right]$$

130

$$\frac{d\left[\text{pC3bBbPCFHR5}\right]}{dt}=k_{\text{C3bP}}^{+}\left[\text{pC3bBbCFHR5}\right]\left[\text{P}\right]-k_{\text{C3bP}}^{-}\left[\text{pC3bBbPCFHR5}\right]-k_{\text{C3bBbP}}^{-}\left[\text{pC3bBbPCFHR5}\right]$$

$$-k_{\text{pCFHR5}}^{-}\left[\text{pC3bBbPCFHR5}\right]$$

131

$$\frac{d\left[\text{Pathogen Surface}\right]}{dt}=-k_{\text{pIgG}}^{+}\left[\text{IgG}_1\right]\left[\text{Pathogen Surface}\right]+k_{\text{pIgG}}^{-}\left[\text{pIgG}_1\right]-k_{\text{pIgM}}^{+}\left[\text{IgM}\right]\left[\text{Pathogen Surface}\right]$$

$$+k_{\text{pIgM}}^{-}\left[\text{pIgM}\right]-k_{\text{pC4b}}^{+}\left[\text{nfC4b}\right]\left[\text{Pathogen Surface}\right]-k_{\text{pC3b}}^{+}\left[\text{nfC3b}\right]\left[\text{Pathogen Surface}\right]$$

$$-k_{\text{C5b7}_{\text{surface}}}^{+}\left[\text{fC5b7}\right]\left[\text{Pathogen Surface}\right]\times 4-k_{\text{pMBL}_3}^{+}\left[\text{MBL}_3\right]\left[\text{Pathogen Surface}\right]+k_{\text{pMBL}_3}^{-}\left[\text{pMBL}_3\right]$$

$$-k_{\text{pMBL}_3}^{+}\left[\text{fMBL}_3\text{MASP1}_{\text{zym}}\right]\left[\text{Pathogen Surface}\right]+k_{\text{pMBL}_3}^{-}\left[\text{pMBL}_3\text{MASP1}_{\text{zym}}\right]$$

$$-k_{\text{pMBL}_3}^{+}\left[\text{fMBL}_3\text{MASP2}_{\text{zym}}\right]\left[\text{Pathogen Surface}\right]+k_{\text{pMBL}_3}^{-}\left[\text{pMBL}_3\text{MASP2}_{\text{zym}}\right]$$

$$-k_{\text{pMBL}_3}^{+}\left[\text{fMBL}_3\text{MASP3}_{\text{zym}}\right]\left[\text{Pathogen Surface}\right]+k_{\text{pMBL}_3}^{-}\left[\text{pMBL}_3\text{MASP3}_{\text{zym}}\right]$$

$$-k_{\text{pMBL}_3}^{+}\left[\text{fMBL}_3\text{MAp19}\right]\left[\text{Pathogen Surface}\right]+k_{\text{pMBL}_3}^{-}\left[\text{pMBL}_3\text{MAp19}\right]-k_{\text{pMBL}_3}^{+}\left[\text{fMBL}_3\text{MAp44}\right]\left[\text{Pathogen Surface}\right]$$

$$+k_{\text{pMBL}_3}^{-}\left[\text{pMBL}_3\text{MAp44}\right]-k_{\text{pMBL}_3}^{+}\left[\text{fMBL}_3\text{MASP1}^{*}\right]\left[\text{Pathogen Surface}\right]+k_{\text{pMBL}_3}^{-}\left[\text{pMBL}_3\text{MASP1}^{*}\right]$$

$$-k_{\text{pMBL}_3}^{+}\left[\text{fMBL}_3\text{MASP2}^{*}\right]\left[\text{Pathogen Surface}\right]+k_{\text{pMBL}_3}^{-}\left[\text{pMBL}_3\text{MASP2}^{*}\right]$$

$$-k_{\text{pMBL}_3}^{+}\left[\text{fMBL}_3\text{MASP3}^{*}\right]\left[\text{Pathogen Surface}\right]+k_{\text{pMBL}_3}^{-}\left[\text{pMBL}_3\text{MASP3}^{*}\right]-k_{\text{pMBL}_4}^{+}\left[\text{MBL}_4\right]\left[\text{Pathogen Surface}\right]$$

$$+k_{\text{pMBL}_4}^{-}\left[\text{pMBL}_4\right]-k_{\text{pMBL}_4}^{+}\left[\text{fMBL}_4\text{MASP1}_{\text{zym}}\right]\left[\text{Pathogen Surface}\right]+k_{\text{pMBL}_4}^{-}\left[\text{pMBL}_4\text{MASP1}_{\text{zym}}\right]$$

$$-k_{\text{pMBL}_4}^{+}\left[\text{fMBL}_4\text{MASP2}_{\text{zym}}\right]\left[\text{Pathogen Surface}\right]+k_{\text{pMBL}_4}^{-}\left[\text{pMBL}_4\text{MASP2}_{\text{zym}}\right]$$

$$-k_{\text{pMBL}_4}^{+}\left[\text{fMBL}_4\text{MASP3}_{\text{zym}}\right]\left[\text{Pathogen Surface}\right]+k_{\text{pMBL}_4}^{-}\left[\text{pMBL}_4\text{MASP3}_{\text{zym}}\right]$$

$$-k_{\text{pMBL}_4}^{+}\left[\text{fMBL}_4\text{MAp19}\right]\left[\text{Pathogen Surface}\right]+k_{\text{pMBL}_4}^{-}\left[\text{pMBL}_4\text{MAp19}\right]-k_{\text{pMBL}_4}^{+}\left[\text{fMBL}_4\text{MAp44}\right]\left[\text{Pathogen Surface}\right]$$

$$+k_{\text{pMBL}_4}^{-}\left[\text{pMBL}_4\text{MAp44}\right]-k_{\text{pMBL}_4}^{+}\left[\text{fMBL}_4\text{MASP1}^{*}\right]\left[\text{Pathogen Surface}\right]+k_{\text{pMBL}_4}^{-}\left[\text{pMBL}_4\text{MASP1}^{*}\right]$$

$$-k_{\text{pMBL}_4}^{+}\left[\text{fMBL}_4\text{MASP2}^{*}\right]\left[\text{Pathogen Surface}\right]+k_{\text{pMBL}_4}^{-}\left[\text{pMBL}_4\text{MASP2}^{*}\right]$$

$$-k_{\text{pMBL}_4}^{+}\left[\text{fMBL}_4\text{MASP3}^{*}\right]\left[\text{Pathogen Surface}\right]+k_{\text{pMBL}_4}^{-}\left[\text{pMBL}_4\text{MASP3}^{*}\right]-k_{\text{pCLLK}}^{+}\left[\text{CLLK}\right]\left[\text{Pathogen Surface}\right]$$

$$+k_{\mathrm{pCLLK}}^{-}\left[\mathrm{pCLLK}\right]-k_{\mathrm{pCLLK}}^{+}\left[\mathrm{fCLLKMASP1_{zym}}\right]\left[\mathrm{Pathogen\ Surface}\right]+k_{\mathrm{pCLLK}}^{-}\left[\mathrm{pCLLKMASP1_{zym}}\right]$$
$$-k_{\mathrm{pCLLK}}^{+}\left[\mathrm{fCLLKMASP2_{zym}}\right]\left[\mathrm{Pathogen\ Surface}\right]+k_{\mathrm{pCLLK}}^{-}\left[\mathrm{pCLLKMASP2_{zym}}\right]$$
$$-k_{\mathrm{pCLLK}}^{+}\left[\mathrm{fCLLKMASP3_{zym}}\right]\left[\mathrm{Pathogen\ Surface}\right]+k_{\mathrm{pCLLK}}^{-}\left[\mathrm{pCLLKMASP3_{zym}}\right]$$
$$-k_{\mathrm{pCLLK}}^{+}\left[\mathrm{fCLLKMAp19}\right]\left[\mathrm{Pathogen\ Surface}\right]+k_{\mathrm{pCLLK}}^{-}\left[\mathrm{pCLLKMAp19}\right]-k_{\mathrm{pCLLK}}^{+}\left[\mathrm{fCLLKMAp44}\right]\left[\mathrm{Pathogen\ Surface}\right]$$
$$+k_{\mathrm{pCLLK}}^{-}\left[\mathrm{pCLLKMAp44}\right]-k_{\mathrm{pCLLK}}^{+}\left[\mathrm{fCLLKMASP1^{*}}\right]\left[\mathrm{Pathogen\ Surface}\right]+k_{\mathrm{pCLLK}}^{-}\left[\mathrm{pCLLKMASP1^{*}}\right]$$
$$-k_{\mathrm{pCLLK}}^{+}\left[\mathrm{fCLLKMASP2^{*}}\right]\left[\mathrm{Pathogen\ Surface}\right]+k_{\mathrm{pCLLK}}^{-}\left[\mathrm{pCLLKMASP2^{*}}\right]$$
$$-k_{\mathrm{pCLLK}}^{+}\left[\mathrm{fCLLKMASP3^{*}}\right]\left[\mathrm{Pathogen\ Surface}\right]+k_{\mathrm{pCLLK}}^{-}\left[\mathrm{pCLLKMASP3^{*}}\right]-k_{\mathrm{pCLK1}}^{+}\left[\mathrm{CLK1}\right]\left[\mathrm{Pathogen\ Surface}\right]$$
$$+k_{\mathrm{pCLK1}}^{-}\left[\mathrm{pCLK1}\right]-k_{\mathrm{pCLK1}}^{+}\left[\mathrm{fCLK1MASP1_{zym}}\right]\left[\mathrm{Pathogen\ Surface}\right]+k_{\mathrm{pCLK1}}^{-}\left[\mathrm{pCLK1MASP1_{zym}}\right]$$
$$-k_{\mathrm{pCLK1}}^{+}\left[\mathrm{fCLK1MASP2_{zym}}\right]\left[\mathrm{Pathogen\ Surface}\right]+k_{\mathrm{pCLK1}}^{-}\left[\mathrm{pCLK1MASP2_{zym}}\right]$$
$$-k_{\mathrm{pCLK1}}^{+}\left[\mathrm{fCLK1MASP3_{zym}}\right]\left[\mathrm{Pathogen\ Surface}\right]+k_{\mathrm{pCLK1}}^{-}\left[\mathrm{pCLK1MASP3_{zym}}\right]$$
$$-k_{\mathrm{pCLK1}}^{+}\left[\mathrm{fCLK1MAp19}\right]\left[\mathrm{Pathogen\ Surface}\right]+k_{\mathrm{pCLK1}}^{-}\left[\mathrm{pCLK1MAp19}\right]-k_{\mathrm{pCLK1}}^{+}\left[\mathrm{fCLK1MAp44}\right]\left[\mathrm{Pathogen\ Surface}\right]$$
$$+k_{\mathrm{pCLK1}}^{-}\left[\mathrm{pCLK1MAp44}\right]-k_{\mathrm{pCLK1}}^{+}\left[\mathrm{fCLK1MASP1^{*}}\right]\left[\mathrm{Pathogen\ Surface}\right]+k_{\mathrm{pCLK1}}^{-}\left[\mathrm{pCLK1MASP1^{*}}\right]$$
$$-k_{\mathrm{pCLK1}}^{+}\left[\mathrm{fCLK1MASP2^{*}}\right]\left[\mathrm{Pathogen\ Surface}\right]+k_{\mathrm{pCLK1}}^{-}\left[\mathrm{pCLK1MASP2^{*}}\right]$$
$$-k_{\mathrm{pCLK1}}^{+}\left[\mathrm{fCLK1MASP3^{*}}\right]\left[\mathrm{Pathogen\ Surface}\right]+k_{\mathrm{pCLK1}}^{-}\left[\mathrm{pCLK1MASP3^{*}}\right]-k_{\mathrm{pCLL1}}^{+}\left[\mathrm{CLL1}\right]\left[\mathrm{Pathogen\ Surface}\right]$$
$$+k_{\mathrm{pCLL1}}^{-}\left[\mathrm{pCLL1}\right]-k_{\mathrm{pCLL1}}^{+}\left[\mathrm{fCLL1MASP1_{zym}}\right]\left[\mathrm{Pathogen\ Surface}\right]+k_{\mathrm{pCLL1}}^{-}\left[\mathrm{pCLL1MASP1_{zym}}\right]$$
$$-k_{\mathrm{pCLL1}}^{+}\left[\mathrm{fCLL1MASP2_{zym}}\right]\left[\mathrm{Pathogen\ Surface}\right]+k_{\mathrm{pCLL1}}^{-}\left[\mathrm{pCLL1MASP2_{zym}}\right]$$
$$-k_{\mathrm{pCLL1}}^{+}\left[\mathrm{fCLL1MASP3_{zym}}\right]\left[\mathrm{Pathogen\ Surface}\right]+k_{\mathrm{pCLL1}}^{-}\left[\mathrm{pCLL1MASP3_{zym}}\right]$$
$$-k_{\mathrm{pCLL1}}^{+}\left[\mathrm{fCLL1MAp19}\right]\left[\mathrm{Pathogen\ Surface}\right]+k_{\mathrm{pCLL1}}^{-}\left[\mathrm{pCLL1MAp19}\right]-k_{\mathrm{pCLL1}}^{+}\left[\mathrm{fCLL1MAp44}\right]\left[\mathrm{Pathogen\ Surface}\right]$$
$$+k_{\mathrm{pCLL1}}^{-}\left[\mathrm{pCLL1MAp44}\right]-k_{\mathrm{pCLL1}}^{+}\left[\mathrm{fCLL1MASP1^{*}}\right]\left[\mathrm{Pathogen\ Surface}\right]+k_{\mathrm{pCLL1}}^{-}\left[\mathrm{pCLL1MASP1^{*}}\right]$$
$$-k_{\mathrm{pCLL1}}^{+}\left[\mathrm{fCLL1MASP2^{*}}\right]\left[\mathrm{Pathogen\ Surface}\right]+k_{\mathrm{pCLL1}}^{-}\left[\mathrm{pCLL1MASP2^{*}}\right]$$
$$-k_{\mathrm{pCLL1}}^{+}\left[\mathrm{fCLL1MASP3^{*}}\right]\left[\mathrm{Pathogen\ Surface}\right]+k_{\mathrm{pCLL1}}^{-}\left[\mathrm{pCLL1MASP3^{*}}\right]-k_{\mathrm{pLF}}^{+}\left[\mathrm{LF}\right]\left[\mathrm{Pathogen\ Surface}\right]+k_{\mathrm{pLF}}^{-}\left[\mathrm{pLF}\right]$$
$$-k_{\mathrm{pLF}}^{+}\left[\mathrm{fLFMASP1_{zym}}\right]\left[\mathrm{Pathogen\ Surface}\right]+k_{\mathrm{pLF}}^{-}\left[\mathrm{pLFMASP1_{zym}}\right]-k_{\mathrm{pLF}}^{+}\left[\mathrm{fLFMASP2_{zym}}\right]\left[\mathrm{Pathogen\ Surface}\right]$$
$$+k_{\mathrm{pLF}}^{-}\left[\mathrm{pLFMASP2_{zym}}\right]-k_{\mathrm{pLF}}^{+}\left[\mathrm{fLFMASP3_{zym}}\right]\left[\mathrm{Pathogen\ Surface}\right]+k_{\mathrm{pLF}}^{-}\left[\mathrm{pLFMASP3_{zym}}\right]$$
$$-k_{\mathrm{pLF}}^{+}\left[\mathrm{fLFMAp19}\right]\left[\mathrm{Pathogen\ Surface}\right]+k_{\mathrm{pLF}}^{-}\left[\mathrm{pLFMAp19}\right]-k_{\mathrm{pLF}}^{+}\left[\mathrm{fLFMAp44}\right]\left[\mathrm{Pathogen\ Surface}\right]$$

$$+k_{\text{pLF}}^{-}\left[\text{pLFMAp44}\right]-k_{\text{pLF}}^{+}\left[\text{fLFMASP1}^{*}\right]\left[\text{Pathogen Surface}\right]+k_{\text{pLF}}^{-}\left[\text{pLFMASP1}^{*}\right]$$

$$-k_{\text{pLF}}^{+}\left[\text{fLFMASP2}^{*}\right]\left[\text{Pathogen Surface}\right]+k_{\text{pLF}}^{-}\left[\text{pLFMASP2}^{*}\right]-k_{\text{pLF}}^{+}\left[\text{fLFMASP3}^{*}\right]\left[\text{Pathogen Surface}\right]$$

$$+k_{\text{pLF}}^{-}\left[\text{pLFMASP3}^{*}\right]-k_{\text{pHF}}^{+}\left[\text{HF}\right]\left[\text{Pathogen Surface}\right]+k_{\text{pHF}}^{-}\left[\text{pHF}\right]-k_{\text{pHF}}^{+}\left[\text{fHFMASP1}_{\text{zym}}\right]\left[\text{Pathogen Surface}\right]$$

$$+k_{\text{pHF}}^{-}\left[\text{pHFMASP1}_{\text{zym}}\right]-k_{\text{pHF}}^{+}\left[\text{fHFMASP2}_{\text{zym}}\right]\left[\text{Pathogen Surface}\right]+k_{\text{pHF}}^{-}\left[\text{pHFMASP2}_{\text{zym}}\right]$$

$$-k_{\text{pHF}}^{+}\left[\text{fHFMASP3}_{\text{zym}}\right]\left[\text{Pathogen Surface}\right]+k_{\text{pHF}}^{-}\left[\text{pHFMASP3}_{\text{zym}}\right]-k_{\text{pHF}}^{+}\left[\text{fHFMAp19}\right]\left[\text{Pathogen Surface}\right]$$

$$+k_{\text{pHF}}^{-}\left[\text{pHFMAp19}\right]-k_{\text{pHF}}^{+}\left[\text{fHFMAp44}\right]\left[\text{Pathogen Surface}\right]+k_{\text{pHF}}^{-}\left[\text{pHFMAp44}\right]$$

$$-k_{\text{pHF}}^{+}\left[\text{fHFMASP1}^{*}\right]\left[\text{Pathogen Surface}\right]+k_{\text{pHF}}^{-}\left[\text{pHFMASP1}^{*}\right]-k_{\text{pHF}}^{+}\left[\text{fHFMASP2}^{*}\right]\left[\text{Pathogen Surface}\right]$$

$$+k_{\text{pHF}}^{-}\left[\text{pHFMASP2}^{*}\right]-k_{\text{pHF}}^{+}\left[\text{fHFMASP3}^{*}\right]\left[\text{Pathogen Surface}\right]+k_{\text{pHF}}^{-}\left[\text{pHFMASP3}^{*}\right]-k_{\text{pMF}}^{+}\left[\text{MF}\right]\left[\text{Pathogen Surface}\right]$$

$$+k_{\text{pMF}}^{-}\left[\text{pMF}\right]-k_{\text{pMF}}^{+}\left[\text{fMFMASP1}_{\text{zym}}\right]\left[\text{Pathogen Surface}\right]+k_{\text{pMF}}^{-}\left[\text{pMFMASP1}_{\text{zym}}\right]$$

$$-k_{\text{pMF}}^{+}\left[\text{fMFMASP2}_{\text{zym}}\right]\left[\text{Pathogen Surface}\right]+k_{\text{pMF}}^{-}\left[\text{pMFMASP2}_{\text{zym}}\right]-k_{\text{pMF}}^{+}\left[\text{fMFMASP3}_{\text{zym}}\right]\left[\text{Pathogen Surface}\right]$$

$$+k_{\text{pMF}}^{-}\left[\text{pMFMASP3}_{\text{zym}}\right]-k_{\text{pMF}}^{+}\left[\text{fMFMAp19}\right]\left[\text{Pathogen Surface}\right]+k_{\text{pMF}}^{-}\left[\text{pMFMAp19}\right]$$

$$-k_{\text{pMF}}^{+}\left[\text{fMFMAp44}\right]\left[\text{Pathogen Surface}\right]+k_{\text{pMF}}^{-}\left[\text{pMFMAp44}\right]-k_{\text{pMF}}^{+}\left[\text{fMFMASP1}^{*}\right]\left[\text{Pathogen Surface}\right]$$

$$+k_{\text{pMF}}^{-}\left[\text{pMFMASP1}^{*}\right]-k_{\text{pMF}}^{+}\left[\text{fMFMASP2}^{*}\right]\left[\text{Pathogen Surface}\right]+k_{\text{pMF}}^{-}\left[\text{pMFMASP2}^{*}\right]$$

$$-k_{\text{pMF}}^{+}\left[\text{fMFMASP3}^{*}\right]\left[\text{Pathogen Surface}\right]+k_{\text{pMF}}^{-}\left[\text{pMFMASP3}^{*}\right]-k_{\text{pCFHR1}}^{+}\left[\text{CFHR1}\right]\left[\text{Pathogen Surface}\right]$$

$$+k_{\text{pCFHR1}}^{-}\left[\text{pCFHR1}\right]+k_{\text{pCFHR1}}^{-}\left[\text{fC3bCFHR1}\right]+k_{\text{pCFHR1}}^{-}\left[\text{fC3bBCFHR1}\right]+k_{\text{pCFHR1}}^{-}\left[\text{fC3bBbCFHR1}\right]$$

$$+k_{\text{pCFHR1}}^{-}\left[\text{fC3bBbPCFHR1}\right]-k_{\text{pCFHR2}}^{+}\left[\text{CFHR2}\right]\left[\text{Pathogen Surface}\right]+k_{\text{pCFHR2}}^{-}\left[\text{pCFHR2}\right]+k_{\text{pCFHR2}}^{-}\left[\text{fC3bCFHR2}\right]$$

$$+k_{\text{pCFHR2}}^{-}\left[\text{fC3bBCFHR2}\right]+k_{\text{pCFHR2}}^{-}\left[\text{fC3bBbCFHR2}\right]+k_{\text{pCFHR2}}^{-}\left[\text{fC3bBbPCFHR2}\right]$$

$$-k_{\text{pCFHR3}}^{+}\left[\text{CFHR3}\right]\left[\text{Pathogen Surface}\right]+k_{\text{pCFHR3}}^{-}\left[\text{pCFHR3}\right]+k_{\text{pCFHR3}}^{-}\left[\text{fC3bCFHR3}\right]+k_{\text{pCFHR3}}^{-}\left[\text{fC3bBCFHR3}\right]$$

$$+k_{\text{pCFHR3}}^{-}\left[\text{fC3bBbCFHR3}\right]+k_{\text{pCFHR3}}^{-}\left[\text{fC3bBbPCFHR3}\right]-k_{\text{pCFHR4}}^{+}\left[\text{CFHR4}\right]\left[\text{Pathogen Surface}\right]+k_{\text{pCFHR4}}^{-}\left[\text{pCFHR4}\right]$$

$$+k_{\text{pCFHR4}}^{-}\left[\text{fC3bCFHR4}\right]+k_{\text{pCFHR4}}^{-}\left[\text{fC3bBCFHR4}\right]+k_{\text{pCFHR4}}^{-}\left[\text{fC3bBbCFHR4}\right]+k_{\text{pCFHR4}}^{-}\left[\text{fC3bBbPCFHR4}\right]$$

$$-k_{\text{pCFHR5}}^{+}\left[\text{CFHR5}\right]\left[\text{Pathogen Surface}\right]+k_{\text{pCFHR5}}^{-}\left[\text{pCFHR5}\right]+k_{\text{pCFHR5}}^{-}\left[\text{fC3bCFHR5}\right]+k_{\text{pCFHR5}}^{-}\left[\text{fC3bBCFHR5}\right]$$

$$+k_{\text{pCFHR5}}^{-}\left[\text{fC3bBbCFHR5}\right]+k_{\text{pCFHR5}}^{-}\left[\text{fC3bBbPCFHR5}\right]-k_{\text{pPTX3}}^{+}\left[\text{PTX3}\right]\left[\text{Pathogen Surface}\right]+k_{\text{pPTX3}}^{-}\left[\text{pPTX3}\right]$$

$$+k_{\text{pPTX3}}^{-}\left[\text{pC4BPPTX3}\right]+k_{\text{pPTX3}}^{-}\left[\text{pFHPTX3}\right]+k_{\text{pPTX3}}^{-}\left[\text{pFHLPTX3}\right]+k_{\text{pMBL}_3}^{-}\left[\text{pMBL}_3\text{SAP}\right]+k_{\text{pMBL}_3}^{-}\left[\text{pMBL}_3\text{PTX3}\right]$$

$$+k_{\text{pMBL}_3}^{-}\left[\text{pMBL}_3\text{PTX3C1q}\right]+k_{\text{pMBL}_3}^{-}\left[\text{pMBL}_3\text{PTX3C1}\right]+k_{\text{pMBL}_3}^{-}\left[\text{pMBL}_3\text{PTX3C1}^{*}\right]+k_{\text{pMBL}_4}^{-}\left[\text{pMBL}_4\text{SAP}\right]$$

$$+k_{\text{pMBL}_4}^{-}\left[\text{pMBL}_4\text{PTX3}\right]+k_{\text{pMBL}_4}^{-}\left[\text{pMBL}_4\text{PTX3C1q}\right]+k_{\text{pMBL}_4}^{-}\left[\text{pMBL}_4\text{PTX3C1}\right]+k_{\text{pMBL}_4}^{-}\left[\text{pMBL}_4\text{PTX3C1}^{*}\right]$$

$$+k_{\text{pPTX3}}^{-}\left[\text{pPTX3LF}\right]+k_{\text{pPTX3}}^{-}\left[\text{pPTX3LFMASP1}_{\text{zym}}\right]+k_{\text{pPTX3}}^{-}\left[\text{pPTX3LFMASP2}_{\text{zym}}\right]+k_{\text{pPTX3}}^{-}\left[\text{pPTX3LFMASP3}_{\text{zym}}\right]$$

$$+k_{\text{pPTX3}}^{-}\left[\text{pPTX3LFMASP1}^{*}\right]+k_{\text{pPTX3}}^{-}\left[\text{pPTX3LFMASP2}^{*}\right]+k_{\text{pPTX3}}^{-}\left[\text{pPTX3LFMASP3}^{*}\right]$$

$$+k_{\text{pPTX3}}^{-}\left[\text{pPTX3LFMAp19}\right]+k_{\text{pPTX3}}^{-}\left[\text{pPTX3LFMAp44}\right]+k_{\text{pCFHR5}}^{-}\left[\text{pCFHR5PTX3}\right]+k_{\text{pPTX3}}^{-}\left[\text{pPTX3CFHR1}\right]$$

$$-k_{\text{pSAP}}^{+}\left[\text{SAP}\right]\left[\text{Pathogen Surface}\right]+k_{\text{pSAP}}^{-}\left[\text{pSAP}\right]-k_{\text{pCRP}}^{+}\left[\text{CRP}\right]\left[\text{Pathogen Surface}\right]+k_{\text{pCRP}}^{-}\left[\text{pCRP}\right]$$

$$+k_{\text{pCFHR4}}^{-}\left[\text{pCFHR4CRP}\right]-k_{\text{pFH}}^{+}\left[\text{FH}\right]\left[\text{Pathogen Surface}\right]+k_{\text{pFH}}^{-}\left[\text{pFH}\right]-k_{\text{FhbpFHL1}}^{+}\left[\text{FHL1}\right]\left[\text{Pathogen Surface}\right]$$

$$+k_{\text{FhbpFHL1}}^{-}\left[\text{pFHL1}\right]-k_{\text{pC4BP}}^{+}\left[\text{C4BP}\right]\left[\text{Pathogen Surface}\right]+k_{\text{pC4BP}}^{-}\left[\text{pC4BP}\right]-k_{\text{pVn}}^{+}\left[\text{Vn}\right]\left[\text{Pathogen Surface}\right]+k_{\text{pVn}}^{-}\left[\text{pVn}\right]$$

132

$$\frac{d[\mathrm{IgG}]}{dt} = -k^{+}_{\mathrm{pIgG}}[\mathrm{IgG}_1][\mathrm{Pathogen\ Surface}] + k^{-}_{\mathrm{pIgG}}[\mathrm{pIgG}_1] - k^{+}_{\mathrm{pIgG}}[\mathrm{IgG}_1][\mathrm{pIgG}_1] + k^{-}_{\mathrm{pIgG}}[\mathrm{pIgG}_2]$$
$$-k^{+}_{\mathrm{pIgG}}[\mathrm{IgG}_1][\mathrm{pIgG}_2] + k^{-}_{\mathrm{pIgG}}[\mathrm{pIgG}_3] - k^{+}_{\mathrm{pIgG}}[\mathrm{IgG}_1][\mathrm{pIgG}_3] + k^{-}_{\mathrm{pIgG}}[\mathrm{pIgG}_4] - k^{+}_{\mathrm{pIgG}}[\mathrm{IgG}_1][\mathrm{pIgG}_4] + k^{-}_{\mathrm{pIgG}}[\mathrm{pIgG}_5]$$
$$-k^{+}_{\mathrm{pIgG}}[\mathrm{IgG}_1][\mathrm{pIgG}_5] + k^{-}_{\mathrm{pIgG}}[\mathrm{pIgG}_6]$$

133

$$\frac{d[\mathrm{IgM}]}{dt} = -k^{+}_{\mathrm{pIgM}}[\mathrm{IgM}][\mathrm{Pathogen\ Surface}] + k^{-}_{\mathrm{pIgM}}[\mathrm{pIgM}]$$

134

$$\frac{d[\mathrm{pIgG}_1]}{dt} = k^{+}_{\mathrm{pIgG}}[\mathrm{IgG}_1][\mathrm{Pathogen\ Surface}] - k^{-}_{\mathrm{pIgG}}[\mathrm{pIgG}_1] - k^{+}_{\mathrm{pIgG}}[\mathrm{IgG}_1][\mathrm{pIgG}_1] + k^{-}_{\mathrm{pIgG}}[\mathrm{pIgG}_2]$$

135

$$\frac{d[\mathrm{pIgG}_2]}{dt} = k^{+}_{\mathrm{pIgG}}[\mathrm{IgG}_1][\mathrm{pIgG}_1] - k^{-}_{\mathrm{pIgG}}[\mathrm{pIgG}_2] - k^{+}_{\mathrm{pIgG}}[\mathrm{IgG}_1][\mathrm{pIgG}_2] + k^{-}_{\mathrm{pIgG}}[\mathrm{pIgG}_3]$$

136

$$\frac{d[\mathrm{pIgG}_3]}{dt} = k^{+}_{\mathrm{pIgG}}[\mathrm{IgG}_1][\mathrm{pIgG}_2] - k^{-}_{\mathrm{pIgG}}[\mathrm{pIgG}_3] - k^{+}_{\mathrm{pIgG}}[\mathrm{IgG}_1][\mathrm{pIgG}_3] + k^{-}_{\mathrm{pIgG}}[\mathrm{pIgG}_4]$$

137

$$\frac{d[\mathrm{pIgG}_4]}{dt} = k^{+}_{\mathrm{pIgG}}[\mathrm{IgG}_1][\mathrm{pIgG}_3] - k^{-}_{\mathrm{pIgG}}[\mathrm{pIgG}_4] - k^{+}_{\mathrm{pIgG}}[\mathrm{IgG}_1][\mathrm{pIgG}_4] + k^{-}_{\mathrm{pIgG}}[\mathrm{pIgG}_5]$$

138

$$\frac{d[\mathrm{pIgG}_5]}{dt} = k^{+}_{\mathrm{pIgG}}[\mathrm{IgG}_1][\mathrm{pIgG}_4] - k^{-}_{\mathrm{pIgG}}[\mathrm{pIgG}_5] - k^{+}_{\mathrm{pIgG}}[\mathrm{IgG}_1][\mathrm{pIgG}_5] + k^{-}_{\mathrm{pIgG}}[\mathrm{pIgG}_6]$$

139

$$\frac{d[\mathrm{pIgG}_6]}{dt} = k^{+}_{\mathrm{pIgG}}[\mathrm{IgG}_1][\mathrm{pIgG}_5] - k^{-}_{\mathrm{pIgG}}[\mathrm{pIgG}_6] - k^{+}_{\mathrm{C1qIgG}}[\mathrm{C1}][\mathrm{pIgG}_6] + k^{-}_{\mathrm{C1qIgG}}[\mathrm{pIgG}_6\mathrm{C1}] - k^{+}_{\mathrm{C1qIgG}}[\mathrm{C1q}][\mathrm{pIgG}_6]$$
$$+k^{-}_{\mathrm{C1qIgG}}[\mathrm{pIgG}_6\mathrm{C1q}] - k^{+}_{\mathrm{C1qIgG}}\left[\mathrm{C1}^{*}\right][\mathrm{pIgG}_6] + k^{-}_{\mathrm{C1qIgG}}\left[\mathrm{pIgG}_6\mathrm{C1}^{*}\right]$$

140

$$\frac{d[\mathrm{pIgG}_6\mathrm{C1q}]}{dt} = k^{+}_{\mathrm{C1qIgG}}[\mathrm{C1q}][\mathrm{pIgG}_6] - k^{-}_{\mathrm{C1qIgG}}[\mathrm{pIgG}_6\mathrm{C1q}] - k^{+}_{\mathrm{C1}}\left[(\mathrm{C1rC1s})_2\right][\mathrm{pIgG}_6\mathrm{C1q}] + k^{-}_{\mathrm{C1}}[\mathrm{pIgG}_6\mathrm{C1}]$$
$$+k^{-}_{\mathrm{C1}}\left[\mathrm{pIgG}_6\mathrm{C1}^{*}\right] - k^{+}_{\mathrm{C1}}[\mathrm{pIgG}_6\mathrm{C1q}]\left[(\mathrm{C1rC1s})_2^{*}\right] + k^{+}_{\mathrm{C1*C1\text{-}INH}}[\mathrm{C1\text{-}INH}]\left[\mathrm{pIgG}_6\mathrm{C1}^{*}\right]$$

141

$$\frac{d[\text{pIgG}_6\text{C1}]}{dt} = k^{+}_{\text{C1}}\left[\left(\text{C1rC1s}\right)_2\right][\text{pIgG}_6\text{C1q}] - k^{-}_{\text{C1}}[\text{pIgG}_6\text{C1}] - k^{+}_{\text{activation}}[\text{pIgG}_6\text{C1}] + k^{+}_{\text{C1qIgG}}[\text{C1}][\text{pIgG}_6]$$
$$-k^{-}_{\text{C1qIgG}}[\text{pIgG}_6\text{C1}]$$

142

$$\frac{d\left[\text{pIgG}_6\text{C1}^*\right]}{dt} = k^{+}_{\text{activation}}[\text{pIgG}_6\text{C1}] + k^{+}_{\text{C1}}[\text{pIgG}_6\text{C1q}]\left[\left(\text{C1rC1s}\right)^*_2\right] - k^{-}_{\text{C1}}\left[\text{pIgG}_6\text{C1}^*\right] + k^{+}_{\text{C1qIgG}}\left[\text{C1}^*\right][\text{pIgG}_6]$$
$$-k^{-}_{\text{C1qIgG}}\left[\text{pIgG}_6\text{C1}^*\right] - k^{+}_{\text{C1*C1-INH}}[\text{C1-INH}]\left[\text{pIgG}_6\text{C1}^*\right]$$

143

$$\frac{d[\text{pIgM}]}{dt} = k^{+}_{\text{pIgM}}[\text{IgM}][\text{Pathogen Surface}] - k^{-}_{\text{pIgM}}[\text{pIgM}] - k^{+}_{\text{C1qIgM}}[\text{C1}][\text{pIgM}] + k^{-}_{\text{C1qIgM}}[\text{pIgMC1}]$$
$$-k^{+}_{\text{C1qIgM}}[\text{C1q}][\text{pIgM}] + k^{-}_{\text{C1qIgM}}[\text{pIgMC1q}] - k^{+}_{\text{C1qIgM}}\left[\text{C1}^*\right][\text{pIgM}] + k^{-}_{\text{C1qIgM}}\left[\text{pIgMC1}^*\right]$$

144

$$\frac{d[\text{pIgMC1q}]}{dt} = k^{+}_{\text{C1qIgM}}[\text{C1q}][\text{pIgM}] - k^{-}_{\text{C1qIgM}}[\text{pIgMC1q}] - k^{+}_{\text{C1}}\left[\left(\text{C1rC1s}\right)_2\right][\text{pIgMC1q}] + k^{-}_{\text{C1}}[\text{pIgMC1}]$$
$$-k^{+}_{\text{C1}}[\text{pIgMC1q}]\left[\left(\text{C1rC1s}\right)^*_2\right] + k^{-}_{\text{C1}}\left[\text{pIgMC1}^*\right] + k^{+}_{\text{C1*C1-INH}}[\text{C1-INH}]\left[\text{pIgMC1}^*\right]$$

145

$$\frac{d[\text{pIgMC1}]}{dt} = k^{+}_{\text{C1}}\left[\left(\text{C1rC1s}\right)_2\right][\text{pIgMC1q}] - k^{-}_{\text{C1}}[\text{pIgMC1}] - k^{+}_{\text{activation}}[\text{pIgMC1}] + k^{+}_{\text{C1qIgM}}[\text{C1}][\text{pIgM}]$$
$$-k^{-}_{\text{C1qIgM}}[\text{pIgMC1}]$$

146

$$\frac{d\left[\text{pIgMC1}^*\right]}{dt} = k^{+}_{\text{activation}}[\text{pIgMC1}] + k^{+}_{\text{C1}}[\text{pIgMC1q}]\left[\left(\text{C1rC1s}\right)^*_2\right] - k^{-}_{\text{C1}}\left[\text{pIgMC1}^*\right] + k^{+}_{\text{C1qIgM}}\left[\text{C1}^*\right][\text{pIgM}]$$
$$-k^{-}_{\text{C1qIgM}}\left[\text{pIgMC1}^*\right] - k^{+}_{\text{C1*C1-INH}}[\text{C1-INH}]\left[\text{pIgMC1}^*\right]$$

147

$$\frac{d[\text{pC4b}]}{dt} = k^{+}_{\text{pC4b}}[\text{nfC4b}][\text{Pathogen Surface}] - k^{+}_{\text{C4bC4b}}[\text{nfC4b}][\text{pC4b}] - k^{+}_{\text{C3bC4b}}[\text{nfC3b}][\text{pC4b}] - k^{+}_{\text{C4bC2}}[\text{pC4b}][\text{C2}]$$
$$+k^{-}_{\text{C4bC2}}[\text{pC4bC2}] + k^{-}_{\text{C4bC2a}}[\text{pC4bC2a}] - k^{+}_{\text{C4bC4BP}}[\text{pC4b}][\text{C4BP}] + k^{-}_{\text{C4bC4BP}}[\text{pC4bC4BP}]$$
$$+k^{-}_{\text{C4bC2aC4BP}_{\text{decay}}}[\text{pC4bC2aC4BP}] - k^{+}_{\text{C4bC4BP}}[\text{pC4b}][\text{pC4BPPTX3}] + k^{-}_{\text{C4bC4BP}}[\text{pC4bC4BPPTX3}]$$
$$+k^{-}_{\text{C4bC2aC4BP}_{\text{decay}}}[\text{pC4bC2aC4BPPTX3}] - k^{+}_{\text{C4bC4BP}}[\text{pC4b}][\text{pC4BP}] + k^{-}_{\text{C4bC4BP}}[\text{pC4bpC4BP}]$$
$$+k^{-}_{\text{C4bC2aC4BP}_{\text{decay}}}[\text{pC4bC2apC4BP}]$$

148

$$
\begin{aligned}
\frac{d\left[\mathrm{pC4bC2}\right]}{dt} &= k_{\mathrm{C4bC2}}^{+}\left[\mathrm{pC4b}\right]\left[\mathrm{C2}\right]-k_{\mathrm{C4bC2}}^{-}\left[\mathrm{pC4bC2}\right] \\
&-\frac{k_{\mathrm{cat\,C1^*}}^{\mathrm{C2}}\left[\mathrm{pIgG_6C1^*}\right]\left[\mathrm{pC4bC2}\right]}{K_{\mathrm{m\,C1^*}}^{\mathrm{C2}}\left(1+\frac{\left[\mathrm{C4}\right]}{K_{\mathrm{m\,C1^*}}^{\mathrm{C4}}}+\frac{\left[\mathrm{fC4bC2}\right]}{K_{\mathrm{m\,C1^*}}^{\mathrm{C2}}}+\frac{\left[\mathrm{fC4bC4bC2}\right]}{K_{\mathrm{m\,C1^*}}^{\mathrm{C2}}}+\frac{\left[\mathrm{fC3bC4bC2}\right]}{K_{\mathrm{m\,C1^*}}^{\mathrm{C2}}}+\frac{\left[\mathrm{pC4bC2}\right]}{K_{\mathrm{m\,C1^*}}^{\mathrm{C2}}}+\frac{\left[\mathrm{pC4bC4bC2}\right]}{K_{\mathrm{m\,C1^*}}^{\mathrm{C2}}}+\frac{\left[\mathrm{pC3bC4bC2}\right]}{K_{\mathrm{m\,C1^*}}^{\mathrm{C2}}}\right)} \\
&-\frac{k_{\mathrm{cat\,C1^*}}^{\mathrm{C2}}\left[\mathrm{pIgMC1^*}\right]\left[\mathrm{pC4bC2}\right]}{K_{\mathrm{m\,C1^*}}^{\mathrm{C2}}\left(1+\frac{\left[\mathrm{C4}\right]}{K_{\mathrm{m\,C1^*}}^{\mathrm{C4}}}+\frac{\left[\mathrm{fC4bC2}\right]}{K_{\mathrm{m\,C1^*}}^{\mathrm{C2}}}+\frac{\left[\mathrm{fC4bC4bC2}\right]}{K_{\mathrm{m\,C1^*}}^{\mathrm{C2}}}+\frac{\left[\mathrm{fC3bC4bC2}\right]}{K_{\mathrm{m\,C1^*}}^{\mathrm{C2}}}+\frac{\left[\mathrm{pC4bC2}\right]}{K_{\mathrm{m\,C1^*}}^{\mathrm{C2}}}+\frac{\left[\mathrm{pC4bC4bC2}\right]}{K_{\mathrm{m\,C1^*}}^{\mathrm{C2}}}+\frac{\left[\mathrm{pC3bC4bC2}\right]}{K_{\mathrm{m\,C1^*}}^{\mathrm{C2}}}\right)} \\
&-\frac{k_{\mathrm{cat\,MASP1^*}}^{\mathrm{C2}}\left[\mathrm{pMBL_3MASP1^*}\right]\left[\mathrm{pC4bC2}\right]}{K_{\mathrm{m\,MASP1^*}}^{\mathrm{C2}}\left(\begin{aligned}
&1+\frac{\left[\mathrm{C4}\right]}{K_{\mathrm{m\,MASP1^*}}^{\mathrm{C4}}}+\frac{\left[\mathrm{fC4bC2}\right]}{K_{\mathrm{m\,MASP1^*}}^{\mathrm{C2}}}+\frac{\left[\mathrm{fC4bC4bC2}\right]}{K_{\mathrm{m\,MASP1^*}}^{\mathrm{C2}}}+\frac{\left[\mathrm{fC3bC4bC2}\right]}{K_{\mathrm{m\,MASP1^*}}^{\mathrm{C2}}}+\frac{\left[\mathrm{pC4bC2}\right]}{K_{\mathrm{m\,MASP1^*}}^{\mathrm{C2}}}+\frac{\left[\mathrm{pC4bC4bC2}\right]}{K_{\mathrm{m\,MASP1^*}}^{\mathrm{C2}}} \\
&+\frac{\left[\mathrm{pC3bC4bC2}\right]}{K_{\mathrm{m\,MASP1^*}}^{\mathrm{C2}}}+\frac{\left[\mathrm{pMBL_3MASP1_{zym}}\right]}{K_{\mathrm{m\,MASP1^*}}^{\mathrm{MASP1_{zym}}}}+\frac{\left[\mathrm{pMBL_3MASP2_{zym}}\right]}{K_{\mathrm{m\,MASP1^*}}^{\mathrm{MASP2_{zym}}}}+\frac{\left[\mathrm{pMBL_3MASP3_{zym}}\right]}{K_{\mathrm{m\,MASP1^*}}^{\mathrm{MASP3_{zym}}}} \\
&+\frac{\left[\mathrm{pMBL_4MASP1_{zym}}\right]}{K_{\mathrm{m\,MASP1^*}}^{\mathrm{MASP1_{zym}}}}+\frac{\left[\mathrm{pMBL_4MASP2_{zym}}\right]}{K_{\mathrm{m\,MASP1^*}}^{\mathrm{MASP2_{zym}}}}+\frac{\left[\mathrm{pMBL_4MASP3_{zym}}\right]}{K_{\mathrm{m\,MASP1^*}}^{\mathrm{MASP3_{zym}}}}+\frac{\left[\mathrm{pCLLKMASP1_{zym}}\right]}{K_{\mathrm{m\,MASP1^*}}^{\mathrm{MASP1_{zym}}}} \\
&+\frac{\left[\mathrm{pCLLKMASP2_{zym}}\right]}{K_{\mathrm{m\,MASP1^*}}^{\mathrm{MASP2_{zym}}}}+\frac{\left[\mathrm{pCLLKMASP3_{zym}}\right]}{K_{\mathrm{m\,MASP1^*}}^{\mathrm{MASP3_{zym}}}}+\frac{\left[\mathrm{pCLK1MASP1_{zym}}\right]}{K_{\mathrm{m\,MASP1^*}}^{\mathrm{MASP1_{zym}}}}+\frac{\left[\mathrm{pCLK1MASP2_{zym}}\right]}{K_{\mathrm{m\,MASP1^*}}^{\mathrm{MASP2_{zym}}}} \\
&+\frac{\left[\mathrm{pCLK1MASP3_{zym}}\right]}{K_{\mathrm{m\,MASP1^*}}^{\mathrm{MASP3_{zym}}}}+\frac{\left[\mathrm{pCLL1MASP1_{zym}}\right]}{K_{\mathrm{m\,MASP1^*}}^{\mathrm{MASP1_{zym}}}}+\frac{\left[\mathrm{pCLL1MASP2_{zym}}\right]}{K_{\mathrm{m\,MASP1^*}}^{\mathrm{MASP2_{zym}}}}+\frac{\left[\mathrm{pCLL1MASP3_{zym}}\right]}{K_{\mathrm{m\,MASP1^*}}^{\mathrm{MASP3_{zym}}}} \\
&+\frac{\left[\mathrm{pLFMASP1_{zym}}\right]}{K_{\mathrm{m\,MASP1^*}}^{\mathrm{MASP1_{zym}}}}+\frac{\left[\mathrm{pLFMASP2_{zym}}\right]}{K_{\mathrm{m\,MASP1^*}}^{\mathrm{MASP2_{zym}}}}+\frac{\left[\mathrm{pLFMASP3_{zym}}\right]}{K_{\mathrm{m\,MASP1^*}}^{\mathrm{MASP3_{zym}}}}+\frac{\left[\mathrm{pHFMASP1_{zym}}\right]}{K_{\mathrm{m\,MASP1^*}}^{\mathrm{MASP1_{zym}}}}+\frac{\left[\mathrm{pHFMASP2_{zym}}\right]}{K_{\mathrm{m\,MASP1^*}}^{\mathrm{MASP2_{zym}}}} \\
&+\frac{\left[\mathrm{pHFMASP3_{zym}}\right]}{K_{\mathrm{m\,MASP1^*}}^{\mathrm{MASP3_{zym}}}}+\frac{\left[\mathrm{pMFMASP1_{zym}}\right]}{K_{\mathrm{m\,MASP1^*}}^{\mathrm{MASP1_{zym}}}}+\frac{\left[\mathrm{pMFMASP2_{zym}}\right]}{K_{\mathrm{m\,MASP1^*}}^{\mathrm{MASP2_{zym}}}}+\frac{\left[\mathrm{pMFMASP3_{zym}}\right]}{K_{\mathrm{m\,MASP1^*}}^{\mathrm{MASP3_{zym}}}}
\end{aligned}\right)}
\end{aligned}
$$

$$
-\frac{k_{\mathrm{cat\,MASP1^*}}^{\mathrm{C2}}\left[\mathrm{pMBL_4MASP1^*}\right]\left[\mathrm{pC4bC2}\right]}{K_{\mathrm{m\,MASP1^*}}^{\mathrm{C2}}\left(\begin{array}{l}1+\frac{[\mathrm{C4}]}{K_{\mathrm{m\,MASP1^*}}^{\mathrm{C4}}}+\frac{[\mathrm{fC4bC2}]}{K_{\mathrm{m\,MASP1^*}}^{\mathrm{C2}}}+\frac{[\mathrm{fC4bC4bC2}]}{K_{\mathrm{m\,MASP1^*}}^{\mathrm{C2}}}+\frac{[\mathrm{fC3bC4bC2}]}{K_{\mathrm{m\,MASP1^*}}^{\mathrm{C2}}}+\frac{[\mathrm{pC4bC2}]}{K_{\mathrm{m\,MASP1^*}}^{\mathrm{C2}}}+\frac{[\mathrm{pC4bC4bC2}]}{K_{\mathrm{m\,MASP1^*}}^{\mathrm{C2}}}\\
+\frac{[\mathrm{pC3bC4bC2}]}{K_{\mathrm{m\,MASP1^*}}^{\mathrm{C2}}}+\frac{\left[\mathrm{pMBL_3MASP1_{zym}}\right]}{K_{\mathrm{m\,MASP1^*}}^{\mathrm{MASP1_{zym}}}}+\frac{\left[\mathrm{pMBL_3MASP2_{zym}}\right]}{K_{\mathrm{m\,MASP1^*}}^{\mathrm{MASP2_{zym}}}}+\frac{\left[\mathrm{pMBL_3MASP3_{zym}}\right]}{K_{\mathrm{m\,MASP1^*}}^{\mathrm{MASP3_{zym}}}}\\
+\frac{\left[\mathrm{pMBL_4MASP1_{zym}}\right]}{K_{\mathrm{m\,MASP1^*}}^{\mathrm{MASP1_{zym}}}}+\frac{\left[\mathrm{pMBL_4MASP2_{zym}}\right]}{K_{\mathrm{m\,MASP1^*}}^{\mathrm{MASP2_{zym}}}}+\frac{\left[\mathrm{pMBL_4MASP3_{zym}}\right]}{K_{\mathrm{m\,MASP1^*}}^{\mathrm{MASP3_{zym}}}}+\frac{\left[\mathrm{pCLLKMASP1_{zym}}\right]}{K_{\mathrm{m\,MASP1^*}}^{\mathrm{MASP1_{zym}}}}\\
+\frac{\left[\mathrm{pCLLKMASP2_{zym}}\right]}{K_{\mathrm{m\,MASP1^*}}^{\mathrm{MASP2_{zym}}}}+\frac{\left[\mathrm{pCLLKMASP3_{zym}}\right]}{K_{\mathrm{m\,MASP1^*}}^{\mathrm{MASP3_{zym}}}}+\frac{\left[\mathrm{pCLK1MASP1_{zym}}\right]}{K_{\mathrm{m\,MASP1^*}}^{\mathrm{MASP1_{zym}}}}+\frac{\left[\mathrm{pCLK1MASP2_{zym}}\right]}{K_{\mathrm{m\,MASP1^*}}^{\mathrm{MASP2_{zym}}}}\\
+\frac{\left[\mathrm{pCLK1MASP3_{zym}}\right]}{K_{\mathrm{m\,MASP1^*}}^{\mathrm{MASP3_{zym}}}}+\frac{\left[\mathrm{pCLL1MASP1_{zym}}\right]}{K_{\mathrm{m\,MASP1^*}}^{\mathrm{MASP1_{zym}}}}+\frac{\left[\mathrm{pCLL1MASP2_{zym}}\right]}{K_{\mathrm{m\,MASP1^*}}^{\mathrm{MASP2_{zym}}}}+\frac{\left[\mathrm{pCLL1MASP3_{zym}}\right]}{K_{\mathrm{m\,MASP1^*}}^{\mathrm{MASP3_{zym}}}}\\
+\frac{\left[\mathrm{pLFMASP1_{zym}}\right]}{K_{\mathrm{m\,MASP1^*}}^{\mathrm{MASP1_{zym}}}}+\frac{\left[\mathrm{pLFMASP2_{zym}}\right]}{K_{\mathrm{m\,MASP1^*}}^{\mathrm{MASP2_{zym}}}}+\frac{\left[\mathrm{pLFMASP3_{zym}}\right]}{K_{\mathrm{m\,MASP1^*}}^{\mathrm{MASP3_{zym}}}}+\frac{\left[\mathrm{pHFMASP1_{zym}}\right]}{K_{\mathrm{m\,MASP1^*}}^{\mathrm{MASP1_{zym}}}}+\frac{\left[\mathrm{pHFMASP2_{zym}}\right]}{K_{\mathrm{m\,MASP1^*}}^{\mathrm{MASP2_{zym}}}}\\
+\frac{\left[\mathrm{pHFMASP3_{zym}}\right]}{K_{\mathrm{m\,MASP1^*}}^{\mathrm{MASP3_{zym}}}}+\frac{\left[\mathrm{pMFMASP1_{zym}}\right]}{K_{\mathrm{m\,MASP1^*}}^{\mathrm{MASP1_{zym}}}}+\frac{\left[\mathrm{pMFMASP2_{zym}}\right]}{K_{\mathrm{m\,MASP1^*}}^{\mathrm{MASP2_{zym}}}}+\frac{\left[\mathrm{pMFMASP3_{zym}}\right]}{K_{\mathrm{m\,MASP1^*}}^{\mathrm{MASP3_{zym}}}}\end{array}\right)}
$$

$$
-\frac{k_{\mathrm{cat\,MASP1^*}}^{\mathrm{C2}}\left[\mathrm{pCLLKMASP1^*}\right]\left[\mathrm{pC4bC2}\right]}{K_{\mathrm{m\,MASP1^*}}^{\mathrm{C2}}\left(\begin{array}{l}1+\frac{[\mathrm{C4}]}{K_{\mathrm{m\,MASP1^*}}^{\mathrm{C4}}}+\frac{[\mathrm{fC4bC2}]}{K_{\mathrm{m\,MASP1^*}}^{\mathrm{C2}}}+\frac{[\mathrm{fC4bC4bC2}]}{K_{\mathrm{m\,MASP1^*}}^{\mathrm{C2}}}+\frac{[\mathrm{fC3bC4bC2}]}{K_{\mathrm{m\,MASP1^*}}^{\mathrm{C2}}}+\frac{[\mathrm{pC4bC2}]}{K_{\mathrm{m\,MASP1^*}}^{\mathrm{C2}}}+\frac{[\mathrm{pC4bC4bC2}]}{K_{\mathrm{m\,MASP1^*}}^{\mathrm{C2}}}\\
+\frac{[\mathrm{pC3bC4bC2}]}{K_{\mathrm{m\,MASP1^*}}^{\mathrm{C2}}}+\frac{\left[\mathrm{pMBL_3MASP1_{zym}}\right]}{K_{\mathrm{m\,MASP1^*}}^{\mathrm{MASP1_{zym}}}}+\frac{\left[\mathrm{pMBL_3MASP2_{zym}}\right]}{K_{\mathrm{m\,MASP1^*}}^{\mathrm{MASP2_{zym}}}}+\frac{\left[\mathrm{pMBL_3MASP3_{zym}}\right]}{K_{\mathrm{m\,MASP1^*}}^{\mathrm{MASP3_{zym}}}}\\
+\frac{\left[\mathrm{pMBL_4MASP1_{zym}}\right]}{K_{\mathrm{m\,MASP1^*}}^{\mathrm{MASP1_{zym}}}}+\frac{\left[\mathrm{pMBL_4MASP2_{zym}}\right]}{K_{\mathrm{m\,MASP1^*}}^{\mathrm{MASP2_{zym}}}}+\frac{\left[\mathrm{pMBL_4MASP3_{zym}}\right]}{K_{\mathrm{m\,MASP1^*}}^{\mathrm{MASP3_{zym}}}}+\frac{\left[\mathrm{pCLLKMASP1_{zym}}\right]}{K_{\mathrm{m\,MASP1^*}}^{\mathrm{MASP1_{zym}}}}\\
+\frac{\left[\mathrm{pCLLKMASP2_{zym}}\right]}{K_{\mathrm{m\,MASP1^*}}^{\mathrm{MASP2_{zym}}}}+\frac{\left[\mathrm{pCLLKMASP3_{zym}}\right]}{K_{\mathrm{m\,MASP1^*}}^{\mathrm{MASP3_{zym}}}}+\frac{\left[\mathrm{pCLK1MASP1_{zym}}\right]}{K_{\mathrm{m\,MASP1^*}}^{\mathrm{MASP1_{zym}}}}+\frac{\left[\mathrm{pCLK1MASP2_{zym}}\right]}{K_{\mathrm{m\,MASP1^*}}^{\mathrm{MASP2_{zym}}}}\\
+\frac{\left[\mathrm{pCLK1MASP3_{zym}}\right]}{K_{\mathrm{m\,MASP1^*}}^{\mathrm{MASP3_{zym}}}}+\frac{\left[\mathrm{pCLL1MASP1_{zym}}\right]}{K_{\mathrm{m\,MASP1^*}}^{\mathrm{MASP1_{zym}}}}+\frac{\left[\mathrm{pCLL1MASP2_{zym}}\right]}{K_{\mathrm{m\,MASP1^*}}^{\mathrm{MASP2_{zym}}}}+\frac{\left[\mathrm{pCLL1MASP3_{zym}}\right]}{K_{\mathrm{m\,MASP1^*}}^{\mathrm{MASP3_{zym}}}}\\
+\frac{\left[\mathrm{pLFMASP1_{zym}}\right]}{K_{\mathrm{m\,MASP1^*}}^{\mathrm{MASP1_{zym}}}}+\frac{\left[\mathrm{pLFMASP2_{zym}}\right]}{K_{\mathrm{m\,MASP1^*}}^{\mathrm{MASP2_{zym}}}}+\frac{\left[\mathrm{pLFMASP3_{zym}}\right]}{K_{\mathrm{m\,MASP1^*}}^{\mathrm{MASP3_{zym}}}}+\frac{\left[\mathrm{pHFMASP1_{zym}}\right]}{K_{\mathrm{m\,MASP1^*}}^{\mathrm{MASP1_{zym}}}}+\frac{\left[\mathrm{pHFMASP2_{zym}}\right]}{K_{\mathrm{m\,MASP1^*}}^{\mathrm{MASP2_{zym}}}}\\
+\frac{\left[\mathrm{pHFMASP3_{zym}}\right]}{K_{\mathrm{m\,MASP1^*}}^{\mathrm{MASP3_{zym}}}}+\frac{\left[\mathrm{pMFMASP1_{zym}}\right]}{K_{\mathrm{m\,MASP1^*}}^{\mathrm{MASP1_{zym}}}}+\frac{\left[\mathrm{pMFMASP2_{zym}}\right]}{K_{\mathrm{m\,MASP1^*}}^{\mathrm{MASP2_{zym}}}}+\frac{\left[\mathrm{pMFMASP3_{zym}}\right]}{K_{\mathrm{m\,MASP1^*}}^{\mathrm{MASP3_{zym}}}}\end{array}\right)}
$$

$$
-\frac{k^{\mathrm{C2}}_{\mathrm{cat\,MASP1^*}}\left[\mathrm{pCLK1MASP1^*}\right]\left[\mathrm{pC4bC2}\right]}{K^{\mathrm{C2}}_{\mathrm{m\,MASP1^*}}\left(\begin{aligned}&1+\frac{[\mathrm{C4}]}{K^{\mathrm{C4}}_{\mathrm{m\,MASP1^*}}}+\frac{[\mathrm{fC4bC2}]}{K^{\mathrm{C2}}_{\mathrm{m\,MASP1^*}}}+\frac{[\mathrm{fC4bC4bC2}]}{K^{\mathrm{C2}}_{\mathrm{m\,MASP1^*}}}+\frac{[\mathrm{fC3bC4bC2}]}{K^{\mathrm{C2}}_{\mathrm{m\,MASP1^*}}}+\frac{[\mathrm{pC4bC2}]}{K^{\mathrm{C2}}_{\mathrm{m\,MASP1^*}}}+\frac{[\mathrm{pC4bC4bC2}]}{K^{\mathrm{C2}}_{\mathrm{m\,MASP1^*}}}\\
&+\frac{[\mathrm{pC3bC4bC2}]}{K^{\mathrm{C2}}_{\mathrm{m\,MASP1^*}}}+\frac{[\mathrm{pMBL_3MASP1_{zym}}]}{K^{\mathrm{MASP1_{zym}}}_{\mathrm{m\,MASP1^*}}}+\frac{[\mathrm{pMBL_3MASP2_{zym}}]}{K^{\mathrm{MASP2_{zym}}}_{\mathrm{m\,MASP1^*}}}+\frac{[\mathrm{pMBL_3MASP3_{zym}}]}{K^{\mathrm{MASP3_{zym}}}_{\mathrm{m\,MASP1^*}}}\\
&+\frac{[\mathrm{pMBL_4MASP1_{zym}}]}{K^{\mathrm{MASP1_{zym}}}_{\mathrm{m\,MASP1^*}}}+\frac{[\mathrm{pMBL_4MASP2_{zym}}]}{K^{\mathrm{MASP2_{zym}}}_{\mathrm{m\,MASP1^*}}}+\frac{[\mathrm{pMBL_4MASP3_{zym}}]}{K^{\mathrm{MASP3_{zym}}}_{\mathrm{m\,MASP1^*}}}+\frac{[\mathrm{pCLLKMASP1_{zym}}]}{K^{\mathrm{MASP1_{zym}}}_{\mathrm{m\,MASP1^*}}}\\
&+\frac{[\mathrm{pCLLKMASP2_{zym}}]}{K^{\mathrm{MASP2_{zym}}}_{\mathrm{m\,MASP1^*}}}+\frac{[\mathrm{pCLLKMASP3_{zym}}]}{K^{\mathrm{MASP3_{zym}}}_{\mathrm{m\,MASP1^*}}}+\frac{[\mathrm{pCLK1MASP1_{zym}}]}{K^{\mathrm{MASP1_{zym}}}_{\mathrm{m\,MASP1^*}}}+\frac{[\mathrm{pCLK1MASP2_{zym}}]}{K^{\mathrm{MASP2_{zym}}}_{\mathrm{m\,MASP1^*}}}\\
&+\frac{[\mathrm{pCLK1MASP3_{zym}}]}{K^{\mathrm{MASP3_{zym}}}_{\mathrm{m\,MASP1^*}}}+\frac{[\mathrm{pCLL1MASP1_{zym}}]}{K^{\mathrm{MASP1_{zym}}}_{\mathrm{m\,MASP1^*}}}+\frac{[\mathrm{pCLL1MASP2_{zym}}]}{K^{\mathrm{MASP2_{zym}}}_{\mathrm{m\,MASP1^*}}}+\frac{[\mathrm{pCLL1MASP3_{zym}}]}{K^{\mathrm{MASP3_{zym}}}_{\mathrm{m\,MASP1^*}}}\\
&+\frac{[\mathrm{pLFMASP1_{zym}}]}{K^{\mathrm{MASP1_{zym}}}_{\mathrm{m\,MASP1^*}}}+\frac{[\mathrm{pLFMASP2_{zym}}]}{K^{\mathrm{MASP2_{zym}}}_{\mathrm{m\,MASP1^*}}}+\frac{[\mathrm{pLFMASP3_{zym}}]}{K^{\mathrm{MASP3_{zym}}}_{\mathrm{m\,MASP1^*}}}+\frac{[\mathrm{pHFMASP1_{zym}}]}{K^{\mathrm{MASP1_{zym}}}_{\mathrm{m\,MASP1^*}}}+\frac{[\mathrm{pHFMASP2_{zym}}]}{K^{\mathrm{MASP2_{zym}}}_{\mathrm{m\,MASP1^*}}}\\
&+\frac{[\mathrm{pHFMASP3_{zym}}]}{K^{\mathrm{MASP3_{zym}}}_{\mathrm{m\,MASP1^*}}}+\frac{[\mathrm{pMFMASP1_{zym}}]}{K^{\mathrm{MASP1_{zym}}}_{\mathrm{m\,MASP1^*}}}+\frac{[\mathrm{pMFMASP2_{zym}}]}{K^{\mathrm{MASP2_{zym}}}_{\mathrm{m\,MASP1^*}}}+\frac{[\mathrm{pMFMASP3_{zym}}]}{K^{\mathrm{MASP3_{zym}}}_{\mathrm{m\,MASP1^*}}}\end{aligned}\right)}
$$

$$
-\frac{k^{\mathrm{C2}}_{\mathrm{cat\,MASP1^*}}\left[\mathrm{pCLL1MASP1^*}\right]\left[\mathrm{pC4bC2}\right]}{K^{\mathrm{C2}}_{\mathrm{m\,MASP1^*}}\left(\begin{aligned}&1+\frac{[\mathrm{C4}]}{K^{\mathrm{C4}}_{\mathrm{m\,MASP1^*}}}+\frac{[\mathrm{fC4bC2}]}{K^{\mathrm{C2}}_{\mathrm{m\,MASP1^*}}}+\frac{[\mathrm{fC4bC4bC2}]}{K^{\mathrm{C2}}_{\mathrm{m\,MASP1^*}}}+\frac{[\mathrm{fC3bC4bC2}]}{K^{\mathrm{C2}}_{\mathrm{m\,MASP1^*}}}+\frac{[\mathrm{pC4bC2}]}{K^{\mathrm{C2}}_{\mathrm{m\,MASP1^*}}}+\frac{[\mathrm{pC4bC4bC2}]}{K^{\mathrm{C2}}_{\mathrm{m\,MASP1^*}}}\\
&+\frac{[\mathrm{pC3bC4bC2}]}{K^{\mathrm{C2}}_{\mathrm{m\,MASP1^*}}}+\frac{[\mathrm{pMBL_3MASP1_{zym}}]}{K^{\mathrm{MASP1_{zym}}}_{\mathrm{m\,MASP1^*}}}+\frac{[\mathrm{pMBL_3MASP2_{zym}}]}{K^{\mathrm{MASP2_{zym}}}_{\mathrm{m\,MASP1^*}}}+\frac{[\mathrm{pMBL_3MASP3_{zym}}]}{K^{\mathrm{MASP3_{zym}}}_{\mathrm{m\,MASP1^*}}}\\
&+\frac{[\mathrm{pMBL_4MASP1_{zym}}]}{K^{\mathrm{MASP1_{zym}}}_{\mathrm{m\,MASP1^*}}}+\frac{[\mathrm{pMBL_4MASP2_{zym}}]}{K^{\mathrm{MASP2_{zym}}}_{\mathrm{m\,MASP1^*}}}+\frac{[\mathrm{pMBL_4MASP3_{zym}}]}{K^{\mathrm{MASP3_{zym}}}_{\mathrm{m\,MASP1^*}}}+\frac{[\mathrm{pCLLKMASP1_{zym}}]}{K^{\mathrm{MASP1_{zym}}}_{\mathrm{m\,MASP1^*}}}\\
&+\frac{[\mathrm{pCLLKMASP2_{zym}}]}{K^{\mathrm{MASP2_{zym}}}_{\mathrm{m\,MASP1^*}}}+\frac{[\mathrm{pCLLKMASP3_{zym}}]}{K^{\mathrm{MASP3_{zym}}}_{\mathrm{m\,MASP1^*}}}+\frac{[\mathrm{pCLK1MASP1_{zym}}]}{K^{\mathrm{MASP1_{zym}}}_{\mathrm{m\,MASP1^*}}}+\frac{[\mathrm{pCLK1MASP2_{zym}}]}{K^{\mathrm{MASP2_{zym}}}_{\mathrm{m\,MASP1^*}}}\\
&+\frac{[\mathrm{pCLK1MASP3_{zym}}]}{K^{\mathrm{MASP3_{zym}}}_{\mathrm{m\,MASP1^*}}}+\frac{[\mathrm{pCLL1MASP1_{zym}}]}{K^{\mathrm{MASP1_{zym}}}_{\mathrm{m\,MASP1^*}}}+\frac{[\mathrm{pCLL1MASP2_{zym}}]}{K^{\mathrm{MASP2_{zym}}}_{\mathrm{m\,MASP1^*}}}+\frac{[\mathrm{pCLL1MASP3_{zym}}]}{K^{\mathrm{MASP3_{zym}}}_{\mathrm{m\,MASP1^*}}}\\
&+\frac{[\mathrm{pLFMASP1_{zym}}]}{K^{\mathrm{MASP1_{zym}}}_{\mathrm{m\,MASP1^*}}}+\frac{[\mathrm{pLFMASP2_{zym}}]}{K^{\mathrm{MASP2_{zym}}}_{\mathrm{m\,MASP1^*}}}+\frac{[\mathrm{pLFMASP3_{zym}}]}{K^{\mathrm{MASP3_{zym}}}_{\mathrm{m\,MASP1^*}}}+\frac{[\mathrm{pHFMASP1_{zym}}]}{K^{\mathrm{MASP1_{zym}}}_{\mathrm{m\,MASP1^*}}}+\frac{[\mathrm{pHFMASP2_{zym}}]}{K^{\mathrm{MASP2_{zym}}}_{\mathrm{m\,MASP1^*}}}\\
&+\frac{[\mathrm{pHFMASP3_{zym}}]}{K^{\mathrm{MASP3_{zym}}}_{\mathrm{m\,MASP1^*}}}+\frac{[\mathrm{pMFMASP1_{zym}}]}{K^{\mathrm{MASP1_{zym}}}_{\mathrm{m\,MASP1^*}}}+\frac{[\mathrm{pMFMASP2_{zym}}]}{K^{\mathrm{MASP2_{zym}}}_{\mathrm{m\,MASP1^*}}}+\frac{[\mathrm{pMFMASP3_{zym}}]}{K^{\mathrm{MASP3_{zym}}}_{\mathrm{m\,MASP1^*}}}\end{aligned}\right)}
$$

$$
-\frac{k_{\mathrm{cat\,MASP1^{*}}}^{\mathrm{C2}}\left[\mathrm{pLFMASP1^{*}}\right]\left[\mathrm{pC4bC2}\right]}{K_{\mathrm{m\,MASP1^{*}}}^{\mathrm{C2}}\left(\begin{array}{l}1+\frac{[\mathrm{C4}]}{K_{\mathrm{m\,MASP1^{*}}}^{\mathrm{C4}}}+\frac{[\mathrm{fC4bC2}]}{K_{\mathrm{m\,MASP1^{*}}}^{\mathrm{C2}}}+\frac{[\mathrm{fC4bC4bC2}]}{K_{\mathrm{m\,MASP1^{*}}}^{\mathrm{C2}}}+\frac{[\mathrm{fC3bC4bC2}]}{K_{\mathrm{m\,MASP1^{*}}}^{\mathrm{C2}}}+\frac{[\mathrm{pC4bC2}]}{K_{\mathrm{m\,MASP1^{*}}}^{\mathrm{C2}}}+\frac{[\mathrm{pC4bC4bC2}]}{K_{\mathrm{m\,MASP1^{*}}}^{\mathrm{C2}}}\\
+\frac{[\mathrm{pC3bC4bC2}]}{K_{\mathrm{m\,MASP1^{*}}}^{\mathrm{C2}}}+\frac{\left[\mathrm{pMBL_{3}MASP1_{zym}}\right]}{K_{\mathrm{m\,MASP1^{*}}}^{\mathrm{MASP1_{zym}}}}+\frac{\left[\mathrm{pMBL_{3}MASP2_{zym}}\right]}{K_{\mathrm{m\,MASP1^{*}}}^{\mathrm{MASP2_{zym}}}}+\frac{\left[\mathrm{pMBL_{3}MASP3_{zym}}\right]}{K_{\mathrm{m\,MASP1^{*}}}^{\mathrm{MASP3_{zym}}}}\\
+\frac{\left[\mathrm{pMBL_{4}MASP1_{zym}}\right]}{K_{\mathrm{m\,MASP1^{*}}}^{\mathrm{MASP1_{zym}}}}+\frac{\left[\mathrm{pMBL_{4}MASP2_{zym}}\right]}{K_{\mathrm{m\,MASP1^{*}}}^{\mathrm{MASP2_{zym}}}}+\frac{\left[\mathrm{pMBL_{4}MASP3_{zym}}\right]}{K_{\mathrm{m\,MASP1^{*}}}^{\mathrm{MASP3_{zym}}}}+\frac{\left[\mathrm{pCLLKMASP1_{zym}}\right]}{K_{\mathrm{m\,MASP1^{*}}}^{\mathrm{MASP1_{zym}}}}\\
+\frac{\left[\mathrm{pCLLKMASP2_{zym}}\right]}{K_{\mathrm{m\,MASP1^{*}}}^{\mathrm{MASP2_{zym}}}}+\frac{\left[\mathrm{pCLLKMASP3_{zym}}\right]}{K_{\mathrm{m\,MASP1^{*}}}^{\mathrm{MASP3_{zym}}}}+\frac{\left[\mathrm{pCLK1MASP1_{zym}}\right]}{K_{\mathrm{m\,MASP1^{*}}}^{\mathrm{MASP1_{zym}}}}+\frac{\left[\mathrm{pCLK1MASP2_{zym}}\right]}{K_{\mathrm{m\,MASP1^{*}}}^{\mathrm{MASP2_{zym}}}}\\
+\frac{\left[\mathrm{pCLK1MASP3_{zym}}\right]}{K_{\mathrm{m\,MASP1^{*}}}^{\mathrm{MASP3_{zym}}}}+\frac{\left[\mathrm{pCLL1MASP1_{zym}}\right]}{K_{\mathrm{m\,MASP1^{*}}}^{\mathrm{MASP1_{zym}}}}+\frac{\left[\mathrm{pCLL1MASP2_{zym}}\right]}{K_{\mathrm{m\,MASP1^{*}}}^{\mathrm{MASP2_{zym}}}}+\frac{\left[\mathrm{pCLL1MASP3_{zym}}\right]}{K_{\mathrm{m\,MASP1^{*}}}^{\mathrm{MASP3_{zym}}}}\\
+\frac{\left[\mathrm{pLFMASP1_{zym}}\right]}{K_{\mathrm{m\,MASP1^{*}}}^{\mathrm{MASP1_{zym}}}}+\frac{\left[\mathrm{pLFMASP2_{zym}}\right]}{K_{\mathrm{m\,MASP1^{*}}}^{\mathrm{MASP2_{zym}}}}+\frac{\left[\mathrm{pLFMASP3_{zym}}\right]}{K_{\mathrm{m\,MASP1^{*}}}^{\mathrm{MASP3_{zym}}}}+\frac{\left[\mathrm{pHFMASP1_{zym}}\right]}{K_{\mathrm{m\,MASP1^{*}}}^{\mathrm{MASP1_{zym}}}}+\frac{\left[\mathrm{pHFMASP2_{zym}}\right]}{K_{\mathrm{m\,MASP1^{*}}}^{\mathrm{MASP2_{zym}}}}\\
+\frac{\left[\mathrm{pHFMASP3_{zym}}\right]}{K_{\mathrm{m\,MASP1^{*}}}^{\mathrm{MASP3_{zym}}}}+\frac{\left[\mathrm{pMFMASP1_{zym}}\right]}{K_{\mathrm{m\,MASP1^{*}}}^{\mathrm{MASP1_{zym}}}}+\frac{\left[\mathrm{pMFMASP2_{zym}}\right]}{K_{\mathrm{m\,MASP1^{*}}}^{\mathrm{MASP2_{zym}}}}+\frac{\left[\mathrm{pMFMASP3_{zym}}\right]}{K_{\mathrm{m\,MASP1^{*}}}^{\mathrm{MASP3_{zym}}}}\end{array}\right)}
$$

$$
-\frac{k_{\mathrm{cat\,MASP1^{*}}}^{\mathrm{C2}}\left[\mathrm{pHFMASP1^{*}}\right]\left[\mathrm{pC4bC2}\right]}{K_{\mathrm{m\,MASP1^{*}}}^{\mathrm{C2}}\left(\begin{array}{l}1+\frac{[\mathrm{C4}]}{K_{\mathrm{m\,MASP1^{*}}}^{\mathrm{C4}}}+\frac{[\mathrm{fC4bC2}]}{K_{\mathrm{m\,MASP1^{*}}}^{\mathrm{C2}}}+\frac{[\mathrm{fC4bC4bC2}]}{K_{\mathrm{m\,MASP1^{*}}}^{\mathrm{C2}}}+\frac{[\mathrm{fC3bC4bC2}]}{K_{\mathrm{m\,MASP1^{*}}}^{\mathrm{C2}}}+\frac{[\mathrm{pC4bC2}]}{K_{\mathrm{m\,MASP1^{*}}}^{\mathrm{C2}}}+\frac{[\mathrm{pC4bC4bC2}]}{K_{\mathrm{m\,MASP1^{*}}}^{\mathrm{C2}}}\\
+\frac{[\mathrm{pC3bC4bC2}]}{K_{\mathrm{m\,MASP1^{*}}}^{\mathrm{C2}}}+\frac{\left[\mathrm{pMBL_{3}MASP1_{zym}}\right]}{K_{\mathrm{m\,MASP1^{*}}}^{\mathrm{MASP1_{zym}}}}+\frac{\left[\mathrm{pMBL_{3}MASP2_{zym}}\right]}{K_{\mathrm{m\,MASP1^{*}}}^{\mathrm{MASP2_{zym}}}}+\frac{\left[\mathrm{pMBL_{3}MASP3_{zym}}\right]}{K_{\mathrm{m\,MASP1^{*}}}^{\mathrm{MASP3_{zym}}}}\\
+\frac{\left[\mathrm{pMBL_{4}MASP1_{zym}}\right]}{K_{\mathrm{m\,MASP1^{*}}}^{\mathrm{MASP1_{zym}}}}+\frac{\left[\mathrm{pMBL_{4}MASP2_{zym}}\right]}{K_{\mathrm{m\,MASP1^{*}}}^{\mathrm{MASP2_{zym}}}}+\frac{\left[\mathrm{pMBL_{4}MASP3_{zym}}\right]}{K_{\mathrm{m\,MASP1^{*}}}^{\mathrm{MASP3_{zym}}}}+\frac{\left[\mathrm{pCLLKMASP1_{zym}}\right]}{K_{\mathrm{m\,MASP1^{*}}}^{\mathrm{MASP1_{zym}}}}\\
+\frac{\left[\mathrm{pCLLKMASP2_{zym}}\right]}{K_{\mathrm{m\,MASP1^{*}}}^{\mathrm{MASP2_{zym}}}}+\frac{\left[\mathrm{pCLLKMASP3_{zym}}\right]}{K_{\mathrm{m\,MASP1^{*}}}^{\mathrm{MASP3_{zym}}}}+\frac{\left[\mathrm{pCLK1MASP1_{zym}}\right]}{K_{\mathrm{m\,MASP1^{*}}}^{\mathrm{MASP1_{zym}}}}+\frac{\left[\mathrm{pCLK1MASP2_{zym}}\right]}{K_{\mathrm{m\,MASP1^{*}}}^{\mathrm{MASP2_{zym}}}}\\
+\frac{\left[\mathrm{pCLK1MASP3_{zym}}\right]}{K_{\mathrm{m\,MASP1^{*}}}^{\mathrm{MASP3_{zym}}}}+\frac{\left[\mathrm{pCLL1MASP1_{zym}}\right]}{K_{\mathrm{m\,MASP1^{*}}}^{\mathrm{MASP1_{zym}}}}+\frac{\left[\mathrm{pCLL1MASP2_{zym}}\right]}{K_{\mathrm{m\,MASP1^{*}}}^{\mathrm{MASP2_{zym}}}}+\frac{\left[\mathrm{pCLL1MASP3_{zym}}\right]}{K_{\mathrm{m\,MASP1^{*}}}^{\mathrm{MASP3_{zym}}}}\\
+\frac{\left[\mathrm{pLFMASP1_{zym}}\right]}{K_{\mathrm{m\,MASP1^{*}}}^{\mathrm{MASP1_{zym}}}}+\frac{\left[\mathrm{pLFMASP2_{zym}}\right]}{K_{\mathrm{m\,MASP1^{*}}}^{\mathrm{MASP2_{zym}}}}+\frac{\left[\mathrm{pLFMASP3_{zym}}\right]}{K_{\mathrm{m\,MASP1^{*}}}^{\mathrm{MASP3_{zym}}}}+\frac{\left[\mathrm{pHFMASP1_{zym}}\right]}{K_{\mathrm{m\,MASP1^{*}}}^{\mathrm{MASP1_{zym}}}}+\frac{\left[\mathrm{pHFMASP2_{zym}}\right]}{K_{\mathrm{m\,MASP1^{*}}}^{\mathrm{MASP2_{zym}}}}\\
+\frac{\left[\mathrm{pHFMASP3_{zym}}\right]}{K_{\mathrm{m\,MASP1^{*}}}^{\mathrm{MASP3_{zym}}}}+\frac{\left[\mathrm{pMFMASP1_{zym}}\right]}{K_{\mathrm{m\,MASP1^{*}}}^{\mathrm{MASP1_{zym}}}}+\frac{\left[\mathrm{pMFMASP2_{zym}}\right]}{K_{\mathrm{m\,MASP1^{*}}}^{\mathrm{MASP2_{zym}}}}+\frac{\left[\mathrm{pMFMASP3_{zym}}\right]}{K_{\mathrm{m\,MASP1^{*}}}^{\mathrm{MASP3_{zym}}}}\end{array}\right)}
$$

$$-\frac{k^{\mathrm{C2}}_{\mathrm{cat\,MASP1^*}}\left[\mathrm{pMFMASP1^*}\right]\left[\mathrm{fC4bC2}\right]}{K^{\mathrm{C2}}_{\mathrm{m\,MASP1^*}}\left(\begin{array}{l}1+\frac{[\mathrm{C4}]}{K^{\mathrm{C4}}_{\mathrm{m\,MASP1^*}}}+\frac{[\mathrm{fC4bC2}]}{K^{\mathrm{C2}}_{\mathrm{m\,MASP1^*}}}+\frac{[\mathrm{fC4bC4bC2}]}{K^{\mathrm{C2}}_{\mathrm{m\,MASP1^*}}}+\frac{[\mathrm{fC3bC4bC2}]}{K^{\mathrm{C2}}_{\mathrm{m\,MASP1^*}}}+\frac{[\mathrm{pC4bC2}]}{K^{\mathrm{C2}}_{\mathrm{m\,MASP1^*}}}+\frac{[\mathrm{pC4bC4bC2}]}{K^{\mathrm{C2}}_{\mathrm{m\,MASP1^*}}}\\
+\frac{[\mathrm{pC3bC4bC2}]}{K^{\mathrm{C2}}_{\mathrm{m\,MASP1^*}}}+\frac{[\mathrm{pMBL_3MASP1_{zym}}]}{K^{\mathrm{MASP1_{zym}}}_{\mathrm{m\,MASP1^*}}}+\frac{[\mathrm{pMBL_3MASP2_{zym}}]}{K^{\mathrm{MASP2_{zym}}}_{\mathrm{m\,MASP1^*}}}+\frac{[\mathrm{pMBL_3MASP3_{zym}}]}{K^{\mathrm{MASP3_{zym}}}_{\mathrm{m\,MASP1^*}}}\\
+\frac{[\mathrm{pMBL_4MASP1_{zym}}]}{K^{\mathrm{MASP1_{zym}}}_{\mathrm{m\,MASP1^*}}}+\frac{[\mathrm{pMBL_4MASP2_{zym}}]}{K^{\mathrm{MASP2_{zym}}}_{\mathrm{m\,MASP1^*}}}+\frac{[\mathrm{pMBL_4MASP3_{zym}}]}{K^{\mathrm{MASP3_{zym}}}_{\mathrm{m\,MASP1^*}}}+\frac{[\mathrm{pCLLKMASP1_{zym}}]}{K^{\mathrm{MASP1_{zym}}}_{\mathrm{m\,MASP1^*}}}\\
+\frac{[\mathrm{pCLLKMASP2_{zym}}]}{K^{\mathrm{MASP2_{zym}}}_{\mathrm{m\,MASP1^*}}}+\frac{[\mathrm{pCLLKMASP3_{zym}}]}{K^{\mathrm{MASP3_{zym}}}_{\mathrm{m\,MASP1^*}}}+\frac{[\mathrm{pCLK1MASP1_{zym}}]}{K^{\mathrm{MASP1_{zym}}}_{\mathrm{m\,MASP1^*}}}+\frac{[\mathrm{pCLK1MASP2_{zym}}]}{K^{\mathrm{MASP2_{zym}}}_{\mathrm{m\,MASP1^*}}}\\
+\frac{[\mathrm{pCLK1MASP3_{zym}}]}{K^{\mathrm{MASP3_{zym}}}_{\mathrm{m\,MASP1^*}}}+\frac{[\mathrm{pCLL1MASP1_{zym}}]}{K^{\mathrm{MASP1_{zym}}}_{\mathrm{m\,MASP1^*}}}+\frac{[\mathrm{pCLL1MASP2_{zym}}]}{K^{\mathrm{MASP2_{zym}}}_{\mathrm{m\,MASP1^*}}}+\frac{[\mathrm{pCLL1MASP3_{zym}}]}{K^{\mathrm{MASP3_{zym}}}_{\mathrm{m\,MASP1^*}}}\\
+\frac{[\mathrm{pLFMASP1_{zym}}]}{K^{\mathrm{MASP1_{zym}}}_{\mathrm{m\,MASP1^*}}}+\frac{[\mathrm{pLFMASP2_{zym}}]}{K^{\mathrm{MASP2_{zym}}}_{\mathrm{m\,MASP1^*}}}+\frac{[\mathrm{pLFMASP3_{zym}}]}{K^{\mathrm{MASP3_{zym}}}_{\mathrm{m\,MASP1^*}}}+\frac{[\mathrm{pHFMASP1_{zym}}]}{K^{\mathrm{MASP1_{zym}}}_{\mathrm{m\,MASP1^*}}}+\frac{[\mathrm{pHFMASP2_{zym}}]}{K^{\mathrm{MASP2_{zym}}}_{\mathrm{m\,MASP1^*}}}\\
+\frac{[\mathrm{pHFMASP3_{zym}}]}{K^{\mathrm{MASP3_{zym}}}_{\mathrm{m\,MASP1^*}}}+\frac{[\mathrm{pMFMASP1_{zym}}]}{K^{\mathrm{MASP1_{zym}}}_{\mathrm{m\,MASP1^*}}}+\frac{[\mathrm{pMFMASP2_{zym}}]}{K^{\mathrm{MASP2_{zym}}}_{\mathrm{m\,MASP1^*}}}+\frac{[\mathrm{pMFMASP3_{zym}}]}{K^{\mathrm{MASP3_{zym}}}_{\mathrm{m\,MASP1^*}}}\end{array}\right)}$$

$$-\frac{k^{\mathrm{C2}}_{\mathrm{cat\,MASP2^*}}\left[\mathrm{pMBL_3MASP2^*}\right]\left[\mathrm{pC4bC2}\right]}{K^{\mathrm{C2}}_{\mathrm{m\,MASP2^*}}\left(\begin{array}{l}1+\frac{[\mathrm{C4}]}{K^{\mathrm{C4}}_{\mathrm{m\,MASP2^*}}}+\frac{[\mathrm{fC4bC2}]}{K^{\mathrm{C2}}_{\mathrm{m\,MASP2^*}}}+\frac{[\mathrm{fC4bC4bC2}]}{K^{\mathrm{C2}}_{\mathrm{m\,MASP2^*}}}+\frac{[\mathrm{fC3bC4bC2}]}{K^{\mathrm{C2}}_{\mathrm{m\,MASP2^*}}}+\frac{[\mathrm{pC4bC2}]}{K^{\mathrm{C2}}_{\mathrm{m\,MASP2^*}}}+\frac{[\mathrm{pC4bC4bC2}]}{K^{\mathrm{C2}}_{\mathrm{m\,MASP2^*}}}\\
+\frac{[\mathrm{pC3bC4bC2}]}{K^{\mathrm{C2}}_{\mathrm{m\,MASP2^*}}}+\frac{[\mathrm{pMBL_3MASP1_{zym}}]}{K^{\mathrm{MASP1_{zym}}}_{\mathrm{m\,MASP2^*}}}+\frac{[\mathrm{pMBL_3MASP2_{zym}}]}{K^{\mathrm{MASP2_{zym}}}_{\mathrm{m\,MASP2^*}}}+\frac{[\mathrm{pMBL_4MASP1_{zym}}]}{K^{\mathrm{MASP1_{zym}}}_{\mathrm{m\,MASP2^*}}}\\
+\frac{[\mathrm{pMBL_4MASP2_{zym}}]}{K^{\mathrm{MASP2_{zym}}}_{\mathrm{m\,MASP2^*}}}+\frac{[\mathrm{pCLLKMASP1_{zym}}]}{K^{\mathrm{MASP1_{zym}}}_{\mathrm{m\,MASP2^*}}}+\frac{[\mathrm{pCLLKMASP2_{zym}}]}{K^{\mathrm{MASP2_{zym}}}_{\mathrm{m\,MASP2^*}}}+\frac{[\mathrm{pCLK1MASP1_{zym}}]}{K^{\mathrm{MASP1_{zym}}}_{\mathrm{m\,MASP2^*}}}\\
+\frac{[\mathrm{pCLK1MASP2_{zym}}]}{K^{\mathrm{MASP2_{zym}}}_{\mathrm{m\,MASP2^*}}}+\frac{[\mathrm{pCLL1MASP1_{zym}}]}{K^{\mathrm{MASP1_{zym}}}_{\mathrm{m\,MASP2^*}}}+\frac{[\mathrm{pCLL1MASP2_{zym}}]}{K^{\mathrm{MASP2_{zym}}}_{\mathrm{m\,MASP2^*}}}+\frac{[\mathrm{pLFMASP1_{zym}}]}{K^{\mathrm{MASP1_{zym}}}_{\mathrm{m\,MASP2^*}}}\\
+\frac{[\mathrm{pLFMASP2_{zym}}]}{K^{\mathrm{MASP2_{zym}}}_{\mathrm{m\,MASP2^*}}}+\frac{[\mathrm{pHFMASP1_{zym}}]}{K^{\mathrm{MASP1_{zym}}}_{\mathrm{m\,MASP2^*}}}+\frac{[\mathrm{pHFMASP2_{zym}}]}{K^{\mathrm{MASP2_{zym}}}_{\mathrm{m\,MASP2^*}}}+\frac{[\mathrm{pMFMASP1_{zym}}]}{K^{\mathrm{MASP1_{zym}}}_{\mathrm{m\,MASP2^*}}}+\frac{[\mathrm{pMFMASP2_{zym}}]}{K^{\mathrm{MASP2_{zym}}}_{\mathrm{m\,MASP2^*}}}\end{array}\right)}$$

$$
-\frac{k_{\text{cat MASP2}^*}^{\text{C2}}\left[\text{pMBL}_4\text{MASP2}^*\right]\left[\text{pC4bC2}\right]}{K_{\text{m MASP2}^*}^{\text{C2}}\left(\begin{array}{l}1+\frac{[\text{C4}]}{K_{\text{m MASP2}^*}^{\text{C4}}}+\frac{[\text{fC4bC2}]}{K_{\text{m MASP2}^*}^{\text{C2}}}+\frac{[\text{fC4bC4bC2}]}{K_{\text{m MASP2}^*}^{\text{C2}}}+\frac{[\text{fC3bC4bC2}]}{K_{\text{m MASP2}^*}^{\text{C2}}}+\frac{[\text{pC4bC2}]}{K_{\text{m MASP2}^*}^{\text{C2}}}+\frac{[\text{pC4bC4bC2}]}{K_{\text{m MASP2}^*}^{\text{C2}}}\\+\frac{[\text{pC3bC4bC2}]}{K_{\text{m MASP2}^*}^{\text{C2}}}+\frac{\left[\text{pMBL}_3\text{MASP1}_{\text{zym}}\right]}{K_{\text{m MASP2}^*}^{\text{MASP1}_{\text{zym}}}}+\frac{\left[\text{pMBL}_3\text{MASP2}_{\text{zym}}\right]}{K_{\text{m MASP2}^*}^{\text{MASP2}_{\text{zym}}}}+\frac{\left[\text{pMBL}_4\text{MASP1}_{\text{zym}}\right]}{K_{\text{m MASP2}^*}^{\text{MASP1}_{\text{zym}}}}\\+\frac{\left[\text{pMBL}_4\text{MASP2}_{\text{zym}}\right]}{K_{\text{m MASP2}^*}^{\text{MASP2}_{\text{zym}}}}+\frac{\left[\text{pCLLKMASP1}_{\text{zym}}\right]}{K_{\text{m MASP2}^*}^{\text{MASP1}_{\text{zym}}}}+\frac{\left[\text{pCLLKMASP2}_{\text{zym}}\right]}{K_{\text{m MASP2}^*}^{\text{MASP2}_{\text{zym}}}}+\frac{\left[\text{pCLK1MASP1}_{\text{zym}}\right]}{K_{\text{m MASP2}^*}^{\text{MASP1}_{\text{zym}}}}\\+\frac{\left[\text{pCLK1MASP2}_{\text{zym}}\right]}{K_{\text{m MASP2}^*}^{\text{MASP2}_{\text{zym}}}}+\frac{\left[\text{pCLL1MASP1}_{\text{zym}}\right]}{K_{\text{m MASP2}^*}^{\text{MASP1}_{\text{zym}}}}+\frac{\left[\text{pCLL1MASP2}_{\text{zym}}\right]}{K_{\text{m MASP2}^*}^{\text{MASP2}_{\text{zym}}}}+\frac{\left[\text{pLFMASP1}_{\text{zym}}\right]}{K_{\text{m MASP2}^*}^{\text{MASP1}_{\text{zym}}}}\\+\frac{\left[\text{pLFMASP2}_{\text{zym}}\right]}{K_{\text{m MASP2}^*}^{\text{MASP2}_{\text{zym}}}}+\frac{\left[\text{pHFMASP1}_{\text{zym}}\right]}{K_{\text{m MASP2}^*}^{\text{MASP1}_{\text{zym}}}}+\frac{\left[\text{pHFMASP2}_{\text{zym}}\right]}{K_{\text{m MASP2}^*}^{\text{MASP2}_{\text{zym}}}}+\frac{\left[\text{pMFMASP1}_{\text{zym}}\right]}{K_{\text{m MASP2}^*}^{\text{MASP1}_{\text{zym}}}}+\frac{\left[\text{pMFMASP2}_{\text{zym}}\right]}{K_{\text{m MASP2}^*}^{\text{MASP2}_{\text{zym}}}}\end{array}\right)}
$$

$$
-\frac{k_{\text{cat MASP2}^*}^{\text{C2}}\left[\text{pCLLKMASP2}^*\right]\left[\text{pC4bC2}\right]}{K_{\text{m MASP2}^*}^{\text{C2}}\left(\begin{array}{l}1+\frac{[\text{C4}]}{K_{\text{m MASP2}^*}^{\text{C4}}}+\frac{[\text{fC4bC2}]}{K_{\text{m MASP2}^*}^{\text{C2}}}+\frac{[\text{fC4bC4bC2}]}{K_{\text{m MASP2}^*}^{\text{C2}}}+\frac{[\text{fC3bC4bC2}]}{K_{\text{m MASP2}^*}^{\text{C2}}}+\frac{[\text{pC4bC2}]}{K_{\text{m MASP2}^*}^{\text{C2}}}+\frac{[\text{pC4bC4bC2}]}{K_{\text{m MASP2}^*}^{\text{C2}}}\\+\frac{[\text{pC3bC4bC2}]}{K_{\text{m MASP2}^*}^{\text{C2}}}+\frac{\left[\text{pMBL}_3\text{MASP1}_{\text{zym}}\right]}{K_{\text{m MASP2}^*}^{\text{MASP1}_{\text{zym}}}}+\frac{\left[\text{pMBL}_3\text{MASP2}_{\text{zym}}\right]}{K_{\text{m MASP2}^*}^{\text{MASP2}_{\text{zym}}}}+\frac{\left[\text{pMBL}_4\text{MASP1}_{\text{zym}}\right]}{K_{\text{m MASP2}^*}^{\text{MASP1}_{\text{zym}}}}\\+\frac{\left[\text{pMBL}_4\text{MASP2}_{\text{zym}}\right]}{K_{\text{m MASP2}^*}^{\text{MASP2}_{\text{zym}}}}+\frac{\left[\text{pCLLKMASP1}_{\text{zym}}\right]}{K_{\text{m MASP2}^*}^{\text{MASP1}_{\text{zym}}}}+\frac{\left[\text{pCLLKMASP2}_{\text{zym}}\right]}{K_{\text{m MASP2}^*}^{\text{MASP2}_{\text{zym}}}}+\frac{\left[\text{pCLK1MASP1}_{\text{zym}}\right]}{K_{\text{m MASP2}^*}^{\text{MASP1}_{\text{zym}}}}\\+\frac{\left[\text{pCLK1MASP2}_{\text{zym}}\right]}{K_{\text{m MASP2}^*}^{\text{MASP2}_{\text{zym}}}}+\frac{\left[\text{pCLL1MASP1}_{\text{zym}}\right]}{K_{\text{m MASP2}^*}^{\text{MASP1}_{\text{zym}}}}+\frac{\left[\text{pCLL1MASP2}_{\text{zym}}\right]}{K_{\text{m MASP2}^*}^{\text{MASP2}_{\text{zym}}}}+\frac{\left[\text{pLFMASP1}_{\text{zym}}\right]}{K_{\text{m MASP2}^*}^{\text{MASP1}_{\text{zym}}}}\\+\frac{\left[\text{pLFMASP2}_{\text{zym}}\right]}{K_{\text{m MASP2}^*}^{\text{MASP2}_{\text{zym}}}}+\frac{\left[\text{pHFMASP1}_{\text{zym}}\right]}{K_{\text{m MASP2}^*}^{\text{MASP1}_{\text{zym}}}}+\frac{\left[\text{pHFMASP2}_{\text{zym}}\right]}{K_{\text{m MASP2}^*}^{\text{MASP2}_{\text{zym}}}}+\frac{\left[\text{pMFMASP1}_{\text{zym}}\right]}{K_{\text{m MASP2}^*}^{\text{MASP1}_{\text{zym}}}}+\frac{\left[\text{pMFMASP2}_{\text{zym}}\right]}{K_{\text{m MASP2}^*}^{\text{MASP2}_{\text{zym}}}}\end{array}\right)}
$$

$$
-\frac{k_{\text{cat MASP2}^*}^{\text{C2}}\left[\text{pCLK1MASP2}^*\right]\left[\text{pC4bC2}\right]}{K_{\text{m MASP2}^*}^{\text{C2}}\left(\begin{array}{l}1+\frac{[\text{C4}]}{K_{\text{m MASP2}^*}^{\text{C4}}}+\frac{[\text{fC4bC2}]}{K_{\text{m MASP2}^*}^{\text{C2}}}+\frac{[\text{fC4bC4bC2}]}{K_{\text{m MASP2}^*}^{\text{C2}}}+\frac{[\text{fC3bC4bC2}]}{K_{\text{m MASP2}^*}^{\text{C2}}}+\frac{[\text{pC4bC2}]}{K_{\text{m MASP2}^*}^{\text{C2}}}+\frac{[\text{pC4bC4bC2}]}{K_{\text{m MASP2}^*}^{\text{C2}}}\\+\frac{[\text{pC3bC4bC2}]}{K_{\text{m MASP2}^*}^{\text{C2}}}+\frac{\left[\text{pMBL}_3\text{MASP1}_{\text{zym}}\right]}{K_{\text{m MASP2}^*}^{\text{MASP1}_{\text{zym}}}}+\frac{\left[\text{pMBL}_3\text{MASP2}_{\text{zym}}\right]}{K_{\text{m MASP2}^*}^{\text{MASP2}_{\text{zym}}}}+\frac{\left[\text{pMBL}_4\text{MASP1}_{\text{zym}}\right]}{K_{\text{m MASP2}^*}^{\text{MASP1}_{\text{zym}}}}\\+\frac{\left[\text{pMBL}_4\text{MASP2}_{\text{zym}}\right]}{K_{\text{m MASP2}^*}^{\text{MASP2}_{\text{zym}}}}+\frac{\left[\text{pCLLKMASP1}_{\text{zym}}\right]}{K_{\text{m MASP2}^*}^{\text{MASP1}_{\text{zym}}}}+\frac{\left[\text{pCLLKMASP2}_{\text{zym}}\right]}{K_{\text{m MASP2}^*}^{\text{MASP2}_{\text{zym}}}}+\frac{\left[\text{pCLK1MASP1}_{\text{zym}}\right]}{K_{\text{m MASP2}^*}^{\text{MASP1}_{\text{zym}}}}\\+\frac{\left[\text{pCLK1MASP2}_{\text{zym}}\right]}{K_{\text{m MASP2}^*}^{\text{MASP2}_{\text{zym}}}}+\frac{\left[\text{pCLL1MASP1}_{\text{zym}}\right]}{K_{\text{m MASP2}^*}^{\text{MASP1}_{\text{zym}}}}+\frac{\left[\text{pCLL1MASP2}_{\text{zym}}\right]}{K_{\text{m MASP2}^*}^{\text{MASP2}_{\text{zym}}}}+\frac{\left[\text{pLFMASP1}_{\text{zym}}\right]}{K_{\text{m MASP2}^*}^{\text{MASP1}_{\text{zym}}}}\\+\frac{\left[\text{pLFMASP2}_{\text{zym}}\right]}{K_{\text{m MASP2}^*}^{\text{MASP2}_{\text{zym}}}}+\frac{\left[\text{pHFMASP1}_{\text{zym}}\right]}{K_{\text{m MASP2}^*}^{\text{MASP1}_{\text{zym}}}}+\frac{\left[\text{pHFMASP2}_{\text{zym}}\right]}{K_{\text{m MASP2}^*}^{\text{MASP2}_{\text{zym}}}}+\frac{\left[\text{pMFMASP1}_{\text{zym}}\right]}{K_{\text{m MASP2}^*}^{\text{MASP1}_{\text{zym}}}}+\frac{\left[\text{pMFMASP2}_{\text{zym}}\right]}{K_{\text{m MASP2}^*}^{\text{MASP2}_{\text{zym}}}}\end{array}\right)}
$$

$$-\frac{k^{\mathrm{C2}}_{\mathrm{cat\ MASP2^*}}\left[\mathrm{pCLL1MASP2^*}\right]\left[\mathrm{pC4bC2}\right]}{K^{\mathrm{C2}}_{\mathrm{m\ MASP2^*}}\left(\begin{array}{l}1+\frac{[\mathrm{C4}]}{K^{\mathrm{C4}}_{\mathrm{m\ MASP2^*}}}+\frac{[\mathrm{fC4bC2}]}{K^{\mathrm{C2}}_{\mathrm{m\ MASP2^*}}}+\frac{[\mathrm{fC4bC4bC2}]}{K^{\mathrm{C2}}_{\mathrm{m\ MASP2^*}}}+\frac{[\mathrm{fC3bC4bC2}]}{K^{\mathrm{C2}}_{\mathrm{m\ MASP2^*}}}+\frac{[\mathrm{pC4bC2}]}{K^{\mathrm{C2}}_{\mathrm{m\ MASP2^*}}}+\frac{[\mathrm{pC4bC4bC2}]}{K^{\mathrm{C2}}_{\mathrm{m\ MASP2^*}}}\\+\frac{[\mathrm{pC3bC4bC2}]}{K^{\mathrm{C2}}_{\mathrm{m\ MASP2^*}}}+\frac{[\mathrm{pMBL_3MASP1_{zym}}]}{K^{\mathrm{MASP1_{zym}}}_{\mathrm{m\ MASP2^*}}}+\frac{[\mathrm{pMBL_3MASP2_{zym}}]}{K^{\mathrm{MASP2_{zym}}}_{\mathrm{m\ MASP2^*}}}+\frac{[\mathrm{pMBL_4MASP1_{zym}}]}{K^{\mathrm{MASP1_{zym}}}_{\mathrm{m\ MASP2^*}}}\\+\frac{[\mathrm{pMBL_4MASP2_{zym}}]}{K^{\mathrm{MASP2_{zym}}}_{\mathrm{m\ MASP2^*}}}+\frac{[\mathrm{pCLLKMASP1_{zym}}]}{K^{\mathrm{MASP1_{zym}}}_{\mathrm{m\ MASP2^*}}}+\frac{[\mathrm{pCLLKMASP2_{zym}}]}{K^{\mathrm{MASP2_{zym}}}_{\mathrm{m\ MASP2^*}}}+\frac{[\mathrm{pCLK1MASP1_{zym}}]}{K^{\mathrm{MASP1_{zym}}}_{\mathrm{m\ MASP2^*}}}\\+\frac{[\mathrm{pCLK1MASP2_{zym}}]}{K^{\mathrm{MASP2_{zym}}}_{\mathrm{m\ MASP2^*}}}+\frac{[\mathrm{pCLL1MASP1_{zym}}]}{K^{\mathrm{MASP1_{zym}}}_{\mathrm{m\ MASP2^*}}}+\frac{[\mathrm{pCLL1MASP2_{zym}}]}{K^{\mathrm{MASP2_{zym}}}_{\mathrm{m\ MASP2^*}}}+\frac{[\mathrm{pLFMASP1_{zym}}]}{K^{\mathrm{MASP1_{zym}}}_{\mathrm{m\ MASP2^*}}}\\+\frac{[\mathrm{pLFMASP2_{zym}}]}{K^{\mathrm{MASP2_{zym}}}_{\mathrm{m\ MASP2^*}}}+\frac{[\mathrm{pHFMASP1_{zym}}]}{K^{\mathrm{MASP1_{zym}}}_{\mathrm{m\ MASP2^*}}}+\frac{[\mathrm{pHFMASP2_{zym}}]}{K^{\mathrm{MASP2_{zym}}}_{\mathrm{m\ MASP2^*}}}+\frac{[\mathrm{pMFMASP1_{zym}}]}{K^{\mathrm{MASP1_{zym}}}_{\mathrm{m\ MASP2^*}}}+\frac{[\mathrm{pMFMASP2_{zym}}]}{K^{\mathrm{MASP2_{zym}}}_{\mathrm{m\ MASP2^*}}}\end{array}\right)}$$

$$-\frac{k^{\mathrm{C2}}_{\mathrm{cat\ MASP2^*}}\left[\mathrm{pLFMASP2^*}\right]\left[\mathrm{pC4bC2}\right]}{K^{\mathrm{C2}}_{\mathrm{m\ MASP2^*}}\left(\begin{array}{l}1+\frac{[\mathrm{C4}]}{K^{\mathrm{C4}}_{\mathrm{m\ MASP2^*}}}+\frac{[\mathrm{fC4bC2}]}{K^{\mathrm{C2}}_{\mathrm{m\ MASP2^*}}}+\frac{[\mathrm{fC4bC4bC2}]}{K^{\mathrm{C2}}_{\mathrm{m\ MASP2^*}}}+\frac{[\mathrm{fC3bC4bC2}]}{K^{\mathrm{C2}}_{\mathrm{m\ MASP2^*}}}+\frac{[\mathrm{pC4bC2}]}{K^{\mathrm{C2}}_{\mathrm{m\ MASP2^*}}}+\frac{[\mathrm{pC4bC4bC2}]}{K^{\mathrm{C2}}_{\mathrm{m\ MASP2^*}}}\\+\frac{[\mathrm{pC3bC4bC2}]}{K^{\mathrm{C2}}_{\mathrm{m\ MASP2^*}}}+\frac{[\mathrm{pMBL_3MASP1_{zym}}]}{K^{\mathrm{MASP1_{zym}}}_{\mathrm{m\ MASP2^*}}}+\frac{[\mathrm{pMBL_3MASP2_{zym}}]}{K^{\mathrm{MASP2_{zym}}}_{\mathrm{m\ MASP2^*}}}+\frac{[\mathrm{pMBL_4MASP1_{zym}}]}{K^{\mathrm{MASP1_{zym}}}_{\mathrm{m\ MASP2^*}}}\\+\frac{[\mathrm{pMBL_4MASP2_{zym}}]}{K^{\mathrm{MASP2_{zym}}}_{\mathrm{m\ MASP2^*}}}+\frac{[\mathrm{pCLLKMASP1_{zym}}]}{K^{\mathrm{MASP1_{zym}}}_{\mathrm{m\ MASP2^*}}}+\frac{[\mathrm{pCLLKMASP2_{zym}}]}{K^{\mathrm{MASP2_{zym}}}_{\mathrm{m\ MASP2^*}}}+\frac{[\mathrm{pCLK1MASP1_{zym}}]}{K^{\mathrm{MASP1_{zym}}}_{\mathrm{m\ MASP2^*}}}\\+\frac{[\mathrm{pCLK1MASP2_{zym}}]}{K^{\mathrm{MASP2_{zym}}}_{\mathrm{m\ MASP2^*}}}+\frac{[\mathrm{pCLL1MASP1_{zym}}]}{K^{\mathrm{MASP1_{zym}}}_{\mathrm{m\ MASP2^*}}}+\frac{[\mathrm{pCLL1MASP2_{zym}}]}{K^{\mathrm{MASP2_{zym}}}_{\mathrm{m\ MASP2^*}}}+\frac{[\mathrm{pLFMASP1_{zym}}]}{K^{\mathrm{MASP1_{zym}}}_{\mathrm{m\ MASP2^*}}}\\+\frac{[\mathrm{pLFMASP2_{zym}}]}{K^{\mathrm{MASP2_{zym}}}_{\mathrm{m\ MASP2^*}}}+\frac{[\mathrm{pHFMASP1_{zym}}]}{K^{\mathrm{MASP1_{zym}}}_{\mathrm{m\ MASP2^*}}}+\frac{[\mathrm{pHFMASP2_{zym}}]}{K^{\mathrm{MASP2_{zym}}}_{\mathrm{m\ MASP2^*}}}+\frac{[\mathrm{pMFMASP1_{zym}}]}{K^{\mathrm{MASP1_{zym}}}_{\mathrm{m\ MASP2^*}}}+\frac{[\mathrm{pMFMASP2_{zym}}]}{K^{\mathrm{MASP2_{zym}}}_{\mathrm{m\ MASP2^*}}}\end{array}\right)}$$

$$-\frac{k^{\mathrm{C2}}_{\mathrm{cat\ MASP2^*}}\left[\mathrm{pHFMASP2^*}\right]\left[\mathrm{pC4bC2}\right]}{K^{\mathrm{C2}}_{\mathrm{m\ MASP2^*}}\left(\begin{array}{l}1+\frac{[\mathrm{C4}]}{K^{\mathrm{C4}}_{\mathrm{m\ MASP2^*}}}+\frac{[\mathrm{fC4bC2}]}{K^{\mathrm{C2}}_{\mathrm{m\ MASP2^*}}}+\frac{[\mathrm{fC4bC4bC2}]}{K^{\mathrm{C2}}_{\mathrm{m\ MASP2^*}}}+\frac{[\mathrm{fC3bC4bC2}]}{K^{\mathrm{C2}}_{\mathrm{m\ MASP2^*}}}+\frac{[\mathrm{pC4bC2}]}{K^{\mathrm{C2}}_{\mathrm{m\ MASP2^*}}}+\frac{[\mathrm{pC4bC4bC2}]}{K^{\mathrm{C2}}_{\mathrm{m\ MASP2^*}}}\\+\frac{[\mathrm{pC3bC4bC2}]}{K^{\mathrm{C2}}_{\mathrm{m\ MASP2^*}}}+\frac{[\mathrm{pMBL_3MASP1_{zym}}]}{K^{\mathrm{MASP1_{zym}}}_{\mathrm{m\ MASP2^*}}}+\frac{[\mathrm{pMBL_3MASP2_{zym}}]}{K^{\mathrm{MASP2_{zym}}}_{\mathrm{m\ MASP2^*}}}+\frac{[\mathrm{pMBL_4MASP1_{zym}}]}{K^{\mathrm{MASP1_{zym}}}_{\mathrm{m\ MASP2^*}}}\\+\frac{[\mathrm{pMBL_4MASP2_{zym}}]}{K^{\mathrm{MASP2_{zym}}}_{\mathrm{m\ MASP2^*}}}+\frac{[\mathrm{pCLLKMASP1_{zym}}]}{K^{\mathrm{MASP1_{zym}}}_{\mathrm{m\ MASP2^*}}}+\frac{[\mathrm{pCLLKMASP2_{zym}}]}{K^{\mathrm{MASP2_{zym}}}_{\mathrm{m\ MASP2^*}}}+\frac{[\mathrm{pCLK1MASP1_{zym}}]}{K^{\mathrm{MASP1_{zym}}}_{\mathrm{m\ MASP2^*}}}\\+\frac{[\mathrm{pCLK1MASP2_{zym}}]}{K^{\mathrm{MASP2_{zym}}}_{\mathrm{m\ MASP2^*}}}+\frac{[\mathrm{pCLL1MASP1_{zym}}]}{K^{\mathrm{MASP1_{zym}}}_{\mathrm{m\ MASP2^*}}}+\frac{[\mathrm{pCLL1MASP2_{zym}}]}{K^{\mathrm{MASP2_{zym}}}_{\mathrm{m\ MASP2^*}}}+\frac{[\mathrm{pLFMASP1_{zym}}]}{K^{\mathrm{MASP1_{zym}}}_{\mathrm{m\ MASP2^*}}}\\+\frac{[\mathrm{pLFMASP2_{zym}}]}{K^{\mathrm{MASP2_{zym}}}_{\mathrm{m\ MASP2^*}}}+\frac{[\mathrm{pHFMASP1_{zym}}]}{K^{\mathrm{MASP1_{zym}}}_{\mathrm{m\ MASP2^*}}}+\frac{[\mathrm{pHFMASP2_{zym}}]}{K^{\mathrm{MASP2_{zym}}}_{\mathrm{m\ MASP2^*}}}+\frac{[\mathrm{pMFMASP1_{zym}}]}{K^{\mathrm{MASP1_{zym}}}_{\mathrm{m\ MASP2^*}}}+\frac{[\mathrm{pMFMASP2_{zym}}]}{K^{\mathrm{MASP2_{zym}}}_{\mathrm{m\ MASP2^*}}}\end{array}\right)}$$

$$-\frac{k_{\text{cat MASP2}^*}^{\text{C2}}\left[\text{pMFMASP2}^*\right]\left[\text{pC4bC2}\right]}{K_{\text{m MASP2}^*}^{\text{C2}}\left(\begin{array}{l}1+\frac{[\text{C4}]}{K_{\text{m MASP2}^*}^{\text{C4}}}+\frac{[\text{fC4bC2}]}{K_{\text{m MASP2}^*}^{\text{C2}}}+\frac{[\text{fC4bC4bC2}]}{K_{\text{m MASP2}^*}^{\text{C2}}}+\frac{[\text{fC3bC4bC2}]}{K_{\text{m MASP2}^*}^{\text{C2}}}+\frac{[\text{pC4bC2}]}{K_{\text{m MASP2}^*}^{\text{C2}}}+\frac{[\text{pC4bC4bC2}]}{K_{\text{m MASP2}^*}^{\text{C2}}}\\ +\frac{[\text{pC3bC4bC2}]}{K_{\text{m MASP2}^*}^{\text{C2}}}+\frac{[\text{pMBL}_3\text{MASP1}_{\text{zym}}]}{K_{\text{m MASP2}^*}^{\text{MASP1}_{\text{zym}}}}+\frac{[\text{pMBL}_3\text{MASP2}_{\text{zym}}]}{K_{\text{m MASP2}^*}^{\text{MASP2}_{\text{zym}}}}+\frac{[\text{pMBL}_4\text{MASP1}_{\text{zym}}]}{K_{\text{m MASP2}^*}^{\text{MASP1}_{\text{zym}}}}\\ +\frac{[\text{pMBL}_4\text{MASP2}_{\text{zym}}]}{K_{\text{m MASP2}^*}^{\text{MASP2}_{\text{zym}}}}+\frac{[\text{pCLLKMASP1}_{\text{zym}}]}{K_{\text{m MASP2}^*}^{\text{MASP1}_{\text{zym}}}}+\frac{[\text{pCLLKMASP2}_{\text{zym}}]}{K_{\text{m MASP2}^*}^{\text{MASP2}_{\text{zym}}}}+\frac{[\text{pCLK1MASP1}_{\text{zym}}]}{K_{\text{m MASP2}^*}^{\text{MASP1}_{\text{zym}}}}\\ +\frac{[\text{pCLK1MASP2}_{\text{zym}}]}{K_{\text{m MASP2}^*}^{\text{MASP2}_{\text{zym}}}}+\frac{[\text{pCLL1MASP1}_{\text{zym}}]}{K_{\text{m MASP2}^*}^{\text{MASP1}_{\text{zym}}}}+\frac{[\text{pCLL1MASP2}_{\text{zym}}]}{K_{\text{m MASP2}^*}^{\text{MASP2}_{\text{zym}}}}+\frac{[\text{pLFMASP1}_{\text{zym}}]}{K_{\text{m MASP2}^*}^{\text{MASP1}_{\text{zym}}}}\\ +\frac{[\text{pLFMASP2}_{\text{zym}}]}{K_{\text{m MASP2}^*}^{\text{MASP2}_{\text{zym}}}}+\frac{[\text{pHFMASP1}_{\text{zym}}]}{K_{\text{m MASP2}^*}^{\text{MASP1}_{\text{zym}}}}+\frac{[\text{pHFMASP2}_{\text{zym}}]}{K_{\text{m MASP2}^*}^{\text{MASP2}_{\text{zym}}}}+\frac{[\text{pMFMASP1}_{\text{zym}}]}{K_{\text{m MASP2}^*}^{\text{MASP1}_{\text{zym}}}}+\frac{[\text{pMFMASP2}_{\text{zym}}]}{K_{\text{m MASP2}^*}^{\text{MASP2}_{\text{zym}}}}\end{array}\right)}$$

$$-\frac{k_{\text{cat C1}^*}^{\text{C2}}\left[\text{pMBL}_3\text{PTX3C1}^*\right]\left[\text{pC4bC2}\right]}{K_{\text{m C1}^*}^{\text{C2}}\left(1+\frac{[\text{C4}]}{K_{\text{m C1}^*}^{\text{C4}}}+\frac{[\text{fC4bC2}]}{K_{\text{m C1}^*}^{\text{C2}}}+\frac{[\text{fC4bC4bC2}]}{K_{\text{m C1}^*}^{\text{C2}}}+\frac{[\text{fC3bC4bC2}]}{K_{\text{m C1}^*}^{\text{C2}}}+\frac{[\text{pC4bC2}]}{K_{\text{m C1}^*}^{\text{C2}}}+\frac{[\text{pC4bC4bC2}]}{K_{\text{m C1}^*}^{\text{C2}}}+\frac{[\text{pC3bC4bC2}]}{K_{\text{m C1}^*}^{\text{C2}}}\right)}$$

$$-\frac{k_{\text{cat C1}^*}^{\text{C2}}\left[\text{pMBL}_4\text{PTX3C1}^*\right]\left[\text{pC4bC2}\right]}{K_{\text{m C1}^*}^{\text{C2}}\left(1+\frac{[\text{C4}]}{K_{\text{m C1}^*}^{\text{C4}}}+\frac{[\text{fC4bC2}]}{K_{\text{m C1}^*}^{\text{C2}}}+\frac{[\text{fC4bC4bC2}]}{K_{\text{m C1}^*}^{\text{C2}}}+\frac{[\text{fC3bC4bC2}]}{K_{\text{m C1}^*}^{\text{C2}}}+\frac{[\text{pC4bC2}]}{K_{\text{m C1}^*}^{\text{C2}}}+\frac{[\text{pC4bC4bC2}]}{K_{\text{m C1}^*}^{\text{C2}}}+\frac{[\text{pC3bC4bC2}]}{K_{\text{m C1}^*}^{\text{C2}}}\right)}$$

149

$$\frac{d[\mathrm{pC4bC2a}]}{dt}=$$

$$\frac{k_{\mathrm{cat\,C1^*}}^{\mathrm{C2}}\left[\mathrm{pIgG_6C1^*}\right][\mathrm{pC4bC2}]}{K_{\mathrm{m\,C1^*}}^{\mathrm{C2}}\left(1+\frac{[\mathrm{C4}]}{K_{\mathrm{m\,C1^*}}^{\mathrm{C4}}}+\frac{[\mathrm{fC4bC2}]}{K_{\mathrm{m\,C1^*}}^{\mathrm{C2}}}+\frac{[\mathrm{fC4bC4bC2}]}{K_{\mathrm{m\,C1^*}}^{\mathrm{C2}}}+\frac{[\mathrm{fC3bC4bC2}]}{K_{\mathrm{m\,C1^*}}^{\mathrm{C2}}}+\frac{[\mathrm{pC4bC2}]}{K_{\mathrm{m\,C1^*}}^{\mathrm{C2}}}+\frac{[\mathrm{pC4bC4bC2}]}{K_{\mathrm{m\,C1^*}}^{\mathrm{C2}}}+\frac{[\mathrm{pC3bC4bC2}]}{K_{\mathrm{m\,C1^*}}^{\mathrm{C2}}}\right)}$$

$$+\frac{k_{\mathrm{cat\,C1^*}}^{\mathrm{C2}}\left[\mathrm{pIgMC1^*}\right][\mathrm{pC4bC2}]}{K_{\mathrm{m\,C1^*}}^{\mathrm{C2}}\left(1+\frac{[\mathrm{C4}]}{K_{\mathrm{m\,C1^*}}^{\mathrm{C4}}}+\frac{[\mathrm{fC4bC2}]}{K_{\mathrm{m\,C1^*}}^{\mathrm{C2}}}+\frac{[\mathrm{fC4bC4bC2}]}{K_{\mathrm{m\,C1^*}}^{\mathrm{C2}}}+\frac{[\mathrm{fC3bC4bC2}]}{K_{\mathrm{m\,C1^*}}^{\mathrm{C2}}}+\frac{[\mathrm{pC4bC2}]}{K_{\mathrm{m\,C1^*}}^{\mathrm{C2}}}+\frac{[\mathrm{pC4bC4bC2}]}{K_{\mathrm{m\,C1^*}}^{\mathrm{C2}}}+\frac{[\mathrm{pC3bC4bC2}]}{K_{\mathrm{m\,C1^*}}^{\mathrm{C2}}}\right)}$$

$$-k_{\mathrm{C4bC4b}}^{+}[\mathrm{nfC4b}][\mathrm{pC4bC2a}]-k_{\mathrm{C3bC4b}}^{+}[\mathrm{nfC3b}][\mathrm{pC4bC2a}]-k_{\mathrm{C4bC2a}}^{-}[\mathrm{pC4bC2a}]-k_{\mathrm{C4bC4BP}}^{+}[\mathrm{pC4bC2a}][\mathrm{C4BP}]$$

$$+k_{\mathrm{C4bC4BP}}^{-}[\mathrm{pC4bC2aC4BP}]$$

$$+\frac{k_{\mathrm{cat\,MASP1^*}}^{\mathrm{C2}}\left[\mathrm{pMBL_3MASP1^*}\right][\mathrm{pC4bC2}]}{K_{\mathrm{m\,MASP1^*}}^{\mathrm{C2}}\left(\begin{array}{l}1+\frac{[\mathrm{C4}]}{K_{\mathrm{m\,MASP1^*}}^{\mathrm{C4}}}+\frac{[\mathrm{fC4bC2}]}{K_{\mathrm{m\,MASP1^*}}^{\mathrm{C2}}}+\frac{[\mathrm{fC4bC4bC2}]}{K_{\mathrm{m\,MASP1^*}}^{\mathrm{C2}}}+\frac{[\mathrm{fC3bC4bC2}]}{K_{\mathrm{m\,MASP1^*}}^{\mathrm{C2}}}+\frac{[\mathrm{pC4bC2}]}{K_{\mathrm{m\,MASP1^*}}^{\mathrm{C2}}}+\frac{[\mathrm{pC4bC4bC2}]}{K_{\mathrm{m\,MASP1^*}}^{\mathrm{C2}}}\\
+\frac{[\mathrm{pC3bC4bC2}]}{K_{\mathrm{m\,MASP1^*}}^{\mathrm{C2}}}+\frac{\left[\mathrm{pMBL_3MASP1_{zym}}\right]}{K_{\mathrm{m\,MASP1^*}}^{\mathrm{MASP1_{zym}}}}+\frac{\left[\mathrm{pMBL_3MASP2_{zym}}\right]}{K_{\mathrm{m\,MASP1^*}}^{\mathrm{MASP2_{zym}}}}+\frac{\left[\mathrm{pMBL_3MASP3_{zym}}\right]}{K_{\mathrm{m\,MASP1^*}}^{\mathrm{MASP3_{zym}}}}\\
+\frac{\left[\mathrm{pMBL_4MASP1_{zym}}\right]}{K_{\mathrm{m\,MASP1^*}}^{\mathrm{MASP1_{zym}}}}+\frac{\left[\mathrm{pMBL_4MASP2_{zym}}\right]}{K_{\mathrm{m\,MASP1^*}}^{\mathrm{MASP2_{zym}}}}+\frac{\left[\mathrm{pMBL_4MASP3_{zym}}\right]}{K_{\mathrm{m\,MASP1^*}}^{\mathrm{MASP3_{zym}}}}+\frac{\left[\mathrm{pCLLKMASP1_{zym}}\right]}{K_{\mathrm{m\,MASP1^*}}^{\mathrm{MASP1_{zym}}}}\\
+\frac{\left[\mathrm{pCLLKMASP2_{zym}}\right]}{K_{\mathrm{m\,MASP1^*}}^{\mathrm{MASP2_{zym}}}}+\frac{\left[\mathrm{pCLLKMASP3_{zym}}\right]}{K_{\mathrm{m\,MASP1^*}}^{\mathrm{MASP3_{zym}}}}+\frac{\left[\mathrm{pCLK1MASP1_{zym}}\right]}{K_{\mathrm{m\,MASP1^*}}^{\mathrm{MASP1_{zym}}}}+\frac{\left[\mathrm{pCLK1MASP2_{zym}}\right]}{K_{\mathrm{m\,MASP1^*}}^{\mathrm{MASP2_{zym}}}}\\
+\frac{\left[\mathrm{pCLK1MASP3_{zym}}\right]}{K_{\mathrm{m\,MASP1^*}}^{\mathrm{MASP3_{zym}}}}+\frac{\left[\mathrm{pCLL1MASP1_{zym}}\right]}{K_{\mathrm{m\,MASP1^*}}^{\mathrm{MASP1_{zym}}}}+\frac{\left[\mathrm{pCLL1MASP2_{zym}}\right]}{K_{\mathrm{m\,MASP1^*}}^{\mathrm{MASP2_{zym}}}}+\frac{\left[\mathrm{pCLL1MASP3_{zym}}\right]}{K_{\mathrm{m\,MASP1^*}}^{\mathrm{MASP3_{zym}}}}\\
+\frac{\left[\mathrm{pLFMASP1_{zym}}\right]}{K_{\mathrm{m\,MASP1^*}}^{\mathrm{MASP1_{zym}}}}+\frac{\left[\mathrm{pLFMASP2_{zym}}\right]}{K_{\mathrm{m\,MASP1^*}}^{\mathrm{MASP2_{zym}}}}+\frac{\left[\mathrm{pLFMASP3_{zym}}\right]}{K_{\mathrm{m\,MASP1^*}}^{\mathrm{MASP3_{zym}}}}+\frac{\left[\mathrm{pHFMASP1_{zym}}\right]}{K_{\mathrm{m\,MASP1^*}}^{\mathrm{MASP1_{zym}}}}+\frac{\left[\mathrm{pHFMASP2_{zym}}\right]}{K_{\mathrm{m\,MASP1^*}}^{\mathrm{MASP2_{zym}}}}\\
+\frac{\left[\mathrm{pHFMASP3_{zym}}\right]}{K_{\mathrm{m\,MASP1^*}}^{\mathrm{MASP3_{zym}}}}+\frac{\left[\mathrm{pMFMASP1_{zym}}\right]}{K_{\mathrm{m\,MASP1^*}}^{\mathrm{MASP1_{zym}}}}+\frac{\left[\mathrm{pMFMASP2_{zym}}\right]}{K_{\mathrm{m\,MASP1^*}}^{\mathrm{MASP2_{zym}}}}+\frac{\left[\mathrm{pMFMASP3_{zym}}\right]}{K_{\mathrm{m\,MASP1^*}}^{\mathrm{MASP3_{zym}}}}\end{array}\right)}$$

$$
+\frac{k_{\text{cat MASP1}^*}^{\text{C2}}\left[\text{pMBL}_4\text{MASP1}^*\right]\left[\text{pC4bC2}\right]}{K_{\text{m MASP1}^*}^{\text{C2}}\left(\begin{array}{l}1+\frac{[\text{C4}]}{K_{\text{m MASP1}^*}^{\text{C4}}}+\frac{[\text{fC4bC2}]}{K_{\text{m MASP1}^*}^{\text{C2}}}+\frac{[\text{fC4bC4bC2}]}{K_{\text{m MASP1}^*}^{\text{C2}}}+\frac{[\text{fC3bC4bC2}]}{K_{\text{m MASP1}^*}^{\text{C2}}}+\frac{[\text{pC4bC2}]}{K_{\text{m MASP1}^*}^{\text{C2}}}+\frac{[\text{pC4bC4bC2}]}{K_{\text{m MASP1}^*}^{\text{C2}}}\\
+\frac{[\text{pC3bC4bC2}]}{K_{\text{m MASP1}^*}^{\text{C2}}}+\frac{\left[\text{pMBL}_3\text{MASP1}_{\text{zym}}\right]}{K_{\text{m MASP1}^*}^{\text{MASP1}_{\text{zym}}}}+\frac{\left[\text{pMBL}_3\text{MASP2}_{\text{zym}}\right]}{K_{\text{m MASP1}^*}^{\text{MASP2}_{\text{zym}}}}+\frac{\left[\text{pMBL}_3\text{MASP3}_{\text{zym}}\right]}{K_{\text{m MASP1}^*}^{\text{MASP3}_{\text{zym}}}}\\
+\frac{\left[\text{pMBL}_4\text{MASP1}_{\text{zym}}\right]}{K_{\text{m MASP1}^*}^{\text{MASP1}_{\text{zym}}}}+\frac{\left[\text{pMBL}_4\text{MASP2}_{\text{zym}}\right]}{K_{\text{m MASP1}^*}^{\text{MASP2}_{\text{zym}}}}+\frac{\left[\text{pMBL}_4\text{MASP3}_{\text{zym}}\right]}{K_{\text{m MASP1}^*}^{\text{MASP3}_{\text{zym}}}}+\frac{\left[\text{pCLLKMASP1}_{\text{zym}}\right]}{K_{\text{m MASP1}^*}^{\text{MASP1}_{\text{zym}}}}\\
+\frac{\left[\text{pCLLKMASP2}_{\text{zym}}\right]}{K_{\text{m MASP1}^*}^{\text{MASP2}_{\text{zym}}}}+\frac{\left[\text{pCLLKMASP3}_{\text{zym}}\right]}{K_{\text{m MASP1}^*}^{\text{MASP3}_{\text{zym}}}}+\frac{\left[\text{pCLK1MASP1}_{\text{zym}}\right]}{K_{\text{m MASP1}^*}^{\text{MASP1}_{\text{zym}}}}+\frac{\left[\text{pCLK1MASP2}_{\text{zym}}\right]}{K_{\text{m MASP1}^*}^{\text{MASP2}_{\text{zym}}}}\\
+\frac{\left[\text{pCLK1MASP3}_{\text{zym}}\right]}{K_{\text{m MASP1}^*}^{\text{MASP3}_{\text{zym}}}}+\frac{\left[\text{pCLL1MASP1}_{\text{zym}}\right]}{K_{\text{m MASP1}^*}^{\text{MASP1}_{\text{zym}}}}+\frac{\left[\text{pCLL1MASP2}_{\text{zym}}\right]}{K_{\text{m MASP1}^*}^{\text{MASP2}_{\text{zym}}}}+\frac{\left[\text{pCLL1MASP3}_{\text{zym}}\right]}{K_{\text{m MASP1}^*}^{\text{MASP3}_{\text{zym}}}}\\
+\frac{\left[\text{pLFMASP1}_{\text{zym}}\right]}{K_{\text{m MASP1}^*}^{\text{MASP1}_{\text{zym}}}}+\frac{\left[\text{pLFMASP2}_{\text{zym}}\right]}{K_{\text{m MASP1}^*}^{\text{MASP2}_{\text{zym}}}}+\frac{\left[\text{pLFMASP3}_{\text{zym}}\right]}{K_{\text{m MASP1}^*}^{\text{MASP3}_{\text{zym}}}}+\frac{\left[\text{pHFMASP1}_{\text{zym}}\right]}{K_{\text{m MASP1}^*}^{\text{MASP1}_{\text{zym}}}}+\frac{\left[\text{pHFMASP2}_{\text{zym}}\right]}{K_{\text{m MASP1}^*}^{\text{MASP2}_{\text{zym}}}}\\
+\frac{\left[\text{pHFMASP3}_{\text{zym}}\right]}{K_{\text{m MASP1}^*}^{\text{MASP3}_{\text{zym}}}}+\frac{\left[\text{pMFMASP1}_{\text{zym}}\right]}{K_{\text{m MASP1}^*}^{\text{MASP1}_{\text{zym}}}}+\frac{\left[\text{pMFMASP2}_{\text{zym}}\right]}{K_{\text{m MASP1}^*}^{\text{MASP2}_{\text{zym}}}}+\frac{\left[\text{pMFMASP3}_{\text{zym}}\right]}{K_{\text{m MASP1}^*}^{\text{MASP3}_{\text{zym}}}}\end{array}\right)}
$$

$$
+\frac{k_{\text{cat MASP1}^*}^{\text{C2}}\left[\text{pCLLKMASP1}^*\right]\left[\text{pC4bC2}\right]}{K_{\text{m MASP1}^*}^{\text{C2}}\left(\begin{array}{l}1+\frac{[\text{C4}]}{K_{\text{m MASP1}^*}^{\text{C4}}}+\frac{[\text{fC4bC2}]}{K_{\text{m MASP1}^*}^{\text{C2}}}+\frac{[\text{fC4bC4bC2}]}{K_{\text{m MASP1}^*}^{\text{C2}}}+\frac{[\text{fC3bC4bC2}]}{K_{\text{m MASP1}^*}^{\text{C2}}}+\frac{[\text{pC4bC2}]}{K_{\text{m MASP1}^*}^{\text{C2}}}+\frac{[\text{pC4bC4bC2}]}{K_{\text{m MASP1}^*}^{\text{C2}}}\\
+\frac{[\text{pC3bC4bC2}]}{K_{\text{m MASP1}^*}^{\text{C2}}}+\frac{\left[\text{pMBL}_3\text{MASP1}_{\text{zym}}\right]}{K_{\text{m MASP1}^*}^{\text{MASP1}_{\text{zym}}}}+\frac{\left[\text{pMBL}_3\text{MASP2}_{\text{zym}}\right]}{K_{\text{m MASP1}^*}^{\text{MASP2}_{\text{zym}}}}+\frac{\left[\text{pMBL}_3\text{MASP3}_{\text{zym}}\right]}{K_{\text{m MASP1}^*}^{\text{MASP3}_{\text{zym}}}}\\
+\frac{\left[\text{pMBL}_4\text{MASP1}_{\text{zym}}\right]}{K_{\text{m MASP1}^*}^{\text{MASP1}_{\text{zym}}}}+\frac{\left[\text{pMBL}_4\text{MASP2}_{\text{zym}}\right]}{K_{\text{m MASP1}^*}^{\text{MASP2}_{\text{zym}}}}+\frac{\left[\text{pMBL}_4\text{MASP3}_{\text{zym}}\right]}{K_{\text{m MASP1}^*}^{\text{MASP3}_{\text{zym}}}}+\frac{\left[\text{pCLLKMASP1}_{\text{zym}}\right]}{K_{\text{m MASP1}^*}^{\text{MASP1}_{\text{zym}}}}\\
+\frac{\left[\text{pCLLKMASP2}_{\text{zym}}\right]}{K_{\text{m MASP1}^*}^{\text{MASP2}_{\text{zym}}}}+\frac{\left[\text{pCLLKMASP3}_{\text{zym}}\right]}{K_{\text{m MASP1}^*}^{\text{MASP3}_{\text{zym}}}}+\frac{\left[\text{pCLK1MASP1}_{\text{zym}}\right]}{K_{\text{m MASP1}^*}^{\text{MASP1}_{\text{zym}}}}+\frac{\left[\text{pCLK1MASP2}_{\text{zym}}\right]}{K_{\text{m MASP1}^*}^{\text{MASP2}_{\text{zym}}}}\\
+\frac{\left[\text{pCLK1MASP3}_{\text{zym}}\right]}{K_{\text{m MASP1}^*}^{\text{MASP3}_{\text{zym}}}}+\frac{\left[\text{pCLL1MASP1}_{\text{zym}}\right]}{K_{\text{m MASP1}^*}^{\text{MASP1}_{\text{zym}}}}+\frac{\left[\text{pCLL1MASP2}_{\text{zym}}\right]}{K_{\text{m MASP1}^*}^{\text{MASP2}_{\text{zym}}}}+\frac{\left[\text{pCLL1MASP3}_{\text{zym}}\right]}{K_{\text{m MASP1}^*}^{\text{MASP3}_{\text{zym}}}}\\
+\frac{\left[\text{pLFMASP1}_{\text{zym}}\right]}{K_{\text{m MASP1}^*}^{\text{MASP1}_{\text{zym}}}}+\frac{\left[\text{pLFMASP2}_{\text{zym}}\right]}{K_{\text{m MASP1}^*}^{\text{MASP2}_{\text{zym}}}}+\frac{\left[\text{pLFMASP3}_{\text{zym}}\right]}{K_{\text{m MASP1}^*}^{\text{MASP3}_{\text{zym}}}}+\frac{\left[\text{pHFMASP1}_{\text{zym}}\right]}{K_{\text{m MASP1}^*}^{\text{MASP1}_{\text{zym}}}}+\frac{\left[\text{pHFMASP2}_{\text{zym}}\right]}{K_{\text{m MASP1}^*}^{\text{MASP2}_{\text{zym}}}}\\
+\frac{\left[\text{pHFMASP3}_{\text{zym}}\right]}{K_{\text{m MASP1}^*}^{\text{MASP3}_{\text{zym}}}}+\frac{\left[\text{pMFMASP1}_{\text{zym}}\right]}{K_{\text{m MASP1}^*}^{\text{MASP1}_{\text{zym}}}}+\frac{\left[\text{pMFMASP2}_{\text{zym}}\right]}{K_{\text{m MASP1}^*}^{\text{MASP2}_{\text{zym}}}}+\frac{\left[\text{pMFMASP3}_{\text{zym}}\right]}{K_{\text{m MASP1}^*}^{\text{MASP3}_{\text{zym}}}}\end{array}\right)}
$$

$$+\frac{k_{\mathrm{cat\,MASP1^*}}^{\mathrm{C2}}\left[\mathrm{pCLK1MASP1^*}\right]\left[\mathrm{pC4bC2}\right]}{K_{\mathrm{m\,MASP1^*}}^{\mathrm{C2}}\left(\begin{array}{l}1+\frac{[\mathrm{C4}]}{K_{\mathrm{m\,MASP1^*}}^{\mathrm{C4}}}+\frac{[\mathrm{fC4bC2}]}{K_{\mathrm{m\,MASP1^*}}^{\mathrm{C2}}}+\frac{[\mathrm{fC4bC4bC2}]}{K_{\mathrm{m\,MASP1^*}}^{\mathrm{C2}}}+\frac{[\mathrm{fC3bC4bC2}]}{K_{\mathrm{m\,MASP1^*}}^{\mathrm{C2}}}+\frac{[\mathrm{pC4bC2}]}{K_{\mathrm{m\,MASP1^*}}^{\mathrm{C2}}}+\frac{[\mathrm{pC4bC4bC2}]}{K_{\mathrm{m\,MASP1^*}}^{\mathrm{C2}}}\\+\frac{[\mathrm{pC3bC4bC2}]}{K_{\mathrm{m\,MASP1^*}}^{\mathrm{C2}}}+\frac{[\mathrm{pMBL_3MASP1_{zym}}]}{K_{\mathrm{m\,MASP1^*}}^{\mathrm{MASP1_{zym}}}}+\frac{[\mathrm{pMBL_3MASP2_{zym}}]}{K_{\mathrm{m\,MASP1^*}}^{\mathrm{MASP2_{zym}}}}+\frac{[\mathrm{pMBL_3MASP3_{zym}}]}{K_{\mathrm{m\,MASP1^*}}^{\mathrm{MASP3_{zym}}}}\\+\frac{[\mathrm{pMBL_4MASP1_{zym}}]}{K_{\mathrm{m\,MASP1^*}}^{\mathrm{MASP1_{zym}}}}+\frac{[\mathrm{pMBL_4MASP2_{zym}}]}{K_{\mathrm{m\,MASP1^*}}^{\mathrm{MASP2_{zym}}}}+\frac{[\mathrm{pMBL_4MASP3_{zym}}]}{K_{\mathrm{m\,MASP1^*}}^{\mathrm{MASP3_{zym}}}}+\frac{[\mathrm{pCLLKMASP1_{zym}}]}{K_{\mathrm{m\,MASP1^*}}^{\mathrm{MASP1_{zym}}}}\\+\frac{[\mathrm{pCLLKMASP2_{zym}}]}{K_{\mathrm{m\,MASP1^*}}^{\mathrm{MASP2_{zym}}}}+\frac{[\mathrm{pCLLKMASP3_{zym}}]}{K_{\mathrm{m\,MASP1^*}}^{\mathrm{MASP3_{zym}}}}+\frac{[\mathrm{pCLK1MASP1_{zym}}]}{K_{\mathrm{m\,MASP1^*}}^{\mathrm{MASP1_{zym}}}}+\frac{[\mathrm{pCLK1MASP2_{zym}}]}{K_{\mathrm{m\,MASP1^*}}^{\mathrm{MASP2_{zym}}}}\\+\frac{[\mathrm{pCLK1MASP3_{zym}}]}{K_{\mathrm{m\,MASP1^*}}^{\mathrm{MASP3_{zym}}}}+\frac{[\mathrm{pCLL1MASP1_{zym}}]}{K_{\mathrm{m\,MASP1^*}}^{\mathrm{MASP1_{zym}}}}+\frac{[\mathrm{pCLL1MASP2_{zym}}]}{K_{\mathrm{m\,MASP1^*}}^{\mathrm{MASP2_{zym}}}}+\frac{[\mathrm{pCLL1MASP3_{zym}}]}{K_{\mathrm{m\,MASP1^*}}^{\mathrm{MASP3_{zym}}}}\\+\frac{[\mathrm{pLFMASP1_{zym}}]}{K_{\mathrm{m\,MASP1^*}}^{\mathrm{MASP1_{zym}}}}+\frac{[\mathrm{pLFMASP2_{zym}}]}{K_{\mathrm{m\,MASP1^*}}^{\mathrm{MASP2_{zym}}}}+\frac{[\mathrm{pLFMASP3_{zym}}]}{K_{\mathrm{m\,MASP1^*}}^{\mathrm{MASP3_{zym}}}}+\frac{[\mathrm{pHFMASP1_{zym}}]}{K_{\mathrm{m\,MASP1^*}}^{\mathrm{MASP1_{zym}}}}+\frac{[\mathrm{pHFMASP2_{zym}}]}{K_{\mathrm{m\,MASP1^*}}^{\mathrm{MASP2_{zym}}}}\\+\frac{[\mathrm{pHFMASP3_{zym}}]}{K_{\mathrm{m\,MASP1^*}}^{\mathrm{MASP3_{zym}}}}+\frac{[\mathrm{pMFMASP1_{zym}}]}{K_{\mathrm{m\,MASP1^*}}^{\mathrm{MASP1_{zym}}}}+\frac{[\mathrm{pMFMASP2_{zym}}]}{K_{\mathrm{m\,MASP1^*}}^{\mathrm{MASP2_{zym}}}}+\frac{[\mathrm{pMFMASP3_{zym}}]}{K_{\mathrm{m\,MASP1^*}}^{\mathrm{MASP3_{zym}}}}\end{array}\right)}$$

$$+\frac{k_{\mathrm{cat\,MASP1^*}}^{\mathrm{C2}}\left[\mathrm{pCLL1MASP1^*}\right]\left[\mathrm{pC4bC2}\right]}{K_{\mathrm{m\,MASP1^*}}^{\mathrm{C2}}\left(\begin{array}{l}1+\frac{[\mathrm{C4}]}{K_{\mathrm{m\,MASP1^*}}^{\mathrm{C4}}}+\frac{[\mathrm{fC4bC2}]}{K_{\mathrm{m\,MASP1^*}}^{\mathrm{C2}}}+\frac{[\mathrm{fC4bC4bC2}]}{K_{\mathrm{m\,MASP1^*}}^{\mathrm{C2}}}+\frac{[\mathrm{fC3bC4bC2}]}{K_{\mathrm{m\,MASP1^*}}^{\mathrm{C2}}}+\frac{[\mathrm{pC4bC2}]}{K_{\mathrm{m\,MASP1^*}}^{\mathrm{C2}}}+\frac{[\mathrm{pC4bC4bC2}]}{K_{\mathrm{m\,MASP1^*}}^{\mathrm{C2}}}\\+\frac{[\mathrm{pC3bC4bC2}]}{K_{\mathrm{m\,MASP1^*}}^{\mathrm{C2}}}+\frac{[\mathrm{pMBL_3MASP1_{zym}}]}{K_{\mathrm{m\,MASP1^*}}^{\mathrm{MASP1_{zym}}}}+\frac{[\mathrm{pMBL_3MASP2_{zym}}]}{K_{\mathrm{m\,MASP1^*}}^{\mathrm{MASP2_{zym}}}}+\frac{[\mathrm{pMBL_3MASP3_{zym}}]}{K_{\mathrm{m\,MASP1^*}}^{\mathrm{MASP3_{zym}}}}\\+\frac{[\mathrm{pMBL_4MASP1_{zym}}]}{K_{\mathrm{m\,MASP1^*}}^{\mathrm{MASP1_{zym}}}}+\frac{[\mathrm{pMBL_4MASP2_{zym}}]}{K_{\mathrm{m\,MASP1^*}}^{\mathrm{MASP2_{zym}}}}+\frac{[\mathrm{pMBL_4MASP3_{zym}}]}{K_{\mathrm{m\,MASP1^*}}^{\mathrm{MASP3_{zym}}}}+\frac{[\mathrm{pCLLKMASP1_{zym}}]}{K_{\mathrm{m\,MASP1^*}}^{\mathrm{MASP1_{zym}}}}\\+\frac{[\mathrm{pCLLKMASP2_{zym}}]}{K_{\mathrm{m\,MASP1^*}}^{\mathrm{MASP2_{zym}}}}+\frac{[\mathrm{pCLLKMASP3_{zym}}]}{K_{\mathrm{m\,MASP1^*}}^{\mathrm{MASP3_{zym}}}}+\frac{[\mathrm{pCLK1MASP1_{zym}}]}{K_{\mathrm{m\,MASP1^*}}^{\mathrm{MASP1_{zym}}}}+\frac{[\mathrm{pCLK1MASP2_{zym}}]}{K_{\mathrm{m\,MASP1^*}}^{\mathrm{MASP2_{zym}}}}\\+\frac{[\mathrm{pCLK1MASP3_{zym}}]}{K_{\mathrm{m\,MASP1^*}}^{\mathrm{MASP3_{zym}}}}+\frac{[\mathrm{pCLL1MASP1_{zym}}]}{K_{\mathrm{m\,MASP1^*}}^{\mathrm{MASP1_{zym}}}}+\frac{[\mathrm{pCLL1MASP2_{zym}}]}{K_{\mathrm{m\,MASP1^*}}^{\mathrm{MASP2_{zym}}}}+\frac{[\mathrm{pCLL1MASP3_{zym}}]}{K_{\mathrm{m\,MASP1^*}}^{\mathrm{MASP3_{zym}}}}\\+\frac{[\mathrm{pLFMASP1_{zym}}]}{K_{\mathrm{m\,MASP1^*}}^{\mathrm{MASP1_{zym}}}}+\frac{[\mathrm{pLFMASP2_{zym}}]}{K_{\mathrm{m\,MASP1^*}}^{\mathrm{MASP2_{zym}}}}+\frac{[\mathrm{pLFMASP3_{zym}}]}{K_{\mathrm{m\,MASP1^*}}^{\mathrm{MASP3_{zym}}}}+\frac{[\mathrm{pHFMASP1_{zym}}]}{K_{\mathrm{m\,MASP1^*}}^{\mathrm{MASP1_{zym}}}}+\frac{[\mathrm{pHFMASP2_{zym}}]}{K_{\mathrm{m\,MASP1^*}}^{\mathrm{MASP2_{zym}}}}\\+\frac{[\mathrm{pHFMASP3_{zym}}]}{K_{\mathrm{m\,MASP1^*}}^{\mathrm{MASP3_{zym}}}}+\frac{[\mathrm{pMFMASP1_{zym}}]}{K_{\mathrm{m\,MASP1^*}}^{\mathrm{MASP1_{zym}}}}+\frac{[\mathrm{pMFMASP2_{zym}}]}{K_{\mathrm{m\,MASP1^*}}^{\mathrm{MASP2_{zym}}}}+\frac{[\mathrm{pMFMASP3_{zym}}]}{K_{\mathrm{m\,MASP1^*}}^{\mathrm{MASP3_{zym}}}}\end{array}\right)}$$

$$+\frac{k_{\text{cat MASP1}^*}^{\text{C2}}\left[\text{pLFMASP1}^*\right]\left[\text{pC4bC2}\right]}{K_{\text{m MASP1}^*}^{\text{C2}}\left(\begin{array}{l}1+\frac{[\text{C4}]}{K_{\text{m MASP1}^*}^{\text{C4}}}+\frac{[\text{fC4bC2}]}{K_{\text{m MASP1}^*}^{\text{C2}}}+\frac{[\text{fC4bC4bC2}]}{K_{\text{m MASP1}^*}^{\text{C2}}}+\frac{[\text{fC3bC4bC2}]}{K_{\text{m MASP1}^*}^{\text{C2}}}+\frac{[\text{pC4bC2}]}{K_{\text{m MASP1}^*}^{\text{C2}}}+\frac{[\text{pC4bC4bC2}]}{K_{\text{m MASP1}^*}^{\text{C2}}}\\+\frac{[\text{pC3bC4bC2}]}{K_{\text{m MASP1}^*}^{\text{C2}}}+\frac{[\text{pMBL}_3\text{MASP1}_{\text{zym}}]}{K_{\text{m MASP1}^*}^{\text{MASP1}_{\text{zym}}}}+\frac{[\text{pMBL}_3\text{MASP2}_{\text{zym}}]}{K_{\text{m MASP1}^*}^{\text{MASP2}_{\text{zym}}}}+\frac{[\text{pMBL}_3\text{MASP3}_{\text{zym}}]}{K_{\text{m MASP1}^*}^{\text{MASP3}_{\text{zym}}}}\\+\frac{[\text{pMBL}_4\text{MASP1}_{\text{zym}}]}{K_{\text{m MASP1}^*}^{\text{MASP1}_{\text{zym}}}}+\frac{[\text{pMBL}_4\text{MASP2}_{\text{zym}}]}{K_{\text{m MASP1}^*}^{\text{MASP2}_{\text{zym}}}}+\frac{[\text{pMBL}_4\text{MASP3}_{\text{zym}}]}{K_{\text{m MASP1}^*}^{\text{MASP3}_{\text{zym}}}}+\frac{[\text{pCLLKMASP1}_{\text{zym}}]}{K_{\text{m MASP1}^*}^{\text{MASP1}_{\text{zym}}}}\\+\frac{[\text{pCLLKMASP2}_{\text{zym}}]}{K_{\text{m MASP1}^*}^{\text{MASP2}_{\text{zym}}}}+\frac{[\text{pCLLKMASP3}_{\text{zym}}]}{K_{\text{m MASP1}^*}^{\text{MASP3}_{\text{zym}}}}+\frac{[\text{pCLK1MASP1}_{\text{zym}}]}{K_{\text{m MASP1}^*}^{\text{MASP1}_{\text{zym}}}}+\frac{[\text{pCLK1MASP2}_{\text{zym}}]}{K_{\text{m MASP1}^*}^{\text{MASP2}_{\text{zym}}}}\\+\frac{[\text{pCLK1MASP3}_{\text{zym}}]}{K_{\text{m MASP1}^*}^{\text{MASP3}_{\text{zym}}}}+\frac{[\text{pCLL1MASP1}_{\text{zym}}]}{K_{\text{m MASP1}^*}^{\text{MASP1}_{\text{zym}}}}+\frac{[\text{pCLL1MASP2}_{\text{zym}}]}{K_{\text{m MASP1}^*}^{\text{MASP2}_{\text{zym}}}}+\frac{[\text{pCLL1MASP3}_{\text{zym}}]}{K_{\text{m MASP1}^*}^{\text{MASP3}_{\text{zym}}}}\\+\frac{[\text{pLFMASP1}_{\text{zym}}]}{K_{\text{m MASP1}^*}^{\text{MASP1}_{\text{zym}}}}+\frac{[\text{pLFMASP2}_{\text{zym}}]}{K_{\text{m MASP1}^*}^{\text{MASP2}_{\text{zym}}}}+\frac{[\text{pLFMASP3}_{\text{zym}}]}{K_{\text{m MASP1}^*}^{\text{MASP3}_{\text{zym}}}}+\frac{[\text{pHFMASP1}_{\text{zym}}]}{K_{\text{m MASP1}^*}^{\text{MASP1}_{\text{zym}}}}+\frac{[\text{pHFMASP2}_{\text{zym}}]}{K_{\text{m MASP1}^*}^{\text{MASP2}_{\text{zym}}}}\\+\frac{[\text{pHFMASP3}_{\text{zym}}]}{K_{\text{m MASP1}^*}^{\text{MASP3}_{\text{zym}}}}+\frac{[\text{pMFMASP1}_{\text{zym}}]}{K_{\text{m MASP1}^*}^{\text{MASP1}_{\text{zym}}}}+\frac{[\text{pMFMASP2}_{\text{zym}}]}{K_{\text{m MASP1}^*}^{\text{MASP2}_{\text{zym}}}}+\frac{[\text{pMFMASP3}_{\text{zym}}]}{K_{\text{m MASP1}^*}^{\text{MASP3}_{\text{zym}}}}\end{array}\right)}$$

$$+\frac{k_{\text{cat MASP1}^*}^{\text{C2}}\left[\text{pHFMASP1}^*\right]\left[\text{pC4bC2}\right]}{K_{\text{m MASP1}^*}^{\text{C2}}\left(\begin{array}{l}1+\frac{[\text{C4}]}{K_{\text{m MASP1}^*}^{\text{C4}}}+\frac{[\text{fC4bC2}]}{K_{\text{m MASP1}^*}^{\text{C2}}}+\frac{[\text{fC4bC4bC2}]}{K_{\text{m MASP1}^*}^{\text{C2}}}+\frac{[\text{fC3bC4bC2}]}{K_{\text{m MASP1}^*}^{\text{C2}}}+\frac{[\text{pC4bC2}]}{K_{\text{m MASP1}^*}^{\text{C2}}}+\frac{[\text{pC4bC4bC2}]}{K_{\text{m MASP1}^*}^{\text{C2}}}\\+\frac{[\text{pC3bC4bC2}]}{K_{\text{m MASP1}^*}^{\text{C2}}}+\frac{[\text{pMBL}_3\text{MASP1}_{\text{zym}}]}{K_{\text{m MASP1}^*}^{\text{MASP1}_{\text{zym}}}}+\frac{[\text{pMBL}_3\text{MASP2}_{\text{zym}}]}{K_{\text{m MASP1}^*}^{\text{MASP2}_{\text{zym}}}}+\frac{[\text{pMBL}_3\text{MASP3}_{\text{zym}}]}{K_{\text{m MASP1}^*}^{\text{MASP3}_{\text{zym}}}}\\+\frac{[\text{pMBL}_4\text{MASP1}_{\text{zym}}]}{K_{\text{m MASP1}^*}^{\text{MASP1}_{\text{zym}}}}+\frac{[\text{pMBL}_4\text{MASP2}_{\text{zym}}]}{K_{\text{m MASP1}^*}^{\text{MASP2}_{\text{zym}}}}+\frac{[\text{pMBL}_4\text{MASP3}_{\text{zym}}]}{K_{\text{m MASP1}^*}^{\text{MASP3}_{\text{zym}}}}+\frac{[\text{pCLLKMASP1}_{\text{zym}}]}{K_{\text{m MASP1}^*}^{\text{MASP1}_{\text{zym}}}}\\+\frac{[\text{pCLLKMASP2}_{\text{zym}}]}{K_{\text{m MASP1}^*}^{\text{MASP2}_{\text{zym}}}}+\frac{[\text{pCLLKMASP3}_{\text{zym}}]}{K_{\text{m MASP1}^*}^{\text{MASP3}_{\text{zym}}}}+\frac{[\text{pCLK1MASP1}_{\text{zym}}]}{K_{\text{m MASP1}^*}^{\text{MASP1}_{\text{zym}}}}+\frac{[\text{pCLK1MASP2}_{\text{zym}}]}{K_{\text{m MASP1}^*}^{\text{MASP2}_{\text{zym}}}}\\+\frac{[\text{pCLK1MASP3}_{\text{zym}}]}{K_{\text{m MASP1}^*}^{\text{MASP3}_{\text{zym}}}}+\frac{[\text{pCLL1MASP1}_{\text{zym}}]}{K_{\text{m MASP1}^*}^{\text{MASP1}_{\text{zym}}}}+\frac{[\text{pCLL1MASP2}_{\text{zym}}]}{K_{\text{m MASP1}^*}^{\text{MASP2}_{\text{zym}}}}+\frac{[\text{pCLL1MASP3}_{\text{zym}}]}{K_{\text{m MASP1}^*}^{\text{MASP3}_{\text{zym}}}}\\+\frac{[\text{pLFMASP1}_{\text{zym}}]}{K_{\text{m MASP1}^*}^{\text{MASP1}_{\text{zym}}}}+\frac{[\text{pLFMASP2}_{\text{zym}}]}{K_{\text{m MASP1}^*}^{\text{MASP2}_{\text{zym}}}}+\frac{[\text{pLFMASP3}_{\text{zym}}]}{K_{\text{m MASP1}^*}^{\text{MASP3}_{\text{zym}}}}+\frac{[\text{pHFMASP1}_{\text{zym}}]}{K_{\text{m MASP1}^*}^{\text{MASP1}_{\text{zym}}}}+\frac{[\text{pHFMASP2}_{\text{zym}}]}{K_{\text{m MASP1}^*}^{\text{MASP2}_{\text{zym}}}}\\+\frac{[\text{pHFMASP3}_{\text{zym}}]}{K_{\text{m MASP1}^*}^{\text{MASP3}_{\text{zym}}}}+\frac{[\text{pMFMASP1}_{\text{zym}}]}{K_{\text{m MASP1}^*}^{\text{MASP1}_{\text{zym}}}}+\frac{[\text{pMFMASP2}_{\text{zym}}]}{K_{\text{m MASP1}^*}^{\text{MASP2}_{\text{zym}}}}+\frac{[\text{pMFMASP3}_{\text{zym}}]}{K_{\text{m MASP1}^*}^{\text{MASP3}_{\text{zym}}}}\end{array}\right)}$$

$$
+\frac{k^{\mathrm{C2}}_{\mathrm{cat\ MASP1^{*}}}\left[\mathrm{pMFMASP1^{*}}\right]\left[\mathrm{pC4bC2}\right]}{K^{\mathrm{C2}}_{\mathrm{m\ MASP1^{*}}}\left(\begin{array}{l}
1+\frac{[\mathrm{C4}]}{K^{\mathrm{C4}}_{\mathrm{m\ MASP1^{*}}}}+\frac{[\mathrm{fC4bC2}]}{K^{\mathrm{C2}}_{\mathrm{m\ MASP1^{*}}}}+\frac{[\mathrm{fC4bC4bC2}]}{K^{\mathrm{C2}}_{\mathrm{m\ MASP1^{*}}}}+\frac{[\mathrm{fC3bC4bC2}]}{K^{\mathrm{C2}}_{\mathrm{m\ MASP1^{*}}}}+\frac{[\mathrm{pC4bC2}]}{K^{\mathrm{C2}}_{\mathrm{m\ MASP1^{*}}}}+\frac{[\mathrm{pC4bC4bC2}]}{K^{\mathrm{C2}}_{\mathrm{m\ MASP1^{*}}}}\\
+\frac{[\mathrm{pC3bC4bC2}]}{K^{\mathrm{C2}}_{\mathrm{m\ MASP1^{*}}}}+\frac{\left[\mathrm{pMBL_{3}MASP1_{zym}}\right]}{K^{\mathrm{MASP1_{zym}}}_{\mathrm{m\ MASP1^{*}}}}+\frac{\left[\mathrm{pMBL_{3}MASP2_{zym}}\right]}{K^{\mathrm{MASP2_{zym}}}_{\mathrm{m\ MASP1^{*}}}}+\frac{\left[\mathrm{pMBL_{3}MASP3_{zym}}\right]}{K^{\mathrm{MASP3_{zym}}}_{\mathrm{m\ MASP1^{*}}}}\\
+\frac{\left[\mathrm{pMBL_{4}MASP1_{zym}}\right]}{K^{\mathrm{MASP1_{zym}}}_{\mathrm{m\ MASP1^{*}}}}+\frac{\left[\mathrm{pMBL_{4}MASP2_{zym}}\right]}{K^{\mathrm{MASP2_{zym}}}_{\mathrm{m\ MASP1^{*}}}}+\frac{\left[\mathrm{pMBL_{4}MASP3_{zym}}\right]}{K^{\mathrm{MASP3_{zym}}}_{\mathrm{m\ MASP1^{*}}}}+\frac{\left[\mathrm{pCLLKMASP1_{zym}}\right]}{K^{\mathrm{MASP1_{zym}}}_{\mathrm{m\ MASP1^{*}}}}\\
+\frac{\left[\mathrm{pCLLKMASP2_{zym}}\right]}{K^{\mathrm{MASP2_{zym}}}_{\mathrm{m\ MASP1^{*}}}}+\frac{\left[\mathrm{pCLLKMASP3_{zym}}\right]}{K^{\mathrm{MASP3_{zym}}}_{\mathrm{m\ MASP1^{*}}}}+\frac{\left[\mathrm{pCLK1MASP1_{zym}}\right]}{K^{\mathrm{MASP1_{zym}}}_{\mathrm{m\ MASP1^{*}}}}+\frac{\left[\mathrm{pCLK1MASP2_{zym}}\right]}{K^{\mathrm{MASP2_{zym}}}_{\mathrm{m\ MASP1^{*}}}}\\
+\frac{\left[\mathrm{pCLK1MASP3_{zym}}\right]}{K^{\mathrm{MASP3_{zym}}}_{\mathrm{m\ MASP1^{*}}}}+\frac{\left[\mathrm{pCLL1MASP1_{zym}}\right]}{K^{\mathrm{MASP1_{zym}}}_{\mathrm{m\ MASP1^{*}}}}+\frac{\left[\mathrm{pCLL1MASP2_{zym}}\right]}{K^{\mathrm{MASP2_{zym}}}_{\mathrm{m\ MASP1^{*}}}}+\frac{\left[\mathrm{pCLL1MASP3_{zym}}\right]}{K^{\mathrm{MASP3_{zym}}}_{\mathrm{m\ MASP1^{*}}}}\\
+\frac{\left[\mathrm{pLFMASP1_{zym}}\right]}{K^{\mathrm{MASP1_{zym}}}_{\mathrm{m\ MASP1^{*}}}}+\frac{\left[\mathrm{pLFMASP2_{zym}}\right]}{K^{\mathrm{MASP2_{zym}}}_{\mathrm{m\ MASP1^{*}}}}+\frac{\left[\mathrm{pLFMASP3_{zym}}\right]}{K^{\mathrm{MASP3_{zym}}}_{\mathrm{m\ MASP1^{*}}}}+\frac{\left[\mathrm{pHFMASP1_{zym}}\right]}{K^{\mathrm{MASP1_{zym}}}_{\mathrm{m\ MASP1^{*}}}}+\frac{\left[\mathrm{pHFMASP2_{zym}}\right]}{K^{\mathrm{MASP2_{zym}}}_{\mathrm{m\ MASP1^{*}}}}\\
+\frac{\left[\mathrm{pHFMASP3_{zym}}\right]}{K^{\mathrm{MASP3_{zym}}}_{\mathrm{m\ MASP1^{*}}}}+\frac{\left[\mathrm{pMFMASP1_{zym}}\right]}{K^{\mathrm{MASP1_{zym}}}_{\mathrm{m\ MASP1^{*}}}}+\frac{\left[\mathrm{pMFMASP2_{zym}}\right]}{K^{\mathrm{MASP2_{zym}}}_{\mathrm{m\ MASP1^{*}}}}+\frac{\left[\mathrm{pMFMASP3_{zym}}\right]}{K^{\mathrm{MASP3_{zym}}}_{\mathrm{m\ MASP1^{*}}}}
\end{array}\right)}
$$

$$
+\frac{k^{\mathrm{C2}}_{\mathrm{cat\ MASP2^{*}}}\left[\mathrm{pMBL_{3}MASP2^{*}}\right]\left[\mathrm{pC4bC2}\right]}{K^{\mathrm{C2}}_{\mathrm{m\ MASP2^{*}}}\left(\begin{array}{l}
1+\frac{[\mathrm{C4}]}{K^{\mathrm{C4}}_{\mathrm{m\ MASP2^{*}}}}+\frac{[\mathrm{fC4bC2}]}{K^{\mathrm{C2}}_{\mathrm{m\ MASP2^{*}}}}+\frac{[\mathrm{fC4bC4bC2}]}{K^{\mathrm{C2}}_{\mathrm{m\ MASP2^{*}}}}+\frac{[\mathrm{fC3bC4bC2}]}{K^{\mathrm{C2}}_{\mathrm{m\ MASP2^{*}}}}+\frac{[\mathrm{pC4bC2}]}{K^{\mathrm{C2}}_{\mathrm{m\ MASP2^{*}}}}+\frac{[\mathrm{pC4bC4bC2}]}{K^{\mathrm{C2}}_{\mathrm{m\ MASP2^{*}}}}\\
+\frac{[\mathrm{pC3bC4bC2}]}{K^{\mathrm{C2}}_{\mathrm{m\ MASP2^{*}}}}+\frac{\left[\mathrm{pMBL_{3}MASP1_{zym}}\right]}{K^{\mathrm{MASP1_{zym}}}_{\mathrm{m\ MASP2^{*}}}}+\frac{\left[\mathrm{pMBL_{3}MASP2_{zym}}\right]}{K^{\mathrm{MASP2_{zym}}}_{\mathrm{m\ MASP2^{*}}}}+\frac{\left[\mathrm{pMBL_{4}MASP1_{zym}}\right]}{K^{\mathrm{MASP1_{zym}}}_{\mathrm{m\ MASP2^{*}}}}\\
+\frac{\left[\mathrm{pMBL_{4}MASP2_{zym}}\right]}{K^{\mathrm{MASP2_{zym}}}_{\mathrm{m\ MASP2^{*}}}}+\frac{\left[\mathrm{pCLLKMASP1_{zym}}\right]}{K^{\mathrm{MASP1_{zym}}}_{\mathrm{m\ MASP2^{*}}}}+\frac{\left[\mathrm{pCLLKMASP2_{zym}}\right]}{K^{\mathrm{MASP2_{zym}}}_{\mathrm{m\ MASP2^{*}}}}+\frac{\left[\mathrm{pCLK1MASP1_{zym}}\right]}{K^{\mathrm{MASP1_{zym}}}_{\mathrm{m\ MASP2^{*}}}}\\
+\frac{\left[\mathrm{pCLK1MASP2_{zym}}\right]}{K^{\mathrm{MASP2_{zym}}}_{\mathrm{m\ MASP2^{*}}}}+\frac{\left[\mathrm{pCLL1MASP1_{zym}}\right]}{K^{\mathrm{MASP1_{zym}}}_{\mathrm{m\ MASP2^{*}}}}+\frac{\left[\mathrm{pCLL1MASP2_{zym}}\right]}{K^{\mathrm{MASP2_{zym}}}_{\mathrm{m\ MASP2^{*}}}}+\frac{\left[\mathrm{pLFMASP1_{zym}}\right]}{K^{\mathrm{MASP1_{zym}}}_{\mathrm{m\ MASP2^{*}}}}\\
+\frac{\left[\mathrm{pLFMASP2_{zym}}\right]}{K^{\mathrm{MASP2_{zym}}}_{\mathrm{m\ MASP2^{*}}}}+\frac{\left[\mathrm{pHFMASP1_{zym}}\right]}{K^{\mathrm{MASP1_{zym}}}_{\mathrm{m\ MASP2^{*}}}}+\frac{\left[\mathrm{pHFMASP2_{zym}}\right]}{K^{\mathrm{MASP2_{zym}}}_{\mathrm{m\ MASP2^{*}}}}+\frac{\left[\mathrm{pMFMASP1_{zym}}\right]}{K^{\mathrm{MASP1_{zym}}}_{\mathrm{m\ MASP2^{*}}}}+\frac{\left[\mathrm{pMFMASP2_{zym}}\right]}{K^{\mathrm{MASP2_{zym}}}_{\mathrm{m\ MASP2^{*}}}}
\end{array}\right)}
$$

$$
+\frac{k^{\mathrm{C2}}_{\mathrm{cat\,MASP2^*}}\left[\mathrm{pMBL_4MASP2^*}\right]\left[\mathrm{pC4bC2}\right]}{K^{\mathrm{C2}}_{\mathrm{m\,MASP2^*}}\left(\begin{aligned}&1+\frac{[\mathrm{C4}]}{K^{\mathrm{C4}}_{\mathrm{m\,MASP2^*}}}+\frac{[\mathrm{fC4bC2}]}{K^{\mathrm{C2}}_{\mathrm{m\,MASP2^*}}}+\frac{[\mathrm{fC4bC4bC2}]}{K^{\mathrm{C2}}_{\mathrm{m\,MASP2^*}}}+\frac{[\mathrm{fC3bC4bC2}]}{K^{\mathrm{C2}}_{\mathrm{m\,MASP2^*}}}+\frac{[\mathrm{pC4bC2}]}{K^{\mathrm{C2}}_{\mathrm{m\,MASP2^*}}}+\frac{[\mathrm{pC4bC4bC2}]}{K^{\mathrm{C2}}_{\mathrm{m\,MASP2^*}}}\\&+\frac{[\mathrm{pC3bC4bC2}]}{K^{\mathrm{C2}}_{\mathrm{m\,MASP2^*}}}+\frac{[\mathrm{pMBL_3MASP1_{zym}}]}{K^{\mathrm{MASP1_{zym}}}_{\mathrm{m\,MASP2^*}}}+\frac{[\mathrm{pMBL_3MASP2_{zym}}]}{K^{\mathrm{MASP2_{zym}}}_{\mathrm{m\,MASP2^*}}}+\frac{[\mathrm{pMBL_4MASP1_{zym}}]}{K^{\mathrm{MASP1_{zym}}}_{\mathrm{m\,MASP2^*}}}\\&+\frac{[\mathrm{pMBL_4MASP2_{zym}}]}{K^{\mathrm{MASP2_{zym}}}_{\mathrm{m\,MASP2^*}}}+\frac{[\mathrm{pCLLKMASP1_{zym}}]}{K^{\mathrm{MASP1_{zym}}}_{\mathrm{m\,MASP2^*}}}+\frac{[\mathrm{pCLLKMASP2_{zym}}]}{K^{\mathrm{MASP2_{zym}}}_{\mathrm{m\,MASP2^*}}}+\frac{[\mathrm{pCLK1MASP1_{zym}}]}{K^{\mathrm{MASP1_{zym}}}_{\mathrm{m\,MASP2^*}}}\\&+\frac{[\mathrm{pCLK1MASP2_{zym}}]}{K^{\mathrm{MASP2_{zym}}}_{\mathrm{m\,MASP2^*}}}+\frac{[\mathrm{pCLL1MASP1_{zym}}]}{K^{\mathrm{MASP1_{zym}}}_{\mathrm{m\,MASP2^*}}}+\frac{[\mathrm{pCLL1MASP2_{zym}}]}{K^{\mathrm{MASP2_{zym}}}_{\mathrm{m\,MASP2^*}}}+\frac{[\mathrm{pLFMASP1_{zym}}]}{K^{\mathrm{MASP1_{zym}}}_{\mathrm{m\,MASP2^*}}}\\&+\frac{[\mathrm{pLFMASP2_{zym}}]}{K^{\mathrm{MASP2_{zym}}}_{\mathrm{m\,MASP2^*}}}+\frac{[\mathrm{pHFMASP1_{zym}}]}{K^{\mathrm{MASP1_{zym}}}_{\mathrm{m\,MASP2^*}}}+\frac{[\mathrm{pHFMASP2_{zym}}]}{K^{\mathrm{MASP2_{zym}}}_{\mathrm{m\,MASP2^*}}}+\frac{[\mathrm{pMFMASP1_{zym}}]}{K^{\mathrm{MASP1_{zym}}}_{\mathrm{m\,MASP2^*}}}+\frac{[\mathrm{pMFMASP2_{zym}}]}{K^{\mathrm{MASP2_{zym}}}_{\mathrm{m\,MASP2^*}}}\end{aligned}\right)}
$$

$$
+\frac{k^{\mathrm{C2}}_{\mathrm{cat\,MASP2^*}}\left[\mathrm{pCLLKMASP2^*}\right]\left[\mathrm{pC4bC2}\right]}{K^{\mathrm{C2}}_{\mathrm{m\,MASP2^*}}\left(\begin{aligned}&1+\frac{[\mathrm{C4}]}{K^{\mathrm{C4}}_{\mathrm{m\,MASP2^*}}}+\frac{[\mathrm{fC4bC2}]}{K^{\mathrm{C2}}_{\mathrm{m\,MASP2^*}}}+\frac{[\mathrm{fC4bC4bC2}]}{K^{\mathrm{C2}}_{\mathrm{m\,MASP2^*}}}+\frac{[\mathrm{fC3bC4bC2}]}{K^{\mathrm{C2}}_{\mathrm{m\,MASP2^*}}}+\frac{[\mathrm{pC4bC2}]}{K^{\mathrm{C2}}_{\mathrm{m\,MASP2^*}}}+\frac{[\mathrm{pC4bC4bC2}]}{K^{\mathrm{C2}}_{\mathrm{m\,MASP2^*}}}\\&+\frac{[\mathrm{pC3bC4bC2}]}{K^{\mathrm{C2}}_{\mathrm{m\,MASP2^*}}}+\frac{[\mathrm{pMBL_3MASP1_{zym}}]}{K^{\mathrm{MASP1_{zym}}}_{\mathrm{m\,MASP2^*}}}+\frac{[\mathrm{pMBL_3MASP2_{zym}}]}{K^{\mathrm{MASP2_{zym}}}_{\mathrm{m\,MASP2^*}}}+\frac{[\mathrm{pMBL_4MASP1_{zym}}]}{K^{\mathrm{MASP1_{zym}}}_{\mathrm{m\,MASP2^*}}}\\&+\frac{[\mathrm{pMBL_4MASP2_{zym}}]}{K^{\mathrm{MASP2_{zym}}}_{\mathrm{m\,MASP2^*}}}+\frac{[\mathrm{pCLLKMASP1_{zym}}]}{K^{\mathrm{MASP1_{zym}}}_{\mathrm{m\,MASP2^*}}}+\frac{[\mathrm{pCLLKMASP2_{zym}}]}{K^{\mathrm{MASP2_{zym}}}_{\mathrm{m\,MASP2^*}}}+\frac{[\mathrm{pCLK1MASP1_{zym}}]}{K^{\mathrm{MASP1_{zym}}}_{\mathrm{m\,MASP2^*}}}\\&+\frac{[\mathrm{pCLK1MASP2_{zym}}]}{K^{\mathrm{MASP2_{zym}}}_{\mathrm{m\,MASP2^*}}}+\frac{[\mathrm{pCLL1MASP1_{zym}}]}{K^{\mathrm{MASP1_{zym}}}_{\mathrm{m\,MASP2^*}}}+\frac{[\mathrm{pCLL1MASP2_{zym}}]}{K^{\mathrm{MASP2_{zym}}}_{\mathrm{m\,MASP2^*}}}+\frac{[\mathrm{pLFMASP1_{zym}}]}{K^{\mathrm{MASP1_{zym}}}_{\mathrm{m\,MASP2^*}}}\\&+\frac{[\mathrm{pLFMASP2_{zym}}]}{K^{\mathrm{MASP2_{zym}}}_{\mathrm{m\,MASP2^*}}}+\frac{[\mathrm{pHFMASP1_{zym}}]}{K^{\mathrm{MASP1_{zym}}}_{\mathrm{m\,MASP2^*}}}+\frac{[\mathrm{pHFMASP2_{zym}}]}{K^{\mathrm{MASP2_{zym}}}_{\mathrm{m\,MASP2^*}}}+\frac{[\mathrm{pMFMASP1_{zym}}]}{K^{\mathrm{MASP1_{zym}}}_{\mathrm{m\,MASP2^*}}}+\frac{[\mathrm{pMFMASP2_{zym}}]}{K^{\mathrm{MASP2_{zym}}}_{\mathrm{m\,MASP2^*}}}\end{aligned}\right)}
$$

$$
+\frac{k^{\mathrm{C2}}_{\mathrm{cat\,MASP2^*}}\left[\mathrm{pCLK1MASP2^*}\right]\left[\mathrm{pC4bC2}\right]}{K^{\mathrm{C2}}_{\mathrm{m\,MASP2^*}}\left(\begin{aligned}&1+\frac{[\mathrm{C4}]}{K^{\mathrm{C4}}_{\mathrm{m\,MASP2^*}}}+\frac{[\mathrm{fC4bC2}]}{K^{\mathrm{C2}}_{\mathrm{m\,MASP2^*}}}+\frac{[\mathrm{fC4bC4bC2}]}{K^{\mathrm{C2}}_{\mathrm{m\,MASP2^*}}}+\frac{[\mathrm{fC3bC4bC2}]}{K^{\mathrm{C2}}_{\mathrm{m\,MASP2^*}}}+\frac{[\mathrm{pC4bC2}]}{K^{\mathrm{C2}}_{\mathrm{m\,MASP2^*}}}+\frac{[\mathrm{pC4bC4bC2}]}{K^{\mathrm{C2}}_{\mathrm{m\,MASP2^*}}}\\&+\frac{[\mathrm{pC3bC4bC2}]}{K^{\mathrm{C2}}_{\mathrm{m\,MASP2^*}}}+\frac{[\mathrm{pMBL_3MASP1_{zym}}]}{K^{\mathrm{MASP1_{zym}}}_{\mathrm{m\,MASP2^*}}}+\frac{[\mathrm{pMBL_3MASP2_{zym}}]}{K^{\mathrm{MASP2_{zym}}}_{\mathrm{m\,MASP2^*}}}+\frac{[\mathrm{pMBL_4MASP1_{zym}}]}{K^{\mathrm{MASP1_{zym}}}_{\mathrm{m\,MASP2^*}}}\\&+\frac{[\mathrm{pMBL_4MASP2_{zym}}]}{K^{\mathrm{MASP2_{zym}}}_{\mathrm{m\,MASP2^*}}}+\frac{[\mathrm{pCLLKMASP1_{zym}}]}{K^{\mathrm{MASP1_{zym}}}_{\mathrm{m\,MASP2^*}}}+\frac{[\mathrm{pCLLKMASP2_{zym}}]}{K^{\mathrm{MASP2_{zym}}}_{\mathrm{m\,MASP2^*}}}+\frac{[\mathrm{pCLK1MASP1_{zym}}]}{K^{\mathrm{MASP1_{zym}}}_{\mathrm{m\,MASP2^*}}}\\&+\frac{[\mathrm{pCLK1MASP2_{zym}}]}{K^{\mathrm{MASP2_{zym}}}_{\mathrm{m\,MASP2^*}}}+\frac{[\mathrm{pCLL1MASP1_{zym}}]}{K^{\mathrm{MASP1_{zym}}}_{\mathrm{m\,MASP2^*}}}+\frac{[\mathrm{pCLL1MASP2_{zym}}]}{K^{\mathrm{MASP2_{zym}}}_{\mathrm{m\,MASP2^*}}}+\frac{[\mathrm{pLFMASP1_{zym}}]}{K^{\mathrm{MASP1_{zym}}}_{\mathrm{m\,MASP2^*}}}\\&+\frac{[\mathrm{pLFMASP2_{zym}}]}{K^{\mathrm{MASP2_{zym}}}_{\mathrm{m\,MASP2^*}}}+\frac{[\mathrm{pHFMASP1_{zym}}]}{K^{\mathrm{MASP1_{zym}}}_{\mathrm{m\,MASP2^*}}}+\frac{[\mathrm{pHFMASP2_{zym}}]}{K^{\mathrm{MASP2_{zym}}}_{\mathrm{m\,MASP2^*}}}+\frac{[\mathrm{pMFMASP1_{zym}}]}{K^{\mathrm{MASP1_{zym}}}_{\mathrm{m\,MASP2^*}}}+\frac{[\mathrm{pMFMASP2_{zym}}]}{K^{\mathrm{MASP2_{zym}}}_{\mathrm{m\,MASP2^*}}}\end{aligned}\right)}
$$

$$+\frac{k_{\text{cat MASP2}^*}^{\text{C2}}\left[\text{pCLL1MASP2}^*\right]\left[\text{pC4bC2}\right]}{K_{\text{m MASP2}^*}^{\text{C2}}\left(1+\frac{[\text{C4}]}{K_{\text{m MASP2}^*}^{\text{C4}}}+\frac{[\text{fC4bC2}]}{K_{\text{m MASP2}^*}^{\text{C2}}}+\frac{[\text{fC4bC4bC2}]}{K_{\text{m MASP2}^*}^{\text{C2}}}+\frac{[\text{fC3bC4bC2}]}{K_{\text{m MASP2}^*}^{\text{C2}}}+\frac{[\text{pC4bC2}]}{K_{\text{m MASP2}^*}^{\text{C2}}}+\frac{[\text{pC4bC4bC2}]}{K_{\text{m MASP2}^*}^{\text{C2}}}+\frac{[\text{pC3bC4bC2}]}{K_{\text{m MASP2}^*}^{\text{C2}}}+\frac{[\text{pMBL}_3\text{MASP1}_{\text{zym}}]}{K_{\text{m MASP2}^*}^{\text{MASP1}_{\text{zym}}}}+\frac{[\text{pMBL}_3\text{MASP2}_{\text{zym}}]}{K_{\text{m MASP2}^*}^{\text{MASP2}_{\text{zym}}}}+\frac{[\text{pMBL}_4\text{MASP1}_{\text{zym}}]}{K_{\text{m MASP2}^*}^{\text{MASP1}_{\text{zym}}}}+\frac{[\text{pMBL}_4\text{MASP2}_{\text{zym}}]}{K_{\text{m MASP2}^*}^{\text{MASP2}_{\text{zym}}}}+\frac{[\text{pCLLKMASP1}_{\text{zym}}]}{K_{\text{m MASP2}^*}^{\text{MASP1}_{\text{zym}}}}+\frac{[\text{pCLLKMASP2}_{\text{zym}}]}{K_{\text{m MASP2}^*}^{\text{MASP2}_{\text{zym}}}}+\frac{[\text{pCLK1MASP1}_{\text{zym}}]}{K_{\text{m MASP2}^*}^{\text{MASP1}_{\text{zym}}}}+\frac{[\text{pCLK1MASP2}_{\text{zym}}]}{K_{\text{m MASP2}^*}^{\text{MASP2}_{\text{zym}}}}+\frac{[\text{pCLL1MASP1}_{\text{zym}}]}{K_{\text{m MASP2}^*}^{\text{MASP1}_{\text{zym}}}}+\frac{[\text{pCLL1MASP2}_{\text{zym}}]}{K_{\text{m MASP2}^*}^{\text{MASP2}_{\text{zym}}}}+\frac{[\text{pLFMASP1}_{\text{zym}}]}{K_{\text{m MASP2}^*}^{\text{MASP1}_{\text{zym}}}}+\frac{[\text{pLFMASP2}_{\text{zym}}]}{K_{\text{m MASP2}^*}^{\text{MASP2}_{\text{zym}}}}+\frac{[\text{pHFMASP1}_{\text{zym}}]}{K_{\text{m MASP2}^*}^{\text{MASP1}_{\text{zym}}}}+\frac{[\text{pHFMASP2}_{\text{zym}}]}{K_{\text{m MASP2}^*}^{\text{MASP2}_{\text{zym}}}}+\frac{[\text{pMFMASP1}_{\text{zym}}]}{K_{\text{m MASP2}^*}^{\text{MASP1}_{\text{zym}}}}+\frac{[\text{pMFMASP2}_{\text{zym}}]}{K_{\text{m MASP2}^*}^{\text{MASP2}_{\text{zym}}}}\right)}$$

$$+\frac{k_{\text{cat MASP2}^*}^{\text{C2}}\left[\text{pLFMASP2}^*\right]\left[\text{pC4bC2}\right]}{K_{\text{m MASP2}^*}^{\text{C2}}\left(1+\frac{[\text{C4}]}{K_{\text{m MASP2}^*}^{\text{C4}}}+\frac{[\text{fC4bC2}]}{K_{\text{m MASP2}^*}^{\text{C2}}}+\frac{[\text{fC4bC4bC2}]}{K_{\text{m MASP2}^*}^{\text{C2}}}+\frac{[\text{fC3bC4bC2}]}{K_{\text{m MASP2}^*}^{\text{C2}}}+\frac{[\text{pC4bC2}]}{K_{\text{m MASP2}^*}^{\text{C2}}}+\frac{[\text{pC4bC4bC2}]}{K_{\text{m MASP2}^*}^{\text{C2}}}+\frac{[\text{pC3bC4bC2}]}{K_{\text{m MASP2}^*}^{\text{C2}}}+\frac{[\text{pMBL}_3\text{MASP1}_{\text{zym}}]}{K_{\text{m MASP2}^*}^{\text{MASP1}_{\text{zym}}}}+\frac{[\text{pMBL}_3\text{MASP2}_{\text{zym}}]}{K_{\text{m MASP2}^*}^{\text{MASP2}_{\text{zym}}}}+\frac{[\text{pMBL}_4\text{MASP1}_{\text{zym}}]}{K_{\text{m MASP2}^*}^{\text{MASP1}_{\text{zym}}}}+\frac{[\text{pMBL}_4\text{MASP2}_{\text{zym}}]}{K_{\text{m MASP2}^*}^{\text{MASP2}_{\text{zym}}}}+\frac{[\text{pCLLKMASP1}_{\text{zym}}]}{K_{\text{m MASP2}^*}^{\text{MASP1}_{\text{zym}}}}+\frac{[\text{pCLLKMASP2}_{\text{zym}}]}{K_{\text{m MASP2}^*}^{\text{MASP2}_{\text{zym}}}}+\frac{[\text{pCLK1MASP1}_{\text{zym}}]}{K_{\text{m MASP2}^*}^{\text{MASP1}_{\text{zym}}}}+\frac{[\text{pCLK1MASP2}_{\text{zym}}]}{K_{\text{m MASP2}^*}^{\text{MASP2}_{\text{zym}}}}+\frac{[\text{pCLL1MASP1}_{\text{zym}}]}{K_{\text{m MASP2}^*}^{\text{MASP1}_{\text{zym}}}}+\frac{[\text{pCLL1MASP2}_{\text{zym}}]}{K_{\text{m MASP2}^*}^{\text{MASP2}_{\text{zym}}}}+\frac{[\text{pLFMASP1}_{\text{zym}}]}{K_{\text{m MASP2}^*}^{\text{MASP1}_{\text{zym}}}}+\frac{[\text{pLFMASP2}_{\text{zym}}]}{K_{\text{m MASP2}^*}^{\text{MASP2}_{\text{zym}}}}+\frac{[\text{pHFMASP1}_{\text{zym}}]}{K_{\text{m MASP2}^*}^{\text{MASP1}_{\text{zym}}}}+\frac{[\text{pHFMASP2}_{\text{zym}}]}{K_{\text{m MASP2}^*}^{\text{MASP2}_{\text{zym}}}}+\frac{[\text{pMFMASP1}_{\text{zym}}]}{K_{\text{m MASP2}^*}^{\text{MASP1}_{\text{zym}}}}+\frac{[\text{pMFMASP2}_{\text{zym}}]}{K_{\text{m MASP2}^*}^{\text{MASP2}_{\text{zym}}}}\right)}$$

$$+\frac{k_{\text{cat MASP2}^*}^{\text{C2}}\left[\text{pHFMASP2}^*\right]\left[\text{pC4bC2}\right]}{K_{\text{m MASP2}^*}^{\text{C2}}\left(1+\frac{[\text{C4}]}{K_{\text{m MASP2}^*}^{\text{C4}}}+\frac{[\text{fC4bC2}]}{K_{\text{m MASP2}^*}^{\text{C2}}}+\frac{[\text{fC4bC4bC2}]}{K_{\text{m MASP2}^*}^{\text{C2}}}+\frac{[\text{fC3bC4bC2}]}{K_{\text{m MASP2}^*}^{\text{C2}}}+\frac{[\text{pC4bC2}]}{K_{\text{m MASP2}^*}^{\text{C2}}}+\frac{[\text{pC4bC4bC2}]}{K_{\text{m MASP2}^*}^{\text{C2}}}+\frac{[\text{pC3bC4bC2}]}{K_{\text{m MASP2}^*}^{\text{C2}}}+\frac{[\text{pMBL}_3\text{MASP1}_{\text{zym}}]}{K_{\text{m MASP2}^*}^{\text{MASP1}_{\text{zym}}}}+\frac{[\text{pMBL}_3\text{MASP2}_{\text{zym}}]}{K_{\text{m MASP2}^*}^{\text{MASP2}_{\text{zym}}}}+\frac{[\text{pMBL}_4\text{MASP1}_{\text{zym}}]}{K_{\text{m MASP2}^*}^{\text{MASP1}_{\text{zym}}}}+\frac{[\text{pMBL}_4\text{MASP2}_{\text{zym}}]}{K_{\text{m MASP2}^*}^{\text{MASP2}_{\text{zym}}}}+\frac{[\text{pCLLKMASP1}_{\text{zym}}]}{K_{\text{m MASP2}^*}^{\text{MASP1}_{\text{zym}}}}+\frac{[\text{pCLLKMASP2}_{\text{zym}}]}{K_{\text{m MASP2}^*}^{\text{MASP2}_{\text{zym}}}}+\frac{[\text{pCLK1MASP1}_{\text{zym}}]}{K_{\text{m MASP2}^*}^{\text{MASP1}_{\text{zym}}}}+\frac{[\text{pCLK1MASP2}_{\text{zym}}]}{K_{\text{m MASP2}^*}^{\text{MASP2}_{\text{zym}}}}+\frac{[\text{pCLL1MASP1}_{\text{zym}}]}{K_{\text{m MASP2}^*}^{\text{MASP1}_{\text{zym}}}}+\frac{[\text{pCLL1MASP2}_{\text{zym}}]}{K_{\text{m MASP2}^*}^{\text{MASP2}_{\text{zym}}}}+\frac{[\text{pLFMASP1}_{\text{zym}}]}{K_{\text{m MASP2}^*}^{\text{MASP1}_{\text{zym}}}}+\frac{[\text{pLFMASP2}_{\text{zym}}]}{K_{\text{m MASP2}^*}^{\text{MASP2}_{\text{zym}}}}+\frac{[\text{pHFMASP1}_{\text{zym}}]}{K_{\text{m MASP2}^*}^{\text{MASP1}_{\text{zym}}}}+\frac{[\text{pHFMASP2}_{\text{zym}}]}{K_{\text{m MASP2}^*}^{\text{MASP2}_{\text{zym}}}}+\frac{[\text{pMFMASP1}_{\text{zym}}]}{K_{\text{m MASP2}^*}^{\text{MASP1}_{\text{zym}}}}+\frac{[\text{pMFMASP2}_{\text{zym}}]}{K_{\text{m MASP2}^*}^{\text{MASP2}_{\text{zym}}}}\right)}$$

$$+\frac{k_{\text{cat MASP2}^*}^{\text{C2}}\left[\text{pMFMASP2}^*\right]\left[\text{pC4bC2}\right]}{K_{\text{m MASP2}^*}^{\text{C2}}\left(\begin{array}{l}1+\frac{[\text{C4}]}{K_{\text{m MASP2}^*}^{\text{C4}}}+\frac{[\text{fC4bC2}]}{K_{\text{m MASP2}^*}^{\text{C2}}}+\frac{[\text{fC4bC4bC2}]}{K_{\text{m MASP2}^*}^{\text{C2}}}+\frac{[\text{fC3bC4bC2}]}{K_{\text{m MASP2}^*}^{\text{C2}}}+\frac{[\text{pC4bC2}]}{K_{\text{m MASP2}^*}^{\text{C2}}}+\frac{[\text{pC4bC4bC2}]}{K_{\text{m MASP2}^*}^{\text{C2}}}\\+\frac{[\text{pC3bC4bC2}]}{K_{\text{m MASP2}^*}^{\text{C2}}}+\frac{[\text{pMBL}_3\text{MASP1}_{\text{zym}}]}{K_{\text{m MASP2}^*}^{\text{MASP1}_{\text{zym}}}}+\frac{[\text{pMBL}_3\text{MASP2}_{\text{zym}}]}{K_{\text{m MASP2}^*}^{\text{MASP2}_{\text{zym}}}}+\frac{[\text{pMBL}_4\text{MASP1}_{\text{zym}}]}{K_{\text{m MASP2}^*}^{\text{MASP1}_{\text{zym}}}}\\+\frac{[\text{pMBL}_4\text{MASP2}_{\text{zym}}]}{K_{\text{m MASP2}^*}^{\text{MASP2}_{\text{zym}}}}+\frac{[\text{pCLLKMASP1}_{\text{zym}}]}{K_{\text{m MASP2}^*}^{\text{MASP1}_{\text{zym}}}}+\frac{[\text{pCLLKMASP2}_{\text{zym}}]}{K_{\text{m MASP2}^*}^{\text{MASP2}_{\text{zym}}}}+\frac{[\text{pCLK1MASP1}_{\text{zym}}]}{K_{\text{m MASP2}^*}^{\text{MASP1}_{\text{zym}}}}\\+\frac{[\text{pCLK1MASP2}_{\text{zym}}]}{K_{\text{m MASP2}^*}^{\text{MASP2}_{\text{zym}}}}+\frac{[\text{pCLL1MASP1}_{\text{zym}}]}{K_{\text{m MASP2}^*}^{\text{MASP1}_{\text{zym}}}}+\frac{[\text{pCLL1MASP2}_{\text{zym}}]}{K_{\text{m MASP2}^*}^{\text{MASP2}_{\text{zym}}}}+\frac{[\text{pLFMASP1}_{\text{zym}}]}{K_{\text{m MASP2}^*}^{\text{MASP1}_{\text{zym}}}}\\+\frac{[\text{pLFMASP2}_{\text{zym}}]}{K_{\text{m MASP2}^*}^{\text{MASP2}_{\text{zym}}}}+\frac{[\text{pHFMASP1}_{\text{zym}}]}{K_{\text{m MASP2}^*}^{\text{MASP1}_{\text{zym}}}}+\frac{[\text{pHFMASP2}_{\text{zym}}]}{K_{\text{m MASP2}^*}^{\text{MASP2}_{\text{zym}}}}+\frac{[\text{pMFMASP1}_{\text{zym}}]}{K_{\text{m MASP2}^*}^{\text{MASP1}_{\text{zym}}}}+\frac{[\text{pMFMASP2}_{\text{zym}}]}{K_{\text{m MASP2}^*}^{\text{MASP2}_{\text{zym}}}}\end{array}\right)}$$

$$-k_{\text{C4bC4BP}}^{+}[\text{pC4bC2a}][\text{pC4BPPTX3}]+k_{\text{C4bC4BP}}^{-}[\text{pC4bC2aC4BPPTX3}]-k_{\text{C4bC4BP}}^{+}[\text{pC4bC2a}][\text{pC4BP}]$$

$$+k_{\text{C4bC4BP}}^{-}[\text{pC4bC2apC4BP}]$$

$$+\frac{k_{\text{cat C1}^*}^{\text{C2}}\left[\text{pMBL}_3\text{PTX3C1}^*\right][\text{pC4bC2}]}{K_{\text{m C1}^*}^{\text{C2}}\left(1+\frac{[\text{C4}]}{K_{\text{m C1}^*}^{\text{C4}}}+\frac{[\text{fC4bC2}]}{K_{\text{m C1}^*}^{\text{C2}}}+\frac{[\text{fC4bC4bC2}]}{K_{\text{m C1}^*}^{\text{C2}}}+\frac{[\text{fC3bC4bC2}]}{K_{\text{m C1}^*}^{\text{C2}}}+\frac{[\text{pC4bC2}]}{K_{\text{m C1}^*}^{\text{C2}}}+\frac{[\text{pC4bC4bC2}]}{K_{\text{m C1}^*}^{\text{C2}}}+\frac{[\text{pC3bC4bC2}]}{K_{\text{m C1}^*}^{\text{C2}}}\right)}$$

$$+\frac{k_{\text{cat C1}^*}^{\text{C2}}\left[\text{pMBL}_4\text{PTX3C1}^*\right][\text{pC4bC2}]}{K_{\text{m C1}^*}^{\text{C2}}\left(1+\frac{[\text{C4}]}{K_{\text{m C1}^*}^{\text{C4}}}+\frac{[\text{fC4bC2}]}{K_{\text{m C1}^*}^{\text{C2}}}+\frac{[\text{fC4bC4bC2}]}{K_{\text{m C1}^*}^{\text{C2}}}+\frac{[\text{fC3bC4bC2}]}{K_{\text{m C1}^*}^{\text{C2}}}+\frac{[\text{pC4bC2}]}{K_{\text{m C1}^*}^{\text{C2}}}+\frac{[\text{pC4bC4bC2}]}{K_{\text{m C1}^*}^{\text{C2}}}+\frac{[\text{pC3bC4bC2}]}{K_{\text{m C1}^*}^{\text{C2}}}\right)}$$

150

$$\frac{d[\text{pC4bC4b}]}{dt}=k_{\text{C4bC4b}}^{+}[\text{nfC4b}][\text{pC4b}]-k_{\text{C4bC2}}^{+}[\text{pC4bC4b}][\text{C2}]+k_{\text{C4bC2}}^{-}[\text{pC4bC4bC2}]+k_{\text{C4bC4bC2a}}^{-}[\text{pC4bC4bC2a}]$$

$$-k_{\text{C4bC4BP}}^{+}[\text{pC4bC4b}][\text{C4BP}]+k_{\text{C4bC4BP}}^{-}[\text{pC4bC4bC4BP}]+k_{\text{C4bC2aC4BP}_{\text{decay}}}^{-}[\text{pC4bC4bC2aC4BP}]$$

$$-k_{\text{C4bC4BP}}^{+}[\text{pC4bC4b}][\text{pC4BPPTX3}]+k_{\text{C4bC4BP}}^{-}[\text{pC4bC4bC4BPPTX3}]+k_{\text{C4bC2aC4BP}_{\text{decay}}}^{-}[\text{pC4bC4bC2aC4BPPTX3}]$$

$$-k_{\text{C4bC4BP}}^{+}[\text{pC4bC4b}][\text{pC4BP}]+k_{\text{C4bC4BP}}^{-}[\text{pC4bC4bpC4BP}]+k_{\text{C4bC2aC4BP}_{\text{decay}}}^{-}[\text{pC4bC4bC2apC4BP}]$$

151

$$
\begin{aligned}
\frac{d\left[\mathrm{pC4bC4bC2}\right]}{dt} &= k^{+}_{\mathrm{C4bC2}}\left[\mathrm{pC4bC4b}\right]\left[\mathrm{C2}\right]-k^{-}_{\mathrm{C4bC2}}\left[\mathrm{pC4bC4bC2}\right] \\
&-\frac{k^{\mathrm{C2}}_{\mathrm{cat\,C1^*}}\left[\mathrm{pIgG_6C1^*}\right]\left[\mathrm{pC4bC4bC2}\right]}{K^{\mathrm{C2}}_{\mathrm{m\,C1^*}}\left(1+\frac{\left[\mathrm{C4}\right]}{K^{\mathrm{C4}}_{\mathrm{m\,C1^*}}}+\frac{\left[\mathrm{fC4bC2}\right]}{K^{\mathrm{C2}}_{\mathrm{m\,C1^*}}}+\frac{\left[\mathrm{fC4bC4bC2}\right]}{K^{\mathrm{C2}}_{\mathrm{m\,C1^*}}}+\frac{\left[\mathrm{fC3bC4bC2}\right]}{K^{\mathrm{C2}}_{\mathrm{m\,C1^*}}}+\frac{\left[\mathrm{pC4bC2}\right]}{K^{\mathrm{C2}}_{\mathrm{m\,C1^*}}}+\frac{\left[\mathrm{pC4bC4bC2}\right]}{K^{\mathrm{C2}}_{\mathrm{m\,C1^*}}}+\frac{\left[\mathrm{pC3bC4bC2}\right]}{K^{\mathrm{C2}}_{\mathrm{m\,C1^*}}}\right)} \\
&-\frac{k^{\mathrm{C2}}_{\mathrm{cat\,C1^*}}\left[\mathrm{pIgMC1^*}\right]\left[\mathrm{pC4bC4bC2}\right]}{K^{\mathrm{C2}}_{\mathrm{m\,C1^*}}\left(1+\frac{\left[\mathrm{C4}\right]}{K^{\mathrm{C4}}_{\mathrm{m\,C1^*}}}+\frac{\left[\mathrm{fC4bC2}\right]}{K^{\mathrm{C2}}_{\mathrm{m\,C1^*}}}+\frac{\left[\mathrm{fC4bC4bC2}\right]}{K^{\mathrm{C2}}_{\mathrm{m\,C1^*}}}+\frac{\left[\mathrm{fC3bC4bC2}\right]}{K^{\mathrm{C2}}_{\mathrm{m\,C1^*}}}+\frac{\left[\mathrm{pC4bC2}\right]}{K^{\mathrm{C2}}_{\mathrm{m\,C1^*}}}+\frac{\left[\mathrm{pC4bC4bC2}\right]}{K^{\mathrm{C2}}_{\mathrm{m\,C1^*}}}+\frac{\left[\mathrm{pC3bC4bC2}\right]}{K^{\mathrm{C2}}_{\mathrm{m\,C1^*}}}\right)} \\
&-\frac{k^{\mathrm{C2}}_{\mathrm{cat\,MASP1^*}}\left[\mathrm{pMBL_3MASP1^*}\right]\left[\mathrm{pC4bC4bC2}\right]}{K^{\mathrm{C2}}_{\mathrm{m\,MASP1^*}}\left(\begin{aligned}&1+\frac{\left[\mathrm{C4}\right]}{K^{\mathrm{C4}}_{\mathrm{m\,MASP1^*}}}+\frac{\left[\mathrm{fC4bC2}\right]}{K^{\mathrm{C2}}_{\mathrm{m\,MASP1^*}}}+\frac{\left[\mathrm{fC4bC4bC2}\right]}{K^{\mathrm{C2}}_{\mathrm{m\,MASP1^*}}}+\frac{\left[\mathrm{fC3bC4bC2}\right]}{K^{\mathrm{C2}}_{\mathrm{m\,MASP1^*}}}+\frac{\left[\mathrm{pC4bC2}\right]}{K^{\mathrm{C2}}_{\mathrm{m\,MASP1^*}}}+\frac{\left[\mathrm{pC4bC4bC2}\right]}{K^{\mathrm{C2}}_{\mathrm{m\,MASP1^*}}}\\
&+\frac{\left[\mathrm{pC3bC4bC2}\right]}{K^{\mathrm{C2}}_{\mathrm{m\,MASP1^*}}}+\frac{\left[\mathrm{pMBL_3MASP1_{zym}}\right]}{K^{\mathrm{MASP1_{zym}}}_{\mathrm{m\,MASP1^*}}}+\frac{\left[\mathrm{pMBL_3MASP2_{zym}}\right]}{K^{\mathrm{MASP2_{zym}}}_{\mathrm{m\,MASP1^*}}}+\frac{\left[\mathrm{pMBL_3MASP3_{zym}}\right]}{K^{\mathrm{MASP3_{zym}}}_{\mathrm{m\,MASP1^*}}}\\
&+\frac{\left[\mathrm{pMBL_4MASP1_{zym}}\right]}{K^{\mathrm{MASP1_{zym}}}_{\mathrm{m\,MASP1^*}}}+\frac{\left[\mathrm{pMBL_4MASP2_{zym}}\right]}{K^{\mathrm{MASP2_{zym}}}_{\mathrm{m\,MASP1^*}}}+\frac{\left[\mathrm{pMBL_4MASP3_{zym}}\right]}{K^{\mathrm{MASP3_{zym}}}_{\mathrm{m\,MASP1^*}}}+\frac{\left[\mathrm{pCLLKMASP1_{zym}}\right]}{K^{\mathrm{MASP1_{zym}}}_{\mathrm{m\,MASP1^*}}}\\
&+\frac{\left[\mathrm{pCLLKMASP2_{zym}}\right]}{K^{\mathrm{MASP2_{zym}}}_{\mathrm{m\,MASP1^*}}}+\frac{\left[\mathrm{pCLLKMASP3_{zym}}\right]}{K^{\mathrm{MASP3_{zym}}}_{\mathrm{m\,MASP1^*}}}+\frac{\left[\mathrm{pCLK1MASP1_{zym}}\right]}{K^{\mathrm{MASP1_{zym}}}_{\mathrm{m\,MASP1^*}}}+\frac{\left[\mathrm{pCLK1MASP2_{zym}}\right]}{K^{\mathrm{MASP2_{zym}}}_{\mathrm{m\,MASP1^*}}}\\
&+\frac{\left[\mathrm{pCLK1MASP3_{zym}}\right]}{K^{\mathrm{MASP3_{zym}}}_{\mathrm{m\,MASP1^*}}}+\frac{\left[\mathrm{pCLL1MASP1_{zym}}\right]}{K^{\mathrm{MASP1_{zym}}}_{\mathrm{m\,MASP1^*}}}+\frac{\left[\mathrm{pCLL1MASP2_{zym}}\right]}{K^{\mathrm{MASP2_{zym}}}_{\mathrm{m\,MASP1^*}}}+\frac{\left[\mathrm{pCLL1MASP3_{zym}}\right]}{K^{\mathrm{MASP3_{zym}}}_{\mathrm{m\,MASP1^*}}}\\
&+\frac{\left[\mathrm{pLFMASP1_{zym}}\right]}{K^{\mathrm{MASP1_{zym}}}_{\mathrm{m\,MASP1^*}}}+\frac{\left[\mathrm{pLFMASP2_{zym}}\right]}{K^{\mathrm{MASP2_{zym}}}_{\mathrm{m\,MASP1^*}}}+\frac{\left[\mathrm{pLFMASP3_{zym}}\right]}{K^{\mathrm{MASP3_{zym}}}_{\mathrm{m\,MASP1^*}}}+\frac{\left[\mathrm{pHFMASP1_{zym}}\right]}{K^{\mathrm{MASP1_{zym}}}_{\mathrm{m\,MASP1^*}}}+\frac{\left[\mathrm{pHFMASP2_{zym}}\right]}{K^{\mathrm{MASP2_{zym}}}_{\mathrm{m\,MASP1^*}}}\\
&+\frac{\left[\mathrm{pHFMASP3_{zym}}\right]}{K^{\mathrm{MASP3_{zym}}}_{\mathrm{m\,MASP1^*}}}+\frac{\left[\mathrm{pMFMASP1_{zym}}\right]}{K^{\mathrm{MASP1_{zym}}}_{\mathrm{m\,MASP1^*}}}+\frac{\left[\mathrm{pMFMASP2_{zym}}\right]}{K^{\mathrm{MASP2_{zym}}}_{\mathrm{m\,MASP1^*}}}+\frac{\left[\mathrm{pMFMASP3_{zym}}\right]}{K^{\mathrm{MASP3_{zym}}}_{\mathrm{m\,MASP1^*}}}\end{aligned}\right)}
\end{aligned}
$$

$$
-\frac{k_{\mathrm{cat\,MASP1^*}}^{\mathrm{C2}}\left[\mathrm{pMBL_4MASP1^*}\right]\left[\mathrm{pC4bC4bC2}\right]}{K_{\mathrm{m\,MASP1^*}}^{\mathrm{C2}}\left(\begin{array}{l}
1+\frac{[\mathrm{C4}]}{K_{\mathrm{m\,MASP1^*}}^{\mathrm{C4}}}+\frac{[\mathrm{fC4bC2}]}{K_{\mathrm{m\,MASP1^*}}^{\mathrm{C2}}}+\frac{[\mathrm{fC4bC4bC2}]}{K_{\mathrm{m\,MASP1^*}}^{\mathrm{C2}}}+\frac{[\mathrm{fC3bC4bC2}]}{K_{\mathrm{m\,MASP1^*}}^{\mathrm{C2}}}+\frac{[\mathrm{pC4bC2}]}{K_{\mathrm{m\,MASP1^*}}^{\mathrm{C2}}}+\frac{[\mathrm{pC4bC4bC2}]}{K_{\mathrm{m\,MASP1^*}}^{\mathrm{C2}}}\\
+\frac{[\mathrm{pC3bC4bC2}]}{K_{\mathrm{m\,MASP1^*}}^{\mathrm{C2}}}+\frac{\left[\mathrm{pMBL_3MASP1_{zym}}\right]}{K_{\mathrm{m\,MASP1^*}}^{\mathrm{MASP1_{zym}}}}+\frac{\left[\mathrm{pMBL_3MASP2_{zym}}\right]}{K_{\mathrm{m\,MASP1^*}}^{\mathrm{MASP2_{zym}}}}+\frac{\left[\mathrm{pMBL_3MASP3_{zym}}\right]}{K_{\mathrm{m\,MASP1^*}}^{\mathrm{MASP3_{zym}}}}\\
+\frac{\left[\mathrm{pMBL_4MASP1_{zym}}\right]}{K_{\mathrm{m\,MASP1^*}}^{\mathrm{MASP1_{zym}}}}+\frac{\left[\mathrm{pMBL_4MASP2_{zym}}\right]}{K_{\mathrm{m\,MASP1^*}}^{\mathrm{MASP2_{zym}}}}+\frac{\left[\mathrm{pMBL_4MASP3_{zym}}\right]}{K_{\mathrm{m\,MASP1^*}}^{\mathrm{MASP3_{zym}}}}+\frac{\left[\mathrm{pCLLKMASP1_{zym}}\right]}{K_{\mathrm{m\,MASP1^*}}^{\mathrm{MASP1_{zym}}}}\\
+\frac{\left[\mathrm{pCLLKMASP2_{zym}}\right]}{K_{\mathrm{m\,MASP1^*}}^{\mathrm{MASP2_{zym}}}}+\frac{\left[\mathrm{pCLLKMASP3_{zym}}\right]}{K_{\mathrm{m\,MASP1^*}}^{\mathrm{MASP3_{zym}}}}+\frac{\left[\mathrm{pCLK1MASP1_{zym}}\right]}{K_{\mathrm{m\,MASP1^*}}^{\mathrm{MASP1_{zym}}}}+\frac{\left[\mathrm{pCLK1MASP2_{zym}}\right]}{K_{\mathrm{m\,MASP1^*}}^{\mathrm{MASP2_{zym}}}}\\
+\frac{\left[\mathrm{pCLK1MASP3_{zym}}\right]}{K_{\mathrm{m\,MASP1^*}}^{\mathrm{MASP3_{zym}}}}+\frac{\left[\mathrm{pCLL1MASP1_{zym}}\right]}{K_{\mathrm{m\,MASP1^*}}^{\mathrm{MASP1_{zym}}}}+\frac{\left[\mathrm{pCLL1MASP2_{zym}}\right]}{K_{\mathrm{m\,MASP1^*}}^{\mathrm{MASP2_{zym}}}}+\frac{\left[\mathrm{pCLL1MASP3_{zym}}\right]}{K_{\mathrm{m\,MASP1^*}}^{\mathrm{MASP3_{zym}}}}\\
+\frac{\left[\mathrm{pLFMASP1_{zym}}\right]}{K_{\mathrm{m\,MASP1^*}}^{\mathrm{MASP1_{zym}}}}+\frac{\left[\mathrm{pLFMASP2_{zym}}\right]}{K_{\mathrm{m\,MASP1^*}}^{\mathrm{MASP2_{zym}}}}+\frac{\left[\mathrm{pLFMASP3_{zym}}\right]}{K_{\mathrm{m\,MASP1^*}}^{\mathrm{MASP3_{zym}}}}+\frac{\left[\mathrm{pHFMASP1_{zym}}\right]}{K_{\mathrm{m\,MASP1^*}}^{\mathrm{MASP1_{zym}}}}+\frac{\left[\mathrm{pHFMASP2_{zym}}\right]}{K_{\mathrm{m\,MASP1^*}}^{\mathrm{MASP2_{zym}}}}\\
+\frac{\left[\mathrm{pHFMASP3_{zym}}\right]}{K_{\mathrm{m\,MASP1^*}}^{\mathrm{MASP3_{zym}}}}+\frac{\left[\mathrm{pMFMASP1_{zym}}\right]}{K_{\mathrm{m\,MASP1^*}}^{\mathrm{MASP1_{zym}}}}+\frac{\left[\mathrm{pMFMASP2_{zym}}\right]}{K_{\mathrm{m\,MASP1^*}}^{\mathrm{MASP2_{zym}}}}+\frac{\left[\mathrm{pMFMASP3_{zym}}\right]}{K_{\mathrm{m\,MASP1^*}}^{\mathrm{MASP3_{zym}}}}
\end{array}\right)}
$$

$$
-\frac{k_{\mathrm{cat\,MASP1^*}}^{\mathrm{C2}}\left[\mathrm{pCLLKMASP1^*}\right]\left[\mathrm{pC4bC4bC2}\right]}{K_{\mathrm{m\,MASP1^*}}^{\mathrm{C2}}\left(\begin{array}{l}
1+\frac{[\mathrm{C4}]}{K_{\mathrm{m\,MASP1^*}}^{\mathrm{C4}}}+\frac{[\mathrm{fC4bC2}]}{K_{\mathrm{m\,MASP1^*}}^{\mathrm{C2}}}+\frac{[\mathrm{fC4bC4bC2}]}{K_{\mathrm{m\,MASP1^*}}^{\mathrm{C2}}}+\frac{[\mathrm{fC3bC4bC2}]}{K_{\mathrm{m\,MASP1^*}}^{\mathrm{C2}}}+\frac{[\mathrm{pC4bC2}]}{K_{\mathrm{m\,MASP1^*}}^{\mathrm{C2}}}+\frac{[\mathrm{pC4bC4bC2}]}{K_{\mathrm{m\,MASP1^*}}^{\mathrm{C2}}}\\
+\frac{[\mathrm{pC3bC4bC2}]}{K_{\mathrm{m\,MASP1^*}}^{\mathrm{C2}}}+\frac{\left[\mathrm{pMBL_3MASP1_{zym}}\right]}{K_{\mathrm{m\,MASP1^*}}^{\mathrm{MASP1_{zym}}}}+\frac{\left[\mathrm{pMBL_3MASP2_{zym}}\right]}{K_{\mathrm{m\,MASP1^*}}^{\mathrm{MASP2_{zym}}}}+\frac{\left[\mathrm{pMBL_3MASP3_{zym}}\right]}{K_{\mathrm{m\,MASP1^*}}^{\mathrm{MASP3_{zym}}}}\\
+\frac{\left[\mathrm{pMBL_4MASP1_{zym}}\right]}{K_{\mathrm{m\,MASP1^*}}^{\mathrm{MASP1_{zym}}}}+\frac{\left[\mathrm{pMBL_4MASP2_{zym}}\right]}{K_{\mathrm{m\,MASP1^*}}^{\mathrm{MASP2_{zym}}}}+\frac{\left[\mathrm{pMBL_4MASP3_{zym}}\right]}{K_{\mathrm{m\,MASP1^*}}^{\mathrm{MASP3_{zym}}}}+\frac{\left[\mathrm{pCLLKMASP1_{zym}}\right]}{K_{\mathrm{m\,MASP1^*}}^{\mathrm{MASP1_{zym}}}}\\
+\frac{\left[\mathrm{pCLLKMASP2_{zym}}\right]}{K_{\mathrm{m\,MASP1^*}}^{\mathrm{MASP2_{zym}}}}+\frac{\left[\mathrm{pCLLKMASP3_{zym}}\right]}{K_{\mathrm{m\,MASP1^*}}^{\mathrm{MASP3_{zym}}}}+\frac{\left[\mathrm{pCLK1MASP1_{zym}}\right]}{K_{\mathrm{m\,MASP1^*}}^{\mathrm{MASP1_{zym}}}}+\frac{\left[\mathrm{pCLK1MASP2_{zym}}\right]}{K_{\mathrm{m\,MASP1^*}}^{\mathrm{MASP2_{zym}}}}\\
+\frac{\left[\mathrm{pCLK1MASP3_{zym}}\right]}{K_{\mathrm{m\,MASP1^*}}^{\mathrm{MASP3_{zym}}}}+\frac{\left[\mathrm{pCLL1MASP1_{zym}}\right]}{K_{\mathrm{m\,MASP1^*}}^{\mathrm{MASP1_{zym}}}}+\frac{\left[\mathrm{pCLL1MASP2_{zym}}\right]}{K_{\mathrm{m\,MASP1^*}}^{\mathrm{MASP2_{zym}}}}+\frac{\left[\mathrm{pCLL1MASP3_{zym}}\right]}{K_{\mathrm{m\,MASP1^*}}^{\mathrm{MASP3_{zym}}}}\\
+\frac{\left[\mathrm{pLFMASP1_{zym}}\right]}{K_{\mathrm{m\,MASP1^*}}^{\mathrm{MASP1_{zym}}}}+\frac{\left[\mathrm{pLFMASP2_{zym}}\right]}{K_{\mathrm{m\,MASP1^*}}^{\mathrm{MASP2_{zym}}}}+\frac{\left[\mathrm{pLFMASP3_{zym}}\right]}{K_{\mathrm{m\,MASP1^*}}^{\mathrm{MASP3_{zym}}}}+\frac{\left[\mathrm{pHFMASP1_{zym}}\right]}{K_{\mathrm{m\,MASP1^*}}^{\mathrm{MASP1_{zym}}}}+\frac{\left[\mathrm{pHFMASP2_{zym}}\right]}{K_{\mathrm{m\,MASP1^*}}^{\mathrm{MASP2_{zym}}}}\\
+\frac{\left[\mathrm{pHFMASP3_{zym}}\right]}{K_{\mathrm{m\,MASP1^*}}^{\mathrm{MASP3_{zym}}}}+\frac{\left[\mathrm{pMFMASP1_{zym}}\right]}{K_{\mathrm{m\,MASP1^*}}^{\mathrm{MASP1_{zym}}}}+\frac{\left[\mathrm{pMFMASP2_{zym}}\right]}{K_{\mathrm{m\,MASP1^*}}^{\mathrm{MASP2_{zym}}}}+\frac{\left[\mathrm{pMFMASP3_{zym}}\right]}{K_{\mathrm{m\,MASP1^*}}^{\mathrm{MASP3_{zym}}}}
\end{array}\right)}
$$

$$
-\frac{k_{\text{cat MASP1}^*}^{\text{C2}}\left[\text{pCLK1MASP1}^*\right]\left[\text{pC4bC4bC2}\right]}{K_{\text{m MASP1}^*}^{\text{C2}}\left(\begin{array}{l}1+\frac{[\text{C4}]}{K_{\text{m MASP1}^*}^{\text{C4}}}+\frac{[\text{fC4bC2}]}{K_{\text{m MASP1}^*}^{\text{C2}}}+\frac{[\text{fC4bC4bC2}]}{K_{\text{m MASP1}^*}^{\text{C2}}}+\frac{[\text{fC3bC4bC2}]}{K_{\text{m MASP1}^*}^{\text{C2}}}+\frac{[\text{pC4bC2}]}{K_{\text{m MASP1}^*}^{\text{C2}}}+\frac{[\text{pC4bC4bC2}]}{K_{\text{m MASP1}^*}^{\text{C2}}}\\
+\frac{[\text{pC3bC4bC2}]}{K_{\text{m MASP1}^*}^{\text{C2}}}+\frac{\left[\text{pMBL}_3\text{MASP1}_{\text{zym}}\right]}{K_{\text{m MASP1}^*}^{\text{MASP1}_{\text{zym}}}}+\frac{\left[\text{pMBL}_3\text{MASP2}_{\text{zym}}\right]}{K_{\text{m MASP1}^*}^{\text{MASP2}_{\text{zym}}}}+\frac{\left[\text{pMBL}_3\text{MASP3}_{\text{zym}}\right]}{K_{\text{m MASP1}^*}^{\text{MASP3}_{\text{zym}}}}\\
+\frac{\left[\text{pMBL}_4\text{MASP1}_{\text{zym}}\right]}{K_{\text{m MASP1}^*}^{\text{MASP1}_{\text{zym}}}}+\frac{\left[\text{pMBL}_4\text{MASP2}_{\text{zym}}\right]}{K_{\text{m MASP1}^*}^{\text{MASP2}_{\text{zym}}}}+\frac{\left[\text{pMBL}_4\text{MASP3}_{\text{zym}}\right]}{K_{\text{m MASP1}^*}^{\text{MASP3}_{\text{zym}}}}+\frac{\left[\text{pCLLKMASP1}_{\text{zym}}\right]}{K_{\text{m MASP1}^*}^{\text{MASP1}_{\text{zym}}}}\\
+\frac{\left[\text{pCLLKMASP2}_{\text{zym}}\right]}{K_{\text{m MASP1}^*}^{\text{MASP2}_{\text{zym}}}}+\frac{\left[\text{pCLLKMASP3}_{\text{zym}}\right]}{K_{\text{m MASP1}^*}^{\text{MASP3}_{\text{zym}}}}+\frac{\left[\text{pCLK1MASP1}_{\text{zym}}\right]}{K_{\text{m MASP1}^*}^{\text{MASP1}_{\text{zym}}}}+\frac{\left[\text{pCLK1MASP2}_{\text{zym}}\right]}{K_{\text{m MASP1}^*}^{\text{MASP2}_{\text{zym}}}}\\
+\frac{\left[\text{pCLK1MASP3}_{\text{zym}}\right]}{K_{\text{m MASP1}^*}^{\text{MASP3}_{\text{zym}}}}+\frac{\left[\text{pCLL1MASP1}_{\text{zym}}\right]}{K_{\text{m MASP1}^*}^{\text{MASP1}_{\text{zym}}}}+\frac{\left[\text{pCLL1MASP2}_{\text{zym}}\right]}{K_{\text{m MASP1}^*}^{\text{MASP2}_{\text{zym}}}}+\frac{\left[\text{pCLL1MASP3}_{\text{zym}}\right]}{K_{\text{m MASP1}^*}^{\text{MASP3}_{\text{zym}}}}\\
+\frac{\left[\text{pLFMASP1}_{\text{zym}}\right]}{K_{\text{m MASP1}^*}^{\text{MASP1}_{\text{zym}}}}+\frac{\left[\text{pLFMASP2}_{\text{zym}}\right]}{K_{\text{m MASP1}^*}^{\text{MASP2}_{\text{zym}}}}+\frac{\left[\text{pLFMASP3}_{\text{zym}}\right]}{K_{\text{m MASP1}^*}^{\text{MASP3}_{\text{zym}}}}+\frac{\left[\text{pHFMASP1}_{\text{zym}}\right]}{K_{\text{m MASP1}^*}^{\text{MASP1}_{\text{zym}}}}+\frac{\left[\text{pHFMASP2}_{\text{zym}}\right]}{K_{\text{m MASP1}^*}^{\text{MASP2}_{\text{zym}}}}\\
+\frac{\left[\text{pHFMASP3}_{\text{zym}}\right]}{K_{\text{m MASP1}^*}^{\text{MASP3}_{\text{zym}}}}+\frac{\left[\text{pMFMASP1}_{\text{zym}}\right]}{K_{\text{m MASP1}^*}^{\text{MASP1}_{\text{zym}}}}+\frac{\left[\text{pMFMASP2}_{\text{zym}}\right]}{K_{\text{m MASP1}^*}^{\text{MASP2}_{\text{zym}}}}+\frac{\left[\text{pMFMASP3}_{\text{zym}}\right]}{K_{\text{m MASP1}^*}^{\text{MASP3}_{\text{zym}}}}\end{array}\right)}
$$

$$
-\frac{k_{\text{cat MASP1}^*}^{\text{C2}}\left[\text{pCLL1MASP1}^*\right]\left[\text{pC4bC4bC2}\right]}{K_{\text{m MASP1}^*}^{\text{C2}}\left(\begin{array}{l}1+\frac{[\text{C4}]}{K_{\text{m MASP1}^*}^{\text{C4}}}+\frac{[\text{fC4bC2}]}{K_{\text{m MASP1}^*}^{\text{C2}}}+\frac{[\text{fC4bC4bC2}]}{K_{\text{m MASP1}^*}^{\text{C2}}}+\frac{[\text{fC3bC4bC2}]}{K_{\text{m MASP1}^*}^{\text{C2}}}+\frac{[\text{pC4bC2}]}{K_{\text{m MASP1}^*}^{\text{C2}}}+\frac{[\text{pC4bC4bC2}]}{K_{\text{m MASP1}^*}^{\text{C2}}}\\
+\frac{[\text{pC3bC4bC2}]}{K_{\text{m MASP1}^*}^{\text{C2}}}+\frac{\left[\text{pMBL}_3\text{MASP1}_{\text{zym}}\right]}{K_{\text{m MASP1}^*}^{\text{MASP1}_{\text{zym}}}}+\frac{\left[\text{pMBL}_3\text{MASP2}_{\text{zym}}\right]}{K_{\text{m MASP1}^*}^{\text{MASP2}_{\text{zym}}}}+\frac{\left[\text{pMBL}_3\text{MASP3}_{\text{zym}}\right]}{K_{\text{m MASP1}^*}^{\text{MASP3}_{\text{zym}}}}\\
+\frac{\left[\text{pMBL}_4\text{MASP1}_{\text{zym}}\right]}{K_{\text{m MASP1}^*}^{\text{MASP1}_{\text{zym}}}}+\frac{\left[\text{pMBL}_4\text{MASP2}_{\text{zym}}\right]}{K_{\text{m MASP1}^*}^{\text{MASP2}_{\text{zym}}}}+\frac{\left[\text{pMBL}_4\text{MASP3}_{\text{zym}}\right]}{K_{\text{m MASP1}^*}^{\text{MASP3}_{\text{zym}}}}+\frac{\left[\text{pCLLKMASP1}_{\text{zym}}\right]}{K_{\text{m MASP1}^*}^{\text{MASP1}_{\text{zym}}}}\\
+\frac{\left[\text{pCLLKMASP2}_{\text{zym}}\right]}{K_{\text{m MASP1}^*}^{\text{MASP2}_{\text{zym}}}}+\frac{\left[\text{pCLLKMASP3}_{\text{zym}}\right]}{K_{\text{m MASP1}^*}^{\text{MASP3}_{\text{zym}}}}+\frac{\left[\text{pCLK1MASP1}_{\text{zym}}\right]}{K_{\text{m MASP1}^*}^{\text{MASP1}_{\text{zym}}}}+\frac{\left[\text{pCLK1MASP2}_{\text{zym}}\right]}{K_{\text{m MASP1}^*}^{\text{MASP2}_{\text{zym}}}}\\
+\frac{\left[\text{pCLK1MASP3}_{\text{zym}}\right]}{K_{\text{m MASP1}^*}^{\text{MASP3}_{\text{zym}}}}+\frac{\left[\text{pCLL1MASP1}_{\text{zym}}\right]}{K_{\text{m MASP1}^*}^{\text{MASP1}_{\text{zym}}}}+\frac{\left[\text{pCLL1MASP2}_{\text{zym}}\right]}{K_{\text{m MASP1}^*}^{\text{MASP2}_{\text{zym}}}}+\frac{\left[\text{pCLL1MASP3}_{\text{zym}}\right]}{K_{\text{m MASP1}^*}^{\text{MASP3}_{\text{zym}}}}\\
+\frac{\left[\text{pLFMASP1}_{\text{zym}}\right]}{K_{\text{m MASP1}^*}^{\text{MASP1}_{\text{zym}}}}+\frac{\left[\text{pLFMASP2}_{\text{zym}}\right]}{K_{\text{m MASP1}^*}^{\text{MASP2}_{\text{zym}}}}+\frac{\left[\text{pLFMASP3}_{\text{zym}}\right]}{K_{\text{m MASP1}^*}^{\text{MASP3}_{\text{zym}}}}+\frac{\left[\text{pHFMASP1}_{\text{zym}}\right]}{K_{\text{m MASP1}^*}^{\text{MASP1}_{\text{zym}}}}+\frac{\left[\text{pHFMASP2}_{\text{zym}}\right]}{K_{\text{m MASP1}^*}^{\text{MASP2}_{\text{zym}}}}\\
+\frac{\left[\text{pHFMASP3}_{\text{zym}}\right]}{K_{\text{m MASP1}^*}^{\text{MASP3}_{\text{zym}}}}+\frac{\left[\text{pMFMASP1}_{\text{zym}}\right]}{K_{\text{m MASP1}^*}^{\text{MASP1}_{\text{zym}}}}+\frac{\left[\text{pMFMASP2}_{\text{zym}}\right]}{K_{\text{m MASP1}^*}^{\text{MASP2}_{\text{zym}}}}+\frac{\left[\text{pMFMASP3}_{\text{zym}}\right]}{K_{\text{m MASP1}^*}^{\text{MASP3}_{\text{zym}}}}\end{array}\right)}
$$

$$
-\frac{k_{\mathrm{cat\,MASP1^*}}^{\mathrm{C2}}\left[\mathrm{pLFMASP1^*}\right]\left[\mathrm{pC4bC4bC2}\right]}{K_{\mathrm{m\,MASP1^*}}^{\mathrm{C2}}\left(\begin{array}{l}
1+\frac{[\mathrm{C4}]}{K_{\mathrm{m\,MASP1^*}}^{\mathrm{C4}}}+\frac{[\mathrm{fC4bC2}]}{K_{\mathrm{m\,MASP1^*}}^{\mathrm{C2}}}+\frac{[\mathrm{fC4bC4bC2}]}{K_{\mathrm{m\,MASP1^*}}^{\mathrm{C2}}}+\frac{[\mathrm{fC3bC4bC2}]}{K_{\mathrm{m\,MASP1^*}}^{\mathrm{C2}}}+\frac{[\mathrm{pC4bC2}]}{K_{\mathrm{m\,MASP1^*}}^{\mathrm{C2}}}+\frac{[\mathrm{pC4bC4bC2}]}{K_{\mathrm{m\,MASP1^*}}^{\mathrm{C2}}}\\
+\frac{[\mathrm{pC3bC4bC2}]}{K_{\mathrm{m\,MASP1^*}}^{\mathrm{C2}}}+\frac{[\mathrm{pMBL_3MASP1_{zym}}]}{K_{\mathrm{m\,MASP1^*}}^{\mathrm{MASP1_{zym}}}}+\frac{[\mathrm{pMBL_3MASP2_{zym}}]}{K_{\mathrm{m\,MASP1^*}}^{\mathrm{MASP2_{zym}}}}+\frac{[\mathrm{pMBL_3MASP3_{zym}}]}{K_{\mathrm{m\,MASP1^*}}^{\mathrm{MASP3_{zym}}}}\\
+\frac{[\mathrm{pMBL_4MASP1_{zym}}]}{K_{\mathrm{m\,MASP1^*}}^{\mathrm{MASP1_{zym}}}}+\frac{[\mathrm{pMBL_4MASP2_{zym}}]}{K_{\mathrm{m\,MASP1^*}}^{\mathrm{MASP2_{zym}}}}+\frac{[\mathrm{pMBL_4MASP3_{zym}}]}{K_{\mathrm{m\,MASP1^*}}^{\mathrm{MASP3_{zym}}}}+\frac{[\mathrm{pCLLKMASP1_{zym}}]}{K_{\mathrm{m\,MASP1^*}}^{\mathrm{MASP1_{zym}}}}\\
+\frac{[\mathrm{pCLLKMASP2_{zym}}]}{K_{\mathrm{m\,MASP1^*}}^{\mathrm{MASP2_{zym}}}}+\frac{[\mathrm{pCLLKMASP3_{zym}}]}{K_{\mathrm{m\,MASP1^*}}^{\mathrm{MASP3_{zym}}}}+\frac{[\mathrm{pCLK1MASP1_{zym}}]}{K_{\mathrm{m\,MASP1^*}}^{\mathrm{MASP1_{zym}}}}+\frac{[\mathrm{pCLK1MASP2_{zym}}]}{K_{\mathrm{m\,MASP1^*}}^{\mathrm{MASP2_{zym}}}}\\
+\frac{[\mathrm{pCLK1MASP3_{zym}}]}{K_{\mathrm{m\,MASP1^*}}^{\mathrm{MASP3_{zym}}}}+\frac{[\mathrm{pCLL1MASP1_{zym}}]}{K_{\mathrm{m\,MASP1^*}}^{\mathrm{MASP1_{zym}}}}+\frac{[\mathrm{pCLL1MASP2_{zym}}]}{K_{\mathrm{m\,MASP1^*}}^{\mathrm{MASP2_{zym}}}}+\frac{[\mathrm{pCLL1MASP3_{zym}}]}{K_{\mathrm{m\,MASP1^*}}^{\mathrm{MASP3_{zym}}}}\\
+\frac{[\mathrm{pLFMASP1_{zym}}]}{K_{\mathrm{m\,MASP1^*}}^{\mathrm{MASP1_{zym}}}}+\frac{[\mathrm{pLFMASP2_{zym}}]}{K_{\mathrm{m\,MASP1^*}}^{\mathrm{MASP2_{zym}}}}+\frac{[\mathrm{pLFMASP3_{zym}}]}{K_{\mathrm{m\,MASP1^*}}^{\mathrm{MASP3_{zym}}}}+\frac{[\mathrm{pHFMASP1_{zym}}]}{K_{\mathrm{m\,MASP1^*}}^{\mathrm{MASP1_{zym}}}}+\frac{[\mathrm{pHFMASP2_{zym}}]}{K_{\mathrm{m\,MASP1^*}}^{\mathrm{MASP2_{zym}}}}\\
+\frac{[\mathrm{pHFMASP3_{zym}}]}{K_{\mathrm{m\,MASP1^*}}^{\mathrm{MASP3_{zym}}}}+\frac{[\mathrm{pMFMASP1_{zym}}]}{K_{\mathrm{m\,MASP1^*}}^{\mathrm{MASP1_{zym}}}}+\frac{[\mathrm{pMFMASP2_{zym}}]}{K_{\mathrm{m\,MASP1^*}}^{\mathrm{MASP2_{zym}}}}+\frac{[\mathrm{pMFMASP3_{zym}}]}{K_{\mathrm{m\,MASP1^*}}^{\mathrm{MASP3_{zym}}}}
\end{array}\right)}
$$

$$
-\frac{k_{\mathrm{cat\,MASP1^*}}^{\mathrm{C2}}\left[\mathrm{pHFMASP1^*}\right]\left[\mathrm{pC4bC4bC2}\right]}{K_{\mathrm{m\,MASP1^*}}^{\mathrm{C2}}\left(\begin{array}{l}
1+\frac{[\mathrm{C4}]}{K_{\mathrm{m\,MASP1^*}}^{\mathrm{C4}}}+\frac{[\mathrm{fC4bC2}]}{K_{\mathrm{m\,MASP1^*}}^{\mathrm{C2}}}+\frac{[\mathrm{fC4bC4bC2}]}{K_{\mathrm{m\,MASP1^*}}^{\mathrm{C2}}}+\frac{[\mathrm{fC3bC4bC2}]}{K_{\mathrm{m\,MASP1^*}}^{\mathrm{C2}}}+\frac{[\mathrm{pC4bC2}]}{K_{\mathrm{m\,MASP1^*}}^{\mathrm{C2}}}+\frac{[\mathrm{pC4bC4bC2}]}{K_{\mathrm{m\,MASP1^*}}^{\mathrm{C2}}}\\
+\frac{[\mathrm{pC3bC4bC2}]}{K_{\mathrm{m\,MASP1^*}}^{\mathrm{C2}}}+\frac{[\mathrm{pMBL_3MASP1_{zym}}]}{K_{\mathrm{m\,MASP1^*}}^{\mathrm{MASP1_{zym}}}}+\frac{[\mathrm{pMBL_3MASP2_{zym}}]}{K_{\mathrm{m\,MASP1^*}}^{\mathrm{MASP2_{zym}}}}+\frac{[\mathrm{pMBL_3MASP3_{zym}}]}{K_{\mathrm{m\,MASP1^*}}^{\mathrm{MASP3_{zym}}}}\\
+\frac{[\mathrm{pMBL_4MASP1_{zym}}]}{K_{\mathrm{m\,MASP1^*}}^{\mathrm{MASP1_{zym}}}}+\frac{[\mathrm{pMBL_4MASP2_{zym}}]}{K_{\mathrm{m\,MASP1^*}}^{\mathrm{MASP2_{zym}}}}+\frac{[\mathrm{pMBL_4MASP3_{zym}}]}{K_{\mathrm{m\,MASP1^*}}^{\mathrm{MASP3_{zym}}}}+\frac{[\mathrm{pCLLKMASP1_{zym}}]}{K_{\mathrm{m\,MASP1^*}}^{\mathrm{MASP1_{zym}}}}\\
+\frac{[\mathrm{pCLLKMASP2_{zym}}]}{K_{\mathrm{m\,MASP1^*}}^{\mathrm{MASP2_{zym}}}}+\frac{[\mathrm{pCLLKMASP3_{zym}}]}{K_{\mathrm{m\,MASP1^*}}^{\mathrm{MASP3_{zym}}}}+\frac{[\mathrm{pCLK1MASP1_{zym}}]}{K_{\mathrm{m\,MASP1^*}}^{\mathrm{MASP1_{zym}}}}+\frac{[\mathrm{pCLK1MASP2_{zym}}]}{K_{\mathrm{m\,MASP1^*}}^{\mathrm{MASP2_{zym}}}}\\
+\frac{[\mathrm{pCLK1MASP3_{zym}}]}{K_{\mathrm{m\,MASP1^*}}^{\mathrm{MASP3_{zym}}}}+\frac{[\mathrm{pCLL1MASP1_{zym}}]}{K_{\mathrm{m\,MASP1^*}}^{\mathrm{MASP1_{zym}}}}+\frac{[\mathrm{pCLL1MASP2_{zym}}]}{K_{\mathrm{m\,MASP1^*}}^{\mathrm{MASP2_{zym}}}}+\frac{[\mathrm{pCLL1MASP3_{zym}}]}{K_{\mathrm{m\,MASP1^*}}^{\mathrm{MASP3_{zym}}}}\\
+\frac{[\mathrm{pLFMASP1_{zym}}]}{K_{\mathrm{m\,MASP1^*}}^{\mathrm{MASP1_{zym}}}}+\frac{[\mathrm{pLFMASP2_{zym}}]}{K_{\mathrm{m\,MASP1^*}}^{\mathrm{MASP2_{zym}}}}+\frac{[\mathrm{pLFMASP3_{zym}}]}{K_{\mathrm{m\,MASP1^*}}^{\mathrm{MASP3_{zym}}}}+\frac{[\mathrm{pHFMASP1_{zym}}]}{K_{\mathrm{m\,MASP1^*}}^{\mathrm{MASP1_{zym}}}}+\frac{[\mathrm{pHFMASP2_{zym}}]}{K_{\mathrm{m\,MASP1^*}}^{\mathrm{MASP2_{zym}}}}\\
+\frac{[\mathrm{pHFMASP3_{zym}}]}{K_{\mathrm{m\,MASP1^*}}^{\mathrm{MASP3_{zym}}}}+\frac{[\mathrm{pMFMASP1_{zym}}]}{K_{\mathrm{m\,MASP1^*}}^{\mathrm{MASP1_{zym}}}}+\frac{[\mathrm{pMFMASP2_{zym}}]}{K_{\mathrm{m\,MASP1^*}}^{\mathrm{MASP2_{zym}}}}+\frac{[\mathrm{pMFMASP3_{zym}}]}{K_{\mathrm{m\,MASP1^*}}^{\mathrm{MASP3_{zym}}}}
\end{array}\right)}
$$

$$
-\frac{k_{\text{cat MASP1}^*}^{\text{C2}}\left[\text{pMFMASP1}^*\right]\left[\text{pC4bC4bC2}\right]}{K_{\text{m MASP1}^*}^{\text{C2}}\left(\begin{array}{l}1+\frac{[\text{C4}]}{K_{\text{m MASP1}^*}^{\text{C4}}}+\frac{[\text{fC4bC2}]}{K_{\text{m MASP1}^*}^{\text{C2}}}+\frac{[\text{fC4bC4bC2}]}{K_{\text{m MASP1}^*}^{\text{C2}}}+\frac{[\text{fC3bC4bC2}]}{K_{\text{m MASP1}^*}^{\text{C2}}}+\frac{[\text{pC4bC2}]}{K_{\text{m MASP1}^*}^{\text{C2}}}+\frac{[\text{pC4bC4bC2}]}{K_{\text{m MASP1}^*}^{\text{C2}}}\\
+\frac{[\text{pC3bC4bC2}]}{K_{\text{m MASP1}^*}^{\text{C2}}}+\frac{\left[\text{pMBL}_3\text{MASP1}_{\text{zym}}\right]}{K_{\text{m MASP1}^*}^{\text{MASP1}_{\text{zym}}}}+\frac{\left[\text{pMBL}_3\text{MASP2}_{\text{zym}}\right]}{K_{\text{m MASP1}^*}^{\text{MASP2}_{\text{zym}}}}+\frac{\left[\text{pMBL}_3\text{MASP3}_{\text{zym}}\right]}{K_{\text{m MASP1}^*}^{\text{MASP3}_{\text{zym}}}}\\
+\frac{\left[\text{pMBL}_4\text{MASP1}_{\text{zym}}\right]}{K_{\text{m MASP1}^*}^{\text{MASP1}_{\text{zym}}}}+\frac{\left[\text{pMBL}_4\text{MASP2}_{\text{zym}}\right]}{K_{\text{m MASP1}^*}^{\text{MASP2}_{\text{zym}}}}+\frac{\left[\text{pMBL}_4\text{MASP3}_{\text{zym}}\right]}{K_{\text{m MASP1}^*}^{\text{MASP3}_{\text{zym}}}}+\frac{\left[\text{pCLLKMASP1}_{\text{zym}}\right]}{K_{\text{m MASP1}^*}^{\text{MASP1}_{\text{zym}}}}\\
+\frac{\left[\text{pCLLKMASP2}_{\text{zym}}\right]}{K_{\text{m MASP1}^*}^{\text{MASP2}_{\text{zym}}}}+\frac{\left[\text{pCLLKMASP3}_{\text{zym}}\right]}{K_{\text{m MASP1}^*}^{\text{MASP3}_{\text{zym}}}}+\frac{\left[\text{pCLK1MASP1}_{\text{zym}}\right]}{K_{\text{m MASP1}^*}^{\text{MASP1}_{\text{zym}}}}+\frac{\left[\text{pCLK1MASP2}_{\text{zym}}\right]}{K_{\text{m MASP1}^*}^{\text{MASP2}_{\text{zym}}}}\\
+\frac{\left[\text{pCLK1MASP3}_{\text{zym}}\right]}{K_{\text{m MASP1}^*}^{\text{MASP3}_{\text{zym}}}}+\frac{\left[\text{pCLL1MASP1}_{\text{zym}}\right]}{K_{\text{m MASP1}^*}^{\text{MASP1}_{\text{zym}}}}+\frac{\left[\text{pCLL1MASP2}_{\text{zym}}\right]}{K_{\text{m MASP1}^*}^{\text{MASP2}_{\text{zym}}}}+\frac{\left[\text{pCLL1MASP3}_{\text{zym}}\right]}{K_{\text{m MASP1}^*}^{\text{MASP3}_{\text{zym}}}}\\
+\frac{\left[\text{pLFMASP1}_{\text{zym}}\right]}{K_{\text{m MASP1}^*}^{\text{MASP1}_{\text{zym}}}}+\frac{\left[\text{pLFMASP2}_{\text{zym}}\right]}{K_{\text{m MASP1}^*}^{\text{MASP2}_{\text{zym}}}}+\frac{\left[\text{pLFMASP3}_{\text{zym}}\right]}{K_{\text{m MASP1}^*}^{\text{MASP3}_{\text{zym}}}}+\frac{\left[\text{pHFMASP1}_{\text{zym}}\right]}{K_{\text{m MASP1}^*}^{\text{MASP1}_{\text{zym}}}}+\frac{\left[\text{pHFMASP2}_{\text{zym}}\right]}{K_{\text{m MASP1}^*}^{\text{MASP2}_{\text{zym}}}}\\
+\frac{\left[\text{pHFMASP3}_{\text{zym}}\right]}{K_{\text{m MASP1}^*}^{\text{MASP3}_{\text{zym}}}}+\frac{\left[\text{pMFMASP1}_{\text{zym}}\right]}{K_{\text{m MASP1}^*}^{\text{MASP1}_{\text{zym}}}}+\frac{\left[\text{pMFMASP2}_{\text{zym}}\right]}{K_{\text{m MASP1}^*}^{\text{MASP2}_{\text{zym}}}}+\frac{\left[\text{pMFMASP3}_{\text{zym}}\right]}{K_{\text{m MASP1}^*}^{\text{MASP3}_{\text{zym}}}}\end{array}\right)}
$$

$$
-\frac{k_{\text{cat MASP2}^*}^{\text{C2}}\left[\text{pMBL}_3\text{MASP2}^*\right]\left[\text{pC4bC4bC2}\right]}{K_{\text{m MASP2}^*}^{\text{C2}}\left(\begin{array}{l}1+\frac{[\text{C4}]}{K_{\text{m MASP2}^*}^{\text{C4}}}+\frac{[\text{fC4bC2}]}{K_{\text{m MASP2}^*}^{\text{C2}}}+\frac{[\text{fC4bC4bC2}]}{K_{\text{m MASP2}^*}^{\text{C2}}}+\frac{[\text{fC3bC4bC2}]}{K_{\text{m MASP2}^*}^{\text{C2}}}+\frac{[\text{pC4bC2}]}{K_{\text{m MASP2}^*}^{\text{C2}}}+\frac{[\text{pC4bC4bC2}]}{K_{\text{m MASP2}^*}^{\text{C2}}}\\
+\frac{[\text{pC3bC4bC2}]}{K_{\text{m MASP2}^*}^{\text{C2}}}+\frac{\left[\text{pMBL}_3\text{MASP1}_{\text{zym}}\right]}{K_{\text{m MASP2}^*}^{\text{MASP1}_{\text{zym}}}}+\frac{\left[\text{pMBL}_3\text{MASP2}_{\text{zym}}\right]}{K_{\text{m MASP2}^*}^{\text{MASP2}_{\text{zym}}}}+\frac{\left[\text{pMBL}_4\text{MASP1}_{\text{zym}}\right]}{K_{\text{m MASP2}^*}^{\text{MASP1}_{\text{zym}}}}\\
+\frac{\left[\text{pMBL}_4\text{MASP2}_{\text{zym}}\right]}{K_{\text{m MASP2}^*}^{\text{MASP2}_{\text{zym}}}}+\frac{\left[\text{pCLLKMASP1}_{\text{zym}}\right]}{K_{\text{m MASP2}^*}^{\text{MASP1}_{\text{zym}}}}+\frac{\left[\text{pCLLKMASP2}_{\text{zym}}\right]}{K_{\text{m MASP2}^*}^{\text{MASP2}_{\text{zym}}}}+\frac{\left[\text{pCLK1MASP1}_{\text{zym}}\right]}{K_{\text{m MASP2}^*}^{\text{MASP1}_{\text{zym}}}}\\
+\frac{\left[\text{pCLK1MASP2}_{\text{zym}}\right]}{K_{\text{m MASP2}^*}^{\text{MASP2}_{\text{zym}}}}+\frac{\left[\text{pCLL1MASP1}_{\text{zym}}\right]}{K_{\text{m MASP2}^*}^{\text{MASP1}_{\text{zym}}}}+\frac{\left[\text{pCLL1MASP2}_{\text{zym}}\right]}{K_{\text{m MASP2}^*}^{\text{MASP2}_{\text{zym}}}}+\frac{\left[\text{pLFMASP1}_{\text{zym}}\right]}{K_{\text{m MASP2}^*}^{\text{MASP1}_{\text{zym}}}}\\
+\frac{\left[\text{pLFMASP2}_{\text{zym}}\right]}{K_{\text{m MASP2}^*}^{\text{MASP2}_{\text{zym}}}}+\frac{\left[\text{pHFMASP1}_{\text{zym}}\right]}{K_{\text{m MASP2}^*}^{\text{MASP1}_{\text{zym}}}}+\frac{\left[\text{pHFMASP2}_{\text{zym}}\right]}{K_{\text{m MASP2}^*}^{\text{MASP2}_{\text{zym}}}}+\frac{\left[\text{pMFMASP1}_{\text{zym}}\right]}{K_{\text{m MASP2}^*}^{\text{MASP1}_{\text{zym}}}}+\frac{\left[\text{pMFMASP2}_{\text{zym}}\right]}{K_{\text{m MASP2}^*}^{\text{MASP2}_{\text{zym}}}}\end{array}\right)}
$$

$$-\frac{k_{\mathrm{cat\,MASP2^*}}^{\mathrm{C2}}\left[\mathrm{pMBL_4MASP2^*}\right]\left[\mathrm{pC4bC4bC2}\right]}{K_{\mathrm{m\,MASP2^*}}^{\mathrm{C2}}\left(\begin{array}{l}1+\frac{[\mathrm{C4}]}{K_{\mathrm{m\,MASP2^*}}^{\mathrm{C4}}}+\frac{[\mathrm{fC4bC2}]}{K_{\mathrm{m\,MASP2^*}}^{\mathrm{C2}}}+\frac{[\mathrm{fC4bC4bC2}]}{K_{\mathrm{m\,MASP2^*}}^{\mathrm{C2}}}+\frac{[\mathrm{fC3bC4bC2}]}{K_{\mathrm{m\,MASP2^*}}^{\mathrm{C2}}}+\frac{[\mathrm{pC4bC2}]}{K_{\mathrm{m\,MASP2^*}}^{\mathrm{C2}}}+\frac{[\mathrm{pC4bC4bC2}]}{K_{\mathrm{m\,MASP2^*}}^{\mathrm{C2}}}\\+\frac{[\mathrm{pC3bC4bC2}]}{K_{\mathrm{m\,MASP2^*}}^{\mathrm{C2}}}+\frac{\left[\mathrm{pMBL_3MASP1_{zym}}\right]}{K_{\mathrm{m\,MASP2^*}}^{\mathrm{MASP1_{zym}}}}+\frac{\left[\mathrm{pMBL_3MASP2_{zym}}\right]}{K_{\mathrm{m\,MASP2^*}}^{\mathrm{MASP2_{zym}}}}+\frac{\left[\mathrm{pMBL_4MASP1_{zym}}\right]}{K_{\mathrm{m\,MASP2^*}}^{\mathrm{MASP1_{zym}}}}\\+\frac{\left[\mathrm{pMBL_4MASP2_{zym}}\right]}{K_{\mathrm{m\,MASP2^*}}^{\mathrm{MASP2_{zym}}}}+\frac{\left[\mathrm{pCLLKMASP1_{zym}}\right]}{K_{\mathrm{m\,MASP2^*}}^{\mathrm{MASP1_{zym}}}}+\frac{\left[\mathrm{pCLLKMASP2_{zym}}\right]}{K_{\mathrm{m\,MASP2^*}}^{\mathrm{MASP2_{zym}}}}+\frac{\left[\mathrm{pCLK1MASP1_{zym}}\right]}{K_{\mathrm{m\,MASP2^*}}^{\mathrm{MASP1_{zym}}}}\\+\frac{\left[\mathrm{pCLK1MASP2_{zym}}\right]}{K_{\mathrm{m\,MASP2^*}}^{\mathrm{MASP2_{zym}}}}+\frac{\left[\mathrm{pCLL1MASP1_{zym}}\right]}{K_{\mathrm{m\,MASP2^*}}^{\mathrm{MASP1_{zym}}}}+\frac{\left[\mathrm{pCLL1MASP2_{zym}}\right]}{K_{\mathrm{m\,MASP2^*}}^{\mathrm{MASP2_{zym}}}}+\frac{\left[\mathrm{pLFMASP1_{zym}}\right]}{K_{\mathrm{m\,MASP2^*}}^{\mathrm{MASP1_{zym}}}}\\+\frac{\left[\mathrm{pLFMASP2_{zym}}\right]}{K_{\mathrm{m\,MASP2^*}}^{\mathrm{MASP2_{zym}}}}+\frac{\left[\mathrm{pHFMASP1_{zym}}\right]}{K_{\mathrm{m\,MASP2^*}}^{\mathrm{MASP1_{zym}}}}+\frac{\left[\mathrm{pHFMASP2_{zym}}\right]}{K_{\mathrm{m\,MASP2^*}}^{\mathrm{MASP2_{zym}}}}+\frac{\left[\mathrm{pMFMASP1_{zym}}\right]}{K_{\mathrm{m\,MASP2^*}}^{\mathrm{MASP1_{zym}}}}+\frac{\left[\mathrm{pMFMASP2_{zym}}\right]}{K_{\mathrm{m\,MASP2^*}}^{\mathrm{MASP2_{zym}}}}\end{array}\right)}$$

$$-\frac{k_{\mathrm{cat\,MASP2^*}}^{\mathrm{C2}}\left[\mathrm{pCLLKMASP2^*}\right]\left[\mathrm{pC4bC4bC2}\right]}{K_{\mathrm{m\,MASP2^*}}^{\mathrm{C2}}\left(\begin{array}{l}1+\frac{[\mathrm{C4}]}{K_{\mathrm{m\,MASP2^*}}^{\mathrm{C4}}}+\frac{[\mathrm{fC4bC2}]}{K_{\mathrm{m\,MASP2^*}}^{\mathrm{C2}}}+\frac{[\mathrm{fC4bC4bC2}]}{K_{\mathrm{m\,MASP2^*}}^{\mathrm{C2}}}+\frac{[\mathrm{fC3bC4bC2}]}{K_{\mathrm{m\,MASP2^*}}^{\mathrm{C2}}}+\frac{[\mathrm{pC4bC2}]}{K_{\mathrm{m\,MASP2^*}}^{\mathrm{C2}}}+\frac{[\mathrm{pC4bC4bC2}]}{K_{\mathrm{m\,MASP2^*}}^{\mathrm{C2}}}\\+\frac{[\mathrm{pC3bC4bC2}]}{K_{\mathrm{m\,MASP2^*}}^{\mathrm{C2}}}+\frac{\left[\mathrm{pMBL_3MASP1_{zym}}\right]}{K_{\mathrm{m\,MASP2^*}}^{\mathrm{MASP1_{zym}}}}+\frac{\left[\mathrm{pMBL_3MASP2_{zym}}\right]}{K_{\mathrm{m\,MASP2^*}}^{\mathrm{MASP2_{zym}}}}+\frac{\left[\mathrm{pMBL_4MASP1_{zym}}\right]}{K_{\mathrm{m\,MASP2^*}}^{\mathrm{MASP1_{zym}}}}\\+\frac{\left[\mathrm{pMBL_4MASP2_{zym}}\right]}{K_{\mathrm{m\,MASP2^*}}^{\mathrm{MASP2_{zym}}}}+\frac{\left[\mathrm{pCLLKMASP1_{zym}}\right]}{K_{\mathrm{m\,MASP2^*}}^{\mathrm{MASP1_{zym}}}}+\frac{\left[\mathrm{pCLLKMASP2_{zym}}\right]}{K_{\mathrm{m\,MASP2^*}}^{\mathrm{MASP2_{zym}}}}+\frac{\left[\mathrm{pCLK1MASP1_{zym}}\right]}{K_{\mathrm{m\,MASP2^*}}^{\mathrm{MASP1_{zym}}}}\\+\frac{\left[\mathrm{pCLK1MASP2_{zym}}\right]}{K_{\mathrm{m\,MASP2^*}}^{\mathrm{MASP2_{zym}}}}+\frac{\left[\mathrm{pCLL1MASP1_{zym}}\right]}{K_{\mathrm{m\,MASP2^*}}^{\mathrm{MASP1_{zym}}}}+\frac{\left[\mathrm{pCLL1MASP2_{zym}}\right]}{K_{\mathrm{m\,MASP2^*}}^{\mathrm{MASP2_{zym}}}}+\frac{\left[\mathrm{pLFMASP1_{zym}}\right]}{K_{\mathrm{m\,MASP2^*}}^{\mathrm{MASP1_{zym}}}}\\+\frac{\left[\mathrm{pLFMASP2_{zym}}\right]}{K_{\mathrm{m\,MASP2^*}}^{\mathrm{MASP2_{zym}}}}+\frac{\left[\mathrm{pHFMASP1_{zym}}\right]}{K_{\mathrm{m\,MASP2^*}}^{\mathrm{MASP1_{zym}}}}+\frac{\left[\mathrm{pHFMASP2_{zym}}\right]}{K_{\mathrm{m\,MASP2^*}}^{\mathrm{MASP2_{zym}}}}+\frac{\left[\mathrm{pMFMASP1_{zym}}\right]}{K_{\mathrm{m\,MASP2^*}}^{\mathrm{MASP1_{zym}}}}+\frac{\left[\mathrm{pMFMASP2_{zym}}\right]}{K_{\mathrm{m\,MASP2^*}}^{\mathrm{MASP2_{zym}}}}\end{array}\right)}$$

$$-\frac{k_{\mathrm{cat\,MASP2^*}}^{\mathrm{C2}}\left[\mathrm{pCLK1MASP2^*}\right]\left[\mathrm{pC4bC4bC2}\right]}{K_{\mathrm{m\,MASP2^*}}^{\mathrm{C2}}\left(\begin{array}{l}1+\frac{[\mathrm{C4}]}{K_{\mathrm{m\,MASP2^*}}^{\mathrm{C4}}}+\frac{[\mathrm{fC4bC2}]}{K_{\mathrm{m\,MASP2^*}}^{\mathrm{C2}}}+\frac{[\mathrm{fC4bC4bC2}]}{K_{\mathrm{m\,MASP2^*}}^{\mathrm{C2}}}+\frac{[\mathrm{fC3bC4bC2}]}{K_{\mathrm{m\,MASP2^*}}^{\mathrm{C2}}}+\frac{[\mathrm{pC4bC2}]}{K_{\mathrm{m\,MASP2^*}}^{\mathrm{C2}}}+\frac{[\mathrm{pC4bC4bC2}]}{K_{\mathrm{m\,MASP2^*}}^{\mathrm{C2}}}\\+\frac{[\mathrm{pC3bC4bC2}]}{K_{\mathrm{m\,MASP2^*}}^{\mathrm{C2}}}+\frac{\left[\mathrm{pMBL_3MASP1_{zym}}\right]}{K_{\mathrm{m\,MASP2^*}}^{\mathrm{MASP1_{zym}}}}+\frac{\left[\mathrm{pMBL_3MASP2_{zym}}\right]}{K_{\mathrm{m\,MASP2^*}}^{\mathrm{MASP2_{zym}}}}+\frac{\left[\mathrm{pMBL_4MASP1_{zym}}\right]}{K_{\mathrm{m\,MASP2^*}}^{\mathrm{MASP1_{zym}}}}\\+\frac{\left[\mathrm{pMBL_4MASP2_{zym}}\right]}{K_{\mathrm{m\,MASP2^*}}^{\mathrm{MASP2_{zym}}}}+\frac{\left[\mathrm{pCLLKMASP1_{zym}}\right]}{K_{\mathrm{m\,MASP2^*}}^{\mathrm{MASP1_{zym}}}}+\frac{\left[\mathrm{pCLLKMASP2_{zym}}\right]}{K_{\mathrm{m\,MASP2^*}}^{\mathrm{MASP2_{zym}}}}+\frac{\left[\mathrm{pCLK1MASP1_{zym}}\right]}{K_{\mathrm{m\,MASP2^*}}^{\mathrm{MASP1_{zym}}}}\\+\frac{\left[\mathrm{pCLK1MASP2_{zym}}\right]}{K_{\mathrm{m\,MASP2^*}}^{\mathrm{MASP2_{zym}}}}+\frac{\left[\mathrm{pCLL1MASP1_{zym}}\right]}{K_{\mathrm{m\,MASP2^*}}^{\mathrm{MASP1_{zym}}}}+\frac{\left[\mathrm{pCLL1MASP2_{zym}}\right]}{K_{\mathrm{m\,MASP2^*}}^{\mathrm{MASP2_{zym}}}}+\frac{\left[\mathrm{pLFMASP1_{zym}}\right]}{K_{\mathrm{m\,MASP2^*}}^{\mathrm{MASP1_{zym}}}}\\+\frac{\left[\mathrm{pLFMASP2_{zym}}\right]}{K_{\mathrm{m\,MASP2^*}}^{\mathrm{MASP2_{zym}}}}+\frac{\left[\mathrm{pHFMASP1_{zym}}\right]}{K_{\mathrm{m\,MASP2^*}}^{\mathrm{MASP1_{zym}}}}+\frac{\left[\mathrm{pHFMASP2_{zym}}\right]}{K_{\mathrm{m\,MASP2^*}}^{\mathrm{MASP2_{zym}}}}+\frac{\left[\mathrm{pMFMASP1_{zym}}\right]}{K_{\mathrm{m\,MASP2^*}}^{\mathrm{MASP1_{zym}}}}+\frac{\left[\mathrm{pMFMASP2_{zym}}\right]}{K_{\mathrm{m\,MASP2^*}}^{\mathrm{MASP2_{zym}}}}\end{array}\right)}$$

$$
-\frac{k_{\text{cat MASP2}^*}^{\text{C2}}\left[\text{pCLL1MASP2}^*\right]\left[\text{pC4bC4bC2}\right]}{K_{\text{m MASP2}^*}^{\text{C2}}\left(\begin{array}{l}
1+\frac{[\text{C4}]}{K_{\text{m MASP2}^*}^{\text{C4}}}+\frac{[\text{fC4bC2}]}{K_{\text{m MASP2}^*}^{\text{C2}}}+\frac{[\text{fC4bC4bC2}]}{K_{\text{m MASP2}^*}^{\text{C2}}}+\frac{[\text{fC3bC4bC2}]}{K_{\text{m MASP2}^*}^{\text{C2}}}+\frac{[\text{pC4bC2}]}{K_{\text{m MASP2}^*}^{\text{C2}}}+\frac{[\text{pC4bC4bC2}]}{K_{\text{m MASP2}^*}^{\text{C2}}}\\
+\frac{[\text{pC3bC4bC2}]}{K_{\text{m MASP2}^*}^{\text{C2}}}+\frac{\left[\text{pMBL}_3\text{MASP1}_{\text{zym}}\right]}{K_{\text{m MASP2}^*}^{\text{MASP1}_{\text{zym}}}}+\frac{\left[\text{pMBL}_3\text{MASP2}_{\text{zym}}\right]}{K_{\text{m MASP2}^*}^{\text{MASP2}_{\text{zym}}}}+\frac{\left[\text{pMBL}_4\text{MASP1}_{\text{zym}}\right]}{K_{\text{m MASP2}^*}^{\text{MASP1}_{\text{zym}}}}\\
+\frac{\left[\text{pMBL}_4\text{MASP2}_{\text{zym}}\right]}{K_{\text{m MASP2}^*}^{\text{MASP2}_{\text{zym}}}}+\frac{\left[\text{pCLLKMASP1}_{\text{zym}}\right]}{K_{\text{m MASP2}^*}^{\text{MASP1}_{\text{zym}}}}+\frac{\left[\text{pCLLKMASP2}_{\text{zym}}\right]}{K_{\text{m MASP2}^*}^{\text{MASP2}_{\text{zym}}}}+\frac{\left[\text{pCLK1MASP1}_{\text{zym}}\right]}{K_{\text{m MASP2}^*}^{\text{MASP1}_{\text{zym}}}}\\
+\frac{\left[\text{pCLK1MASP2}_{\text{zym}}\right]}{K_{\text{m MASP2}^*}^{\text{MASP2}_{\text{zym}}}}+\frac{\left[\text{pCLL1MASP1}_{\text{zym}}\right]}{K_{\text{m MASP2}^*}^{\text{MASP1}_{\text{zym}}}}+\frac{\left[\text{pCLL1MASP2}_{\text{zym}}\right]}{K_{\text{m MASP2}^*}^{\text{MASP2}_{\text{zym}}}}+\frac{\left[\text{pLFMASP1}_{\text{zym}}\right]}{K_{\text{m MASP2}^*}^{\text{MASP1}_{\text{zym}}}}\\
+\frac{\left[\text{pLFMASP2}_{\text{zym}}\right]}{K_{\text{m MASP2}^*}^{\text{MASP2}_{\text{zym}}}}+\frac{\left[\text{pHFMASP1}_{\text{zym}}\right]}{K_{\text{m MASP2}^*}^{\text{MASP1}_{\text{zym}}}}+\frac{\left[\text{pHFMASP2}_{\text{zym}}\right]}{K_{\text{m MASP2}^*}^{\text{MASP2}_{\text{zym}}}}+\frac{\left[\text{pMFMASP1}_{\text{zym}}\right]}{K_{\text{m MASP2}^*}^{\text{MASP1}_{\text{zym}}}}+\frac{\left[\text{pMFMASP2}_{\text{zym}}\right]}{K_{\text{m MASP2}^*}^{\text{MASP2}_{\text{zym}}}}
\end{array}\right)}
$$

$$
-\frac{k_{\text{cat MASP2}^*}^{\text{C2}}\left[\text{pLFMASP2}^*\right]\left[\text{pC4bC4bC2}\right]}{K_{\text{m MASP2}^*}^{\text{C2}}\left(\begin{array}{l}
1+\frac{[\text{C4}]}{K_{\text{m MASP2}^*}^{\text{C4}}}+\frac{[\text{fC4bC2}]}{K_{\text{m MASP2}^*}^{\text{C2}}}+\frac{[\text{fC4bC4bC2}]}{K_{\text{m MASP2}^*}^{\text{C2}}}+\frac{[\text{fC3bC4bC2}]}{K_{\text{m MASP2}^*}^{\text{C2}}}+\frac{[\text{pC4bC2}]}{K_{\text{m MASP2}^*}^{\text{C2}}}+\frac{[\text{pC4bC4bC2}]}{K_{\text{m MASP2}^*}^{\text{C2}}}\\
+\frac{[\text{pC3bC4bC2}]}{K_{\text{m MASP2}^*}^{\text{C2}}}+\frac{\left[\text{pMBL}_3\text{MASP1}_{\text{zym}}\right]}{K_{\text{m MASP2}^*}^{\text{MASP1}_{\text{zym}}}}+\frac{\left[\text{pMBL}_3\text{MASP2}_{\text{zym}}\right]}{K_{\text{m MASP2}^*}^{\text{MASP2}_{\text{zym}}}}+\frac{\left[\text{pMBL}_4\text{MASP1}_{\text{zym}}\right]}{K_{\text{m MASP2}^*}^{\text{MASP1}_{\text{zym}}}}\\
+\frac{\left[\text{pMBL}_4\text{MASP2}_{\text{zym}}\right]}{K_{\text{m MASP2}^*}^{\text{MASP2}_{\text{zym}}}}+\frac{\left[\text{pCLLKMASP1}_{\text{zym}}\right]}{K_{\text{m MASP2}^*}^{\text{MASP1}_{\text{zym}}}}+\frac{\left[\text{pCLLKMASP2}_{\text{zym}}\right]}{K_{\text{m MASP2}^*}^{\text{MASP2}_{\text{zym}}}}+\frac{\left[\text{pCLK1MASP1}_{\text{zym}}\right]}{K_{\text{m MASP2}^*}^{\text{MASP1}_{\text{zym}}}}\\
+\frac{\left[\text{pCLK1MASP2}_{\text{zym}}\right]}{K_{\text{m MASP2}^*}^{\text{MASP2}_{\text{zym}}}}+\frac{\left[\text{pCLL1MASP1}_{\text{zym}}\right]}{K_{\text{m MASP2}^*}^{\text{MASP1}_{\text{zym}}}}+\frac{\left[\text{pCLL1MASP2}_{\text{zym}}\right]}{K_{\text{m MASP2}^*}^{\text{MASP2}_{\text{zym}}}}+\frac{\left[\text{pLFMASP1}_{\text{zym}}\right]}{K_{\text{m MASP2}^*}^{\text{MASP1}_{\text{zym}}}}\\
+\frac{\left[\text{pLFMASP2}_{\text{zym}}\right]}{K_{\text{m MASP2}^*}^{\text{MASP2}_{\text{zym}}}}+\frac{\left[\text{pHFMASP1}_{\text{zym}}\right]}{K_{\text{m MASP2}^*}^{\text{MASP1}_{\text{zym}}}}+\frac{\left[\text{pHFMASP2}_{\text{zym}}\right]}{K_{\text{m MASP2}^*}^{\text{MASP2}_{\text{zym}}}}+\frac{\left[\text{pMFMASP1}_{\text{zym}}\right]}{K_{\text{m MASP2}^*}^{\text{MASP1}_{\text{zym}}}}+\frac{\left[\text{pMFMASP2}_{\text{zym}}\right]}{K_{\text{m MASP2}^*}^{\text{MASP2}_{\text{zym}}}}
\end{array}\right)}
$$

$$
-\frac{k_{\text{cat MASP2}^*}^{\text{C2}}\left[\text{pHFMASP2}^*\right]\left[\text{pC4bC4bC2}\right]}{K_{\text{m MASP2}^*}^{\text{C2}}\left(\begin{array}{l}
1+\frac{[\text{C4}]}{K_{\text{m MASP2}^*}^{\text{C4}}}+\frac{[\text{fC4bC2}]}{K_{\text{m MASP2}^*}^{\text{C2}}}+\frac{[\text{fC4bC4bC2}]}{K_{\text{m MASP2}^*}^{\text{C2}}}+\frac{[\text{fC3bC4bC2}]}{K_{\text{m MASP2}^*}^{\text{C2}}}+\frac{[\text{pC4bC2}]}{K_{\text{m MASP2}^*}^{\text{C2}}}+\frac{[\text{pC4bC4bC2}]}{K_{\text{m MASP2}^*}^{\text{C2}}}\\
+\frac{[\text{pC3bC4bC2}]}{K_{\text{m MASP2}^*}^{\text{C2}}}+\frac{\left[\text{pMBL}_3\text{MASP1}_{\text{zym}}\right]}{K_{\text{m MASP2}^*}^{\text{MASP1}_{\text{zym}}}}+\frac{\left[\text{pMBL}_3\text{MASP2}_{\text{zym}}\right]}{K_{\text{m MASP2}^*}^{\text{MASP2}_{\text{zym}}}}+\frac{\left[\text{pMBL}_4\text{MASP1}_{\text{zym}}\right]}{K_{\text{m MASP2}^*}^{\text{MASP1}_{\text{zym}}}}\\
+\frac{\left[\text{pMBL}_4\text{MASP2}_{\text{zym}}\right]}{K_{\text{m MASP2}^*}^{\text{MASP2}_{\text{zym}}}}+\frac{\left[\text{pCLLKMASP1}_{\text{zym}}\right]}{K_{\text{m MASP2}^*}^{\text{MASP1}_{\text{zym}}}}+\frac{\left[\text{pCLLKMASP2}_{\text{zym}}\right]}{K_{\text{m MASP2}^*}^{\text{MASP2}_{\text{zym}}}}+\frac{\left[\text{pCLK1MASP1}_{\text{zym}}\right]}{K_{\text{m MASP2}^*}^{\text{MASP1}_{\text{zym}}}}\\
+\frac{\left[\text{pCLK1MASP2}_{\text{zym}}\right]}{K_{\text{m MASP2}^*}^{\text{MASP2}_{\text{zym}}}}+\frac{\left[\text{pCLL1MASP1}_{\text{zym}}\right]}{K_{\text{m MASP2}^*}^{\text{MASP1}_{\text{zym}}}}+\frac{\left[\text{pCLL1MASP2}_{\text{zym}}\right]}{K_{\text{m MASP2}^*}^{\text{MASP2}_{\text{zym}}}}+\frac{\left[\text{pLFMASP1}_{\text{zym}}\right]}{K_{\text{m MASP2}^*}^{\text{MASP1}_{\text{zym}}}}\\
+\frac{\left[\text{pLFMASP2}_{\text{zym}}\right]}{K_{\text{m MASP2}^*}^{\text{MASP2}_{\text{zym}}}}+\frac{\left[\text{pHFMASP1}_{\text{zym}}\right]}{K_{\text{m MASP2}^*}^{\text{MASP1}_{\text{zym}}}}+\frac{\left[\text{pHFMASP2}_{\text{zym}}\right]}{K_{\text{m MASP2}^*}^{\text{MASP2}_{\text{zym}}}}+\frac{\left[\text{pMFMASP1}_{\text{zym}}\right]}{K_{\text{m MASP2}^*}^{\text{MASP1}_{\text{zym}}}}+\frac{\left[\text{pMFMASP2}_{\text{zym}}\right]}{K_{\text{m MASP2}^*}^{\text{MASP2}_{\text{zym}}}}
\end{array}\right)}
$$

$$-\frac{k_{\text{cat MASP2}^*}^{\text{C2}}\left[\text{pMFMASP2}^*\right]\left[\text{pC4bC4bC2}\right]}{K_{\text{m MASP2}^*}^{\text{C2}}\left(\begin{array}{l}1+\frac{[\text{C4}]}{K_{\text{m MASP2}^*}^{\text{C4}}}+\frac{[\text{fC4bC2}]}{K_{\text{m MASP2}^*}^{\text{C2}}}+\frac{[\text{fC4bC4bC2}]}{K_{\text{m MASP2}^*}^{\text{C2}}}+\frac{[\text{fC3bC4bC2}]}{K_{\text{m MASP2}^*}^{\text{C2}}}+\frac{[\text{pC4bC2}]}{K_{\text{m MASP2}^*}^{\text{C2}}}+\frac{[\text{pC4bC4bC2}]}{K_{\text{m MASP2}^*}^{\text{C2}}}\\+\frac{[\text{pC3bC4bC2}]}{K_{\text{m MASP2}^*}^{\text{C2}}}+\frac{\left[\text{pMBL}_3\text{MASP1}_{\text{zym}}\right]}{K_{\text{m MASP2}^*}^{\text{MASP1}_{\text{zym}}}}+\frac{\left[\text{pMBL}_3\text{MASP2}_{\text{zym}}\right]}{K_{\text{m MASP2}^*}^{\text{MASP2}_{\text{zym}}}}+\frac{\left[\text{pMBL}_4\text{MASP1}_{\text{zym}}\right]}{K_{\text{m MASP2}^*}^{\text{MASP1}_{\text{zym}}}}\\+\frac{\left[\text{pMBL}_4\text{MASP2}_{\text{zym}}\right]}{K_{\text{m MASP2}^*}^{\text{MASP2}_{\text{zym}}}}+\frac{\left[\text{pCLLKMASP1}_{\text{zym}}\right]}{K_{\text{m MASP2}^*}^{\text{MASP1}_{\text{zym}}}}+\frac{\left[\text{pCLLKMASP2}_{\text{zym}}\right]}{K_{\text{m MASP2}^*}^{\text{MASP2}_{\text{zym}}}}+\frac{\left[\text{pCLK1MASP1}_{\text{zym}}\right]}{K_{\text{m MASP2}^*}^{\text{MASP1}_{\text{zym}}}}\\+\frac{\left[\text{pCLK1MASP2}_{\text{zym}}\right]}{K_{\text{m MASP2}^*}^{\text{MASP2}_{\text{zym}}}}+\frac{\left[\text{pCLL1MASP1}_{\text{zym}}\right]}{K_{\text{m MASP2}^*}^{\text{MASP1}_{\text{zym}}}}+\frac{\left[\text{pCLL1MASP2}_{\text{zym}}\right]}{K_{\text{m MASP2}^*}^{\text{MASP2}_{\text{zym}}}}+\frac{\left[\text{pLFMASP1}_{\text{zym}}\right]}{K_{\text{m MASP2}^*}^{\text{MASP1}_{\text{zym}}}}\\+\frac{\left[\text{pLFMASP2}_{\text{zym}}\right]}{K_{\text{m MASP2}^*}^{\text{MASP2}_{\text{zym}}}}+\frac{\left[\text{pHFMASP1}_{\text{zym}}\right]}{K_{\text{m MASP2}^*}^{\text{MASP1}_{\text{zym}}}}+\frac{\left[\text{pHFMASP2}_{\text{zym}}\right]}{K_{\text{m MASP2}^*}^{\text{MASP2}_{\text{zym}}}}+\frac{\left[\text{pMFMASP1}_{\text{zym}}\right]}{K_{\text{m MASP2}^*}^{\text{MASP1}_{\text{zym}}}}+\frac{\left[\text{pMFMASP2}_{\text{zym}}\right]}{K_{\text{m MASP2}^*}^{\text{MASP2}_{\text{zym}}}}\end{array}\right)}$$

$$-\frac{k_{\text{cat C1}^*}^{\text{C2}}\left[\text{pMBL}_3\text{PTX3C1}^*\right]\left[\text{pC4bC4bC2}\right]}{K_{\text{m C1}^*}^{\text{C2}}\left(1+\frac{[\text{C4}]}{K_{\text{m C1}^*}^{\text{C4}}}+\frac{[\text{fC4bC2}]}{K_{\text{m C1}^*}^{\text{C2}}}+\frac{[\text{fC4bC4bC2}]}{K_{\text{m C1}^*}^{\text{C2}}}+\frac{[\text{fC3bC4bC2}]}{K_{\text{m C1}^*}^{\text{C2}}}+\frac{[\text{pC4bC2}]}{K_{\text{m C1}^*}^{\text{C2}}}+\frac{[\text{pC4bC4bC2}]}{K_{\text{m C1}^*}^{\text{C2}}}+\frac{[\text{pC3bC4bC2}]}{K_{\text{m C1}^*}^{\text{C2}}}\right)}$$

$$-\frac{k_{\text{cat C1}^*}^{\text{C2}}\left[\text{pMBL}_4\text{PTX3C1}^*\right]\left[\text{pC4bC4bC2}\right]}{K_{\text{m C1}^*}^{\text{C2}}\left(1+\frac{[\text{C4}]}{K_{\text{m C1}^*}^{\text{C4}}}+\frac{[\text{fC4bC2}]}{K_{\text{m C1}^*}^{\text{C2}}}+\frac{[\text{fC4bC4bC2}]}{K_{\text{m C1}^*}^{\text{C2}}}+\frac{[\text{fC3bC4bC2}]}{K_{\text{m C1}^*}^{\text{C2}}}+\frac{[\text{pC4bC2}]}{K_{\text{m C1}^*}^{\text{C2}}}+\frac{[\text{pC4bC4bC2}]}{K_{\text{m C1}^*}^{\text{C2}}}+\frac{[\text{pC3bC4bC2}]}{K_{\text{m C1}^*}^{\text{C2}}}\right)}$$

152

$$\frac{d[\mathrm{pC4bC4bC2a}]}{dt}=$$

$$\frac{k_{\mathrm{cat\,C1^*}}^{\mathrm{C2}}[\mathrm{pIgG_6C1^*}][\mathrm{pC4bC4bC2}]}{K_{\mathrm{m\,C1^*}}^{\mathrm{C2}}\left(1+\frac{[\mathrm{C4}]}{K_{\mathrm{m\,C1^*}}^{\mathrm{C4}}}+\frac{[\mathrm{fC4bC2}]}{K_{\mathrm{m\,C1^*}}^{\mathrm{C2}}}+\frac{[\mathrm{fC4bC4bC2}]}{K_{\mathrm{m\,C1^*}}^{\mathrm{C2}}}+\frac{[\mathrm{fC3bC4bC2}]}{K_{\mathrm{m\,C1^*}}^{\mathrm{C2}}}+\frac{[\mathrm{pC4bC2}]}{K_{\mathrm{m\,C1^*}}^{\mathrm{C2}}}+\frac{[\mathrm{pC4bC4bC2}]}{K_{\mathrm{m\,C1^*}}^{\mathrm{C2}}}+\frac{[\mathrm{pC3bC4bC2}]}{K_{\mathrm{m\,C1^*}}^{\mathrm{C2}}}\right)}$$

$$+\frac{k_{\mathrm{cat\,C1^*}}^{\mathrm{C2}}[\mathrm{pIgMC1^*}][\mathrm{pC4bC4bC2}]}{K_{\mathrm{m\,C1^*}}^{\mathrm{C2}}\left(1+\frac{[\mathrm{C4}]}{K_{\mathrm{m\,C1^*}}^{\mathrm{C4}}}+\frac{[\mathrm{fC4bC2}]}{K_{\mathrm{m\,C1^*}}^{\mathrm{C2}}}+\frac{[\mathrm{fC4bC4bC2}]}{K_{\mathrm{m\,C1^*}}^{\mathrm{C2}}}+\frac{[\mathrm{fC3bC4bC2}]}{K_{\mathrm{m\,C1^*}}^{\mathrm{C2}}}+\frac{[\mathrm{pC4bC2}]}{K_{\mathrm{m\,C1^*}}^{\mathrm{C2}}}+\frac{[\mathrm{pC4bC4bC2}]}{K_{\mathrm{m\,C1^*}}^{\mathrm{C2}}}+\frac{[\mathrm{pC3bC4bC2}]}{K_{\mathrm{m\,C1^*}}^{\mathrm{C2}}}\right)}$$

$$+k_{\mathrm{C4bC4b}}^{+}[\mathrm{nfC4b}][\mathrm{pC4bC2a}]-k_{\mathrm{C4bC4bC2a}}^{-}[\mathrm{pC4bC4bC2a}]+k_{\mathrm{C5b^*}}^{-}[\mathrm{pC4bC4bC2aC5b}]+k_{\mathrm{C5b7}}^{+}[\mathrm{pC4bC4bC2aC5b}][\mathrm{C7}]$$

$$-k_{\mathrm{C4bC4BP}}^{+}[\mathrm{pC4bC4bC2a}][\mathrm{C4BP}]+k_{\mathrm{C4bC4BP}}^{-}[\mathrm{pC4bC4bC2aC4BP}]$$

$$+\frac{k_{\mathrm{cat\,MASP1^*}}^{\mathrm{C2}}[\mathrm{pMBL_3MASP1^*}][\mathrm{pC4bC4bC2}]}{K_{\mathrm{m\,MASP1^*}}^{\mathrm{C2}}\left(\begin{array}{l}1+\frac{[\mathrm{C4}]}{K_{\mathrm{m\,MASP1^*}}^{\mathrm{C4}}}+\frac{[\mathrm{fC4bC2}]}{K_{\mathrm{m\,MASP1^*}}^{\mathrm{C2}}}+\frac{[\mathrm{fC4bC4bC2}]}{K_{\mathrm{m\,MASP1^*}}^{\mathrm{C2}}}+\frac{[\mathrm{fC3bC4bC2}]}{K_{\mathrm{m\,MASP1^*}}^{\mathrm{C2}}}+\frac{[\mathrm{pC4bC2}]}{K_{\mathrm{m\,MASP1^*}}^{\mathrm{C2}}}+\frac{[\mathrm{pC4bC4bC2}]}{K_{\mathrm{m\,MASP1^*}}^{\mathrm{C2}}}\\
+\frac{[\mathrm{pC3bC4bC2}]}{K_{\mathrm{m\,MASP1^*}}^{\mathrm{C2}}}+\frac{[\mathrm{pMBL_3MASP1_{zym}}]}{K_{\mathrm{m\,MASP1^*}}^{\mathrm{MASP1_{zym}}}}+\frac{[\mathrm{pMBL_3MASP2_{zym}}]}{K_{\mathrm{m\,MASP1^*}}^{\mathrm{MASP2_{zym}}}}+\frac{[\mathrm{pMBL_3MASP3_{zym}}]}{K_{\mathrm{m\,MASP1^*}}^{\mathrm{MASP3_{zym}}}}\\
+\frac{[\mathrm{pMBL_4MASP1_{zym}}]}{K_{\mathrm{m\,MASP1^*}}^{\mathrm{MASP1_{zym}}}}+\frac{[\mathrm{pMBL_4MASP2_{zym}}]}{K_{\mathrm{m\,MASP1^*}}^{\mathrm{MASP2_{zym}}}}+\frac{[\mathrm{pMBL_4MASP3_{zym}}]}{K_{\mathrm{m\,MASP1^*}}^{\mathrm{MASP3_{zym}}}}+\frac{[\mathrm{pCLLKMASP1_{zym}}]}{K_{\mathrm{m\,MASP1^*}}^{\mathrm{MASP1_{zym}}}}\\
+\frac{[\mathrm{pCLLKMASP2_{zym}}]}{K_{\mathrm{m\,MASP1^*}}^{\mathrm{MASP2_{zym}}}}+\frac{[\mathrm{pCLLKMASP3_{zym}}]}{K_{\mathrm{m\,MASP1^*}}^{\mathrm{MASP3_{zym}}}}+\frac{[\mathrm{pCLK1MASP1_{zym}}]}{K_{\mathrm{m\,MASP1^*}}^{\mathrm{MASP1_{zym}}}}+\frac{[\mathrm{pCLK1MASP2_{zym}}]}{K_{\mathrm{m\,MASP1^*}}^{\mathrm{MASP2_{zym}}}}\\
+\frac{[\mathrm{pCLK1MASP3_{zym}}]}{K_{\mathrm{m\,MASP1^*}}^{\mathrm{MASP3_{zym}}}}+\frac{[\mathrm{pCLL1MASP1_{zym}}]}{K_{\mathrm{m\,MASP1^*}}^{\mathrm{MASP1_{zym}}}}+\frac{[\mathrm{pCLL1MASP2_{zym}}]}{K_{\mathrm{m\,MASP1^*}}^{\mathrm{MASP2_{zym}}}}+\frac{[\mathrm{pCLL1MASP3_{zym}}]}{K_{\mathrm{m\,MASP1^*}}^{\mathrm{MASP3_{zym}}}}\\
+\frac{[\mathrm{pLFMASP1_{zym}}]}{K_{\mathrm{m\,MASP1^*}}^{\mathrm{MASP1_{zym}}}}+\frac{[\mathrm{pLFMASP2_{zym}}]}{K_{\mathrm{m\,MASP1^*}}^{\mathrm{MASP2_{zym}}}}+\frac{[\mathrm{pLFMASP3_{zym}}]}{K_{\mathrm{m\,MASP1^*}}^{\mathrm{MASP3_{zym}}}}+\frac{[\mathrm{pHFMASP1_{zym}}]}{K_{\mathrm{m\,MASP1^*}}^{\mathrm{MASP1_{zym}}}}+\frac{[\mathrm{pHFMASP2_{zym}}]}{K_{\mathrm{m\,MASP1^*}}^{\mathrm{MASP2_{zym}}}}\\
+\frac{[\mathrm{pHFMASP3_{zym}}]}{K_{\mathrm{m\,MASP1^*}}^{\mathrm{MASP3_{zym}}}}+\frac{[\mathrm{pMFMASP1_{zym}}]}{K_{\mathrm{m\,MASP1^*}}^{\mathrm{MASP1_{zym}}}}+\frac{[\mathrm{pMFMASP2_{zym}}]}{K_{\mathrm{m\,MASP1^*}}^{\mathrm{MASP2_{zym}}}}+\frac{[\mathrm{pMFMASP3_{zym}}]}{K_{\mathrm{m\,MASP1^*}}^{\mathrm{MASP3_{zym}}}}\end{array}\right)}$$

$$+\frac{k_{\mathrm{cat\,MASP1^*}}^{\mathrm{C2}}\left[\mathrm{pMBL_4MASP1^*}\right]\left[\mathrm{pC4bC4bC2}\right]}{K_{\mathrm{m\,MASP1^*}}^{\mathrm{C2}}\left(\begin{aligned}&1+\frac{[\mathrm{C4}]}{K_{\mathrm{m\,MASP1^*}}^{\mathrm{C4}}}+\frac{[\mathrm{fC4bC2}]}{K_{\mathrm{m\,MASP1^*}}^{\mathrm{C2}}}+\frac{[\mathrm{fC4bC4bC2}]}{K_{\mathrm{m\,MASP1^*}}^{\mathrm{C2}}}+\frac{[\mathrm{fC3bC4bC2}]}{K_{\mathrm{m\,MASP1^*}}^{\mathrm{C2}}}+\frac{[\mathrm{pC4bC2}]}{K_{\mathrm{m\,MASP1^*}}^{\mathrm{C2}}}+\frac{[\mathrm{pC4bC4bC2}]}{K_{\mathrm{m\,MASP1^*}}^{\mathrm{C2}}}\\&+\frac{[\mathrm{pC3bC4bC2}]}{K_{\mathrm{m\,MASP1^*}}^{\mathrm{C2}}}+\frac{[\mathrm{pMBL_3MASP1_{zym}}]}{K_{\mathrm{m\,MASP1^*}}^{\mathrm{MASP1_{zym}}}}+\frac{[\mathrm{pMBL_3MASP2_{zym}}]}{K_{\mathrm{m\,MASP1^*}}^{\mathrm{MASP2_{zym}}}}+\frac{[\mathrm{pMBL_3MASP3_{zym}}]}{K_{\mathrm{m\,MASP1^*}}^{\mathrm{MASP3_{zym}}}}\\&+\frac{[\mathrm{pMBL_4MASP1_{zym}}]}{K_{\mathrm{m\,MASP1^*}}^{\mathrm{MASP1_{zym}}}}+\frac{[\mathrm{pMBL_4MASP2_{zym}}]}{K_{\mathrm{m\,MASP1^*}}^{\mathrm{MASP2_{zym}}}}+\frac{[\mathrm{pMBL_4MASP3_{zym}}]}{K_{\mathrm{m\,MASP1^*}}^{\mathrm{MASP3_{zym}}}}+\frac{[\mathrm{pCLLKMASP1_{zym}}]}{K_{\mathrm{m\,MASP1^*}}^{\mathrm{MASP1_{zym}}}}\\&+\frac{[\mathrm{pCLLKMASP2_{zym}}]}{K_{\mathrm{m\,MASP1^*}}^{\mathrm{MASP2_{zym}}}}+\frac{[\mathrm{pCLLKMASP3_{zym}}]}{K_{\mathrm{m\,MASP1^*}}^{\mathrm{MASP3_{zym}}}}+\frac{[\mathrm{pCLK1MASP1_{zym}}]}{K_{\mathrm{m\,MASP1^*}}^{\mathrm{MASP1_{zym}}}}+\frac{[\mathrm{pCLK1MASP2_{zym}}]}{K_{\mathrm{m\,MASP1^*}}^{\mathrm{MASP2_{zym}}}}\\&+\frac{[\mathrm{pCLK1MASP3_{zym}}]}{K_{\mathrm{m\,MASP1^*}}^{\mathrm{MASP3_{zym}}}}+\frac{[\mathrm{pCLL1MASP1_{zym}}]}{K_{\mathrm{m\,MASP1^*}}^{\mathrm{MASP1_{zym}}}}+\frac{[\mathrm{pCLL1MASP2_{zym}}]}{K_{\mathrm{m\,MASP1^*}}^{\mathrm{MASP2_{zym}}}}+\frac{[\mathrm{pCLL1MASP3_{zym}}]}{K_{\mathrm{m\,MASP1^*}}^{\mathrm{MASP3_{zym}}}}\\&+\frac{[\mathrm{pLFMASP1_{zym}}]}{K_{\mathrm{m\,MASP1^*}}^{\mathrm{MASP1_{zym}}}}+\frac{[\mathrm{pLFMASP2_{zym}}]}{K_{\mathrm{m\,MASP1^*}}^{\mathrm{MASP2_{zym}}}}+\frac{[\mathrm{pLFMASP3_{zym}}]}{K_{\mathrm{m\,MASP1^*}}^{\mathrm{MASP3_{zym}}}}+\frac{[\mathrm{pHFMASP1_{zym}}]}{K_{\mathrm{m\,MASP1^*}}^{\mathrm{MASP1_{zym}}}}+\frac{[\mathrm{pHFMASP2_{zym}}]}{K_{\mathrm{m\,MASP1^*}}^{\mathrm{MASP2_{zym}}}}\\&+\frac{[\mathrm{pHFMASP3_{zym}}]}{K_{\mathrm{m\,MASP1^*}}^{\mathrm{MASP3_{zym}}}}+\frac{[\mathrm{pMFMASP1_{zym}}]}{K_{\mathrm{m\,MASP1^*}}^{\mathrm{MASP1_{zym}}}}+\frac{[\mathrm{pMFMASP2_{zym}}]}{K_{\mathrm{m\,MASP1^*}}^{\mathrm{MASP2_{zym}}}}+\frac{[\mathrm{pMFMASP3_{zym}}]}{K_{\mathrm{m\,MASP1^*}}^{\mathrm{MASP3_{zym}}}}\end{aligned}\right)}$$

$$+\frac{k_{\mathrm{cat\,MASP1^*}}^{\mathrm{C2}}\left[\mathrm{pCLLKMASP1^*}\right]\left[\mathrm{pC4bC4bC2}\right]}{K_{\mathrm{m\,MASP1^*}}^{\mathrm{C2}}\left(\begin{aligned}&1+\frac{[\mathrm{C4}]}{K_{\mathrm{m\,MASP1^*}}^{\mathrm{C4}}}+\frac{[\mathrm{fC4bC2}]}{K_{\mathrm{m\,MASP1^*}}^{\mathrm{C2}}}+\frac{[\mathrm{fC4bC4bC2}]}{K_{\mathrm{m\,MASP1^*}}^{\mathrm{C2}}}+\frac{[\mathrm{fC3bC4bC2}]}{K_{\mathrm{m\,MASP1^*}}^{\mathrm{C2}}}+\frac{[\mathrm{pC4bC2}]}{K_{\mathrm{m\,MASP1^*}}^{\mathrm{C2}}}+\frac{[\mathrm{pC4bC4bC2}]}{K_{\mathrm{m\,MASP1^*}}^{\mathrm{C2}}}\\&+\frac{[\mathrm{pC3bC4bC2}]}{K_{\mathrm{m\,MASP1^*}}^{\mathrm{C2}}}+\frac{[\mathrm{pMBL_3MASP1_{zym}}]}{K_{\mathrm{m\,MASP1^*}}^{\mathrm{MASP1_{zym}}}}+\frac{[\mathrm{pMBL_3MASP2_{zym}}]}{K_{\mathrm{m\,MASP1^*}}^{\mathrm{MASP2_{zym}}}}+\frac{[\mathrm{pMBL_3MASP3_{zym}}]}{K_{\mathrm{m\,MASP1^*}}^{\mathrm{MASP3_{zym}}}}\\&+\frac{[\mathrm{pMBL_4MASP1_{zym}}]}{K_{\mathrm{m\,MASP1^*}}^{\mathrm{MASP1_{zym}}}}+\frac{[\mathrm{pMBL_4MASP2_{zym}}]}{K_{\mathrm{m\,MASP1^*}}^{\mathrm{MASP2_{zym}}}}+\frac{[\mathrm{pMBL_4MASP3_{zym}}]}{K_{\mathrm{m\,MASP1^*}}^{\mathrm{MASP3_{zym}}}}+\frac{[\mathrm{pCLLKMASP1_{zym}}]}{K_{\mathrm{m\,MASP1^*}}^{\mathrm{MASP1_{zym}}}}\\&+\frac{[\mathrm{pCLLKMASP2_{zym}}]}{K_{\mathrm{m\,MASP1^*}}^{\mathrm{MASP2_{zym}}}}+\frac{[\mathrm{pCLLKMASP3_{zym}}]}{K_{\mathrm{m\,MASP1^*}}^{\mathrm{MASP3_{zym}}}}+\frac{[\mathrm{pCLK1MASP1_{zym}}]}{K_{\mathrm{m\,MASP1^*}}^{\mathrm{MASP1_{zym}}}}+\frac{[\mathrm{pCLK1MASP2_{zym}}]}{K_{\mathrm{m\,MASP1^*}}^{\mathrm{MASP2_{zym}}}}\\&+\frac{[\mathrm{pCLK1MASP3_{zym}}]}{K_{\mathrm{m\,MASP1^*}}^{\mathrm{MASP3_{zym}}}}+\frac{[\mathrm{pCLL1MASP1_{zym}}]}{K_{\mathrm{m\,MASP1^*}}^{\mathrm{MASP1_{zym}}}}+\frac{[\mathrm{pCLL1MASP2_{zym}}]}{K_{\mathrm{m\,MASP1^*}}^{\mathrm{MASP2_{zym}}}}+\frac{[\mathrm{pCLL1MASP3_{zym}}]}{K_{\mathrm{m\,MASP1^*}}^{\mathrm{MASP3_{zym}}}}\\&+\frac{[\mathrm{pLFMASP1_{zym}}]}{K_{\mathrm{m\,MASP1^*}}^{\mathrm{MASP1_{zym}}}}+\frac{[\mathrm{pLFMASP2_{zym}}]}{K_{\mathrm{m\,MASP1^*}}^{\mathrm{MASP2_{zym}}}}+\frac{[\mathrm{pLFMASP3_{zym}}]}{K_{\mathrm{m\,MASP1^*}}^{\mathrm{MASP3_{zym}}}}+\frac{[\mathrm{pHFMASP1_{zym}}]}{K_{\mathrm{m\,MASP1^*}}^{\mathrm{MASP1_{zym}}}}+\frac{[\mathrm{pHFMASP2_{zym}}]}{K_{\mathrm{m\,MASP1^*}}^{\mathrm{MASP2_{zym}}}}\\&+\frac{[\mathrm{pHFMASP3_{zym}}]}{K_{\mathrm{m\,MASP1^*}}^{\mathrm{MASP3_{zym}}}}+\frac{[\mathrm{pMFMASP1_{zym}}]}{K_{\mathrm{m\,MASP1^*}}^{\mathrm{MASP1_{zym}}}}+\frac{[\mathrm{pMFMASP2_{zym}}]}{K_{\mathrm{m\,MASP1^*}}^{\mathrm{MASP2_{zym}}}}+\frac{[\mathrm{pMFMASP3_{zym}}]}{K_{\mathrm{m\,MASP1^*}}^{\mathrm{MASP3_{zym}}}}\end{aligned}\right)}$$

$$
+\frac{k_{\mathrm{cat\,MASP1^{*}}}^{\mathrm{C2}}\left[\mathrm{pCLK1MASP1^{*}}\right]\left[\mathrm{pC4bC4bC2}\right]}{K_{\mathrm{m\,MASP1^{*}}}^{\mathrm{C2}}\left(\begin{array}{l}
1+\frac{[\mathrm{C4}]}{K_{\mathrm{m\,MASP1^{*}}}^{\mathrm{C4}}}+\frac{[\mathrm{fC4bC2}]}{K_{\mathrm{m\,MASP1^{*}}}^{\mathrm{C2}}}+\frac{[\mathrm{fC4bC4bC2}]}{K_{\mathrm{m\,MASP1^{*}}}^{\mathrm{C2}}}+\frac{[\mathrm{fC3bC4bC2}]}{K_{\mathrm{m\,MASP1^{*}}}^{\mathrm{C2}}}+\frac{[\mathrm{pC4bC2}]}{K_{\mathrm{m\,MASP1^{*}}}^{\mathrm{C2}}}+\frac{[\mathrm{pC4bC4bC2}]}{K_{\mathrm{m\,MASP1^{*}}}^{\mathrm{C2}}}\\
+\frac{[\mathrm{pC3bC4bC2}]}{K_{\mathrm{m\,MASP1^{*}}}^{\mathrm{C2}}}+\frac{\left[\mathrm{pMBL_{3}MASP1_{zym}}\right]}{K_{\mathrm{m\,MASP1^{*}}}^{\mathrm{MASP1_{zym}}}}+\frac{\left[\mathrm{pMBL_{3}MASP2_{zym}}\right]}{K_{\mathrm{m\,MASP1^{*}}}^{\mathrm{MASP2_{zym}}}}+\frac{\left[\mathrm{pMBL_{3}MASP3_{zym}}\right]}{K_{\mathrm{m\,MASP1^{*}}}^{\mathrm{MASP3_{zym}}}}\\
+\frac{\left[\mathrm{pMBL_{4}MASP1_{zym}}\right]}{K_{\mathrm{m\,MASP1^{*}}}^{\mathrm{MASP1_{zym}}}}+\frac{\left[\mathrm{pMBL_{4}MASP2_{zym}}\right]}{K_{\mathrm{m\,MASP1^{*}}}^{\mathrm{MASP2_{zym}}}}+\frac{\left[\mathrm{pMBL_{4}MASP3_{zym}}\right]}{K_{\mathrm{m\,MASP1^{*}}}^{\mathrm{MASP3_{zym}}}}+\frac{\left[\mathrm{pCLLKMASP1_{zym}}\right]}{K_{\mathrm{m\,MASP1^{*}}}^{\mathrm{MASP1_{zym}}}}\\
+\frac{\left[\mathrm{pCLLKMASP2_{zym}}\right]}{K_{\mathrm{m\,MASP1^{*}}}^{\mathrm{MASP2_{zym}}}}+\frac{\left[\mathrm{pCLLKMASP3_{zym}}\right]}{K_{\mathrm{m\,MASP1^{*}}}^{\mathrm{MASP3_{zym}}}}+\frac{\left[\mathrm{pCLK1MASP1_{zym}}\right]}{K_{\mathrm{m\,MASP1^{*}}}^{\mathrm{MASP1_{zym}}}}+\frac{\left[\mathrm{pCLK1MASP2_{zym}}\right]}{K_{\mathrm{m\,MASP1^{*}}}^{\mathrm{MASP2_{zym}}}}\\
+\frac{\left[\mathrm{pCLK1MASP3_{zym}}\right]}{K_{\mathrm{m\,MASP1^{*}}}^{\mathrm{MASP3_{zym}}}}+\frac{\left[\mathrm{pCLL1MASP1_{zym}}\right]}{K_{\mathrm{m\,MASP1^{*}}}^{\mathrm{MASP1_{zym}}}}+\frac{\left[\mathrm{pCLL1MASP2_{zym}}\right]}{K_{\mathrm{m\,MASP1^{*}}}^{\mathrm{MASP2_{zym}}}}+\frac{\left[\mathrm{pCLL1MASP3_{zym}}\right]}{K_{\mathrm{m\,MASP1^{*}}}^{\mathrm{MASP3_{zym}}}}\\
+\frac{\left[\mathrm{pLFMASP1_{zym}}\right]}{K_{\mathrm{m\,MASP1^{*}}}^{\mathrm{MASP1_{zym}}}}+\frac{\left[\mathrm{pLFMASP2_{zym}}\right]}{K_{\mathrm{m\,MASP1^{*}}}^{\mathrm{MASP2_{zym}}}}+\frac{\left[\mathrm{pLFMASP3_{zym}}\right]}{K_{\mathrm{m\,MASP1^{*}}}^{\mathrm{MASP3_{zym}}}}+\frac{\left[\mathrm{pHFMASP1_{zym}}\right]}{K_{\mathrm{m\,MASP1^{*}}}^{\mathrm{MASP1_{zym}}}}+\frac{\left[\mathrm{pHFMASP2_{zym}}\right]}{K_{\mathrm{m\,MASP1^{*}}}^{\mathrm{MASP2_{zym}}}}\\
+\frac{\left[\mathrm{pHFMASP3_{zym}}\right]}{K_{\mathrm{m\,MASP1^{*}}}^{\mathrm{MASP3_{zym}}}}+\frac{\left[\mathrm{pMFMASP1_{zym}}\right]}{K_{\mathrm{m\,MASP1^{*}}}^{\mathrm{MASP1_{zym}}}}+\frac{\left[\mathrm{pMFMASP2_{zym}}\right]}{K_{\mathrm{m\,MASP1^{*}}}^{\mathrm{MASP2_{zym}}}}+\frac{\left[\mathrm{pMFMASP3_{zym}}\right]}{K_{\mathrm{m\,MASP1^{*}}}^{\mathrm{MASP3_{zym}}}}
\end{array}\right)}
$$

$$
+\frac{k_{\mathrm{cat\,MASP1^{*}}}^{\mathrm{C2}}\left[\mathrm{pCLL1MASP1^{*}}\right]\left[\mathrm{pC4bC4bC2}\right]}{K_{\mathrm{m\,MASP1^{*}}}^{\mathrm{C2}}\left(\begin{array}{l}
1+\frac{[\mathrm{C4}]}{K_{\mathrm{m\,MASP1^{*}}}^{\mathrm{C4}}}+\frac{[\mathrm{fC4bC2}]}{K_{\mathrm{m\,MASP1^{*}}}^{\mathrm{C2}}}+\frac{[\mathrm{fC4bC4bC2}]}{K_{\mathrm{m\,MASP1^{*}}}^{\mathrm{C2}}}+\frac{[\mathrm{fC3bC4bC2}]}{K_{\mathrm{m\,MASP1^{*}}}^{\mathrm{C2}}}+\frac{[\mathrm{pC4bC2}]}{K_{\mathrm{m\,MASP1^{*}}}^{\mathrm{C2}}}+\frac{[\mathrm{pC4bC4bC2}]}{K_{\mathrm{m\,MASP1^{*}}}^{\mathrm{C2}}}\\
+\frac{[\mathrm{pC3bC4bC2}]}{K_{\mathrm{m\,MASP1^{*}}}^{\mathrm{C2}}}+\frac{\left[\mathrm{pMBL_{3}MASP1_{zym}}\right]}{K_{\mathrm{m\,MASP1^{*}}}^{\mathrm{MASP1_{zym}}}}+\frac{\left[\mathrm{pMBL_{3}MASP2_{zym}}\right]}{K_{\mathrm{m\,MASP1^{*}}}^{\mathrm{MASP2_{zym}}}}+\frac{\left[\mathrm{pMBL_{3}MASP3_{zym}}\right]}{K_{\mathrm{m\,MASP1^{*}}}^{\mathrm{MASP3_{zym}}}}\\
+\frac{\left[\mathrm{pMBL_{4}MASP1_{zym}}\right]}{K_{\mathrm{m\,MASP1^{*}}}^{\mathrm{MASP1_{zym}}}}+\frac{\left[\mathrm{pMBL_{4}MASP2_{zym}}\right]}{K_{\mathrm{m\,MASP1^{*}}}^{\mathrm{MASP2_{zym}}}}+\frac{\left[\mathrm{pMBL_{4}MASP3_{zym}}\right]}{K_{\mathrm{m\,MASP1^{*}}}^{\mathrm{MASP3_{zym}}}}+\frac{\left[\mathrm{pCLLKMASP1_{zym}}\right]}{K_{\mathrm{m\,MASP1^{*}}}^{\mathrm{MASP1_{zym}}}}\\
+\frac{\left[\mathrm{pCLLKMASP2_{zym}}\right]}{K_{\mathrm{m\,MASP1^{*}}}^{\mathrm{MASP2_{zym}}}}+\frac{\left[\mathrm{pCLLKMASP3_{zym}}\right]}{K_{\mathrm{m\,MASP1^{*}}}^{\mathrm{MASP3_{zym}}}}+\frac{\left[\mathrm{pCLK1MASP1_{zym}}\right]}{K_{\mathrm{m\,MASP1^{*}}}^{\mathrm{MASP1_{zym}}}}+\frac{\left[\mathrm{pCLK1MASP2_{zym}}\right]}{K_{\mathrm{m\,MASP1^{*}}}^{\mathrm{MASP2_{zym}}}}\\
+\frac{\left[\mathrm{pCLK1MASP3_{zym}}\right]}{K_{\mathrm{m\,MASP1^{*}}}^{\mathrm{MASP3_{zym}}}}+\frac{\left[\mathrm{pCLL1MASP1_{zym}}\right]}{K_{\mathrm{m\,MASP1^{*}}}^{\mathrm{MASP1_{zym}}}}+\frac{\left[\mathrm{pCLL1MASP2_{zym}}\right]}{K_{\mathrm{m\,MASP1^{*}}}^{\mathrm{MASP2_{zym}}}}+\frac{\left[\mathrm{pCLL1MASP3_{zym}}\right]}{K_{\mathrm{m\,MASP1^{*}}}^{\mathrm{MASP3_{zym}}}}\\
+\frac{\left[\mathrm{pLFMASP1_{zym}}\right]}{K_{\mathrm{m\,MASP1^{*}}}^{\mathrm{MASP1_{zym}}}}+\frac{\left[\mathrm{pLFMASP2_{zym}}\right]}{K_{\mathrm{m\,MASP1^{*}}}^{\mathrm{MASP2_{zym}}}}+\frac{\left[\mathrm{pLFMASP3_{zym}}\right]}{K_{\mathrm{m\,MASP1^{*}}}^{\mathrm{MASP3_{zym}}}}+\frac{\left[\mathrm{pHFMASP1_{zym}}\right]}{K_{\mathrm{m\,MASP1^{*}}}^{\mathrm{MASP1_{zym}}}}+\frac{\left[\mathrm{pHFMASP2_{zym}}\right]}{K_{\mathrm{m\,MASP1^{*}}}^{\mathrm{MASP2_{zym}}}}\\
+\frac{\left[\mathrm{pHFMASP3_{zym}}\right]}{K_{\mathrm{m\,MASP1^{*}}}^{\mathrm{MASP3_{zym}}}}+\frac{\left[\mathrm{pMFMASP1_{zym}}\right]}{K_{\mathrm{m\,MASP1^{*}}}^{\mathrm{MASP1_{zym}}}}+\frac{\left[\mathrm{pMFMASP2_{zym}}\right]}{K_{\mathrm{m\,MASP1^{*}}}^{\mathrm{MASP2_{zym}}}}+\frac{\left[\mathrm{pMFMASP3_{zym}}\right]}{K_{\mathrm{m\,MASP1^{*}}}^{\mathrm{MASP3_{zym}}}}
\end{array}\right)}
$$

$$
+\frac{k_{\text{cat MASP1}^*}^{\text{C2}}\left[\text{pLFMASP1}^*\right]\left[\text{pC4bC4bC2}\right]}{K_{\text{m MASP1}^*}^{\text{C2}}\left(\begin{aligned}&1+\frac{[\text{C4}]}{K_{\text{m MASP1}^*}^{\text{C4}}}+\frac{[\text{fC4bC2}]}{K_{\text{m MASP1}^*}^{\text{C2}}}+\frac{[\text{fC4bC4bC2}]}{K_{\text{m MASP1}^*}^{\text{C2}}}+\frac{[\text{fC3bC4bC2}]}{K_{\text{m MASP1}^*}^{\text{C2}}}+\frac{[\text{pC4bC2}]}{K_{\text{m MASP1}^*}^{\text{C2}}}+\frac{[\text{pC4bC4bC2}]}{K_{\text{m MASP1}^*}^{\text{C2}}}\\&+\frac{[\text{pC3bC4bC2}]}{K_{\text{m MASP1}^*}^{\text{C2}}}+\frac{\left[\text{pMBL}_3\text{MASP1}_{\text{zym}}\right]}{K_{\text{m MASP1}^*}^{\text{MASP1}_{\text{zym}}}}+\frac{\left[\text{pMBL}_3\text{MASP2}_{\text{zym}}\right]}{K_{\text{m MASP1}^*}^{\text{MASP2}_{\text{zym}}}}+\frac{\left[\text{pMBL}_3\text{MASP3}_{\text{zym}}\right]}{K_{\text{m MASP1}^*}^{\text{MASP3}_{\text{zym}}}}\\&+\frac{\left[\text{pMBL}_4\text{MASP1}_{\text{zym}}\right]}{K_{\text{m MASP1}^*}^{\text{MASP1}_{\text{zym}}}}+\frac{\left[\text{pMBL}_4\text{MASP2}_{\text{zym}}\right]}{K_{\text{m MASP1}^*}^{\text{MASP2}_{\text{zym}}}}+\frac{\left[\text{pMBL}_4\text{MASP3}_{\text{zym}}\right]}{K_{\text{m MASP1}^*}^{\text{MASP3}_{\text{zym}}}}+\frac{\left[\text{pCLLKMASP1}_{\text{zym}}\right]}{K_{\text{m MASP1}^*}^{\text{MASP1}_{\text{zym}}}}\\&+\frac{\left[\text{pCLLKMASP2}_{\text{zym}}\right]}{K_{\text{m MASP1}^*}^{\text{MASP2}_{\text{zym}}}}+\frac{\left[\text{pCLLKMASP3}_{\text{zym}}\right]}{K_{\text{m MASP1}^*}^{\text{MASP3}_{\text{zym}}}}+\frac{\left[\text{pCLK1MASP1}_{\text{zym}}\right]}{K_{\text{m MASP1}^*}^{\text{MASP1}_{\text{zym}}}}+\frac{\left[\text{pCLK1MASP2}_{\text{zym}}\right]}{K_{\text{m MASP1}^*}^{\text{MASP2}_{\text{zym}}}}\\&+\frac{\left[\text{pCLK1MASP3}_{\text{zym}}\right]}{K_{\text{m MASP1}^*}^{\text{MASP3}_{\text{zym}}}}+\frac{\left[\text{pCLL1MASP1}_{\text{zym}}\right]}{K_{\text{m MASP1}^*}^{\text{MASP1}_{\text{zym}}}}+\frac{\left[\text{pCLL1MASP2}_{\text{zym}}\right]}{K_{\text{m MASP1}^*}^{\text{MASP2}_{\text{zym}}}}+\frac{\left[\text{pCLL1MASP3}_{\text{zym}}\right]}{K_{\text{m MASP1}^*}^{\text{MASP3}_{\text{zym}}}}\\&+\frac{\left[\text{pLFMASP1}_{\text{zym}}\right]}{K_{\text{m MASP1}^*}^{\text{MASP1}_{\text{zym}}}}+\frac{\left[\text{pLFMASP2}_{\text{zym}}\right]}{K_{\text{m MASP1}^*}^{\text{MASP2}_{\text{zym}}}}+\frac{\left[\text{pLFMASP3}_{\text{zym}}\right]}{K_{\text{m MASP1}^*}^{\text{MASP3}_{\text{zym}}}}+\frac{\left[\text{pHFMASP1}_{\text{zym}}\right]}{K_{\text{m MASP1}^*}^{\text{MASP1}_{\text{zym}}}}+\frac{\left[\text{pHFMASP2}_{\text{zym}}\right]}{K_{\text{m MASP1}^*}^{\text{MASP2}_{\text{zym}}}}\\&+\frac{\left[\text{pHFMASP3}_{\text{zym}}\right]}{K_{\text{m MASP1}^*}^{\text{MASP3}_{\text{zym}}}}+\frac{\left[\text{pMFMASP1}_{\text{zym}}\right]}{K_{\text{m MASP1}^*}^{\text{MASP1}_{\text{zym}}}}+\frac{\left[\text{pMFMASP2}_{\text{zym}}\right]}{K_{\text{m MASP1}^*}^{\text{MASP2}_{\text{zym}}}}+\frac{\left[\text{pMFMASP3}_{\text{zym}}\right]}{K_{\text{m MASP1}^*}^{\text{MASP3}_{\text{zym}}}}\end{aligned}\right)}
$$

$$
+\frac{k_{\text{cat MASP1}^*}^{\text{C2}}\left[\text{pHFMASP1}^*\right]\left[\text{pC4bC4bC2}\right]}{K_{\text{m MASP1}^*}^{\text{C2}}\left(\begin{aligned}&1+\frac{[\text{C4}]}{K_{\text{m MASP1}^*}^{\text{C4}}}+\frac{[\text{fC4bC2}]}{K_{\text{m MASP1}^*}^{\text{C2}}}+\frac{[\text{fC4bC4bC2}]}{K_{\text{m MASP1}^*}^{\text{C2}}}+\frac{[\text{fC3bC4bC2}]}{K_{\text{m MASP1}^*}^{\text{C2}}}+\frac{[\text{pC4bC2}]}{K_{\text{m MASP1}^*}^{\text{C2}}}+\frac{[\text{pC4bC4bC2}]}{K_{\text{m MASP1}^*}^{\text{C2}}}\\&+\frac{[\text{pC3bC4bC2}]}{K_{\text{m MASP1}^*}^{\text{C2}}}+\frac{\left[\text{pMBL}_3\text{MASP1}_{\text{zym}}\right]}{K_{\text{m MASP1}^*}^{\text{MASP1}_{\text{zym}}}}+\frac{\left[\text{pMBL}_3\text{MASP2}_{\text{zym}}\right]}{K_{\text{m MASP1}^*}^{\text{MASP2}_{\text{zym}}}}+\frac{\left[\text{pMBL}_3\text{MASP3}_{\text{zym}}\right]}{K_{\text{m MASP1}^*}^{\text{MASP3}_{\text{zym}}}}\\&+\frac{\left[\text{pMBL}_4\text{MASP1}_{\text{zym}}\right]}{K_{\text{m MASP1}^*}^{\text{MASP1}_{\text{zym}}}}+\frac{\left[\text{pMBL}_4\text{MASP2}_{\text{zym}}\right]}{K_{\text{m MASP1}^*}^{\text{MASP2}_{\text{zym}}}}+\frac{\left[\text{pMBL}_4\text{MASP3}_{\text{zym}}\right]}{K_{\text{m MASP1}^*}^{\text{MASP3}_{\text{zym}}}}+\frac{\left[\text{pCLLKMASP1}_{\text{zym}}\right]}{K_{\text{m MASP1}^*}^{\text{MASP1}_{\text{zym}}}}\\&+\frac{\left[\text{pCLLKMASP2}_{\text{zym}}\right]}{K_{\text{m MASP1}^*}^{\text{MASP2}_{\text{zym}}}}+\frac{\left[\text{pCLLKMASP3}_{\text{zym}}\right]}{K_{\text{m MASP1}^*}^{\text{MASP3}_{\text{zym}}}}+\frac{\left[\text{pCLK1MASP1}_{\text{zym}}\right]}{K_{\text{m MASP1}^*}^{\text{MASP1}_{\text{zym}}}}+\frac{\left[\text{pCLK1MASP2}_{\text{zym}}\right]}{K_{\text{m MASP1}^*}^{\text{MASP2}_{\text{zym}}}}\\&+\frac{\left[\text{pCLK1MASP3}_{\text{zym}}\right]}{K_{\text{m MASP1}^*}^{\text{MASP3}_{\text{zym}}}}+\frac{\left[\text{pCLL1MASP1}_{\text{zym}}\right]}{K_{\text{m MASP1}^*}^{\text{MASP1}_{\text{zym}}}}+\frac{\left[\text{pCLL1MASP2}_{\text{zym}}\right]}{K_{\text{m MASP1}^*}^{\text{MASP2}_{\text{zym}}}}+\frac{\left[\text{pCLL1MASP3}_{\text{zym}}\right]}{K_{\text{m MASP1}^*}^{\text{MASP3}_{\text{zym}}}}\\&+\frac{\left[\text{pLFMASP1}_{\text{zym}}\right]}{K_{\text{m MASP1}^*}^{\text{MASP1}_{\text{zym}}}}+\frac{\left[\text{pLFMASP2}_{\text{zym}}\right]}{K_{\text{m MASP1}^*}^{\text{MASP2}_{\text{zym}}}}+\frac{\left[\text{pLFMASP3}_{\text{zym}}\right]}{K_{\text{m MASP1}^*}^{\text{MASP3}_{\text{zym}}}}+\frac{\left[\text{pHFMASP1}_{\text{zym}}\right]}{K_{\text{m MASP1}^*}^{\text{MASP1}_{\text{zym}}}}+\frac{\left[\text{pHFMASP2}_{\text{zym}}\right]}{K_{\text{m MASP1}^*}^{\text{MASP2}_{\text{zym}}}}\\&+\frac{\left[\text{pHFMASP3}_{\text{zym}}\right]}{K_{\text{m MASP1}^*}^{\text{MASP3}_{\text{zym}}}}+\frac{\left[\text{pMFMASP1}_{\text{zym}}\right]}{K_{\text{m MASP1}^*}^{\text{MASP1}_{\text{zym}}}}+\frac{\left[\text{pMFMASP2}_{\text{zym}}\right]}{K_{\text{m MASP1}^*}^{\text{MASP2}_{\text{zym}}}}+\frac{\left[\text{pMFMASP3}_{\text{zym}}\right]}{K_{\text{m MASP1}^*}^{\text{MASP3}_{\text{zym}}}}\end{aligned}\right)}
$$

$$
+\frac{k_{\mathrm{cat\ MASP1^{*}}}^{\mathrm{C2}}\left[\mathrm{pMFMASP1^{*}}\right]\left[\mathrm{pC4bC4bC2}\right]}{K_{\mathrm{m\ MASP1^{*}}}^{\mathrm{C2}}\left(\begin{aligned}
&1+\frac{[\mathrm{C4}]}{K_{\mathrm{m\ MASP1^{*}}}^{\mathrm{C4}}}+\frac{[\mathrm{fC4bC2}]}{K_{\mathrm{m\ MASP1^{*}}}^{\mathrm{C2}}}+\frac{[\mathrm{fC4bC4bC2}]}{K_{\mathrm{m\ MASP1^{*}}}^{\mathrm{C2}}}+\frac{[\mathrm{fC3bC4bC2}]}{K_{\mathrm{m\ MASP1^{*}}}^{\mathrm{C2}}}+\frac{[\mathrm{pC4bC2}]}{K_{\mathrm{m\ MASP1^{*}}}^{\mathrm{C2}}}+\frac{[\mathrm{pC4bC4bC2}]}{K_{\mathrm{m\ MASP1^{*}}}^{\mathrm{C2}}}\\
&+\frac{[\mathrm{pC3bC4bC2}]}{K_{\mathrm{m\ MASP1^{*}}}^{\mathrm{C2}}}+\frac{\left[\mathrm{pMBL_{3}MASP1_{zym}}\right]}{K_{\mathrm{m\ MASP1^{*}}}^{\mathrm{MASP1_{zym}}}}+\frac{\left[\mathrm{pMBL_{3}MASP2_{zym}}\right]}{K_{\mathrm{m\ MASP1^{*}}}^{\mathrm{MASP2_{zym}}}}+\frac{\left[\mathrm{pMBL_{3}MASP3_{zym}}\right]}{K_{\mathrm{m\ MASP1^{*}}}^{\mathrm{MASP3_{zym}}}}\\
&+\frac{\left[\mathrm{pMBL_{4}MASP1_{zym}}\right]}{K_{\mathrm{m\ MASP1^{*}}}^{\mathrm{MASP1_{zym}}}}+\frac{\left[\mathrm{pMBL_{4}MASP2_{zym}}\right]}{K_{\mathrm{m\ MASP1^{*}}}^{\mathrm{MASP2_{zym}}}}+\frac{\left[\mathrm{pMBL_{4}MASP3_{zym}}\right]}{K_{\mathrm{m\ MASP1^{*}}}^{\mathrm{MASP3_{zym}}}}+\frac{\left[\mathrm{pCLLKMASP1_{zym}}\right]}{K_{\mathrm{m\ MASP1^{*}}}^{\mathrm{MASP1_{zym}}}}\\
&+\frac{\left[\mathrm{pCLLKMASP2_{zym}}\right]}{K_{\mathrm{m\ MASP1^{*}}}^{\mathrm{MASP2_{zym}}}}+\frac{\left[\mathrm{pCLLKMASP3_{zym}}\right]}{K_{\mathrm{m\ MASP1^{*}}}^{\mathrm{MASP3_{zym}}}}+\frac{\left[\mathrm{pCLK1MASP1_{zym}}\right]}{K_{\mathrm{m\ MASP1^{*}}}^{\mathrm{MASP1_{zym}}}}+\frac{\left[\mathrm{pCLK1MASP2_{zym}}\right]}{K_{\mathrm{m\ MASP1^{*}}}^{\mathrm{MASP2_{zym}}}}\\
&+\frac{\left[\mathrm{pCLK1MASP3_{zym}}\right]}{K_{\mathrm{m\ MASP1^{*}}}^{\mathrm{MASP3_{zym}}}}+\frac{\left[\mathrm{pCLL1MASP1_{zym}}\right]}{K_{\mathrm{m\ MASP1^{*}}}^{\mathrm{MASP1_{zym}}}}+\frac{\left[\mathrm{pCLL1MASP2_{zym}}\right]}{K_{\mathrm{m\ MASP1^{*}}}^{\mathrm{MASP2_{zym}}}}+\frac{\left[\mathrm{pCLL1MASP3_{zym}}\right]}{K_{\mathrm{m\ MASP1^{*}}}^{\mathrm{MASP3_{zym}}}}\\
&+\frac{\left[\mathrm{pLFMASP1_{zym}}\right]}{K_{\mathrm{m\ MASP1^{*}}}^{\mathrm{MASP1_{zym}}}}+\frac{\left[\mathrm{pLFMASP2_{zym}}\right]}{K_{\mathrm{m\ MASP1^{*}}}^{\mathrm{MASP2_{zym}}}}+\frac{\left[\mathrm{pLFMASP3_{zym}}\right]}{K_{\mathrm{m\ MASP1^{*}}}^{\mathrm{MASP3_{zym}}}}+\frac{\left[\mathrm{pHFMASP1_{zym}}\right]}{K_{\mathrm{m\ MASP1^{*}}}^{\mathrm{MASP1_{zym}}}}+\frac{\left[\mathrm{pHFMASP2_{zym}}\right]}{K_{\mathrm{m\ MASP1^{*}}}^{\mathrm{MASP2_{zym}}}}\\
&+\frac{\left[\mathrm{pHFMASP3_{zym}}\right]}{K_{\mathrm{m\ MASP1^{*}}}^{\mathrm{MASP3_{zym}}}}+\frac{\left[\mathrm{pMFMASP1_{zym}}\right]}{K_{\mathrm{m\ MASP1^{*}}}^{\mathrm{MASP1_{zym}}}}+\frac{\left[\mathrm{pMFMASP2_{zym}}\right]}{K_{\mathrm{m\ MASP1^{*}}}^{\mathrm{MASP2_{zym}}}}+\frac{\left[\mathrm{pMFMASP3_{zym}}\right]}{K_{\mathrm{m\ MASP1^{*}}}^{\mathrm{MASP3_{zym}}}}
\end{aligned}\right)}
$$

$$
+\frac{k_{\mathrm{cat\ MASP2^{*}}}^{\mathrm{C2}}\left[\mathrm{pMBL_{3}MASP2^{*}}\right]\left[\mathrm{pC4bC4bC2}\right]}{K_{\mathrm{m\ MASP2^{*}}}^{\mathrm{C2}}\left(\begin{aligned}
&1+\frac{[\mathrm{C4}]}{K_{\mathrm{m\ MASP2^{*}}}^{\mathrm{C4}}}+\frac{[\mathrm{fC4bC2}]}{K_{\mathrm{m\ MASP2^{*}}}^{\mathrm{C2}}}+\frac{[\mathrm{fC4bC4bC2}]}{K_{\mathrm{m\ MASP2^{*}}}^{\mathrm{C2}}}+\frac{[\mathrm{fC3bC4bC2}]}{K_{\mathrm{m\ MASP2^{*}}}^{\mathrm{C2}}}+\frac{[\mathrm{pC4bC2}]}{K_{\mathrm{m\ MASP2^{*}}}^{\mathrm{C2}}}+\frac{[\mathrm{pC4bC4bC2}]}{K_{\mathrm{m\ MASP2^{*}}}^{\mathrm{C2}}}\\
&+\frac{[\mathrm{pC3bC4bC2}]}{K_{\mathrm{m\ MASP2^{*}}}^{\mathrm{C2}}}+\frac{\left[\mathrm{pMBL_{3}MASP1_{zym}}\right]}{K_{\mathrm{m\ MASP2^{*}}}^{\mathrm{MASP1_{zym}}}}+\frac{\left[\mathrm{pMBL_{3}MASP2_{zym}}\right]}{K_{\mathrm{m\ MASP2^{*}}}^{\mathrm{MASP2_{zym}}}}+\frac{\left[\mathrm{pMBL_{4}MASP1_{zym}}\right]}{K_{\mathrm{m\ MASP2^{*}}}^{\mathrm{MASP1_{zym}}}}\\
&+\frac{\left[\mathrm{pMBL_{4}MASP2_{zym}}\right]}{K_{\mathrm{m\ MASP2^{*}}}^{\mathrm{MASP2_{zym}}}}+\frac{\left[\mathrm{pCLLKMASP1_{zym}}\right]}{K_{\mathrm{m\ MASP2^{*}}}^{\mathrm{MASP1_{zym}}}}+\frac{\left[\mathrm{pCLLKMASP2_{zym}}\right]}{K_{\mathrm{m\ MASP2^{*}}}^{\mathrm{MASP2_{zym}}}}+\frac{\left[\mathrm{pCLK1MASP1_{zym}}\right]}{K_{\mathrm{m\ MASP2^{*}}}^{\mathrm{MASP1_{zym}}}}\\
&+\frac{\left[\mathrm{pCLK1MASP2_{zym}}\right]}{K_{\mathrm{m\ MASP2^{*}}}^{\mathrm{MASP2_{zym}}}}+\frac{\left[\mathrm{pCLL1MASP1_{zym}}\right]}{K_{\mathrm{m\ MASP2^{*}}}^{\mathrm{MASP1_{zym}}}}+\frac{\left[\mathrm{pCLL1MASP2_{zym}}\right]}{K_{\mathrm{m\ MASP2^{*}}}^{\mathrm{MASP2_{zym}}}}+\frac{\left[\mathrm{pLFMASP1_{zym}}\right]}{K_{\mathrm{m\ MASP2^{*}}}^{\mathrm{MASP1_{zym}}}}\\
&+\frac{\left[\mathrm{pLFMASP2_{zym}}\right]}{K_{\mathrm{m\ MASP2^{*}}}^{\mathrm{MASP2_{zym}}}}+\frac{\left[\mathrm{pHFMASP1_{zym}}\right]}{K_{\mathrm{m\ MASP2^{*}}}^{\mathrm{MASP1_{zym}}}}+\frac{\left[\mathrm{pHFMASP2_{zym}}\right]}{K_{\mathrm{m\ MASP2^{*}}}^{\mathrm{MASP2_{zym}}}}+\frac{\left[\mathrm{pMFMASP1_{zym}}\right]}{K_{\mathrm{m\ MASP2^{*}}}^{\mathrm{MASP1_{zym}}}}+\frac{\left[\mathrm{pMFMASP2_{zym}}\right]}{K_{\mathrm{m\ MASP2^{*}}}^{\mathrm{MASP2_{zym}}}}
\end{aligned}\right)}
$$

$$
\begin{aligned}
&+\frac{k^{\mathrm{C2}}_{\mathrm{cat\,MASP2^*}}\left[\mathrm{pMBL_4MASP2^*}\right]\left[\mathrm{pC4bC4bC2}\right]}{K^{\mathrm{C2}}_{\mathrm{m\,MASP2^*}}\left(\begin{array}{l}1+\frac{[\mathrm{C4}]}{K^{\mathrm{C4}}_{\mathrm{m\,MASP2^*}}}+\frac{[\mathrm{fC4bC2}]}{K^{\mathrm{C2}}_{\mathrm{m\,MASP2^*}}}+\frac{[\mathrm{fC4bC4bC2}]}{K^{\mathrm{C2}}_{\mathrm{m\,MASP2^*}}}+\frac{[\mathrm{fC3bC4bC2}]}{K^{\mathrm{C2}}_{\mathrm{m\,MASP2^*}}}+\frac{[\mathrm{pC4bC2}]}{K^{\mathrm{C2}}_{\mathrm{m\,MASP2^*}}}+\frac{[\mathrm{pC4bC4bC2}]}{K^{\mathrm{C2}}_{\mathrm{m\,MASP2^*}}}\\
+\frac{[\mathrm{pC3bC4bC2}]}{K^{\mathrm{C2}}_{\mathrm{m\,MASP2^*}}}+\frac{[\mathrm{pMBL_3MASP1_{zym}}]}{K^{\mathrm{MASP1_{zym}}}_{\mathrm{m\,MASP2^*}}}+\frac{[\mathrm{pMBL_3MASP2_{zym}}]}{K^{\mathrm{MASP2_{zym}}}_{\mathrm{m\,MASP2^*}}}+\frac{[\mathrm{pMBL_4MASP1_{zym}}]}{K^{\mathrm{MASP1_{zym}}}_{\mathrm{m\,MASP2^*}}}\\
+\frac{[\mathrm{pMBL_4MASP2_{zym}}]}{K^{\mathrm{MASP2_{zym}}}_{\mathrm{m\,MASP2^*}}}+\frac{[\mathrm{pCLLKMASP1_{zym}}]}{K^{\mathrm{MASP1_{zym}}}_{\mathrm{m\,MASP2^*}}}+\frac{[\mathrm{pCLLKMASP2_{zym}}]}{K^{\mathrm{MASP2_{zym}}}_{\mathrm{m\,MASP2^*}}}+\frac{[\mathrm{pCLK1MASP1_{zym}}]}{K^{\mathrm{MASP1_{zym}}}_{\mathrm{m\,MASP2^*}}}\\
+\frac{[\mathrm{pCLK1MASP2_{zym}}]}{K^{\mathrm{MASP2_{zym}}}_{\mathrm{m\,MASP2^*}}}+\frac{[\mathrm{pCLL1MASP1_{zym}}]}{K^{\mathrm{MASP1_{zym}}}_{\mathrm{m\,MASP2^*}}}+\frac{[\mathrm{pCLL1MASP2_{zym}}]}{K^{\mathrm{MASP2_{zym}}}_{\mathrm{m\,MASP2^*}}}+\frac{[\mathrm{pLFMASP1_{zym}}]}{K^{\mathrm{MASP1_{zym}}}_{\mathrm{m\,MASP2^*}}}\\
+\frac{[\mathrm{pLFMASP2_{zym}}]}{K^{\mathrm{MASP2_{zym}}}_{\mathrm{m\,MASP2^*}}}+\frac{[\mathrm{pHFMASP1_{zym}}]}{K^{\mathrm{MASP1_{zym}}}_{\mathrm{m\,MASP2^*}}}+\frac{[\mathrm{pHFMASP2_{zym}}]}{K^{\mathrm{MASP2_{zym}}}_{\mathrm{m\,MASP2^*}}}+\frac{[\mathrm{pMFMASP1_{zym}}]}{K^{\mathrm{MASP1_{zym}}}_{\mathrm{m\,MASP2^*}}}+\frac{[\mathrm{pMFMASP2_{zym}}]}{K^{\mathrm{MASP2_{zym}}}_{\mathrm{m\,MASP2^*}}}\end{array}\right)}\\
&+\frac{k^{\mathrm{C2}}_{\mathrm{cat\,MASP2^*}}\left[\mathrm{pCLLKMASP2^*}\right]\left[\mathrm{pC4bC4bC2}\right]}{K^{\mathrm{C2}}_{\mathrm{m\,MASP2^*}}\left(\begin{array}{l}1+\frac{[\mathrm{C4}]}{K^{\mathrm{C4}}_{\mathrm{m\,MASP2^*}}}+\frac{[\mathrm{fC4bC2}]}{K^{\mathrm{C2}}_{\mathrm{m\,MASP2^*}}}+\frac{[\mathrm{fC4bC4bC2}]}{K^{\mathrm{C2}}_{\mathrm{m\,MASP2^*}}}+\frac{[\mathrm{fC3bC4bC2}]}{K^{\mathrm{C2}}_{\mathrm{m\,MASP2^*}}}+\frac{[\mathrm{pC4bC2}]}{K^{\mathrm{C2}}_{\mathrm{m\,MASP2^*}}}+\frac{[\mathrm{pC4bC4bC2}]}{K^{\mathrm{C2}}_{\mathrm{m\,MASP2^*}}}\\
+\frac{[\mathrm{pC3bC4bC2}]}{K^{\mathrm{C2}}_{\mathrm{m\,MASP2^*}}}+\frac{[\mathrm{pMBL_3MASP1_{zym}}]}{K^{\mathrm{MASP1_{zym}}}_{\mathrm{m\,MASP2^*}}}+\frac{[\mathrm{pMBL_3MASP2_{zym}}]}{K^{\mathrm{MASP2_{zym}}}_{\mathrm{m\,MASP2^*}}}+\frac{[\mathrm{pMBL_4MASP1_{zym}}]}{K^{\mathrm{MASP1_{zym}}}_{\mathrm{m\,MASP2^*}}}\\
+\frac{[\mathrm{pMBL_4MASP2_{zym}}]}{K^{\mathrm{MASP2_{zym}}}_{\mathrm{m\,MASP2^*}}}+\frac{[\mathrm{pCLLKMASP1_{zym}}]}{K^{\mathrm{MASP1_{zym}}}_{\mathrm{m\,MASP2^*}}}+\frac{[\mathrm{pCLLKMASP2_{zym}}]}{K^{\mathrm{MASP2_{zym}}}_{\mathrm{m\,MASP2^*}}}+\frac{[\mathrm{pCLK1MASP1_{zym}}]}{K^{\mathrm{MASP1_{zym}}}_{\mathrm{m\,MASP2^*}}}\\
+\frac{[\mathrm{pCLK1MASP2_{zym}}]}{K^{\mathrm{MASP2_{zym}}}_{\mathrm{m\,MASP2^*}}}+\frac{[\mathrm{pCLL1MASP1_{zym}}]}{K^{\mathrm{MASP1_{zym}}}_{\mathrm{m\,MASP2^*}}}+\frac{[\mathrm{pCLL1MASP2_{zym}}]}{K^{\mathrm{MASP2_{zym}}}_{\mathrm{m\,MASP2^*}}}+\frac{[\mathrm{pLFMASP1_{zym}}]}{K^{\mathrm{MASP1_{zym}}}_{\mathrm{m\,MASP2^*}}}\\
+\frac{[\mathrm{pLFMASP2_{zym}}]}{K^{\mathrm{MASP2_{zym}}}_{\mathrm{m\,MASP2^*}}}+\frac{[\mathrm{pHFMASP1_{zym}}]}{K^{\mathrm{MASP1_{zym}}}_{\mathrm{m\,MASP2^*}}}+\frac{[\mathrm{pHFMASP2_{zym}}]}{K^{\mathrm{MASP2_{zym}}}_{\mathrm{m\,MASP2^*}}}+\frac{[\mathrm{pMFMASP1_{zym}}]}{K^{\mathrm{MASP1_{zym}}}_{\mathrm{m\,MASP2^*}}}+\frac{[\mathrm{pMFMASP2_{zym}}]}{K^{\mathrm{MASP2_{zym}}}_{\mathrm{m\,MASP2^*}}}\end{array}\right)}\\
&+\frac{k^{\mathrm{C2}}_{\mathrm{cat\,MASP2^*}}\left[\mathrm{pCLK1MASP2^*}\right]\left[\mathrm{pC4bC4bC2}\right]}{K^{\mathrm{C2}}_{\mathrm{m\,MASP2^*}}\left(\begin{array}{l}1+\frac{[\mathrm{C4}]}{K^{\mathrm{C4}}_{\mathrm{m\,MASP2^*}}}+\frac{[\mathrm{fC4bC2}]}{K^{\mathrm{C2}}_{\mathrm{m\,MASP2^*}}}+\frac{[\mathrm{fC4bC4bC2}]}{K^{\mathrm{C2}}_{\mathrm{m\,MASP2^*}}}+\frac{[\mathrm{fC3bC4bC2}]}{K^{\mathrm{C2}}_{\mathrm{m\,MASP2^*}}}+\frac{[\mathrm{pC4bC2}]}{K^{\mathrm{C2}}_{\mathrm{m\,MASP2^*}}}+\frac{[\mathrm{pC4bC4bC2}]}{K^{\mathrm{C2}}_{\mathrm{m\,MASP2^*}}}\\
+\frac{[\mathrm{pC3bC4bC2}]}{K^{\mathrm{C2}}_{\mathrm{m\,MASP2^*}}}+\frac{[\mathrm{pMBL_3MASP1_{zym}}]}{K^{\mathrm{MASP1_{zym}}}_{\mathrm{m\,MASP2^*}}}+\frac{[\mathrm{pMBL_3MASP2_{zym}}]}{K^{\mathrm{MASP2_{zym}}}_{\mathrm{m\,MASP2^*}}}+\frac{[\mathrm{pMBL_4MASP1_{zym}}]}{K^{\mathrm{MASP1_{zym}}}_{\mathrm{m\,MASP2^*}}}\\
+\frac{[\mathrm{pMBL_4MASP2_{zym}}]}{K^{\mathrm{MASP2_{zym}}}_{\mathrm{m\,MASP2^*}}}+\frac{[\mathrm{pCLLKMASP1_{zym}}]}{K^{\mathrm{MASP1_{zym}}}_{\mathrm{m\,MASP2^*}}}+\frac{[\mathrm{pCLLKMASP2_{zym}}]}{K^{\mathrm{MASP2_{zym}}}_{\mathrm{m\,MASP2^*}}}+\frac{[\mathrm{pCLK1MASP1_{zym}}]}{K^{\mathrm{MASP1_{zym}}}_{\mathrm{m\,MASP2^*}}}\\
+\frac{[\mathrm{pCLK1MASP2_{zym}}]}{K^{\mathrm{MASP2_{zym}}}_{\mathrm{m\,MASP2^*}}}+\frac{[\mathrm{pCLL1MASP1_{zym}}]}{K^{\mathrm{MASP1_{zym}}}_{\mathrm{m\,MASP2^*}}}+\frac{[\mathrm{pCLL1MASP2_{zym}}]}{K^{\mathrm{MASP2_{zym}}}_{\mathrm{m\,MASP2^*}}}+\frac{[\mathrm{pLFMASP1_{zym}}]}{K^{\mathrm{MASP1_{zym}}}_{\mathrm{m\,MASP2^*}}}\\
+\frac{[\mathrm{pLFMASP2_{zym}}]}{K^{\mathrm{MASP2_{zym}}}_{\mathrm{m\,MASP2^*}}}+\frac{[\mathrm{pHFMASP1_{zym}}]}{K^{\mathrm{MASP1_{zym}}}_{\mathrm{m\,MASP2^*}}}+\frac{[\mathrm{pHFMASP2_{zym}}]}{K^{\mathrm{MASP2_{zym}}}_{\mathrm{m\,MASP2^*}}}+\frac{[\mathrm{pMFMASP1_{zym}}]}{K^{\mathrm{MASP1_{zym}}}_{\mathrm{m\,MASP2^*}}}+\frac{[\mathrm{pMFMASP2_{zym}}]}{K^{\mathrm{MASP2_{zym}}}_{\mathrm{m\,MASP2^*}}}\end{array}\right)}
\end{aligned}
$$

$$+\frac{k^{\mathrm{C2}}_{\mathrm{cat\,MASP2^*}}\left[\mathrm{pCLL1MASP2^*}\right]\left[\mathrm{pC4bC4bC2}\right]}{K^{\mathrm{C2}}_{\mathrm{m\,MASP2^*}}\left(1+\frac{[\mathrm{C4}]}{K^{\mathrm{C4}}_{\mathrm{m\,MASP2^*}}}+\frac{[\mathrm{fC4bC2}]}{K^{\mathrm{C2}}_{\mathrm{m\,MASP2^*}}}+\frac{[\mathrm{fC4bC4bC2}]}{K^{\mathrm{C2}}_{\mathrm{m\,MASP2^*}}}+\frac{[\mathrm{fC3bC4bC2}]}{K^{\mathrm{C2}}_{\mathrm{m\,MASP2^*}}}+\frac{[\mathrm{pC4bC2}]}{K^{\mathrm{C2}}_{\mathrm{m\,MASP2^*}}}+\frac{[\mathrm{pC4bC4bC2}]}{K^{\mathrm{C2}}_{\mathrm{m\,MASP2^*}}}+\frac{[\mathrm{pC3bC4bC2}]}{K^{\mathrm{C2}}_{\mathrm{m\,MASP2^*}}}+\frac{[\mathrm{pMBL_3MASP1_{zym}}]}{K^{\mathrm{MASP1_{zym}}}_{\mathrm{m\,MASP2^*}}}+\frac{[\mathrm{pMBL_3MASP2_{zym}}]}{K^{\mathrm{MASP2_{zym}}}_{\mathrm{m\,MASP2^*}}}+\frac{[\mathrm{pMBL_4MASP1_{zym}}]}{K^{\mathrm{MASP1_{zym}}}_{\mathrm{m\,MASP2^*}}}+\frac{[\mathrm{pMBL_4MASP2_{zym}}]}{K^{\mathrm{MASP2_{zym}}}_{\mathrm{m\,MASP2^*}}}+\frac{[\mathrm{pCLLKMASP1_{zym}}]}{K^{\mathrm{MASP1_{zym}}}_{\mathrm{m\,MASP2^*}}}+\frac{[\mathrm{pCLLKMASP2_{zym}}]}{K^{\mathrm{MASP2_{zym}}}_{\mathrm{m\,MASP2^*}}}+\frac{[\mathrm{pCLK1MASP1_{zym}}]}{K^{\mathrm{MASP1_{zym}}}_{\mathrm{m\,MASP2^*}}}+\frac{[\mathrm{pCLK1MASP2_{zym}}]}{K^{\mathrm{MASP2_{zym}}}_{\mathrm{m\,MASP2^*}}}+\frac{[\mathrm{pCLL1MASP1_{zym}}]}{K^{\mathrm{MASP1_{zym}}}_{\mathrm{m\,MASP2^*}}}+\frac{[\mathrm{pCLL1MASP2_{zym}}]}{K^{\mathrm{MASP2_{zym}}}_{\mathrm{m\,MASP2^*}}}+\frac{[\mathrm{pLFMASP1_{zym}}]}{K^{\mathrm{MASP1_{zym}}}_{\mathrm{m\,MASP2^*}}}+\frac{[\mathrm{pLFMASP2_{zym}}]}{K^{\mathrm{MASP2_{zym}}}_{\mathrm{m\,MASP2^*}}}+\frac{[\mathrm{pHFMASP1_{zym}}]}{K^{\mathrm{MASP1_{zym}}}_{\mathrm{m\,MASP2^*}}}+\frac{[\mathrm{pHFMASP2_{zym}}]}{K^{\mathrm{MASP2_{zym}}}_{\mathrm{m\,MASP2^*}}}+\frac{[\mathrm{pMFMASP1_{zym}}]}{K^{\mathrm{MASP1_{zym}}}_{\mathrm{m\,MASP2^*}}}+\frac{[\mathrm{pMFMASP2_{zym}}]}{K^{\mathrm{MASP2_{zym}}}_{\mathrm{m\,MASP2^*}}}\right)}$$

$$+\frac{k^{\mathrm{C2}}_{\mathrm{cat\,MASP2^*}}\left[\mathrm{pLFMASP2^*}\right]\left[\mathrm{pC4bC4bC2}\right]}{K^{\mathrm{C2}}_{\mathrm{m\,MASP2^*}}\left(1+\frac{[\mathrm{C4}]}{K^{\mathrm{C4}}_{\mathrm{m\,MASP2^*}}}+\frac{[\mathrm{fC4bC2}]}{K^{\mathrm{C2}}_{\mathrm{m\,MASP2^*}}}+\frac{[\mathrm{fC4bC4bC2}]}{K^{\mathrm{C2}}_{\mathrm{m\,MASP2^*}}}+\frac{[\mathrm{fC3bC4bC2}]}{K^{\mathrm{C2}}_{\mathrm{m\,MASP2^*}}}+\frac{[\mathrm{pC4bC2}]}{K^{\mathrm{C2}}_{\mathrm{m\,MASP2^*}}}+\frac{[\mathrm{pC4bC4bC2}]}{K^{\mathrm{C2}}_{\mathrm{m\,MASP2^*}}}+\frac{[\mathrm{pC3bC4bC2}]}{K^{\mathrm{C2}}_{\mathrm{m\,MASP2^*}}}+\frac{[\mathrm{pMBL_3MASP1_{zym}}]}{K^{\mathrm{MASP1_{zym}}}_{\mathrm{m\,MASP2^*}}}+\frac{[\mathrm{pMBL_3MASP2_{zym}}]}{K^{\mathrm{MASP2_{zym}}}_{\mathrm{m\,MASP2^*}}}+\frac{[\mathrm{pMBL_4MASP1_{zym}}]}{K^{\mathrm{MASP1_{zym}}}_{\mathrm{m\,MASP2^*}}}+\frac{[\mathrm{pMBL_4MASP2_{zym}}]}{K^{\mathrm{MASP2_{zym}}}_{\mathrm{m\,MASP2^*}}}+\frac{[\mathrm{pCLLKMASP1_{zym}}]}{K^{\mathrm{MASP1_{zym}}}_{\mathrm{m\,MASP2^*}}}+\frac{[\mathrm{pCLLKMASP2_{zym}}]}{K^{\mathrm{MASP2_{zym}}}_{\mathrm{m\,MASP2^*}}}+\frac{[\mathrm{pCLK1MASP1_{zym}}]}{K^{\mathrm{MASP1_{zym}}}_{\mathrm{m\,MASP2^*}}}+\frac{[\mathrm{pCLK1MASP2_{zym}}]}{K^{\mathrm{MASP2_{zym}}}_{\mathrm{m\,MASP2^*}}}+\frac{[\mathrm{pCLL1MASP1_{zym}}]}{K^{\mathrm{MASP1_{zym}}}_{\mathrm{m\,MASP2^*}}}+\frac{[\mathrm{pCLL1MASP2_{zym}}]}{K^{\mathrm{MASP2_{zym}}}_{\mathrm{m\,MASP2^*}}}+\frac{[\mathrm{pLFMASP1_{zym}}]}{K^{\mathrm{MASP1_{zym}}}_{\mathrm{m\,MASP2^*}}}+\frac{[\mathrm{pLFMASP2_{zym}}]}{K^{\mathrm{MASP2_{zym}}}_{\mathrm{m\,MASP2^*}}}+\frac{[\mathrm{pHFMASP1_{zym}}]}{K^{\mathrm{MASP1_{zym}}}_{\mathrm{m\,MASP2^*}}}+\frac{[\mathrm{pHFMASP2_{zym}}]}{K^{\mathrm{MASP2_{zym}}}_{\mathrm{m\,MASP2^*}}}+\frac{[\mathrm{pMFMASP1_{zym}}]}{K^{\mathrm{MASP1_{zym}}}_{\mathrm{m\,MASP2^*}}}+\frac{[\mathrm{pMFMASP2_{zym}}]}{K^{\mathrm{MASP2_{zym}}}_{\mathrm{m\,MASP2^*}}}\right)}$$

$$+\frac{k^{\mathrm{C2}}_{\mathrm{cat\,MASP2^*}}\left[\mathrm{pHFMASP2^*}\right]\left[\mathrm{pC4bC4bC2}\right]}{K^{\mathrm{C2}}_{\mathrm{m\,MASP2^*}}\left(1+\frac{[\mathrm{C4}]}{K^{\mathrm{C4}}_{\mathrm{m\,MASP2^*}}}+\frac{[\mathrm{fC4bC2}]}{K^{\mathrm{C2}}_{\mathrm{m\,MASP2^*}}}+\frac{[\mathrm{fC4bC4bC2}]}{K^{\mathrm{C2}}_{\mathrm{m\,MASP2^*}}}+\frac{[\mathrm{fC3bC4bC2}]}{K^{\mathrm{C2}}_{\mathrm{m\,MASP2^*}}}+\frac{[\mathrm{pC4bC2}]}{K^{\mathrm{C2}}_{\mathrm{m\,MASP2^*}}}+\frac{[\mathrm{pC4bC4bC2}]}{K^{\mathrm{C2}}_{\mathrm{m\,MASP2^*}}}+\frac{[\mathrm{pC3bC4bC2}]}{K^{\mathrm{C2}}_{\mathrm{m\,MASP2^*}}}+\frac{[\mathrm{pMBL_3MASP1_{zym}}]}{K^{\mathrm{MASP1_{zym}}}_{\mathrm{m\,MASP2^*}}}+\frac{[\mathrm{pMBL_3MASP2_{zym}}]}{K^{\mathrm{MASP2_{zym}}}_{\mathrm{m\,MASP2^*}}}+\frac{[\mathrm{pMBL_4MASP1_{zym}}]}{K^{\mathrm{MASP1_{zym}}}_{\mathrm{m\,MASP2^*}}}+\frac{[\mathrm{pMBL_4MASP2_{zym}}]}{K^{\mathrm{MASP2_{zym}}}_{\mathrm{m\,MASP2^*}}}+\frac{[\mathrm{pCLLKMASP1_{zym}}]}{K^{\mathrm{MASP1_{zym}}}_{\mathrm{m\,MASP2^*}}}+\frac{[\mathrm{pCLLKMASP2_{zym}}]}{K^{\mathrm{MASP2_{zym}}}_{\mathrm{m\,MASP2^*}}}+\frac{[\mathrm{pCLK1MASP1_{zym}}]}{K^{\mathrm{MASP1_{zym}}}_{\mathrm{m\,MASP2^*}}}+\frac{[\mathrm{pCLK1MASP2_{zym}}]}{K^{\mathrm{MASP2_{zym}}}_{\mathrm{m\,MASP2^*}}}+\frac{[\mathrm{pCLL1MASP1_{zym}}]}{K^{\mathrm{MASP1_{zym}}}_{\mathrm{m\,MASP2^*}}}+\frac{[\mathrm{pCLL1MASP2_{zym}}]}{K^{\mathrm{MASP2_{zym}}}_{\mathrm{m\,MASP2^*}}}+\frac{[\mathrm{pLFMASP1_{zym}}]}{K^{\mathrm{MASP1_{zym}}}_{\mathrm{m\,MASP2^*}}}+\frac{[\mathrm{pLFMASP2_{zym}}]}{K^{\mathrm{MASP2_{zym}}}_{\mathrm{m\,MASP2^*}}}+\frac{[\mathrm{pHFMASP1_{zym}}]}{K^{\mathrm{MASP1_{zym}}}_{\mathrm{m\,MASP2^*}}}+\frac{[\mathrm{pHFMASP2_{zym}}]}{K^{\mathrm{MASP2_{zym}}}_{\mathrm{m\,MASP2^*}}}+\frac{[\mathrm{pMFMASP1_{zym}}]}{K^{\mathrm{MASP1_{zym}}}_{\mathrm{m\,MASP2^*}}}+\frac{[\mathrm{pMFMASP2_{zym}}]}{K^{\mathrm{MASP2_{zym}}}_{\mathrm{m\,MASP2^*}}}\right)}$$

$$+\frac{k_{\mathrm{cat\ MASP2^*}}^{\mathrm{C2}}\left[\mathrm{pMFMASP2^*}\right]\left[\mathrm{pC4bC4bC2}\right]}{K_{\mathrm{m\ MASP2^*}}^{\mathrm{C2}}\left(\begin{array}{l}1+\frac{[\mathrm{C4}]}{K_{\mathrm{m\ MASP2^*}}^{\mathrm{C4}}}+\frac{[\mathrm{fC4bC2}]}{K_{\mathrm{m\ MASP2^*}}^{\mathrm{C2}}}+\frac{[\mathrm{fC4bC4bC2}]}{K_{\mathrm{m\ MASP2^*}}^{\mathrm{C2}}}+\frac{[\mathrm{fC3bC4bC2}]}{K_{\mathrm{m\ MASP2^*}}^{\mathrm{C2}}}+\frac{[\mathrm{pC4bC2}]}{K_{\mathrm{m\ MASP2^*}}^{\mathrm{C2}}}+\frac{[\mathrm{pC4bC4bC2}]}{K_{\mathrm{m\ MASP2^*}}^{\mathrm{C2}}}\\+\frac{[\mathrm{pC3bC4bC2}]}{K_{\mathrm{m\ MASP2^*}}^{\mathrm{C2}}}+\frac{[\mathrm{pMBL_3MASP1_{zym}}]}{K_{\mathrm{m\ MASP2^*}}^{\mathrm{MASP1_{zym}}}}+\frac{[\mathrm{pMBL_3MASP2_{zym}}]}{K_{\mathrm{m\ MASP2^*}}^{\mathrm{MASP2_{zym}}}}+\frac{[\mathrm{pMBL_4MASP1_{zym}}]}{K_{\mathrm{m\ MASP2^*}}^{\mathrm{MASP1_{zym}}}}\\+\frac{[\mathrm{pMBL_4MASP2_{zym}}]}{K_{\mathrm{m\ MASP2^*}}^{\mathrm{MASP2_{zym}}}}+\frac{[\mathrm{pCLLKMASP1_{zym}}]}{K_{\mathrm{m\ MASP2^*}}^{\mathrm{MASP1_{zym}}}}+\frac{[\mathrm{pCLLKMASP2_{zym}}]}{K_{\mathrm{m\ MASP2^*}}^{\mathrm{MASP2_{zym}}}}+\frac{[\mathrm{pCLK1MASP1_{zym}}]}{K_{\mathrm{m\ MASP2^*}}^{\mathrm{MASP1_{zym}}}}\\+\frac{[\mathrm{pCLK1MASP2_{zym}}]}{K_{\mathrm{m\ MASP2^*}}^{\mathrm{MASP2_{zym}}}}+\frac{[\mathrm{pCLL1MASP1_{zym}}]}{K_{\mathrm{m\ MASP2^*}}^{\mathrm{MASP1_{zym}}}}+\frac{[\mathrm{pCLL1MASP2_{zym}}]}{K_{\mathrm{m\ MASP2^*}}^{\mathrm{MASP2_{zym}}}}+\frac{[\mathrm{pLFMASP1_{zym}}]}{K_{\mathrm{m\ MASP2^*}}^{\mathrm{MASP1_{zym}}}}\\+\frac{[\mathrm{pLFMASP2_{zym}}]}{K_{\mathrm{m\ MASP2^*}}^{\mathrm{MASP2_{zym}}}}+\frac{[\mathrm{pHFMASP1_{zym}}]}{K_{\mathrm{m\ MASP2^*}}^{\mathrm{MASP1_{zym}}}}+\frac{[\mathrm{pHFMASP2_{zym}}]}{K_{\mathrm{m\ MASP2^*}}^{\mathrm{MASP2_{zym}}}}+\frac{[\mathrm{pMFMASP1_{zym}}]}{K_{\mathrm{m\ MASP2^*}}^{\mathrm{MASP1_{zym}}}}+\frac{[\mathrm{pMFMASP2_{zym}}]}{K_{\mathrm{m\ MASP2^*}}^{\mathrm{MASP2_{zym}}}}\end{array}\right)}$$

$$-k_{\mathrm{C4bC4BP}}^{+}\left[\mathrm{pC4bC4bC2a}\right]\left[\mathrm{pC4BPPTX3}\right]+k_{\mathrm{C4bC4BP}}^{-}\left[\mathrm{pC4bC4bC2aC4BPPTX3}\right]-k_{\mathrm{C4bC4BP}}^{+}\left[\mathrm{pC4bC4bC2a}\right]\left[\mathrm{pC4BP}\right]$$

$$+k_{\mathrm{C4bC4BP}}^{-}\left[\mathrm{pC4bC4bC2apC4BP}\right]$$

$$+\frac{k_{\mathrm{cat\ C1^*}}^{\mathrm{C2}}\left[\mathrm{pMBL_3PTX3C1^*}\right]\left[\mathrm{pC4bC4bC2}\right]}{K_{\mathrm{m\ C1^*}}^{\mathrm{C2}}\left(1+\frac{[\mathrm{C4}]}{K_{\mathrm{m\ C1^*}}^{\mathrm{C4}}}+\frac{[\mathrm{fC4bC2}]}{K_{\mathrm{m\ C1^*}}^{\mathrm{C2}}}+\frac{[\mathrm{fC4bC4bC2}]}{K_{\mathrm{m\ C1^*}}^{\mathrm{C2}}}+\frac{[\mathrm{fC3bC4bC2}]}{K_{\mathrm{m\ C1^*}}^{\mathrm{C2}}}+\frac{[\mathrm{pC4bC2}]}{K_{\mathrm{m\ C1^*}}^{\mathrm{C2}}}+\frac{[\mathrm{pC4bC4bC2}]}{K_{\mathrm{m\ C1^*}}^{\mathrm{C2}}}+\frac{[\mathrm{pC3bC4bC2}]}{K_{\mathrm{m\ C1^*}}^{\mathrm{C2}}}\right)}$$

$$+\frac{k_{\mathrm{cat\ C1^*}}^{\mathrm{C2}}\left[\mathrm{pMBL_4PTX3C1^*}\right]\left[\mathrm{pC4bC4bC2}\right]}{K_{\mathrm{m\ C1^*}}^{\mathrm{C2}}\left(1+\frac{[\mathrm{C4}]}{K_{\mathrm{m\ C1^*}}^{\mathrm{C4}}}+\frac{[\mathrm{fC4bC2}]}{K_{\mathrm{m\ C1^*}}^{\mathrm{C2}}}+\frac{[\mathrm{fC4bC4bC2}]}{K_{\mathrm{m\ C1^*}}^{\mathrm{C2}}}+\frac{[\mathrm{fC3bC4bC2}]}{K_{\mathrm{m\ C1^*}}^{\mathrm{C2}}}+\frac{[\mathrm{pC4bC2}]}{K_{\mathrm{m\ C1^*}}^{\mathrm{C2}}}+\frac{[\mathrm{pC4bC4bC2}]}{K_{\mathrm{m\ C1^*}}^{\mathrm{C2}}}+\frac{[\mathrm{pC3bC4bC2}]}{K_{\mathrm{m\ C1^*}}^{\mathrm{C2}}}\right)}$$

153

$$\frac{d\left[\mathrm{pC4bC4bC2aC5b}\right]}{dt}=\frac{k_{\mathrm{cat\ C4bC4bC2a}}^{\mathrm{C5}}\left[\mathrm{C5}\right]\left[\mathrm{pC4bC4bC2a}\right]}{K_{\mathrm{m\ C4bC4bC2a}}^{\mathrm{C5}}\left(1+\frac{[\mathrm{C3}]}{K_{\mathrm{m\ C3bBb}}^{\mathrm{C3}}}+\frac{[\mathrm{C5}]}{K_{\mathrm{m\ C4bC4bC2a}}^{\mathrm{C5}}}\right)}-k_{\mathrm{C5b6}}^{+}\left[\mathrm{pC4bC4bC2aC5b}\right]\left[\mathrm{C6}\right]$$

$$+k_{\mathrm{C5b6}}^{-}\left[\mathrm{pC4bC4bC2aC5b6}\right]-k_{\mathrm{C5b^*}}^{-}\left[\mathrm{pC4bC4bC2aC5b}\right]$$

154

$$\frac{d\left[\mathrm{pC4bC4bC2aC5b6}\right]}{dt}=k_{\mathrm{C5b6}}^{+}\left[\mathrm{pC4bC4bC2aC5b}\right]\left[\mathrm{C6}\right]-k_{\mathrm{C5b6}}^{-}\left[\mathrm{pC4bC4bC2aC5b6}\right]-k_{\mathrm{C5b7}}^{+}\left[\mathrm{pC4bC4bC2aC5b6}\right]\left[\mathrm{C7}\right]$$

155

$$\frac{d[\text{pC3bC4b}]}{dt} = k^{+}_{\text{C3bC4b}}[\text{nfC3b}][\text{pC4b}] - k^{+}_{\text{C4bC2}}[\text{pC3bC4b}][\text{C2}] + k^{-}_{\text{C4bC2}}[\text{pC3bC4bC2}] - k^{+}_{\text{C3bP}}[\text{pC3bC4b}][\text{P}]$$
$$+k^{-}_{\text{C3bP}}[\text{pC3bC4bP}] - k^{+}_{\text{C3bB}}[\text{pC3bC4b}][\text{FB}] + k^{-}_{\text{C3bB}}[\text{pC4bC3bB}] + k^{-}_{\text{C3bC4bC2a}}[\text{pC3bC4bC2a}] + k^{-}_{\text{C3bC4bBb}}[\text{pC4bC3bBb}]$$
$$+k^{-}_{\text{C3bC4bBbP}}[\text{pC4bC3bBbP}] + k^{+}_{\text{C3bC4b}}[\text{pC3b}][\text{nfC4b}] - k^{+}_{\text{C3bH}}[\text{pC3bC4b}][\text{FH}] + k^{-}_{\text{C3bH}}[\text{pC3bC4bFH}]$$
$$-k^{+}_{\text{C3bFHL1}}[\text{pC3bC4b}][\text{FHL1}] + k^{-}_{\text{C3bFHL1}}[\text{pC3bC4bFHL1}] - k^{+}_{\text{C4bC4BP}}[\text{pC3bC4b}][\text{C4BP}] + k^{-}_{\text{C4bC4BP}}[\text{pC3bC4bC4BP}]$$
$$+k^{-}_{\text{C3bBbH}_{\text{decay}}}[\text{pC4bC3bBbFH}] + k^{-}_{\text{C3bBbFHL1}_{\text{decay}}}[\text{pC4bC3bBbFHL1}] + k^{-}_{\text{C4bC2aC4BP}_{\text{decay}}}[\text{pC3bC4bC2aC4BP}]$$
$$-k^{+}_{\text{C3bFHL1}}[\text{pC3bC4b}][\text{pFHL1PTX3}] + k^{-}_{\text{C3bFHL1}}[\text{pC3bC4bFHL1PTX3}] + k^{-}_{\text{C4bC2aC4BP}_{\text{decay}}}[\text{pC3bC4bC2aC4BPPTX3}]$$
$$+k^{-}_{\text{C3bBbH}_{\text{decay}}}[\text{pC4bC3bBbFHPTX3}] + k^{-}_{\text{C3bBbFHL1}_{\text{decay}}}[\text{pC4bC3bBbFHL1PTX3}] - k^{+}_{\text{C3bH}}[\text{pC3bC4b}][\text{pFHPTX3}]$$
$$+k^{-}_{\text{C3bH}}[\text{pC3bC4bFHPTX3}] - k^{+}_{\text{C4bC4BP}}[\text{pC3bC4b}][\text{pC4BPPTX3}] + k^{-}_{\text{C4bC4BP}}[\text{pC3bC4bC4BPPTX3}]$$
$$-k^{+}_{\text{C3bFHL1}}[\text{pC3bC4b}][\text{pFHL1}] + k^{-}_{\text{C3bFHL1}}[\text{pC3bC4bpFHL1}] + k^{-}_{\text{C4bC2aC4BP}_{\text{decay}}}[\text{pC3bC4bC2apC4BP}]$$
$$-k^{+}_{\text{C4bC4BP}}[\text{pC3bC4b}][\text{pC4BP}] + k^{-}_{\text{C4bC4BP}}[\text{pC3bC4bpC4BP}] + k^{-}_{\text{C3bBbH}_{\text{decay}}}[\text{pC4bC3bBbpFH}]$$
$$+k^{-}_{\text{C3bBbFHL1}_{\text{decay}}}[\text{pC4bC3bBbpFHL1}] - k^{+}_{\text{C3bH}}[\text{pC3bC4b}][\text{pFH}] + k^{-}_{\text{C3bH}}[\text{pC3bC4bpFH}]$$

156

$$\frac{d[\text{pC3bC4bC2}]}{dt} = k^{+}_{\text{C4bC2}}[\text{pC3bC4b}][\text{C2}] - k^{-}_{\text{C4bC2}}[\text{pC3bC4bC2}]$$
$$-\frac{k^{\text{C2}}_{\text{cat C1}^*}\left[\text{pIgG}_6\text{C1}^*\right][\text{pC3bC4bC2}]}{K^{\text{C2}}_{\text{m C1}^*}\left(1+\frac{[\text{C4}]}{K^{\text{C4}}_{\text{m C1}^*}}+\frac{[\text{fC4bC2}]}{K^{\text{C2}}_{\text{m C1}^*}}+\frac{[\text{fC4bC4bC2}]}{K^{\text{C2}}_{\text{m C1}^*}}+\frac{[\text{fC3bC4bC2}]}{K^{\text{C2}}_{\text{m C1}^*}}+\frac{[\text{pC4bC2}]}{K^{\text{C2}}_{\text{m C1}^*}}+\frac{[\text{pC4bC4bC2}]}{K^{\text{C2}}_{\text{m C1}^*}}+\frac{[\text{pC3bC4bC2}]}{K^{\text{C2}}_{\text{m C1}^*}}\right)}$$
$$-\frac{k^{\text{C2}}_{\text{cat C1}^*}\left[\text{pIgMC1}^*\right][\text{pC3bC4bC2}]}{K^{\text{C2}}_{\text{m C1}^*}\left(1+\frac{[\text{C4}]}{K^{\text{C4}}_{\text{m C1}^*}}+\frac{[\text{fC4bC2}]}{K^{\text{C2}}_{\text{m C1}^*}}+\frac{[\text{fC4bC4bC2}]}{K^{\text{C2}}_{\text{m C1}^*}}+\frac{[\text{fC3bC4bC2}]}{K^{\text{C2}}_{\text{m C1}^*}}+\frac{[\text{pC4bC2}]}{K^{\text{C2}}_{\text{m C1}^*}}+\frac{[\text{pC4bC4bC2}]}{K^{\text{C2}}_{\text{m C1}^*}}+\frac{[\text{pC3bC4bC2}]}{K^{\text{C2}}_{\text{m C1}^*}}\right)}$$

$$
-\frac{k_{\mathrm{cat\,MASP1^*}}^{\mathrm{C2}}\left[\mathrm{pMBL_3MASP1^*}\right]\left[\mathrm{pC3bC4bC2}\right]}{K_{\mathrm{m\,MASP1^*}}^{\mathrm{C2}}\left(\begin{array}{l}
1+\frac{[\mathrm{C4}]}{K_{\mathrm{m\,MASP1^*}}^{\mathrm{C4}}}+\frac{[\mathrm{fC4bC2}]}{K_{\mathrm{m\,MASP1^*}}^{\mathrm{C2}}}+\frac{[\mathrm{fC4bC4bC2}]}{K_{\mathrm{m\,MASP1^*}}^{\mathrm{C2}}}+\frac{[\mathrm{fC3bC4bC2}]}{K_{\mathrm{m\,MASP1^*}}^{\mathrm{C2}}}+\frac{[\mathrm{pC4bC2}]}{K_{\mathrm{m\,MASP1^*}}^{\mathrm{C2}}}+\frac{[\mathrm{pC4bC4bC2}]}{K_{\mathrm{m\,MASP1^*}}^{\mathrm{C2}}}\\
+\frac{[\mathrm{pC3bC4bC2}]}{K_{\mathrm{m\,MASP1^*}}^{\mathrm{C2}}}+\frac{[\mathrm{pMBL_3MASP1_{zym}}]}{K_{\mathrm{m\,MASP1^*}}^{\mathrm{MASP1_{zym}}}}+\frac{[\mathrm{pMBL_3MASP2_{zym}}]}{K_{\mathrm{m\,MASP1^*}}^{\mathrm{MASP2_{zym}}}}+\frac{[\mathrm{pMBL_3MASP3_{zym}}]}{K_{\mathrm{m\,MASP1^*}}^{\mathrm{MASP3_{zym}}}}\\
+\frac{[\mathrm{pMBL_4MASP1_{zym}}]}{K_{\mathrm{m\,MASP1^*}}^{\mathrm{MASP1_{zym}}}}+\frac{[\mathrm{pMBL_4MASP2_{zym}}]}{K_{\mathrm{m\,MASP1^*}}^{\mathrm{MASP2_{zym}}}}+\frac{[\mathrm{pMBL_4MASP3_{zym}}]}{K_{\mathrm{m\,MASP1^*}}^{\mathrm{MASP3_{zym}}}}+\frac{[\mathrm{pCLLKMASP1_{zym}}]}{K_{\mathrm{m\,MASP1^*}}^{\mathrm{MASP1_{zym}}}}\\
+\frac{[\mathrm{pCLLKMASP2_{zym}}]}{K_{\mathrm{m\,MASP1^*}}^{\mathrm{MASP2_{zym}}}}+\frac{[\mathrm{pCLLKMASP3_{zym}}]}{K_{\mathrm{m\,MASP1^*}}^{\mathrm{MASP3_{zym}}}}+\frac{[\mathrm{pCLK1MASP1_{zym}}]}{K_{\mathrm{m\,MASP1^*}}^{\mathrm{MASP1_{zym}}}}+\frac{[\mathrm{pCLK1MASP2_{zym}}]}{K_{\mathrm{m\,MASP1^*}}^{\mathrm{MASP2_{zym}}}}\\
+\frac{[\mathrm{pCLK1MASP3_{zym}}]}{K_{\mathrm{m\,MASP1^*}}^{\mathrm{MASP3_{zym}}}}+\frac{[\mathrm{pCLL1MASP1_{zym}}]}{K_{\mathrm{m\,MASP1^*}}^{\mathrm{MASP1_{zym}}}}+\frac{[\mathrm{pCLL1MASP2_{zym}}]}{K_{\mathrm{m\,MASP1^*}}^{\mathrm{MASP2_{zym}}}}+\frac{[\mathrm{pCLL1MASP3_{zym}}]}{K_{\mathrm{m\,MASP1^*}}^{\mathrm{MASP3_{zym}}}}\\
+\frac{[\mathrm{pLFMASP1_{zym}}]}{K_{\mathrm{m\,MASP1^*}}^{\mathrm{MASP1_{zym}}}}+\frac{[\mathrm{pLFMASP2_{zym}}]}{K_{\mathrm{m\,MASP1^*}}^{\mathrm{MASP2_{zymogen}}}}+\frac{[\mathrm{pLFMASP3_{zym}}]}{K_{\mathrm{m\,MASP1^*}}^{\mathrm{MASP3_{zym}}}}+\frac{[\mathrm{pHFMASP1_{zym}}]}{K_{\mathrm{m\,MASP1^*}}^{\mathrm{MASP1_{zym}}}}+\frac{[\mathrm{pHFMASP2_{zym}}]}{K_{\mathrm{m\,MASP1^*}}^{\mathrm{MASP2_{zym}}}}\\
+\frac{[\mathrm{pHFMASP3_{zym}}]}{K_{\mathrm{m\,MASP1^*}}^{\mathrm{MASP3_{zym}}}}+\frac{[\mathrm{pMFMASP1_{zym}}]}{K_{\mathrm{m\,MASP1^*}}^{\mathrm{MASP1_{zym}}}}+\frac{[\mathrm{pMFMASP2_{zym}}]}{K_{\mathrm{m\,MASP1^*}}^{\mathrm{MASP2_{zym}}}}+\frac{[\mathrm{pMFMASP3_{zym}}]}{K_{\mathrm{m\,MASP1^*}}^{\mathrm{MASP3_{zym}}}}
\end{array}\right)}
$$

$$
-\frac{k_{\mathrm{cat\,MASP1^*}}^{\mathrm{C2}}\left[\mathrm{pMBL_4MASP1^*}\right]\left[\mathrm{pC3bC4bC2}\right]}{K_{\mathrm{m\,MASP1^*}}^{\mathrm{C2}}\left(\begin{array}{l}
1+\frac{[\mathrm{C4}]}{K_{\mathrm{m\,MASP1^*}}^{\mathrm{C4}}}+\frac{[\mathrm{fC4bC2}]}{K_{\mathrm{m\,MASP1^*}}^{\mathrm{C2}}}+\frac{[\mathrm{fC4bC4bC2}]}{K_{\mathrm{m\,MASP1^*}}^{\mathrm{C2}}}+\frac{[\mathrm{fC3bC4bC2}]}{K_{\mathrm{m\,MASP1^*}}^{\mathrm{C2}}}+\frac{[\mathrm{pC4bC2}]}{K_{\mathrm{m\,MASP1^*}}^{\mathrm{C2}}}+\frac{[\mathrm{pC4bC4bC2}]}{K_{\mathrm{m\,MASP1^*}}^{\mathrm{C2}}}\\
+\frac{[\mathrm{pC3bC4bC2}]}{K_{\mathrm{m\,MASP1^*}}^{\mathrm{C2}}}+\frac{[\mathrm{pMBL_3MASP1_{zym}}]}{K_{\mathrm{m\,MASP1^*}}^{\mathrm{MASP1_{zym}}}}+\frac{[\mathrm{pMBL_3MASP2_{zym}}]}{K_{\mathrm{m\,MASP1^*}}^{\mathrm{MASP2_{zym}}}}+\frac{[\mathrm{pMBL_3MASP3_{zym}}]}{K_{\mathrm{m\,MASP1^*}}^{\mathrm{MASP3_{zym}}}}\\
+\frac{[\mathrm{pMBL_4MASP1_{zym}}]}{K_{\mathrm{m\,MASP1^*}}^{\mathrm{MASP1_{zym}}}}+\frac{[\mathrm{pMBL_4MASP2_{zym}}]}{K_{\mathrm{m\,MASP1^*}}^{\mathrm{MASP2_{zym}}}}+\frac{[\mathrm{pMBL_4MASP3_{zym}}]}{K_{\mathrm{m\,MASP1^*}}^{\mathrm{MASP3_{zym}}}}+\frac{[\mathrm{pCLLKMASP1_{zym}}]}{K_{\mathrm{m\,MASP1^*}}^{\mathrm{MASP1_{zym}}}}\\
+\frac{[\mathrm{pCLLKMASP2_{zym}}]}{K_{\mathrm{m\,MASP1^*}}^{\mathrm{MASP2_{zym}}}}+\frac{[\mathrm{pCLLKMASP3_{zym}}]}{K_{\mathrm{m\,MASP1^*}}^{\mathrm{MASP3_{zym}}}}+\frac{[\mathrm{pCLK1MASP1_{zym}}]}{K_{\mathrm{m\,MASP1^*}}^{\mathrm{MASP1_{zym}}}}+\frac{[\mathrm{pCLK1MASP2_{zym}}]}{K_{\mathrm{m\,MASP1^*}}^{\mathrm{MASP2_{zym}}}}\\
+\frac{[\mathrm{pCLK1MASP3_{zym}}]}{K_{\mathrm{m\,MASP1^*}}^{\mathrm{MASP3_{zym}}}}+\frac{[\mathrm{pCLL1MASP1_{zym}}]}{K_{\mathrm{m\,MASP1^*}}^{\mathrm{MASP1_{zym}}}}+\frac{[\mathrm{pCLL1MASP2_{zym}}]}{K_{\mathrm{m\,MASP1^*}}^{\mathrm{MASP2_{zym}}}}+\frac{[\mathrm{pCLL1MASP3_{zym}}]}{K_{\mathrm{m\,MASP1^*}}^{\mathrm{MASP3_{zym}}}}\\
+\frac{[\mathrm{pLFMASP1_{zym}}]}{K_{\mathrm{m\,MASP1^*}}^{\mathrm{MASP1_{zym}}}}+\frac{[\mathrm{pLFMASP2_{zym}}]}{K_{\mathrm{m\,MASP1^*}}^{\mathrm{MASP2_{zym}}}}+\frac{[\mathrm{pLFMASP3_{zym}}]}{K_{\mathrm{m\,MASP1^*}}^{\mathrm{MASP3_{zym}}}}+\frac{[\mathrm{pHFMASP1_{zym}}]}{K_{\mathrm{m\,MASP1^*}}^{\mathrm{MASP1_{zym}}}}+\frac{[\mathrm{pHFMASP2_{zym}}]}{K_{\mathrm{m\,MASP1^*}}^{\mathrm{MASP2_{zym}}}}\\
+\frac{[\mathrm{pHFMASP3_{zym}}]}{K_{\mathrm{m\,MASP1^*}}^{\mathrm{MASP3_{zym}}}}+\frac{[\mathrm{pMFMASP1_{zym}}]}{K_{\mathrm{m\,MASP1^*}}^{\mathrm{MASP1_{zym}}}}+\frac{[\mathrm{pMFMASP2_{zym}}]}{K_{\mathrm{m\,MASP1^*}}^{\mathrm{MASP2_{zym}}}}+\frac{[\mathrm{pMFMASP3_{zym}}]}{K_{\mathrm{m\,MASP1^*}}^{\mathrm{MASP3_{zym}}}}
\end{array}\right)}
$$

$$
-\frac{k_{\mathrm{cat\,MASP1^*}}^{\mathrm{C2}}\left[\mathrm{pCLLKMASP1^*}\right]\left[\mathrm{pC3bC4bC2}\right]}{K_{\mathrm{m\,MASP1^*}}^{\mathrm{C2}}\left(\begin{array}{l}1+\frac{[\mathrm{C4}]}{K_{\mathrm{m\,MASP1^*}}^{\mathrm{C4}}}+\frac{[\mathrm{fC4bC2}]}{K_{\mathrm{m\,MASP1^*}}^{\mathrm{C2}}}+\frac{[\mathrm{fC4bC4bC2}]}{K_{\mathrm{m\,MASP1^*}}^{\mathrm{C2}}}+\frac{[\mathrm{fC3bC4bC2}]}{K_{\mathrm{m\,MASP1^*}}^{\mathrm{C2}}}+\frac{[\mathrm{pC4bC2}]}{K_{\mathrm{m\,MASP1^*}}^{\mathrm{C2}}}+\frac{[\mathrm{pC4bC4bC2}]}{K_{\mathrm{m\,MASP1^*}}^{\mathrm{C2}}}\\
+\frac{[\mathrm{pC3bC4bC2}]}{K_{\mathrm{m\,MASP1^*}}^{\mathrm{C2}}}+\frac{[\mathrm{pMBL_3MASP1_{zym}}]}{K_{\mathrm{m\,MASP1^*}}^{\mathrm{MASP1_{zym}}}}+\frac{[\mathrm{pMBL_3MASP2_{zym}}]}{K_{\mathrm{m\,MASP1^*}}^{\mathrm{MASP2_{zym}}}}+\frac{[\mathrm{pMBL_3MASP3_{zym}}]}{K_{\mathrm{m\,MASP1^*}}^{\mathrm{MASP3_{zym}}}}\\
+\frac{[\mathrm{pMBL_4MASP1_{zym}}]}{K_{\mathrm{m\,MASP1^*}}^{\mathrm{MASP1_{zym}}}}+\frac{[\mathrm{pMBL_4MASP2_{zym}}]}{K_{\mathrm{m\,MASP1^*}}^{\mathrm{MASP2_{zym}}}}+\frac{[\mathrm{pMBL_4MASP3_{zym}}]}{K_{\mathrm{m\,MASP1^*}}^{\mathrm{MASP3_{zym}}}}+\frac{[\mathrm{pCLLKMASP1_{zym}}]}{K_{\mathrm{m\,MASP1^*}}^{\mathrm{MASP1_{zym}}}}\\
+\frac{[\mathrm{pCLLKMASP2_{zym}}]}{K_{\mathrm{m\,MASP1^*}}^{\mathrm{MASP2_{zym}}}}+\frac{[\mathrm{pCLLKMASP3_{zym}}]}{K_{\mathrm{m\,MASP1^*}}^{\mathrm{MASP3_{zym}}}}+\frac{[\mathrm{pCLK1MASP1_{zym}}]}{K_{\mathrm{m\,MASP1^*}}^{\mathrm{MASP1_{zym}}}}+\frac{[\mathrm{pCLK1MASP2_{zym}}]}{K_{\mathrm{m\,MASP1^*}}^{\mathrm{MASP2_{zym}}}}\\
+\frac{[\mathrm{pCLK1MASP3_{zym}}]}{K_{\mathrm{m\,MASP1^*}}^{\mathrm{MASP3_{zym}}}}+\frac{[\mathrm{pCLL1MASP1_{zym}}]}{K_{\mathrm{m\,MASP1^*}}^{\mathrm{MASP1_{zym}}}}+\frac{[\mathrm{pCLL1MASP2_{zym}}]}{K_{\mathrm{m\,MASP1^*}}^{\mathrm{MASP2_{zym}}}}+\frac{[\mathrm{pCLL1MASP3_{zym}}]}{K_{\mathrm{m\,MASP1^*}}^{\mathrm{MASP3_{zym}}}}\\
+\frac{[\mathrm{pLFMASP1_{zym}}]}{K_{\mathrm{m\,MASP1^*}}^{\mathrm{MASP1_{zym}}}}+\frac{[\mathrm{pLFMASP2_{zym}}]}{K_{\mathrm{m\,MASP1^*}}^{\mathrm{MASP2_{zym}}}}+\frac{[\mathrm{pLFMASP3_{zym}}]}{K_{\mathrm{m\,MASP1^*}}^{\mathrm{MASP3_{zym}}}}+\frac{[\mathrm{pHFMASP1_{zym}}]}{K_{\mathrm{m\,MASP1^*}}^{\mathrm{MASP1_{zym}}}}+\frac{[\mathrm{pHFMASP2_{zym}}]}{K_{\mathrm{m\,MASP1^*}}^{\mathrm{MASP2_{zym}}}}\\
+\frac{[\mathrm{pHFMASP3_{zym}}]}{K_{\mathrm{m\,MASP1^*}}^{\mathrm{MASP3_{zym}}}}+\frac{[\mathrm{pMFMASP1_{zym}}]}{K_{\mathrm{m\,MASP1^*}}^{\mathrm{MASP1_{zym}}}}+\frac{[\mathrm{pMFMASP2_{zym}}]}{K_{\mathrm{m\,MASP1^*}}^{\mathrm{MASP2_{zym}}}}+\frac{[\mathrm{pMFMASP3_{zym}}]}{K_{\mathrm{m\,MASP1^*}}^{\mathrm{MASP3_{zym}}}}\end{array}\right)}
$$

$$
-\frac{k_{\mathrm{cat\,MASP1^*}}^{\mathrm{C2}}\left[\mathrm{pCLK1MASP1^*}\right]\left[\mathrm{pC3bC4bC2}\right]}{K_{\mathrm{m\,MASP1^*}}^{\mathrm{C2}}\left(\begin{array}{l}1+\frac{[\mathrm{C4}]}{K_{\mathrm{m\,MASP1^*}}^{\mathrm{C4}}}+\frac{[\mathrm{fC4bC2}]}{K_{\mathrm{m\,MASP1^*}}^{\mathrm{C2}}}+\frac{[\mathrm{fC4bC4bC2}]}{K_{\mathrm{m\,MASP1^*}}^{\mathrm{C2}}}+\frac{[\mathrm{fC3bC4bC2}]}{K_{\mathrm{m\,MASP1^*}}^{\mathrm{C2}}}+\frac{[\mathrm{pC4bC2}]}{K_{\mathrm{m\,MASP1^*}}^{\mathrm{C2}}}+\frac{[\mathrm{pC4bC4bC2}]}{K_{\mathrm{m\,MASP1^*}}^{\mathrm{C2}}}\\
+\frac{[\mathrm{pC3bC4bC2}]}{K_{\mathrm{m\,MASP1^*}}^{\mathrm{C2}}}+\frac{[\mathrm{pMBL_3MASP1_{zym}}]}{K_{\mathrm{m\,MASP1^*}}^{\mathrm{MASP1_{zym}}}}+\frac{[\mathrm{pMBL_3MASP2_{zym}}]}{K_{\mathrm{m\,MASP1^*}}^{\mathrm{MASP2_{zym}}}}+\frac{[\mathrm{pMBL_3MASP3_{zym}}]}{K_{\mathrm{m\,MASP1^*}}^{\mathrm{MASP3_{zym}}}}\\
+\frac{[\mathrm{pMBL_4MASP1_{zym}}]}{K_{\mathrm{m\,MASP1^*}}^{\mathrm{MASP1_{zym}}}}+\frac{[\mathrm{pMBL_4MASP2_{zym}}]}{K_{\mathrm{m\,MASP1^*}}^{\mathrm{MASP2_{zym}}}}+\frac{[\mathrm{pMBL_4MASP3_{zym}}]}{K_{\mathrm{m\,MASP1^*}}^{\mathrm{MASP3_{zym}}}}+\frac{[\mathrm{pCLLKMASP1_{zym}}]}{K_{\mathrm{m\,MASP1^*}}^{\mathrm{MASP1_{zym}}}}\\
+\frac{[\mathrm{pCLLKMASP2_{zym}}]}{K_{\mathrm{m\,MASP1^*}}^{\mathrm{MASP2_{zym}}}}+\frac{[\mathrm{pCLLKMASP3_{zym}}]}{K_{\mathrm{m\,MASP1^*}}^{\mathrm{MASP3_{zym}}}}+\frac{[\mathrm{pCLK1MASP1_{zym}}]}{K_{\mathrm{m\,MASP1^*}}^{\mathrm{MASP1_{zym}}}}+\frac{[\mathrm{pCLK1MASP2_{zym}}]}{K_{\mathrm{m\,MASP1^*}}^{\mathrm{MASP2_{zym}}}}\\
+\frac{[\mathrm{pCLK1MASP3_{zym}}]}{K_{\mathrm{m\,MASP1^*}}^{\mathrm{MASP3_{zym}}}}+\frac{[\mathrm{pCLL1MASP1_{zym}}]}{K_{\mathrm{m\,MASP1^*}}^{\mathrm{MASP1_{zym}}}}+\frac{[\mathrm{pCLL1MASP2_{zym}}]}{K_{\mathrm{m\,MASP1^*}}^{\mathrm{MASP2_{zym}}}}+\frac{[\mathrm{pCLL1MASP3_{zym}}]}{K_{\mathrm{m\,MASP1^*}}^{\mathrm{MASP3_{zym}}}}\\
+\frac{[\mathrm{pLFMASP1_{zym}}]}{K_{\mathrm{m\,MASP1^*}}^{\mathrm{MASP1_{zym}}}}+\frac{[\mathrm{pLFMASP2_{zym}}]}{K_{\mathrm{m\,MASP1^*}}^{\mathrm{MASP2_{zym}}}}+\frac{[\mathrm{pLFMASP3_{zym}}]}{K_{\mathrm{m\,MASP1^*}}^{\mathrm{MASP3_{zym}}}}+\frac{[\mathrm{pHFMASP1_{zym}}]}{K_{\mathrm{m\,MASP1^*}}^{\mathrm{MASP1_{zym}}}}+\frac{[\mathrm{pHFMASP2_{zym}}]}{K_{\mathrm{m\,MASP1^*}}^{\mathrm{MASP2_{zym}}}}\\
+\frac{[\mathrm{pHFMASP3_{zym}}]}{K_{\mathrm{m\,MASP1^*}}^{\mathrm{MASP3_{zym}}}}+\frac{[\mathrm{pMFMASP1_{zym}}]}{K_{\mathrm{m\,MASP1^*}}^{\mathrm{MASP1_{zym}}}}+\frac{[\mathrm{pMFMASP2_{zym}}]}{K_{\mathrm{m\,MASP1^*}}^{\mathrm{MASP2_{zym}}}}+\frac{[\mathrm{pMFMASP3_{zym}}]}{K_{\mathrm{m\,MASP1^*}}^{\mathrm{MASP3_{zym}}}}\end{array}\right)}
$$

$$-\frac{k^{\mathrm{C2}}_{\mathrm{cat\,MASP1^*}}\left[\mathrm{pCLL1MASP1^*}\right]\left[\mathrm{pC3bC4bC2}\right]}{K^{\mathrm{C2}}_{\mathrm{m\,MASP1^*}}\left(\begin{aligned}&1+\frac{[\mathrm{C4}]}{K^{\mathrm{C4}}_{\mathrm{m\,MASP1^*}}}+\frac{[\mathrm{fC4bC2}]}{K^{\mathrm{C2}}_{\mathrm{m\,MASP1^*}}}+\frac{[\mathrm{fC4bC4bC2}]}{K^{\mathrm{C2}}_{\mathrm{m\,MASP1^*}}}+\frac{[\mathrm{fC3bC4bC2}]}{K^{\mathrm{C2}}_{\mathrm{m\,MASP1^*}}}+\frac{[\mathrm{pC4bC2}]}{K^{\mathrm{C2}}_{\mathrm{m\,MASP1^*}}}+\frac{[\mathrm{pC4bC4bC2}]}{K^{\mathrm{C2}}_{\mathrm{m\,MASP1^*}}}\\&+\frac{[\mathrm{pC3bC4bC2}]}{K^{\mathrm{C2}}_{\mathrm{m\,MASP1^*}}}+\frac{\left[\mathrm{pMBL_3MASP1_{zym}}\right]}{K^{\mathrm{MASP1_{zym}}}_{\mathrm{m\,MASP1^*}}}+\frac{\left[\mathrm{pMBL_3MASP2_{zym}}\right]}{K^{\mathrm{MASP2_{zym}}}_{\mathrm{m\,MASP1^*}}}+\frac{\left[\mathrm{pMBL_3MASP3_{zym}}\right]}{K^{\mathrm{MASP3_{zym}}}_{\mathrm{m\,MASP1^*}}}\\&+\frac{\left[\mathrm{pMBL_4MASP1_{zym}}\right]}{K^{\mathrm{MASP1_{zym}}}_{\mathrm{m\,MASP1^*}}}+\frac{\left[\mathrm{pMBL_4MASP2_{zym}}\right]}{K^{\mathrm{MASP2_{zym}}}_{\mathrm{m\,MASP1^*}}}+\frac{\left[\mathrm{pMBL_4MASP3_{zym}}\right]}{K^{\mathrm{MASP3_{zym}}}_{\mathrm{m\,MASP1^*}}}+\frac{\left[\mathrm{pCLLKMASP1_{zym}}\right]}{K^{\mathrm{MASP1_{zym}}}_{\mathrm{m\,MASP1^*}}}\\&+\frac{\left[\mathrm{pCLLKMASP2_{zym}}\right]}{K^{\mathrm{MASP2_{zym}}}_{\mathrm{m\,MASP1^*}}}+\frac{\left[\mathrm{pCLLKMASP3_{zym}}\right]}{K^{\mathrm{MASP3_{zym}}}_{\mathrm{m\,MASP1^*}}}+\frac{\left[\mathrm{pCLK1MASP1_{zym}}\right]}{K^{\mathrm{MASP1_{zym}}}_{\mathrm{m\,MASP1^*}}}+\frac{\left[\mathrm{pCLK1MASP2_{zym}}\right]}{K^{\mathrm{MASP2_{zym}}}_{\mathrm{m\,MASP1^*}}}\\&+\frac{\left[\mathrm{pCLK1MASP3_{zym}}\right]}{K^{\mathrm{MASP3_{zym}}}_{\mathrm{m\,MASP1^*}}}+\frac{\left[\mathrm{pCLL1MASP1_{zym}}\right]}{K^{\mathrm{MASP1_{zym}}}_{\mathrm{m\,MASP1^*}}}+\frac{\left[\mathrm{pCLL1MASP2_{zym}}\right]}{K^{\mathrm{MASP2_{zym}}}_{\mathrm{m\,MASP1^*}}}+\frac{\left[\mathrm{pCLL1MASP3_{zym}}\right]}{K^{\mathrm{MASP3_{zym}}}_{\mathrm{m\,MASP1^*}}}\\&+\frac{\left[\mathrm{pLFMASP1_{zym}}\right]}{K^{\mathrm{MASP1_{zym}}}_{\mathrm{m\,MASP1^*}}}+\frac{\left[\mathrm{pLFMASP2_{zym}}\right]}{K^{\mathrm{MASP2_{zym}}}_{\mathrm{m\,MASP1^*}}}+\frac{\left[\mathrm{pLFMASP3_{zym}}\right]}{K^{\mathrm{MASP3_{zym}}}_{\mathrm{m\,MASP1^*}}}+\frac{\left[\mathrm{pHFMASP1_{zym}}\right]}{K^{\mathrm{MASP1_{zym}}}_{\mathrm{m\,MASP1^*}}}+\frac{\left[\mathrm{pHFMASP2_{zym}}\right]}{K^{\mathrm{MASP2_{zym}}}_{\mathrm{m\,MASP1^*}}}\\&+\frac{\left[\mathrm{pHFMASP3_{zym}}\right]}{K^{\mathrm{MASP3_{zym}}}_{\mathrm{m\,MASP1^*}}}+\frac{\left[\mathrm{pMFMASP1_{zym}}\right]}{K^{\mathrm{MASP1_{zym}}}_{\mathrm{m\,MASP1^*}}}+\frac{\left[\mathrm{pMFMASP2_{zym}}\right]}{K^{\mathrm{MASP2_{zym}}}_{\mathrm{m\,MASP1^*}}}+\frac{\left[\mathrm{pMFMASP3_{zym}}\right]}{K^{\mathrm{MASP3_{zym}}}_{\mathrm{m\,MASP1^*}}}\end{aligned}\right)}$$

$$-\frac{k^{\mathrm{C2}}_{\mathrm{cat\,MASP1^*}}\left[\mathrm{pLFMASP1^*}\right]\left[\mathrm{pC3bC4bC2}\right]}{K^{\mathrm{C2}}_{\mathrm{m\,MASP1^*}}\left(\begin{aligned}&1+\frac{[\mathrm{C4}]}{K^{\mathrm{C4}}_{\mathrm{m\,MASP1^*}}}+\frac{[\mathrm{fC4bC2}]}{K^{\mathrm{C2}}_{\mathrm{m\,MASP1^*}}}+\frac{[\mathrm{fC4bC4bC2}]}{K^{\mathrm{C2}}_{\mathrm{m\,MASP1^*}}}+\frac{[\mathrm{fC3bC4bC2}]}{K^{\mathrm{C2}}_{\mathrm{m\,MASP1^*}}}+\frac{[\mathrm{pC4bC2}]}{K^{\mathrm{C2}}_{\mathrm{m\,MASP1^*}}}+\frac{[\mathrm{pC4bC4bC2}]}{K^{\mathrm{C2}}_{\mathrm{m\,MASP1^*}}}\\&+\frac{[\mathrm{pC3bC4bC2}]}{K^{\mathrm{C2}}_{\mathrm{m\,MASP1^*}}}+\frac{\left[\mathrm{pMBL_3MASP1_{zym}}\right]}{K^{\mathrm{MASP1_{zym}}}_{\mathrm{m\,MASP1^*}}}+\frac{\left[\mathrm{pMBL_3MASP2_{zym}}\right]}{K^{\mathrm{MASP2_{zym}}}_{\mathrm{m\,MASP1^*}}}+\frac{\left[\mathrm{pMBL_3MASP3_{zym}}\right]}{K^{\mathrm{MASP3_{zym}}}_{\mathrm{m\,MASP1^*}}}\\&+\frac{\left[\mathrm{pMBL_4MASP1_{zym}}\right]}{K^{\mathrm{MASP1_{zym}}}_{\mathrm{m\,MASP1^*}}}+\frac{\left[\mathrm{pMBL_4MASP2_{zym}}\right]}{K^{\mathrm{MASP2_{zym}}}_{\mathrm{m\,MASP1^*}}}+\frac{\left[\mathrm{pMBL_4MASP3_{zym}}\right]}{K^{\mathrm{MASP3_{zym}}}_{\mathrm{m\,MASP1^*}}}+\frac{\left[\mathrm{pCLLKMASP1_{zym}}\right]}{K^{\mathrm{MASP1_{zym}}}_{\mathrm{m\,MASP1^*}}}\\&+\frac{\left[\mathrm{pCLLKMASP2_{zym}}\right]}{K^{\mathrm{MASP2_{zym}}}_{\mathrm{m\,MASP1^*}}}+\frac{\left[\mathrm{pCLLKMASP3_{zym}}\right]}{K^{\mathrm{MASP3_{zym}}}_{\mathrm{m\,MASP1^*}}}+\frac{\left[\mathrm{pCLK1MASP1_{zym}}\right]}{K^{\mathrm{MASP1_{zym}}}_{\mathrm{m\,MASP1^*}}}+\frac{\left[\mathrm{pCLK1MASP2_{zym}}\right]}{K^{\mathrm{MASP2_{zym}}}_{\mathrm{m\,MASP1^*}}}\\&+\frac{\left[\mathrm{pCLK1MASP3_{zym}}\right]}{K^{\mathrm{MASP3_{zym}}}_{\mathrm{m\,MASP1^*}}}+\frac{\left[\mathrm{pCLL1MASP1_{zym}}\right]}{K^{\mathrm{MASP1_{zym}}}_{\mathrm{m\,MASP1^*}}}+\frac{\left[\mathrm{pCLL1MASP2_{zym}}\right]}{K^{\mathrm{MASP2_{zym}}}_{\mathrm{m\,MASP1^*}}}+\frac{\left[\mathrm{pCLL1MASP3_{zym}}\right]}{K^{\mathrm{MASP3_{zym}}}_{\mathrm{m\,MASP1^*}}}\\&+\frac{\left[\mathrm{pLFMASP1_{zym}}\right]}{K^{\mathrm{MASP1_{zym}}}_{\mathrm{m\,MASP1^*}}}+\frac{\left[\mathrm{pLFMASP2_{zym}}\right]}{K^{\mathrm{MASP2_{zym}}}_{\mathrm{m\,MASP1^*}}}+\frac{\left[\mathrm{pLFMASP3_{zym}}\right]}{K^{\mathrm{MASP3_{zym}}}_{\mathrm{m\,MASP1^*}}}+\frac{\left[\mathrm{pHFMASP1_{zym}}\right]}{K^{\mathrm{MASP1_{zym}}}_{\mathrm{m\,MASP1^*}}}+\frac{\left[\mathrm{pHFMASP2_{zym}}\right]}{K^{\mathrm{MASP2_{zym}}}_{\mathrm{m\,MASP1^*}}}\\&+\frac{\left[\mathrm{pHFMASP3_{zym}}\right]}{K^{\mathrm{MASP3_{zym}}}_{\mathrm{m\,MASP1^*}}}+\frac{\left[\mathrm{pMFMASP1_{zym}}\right]}{K^{\mathrm{MASP1_{zym}}}_{\mathrm{m\,MASP1^*}}}+\frac{\left[\mathrm{pMFMASP2_{zym}}\right]}{K^{\mathrm{MASP2_{zym}}}_{\mathrm{m\,MASP1^*}}}+\frac{\left[\mathrm{pMFMASP3_{zym}}\right]}{K^{\mathrm{MASP3_{zym}}}_{\mathrm{m\,MASP1^*}}}\end{aligned}\right)}$$

$$
-\frac{k^{\mathrm{C2}}_{\mathrm{cat\ MASP1^*}}\left[\mathrm{pHFMASP1^*}\right]\left[\mathrm{pC3bC4bC2}\right]}{K^{\mathrm{C2}}_{\mathrm{m\ MASP1^*}}\left(\begin{aligned}&1+\frac{[\mathrm{C4}]}{K^{\mathrm{C4}}_{\mathrm{m\ MASP1^*}}}+\frac{[\mathrm{fC4bC2}]}{K^{\mathrm{C2}}_{\mathrm{m\ MASP1^*}}}+\frac{[\mathrm{fC4bC4bC2}]}{K^{\mathrm{C2}}_{\mathrm{m\ MASP1^*}}}+\frac{[\mathrm{fC3bC4bC2}]}{K^{\mathrm{C2}}_{\mathrm{m\ MASP1^*}}}+\frac{[\mathrm{pC4bC2}]}{K^{\mathrm{C2}}_{\mathrm{m\ MASP1^*}}}+\frac{[\mathrm{pC4bC4bC2}]}{K^{\mathrm{C2}}_{\mathrm{m\ MASP1^*}}}\\
&+\frac{[\mathrm{pC3bC4bC2}]}{K^{\mathrm{C2}}_{\mathrm{m\ MASP1^*}}}+\frac{[\mathrm{pMBL_3MASP1_{zym}}]}{K^{\mathrm{MASP1_{zym}}}_{\mathrm{m\ MASP1^*}}}+\frac{[\mathrm{pMBL_3MASP2_{zym}}]}{K^{\mathrm{MASP2_{zym}}}_{\mathrm{m\ MASP1^*}}}+\frac{[\mathrm{pMBL_3MASP3_{zym}}]}{K^{\mathrm{MASP3_{zym}}}_{\mathrm{m\ MASP1^*}}}\\
&+\frac{[\mathrm{pMBL_4MASP1_{zym}}]}{K^{\mathrm{MASP1_{zym}}}_{\mathrm{m\ MASP1^*}}}+\frac{[\mathrm{pMBL_4MASP2_{zym}}]}{K^{\mathrm{MASP2_{zym}}}_{\mathrm{m\ MASP1^*}}}+\frac{[\mathrm{pMBL_4MASP3_{zym}}]}{K^{\mathrm{MASP3_{zym}}}_{\mathrm{m\ MASP1^*}}}+\frac{[\mathrm{pCLLKMASP1_{zym}}]}{K^{\mathrm{MASP1_{zym}}}_{\mathrm{m\ MASP1^*}}}\\
&+\frac{[\mathrm{pCLLKMASP2_{zym}}]}{K^{\mathrm{MASP2_{zym}}}_{\mathrm{m\ MASP1^*}}}+\frac{[\mathrm{pCLLKMASP3_{zym}}]}{K^{\mathrm{MASP3_{zym}}}_{\mathrm{m\ MASP1^*}}}+\frac{[\mathrm{pCLK1MASP1_{zym}}]}{K^{\mathrm{MASP1_{zym}}}_{\mathrm{m\ MASP1^*}}}+\frac{[\mathrm{pCLK1MASP2_{zym}}]}{K^{\mathrm{MASP2_{zym}}}_{\mathrm{m\ MASP1^*}}}\\
&+\frac{[\mathrm{pCLK1MASP3_{zym}}]}{K^{\mathrm{MASP3_{zym}}}_{\mathrm{m\ MASP1^*}}}+\frac{[\mathrm{pCLL1MASP1_{zym}}]}{K^{\mathrm{MASP1_{zym}}}_{\mathrm{m\ MASP1^*}}}+\frac{[\mathrm{pCLL1MASP2_{zym}}]}{K^{\mathrm{MASP2_{zym}}}_{\mathrm{m\ MASP1^*}}}+\frac{[\mathrm{pCLL1MASP3_{zym}}]}{K^{\mathrm{MASP3_{zym}}}_{\mathrm{m\ MASP1^*}}}\\
&+\frac{[\mathrm{pLFMASP1_{zym}}]}{K^{\mathrm{MASP1_{zym}}}_{\mathrm{m\ MASP1^*}}}+\frac{[\mathrm{pLFMASP2_{zym}}]}{K^{\mathrm{MASP2_{zym}}}_{\mathrm{m\ MASP1^*}}}+\frac{[\mathrm{pLFMASP3_{zym}}]}{K^{\mathrm{MASP3_{zym}}}_{\mathrm{m\ MASP1^*}}}+\frac{[\mathrm{pHFMASP1_{zym}}]}{K^{\mathrm{MASP1_{zym}}}_{\mathrm{m\ MASP1^*}}}+\frac{[\mathrm{pHFMASP2_{zym}}]}{K^{\mathrm{MASP2_{zym}}}_{\mathrm{m\ MASP1^*}}}\\
&+\frac{[\mathrm{pHFMASP3_{zym}}]}{K^{\mathrm{MASP3_{zym}}}_{\mathrm{m\ MASP1^*}}}+\frac{[\mathrm{pMFMASP1_{zym}}]}{K^{\mathrm{MASP1_{zym}}}_{\mathrm{m\ MASP1^*}}}+\frac{[\mathrm{pMFMASP2_{zym}}]}{K^{\mathrm{MASP2_{zym}}}_{\mathrm{m\ MASP1^*}}}+\frac{[\mathrm{pMFMASP3_{zym}}]}{K^{\mathrm{MASP3_{zym}}}_{\mathrm{m\ MASP1^*}}}\end{aligned}\right)}
$$

$$
-\frac{k^{\mathrm{C2}}_{\mathrm{cat\ MASP1^*}}\left[\mathrm{pMFMASP1^*}\right]\left[\mathrm{pC3bC4bC2}\right]}{K^{\mathrm{C2}}_{\mathrm{m\ MASP1^*}}\left(\begin{aligned}&1+\frac{[\mathrm{C4}]}{K^{\mathrm{C4}}_{\mathrm{m\ MASP1^*}}}+\frac{[\mathrm{fC4bC2}]}{K^{\mathrm{C2}}_{\mathrm{m\ MASP1^*}}}+\frac{[\mathrm{fC4bC4bC2}]}{K^{\mathrm{C2}}_{\mathrm{m\ MASP1^*}}}+\frac{[\mathrm{fC3bC4bC2}]}{K^{\mathrm{C2}}_{\mathrm{m\ MASP1^*}}}+\frac{[\mathrm{pC4bC2}]}{K^{\mathrm{C2}}_{\mathrm{m\ MASP1^*}}}+\frac{[\mathrm{pC4bC4bC2}]}{K^{\mathrm{C2}}_{\mathrm{m\ MASP1^*}}}\\
&+\frac{[\mathrm{pC3bC4bC2}]}{K^{\mathrm{C2}}_{\mathrm{m\ MASP1^*}}}+\frac{[\mathrm{pMBL_3MASP1_{zym}}]}{K^{\mathrm{MASP1_{zym}}}_{\mathrm{m\ MASP1^*}}}+\frac{[\mathrm{pMBL_3MASP2_{zym}}]}{K^{\mathrm{MASP2_{zym}}}_{\mathrm{m\ MASP1^*}}}+\frac{[\mathrm{pMBL_3MASP3_{zym}}]}{K^{\mathrm{MASP3_{zym}}}_{\mathrm{m\ MASP1^*}}}\\
&+\frac{[\mathrm{pMBL_4MASP1_{zym}}]}{K^{\mathrm{MASP1_{zym}}}_{\mathrm{m\ MASP1^*}}}+\frac{[\mathrm{pMBL_4MASP2_{zym}}]}{K^{\mathrm{MASP2_{zym}}}_{\mathrm{m\ MASP1^*}}}+\frac{[\mathrm{pMBL_4MASP3_{zym}}]}{K^{\mathrm{MASP3_{zym}}}_{\mathrm{m\ MASP1^*}}}+\frac{[\mathrm{pCLLKMASP1_{zym}}]}{K^{\mathrm{MASP1_{zym}}}_{\mathrm{m\ MASP1^*}}}\\
&+\frac{[\mathrm{pCLLKMASP2_{zym}}]}{K^{\mathrm{MASP2_{zym}}}_{\mathrm{m\ MASP1^*}}}+\frac{[\mathrm{pCLLKMASP3_{zym}}]}{K^{\mathrm{MASP3_{zym}}}_{\mathrm{m\ MASP1^*}}}+\frac{[\mathrm{pCLK1MASP1_{zym}}]}{K^{\mathrm{MASP1_{zym}}}_{\mathrm{m\ MASP1^*}}}+\frac{[\mathrm{pCLK1MASP2_{zym}}]}{K^{\mathrm{MASP2_{zym}}}_{\mathrm{m\ MASP1^*}}}\\
&+\frac{[\mathrm{pCLK1MASP3_{zym}}]}{K^{\mathrm{MASP3_{zym}}}_{\mathrm{m\ MASP1^*}}}+\frac{[\mathrm{pCLL1MASP1_{zym}}]}{K^{\mathrm{MASP1_{zym}}}_{\mathrm{m\ MASP1^*}}}+\frac{[\mathrm{pCLL1MASP2_{zym}}]}{K^{\mathrm{MASP2_{zym}}}_{\mathrm{m\ MASP1^*}}}+\frac{[\mathrm{pCLL1MASP3_{zym}}]}{K^{\mathrm{MASP3_{zym}}}_{\mathrm{m\ MASP1^*}}}\\
&+\frac{[\mathrm{pLFMASP1_{zym}}]}{K^{\mathrm{MASP1_{zym}}}_{\mathrm{m\ MASP1^*}}}+\frac{[\mathrm{pLFMASP2_{zym}}]}{K^{\mathrm{MASP2_{zym}}}_{\mathrm{m\ MASP1^*}}}+\frac{[\mathrm{pLFMASP3_{zym}}]}{K^{\mathrm{MASP3_{zym}}}_{\mathrm{m\ MASP1^*}}}+\frac{[\mathrm{pHFMASP1_{zym}}]}{K^{\mathrm{MASP1_{zym}}}_{\mathrm{m\ MASP1^*}}}+\frac{[\mathrm{pHFMASP2_{zym}}]}{K^{\mathrm{MASP2_{zym}}}_{\mathrm{m\ MASP1^*}}}\\
&+\frac{[\mathrm{pHFMASP3_{zym}}]}{K^{\mathrm{MASP3_{zym}}}_{\mathrm{m\ MASP1^*}}}+\frac{[\mathrm{pMFMASP1_{zym}}]}{K^{\mathrm{MASP1_{zym}}}_{\mathrm{m\ MASP1^*}}}+\frac{[\mathrm{pMFMASP2_{zym}}]}{K^{\mathrm{MASP2_{zym}}}_{\mathrm{m\ MASP1^*}}}+\frac{[\mathrm{pMFMASP3_{zym}}]}{K^{\mathrm{MASP3_{zym}}}_{\mathrm{m\ MASP1^*}}}\end{aligned}\right)}
$$

$$
-\frac{k_{\mathrm{cat\,MASP2^*}}^{\mathrm{C2}}\left[\mathrm{pMBL_3MASP2^*}\right]\left[\mathrm{pC3bC4bC2}\right]}{K_{\mathrm{m\,MASP2^*}}^{\mathrm{C2}}\left(\begin{array}{l}1+\frac{[\mathrm{C4}]}{K_{\mathrm{m\,MASP2^*}}^{\mathrm{C4}}}+\frac{[\mathrm{fC4bC2}]}{K_{\mathrm{m\,MASP2^*}}^{\mathrm{C2}}}+\frac{[\mathrm{fC4bC4bC2}]}{K_{\mathrm{m\,MASP2^*}}^{\mathrm{C2}}}+\frac{[\mathrm{fC3bC4bC2}]}{K_{\mathrm{m\,MASP2^*}}^{\mathrm{C2}}}+\frac{[\mathrm{pC4bC2}]}{K_{\mathrm{m\,MASP2^*}}^{\mathrm{C2}}}+\frac{[\mathrm{pC4bC4bC2}]}{K_{\mathrm{m\,MASP2^*}}^{\mathrm{C2}}}\\
+\frac{[\mathrm{pC3bC4bC2}]}{K_{\mathrm{m\,MASP2^*}}^{\mathrm{C2}}}+\frac{[\mathrm{pMBL_3MASP1_{zym}}]}{K_{\mathrm{m\,MASP2^*}}^{\mathrm{MASP1_{zym}}}}+\frac{[\mathrm{pMBL_3MASP2_{zym}}]}{K_{\mathrm{m\,MASP2^*}}^{\mathrm{MASP2_{zym}}}}+\frac{[\mathrm{pMBL_4MASP1_{zym}}]}{K_{\mathrm{m\,MASP2^*}}^{\mathrm{MASP1_{zym}}}}\\
+\frac{[\mathrm{pMBL_4MASP2_{zym}}]}{K_{\mathrm{m\,MASP2^*}}^{\mathrm{MASP2_{zym}}}}+\frac{[\mathrm{pCLLKMASP1_{zym}}]}{K_{\mathrm{m\,MASP2^*}}^{\mathrm{MASP1_{zym}}}}+\frac{[\mathrm{pCLLKMASP2_{zym}}]}{K_{\mathrm{m\,MASP2^*}}^{\mathrm{MASP2_{zym}}}}+\frac{[\mathrm{pCLK1MASP1_{zym}}]}{K_{\mathrm{m\,MASP2^*}}^{\mathrm{MASP1_{zym}}}}\\
+\frac{[\mathrm{pCLK1MASP2_{zym}}]}{K_{\mathrm{m\,MASP2^*}}^{\mathrm{MASP2_{zym}}}}+\frac{[\mathrm{pCLL1MASP1_{zym}}]}{K_{\mathrm{m\,MASP2^*}}^{\mathrm{MASP1_{zym}}}}+\frac{[\mathrm{pCLL1MASP2_{zym}}]}{K_{\mathrm{m\,MASP2^*}}^{\mathrm{MASP2_{zym}}}}+\frac{[\mathrm{pLFMASP1_{zym}}]}{K_{\mathrm{m\,MASP2^*}}^{\mathrm{MASP1_{zym}}}}\\
+\frac{[\mathrm{pLFMASP2_{zym}}]}{K_{\mathrm{m\,MASP2^*}}^{\mathrm{MASP2_{zym}}}}+\frac{[\mathrm{pHFMASP1_{zym}}]}{K_{\mathrm{m\,MASP2^*}}^{\mathrm{MASP1_{zym}}}}+\frac{[\mathrm{pHFMASP2_{zym}}]}{K_{\mathrm{m\,MASP2^*}}^{\mathrm{MASP2_{zym}}}}+\frac{[\mathrm{pMFMASP1_{zym}}]}{K_{\mathrm{m\,MASP2^*}}^{\mathrm{MASP1_{zym}}}}+\frac{[\mathrm{pMFMASP2_{zym}}]}{K_{\mathrm{m\,MASP2^*}}^{\mathrm{MASP2_{zym}}}}\end{array}\right)}
$$

$$
-\frac{k_{\mathrm{cat\,MASP2^*}}^{\mathrm{C2}}\left[\mathrm{pMBL_4MASP2^*}\right]\left[\mathrm{pC3bC4bC2}\right]}{K_{\mathrm{m\,MASP2^*}}^{\mathrm{C2}}\left(\begin{array}{l}1+\frac{[\mathrm{C4}]}{K_{\mathrm{m\,MASP2^*}}^{\mathrm{C4}}}+\frac{[\mathrm{fC4bC2}]}{K_{\mathrm{m\,MASP2^*}}^{\mathrm{C2}}}+\frac{[\mathrm{fC4bC4bC2}]}{K_{\mathrm{m\,MASP2^*}}^{\mathrm{C2}}}+\frac{[\mathrm{fC3bC4bC2}]}{K_{\mathrm{m\,MASP2^*}}^{\mathrm{C2}}}+\frac{[\mathrm{pC4bC2}]}{K_{\mathrm{m\,MASP2^*}}^{\mathrm{C2}}}+\frac{[\mathrm{pC4bC4bC2}]}{K_{\mathrm{m\,MASP2^*}}^{\mathrm{C2}}}\\
+\frac{[\mathrm{pC3bC4bC2}]}{K_{\mathrm{m\,MASP2^*}}^{\mathrm{C2}}}+\frac{[\mathrm{pMBL_3MASP1_{zym}}]}{K_{\mathrm{m\,MASP2^*}}^{\mathrm{MASP1_{zym}}}}+\frac{[\mathrm{pMBL_3MASP2_{zym}}]}{K_{\mathrm{m\,MASP2^*}}^{\mathrm{MASP2_{zym}}}}+\frac{[\mathrm{pMBL_4MASP1_{zym}}]}{K_{\mathrm{m\,MASP2^*}}^{\mathrm{MASP1_{zym}}}}\\
+\frac{[\mathrm{pMBL_4MASP2_{zym}}]}{K_{\mathrm{m\,MASP2^*}}^{\mathrm{MASP2_{zym}}}}+\frac{[\mathrm{pCLLKMASP1_{zym}}]}{K_{\mathrm{m\,MASP2^*}}^{\mathrm{MASP1_{zym}}}}+\frac{[\mathrm{pCLLKMASP2_{zym}}]}{K_{\mathrm{m\,MASP2^*}}^{\mathrm{MASP2_{zym}}}}+\frac{[\mathrm{pCLK1MASP1_{zym}}]}{K_{\mathrm{m\,MASP2^*}}^{\mathrm{MASP1_{zym}}}}\\
+\frac{[\mathrm{pCLK1MASP2_{zym}}]}{K_{\mathrm{m\,MASP2^*}}^{\mathrm{MASP2_{zym}}}}+\frac{[\mathrm{pCLL1MASP1_{zym}}]}{K_{\mathrm{m\,MASP2^*}}^{\mathrm{MASP1_{zym}}}}+\frac{[\mathrm{pCLL1MASP2_{zym}}]}{K_{\mathrm{m\,MASP2^*}}^{\mathrm{MASP2_{zym}}}}+\frac{[\mathrm{pLFMASP1_{zym}}]}{K_{\mathrm{m\,MASP2^*}}^{\mathrm{MASP1_{zym}}}}\\
+\frac{[\mathrm{pLFMASP2_{zym}}]}{K_{\mathrm{m\,MASP2^*}}^{\mathrm{MASP2_{zym}}}}+\frac{[\mathrm{pHFMASP1_{zym}}]}{K_{\mathrm{m\,MASP2^*}}^{\mathrm{MASP1_{zym}}}}+\frac{[\mathrm{pHFMASP2_{zym}}]}{K_{\mathrm{m\,MASP2^*}}^{\mathrm{MASP2_{zym}}}}+\frac{[\mathrm{pMFMASP1_{zym}}]}{K_{\mathrm{m\,MASP2^*}}^{\mathrm{MASP1_{zym}}}}+\frac{[\mathrm{pMFMASP2_{zym}}]}{K_{\mathrm{m\,MASP2^*}}^{\mathrm{MASP2_{zym}}}}\end{array}\right)}
$$

$$
-\frac{k_{\mathrm{cat\,MASP2^*}}^{\mathrm{C2}}\left[\mathrm{pCLLKMASP2^*}\right]\left[\mathrm{pC3bC4bC2}\right]}{K_{\mathrm{m\,MASP2^*}}^{\mathrm{C2}}\left(\begin{array}{l}1+\frac{[\mathrm{C4}]}{K_{\mathrm{m\,MASP2^*}}^{\mathrm{C4}}}+\frac{[\mathrm{fC4bC2}]}{K_{\mathrm{m\,MASP2^*}}^{\mathrm{C2}}}+\frac{[\mathrm{fC4bC4bC2}]}{K_{\mathrm{m\,MASP2^*}}^{\mathrm{C2}}}+\frac{[\mathrm{fC3bC4bC2}]}{K_{\mathrm{m\,MASP2^*}}^{\mathrm{C2}}}+\frac{[\mathrm{pC4bC2}]}{K_{\mathrm{m\,MASP2^*}}^{\mathrm{C2}}}+\frac{[\mathrm{pC4bC4bC2}]}{K_{\mathrm{m\,MASP2^*}}^{\mathrm{C2}}}\\
+\frac{[\mathrm{pC3bC4bC2}]}{K_{\mathrm{m\,MASP2^*}}^{\mathrm{C2}}}+\frac{[\mathrm{pMBL_3MASP1_{zym}}]}{K_{\mathrm{m\,MASP2^*}}^{\mathrm{MASP1_{zym}}}}+\frac{[\mathrm{pMBL_3MASP2_{zym}}]}{K_{\mathrm{m\,MASP2^*}}^{\mathrm{MASP2_{zym}}}}+\frac{[\mathrm{pMBL_4MASP1_{zym}}]}{K_{\mathrm{m\,MASP2^*}}^{\mathrm{MASP1_{zym}}}}\\
+\frac{[\mathrm{pMBL_4MASP2_{zym}}]}{K_{\mathrm{m\,MASP2^*}}^{\mathrm{MASP2_{zym}}}}+\frac{[\mathrm{pCLLKMASP1_{zym}}]}{K_{\mathrm{m\,MASP2^*}}^{\mathrm{MASP1_{zym}}}}+\frac{[\mathrm{pCLLKMASP2_{zym}}]}{K_{\mathrm{m\,MASP2^*}}^{\mathrm{MASP2_{zym}}}}+\frac{[\mathrm{pCLK1MASP1_{zym}}]}{K_{\mathrm{m\,MASP2^*}}^{\mathrm{MASP1_{zym}}}}\\
+\frac{[\mathrm{pCLK1MASP2_{zym}}]}{K_{\mathrm{m\,MASP2^*}}^{\mathrm{MASP2_{zym}}}}+\frac{[\mathrm{pCLL1MASP1_{zym}}]}{K_{\mathrm{m\,MASP2^*}}^{\mathrm{MASP1_{zym}}}}+\frac{[\mathrm{pCLL1MASP2_{zym}}]}{K_{\mathrm{m\,MASP2^*}}^{\mathrm{MASP2_{zym}}}}+\frac{[\mathrm{pLFMASP1_{zym}}]}{K_{\mathrm{m\,MASP2^*}}^{\mathrm{MASP1_{zym}}}}\\
+\frac{[\mathrm{pLFMASP2_{zym}}]}{K_{\mathrm{m\,MASP2^*}}^{\mathrm{MASP2_{zym}}}}+\frac{[\mathrm{pHFMASP1_{zym}}]}{K_{\mathrm{m\,MASP2^*}}^{\mathrm{MASP1_{zym}}}}+\frac{[\mathrm{pHFMASP2_{zym}}]}{K_{\mathrm{m\,MASP2^*}}^{\mathrm{MASP2_{zym}}}}+\frac{[\mathrm{pMFMASP1_{zym}}]}{K_{\mathrm{m\,MASP2^*}}^{\mathrm{MASP1_{zym}}}}+\frac{[\mathrm{pMFMASP2_{zym}}]}{K_{\mathrm{m\,MASP2^*}}^{\mathrm{MASP2_{zym}}}}\end{array}\right)}
$$

$$-\frac{k^{\mathrm{C2}}_{\mathrm{cat\,MASP2^*}}\left[\mathrm{pCLK1MASP2^*}\right]\left[\mathrm{pC3bC4bC2}\right]}{K^{\mathrm{C2}}_{\mathrm{m\,MASP2^*}}\left(\begin{array}{l}1+\frac{[\mathrm{C4}]}{K^{\mathrm{C4}}_{\mathrm{m\,MASP2^*}}}+\frac{[\mathrm{fC4bC2}]}{K^{\mathrm{C2}}_{\mathrm{m\,MASP2^*}}}+\frac{[\mathrm{fC4bC4bC2}]}{K^{\mathrm{C2}}_{\mathrm{m\,MASP2^*}}}+\frac{[\mathrm{fC3bC4bC2}]}{K^{\mathrm{C2}}_{\mathrm{m\,MASP2^*}}}+\frac{[\mathrm{pC4bC2}]}{K^{\mathrm{C2}}_{\mathrm{m\,MASP2^*}}}+\frac{[\mathrm{pC4bC4bC2}]}{K^{\mathrm{C2}}_{\mathrm{m\,MASP2^*}}}\\+\frac{[\mathrm{pC3bC4bC2}]}{K^{\mathrm{C2}}_{\mathrm{m\,MASP2^*}}}+\frac{[\mathrm{pMBL_3MASP1_{zym}}]}{K^{\mathrm{MASP1_{zym}}}_{\mathrm{m\,MASP2^*}}}+\frac{[\mathrm{pMBL_3MASP2_{zym}}]}{K^{\mathrm{MASP2_{zym}}}_{\mathrm{m\,MASP2^*}}}+\frac{[\mathrm{pMBL_4MASP1_{zym}}]}{K^{\mathrm{MASP1_{zym}}}_{\mathrm{m\,MASP2^*}}}\\+\frac{[\mathrm{pMBL_4MASP2_{zym}}]}{K^{\mathrm{MASP2_{zym}}}_{\mathrm{m\,MASP2^*}}}+\frac{[\mathrm{pCLLKMASP1_{zym}}]}{K^{\mathrm{MASP1_{zym}}}_{\mathrm{m\,MASP2^*}}}+\frac{[\mathrm{pCLLKMASP2_{zym}}]}{K^{\mathrm{MASP2_{zym}}}_{\mathrm{m\,MASP2^*}}}+\frac{[\mathrm{pCLK1MASP1_{zym}}]}{K^{\mathrm{MASP1_{zym}}}_{\mathrm{m\,MASP2^*}}}\\+\frac{[\mathrm{pCLK1MASP2_{zym}}]}{K^{\mathrm{MASP2_{zym}}}_{\mathrm{m\,MASP2^*}}}+\frac{[\mathrm{pCLL1MASP1_{zym}}]}{K^{\mathrm{MASP1_{zym}}}_{\mathrm{m\,MASP2^*}}}+\frac{[\mathrm{pCLL1MASP2_{zym}}]}{K^{\mathrm{MASP2_{zym}}}_{\mathrm{m\,MASP2^*}}}+\frac{[\mathrm{pLFMASP1_{zym}}]}{K^{\mathrm{MASP1_{zym}}}_{\mathrm{m\,MASP2^*}}}\\+\frac{[\mathrm{pLFMASP2_{zym}}]}{K^{\mathrm{MASP2_{zym}}}_{\mathrm{m\,MASP2^*}}}+\frac{[\mathrm{pHFMASP1_{zym}}]}{K^{\mathrm{MASP1_{zym}}}_{\mathrm{m\,MASP2^*}}}+\frac{[\mathrm{pHFMASP2_{zym}}]}{K^{\mathrm{MASP2_{zym}}}_{\mathrm{m\,MASP2^*}}}+\frac{[\mathrm{pMFMASP1_{zym}}]}{K^{\mathrm{MASP1_{zym}}}_{\mathrm{m\,MASP2^*}}}+\frac{[\mathrm{pMFMASP2_{zym}}]}{K^{\mathrm{MASP2_{zym}}}_{\mathrm{m\,MASP2^*}}}\end{array}\right)}$$

$$-\frac{k^{\mathrm{C2}}_{\mathrm{cat\,MASP2^*}}\left[\mathrm{pCLL1MASP2^*}\right]\left[\mathrm{pC3bC4bC2}\right]}{K^{\mathrm{C2}}_{\mathrm{m\,MASP2^*}}\left(\begin{array}{l}1+\frac{[\mathrm{C4}]}{K^{\mathrm{C4}}_{\mathrm{m\,MASP2^*}}}+\frac{[\mathrm{fC4bC2}]}{K^{\mathrm{C2}}_{\mathrm{m\,MASP2^*}}}+\frac{[\mathrm{fC4bC4bC2}]}{K^{\mathrm{C2}}_{\mathrm{m\,MASP2^*}}}+\frac{[\mathrm{fC3bC4bC2}]}{K^{\mathrm{C2}}_{\mathrm{m\,MASP2^*}}}+\frac{[\mathrm{pC4bC2}]}{K^{\mathrm{C2}}_{\mathrm{m\,MASP2^*}}}+\frac{[\mathrm{pC4bC4bC2}]}{K^{\mathrm{C2}}_{\mathrm{m\,MASP2^*}}}\\+\frac{[\mathrm{pC3bC4bC2}]}{K^{\mathrm{C2}}_{\mathrm{m\,MASP2^*}}}+\frac{[\mathrm{pMBL_3MASP1_{zym}}]}{K^{\mathrm{MASP1_{zym}}}_{\mathrm{m\,MASP2^*}}}+\frac{[\mathrm{pMBL_3MASP2_{zym}}]}{K^{\mathrm{MASP2_{zym}}}_{\mathrm{m\,MASP2^*}}}+\frac{[\mathrm{pMBL_4MASP1_{zym}}]}{K^{\mathrm{MASP1_{zym}}}_{\mathrm{m\,MASP2^*}}}\\+\frac{[\mathrm{pMBL_4MASP2_{zym}}]}{K^{\mathrm{MASP2_{zym}}}_{\mathrm{m\,MASP2^*}}}+\frac{[\mathrm{pCLLKMASP1_{zym}}]}{K^{\mathrm{MASP1_{zym}}}_{\mathrm{m\,MASP2^*}}}+\frac{[\mathrm{pCLLKMASP2_{zym}}]}{K^{\mathrm{MASP2_{zym}}}_{\mathrm{m\,MASP2^*}}}+\frac{[\mathrm{pCLK1MASP1_{zym}}]}{K^{\mathrm{MASP1_{zym}}}_{\mathrm{m\,MASP2^*}}}\\+\frac{[\mathrm{pCLK1MASP2_{zym}}]}{K^{\mathrm{MASP2_{zym}}}_{\mathrm{m\,MASP2^*}}}+\frac{[\mathrm{pCLL1MASP1_{zym}}]}{K^{\mathrm{MASP1_{zym}}}_{\mathrm{m\,MASP2^*}}}+\frac{[\mathrm{pCLL1MASP2_{zym}}]}{K^{\mathrm{MASP2_{zym}}}_{\mathrm{m\,MASP2^*}}}+\frac{[\mathrm{pLFMASP1_{zym}}]}{K^{\mathrm{MASP1_{zym}}}_{\mathrm{m\,MASP2^*}}}\\+\frac{[\mathrm{pLFMASP2_{zym}}]}{K^{\mathrm{MASP2_{zym}}}_{\mathrm{m\,MASP2^*}}}+\frac{[\mathrm{pHFMASP1_{zym}}]}{K^{\mathrm{MASP1_{zym}}}_{\mathrm{m\,MASP2^*}}}+\frac{[\mathrm{pHFMASP2_{zym}}]}{K^{\mathrm{MASP2_{zym}}}_{\mathrm{m\,MASP2^*}}}+\frac{[\mathrm{pMFMASP1_{zym}}]}{K^{\mathrm{MASP1_{zym}}}_{\mathrm{m\,MASP2^*}}}+\frac{[\mathrm{pMFMASP2_{zym}}]}{K^{\mathrm{MASP2_{zym}}}_{\mathrm{m\,MASP2^*}}}\end{array}\right)}$$

$$-\frac{k^{\mathrm{C2}}_{\mathrm{cat\,MASP2^*}}\left[\mathrm{pLFMASP2^*}\right]\left[\mathrm{pC3bC4bC2}\right]}{K^{\mathrm{C2}}_{\mathrm{m\,MASP2^*}}\left(\begin{array}{l}1+\frac{[\mathrm{C4}]}{K^{\mathrm{C4}}_{\mathrm{m\,MASP2^*}}}+\frac{[\mathrm{fC4bC2}]}{K^{\mathrm{C2}}_{\mathrm{m\,MASP2^*}}}+\frac{[\mathrm{fC4bC4bC2}]}{K^{\mathrm{C2}}_{\mathrm{m\,MASP2^*}}}+\frac{[\mathrm{fC3bC4bC2}]}{K^{\mathrm{C2}}_{\mathrm{m\,MASP2^*}}}+\frac{[\mathrm{pC4bC2}]}{K^{\mathrm{C2}}_{\mathrm{m\,MASP2^*}}}+\frac{[\mathrm{pC4bC4bC2}]}{K^{\mathrm{C2}}_{\mathrm{m\,MASP2^*}}}\\+\frac{[\mathrm{pC3bC4bC2}]}{K^{\mathrm{C2}}_{\mathrm{m\,MASP2^*}}}+\frac{[\mathrm{pMBL_3MASP1_{zym}}]}{K^{\mathrm{MASP1_{zym}}}_{\mathrm{m\,MASP2^*}}}+\frac{[\mathrm{pMBL_3MASP2_{zym}}]}{K^{\mathrm{MASP2_{zym}}}_{\mathrm{m\,MASP2^*}}}+\frac{[\mathrm{pMBL_4MASP1_{zym}}]}{K^{\mathrm{MASP1_{zym}}}_{\mathrm{m\,MASP2^*}}}\\+\frac{[\mathrm{pMBL_4MASP2_{zym}}]}{K^{\mathrm{MASP2_{zym}}}_{\mathrm{m\,MASP2^*}}}+\frac{[\mathrm{pCLLKMASP1_{zym}}]}{K^{\mathrm{MASP1_{zym}}}_{\mathrm{m\,MASP2^*}}}+\frac{[\mathrm{pCLLKMASP2_{zym}}]}{K^{\mathrm{MASP2_{zym}}}_{\mathrm{m\,MASP2^*}}}+\frac{[\mathrm{pCLK1MASP1_{zym}}]}{K^{\mathrm{MASP1_{zym}}}_{\mathrm{m\,MASP2^*}}}\\+\frac{[\mathrm{pCLK1MASP2_{zym}}]}{K^{\mathrm{MASP2_{zym}}}_{\mathrm{m\,MASP2^*}}}+\frac{[\mathrm{pCLL1MASP1_{zym}}]}{K^{\mathrm{MASP1_{zym}}}_{\mathrm{m\,MASP2^*}}}+\frac{[\mathrm{pCLL1MASP2_{zym}}]}{K^{\mathrm{MASP2_{zym}}}_{\mathrm{m\,MASP2^*}}}+\frac{[\mathrm{pLFMASP1_{zym}}]}{K^{\mathrm{MASP1_{zym}}}_{\mathrm{m\,MASP2^*}}}\\+\frac{[\mathrm{pLFMASP2_{zym}}]}{K^{\mathrm{MASP2_{zym}}}_{\mathrm{m\,MASP2^*}}}+\frac{[\mathrm{pHFMASP1_{zym}}]}{K^{\mathrm{MASP1_{zym}}}_{\mathrm{m\,MASP2^*}}}+\frac{[\mathrm{pHFMASP2_{zym}}]}{K^{\mathrm{MASP2_{zym}}}_{\mathrm{m\,MASP2^*}}}+\frac{[\mathrm{pMFMASP1_{zym}}]}{K^{\mathrm{MASP1_{zym}}}_{\mathrm{m\,MASP2^*}}}+\frac{[\mathrm{pMFMASP2_{zym}}]}{K^{\mathrm{MASP2_{zym}}}_{\mathrm{m\,MASP2^*}}}\end{array}\right)}$$

$$-\frac{k^{\mathrm{C2}}_{\mathrm{cat\ MASP2^*}}\left[\mathrm{pHFMASP2^*}\right]\left[\mathrm{pC3bC4bC2}\right]}{K^{\mathrm{C2}}_{\mathrm{m\ MASP2^*}}\left(\begin{aligned}&1+\frac{[\mathrm{C4}]}{K^{\mathrm{C4}}_{\mathrm{m\ MASP2^*}}}+\frac{[\mathrm{fC4bC2}]}{K^{\mathrm{C2}}_{\mathrm{m\ MASP2^*}}}+\frac{[\mathrm{fC4bC4bC2}]}{K^{\mathrm{C2}}_{\mathrm{m\ MASP2^*}}}+\frac{[\mathrm{fC3bC4bC2}]}{K^{\mathrm{C2}}_{\mathrm{m\ MASP2^*}}}+\frac{[\mathrm{pC4bC2}]}{K^{\mathrm{C2}}_{\mathrm{m\ MASP2^*}}}+\frac{[\mathrm{pC4bC4bC2}]}{K^{\mathrm{C2}}_{\mathrm{m\ MASP2^*}}}\\&+\frac{[\mathrm{pC3bC4bC2}]}{K^{\mathrm{C2}}_{\mathrm{m\ MASP2^*}}}+\frac{[\mathrm{pMBL_3MASP1_{zym}}]}{K^{\mathrm{MASP1_{zym}}}_{\mathrm{m\ MASP2^*}}}+\frac{[\mathrm{pMBL_3MASP2_{zym}}]}{K^{\mathrm{MASP2_{zym}}}_{\mathrm{m\ MASP2^*}}}+\frac{[\mathrm{pMBL_4MASP1_{zym}}]}{K^{\mathrm{MASP1_{zym}}}_{\mathrm{m\ MASP2^*}}}\\&+\frac{[\mathrm{pMBL_4MASP2_{zym}}]}{K^{\mathrm{MASP2_{zym}}}_{\mathrm{m\ MASP2^*}}}+\frac{[\mathrm{pCLLKMASP1_{zym}}]}{K^{\mathrm{MASP1_{zym}}}_{\mathrm{m\ MASP2^*}}}+\frac{[\mathrm{pCLLKMASP2_{zym}}]}{K^{\mathrm{MASP2_{zym}}}_{\mathrm{m\ MASP2^*}}}+\frac{[\mathrm{pCLK1MASP1_{zym}}]}{K^{\mathrm{MASP1_{zym}}}_{\mathrm{m\ MASP2^*}}}\\&+\frac{[\mathrm{pCLK1MASP2_{zym}}]}{K^{\mathrm{MASP2_{zym}}}_{\mathrm{m\ MASP2^*}}}+\frac{[\mathrm{pCLL1MASP1_{zym}}]}{K^{\mathrm{MASP1_{zym}}}_{\mathrm{m\ MASP2^*}}}+\frac{[\mathrm{pCLL1MASP2_{zym}}]}{K^{\mathrm{MASP2_{zym}}}_{\mathrm{m\ MASP2^*}}}+\frac{[\mathrm{pLFMASP1_{zym}}]}{K^{\mathrm{MASP1_{zym}}}_{\mathrm{m\ MASP2^*}}}\\&+\frac{[\mathrm{pLFMASP2_{zym}}]}{K^{\mathrm{MASP2_{zym}}}_{\mathrm{m\ MASP2^*}}}+\frac{[\mathrm{pHFMASP1_{zym}}]}{K^{\mathrm{MASP1_{zym}}}_{\mathrm{m\ MASP2^*}}}+\frac{[\mathrm{pHFMASP2_{zym}}]}{K^{\mathrm{MASP2_{zym}}}_{\mathrm{m\ MASP2^*}}}+\frac{[\mathrm{pMFMASP1_{zym}}]}{K^{\mathrm{MASP1_{zym}}}_{\mathrm{m\ MASP2^*}}}+\frac{[\mathrm{pMFMASP2_{zym}}]}{K^{\mathrm{MASP2_{zym}}}_{\mathrm{m\ MASP2^*}}}\end{aligned}\right)}$$

$$-\frac{k^{\mathrm{C2}}_{\mathrm{cat\ MASP2^*}}\left[\mathrm{pMFMASP2^*}\right]\left[\mathrm{pC3bC4bC2}\right]}{K^{\mathrm{C2}}_{\mathrm{m\ MASP2^*}}\left(\begin{aligned}&1+\frac{[\mathrm{C4}]}{K^{\mathrm{C4}}_{\mathrm{m\ MASP2^*}}}+\frac{[\mathrm{fC4bC2}]}{K^{\mathrm{C2}}_{\mathrm{m\ MASP2^*}}}+\frac{[\mathrm{fC4bC4bC2}]}{K^{\mathrm{C2}}_{\mathrm{m\ MASP2^*}}}+\frac{[\mathrm{fC3bC4bC2}]}{K^{\mathrm{C2}}_{\mathrm{m\ MASP2^*}}}+\frac{[\mathrm{pC4bC2}]}{K^{\mathrm{C2}}_{\mathrm{m\ MASP2^*}}}+\frac{[\mathrm{pC4bC4bC2}]}{K^{\mathrm{C2}}_{\mathrm{m\ MASP2^*}}}\\&+\frac{[\mathrm{pC3bC4bC2}]}{K^{\mathrm{C2}}_{\mathrm{m\ MASP2^*}}}+\frac{[\mathrm{pMBL_3MASP1_{zym}}]}{K^{\mathrm{MASP1_{zym}}}_{\mathrm{m\ MASP2^*}}}+\frac{[\mathrm{pMBL_3MASP2_{zym}}]}{K^{\mathrm{MASP2_{zym}}}_{\mathrm{m\ MASP2^*}}}+\frac{[\mathrm{pMBL_4MASP1_{zym}}]}{K^{\mathrm{MASP1_{zym}}}_{\mathrm{m\ MASP2^*}}}\\&+\frac{[\mathrm{pMBL_4MASP2_{zym}}]}{K^{\mathrm{MASP2_{zym}}}_{\mathrm{m\ MASP2^*}}}+\frac{[\mathrm{pCLLKMASP1_{zym}}]}{K^{\mathrm{MASP1_{zym}}}_{\mathrm{m\ MASP2^*}}}+\frac{[\mathrm{pCLLKMASP2_{zym}}]}{K^{\mathrm{MASP2_{zym}}}_{\mathrm{m\ MASP2^*}}}+\frac{[\mathrm{pCLK1MASP1_{zym}}]}{K^{\mathrm{MASP1_{zym}}}_{\mathrm{m\ MASP2^*}}}\\&+\frac{[\mathrm{pCLK1MASP2_{zym}}]}{K^{\mathrm{MASP2_{zym}}}_{\mathrm{m\ MASP2^*}}}+\frac{[\mathrm{pCLL1MASP1_{zym}}]}{K^{\mathrm{MASP1_{zym}}}_{\mathrm{m\ MASP2^*}}}+\frac{[\mathrm{pCLL1MASP2_{zym}}]}{K^{\mathrm{MASP2_{zym}}}_{\mathrm{m\ MASP2^*}}}+\frac{[\mathrm{pLFMASP1_{zym}}]}{K^{\mathrm{MASP1_{zym}}}_{\mathrm{m\ MASP2^*}}}\\&+\frac{[\mathrm{pLFMASP2_{zym}}]}{K^{\mathrm{MASP2_{zym}}}_{\mathrm{m\ MASP2^*}}}+\frac{[\mathrm{pHFMASP1_{zym}}]}{K^{\mathrm{MASP1_{zym}}}_{\mathrm{m\ MASP2^*}}}+\frac{[\mathrm{pHFMASP2_{zym}}]}{K^{\mathrm{MASP2_{zym}}}_{\mathrm{m\ MASP2^*}}}+\frac{[\mathrm{pMFMASP1_{zym}}]}{K^{\mathrm{MASP1_{zym}}}_{\mathrm{m\ MASP2^*}}}+\frac{[\mathrm{pMFMASP2_{zym}}]}{K^{\mathrm{MASP2_{zym}}}_{\mathrm{m\ MASP2^*}}}\end{aligned}\right)}$$

$$-\frac{k^{\mathrm{C2}}_{\mathrm{cat\ C1^*}}\left[\mathrm{pMBL_3PTX3C1^*}\right]\left[\mathrm{pC3bC4bC2}\right]}{K^{\mathrm{C2}}_{\mathrm{m\ C1^*}}\left(1+\frac{[\mathrm{C4}]}{K^{\mathrm{C4}}_{\mathrm{m\ C1^*}}}+\frac{[\mathrm{fC4bC2}]}{K^{\mathrm{C2}}_{\mathrm{m\ C1^*}}}+\frac{[\mathrm{fC4bC4bC2}]}{K^{\mathrm{C2}}_{\mathrm{m\ C1^*}}}+\frac{[\mathrm{fC3bC4bC2}]}{K^{\mathrm{C2}}_{\mathrm{m\ C1^*}}}+\frac{[\mathrm{pC4bC2}]}{K^{\mathrm{C2}}_{\mathrm{m\ C1^*}}}+\frac{[\mathrm{pC4bC4bC2}]}{K^{\mathrm{C2}}_{\mathrm{m\ C1^*}}}+\frac{[\mathrm{pC3bC4bC2}]}{K^{\mathrm{C2}}_{\mathrm{m\ C1^*}}}\right)}$$

$$-\frac{k^{\mathrm{C2}}_{\mathrm{cat\ C1^*}}\left[\mathrm{pMBL_4PTX3C1^*}\right]\left[\mathrm{pC3bC4bC2}\right]}{K^{\mathrm{C2}}_{\mathrm{m\ C1^*}}\left(1+\frac{[\mathrm{C4}]}{K^{\mathrm{C4}}_{\mathrm{m\ C1^*}}}+\frac{[\mathrm{fC4bC2}]}{K^{\mathrm{C2}}_{\mathrm{m\ C1^*}}}+\frac{[\mathrm{fC4bC4bC2}]}{K^{\mathrm{C2}}_{\mathrm{m\ C1^*}}}+\frac{[\mathrm{fC3bC4bC2}]}{K^{\mathrm{C2}}_{\mathrm{m\ C1^*}}}+\frac{[\mathrm{pC4bC2}]}{K^{\mathrm{C2}}_{\mathrm{m\ C1^*}}}+\frac{[\mathrm{pC4bC4bC2}]}{K^{\mathrm{C2}}_{\mathrm{m\ C1^*}}}+\frac{[\mathrm{pC3bC4bC2}]}{K^{\mathrm{C2}}_{\mathrm{m\ C1^*}}}\right)}$$

157

$$
\begin{aligned}
&\frac{d\left[\mathrm{pC3bC4bC2a}\right]}{dt}= \\
&\frac{k_{\mathrm{cat\,C1^*}}^{\mathrm{C2}}\left[\mathrm{pIgG_6C1^*}\right]\left[\mathrm{pC3bC4bC2}\right]}{K_{\mathrm{m\,C1^*}}^{\mathrm{C2}}\left(1+\frac{[\mathrm{C4}]}{K_{\mathrm{m\,C1^*}}^{\mathrm{C4}}}+\frac{[\mathrm{fC4bC2}]}{K_{\mathrm{m\,C1^*}}^{\mathrm{C2}}}+\frac{[\mathrm{fC4bC4bC2}]}{K_{\mathrm{m\,C1^*}}^{\mathrm{C2}}}+\frac{[\mathrm{fC3bC4bC2}]}{K_{\mathrm{m\,C1^*}}^{\mathrm{C2}}}+\frac{[\mathrm{pC4bC2}]}{K_{\mathrm{m\,C1^*}}^{\mathrm{C2}}}+\frac{[\mathrm{pC4bC4bC2}]}{K_{\mathrm{m\,C1^*}}^{\mathrm{C2}}}+\frac{[\mathrm{pC3bC4bC2}]}{K_{\mathrm{m\,C1^*}}^{\mathrm{C2}}}\right)} \\
&+\frac{k_{\mathrm{cat\,C1^*}}^{\mathrm{C2}}\left[\mathrm{pIgMC1^*}\right]\left[\mathrm{pC3bC4bC2}\right]}{K_{\mathrm{m\,C1^*}}^{\mathrm{C2}}\left(1+\frac{[\mathrm{C4}]}{K_{\mathrm{m\,C1^*}}^{\mathrm{C4}}}+\frac{[\mathrm{fC4bC2}]}{K_{\mathrm{m\,C1^*}}^{\mathrm{C2}}}+\frac{[\mathrm{fC4bC4bC2}]}{K_{\mathrm{m\,C1^*}}^{\mathrm{C2}}}+\frac{[\mathrm{fC3bC4bC2}]}{K_{\mathrm{m\,C1^*}}^{\mathrm{C2}}}+\frac{[\mathrm{pC4bC2}]}{K_{\mathrm{m\,C1^*}}^{\mathrm{C2}}}+\frac{[\mathrm{pC4bC4bC2}]}{K_{\mathrm{m\,C1^*}}^{\mathrm{C2}}}+\frac{[\mathrm{pC3bC4bC2}]}{K_{\mathrm{m\,C1^*}}^{\mathrm{C2}}}\right)} \\
&+k_{\mathrm{C3bC4b}}^{+}\left[\mathrm{nfC3b}\right]\left[\mathrm{pC4bC2a}\right]-k_{\mathrm{C3bC4bC2a}}^{-}\left[\mathrm{pC3bC4bC2a}\right]+k_{\mathrm{C5b^*}}^{-}\left[\mathrm{pC3bC4bC2aC5b}\right]+k_{\mathrm{C5b7}}^{+}\left[\mathrm{pC3bC4bC2aC5b}\right]\left[\mathrm{C7}\right] \\
&-k_{\mathrm{C4bC4BP}}^{+}\left[\mathrm{pC3bC4bC2a}\right]\left[\mathrm{C4BP}\right]+k_{\mathrm{C4bC4BP}}^{-}\left[\mathrm{pC3bC4bC2aC4BP}\right] \\
&+\frac{k_{\mathrm{cat\,MASP1^*}}^{\mathrm{C2}}\left[\mathrm{pMBL_3MASP1^*}\right]\left[\mathrm{pC3bC4bC2}\right]}{K_{\mathrm{m\,MASP1^*}}^{\mathrm{C2}}\left(\begin{aligned}&1+\frac{[\mathrm{C4}]}{K_{\mathrm{m\,MASP1^*}}^{\mathrm{C4}}}+\frac{[\mathrm{fC4bC2}]}{K_{\mathrm{m\,MASP1^*}}^{\mathrm{C2}}}+\frac{[\mathrm{fC4bC4bC2}]}{K_{\mathrm{m\,MASP1^*}}^{\mathrm{C2}}}+\frac{[\mathrm{fC3bC4bC2}]}{K_{\mathrm{m\,MASP1^*}}^{\mathrm{C2}}}+\frac{[\mathrm{pC4bC2}]}{K_{\mathrm{m\,MASP1^*}}^{\mathrm{C2}}}+\frac{[\mathrm{pC4bC4bC2}]}{K_{\mathrm{m\,MASP1^*}}^{\mathrm{C2}}}\\
&+\frac{[\mathrm{pC3bC4bC2}]}{K_{\mathrm{m\,MASP1^*}}^{\mathrm{C2}}}+\frac{[\mathrm{pMBL_3MASP1_{zym}}]}{K_{\mathrm{m\,MASP1^*}}^{\mathrm{MASP1_{zym}}}}+\frac{[\mathrm{pMBL_3MASP2_{zym}}]}{K_{\mathrm{m\,MASP1^*}}^{\mathrm{MASP2_{zym}}}}+\frac{[\mathrm{pMBL_3MASP3_{zym}}]}{K_{\mathrm{m\,MASP1^*}}^{\mathrm{MASP3_{zym}}}}\\
&+\frac{[\mathrm{pMBL_4MASP1_{zym}}]}{K_{\mathrm{m\,MASP1^*}}^{\mathrm{MASP1_{zym}}}}+\frac{[\mathrm{pMBL_4MASP2_{zym}}]}{K_{\mathrm{m\,MASP1^*}}^{\mathrm{MASP2_{zym}}}}+\frac{[\mathrm{pMBL_4MASP3_{zym}}]}{K_{\mathrm{m\,MASP1^*}}^{\mathrm{MASP3_{zym}}}}+\frac{[\mathrm{pCLLKMASP1_{zym}}]}{K_{\mathrm{m\,MASP1^*}}^{\mathrm{MASP1_{zym}}}}\\
&+\frac{[\mathrm{pCLLKMASP2_{zym}}]}{K_{\mathrm{m\,MASP1^*}}^{\mathrm{MASP2_{zym}}}}+\frac{[\mathrm{pCLLKMASP3_{zym}}]}{K_{\mathrm{m\,MASP1^*}}^{\mathrm{MASP3_{zym}}}}+\frac{[\mathrm{pCLK1MASP1_{zym}}]}{K_{\mathrm{m\,MASP1^*}}^{\mathrm{MASP1_{zym}}}}+\frac{[\mathrm{pCLK1MASP2_{zym}}]}{K_{\mathrm{m\,MASP1^*}}^{\mathrm{MASP2_{zym}}}}\\
&+\frac{[\mathrm{pCLK1MASP3_{zym}}]}{K_{\mathrm{m\,MASP1^*}}^{\mathrm{MASP3_{zym}}}}+\frac{[\mathrm{pCLL1MASP1_{zym}}]}{K_{\mathrm{m\,MASP1^*}}^{\mathrm{MASP1_{zym}}}}+\frac{[\mathrm{pCLL1MASP2_{zym}}]}{K_{\mathrm{m\,MASP1^*}}^{\mathrm{MASP2_{zym}}}}+\frac{[\mathrm{pCLL1MASP3_{zym}}]}{K_{\mathrm{m\,MASP1^*}}^{\mathrm{MASP3_{zym}}}}\\
&+\frac{[\mathrm{pLFMASP1_{zym}}]}{K_{\mathrm{m\,MASP1^*}}^{\mathrm{MASP1_{zym}}}}+\frac{[\mathrm{pLFMASP2_{zym}}]}{K_{\mathrm{m\,MASP1^*}}^{\mathrm{MASP2_{zym}}}}+\frac{[\mathrm{pLFMASP3_{zym}}]}{K_{\mathrm{m\,MASP1^*}}^{\mathrm{MASP3_{zym}}}}+\frac{[\mathrm{pHFMASP1_{zym}}]}{K_{\mathrm{m\,MASP1^*}}^{\mathrm{MASP1_{zym}}}}+\frac{[\mathrm{pHFMASP2_{zym}}]}{K_{\mathrm{m\,MASP1^*}}^{\mathrm{MASP2_{zym}}}}\\
&+\frac{[\mathrm{pHFMASP3_{zym}}]}{K_{\mathrm{m\,MASP1^*}}^{\mathrm{MASP3_{zym}}}}+\frac{[\mathrm{pMFMASP1_{zym}}]}{K_{\mathrm{m\,MASP1^*}}^{\mathrm{MASP1_{zym}}}}+\frac{[\mathrm{pMFMASP2_{zym}}]}{K_{\mathrm{m\,MASP1^*}}^{\mathrm{MASP2_{zym}}}}+\frac{[\mathrm{pMFMASP3_{zym}}]}{K_{\mathrm{m\,MASP1^*}}^{\mathrm{MASP3_{zym}}}}\end{aligned}\right)}
\end{aligned}
$$

$$+\frac{k_{\mathrm{cat\,MASP1^{*}}}^{\mathrm{C2}}\left[\mathrm{pMBL_{4}MASP1^{*}}\right]\left[\mathrm{pC3bC4bC2}\right]}{K_{\mathrm{m\,MASP1^{*}}}^{\mathrm{C2}}\left(\begin{array}{l}1+\frac{[\mathrm{C4}]}{K_{\mathrm{m\,MASP1^{*}}}^{\mathrm{C4}}}+\frac{[\mathrm{fC4bC2}]}{K_{\mathrm{m\,MASP1^{*}}}^{\mathrm{C2}}}+\frac{[\mathrm{fC4bC4bC2}]}{K_{\mathrm{m\,MASP1^{*}}}^{\mathrm{C2}}}+\frac{[\mathrm{fC3bC4bC2}]}{K_{\mathrm{m\,MASP1^{*}}}^{\mathrm{C2}}}+\frac{[\mathrm{pC4bC2}]}{K_{\mathrm{m\,MASP1^{*}}}^{\mathrm{C2}}}+\frac{[\mathrm{pC4bC4bC2}]}{K_{\mathrm{m\,MASP1^{*}}}^{\mathrm{C2}}}\\
+\frac{[\mathrm{pC3bC4bC2}]}{K_{\mathrm{m\,MASP1^{*}}}^{\mathrm{C2}}}+\frac{\left[\mathrm{pMBL_{3}MASP1_{zym}}\right]}{K_{\mathrm{m\,MASP1^{*}}}^{\mathrm{MASP1_{zym}}}}+\frac{\left[\mathrm{pMBL_{3}MASP2_{zym}}\right]}{K_{\mathrm{m\,MASP1^{*}}}^{\mathrm{MASP2_{zym}}}}+\frac{\left[\mathrm{pMBL_{3}MASP3_{zym}}\right]}{K_{\mathrm{m\,MASP1^{*}}}^{\mathrm{MASP3_{zym}}}}\\
+\frac{\left[\mathrm{pMBL_{4}MASP1_{zym}}\right]}{K_{\mathrm{m\,MASP1^{*}}}^{\mathrm{MASP1_{zym}}}}+\frac{\left[\mathrm{pMBL_{4}MASP2_{zym}}\right]}{K_{\mathrm{m\,MASP1^{*}}}^{\mathrm{MASP2_{zym}}}}+\frac{\left[\mathrm{pMBL_{4}MASP3_{zym}}\right]}{K_{\mathrm{m\,MASP1^{*}}}^{\mathrm{MASP3_{zym}}}}+\frac{\left[\mathrm{pCLLKMASP1_{zym}}\right]}{K_{\mathrm{m\,MASP1^{*}}}^{\mathrm{MASP1_{zym}}}}\\
+\frac{\left[\mathrm{pCLLKMASP2_{zym}}\right]}{K_{\mathrm{m\,MASP1^{*}}}^{\mathrm{MASP2_{zym}}}}+\frac{\left[\mathrm{pCLLKMASP3_{zym}}\right]}{K_{\mathrm{m\,MASP1^{*}}}^{\mathrm{MASP3_{zym}}}}+\frac{\left[\mathrm{pCLK1MASP1_{zym}}\right]}{K_{\mathrm{m\,MASP1^{*}}}^{\mathrm{MASP1_{zym}}}}+\frac{\left[\mathrm{pCLK1MASP2_{zym}}\right]}{K_{\mathrm{m\,MASP1^{*}}}^{\mathrm{MASP2_{zym}}}}\\
+\frac{\left[\mathrm{pCLK1MASP3_{zym}}\right]}{K_{\mathrm{m\,MASP1^{*}}}^{\mathrm{MASP3_{zym}}}}+\frac{\left[\mathrm{pCLL1MASP1_{zym}}\right]}{K_{\mathrm{m\,MASP1^{*}}}^{\mathrm{MASP1_{zym}}}}+\frac{\left[\mathrm{pCLL1MASP2_{zym}}\right]}{K_{\mathrm{m\,MASP1^{*}}}^{\mathrm{MASP2_{zym}}}}+\frac{\left[\mathrm{pCLL1MASP3_{zym}}\right]}{K_{\mathrm{m\,MASP1^{*}}}^{\mathrm{MASP3_{zym}}}}\\
+\frac{\left[\mathrm{pLFMASP1_{zym}}\right]}{K_{\mathrm{m\,MASP1^{*}}}^{\mathrm{MASP1_{zym}}}}+\frac{\left[\mathrm{pLFMASP2_{zym}}\right]}{K_{\mathrm{m\,MASP1^{*}}}^{\mathrm{MASP2_{zym}}}}+\frac{\left[\mathrm{pLFMASP3_{zym}}\right]}{K_{\mathrm{m\,MASP1^{*}}}^{\mathrm{MASP3_{zym}}}}+\frac{\left[\mathrm{pHFMASP1_{zym}}\right]}{K_{\mathrm{m\,MASP1^{*}}}^{\mathrm{MASP1_{zym}}}}+\frac{\left[\mathrm{pHFMASP2_{zym}}\right]}{K_{\mathrm{m\,MASP1^{*}}}^{\mathrm{MASP2_{zym}}}}\\
+\frac{\left[\mathrm{pHFMASP3_{zym}}\right]}{K_{\mathrm{m\,MASP1^{*}}}^{\mathrm{MASP3_{zym}}}}+\frac{\left[\mathrm{pMFMASP1_{zym}}\right]}{K_{\mathrm{m\,MASP1^{*}}}^{\mathrm{MASP1_{zym}}}}+\frac{\left[\mathrm{pMFMASP2_{zym}}\right]}{K_{\mathrm{m\,MASP1^{*}}}^{\mathrm{MASP2_{zym}}}}+\frac{\left[\mathrm{pMFMASP3_{zym}}\right]}{K_{\mathrm{m\,MASP1^{*}}}^{\mathrm{MASP3_{zym}}}}\end{array}\right)}$$

$$+\frac{k_{\mathrm{cat\,MASP1^{*}}}^{\mathrm{C2}}\left[\mathrm{pCLLKMASP1^{*}}\right]\left[\mathrm{pC3bC4bC2}\right]}{K_{\mathrm{m\,MASP1^{*}}}^{\mathrm{C2}}\left(\begin{array}{l}1+\frac{[\mathrm{C4}]}{K_{\mathrm{m\,MASP1^{*}}}^{\mathrm{C4}}}+\frac{[\mathrm{fC4bC2}]}{K_{\mathrm{m\,MASP1^{*}}}^{\mathrm{C2}}}+\frac{[\mathrm{fC4bC4bC2}]}{K_{\mathrm{m\,MASP1^{*}}}^{\mathrm{C2}}}+\frac{[\mathrm{fC3bC4bC2}]}{K_{\mathrm{m\,MASP1^{*}}}^{\mathrm{C2}}}+\frac{[\mathrm{pC4bC2}]}{K_{\mathrm{m\,MASP1^{*}}}^{\mathrm{C2}}}+\frac{[\mathrm{pC4bC4bC2}]}{K_{\mathrm{m\,MASP1^{*}}}^{\mathrm{C2}}}\\
+\frac{[\mathrm{pC3bC4bC2}]}{K_{\mathrm{m\,MASP1^{*}}}^{\mathrm{C2}}}+\frac{\left[\mathrm{pMBL_{3}MASP1_{zym}}\right]}{K_{\mathrm{m\,MASP1^{*}}}^{\mathrm{MASP1_{zym}}}}+\frac{\left[\mathrm{pMBL_{3}MASP2_{zym}}\right]}{K_{\mathrm{m\,MASP1^{*}}}^{\mathrm{MASP2_{zym}}}}+\frac{\left[\mathrm{pMBL_{3}MASP3_{zym}}\right]}{K_{\mathrm{m\,MASP1^{*}}}^{\mathrm{MASP3_{zym}}}}\\
+\frac{\left[\mathrm{pMBL_{4}MASP1_{zym}}\right]}{K_{\mathrm{m\,MASP1^{*}}}^{\mathrm{MASP1_{zym}}}}+\frac{\left[\mathrm{pMBL_{4}MASP2_{zym}}\right]}{K_{\mathrm{m\,MASP1^{*}}}^{\mathrm{MASP2_{zym}}}}+\frac{\left[\mathrm{pMBL_{4}MASP3_{zym}}\right]}{K_{\mathrm{m\,MASP1^{*}}}^{\mathrm{MASP3_{zym}}}}+\frac{\left[\mathrm{pCLLKMASP1_{zym}}\right]}{K_{\mathrm{m\,MASP1^{*}}}^{\mathrm{MASP1_{zym}}}}\\
+\frac{\left[\mathrm{pCLLKMASP2_{zym}}\right]}{K_{\mathrm{m\,MASP1^{*}}}^{\mathrm{MASP2_{zym}}}}+\frac{\left[\mathrm{pCLLKMASP3_{zym}}\right]}{K_{\mathrm{m\,MASP1^{*}}}^{\mathrm{MASP3_{zym}}}}+\frac{\left[\mathrm{pCLK1MASP1_{zym}}\right]}{K_{\mathrm{m\,MASP1^{*}}}^{\mathrm{MASP1_{zym}}}}+\frac{\left[\mathrm{pCLK1MASP2_{zym}}\right]}{K_{\mathrm{m\,MASP1^{*}}}^{\mathrm{MASP2_{zym}}}}\\
+\frac{\left[\mathrm{pCLK1MASP3_{zym}}\right]}{K_{\mathrm{m\,MASP1^{*}}}^{\mathrm{MASP3_{zym}}}}+\frac{\left[\mathrm{pCLL1MASP1_{zym}}\right]}{K_{\mathrm{m\,MASP1^{*}}}^{\mathrm{MASP1_{zym}}}}+\frac{\left[\mathrm{pCLL1MASP2_{zym}}\right]}{K_{\mathrm{m\,MASP1^{*}}}^{\mathrm{MASP2_{zym}}}}+\frac{\left[\mathrm{pCLL1MASP3_{zym}}\right]}{K_{\mathrm{m\,MASP1^{*}}}^{\mathrm{MASP3_{zym}}}}\\
+\frac{\left[\mathrm{pLFMASP1_{zym}}\right]}{K_{\mathrm{m\,MASP1^{*}}}^{\mathrm{MASP1_{zym}}}}+\frac{\left[\mathrm{pLFMASP2_{zym}}\right]}{K_{\mathrm{m\,MASP1^{*}}}^{\mathrm{MASP2_{zym}}}}+\frac{\left[\mathrm{pLFMASP3_{zym}}\right]}{K_{\mathrm{m\,MASP1^{*}}}^{\mathrm{MASP3_{zym}}}}+\frac{\left[\mathrm{pHFMASP1_{zym}}\right]}{K_{\mathrm{m\,MASP1^{*}}}^{\mathrm{MASP1_{zym}}}}+\frac{\left[\mathrm{pHFMASP2_{zym}}\right]}{K_{\mathrm{m\,MASP1^{*}}}^{\mathrm{MASP2_{zym}}}}\\
+\frac{\left[\mathrm{pHFMASP3_{zym}}\right]}{K_{\mathrm{m\,MASP1^{*}}}^{\mathrm{MASP3_{zym}}}}+\frac{\left[\mathrm{pMFMASP1_{zym}}\right]}{K_{\mathrm{m\,MASP1^{*}}}^{\mathrm{MASP1_{zym}}}}+\frac{\left[\mathrm{pMFMASP2_{zym}}\right]}{K_{\mathrm{m\,MASP1^{*}}}^{\mathrm{MASP2_{zym}}}}+\frac{\left[\mathrm{pMFMASP3_{zym}}\right]}{K_{\mathrm{m\,MASP1^{*}}}^{\mathrm{MASP3_{zym}}}}\end{array}\right)}$$

$$
+\frac{k^{\mathrm{C2}}_{\mathrm{cat\,MASP1^*}}\left[\mathrm{pCLK1MASP1^*}\right]\left[\mathrm{pC3bC4bC2}\right]}{K^{\mathrm{C2}}_{\mathrm{m\,MASP1^*}}\left(\begin{array}{l}1+\frac{[\mathrm{C4}]}{K^{\mathrm{C4}}_{\mathrm{m\,MASP1^*}}}+\frac{[\mathrm{fC4bC2}]}{K^{\mathrm{C2}}_{\mathrm{m\,MASP1^*}}}+\frac{[\mathrm{fC4bC4bC2}]}{K^{\mathrm{C2}}_{\mathrm{m\,MASP1^*}}}+\frac{[\mathrm{fC3bC4bC2}]}{K^{\mathrm{C2}}_{\mathrm{m\,MASP1^*}}}+\frac{[\mathrm{pC4bC2}]}{K^{\mathrm{C2}}_{\mathrm{m\,MASP1^*}}}+\frac{[\mathrm{pC4bC4bC2}]}{K^{\mathrm{C2}}_{\mathrm{m\,MASP1^*}}}\\
+\frac{[\mathrm{pC3bC4bC2}]}{K^{\mathrm{C2}}_{\mathrm{m\,MASP1^*}}}+\frac{[\mathrm{pMBL_3MASP1_{zym}}]}{K^{\mathrm{MASP1_{zym}}}_{\mathrm{m\,MASP1^*}}}+\frac{[\mathrm{pMBL_3MASP2_{zym}}]}{K^{\mathrm{MASP2_{zym}}}_{\mathrm{m\,MASP1^*}}}+\frac{[\mathrm{pMBL_3MASP3_{zym}}]}{K^{\mathrm{MASP3_{zym}}}_{\mathrm{m\,MASP1^*}}}\\
+\frac{[\mathrm{pMBL_4MASP1_{zym}}]}{K^{\mathrm{MASP1_{zym}}}_{\mathrm{m\,MASP1^*}}}+\frac{[\mathrm{pMBL_4MASP2_{zym}}]}{K^{\mathrm{MASP2_{zym}}}_{\mathrm{m\,MASP1^*}}}+\frac{[\mathrm{pMBL_4MASP3_{zym}}]}{K^{\mathrm{MASP3_{zym}}}_{\mathrm{m\,MASP1^*}}}+\frac{[\mathrm{pCLLKMASP1_{zym}}]}{K^{\mathrm{MASP1_{zym}}}_{\mathrm{m\,MASP1^*}}}\\
+\frac{[\mathrm{pCLLKMASP2_{zym}}]}{K^{\mathrm{MASP2_{zym}}}_{\mathrm{m\,MASP1^*}}}+\frac{[\mathrm{pCLLKMASP3_{zym}}]}{K^{\mathrm{MASP3_{zym}}}_{\mathrm{m\,MASP1^*}}}+\frac{[\mathrm{pCLK1MASP1_{zym}}]}{K^{\mathrm{MASP1_{zym}}}_{\mathrm{m\,MASP1^*}}}+\frac{[\mathrm{pCLK1MASP2_{zym}}]}{K^{\mathrm{MASP2_{zym}}}_{\mathrm{m\,MASP1^*}}}\\
+\frac{[\mathrm{pCLK1MASP3_{zym}}]}{K^{\mathrm{MASP3_{zym}}}_{\mathrm{m\,MASP1^*}}}+\frac{[\mathrm{pCLL1MASP1_{zym}}]}{K^{\mathrm{MASP1_{zym}}}_{\mathrm{m\,MASP1^*}}}+\frac{[\mathrm{pCLL1MASP2_{zym}}]}{K^{\mathrm{MASP2_{zym}}}_{\mathrm{m\,MASP1^*}}}+\frac{[\mathrm{pCLL1MASP3_{zym}}]}{K^{\mathrm{MASP3_{zym}}}_{\mathrm{m\,MASP1^*}}}\\
+\frac{[\mathrm{pLFMASP1_{zym}}]}{K^{\mathrm{MASP1_{zym}}}_{\mathrm{m\,MASP1^*}}}+\frac{[\mathrm{pLFMASP2_{zym}}]}{K^{\mathrm{MASP2_{zym}}}_{\mathrm{m\,MASP1^*}}}+\frac{[\mathrm{pLFMASP3_{zym}}]}{K^{\mathrm{MASP3_{zym}}}_{\mathrm{m\,MASP1^*}}}+\frac{[\mathrm{pHFMASP1_{zym}}]}{K^{\mathrm{MASP1_{zym}}}_{\mathrm{m\,MASP1^*}}}+\frac{[\mathrm{pHFMASP2_{zym}}]}{K^{\mathrm{MASP2_{zym}}}_{\mathrm{m\,MASP1^*}}}\\
+\frac{[\mathrm{pHFMASP3_{zym}}]}{K^{\mathrm{MASP3_{zym}}}_{\mathrm{m\,MASP1^*}}}+\frac{[\mathrm{pMFMASP1_{zym}}]}{K^{\mathrm{MASP1_{zym}}}_{\mathrm{m\,MASP1^*}}}+\frac{[\mathrm{pMFMASP2_{zym}}]}{K^{\mathrm{MASP2_{zym}}}_{\mathrm{m\,MASP1^*}}}+\frac{[\mathrm{pMFMASP3_{zym}}]}{K^{\mathrm{MASP3_{zym}}}_{\mathrm{m\,MASP1^*}}}\end{array}\right)}
$$

$$
+\frac{k^{\mathrm{C2}}_{\mathrm{cat\,MASP1^*}}\left[\mathrm{pCLL1MASP1^*}\right]\left[\mathrm{pC3bC4bC2}\right]}{K^{\mathrm{C2}}_{\mathrm{m\,MASP1^*}}\left(\begin{array}{l}1+\frac{[\mathrm{C4}]}{K^{\mathrm{C4}}_{\mathrm{m\,MASP1^*}}}+\frac{[\mathrm{fC4bC2}]}{K^{\mathrm{C2}}_{\mathrm{m\,MASP1^*}}}+\frac{[\mathrm{fC4bC4bC2}]}{K^{\mathrm{C2}}_{\mathrm{m\,MASP1^*}}}+\frac{[\mathrm{fC3bC4bC2}]}{K^{\mathrm{C2}}_{\mathrm{m\,MASP1^*}}}+\frac{[\mathrm{pC4bC2}]}{K^{\mathrm{C2}}_{\mathrm{m\,MASP1^*}}}+\frac{[\mathrm{pC4bC4bC2}]}{K^{\mathrm{C2}}_{\mathrm{m\,MASP1^*}}}\\
+\frac{[\mathrm{pC3bC4bC2}]}{K^{\mathrm{C2}}_{\mathrm{m\,MASP1^*}}}+\frac{[\mathrm{pMBL_3MASP1_{zym}}]}{K^{\mathrm{MASP1_{zym}}}_{\mathrm{m\,MASP1^*}}}+\frac{[\mathrm{pMBL_3MASP2_{zym}}]}{K^{\mathrm{MASP2_{zym}}}_{\mathrm{m\,MASP1^*}}}+\frac{[\mathrm{pMBL_3MASP3_{zym}}]}{K^{\mathrm{MASP3_{zym}}}_{\mathrm{m\,MASP1^*}}}\\
+\frac{[\mathrm{pMBL_4MASP1_{zym}}]}{K^{\mathrm{MASP1_{zym}}}_{\mathrm{m\,MASP1^*}}}+\frac{[\mathrm{pMBL_4MASP2_{zym}}]}{K^{\mathrm{MASP2_{zym}}}_{\mathrm{m\,MASP1^*}}}+\frac{[\mathrm{pMBL_4MASP3_{zym}}]}{K^{\mathrm{MASP3_{zym}}}_{\mathrm{m\,MASP1^*}}}+\frac{[\mathrm{pCLLKMASP1_{zym}}]}{K^{\mathrm{MASP1_{zym}}}_{\mathrm{m\,MASP1^*}}}\\
+\frac{[\mathrm{pCLLKMASP2_{zym}}]}{K^{\mathrm{MASP2_{zym}}}_{\mathrm{m\,MASP1^*}}}+\frac{[\mathrm{pCLLKMASP3_{zym}}]}{K^{\mathrm{MASP3_{zym}}}_{\mathrm{m\,MASP1^*}}}+\frac{[\mathrm{pCLK1MASP1_{zym}}]}{K^{\mathrm{MASP1_{zym}}}_{\mathrm{m\,MASP1^*}}}+\frac{[\mathrm{pCLK1MASP2_{zym}}]}{K^{\mathrm{MASP2_{zym}}}_{\mathrm{m\,MASP1^*}}}\\
+\frac{[\mathrm{pCLK1MASP3_{zym}}]}{K^{\mathrm{MASP3_{zym}}}_{\mathrm{m\,MASP1^*}}}+\frac{[\mathrm{pCLL1MASP1_{zym}}]}{K^{\mathrm{MASP1_{zym}}}_{\mathrm{m\,MASP1^*}}}+\frac{[\mathrm{pCLL1MASP2_{zym}}]}{K^{\mathrm{MASP2_{zym}}}_{\mathrm{m\,MASP1^*}}}+\frac{[\mathrm{pCLL1MASP3_{zym}}]}{K^{\mathrm{MASP3_{zym}}}_{\mathrm{m\,MASP1^*}}}\\
+\frac{[\mathrm{pLFMASP1_{zym}}]}{K^{\mathrm{MASP1_{zym}}}_{\mathrm{m\,MASP1^*}}}+\frac{[\mathrm{pLFMASP2_{zym}}]}{K^{\mathrm{MASP2_{zym}}}_{\mathrm{m\,MASP1^*}}}+\frac{[\mathrm{pLFMASP3_{zym}}]}{K^{\mathrm{MASP3_{zym}}}_{\mathrm{m\,MASP1^*}}}+\frac{[\mathrm{pHFMASP1_{zym}}]}{K^{\mathrm{MASP1_{zym}}}_{\mathrm{m\,MASP1^*}}}+\frac{[\mathrm{pHFMASP2_{zym}}]}{K^{\mathrm{MASP2_{zym}}}_{\mathrm{m\,MASP1^*}}}\\
+\frac{[\mathrm{pHFMASP3_{zym}}]}{K^{\mathrm{MASP3_{zym}}}_{\mathrm{m\,MASP1^*}}}+\frac{[\mathrm{pMFMASP1_{zym}}]}{K^{\mathrm{MASP1_{zym}}}_{\mathrm{m\,MASP1^*}}}+\frac{[\mathrm{pMFMASP2_{zym}}]}{K^{\mathrm{MASP2_{zym}}}_{\mathrm{m\,MASP1^*}}}+\frac{[\mathrm{pMFMASP3_{zym}}]}{K^{\mathrm{MASP3_{zym}}}_{\mathrm{m\,MASP1^*}}}\end{array}\right)}
$$

$$
+\frac{k_{\text{cat MASP1}^*}^{\text{C2}}\left[\text{pLFMASP1}^*\right]\left[\text{pC3bC4bC2}\right]}{K_{\text{m MASP1}^*}^{\text{C2}}\left(\begin{aligned}&1+\frac{[\text{C4}]}{K_{\text{m MASP1}^*}^{\text{C4}}}+\frac{[\text{fC4bC2}]}{K_{\text{m MASP1}^*}^{\text{C2}}}+\frac{[\text{fC4bC4bC2}]}{K_{\text{m MASP1}^*}^{\text{C2}}}+\frac{[\text{fC3bC4bC2}]}{K_{\text{m MASP1}^*}^{\text{C2}}}+\frac{[\text{pC4bC2}]}{K_{\text{m MASP1}^*}^{\text{C2}}}+\frac{[\text{pC4bC4bC2}]}{K_{\text{m MASP1}^*}^{\text{C2}}}\\
&+\frac{[\text{pC3bC4bC2}]}{K_{\text{m MASP1}^*}^{\text{C2}}}+\frac{\left[\text{pMBL}_3\text{MASP1}_{\text{zym}}\right]}{K_{\text{m MASP1}^*}^{\text{MASP1}_{\text{zym}}}}+\frac{\left[\text{pMBL}_3\text{MASP2}_{\text{zym}}\right]}{K_{\text{m MASP1}^*}^{\text{MASP2}_{\text{zym}}}}+\frac{\left[\text{pMBL}_3\text{MASP3}_{\text{zym}}\right]}{K_{\text{m MASP1}^*}^{\text{MASP3}_{\text{zym}}}}\\
&+\frac{\left[\text{pMBL}_4\text{MASP1}_{\text{zym}}\right]}{K_{\text{m MASP1}^*}^{\text{MASP1}_{\text{zym}}}}+\frac{\left[\text{pMBL}_4\text{MASP2}_{\text{zym}}\right]}{K_{\text{m MASP1}^*}^{\text{MASP2}_{\text{zym}}}}+\frac{\left[\text{pMBL}_4\text{MASP3}_{\text{zym}}\right]}{K_{\text{m MASP1}^*}^{\text{MASP3}_{\text{zym}}}}+\frac{\left[\text{pCLLKMASP1}_{\text{zym}}\right]}{K_{\text{m MASP1}^*}^{\text{MASP1}_{\text{zym}}}}\\
&+\frac{\left[\text{pCLLKMASP2}_{\text{zym}}\right]}{K_{\text{m MASP1}^*}^{\text{MASP2}_{\text{zym}}}}+\frac{\left[\text{pCLLKMASP3}_{\text{zym}}\right]}{K_{\text{m MASP1}^*}^{\text{MASP3}_{\text{zym}}}}+\frac{\left[\text{pCLK1MASP1}_{\text{zym}}\right]}{K_{\text{m MASP1}^*}^{\text{MASP1}_{\text{zym}}}}+\frac{\left[\text{pCLK1MASP2}_{\text{zym}}\right]}{K_{\text{m MASP1}^*}^{\text{MASP2}_{\text{zym}}}}\\
&+\frac{\left[\text{pCLK1MASP3}_{\text{zym}}\right]}{K_{\text{m MASP1}^*}^{\text{MASP3}_{\text{zym}}}}+\frac{\left[\text{pCLL1MASP1}_{\text{zym}}\right]}{K_{\text{m MASP1}^*}^{\text{MASP1}_{\text{zym}}}}+\frac{\left[\text{pCLL1MASP2}_{\text{zym}}\right]}{K_{\text{m MASP1}^*}^{\text{MASP2}_{\text{zym}}}}+\frac{\left[\text{pCLL1MASP3}_{\text{zym}}\right]}{K_{\text{m MASP1}^*}^{\text{MASP3}_{\text{zym}}}}\\
&+\frac{\left[\text{pLFMASP1}_{\text{zym}}\right]}{K_{\text{m MASP1}^*}^{\text{MASP1}_{\text{zym}}}}+\frac{\left[\text{pLFMASP2}_{\text{zym}}\right]}{K_{\text{m MASP1}^*}^{\text{MASP2}_{\text{zym}}}}+\frac{\left[\text{pLFMASP3}_{\text{zym}}\right]}{K_{\text{m MASP1}^*}^{\text{MASP3}_{\text{zym}}}}+\frac{\left[\text{pHFMASP1}_{\text{zym}}\right]}{K_{\text{m MASP1}^*}^{\text{MASP1}_{\text{zym}}}}+\frac{\left[\text{pHFMASP2}_{\text{zym}}\right]}{K_{\text{m MASP1}^*}^{\text{MASP2}_{\text{zym}}}}\\
&+\frac{\left[\text{pHFMASP3}_{\text{zym}}\right]}{K_{\text{m MASP1}^*}^{\text{MASP3}_{\text{zym}}}}+\frac{\left[\text{pMFMASP1}_{\text{zym}}\right]}{K_{\text{m MASP1}^*}^{\text{MASP1}_{\text{zym}}}}+\frac{\left[\text{pMFMASP2}_{\text{zym}}\right]}{K_{\text{m MASP1}^*}^{\text{MASP2}_{\text{zym}}}}+\frac{\left[\text{pMFMASP3}_{\text{zym}}\right]}{K_{\text{m MASP1}^*}^{\text{MASP3}_{\text{zym}}}}\end{aligned}\right)}
$$

$$
+\frac{k_{\text{cat MASP1}^*}^{\text{C2}}\left[\text{pHFMASP1}^*\right]\left[\text{pC3bC4bC2}\right]}{K_{\text{m MASP1}^*}^{\text{C2}}\left(\begin{aligned}&1+\frac{[\text{C4}]}{K_{\text{m MASP1}^*}^{\text{C4}}}+\frac{[\text{fC4bC2}]}{K_{\text{m MASP1}^*}^{\text{C2}}}+\frac{[\text{fC4bC4bC2}]}{K_{\text{m MASP1}^*}^{\text{C2}}}+\frac{[\text{fC3bC4bC2}]}{K_{\text{m MASP1}^*}^{\text{C2}}}+\frac{[\text{pC4bC2}]}{K_{\text{m MASP1}^*}^{\text{C2}}}+\frac{[\text{pC4bC4bC2}]}{K_{\text{m MASP1}^*}^{\text{C2}}}\\
&+\frac{[\text{pC3bC4bC2}]}{K_{\text{m MASP1}^*}^{\text{C2}}}+\frac{\left[\text{pMBL}_3\text{MASP1}_{\text{zym}}\right]}{K_{\text{m MASP1}^*}^{\text{MASP1}_{\text{zym}}}}+\frac{\left[\text{pMBL}_3\text{MASP2}_{\text{zym}}\right]}{K_{\text{m MASP1}^*}^{\text{MASP2}_{\text{zym}}}}+\frac{\left[\text{pMBL}_3\text{MASP3}_{\text{zym}}\right]}{K_{\text{m MASP1}^*}^{\text{MASP3}_{\text{zym}}}}\\
&+\frac{\left[\text{pMBL}_4\text{MASP1}_{\text{zym}}\right]}{K_{\text{m MASP1}^*}^{\text{MASP1}_{\text{zym}}}}+\frac{\left[\text{pMBL}_4\text{MASP2}_{\text{zym}}\right]}{K_{\text{m MASP1}^*}^{\text{MASP2}_{\text{zym}}}}+\frac{\left[\text{pMBL}_4\text{MASP3}_{\text{zym}}\right]}{K_{\text{m MASP1}^*}^{\text{MASP3}_{\text{zym}}}}+\frac{\left[\text{pCLLKMASP1}_{\text{zym}}\right]}{K_{\text{m MASP1}^*}^{\text{MASP1}_{\text{zym}}}}\\
&+\frac{\left[\text{pCLLKMASP2}_{\text{zym}}\right]}{K_{\text{m MASP1}^*}^{\text{MASP2}_{\text{zym}}}}+\frac{\left[\text{pCLLKMASP3}_{\text{zym}}\right]}{K_{\text{m MASP1}^*}^{\text{MASP3}_{\text{zym}}}}+\frac{\left[\text{pCLK1MASP1}_{\text{zym}}\right]}{K_{\text{m MASP1}^*}^{\text{MASP1}_{\text{zym}}}}+\frac{\left[\text{pCLK1MASP2}_{\text{zym}}\right]}{K_{\text{m MASP1}^*}^{\text{MASP2}_{\text{zym}}}}\\
&+\frac{\left[\text{pCLK1MASP3}_{\text{zym}}\right]}{K_{\text{m MASP1}^*}^{\text{MASP3}_{\text{zym}}}}+\frac{\left[\text{pCLL1MASP1}_{\text{zym}}\right]}{K_{\text{m MASP1}^*}^{\text{MASP1}_{\text{zym}}}}+\frac{\left[\text{pCLL1MASP2}_{\text{zym}}\right]}{K_{\text{m MASP1}^*}^{\text{MASP2}_{\text{zym}}}}+\frac{\left[\text{pCLL1MASP3}_{\text{zym}}\right]}{K_{\text{m MASP1}^*}^{\text{MASP3}_{\text{zym}}}}\\
&+\frac{\left[\text{pLFMASP1}_{\text{zym}}\right]}{K_{\text{m MASP1}^*}^{\text{MASP1}_{\text{zym}}}}+\frac{\left[\text{pLFMASP2}_{\text{zym}}\right]}{K_{\text{m MASP1}^*}^{\text{MASP2}_{\text{zym}}}}+\frac{\left[\text{pLFMASP3}_{\text{zym}}\right]}{K_{\text{m MASP1}^*}^{\text{MASP3}_{\text{zym}}}}+\frac{\left[\text{pHFMASP1}_{\text{zym}}\right]}{K_{\text{m MASP1}^*}^{\text{MASP1}_{\text{zym}}}}+\frac{\left[\text{pHFMASP2}_{\text{zym}}\right]}{K_{\text{m MASP1}^*}^{\text{MASP2}_{\text{zym}}}}\\
&+\frac{\left[\text{pHFMASP3}_{\text{zym}}\right]}{K_{\text{m MASP1}^*}^{\text{MASP3}_{\text{zym}}}}+\frac{\left[\text{pMFMASP1}_{\text{zym}}\right]}{K_{\text{m MASP1}^*}^{\text{MASP1}_{\text{zym}}}}+\frac{\left[\text{pMFMASP2}_{\text{zym}}\right]}{K_{\text{m MASP1}^*}^{\text{MASP2}_{\text{zym}}}}+\frac{\left[\text{pMFMASP3}_{\text{zym}}\right]}{K_{\text{m MASP1}^*}^{\text{MASP3}_{\text{zym}}}}\end{aligned}\right)}
$$

$$+\frac{k_{\mathrm{cat\,MASP1^*}}^{\mathrm{C2}}\left[\mathrm{pMFMASP1^*}\right]\left[\mathrm{pC3bC4bC2}\right]}{K_{\mathrm{m\,MASP1^*}}^{\mathrm{C2}}\left(\begin{array}{l}1+\frac{[\mathrm{C4}]}{K_{\mathrm{m\,MASP1^*}}^{\mathrm{C4}}}+\frac{[\mathrm{fC4bC2}]}{K_{\mathrm{m\,MASP1^*}}^{\mathrm{C2}}}+\frac{[\mathrm{fC4bC4bC2}]}{K_{\mathrm{m\,MASP1^*}}^{\mathrm{C2}}}+\frac{[\mathrm{fC3bC4bC2}]}{K_{\mathrm{m\,MASP1^*}}^{\mathrm{C2}}}+\frac{[\mathrm{pC4bC2}]}{K_{\mathrm{m\,MASP1^*}}^{\mathrm{C2}}}+\frac{[\mathrm{pC4bC4bC2}]}{K_{\mathrm{m\,MASP1^*}}^{\mathrm{C2}}}\\
+\frac{[\mathrm{pC3bC4bC2}]}{K_{\mathrm{m\,MASP1^*}}^{\mathrm{C2}}}+\frac{\left[\mathrm{pMBL_3MASP1_{zym}}\right]}{K_{\mathrm{m\,MASP1^*}}^{\mathrm{MASP1_{zym}}}}+\frac{\left[\mathrm{pMBL_3MASP2_{zym}}\right]}{K_{\mathrm{m\,MASP1^*}}^{\mathrm{MASP2_{zym}}}}+\frac{\left[\mathrm{pMBL_3MASP3_{zym}}\right]}{K_{\mathrm{m\,MASP1^*}}^{\mathrm{MASP3_{zym}}}}\\
+\frac{\left[\mathrm{pMBL_4MASP1_{zym}}\right]}{K_{\mathrm{m\,MASP1^*}}^{\mathrm{MASP1_{zym}}}}+\frac{\left[\mathrm{pMBL_4MASP2_{zym}}\right]}{K_{\mathrm{m\,MASP1^*}}^{\mathrm{MASP2_{zym}}}}+\frac{\left[\mathrm{pMBL_4MASP3_{zym}}\right]}{K_{\mathrm{m\,MASP1^*}}^{\mathrm{MASP3_{zym}}}}+\frac{\left[\mathrm{pCLLKMASP1_{zym}}\right]}{K_{\mathrm{m\,MASP1^*}}^{\mathrm{MASP1_{zym}}}}\\
+\frac{\left[\mathrm{pCLLKMASP2_{zym}}\right]}{K_{\mathrm{m\,MASP1^*}}^{\mathrm{MASP2_{zym}}}}+\frac{\left[\mathrm{pCLLKMASP3_{zym}}\right]}{K_{\mathrm{m\,MASP1^*}}^{\mathrm{MASP3_{zym}}}}+\frac{\left[\mathrm{pCLK1MASP1_{zym}}\right]}{K_{\mathrm{m\,MASP1^*}}^{\mathrm{MASP1_{zym}}}}+\frac{\left[\mathrm{pCLK1MASP2_{zym}}\right]}{K_{\mathrm{m\,MASP1^*}}^{\mathrm{MASP2_{zym}}}}\\
+\frac{\left[\mathrm{pCLK1MASP3_{zym}}\right]}{K_{\mathrm{m\,MASP1^*}}^{\mathrm{MASP3_{zym}}}}+\frac{\left[\mathrm{pCLL1MASP1_{zym}}\right]}{K_{\mathrm{m\,MASP1^*}}^{\mathrm{MASP1_{zym}}}}+\frac{\left[\mathrm{pCLL1MASP2_{zym}}\right]}{K_{\mathrm{m\,MASP1^*}}^{\mathrm{MASP2_{zym}}}}+\frac{\left[\mathrm{pCLL1MASP3_{zym}}\right]}{K_{\mathrm{m\,MASP1^*}}^{\mathrm{MASP3_{zym}}}}\\
+\frac{\left[\mathrm{pLFMASP1_{zym}}\right]}{K_{\mathrm{m\,MASP1^*}}^{\mathrm{MASP1_{zym}}}}+\frac{\left[\mathrm{pLFMASP2_{zym}}\right]}{K_{\mathrm{m\,MASP1^*}}^{\mathrm{MASP2_{zym}}}}+\frac{\left[\mathrm{pLFMASP3_{zym}}\right]}{K_{\mathrm{m\,MASP1^*}}^{\mathrm{MASP3_{zym}}}}+\frac{\left[\mathrm{pHFMASP1_{zym}}\right]}{K_{\mathrm{m\,MASP1^*}}^{\mathrm{MASP1_{zym}}}}+\frac{\left[\mathrm{pHFMASP2_{zym}}\right]}{K_{\mathrm{m\,MASP1^*}}^{\mathrm{MASP2_{zym}}}}\\
+\frac{\left[\mathrm{pHFMASP3_{zym}}\right]}{K_{\mathrm{m\,MASP1^*}}^{\mathrm{MASP3_{zym}}}}+\frac{\left[\mathrm{pMFMASP1_{zym}}\right]}{K_{\mathrm{m\,MASP1^*}}^{\mathrm{MASP1_{zym}}}}+\frac{\left[\mathrm{pMFMASP2_{zym}}\right]}{K_{\mathrm{m\,MASP1^*}}^{\mathrm{MASP2_{zym}}}}+\frac{\left[\mathrm{pMFMASP3_{zym}}\right]}{K_{\mathrm{m\,MASP1^*}}^{\mathrm{MASP3_{zym}}}}\end{array}\right)}$$

$$+\frac{k_{\mathrm{cat\,MASP2^*}}^{\mathrm{C2}}\left[\mathrm{pMBL_3MASP2^*}\right]\left[\mathrm{pC3bC4bC2}\right]}{K_{\mathrm{m\,MASP2^*}}^{\mathrm{C2}}\left(\begin{array}{l}1+\frac{[\mathrm{C4}]}{K_{\mathrm{m\,MASP2^*}}^{\mathrm{C4}}}+\frac{[\mathrm{fC4bC2}]}{K_{\mathrm{m\,MASP2^*}}^{\mathrm{C2}}}+\frac{[\mathrm{fC4bC4bC2}]}{K_{\mathrm{m\,MASP2^*}}^{\mathrm{C2}}}+\frac{[\mathrm{fC3bC4bC2}]}{K_{\mathrm{m\,MASP2^*}}^{\mathrm{C2}}}+\frac{[\mathrm{pC4bC2}]}{K_{\mathrm{m\,MASP2^*}}^{\mathrm{C2}}}+\frac{[\mathrm{pC4bC4bC2}]}{K_{\mathrm{m\,MASP2^*}}^{\mathrm{C2}}}\\
+\frac{[\mathrm{pC3bC4bC2}]}{K_{\mathrm{m\,MASP2^*}}^{\mathrm{C2}}}+\frac{\left[\mathrm{pMBL_3MASP1_{zym}}\right]}{K_{\mathrm{m\,MASP2^*}}^{\mathrm{MASP1_{zym}}}}+\frac{\left[\mathrm{pMBL_3MASP2_{zym}}\right]}{K_{\mathrm{m\,MASP2^*}}^{\mathrm{MASP2_{zym}}}}+\frac{\left[\mathrm{pMBL_4MASP1_{zym}}\right]}{K_{\mathrm{m\,MASP2^*}}^{\mathrm{MASP1_{zym}}}}\\
+\frac{\left[\mathrm{pMBL_4MASP2_{zym}}\right]}{K_{\mathrm{m\,MASP2^*}}^{\mathrm{MASP2_{zym}}}}+\frac{\left[\mathrm{pCLLKMASP1_{zym}}\right]}{K_{\mathrm{m\,MASP2^*}}^{\mathrm{MASP1_{zym}}}}+\frac{\left[\mathrm{pCLLKMASP2_{zym}}\right]}{K_{\mathrm{m\,MASP2^*}}^{\mathrm{MASP2_{zym}}}}+\frac{\left[\mathrm{pCLK1MASP1_{zym}}\right]}{K_{\mathrm{m\,MASP2^*}}^{\mathrm{MASP1_{zym}}}}\\
+\frac{\left[\mathrm{pCLK1MASP2_{zym}}\right]}{K_{\mathrm{m\,MASP2^*}}^{\mathrm{MASP2_{zym}}}}+\frac{\left[\mathrm{pCLL1MASP1_{zym}}\right]}{K_{\mathrm{m\,MASP2^*}}^{\mathrm{MASP1_{zym}}}}+\frac{\left[\mathrm{pCLL1MASP2_{zym}}\right]}{K_{\mathrm{m\,MASP2^*}}^{\mathrm{MASP2_{zym}}}}+\frac{\left[\mathrm{pLFMASP1_{zym}}\right]}{K_{\mathrm{m\,MASP2^*}}^{\mathrm{MASP1_{zym}}}}\\
+\frac{\left[\mathrm{pLFMASP2_{zym}}\right]}{K_{\mathrm{m\,MASP2^*}}^{\mathrm{MASP2_{zym}}}}+\frac{\left[\mathrm{pHFMASP1_{zym}}\right]}{K_{\mathrm{m\,MASP2^*}}^{\mathrm{MASP1_{zym}}}}+\frac{\left[\mathrm{pHFMASP2_{zym}}\right]}{K_{\mathrm{m\,MASP2^*}}^{\mathrm{MASP2_{zym}}}}+\frac{\left[\mathrm{pMFMASP1_{zym}}\right]}{K_{\mathrm{m\,MASP2^*}}^{\mathrm{MASP1_{zym}}}}+\frac{\left[\mathrm{pMFMASP2_{zym}}\right]}{K_{\mathrm{m\,MASP2^*}}^{\mathrm{MASP2_{zym}}}}\end{array}\right)}$$

$$+\frac{k_{\mathrm{cat\,MASP2^*}}^{\mathrm{C2}}\left[\mathrm{pMBL_4MASP2^*}\right]\left[\mathrm{pC3bC4bC2}\right]}{K_{\mathrm{m\,MASP2^*}}^{\mathrm{C2}}\left(\begin{aligned}&1+\frac{[\mathrm{C4}]}{K_{\mathrm{m\,MASP2^*}}^{\mathrm{C4}}}+\frac{[\mathrm{fC4bC2}]}{K_{\mathrm{m\,MASP2^*}}^{\mathrm{C2}}}+\frac{[\mathrm{fC4bC4bC2}]}{K_{\mathrm{m\,MASP2^*}}^{\mathrm{C2}}}+\frac{[\mathrm{fC3bC4bC2}]}{K_{\mathrm{m\,MASP2^*}}^{\mathrm{C2}}}+\frac{[\mathrm{pC4bC2}]}{K_{\mathrm{m\,MASP2^*}}^{\mathrm{C2}}}+\frac{[\mathrm{pC4bC4bC2}]}{K_{\mathrm{m\,MASP2^*}}^{\mathrm{C2}}}\\&+\frac{[\mathrm{pC3bC4bC2}]}{K_{\mathrm{m\,MASP2^*}}^{\mathrm{C2}}}+\frac{\left[\mathrm{pMBL_3MASP1_{zym}}\right]}{K_{\mathrm{m\,MASP2^*}}^{\mathrm{MASP1_{zym}}}}+\frac{\left[\mathrm{pMBL_3MASP2_{zym}}\right]}{K_{\mathrm{m\,MASP2^*}}^{\mathrm{MASP2_{zym}}}}+\frac{\left[\mathrm{pMBL_4MASP1_{zym}}\right]}{K_{\mathrm{m\,MASP2^*}}^{\mathrm{MASP1_{zym}}}}\\&+\frac{\left[\mathrm{pMBL_4MASP2_{zym}}\right]}{K_{\mathrm{m\,MASP2^*}}^{\mathrm{MASP2_{zym}}}}+\frac{\left[\mathrm{pCLLKMASP1_{zym}}\right]}{K_{\mathrm{m\,MASP2^*}}^{\mathrm{MASP1_{zym}}}}+\frac{\left[\mathrm{pCLLKMASP2_{zym}}\right]}{K_{\mathrm{m\,MASP2^*}}^{\mathrm{MASP2_{zym}}}}+\frac{\left[\mathrm{pCLK1MASP1_{zym}}\right]}{K_{\mathrm{m\,MASP2^*}}^{\mathrm{MASP1_{zym}}}}\\&+\frac{\left[\mathrm{pCLK1MASP2_{zym}}\right]}{K_{\mathrm{m\,MASP2^*}}^{\mathrm{MASP2_{zym}}}}+\frac{\left[\mathrm{pCLL1MASP1_{zym}}\right]}{K_{\mathrm{m\,MASP2^*}}^{\mathrm{MASP1_{zym}}}}+\frac{\left[\mathrm{pCLL1MASP2_{zym}}\right]}{K_{\mathrm{m\,MASP2^*}}^{\mathrm{MASP2_{zym}}}}+\frac{\left[\mathrm{pLFMASP1_{zym}}\right]}{K_{\mathrm{m\,MASP2^*}}^{\mathrm{MASP1_{zym}}}}\\&+\frac{\left[\mathrm{pLFMASP2_{zym}}\right]}{K_{\mathrm{m\,MASP2^*}}^{\mathrm{MASP2_{zym}}}}+\frac{\left[\mathrm{pHFMASP1_{zym}}\right]}{K_{\mathrm{m\,MASP2^*}}^{\mathrm{MASP1_{zym}}}}+\frac{\left[\mathrm{pHFMASP2_{zym}}\right]}{K_{\mathrm{m\,MASP2^*}}^{\mathrm{MASP2_{zym}}}}+\frac{\left[\mathrm{pMFMASP1_{zym}}\right]}{K_{\mathrm{m\,MASP2^*}}^{\mathrm{MASP1_{zym}}}}+\frac{\left[\mathrm{pMFMASP2_{zym}}\right]}{K_{\mathrm{m\,MASP2^*}}^{\mathrm{MASP2_{zym}}}}\end{aligned}\right)}$$

$$+\frac{k_{\mathrm{cat\,MASP2^*}}^{\mathrm{C2}}\left[\mathrm{pCLLKMASP2^*}\right]\left[\mathrm{pC3bC4bC2}\right]}{K_{\mathrm{m\,MASP2^*}}^{\mathrm{C2}}\left(\begin{aligned}&1+\frac{[\mathrm{C4}]}{K_{\mathrm{m\,MASP2^*}}^{\mathrm{C4}}}+\frac{[\mathrm{fC4bC2}]}{K_{\mathrm{m\,MASP2^*}}^{\mathrm{C2}}}+\frac{[\mathrm{fC4bC4bC2}]}{K_{\mathrm{m\,MASP2^*}}^{\mathrm{C2}}}+\frac{[\mathrm{fC3bC4bC2}]}{K_{\mathrm{m\,MASP2^*}}^{\mathrm{C2}}}+\frac{[\mathrm{pC4bC2}]}{K_{\mathrm{m\,MASP2^*}}^{\mathrm{C2}}}+\frac{[\mathrm{pC4bC4bC2}]}{K_{\mathrm{m\,MASP2^*}}^{\mathrm{C2}}}\\&+\frac{[\mathrm{pC3bC4bC2}]}{K_{\mathrm{m\,MASP2^*}}^{\mathrm{C2}}}+\frac{\left[\mathrm{pMBL_3MASP1_{zym}}\right]}{K_{\mathrm{m\,MASP2^*}}^{\mathrm{MASP1_{zym}}}}+\frac{\left[\mathrm{pMBL_3MASP2_{zym}}\right]}{K_{\mathrm{m\,MASP2^*}}^{\mathrm{MASP2_{zym}}}}+\frac{\left[\mathrm{pMBL_4MASP1_{zym}}\right]}{K_{\mathrm{m\,MASP2^*}}^{\mathrm{MASP1_{zym}}}}\\&+\frac{\left[\mathrm{pMBL_4MASP2_{zym}}\right]}{K_{\mathrm{m\,MASP2^*}}^{\mathrm{MASP2_{zym}}}}+\frac{\left[\mathrm{pCLLKMASP1_{zym}}\right]}{K_{\mathrm{m\,MASP2^*}}^{\mathrm{MASP1_{zym}}}}+\frac{\left[\mathrm{pCLLKMASP2_{zym}}\right]}{K_{\mathrm{m\,MASP2^*}}^{\mathrm{MASP2_{zym}}}}+\frac{\left[\mathrm{pCLK1MASP1_{zym}}\right]}{K_{\mathrm{m\,MASP2^*}}^{\mathrm{MASP1_{zym}}}}\\&+\frac{\left[\mathrm{pCLK1MASP2_{zym}}\right]}{K_{\mathrm{m\,MASP2^*}}^{\mathrm{MASP2_{zym}}}}+\frac{\left[\mathrm{pCLL1MASP1_{zym}}\right]}{K_{\mathrm{m\,MASP2^*}}^{\mathrm{MASP1_{zym}}}}+\frac{\left[\mathrm{pCLL1MASP2_{zym}}\right]}{K_{\mathrm{m\,MASP2^*}}^{\mathrm{MASP2_{zym}}}}+\frac{\left[\mathrm{pLFMASP1_{zym}}\right]}{K_{\mathrm{m\,MASP2^*}}^{\mathrm{MASP1_{zym}}}}\\&+\frac{\left[\mathrm{pLFMASP2_{zym}}\right]}{K_{\mathrm{m\,MASP2^*}}^{\mathrm{MASP2_{zym}}}}+\frac{\left[\mathrm{pHFMASP1_{zym}}\right]}{K_{\mathrm{m\,MASP2^*}}^{\mathrm{MASP1_{zym}}}}+\frac{\left[\mathrm{pHFMASP2_{zym}}\right]}{K_{\mathrm{m\,MASP2^*}}^{\mathrm{MASP2_{zym}}}}+\frac{\left[\mathrm{pMFMASP1_{zym}}\right]}{K_{\mathrm{m\,MASP2^*}}^{\mathrm{MASP1_{zym}}}}+\frac{\left[\mathrm{pMFMASP2_{zym}}\right]}{K_{\mathrm{m\,MASP2^*}}^{\mathrm{MASP2_{zym}}}}\end{aligned}\right)}$$

$$+\frac{k_{\mathrm{cat\,MASP2^*}}^{\mathrm{C2}}\left[\mathrm{pCLK1MASP2^*}\right]\left[\mathrm{pC3bC4bC2}\right]}{K_{\mathrm{m\,MASP2^*}}^{\mathrm{C2}}\left(\begin{aligned}&1+\frac{[\mathrm{C4}]}{K_{\mathrm{m\,MASP2^*}}^{\mathrm{C4}}}+\frac{[\mathrm{fC4bC2}]}{K_{\mathrm{m\,MASP2^*}}^{\mathrm{C2}}}+\frac{[\mathrm{fC4bC4bC2}]}{K_{\mathrm{m\,MASP2^*}}^{\mathrm{C2}}}+\frac{[\mathrm{fC3bC4bC2}]}{K_{\mathrm{m\,MASP2^*}}^{\mathrm{C2}}}+\frac{[\mathrm{pC4bC2}]}{K_{\mathrm{m\,MASP2^*}}^{\mathrm{C2}}}+\frac{[\mathrm{pC4bC4bC2}]}{K_{\mathrm{m\,MASP2^*}}^{\mathrm{C2}}}\\&+\frac{[\mathrm{pC3bC4bC2}]}{K_{\mathrm{m\,MASP2^*}}^{\mathrm{C2}}}+\frac{\left[\mathrm{pMBL_3MASP1_{zym}}\right]}{K_{\mathrm{m\,MASP2^*}}^{\mathrm{MASP1_{zym}}}}+\frac{\left[\mathrm{pMBL_3MASP2_{zym}}\right]}{K_{\mathrm{m\,MASP2^*}}^{\mathrm{MASP2_{zym}}}}+\frac{\left[\mathrm{pMBL_4MASP1_{zym}}\right]}{K_{\mathrm{m\,MASP2^*}}^{\mathrm{MASP1_{zym}}}}\\&+\frac{\left[\mathrm{pMBL_4MASP2_{zym}}\right]}{K_{\mathrm{m\,MASP2^*}}^{\mathrm{MASP2_{zym}}}}+\frac{\left[\mathrm{pCLLKMASP1_{zym}}\right]}{K_{\mathrm{m\,MASP2^*}}^{\mathrm{MASP1_{zym}}}}+\frac{\left[\mathrm{pCLLKMASP2_{zym}}\right]}{K_{\mathrm{m\,MASP2^*}}^{\mathrm{MASP2_{zym}}}}+\frac{\left[\mathrm{pCLK1MASP1_{zym}}\right]}{K_{\mathrm{m\,MASP2^*}}^{\mathrm{MASP1_{zym}}}}\\&+\frac{\left[\mathrm{pCLK1MASP2_{zym}}\right]}{K_{\mathrm{m\,MASP2^*}}^{\mathrm{MASP2_{zym}}}}+\frac{\left[\mathrm{pCLL1MASP1_{zym}}\right]}{K_{\mathrm{m\,MASP2^*}}^{\mathrm{MASP1_{zym}}}}+\frac{\left[\mathrm{pCLL1MASP2_{zym}}\right]}{K_{\mathrm{m\,MASP2^*}}^{\mathrm{MASP2_{zym}}}}+\frac{\left[\mathrm{pLFMASP1_{zym}}\right]}{K_{\mathrm{m\,MASP2^*}}^{\mathrm{MASP1_{zym}}}}\\&+\frac{\left[\mathrm{pLFMASP2_{zym}}\right]}{K_{\mathrm{m\,MASP2^*}}^{\mathrm{MASP2_{zym}}}}+\frac{\left[\mathrm{pHFMASP1_{zym}}\right]}{K_{\mathrm{m\,MASP2^*}}^{\mathrm{MASP1_{zym}}}}+\frac{\left[\mathrm{pHFMASP2_{zym}}\right]}{K_{\mathrm{m\,MASP2^*}}^{\mathrm{MASP2_{zym}}}}+\frac{\left[\mathrm{pMFMASP1_{zym}}\right]}{K_{\mathrm{m\,MASP2^*}}^{\mathrm{MASP1_{zym}}}}+\frac{\left[\mathrm{pMFMASP2_{zym}}\right]}{K_{\mathrm{m\,MASP2^*}}^{\mathrm{MASP2_{zym}}}}\end{aligned}\right)}$$

$$
+\frac{k_{\mathrm{cat\,MASP2^*}}^{\mathrm{C2}}\left[\mathrm{pCLL1MASP2^*}\right]\left[\mathrm{pC3bC4bC2}\right]}{K_{\mathrm{m\,MASP2^*}}^{\mathrm{C2}}\left(\begin{array}{l}1+\frac{\left[\mathrm{C4}\right]}{K_{\mathrm{m\,MASP2^*}}^{\mathrm{C4}}}+\frac{\left[\mathrm{fC4bC2}\right]}{K_{\mathrm{m\,MASP2^*}}^{\mathrm{C2}}}+\frac{\left[\mathrm{fC4bC4bC2}\right]}{K_{\mathrm{m\,MASP2^*}}^{\mathrm{C2}}}+\frac{\left[\mathrm{fC3bC4bC2}\right]}{K_{\mathrm{m\,MASP2^*}}^{\mathrm{C2}}}+\frac{\left[\mathrm{pC4bC2}\right]}{K_{\mathrm{m\,MASP2^*}}^{\mathrm{C2}}}+\frac{\left[\mathrm{pC4bC4bC2}\right]}{K_{\mathrm{m\,MASP2^*}}^{\mathrm{C2}}}\\+\frac{\left[\mathrm{pC3bC4bC2}\right]}{K_{\mathrm{m\,MASP2^*}}^{\mathrm{C2}}}+\frac{\left[\mathrm{pMBL_3MASP1_{zym}}\right]}{K_{\mathrm{m\,MASP2^*}}^{\mathrm{MASP1_{zym}}}}+\frac{\left[\mathrm{pMBL_3MASP2_{zym}}\right]}{K_{\mathrm{m\,MASP2^*}}^{\mathrm{MASP2_{zym}}}}+\frac{\left[\mathrm{pMBL_4MASP1_{zym}}\right]}{K_{\mathrm{m\,MASP2^*}}^{\mathrm{MASP1_{zym}}}}\\+\frac{\left[\mathrm{pMBL_4MASP2_{zym}}\right]}{K_{\mathrm{m\,MASP2^*}}^{\mathrm{MASP2_{zym}}}}+\frac{\left[\mathrm{pCLLKMASP1_{zym}}\right]}{K_{\mathrm{m\,MASP2^*}}^{\mathrm{MASP1_{zym}}}}+\frac{\left[\mathrm{pCLLKMASP2_{zym}}\right]}{K_{\mathrm{m\,MASP2^*}}^{\mathrm{MASP2_{zym}}}}+\frac{\left[\mathrm{pCLK1MASP1_{zym}}\right]}{K_{\mathrm{m\,MASP2^*}}^{\mathrm{MASP1_{zym}}}}\\+\frac{\left[\mathrm{pCLK1MASP2_{zym}}\right]}{K_{\mathrm{m\,MASP2^*}}^{\mathrm{MASP2_{zym}}}}+\frac{\left[\mathrm{pCLL1MASP1_{zym}}\right]}{K_{\mathrm{m\,MASP2^*}}^{\mathrm{MASP1_{zym}}}}+\frac{\left[\mathrm{pCLL1MASP2_{zym}}\right]}{K_{\mathrm{m\,MASP2^*}}^{\mathrm{MASP2_{zym}}}}+\frac{\left[\mathrm{pLFMASP1_{zym}}\right]}{K_{\mathrm{m\,MASP2^*}}^{\mathrm{MASP1_{zym}}}}\\+\frac{\left[\mathrm{pLFMASP2_{zym}}\right]}{K_{\mathrm{m\,MASP2^*}}^{\mathrm{MASP2_{zym}}}}+\frac{\left[\mathrm{pHFMASP1_{zym}}\right]}{K_{\mathrm{m\,MASP2^*}}^{\mathrm{MASP1_{zym}}}}+\frac{\left[\mathrm{pHFMASP2_{zym}}\right]}{K_{\mathrm{m\,MASP2^*}}^{\mathrm{MASP2_{zym}}}}+\frac{\left[\mathrm{pMFMASP1_{zym}}\right]}{K_{\mathrm{m\,MASP2^*}}^{\mathrm{MASP1_{zym}}}}+\frac{\left[\mathrm{pMFMASP2_{zym}}\right]}{K_{\mathrm{m\,MASP2^*}}^{\mathrm{MASP2_{zym}}}}\end{array}\right)}
$$

$$
+\frac{k_{\mathrm{cat\,MASP2^*}}^{\mathrm{C2}}\left[\mathrm{pLFMASP2^*}\right]\left[\mathrm{pC3bC4bC2}\right]}{K_{\mathrm{m\,MASP2^*}}^{\mathrm{C2}}\left(\begin{array}{l}1+\frac{\left[\mathrm{C4}\right]}{K_{\mathrm{m\,MASP2^*}}^{\mathrm{C4}}}+\frac{\left[\mathrm{fC4bC2}\right]}{K_{\mathrm{m\,MASP2^*}}^{\mathrm{C2}}}+\frac{\left[\mathrm{fC4bC4bC2}\right]}{K_{\mathrm{m\,MASP2^*}}^{\mathrm{C2}}}+\frac{\left[\mathrm{fC3bC4bC2}\right]}{K_{\mathrm{m\,MASP2^*}}^{\mathrm{C2}}}+\frac{\left[\mathrm{pC4bC2}\right]}{K_{\mathrm{m\,MASP2^*}}^{\mathrm{C2}}}+\frac{\left[\mathrm{pC4bC4bC2}\right]}{K_{\mathrm{m\,MASP2^*}}^{\mathrm{C2}}}\\+\frac{\left[\mathrm{pC3bC4bC2}\right]}{K_{\mathrm{m\,MASP2^*}}^{\mathrm{C2}}}+\frac{\left[\mathrm{pMBL_3MASP1_{zym}}\right]}{K_{\mathrm{m\,MASP2^*}}^{\mathrm{MASP1_{zym}}}}+\frac{\left[\mathrm{pMBL_3MASP2_{zym}}\right]}{K_{\mathrm{m\,MASP2^*}}^{\mathrm{MASP2_{zym}}}}+\frac{\left[\mathrm{pMBL_4MASP1_{zym}}\right]}{K_{\mathrm{m\,MASP2^*}}^{\mathrm{MASP1_{zym}}}}\\+\frac{\left[\mathrm{pMBL_4MASP2_{zym}}\right]}{K_{\mathrm{m\,MASP2^*}}^{\mathrm{MASP2_{zym}}}}+\frac{\left[\mathrm{pCLLKMASP1_{zym}}\right]}{K_{\mathrm{m\,MASP2^*}}^{\mathrm{MASP1_{zym}}}}+\frac{\left[\mathrm{pCLLKMASP2_{zym}}\right]}{K_{\mathrm{m\,MASP2^*}}^{\mathrm{MASP2_{zym}}}}+\frac{\left[\mathrm{pCLK1MASP1_{zym}}\right]}{K_{\mathrm{m\,MASP2^*}}^{\mathrm{MASP1_{zym}}}}\\+\frac{\left[\mathrm{pCLK1MASP2_{zym}}\right]}{K_{\mathrm{m\,MASP2^*}}^{\mathrm{MASP2_{zym}}}}+\frac{\left[\mathrm{pCLL1MASP1_{zym}}\right]}{K_{\mathrm{m\,MASP2^*}}^{\mathrm{MASP1_{zym}}}}+\frac{\left[\mathrm{pCLL1MASP2_{zym}}\right]}{K_{\mathrm{m\,MASP2^*}}^{\mathrm{MASP2_{zym}}}}+\frac{\left[\mathrm{pLFMASP1_{zym}}\right]}{K_{\mathrm{m\,MASP2^*}}^{\mathrm{MASP1_{zym}}}}\\+\frac{\left[\mathrm{pLFMASP2_{zym}}\right]}{K_{\mathrm{m\,MASP2^*}}^{\mathrm{MASP2_{zym}}}}+\frac{\left[\mathrm{pHFMASP1_{zym}}\right]}{K_{\mathrm{m\,MASP2^*}}^{\mathrm{MASP1_{zym}}}}+\frac{\left[\mathrm{pHFMASP2_{zym}}\right]}{K_{\mathrm{m\,MASP2^*}}^{\mathrm{MASP2_{zym}}}}+\frac{\left[\mathrm{pMFMASP1_{zym}}\right]}{K_{\mathrm{m\,MASP2^*}}^{\mathrm{MASP1_{zym}}}}+\frac{\left[\mathrm{pMFMASP2_{zym}}\right]}{K_{\mathrm{m\,MASP2^*}}^{\mathrm{MASP2_{zym}}}}\end{array}\right)}
$$

$$
+\frac{k_{\mathrm{cat\,MASP2^*}}^{\mathrm{C2}}\left[\mathrm{pHFMASP2^*}\right]\left[\mathrm{pC3bC4bC2}\right]}{K_{\mathrm{m\,MASP2^*}}^{\mathrm{C2}}\left(\begin{array}{l}1+\frac{\left[\mathrm{C4}\right]}{K_{\mathrm{m\,MASP2^*}}^{\mathrm{C4}}}+\frac{\left[\mathrm{fC4bC2}\right]}{K_{\mathrm{m\,MASP2^*}}^{\mathrm{C2}}}+\frac{\left[\mathrm{fC4bC4bC2}\right]}{K_{\mathrm{m\,MASP2^*}}^{\mathrm{C2}}}+\frac{\left[\mathrm{fC3bC4bC2}\right]}{K_{\mathrm{m\,MASP2^*}}^{\mathrm{C2}}}+\frac{\left[\mathrm{pC4bC2}\right]}{K_{\mathrm{m\,MASP2^*}}^{\mathrm{C2}}}+\frac{\left[\mathrm{pC4bC4bC2}\right]}{K_{\mathrm{m\,MASP2^*}}^{\mathrm{C2}}}\\+\frac{\left[\mathrm{pC3bC4bC2}\right]}{K_{\mathrm{m\,MASP2^*}}^{\mathrm{C2}}}+\frac{\left[\mathrm{pMBL_3MASP1_{zym}}\right]}{K_{\mathrm{m\,MASP2^*}}^{\mathrm{MASP1_{zym}}}}+\frac{\left[\mathrm{pMBL_3MASP2_{zym}}\right]}{K_{\mathrm{m\,MASP2^*}}^{\mathrm{MASP2_{zym}}}}+\frac{\left[\mathrm{pMBL_4MASP1_{zym}}\right]}{K_{\mathrm{m\,MASP2^*}}^{\mathrm{MASP1_{zym}}}}\\+\frac{\left[\mathrm{pMBL_4MASP2_{zym}}\right]}{K_{\mathrm{m\,MASP2^*}}^{\mathrm{MASP2_{zym}}}}+\frac{\left[\mathrm{pCLLKMASP1_{zym}}\right]}{K_{\mathrm{m\,MASP2^*}}^{\mathrm{MASP1_{zym}}}}+\frac{\left[\mathrm{pCLLKMASP2_{zym}}\right]}{K_{\mathrm{m\,MASP2^*}}^{\mathrm{MASP2_{zym}}}}+\frac{\left[\mathrm{pCLK1MASP1_{zym}}\right]}{K_{\mathrm{m\,MASP2^*}}^{\mathrm{MASP1_{zym}}}}\\+\frac{\left[\mathrm{pCLK1MASP2_{zym}}\right]}{K_{\mathrm{m\,MASP2^*}}^{\mathrm{MASP2_{zym}}}}+\frac{\left[\mathrm{pCLL1MASP1_{zym}}\right]}{K_{\mathrm{m\,MASP2^*}}^{\mathrm{MASP1_{zym}}}}+\frac{\left[\mathrm{pCLL1MASP2_{zym}}\right]}{K_{\mathrm{m\,MASP2^*}}^{\mathrm{MASP2_{zym}}}}+\frac{\left[\mathrm{pLFMASP1_{zym}}\right]}{K_{\mathrm{m\,MASP2^*}}^{\mathrm{MASP1_{zym}}}}\\+\frac{\left[\mathrm{pLFMASP2_{zym}}\right]}{K_{\mathrm{m\,MASP2^*}}^{\mathrm{MASP2_{zym}}}}+\frac{\left[\mathrm{pHFMASP1_{zym}}\right]}{K_{\mathrm{m\,MASP2^*}}^{\mathrm{MASP1_{zym}}}}+\frac{\left[\mathrm{pHFMASP2_{zym}}\right]}{K_{\mathrm{m\,MASP2^*}}^{\mathrm{MASP2_{zym}}}}+\frac{\left[\mathrm{pMFMASP1_{zym}}\right]}{K_{\mathrm{m\,MASP2^*}}^{\mathrm{MASP1_{zym}}}}+\frac{\left[\mathrm{pMFMASP2_{zym}}\right]}{K_{\mathrm{m\,MASP2^*}}^{\mathrm{MASP2_{zym}}}}\end{array}\right)}
$$

$$+\frac{k_{\text{cat MASP2}^*}^{\text{C2}}\left[\text{pMFMASP2}^*\right]\left[\text{pC3bC4bC2}\right]}{K_{\text{m MASP2}^*}^{\text{C2}}\left(\begin{array}{l}1+\frac{[\text{C4}]}{K_{\text{m MASP2}^*}^{\text{C4}}}+\frac{[\text{fC4bC2}]}{K_{\text{m MASP2}^*}^{\text{C2}}}+\frac{[\text{fC4bC4bC2}]}{K_{\text{m MASP2}^*}^{\text{C2}}}+\frac{[\text{fC3bC4bC2}]}{K_{\text{m MASP2}^*}^{\text{C2}}}+\frac{[\text{pC4bC2}]}{K_{\text{m MASP2}^*}^{\text{C2}}}+\frac{[\text{pC4bC4bC2}]}{K_{\text{m MASP2}^*}^{\text{C2}}}\\+\frac{[\text{pC3bC4bC2}]}{K_{\text{m MASP2}^*}^{\text{C2}}}+\frac{[\text{pMBL}_3\text{MASP1}_{\text{zym}}]}{K_{\text{m MASP2}^*}^{\text{MASP1}_{\text{zym}}}}+\frac{[\text{pMBL}_3\text{MASP2}_{\text{zym}}]}{K_{\text{m MASP2}^*}^{\text{MASP2}_{\text{zym}}}}+\frac{[\text{pMBL}_4\text{MASP1}_{\text{zym}}]}{K_{\text{m MASP2}^*}^{\text{MASP1}_{\text{zym}}}}\\+\frac{[\text{pMBL}_4\text{MASP2}_{\text{zym}}]}{K_{\text{m MASP2}^*}^{\text{MASP2}_{\text{zym}}}}+\frac{[\text{pCLLKMASP1}_{\text{zym}}]}{K_{\text{m MASP2}^*}^{\text{MASP1}_{\text{zym}}}}+\frac{[\text{pCLLKMASP2}_{\text{zym}}]}{K_{\text{m MASP2}^*}^{\text{MASP2}_{\text{zym}}}}+\frac{[\text{pCLK1MASP1}_{\text{zym}}]}{K_{\text{m MASP2}^*}^{\text{MASP1}_{\text{zym}}}}\\+\frac{[\text{pCLK1MASP2}_{\text{zym}}]}{K_{\text{m MASP2}^*}^{\text{MASP2}_{\text{zym}}}}+\frac{[\text{pCLL1MASP1}_{\text{zym}}]}{K_{\text{m MASP2}^*}^{\text{MASP1}_{\text{zym}}}}+\frac{[\text{pCLL1MASP2}_{\text{zym}}]}{K_{\text{m MASP2}^*}^{\text{MASP2}_{\text{zym}}}}+\frac{[\text{pLFMASP1}_{\text{zym}}]}{K_{\text{m MASP2}^*}^{\text{MASP1}_{\text{zym}}}}\\+\frac{[\text{pLFMASP2}_{\text{zym}}]}{K_{\text{m MASP2}^*}^{\text{MASP2}_{\text{zym}}}}+\frac{[\text{pHFMASP1}_{\text{zym}}]}{K_{\text{m MASP2}^*}^{\text{MASP1}_{\text{zym}}}}+\frac{[\text{pHFMASP2}_{\text{zym}}]}{K_{\text{m MASP2}^*}^{\text{MASP2}_{\text{zym}}}}+\frac{[\text{pMFMASP1}_{\text{zym}}]}{K_{\text{m MASP2}^*}^{\text{MASP1}_{\text{zym}}}}+\frac{[\text{pMFMASP2}_{\text{zym}}]}{K_{\text{m MASP2}^*}^{\text{MASP2}_{\text{zym}}}}\end{array}\right)}$$

$$-k_{\text{C4bC4BP}}^{+}[\text{pC3bC4bC2a}][\text{pC4BPPTX3}]+k_{\text{C4bC4BP}}^{-}[\text{pC3bC4bC2aC4BPPTX3}]-k_{\text{C4bC4BP}}^{+}[\text{pC3bC4bC2a}][\text{pC4BP}]$$
$$+k_{\text{C4bC4BP}}^{-}[\text{pC3bC4bC2apC4BP}]$$

$$+\frac{k_{\text{cat C1}^*}^{\text{C2}}\left[\text{pMBL}_3\text{PTX3C1}^*\right][\text{pC3bC4bC2}]}{K_{\text{m C1}^*}^{\text{C2}}\left(1+\frac{[\text{C4}]}{K_{\text{m C1}^*}^{\text{C4}}}+\frac{[\text{fC4bC2}]}{K_{\text{m C1}^*}^{\text{C2}}}+\frac{[\text{fC4bC4bC2}]}{K_{\text{m C1}^*}^{\text{C2}}}+\frac{[\text{fC3bC4bC2}]}{K_{\text{m C1}^*}^{\text{C2}}}+\frac{[\text{pC4bC2}]}{K_{\text{m C1}^*}^{\text{C2}}}+\frac{[\text{pC4bC4bC2}]}{K_{\text{m C1}^*}^{\text{C2}}}+\frac{[\text{pC3bC4bC2}]}{K_{\text{m C1}^*}^{\text{C2}}}\right)}$$

$$+\frac{k_{\text{cat C1}^*}^{\text{C2}}\left[\text{pMBL}_4\text{PTX3C1}^*\right][\text{pC3bC4bC2}]}{K_{\text{m C1}^*}^{\text{C2}}\left(1+\frac{[\text{C4}]}{K_{\text{m C1}^*}^{\text{C4}}}+\frac{[\text{fC4bC2}]}{K_{\text{m C1}^*}^{\text{C2}}}+\frac{[\text{fC4bC4bC2}]}{K_{\text{m C1}^*}^{\text{C2}}}+\frac{[\text{fC3bC4bC2}]}{K_{\text{m C1}^*}^{\text{C2}}}+\frac{[\text{pC4bC2}]}{K_{\text{m C1}^*}^{\text{C2}}}+\frac{[\text{pC4bC4bC2}]}{K_{\text{m C1}^*}^{\text{C2}}}+\frac{[\text{pC3bC4bC2}]}{K_{\text{m C1}^*}^{\text{C2}}}\right)}$$

158

$$\frac{d[\text{pC3bC4bC2aC5b}]}{dt}=\frac{k_{\text{cat C4bC4bC2a}}^{\text{C5}}[\text{C5}][\text{pC3bC4bC2a}]}{K_{\text{m C4bC4bC2a}}^{\text{C5}}\left(1+\frac{[\text{C3}]}{K_{\text{m C3bBb}}^{\text{C3}}}+\frac{[\text{C5}]}{K_{\text{m C4bC4bC2a}}^{\text{C5}}}\right)}-k_{\text{C5b}^*}^{-}[\text{pC3bC4bC2aC5b}]$$
$$-k_{\text{C5b6}}^{+}[\text{pC3bC4bC2aC5b}][\text{C6}]+k_{\text{C5b6}}^{-}[\text{pC3bC4bC2aC5b6}]$$

159

$$\frac{d[\text{pC3bC4bC2aC5b6}]}{dt}=k_{\text{C5b6}}^{+}[\text{pC3bC4bC2aC5b}][\text{C6}]-k_{\text{C5b6}}^{-}[\text{pC3bC4bC2aC5b6}]$$
$$-k_{\text{C5b7}}^{+}[\text{pC3bC4bC2aC5b6}][\text{C7}]$$

160

$$\frac{d[\text{pC4bC3bB}]}{dt}=k_{\text{C3bB}}^{+}[\text{pC3bC4b}][\text{FB}]-k_{\text{C3bB}}^{-}[\text{pC4bC3bB}]$$

$$-\frac{k_{\text{cat C3(H}_2\text{O)B}}^{\text{FD}}[\text{FD}][\text{pC4bC3bB}]}{\begin{pmatrix}K_{\text{m C3(H}_2\text{O)B}}^{\text{D}}+[\text{C3(H}_2\text{O)B}]+[\text{fC3bB}]+[\text{fC3bC3bB}]+[\text{fC3bC3bBP}]+[\text{IgGC3bC3bB}]+[\text{IgGC3bC3bBP}]\\+[\text{fC4bC3bB}]+[\text{fC4bC3bBP}]+[\text{pC3bBCFHR1}]+[\text{pC3bBCFHR2}]+[\text{pC3bBCFHR3}]+[\text{pC3bBCFHR4}]\\+[\text{pC3bBCFHR5}]+[\text{hC4bC3bBP}]+[\text{hC4bC3bB}]+[\text{hC3bC3bB}]+[\text{hC3bC3bBP}]+[\text{hC3bB}]+[\text{pC4bC3bB}]\\+[\text{pC4bC3bBP}]+[\text{pC3bC3bB}]+[\text{pC3bC3bBP}]+[\text{pC3bB}]\end{pmatrix}}$$

161

$$\frac{d[\text{pC4bC3bBb}]}{dt}=$$

$$\frac{k_{\text{cat C3(H}_2\text{O)B}}^{\text{FD}}[\text{FD}][\text{pC4bC3bB}]}{\begin{pmatrix}K_{\text{m C3(H}_2\text{O)B}}^{\text{D}}+[\text{C3(H}_2\text{O)B}]+[\text{fC3bB}]+[\text{fC3bC3bB}]+[\text{fC3bC3bBP}]+[\text{IgGC3bC3bB}]+[\text{IgGC3bC3bBP}]\\+[\text{fC4bC3bB}]+[\text{fC4bC3bBP}]+[\text{pC3bBCFHR1}]+[\text{pC3bBCFHR2}]+[\text{pC3bBCFHR3}]+[\text{pC3bBCFHR4}]\\+[\text{pC3bBCFHR5}]+[\text{hC4bC3bBP}]+[\text{hC4bC3bB}]+[\text{hC3bC3bB}]+[\text{hC3bC3bBP}]+[\text{hC3bB}]+[\text{pC4bC3bB}]\\+[\text{pC4bC3bBP}]+[\text{pC3bC3bB}]+[\text{pC3bC3bBP}]+[\text{pC3bB}]\end{pmatrix}}$$

$$-k_{\text{C3bC4bBb}}^{-}[\text{pC4bC3bBb}]+k_{\text{C5b}^*}^{-}[\text{pC4bC3bBbC5b}]+k_{\text{C5b7}}^{+}[\text{pC4bC3bBbC5b6}][\text{C7}]-k_{\text{C3bP}}^{+}[\text{pC4bC3bBb}][\text{P}]$$
$$+k_{\text{C3bP}}^{-}[\text{pC4bC3bBbP}]+k_{\text{C3bC4b}}^{+}[\text{pC3bBb}][\text{nfC4b}]-k_{\text{C3bH}}^{+}[\text{pC4bC3bBb}][\text{FH}]+k_{\text{C3bH}}^{-}[\text{pC4bC3bBbFH}]$$
$$-k_{\text{C3bFHL1}}^{+}[\text{pC4bC3bBb}][\text{FHL1}]+k_{\text{C3bFHL1}}^{-}[\text{pC4bC3bBbFHL1}]-k_{\text{C3bH}}^{+}[\text{pC4bC3bBb}][\text{pFHPTX3}]$$
$$+k_{\text{C3bH}}^{-}[\text{pC4bC3bBbFHPTX3}]-k_{\text{C3bFHL1}}^{+}[\text{pC4bC3bBb}][\text{pFHL1PTX3}]+k_{\text{C3bFHL1}}^{-}[\text{pC4bC3bBbFHL1PTX3}]$$
$$-k_{\text{C3bH}}^{+}[\text{pC4bC3bBb}][\text{pFH}]+k_{\text{C3bH}}^{-}[\text{pC4bC3bBbpFH}]-k_{\text{C3bFHL1}}^{+}[\text{pC4bC3bBb}][\text{pFHL1}]$$
$$+k_{\text{C3bFHL1}}^{-}[\text{pC4bC3bBbpFHL1}]$$

162

$$\frac{d[\text{pC3bC4bP}]}{dt}=k_{\text{C3bP}}^{+}[\text{pC3bC4b}][\text{P}]-k_{\text{C3bP}}^{-}[\text{pC3bC4bP}]-k_{\text{C3bB}}^{+}[\text{pC3bC4bP}][\text{FB}]+k_{\text{C3bB}}^{-}[\text{pC4bC3bBP}]$$

163

$$\frac{d\left[\mathrm{pC4bC3bBP}\right]}{dt}=k_{\mathrm{C3bB}}^{+}\left[\mathrm{pC3bC4bP}\right]\left[\mathrm{FB}\right]-k_{\mathrm{C3bB}}^{-}\left[\mathrm{pC4bC3bBP}\right]$$
$$-\frac{k_{\mathrm{cat\ C3(H_2O)B}}^{\mathrm{FD}}\left[\mathrm{FD}\right]\left[\mathrm{pC4bC3bBP}\right]}{\left(\begin{array}{l}K_{\mathrm{m\ C3(H_2O)B}}^{\mathrm{D}}+\left[\mathrm{C3(H_2O)B}\right]+\left[\mathrm{fC3bB}\right]+\left[\mathrm{fC3bC3bB}\right]+\left[\mathrm{fC3bC3bBP}\right]+\left[\mathrm{IgGC3bC3bB}\right]+\left[\mathrm{IgGC3bC3bBP}\right]\\+\left[\mathrm{fC4bC3bB}\right]+\left[\mathrm{fC4bC3bBP}\right]+\left[\mathrm{pC3bBCFHR1}\right]+\left[\mathrm{pC3bBCFHR2}\right]+\left[\mathrm{pC3bBCFHR3}\right]+\left[\mathrm{pC3bBCFHR4}\right]\\+\left[\mathrm{pC3bBCFHR5}\right]+\left[\mathrm{hC4bC3bBP}\right]+\left[\mathrm{hC4bC3bB}\right]+\left[\mathrm{hC3bC3bB}\right]+\left[\mathrm{hC3bC3bBP}\right]+\left[\mathrm{hC3bB}\right]+\left[\mathrm{pC4bC3bB}\right]\\+\left[\mathrm{pC4bC3bBP}\right]+\left[\mathrm{pC3bC3bB}\right]+\left[\mathrm{pC3bC3bBP}\right]+\left[\mathrm{pC3bB}\right]\end{array}\right)}$$

164

$$\frac{d\left[\mathrm{pC4bC3bBbP}\right]}{dt}=$$
$$\frac{k_{\mathrm{cat\ C3(H_2O)B}}^{\mathrm{FD}}\left[\mathrm{FD}\right]\left[\mathrm{pC4bC3bBP}\right]}{\left(\begin{array}{l}K_{\mathrm{m\ C3(H_2O)B}}^{\mathrm{D}}+\left[\mathrm{C3(H_2O)B}\right]+\left[\mathrm{fC3bB}\right]+\left[\mathrm{fC3bC3bB}\right]+\left[\mathrm{fC3bC3bBP}\right]+\left[\mathrm{IgGC3bC3bB}\right]+\left[\mathrm{IgGC3bC3bBP}\right]\\+\left[\mathrm{fC4bC3bB}\right]+\left[\mathrm{fC4bC3bBP}\right]+\left[\mathrm{pC3bBCFHR1}\right]+\left[\mathrm{pC3bBCFHR2}\right]+\left[\mathrm{pC3bBCFHR3}\right]+\left[\mathrm{pC3bBCFHR4}\right]\\+\left[\mathrm{pC3bBCFHR5}\right]+\left[\mathrm{hC4bC3bBP}\right]+\left[\mathrm{hC4bC3bB}\right]+\left[\mathrm{hC3bC3bB}\right]+\left[\mathrm{hC3bC3bBP}\right]+\left[\mathrm{hC3bB}\right]+\left[\mathrm{pC4bC3bB}\right]\\+\left[\mathrm{pC4bC3bBP}\right]+\left[\mathrm{pC3bC3bB}\right]+\left[\mathrm{pC3bC3bBP}\right]+\left[\mathrm{pC3bB}\right]\end{array}\right)}$$
$$-k_{\mathrm{C3bC4bBbP}}^{-}\left[\mathrm{pC4bC3bBbP}\right]+k_{\mathrm{C3bP}}^{+}\left[\mathrm{pC4bC3bBb}\right]\left[\mathrm{P}\right]-k_{\mathrm{C3bP}}^{-}\left[\mathrm{pC4bC3bBbP}\right]+k_{\mathrm{C5b^*}}^{-}\left[\mathrm{pC4bC3bBbPC5b}\right]$$
$$+k_{\mathrm{C5b7}}^{+}\left[\mathrm{pC4bC3bBbPC5b6}\right]\left[\mathrm{C7}\right]+k_{\mathrm{C3bC4b}}^{+}\left[\mathrm{pC3bBbP}\right]\left[\mathrm{nfC4b}\right]$$

165

$$\frac{d\left[\mathrm{pC3b}\right]}{dt}=k_{\mathrm{pC3b}}^{+}\left[\mathrm{nfC3b}\right]\left[\mathrm{Pathogen\ Surface}\right]-k_{\mathrm{C3bC3b}}^{+}\left[\mathrm{nfC3b}\right]\left[\mathrm{pC3b}\right]-k_{\mathrm{C3bC4b}}^{+}\left[\mathrm{pC3b}\right]\left[\mathrm{nfC4b}\right]-k_{\mathrm{C3bB}}^{+}\left[\mathrm{pC3b}\right]\left[\mathrm{FB}\right]$$
$$+k_{\mathrm{C3bB}}^{-}\left[\mathrm{pC3bB}\right]+k_{\mathrm{C3bBb}}^{-}\left[\mathrm{pC3bBb}\right]+k_{\mathrm{C3bBbP}}^{-}\left[\mathrm{pC3bBbP}\right]-k_{\mathrm{C3bH}}^{+}\left[\mathrm{pC3b}\right]\left[\mathrm{FH}\right]+k_{\mathrm{C3bH}}^{-}\left[\mathrm{pC3bFH}\right]$$
$$+k_{\mathrm{C3bBbH_{decay}}}^{-}\left[\mathrm{pC3bBbFH}\right]-k_{\mathrm{C3bFHL1}}^{+}\left[\mathrm{pC3b}\right]\left[\mathrm{FHL1}\right]+k_{\mathrm{C3bFHL1}}^{-}\left[\mathrm{pC3bFHL1}\right]+k_{\mathrm{C3bBbFHL1_{decay}}}^{-}\left[\mathrm{pC3bBbFHL1}\right]$$
$$-k_{\mathrm{C3bH}}^{+}\left[\mathrm{pC3b}\right]\left[\mathrm{pFHPTX3}\right]+k_{\mathrm{C3bH}}^{-}\left[\mathrm{pC3bFHPTX3}\right]+k_{\mathrm{C3bBbH_{decay}}}^{-}\left[\mathrm{pC3bBbFHPTX3}\right]-k_{\mathrm{C3bFHL1}}^{+}\left[\mathrm{pC3b}\right]\left[\mathrm{pFHL1PTX3}\right]$$
$$+k_{\mathrm{C3bFHL1}}^{-}\left[\mathrm{pC3bFHL1PTX3}\right]+k_{\mathrm{C3bBbFHL1_{decay}}}^{-}\left[\mathrm{pC3bBbFHL1PTX3}\right]-k_{\mathrm{C3bH}}^{+}\left[\mathrm{pC3b}\right]\left[\mathrm{pFH}\right]+k_{\mathrm{C3bH}}^{-}\left[\mathrm{pC3bpFH}\right]$$
$$+k_{\mathrm{C3bBbH_{decay}}}^{-}\left[\mathrm{pC3bBbpFH}\right]-k_{\mathrm{C3bFHL1}}^{+}\left[\mathrm{pC3b}\right]\left[\mathrm{pFHL1}\right]+k_{\mathrm{C3bFHL1}}^{-}\left[\mathrm{pC3bpFHL1}\right]+k_{\mathrm{C3bBbFHL1_{decay}}}^{-}\left[\mathrm{pC3bBbpFHL1}\right]$$

166

$$\frac{d[\mathrm{pC3bC3b}]}{dt}=k^{+}_{\mathrm{C3bC3b}}[\mathrm{nfC3b}][\mathrm{pC3b}]-k^{+}_{\mathrm{C3bB}}[\mathrm{pC3bC3b}][\mathrm{FB}]+k^{-}_{\mathrm{C3bB}}[\mathrm{pC3bC3bB}]-k^{+}_{\mathrm{C3bP}}[\mathrm{pC3bC3b}][\mathrm{P}]$$
$$+k^{-}_{\mathrm{C3bP}}[\mathrm{pC3bC3bP}]+k^{-}_{\mathrm{C3bC3bBb}}[\mathrm{pC3bC3bBb}]+k^{-}_{\mathrm{C3bC3bBbP}}[\mathrm{pC3bC3bBbP}]-k^{+}_{\mathrm{C3bH}}[\mathrm{pC3bC3b}][\mathrm{FH}]$$
$$+k^{-}_{\mathrm{C3bH}}[\mathrm{pC3bC3bFH}]+k^{-}_{\mathrm{C3bBbH_{decay}}}[\mathrm{pC3bC3bBbFH}]-k^{+}_{\mathrm{C3bFHL1}}[\mathrm{pC3bC3b}][\mathrm{FHL1}]+k^{-}_{\mathrm{C3bFHL1}}[\mathrm{pC3bC3bFHL1}]$$
$$+k^{-}_{\mathrm{C3bBbFHL1_{decay}}}[\mathrm{pC3bC3bBbFHL1}]+k^{-}_{\mathrm{C3bBbH_{decay}}}[\mathrm{pC3bC3bBbFHPTX3}]+k^{-}_{\mathrm{C3bBbFHL1_{decay}}}[\mathrm{pC3bC3bBbFHL1PTX3}]$$
$$-k^{+}_{\mathrm{C3bH}}[\mathrm{pC3bC3b}][\mathrm{pFHPTX3}]+k^{-}_{\mathrm{C3bH}}[\mathrm{pC3bC3bFHPTX3}]-k^{+}_{\mathrm{C3bFHL1}}[\mathrm{pC3bC3b}][\mathrm{pFHL1PTX3}]$$
$$+k^{-}_{\mathrm{C3bFHL1}}[\mathrm{pC3bC3bFHL1PTX3}]+k^{-}_{\mathrm{C3bBbH_{decay}}}[\mathrm{pC3bC3bBbpFH}]+k^{-}_{\mathrm{C3bBbFHL1_{decay}}}[\mathrm{pC3bC3bBbpFHL1}]$$
$$-k^{+}_{\mathrm{C3bH}}[\mathrm{pC3bC3b}][\mathrm{pFH}]+k^{-}_{\mathrm{C3bH}}[\mathrm{pC3bC3bpFH}]-k^{+}_{\mathrm{C3bFHL1}}[\mathrm{pC3bC3b}][\mathrm{pFHL1}]+k^{-}_{\mathrm{C3bFHL1}}[\mathrm{pC3bC3bpFHL1}]$$

167

$$\frac{d[\mathrm{pC3bC3bB}]}{dt}=k^{+}_{\mathrm{C3bB}}[\mathrm{pC3bC3b}][\mathrm{FB}]-k^{-}_{\mathrm{C3bB}}[\mathrm{pC3bC3bB}]$$
$$-\frac{k^{\mathrm{FD}}_{\mathrm{cat\ C3(H_2O)B}}[\mathrm{FD}][\mathrm{pC3bC3bB}]}{\left(\begin{array}{l}K^{\mathrm{D}}_{\mathrm{m\ C3(H_2O)B}}+[\mathrm{C3(H_2O)B}]+[\mathrm{fC3bB}]+[\mathrm{fC3bC3bB}]+[\mathrm{fC3bC3bBP}]+[\mathrm{IgGC3bC3bB}]+[\mathrm{IgGC3bC3bBP}]\\+[\mathrm{fC4bC3bB}]+[\mathrm{fC4bC3bBP}]+[\mathrm{pC3bBCFHR1}]+[\mathrm{pC3bBCFHR2}]+[\mathrm{pC3bBCFHR3}]+[\mathrm{pC3bBCFHR4}]\\+[\mathrm{pC3bBCFHR5}]+[\mathrm{hC4bC3bBP}]+[\mathrm{hC4bC3bB}]+[\mathrm{hC3bC3bB}]+[\mathrm{hC3bC3bBP}]+[\mathrm{hC3bB}]+[\mathrm{pC4bC3bB}]\\+[\mathrm{pC4bC3bBP}]+[\mathrm{pC3bC3bB}]+[\mathrm{pC3bC3bBP}]+[\mathrm{pC3bB}]\end{array}\right)}$$

168

$$\frac{d[\mathrm{pC3bC3bBb}]}{dt}=$$
$$\frac{k^{\mathrm{FD}}_{\mathrm{cat\ C3(H_2O)B}}[\mathrm{FD}][\mathrm{pC3bC3bB}]}{\left(\begin{array}{l}K^{\mathrm{D}}_{\mathrm{m\ C3(H_2O)B}}+[\mathrm{C3(H_2O)B}]+[\mathrm{fC3bB}]+[\mathrm{fC3bC3bB}]+[\mathrm{fC3bC3bBP}]+[\mathrm{IgGC3bC3bB}]+[\mathrm{IgGC3bC3bBP}]\\+[\mathrm{fC4bC3bB}]+[\mathrm{fC4bC3bBP}]+[\mathrm{pC3bBCFHR1}]+[\mathrm{pC3bBCFHR2}]+[\mathrm{pC3bBCFHR3}]+[\mathrm{pC3bBCFHR4}]\\+[\mathrm{pC3bBCFHR5}]+[\mathrm{hC4bC3bBP}]+[\mathrm{hC4bC3bB}]+[\mathrm{hC3bC3bB}]+[\mathrm{hC3bC3bBP}]+[\mathrm{hC3bB}]+[\mathrm{pC4bC3bB}]\\+[\mathrm{pC4bC3bBP}]+[\mathrm{pC3bC3bB}]+[\mathrm{pC3bC3bBP}]+[\mathrm{pC3bB}]\end{array}\right)}$$
$$+k^{+}_{\mathrm{C3bC3b}}[\mathrm{pC3bBb}][\mathrm{nfC3b}]-k^{-}_{\mathrm{C3bC3bBb}}[\mathrm{pC3bC3bBb}]-k^{+}_{\mathrm{C3bP}}[\mathrm{pC3bC3bBb}][\mathrm{P}]+k^{-}_{\mathrm{C3bP}}[\mathrm{pC3bC3bBbP}]$$
$$+k^{-}_{\mathrm{C5b^*}}[\mathrm{pC3bC3bBbC5b}]+k^{+}_{\mathrm{C5b7}}[\mathrm{pC3bC3bBbC5b6}][\mathrm{C7}]-k^{+}_{\mathrm{C3bH}}[\mathrm{pC3bC3bBb}][\mathrm{FH}]+k^{-}_{\mathrm{C3bH}}[\mathrm{pC3bC3bBbFH}]$$
$$-k^{+}_{\mathrm{C3bFHL1}}[\mathrm{pC3bC3bBb}][\mathrm{FHL1}]+k^{-}_{\mathrm{C3bFHL1}}[\mathrm{pC3bC3bBbFHL1}]-k^{+}_{\mathrm{C3bH}}[\mathrm{pC3bC3bBb}][\mathrm{pFHPTX3}]$$
$$+k^{-}_{\mathrm{C3bH}}[\mathrm{pC3bC3bBbFHPTX3}]-k^{+}_{\mathrm{C3bFHL1}}[\mathrm{pC3bC3bBb}][\mathrm{pFHL1PTX3}]+k^{-}_{\mathrm{C3bFHL1}}[\mathrm{pC3bC3bBbFHL1PTX3}]$$
$$-k^{+}_{\mathrm{C3bH}}[\mathrm{pC3bC3bBb}][\mathrm{pFH}]+k^{-}_{\mathrm{C3bH}}[\mathrm{pC3bC3bBbpFH}]-k^{+}_{\mathrm{C3bFHL1}}[\mathrm{pC3bC3bBb}][\mathrm{pFHL1}]$$
$$+k^{-}_{\mathrm{C3bFHL1}}[\mathrm{pC3bC3bBbpFHL1}]$$

169

$$\frac{d[\text{pC3bC3bP}]}{dt}=k^{+}_{\text{C3bP}}[\text{pC3bC3b}][\text{P}]-k^{-}_{\text{C3bP}}[\text{pC3bC3bP}]-k^{+}_{\text{C3bB}}[\text{pC3bC3bP}][\text{FB}]+k^{-}_{\text{C3bB}}[\text{pC3bC3bBP}]$$

170

$$\frac{d[\text{pC3bC3bBP}]}{dt}=k^{+}_{\text{C3bB}}[\text{pC3bC3bP}][\text{FB}]-k^{-}_{\text{C3bB}}[\text{pC3bC3bBP}]$$
$$-\frac{k^{\text{FD}}_{\text{cat C3(H}_2\text{O)B}}[\text{FD}][\text{pC3bC3bBP}]}{\left(\begin{array}{l}K^{\text{D}}_{\text{m C3(H}_2\text{O)B}}+[\text{C3(H}_2\text{O)B}]+[\text{fC3bB}]+[\text{fC3bC3bB}]+[\text{fC3bC3bBP}]+[\text{IgGC3bC3bB}]+[\text{IgGC3bC3bBP}]\\+[\text{fC4bC3bB}]+[\text{fC4bC3bBP}]+[\text{pC3bBCFHR1}]+[\text{pC3bBCFHR2}]+[\text{pC3bBCFHR3}]+[\text{pC3bBCFHR4}]\\+[\text{pC3bBCFHR5}]+[\text{hC4bC3bBP}]+[\text{hC4bC3bB}]+[\text{hC3bC3bB}]+[\text{hC3bC3bBP}]+[\text{hC3bB}]+[\text{pC4bC3bB}]\\+[\text{pC4bC3bBP}]+[\text{pC3bC3bB}]+[\text{pC3bC3bBP}]+[\text{pC3bB}]\end{array}\right)}$$

171

$$\frac{d[\text{pC3bC3bBbP}]}{dt}=$$
$$\frac{k^{\text{FD}}_{\text{cat C3(H}_2\text{O)B}}[\text{FD}][\text{pC3bC3bBP}]}{\left(\begin{array}{l}K^{\text{D}}_{\text{m C3(H}_2\text{O)B}}+[\text{C3(H}_2\text{O)B}]+[\text{fC3bB}]+[\text{fC3bC3bB}]+[\text{fC3bC3bBP}]+[\text{IgGC3bC3bB}]+[\text{IgGC3bC3bBP}]\\+[\text{fC4bC3bB}]+[\text{fC4bC3bBP}]+[\text{pC3bBCFHR1}]+[\text{pC3bBCFHR2}]+[\text{pC3bBCFHR3}]+[\text{pC3bBCFHR4}]\\+[\text{pC3bBCFHR5}]+[\text{hC4bC3bBP}]+[\text{hC4bC3bB}]+[\text{hC3bC3bB}]+[\text{hC3bC3bBP}]+[\text{hC3bB}]+[\text{pC4bC3bB}]\\+[\text{pC4bC3bBP}]+[\text{pC3bC3bB}]+[\text{pC3bC3bBP}]+[\text{pC3bB}]\end{array}\right)}$$
$$+k^{+}_{\text{C3bC3b}}[\text{nfC3b}][\text{pC3bBbP}]+k^{+}_{\text{C3bP}}[\text{pC3bC3bBb}][\text{P}]-k^{-}_{\text{C3bP}}[\text{pC3bC3bBbP}]-k^{-}_{\text{C3bC3bBbP}}[\text{pC3bC3bBbP}]$$
$$+k^{-}_{\text{C5b}^*}[\text{pC3bC3bBbPC5b}]+k^{+}_{\text{C5b7}}[\text{pC3bC3bBbPC5b6}][\text{C7}]$$

172

$$\frac{d[\text{pC3bB}]}{dt}=k^{+}_{\text{C3bB}}[\text{pC3b}][\text{FB}]-k^{-}_{\text{C3bB}}[\text{pC3bB}]$$
$$-\frac{k^{\text{FD}}_{\text{cat C3(H}_2\text{O)B}}[\text{FD}][\text{pC3bB}]}{\left(\begin{array}{l}K^{\text{D}}_{\text{m C3(H}_2\text{O)B}}+[\text{C3(H}_2\text{O)B}]+[\text{fC3bB}]+[\text{fC3bC3bB}]+[\text{fC3bC3bBP}]+[\text{IgGC3bC3bB}]+[\text{IgGC3bC3bBP}]\\+[\text{fC4bC3bB}]+[\text{fC4bC3bBP}]+[\text{pC3bBCFHR1}]+[\text{pC3bBCFHR2}]+[\text{pC3bBCFHR3}]+[\text{pC3bBCFHR4}]\\+[\text{pC3bBCFHR5}]+[\text{hC4bC3bBP}]+[\text{hC4bC3bB}]+[\text{hC3bC3bB}]+[\text{hC3bC3bBP}]+[\text{hC3bB}]+[\text{pC4bC3bB}]\\+[\text{pC4bC3bBP}]+[\text{pC3bC3bB}]+[\text{pC3bC3bBP}]+[\text{pC3bB}]\end{array}\right)}$$

173

$$\frac{d[\text{pC3bBb}]}{dt}=$$

$$\frac{k^{\text{FD}}_{\text{cat C3(H}_2\text{O)B}}[\text{FD}][\text{pC3bB}]}{\left(\begin{array}{l}K^{\text{D}}_{\text{m C3(H}_2\text{O)B}}+[\text{C3(H}_2\text{O)B}]+[\text{fC3bB}]+[\text{fC3bC3bB}]+[\text{fC3bC3bBP}]+[\text{IgGC3bC3bB}]+[\text{IgGC3bC3bBP}]\\+[\text{fC4bC3bB}]+[\text{fC4bC3bBP}]+[\text{pC3bBCFHR1}]+[\text{pC3bBCFHR2}]+[\text{pC3bBCFHR3}]+[\text{pC3bBCFHR4}]\\+[\text{pC3bBCFHR5}]+[\text{hC4bC3bBP}]+[\text{hC4bC3bB}]+[\text{hC3bC3bB}]+[\text{hC3bC3bBP}]+[\text{hC3bB}]+[\text{pC4bC3bB}]\\+[\text{pC4bC3bBP}]+[\text{pC3bC3bB}]+[\text{pC3bC3bBP}]+[\text{pC3bB}]\end{array}\right)}$$

$$-k^{+}_{\text{C3bC3b}}[\text{nfC3b}][\text{pC3bBb}]-k^{+}_{\text{C3bC4b}}[\text{pC3bBb}][\text{nfC4b}]-k^{+}_{\text{C3bP}}[\text{pC3bBb}][\text{P}]+k^{-}_{\text{C3bP}}[\text{pC3bBbP}]-k^{-}_{\text{C3bBb}}[\text{pC3bBb}]$$
$$-k^{+}_{\text{C3bH}}[\text{pC3bBb}][\text{FH}]+k^{-}_{\text{C3bH}}[\text{pC3bBbFH}]-k^{+}_{\text{C3bFHL1}}[\text{pC3bBb}][\text{FHL1}]+k^{-}_{\text{C3bFHL1}}[\text{pC3bBbFHL1}]$$
$$-k^{+}_{\text{C3bH}}[\text{pC3bBb}][\text{pFHPTX3}]+k^{-}_{\text{C3bH}}[\text{pC3bBbFHPTX3}]-k^{+}_{\text{C3bFHL1}}[\text{pC3bBb}][\text{pFHL1PTX3}]$$
$$+k^{-}_{\text{C3bFHL1}}[\text{pC3bBbFHL1PTX3}]-k^{+}_{\text{C3bH}}[\text{pC3bBb}][\text{pFH}]+k^{-}_{\text{C3bH}}[\text{pC3bBbpFH}]-k^{+}_{\text{C3bFHL1}}[\text{pC3bBb}][\text{pFHL1}]$$
$$+k^{-}_{\text{C3bFHL1}}[\text{pC3bBbpFHL1}]$$

174

$$\frac{d[\text{pC3bBbP}]}{dt}=k^{+}_{\text{C3bP}}[\text{pC3bBb}][\text{P}]-k^{-}_{\text{C3bP}}[\text{pC3bBbP}]-k^{-}_{\text{C3bBbP}}[\text{pC3bBbP}]-k^{+}_{\text{C3bC3b}}[\text{nfC3b}][\text{pC3bBbP}]$$
$$-k^{+}_{\text{C3bC4b}}[\text{pC3bBbP}][\text{nfC4b}]$$

175

$$\frac{d[\text{pC4bC3bBbC5b}]}{dt}=\frac{k^{\text{C5}}_{\text{cat C3bC4bBb}}[\text{C5}][\text{pC4bC3bBb}]}{K^{\text{C5}}_{\text{m C3bC4bBb}}\left(1+\frac{[\text{C3}]}{K^{\text{C3}}_{\text{m C3bBb}}}+\frac{[\text{C5}]}{K^{\text{C5}}_{\text{m C3bC4bBb}}}\right)}-k^{-}_{\text{C5b}^*}[\text{pC4bC3bBbC5b}]$$
$$-k^{+}_{\text{C5b6}}[\text{pC4bC3bBbC5b}][\text{C6}]+k^{-}_{\text{C5b6}}[\text{pC4bC3bBbC5b6}]$$

176

$$\frac{d[\text{pC4bC3bBbC5b6}]}{dt}=k^{+}_{\text{C5b6}}[\text{pC4bC3bBbC5b}][\text{C6}]-k^{-}_{\text{C5b6}}[\text{pC4bC3bBbC5b6}]-k^{+}_{\text{C5b7}}[\text{pC4bC3bBbC5b6}][\text{C7}]$$

177

$$\frac{d[\text{pC4bC3bBbPC5b}]}{dt}=\frac{k^{\text{C5}}_{\text{cat C3bC4bBb}}[\text{C5}][\text{pC4bC3bBbP}]}{K^{\text{C5}}_{\text{m C3bC4bBb}}\left(1+\frac{[\text{C3}]}{K^{\text{C3}}_{\text{m C3bBb}}}+\frac{[\text{C5}]}{K^{\text{C5}}_{\text{m C3bC4bBb}}}\right)}-k^{-}_{\text{C5b}^*}[\text{pC4bC3bBbPC5b}]$$
$$-k^{+}_{\text{C5b6}}[\text{pC4bC3bBbPC5b}][\text{C6}]+k^{-}_{\text{C5b6}}[\text{pC4bC3bBbPC5b6}]$$

178

$$\frac{d[\text{pC4bC3bBbPC5b6}]}{dt}=k^{+}_{\text{C5b6}}[\text{pC4bC3bBbPC5b}][\text{C6}]-k^{-}_{\text{C5b6}}[\text{pC4bC3bBbPC5b6}]$$
$$-k^{+}_{\text{C5b7}}[\text{pC4bC3bBbPC5b6}][\text{C7}]$$

179

$$\frac{d\left[\text{pC3bC3bBbC5b}\right]}{dt}=\frac{k_{\text{cat C3bC3bBb}}^{\text{C5}}\left[\text{C5}\right]\left[\text{pC3bC3bBb}\right]}{K_{\text{m C3bC3bBb}}^{\text{C5}}\left(1+\frac{\left[\text{C3}\right]}{K_{\text{m C3bBb}}^{\text{C3}}}+\frac{\left[\text{C5}\right]}{K_{\text{m C3bC3bBb}}^{\text{C5}}}\right)}-k_{\text{C5b}^*}^{-}\left[\text{pC3bC3bBbC5b}\right]$$

$$-k_{\text{C5b6}}^{+}\left[\text{pC3bC3bBbC5b}\right]\left[\text{C6}\right]+k_{\text{C5b6}}^{-}\left[\text{pC3bC3bBbC5b6}\right]$$

180

$$\frac{d\left[\text{pC3bC3bBbC5b6}\right]}{dt}=k_{\text{C5b6}}^{+}\left[\text{pC3bC3bBbC5b}\right]\left[\text{C6}\right]-k_{\text{C5b6}}^{-}\left[\text{pC3bC3bBbC5b6}\right]-k_{\text{C5b7}}^{+}\left[\text{pC3bC3bBbC5b6}\right]\left[\text{C7}\right]$$

181

$$\frac{d\left[\text{pC3bC3bBbPC5b}\right]}{dt}=\frac{k_{\text{cat C3bC3bBb}}^{\text{C5}}\left[\text{C5}\right]\left[\text{pC3bC3bBbP}\right]}{K_{\text{m C3bC3bBb}}^{\text{C5}}\left(1+\frac{\left[\text{C3}\right]}{K_{\text{m C3bBb}}^{\text{C3}}}+\frac{\left[\text{C5}\right]}{K_{\text{m C3bC3bBb}}^{\text{C5}}}\right)}-k_{\text{C5b}^*}^{-}\left[\text{pC3bC3bBbPC5b}\right]$$

$$-k_{\text{C5b6}}^{+}\left[\text{pC3bC3bBbPC5b}\right]\left[\text{C6}\right]+k_{\text{C5b6}}^{-}\left[\text{pC3bC3bBbPC5b6}\right]$$

182

$$\frac{d\left[\text{pC3bC3bBbPC5b6}\right]}{dt}=k_{\text{C5b6}}^{+}\left[\text{pC3bC3bBbPC5b}\right]\left[\text{C6}\right]-k_{\text{C5b6}}^{-}\left[\text{pC3bC3bBbPC5b6}\right]$$

$$-k_{\text{C5b7}}^{+}\left[\text{pC3bC3bBbPC5b6}\right]\left[\text{C7}\right]$$

183

$$\frac{d\left[\text{pC5b7}\right]}{dt}=k_{\text{C5b7}_{\text{surface}}}^{+}\left[\text{fC5b7}\right]\left[\text{Pathogen Surface}\right]\times 4-k_{\text{C5b8}}^{+}\left[\text{pC5b7}\right]\left[\text{C8}\right]-k_{\text{VnC5b7}}^{+}\left[\text{pVn}\right]\left[\text{pC5b7}\right]$$

$$+k_{\text{VnC5b7}}^{-}\left[\text{pC5b7pVn}\right]$$

184

$$\frac{d\left[\text{pC5b8}\right]}{dt}=k_{\text{C5b8}}^{+}\left[\text{pC5b7}\right]\left[\text{C8}\right]-k_{\text{C5b9}_1}^{+}\left[\text{pC5b8}\right]\left[\text{C9}_1\right]$$

185

$$\frac{d\left[\text{pC5b9}_1\right]}{dt}=k_{\text{C5b9}_1}^{+}\left[\text{pC5b8}\right]\left[\text{C9}_1\right]-k_{\text{C5b9}_{\text{poly}}}^{+}\left[\text{pC5b9}_1\right]\left[\text{C9}_2\right]$$

186

$$\frac{d\left[\text{pC5b9}_2\right]}{dt}=k_{\text{C5b9}_{\text{poly}}}^{+}\left[\text{pC5b9}_1\right]\left[\text{C9}_2\right]-k_{\text{C5b9}_{\text{poly}}}^{+}\left[\text{pC5b9}_2\right]\left[\text{C9}_3\right]$$

187

$$\frac{d\left[\text{pC5b9}_3\right]}{dt}=k_{\text{C5b9}_{\text{poly}}}^{+}\left[\text{pC5b9}_2\right]\left[\text{C9}_3\right]-k_{\text{C5b9}_{\text{poly}}}^{+}\left[\text{pC5b9}_3\right]\left[\text{C9}_4\right]$$

188
$$\frac{d\left[\mathrm{pC5b9}_{4}\right]}{dt}=k_{\mathrm{C5b9}_{\mathrm{poly}}}^{+}\left[\mathrm{pC5b9}_{3}\right]\left[\mathrm{C9}_{4}\right]-k_{\mathrm{C5b9}_{\mathrm{poly}}}^{+}\left[\mathrm{pC5b9}_{4}\right]\left[\mathrm{C9}_{5}\right]$$

189
$$\frac{d\left[\mathrm{pC5b9}_{5}\right]}{dt}=k_{\mathrm{C5b9}_{\mathrm{poly}}}^{+}\left[\mathrm{pC5b9}_{4}\right]\left[\mathrm{C9}_{5}\right]-k_{\mathrm{C5b9}_{\mathrm{poly}}}^{+}\left[\mathrm{pC5b9}_{5}\right]\left[\mathrm{C9}_{6}\right]$$

190
$$\frac{d\left[\mathrm{pC5b9}_{6}\right]}{dt}=k_{\mathrm{C5b9}_{\mathrm{poly}}}^{+}\left[\mathrm{pC5b9}_{5}\right]\left[\mathrm{C9}_{6}\right]-k_{\mathrm{C5b9}_{\mathrm{poly}}}^{+}\left[\mathrm{pC5b9}_{6}\right]\left[\mathrm{C9}_{7}\right]$$

191
$$\frac{d\left[\mathrm{pC5b9}_{7}\right]}{dt}=k_{\mathrm{C5b9}_{\mathrm{poly}}}^{+}\left[\mathrm{pC5b9}_{6}\right]\left[\mathrm{C9}_{7}\right]-k_{\mathrm{C5b9}_{\mathrm{poly}}}^{+}\left[\mathrm{pC5b9}_{7}\right]\left[\mathrm{C9}_{8}\right]$$

192
$$\frac{d\left[\mathrm{pC5b9}_{8}\right]}{dt}=k_{\mathrm{C5b9}_{\mathrm{poly}}}^{+}\left[\mathrm{pC5b9}_{7}\right]\left[\mathrm{C9}_{8}\right]-k_{\mathrm{C5b9}_{\mathrm{poly}}}^{+}\left[\mathrm{pC5b9}_{8}\right]\left[\mathrm{C9}_{9}\right]$$

193
$$\frac{d\left[\mathrm{pC5b9}_{9}\right]}{dt}=k_{\mathrm{C5b9}_{\mathrm{poly}}}^{+}\left[\mathrm{pC5b9}_{8}\right]\left[\mathrm{C9}_{9}\right]-k_{\mathrm{C5b9}_{\mathrm{poly}}}^{+}\left[\mathrm{pC5b9}_{9}\right]\left[\mathrm{C9}_{10}\right]$$

194
$$\frac{d\left[\mathrm{pC5b9}_{10}\right]}{dt}=k_{\mathrm{C5b9}_{\mathrm{poly}}}^{+}\left[\mathrm{pC5b9}_{9}\right]\left[\mathrm{C9}_{10}\right]-k_{\mathrm{C5b9}_{\mathrm{poly}}}^{+}\left[\mathrm{pC5b9}_{10}\right]\left[\mathrm{C9}_{11}\right]$$

195
$$\frac{d\left[\mathrm{pC5b9}_{11}\right]}{dt}=k_{\mathrm{C5b9}_{\mathrm{poly}}}^{+}\left[\mathrm{pC5b9}_{10}\right]\left[\mathrm{C9}_{11}\right]-k_{\mathrm{C5b9}_{\mathrm{poly}}}^{+}\left[\mathrm{pC5b9}_{11}\right]\left[\mathrm{C9}_{12}\right]$$

196
$$\frac{d\left[\mathrm{pC5b9}_{12}\right]}{dt}=k_{\mathrm{C5b9}_{\mathrm{poly}}}^{+}\left[\mathrm{pC5b9}_{11}\right]\left[\mathrm{C9}_{12}\right]-k_{\mathrm{C5b9}_{\mathrm{poly}}}^{+}\left[\mathrm{pC5b9}_{12}\right]\left[\mathrm{C9}_{13}\right]$$

197
$$\frac{d\left[\mathrm{pC5b9}_{13}\right]}{dt}=k_{\mathrm{C5b9}_{\mathrm{poly}}}^{+}\left[\mathrm{pC5b9}_{12}\right]\left[\mathrm{C9}_{13}\right]-k_{\mathrm{C5b9}_{\mathrm{poly}}}^{+}\left[\mathrm{pC5b9}_{13}\right]\left[\mathrm{C9}_{14}\right]$$

198
$$\frac{d\left[\mathrm{pC5b9}_{14}\right]}{dt}=k_{\mathrm{C5b9}_{\mathrm{poly}}}^{+}\left[\mathrm{pC5b9}_{13}\right]\left[\mathrm{C9}_{14}\right]-k_{\mathrm{C5b9}_{\mathrm{poly}}}^{+}\left[\mathrm{pC5b9}_{14}\right]\left[\mathrm{C9}_{15}\right]$$

199

$$\frac{d\left[\mathrm{pC5b9}_{15}\right]}{dt}=k^{+}_{\mathrm{C5b9}_{\mathrm{poly}}}\left[\mathrm{pC5b9}_{14}\right]\left[\mathrm{C9}_{15}\right]-k^{+}_{\mathrm{C5b9}_{\mathrm{poly}}}\left[\mathrm{pC5b9}_{15}\right]\left[\mathrm{C9}_{16}\right]$$

200

$$\frac{d\left[\mathrm{pC5b9}_{16}\right]}{dt}=k^{+}_{\mathrm{C5b9}_{\mathrm{poly}}}\left[\mathrm{pC5b9}_{15}\right]\left[\mathrm{C9}_{16}\right]-k^{+}_{\mathrm{C5b9}_{\mathrm{poly}}}\left[\mathrm{pC5b9}_{16}\right]\left[\mathrm{C9}_{17}\right]$$

201

$$\frac{d\left[\mathrm{pC5b9}_{17}\right]}{dt}=k^{+}_{\mathrm{C5b9}_{\mathrm{poly}}}\left[\mathrm{pC5b9}_{16}\right]\left[\mathrm{C9}_{17}\right]-k^{+}_{\mathrm{C5b9}_{\mathrm{poly}}}\left[\mathrm{pC5b9}_{17}\right]\left[\mathrm{C9}_{18}\right]$$

202

$$\frac{d\left[\mathrm{pC5b9}_{18}\right]}{dt}=k^{+}_{\mathrm{C5b9}_{\mathrm{poly}}}\left[\mathrm{pC5b9}_{17}\right]\left[\mathrm{C9}_{18}\right]$$

203

$$\frac{d\left[\mathrm{pC4bC4BP}\right]}{dt}=k_{\mathrm{C4bC4BP}}^{+}\left[\mathrm{pC4b}\right]\left[\mathrm{C4BP}\right]-k_{\mathrm{C4bC4BP}}^{-}\left[\mathrm{pC4bC4BP}\right]$$

$$-\frac{k_{\mathrm{cat\ C3bH}}^{\mathrm{FI}}\left[\mathrm{FI}\right]\left[\mathrm{pC4bC4BP}\right]}{\left(\begin{array}{l}
K_{\mathrm{m\ C3bH}}^{\mathrm{FI}}+\left[\mathrm{C3(H_2O)FH}\right]+\left[\mathrm{C3(H_2O)FHL1}\right]+\left[\mathrm{fC3bFH}\right]+\left[\mathrm{fC3bFHL1}\right]+\left[\mathrm{fC3bCR1}\right]+\left[\mathrm{fiC3bCR1}\right]\\
+\left[\mathrm{IgGC3bC3bFH}\right]+\left[\mathrm{IgGC3biC3bFH}\right]+\left[\mathrm{IgGC3bC3bFHL1}\right]+\left[\mathrm{IgGC3biC3bFHL1}\right]+\left[\mathrm{IgGC3bC3bCR1}\right]\\
+\left[\mathrm{IgGC3biC3bCR1}\right]+\left[\mathrm{IgGiC3biC3bCR1}\right]+\left[\mathrm{IgGiC3bC3dgCR1}\right]+\left[\mathrm{fC4bC3bFH}\right]+\left[\mathrm{fC4bC3bFHL1}\right]\\
+\left[\mathrm{fC4bC3bCR1}\right]+\left[\mathrm{fC4bC3bC4BP}\right]+\left[\mathrm{fC4dC3bFH}\right]+\left[\mathrm{fC4dC3bFHL1}\right]+\left[\mathrm{fC4dC3bCR1}\right]+\left[\mathrm{fC4diC3bCR1}\right]\\
+\left[\mathrm{fC4biC3bCR1}\right]+\left[\mathrm{fC4bC3dgCR1}\right]+\left[\mathrm{fC4bC3dgC4BP}\right]+\left[\mathrm{fC3bC3bFH}\right]+\left[\mathrm{fC3bC3bFHL1}\right]+\left[\mathrm{fC3bC3bCR1}\right]\\
+\left[\mathrm{fC3biC3bFH}\right]+\left[\mathrm{fC3biC3bFHL1}\right]+\left[\mathrm{fC3biC3bCR1}\right]+\left[\mathrm{fiC3biC3bCR1}\right]+\left[\mathrm{fiC3bC3dgCR1}\right]\\
+\left[\mathrm{fC4biC3bC4BP}\right]+\left[\mathrm{nfC4bC4BP}\right]+\left[\mathrm{fC4bC4BP}\right]+\left[\mathrm{fC4bCR1}\right]+\left[\mathrm{fC4bC4bC4BP}\right]+\left[\mathrm{fC4bC4dC4BP}\right]\\
+\left[\mathrm{fC4bC4bCR1}\right]+\left[\mathrm{fC4bC4dCR1}\right]+\left[\mathrm{hC4bCR1}\right]+\left[\mathrm{hC4bC4BP}\right]+\left[\mathrm{hC4bC4bCR1}\right]+\left[\mathrm{hC4bC4bC4BP}\right]\\
+\left[\mathrm{hC4bC4dCR1}\right]+\left[\mathrm{hC4bC4dC4BP}\right]+\left[\mathrm{hC4bC3bFH}\right]+\left[\mathrm{hC4bC3bFHL1}\right]+\left[\mathrm{hC4bC3bCR1}\right]\\
+\left[\mathrm{hC4bC3bC4BP}\right]+\left[\mathrm{hC4biC3bCR1}\right]+\left[\mathrm{hC4biC3bC4BP}\right]+\left[\mathrm{hC4bC3dgCR1}\right]+\left[\mathrm{hC4bC3dgC4BP}\right]\\
+\left[\mathrm{hC4dC3bFH}\right]+\left[\mathrm{hC4dC3bFHL1}\right]+\left[\mathrm{hC4dC3bCR1}\right]+\left[\mathrm{hC4diC3bCR1}\right]+\left[\mathrm{hC3bFH}\right]+\left[\mathrm{hC3bFHL1}\right]\\
+\left[\mathrm{hC3bCR1}\right]+\left[\mathrm{hiC3bCR1}\right]+\left[\mathrm{hC3bC3bFH}\right]+\left[\mathrm{hC3bC3bFHL1}\right]+\left[\mathrm{hC3bC3bCR1}\right]+\left[\mathrm{hC3biC3bFH}\right]\\
+\left[\mathrm{hC3biC3bFHL1}\right]+\left[\mathrm{hC3biC3bCR1}\right]+\left[\mathrm{hiC3biC3bCR1}\right]+\left[\mathrm{hiC3biC3dgCR1}\right]+\left[\mathrm{pC4bC4BP}\right]\\
+\left[\mathrm{pC4bC4bC4BP}\right]+\left[\mathrm{pC4bC4dC4BP}\right]+\left[\mathrm{pC4bC3bC4BP}\right]+\left[\mathrm{pC4biC3bC4BP}\right]+\left[\mathrm{pC4bC3bFH}\right]\\
+\left[\mathrm{pC4bC3bFHL1}\right]+\left[\mathrm{pC4dC3bFH}\right]+\left[\mathrm{pC4dC3bFHL1}\right]+\left[\mathrm{pC3bFH}\right]+\left[\mathrm{pC3bFHL1}\right]+\left[\mathrm{pC3bC3bFH}\right]\\
+\left[\mathrm{pC3bC3bFHL1}\right]+\left[\mathrm{pC3biC3bFH}\right]+\left[\mathrm{pC3biC3bFHL1}\right]+\left[\mathrm{pC4bC4BPPTX3}\right]+\left[\mathrm{pC4bC4bC4BPPTX3}\right]\\
+\left[\mathrm{pC4bC4dC4BPPTX3}\right]+\left[\mathrm{pC4bC3bFHPTX3}\right]+\left[\mathrm{pC4bC3bFHL1PTX3}\right]+\left[\mathrm{pC4bC3bC4BPPTX3}\right]\\
+\left[\mathrm{pC4dC3bFHPTX3}\right]+\left[\mathrm{pC4dC3bFHL1PTX3}\right]+\left[\mathrm{pC4biC3bC4BPPTX3}\right]+\left[\mathrm{pC3bFHPTX3}\right]\\
+\left[\mathrm{pC3bFHL1PTX3}\right]+\left[\mathrm{pC3bC3bFHPTX3}\right]+\left[\mathrm{pC3bC3bFHL1PTX3}\right]+\left[\mathrm{pC3biC3bFHPTX3}\right]\\
+\left[\mathrm{pC3biC3bFHL1PTX3}\right]+\left[\mathrm{pC4bpC4BP}\right]+\left[\mathrm{pC4bC4bpC4BP}\right]+\left[\mathrm{pC4bC4dpC4BP}\right]+\left[\mathrm{pC4bC3bpC4BP}\right]\\
+\left[\mathrm{pC4biC3bpC4BP}\right]+\left[\mathrm{pC4bC3bpFH}\right]+\left[\mathrm{pC4bC3bpFHL1}\right]+\left[\mathrm{pC4dC3bpFH}\right]+\left[\mathrm{pC4dC3bpFHL1}\right]\\
+\left[\mathrm{pC3bpFH}\right]+\left[\mathrm{pC3bpFHL1}\right]+\left[\mathrm{pC3bC3bpFH}\right]+\left[\mathrm{pC3bC3bpFHL1}\right]+\left[\mathrm{pC3biC3bpFH}\right]+\left[\mathrm{pC3biC3bpFHL1}\right]
\end{array}\right)}$$

204

$$\frac{d[\mathrm{pC4d}]}{dt}=$$

$$\frac{k_{\mathrm{cat\ C3bH}}^{\mathrm{FI}}[\mathrm{FI}][\mathrm{pC4bC4BP}]}{\left(\begin{aligned}&K_{\mathrm{m\ C3bH}}^{\mathrm{FI}}+[\mathrm{C3(H_2O)FH}]+[\mathrm{C3(H_2O)FHL1}]+[\mathrm{fC3bFH}]+[\mathrm{fC3bFHL1}]+[\mathrm{fC3bCR1}]+[\mathrm{fiC3bCR1}]\\&+[\mathrm{IgGC3bC3bFH}]+[\mathrm{IgGC3biC3bFH}]+[\mathrm{IgGC3bC3bFHL1}]+[\mathrm{IgGC3biC3bFHL1}]+[\mathrm{IgGC3bC3bCR1}]\\&+[\mathrm{IgGC3biC3bCR1}]+[\mathrm{IgGiC3biC3bCR1}]+[\mathrm{IgGiC3bC3dgCR1}]+[\mathrm{fC4bC3bFH}]+[\mathrm{fC4bC3bFHL1}]\\&+[\mathrm{fC4bC3bCR1}]+[\mathrm{fC4bC3bC4BP}]+[\mathrm{fC4dC3bFH}]+[\mathrm{fC4dC3bFHL1}]+[\mathrm{fC4dC3bCR1}]+[\mathrm{fC4diC3bCR1}]\\&+[\mathrm{fC4biC3bCR1}]+[\mathrm{fC4bC3dgCR1}]+[\mathrm{fC4bC3dgC4BP}]+[\mathrm{fC3bC3bFH}]+[\mathrm{fC3bC3bFHL1}]+[\mathrm{fC3bC3bCR1}]\\&+[\mathrm{fC3biC3bFH}]+[\mathrm{fC3biC3bFHL1}]+[\mathrm{fC3biC3bCR1}]+[\mathrm{fiC3biC3bCR1}]+[\mathrm{fiC3bC3dgCR1}]\\&+[\mathrm{fC4biC3bC4BP}]+[\mathrm{nfC4bC4BP}]+[\mathrm{fC4bC4BP}]+[\mathrm{fC4bCR1}]+[\mathrm{fC4bC4bC4BP}]+[\mathrm{fC4bC4dC4BP}]\\&+[\mathrm{fC4bC4bCR1}]+[\mathrm{fC4bC4dCR1}]+[\mathrm{hC4bCR1}]+[\mathrm{hC4bC4BP}]+[\mathrm{hC4bC4bCR1}]+[\mathrm{hC4bC4bC4BP}]\\&+[\mathrm{hC4bC4dCR1}]+[\mathrm{hC4bC4dC4BP}]+[\mathrm{hC4bC3bFH}]+[\mathrm{hC4bC3bFHL1}]+[\mathrm{hC4bC3bCR1}]\\&+[\mathrm{hC4bC3bC4BP}]+[\mathrm{hC4biC3bCR1}]+[\mathrm{hC4biC3bC4BP}]+[\mathrm{hC4bC3dgCR1}]+[\mathrm{hC4bC3dgC4BP}]\\&+[\mathrm{hC4dC3bFH}]+[\mathrm{hC4dC3bFHL1}]+[\mathrm{hC4dC3bCR1}]+[\mathrm{hC4diC3bCR1}]+[\mathrm{hC3bFH}]+[\mathrm{hC3bFHL1}]\\&+[\mathrm{hC3bCR1}]+[\mathrm{hiC3bCR1}]+[\mathrm{hC3bC3bFH}]+[\mathrm{hC3bC3bFHL1}]+[\mathrm{hC3bC3bCR1}]+[\mathrm{hC3biC3bFH}]\\&+[\mathrm{hC3biC3bFHL1}]+[\mathrm{hC3biC3bCR1}]+[\mathrm{hiC3biC3bCR1}]+[\mathrm{hiC3biC3dgCR1}]+[\mathrm{pC4bC4BP}]\\&+[\mathrm{pC4bC4bC4BP}]+[\mathrm{pC4bC4dC4BP}]+[\mathrm{pC4bC3bC4BP}]+[\mathrm{pC4biC3bC4BP}]+[\mathrm{pC4bC3bFH}]\\&+[\mathrm{pC4bC3bFHL1}]+[\mathrm{pC4dC3bFH}]+[\mathrm{pC4dC3bFHL1}]+[\mathrm{pC3bFH}]+[\mathrm{pC3bFHL1}]+[\mathrm{pC3bC3bFH}]\\&+[\mathrm{pC3bC3bFHL1}]+[\mathrm{pC3biC3bFH}]+[\mathrm{pC3biC3bFHL1}]+[\mathrm{pC4bC4BPPTX3}]+[\mathrm{pC4bC4bC4BPPTX3}]\\&+[\mathrm{pC4bC4dC4BPPTX3}]+[\mathrm{pC4bC3bFHPTX3}]+[\mathrm{pC4bC3bFHL1PTX3}]+[\mathrm{pC4bC3bC4BPPTX3}]\\&+[\mathrm{pC4dC3bFHPTX3}]+[\mathrm{pC4dC3bFHL1PTX3}]+[\mathrm{pC4biC3bC4BPPTX3}]+[\mathrm{pC3bFHPTX3}]\\&+[\mathrm{pC3bFHL1PTX3}]+[\mathrm{pC3bC3bFHPTX3}]+[\mathrm{pC3bC3bFHL1PTX3}]+[\mathrm{pC3biC3bFHPTX3}]\\&+[\mathrm{pC3biC3bFHL1PTX3}]+[\mathrm{pC4bpC4BP}]+[\mathrm{pC4bC4bpC4BP}]+[\mathrm{pC4bC4dpC4BP}]+[\mathrm{pC4bC3bpC4BP}]\\&+[\mathrm{pC4biC3bpC4BP}]+[\mathrm{pC4bC3bpFH}]+[\mathrm{pC4bC3bpFHL1}]+[\mathrm{pC4dC3bpFH}]+[\mathrm{pC4dC3bpFHL1}]\\&+[\mathrm{pC3bpFH}]+[\mathrm{pC3bpFHL1}]+[\mathrm{pC3bC3bpFH}]+[\mathrm{pC3bC3bpFHL1}]+[\mathrm{pC3biC3bpFH}]+[\mathrm{pC3biC3bpFHL1}]\end{aligned}\right)}$$

$$
+\frac{k_{\text{cat C3bH}}^{\text{FI}}[\text{FI}][\text{pC4bC4BPPTX3}]}{\left(\begin{array}{l}K_{\text{m C3bH}}^{\text{FI}}+[\text{C3(H}_2\text{O)FH}]+[\text{C3(H}_2\text{O)FHL1}]+[\text{fC3bFH}]+[\text{fC3bFHL1}]+[\text{fC3bCR1}]+[\text{fiC3bCR1}]\\
+[\text{IgGC3bC3bFH}]+[\text{IgGC3biC3bFH}]+[\text{IgGC3bC3bFHL1}]+[\text{IgGC3biC3bFHL1}]+[\text{IgGC3bC3bCR1}]\\
+[\text{IgGC3biC3bCR1}]+[\text{IgGiC3biC3bCR1}]+[\text{IgGiC3bC3dgCR1}]+[\text{fC4bC3bFH}]+[\text{fC4bC3bFHL1}]\\
+[\text{fC4bC3bCR1}]+[\text{fC4bC3bC4BP}]+[\text{fC4dC3bFH}]+[\text{fC4dC3bFHL1}]+[\text{fC4dC3bCR1}]+[\text{fC4diC3bCR1}]\\
+[\text{fC4biC3bCR1}]+[\text{fC4bC3dgCR1}]+[\text{fC4bC3dgC4BP}]+[\text{fC3bC3bFH}]+[\text{fC3bC3bFHL1}]+[\text{fC3bC3bCR1}]\\
+[\text{fC3biC3bFH}]+[\text{fC3biC3bFHL1}]+[\text{fC3biC3bCR1}]+[\text{fiC3biC3bCR1}]+[\text{fiC3bC3dgCR1}]\\
+[\text{fC4biC3bC4BP}]+[\text{nfC4bC4BP}]+[\text{fC4bC4BP}]+[\text{fC4bCR1}]+[\text{fC4bC4bC4BP}]+[\text{fC4bC4dC4BP}]\\
+[\text{fC4bC4bCR1}]+[\text{fC4bC4dCR1}]+[\text{hC4bCR1}]+[\text{hC4bC4BP}]+[\text{hC4bC4bCR1}]+[\text{hC4bC4bC4BP}]\\
+[\text{hC4bC4dCR1}]+[\text{hC4bC4dC4BP}]+[\text{hC4bC3bFH}]+[\text{hC4bC3bFHL1}]+[\text{hC4bC3bCR1}]\\
+[\text{hC4bC3bC4BP}]+[\text{hC4biC3bCR1}]+[\text{hC4biC3bC4BP}]+[\text{hC4bC3dgCR1}]+[\text{hC4bC3dgC4BP}]\\
+[\text{hC4dC3bFH}]+[\text{hC4dC3bFHL1}]+[\text{hC4dC3bCR1}]+[\text{hC4diC3bCR1}]+[\text{hC3bFH}]+[\text{hC3bFHL1}]\\
+[\text{hC3bCR1}]+[\text{hiC3bCR1}]+[\text{hC3bC3bFH}]+[\text{hC3bC3bFHL1}]+[\text{hC3bC3bCR1}]+[\text{hC3biC3bFH}]\\
+[\text{hC3biC3bFHL1}]+[\text{hC3biC3bCR1}]+[\text{hiC3biC3bCR1}]+[\text{hiC3biC3dgCR1}]+[\text{pC4bC4BP}]\\
+[\text{pC4bC4bC4BP}]+[\text{pC4bC4dC4BP}]+[\text{pC4bC3bC4BP}]+[\text{pC4biC3bC4BP}]+[\text{pC4bC3bFH}]\\
+[\text{pC4bC3bFHL1}]+[\text{pC4dC3bFH}]+[\text{pC4dC3bFHL1}]+[\text{pC3bFH}]+[\text{pC3bFHL1}]+[\text{pC3bC3bFH}]\\
+[\text{pC3bC3bFHL1}]+[\text{pC3biC3bFH}]+[\text{pC3biC3bFHL1}]+[\text{pC4bC4BPPTX3}]+[\text{pC4bC4bC4BPPTX3}]\\
+[\text{pC4bC4dC4BPPTX3}]+[\text{pC4bC3bFHPTX3}]+[\text{pC4bC3bFHL1PTX3}]+[\text{pC4bC3bC4BPPTX3}]\\
+[\text{pC4dC3bFHPTX3}]+[\text{pC4dC3bFHL1PTX3}]+[\text{pC4biC3bC4BPPTX3}]+[\text{pC3bFHPTX3}]\\
+[\text{pC3bFHL1PTX3}]+[\text{pC3bC3bFHPTX3}]+[\text{pC3bC3bFHL1PTX3}]+[\text{pC3biC3bFHPTX3}]\\
+[\text{pC3biC3bFHL1PTX3}]+[\text{pC4bpC4BP}]+[\text{pC4bC4bpC4BP}]+[\text{pC4bC4dpC4BP}]+[\text{pC4bC3bpC4BP}]\\
+[\text{pC4biC3bpC4BP}]+[\text{pC4bC3bpFH}]+[\text{pC4bC3bpFHL1}]+[\text{pC4dC3bpFH}]+[\text{pC4dC3bpFHL1}]\\
+[\text{pC3bpFH}]+[\text{pC3bpFHL1}]+[\text{pC3bC3bpFH}]+[\text{pC3bC3bpFHL1}]+[\text{pC3biC3bpFH}]+[\text{pC3biC3bpFHL1}]\end{array}\right)}
$$

$$+\frac{k_{\text{cat C3bH}}^{\text{FI}}[\text{FI}][\text{pC4bpC4BP}]}{\left(\begin{array}{l}K_{\text{m C3bH}}^{\text{FI}}+[\text{C3(H}_2\text{O)FH}]+[\text{C3(H}_2\text{O)FHL1}]+[\text{fC3bFH}]+[\text{fC3bFHL1}]+[\text{fC3bCR1}]+[\text{fiC3bCR1}]\\+[\text{IgGC3bC3bFH}]+[\text{IgGC3biC3bFH}]+[\text{IgGC3bC3bFHL1}]+[\text{IgGC3biC3bFHL1}]+[\text{IgGC3bC3bCR1}]\\+[\text{IgGC3biC3bCR1}]+[\text{IgGiC3biC3bCR1}]+[\text{IgGiC3bC3dgCR1}]+[\text{fC4bC3bFH}]+[\text{fC4bC3bFHL1}]\\+[\text{fC4bC3bCR1}]+[\text{fC4bC3bC4BP}]+[\text{fC4dC3bFH}]+[\text{fC4dC3bFHL1}]+[\text{fC4dC3bCR1}]+[\text{fC4diC3bCR1}]\\+[\text{fC4biC3bCR1}]+[\text{fC4bC3dgCR1}]+[\text{fC4bC3dgC4BP}]+[\text{fC3bC3bFH}]+[\text{fC3bC3bFHL1}]+[\text{fC3bC3bCR1}]\\+[\text{fC3biC3bFH}]+[\text{fC3biC3bFHL1}]+[\text{fC3biC3bCR1}]+[\text{fiC3biC3bCR1}]+[\text{fiC3bC3dgCR1}]\\+[\text{fC4biC3bC4BP}]+[\text{nfC4bC4BP}]+[\text{fC4bC4BP}]+[\text{fC4bCR1}]+[\text{fC4bC4bC4BP}]+[\text{fC4bC4dC4BP}]\\+[\text{fC4bC4bCR1}]+[\text{fC4bC4dCR1}]+[\text{hC4bCR1}]+[\text{hC4bC4BP}]+[\text{hC4bC4bCR1}]+[\text{hC4bC4bC4BP}]\\+[\text{hC4bC4dCR1}]+[\text{hC4bC4dC4BP}]+[\text{hC4bC3bFH}]+[\text{hC4bC3bFHL1}]+[\text{hC4bC3bCR1}]\\+[\text{hC4bC3bC4BP}]+[\text{hC4biC3bCR1}]+[\text{hC4biC3bC4BP}]+[\text{hC4bC3dgCR1}]+[\text{hC4bC3dgC4BP}]\\+[\text{hC4dC3bFH}]+[\text{hC4dC3bFHL1}]+[\text{hC4dC3bCR1}]+[\text{hC4diC3bCR1}]+[\text{hC3bFH}]+[\text{hC3bFHL1}]\\+[\text{hC3bCR1}]+[\text{hiC3bCR1}]+[\text{hC3bC3bFH}]+[\text{hC3bC3bFHL1}]+[\text{hC3bC3bCR1}]+[\text{hC3biC3bFH}]\\+[\text{hC3biC3bFHL1}]+[\text{hC3biC3bCR1}]+[\text{hiC3biC3bCR1}]+[\text{hiC3biC3dgCR1}]+[\text{pC4bC4BP}]\\+[\text{pC4bC4bC4BP}]+[\text{pC4bC4dC4BP}]+[\text{pC4bC3bC4BP}]+[\text{pC4biC3bC4BP}]+[\text{pC4bC3bFH}]\\+[\text{pC4bC3bFHL1}]+[\text{pC4dC3bFH}]+[\text{pC4dC3bFHL1}]+[\text{pC3bFH}]+[\text{pC3bFHL1}]+[\text{pC3bC3bFH}]\\+[\text{pC3bC3bFHL1}]+[\text{pC3biC3bFH}]+[\text{pC3biC3bFHL1}]+[\text{pC4bC4BPPTX3}]+[\text{pC4bC4bC4BPPTX3}]\\+[\text{pC4bC4dC4BPPTX3}]+[\text{pC4bC3bFHPTX3}]+[\text{pC4bC3bFHL1PTX3}]+[\text{pC4bC3bC4BPPTX3}]\\+[\text{pC4dC3bFHPTX3}]+[\text{pC4dC3bFHL1PTX3}]+[\text{pC4biC3bC4BPPTX3}]+[\text{pC3bFHPTX3}]\\+[\text{pC3bFHL1PTX3}]+[\text{pC3bC3bFHPTX3}]+[\text{pC3bC3bFHL1PTX3}]+[\text{pC3biC3bFHPTX3}]\\+[\text{pC3biC3bFHL1PTX3}]+[\text{pC4bpC4BP}]+[\text{pC4bC4bpC4BP}]+[\text{pC4bC4dpC4BP}]+[\text{pC4bC3bpC4BP}]\\+[\text{pC4biC3bpC4BP}]+[\text{pC4bC3bpFH}]+[\text{pC4bC3bpFHL1}]+[\text{pC4dC3bpFH}]+[\text{pC4dC3bpFHL1}]\\+[\text{pC3bpFH}]+[\text{pC3bpFHL1}]+[\text{pC3bC3bpFH}]+[\text{pC3bC3bpFHL1}]+[\text{pC3biC3bpFH}]+[\text{pC3biC3bpFHL1}]\end{array}\right)}$$

205

$$\frac{d[\text{pC4bC2aC4BP}]}{dt}=k_{\text{C4bC4BP}}^{+}[\text{pC4bC2a}][\text{C4BP}]-k_{\text{C4bC4BP}}^{-}[\text{pC4bC2aC4BP}]-k_{\text{C4bC2aC4BP}_{\text{decay}}}^{-}[\text{pC4bC2aC4BP}]$$

206

$$\frac{d[\text{pC4bC4bC2aC4BP}]}{dt}=k_{\text{C4bC4BP}}^{+}[\text{pC4bC4bC2a}][\text{C4BP}]-k_{\text{C4bC4BP}}^{-}[\text{pC4bC4bC2aC4BP}]$$
$$-k_{\text{C4bC2aC4BP}_{\text{decay}}}^{-}[\text{pC4bC4bC2aC4BP}]$$

207

$$\frac{d\left[\text{pC4bC4bC4BP}\right]}{dt}=k_{\text{C4bC4BP}}^{+}\left[\text{pC4bC4b}\right]\left[\text{C4BP}\right]-k_{\text{C4bC4BP}}^{-}\left[\text{pC4bC4bC4BP}\right]$$

$$-\frac{k_{\text{cat C3bH}}^{\text{FI}}\left[\text{FI}\right]\left[\text{pC4bC4bC4BP}\right]}{\left(\begin{array}{l}K_{\text{m C3bH}}^{\text{FI}}+\left[\text{C3(H}_2\text{O)FH}\right]+\left[\text{C3(H}_2\text{O)FHL1}\right]+\left[\text{fC3bFH}\right]+\left[\text{fC3bFHL1}\right]+\left[\text{fC3bCR1}\right]+\left[\text{fiC3bCR1}\right]\\+\left[\text{IgGC3bC3bFH}\right]+\left[\text{IgGC3biC3bFH}\right]+\left[\text{IgGC3bC3bFHL1}\right]+\left[\text{IgGC3biC3bFHL1}\right]+\left[\text{IgGC3bC3bCR1}\right]\\+\left[\text{IgGC3biC3bCR1}\right]+\left[\text{IgGiC3biC3bCR1}\right]+\left[\text{IgGiC3bC3dgCR1}\right]+\left[\text{fC4bC3bFH}\right]+\left[\text{fC4bC3bFHL1}\right]\\+\left[\text{fC4bC3bCR1}\right]+\left[\text{fC4bC3bC4BP}\right]+\left[\text{fC4dC3bFH}\right]+\left[\text{fC4dC3bFHL1}\right]+\left[\text{fC4dC3bCR1}\right]+\left[\text{fC4diC3bCR1}\right]\\+\left[\text{fC4biC3bCR1}\right]+\left[\text{fC4bC3dgCR1}\right]+\left[\text{fC4bC3dgC4BP}\right]+\left[\text{fC3bC3bFH}\right]+\left[\text{fC3bC3bFHL1}\right]+\left[\text{fC3bC3bCR1}\right]\\+\left[\text{fC3biC3bFH}\right]+\left[\text{fC3biC3bFHL1}\right]+\left[\text{fC3biC3bCR1}\right]+\left[\text{fiC3biC3bCR1}\right]+\left[\text{fiC3bC3dgCR1}\right]\\+\left[\text{fC4biC3bC4BP}\right]+\left[\text{nfC4bC4BP}\right]+\left[\text{fC4bC4BP}\right]+\left[\text{fC4bCR1}\right]+\left[\text{fC4bC4bC4BP}\right]+\left[\text{fC4bC4dC4BP}\right]\\+\left[\text{fC4bC4bCR1}\right]+\left[\text{fC4bC4dCR1}\right]+\left[\text{hC4bCR1}\right]+\left[\text{hC4bC4BP}\right]+\left[\text{hC4bC4bCR1}\right]+\left[\text{hC4bC4bC4BP}\right]\\+\left[\text{hC4bC4dCR1}\right]+\left[\text{hC4bC4dC4BP}\right]+\left[\text{hC4bC3bFH}\right]+\left[\text{hC4bC3bFHL1}\right]+\left[\text{hC4bC3bCR1}\right]\\+\left[\text{hC4bC3bC4BP}\right]+\left[\text{hC4biC3bCR1}\right]+\left[\text{hC4biC3bC4BP}\right]+\left[\text{hC4bC3dgCR1}\right]+\left[\text{hC4bC3dgC4BP}\right]\\+\left[\text{hC4dC3bFH}\right]+\left[\text{hC4dC3bFHL1}\right]+\left[\text{hC4dC3bCR1}\right]+\left[\text{hC4diC3bCR1}\right]+\left[\text{hC3bFH}\right]+\left[\text{hC3bFHL1}\right]\\+\left[\text{hC3bCR1}\right]+\left[\text{hiC3bCR1}\right]+\left[\text{hC3bC3bFH}\right]+\left[\text{hC3bC3bFHL1}\right]+\left[\text{hC3bC3bCR1}\right]+\left[\text{hC3biC3bFH}\right]\\+\left[\text{hC3biC3bFHL1}\right]+\left[\text{hC3biC3bCR1}\right]+\left[\text{hiC3biC3bCR1}\right]+\left[\text{hiC3biC3dgCR1}\right]+\left[\text{pC4bC4BP}\right]\\+\left[\text{pC4bC4bC4BP}\right]+\left[\text{pC4bC4dC4BP}\right]+\left[\text{pC4bC3bC4BP}\right]+\left[\text{pC4biC3bC4BP}\right]+\left[\text{pC4bC3bFH}\right]\\+\left[\text{pC4bC3bFHL1}\right]+\left[\text{pC4dC3bFH}\right]+\left[\text{pC4dC3bFHL1}\right]+\left[\text{pC3bFH}\right]+\left[\text{pC3bFHL1}\right]+\left[\text{pC3bC3bFH}\right]\\+\left[\text{pC3bC3bFHL1}\right]+\left[\text{pC3biC3bFH}\right]+\left[\text{pC3biC3bFHL1}\right]+\left[\text{pC4bC4BPPTX3}\right]+\left[\text{pC4bC4bC4BPPTX3}\right]\\+\left[\text{pC4bC4dC4BPPTX3}\right]+\left[\text{pC4bC3bFHPTX3}\right]+\left[\text{pC4bC3bFHL1PTX3}\right]+\left[\text{pC4bC3bC4BPPTX3}\right]\\+\left[\text{pC4dC3bFHPTX3}\right]+\left[\text{pC4dC3bFHL1PTX3}\right]+\left[\text{pC4biC3bC4BPPTX3}\right]+\left[\text{pC3bFHPTX3}\right]\\+\left[\text{pC3bFHL1PTX3}\right]+\left[\text{pC3bC3bFHPTX3}\right]+\left[\text{pC3bC3bFHL1PTX3}\right]+\left[\text{pC3biC3bFHPTX3}\right]\\+\left[\text{pC3biC3bFHL1PTX3}\right]+\left[\text{pC4bpC4BP}\right]+\left[\text{pC4bC4bpC4BP}\right]+\left[\text{pC4bC4dpC4BP}\right]+\left[\text{pC4bC3bpC4BP}\right]\\+\left[\text{pC4biC3bpC4BP}\right]+\left[\text{pC4bC3bpFH}\right]+\left[\text{pC4bC3bpFHL1}\right]+\left[\text{pC4dC3bpFH}\right]+\left[\text{pC4dC3bpFHL1}\right]\\+\left[\text{pC3bpFH}\right]+\left[\text{pC3bpFHL1}\right]+\left[\text{pC3bC3bpFH}\right]+\left[\text{pC3bC3bpFHL1}\right]+\left[\text{pC3biC3bpFH}\right]+\left[\text{pC3biC3bpFHL1}\right]\end{array}\right)}$$

208

$$\frac{d\left[\mathrm{pC4bC4d}\right]}{dt}=$$

$$\frac{k_{\mathrm{cat\ C3bH}}^{\mathrm{FI}}\left[\mathrm{FI}\right]\left[\mathrm{pC4bC4bC4BP}\right]}{\left(\begin{array}{l}K_{\mathrm{m\ C3bH}}^{\mathrm{FI}}+\left[\mathrm{C3(H_2O)FH}\right]+\left[\mathrm{C3(H_2O)FHL1}\right]+\left[\mathrm{fC3bFH}\right]+\left[\mathrm{fC3bFHL1}\right]+\left[\mathrm{fC3bCR1}\right]+\left[\mathrm{fiC3bCR1}\right]\\
+\left[\mathrm{IgGC3bC3bFH}\right]+\left[\mathrm{IgGC3biC3bFH}\right]+\left[\mathrm{IgGC3bC3bFHL1}\right]+\left[\mathrm{IgGC3biC3bFHL1}\right]+\left[\mathrm{IgGC3bC3bCR1}\right]\\
+\left[\mathrm{IgGC3biC3bCR1}\right]+\left[\mathrm{IgGiC3biC3bCR1}\right]+\left[\mathrm{IgGiC3bC3dgCR1}\right]+\left[\mathrm{fC4bC3bFH}\right]+\left[\mathrm{fC4bC3bFHL1}\right]\\
+\left[\mathrm{fC4bC3bCR1}\right]+\left[\mathrm{fC4bC3bC4BP}\right]+\left[\mathrm{fC4dC3bFH}\right]+\left[\mathrm{fC4dC3bFHL1}\right]+\left[\mathrm{fC4dC3bCR1}\right]+\left[\mathrm{fC4diC3bCR1}\right]\\
+\left[\mathrm{fC4biC3bCR1}\right]+\left[\mathrm{fC4bC3dgCR1}\right]+\left[\mathrm{fC4bC3dgC4BP}\right]+\left[\mathrm{fC3bC3bFH}\right]+\left[\mathrm{fC3bC3bFHL1}\right]+\left[\mathrm{fC3bC3bCR1}\right]\\
+\left[\mathrm{fC3biC3bFH}\right]+\left[\mathrm{fC3biC3bFHL1}\right]+\left[\mathrm{fC3biC3bCR1}\right]+\left[\mathrm{fiC3biC3bCR1}\right]+\left[\mathrm{fiC3bC3dgCR1}\right]\\
+\left[\mathrm{fC4biC3bC4BP}\right]+\left[\mathrm{nfC4bC4BP}\right]+\left[\mathrm{fC4bC4BP}\right]+\left[\mathrm{fC4bCR1}\right]+\left[\mathrm{fC4bC4bC4BP}\right]+\left[\mathrm{fC4bC4dC4BP}\right]\\
+\left[\mathrm{fC4bC4bCR1}\right]+\left[\mathrm{fC4bC4dCR1}\right]+\left[\mathrm{hC4bCR1}\right]+\left[\mathrm{hC4bC4BP}\right]+\left[\mathrm{hC4bC4bCR1}\right]+\left[\mathrm{hC4bC4bC4BP}\right]\\
+\left[\mathrm{hC4bC4dCR1}\right]+\left[\mathrm{hC4bC4dC4BP}\right]+\left[\mathrm{hC4bC3bFH}\right]+\left[\mathrm{hC4bC3bFHL1}\right]+\left[\mathrm{hC4bC3bCR1}\right]\\
+\left[\mathrm{hC4bC3bC4BP}\right]+\left[\mathrm{hC4biC3bCR1}\right]+\left[\mathrm{hC4biC3bC4BP}\right]+\left[\mathrm{hC4bC3dgCR1}\right]+\left[\mathrm{hC4bC3dgC4BP}\right]\\
+\left[\mathrm{hC4dC3bFH}\right]+\left[\mathrm{hC4dC3bFHL1}\right]+\left[\mathrm{hC4dC3bCR1}\right]+\left[\mathrm{hC4diC3bCR1}\right]+\left[\mathrm{hC3bFH}\right]+\left[\mathrm{hC3bFHL1}\right]\\
+\left[\mathrm{hC3bCR1}\right]+\left[\mathrm{hiC3bCR1}\right]+\left[\mathrm{hC3bC3bFH}\right]+\left[\mathrm{hC3bC3bFHL1}\right]+\left[\mathrm{hC3bC3bCR1}\right]+\left[\mathrm{hC3biC3bFH}\right]\\
+\left[\mathrm{hC3biC3bFHL1}\right]+\left[\mathrm{hC3biC3bCR1}\right]+\left[\mathrm{hiC3biC3bCR1}\right]+\left[\mathrm{hiC3biC3dgCR1}\right]+\left[\mathrm{pC4bC4BP}\right]\\
+\left[\mathrm{pC4bC4bC4BP}\right]+\left[\mathrm{pC4bC4dC4BP}\right]+\left[\mathrm{pC4bC3bC4BP}\right]+\left[\mathrm{pC4biC3bC4BP}\right]+\left[\mathrm{pC4bC3bFH}\right]\\
+\left[\mathrm{pC4bC3bFHL1}\right]+\left[\mathrm{pC4dC3bFH}\right]+\left[\mathrm{pC4dC3bFHL1}\right]+\left[\mathrm{pC3bFH}\right]+\left[\mathrm{pC3bFHL1}\right]+\left[\mathrm{pC3bC3bFH}\right]\\
+\left[\mathrm{pC3bC3bFHL1}\right]+\left[\mathrm{pC3biC3bFH}\right]+\left[\mathrm{pC3biC3bFHL1}\right]+\left[\mathrm{pC4bC4BPPTX3}\right]+\left[\mathrm{pC4bC4bC4BPPTX3}\right]\\
+\left[\mathrm{pC4bC4dC4BPPTX3}\right]+\left[\mathrm{pC4bC3bFHPTX3}\right]+\left[\mathrm{pC4bC3bFHL1PTX3}\right]+\left[\mathrm{pC4bC3bC4BPPTX3}\right]\\
+\left[\mathrm{pC4dC3bFHPTX3}\right]+\left[\mathrm{pC4dC3bFHL1PTX3}\right]+\left[\mathrm{pC4biC3bC4BPPTX3}\right]+\left[\mathrm{pC3bFHPTX3}\right]\\
+\left[\mathrm{pC3bFHL1PTX3}\right]+\left[\mathrm{pC3bC3bFHPTX3}\right]+\left[\mathrm{pC3bC3bFHL1PTX3}\right]+\left[\mathrm{pC3biC3bFHPTX3}\right]\\
+\left[\mathrm{pC3biC3bFHL1PTX3}\right]+\left[\mathrm{pC4bpC4BP}\right]+\left[\mathrm{pC4bC4bpC4BP}\right]+\left[\mathrm{pC4bC4dpC4BP}\right]+\left[\mathrm{pC4bC3bpC4BP}\right]\\
+\left[\mathrm{pC4biC3bpC4BP}\right]+\left[\mathrm{pC4bC3bpFH}\right]+\left[\mathrm{pC4bC3bpFHL1}\right]+\left[\mathrm{pC4dC3bpFH}\right]+\left[\mathrm{pC4dC3bpFHL1}\right]\\
+\left[\mathrm{pC3bpFH}\right]+\left[\mathrm{pC3bpFHL1}\right]+\left[\mathrm{pC3bC3bpFH}\right]+\left[\mathrm{pC3bC3bpFHL1}\right]+\left[\mathrm{pC3biC3bpFH}\right]+\left[\mathrm{pC3biC3bpFHL1}\right]\end{array}\right)}$$

$$-k_{\mathrm{C4bC4BP}}^{+}\left[\mathrm{pC4bC4d}\right]\left[\mathrm{C4BP}\right]+k_{\mathrm{C4bC4BP}}^{-}\left[\mathrm{pC4bC4dC4BP}\right]$$

$$+\frac{k_{\mathrm{cat\ C3bH}}^{\mathrm{FI}}[\mathrm{FI}][\mathrm{pC4bC4bC4BPPTX3}]}{\left(\begin{array}{l}K_{\mathrm{m\ C3bH}}^{\mathrm{FI}}+[\mathrm{C3(H_2O)FH}]+[\mathrm{C3(H_2O)FHL1}]+[\mathrm{fC3bFH}]+[\mathrm{fC3bFHL1}]+[\mathrm{fC3bCR1}]+[\mathrm{fiC3bCR1}]\\
+[\mathrm{IgGC3bC3bFH}]+[\mathrm{IgGC3biC3bFH}]+[\mathrm{IgGC3bC3bFHL1}]+[\mathrm{IgGC3biC3bFHL1}]+[\mathrm{IgGC3bC3bCR1}]\\
+[\mathrm{IgGC3biC3bCR1}]+[\mathrm{IgGiC3biC3bCR1}]+[\mathrm{IgGiC3bC3dgCR1}]+[\mathrm{fC4bC3bFH}]+[\mathrm{fC4bC3bFHL1}]\\
+[\mathrm{fC4bC3bCR1}]+[\mathrm{fC4bC3bC4BP}]+[\mathrm{fC4dC3bFH}]+[\mathrm{fC4dC3bFHL1}]+[\mathrm{fC4dC3bCR1}]+[\mathrm{fC4diC3bCR1}]\\
+[\mathrm{fC4biC3bCR1}]+[\mathrm{fC4bC3dgCR1}]+[\mathrm{fC4bC3dgC4BP}]+[\mathrm{fC3bC3bFH}]+[\mathrm{fC3bC3bFHL1}]+[\mathrm{fC3bC3bCR1}]\\
+[\mathrm{fC3biC3bFH}]+[\mathrm{fC3biC3bFHL1}]+[\mathrm{fC3biC3bCR1}]+[\mathrm{fiC3biC3bCR1}]+[\mathrm{fiC3bC3dgCR1}]\\
+[\mathrm{fC4biC3bC4BP}]+[\mathrm{nfC4bC4BP}]+[\mathrm{fC4bC4BP}]+[\mathrm{fC4bCR1}]+[\mathrm{fC4bC4bC4BP}]+[\mathrm{fC4bC4dC4BP}]\\
+[\mathrm{fC4bC4bCR1}]+[\mathrm{fC4bC4dCR1}]+[\mathrm{hC4bCR1}]+[\mathrm{hC4bC4BP}]+[\mathrm{hC4bC4bCR1}]+[\mathrm{hC4bC4bC4BP}]\\
+[\mathrm{hC4bC4dCR1}]+[\mathrm{hC4bC4dC4BP}]+[\mathrm{hC4bC3bFH}]+[\mathrm{hC4bC3bFHL1}]+[\mathrm{hC4bC3bCR1}]\\
+[\mathrm{hC4bC3bC4BP}]+[\mathrm{hC4biC3bCR1}]+[\mathrm{hC4biC3bC4BP}]+[\mathrm{hC4bC3dgCR1}]+[\mathrm{hC4bC3dgC4BP}]\\
+[\mathrm{hC4dC3bFH}]+[\mathrm{hC4dC3bFHL1}]+[\mathrm{hC4dC3bCR1}]+[\mathrm{hC4diC3bCR1}]+[\mathrm{hC3bFH}]+[\mathrm{hC3bFHL1}]\\
+[\mathrm{hC3bCR1}]+[\mathrm{hiC3bCR1}]+[\mathrm{hC3bC3bFH}]+[\mathrm{hC3bC3bFHL1}]+[\mathrm{hC3bC3bCR1}]+[\mathrm{hC3biC3bFH}]\\
+[\mathrm{hC3biC3bFHL1}]+[\mathrm{hC3biC3bCR1}]+[\mathrm{hiC3biC3bCR1}]+[\mathrm{hiC3biC3dgCR1}]+[\mathrm{pC4bC4BP}]\\
+[\mathrm{pC4bC4bC4BP}]+[\mathrm{pC4bC4dC4BP}]+[\mathrm{pC4bC3bC4BP}]+[\mathrm{pC4biC3bC4BP}]+[\mathrm{pC4bC3bFH}]\\
+[\mathrm{pC4bC3bFHL1}]+[\mathrm{pC4dC3bFH}]+[\mathrm{pC4dC3bFHL1}]+[\mathrm{pC3bFH}]+[\mathrm{pC3bFHL1}]+[\mathrm{pC3bC3bFH}]\\
+[\mathrm{pC3bC3bFHL1}]+[\mathrm{pC3biC3bFH}]+[\mathrm{pC3biC3bFHL1}]+[\mathrm{pC4bC4BPPTX3}]+[\mathrm{pC4bC4bC4BPPTX3}]\\
+[\mathrm{pC4bC4dC4BPPTX3}]+[\mathrm{pC4bC3bFHPTX3}]+[\mathrm{pC4bC3bFHL1PTX3}]+[\mathrm{pC4bC3bC4BPPTX3}]\\
+[\mathrm{pC4dC3bFHPTX3}]+[\mathrm{pC4dC3bFHL1PTX3}]+[\mathrm{pC4biC3bC4BPPTX3}]+[\mathrm{pC3bFHPTX3}]\\
+[\mathrm{pC3bFHL1PTX3}]+[\mathrm{pC3bC3bFHPTX3}]+[\mathrm{pC3bC3bFHL1PTX3}]+[\mathrm{pC3biC3bFHPTX3}]\\
+[\mathrm{pC3biC3bFHL1PTX3}]+[\mathrm{pC4bpC4BP}]+[\mathrm{pC4bC4bpC4BP}]+[\mathrm{pC4bC4dpC4BP}]+[\mathrm{pC4bC3bpC4BP}]\\
+[\mathrm{pC4biC3bpC4BP}]+[\mathrm{pC4bC3bpFH}]+[\mathrm{pC4bC3bpFHL1}]+[\mathrm{pC4dC3bpFH}]+[\mathrm{pC4dC3bpFHL1}]\\
+[\mathrm{pC3bpFH}]+[\mathrm{pC3bpFHL1}]+[\mathrm{pC3bC3bpFH}]+[\mathrm{pC3bC3bpFHL1}]+[\mathrm{pC3biC3bpFH}]+[\mathrm{pC3biC3bpFHL1}]\end{array}\right)}$$

$$-k_{\mathrm{C4bC4BP}}^{+}[\mathrm{pC4bC4d}][\mathrm{pC4BPPTX3}]+k_{\mathrm{C4bC4BP}}^{-}[\mathrm{pC4bC4dC4BPPTX3}]$$

$$
+\frac{k_{\mathrm{cat\,C3bH}}^{\mathrm{FI}}[\mathrm{FI}][\mathrm{pC4bC4bpC4BP}]}{\left(\begin{array}{l}
K_{\mathrm{m\,C3bH}}^{\mathrm{FI}}+[\mathrm{C3(H_2O)FH}]+[\mathrm{C3(H_2O)FHL1}]+[\mathrm{fC3bFH}]+[\mathrm{fC3bFHL1}]+[\mathrm{fC3bCR1}]+[\mathrm{fiC3bCR1}]\\
+[\mathrm{IgGC3bC3bFH}]+[\mathrm{IgGC3biC3bFH}]+[\mathrm{IgGC3bC3bFHL1}]+[\mathrm{IgGC3biC3bFHL1}]+[\mathrm{IgGC3bC3bCR1}]\\
+[\mathrm{IgGC3biC3bCR1}]+[\mathrm{IgGiC3biC3bCR1}]+[\mathrm{IgGiC3bC3dgCR1}]+[\mathrm{fC4bC3bFH}]+[\mathrm{fC4bC3bFHL1}]\\
+[\mathrm{fC4bC3bCR1}]+[\mathrm{fC4bC3bC4BP}]+[\mathrm{fC4dC3bFH}]+[\mathrm{fC4dC3bFHL1}]+[\mathrm{fC4dC3bCR1}]+[\mathrm{fC4diC3bCR1}]\\
+[\mathrm{fC4biC3bCR1}]+[\mathrm{fC4bC3dgCR1}]+[\mathrm{fC4bC3dgC4BP}]+[\mathrm{fC3bC3bFH}]+[\mathrm{fC3bC3bFHL1}]+[\mathrm{fC3bC3bCR1}]\\
+[\mathrm{fC3biC3bFH}]+[\mathrm{fC3biC3bFHL1}]+[\mathrm{fC3biC3bCR1}]+[\mathrm{fiC3biC3bCR1}]+[\mathrm{fiC3bC3dgCR1}]\\
+[\mathrm{fC4biC3bC4BP}]+[\mathrm{nfC4bC4BP}]+[\mathrm{fC4bC4BP}]+[\mathrm{fC4bCR1}]+[\mathrm{fC4bC4bC4BP}]+[\mathrm{fC4bC4dC4BP}]\\
+[\mathrm{fC4bC4bCR1}]+[\mathrm{fC4bC4dCR1}]+[\mathrm{hC4bCR1}]+[\mathrm{hC4bC4BP}]+[\mathrm{hC4bC4bCR1}]+[\mathrm{hC4bC4bC4BP}]\\
+[\mathrm{hC4bC4dCR1}]+[\mathrm{hC4bC4dC4BP}]+[\mathrm{hC4bC3bFH}]+[\mathrm{hC4bC3bFHL1}]+[\mathrm{hC4bC3bCR1}]\\
+[\mathrm{hC4bC3bC4BP}]+[\mathrm{hC4biC3bCR1}]+[\mathrm{hC4biC3bC4BP}]+[\mathrm{hC4bC3dgCR1}]+[\mathrm{hC4bC3dgC4BP}]\\
+[\mathrm{hC4dC3bFH}]+[\mathrm{hC4dC3bFHL1}]+[\mathrm{hC4dC3bCR1}]+[\mathrm{hC4diC3bCR1}]+[\mathrm{hC3bFH}]+[\mathrm{hC3bFHL1}]\\
+[\mathrm{hC3bCR1}]+[\mathrm{hiC3bCR1}]+[\mathrm{hC3bC3bFH}]+[\mathrm{hC3bC3bFHL1}]+[\mathrm{hC3bC3bCR1}]+[\mathrm{hC3biC3bFH}]\\
+[\mathrm{hC3biC3bFHL1}]+[\mathrm{hC3biC3bCR1}]+[\mathrm{hiC3biC3bCR1}]+[\mathrm{hiC3biC3dgCR1}]+[\mathrm{pC4bC4BP}]\\
+[\mathrm{pC4bC4bC4BP}]+[\mathrm{pC4bC4dC4BP}]+[\mathrm{pC4bC3bC4BP}]+[\mathrm{pC4biC3bC4BP}]+[\mathrm{pC4bC3bFH}]\\
+[\mathrm{pC4bC3bFHL1}]+[\mathrm{pC4dC3bFH}]+[\mathrm{pC4dC3bFHL1}]+[\mathrm{pC3bFH}]+[\mathrm{pC3bFHL1}]+[\mathrm{pC3bC3bFH}]\\
+[\mathrm{pC3bC3bFHL1}]+[\mathrm{pC3biC3bFH}]+[\mathrm{pC3biC3bFHL1}]+[\mathrm{pC4bC4BPPTX3}]+[\mathrm{pC4bC4bC4BPPTX3}]\\
+[\mathrm{pC4bC4dC4BPPTX3}]+[\mathrm{pC4bC3bFHPTX3}]+[\mathrm{pC4bC3bFHL1PTX3}]+[\mathrm{pC4bC3bC4BPPTX3}]\\
+[\mathrm{pC4dC3bFHPTX3}]+[\mathrm{pC4dC3bFHL1PTX3}]+[\mathrm{pC4biC3bC4BPPTX3}]+[\mathrm{pC3bFHPTX3}]\\
+[\mathrm{pC3bFHL1PTX3}]+[\mathrm{pC3bC3bFHPTX3}]+[\mathrm{pC3bC3bFHL1PTX3}]+[\mathrm{pC3biC3bFHPTX3}]\\
+[\mathrm{pC3biC3bFHL1PTX3}]+[\mathrm{pC4bpC4BP}]+[\mathrm{pC4bC4bpC4BP}]+[\mathrm{pC4bC4dpC4BP}]+[\mathrm{pC4bC3bpC4BP}]\\
+[\mathrm{pC4biC3bpC4BP}]+[\mathrm{pC4bC3bpFH}]+[\mathrm{pC4bC3bpFHL1}]+[\mathrm{pC4dC3bpFH}]+[\mathrm{pC4dC3bpFHL1}]\\
+[\mathrm{pC3bpFH}]+[\mathrm{pC3bpFHL1}]+[\mathrm{pC3bC3bpFH}]+[\mathrm{pC3bC3bpFHL1}]+[\mathrm{pC3biC3bpFH}]+[\mathrm{pC3biC3bpFHL1}]
\end{array}\right)}
$$

$$
-k_{\mathrm{C4bC4BP}}^{+}[\mathrm{pC4bC4d}][\mathrm{pC4BP}]+k_{\mathrm{C4bC4BP}}^{-}[\mathrm{pC4bC4dpC4BP}]
$$

209

$$\frac{d\left[\mathrm{pC4bC4dC4BP}\right]}{dt}=k_{\mathrm{C4bC4BP}}^{+}\left[\mathrm{pC4bC4d}\right]\left[\mathrm{C4BP}\right]-k_{\mathrm{C4bC4BP}}^{-}\left[\mathrm{pC4bC4dC4BP}\right]$$

$$-\frac{k_{\mathrm{cat\ C3bH}}^{\mathrm{FI}}\left[\mathrm{FI}\right]\left[\mathrm{pC4bC4dC4BP}\right]}{\left(\begin{array}{l}K_{\mathrm{m\ C3bH}}^{\mathrm{FI}}+\left[\mathrm{C3(H_2O)FH}\right]+\left[\mathrm{C3(H_2O)FHL1}\right]+\left[\mathrm{fC3bFH}\right]+\left[\mathrm{fC3bFHL1}\right]+\left[\mathrm{fC3bCR1}\right]+\left[\mathrm{fiC3bCR1}\right]\\
+\left[\mathrm{IgGC3bC3bFH}\right]+\left[\mathrm{IgGC3biC3bFH}\right]+\left[\mathrm{IgGC3bC3bFHL1}\right]+\left[\mathrm{IgGC3biC3bFHL1}\right]+\left[\mathrm{IgGC3bC3bCR1}\right]\\
+\left[\mathrm{IgGC3biC3bCR1}\right]+\left[\mathrm{IgGiC3biC3bCR1}\right]+\left[\mathrm{IgGiC3bC3dgCR1}\right]+\left[\mathrm{fC4bC3bFH}\right]+\left[\mathrm{fC4bC3bFHL1}\right]\\
+\left[\mathrm{fC4bC3bCR1}\right]+\left[\mathrm{fC4bC3bC4BP}\right]+\left[\mathrm{fC4dC3bFH}\right]+\left[\mathrm{fC4dC3bFHL1}\right]+\left[\mathrm{fC4dC3bCR1}\right]+\left[\mathrm{fC4diC3bCR1}\right]\\
+\left[\mathrm{fC4biC3bCR1}\right]+\left[\mathrm{fC4bC3dgCR1}\right]+\left[\mathrm{fC4bC3dgC4BP}\right]+\left[\mathrm{fC3bC3bFH}\right]+\left[\mathrm{fC3bC3bFHL1}\right]+\left[\mathrm{fC3bC3bCR1}\right]\\
+\left[\mathrm{fC3biC3bFH}\right]+\left[\mathrm{fC3biC3bFHL1}\right]+\left[\mathrm{fC3biC3bCR1}\right]+\left[\mathrm{fiC3biC3bCR1}\right]+\left[\mathrm{fiC3bC3dgCR1}\right]\\
+\left[\mathrm{fC4biC3bC4BP}\right]+\left[\mathrm{nfC4bC4BP}\right]+\left[\mathrm{fC4bC4BP}\right]+\left[\mathrm{fC4bCR1}\right]+\left[\mathrm{fC4bC4bC4BP}\right]+\left[\mathrm{fC4bC4dC4BP}\right]\\
+\left[\mathrm{fC4bC4bCR1}\right]+\left[\mathrm{fC4bC4dCR1}\right]+\left[\mathrm{hC4bCR1}\right]+\left[\mathrm{hC4bC4BP}\right]+\left[\mathrm{hC4bC4bCR1}\right]+\left[\mathrm{hC4bC4bC4BP}\right]\\
+\left[\mathrm{hC4bC4dCR1}\right]+\left[\mathrm{hC4bC4dC4BP}\right]+\left[\mathrm{hC4bC3bFH}\right]+\left[\mathrm{hC4bC3bFHL1}\right]+\left[\mathrm{hC4bC3bCR1}\right]\\
+\left[\mathrm{hC4bC3bC4BP}\right]+\left[\mathrm{hC4biC3bCR1}\right]+\left[\mathrm{hC4biC3bC4BP}\right]+\left[\mathrm{hC4bC3dgCR1}\right]+\left[\mathrm{hC4bC3dgC4BP}\right]\\
+\left[\mathrm{hC4dC3bFH}\right]+\left[\mathrm{hC4dC3bFHL1}\right]+\left[\mathrm{hC4dC3bCR1}\right]+\left[\mathrm{hC4diC3bCR1}\right]+\left[\mathrm{hC3bFH}\right]+\left[\mathrm{hC3bFHL1}\right]\\
+\left[\mathrm{hC3bCR1}\right]+\left[\mathrm{hiC3bCR1}\right]+\left[\mathrm{hC3bC3bFH}\right]+\left[\mathrm{hC3bC3bFHL1}\right]+\left[\mathrm{hC3bC3bCR1}\right]+\left[\mathrm{hC3biC3bFH}\right]\\
+\left[\mathrm{hC3biC3bFHL1}\right]+\left[\mathrm{hC3biC3bCR1}\right]+\left[\mathrm{hiC3biC3bCR1}\right]+\left[\mathrm{hiC3biC3dgCR1}\right]+\left[\mathrm{pC4bC4BP}\right]\\
+\left[\mathrm{pC4bC4bC4BP}\right]+\left[\mathrm{pC4bC4dC4BP}\right]+\left[\mathrm{pC4bC3bC4BP}\right]+\left[\mathrm{pC4biC3bC4BP}\right]+\left[\mathrm{pC4bC3bFH}\right]\\
+\left[\mathrm{pC4bC3bFHL1}\right]+\left[\mathrm{pC4dC3bFH}\right]+\left[\mathrm{pC4dC3bFHL1}\right]+\left[\mathrm{pC3bFH}\right]+\left[\mathrm{pC3bFHL1}\right]+\left[\mathrm{pC3bC3bFH}\right]\\
+\left[\mathrm{pC3bC3bFHL1}\right]+\left[\mathrm{pC3biC3bFH}\right]+\left[\mathrm{pC3biC3bFHL1}\right]+\left[\mathrm{pC4bC4BPPTX3}\right]+\left[\mathrm{pC4bC4bC4BPPTX3}\right]\\
+\left[\mathrm{pC4bC4dC4BPPTX3}\right]+\left[\mathrm{pC4bC3bFHPTX3}\right]+\left[\mathrm{pC4bC3bFHL1PTX3}\right]+\left[\mathrm{pC4bC3bC4BPPTX3}\right]\\
+\left[\mathrm{pC4dC3bFHPTX3}\right]+\left[\mathrm{pC4dC3bFHL1PTX3}\right]+\left[\mathrm{pC4biC3bC4BPPTX3}\right]+\left[\mathrm{pC3bFHPTX3}\right]\\
+\left[\mathrm{pC3bFHL1PTX3}\right]+\left[\mathrm{pC3bC3bFHPTX3}\right]+\left[\mathrm{pC3bC3bFHL1PTX3}\right]+\left[\mathrm{pC3biC3bFHPTX3}\right]\\
+\left[\mathrm{pC3biC3bFHL1PTX3}\right]+\left[\mathrm{pC4bpC4BP}\right]+\left[\mathrm{pC4bC4bpC4BP}\right]+\left[\mathrm{pC4bC4dpC4BP}\right]+\left[\mathrm{pC4bC3bpC4BP}\right]\\
+\left[\mathrm{pC4biC3bpC4BP}\right]+\left[\mathrm{pC4bC3bpFH}\right]+\left[\mathrm{pC4bC3bpFHL1}\right]+\left[\mathrm{pC4dC3bpFH}\right]+\left[\mathrm{pC4dC3bpFHL1}\right]\\
+\left[\mathrm{pC3bpFH}\right]+\left[\mathrm{pC3bpFHL1}\right]+\left[\mathrm{pC3bC3bpFH}\right]+\left[\mathrm{pC3bC3bpFHL1}\right]+\left[\mathrm{pC3biC3bpFH}\right]+\left[\mathrm{pC3biC3bpFHL1}\right]\end{array}\right)}$$

210

$$\frac{d\left[\mathrm{pC4dC4d}\right]}{dt}=$$

$$\frac{k_{\mathrm{cat\ C3bH}}^{\mathrm{FI}}\left[\mathrm{FI}\right]\left[\mathrm{pC4bC4dC4BP}\right]}{\left(\begin{array}{l}
K_{\mathrm{m\ C3bH}}^{\mathrm{FI}}+\left[\mathrm{C3(H_2O)FH}\right]+\left[\mathrm{C3(H_2O)FHL1}\right]+\left[\mathrm{fC3bFH}\right]+\left[\mathrm{fC3bFHL1}\right]+\left[\mathrm{fC3bCR1}\right]+\left[\mathrm{fiC3bCR1}\right]\\
+\left[\mathrm{IgGC3bC3bFH}\right]+\left[\mathrm{IgGC3biC3bFH}\right]+\left[\mathrm{IgGC3bC3bFHL1}\right]+\left[\mathrm{IgGC3biC3bFHL1}\right]+\left[\mathrm{IgGC3bC3bCR1}\right]\\
+\left[\mathrm{IgGC3biC3bCR1}\right]+\left[\mathrm{IgGiC3biC3bCR1}\right]+\left[\mathrm{IgGiC3bC3dgCR1}\right]+\left[\mathrm{fC4bC3bFH}\right]+\left[\mathrm{fC4bC3bFHL1}\right]\\
+\left[\mathrm{fC4bC3bCR1}\right]+\left[\mathrm{fC4bC3bC4BP}\right]+\left[\mathrm{fC4dC3bFH}\right]+\left[\mathrm{fC4dC3bFHL1}\right]+\left[\mathrm{fC4dC3bCR1}\right]+\left[\mathrm{fC4diC3bCR1}\right]\\
+\left[\mathrm{fC4biC3bCR1}\right]+\left[\mathrm{fC4bC3dgCR1}\right]+\left[\mathrm{fC4bC3dgC4BP}\right]+\left[\mathrm{fC3bC3bFH}\right]+\left[\mathrm{fC3bC3bFHL1}\right]+\left[\mathrm{fC3bC3bCR1}\right]\\
+\left[\mathrm{fC3biC3bFH}\right]+\left[\mathrm{fC3biC3bFHL1}\right]+\left[\mathrm{fC3biC3bCR1}\right]+\left[\mathrm{fiC3biC3bCR1}\right]+\left[\mathrm{fiC3bC3dgCR1}\right]\\
+\left[\mathrm{fC4biC3bC4BP}\right]+\left[\mathrm{nfC4bC4BP}\right]+\left[\mathrm{fC4bC4BP}\right]+\left[\mathrm{fC4bCR1}\right]+\left[\mathrm{fC4bC4bC4BP}\right]+\left[\mathrm{fC4bC4dC4BP}\right]\\
+\left[\mathrm{fC4bC4bCR1}\right]+\left[\mathrm{fC4bC4dCR1}\right]+\left[\mathrm{hC4bCR1}\right]+\left[\mathrm{hC4bC4BP}\right]+\left[\mathrm{hC4bC4bCR1}\right]+\left[\mathrm{hC4bC4bC4BP}\right]\\
+\left[\mathrm{hC4bC4dCR1}\right]+\left[\mathrm{hC4bC4dC4BP}\right]+\left[\mathrm{hC4bC3bFH}\right]+\left[\mathrm{hC4bC3bFHL1}\right]+\left[\mathrm{hC4bC3bCR1}\right]\\
+\left[\mathrm{hC4bC3bC4BP}\right]+\left[\mathrm{hC4biC3bCR1}\right]+\left[\mathrm{hC4biC3bC4BP}\right]+\left[\mathrm{hC4bC3dgCR1}\right]+\left[\mathrm{hC4bC3dgC4BP}\right]\\
+\left[\mathrm{hC4dC3bFH}\right]+\left[\mathrm{hC4dC3bFHL1}\right]+\left[\mathrm{hC4dC3bCR1}\right]+\left[\mathrm{hC4diC3bCR1}\right]+\left[\mathrm{hC3bFH}\right]+\left[\mathrm{hC3bFHL1}\right]\\
+\left[\mathrm{hC3bCR1}\right]+\left[\mathrm{hiC3bCR1}\right]+\left[\mathrm{hC3bC3bFH}\right]+\left[\mathrm{hC3bC3bFHL1}\right]+\left[\mathrm{hC3bC3bCR1}\right]+\left[\mathrm{hC3biC3bFH}\right]\\
+\left[\mathrm{hC3biC3bFHL1}\right]+\left[\mathrm{hC3biC3bCR1}\right]+\left[\mathrm{hiC3biC3bCR1}\right]+\left[\mathrm{hiC3biC3dgCR1}\right]+\left[\mathrm{pC4bC4BP}\right]\\
+\left[\mathrm{pC4bC4bC4BP}\right]+\left[\mathrm{pC4bC4dC4BP}\right]+\left[\mathrm{pC4bC3bC4BP}\right]+\left[\mathrm{pC4biC3bC4BP}\right]+\left[\mathrm{pC4bC3bFH}\right]\\
+\left[\mathrm{pC4bC3bFHL1}\right]+\left[\mathrm{pC4dC3bFH}\right]+\left[\mathrm{pC4dC3bFHL1}\right]+\left[\mathrm{pC3bFH}\right]+\left[\mathrm{pC3bFHL1}\right]+\left[\mathrm{pC3bC3bFH}\right]\\
+\left[\mathrm{pC3bC3bFHL1}\right]+\left[\mathrm{pC3biC3bFH}\right]+\left[\mathrm{pC3biC3bFHL1}\right]+\left[\mathrm{pC4bC4BPPTX3}\right]+\left[\mathrm{pC4bC4bC4BPPTX3}\right]\\
+\left[\mathrm{pC4bC4dC4BPPTX3}\right]+\left[\mathrm{pC4bC3bFHPTX3}\right]+\left[\mathrm{pC4bC3bFHL1PTX3}\right]+\left[\mathrm{pC4bC3bC4BPPTX3}\right]\\
+\left[\mathrm{pC4dC3bFHPTX3}\right]+\left[\mathrm{pC4dC3bFHL1PTX3}\right]+\left[\mathrm{pC4biC3bC4BPPTX3}\right]+\left[\mathrm{pC3bFHPTX3}\right]\\
+\left[\mathrm{pC3bFHL1PTX3}\right]+\left[\mathrm{pC3bC3bFHPTX3}\right]+\left[\mathrm{pC3bC3bFHL1PTX3}\right]+\left[\mathrm{pC3biC3bFHPTX3}\right]\\
+\left[\mathrm{pC3biC3bFHL1PTX3}\right]+\left[\mathrm{pC4bpC4BP}\right]+\left[\mathrm{pC4bC4bpC4BP}\right]+\left[\mathrm{pC4bC4dpC4BP}\right]+\left[\mathrm{pC4bC3bpC4BP}\right]\\
+\left[\mathrm{pC4biC3bpC4BP}\right]+\left[\mathrm{pC4bC3bpFH}\right]+\left[\mathrm{pC4bC3bpFHL1}\right]+\left[\mathrm{pC4dC3bpFH}\right]+\left[\mathrm{pC4dC3bpFHL1}\right]\\
+\left[\mathrm{pC3bpFH}\right]+\left[\mathrm{pC3bpFHL1}\right]+\left[\mathrm{pC3bC3bpFH}\right]+\left[\mathrm{pC3bC3bpFHL1}\right]+\left[\mathrm{pC3biC3bpFH}\right]+\left[\mathrm{pC3biC3bpFHL1}\right]
\end{array}\right)}$$

$$
+\frac{k_{\text{cat C3bH}}^{\text{FI}}[\text{FI}][\text{pC4bC4dC4BPPTX3}]}{\left(\begin{array}{l}
K_{\text{m C3bH}}^{\text{FI}}+[\text{C3(H}_2\text{O)FH}]+[\text{C3(H}_2\text{O)FHL1}]+[\text{fC3bFH}]+[\text{fC3bFHL1}]+[\text{fC3bCR1}]+[\text{fiC3bCR1}] \\
+[\text{IgGC3bC3bFH}]+[\text{IgGC3biC3bFH}]+[\text{IgGC3bC3bFHL1}]+[\text{IgGC3biC3bFHL1}]+[\text{IgGC3bC3bCR1}] \\
+[\text{IgGC3biC3bCR1}]+[\text{IgGiC3biC3bCR1}]+[\text{IgGiC3bC3dgCR1}]+[\text{fC4bC3bFH}]+[\text{fC4bC3bFHL1}] \\
+[\text{fC4bC3bCR1}]+[\text{fC4bC3bC4BP}]+[\text{fC4dC3bFH}]+[\text{fC4dC3bFHL1}]+[\text{fC4dC3bCR1}]+[\text{fC4diC3bCR1}] \\
+[\text{fC4biC3bCR1}]+[\text{fC4bC3dgCR1}]+[\text{fC4bC3dgC4BP}]+[\text{fC3bC3bFH}]+[\text{fC3bC3bFHL1}]+[\text{fC3bC3bCR1}] \\
+[\text{fC3biC3bFH}]+[\text{fC3biC3bFHL1}]+[\text{fC3biC3bCR1}]+[\text{fiC3biC3bCR1}]+[\text{fiC3bC3dgCR1}] \\
+[\text{fC4biC3bC4BP}]+[\text{nfC4bC4BP}]+[\text{fC4bC4BP}]+[\text{fC4bCR1}]+[\text{fC4bC4bC4BP}]+[\text{fC4bC4dC4BP}] \\
+[\text{fC4bC4bCR1}]+[\text{fC4bC4dCR1}]+[\text{hC4bCR1}]+[\text{hC4bC4BP}]+[\text{hC4bC4bCR1}]+[\text{hC4bC4bC4BP}] \\
+[\text{hC4bC4dCR1}]+[\text{hC4bC4dC4BP}]+[\text{hC4bC3bFH}]+[\text{hC4bC3bFHL1}]+[\text{hC4bC3bCR1}] \\
+[\text{hC4bC3bC4BP}]+[\text{hC4biC3bCR1}]+[\text{hC4biC3bC4BP}]+[\text{hC4bC3dgCR1}]+[\text{hC4bC3dgC4BP}] \\
+[\text{hC4dC3bFH}]+[\text{hC4dC3bFHL1}]+[\text{hC4dC3bCR1}]+[\text{hC4diC3bCR1}]+[\text{hC3bFH}]+[\text{hC3bFHL1}] \\
+[\text{hC3bCR1}]+[\text{hiC3bCR1}]+[\text{hC3bC3bFH}]+[\text{hC3bC3bFHL1}]+[\text{hC3bC3bCR1}]+[\text{hC3biC3bFH}] \\
+[\text{hC3biC3bFHL1}]+[\text{hC3biC3bCR1}]+[\text{hiC3biC3bCR1}]+[\text{hiC3biC3dgCR1}]+[\text{pC4bC4BP}] \\
+[\text{pC4bC4bC4BP}]+[\text{pC4bC4dC4BP}]+[\text{pC4bC3bC4BP}]+[\text{pC4biC3bC4BP}]+[\text{pC4bC3bFH}] \\
+[\text{pC4bC3bFHL1}]+[\text{pC4dC3bFH}]+[\text{pC4dC3bFHL1}]+[\text{pC3bFH}]+[\text{pC3bFHL1}]+[\text{pC3bC3bFH}] \\
+[\text{pC3bC3bFHL1}]+[\text{pC3biC3bFH}]+[\text{pC3biC3bFHL1}]+[\text{pC4bC4BPPTX3}]+[\text{pC4bC4bC4BPPTX3}] \\
+[\text{pC4bC4dC4BPPTX3}]+[\text{pC4bC3bFHPTX3}]+[\text{pC4bC3bFHL1PTX3}]+[\text{pC4bC3bC4BPPTX3}] \\
+[\text{pC4dC3bFHPTX3}]+[\text{pC4dC3bFHL1PTX3}]+[\text{pC4biC3bC4BPPTX3}]+[\text{pC3bFHPTX3}] \\
+[\text{pC3bFHL1PTX3}]+[\text{pC3bC3bFHPTX3}]+[\text{pC3bC3bFHL1PTX3}]+[\text{pC3biC3bFHPTX3}] \\
+[\text{pC3biC3bFHL1PTX3}]+[\text{pC4bpC4BP}]+[\text{pC4bC4bpC4BP}]+[\text{pC4bC4dpC4BP}]+[\text{pC4bC3bpC4BP}] \\
+[\text{pC4biC3bpC4BP}]+[\text{pC4bC3bpFH}]+[\text{pC4bC3bpFHL1}]+[\text{pC4dC3bpFH}]+[\text{pC4dC3bpFHL1}] \\
+[\text{pC3bpFH}]+[\text{pC3bpFHL1}]+[\text{pC3bC3bpFH}]+[\text{pC3bC3bpFHL1}]+[\text{pC3biC3bpFH}]+[\text{pC3biC3bpFHL1}]
\end{array}\right)}
$$

$$+\frac{k_{\text{cat C3bH}}^{\text{FI}}[\text{FI}][\text{pC4bC4dC4BPPTX3}]}{\left(\begin{array}{l}K_{\text{m C3bH}}^{\text{FI}}+[\text{C3(H}_2\text{O)FH}]+[\text{C3(H}_2\text{O)FHL1}]+[\text{fC3bFH}]+[\text{fC3bFHL1}]+[\text{fC3bCR1}]+[\text{fiC3bCR1}]\\+[\text{IgGC3bC3bFH}]+[\text{IgGC3biC3bFH}]+[\text{IgGC3bC3bFHL1}]+[\text{IgGC3biC3bFHL1}]+[\text{IgGC3bC3bCR1}]\\+[\text{IgGC3biC3bCR1}]+[\text{IgGiC3biC3bCR1}]+[\text{IgGiC3bC3dgCR1}]+[\text{fC4bC3bFH}]+[\text{fC4bC3bFHL1}]\\+[\text{fC4bC3bCR1}]+[\text{fC4bC3bC4BP}]+[\text{fC4dC3bFH}]+[\text{fC4dC3bFHL1}]+[\text{fC4dC3bCR1}]+[\text{fC4diC3bCR1}]\\+[\text{fC4biC3bCR1}]+[\text{fC4bC3dgCR1}]+[\text{fC4bC3dgC4BP}]+[\text{fC3bC3bFH}]+[\text{fC3bC3bFHL1}]+[\text{fC3bC3bCR1}]\\+[\text{fC3biC3bFH}]+[\text{fC3biC3bFHL1}]+[\text{fC3biC3bCR1}]+[\text{fiC3biC3bCR1}]+[\text{fiC3bC3dgCR1}]\\+[\text{fC4biC3bC4BP}]+[\text{nfC4bC4BP}]+[\text{fC4bC4BP}]+[\text{fC4bCR1}]+[\text{fC4bC4bC4BP}]+[\text{fC4bC4dC4BP}]\\+[\text{fC4bC4bCR1}]+[\text{fC4bC4dCR1}]+[\text{hC4bCR1}]+[\text{hC4bC4BP}]+[\text{hC4bC4bCR1}]+[\text{hC4bC4bC4BP}]\\+[\text{hC4bC4dCR1}]+[\text{hC4bC4dC4BP}]+[\text{hC4bC3bFH}]+[\text{hC4bC3bFHL1}]+[\text{hC4bC3bCR1}]\\+[\text{hC4bC3bC4BP}]+[\text{hC4biC3bCR1}]+[\text{hC4biC3bC4BP}]+[\text{hC4bC3dgCR1}]+[\text{hC4bC3dgC4BP}]\\+[\text{hC4dC3bFH}]+[\text{hC4dC3bFHL1}]+[\text{hC4dC3bCR1}]+[\text{hC4diC3bCR1}]+[\text{hC3bFH}]+[\text{hC3bFHL1}]\\+[\text{hC3bCR1}]+[\text{hiC3bCR1}]+[\text{hC3bC3bFH}]+[\text{hC3bC3bFHL1}]+[\text{hC3bC3bCR1}]+[\text{hC3biC3bFH}]\\+[\text{hC3biC3bFHL1}]+[\text{hC3biC3bCR1}]+[\text{hiC3biC3bCR1}]+[\text{hiC3biC3dgCR1}]+[\text{pC4bC4BP}]\\+[\text{pC4bC4bC4BP}]+[\text{pC4bC4dC4BP}]+[\text{pC4bC3bC4BP}]+[\text{pC4biC3bC4BP}]+[\text{pC4bC3bFH}]\\+[\text{pC4bC3bFHL1}]+[\text{pC4dC3bFH}]+[\text{pC4dC3bFHL1}]+[\text{pC3bFH}]+[\text{pC3bFHL1}]+[\text{pC3bC3bFH}]\\+[\text{pC3bC3bFHL1}]+[\text{pC3biC3bFH}]+[\text{pC3biC3bFHL1}]+[\text{pC4bC4BPPTX3}]+[\text{pC4bC4bC4BPPTX3}]\\+[\text{pC4bC4dC4BPPTX3}]+[\text{pC4bC3bFHPTX3}]+[\text{pC4bC3bFHL1PTX3}]+[\text{pC4bC3bC4BPPTX3}]\\+[\text{pC4dC3bFHPTX3}]+[\text{pC4dC3bFHL1PTX3}]+[\text{pC4biC3bC4BPPTX3}]+[\text{pC3bFHPTX3}]\\+[\text{pC3bFHL1PTX3}]+[\text{pC3bC3bFHPTX3}]+[\text{pC3bC3bFHL1PTX3}]+[\text{pC3biC3bFHPTX3}]\\+[\text{pC3biC3bFHL1PTX3}]+[\text{pC4bpC4BP}]+[\text{pC4bC4bpC4BP}]+[\text{pC4bC4dpC4BP}]+[\text{pC4bC3bpC4BP}]\\+[\text{pC4biC3bpC4BP}]+[\text{pC4bC3bpFH}]+[\text{pC4bC3bpFHL1}]+[\text{pC4dC3bpFH}]+[\text{pC4dC3bpFHL1}]\\+[\text{pC3bpFH}]+[\text{pC3bpFHL1}]+[\text{pC3bC3bpFH}]+[\text{pC3bC3bpFHL1}]+[\text{pC3biC3bpFH}]+[\text{pC3biC3bpFHL1}]\end{array}\right)}$$

$$+\frac{k_{\text{cat C3bH}}^{\text{FI}}[\text{FI}][\text{pC4bC4dpC4BP}]}{\left(\begin{array}{l}K_{\text{m C3bH}}^{\text{FI}}+[\text{C3(H}_2\text{O)FH}]+[\text{C3(H}_2\text{O)FHL1}]+[\text{fC3bFH}]+[\text{fC3bFHL1}]+[\text{fC3bCR1}]+[\text{fiC3bCR1}]\\ +[\text{IgGC3bC3bFH}]+[\text{IgGC3biC3bFH}]+[\text{IgGC3bC3bFHL1}]+[\text{IgGC3biC3bFHL1}]+[\text{IgGC3bC3bCR1}]\\ +[\text{IgGC3biC3bCR1}]+[\text{IgGiC3biC3bCR1}]+[\text{IgGiC3bC3dgCR1}]+[\text{fC4bC3bFH}]+[\text{fC4bC3bFHL1}]\\ +[\text{fC4bC3bCR1}]+[\text{fC4bC3bC4BP}]+[\text{fC4dC3bFH}]+[\text{fC4dC3bFHL1}]+[\text{fC4dC3bCR1}]+[\text{fC4diC3bCR1}]\\ +[\text{fC4biC3bCR1}]+[\text{fC4bC3dgCR1}]+[\text{fC4bC3dgC4BP}]+[\text{fC3bC3bFH}]+[\text{fC3bC3bFHL1}]+[\text{fC3bC3bCR1}]\\ +[\text{fC3biC3bFH}]+[\text{fC3biC3bFHL1}]+[\text{fC3biC3bCR1}]+[\text{fiC3biC3bCR1}]+[\text{fiC3bC3dgCR1}]\\ +[\text{fC4biC3bC4BP}]+[\text{nfC4bC4BP}]+[\text{fC4bC4BP}]+[\text{fC4bCR1}]+[\text{fC4bC4bC4BP}]+[\text{fC4bC4dC4BP}]\\ +[\text{fC4bC4bCR1}]+[\text{fC4bC4dCR1}]+[\text{hC4bCR1}]+[\text{hC4bC4BP}]+[\text{hC4bC4bCR1}]+[\text{hC4bC4bC4BP}]\\ +[\text{hC4bC4dCR1}]+[\text{hC4bC4dC4BP}]+[\text{hC4bC3bFH}]+[\text{hC4bC3bFHL1}]+[\text{hC4bC3bCR1}]\\ +[\text{hC4bC3bC4BP}]+[\text{hC4biC3bCR1}]+[\text{hC4biC3bC4BP}]+[\text{hC4bC3dgCR1}]+[\text{hC4bC3dgC4BP}]\\ +[\text{hC4dC3bFH}]+[\text{hC4dC3bFHL1}]+[\text{hC4dC3bCR1}]+[\text{hC4diC3bCR1}]+[\text{hC3bFH}]+[\text{hC3bFHL1}]\\ +[\text{hC3bCR1}]+[\text{hiC3bCR1}]+[\text{hC3bC3bFH}]+[\text{hC3bC3bFHL1}]+[\text{hC3bC3bCR1}]+[\text{hC3biC3bFH}]\\ +[\text{hC3biC3bFHL1}]+[\text{hC3biC3bCR1}]+[\text{hiC3biC3bCR1}]+[\text{hiC3biC3dgCR1}]+[\text{pC4bC4BP}]\\ +[\text{pC4bC4bC4BP}]+[\text{pC4bC4dC4BP}]+[\text{pC4bC3bC4BP}]+[\text{pC4biC3bC4BP}]+[\text{pC4bC3bFH}]\\ +[\text{pC4bC3bFHL1}]+[\text{pC4dC3bFH}]+[\text{pC4dC3bFHL1}]+[\text{pC3bFH}]+[\text{pC3bFHL1}]+[\text{pC3bC3bFH}]\\ +[\text{pC3bC3bFHL1}]+[\text{pC3biC3bFH}]+[\text{pC3biC3bFHL1}]+[\text{pC4bC4BPPTX3}]+[\text{pC4bC4bC4BPPTX3}]\\ +[\text{pC4bC4dC4BPPTX3}]+[\text{pC4bC3bFHPTX3}]+[\text{pC4bC3bFHL1PTX3}]+[\text{pC4bC3bC4BPPTX3}]\\ +[\text{pC4dC3bFHPTX3}]+[\text{pC4dC3bFHL1PTX3}]+[\text{pC4biC3bC4BPPTX3}]+[\text{pC3bFHPTX3}]\\ +[\text{pC3bFHL1PTX3}]+[\text{pC3bC3bFHPTX3}]+[\text{pC3bC3bFHL1PTX3}]+[\text{pC3biC3bFHPTX3}]\\ +[\text{pC3biC3bFHL1PTX3}]+[\text{pC4bpC4BP}]+[\text{pC4bC4bpC4BP}]+[\text{pC4bC4dpC4BP}]+[\text{pC4bC3bpC4BP}]\\ +[\text{pC4biC3bpC4BP}]+[\text{pC4bC3bpFH}]+[\text{pC4bC3bpFHL1}]+[\text{pC4dC3bpFH}]+[\text{pC4dC3bpFHL1}]\\ +[\text{pC3bpFH}]+[\text{pC3bpFHL1}]+[\text{pC3bC3bpFH}]+[\text{pC3bC3bpFHL1}]+[\text{pC3biC3bpFH}]+[\text{pC3biC3bpFHL1}]\end{array}\right)}$$

211

$$\frac{d[\text{pC3bC4bC2aC4BP}]}{dt}=k_{\text{C4bC4BP}}^{+}[\text{pC3bC4bC2a}][\text{C4BP}]-k_{\text{C4bC4BP}}^{-}[\text{pC3bC4bC2aC4BP}]$$
$$-k_{\text{C4bC2aC4BP}_{\text{decay}}}^{-}[\text{pC3bC4bC2aC4BP}]$$

212

$$\frac{d[\text{pC4bC3bBbFH}]}{dt}=k_{\text{C3bH}}^{+}[\text{pC4bC3bBb}][\text{FH}]-k_{\text{C3bH}}^{-}[\text{pC4bC3bBbFH}]-k_{\text{C3bBbH}_{\text{decay}}}^{-}[\text{pC4bC3bBbFH}]$$

213

$$\frac{d[\text{pC4bC3bBbFHL1}]}{dt}=k_{\text{C3bFHL1}}^{+}[\text{pC4bC3bBb}][\text{FHL1}]-k_{\text{C3bFHL1}}^{-}[\text{pC4bC3bBbFHL1}]$$
$$-k_{\text{C3bBbFHL1}_{\text{decay}}}^{-}[\text{pC4bC3bBbFHL1}]$$

214

$$
\begin{aligned}
\frac{d\left[\mathrm{pC3bC4bFH}\right]}{dt} &= k_{\mathrm{C3bH}}^{+}\left[\mathrm{pC3bC4b}\right]\left[\mathrm{FH}\right]-k_{\mathrm{C3bH}}^{-}\left[\mathrm{pC3bC4bFH}\right] \\
&-\frac{k_{\mathrm{cat\ C3bH}}^{\mathrm{FI}}\left[\mathrm{FI}\right]\left[\mathrm{pC3bC4bFH}\right]}{\left(\begin{array}{l}
K_{\mathrm{m\ C3bH}}^{\mathrm{FI}}+\left[\mathrm{C3(H_2O)FH}\right]+\left[\mathrm{C3(H_2O)FHL1}\right]+\left[\mathrm{fC3bFH}\right]+\left[\mathrm{fC3bFHL1}\right]+\left[\mathrm{fC3bCR1}\right]+\left[\mathrm{fiC3bCR1}\right] \\
+\left[\mathrm{IgGC3bC3bFH}\right]+\left[\mathrm{IgGC3biC3bFH}\right]+\left[\mathrm{IgGC3bC3bFHL1}\right]+\left[\mathrm{IgGC3biC3bFHL1}\right]+\left[\mathrm{IgGC3bC3bCR1}\right] \\
+\left[\mathrm{IgGC3biC3bCR1}\right]+\left[\mathrm{IgGiC3biC3bCR1}\right]+\left[\mathrm{IgGiC3bC3dgCR1}\right]+\left[\mathrm{fC4bC3bFH}\right]+\left[\mathrm{fC4bC3bFHL1}\right] \\
+\left[\mathrm{fC4bC3bCR1}\right]+\left[\mathrm{fC4bC3bC4BP}\right]+\left[\mathrm{fC4dC3bFH}\right]+\left[\mathrm{fC4dC3bFHL1}\right]+\left[\mathrm{fC4dC3bCR1}\right]+\left[\mathrm{fC4diC3bCR1}\right] \\
+\left[\mathrm{fC4biC3bCR1}\right]+\left[\mathrm{fC4bC3dgCR1}\right]+\left[\mathrm{fC4bC3dgC4BP}\right]+\left[\mathrm{fC3bC3bFH}\right]+\left[\mathrm{fC3bC3bFHL1}\right]+\left[\mathrm{fC3bC3bCR1}\right] \\
+\left[\mathrm{fC3biC3bFH}\right]+\left[\mathrm{fC3biC3bFHL1}\right]+\left[\mathrm{fC3biC3bCR1}\right]+\left[\mathrm{fiC3biC3bCR1}\right]+\left[\mathrm{fiC3bC3dgCR1}\right] \\
+\left[\mathrm{fC4biC3bC4BP}\right]+\left[\mathrm{nfC4bC4BP}\right]+\left[\mathrm{fC4bC4BP}\right]+\left[\mathrm{fC4bCR1}\right]+\left[\mathrm{fC4bC4bC4BP}\right]+\left[\mathrm{fC4bC4dC4BP}\right] \\
+\left[\mathrm{fC4bC4bCR1}\right]+\left[\mathrm{fC4bC4dCR1}\right]+\left[\mathrm{hC4bCR1}\right]+\left[\mathrm{hC4bC4BP}\right]+\left[\mathrm{hC4bC4bCR1}\right]+\left[\mathrm{hC4bC4bC4BP}\right] \\
+\left[\mathrm{hC4bC4dCR1}\right]+\left[\mathrm{hC4bC4dC4BP}\right]+\left[\mathrm{hC4bC3bFH}\right]+\left[\mathrm{hC4bC3bFHL1}\right]+\left[\mathrm{hC4bC3bCR1}\right] \\
+\left[\mathrm{hC4bC3bC4BP}\right]+\left[\mathrm{hC4biC3bCR1}\right]+\left[\mathrm{hC4biC3bC4BP}\right]+\left[\mathrm{hC4bC3dgCR1}\right]+\left[\mathrm{hC4bC3dgC4BP}\right] \\
+\left[\mathrm{hC4dC3bFH}\right]+\left[\mathrm{hC4dC3bFHL1}\right]+\left[\mathrm{hC4dC3bCR1}\right]+\left[\mathrm{hC4diC3bCR1}\right]+\left[\mathrm{hC3bFH}\right]+\left[\mathrm{hC3bFHL1}\right] \\
+\left[\mathrm{hC3bCR1}\right]+\left[\mathrm{hiC3bCR1}\right]+\left[\mathrm{hC3bC3bFH}\right]+\left[\mathrm{hC3bC3bFHL1}\right]+\left[\mathrm{hC3bC3bCR1}\right]+\left[\mathrm{hC3biC3bFH}\right] \\
+\left[\mathrm{hC3biC3bFHL1}\right]+\left[\mathrm{hC3biC3bCR1}\right]+\left[\mathrm{hiC3biC3bCR1}\right]+\left[\mathrm{hiC3biC3dgCR1}\right]+\left[\mathrm{pC4bC4BP}\right] \\
+\left[\mathrm{pC4bC4bC4BP}\right]+\left[\mathrm{pC4bC4dC4BP}\right]+\left[\mathrm{pC4bC3bC4BP}\right]+\left[\mathrm{pC4biC3bC4BP}\right]+\left[\mathrm{pC4bC3bFH}\right] \\
+\left[\mathrm{pC4bC3bFHL1}\right]+\left[\mathrm{pC4dC3bFH}\right]+\left[\mathrm{pC4dC3bFHL1}\right]+\left[\mathrm{pC3bFH}\right]+\left[\mathrm{pC3bFHL1}\right]+\left[\mathrm{pC3bC3bFH}\right] \\
+\left[\mathrm{pC3bC3bFHL1}\right]+\left[\mathrm{pC3biC3bFH}\right]+\left[\mathrm{pC3biC3bFHL1}\right]+\left[\mathrm{pC4bC4BPPTX3}\right]+\left[\mathrm{pC4bC4bC4BPPTX3}\right] \\
+\left[\mathrm{pC4bC4dC4BPPTX3}\right]+\left[\mathrm{pC4bC3bFHPTX3}\right]+\left[\mathrm{pC4bC3bFHL1PTX3}\right]+\left[\mathrm{pC4bC3bC4BPPTX3}\right] \\
+\left[\mathrm{pC4dC3bFHPTX3}\right]+\left[\mathrm{pC4dC3bFHL1PTX3}\right]+\left[\mathrm{pC4biC3bC4BPPTX3}\right]+\left[\mathrm{pC3bFHPTX3}\right] \\
+\left[\mathrm{pC3bFHL1PTX3}\right]+\left[\mathrm{pC3bC3bFHPTX3}\right]+\left[\mathrm{pC3bC3bFHL1PTX3}\right]+\left[\mathrm{pC3biC3bFHPTX3}\right] \\
+\left[\mathrm{pC3biC3bFHL1PTX3}\right]+\left[\mathrm{pC4bpC4BP}\right]+\left[\mathrm{pC4bC4bpC4BP}\right]+\left[\mathrm{pC4bC4dpC4BP}\right]+\left[\mathrm{pC4bC3bpC4BP}\right] \\
+\left[\mathrm{pC4biC3bpC4BP}\right]+\left[\mathrm{pC4bC3bpFH}\right]+\left[\mathrm{pC4bC3bpFHL1}\right]+\left[\mathrm{pC4dC3bpFH}\right]+\left[\mathrm{pC4dC3bpFHL1}\right] \\
+\left[\mathrm{pC3bpFH}\right]+\left[\mathrm{pC3bpFHL1}\right]+\left[\mathrm{pC3bC3bpFH}\right]+\left[\mathrm{pC3bC3bpFHL1}\right]+\left[\mathrm{pC3biC3bpFH}\right]+\left[\mathrm{pC3biC3bpFHL1}\right]
\end{array}\right)}
\end{aligned}
$$

215

$$\frac{d\left[\mathrm{pC3bC4bFHL1}\right]}{dt}=k_{\mathrm{C3bFHL1}}^{+}\left[\mathrm{pC3bC4b}\right]\left[\mathrm{FHL1}\right]-k_{\mathrm{C3bFHL1}}^{-}\left[\mathrm{pC3bC4bFHL1}\right]$$

$$-\frac{k_{\mathrm{cat\ C3bH}}^{\mathrm{FI}}\left[\mathrm{FI}\right]\left[\mathrm{pC3bC4bFHL1}\right]}{\left(\begin{array}{l}K_{\mathrm{m\ C3bH}}^{\mathrm{FI}}+\left[\mathrm{C3(H_2O)FH}\right]+\left[\mathrm{C3(H_2O)FHL1}\right]+\left[\mathrm{fC3bFH}\right]+\left[\mathrm{fC3bFHL1}\right]+\left[\mathrm{fC3bCR1}\right]+\left[\mathrm{fiC3bCR1}\right]\\
+\left[\mathrm{IgGC3bC3bFH}\right]+\left[\mathrm{IgGC3biC3bFH}\right]+\left[\mathrm{IgGC3bC3bFHL1}\right]+\left[\mathrm{IgGC3biC3bFHL1}\right]+\left[\mathrm{IgGC3bC3bCR1}\right]\\
+\left[\mathrm{IgGC3biC3bCR1}\right]+\left[\mathrm{IgGiC3biC3bCR1}\right]+\left[\mathrm{IgGiC3bC3dgCR1}\right]+\left[\mathrm{fC4bC3bFH}\right]+\left[\mathrm{fC4bC3bFHL1}\right]\\
+\left[\mathrm{fC4bC3bCR1}\right]+\left[\mathrm{fC4bC3bC4BP}\right]+\left[\mathrm{fC4dC3bFH}\right]+\left[\mathrm{fC4dC3bFHL1}\right]+\left[\mathrm{fC4dC3bCR1}\right]+\left[\mathrm{fC4diC3bCR1}\right]\\
+\left[\mathrm{fC4biC3bCR1}\right]+\left[\mathrm{fC4bC3dgCR1}\right]+\left[\mathrm{fC4bC3dgC4BP}\right]+\left[\mathrm{fC3bC3bFH}\right]+\left[\mathrm{fC3bC3bFHL1}\right]+\left[\mathrm{fC3bC3bCR1}\right]\\
+\left[\mathrm{fC3biC3bFH}\right]+\left[\mathrm{fC3biC3bFHL1}\right]+\left[\mathrm{fC3biC3bCR1}\right]+\left[\mathrm{fiC3biC3bCR1}\right]+\left[\mathrm{fiC3bC3dgCR1}\right]\\
+\left[\mathrm{fC4biC3bC4BP}\right]+\left[\mathrm{nfC4bC4BP}\right]+\left[\mathrm{fC4bC4BP}\right]+\left[\mathrm{fC4bCR1}\right]+\left[\mathrm{fC4bC4bC4BP}\right]+\left[\mathrm{fC4bC4dC4BP}\right]\\
+\left[\mathrm{fC4bC4bCR1}\right]+\left[\mathrm{fC4bC4dCR1}\right]+\left[\mathrm{hC4bCR1}\right]+\left[\mathrm{hC4bC4BP}\right]+\left[\mathrm{hC4bC4bCR1}\right]+\left[\mathrm{hC4bC4bC4BP}\right]\\
+\left[\mathrm{hC4bC4dCR1}\right]+\left[\mathrm{hC4bC4dC4BP}\right]+\left[\mathrm{hC4bC3bFH}\right]+\left[\mathrm{hC4bC3bFHL1}\right]+\left[\mathrm{hC4bC3bCR1}\right]\\
+\left[\mathrm{hC4bC3bC4BP}\right]+\left[\mathrm{hC4biC3bCR1}\right]+\left[\mathrm{hC4biC3bC4BP}\right]+\left[\mathrm{hC4bC3dgCR1}\right]+\left[\mathrm{hC4bC3dgC4BP}\right]\\
+\left[\mathrm{hC4dC3bFH}\right]+\left[\mathrm{hC4dC3bFHL1}\right]+\left[\mathrm{hC4dC3bCR1}\right]+\left[\mathrm{hC4diC3bCR1}\right]+\left[\mathrm{hC3bFH}\right]+\left[\mathrm{hC3bFHL1}\right]\\
+\left[\mathrm{hC3bCR1}\right]+\left[\mathrm{hiC3bCR1}\right]+\left[\mathrm{hC3bC3bFH}\right]+\left[\mathrm{hC3bC3bFHL1}\right]+\left[\mathrm{hC3bC3bCR1}\right]+\left[\mathrm{hC3biC3bFH}\right]\\
+\left[\mathrm{hC3biC3bFHL1}\right]+\left[\mathrm{hC3biC3bCR1}\right]+\left[\mathrm{hiC3biC3bCR1}\right]+\left[\mathrm{hiC3biC3dgCR1}\right]+\left[\mathrm{pC4bC4BP}\right]\\
+\left[\mathrm{pC4bC4bC4BP}\right]+\left[\mathrm{pC4bC4dC4BP}\right]+\left[\mathrm{pC4bC3bC4BP}\right]+\left[\mathrm{pC4biC3bC4BP}\right]+\left[\mathrm{pC4bC3bFH}\right]\\
+\left[\mathrm{pC4bC3bFHL1}\right]+\left[\mathrm{pC4dC3bFH}\right]+\left[\mathrm{pC4dC3bFHL1}\right]+\left[\mathrm{pC3bFH}\right]+\left[\mathrm{pC3bFHL1}\right]+\left[\mathrm{pC3bC3bFH}\right]\\
+\left[\mathrm{pC3bC3bFHL1}\right]+\left[\mathrm{pC3biC3bFH}\right]+\left[\mathrm{pC3biC3bFHL1}\right]+\left[\mathrm{pC4bC4BPPTX3}\right]+\left[\mathrm{pC4bC4bC4BPPTX3}\right]\\
+\left[\mathrm{pC4bC4dC4BPPTX3}\right]+\left[\mathrm{pC4bC3bFHPTX3}\right]+\left[\mathrm{pC4bC3bFHL1PTX3}\right]+\left[\mathrm{pC4bC3bC4BPPTX3}\right]\\
+\left[\mathrm{pC4dC3bFHPTX3}\right]+\left[\mathrm{pC4dC3bFHL1PTX3}\right]+\left[\mathrm{pC4biC3bC4BPPTX3}\right]+\left[\mathrm{pC3bFHPTX3}\right]\\
+\left[\mathrm{pC3bFHL1PTX3}\right]+\left[\mathrm{pC3bC3bFHPTX3}\right]+\left[\mathrm{pC3bC3bFHL1PTX3}\right]+\left[\mathrm{pC3biC3bFHPTX3}\right]\\
+\left[\mathrm{pC3biC3bFHL1PTX3}\right]+\left[\mathrm{pC4bpC4BP}\right]+\left[\mathrm{pC4bC4bpC4BP}\right]+\left[\mathrm{pC4bC4dpC4BP}\right]+\left[\mathrm{pC4bC3bpC4BP}\right]\\
+\left[\mathrm{pC4biC3bpC4BP}\right]+\left[\mathrm{pC4bC3bpFH}\right]+\left[\mathrm{pC4bC3bpFHL1}\right]+\left[\mathrm{pC4dC3bpFH}\right]+\left[\mathrm{pC4dC3bpFHL1}\right]\\
+\left[\mathrm{pC3bpFH}\right]+\left[\mathrm{pC3bpFHL1}\right]+\left[\mathrm{pC3bC3bpFH}\right]+\left[\mathrm{pC3bC3bpFHL1}\right]+\left[\mathrm{pC3biC3bpFH}\right]+\left[\mathrm{pC3biC3bpFHL1}\right]\end{array}\right)}$$

216

$$\frac{d\left[\mathrm{pC3bC4bC4BP}\right]}{dt}=k_{\mathrm{C4bC4BP}}^{+}\left[\mathrm{pC3bC4b}\right]\left[\mathrm{C4BP}\right]-k_{\mathrm{C4bC4BP}}^{-}\left[\mathrm{pC3bC4bC4BP}\right]$$

$$-\frac{k_{\mathrm{cat\ C3bH}}^{\mathrm{FI}}\left[\mathrm{FI}\right]\left[\mathrm{pC3bC4bC4BP}\right]}{\left(\begin{array}{l}K_{\mathrm{m\ C3bH}}^{\mathrm{FI}}+\left[\mathrm{C3(H_2O)FH}\right]+\left[\mathrm{C3(H_2O)FHL1}\right]+\left[\mathrm{fC3bFH}\right]+\left[\mathrm{fC3bFHL1}\right]+\left[\mathrm{fC3bCR1}\right]+\left[\mathrm{fiC3bCR1}\right]\\
+\left[\mathrm{IgGC3bC3bFH}\right]+\left[\mathrm{IgGC3biC3bFH}\right]+\left[\mathrm{IgGC3bC3bFHL1}\right]+\left[\mathrm{IgGC3biC3bFHL1}\right]+\left[\mathrm{IgGC3bC3bCR1}\right]\\
+\left[\mathrm{IgGC3biC3bCR1}\right]+\left[\mathrm{IgGiC3biC3bCR1}\right]+\left[\mathrm{IgGiC3bC3dgCR1}\right]+\left[\mathrm{fC4bC3bFH}\right]+\left[\mathrm{fC4bC3bFHL1}\right]\\
+\left[\mathrm{fC4bC3bCR1}\right]+\left[\mathrm{fC4bC3bC4BP}\right]+\left[\mathrm{fC4dC3bFH}\right]+\left[\mathrm{fC4dC3bFHL1}\right]+\left[\mathrm{fC4dC3bCR1}\right]+\left[\mathrm{fC4diC3bCR1}\right]\\
+\left[\mathrm{fC4biC3bCR1}\right]+\left[\mathrm{fC4bC3dgCR1}\right]+\left[\mathrm{fC4bC3dgC4BP}\right]+\left[\mathrm{fC3bC3bFH}\right]+\left[\mathrm{fC3bC3bFHL1}\right]+\left[\mathrm{fC3bC3bCR1}\right]\\
+\left[\mathrm{fC3biC3bFH}\right]+\left[\mathrm{fC3biC3bFHL1}\right]+\left[\mathrm{fC3biC3bCR1}\right]+\left[\mathrm{fiC3biC3bCR1}\right]+\left[\mathrm{fiC3bC3dgCR1}\right]\\
+\left[\mathrm{fC4biC3bC4BP}\right]+\left[\mathrm{nfC4bC4BP}\right]+\left[\mathrm{fC4bC4BP}\right]+\left[\mathrm{fC4bCR1}\right]+\left[\mathrm{fC4bC4bC4BP}\right]+\left[\mathrm{fC4bC4dC4BP}\right]\\
+\left[\mathrm{fC4bC4bCR1}\right]+\left[\mathrm{fC4bC4dCR1}\right]+\left[\mathrm{hC4bCR1}\right]+\left[\mathrm{hC4bC4BP}\right]+\left[\mathrm{hC4bC4bCR1}\right]+\left[\mathrm{hC4bC4bC4BP}\right]\\
+\left[\mathrm{hC4bC4dCR1}\right]+\left[\mathrm{hC4bC4dC4BP}\right]+\left[\mathrm{hC4bC3bFH}\right]+\left[\mathrm{hC4bC3bFHL1}\right]+\left[\mathrm{hC4bC3bCR1}\right]\\
+\left[\mathrm{hC4bC3bC4BP}\right]+\left[\mathrm{hC4biC3bCR1}\right]+\left[\mathrm{hC4biC3bC4BP}\right]+\left[\mathrm{hC4bC3dgCR1}\right]+\left[\mathrm{hC4bC3dgC4BP}\right]\\
+\left[\mathrm{hC4dC3bFH}\right]+\left[\mathrm{hC4dC3bFHL1}\right]+\left[\mathrm{hC4dC3bCR1}\right]+\left[\mathrm{hC4diC3bCR1}\right]+\left[\mathrm{hC3bFH}\right]+\left[\mathrm{hC3bFHL1}\right]\\
+\left[\mathrm{hC3bCR1}\right]+\left[\mathrm{hiC3bCR1}\right]+\left[\mathrm{hC3bC3bFH}\right]+\left[\mathrm{hC3bC3bFHL1}\right]+\left[\mathrm{hC3bC3bCR1}\right]+\left[\mathrm{hC3biC3bFH}\right]\\
+\left[\mathrm{hC3biC3bFHL1}\right]+\left[\mathrm{hC3biC3bCR1}\right]+\left[\mathrm{hiC3biC3bCR1}\right]+\left[\mathrm{hiC3biC3dgCR1}\right]+\left[\mathrm{pC4bC4BP}\right]\\
+\left[\mathrm{pC4bC4bC4BP}\right]+\left[\mathrm{pC4bC4dC4BP}\right]+\left[\mathrm{pC4bC3bC4BP}\right]+\left[\mathrm{pC4biC3bC4BP}\right]+\left[\mathrm{pC4bC3bFH}\right]\\
+\left[\mathrm{pC4bC3bFHL1}\right]+\left[\mathrm{pC4dC3bFH}\right]+\left[\mathrm{pC4dC3bFHL1}\right]+\left[\mathrm{pC3bFH}\right]+\left[\mathrm{pC3bFHL1}\right]+\left[\mathrm{pC3bC3bFH}\right]\\
+\left[\mathrm{pC3bC3bFHL1}\right]+\left[\mathrm{pC3biC3bFH}\right]+\left[\mathrm{pC3biC3bFHL1}\right]+\left[\mathrm{pC4bC4BPPTX3}\right]+\left[\mathrm{pC4bC4bC4BPPTX3}\right]\\
+\left[\mathrm{pC4bC4dC4BPPTX3}\right]+\left[\mathrm{pC4bC3bFHPTX3}\right]+\left[\mathrm{pC4bC3bFHL1PTX3}\right]+\left[\mathrm{pC4bC3bC4BPPTX3}\right]\\
+\left[\mathrm{pC4dC3bFHPTX3}\right]+\left[\mathrm{pC4dC3bFHL1PTX3}\right]+\left[\mathrm{pC4biC3bC4BPPTX3}\right]+\left[\mathrm{pC3bFHPTX3}\right]\\
+\left[\mathrm{pC3bFHL1PTX3}\right]+\left[\mathrm{pC3bC3bFHPTX3}\right]+\left[\mathrm{pC3bC3bFHL1PTX3}\right]+\left[\mathrm{pC3biC3bFHPTX3}\right]\\
+\left[\mathrm{pC3biC3bFHL1PTX3}\right]+\left[\mathrm{pC4bpC4BP}\right]+\left[\mathrm{pC4bC4bpC4BP}\right]+\left[\mathrm{pC4bC4dpC4BP}\right]+\left[\mathrm{pC4bC3bpC4BP}\right]\\
+\left[\mathrm{pC4biC3bpC4BP}\right]+\left[\mathrm{pC4bC3bpFH}\right]+\left[\mathrm{pC4bC3bpFHL1}\right]+\left[\mathrm{pC4dC3bpFH}\right]+\left[\mathrm{pC4dC3bpFHL1}\right]\\
+\left[\mathrm{pC3bpFH}\right]+\left[\mathrm{pC3bpFHL1}\right]+\left[\mathrm{pC3bC3bpFH}\right]+\left[\mathrm{pC3bC3bpFHL1}\right]+\left[\mathrm{pC3biC3bpFH}\right]+\left[\mathrm{pC3biC3bpFHL1}\right]\end{array}\right)}$$

217

$$\frac{d[\mathrm{piC3bC4b}]}{dt}=$$

$$\frac{k_{\mathrm{cat\ C3bH}}^{\mathrm{FI}}[\mathrm{FI}][\mathrm{pC3bC4bFH}]}{\left(\begin{array}{l} K_{\mathrm{m\ C3bH}}^{\mathrm{FI}}+[\mathrm{C3(H_2O)FH}]+[\mathrm{C3(H_2O)FHL1}]+[\mathrm{fC3bFH}]+[\mathrm{fC3bFHL1}]+[\mathrm{fC3bCR1}]+[\mathrm{fiC3bCR1}] \\ +[\mathrm{IgGC3bC3bFH}]+[\mathrm{IgGC3biC3bFH}]+[\mathrm{IgGC3bC3bFHL1}]+[\mathrm{IgGC3biC3bFHL1}]+[\mathrm{IgGC3bC3bCR1}] \\ +[\mathrm{IgGC3biC3bCR1}]+[\mathrm{IgGiC3biC3bCR1}]+[\mathrm{IgGiC3bC3dgCR1}]+[\mathrm{fC4bC3bFH}]+[\mathrm{fC4bC3bFHL1}] \\ +[\mathrm{fC4bC3bCR1}]+[\mathrm{fC4bC3bC4BP}]+[\mathrm{fC4dC3bFH}]+[\mathrm{fC4dC3bFHL1}]+[\mathrm{fC4dC3bCR1}]+[\mathrm{fC4diC3bCR1}] \\ +[\mathrm{fC4biC3bCR1}]+[\mathrm{fC4bC3dgCR1}]+[\mathrm{fC4bC3dgC4BP}]+[\mathrm{fC3bC3bFH}]+[\mathrm{fC3bC3bFHL1}]+[\mathrm{fC3bC3bCR1}] \\ +[\mathrm{fC3biC3bFH}]+[\mathrm{fC3biC3bFHL1}]+[\mathrm{fC3biC3bCR1}]+[\mathrm{fiC3biC3bCR1}]+[\mathrm{fiC3bC3dgCR1}] \\ +[\mathrm{fC4biC3bC4BP}]+[\mathrm{nfC4bC4BP}]+[\mathrm{fC4bC4BP}]+[\mathrm{fC4bCR1}]+[\mathrm{fC4bC4bC4BP}]+[\mathrm{fC4bC4dC4BP}] \\ +[\mathrm{fC4bC4bCR1}]+[\mathrm{fC4bC4dCR1}]+[\mathrm{hC4bCR1}]+[\mathrm{hC4bC4BP}]+[\mathrm{hC4bC4bCR1}]+[\mathrm{hC4bC4bC4BP}] \\ +[\mathrm{hC4bC4dCR1}]+[\mathrm{hC4bC4dC4BP}]+[\mathrm{hC4bC3bFH}]+[\mathrm{hC4bC3bFHL1}]+[\mathrm{hC4bC3bCR1}] \\ +[\mathrm{hC4bC3bC4BP}]+[\mathrm{hC4biC3bCR1}]+[\mathrm{hC4biC3bC4BP}]+[\mathrm{hC4bC3dgCR1}]+[\mathrm{hC4bC3dgC4BP}] \\ +[\mathrm{hC4dC3bFH}]+[\mathrm{hC4dC3bFHL1}]+[\mathrm{hC4dC3bCR1}]+[\mathrm{hC4diC3bCR1}]+[\mathrm{hC3bFH}]+[\mathrm{hC3bFHL1}] \\ +[\mathrm{hC3bCR1}]+[\mathrm{hiC3bCR1}]+[\mathrm{hC3bC3bFH}]+[\mathrm{hC3bC3bFHL1}]+[\mathrm{hC3bC3bCR1}]+[\mathrm{hC3biC3bFH}] \\ +[\mathrm{hC3biC3bFHL1}]+[\mathrm{hC3biC3bCR1}]+[\mathrm{hiC3biC3bCR1}]+[\mathrm{hiC3biC3dgCR1}]+[\mathrm{pC4bC4BP}] \\ +[\mathrm{pC4bC4bC4BP}]+[\mathrm{pC4bC4dC4BP}]+[\mathrm{pC4bC3bC4BP}]+[\mathrm{pC4biC3bC4BP}]+[\mathrm{pC4bC3bFH}] \\ +[\mathrm{pC4bC3bFHL1}]+[\mathrm{pC4dC3bFH}]+[\mathrm{pC4dC3bFHL1}]+[\mathrm{pC3bFH}]+[\mathrm{pC3bFHL1}]+[\mathrm{pC3bC3bFH}] \\ +[\mathrm{pC3bC3bFHL1}]+[\mathrm{pC3biC3bFH}]+[\mathrm{pC3biC3bFHL1}]+[\mathrm{pC4bC4BPPTX3}]+[\mathrm{pC4bC4bC4BPPTX3}] \\ +[\mathrm{pC4bC4dC4BPPTX3}]+[\mathrm{pC4bC3bFHPTX3}]+[\mathrm{pC4bC3bFHL1PTX3}]+[\mathrm{pC4bC3bC4BPPTX3}] \\ +[\mathrm{pC4dC3bFHPTX3}]+[\mathrm{pC4dC3bFHL1PTX3}]+[\mathrm{pC4biC3bC4BPPTX3}]+[\mathrm{pC3bFHPTX3}] \\ +[\mathrm{pC3bFHL1PTX3}]+[\mathrm{pC3bC3bFHPTX3}]+[\mathrm{pC3bC3bFHL1PTX3}]+[\mathrm{pC3biC3bFHPTX3}] \\ +[\mathrm{pC3biC3bFHL1PTX3}]+[\mathrm{pC4bpC4BP}]+[\mathrm{pC4bC4bpC4BP}]+[\mathrm{pC4bC4dpC4BP}]+[\mathrm{pC4bC3bpC4BP}] \\ +[\mathrm{pC4biC3bpC4BP}]+[\mathrm{pC4bC3bpFH}]+[\mathrm{pC4bC3bpFHL1}]+[\mathrm{pC4dC3bpFH}]+[\mathrm{pC4dC3bpFHL1}] \\ +[\mathrm{pC3bpFH}]+[\mathrm{pC3bpFHL1}]+[\mathrm{pC3bC3bpFH}]+[\mathrm{pC3bC3bpFHL1}]+[\mathrm{pC3biC3bpFH}]+[\mathrm{pC3biC3bpFHL1}] \end{array}\right)}$$

$$+\frac{k_{\text{cat C3bH}}^{\text{FI}}[\text{FI}][\text{pC3bC4bFHL1}]}{\left(\begin{array}{l}K_{\text{m C3bH}}^{\text{FI}}+[\text{C3(H}_2\text{O)FH}]+[\text{C3(H}_2\text{O)FHL1}]+[\text{fC3bFH}]+[\text{fC3bFHL1}]+[\text{fC3bCR1}]+[\text{fiC3bCR1}]\\
+[\text{IgGC3bC3bFH}]+[\text{IgGC3biC3bFH}]+[\text{IgGC3bC3bFHL1}]+[\text{IgGC3biC3bFHL1}]+[\text{IgGC3bC3bCR1}]\\
+[\text{IgGC3biC3bCR1}]+[\text{IgGiC3biC3bCR1}]+[\text{IgGiC3bC3dgCR1}]+[\text{fC4bC3bFH}]+[\text{fC4bC3bFHL1}]\\
+[\text{fC4bC3bCR1}]+[\text{fC4bC3bC4BP}]+[\text{fC4dC3bFH}]+[\text{fC4dC3bFHL1}]+[\text{fC4dC3bCR1}]+[\text{fC4diC3bCR1}]\\
+[\text{fC4biC3bCR1}]+[\text{fC4bC3dgCR1}]+[\text{fC4bC3dgC4BP}]+[\text{fC3bC3bFH}]+[\text{fC3bC3bFHL1}]+[\text{fC3bC3bCR1}]\\
+[\text{fC3biC3bFH}]+[\text{fC3biC3bFHL1}]+[\text{fC3biC3bCR1}]+[\text{fiC3biC3bCR1}]+[\text{fiC3bC3dgCR1}]\\
+[\text{fC4biC3bC4BP}]+[\text{nfC4bC4BP}]+[\text{fC4bC4BP}]+[\text{fC4bCR1}]+[\text{fC4bC4bC4BP}]+[\text{fC4bC4dC4BP}]\\
+[\text{fC4bC4bCR1}]+[\text{fC4bC4dCR1}]+[\text{hC4bCR1}]+[\text{hC4bC4BP}]+[\text{hC4bC4bCR1}]+[\text{hC4bC4bC4BP}]\\
+[\text{hC4bC4dCR1}]+[\text{hC4bC4dC4BP}]+[\text{hC4bC3bFH}]+[\text{hC4bC3bFHL1}]+[\text{hC4bC3bCR1}]\\
+[\text{hC4bC3bC4BP}]+[\text{hC4biC3bCR1}]+[\text{hC4biC3bC4BP}]+[\text{hC4bC3dgCR1}]+[\text{hC4bC3dgC4BP}]\\
+[\text{hC4dC3bFH}]+[\text{hC4dC3bFHL1}]+[\text{hC4dC3bCR1}]+[\text{hC4diC3bCR1}]+[\text{hC3bFH}]+[\text{hC3bFHL1}]\\
+[\text{hC3bCR1}]+[\text{hiC3bCR1}]+[\text{hC3bC3bFH}]+[\text{hC3bC3bFHL1}]+[\text{hC3bC3bCR1}]+[\text{hC3biC3bFH}]\\
+[\text{hC3biC3bFHL1}]+[\text{hC3biC3bCR1}]+[\text{hiC3biC3bCR1}]+[\text{hiC3biC3dgCR1}]+[\text{pC4bC4BP}]\\
+[\text{pC4bC4bC4BP}]+[\text{pC4bC4dC4BP}]+[\text{pC4bC3bC4BP}]+[\text{pC4biC3bC4BP}]+[\text{pC4bC3bFH}]\\
+[\text{pC4bC3bFHL1}]+[\text{pC4dC3bFH}]+[\text{pC4dC3bFHL1}]+[\text{pC3bFH}]+[\text{pC3bFHL1}]+[\text{pC3bC3bFH}]\\
+[\text{pC3bC3bFHL1}]+[\text{pC3biC3bFH}]+[\text{pC3biC3bFHL1}]+[\text{pC4bC4BPPTX3}]+[\text{pC4bC4bC4BPPTX3}]\\
+[\text{pC4bC4dC4BPPTX3}]+[\text{pC4bC3bFHPTX3}]+[\text{pC4bC3bFHL1PTX3}]+[\text{pC4bC3bC4BPPTX3}]\\
+[\text{pC4dC3bFHPTX3}]+[\text{pC4dC3bFHL1PTX3}]+[\text{pC4biC3bC4BPPTX3}]+[\text{pC3bFHPTX3}]\\
+[\text{pC3bFHL1PTX3}]+[\text{pC3bC3bFHPTX3}]+[\text{pC3bC3bFHL1PTX3}]+[\text{pC3biC3bFHPTX3}]\\
+[\text{pC3biC3bFHL1PTX3}]+[\text{pC4bpC4BP}]+[\text{pC4bC4bpC4BP}]+[\text{pC4bC4dpC4BP}]+[\text{pC4bC3bpC4BP}]\\
+[\text{pC4biC3bpC4BP}]+[\text{pC4bC3bpFH}]+[\text{pC4bC3bpFHL1}]+[\text{pC4dC3bpFH}]+[\text{pC4dC3bpFHL1}]\\
+[\text{pC3bpFH}]+[\text{pC3bpFHL1}]+[\text{pC3bC3bpFH}]+[\text{pC3bC3bpFHL1}]+[\text{pC3biC3bpFH}]+[\text{pC3biC3bpFHL1}]\end{array}\right)}$$

$$
-k_{\mathrm{C4bC4BP}}^{+}\left[\mathrm{piC3bC4b}\right]\left[\mathrm{C4BP}\right]+k_{\mathrm{C4bC4BP}}^{-}\left[\mathrm{piC3bC4bC4BP}\right]
$$

$$
+\frac{k_{\mathrm{cat\ C3bH}}^{\mathrm{FI}}\left[\mathrm{FI}\right]\left[\mathrm{pC3bC4bFHPTX3}\right]}{\left(\begin{array}{l}
K_{\mathrm{m\ C3bH}}^{\mathrm{FI}}+\left[\mathrm{C3(H_2O)FH}\right]+\left[\mathrm{C3(H_2O)FHL1}\right]+\left[\mathrm{fC3bFH}\right]+\left[\mathrm{fC3bFHL1}\right]+\left[\mathrm{fC3bCR1}\right]+\left[\mathrm{fiC3bCR1}\right]\\
+\left[\mathrm{IgGC3bC3bFH}\right]+\left[\mathrm{IgGC3biC3bFH}\right]+\left[\mathrm{IgGC3bC3bFHL1}\right]+\left[\mathrm{IgGC3biC3bFHL1}\right]+\left[\mathrm{IgGC3bC3bCR1}\right]\\
+\left[\mathrm{IgGC3biC3bCR1}\right]+\left[\mathrm{IgGiC3biC3bCR1}\right]+\left[\mathrm{IgGiC3bC3dgCR1}\right]+\left[\mathrm{fC4bC3bFH}\right]+\left[\mathrm{fC4bC3bFHL1}\right]\\
+\left[\mathrm{fC4bC3bCR1}\right]+\left[\mathrm{fC4bC3bC4BP}\right]+\left[\mathrm{fC4dC3bFH}\right]+\left[\mathrm{fC4dC3bFHL1}\right]+\left[\mathrm{fC4dC3bCR1}\right]+\left[\mathrm{fC4diC3bCR1}\right]\\
+\left[\mathrm{fC4biC3bCR1}\right]+\left[\mathrm{fC4bC3dgCR1}\right]+\left[\mathrm{fC4bC3dgC4BP}\right]+\left[\mathrm{fC3bC3bFH}\right]+\left[\mathrm{fC3bC3bFHL1}\right]+\left[\mathrm{fC3bC3bCR1}\right]\\
+\left[\mathrm{fC3biC3bFH}\right]+\left[\mathrm{fC3biC3bFHL1}\right]+\left[\mathrm{fC3biC3bCR1}\right]+\left[\mathrm{fiC3biC3bCR1}\right]+\left[\mathrm{fiC3bC3dgCR1}\right]\\
+\left[\mathrm{fC4biC3bC4BP}\right]+\left[\mathrm{nfC4bC4BP}\right]+\left[\mathrm{fC4bC4BP}\right]+\left[\mathrm{fC4bCR1}\right]+\left[\mathrm{fC4bC4bC4BP}\right]+\left[\mathrm{fC4bC4dC4BP}\right]\\
+\left[\mathrm{fC4bC4bCR1}\right]+\left[\mathrm{fC4bC4dCR1}\right]+\left[\mathrm{hC4bCR1}\right]+\left[\mathrm{hC4bC4BP}\right]+\left[\mathrm{hC4bC4bCR1}\right]+\left[\mathrm{hC4bC4bC4BP}\right]\\
+\left[\mathrm{hC4bC4dCR1}\right]+\left[\mathrm{hC4bC4dC4BP}\right]+\left[\mathrm{hC4bC3bFH}\right]+\left[\mathrm{hC4bC3bFHL1}\right]+\left[\mathrm{hC4bC3bCR1}\right]\\
+\left[\mathrm{hC4bC3bC4BP}\right]+\left[\mathrm{hC4biC3bCR1}\right]+\left[\mathrm{hC4biC3bC4BP}\right]+\left[\mathrm{hC4bC3dgCR1}\right]+\left[\mathrm{hC4bC3dgC4BP}\right]\\
+\left[\mathrm{hC4dC3bFH}\right]+\left[\mathrm{hC4dC3bFHL1}\right]+\left[\mathrm{hC4dC3bCR1}\right]+\left[\mathrm{hC4diC3bCR1}\right]+\left[\mathrm{hC3bFH}\right]+\left[\mathrm{hC3bFHL1}\right]\\
+\left[\mathrm{hC3bCR1}\right]+\left[\mathrm{hiC3bCR1}\right]+\left[\mathrm{hC3bC3bFH}\right]+\left[\mathrm{hC3bC3bFHL1}\right]+\left[\mathrm{hC3bC3bCR1}\right]+\left[\mathrm{hC3biC3bFH}\right]\\
+\left[\mathrm{hC3biC3bFHL1}\right]+\left[\mathrm{hC3biC3bCR1}\right]+\left[\mathrm{hiC3biC3bCR1}\right]+\left[\mathrm{hiC3biC3dgCR1}\right]+\left[\mathrm{pC4bC4BP}\right]\\
+\left[\mathrm{pC4bC4bC4BP}\right]+\left[\mathrm{pC4bC4dC4BP}\right]+\left[\mathrm{pC4bC3bC4BP}\right]+\left[\mathrm{pC4biC3bC4BP}\right]+\left[\mathrm{pC4bC3bFH}\right]\\
+\left[\mathrm{pC4bC3bFHL1}\right]+\left[\mathrm{pC4dC3bFH}\right]+\left[\mathrm{pC4dC3bFHL1}\right]+\left[\mathrm{pC3bFH}\right]+\left[\mathrm{pC3bFHL1}\right]+\left[\mathrm{pC3bC3bFH}\right]\\
+\left[\mathrm{pC3bC3bFHL1}\right]+\left[\mathrm{pC3biC3bFH}\right]+\left[\mathrm{pC3biC3bFHL1}\right]+\left[\mathrm{pC4bC4BPPTX3}\right]+\left[\mathrm{pC4bC4bC4BPPTX3}\right]\\
+\left[\mathrm{pC4bC4dC4BPPTX3}\right]+\left[\mathrm{pC4bC3bFHPTX3}\right]+\left[\mathrm{pC4bC3bFHL1PTX3}\right]+\left[\mathrm{pC4bC3bC4BPPTX3}\right]\\
+\left[\mathrm{pC4dC3bFHPTX3}\right]+\left[\mathrm{pC4dC3bFHL1PTX3}\right]+\left[\mathrm{pC4biC3bC4BPPTX3}\right]+\left[\mathrm{pC3bFHPTX3}\right]\\
+\left[\mathrm{pC3bFHL1PTX3}\right]+\left[\mathrm{pC3bC3bFHPTX3}\right]+\left[\mathrm{pC3bC3bFHL1PTX3}\right]+\left[\mathrm{pC3biC3bFHPTX3}\right]\\
+\left[\mathrm{pC3biC3bFHL1PTX3}\right]+\left[\mathrm{pC4bpC4BP}\right]+\left[\mathrm{pC4bC4bpC4BP}\right]+\left[\mathrm{pC4bC4dpC4BP}\right]+\left[\mathrm{pC4bC3bpC4BP}\right]\\
+\left[\mathrm{pC4biC3bpC4BP}\right]+\left[\mathrm{pC4bC3bpFH}\right]+\left[\mathrm{pC4bC3bpFHL1}\right]+\left[\mathrm{pC4dC3bpFH}\right]+\left[\mathrm{pC4dC3bpFHL1}\right]\\
+\left[\mathrm{pC3bpFH}\right]+\left[\mathrm{pC3bpFHL1}\right]+\left[\mathrm{pC3bC3bpFH}\right]+\left[\mathrm{pC3bC3bpFHL1}\right]+\left[\mathrm{pC3biC3bpFH}\right]+\left[\mathrm{pC3biC3bpFHL1}\right]
\end{array}\right)}
$$

$$+\frac{k_{\mathrm{cat\ C3bH}}^{\mathrm{FI}}[\mathrm{FI}][\mathrm{pC3bC4bFHL1PTX3}]}{\left(\begin{array}{l}K_{\mathrm{m\ C3bH}}^{\mathrm{FI}}+[\mathrm{C3(H_2O)FH}]+[\mathrm{C3(H_2O)FHL1}]+[\mathrm{fC3bFH}]+[\mathrm{fC3bFHL1}]+[\mathrm{fC3bCR1}]+[\mathrm{fiC3bCR1}]\\
+[\mathrm{IgGC3bC3bFH}]+[\mathrm{IgGC3biC3bFH}]+[\mathrm{IgGC3bC3bFHL1}]+[\mathrm{IgGC3biC3bFHL1}]+[\mathrm{IgGC3bC3bCR1}]\\
+[\mathrm{IgGC3biC3bCR1}]+[\mathrm{IgGiC3biC3bCR1}]+[\mathrm{IgGiC3bC3dgCR1}]+[\mathrm{fC4bC3bFH}]+[\mathrm{fC4bC3bFHL1}]\\
+[\mathrm{fC4bC3bCR1}]+[\mathrm{fC4bC3bC4BP}]+[\mathrm{fC4dC3bFH}]+[\mathrm{fC4dC3bFHL1}]+[\mathrm{fC4dC3bCR1}]+[\mathrm{fC4diC3bCR1}]\\
+[\mathrm{fC4biC3bCR1}]+[\mathrm{fC4bC3dgCR1}]+[\mathrm{fC4bC3dgC4BP}]+[\mathrm{fC3bC3bFH}]+[\mathrm{fC3bC3bFHL1}]+[\mathrm{fC3bC3bCR1}]\\
+[\mathrm{fC3biC3bFH}]+[\mathrm{fC3biC3bFHL1}]+[\mathrm{fC3biC3bCR1}]+[\mathrm{fiC3biC3bCR1}]+[\mathrm{fiC3bC3dgCR1}]\\
+[\mathrm{fC4biC3bC4BP}]+[\mathrm{nfC4bC4BP}]+[\mathrm{fC4bC4BP}]+[\mathrm{fC4bCR1}]+[\mathrm{fC4bC4bC4BP}]+[\mathrm{fC4bC4dC4BP}]\\
+[\mathrm{fC4bC4bCR1}]+[\mathrm{fC4bC4dCR1}]+[\mathrm{hC4bCR1}]+[\mathrm{hC4bC4BP}]+[\mathrm{hC4bC4bCR1}]+[\mathrm{hC4bC4bC4BP}]\\
+[\mathrm{hC4bC4dCR1}]+[\mathrm{hC4bC4dC4BP}]+[\mathrm{hC4bC3bFH}]+[\mathrm{hC4bC3bFHL1}]+[\mathrm{hC4bC3bCR1}]\\
+[\mathrm{hC4bC3bC4BP}]+[\mathrm{hC4biC3bCR1}]+[\mathrm{hC4biC3bC4BP}]+[\mathrm{hC4bC3dgCR1}]+[\mathrm{hC4bC3dgC4BP}]\\
+[\mathrm{hC4dC3bFH}]+[\mathrm{hC4dC3bFHL1}]+[\mathrm{hC4dC3bCR1}]+[\mathrm{hC4diC3bCR1}]+[\mathrm{hC3bFH}]+[\mathrm{hC3bFHL1}]\\
+[\mathrm{hC3bCR1}]+[\mathrm{hiC3bCR1}]+[\mathrm{hC3bC3bFH}]+[\mathrm{hC3bC3bFHL1}]+[\mathrm{hC3bC3bCR1}]+[\mathrm{hC3biC3bFH}]\\
+[\mathrm{hC3biC3bFHL1}]+[\mathrm{hC3biC3bCR1}]+[\mathrm{hiC3biC3bCR1}]+[\mathrm{hiC3biC3dgCR1}]+[\mathrm{pC4bC4BP}]\\
+[\mathrm{pC4bC4bC4BP}]+[\mathrm{pC4bC4dC4BP}]+[\mathrm{pC4bC3bC4BP}]+[\mathrm{pC4biC3bC4BP}]+[\mathrm{pC4bC3bFH}]\\
+[\mathrm{pC4bC3bFHL1}]+[\mathrm{pC4dC3bFH}]+[\mathrm{pC4dC3bFHL1}]+[\mathrm{pC3bFH}]+[\mathrm{pC3bFHL1}]+[\mathrm{pC3bC3bFH}]\\
+[\mathrm{pC3bC3bFHL1}]+[\mathrm{pC3biC3bFH}]+[\mathrm{pC3biC3bFHL1}]+[\mathrm{pC4bC4BPPTX3}]+[\mathrm{pC4bC4bC4BPPTX3}]\\
+[\mathrm{pC4bC4dC4BPPTX3}]+[\mathrm{pC4bC3bFHPTX3}]+[\mathrm{pC4bC3bFHL1PTX3}]+[\mathrm{pC4bC3bC4BPPTX3}]\\
+[\mathrm{pC4dC3bFHPTX3}]+[\mathrm{pC4dC3bFHL1PTX3}]+[\mathrm{pC4biC3bC4BPPTX3}]+[\mathrm{pC3bFHPTX3}]\\
+[\mathrm{pC3bFHL1PTX3}]+[\mathrm{pC3bC3bFHPTX3}]+[\mathrm{pC3bC3bFHL1PTX3}]+[\mathrm{pC3biC3bFHPTX3}]\\
+[\mathrm{pC3biC3bFHL1PTX3}]+[\mathrm{pC4bpC4BP}]+[\mathrm{pC4bC4bpC4BP}]+[\mathrm{pC4bC4dpC4BP}]+[\mathrm{pC4bC3bpC4BP}]\\
+[\mathrm{pC4biC3bpC4BP}]+[\mathrm{pC4bC3bpFH}]+[\mathrm{pC4bC3bpFHL1}]+[\mathrm{pC4dC3bpFH}]+[\mathrm{pC4dC3bpFHL1}]\\
+[\mathrm{pC3bpFH}]+[\mathrm{pC3bpFHL1}]+[\mathrm{pC3bC3bpFH}]+[\mathrm{pC3bC3bpFHL1}]+[\mathrm{pC3biC3bpFH}]+[\mathrm{pC3biC3bpFHL1}]\end{array}\right)}$$

$$-k_{\mathrm{C4bC4BP}}^{+}[\mathrm{piC3bC4b}][\mathrm{pC4BPPTX3}]+k_{\mathrm{C4bC4BP}}^{-}[\mathrm{piC3bC4bC4BPPTX3}]-k_{\mathrm{C4bC4BP}}^{+}[\mathrm{piC3bC4b}][\mathrm{pC4BP}]$$
$$+k_{\mathrm{C4bC4BP}}^{-}[\mathrm{piC3bC4bpC4BP}]$$

$$
+\frac{k_{\text{cat C3bH}}^{\text{FI}}\left[\text{FI}\right]\left[\text{pC3bC4bpFH}\right]}{\left(\begin{array}{l}
K_{\text{m C3bH}}^{\text{FI}}+\left[\text{C3(H}_2\text{O)FH}\right]+\left[\text{C3(H}_2\text{O)FHL1}\right]+\left[\text{fC3bFH}\right]+\left[\text{fC3bFHL1}\right]+\left[\text{fC3bCR1}\right]+\left[\text{fiC3bCR1}\right]\\
+\left[\text{IgGC3bC3bFH}\right]+\left[\text{IgGC3biC3bFH}\right]+\left[\text{IgGC3bC3bFHL1}\right]+\left[\text{IgGC3biC3bFHL1}\right]+\left[\text{IgGC3bC3bCR1}\right]\\
+\left[\text{IgGC3biC3bCR1}\right]+\left[\text{IgGiC3biC3bCR1}\right]+\left[\text{IgGiC3bC3dgCR1}\right]+\left[\text{fC4bC3bFH}\right]+\left[\text{fC4bC3bFHL1}\right]\\
+\left[\text{fC4bC3bCR1}\right]+\left[\text{fC4bC3bC4BP}\right]+\left[\text{fC4dC3bFH}\right]+\left[\text{fC4dC3bFHL1}\right]+\left[\text{fC4dC3bCR1}\right]+\left[\text{fC4diC3bCR1}\right]\\
+\left[\text{fC4biC3bCR1}\right]+\left[\text{fC4bC3dgCR1}\right]+\left[\text{fC4bC3dgC4BP}\right]+\left[\text{fC3bC3bFH}\right]+\left[\text{fC3bC3bFHL1}\right]+\left[\text{fC3bC3bCR1}\right]\\
+\left[\text{fC3biC3bFH}\right]+\left[\text{fC3biC3bFHL1}\right]+\left[\text{fC3biC3bCR1}\right]+\left[\text{fiC3biC3bCR1}\right]+\left[\text{fiC3bC3dgCR1}\right]\\
+\left[\text{fC4biC3bC4BP}\right]+\left[\text{nfC4bC4BP}\right]+\left[\text{fC4bC4BP}\right]+\left[\text{fC4bCR1}\right]+\left[\text{fC4bC4bC4BP}\right]+\left[\text{fC4bC4dC4BP}\right]\\
+\left[\text{fC4bC4bCR1}\right]+\left[\text{fC4bC4dCR1}\right]+\left[\text{hC4bCR1}\right]+\left[\text{hC4bC4BP}\right]+\left[\text{hC4bC4bCR1}\right]+\left[\text{hC4bC4bC4BP}\right]\\
+\left[\text{hC4bC4dCR1}\right]+\left[\text{hC4bC4dC4BP}\right]+\left[\text{hC4bC3bFH}\right]+\left[\text{hC4bC3bFHL1}\right]+\left[\text{hC4bC3bCR1}\right]\\
+\left[\text{hC4bC3bC4BP}\right]+\left[\text{hC4biC3bCR1}\right]+\left[\text{hC4biC3bC4BP}\right]+\left[\text{hC4bC3dgCR1}\right]+\left[\text{hC4bC3dgC4BP}\right]\\
+\left[\text{hC4dC3bFH}\right]+\left[\text{hC4dC3bFHL1}\right]+\left[\text{hC4dC3bCR1}\right]+\left[\text{hC4diC3bCR1}\right]+\left[\text{hC3bFH}\right]+\left[\text{hC3bFHL1}\right]\\
+\left[\text{hC3bCR1}\right]+\left[\text{hiC3bCR1}\right]+\left[\text{hC3bC3bFH}\right]+\left[\text{hC3bC3bFHL1}\right]+\left[\text{hC3bC3bCR1}\right]+\left[\text{hC3biC3bFH}\right]\\
+\left[\text{hC3biC3bFHL1}\right]+\left[\text{hC3biC3bCR1}\right]+\left[\text{hiC3biC3bCR1}\right]+\left[\text{hiC3biC3dgCR1}\right]+\left[\text{pC4bC4BP}\right]\\
+\left[\text{pC4bC4bC4BP}\right]+\left[\text{pC4bC4dC4BP}\right]+\left[\text{pC4bC3bC4BP}\right]+\left[\text{pC4biC3bC4BP}\right]+\left[\text{pC4bC3bFH}\right]\\
+\left[\text{pC4bC3bFHL1}\right]+\left[\text{pC4dC3bFH}\right]+\left[\text{pC4dC3bFHL1}\right]+\left[\text{pC3bFH}\right]+\left[\text{pC3bFHL1}\right]+\left[\text{pC3bC3bFH}\right]\\
+\left[\text{pC3bC3bFHL1}\right]+\left[\text{pC3biC3bFH}\right]+\left[\text{pC3biC3bFHL1}\right]+\left[\text{pC4bC4BPPTX3}\right]+\left[\text{pC4bC4bC4BPPTX3}\right]\\
+\left[\text{pC4bC4dC4BPPTX3}\right]+\left[\text{pC4bC3bFHPTX3}\right]+\left[\text{pC4bC3bFHL1PTX3}\right]+\left[\text{pC4bC3bC4BPPTX3}\right]\\
+\left[\text{pC4dC3bFHPTX3}\right]+\left[\text{pC4dC3bFHL1PTX3}\right]+\left[\text{pC4biC3bC4BPPTX3}\right]+\left[\text{pC3bFHPTX3}\right]\\
+\left[\text{pC3bFHL1PTX3}\right]+\left[\text{pC3bC3bFHPTX3}\right]+\left[\text{pC3bC3bFHL1PTX3}\right]+\left[\text{pC3biC3bFHPTX3}\right]\\
+\left[\text{pC3biC3bFHL1PTX3}\right]+\left[\text{pC4bpC4BP}\right]+\left[\text{pC4bC4bpC4BP}\right]+\left[\text{pC4bC4dpC4BP}\right]+\left[\text{pC4bC3bpC4BP}\right]\\
+\left[\text{pC4biC3bpC4BP}\right]+\left[\text{pC4bC3bpFH}\right]+\left[\text{pC4bC3bpFHL1}\right]+\left[\text{pC4dC3bpFH}\right]+\left[\text{pC4dC3bpFHL1}\right]\\
+\left[\text{pC3bpFH}\right]+\left[\text{pC3bpFHL1}\right]+\left[\text{pC3bC3bpFH}\right]+\left[\text{pC3bC3bpFHL1}\right]+\left[\text{pC3biC3bpFH}\right]+\left[\text{pC3biC3bpFHL1}\right]
\end{array}\right)}
$$

$$+\frac{k_{\mathrm{cat\ C3bH}}^{\mathrm{FI}}[\mathrm{FI}][\mathrm{pC3bC4bpFHL1}]}{\left(\begin{array}{l} K_{\mathrm{m\ C3bH}}^{\mathrm{FI}}+[\mathrm{C3(H_2O)FH}]+[\mathrm{C3(H_2O)FHL1}]+[\mathrm{fC3bFH}]+[\mathrm{fC3bFHL1}]+[\mathrm{fC3bCR1}]+[\mathrm{fiC3bCR1}] \\ +[\mathrm{IgGC3bC3bFH}]+[\mathrm{IgGC3biC3bFH}]+[\mathrm{IgGC3bC3bFHL1}]+[\mathrm{IgGC3biC3bFHL1}]+[\mathrm{IgGC3bC3bCR1}] \\ +[\mathrm{IgGC3biC3bCR1}]+[\mathrm{IgGiC3biC3bCR1}]+[\mathrm{IgGiC3bC3dgCR1}]+[\mathrm{fC4bC3bFH}]+[\mathrm{fC4bC3bFHL1}] \\ +[\mathrm{fC4bC3bCR1}]+[\mathrm{fC4bC3bC4BP}]+[\mathrm{fC4dC3bFH}]+[\mathrm{fC4dC3bFHL1}]+[\mathrm{fC4dC3bCR1}]+[\mathrm{fC4diC3bCR1}] \\ +[\mathrm{fC4biC3bCR1}]+[\mathrm{fC4bC3dgCR1}]+[\mathrm{fC4bC3dgC4BP}]+[\mathrm{fC3bC3bFH}]+[\mathrm{fC3bC3bFHL1}]+[\mathrm{fC3bC3bCR1}] \\ +[\mathrm{fC3biC3bFH}]+[\mathrm{fC3biC3bFHL1}]+[\mathrm{fC3biC3bCR1}]+[\mathrm{fiC3biC3bCR1}]+[\mathrm{fiC3bC3dgCR1}] \\ +[\mathrm{fC4biC3bC4BP}]+[\mathrm{nfC4bC4BP}]+[\mathrm{fC4bC4BP}]+[\mathrm{fC4bCR1}]+[\mathrm{fC4bC4bC4BP}]+[\mathrm{fC4bC4dC4BP}] \\ +[\mathrm{fC4bC4bCR1}]+[\mathrm{fC4bC4dCR1}]+[\mathrm{hC4bCR1}]+[\mathrm{hC4bC4BP}]+[\mathrm{hC4bC4bCR1}]+[\mathrm{hC4bC4bC4BP}] \\ +[\mathrm{hC4bC4dCR1}]+[\mathrm{hC4bC4dC4BP}]+[\mathrm{hC4bC3bFH}]+[\mathrm{hC4bC3bFHL1}]+[\mathrm{hC4bC3bCR1}] \\ +[\mathrm{hC4bC3bC4BP}]+[\mathrm{hC4biC3bCR1}]+[\mathrm{hC4biC3bC4BP}]+[\mathrm{hC4bC3dgCR1}]+[\mathrm{hC4bC3dgC4BP}] \\ +[\mathrm{hC4dC3bFH}]+[\mathrm{hC4dC3bFHL1}]+[\mathrm{hC4dC3bCR1}]+[\mathrm{hC4diC3bCR1}]+[\mathrm{hC3bFH}]+[\mathrm{hC3bFHL1}] \\ +[\mathrm{hC3bCR1}]+[\mathrm{hiC3bCR1}]+[\mathrm{hC3bC3bFH}]+[\mathrm{hC3bC3bFHL1}]+[\mathrm{hC3bC3bCR1}]+[\mathrm{hC3biC3bFH}] \\ +[\mathrm{hC3biC3bFHL1}]+[\mathrm{hC3biC3bCR1}]+[\mathrm{hiC3biC3bCR1}]+[\mathrm{hiC3biC3dgCR1}]+[\mathrm{pC4bC4BP}] \\ +[\mathrm{pC4bC4bC4BP}]+[\mathrm{pC4bC4dC4BP}]+[\mathrm{pC4bC3bC4BP}]+[\mathrm{pC4biC3bC4BP}]+[\mathrm{pC4bC3bFH}] \\ +[\mathrm{pC4bC3bFHL1}]+[\mathrm{pC4dC3bFH}]+[\mathrm{pC4dC3bFHL1}]+[\mathrm{pC3bFH}]+[\mathrm{pC3bFHL1}]+[\mathrm{pC3bC3bFH}] \\ +[\mathrm{pC3bC3bFHL1}]+[\mathrm{pC3biC3bFH}]+[\mathrm{pC3biC3bFHL1}]+[\mathrm{pC4bC4BPPTX3}]+[\mathrm{pC4bC4bC4BPPTX3}] \\ +[\mathrm{pC4bC4dC4BPPTX3}]+[\mathrm{pC4bC3bFHPTX3}]+[\mathrm{pC4bC3bFHL1PTX3}]+[\mathrm{pC4bC3bC4BPPTX3}] \\ +[\mathrm{pC4dC3bFHPTX3}]+[\mathrm{pC4dC3bFHL1PTX3}]+[\mathrm{pC4biC3bC4BPPTX3}]+[\mathrm{pC3bFHPTX3}] \\ +[\mathrm{pC3bFHL1PTX3}]+[\mathrm{pC3bC3bFHPTX3}]+[\mathrm{pC3bC3bFHL1PTX3}]+[\mathrm{pC3biC3bFHPTX3}] \\ +[\mathrm{pC3biC3bFHL1PTX3}]+[\mathrm{pC4bpC4BP}]+[\mathrm{pC4bC4bpC4BP}]+[\mathrm{pC4bC4dpC4BP}]+[\mathrm{pC4bC3bpC4BP}] \\ +[\mathrm{pC4biC3bpC4BP}]+[\mathrm{pC4bC3bpFH}]+[\mathrm{pC4bC3bpFHL1}]+[\mathrm{pC4dC3bpFH}]+[\mathrm{pC4dC3bpFHL1}] \\ +[\mathrm{pC3bpFH}]+[\mathrm{pC3bpFHL1}]+[\mathrm{pC3bC3bpFH}]+[\mathrm{pC3bC3bpFHL1}]+[\mathrm{pC3biC3bpFH}]+[\mathrm{pC3biC3bpFHL1}] \end{array}\right)}$$

218

$$\frac{d[\mathrm{pC3bC4d}]}{dt}=$$

$$\frac{k_{\mathrm{cat\ C3bH}}^{\mathrm{FI}}[\mathrm{FI}][\mathrm{pC3bC4bC4BP}]}{\left(\begin{array}{l}K_{\mathrm{m\ C3bH}}^{\mathrm{FI}}+[\mathrm{C3(H_2O)FH}]+[\mathrm{C3(H_2O)FHL1}]+[\mathrm{fC3bFH}]+[\mathrm{fC3bFHL1}]+[\mathrm{fC3bCR1}]+[\mathrm{fiC3bCR1}]\\+[\mathrm{IgGC3bC3bFH}]+[\mathrm{IgGC3biC3bFH}]+[\mathrm{IgGC3bC3bFHL1}]+[\mathrm{IgGC3biC3bFHL1}]+[\mathrm{IgGC3bC3bCR1}]\\+[\mathrm{IgGC3biC3bCR1}]+[\mathrm{IgGiC3biC3bCR1}]+[\mathrm{IgGiC3bC3dgCR1}]+[\mathrm{fC4bC3bFH}]+[\mathrm{fC4bC3bFHL1}]\\+[\mathrm{fC4bC3bCR1}]+[\mathrm{fC4bC3bC4BP}]+[\mathrm{fC4dC3bFH}]+[\mathrm{fC4dC3bFHL1}]+[\mathrm{fC4dC3bCR1}]+[\mathrm{fC4diC3bCR1}]\\+[\mathrm{fC4biC3bCR1}]+[\mathrm{fC4bC3dgCR1}]+[\mathrm{fC4bC3dgC4BP}]+[\mathrm{fC3bC3bFH}]+[\mathrm{fC3bC3bFHL1}]+[\mathrm{fC3bC3bCR1}]\\+[\mathrm{fC3biC3bFH}]+[\mathrm{fC3biC3bFHL1}]+[\mathrm{fC3biC3bCR1}]+[\mathrm{fiC3biC3bCR1}]+[\mathrm{fiC3bC3dgCR1}]\\+[\mathrm{fC4biC3bC4BP}]+[\mathrm{nfC4bC4BP}]+[\mathrm{fC4bC4BP}]+[\mathrm{fC4bCR1}]+[\mathrm{fC4bC4bC4BP}]+[\mathrm{fC4bC4dC4BP}]\\+[\mathrm{fC4bC4bCR1}]+[\mathrm{fC4bC4dCR1}]+[\mathrm{hC4bCR1}]+[\mathrm{hC4bC4BP}]+[\mathrm{hC4bC4bCR1}]+[\mathrm{hC4bC4bC4BP}]\\+[\mathrm{hC4bC4dCR1}]+[\mathrm{hC4bC4dC4BP}]+[\mathrm{hC4bC3bFH}]+[\mathrm{hC4bC3bFHL1}]+[\mathrm{hC4bC3bCR1}]\\+[\mathrm{hC4bC3bC4BP}]+[\mathrm{hC4biC3bCR1}]+[\mathrm{hC4biC3bC4BP}]+[\mathrm{hC4bC3dgCR1}]+[\mathrm{hC4bC3dgC4BP}]\\+[\mathrm{hC4dC3bFH}]+[\mathrm{hC4dC3bFHL1}]+[\mathrm{hC4dC3bCR1}]+[\mathrm{hC4diC3bCR1}]+[\mathrm{hC3bFH}]+[\mathrm{hC3bFHL1}]\\+[\mathrm{hC3bCR1}]+[\mathrm{hiC3bCR1}]+[\mathrm{hC3bC3bFH}]+[\mathrm{hC3bC3bFHL1}]+[\mathrm{hC3bC3bCR1}]+[\mathrm{hC3biC3bFH}]\\+[\mathrm{hC3biC3bFHL1}]+[\mathrm{hC3biC3bCR1}]+[\mathrm{hiC3biC3bCR1}]+[\mathrm{hiC3biC3dgCR1}]+[\mathrm{pC4bC4BP}]\\+[\mathrm{pC4bC4bC4BP}]+[\mathrm{pC4bC4dC4BP}]+[\mathrm{pC4bC3bC4BP}]+[\mathrm{pC4biC3bC4BP}]+[\mathrm{pC4bC3bFH}]\\+[\mathrm{pC4bC3bFHL1}]+[\mathrm{pC4dC3bFH}]+[\mathrm{pC4dC3bFHL1}]+[\mathrm{pC3bFH}]+[\mathrm{pC3bFHL1}]+[\mathrm{pC3bC3bFH}]\\+[\mathrm{pC3bC3bFHL1}]+[\mathrm{pC3biC3bFH}]+[\mathrm{pC3biC3bFHL1}]+[\mathrm{pC4bC4BPPTX3}]+[\mathrm{pC4bC4bC4BPPTX3}]\\+[\mathrm{pC4bC4dC4BPPTX3}]+[\mathrm{pC4bC3bFHPTX3}]+[\mathrm{pC4bC3bFHL1PTX3}]+[\mathrm{pC4bC3bC4BPPTX3}]\\+[\mathrm{pC4dC3bFHPTX3}]+[\mathrm{pC4dC3bFHL1PTX3}]+[\mathrm{pC4biC3bC4BPPTX3}]+[\mathrm{pC3bFHPTX3}]\\+[\mathrm{pC3bFHL1PTX3}]+[\mathrm{pC3bC3bFHPTX3}]+[\mathrm{pC3bC3bFHL1PTX3}]+[\mathrm{pC3biC3bFHPTX3}]\\+[\mathrm{pC3biC3bFHL1PTX3}]+[\mathrm{pC4bpC4BP}]+[\mathrm{pC4bC4bpC4BP}]+[\mathrm{pC4bC4dpC4BP}]+[\mathrm{pC4bC3bpC4BP}]\\+[\mathrm{pC4biC3bpC4BP}]+[\mathrm{pC4bC3bpFH}]+[\mathrm{pC4bC3bpFHL1}]+[\mathrm{pC4dC3bpFH}]+[\mathrm{pC4dC3bpFHL1}]\\+[\mathrm{pC3bpFH}]+[\mathrm{pC3bpFHL1}]+[\mathrm{pC3bC3bpFH}]+[\mathrm{pC3bC3bpFHL1}]+[\mathrm{pC3biC3bpFH}]+[\mathrm{pC3biC3bpFHL1}]\end{array}\right)}$$

$$-k_{\mathrm{C3bH}}^{+}[\mathrm{pC3bC4d}][\mathrm{FH}]+k_{\mathrm{C3bH}}^{-}[\mathrm{pC3bC4dFH}]-k_{\mathrm{C3bFHL1}}^{+}[\mathrm{pC3bC4d}][\mathrm{FHL1}]+k_{\mathrm{C3bFHL1}}^{-}[\mathrm{pC3bC4dFHL1}]$$

$$
+\frac{k_{\mathrm{cat\,C3bH}}^{\mathrm{FI}}[\mathrm{FI}][\mathrm{pC3bC4bC4BPPTX3}]}{\left(\begin{array}{l}
K_{\mathrm{m\,C3bH}}^{\mathrm{FI}}+[\mathrm{C3(H_2O)FH}]+[\mathrm{C3(H_2O)FHL1}]+[\mathrm{fC3bFH}]+[\mathrm{fC3bFHL1}]+[\mathrm{fC3bCR1}]+[\mathrm{fiC3bCR1}]\\
+[\mathrm{IgGC3bC3bFH}]+[\mathrm{IgGC3biC3bFH}]+[\mathrm{IgGC3bC3bFHL1}]+[\mathrm{IgGC3biC3bFHL1}]+[\mathrm{IgGC3bC3bCR1}]\\
+[\mathrm{IgGC3biC3bCR1}]+[\mathrm{IgGiC3biC3bCR1}]+[\mathrm{IgGiC3bC3dgCR1}]+[\mathrm{fC4bC3bFH}]+[\mathrm{fC4bC3bFHL1}]\\
+[\mathrm{fC4bC3bCR1}]+[\mathrm{fC4bC3bC4BP}]+[\mathrm{fC4dC3bFH}]+[\mathrm{fC4dC3bFHL1}]+[\mathrm{fC4dC3bCR1}]+[\mathrm{fC4diC3bCR1}]\\
+[\mathrm{fC4biC3bCR1}]+[\mathrm{fC4bC3dgCR1}]+[\mathrm{fC4bC3dgC4BP}]+[\mathrm{fC3bC3bFH}]+[\mathrm{fC3bC3bFHL1}]+[\mathrm{fC3bC3bCR1}]\\
+[\mathrm{fC3biC3bFH}]+[\mathrm{fC3biC3bFHL1}]+[\mathrm{fC3biC3bCR1}]+[\mathrm{fiC3biC3bCR1}]+[\mathrm{fiC3bC3dgCR1}]\\
+[\mathrm{fC4biC3bC4BP}]+[\mathrm{nfC4bC4BP}]+[\mathrm{fC4bC4BP}]+[\mathrm{fC4bCR1}]+[\mathrm{fC4bC4bC4BP}]+[\mathrm{fC4bC4dC4BP}]\\
+[\mathrm{fC4bC4bCR1}]+[\mathrm{fC4bC4dCR1}]+[\mathrm{hC4bCR1}]+[\mathrm{hC4bC4BP}]+[\mathrm{hC4bC4bCR1}]+[\mathrm{hC4bC4bC4BP}]\\
+[\mathrm{hC4bC4dCR1}]+[\mathrm{hC4bC4dC4BP}]+[\mathrm{hC4bC3bFH}]+[\mathrm{hC4bC3bFHL1}]+[\mathrm{hC4bC3bCR1}]\\
+[\mathrm{hC4bC3bC4BP}]+[\mathrm{hC4biC3bCR1}]+[\mathrm{hC4biC3bC4BP}]+[\mathrm{hC4bC3dgCR1}]+[\mathrm{hC4bC3dgC4BP}]\\
+[\mathrm{hC4dC3bFH}]+[\mathrm{hC4dC3bFHL1}]+[\mathrm{hC4dC3bCR1}]+[\mathrm{hC4diC3bCR1}]+[\mathrm{hC3bFH}]+[\mathrm{hC3bFHL1}]\\
+[\mathrm{hC3bCR1}]+[\mathrm{hiC3bCR1}]+[\mathrm{hC3bC3bFH}]+[\mathrm{hC3bC3bFHL1}]+[\mathrm{hC3bC3bCR1}]+[\mathrm{hC3biC3bFH}]\\
+[\mathrm{hC3biC3bFHL1}]+[\mathrm{hC3biC3bCR1}]+[\mathrm{hiC3biC3bCR1}]+[\mathrm{hiC3biC3dgCR1}]+[\mathrm{pC4bC4BP}]\\
+[\mathrm{pC4bC4bC4BP}]+[\mathrm{pC4bC4dC4BP}]+[\mathrm{pC4bC3bC4BP}]+[\mathrm{pC4biC3bC4BP}]+[\mathrm{pC4bC3bFH}]\\
+[\mathrm{pC4bC3bFHL1}]+[\mathrm{pC4dC3bFH}]+[\mathrm{pC4dC3bFHL1}]+[\mathrm{pC3bFH}]+[\mathrm{pC3bFHL1}]+[\mathrm{pC3bC3bFH}]\\
+[\mathrm{pC3bC3bFHL1}]+[\mathrm{pC3biC3bFH}]+[\mathrm{pC3biC3bFHL1}]+[\mathrm{pC4bC4BPPTX3}]+[\mathrm{pC4bC4bC4BPPTX3}]\\
+[\mathrm{pC4bC4dC4BPPTX3}]+[\mathrm{pC4bC3bFHPTX3}]+[\mathrm{pC4bC3bFHL1PTX3}]+[\mathrm{pC4bC3bC4BPPTX3}]\\
+[\mathrm{pC4dC3bFHPTX3}]+[\mathrm{pC4dC3bFHL1PTX3}]+[\mathrm{pC4biC3bC4BPPTX3}]+[\mathrm{pC3bFHPTX3}]\\
+[\mathrm{pC3bFHL1PTX3}]+[\mathrm{pC3bC3bFHPTX3}]+[\mathrm{pC3bC3bFHL1PTX3}]+[\mathrm{pC3biC3bFHPTX3}]\\
+[\mathrm{pC3biC3bFHL1PTX3}]+[\mathrm{pC4bpC4BP}]+[\mathrm{pC4bC4bpC4BP}]+[\mathrm{pC4bC4dpC4BP}]+[\mathrm{pC4bC3bpC4BP}]\\
+[\mathrm{pC4biC3bpC4BP}]+[\mathrm{pC4bC3bpFH}]+[\mathrm{pC4bC3bpFHL1}]+[\mathrm{pC4dC3bpFH}]+[\mathrm{pC4dC3bpFHL1}]\\
+[\mathrm{pC3bpFH}]+[\mathrm{pC3bpFHL1}]+[\mathrm{pC3bC3bpFH}]+[\mathrm{pC3bC3bpFHL1}]+[\mathrm{pC3biC3bpFH}]+[\mathrm{pC3biC3bpFHL1}]
\end{array}\right)}
$$

$$
-k_{\mathrm{C3bH}}^{+}[\mathrm{pC3bC4d}][\mathrm{pFHPTX3}]+k_{\mathrm{C3bH}}^{-}[\mathrm{pC3bC4dFHPTX3}]-k_{\mathrm{C3bFHL1}}^{+}[\mathrm{pC3bC4d}][\mathrm{pFHL1PTX3}]
$$

$$
+k_{\mathrm{C3bFHL1}}^{-}[\mathrm{pC3bC4dFHL1PTX3}]
$$

$$
\begin{aligned}
&+\frac{k^{\mathrm{FI}}_{\mathrm{cat\ C3bH}}[\mathrm{FI}][\mathrm{pC3bC4bpC4BP}]}{\left(\begin{array}{l}
K^{\mathrm{FI}}_{\mathrm{m\ C3bH}}+[\mathrm{C3(H_2O)FH}]+[\mathrm{C3(H_2O)FHL1}]+[\mathrm{fC3bFH}]+[\mathrm{fC3bFHL1}]+[\mathrm{fC3bCR1}]+[\mathrm{fiC3bCR1}]\\
+[\mathrm{IgGC3bC3bFH}]+[\mathrm{IgGC3biC3bFH}]+[\mathrm{IgGC3bC3bFHL1}]+[\mathrm{IgGC3biC3bFHL1}]+[\mathrm{IgGC3bC3bCR1}]\\
+[\mathrm{IgGC3biC3bCR1}]+[\mathrm{IgGiC3biC3bCR1}]+[\mathrm{IgGiC3bC3dgCR1}]+[\mathrm{fC4bC3bFH}]+[\mathrm{fC4bC3bFHL1}]\\
+[\mathrm{fC4bC3bCR1}]+[\mathrm{fC4bC3bC4BP}]+[\mathrm{fC4dC3bFH}]+[\mathrm{fC4dC3bFHL1}]+[\mathrm{fC4dC3bCR1}]+[\mathrm{fC4diC3bCR1}]\\
+[\mathrm{fC4biC3bCR1}]+[\mathrm{fC4bC3dgCR1}]+[\mathrm{fC4bC3dgC4BP}]+[\mathrm{fC3bC3bFH}]+[\mathrm{fC3bC3bFHL1}]+[\mathrm{fC3bC3bCR1}]\\
+[\mathrm{fC3biC3bFH}]+[\mathrm{fC3biC3bFHL1}]+[\mathrm{fC3biC3bCR1}]+[\mathrm{fiC3biC3bCR1}]+[\mathrm{fiC3bC3dgCR1}]\\
+[\mathrm{fC4biC3bC4BP}]+[\mathrm{nfC4bC4BP}]+[\mathrm{fC4bC4BP}]+[\mathrm{fC4bCR1}]+[\mathrm{fC4bC4bC4BP}]+[\mathrm{fC4bC4dC4BP}]\\
+[\mathrm{fC4bC4bCR1}]+[\mathrm{fC4bC4dCR1}]+[\mathrm{hC4bCR1}]+[\mathrm{hC4bC4BP}]+[\mathrm{hC4bC4bCR1}]+[\mathrm{hC4bC4bC4BP}]\\
+[\mathrm{hC4bC4dCR1}]+[\mathrm{hC4bC4dC4BP}]+[\mathrm{hC4bC3bFH}]+[\mathrm{hC4bC3bFHL1}]+[\mathrm{hC4bC3bCR1}]\\
+[\mathrm{hC4bC3bC4BP}]+[\mathrm{hC4biC3bCR1}]+[\mathrm{hC4biC3bC4BP}]+[\mathrm{hC4bC3dgCR1}]+[\mathrm{hC4bC3dgC4BP}]\\
+[\mathrm{hC4dC3bFH}]+[\mathrm{hC4dC3bFHL1}]+[\mathrm{hC4dC3bCR1}]+[\mathrm{hC4diC3bCR1}]+[\mathrm{hC3bFH}]+[\mathrm{hC3bFHL1}]\\
+[\mathrm{hC3bCR1}]+[\mathrm{hiC3bCR1}]+[\mathrm{hC3bC3bFH}]+[\mathrm{hC3bC3bFHL1}]+[\mathrm{hC3bC3bCR1}]+[\mathrm{hC3biC3bFH}]\\
+[\mathrm{hC3biC3bFHL1}]+[\mathrm{hC3biC3bCR1}]+[\mathrm{hiC3biC3bCR1}]+[\mathrm{hiC3biC3dgCR1}]+[\mathrm{pC4bC4BP}]\\
+[\mathrm{pC4bC4bC4BP}]+[\mathrm{pC4bC4dC4BP}]+[\mathrm{pC4bC3bC4BP}]+[\mathrm{pC4biC3bC4BP}]+[\mathrm{pC4bC3bFH}]\\
+[\mathrm{pC4bC3bFHL1}]+[\mathrm{pC4dC3bFH}]+[\mathrm{pC4dC3bFHL1}]+[\mathrm{pC3bFH}]+[\mathrm{pC3bFHL1}]+[\mathrm{pC3bC3bFH}]\\
+[\mathrm{pC3bC3bFHL1}]+[\mathrm{pC3biC3bFH}]+[\mathrm{pC3biC3bFHL1}]+[\mathrm{pC4bC4BPPTX3}]+[\mathrm{pC4bC4bC4BPPTX3}]\\
+[\mathrm{pC4bC4dC4BPPTX3}]+[\mathrm{pC4bC3bFHPTX3}]+[\mathrm{pC4bC3bFHL1PTX3}]+[\mathrm{pC4bC3bC4BPPTX3}]\\
+[\mathrm{pC4dC3bFHPTX3}]+[\mathrm{pC4dC3bFHL1PTX3}]+[\mathrm{pC4biC3bC4BPPTX3}]+[\mathrm{pC3bFHPTX3}]\\
+[\mathrm{pC3bFHL1PTX3}]+[\mathrm{pC3bC3bFHPTX3}]+[\mathrm{pC3bC3bFHL1PTX3}]+[\mathrm{pC3biC3bFHPTX3}]\\
+[\mathrm{pC3biC3bFHL1PTX3}]+[\mathrm{pC4bpC4BP}]+[\mathrm{pC4bC4bpC4BP}]+[\mathrm{pC4bC4dpC4BP}]+[\mathrm{pC4bC3bpC4BP}]\\
+[\mathrm{pC4biC3bpC4BP}]+[\mathrm{pC4bC3bpFH}]+[\mathrm{pC4bC3bpFHL1}]+[\mathrm{pC4dC3bpFH}]+[\mathrm{pC4dC3bpFHL1}]\\
+[\mathrm{pC3bpFH}]+[\mathrm{pC3bpFHL1}]+[\mathrm{pC3bC3bpFH}]+[\mathrm{pC3bC3bpFHL1}]+[\mathrm{pC3biC3bpFH}]+[\mathrm{pC3biC3bpFHL1}]
\end{array}\right)}\\
&-k^{+}_{\mathrm{C3bH}}[\mathrm{pC3bC4d}][\mathrm{pFH}]+k^{-}_{\mathrm{C3bH}}[\mathrm{pC3bC4dpFH}]-k^{+}_{\mathrm{C3bFHL1}}[\mathrm{pC3bC4d}][\mathrm{pFHL1}]+k^{-}_{\mathrm{C3bFHL1}}[\mathrm{pC3bC4dpFHL1}]
\end{aligned}
$$

219

$$
\begin{aligned}
\frac{d\left[\mathrm{pC3bC4dFH}\right]}{dt} &= k_{\mathrm{C3bH}}^{+}\left[\mathrm{pC3bC4d}\right]\left[\mathrm{FH}\right]-k_{\mathrm{C3bH}}^{-}\left[\mathrm{pC3bC4dFH}\right] \\
&- \frac{k_{\mathrm{cat\ C3bH}}^{\mathrm{FI}}\left[\mathrm{FI}\right]\left[\mathrm{pC3bC4dFH}\right]}{\left(\begin{array}{l}
K_{\mathrm{m\ C3bH}}^{\mathrm{FI}}+\left[\mathrm{C3(H_2O)FH}\right]+\left[\mathrm{C3(H_2O)FHL1}\right]+\left[\mathrm{fC3bFH}\right]+\left[\mathrm{fC3bFHL1}\right]+\left[\mathrm{fC3bCR1}\right]+\left[\mathrm{fiC3bCR1}\right] \\
+\left[\mathrm{IgGC3bC3bFH}\right]+\left[\mathrm{IgGC3biC3bFH}\right]+\left[\mathrm{IgGC3bC3bFHL1}\right]+\left[\mathrm{IgGC3biC3bFHL1}\right]+\left[\mathrm{IgGC3bC3bCR1}\right] \\
+\left[\mathrm{IgGC3biC3bCR1}\right]+\left[\mathrm{IgGiC3biC3bCR1}\right]+\left[\mathrm{IgGiC3bC3dgCR1}\right]+\left[\mathrm{fC4bC3bFH}\right]+\left[\mathrm{fC4bC3bFHL1}\right] \\
+\left[\mathrm{fC4bC3bCR1}\right]+\left[\mathrm{fC4bC3bC4BP}\right]+\left[\mathrm{fC4dC3bFH}\right]+\left[\mathrm{fC4dC3bFHL1}\right]+\left[\mathrm{fC4dC3bCR1}\right]+\left[\mathrm{fC4diC3bCR1}\right] \\
+\left[\mathrm{fC4biC3bCR1}\right]+\left[\mathrm{fC4bC3dgCR1}\right]+\left[\mathrm{fC4bC3dgC4BP}\right]+\left[\mathrm{fC3bC3bFH}\right]+\left[\mathrm{fC3bC3bFHL1}\right]+\left[\mathrm{fC3bC3bCR1}\right] \\
+\left[\mathrm{fC3biC3bFH}\right]+\left[\mathrm{fC3biC3bFHL1}\right]+\left[\mathrm{fC3biC3bCR1}\right]+\left[\mathrm{fiC3biC3bCR1}\right]+\left[\mathrm{fiC3bC3dgCR1}\right] \\
+\left[\mathrm{fC4biC3bC4BP}\right]+\left[\mathrm{nfC4bC4BP}\right]+\left[\mathrm{fC4bC4BP}\right]+\left[\mathrm{fC4bCR1}\right]+\left[\mathrm{fC4bC4bC4BP}\right]+\left[\mathrm{fC4bC4dC4BP}\right] \\
+\left[\mathrm{fC4bC4bCR1}\right]+\left[\mathrm{fC4bC4dCR1}\right]+\left[\mathrm{hC4bCR1}\right]+\left[\mathrm{hC4bC4BP}\right]+\left[\mathrm{hC4bC4bCR1}\right]+\left[\mathrm{hC4bC4bC4BP}\right] \\
+\left[\mathrm{hC4bC4dCR1}\right]+\left[\mathrm{hC4bC4dC4BP}\right]+\left[\mathrm{hC4bC3bFH}\right]+\left[\mathrm{hC4bC3bFHL1}\right]+\left[\mathrm{hC4bC3bCR1}\right] \\
+\left[\mathrm{hC4bC3bC4BP}\right]+\left[\mathrm{hC4biC3bCR1}\right]+\left[\mathrm{hC4biC3bC4BP}\right]+\left[\mathrm{hC4bC3dgCR1}\right]+\left[\mathrm{hC4bC3dgC4BP}\right] \\
+\left[\mathrm{hC4dC3bFH}\right]+\left[\mathrm{hC4dC3bFHL1}\right]+\left[\mathrm{hC4dC3bCR1}\right]+\left[\mathrm{hC4diC3bCR1}\right]+\left[\mathrm{hC3bFH}\right]+\left[\mathrm{hC3bFHL1}\right] \\
+\left[\mathrm{hC3bCR1}\right]+\left[\mathrm{hiC3bCR1}\right]+\left[\mathrm{hC3bC3bFH}\right]+\left[\mathrm{hC3bC3bFHL1}\right]+\left[\mathrm{hC3bC3bCR1}\right]+\left[\mathrm{hC3biC3bFH}\right] \\
+\left[\mathrm{hC3biC3bFHL1}\right]+\left[\mathrm{hC3biC3bCR1}\right]+\left[\mathrm{hiC3biC3bCR1}\right]+\left[\mathrm{hiC3biC3dgCR1}\right]+\left[\mathrm{pC4bC4BP}\right] \\
+\left[\mathrm{pC4bC4bC4BP}\right]+\left[\mathrm{pC4bC4dC4BP}\right]+\left[\mathrm{pC4bC3bC4BP}\right]+\left[\mathrm{pC4biC3bC4BP}\right]+\left[\mathrm{pC4bC3bFH}\right] \\
+\left[\mathrm{pC4bC3bFHL1}\right]+\left[\mathrm{pC4dC3bFH}\right]+\left[\mathrm{pC4dC3bFHL1}\right]+\left[\mathrm{pC3bFH}\right]+\left[\mathrm{pC3bFHL1}\right]+\left[\mathrm{pC3bC3bFH}\right] \\
+\left[\mathrm{pC3bC3bFHL1}\right]+\left[\mathrm{pC3biC3bFH}\right]+\left[\mathrm{pC3biC3bFHL1}\right]+\left[\mathrm{pC4bC4BPPTX3}\right]+\left[\mathrm{pC4bC4bC4BPPTX3}\right] \\
+\left[\mathrm{pC4bC4dC4BPPTX3}\right]+\left[\mathrm{pC4bC3bFHPTX3}\right]+\left[\mathrm{pC4bC3bFHL1PTX3}\right]+\left[\mathrm{pC4bC3bC4BPPTX3}\right] \\
+\left[\mathrm{pC4dC3bFHPTX3}\right]+\left[\mathrm{pC4dC3bFHL1PTX3}\right]+\left[\mathrm{pC4biC3bC4BPPTX3}\right]+\left[\mathrm{pC3bFHPTX3}\right] \\
+\left[\mathrm{pC3bFHL1PTX3}\right]+\left[\mathrm{pC3bC3bFHPTX3}\right]+\left[\mathrm{pC3bC3bFHL1PTX3}\right]+\left[\mathrm{pC3biC3bFHPTX3}\right] \\
+\left[\mathrm{pC3biC3bFHL1PTX3}\right]+\left[\mathrm{pC4bpC4BP}\right]+\left[\mathrm{pC4bC4bpC4BP}\right]+\left[\mathrm{pC4bC4dpC4BP}\right]+\left[\mathrm{pC4bC3bpC4BP}\right] \\
+\left[\mathrm{pC4biC3bpC4BP}\right]+\left[\mathrm{pC4bC3bpFH}\right]+\left[\mathrm{pC4bC3bpFHL1}\right]+\left[\mathrm{pC4dC3bpFH}\right]+\left[\mathrm{pC4dC3bpFHL1}\right] \\
+\left[\mathrm{pC3bpFH}\right]+\left[\mathrm{pC3bpFHL1}\right]+\left[\mathrm{pC3bC3bpFH}\right]+\left[\mathrm{pC3bC3bpFHL1}\right]+\left[\mathrm{pC3biC3bpFH}\right]+\left[\mathrm{pC3biC3bpFHL1}\right]
\end{array}\right)}
\end{aligned}
$$

220

$$
\begin{aligned}
\frac{d\left[\mathrm{pC3bC4dFHL1}\right]}{dt} &= k^{+}_{\mathrm{C3bFHL1}}\left[\mathrm{pC3bC4d}\right]\left[\mathrm{FHL1}\right]-k^{-}_{\mathrm{C3bFHL1}}\left[\mathrm{pC3bC4dFHL1}\right] \\
&-\frac{k^{\mathrm{FI}}_{\mathrm{cat\ C3bH}}\left[\mathrm{FI}\right]\left[\mathrm{pC3bC4dFHL1}\right]}{\left(\begin{aligned}
&K^{\mathrm{FI}}_{\mathrm{m\ C3bH}}+\left[\mathrm{C3(H_2O)FH}\right]+\left[\mathrm{C3(H_2O)FHL1}\right]+\left[\mathrm{fC3bFH}\right]+\left[\mathrm{fC3bFHL1}\right]+\left[\mathrm{fC3bCR1}\right]+\left[\mathrm{fiC3bCR1}\right]\\
&+\left[\mathrm{IgGC3bC3bFH}\right]+\left[\mathrm{IgGC3biC3bFH}\right]+\left[\mathrm{IgGC3bC3bFHL1}\right]+\left[\mathrm{IgGC3biC3bFHL1}\right]+\left[\mathrm{IgGC3bC3bCR1}\right]\\
&+\left[\mathrm{IgGC3biC3bCR1}\right]+\left[\mathrm{IgGiC3biC3bCR1}\right]+\left[\mathrm{IgGiC3bC3dgCR1}\right]+\left[\mathrm{fC4bC3bFH}\right]+\left[\mathrm{fC4bC3bFHL1}\right]\\
&+\left[\mathrm{fC4bC3bCR1}\right]+\left[\mathrm{fC4bC3bC4BP}\right]+\left[\mathrm{fC4dC3bFH}\right]+\left[\mathrm{fC4dC3bFHL1}\right]+\left[\mathrm{fC4dC3bCR1}\right]+\left[\mathrm{fC4diC3bCR1}\right]\\
&+\left[\mathrm{fC4biC3bCR1}\right]+\left[\mathrm{fC4bC3dgCR1}\right]+\left[\mathrm{fC4bC3dgC4BP}\right]+\left[\mathrm{fC3bC3bFH}\right]+\left[\mathrm{fC3bC3bFHL1}\right]+\left[\mathrm{fC3bC3bCR1}\right]\\
&+\left[\mathrm{fC3biC3bFH}\right]+\left[\mathrm{fC3biC3bFHL1}\right]+\left[\mathrm{fC3biC3bCR1}\right]+\left[\mathrm{fiC3biC3bCR1}\right]+\left[\mathrm{fiC3bC3dgCR1}\right]\\
&+\left[\mathrm{fC4biC3bC4BP}\right]+\left[\mathrm{nfC4bC4BP}\right]+\left[\mathrm{fC4bC4BP}\right]+\left[\mathrm{fC4bCR1}\right]+\left[\mathrm{fC4bC4bC4BP}\right]+\left[\mathrm{fC4bC4dC4BP}\right]\\
&+\left[\mathrm{fC4bC4bCR1}\right]+\left[\mathrm{fC4bC4dCR1}\right]+\left[\mathrm{hC4bCR1}\right]+\left[\mathrm{hC4bC4BP}\right]+\left[\mathrm{hC4bC4bCR1}\right]+\left[\mathrm{hC4bC4bC4BP}\right]\\
&+\left[\mathrm{hC4bC4dCR1}\right]+\left[\mathrm{hC4bC4dC4BP}\right]+\left[\mathrm{hC4bC3bFH}\right]+\left[\mathrm{hC4bC3bFHL1}\right]+\left[\mathrm{hC4bC3bCR1}\right]\\
&+\left[\mathrm{hC4bC3bC4BP}\right]+\left[\mathrm{hC4biC3bCR1}\right]+\left[\mathrm{hC4biC3bC4BP}\right]+\left[\mathrm{hC4bC3dgCR1}\right]+\left[\mathrm{hC4bC3dgC4BP}\right]\\
&+\left[\mathrm{hC4dC3bFH}\right]+\left[\mathrm{hC4dC3bFHL1}\right]+\left[\mathrm{hC4dC3bCR1}\right]+\left[\mathrm{hC4diC3bCR1}\right]+\left[\mathrm{hC3bFH}\right]+\left[\mathrm{hC3bFHL1}\right]\\
&+\left[\mathrm{hC3bCR1}\right]+\left[\mathrm{hiC3bCR1}\right]+\left[\mathrm{hC3bC3bFH}\right]+\left[\mathrm{hC3bC3bFHL1}\right]+\left[\mathrm{hC3bC3bCR1}\right]+\left[\mathrm{hC3biC3bFH}\right]\\
&+\left[\mathrm{hC3biC3bFHL1}\right]+\left[\mathrm{hC3biC3bCR1}\right]+\left[\mathrm{hiC3biC3bCR1}\right]+\left[\mathrm{hiC3biC3dgCR1}\right]+\left[\mathrm{pC4bC4BP}\right]\\
&+\left[\mathrm{pC4bC4bC4BP}\right]+\left[\mathrm{pC4bC4dC4BP}\right]+\left[\mathrm{pC4bC3bC4BP}\right]+\left[\mathrm{pC4biC3bC4BP}\right]+\left[\mathrm{pC4bC3bFH}\right]\\
&+\left[\mathrm{pC4bC3bFHL1}\right]+\left[\mathrm{pC4dC3bFH}\right]+\left[\mathrm{pC4dC3bFHL1}\right]+\left[\mathrm{pC3bFH}\right]+\left[\mathrm{pC3bFHL1}\right]+\left[\mathrm{pC3bC3bFH}\right]\\
&+\left[\mathrm{pC3bC3bFHL1}\right]+\left[\mathrm{pC3biC3bFH}\right]+\left[\mathrm{pC3biC3bFHL1}\right]+\left[\mathrm{pC4bC4BPPTX3}\right]+\left[\mathrm{pC4bC4bC4BPPTX3}\right]\\
&+\left[\mathrm{pC4bC4dC4BPPTX3}\right]+\left[\mathrm{pC4bC3bFHPTX3}\right]+\left[\mathrm{pC4bC3bFHL1PTX3}\right]+\left[\mathrm{pC4bC3bC4BPPTX3}\right]\\
&+\left[\mathrm{pC4dC3bFHPTX3}\right]+\left[\mathrm{pC4dC3bFHL1PTX3}\right]+\left[\mathrm{pC4biC3bC4BPPTX3}\right]+\left[\mathrm{pC3bFHPTX3}\right]\\
&+\left[\mathrm{pC3bFHL1PTX3}\right]+\left[\mathrm{pC3bC3bFHPTX3}\right]+\left[\mathrm{pC3bC3bFHL1PTX3}\right]+\left[\mathrm{pC3biC3bFHPTX3}\right]\\
&+\left[\mathrm{pC3biC3bFHL1PTX3}\right]+\left[\mathrm{pC4bpC4BP}\right]+\left[\mathrm{pC4bC4bpC4BP}\right]+\left[\mathrm{pC4bC4dpC4BP}\right]+\left[\mathrm{pC4bC3bpC4BP}\right]\\
&+\left[\mathrm{pC4biC3bpC4BP}\right]+\left[\mathrm{pC4bC3bpFH}\right]+\left[\mathrm{pC4bC3bpFHL1}\right]+\left[\mathrm{pC4dC3bpFH}\right]+\left[\mathrm{pC4dC3bpFHL1}\right]\\
&+\left[\mathrm{pC3bpFH}\right]+\left[\mathrm{pC3bpFHL1}\right]+\left[\mathrm{pC3bC3bpFH}\right]+\left[\mathrm{pC3bC3bpFHL1}\right]+\left[\mathrm{pC3biC3bpFH}\right]+\left[\mathrm{pC3biC3bpFHL1}\right]
\end{aligned}\right)}
\end{aligned}
$$

221

$$\frac{d[\mathrm{piC3bC4bC4BP}]}{dt} = k^{+}_{\mathrm{C4bC4BP}}[\mathrm{piC3bC4b}][\mathrm{C4BP}] - k^{-}_{\mathrm{C4bC4BP}}[\mathrm{piC3bC4bC4BP}]$$

$$- \frac{k^{\mathrm{FI}}_{\mathrm{cat\ C3bH}}[\mathrm{FI}][\mathrm{piC3bC4bC4BP}]}{\left(\begin{array}{l} K^{\mathrm{FI}}_{\mathrm{m\ C3bH}} + [\mathrm{C3(H_2O)FH}] + [\mathrm{C3(H_2O)FHL1}] + [\mathrm{fC3bFH}] + [\mathrm{fC3bFHL1}] + [\mathrm{fC3bCR1}] + [\mathrm{fiC3bCR1}] \\ + [\mathrm{IgGC3bC3bFH}] + [\mathrm{IgGC3biC3bFH}] + [\mathrm{IgGC3bC3bFHL1}] + [\mathrm{IgGC3biC3bFHL1}] + [\mathrm{IgGC3bC3bCR1}] \\ + [\mathrm{IgGC3biC3bCR1}] + [\mathrm{IgGiC3biC3bCR1}] + [\mathrm{IgGiC3bC3dgCR1}] + [\mathrm{fC4bC3bFH}] + [\mathrm{fC4bC3bFHL1}] \\ + [\mathrm{fC4bC3bCR1}] + [\mathrm{fC4bC3bC4BP}] + [\mathrm{fC4dC3bFH}] + [\mathrm{fC4dC3bFHL1}] + [\mathrm{fC4dC3bCR1}] + [\mathrm{fC4diC3bCR1}] \\ + [\mathrm{fC4biC3bCR1}] + [\mathrm{fC4bC3dgCR1}] + [\mathrm{fC4bC3dgC4BP}] + [\mathrm{fC3bC3bFH}] + [\mathrm{fC3bC3bFHL1}] + [\mathrm{fC3bC3bCR1}] \\ + [\mathrm{fC3biC3bFH}] + [\mathrm{fC3biC3bFHL1}] + [\mathrm{fC3biC3bCR1}] + [\mathrm{fiC3biC3bCR1}] + [\mathrm{fiC3bC3dgCR1}] \\ + [\mathrm{fC4biC3bC4BP}] + [\mathrm{nfC4bC4BP}] + [\mathrm{fC4bC4BP}] + [\mathrm{fC4bCR1}] + [\mathrm{fC4bC4bC4BP}] + [\mathrm{fC4bC4dC4BP}] \\ + [\mathrm{fC4bC4bCR1}] + [\mathrm{fC4bC4dCR1}] + [\mathrm{hC4bCR1}] + [\mathrm{hC4bC4BP}] + [\mathrm{hC4bC4bCR1}] + [\mathrm{hC4bC4bC4BP}] \\ + [\mathrm{hC4bC4dCR1}] + [\mathrm{hC4bC4dC4BP}] + [\mathrm{hC4bC3bFH}] + [\mathrm{hC4bC3bFHL1}] + [\mathrm{hC4bC3bCR1}] \\ + [\mathrm{hC4bC3bC4BP}] + [\mathrm{hC4biC3bCR1}] + [\mathrm{hC4biC3bC4BP}] + [\mathrm{hC4bC3dgCR1}] + [\mathrm{hC4bC3dgC4BP}] \\ + [\mathrm{hC4dC3bFH}] + [\mathrm{hC4dC3bFHL1}] + [\mathrm{hC4dC3bCR1}] + [\mathrm{hC4diC3bCR1}] + [\mathrm{hC3bFH}] + [\mathrm{hC3bFHL1}] \\ + [\mathrm{hC3bCR1}] + [\mathrm{hiC3bCR1}] + [\mathrm{hC3bC3bFH}] + [\mathrm{hC3bC3bFHL1}] + [\mathrm{hC3bC3bCR1}] + [\mathrm{hC3biC3bFH}] \\ + [\mathrm{hC3biC3bFHL1}] + [\mathrm{hC3biC3bCR1}] + [\mathrm{hiC3biC3bCR1}] + [\mathrm{hiC3biC3dgCR1}] + [\mathrm{pC4bC4BP}] \\ + [\mathrm{pC4bC4bC4BP}] + [\mathrm{pC4bC4dC4BP}] + [\mathrm{pC4bC3bC4BP}] + [\mathrm{pC4biC3bC4BP}] + [\mathrm{pC4bC3bFH}] \\ + [\mathrm{pC4bC3bFHL1}] + [\mathrm{pC4dC3bFH}] + [\mathrm{pC4dC3bFHL1}] + [\mathrm{pC3bFH}] + [\mathrm{pC3bFHL1}] + [\mathrm{pC3bC3bFH}] \\ + [\mathrm{pC3bC3bFHL1}] + [\mathrm{pC3biC3bFH}] + [\mathrm{pC3biC3bFHL1}] + [\mathrm{pC4bC4BPPTX3}] + [\mathrm{pC4bC4bC4BPPTX3}] \\ + [\mathrm{pC4bC4dC4BPPTX3}] + [\mathrm{pC4bC3bFHPTX3}] + [\mathrm{pC4bC3bFHL1PTX3}] + [\mathrm{pC4bC3bC4BPPTX3}] \\ + [\mathrm{pC4dC3bFHPTX3}] + [\mathrm{pC4dC3bFHL1PTX3}] + [\mathrm{pC4biC3bC4BPPTX3}] + [\mathrm{pC3bFHPTX3}] \\ + [\mathrm{pC3bFHL1PTX3}] + [\mathrm{pC3bC3bFHPTX3}] + [\mathrm{pC3bC3bFHL1PTX3}] + [\mathrm{pC3biC3bFHPTX3}] \\ + [\mathrm{pC3biC3bFHL1PTX3}] + [\mathrm{pC4bpC4BP}] + [\mathrm{pC4bC4bpC4BP}] + [\mathrm{pC4bC4dpC4BP}] + [\mathrm{pC4bC3bpC4BP}] \\ + [\mathrm{pC4biC3bpC4BP}] + [\mathrm{pC4bC3bpFH}] + [\mathrm{pC4bC3bpFHL1}] + [\mathrm{pC4dC3bpFH}] + [\mathrm{pC4dC3bpFHL1}] \\ + [\mathrm{pC3bpFH}] + [\mathrm{pC3bpFHL1}] + [\mathrm{pC3bC3bpFH}] + [\mathrm{pC3bC3bpFHL1}] + [\mathrm{pC3biC3bpFH}] + [\mathrm{pC3biC3bpFHL1}] \end{array}\right)}$$

222

$$\frac{d\left[\text{piC3bC4d}\right]}{dt}=$$

$$\frac{k_{\text{cat C3bH}}^{\text{FI}}\left[\text{FI}\right]\left[\text{piC3bC4bC4BP}\right]}{\left(\begin{array}{l}K_{\text{m C3bH}}^{\text{FI}}+\left[\text{C3(H}_2\text{O)FH}\right]+\left[\text{C3(H}_2\text{O)FHL1}\right]+\left[\text{fC3bFH}\right]+\left[\text{fC3bFHL1}\right]+\left[\text{fC3bCR1}\right]+\left[\text{fiC3bCR1}\right]\\+\left[\text{IgGC3bC3bFH}\right]+\left[\text{IgGC3biC3bFH}\right]+\left[\text{IgGC3bC3bFHL1}\right]+\left[\text{IgGC3biC3bFHL1}\right]+\left[\text{IgGC3bC3bCR1}\right]\\+\left[\text{IgGC3biC3bCR1}\right]+\left[\text{IgGiC3biC3bCR1}\right]+\left[\text{IgGiC3bC3dgCR1}\right]+\left[\text{fC4bC3bFH}\right]+\left[\text{fC4bC3bFHL1}\right]\\+\left[\text{fC4bC3bCR1}\right]+\left[\text{fC4bC3bC4BP}\right]+\left[\text{fC4dC3bFH}\right]+\left[\text{fC4dC3bFHL1}\right]+\left[\text{fC4dC3bCR1}\right]+\left[\text{fC4diC3bCR1}\right]\\+\left[\text{fC4biC3bCR1}\right]+\left[\text{fC4bC3dgCR1}\right]+\left[\text{fC4bC3dgC4BP}\right]+\left[\text{fC3bC3bFH}\right]+\left[\text{fC3bC3bFHL1}\right]+\left[\text{fC3bC3bCR1}\right]\\+\left[\text{fC3biC3bFH}\right]+\left[\text{fC3biC3bFHL1}\right]+\left[\text{fC3biC3bCR1}\right]+\left[\text{fiC3biC3bCR1}\right]+\left[\text{fiC3bC3dgCR1}\right]\\+\left[\text{fC4biC3bC4BP}\right]+\left[\text{nfC4bC4BP}\right]+\left[\text{fC4bC4BP}\right]+\left[\text{fC4bCR1}\right]+\left[\text{fC4bC4bC4BP}\right]+\left[\text{fC4bC4dC4BP}\right]\\+\left[\text{fC4bC4bCR1}\right]+\left[\text{fC4bC4dCR1}\right]+\left[\text{hC4bCR1}\right]+\left[\text{hC4bC4BP}\right]+\left[\text{hC4bC4bCR1}\right]+\left[\text{hC4bC4bC4BP}\right]\\+\left[\text{hC4bC4dCR1}\right]+\left[\text{hC4bC4dC4BP}\right]+\left[\text{hC4bC3bFH}\right]+\left[\text{hC4bC3bFHL1}\right]+\left[\text{hC4bC3bCR1}\right]\\+\left[\text{hC4bC3bC4BP}\right]+\left[\text{hC4biC3bCR1}\right]+\left[\text{hC4biC3bC4BP}\right]+\left[\text{hC4bC3dgCR1}\right]+\left[\text{hC4bC3dgC4BP}\right]\\+\left[\text{hC4dC3bFH}\right]+\left[\text{hC4dC3bFHL1}\right]+\left[\text{hC4dC3bCR1}\right]+\left[\text{hC4diC3bCR1}\right]+\left[\text{hC3bFH}\right]+\left[\text{hC3bFHL1}\right]\\+\left[\text{hC3bCR1}\right]+\left[\text{hiC3bCR1}\right]+\left[\text{hC3bC3bFH}\right]+\left[\text{hC3bC3bFHL1}\right]+\left[\text{hC3bC3bCR1}\right]+\left[\text{hC3biC3bFH}\right]\\+\left[\text{hC3biC3bFHL1}\right]+\left[\text{hC3biC3bCR1}\right]+\left[\text{hiC3biC3bCR1}\right]+\left[\text{hiC3biC3dgCR1}\right]+\left[\text{pC4bC4BP}\right]\\+\left[\text{pC4bC4bC4BP}\right]+\left[\text{pC4bC4dC4BP}\right]+\left[\text{pC4bC3bC4BP}\right]+\left[\text{pC4biC3bC4BP}\right]+\left[\text{pC4bC3bFH}\right]\\+\left[\text{pC4bC3bFHL1}\right]+\left[\text{pC4dC3bFH}\right]+\left[\text{pC4dC3bFHL1}\right]+\left[\text{pC3bFH}\right]+\left[\text{pC3bFHL1}\right]+\left[\text{pC3bC3bFH}\right]\\+\left[\text{pC3bC3bFHL1}\right]+\left[\text{pC3biC3bFH}\right]+\left[\text{pC3biC3bFHL1}\right]+\left[\text{pC4bC4BPPTX3}\right]+\left[\text{pC4bC4bC4BPPTX3}\right]\\+\left[\text{pC4bC4dC4BPPTX3}\right]+\left[\text{pC4bC3bFHPTX3}\right]+\left[\text{pC4bC3bFHL1PTX3}\right]+\left[\text{pC4bC3bC4BPPTX3}\right]\\+\left[\text{pC4dC3bFHPTX3}\right]+\left[\text{pC4dC3bFHL1PTX3}\right]+\left[\text{pC4biC3bC4BPPTX3}\right]+\left[\text{pC3bFHPTX3}\right]\\+\left[\text{pC3bFHL1PTX3}\right]+\left[\text{pC3bC3bFHPTX3}\right]+\left[\text{pC3bC3bFHL1PTX3}\right]+\left[\text{pC3biC3bFHPTX3}\right]\\+\left[\text{pC3biC3bFHL1PTX3}\right]+\left[\text{pC4bpC4BP}\right]+\left[\text{pC4bC4bpC4BP}\right]+\left[\text{pC4bC4dpC4BP}\right]+\left[\text{pC4bC3bpC4BP}\right]\\+\left[\text{pC4biC3bpC4BP}\right]+\left[\text{pC4bC3bpFH}\right]+\left[\text{pC4bC3bpFHL1}\right]+\left[\text{pC4dC3bpFH}\right]+\left[\text{pC4dC3bpFHL1}\right]\\+\left[\text{pC3bpFH}\right]+\left[\text{pC3bpFHL1}\right]+\left[\text{pC3bC3bpFH}\right]+\left[\text{pC3bC3bpFHL1}\right]+\left[\text{pC3biC3bpFH}\right]+\left[\text{pC3biC3bpFHL1}\right]\end{array}\right)}$$

$$+\frac{k_{\text{cat C3bH}}^{\text{FI}}[\text{FI}][\text{pC3bC4dFH}]}{\left(\begin{array}{l}K_{\text{m C3bH}}^{\text{FI}}+[\text{C3(H}_2\text{O)FH}]+[\text{C3(H}_2\text{O)FHL1}]+[\text{fC3bFH}]+[\text{fC3bFHL1}]+[\text{fC3bCR1}]+[\text{fiC3bCR1}]\\+[\text{IgGC3bC3bFH}]+[\text{IgGC3biC3bFH}]+[\text{IgGC3bC3bFHL1}]+[\text{IgGC3biC3bFHL1}]+[\text{IgGC3bC3bCR1}]\\+[\text{IgGC3biC3bCR1}]+[\text{IgGiC3biC3bCR1}]+[\text{IgGiC3bC3dgCR1}]+[\text{fC4bC3bFH}]+[\text{fC4bC3bFHL1}]\\+[\text{fC4bC3bCR1}]+[\text{fC4bC3bC4BP}]+[\text{fC4dC3bFH}]+[\text{fC4dC3bFHL1}]+[\text{fC4dC3bCR1}]+[\text{fC4diC3bCR1}]\\+[\text{fC4biC3bCR1}]+[\text{fC4bC3dgCR1}]+[\text{fC4bC3dgC4BP}]+[\text{fC3bC3bFH}]+[\text{fC3bC3bFHL1}]+[\text{fC3bC3bCR1}]\\+[\text{fC3biC3bFH}]+[\text{fC3biC3bFHL1}]+[\text{fC3biC3bCR1}]+[\text{fiC3biC3bCR1}]+[\text{fiC3bC3dgCR1}]\\+[\text{fC4biC3bC4BP}]+[\text{nfC4bC4BP}]+[\text{fC4bC4BP}]+[\text{fC4bCR1}]+[\text{fC4bC4bC4BP}]+[\text{fC4bC4dC4BP}]\\+[\text{fC4bC4bCR1}]+[\text{fC4bC4dCR1}]+[\text{hC4bCR1}]+[\text{hC4bC4BP}]+[\text{hC4bC4bCR1}]+[\text{hC4bC4bC4BP}]\\+[\text{hC4bC4dCR1}]+[\text{hC4bC4dC4BP}]+[\text{hC4bC3bFH}]+[\text{hC4bC3bFHL1}]+[\text{hC4bC3bCR1}]\\+[\text{hC4bC3bC4BP}]+[\text{hC4biC3bCR1}]+[\text{hC4biC3bC4BP}]+[\text{hC4bC3dgCR1}]+[\text{hC4bC3dgC4BP}]\\+[\text{hC4dC3bFH}]+[\text{hC4dC3bFHL1}]+[\text{hC4dC3bCR1}]+[\text{hC4diC3bCR1}]+[\text{hC3bFH}]+[\text{hC3bFHL1}]\\+[\text{hC3bCR1}]+[\text{hiC3bCR1}]+[\text{hC3bC3bFH}]+[\text{hC3bC3bFHL1}]+[\text{hC3bC3bCR1}]+[\text{hC3biC3bFH}]\\+[\text{hC3biC3bFHL1}]+[\text{hC3biC3bCR1}]+[\text{hiC3biC3bCR1}]+[\text{hiC3biC3dgCR1}]+[\text{pC4bC4BP}]\\+[\text{pC4bC4bC4BP}]+[\text{pC4bC4dC4BP}]+[\text{pC4bC3bC4BP}]+[\text{pC4biC3bC4BP}]+[\text{pC4bC3bFH}]\\+[\text{pC4bC3bFHL1}]+[\text{pC4dC3bFH}]+[\text{pC4dC3bFHL1}]+[\text{pC3bFH}]+[\text{pC3bFHL1}]+[\text{pC3bC3bFH}]\\+[\text{pC3bC3bFHL1}]+[\text{pC3biC3bFH}]+[\text{pC3biC3bFHL1}]+[\text{pC4bC4BPPTX3}]+[\text{pC4bC4bC4BPPTX3}]\\+[\text{pC4bC4dC4BPPTX3}]+[\text{pC4bC3bFHPTX3}]+[\text{pC4bC3bFHL1PTX3}]+[\text{pC4bC3bC4BPPTX3}]\\+[\text{pC4dC3bFHPTX3}]+[\text{pC4dC3bFHL1PTX3}]+[\text{pC4biC3bC4BPPTX3}]+[\text{pC3bFHPTX3}]\\+[\text{pC3bFHL1PTX3}]+[\text{pC3bC3bFHPTX3}]+[\text{pC3bC3bFHL1PTX3}]+[\text{pC3biC3bFHPTX3}]\\+[\text{pC3biC3bFHL1PTX3}]+[\text{pC4bpC4BP}]+[\text{pC4bC4bpC4BP}]+[\text{pC4bC4dpC4BP}]+[\text{pC4bC3bpC4BP}]\\+[\text{pC4biC3bpC4BP}]+[\text{pC4bC3bpFH}]+[\text{pC4bC3bpFHL1}]+[\text{pC4dC3bpFH}]+[\text{pC4dC3bpFHL1}]\\+[\text{pC3bpFH}]+[\text{pC3bpFHL1}]+[\text{pC3bC3bpFH}]+[\text{pC3bC3bpFHL1}]+[\text{pC3biC3bpFH}]+[\text{pC3biC3bpFHL1}]\end{array}\right)}$$

$$+\frac{k_{\mathrm{cat\ C3bH}}^{\mathrm{FI}}[\mathrm{FI}][\mathrm{pC3bC4dFHL1}]}{\left(\begin{aligned}&K_{\mathrm{m\ C3bH}}^{\mathrm{FI}}+[\mathrm{C3(H_2O)FH}]+[\mathrm{C3(H_2O)FHL1}]+[\mathrm{fC3bFH}]+[\mathrm{fC3bFHL1}]+[\mathrm{fC3bCR1}]+[\mathrm{fiC3bCR1}]\\
&+[\mathrm{IgGC3bC3bFH}]+[\mathrm{IgGC3biC3bFH}]+[\mathrm{IgGC3bC3bFHL1}]+[\mathrm{IgGC3biC3bFHL1}]+[\mathrm{IgGC3bC3bCR1}]\\
&+[\mathrm{IgGC3biC3bCR1}]+[\mathrm{IgGiC3biC3bCR1}]+[\mathrm{IgGiC3bC3dgCR1}]+[\mathrm{fC4bC3bFH}]+[\mathrm{fC4bC3bFHL1}]\\
&+[\mathrm{fC4bC3bCR1}]+[\mathrm{fC4bC3bC4BP}]+[\mathrm{fC4dC3bFH}]+[\mathrm{fC4dC3bFHL1}]+[\mathrm{fC4dC3bCR1}]+[\mathrm{fC4diC3bCR1}]\\
&+[\mathrm{fC4biC3bCR1}]+[\mathrm{fC4bC3dgCR1}]+[\mathrm{fC4bC3dgC4BP}]+[\mathrm{fC3bC3bFH}]+[\mathrm{fC3bC3bFHL1}]+[\mathrm{fC3bC3bCR1}]\\
&+[\mathrm{fC3biC3bFH}]+[\mathrm{fC3biC3bFHL1}]+[\mathrm{fC3biC3bCR1}]+[\mathrm{fiC3biC3bCR1}]+[\mathrm{fiC3bC3dgCR1}]\\
&+[\mathrm{fC4biC3bC4BP}]+[\mathrm{nfC4bC4BP}]+[\mathrm{fC4bC4BP}]+[\mathrm{fC4bCR1}]+[\mathrm{fC4bC4bC4BP}]+[\mathrm{fC4bC4dC4BP}]\\
&+[\mathrm{fC4bC4bCR1}]+[\mathrm{fC4bC4dCR1}]+[\mathrm{hC4bCR1}]+[\mathrm{hC4bC4BP}]+[\mathrm{hC4bC4bCR1}]+[\mathrm{hC4bC4bC4BP}]\\
&+[\mathrm{hC4bC4dCR1}]+[\mathrm{hC4bC4dC4BP}]+[\mathrm{hC4bC3bFH}]+[\mathrm{hC4bC3bFHL1}]+[\mathrm{hC4bC3bCR1}]\\
&+[\mathrm{hC4bC3bC4BP}]+[\mathrm{hC4biC3bCR1}]+[\mathrm{hC4biC3bC4BP}]+[\mathrm{hC4bC3dgCR1}]+[\mathrm{hC4bC3dgC4BP}]\\
&+[\mathrm{hC4dC3bFH}]+[\mathrm{hC4dC3bFHL1}]+[\mathrm{hC4dC3bCR1}]+[\mathrm{hC4diC3bCR1}]+[\mathrm{hC3bFH}]+[\mathrm{hC3bFHL1}]\\
&+[\mathrm{hC3bCR1}]+[\mathrm{hiC3bCR1}]+[\mathrm{hC3bC3bFH}]+[\mathrm{hC3bC3bFHL1}]+[\mathrm{hC3bC3bCR1}]+[\mathrm{hC3biC3bFH}]\\
&+[\mathrm{hC3biC3bFHL1}]+[\mathrm{hC3biC3bCR1}]+[\mathrm{hiC3biC3bCR1}]+[\mathrm{hiC3biC3dgCR1}]+[\mathrm{pC4bC4BP}]\\
&+[\mathrm{pC4bC4bC4BP}]+[\mathrm{pC4bC4dC4BP}]+[\mathrm{pC4bC3bC4BP}]+[\mathrm{pC4biC3bC4BP}]+[\mathrm{pC4bC3bFH}]\\
&+[\mathrm{pC4bC3bFHL1}]+[\mathrm{pC4dC3bFH}]+[\mathrm{pC4dC3bFHL1}]+[\mathrm{pC3bFH}]+[\mathrm{pC3bFHL1}]+[\mathrm{pC3bC3bFH}]\\
&+[\mathrm{pC3bC3bFHL1}]+[\mathrm{pC3biC3bFH}]+[\mathrm{pC3biC3bFHL1}]+[\mathrm{pC4bC4BPPTX3}]+[\mathrm{pC4bC4bC4BPPTX3}]\\
&+[\mathrm{pC4bC4dC4BPPTX3}]+[\mathrm{pC4bC3bFHPTX3}]+[\mathrm{pC4bC3bFHL1PTX3}]+[\mathrm{pC4bC3bC4BPPTX3}]\\
&+[\mathrm{pC4dC3bFHPTX3}]+[\mathrm{pC4dC3bFHL1PTX3}]+[\mathrm{pC4biC3bC4BPPTX3}]+[\mathrm{pC3bFHPTX3}]\\
&+[\mathrm{pC3bFHL1PTX3}]+[\mathrm{pC3bC3bFHPTX3}]+[\mathrm{pC3bC3bFHL1PTX3}]+[\mathrm{pC3biC3bFHPTX3}]\\
&+[\mathrm{pC3biC3bFHL1PTX3}]+[\mathrm{pC4bpC4BP}]+[\mathrm{pC4bC4bpC4BP}]+[\mathrm{pC4bC4dpC4BP}]+[\mathrm{pC4bC3bpC4BP}]\\
&+[\mathrm{pC4biC3bpC4BP}]+[\mathrm{pC4bC3bpFH}]+[\mathrm{pC4bC3bpFHL1}]+[\mathrm{pC4dC3bpFH}]+[\mathrm{pC4dC3bpFHL1}]\\
&+[\mathrm{pC3bpFH}]+[\mathrm{pC3bpFHL1}]+[\mathrm{pC3bC3bpFH}]+[\mathrm{pC3bC3bpFHL1}]+[\mathrm{pC3biC3bpFH}]+[\mathrm{pC3biC3bpFHL1}]\end{aligned}\right)}$$

$$+\frac{k_{\text{cat C3bH}}^{\text{FI}}[\text{FI}][\text{pC3bC4dFHPTX3}]}{\left(\begin{array}{l}K_{\text{m C3bH}}^{\text{FI}}+[\text{C3(H}_2\text{O)FH}]+[\text{C3(H}_2\text{O)FHL1}]+[\text{fC3bFH}]+[\text{fC3bFHL1}]+[\text{fC3bCR1}]+[\text{fiC3bCR1}]\\+[\text{IgGC3bC3bFH}]+[\text{IgGC3biC3bFH}]+[\text{IgGC3bC3bFHL1}]+[\text{IgGC3biC3bFHL1}]+[\text{IgGC3bC3bCR1}]\\+[\text{IgGC3biC3bCR1}]+[\text{IgGiC3biC3bCR1}]+[\text{IgGiC3bC3dgCR1}]+[\text{fC4bC3bFH}]+[\text{fC4bC3bFHL1}]\\+[\text{fC4bC3bCR1}]+[\text{fC4bC3bC4BP}]+[\text{fC4dC3bFH}]+[\text{fC4dC3bFHL1}]+[\text{fC4dC3bCR1}]+[\text{fC4diC3bCR1}]\\+[\text{fC4biC3bCR1}]+[\text{fC4bC3dgCR1}]+[\text{fC4bC3dgC4BP}]+[\text{fC3bC3bFH}]+[\text{fC3bC3bFHL1}]+[\text{fC3bC3bCR1}]\\+[\text{fC3biC3bFH}]+[\text{fC3biC3bFHL1}]+[\text{fC3biC3bCR1}]+[\text{fiC3biC3bCR1}]+[\text{fiC3bC3dgCR1}]\\+[\text{fC4biC3bC4BP}]+[\text{nfC4bC4BP}]+[\text{fC4bC4BP}]+[\text{fC4bCR1}]+[\text{fC4bC4bC4BP}]+[\text{fC4bC4dC4BP}]\\+[\text{fC4bC4bCR1}]+[\text{fC4bC4dCR1}]+[\text{hC4bCR1}]+[\text{hC4bC4BP}]+[\text{hC4bC4bCR1}]+[\text{hC4bC4bC4BP}]\\+[\text{hC4bC4dCR1}]+[\text{hC4bC4dC4BP}]+[\text{hC4bC3bFH}]+[\text{hC4bC3bFHL1}]+[\text{hC4bC3bCR1}]\\+[\text{hC4bC3bC4BP}]+[\text{hC4biC3bCR1}]+[\text{hC4biC3bC4BP}]+[\text{hC4bC3dgCR1}]+[\text{hC4bC3dgC4BP}]\\+[\text{hC4dC3bFH}]+[\text{hC4dC3bFHL1}]+[\text{hC4dC3bCR1}]+[\text{hC4diC3bCR1}]+[\text{hC3bFH}]+[\text{hC3bFHL1}]\\+[\text{hC3bCR1}]+[\text{hiC3bCR1}]+[\text{hC3bC3bFH}]+[\text{hC3bC3bFHL1}]+[\text{hC3bC3bCR1}]+[\text{hC3biC3bFH}]\\+[\text{hC3biC3bFHL1}]+[\text{hC3biC3bCR1}]+[\text{hiC3biC3bCR1}]+[\text{hiC3biC3dgCR1}]+[\text{pC4bC4BP}]\\+[\text{pC4bC4bC4BP}]+[\text{pC4bC4dC4BP}]+[\text{pC4bC3bC4BP}]+[\text{pC4biC3bC4BP}]+[\text{pC4bC3bFH}]\\+[\text{pC4bC3bFHL1}]+[\text{pC4dC3bFH}]+[\text{pC4dC3bFHL1}]+[\text{pC3bFH}]+[\text{pC3bFHL1}]+[\text{pC3bC3bFH}]\\+[\text{pC3bC3bFHL1}]+[\text{pC3biC3bFH}]+[\text{pC3biC3bFHL1}]+[\text{pC4bC4BPPTX3}]+[\text{pC4bC4bC4BPPTX3}]\\+[\text{pC4bC4dC4BPPTX3}]+[\text{pC4bC3bFHPTX3}]+[\text{pC4bC3bFHL1PTX3}]+[\text{pC4bC3bC4BPPTX3}]\\+[\text{pC4dC3bFHPTX3}]+[\text{pC4dC3bFHL1PTX3}]+[\text{pC4biC3bC4BPPTX3}]+[\text{pC3bFHPTX3}]\\+[\text{pC3bFHL1PTX3}]+[\text{pC3bC3bFHPTX3}]+[\text{pC3bC3bFHL1PTX3}]+[\text{pC3biC3bFHPTX3}]\\+[\text{pC3biC3bFHL1PTX3}]+[\text{pC4bpC4BP}]+[\text{pC4bC4bpC4BP}]+[\text{pC4bC4dpC4BP}]+[\text{pC4bC3bpC4BP}]\\+[\text{pC4biC3bpC4BP}]+[\text{pC4bC3bpFH}]+[\text{pC4bC3bpFHL1}]+[\text{pC4dC3bpFH}]+[\text{pC4dC3bpFHL1}]\\+[\text{pC3bpFH}]+[\text{pC3bpFHL1}]+[\text{pC3bC3bpFH}]+[\text{pC3bC3bpFHL1}]+[\text{pC3biC3bpFH}]+[\text{pC3biC3bpFHL1}]\end{array}\right)}$$

$$+\frac{k_{\text{cat C3bH}}^{\text{FI}}[\text{FI}][\text{pC3bC4dFHL1PTX3}]}{\left(\begin{array}{l}K_{\text{m C3bH}}^{\text{FI}}+[\text{C3(H}_2\text{O)FH}]+[\text{C3(H}_2\text{O)FHL1}]+[\text{fC3bFH}]+[\text{fC3bFHL1}]+[\text{fC3bCR1}]+[\text{fiC3bCR1}]\\+[\text{IgGC3bC3bFH}]+[\text{IgGC3biC3bFH}]+[\text{IgGC3bC3bFHL1}]+[\text{IgGC3biC3bFHL1}]+[\text{IgGC3bC3bCR1}]\\+[\text{IgGC3biC3bCR1}]+[\text{IgGiC3biC3bCR1}]+[\text{IgGiC3bC3dgCR1}]+[\text{fC4bC3bFH}]+[\text{fC4bC3bFHL1}]\\+[\text{fC4bC3bCR1}]+[\text{fC4bC3bC4BP}]+[\text{fC4dC3bFH}]+[\text{fC4dC3bFHL1}]+[\text{fC4dC3bCR1}]+[\text{fC4diC3bCR1}]\\+[\text{fC4biC3bCR1}]+[\text{fC4bC3dgCR1}]+[\text{fC4bC3dgC4BP}]+[\text{fC3bC3bFH}]+[\text{fC3bC3bFHL1}]+[\text{fC3bC3bCR1}]\\+[\text{fC3biC3bFH}]+[\text{fC3biC3bFHL1}]+[\text{fC3biC3bCR1}]+[\text{fiC3biC3bCR1}]+[\text{fiC3bC3dgCR1}]\\+[\text{fC4biC3bC4BP}]+[\text{nfC4bC4BP}]+[\text{fC4bC4BP}]+[\text{fC4bCR1}]+[\text{fC4bC4bC4BP}]+[\text{fC4bC4dC4BP}]\\+[\text{fC4bC4bCR1}]+[\text{fC4bC4dCR1}]+[\text{hC4bCR1}]+[\text{hC4bC4BP}]+[\text{hC4bC4bCR1}]+[\text{hC4bC4bC4BP}]\\+[\text{hC4bC4dCR1}]+[\text{hC4bC4dC4BP}]+[\text{hC4bC3bFH}]+[\text{hC4bC3bFHL1}]+[\text{hC4bC3bCR1}]\\+[\text{hC4bC3bC4BP}]+[\text{hC4biC3bCR1}]+[\text{hC4biC3bC4BP}]+[\text{hC4bC3dgCR1}]+[\text{hC4bC3dgC4BP}]\\+[\text{hC4dC3bFH}]+[\text{hC4dC3bFHL1}]+[\text{hC4dC3bCR1}]+[\text{hC4diC3bCR1}]+[\text{hC3bFH}]+[\text{hC3bFHL1}]\\+[\text{hC3bCR1}]+[\text{hiC3bCR1}]+[\text{hC3bC3bFH}]+[\text{hC3bC3bFHL1}]+[\text{hC3bC3bCR1}]+[\text{hC3biC3bFH}]\\+[\text{hC3biC3bFHL1}]+[\text{hC3biC3bCR1}]+[\text{hiC3biC3bCR1}]+[\text{hiC3biC3dgCR1}]+[\text{pC4bC4BP}]\\+[\text{pC4bC4bC4BP}]+[\text{pC4bC4dC4BP}]+[\text{pC4bC3bC4BP}]+[\text{pC4biC3bC4BP}]+[\text{pC4bC3bFH}]\\+[\text{pC4bC3bFHL1}]+[\text{pC4dC3bFH}]+[\text{pC4dC3bFHL1}]+[\text{pC3bFH}]+[\text{pC3bFHL1}]+[\text{pC3bC3bFH}]\\+[\text{pC3bC3bFHL1}]+[\text{pC3biC3bFH}]+[\text{pC3biC3bFHL1}]+[\text{pC4bC4BPPTX3}]+[\text{pC4bC4bC4BPPTX3}]\\+[\text{pC4bC4dC4BPPTX3}]+[\text{pC4bC3bFHPTX3}]+[\text{pC4bC3bFHL1PTX3}]+[\text{pC4bC3bC4BPPTX3}]\\+[\text{pC4dC3bFHPTX3}]+[\text{pC4dC3bFHL1PTX3}]+[\text{pC4biC3bC4BPPTX3}]+[\text{pC3bFHPTX3}]\\+[\text{pC3bFHL1PTX3}]+[\text{pC3bC3bFHPTX3}]+[\text{pC3bC3bFHL1PTX3}]+[\text{pC3biC3bFHPTX3}]\\+[\text{pC3biC3bFHL1PTX3}]+[\text{pC4bpC4BP}]+[\text{pC4bC4bpC4BP}]+[\text{pC4bC4dpC4BP}]+[\text{pC4bC3bpC4BP}]\\+[\text{pC4biC3bpC4BP}]+[\text{pC4bC3bpFH}]+[\text{pC4bC3bpFHL1}]+[\text{pC4dC3bpFH}]+[\text{pC4dC3bpFHL1}]\\+[\text{pC3bpFH}]+[\text{pC3bpFHL1}]+[\text{pC3bC3bpFH}]+[\text{pC3bC3bpFHL1}]+[\text{pC3biC3bpFH}]+[\text{pC3biC3bpFHL1}]\end{array}\right)}$$

$$+\frac{k_{\mathrm{cat\ C3bH}}^{\mathrm{FI}}[\mathrm{FI}][\mathrm{piC3bC4bC4BPPTX3}]}{\left(\begin{array}{l}K_{\mathrm{m\ C3bH}}^{\mathrm{FI}}+[\mathrm{C3(H_2O)FH}]+[\mathrm{C3(H_2O)FHL1}]+[\mathrm{fC3bFH}]+[\mathrm{fC3bFHL1}]+[\mathrm{fC3bCR1}]+[\mathrm{fiC3bCR1}]\\
+[\mathrm{IgGC3bC3bFH}]+[\mathrm{IgGC3biC3bFH}]+[\mathrm{IgGC3bC3bFHL1}]+[\mathrm{IgGC3biC3bFHL1}]+[\mathrm{IgGC3bC3bCR1}]\\
+[\mathrm{IgGC3biC3bCR1}]+[\mathrm{IgGiC3biC3bCR1}]+[\mathrm{IgGiC3bC3dgCR1}]+[\mathrm{fC4bC3bFH}]+[\mathrm{fC4bC3bFHL1}]\\
+[\mathrm{fC4bC3bCR1}]+[\mathrm{fC4bC3bC4BP}]+[\mathrm{fC4dC3bFH}]+[\mathrm{fC4dC3bFHL1}]+[\mathrm{fC4dC3bCR1}]+[\mathrm{fC4diC3bCR1}]\\
+[\mathrm{fC4biC3bCR1}]+[\mathrm{fC4bC3dgCR1}]+[\mathrm{fC4bC3dgC4BP}]+[\mathrm{fC3bC3bFH}]+[\mathrm{fC3bC3bFHL1}]+[\mathrm{fC3bC3bCR1}]\\
+[\mathrm{fC3biC3bFH}]+[\mathrm{fC3biC3bFHL1}]+[\mathrm{fC3biC3bCR1}]+[\mathrm{fiC3biC3bCR1}]+[\mathrm{fiC3bC3dgCR1}]\\
+[\mathrm{fC4biC3bC4BP}]+[\mathrm{nfC4bC4BP}]+[\mathrm{fC4bC4BP}]+[\mathrm{fC4bCR1}]+[\mathrm{fC4bC4bC4BP}]+[\mathrm{fC4bC4dC4BP}]\\
+[\mathrm{fC4bC4bCR1}]+[\mathrm{fC4bC4dCR1}]+[\mathrm{hC4bCR1}]+[\mathrm{hC4bC4BP}]+[\mathrm{hC4bC4bCR1}]+[\mathrm{hC4bC4bC4BP}]\\
+[\mathrm{hC4bC4dCR1}]+[\mathrm{hC4bC4dC4BP}]+[\mathrm{hC4bC3bFH}]+[\mathrm{hC4bC3bFHL1}]+[\mathrm{hC4bC3bCR1}]\\
+[\mathrm{hC4bC3bC4BP}]+[\mathrm{hC4biC3bCR1}]+[\mathrm{hC4biC3bC4BP}]+[\mathrm{hC4bC3dgCR1}]+[\mathrm{hC4bC3dgC4BP}]\\
+[\mathrm{hC4dC3bFH}]+[\mathrm{hC4dC3bFHL1}]+[\mathrm{hC4dC3bCR1}]+[\mathrm{hC4diC3bCR1}]+[\mathrm{hC3bFH}]+[\mathrm{hC3bFHL1}]\\
+[\mathrm{hC3bCR1}]+[\mathrm{hiC3bCR1}]+[\mathrm{hC3bC3bFH}]+[\mathrm{hC3bC3bFHL1}]+[\mathrm{hC3bC3bCR1}]+[\mathrm{hC3biC3bFH}]\\
+[\mathrm{hC3biC3bFHL1}]+[\mathrm{hC3biC3bCR1}]+[\mathrm{hiC3biC3bCR1}]+[\mathrm{hiC3biC3dgCR1}]+[\mathrm{pC4bC4BP}]\\
+[\mathrm{pC4bC4bC4BP}]+[\mathrm{pC4bC4dC4BP}]+[\mathrm{pC4bC3bC4BP}]+[\mathrm{pC4biC3bC4BP}]+[\mathrm{pC4bC3bFH}]\\
+[\mathrm{pC4bC3bFHL1}]+[\mathrm{pC4dC3bFH}]+[\mathrm{pC4dC3bFHL1}]+[\mathrm{pC3bFH}]+[\mathrm{pC3bFHL1}]+[\mathrm{pC3bC3bFH}]\\
+[\mathrm{pC3bC3bFHL1}]+[\mathrm{pC3biC3bFH}]+[\mathrm{pC3biC3bFHL1}]+[\mathrm{pC4bC4BPPTX3}]+[\mathrm{pC4bC4bC4BPPTX3}]\\
+[\mathrm{pC4bC4dC4BPPTX3}]+[\mathrm{pC4bC3bFHPTX3}]+[\mathrm{pC4bC3bFHL1PTX3}]+[\mathrm{pC4bC3bC4BPPTX3}]\\
+[\mathrm{pC4dC3bFHPTX3}]+[\mathrm{pC4dC3bFHL1PTX3}]+[\mathrm{pC4biC3bC4BPPTX3}]+[\mathrm{pC3bFHPTX3}]\\
+[\mathrm{pC3bFHL1PTX3}]+[\mathrm{pC3bC3bFHPTX3}]+[\mathrm{pC3bC3bFHL1PTX3}]+[\mathrm{pC3biC3bFHPTX3}]\\
+[\mathrm{pC3biC3bFHL1PTX3}]+[\mathrm{pC4bpC4BP}]+[\mathrm{pC4bC4bpC4BP}]+[\mathrm{pC4bC4dpC4BP}]+[\mathrm{pC4bC3bpC4BP}]\\
+[\mathrm{pC4biC3bpC4BP}]+[\mathrm{pC4bC3bpFH}]+[\mathrm{pC4bC3bpFHL1}]+[\mathrm{pC4dC3bpFH}]+[\mathrm{pC4dC3bpFHL1}]\\
+[\mathrm{pC3bpFH}]+[\mathrm{pC3bpFHL1}]+[\mathrm{pC3bC3bpFH}]+[\mathrm{pC3bC3bpFHL1}]+[\mathrm{pC3biC3bpFH}]+[\mathrm{pC3biC3bpFHL1}]\end{array}\right)}$$

$$+\frac{k_{\text{cat C3bH}}^{\text{FI}}[\text{FI}][\text{piC3bC4bpC4BP}]}{\left(\begin{array}{l}K_{\text{m C3bH}}^{\text{FI}}+[\text{C3(H}_2\text{O)FH}]+[\text{C3(H}_2\text{O)FHL1}]+[\text{fC3bFH}]+[\text{fC3bFHL1}]+[\text{fC3bCR1}]+[\text{fiC3bCR1}]\\
+[\text{IgGC3bC3bFH}]+[\text{IgGC3biC3bFH}]+[\text{IgGC3bC3bFHL1}]+[\text{IgGC3biC3bFHL1}]+[\text{IgGC3bC3bCR1}]\\
+[\text{IgGC3biC3bCR1}]+[\text{IgGiC3biC3bCR1}]+[\text{IgGiC3bC3dgCR1}]+[\text{fC4bC3bFH}]+[\text{fC4bC3bFHL1}]\\
+[\text{fC4bC3bCR1}]+[\text{fC4bC3bC4BP}]+[\text{fC4dC3bFH}]+[\text{fC4dC3bFHL1}]+[\text{fC4dC3bCR1}]+[\text{fC4diC3bCR1}]\\
+[\text{fC4biC3bCR1}]+[\text{fC4bC3dgCR1}]+[\text{fC4bC3dgC4BP}]+[\text{fC3bC3bFH}]+[\text{fC3bC3bFHL1}]+[\text{fC3bC3bCR1}]\\
+[\text{fC3biC3bFH}]+[\text{fC3biC3bFHL1}]+[\text{fC3biC3bCR1}]+[\text{fiC3biC3bCR1}]+[\text{fiC3bC3dgCR1}]\\
+[\text{fC4biC3bC4BP}]+[\text{nfC4bC4BP}]+[\text{fC4bC4BP}]+[\text{fC4bCR1}]+[\text{fC4bC4bC4BP}]+[\text{fC4bC4dC4BP}]\\
+[\text{fC4bC4bCR1}]+[\text{fC4bC4dCR1}]+[\text{hC4bCR1}]+[\text{hC4bC4BP}]+[\text{hC4bC4bCR1}]+[\text{hC4bC4bC4BP}]\\
+[\text{hC4bC4dCR1}]+[\text{hC4bC4dC4BP}]+[\text{hC4bC3bFH}]+[\text{hC4bC3bFHL1}]+[\text{hC4bC3bCR1}]\\
+[\text{hC4bC3bC4BP}]+[\text{hC4biC3bCR1}]+[\text{hC4biC3bC4BP}]+[\text{hC4bC3dgCR1}]+[\text{hC4bC3dgC4BP}]\\
+[\text{hC4dC3bFH}]+[\text{hC4dC3bFHL1}]+[\text{hC4dC3bCR1}]+[\text{hC4diC3bCR1}]+[\text{hC3bFH}]+[\text{hC3bFHL1}]\\
+[\text{hC3bCR1}]+[\text{hiC3bCR1}]+[\text{hC3bC3bFH}]+[\text{hC3bC3bFHL1}]+[\text{hC3bC3bCR1}]+[\text{hC3biC3bFH}]\\
+[\text{hC3biC3bFHL1}]+[\text{hC3biC3bCR1}]+[\text{hiC3biC3bCR1}]+[\text{hiC3biC3dgCR1}]+[\text{pC4bC4BP}]\\
+[\text{pC4bC4bC4BP}]+[\text{pC4bC4dC4BP}]+[\text{pC4bC3bC4BP}]+[\text{pC4biC3bC4BP}]+[\text{pC4bC3bFH}]\\
+[\text{pC4bC3bFHL1}]+[\text{pC4dC3bFH}]+[\text{pC4dC3bFHL1}]+[\text{pC3bFH}]+[\text{pC3bFHL1}]+[\text{pC3bC3bFH}]\\
+[\text{pC3bC3bFHL1}]+[\text{pC3biC3bFH}]+[\text{pC3biC3bFHL1}]+[\text{pC4bC4BPPTX3}]+[\text{pC4bC4bC4BPPTX3}]\\
+[\text{pC4bC4dC4BPPTX3}]+[\text{pC4bC3bFHPTX3}]+[\text{pC4bC3bFHL1PTX3}]+[\text{pC4bC3bC4BPPTX3}]\\
+[\text{pC4dC3bFHPTX3}]+[\text{pC4dC3bFHL1PTX3}]+[\text{pC4biC3bC4BPPTX3}]+[\text{pC3bFHPTX3}]\\
+[\text{pC3bFHL1PTX3}]+[\text{pC3bC3bFHPTX3}]+[\text{pC3bC3bFHL1PTX3}]+[\text{pC3biC3bFHPTX3}]\\
+[\text{pC3biC3bFHL1PTX3}]+[\text{pC4bpC4BP}]+[\text{pC4bC4bpC4BP}]+[\text{pC4bC4dpC4BP}]+[\text{pC4bC3bpC4BP}]\\
+[\text{pC4biC3bpC4BP}]+[\text{pC4bC3bpFH}]+[\text{pC4bC3bpFHL1}]+[\text{pC4dC3bpFH}]+[\text{pC4dC3bpFHL1}]\\
+[\text{pC3bpFH}]+[\text{pC3bpFHL1}]+[\text{pC3bC3bpFH}]+[\text{pC3bC3bpFHL1}]+[\text{pC3biC3bpFH}]+[\text{pC3biC3bpFHL1}]\end{array}\right)}$$

$$
+\frac{k^{\mathrm{FI}}_{\mathrm{cat\,C3bH}}\left[\mathrm{FI}\right]\left[\mathrm{pC3bC4dpFH}\right]}{\left(\begin{aligned}
&K^{\mathrm{FI}}_{\mathrm{m\,C3bH}}+\left[\mathrm{C3(H_2O)FH}\right]+\left[\mathrm{C3(H_2O)FHL1}\right]+\left[\mathrm{fC3bFH}\right]+\left[\mathrm{fC3bFHL1}\right]+\left[\mathrm{fC3bCR1}\right]+\left[\mathrm{fiC3bCR1}\right]\\
&+\left[\mathrm{IgGC3bC3bFH}\right]+\left[\mathrm{IgGC3biC3bFH}\right]+\left[\mathrm{IgGC3bC3bFHL1}\right]+\left[\mathrm{IgGC3biC3bFHL1}\right]+\left[\mathrm{IgGC3bC3bCR1}\right]\\
&+\left[\mathrm{IgGC3biC3bCR1}\right]+\left[\mathrm{IgGiC3biC3bCR1}\right]+\left[\mathrm{IgGiC3bC3dgCR1}\right]+\left[\mathrm{fC4bC3bFH}\right]+\left[\mathrm{fC4bC3bFHL1}\right]\\
&+\left[\mathrm{fC4bC3bCR1}\right]+\left[\mathrm{fC4bC3bC4BP}\right]+\left[\mathrm{fC4dC3bFH}\right]+\left[\mathrm{fC4dC3bFHL1}\right]+\left[\mathrm{fC4dC3bCR1}\right]+\left[\mathrm{fC4diC3bCR1}\right]\\
&+\left[\mathrm{fC4biC3bCR1}\right]+\left[\mathrm{fC4bC3dgCR1}\right]+\left[\mathrm{fC4bC3dgC4BP}\right]+\left[\mathrm{fC3bC3bFH}\right]+\left[\mathrm{fC3bC3bFHL1}\right]+\left[\mathrm{fC3bC3bCR1}\right]\\
&+\left[\mathrm{fC3biC3bFH}\right]+\left[\mathrm{fC3biC3bFHL1}\right]+\left[\mathrm{fC3biC3bCR1}\right]+\left[\mathrm{fiC3biC3bCR1}\right]+\left[\mathrm{fiC3bC3dgCR1}\right]\\
&+\left[\mathrm{fC4biC3bC4BP}\right]+\left[\mathrm{nfC4bC4BP}\right]+\left[\mathrm{fC4bC4BP}\right]+\left[\mathrm{fC4bCR1}\right]+\left[\mathrm{fC4bC4bC4BP}\right]+\left[\mathrm{fC4bC4dC4BP}\right]\\
&+\left[\mathrm{fC4bC4bCR1}\right]+\left[\mathrm{fC4bC4dCR1}\right]+\left[\mathrm{hC4bCR1}\right]+\left[\mathrm{hC4bC4BP}\right]+\left[\mathrm{hC4bC4bCR1}\right]+\left[\mathrm{hC4bC4bC4BP}\right]\\
&+\left[\mathrm{hC4bC4dCR1}\right]+\left[\mathrm{hC4bC4dC4BP}\right]+\left[\mathrm{hC4bC3bFH}\right]+\left[\mathrm{hC4bC3bFHL1}\right]+\left[\mathrm{hC4bC3bCR1}\right]\\
&+\left[\mathrm{hC4bC3bC4BP}\right]+\left[\mathrm{hC4biC3bCR1}\right]+\left[\mathrm{hC4biC3bC4BP}\right]+\left[\mathrm{hC4bC3dgCR1}\right]+\left[\mathrm{hC4bC3dgC4BP}\right]\\
&+\left[\mathrm{hC4dC3bFH}\right]+\left[\mathrm{hC4dC3bFHL1}\right]+\left[\mathrm{hC4dC3bCR1}\right]+\left[\mathrm{hC4diC3bCR1}\right]+\left[\mathrm{hC3bFH}\right]+\left[\mathrm{hC3bFHL1}\right]\\
&+\left[\mathrm{hC3bCR1}\right]+\left[\mathrm{hiC3bCR1}\right]+\left[\mathrm{hC3bC3bFH}\right]+\left[\mathrm{hC3bC3bFHL1}\right]+\left[\mathrm{hC3bC3bCR1}\right]+\left[\mathrm{hC3biC3bFH}\right]\\
&+\left[\mathrm{hC3biC3bFHL1}\right]+\left[\mathrm{hC3biC3bCR1}\right]+\left[\mathrm{hiC3biC3bCR1}\right]+\left[\mathrm{hiC3biC3dgCR1}\right]+\left[\mathrm{pC4bC4BP}\right]\\
&+\left[\mathrm{pC4bC4bC4BP}\right]+\left[\mathrm{pC4bC4dC4BP}\right]+\left[\mathrm{pC4bC3bC4BP}\right]+\left[\mathrm{pC4biC3bC4BP}\right]+\left[\mathrm{pC4bC3bFH}\right]\\
&+\left[\mathrm{pC4bC3bFHL1}\right]+\left[\mathrm{pC4dC3bFH}\right]+\left[\mathrm{pC4dC3bFHL1}\right]+\left[\mathrm{pC3bFH}\right]+\left[\mathrm{pC3bFHL1}\right]+\left[\mathrm{pC3bC3bFH}\right]\\
&+\left[\mathrm{pC3bC3bFHL1}\right]+\left[\mathrm{pC3biC3bFH}\right]+\left[\mathrm{pC3biC3bFHL1}\right]+\left[\mathrm{pC4bC4BPPTX3}\right]+\left[\mathrm{pC4bC4bC4BPPTX3}\right]\\
&+\left[\mathrm{pC4bC4dC4BPPTX3}\right]+\left[\mathrm{pC4bC3bFHPTX3}\right]+\left[\mathrm{pC4bC3bFHL1PTX3}\right]+\left[\mathrm{pC4bC3bC4BPPTX3}\right]\\
&+\left[\mathrm{pC4dC3bFHPTX3}\right]+\left[\mathrm{pC4dC3bFHL1PTX3}\right]+\left[\mathrm{pC4biC3bC4BPPTX3}\right]+\left[\mathrm{pC3bFHPTX3}\right]\\
&+\left[\mathrm{pC3bFHL1PTX3}\right]+\left[\mathrm{pC3bC3bFHPTX3}\right]+\left[\mathrm{pC3bC3bFHL1PTX3}\right]+\left[\mathrm{pC3biC3bFHPTX3}\right]\\
&+\left[\mathrm{pC3biC3bFHL1PTX3}\right]+\left[\mathrm{pC4bpC4BP}\right]+\left[\mathrm{pC4bC4bpC4BP}\right]+\left[\mathrm{pC4bC4dpC4BP}\right]+\left[\mathrm{pC4bC3bpC4BP}\right]\\
&+\left[\mathrm{pC4biC3bpC4BP}\right]+\left[\mathrm{pC4bC3bpFH}\right]+\left[\mathrm{pC4bC3bpFHL1}\right]+\left[\mathrm{pC4dC3bpFH}\right]+\left[\mathrm{pC4dC3bpFHL1}\right]\\
&+\left[\mathrm{pC3bpFH}\right]+\left[\mathrm{pC3bpFHL1}\right]+\left[\mathrm{pC3bC3bpFH}\right]+\left[\mathrm{pC3bC3bpFHL1}\right]+\left[\mathrm{pC3biC3bpFH}\right]+\left[\mathrm{pC3biC3bpFHL1}\right]
\end{aligned}\right)}
$$

$$+\frac{k_{\mathrm{cat\ C3bH}}^{\mathrm{FI}}[\mathrm{FI}][\mathrm{pC3bC4dpFHL1}]}{\left(\begin{array}{l}K_{\mathrm{m\ C3bH}}^{\mathrm{FI}}+[\mathrm{C3(H_2O)FH}]+[\mathrm{C3(H_2O)FHL1}]+[\mathrm{fC3bFH}]+[\mathrm{fC3bFHL1}]+[\mathrm{fC3bCR1}]+[\mathrm{fiC3bCR1}]\\
+[\mathrm{IgGC3bC3bFH}]+[\mathrm{IgGC3biC3bFH}]+[\mathrm{IgGC3bC3bFHL1}]+[\mathrm{IgGC3biC3bFHL1}]+[\mathrm{IgGC3bC3bCR1}]\\
+[\mathrm{IgGC3biC3bCR1}]+[\mathrm{IgGiC3biC3bCR1}]+[\mathrm{IgGiC3bC3dgCR1}]+[\mathrm{fC4bC3bFH}]+[\mathrm{fC4bC3bFHL1}]\\
+[\mathrm{fC4bC3bCR1}]+[\mathrm{fC4bC3bC4BP}]+[\mathrm{fC4dC3bFH}]+[\mathrm{fC4dC3bFHL1}]+[\mathrm{fC4dC3bCR1}]+[\mathrm{fC4diC3bCR1}]\\
+[\mathrm{fC4biC3bCR1}]+[\mathrm{fC4bC3dgCR1}]+[\mathrm{fC4bC3dgC4BP}]+[\mathrm{fC3bC3bFH}]+[\mathrm{fC3bC3bFHL1}]+[\mathrm{fC3bC3bCR1}]\\
+[\mathrm{fC3biC3bFH}]+[\mathrm{fC3biC3bFHL1}]+[\mathrm{fC3biC3bCR1}]+[\mathrm{fiC3biC3bCR1}]+[\mathrm{fiC3bC3dgCR1}]\\
+[\mathrm{fC4biC3bC4BP}]+[\mathrm{nfC4bC4BP}]+[\mathrm{fC4bC4BP}]+[\mathrm{fC4bCR1}]+[\mathrm{fC4bC4bC4BP}]+[\mathrm{fC4bC4dC4BP}]\\
+[\mathrm{fC4bC4bCR1}]+[\mathrm{fC4bC4dCR1}]+[\mathrm{hC4bCR1}]+[\mathrm{hC4bC4BP}]+[\mathrm{hC4bC4bCR1}]+[\mathrm{hC4bC4bC4BP}]\\
+[\mathrm{hC4bC4dCR1}]+[\mathrm{hC4bC4dC4BP}]+[\mathrm{hC4bC3bFH}]+[\mathrm{hC4bC3bFHL1}]+[\mathrm{hC4bC3bCR1}]\\
+[\mathrm{hC4bC3bC4BP}]+[\mathrm{hC4biC3bCR1}]+[\mathrm{hC4biC3bC4BP}]+[\mathrm{hC4bC3dgCR1}]+[\mathrm{hC4bC3dgC4BP}]\\
+[\mathrm{hC4dC3bFH}]+[\mathrm{hC4dC3bFHL1}]+[\mathrm{hC4dC3bCR1}]+[\mathrm{hC4diC3bCR1}]+[\mathrm{hC3bFH}]+[\mathrm{hC3bFHL1}]\\
+[\mathrm{hC3bCR1}]+[\mathrm{hiC3bCR1}]+[\mathrm{hC3bC3bFH}]+[\mathrm{hC3bC3bFHL1}]+[\mathrm{hC3bC3bCR1}]+[\mathrm{hC3biC3bFH}]\\
+[\mathrm{hC3biC3bFHL1}]+[\mathrm{hC3biC3bCR1}]+[\mathrm{hiC3biC3bCR1}]+[\mathrm{hiC3biC3dgCR1}]+[\mathrm{pC4bC4BP}]\\
+[\mathrm{pC4bC4bC4BP}]+[\mathrm{pC4bC4dC4BP}]+[\mathrm{pC4bC3bC4BP}]+[\mathrm{pC4biC3bC4BP}]+[\mathrm{pC4bC3bFH}]\\
+[\mathrm{pC4bC3bFHL1}]+[\mathrm{pC4dC3bFH}]+[\mathrm{pC4dC3bFHL1}]+[\mathrm{pC3bFH}]+[\mathrm{pC3bFHL1}]+[\mathrm{pC3bC3bFH}]\\
+[\mathrm{pC3bC3bFHL1}]+[\mathrm{pC3biC3bFH}]+[\mathrm{pC3biC3bFHL1}]+[\mathrm{pC4bC4BPPTX3}]+[\mathrm{pC4bC4bC4BPPTX3}]\\
+[\mathrm{pC4bC4dC4BPPTX3}]+[\mathrm{pC4bC3bFHPTX3}]+[\mathrm{pC4bC3bFHL1PTX3}]+[\mathrm{pC4bC3bC4BPPTX3}]\\
+[\mathrm{pC4dC3bFHPTX3}]+[\mathrm{pC4dC3bFHL1PTX3}]+[\mathrm{pC4biC3bC4BPPTX3}]+[\mathrm{pC3bFHPTX3}]\\
+[\mathrm{pC3bFHL1PTX3}]+[\mathrm{pC3bC3bFHPTX3}]+[\mathrm{pC3bC3bFHL1PTX3}]+[\mathrm{pC3biC3bFHPTX3}]\\
+[\mathrm{pC3biC3bFHL1PTX3}]+[\mathrm{pC4bpC4BP}]+[\mathrm{pC4bC4bpC4BP}]+[\mathrm{pC4bC4dpC4BP}]+[\mathrm{pC4bC3bpC4BP}]\\
+[\mathrm{pC4biC3bpC4BP}]+[\mathrm{pC4bC3bpFH}]+[\mathrm{pC4bC3bpFHL1}]+[\mathrm{pC4dC3bpFH}]+[\mathrm{pC4dC3bpFHL1}]\\
+[\mathrm{pC3bpFH}]+[\mathrm{pC3bpFHL1}]+[\mathrm{pC3bC3bpFH}]+[\mathrm{pC3bC3bpFHL1}]+[\mathrm{pC3biC3bpFH}]+[\mathrm{pC3biC3bpFHL1}]\end{array}\right)}$$

223

$$
\begin{aligned}
\frac{d\left[\mathrm{pC3bFH}\right]}{dt} &= k_{\mathrm{C3bH}}^{+}\left[\mathrm{pC3b}\right]\left[\mathrm{FH}\right]-k_{\mathrm{C3bH}}^{-}\left[\mathrm{pC3bFH}\right] \\
&-\frac{k_{\mathrm{cat\ C3bH}}^{\mathrm{FI}}\left[\mathrm{FI}\right]\left[\mathrm{pC3bFH}\right]}{\left(\begin{array}{l}
K_{\mathrm{m\ C3bH}}^{\mathrm{FI}}+\left[\mathrm{C3(H_2O)FH}\right]+\left[\mathrm{C3(H_2O)FHL1}\right]+\left[\mathrm{fC3bFH}\right]+\left[\mathrm{fC3bFHL1}\right]+\left[\mathrm{fC3bCR1}\right]+\left[\mathrm{fiC3bCR1}\right] \\
+\left[\mathrm{IgGC3bC3bFH}\right]+\left[\mathrm{IgGC3biC3bFH}\right]+\left[\mathrm{IgGC3bC3bFHL1}\right]+\left[\mathrm{IgGC3biC3bFHL1}\right]+\left[\mathrm{IgGC3bC3bCR1}\right] \\
+\left[\mathrm{IgGC3biC3bCR1}\right]+\left[\mathrm{IgGiC3biC3bCR1}\right]+\left[\mathrm{IgGiC3bC3dgCR1}\right]+\left[\mathrm{fC4bC3bFH}\right]+\left[\mathrm{fC4bC3bFHL1}\right] \\
+\left[\mathrm{fC4bC3bCR1}\right]+\left[\mathrm{fC4bC3bC4BP}\right]+\left[\mathrm{fC4dC3bFH}\right]+\left[\mathrm{fC4dC3bFHL1}\right]+\left[\mathrm{fC4dC3bCR1}\right]+\left[\mathrm{fC4diC3bCR1}\right] \\
+\left[\mathrm{fC4biC3bCR1}\right]+\left[\mathrm{fC4bC3dgCR1}\right]+\left[\mathrm{fC4bC3dgC4BP}\right]+\left[\mathrm{fC3bC3bFH}\right]+\left[\mathrm{fC3bC3bFHL1}\right]+\left[\mathrm{fC3bC3bCR1}\right] \\
+\left[\mathrm{fC3biC3bFH}\right]+\left[\mathrm{fC3biC3bFHL1}\right]+\left[\mathrm{fC3biC3bCR1}\right]+\left[\mathrm{fiC3biC3bCR1}\right]+\left[\mathrm{fiC3bC3dgCR1}\right] \\
+\left[\mathrm{fC4biC3bC4BP}\right]+\left[\mathrm{nfC4bC4BP}\right]+\left[\mathrm{fC4bC4BP}\right]+\left[\mathrm{fC4bCR1}\right]+\left[\mathrm{fC4bC4bC4BP}\right]+\left[\mathrm{fC4bC4dC4BP}\right] \\
+\left[\mathrm{fC4bC4bCR1}\right]+\left[\mathrm{fC4bC4dCR1}\right]+\left[\mathrm{hC4bCR1}\right]+\left[\mathrm{hC4bC4BP}\right]+\left[\mathrm{hC4bC4bCR1}\right]+\left[\mathrm{hC4bC4bC4BP}\right] \\
+\left[\mathrm{hC4bC4dCR1}\right]+\left[\mathrm{hC4bC4dC4BP}\right]+\left[\mathrm{hC4bC3bFH}\right]+\left[\mathrm{hC4bC3bFHL1}\right]+\left[\mathrm{hC4bC3bCR1}\right] \\
+\left[\mathrm{hC4bC3bC4BP}\right]+\left[\mathrm{hC4biC3bCR1}\right]+\left[\mathrm{hC4biC3bC4BP}\right]+\left[\mathrm{hC4bC3dgCR1}\right]+\left[\mathrm{hC4bC3dgC4BP}\right] \\
+\left[\mathrm{hC4dC3bFH}\right]+\left[\mathrm{hC4dC3bFHL1}\right]+\left[\mathrm{hC4dC3bCR1}\right]+\left[\mathrm{hC4diC3bCR1}\right]+\left[\mathrm{hC3bFH}\right]+\left[\mathrm{hC3bFHL1}\right] \\
+\left[\mathrm{hC3bCR1}\right]+\left[\mathrm{hiC3bCR1}\right]+\left[\mathrm{hC3bC3bFH}\right]+\left[\mathrm{hC3bC3bFHL1}\right]+\left[\mathrm{hC3bC3bCR1}\right]+\left[\mathrm{hC3biC3bFH}\right] \\
+\left[\mathrm{hC3biC3bFHL1}\right]+\left[\mathrm{hC3biC3bCR1}\right]+\left[\mathrm{hiC3biC3bCR1}\right]+\left[\mathrm{hiC3biC3dgCR1}\right]+\left[\mathrm{pC4bC4BP}\right] \\
+\left[\mathrm{pC4bC4bC4BP}\right]+\left[\mathrm{pC4bC4dC4BP}\right]+\left[\mathrm{pC4bC3bC4BP}\right]+\left[\mathrm{pC4biC3bC4BP}\right]+\left[\mathrm{pC4bC3bFH}\right] \\
+\left[\mathrm{pC4bC3bFHL1}\right]+\left[\mathrm{pC4dC3bFH}\right]+\left[\mathrm{pC4dC3bFHL1}\right]+\left[\mathrm{pC3bFH}\right]+\left[\mathrm{pC3bFHL1}\right]+\left[\mathrm{pC3bC3bFH}\right] \\
+\left[\mathrm{pC3bC3bFHL1}\right]+\left[\mathrm{pC3biC3bFH}\right]+\left[\mathrm{pC3biC3bFHL1}\right]+\left[\mathrm{pC4bC4BPPTX3}\right]+\left[\mathrm{pC4bC4bC4BPPTX3}\right] \\
+\left[\mathrm{pC4bC4dC4BPPTX3}\right]+\left[\mathrm{pC4bC3bFHPTX3}\right]+\left[\mathrm{pC4bC3bFHL1PTX3}\right]+\left[\mathrm{pC4bC3bC4BPPTX3}\right] \\
+\left[\mathrm{pC4dC3bFHPTX3}\right]+\left[\mathrm{pC4dC3bFHL1PTX3}\right]+\left[\mathrm{pC4biC3bC4BPPTX3}\right]+\left[\mathrm{pC3bFHPTX3}\right] \\
+\left[\mathrm{pC3bFHL1PTX3}\right]+\left[\mathrm{pC3bC3bFHPTX3}\right]+\left[\mathrm{pC3bC3bFHL1PTX3}\right]+\left[\mathrm{pC3biC3bFHPTX3}\right] \\
+\left[\mathrm{pC3biC3bFHL1PTX3}\right]+\left[\mathrm{pC4bpC4BP}\right]+\left[\mathrm{pC4bC4bpC4BP}\right]+\left[\mathrm{pC4bC4dpC4BP}\right]+\left[\mathrm{pC4bC3bpC4BP}\right] \\
+\left[\mathrm{pC4biC3bpC4BP}\right]+\left[\mathrm{pC4bC3bpFH}\right]+\left[\mathrm{pC4bC3bpFHL1}\right]+\left[\mathrm{pC4dC3bpFH}\right]+\left[\mathrm{pC4dC3bpFHL1}\right] \\
+\left[\mathrm{pC3bpFH}\right]+\left[\mathrm{pC3bpFHL1}\right]+\left[\mathrm{pC3bC3bpFH}\right]+\left[\mathrm{pC3bC3bpFHL1}\right]+\left[\mathrm{pC3biC3bpFH}\right]+\left[\mathrm{pC3biC3bpFHL1}\right]
\end{array}\right)}
\end{aligned}
$$

224

$$\frac{d[\mathrm{pC3bFHL1}]}{dt} = k^{+}_{\mathrm{C3bFHL1}}[\mathrm{pC3b}][\mathrm{FHL1}] - k^{-}_{\mathrm{C3bFHL1}}[\mathrm{pC3bFHL1}]$$

$$-\frac{k^{\mathrm{FI}}_{\mathrm{cat\ C3bH}}[\mathrm{FI}][\mathrm{pC3bFHL1}]}{\left(\begin{array}{l} K^{\mathrm{FI}}_{\mathrm{m\ C3bH}} + [\mathrm{C3(H_2O)FH}] + [\mathrm{C3(H_2O)FHL1}] + [\mathrm{fC3bFH}] + [\mathrm{fC3bFHL1}] + [\mathrm{fC3bCR1}] + [\mathrm{fiC3bCR1}] \\ + [\mathrm{IgGC3bC3bFH}] + [\mathrm{IgGC3biC3bFH}] + [\mathrm{IgGC3bC3bFHL1}] + [\mathrm{IgGC3biC3bFHL1}] + [\mathrm{IgGC3bC3bCR1}] \\ + [\mathrm{IgGC3biC3bCR1}] + [\mathrm{IgGiC3biC3bCR1}] + [\mathrm{IgGiC3bC3dgCR1}] + [\mathrm{fC4bC3bFH}] + [\mathrm{fC4bC3bFHL1}] \\ + [\mathrm{fC4bC3bCR1}] + [\mathrm{fC4bC3bC4BP}] + [\mathrm{fC4dC3bFH}] + [\mathrm{fC4dC3bFHL1}] + [\mathrm{fC4dC3bCR1}] + [\mathrm{fC4diC3bCR1}] \\ + [\mathrm{fC4biC3bCR1}] + [\mathrm{fC4bC3dgCR1}] + [\mathrm{fC4bC3dgC4BP}] + [\mathrm{fC3bC3bFH}] + [\mathrm{fC3bC3bFHL1}] + [\mathrm{fC3bC3bCR1}] \\ + [\mathrm{fC3biC3bFH}] + [\mathrm{fC3biC3bFHL1}] + [\mathrm{fC3biC3bCR1}] + [\mathrm{fiC3biC3bCR1}] + [\mathrm{fiC3bC3dgCR1}] \\ + [\mathrm{fC4biC3bC4BP}] + [\mathrm{nfC4bC4BP}] + [\mathrm{fC4bC4BP}] + [\mathrm{fC4bCR1}] + [\mathrm{fC4bC4bC4BP}] + [\mathrm{fC4bC4dC4BP}] \\ + [\mathrm{fC4bC4bCR1}] + [\mathrm{fC4bC4dCR1}] + [\mathrm{hC4bCR1}] + [\mathrm{hC4bC4BP}] + [\mathrm{hC4bC4bCR1}] + [\mathrm{hC4bC4bC4BP}] \\ + [\mathrm{hC4bC4dCR1}] + [\mathrm{hC4bC4dC4BP}] + [\mathrm{hC4bC3bFH}] + [\mathrm{hC4bC3bFHL1}] + [\mathrm{hC4bC3bCR1}] \\ + [\mathrm{hC4bC3bC4BP}] + [\mathrm{hC4biC3bCR1}] + [\mathrm{hC4biC3bC4BP}] + [\mathrm{hC4bC3dgCR1}] + [\mathrm{hC4bC3dgC4BP}] \\ + [\mathrm{hC4dC3bFH}] + [\mathrm{hC4dC3bFHL1}] + [\mathrm{hC4dC3bCR1}] + [\mathrm{hC4diC3bCR1}] + [\mathrm{hC3bFH}] + [\mathrm{hC3bFHL1}] \\ + [\mathrm{hC3bCR1}] + [\mathrm{hiC3bCR1}] + [\mathrm{hC3bC3bFH}] + [\mathrm{hC3bC3bFHL1}] + [\mathrm{hC3bC3bCR1}] + [\mathrm{hC3biC3bFH}] \\ + [\mathrm{hC3biC3bFHL1}] + [\mathrm{hC3biC3bCR1}] + [\mathrm{hiC3biC3bCR1}] + [\mathrm{hiC3biC3dgCR1}] + [\mathrm{pC4bC4BP}] \\ + [\mathrm{pC4bC4bC4BP}] + [\mathrm{pC4bC4dC4BP}] + [\mathrm{pC4bC3bC4BP}] + [\mathrm{pC4biC3bC4BP}] + [\mathrm{pC4bC3bFH}] \\ + [\mathrm{pC4bC3bFHL1}] + [\mathrm{pC4dC3bFH}] + [\mathrm{pC4dC3bFHL1}] + [\mathrm{pC3bFH}] + [\mathrm{pC3bFHL1}] + [\mathrm{pC3bC3bFH}] \\ + [\mathrm{pC3bC3bFHL1}] + [\mathrm{pC3biC3bFH}] + [\mathrm{pC3biC3bFHL1}] + [\mathrm{pC4bC4BPPTX3}] + [\mathrm{pC4bC4bC4BPPTX3}] \\ + [\mathrm{pC4bC4dC4BPPTX3}] + [\mathrm{pC4bC3bFHPTX3}] + [\mathrm{pC4bC3bFHL1PTX3}] + [\mathrm{pC4bC3bC4BPPTX3}] \\ + [\mathrm{pC4dC3bFHPTX3}] + [\mathrm{pC4dC3bFHL1PTX3}] + [\mathrm{pC4biC3bC4BPPTX3}] + [\mathrm{pC3bFHPTX3}] \\ + [\mathrm{pC3bFHL1PTX3}] + [\mathrm{pC3bC3bFHPTX3}] + [\mathrm{pC3bC3bFHL1PTX3}] + [\mathrm{pC3biC3bFHPTX3}] \\ + [\mathrm{pC3biC3bFHL1PTX3}] + [\mathrm{pC4bpC4BP}] + [\mathrm{pC4bC4bpC4BP}] + [\mathrm{pC4bC4dpC4BP}] + [\mathrm{pC4bC3bpC4BP}] \\ + [\mathrm{pC4biC3bpC4BP}] + [\mathrm{pC4bC3bpFH}] + [\mathrm{pC4bC3bpFHL1}] + [\mathrm{pC4dC3bpFH}] + [\mathrm{pC4dC3bpFHL1}] \\ + [\mathrm{pC3bpFH}] + [\mathrm{pC3bpFHL1}] + [\mathrm{pC3bC3bpFH}] + [\mathrm{pC3bC3bpFHL1}] + [\mathrm{pC3biC3bpFH}] + [\mathrm{pC3biC3bpFHL1}] \end{array}\right)}$$

225

$$\frac{d[\mathrm{piC3b}]}{dt}=$$

$$\frac{k_{\mathrm{cat\ C3bH}}^{\mathrm{FI}}[\mathrm{FI}][\mathrm{pC3bFH}]}{\left(\begin{array}{l}K_{\mathrm{m\ C3bH}}^{\mathrm{FI}}+[\mathrm{C3(H_2O)FH}]+[\mathrm{C3(H_2O)FHL1}]+[\mathrm{fC3bFH}]+[\mathrm{fC3bFHL1}]+[\mathrm{fC3bCR1}]+[\mathrm{fiC3bCR1}]\\
+[\mathrm{IgGC3bC3bFH}]+[\mathrm{IgGC3biC3bFH}]+[\mathrm{IgGC3bC3bFHL1}]+[\mathrm{IgGC3biC3bFHL1}]+[\mathrm{IgGC3bC3bCR1}]\\
+[\mathrm{IgGC3biC3bCR1}]+[\mathrm{IgGiC3biC3bCR1}]+[\mathrm{IgGiC3bC3dgCR1}]+[\mathrm{fC4bC3bFH}]+[\mathrm{fC4bC3bFHL1}]\\
+[\mathrm{fC4bC3bCR1}]+[\mathrm{fC4bC3bC4BP}]+[\mathrm{fC4dC3bFH}]+[\mathrm{fC4dC3bFHL1}]+[\mathrm{fC4dC3bCR1}]+[\mathrm{fC4diC3bCR1}]\\
+[\mathrm{fC4biC3bCR1}]+[\mathrm{fC4bC3dgCR1}]+[\mathrm{fC4bC3dgC4BP}]+[\mathrm{fC3bC3bFH}]+[\mathrm{fC3bC3bFHL1}]+[\mathrm{fC3bC3bCR1}]\\
+[\mathrm{fC3biC3bFH}]+[\mathrm{fC3biC3bFHL1}]+[\mathrm{fC3biC3bCR1}]+[\mathrm{fiC3biC3bCR1}]+[\mathrm{fiC3bC3dgCR1}]\\
+[\mathrm{fC4biC3bC4BP}]+[\mathrm{nfC4bC4BP}]+[\mathrm{fC4bC4BP}]+[\mathrm{fC4bCR1}]+[\mathrm{fC4bC4bC4BP}]+[\mathrm{fC4bC4dC4BP}]\\
+[\mathrm{fC4bC4bCR1}]+[\mathrm{fC4bC4dCR1}]+[\mathrm{hC4bCR1}]+[\mathrm{hC4bC4BP}]+[\mathrm{hC4bC4bCR1}]+[\mathrm{hC4bC4bC4BP}]\\
+[\mathrm{hC4bC4dCR1}]+[\mathrm{hC4bC4dC4BP}]+[\mathrm{hC4bC3bFH}]+[\mathrm{hC4bC3bFHL1}]+[\mathrm{hC4bC3bCR1}]\\
+[\mathrm{hC4bC3bC4BP}]+[\mathrm{hC4biC3bCR1}]+[\mathrm{hC4biC3bC4BP}]+[\mathrm{hC4bC3dgCR1}]+[\mathrm{hC4bC3dgC4BP}]\\
+[\mathrm{hC4dC3bFH}]+[\mathrm{hC4dC3bFHL1}]+[\mathrm{hC4dC3bCR1}]+[\mathrm{hC4diC3bCR1}]+[\mathrm{hC3bFH}]+[\mathrm{hC3bFHL1}]\\
+[\mathrm{hC3bCR1}]+[\mathrm{hiC3bCR1}]+[\mathrm{hC3bC3bFH}]+[\mathrm{hC3bC3bFHL1}]+[\mathrm{hC3bC3bCR1}]+[\mathrm{hC3biC3bFH}]\\
+[\mathrm{hC3biC3bFHL1}]+[\mathrm{hC3biC3bCR1}]+[\mathrm{hiC3biC3bCR1}]+[\mathrm{hiC3biC3dgCR1}]+[\mathrm{pC4bC4BP}]\\
+[\mathrm{pC4bC4bC4BP}]+[\mathrm{pC4bC4dC4BP}]+[\mathrm{pC4bC3bC4BP}]+[\mathrm{pC4biC3bC4BP}]+[\mathrm{pC4bC3bFH}]\\
+[\mathrm{pC4bC3bFHL1}]+[\mathrm{pC4dC3bFH}]+[\mathrm{pC4dC3bFHL1}]+[\mathrm{pC3bFH}]+[\mathrm{pC3bFHL1}]+[\mathrm{pC3bC3bFH}]\\
+[\mathrm{pC3bC3bFHL1}]+[\mathrm{pC3biC3bFH}]+[\mathrm{pC3biC3bFHL1}]+[\mathrm{pC4bC4BPPTX3}]+[\mathrm{pC4bC4bC4BPPTX3}]\\
+[\mathrm{pC4bC4dC4BPPTX3}]+[\mathrm{pC4bC3bFHPTX3}]+[\mathrm{pC4bC3bFHL1PTX3}]+[\mathrm{pC4bC3bC4BPPTX3}]\\
+[\mathrm{pC4dC3bFHPTX3}]+[\mathrm{pC4dC3bFHL1PTX3}]+[\mathrm{pC4biC3bC4BPPTX3}]+[\mathrm{pC3bFHPTX3}]\\
+[\mathrm{pC3bFHL1PTX3}]+[\mathrm{pC3bC3bFHPTX3}]+[\mathrm{pC3bC3bFHL1PTX3}]+[\mathrm{pC3biC3bFHPTX3}]\\
+[\mathrm{pC3biC3bFHL1PTX3}]+[\mathrm{pC4bpC4BP}]+[\mathrm{pC4bC4bpC4BP}]+[\mathrm{pC4bC4dpC4BP}]+[\mathrm{pC4bC3bpC4BP}]\\
+[\mathrm{pC4biC3bpC4BP}]+[\mathrm{pC4bC3bpFH}]+[\mathrm{pC4bC3bpFHL1}]+[\mathrm{pC4dC3bpFH}]+[\mathrm{pC4dC3bpFHL1}]\\
+[\mathrm{pC3bpFH}]+[\mathrm{pC3bpFHL1}]+[\mathrm{pC3bC3bpFH}]+[\mathrm{pC3bC3bpFHL1}]+[\mathrm{pC3biC3bpFH}]+[\mathrm{pC3biC3bpFHL1}]\end{array}\right)}$$

$$+\frac{k_{\text{cat C3bH}}^{\text{FI}}[\text{FI}][\text{pC3bFHL1}]}{\left(\begin{array}{l}K_{\text{m C3bH}}^{\text{FI}}+[\text{C3(H}_2\text{O)FH}]+[\text{C3(H}_2\text{O)FHL1}]+[\text{fC3bFH}]+[\text{fC3bFHL1}]+[\text{fC3bCR1}]+[\text{fiC3bCR1}]\\+[\text{IgGC3bC3bFH}]+[\text{IgGC3biC3bFH}]+[\text{IgGC3bC3bFHL1}]+[\text{IgGC3biC3bFHL1}]+[\text{IgGC3bC3bCR1}]\\+[\text{IgGC3biC3bCR1}]+[\text{IgGiC3biC3bCR1}]+[\text{IgGiC3bC3dgCR1}]+[\text{fC4bC3bFH}]+[\text{fC4bC3bFHL1}]\\+[\text{fC4bC3bCR1}]+[\text{fC4bC3bC4BP}]+[\text{fC4dC3bFH}]+[\text{fC4dC3bFHL1}]+[\text{fC4dC3bCR1}]+[\text{fC4diC3bCR1}]\\+[\text{fC4biC3bCR1}]+[\text{fC4bC3dgCR1}]+[\text{fC4bC3dgC4BP}]+[\text{fC3bC3bFH}]+[\text{fC3bC3bFHL1}]+[\text{fC3bC3bCR1}]\\+[\text{fC3biC3bFH}]+[\text{fC3biC3bFHL1}]+[\text{fC3biC3bCR1}]+[\text{fiC3biC3bCR1}]+[\text{fiC3bC3dgCR1}]\\+[\text{fC4biC3bC4BP}]+[\text{nfC4bC4BP}]+[\text{fC4bC4BP}]+[\text{fC4bCR1}]+[\text{fC4bC4bC4BP}]+[\text{fC4bC4dC4BP}]\\+[\text{fC4bC4bCR1}]+[\text{fC4bC4dCR1}]+[\text{hC4bCR1}]+[\text{hC4bC4BP}]+[\text{hC4bC4bCR1}]+[\text{hC4bC4bC4BP}]\\+[\text{hC4bC4dCR1}]+[\text{hC4bC4dC4BP}]+[\text{hC4bC3bFH}]+[\text{hC4bC3bFHL1}]+[\text{hC4bC3bCR1}]\\+[\text{hC4bC3bC4BP}]+[\text{hC4biC3bCR1}]+[\text{hC4biC3bC4BP}]+[\text{hC4bC3dgCR1}]+[\text{hC4bC3dgC4BP}]\\+[\text{hC4dC3bFH}]+[\text{hC4dC3bFHL1}]+[\text{hC4dC3bCR1}]+[\text{hC4diC3bCR1}]+[\text{hC3bFH}]+[\text{hC3bFHL1}]\\+[\text{hC3bCR1}]+[\text{hiC3bCR1}]+[\text{hC3bC3bFH}]+[\text{hC3bC3bFHL1}]+[\text{hC3bC3bCR1}]+[\text{hC3biC3bFH}]\\+[\text{hC3biC3bFHL1}]+[\text{hC3biC3bCR1}]+[\text{hiC3biC3bCR1}]+[\text{hiC3biC3dgCR1}]+[\text{pC4bC4BP}]\\+[\text{pC4bC4bC4BP}]+[\text{pC4bC4dC4BP}]+[\text{pC4bC3bC4BP}]+[\text{pC4biC3bC4BP}]+[\text{pC4bC3bFH}]\\+[\text{pC4bC3bFHL1}]+[\text{pC4dC3bFH}]+[\text{pC4dC3bFHL1}]+[\text{pC3bFH}]+[\text{pC3bFHL1}]+[\text{pC3bC3bFH}]\\+[\text{pC3bC3bFHL1}]+[\text{pC3biC3bFH}]+[\text{pC3biC3bFHL1}]+[\text{pC4bC4BPPTX3}]+[\text{pC4bC4bC4BPPTX3}]\\+[\text{pC4bC4dC4BPPTX3}]+[\text{pC4bC3bFHPTX3}]+[\text{pC4bC3bFHL1PTX3}]+[\text{pC4bC3bC4BPPTX3}]\\+[\text{pC4dC3bFHPTX3}]+[\text{pC4dC3bFHL1PTX3}]+[\text{pC4biC3bC4BPPTX3}]+[\text{pC3bFHPTX3}]\\+[\text{pC3bFHL1PTX3}]+[\text{pC3bC3bFHPTX3}]+[\text{pC3bC3bFHL1PTX3}]+[\text{pC3biC3bFHPTX3}]\\+[\text{pC3biC3bFHL1PTX3}]+[\text{pC4bpC4BP}]+[\text{pC4bC4bpC4BP}]+[\text{pC4bC4dpC4BP}]+[\text{pC4bC3bpC4BP}]\\+[\text{pC4biC3bpC4BP}]+[\text{pC4bC3bpFH}]+[\text{pC4bC3bpFHL1}]+[\text{pC4dC3bpFH}]+[\text{pC4dC3bpFHL1}]\\+[\text{pC3bpFH}]+[\text{pC3bpFHL1}]+[\text{pC3bC3bpFH}]+[\text{pC3bC3bpFHL1}]+[\text{pC3biC3bpFH}]+[\text{pC3biC3bpFHL1}]\end{array}\right)}$$

$$+\frac{k_{\mathrm{cat\ C3bH}}^{\mathrm{FI}}[\mathrm{FI}][\mathrm{pC3bFHPTX3}]}{\left(\begin{array}{l}K_{\mathrm{m\ C3bH}}^{\mathrm{FI}}+[\mathrm{C3(H_2O)FH}]+[\mathrm{C3(H_2O)FHL1}]+[\mathrm{fC3bFH}]+[\mathrm{fC3bFHL1}]+[\mathrm{fC3bCR1}]+[\mathrm{fiC3bCR1}]\\
+[\mathrm{IgGC3bC3bFH}]+[\mathrm{IgGC3biC3bFH}]+[\mathrm{IgGC3bC3bFHL1}]+[\mathrm{IgGC3biC3bFHL1}]+[\mathrm{IgGC3bC3bCR1}]\\
+[\mathrm{IgGC3biC3bCR1}]+[\mathrm{IgGiC3biC3bCR1}]+[\mathrm{IgGiC3bC3dgCR1}]+[\mathrm{fC4bC3bFH}]+[\mathrm{fC4bC3bFHL1}]\\
+[\mathrm{fC4bC3bCR1}]+[\mathrm{fC4bC3bC4BP}]+[\mathrm{fC4dC3bFH}]+[\mathrm{fC4dC3bFHL1}]+[\mathrm{fC4dC3bCR1}]+[\mathrm{fC4diC3bCR1}]\\
+[\mathrm{fC4biC3bCR1}]+[\mathrm{fC4bC3dgCR1}]+[\mathrm{fC4bC3dgC4BP}]+[\mathrm{fC3bC3bFH}]+[\mathrm{fC3bC3bFHL1}]+[\mathrm{fC3bC3bCR1}]\\
+[\mathrm{fC3biC3bFH}]+[\mathrm{fC3biC3bFHL1}]+[\mathrm{fC3biC3bCR1}]+[\mathrm{fiC3biC3bCR1}]+[\mathrm{fiC3bC3dgCR1}]\\
+[\mathrm{fC4biC3bC4BP}]+[\mathrm{nfC4bC4BP}]+[\mathrm{fC4bC4BP}]+[\mathrm{fC4bCR1}]+[\mathrm{fC4bC4bC4BP}]+[\mathrm{fC4bC4dC4BP}]\\
+[\mathrm{fC4bC4bCR1}]+[\mathrm{fC4bC4dCR1}]+[\mathrm{hC4bCR1}]+[\mathrm{hC4bC4BP}]+[\mathrm{hC4bC4bCR1}]+[\mathrm{hC4bC4bC4BP}]\\
+[\mathrm{hC4bC4dCR1}]+[\mathrm{hC4bC4dC4BP}]+[\mathrm{hC4bC3bFH}]+[\mathrm{hC4bC3bFHL1}]+[\mathrm{hC4bC3bCR1}]\\
+[\mathrm{hC4bC3bC4BP}]+[\mathrm{hC4biC3bCR1}]+[\mathrm{hC4biC3bC4BP}]+[\mathrm{hC4bC3dgCR1}]+[\mathrm{hC4bC3dgC4BP}]\\
+[\mathrm{hC4dC3bFH}]+[\mathrm{hC4dC3bFHL1}]+[\mathrm{hC4dC3bCR1}]+[\mathrm{hC4diC3bCR1}]+[\mathrm{hC3bFH}]+[\mathrm{hC3bFHL1}]\\
+[\mathrm{hC3bCR1}]+[\mathrm{hiC3bCR1}]+[\mathrm{hC3bC3bFH}]+[\mathrm{hC3bC3bFHL1}]+[\mathrm{hC3bC3bCR1}]+[\mathrm{hC3biC3bFH}]\\
+[\mathrm{hC3biC3bFHL1}]+[\mathrm{hC3biC3bCR1}]+[\mathrm{hiC3biC3bCR1}]+[\mathrm{hiC3biC3dgCR1}]+[\mathrm{pC4bC4BP}]\\
+[\mathrm{pC4bC4bC4BP}]+[\mathrm{pC4bC4dC4BP}]+[\mathrm{pC4bC3bC4BP}]+[\mathrm{pC4biC3bC4BP}]+[\mathrm{pC4bC3bFH}]\\
+[\mathrm{pC4bC3bFHL1}]+[\mathrm{pC4dC3bFH}]+[\mathrm{pC4dC3bFHL1}]+[\mathrm{pC3bFH}]+[\mathrm{pC3bFHL1}]+[\mathrm{pC3bC3bFH}]\\
+[\mathrm{pC3bC3bFHL1}]+[\mathrm{pC3biC3bFH}]+[\mathrm{pC3biC3bFHL1}]+[\mathrm{pC4bC4BPPTX3}]+[\mathrm{pC4bC4bC4BPPTX3}]\\
+[\mathrm{pC4bC4dC4BPPTX3}]+[\mathrm{pC4bC3bFHPTX3}]+[\mathrm{pC4bC3bFHL1PTX3}]+[\mathrm{pC4bC3bC4BPPTX3}]\\
+[\mathrm{pC4dC3bFHPTX3}]+[\mathrm{pC4dC3bFHL1PTX3}]+[\mathrm{pC4biC3bC4BPPTX3}]+[\mathrm{pC3bFHPTX3}]\\
+[\mathrm{pC3bFHL1PTX3}]+[\mathrm{pC3bC3bFHPTX3}]+[\mathrm{pC3bC3bFHL1PTX3}]+[\mathrm{pC3biC3bFHPTX3}]\\
+[\mathrm{pC3biC3bFHL1PTX3}]+[\mathrm{pC4bpC4BP}]+[\mathrm{pC4bC4bpC4BP}]+[\mathrm{pC4bC4dpC4BP}]+[\mathrm{pC4bC3bpC4BP}]\\
+[\mathrm{pC4biC3bpC4BP}]+[\mathrm{pC4bC3bpFH}]+[\mathrm{pC4bC3bpFHL1}]+[\mathrm{pC4dC3bpFH}]+[\mathrm{pC4dC3bpFHL1}]\\
+[\mathrm{pC3bpFH}]+[\mathrm{pC3bpFHL1}]+[\mathrm{pC3bC3bpFH}]+[\mathrm{pC3bC3bpFHL1}]+[\mathrm{pC3biC3bpFH}]+[\mathrm{pC3biC3bpFHL1}]\end{array}\right)}$$

$$+\frac{k_{\text{cat C3bH}}^{\text{FI}}[\text{FI}][\text{pC3bFHL1PTX3}]}{\left(\begin{array}{l}K_{\text{m C3bH}}^{\text{FI}}+[\text{C3(H}_2\text{O)FH}]+[\text{C3(H}_2\text{O)FHL1}]+[\text{fC3bFH}]+[\text{fC3bFHL1}]+[\text{fC3bCR1}]+[\text{fiC3bCR1}]\\+[\text{IgGC3bC3bFH}]+[\text{IgGC3biC3bFH}]+[\text{IgGC3bC3bFHL1}]+[\text{IgGC3biC3bFHL1}]+[\text{IgGC3bC3bCR1}]\\+[\text{IgGC3biC3bCR1}]+[\text{IgGiC3biC3bCR1}]+[\text{IgGiC3bC3dgCR1}]+[\text{fC4bC3bFH}]+[\text{fC4bC3bFHL1}]\\+[\text{fC4bC3bCR1}]+[\text{fC4bC3bC4BP}]+[\text{fC4dC3bFH}]+[\text{fC4dC3bFHL1}]+[\text{fC4dC3bCR1}]+[\text{fC4diC3bCR1}]\\+[\text{fC4biC3bCR1}]+[\text{fC4bC3dgCR1}]+[\text{fC4bC3dgC4BP}]+[\text{fC3bC3bFH}]+[\text{fC3bC3bFHL1}]+[\text{fC3bC3bCR1}]\\+[\text{fC3biC3bFH}]+[\text{fC3biC3bFHL1}]+[\text{fC3biC3bCR1}]+[\text{fiC3biC3bCR1}]+[\text{fiC3bC3dgCR1}]\\+[\text{fC4biC3bC4BP}]+[\text{nfC4bC4BP}]+[\text{fC4bC4BP}]+[\text{fC4bCR1}]+[\text{fC4bC4bC4BP}]+[\text{fC4bC4dC4BP}]\\+[\text{fC4bC4bCR1}]+[\text{fC4bC4dCR1}]+[\text{hC4bCR1}]+[\text{hC4bC4BP}]+[\text{hC4bC4bCR1}]+[\text{hC4bC4bC4BP}]\\+[\text{hC4bC4dCR1}]+[\text{hC4bC4dC4BP}]+[\text{hC4bC3bFH}]+[\text{hC4bC3bFHL1}]+[\text{hC4bC3bCR1}]\\+[\text{hC4bC3bC4BP}]+[\text{hC4biC3bCR1}]+[\text{hC4biC3bC4BP}]+[\text{hC4bC3dgCR1}]+[\text{hC4bC3dgC4BP}]\\+[\text{hC4dC3bFH}]+[\text{hC4dC3bFHL1}]+[\text{hC4dC3bCR1}]+[\text{hC4diC3bCR1}]+[\text{hC3bFH}]+[\text{hC3bFHL1}]\\+[\text{hC3bCR1}]+[\text{hiC3bCR1}]+[\text{hC3bC3bFH}]+[\text{hC3bC3bFHL1}]+[\text{hC3bC3bCR1}]+[\text{hC3biC3bFH}]\\+[\text{hC3biC3bFHL1}]+[\text{hC3biC3bCR1}]+[\text{hiC3biC3bCR1}]+[\text{hiC3biC3dgCR1}]+[\text{pC4bC4BP}]\\+[\text{pC4bC4bC4BP}]+[\text{pC4bC4dC4BP}]+[\text{pC4bC3bC4BP}]+[\text{pC4biC3bC4BP}]+[\text{pC4bC3bFH}]\\+[\text{pC4bC3bFHL1}]+[\text{pC4dC3bFH}]+[\text{pC4dC3bFHL1}]+[\text{pC3bFH}]+[\text{pC3bFHL1}]+[\text{pC3bC3bFH}]\\+[\text{pC3bC3bFHL1}]+[\text{pC3biC3bFH}]+[\text{pC3biC3bFHL1}]+[\text{pC4bC4BPPTX3}]+[\text{pC4bC4bC4BPPTX3}]\\+[\text{pC4bC4dC4BPPTX3}]+[\text{pC4bC3bFHPTX3}]+[\text{pC4bC3bFHL1PTX3}]+[\text{pC4bC3bC4BPPTX3}]\\+[\text{pC4dC3bFHPTX3}]+[\text{pC4dC3bFHL1PTX3}]+[\text{pC4biC3bC4BPPTX3}]+[\text{pC3bFHPTX3}]\\+[\text{pC3bFHL1PTX3}]+[\text{pC3bC3bFHPTX3}]+[\text{pC3bC3bFHL1PTX3}]+[\text{pC3biC3bFHPTX3}]\\+[\text{pC3biC3bFHL1PTX3}]+[\text{pC4bpC4BP}]+[\text{pC4bC4bpC4BP}]+[\text{pC4bC4dpC4BP}]+[\text{pC4bC3bpC4BP}]\\+[\text{pC4biC3bpC4BP}]+[\text{pC4bC3bpFH}]+[\text{pC4bC3bpFHL1}]+[\text{pC4dC3bpFH}]+[\text{pC4dC3bpFHL1}]\\+[\text{pC3bpFH}]+[\text{pC3bpFHL1}]+[\text{pC3bC3bpFH}]+[\text{pC3bC3bpFHL1}]+[\text{pC3biC3bpFH}]+[\text{pC3biC3bpFHL1}]\end{array}\right)}$$

$$
+\frac{k^{\mathrm{FI}}_{\mathrm{cat\ C3bH}}[\mathrm{FI}][\mathrm{pC3bpFH}]}{\left(\begin{aligned}
&K^{\mathrm{FI}}_{\mathrm{m\ C3bH}}+[\mathrm{C3(H_2O)FH}]+[\mathrm{C3(H_2O)FHL1}]+[\mathrm{fC3bFH}]+[\mathrm{fC3bFHL1}]+[\mathrm{fC3bCR1}]+[\mathrm{fiC3bCR1}]\\
&+[\mathrm{IgGC3bC3bFH}]+[\mathrm{IgGC3biC3bFH}]+[\mathrm{IgGC3bC3bFHL1}]+[\mathrm{IgGC3biC3bFHL1}]+[\mathrm{IgGC3bC3bCR1}]\\
&+[\mathrm{IgGC3biC3bCR1}]+[\mathrm{IgGiC3biC3bCR1}]+[\mathrm{IgGiC3bC3dgCR1}]+[\mathrm{fC4bC3bFH}]+[\mathrm{fC4bC3bFHL1}]\\
&+[\mathrm{fC4bC3bCR1}]+[\mathrm{fC4bC3bC4BP}]+[\mathrm{fC4dC3bFH}]+[\mathrm{fC4dC3bFHL1}]+[\mathrm{fC4dC3bCR1}]+[\mathrm{fC4diC3bCR1}]\\
&+[\mathrm{fC4biC3bCR1}]+[\mathrm{fC4bC3dgCR1}]+[\mathrm{fC4bC3dgC4BP}]+[\mathrm{fC3bC3bFH}]+[\mathrm{fC3bC3bFHL1}]+[\mathrm{fC3bC3bCR1}]\\
&+[\mathrm{fC3biC3bFH}]+[\mathrm{fC3biC3bFHL1}]+[\mathrm{fC3biC3bCR1}]+[\mathrm{fiC3biC3bCR1}]+[\mathrm{fiC3bC3dgCR1}]\\
&+[\mathrm{fC4biC3bC4BP}]+[\mathrm{nfC4bC4BP}]+[\mathrm{fC4bC4BP}]+[\mathrm{fC4bCR1}]+[\mathrm{fC4bC4bC4BP}]+[\mathrm{fC4bC4dC4BP}]\\
&+[\mathrm{fC4bC4bCR1}]+[\mathrm{fC4bC4dCR1}]+[\mathrm{hC4bCR1}]+[\mathrm{hC4bC4BP}]+[\mathrm{hC4bC4bCR1}]+[\mathrm{hC4bC4bC4BP}]\\
&+[\mathrm{hC4bC4dCR1}]+[\mathrm{hC4bC4dC4BP}]+[\mathrm{hC4bC3bFH}]+[\mathrm{hC4bC3bFHL1}]+[\mathrm{hC4bC3bCR1}]\\
&+[\mathrm{hC4bC3bC4BP}]+[\mathrm{hC4biC3bCR1}]+[\mathrm{hC4biC3bC4BP}]+[\mathrm{hC4bC3dgCR1}]+[\mathrm{hC4bC3dgC4BP}]\\
&+[\mathrm{hC4dC3bFH}]+[\mathrm{hC4dC3bFHL1}]+[\mathrm{hC4dC3bCR1}]+[\mathrm{hC4diC3bCR1}]+[\mathrm{hC3bFH}]+[\mathrm{hC3bFHL1}]\\
&+[\mathrm{hC3bCR1}]+[\mathrm{hiC3bCR1}]+[\mathrm{hC3bC3bFH}]+[\mathrm{hC3bC3bFHL1}]+[\mathrm{hC3bC3bCR1}]+[\mathrm{hC3biC3bFH}]\\
&+[\mathrm{hC3biC3bFHL1}]+[\mathrm{hC3biC3bCR1}]+[\mathrm{hiC3biC3bCR1}]+[\mathrm{hiC3biC3dgCR1}]+[\mathrm{pC4bC4BP}]\\
&+[\mathrm{pC4bC4bC4BP}]+[\mathrm{pC4bC4dC4BP}]+[\mathrm{pC4bC3bC4BP}]+[\mathrm{pC4biC3bC4BP}]+[\mathrm{pC4bC3bFH}]\\
&+[\mathrm{pC4bC3bFHL1}]+[\mathrm{pC4dC3bFH}]+[\mathrm{pC4dC3bFHL1}]+[\mathrm{pC3bFH}]+[\mathrm{pC3bFHL1}]+[\mathrm{pC3bC3bFH}]\\
&+[\mathrm{pC3bC3bFHL1}]+[\mathrm{pC3biC3bFH}]+[\mathrm{pC3biC3bFHL1}]+[\mathrm{pC4bC4BPPTX3}]+[\mathrm{pC4bC4bC4BPPTX3}]\\
&+[\mathrm{pC4bC4dC4BPPTX3}]+[\mathrm{pC4bC3bFHPTX3}]+[\mathrm{pC4bC3bFHL1PTX3}]+[\mathrm{pC4bC3bC4BPPTX3}]\\
&+[\mathrm{pC4dC3bFHPTX3}]+[\mathrm{pC4dC3bFHL1PTX3}]+[\mathrm{pC4biC3bC4BPPTX3}]+[\mathrm{pC3bFHPTX3}]\\
&+[\mathrm{pC3bFHL1PTX3}]+[\mathrm{pC3bC3bFHPTX3}]+[\mathrm{pC3bC3bFHL1PTX3}]+[\mathrm{pC3biC3bFHPTX3}]\\
&+[\mathrm{pC3biC3bFHL1PTX3}]+[\mathrm{pC4bpC4BP}]+[\mathrm{pC4bC4bpC4BP}]+[\mathrm{pC4bC4dpC4BP}]+[\mathrm{pC4bC3bpC4BP}]\\
&+[\mathrm{pC4biC3bpC4BP}]+[\mathrm{pC4bC3bpFH}]+[\mathrm{pC4bC3bpFHL1}]+[\mathrm{pC4dC3bpFH}]+[\mathrm{pC4dC3bpFHL1}]\\
&+[\mathrm{pC3bpFH}]+[\mathrm{pC3bpFHL1}]+[\mathrm{pC3bC3bpFH}]+[\mathrm{pC3bC3bpFHL1}]+[\mathrm{pC3biC3bpFH}]+[\mathrm{pC3biC3bpFHL1}]
\end{aligned}\right)}
$$

$$+\frac{k_{\text{cat C3bH}}^{\text{FI}}[\text{FI}][\text{pC3bpFHL1}]}{\left(\begin{array}{l}K_{\text{m C3bH}}^{\text{FI}}+[\text{C3(H}_2\text{O)FH}]+[\text{C3(H}_2\text{O)FHL1}]+[\text{fC3bFH}]+[\text{fC3bFHL1}]+[\text{fC3bCR1}]+[\text{fiC3bCR1}]\\+[\text{IgGC3bC3bFH}]+[\text{IgGC3biC3bFH}]+[\text{IgGC3bC3bFHL1}]+[\text{IgGC3biC3bFHL1}]+[\text{IgGC3bC3bCR1}]\\+[\text{IgGC3biC3bCR1}]+[\text{IgGiC3biC3bCR1}]+[\text{IgGiC3bC3dgCR1}]+[\text{fC4bC3bFH}]+[\text{fC4bC3bFHL1}]\\+[\text{fC4bC3bCR1}]+[\text{fC4bC3bC4BP}]+[\text{fC4dC3bFH}]+[\text{fC4dC3bFHL1}]+[\text{fC4dC3bCR1}]+[\text{fC4diC3bCR1}]\\+[\text{fC4biC3bCR1}]+[\text{fC4bC3dgCR1}]+[\text{fC4bC3dgC4BP}]+[\text{fC3bC3bFH}]+[\text{fC3bC3bFHL1}]+[\text{fC3bC3bCR1}]\\+[\text{fC3biC3bFH}]+[\text{fC3biC3bFHL1}]+[\text{fC3biC3bCR1}]+[\text{fiC3biC3bCR1}]+[\text{fiC3bC3dgCR1}]\\+[\text{fC4biC3bC4BP}]+[\text{nfC4bC4BP}]+[\text{fC4bC4BP}]+[\text{fC4bCR1}]+[\text{fC4bC4bC4BP}]+[\text{fC4bC4dC4BP}]\\+[\text{fC4bC4bCR1}]+[\text{fC4bC4dCR1}]+[\text{hC4bCR1}]+[\text{hC4bC4BP}]+[\text{hC4bC4bCR1}]+[\text{hC4bC4bC4BP}]\\+[\text{hC4bC4dCR1}]+[\text{hC4bC4dC4BP}]+[\text{hC4bC3bFH}]+[\text{hC4bC3bFHL1}]+[\text{hC4bC3bCR1}]\\+[\text{hC4bC3bC4BP}]+[\text{hC4biC3bCR1}]+[\text{hC4biC3bC4BP}]+[\text{hC4bC3dgCR1}]+[\text{hC4bC3dgC4BP}]\\+[\text{hC4dC3bFH}]+[\text{hC4dC3bFHL1}]+[\text{hC4dC3bCR1}]+[\text{hC4diC3bCR1}]+[\text{hC3bFH}]+[\text{hC3bFHL1}]\\+[\text{hC3bCR1}]+[\text{hiC3bCR1}]+[\text{hC3bC3bFH}]+[\text{hC3bC3bFHL1}]+[\text{hC3bC3bCR1}]+[\text{hC3biC3bFH}]\\+[\text{hC3biC3bFHL1}]+[\text{hC3biC3bCR1}]+[\text{hiC3biC3bCR1}]+[\text{hiC3biC3dgCR1}]+[\text{pC4bC4BP}]\\+[\text{pC4bC4bC4BP}]+[\text{pC4bC4dC4BP}]+[\text{pC4bC3bC4BP}]+[\text{pC4biC3bC4BP}]+[\text{pC4bC3bFH}]\\+[\text{pC4bC3bFHL1}]+[\text{pC4dC3bFH}]+[\text{pC4dC3bFHL1}]+[\text{pC3bFH}]+[\text{pC3bFHL1}]+[\text{pC3bC3bFH}]\\+[\text{pC3bC3bFHL1}]+[\text{pC3biC3bFH}]+[\text{pC3biC3bFHL1}]+[\text{pC4bC4BPPTX3}]+[\text{pC4bC4bC4BPPTX3}]\\+[\text{pC4bC4dC4BPPTX3}]+[\text{pC4bC3bFHPTX3}]+[\text{pC4bC3bFHL1PTX3}]+[\text{pC4bC3bC4BPPTX3}]\\+[\text{pC4dC3bFHPTX3}]+[\text{pC4dC3bFHL1PTX3}]+[\text{pC4biC3bC4BPPTX3}]+[\text{pC3bFHPTX3}]\\+[\text{pC3bFHL1PTX3}]+[\text{pC3bC3bFHPTX3}]+[\text{pC3bC3bFHL1PTX3}]+[\text{pC3biC3bFHPTX3}]\\+[\text{pC3biC3bFHL1PTX3}]+[\text{pC4bpC4BP}]+[\text{pC4bC4bpC4BP}]+[\text{pC4bC4dpC4BP}]+[\text{pC4bC3bpC4BP}]\\+[\text{pC4biC3bpC4BP}]+[\text{pC4bC3bpFH}]+[\text{pC4bC3bpFHL1}]+[\text{pC4dC3bpFH}]+[\text{pC4dC3bpFHL1}]\\+[\text{pC3bpFH}]+[\text{pC3bpFHL1}]+[\text{pC3bC3bpFH}]+[\text{pC3bC3bpFHL1}]+[\text{pC3biC3bpFH}]+[\text{pC3biC3bpFHL1}]\end{array}\right)}$$

226

$$\frac{d[\text{pC3bBbFH}]}{dt}=k_{\text{C3bH}}^{+}[\text{pC3bBb}][\text{FH}]-k_{\text{C3bH}}^{-}[\text{pC3bBbFH}]-k_{\text{C3bBbH}_{\text{decay}}}^{-}[\text{pC3bBbFH}]$$

227

$$\frac{d[\text{pC3bBbFHL1}]}{dt}=k_{\text{C3bFHL1}}^{+}[\text{pC3bBb}][\text{FHL1}]-k_{\text{C3bFHL1}}^{-}[\text{pC3bBbFHL1}]-k_{\text{C3bBbFHL1}_{\text{decay}}}^{-}[\text{pC3bBbFHL1}]$$

228

$$\frac{d[\text{pC3bC3bBbFH}]}{dt}=k_{\text{C3bH}}^{+}[\text{pC3bC3bBb}][\text{FH}]-k_{\text{C3bH}}^{-}[\text{pC3bC3bBbFH}]-k_{\text{C3bBbH}_{\text{decay}}}^{-}[\text{pC3bC3bBbFH}]$$

229

$$\frac{d[\mathrm{pC3bC3bBbFHL1}]}{dt} = k^{+}_{\mathrm{C3bFHL1}}[\mathrm{pC3bC3bBb}][\mathrm{FHL1}] - k^{-}_{\mathrm{C3bFHL1}}[\mathrm{pC3bC3bBbFHL1}]$$
$$-k^{-}_{\mathrm{C3bBbFHL1}_{\mathrm{decay}}}[\mathrm{pC3bC3bBbFHL1}]$$

230

$$\frac{d[\mathrm{pC3bC3bFH}]}{dt} = k^{+}_{\mathrm{C3bH}}[\mathrm{pC3bC3b}][\mathrm{FH}] - k^{-}_{\mathrm{C3bH}}[\mathrm{pC3bC3bFH}]$$
$$-\frac{k^{\mathrm{FI}}_{\mathrm{cat\ C3bH}}[\mathrm{FI}][\mathrm{pC3bC3bFH}]}{\left(\begin{array}{l} K^{\mathrm{FI}}_{\mathrm{m\ C3bH}} + [\mathrm{C3(H_2O)FH}] + [\mathrm{C3(H_2O)FHL1}] + [\mathrm{fC3bFH}] + [\mathrm{fC3bFHL1}] + [\mathrm{fC3bCR1}] + [\mathrm{fiC3bCR1}] \\ + [\mathrm{IgGC3bC3bFH}] + [\mathrm{IgGC3biC3bFH}] + [\mathrm{IgGC3bC3bFHL1}] + [\mathrm{IgGC3biC3bFHL1}] + [\mathrm{IgGC3bC3bCR1}] \\ + [\mathrm{IgGC3biC3bCR1}] + [\mathrm{IgGiC3biC3bCR1}] + [\mathrm{IgGiC3bC3dgCR1}] + [\mathrm{fC4bC3bFH}] + [\mathrm{fC4bC3bFHL1}] \\ + [\mathrm{fC4bC3bCR1}] + [\mathrm{fC4bC3bC4BP}] + [\mathrm{fC4dC3bFH}] + [\mathrm{fC4dC3bFHL1}] + [\mathrm{fC4dC3bCR1}] + [\mathrm{fC4diC3bCR1}] \\ + [\mathrm{fC4biC3bCR1}] + [\mathrm{fC4bC3dgCR1}] + [\mathrm{fC4bC3dgC4BP}] + [\mathrm{fC3bC3bFH}] + [\mathrm{fC3bC3bFHL1}] + [\mathrm{fC3bC3bCR1}] \\ + [\mathrm{fC3biC3bFH}] + [\mathrm{fC3biC3bFHL1}] + [\mathrm{fC3biC3bCR1}] + [\mathrm{fiC3biC3bCR1}] + [\mathrm{fiC3bC3dgCR1}] \\ + [\mathrm{fC4biC3bC4BP}] + [\mathrm{nfC4bC4BP}] + [\mathrm{fC4bC4BP}] + [\mathrm{fC4bCR1}] + [\mathrm{fC4bC4bC4BP}] + [\mathrm{fC4bC4dC4BP}] \\ + [\mathrm{fC4bC4bCR1}] + [\mathrm{fC4bC4dCR1}] + [\mathrm{hC4bCR1}] + [\mathrm{hC4bC4BP}] + [\mathrm{hC4bC4bCR1}] + [\mathrm{hC4bC4bC4BP}] \\ + [\mathrm{hC4bC4dCR1}] + [\mathrm{hC4bC4dC4BP}] + [\mathrm{hC4bC3bFH}] + [\mathrm{hC4bC3bFHL1}] + [\mathrm{hC4bC3bCR1}] \\ + [\mathrm{hC4bC3bC4BP}] + [\mathrm{hC4biC3bCR1}] + [\mathrm{hC4biC3bC4BP}] + [\mathrm{hC4bC3dgCR1}] + [\mathrm{hC4bC3dgC4BP}] \\ + [\mathrm{hC4dC3bFH}] + [\mathrm{hC4dC3bFHL1}] + [\mathrm{hC4dC3bCR1}] + [\mathrm{hC4diC3bCR1}] + [\mathrm{hC3bFH}] + [\mathrm{hC3bFHL1}] \\ + [\mathrm{hC3bCR1}] + [\mathrm{hiC3bCR1}] + [\mathrm{hC3bC3bFH}] + [\mathrm{hC3bC3bFHL1}] + [\mathrm{hC3bC3bCR1}] + [\mathrm{hC3biC3bFH}] \\ + [\mathrm{hC3biC3bFHL1}] + [\mathrm{hC3biC3bCR1}] + [\mathrm{hiC3biC3bCR1}] + [\mathrm{hiC3biC3dgCR1}] + [\mathrm{pC4bC4BP}] \\ + [\mathrm{pC4bC4bC4BP}] + [\mathrm{pC4bC4dC4BP}] + [\mathrm{pC4bC3bC4BP}] + [\mathrm{pC4biC3bC4BP}] + [\mathrm{pC4bC3bFH}] \\ + [\mathrm{pC4bC3bFHL1}] + [\mathrm{pC4dC3bFH}] + [\mathrm{pC4dC3bFHL1}] + [\mathrm{pC3bFH}] + [\mathrm{pC3bFHL1}] + [\mathrm{pC3bC3bFH}] \\ + [\mathrm{pC3bC3bFHL1}] + [\mathrm{pC3biC3bFH}] + [\mathrm{pC3biC3bFHL1}] + [\mathrm{pC4bC4BPPTX3}] + [\mathrm{pC4bC4bC4BPPTX3}] \\ + [\mathrm{pC4bC4dC4BPPTX3}] + [\mathrm{pC4bC3bFHPTX3}] + [\mathrm{pC4bC3bFHL1PTX3}] + [\mathrm{pC4bC3bC4BPPTX3}] \\ + [\mathrm{pC4dC3bFHPTX3}] + [\mathrm{pC4dC3bFHL1PTX3}] + [\mathrm{pC4biC3bC4BPPTX3}] + [\mathrm{pC3bFHPTX3}] \\ + [\mathrm{pC3bFHL1PTX3}] + [\mathrm{pC3bC3bFHPTX3}] + [\mathrm{pC3bC3bFHL1PTX3}] + [\mathrm{pC3biC3bFHPTX3}] \\ + [\mathrm{pC3biC3bFHL1PTX3}] + [\mathrm{pC4bpC4BP}] + [\mathrm{pC4bC4bpC4BP}] + [\mathrm{pC4bC4dpC4BP}] + [\mathrm{pC4bC3bpC4BP}] \\ + [\mathrm{pC4biC3bpC4BP}] + [\mathrm{pC4bC3bpFH}] + [\mathrm{pC4bC3bpFHL1}] + [\mathrm{pC4dC3bpFH}] + [\mathrm{pC4dC3bpFHL1}] \\ + [\mathrm{pC3bpFH}] + [\mathrm{pC3bpFHL1}] + [\mathrm{pC3bC3bpFH}] + [\mathrm{pC3bC3bpFHL1}] + [\mathrm{pC3biC3bpFH}] + [\mathrm{pC3biC3bpFHL1}] \end{array}\right)}$$

231

$$\frac{d[\mathrm{pC3bC3bFHL1}]}{dt}=k^{+}_{\mathrm{C3bFHL1}}[\mathrm{pC3bC3b}][\mathrm{FHL1}]-k^{-}_{\mathrm{C3bFHL1}}[\mathrm{pC3bC3bFHL1}]$$

$$-\frac{k^{\mathrm{FI}}_{\mathrm{cat\ C3bH}}[\mathrm{FI}][\mathrm{pC3bC3bFHL1}]}{\left(\begin{array}{l}K^{\mathrm{FI}}_{\mathrm{m\ C3bH}}+[\mathrm{C3(H_2O)FH}]+[\mathrm{C3(H_2O)FHL1}]+[\mathrm{fC3bFH}]+[\mathrm{fC3bFHL1}]+[\mathrm{fC3bCR1}]+[\mathrm{fiC3bCR1}]\\
+[\mathrm{IgGC3bC3bFH}]+[\mathrm{IgGC3biC3bFH}]+[\mathrm{IgGC3bC3bFHL1}]+[\mathrm{IgGC3biC3bFHL1}]+[\mathrm{IgGC3bC3bCR1}]\\
+[\mathrm{IgGC3biC3bCR1}]+[\mathrm{IgGiC3biC3bCR1}]+[\mathrm{IgGiC3bC3dgCR1}]+[\mathrm{fC4bC3bFH}]+[\mathrm{fC4bC3bFHL1}]\\
+[\mathrm{fC4bC3bCR1}]+[\mathrm{fC4bC3bC4BP}]+[\mathrm{fC4dC3bFH}]+[\mathrm{fC4dC3bFHL1}]+[\mathrm{fC4dC3bCR1}]+[\mathrm{fC4diC3bCR1}]\\
+[\mathrm{fC4biC3bCR1}]+[\mathrm{fC4bC3dgCR1}]+[\mathrm{fC4bC3dgC4BP}]+[\mathrm{fC3bC3bFH}]+[\mathrm{fC3bC3bFHL1}]+[\mathrm{fC3bC3bCR1}]\\
+[\mathrm{fC3biC3bFH}]+[\mathrm{fC3biC3bFHL1}]+[\mathrm{fC3biC3bCR1}]+[\mathrm{fiC3biC3bCR1}]+[\mathrm{fiC3bC3dgCR1}]\\
+[\mathrm{fC4biC3bC4BP}]+[\mathrm{nfC4bC4BP}]+[\mathrm{fC4bC4BP}]+[\mathrm{fC4bCR1}]+[\mathrm{fC4bC4bC4BP}]+[\mathrm{fC4bC4dC4BP}]\\
+[\mathrm{fC4bC4bCR1}]+[\mathrm{fC4bC4dCR1}]+[\mathrm{hC4bCR1}]+[\mathrm{hC4bC4BP}]+[\mathrm{hC4bC4bCR1}]+[\mathrm{hC4bC4bC4BP}]\\
+[\mathrm{hC4bC4dCR1}]+[\mathrm{hC4bC4dC4BP}]+[\mathrm{hC4bC3bFH}]+[\mathrm{hC4bC3bFHL1}]+[\mathrm{hC4bC3bCR1}]\\
+[\mathrm{hC4bC3bC4BP}]+[\mathrm{hC4biC3bCR1}]+[\mathrm{hC4biC3bC4BP}]+[\mathrm{hC4bC3dgCR1}]+[\mathrm{hC4bC3dgC4BP}]\\
+[\mathrm{hC4dC3bFH}]+[\mathrm{hC4dC3bFHL1}]+[\mathrm{hC4dC3bCR1}]+[\mathrm{hC4diC3bCR1}]+[\mathrm{hC3bFH}]+[\mathrm{hC3bFHL1}]\\
+[\mathrm{hC3bCR1}]+[\mathrm{hiC3bCR1}]+[\mathrm{hC3bC3bFH}]+[\mathrm{hC3bC3bFHL1}]+[\mathrm{hC3bC3bCR1}]+[\mathrm{hC3biC3bFH}]\\
+[\mathrm{hC3biC3bFHL1}]+[\mathrm{hC3biC3bCR1}]+[\mathrm{hiC3biC3bCR1}]+[\mathrm{hiC3biC3dgCR1}]+[\mathrm{pC4bC4BP}]\\
+[\mathrm{pC4bC4bC4BP}]+[\mathrm{pC4bC4dC4BP}]+[\mathrm{pC4bC3bC4BP}]+[\mathrm{pC4biC3bC4BP}]+[\mathrm{pC4bC3bFH}]\\
+[\mathrm{pC4bC3bFHL1}]+[\mathrm{pC4dC3bFH}]+[\mathrm{pC4dC3bFHL1}]+[\mathrm{pC3bFH}]+[\mathrm{pC3bFHL1}]+[\mathrm{pC3bC3bFH}]\\
+[\mathrm{pC3bC3bFHL1}]+[\mathrm{pC3biC3bFH}]+[\mathrm{pC3biC3bFHL1}]+[\mathrm{pC4bC4BPPTX3}]+[\mathrm{pC4bC4bC4BPPTX3}]\\
+[\mathrm{pC4bC4dC4BPPTX3}]+[\mathrm{pC4bC3bFHPTX3}]+[\mathrm{pC4bC3bFHL1PTX3}]+[\mathrm{pC4bC3bC4BPPTX3}]\\
+[\mathrm{pC4dC3bFHPTX3}]+[\mathrm{pC4dC3bFHL1PTX3}]+[\mathrm{pC4biC3bC4BPPTX3}]+[\mathrm{pC3bFHPTX3}]\\
+[\mathrm{pC3bFHL1PTX3}]+[\mathrm{pC3bC3bFHPTX3}]+[\mathrm{pC3bC3bFHL1PTX3}]+[\mathrm{pC3biC3bFHPTX3}]\\
+[\mathrm{pC3biC3bFHL1PTX3}]+[\mathrm{pC4bpC4BP}]+[\mathrm{pC4bC4bpC4BP}]+[\mathrm{pC4bC4dpC4BP}]+[\mathrm{pC4bC3bpC4BP}]\\
+[\mathrm{pC4biC3bpC4BP}]+[\mathrm{pC4bC3bpFH}]+[\mathrm{pC4bC3bpFHL1}]+[\mathrm{pC4dC3bpFH}]+[\mathrm{pC4dC3bpFHL1}]\\
+[\mathrm{pC3bpFH}]+[\mathrm{pC3bpFHL1}]+[\mathrm{pC3bC3bpFH}]+[\mathrm{pC3bC3bpFHL1}]+[\mathrm{pC3biC3bpFH}]+[\mathrm{pC3biC3bpFHL1}]\end{array}\right)}$$

232

$$\frac{d\left[\mathrm{pC3biC3b}\right]}{dt}=$$

$$\frac{k_{\mathrm{cat\ C3bH}}^{\mathrm{FI}}\left[\mathrm{FI}\right]\left[\mathrm{pC3bC3bFH}\right]}{\left(\begin{array}{l}
K_{\mathrm{m\ C3bH}}^{\mathrm{FI}}+\left[\mathrm{C3(H_2O)FH}\right]+\left[\mathrm{C3(H_2O)FHL1}\right]+\left[\mathrm{fC3bFH}\right]+\left[\mathrm{fC3bFHL1}\right]+\left[\mathrm{fC3bCR1}\right]+\left[\mathrm{fiC3bCR1}\right]\\
+\left[\mathrm{IgGC3bC3bFH}\right]+\left[\mathrm{IgGC3biC3bFH}\right]+\left[\mathrm{IgGC3bC3bFHL1}\right]+\left[\mathrm{IgGC3biC3bFHL1}\right]+\left[\mathrm{IgGC3bC3bCR1}\right]\\
+\left[\mathrm{IgGC3biC3bCR1}\right]+\left[\mathrm{IgGiC3biC3bCR1}\right]+\left[\mathrm{IgGiC3bC3dgCR1}\right]+\left[\mathrm{fC4bC3bFH}\right]+\left[\mathrm{fC4bC3bFHL1}\right]\\
+\left[\mathrm{fC4bC3bCR1}\right]+\left[\mathrm{fC4bC3bC4BP}\right]+\left[\mathrm{fC4dC3bFH}\right]+\left[\mathrm{fC4dC3bFHL1}\right]+\left[\mathrm{fC4dC3bCR1}\right]+\left[\mathrm{fC4diC3bCR1}\right]\\
+\left[\mathrm{fC4biC3bCR1}\right]+\left[\mathrm{fC4bC3dgCR1}\right]+\left[\mathrm{fC4bC3dgC4BP}\right]+\left[\mathrm{fC3bC3bFH}\right]+\left[\mathrm{fC3bC3bFHL1}\right]+\left[\mathrm{fC3bC3bCR1}\right]\\
+\left[\mathrm{fC3biC3bFH}\right]+\left[\mathrm{fC3biC3bFHL1}\right]+\left[\mathrm{fC3biC3bCR1}\right]+\left[\mathrm{fiC3biC3bCR1}\right]+\left[\mathrm{fiC3bC3dgCR1}\right]\\
+\left[\mathrm{fC4biC3bC4BP}\right]+\left[\mathrm{nfC4bC4BP}\right]+\left[\mathrm{fC4bC4BP}\right]+\left[\mathrm{fC4bCR1}\right]+\left[\mathrm{fC4bC4bC4BP}\right]+\left[\mathrm{fC4bC4dC4BP}\right]\\
+\left[\mathrm{fC4bC4bCR1}\right]+\left[\mathrm{fC4bC4dCR1}\right]+\left[\mathrm{hC4bCR1}\right]+\left[\mathrm{hC4bC4BP}\right]+\left[\mathrm{hC4bC4bCR1}\right]+\left[\mathrm{hC4bC4bC4BP}\right]\\
+\left[\mathrm{hC4bC4dCR1}\right]+\left[\mathrm{hC4bC4dC4BP}\right]+\left[\mathrm{hC4bC3bFH}\right]+\left[\mathrm{hC4bC3bFHL1}\right]+\left[\mathrm{hC4bC3bCR1}\right]\\
+\left[\mathrm{hC4bC3bC4BP}\right]+\left[\mathrm{hC4biC3bCR1}\right]+\left[\mathrm{hC4biC3bC4BP}\right]+\left[\mathrm{hC4bC3dgCR1}\right]+\left[\mathrm{hC4bC3dgC4BP}\right]\\
+\left[\mathrm{hC4dC3bFH}\right]+\left[\mathrm{hC4dC3bFHL1}\right]+\left[\mathrm{hC4dC3bCR1}\right]+\left[\mathrm{hC4diC3bCR1}\right]+\left[\mathrm{hC3bFH}\right]+\left[\mathrm{hC3bFHL1}\right]\\
+\left[\mathrm{hC3bCR1}\right]+\left[\mathrm{hiC3bCR1}\right]+\left[\mathrm{hC3bC3bFH}\right]+\left[\mathrm{hC3bC3bFHL1}\right]+\left[\mathrm{hC3bC3bCR1}\right]+\left[\mathrm{hC3biC3bFH}\right]\\
+\left[\mathrm{hC3biC3bFHL1}\right]+\left[\mathrm{hC3biC3bCR1}\right]+\left[\mathrm{hiC3biC3bCR1}\right]+\left[\mathrm{hiC3biC3dgCR1}\right]+\left[\mathrm{pC4bC4BP}\right]\\
+\left[\mathrm{pC4bC4bC4BP}\right]+\left[\mathrm{pC4bC4dC4BP}\right]+\left[\mathrm{pC4bC3bC4BP}\right]+\left[\mathrm{pC4biC3bC4BP}\right]+\left[\mathrm{pC4bC3bFH}\right]\\
+\left[\mathrm{pC4bC3bFHL1}\right]+\left[\mathrm{pC4dC3bFH}\right]+\left[\mathrm{pC4dC3bFHL1}\right]+\left[\mathrm{pC3bFH}\right]+\left[\mathrm{pC3bFHL1}\right]+\left[\mathrm{pC3bC3bFH}\right]\\
+\left[\mathrm{pC3bC3bFHL1}\right]+\left[\mathrm{pC3biC3bFH}\right]+\left[\mathrm{pC3biC3bFHL1}\right]+\left[\mathrm{pC4bC4BPPTX3}\right]+\left[\mathrm{pC4bC4bC4BPPTX3}\right]\\
+\left[\mathrm{pC4bC4dC4BPPTX3}\right]+\left[\mathrm{pC4bC3bFHPTX3}\right]+\left[\mathrm{pC4bC3bFHL1PTX3}\right]+\left[\mathrm{pC4bC3bC4BPPTX3}\right]\\
+\left[\mathrm{pC4dC3bFHPTX3}\right]+\left[\mathrm{pC4dC3bFHL1PTX3}\right]+\left[\mathrm{pC4biC3bC4BPPTX3}\right]+\left[\mathrm{pC3bFHPTX3}\right]\\
+\left[\mathrm{pC3bFHL1PTX3}\right]+\left[\mathrm{pC3bC3bFHPTX3}\right]+\left[\mathrm{pC3bC3bFHL1PTX3}\right]+\left[\mathrm{pC3biC3bFHPTX3}\right]\\
+\left[\mathrm{pC3biC3bFHL1PTX3}\right]+\left[\mathrm{pC4bpC4BP}\right]+\left[\mathrm{pC4bC4bpC4BP}\right]+\left[\mathrm{pC4bC4dpC4BP}\right]+\left[\mathrm{pC4bC3bpC4BP}\right]\\
+\left[\mathrm{pC4biC3bpC4BP}\right]+\left[\mathrm{pC4bC3bpFH}\right]+\left[\mathrm{pC4bC3bpFHL1}\right]+\left[\mathrm{pC4dC3bpFH}\right]+\left[\mathrm{pC4dC3bpFHL1}\right]\\
+\left[\mathrm{pC3bpFH}\right]+\left[\mathrm{pC3bpFHL1}\right]+\left[\mathrm{pC3bC3bpFH}\right]+\left[\mathrm{pC3bC3bpFHL1}\right]+\left[\mathrm{pC3biC3bpFH}\right]+\left[\mathrm{pC3biC3bpFHL1}\right]
\end{array}\right)}$$

$$+\frac{k_{\mathrm{cat\ C3bH}}^{\mathrm{FI}}[\mathrm{FI}][\mathrm{pC3bC3bFHL1}]}{\left(\begin{array}{l}K_{\mathrm{m\ C3bH}}^{\mathrm{FI}}+[\mathrm{C3(H_2O)FH}]+[\mathrm{C3(H_2O)FHL1}]+[\mathrm{fC3bFH}]+[\mathrm{fC3bFHL1}]+[\mathrm{fC3bCR1}]+[\mathrm{fiC3bCR1}]\\
+[\mathrm{IgGC3bC3bFH}]+[\mathrm{IgGC3biC3bFH}]+[\mathrm{IgGC3bC3bFHL1}]+[\mathrm{IgGC3biC3bFHL1}]+[\mathrm{IgGC3bC3bCR1}]\\
+[\mathrm{IgGC3biC3bCR1}]+[\mathrm{IgGiC3biC3bCR1}]+[\mathrm{IgGiC3bC3dgCR1}]+[\mathrm{fC4bC3bFH}]+[\mathrm{fC4bC3bFHL1}]\\
+[\mathrm{fC4bC3bCR1}]+[\mathrm{fC4bC3bC4BP}]+[\mathrm{fC4dC3bFH}]+[\mathrm{fC4dC3bFHL1}]+[\mathrm{fC4dC3bCR1}]+[\mathrm{fC4diC3bCR1}]\\
+[\mathrm{fC4biC3bCR1}]+[\mathrm{fC4bC3dgCR1}]+[\mathrm{fC4bC3dgC4BP}]+[\mathrm{fC3bC3bFH}]+[\mathrm{fC3bC3bFHL1}]+[\mathrm{fC3bC3bCR1}]\\
+[\mathrm{fC3biC3bFH}]+[\mathrm{fC3biC3bFHL1}]+[\mathrm{fC3biC3bCR1}]+[\mathrm{fiC3biC3bCR1}]+[\mathrm{fiC3bC3dgCR1}]\\
+[\mathrm{fC4biC3bC4BP}]+[\mathrm{nfC4bC4BP}]+[\mathrm{fC4bC4BP}]+[\mathrm{fC4bCR1}]+[\mathrm{fC4bC4bC4BP}]+[\mathrm{fC4bC4dC4BP}]\\
+[\mathrm{fC4bC4bCR1}]+[\mathrm{fC4bC4dCR1}]+[\mathrm{hC4bCR1}]+[\mathrm{hC4bC4BP}]+[\mathrm{hC4bC4bCR1}]+[\mathrm{hC4bC4bC4BP}]\\
+[\mathrm{hC4bC4dCR1}]+[\mathrm{hC4bC4dC4BP}]+[\mathrm{hC4bC3bFH}]+[\mathrm{hC4bC3bFHL1}]+[\mathrm{hC4bC3bCR1}]\\
+[\mathrm{hC4bC3bC4BP}]+[\mathrm{hC4biC3bCR1}]+[\mathrm{hC4biC3bC4BP}]+[\mathrm{hC4bC3dgCR1}]+[\mathrm{hC4bC3dgC4BP}]\\
+[\mathrm{hC4dC3bFH}]+[\mathrm{hC4dC3bFHL1}]+[\mathrm{hC4dC3bCR1}]+[\mathrm{hC4diC3bCR1}]+[\mathrm{hC3bFH}]+[\mathrm{hC3bFHL1}]\\
+[\mathrm{hC3bCR1}]+[\mathrm{hiC3bCR1}]+[\mathrm{hC3bC3bFH}]+[\mathrm{hC3bC3bFHL1}]+[\mathrm{hC3bC3bCR1}]+[\mathrm{hC3biC3bFH}]\\
+[\mathrm{hC3biC3bFHL1}]+[\mathrm{hC3biC3bCR1}]+[\mathrm{hiC3biC3bCR1}]+[\mathrm{hiC3biC3dgCR1}]+[\mathrm{pC4bC4BP}]\\
+[\mathrm{pC4bC4bC4BP}]+[\mathrm{pC4bC4dC4BP}]+[\mathrm{pC4bC3bC4BP}]+[\mathrm{pC4biC3bC4BP}]+[\mathrm{pC4bC3bFH}]\\
+[\mathrm{pC4bC3bFHL1}]+[\mathrm{pC4dC3bFH}]+[\mathrm{pC4dC3bFHL1}]+[\mathrm{pC3bFH}]+[\mathrm{pC3bFHL1}]+[\mathrm{pC3bC3bFH}]\\
+[\mathrm{pC3bC3bFHL1}]+[\mathrm{pC3biC3bFH}]+[\mathrm{pC3biC3bFHL1}]+[\mathrm{pC4bC4BPPTX3}]+[\mathrm{pC4bC4bC4BPPTX3}]\\
+[\mathrm{pC4bC4dC4BPPTX3}]+[\mathrm{pC4bC3bFHPTX3}]+[\mathrm{pC4bC3bFHL1PTX3}]+[\mathrm{pC4bC3bC4BPPTX3}]\\
+[\mathrm{pC4dC3bFHPTX3}]+[\mathrm{pC4dC3bFHL1PTX3}]+[\mathrm{pC4biC3bC4BPPTX3}]+[\mathrm{pC3bFHPTX3}]\\
+[\mathrm{pC3bFHL1PTX3}]+[\mathrm{pC3bC3bFHPTX3}]+[\mathrm{pC3bC3bFHL1PTX3}]+[\mathrm{pC3biC3bFHPTX3}]\\
+[\mathrm{pC3biC3bFHL1PTX3}]+[\mathrm{pC4bpC4BP}]+[\mathrm{pC4bC4bpC4BP}]+[\mathrm{pC4bC4dpC4BP}]+[\mathrm{pC4bC3bpC4BP}]\\
+[\mathrm{pC4biC3bpC4BP}]+[\mathrm{pC4bC3bpFH}]+[\mathrm{pC4bC3bpFHL1}]+[\mathrm{pC4dC3bpFH}]+[\mathrm{pC4dC3bpFHL1}]\\
+[\mathrm{pC3bpFH}]+[\mathrm{pC3bpFHL1}]+[\mathrm{pC3bC3bpFH}]+[\mathrm{pC3bC3bpFHL1}]+[\mathrm{pC3biC3bpFH}]+[\mathrm{pC3biC3bpFHL1}]\end{array}\right)}$$

$$-k_{\mathrm{C3bH}}^{+}[\mathrm{pC3biC3b}][\mathrm{FH}]+k_{\mathrm{C3bH}}^{-}[\mathrm{pC3biC3bFH}]-k_{\mathrm{C3bFHL1}}^{+}[\mathrm{pC3biC3b}][\mathrm{FHL1}]+k_{\mathrm{C3bFHL1}}^{-}[\mathrm{pC3biC3bFHL1}]$$

$$+\frac{k_{\mathrm{cat\ C3bH}}^{\mathrm{FI}}[\mathrm{FI}][\mathrm{pC3bC3bFHPTX3}]}{\left(\begin{array}{l}K_{\mathrm{m\ C3bH}}^{\mathrm{FI}}+[\mathrm{C3(H_2O)FH}]+[\mathrm{C3(H_2O)FHL1}]+[\mathrm{fC3bFH}]+[\mathrm{fC3bFHL1}]+[\mathrm{fC3bCR1}]+[\mathrm{fiC3bCR1}]\\
+[\mathrm{IgGC3bC3bFH}]+[\mathrm{IgGC3biC3bFH}]+[\mathrm{IgGC3bC3bFHL1}]+[\mathrm{IgGC3biC3bFHL1}]+[\mathrm{IgGC3bC3bCR1}]\\
+[\mathrm{IgGC3biC3bCR1}]+[\mathrm{IgGiC3biC3bCR1}]+[\mathrm{IgGiC3bC3dgCR1}]+[\mathrm{fC4bC3bFH}]+[\mathrm{fC4bC3bFHL1}]\\
+[\mathrm{fC4bC3bCR1}]+[\mathrm{fC4bC3bC4BP}]+[\mathrm{fC4dC3bFH}]+[\mathrm{fC4dC3bFHL1}]+[\mathrm{fC4dC3bCR1}]+[\mathrm{fC4diC3bCR1}]\\
+[\mathrm{fC4biC3bCR1}]+[\mathrm{fC4bC3dgCR1}]+[\mathrm{fC4bC3dgC4BP}]+[\mathrm{fC3bC3bFH}]+[\mathrm{fC3bC3bFHL1}]+[\mathrm{fC3bC3bCR1}]\\
+[\mathrm{fC3biC3bFH}]+[\mathrm{fC3biC3bFHL1}]+[\mathrm{fC3biC3bCR1}]+[\mathrm{fiC3biC3bCR1}]+[\mathrm{fiC3bC3dgCR1}]\\
+[\mathrm{fC4biC3bC4BP}]+[\mathrm{nfC4bC4BP}]+[\mathrm{fC4bC4BP}]+[\mathrm{fC4bCR1}]+[\mathrm{fC4bC4bC4BP}]+[\mathrm{fC4bC4dC4BP}]\\
+[\mathrm{fC4bC4bCR1}]+[\mathrm{fC4bC4dCR1}]+[\mathrm{hC4bCR1}]+[\mathrm{hC4bC4BP}]+[\mathrm{hC4bC4bCR1}]+[\mathrm{hC4bC4bC4BP}]\\
+[\mathrm{hC4bC4dCR1}]+[\mathrm{hC4bC4dC4BP}]+[\mathrm{hC4bC3bFH}]+[\mathrm{hC4bC3bFHL1}]+[\mathrm{hC4bC3bCR1}]\\
+[\mathrm{hC4bC3bC4BP}]+[\mathrm{hC4biC3bCR1}]+[\mathrm{hC4biC3bC4BP}]+[\mathrm{hC4bC3dgCR1}]+[\mathrm{hC4bC3dgC4BP}]\\
+[\mathrm{hC4dC3bFH}]+[\mathrm{hC4dC3bFHL1}]+[\mathrm{hC4dC3bCR1}]+[\mathrm{hC4diC3bCR1}]+[\mathrm{hC3bFH}]+[\mathrm{hC3bFHL1}]\\
+[\mathrm{hC3bCR1}]+[\mathrm{hiC3bCR1}]+[\mathrm{hC3bC3bFH}]+[\mathrm{hC3bC3bFHL1}]+[\mathrm{hC3bC3bCR1}]+[\mathrm{hC3biC3bFH}]\\
+[\mathrm{hC3biC3bFHL1}]+[\mathrm{hC3biC3bCR1}]+[\mathrm{hiC3biC3bCR1}]+[\mathrm{hiC3biC3dgCR1}]+[\mathrm{pC4bC4BP}]\\
+[\mathrm{pC4bC4bC4BP}]+[\mathrm{pC4bC4dC4BP}]+[\mathrm{pC4bC3bC4BP}]+[\mathrm{pC4biC3bC4BP}]+[\mathrm{pC4bC3bFH}]\\
+[\mathrm{pC4bC3bFHL1}]+[\mathrm{pC4dC3bFH}]+[\mathrm{pC4dC3bFHL1}]+[\mathrm{pC3bFH}]+[\mathrm{pC3bFHL1}]+[\mathrm{pC3bC3bFH}]\\
+[\mathrm{pC3bC3bFHL1}]+[\mathrm{pC3biC3bFH}]+[\mathrm{pC3biC3bFHL1}]+[\mathrm{pC4bC4BPPTX3}]+[\mathrm{pC4bC4bC4BPPTX3}]\\
+[\mathrm{pC4bC4dC4BPPTX3}]+[\mathrm{pC4bC3bFHPTX3}]+[\mathrm{pC4bC3bFHL1PTX3}]+[\mathrm{pC4bC3bC4BPPTX3}]\\
+[\mathrm{pC4dC3bFHPTX3}]+[\mathrm{pC4dC3bFHL1PTX3}]+[\mathrm{pC4biC3bC4BPPTX3}]+[\mathrm{pC3bFHPTX3}]\\
+[\mathrm{pC3bFHL1PTX3}]+[\mathrm{pC3bC3bFHPTX3}]+[\mathrm{pC3bC3bFHL1PTX3}]+[\mathrm{pC3biC3bFHPTX3}]\\
+[\mathrm{pC3biC3bFHL1PTX3}]+[\mathrm{pC4bpC4BP}]+[\mathrm{pC4bC4bpC4BP}]+[\mathrm{pC4bC4dpC4BP}]+[\mathrm{pC4bC3bpC4BP}]\\
+[\mathrm{pC4biC3bpC4BP}]+[\mathrm{pC4bC3bpFH}]+[\mathrm{pC4bC3bpFHL1}]+[\mathrm{pC4dC3bpFH}]+[\mathrm{pC4dC3bpFHL1}]\\
+[\mathrm{pC3bpFH}]+[\mathrm{pC3bpFHL1}]+[\mathrm{pC3bC3bpFH}]+[\mathrm{pC3bC3bpFHL1}]+[\mathrm{pC3biC3bpFH}]+[\mathrm{pC3biC3bpFHL1}]\end{array}\right)}$$

$$+\frac{k_{\text{cat C3bH}}^{\text{FI}}[\text{FI}][\text{pC3bC3bFHL1PTX3}]}{\left(\begin{aligned}&K_{\text{m C3bH}}^{\text{FI}}+[\text{C3(H}_2\text{O)FH}]+[\text{C3(H}_2\text{O)FHL1}]+[\text{fC3bFH}]+[\text{fC3bFHL1}]+[\text{fC3bCR1}]+[\text{fiC3bCR1}]\\&+[\text{IgGC3bC3bFH}]+[\text{IgGC3biC3bFH}]+[\text{IgGC3bC3bFHL1}]+[\text{IgGC3biC3bFHL1}]+[\text{IgGC3bC3bCR1}]\\&+[\text{IgGC3biC3bCR1}]+[\text{IgGiC3biC3bCR1}]+[\text{IgGiC3bC3dgCR1}]+[\text{fC4bC3bFH}]+[\text{fC4bC3bFHL1}]\\&+[\text{fC4bC3bCR1}]+[\text{fC4bC3bC4BP}]+[\text{fC4dC3bFH}]+[\text{fC4dC3bFHL1}]+[\text{fC4dC3bCR1}]+[\text{fC4diC3bCR1}]\\&+[\text{fC4biC3bCR1}]+[\text{fC4bC3dgCR1}]+[\text{fC4bC3dgC4BP}]+[\text{fC3bC3bFH}]+[\text{fC3bC3bFHL1}]+[\text{fC3bC3bCR1}]\\&+[\text{fC3biC3bFH}]+[\text{fC3biC3bFHL1}]+[\text{fC3biC3bCR1}]+[\text{fiC3biC3bCR1}]+[\text{fiC3bC3dgCR1}]\\&+[\text{fC4biC3bC4BP}]+[\text{nfC4bC4BP}]+[\text{fC4bC4BP}]+[\text{fC4bCR1}]+[\text{fC4bC4bC4BP}]+[\text{fC4bC4dC4BP}]\\&+[\text{fC4bC4bCR1}]+[\text{fC4bC4dCR1}]+[\text{hC4bCR1}]+[\text{hC4bC4BP}]+[\text{hC4bC4bCR1}]+[\text{hC4bC4bC4BP}]\\&+[\text{hC4bC4dCR1}]+[\text{hC4bC4dC4BP}]+[\text{hC4bC3bFH}]+[\text{hC4bC3bFHL1}]+[\text{hC4bC3bCR1}]\\&+[\text{hC4bC3bC4BP}]+[\text{hC4biC3bCR1}]+[\text{hC4biC3bC4BP}]+[\text{hC4bC3dgCR1}]+[\text{hC4bC3dgC4BP}]\\&+[\text{hC4dC3bFH}]+[\text{hC4dC3bFHL1}]+[\text{hC4dC3bCR1}]+[\text{hC4diC3bCR1}]+[\text{hC3bFH}]+[\text{hC3bFHL1}]\\&+[\text{hC3bCR1}]+[\text{hiC3bCR1}]+[\text{hC3bC3bFH}]+[\text{hC3bC3bFHL1}]+[\text{hC3bC3bCR1}]+[\text{hC3biC3bFH}]\\&+[\text{hC3biC3bFHL1}]+[\text{hC3biC3bCR1}]+[\text{hiC3biC3bCR1}]+[\text{hiC3biC3dgCR1}]+[\text{pC4bC4BP}]\\&+[\text{pC4bC4bC4BP}]+[\text{pC4bC4dC4BP}]+[\text{pC4bC3bC4BP}]+[\text{pC4biC3bC4BP}]+[\text{pC4bC3bFH}]\\&+[\text{pC4bC3bFHL1}]+[\text{pC4dC3bFH}]+[\text{pC4dC3bFHL1}]+[\text{pC3bFH}]+[\text{pC3bFHL1}]+[\text{pC3bC3bFH}]\\&+[\text{pC3bC3bFHL1}]+[\text{pC3biC3bFH}]+[\text{pC3biC3bFHL1}]+[\text{pC4bC4BPPTX3}]+[\text{pC4bC4bC4BPPTX3}]\\&+[\text{pC4bC4dC4BPPTX3}]+[\text{pC4bC3bFHPTX3}]+[\text{pC4bC3bFHL1PTX3}]+[\text{pC4bC3bC4BPPTX3}]\\&+[\text{pC4dC3bFHPTX3}]+[\text{pC4dC3bFHL1PTX3}]+[\text{pC4biC3bC4BPPTX3}]+[\text{pC3bFHPTX3}]\\&+[\text{pC3bFHL1PTX3}]+[\text{pC3bC3bFHPTX3}]+[\text{pC3bC3bFHL1PTX3}]+[\text{pC3biC3bFHPTX3}]\\&+[\text{pC3biC3bFHL1PTX3}]+[\text{pC4bpC4BP}]+[\text{pC4bC4bpC4BP}]+[\text{pC4bC4dpC4BP}]+[\text{pC4bC3bpC4BP}]\\&+[\text{pC4biC3bpC4BP}]+[\text{pC4bC3bpFH}]+[\text{pC4bC3bpFHL1}]+[\text{pC4dC3bpFH}]+[\text{pC4dC3bpFHL1}]\\&+[\text{pC3bpFH}]+[\text{pC3bpFHL1}]+[\text{pC3bC3bpFH}]+[\text{pC3bC3bpFHL1}]+[\text{pC3biC3bpFH}]+[\text{pC3biC3bpFHL1}]\end{aligned}\right)}$$

$$-k_{\text{C3bH}}^{+}[\text{pC3biC3b}][\text{pFHPTX3}]+k_{\text{C3bH}}^{-}[\text{pC3biC3bFHPTX3}]-k_{\text{C3bFHL1}}^{+}[\text{pC3biC3b}][\text{pFHL1PTX3}]$$
$$+k_{\text{C3bFHL1}}^{-}[\text{pC3biC3bFHL1PTX3}]$$

$$
+\frac{k_{\text{cat C3bH}}^{\text{FI}}\left[\text{FI}\right]\left[\text{pC3bC3bpFH}\right]}{\left(\begin{array}{l}
K_{\text{m C3bH}}^{\text{FI}}+\left[\text{C3(H}_2\text{O)FH}\right]+\left[\text{C3(H}_2\text{O)FHL1}\right]+\left[\text{fC3bFH}\right]+\left[\text{fC3bFHL1}\right]+\left[\text{fC3bCR1}\right]+\left[\text{fiC3bCR1}\right]\\
+\left[\text{IgGC3bC3bFH}\right]+\left[\text{IgGC3biC3bFH}\right]+\left[\text{IgGC3bC3bFHL1}\right]+\left[\text{IgGC3biC3bFHL1}\right]+\left[\text{IgGC3bC3bCR1}\right]\\
+\left[\text{IgGC3biC3bCR1}\right]+\left[\text{IgGiC3biC3bCR1}\right]+\left[\text{IgGiC3bC3dgCR1}\right]+\left[\text{fC4bC3bFH}\right]+\left[\text{fC4bC3bFHL1}\right]\\
+\left[\text{fC4bC3bCR1}\right]+\left[\text{fC4bC3bC4BP}\right]+\left[\text{fC4dC3bFH}\right]+\left[\text{fC4dC3bFHL1}\right]+\left[\text{fC4dC3bCR1}\right]+\left[\text{fC4diC3bCR1}\right]\\
+\left[\text{fC4biC3bCR1}\right]+\left[\text{fC4bC3dgCR1}\right]+\left[\text{fC4bC3dgC4BP}\right]+\left[\text{fC3bC3bFH}\right]+\left[\text{fC3bC3bFHL1}\right]+\left[\text{fC3bC3bCR1}\right]\\
+\left[\text{fC3biC3bFH}\right]+\left[\text{fC3biC3bFHL1}\right]+\left[\text{fC3biC3bCR1}\right]+\left[\text{fiC3biC3bCR1}\right]+\left[\text{fiC3bC3dgCR1}\right]\\
+\left[\text{fC4biC3bC4BP}\right]+\left[\text{nfC4bC4BP}\right]+\left[\text{fC4bC4BP}\right]+\left[\text{fC4bCR1}\right]+\left[\text{fC4bC4bC4BP}\right]+\left[\text{fC4bC4dC4BP}\right]\\
+\left[\text{fC4bC4bCR1}\right]+\left[\text{fC4bC4dCR1}\right]+\left[\text{hC4bCR1}\right]+\left[\text{hC4bC4BP}\right]+\left[\text{hC4bC4bCR1}\right]+\left[\text{hC4bC4bC4BP}\right]\\
+\left[\text{hC4bC4dCR1}\right]+\left[\text{hC4bC4dC4BP}\right]+\left[\text{hC4bC3bFH}\right]+\left[\text{hC4bC3bFHL1}\right]+\left[\text{hC4bC3bCR1}\right]\\
+\left[\text{hC4bC3bC4BP}\right]+\left[\text{hC4biC3bCR1}\right]+\left[\text{hC4biC3bC4BP}\right]+\left[\text{hC4bC3dgCR1}\right]+\left[\text{hC4bC3dgC4BP}\right]\\
+\left[\text{hC4dC3bFH}\right]+\left[\text{hC4dC3bFHL1}\right]+\left[\text{hC4dC3bCR1}\right]+\left[\text{hC4diC3bCR1}\right]+\left[\text{hC3bFH}\right]+\left[\text{hC3bFHL1}\right]\\
+\left[\text{hC3bCR1}\right]+\left[\text{hiC3bCR1}\right]+\left[\text{hC3bC3bFH}\right]+\left[\text{hC3bC3bFHL1}\right]+\left[\text{hC3bC3bCR1}\right]+\left[\text{hC3biC3bFH}\right]\\
+\left[\text{hC3biC3bFHL1}\right]+\left[\text{hC3biC3bCR1}\right]+\left[\text{hiC3biC3bCR1}\right]+\left[\text{hiC3biC3dgCR1}\right]+\left[\text{pC4bC4BP}\right]\\
+\left[\text{pC4bC4bC4BP}\right]+\left[\text{pC4bC4dC4BP}\right]+\left[\text{pC4bC3bC4BP}\right]+\left[\text{pC4biC3bC4BP}\right]+\left[\text{pC4bC3bFH}\right]\\
+\left[\text{pC4bC3bFHL1}\right]+\left[\text{pC4dC3bFH}\right]+\left[\text{pC4dC3bFHL1}\right]+\left[\text{pC3bFH}\right]+\left[\text{pC3bFHL1}\right]+\left[\text{pC3bC3bFH}\right]\\
+\left[\text{pC3bC3bFHL1}\right]+\left[\text{pC3biC3bFH}\right]+\left[\text{pC3biC3bFHL1}\right]+\left[\text{pC4bC4BPPTX3}\right]+\left[\text{pC4bC4bC4BPPTX3}\right]\\
+\left[\text{pC4bC4dC4BPPTX3}\right]+\left[\text{pC4bC3bFHPTX3}\right]+\left[\text{pC4bC3bFHL1PTX3}\right]+\left[\text{pC4bC3bC4BPPTX3}\right]\\
+\left[\text{pC4dC3bFHPTX3}\right]+\left[\text{pC4dC3bFHL1PTX3}\right]+\left[\text{pC4biC3bC4BPPTX3}\right]+\left[\text{pC3bFHPTX3}\right]\\
+\left[\text{pC3bFHL1PTX3}\right]+\left[\text{pC3bC3bFHPTX3}\right]+\left[\text{pC3bC3bFHL1PTX3}\right]+\left[\text{pC3biC3bFHPTX3}\right]\\
+\left[\text{pC3biC3bFHL1PTX3}\right]+\left[\text{pC4bpC4BP}\right]+\left[\text{pC4bC4bpC4BP}\right]+\left[\text{pC4bC4dpC4BP}\right]+\left[\text{pC4bC3bpC4BP}\right]\\
+\left[\text{pC4biC3bpC4BP}\right]+\left[\text{pC4bC3bpFH}\right]+\left[\text{pC4bC3bpFHL1}\right]+\left[\text{pC4dC3bpFH}\right]+\left[\text{pC4dC3bpFHL1}\right]\\
+\left[\text{pC3bpFH}\right]+\left[\text{pC3bpFHL1}\right]+\left[\text{pC3bC3bpFH}\right]+\left[\text{pC3bC3bpFHL1}\right]+\left[\text{pC3biC3bpFH}\right]+\left[\text{pC3biC3bpFHL1}\right]
\end{array}\right)}
$$

$$+\frac{k_{\text{cat C3bH}}^{\text{FI}}[\text{FI}][\text{pC3bC3bpFHL1}]}{\left(\begin{array}{l} K_{\text{m C3bH}}^{\text{FI}}+[\text{C3(H}_2\text{O)FH}]+[\text{C3(H}_2\text{O)FHL1}]+[\text{fC3bFH}]+[\text{fC3bFHL1}]+[\text{fC3bCR1}]+[\text{fiC3bCR1}] \\ +[\text{IgGC3bC3bFH}]+[\text{IgGC3biC3bFH}]+[\text{IgGC3bC3bFHL1}]+[\text{IgGC3biC3bFHL1}]+[\text{IgGC3bC3bCR1}] \\ +[\text{IgGC3biC3bCR1}]+[\text{IgGiC3biC3bCR1}]+[\text{IgGiC3bC3dgCR1}]+[\text{fC4bC3bFH}]+[\text{fC4bC3bFHL1}] \\ +[\text{fC4bC3bCR1}]+[\text{fC4bC3bC4BP}]+[\text{fC4dC3bFH}]+[\text{fC4dC3bFHL1}]+[\text{fC4dC3bCR1}]+[\text{fC4diC3bCR1}] \\ +[\text{fC4biC3bCR1}]+[\text{fC4bC3dgCR1}]+[\text{fC4bC3dgC4BP}]+[\text{fC3bC3bFH}]+[\text{fC3bC3bFHL1}]+[\text{fC3bC3bCR1}] \\ +[\text{fC3biC3bFH}]+[\text{fC3biC3bFHL1}]+[\text{fC3biC3bCR1}]+[\text{fiC3biC3bCR1}]+[\text{fiC3bC3dgCR1}] \\ +[\text{fC4biC3bC4BP}]+[\text{nfC4bC4BP}]+[\text{fC4bC4BP}]+[\text{fC4bCR1}]+[\text{fC4bC4bC4BP}]+[\text{fC4bC4dC4BP}] \\ +[\text{fC4bC4bCR1}]+[\text{fC4bC4dCR1}]+[\text{hC4bCR1}]+[\text{hC4bC4BP}]+[\text{hC4bC4bCR1}]+[\text{hC4bC4bC4BP}] \\ +[\text{hC4bC4dCR1}]+[\text{hC4bC4dC4BP}]+[\text{hC4bC3bFH}]+[\text{hC4bC3bFHL1}]+[\text{hC4bC3bCR1}] \\ +[\text{hC4bC3bC4BP}]+[\text{hC4biC3bCR1}]+[\text{hC4biC3bC4BP}]+[\text{hC4bC3dgCR1}]+[\text{hC4bC3dgC4BP}] \\ +[\text{hC4dC3bFH}]+[\text{hC4dC3bFHL1}]+[\text{hC4dC3bCR1}]+[\text{hC4diC3bCR1}]+[\text{hC3bFH}]+[\text{hC3bFHL1}] \\ +[\text{hC3bCR1}]+[\text{hiC3bCR1}]+[\text{hC3bC3bFH}]+[\text{hC3bC3bFHL1}]+[\text{hC3bC3bCR1}]+[\text{hC3biC3bFH}] \\ +[\text{hC3biC3bFHL1}]+[\text{hC3biC3bCR1}]+[\text{hiC3biC3bCR1}]+[\text{hiC3biC3dgCR1}]+[\text{pC4bC4BP}] \\ +[\text{pC4bC4bC4BP}]+[\text{pC4bC4dC4BP}]+[\text{pC4bC3bC4BP}]+[\text{pC4biC3bC4BP}]+[\text{pC4bC3bFH}] \\ +[\text{pC4bC3bFHL1}]+[\text{pC4dC3bFH}]+[\text{pC4dC3bFHL1}]+[\text{pC3bFH}]+[\text{pC3bFHL1}]+[\text{pC3bC3bFH}] \\ +[\text{pC3bC3bFHL1}]+[\text{pC3biC3bFH}]+[\text{pC3biC3bFHL1}]+[\text{pC4bC4BPPTX3}]+[\text{pC4bC4bC4BPPTX3}] \\ +[\text{pC4bC4dC4BPPTX3}]+[\text{pC4bC3bFHPTX3}]+[\text{pC4bC3bFHL1PTX3}]+[\text{pC4bC3bC4BPPTX3}] \\ +[\text{pC4dC3bFHPTX3}]+[\text{pC4dC3bFHL1PTX3}]+[\text{pC4biC3bC4BPPTX3}]+[\text{pC3bFHPTX3}] \\ +[\text{pC3bFHL1PTX3}]+[\text{pC3bC3bFHPTX3}]+[\text{pC3bC3bFHL1PTX3}]+[\text{pC3biC3bFHPTX3}] \\ +[\text{pC3biC3bFHL1PTX3}]+[\text{pC4bpC4BP}]+[\text{pC4bC4bpC4BP}]+[\text{pC4bC4dpC4BP}]+[\text{pC4bC3bpC4BP}] \\ +[\text{pC4biC3bpC4BP}]+[\text{pC4bC3bpFH}]+[\text{pC4bC3bpFHL1}]+[\text{pC4dC3bpFH}]+[\text{pC4dC3bpFHL1}] \\ +[\text{pC3bpFH}]+[\text{pC3bpFHL1}]+[\text{pC3bC3bpFH}]+[\text{pC3bC3bpFHL1}]+[\text{pC3biC3bpFH}]+[\text{pC3biC3bpFHL1}] \end{array}\right)}$$

$$-k_{\text{C3bH}}^{+}[\text{pC3biC3b}][\text{pFH}]+k_{\text{C3bH}}^{-}[\text{pC3biC3bpFH}]-k_{\text{C3bFHL1}}^{+}[\text{pC3biC3b}][\text{pFHL1}]+k_{\text{C3bFHL1}}^{-}[\text{pC3biC3bpFHL1}]$$

233

$$
\begin{aligned}
\frac{d\left[\mathrm{pC3biC3bFH}\right]}{dt} &= k_{\mathrm{C3bH}}^{+}\left[\mathrm{pC3biC3b}\right]\left[\mathrm{FH}\right]-k_{\mathrm{C3bH}}^{-}\left[\mathrm{pC3biC3bFH}\right] \\
&- \frac{k_{\mathrm{cat\ C3bH}}^{\mathrm{FI}}\left[\mathrm{FI}\right]\left[\mathrm{pC3biC3bFH}\right]}{\left(\begin{array}{l}
K_{\mathrm{m\ C3bH}}^{\mathrm{FI}}+\left[\mathrm{C3(H_2O)FH}\right]+\left[\mathrm{C3(H_2O)FHL1}\right]+\left[\mathrm{fC3bFH}\right]+\left[\mathrm{fC3bFHL1}\right]+\left[\mathrm{fC3bCR1}\right]+\left[\mathrm{fiC3bCR1}\right] \\
+\left[\mathrm{IgGC3bC3bFH}\right]+\left[\mathrm{IgGC3biC3bFH}\right]+\left[\mathrm{IgGC3bC3bFHL1}\right]+\left[\mathrm{IgGC3biC3bFHL1}\right]+\left[\mathrm{IgGC3bC3bCR1}\right] \\
+\left[\mathrm{IgGC3biC3bCR1}\right]+\left[\mathrm{IgGiC3biC3bCR1}\right]+\left[\mathrm{IgGiC3bC3dgCR1}\right]+\left[\mathrm{fC4bC3bFH}\right]+\left[\mathrm{fC4bC3bFHL1}\right] \\
+\left[\mathrm{fC4bC3bCR1}\right]+\left[\mathrm{fC4bC3bC4BP}\right]+\left[\mathrm{fC4dC3bFH}\right]+\left[\mathrm{fC4dC3bFHL1}\right]+\left[\mathrm{fC4dC3bCR1}\right]+\left[\mathrm{fC4diC3bCR1}\right] \\
+\left[\mathrm{fC4biC3bCR1}\right]+\left[\mathrm{fC4bC3dgCR1}\right]+\left[\mathrm{fC4bC3dgC4BP}\right]+\left[\mathrm{fC3bC3bFH}\right]+\left[\mathrm{fC3bC3bFHL1}\right]+\left[\mathrm{fC3bC3bCR1}\right] \\
+\left[\mathrm{fC3biC3bFH}\right]+\left[\mathrm{fC3biC3bFHL1}\right]+\left[\mathrm{fC3biC3bCR1}\right]+\left[\mathrm{fiC3biC3bCR1}\right]+\left[\mathrm{fiC3bC3dgCR1}\right] \\
+\left[\mathrm{fC4biC3bC4BP}\right]+\left[\mathrm{nfC4bC4BP}\right]+\left[\mathrm{fC4bC4BP}\right]+\left[\mathrm{fC4bCR1}\right]+\left[\mathrm{fC4bC4bC4BP}\right]+\left[\mathrm{fC4bC4dC4BP}\right] \\
+\left[\mathrm{fC4bC4bCR1}\right]+\left[\mathrm{fC4bC4dCR1}\right]+\left[\mathrm{hC4bCR1}\right]+\left[\mathrm{hC4bC4BP}\right]+\left[\mathrm{hC4bC4bCR1}\right]+\left[\mathrm{hC4bC4bC4BP}\right] \\
+\left[\mathrm{hC4bC4dCR1}\right]+\left[\mathrm{hC4bC4dC4BP}\right]+\left[\mathrm{hC4bC3bFH}\right]+\left[\mathrm{hC4bC3bFHL1}\right]+\left[\mathrm{hC4bC3bCR1}\right] \\
+\left[\mathrm{hC4bC3bC4BP}\right]+\left[\mathrm{hC4biC3bCR1}\right]+\left[\mathrm{hC4biC3bC4BP}\right]+\left[\mathrm{hC4bC3dgCR1}\right]+\left[\mathrm{hC4bC3dgC4BP}\right] \\
+\left[\mathrm{hC4dC3bFH}\right]+\left[\mathrm{hC4dC3bFHL1}\right]+\left[\mathrm{hC4dC3bCR1}\right]+\left[\mathrm{hC4diC3bCR1}\right]+\left[\mathrm{hC3bFH}\right]+\left[\mathrm{hC3bFHL1}\right] \\
+\left[\mathrm{hC3bCR1}\right]+\left[\mathrm{hiC3bCR1}\right]+\left[\mathrm{hC3bC3bFH}\right]+\left[\mathrm{hC3bC3bFHL1}\right]+\left[\mathrm{hC3bC3bCR1}\right]+\left[\mathrm{hC3biC3bFH}\right] \\
+\left[\mathrm{hC3biC3bFHL1}\right]+\left[\mathrm{hC3biC3bCR1}\right]+\left[\mathrm{hiC3biC3bCR1}\right]+\left[\mathrm{hiC3biC3dgCR1}\right]+\left[\mathrm{pC4bC4BP}\right] \\
+\left[\mathrm{pC4bC4bC4BP}\right]+\left[\mathrm{pC4bC4dC4BP}\right]+\left[\mathrm{pC4bC3bC4BP}\right]+\left[\mathrm{pC4biC3bC4BP}\right]+\left[\mathrm{pC4bC3bFH}\right] \\
+\left[\mathrm{pC4bC3bFHL1}\right]+\left[\mathrm{pC4dC3bFH}\right]+\left[\mathrm{pC4dC3bFHL1}\right]+\left[\mathrm{pC3bFH}\right]+\left[\mathrm{pC3bFHL1}\right]+\left[\mathrm{pC3bC3bFH}\right] \\
+\left[\mathrm{pC3bC3bFHL1}\right]+\left[\mathrm{pC3biC3bFH}\right]+\left[\mathrm{pC3biC3bFHL1}\right]+\left[\mathrm{pC4bC4BPPTX3}\right]+\left[\mathrm{pC4bC4bC4BPPTX3}\right] \\
+\left[\mathrm{pC4bC4dC4BPPTX3}\right]+\left[\mathrm{pC4bC3bFHPTX3}\right]+\left[\mathrm{pC4bC3bFHL1PTX3}\right]+\left[\mathrm{pC4bC3bC4BPPTX3}\right] \\
+\left[\mathrm{pC4dC3bFHPTX3}\right]+\left[\mathrm{pC4dC3bFHL1PTX3}\right]+\left[\mathrm{pC4biC3bC4BPPTX3}\right]+\left[\mathrm{pC3bFHPTX3}\right] \\
+\left[\mathrm{pC3bFHL1PTX3}\right]+\left[\mathrm{pC3bC3bFHPTX3}\right]+\left[\mathrm{pC3bC3bFHL1PTX3}\right]+\left[\mathrm{pC3biC3bFHPTX3}\right] \\
+\left[\mathrm{pC3biC3bFHL1PTX3}\right]+\left[\mathrm{pC4bpC4BP}\right]+\left[\mathrm{pC4bC4bpC4BP}\right]+\left[\mathrm{pC4bC4dpC4BP}\right]+\left[\mathrm{pC4bC3bpC4BP}\right] \\
+\left[\mathrm{pC4biC3bpC4BP}\right]+\left[\mathrm{pC4bC3bpFH}\right]+\left[\mathrm{pC4bC3bpFHL1}\right]+\left[\mathrm{pC4dC3bpFH}\right]+\left[\mathrm{pC4dC3bpFHL1}\right] \\
+\left[\mathrm{pC3bpFH}\right]+\left[\mathrm{pC3bpFHL1}\right]+\left[\mathrm{pC3bC3bpFH}\right]+\left[\mathrm{pC3bC3bpFHL1}\right]+\left[\mathrm{pC3biC3bpFH}\right]+\left[\mathrm{pC3biC3bpFHL1}\right]
\end{array}\right)}
\end{aligned}
$$

234

$$\frac{d\left[\mathrm{pC3biC3bFHL1}\right]}{dt} = k^{+}_{\mathrm{C3bFHL1}}\left[\mathrm{pC3biC3b}\right]\left[\mathrm{FHL1}\right] - k^{-}_{\mathrm{C3bFHL1}}\left[\mathrm{pC3biC3bFHL1}\right]$$

$$-\frac{k^{\mathrm{FI}}_{\mathrm{cat\ C3bH}}\left[\mathrm{FI}\right]\left[\mathrm{pC3biC3bFHL1}\right]}{\left(\begin{aligned}
&K^{\mathrm{FI}}_{\mathrm{m\ C3bH}}+\left[\mathrm{C3(H_2O)FH}\right]+\left[\mathrm{C3(H_2O)FHL1}\right]+\left[\mathrm{fC3bFH}\right]+\left[\mathrm{fC3bFHL1}\right]+\left[\mathrm{fC3bCR1}\right]+\left[\mathrm{fiC3bCR1}\right]\\
&+\left[\mathrm{IgGC3bC3bFH}\right]+\left[\mathrm{IgGC3biC3bFH}\right]+\left[\mathrm{IgGC3bC3bFHL1}\right]+\left[\mathrm{IgGC3biC3bFHL1}\right]+\left[\mathrm{IgGC3bC3bCR1}\right]\\
&+\left[\mathrm{IgGC3biC3bCR1}\right]+\left[\mathrm{IgGiC3biC3bCR1}\right]+\left[\mathrm{IgGiC3bC3dgCR1}\right]+\left[\mathrm{fC4bC3bFH}\right]+\left[\mathrm{fC4bC3bFHL1}\right]\\
&+\left[\mathrm{fC4bC3bCR1}\right]+\left[\mathrm{fC4bC3bC4BP}\right]+\left[\mathrm{fC4dC3bFH}\right]+\left[\mathrm{fC4dC3bFHL1}\right]+\left[\mathrm{fC4dC3bCR1}\right]+\left[\mathrm{fC4diC3bCR1}\right]\\
&+\left[\mathrm{fC4biC3bCR1}\right]+\left[\mathrm{fC4bC3dgCR1}\right]+\left[\mathrm{fC4bC3dgC4BP}\right]+\left[\mathrm{fC3bC3bFH}\right]+\left[\mathrm{fC3bC3bFHL1}\right]+\left[\mathrm{fC3bC3bCR1}\right]\\
&+\left[\mathrm{fC3biC3bFH}\right]+\left[\mathrm{fC3biC3bFHL1}\right]+\left[\mathrm{fC3biC3bCR1}\right]+\left[\mathrm{fiC3biC3bCR1}\right]+\left[\mathrm{fiC3bC3dgCR1}\right]\\
&+\left[\mathrm{fC4biC3bC4BP}\right]+\left[\mathrm{nfC4bC4BP}\right]+\left[\mathrm{fC4bC4BP}\right]+\left[\mathrm{fC4bCR1}\right]+\left[\mathrm{fC4bC4bC4BP}\right]+\left[\mathrm{fC4bC4dC4BP}\right]\\
&+\left[\mathrm{fC4bC4bCR1}\right]+\left[\mathrm{fC4bC4dCR1}\right]+\left[\mathrm{hC4bCR1}\right]+\left[\mathrm{hC4bC4BP}\right]+\left[\mathrm{hC4bC4bCR1}\right]+\left[\mathrm{hC4bC4bC4BP}\right]\\
&+\left[\mathrm{hC4bC4dCR1}\right]+\left[\mathrm{hC4bC4dC4BP}\right]+\left[\mathrm{hC4bC3bFH}\right]+\left[\mathrm{hC4bC3bFHL1}\right]+\left[\mathrm{hC4bC3bCR1}\right]\\
&+\left[\mathrm{hC4bC3bC4BP}\right]+\left[\mathrm{hC4biC3bCR1}\right]+\left[\mathrm{hC4biC3bC4BP}\right]+\left[\mathrm{hC4bC3dgCR1}\right]+\left[\mathrm{hC4bC3dgC4BP}\right]\\
&+\left[\mathrm{hC4dC3bFH}\right]+\left[\mathrm{hC4dC3bFHL1}\right]+\left[\mathrm{hC4dC3bCR1}\right]+\left[\mathrm{hC4diC3bCR1}\right]+\left[\mathrm{hC3bFH}\right]+\left[\mathrm{hC3bFHL1}\right]\\
&+\left[\mathrm{hC3bCR1}\right]+\left[\mathrm{hiC3bCR1}\right]+\left[\mathrm{hC3bC3bFH}\right]+\left[\mathrm{hC3bC3bFHL1}\right]+\left[\mathrm{hC3bC3bCR1}\right]+\left[\mathrm{hC3biC3bFH}\right]\\
&+\left[\mathrm{hC3biC3bFHL1}\right]+\left[\mathrm{hC3biC3bCR1}\right]+\left[\mathrm{hiC3biC3bCR1}\right]+\left[\mathrm{hiC3biC3dgCR1}\right]+\left[\mathrm{pC4bC4BP}\right]\\
&+\left[\mathrm{pC4bC4bC4BP}\right]+\left[\mathrm{pC4bC4dC4BP}\right]+\left[\mathrm{pC4bC3bC4BP}\right]+\left[\mathrm{pC4biC3bC4BP}\right]+\left[\mathrm{pC4bC3bFH}\right]\\
&+\left[\mathrm{pC4bC3bFHL1}\right]+\left[\mathrm{pC4dC3bFH}\right]+\left[\mathrm{pC4dC3bFHL1}\right]+\left[\mathrm{pC3bFH}\right]+\left[\mathrm{pC3bFHL1}\right]+\left[\mathrm{pC3bC3bFH}\right]\\
&+\left[\mathrm{pC3bC3bFHL1}\right]+\left[\mathrm{pC3biC3bFH}\right]+\left[\mathrm{pC3biC3bFHL1}\right]+\left[\mathrm{pC4bC4BPPTX3}\right]+\left[\mathrm{pC4bC4bC4BPPTX3}\right]\\
&+\left[\mathrm{pC4bC4dC4BPPTX3}\right]+\left[\mathrm{pC4bC3bFHPTX3}\right]+\left[\mathrm{pC4bC3bFHL1PTX3}\right]+\left[\mathrm{pC4bC3bC4BPPTX3}\right]\\
&+\left[\mathrm{pC4dC3bFHPTX3}\right]+\left[\mathrm{pC4dC3bFHL1PTX3}\right]+\left[\mathrm{pC4biC3bC4BPPTX3}\right]+\left[\mathrm{pC3bFHPTX3}\right]\\
&+\left[\mathrm{pC3bFHL1PTX3}\right]+\left[\mathrm{pC3bC3bFHPTX3}\right]+\left[\mathrm{pC3bC3bFHL1PTX3}\right]+\left[\mathrm{pC3biC3bFHPTX3}\right]\\
&+\left[\mathrm{pC3biC3bFHL1PTX3}\right]+\left[\mathrm{pC4bpC4BP}\right]+\left[\mathrm{pC4bC4bpC4BP}\right]+\left[\mathrm{pC4bC4dpC4BP}\right]+\left[\mathrm{pC4bC3bpC4BP}\right]\\
&+\left[\mathrm{pC4biC3bpC4BP}\right]+\left[\mathrm{pC4bC3bpFH}\right]+\left[\mathrm{pC4bC3bpFHL1}\right]+\left[\mathrm{pC4dC3bpFH}\right]+\left[\mathrm{pC4dC3bpFHL1}\right]\\
&+\left[\mathrm{pC3bpFH}\right]+\left[\mathrm{pC3bpFHL1}\right]+\left[\mathrm{pC3bC3bpFH}\right]+\left[\mathrm{pC3bC3bpFHL1}\right]+\left[\mathrm{pC3biC3bpFH}\right]+\left[\mathrm{pC3biC3bpFHL1}\right]
\end{aligned}\right)}$$

235

$$\frac{d\left[\mathrm{piC3biC3b}\right]}{dt}=$$

$$\frac{k_{\mathrm{cat\ C3bH}}^{\mathrm{FI}}\left[\mathrm{FI}\right]\left[\mathrm{pC3biC3bFH}\right]}{\left(\begin{array}{l} K_{\mathrm{m\ C3bH}}^{\mathrm{FI}}+\left[\mathrm{C3(H_2O)FH}\right]+\left[\mathrm{C3(H_2O)FHL1}\right]+\left[\mathrm{fC3bFH}\right]+\left[\mathrm{fC3bFHL1}\right]+\left[\mathrm{fC3bCR1}\right]+\left[\mathrm{fiC3bCR1}\right] \\ +\left[\mathrm{IgGC3bC3bFH}\right]+\left[\mathrm{IgGC3biC3bFH}\right]+\left[\mathrm{IgGC3bC3bFHL1}\right]+\left[\mathrm{IgGC3biC3bFHL1}\right]+\left[\mathrm{IgGC3bC3bCR1}\right] \\ +\left[\mathrm{IgGC3biC3bCR1}\right]+\left[\mathrm{IgGiC3biC3bCR1}\right]+\left[\mathrm{IgGiC3bC3dgCR1}\right]+\left[\mathrm{fC4bC3bFH}\right]+\left[\mathrm{fC4bC3bFHL1}\right] \\ +\left[\mathrm{fC4bC3bCR1}\right]+\left[\mathrm{fC4bC3bC4BP}\right]+\left[\mathrm{fC4dC3bFH}\right]+\left[\mathrm{fC4dC3bFHL1}\right]+\left[\mathrm{fC4dC3bCR1}\right]+\left[\mathrm{fC4diC3bCR1}\right] \\ +\left[\mathrm{fC4biC3bCR1}\right]+\left[\mathrm{fC4bC3dgCR1}\right]+\left[\mathrm{fC4bC3dgC4BP}\right]+\left[\mathrm{fC3bC3bFH}\right]+\left[\mathrm{fC3bC3bFHL1}\right]+\left[\mathrm{fC3bC3bCR1}\right] \\ +\left[\mathrm{fC3biC3bFH}\right]+\left[\mathrm{fC3biC3bFHL1}\right]+\left[\mathrm{fC3biC3bCR1}\right]+\left[\mathrm{fiC3biC3bCR1}\right]+\left[\mathrm{fiC3bC3dgCR1}\right] \\ +\left[\mathrm{fC4biC3bC4BP}\right]+\left[\mathrm{nfC4bC4BP}\right]+\left[\mathrm{fC4bC4BP}\right]+\left[\mathrm{fC4bCR1}\right]+\left[\mathrm{fC4bC4bC4BP}\right]+\left[\mathrm{fC4bC4dC4BP}\right] \\ +\left[\mathrm{fC4bC4bCR1}\right]+\left[\mathrm{fC4bC4dCR1}\right]+\left[\mathrm{hC4bCR1}\right]+\left[\mathrm{hC4bC4BP}\right]+\left[\mathrm{hC4bC4bCR1}\right]+\left[\mathrm{hC4bC4bC4BP}\right] \\ +\left[\mathrm{hC4bC4dCR1}\right]+\left[\mathrm{hC4bC4dC4BP}\right]+\left[\mathrm{hC4bC3bFH}\right]+\left[\mathrm{hC4bC3bFHL1}\right]+\left[\mathrm{hC4bC3bCR1}\right] \\ +\left[\mathrm{hC4bC3bC4BP}\right]+\left[\mathrm{hC4biC3bCR1}\right]+\left[\mathrm{hC4biC3bC4BP}\right]+\left[\mathrm{hC4bC3dgCR1}\right]+\left[\mathrm{hC4bC3dgC4BP}\right] \\ +\left[\mathrm{hC4dC3bFH}\right]+\left[\mathrm{hC4dC3bFHL1}\right]+\left[\mathrm{hC4dC3bCR1}\right]+\left[\mathrm{hC4diC3bCR1}\right]+\left[\mathrm{hC3bFH}\right]+\left[\mathrm{hC3bFHL1}\right] \\ +\left[\mathrm{hC3bCR1}\right]+\left[\mathrm{hiC3bCR1}\right]+\left[\mathrm{hC3bC3bFH}\right]+\left[\mathrm{hC3bC3bFHL1}\right]+\left[\mathrm{hC3bC3bCR1}\right]+\left[\mathrm{hC3biC3bFH}\right] \\ +\left[\mathrm{hC3biC3bFHL1}\right]+\left[\mathrm{hC3biC3bCR1}\right]+\left[\mathrm{hiC3biC3bCR1}\right]+\left[\mathrm{hiC3biC3dgCR1}\right]+\left[\mathrm{pC4bC4BP}\right] \\ +\left[\mathrm{pC4bC4bC4BP}\right]+\left[\mathrm{pC4bC4dC4BP}\right]+\left[\mathrm{pC4bC3bC4BP}\right]+\left[\mathrm{pC4biC3bC4BP}\right]+\left[\mathrm{pC4bC3bFH}\right] \\ +\left[\mathrm{pC4bC3bFHL1}\right]+\left[\mathrm{pC4dC3bFH}\right]+\left[\mathrm{pC4dC3bFHL1}\right]+\left[\mathrm{pC3bFH}\right]+\left[\mathrm{pC3bFHL1}\right]+\left[\mathrm{pC3bC3bFH}\right] \\ +\left[\mathrm{pC3bC3bFHL1}\right]+\left[\mathrm{pC3biC3bFH}\right]+\left[\mathrm{pC3biC3bFHL1}\right]+\left[\mathrm{pC4bC4BPPTX3}\right]+\left[\mathrm{pC4bC4bC4BPPTX3}\right] \\ +\left[\mathrm{pC4bC4dC4BPPTX3}\right]+\left[\mathrm{pC4bC3bFHPTX3}\right]+\left[\mathrm{pC4bC3bFHL1PTX3}\right]+\left[\mathrm{pC4bC3bC4BPPTX3}\right] \\ +\left[\mathrm{pC4dC3bFHPTX3}\right]+\left[\mathrm{pC4dC3bFHL1PTX3}\right]+\left[\mathrm{pC4biC3bC4BPPTX3}\right]+\left[\mathrm{pC3bFHPTX3}\right] \\ +\left[\mathrm{pC3bFHL1PTX3}\right]+\left[\mathrm{pC3bC3bFHPTX3}\right]+\left[\mathrm{pC3bC3bFHL1PTX3}\right]+\left[\mathrm{pC3biC3bFHPTX3}\right] \\ +\left[\mathrm{pC3biC3bFHL1PTX3}\right]+\left[\mathrm{pC4bpC4BP}\right]+\left[\mathrm{pC4bC4bpC4BP}\right]+\left[\mathrm{pC4bC4dpC4BP}\right]+\left[\mathrm{pC4bC3bpC4BP}\right] \\ +\left[\mathrm{pC4biC3bpC4BP}\right]+\left[\mathrm{pC4bC3bpFH}\right]+\left[\mathrm{pC4bC3bpFHL1}\right]+\left[\mathrm{pC4dC3bpFH}\right]+\left[\mathrm{pC4dC3bpFHL1}\right] \\ +\left[\mathrm{pC3bpFH}\right]+\left[\mathrm{pC3bpFHL1}\right]+\left[\mathrm{pC3bC3bpFH}\right]+\left[\mathrm{pC3bC3bpFHL1}\right]+\left[\mathrm{pC3biC3bpFH}\right]+\left[\mathrm{pC3biC3bpFHL1}\right] \end{array}\right)}$$

$$+\frac{k_{\mathrm{cat\ C3bH}}^{\mathrm{FI}}[\mathrm{FI}][\mathrm{pC3biC3bFHL1}]}{\left(\begin{array}{l}K_{\mathrm{m\ C3bH}}^{\mathrm{FI}}+[\mathrm{C3(H_2O)FH}]+[\mathrm{C3(H_2O)FHL1}]+[\mathrm{fC3bFH}]+[\mathrm{fC3bFHL1}]+[\mathrm{fC3bCR1}]+[\mathrm{fiC3bCR1}]\\
+[\mathrm{IgGC3bC3bFH}]+[\mathrm{IgGC3biC3bFH}]+[\mathrm{IgGC3bC3bFHL1}]+[\mathrm{IgGC3biC3bFHL1}]+[\mathrm{IgGC3bC3bCR1}]\\
+[\mathrm{IgGC3biC3bCR1}]+[\mathrm{IgGiC3biC3bCR1}]+[\mathrm{IgGiC3bC3dgCR1}]+[\mathrm{fC4bC3bFH}]+[\mathrm{fC4bC3bFHL1}]\\
+[\mathrm{fC4bC3bCR1}]+[\mathrm{fC4bC3bC4BP}]+[\mathrm{fC4dC3bFH}]+[\mathrm{fC4dC3bFHL1}]+[\mathrm{fC4dC3bCR1}]+[\mathrm{fC4diC3bCR1}]\\
+[\mathrm{fC4biC3bCR1}]+[\mathrm{fC4bC3dgCR1}]+[\mathrm{fC4bC3dgC4BP}]+[\mathrm{fC3bC3bFH}]+[\mathrm{fC3bC3bFHL1}]+[\mathrm{fC3bC3bCR1}]\\
+[\mathrm{fC3biC3bFH}]+[\mathrm{fC3biC3bFHL1}]+[\mathrm{fC3biC3bCR1}]+[\mathrm{fiC3biC3bCR1}]+[\mathrm{fiC3bC3dgCR1}]\\
+[\mathrm{fC4biC3bC4BP}]+[\mathrm{nfC4bC4BP}]+[\mathrm{fC4bC4BP}]+[\mathrm{fC4bCR1}]+[\mathrm{fC4bC4bC4BP}]+[\mathrm{fC4bC4dC4BP}]\\
+[\mathrm{fC4bC4bCR1}]+[\mathrm{fC4bC4dCR1}]+[\mathrm{hC4bCR1}]+[\mathrm{hC4bC4BP}]+[\mathrm{hC4bC4bCR1}]+[\mathrm{hC4bC4bC4BP}]\\
+[\mathrm{hC4bC4dCR1}]+[\mathrm{hC4bC4dC4BP}]+[\mathrm{hC4bC3bFH}]+[\mathrm{hC4bC3bFHL1}]+[\mathrm{hC4bC3bCR1}]\\
+[\mathrm{hC4bC3bC4BP}]+[\mathrm{hC4biC3bCR1}]+[\mathrm{hC4biC3bC4BP}]+[\mathrm{hC4bC3dgCR1}]+[\mathrm{hC4bC3dgC4BP}]\\
+[\mathrm{hC4dC3bFH}]+[\mathrm{hC4dC3bFHL1}]+[\mathrm{hC4dC3bCR1}]+[\mathrm{hC4diC3bCR1}]+[\mathrm{hC3bFH}]+[\mathrm{hC3bFHL1}]\\
+[\mathrm{hC3bCR1}]+[\mathrm{hiC3bCR1}]+[\mathrm{hC3bC3bFH}]+[\mathrm{hC3bC3bFHL1}]+[\mathrm{hC3bC3bCR1}]+[\mathrm{hC3biC3bFH}]\\
+[\mathrm{hC3biC3bFHL1}]+[\mathrm{hC3biC3bCR1}]+[\mathrm{hiC3biC3bCR1}]+[\mathrm{hiC3biC3dgCR1}]+[\mathrm{pC4bC4BP}]\\
+[\mathrm{pC4bC4bC4BP}]+[\mathrm{pC4bC4dC4BP}]+[\mathrm{pC4bC3bC4BP}]+[\mathrm{pC4biC3bC4BP}]+[\mathrm{pC4bC3bFH}]\\
+[\mathrm{pC4bC3bFHL1}]+[\mathrm{pC4dC3bFH}]+[\mathrm{pC4dC3bFHL1}]+[\mathrm{pC3bFH}]+[\mathrm{pC3bFHL1}]+[\mathrm{pC3bC3bFH}]\\
+[\mathrm{pC3bC3bFHL1}]+[\mathrm{pC3biC3bFH}]+[\mathrm{pC3biC3bFHL1}]+[\mathrm{pC4bC4BPPTX3}]+[\mathrm{pC4bC4bC4BPPTX3}]\\
+[\mathrm{pC4bC4dC4BPPTX3}]+[\mathrm{pC4bC3bFHPTX3}]+[\mathrm{pC4bC3bFHL1PTX3}]+[\mathrm{pC4bC3bC4BPPTX3}]\\
+[\mathrm{pC4dC3bFHPTX3}]+[\mathrm{pC4dC3bFHL1PTX3}]+[\mathrm{pC4biC3bC4BPPTX3}]+[\mathrm{pC3bFHPTX3}]\\
+[\mathrm{pC3bFHL1PTX3}]+[\mathrm{pC3bC3bFHPTX3}]+[\mathrm{pC3bC3bFHL1PTX3}]+[\mathrm{pC3biC3bFHPTX3}]\\
+[\mathrm{pC3biC3bFHL1PTX3}]+[\mathrm{pC4bpC4BP}]+[\mathrm{pC4bC4bpC4BP}]+[\mathrm{pC4bC4dpC4BP}]+[\mathrm{pC4bC3bpC4BP}]\\
+[\mathrm{pC4biC3bpC4BP}]+[\mathrm{pC4bC3bpFH}]+[\mathrm{pC4bC3bpFHL1}]+[\mathrm{pC4dC3bpFH}]+[\mathrm{pC4dC3bpFHL1}]\\
+[\mathrm{pC3bpFH}]+[\mathrm{pC3bpFHL1}]+[\mathrm{pC3bC3bpFH}]+[\mathrm{pC3bC3bpFHL1}]+[\mathrm{pC3biC3bpFH}]+[\mathrm{pC3biC3bpFHL1}]\end{array}\right)}$$

$$+\frac{k_{\text{cat C3bH}}^{\text{FI}}[\text{FI}][\text{pC3biC3bFHPTX3}]}{\left(\begin{array}{l}K_{\text{m C3bH}}^{\text{FI}}+[\text{C3(H}_2\text{O)FH}]+[\text{C3(H}_2\text{O)FHL1}]+[\text{fC3bFH}]+[\text{fC3bFHL1}]+[\text{fC3bCR1}]+[\text{fiC3bCR1}]\\+[\text{IgGC3bC3bFH}]+[\text{IgGC3biC3bFH}]+[\text{IgGC3bC3bFHL1}]+[\text{IgGC3biC3bFHL1}]+[\text{IgGC3bC3bCR1}]\\+[\text{IgGC3biC3bCR1}]+[\text{IgGiC3biC3bCR1}]+[\text{IgGiC3bC3dgCR1}]+[\text{fC4bC3bFH}]+[\text{fC4bC3bFHL1}]\\+[\text{fC4bC3bCR1}]+[\text{fC4bC3bC4BP}]+[\text{fC4dC3bFH}]+[\text{fC4dC3bFHL1}]+[\text{fC4dC3bCR1}]+[\text{fC4diC3bCR1}]\\+[\text{fC4biC3bCR1}]+[\text{fC4bC3dgCR1}]+[\text{fC4bC3dgC4BP}]+[\text{fC3bC3bFH}]+[\text{fC3bC3bFHL1}]+[\text{fC3bC3bCR1}]\\+[\text{fC3biC3bFH}]+[\text{fC3biC3bFHL1}]+[\text{fC3biC3bCR1}]+[\text{fiC3biC3bCR1}]+[\text{fiC3bC3dgCR1}]\\+[\text{fC4biC3bC4BP}]+[\text{nfC4bC4BP}]+[\text{fC4bC4BP}]+[\text{fC4bCR1}]+[\text{fC4bC4bC4BP}]+[\text{fC4bC4dC4BP}]\\+[\text{fC4bC4bCR1}]+[\text{fC4bC4dCR1}]+[\text{hC4bCR1}]+[\text{hC4bC4BP}]+[\text{hC4bC4bCR1}]+[\text{hC4bC4bC4BP}]\\+[\text{hC4bC4dCR1}]+[\text{hC4bC4dC4BP}]+[\text{hC4bC3bFH}]+[\text{hC4bC3bFHL1}]+[\text{hC4bC3bCR1}]\\+[\text{hC4bC3bC4BP}]+[\text{hC4biC3bCR1}]+[\text{hC4biC3bC4BP}]+[\text{hC4bC3dgCR1}]+[\text{hC4bC3dgC4BP}]\\+[\text{hC4dC3bFH}]+[\text{hC4dC3bFHL1}]+[\text{hC4dC3bCR1}]+[\text{hC4diC3bCR1}]+[\text{hC3bFH}]+[\text{hC3bFHL1}]\\+[\text{hC3bCR1}]+[\text{hiC3bCR1}]+[\text{hC3bC3bFH}]+[\text{hC3bC3bFHL1}]+[\text{hC3bC3bCR1}]+[\text{hC3biC3bFH}]\\+[\text{hC3biC3bFHL1}]+[\text{hC3biC3bCR1}]+[\text{hiC3biC3bCR1}]+[\text{hiC3biC3dgCR1}]+[\text{pC4bC4BP}]\\+[\text{pC4bC4bC4BP}]+[\text{pC4bC4dC4BP}]+[\text{pC4bC3bC4BP}]+[\text{pC4biC3bC4BP}]+[\text{pC4bC3bFH}]\\+[\text{pC4bC3bFHL1}]+[\text{pC4dC3bFH}]+[\text{pC4dC3bFHL1}]+[\text{pC3bFH}]+[\text{pC3bFHL1}]+[\text{pC3bC3bFH}]\\+[\text{pC3bC3bFHL1}]+[\text{pC3biC3bFH}]+[\text{pC3biC3bFHL1}]+[\text{pC4bC4BPPTX3}]+[\text{pC4bC4bC4BPPTX3}]\\+[\text{pC4bC4dC4BPPTX3}]+[\text{pC4bC3bFHPTX3}]+[\text{pC4bC3bFHL1PTX3}]+[\text{pC4bC3bC4BPPTX3}]\\+[\text{pC4dC3bFHPTX3}]+[\text{pC4dC3bFHL1PTX3}]+[\text{pC4biC3bC4BPPTX3}]+[\text{pC3bFHPTX3}]\\+[\text{pC3bFHL1PTX3}]+[\text{pC3bC3bFHPTX3}]+[\text{pC3bC3bFHL1PTX3}]+[\text{pC3biC3bFHPTX3}]\\+[\text{pC3biC3bFHL1PTX3}]+[\text{pC4bpC4BP}]+[\text{pC4bC4bpC4BP}]+[\text{pC4bC4dpC4BP}]+[\text{pC4bC3bpC4BP}]\\+[\text{pC4biC3bpC4BP}]+[\text{pC4bC3bpFH}]+[\text{pC4bC3bpFHL1}]+[\text{pC4dC3bpFH}]+[\text{pC4dC3bpFHL1}]\\+[\text{pC3bpFH}]+[\text{pC3bpFHL1}]+[\text{pC3bC3bpFH}]+[\text{pC3bC3bpFHL1}]+[\text{pC3biC3bpFH}]+[\text{pC3biC3bpFHL1}]\end{array}\right)}$$

$$+\frac{k_{\text{cat C3bH}}^{\text{FI}}[\text{FI}][\text{pC3biC3bFHL1PTX3}]}{\begin{pmatrix} K_{\text{m C3bH}}^{\text{FI}}+[\text{C3(H}_2\text{O)FH}]+[\text{C3(H}_2\text{O)FHL1}]+[\text{fC3bFH}]+[\text{fC3bFHL1}]+[\text{fC3bCR1}]+[\text{fiC3bCR1}] \\ +[\text{IgGC3bC3bFH}]+[\text{IgGC3biC3bFH}]+[\text{IgGC3bC3bFHL1}]+[\text{IgGC3biC3bFHL1}]+[\text{IgGC3bC3bCR1}] \\ +[\text{IgGC3biC3bCR1}]+[\text{IgGiC3biC3bCR1}]+[\text{IgGiC3bC3dgCR1}]+[\text{fC4bC3bFH}]+[\text{fC4bC3bFHL1}] \\ +[\text{fC4bC3bCR1}]+[\text{fC4bC3bC4BP}]+[\text{fC4dC3bFH}]+[\text{fC4dC3bFHL1}]+[\text{fC4dC3bCR1}]+[\text{fC4diC3bCR1}] \\ +[\text{fC4biC3bCR1}]+[\text{fC4bC3dgCR1}]+[\text{fC4bC3dgC4BP}]+[\text{fC3bC3bFH}]+[\text{fC3bC3bFHL1}]+[\text{fC3bC3bCR1}] \\ +[\text{fC3biC3bFH}]+[\text{fC3biC3bFHL1}]+[\text{fC3biC3bCR1}]+[\text{fiC3biC3bCR1}]+[\text{fiC3bC3dgCR1}] \\ +[\text{fC4biC3bC4BP}]+[\text{nfC4bC4BP}]+[\text{fC4bC4BP}]+[\text{fC4bCR1}]+[\text{fC4bC4bC4BP}]+[\text{fC4bC4dC4BP}] \\ +[\text{fC4bC4bCR1}]+[\text{fC4bC4dCR1}]+[\text{hC4bCR1}]+[\text{hC4bC4BP}]+[\text{hC4bC4bCR1}]+[\text{hC4bC4bC4BP}] \\ +[\text{hC4bC4dCR1}]+[\text{hC4bC4dC4BP}]+[\text{hC4bC3bFH}]+[\text{hC4bC3bFHL1}]+[\text{hC4bC3bCR1}] \\ +[\text{hC4bC3bC4BP}]+[\text{hC4biC3bCR1}]+[\text{hC4biC3bC4BP}]+[\text{hC4bC3dgCR1}]+[\text{hC4bC3dgC4BP}] \\ +[\text{hC4dC3bFH}]+[\text{hC4dC3bFHL1}]+[\text{hC4dC3bCR1}]+[\text{hC4diC3bCR1}]+[\text{hC3bFH}]+[\text{hC3bFHL1}] \\ +[\text{hC3bCR1}]+[\text{hiC3bCR1}]+[\text{hC3bC3bFH}]+[\text{hC3bC3bFHL1}]+[\text{hC3bC3bCR1}]+[\text{hC3biC3bFH}] \\ +[\text{hC3biC3bFHL1}]+[\text{hC3biC3bCR1}]+[\text{hiC3biC3bCR1}]+[\text{hiC3biC3dgCR1}]+[\text{pC4bC4BP}] \\ +[\text{pC4bC4bC4BP}]+[\text{pC4bC4dC4BP}]+[\text{pC4bC3bC4BP}]+[\text{pC4biC3bC4BP}]+[\text{pC4bC3bFH}] \\ +[\text{pC4bC3bFHL1}]+[\text{pC4dC3bFH}]+[\text{pC4dC3bFHL1}]+[\text{pC3bFH}]+[\text{pC3bFHL1}]+[\text{pC3bC3bFH}] \\ +[\text{pC3bC3bFHL1}]+[\text{pC3biC3bFH}]+[\text{pC3biC3bFHL1}]+[\text{pC4bC4BPPTX3}]+[\text{pC4bC4bC4BPPTX3}] \\ +[\text{pC4bC4dC4BPPTX3}]+[\text{pC4bC3bFHPTX3}]+[\text{pC4bC3bFHL1PTX3}]+[\text{pC4bC3bC4BPPTX3}] \\ +[\text{pC4dC3bFHPTX3}]+[\text{pC4dC3bFHL1PTX3}]+[\text{pC4biC3bC4BPPTX3}]+[\text{pC3bFHPTX3}] \\ +[\text{pC3bFHL1PTX3}]+[\text{pC3bC3bFHPTX3}]+[\text{pC3bC3bFHL1PTX3}]+[\text{pC3biC3bFHPTX3}] \\ +[\text{pC3biC3bFHL1PTX3}]+[\text{pC4bpC4BP}]+[\text{pC4bC4bpC4BP}]+[\text{pC4bC4dpC4BP}]+[\text{pC4bC3bpC4BP}] \\ +[\text{pC4biC3bpC4BP}]+[\text{pC4bC3bpFH}]+[\text{pC4bC3bpFHL1}]+[\text{pC4dC3bpFH}]+[\text{pC4dC3bpFHL1}] \\ +[\text{pC3bpFH}]+[\text{pC3bpFHL1}]+[\text{pC3bC3bpFH}]+[\text{pC3bC3bpFHL1}]+[\text{pC3biC3bpFH}]+[\text{pC3biC3bpFHL1}] \end{pmatrix}}$$

$$
+\frac{k_{\mathrm{cat\ C3bH}}^{\mathrm{FI}}[\mathrm{FI}][\mathrm{pC3biC3bpFH}]}{\left(\begin{array}{l}
K_{\mathrm{m\ C3bH}}^{\mathrm{FI}}+[\mathrm{C3(H_2O)FH}]+[\mathrm{C3(H_2O)FHL1}]+[\mathrm{fC3bFH}]+[\mathrm{fC3bFHL1}]+[\mathrm{fC3bCR1}]+[\mathrm{fiC3bCR1}]\\
+[\mathrm{IgGC3bC3bFH}]+[\mathrm{IgGC3biC3bFH}]+[\mathrm{IgGC3bC3bFHL1}]+[\mathrm{IgGC3biC3bFHL1}]+[\mathrm{IgGC3bC3bCR1}]\\
+[\mathrm{IgGC3biC3bCR1}]+[\mathrm{IgGiC3biC3bCR1}]+[\mathrm{IgGiC3bC3dgCR1}]+[\mathrm{fC4bC3bFH}]+[\mathrm{fC4bC3bFHL1}]\\
+[\mathrm{fC4bC3bCR1}]+[\mathrm{fC4bC3bC4BP}]+[\mathrm{fC4dC3bFH}]+[\mathrm{fC4dC3bFHL1}]+[\mathrm{fC4dC3bCR1}]+[\mathrm{fC4diC3bCR1}]\\
+[\mathrm{fC4biC3bCR1}]+[\mathrm{fC4bC3dgCR1}]+[\mathrm{fC4bC3dgC4BP}]+[\mathrm{fC3bC3bFH}]+[\mathrm{fC3bC3bFHL1}]+[\mathrm{fC3bC3bCR1}]\\
+[\mathrm{fC3biC3bFH}]+[\mathrm{fC3biC3bFHL1}]+[\mathrm{fC3biC3bCR1}]+[\mathrm{fiC3biC3bCR1}]+[\mathrm{fiC3bC3dgCR1}]\\
+[\mathrm{fC4biC3bC4BP}]+[\mathrm{nfC4bC4BP}]+[\mathrm{fC4bC4BP}]+[\mathrm{fC4bCR1}]+[\mathrm{fC4bC4bC4BP}]+[\mathrm{fC4bC4dC4BP}]\\
+[\mathrm{fC4bC4bCR1}]+[\mathrm{fC4bC4dCR1}]+[\mathrm{hC4bCR1}]+[\mathrm{hC4bC4BP}]+[\mathrm{hC4bC4bCR1}]+[\mathrm{hC4bC4bC4BP}]\\
+[\mathrm{hC4bC4dCR1}]+[\mathrm{hC4bC4dC4BP}]+[\mathrm{hC4bC3bFH}]+[\mathrm{hC4bC3bFHL1}]+[\mathrm{hC4bC3bCR1}]\\
+[\mathrm{hC4bC3bC4BP}]+[\mathrm{hC4biC3bCR1}]+[\mathrm{hC4biC3bC4BP}]+[\mathrm{hC4bC3dgCR1}]+[\mathrm{hC4bC3dgC4BP}]\\
+[\mathrm{hC4dC3bFH}]+[\mathrm{hC4dC3bFHL1}]+[\mathrm{hC4dC3bCR1}]+[\mathrm{hC4diC3bCR1}]+[\mathrm{hC3bFH}]+[\mathrm{hC3bFHL1}]\\
+[\mathrm{hC3bCR1}]+[\mathrm{hiC3bCR1}]+[\mathrm{hC3bC3bFH}]+[\mathrm{hC3bC3bFHL1}]+[\mathrm{hC3bC3bCR1}]+[\mathrm{hC3biC3bFH}]\\
+[\mathrm{hC3biC3bFHL1}]+[\mathrm{hC3biC3bCR1}]+[\mathrm{hiC3biC3bCR1}]+[\mathrm{hiC3biC3dgCR1}]+[\mathrm{pC4bC4BP}]\\
+[\mathrm{pC4bC4bC4BP}]+[\mathrm{pC4bC4dC4BP}]+[\mathrm{pC4bC3bC4BP}]+[\mathrm{pC4biC3bC4BP}]+[\mathrm{pC4bC3bFH}]\\
+[\mathrm{pC4bC3bFHL1}]+[\mathrm{pC4dC3bFH}]+[\mathrm{pC4dC3bFHL1}]+[\mathrm{pC3bFH}]+[\mathrm{pC3bFHL1}]+[\mathrm{pC3bC3bFH}]\\
+[\mathrm{pC3bC3bFHL1}]+[\mathrm{pC3biC3bFH}]+[\mathrm{pC3biC3bFHL1}]+[\mathrm{pC4bC4BPPTX3}]+[\mathrm{pC4bC4bC4BPPTX3}]\\
+[\mathrm{pC4bC4dC4BPPTX3}]+[\mathrm{pC4bC3bFHPTX3}]+[\mathrm{pC4bC3bFHL1PTX3}]+[\mathrm{pC4bC3bC4BPPTX3}]\\
+[\mathrm{pC4dC3bFHPTX3}]+[\mathrm{pC4dC3bFHL1PTX3}]+[\mathrm{pC4biC3bC4BPPTX3}]+[\mathrm{pC3bFHPTX3}]\\
+[\mathrm{pC3bFHL1PTX3}]+[\mathrm{pC3bC3bFHPTX3}]+[\mathrm{pC3bC3bFHL1PTX3}]+[\mathrm{pC3biC3bFHPTX3}]\\
+[\mathrm{pC3biC3bFHL1PTX3}]+[\mathrm{pC4bpC4BP}]+[\mathrm{pC4bC4bpC4BP}]+[\mathrm{pC4bC4dpC4BP}]+[\mathrm{pC4bC3bpC4BP}]\\
+[\mathrm{pC4biC3bpC4BP}]+[\mathrm{pC4bC3bpFH}]+[\mathrm{pC4bC3bpFHL1}]+[\mathrm{pC4dC3bpFH}]+[\mathrm{pC4dC3bpFHL1}]\\
+[\mathrm{pC3bpFH}]+[\mathrm{pC3bpFHL1}]+[\mathrm{pC3bC3bpFH}]+[\mathrm{pC3bC3bpFHL1}]+[\mathrm{pC3biC3bpFH}]+[\mathrm{pC3biC3bpFHL1}]
\end{array}\right)}
$$

$$+\frac{k_{\text{cat C3bH}}^{\text{FI}}[\text{FI}][\text{pC3biC3bpFHL1}]}{\left(\begin{array}{l}K_{\text{m C3bH}}^{\text{FI}}+[\text{C3(H}_2\text{O)FH}]+[\text{C3(H}_2\text{O)FHL1}]+[\text{fC3bFH}]+[\text{fC3bFHL1}]+[\text{fC3bCR1}]+[\text{fiC3bCR1}]\\+[\text{IgGC3bC3bFH}]+[\text{IgGC3biC3bFH}]+[\text{IgGC3bC3bFHL1}]+[\text{IgGC3biC3bFHL1}]+[\text{IgGC3bC3bCR1}]\\+[\text{IgGC3biC3bCR1}]+[\text{IgGiC3biC3bCR1}]+[\text{IgGiC3bC3dgCR1}]+[\text{fC4bC3bFH}]+[\text{fC4bC3bFHL1}]\\+[\text{fC4bC3bCR1}]+[\text{fC4bC3bC4BP}]+[\text{fC4dC3bFH}]+[\text{fC4dC3bFHL1}]+[\text{fC4dC3bCR1}]+[\text{fC4diC3bCR1}]\\+[\text{fC4biC3bCR1}]+[\text{fC4bC3dgCR1}]+[\text{fC4bC3dgC4BP}]+[\text{fC3bC3bFH}]+[\text{fC3bC3bFHL1}]+[\text{fC3bC3bCR1}]\\+[\text{fC3biC3bFH}]+[\text{fC3biC3bFHL1}]+[\text{fC3biC3bCR1}]+[\text{fiC3biC3bCR1}]+[\text{fiC3bC3dgCR1}]\\+[\text{fC4biC3bC4BP}]+[\text{nfC4bC4BP}]+[\text{fC4bC4BP}]+[\text{fC4bCR1}]+[\text{fC4bC4bC4BP}]+[\text{fC4bC4dC4BP}]\\+[\text{fC4bC4bCR1}]+[\text{fC4bC4dCR1}]+[\text{hC4bCR1}]+[\text{hC4bC4BP}]+[\text{hC4bC4bCR1}]+[\text{hC4bC4bC4BP}]\\+[\text{hC4bC4dCR1}]+[\text{hC4bC4dC4BP}]+[\text{hC4bC3bFH}]+[\text{hC4bC3bFHL1}]+[\text{hC4bC3bCR1}]\\+[\text{hC4bC3bC4BP}]+[\text{hC4biC3bCR1}]+[\text{hC4biC3bC4BP}]+[\text{hC4bC3dgCR1}]+[\text{hC4bC3dgC4BP}]\\+[\text{hC4dC3bFH}]+[\text{hC4dC3bFHL1}]+[\text{hC4dC3bCR1}]+[\text{hC4diC3bCR1}]+[\text{hC3bFH}]+[\text{hC3bFHL1}]\\+[\text{hC3bCR1}]+[\text{hiC3bCR1}]+[\text{hC3bC3bFH}]+[\text{hC3bC3bFHL1}]+[\text{hC3bC3bCR1}]+[\text{hC3biC3bFH}]\\+[\text{hC3biC3bFHL1}]+[\text{hC3biC3bCR1}]+[\text{hiC3biC3bCR1}]+[\text{hiC3biC3dgCR1}]+[\text{pC4bC4BP}]\\+[\text{pC4bC4bC4BP}]+[\text{pC4bC4dC4BP}]+[\text{pC4bC3bC4BP}]+[\text{pC4biC3bC4BP}]+[\text{pC4bC3bFH}]\\+[\text{pC4bC3bFHL1}]+[\text{pC4dC3bFH}]+[\text{pC4dC3bFHL1}]+[\text{pC3bFH}]+[\text{pC3bFHL1}]+[\text{pC3bC3bFH}]\\+[\text{pC3bC3bFHL1}]+[\text{pC3biC3bFH}]+[\text{pC3biC3bFHL1}]+[\text{pC4bC4BPPTX3}]+[\text{pC4bC4bC4BPPTX3}]\\+[\text{pC4bC4dC4BPPTX3}]+[\text{pC4bC3bFHPTX3}]+[\text{pC4bC3bFHL1PTX3}]+[\text{pC4bC3bC4BPPTX3}]\\+[\text{pC4dC3bFHPTX3}]+[\text{pC4dC3bFHL1PTX3}]+[\text{pC4biC3bC4BPPTX3}]+[\text{pC3bFHPTX3}]\\+[\text{pC3bFHL1PTX3}]+[\text{pC3bC3bFHPTX3}]+[\text{pC3bC3bFHL1PTX3}]+[\text{pC3biC3bFHPTX3}]\\+[\text{pC3biC3bFHL1PTX3}]+[\text{pC4bpC4BP}]+[\text{pC4bC4bpC4BP}]+[\text{pC4bC4dpC4BP}]+[\text{pC4bC3bpC4BP}]\\+[\text{pC4biC3bpC4BP}]+[\text{pC4bC3bpFH}]+[\text{pC4bC3bpFHL1}]+[\text{pC4dC3bpFH}]+[\text{pC4dC3bpFHL1}]\\+[\text{pC3bpFH}]+[\text{pC3bpFHL1}]+[\text{pC3bC3bpFH}]+[\text{pC3bC3bpFHL1}]+[\text{pC3biC3bpFH}]+[\text{pC3biC3bpFHL1}]\end{array}\right)}$$

236

$$\begin{array}{l}\frac{d[\text{MBL}_3]}{dt}=-k_{\text{MBL}_3\text{MASP1}}^{+}[\text{MBL}_3][\text{MASP1}]+k_{\text{MBL}_3\text{MASP1}}^{-}[\text{fMBL}_3\text{MASP1}_{\text{zym}}]-k_{\text{MBL}_3\text{MASP2}}^{+}[\text{MBL}_3][\text{MASP2}]\\+k_{\text{MBL}_3\text{MASP2}}^{-}[\text{fMBL}_3\text{MASP2}_{\text{zym}}]-k_{\text{MBL}_3\text{MASP3}}^{+}[\text{MBL}_3][\text{MASP3}]+k_{\text{MBL}_3\text{MASP3}}^{-}[\text{fMBL}_3\text{MASP3}_{\text{zym}}]\\-k_{\text{MBL}_3\text{MAp19}}^{+}[\text{MBL}_3][\text{MAp19}]+k_{\text{MBL}_3\text{MAp19}}^{-}[\text{fMBL}_3\text{MAp19}]-k_{\text{MBL}_3\text{MAp44}}^{+}[\text{MBL}_3][\text{MAp44}]\\+k_{\text{MBL}_3\text{MAp44}}^{-}[\text{fMBL}_3\text{MAp44}]-k_{\text{pMBL}_3}^{+}[\text{MBL}_3][\text{Pathogen Surface}]+k_{\text{pMBL}_3}^{-}[\text{pMBL}_3]-k_{\text{MBL}_3\text{MASP1}}^{+}[\text{MBL}_3][\text{MASP1}^{*}]\\+k_{\text{MBL}_3\text{MASP1}}^{-}[\text{fMBL}_3\text{MASP1}^{*}]-k_{\text{MBL}_3\text{MASP2}}^{+}[\text{MBL}_3][\text{MASP2}^{*}]+k_{\text{MBL}_3\text{MASP2}}^{-}[\text{fMBL}_3\text{MASP2}^{*}]\\-k_{\text{MBL}_3\text{MASP3}}^{+}[\text{MBL}_3][\text{MASP3}^{*}]+k_{\text{MBL}_3\text{MASP3}}^{-}[\text{fMBL}_3\text{MASP3}^{*}]+k_{\text{C1INHMASP1}}^{+}[\text{C1-INH}][\text{fMBL}_3\text{MASP1}^{*}]\\+k_{\text{C1INHMASP2}}^{+}[\text{C1-INH}][\text{fMBL}_3\text{MASP2}^{*}]+k_{\text{pMBL}_3}^{-}[\text{pMBL}_3\text{SAP}]+k_{\text{pMBL}_3}^{-}[\text{pMBL}_3\text{PTX3}]+k_{\text{pMBL}_3}^{-}[\text{pMBL}_3\text{PTX3C1q}]\\+k_{\text{pMBL}_3}^{-}[\text{pMBL}_3\text{PTX3C1}]+k_{\text{pMBL}_3}^{-}[\text{pMBL}_3\text{PTX3C1}^{*}]\end{array}$$

237

$$\frac{d[\mathrm{MBL_4}]}{dt}=-k^{+}_{\mathrm{MBL_4MASP1}}[\mathrm{MBL_4}][\mathrm{MASP1}]+k^{-}_{\mathrm{MBL_4MASP1}}\left[\mathrm{fMBL_4MASP1_{zym}}\right]-k^{+}_{\mathrm{MBL_4MASP2}}[\mathrm{MBL_4}][\mathrm{MASP2}]$$
$$+k^{-}_{\mathrm{MBL_4MASP2}}\left[\mathrm{fMBL_4MASP2_{zym}}\right]-k^{+}_{\mathrm{MBL_4MASP3}}[\mathrm{MBL_4}][\mathrm{MASP3}]+k^{-}_{\mathrm{MBL_4MASP3}}\left[\mathrm{fMBL_4MASP3_{zym}}\right]$$
$$-k^{+}_{\mathrm{MBL_4MAp19}}[\mathrm{MBL_4}][\mathrm{MAp19}]+k^{-}_{\mathrm{MBL_4MAp19}}[\mathrm{fMBL_4MAp19}]-k^{+}_{\mathrm{MBL_4MAp44}}[\mathrm{MBL_4}][\mathrm{MAp44}]$$
$$+k^{-}_{\mathrm{MBL_4MAp44}}[\mathrm{fMBL_4MAp44}]-k^{+}_{\mathrm{pMBL_4}}[\mathrm{MBL_4}][\text{Pathogen Surface}]+k^{-}_{\mathrm{pMBL_4}}[\mathrm{pMBL_4}]-k^{+}_{\mathrm{MBL_4MASP1}}[\mathrm{MBL_4}]\left[\mathrm{MASP1^*}\right]$$
$$+k^{-}_{\mathrm{MBL_4MASP1}}\left[\mathrm{fMBL_4MASP1^*}\right]-k^{+}_{\mathrm{MBL_4MASP2}}[\mathrm{MBL_4}]\left[\mathrm{MASP2^*}\right]+k^{-}_{\mathrm{MBL_4MASP2}}\left[\mathrm{fMBL_4MASP2^*}\right]$$
$$-k^{+}_{\mathrm{MBL_4MASP3}}[\mathrm{MBL_4}]\left[\mathrm{MASP3^*}\right]+k^{-}_{\mathrm{MBL_4MASP3}}\left[\mathrm{fMBL_4MASP3^*}\right]+k^{+}_{\mathrm{C1INHMASP1}}[\mathrm{C1\text{-}INH}]\left[\mathrm{fMBL_4MASP1^*}\right]$$
$$+k^{+}_{\mathrm{C1INHMASP2}}[\mathrm{C1\text{-}INH}]\left[\mathrm{fMBL_4MASP2^*}\right]+k^{-}_{\mathrm{pMBL_4}}[\mathrm{pMBL_4SAP}]+k^{-}_{\mathrm{pMBL_4}}[\mathrm{pMBL_4PTX3}]+k^{-}_{\mathrm{pMBL_4}}[\mathrm{pMBL_4PTX3C1q}]$$
$$+k^{-}_{\mathrm{pMBL_4}}[\mathrm{pMBL_4PTX3C1}]+k^{-}_{\mathrm{pMBL_4}}\left[\mathrm{pMBL_4PTX3C1^*}\right]$$

238

$$\frac{d[\mathrm{MASP1}]}{dt}=-k^{+}_{\mathrm{MBL_3MASP1}}[\mathrm{MBL_3}][\mathrm{MASP1}]+k^{-}_{\mathrm{MBL_3MASP1}}\left[\mathrm{fMBL_3MASP1_{zym}}\right]-k^{+}_{\mathrm{MBL_3MASP1}}[\mathrm{pMBL_3}][\mathrm{MASP1}]$$
$$+k^{-}_{\mathrm{MBL_3MASP1}}\left[\mathrm{pMBL_3MASP1_{zym}}\right]-k^{+}_{\mathrm{MBL_4MASP1}}[\mathrm{MBL_4}][\mathrm{MASP1}]+k^{-}_{\mathrm{MBL_4MASP1}}\left[\mathrm{fMBL_4MASP1_{zym}}\right]$$
$$-k^{+}_{\mathrm{MBL_4MASP1}}[\mathrm{pMBL_4}][\mathrm{MASP1}]+k^{-}_{\mathrm{MBL_4MASP1}}\left[\mathrm{pMBL_4MASP1_{zym}}\right]-k^{+}_{\mathrm{CLLKMASP1}}[\mathrm{CLLK}][\mathrm{MASP1}]$$
$$+k^{-}_{\mathrm{CLLKMASP1}}\left[\mathrm{fCLLKMASP1_{zym}}\right]-k^{+}_{\mathrm{CLLKMASP1}}[\mathrm{pCLLK}][\mathrm{MASP1}]+k^{-}_{\mathrm{CLLKMASP1}}\left[\mathrm{pCLLKMASP1_{zym}}\right]$$
$$-k^{+}_{\mathrm{CLK1MASP1}}[\mathrm{CLK1}][\mathrm{MASP1}]+k^{-}_{\mathrm{CLK1MASP1}}\left[\mathrm{fCLK1MASP1_{zym}}\right]-k^{+}_{\mathrm{CLK1MASP1}}[\mathrm{pCLK1}][\mathrm{MASP1}]$$
$$+k^{-}_{\mathrm{CLK1MASP1}}\left[\mathrm{pCLK1MASP1_{zym}}\right]-k^{+}_{\mathrm{CLL1MASP1}}[\mathrm{CLL1}][\mathrm{MASP1}]+k^{-}_{\mathrm{CLL1MASP1}}\left[\mathrm{fCLL1MASP1_{zym}}\right]$$
$$-k^{+}_{\mathrm{CLL1MASP1}}[\mathrm{pCLL1}][\mathrm{MASP1}]+k^{-}_{\mathrm{CLL1MASP1}}\left[\mathrm{pCLL1MASP1_{zym}}\right]-k^{+}_{\mathrm{LFMASP1}}[\mathrm{LF}][\mathrm{MASP1}]+k^{-}_{\mathrm{LFMASP1}}\left[\mathrm{fLFMASP1_{zym}}\right]$$
$$-k^{+}_{\mathrm{LFMASP1}}[\mathrm{pLF}][\mathrm{MASP1}]+k^{-}_{\mathrm{LFMASP1}}\left[\mathrm{pLFMASP1_{zym}}\right]-k^{+}_{\mathrm{HFMASP1}}[\mathrm{HF}][\mathrm{MASP1}]+k^{-}_{\mathrm{HFMASP1}}\left[\mathrm{fHFMASP1_{zym}}\right]$$
$$-k^{+}_{\mathrm{HFMASP1}}[\mathrm{pHF}][\mathrm{MASP1}]+k^{-}_{\mathrm{HFMASP1}}\left[\mathrm{pHFMASP1_{zym}}\right]-k^{+}_{\mathrm{MFMASP1}}[\mathrm{MF}][\mathrm{MASP1}]+k^{-}_{\mathrm{MFMASP1}}\left[\mathrm{fMFMASP1_{zym}}\right]$$
$$-k^{+}_{\mathrm{MFMASP1}}[\mathrm{pMF}][\mathrm{MASP1}]+k^{-}_{\mathrm{MFMASP1}}\left[\mathrm{pMFMASP1_{zym}}\right]-k^{+}_{\mathrm{LFMASP1}}[\mathrm{pPTX3LF}][\mathrm{MASP1}]$$
$$+k^{-}_{\mathrm{LFMASP1}}\left[\mathrm{pPTX3LFMASP1_{zym}}\right]$$

239

$$\frac{d[\text{MASP2}]}{dt} = -k^{+}_{\text{MBL}_3\text{MASP2}}[\text{MBL}_3][\text{MASP2}] + k^{-}_{\text{MBL}_3\text{MASP2}}\left[\text{fMBL}_3\text{MASP2}_{\text{zym}}\right] - k^{+}_{\text{MBL}_3\text{MASP2}}[\text{pMBL}_3][\text{MASP2}]$$
$$+k^{-}_{\text{MBL}_3\text{MASP2}}\left[\text{pMBL}_3\text{MASP2}_{\text{zym}}\right] - k^{+}_{\text{MBL}_4\text{MASP2}}[\text{MBL}_4][\text{MASP2}] + k^{-}_{\text{MBL}_4\text{MASP2}}\left[\text{fMBL}_4\text{MASP2}_{\text{zym}}\right]$$
$$-k^{+}_{\text{MBL}_4\text{MASP2}}[\text{pMBL}_4][\text{MASP2}] + k^{-}_{\text{MBL}_4\text{MASP2}}\left[\text{pMBL}_4\text{MASP2}_{\text{zym}}\right] - k^{+}_{\text{CLLKMASP2}}[\text{CLLK}][\text{MASP2}]$$
$$+k^{-}_{\text{CLLKMASP2}}\left[\text{fCLLKMASP2}_{\text{zym}}\right] - k^{+}_{\text{CLLKMASP2}}[\text{pCLLK}][\text{MASP2}] + k^{-}_{\text{CLLKMASP2}}\left[\text{pCLLKMASP2}_{\text{zym}}\right]$$
$$-k^{+}_{\text{CLK1MASP2}}[\text{CLK1}][\text{MASP2}] + k^{-}_{\text{CLK1MASP2}}\left[\text{fCLK1MASP2}_{\text{zym}}\right] - k^{+}_{\text{CLK1MASP2}}[\text{pCLK1}][\text{MASP2}]$$
$$+k^{-}_{\text{CLK1MASP2}}\left[\text{pCLK1MASP2}_{\text{zym}}\right] - k^{+}_{\text{CLL1MASP2}}[\text{CLL1}][\text{MASP2}] + k^{-}_{\text{CLL1MASP2}}\left[\text{fCLL1MASP2}_{\text{zym}}\right]$$
$$-k^{+}_{\text{CLL1MASP2}}[\text{pCLL1}][\text{MASP2}] + k^{-}_{\text{CLL1MASP2}}\left[\text{pCLL1MASP2}_{\text{zym}}\right] - k^{+}_{\text{LFMASP2}}[\text{LF}][\text{MASP2}] + k^{-}_{\text{LFMASP2}}\left[\text{fLFMASP2}_{\text{zym}}\right]$$
$$-k^{+}_{\text{LFMASP2}}[\text{pLF}][\text{MASP2}] + k^{-}_{\text{LFMASP2}}\left[\text{pLFMASP2}_{\text{zym}}\right] - k^{+}_{\text{HFMASP2}}[\text{HF}][\text{MASP2}] + k^{-}_{\text{HFMASP2}}\left[\text{fHFMASP2}_{\text{zym}}\right]$$
$$-k^{+}_{\text{HFMASP2}}[\text{pHF}][\text{MASP2}] + k^{-}_{\text{HFMASP2}}\left[\text{pHFMASP2}_{\text{zym}}\right] - k^{+}_{\text{MFMASP2}}[\text{MF}][\text{MASP2}] + k^{-}_{\text{MFMASP2}}\left[\text{fMFMASP2}_{\text{zym}}\right]$$
$$-k^{+}_{\text{MFMASP2}}[\text{pMF}][\text{MASP2}] + k^{-}_{\text{MFMASP2}}\left[\text{pMFMASP2}_{\text{zym}}\right] - k^{+}_{\text{LFMASP2}}[\text{pPTX3LF}][\text{MASP2}]$$
$$+k^{-}_{\text{LFMASP2}}\left[\text{pPTX3LFMASP2}_{\text{zym}}\right]$$

240

$$\frac{d[\text{MASP3}]}{dt} = -k^{+}_{\text{MBL}_3\text{MASP3}}[\text{MBL}_3][\text{MASP3}] + k^{-}_{\text{MBL}_3\text{MASP3}}\left[\text{fMBL}_3\text{MASP3}_{\text{zym}}\right] - k^{+}_{\text{MBL}_3\text{MASP3}}[\text{pMBL}_3][\text{MASP3}]$$
$$+k^{-}_{\text{MBL}_3\text{MASP3}}\left[\text{pMBL}_3\text{MASP3}_{\text{zym}}\right] - k^{+}_{\text{MBL}_4\text{MASP3}}[\text{MBL}_4][\text{MASP3}] + k^{-}_{\text{MBL}_4\text{MASP3}}\left[\text{fMBL}_4\text{MASP3}_{\text{zym}}\right]$$
$$-k^{+}_{\text{MBL}_4\text{MASP3}}[\text{pMBL}_4][\text{MASP3}] + k^{-}_{\text{MBL}_4\text{MASP3}}\left[\text{pMBL}_4\text{MASP3}_{\text{zym}}\right] - k^{+}_{\text{CLLKMASP3}}[\text{CLLK}][\text{MASP3}]$$
$$+k^{-}_{\text{CLLKMASP3}}\left[\text{fCLLKMASP3}_{\text{zym}}\right] - k^{+}_{\text{CLLKMASP3}}[\text{pCLLK}][\text{MASP3}] + k^{-}_{\text{CLLKMASP3}}\left[\text{pCLLKMASP3}_{\text{zym}}\right]$$
$$-k^{+}_{\text{CLK1MASP3}}[\text{CLK1}][\text{MASP3}] + k^{-}_{\text{CLK1MASP3}}\left[\text{fCLK1MASP3}_{\text{zym}}\right] - k^{+}_{\text{CLK1MASP3}}[\text{pCLK1}][\text{MASP3}]$$
$$+k^{-}_{\text{CLK1MASP3}}\left[\text{pCLK1MASP3}_{\text{zym}}\right] - k^{+}_{\text{CLL1MASP3}}[\text{CLL1}][\text{MASP3}] + k^{-}_{\text{CLL1MASP3}}\left[\text{fCLL1MASP3}_{\text{zym}}\right]$$
$$-k^{+}_{\text{CLL1MASP3}}[\text{pCLL1}][\text{MASP3}] + k^{-}_{\text{CLL1MASP3}}\left[\text{pCLL1MASP3}_{\text{zym}}\right] - k^{+}_{\text{LFMASP3}}[\text{LF}][\text{MASP3}] + k^{-}_{\text{LFMASP3}}\left[\text{fLFMASP3}_{\text{zym}}\right]$$
$$-k^{+}_{\text{LFMASP3}}[\text{pLF}][\text{MASP3}] + k^{-}_{\text{LFMASP3}}\left[\text{pLFMASP3}_{\text{zym}}\right] - k^{+}_{\text{HFMASP3}}[\text{HF}][\text{MASP3}] + k^{-}_{\text{HFMASP3}}\left[\text{fHFMASP3}_{\text{zym}}\right]$$
$$-k^{+}_{\text{HFMASP3}}[\text{pHF}][\text{MASP3}] + k^{-}_{\text{HFMASP3}}\left[\text{pHFMASP3}_{\text{zym}}\right] - k^{+}_{\text{MFMASP3}}[\text{MF}][\text{MASP3}] + k^{-}_{\text{MFMASP3}}\left[\text{fMFMASP3}_{\text{zym}}\right]$$
$$-k^{+}_{\text{MFMASP3}}[\text{pMF}][\text{MASP3}] + k^{-}_{\text{MFMASP3}}\left[\text{pMFMASP3}_{\text{zym}}\right] - k^{+}_{\text{LFMASP3}}[\text{pPTX3LF}][\text{MASP3}]$$
$$+k^{-}_{\text{LFMASP3}}\left[\text{pPTX3LFMASP3}_{\text{zym}}\right]$$

241

$$\frac{d[\text{MAp19}]}{dt}=-k^{+}_{\text{MBL}_3\text{MAp19}}[\text{MBL}_3][\text{MAp19}]+k^{-}_{\text{MBL}_3\text{MAp19}}[\text{fMBL}_3\text{MAp19}]-k^{+}_{\text{MBL}_3\text{MAp19}}[\text{pMBL}_3][\text{MAp19}]$$
$$+k^{-}_{\text{MBL}_3\text{MAp19}}[\text{pMBL}_3\text{MAp19}]-k^{+}_{\text{MBL}_4\text{MAp19}}[\text{MBL}_4][\text{MAp19}]+k^{-}_{\text{MBL}_4\text{MAp19}}[\text{fMBL}_4\text{MAp19}]$$
$$-k^{+}_{\text{MBL}_4\text{MAp19}}[\text{pMBL}_4][\text{MAp19}]+k^{-}_{\text{MBL}_4\text{MAp19}}[\text{pMBL}_4\text{MAp19}]-k^{+}_{\text{CLLKMAp19}}[\text{CLLK}][\text{MAp19}]$$
$$+k^{-}_{\text{CLLKMAp19}}[\text{fCLLKMAp19}]-k^{+}_{\text{CLLKMAp19}}[\text{pCLLK}][\text{MAp19}]+k^{-}_{\text{CLLKMAp19}}[\text{pCLLKMAp19}]$$
$$-k^{+}_{\text{CLK1MAp19}}[\text{CLK1}][\text{MAp19}]+k^{-}_{\text{CLK1MAp19}}[\text{fCLK1MAp19}]-k^{+}_{\text{CLK1MAp19}}[\text{pCLK1}][\text{MAp19}]$$
$$+k^{-}_{\text{CLK1MAp19}}[\text{pCLK1MAp19}]-k^{+}_{\text{CLL1MAp19}}[\text{CLL1}][\text{MAp19}]+k^{-}_{\text{CLL1MAp19}}[\text{fCLL1MAp19}]$$
$$-k^{+}_{\text{CLL1MAp19}}[\text{pCLL1}][\text{MAp19}]+k^{-}_{\text{CLL1MAp19}}[\text{pCLL1MAp19}]-k^{+}_{\text{LFMAp19}}[\text{LF}][\text{MAp19}]+k^{-}_{\text{LFMAp19}}[\text{fLFMAp19}]$$
$$-k^{+}_{\text{LFMAp19}}[\text{pLF}][\text{MAp19}]+k^{-}_{\text{LFMAp19}}[\text{pLFMAp19}]-k^{+}_{\text{HFMAp19}}[\text{HF}][\text{MAp19}]+k^{-}_{\text{HFMAp19}}[\text{fHFMAp19}]$$
$$-k^{+}_{\text{HFMAp19}}[\text{pHF}][\text{MAp19}]+k^{-}_{\text{HFMAp19}}[\text{pHFMAp19}]-k^{+}_{\text{MFMAp19}}[\text{MF}][\text{MAp19}]+k^{-}_{\text{MFMAp19}}[\text{fMFMAp19}]$$
$$-k^{+}_{\text{MFMAp19}}[\text{pMF}][\text{MAp19}]+k^{-}_{\text{MFMAp19}}[\text{pMFMAp19}]-k^{+}_{\text{LFMAp19}}[\text{pPTX3LF}][\text{MAp19}]$$
$$+k^{-}_{\text{LFMAp19}}[\text{pPTX3LFMAp19}]$$

242

$$\frac{d[\text{MAp44}]}{dt}=-k^{+}_{\text{MBL}_3\text{MAp44}}[\text{MBL}_3][\text{MAp44}]+k^{-}_{\text{MBL}_3\text{MAp44}}[\text{fMBL}_3\text{MAp44}]-k^{+}_{\text{MBL}_3\text{MAp44}}[\text{pMBL}_3][\text{MAp44}]$$
$$+k^{-}_{\text{MBL}_3\text{MAp44}}[\text{pMBL}_3\text{MAp44}]-k^{+}_{\text{MBL}_4\text{MAp44}}[\text{MBL}_4][\text{MAp44}]+k^{-}_{\text{MBL}_4\text{MAp44}}[\text{fMBL}_4\text{MAp44}]$$
$$-k^{+}_{\text{MBL}_4\text{MAp44}}[\text{pMBL}_4][\text{MAp44}]+k^{-}_{\text{MBL}_4\text{MAp44}}[\text{pMBL}_4\text{MAp44}]-k^{+}_{\text{CLLKMAp44}}[\text{CLLK}][\text{MAp44}]$$
$$+k^{-}_{\text{CLLKMAp44}}[\text{fCLLKMAp44}]-k^{+}_{\text{CLLKMAp44}}[\text{pCLLK}][\text{MAp44}]+k^{-}_{\text{CLLKMAp44}}[\text{pCLLKMAp44}]$$
$$-k^{+}_{\text{CLK1MAp44}}[\text{CLK1}][\text{MAp44}]+k^{-}_{\text{CLK1MAp44}}[\text{fCLK1MAp44}]-k^{+}_{\text{CLK1MAp44}}[\text{pCLK1}][\text{MAp44}]$$
$$+k^{-}_{\text{CLK1MAp44}}[\text{pCLK1MAp44}]-k^{+}_{\text{CLL1MAp19}}[\text{CLL1}][\text{MAp44}]+k^{-}_{\text{CLL1MAp44}}[\text{fCLL1MAp44}]$$
$$-k^{+}_{\text{CLL1MAp44}}[\text{pCLL1}][\text{MAp44}]+k^{-}_{\text{CLL1MAp44}}[\text{pCLL1MAp44}]-k^{+}_{\text{LFMAp44}}[\text{LF}][\text{MAp44}]+k^{-}_{\text{LFMAp44}}[\text{fLFMAp44}]$$
$$-k^{+}_{\text{LFMAp44}}[\text{pLF}][\text{MAp44}]+k^{-}_{\text{LFMAp44}}[\text{pLFMAp44}]-k^{+}_{\text{HFMAp44}}[\text{HF}][\text{MAp44}]+k^{-}_{\text{HFMAp44}}[\text{fHFMAp44}]$$
$$-k^{+}_{\text{HFMAp44}}[\text{pHF}][\text{MAp44}]+k^{-}_{\text{HFMAp44}}[\text{pHFMAp44}]-k^{+}_{\text{MFMAp44}}[\text{MF}][\text{MAp44}]+k^{-}_{\text{MFMAp44}}[\text{fMFMAp44}]$$
$$-k^{+}_{\text{MFMAp44}}[\text{pMF}][\text{MAp44}]+k^{-}_{\text{MFMAp44}}[\text{pMFMAp44}]-k^{+}_{\text{LFMAp44}}[\text{pPTX3LF}][\text{MAp44}]$$
$$+k^{-}_{\text{LFMAp44}}[\text{pPTX3LFMAp44}]$$

243

$$\frac{d\left[\text{fMBL}_3\text{MASP1}_{\text{zym}}\right]}{dt}=k^{+}_{\text{MBL}_3\text{MASP1}}[\text{MBL}_3][\text{MASP1}]-k^{-}_{\text{MBL}_3\text{MASP1}}\left[\text{fMBL}_3\text{MASP1}_{\text{zym}}\right]$$
$$-k^{+}_{\text{pMBL}_3}\left[\text{fMBL}_3\text{MASP1}_{\text{zym}}\right][\text{Pathogen Surface}]+k^{-}_{\text{pMBL}_3}\left[\text{pMBL}_3\text{MASP1}_{\text{zym}}\right]$$

244

$$\frac{d\left[\text{pMBL}_3\text{MASP1}_{\text{zym}}\right]}{dt}=k^{+}_{\text{pMBL}_3}\left[\text{fMBL}_3\text{MASP1}_{\text{zym}}\right]\left[\text{Pathogen Surface}\right]-k^{-}_{\text{pMBL}_3}\left[\text{pMBL}_3\text{MASP1}_{\text{zym}}\right]$$

$$+k^{+}_{\text{MBL}_3\text{MASP1}}\left[\text{pMBL}_3\right]\left[\text{MASP1}\right]-k^{-}_{\text{MBL}_3\text{MASP1}}\left[\text{pMBL}_3\text{MASP1}_{\text{zym}}\right]$$

$$-\frac{k^{\text{MASP1}_{\text{zym}}}_{\text{cat MASP1}_{\text{zym}}}\left[\text{pMBL}_3\text{MASP1}_{\text{zym}}\right]\left[\text{pMBL}_3\text{MASP1}_{\text{zym}}\right]}{K^{\text{MASP1}_{\text{zym}}}_{\text{m MASP1}_{\text{zym}}}\left(\begin{array}{l}1+\frac{\left[\text{pMBL}_3\text{MASP1}_{\text{zym}}\right]}{K^{\text{MASP1}_{\text{zym}}}_{\text{m MASP1}_{\text{zym}}}}+\frac{\left[\text{pMBL}_3\text{MASP2}_{\text{zym}}\right]}{K^{\text{MASP2}_{\text{zym}}}_{\text{m MASP1}_{\text{zym}}}}+\frac{\left[\text{pMBL}_4\text{MASP1}_{\text{zym}}\right]}{K^{\text{MASP1}_{\text{zym}}}_{\text{m MASP1}_{\text{zym}}}}+\frac{\left[\text{pMBL}_4\text{MASP2}_{\text{zym}}\right]}{K^{\text{MASP2}_{\text{zym}}}_{\text{m MASP1}_{\text{zym}}}}\\+\frac{\left[\text{pCLLKMASP1}_{\text{zym}}\right]}{K^{\text{MASP1}_{\text{zym}}}_{\text{m MASP1}_{\text{zym}}}}+\frac{\left[\text{pCLLKMASP2}_{\text{zym}}\right]}{K^{\text{MASP2}_{\text{zym}}}_{\text{m MASP1}_{\text{zym}}}}+\frac{\left[\text{pCLK1MASP1}_{\text{zym}}\right]}{K^{\text{MASP1}_{\text{zym}}}_{\text{m MASP1}_{\text{zym}}}}+\frac{\left[\text{pCLK1MASP2}_{\text{zym}}\right]}{K^{\text{MASP2}_{\text{zym}}}_{\text{m MASP1}_{\text{zym}}}}\\+\frac{\left[\text{pCLL1MASP1}_{\text{zym}}\right]}{K^{\text{MASP1}_{\text{zym}}}_{\text{m MASP1}_{\text{zym}}}}+\frac{\left[\text{pCLL1MASP2}_{\text{zym}}\right]}{K^{\text{MASP2}_{\text{zym}}}_{\text{m MASP1}_{\text{zym}}}}+\frac{\left[\text{pLFMASP1}_{\text{zym}}\right]}{K^{\text{MASP1}_{\text{zym}}}_{\text{m MASP1}_{\text{zym}}}}+\frac{\left[\text{pLFMASP2}_{\text{zym}}\right]}{K^{\text{MASP2}_{\text{zym}}}_{\text{m MASP1}_{\text{zym}}}}\\+\frac{\left[\text{pHFMASP1}_{\text{zym}}\right]}{K^{\text{MASP1}_{\text{zym}}}_{\text{m MASP1}_{\text{zym}}}}+\frac{\left[\text{pHFMASP2}_{\text{zym}}\right]}{K^{\text{MASP2}_{\text{zym}}}_{\text{m MASP1}_{\text{zym}}}}+\frac{\left[\text{pMFMASP1}_{\text{zym}}\right]}{K^{\text{MASP1}_{\text{zym}}}_{\text{m MASP1}_{\text{zym}}}}+\frac{\left[\text{pMFMASP2}_{\text{zym}}\right]}{K^{\text{MASP2}_{\text{zym}}}_{\text{m MASP1}_{\text{zym}}}}\end{array}\right)}$$

$$-\frac{k^{\text{MASP1}_{\text{zym}}}_{\text{cat MASP1}_{\text{zym}}}\left[\text{pMBL}_4\text{MASP1}_{\text{zym}}\right]\left[\text{pMBL}_3\text{MASP1}_{\text{zym}}\right]}{K^{\text{MASP1}_{\text{zym}}}_{\text{m MASP1}_{\text{zym}}}\left(\begin{array}{l}1+\frac{\left[\text{pMBL}_3\text{MASP1}_{\text{zym}}\right]}{K^{\text{MASP1}_{\text{zym}}}_{\text{m MASP1}_{\text{zym}}}}+\frac{\left[\text{pMBL}_3\text{MASP2}_{\text{zym}}\right]}{K^{\text{MASP2}_{\text{zym}}}_{\text{m MASP1}_{\text{zym}}}}+\frac{\left[\text{pMBL}_4\text{MASP1}_{\text{zym}}\right]}{K^{\text{MASP1}_{\text{zym}}}_{\text{m MASP1}_{\text{zym}}}}+\frac{\left[\text{pMBL}_4\text{MASP2}_{\text{zym}}\right]}{K^{\text{MASP2}_{\text{zym}}}_{\text{m MASP1}_{\text{zym}}}}\\+\frac{\left[\text{pCLLKMASP1}_{\text{zym}}\right]}{K^{\text{MASP1}_{\text{zym}}}_{\text{m MASP1}_{\text{zym}}}}+\frac{\left[\text{pCLLKMASP2}_{\text{zym}}\right]}{K^{\text{MASP2}_{\text{zym}}}_{\text{m MASP1}_{\text{zym}}}}+\frac{\left[\text{pCLK1MASP1}_{\text{zym}}\right]}{K^{\text{MASP1}_{\text{zym}}}_{\text{m MASP1}_{\text{zym}}}}+\frac{\left[\text{pCLK1MASP2}_{\text{zym}}\right]}{K^{\text{MASP2}_{\text{zym}}}_{\text{m MASP1}_{\text{zym}}}}\\+\frac{\left[\text{pCLL1MASP1}_{\text{zym}}\right]}{K^{\text{MASP1}_{\text{zym}}}_{\text{m MASP1}_{\text{zym}}}}+\frac{\left[\text{pCLL1MASP2}_{\text{zym}}\right]}{K^{\text{MASP2}_{\text{zym}}}_{\text{m MASP1}_{\text{zym}}}}+\frac{\left[\text{pLFMASP1}_{\text{zym}}\right]}{K^{\text{MASP1}_{\text{zym}}}_{\text{m MASP1}_{\text{zym}}}}+\frac{\left[\text{pLFMASP2}_{\text{zym}}\right]}{K^{\text{MASP2}_{\text{zym}}}_{\text{m MASP1}_{\text{zym}}}}\\+\frac{\left[\text{pHFMASP1}_{\text{zym}}\right]}{K^{\text{MASP1}_{\text{zym}}}_{\text{m MASP1}_{\text{zym}}}}+\frac{\left[\text{pHFMASP2}_{\text{zym}}\right]}{K^{\text{MASP2}_{\text{zym}}}_{\text{m MASP1}_{\text{zym}}}}+\frac{\left[\text{pMFMASP1}_{\text{zym}}\right]}{K^{\text{MASP1}_{\text{zym}}}_{\text{m MASP1}_{\text{zym}}}}+\frac{\left[\text{pMFMASP2}_{\text{zym}}\right]}{K^{\text{MASP2}_{\text{zym}}}_{\text{m MASP1}_{\text{zym}}}}\end{array}\right)}$$

$$-\frac{k^{\text{MASP1}_{\text{zym}}}_{\text{cat MASP1}_{\text{zym}}}\left[\text{pCLLKMASP1}_{\text{zym}}\right]\left[\text{pMBL}_3\text{MASP1}_{\text{zym}}\right]}{K^{\text{MASP1}_{\text{zym}}}_{\text{m MASP1}_{\text{zym}}}\left(\begin{array}{l}1+\frac{\left[\text{pMBL}_3\text{MASP1}_{\text{zym}}\right]}{K^{\text{MASP1}_{\text{zym}}}_{\text{m MASP1}_{\text{zym}}}}+\frac{\left[\text{pMBL}_3\text{MASP2}_{\text{zym}}\right]}{K^{\text{MASP2}_{\text{zym}}}_{\text{m MASP1}_{\text{zym}}}}+\frac{\left[\text{pMBL}_4\text{MASP1}_{\text{zym}}\right]}{K^{\text{MASP1}_{\text{zym}}}_{\text{m MASP1}_{\text{zym}}}}+\frac{\left[\text{pMBL}_4\text{MASP2}_{\text{zym}}\right]}{K^{\text{MASP2}_{\text{zym}}}_{\text{m MASP1}_{\text{zym}}}}\\+\frac{\left[\text{pCLLKMASP1}_{\text{zym}}\right]}{K^{\text{MASP1}_{\text{zym}}}_{\text{m MASP1}_{\text{zym}}}}+\frac{\left[\text{pCLLKMASP2}_{\text{zym}}\right]}{K^{\text{MASP2}_{\text{zym}}}_{\text{m MASP1}_{\text{zym}}}}+\frac{\left[\text{pCLK1MASP1}_{\text{zym}}\right]}{K^{\text{MASP1}_{\text{zym}}}_{\text{m MASP1}_{\text{zym}}}}+\frac{\left[\text{pCLK1MASP2}_{\text{zym}}\right]}{K^{\text{MASP2}_{\text{zym}}}_{\text{m MASP1}_{\text{zym}}}}\\+\frac{\left[\text{pCLL1MASP1}_{\text{zym}}\right]}{K^{\text{MASP1}_{\text{zym}}}_{\text{m MASP1}_{\text{zym}}}}+\frac{\left[\text{pCLL1MASP2}_{\text{zym}}\right]}{K^{\text{MASP2}_{\text{zym}}}_{\text{m MASP1}_{\text{zym}}}}+\frac{\left[\text{pLFMASP1}_{\text{zym}}\right]}{K^{\text{MASP1}_{\text{zym}}}_{\text{m MASP1}_{\text{zym}}}}+\frac{\left[\text{pLFMASP2}_{\text{zym}}\right]}{K^{\text{MASP2}_{\text{zym}}}_{\text{m MASP1}_{\text{zym}}}}\\+\frac{\left[\text{pHFMASP1}_{\text{zym}}\right]}{K^{\text{MASP1}_{\text{zym}}}_{\text{m MASP1}_{\text{zym}}}}+\frac{\left[\text{pHFMASP2}_{\text{zym}}\right]}{K^{\text{MASP2}_{\text{zym}}}_{\text{m MASP1}_{\text{zym}}}}+\frac{\left[\text{pMFMASP1}_{\text{zym}}\right]}{K^{\text{MASP1}_{\text{zym}}}_{\text{m MASP1}_{\text{zym}}}}+\frac{\left[\text{pMFMASP2}_{\text{zym}}\right]}{K^{\text{MASP2}_{\text{zym}}}_{\text{m MASP1}_{\text{zym}}}}\end{array}\right)}$$

$$
-\frac{k_{\text{cat MASP1}_{\text{zym}}}^{\text{MASP1}_{\text{zym}}}\left[\text{pCLK1MASP1}_{\text{zym}}\right]\left[\text{pMBL}_3\text{MASP1}_{\text{zym}}\right]}{K_{\text{m MASP1}_{\text{zym}}}^{\text{MASP1}_{\text{zym}}}\left(\begin{aligned}&1+\frac{\left[\text{pMBL}_3\text{MASP1}_{\text{zym}}\right]}{K_{\text{m MASP1}_{\text{zym}}}^{\text{MASP1}_{\text{zym}}}}+\frac{\left[\text{pMBL}_3\text{MASP2}_{\text{zym}}\right]}{K_{\text{m MASP1}_{\text{zym}}}^{\text{MASP2}_{\text{zym}}}}+\frac{\left[\text{pMBL}_4\text{MASP1}_{\text{zym}}\right]}{K_{\text{m MASP1}_{\text{zym}}}^{\text{MASP1}_{\text{zym}}}}+\frac{\left[\text{pMBL}_4\text{MASP2}_{\text{zym}}\right]}{K_{\text{m MASP1}_{\text{zym}}}^{\text{MASP2}_{\text{zym}}}}\\&+\frac{\left[\text{pCLLKMASP1}_{\text{zym}}\right]}{K_{\text{m MASP1}_{\text{zym}}}^{\text{MASP1}_{\text{zym}}}}+\frac{\left[\text{pCLLKMASP2}_{\text{zym}}\right]}{K_{\text{m MASP1}_{\text{zym}}}^{\text{MASP2}_{\text{zym}}}}+\frac{\left[\text{pCLK1MASP1}_{\text{zym}}\right]}{K_{\text{m MASP1}_{\text{zym}}}^{\text{MASP1}_{\text{zym}}}}+\frac{\left[\text{pCLK1MASP2}_{\text{zym}}\right]}{K_{\text{m MASP1}_{\text{zym}}}^{\text{MASP2}_{\text{zym}}}}\\&+\frac{\left[\text{pCLL1MASP1}_{\text{zym}}\right]}{K_{\text{m MASP1}_{\text{zym}}}^{\text{MASP1}_{\text{zym}}}}+\frac{\left[\text{pCLL1MASP2}_{\text{zym}}\right]}{K_{\text{m MASP1}_{\text{zym}}}^{\text{MASP2}_{\text{zym}}}}+\frac{\left[\text{pLFMASP1}_{\text{zym}}\right]}{K_{\text{m MASP1}_{\text{zym}}}^{\text{MASP1}_{\text{zym}}}}+\frac{\left[\text{pLFMASP2}_{\text{zym}}\right]}{K_{\text{m MASP1}_{\text{zym}}}^{\text{MASP2}_{\text{zym}}}}\\&+\frac{\left[\text{pHFMASP1}_{\text{zym}}\right]}{K_{\text{m MASP1}_{\text{zym}}}^{\text{MASP1}_{\text{zym}}}}+\frac{\left[\text{pHFMASP2}_{\text{zym}}\right]}{K_{\text{m MASP1}_{\text{zym}}}^{\text{MASP2}_{\text{zym}}}}+\frac{\left[\text{pMFMASP1}_{\text{zym}}\right]}{K_{\text{m MASP1}_{\text{zym}}}^{\text{MASP1}_{\text{zym}}}}+\frac{\left[\text{pMFMASP2}_{\text{zym}}\right]}{K_{\text{m MASP1}_{\text{zym}}}^{\text{MASP2}_{\text{zym}}}}\end{aligned}\right)}
$$

$$
-\frac{k_{\text{cat MASP1}_{\text{zym}}}^{\text{MASP1}_{\text{zym}}}\left[\text{pCLL1MASP1}_{\text{zym}}\right]\left[\text{pMBL}_3\text{MASP1}_{\text{zym}}\right]}{K_{\text{m MASP1}_{\text{zym}}}^{\text{MASP1}_{\text{zym}}}\left(\begin{aligned}&1+\frac{\left[\text{pMBL}_3\text{MASP1}_{\text{zym}}\right]}{K_{\text{m MASP1}_{\text{zym}}}^{\text{MASP1}_{\text{zym}}}}+\frac{\left[\text{pMBL}_3\text{MASP2}_{\text{zym}}\right]}{K_{\text{m MASP1}_{\text{zym}}}^{\text{MASP2}_{\text{zym}}}}+\frac{\left[\text{pMBL}_4\text{MASP1}_{\text{zym}}\right]}{K_{\text{m MASP1}_{\text{zym}}}^{\text{MASP1}_{\text{zym}}}}+\frac{\left[\text{pMBL}_4\text{MASP2}_{\text{zym}}\right]}{K_{\text{m MASP1}_{\text{zym}}}^{\text{MASP2}_{\text{zym}}}}\\&+\frac{\left[\text{pCLLKMASP1}_{\text{zym}}\right]}{K_{\text{m MASP1}_{\text{zym}}}^{\text{MASP1}_{\text{zym}}}}+\frac{\left[\text{pCLLKMASP2}_{\text{zym}}\right]}{K_{\text{m MASP1}_{\text{zym}}}^{\text{MASP2}_{\text{zym}}}}+\frac{\left[\text{pCLK1MASP1}_{\text{zym}}\right]}{K_{\text{m MASP1}_{\text{zym}}}^{\text{MASP1}_{\text{zym}}}}+\frac{\left[\text{pCLK1MASP2}_{\text{zym}}\right]}{K_{\text{m MASP1}_{\text{zym}}}^{\text{MASP2}_{\text{zym}}}}\\&+\frac{\left[\text{pCLL1MASP1}_{\text{zym}}\right]}{K_{\text{m MASP1}_{\text{zym}}}^{\text{MASP1}_{\text{zym}}}}+\frac{\left[\text{pCLL1MASP2}_{\text{zym}}\right]}{K_{\text{m MASP1}_{\text{zym}}}^{\text{MASP2}_{\text{zym}}}}+\frac{\left[\text{pLFMASP1}_{\text{zym}}\right]}{K_{\text{m MASP1}_{\text{zym}}}^{\text{MASP1}_{\text{zym}}}}+\frac{\left[\text{pLFMASP2}_{\text{zym}}\right]}{K_{\text{m MASP1}_{\text{zym}}}^{\text{MASP2}_{\text{zym}}}}\\&+\frac{\left[\text{pHFMASP1}_{\text{zym}}\right]}{K_{\text{m MASP1}_{\text{zym}}}^{\text{MASP1}_{\text{zym}}}}+\frac{\left[\text{pHFMASP2}_{\text{zym}}\right]}{K_{\text{m MASP1}_{\text{zym}}}^{\text{MASP2}_{\text{zym}}}}+\frac{\left[\text{pMFMASP1}_{\text{zym}}\right]}{K_{\text{m MASP1}_{\text{zym}}}^{\text{MASP1}_{\text{zym}}}}+\frac{\left[\text{pMFMASP2}_{\text{zym}}\right]}{K_{\text{m MASP1}_{\text{zym}}}^{\text{MASP2}_{\text{zym}}}}\end{aligned}\right)}
$$

$$
-\frac{k_{\text{cat MASP1}_{\text{zym}}}^{\text{MASP1}_{\text{zym}}}\left[\text{pLFMASP1}_{\text{zym}}\right]\left[\text{pMBL}_3\text{MASP1}_{\text{zym}}\right]}{K_{\text{m MASP1}_{\text{zym}}}^{\text{MASP1}_{\text{zym}}}\left(\begin{aligned}&1+\frac{\left[\text{pMBL}_3\text{MASP1}_{\text{zym}}\right]}{K_{\text{m MASP1}_{\text{zym}}}^{\text{MASP1}_{\text{zym}}}}+\frac{\left[\text{pMBL}_3\text{MASP2}_{\text{zym}}\right]}{K_{\text{m MASP1}_{\text{zym}}}^{\text{MASP2}_{\text{zym}}}}+\frac{\left[\text{pMBL}_4\text{MASP1}_{\text{zym}}\right]}{K_{\text{m MASP1}_{\text{zym}}}^{\text{MASP1}_{\text{zym}}}}+\frac{\left[\text{pMBL}_4\text{MASP2}_{\text{zym}}\right]}{K_{\text{m MASP1}_{\text{zym}}}^{\text{MASP2}_{\text{zym}}}}\\&+\frac{\left[\text{pCLLKMASP1}_{\text{zym}}\right]}{K_{\text{m MASP1}_{\text{zym}}}^{\text{MASP1}_{\text{zym}}}}+\frac{\left[\text{pCLLKMASP2}_{\text{zym}}\right]}{K_{\text{m MASP1}_{\text{zym}}}^{\text{MASP2}_{\text{zym}}}}+\frac{\left[\text{pCLK1MASP1}_{\text{zym}}\right]}{K_{\text{m MASP1}_{\text{zym}}}^{\text{MASP1}_{\text{zym}}}}+\frac{\left[\text{pCLK1MASP2}_{\text{zym}}\right]}{K_{\text{m MASP1}_{\text{zym}}}^{\text{MASP2}_{\text{zym}}}}\\&+\frac{\left[\text{pCLL1MASP1}_{\text{zym}}\right]}{K_{\text{m MASP1}_{\text{zym}}}^{\text{MASP1}_{\text{zym}}}}+\frac{\left[\text{pCLL1MASP2}_{\text{zym}}\right]}{K_{\text{m MASP1}_{\text{zym}}}^{\text{MASP2}_{\text{zym}}}}+\frac{\left[\text{pLFMASP1}_{\text{zym}}\right]}{K_{\text{m MASP1}_{\text{zym}}}^{\text{MASP1}_{\text{zym}}}}+\frac{\left[\text{pLFMASP2}_{\text{zym}}\right]}{K_{\text{m MASP1}_{\text{zym}}}^{\text{MASP2}_{\text{zym}}}}\\&+\frac{\left[\text{pHFMASP1}_{\text{zym}}\right]}{K_{\text{m MASP1}_{\text{zym}}}^{\text{MASP1}_{\text{zym}}}}+\frac{\left[\text{pHFMASP2}_{\text{zym}}\right]}{K_{\text{m MASP1}_{\text{zym}}}^{\text{MASP2}_{\text{zym}}}}+\frac{\left[\text{pMFMASP1}_{\text{zym}}\right]}{K_{\text{m MASP1}_{\text{zym}}}^{\text{MASP1}_{\text{zym}}}}+\frac{\left[\text{pMFMASP2}_{\text{zym}}\right]}{K_{\text{m MASP1}_{\text{zym}}}^{\text{MASP2}_{\text{zym}}}}\end{aligned}\right)}
$$

$$
-\frac{k_{\mathrm{cat\ MASP1_{zym}}}^{\mathrm{MASP1_{zym}}}\left[\mathrm{pHFMASP1_{zym}}\right]\left[\mathrm{pMBL_3MASP1_{zym}}\right]}{K_{\mathrm{m\ MASP1_{zym}}}^{\mathrm{MASP1_{zym}}}\left(\begin{array}{l}1+\frac{\left[\mathrm{pMBL_3MASP1_{zym}}\right]}{K_{\mathrm{m\ MASP1_{zym}}}^{\mathrm{MASP1_{zym}}}}+\frac{\left[\mathrm{pMBL_3MASP2_{zym}}\right]}{K_{\mathrm{m\ MASP1_{zym}}}^{\mathrm{MASP2_{zym}}}}+\frac{\left[\mathrm{pMBL_4MASP1_{zym}}\right]}{K_{\mathrm{m\ MASP1_{zym}}}^{\mathrm{MASP1_{zym}}}}+\frac{\left[\mathrm{pMBL_4MASP2_{zym}}\right]}{K_{\mathrm{m\ MASP1_{zym}}}^{\mathrm{MASP2_{zym}}}}\\+\frac{\left[\mathrm{pCLLKMASP1_{zym}}\right]}{K_{\mathrm{m\ MASP1_{zym}}}^{\mathrm{MASP1_{zym}}}}+\frac{\left[\mathrm{pCLLKMASP2_{zym}}\right]}{K_{\mathrm{m\ MASP1_{zym}}}^{\mathrm{MASP2_{zym}}}}+\frac{\left[\mathrm{pCLK1MASP1_{zym}}\right]}{K_{\mathrm{m\ MASP1_{zym}}}^{\mathrm{MASP1_{zym}}}}+\frac{\left[\mathrm{pCLK1MASP2_{zym}}\right]}{K_{\mathrm{m\ MASP1_{zym}}}^{\mathrm{MASP2_{zym}}}}\\+\frac{\left[\mathrm{pCLL1MASP1_{zym}}\right]}{K_{\mathrm{m\ MASP1_{zym}}}^{\mathrm{MASP1_{zym}}}}+\frac{\left[\mathrm{pCLL1MASP2_{zym}}\right]}{K_{\mathrm{m\ MASP1_{zym}}}^{\mathrm{MASP2_{zym}}}}+\frac{\left[\mathrm{pLFMASP1_{zym}}\right]}{K_{\mathrm{m\ MASP1_{zym}}}^{\mathrm{MASP1_{zym}}}}+\frac{\left[\mathrm{pLFMASP2_{zym}}\right]}{K_{\mathrm{m\ MASP1_{zym}}}^{\mathrm{MASP2_{zym}}}}\\+\frac{\left[\mathrm{pHFMASP1_{zym}}\right]}{K_{\mathrm{m\ MASP1_{zym}}}^{\mathrm{MASP1_{zym}}}}+\frac{\left[\mathrm{pHFMASP2_{zym}}\right]}{K_{\mathrm{m\ MASP1_{zym}}}^{\mathrm{MASP2_{zym}}}}+\frac{\left[\mathrm{pMFMASP1_{zym}}\right]}{K_{\mathrm{m\ MASP1_{zym}}}^{\mathrm{MASP1_{zym}}}}+\frac{\left[\mathrm{pMFMASP2_{zym}}\right]}{K_{\mathrm{m\ MASP1_{zym}}}^{\mathrm{MASP2_{zym}}}}\end{array}\right)}
$$

$$
-\frac{k_{\mathrm{cat\ MASP1_{zym}}}^{\mathrm{MASP1_{zym}}}\left[\mathrm{pMFMASP1_{zym}}\right]\left[\mathrm{pMBL_3MASP1_{zym}}\right]}{K_{\mathrm{m\ MASP1_{zym}}}^{\mathrm{MASP1_{zym}}}\left(\begin{array}{l}1+\frac{\left[\mathrm{pMBL_3MASP1_{zym}}\right]}{K_{\mathrm{m\ MASP1_{zym}}}^{\mathrm{MASP1_{zym}}}}+\frac{\left[\mathrm{pMBL_3MASP2_{zym}}\right]}{K_{\mathrm{m\ MASP1_{zym}}}^{\mathrm{MASP2_{zym}}}}+\frac{\left[\mathrm{pMBL_4MASP1_{zym}}\right]}{K_{\mathrm{m\ MASP1_{zym}}}^{\mathrm{MASP1_{zym}}}}+\frac{\left[\mathrm{pMBL_4MASP2_{zym}}\right]}{K_{\mathrm{m\ MASP1_{zym}}}^{\mathrm{MASP2_{zym}}}}\\+\frac{\left[\mathrm{pCLLKMASP1_{zym}}\right]}{K_{\mathrm{m\ MASP1_{zym}}}^{\mathrm{MASP1_{zym}}}}+\frac{\left[\mathrm{pCLLKMASP2_{zym}}\right]}{K_{\mathrm{m\ MASP1_{zym}}}^{\mathrm{MASP2_{zym}}}}+\frac{\left[\mathrm{pCLK1MASP1_{zym}}\right]}{K_{\mathrm{m\ MASP1_{zym}}}^{\mathrm{MASP1_{zym}}}}+\frac{\left[\mathrm{pCLK1MASP2_{zym}}\right]}{K_{\mathrm{m\ MASP1_{zym}}}^{\mathrm{MASP2_{zym}}}}\\+\frac{\left[\mathrm{pCLL1MASP1_{zym}}\right]}{K_{\mathrm{m\ MASP1_{zym}}}^{\mathrm{MASP1_{zym}}}}+\frac{\left[\mathrm{pCLL1MASP2_{zym}}\right]}{K_{\mathrm{m\ MASP1_{zym}}}^{\mathrm{MASP2_{zym}}}}+\frac{\left[\mathrm{pLFMASP1_{zym}}\right]}{K_{\mathrm{m\ MASP1_{zym}}}^{\mathrm{MASP1_{zym}}}}+\frac{\left[\mathrm{pLFMASP2_{zym}}\right]}{K_{\mathrm{m\ MASP1_{zym}}}^{\mathrm{MASP2_{zym}}}}\\+\frac{\left[\mathrm{pHFMASP1_{zym}}\right]}{K_{\mathrm{m\ MASP1_{zym}}}^{\mathrm{MASP1_{zym}}}}+\frac{\left[\mathrm{pHFMASP2_{zym}}\right]}{K_{\mathrm{m\ MASP1_{zym}}}^{\mathrm{MASP2_{zym}}}}+\frac{\left[\mathrm{pMFMASP1_{zym}}\right]}{K_{\mathrm{m\ MASP1_{zym}}}^{\mathrm{MASP1_{zym}}}}+\frac{\left[\mathrm{pMFMASP2_{zym}}\right]}{K_{\mathrm{m\ MASP1_{zym}}}^{\mathrm{MASP2_{zym}}}}\end{array}\right)}
$$

$$
-\frac{k_{\mathrm{cat\ MASP1_{zym}}}^{\mathrm{MASP1_{zym}}}\left[\mathrm{pPTX3LFMASP1_{zym}}\right]\left[\mathrm{pMBL_3MASP1_{zym}}\right]}{K_{\mathrm{m\ MASP1_{zym}}}^{\mathrm{MASP1_{zym}}}\left(\begin{array}{l}1+\frac{\left[\mathrm{pMBL_3MASP1_{zym}}\right]}{K_{\mathrm{m\ MASP1_{zym}}}^{\mathrm{MASP1_{zym}}}}+\frac{\left[\mathrm{pMBL_3MASP2_{zym}}\right]}{K_{\mathrm{m\ MASP1_{zym}}}^{\mathrm{MASP2_{zym}}}}+\frac{\left[\mathrm{pMBL_4MASP1_{zym}}\right]}{K_{\mathrm{m\ MASP1_{zym}}}^{\mathrm{MASP1_{zym}}}}+\frac{\left[\mathrm{pMBL_4MASP2_{zym}}\right]}{K_{\mathrm{m\ MASP1_{zym}}}^{\mathrm{MASP2_{zym}}}}\\+\frac{\left[\mathrm{pCLLKMASP1_{zym}}\right]}{K_{\mathrm{m\ MASP1_{zym}}}^{\mathrm{MASP1_{zym}}}}+\frac{\left[\mathrm{pCLLKMASP2_{zym}}\right]}{K_{\mathrm{m\ MASP1_{zym}}}^{\mathrm{MASP2_{zym}}}}+\frac{\left[\mathrm{pCLK1MASP1_{zym}}\right]}{K_{\mathrm{m\ MASP1_{zym}}}^{\mathrm{MASP1_{zym}}}}+\frac{\left[\mathrm{pCLK1MASP2_{zym}}\right]}{K_{\mathrm{m\ MASP1_{zym}}}^{\mathrm{MASP2_{zym}}}}\\+\frac{\left[\mathrm{pCLL1MASP1_{zym}}\right]}{K_{\mathrm{m\ MASP1_{zym}}}^{\mathrm{MASP1_{zym}}}}+\frac{\left[\mathrm{pCLL1MASP2_{zym}}\right]}{K_{\mathrm{m\ MASP1_{zym}}}^{\mathrm{MASP2_{zym}}}}+\frac{\left[\mathrm{pLFMASP1_{zym}}\right]}{K_{\mathrm{m\ MASP1_{zym}}}^{\mathrm{MASP1_{zym}}}}+\frac{\left[\mathrm{pLFMASP2_{zym}}\right]}{K_{\mathrm{m\ MASP1_{zym}}}^{\mathrm{MASP2_{zym}}}}\\+\frac{\left[\mathrm{pHFMASP1_{zym}}\right]}{K_{\mathrm{m\ MASP1_{zym}}}^{\mathrm{MASP1_{zym}}}}+\frac{\left[\mathrm{pHFMASP2_{zym}}\right]}{K_{\mathrm{m\ MASP1_{zym}}}^{\mathrm{MASP2_{zym}}}}+\frac{\left[\mathrm{pMFMASP1_{zym}}\right]}{K_{\mathrm{m\ MASP1_{zym}}}^{\mathrm{MASP1_{zym}}}}+\frac{\left[\mathrm{pMFMASP2_{zym}}\right]}{K_{\mathrm{m\ MASP1_{zym}}}^{\mathrm{MASP2_{zym}}}}\\+\frac{\left[\mathrm{pPTX3LFMASP1_{zym}}\right]}{K_{\mathrm{m\ MASP1_{zym}}}^{\mathrm{MASP1_{zym}}}}+\frac{\left[\mathrm{pPTX3LFMASP2_{zym}}\right]}{K_{\mathrm{m\ MASP1_{zym}}}^{\mathrm{MASP2_{zym}}}}\end{array}\right)}
$$

$$
-\frac{k_{\mathrm{cat\ MASP1}^*}^{\mathrm{MASP1}_{\mathrm{zym}}}\left[\mathrm{pMBL}_3\mathrm{MASP1}^*\right]\left[\mathrm{pMBL}_3\mathrm{MASP1}_{\mathrm{zym}}\right]}{K_{\mathrm{m\ MASP1}^*}^{\mathrm{MASP1}_{\mathrm{zym}}}\left(\begin{array}{l}
1+\frac{[\mathrm{C4}]}{K_{\mathrm{m\ MASP1}^*}^{\mathrm{C4}}}+\frac{[\mathrm{fC4bC2}]}{K_{\mathrm{m\ MASP1}^*}^{\mathrm{C2}}}+\frac{[\mathrm{fC4bC4bC2}]}{K_{\mathrm{m\ MASP1}^*}^{\mathrm{C2}}}+\frac{[\mathrm{fC3bC4bC2}]}{K_{\mathrm{m\ MASP1}^*}^{\mathrm{C2}}}+\frac{[\mathrm{pC4bC2}]}{K_{\mathrm{m\ MASP1}^*}^{\mathrm{C2}}}+\frac{[\mathrm{pC4bC4bC2}]}{K_{\mathrm{m\ MASP1}^*}^{\mathrm{C2}}}\\
+\frac{[\mathrm{pC3bC4bC2}]}{K_{\mathrm{m\ MASP1}^*}^{\mathrm{C2}}}+\frac{\left[\mathrm{pMBL}_3\mathrm{MASP1}_{\mathrm{zym}}\right]}{K_{\mathrm{m\ MASP1}^*}^{\mathrm{MASP1}_{\mathrm{zym}}}}+\frac{\left[\mathrm{pMBL}_3\mathrm{MASP2}_{\mathrm{zym}}\right]}{K_{\mathrm{m\ MASP1}^*}^{\mathrm{MASP2}_{\mathrm{zym}}}}+\frac{\left[\mathrm{pMBL}_3\mathrm{MASP3}_{\mathrm{zym}}\right]}{K_{\mathrm{m\ MASP1}^*}^{\mathrm{MASP3}_{\mathrm{zym}}}}\\
+\frac{\left[\mathrm{pMBL}_4\mathrm{MASP1}_{\mathrm{zym}}\right]}{K_{\mathrm{m\ MASP1}^*}^{\mathrm{MASP1}_{\mathrm{zym}}}}+\frac{\left[\mathrm{pMBL}_4\mathrm{MASP2}_{\mathrm{zym}}\right]}{K_{\mathrm{m\ MASP1}^*}^{\mathrm{MASP2}_{\mathrm{zym}}}}+\frac{\left[\mathrm{pMBL}_4\mathrm{MASP3}_{\mathrm{zym}}\right]}{K_{\mathrm{m\ MASP1}^*}^{\mathrm{MASP3}_{\mathrm{zym}}}}+\frac{\left[\mathrm{pCLLKMASP1}_{\mathrm{zym}}\right]}{K_{\mathrm{m\ MASP1}^*}^{\mathrm{MASP1}_{\mathrm{zym}}}}\\
+\frac{\left[\mathrm{pCLLKMASP2}_{\mathrm{zym}}\right]}{K_{\mathrm{m\ MASP1}^*}^{\mathrm{MASP2}_{\mathrm{zym}}}}+\frac{\left[\mathrm{pCLLKMASP3}_{\mathrm{zym}}\right]}{K_{\mathrm{m\ MASP1}^*}^{\mathrm{MASP3}_{\mathrm{zym}}}}+\frac{\left[\mathrm{pCLK1MASP1}_{\mathrm{zym}}\right]}{K_{\mathrm{m\ MASP1}^*}^{\mathrm{MASP1}_{\mathrm{zym}}}}+\frac{\left[\mathrm{pCLK1MASP2}_{\mathrm{zym}}\right]}{K_{\mathrm{m\ MASP1}^*}^{\mathrm{MASP2}_{\mathrm{zym}}}}\\
+\frac{\left[\mathrm{pCLK1MASP3}_{\mathrm{zym}}\right]}{K_{\mathrm{m\ MASP1}^*}^{\mathrm{MASP3}_{\mathrm{zym}}}}+\frac{\left[\mathrm{pCLL1MASP1}_{\mathrm{zym}}\right]}{K_{\mathrm{m\ MASP1}^*}^{\mathrm{MASP1}_{\mathrm{zym}}}}+\frac{\left[\mathrm{pCLL1MASP2}_{\mathrm{zym}}\right]}{K_{\mathrm{m\ MASP1}^*}^{\mathrm{MASP2}_{\mathrm{zym}}}}+\frac{\left[\mathrm{pCLL1MASP3}_{\mathrm{zym}}\right]}{K_{\mathrm{m\ MASP1}^*}^{\mathrm{MASP3}_{\mathrm{zym}}}}\\
+\frac{\left[\mathrm{pLFMASP1}_{\mathrm{zym}}\right]}{K_{\mathrm{m\ MASP1}^*}^{\mathrm{MASP1}_{\mathrm{zym}}}}+\frac{\left[\mathrm{pLFMASP2}_{\mathrm{zym}}\right]}{K_{\mathrm{m\ MASP1}^*}^{\mathrm{MASP2}_{\mathrm{zym}}}}+\frac{\left[\mathrm{pLFMASP3}_{\mathrm{zym}}\right]}{K_{\mathrm{m\ MASP1}^*}^{\mathrm{MASP3}_{\mathrm{zym}}}}+\frac{\left[\mathrm{pHFMASP1}_{\mathrm{zym}}\right]}{K_{\mathrm{m\ MASP1}^*}^{\mathrm{MASP1}_{\mathrm{zym}}}}+\frac{\left[\mathrm{pHFMASP2}_{\mathrm{zym}}\right]}{K_{\mathrm{m\ MASP1}^*}^{\mathrm{MASP2}_{\mathrm{zym}}}}\\
+\frac{\left[\mathrm{pHFMASP3}_{\mathrm{zym}}\right]}{K_{\mathrm{m\ MASP1}^*}^{\mathrm{MASP3}_{\mathrm{zym}}}}+\frac{\left[\mathrm{pMFMASP1}_{\mathrm{zym}}\right]}{K_{\mathrm{m\ MASP1}^*}^{\mathrm{MASP1}_{\mathrm{zym}}}}+\frac{\left[\mathrm{pMFMASP2}_{\mathrm{zym}}\right]}{K_{\mathrm{m\ MASP1}^*}^{\mathrm{MASP2}_{\mathrm{zym}}}}+\frac{\left[\mathrm{pMFMASP3}_{\mathrm{zym}}\right]}{K_{\mathrm{m\ MASP1}^*}^{\mathrm{MASP3}_{\mathrm{zym}}}}
\end{array}\right)}
$$

$$
-\frac{k_{\mathrm{cat\ MASP1}^*}^{\mathrm{MASP1}_{\mathrm{zym}}}\left[\mathrm{pMBL}_4\mathrm{MASP1}^*\right]\left[\mathrm{pMBL}_3\mathrm{MASP1}_{\mathrm{zym}}\right]}{K_{\mathrm{m\ MASP1}^*}^{\mathrm{MASP1}_{\mathrm{zym}}}\left(\begin{array}{l}
1+\frac{[\mathrm{C4}]}{K_{\mathrm{m\ MASP1}^*}^{\mathrm{C4}}}+\frac{[\mathrm{fC4bC2}]}{K_{\mathrm{m\ MASP1}^*}^{\mathrm{C2}}}+\frac{[\mathrm{fC4bC4bC2}]}{K_{\mathrm{m\ MASP1}^*}^{\mathrm{C2}}}+\frac{[\mathrm{fC3bC4bC2}]}{K_{\mathrm{m\ MASP1}^*}^{\mathrm{C2}}}+\frac{[\mathrm{pC4bC2}]}{K_{\mathrm{m\ MASP1}^*}^{\mathrm{C2}}}+\frac{[\mathrm{pC4bC4bC2}]}{K_{\mathrm{m\ MASP1}^*}^{\mathrm{C2}}}\\
+\frac{[\mathrm{pC3bC4bC2}]}{K_{\mathrm{m\ MASP1}^*}^{\mathrm{C2}}}+\frac{\left[\mathrm{pMBL}_3\mathrm{MASP1}_{\mathrm{zym}}\right]}{K_{\mathrm{m\ MASP1}^*}^{\mathrm{MASP1}_{\mathrm{zym}}}}+\frac{\left[\mathrm{pMBL}_3\mathrm{MASP2}_{\mathrm{zym}}\right]}{K_{\mathrm{m\ MASP1}^*}^{\mathrm{MASP2}_{\mathrm{zym}}}}+\frac{\left[\mathrm{pMBL}_3\mathrm{MASP3}_{\mathrm{zym}}\right]}{K_{\mathrm{m\ MASP1}^*}^{\mathrm{MASP3}_{\mathrm{zym}}}}\\
+\frac{\left[\mathrm{pMBL}_4\mathrm{MASP1}_{\mathrm{zym}}\right]}{K_{\mathrm{m\ MASP1}^*}^{\mathrm{MASP1}_{\mathrm{zym}}}}+\frac{\left[\mathrm{pMBL}_4\mathrm{MASP2}_{\mathrm{zym}}\right]}{K_{\mathrm{m\ MASP1}^*}^{\mathrm{MASP2}_{\mathrm{zym}}}}+\frac{\left[\mathrm{pMBL}_4\mathrm{MASP3}_{\mathrm{zym}}\right]}{K_{\mathrm{m\ MASP1}^*}^{\mathrm{MASP3}_{\mathrm{zym}}}}+\frac{\left[\mathrm{pCLLKMASP1}_{\mathrm{zym}}\right]}{K_{\mathrm{m\ MASP1}^*}^{\mathrm{MASP1}_{\mathrm{zym}}}}\\
+\frac{\left[\mathrm{pCLLKMASP2}_{\mathrm{zym}}\right]}{K_{\mathrm{m\ MASP1}^*}^{\mathrm{MASP2}_{\mathrm{zym}}}}+\frac{\left[\mathrm{pCLLKMASP3}_{\mathrm{zym}}\right]}{K_{\mathrm{m\ MASP1}^*}^{\mathrm{MASP3}_{\mathrm{zym}}}}+\frac{\left[\mathrm{pCLK1MASP1}_{\mathrm{zym}}\right]}{K_{\mathrm{m\ MASP1}^*}^{\mathrm{MASP1}_{\mathrm{zym}}}}+\frac{\left[\mathrm{pCLK1MASP2}_{\mathrm{zym}}\right]}{K_{\mathrm{m\ MASP1}^*}^{\mathrm{MASP2}_{\mathrm{zym}}}}\\
+\frac{\left[\mathrm{pCLK1MASP3}_{\mathrm{zym}}\right]}{K_{\mathrm{m\ MASP1}^*}^{\mathrm{MASP3}_{\mathrm{zym}}}}+\frac{\left[\mathrm{pCLL1MASP1}_{\mathrm{zym}}\right]}{K_{\mathrm{m\ MASP1}^*}^{\mathrm{MASP1}_{\mathrm{zym}}}}+\frac{\left[\mathrm{pCLL1MASP2}_{\mathrm{zym}}\right]}{K_{\mathrm{m\ MASP1}^*}^{\mathrm{MASP2}_{\mathrm{zym}}}}+\frac{\left[\mathrm{pCLL1MASP3}_{\mathrm{zym}}\right]}{K_{\mathrm{m\ MASP1}^*}^{\mathrm{MASP3}_{\mathrm{zym}}}}\\
+\frac{\left[\mathrm{pLFMASP1}_{\mathrm{zym}}\right]}{K_{\mathrm{m\ MASP1}^*}^{\mathrm{MASP1}_{\mathrm{zym}}}}+\frac{\left[\mathrm{pLFMASP2}_{\mathrm{zym}}\right]}{K_{\mathrm{m\ MASP1}^*}^{\mathrm{MASP2}_{\mathrm{zym}}}}+\frac{\left[\mathrm{pLFMASP3}_{\mathrm{zym}}\right]}{K_{\mathrm{m\ MASP1}^*}^{\mathrm{MASP3}_{\mathrm{zym}}}}+\frac{\left[\mathrm{pHFMASP1}_{\mathrm{zym}}\right]}{K_{\mathrm{m\ MASP1}^*}^{\mathrm{MASP1}_{\mathrm{zym}}}}+\frac{\left[\mathrm{pHFMASP2}_{\mathrm{zym}}\right]}{K_{\mathrm{m\ MASP1}^*}^{\mathrm{MASP2}_{\mathrm{zym}}}}\\
+\frac{\left[\mathrm{pHFMASP3}_{\mathrm{zym}}\right]}{K_{\mathrm{m\ MASP1}^*}^{\mathrm{MASP3}_{\mathrm{zym}}}}+\frac{\left[\mathrm{pMFMASP1}_{\mathrm{zym}}\right]}{K_{\mathrm{m\ MASP1}^*}^{\mathrm{MASP1}_{\mathrm{zym}}}}+\frac{\left[\mathrm{pMFMASP2}_{\mathrm{zym}}\right]}{K_{\mathrm{m\ MASP1}^*}^{\mathrm{MASP2}_{\mathrm{zym}}}}+\frac{\left[\mathrm{pMFMASP3}_{\mathrm{zym}}\right]}{K_{\mathrm{m\ MASP1}^*}^{\mathrm{MASP3}_{\mathrm{zym}}}}
\end{array}\right)}
$$

$$-\frac{k_{\text{cat MASP1}^*}^{\text{MASP1}_{\text{zym}}}\left[\text{pCLLKMASP1}^*\right]\left[\text{pMBL}_3\text{MASP1}_{\text{zym}}\right]}{K_{\text{m MASP1}^*}^{\text{MASP1}_{\text{zym}}}\left(\begin{array}{l}1+\frac{[\text{C4}]}{K_{\text{m MASP1}^*}^{\text{C4}}}+\frac{[\text{fC4bC2}]}{K_{\text{m MASP1}^*}^{\text{C2}}}+\frac{[\text{fC4bC4bC2}]}{K_{\text{m MASP1}^*}^{\text{C2}}}+\frac{[\text{fC3bC4bC2}]}{K_{\text{m MASP1}^*}^{\text{C2}}}+\frac{[\text{pC4bC2}]}{K_{\text{m MASP1}^*}^{\text{C2}}}+\frac{[\text{pC4bC4bC2}]}{K_{\text{m MASP1}^*}^{\text{C2}}}\\+\frac{[\text{pC3bC4bC2}]}{K_{\text{m MASP1}^*}^{\text{C2}}}+\frac{\left[\text{pMBL}_3\text{MASP1}_{\text{zym}}\right]}{K_{\text{m MASP1}^*}^{\text{MASP1}_{\text{zym}}}}+\frac{\left[\text{pMBL}_3\text{MASP2}_{\text{zym}}\right]}{K_{\text{m MASP1}^*}^{\text{MASP2}_{\text{zym}}}}+\frac{\left[\text{pMBL}_3\text{MASP3}_{\text{zym}}\right]}{K_{\text{m MASP1}^*}^{\text{MASP3}_{\text{zym}}}}\\+\frac{\left[\text{pMBL}_4\text{MASP1}_{\text{zym}}\right]}{K_{\text{m MASP1}^*}^{\text{MASP1}_{\text{zym}}}}+\frac{\left[\text{pMBL}_4\text{MASP2}_{\text{zym}}\right]}{K_{\text{m MASP1}^*}^{\text{MASP2}_{\text{zym}}}}+\frac{\left[\text{pMBL}_4\text{MASP3}_{\text{zym}}\right]}{K_{\text{m MASP1}^*}^{\text{MASP3}_{\text{zym}}}}+\frac{\left[\text{pCLLKMASP1}_{\text{zym}}\right]}{K_{\text{m MASP1}^*}^{\text{MASP1}_{\text{zym}}}}\\+\frac{\left[\text{pCLLKMASP2}_{\text{zym}}\right]}{K_{\text{m MASP1}^*}^{\text{MASP2}_{\text{zym}}}}+\frac{\left[\text{pCLLKMASP3}_{\text{zym}}\right]}{K_{\text{m MASP1}^*}^{\text{MASP3}_{\text{zym}}}}+\frac{\left[\text{pCLK1MASP1}_{\text{zym}}\right]}{K_{\text{m MASP1}^*}^{\text{MASP1}_{\text{zym}}}}+\frac{\left[\text{pCLK1MASP2}_{\text{zym}}\right]}{K_{\text{m MASP1}^*}^{\text{MASP2}_{\text{zym}}}}\\+\frac{\left[\text{pCLK1MASP3}_{\text{zym}}\right]}{K_{\text{m MASP1}^*}^{\text{MASP3}_{\text{zym}}}}+\frac{\left[\text{pCLL1MASP1}_{\text{zym}}\right]}{K_{\text{m MASP1}^*}^{\text{MASP1}_{\text{zym}}}}+\frac{\left[\text{pCLL1MASP2}_{\text{zym}}\right]}{K_{\text{m MASP1}^*}^{\text{MASP2}_{\text{zym}}}}+\frac{\left[\text{pCLL1MASP3}_{\text{zym}}\right]}{K_{\text{m MASP1}^*}^{\text{MASP3}_{\text{zym}}}}\\+\frac{\left[\text{pLFMASP1}_{\text{zym}}\right]}{K_{\text{m MASP1}^*}^{\text{MASP1}_{\text{zym}}}}+\frac{\left[\text{pLFMASP2}_{\text{zym}}\right]}{K_{\text{m MASP1}^*}^{\text{MASP2}_{\text{zym}}}}+\frac{\left[\text{pLFMASP3}_{\text{zym}}\right]}{K_{\text{m MASP1}^*}^{\text{MASP3}_{\text{zym}}}}+\frac{\left[\text{pHFMASP1}_{\text{zym}}\right]}{K_{\text{m MASP1}^*}^{\text{MASP1}_{\text{zym}}}}+\frac{\left[\text{pHFMASP2}_{\text{zym}}\right]}{K_{\text{m MASP1}^*}^{\text{MASP2}_{\text{zym}}}}\\+\frac{\left[\text{pHFMASP3}_{\text{zym}}\right]}{K_{\text{m MASP1}^*}^{\text{MASP3}_{\text{zym}}}}+\frac{\left[\text{pMFMASP1}_{\text{zym}}\right]}{K_{\text{m MASP1}^*}^{\text{MASP1}_{\text{zym}}}}+\frac{\left[\text{pMFMASP2}_{\text{zym}}\right]}{K_{\text{m MASP1}^*}^{\text{MASP2}_{\text{zym}}}}+\frac{\left[\text{pMFMASP3}_{\text{zym}}\right]}{K_{\text{m MASP1}^*}^{\text{MASP3}_{\text{zym}}}}\end{array}\right)}$$

$$-\frac{k_{\text{cat MASP1}^*}^{\text{MASP1}_{\text{zym}}}\left[\text{pCLK1MASP1}^*\right]\left[\text{pMBL}_3\text{MASP1}_{\text{zym}}\right]}{K_{\text{m MASP1}^*}^{\text{MASP1}_{\text{zym}}}\left(\begin{array}{l}1+\frac{[\text{C4}]}{K_{\text{m MASP1}^*}^{\text{C4}}}+\frac{[\text{fC4bC2}]}{K_{\text{m MASP1}^*}^{\text{C2}}}+\frac{[\text{fC4bC4bC2}]}{K_{\text{m MASP1}^*}^{\text{C2}}}+\frac{[\text{fC3bC4bC2}]}{K_{\text{m MASP1}^*}^{\text{C2}}}+\frac{[\text{pC4bC2}]}{K_{\text{m MASP1}^*}^{\text{C2}}}+\frac{[\text{pC4bC4bC2}]}{K_{\text{m MASP1}^*}^{\text{C2}}}\\+\frac{[\text{pC3bC4bC2}]}{K_{\text{m MASP1}^*}^{\text{C2}}}+\frac{\left[\text{pMBL}_3\text{MASP1}_{\text{zym}}\right]}{K_{\text{m MASP1}^*}^{\text{MASP1}_{\text{zym}}}}+\frac{\left[\text{pMBL}_3\text{MASP2}_{\text{zym}}\right]}{K_{\text{m MASP1}^*}^{\text{MASP2}_{\text{zym}}}}+\frac{\left[\text{pMBL}_3\text{MASP3}_{\text{zym}}\right]}{K_{\text{m MASP1}^*}^{\text{MASP3}_{\text{zym}}}}\\+\frac{\left[\text{pMBL}_4\text{MASP1}_{\text{zym}}\right]}{K_{\text{m MASP1}^*}^{\text{MASP1}_{\text{zym}}}}+\frac{\left[\text{pMBL}_4\text{MASP2}_{\text{zym}}\right]}{K_{\text{m MASP1}^*}^{\text{MASP2}_{\text{zym}}}}+\frac{\left[\text{pMBL}_4\text{MASP3}_{\text{zym}}\right]}{K_{\text{m MASP1}^*}^{\text{MASP3}_{\text{zym}}}}+\frac{\left[\text{pCLLKMASP1}_{\text{zym}}\right]}{K_{\text{m MASP1}^*}^{\text{MASP1}_{\text{zym}}}}\\+\frac{\left[\text{pCLLKMASP2}_{\text{zym}}\right]}{K_{\text{m MASP1}^*}^{\text{MASP2}_{\text{zym}}}}+\frac{\left[\text{pCLLKMASP3}_{\text{zym}}\right]}{K_{\text{m MASP1}^*}^{\text{MASP3}_{\text{zym}}}}+\frac{\left[\text{pCLK1MASP1}_{\text{zym}}\right]}{K_{\text{m MASP1}^*}^{\text{MASP1}_{\text{zym}}}}+\frac{\left[\text{pCLK1MASP2}_{\text{zym}}\right]}{K_{\text{m MASP1}^*}^{\text{MASP2}_{\text{zym}}}}\\+\frac{\left[\text{pCLK1MASP3}_{\text{zym}}\right]}{K_{\text{m MASP1}^*}^{\text{MASP3}_{\text{zym}}}}+\frac{\left[\text{pCLL1MASP1}_{\text{zym}}\right]}{K_{\text{m MASP1}^*}^{\text{MASP1}_{\text{zym}}}}+\frac{\left[\text{pCLL1MASP2}_{\text{zym}}\right]}{K_{\text{m MASP1}^*}^{\text{MASP2}_{\text{zym}}}}+\frac{\left[\text{pCLL1MASP3}_{\text{zym}}\right]}{K_{\text{m MASP1}^*}^{\text{MASP3}_{\text{zym}}}}\\+\frac{\left[\text{pLFMASP1}_{\text{zym}}\right]}{K_{\text{m MASP1}^*}^{\text{MASP1}_{\text{zym}}}}+\frac{\left[\text{pLFMASP2}_{\text{zym}}\right]}{K_{\text{m MASP1}^*}^{\text{MASP2}_{\text{zym}}}}+\frac{\left[\text{pLFMASP3}_{\text{zym}}\right]}{K_{\text{m MASP1}^*}^{\text{MASP3}_{\text{zym}}}}+\frac{\left[\text{pHFMASP1}_{\text{zym}}\right]}{K_{\text{m MASP1}^*}^{\text{MASP1}_{\text{zym}}}}+\frac{\left[\text{pHFMASP2}_{\text{zym}}\right]}{K_{\text{m MASP1}^*}^{\text{MASP2}_{\text{zym}}}}\\+\frac{\left[\text{pHFMASP3}_{\text{zym}}\right]}{K_{\text{m MASP1}^*}^{\text{MASP3}_{\text{zym}}}}+\frac{\left[\text{pMFMASP1}_{\text{zym}}\right]}{K_{\text{m MASP1}^*}^{\text{MASP1}_{\text{zym}}}}+\frac{\left[\text{pMFMASP2}_{\text{zym}}\right]}{K_{\text{m MASP1}^*}^{\text{MASP2}_{\text{zym}}}}+\frac{\left[\text{pMFMASP3}_{\text{zym}}\right]}{K_{\text{m MASP1}^*}^{\text{MASP3}_{\text{zym}}}}\end{array}\right)}$$

$$
-\frac{k_{\mathrm{cat\ MASP1^{*}}}^{\mathrm{MASP1_{zym}}}\left[\mathrm{pCLL1MASP1^{*}}\right]\left[\mathrm{pMBL_{3}MASP1_{zym}}\right]}{K_{\mathrm{m\ MASP1^{*}}}^{\mathrm{MASP1_{zym}}}\left(\begin{array}{l}
1+\frac{[\mathrm{C4}]}{K_{\mathrm{m\ MASP1^{*}}}^{\mathrm{C4}}}+\frac{[\mathrm{fC4bC2}]}{K_{\mathrm{m\ MASP1^{*}}}^{\mathrm{C2}}}+\frac{[\mathrm{fC4bC4bC2}]}{K_{\mathrm{m\ MASP1^{*}}}^{\mathrm{C2}}}+\frac{[\mathrm{fC3bC4bC2}]}{K_{\mathrm{m\ MASP1^{*}}}^{\mathrm{C2}}}+\frac{[\mathrm{pC4bC2}]}{K_{\mathrm{m\ MASP1^{*}}}^{\mathrm{C2}}}+\frac{[\mathrm{pC4bC4bC2}]}{K_{\mathrm{m\ MASP1^{*}}}^{\mathrm{C2}}}\\
+\frac{[\mathrm{pC3bC4bC2}]}{K_{\mathrm{m\ MASP1^{*}}}^{\mathrm{C2}}}+\frac{\left[\mathrm{pMBL_{3}MASP1_{zym}}\right]}{K_{\mathrm{m\ MASP1^{*}}}^{\mathrm{MASP1_{zym}}}}+\frac{\left[\mathrm{pMBL_{3}MASP2_{zym}}\right]}{K_{\mathrm{m\ MASP1^{*}}}^{\mathrm{MASP2_{zym}}}}+\frac{\left[\mathrm{pMBL_{3}MASP3_{zym}}\right]}{K_{\mathrm{m\ MASP1^{*}}}^{\mathrm{MASP3_{zym}}}}\\
+\frac{\left[\mathrm{pMBL_{4}MASP1_{zym}}\right]}{K_{\mathrm{m\ MASP1^{*}}}^{\mathrm{MASP1_{zym}}}}+\frac{\left[\mathrm{pMBL_{4}MASP2_{zym}}\right]}{K_{\mathrm{m\ MASP1^{*}}}^{\mathrm{MASP2_{zym}}}}+\frac{\left[\mathrm{pMBL_{4}MASP3_{zym}}\right]}{K_{\mathrm{m\ MASP1^{*}}}^{\mathrm{MASP3_{zym}}}}+\frac{\left[\mathrm{pCLLKMASP1_{zym}}\right]}{K_{\mathrm{m\ MASP1^{*}}}^{\mathrm{MASP1_{zym}}}}\\
+\frac{\left[\mathrm{pCLLKMASP2_{zym}}\right]}{K_{\mathrm{m\ MASP1^{*}}}^{\mathrm{MASP2_{zym}}}}+\frac{\left[\mathrm{pCLLKMASP3_{zym}}\right]}{K_{\mathrm{m\ MASP1^{*}}}^{\mathrm{MASP3_{zym}}}}+\frac{\left[\mathrm{pCLK1MASP1_{zym}}\right]}{K_{\mathrm{m\ MASP1^{*}}}^{\mathrm{MASP1_{zym}}}}+\frac{\left[\mathrm{pCLK1MASP2_{zym}}\right]}{K_{\mathrm{m\ MASP1^{*}}}^{\mathrm{MASP2_{zym}}}}\\
+\frac{\left[\mathrm{pCLK1MASP3_{zym}}\right]}{K_{\mathrm{m\ MASP1^{*}}}^{\mathrm{MASP3_{zym}}}}+\frac{\left[\mathrm{pCLL1MASP1_{zym}}\right]}{K_{\mathrm{m\ MASP1^{*}}}^{\mathrm{MASP1_{zym}}}}+\frac{\left[\mathrm{pCLL1MASP2_{zym}}\right]}{K_{\mathrm{m\ MASP1^{*}}}^{\mathrm{MASP2_{zym}}}}+\frac{\left[\mathrm{pCLL1MASP3_{zym}}\right]}{K_{\mathrm{m\ MASP1^{*}}}^{\mathrm{MASP3_{zym}}}}\\
+\frac{\left[\mathrm{pLFMASP1_{zym}}\right]}{K_{\mathrm{m\ MASP1^{*}}}^{\mathrm{MASP1_{zym}}}}+\frac{\left[\mathrm{pLFMASP2_{zym}}\right]}{K_{\mathrm{m\ MASP1^{*}}}^{\mathrm{MASP2_{zym}}}}+\frac{\left[\mathrm{pLFMASP3_{zym}}\right]}{K_{\mathrm{m\ MASP1^{*}}}^{\mathrm{MASP3_{zym}}}}+\frac{\left[\mathrm{pHFMASP1_{zym}}\right]}{K_{\mathrm{m\ MASP1^{*}}}^{\mathrm{MASP1_{zym}}}}+\frac{\left[\mathrm{pHFMASP2_{zym}}\right]}{K_{\mathrm{m\ MASP1^{*}}}^{\mathrm{MASP2_{zym}}}}\\
+\frac{\left[\mathrm{pHFMASP3_{zym}}\right]}{K_{\mathrm{m\ MASP1^{*}}}^{\mathrm{MASP3_{zym}}}}+\frac{\left[\mathrm{pMFMASP1_{zym}}\right]}{K_{\mathrm{m\ MASP1^{*}}}^{\mathrm{MASP1_{zym}}}}+\frac{\left[\mathrm{pMFMASP2_{zym}}\right]}{K_{\mathrm{m\ MASP1^{*}}}^{\mathrm{MASP2_{zym}}}}+\frac{\left[\mathrm{pMFMASP3_{zym}}\right]}{K_{\mathrm{m\ MASP1^{*}}}^{\mathrm{MASP3_{zym}}}}
\end{array}\right)}
$$

$$
-\frac{k_{\mathrm{cat\ MASP1^{*}}}^{\mathrm{MASP1_{zym}}}\left[\mathrm{pLFMASP1^{*}}\right]\left[\mathrm{pMBL_{3}MASP1_{zym}}\right]}{K_{\mathrm{m\ MASP1^{*}}}^{\mathrm{MASP1_{zym}}}\left(\begin{array}{l}
1+\frac{[\mathrm{C4}]}{K_{\mathrm{m\ MASP1^{*}}}^{\mathrm{C4}}}+\frac{[\mathrm{fC4bC2}]}{K_{\mathrm{m\ MASP1^{*}}}^{\mathrm{C2}}}+\frac{[\mathrm{fC4bC4bC2}]}{K_{\mathrm{m\ MASP1^{*}}}^{\mathrm{C2}}}+\frac{[\mathrm{fC3bC4bC2}]}{K_{\mathrm{m\ MASP1^{*}}}^{\mathrm{C2}}}+\frac{[\mathrm{pC4bC2}]}{K_{\mathrm{m\ MASP1^{*}}}^{\mathrm{C2}}}+\frac{[\mathrm{pC4bC4bC2}]}{K_{\mathrm{m\ MASP1^{*}}}^{\mathrm{C2}}}\\
+\frac{[\mathrm{pC3bC4bC2}]}{K_{\mathrm{m\ MASP1^{*}}}^{\mathrm{C2}}}+\frac{\left[\mathrm{pMBL_{3}MASP1_{zym}}\right]}{K_{\mathrm{m\ MASP1^{*}}}^{\mathrm{MASP1_{zym}}}}+\frac{\left[\mathrm{pMBL_{3}MASP2_{zym}}\right]}{K_{\mathrm{m\ MASP1^{*}}}^{\mathrm{MASP2_{zym}}}}+\frac{\left[\mathrm{pMBL_{3}MASP3_{zym}}\right]}{K_{\mathrm{m\ MASP1^{*}}}^{\mathrm{MASP3_{zym}}}}\\
+\frac{\left[\mathrm{pMBL_{4}MASP1_{zym}}\right]}{K_{\mathrm{m\ MASP1^{*}}}^{\mathrm{MASP1_{zym}}}}+\frac{\left[\mathrm{pMBL_{4}MASP2_{zym}}\right]}{K_{\mathrm{m\ MASP1^{*}}}^{\mathrm{MASP2_{zym}}}}+\frac{\left[\mathrm{pMBL_{4}MASP3_{zym}}\right]}{K_{\mathrm{m\ MASP1^{*}}}^{\mathrm{MASP3_{zym}}}}+\frac{\left[\mathrm{pCLLKMASP1_{zym}}\right]}{K_{\mathrm{m\ MASP1^{*}}}^{\mathrm{MASP1_{zym}}}}\\
+\frac{\left[\mathrm{pCLLKMASP2_{zym}}\right]}{K_{\mathrm{m\ MASP1^{*}}}^{\mathrm{MASP2_{zym}}}}+\frac{\left[\mathrm{pCLLKMASP3_{zym}}\right]}{K_{\mathrm{m\ MASP1^{*}}}^{\mathrm{MASP3_{zym}}}}+\frac{\left[\mathrm{pCLK1MASP1_{zym}}\right]}{K_{\mathrm{m\ MASP1^{*}}}^{\mathrm{MASP1_{zym}}}}+\frac{\left[\mathrm{pCLK1MASP2_{zym}}\right]}{K_{\mathrm{m\ MASP1^{*}}}^{\mathrm{MASP2_{zym}}}}\\
+\frac{\left[\mathrm{pCLK1MASP3_{zym}}\right]}{K_{\mathrm{m\ MASP1^{*}}}^{\mathrm{MASP3_{zym}}}}+\frac{\left[\mathrm{pCLL1MASP1_{zym}}\right]}{K_{\mathrm{m\ MASP1^{*}}}^{\mathrm{MASP1_{zym}}}}+\frac{\left[\mathrm{pCLL1MASP2_{zym}}\right]}{K_{\mathrm{m\ MASP1^{*}}}^{\mathrm{MASP2_{zym}}}}+\frac{\left[\mathrm{pCLL1MASP3_{zym}}\right]}{K_{\mathrm{m\ MASP1^{*}}}^{\mathrm{MASP3_{zym}}}}\\
+\frac{\left[\mathrm{pLFMASP1_{zym}}\right]}{K_{\mathrm{m\ MASP1^{*}}}^{\mathrm{MASP1_{zym}}}}+\frac{\left[\mathrm{pLFMASP2_{zym}}\right]}{K_{\mathrm{m\ MASP1^{*}}}^{\mathrm{MASP2_{zym}}}}+\frac{\left[\mathrm{pLFMASP3_{zym}}\right]}{K_{\mathrm{m\ MASP1^{*}}}^{\mathrm{MASP3_{zym}}}}+\frac{\left[\mathrm{pHFMASP1_{zym}}\right]}{K_{\mathrm{m\ MASP1^{*}}}^{\mathrm{MASP1_{zym}}}}+\frac{\left[\mathrm{pHFMASP2_{zym}}\right]}{K_{\mathrm{m\ MASP1^{*}}}^{\mathrm{MASP2_{zym}}}}\\
+\frac{\left[\mathrm{pHFMASP3_{zym}}\right]}{K_{\mathrm{m\ MASP1^{*}}}^{\mathrm{MASP3_{zym}}}}+\frac{\left[\mathrm{pMFMASP1_{zym}}\right]}{K_{\mathrm{m\ MASP1^{*}}}^{\mathrm{MASP1_{zym}}}}+\frac{\left[\mathrm{pMFMASP2_{zym}}\right]}{K_{\mathrm{m\ MASP1^{*}}}^{\mathrm{MASP2_{zym}}}}+\frac{\left[\mathrm{pMFMASP3_{zym}}\right]}{K_{\mathrm{m\ MASP1^{*}}}^{\mathrm{MASP3_{zym}}}}
\end{array}\right)}
$$

$$
-\frac{k_{\mathrm{cat\,MASP1^*}}^{\mathrm{MASP1_{zym}}}\left[\mathrm{pHFMASP1^*}\right]\left[\mathrm{pMBL_3MASP1_{zym}}\right]}{K_{\mathrm{m\,MASP1^*}}^{\mathrm{MASP1_{zym}}}\left(\begin{aligned}
&1+\frac{[\mathrm{C4}]}{K_{\mathrm{m\,MASP1^*}}^{\mathrm{C4}}}+\frac{[\mathrm{fC4bC2}]}{K_{\mathrm{m\,MASP1^*}}^{\mathrm{C2}}}+\frac{[\mathrm{fC4bC4bC2}]}{K_{\mathrm{m\,MASP1^*}}^{\mathrm{C2}}}+\frac{[\mathrm{fC3bC4bC2}]}{K_{\mathrm{m\,MASP1^*}}^{\mathrm{C2}}}+\frac{[\mathrm{pC4bC2}]}{K_{\mathrm{m\,MASP1^*}}^{\mathrm{C2}}}+\frac{[\mathrm{pC4bC4bC2}]}{K_{\mathrm{m\,MASP1^*}}^{\mathrm{C2}}}\\
&+\frac{[\mathrm{pC3bC4bC2}]}{K_{\mathrm{m\,MASP1^*}}^{\mathrm{C2}}}+\frac{[\mathrm{pMBL_3MASP1_{zym}}]}{K_{\mathrm{m\,MASP1^*}}^{\mathrm{MASP1_{zym}}}}+\frac{[\mathrm{pMBL_3MASP2_{zym}}]}{K_{\mathrm{m\,MASP1^*}}^{\mathrm{MASP2_{zym}}}}+\frac{[\mathrm{pMBL_3MASP3_{zym}}]}{K_{\mathrm{m\,MASP1^*}}^{\mathrm{MASP3_{zym}}}}\\
&+\frac{[\mathrm{pMBL_4MASP1_{zym}}]}{K_{\mathrm{m\,MASP1^*}}^{\mathrm{MASP1_{zym}}}}+\frac{[\mathrm{pMBL_4MASP2_{zym}}]}{K_{\mathrm{m\,MASP1^*}}^{\mathrm{MASP2_{zym}}}}+\frac{[\mathrm{pMBL_4MASP3_{zym}}]}{K_{\mathrm{m\,MASP1^*}}^{\mathrm{MASP3_{zym}}}}+\frac{[\mathrm{pCLLKMASP1_{zym}}]}{K_{\mathrm{m\,MASP1^*}}^{\mathrm{MASP1_{zym}}}}\\
&+\frac{[\mathrm{pCLLKMASP2_{zym}}]}{K_{\mathrm{m\,MASP1^*}}^{\mathrm{MASP2_{zym}}}}+\frac{[\mathrm{pCLLKMASP3_{zym}}]}{K_{\mathrm{m\,MASP1^*}}^{\mathrm{MASP3_{zym}}}}+\frac{[\mathrm{pCLK1MASP1_{zym}}]}{K_{\mathrm{m\,MASP1^*}}^{\mathrm{MASP1_{zym}}}}+\frac{[\mathrm{pCLK1MASP2_{zym}}]}{K_{\mathrm{m\,MASP1^*}}^{\mathrm{MASP2_{zym}}}}\\
&+\frac{[\mathrm{pCLK1MASP3_{zym}}]}{K_{\mathrm{m\,MASP1^*}}^{\mathrm{MASP3_{zym}}}}+\frac{[\mathrm{pCLL1MASP1_{zym}}]}{K_{\mathrm{m\,MASP1^*}}^{\mathrm{MASP1_{zym}}}}+\frac{[\mathrm{pCLL1MASP2_{zym}}]}{K_{\mathrm{m\,MASP1^*}}^{\mathrm{MASP2_{zym}}}}+\frac{[\mathrm{pCLL1MASP3_{zym}}]}{K_{\mathrm{m\,MASP1^*}}^{\mathrm{MASP3_{zym}}}}\\
&+\frac{[\mathrm{pLFMASP1_{zym}}]}{K_{\mathrm{m\,MASP1^*}}^{\mathrm{MASP1_{zym}}}}+\frac{[\mathrm{pLFMASP2_{zym}}]}{K_{\mathrm{m\,MASP1^*}}^{\mathrm{MASP2_{zym}}}}+\frac{[\mathrm{pLFMASP3_{zym}}]}{K_{\mathrm{m\,MASP1^*}}^{\mathrm{MASP3_{zym}}}}+\frac{[\mathrm{pHFMASP1_{zym}}]}{K_{\mathrm{m\,MASP1^*}}^{\mathrm{MASP1_{zym}}}}+\frac{[\mathrm{pHFMASP2_{zym}}]}{K_{\mathrm{m\,MASP1^*}}^{\mathrm{MASP2_{zym}}}}\\
&+\frac{[\mathrm{pHFMASP3_{zym}}]}{K_{\mathrm{m\,MASP1^*}}^{\mathrm{MASP3_{zym}}}}+\frac{[\mathrm{pMFMASP1_{zym}}]}{K_{\mathrm{m\,MASP1^*}}^{\mathrm{MASP1_{zym}}}}+\frac{[\mathrm{pMFMASP2_{zym}}]}{K_{\mathrm{m\,MASP1^*}}^{\mathrm{MASP2_{zym}}}}+\frac{[\mathrm{pMFMASP3_{zym}}]}{K_{\mathrm{m\,MASP1^*}}^{\mathrm{MASP3_{zym}}}}
\end{aligned}\right)}
$$

$$
-\frac{k_{\mathrm{cat\,MASP1^*}}^{\mathrm{MASP1_{zym}}}\left[\mathrm{pMFMASP1^*}\right]\left[\mathrm{pMBL_3MASP1_{zym}}\right]}{K_{\mathrm{m\,MASP1^*}}^{\mathrm{MASP1_{zym}}}\left(\begin{aligned}
&1+\frac{[\mathrm{C4}]}{K_{\mathrm{m\,MASP1^*}}^{\mathrm{C4}}}+\frac{[\mathrm{fC4bC2}]}{K_{\mathrm{m\,MASP1^*}}^{\mathrm{C2}}}+\frac{[\mathrm{fC4bC4bC2}]}{K_{\mathrm{m\,MASP1^*}}^{\mathrm{C2}}}+\frac{[\mathrm{fC3bC4bC2}]}{K_{\mathrm{m\,MASP1^*}}^{\mathrm{C2}}}+\frac{[\mathrm{pC4bC2}]}{K_{\mathrm{m\,MASP1^*}}^{\mathrm{C2}}}+\frac{[\mathrm{pC4bC4bC2}]}{K_{\mathrm{m\,MASP1^*}}^{\mathrm{C2}}}\\
&+\frac{[\mathrm{pC3bC4bC2}]}{K_{\mathrm{m\,MASP1^*}}^{\mathrm{C2}}}+\frac{[\mathrm{pMBL_3MASP1_{zym}}]}{K_{\mathrm{m\,MASP1^*}}^{\mathrm{MASP1_{zym}}}}+\frac{[\mathrm{pMBL_3MASP2_{zym}}]}{K_{\mathrm{m\,MASP1^*}}^{\mathrm{MASP2_{zym}}}}+\frac{[\mathrm{pMBL_3MASP3_{zym}}]}{K_{\mathrm{m\,MASP1^*}}^{\mathrm{MASP3_{zym}}}}\\
&+\frac{[\mathrm{pMBL_4MASP1_{zym}}]}{K_{\mathrm{m\,MASP1^*}}^{\mathrm{MASP1_{zym}}}}+\frac{[\mathrm{pMBL_4MASP2_{zym}}]}{K_{\mathrm{m\,MASP1^*}}^{\mathrm{MASP2_{zym}}}}+\frac{[\mathrm{pMBL_4MASP3_{zym}}]}{K_{\mathrm{m\,MASP1^*}}^{\mathrm{MASP3_{zym}}}}+\frac{[\mathrm{pCLLKMASP1_{zym}}]}{K_{\mathrm{m\,MASP1^*}}^{\mathrm{MASP1_{zym}}}}\\
&+\frac{[\mathrm{pCLLKMASP2_{zym}}]}{K_{\mathrm{m\,MASP1^*}}^{\mathrm{MASP2_{zym}}}}+\frac{[\mathrm{pCLLKMASP3_{zym}}]}{K_{\mathrm{m\,MASP1^*}}^{\mathrm{MASP3_{zym}}}}+\frac{[\mathrm{pCLK1MASP1_{zym}}]}{K_{\mathrm{m\,MASP1^*}}^{\mathrm{MASP1_{zym}}}}+\frac{[\mathrm{pCLK1MASP2_{zym}}]}{K_{\mathrm{m\,MASP1^*}}^{\mathrm{MASP2_{zym}}}}\\
&+\frac{[\mathrm{pCLK1MASP3_{zym}}]}{K_{\mathrm{m\,MASP1^*}}^{\mathrm{MASP3_{zym}}}}+\frac{[\mathrm{pCLL1MASP1_{zym}}]}{K_{\mathrm{m\,MASP1^*}}^{\mathrm{MASP1_{zym}}}}+\frac{[\mathrm{pCLL1MASP2_{zym}}]}{K_{\mathrm{m\,MASP1^*}}^{\mathrm{MASP2_{zym}}}}+\frac{[\mathrm{pCLL1MASP3_{zym}}]}{K_{\mathrm{m\,MASP1^*}}^{\mathrm{MASP3_{zym}}}}\\
&+\frac{[\mathrm{pLFMASP1_{zym}}]}{K_{\mathrm{m\,MASP1^*}}^{\mathrm{MASP1_{zym}}}}+\frac{[\mathrm{pLFMASP2_{zym}}]}{K_{\mathrm{m\,MASP1^*}}^{\mathrm{MASP2_{zym}}}}+\frac{[\mathrm{pLFMASP3_{zym}}]}{K_{\mathrm{m\,MASP1^*}}^{\mathrm{MASP3_{zym}}}}+\frac{[\mathrm{pHFMASP1_{zym}}]}{K_{\mathrm{m\,MASP1^*}}^{\mathrm{MASP1_{zym}}}}+\frac{[\mathrm{pHFMASP2_{zym}}]}{K_{\mathrm{m\,MASP1^*}}^{\mathrm{MASP2_{zym}}}}\\
&+\frac{[\mathrm{pHFMASP3_{zym}}]}{K_{\mathrm{m\,MASP1^*}}^{\mathrm{MASP3_{zym}}}}+\frac{[\mathrm{pMFMASP1_{zym}}]}{K_{\mathrm{m\,MASP1^*}}^{\mathrm{MASP1_{zym}}}}+\frac{[\mathrm{pMFMASP2_{zym}}]}{K_{\mathrm{m\,MASP1^*}}^{\mathrm{MASP2_{zym}}}}+\frac{[\mathrm{pMFMASP3_{zym}}]}{K_{\mathrm{m\,MASP1^*}}^{\mathrm{MASP3_{zym}}}}
\end{aligned}\right)}
$$

$$
-\frac{k_{\text{cat MASP1}^*}^{\text{MASP1}_{\text{zym}}}\left[\text{pPTX3LFMASP1}^*\right]\left[\text{pMBL}_3\text{MASP1}_{\text{zym}}\right]}{K_{\text{m MASP1}^*}^{\text{MASP1}_{\text{zym}}}\left(\begin{array}{l}1+\frac{[\text{C4}]}{K_{\text{m MASP1}^*}^{\text{C4}}}+\frac{[\text{fC4bC2}]}{K_{\text{m MASP1}^*}^{\text{C2}}}+\frac{[\text{fC4bC4bC2}]}{K_{\text{m MASP1}^*}^{\text{C2}}}+\frac{[\text{fC3bC4bC2}]}{K_{\text{m MASP1}^*}^{\text{C2}}}+\frac{[\text{pC4bC2}]}{K_{\text{m MASP1}^*}^{\text{C2}}}+\frac{[\text{pC4bC4bC2}]}{K_{\text{m MASP1}^*}^{\text{C2}}}\\
+\frac{[\text{pC3bC4bC2}]}{K_{\text{m MASP1}^*}^{\text{C2}}}+\frac{[\text{pMBL}_3\text{MASP1}_{\text{zym}}]}{K_{\text{m MASP1}^*}^{\text{MASP1}_{\text{zym}}}}+\frac{[\text{pMBL}_3\text{MASP2}_{\text{zym}}]}{K_{\text{m MASP1}^*}^{\text{MASP2}_{\text{zym}}}}+\frac{[\text{pMBL}_3\text{MASP3}_{\text{zym}}]}{K_{\text{m MASP1}^*}^{\text{MASP3}_{\text{zym}}}}\\
+\frac{[\text{pMBL}_4\text{MASP1}_{\text{zym}}]}{K_{\text{m MASP1}^*}^{\text{MASP1}_{\text{zym}}}}+\frac{[\text{pMBL}_4\text{MASP2}_{\text{zym}}]}{K_{\text{m MASP1}^*}^{\text{MASP2}_{\text{zym}}}}+\frac{[\text{pMBL}_4\text{MASP3}_{\text{zym}}]}{K_{\text{m MASP1}^*}^{\text{MASP3}_{\text{zym}}}}+\frac{[\text{pCLLKMASP1}_{\text{zym}}]}{K_{\text{m MASP1}^*}^{\text{MASP1}_{\text{zym}}}}\\
+\frac{[\text{pCLLKMASP2}_{\text{zym}}]}{K_{\text{m MASP1}^*}^{\text{MASP2}_{\text{zym}}}}+\frac{[\text{pCLLKMASP3}_{\text{zym}}]}{K_{\text{m MASP1}^*}^{\text{MASP3}_{\text{zym}}}}+\frac{[\text{pCLK1MASP1}_{\text{zym}}]}{K_{\text{m MASP1}^*}^{\text{MASP1}_{\text{zym}}}}+\frac{[\text{pCLK1MASP2}_{\text{zym}}]}{K_{\text{m MASP1}^*}^{\text{MASP2}_{\text{zym}}}}\\
+\frac{[\text{pCLK1MASP3}_{\text{zym}}]}{K_{\text{m MASP1}^*}^{\text{MASP3}_{\text{zym}}}}+\frac{[\text{pCLL1MASP1}_{\text{zym}}]}{K_{\text{m MASP1}^*}^{\text{MASP1}_{\text{zym}}}}+\frac{[\text{pCLL1MASP2}_{\text{zym}}]}{K_{\text{m MASP1}^*}^{\text{MASP2}_{\text{zym}}}}+\frac{[\text{pCLL1MASP3}_{\text{zym}}]}{K_{\text{m MASP1}^*}^{\text{MASP3}_{\text{zym}}}}\\
+\frac{[\text{pLFMASP1}_{\text{zym}}]}{K_{\text{m MASP1}^*}^{\text{MASP1}_{\text{zym}}}}+\frac{[\text{pLFMASP2}_{\text{zym}}]}{K_{\text{m MASP1}^*}^{\text{MASP2}_{\text{zym}}}}+\frac{[\text{pLFMASP3}_{\text{zym}}]}{K_{\text{m MASP1}^*}^{\text{MASP3}_{\text{zym}}}}+\frac{[\text{pHFMASP1}_{\text{zym}}]}{K_{\text{m MASP1}^*}^{\text{MASP1}_{\text{zym}}}}+\frac{[\text{pHFMASP2}_{\text{zym}}]}{K_{\text{m MASP1}^*}^{\text{MASP2}_{\text{zym}}}}\\
+\frac{[\text{pHFMASP3}_{\text{zym}}]}{K_{\text{m MASP1}^*}^{\text{MASP3}_{\text{zym}}}}+\frac{[\text{pMFMASP1}_{\text{zym}}]}{K_{\text{m MASP1}^*}^{\text{MASP1}_{\text{zym}}}}+\frac{[\text{pMFMASP2}_{\text{zym}}]}{K_{\text{m MASP1}^*}^{\text{MASP2}_{\text{zym}}}}+\frac{[\text{pMFMASP3}_{\text{zym}}]}{K_{\text{m MASP1}^*}^{\text{MASP3}_{\text{zym}}}}\\
+\frac{[\text{pPTX3LFMASP1}_{\text{zym}}]}{K_{\text{m MASP1}^*}^{\text{MASP1}_{\text{zym}}}}+\frac{[\text{pPXT3LFMASP2}_{\text{zym}}]}{K_{\text{m MASP1}^*}^{\text{MASP2}_{\text{zym}}}}+\frac{[\text{pPTX3LFMASP3}_{\text{zym}}]}{K_{\text{m MASP1}^*}^{\text{MASP3}_{\text{zym}}}}\end{array}\right)}
$$

$$
-\frac{k_{\text{cat MASP2}^*}^{\text{MASP1}_{\text{zym}}}\left[\text{pMBL}_3\text{MASP2}^*\right]\left[\text{pMBL}_3\text{MASP1}_{\text{zym}}\right]}{K_{\text{m MASP2}^*}^{\text{MASP1}_{\text{zym}}}\left(\begin{array}{l}1+\frac{[\text{C4}]}{K_{\text{m MASP2}^*}^{\text{C4}}}+\frac{[\text{fC4bC2}]}{K_{\text{m MASP2}^*}^{\text{C2}}}+\frac{[\text{fC4bC4bC2}]}{K_{\text{m MASP2}^*}^{\text{C2}}}+\frac{[\text{fC3bC4bC2}]}{K_{\text{m MASP2}^*}^{\text{C2}}}+\frac{[\text{pC4bC2}]}{K_{\text{m MASP2}^*}^{\text{C2}}}+\frac{[\text{pC4bC4bC2}]}{K_{\text{m MASP2}^*}^{\text{C2}}}\\
+\frac{[\text{pC3bC4bC2}]}{K_{\text{m MASP2}^*}^{\text{C2}}}+\frac{[\text{pMBL}_3\text{MASP1}_{\text{zym}}]}{K_{\text{m MASP2}^*}^{\text{MASP1}_{\text{zym}}}}+\frac{[\text{pMBL}_3\text{MASP2}_{\text{zym}}]}{K_{\text{m MASP2}^*}^{\text{MASP2}_{\text{zym}}}}+\frac{[\text{pMBL}_4\text{MASP1}_{\text{zym}}]}{K_{\text{m MASP2}^*}^{\text{MASP1}_{\text{zym}}}}\\
+\frac{[\text{pMBL}_4\text{MASP2}_{\text{zym}}]}{K_{\text{m MASP2}^*}^{\text{MASP2}_{\text{zym}}}}+\frac{[\text{pCLLKMASP1}_{\text{zym}}]}{K_{\text{m MASP2}^*}^{\text{MASP1}_{\text{zym}}}}+\frac{[\text{pCLLKMASP2}_{\text{zym}}]}{K_{\text{m MASP2}^*}^{\text{MASP2}_{\text{zym}}}}+\frac{[\text{pCLK1MASP1}_{\text{zym}}]}{K_{\text{m MASP2}^*}^{\text{MASP1}_{\text{zym}}}}\\
+\frac{[\text{pCLK1MASP2}_{\text{zym}}]}{K_{\text{m MASP2}^*}^{\text{MASP2}_{\text{zym}}}}+\frac{[\text{pCLL1MASP1}_{\text{zym}}]}{K_{\text{m MASP2}^*}^{\text{MASP1}_{\text{zym}}}}+\frac{[\text{pCLL1MASP2}_{\text{zym}}]}{K_{\text{m MASP2}^*}^{\text{MASP2}_{\text{zym}}}}+\frac{[\text{pLFMASP1}_{\text{zym}}]}{K_{\text{m MASP2}^*}^{\text{MASP1}_{\text{zym}}}}\\
+\frac{[\text{pLFMASP2}_{\text{zym}}]}{K_{\text{m MASP2}^*}^{\text{MASP2}_{\text{zym}}}}+\frac{[\text{pHFMASP1}_{\text{zym}}]}{K_{\text{m MASP2}^*}^{\text{MASP1}_{\text{zym}}}}+\frac{[\text{pHFMASP2}_{\text{zym}}]}{K_{\text{m MASP2}^*}^{\text{MASP2}_{\text{zym}}}}+\frac{[\text{pMFMASP1}_{\text{zym}}]}{K_{\text{m MASP2}^*}^{\text{MASP1}_{\text{zym}}}}+\frac{[\text{pMFMASP2}_{\text{zym}}]}{K_{\text{m MASP2}^*}^{\text{MASP2}_{\text{zym}}}}\end{array}\right)}
$$

$$-\frac{k_{\mathrm{cat\,MASP2^*}}^{\mathrm{MASP1_{zym}}}\left[\mathrm{pMBL_4MASP2^*}\right]\left[\mathrm{pMBL_3MASP1_{zym}}\right]}{K_{\mathrm{m\,MASP2^*}}^{\mathrm{MASP1_{zym}}}\left(\begin{array}{l}1+\frac{[\mathrm{C4}]}{K_{\mathrm{m\,MASP2^*}}^{\mathrm{C4}}}+\frac{[\mathrm{fC4bC2}]}{K_{\mathrm{m\,MASP2^*}}^{\mathrm{C2}}}+\frac{[\mathrm{fC4bC4bC2}]}{K_{\mathrm{m\,MASP2^*}}^{\mathrm{C2}}}+\frac{[\mathrm{fC3bC4bC2}]}{K_{\mathrm{m\,MASP2^*}}^{\mathrm{C2}}}+\frac{[\mathrm{pC4bC2}]}{K_{\mathrm{m\,MASP2^*}}^{\mathrm{C2}}}+\frac{[\mathrm{pC4bC4bC2}]}{K_{\mathrm{m\,MASP2^*}}^{\mathrm{C2}}}\\ +\frac{[\mathrm{pC3bC4bC2}]}{K_{\mathrm{m\,MASP2^*}}^{\mathrm{C2}}}+\frac{[\mathrm{pMBL_3MASP1_{zym}}]}{K_{\mathrm{m\,MASP2^*}}^{\mathrm{MASP1_{zym}}}}+\frac{[\mathrm{pMBL_3MASP2_{zym}}]}{K_{\mathrm{m\,MASP2^*}}^{\mathrm{MASP2_{zym}}}}+\frac{[\mathrm{pMBL_4MASP1_{zym}}]}{K_{\mathrm{m\,MASP2^*}}^{\mathrm{MASP1_{zym}}}}\\ +\frac{[\mathrm{pMBL_4MASP2_{zym}}]}{K_{\mathrm{m\,MASP2^*}}^{\mathrm{MASP2_{zym}}}}+\frac{[\mathrm{pCLLKMASP1_{zym}}]}{K_{\mathrm{m\,MASP2^*}}^{\mathrm{MASP1_{zym}}}}+\frac{[\mathrm{pCLLKMASP2_{zym}}]}{K_{\mathrm{m\,MASP2^*}}^{\mathrm{MASP2_{zym}}}}+\frac{[\mathrm{pCLK1MASP1_{zym}}]}{K_{\mathrm{m\,MASP2^*}}^{\mathrm{MASP1_{zym}}}}\\ +\frac{[\mathrm{pCLK1MASP2_{zym}}]}{K_{\mathrm{m\,MASP2^*}}^{\mathrm{MASP2_{zym}}}}+\frac{[\mathrm{pCLL1MASP1_{zym}}]}{K_{\mathrm{m\,MASP2^*}}^{\mathrm{MASP1_{zym}}}}+\frac{[\mathrm{pCLL1MASP2_{zym}}]}{K_{\mathrm{m\,MASP2^*}}^{\mathrm{MASP2_{zym}}}}+\frac{[\mathrm{pLFMASP1_{zym}}]}{K_{\mathrm{m\,MASP2^*}}^{\mathrm{MASP1_{zym}}}}\\ +\frac{[\mathrm{pLFMASP2_{zym}}]}{K_{\mathrm{m\,MASP2^*}}^{\mathrm{MASP2_{zym}}}}+\frac{[\mathrm{pHFMASP1_{zym}}]}{K_{\mathrm{m\,MASP2^*}}^{\mathrm{MASP1_{zym}}}}+\frac{[\mathrm{pHFMASP2_{zym}}]}{K_{\mathrm{m\,MASP2^*}}^{\mathrm{MASP2_{zym}}}}+\frac{[\mathrm{pMFMASP1_{zym}}]}{K_{\mathrm{m\,MASP2^*}}^{\mathrm{MASP1_{zym}}}}+\frac{[\mathrm{pMFMASP2_{zym}}]}{K_{\mathrm{m\,MASP2^*}}^{\mathrm{MASP2_{zym}}}}\end{array}\right)}$$

$$-\frac{k_{\mathrm{cat\,MASP2^*}}^{\mathrm{MASP1_{zym}}}\left[\mathrm{pCLLKMASP2^*}\right]\left[\mathrm{pMBL_3MASP1_{zym}}\right]}{K_{\mathrm{m\,MASP2^*}}^{\mathrm{MASP1_{zym}}}\left(\begin{array}{l}1+\frac{[\mathrm{C4}]}{K_{\mathrm{m\,MASP2^*}}^{\mathrm{C4}}}+\frac{[\mathrm{fC4bC2}]}{K_{\mathrm{m\,MASP2^*}}^{\mathrm{C2}}}+\frac{[\mathrm{fC4bC4bC2}]}{K_{\mathrm{m\,MASP2^*}}^{\mathrm{C2}}}+\frac{[\mathrm{fC3bC4bC2}]}{K_{\mathrm{m\,MASP2^*}}^{\mathrm{C2}}}+\frac{[\mathrm{pC4bC2}]}{K_{\mathrm{m\,MASP2^*}}^{\mathrm{C2}}}+\frac{[\mathrm{pC4bC4bC2}]}{K_{\mathrm{m\,MASP2^*}}^{\mathrm{C2}}}\\ +\frac{[\mathrm{pC3bC4bC2}]}{K_{\mathrm{m\,MASP2^*}}^{\mathrm{C2}}}+\frac{[\mathrm{pMBL_3MASP1_{zym}}]}{K_{\mathrm{m\,MASP2^*}}^{\mathrm{MASP1_{zym}}}}+\frac{[\mathrm{pMBL_3MASP2_{zym}}]}{K_{\mathrm{m\,MASP2^*}}^{\mathrm{MASP2_{zym}}}}+\frac{[\mathrm{pMBL_4MASP1_{zym}}]}{K_{\mathrm{m\,MASP2^*}}^{\mathrm{MASP1_{zym}}}}\\ +\frac{[\mathrm{pMBL_4MASP2_{zym}}]}{K_{\mathrm{m\,MASP2^*}}^{\mathrm{MASP2_{zym}}}}+\frac{[\mathrm{pCLLKMASP1_{zym}}]}{K_{\mathrm{m\,MASP2^*}}^{\mathrm{MASP1_{zym}}}}+\frac{[\mathrm{pCLLKMASP2_{zym}}]}{K_{\mathrm{m\,MASP2^*}}^{\mathrm{MASP2_{zym}}}}+\frac{[\mathrm{pCLK1MASP1_{zym}}]}{K_{\mathrm{m\,MASP2^*}}^{\mathrm{MASP1_{zym}}}}\\ +\frac{[\mathrm{pCLK1MASP2_{zym}}]}{K_{\mathrm{m\,MASP2^*}}^{\mathrm{MASP2_{zym}}}}+\frac{[\mathrm{pCLL1MASP1_{zym}}]}{K_{\mathrm{m\,MASP2^*}}^{\mathrm{MASP1_{zym}}}}+\frac{[\mathrm{pCLL1MASP2_{zym}}]}{K_{\mathrm{m\,MASP2^*}}^{\mathrm{MASP2_{zym}}}}+\frac{[\mathrm{pLFMASP1_{zym}}]}{K_{\mathrm{m\,MASP2^*}}^{\mathrm{MASP1_{zym}}}}\\ +\frac{[\mathrm{pLFMASP2_{zym}}]}{K_{\mathrm{m\,MASP2^*}}^{\mathrm{MASP2_{zym}}}}+\frac{[\mathrm{pHFMASP1_{zym}}]}{K_{\mathrm{m\,MASP2^*}}^{\mathrm{MASP1_{zym}}}}+\frac{[\mathrm{pHFMASP2_{zym}}]}{K_{\mathrm{m\,MASP2^*}}^{\mathrm{MASP2_{zym}}}}+\frac{[\mathrm{pMFMASP1_{zym}}]}{K_{\mathrm{m\,MASP2^*}}^{\mathrm{MASP1_{zym}}}}+\frac{[\mathrm{pMFMASP2_{zym}}]}{K_{\mathrm{m\,MASP2^*}}^{\mathrm{MASP2_{zym}}}}\end{array}\right)}$$

$$-\frac{k_{\mathrm{cat\,MASP2^*}}^{\mathrm{MASP1_{zym}}}\left[\mathrm{pCLK1MASP2^*}\right]\left[\mathrm{pMBL_3MASP1_{zym}}\right]}{K_{\mathrm{m\,MASP2^*}}^{\mathrm{MASP1_{zym}}}\left(\begin{array}{l}1+\frac{[\mathrm{C4}]}{K_{\mathrm{m\,MASP2^*}}^{\mathrm{C4}}}+\frac{[\mathrm{fC4bC2}]}{K_{\mathrm{m\,MASP2^*}}^{\mathrm{C2}}}+\frac{[\mathrm{fC4bC4bC2}]}{K_{\mathrm{m\,MASP2^*}}^{\mathrm{C2}}}+\frac{[\mathrm{fC3bC4bC2}]}{K_{\mathrm{m\,MASP2^*}}^{\mathrm{C2}}}+\frac{[\mathrm{pC4bC2}]}{K_{\mathrm{m\,MASP2^*}}^{\mathrm{C2}}}+\frac{[\mathrm{pC4bC4bC2}]}{K_{\mathrm{m\,MASP2^*}}^{\mathrm{C2}}}\\ +\frac{[\mathrm{pC3bC4bC2}]}{K_{\mathrm{m\,MASP2^*}}^{\mathrm{C2}}}+\frac{[\mathrm{pMBL_3MASP1_{zym}}]}{K_{\mathrm{m\,MASP2^*}}^{\mathrm{MASP1_{zym}}}}+\frac{[\mathrm{pMBL_3MASP2_{zym}}]}{K_{\mathrm{m\,MASP2^*}}^{\mathrm{MASP2_{zym}}}}+\frac{[\mathrm{pMBL_4MASP1_{zym}}]}{K_{\mathrm{m\,MASP2^*}}^{\mathrm{MASP1_{zym}}}}\\ +\frac{[\mathrm{pMBL_4MASP2_{zym}}]}{K_{\mathrm{m\,MASP2^*}}^{\mathrm{MASP2_{zym}}}}+\frac{[\mathrm{pCLLKMASP1_{zym}}]}{K_{\mathrm{m\,MASP2^*}}^{\mathrm{MASP1_{zym}}}}+\frac{[\mathrm{pCLLKMASP2_{zym}}]}{K_{\mathrm{m\,MASP2^*}}^{\mathrm{MASP2_{zym}}}}+\frac{[\mathrm{pCLK1MASP1_{zym}}]}{K_{\mathrm{m\,MASP2^*}}^{\mathrm{MASP1_{zym}}}}\\ +\frac{[\mathrm{pCLK1MASP2_{zym}}]}{K_{\mathrm{m\,MASP2^*}}^{\mathrm{MASP2_{zym}}}}+\frac{[\mathrm{pCLL1MASP1_{zym}}]}{K_{\mathrm{m\,MASP2^*}}^{\mathrm{MASP1_{zym}}}}+\frac{[\mathrm{pCLL1MASP2_{zym}}]}{K_{\mathrm{m\,MASP2^*}}^{\mathrm{MASP2_{zym}}}}+\frac{[\mathrm{pLFMASP1_{zym}}]}{K_{\mathrm{m\,MASP2^*}}^{\mathrm{MASP1_{zym}}}}\\ +\frac{[\mathrm{pLFMASP2_{zym}}]}{K_{\mathrm{m\,MASP2^*}}^{\mathrm{MASP2_{zym}}}}+\frac{[\mathrm{pHFMASP1_{zym}}]}{K_{\mathrm{m\,MASP2^*}}^{\mathrm{MASP1_{zym}}}}+\frac{[\mathrm{pHFMASP2_{zym}}]}{K_{\mathrm{m\,MASP2^*}}^{\mathrm{MASP2_{zym}}}}+\frac{[\mathrm{pMFMASP1_{zym}}]}{K_{\mathrm{m\,MASP2^*}}^{\mathrm{MASP1_{zym}}}}+\frac{[\mathrm{pMFMASP2_{zym}}]}{K_{\mathrm{m\,MASP2^*}}^{\mathrm{MASP2_{zym}}}}\end{array}\right)}$$

$$
-\frac{k_{\text{cat MASP2}^*}^{\text{MASP1}_{\text{zym}}}\left[\text{pCLL1MASP2}^*\right]\left[\text{pMBL}_3\text{MASP1}_{\text{zym}}\right]}{K_{\text{m MASP2}^*}^{\text{MASP1}_{\text{zym}}}\left(\begin{aligned}&1+\frac{[\text{C4}]}{K_{\text{m MASP2}^*}^{\text{C4}}}+\frac{[\text{fC4bC2}]}{K_{\text{m MASP2}^*}^{\text{C2}}}+\frac{[\text{fC4bC4bC2}]}{K_{\text{m MASP2}^*}^{\text{C2}}}+\frac{[\text{fC3bC4bC2}]}{K_{\text{m MASP2}^*}^{\text{C2}}}+\frac{[\text{pC4bC2}]}{K_{\text{m MASP2}^*}^{\text{C2}}}+\frac{[\text{pC4bC4bC2}]}{K_{\text{m MASP2}^*}^{\text{C2}}}\\&+\frac{[\text{pC3bC4bC2}]}{K_{\text{m MASP2}^*}^{\text{C2}}}+\frac{\left[\text{pMBL}_3\text{MASP1}_{\text{zym}}\right]}{K_{\text{m MASP2}^*}^{\text{MASP1}_{\text{zym}}}}+\frac{\left[\text{pMBL}_3\text{MASP2}_{\text{zym}}\right]}{K_{\text{m MASP2}^*}^{\text{MASP2}_{\text{zym}}}}+\frac{\left[\text{pMBL}_4\text{MASP1}_{\text{zym}}\right]}{K_{\text{m MASP2}^*}^{\text{MASP1}_{\text{zym}}}}\\&+\frac{\left[\text{pMBL}_4\text{MASP2}_{\text{zym}}\right]}{K_{\text{m MASP2}^*}^{\text{MASP2}_{\text{zym}}}}+\frac{\left[\text{pCLLKMASP1}_{\text{zym}}\right]}{K_{\text{m MASP2}^*}^{\text{MASP1}_{\text{zym}}}}+\frac{\left[\text{pCLLKMASP2}_{\text{zym}}\right]}{K_{\text{m MASP2}^*}^{\text{MASP2}_{\text{zym}}}}+\frac{\left[\text{pCLK1MASP1}_{\text{zym}}\right]}{K_{\text{m MASP2}^*}^{\text{MASP1}_{\text{zym}}}}\\&+\frac{\left[\text{pCLK1MASP2}_{\text{zym}}\right]}{K_{\text{m MASP2}^*}^{\text{MASP2}_{\text{zym}}}}+\frac{\left[\text{pCLL1MASP1}_{\text{zym}}\right]}{K_{\text{m MASP2}^*}^{\text{MASP1}_{\text{zym}}}}+\frac{\left[\text{pCLL1MASP2}_{\text{zym}}\right]}{K_{\text{m MASP2}^*}^{\text{MASP2}_{\text{zym}}}}+\frac{\left[\text{pLFMASP1}_{\text{zym}}\right]}{K_{\text{m MASP2}^*}^{\text{MASP1}_{\text{zym}}}}\\&+\frac{\left[\text{pLFMASP2}_{\text{zym}}\right]}{K_{\text{m MASP2}^*}^{\text{MASP2}_{\text{zym}}}}+\frac{\left[\text{pHFMASP1}_{\text{zym}}\right]}{K_{\text{m MASP2}^*}^{\text{MASP1}_{\text{zym}}}}+\frac{\left[\text{pHFMASP2}_{\text{zym}}\right]}{K_{\text{m MASP2}^*}^{\text{MASP2}_{\text{zym}}}}+\frac{\left[\text{pMFMASP1}_{\text{zym}}\right]}{K_{\text{m MASP2}^*}^{\text{MASP1}_{\text{zym}}}}+\frac{\left[\text{pMFMASP2}_{\text{zym}}\right]}{K_{\text{m MASP2}^*}^{\text{MASP2}_{\text{zym}}}}\end{aligned}\right)}
$$

$$
-\frac{k_{\text{cat MASP2}^*}^{\text{MASP1}_{\text{zym}}}\left[\text{pLFMASP2}^*\right]\left[\text{pMBL}_3\text{MASP1}_{\text{zym}}\right]}{K_{\text{m MASP2}^*}^{\text{MASP1}_{\text{zym}}}\left(\begin{aligned}&1+\frac{[\text{C4}]}{K_{\text{m MASP2}^*}^{\text{C4}}}+\frac{[\text{fC4bC2}]}{K_{\text{m MASP2}^*}^{\text{C2}}}+\frac{[\text{fC4bC4bC2}]}{K_{\text{m MASP2}^*}^{\text{C2}}}+\frac{[\text{fC3bC4bC2}]}{K_{\text{m MASP2}^*}^{\text{C2}}}+\frac{[\text{pC4bC2}]}{K_{\text{m MASP2}^*}^{\text{C2}}}+\frac{[\text{pC4bC4bC2}]}{K_{\text{m MASP2}^*}^{\text{C2}}}\\&+\frac{[\text{pC3bC4bC2}]}{K_{\text{m MASP2}^*}^{\text{C2}}}+\frac{\left[\text{pMBL}_3\text{MASP1}_{\text{zym}}\right]}{K_{\text{m MASP2}^*}^{\text{MASP1}_{\text{zym}}}}+\frac{\left[\text{pMBL}_3\text{MASP2}_{\text{zym}}\right]}{K_{\text{m MASP2}^*}^{\text{MASP2}_{\text{zym}}}}+\frac{\left[\text{pMBL}_4\text{MASP1}_{\text{zym}}\right]}{K_{\text{m MASP2}^*}^{\text{MASP1}_{\text{zym}}}}\\&+\frac{\left[\text{pMBL}_4\text{MASP2}_{\text{zym}}\right]}{K_{\text{m MASP2}^*}^{\text{MASP2}_{\text{zym}}}}+\frac{\left[\text{pCLLKMASP1}_{\text{zym}}\right]}{K_{\text{m MASP2}^*}^{\text{MASP1}_{\text{zym}}}}+\frac{\left[\text{pCLLKMASP2}_{\text{zym}}\right]}{K_{\text{m MASP2}^*}^{\text{MASP2}_{\text{zym}}}}+\frac{\left[\text{pCLK1MASP1}_{\text{zym}}\right]}{K_{\text{m MASP2}^*}^{\text{MASP1}_{\text{zym}}}}\\&+\frac{\left[\text{pCLK1MASP2}_{\text{zym}}\right]}{K_{\text{m MASP2}^*}^{\text{MASP2}_{\text{zym}}}}+\frac{\left[\text{pCLL1MASP1}_{\text{zym}}\right]}{K_{\text{m MASP2}^*}^{\text{MASP1}_{\text{zym}}}}+\frac{\left[\text{pCLL1MASP2}_{\text{zym}}\right]}{K_{\text{m MASP2}^*}^{\text{MASP2}_{\text{zym}}}}+\frac{\left[\text{pLFMASP1}_{\text{zym}}\right]}{K_{\text{m MASP2}^*}^{\text{MASP1}_{\text{zym}}}}\\&+\frac{\left[\text{pLFMASP2}_{\text{zym}}\right]}{K_{\text{m MASP2}^*}^{\text{MASP2}_{\text{zym}}}}+\frac{\left[\text{pHFMASP1}_{\text{zym}}\right]}{K_{\text{m MASP2}^*}^{\text{MASP1}_{\text{zym}}}}+\frac{\left[\text{pHFMASP2}_{\text{zym}}\right]}{K_{\text{m MASP2}^*}^{\text{MASP2}_{\text{zym}}}}+\frac{\left[\text{pMFMASP1}_{\text{zym}}\right]}{K_{\text{m MASP2}^*}^{\text{MASP1}_{\text{zym}}}}+\frac{\left[\text{pMFMASP2}_{\text{zym}}\right]}{K_{\text{m MASP2}^*}^{\text{MASP2}_{\text{zym}}}}\end{aligned}\right)}
$$

$$
-\frac{k_{\text{cat MASP2}^*}^{\text{MASP1}_{\text{zym}}}\left[\text{pHFMASP2}^*\right]\left[\text{pMBL}_3\text{MASP1}_{\text{zym}}\right]}{K_{\text{m MASP2}^*}^{\text{MASP1}_{\text{zym}}}\left(\begin{aligned}&1+\frac{[\text{C4}]}{K_{\text{m MASP2}^*}^{\text{C4}}}+\frac{[\text{fC4bC2}]}{K_{\text{m MASP2}^*}^{\text{C2}}}+\frac{[\text{fC4bC4bC2}]}{K_{\text{m MASP2}^*}^{\text{C2}}}+\frac{[\text{fC3bC4bC2}]}{K_{\text{m MASP2}^*}^{\text{C2}}}+\frac{[\text{pC4bC2}]}{K_{\text{m MASP2}^*}^{\text{C2}}}+\frac{[\text{pC4bC4bC2}]}{K_{\text{m MASP2}^*}^{\text{C2}}}\\&+\frac{[\text{pC3bC4bC2}]}{K_{\text{m MASP2}^*}^{\text{C2}}}+\frac{\left[\text{pMBL}_3\text{MASP1}_{\text{zym}}\right]}{K_{\text{m MASP2}^*}^{\text{MASP1}_{\text{zym}}}}+\frac{\left[\text{pMBL}_3\text{MASP2}_{\text{zym}}\right]}{K_{\text{m MASP2}^*}^{\text{MASP2}_{\text{zym}}}}+\frac{\left[\text{pMBL}_4\text{MASP1}_{\text{zym}}\right]}{K_{\text{m MASP2}^*}^{\text{MASP1}_{\text{zym}}}}\\&+\frac{\left[\text{pMBL}_4\text{MASP2}_{\text{zym}}\right]}{K_{\text{m MASP2}^*}^{\text{MASP2}_{\text{zym}}}}+\frac{\left[\text{pCLLKMASP1}_{\text{zym}}\right]}{K_{\text{m MASP2}^*}^{\text{MASP1}_{\text{zym}}}}+\frac{\left[\text{pCLLKMASP2}_{\text{zym}}\right]}{K_{\text{m MASP2}^*}^{\text{MASP2}_{\text{zym}}}}+\frac{\left[\text{pCLK1MASP1}_{\text{zym}}\right]}{K_{\text{m MASP2}^*}^{\text{MASP1}_{\text{zym}}}}\\&+\frac{\left[\text{pCLK1MASP2}_{\text{zym}}\right]}{K_{\text{m MASP2}^*}^{\text{MASP2}_{\text{zym}}}}+\frac{\left[\text{pCLL1MASP1}_{\text{zym}}\right]}{K_{\text{m MASP2}^*}^{\text{MASP1}_{\text{zym}}}}+\frac{\left[\text{pCLL1MASP2}_{\text{zym}}\right]}{K_{\text{m MASP2}^*}^{\text{MASP2}_{\text{zym}}}}+\frac{\left[\text{pLFMASP1}_{\text{zym}}\right]}{K_{\text{m MASP2}^*}^{\text{MASP1}_{\text{zym}}}}\\&+\frac{\left[\text{pLFMASP2}_{\text{zym}}\right]}{K_{\text{m MASP2}^*}^{\text{MASP2}_{\text{zym}}}}+\frac{\left[\text{pHFMASP1}_{\text{zym}}\right]}{K_{\text{m MASP2}^*}^{\text{MASP1}_{\text{zym}}}}+\frac{\left[\text{pHFMASP2}_{\text{zym}}\right]}{K_{\text{m MASP2}^*}^{\text{MASP2}_{\text{zym}}}}+\frac{\left[\text{pMFMASP1}_{\text{zym}}\right]}{K_{\text{m MASP2}^*}^{\text{MASP1}_{\text{zym}}}}+\frac{\left[\text{pMFMASP2}_{\text{zym}}\right]}{K_{\text{m MASP2}^*}^{\text{MASP2}_{\text{zym}}}}\end{aligned}\right)}
$$

$$
-\frac{k_{\text{cat MASP2}^{*}}^{\text{MASP1}_{\text{zym}}}\left[\text{pMFMASP2}^{*}\right]\left[\text{pMBL}_{3}\text{MASP1}_{\text{zym}}\right]}{K_{\text{m MASP2}^{*}}^{\text{MASP1}_{\text{zym}}}\left(\begin{array}{l}1+\frac{[\text{C4}]}{K_{\text{m MASP2}^{*}}^{\text{C4}}}+\frac{[\text{fC4bC2}]}{K_{\text{m MASP2}^{*}}^{\text{C2}}}+\frac{[\text{fC4bC4bC2}]}{K_{\text{m MASP2}^{*}}^{\text{C2}}}+\frac{[\text{fC3bC4bC2}]}{K_{\text{m MASP2}^{*}}^{\text{C2}}}+\frac{[\text{pC4bC2}]}{K_{\text{m MASP2}^{*}}^{\text{C2}}}+\frac{[\text{pC4bC4bC2}]}{K_{\text{m MASP2}^{*}}^{\text{C2}}}\\+\frac{[\text{pC3bC4bC2}]}{K_{\text{m MASP2}^{*}}^{\text{C2}}}+\frac{\left[\text{pMBL}_{3}\text{MASP1}_{\text{zym}}\right]}{K_{\text{m MASP2}^{*}}^{\text{MASP1}_{\text{zym}}}}+\frac{\left[\text{pMBL}_{3}\text{MASP2}_{\text{zym}}\right]}{K_{\text{m MASP2}^{*}}^{\text{MASP2}_{\text{zym}}}}+\frac{\left[\text{pMBL}_{4}\text{MASP1}_{\text{zym}}\right]}{K_{\text{m MASP2}^{*}}^{\text{MASP1}_{\text{zym}}}}\\+\frac{\left[\text{pMBL}_{4}\text{MASP2}_{\text{zym}}\right]}{K_{\text{m MASP2}^{*}}^{\text{MASP2}_{\text{zym}}}}+\frac{\left[\text{pCLLKMASP1}_{\text{zym}}\right]}{K_{\text{m MASP2}^{*}}^{\text{MASP1}_{\text{zym}}}}+\frac{\left[\text{pCLLKMASP2}_{\text{zym}}\right]}{K_{\text{m MASP2}^{*}}^{\text{MASP2}_{\text{zym}}}}+\frac{\left[\text{pCLK1MASP1}_{\text{zym}}\right]}{K_{\text{m MASP2}^{*}}^{\text{MASP1}_{\text{zym}}}}\\+\frac{\left[\text{pCLK1MASP2}_{\text{zym}}\right]}{K_{\text{m MASP2}^{*}}^{\text{MASP2}_{\text{zym}}}}+\frac{\left[\text{pCLL1MASP1}_{\text{zym}}\right]}{K_{\text{m MASP2}^{*}}^{\text{MASP1}_{\text{zym}}}}+\frac{\left[\text{pCLL1MASP2}_{\text{zym}}\right]}{K_{\text{m MASP2}^{*}}^{\text{MASP2}_{\text{zym}}}}+\frac{\left[\text{pLFMASP1}_{\text{zym}}\right]}{K_{\text{m MASP2}^{*}}^{\text{MASP1}_{\text{zym}}}}\\+\frac{\left[\text{pLFMASP2}_{\text{zym}}\right]}{K_{\text{m MASP2}^{*}}^{\text{MASP2}_{\text{zym}}}}+\frac{\left[\text{pHFMASP1}_{\text{zym}}\right]}{K_{\text{m MASP2}^{*}}^{\text{MASP1}_{\text{zym}}}}+\frac{\left[\text{pHFMASP2}_{\text{zym}}\right]}{K_{\text{m MASP2}^{*}}^{\text{MASP2}_{\text{zym}}}}+\frac{\left[\text{pMFMASP1}_{\text{zym}}\right]}{K_{\text{m MASP2}^{*}}^{\text{MASP1}_{\text{zym}}}}+\frac{\left[\text{pMFMASP2}_{\text{zym}}\right]}{K_{\text{m MASP2}^{*}}^{\text{MASP2}_{\text{zym}}}}\end{array}\right)}
$$

$$
-\frac{k_{\text{cat MASP2}^{*}}^{\text{MASP1}_{\text{zym}}}\left[\text{pPTX3LFMASP2}^{*}\right]\left[\text{pMBL}_{3}\text{MASP1}_{\text{zym}}\right]}{K_{\text{m MASP2}^{*}}^{\text{MASP1}_{\text{zym}}}\left(\begin{array}{l}1+\frac{[\text{C4}]}{K_{\text{m MASP2}^{*}}^{\text{C4}}}+\frac{[\text{fC4bC2}]}{K_{\text{m MASP2}^{*}}^{\text{C2}}}+\frac{[\text{fC4bC4bC2}]}{K_{\text{m MASP2}^{*}}^{\text{C2}}}+\frac{[\text{fC3bC4bC2}]}{K_{\text{m MASP2}^{*}}^{\text{C2}}}+\frac{[\text{pC4bC2}]}{K_{\text{m MASP2}^{*}}^{\text{C2}}}+\frac{[\text{pC4bC4bC2}]}{K_{\text{m MASP2}^{*}}^{\text{C2}}}\\+\frac{[\text{pC3bC4bC2}]}{K_{\text{m MASP2}^{*}}^{\text{C2}}}+\frac{\left[\text{pMBL}_{3}\text{MASP1}_{\text{zym}}\right]}{K_{\text{m MASP2}^{*}}^{\text{MASP1}_{\text{zym}}}}+\frac{\left[\text{pMBL}_{3}\text{MASP2}_{\text{zym}}\right]}{K_{\text{m MASP2}^{*}}^{\text{MASP2}_{\text{zym}}}}+\frac{\left[\text{pMBL}_{4}\text{MASP1}_{\text{zym}}\right]}{K_{\text{m MASP2}^{*}}^{\text{MASP1}_{\text{zym}}}}\\+\frac{\left[\text{pMBL}_{4}\text{MASP2}_{\text{zym}}\right]}{K_{\text{m MASP2}^{*}}^{\text{MASP2}_{\text{zym}}}}+\frac{\left[\text{pCLLKMASP1}_{\text{zym}}\right]}{K_{\text{m MASP2}^{*}}^{\text{MASP1}_{\text{zym}}}}+\frac{\left[\text{pCLLKMASP2}_{\text{zym}}\right]}{K_{\text{m MASP2}^{*}}^{\text{MASP2}_{\text{zym}}}}+\frac{\left[\text{pCLK1MASP1}_{\text{zym}}\right]}{K_{\text{m MASP2}^{*}}^{\text{MASP1}_{\text{zym}}}}\\+\frac{\left[\text{pCLK1MASP2}_{\text{zym}}\right]}{K_{\text{m MASP2}^{*}}^{\text{MASP2}_{\text{zym}}}}+\frac{\left[\text{pCLL1MASP1}_{\text{zym}}\right]}{K_{\text{m MASP2}^{*}}^{\text{MASP1}_{\text{zym}}}}+\frac{\left[\text{pCLL1MASP2}_{\text{zym}}\right]}{K_{\text{m MASP2}^{*}}^{\text{MASP2}_{\text{zym}}}}+\frac{\left[\text{pLFMASP1}_{\text{zym}}\right]}{K_{\text{m MASP2}^{*}}^{\text{MASP1}_{\text{zym}}}}\\+\frac{\left[\text{pLFMASP2}_{\text{zym}}\right]}{K_{\text{m MASP2}^{*}}^{\text{MASP2}_{\text{zym}}}}+\frac{\left[\text{pHFMASP1}_{\text{zym}}\right]}{K_{\text{m MASP2}^{*}}^{\text{MASP1}_{\text{zym}}}}+\frac{\left[\text{pHFMASP2}_{\text{zym}}\right]}{K_{\text{m MASP2}^{*}}^{\text{MASP2}_{\text{zym}}}}+\frac{\left[\text{pMFMASP1}_{\text{zym}}\right]}{K_{\text{m MASP2}^{*}}^{\text{MASP1}_{\text{zym}}}}+\frac{\left[\text{pMFMASP2}_{\text{zym}}\right]}{K_{\text{m MASP2}^{*}}^{\text{MASP2}_{\text{zym}}}}\\+\frac{\left[\text{pPTX3LFMASP1}_{\text{zym}}\right]}{K_{\text{m MASP2}^{*}}^{\text{MASP1}_{\text{zym}}}}+\frac{\left[\text{pPTX3LFMASP2}_{\text{zym}}\right]}{K_{\text{m MASP2}^{*}}^{\text{MASP2}_{\text{zym}}}}\end{array}\right)}
$$

245

$$\begin{aligned}
\frac{d\left[\mathrm{pMBL_3}\right]}{dt} &= k^{+}_{\mathrm{pMBL_3}}\left[\mathrm{MBL_3}\right]\left[\text{Pathogen Surface}\right] - k^{-}_{\mathrm{pMBL_3}}\left[\mathrm{pMBL_3}\right] - k^{+}_{\mathrm{MBL_3MASP1}}\left[\mathrm{pMBL_3}\right]\left[\mathrm{MASP1}\right] \\
&+ k^{-}_{\mathrm{MBL_3MASP1}}\left[\mathrm{pMBL_3MASP1_{zym}}\right] - k^{+}_{\mathrm{MBL_3MASP2}}\left[\mathrm{pMBL_3}\right]\left[\mathrm{MASP2}\right] + k^{-}_{\mathrm{MBL_3MASP2}}\left[\mathrm{pMBL_3MASP2_{zym}}\right] \\
&- k^{+}_{\mathrm{MBL_3MASP3}}\left[\mathrm{pMBL_3}\right]\left[\mathrm{MASP3}\right] + k^{-}_{\mathrm{MBL_3MASP3}}\left[\mathrm{pMBL_3MASP3_{zym}}\right] - k^{+}_{\mathrm{MBL_3MASP1}}\left[\mathrm{pMBL_3}\right]\left[\mathrm{MASP1^*}\right] \\
&+ k^{-}_{\mathrm{MBL_3MASP1}}\left[\mathrm{pMBL_3MASP1^*}\right] - k^{+}_{\mathrm{MBL_3MASP2}}\left[\mathrm{pMBL_3}\right]\left[\mathrm{MASP2^*}\right] + k^{-}_{\mathrm{MBL_3MASP2}}\left[\mathrm{pMBL_3MASP2^*}\right] \\
&- k^{+}_{\mathrm{MBL_3MASP3}}\left[\mathrm{pMBL_3}\right]\left[\mathrm{MASP3^*}\right] + k^{-}_{\mathrm{MBL_3MASP3}}\left[\mathrm{pMBL_3MASP3^*}\right] - k^{+}_{\mathrm{MBL_3MAp19}}\left[\mathrm{pMBL_3}\right]\left[\mathrm{MAp19}\right] \\
&+ k^{-}_{\mathrm{MBL_3MAp19}}\left[\mathrm{pMBL_3MAp19}\right] - k^{+}_{\mathrm{MBL_3MAp44}}\left[\mathrm{pMBL_3}\right]\left[\mathrm{MAp44}\right] + k^{-}_{\mathrm{MBL_3MAp44}}\left[\mathrm{pMBL_3MAp44}\right] \\
&+ k^{+}_{\mathrm{C1INHMASP1}}\left[\mathrm{C1\text{-}INH}\right]\left[\mathrm{pMBL_3MASP1^*}\right] + k^{+}_{\mathrm{C1INHMASP2}}\left[\mathrm{C1\text{-}INH}\right]\left[\mathrm{pMBL_3MASP2^*}\right] - k^{+}_{\mathrm{MBL_3PTX3}}\left[\mathrm{pMBL_3}\right]\left[\mathrm{PTX3}\right] \\
&+ k^{-}_{\mathrm{MBL_3PTX3}}\left[\mathrm{pMBL_3PTX3}\right] - k^{+}_{\mathrm{MBL_3SAP}}\left[\mathrm{pMBL_3}\right]\left[\mathrm{SAP}\right] + k^{-}_{\mathrm{MBL_3SAP}}\left[\mathrm{pMBL_3SAP}\right] + k^{-}_{\mathrm{MBL_3PTX3}}\left[\mathrm{pMBL_3PTX3C1q}\right] \\
&+ k^{-}_{\mathrm{MBL_3PTX3}}\left[\mathrm{pMBL_3PTX3C1}\right] + k^{-}_{\mathrm{MBL_3PTX3}}\left[\mathrm{pMBL_3PTX3C1^*}\right]
\end{aligned}$$

246

$$\begin{aligned}
\frac{d\left[\mathrm{fMBL_3MASP1^*}\right]}{dt} &= -k^{+}_{\mathrm{pMBL_3}}\left[\mathrm{fMBL_3MASP1^*}\right]\left[\text{Pathogen Surface}\right] + k^{-}_{\mathrm{pMBL_3}}\left[\mathrm{pMBL_3MASP1^*}\right] \\
&+ k^{+}_{\mathrm{MBL_3MASP1}}\left[\mathrm{MBL_3}\right]\left[\mathrm{MASP1^*}\right] - k^{-}_{\mathrm{MBL_3MASP1}}\left[\mathrm{fMBL_3MASP1^*}\right] - k^{+}_{\mathrm{C1INHMASP1}}\left[\mathrm{C1\text{-}INH}\right]\left[\mathrm{fMBL_3MASP1^*}\right]
\end{aligned}$$

247

$$\begin{aligned}
\frac{d\left[\mathrm{pMBL_3MASP1^*}\right]}{dt} &= k^{+}_{\mathrm{pMBL_3}}\left[\mathrm{fMBL_3MASP1^*}\right]\left[\text{Pathogen Surface}\right] - k^{-}_{\mathrm{pMBL_3}}\left[\mathrm{pMBL_3MASP1^*}\right] \\
&+ k^{+}_{\mathrm{MBL_3MASP1}}\left[\mathrm{pMBL_3}\right]\left[\mathrm{MASP1^*}\right] - k^{-}_{\mathrm{MBL_3MASP1}}\left[\mathrm{pMBL_3MASP1^*}\right] \\
&+ \frac{k^{\mathrm{MASP1_{zym}}}_{\mathrm{cat\ MASP1_{zym}}}\left[\mathrm{pMBL_3MASP1_{zym}}\right]\left[\mathrm{pMBL_3MASP1_{zym}}\right]}{K^{\mathrm{MASP1_{zym}}}_{\mathrm{m\ MASP1_{zym}}}\left(\begin{aligned}
&1 + \frac{\left[\mathrm{pMBL_3MASP1_{zym}}\right]}{K^{\mathrm{MASP1_{zym}}}_{\mathrm{m\ MASP1_{zym}}}} + \frac{\left[\mathrm{pMBL_3MASP2_{zym}}\right]}{K^{\mathrm{MASP2_{zym}}}_{\mathrm{m\ MASP1_{zym}}}} + \frac{\left[\mathrm{pMBL_4MASP1_{zym}}\right]}{K^{\mathrm{MASP1_{zym}}}_{\mathrm{m\ MASP1_{zym}}}} + \frac{\left[\mathrm{pMBL_4MASP2_{zym}}\right]}{K^{\mathrm{MASP2_{zym}}}_{\mathrm{m\ MASP1_{zym}}}} \\
&+ \frac{\left[\mathrm{pCLLKMASP1_{zym}}\right]}{K^{\mathrm{MASP1_{zym}}}_{\mathrm{m\ MASP1_{zym}}}} + \frac{\left[\mathrm{pCLLKMASP2_{zym}}\right]}{K^{\mathrm{MASP2_{zym}}}_{\mathrm{m\ MASP1_{zym}}}} + \frac{\left[\mathrm{pCLK1MASP1_{zym}}\right]}{K^{\mathrm{MASP1_{zym}}}_{\mathrm{m\ MASP1_{zym}}}} + \frac{\left[\mathrm{pCLK1MASP2_{zym}}\right]}{K^{\mathrm{MASP2_{zym}}}_{\mathrm{m\ MASP1_{zym}}}} \\
&+ \frac{\left[\mathrm{pCLL1MASP1_{zym}}\right]}{K^{\mathrm{MASP1_{zym}}}_{\mathrm{m\ MASP1_{zym}}}} + \frac{\left[\mathrm{pCLL1MASP2_{zym}}\right]}{K^{\mathrm{MASP2_{zym}}}_{\mathrm{m\ MASP1_{zym}}}} + \frac{\left[\mathrm{pLFMASP1_{zym}}\right]}{K^{\mathrm{MASP1_{zym}}}_{\mathrm{m\ MASP1_{zym}}}} + \frac{\left[\mathrm{pLFMASP2_{zym}}\right]}{K^{\mathrm{MASP2_{zym}}}_{\mathrm{m\ MASP1_{zym}}}} \\
&+ \frac{\left[\mathrm{pHFMASP1_{zym}}\right]}{K^{\mathrm{MASP1_{zym}}}_{\mathrm{m\ MASP1_{zym}}}} + \frac{\left[\mathrm{pHFMASP2_{zym}}\right]}{K^{\mathrm{MASP2_{zym}}}_{\mathrm{m\ MASP1_{zym}}}} + \frac{\left[\mathrm{pMFMASP1_{zym}}\right]}{K^{\mathrm{MASP1_{zym}}}_{\mathrm{m\ MASP1_{zym}}}} + \frac{\left[\mathrm{pMFMASP2_{zym}}\right]}{K^{\mathrm{MASP2_{zym}}}_{\mathrm{m\ MASP1_{zym}}}}
\end{aligned}\right)}
\end{aligned}$$

$$
+\frac{k_{\mathrm{cat\ MASP1_{zym}}}^{\mathrm{MASP1_{zym}}}\left[\mathrm{pMBL_4MASP1_{zym}}\right]\left[\mathrm{pMBL_3MASP1_{zym}}\right]}{K_{\mathrm{m\ MASP1_{zym}}}^{\mathrm{MASP1_{zym}}}\left(\begin{array}{l}1+\frac{\left[\mathrm{pMBL_3MASP1_{zym}}\right]}{K_{\mathrm{m\ MASP1_{zym}}}^{\mathrm{MASP1_{zym}}}}+\frac{\left[\mathrm{pMBL_3MASP2_{zym}}\right]}{K_{\mathrm{m\ MASP1_{zym}}}^{\mathrm{MASP2_{zym}}}}+\frac{\left[\mathrm{pMBL_4MASP1_{zym}}\right]}{K_{\mathrm{m\ MASP1_{zym}}}^{\mathrm{MASP1_{zym}}}}+\frac{\left[\mathrm{pMBL_4MASP2_{zym}}\right]}{K_{\mathrm{m\ MASP1_{zym}}}^{\mathrm{MASP2_{zym}}}}\\+\frac{\left[\mathrm{pCLLKMASP1_{zym}}\right]}{K_{\mathrm{m\ MASP1_{zym}}}^{\mathrm{MASP1_{zym}}}}+\frac{\left[\mathrm{pCLLKMASP2_{zym}}\right]}{K_{\mathrm{m\ MASP1_{zym}}}^{\mathrm{MASP2_{zym}}}}+\frac{\left[\mathrm{pCLK1MASP1_{zym}}\right]}{K_{\mathrm{m\ MASP1_{zym}}}^{\mathrm{MASP1_{zym}}}}+\frac{\left[\mathrm{pCLK1MASP2_{zym}}\right]}{K_{\mathrm{m\ MASP1_{zym}}}^{\mathrm{MASP2_{zym}}}}\\+\frac{\left[\mathrm{pCLL1MASP1_{zym}}\right]}{K_{\mathrm{m\ MASP1_{zym}}}^{\mathrm{MASP1_{zym}}}}+\frac{\left[\mathrm{pCLL1MASP2_{zym}}\right]}{K_{\mathrm{m\ MASP1_{zym}}}^{\mathrm{MASP2_{zym}}}}+\frac{\left[\mathrm{pLFMASP1_{zym}}\right]}{K_{\mathrm{m\ MASP1_{zym}}}^{\mathrm{MASP1_{zym}}}}+\frac{\left[\mathrm{pLFMASP2_{zym}}\right]}{K_{\mathrm{m\ MASP1_{zym}}}^{\mathrm{MASP2_{zym}}}}\\+\frac{\left[\mathrm{pHFMASP1_{zym}}\right]}{K_{\mathrm{m\ MASP1_{zym}}}^{\mathrm{MASP1_{zym}}}}+\frac{\left[\mathrm{pHFMASP2_{zym}}\right]}{K_{\mathrm{m\ MASP1_{zym}}}^{\mathrm{MASP2_{zym}}}}+\frac{\left[\mathrm{pMFMASP1_{zym}}\right]}{K_{\mathrm{m\ MASP1_{zym}}}^{\mathrm{MASP1_{zym}}}}+\frac{\left[\mathrm{pMFMASP2_{zym}}\right]}{K_{\mathrm{m\ MASP1_{zym}}}^{\mathrm{MASP2_{zym}}}}\end{array}\right)}
$$

$$
+\frac{k_{\mathrm{cat\ MASP1_{zym}}}^{\mathrm{MASP1_{zym}}}\left[\mathrm{pCLLKMASP1_{zym}}\right]\left[\mathrm{pMBL_3MASP1_{zym}}\right]}{K_{\mathrm{m\ MASP1_{zym}}}^{\mathrm{MASP1_{zym}}}\left(\begin{array}{l}1+\frac{\left[\mathrm{pMBL_3MASP1_{zym}}\right]}{K_{\mathrm{m\ MASP1_{zym}}}^{\mathrm{MASP1_{zym}}}}+\frac{\left[\mathrm{pMBL_3MASP2_{zym}}\right]}{K_{\mathrm{m\ MASP1_{zym}}}^{\mathrm{MASP2_{zym}}}}+\frac{\left[\mathrm{pMBL_4MASP1_{zym}}\right]}{K_{\mathrm{m\ MASP1_{zym}}}^{\mathrm{MASP1_{zym}}}}+\frac{\left[\mathrm{pMBL_4MASP2_{zym}}\right]}{K_{\mathrm{m\ MASP1_{zym}}}^{\mathrm{MASP2_{zym}}}}\\+\frac{\left[\mathrm{pCLLKMASP1_{zym}}\right]}{K_{\mathrm{m\ MASP1_{zym}}}^{\mathrm{MASP1_{zym}}}}+\frac{\left[\mathrm{pCLLKMASP2_{zym}}\right]}{K_{\mathrm{m\ MASP1_{zym}}}^{\mathrm{MASP2_{zym}}}}+\frac{\left[\mathrm{pCLK1MASP1_{zym}}\right]}{K_{\mathrm{m\ MASP1_{zym}}}^{\mathrm{MASP1_{zym}}}}+\frac{\left[\mathrm{pCLK1MASP2_{zym}}\right]}{K_{\mathrm{m\ MASP1_{zym}}}^{\mathrm{MASP2_{zym}}}}\\+\frac{\left[\mathrm{pCLL1MASP1_{zym}}\right]}{K_{\mathrm{m\ MASP1_{zym}}}^{\mathrm{MASP1_{zym}}}}+\frac{\left[\mathrm{pCLL1MASP2_{zym}}\right]}{K_{\mathrm{m\ MASP1_{zym}}}^{\mathrm{MASP2_{zym}}}}+\frac{\left[\mathrm{pLFMASP1_{zym}}\right]}{K_{\mathrm{m\ MASP1_{zym}}}^{\mathrm{MASP1_{zym}}}}+\frac{\left[\mathrm{pLFMASP2_{zym}}\right]}{K_{\mathrm{m\ MASP1_{zym}}}^{\mathrm{MASP2_{zym}}}}\\+\frac{\left[\mathrm{pHFMASP1_{zym}}\right]}{K_{\mathrm{m\ MASP1_{zym}}}^{\mathrm{MASP1_{zym}}}}+\frac{\left[\mathrm{pHFMASP2_{zym}}\right]}{K_{\mathrm{m\ MASP1_{zym}}}^{\mathrm{MASP2_{zym}}}}+\frac{\left[\mathrm{pMFMASP1_{zym}}\right]}{K_{\mathrm{m\ MASP1_{zym}}}^{\mathrm{MASP1_{zym}}}}+\frac{\left[\mathrm{pMFMASP2_{zym}}\right]}{K_{\mathrm{m\ MASP1_{zym}}}^{\mathrm{MASP2_{zym}}}}\end{array}\right)}
$$

$$
+\frac{k_{\mathrm{cat\ MASP1_{zym}}}^{\mathrm{MASP1_{zym}}}\left[\mathrm{pCLK1MASP1_{zym}}\right]\left[\mathrm{pMBL_3MASP1_{zym}}\right]}{K_{\mathrm{m\ MASP1_{zym}}}^{\mathrm{MASP1_{zym}}}\left(\begin{array}{l}1+\frac{\left[\mathrm{pMBL_3MASP1_{zym}}\right]}{K_{\mathrm{m\ MASP1_{zym}}}^{\mathrm{MASP1_{zym}}}}+\frac{\left[\mathrm{pMBL_3MASP2_{zym}}\right]}{K_{\mathrm{m\ MASP1_{zym}}}^{\mathrm{MASP2_{zym}}}}+\frac{\left[\mathrm{pMBL_4MASP1_{zym}}\right]}{K_{\mathrm{m\ MASP1_{zym}}}^{\mathrm{MASP1_{zym}}}}+\frac{\left[\mathrm{pMBL_4MASP2_{zym}}\right]}{K_{\mathrm{m\ MASP1_{zym}}}^{\mathrm{MASP2_{zym}}}}\\+\frac{\left[\mathrm{pCLLKMASP1_{zym}}\right]}{K_{\mathrm{m\ MASP1_{zym}}}^{\mathrm{MASP1_{zym}}}}+\frac{\left[\mathrm{pCLLKMASP2_{zym}}\right]}{K_{\mathrm{m\ MASP1_{zym}}}^{\mathrm{MASP2_{zym}}}}+\frac{\left[\mathrm{pCLK1MASP1_{zym}}\right]}{K_{\mathrm{m\ MASP1_{zym}}}^{\mathrm{MASP1_{zym}}}}+\frac{\left[\mathrm{pCLK1MASP2_{zym}}\right]}{K_{\mathrm{m\ MASP1_{zym}}}^{\mathrm{MASP2_{zym}}}}\\+\frac{\left[\mathrm{pCLL1MASP1_{zym}}\right]}{K_{\mathrm{m\ MASP1_{zym}}}^{\mathrm{MASP1_{zym}}}}+\frac{\left[\mathrm{pCLL1MASP2_{zym}}\right]}{K_{\mathrm{m\ MASP1_{zym}}}^{\mathrm{MASP2_{zym}}}}+\frac{\left[\mathrm{pLFMASP1_{zym}}\right]}{K_{\mathrm{m\ MASP1_{zym}}}^{\mathrm{MASP1_{zym}}}}+\frac{\left[\mathrm{pLFMASP2_{zym}}\right]}{K_{\mathrm{m\ MASP1_{zym}}}^{\mathrm{MASP2_{zym}}}}\\+\frac{\left[\mathrm{pHFMASP1_{zym}}\right]}{K_{\mathrm{m\ MASP1_{zym}}}^{\mathrm{MASP1_{zym}}}}+\frac{\left[\mathrm{pHFMASP2_{zym}}\right]}{K_{\mathrm{m\ MASP1_{zym}}}^{\mathrm{MASP2_{zym}}}}+\frac{\left[\mathrm{pMFMASP1_{zym}}\right]}{K_{\mathrm{m\ MASP1_{zym}}}^{\mathrm{MASP1_{zym}}}}+\frac{\left[\mathrm{pMFMASP2_{zym}}\right]}{K_{\mathrm{m\ MASP1_{zym}}}^{\mathrm{MASP2_{zym}}}}\end{array}\right)}
$$

$$+\frac{k_{\mathrm{cat\ MASP1_{zym}}}^{\mathrm{MASP1_{zym}}}\left[\mathrm{pCLL1MASP1_{zym}}\right]\left[\mathrm{pMBL_3MASP1_{zym}}\right]}{K_{\mathrm{m\ MASP1_{zym}}}^{\mathrm{MASP1_{zym}}}\left(\begin{array}{l}1+\frac{\left[\mathrm{pMBL_3MASP1_{zym}}\right]}{K_{\mathrm{m\ MASP1_{zym}}}^{\mathrm{MASP1_{zym}}}}+\frac{\left[\mathrm{pMBL_3MASP2_{zym}}\right]}{K_{\mathrm{m\ MASP1_{zym}}}^{\mathrm{MASP2_{zym}}}}+\frac{\left[\mathrm{pMBL_4MASP1_{zym}}\right]}{K_{\mathrm{m\ MASP1_{zym}}}^{\mathrm{MASP1_{zym}}}}+\frac{\left[\mathrm{pMBL_4MASP2_{zym}}\right]}{K_{\mathrm{m\ MASP1_{zym}}}^{\mathrm{MASP2_{zym}}}}\\+\frac{\left[\mathrm{pCLLKMASP1_{zym}}\right]}{K_{\mathrm{m\ MASP1_{zym}}}^{\mathrm{MASP1_{zym}}}}+\frac{\left[\mathrm{pCLLKMASP2_{zym}}\right]}{K_{\mathrm{m\ MASP1_{zym}}}^{\mathrm{MASP2_{zym}}}}+\frac{\left[\mathrm{pCLK1MASP1_{zym}}\right]}{K_{\mathrm{m\ MASP1_{zym}}}^{\mathrm{MASP1_{zym}}}}+\frac{\left[\mathrm{pCLK1MASP2_{zym}}\right]}{K_{\mathrm{m\ MASP1_{zym}}}^{\mathrm{MASP2_{zym}}}}\\+\frac{\left[\mathrm{pCLL1MASP1_{zym}}\right]}{K_{\mathrm{m\ MASP1_{zym}}}^{\mathrm{MASP1_{zym}}}}+\frac{\left[\mathrm{pCLL1MASP2_{zym}}\right]}{K_{\mathrm{m\ MASP1_{zym}}}^{\mathrm{MASP2_{zym}}}}+\frac{\left[\mathrm{pLFMASP1_{zym}}\right]}{K_{\mathrm{m\ MASP1_{zym}}}^{\mathrm{MASP1_{zym}}}}+\frac{\left[\mathrm{pLFMASP2_{zym}}\right]}{K_{\mathrm{m\ MASP1_{zym}}}^{\mathrm{MASP2_{zym}}}}\\+\frac{\left[\mathrm{pHFMASP1_{zym}}\right]}{K_{\mathrm{m\ MASP1_{zym}}}^{\mathrm{MASP1_{zym}}}}+\frac{\left[\mathrm{pHFMASP2_{zym}}\right]}{K_{\mathrm{m\ MASP1_{zym}}}^{\mathrm{MASP2_{zym}}}}+\frac{\left[\mathrm{pMFMASP1_{zym}}\right]}{K_{\mathrm{m\ MASP1_{zym}}}^{\mathrm{MASP1_{zym}}}}+\frac{\left[\mathrm{pMFMASP2_{zym}}\right]}{K_{\mathrm{m\ MASP1_{zym}}}^{\mathrm{MASP2_{zym}}}}\end{array}\right)}$$

$$+\frac{k_{\mathrm{cat\ MASP1_{zym}}}^{\mathrm{MASP1_{zym}}}\left[\mathrm{pLFMASP1_{zym}}\right]\left[\mathrm{pMBL_3MASP1_{zym}}\right]}{K_{\mathrm{m\ MASP1_{zym}}}^{\mathrm{MASP1_{zym}}}\left(\begin{array}{l}1+\frac{\left[\mathrm{pMBL_3MASP1_{zym}}\right]}{K_{\mathrm{m\ MASP1_{zym}}}^{\mathrm{MASP1_{zym}}}}+\frac{\left[\mathrm{pMBL_3MASP2_{zym}}\right]}{K_{\mathrm{m\ MASP1_{zym}}}^{\mathrm{MASP2_{zym}}}}+\frac{\left[\mathrm{pMBL_4MASP1_{zym}}\right]}{K_{\mathrm{m\ MASP1_{zym}}}^{\mathrm{MASP1_{zym}}}}+\frac{\left[\mathrm{pMBL_4MASP2_{zym}}\right]}{K_{\mathrm{m\ MASP1_{zym}}}^{\mathrm{MASP2_{zym}}}}\\+\frac{\left[\mathrm{pCLLKMASP1_{zym}}\right]}{K_{\mathrm{m\ MASP1_{zym}}}^{\mathrm{MASP1_{zym}}}}+\frac{\left[\mathrm{pCLLKMASP2_{zym}}\right]}{K_{\mathrm{m\ MASP1_{zym}}}^{\mathrm{MASP2_{zym}}}}+\frac{\left[\mathrm{pCLK1MASP1_{zym}}\right]}{K_{\mathrm{m\ MASP1_{zym}}}^{\mathrm{MASP1_{zym}}}}+\frac{\left[\mathrm{pCLK1MASP2_{zym}}\right]}{K_{\mathrm{m\ MASP1_{zym}}}^{\mathrm{MASP2_{zym}}}}\\+\frac{\left[\mathrm{pCLL1MASP1_{zym}}\right]}{K_{\mathrm{m\ MASP1_{zym}}}^{\mathrm{MASP1_{zym}}}}+\frac{\left[\mathrm{pCLL1MASP2_{zym}}\right]}{K_{\mathrm{m\ MASP1_{zym}}}^{\mathrm{MASP2_{zym}}}}+\frac{\left[\mathrm{pLFMASP1_{zym}}\right]}{K_{\mathrm{m\ MASP1_{zym}}}^{\mathrm{MASP1_{zym}}}}+\frac{\left[\mathrm{pLFMASP2_{zym}}\right]}{K_{\mathrm{m\ MASP1_{zym}}}^{\mathrm{MASP2_{zym}}}}\\+\frac{\left[\mathrm{pHFMASP1_{zym}}\right]}{K_{\mathrm{m\ MASP1_{zym}}}^{\mathrm{MASP1_{zym}}}}+\frac{\left[\mathrm{pHFMASP2_{zym}}\right]}{K_{\mathrm{m\ MASP1_{zym}}}^{\mathrm{MASP2_{zym}}}}+\frac{\left[\mathrm{pMFMASP1_{zym}}\right]}{K_{\mathrm{m\ MASP1_{zym}}}^{\mathrm{MASP1_{zym}}}}+\frac{\left[\mathrm{pMFMASP2_{zym}}\right]}{K_{\mathrm{m\ MASP1_{zym}}}^{\mathrm{MASP2_{zym}}}}\end{array}\right)}$$

$$+\frac{k_{\mathrm{cat\ MASP1_{zym}}}^{\mathrm{MASP1_{zym}}}\left[\mathrm{pHFMASP1_{zym}}\right]\left[\mathrm{pMBL_3MASP1_{zym}}\right]}{K_{\mathrm{m\ MASP1_{zym}}}^{\mathrm{MASP1_{zym}}}\left(\begin{array}{l}1+\frac{\left[\mathrm{pMBL_3MASP1_{zym}}\right]}{K_{\mathrm{m\ MASP1_{zym}}}^{\mathrm{MASP1_{zym}}}}+\frac{\left[\mathrm{pMBL_3MASP2_{zym}}\right]}{K_{\mathrm{m\ MASP1_{zym}}}^{\mathrm{MASP2_{zym}}}}+\frac{\left[\mathrm{pMBL_4MASP1_{zym}}\right]}{K_{\mathrm{m\ MASP1_{zym}}}^{\mathrm{MASP1_{zym}}}}+\frac{\left[\mathrm{pMBL_4MASP2_{zym}}\right]}{K_{\mathrm{m\ MASP1_{zym}}}^{\mathrm{MASP2_{zym}}}}\\+\frac{\left[\mathrm{pCLLKMASP1_{zym}}\right]}{K_{\mathrm{m\ MASP1_{zym}}}^{\mathrm{MASP1_{zym}}}}+\frac{\left[\mathrm{pCLLKMASP2_{zym}}\right]}{K_{\mathrm{m\ MASP1_{zym}}}^{\mathrm{MASP2_{zym}}}}+\frac{\left[\mathrm{pCLK1MASP1_{zym}}\right]}{K_{\mathrm{m\ MASP1_{zym}}}^{\mathrm{MASP1_{zym}}}}+\frac{\left[\mathrm{pCLK1MASP2_{zym}}\right]}{K_{\mathrm{m\ MASP1_{zym}}}^{\mathrm{MASP2_{zym}}}}\\+\frac{\left[\mathrm{pCLL1MASP1_{zym}}\right]}{K_{\mathrm{m\ MASP1_{zym}}}^{\mathrm{MASP1_{zym}}}}+\frac{\left[\mathrm{pCLL1MASP2_{zym}}\right]}{K_{\mathrm{m\ MASP1_{zym}}}^{\mathrm{MASP2_{zym}}}}+\frac{\left[\mathrm{pLFMASP1_{zym}}\right]}{K_{\mathrm{m\ MASP1_{zym}}}^{\mathrm{MASP1_{zym}}}}+\frac{\left[\mathrm{pLFMASP2_{zym}}\right]}{K_{\mathrm{m\ MASP1_{zym}}}^{\mathrm{MASP2_{zym}}}}\\+\frac{\left[\mathrm{pHFMASP1_{zym}}\right]}{K_{\mathrm{m\ MASP1_{zym}}}^{\mathrm{MASP1_{zym}}}}+\frac{\left[\mathrm{pHFMASP2_{zym}}\right]}{K_{\mathrm{m\ MASP1_{zym}}}^{\mathrm{MASP2_{zym}}}}+\frac{\left[\mathrm{pMFMASP1_{zym}}\right]}{K_{\mathrm{m\ MASP1_{zym}}}^{\mathrm{MASP1_{zym}}}}+\frac{\left[\mathrm{pMFMASP2_{zym}}\right]}{K_{\mathrm{m\ MASP1_{zym}}}^{\mathrm{MASP2_{zym}}}}\end{array}\right)}$$

$$
+\frac{k_{\mathrm{cat\,MASP1_{zym}}}^{\mathrm{MASP1_{zym}}}\left[\mathrm{pMFMASP1_{zym}}\right]\left[\mathrm{pMBL_3MASP1_{zym}}\right]}{K_{\mathrm{m\,MASP1_{zym}}}^{\mathrm{MASP1_{zym}}}\left(\begin{array}{l}1+\frac{\left[\mathrm{pMBL_3MASP1_{zym}}\right]}{K_{\mathrm{m\,MASP1_{zym}}}^{\mathrm{MASP1_{zym}}}}+\frac{\left[\mathrm{pMBL_3MASP2_{zym}}\right]}{K_{\mathrm{m\,MASP1_{zym}}}^{\mathrm{MASP2_{zym}}}}+\frac{\left[\mathrm{pMBL_4MASP1_{zym}}\right]}{K_{\mathrm{m\,MASP1_{zym}}}^{\mathrm{MASP1_{zym}}}}+\frac{\left[\mathrm{pMBL_4MASP2_{zym}}\right]}{K_{\mathrm{m\,MASP1_{zym}}}^{\mathrm{MASP2_{zym}}}}\\+\frac{\left[\mathrm{pCLLKMASP1_{zym}}\right]}{K_{\mathrm{m\,MASP1_{zym}}}^{\mathrm{MASP1_{zym}}}}+\frac{\left[\mathrm{pCLLKMASP2_{zym}}\right]}{K_{\mathrm{m\,MASP1_{zym}}}^{\mathrm{MASP2_{zym}}}}+\frac{\left[\mathrm{pCLK1MASP1_{zym}}\right]}{K_{\mathrm{m\,MASP1_{zym}}}^{\mathrm{MASP1_{zym}}}}+\frac{\left[\mathrm{pCLK1MASP2_{zym}}\right]}{K_{\mathrm{m\,MASP1_{zym}}}^{\mathrm{MASP2_{zym}}}}\\+\frac{\left[\mathrm{pCLL1MASP1_{zym}}\right]}{K_{\mathrm{m\,MASP1_{zym}}}^{\mathrm{MASP1_{zym}}}}+\frac{\left[\mathrm{pCLL1MASP2_{zym}}\right]}{K_{\mathrm{m\,MASP1_{zym}}}^{\mathrm{MASP2_{zym}}}}+\frac{\left[\mathrm{pLFMASP1_{zym}}\right]}{K_{\mathrm{m\,MASP1_{zym}}}^{\mathrm{MASP1_{zym}}}}+\frac{\left[\mathrm{pLFMASP2_{zym}}\right]}{K_{\mathrm{m\,MASP1_{zym}}}^{\mathrm{MASP2_{zym}}}}\\+\frac{\left[\mathrm{pHFMASP1_{zym}}\right]}{K_{\mathrm{m\,MASP1_{zym}}}^{\mathrm{MASP1_{zym}}}}+\frac{\left[\mathrm{pHFMASP2_{zym}}\right]}{K_{\mathrm{m\,MASP1_{zym}}}^{\mathrm{MASP2_{zym}}}}+\frac{\left[\mathrm{pMFMASP1_{zym}}\right]}{K_{\mathrm{m\,MASP1_{zym}}}^{\mathrm{MASP1_{zym}}}}+\frac{\left[\mathrm{pMFMASP2_{zym}}\right]}{K_{\mathrm{m\,MASP1_{zym}}}^{\mathrm{MASP2_{zym}}}}\end{array}\right)}
$$

$$
+\frac{k_{\mathrm{cat\,MASP1_{zym}}}^{\mathrm{MASP1_{zym}}}\left[\mathrm{pPTX3LFMASP1_{zym}}\right]\left[\mathrm{pMBL_3MASP1_{zym}}\right]}{K_{\mathrm{m\,MASP1_{zym}}}^{\mathrm{MASP1_{zym}}}\left(\begin{array}{l}1+\frac{\left[\mathrm{pMBL_3MASP1_{zym}}\right]}{K_{\mathrm{m\,MASP1_{zym}}}^{\mathrm{MASP1_{zym}}}}+\frac{\left[\mathrm{pMBL_3MASP2_{zym}}\right]}{K_{\mathrm{m\,MASP1_{zym}}}^{\mathrm{MASP2_{zym}}}}+\frac{\left[\mathrm{pMBL_4MASP1_{zym}}\right]}{K_{\mathrm{m\,MASP1_{zym}}}^{\mathrm{MASP1_{zym}}}}+\frac{\left[\mathrm{pMBL_4MASP2_{zym}}\right]}{K_{\mathrm{m\,MASP1_{zym}}}^{\mathrm{MASP2_{zym}}}}\\+\frac{\left[\mathrm{pCLLKMASP1_{zym}}\right]}{K_{\mathrm{m\,MASP1_{zym}}}^{\mathrm{MASP1_{zym}}}}+\frac{\left[\mathrm{pCLLKMASP2_{zym}}\right]}{K_{\mathrm{m\,MASP1_{zym}}}^{\mathrm{MASP2_{zym}}}}+\frac{\left[\mathrm{pCLK1MASP1_{zym}}\right]}{K_{\mathrm{m\,MASP1_{zym}}}^{\mathrm{MASP1_{zym}}}}+\frac{\left[\mathrm{pCLK1MASP2_{zym}}\right]}{K_{\mathrm{m\,MASP1_{zym}}}^{\mathrm{MASP2_{zym}}}}\\+\frac{\left[\mathrm{pCLL1MASP1_{zym}}\right]}{K_{\mathrm{m\,MASP1_{zym}}}^{\mathrm{MASP1_{zym}}}}+\frac{\left[\mathrm{pCLL1MASP2_{zym}}\right]}{K_{\mathrm{m\,MASP1_{zym}}}^{\mathrm{MASP2_{zym}}}}+\frac{\left[\mathrm{pLFMASP1_{zym}}\right]}{K_{\mathrm{m\,MASP1_{zym}}}^{\mathrm{MASP1_{zym}}}}+\frac{\left[\mathrm{pLFMASP2_{zym}}\right]}{K_{\mathrm{m\,MASP1_{zym}}}^{\mathrm{MASP2_{zym}}}}\\+\frac{\left[\mathrm{pHFMASP1_{zym}}\right]}{K_{\mathrm{m\,MASP1_{zym}}}^{\mathrm{MASP1_{zym}}}}+\frac{\left[\mathrm{pHFMASP2_{zym}}\right]}{K_{\mathrm{m\,MASP1_{zym}}}^{\mathrm{MASP2_{zym}}}}+\frac{\left[\mathrm{pMFMASP1_{zym}}\right]}{K_{\mathrm{m\,MASP1_{zym}}}^{\mathrm{MASP1_{zym}}}}+\frac{\left[\mathrm{pMFMASP2_{zym}}\right]}{K_{\mathrm{m\,MASP1_{zym}}}^{\mathrm{MASP2_{zym}}}}\\+\frac{\left[\mathrm{pPTX3LFMASP1_{zym}}\right]}{K_{\mathrm{m\,MASP1_{zym}}}^{\mathrm{MASP1_{zym}}}}+\frac{\left[\mathrm{pPTX3LFMASP2_{zym}}\right]}{K_{\mathrm{m\,MASP1_{zym}}}^{\mathrm{MASP2_{zym}}}}\end{array}\right)}
$$

$$
+\frac{k_{\mathrm{cat\,MASP1^*}}^{\mathrm{MASP1_{zym}}}\left[\mathrm{pMBL_3MASP1^*}\right]\left[\mathrm{pMBL_3MASP1_{zym}}\right]}{K_{\mathrm{m\,MASP1^*}}^{\mathrm{MASP1_{zym}}}\left(\begin{aligned}&1+\frac{[\mathrm{C4}]}{K_{\mathrm{m\,MASP1^*}}^{\mathrm{C4}}}+\frac{[\mathrm{fC4bC2}]}{K_{\mathrm{m\,MASP1^*}}^{\mathrm{C2}}}+\frac{[\mathrm{fC4bC4bC2}]}{K_{\mathrm{m\,MASP1^*}}^{\mathrm{C2}}}+\frac{[\mathrm{fC3bC4bC2}]}{K_{\mathrm{m\,MASP1^*}}^{\mathrm{C2}}}+\frac{[\mathrm{pC4bC2}]}{K_{\mathrm{m\,MASP1^*}}^{\mathrm{C2}}}+\frac{[\mathrm{pC4bC4bC2}]}{K_{\mathrm{m\,MASP1^*}}^{\mathrm{C2}}}\\
&+\frac{[\mathrm{pC3bC4bC2}]}{K_{\mathrm{m\,MASP1^*}}^{\mathrm{C2}}}+\frac{[\mathrm{pMBL_3MASP1_{zym}}]}{K_{\mathrm{m\,MASP1^*}}^{\mathrm{MASP1_{zym}}}}+\frac{[\mathrm{pMBL_3MASP2_{zym}}]}{K_{\mathrm{m\,MASP1^*}}^{\mathrm{MASP2_{zym}}}}+\frac{[\mathrm{pMBL_3MASP3_{zym}}]}{K_{\mathrm{m\,MASP1^*}}^{\mathrm{MASP3_{zym}}}}\\
&+\frac{[\mathrm{pMBL_4MASP1_{zym}}]}{K_{\mathrm{m\,MASP1^*}}^{\mathrm{MASP1_{zym}}}}+\frac{[\mathrm{pMBL_4MASP2_{zym}}]}{K_{\mathrm{m\,MASP1^*}}^{\mathrm{MASP2_{zym}}}}+\frac{[\mathrm{pMBL_4MASP3_{zym}}]}{K_{\mathrm{m\,MASP1^*}}^{\mathrm{MASP3_{zym}}}}+\frac{[\mathrm{pCLLKMASP1_{zym}}]}{K_{\mathrm{m\,MASP1^*}}^{\mathrm{MASP1_{zym}}}}\\
&+\frac{[\mathrm{pCLLKMASP2_{zym}}]}{K_{\mathrm{m\,MASP1^*}}^{\mathrm{MASP2_{zym}}}}+\frac{[\mathrm{pCLLKMASP3_{zym}}]}{K_{\mathrm{m\,MASP1^*}}^{\mathrm{MASP3_{zym}}}}+\frac{[\mathrm{pCLK1MASP1_{zym}}]}{K_{\mathrm{m\,MASP1^*}}^{\mathrm{MASP1_{zym}}}}+\frac{[\mathrm{pCLK1MASP2_{zym}}]}{K_{\mathrm{m\,MASP1^*}}^{\mathrm{MASP2_{zym}}}}\\
&+\frac{[\mathrm{pCLK1MASP3_{zym}}]}{K_{\mathrm{m\,MASP1^*}}^{\mathrm{MASP3_{zym}}}}+\frac{[\mathrm{pCLL1MASP1_{zym}}]}{K_{\mathrm{m\,MASP1^*}}^{\mathrm{MASP1_{zym}}}}+\frac{[\mathrm{pCLL1MASP2_{zym}}]}{K_{\mathrm{m\,MASP1^*}}^{\mathrm{MASP2_{zym}}}}+\frac{[\mathrm{pCLL1MASP3_{zym}}]}{K_{\mathrm{m\,MASP1^*}}^{\mathrm{MASP3_{zym}}}}\\
&+\frac{[\mathrm{pLFMASP1_{zym}}]}{K_{\mathrm{m\,MASP1^*}}^{\mathrm{MASP1_{zym}}}}+\frac{[\mathrm{pLFMASP2_{zym}}]}{K_{\mathrm{m\,MASP1^*}}^{\mathrm{MASP2_{zym}}}}+\frac{[\mathrm{pLFMASP3_{zym}}]}{K_{\mathrm{m\,MASP1^*}}^{\mathrm{MASP3_{zym}}}}+\frac{[\mathrm{pHFMASP1_{zym}}]}{K_{\mathrm{m\,MASP1^*}}^{\mathrm{MASP1_{zym}}}}+\frac{[\mathrm{pHFMASP2_{zym}}]}{K_{\mathrm{m\,MASP1^*}}^{\mathrm{MASP2_{zym}}}}\\
&+\frac{[\mathrm{pHFMASP3_{zym}}]}{K_{\mathrm{m\,MASP1^*}}^{\mathrm{MASP3_{zym}}}}+\frac{[\mathrm{pMFMASP1_{zym}}]}{K_{\mathrm{m\,MASP1^*}}^{\mathrm{MASP1_{zym}}}}+\frac{[\mathrm{pMFMASP2_{zym}}]}{K_{\mathrm{m\,MASP1^*}}^{\mathrm{MASP2_{zym}}}}+\frac{[\mathrm{pMFMASP3_{zym}}]}{K_{\mathrm{m\,MASP1^*}}^{\mathrm{MASP3_{zym}}}}\end{aligned}\right)}
$$

$$
+\frac{k_{\mathrm{cat\,MASP1^*}}^{\mathrm{MASP1_{zym}}}\left[\mathrm{pMBL_4MASP1^*}\right]\left[\mathrm{pMBL_3MASP1_{zym}}\right]}{K_{\mathrm{m\,MASP1^*}}^{\mathrm{MASP1_{zym}}}\left(\begin{aligned}&1+\frac{[\mathrm{C4}]}{K_{\mathrm{m\,MASP1^*}}^{\mathrm{C4}}}+\frac{[\mathrm{fC4bC2}]}{K_{\mathrm{m\,MASP1^*}}^{\mathrm{C2}}}+\frac{[\mathrm{fC4bC4bC2}]}{K_{\mathrm{m\,MASP1^*}}^{\mathrm{C2}}}+\frac{[\mathrm{fC3bC4bC2}]}{K_{\mathrm{m\,MASP1^*}}^{\mathrm{C2}}}+\frac{[\mathrm{pC4bC2}]}{K_{\mathrm{m\,MASP1^*}}^{\mathrm{C2}}}+\frac{[\mathrm{pC4bC4bC2}]}{K_{\mathrm{m\,MASP1^*}}^{\mathrm{C2}}}\\
&+\frac{[\mathrm{pC3bC4bC2}]}{K_{\mathrm{m\,MASP1^*}}^{\mathrm{C2}}}+\frac{[\mathrm{pMBL_3MASP1_{zym}}]}{K_{\mathrm{m\,MASP1^*}}^{\mathrm{MASP1_{zym}}}}+\frac{[\mathrm{pMBL_3MASP2_{zym}}]}{K_{\mathrm{m\,MASP1^*}}^{\mathrm{MASP2_{zym}}}}+\frac{[\mathrm{pMBL_3MASP3_{zym}}]}{K_{\mathrm{m\,MASP1^*}}^{\mathrm{MASP3_{zym}}}}\\
&+\frac{[\mathrm{pMBL_4MASP1_{zym}}]}{K_{\mathrm{m\,MASP1^*}}^{\mathrm{MASP1_{zym}}}}+\frac{[\mathrm{pMBL_4MASP2_{zym}}]}{K_{\mathrm{m\,MASP1^*}}^{\mathrm{MASP2_{zym}}}}+\frac{[\mathrm{pMBL_4MASP3_{zym}}]}{K_{\mathrm{m\,MASP1^*}}^{\mathrm{MASP3_{zym}}}}+\frac{[\mathrm{pCLLKMASP1_{zym}}]}{K_{\mathrm{m\,MASP1^*}}^{\mathrm{MASP1_{zym}}}}\\
&+\frac{[\mathrm{pCLLKMASP2_{zym}}]}{K_{\mathrm{m\,MASP1^*}}^{\mathrm{MASP2_{zym}}}}+\frac{[\mathrm{pCLLKMASP3_{zym}}]}{K_{\mathrm{m\,MASP1^*}}^{\mathrm{MASP3_{zym}}}}+\frac{[\mathrm{pCLK1MASP1_{zym}}]}{K_{\mathrm{m\,MASP1^*}}^{\mathrm{MASP1_{zym}}}}+\frac{[\mathrm{pCLK1MASP2_{zym}}]}{K_{\mathrm{m\,MASP1^*}}^{\mathrm{MASP2_{zym}}}}\\
&+\frac{[\mathrm{pCLK1MASP3_{zym}}]}{K_{\mathrm{m\,MASP1^*}}^{\mathrm{MASP3_{zym}}}}+\frac{[\mathrm{pCLL1MASP1_{zym}}]}{K_{\mathrm{m\,MASP1^*}}^{\mathrm{MASP1_{zym}}}}+\frac{[\mathrm{pCLL1MASP2_{zym}}]}{K_{\mathrm{m\,MASP1^*}}^{\mathrm{MASP2_{zym}}}}+\frac{[\mathrm{pCLL1MASP3_{zym}}]}{K_{\mathrm{m\,MASP1^*}}^{\mathrm{MASP3_{zym}}}}\\
&+\frac{[\mathrm{pLFMASP1_{zym}}]}{K_{\mathrm{m\,MASP1^*}}^{\mathrm{MASP1_{zym}}}}+\frac{[\mathrm{pLFMASP2_{zym}}]}{K_{\mathrm{m\,MASP1^*}}^{\mathrm{MASP2_{zym}}}}+\frac{[\mathrm{pLFMASP3_{zym}}]}{K_{\mathrm{m\,MASP1^*}}^{\mathrm{MASP3_{zym}}}}+\frac{[\mathrm{pHFMASP1_{zym}}]}{K_{\mathrm{m\,MASP1^*}}^{\mathrm{MASP1_{zym}}}}+\frac{[\mathrm{pHFMASP2_{zym}}]}{K_{\mathrm{m\,MASP1^*}}^{\mathrm{MASP2_{zym}}}}\\
&+\frac{[\mathrm{pHFMASP3_{zym}}]}{K_{\mathrm{m\,MASP1^*}}^{\mathrm{MASP3_{zym}}}}+\frac{[\mathrm{pMFMASP1_{zym}}]}{K_{\mathrm{m\,MASP1^*}}^{\mathrm{MASP1_{zym}}}}+\frac{[\mathrm{pMFMASP2_{zym}}]}{K_{\mathrm{m\,MASP1^*}}^{\mathrm{MASP2_{zym}}}}+\frac{[\mathrm{pMFMASP3_{zym}}]}{K_{\mathrm{m\,MASP1^*}}^{\mathrm{MASP3_{zym}}}}\end{aligned}\right)}
$$

$$+\frac{k_{\mathrm{cat\,MASP1^{*}}}^{\mathrm{MASP1_{zym}}}\left[\mathrm{pCLLKMASP1^{*}}\right]\left[\mathrm{pMBL_{3}MASP1_{zym}}\right]}{K_{\mathrm{m\,MASP1^{*}}}^{\mathrm{MASP1_{zym}}}\left(\begin{array}{l}1+\frac{[\mathrm{C4}]}{K_{\mathrm{m\,MASP1^{*}}}^{\mathrm{C4}}}+\frac{[\mathrm{fC4bC2}]}{K_{\mathrm{m\,MASP1^{*}}}^{\mathrm{C2}}}+\frac{[\mathrm{fC4bC4bC2}]}{K_{\mathrm{m\,MASP1^{*}}}^{\mathrm{C2}}}+\frac{[\mathrm{fC3bC4bC2}]}{K_{\mathrm{m\,MASP1^{*}}}^{\mathrm{C2}}}+\frac{[\mathrm{pC4bC2}]}{K_{\mathrm{m\,MASP1^{*}}}^{\mathrm{C2}}}+\frac{[\mathrm{pC4bC4bC2}]}{K_{\mathrm{m\,MASP1^{*}}}^{\mathrm{C2}}}\\+\frac{[\mathrm{pC3bC4bC2}]}{K_{\mathrm{m\,MASP1^{*}}}^{\mathrm{C2}}}+\frac{[\mathrm{pMBL_{3}MASP1_{zym}}]}{K_{\mathrm{m\,MASP1^{*}}}^{\mathrm{MASP1_{zym}}}}+\frac{[\mathrm{pMBL_{3}MASP2_{zym}}]}{K_{\mathrm{m\,MASP1^{*}}}^{\mathrm{MASP2_{zym}}}}+\frac{[\mathrm{pMBL_{3}MASP3_{zym}}]}{K_{\mathrm{m\,MASP1^{*}}}^{\mathrm{MASP3_{zym}}}}\\+\frac{[\mathrm{pMBL_{4}MASP1_{zym}}]}{K_{\mathrm{m\,MASP1^{*}}}^{\mathrm{MASP1_{zym}}}}+\frac{[\mathrm{pMBL_{4}MASP2_{zym}}]}{K_{\mathrm{m\,MASP1^{*}}}^{\mathrm{MASP2_{zym}}}}+\frac{[\mathrm{pMBL_{4}MASP3_{zym}}]}{K_{\mathrm{m\,MASP1^{*}}}^{\mathrm{MASP3_{zym}}}}+\frac{[\mathrm{pCLLKMASP1_{zym}}]}{K_{\mathrm{m\,MASP1^{*}}}^{\mathrm{MASP1_{zym}}}}\\+\frac{[\mathrm{pCLLKMASP2_{zym}}]}{K_{\mathrm{m\,MASP1^{*}}}^{\mathrm{MASP2_{zym}}}}+\frac{[\mathrm{pCLLKMASP3_{zym}}]}{K_{\mathrm{m\,MASP1^{*}}}^{\mathrm{MASP3_{zym}}}}+\frac{[\mathrm{pCLK1MASP1_{zym}}]}{K_{\mathrm{m\,MASP1^{*}}}^{\mathrm{MASP1_{zym}}}}+\frac{[\mathrm{pCLK1MASP2_{zym}}]}{K_{\mathrm{m\,MASP1^{*}}}^{\mathrm{MASP2_{zym}}}}\\+\frac{[\mathrm{pCLK1MASP3_{zym}}]}{K_{\mathrm{m\,MASP1^{*}}}^{\mathrm{MASP3_{zym}}}}+\frac{[\mathrm{pCLL1MASP1_{zym}}]}{K_{\mathrm{m\,MASP1^{*}}}^{\mathrm{MASP1_{zym}}}}+\frac{[\mathrm{pCLL1MASP2_{zym}}]}{K_{\mathrm{m\,MASP1^{*}}}^{\mathrm{MASP2_{zym}}}}+\frac{[\mathrm{pCLL1MASP3_{zym}}]}{K_{\mathrm{m\,MASP1^{*}}}^{\mathrm{MASP3_{zym}}}}\\+\frac{[\mathrm{pLFMASP1_{zym}}]}{K_{\mathrm{m\,MASP1^{*}}}^{\mathrm{MASP1_{zym}}}}+\frac{[\mathrm{pLFMASP2_{zym}}]}{K_{\mathrm{m\,MASP1^{*}}}^{\mathrm{MASP2_{zym}}}}+\frac{[\mathrm{pLFMASP3_{zym}}]}{K_{\mathrm{m\,MASP1^{*}}}^{\mathrm{MASP3_{zym}}}}+\frac{[\mathrm{pHFMASP1_{zym}}]}{K_{\mathrm{m\,MASP1^{*}}}^{\mathrm{MASP1_{zym}}}}+\frac{[\mathrm{pHFMASP2_{zym}}]}{K_{\mathrm{m\,MASP1^{*}}}^{\mathrm{MASP2_{zym}}}}\\+\frac{[\mathrm{pHFMASP3_{zym}}]}{K_{\mathrm{m\,MASP1^{*}}}^{\mathrm{MASP3_{zym}}}}+\frac{[\mathrm{pMFMASP1_{zym}}]}{K_{\mathrm{m\,MASP1^{*}}}^{\mathrm{MASP1_{zym}}}}+\frac{[\mathrm{pMFMASP2_{zym}}]}{K_{\mathrm{m\,MASP1^{*}}}^{\mathrm{MASP2_{zym}}}}+\frac{[\mathrm{pMFMASP3_{zym}}]}{K_{\mathrm{m\,MASP1^{*}}}^{\mathrm{MASP3_{zym}}}}\end{array}\right)}$$

$$+\frac{k_{\mathrm{cat\,MASP1^{*}}}^{\mathrm{MASP1_{zym}}}\left[\mathrm{pCLK1MASP1^{*}}\right]\left[\mathrm{pMBL_{3}MASP1_{zym}}\right]}{K_{\mathrm{m\,MASP1^{*}}}^{\mathrm{MASP1_{zym}}}\left(\begin{array}{l}1+\frac{[\mathrm{C4}]}{K_{\mathrm{m\,MASP1^{*}}}^{\mathrm{C4}}}+\frac{[\mathrm{fC4bC2}]}{K_{\mathrm{m\,MASP1^{*}}}^{\mathrm{C2}}}+\frac{[\mathrm{fC4bC4bC2}]}{K_{\mathrm{m\,MASP1^{*}}}^{\mathrm{C2}}}+\frac{[\mathrm{fC3bC4bC2}]}{K_{\mathrm{m\,MASP1^{*}}}^{\mathrm{C2}}}+\frac{[\mathrm{pC4bC2}]}{K_{\mathrm{m\,MASP1^{*}}}^{\mathrm{C2}}}+\frac{[\mathrm{pC4bC4bC2}]}{K_{\mathrm{m\,MASP1^{*}}}^{\mathrm{C2}}}\\+\frac{[\mathrm{pC3bC4bC2}]}{K_{\mathrm{m\,MASP1^{*}}}^{\mathrm{C2}}}+\frac{[\mathrm{pMBL_{3}MASP1_{zym}}]}{K_{\mathrm{m\,MASP1^{*}}}^{\mathrm{MASP1_{zym}}}}+\frac{[\mathrm{pMBL_{3}MASP2_{zym}}]}{K_{\mathrm{m\,MASP1^{*}}}^{\mathrm{MASP2_{zym}}}}+\frac{[\mathrm{pMBL_{3}MASP3_{zym}}]}{K_{\mathrm{m\,MASP1^{*}}}^{\mathrm{MASP3_{zym}}}}\\+\frac{[\mathrm{pMBL_{4}MASP1_{zym}}]}{K_{\mathrm{m\,MASP1^{*}}}^{\mathrm{MASP1_{zym}}}}+\frac{[\mathrm{pMBL_{4}MASP2_{zym}}]}{K_{\mathrm{m\,MASP1^{*}}}^{\mathrm{MASP2_{zym}}}}+\frac{[\mathrm{pMBL_{4}MASP3_{zym}}]}{K_{\mathrm{m\,MASP1^{*}}}^{\mathrm{MASP3_{zym}}}}+\frac{[\mathrm{pCLLKMASP1_{zym}}]}{K_{\mathrm{m\,MASP1^{*}}}^{\mathrm{MASP1_{zym}}}}\\+\frac{[\mathrm{pCLLKMASP2_{zym}}]}{K_{\mathrm{m\,MASP1^{*}}}^{\mathrm{MASP2_{zym}}}}+\frac{[\mathrm{pCLLKMASP3_{zym}}]}{K_{\mathrm{m\,MASP1^{*}}}^{\mathrm{MASP3_{zym}}}}+\frac{[\mathrm{pCLK1MASP1_{zym}}]}{K_{\mathrm{m\,MASP1^{*}}}^{\mathrm{MASP1_{zym}}}}+\frac{[\mathrm{pCLK1MASP2_{zym}}]}{K_{\mathrm{m\,MASP1^{*}}}^{\mathrm{MASP2_{zym}}}}\\+\frac{[\mathrm{pCLK1MASP3_{zym}}]}{K_{\mathrm{m\,MASP1^{*}}}^{\mathrm{MASP3_{zym}}}}+\frac{[\mathrm{pCLL1MASP1_{zym}}]}{K_{\mathrm{m\,MASP1^{*}}}^{\mathrm{MASP1_{zym}}}}+\frac{[\mathrm{pCLL1MASP2_{zym}}]}{K_{\mathrm{m\,MASP1^{*}}}^{\mathrm{MASP2_{zym}}}}+\frac{[\mathrm{pCLL1MASP3_{zym}}]}{K_{\mathrm{m\,MASP1^{*}}}^{\mathrm{MASP3_{zym}}}}\\+\frac{[\mathrm{pLFMASP1_{zym}}]}{K_{\mathrm{m\,MASP1^{*}}}^{\mathrm{MASP1_{zym}}}}+\frac{[\mathrm{pLFMASP2_{zym}}]}{K_{\mathrm{m\,MASP1^{*}}}^{\mathrm{MASP2_{zym}}}}+\frac{[\mathrm{pLFMASP3_{zym}}]}{K_{\mathrm{m\,MASP1^{*}}}^{\mathrm{MASP3_{zym}}}}+\frac{[\mathrm{pHFMASP1_{zym}}]}{K_{\mathrm{m\,MASP1^{*}}}^{\mathrm{MASP1_{zym}}}}+\frac{[\mathrm{pHFMASP2_{zym}}]}{K_{\mathrm{m\,MASP1^{*}}}^{\mathrm{MASP2_{zym}}}}\\+\frac{[\mathrm{pHFMASP3_{zym}}]}{K_{\mathrm{m\,MASP1^{*}}}^{\mathrm{MASP3_{zym}}}}+\frac{[\mathrm{pMFMASP1_{zym}}]}{K_{\mathrm{m\,MASP1^{*}}}^{\mathrm{MASP1_{zym}}}}+\frac{[\mathrm{pMFMASP2_{zym}}]}{K_{\mathrm{m\,MASP1^{*}}}^{\mathrm{MASP2_{zym}}}}+\frac{[\mathrm{pMFMASP3_{zym}}]}{K_{\mathrm{m\,MASP1^{*}}}^{\mathrm{MASP3_{zym}}}}\end{array}\right)}$$

$$+\frac{k_{\mathrm{cat\ MASP1^*}}^{\mathrm{MASP1_{zym}}}\left[\mathrm{pCLL1MASP1^*}\right]\left[\mathrm{pMBL_3MASP1_{zym}}\right]}{K_{\mathrm{m\ MASP1^*}}^{\mathrm{MASP1_{zym}}}\left(\begin{array}{l}1+\frac{[\mathrm{C4}]}{K_{\mathrm{m\ MASP1^*}}^{\mathrm{C4}}}+\frac{[\mathrm{fC4bC2}]}{K_{\mathrm{m\ MASP1^*}}^{\mathrm{C2}}}+\frac{[\mathrm{fC4bC4bC2}]}{K_{\mathrm{m\ MASP1^*}}^{\mathrm{C2}}}+\frac{[\mathrm{fC3bC4bC2}]}{K_{\mathrm{m\ MASP1^*}}^{\mathrm{C2}}}+\frac{[\mathrm{pC4bC2}]}{K_{\mathrm{m\ MASP1^*}}^{\mathrm{C2}}}+\frac{[\mathrm{pC4bC4bC2}]}{K_{\mathrm{m\ MASP1^*}}^{\mathrm{C2}}}\\
+\frac{[\mathrm{pC3bC4bC2}]}{K_{\mathrm{m\ MASP1^*}}^{\mathrm{C2}}}+\frac{[\mathrm{pMBL_3MASP1_{zym}}]}{K_{\mathrm{m\ MASP1^*}}^{\mathrm{MASP1_{zym}}}}+\frac{[\mathrm{pMBL_3MASP2_{zym}}]}{K_{\mathrm{m\ MASP1^*}}^{\mathrm{MASP2_{zym}}}}+\frac{[\mathrm{pMBL_3MASP3_{zym}}]}{K_{\mathrm{m\ MASP1^*}}^{\mathrm{MASP3_{zym}}}}\\
+\frac{[\mathrm{pMBL_4MASP1_{zym}}]}{K_{\mathrm{m\ MASP1^*}}^{\mathrm{MASP1_{zym}}}}+\frac{[\mathrm{pMBL_4MASP2_{zym}}]}{K_{\mathrm{m\ MASP1^*}}^{\mathrm{MASP2_{zym}}}}+\frac{[\mathrm{pMBL_4MASP3_{zym}}]}{K_{\mathrm{m\ MASP1^*}}^{\mathrm{MASP3_{zym}}}}+\frac{[\mathrm{pCLLKMASP1_{zym}}]}{K_{\mathrm{m\ MASP1^*}}^{\mathrm{MASP1_{zym}}}}\\
+\frac{[\mathrm{pCLLKMASP2_{zym}}]}{K_{\mathrm{m\ MASP1^*}}^{\mathrm{MASP2_{zym}}}}+\frac{[\mathrm{pCLLKMASP3_{zym}}]}{K_{\mathrm{m\ MASP1^*}}^{\mathrm{MASP3_{zym}}}}+\frac{[\mathrm{pCLK1MASP1_{zym}}]}{K_{\mathrm{m\ MASP1^*}}^{\mathrm{MASP1_{zym}}}}+\frac{[\mathrm{pCLK1MASP2_{zym}}]}{K_{\mathrm{m\ MASP1^*}}^{\mathrm{MASP2_{zym}}}}\\
+\frac{[\mathrm{pCLK1MASP3_{zym}}]}{K_{\mathrm{m\ MASP1^*}}^{\mathrm{MASP3_{zym}}}}+\frac{[\mathrm{pCLL1MASP1_{zym}}]}{K_{\mathrm{m\ MASP1^*}}^{\mathrm{MASP1_{zym}}}}+\frac{[\mathrm{pCLL1MASP2_{zym}}]}{K_{\mathrm{m\ MASP1^*}}^{\mathrm{MASP2_{zym}}}}+\frac{[\mathrm{pCLL1MASP3_{zym}}]}{K_{\mathrm{m\ MASP1^*}}^{\mathrm{MASP3_{zym}}}}\\
+\frac{[\mathrm{pLFMASP1_{zym}}]}{K_{\mathrm{m\ MASP1^*}}^{\mathrm{MASP1_{zym}}}}+\frac{[\mathrm{pLFMASP2_{zym}}]}{K_{\mathrm{m\ MASP1^*}}^{\mathrm{MASP2_{zym}}}}+\frac{[\mathrm{pLFMASP3_{zym}}]}{K_{\mathrm{m\ MASP1^*}}^{\mathrm{MASP3_{zym}}}}+\frac{[\mathrm{pHFMASP1_{zym}}]}{K_{\mathrm{m\ MASP1^*}}^{\mathrm{MASP1_{zym}}}}+\frac{[\mathrm{pHFMASP2_{zym}}]}{K_{\mathrm{m\ MASP1^*}}^{\mathrm{MASP2_{zym}}}}\\
+\frac{[\mathrm{pHFMASP3_{zym}}]}{K_{\mathrm{m\ MASP1^*}}^{\mathrm{MASP3_{zym}}}}+\frac{[\mathrm{pMFMASP1_{zym}}]}{K_{\mathrm{m\ MASP1^*}}^{\mathrm{MASP1_{zym}}}}+\frac{[\mathrm{pMFMASP2_{zym}}]}{K_{\mathrm{m\ MASP1^*}}^{\mathrm{MASP2_{zym}}}}+\frac{[\mathrm{pMFMASP3_{zym}}]}{K_{\mathrm{m\ MASP1^*}}^{\mathrm{MASP3_{zym}}}}\end{array}\right)}$$

$$+\frac{k_{\mathrm{cat\ MASP1^*}}^{\mathrm{MASP1_{zym}}}\left[\mathrm{pLFMASP1^*}\right]\left[\mathrm{pMBL_3MASP1_{zym}}\right]}{K_{\mathrm{m\ MASP1^*}}^{\mathrm{MASP1_{zym}}}\left(\begin{array}{l}1+\frac{[\mathrm{C4}]}{K_{\mathrm{m\ MASP1^*}}^{\mathrm{C4}}}+\frac{[\mathrm{fC4bC2}]}{K_{\mathrm{m\ MASP1^*}}^{\mathrm{C2}}}+\frac{[\mathrm{fC4bC4bC2}]}{K_{\mathrm{m\ MASP1^*}}^{\mathrm{C2}}}+\frac{[\mathrm{fC3bC4bC2}]}{K_{\mathrm{m\ MASP1^*}}^{\mathrm{C2}}}+\frac{[\mathrm{pC4bC2}]}{K_{\mathrm{m\ MASP1^*}}^{\mathrm{C2}}}+\frac{[\mathrm{pC4bC4bC2}]}{K_{\mathrm{m\ MASP1^*}}^{\mathrm{C2}}}\\
+\frac{[\mathrm{pC3bC4bC2}]}{K_{\mathrm{m\ MASP1^*}}^{\mathrm{C2}}}+\frac{[\mathrm{pMBL_3MASP1_{zym}}]}{K_{\mathrm{m\ MASP1^*}}^{\mathrm{MASP1_{zym}}}}+\frac{[\mathrm{pMBL_3MASP2_{zym}}]}{K_{\mathrm{m\ MASP1^*}}^{\mathrm{MASP2_{zym}}}}+\frac{[\mathrm{pMBL_3MASP3_{zym}}]}{K_{\mathrm{m\ MASP1^*}}^{\mathrm{MASP3_{zym}}}}\\
+\frac{[\mathrm{pMBL_4MASP1_{zym}}]}{K_{\mathrm{m\ MASP1^*}}^{\mathrm{MASP1_{zym}}}}+\frac{[\mathrm{pMBL_4MASP2_{zym}}]}{K_{\mathrm{m\ MASP1^*}}^{\mathrm{MASP2_{zym}}}}+\frac{[\mathrm{pMBL_4MASP3_{zym}}]}{K_{\mathrm{m\ MASP1^*}}^{\mathrm{MASP3_{zym}}}}+\frac{[\mathrm{pCLLKMASP1_{zym}}]}{K_{\mathrm{m\ MASP1^*}}^{\mathrm{MASP1_{zym}}}}\\
+\frac{[\mathrm{pCLLKMASP2_{zym}}]}{K_{\mathrm{m\ MASP1^*}}^{\mathrm{MASP2_{zym}}}}+\frac{[\mathrm{pCLLKMASP3_{zym}}]}{K_{\mathrm{m\ MASP1^*}}^{\mathrm{MASP3_{zym}}}}+\frac{[\mathrm{pCLK1MASP1_{zym}}]}{K_{\mathrm{m\ MASP1^*}}^{\mathrm{MASP1_{zym}}}}+\frac{[\mathrm{pCLK1MASP2_{zym}}]}{K_{\mathrm{m\ MASP1^*}}^{\mathrm{MASP2_{zym}}}}\\
+\frac{[\mathrm{pCLK1MASP3_{zym}}]}{K_{\mathrm{m\ MASP1^*}}^{\mathrm{MASP3_{zym}}}}+\frac{[\mathrm{pCLL1MASP1_{zym}}]}{K_{\mathrm{m\ MASP1^*}}^{\mathrm{MASP1_{zym}}}}+\frac{[\mathrm{pCLL1MASP2_{zym}}]}{K_{\mathrm{m\ MASP1^*}}^{\mathrm{MASP2_{zym}}}}+\frac{[\mathrm{pCLL1MASP3_{zym}}]}{K_{\mathrm{m\ MASP1^*}}^{\mathrm{MASP3_{zym}}}}\\
+\frac{[\mathrm{pLFMASP1_{zym}}]}{K_{\mathrm{m\ MASP1^*}}^{\mathrm{MASP1_{zym}}}}+\frac{[\mathrm{pLFMASP2_{zym}}]}{K_{\mathrm{m\ MASP1^*}}^{\mathrm{MASP2_{zym}}}}+\frac{[\mathrm{pLFMASP3_{zym}}]}{K_{\mathrm{m\ MASP1^*}}^{\mathrm{MASP3_{zym}}}}+\frac{[\mathrm{pHFMASP1_{zym}}]}{K_{\mathrm{m\ MASP1^*}}^{\mathrm{MASP1_{zym}}}}+\frac{[\mathrm{pHFMASP2_{zym}}]}{K_{\mathrm{m\ MASP1^*}}^{\mathrm{MASP2_{zym}}}}\\
+\frac{[\mathrm{pHFMASP3_{zym}}]}{K_{\mathrm{m\ MASP1^*}}^{\mathrm{MASP3_{zym}}}}+\frac{[\mathrm{pMFMASP1_{zym}}]}{K_{\mathrm{m\ MASP1^*}}^{\mathrm{MASP1_{zym}}}}+\frac{[\mathrm{pMFMASP2_{zym}}]}{K_{\mathrm{m\ MASP1^*}}^{\mathrm{MASP2_{zym}}}}+\frac{[\mathrm{pMFMASP3_{zym}}]}{K_{\mathrm{m\ MASP1^*}}^{\mathrm{MASP3_{zym}}}}\end{array}\right)}$$

$$
+\frac{k_{\text{cat MASP1}^*}^{\text{MASP1}_{\text{zym}}}\left[\text{pHFMASP1}^*\right]\left[\text{pMBL}_3\text{MASP1}_{\text{zym}}\right]}{K_{\text{m MASP1}^*}^{\text{MASP1}_{\text{zym}}}\left(\begin{aligned}&1+\frac{[\text{C4}]}{K_{\text{m MASP1}^*}^{\text{C4}}}+\frac{[\text{fC4bC2}]}{K_{\text{m MASP1}^*}^{\text{C2}}}+\frac{[\text{fC4bC4bC2}]}{K_{\text{m MASP1}^*}^{\text{C2}}}+\frac{[\text{fC3bC4bC2}]}{K_{\text{m MASP1}^*}^{\text{C2}}}+\frac{[\text{pC4bC2}]}{K_{\text{m MASP1}^*}^{\text{C2}}}+\frac{[\text{pC4bC4bC2}]}{K_{\text{m MASP1}^*}^{\text{C2}}}\\
&+\frac{[\text{pC3bC4bC2}]}{K_{\text{m MASP1}^*}^{\text{C2}}}+\frac{\left[\text{pMBL}_3\text{MASP1}_{\text{zym}}\right]}{K_{\text{m MASP1}^*}^{\text{MASP1}_{\text{zym}}}}+\frac{\left[\text{pMBL}_3\text{MASP2}_{\text{zym}}\right]}{K_{\text{m MASP1}^*}^{\text{MASP2}_{\text{zym}}}}+\frac{\left[\text{pMBL}_3\text{MASP3}_{\text{zym}}\right]}{K_{\text{m MASP1}^*}^{\text{MASP3}_{\text{zym}}}}\\
&+\frac{\left[\text{pMBL}_4\text{MASP1}_{\text{zym}}\right]}{K_{\text{m MASP1}^*}^{\text{MASP1}_{\text{zym}}}}+\frac{\left[\text{pMBL}_4\text{MASP2}_{\text{zym}}\right]}{K_{\text{m MASP1}^*}^{\text{MASP2}_{\text{zym}}}}+\frac{\left[\text{pMBL}_4\text{MASP3}_{\text{zym}}\right]}{K_{\text{m MASP1}^*}^{\text{MASP3}_{\text{zym}}}}+\frac{\left[\text{pCLLKMASP1}_{\text{zym}}\right]}{K_{\text{m MASP1}^*}^{\text{MASP1}_{\text{zym}}}}\\
&+\frac{\left[\text{pCLLKMASP2}_{\text{zym}}\right]}{K_{\text{m MASP1}^*}^{\text{MASP2}_{\text{zym}}}}+\frac{\left[\text{pCLLKMASP3}_{\text{zym}}\right]}{K_{\text{m MASP1}^*}^{\text{MASP3}_{\text{zym}}}}+\frac{\left[\text{pCLK1MASP1}_{\text{zym}}\right]}{K_{\text{m MASP1}^*}^{\text{MASP1}_{\text{zym}}}}+\frac{\left[\text{pCLK1MASP2}_{\text{zym}}\right]}{K_{\text{m MASP1}^*}^{\text{MASP2}_{\text{zym}}}}\\
&+\frac{\left[\text{pCLK1MASP3}_{\text{zym}}\right]}{K_{\text{m MASP1}^*}^{\text{MASP3}_{\text{zym}}}}+\frac{\left[\text{pCLL1MASP1}_{\text{zym}}\right]}{K_{\text{m MASP1}^*}^{\text{MASP1}_{\text{zym}}}}+\frac{\left[\text{pCLL1MASP2}_{\text{zym}}\right]}{K_{\text{m MASP1}^*}^{\text{MASP2}_{\text{zym}}}}+\frac{\left[\text{pCLL1MASP3}_{\text{zym}}\right]}{K_{\text{m MASP1}^*}^{\text{MASP3}_{\text{zym}}}}\\
&+\frac{\left[\text{pLFMASP1}_{\text{zym}}\right]}{K_{\text{m MASP1}^*}^{\text{MASP1}_{\text{zym}}}}+\frac{\left[\text{pLFMASP2}_{\text{zym}}\right]}{K_{\text{m MASP1}^*}^{\text{MASP2}_{\text{zym}}}}+\frac{\left[\text{pLFMASP3}_{\text{zym}}\right]}{K_{\text{m MASP1}^*}^{\text{MASP3}_{\text{zym}}}}+\frac{\left[\text{pHFMASP1}_{\text{zym}}\right]}{K_{\text{m MASP1}^*}^{\text{MASP1}_{\text{zym}}}}+\frac{\left[\text{pHFMASP2}_{\text{zym}}\right]}{K_{\text{m MASP1}^*}^{\text{MASP2}_{\text{zym}}}}\\
&+\frac{\left[\text{pHFMASP3}_{\text{zym}}\right]}{K_{\text{m MASP1}^*}^{\text{MASP3}_{\text{zym}}}}+\frac{\left[\text{pMFMASP1}_{\text{zym}}\right]}{K_{\text{m MASP1}^*}^{\text{MASP1}_{\text{zym}}}}+\frac{\left[\text{pMFMASP2}_{\text{zym}}\right]}{K_{\text{m MASP1}^*}^{\text{MASP2}_{\text{zym}}}}+\frac{\left[\text{pMFMASP3}_{\text{zym}}\right]}{K_{\text{m MASP1}^*}^{\text{MASP3}_{\text{zym}}}}\end{aligned}\right)}
$$

$$
+\frac{k_{\text{cat MASP1}^*}^{\text{MASP1}_{\text{zym}}}\left[\text{pMFMASP1}^*\right]\left[\text{pMBL}_3\text{MASP1}_{\text{zym}}\right]}{K_{\text{m MASP1}^*}^{\text{MASP1}_{\text{zym}}}\left(\begin{aligned}&1+\frac{[\text{C4}]}{K_{\text{m MASP1}^*}^{\text{C4}}}+\frac{[\text{fC4bC2}]}{K_{\text{m MASP1}^*}^{\text{C2}}}+\frac{[\text{fC4bC4bC2}]}{K_{\text{m MASP1}^*}^{\text{C2}}}+\frac{[\text{fC3bC4bC2}]}{K_{\text{m MASP1}^*}^{\text{C2}}}+\frac{[\text{pC4bC2}]}{K_{\text{m MASP1}^*}^{\text{C2}}}+\frac{[\text{pC4bC4bC2}]}{K_{\text{m MASP1}^*}^{\text{C2}}}\\
&+\frac{[\text{pC3bC4bC2}]}{K_{\text{m MASP1}^*}^{\text{C2}}}+\frac{\left[\text{pMBL}_3\text{MASP1}_{\text{zym}}\right]}{K_{\text{m MASP1}^*}^{\text{MASP1}_{\text{zym}}}}+\frac{\left[\text{pMBL}_3\text{MASP2}_{\text{zym}}\right]}{K_{\text{m MASP1}^*}^{\text{MASP2}_{\text{zym}}}}+\frac{\left[\text{pMBL}_3\text{MASP3}_{\text{zym}}\right]}{K_{\text{m MASP1}^*}^{\text{MASP3}_{\text{zym}}}}\\
&+\frac{\left[\text{pMBL}_4\text{MASP1}_{\text{zym}}\right]}{K_{\text{m MASP1}^*}^{\text{MASP1}_{\text{zym}}}}+\frac{\left[\text{pMBL}_4\text{MASP2}_{\text{zym}}\right]}{K_{\text{m MASP1}^*}^{\text{MASP2}_{\text{zym}}}}+\frac{\left[\text{pMBL}_4\text{MASP3}_{\text{zym}}\right]}{K_{\text{m MASP1}^*}^{\text{MASP3}_{\text{zym}}}}+\frac{\left[\text{pCLLKMASP1}_{\text{zym}}\right]}{K_{\text{m MASP1}^*}^{\text{MASP1}_{\text{zym}}}}\\
&+\frac{\left[\text{pCLLKMASP2}_{\text{zym}}\right]}{K_{\text{m MASP1}^*}^{\text{MASP2}_{\text{zym}}}}+\frac{\left[\text{pCLLKMASP3}_{\text{zym}}\right]}{K_{\text{m MASP1}^*}^{\text{MASP3}_{\text{zym}}}}+\frac{\left[\text{pCLK1MASP1}_{\text{zym}}\right]}{K_{\text{m MASP1}^*}^{\text{MASP1}_{\text{zym}}}}+\frac{\left[\text{pCLK1MASP2}_{\text{zym}}\right]}{K_{\text{m MASP1}^*}^{\text{MASP2}_{\text{zym}}}}\\
&+\frac{\left[\text{pCLK1MASP3}_{\text{zym}}\right]}{K_{\text{m MASP1}^*}^{\text{MASP3}_{\text{zym}}}}+\frac{\left[\text{pCLL1MASP1}_{\text{zym}}\right]}{K_{\text{m MASP1}^*}^{\text{MASP1}_{\text{zym}}}}+\frac{\left[\text{pCLL1MASP2}_{\text{zym}}\right]}{K_{\text{m MASP1}^*}^{\text{MASP2}_{\text{zym}}}}+\frac{\left[\text{pCLL1MASP3}_{\text{zym}}\right]}{K_{\text{m MASP1}^*}^{\text{MASP3}_{\text{zym}}}}\\
&+\frac{\left[\text{pLFMASP1}_{\text{zym}}\right]}{K_{\text{m MASP1}^*}^{\text{MASP1}_{\text{zym}}}}+\frac{\left[\text{pLFMASP2}_{\text{zym}}\right]}{K_{\text{m MASP1}^*}^{\text{MASP2}_{\text{zym}}}}+\frac{\left[\text{pLFMASP3}_{\text{zym}}\right]}{K_{\text{m MASP1}^*}^{\text{MASP3}_{\text{zym}}}}+\frac{\left[\text{pHFMASP1}_{\text{zym}}\right]}{K_{\text{m MASP1}^*}^{\text{MASP1}_{\text{zym}}}}+\frac{\left[\text{pHFMASP2}_{\text{zym}}\right]}{K_{\text{m MASP1}^*}^{\text{MASP2}_{\text{zym}}}}\\
&+\frac{\left[\text{pHFMASP3}_{\text{zym}}\right]}{K_{\text{m MASP1}^*}^{\text{MASP3}_{\text{zym}}}}+\frac{\left[\text{pMFMASP1}_{\text{zym}}\right]}{K_{\text{m MASP1}^*}^{\text{MASP1}_{\text{zym}}}}+\frac{\left[\text{pMFMASP2}_{\text{zym}}\right]}{K_{\text{m MASP1}^*}^{\text{MASP2}_{\text{zym}}}}+\frac{\left[\text{pMFMASP3}_{\text{zym}}\right]}{K_{\text{m MASP1}^*}^{\text{MASP3}_{\text{zym}}}}\end{aligned}\right)}
$$

$$
+\frac{k_{\mathrm{cat\ MASP1^*}}^{\mathrm{MASP1_{zym}}}\left[\mathrm{pPTX3LFMASP1^*}\right]\left[\mathrm{pMBL_3MASP1_{zym}}\right]}{K_{\mathrm{m\ MASP1^*}}^{\mathrm{MASP1_{zym}}}\left(\begin{array}{l}
1+\frac{[\mathrm{C4}]}{K_{\mathrm{m\ MASP1^*}}^{\mathrm{C4}}}+\frac{[\mathrm{fC4bC2}]}{K_{\mathrm{m\ MASP1^*}}^{\mathrm{C2}}}+\frac{[\mathrm{fC4bC4bC2}]}{K_{\mathrm{m\ MASP1^*}}^{\mathrm{C2}}}+\frac{[\mathrm{fC3bC4bC2}]}{K_{\mathrm{m\ MASP1^*}}^{\mathrm{C2}}}+\frac{[\mathrm{pC4bC2}]}{K_{\mathrm{m\ MASP1^*}}^{\mathrm{C2}}}+\frac{[\mathrm{pC4bC4bC2}]}{K_{\mathrm{m\ MASP1^*}}^{\mathrm{C2}}} \\
+\frac{[\mathrm{pC3bC4bC2}]}{K_{\mathrm{m\ MASP1^*}}^{\mathrm{C2}}}+\frac{[\mathrm{pMBL_3MASP1_{zym}}]}{K_{\mathrm{m\ MASP1^*}}^{\mathrm{MASP1_{zym}}}}+\frac{[\mathrm{pMBL_3MASP2_{zym}}]}{K_{\mathrm{m\ MASP1^*}}^{\mathrm{MASP2_{zym}}}}+\frac{[\mathrm{pMBL_3MASP3_{zym}}]}{K_{\mathrm{m\ MASP1^*}}^{\mathrm{MASP3_{zym}}}} \\
+\frac{[\mathrm{pMBL_4MASP1_{zym}}]}{K_{\mathrm{m\ MASP1^*}}^{\mathrm{MASP1_{zym}}}}+\frac{[\mathrm{pMBL_4MASP2_{zym}}]}{K_{\mathrm{m\ MASP1^*}}^{\mathrm{MASP2_{zym}}}}+\frac{[\mathrm{pMBL_4MASP3_{zym}}]}{K_{\mathrm{m\ MASP1^*}}^{\mathrm{MASP3_{zym}}}}+\frac{[\mathrm{pCLLKMASP1_{zym}}]}{K_{\mathrm{m\ MASP1^*}}^{\mathrm{MASP1_{zym}}}} \\
+\frac{[\mathrm{pCLLKMASP2_{zym}}]}{K_{\mathrm{m\ MASP1^*}}^{\mathrm{MASP2_{zym}}}}+\frac{[\mathrm{pCLLKMASP3_{zym}}]}{K_{\mathrm{m\ MASP1^*}}^{\mathrm{MASP3_{zym}}}}+\frac{[\mathrm{pCLK1MASP1_{zym}}]}{K_{\mathrm{m\ MASP1^*}}^{\mathrm{MASP1_{zym}}}}+\frac{[\mathrm{pCLK1MASP2_{zym}}]}{K_{\mathrm{m\ MASP1^*}}^{\mathrm{MASP2_{zym}}}} \\
+\frac{[\mathrm{pCLK1MASP3_{zym}}]}{K_{\mathrm{m\ MASP1^*}}^{\mathrm{MASP3_{zym}}}}+\frac{[\mathrm{pCLL1MASP1_{zym}}]}{K_{\mathrm{m\ MASP1^*}}^{\mathrm{MASP1_{zym}}}}+\frac{[\mathrm{pCLL1MASP2_{zym}}]}{K_{\mathrm{m\ MASP1^*}}^{\mathrm{MASP2_{zym}}}}+\frac{[\mathrm{pCLL1MASP3_{zym}}]}{K_{\mathrm{m\ MASP1^*}}^{\mathrm{MASP3_{zym}}}} \\
+\frac{[\mathrm{pLFMASP1_{zym}}]}{K_{\mathrm{m\ MASP1^*}}^{\mathrm{MASP1_{zym}}}}+\frac{[\mathrm{pLFMASP2_{zym}}]}{K_{\mathrm{m\ MASP1^*}}^{\mathrm{MASP2_{zym}}}}+\frac{[\mathrm{pLFMASP3_{zym}}]}{K_{\mathrm{m\ MASP1^*}}^{\mathrm{MASP3_{zym}}}}+\frac{[\mathrm{pHFMASP1_{zym}}]}{K_{\mathrm{m\ MASP1^*}}^{\mathrm{MASP1_{zym}}}}+\frac{[\mathrm{pHFMASP2_{zym}}]}{K_{\mathrm{m\ MASP1^*}}^{\mathrm{MASP2_{zym}}}} \\
+\frac{[\mathrm{pHFMASP3_{zym}}]}{K_{\mathrm{m\ MASP1^*}}^{\mathrm{MASP3_{zym}}}}+\frac{[\mathrm{pMFMASP1_{zym}}]}{K_{\mathrm{m\ MASP1^*}}^{\mathrm{MASP1_{zym}}}}+\frac{[\mathrm{pMFMASP2_{zym}}]}{K_{\mathrm{m\ MASP1^*}}^{\mathrm{MASP2_{zym}}}}+\frac{[\mathrm{pMFMASP3_{zym}}]}{K_{\mathrm{m\ MASP1^*}}^{\mathrm{MASP3_{zym}}}} \\
+\frac{[\mathrm{pPTX3LFMASP1_{zym}}]}{K_{\mathrm{m\ MASP1^*}}^{\mathrm{MASP1_{zym}}}}+\frac{[\mathrm{pPXT3LFMASP2_{zym}}]}{K_{\mathrm{m\ MASP1^*}}^{\mathrm{MASP2_{zym}}}}+\frac{[\mathrm{pPTX3LFMASP3_{zym}}]}{K_{\mathrm{m\ MASP1^*}}^{\mathrm{MASP3_{zym}}}}
\end{array}\right)}
$$

$$
+\frac{k_{\mathrm{cat\ MASP2^*}}^{\mathrm{MASP1_{zym}}}\left[\mathrm{pMBL_3MASP2^*}\right]\left[\mathrm{pMBL_3MASP1_{zym}}\right]}{K_{\mathrm{m\ MASP2^*}}^{\mathrm{MASP1_{zym}}}\left(\begin{array}{l}
1+\frac{[\mathrm{C4}]}{K_{\mathrm{m\ MASP2^*}}^{\mathrm{C4}}}+\frac{[\mathrm{fC4bC2}]}{K_{\mathrm{m\ MASP2^*}}^{\mathrm{C2}}}+\frac{[\mathrm{fC4bC4bC2}]}{K_{\mathrm{m\ MASP2^*}}^{\mathrm{C2}}}+\frac{[\mathrm{fC3bC4bC2}]}{K_{\mathrm{m\ MASP2^*}}^{\mathrm{C2}}}+\frac{[\mathrm{pC4bC2}]}{K_{\mathrm{m\ MASP2^*}}^{\mathrm{C2}}}+\frac{[\mathrm{pC4bC4bC2}]}{K_{\mathrm{m\ MASP2^*}}^{\mathrm{C2}}} \\
+\frac{[\mathrm{pC3bC4bC2}]}{K_{\mathrm{m\ MASP2^*}}^{\mathrm{C2}}}+\frac{[\mathrm{pMBL_3MASP1_{zym}}]}{K_{\mathrm{m\ MASP2^*}}^{\mathrm{MASP1_{zym}}}}+\frac{[\mathrm{pMBL_3MASP2_{zym}}]}{K_{\mathrm{m\ MASP2^*}}^{\mathrm{MASP2_{zym}}}}+\frac{[\mathrm{pMBL_4MASP1_{zym}}]}{K_{\mathrm{m\ MASP2^*}}^{\mathrm{MASP1_{zym}}}} \\
+\frac{[\mathrm{pMBL_4MASP2_{zym}}]}{K_{\mathrm{m\ MASP2^*}}^{\mathrm{MASP2_{zym}}}}+\frac{[\mathrm{pCLLKMASP1_{zym}}]}{K_{\mathrm{m\ MASP2^*}}^{\mathrm{MASP1_{zym}}}}+\frac{[\mathrm{pCLLKMASP2_{zym}}]}{K_{\mathrm{m\ MASP2^*}}^{\mathrm{MASP2_{zym}}}}+\frac{[\mathrm{pCLK1MASP1_{zym}}]}{K_{\mathrm{m\ MASP2^*}}^{\mathrm{MASP1_{zym}}}} \\
+\frac{[\mathrm{pCLK1MASP2_{zym}}]}{K_{\mathrm{m\ MASP2^*}}^{\mathrm{MASP2_{zym}}}}+\frac{[\mathrm{pCLL1MASP1_{zym}}]}{K_{\mathrm{m\ MASP2^*}}^{\mathrm{MASP1_{zym}}}}+\frac{[\mathrm{pCLL1MASP2_{zym}}]}{K_{\mathrm{m\ MASP2^*}}^{\mathrm{MASP2_{zym}}}}+\frac{[\mathrm{pLFMASP1_{zym}}]}{K_{\mathrm{m\ MASP2^*}}^{\mathrm{MASP1_{zym}}}} \\
+\frac{[\mathrm{pLFMASP2_{zym}}]}{K_{\mathrm{m\ MASP2^*}}^{\mathrm{MASP2_{zym}}}}+\frac{[\mathrm{pHFMASP1_{zym}}]}{K_{\mathrm{m\ MASP2^*}}^{\mathrm{MASP1_{zym}}}}+\frac{[\mathrm{pHFMASP2_{zym}}]}{K_{\mathrm{m\ MASP2^*}}^{\mathrm{MASP2_{zym}}}}+\frac{[\mathrm{pMFMASP1_{zym}}]}{K_{\mathrm{m\ MASP2^*}}^{\mathrm{MASP1_{zym}}}}+\frac{[\mathrm{pMFMASP2_{zym}}]}{K_{\mathrm{m\ MASP2^*}}^{\mathrm{MASP2_{zym}}}}
\end{array}\right)}
$$

$$
+\frac{k_{\mathrm{cat\ MASP2^*}}^{\mathrm{MASP1_{zym}}}\left[\mathrm{pMBL_4MASP2^*}\right]\left[\mathrm{pMBL_3MASP1_{zym}}\right]}{K_{\mathrm{m\ MASP2^*}}^{\mathrm{MASP1_{zym}}}\left(\begin{aligned}
&1+\frac{[\mathrm{C4}]}{K_{\mathrm{m\ MASP2^*}}^{\mathrm{C4}}}+\frac{[\mathrm{fC4bC2}]}{K_{\mathrm{m\ MASP2^*}}^{\mathrm{C2}}}+\frac{[\mathrm{fC4bC4bC2}]}{K_{\mathrm{m\ MASP2^*}}^{\mathrm{C2}}}+\frac{[\mathrm{fC3bC4bC2}]}{K_{\mathrm{m\ MASP2^*}}^{\mathrm{C2}}}+\frac{[\mathrm{pC4bC2}]}{K_{\mathrm{m\ MASP2^*}}^{\mathrm{C2}}}+\frac{[\mathrm{pC4bC4bC2}]}{K_{\mathrm{m\ MASP2^*}}^{\mathrm{C2}}}\\
&+\frac{[\mathrm{pC3bC4bC2}]}{K_{\mathrm{m\ MASP2^*}}^{\mathrm{C2}}}+\frac{\left[\mathrm{pMBL_3MASP1_{zym}}\right]}{K_{\mathrm{m\ MASP2^*}}^{\mathrm{MASP1_{zym}}}}+\frac{\left[\mathrm{pMBL_3MASP2_{zym}}\right]}{K_{\mathrm{m\ MASP2^*}}^{\mathrm{MASP2_{zym}}}}+\frac{\left[\mathrm{pMBL_4MASP1_{zym}}\right]}{K_{\mathrm{m\ MASP2^*}}^{\mathrm{MASP1_{zym}}}}\\
&+\frac{\left[\mathrm{pMBL_4MASP2_{zym}}\right]}{K_{\mathrm{m\ MASP2^*}}^{\mathrm{MASP2_{zym}}}}+\frac{\left[\mathrm{pCLLKMASP1_{zym}}\right]}{K_{\mathrm{m\ MASP2^*}}^{\mathrm{MASP1_{zym}}}}+\frac{\left[\mathrm{pCLLKMASP2_{zym}}\right]}{K_{\mathrm{m\ MASP2^*}}^{\mathrm{MASP2_{zym}}}}+\frac{\left[\mathrm{pCLK1MASP1_{zym}}\right]}{K_{\mathrm{m\ MASP2^*}}^{\mathrm{MASP1_{zym}}}}\\
&+\frac{\left[\mathrm{pCLK1MASP2_{zym}}\right]}{K_{\mathrm{m\ MASP2^*}}^{\mathrm{MASP2_{zym}}}}+\frac{\left[\mathrm{pCLL1MASP1_{zym}}\right]}{K_{\mathrm{m\ MASP2^*}}^{\mathrm{MASP1_{zym}}}}+\frac{\left[\mathrm{pCLL1MASP2_{zym}}\right]}{K_{\mathrm{m\ MASP2^*}}^{\mathrm{MASP2_{zym}}}}+\frac{\left[\mathrm{pLFMASP1_{zym}}\right]}{K_{\mathrm{m\ MASP2^*}}^{\mathrm{MASP1_{zym}}}}\\
&+\frac{\left[\mathrm{pLFMASP2_{zym}}\right]}{K_{\mathrm{m\ MASP2^*}}^{\mathrm{MASP2_{zym}}}}+\frac{\left[\mathrm{pHFMASP1_{zym}}\right]}{K_{\mathrm{m\ MASP2^*}}^{\mathrm{MASP1_{zym}}}}+\frac{\left[\mathrm{pHFMASP2_{zym}}\right]}{K_{\mathrm{m\ MASP2^*}}^{\mathrm{MASP2_{zym}}}}+\frac{\left[\mathrm{pMFMASP1_{zym}}\right]}{K_{\mathrm{m\ MASP2^*}}^{\mathrm{MASP1_{zym}}}}+\frac{\left[\mathrm{pMFMASP2_{zym}}\right]}{K_{\mathrm{m\ MASP2^*}}^{\mathrm{MASP2_{zym}}}}
\end{aligned}\right)}
$$

$$
+\frac{k_{\mathrm{cat\ MASP2^*}}^{\mathrm{MASP1_{zym}}}\left[\mathrm{pCLLKMASP2^*}\right]\left[\mathrm{pMBL_3MASP1_{zym}}\right]}{K_{\mathrm{m\ MASP2^*}}^{\mathrm{MASP1_{zym}}}\left(\begin{aligned}
&1+\frac{[\mathrm{C4}]}{K_{\mathrm{m\ MASP2^*}}^{\mathrm{C4}}}+\frac{[\mathrm{fC4bC2}]}{K_{\mathrm{m\ MASP2^*}}^{\mathrm{C2}}}+\frac{[\mathrm{fC4bC4bC2}]}{K_{\mathrm{m\ MASP2^*}}^{\mathrm{C2}}}+\frac{[\mathrm{fC3bC4bC2}]}{K_{\mathrm{m\ MASP2^*}}^{\mathrm{C2}}}+\frac{[\mathrm{pC4bC2}]}{K_{\mathrm{m\ MASP2^*}}^{\mathrm{C2}}}+\frac{[\mathrm{pC4bC4bC2}]}{K_{\mathrm{m\ MASP2^*}}^{\mathrm{C2}}}\\
&+\frac{[\mathrm{pC3bC4bC2}]}{K_{\mathrm{m\ MASP2^*}}^{\mathrm{C2}}}+\frac{\left[\mathrm{pMBL_3MASP1_{zym}}\right]}{K_{\mathrm{m\ MASP2^*}}^{\mathrm{MASP1_{zym}}}}+\frac{\left[\mathrm{pMBL_3MASP2_{zym}}\right]}{K_{\mathrm{m\ MASP2^*}}^{\mathrm{MASP2_{zym}}}}+\frac{\left[\mathrm{pMBL_4MASP1_{zym}}\right]}{K_{\mathrm{m\ MASP2^*}}^{\mathrm{MASP1_{zym}}}}\\
&+\frac{\left[\mathrm{pMBL_4MASP2_{zym}}\right]}{K_{\mathrm{m\ MASP2^*}}^{\mathrm{MASP2_{zym}}}}+\frac{\left[\mathrm{pCLLKMASP1_{zym}}\right]}{K_{\mathrm{m\ MASP2^*}}^{\mathrm{MASP1_{zym}}}}+\frac{\left[\mathrm{pCLLKMASP2_{zym}}\right]}{K_{\mathrm{m\ MASP2^*}}^{\mathrm{MASP2_{zym}}}}+\frac{\left[\mathrm{pCLK1MASP1_{zym}}\right]}{K_{\mathrm{m\ MASP2^*}}^{\mathrm{MASP1_{zym}}}}\\
&+\frac{\left[\mathrm{pCLK1MASP2_{zym}}\right]}{K_{\mathrm{m\ MASP2^*}}^{\mathrm{MASP2_{zym}}}}+\frac{\left[\mathrm{pCLL1MASP1_{zym}}\right]}{K_{\mathrm{m\ MASP2^*}}^{\mathrm{MASP1_{zym}}}}+\frac{\left[\mathrm{pCLL1MASP2_{zym}}\right]}{K_{\mathrm{m\ MASP2^*}}^{\mathrm{MASP2_{zym}}}}+\frac{\left[\mathrm{pLFMASP1_{zym}}\right]}{K_{\mathrm{m\ MASP2^*}}^{\mathrm{MASP1_{zym}}}}\\
&+\frac{\left[\mathrm{pLFMASP2_{zym}}\right]}{K_{\mathrm{m\ MASP2^*}}^{\mathrm{MASP2_{zym}}}}+\frac{\left[\mathrm{pHFMASP1_{zym}}\right]}{K_{\mathrm{m\ MASP2^*}}^{\mathrm{MASP1_{zym}}}}+\frac{\left[\mathrm{pHFMASP2_{zym}}\right]}{K_{\mathrm{m\ MASP2^*}}^{\mathrm{MASP2_{zym}}}}+\frac{\left[\mathrm{pMFMASP1_{zym}}\right]}{K_{\mathrm{m\ MASP2^*}}^{\mathrm{MASP1_{zym}}}}+\frac{\left[\mathrm{pMFMASP2_{zym}}\right]}{K_{\mathrm{m\ MASP2^*}}^{\mathrm{MASP2_{zym}}}}
\end{aligned}\right)}
$$

$$
+\frac{k_{\mathrm{cat\ MASP2^*}}^{\mathrm{MASP1_{zym}}}\left[\mathrm{pCLK1MASP2^*}\right]\left[\mathrm{pMBL_3MASP1_{zym}}\right]}{K_{\mathrm{m\ MASP2^*}}^{\mathrm{MASP1_{zym}}}\left(\begin{aligned}
&1+\frac{[\mathrm{C4}]}{K_{\mathrm{m\ MASP2^*}}^{\mathrm{C4}}}+\frac{[\mathrm{fC4bC2}]}{K_{\mathrm{m\ MASP2^*}}^{\mathrm{C2}}}+\frac{[\mathrm{fC4bC4bC2}]}{K_{\mathrm{m\ MASP2^*}}^{\mathrm{C2}}}+\frac{[\mathrm{fC3bC4bC2}]}{K_{\mathrm{m\ MASP2^*}}^{\mathrm{C2}}}+\frac{[\mathrm{pC4bC2}]}{K_{\mathrm{m\ MASP2^*}}^{\mathrm{C2}}}+\frac{[\mathrm{pC4bC4bC2}]}{K_{\mathrm{m\ MASP2^*}}^{\mathrm{C2}}}\\
&+\frac{[\mathrm{pC3bC4bC2}]}{K_{\mathrm{m\ MASP2^*}}^{\mathrm{C2}}}+\frac{\left[\mathrm{pMBL_3MASP1_{zym}}\right]}{K_{\mathrm{m\ MASP2^*}}^{\mathrm{MASP1_{zym}}}}+\frac{\left[\mathrm{pMBL_3MASP2_{zym}}\right]}{K_{\mathrm{m\ MASP2^*}}^{\mathrm{MASP2_{zym}}}}+\frac{\left[\mathrm{pMBL_4MASP1_{zym}}\right]}{K_{\mathrm{m\ MASP2^*}}^{\mathrm{MASP1_{zym}}}}\\
&+\frac{\left[\mathrm{pMBL_4MASP2_{zym}}\right]}{K_{\mathrm{m\ MASP2^*}}^{\mathrm{MASP2_{zym}}}}+\frac{\left[\mathrm{pCLLKMASP1_{zym}}\right]}{K_{\mathrm{m\ MASP2^*}}^{\mathrm{MASP1_{zym}}}}+\frac{\left[\mathrm{pCLLKMASP2_{zym}}\right]}{K_{\mathrm{m\ MASP2^*}}^{\mathrm{MASP2_{zym}}}}+\frac{\left[\mathrm{pCLK1MASP1_{zym}}\right]}{K_{\mathrm{m\ MASP2^*}}^{\mathrm{MASP1_{zym}}}}\\
&+\frac{\left[\mathrm{pCLK1MASP2_{zym}}\right]}{K_{\mathrm{m\ MASP2^*}}^{\mathrm{MASP2_{zym}}}}+\frac{\left[\mathrm{pCLL1MASP1_{zym}}\right]}{K_{\mathrm{m\ MASP2^*}}^{\mathrm{MASP1_{zym}}}}+\frac{\left[\mathrm{pCLL1MASP2_{zym}}\right]}{K_{\mathrm{m\ MASP2^*}}^{\mathrm{MASP2_{zym}}}}+\frac{\left[\mathrm{pLFMASP1_{zym}}\right]}{K_{\mathrm{m\ MASP2^*}}^{\mathrm{MASP1_{zym}}}}\\
&+\frac{\left[\mathrm{pLFMASP2_{zym}}\right]}{K_{\mathrm{m\ MASP2^*}}^{\mathrm{MASP2_{zym}}}}+\frac{\left[\mathrm{pHFMASP1_{zym}}\right]}{K_{\mathrm{m\ MASP2^*}}^{\mathrm{MASP1_{zym}}}}+\frac{\left[\mathrm{pHFMASP2_{zym}}\right]}{K_{\mathrm{m\ MASP2^*}}^{\mathrm{MASP2_{zym}}}}+\frac{\left[\mathrm{pMFMASP1_{zym}}\right]}{K_{\mathrm{m\ MASP2^*}}^{\mathrm{MASP1_{zym}}}}+\frac{\left[\mathrm{pMFMASP2_{zym}}\right]}{K_{\mathrm{m\ MASP2^*}}^{\mathrm{MASP2_{zym}}}}
\end{aligned}\right)}
$$

$$+\frac{k_{\mathrm{cat\ MASP2^*}}^{\mathrm{MASP1_{zym}}}\left[\mathrm{pCLL1MASP2^*}\right]\left[\mathrm{pMBL_3MASP1_{zym}}\right]}{K_{\mathrm{m\ MASP2^*}}^{\mathrm{MASP1_{zym}}}\left(\begin{array}{l}1+\frac{[\mathrm{C4}]}{K_{\mathrm{m\ MASP2^*}}^{\mathrm{C4}}}+\frac{[\mathrm{fC4bC2}]}{K_{\mathrm{m\ MASP2^*}}^{\mathrm{C2}}}+\frac{[\mathrm{fC4bC4bC2}]}{K_{\mathrm{m\ MASP2^*}}^{\mathrm{C2}}}+\frac{[\mathrm{fC3bC4bC2}]}{K_{\mathrm{m\ MASP2^*}}^{\mathrm{C2}}}+\frac{[\mathrm{pC4bC2}]}{K_{\mathrm{m\ MASP2^*}}^{\mathrm{C2}}}+\frac{[\mathrm{pC4bC4bC2}]}{K_{\mathrm{m\ MASP2^*}}^{\mathrm{C2}}}\\+\frac{[\mathrm{pC3bC4bC2}]}{K_{\mathrm{m\ MASP2^*}}^{\mathrm{C2}}}+\frac{[\mathrm{pMBL_3MASP1_{zym}}]}{K_{\mathrm{m\ MASP2^*}}^{\mathrm{MASP1_{zym}}}}+\frac{[\mathrm{pMBL_3MASP2_{zym}}]}{K_{\mathrm{m\ MASP2^*}}^{\mathrm{MASP2_{zym}}}}+\frac{[\mathrm{pMBL_4MASP1_{zym}}]}{K_{\mathrm{m\ MASP2^*}}^{\mathrm{MASP1_{zym}}}}\\+\frac{[\mathrm{pMBL_4MASP2_{zym}}]}{K_{\mathrm{m\ MASP2^*}}^{\mathrm{MASP2_{zym}}}}+\frac{[\mathrm{pCLLKMASP1_{zym}}]}{K_{\mathrm{m\ MASP2^*}}^{\mathrm{MASP1_{zym}}}}+\frac{[\mathrm{pCLLKMASP2_{zym}}]}{K_{\mathrm{m\ MASP2^*}}^{\mathrm{MASP2_{zym}}}}+\frac{[\mathrm{pCLK1MASP1_{zym}}]}{K_{\mathrm{m\ MASP2^*}}^{\mathrm{MASP1_{zym}}}}\\+\frac{[\mathrm{pCLK1MASP2_{zym}}]}{K_{\mathrm{m\ MASP2^*}}^{\mathrm{MASP2_{zym}}}}+\frac{[\mathrm{pCLL1MASP1_{zym}}]}{K_{\mathrm{m\ MASP2^*}}^{\mathrm{MASP1_{zym}}}}+\frac{[\mathrm{pCLL1MASP2_{zym}}]}{K_{\mathrm{m\ MASP2^*}}^{\mathrm{MASP2_{zym}}}}+\frac{[\mathrm{pLFMASP1_{zym}}]}{K_{\mathrm{m\ MASP2^*}}^{\mathrm{MASP1_{zym}}}}\\+\frac{[\mathrm{pLFMASP2_{zym}}]}{K_{\mathrm{m\ MASP2^*}}^{\mathrm{MASP2_{zym}}}}+\frac{[\mathrm{pHFMASP1_{zym}}]}{K_{\mathrm{m\ MASP2^*}}^{\mathrm{MASP1_{zym}}}}+\frac{[\mathrm{pHFMASP2_{zym}}]}{K_{\mathrm{m\ MASP2^*}}^{\mathrm{MASP2_{zym}}}}+\frac{[\mathrm{pMFMASP1_{zym}}]}{K_{\mathrm{m\ MASP2^*}}^{\mathrm{MASP1_{zym}}}}+\frac{[\mathrm{pMFMASP2_{zym}}]}{K_{\mathrm{m\ MASP2^*}}^{\mathrm{MASP2_{zym}}}}\end{array}\right)}$$

$$+\frac{k_{\mathrm{cat\ MASP2^*}}^{\mathrm{MASP1_{zym}}}\left[\mathrm{pLFMASP2^*}\right]\left[\mathrm{pMBL_3MASP1_{zym}}\right]}{K_{\mathrm{m\ MASP2^*}}^{\mathrm{MASP1_{zym}}}\left(\begin{array}{l}1+\frac{[\mathrm{C4}]}{K_{\mathrm{m\ MASP2^*}}^{\mathrm{C4}}}+\frac{[\mathrm{fC4bC2}]}{K_{\mathrm{m\ MASP2^*}}^{\mathrm{C2}}}+\frac{[\mathrm{fC4bC4bC2}]}{K_{\mathrm{m\ MASP2^*}}^{\mathrm{C2}}}+\frac{[\mathrm{fC3bC4bC2}]}{K_{\mathrm{m\ MASP2^*}}^{\mathrm{C2}}}+\frac{[\mathrm{pC4bC2}]}{K_{\mathrm{m\ MASP2^*}}^{\mathrm{C2}}}+\frac{[\mathrm{pC4bC4bC2}]}{K_{\mathrm{m\ MASP2^*}}^{\mathrm{C2}}}\\+\frac{[\mathrm{pC3bC4bC2}]}{K_{\mathrm{m\ MASP2^*}}^{\mathrm{C2}}}+\frac{[\mathrm{pMBL_3MASP1_{zym}}]}{K_{\mathrm{m\ MASP2^*}}^{\mathrm{MASP1_{zym}}}}+\frac{[\mathrm{pMBL_3MASP2_{zym}}]}{K_{\mathrm{m\ MASP2^*}}^{\mathrm{MASP2_{zym}}}}+\frac{[\mathrm{pMBL_4MASP1_{zym}}]}{K_{\mathrm{m\ MASP2^*}}^{\mathrm{MASP1_{zym}}}}\\+\frac{[\mathrm{pMBL_4MASP2_{zym}}]}{K_{\mathrm{m\ MASP2^*}}^{\mathrm{MASP2_{zym}}}}+\frac{[\mathrm{pCLLKMASP1_{zym}}]}{K_{\mathrm{m\ MASP2^*}}^{\mathrm{MASP1_{zym}}}}+\frac{[\mathrm{pCLLKMASP2_{zym}}]}{K_{\mathrm{m\ MASP2^*}}^{\mathrm{MASP2_{zym}}}}+\frac{[\mathrm{pCLK1MASP1_{zym}}]}{K_{\mathrm{m\ MASP2^*}}^{\mathrm{MASP1_{zym}}}}\\+\frac{[\mathrm{pCLK1MASP2_{zym}}]}{K_{\mathrm{m\ MASP2^*}}^{\mathrm{MASP2_{zym}}}}+\frac{[\mathrm{pCLL1MASP1_{zym}}]}{K_{\mathrm{m\ MASP2^*}}^{\mathrm{MASP1_{zym}}}}+\frac{[\mathrm{pCLL1MASP2_{zym}}]}{K_{\mathrm{m\ MASP2^*}}^{\mathrm{MASP2_{zym}}}}+\frac{[\mathrm{pLFMASP1_{zym}}]}{K_{\mathrm{m\ MASP2^*}}^{\mathrm{MASP1_{zym}}}}\\+\frac{[\mathrm{pLFMASP2_{zym}}]}{K_{\mathrm{m\ MASP2^*}}^{\mathrm{MASP2_{zym}}}}+\frac{[\mathrm{pHFMASP1_{zym}}]}{K_{\mathrm{m\ MASP2^*}}^{\mathrm{MASP1_{zym}}}}+\frac{[\mathrm{pHFMASP2_{zym}}]}{K_{\mathrm{m\ MASP2^*}}^{\mathrm{MASP2_{zym}}}}+\frac{[\mathrm{pMFMASP1_{zym}}]}{K_{\mathrm{m\ MASP2^*}}^{\mathrm{MASP1_{zym}}}}+\frac{[\mathrm{pMFMASP2_{zym}}]}{K_{\mathrm{m\ MASP2^*}}^{\mathrm{MASP2_{zym}}}}\end{array}\right)}$$

$$+\frac{k_{\mathrm{cat\ MASP2^*}}^{\mathrm{MASP1_{zym}}}\left[\mathrm{pHFMASP2^*}\right]\left[\mathrm{pMBL_3MASP1_{zym}}\right]}{K_{\mathrm{m\ MASP2^*}}^{\mathrm{MASP1_{zym}}}\left(\begin{array}{l}1+\frac{[\mathrm{C4}]}{K_{\mathrm{m\ MASP2^*}}^{\mathrm{C4}}}+\frac{[\mathrm{fC4bC2}]}{K_{\mathrm{m\ MASP2^*}}^{\mathrm{C2}}}+\frac{[\mathrm{fC4bC4bC2}]}{K_{\mathrm{m\ MASP2^*}}^{\mathrm{C2}}}+\frac{[\mathrm{fC3bC4bC2}]}{K_{\mathrm{m\ MASP2^*}}^{\mathrm{C2}}}+\frac{[\mathrm{pC4bC2}]}{K_{\mathrm{m\ MASP2^*}}^{\mathrm{C2}}}+\frac{[\mathrm{pC4bC4bC2}]}{K_{\mathrm{m\ MASP2^*}}^{\mathrm{C2}}}\\+\frac{[\mathrm{pC3bC4bC2}]}{K_{\mathrm{m\ MASP2^*}}^{\mathrm{C2}}}+\frac{[\mathrm{pMBL_3MASP1_{zym}}]}{K_{\mathrm{m\ MASP2^*}}^{\mathrm{MASP1_{zym}}}}+\frac{[\mathrm{pMBL_3MASP2_{zym}}]}{K_{\mathrm{m\ MASP2^*}}^{\mathrm{MASP2_{zym}}}}+\frac{[\mathrm{pMBL_4MASP1_{zym}}]}{K_{\mathrm{m\ MASP2^*}}^{\mathrm{MASP1_{zym}}}}\\+\frac{[\mathrm{pMBL_4MASP2_{zym}}]}{K_{\mathrm{m\ MASP2^*}}^{\mathrm{MASP2_{zym}}}}+\frac{[\mathrm{pCLLKMASP1_{zym}}]}{K_{\mathrm{m\ MASP2^*}}^{\mathrm{MASP1_{zym}}}}+\frac{[\mathrm{pCLLKMASP2_{zym}}]}{K_{\mathrm{m\ MASP2^*}}^{\mathrm{MASP2_{zym}}}}+\frac{[\mathrm{pCLK1MASP1_{zym}}]}{K_{\mathrm{m\ MASP2^*}}^{\mathrm{MASP1_{zym}}}}\\+\frac{[\mathrm{pCLK1MASP2_{zym}}]}{K_{\mathrm{m\ MASP2^*}}^{\mathrm{MASP2_{zym}}}}+\frac{[\mathrm{pCLL1MASP1_{zym}}]}{K_{\mathrm{m\ MASP2^*}}^{\mathrm{MASP1_{zym}}}}+\frac{[\mathrm{pCLL1MASP2_{zym}}]}{K_{\mathrm{m\ MASP2^*}}^{\mathrm{MASP2_{zym}}}}+\frac{[\mathrm{pLFMASP1_{zym}}]}{K_{\mathrm{m\ MASP2^*}}^{\mathrm{MASP1_{zym}}}}\\+\frac{[\mathrm{pLFMASP2_{zym}}]}{K_{\mathrm{m\ MASP2^*}}^{\mathrm{MASP2_{zym}}}}+\frac{[\mathrm{pHFMASP1_{zym}}]}{K_{\mathrm{m\ MASP2^*}}^{\mathrm{MASP1_{zym}}}}+\frac{[\mathrm{pHFMASP2_{zym}}]}{K_{\mathrm{m\ MASP2^*}}^{\mathrm{MASP2_{zym}}}}+\frac{[\mathrm{pMFMASP1_{zym}}]}{K_{\mathrm{m\ MASP2^*}}^{\mathrm{MASP1_{zym}}}}+\frac{[\mathrm{pMFMASP2_{zym}}]}{K_{\mathrm{m\ MASP2^*}}^{\mathrm{MASP2_{zym}}}}\end{array}\right)}$$

$$+\frac{k_{\mathrm{cat\ MASP2}^*}^{\mathrm{MASP1_{zym}}}\left[\mathrm{pMFMASP2}^*\right]\left[\mathrm{pMBL_3MASP1_{zym}}\right]}{K_{\mathrm{m\ MASP2}^*}^{\mathrm{MASP1_{zym}}}\left(\begin{array}{l}1+\frac{[\mathrm{C4}]}{K_{\mathrm{m\ MASP2}^*}^{\mathrm{C4}}}+\frac{[\mathrm{fC4bC2}]}{K_{\mathrm{m\ MASP2}^*}^{\mathrm{C2}}}+\frac{[\mathrm{fC4bC4bC2}]}{K_{\mathrm{m\ MASP2}^*}^{\mathrm{C2}}}+\frac{[\mathrm{fC3bC4bC2}]}{K_{\mathrm{m\ MASP2}^*}^{\mathrm{C2}}}+\frac{[\mathrm{pC4bC2}]}{K_{\mathrm{m\ MASP2}^*}^{\mathrm{C2}}}+\frac{[\mathrm{pC4bC4bC2}]}{K_{\mathrm{m\ MASP2}^*}^{\mathrm{C2}}}\\+\frac{[\mathrm{pC3bC4bC2}]}{K_{\mathrm{m\ MASP2}^*}^{\mathrm{C2}}}+\frac{[\mathrm{pMBL_3MASP1_{zym}}]}{K_{\mathrm{m\ MASP2}^*}^{\mathrm{MASP1_{zym}}}}+\frac{[\mathrm{pMBL_3MASP2_{zym}}]}{K_{\mathrm{m\ MASP2}^*}^{\mathrm{MASP2_{zym}}}}+\frac{[\mathrm{pMBL_4MASP1_{zym}}]}{K_{\mathrm{m\ MASP2}^*}^{\mathrm{MASP1_{zym}}}}\\+\frac{[\mathrm{pMBL_4MASP2_{zym}}]}{K_{\mathrm{m\ MASP2}^*}^{\mathrm{MASP2_{zym}}}}+\frac{[\mathrm{pCLLKMASP1_{zym}}]}{K_{\mathrm{m\ MASP2}^*}^{\mathrm{MASP1_{zym}}}}+\frac{[\mathrm{pCLLKMASP2_{zym}}]}{K_{\mathrm{m\ MASP2}^*}^{\mathrm{MASP2_{zym}}}}+\frac{[\mathrm{pCLK1MASP1_{zym}}]}{K_{\mathrm{m\ MASP2}^*}^{\mathrm{MASP1_{zym}}}}\\+\frac{[\mathrm{pCLK1MASP2_{zym}}]}{K_{\mathrm{m\ MASP2}^*}^{\mathrm{MASP2_{zym}}}}+\frac{[\mathrm{pCLL1MASP1_{zym}}]}{K_{\mathrm{m\ MASP2}^*}^{\mathrm{MASP1_{zym}}}}+\frac{[\mathrm{pCLL1MASP2_{zym}}]}{K_{\mathrm{m\ MASP2}^*}^{\mathrm{MASP2_{zym}}}}+\frac{[\mathrm{pLFMASP1_{zym}}]}{K_{\mathrm{m\ MASP2}^*}^{\mathrm{MASP1_{zym}}}}\\+\frac{[\mathrm{pLFMASP2_{zym}}]}{K_{\mathrm{m\ MASP2}^*}^{\mathrm{MASP2_{zym}}}}+\frac{[\mathrm{pHFMASP1_{zym}}]}{K_{\mathrm{m\ MASP2}^*}^{\mathrm{MASP1_{zym}}}}+\frac{[\mathrm{pHFMASP2_{zym}}]}{K_{\mathrm{m\ MASP2}^*}^{\mathrm{MASP2_{zym}}}}+\frac{[\mathrm{pMFMASP1_{zym}}]}{K_{\mathrm{m\ MASP2}^*}^{\mathrm{MASP1_{zym}}}}+\frac{[\mathrm{pMFMASP2_{zym}}]}{K_{\mathrm{m\ MASP2}^*}^{\mathrm{MASP2_{zym}}}}\end{array}\right)}$$

$$+\frac{k_{\mathrm{cat\ MASP2}^*}^{\mathrm{MASP1_{zym}}}\left[\mathrm{pPTX3LFMASP2}^*\right]\left[\mathrm{pMBL_3MASP1_{zym}}\right]}{K_{\mathrm{m\ MASP2}^*}^{\mathrm{MASP1_{zym}}}\left(\begin{array}{l}1+\frac{[\mathrm{C4}]}{K_{\mathrm{m\ MASP2}^*}^{\mathrm{C4}}}+\frac{[\mathrm{fC4bC2}]}{K_{\mathrm{m\ MASP2}^*}^{\mathrm{C2}}}+\frac{[\mathrm{fC4bC4bC2}]}{K_{\mathrm{m\ MASP2}^*}^{\mathrm{C2}}}+\frac{[\mathrm{fC3bC4bC2}]}{K_{\mathrm{m\ MASP2}^*}^{\mathrm{C2}}}+\frac{[\mathrm{pC4bC2}]}{K_{\mathrm{m\ MASP2}^*}^{\mathrm{C2}}}+\frac{[\mathrm{pC4bC4bC2}]}{K_{\mathrm{m\ MASP2}^*}^{\mathrm{C2}}}\\+\frac{[\mathrm{pC3bC4bC2}]}{K_{\mathrm{m\ MASP2}^*}^{\mathrm{C2}}}+\frac{[\mathrm{pMBL_3MASP1_{zym}}]}{K_{\mathrm{m\ MASP2}^*}^{\mathrm{MASP1_{zym}}}}+\frac{[\mathrm{pMBL_3MASP2_{zym}}]}{K_{\mathrm{m\ MASP2}^*}^{\mathrm{MASP2_{zym}}}}+\frac{[\mathrm{pMBL_4MASP1_{zym}}]}{K_{\mathrm{m\ MASP2}^*}^{\mathrm{MASP1_{zym}}}}\\+\frac{[\mathrm{pMBL_4MASP2_{zym}}]}{K_{\mathrm{m\ MASP2}^*}^{\mathrm{MASP2_{zym}}}}+\frac{[\mathrm{pCLLKMASP1_{zym}}]}{K_{\mathrm{m\ MASP2}^*}^{\mathrm{MASP1_{zym}}}}+\frac{[\mathrm{pCLLKMASP2_{zym}}]}{K_{\mathrm{m\ MASP2}^*}^{\mathrm{MASP2_{zym}}}}+\frac{[\mathrm{pCLK1MASP1_{zym}}]}{K_{\mathrm{m\ MASP2}^*}^{\mathrm{MASP1_{zym}}}}\\+\frac{[\mathrm{pCLK1MASP2_{zym}}]}{K_{\mathrm{m\ MASP2}^*}^{\mathrm{MASP2_{zym}}}}+\frac{[\mathrm{pCLL1MASP1_{zym}}]}{K_{\mathrm{m\ MASP2}^*}^{\mathrm{MASP1_{zym}}}}+\frac{[\mathrm{pCLL1MASP2_{zym}}]}{K_{\mathrm{m\ MASP2}^*}^{\mathrm{MASP2_{zym}}}}+\frac{[\mathrm{pLFMASP1_{zym}}]}{K_{\mathrm{m\ MASP2}^*}^{\mathrm{MASP1_{zym}}}}\\+\frac{[\mathrm{pLFMASP2_{zym}}]}{K_{\mathrm{m\ MASP2}^*}^{\mathrm{MASP2_{zym}}}}+\frac{[\mathrm{pHFMASP1_{zym}}]}{K_{\mathrm{m\ MASP2}^*}^{\mathrm{MASP1_{zym}}}}+\frac{[\mathrm{pHFMASP2_{zym}}]}{K_{\mathrm{m\ MASP2}^*}^{\mathrm{MASP2_{zym}}}}+\frac{[\mathrm{pMFMASP1_{zym}}]}{K_{\mathrm{m\ MASP2}^*}^{\mathrm{MASP1_{zym}}}}+\frac{[\mathrm{pMFMASP2_{zym}}]}{K_{\mathrm{m\ MASP2}^*}^{\mathrm{MASP2_{zym}}}}\\+\frac{[\mathrm{pPTX3LFMASP1_{zym}}]}{K_{\mathrm{m\ MASP2}^*}^{\mathrm{MASP1_{zym}}}}+\frac{[\mathrm{pPTX3LFMASP2_{zym}}]}{K_{\mathrm{m\ MASP2}^*}^{\mathrm{MASP2_{zym}}}}\end{array}\right)}$$

$$-k_{\mathrm{C1INHMASP1}}^{+}[\mathrm{C1\text{-}INH}]\left[\mathrm{pMBL_3MASP1}^*\right]$$

248

$$\frac{d\left[\mathrm{fMBL_3MASP2_{zym}}\right]}{dt}=k_{\mathrm{MBL_3MASP2}}^{+}[\mathrm{MBL_3}][\mathrm{MASP2}]-k_{\mathrm{MBL_3MASP2}}^{-}\left[\mathrm{fMBL_3MASP2_{zym}}\right]$$

$$-k_{\mathrm{pMBL_3}}^{+}\left[\mathrm{fMBL_3MASP2_{zym}}\right][\text{Pathogen Surface}]+k_{\mathrm{pMBL_3}}^{-}\left[\mathrm{pMBL_3MASP2_{zym}}\right]$$

249

$$\frac{d\left[\mathrm{pMBL_3MASP2_{zym}}\right]}{dt}=k^{+}_{\mathrm{pMBL_3}}\left[\mathrm{fMBL_3MASP2_{zym}}\right]\left[\mathrm{Pathogen\ Surface}\right]-k^{-}_{\mathrm{pMBL_3}}\left[\mathrm{pMBL_3MASP2_{zym}}\right]$$

$$+k^{+}_{\mathrm{MBL_3MASP2}}\left[\mathrm{pMBL_3}\right]\left[\mathrm{MASP2}\right]-k^{-}_{\mathrm{MBL_3MASP2}}\left[\mathrm{pMBL_3MASP2_{zym}}\right]$$

$$-\frac{k^{\mathrm{MASP2_{zym}}}_{\mathrm{cat\ MASP1_{zym}}}\left[\mathrm{pMBL_3MASP1_{zym}}\right]\left[\mathrm{pMBL_3MASP2_{zym}}\right]}{K^{\mathrm{MASP2_{zym}}}_{\mathrm{m\ MASP1_{zym}}}\left(\begin{array}{l}1+\frac{\left[\mathrm{pMBL_3MASP1_{zym}}\right]}{K^{\mathrm{MASP1_{zym}}}_{\mathrm{m\ MASP1_{zym}}}}+\frac{\left[\mathrm{pMBL_3MASP2_{zym}}\right]}{K^{\mathrm{MASP2_{zym}}}_{\mathrm{m\ MASP1_{zym}}}}+\frac{\left[\mathrm{pMBL_4MASP1_{zym}}\right]}{K^{\mathrm{MASP1_{zym}}}_{\mathrm{m\ MASP1_{zym}}}}+\frac{\left[\mathrm{pMBL_4MASP2_{zym}}\right]}{K^{\mathrm{MASP2_{zym}}}_{\mathrm{m\ MASP1_{zym}}}}\\+\frac{\left[\mathrm{pCLLKMASP1_{zym}}\right]}{K^{\mathrm{MASP1_{zym}}}_{\mathrm{m\ MASP1_{zym}}}}+\frac{\left[\mathrm{pCLLKMASP2_{zym}}\right]}{K^{\mathrm{MASP2_{zym}}}_{\mathrm{m\ MASP1_{zym}}}}+\frac{\left[\mathrm{pCLK1MASP1_{zym}}\right]}{K^{\mathrm{MASP1_{zym}}}_{\mathrm{m\ MASP1_{zym}}}}+\frac{\left[\mathrm{pCLK1MASP2_{zym}}\right]}{K^{\mathrm{MASP2_{zym}}}_{\mathrm{m\ MASP1_{zym}}}}\\+\frac{\left[\mathrm{pCLL1MASP1_{zym}}\right]}{K^{\mathrm{MASP1_{zym}}}_{\mathrm{m\ MASP1_{zym}}}}+\frac{\left[\mathrm{pCLL1MASP2_{zym}}\right]}{K^{\mathrm{MASP2_{zym}}}_{\mathrm{m\ MASP1_{zym}}}}+\frac{\left[\mathrm{pLFMASP1_{zym}}\right]}{K^{\mathrm{MASP1_{zym}}}_{\mathrm{m\ MASP1_{zym}}}}+\frac{\left[\mathrm{pLFMASP2_{zym}}\right]}{K^{\mathrm{MASP2_{zym}}}_{\mathrm{m\ MASP1_{zym}}}}\\+\frac{\left[\mathrm{pHFMASP1_{zym}}\right]}{K^{\mathrm{MASP1_{zym}}}_{\mathrm{m\ MASP1_{zym}}}}+\frac{\left[\mathrm{pHFMASP2_{zym}}\right]}{K^{\mathrm{MASP2_{zym}}}_{\mathrm{m\ MASP1_{zym}}}}+\frac{\left[\mathrm{pMFMASP1_{zym}}\right]}{K^{\mathrm{MASP1_{zym}}}_{\mathrm{m\ MASP1_{zym}}}}+\frac{\left[\mathrm{pMFMASP2_{zym}}\right]}{K^{\mathrm{MASP2_{zym}}}_{\mathrm{m\ MASP1_{zym}}}}\\+\frac{\left[\mathrm{pPTX3LFMASP1_{zym}}\right]}{K^{\mathrm{MASP1_{zym}}}_{\mathrm{m\ MASP1_{zym}}}}+\frac{\left[\mathrm{pPTX3LFMASP2_{zym}}\right]}{K^{\mathrm{MASP2_{zym}}}_{\mathrm{m\ MASP1_{zym}}}}\end{array}\right)}$$

$$-\frac{k^{\mathrm{MASP2_{zym}}}_{\mathrm{cat\ MASP1_{zym}}}\left[\mathrm{pMBL_4MASP1_{zym}}\right]\left[\mathrm{pMBL_3MASP2_{zym}}\right]}{K^{\mathrm{MASP2_{zym}}}_{\mathrm{m\ MASP1_{zym}}}\left(\begin{array}{l}1+\frac{\left[\mathrm{pMBL_3MASP1_{zym}}\right]}{K^{\mathrm{MASP1_{zym}}}_{\mathrm{m\ MASP1_{zym}}}}+\frac{\left[\mathrm{pMBL_3MASP2_{zym}}\right]}{K^{\mathrm{MASP2_{zym}}}_{\mathrm{m\ MASP1_{zym}}}}+\frac{\left[\mathrm{pMBL_4MASP1_{zym}}\right]}{K^{\mathrm{MASP1_{zym}}}_{\mathrm{m\ MASP1_{zym}}}}+\frac{\left[\mathrm{pMBL_4MASP2_{zym}}\right]}{K^{\mathrm{MASP2_{zym}}}_{\mathrm{m\ MASP1_{zym}}}}\\+\frac{\left[\mathrm{pCLLKMASP1_{zym}}\right]}{K^{\mathrm{MASP1_{zym}}}_{\mathrm{m\ MASP1_{zym}}}}+\frac{\left[\mathrm{pCLLKMASP2_{zym}}\right]}{K^{\mathrm{MASP2_{zym}}}_{\mathrm{m\ MASP1_{zym}}}}+\frac{\left[\mathrm{pCLK1MASP1_{zym}}\right]}{K^{\mathrm{MASP1_{zym}}}_{\mathrm{m\ MASP1_{zym}}}}+\frac{\left[\mathrm{pCLK1MASP2_{zym}}\right]}{K^{\mathrm{MASP2_{zym}}}_{\mathrm{m\ MASP1_{zym}}}}\\+\frac{\left[\mathrm{pCLL1MASP1_{zym}}\right]}{K^{\mathrm{MASP1_{zym}}}_{\mathrm{m\ MASP1_{zym}}}}+\frac{\left[\mathrm{pCLL1MASP2_{zym}}\right]}{K^{\mathrm{MASP2_{zym}}}_{\mathrm{m\ MASP1_{zym}}}}+\frac{\left[\mathrm{pLFMASP1_{zym}}\right]}{K^{\mathrm{MASP1_{zym}}}_{\mathrm{m\ MASP1_{zym}}}}+\frac{\left[\mathrm{pLFMASP2_{zym}}\right]}{K^{\mathrm{MASP2_{zym}}}_{\mathrm{m\ MASP1_{zym}}}}\\+\frac{\left[\mathrm{pHFMASP1_{zym}}\right]}{K^{\mathrm{MASP1_{zym}}}_{\mathrm{m\ MASP1_{zym}}}}+\frac{\left[\mathrm{pHFMASP2_{zym}}\right]}{K^{\mathrm{MASP2_{zym}}}_{\mathrm{m\ MASP1_{zym}}}}+\frac{\left[\mathrm{pMFMASP1_{zym}}\right]}{K^{\mathrm{MASP1_{zym}}}_{\mathrm{m\ MASP1_{zym}}}}+\frac{\left[\mathrm{pMFMASP2_{zym}}\right]}{K^{\mathrm{MASP2_{zym}}}_{\mathrm{m\ MASP1_{zym}}}}\\+\frac{\left[\mathrm{pPTX3LFMASP1_{zym}}\right]}{K^{\mathrm{MASP1_{zym}}}_{\mathrm{m\ MASP1_{zym}}}}+\frac{\left[\mathrm{pPTX3LFMASP2_{zym}}\right]}{K^{\mathrm{MASP2_{zym}}}_{\mathrm{m\ MASP1_{zym}}}}\end{array}\right)}$$

$$-\frac{k_{\mathrm{cat\ MASP1_{zym}}}^{\mathrm{MASP2_{zym}}}\left[\mathrm{pCLLKMASP1_{zym}}\right]\left[\mathrm{pMBL_3MASP2_{zym}}\right]}{K_{\mathrm{m\ MASP1_{zym}}}^{\mathrm{MASP2_{zym}}}\left(\begin{array}{l}1+\frac{\left[\mathrm{pMBL_3MASP1_{zym}}\right]}{K_{\mathrm{m\ MASP1_{zym}}}^{\mathrm{MASP1_{zym}}}}+\frac{\left[\mathrm{pMBL_3MASP2_{zym}}\right]}{K_{\mathrm{m\ MASP1_{zym}}}^{\mathrm{MASP2_{zym}}}}+\frac{\left[\mathrm{pMBL_4MASP1_{zym}}\right]}{K_{\mathrm{m\ MASP1_{zym}}}^{\mathrm{MASP1_{zym}}}}+\frac{\left[\mathrm{pMBL_4MASP2_{zym}}\right]}{K_{\mathrm{m\ MASP1_{zym}}}^{\mathrm{MASP2_{zym}}}}\\+\frac{\left[\mathrm{pCLLKMASP1_{zym}}\right]}{K_{\mathrm{m\ MASP1_{zym}}}^{\mathrm{MASP1_{zym}}}}+\frac{\left[\mathrm{pCLLKMASP2_{zym}}\right]}{K_{\mathrm{m\ MASP1_{zym}}}^{\mathrm{MASP2_{zym}}}}+\frac{\left[\mathrm{pCLK1MASP1_{zym}}\right]}{K_{\mathrm{m\ MASP1_{zym}}}^{\mathrm{MASP1_{zym}}}}+\frac{\left[\mathrm{pCLK1MASP2_{zym}}\right]}{K_{\mathrm{m\ MASP1_{zym}}}^{\mathrm{MASP2_{zym}}}}\\+\frac{\left[\mathrm{pCLL1MASP1_{zym}}\right]}{K_{\mathrm{m\ MASP1_{zym}}}^{\mathrm{MASP1_{zym}}}}+\frac{\left[\mathrm{pCLL1MASP2_{zym}}\right]}{K_{\mathrm{m\ MASP1_{zym}}}^{\mathrm{MASP2_{zym}}}}+\frac{\left[\mathrm{pLFMASP1_{zym}}\right]}{K_{\mathrm{m\ MASP1_{zym}}}^{\mathrm{MASP1_{zym}}}}+\frac{\left[\mathrm{pLFMASP2_{zym}}\right]}{K_{\mathrm{m\ MASP1_{zym}}}^{\mathrm{MASP2_{zym}}}}\\+\frac{\left[\mathrm{pHFMASP1_{zym}}\right]}{K_{\mathrm{m\ MASP1_{zym}}}^{\mathrm{MASP1_{zym}}}}+\frac{\left[\mathrm{pHFMASP2_{zym}}\right]}{K_{\mathrm{m\ MASP1_{zym}}}^{\mathrm{MASP2_{zym}}}}+\frac{\left[\mathrm{pMFMASP1_{zym}}\right]}{K_{\mathrm{m\ MASP1_{zym}}}^{\mathrm{MASP1_{zym}}}}+\frac{\left[\mathrm{pMFMASP2_{zym}}\right]}{K_{\mathrm{m\ MASP1_{zym}}}^{\mathrm{MASP2_{zym}}}}\\+\frac{\left[\mathrm{pPTX3LFMASP1_{zym}}\right]}{K_{\mathrm{m\ MASP1_{zym}}}^{\mathrm{MASP1_{zym}}}}+\frac{\left[\mathrm{pPTX3LFMASP2_{zym}}\right]}{K_{\mathrm{m\ MASP1_{zym}}}^{\mathrm{MASP2_{zym}}}}\end{array}\right)}$$

$$-\frac{k_{\mathrm{cat\ MASP1_{zym}}}^{\mathrm{MASP2_{zym}}}\left[\mathrm{pCLK1MASP1_{zym}}\right]\left[\mathrm{pMBL_3MASP2_{zym}}\right]}{K_{\mathrm{m\ MASP1_{zym}}}^{\mathrm{MASP2_{zym}}}\left(\begin{array}{l}1+\frac{\left[\mathrm{pMBL_3MASP1_{zym}}\right]}{K_{\mathrm{m\ MASP1_{zym}}}^{\mathrm{MASP1_{zym}}}}+\frac{\left[\mathrm{pMBL_3MASP2_{zym}}\right]}{K_{\mathrm{m\ MASP1_{zym}}}^{\mathrm{MASP2_{zym}}}}+\frac{\left[\mathrm{pMBL_4MASP1_{zym}}\right]}{K_{\mathrm{m\ MASP1_{zym}}}^{\mathrm{MASP1_{zym}}}}+\frac{\left[\mathrm{pMBL_4MASP2_{zym}}\right]}{K_{\mathrm{m\ MASP1_{zym}}}^{\mathrm{MASP2_{zym}}}}\\+\frac{\left[\mathrm{pCLLKMASP1_{zym}}\right]}{K_{\mathrm{m\ MASP1_{zym}}}^{\mathrm{MASP1_{zym}}}}+\frac{\left[\mathrm{pCLLKMASP2_{zym}}\right]}{K_{\mathrm{m\ MASP1_{zym}}}^{\mathrm{MASP2_{zym}}}}+\frac{\left[\mathrm{pCLK1MASP1_{zym}}\right]}{K_{\mathrm{m\ MASP1_{zym}}}^{\mathrm{MASP1_{zym}}}}+\frac{\left[\mathrm{pCLK1MASP2_{zym}}\right]}{K_{\mathrm{m\ MASP1_{zym}}}^{\mathrm{MASP2_{zym}}}}\\+\frac{\left[\mathrm{pCLL1MASP1_{zym}}\right]}{K_{\mathrm{m\ MASP1_{zym}}}^{\mathrm{MASP1_{zym}}}}+\frac{\left[\mathrm{pCLL1MASP2_{zym}}\right]}{K_{\mathrm{m\ MASP1_{zym}}}^{\mathrm{MASP2_{zym}}}}+\frac{\left[\mathrm{pLFMASP1_{zym}}\right]}{K_{\mathrm{m\ MASP1_{zym}}}^{\mathrm{MASP1_{zym}}}}+\frac{\left[\mathrm{pLFMASP2_{zym}}\right]}{K_{\mathrm{m\ MASP1_{zym}}}^{\mathrm{MASP2_{zym}}}}\\+\frac{\left[\mathrm{pHFMASP1_{zym}}\right]}{K_{\mathrm{m\ MASP1_{zym}}}^{\mathrm{MASP1_{zym}}}}+\frac{\left[\mathrm{pHFMASP2_{zym}}\right]}{K_{\mathrm{m\ MASP1_{zym}}}^{\mathrm{MASP2_{zym}}}}+\frac{\left[\mathrm{pMFMASP1_{zym}}\right]}{K_{\mathrm{m\ MASP1_{zym}}}^{\mathrm{MASP1_{zym}}}}+\frac{\left[\mathrm{pMFMASP2_{zym}}\right]}{K_{\mathrm{m\ MASP1_{zym}}}^{\mathrm{MASP2_{zym}}}}\\+\frac{\left[\mathrm{pPTX3LFMASP1_{zym}}\right]}{K_{\mathrm{m\ MASP1_{zym}}}^{\mathrm{MASP1_{zym}}}}+\frac{\left[\mathrm{pPTX3LFMASP2_{zym}}\right]}{K_{\mathrm{m\ MASP1_{zym}}}^{\mathrm{MASP2_{zym}}}}\end{array}\right)}$$

$$-\frac{k_{\text{cat MASP1}_{\text{zym}}}^{\text{MASP2}_{\text{zym}}}\left[\text{pCLL1MASP1}_{\text{zym}}\right]\left[\text{pMBL}_3\text{MASP2}_{\text{zym}}\right]}{K_{\text{m MASP1}_{\text{zym}}}^{\text{MASP2}_{\text{zym}}}\left(\begin{array}{l}1+\frac{\left[\text{pMBL}_3\text{MASP1}_{\text{zym}}\right]}{K_{\text{m MASP1}_{\text{zym}}}^{\text{MASP1}_{\text{zym}}}}+\frac{\left[\text{pMBL}_3\text{MASP2}_{\text{zym}}\right]}{K_{\text{m MASP1}_{\text{zym}}}^{\text{MASP2}_{\text{zym}}}}+\frac{\left[\text{pMBL}_4\text{MASP1}_{\text{zym}}\right]}{K_{\text{m MASP1}_{\text{zym}}}^{\text{MASP1}_{\text{zym}}}}+\frac{\left[\text{pMBL}_4\text{MASP2}_{\text{zym}}\right]}{K_{\text{m MASP1}_{\text{zym}}}^{\text{MASP2}_{\text{zym}}}}\\+\frac{\left[\text{pCLLKMASP1}_{\text{zym}}\right]}{K_{\text{m MASP1}_{\text{zym}}}^{\text{MASP1}_{\text{zym}}}}+\frac{\left[\text{pCLLKMASP2}_{\text{zym}}\right]}{K_{\text{m MASP1}_{\text{zym}}}^{\text{MASP2}_{\text{zym}}}}+\frac{\left[\text{pCLK1MASP1}_{\text{zym}}\right]}{K_{\text{m MASP1}_{\text{zym}}}^{\text{MASP1}_{\text{zym}}}}+\frac{\left[\text{pCLK1MASP2}_{\text{zym}}\right]}{K_{\text{m MASP1}_{\text{zym}}}^{\text{MASP2}_{\text{zym}}}}\\+\frac{\left[\text{pCLL1MASP1}_{\text{zym}}\right]}{K_{\text{m MASP1}_{\text{zym}}}^{\text{MASP1}_{\text{zym}}}}+\frac{\left[\text{pCLL1MASP2}_{\text{zym}}\right]}{K_{\text{m MASP1}_{\text{zym}}}^{\text{MASP2}_{\text{zym}}}}+\frac{\left[\text{pLFMASP1}_{\text{zym}}\right]}{K_{\text{m MASP1}_{\text{zym}}}^{\text{MASP1}_{\text{zym}}}}+\frac{\left[\text{pLFMASP2}_{\text{zym}}\right]}{K_{\text{m MASP1}_{\text{zym}}}^{\text{MASP2}_{\text{zym}}}}\\+\frac{\left[\text{pHFMASP1}_{\text{zym}}\right]}{K_{\text{m MASP1}_{\text{zym}}}^{\text{MASP1}_{\text{zym}}}}+\frac{\left[\text{pHFMASP2}_{\text{zym}}\right]}{K_{\text{m MASP1}_{\text{zym}}}^{\text{MASP2}_{\text{zym}}}}+\frac{\left[\text{pMFMASP1}_{\text{zym}}\right]}{K_{\text{m MASP1}_{\text{zym}}}^{\text{MASP1}_{\text{zym}}}}+\frac{\left[\text{pMFMASP2}_{\text{zym}}\right]}{K_{\text{m MASP1}_{\text{zym}}}^{\text{MASP2}_{\text{zym}}}}\\+\frac{\left[\text{pPTX3LFMASP1}_{\text{zym}}\right]}{K_{\text{m MASP1}_{\text{zym}}}^{\text{MASP1}_{\text{zym}}}}+\frac{\left[\text{pPTX3LFMASP2}_{\text{zym}}\right]}{K_{\text{m MASP1}_{\text{zym}}}^{\text{MASP2}_{\text{zym}}}}\end{array}\right)}$$

$$-\frac{k_{\text{cat MASP1}_{\text{zym}}}^{\text{MASP2}_{\text{zym}}}\left[\text{pLFMASP1}_{\text{zym}}\right]\left[\text{pMBL}_3\text{MASP2}_{\text{zym}}\right]}{K_{\text{m MASP1}_{\text{zym}}}^{\text{MASP2}_{\text{zym}}}\left(\begin{array}{l}1+\frac{\left[\text{pMBL}_3\text{MASP1}_{\text{zym}}\right]}{K_{\text{m MASP1}_{\text{zym}}}^{\text{MASP1}_{\text{zym}}}}+\frac{\left[\text{pMBL}_3\text{MASP2}_{\text{zym}}\right]}{K_{\text{m MASP1}_{\text{zym}}}^{\text{MASP2}_{\text{zym}}}}+\frac{\left[\text{pMBL}_4\text{MASP1}_{\text{zym}}\right]}{K_{\text{m MASP1}_{\text{zym}}}^{\text{MASP1}_{\text{zym}}}}+\frac{\left[\text{pMBL}_4\text{MASP2}_{\text{zym}}\right]}{K_{\text{m MASP1}_{\text{zym}}}^{\text{MASP2}_{\text{zym}}}}\\+\frac{\left[\text{pCLLKMASP1}_{\text{zym}}\right]}{K_{\text{m MASP1}_{\text{zym}}}^{\text{MASP1}_{\text{zym}}}}+\frac{\left[\text{pCLLKMASP2}_{\text{zym}}\right]}{K_{\text{m MASP1}_{\text{zym}}}^{\text{MASP2}_{\text{zym}}}}+\frac{\left[\text{pCLK1MASP1}_{\text{zym}}\right]}{K_{\text{m MASP1}_{\text{zym}}}^{\text{MASP1}_{\text{zym}}}}+\frac{\left[\text{pCLK1MASP2}_{\text{zym}}\right]}{K_{\text{m MASP1}_{\text{zym}}}^{\text{MASP2}_{\text{zym}}}}\\+\frac{\left[\text{pCLL1MASP1}_{\text{zym}}\right]}{K_{\text{m MASP1}_{\text{zym}}}^{\text{MASP1}_{\text{zym}}}}+\frac{\left[\text{pCLL1MASP2}_{\text{zym}}\right]}{K_{\text{m MASP1}_{\text{zym}}}^{\text{MASP2}_{\text{zym}}}}+\frac{\left[\text{pLFMASP1}_{\text{zym}}\right]}{K_{\text{m MASP1}_{\text{zym}}}^{\text{MASP1}_{\text{zym}}}}+\frac{\left[\text{pLFMASP2}_{\text{zym}}\right]}{K_{\text{m MASP1}_{\text{zym}}}^{\text{MASP2}_{\text{zym}}}}\\+\frac{\left[\text{pHFMASP1}_{\text{zym}}\right]}{K_{\text{m MASP1}_{\text{zym}}}^{\text{MASP1}_{\text{zym}}}}+\frac{\left[\text{pHFMASP2}_{\text{zym}}\right]}{K_{\text{m MASP1}_{\text{zym}}}^{\text{MASP2}_{\text{zym}}}}+\frac{\left[\text{pMFMASP1}_{\text{zym}}\right]}{K_{\text{m MASP1}_{\text{zym}}}^{\text{MASP1}_{\text{zym}}}}+\frac{\left[\text{pMFMASP2}_{\text{zym}}\right]}{K_{\text{m MASP1}_{\text{zym}}}^{\text{MASP2}_{\text{zym}}}}\\+\frac{\left[\text{pPTX3LFMASP1}_{\text{zym}}\right]}{K_{\text{m MASP1}_{\text{zym}}}^{\text{MASP1}_{\text{zym}}}}+\frac{\left[\text{pPTX3LFMASP2}_{\text{zym}}\right]}{K_{\text{m MASP1}_{\text{zym}}}^{\text{MASP2}_{\text{zym}}}}\end{array}\right)}$$

$$-\frac{k_{\text{cat MASP1}_{\text{zym}}}^{\text{MASP2}_{\text{zym}}}\left[\text{pHFMASP1}_{\text{zym}}\right]\left[\text{pMBL}_3\text{MASP2}_{\text{zym}}\right]}{K_{\text{m MASP1}_{\text{zym}}}^{\text{MASP2}_{\text{zym}}}\left(\begin{array}{l}1+\frac{\left[\text{pMBL}_3\text{MASP1}_{\text{zym}}\right]}{K_{\text{m MASP1}_{\text{zym}}}^{\text{MASP1}_{\text{zym}}}}+\frac{\left[\text{pMBL}_3\text{MASP2}_{\text{zym}}\right]}{K_{\text{m MASP1}_{\text{zym}}}^{\text{MASP2}_{\text{zym}}}}+\frac{\left[\text{pMBL}_4\text{MASP1}_{\text{zym}}\right]}{K_{\text{m MASP1}_{\text{zym}}}^{\text{MASP1}_{\text{zym}}}}+\frac{\left[\text{pMBL}_4\text{MASP2}_{\text{zym}}\right]}{K_{\text{m MASP1}_{\text{zym}}}^{\text{MASP2}_{\text{zym}}}}\\+\frac{\left[\text{pCLLKMASP1}_{\text{zym}}\right]}{K_{\text{m MASP1}_{\text{zym}}}^{\text{MASP1}_{\text{zym}}}}+\frac{\left[\text{pCLLKMASP2}_{\text{zym}}\right]}{K_{\text{m MASP1}_{\text{zym}}}^{\text{MASP2}_{\text{zym}}}}+\frac{\left[\text{pCLK1MASP1}_{\text{zym}}\right]}{K_{\text{m MASP1}_{\text{zym}}}^{\text{MASP1}_{\text{zym}}}}+\frac{\left[\text{pCLK1MASP2}_{\text{zym}}\right]}{K_{\text{m MASP1}_{\text{zym}}}^{\text{MASP2}_{\text{zym}}}}\\+\frac{\left[\text{pCLL1MASP1}_{\text{zym}}\right]}{K_{\text{m MASP1}_{\text{zym}}}^{\text{MASP1}_{\text{zym}}}}+\frac{\left[\text{pCLL1MASP2}_{\text{zym}}\right]}{K_{\text{m MASP1}_{\text{zym}}}^{\text{MASP2}_{\text{zym}}}}+\frac{\left[\text{pLFMASP1}_{\text{zym}}\right]}{K_{\text{m MASP1}_{\text{zym}}}^{\text{MASP1}_{\text{zym}}}}+\frac{\left[\text{pLFMASP2}_{\text{zym}}\right]}{K_{\text{m MASP1}_{\text{zym}}}^{\text{MASP2}_{\text{zym}}}}\\+\frac{\left[\text{pHFMASP1}_{\text{zym}}\right]}{K_{\text{m MASP1}_{\text{zym}}}^{\text{MASP1}_{\text{zym}}}}+\frac{\left[\text{pHFMASP2}_{\text{zym}}\right]}{K_{\text{m MASP1}_{\text{zym}}}^{\text{MASP2}_{\text{zym}}}}+\frac{\left[\text{pMFMASP1}_{\text{zym}}\right]}{K_{\text{m MASP1}_{\text{zym}}}^{\text{MASP1}_{\text{zym}}}}+\frac{\left[\text{pMFMASP2}_{\text{zym}}\right]}{K_{\text{m MASP1}_{\text{zym}}}^{\text{MASP2}_{\text{zym}}}}\\+\frac{\left[\text{pPTX3LFMASP1}_{\text{zym}}\right]}{K_{\text{m MASP1}_{\text{zym}}}^{\text{MASP1}_{\text{zym}}}}+\frac{\left[\text{pPTX3LFMASP2}_{\text{zym}}\right]}{K_{\text{m MASP1}_{\text{zym}}}^{\text{MASP2}_{\text{zym}}}}\end{array}\right)}$$

$$-\frac{k_{\text{cat MASP1}_{\text{zym}}}^{\text{MASP2}_{\text{zym}}}\left[\text{pMFMASP1}_{\text{zym}}\right]\left[\text{pMBL}_3\text{MASP2}_{\text{zym}}\right]}{K_{\text{m MASP1}_{\text{zym}}}^{\text{MASP2}_{\text{zym}}}\left(\begin{array}{l}1+\frac{\left[\text{pMBL}_3\text{MASP1}_{\text{zym}}\right]}{K_{\text{m MASP1}_{\text{zym}}}^{\text{MASP1}_{\text{zym}}}}+\frac{\left[\text{pMBL}_3\text{MASP2}_{\text{zym}}\right]}{K_{\text{m MASP1}_{\text{zym}}}^{\text{MASP2}_{\text{zym}}}}+\frac{\left[\text{pMBL}_4\text{MASP1}_{\text{zym}}\right]}{K_{\text{m MASP1}_{\text{zym}}}^{\text{MASP1}_{\text{zym}}}}+\frac{\left[\text{pMBL}_4\text{MASP2}_{\text{zym}}\right]}{K_{\text{m MASP1}_{\text{zym}}}^{\text{MASP2}_{\text{zym}}}}\\+\frac{\left[\text{pCLLKMASP1}_{\text{zym}}\right]}{K_{\text{m MASP1}_{\text{zym}}}^{\text{MASP1}_{\text{zym}}}}+\frac{\left[\text{pCLLKMASP2}_{\text{zym}}\right]}{K_{\text{m MASP1}_{\text{zym}}}^{\text{MASP2}_{\text{zym}}}}+\frac{\left[\text{pCLK1MASP1}_{\text{zym}}\right]}{K_{\text{m MASP1}_{\text{zym}}}^{\text{MASP1}_{\text{zym}}}}+\frac{\left[\text{pCLK1MASP2}_{\text{zym}}\right]}{K_{\text{m MASP1}_{\text{zym}}}^{\text{MASP2}_{\text{zym}}}}\\+\frac{\left[\text{pCLL1MASP1}_{\text{zym}}\right]}{K_{\text{m MASP1}_{\text{zym}}}^{\text{MASP1}_{\text{zym}}}}+\frac{\left[\text{pCLL1MASP2}_{\text{zym}}\right]}{K_{\text{m MASP1}_{\text{zym}}}^{\text{MASP2}_{\text{zym}}}}+\frac{\left[\text{pLFMASP1}_{\text{zym}}\right]}{K_{\text{m MASP1}_{\text{zym}}}^{\text{MASP1}_{\text{zym}}}}+\frac{\left[\text{pLFMASP2}_{\text{zym}}\right]}{K_{\text{m MASP1}_{\text{zym}}}^{\text{MASP2}_{\text{zym}}}}\\+\frac{\left[\text{pHFMASP1}_{\text{zym}}\right]}{K_{\text{m MASP1}_{\text{zym}}}^{\text{MASP1}_{\text{zym}}}}+\frac{\left[\text{pHFMASP2}_{\text{zym}}\right]}{K_{\text{m MASP1}_{\text{zym}}}^{\text{MASP2}_{\text{zym}}}}+\frac{\left[\text{pMFMASP1}_{\text{zym}}\right]}{K_{\text{m MASP1}_{\text{zym}}}^{\text{MASP1}_{\text{zym}}}}+\frac{\left[\text{pMFMASP2}_{\text{zym}}\right]}{K_{\text{m MASP1}_{\text{zym}}}^{\text{MASP2}_{\text{zym}}}}\\+\frac{\left[\text{pPTX3LFMASP1}_{\text{zym}}\right]}{K_{\text{m MASP1}_{\text{zym}}}^{\text{MASP1}_{\text{zym}}}}+\frac{\left[\text{pPTX3LFMASP2}_{\text{zym}}\right]}{K_{\text{m MASP1}_{\text{zym}}}^{\text{MASP2}_{\text{zym}}}}\end{array}\right)}$$

$$-\frac{k_{\text{cat MASP1}_{\text{zym}}}^{\text{MASP2}_{\text{zym}}}\left[\text{pPTX3LFMASP1}_{\text{zym}}\right]\left[\text{pMBL}_3\text{MASP2}_{\text{zym}}\right]}{K_{\text{m MASP1}_{\text{zym}}}^{\text{MASP2}_{\text{zym}}}\left(\begin{array}{l}1+\frac{\left[\text{pMBL}_3\text{MASP1}_{\text{zym}}\right]}{K_{\text{m MASP1}_{\text{zym}}}^{\text{MASP1}_{\text{zym}}}}+\frac{\left[\text{pMBL}_3\text{MASP2}_{\text{zym}}\right]}{K_{\text{m MASP1}_{\text{zym}}}^{\text{MASP2}_{\text{zym}}}}+\frac{\left[\text{pMBL}_4\text{MASP1}_{\text{zym}}\right]}{K_{\text{m MASP1}_{\text{zym}}}^{\text{MASP1}_{\text{zym}}}}+\frac{\left[\text{pMBL}_4\text{MASP2}_{\text{zym}}\right]}{K_{\text{m MASP1}_{\text{zym}}}^{\text{MASP2}_{\text{zym}}}}\\+\frac{\left[\text{pCLLKMASP1}_{\text{zym}}\right]}{K_{\text{m MASP1}_{\text{zym}}}^{\text{MASP1}_{\text{zym}}}}+\frac{\left[\text{pCLLKMASP2}_{\text{zym}}\right]}{K_{\text{m MASP1}_{\text{zym}}}^{\text{MASP2}_{\text{zym}}}}+\frac{\left[\text{pCLK1MASP1}_{\text{zym}}\right]}{K_{\text{m MASP1}_{\text{zym}}}^{\text{MASP1}_{\text{zym}}}}+\frac{\left[\text{pCLK1MASP2}_{\text{zym}}\right]}{K_{\text{m MASP1}_{\text{zym}}}^{\text{MASP2}_{\text{zym}}}}\\+\frac{\left[\text{pCLL1MASP1}_{\text{zym}}\right]}{K_{\text{m MASP1}_{\text{zym}}}^{\text{MASP1}_{\text{zym}}}}+\frac{\left[\text{pCLL1MASP2}_{\text{zym}}\right]}{K_{\text{m MASP1}_{\text{zym}}}^{\text{MASP2}_{\text{zym}}}}+\frac{\left[\text{pLFMASP1}_{\text{zym}}\right]}{K_{\text{m MASP1}_{\text{zym}}}^{\text{MASP1}_{\text{zym}}}}+\frac{\left[\text{pLFMASP2}_{\text{zym}}\right]}{K_{\text{m MASP1}_{\text{zym}}}^{\text{MASP2}_{\text{zym}}}}\\+\frac{\left[\text{pHFMASP1}_{\text{zym}}\right]}{K_{\text{m MASP1}_{\text{zym}}}^{\text{MASP1}_{\text{zym}}}}+\frac{\left[\text{pHFMASP2}_{\text{zym}}\right]}{K_{\text{m MASP1}_{\text{zym}}}^{\text{MASP2}_{\text{zym}}}}+\frac{\left[\text{pMFMASP1}_{\text{zym}}\right]}{K_{\text{m MASP1}_{\text{zym}}}^{\text{MASP1}_{\text{zym}}}}+\frac{\left[\text{pMFMASP2}_{\text{zym}}\right]}{K_{\text{m MASP1}_{\text{zym}}}^{\text{MASP2}_{\text{zym}}}}\\+\frac{\left[\text{pPTX3LFMASP1}_{\text{zym}}\right]}{K_{\text{m MASP1}_{\text{zym}}}^{\text{MASP1}_{\text{zym}}}}+\frac{\left[\text{pPTX3LFMASP2}_{\text{zym}}\right]}{K_{\text{m MASP1}_{\text{zym}}}^{\text{MASP2}_{\text{zym}}}}\end{array}\right)}$$

$$-\frac{k_{\text{cat MASP2}_{\text{zym}}}^{\text{MASP2}_{\text{zym}}}\left[\text{pMBL}_3\text{MASP2}_{\text{zym}}\right]\left[\text{pMBL}_3\text{MASP2}_{\text{zym}}\right]}{K_{\text{m MASP2}_{\text{zym}}}^{\text{MASP2}_{\text{zym}}}\left(\begin{array}{l}1+\frac{\left[\text{pMBL}_3\text{MASP2}_{\text{zym}}\right]}{K_{\text{m MASP2}_{\text{zym}}}^{\text{MASP2}_{\text{zym}}}}+\frac{\left[\text{pMBL}_4\text{MASP2}_{\text{zym}}\right]}{K_{\text{m MASP2}_{\text{zym}}}^{\text{MASP2}_{\text{zym}}}}+\frac{\left[\text{pCLLKMASP2}_{\text{zym}}\right]}{K_{\text{m MASP2}_{\text{zym}}}^{\text{MASP2}_{\text{zym}}}}+\frac{\left[\text{pCLK1MASP2}_{\text{zym}}\right]}{K_{\text{m MASP2}_{\text{zym}}}^{\text{MASP2}_{\text{zym}}}}\\+\frac{\left[\text{pCLL1MASP2}_{\text{zym}}\right]}{K_{\text{m MASP2}_{\text{zym}}}^{\text{MASP2}_{\text{zym}}}}+\frac{\left[\text{pLFMASP2}_{\text{zym}}\right]}{K_{\text{m MASP2}_{\text{zym}}}^{\text{MASP2}_{\text{zym}}}}+\frac{\left[\text{pHFMASP2}_{\text{zym}}\right]}{K_{\text{m MASP2}_{\text{zym}}}^{\text{MASP2}_{\text{zym}}}}+\frac{\left[\text{pMFMASP2}_{\text{zym}}\right]}{K_{\text{m MASP2}_{\text{zym}}}^{\text{MASP2}_{\text{zym}}}}\\+\frac{\left[\text{pPTX3LFMASP2}_{\text{zym}}\right]}{K_{\text{m MASP2}_{\text{zym}}}^{\text{MASP2}_{\text{zym}}}}\end{array}\right)}$$

$$-\frac{k_{\text{cat MASP2}_{\text{zym}}}^{\text{MASP2}_{\text{zym}}}\left[\text{pMBL}_4\text{MASP2}_{\text{zym}}\right]\left[\text{pMBL}_3\text{MASP2}_{\text{zym}}\right]}{K_{\text{m MASP2}_{\text{zym}}}^{\text{MASP2}_{\text{zym}}}\left(\begin{array}{l}1+\frac{\left[\text{pMBL}_3\text{MASP2}_{\text{zym}}\right]}{K_{\text{m MASP2}_{\text{zym}}}^{\text{MASP2}_{\text{zym}}}}+\frac{\left[\text{pMBL}_4\text{MASP2}_{\text{zym}}\right]}{K_{\text{m MASP2}_{\text{zym}}}^{\text{MASP2}_{\text{zym}}}}+\frac{\left[\text{pCLLKMASP2}_{\text{zym}}\right]}{K_{\text{m MASP2}_{\text{zym}}}^{\text{MASP2}_{\text{zym}}}}+\frac{\left[\text{pCLK1MASP2}_{\text{zym}}\right]}{K_{\text{m MASP2}_{\text{zym}}}^{\text{MASP2}_{\text{zym}}}}\\+\frac{\left[\text{pCLL1MASP2}_{\text{zym}}\right]}{K_{\text{m MASP2}_{\text{zym}}}^{\text{MASP2}_{\text{zym}}}}+\frac{\left[\text{pLFMASP2}_{\text{zym}}\right]}{K_{\text{m MASP2}_{\text{zym}}}^{\text{MASP2}_{\text{zym}}}}+\frac{\left[\text{pHFMASP2}_{\text{zym}}\right]}{K_{\text{m MASP2}_{\text{zym}}}^{\text{MASP2}_{\text{zym}}}}+\frac{\left[\text{pMFMASP2}_{\text{zym}}\right]}{K_{\text{m MASP2}_{\text{zym}}}^{\text{MASP2}_{\text{zym}}}}\\+\frac{\left[\text{pPTX3LFMASP2}_{\text{zym}}\right]}{K_{\text{m MASP2}_{\text{zym}}}^{\text{MASP2}_{\text{zym}}}}\end{array}\right)}$$

$$-\frac{k_{\mathrm{cat\ MASP2_{zym}}}^{\mathrm{MASP2_{zym}}}\left[\mathrm{pCLLKMASP2_{zym}}\right]\left[\mathrm{pMBL_3MASP2_{zym}}\right]}{K_{\mathrm{m\ MASP2_{zym}}}^{\mathrm{MASP2_{zym}}}\left(\begin{array}{l}1+\frac{\left[\mathrm{pMBL_3MASP2_{zym}}\right]}{K_{\mathrm{m\ MASP2_{zym}}}^{\mathrm{MASP2_{zym}}}}+\frac{\left[\mathrm{pMBL_4MASP2_{zym}}\right]}{K_{\mathrm{m\ MASP2_{zym}}}^{\mathrm{MASP2_{zym}}}}+\frac{\left[\mathrm{pCLLKMASP2_{zym}}\right]}{K_{\mathrm{m\ MASP2_{zym}}}^{\mathrm{MASP2_{zym}}}}+\frac{\left[\mathrm{pCLK1MASP2_{zym}}\right]}{K_{\mathrm{m\ MASP2_{zym}}}^{\mathrm{MASP2_{zym}}}}\\+\frac{\left[\mathrm{pCLL1MASP2_{zym}}\right]}{K_{\mathrm{m\ MASP2_{zym}}}^{\mathrm{MASP2_{zym}}}}+\frac{\left[\mathrm{pLFMASP2_{zym}}\right]}{K_{\mathrm{m\ MASP2_{zym}}}^{\mathrm{MASP2_{zym}}}}+\frac{\left[\mathrm{pHFMASP2_{zym}}\right]}{K_{\mathrm{m\ MASP2_{zym}}}^{\mathrm{MASP2_{zym}}}}+\frac{\left[\mathrm{pMFMASP2_{zym}}\right]}{K_{\mathrm{m\ MASP2_{zym}}}^{\mathrm{MASP2_{zym}}}}\\+\frac{\left[\mathrm{pPTX3LFMASP2_{zym}}\right]}{K_{\mathrm{m\ MASP2_{zym}}}^{\mathrm{MASP2_{zym}}}}\end{array}\right)}$$

$$-\frac{k_{\mathrm{cat\ MASP2_{zym}}}^{\mathrm{MASP2_{zym}}}\left[\mathrm{pCLK1MASP2_{zym}}\right]\left[\mathrm{pMBL_3MASP2_{zym}}\right]}{K_{\mathrm{m\ MASP2_{zym}}}^{\mathrm{MASP2_{zym}}}\left(\begin{array}{l}1+\frac{\left[\mathrm{pMBL_3MASP2_{zym}}\right]}{K_{\mathrm{m\ MASP2_{zym}}}^{\mathrm{MASP2_{zym}}}}+\frac{\left[\mathrm{pMBL_4MASP2_{zym}}\right]}{K_{\mathrm{m\ MASP2_{zym}}}^{\mathrm{MASP2_{zym}}}}+\frac{\left[\mathrm{pCLLKMASP2_{zym}}\right]}{K_{\mathrm{m\ MASP2_{zym}}}^{\mathrm{MASP2_{zym}}}}+\frac{\left[\mathrm{pCLK1MASP2_{zym}}\right]}{K_{\mathrm{m\ MASP2_{zym}}}^{\mathrm{MASP2_{zym}}}}\\+\frac{\left[\mathrm{pCLL1MASP2_{zym}}\right]}{K_{\mathrm{m\ MASP2_{zym}}}^{\mathrm{MASP2_{zym}}}}+\frac{\left[\mathrm{pLFMASP2_{zym}}\right]}{K_{\mathrm{m\ MASP2_{zym}}}^{\mathrm{MASP2_{zym}}}}+\frac{\left[\mathrm{pHFMASP2_{zym}}\right]}{K_{\mathrm{m\ MASP2_{zym}}}^{\mathrm{MASP2_{zym}}}}+\frac{\left[\mathrm{pMFMASP2_{zym}}\right]}{K_{\mathrm{m\ MASP2_{zym}}}^{\mathrm{MASP2_{zym}}}}\\+\frac{\left[\mathrm{pPTX3LFMASP2_{zym}}\right]}{K_{\mathrm{m\ MASP2_{zym}}}^{\mathrm{MASP2_{zym}}}}\end{array}\right)}$$

$$-\frac{k_{\mathrm{cat\ MASP2_{zym}}}^{\mathrm{MASP2_{zym}}}\left[\mathrm{pCLL1MASP2_{zym}}\right]\left[\mathrm{pMBL_3MASP2_{zym}}\right]}{K_{\mathrm{m\ MASP2_{zym}}}^{\mathrm{MASP2_{zym}}}\left(\begin{array}{l}1+\frac{\left[\mathrm{pMBL_3MASP2_{zym}}\right]}{K_{\mathrm{m\ MASP2_{zym}}}^{\mathrm{MASP2_{zym}}}}+\frac{\left[\mathrm{pMBL_4MASP2_{zym}}\right]}{K_{\mathrm{m\ MASP2_{zym}}}^{\mathrm{MASP2_{zym}}}}+\frac{\left[\mathrm{pCLLKMASP2_{zym}}\right]}{K_{\mathrm{m\ MASP2_{zym}}}^{\mathrm{MASP2_{zym}}}}+\frac{\left[\mathrm{pCLK1MASP2_{zym}}\right]}{K_{\mathrm{m\ MASP2_{zym}}}^{\mathrm{MASP2_{zym}}}}\\+\frac{\left[\mathrm{pCLL1MASP2_{zym}}\right]}{K_{\mathrm{m\ MASP2_{zym}}}^{\mathrm{MASP2_{zym}}}}+\frac{\left[\mathrm{pLFMASP2_{zym}}\right]}{K_{\mathrm{m\ MASP2_{zym}}}^{\mathrm{MASP2_{zym}}}}+\frac{\left[\mathrm{pHFMASP2_{zym}}\right]}{K_{\mathrm{m\ MASP2_{zym}}}^{\mathrm{MASP2_{zym}}}}+\frac{\left[\mathrm{pMFMASP2_{zym}}\right]}{K_{\mathrm{m\ MASP2_{zym}}}^{\mathrm{MASP2_{zym}}}}\\+\frac{\left[\mathrm{pPTX3LFMASP2_{zym}}\right]}{K_{\mathrm{m\ MASP2_{zym}}}^{\mathrm{MASP2_{zym}}}}\end{array}\right)}$$

$$-\frac{k_{\mathrm{cat\ MASP2_{zym}}}^{\mathrm{MASP2_{zym}}}\left[\mathrm{pLFMASP2_{zym}}\right]\left[\mathrm{pMBL_3MASP2_{zym}}\right]}{K_{\mathrm{m\ MASP2_{zym}}}^{\mathrm{MASP2_{zym}}}\left(\begin{array}{l}1+\frac{\left[\mathrm{pMBL_3MASP2_{zym}}\right]}{K_{\mathrm{m\ MASP2_{zym}}}^{\mathrm{MASP2_{zym}}}}+\frac{\left[\mathrm{pMBL_4MASP2_{zym}}\right]}{K_{\mathrm{m\ MASP2_{zym}}}^{\mathrm{MASP2_{zym}}}}+\frac{\left[\mathrm{pCLLKMASP2_{zym}}\right]}{K_{\mathrm{m\ MASP2_{zym}}}^{\mathrm{MASP2_{zym}}}}+\frac{\left[\mathrm{pCLK1MASP2_{zym}}\right]}{K_{\mathrm{m\ MASP2_{zym}}}^{\mathrm{MASP2_{zym}}}}\\+\frac{\left[\mathrm{pCLL1MASP2_{zym}}\right]}{K_{\mathrm{m\ MASP2_{zym}}}^{\mathrm{MASP2_{zym}}}}+\frac{\left[\mathrm{pLFMASP2_{zym}}\right]}{K_{\mathrm{m\ MASP2_{zym}}}^{\mathrm{MASP2_{zym}}}}+\frac{\left[\mathrm{pHFMASP2_{zym}}\right]}{K_{\mathrm{m\ MASP2_{zym}}}^{\mathrm{MASP2_{zym}}}}+\frac{\left[\mathrm{pMFMASP2_{zym}}\right]}{K_{\mathrm{m\ MASP2_{zym}}}^{\mathrm{MASP2_{zym}}}}\\+\frac{\left[\mathrm{pPTX3LFMASP2_{zym}}\right]}{K_{\mathrm{m\ MASP2_{zym}}}^{\mathrm{MASP2_{zym}}}}\end{array}\right)}$$

$$-\frac{k_{\text{cat MASP2}_{\text{zym}}}^{\text{MASP2}_{\text{zym}}}\left[\text{pHFMASP2}_{\text{zym}}\right]\left[\text{pMBL}_3\text{MASP2}_{\text{zym}}\right]}{K_{\text{m MASP2}_{\text{zym}}}^{\text{MASP2}_{\text{zym}}}\left(\begin{array}{l}1+\frac{\left[\text{pMBL}_3\text{MASP2}_{\text{zym}}\right]}{K_{\text{m MASP2}_{\text{zym}}}^{\text{MASP2}_{\text{zym}}}}+\frac{\left[\text{pMBL}_4\text{MASP2}_{\text{zym}}\right]}{K_{\text{m MASP2}_{\text{zym}}}^{\text{MASP2}_{\text{zym}}}}+\frac{\left[\text{pCLLKMASP2}_{\text{zym}}\right]}{K_{\text{m MASP2}_{\text{zym}}}^{\text{MASP2}_{\text{zym}}}}+\frac{\left[\text{pCLK1MASP2}_{\text{zym}}\right]}{K_{\text{m MASP2}_{\text{zym}}}^{\text{MASP2}_{\text{zym}}}}\\+\frac{\left[\text{pCLL1MASP2}_{\text{zym}}\right]}{K_{\text{m MASP2}_{\text{zym}}}^{\text{MASP2}_{\text{zym}}}}+\frac{\left[\text{pLFMASP2}_{\text{zym}}\right]}{K_{\text{m MASP2}_{\text{zym}}}^{\text{MASP2}_{\text{zym}}}}+\frac{\left[\text{pHFMASP2}_{\text{zym}}\right]}{K_{\text{m MASP2}_{\text{zym}}}^{\text{MASP2}_{\text{zym}}}}+\frac{\left[\text{pMFMASP2}_{\text{zym}}\right]}{K_{\text{m MASP2}_{\text{zym}}}^{\text{MASP2}_{\text{zym}}}}\\+\frac{\left[\text{pPTX3LFMASP2}_{\text{zym}}\right]}{K_{\text{m MASP2}_{\text{zym}}}^{\text{MASP2}_{\text{zym}}}}\end{array}\right)}$$

$$-\frac{k_{\text{cat MASP2}_{\text{zym}}}^{\text{MASP2}_{\text{zym}}}\left[\text{pMFMASP2}_{\text{zym}}\right]\left[\text{pMBL}_3\text{MASP2}_{\text{zym}}\right]}{K_{\text{m MASP2}_{\text{zym}}}^{\text{MASP2}_{\text{zym}}}\left(\begin{array}{l}1+\frac{\left[\text{pMBL}_3\text{MASP2}_{\text{zym}}\right]}{K_{\text{m MASP2}_{\text{zym}}}^{\text{MASP2}_{\text{zym}}}}+\frac{\left[\text{pMBL}_4\text{MASP2}_{\text{zym}}\right]}{K_{\text{m MASP2}_{\text{zym}}}^{\text{MASP2}_{\text{zym}}}}+\frac{\left[\text{pCLLKMASP2}_{\text{zym}}\right]}{K_{\text{m MASP2}_{\text{zym}}}^{\text{MASP2}_{\text{zym}}}}+\frac{\left[\text{pCLK1MASP2}_{\text{zym}}\right]}{K_{\text{m MASP2}_{\text{zym}}}^{\text{MASP2}_{\text{zym}}}}\\+\frac{\left[\text{pCLL1MASP2}_{\text{zym}}\right]}{K_{\text{m MASP2}_{\text{zym}}}^{\text{MASP2}_{\text{zym}}}}+\frac{\left[\text{pLFMASP2}_{\text{zym}}\right]}{K_{\text{m MASP2}_{\text{zym}}}^{\text{MASP2}_{\text{zym}}}}+\frac{\left[\text{pHFMASP2}_{\text{zym}}\right]}{K_{\text{m MASP2}_{\text{zym}}}^{\text{MASP2}_{\text{zym}}}}+\frac{\left[\text{pMFMASP2}_{\text{zym}}\right]}{K_{\text{m MASP2}_{\text{zym}}}^{\text{MASP2}_{\text{zym}}}}\\+\frac{\left[\text{pPTX3LFMASP2}_{\text{zym}}\right]}{K_{\text{m MASP2}_{\text{zym}}}^{\text{MASP2}_{\text{zym}}}}\end{array}\right)}$$

$$-\frac{k_{\text{cat MASP2}_{\text{zym}}}^{\text{MASP2}_{\text{zym}}}\left[\text{pPTX3LFMASP2}_{\text{zym}}\right]\left[\text{pMBL}_3\text{MASP2}_{\text{zym}}\right]}{K_{\text{m MASP2}_{\text{zym}}}^{\text{MASP2}_{\text{zym}}}\left(\begin{array}{l}1+\frac{\left[\text{pMBL}_3\text{MASP2}_{\text{zym}}\right]}{K_{\text{m MASP2}_{\text{zym}}}^{\text{MASP2}_{\text{zym}}}}+\frac{\left[\text{pMBL}_4\text{MASP2}_{\text{zym}}\right]}{K_{\text{m MASP2}_{\text{zym}}}^{\text{MASP2}_{\text{zym}}}}+\frac{\left[\text{pCLLKMASP2}_{\text{zym}}\right]}{K_{\text{m MASP2}_{\text{zym}}}^{\text{MASP2}_{\text{zym}}}}+\frac{\left[\text{pCLK1MASP2}_{\text{zym}}\right]}{K_{\text{m MASP2}_{\text{zym}}}^{\text{MASP2}_{\text{zym}}}}\\+\frac{\left[\text{pCLL1MASP2}_{\text{zym}}\right]}{K_{\text{m MASP2}_{\text{zym}}}^{\text{MASP2}_{\text{zym}}}}+\frac{\left[\text{pLFMASP2}_{\text{zym}}\right]}{K_{\text{m MASP2}_{\text{zym}}}^{\text{MASP2}_{\text{zym}}}}+\frac{\left[\text{pHFMASP2}_{\text{zym}}\right]}{K_{\text{m MASP2}_{\text{zym}}}^{\text{MASP2}_{\text{zym}}}}+\frac{\left[\text{pMFMASP2}_{\text{zym}}\right]}{K_{\text{m MASP2}_{\text{zym}}}^{\text{MASP2}_{\text{zym}}}}\\+\frac{\left[\text{pPTX3LFMASP2}_{\text{zym}}\right]}{K_{\text{m MASP2}_{\text{zym}}}^{\text{MASP2}_{\text{zym}}}}\end{array}\right)}$$

$$
-\frac{k_{\text{cat MASP1}^*}^{\text{MASP2}_{\text{zym}}}\left[\text{pMBL}_3\text{MASP1}^*\right]\left[\text{pMBL}_3\text{MASP2}_{\text{zym}}\right]}{K_{\text{m MASP1}^*}^{\text{MASP2}_{\text{zym}}}\left(\begin{array}{l}
1+\frac{[\text{C4}]}{K_{\text{m MASP1}^*}^{\text{C4}}}+\frac{[\text{fC4bC2}]}{K_{\text{m MASP1}^*}^{\text{C2}}}+\frac{[\text{fC4bC4bC2}]}{K_{\text{m MASP1}^*}^{\text{C2}}}+\frac{[\text{fC3bC4bC2}]}{K_{\text{m MASP1}^*}^{\text{C2}}}+\frac{[\text{pC4bC2}]}{K_{\text{m MASP1}^*}^{\text{C2}}}+\frac{[\text{pC4bC4bC2}]}{K_{\text{m MASP1}^*}^{\text{C2}}}\\
+\frac{[\text{pC3bC4bC2}]}{K_{\text{m MASP1}^*}^{\text{C2}}}+\frac{\left[\text{pMBL}_3\text{MASP1}_{\text{zym}}\right]}{K_{\text{m MASP1}^*}^{\text{MASP1}_{\text{zym}}}}+\frac{\left[\text{pMBL}_3\text{MASP2}_{\text{zym}}\right]}{K_{\text{m MASP1}^*}^{\text{MASP2}_{\text{zym}}}}+\frac{\left[\text{pMBL}_3\text{MASP3}_{\text{zym}}\right]}{K_{\text{m MASP1}^*}^{\text{MASP3}_{\text{zym}}}}\\
+\frac{\left[\text{pMBL}_4\text{MASP1}_{\text{zym}}\right]}{K_{\text{m MASP1}^*}^{\text{MASP1}_{\text{zym}}}}+\frac{\left[\text{pMBL}_4\text{MASP2}_{\text{zym}}\right]}{K_{\text{m MASP1}^*}^{\text{MASP2}_{\text{zym}}}}+\frac{\left[\text{pMBL}_4\text{MASP3}_{\text{zym}}\right]}{K_{\text{m MASP1}^*}^{\text{MASP3}_{\text{zym}}}}+\frac{\left[\text{pCLLKMASP1}_{\text{zym}}\right]}{K_{\text{m MASP1}^*}^{\text{MASP1}_{\text{zym}}}}\\
+\frac{\left[\text{pCLLKMASP2}_{\text{zym}}\right]}{K_{\text{m MASP1}^*}^{\text{MASP2}_{\text{zym}}}}+\frac{\left[\text{pCLLKMASP3}_{\text{zym}}\right]}{K_{\text{m MASP1}^*}^{\text{MASP3}_{\text{zym}}}}+\frac{\left[\text{pCLK1MASP1}_{\text{zym}}\right]}{K_{\text{m MASP1}^*}^{\text{MASP1}_{\text{zym}}}}+\frac{\left[\text{pCLK1MASP2}_{\text{zym}}\right]}{K_{\text{m MASP1}^*}^{\text{MASP2}_{\text{zym}}}}\\
+\frac{\left[\text{pCLK1MASP3}_{\text{zym}}\right]}{K_{\text{m MASP1}^*}^{\text{MASP3}_{\text{zym}}}}+\frac{\left[\text{pCLL1MASP1}_{\text{zym}}\right]}{K_{\text{m MASP1}^*}^{\text{MASP1}_{\text{zym}}}}+\frac{\left[\text{pCLL1MASP2}_{\text{zym}}\right]}{K_{\text{m MASP1}^*}^{\text{MASP2}_{\text{zym}}}}+\frac{\left[\text{pCLL1MASP3}_{\text{zym}}\right]}{K_{\text{m MASP1}^*}^{\text{MASP3}_{\text{zym}}}}\\
+\frac{\left[\text{pLFMASP1}_{\text{zym}}\right]}{K_{\text{m MASP1}^*}^{\text{MASP1}_{\text{zym}}}}+\frac{\left[\text{pLFMASP2}_{\text{zym}}\right]}{K_{\text{m MASP1}^*}^{\text{MASP2}_{\text{zym}}}}+\frac{\left[\text{pLFMASP3}_{\text{zym}}\right]}{K_{\text{m MASP1}^*}^{\text{MASP3}_{\text{zym}}}}+\frac{\left[\text{pHFMASP1}_{\text{zym}}\right]}{K_{\text{m MASP1}^*}^{\text{MASP1}_{\text{zym}}}}+\frac{\left[\text{pHFMASP2}_{\text{zym}}\right]}{K_{\text{m MASP1}^*}^{\text{MASP2}_{\text{zym}}}}\\
+\frac{\left[\text{pHFMASP3}_{\text{zym}}\right]}{K_{\text{m MASP1}^*}^{\text{MASP3}_{\text{zym}}}}+\frac{\left[\text{pMFMASP1}_{\text{zym}}\right]}{K_{\text{m MASP1}^*}^{\text{MASP1}_{\text{zym}}}}+\frac{\left[\text{pMFMASP2}_{\text{zym}}\right]}{K_{\text{m MASP1}^*}^{\text{MASP2}_{\text{zym}}}}+\frac{\left[\text{pMFMASP3}_{\text{zym}}\right]}{K_{\text{m MASP1}^*}^{\text{MASP3}_{\text{zym}}}}\\
+\frac{\left[\text{pPTX3LFMASP1}_{\text{zym}}\right]}{K_{\text{m MASP1}^*}^{\text{MASP1}_{\text{zym}}}}+\frac{\left[\text{pPXT3LFMASP2}_{\text{zym}}\right]}{K_{\text{m MASP1}^*}^{\text{MASP2}_{\text{zym}}}}+\frac{\left[\text{pPTX3LFMASP3}_{\text{zym}}\right]}{K_{\text{m MASP1}^*}^{\text{MASP3}_{\text{zym}}}}
\end{array}\right)}
$$

$$-\frac{k_{\mathrm{cat\ MASP1^*}}^{\mathrm{MASP2_{zym}}}\left[\mathrm{pMBL_4MASP1^*}\right]\left[\mathrm{pMBL_3MASP2_{zym}}\right]}{K_{\mathrm{m\ MASP1^*}}^{\mathrm{MASP2_{zym}}}\left(\begin{array}{l}1+\frac{[\mathrm{C4}]}{K_{\mathrm{m\ MASP1^*}}^{\mathrm{C4}}}+\frac{[\mathrm{fC4bC2}]}{K_{\mathrm{m\ MASP1^*}}^{\mathrm{C2}}}+\frac{[\mathrm{fC4bC4bC2}]}{K_{\mathrm{m\ MASP1^*}}^{\mathrm{C2}}}+\frac{[\mathrm{fC3bC4bC2}]}{K_{\mathrm{m\ MASP1^*}}^{\mathrm{C2}}}+\frac{[\mathrm{pC4bC2}]}{K_{\mathrm{m\ MASP1^*}}^{\mathrm{C2}}}+\frac{[\mathrm{pC4bC4bC2}]}{K_{\mathrm{m\ MASP1^*}}^{\mathrm{C2}}}\\
+\frac{[\mathrm{pC3bC4bC2}]}{K_{\mathrm{m\ MASP1^*}}^{\mathrm{C2}}}+\frac{[\mathrm{pMBL_3MASP1_{zym}}]}{K_{\mathrm{m\ MASP1^*}}^{\mathrm{MASP1_{zym}}}}+\frac{[\mathrm{pMBL_3MASP2_{zym}}]}{K_{\mathrm{m\ MASP1^*}}^{\mathrm{MASP2_{zym}}}}+\frac{[\mathrm{pMBL_3MASP3_{zym}}]}{K_{\mathrm{m\ MASP1^*}}^{\mathrm{MASP3_{zym}}}}\\
+\frac{[\mathrm{pMBL_4MASP1_{zym}}]}{K_{\mathrm{m\ MASP1^*}}^{\mathrm{MASP1_{zym}}}}+\frac{[\mathrm{pMBL_4MASP2_{zym}}]}{K_{\mathrm{m\ MASP1^*}}^{\mathrm{MASP2_{zym}}}}+\frac{[\mathrm{pMBL_4MASP3_{zym}}]}{K_{\mathrm{m\ MASP1^*}}^{\mathrm{MASP3_{zym}}}}+\frac{[\mathrm{pCLLKMASP1_{zym}}]}{K_{\mathrm{m\ MASP1^*}}^{\mathrm{MASP1_{zym}}}}\\
+\frac{[\mathrm{pCLLKMASP2_{zym}}]}{K_{\mathrm{m\ MASP1^*}}^{\mathrm{MASP2_{zym}}}}+\frac{[\mathrm{pCLLKMASP3_{zym}}]}{K_{\mathrm{m\ MASP1^*}}^{\mathrm{MASP3_{zym}}}}+\frac{[\mathrm{pCLK1MASP1_{zym}}]}{K_{\mathrm{m\ MASP1^*}}^{\mathrm{MASP1_{zym}}}}+\frac{[\mathrm{pCLK1MASP2_{zym}}]}{K_{\mathrm{m\ MASP1^*}}^{\mathrm{MASP2_{zym}}}}\\
+\frac{[\mathrm{pCLK1MASP3_{zym}}]}{K_{\mathrm{m\ MASP1^*}}^{\mathrm{MASP3_{zym}}}}+\frac{[\mathrm{pCLL1MASP1_{zym}}]}{K_{\mathrm{m\ MASP1^*}}^{\mathrm{MASP1_{zym}}}}+\frac{[\mathrm{pCLL1MASP2_{zym}}]}{K_{\mathrm{m\ MASP1^*}}^{\mathrm{MASP2_{zym}}}}+\frac{[\mathrm{pCLL1MASP3_{zym}}]}{K_{\mathrm{m\ MASP1^*}}^{\mathrm{MASP3_{zym}}}}\\
+\frac{[\mathrm{pLFMASP1_{zym}}]}{K_{\mathrm{m\ MASP1^*}}^{\mathrm{MASP1_{zym}}}}+\frac{[\mathrm{pLFMASP2_{zym}}]}{K_{\mathrm{m\ MASP1^*}}^{\mathrm{MASP2_{zym}}}}+\frac{[\mathrm{pLFMASP3_{zym}}]}{K_{\mathrm{m\ MASP1^*}}^{\mathrm{MASP3_{zym}}}}+\frac{[\mathrm{pHFMASP1_{zym}}]}{K_{\mathrm{m\ MASP1^*}}^{\mathrm{MASP1_{zym}}}}+\frac{[\mathrm{pHFMASP2_{zym}}]}{K_{\mathrm{m\ MASP1^*}}^{\mathrm{MASP2_{zym}}}}\\
+\frac{[\mathrm{pHFMASP3_{zym}}]}{K_{\mathrm{m\ MASP1^*}}^{\mathrm{MASP3_{zym}}}}+\frac{[\mathrm{pMFMASP1_{zym}}]}{K_{\mathrm{m\ MASP1^*}}^{\mathrm{MASP1_{zym}}}}+\frac{[\mathrm{pMFMASP2_{zym}}]}{K_{\mathrm{m\ MASP1^*}}^{\mathrm{MASP2_{zym}}}}+\frac{[\mathrm{pMFMASP3_{zym}}]}{K_{\mathrm{m\ MASP1^*}}^{\mathrm{MASP3_{zym}}}}\\
+\frac{[\mathrm{pPTX3LFMASP1_{zym}}]}{K_{\mathrm{m\ MASP1^*}}^{\mathrm{MASP1_{zym}}}}+\frac{[\mathrm{pPXT3LFMASP2_{zym}}]}{K_{\mathrm{m\ MASP1^*}}^{\mathrm{MASP2_{zym}}}}+\frac{[\mathrm{pPTX3LFMASP3_{zym}}]}{K_{\mathrm{m\ MASP1^*}}^{\mathrm{MASP3_{zym}}}}\end{array}\right)}$$

$$
-\frac{k_{\text{cat MASP1}^*}^{\text{MASP2}_{\text{zym}}}\left[\text{pCLLKMASP1}^*\right]\left[\text{pMBL}_3\text{MASP2}_{\text{zym}}\right]}{K_{\text{m MASP1}^*}^{\text{MASP2}_{\text{zym}}}\left(\begin{array}{l}
1+\frac{[\text{C4}]}{K_{\text{m MASP1}^*}^{\text{C4}}}+\frac{[\text{fC4bC2}]}{K_{\text{m MASP1}^*}^{\text{C2}}}+\frac{[\text{fC4bC4bC2}]}{K_{\text{m MASP1}^*}^{\text{C2}}}+\frac{[\text{fC3bC4bC2}]}{K_{\text{m MASP1}^*}^{\text{C2}}}+\frac{[\text{pC4bC2}]}{K_{\text{m MASP1}^*}^{\text{C2}}}+\frac{[\text{pC4bC4bC2}]}{K_{\text{m MASP1}^*}^{\text{C2}}} \\
+\frac{[\text{pC3bC4bC2}]}{K_{\text{m MASP1}^*}^{\text{C2}}}+\frac{[\text{pMBL}_3\text{MASP1}_{\text{zym}}]}{K_{\text{m MASP1}^*}^{\text{MASP1}_{\text{zym}}}}+\frac{[\text{pMBL}_3\text{MASP2}_{\text{zym}}]}{K_{\text{m MASP1}^*}^{\text{MASP2}_{\text{zym}}}}+\frac{[\text{pMBL}_3\text{MASP3}_{\text{zym}}]}{K_{\text{m MASP1}^*}^{\text{MASP3}_{\text{zym}}}} \\
+\frac{[\text{pMBL}_4\text{MASP1}_{\text{zym}}]}{K_{\text{m MASP1}^*}^{\text{MASP1}_{\text{zym}}}}+\frac{[\text{pMBL}_4\text{MASP2}_{\text{zym}}]}{K_{\text{m MASP1}^*}^{\text{MASP2}_{\text{zym}}}}+\frac{[\text{pMBL}_4\text{MASP3}_{\text{zym}}]}{K_{\text{m MASP1}^*}^{\text{MASP3}_{\text{zym}}}}+\frac{[\text{pCLLKMASP1}_{\text{zym}}]}{K_{\text{m MASP1}^*}^{\text{MASP1}_{\text{zym}}}} \\
+\frac{[\text{pCLLKMASP2}_{\text{zym}}]}{K_{\text{m MASP1}^*}^{\text{MASP2}_{\text{zym}}}}+\frac{[\text{pCLLKMASP3}_{\text{zym}}]}{K_{\text{m MASP1}^*}^{\text{MASP3}_{\text{zym}}}}+\frac{[\text{pCLK1MASP1}_{\text{zym}}]}{K_{\text{m MASP1}^*}^{\text{MASP1}_{\text{zym}}}}+\frac{[\text{pCLK1MASP2}_{\text{zym}}]}{K_{\text{m MASP1}^*}^{\text{MASP2}_{\text{zym}}}} \\
+\frac{[\text{pCLK1MASP3}_{\text{zym}}]}{K_{\text{m MASP1}^*}^{\text{MASP3}_{\text{zym}}}}+\frac{[\text{pCLL1MASP1}_{\text{zym}}]}{K_{\text{m MASP1}^*}^{\text{MASP1}_{\text{zym}}}}+\frac{[\text{pCLL1MASP2}_{\text{zym}}]}{K_{\text{m MASP1}^*}^{\text{MASP2}_{\text{zym}}}}+\frac{[\text{pCLL1MASP3}_{\text{zym}}]}{K_{\text{m MASP1}^*}^{\text{MASP3}_{\text{zym}}}} \\
+\frac{[\text{pLFMASP1}_{\text{zym}}]}{K_{\text{m MASP1}^*}^{\text{MASP1}_{\text{zym}}}}+\frac{[\text{pLFMASP2}_{\text{zym}}]}{K_{\text{m MASP1}^*}^{\text{MASP2}_{\text{zym}}}}+\frac{[\text{pLFMASP3}_{\text{zym}}]}{K_{\text{m MASP1}^*}^{\text{MASP3}_{\text{zym}}}}+\frac{[\text{pHFMASP1}_{\text{zym}}]}{K_{\text{m MASP1}^*}^{\text{MASP1}_{\text{zym}}}}+\frac{[\text{pHFMASP2}_{\text{zym}}]}{K_{\text{m MASP1}^*}^{\text{MASP2}_{\text{zym}}}} \\
+\frac{[\text{pHFMASP3}_{\text{zym}}]}{K_{\text{m MASP1}^*}^{\text{MASP3}_{\text{zym}}}}+\frac{[\text{pMFMASP1}_{\text{zym}}]}{K_{\text{m MASP1}^*}^{\text{MASP1}_{\text{zym}}}}+\frac{[\text{pMFMASP2}_{\text{zym}}]}{K_{\text{m MASP1}^*}^{\text{MASP2}_{\text{zym}}}}+\frac{[\text{pMFMASP3}_{\text{zym}}]}{K_{\text{m MASP1}^*}^{\text{MASP3}_{\text{zym}}}} \\
+\frac{[\text{pPTX3LFMASP1}_{\text{zym}}]}{K_{\text{m MASP1}^*}^{\text{MASP1}_{\text{zym}}}}+\frac{[\text{pPXT3LFMASP2}_{\text{zym}}]}{K_{\text{m MASP1}^*}^{\text{MASP2}_{\text{zym}}}}+\frac{[\text{pPTX3LFMASP3}_{\text{zym}}]}{K_{\text{m MASP1}^*}^{\text{MASP3}_{\text{zym}}}}
\end{array}\right)}
$$

$$
-\frac{k_{\text{cat MASP1}^*}^{\text{MASP2}_{\text{zym}}}\left[\text{pCLK1MASP1}^*\right]\left[\text{pMBL}_3\text{MASP2}_{\text{zym}}\right]}{K_{\text{m MASP1}^*}^{\text{MASP2}_{\text{zym}}}\left(\begin{aligned}&1+\frac{[\text{C4}]}{K_{\text{m MASP1}^*}^{\text{C4}}}+\frac{[\text{fC4bC2}]}{K_{\text{m MASP1}^*}^{\text{C2}}}+\frac{[\text{fC4bC4bC2}]}{K_{\text{m MASP1}^*}^{\text{C2}}}+\frac{[\text{fC3bC4bC2}]}{K_{\text{m MASP1}^*}^{\text{C2}}}+\frac{[\text{pC4bC2}]}{K_{\text{m MASP1}^*}^{\text{C2}}}+\frac{[\text{pC4bC4bC2}]}{K_{\text{m MASP1}^*}^{\text{C2}}}\\
&+\frac{[\text{pC3bC4bC2}]}{K_{\text{m MASP1}^*}^{\text{C2}}}+\frac{[\text{pMBL}_3\text{MASP1}_{\text{zym}}]}{K_{\text{m MASP1}^*}^{\text{MASP1}_{\text{zym}}}}+\frac{[\text{pMBL}_3\text{MASP2}_{\text{zym}}]}{K_{\text{m MASP1}^*}^{\text{MASP2}_{\text{zym}}}}+\frac{[\text{pMBL}_3\text{MASP3}_{\text{zym}}]}{K_{\text{m MASP1}^*}^{\text{MASP3}_{\text{zym}}}}\\
&+\frac{[\text{pMBL}_4\text{MASP1}_{\text{zym}}]}{K_{\text{m MASP1}^*}^{\text{MASP1}_{\text{zym}}}}+\frac{[\text{pMBL}_4\text{MASP2}_{\text{zym}}]}{K_{\text{m MASP1}^*}^{\text{MASP2}_{\text{zym}}}}+\frac{[\text{pMBL}_4\text{MASP3}_{\text{zym}}]}{K_{\text{m MASP1}^*}^{\text{MASP3}_{\text{zym}}}}+\frac{[\text{pCLLKMASP1}_{\text{zym}}]}{K_{\text{m MASP1}^*}^{\text{MASP1}_{\text{zym}}}}\\
&+\frac{[\text{pCLLKMASP2}_{\text{zym}}]}{K_{\text{m MASP1}^*}^{\text{MASP2}_{\text{zym}}}}+\frac{[\text{pCLLKMASP3}_{\text{zym}}]}{K_{\text{m MASP1}^*}^{\text{MASP3}_{\text{zym}}}}+\frac{[\text{pCLK1MASP1}_{\text{zym}}]}{K_{\text{m MASP1}^*}^{\text{MASP1}_{\text{zym}}}}+\frac{[\text{pCLK1MASP2}_{\text{zym}}]}{K_{\text{m MASP1}^*}^{\text{MASP2}_{\text{zym}}}}\\
&+\frac{[\text{pCLK1MASP3}_{\text{zym}}]}{K_{\text{m MASP1}^*}^{\text{MASP3}_{\text{zym}}}}+\frac{[\text{pCLL1MASP1}_{\text{zym}}]}{K_{\text{m MASP1}^*}^{\text{MASP1}_{\text{zym}}}}+\frac{[\text{pCLL1MASP2}_{\text{zym}}]}{K_{\text{m MASP1}^*}^{\text{MASP2}_{\text{zym}}}}+\frac{[\text{pCLL1MASP3}_{\text{zym}}]}{K_{\text{m MASP1}^*}^{\text{MASP3}_{\text{zym}}}}\\
&+\frac{[\text{pLFMASP1}_{\text{zym}}]}{K_{\text{m MASP1}^*}^{\text{MASP1}_{\text{zym}}}}+\frac{[\text{pLFMASP2}_{\text{zym}}]}{K_{\text{m MASP1}^*}^{\text{MASP2}_{\text{zym}}}}+\frac{[\text{pLFMASP3}_{\text{zym}}]}{K_{\text{m MASP1}^*}^{\text{MASP3}_{\text{zym}}}}+\frac{[\text{pHFMASP1}_{\text{zym}}]}{K_{\text{m MASP1}^*}^{\text{MASP1}_{\text{zym}}}}+\frac{[\text{pHFMASP2}_{\text{zym}}]}{K_{\text{m MASP1}^*}^{\text{MASP2}_{\text{zym}}}}\\
&+\frac{[\text{pHFMASP3}_{\text{zym}}]}{K_{\text{m MASP1}^*}^{\text{MASP3}_{\text{zym}}}}+\frac{[\text{pMFMASP1}_{\text{zym}}]}{K_{\text{m MASP1}^*}^{\text{MASP1}_{\text{zym}}}}+\frac{[\text{pMFMASP2}_{\text{zym}}]}{K_{\text{m MASP1}^*}^{\text{MASP2}_{\text{zym}}}}+\frac{[\text{pMFMASP3}_{\text{zym}}]}{K_{\text{m MASP1}^*}^{\text{MASP3}_{\text{zym}}}}\\
&+\frac{[\text{pPTX3LFMASP1}_{\text{zym}}]}{K_{\text{m MASP1}^*}^{\text{MASP1}_{\text{zym}}}}+\frac{[\text{pPXT3LFMASP2}_{\text{zym}}]}{K_{\text{m MASP1}^*}^{\text{MASP2}_{\text{zym}}}}+\frac{[\text{pPTX3LFMASP3}_{\text{zym}}]}{K_{\text{m MASP1}^*}^{\text{MASP3}_{\text{zym}}}}\end{aligned}\right)}
$$

$$-\frac{k_{\text{cat MASP1}^*}^{\text{MASP2}_{\text{zym}}}\left[\text{pCLL1MASP1}^*\right]\left[\text{pMBL}_3\text{MASP2}_{\text{zym}}\right]}{K_{\text{m MASP1}^*}^{\text{MASP2}_{\text{zym}}}\left(\begin{array}{l}1+\frac{[\text{C4}]}{K_{\text{m MASP1}^*}^{\text{C4}}}+\frac{[\text{fC4bC2}]}{K_{\text{m MASP1}^*}^{\text{C2}}}+\frac{[\text{fC4bC4bC2}]}{K_{\text{m MASP1}^*}^{\text{C2}}}+\frac{[\text{fC3bC4bC2}]}{K_{\text{m MASP1}^*}^{\text{C2}}}+\frac{[\text{pC4bC2}]}{K_{\text{m MASP1}^*}^{\text{C2}}}+\frac{[\text{pC4bC4bC2}]}{K_{\text{m MASP1}^*}^{\text{C2}}}\\
+\frac{[\text{pC3bC4bC2}]}{K_{\text{m MASP1}^*}^{\text{C2}}}+\frac{[\text{pMBL}_3\text{MASP1}_{\text{zym}}]}{K_{\text{m MASP1}^*}^{\text{MASP1}_{\text{zym}}}}+\frac{[\text{pMBL}_3\text{MASP2}_{\text{zym}}]}{K_{\text{m MASP1}^*}^{\text{MASP2}_{\text{zym}}}}+\frac{[\text{pMBL}_3\text{MASP3}_{\text{zym}}]}{K_{\text{m MASP1}^*}^{\text{MASP3}_{\text{zym}}}}\\
+\frac{[\text{pMBL}_4\text{MASP1}_{\text{zym}}]}{K_{\text{m MASP1}^*}^{\text{MASP1}_{\text{zym}}}}+\frac{[\text{pMBL}_4\text{MASP2}_{\text{zym}}]}{K_{\text{m MASP1}^*}^{\text{MASP2}_{\text{zym}}}}+\frac{[\text{pMBL}_4\text{MASP3}_{\text{zym}}]}{K_{\text{m MASP1}^*}^{\text{MASP3}_{\text{zym}}}}+\frac{[\text{pCLLKMASP1}_{\text{zym}}]}{K_{\text{m MASP1}^*}^{\text{MASP1}_{\text{zym}}}}\\
+\frac{[\text{pCLLKMASP2}_{\text{zym}}]}{K_{\text{m MASP1}^*}^{\text{MASP2}_{\text{zym}}}}+\frac{[\text{pCLLKMASP3}_{\text{zym}}]}{K_{\text{m MASP1}^*}^{\text{MASP3}_{\text{zym}}}}+\frac{[\text{pCLK1MASP1}_{\text{zym}}]}{K_{\text{m MASP1}^*}^{\text{MASP1}_{\text{zym}}}}+\frac{[\text{pCLK1MASP2}_{\text{zym}}]}{K_{\text{m MASP1}^*}^{\text{MASP2}_{\text{zym}}}}\\
+\frac{[\text{pCLK1MASP3}_{\text{zym}}]}{K_{\text{m MASP1}^*}^{\text{MASP3}_{\text{zym}}}}+\frac{[\text{pCLL1MASP1}_{\text{zym}}]}{K_{\text{m MASP1}^*}^{\text{MASP1}_{\text{zym}}}}+\frac{[\text{pCLL1MASP2}_{\text{zym}}]}{K_{\text{m MASP1}^*}^{\text{MASP2}_{\text{zym}}}}+\frac{[\text{pCLL1MASP3}_{\text{zym}}]}{K_{\text{m MASP1}^*}^{\text{MASP3}_{\text{zym}}}}\\
+\frac{[\text{pLFMASP1}_{\text{zym}}]}{K_{\text{m MASP1}^*}^{\text{MASP1}_{\text{zym}}}}+\frac{[\text{pLFMASP2}_{\text{zym}}]}{K_{\text{m MASP1}^*}^{\text{MASP2}_{\text{zym}}}}+\frac{[\text{pLFMASP3}_{\text{zym}}]}{K_{\text{m MASP1}^*}^{\text{MASP3}_{\text{zym}}}}+\frac{[\text{pHFMASP1}_{\text{zym}}]}{K_{\text{m MASP1}^*}^{\text{MASP1}_{\text{zym}}}}+\frac{[\text{pHFMASP2}_{\text{zym}}]}{K_{\text{m MASP1}^*}^{\text{MASP2}_{\text{zym}}}}\\
+\frac{[\text{pHFMASP3}_{\text{zym}}]}{K_{\text{m MASP1}^*}^{\text{MASP3}_{\text{zym}}}}+\frac{[\text{pMFMASP1}_{\text{zym}}]}{K_{\text{m MASP1}^*}^{\text{MASP1}_{\text{zym}}}}+\frac{[\text{pMFMASP2}_{\text{zym}}]}{K_{\text{m MASP1}^*}^{\text{MASP2}_{\text{zym}}}}+\frac{[\text{pMFMASP3}_{\text{zym}}]}{K_{\text{m MASP1}^*}^{\text{MASP3}_{\text{zym}}}}\\
+\frac{[\text{pPTX3LFMASP1}_{\text{zym}}]}{K_{\text{m MASP1}^*}^{\text{MASP1}_{\text{zym}}}}+\frac{[\text{pPXT3LFMASP2}_{\text{zym}}]}{K_{\text{m MASP1}^*}^{\text{MASP2}_{\text{zym}}}}+\frac{[\text{pPTX3LFMASP3}_{\text{zym}}]}{K_{\text{m MASP1}^*}^{\text{MASP3}_{\text{zym}}}}\end{array}\right)}$$

$$
-\frac{k_{\mathrm{cat\,MASP1^{*}}}^{\mathrm{MASP2_{zym}}}\left[\mathrm{pLFMASP1^{*}}\right]\left[\mathrm{pMBL_{3}MASP2_{zym}}\right]}{K_{\mathrm{m\,MASP1^{*}}}^{\mathrm{MASP2_{zym}}}\left(\begin{aligned}
&1+\frac{[\mathrm{C4}]}{K_{\mathrm{m\,MASP1^{*}}}^{\mathrm{C4}}}+\frac{[\mathrm{fC4bC2}]}{K_{\mathrm{m\,MASP1^{*}}}^{\mathrm{C2}}}+\frac{[\mathrm{fC4bC4bC2}]}{K_{\mathrm{m\,MASP1^{*}}}^{\mathrm{C2}}}+\frac{[\mathrm{fC3bC4bC2}]}{K_{\mathrm{m\,MASP1^{*}}}^{\mathrm{C2}}}+\frac{[\mathrm{pC4bC2}]}{K_{\mathrm{m\,MASP1^{*}}}^{\mathrm{C2}}}+\frac{[\mathrm{pC4bC4bC2}]}{K_{\mathrm{m\,MASP1^{*}}}^{\mathrm{C2}}}\\
&+\frac{[\mathrm{pC3bC4bC2}]}{K_{\mathrm{m\,MASP1^{*}}}^{\mathrm{C2}}}+\frac{[\mathrm{pMBL_{3}MASP1_{zym}}]}{K_{\mathrm{m\,MASP1^{*}}}^{\mathrm{MASP1_{zym}}}}+\frac{[\mathrm{pMBL_{3}MASP2_{zym}}]}{K_{\mathrm{m\,MASP1^{*}}}^{\mathrm{MASP2_{zym}}}}+\frac{[\mathrm{pMBL_{3}MASP3_{zym}}]}{K_{\mathrm{m\,MASP1^{*}}}^{\mathrm{MASP3_{zym}}}}\\
&+\frac{[\mathrm{pMBL_{4}MASP1_{zym}}]}{K_{\mathrm{m\,MASP1^{*}}}^{\mathrm{MASP1_{zym}}}}+\frac{[\mathrm{pMBL_{4}MASP2_{zym}}]}{K_{\mathrm{m\,MASP1^{*}}}^{\mathrm{MASP2_{zym}}}}+\frac{[\mathrm{pMBL_{4}MASP3_{zym}}]}{K_{\mathrm{m\,MASP1^{*}}}^{\mathrm{MASP3_{zym}}}}+\frac{[\mathrm{pCLLKMASP1_{zym}}]}{K_{\mathrm{m\,MASP1^{*}}}^{\mathrm{MASP1_{zym}}}}\\
&+\frac{[\mathrm{pCLLKMASP2_{zym}}]}{K_{\mathrm{m\,MASP1^{*}}}^{\mathrm{MASP2_{zym}}}}+\frac{[\mathrm{pCLLKMASP3_{zym}}]}{K_{\mathrm{m\,MASP1^{*}}}^{\mathrm{MASP3_{zym}}}}+\frac{[\mathrm{pCLK1MASP1_{zym}}]}{K_{\mathrm{m\,MASP1^{*}}}^{\mathrm{MASP1_{zym}}}}+\frac{[\mathrm{pCLK1MASP2_{zym}}]}{K_{\mathrm{m\,MASP1^{*}}}^{\mathrm{MASP2_{zym}}}}\\
&+\frac{[\mathrm{pCLK1MASP3_{zym}}]}{K_{\mathrm{m\,MASP1^{*}}}^{\mathrm{MASP3_{zym}}}}+\frac{[\mathrm{pCLL1MASP1_{zym}}]}{K_{\mathrm{m\,MASP1^{*}}}^{\mathrm{MASP1_{zym}}}}+\frac{[\mathrm{pCLL1MASP2_{zym}}]}{K_{\mathrm{m\,MASP1^{*}}}^{\mathrm{MASP2_{zym}}}}+\frac{[\mathrm{pCLL1MASP3_{zym}}]}{K_{\mathrm{m\,MASP1^{*}}}^{\mathrm{MASP3_{zym}}}}\\
&+\frac{[\mathrm{pLFMASP1_{zym}}]}{K_{\mathrm{m\,MASP1^{*}}}^{\mathrm{MASP1_{zym}}}}+\frac{[\mathrm{pLFMASP2_{zym}}]}{K_{\mathrm{m\,MASP1^{*}}}^{\mathrm{MASP2_{zym}}}}+\frac{[\mathrm{pLFMASP3_{zym}}]}{K_{\mathrm{m\,MASP1^{*}}}^{\mathrm{MASP3_{zym}}}}+\frac{[\mathrm{pHFMASP1_{zym}}]}{K_{\mathrm{m\,MASP1^{*}}}^{\mathrm{MASP1_{zym}}}}+\frac{[\mathrm{pHFMASP2_{zym}}]}{K_{\mathrm{m\,MASP1^{*}}}^{\mathrm{MASP2_{zym}}}}\\
&+\frac{[\mathrm{pHFMASP3_{zym}}]}{K_{\mathrm{m\,MASP1^{*}}}^{\mathrm{MASP3_{zym}}}}+\frac{[\mathrm{pMFMASP1_{zym}}]}{K_{\mathrm{m\,MASP1^{*}}}^{\mathrm{MASP1_{zym}}}}+\frac{[\mathrm{pMFMASP2_{zym}}]}{K_{\mathrm{m\,MASP1^{*}}}^{\mathrm{MASP2_{zym}}}}+\frac{[\mathrm{pMFMASP3_{zym}}]}{K_{\mathrm{m\,MASP1^{*}}}^{\mathrm{MASP3_{zym}}}}\\
&+\frac{[\mathrm{pPTX3LFMASP1_{zym}}]}{K_{\mathrm{m\,MASP1^{*}}}^{\mathrm{MASP1_{zym}}}}+\frac{[\mathrm{pPXT3LFMASP2_{zym}}]}{K_{\mathrm{m\,MASP1^{*}}}^{\mathrm{MASP2_{zym}}}}+\frac{[\mathrm{pPTX3LFMASP3_{zym}}]}{K_{\mathrm{m\,MASP1^{*}}}^{\mathrm{MASP3_{zym}}}}
\end{aligned}\right)}
$$

$$
-\frac{k_{\mathrm{cat\,MASP1^{*}}}^{\mathrm{MASP2_{zym}}}\left[\mathrm{pHFMASP1^{*}}\right]\left[\mathrm{pMBL_{3}MASP2_{zym}}\right]}{K_{\mathrm{m\,MASP1^{*}}}^{\mathrm{MASP2_{zym}}}\left(\begin{array}{l}
1+\frac{[\mathrm{C4}]}{K_{\mathrm{m\,MASP1^{*}}}^{\mathrm{C4}}}+\frac{[\mathrm{fC4bC2}]}{K_{\mathrm{m\,MASP1^{*}}}^{\mathrm{C2}}}+\frac{[\mathrm{fC4bC4bC2}]}{K_{\mathrm{m\,MASP1^{*}}}^{\mathrm{C2}}}+\frac{[\mathrm{fC3bC4bC2}]}{K_{\mathrm{m\,MASP1^{*}}}^{\mathrm{C2}}}+\frac{[\mathrm{pC4bC2}]}{K_{\mathrm{m\,MASP1^{*}}}^{\mathrm{C2}}}+\frac{[\mathrm{pC4bC4bC2}]}{K_{\mathrm{m\,MASP1^{*}}}^{\mathrm{C2}}}\\
+\frac{[\mathrm{pC3bC4bC2}]}{K_{\mathrm{m\,MASP1^{*}}}^{\mathrm{C2}}}+\frac{\left[\mathrm{pMBL_{3}MASP1_{zym}}\right]}{K_{\mathrm{m\,MASP1^{*}}}^{\mathrm{MASP1_{zym}}}}+\frac{\left[\mathrm{pMBL_{3}MASP2_{zym}}\right]}{K_{\mathrm{m\,MASP1^{*}}}^{\mathrm{MASP2_{zym}}}}+\frac{\left[\mathrm{pMBL_{3}MASP3_{zym}}\right]}{K_{\mathrm{m\,MASP1^{*}}}^{\mathrm{MASP3_{zym}}}}\\
+\frac{\left[\mathrm{pMBL_{4}MASP1_{zym}}\right]}{K_{\mathrm{m\,MASP1^{*}}}^{\mathrm{MASP1_{zym}}}}+\frac{\left[\mathrm{pMBL_{4}MASP2_{zym}}\right]}{K_{\mathrm{m\,MASP1^{*}}}^{\mathrm{MASP2_{zym}}}}+\frac{\left[\mathrm{pMBL_{4}MASP3_{zym}}\right]}{K_{\mathrm{m\,MASP1^{*}}}^{\mathrm{MASP3_{zym}}}}+\frac{\left[\mathrm{pCLLKMASP1_{zym}}\right]}{K_{\mathrm{m\,MASP1^{*}}}^{\mathrm{MASP1_{zym}}}}\\
+\frac{\left[\mathrm{pCLLKMASP2_{zym}}\right]}{K_{\mathrm{m\,MASP1^{*}}}^{\mathrm{MASP2_{zym}}}}+\frac{\left[\mathrm{pCLLKMASP3_{zym}}\right]}{K_{\mathrm{m\,MASP1^{*}}}^{\mathrm{MASP3_{zym}}}}+\frac{\left[\mathrm{pCLK1MASP1_{zym}}\right]}{K_{\mathrm{m\,MASP1^{*}}}^{\mathrm{MASP1_{zym}}}}+\frac{\left[\mathrm{pCLK1MASP2_{zym}}\right]}{K_{\mathrm{m\,MASP1^{*}}}^{\mathrm{MASP2_{zym}}}}\\
+\frac{\left[\mathrm{pCLK1MASP3_{zym}}\right]}{K_{\mathrm{m\,MASP1^{*}}}^{\mathrm{MASP3_{zym}}}}+\frac{\left[\mathrm{pCLL1MASP1_{zym}}\right]}{K_{\mathrm{m\,MASP1^{*}}}^{\mathrm{MASP1_{zym}}}}+\frac{\left[\mathrm{pCLL1MASP2_{zym}}\right]}{K_{\mathrm{m\,MASP1^{*}}}^{\mathrm{MASP2_{zym}}}}+\frac{\left[\mathrm{pCLL1MASP3_{zym}}\right]}{K_{\mathrm{m\,MASP1^{*}}}^{\mathrm{MASP3_{zym}}}}\\
+\frac{\left[\mathrm{pLFMASP1_{zym}}\right]}{K_{\mathrm{m\,MASP1^{*}}}^{\mathrm{MASP1_{zym}}}}+\frac{\left[\mathrm{pLFMASP2_{zym}}\right]}{K_{\mathrm{m\,MASP1^{*}}}^{\mathrm{MASP2_{zym}}}}+\frac{\left[\mathrm{pLFMASP3_{zym}}\right]}{K_{\mathrm{m\,MASP1^{*}}}^{\mathrm{MASP3_{zym}}}}+\frac{\left[\mathrm{pHFMASP1_{zym}}\right]}{K_{\mathrm{m\,MASP1^{*}}}^{\mathrm{MASP1_{zym}}}}+\frac{\left[\mathrm{pHFMASP2_{zym}}\right]}{K_{\mathrm{m\,MASP1^{*}}}^{\mathrm{MASP2_{zym}}}}\\
+\frac{\left[\mathrm{pHFMASP3_{zym}}\right]}{K_{\mathrm{m\,MASP1^{*}}}^{\mathrm{MASP3_{zym}}}}+\frac{\left[\mathrm{pMFMASP1_{zym}}\right]}{K_{\mathrm{m\,MASP1^{*}}}^{\mathrm{MASP1_{zym}}}}+\frac{\left[\mathrm{pMFMASP2_{zym}}\right]}{K_{\mathrm{m\,MASP1^{*}}}^{\mathrm{MASP2_{zym}}}}+\frac{\left[\mathrm{pMFMASP3_{zym}}\right]}{K_{\mathrm{m\,MASP1^{*}}}^{\mathrm{MASP3_{zym}}}}\\
+\frac{\left[\mathrm{pPTX3LFMASP1_{zym}}\right]}{K_{\mathrm{m\,MASP1^{*}}}^{\mathrm{MASP1_{zym}}}}+\frac{\left[\mathrm{pPXT3LFMASP2_{zym}}\right]}{K_{\mathrm{m\,MASP1^{*}}}^{\mathrm{MASP2_{zym}}}}+\frac{\left[\mathrm{pPTX3LFMASP3_{zym}}\right]}{K_{\mathrm{m\,MASP1^{*}}}^{\mathrm{MASP3_{zym}}}}
\end{array}\right)}
$$

$$
-\frac{k_{\mathrm{cat\ MASP1^{*}}}^{\mathrm{MASP2_{zym}}}\left[\mathrm{pMFMASP1^{*}}\right]\left[\mathrm{pMBL_{3}MASP2_{zym}}\right]}{K_{\mathrm{m\ MASP1^{*}}}^{\mathrm{MASP2_{zym}}}\left(\begin{array}{l}
1+\frac{[\mathrm{C4}]}{K_{\mathrm{m\ MASP1^{*}}}^{\mathrm{C4}}}+\frac{[\mathrm{fC4bC2}]}{K_{\mathrm{m\ MASP1^{*}}}^{\mathrm{C2}}}+\frac{[\mathrm{fC4bC4bC2}]}{K_{\mathrm{m\ MASP1^{*}}}^{\mathrm{C2}}}+\frac{[\mathrm{fC3bC4bC2}]}{K_{\mathrm{m\ MASP1^{*}}}^{\mathrm{C2}}}+\frac{[\mathrm{pC4bC2}]}{K_{\mathrm{m\ MASP1^{*}}}^{\mathrm{C2}}}+\frac{[\mathrm{pC4bC4bC2}]}{K_{\mathrm{m\ MASP1^{*}}}^{\mathrm{C2}}}\\
+\frac{[\mathrm{pC3bC4bC2}]}{K_{\mathrm{m\ MASP1^{*}}}^{\mathrm{C2}}}+\frac{[\mathrm{pMBL_{3}MASP1_{zym}}]}{K_{\mathrm{m\ MASP1^{*}}}^{\mathrm{MASP1_{zym}}}}+\frac{[\mathrm{pMBL_{3}MASP2_{zym}}]}{K_{\mathrm{m\ MASP1^{*}}}^{\mathrm{MASP2_{zym}}}}+\frac{[\mathrm{pMBL_{3}MASP3_{zym}}]}{K_{\mathrm{m\ MASP1^{*}}}^{\mathrm{MASP3_{zym}}}}\\
+\frac{[\mathrm{pMBL_{4}MASP1_{zym}}]}{K_{\mathrm{m\ MASP1^{*}}}^{\mathrm{MASP1_{zym}}}}+\frac{[\mathrm{pMBL_{4}MASP2_{zym}}]}{K_{\mathrm{m\ MASP1^{*}}}^{\mathrm{MASP2_{zym}}}}+\frac{[\mathrm{pMBL_{4}MASP3_{zym}}]}{K_{\mathrm{m\ MASP1^{*}}}^{\mathrm{MASP3_{zym}}}}+\frac{[\mathrm{pCLLKMASP1_{zym}}]}{K_{\mathrm{m\ MASP1^{*}}}^{\mathrm{MASP1_{zym}}}}\\
+\frac{[\mathrm{pCLLKMASP2_{zym}}]}{K_{\mathrm{m\ MASP1^{*}}}^{\mathrm{MASP2_{zym}}}}+\frac{[\mathrm{pCLLKMASP3_{zym}}]}{K_{\mathrm{m\ MASP1^{*}}}^{\mathrm{MASP3_{zym}}}}+\frac{[\mathrm{pCLK1MASP1_{zym}}]}{K_{\mathrm{m\ MASP1^{*}}}^{\mathrm{MASP1_{zym}}}}+\frac{[\mathrm{pCLK1MASP2_{zym}}]}{K_{\mathrm{m\ MASP1^{*}}}^{\mathrm{MASP2_{zym}}}}\\
+\frac{[\mathrm{pCLK1MASP3_{zym}}]}{K_{\mathrm{m\ MASP1^{*}}}^{\mathrm{MASP3_{zym}}}}+\frac{[\mathrm{pCLL1MASP1_{zym}}]}{K_{\mathrm{m\ MASP1^{*}}}^{\mathrm{MASP1_{zym}}}}+\frac{[\mathrm{pCLL1MASP2_{zym}}]}{K_{\mathrm{m\ MASP1^{*}}}^{\mathrm{MASP2_{zym}}}}+\frac{[\mathrm{pCLL1MASP3_{zym}}]}{K_{\mathrm{m\ MASP1^{*}}}^{\mathrm{MASP3_{zym}}}}\\
+\frac{[\mathrm{pLFMASP1_{zym}}]}{K_{\mathrm{m\ MASP1^{*}}}^{\mathrm{MASP1_{zym}}}}+\frac{[\mathrm{pLFMASP2_{zym}}]}{K_{\mathrm{m\ MASP1^{*}}}^{\mathrm{MASP2_{zym}}}}+\frac{[\mathrm{pLFMASP3_{zym}}]}{K_{\mathrm{m\ MASP1^{*}}}^{\mathrm{MASP3_{zym}}}}+\frac{[\mathrm{pHFMASP1_{zym}}]}{K_{\mathrm{m\ MASP1^{*}}}^{\mathrm{MASP1_{zym}}}}+\frac{[\mathrm{pHFMASP2_{zym}}]}{K_{\mathrm{m\ MASP1^{*}}}^{\mathrm{MASP2_{zym}}}}\\
+\frac{[\mathrm{pHFMASP3_{zym}}]}{K_{\mathrm{m\ MASP1^{*}}}^{\mathrm{MASP3_{zym}}}}+\frac{[\mathrm{pMFMASP1_{zym}}]}{K_{\mathrm{m\ MASP1^{*}}}^{\mathrm{MASP1_{zym}}}}+\frac{[\mathrm{pMFMASP2_{zym}}]}{K_{\mathrm{m\ MASP1^{*}}}^{\mathrm{MASP2_{zym}}}}+\frac{[\mathrm{pMFMASP3_{zym}}]}{K_{\mathrm{m\ MASP1^{*}}}^{\mathrm{MASP3_{zym}}}}\\
+\frac{[\mathrm{pPTX3LFMASP1_{zym}}]}{K_{\mathrm{m\ MASP1^{*}}}^{\mathrm{MASP1_{zym}}}}+\frac{[\mathrm{pPXT3LFMASP2_{zym}}]}{K_{\mathrm{m\ MASP1^{*}}}^{\mathrm{MASP2_{zym}}}}+\frac{[\mathrm{pPTX3LFMASP3_{zym}}]}{K_{\mathrm{m\ MASP1^{*}}}^{\mathrm{MASP3_{zym}}}}
\end{array}\right)}
$$

$$
-\frac{k_{\text{cat MASP1}^*}^{\text{MASP2}_{\text{zym}}}\left[\text{pPTX3LFMASP1}^*\right]\left[\text{pMBL}_3\text{MASP2}_{\text{zym}}\right]}{K_{\text{m MASP1}^*}^{\text{MASP2}_{\text{zym}}}\left(\begin{array}{l}
1+\frac{[\text{C4}]}{K_{\text{m MASP1}^*}^{\text{C4}}}+\frac{[\text{fC4bC2}]}{K_{\text{m MASP1}^*}^{\text{C2}}}+\frac{[\text{fC4bC4bC2}]}{K_{\text{m MASP1}^*}^{\text{C2}}}+\frac{[\text{fC3bC4bC2}]}{K_{\text{m MASP1}^*}^{\text{C2}}}+\frac{[\text{pC4bC2}]}{K_{\text{m MASP1}^*}^{\text{C2}}}+\frac{[\text{pC4bC4bC2}]}{K_{\text{m MASP1}^*}^{\text{C2}}}\\
+\frac{[\text{pC3bC4bC2}]}{K_{\text{m MASP1}^*}^{\text{C2}}}+\frac{[\text{pMBL}_3\text{MASP1}_{\text{zym}}]}{K_{\text{m MASP1}^*}^{\text{MASP1}_{\text{zym}}}}+\frac{[\text{pMBL}_3\text{MASP2}_{\text{zym}}]}{K_{\text{m MASP1}^*}^{\text{MASP2}_{\text{zym}}}}+\frac{[\text{pMBL}_3\text{MASP3}_{\text{zym}}]}{K_{\text{m MASP1}^*}^{\text{MASP3}_{\text{zym}}}}\\
+\frac{[\text{pMBL}_4\text{MASP1}_{\text{zym}}]}{K_{\text{m MASP1}^*}^{\text{MASP1}_{\text{zym}}}}+\frac{[\text{pMBL}_4\text{MASP2}_{\text{zym}}]}{K_{\text{m MASP1}^*}^{\text{MASP2}_{\text{zym}}}}+\frac{[\text{pMBL}_4\text{MASP3}_{\text{zym}}]}{K_{\text{m MASP1}^*}^{\text{MASP3}_{\text{zym}}}}+\frac{[\text{pCLLKMASP1}_{\text{zym}}]}{K_{\text{m MASP1}^*}^{\text{MASP1}_{\text{zym}}}}\\
+\frac{[\text{pCLLKMASP2}_{\text{zym}}]}{K_{\text{m MASP1}^*}^{\text{MASP2}_{\text{zym}}}}+\frac{[\text{pCLLKMASP3}_{\text{zym}}]}{K_{\text{m MASP1}^*}^{\text{MASP3}_{\text{zym}}}}+\frac{[\text{pCLK1MASP1}_{\text{zym}}]}{K_{\text{m MASP1}^*}^{\text{MASP1}_{\text{zym}}}}+\frac{[\text{pCLK1MASP2}_{\text{zym}}]}{K_{\text{m MASP1}^*}^{\text{MASP2}_{\text{zym}}}}\\
+\frac{[\text{pCLK1MASP3}_{\text{zym}}]}{K_{\text{m MASP1}^*}^{\text{MASP3}_{\text{zym}}}}+\frac{[\text{pCLL1MASP1}_{\text{zym}}]}{K_{\text{m MASP1}^*}^{\text{MASP1}_{\text{zym}}}}+\frac{[\text{pCLL1MASP2}_{\text{zym}}]}{K_{\text{m MASP1}^*}^{\text{MASP2}_{\text{zym}}}}+\frac{[\text{pCLL1MASP3}_{\text{zym}}]}{K_{\text{m MASP1}^*}^{\text{MASP3}_{\text{zym}}}}\\
+\frac{[\text{pLFMASP1}_{\text{zym}}]}{K_{\text{m MASP1}^*}^{\text{MASP1}_{\text{zym}}}}+\frac{[\text{pLFMASP2}_{\text{zym}}]}{K_{\text{m MASP1}^*}^{\text{MASP2}_{\text{zym}}}}+\frac{[\text{pLFMASP3}_{\text{zym}}]}{K_{\text{m MASP1}^*}^{\text{MASP3}_{\text{zym}}}}+\frac{[\text{pHFMASP1}_{\text{zym}}]}{K_{\text{m MASP1}^*}^{\text{MASP1}_{\text{zym}}}}+\frac{[\text{pHFMASP2}_{\text{zym}}]}{K_{\text{m MASP1}^*}^{\text{MASP2}_{\text{zym}}}}\\
+\frac{[\text{pHFMASP3}_{\text{zym}}]}{K_{\text{m MASP1}^*}^{\text{MASP3}_{\text{zym}}}}+\frac{[\text{pMFMASP1}_{\text{zym}}]}{K_{\text{m MASP1}^*}^{\text{MASP1}_{\text{zym}}}}+\frac{[\text{pMFMASP2}_{\text{zym}}]}{K_{\text{m MASP1}^*}^{\text{MASP2}_{\text{zym}}}}+\frac{[\text{pMFMASP3}_{\text{zym}}]}{K_{\text{m MASP1}^*}^{\text{MASP3}_{\text{zym}}}}\\
+\frac{[\text{pPTX3LFMASP1}_{\text{zym}}]}{K_{\text{m MASP1}^*}^{\text{MASP1}_{\text{zym}}}}+\frac{[\text{pPXT3LFMASP2}_{\text{zym}}]}{K_{\text{m MASP1}^*}^{\text{MASP2}_{\text{zym}}}}+\frac{[\text{pPTX3LFMASP3}_{\text{zym}}]}{K_{\text{m MASP1}^*}^{\text{MASP3}_{\text{zym}}}}
\end{array}\right)}
$$

$$
-\frac{k_{\text{cat MASP2}^*}^{\text{MASP2}_{\text{zym}}}\left[\text{pMBL}_3\text{MASP2}^*\right]\left[\text{pMBL}_3\text{MASP2}_{\text{zym}}\right]}{K_{\text{m MASP2}^*}^{\text{MASP2}_{\text{zym}}}\left(\begin{array}{l}
1+\frac{[\text{C4}]}{K_{\text{m MASP2}^*}^{\text{C4}}}+\frac{[\text{fC4bC2}]}{K_{\text{m MASP2}^*}^{\text{C2}}}+\frac{[\text{fC4bC4bC2}]}{K_{\text{m MASP2}^*}^{\text{C2}}}+\frac{[\text{fC3bC4bC2}]}{K_{\text{m MASP2}^*}^{\text{C2}}}+\frac{[\text{pC4bC2}]}{K_{\text{m MASP2}^*}^{\text{C2}}}+\frac{[\text{pC4bC4bC2}]}{K_{\text{m MASP2}^*}^{\text{C2}}}\\
+\frac{[\text{pC3bC4bC2}]}{K_{\text{m MASP2}^*}^{\text{C2}}}+\frac{[\text{pMBL}_3\text{MASP1}_{\text{zym}}]}{K_{\text{m MASP2}^*}^{\text{MASP1}_{\text{zym}}}}+\frac{[\text{pMBL}_3\text{MASP2}_{\text{zym}}]}{K_{\text{m MASP2}^*}^{\text{MASP2}_{\text{zym}}}}+\frac{[\text{pMBL}_4\text{MASP1}_{\text{zym}}]}{K_{\text{m MASP2}^*}^{\text{MASP1}_{\text{zym}}}}\\
+\frac{[\text{pMBL}_4\text{MASP2}_{\text{zym}}]}{K_{\text{m MASP2}^*}^{\text{MASP2}_{\text{zym}}}}+\frac{[\text{pCLLKMASP1}_{\text{zym}}]}{K_{\text{m MASP2}^*}^{\text{MASP1}_{\text{zym}}}}+\frac{[\text{pCLLKMASP2}_{\text{zym}}]}{K_{\text{m MASP2}^*}^{\text{MASP2}_{\text{zym}}}}+\frac{[\text{pCLK1MASP1}_{\text{zym}}]}{K_{\text{m MASP2}^*}^{\text{MASP1}_{\text{zym}}}}\\
+\frac{[\text{pCLK1MASP2}_{\text{zym}}]}{K_{\text{m MASP2}^*}^{\text{MASP2}_{\text{zym}}}}+\frac{[\text{pCLL1MASP1}_{\text{zym}}]}{K_{\text{m MASP2}^*}^{\text{MASP1}_{\text{zym}}}}+\frac{[\text{pCLL1MASP2}_{\text{zym}}]}{K_{\text{m MASP2}^*}^{\text{MASP2}_{\text{zym}}}}+\frac{[\text{pLFMASP1}_{\text{zym}}]}{K_{\text{m MASP2}^*}^{\text{MASP1}_{\text{zym}}}}\\
+\frac{[\text{pLFMASP2}_{\text{zym}}]}{K_{\text{m MASP2}^*}^{\text{MASP2}_{\text{zym}}}}+\frac{[\text{pHFMASP1}_{\text{zym}}]}{K_{\text{m MASP2}^*}^{\text{MASP1}_{\text{zym}}}}+\frac{[\text{pHFMASP2}_{\text{zym}}]}{K_{\text{m MASP2}^*}^{\text{MASP2}_{\text{zym}}}}+\frac{[\text{pMFMASP1}_{\text{zym}}]}{K_{\text{m MASP2}^*}^{\text{MASP1}_{\text{zym}}}}+\frac{[\text{pMFMASP2}_{\text{zym}}]}{K_{\text{m MASP2}^*}^{\text{MASP2}_{\text{zym}}}}\\
+\frac{[\text{pPTX3LFMASP1}_{\text{zym}}]}{K_{\text{m MASP2}^*}^{\text{MASP1}_{\text{zym}}}}+\frac{[\text{pPTX3LFMASP2}_{\text{zym}}]}{K_{\text{m MASP2}^*}^{\text{MASP2}_{\text{zym}}}}
\end{array}\right)}
$$

$$-\frac{k_{\text{cat MASP2}^*}^{\text{MASP2}_{\text{zym}}}\left[\text{pMBL}_4\text{MASP2}^*\right]\left[\text{pMBL}_3\text{MASP2}_{\text{zym}}\right]}{K_{\text{m MASP2}^*}^{\text{MASP2}_{\text{zym}}}\left(\begin{array}{l}1+\frac{[\text{C4}]}{K_{\text{m MASP2}^*}^{\text{C4}}}+\frac{[\text{fC4bC2}]}{K_{\text{m MASP2}^*}^{\text{C2}}}+\frac{[\text{fC4bC4bC2}]}{K_{\text{m MASP2}^*}^{\text{C2}}}+\frac{[\text{fC3bC4bC2}]}{K_{\text{m MASP2}^*}^{\text{C2}}}+\frac{[\text{pC4bC2}]}{K_{\text{m MASP2}^*}^{\text{C2}}}+\frac{[\text{pC4bC4bC2}]}{K_{\text{m MASP2}^*}^{\text{C2}}}\\+\frac{[\text{pC3bC4bC2}]}{K_{\text{m MASP2}^*}^{\text{C2}}}+\frac{[\text{pMBL}_3\text{MASP1}_{\text{zym}}]}{K_{\text{m MASP2}^*}^{\text{MASP1}_{\text{zym}}}}+\frac{[\text{pMBL}_3\text{MASP2}_{\text{zym}}]}{K_{\text{m MASP2}^*}^{\text{MASP2}_{\text{zym}}}}+\frac{[\text{pMBL}_4\text{MASP1}_{\text{zym}}]}{K_{\text{m MASP2}^*}^{\text{MASP1}_{\text{zym}}}}\\+\frac{[\text{pMBL}_4\text{MASP2}_{\text{zym}}]}{K_{\text{m MASP2}^*}^{\text{MASP2}_{\text{zym}}}}+\frac{[\text{pCLLKMASP1}_{\text{zym}}]}{K_{\text{m MASP2}^*}^{\text{MASP1}_{\text{zym}}}}+\frac{[\text{pCLLKMASP2}_{\text{zym}}]}{K_{\text{m MASP2}^*}^{\text{MASP2}_{\text{zym}}}}+\frac{[\text{pCLK1MASP1}_{\text{zym}}]}{K_{\text{m MASP2}^*}^{\text{MASP1}_{\text{zym}}}}\\+\frac{[\text{pCLK1MASP2}_{\text{zym}}]}{K_{\text{m MASP2}^*}^{\text{MASP2}_{\text{zym}}}}+\frac{[\text{pCLL1MASP1}_{\text{zym}}]}{K_{\text{m MASP2}^*}^{\text{MASP1}_{\text{zym}}}}+\frac{[\text{pCLL1MASP2}_{\text{zym}}]}{K_{\text{m MASP2}^*}^{\text{MASP2}_{\text{zym}}}}+\frac{[\text{pLFMASP1}_{\text{zym}}]}{K_{\text{m MASP2}^*}^{\text{MASP1}_{\text{zym}}}}\\+\frac{[\text{pLFMASP2}_{\text{zym}}]}{K_{\text{m MASP2}^*}^{\text{MASP2}_{\text{zym}}}}+\frac{[\text{pHFMASP1}_{\text{zym}}]}{K_{\text{m MASP2}^*}^{\text{MASP1}_{\text{zym}}}}+\frac{[\text{pHFMASP2}_{\text{zym}}]}{K_{\text{m MASP2}^*}^{\text{MASP2}_{\text{zym}}}}+\frac{[\text{pMFMASP1}_{\text{zym}}]}{K_{\text{m MASP2}^*}^{\text{MASP1}_{\text{zym}}}}+\frac{[\text{pMFMASP2}_{\text{zym}}]}{K_{\text{m MASP2}^*}^{\text{MASP2}_{\text{zym}}}}\\+\frac{[\text{pPTX3LFMASP1}_{\text{zym}}]}{K_{\text{m MASP2}^*}^{\text{MASP1}_{\text{zym}}}}+\frac{[\text{pPTX3LFMASP2}_{\text{zym}}]}{K_{\text{m MASP2}^*}^{\text{MASP2}_{\text{zym}}}}\end{array}\right)}$$

$$-\frac{k_{\text{cat MASP2}^*}^{\text{MASP2}_{\text{zym}}}\left[\text{pCLLKMASP2}^*\right]\left[\text{pMBL}_3\text{MASP2}_{\text{zym}}\right]}{K_{\text{m MASP2}^*}^{\text{MASP2}_{\text{zym}}}\left(\begin{array}{l}1+\frac{[\text{C4}]}{K_{\text{m MASP2}^*}^{\text{C4}}}+\frac{[\text{fC4bC2}]}{K_{\text{m MASP2}^*}^{\text{C2}}}+\frac{[\text{fC4bC4bC2}]}{K_{\text{m MASP2}^*}^{\text{C2}}}+\frac{[\text{fC3bC4bC2}]}{K_{\text{m MASP2}^*}^{\text{C2}}}+\frac{[\text{pC4bC2}]}{K_{\text{m MASP2}^*}^{\text{C2}}}+\frac{[\text{pC4bC4bC2}]}{K_{\text{m MASP2}^*}^{\text{C2}}}\\+\frac{[\text{pC3bC4bC2}]}{K_{\text{m MASP2}^*}^{\text{C2}}}+\frac{[\text{pMBL}_3\text{MASP1}_{\text{zym}}]}{K_{\text{m MASP2}^*}^{\text{MASP1}_{\text{zym}}}}+\frac{[\text{pMBL}_3\text{MASP2}_{\text{zym}}]}{K_{\text{m MASP2}^*}^{\text{MASP2}_{\text{zym}}}}+\frac{[\text{pMBL}_4\text{MASP1}_{\text{zym}}]}{K_{\text{m MASP2}^*}^{\text{MASP1}_{\text{zym}}}}\\+\frac{[\text{pMBL}_4\text{MASP2}_{\text{zym}}]}{K_{\text{m MASP2}^*}^{\text{MASP2}_{\text{zym}}}}+\frac{[\text{pCLLKMASP1}_{\text{zym}}]}{K_{\text{m MASP2}^*}^{\text{MASP1}_{\text{zym}}}}+\frac{[\text{pCLLKMASP2}_{\text{zym}}]}{K_{\text{m MASP2}^*}^{\text{MASP2}_{\text{zym}}}}+\frac{[\text{pCLK1MASP1}_{\text{zym}}]}{K_{\text{m MASP2}^*}^{\text{MASP1}_{\text{zym}}}}\\+\frac{[\text{pCLK1MASP2}_{\text{zym}}]}{K_{\text{m MASP2}^*}^{\text{MASP2}_{\text{zym}}}}+\frac{[\text{pCLL1MASP1}_{\text{zym}}]}{K_{\text{m MASP2}^*}^{\text{MASP1}_{\text{zym}}}}+\frac{[\text{pCLL1MASP2}_{\text{zym}}]}{K_{\text{m MASP2}^*}^{\text{MASP2}_{\text{zym}}}}+\frac{[\text{pLFMASP1}_{\text{zym}}]}{K_{\text{m MASP2}^*}^{\text{MASP1}_{\text{zym}}}}\\+\frac{[\text{pLFMASP2}_{\text{zym}}]}{K_{\text{m MASP2}^*}^{\text{MASP2}_{\text{zym}}}}+\frac{[\text{pHFMASP1}_{\text{zym}}]}{K_{\text{m MASP2}^*}^{\text{MASP1}_{\text{zym}}}}+\frac{[\text{pHFMASP2}_{\text{zym}}]}{K_{\text{m MASP2}^*}^{\text{MASP2}_{\text{zym}}}}+\frac{[\text{pMFMASP1}_{\text{zym}}]}{K_{\text{m MASP2}^*}^{\text{MASP1}_{\text{zym}}}}+\frac{[\text{pMFMASP2}_{\text{zym}}]}{K_{\text{m MASP2}^*}^{\text{MASP2}_{\text{zym}}}}\\+\frac{[\text{pPTX3LFMASP1}_{\text{zym}}]}{K_{\text{m MASP2}^*}^{\text{MASP1}_{\text{zym}}}}+\frac{[\text{pPTX3LFMASP2}_{\text{zym}}]}{K_{\text{m MASP2}^*}^{\text{MASP2}_{\text{zym}}}}\end{array}\right)}$$

$$
-\frac{k_{\text{cat MASP2}^*}^{\text{MASP2}_{\text{zym}}}\left[\text{pCLK1MASP2}^*\right]\left[\text{pMBL}_3\text{MASP2}_{\text{zym}}\right]}{K_{\text{m MASP2}^*}^{\text{MASP2}_{\text{zym}}}\left(\begin{array}{l}1+\frac{[\text{C4}]}{K_{\text{m MASP2}^*}^{\text{C4}}}+\frac{[\text{fC4bC2}]}{K_{\text{m MASP2}^*}^{\text{C2}}}+\frac{[\text{fC4bC4bC2}]}{K_{\text{m MASP2}^*}^{\text{C2}}}+\frac{[\text{fC3bC4bC2}]}{K_{\text{m MASP2}^*}^{\text{C2}}}+\frac{[\text{pC4bC2}]}{K_{\text{m MASP2}^*}^{\text{C2}}}+\frac{[\text{pC4bC4bC2}]}{K_{\text{m MASP2}^*}^{\text{C2}}}\\+\frac{[\text{pC3bC4bC2}]}{K_{\text{m MASP2}^*}^{\text{C2}}}+\frac{[\text{pMBL}_3\text{MASP1}_{\text{zym}}]}{K_{\text{m MASP2}^*}^{\text{MASP1}_{\text{zym}}}}+\frac{[\text{pMBL}_3\text{MASP2}_{\text{zym}}]}{K_{\text{m MASP2}^*}^{\text{MASP2}_{\text{zym}}}}+\frac{[\text{pMBL}_4\text{MASP1}_{\text{zym}}]}{K_{\text{m MASP2}^*}^{\text{MASP1}_{\text{zym}}}}\\+\frac{[\text{pMBL}_4\text{MASP2}_{\text{zym}}]}{K_{\text{m MASP2}^*}^{\text{MASP2}_{\text{zym}}}}+\frac{[\text{pCLLKMASP1}_{\text{zym}}]}{K_{\text{m MASP2}^*}^{\text{MASP1}_{\text{zym}}}}+\frac{[\text{pCLLKMASP2}_{\text{zym}}]}{K_{\text{m MASP2}^*}^{\text{MASP2}_{\text{zym}}}}+\frac{[\text{pCLK1MASP1}_{\text{zym}}]}{K_{\text{m MASP2}^*}^{\text{MASP1}_{\text{zym}}}}\\+\frac{[\text{pCLK1MASP2}_{\text{zym}}]}{K_{\text{m MASP2}^*}^{\text{MASP2}_{\text{zym}}}}+\frac{[\text{pCLL1MASP1}_{\text{zym}}]}{K_{\text{m MASP2}^*}^{\text{MASP1}_{\text{zym}}}}+\frac{[\text{pCLL1MASP2}_{\text{zym}}]}{K_{\text{m MASP2}^*}^{\text{MASP2}_{\text{zym}}}}+\frac{[\text{pLFMASP1}_{\text{zym}}]}{K_{\text{m MASP2}^*}^{\text{MASP1}_{\text{zym}}}}\\+\frac{[\text{pLFMASP2}_{\text{zym}}]}{K_{\text{m MASP2}^*}^{\text{MASP2}_{\text{zym}}}}+\frac{[\text{pHFMASP1}_{\text{zym}}]}{K_{\text{m MASP2}^*}^{\text{MASP1}_{\text{zym}}}}+\frac{[\text{pHFMASP2}_{\text{zym}}]}{K_{\text{m MASP2}^*}^{\text{MASP2}_{\text{zym}}}}+\frac{[\text{pMFMASP1}_{\text{zym}}]}{K_{\text{m MASP2}^*}^{\text{MASP1}_{\text{zym}}}}+\frac{[\text{pMFMASP2}_{\text{zym}}]}{K_{\text{m MASP2}^*}^{\text{MASP2}_{\text{zym}}}}\\+\frac{[\text{pPTX3LFMASP1}_{\text{zym}}]}{K_{\text{m MASP2}^*}^{\text{MASP1}_{\text{zym}}}}+\frac{[\text{pPTX3LFMASP2}_{\text{zym}}]}{K_{\text{m MASP2}^*}^{\text{MASP2}_{\text{zym}}}}\end{array}\right)}
$$

$$
-\frac{k_{\text{cat MASP2}^*}^{\text{MASP2}_{\text{zym}}}\left[\text{pCLL1MASP2}^*\right]\left[\text{pMBL}_3\text{MASP2}_{\text{zym}}\right]}{K_{\text{m MASP2}^*}^{\text{MASP2}_{\text{zym}}}\left(\begin{array}{l}1+\frac{[\text{C4}]}{K_{\text{m MASP2}^*}^{\text{C4}}}+\frac{[\text{fC4bC2}]}{K_{\text{m MASP2}^*}^{\text{C2}}}+\frac{[\text{fC4bC4bC2}]}{K_{\text{m MASP2}^*}^{\text{C2}}}+\frac{[\text{fC3bC4bC2}]}{K_{\text{m MASP2}^*}^{\text{C2}}}+\frac{[\text{pC4bC2}]}{K_{\text{m MASP2}^*}^{\text{C2}}}+\frac{[\text{pC4bC4bC2}]}{K_{\text{m MASP2}^*}^{\text{C2}}}\\+\frac{[\text{pC3bC4bC2}]}{K_{\text{m MASP2}^*}^{\text{C2}}}+\frac{[\text{pMBL}_3\text{MASP1}_{\text{zym}}]}{K_{\text{m MASP2}^*}^{\text{MASP1}_{\text{zym}}}}+\frac{[\text{pMBL}_3\text{MASP2}_{\text{zym}}]}{K_{\text{m MASP2}^*}^{\text{MASP2}_{\text{zym}}}}+\frac{[\text{pMBL}_4\text{MASP1}_{\text{zym}}]}{K_{\text{m MASP2}^*}^{\text{MASP1}_{\text{zym}}}}\\+\frac{[\text{pMBL}_4\text{MASP2}_{\text{zym}}]}{K_{\text{m MASP2}^*}^{\text{MASP2}_{\text{zym}}}}+\frac{[\text{pCLLKMASP1}_{\text{zym}}]}{K_{\text{m MASP2}^*}^{\text{MASP1}_{\text{zym}}}}+\frac{[\text{pCLLKMASP2}_{\text{zym}}]}{K_{\text{m MASP2}^*}^{\text{MASP2}_{\text{zym}}}}+\frac{[\text{pCLK1MASP1}_{\text{zym}}]}{K_{\text{m MASP2}^*}^{\text{MASP1}_{\text{zym}}}}\\+\frac{[\text{pCLK1MASP2}_{\text{zym}}]}{K_{\text{m MASP2}^*}^{\text{MASP2}_{\text{zym}}}}+\frac{[\text{pCLL1MASP1}_{\text{zym}}]}{K_{\text{m MASP2}^*}^{\text{MASP1}_{\text{zym}}}}+\frac{[\text{pCLL1MASP2}_{\text{zym}}]}{K_{\text{m MASP2}^*}^{\text{MASP2}_{\text{zym}}}}+\frac{[\text{pLFMASP1}_{\text{zym}}]}{K_{\text{m MASP2}^*}^{\text{MASP1}_{\text{zym}}}}\\+\frac{[\text{pLFMASP2}_{\text{zym}}]}{K_{\text{m MASP2}^*}^{\text{MASP2}_{\text{zym}}}}+\frac{[\text{pHFMASP1}_{\text{zym}}]}{K_{\text{m MASP2}^*}^{\text{MASP1}_{\text{zym}}}}+\frac{[\text{pHFMASP2}_{\text{zym}}]}{K_{\text{m MASP2}^*}^{\text{MASP2}_{\text{zym}}}}+\frac{[\text{pMFMASP1}_{\text{zym}}]}{K_{\text{m MASP2}^*}^{\text{MASP1}_{\text{zym}}}}+\frac{[\text{pMFMASP2}_{\text{zym}}]}{K_{\text{m MASP2}^*}^{\text{MASP2}_{\text{zym}}}}\\+\frac{[\text{pPTX3LFMASP1}_{\text{zym}}]}{K_{\text{m MASP2}^*}^{\text{MASP1}_{\text{zym}}}}+\frac{[\text{pPTX3LFMASP2}_{\text{zym}}]}{K_{\text{m MASP2}^*}^{\text{MASP2}_{\text{zym}}}}\end{array}\right)}
$$

$$
-\frac{k_{\text{cat MASP2}^*}^{\text{MASP2}_{\text{zym}}}\left[\text{pLFMASP2}^*\right]\left[\text{pMBL}_3\text{MASP2}_{\text{zym}}\right]}{K_{\text{m MASP2}^*}^{\text{MASP2}_{\text{zym}}}\left(\begin{array}{l}1+\frac{[\text{C4}]}{K_{\text{m MASP2}^*}^{\text{C4}}}+\frac{[\text{fC4bC2}]}{K_{\text{m MASP2}^*}^{\text{C2}}}+\frac{[\text{fC4bC4bC2}]}{K_{\text{m MASP2}^*}^{\text{C2}}}+\frac{[\text{fC3bC4bC2}]}{K_{\text{m MASP2}^*}^{\text{C2}}}+\frac{[\text{pC4bC2}]}{K_{\text{m MASP2}^*}^{\text{C2}}}+\frac{[\text{pC4bC4bC2}]}{K_{\text{m MASP2}^*}^{\text{C2}}}\\+\frac{[\text{pC3bC4bC2}]}{K_{\text{m MASP2}^*}^{\text{C2}}}+\frac{[\text{pMBL}_3\text{MASP1}_{\text{zym}}]}{K_{\text{m MASP2}^*}^{\text{MASP1}_{\text{zym}}}}+\frac{[\text{pMBL}_3\text{MASP2}_{\text{zym}}]}{K_{\text{m MASP2}^*}^{\text{MASP2}_{\text{zym}}}}+\frac{[\text{pMBL}_4\text{MASP1}_{\text{zym}}]}{K_{\text{m MASP2}^*}^{\text{MASP1}_{\text{zym}}}}\\+\frac{[\text{pMBL}_4\text{MASP2}_{\text{zym}}]}{K_{\text{m MASP2}^*}^{\text{MASP2}_{\text{zym}}}}+\frac{[\text{pCLLKMASP1}_{\text{zym}}]}{K_{\text{m MASP2}^*}^{\text{MASP1}_{\text{zym}}}}+\frac{[\text{pCLLKMASP2}_{\text{zym}}]}{K_{\text{m MASP2}^*}^{\text{MASP2}_{\text{zym}}}}+\frac{[\text{pCLK1MASP1}_{\text{zym}}]}{K_{\text{m MASP2}^*}^{\text{MASP1}_{\text{zym}}}}\\+\frac{[\text{pCLK1MASP2}_{\text{zym}}]}{K_{\text{m MASP2}^*}^{\text{MASP2}_{\text{zym}}}}+\frac{[\text{pCLL1MASP1}_{\text{zym}}]}{K_{\text{m MASP2}^*}^{\text{MASP1}_{\text{zym}}}}+\frac{[\text{pCLL1MASP2}_{\text{zym}}]}{K_{\text{m MASP2}^*}^{\text{MASP2}_{\text{zym}}}}+\frac{[\text{pLFMASP1}_{\text{zym}}]}{K_{\text{m MASP2}^*}^{\text{MASP1}_{\text{zym}}}}\\+\frac{[\text{pLFMASP2}_{\text{zym}}]}{K_{\text{m MASP2}^*}^{\text{MASP2}_{\text{zym}}}}+\frac{[\text{pHFMASP1}_{\text{zym}}]}{K_{\text{m MASP2}^*}^{\text{MASP1}_{\text{zym}}}}+\frac{[\text{pHFMASP2}_{\text{zym}}]}{K_{\text{m MASP2}^*}^{\text{MASP2}_{\text{zym}}}}+\frac{[\text{pMFMASP1}_{\text{zym}}]}{K_{\text{m MASP2}^*}^{\text{MASP1}_{\text{zym}}}}+\frac{[\text{pMFMASP2}_{\text{zym}}]}{K_{\text{m MASP2}^*}^{\text{MASP2}_{\text{zym}}}}\\+\frac{[\text{pPTX3LFMASP1}_{\text{zym}}]}{K_{\text{m MASP2}^*}^{\text{MASP1}_{\text{zym}}}}+\frac{[\text{pPTX3LFMASP2}_{\text{zym}}]}{K_{\text{m MASP2}^*}^{\text{MASP2}_{\text{zym}}}}\end{array}\right)}
$$

$$
-\frac{k_{\text{cat MASP2}^*}^{\text{MASP2}_{\text{zym}}}\left[\text{pHFMASP2}^*\right]\left[\text{pMBL}_3\text{MASP2}_{\text{zym}}\right]}{K_{\text{m MASP2}^*}^{\text{MASP2}_{\text{zym}}}\left(\begin{array}{l}1+\frac{[\text{C4}]}{K_{\text{m MASP2}^*}^{\text{C4}}}+\frac{[\text{fC4bC2}]}{K_{\text{m MASP2}^*}^{\text{C2}}}+\frac{[\text{fC4bC4bC2}]}{K_{\text{m MASP2}^*}^{\text{C2}}}+\frac{[\text{fC3bC4bC2}]}{K_{\text{m MASP2}^*}^{\text{C2}}}+\frac{[\text{pC4bC2}]}{K_{\text{m MASP2}^*}^{\text{C2}}}+\frac{[\text{pC4bC4bC2}]}{K_{\text{m MASP2}^*}^{\text{C2}}}\\+\frac{[\text{pC3bC4bC2}]}{K_{\text{m MASP2}^*}^{\text{C2}}}+\frac{[\text{pMBL}_3\text{MASP1}_{\text{zym}}]}{K_{\text{m MASP2}^*}^{\text{MASP1}_{\text{zym}}}}+\frac{[\text{pMBL}_3\text{MASP2}_{\text{zym}}]}{K_{\text{m MASP2}^*}^{\text{MASP2}_{\text{zym}}}}+\frac{[\text{pMBL}_4\text{MASP1}_{\text{zym}}]}{K_{\text{m MASP2}^*}^{\text{MASP1}_{\text{zym}}}}\\+\frac{[\text{pMBL}_4\text{MASP2}_{\text{zym}}]}{K_{\text{m MASP2}^*}^{\text{MASP2}_{\text{zym}}}}+\frac{[\text{pCLLKMASP1}_{\text{zym}}]}{K_{\text{m MASP2}^*}^{\text{MASP1}_{\text{zym}}}}+\frac{[\text{pCLLKMASP2}_{\text{zym}}]}{K_{\text{m MASP2}^*}^{\text{MASP2}_{\text{zym}}}}+\frac{[\text{pCLK1MASP1}_{\text{zym}}]}{K_{\text{m MASP2}^*}^{\text{MASP1}_{\text{zym}}}}\\+\frac{[\text{pCLK1MASP2}_{\text{zym}}]}{K_{\text{m MASP2}^*}^{\text{MASP2}_{\text{zym}}}}+\frac{[\text{pCLL1MASP1}_{\text{zym}}]}{K_{\text{m MASP2}^*}^{\text{MASP1}_{\text{zym}}}}+\frac{[\text{pCLL1MASP2}_{\text{zym}}]}{K_{\text{m MASP2}^*}^{\text{MASP2}_{\text{zym}}}}+\frac{[\text{pLFMASP1}_{\text{zym}}]}{K_{\text{m MASP2}^*}^{\text{MASP1}_{\text{zym}}}}\\+\frac{[\text{pLFMASP2}_{\text{zym}}]}{K_{\text{m MASP2}^*}^{\text{MASP2}_{\text{zym}}}}+\frac{[\text{pHFMASP1}_{\text{zym}}]}{K_{\text{m MASP2}^*}^{\text{MASP1}_{\text{zym}}}}+\frac{[\text{pHFMASP2}_{\text{zym}}]}{K_{\text{m MASP2}^*}^{\text{MASP2}_{\text{zym}}}}+\frac{[\text{pMFMASP1}_{\text{zym}}]}{K_{\text{m MASP2}^*}^{\text{MASP1}_{\text{zym}}}}+\frac{[\text{pMFMASP2}_{\text{zym}}]}{K_{\text{m MASP2}^*}^{\text{MASP2}_{\text{zym}}}}\\+\frac{[\text{pPTX3LFMASP1}_{\text{zym}}]}{K_{\text{m MASP2}^*}^{\text{MASP1}_{\text{zym}}}}+\frac{[\text{pPTX3LFMASP2}_{\text{zym}}]}{K_{\text{m MASP2}^*}^{\text{MASP2}_{\text{zym}}}}\end{array}\right)}
$$

$$-\frac{k_{\text{cat MASP2}^*}^{\text{MASP2}_{\text{zym}}}\left[\text{pMFMASP2}^*\right]\left[\text{pMBL}_3\text{MASP2}_{\text{zym}}\right]}{K_{\text{m MASP2}^*}^{\text{MASP2}_{\text{zym}}}\left(\begin{array}{l}1+\frac{[\text{C4}]}{K_{\text{m MASP2}^*}^{\text{C4}}}+\frac{[\text{fC4bC2}]}{K_{\text{m MASP2}^*}^{\text{C2}}}+\frac{[\text{fC4bC4bC2}]}{K_{\text{m MASP2}^*}^{\text{C2}}}+\frac{[\text{fC3bC4bC2}]}{K_{\text{m MASP2}^*}^{\text{C2}}}+\frac{[\text{pC4bC2}]}{K_{\text{m MASP2}^*}^{\text{C2}}}+\frac{[\text{pC4bC4bC2}]}{K_{\text{m MASP2}^*}^{\text{C2}}}\\+\frac{[\text{pC3bC4bC2}]}{K_{\text{m MASP2}^*}^{\text{C2}}}+\frac{[\text{pMBL}_3\text{MASP1}_{\text{zym}}]}{K_{\text{m MASP2}^*}^{\text{MASP1}_{\text{zym}}}}+\frac{[\text{pMBL}_3\text{MASP2}_{\text{zym}}]}{K_{\text{m MASP2}^*}^{\text{MASP2}_{\text{zym}}}}+\frac{[\text{pMBL}_4\text{MASP1}_{\text{zym}}]}{K_{\text{m MASP2}^*}^{\text{MASP1}_{\text{zym}}}}\\+\frac{[\text{pMBL}_4\text{MASP2}_{\text{zym}}]}{K_{\text{m MASP2}^*}^{\text{MASP2}_{\text{zym}}}}+\frac{[\text{pCLLKMASP1}_{\text{zym}}]}{K_{\text{m MASP2}^*}^{\text{MASP1}_{\text{zym}}}}+\frac{[\text{pCLLKMASP2}_{\text{zym}}]}{K_{\text{m MASP2}^*}^{\text{MASP2}_{\text{zym}}}}+\frac{[\text{pCLK1MASP1}_{\text{zym}}]}{K_{\text{m MASP2}^*}^{\text{MASP1}_{\text{zym}}}}\\+\frac{[\text{pCLK1MASP2}_{\text{zym}}]}{K_{\text{m MASP2}^*}^{\text{MASP2}_{\text{zym}}}}+\frac{[\text{pCLL1MASP1}_{\text{zym}}]}{K_{\text{m MASP2}^*}^{\text{MASP1}_{\text{zym}}}}+\frac{[\text{pCLL1MASP2}_{\text{zym}}]}{K_{\text{m MASP2}^*}^{\text{MASP2}_{\text{zym}}}}+\frac{[\text{pLFMASP1}_{\text{zym}}]}{K_{\text{m MASP2}^*}^{\text{MASP1}_{\text{zym}}}}\\+\frac{[\text{pLFMASP2}_{\text{zym}}]}{K_{\text{m MASP2}^*}^{\text{MASP2}_{\text{zym}}}}+\frac{[\text{pHFMASP1}_{\text{zym}}]}{K_{\text{m MASP2}^*}^{\text{MASP1}_{\text{zym}}}}+\frac{[\text{pHFMASP2}_{\text{zym}}]}{K_{\text{m MASP2}^*}^{\text{MASP2}_{\text{zym}}}}+\frac{[\text{pMFMASP1}_{\text{zym}}]}{K_{\text{m MASP2}^*}^{\text{MASP1}_{\text{zym}}}}+\frac{[\text{pMFMASP2}_{\text{zym}}]}{K_{\text{m MASP2}^*}^{\text{MASP2}_{\text{zym}}}}\\+\frac{[\text{pPTX3LFMASP1}_{\text{zym}}]}{K_{\text{m MASP2}^*}^{\text{MASP1}_{\text{zym}}}}+\frac{[\text{pPTX3LFMASP2}_{\text{zym}}]}{K_{\text{m MASP2}^*}^{\text{MASP2}_{\text{zym}}}}\end{array}\right)}$$

$$-\frac{k_{\text{cat MASP2}^*}^{\text{MASP2}_{\text{zym}}}\left[\text{pPTX3LFMASP2}^*\right]\left[\text{pMBL}_3\text{MASP2}_{\text{zym}}\right]}{K_{\text{m MASP2}^*}^{\text{MASP2}_{\text{zym}}}\left(\begin{array}{l}1+\frac{[\text{C4}]}{K_{\text{m MASP2}^*}^{\text{C4}}}+\frac{[\text{fC4bC2}]}{K_{\text{m MASP2}^*}^{\text{C2}}}+\frac{[\text{fC4bC4bC2}]}{K_{\text{m MASP2}^*}^{\text{C2}}}+\frac{[\text{fC3bC4bC2}]}{K_{\text{m MASP2}^*}^{\text{C2}}}+\frac{[\text{pC4bC2}]}{K_{\text{m MASP2}^*}^{\text{C2}}}+\frac{[\text{pC4bC4bC2}]}{K_{\text{m MASP2}^*}^{\text{C2}}}\\+\frac{[\text{pC3bC4bC2}]}{K_{\text{m MASP2}^*}^{\text{C2}}}+\frac{[\text{pMBL}_3\text{MASP1}_{\text{zym}}]}{K_{\text{m MASP2}^*}^{\text{MASP1}_{\text{zym}}}}+\frac{[\text{pMBL}_3\text{MASP2}_{\text{zym}}]}{K_{\text{m MASP2}^*}^{\text{MASP2}_{\text{zym}}}}+\frac{[\text{pMBL}_4\text{MASP1}_{\text{zym}}]}{K_{\text{m MASP2}^*}^{\text{MASP1}_{\text{zym}}}}\\+\frac{[\text{pMBL}_4\text{MASP2}_{\text{zym}}]}{K_{\text{m MASP2}^*}^{\text{MASP2}_{\text{zym}}}}+\frac{[\text{pCLLKMASP1}_{\text{zym}}]}{K_{\text{m MASP2}^*}^{\text{MASP1}_{\text{zym}}}}+\frac{[\text{pCLLKMASP2}_{\text{zym}}]}{K_{\text{m MASP2}^*}^{\text{MASP2}_{\text{zym}}}}+\frac{[\text{pCLK1MASP1}_{\text{zym}}]}{K_{\text{m MASP2}^*}^{\text{MASP1}_{\text{zym}}}}\\+\frac{[\text{pCLK1MASP2}_{\text{zym}}]}{K_{\text{m MASP2}^*}^{\text{MASP2}_{\text{zym}}}}+\frac{[\text{pCLL1MASP1}_{\text{zym}}]}{K_{\text{m MASP2}^*}^{\text{MASP1}_{\text{zym}}}}+\frac{[\text{pCLL1MASP2}_{\text{zym}}]}{K_{\text{m MASP2}^*}^{\text{MASP2}_{\text{zym}}}}+\frac{[\text{pLFMASP1}_{\text{zym}}]}{K_{\text{m MASP2}^*}^{\text{MASP1}_{\text{zym}}}}\\+\frac{[\text{pLFMASP2}_{\text{zym}}]}{K_{\text{m MASP2}^*}^{\text{MASP2}_{\text{zym}}}}+\frac{[\text{pHFMASP1}_{\text{zym}}]}{K_{\text{m MASP2}^*}^{\text{MASP1}_{\text{zym}}}}+\frac{[\text{pHFMASP2}_{\text{zym}}]}{K_{\text{m MASP2}^*}^{\text{MASP2}_{\text{zym}}}}+\frac{[\text{pMFMASP1}_{\text{zym}}]}{K_{\text{m MASP2}^*}^{\text{MASP1}_{\text{zym}}}}+\frac{[\text{pMFMASP2}_{\text{zym}}]}{K_{\text{m MASP2}^*}^{\text{MASP2}_{\text{zym}}}}\\+\frac{[\text{pPTX3LFMASP1}_{\text{zym}}]}{K_{\text{m MASP2}^*}^{\text{MASP1}_{\text{zym}}}}+\frac{[\text{pPTX3LFMASP2}_{\text{zym}}]}{K_{\text{m MASP2}^*}^{\text{MASP2}_{\text{zym}}}}\end{array}\right)}$$

250

$$\frac{d\left[\text{fMBL}_3\text{MASP2}^*\right]}{dt}=-k_{\text{pMBL}_3}^{+}\left[\text{fMBL}_3\text{MASP2}^*\right]\left[\text{Pathogen Surface}\right]+k_{\text{pMBL}_3}^{-}\left[\text{pMBL}_3\text{MASP2}^*\right]$$
$$+k_{\text{MBL}_3\text{MASP2}}^{+}\left[\text{MBL}_3\right]\left[\text{MASP2}^*\right]-k_{\text{MBL}_3\text{MASP2}}^{-}\left[\text{fMBL}_3\text{MASP2}^*\right]-k_{\text{C1INHMASP2}}^{+}\left[\text{C1-INH}\right]\left[\text{fMBL}_3\text{MASP2}^*\right]$$

251

$$\frac{d\left[\mathrm{pMBL_3MASP2^*}\right]}{dt}=k^{+}_{\mathrm{pMBL_3}}\left[\mathrm{fMBL_3MASP2^*}\right]\left[\mathrm{Pathogen\ Surface}\right]-k^{-}_{\mathrm{pMBL_3}}\left[\mathrm{pMBL_3MASP2^*}\right]$$

$$+k^{+}_{\mathrm{MBL_3MASP2}}\left[\mathrm{pMBL_3}\right]\left[\mathrm{MASP2^*}\right]-k^{-}_{\mathrm{MBL_3MASP2}}\left[\mathrm{pMBL_3MASP2^*}\right]$$

$$+\frac{k^{\mathrm{MASP2_{zym}}}_{\mathrm{cat\ MASP1_{zym}}}\left[\mathrm{pMBL_3MASP1_{zym}}\right]\left[\mathrm{pMBL_3MASP2_{zym}}\right]}{K^{\mathrm{MASP2_{zym}}}_{\mathrm{m\ MASP1_{zym}}}\left(\begin{array}{l}1+\frac{\left[\mathrm{pMBL_3MASP1_{zym}}\right]}{K^{\mathrm{MASP1_{zym}}}_{\mathrm{m\ MASP1_{zym}}}}+\frac{\left[\mathrm{pMBL_3MASP2_{zym}}\right]}{K^{\mathrm{MASP2_{zym}}}_{\mathrm{m\ MASP1_{zym}}}}+\frac{\left[\mathrm{pMBL_4MASP1_{zym}}\right]}{K^{\mathrm{MASP1_{zym}}}_{\mathrm{m\ MASP1_{zym}}}}+\frac{\left[\mathrm{pMBL_4MASP2_{zym}}\right]}{K^{\mathrm{MASP2_{zym}}}_{\mathrm{m\ MASP1_{zym}}}}\\+\frac{\left[\mathrm{pCLLKMASP1_{zym}}\right]}{K^{\mathrm{MASP1_{zym}}}_{\mathrm{m\ MASP1_{zym}}}}+\frac{\left[\mathrm{pCLLKMASP2_{zym}}\right]}{K^{\mathrm{MASP2_{zym}}}_{\mathrm{m\ MASP1_{zym}}}}+\frac{\left[\mathrm{pCLK1MASP1_{zym}}\right]}{K^{\mathrm{MASP1_{zym}}}_{\mathrm{m\ MASP1_{zym}}}}+\frac{\left[\mathrm{pCLK1MASP2_{zym}}\right]}{K^{\mathrm{MASP2_{zym}}}_{\mathrm{m\ MASP1_{zym}}}}\\+\frac{\left[\mathrm{pCLL1MASP1_{zym}}\right]}{K^{\mathrm{MASP1_{zym}}}_{\mathrm{m\ MASP1_{zym}}}}+\frac{\left[\mathrm{pCLL1MASP2_{zym}}\right]}{K^{\mathrm{MASP2_{zym}}}_{\mathrm{m\ MASP1_{zym}}}}+\frac{\left[\mathrm{pLFMASP1_{zym}}\right]}{K^{\mathrm{MASP1_{zym}}}_{\mathrm{m\ MASP1_{zym}}}}+\frac{\left[\mathrm{pLFMASP2_{zym}}\right]}{K^{\mathrm{MASP2_{zym}}}_{\mathrm{m\ MASP1_{zym}}}}\\+\frac{\left[\mathrm{pHFMASP1_{zym}}\right]}{K^{\mathrm{MASP1_{zym}}}_{\mathrm{m\ MASP1_{zym}}}}+\frac{\left[\mathrm{pHFMASP2_{zym}}\right]}{K^{\mathrm{MASP2_{zym}}}_{\mathrm{m\ MASP1_{zym}}}}+\frac{\left[\mathrm{pMFMASP1_{zym}}\right]}{K^{\mathrm{MASP1_{zym}}}_{\mathrm{m\ MASP1_{zym}}}}+\frac{\left[\mathrm{pMFMASP2_{zym}}\right]}{K^{\mathrm{MASP2_{zym}}}_{\mathrm{m\ MASP1_{zym}}}}\\+\frac{\left[\mathrm{pPTX3LFMASP1_{zym}}\right]}{K^{\mathrm{MASP1_{zym}}}_{\mathrm{m\ MASP1_{zym}}}}+\frac{\left[\mathrm{pPTX3LFMASP2_{zym}}\right]}{K^{\mathrm{MASP2_{zym}}}_{\mathrm{m\ MASP1_{zym}}}}\end{array}\right)}$$

$$+\frac{k^{\mathrm{MASP2_{zym}}}_{\mathrm{cat\ MASP1_{zym}}}\left[\mathrm{pMBL_4MASP1_{zym}}\right]\left[\mathrm{pMBL_3MASP2_{zym}}\right]}{K^{\mathrm{MASP2_{zym}}}_{\mathrm{m\ MASP1_{zym}}}\left(\begin{array}{l}1+\frac{\left[\mathrm{pMBL_3MASP1_{zym}}\right]}{K^{\mathrm{MASP1_{zym}}}_{\mathrm{m\ MASP1_{zym}}}}+\frac{\left[\mathrm{pMBL_3MASP2_{zym}}\right]}{K^{\mathrm{MASP2_{zym}}}_{\mathrm{m\ MASP1_{zym}}}}+\frac{\left[\mathrm{pMBL_4MASP1_{zym}}\right]}{K^{\mathrm{MASP1_{zym}}}_{\mathrm{m\ MASP1_{zym}}}}+\frac{\left[\mathrm{pMBL_4MASP2_{zym}}\right]}{K^{\mathrm{MASP2_{zym}}}_{\mathrm{m\ MASP1_{zym}}}}\\+\frac{\left[\mathrm{pCLLKMASP1_{zym}}\right]}{K^{\mathrm{MASP1_{zym}}}_{\mathrm{m\ MASP1_{zym}}}}+\frac{\left[\mathrm{pCLLKMASP2_{zym}}\right]}{K^{\mathrm{MASP2_{zym}}}_{\mathrm{m\ MASP1_{zym}}}}+\frac{\left[\mathrm{pCLK1MASP1_{zym}}\right]}{K^{\mathrm{MASP1_{zym}}}_{\mathrm{m\ MASP1_{zym}}}}+\frac{\left[\mathrm{pCLK1MASP2_{zym}}\right]}{K^{\mathrm{MASP2_{zym}}}_{\mathrm{m\ MASP1_{zym}}}}\\+\frac{\left[\mathrm{pCLL1MASP1_{zym}}\right]}{K^{\mathrm{MASP1_{zym}}}_{\mathrm{m\ MASP1_{zym}}}}+\frac{\left[\mathrm{pCLL1MASP2_{zym}}\right]}{K^{\mathrm{MASP2_{zym}}}_{\mathrm{m\ MASP1_{zym}}}}+\frac{\left[\mathrm{pLFMASP1_{zym}}\right]}{K^{\mathrm{MASP1_{zym}}}_{\mathrm{m\ MASP1_{zym}}}}+\frac{\left[\mathrm{pLFMASP2_{zym}}\right]}{K^{\mathrm{MASP2_{zym}}}_{\mathrm{m\ MASP1_{zym}}}}\\+\frac{\left[\mathrm{pHFMASP1_{zym}}\right]}{K^{\mathrm{MASP1_{zym}}}_{\mathrm{m\ MASP1_{zym}}}}+\frac{\left[\mathrm{pHFMASP2_{zym}}\right]}{K^{\mathrm{MASP2_{zym}}}_{\mathrm{m\ MASP1_{zym}}}}+\frac{\left[\mathrm{pMFMASP1_{zym}}\right]}{K^{\mathrm{MASP1_{zym}}}_{\mathrm{m\ MASP1_{zym}}}}+\frac{\left[\mathrm{pMFMASP2_{zym}}\right]}{K^{\mathrm{MASP2_{zym}}}_{\mathrm{m\ MASP1_{zym}}}}\\+\frac{\left[\mathrm{pPTX3LFMASP1_{zym}}\right]}{K^{\mathrm{MASP1_{zym}}}_{\mathrm{m\ MASP1_{zym}}}}+\frac{\left[\mathrm{pPTX3LFMASP2_{zym}}\right]}{K^{\mathrm{MASP2_{zym}}}_{\mathrm{m\ MASP1_{zym}}}}\end{array}\right)}$$

$$
+\frac{k_{\text{cat MASP1}_{\text{zym}}}^{\text{MASP2}_{\text{zym}}}\left[\text{pCLLKMASP1}_{\text{zym}}\right]\left[\text{pMBL}_3\text{MASP2}_{\text{zym}}\right]}{K_{\text{m MASP1}_{\text{zym}}}^{\text{MASP2}_{\text{zym}}}\left(\begin{aligned}&1+\frac{\left[\text{pMBL}_3\text{MASP1}_{\text{zym}}\right]}{K_{\text{m MASP1}_{\text{zym}}}^{\text{MASP1}_{\text{zym}}}}+\frac{\left[\text{pMBL}_3\text{MASP2}_{\text{zym}}\right]}{K_{\text{m MASP1}_{\text{zym}}}^{\text{MASP2}_{\text{zym}}}}+\frac{\left[\text{pMBL}_4\text{MASP1}_{\text{zym}}\right]}{K_{\text{m MASP1}_{\text{zym}}}^{\text{MASP1}_{\text{zym}}}}+\frac{\left[\text{pMBL}_4\text{MASP2}_{\text{zym}}\right]}{K_{\text{m MASP1}_{\text{zym}}}^{\text{MASP2}_{\text{zym}}}}\\&+\frac{\left[\text{pCLLKMASP1}_{\text{zym}}\right]}{K_{\text{m MASP1}_{\text{zym}}}^{\text{MASP1}_{\text{zym}}}}+\frac{\left[\text{pCLLKMASP2}_{\text{zym}}\right]}{K_{\text{m MASP1}_{\text{zym}}}^{\text{MASP2}_{\text{zym}}}}+\frac{\left[\text{pCLK1MASP1}_{\text{zym}}\right]}{K_{\text{m MASP1}_{\text{zym}}}^{\text{MASP1}_{\text{zym}}}}+\frac{\left[\text{pCLK1MASP2}_{\text{zym}}\right]}{K_{\text{m MASP1}_{\text{zym}}}^{\text{MASP2}_{\text{zym}}}}\\&+\frac{\left[\text{pCLL1MASP1}_{\text{zym}}\right]}{K_{\text{m MASP1}_{\text{zym}}}^{\text{MASP1}_{\text{zym}}}}+\frac{\left[\text{pCLL1MASP2}_{\text{zym}}\right]}{K_{\text{m MASP1}_{\text{zym}}}^{\text{MASP2}_{\text{zym}}}}+\frac{\left[\text{pLFMASP1}_{\text{zym}}\right]}{K_{\text{m MASP1}_{\text{zym}}}^{\text{MASP1}_{\text{zym}}}}+\frac{\left[\text{pLFMASP2}_{\text{zym}}\right]}{K_{\text{m MASP1}_{\text{zym}}}^{\text{MASP2}_{\text{zym}}}}\\&+\frac{\left[\text{pHFMASP1}_{\text{zym}}\right]}{K_{\text{m MASP1}_{\text{zym}}}^{\text{MASP1}_{\text{zym}}}}+\frac{\left[\text{pHFMASP2}_{\text{zym}}\right]}{K_{\text{m MASP1}_{\text{zym}}}^{\text{MASP2}_{\text{zym}}}}+\frac{\left[\text{pMFMASP1}_{\text{zym}}\right]}{K_{\text{m MASP1}_{\text{zym}}}^{\text{MASP1}_{\text{zym}}}}+\frac{\left[\text{pMFMASP2}_{\text{zym}}\right]}{K_{\text{m MASP1}_{\text{zym}}}^{\text{MASP2}_{\text{zym}}}}\\&+\frac{\left[\text{pPTX3LFMASP1}_{\text{zym}}\right]}{K_{\text{m MASP1}_{\text{zym}}}^{\text{MASP1}_{\text{zym}}}}+\frac{\left[\text{pPTX3LFMASP2}_{\text{zym}}\right]}{K_{\text{m MASP1}_{\text{zym}}}^{\text{MASP2}_{\text{zym}}}}\end{aligned}\right)}
$$

$$
+\frac{k_{\text{cat MASP1}_{\text{zym}}}^{\text{MASP2}_{\text{zym}}}\left[\text{pCLK1MASP1}_{\text{zym}}\right]\left[\text{pMBL}_3\text{MASP2}_{\text{zym}}\right]}{K_{\text{m MASP1}_{\text{zym}}}^{\text{MASP2}_{\text{zym}}}\left(\begin{aligned}&1+\frac{\left[\text{pMBL}_3\text{MASP1}_{\text{zym}}\right]}{K_{\text{m MASP1}_{\text{zym}}}^{\text{MASP1}_{\text{zym}}}}+\frac{\left[\text{pMBL}_3\text{MASP2}_{\text{zym}}\right]}{K_{\text{m MASP1}_{\text{zym}}}^{\text{MASP2}_{\text{zym}}}}+\frac{\left[\text{pMBL}_4\text{MASP1}_{\text{zym}}\right]}{K_{\text{m MASP1}_{\text{zym}}}^{\text{MASP1}_{\text{zym}}}}+\frac{\left[\text{pMBL}_4\text{MASP2}_{\text{zym}}\right]}{K_{\text{m MASP1}_{\text{zym}}}^{\text{MASP2}_{\text{zym}}}}\\&+\frac{\left[\text{pCLLKMASP1}_{\text{zym}}\right]}{K_{\text{m MASP1}_{\text{zym}}}^{\text{MASP1}_{\text{zym}}}}+\frac{\left[\text{pCLLKMASP2}_{\text{zym}}\right]}{K_{\text{m MASP1}_{\text{zym}}}^{\text{MASP2}_{\text{zym}}}}+\frac{\left[\text{pCLK1MASP1}_{\text{zym}}\right]}{K_{\text{m MASP1}_{\text{zym}}}^{\text{MASP1}_{\text{zym}}}}+\frac{\left[\text{pCLK1MASP2}_{\text{zym}}\right]}{K_{\text{m MASP1}_{\text{zym}}}^{\text{MASP2}_{\text{zym}}}}\\&+\frac{\left[\text{pCLL1MASP1}_{\text{zym}}\right]}{K_{\text{m MASP1}_{\text{zym}}}^{\text{MASP1}_{\text{zym}}}}+\frac{\left[\text{pCLL1MASP2}_{\text{zym}}\right]}{K_{\text{m MASP1}_{\text{zym}}}^{\text{MASP2}_{\text{zym}}}}+\frac{\left[\text{pLFMASP1}_{\text{zym}}\right]}{K_{\text{m MASP1}_{\text{zym}}}^{\text{MASP1}_{\text{zym}}}}+\frac{\left[\text{pLFMASP2}_{\text{zym}}\right]}{K_{\text{m MASP1}_{\text{zym}}}^{\text{MASP2}_{\text{zym}}}}\\&+\frac{\left[\text{pHFMASP1}_{\text{zym}}\right]}{K_{\text{m MASP1}_{\text{zym}}}^{\text{MASP1}_{\text{zym}}}}+\frac{\left[\text{pHFMASP2}_{\text{zym}}\right]}{K_{\text{m MASP1}_{\text{zym}}}^{\text{MASP2}_{\text{zym}}}}+\frac{\left[\text{pMFMASP1}_{\text{zym}}\right]}{K_{\text{m MASP1}_{\text{zym}}}^{\text{MASP1}_{\text{zym}}}}+\frac{\left[\text{pMFMASP2}_{\text{zym}}\right]}{K_{\text{m MASP1}_{\text{zym}}}^{\text{MASP2}_{\text{zym}}}}\\&+\frac{\left[\text{pPTX3LFMASP1}_{\text{zym}}\right]}{K_{\text{m MASP1}_{\text{zym}}}^{\text{MASP1}_{\text{zym}}}}+\frac{\left[\text{pPTX3LFMASP2}_{\text{zym}}\right]}{K_{\text{m MASP1}_{\text{zym}}}^{\text{MASP2}_{\text{zym}}}}\end{aligned}\right)}
$$

$$+\frac{k_{\text{cat MASP1}_{\text{zym}}}^{\text{MASP2}_{\text{zym}}}\left[\text{pCLL1MASP1}_{\text{zym}}\right]\left[\text{pMBL}_3\text{MASP2}_{\text{zym}}\right]}{K_{\text{m MASP1}_{\text{zym}}}^{\text{MASP2}_{\text{zym}}}\left(\begin{array}{l}1+\frac{\left[\text{pMBL}_3\text{MASP1}_{\text{zym}}\right]}{K_{\text{m MASP1}_{\text{zym}}}^{\text{MASP1}_{\text{zym}}}}+\frac{\left[\text{pMBL}_3\text{MASP2}_{\text{zym}}\right]}{K_{\text{m MASP1}_{\text{zym}}}^{\text{MASP2}_{\text{zym}}}}+\frac{\left[\text{pMBL}_4\text{MASP1}_{\text{zym}}\right]}{K_{\text{m MASP1}_{\text{zym}}}^{\text{MASP1}_{\text{zym}}}}+\frac{\left[\text{pMBL}_4\text{MASP2}_{\text{zym}}\right]}{K_{\text{m MASP1}_{\text{zym}}}^{\text{MASP2}_{\text{zym}}}}\\+\frac{\left[\text{pCLLKMASP1}_{\text{zym}}\right]}{K_{\text{m MASP1}_{\text{zym}}}^{\text{MASP1}_{\text{zym}}}}+\frac{\left[\text{pCLLKMASP2}_{\text{zym}}\right]}{K_{\text{m MASP1}_{\text{zym}}}^{\text{MASP2}_{\text{zym}}}}+\frac{\left[\text{pCLK1MASP1}_{\text{zym}}\right]}{K_{\text{m MASP1}_{\text{zym}}}^{\text{MASP1}_{\text{zym}}}}+\frac{\left[\text{pCLK1MASP2}_{\text{zym}}\right]}{K_{\text{m MASP1}_{\text{zym}}}^{\text{MASP2}_{\text{zym}}}}\\+\frac{\left[\text{pCLL1MASP1}_{\text{zym}}\right]}{K_{\text{m MASP1}_{\text{zym}}}^{\text{MASP1}_{\text{zym}}}}+\frac{\left[\text{pCLL1MASP2}_{\text{zym}}\right]}{K_{\text{m MASP1}_{\text{zym}}}^{\text{MASP2}_{\text{zym}}}}+\frac{\left[\text{pLFMASP1}_{\text{zym}}\right]}{K_{\text{m MASP1}_{\text{zym}}}^{\text{MASP1}_{\text{zym}}}}+\frac{\left[\text{pLFMASP2}_{\text{zym}}\right]}{K_{\text{m MASP1}_{\text{zym}}}^{\text{MASP2}_{\text{zym}}}}\\+\frac{\left[\text{pHFMASP1}_{\text{zym}}\right]}{K_{\text{m MASP1}_{\text{zym}}}^{\text{MASP1}_{\text{zym}}}}+\frac{\left[\text{pHFMASP2}_{\text{zym}}\right]}{K_{\text{m MASP1}_{\text{zym}}}^{\text{MASP2}_{\text{zym}}}}+\frac{\left[\text{pMFMASP1}_{\text{zym}}\right]}{K_{\text{m MASP1}_{\text{zym}}}^{\text{MASP1}_{\text{zym}}}}+\frac{\left[\text{pMFMASP2}_{\text{zym}}\right]}{K_{\text{m MASP1}_{\text{zym}}}^{\text{MASP2}_{\text{zym}}}}\\+\frac{\left[\text{pPTX3LFMASP1}_{\text{zym}}\right]}{K_{\text{m MASP1}_{\text{zym}}}^{\text{MASP1}_{\text{zym}}}}+\frac{\left[\text{pPTX3LFMASP2}_{\text{zym}}\right]}{K_{\text{m MASP1}_{\text{zym}}}^{\text{MASP2}_{\text{zym}}}}\end{array}\right)}$$

$$+\frac{k_{\text{cat MASP1}_{\text{zym}}}^{\text{MASP2}_{\text{zym}}}\left[\text{pLFMASP1}_{\text{zym}}\right]\left[\text{pMBL}_3\text{MASP2}_{\text{zym}}\right]}{K_{\text{m MASP1}_{\text{zym}}}^{\text{MASP2}_{\text{zym}}}\left(\begin{array}{l}1+\frac{\left[\text{pMBL}_3\text{MASP1}_{\text{zym}}\right]}{K_{\text{m MASP1}_{\text{zym}}}^{\text{MASP1}_{\text{zym}}}}+\frac{\left[\text{pMBL}_3\text{MASP2}_{\text{zym}}\right]}{K_{\text{m MASP1}_{\text{zym}}}^{\text{MASP2}_{\text{zym}}}}+\frac{\left[\text{pMBL}_4\text{MASP1}_{\text{zym}}\right]}{K_{\text{m MASP1}_{\text{zym}}}^{\text{MASP1}_{\text{zym}}}}+\frac{\left[\text{pMBL}_4\text{MASP2}_{\text{zym}}\right]}{K_{\text{m MASP1}_{\text{zym}}}^{\text{MASP2}_{\text{zym}}}}\\+\frac{\left[\text{pCLLKMASP1}_{\text{zym}}\right]}{K_{\text{m MASP1}_{\text{zym}}}^{\text{MASP1}_{\text{zym}}}}+\frac{\left[\text{pCLLKMASP2}_{\text{zym}}\right]}{K_{\text{m MASP1}_{\text{zym}}}^{\text{MASP2}_{\text{zym}}}}+\frac{\left[\text{pCLK1MASP1}_{\text{zym}}\right]}{K_{\text{m MASP1}_{\text{zym}}}^{\text{MASP1}_{\text{zym}}}}+\frac{\left[\text{pCLK1MASP2}_{\text{zym}}\right]}{K_{\text{m MASP1}_{\text{zym}}}^{\text{MASP2}_{\text{zym}}}}\\+\frac{\left[\text{pCLL1MASP1}_{\text{zym}}\right]}{K_{\text{m MASP1}_{\text{zym}}}^{\text{MASP1}_{\text{zym}}}}+\frac{\left[\text{pCLL1MASP2}_{\text{zym}}\right]}{K_{\text{m MASP1}_{\text{zym}}}^{\text{MASP2}_{\text{zym}}}}+\frac{\left[\text{pLFMASP1}_{\text{zym}}\right]}{K_{\text{m MASP1}_{\text{zym}}}^{\text{MASP1}_{\text{zym}}}}+\frac{\left[\text{pLFMASP2}_{\text{zym}}\right]}{K_{\text{m MASP1}_{\text{zym}}}^{\text{MASP2}_{\text{zym}}}}\\+\frac{\left[\text{pHFMASP1}_{\text{zym}}\right]}{K_{\text{m MASP1}_{\text{zym}}}^{\text{MASP1}_{\text{zym}}}}+\frac{\left[\text{pHFMASP2}_{\text{zym}}\right]}{K_{\text{m MASP1}_{\text{zym}}}^{\text{MASP2}_{\text{zym}}}}+\frac{\left[\text{pMFMASP1}_{\text{zym}}\right]}{K_{\text{m MASP1}_{\text{zym}}}^{\text{MASP1}_{\text{zym}}}}+\frac{\left[\text{pMFMASP2}_{\text{zym}}\right]}{K_{\text{m MASP1}_{\text{zym}}}^{\text{MASP2}_{\text{zym}}}}\\+\frac{\left[\text{pPTX3LFMASP1}_{\text{zym}}\right]}{K_{\text{m MASP1}_{\text{zym}}}^{\text{MASP1}_{\text{zym}}}}+\frac{\left[\text{pPTX3LFMASP2}_{\text{zym}}\right]}{K_{\text{m MASP1}_{\text{zym}}}^{\text{MASP2}_{\text{zym}}}}\end{array}\right)}$$

$$
+\frac{k_{\mathrm{cat\ MASP1_{zym}}}^{\mathrm{MASP2_{zym}}}\left[\mathrm{pHFMASP1_{zym}}\right]\left[\mathrm{pMBL_3MASP2_{zym}}\right]}{K_{\mathrm{m\ MASP1_{zym}}}^{\mathrm{MASP2_{zym}}}\left(\begin{array}{l}1+\frac{\left[\mathrm{pMBL_3MASP1_{zym}}\right]}{K_{\mathrm{m\ MASP1_{zym}}}^{\mathrm{MASP1_{zym}}}}+\frac{\left[\mathrm{pMBL_3MASP2_{zym}}\right]}{K_{\mathrm{m\ MASP1_{zym}}}^{\mathrm{MASP2_{zym}}}}+\frac{\left[\mathrm{pMBL_4MASP1_{zym}}\right]}{K_{\mathrm{m\ MASP1_{zym}}}^{\mathrm{MASP1_{zym}}}}+\frac{\left[\mathrm{pMBL_4MASP2_{zym}}\right]}{K_{\mathrm{m\ MASP1_{zym}}}^{\mathrm{MASP2_{zym}}}}\\+\frac{\left[\mathrm{pCLLKMASP1_{zym}}\right]}{K_{\mathrm{m\ MASP1_{zym}}}^{\mathrm{MASP1_{zym}}}}+\frac{\left[\mathrm{pCLLKMASP2_{zym}}\right]}{K_{\mathrm{m\ MASP1_{zym}}}^{\mathrm{MASP2_{zym}}}}+\frac{\left[\mathrm{pCLK1MASP1_{zym}}\right]}{K_{\mathrm{m\ MASP1_{zym}}}^{\mathrm{MASP1_{zym}}}}+\frac{\left[\mathrm{pCLK1MASP2_{zym}}\right]}{K_{\mathrm{m\ MASP1_{zym}}}^{\mathrm{MASP2_{zym}}}}\\+\frac{\left[\mathrm{pCLL1MASP1_{zym}}\right]}{K_{\mathrm{m\ MASP1_{zym}}}^{\mathrm{MASP1_{zym}}}}+\frac{\left[\mathrm{pCLL1MASP2_{zym}}\right]}{K_{\mathrm{m\ MASP1_{zym}}}^{\mathrm{MASP2_{zym}}}}+\frac{\left[\mathrm{pLFMASP1_{zym}}\right]}{K_{\mathrm{m\ MASP1_{zym}}}^{\mathrm{MASP1_{zym}}}}+\frac{\left[\mathrm{pLFMASP2_{zym}}\right]}{K_{\mathrm{m\ MASP1_{zym}}}^{\mathrm{MASP2_{zym}}}}\\+\frac{\left[\mathrm{pHFMASP1_{zym}}\right]}{K_{\mathrm{m\ MASP1_{zym}}}^{\mathrm{MASP1_{zym}}}}+\frac{\left[\mathrm{pHFMASP2_{zym}}\right]}{K_{\mathrm{m\ MASP1_{zym}}}^{\mathrm{MASP2_{zym}}}}+\frac{\left[\mathrm{pMFMASP1_{zym}}\right]}{K_{\mathrm{m\ MASP1_{zym}}}^{\mathrm{MASP1_{zym}}}}+\frac{\left[\mathrm{pMFMASP2_{zym}}\right]}{K_{\mathrm{m\ MASP1_{zym}}}^{\mathrm{MASP2_{zym}}}}\\+\frac{\left[\mathrm{pPTX3LFMASP1_{zym}}\right]}{K_{\mathrm{m\ MASP1_{zym}}}^{\mathrm{MASP1_{zym}}}}+\frac{\left[\mathrm{pPTX3LFMASP2_{zym}}\right]}{K_{\mathrm{m\ MASP1_{zym}}}^{\mathrm{MASP2_{zym}}}}\end{array}\right)}
$$

$$
+\frac{k_{\mathrm{cat\ MASP1_{zym}}}^{\mathrm{MASP2_{zym}}}\left[\mathrm{pMFMASP1_{zym}}\right]\left[\mathrm{pMBL_3MASP2_{zym}}\right]}{K_{\mathrm{m\ MASP1_{zym}}}^{\mathrm{MASP2_{zym}}}\left(\begin{array}{l}1+\frac{\left[\mathrm{pMBL_3MASP1_{zym}}\right]}{K_{\mathrm{m\ MASP1_{zym}}}^{\mathrm{MASP1_{zym}}}}+\frac{\left[\mathrm{pMBL_3MASP2_{zym}}\right]}{K_{\mathrm{m\ MASP1_{zym}}}^{\mathrm{MASP2_{zym}}}}+\frac{\left[\mathrm{pMBL_4MASP1_{zym}}\right]}{K_{\mathrm{m\ MASP1_{zym}}}^{\mathrm{MASP1_{zym}}}}+\frac{\left[\mathrm{pMBL_4MASP2_{zym}}\right]}{K_{\mathrm{m\ MASP1_{zym}}}^{\mathrm{MASP2_{zym}}}}\\+\frac{\left[\mathrm{pCLLKMASP1_{zym}}\right]}{K_{\mathrm{m\ MASP1_{zym}}}^{\mathrm{MASP1_{zym}}}}+\frac{\left[\mathrm{pCLLKMASP2_{zym}}\right]}{K_{\mathrm{m\ MASP1_{zym}}}^{\mathrm{MASP2_{zym}}}}+\frac{\left[\mathrm{pCLK1MASP1_{zym}}\right]}{K_{\mathrm{m\ MASP1_{zym}}}^{\mathrm{MASP1_{zym}}}}+\frac{\left[\mathrm{pCLK1MASP2_{zym}}\right]}{K_{\mathrm{m\ MASP1_{zym}}}^{\mathrm{MASP2_{zym}}}}\\+\frac{\left[\mathrm{pCLL1MASP1_{zym}}\right]}{K_{\mathrm{m\ MASP1_{zym}}}^{\mathrm{MASP1_{zym}}}}+\frac{\left[\mathrm{pCLL1MASP2_{zym}}\right]}{K_{\mathrm{m\ MASP1_{zym}}}^{\mathrm{MASP2_{zym}}}}+\frac{\left[\mathrm{pLFMASP1_{zym}}\right]}{K_{\mathrm{m\ MASP1_{zym}}}^{\mathrm{MASP1_{zym}}}}+\frac{\left[\mathrm{pLFMASP2_{zym}}\right]}{K_{\mathrm{m\ MASP1_{zym}}}^{\mathrm{MASP2_{zym}}}}\\+\frac{\left[\mathrm{pHFMASP1_{zym}}\right]}{K_{\mathrm{m\ MASP1_{zym}}}^{\mathrm{MASP1_{zym}}}}+\frac{\left[\mathrm{pHFMASP2_{zym}}\right]}{K_{\mathrm{m\ MASP1_{zym}}}^{\mathrm{MASP2_{zym}}}}+\frac{\left[\mathrm{pMFMASP1_{zym}}\right]}{K_{\mathrm{m\ MASP1_{zym}}}^{\mathrm{MASP1_{zym}}}}+\frac{\left[\mathrm{pMFMASP2_{zym}}\right]}{K_{\mathrm{m\ MASP1_{zym}}}^{\mathrm{MASP2_{zym}}}}\\+\frac{\left[\mathrm{pPTX3LFMASP1_{zym}}\right]}{K_{\mathrm{m\ MASP1_{zym}}}^{\mathrm{MASP1_{zym}}}}+\frac{\left[\mathrm{pPTX3LFMASP2_{zym}}\right]}{K_{\mathrm{m\ MASP1_{zym}}}^{\mathrm{MASP2_{zym}}}}\end{array}\right)}
$$

$$+\frac{k_{\text{cat MASP1}_{\text{zym}}}^{\text{MASP2}_{\text{zym}}}\left[\text{pPTX3LFMASP1}_{\text{zym}}\right]\left[\text{pMBL}_3\text{MASP2}_{\text{zym}}\right]}{K_{\text{m MASP1}_{\text{zym}}}^{\text{MASP2}_{\text{zym}}}\left(\begin{array}{l}1+\frac{\left[\text{pMBL}_3\text{MASP1}_{\text{zym}}\right]}{K_{\text{m MASP1}_{\text{zym}}}^{\text{MASP1}_{\text{zym}}}}+\frac{\left[\text{pMBL}_3\text{MASP2}_{\text{zym}}\right]}{K_{\text{m MASP1}_{\text{zym}}}^{\text{MASP2}_{\text{zym}}}}+\frac{\left[\text{pMBL}_4\text{MASP1}_{\text{zym}}\right]}{K_{\text{m MASP1}_{\text{zym}}}^{\text{MASP1}_{\text{zym}}}}+\frac{\left[\text{pMBL}_4\text{MASP2}_{\text{zym}}\right]}{K_{\text{m MASP1}_{\text{zym}}}^{\text{MASP2}_{\text{zym}}}}\\+\frac{\left[\text{pCLLKMASP1}_{\text{zym}}\right]}{K_{\text{m MASP1}_{\text{zym}}}^{\text{MASP1}_{\text{zym}}}}+\frac{\left[\text{pCLLKMASP2}_{\text{zym}}\right]}{K_{\text{m MASP1}_{\text{zym}}}^{\text{MASP2}_{\text{zym}}}}+\frac{\left[\text{pCLK1MASP1}_{\text{zym}}\right]}{K_{\text{m MASP1}_{\text{zym}}}^{\text{MASP1}_{\text{zym}}}}+\frac{\left[\text{pCLK1MASP2}_{\text{zym}}\right]}{K_{\text{m MASP1}_{\text{zym}}}^{\text{MASP2}_{\text{zym}}}}\\+\frac{\left[\text{pCLL1MASP1}_{\text{zym}}\right]}{K_{\text{m MASP1}_{\text{zym}}}^{\text{MASP1}_{\text{zym}}}}+\frac{\left[\text{pCLL1MASP2}_{\text{zym}}\right]}{K_{\text{m MASP1}_{\text{zym}}}^{\text{MASP2}_{\text{zym}}}}+\frac{\left[\text{pLFMASP1}_{\text{zym}}\right]}{K_{\text{m MASP1}_{\text{zym}}}^{\text{MASP1}_{\text{zym}}}}+\frac{\left[\text{pLFMASP2}_{\text{zym}}\right]}{K_{\text{m MASP1}_{\text{zym}}}^{\text{MASP2}_{\text{zym}}}}\\+\frac{\left[\text{pHFMASP1}_{\text{zym}}\right]}{K_{\text{m MASP1}_{\text{zym}}}^{\text{MASP1}_{\text{zym}}}}+\frac{\left[\text{pHFMASP2}_{\text{zym}}\right]}{K_{\text{m MASP1}_{\text{zym}}}^{\text{MASP2}_{\text{zym}}}}+\frac{\left[\text{pMFMASP1}_{\text{zym}}\right]}{K_{\text{m MASP1}_{\text{zym}}}^{\text{MASP1}_{\text{zym}}}}+\frac{\left[\text{pMFMASP2}_{\text{zym}}\right]}{K_{\text{m MASP1}_{\text{zym}}}^{\text{MASP2}_{\text{zym}}}}\\+\frac{\left[\text{pPTX3LFMASP1}_{\text{zym}}\right]}{K_{\text{m MASP1}_{\text{zym}}}^{\text{MASP1}_{\text{zym}}}}+\frac{\left[\text{pPTX3LFMASP2}_{\text{zym}}\right]}{K_{\text{m MASP1}_{\text{zym}}}^{\text{MASP2}_{\text{zym}}}}\end{array}\right)}$$

$$+\frac{k_{\text{cat MASP2}_{\text{zym}}}^{\text{MASP2}_{\text{zym}}}\left[\text{pMBL}_3\text{MASP2}_{\text{zym}}\right]\left[\text{pMBL}_3\text{MASP2}_{\text{zym}}\right]}{K_{\text{m MASP2}_{\text{zym}}}^{\text{MASP2}_{\text{zym}}}\left(\begin{array}{l}1+\frac{\left[\text{pMBL}_3\text{MASP2}_{\text{zym}}\right]}{K_{\text{m MASP2}_{\text{zym}}}^{\text{MASP2}_{\text{zym}}}}+\frac{\left[\text{pMBL}_4\text{MASP2}_{\text{zym}}\right]}{K_{\text{m MASP2}_{\text{zym}}}^{\text{MASP2}_{\text{zym}}}}+\frac{\left[\text{pCLLKMASP2}_{\text{zym}}\right]}{K_{\text{m MASP2}_{\text{zym}}}^{\text{MASP2}_{\text{zym}}}}+\frac{\left[\text{pCLK1MASP2}_{\text{zym}}\right]}{K_{\text{m MASP2}_{\text{zym}}}^{\text{MASP2}_{\text{zym}}}}\\+\frac{\left[\text{pCLL1MASP2}_{\text{zym}}\right]}{K_{\text{m MASP2}_{\text{zym}}}^{\text{MASP2}_{\text{zym}}}}+\frac{\left[\text{pLFMASP2}_{\text{zym}}\right]}{K_{\text{m MASP2}_{\text{zym}}}^{\text{MASP2}_{\text{zym}}}}+\frac{\left[\text{pHFMASP2}_{\text{zym}}\right]}{K_{\text{m MASP2}_{\text{zym}}}^{\text{MASP2}_{\text{zym}}}}+\frac{\left[\text{pMFMASP2}_{\text{zym}}\right]}{K_{\text{m MASP2}_{\text{zym}}}^{\text{MASP2}_{\text{zym}}}}\\+\frac{\left[\text{pPTX3LFMASP2}_{\text{zym}}\right]}{K_{\text{m MASP2}_{\text{zym}}}^{\text{MASP2}_{\text{zym}}}}\end{array}\right)}$$

$$+\frac{k_{\text{cat MASP2}_{\text{zym}}}^{\text{MASP2}_{\text{zym}}}\left[\text{pMBL}_4\text{MASP2}_{\text{zym}}\right]\left[\text{pMBL}_3\text{MASP2}_{\text{zym}}\right]}{K_{\text{m MASP2}_{\text{zym}}}^{\text{MASP2}_{\text{zym}}}\left(\begin{array}{l}1+\frac{\left[\text{pMBL}_3\text{MASP2}_{\text{zym}}\right]}{K_{\text{m MASP2}_{\text{zym}}}^{\text{MASP2}_{\text{zym}}}}+\frac{\left[\text{pMBL}_4\text{MASP2}_{\text{zym}}\right]}{K_{\text{m MASP2}_{\text{zym}}}^{\text{MASP2}_{\text{zym}}}}+\frac{\left[\text{pCLLKMASP2}_{\text{zym}}\right]}{K_{\text{m MASP2}_{\text{zym}}}^{\text{MASP2}_{\text{zym}}}}+\frac{\left[\text{pCLK1MASP2}_{\text{zym}}\right]}{K_{\text{m MASP2}_{\text{zym}}}^{\text{MASP2}_{\text{zym}}}}\\+\frac{\left[\text{pCLL1MASP2}_{\text{zym}}\right]}{K_{\text{m MASP2}_{\text{zym}}}^{\text{MASP2}_{\text{zym}}}}+\frac{\left[\text{pLFMASP2}_{\text{zym}}\right]}{K_{\text{m MASP2}_{\text{zym}}}^{\text{MASP2}_{\text{zym}}}}+\frac{\left[\text{pHFMASP2}_{\text{zym}}\right]}{K_{\text{m MASP2}_{\text{zym}}}^{\text{MASP2}_{\text{zym}}}}+\frac{\left[\text{pMFMASP2}_{\text{zym}}\right]}{K_{\text{m MASP2}_{\text{zym}}}^{\text{MASP2}_{\text{zym}}}}\\+\frac{\left[\text{pPTX3LFMASP2}_{\text{zym}}\right]}{K_{\text{m MASP2}_{\text{zym}}}^{\text{MASP2}_{\text{zym}}}}\end{array}\right)}$$

$$+\frac{k_{\text{cat MASP2}_{\text{zym}}}^{\text{MASP2}_{\text{zym}}}\left[\text{pCLLKMASP2}_{\text{zym}}\right]\left[\text{pMBL}_3\text{MASP2}_{\text{zym}}\right]}{K_{\text{m MASP2}_{\text{zym}}}^{\text{MASP2}_{\text{zym}}}\left(\begin{array}{l}1+\frac{\left[\text{pMBL}_3\text{MASP2}_{\text{zym}}\right]}{K_{\text{m MASP2}_{\text{zym}}}^{\text{MASP2}_{\text{zym}}}}+\frac{\left[\text{pMBL}_4\text{MASP2}_{\text{zym}}\right]}{K_{\text{m MASP2}_{\text{zym}}}^{\text{MASP2}_{\text{zym}}}}+\frac{\left[\text{pCLLKMASP2}_{\text{zym}}\right]}{K_{\text{m MASP2}_{\text{zym}}}^{\text{MASP2}_{\text{zym}}}}+\frac{\left[\text{pCLK1MASP2}_{\text{zym}}\right]}{K_{\text{m MASP2}_{\text{zym}}}^{\text{MASP2}_{\text{zym}}}}\\+\frac{\left[\text{pCLL1MASP2}_{\text{zym}}\right]}{K_{\text{m MASP2}_{\text{zym}}}^{\text{MASP2}_{\text{zym}}}}+\frac{\left[\text{pLFMASP2}_{\text{zym}}\right]}{K_{\text{m MASP2}_{\text{zym}}}^{\text{MASP2}_{\text{zym}}}}+\frac{\left[\text{pHFMASP2}_{\text{zym}}\right]}{K_{\text{m MASP2}_{\text{zym}}}^{\text{MASP2}_{\text{zym}}}}+\frac{\left[\text{pMFMASP2}_{\text{zym}}\right]}{K_{\text{m MASP2}_{\text{zym}}}^{\text{MASP2}_{\text{zym}}}}\\+\frac{\left[\text{pPTX3LFMASP2}_{\text{zym}}\right]}{K_{\text{m MASP2}_{\text{zym}}}^{\text{MASP2}_{\text{zym}}}}\end{array}\right)}$$

$$+\frac{k_{\text{cat MASP2}_{\text{zym}}}^{\text{MASP2}_{\text{zym}}}\left[\text{pCLK1MASP2}_{\text{zym}}\right]\left[\text{pMBL}_3\text{MASP2}_{\text{zym}}\right]}{K_{\text{m MASP2}_{\text{zym}}}^{\text{MASP2}_{\text{zym}}}\left(\begin{array}{l}1+\frac{\left[\text{pMBL}_3\text{MASP2}_{\text{zym}}\right]}{K_{\text{m MASP2}_{\text{zym}}}^{\text{MASP2}_{\text{zym}}}}+\frac{\left[\text{pMBL}_4\text{MASP2}_{\text{zym}}\right]}{K_{\text{m MASP2}_{\text{zym}}}^{\text{MASP2}_{\text{zym}}}}+\frac{\left[\text{pCLLKMASP2}_{\text{zym}}\right]}{K_{\text{m MASP2}_{\text{zym}}}^{\text{MASP2}_{\text{zym}}}}+\frac{\left[\text{pCLK1MASP2}_{\text{zym}}\right]}{K_{\text{m MASP2}_{\text{zym}}}^{\text{MASP2}_{\text{zym}}}}\\+\frac{\left[\text{pCLL1MASP2}_{\text{zym}}\right]}{K_{\text{m MASP2}_{\text{zym}}}^{\text{MASP2}_{\text{zym}}}}+\frac{\left[\text{pLFMASP2}_{\text{zym}}\right]}{K_{\text{m MASP2}_{\text{zym}}}^{\text{MASP2}_{\text{zym}}}}+\frac{\left[\text{pHFMASP2}_{\text{zym}}\right]}{K_{\text{m MASP2}_{\text{zym}}}^{\text{MASP2}_{\text{zym}}}}+\frac{\left[\text{pMFMASP2}_{\text{zym}}\right]}{K_{\text{m MASP2}_{\text{zym}}}^{\text{MASP2}_{\text{zym}}}}\\+\frac{\left[\text{pPTX3LFMASP2}_{\text{zym}}\right]}{K_{\text{m MASP2}_{\text{zym}}}^{\text{MASP2}_{\text{zym}}}}\end{array}\right)}$$

$$+\frac{k_{\text{cat MASP2}_{\text{zym}}}^{\text{MASP2}_{\text{zym}}}\left[\text{pCLL1MASP2}_{\text{zym}}\right]\left[\text{pMBL}_3\text{MASP2}_{\text{zym}}\right]}{K_{\text{m MASP2}_{\text{zym}}}^{\text{MASP2}_{\text{zym}}}\left(\begin{array}{l}1+\frac{\left[\text{pMBL}_3\text{MASP2}_{\text{zym}}\right]}{K_{\text{m MASP2}_{\text{zym}}}^{\text{MASP2}_{\text{zym}}}}+\frac{\left[\text{pMBL}_4\text{MASP2}_{\text{zym}}\right]}{K_{\text{m MASP2}_{\text{zym}}}^{\text{MASP2}_{\text{zym}}}}+\frac{\left[\text{pCLLKMASP2}_{\text{zym}}\right]}{K_{\text{m MASP2}_{\text{zym}}}^{\text{MASP2}_{\text{zym}}}}+\frac{\left[\text{pCLK1MASP2}_{\text{zym}}\right]}{K_{\text{m MASP2}_{\text{zym}}}^{\text{MASP2}_{\text{zym}}}}\\+\frac{\left[\text{pCLL1MASP2}_{\text{zym}}\right]}{K_{\text{m MASP2}_{\text{zym}}}^{\text{MASP2}_{\text{zym}}}}+\frac{\left[\text{pLFMASP2}_{\text{zym}}\right]}{K_{\text{m MASP2}_{\text{zym}}}^{\text{MASP2}_{\text{zym}}}}+\frac{\left[\text{pHFMASP2}_{\text{zym}}\right]}{K_{\text{m MASP2}_{\text{zym}}}^{\text{MASP2}_{\text{zym}}}}+\frac{\left[\text{pMFMASP2}_{\text{zym}}\right]}{K_{\text{m MASP2}_{\text{zym}}}^{\text{MASP2}_{\text{zym}}}}\\+\frac{\left[\text{pPTX3LFMASP2}_{\text{zym}}\right]}{K_{\text{m MASP2}_{\text{zym}}}^{\text{MASP2}_{\text{zym}}}}\end{array}\right)}$$

$$+\frac{k_{\text{cat MASP2}_{\text{zym}}}^{\text{MASP2}_{\text{zym}}}\left[\text{pLFMASP2}_{\text{zym}}\right]\left[\text{pMBL}_3\text{MASP2}_{\text{zym}}\right]}{K_{\text{m MASP2}_{\text{zym}}}^{\text{MASP2}_{\text{zym}}}\left(\begin{array}{l}1+\frac{\left[\text{pMBL}_3\text{MASP2}_{\text{zym}}\right]}{K_{\text{m MASP2}_{\text{zym}}}^{\text{MASP2}_{\text{zym}}}}+\frac{\left[\text{pMBL}_4\text{MASP2}_{\text{zym}}\right]}{K_{\text{m MASP2}_{\text{zym}}}^{\text{MASP2}_{\text{zym}}}}+\frac{\left[\text{pCLLKMASP2}_{\text{zym}}\right]}{K_{\text{m MASP2}_{\text{zym}}}^{\text{MASP2}_{\text{zym}}}}+\frac{\left[\text{pCLK1MASP2}_{\text{zym}}\right]}{K_{\text{m MASP2}_{\text{zym}}}^{\text{MASP2}_{\text{zym}}}}\\+\frac{\left[\text{pCLL1MASP2}_{\text{zym}}\right]}{K_{\text{m MASP2}_{\text{zym}}}^{\text{MASP2}_{\text{zym}}}}+\frac{\left[\text{pLFMASP2}_{\text{zym}}\right]}{K_{\text{m MASP2}_{\text{zym}}}^{\text{MASP2}_{\text{zym}}}}+\frac{\left[\text{pHFMASP2}_{\text{zym}}\right]}{K_{\text{m MASP2}_{\text{zym}}}^{\text{MASP2}_{\text{zym}}}}+\frac{\left[\text{pMFMASP2}_{\text{zym}}\right]}{K_{\text{m MASP2}_{\text{zym}}}^{\text{MASP2}_{\text{zym}}}}\\+\frac{\left[\text{pPTX3LFMASP2}_{\text{zym}}\right]}{K_{\text{m MASP2}_{\text{zym}}}^{\text{MASP2}_{\text{zym}}}}\end{array}\right)}$$

$$+\frac{k_{\text{cat MASP2}_{\text{zym}}}^{\text{MASP2}_{\text{zym}}}\left[\text{pHFMASP2}_{\text{zym}}\right]\left[\text{pMBL}_3\text{MASP2}_{\text{zym}}\right]}{K_{\text{m MASP2}_{\text{zym}}}^{\text{MASP2}_{\text{zym}}}\left(\begin{array}{l}1+\frac{\left[\text{pMBL}_3\text{MASP2}_{\text{zym}}\right]}{K_{\text{m MASP2}_{\text{zym}}}^{\text{MASP2}_{\text{zym}}}}+\frac{\left[\text{pMBL}_4\text{MASP2}_{\text{zym}}\right]}{K_{\text{m MASP2}_{\text{zym}}}^{\text{MASP2}_{\text{zym}}}}+\frac{\left[\text{pCLLKMASP2}_{\text{zym}}\right]}{K_{\text{m MASP2}_{\text{zym}}}^{\text{MASP2}_{\text{zym}}}}+\frac{\left[\text{pCLK1MASP2}_{\text{zym}}\right]}{K_{\text{m MASP2}_{\text{zym}}}^{\text{MASP2}_{\text{zym}}}}\\+\frac{\left[\text{pCLL1MASP2}_{\text{zym}}\right]}{K_{\text{m MASP2}_{\text{zym}}}^{\text{MASP2}_{\text{zym}}}}+\frac{\left[\text{pLFMASP2}_{\text{zym}}\right]}{K_{\text{m MASP2}_{\text{zym}}}^{\text{MASP2}_{\text{zym}}}}+\frac{\left[\text{pHFMASP2}_{\text{zym}}\right]}{K_{\text{m MASP2}_{\text{zym}}}^{\text{MASP2}_{\text{zym}}}}+\frac{\left[\text{pMFMASP2}_{\text{zym}}\right]}{K_{\text{m MASP2}_{\text{zym}}}^{\text{MASP2}_{\text{zym}}}}\\+\frac{\left[\text{pPTX3LFMASP2}_{\text{zym}}\right]}{K_{\text{m MASP2}_{\text{zym}}}^{\text{MASP2}_{\text{zym}}}}\end{array}\right)}$$

$$+\frac{k_{\text{cat MASP2}_{\text{zym}}}^{\text{MASP2}_{\text{zym}}}\left[\text{pMFMASP2}_{\text{zym}}\right]\left[\text{pMBL}_3\text{MASP2}_{\text{zym}}\right]}{K_{\text{m MASP2}_{\text{zym}}}^{\text{MASP2}_{\text{zym}}}\left(\begin{array}{l}1+\frac{\left[\text{pMBL}_3\text{MASP2}_{\text{zym}}\right]}{K_{\text{m MASP2}_{\text{zym}}}^{\text{MASP2}_{\text{zym}}}}+\frac{\left[\text{pMBL}_4\text{MASP2}_{\text{zym}}\right]}{K_{\text{m MASP2}_{\text{zym}}}^{\text{MASP2}_{\text{zym}}}}+\frac{\left[\text{pCLLKMASP2}_{\text{zym}}\right]}{K_{\text{m MASP2}_{\text{zym}}}^{\text{MASP2}_{\text{zym}}}}+\frac{\left[\text{pCLK1MASP2}_{\text{zym}}\right]}{K_{\text{m MASP2}_{\text{zym}}}^{\text{MASP2}_{\text{zym}}}}\\+\frac{\left[\text{pCLL1MASP2}_{\text{zym}}\right]}{K_{\text{m MASP2}_{\text{zym}}}^{\text{MASP2}_{\text{zym}}}}+\frac{\left[\text{pLFMASP2}_{\text{zym}}\right]}{K_{\text{m MASP2}_{\text{zym}}}^{\text{MASP2}_{\text{zym}}}}+\frac{\left[\text{pHFMASP2}_{\text{zym}}\right]}{K_{\text{m MASP2}_{\text{zym}}}^{\text{MASP2}_{\text{zym}}}}+\frac{\left[\text{pMFMASP2}_{\text{zym}}\right]}{K_{\text{m MASP2}_{\text{zym}}}^{\text{MASP2}_{\text{zym}}}}\\+\frac{\left[\text{pPTX3LFMASP2}_{\text{zym}}\right]}{K_{\text{m MASP2}_{\text{zym}}}^{\text{MASP2}_{\text{zym}}}}\end{array}\right)}$$

$$+\frac{k_{\text{cat MASP2}_{\text{zym}}}^{\text{MASP2}_{\text{zym}}}\left[\text{pPTX3LFMASP2}_{\text{zym}}\right]\left[\text{pMBL}_3\text{MASP2}_{\text{zym}}\right]}{K_{\text{m MASP2}_{\text{zym}}}^{\text{MASP2}_{\text{zym}}}\left(\begin{array}{l}1+\frac{\left[\text{pMBL}_3\text{MASP2}_{\text{zym}}\right]}{K_{\text{m MASP2}_{\text{zym}}}^{\text{MASP2}_{\text{zym}}}}+\frac{\left[\text{pMBL}_4\text{MASP2}_{\text{zym}}\right]}{K_{\text{m MASP2}_{\text{zym}}}^{\text{MASP2}_{\text{zym}}}}+\frac{\left[\text{pCLLKMASP2}_{\text{zym}}\right]}{K_{\text{m MASP2}_{\text{zym}}}^{\text{MASP2}_{\text{zym}}}}+\frac{\left[\text{pCLK1MASP2}_{\text{zym}}\right]}{K_{\text{m MASP2}_{\text{zym}}}^{\text{MASP2}_{\text{zym}}}}\\+\frac{\left[\text{pCLL1MASP2}_{\text{zym}}\right]}{K_{\text{m MASP2}_{\text{zym}}}^{\text{MASP2}_{\text{zym}}}}+\frac{\left[\text{pLFMASP2}_{\text{zym}}\right]}{K_{\text{m MASP2}_{\text{zym}}}^{\text{MASP2}_{\text{zym}}}}+\frac{\left[\text{pHFMASP2}_{\text{zym}}\right]}{K_{\text{m MASP2}_{\text{zym}}}^{\text{MASP2}_{\text{zym}}}}+\frac{\left[\text{pMFMASP2}_{\text{zym}}\right]}{K_{\text{m MASP2}_{\text{zym}}}^{\text{MASP2}_{\text{zym}}}}\\+\frac{\left[\text{pPTX3LFMASP2}_{\text{zym}}\right]}{K_{\text{m MASP2}_{\text{zym}}}^{\text{MASP2}_{\text{zym}}}}\end{array}\right)}$$

$$
+\frac{k_{\text{cat MASP1}^*}^{\text{MASP2}_{\text{zym}}}\left[\text{pMBL}_3\text{MASP1}^*\right]\left[\text{pMBL}_3\text{MASP2}_{\text{zym}}\right]}{K_{\text{m MASP1}^*}^{\text{MASP2}_{\text{zym}}}\left(\begin{array}{l}
1+\frac{[\text{C4}]}{K_{\text{m MASP1}^*}^{\text{C4}}}+\frac{[\text{fC4bC2}]}{K_{\text{m MASP1}^*}^{\text{C2}}}+\frac{[\text{fC4bC4bC2}]}{K_{\text{m MASP1}^*}^{\text{C2}}}+\frac{[\text{fC3bC4bC2}]}{K_{\text{m MASP1}^*}^{\text{C2}}}+\frac{[\text{pC4bC2}]}{K_{\text{m MASP1}^*}^{\text{C2}}}+\frac{[\text{pC4bC4bC2}]}{K_{\text{m MASP1}^*}^{\text{C2}}}\\
+\frac{[\text{pC3bC4bC2}]}{K_{\text{m MASP1}^*}^{\text{C2}}}+\frac{[\text{pMBL}_3\text{MASP1}_{\text{zym}}]}{K_{\text{m MASP1}^*}^{\text{MASP1}_{\text{zym}}}}+\frac{[\text{pMBL}_3\text{MASP2}_{\text{zym}}]}{K_{\text{m MASP1}^*}^{\text{MASP2}_{\text{zym}}}}+\frac{[\text{pMBL}_3\text{MASP3}_{\text{zym}}]}{K_{\text{m MASP1}^*}^{\text{MASP3}_{\text{zym}}}}\\
+\frac{[\text{pMBL}_4\text{MASP1}_{\text{zym}}]}{K_{\text{m MASP1}^*}^{\text{MASP1}_{\text{zym}}}}+\frac{[\text{pMBL}_4\text{MASP2}_{\text{zym}}]}{K_{\text{m MASP1}^*}^{\text{MASP2}_{\text{zym}}}}+\frac{[\text{pMBL}_4\text{MASP3}_{\text{zym}}]}{K_{\text{m MASP1}^*}^{\text{MASP3}_{\text{zym}}}}+\frac{[\text{pCLLKMASP1}_{\text{zym}}]}{K_{\text{m MASP1}^*}^{\text{MASP1}_{\text{zym}}}}\\
+\frac{[\text{pCLLKMASP2}_{\text{zym}}]}{K_{\text{m MASP1}^*}^{\text{MASP2}_{\text{zym}}}}+\frac{[\text{pCLLKMASP3}_{\text{zym}}]}{K_{\text{m MASP1}^*}^{\text{MASP3}_{\text{zym}}}}+\frac{[\text{pCLK1MASP1}_{\text{zym}}]}{K_{\text{m MASP1}^*}^{\text{MASP1}_{\text{zym}}}}+\frac{[\text{pCLK1MASP2}_{\text{zym}}]}{K_{\text{m MASP1}^*}^{\text{MASP2}_{\text{zym}}}}\\
+\frac{[\text{pCLK1MASP3}_{\text{zym}}]}{K_{\text{m MASP1}^*}^{\text{MASP3}_{\text{zym}}}}+\frac{[\text{pCLL1MASP1}_{\text{zym}}]}{K_{\text{m MASP1}^*}^{\text{MASP1}_{\text{zym}}}}+\frac{[\text{pCLL1MASP2}_{\text{zym}}]}{K_{\text{m MASP1}^*}^{\text{MASP2}_{\text{zym}}}}+\frac{[\text{pCLL1MASP3}_{\text{zym}}]}{K_{\text{m MASP1}^*}^{\text{MASP3}_{\text{zym}}}}\\
+\frac{[\text{pLFMASP1}_{\text{zym}}]}{K_{\text{m MASP1}^*}^{\text{MASP1}_{\text{zym}}}}+\frac{[\text{pLFMASP2}_{\text{zym}}]}{K_{\text{m MASP1}^*}^{\text{MASP2}_{\text{zym}}}}+\frac{[\text{pLFMASP3}_{\text{zym}}]}{K_{\text{m MASP1}^*}^{\text{MASP3}_{\text{zym}}}}+\frac{[\text{pHFMASP1}_{\text{zym}}]}{K_{\text{m MASP1}^*}^{\text{MASP1}_{\text{zym}}}}+\frac{[\text{pHFMASP2}_{\text{zym}}]}{K_{\text{m MASP1}^*}^{\text{MASP2}_{\text{zym}}}}\\
+\frac{[\text{pHFMASP3}_{\text{zym}}]}{K_{\text{m MASP1}^*}^{\text{MASP3}_{\text{zym}}}}+\frac{[\text{pMFMASP1}_{\text{zym}}]}{K_{\text{m MASP1}^*}^{\text{MASP1}_{\text{zym}}}}+\frac{[\text{pMFMASP2}_{\text{zym}}]}{K_{\text{m MASP1}^*}^{\text{MASP2}_{\text{zym}}}}+\frac{[\text{pMFMASP3}_{\text{zym}}]}{K_{\text{m MASP1}^*}^{\text{MASP3}_{\text{zym}}}}\\
+\frac{[\text{pPTX3LFMASP1}_{\text{zym}}]}{K_{\text{m MASP1}^*}^{\text{MASP1}_{\text{zym}}}}+\frac{[\text{pPXT3LFMASP2}_{\text{zym}}]}{K_{\text{m MASP1}^*}^{\text{MASP2}_{\text{zym}}}}+\frac{[\text{pPTX3LFMASP3}_{\text{zym}}]}{K_{\text{m MASP1}^*}^{\text{MASP3}_{\text{zym}}}}
\end{array}\right)}
$$

$$
+\frac{k_{\mathrm{cat\ MASP1^*}}^{\mathrm{MASP2_{zym}}}\left[\mathrm{pMBL_4MASP1^*}\right]\left[\mathrm{pMBL_3MASP2_{zym}}\right]}{K_{\mathrm{m\ MASP1^*}}^{\mathrm{MASP2_{zym}}}\left(\begin{array}{l}
1+\frac{\left[\mathrm{C4}\right]}{K_{\mathrm{m\ MASP1^*}}^{\mathrm{C4}}}+\frac{\left[\mathrm{fC4bC2}\right]}{K_{\mathrm{m\ MASP1^*}}^{\mathrm{C2}}}+\frac{\left[\mathrm{fC4bC4bC2}\right]}{K_{\mathrm{m\ MASP1^*}}^{\mathrm{C2}}}+\frac{\left[\mathrm{fC3bC4bC2}\right]}{K_{\mathrm{m\ MASP1^*}}^{\mathrm{C2}}}+\frac{\left[\mathrm{pC4bC2}\right]}{K_{\mathrm{m\ MASP1^*}}^{\mathrm{C2}}}+\frac{\left[\mathrm{pC4bC4bC2}\right]}{K_{\mathrm{m\ MASP1^*}}^{\mathrm{C2}}}\\
+\frac{\left[\mathrm{pC3bC4bC2}\right]}{K_{\mathrm{m\ MASP1^*}}^{\mathrm{C2}}}+\frac{\left[\mathrm{pMBL_3MASP1_{zym}}\right]}{K_{\mathrm{m\ MASP1^*}}^{\mathrm{MASP1_{zym}}}}+\frac{\left[\mathrm{pMBL_3MASP2_{zym}}\right]}{K_{\mathrm{m\ MASP1^*}}^{\mathrm{MASP2_{zym}}}}+\frac{\left[\mathrm{pMBL_3MASP3_{zym}}\right]}{K_{\mathrm{m\ MASP1^*}}^{\mathrm{MASP3_{zym}}}}\\
+\frac{\left[\mathrm{pMBL_4MASP1_{zym}}\right]}{K_{\mathrm{m\ MASP1^*}}^{\mathrm{MASP1_{zym}}}}+\frac{\left[\mathrm{pMBL_4MASP2_{zym}}\right]}{K_{\mathrm{m\ MASP1^*}}^{\mathrm{MASP2_{zym}}}}+\frac{\left[\mathrm{pMBL_4MASP3_{zym}}\right]}{K_{\mathrm{m\ MASP1^*}}^{\mathrm{MASP3_{zym}}}}+\frac{\left[\mathrm{pCLLKMASP1_{zym}}\right]}{K_{\mathrm{m\ MASP1^*}}^{\mathrm{MASP1_{zym}}}}\\
+\frac{\left[\mathrm{pCLLKMASP2_{zym}}\right]}{K_{\mathrm{m\ MASP1^*}}^{\mathrm{MASP2_{zym}}}}+\frac{\left[\mathrm{pCLLKMASP3_{zym}}\right]}{K_{\mathrm{m\ MASP1^*}}^{\mathrm{MASP3_{zym}}}}+\frac{\left[\mathrm{pCLK1MASP1_{zym}}\right]}{K_{\mathrm{m\ MASP1^*}}^{\mathrm{MASP1_{zym}}}}+\frac{\left[\mathrm{pCLK1MASP2_{zym}}\right]}{K_{\mathrm{m\ MASP1^*}}^{\mathrm{MASP2_{zym}}}}\\
+\frac{\left[\mathrm{pCLK1MASP3_{zym}}\right]}{K_{\mathrm{m\ MASP1^*}}^{\mathrm{MASP3_{zym}}}}+\frac{\left[\mathrm{pCLL1MASP1_{zym}}\right]}{K_{\mathrm{m\ MASP1^*}}^{\mathrm{MASP1_{zym}}}}+\frac{\left[\mathrm{pCLL1MASP2_{zym}}\right]}{K_{\mathrm{m\ MASP1^*}}^{\mathrm{MASP2_{zym}}}}+\frac{\left[\mathrm{pCLL1MASP3_{zym}}\right]}{K_{\mathrm{m\ MASP1^*}}^{\mathrm{MASP3_{zym}}}}\\
+\frac{\left[\mathrm{pLFMASP1_{zym}}\right]}{K_{\mathrm{m\ MASP1^*}}^{\mathrm{MASP1_{zym}}}}+\frac{\left[\mathrm{pLFMASP2_{zym}}\right]}{K_{\mathrm{m\ MASP1^*}}^{\mathrm{MASP2_{zym}}}}+\frac{\left[\mathrm{pLFMASP3_{zym}}\right]}{K_{\mathrm{m\ MASP1^*}}^{\mathrm{MASP3_{zym}}}}+\frac{\left[\mathrm{pHFMASP1_{zym}}\right]}{K_{\mathrm{m\ MASP1^*}}^{\mathrm{MASP1_{zym}}}}+\frac{\left[\mathrm{pHFMASP2_{zym}}\right]}{K_{\mathrm{m\ MASP1^*}}^{\mathrm{MASP2_{zym}}}}\\
+\frac{\left[\mathrm{pHFMASP3_{zym}}\right]}{K_{\mathrm{m\ MASP1^*}}^{\mathrm{MASP3_{zym}}}}+\frac{\left[\mathrm{pMFMASP1_{zym}}\right]}{K_{\mathrm{m\ MASP1^*}}^{\mathrm{MASP1_{zym}}}}+\frac{\left[\mathrm{pMFMASP2_{zym}}\right]}{K_{\mathrm{m\ MASP1^*}}^{\mathrm{MASP2_{zym}}}}+\frac{\left[\mathrm{pMFMASP3_{zym}}\right]}{K_{\mathrm{m\ MASP1^*}}^{\mathrm{MASP3_{zym}}}}\\
+\frac{\left[\mathrm{pPTX3LFMASP1_{zym}}\right]}{K_{\mathrm{m\ MASP1^*}}^{\mathrm{MASP1_{zym}}}}+\frac{\left[\mathrm{pPXT3LFMASP2_{zym}}\right]}{K_{\mathrm{m\ MASP1^*}}^{\mathrm{MASP2_{zym}}}}+\frac{\left[\mathrm{pPTX3LFMASP3_{zym}}\right]}{K_{\mathrm{m\ MASP1^*}}^{\mathrm{MASP3_{zym}}}}
\end{array}\right)}
$$

$$
+\frac{k_{\text{cat MASP1}^*}^{\text{MASP2}_{\text{zym}}}\left[\text{pCLLKMASP1}^*\right]\left[\text{pMBL}_3\text{MASP2}_{\text{zym}}\right]}{K_{\text{m MASP1}^*}^{\text{MASP2}_{\text{zym}}}\left(\begin{array}{l}
1+\frac{[\text{C4}]}{K_{\text{m MASP1}^*}^{\text{C4}}}+\frac{[\text{fC4bC2}]}{K_{\text{m MASP1}^*}^{\text{C2}}}+\frac{[\text{fC4bC4bC2}]}{K_{\text{m MASP1}^*}^{\text{C2}}}+\frac{[\text{fC3bC4bC2}]}{K_{\text{m MASP1}^*}^{\text{C2}}}+\frac{[\text{pC4bC2}]}{K_{\text{m MASP1}^*}^{\text{C2}}}+\frac{[\text{pC4bC4bC2}]}{K_{\text{m MASP1}^*}^{\text{C2}}} \\
+\frac{[\text{pC3bC4bC2}]}{K_{\text{m MASP1}^*}^{\text{C2}}}+\frac{\left[\text{pMBL}_3\text{MASP1}_{\text{zym}}\right]}{K_{\text{m MASP1}^*}^{\text{MASP1}_{\text{zym}}}}+\frac{\left[\text{pMBL}_3\text{MASP2}_{\text{zym}}\right]}{K_{\text{m MASP1}^*}^{\text{MASP2}_{\text{zym}}}}+\frac{\left[\text{pMBL}_3\text{MASP3}_{\text{zym}}\right]}{K_{\text{m MASP1}^*}^{\text{MASP3}_{\text{zym}}}} \\
+\frac{\left[\text{pMBL}_4\text{MASP1}_{\text{zym}}\right]}{K_{\text{m MASP1}^*}^{\text{MASP1}_{\text{zym}}}}+\frac{\left[\text{pMBL}_4\text{MASP2}_{\text{zym}}\right]}{K_{\text{m MASP1}^*}^{\text{MASP2}_{\text{zym}}}}+\frac{\left[\text{pMBL}_4\text{MASP3}_{\text{zym}}\right]}{K_{\text{m MASP1}^*}^{\text{MASP3}_{\text{zym}}}}+\frac{\left[\text{pCLLKMASP1}_{\text{zym}}\right]}{K_{\text{m MASP1}^*}^{\text{MASP1}_{\text{zym}}}} \\
+\frac{\left[\text{pCLLKMASP2}_{\text{zym}}\right]}{K_{\text{m MASP1}^*}^{\text{MASP2}_{\text{zym}}}}+\frac{\left[\text{pCLLKMASP3}_{\text{zym}}\right]}{K_{\text{m MASP1}^*}^{\text{MASP3}_{\text{zym}}}}+\frac{\left[\text{pCLK1MASP1}_{\text{zym}}\right]}{K_{\text{m MASP1}^*}^{\text{MASP1}_{\text{zym}}}}+\frac{\left[\text{pCLK1MASP2}_{\text{zym}}\right]}{K_{\text{m MASP1}^*}^{\text{MASP2}_{\text{zym}}}} \\
+\frac{\left[\text{pCLK1MASP3}_{\text{zym}}\right]}{K_{\text{m MASP1}^*}^{\text{MASP3}_{\text{zym}}}}+\frac{\left[\text{pCLL1MASP1}_{\text{zym}}\right]}{K_{\text{m MASP1}^*}^{\text{MASP1}_{\text{zym}}}}+\frac{\left[\text{pCLL1MASP2}_{\text{zym}}\right]}{K_{\text{m MASP1}^*}^{\text{MASP2}_{\text{zym}}}}+\frac{\left[\text{pCLL1MASP3}_{\text{zym}}\right]}{K_{\text{m MASP1}^*}^{\text{MASP3}_{\text{zym}}}} \\
+\frac{\left[\text{pLFMASP1}_{\text{zym}}\right]}{K_{\text{m MASP1}^*}^{\text{MASP1}_{\text{zym}}}}+\frac{\left[\text{pLFMASP2}_{\text{zym}}\right]}{K_{\text{m MASP1}^*}^{\text{MASP2}_{\text{zym}}}}+\frac{\left[\text{pLFMASP3}_{\text{zym}}\right]}{K_{\text{m MASP1}^*}^{\text{MASP3}_{\text{zym}}}}+\frac{\left[\text{pHFMASP1}_{\text{zym}}\right]}{K_{\text{m MASP1}^*}^{\text{MASP1}_{\text{zym}}}}+\frac{\left[\text{pHFMASP2}_{\text{zym}}\right]}{K_{\text{m MASP1}^*}^{\text{MASP2}_{\text{zym}}}} \\
+\frac{\left[\text{pHFMASP3}_{\text{zym}}\right]}{K_{\text{m MASP1}^*}^{\text{MASP3}_{\text{zym}}}}+\frac{\left[\text{pMFMASP1}_{\text{zym}}\right]}{K_{\text{m MASP1}^*}^{\text{MASP1}_{\text{zym}}}}+\frac{\left[\text{pMFMASP2}_{\text{zym}}\right]}{K_{\text{m MASP1}^*}^{\text{MASP2}_{\text{zym}}}}+\frac{\left[\text{pMFMASP3}_{\text{zym}}\right]}{K_{\text{m MASP1}^*}^{\text{MASP3}_{\text{zym}}}} \\
+\frac{\left[\text{pPTX3LFMASP1}_{\text{zym}}\right]}{K_{\text{m MASP1}^*}^{\text{MASP1}_{\text{zym}}}}+\frac{\left[\text{pPXT3LFMASP2}_{\text{zym}}\right]}{K_{\text{m MASP1}^*}^{\text{MASP2}_{\text{zym}}}}+\frac{\left[\text{pPTX3LFMASP3}_{\text{zym}}\right]}{K_{\text{m MASP1}^*}^{\text{MASP3}_{\text{zym}}}}
\end{array}\right)}
$$

$$
+\frac{k_{\mathrm{cat\ MASP1^*}}^{\mathrm{MASP2_{zym}}}\left[\mathrm{pCLL1MASP1^*}\right]\left[\mathrm{pMBL_3MASP2_{zym}}\right]}{K_{\mathrm{m\ MASP1^*}}^{\mathrm{MASP2_{zym}}}\left(\begin{aligned}
&1+\frac{[\mathrm{C4}]}{K_{\mathrm{m\ MASP1^*}}^{\mathrm{C4}}}+\frac{[\mathrm{fC4bC2}]}{K_{\mathrm{m\ MASP1^*}}^{\mathrm{C2}}}+\frac{[\mathrm{fC4bC4bC2}]}{K_{\mathrm{m\ MASP1^*}}^{\mathrm{C2}}}+\frac{[\mathrm{fC3bC4bC2}]}{K_{\mathrm{m\ MASP1^*}}^{\mathrm{C2}}}+\frac{[\mathrm{pC4bC2}]}{K_{\mathrm{m\ MASP1^*}}^{\mathrm{C2}}}+\frac{[\mathrm{pC4bC4bC2}]}{K_{\mathrm{m\ MASP1^*}}^{\mathrm{C2}}}\\
&+\frac{[\mathrm{pC3bC4bC2}]}{K_{\mathrm{m\ MASP1^*}}^{\mathrm{C2}}}+\frac{[\mathrm{pMBL_3MASP1_{zym}}]}{K_{\mathrm{m\ MASP1^*}}^{\mathrm{MASP1_{zym}}}}+\frac{[\mathrm{pMBL_3MASP2_{zym}}]}{K_{\mathrm{m\ MASP1^*}}^{\mathrm{MASP2_{zym}}}}+\frac{[\mathrm{pMBL_3MASP3_{zym}}]}{K_{\mathrm{m\ MASP1^*}}^{\mathrm{MASP3_{zym}}}}\\
&+\frac{[\mathrm{pMBL_4MASP1_{zym}}]}{K_{\mathrm{m\ MASP1^*}}^{\mathrm{MASP1_{zym}}}}+\frac{[\mathrm{pMBL_4MASP2_{zym}}]}{K_{\mathrm{m\ MASP1^*}}^{\mathrm{MASP2_{zym}}}}+\frac{[\mathrm{pMBL_4MASP3_{zym}}]}{K_{\mathrm{m\ MASP1^*}}^{\mathrm{MASP3_{zym}}}}+\frac{[\mathrm{pCLLKMASP1_{zym}}]}{K_{\mathrm{m\ MASP1^*}}^{\mathrm{MASP1_{zym}}}}\\
&+\frac{[\mathrm{pCLLKMASP2_{zym}}]}{K_{\mathrm{m\ MASP1^*}}^{\mathrm{MASP2_{zym}}}}+\frac{[\mathrm{pCLLKMASP3_{zym}}]}{K_{\mathrm{m\ MASP1^*}}^{\mathrm{MASP3_{zym}}}}+\frac{[\mathrm{pCLK1MASP1_{zym}}]}{K_{\mathrm{m\ MASP1^*}}^{\mathrm{MASP1_{zym}}}}+\frac{[\mathrm{pCLK1MASP2_{zym}}]}{K_{\mathrm{m\ MASP1^*}}^{\mathrm{MASP2_{zym}}}}\\
&+\frac{[\mathrm{pCLK1MASP3_{zym}}]}{K_{\mathrm{m\ MASP1^*}}^{\mathrm{MASP3_{zym}}}}+\frac{[\mathrm{pCLL1MASP1_{zym}}]}{K_{\mathrm{m\ MASP1^*}}^{\mathrm{MASP1_{zym}}}}+\frac{[\mathrm{pCLL1MASP2_{zym}}]}{K_{\mathrm{m\ MASP1^*}}^{\mathrm{MASP2_{zym}}}}+\frac{[\mathrm{pCLL1MASP3_{zym}}]}{K_{\mathrm{m\ MASP1^*}}^{\mathrm{MASP3_{zym}}}}\\
&+\frac{[\mathrm{pLFMASP1_{zym}}]}{K_{\mathrm{m\ MASP1^*}}^{\mathrm{MASP1_{zym}}}}+\frac{[\mathrm{pLFMASP2_{zym}}]}{K_{\mathrm{m\ MASP1^*}}^{\mathrm{MASP2_{zym}}}}+\frac{[\mathrm{pLFMASP3_{zym}}]}{K_{\mathrm{m\ MASP1^*}}^{\mathrm{MASP3_{zym}}}}+\frac{[\mathrm{pHFMASP1_{zym}}]}{K_{\mathrm{m\ MASP1^*}}^{\mathrm{MASP1_{zym}}}}+\frac{[\mathrm{pHFMASP2_{zym}}]}{K_{\mathrm{m\ MASP1^*}}^{\mathrm{MASP2_{zym}}}}\\
&+\frac{[\mathrm{pHFMASP3_{zym}}]}{K_{\mathrm{m\ MASP1^*}}^{\mathrm{MASP3_{zym}}}}+\frac{[\mathrm{pMFMASP1_{zym}}]}{K_{\mathrm{m\ MASP1^*}}^{\mathrm{MASP1_{zym}}}}+\frac{[\mathrm{pMFMASP2_{zym}}]}{K_{\mathrm{m\ MASP1^*}}^{\mathrm{MASP2_{zym}}}}+\frac{[\mathrm{pMFMASP3_{zym}}]}{K_{\mathrm{m\ MASP1^*}}^{\mathrm{MASP3_{zym}}}}\\
&+\frac{[\mathrm{pPTX3LFMASP1_{zym}}]}{K_{\mathrm{m\ MASP1^*}}^{\mathrm{MASP1_{zym}}}}+\frac{[\mathrm{pPXT3LFMASP2_{zym}}]}{K_{\mathrm{m\ MASP1^*}}^{\mathrm{MASP2_{zym}}}}+\frac{[\mathrm{pPTX3LFMASP3_{zym}}]}{K_{\mathrm{m\ MASP1^*}}^{\mathrm{MASP3_{zym}}}}
\end{aligned}\right)}
$$

$$+\frac{k_{\text{cat MASP1}^*}^{\text{MASP2}_{\text{zym}}}\left[\text{pCLK1MASP1}^*\right]\left[\text{pMBL}_3\text{MASP2}_{\text{zym}}\right]}{K_{\text{m MASP1}^*}^{\text{MASP2}_{\text{zym}}}\left(\begin{array}{l}1+\frac{[\text{C4}]}{K_{\text{m MASP1}^*}^{\text{C4}}}+\frac{[\text{fC4bC2}]}{K_{\text{m MASP1}^*}^{\text{C2}}}+\frac{[\text{fC4bC4bC2}]}{K_{\text{m MASP1}^*}^{\text{C2}}}+\frac{[\text{fC3bC4bC2}]}{K_{\text{m MASP1}^*}^{\text{C2}}}+\frac{[\text{pC4bC2}]}{K_{\text{m MASP1}^*}^{\text{C2}}}+\frac{[\text{pC4bC4bC2}]}{K_{\text{m MASP1}^*}^{\text{C2}}}\\ +\frac{[\text{pC3bC4bC2}]}{K_{\text{m MASP1}^*}^{\text{C2}}}+\frac{[\text{pMBL}_3\text{MASP1}_{\text{zym}}]}{K_{\text{m MASP1}^*}^{\text{MASP1}_{\text{zym}}}}+\frac{[\text{pMBL}_3\text{MASP2}_{\text{zym}}]}{K_{\text{m MASP1}^*}^{\text{MASP2}_{\text{zym}}}}+\frac{[\text{pMBL}_3\text{MASP3}_{\text{zym}}]}{K_{\text{m MASP1}^*}^{\text{MASP3}_{\text{zym}}}}\\ +\frac{[\text{pMBL}_4\text{MASP1}_{\text{zym}}]}{K_{\text{m MASP1}^*}^{\text{MASP1}_{\text{zym}}}}+\frac{[\text{pMBL}_4\text{MASP2}_{\text{zym}}]}{K_{\text{m MASP1}^*}^{\text{MASP2}_{\text{zym}}}}+\frac{[\text{pMBL}_4\text{MASP3}_{\text{zym}}]}{K_{\text{m MASP1}^*}^{\text{MASP3}_{\text{zym}}}}+\frac{[\text{pCLLKMASP1}_{\text{zym}}]}{K_{\text{m MASP1}^*}^{\text{MASP1}_{\text{zym}}}}\\ +\frac{[\text{pCLLKMASP2}_{\text{zym}}]}{K_{\text{m MASP1}^*}^{\text{MASP2}_{\text{zym}}}}+\frac{[\text{pCLLKMASP3}_{\text{zym}}]}{K_{\text{m MASP1}^*}^{\text{MASP3}_{\text{zym}}}}+\frac{[\text{pCLK1MASP1}_{\text{zym}}]}{K_{\text{m MASP1}^*}^{\text{MASP1}_{\text{zym}}}}+\frac{[\text{pCLK1MASP2}_{\text{zym}}]}{K_{\text{m MASP1}^*}^{\text{MASP2}_{\text{zym}}}}\\ +\frac{[\text{pCLK1MASP3}_{\text{zym}}]}{K_{\text{m MASP1}^*}^{\text{MASP3}_{\text{zym}}}}+\frac{[\text{pCLL1MASP1}_{\text{zym}}]}{K_{\text{m MASP1}^*}^{\text{MASP1}_{\text{zym}}}}+\frac{[\text{pCLL1MASP2}_{\text{zym}}]}{K_{\text{m MASP1}^*}^{\text{MASP2}_{\text{zym}}}}+\frac{[\text{pCLL1MASP3}_{\text{zym}}]}{K_{\text{m MASP1}^*}^{\text{MASP3}_{\text{zym}}}}\\ +\frac{[\text{pLFMASP1}_{\text{zym}}]}{K_{\text{m MASP1}^*}^{\text{MASP1}_{\text{zym}}}}+\frac{[\text{pLFMASP2}_{\text{zym}}]}{K_{\text{m MASP1}^*}^{\text{MASP2}_{\text{zym}}}}+\frac{[\text{pLFMASP3}_{\text{zym}}]}{K_{\text{m MASP1}^*}^{\text{MASP3}_{\text{zym}}}}+\frac{[\text{pHFMASP1}_{\text{zym}}]}{K_{\text{m MASP1}^*}^{\text{MASP1}_{\text{zym}}}}+\frac{[\text{pHFMASP2}_{\text{zym}}]}{K_{\text{m MASP1}^*}^{\text{MASP2}_{\text{zym}}}}\\ +\frac{[\text{pHFMASP3}_{\text{zym}}]}{K_{\text{m MASP1}^*}^{\text{MASP3}_{\text{zym}}}}+\frac{[\text{pMFMASP1}_{\text{zym}}]}{K_{\text{m MASP1}^*}^{\text{MASP1}_{\text{zym}}}}+\frac{[\text{pMFMASP2}_{\text{zym}}]}{K_{\text{m MASP1}^*}^{\text{MASP2}_{\text{zym}}}}+\frac{[\text{pMFMASP3}_{\text{zym}}]}{K_{\text{m MASP1}^*}^{\text{MASP3}_{\text{zym}}}}\\ +\frac{[\text{pPTX3LFMASP1}_{\text{zym}}]}{K_{\text{m MASP1}^*}^{\text{MASP1}_{\text{zym}}}}+\frac{[\text{pPXT3LFMASP2}_{\text{zym}}]}{K_{\text{m MASP1}^*}^{\text{MASP2}_{\text{zym}}}}+\frac{[\text{pPTX3LFMASP3}_{\text{zym}}]}{K_{\text{m MASP1}^*}^{\text{MASP3}_{\text{zym}}}}\end{array}\right)}$$

$$+\frac{k_{\mathrm{cat\,MASP1^*}}^{\mathrm{MASP2_{zym}}}\left[\mathrm{pLFMASP1^*}\right]\left[\mathrm{pMBL_3MASP2_{zym}}\right]}{K_{\mathrm{m\,MASP1^*}}^{\mathrm{MASP2_{zym}}}\left(\begin{array}{l}1+\frac{[\mathrm{C4}]}{K_{\mathrm{m\,MASP1^*}}^{\mathrm{C4}}}+\frac{[\mathrm{fC4bC2}]}{K_{\mathrm{m\,MASP1^*}}^{\mathrm{C2}}}+\frac{[\mathrm{fC4bC4bC2}]}{K_{\mathrm{m\,MASP1^*}}^{\mathrm{C2}}}+\frac{[\mathrm{fC3bC4bC2}]}{K_{\mathrm{m\,MASP1^*}}^{\mathrm{C2}}}+\frac{[\mathrm{pC4bC2}]}{K_{\mathrm{m\,MASP1^*}}^{\mathrm{C2}}}+\frac{[\mathrm{pC4bC4bC2}]}{K_{\mathrm{m\,MASP1^*}}^{\mathrm{C2}}}\\
+\frac{[\mathrm{pC3bC4bC2}]}{K_{\mathrm{m\,MASP1^*}}^{\mathrm{C2}}}+\frac{[\mathrm{pMBL_3MASP1_{zym}}]}{K_{\mathrm{m\,MASP1^*}}^{\mathrm{MASP1_{zym}}}}+\frac{[\mathrm{pMBL_3MASP2_{zym}}]}{K_{\mathrm{m\,MASP1^*}}^{\mathrm{MASP2_{zym}}}}+\frac{[\mathrm{pMBL_3MASP3_{zym}}]}{K_{\mathrm{m\,MASP1^*}}^{\mathrm{MASP3_{zym}}}}\\
+\frac{[\mathrm{pMBL_4MASP1_{zym}}]}{K_{\mathrm{m\,MASP1^*}}^{\mathrm{MASP1_{zym}}}}+\frac{[\mathrm{pMBL_4MASP2_{zym}}]}{K_{\mathrm{m\,MASP1^*}}^{\mathrm{MASP2_{zym}}}}+\frac{[\mathrm{pMBL_4MASP3_{zym}}]}{K_{\mathrm{m\,MASP1^*}}^{\mathrm{MASP3_{zym}}}}+\frac{[\mathrm{pCLLKMASP1_{zym}}]}{K_{\mathrm{m\,MASP1^*}}^{\mathrm{MASP1_{zym}}}}\\
+\frac{[\mathrm{pCLLKMASP2_{zym}}]}{K_{\mathrm{m\,MASP1^*}}^{\mathrm{MASP2_{zym}}}}+\frac{[\mathrm{pCLLKMASP3_{zym}}]}{K_{\mathrm{m\,MASP1^*}}^{\mathrm{MASP3_{zym}}}}+\frac{[\mathrm{pCLK1MASP1_{zym}}]}{K_{\mathrm{m\,MASP1^*}}^{\mathrm{MASP1_{zym}}}}+\frac{[\mathrm{pCLK1MASP2_{zym}}]}{K_{\mathrm{m\,MASP1^*}}^{\mathrm{MASP2_{zym}}}}\\
+\frac{[\mathrm{pCLK1MASP3_{zym}}]}{K_{\mathrm{m\,MASP1^*}}^{\mathrm{MASP3_{zym}}}}+\frac{[\mathrm{pCLL1MASP1_{zym}}]}{K_{\mathrm{m\,MASP1^*}}^{\mathrm{MASP1_{zym}}}}+\frac{[\mathrm{pCLL1MASP2_{zym}}]}{K_{\mathrm{m\,MASP1^*}}^{\mathrm{MASP2_{zym}}}}+\frac{[\mathrm{pCLL1MASP3_{zym}}]}{K_{\mathrm{m\,MASP1^*}}^{\mathrm{MASP3_{zym}}}}\\
+\frac{[\mathrm{pLFMASP1_{zym}}]}{K_{\mathrm{m\,MASP1^*}}^{\mathrm{MASP1_{zym}}}}+\frac{[\mathrm{pLFMASP2_{zym}}]}{K_{\mathrm{m\,MASP1^*}}^{\mathrm{MASP2_{zym}}}}+\frac{[\mathrm{pLFMASP3_{zym}}]}{K_{\mathrm{m\,MASP1^*}}^{\mathrm{MASP3_{zym}}}}+\frac{[\mathrm{pHFMASP1_{zym}}]}{K_{\mathrm{m\,MASP1^*}}^{\mathrm{MASP1_{zym}}}}+\frac{[\mathrm{pHFMASP2_{zym}}]}{K_{\mathrm{m\,MASP1^*}}^{\mathrm{MASP2_{zym}}}}\\
+\frac{[\mathrm{pHFMASP3_{zym}}]}{K_{\mathrm{m\,MASP1^*}}^{\mathrm{MASP3_{zym}}}}+\frac{[\mathrm{pMFMASP1_{zym}}]}{K_{\mathrm{m\,MASP1^*}}^{\mathrm{MASP1_{zym}}}}+\frac{[\mathrm{pMFMASP2_{zym}}]}{K_{\mathrm{m\,MASP1^*}}^{\mathrm{MASP2_{zym}}}}+\frac{[\mathrm{pMFMASP3_{zym}}]}{K_{\mathrm{m\,MASP1^*}}^{\mathrm{MASP3_{zym}}}}\\
+\frac{[\mathrm{pPTX3LFMASP1_{zym}}]}{K_{\mathrm{m\,MASP1^*}}^{\mathrm{MASP1_{zym}}}}+\frac{[\mathrm{pPXT3LFMASP2_{zym}}]}{K_{\mathrm{m\,MASP1^*}}^{\mathrm{MASP2_{zym}}}}+\frac{[\mathrm{pPTX3LFMASP3_{zym}}]}{K_{\mathrm{m\,MASP1^*}}^{\mathrm{MASP3_{zym}}}}\end{array}\right)}$$

$$
+\frac{k_{\text{cat MASP1}^*}^{\text{MASP2}_{\text{zym}}}\left[\text{pHFMASP1}^*\right]\left[\text{pMBL}_3\text{MASP2}_{\text{zym}}\right]}{K_{\text{m MASP1}^*}^{\text{MASP2}_{\text{zym}}}\left(\begin{array}{l}1+\frac{[\text{C4}]}{K_{\text{m MASP1}^*}^{\text{C4}}}+\frac{[\text{fC4bC2}]}{K_{\text{m MASP1}^*}^{\text{C2}}}+\frac{[\text{fC4bC4bC2}]}{K_{\text{m MASP1}^*}^{\text{C2}}}+\frac{[\text{fC3bC4bC2}]}{K_{\text{m MASP1}^*}^{\text{C2}}}+\frac{[\text{pC4bC2}]}{K_{\text{m MASP1}^*}^{\text{C2}}}+\frac{[\text{pC4bC4bC2}]}{K_{\text{m MASP1}^*}^{\text{C2}}}\\
+\frac{[\text{pC3bC4bC2}]}{K_{\text{m MASP1}^*}^{\text{C2}}}+\frac{[\text{pMBL}_3\text{MASP1}_{\text{zym}}]}{K_{\text{m MASP1}^*}^{\text{MASP1}_{\text{zym}}}}+\frac{[\text{pMBL}_3\text{MASP2}_{\text{zym}}]}{K_{\text{m MASP1}^*}^{\text{MASP2}_{\text{zym}}}}+\frac{[\text{pMBL}_3\text{MASP3}_{\text{zym}}]}{K_{\text{m MASP1}^*}^{\text{MASP3}_{\text{zym}}}}\\
+\frac{[\text{pMBL}_4\text{MASP1}_{\text{zym}}]}{K_{\text{m MASP1}^*}^{\text{MASP1}_{\text{zym}}}}+\frac{[\text{pMBL}_4\text{MASP2}_{\text{zym}}]}{K_{\text{m MASP1}^*}^{\text{MASP2}_{\text{zym}}}}+\frac{[\text{pMBL}_4\text{MASP3}_{\text{zym}}]}{K_{\text{m MASP1}^*}^{\text{MASP3}_{\text{zym}}}}+\frac{[\text{pCLLKMASP1}_{\text{zym}}]}{K_{\text{m MASP1}^*}^{\text{MASP1}_{\text{zym}}}}\\
+\frac{[\text{pCLLKMASP2}_{\text{zym}}]}{K_{\text{m MASP1}^*}^{\text{MASP2}_{\text{zym}}}}+\frac{[\text{pCLLKMASP3}_{\text{zym}}]}{K_{\text{m MASP1}^*}^{\text{MASP3}_{\text{zym}}}}+\frac{[\text{pCLK1MASP1}_{\text{zym}}]}{K_{\text{m MASP1}^*}^{\text{MASP1}_{\text{zym}}}}+\frac{[\text{pCLK1MASP2}_{\text{zym}}]}{K_{\text{m MASP1}^*}^{\text{MASP2}_{\text{zym}}}}\\
+\frac{[\text{pCLK1MASP3}_{\text{zym}}]}{K_{\text{m MASP1}^*}^{\text{MASP3}_{\text{zym}}}}+\frac{[\text{pCLL1MASP1}_{\text{zym}}]}{K_{\text{m MASP1}^*}^{\text{MASP1}_{\text{zym}}}}+\frac{[\text{pCLL1MASP2}_{\text{zym}}]}{K_{\text{m MASP1}^*}^{\text{MASP2}_{\text{zym}}}}+\frac{[\text{pCLL1MASP3}_{\text{zym}}]}{K_{\text{m MASP1}^*}^{\text{MASP3}_{\text{zym}}}}\\
+\frac{[\text{pLFMASP1}_{\text{zym}}]}{K_{\text{m MASP1}^*}^{\text{MASP1}_{\text{zym}}}}+\frac{[\text{pLFMASP2}_{\text{zym}}]}{K_{\text{m MASP1}^*}^{\text{MASP2}_{\text{zym}}}}+\frac{[\text{pLFMASP3}_{\text{zym}}]}{K_{\text{m MASP1}^*}^{\text{MASP3}_{\text{zym}}}}+\frac{[\text{pHFMASP1}_{\text{zym}}]}{K_{\text{m MASP1}^*}^{\text{MASP1}_{\text{zym}}}}+\frac{[\text{pHFMASP2}_{\text{zym}}]}{K_{\text{m MASP1}^*}^{\text{MASP2}_{\text{zym}}}}\\
+\frac{[\text{pHFMASP3}_{\text{zym}}]}{K_{\text{m MASP1}^*}^{\text{MASP3}_{\text{zym}}}}+\frac{[\text{pMFMASP1}_{\text{zym}}]}{K_{\text{m MASP1}^*}^{\text{MASP1}_{\text{zym}}}}+\frac{[\text{pMFMASP2}_{\text{zym}}]}{K_{\text{m MASP1}^*}^{\text{MASP2}_{\text{zym}}}}+\frac{[\text{pMFMASP3}_{\text{zym}}]}{K_{\text{m MASP1}^*}^{\text{MASP3}_{\text{zym}}}}\\
+\frac{[\text{pPTX3LFMASP1}_{\text{zym}}]}{K_{\text{m MASP1}^*}^{\text{MASP1}_{\text{zym}}}}+\frac{[\text{pPXT3LFMASP2}_{\text{zym}}]}{K_{\text{m MASP1}^*}^{\text{MASP2}_{\text{zym}}}}+\frac{[\text{pPTX3LFMASP3}_{\text{zym}}]}{K_{\text{m MASP1}^*}^{\text{MASP3}_{\text{zym}}}}\end{array}\right)}
$$

$$
+\frac{k^{\mathrm{MASP2_{zym}}}_{\mathrm{cat\,MASP1^*}}\left[\mathrm{pMFMASP1^*}\right]\left[\mathrm{pMBL_3MASP2_{zym}}\right]}{K^{\mathrm{MASP2_{zym}}}_{\mathrm{m\,MASP1^*}}\left(\begin{aligned}
&1+\frac{[\mathrm{C4}]}{K^{\mathrm{C4}}_{\mathrm{m\,MASP1^*}}}+\frac{[\mathrm{fC4bC2}]}{K^{\mathrm{C2}}_{\mathrm{m\,MASP1^*}}}+\frac{[\mathrm{fC4bC4bC2}]}{K^{\mathrm{C2}}_{\mathrm{m\,MASP1^*}}}+\frac{[\mathrm{fC3bC4bC2}]}{K^{\mathrm{C2}}_{\mathrm{m\,MASP1^*}}}+\frac{[\mathrm{pC4bC2}]}{K^{\mathrm{C2}}_{\mathrm{m\,MASP1^*}}}+\frac{[\mathrm{pC4bC4bC2}]}{K^{\mathrm{C2}}_{\mathrm{m\,MASP1^*}}}\\
&+\frac{[\mathrm{pC3bC4bC2}]}{K^{\mathrm{C2}}_{\mathrm{m\,MASP1^*}}}+\frac{[\mathrm{pMBL_3MASP1_{zym}}]}{K^{\mathrm{MASP1_{zym}}}_{\mathrm{m\,MASP1^*}}}+\frac{[\mathrm{pMBL_3MASP2_{zym}}]}{K^{\mathrm{MASP2_{zym}}}_{\mathrm{m\,MASP1^*}}}+\frac{[\mathrm{pMBL_3MASP3_{zym}}]}{K^{\mathrm{MASP3_{zym}}}_{\mathrm{m\,MASP1^*}}}\\
&+\frac{[\mathrm{pMBL_4MASP1_{zym}}]}{K^{\mathrm{MASP1_{zym}}}_{\mathrm{m\,MASP1^*}}}+\frac{[\mathrm{pMBL_4MASP2_{zym}}]}{K^{\mathrm{MASP2_{zym}}}_{\mathrm{m\,MASP1^*}}}+\frac{[\mathrm{pMBL_4MASP3_{zym}}]}{K^{\mathrm{MASP3_{zym}}}_{\mathrm{m\,MASP1^*}}}+\frac{[\mathrm{pCLLKMASP1_{zym}}]}{K^{\mathrm{MASP1_{zym}}}_{\mathrm{m\,MASP1^*}}}\\
&+\frac{[\mathrm{pCLLKMASP2_{zym}}]}{K^{\mathrm{MASP2_{zym}}}_{\mathrm{m\,MASP1^*}}}+\frac{[\mathrm{pCLLKMASP3_{zym}}]}{K^{\mathrm{MASP3_{zym}}}_{\mathrm{m\,MASP1^*}}}+\frac{[\mathrm{pCLK1MASP1_{zym}}]}{K^{\mathrm{MASP1_{zym}}}_{\mathrm{m\,MASP1^*}}}+\frac{[\mathrm{pCLK1MASP2_{zym}}]}{K^{\mathrm{MASP2_{zym}}}_{\mathrm{m\,MASP1^*}}}\\
&+\frac{[\mathrm{pCLK1MASP3_{zym}}]}{K^{\mathrm{MASP3_{zym}}}_{\mathrm{m\,MASP1^*}}}+\frac{[\mathrm{pCLL1MASP1_{zym}}]}{K^{\mathrm{MASP1_{zym}}}_{\mathrm{m\,MASP1^*}}}+\frac{[\mathrm{pCLL1MASP2_{zym}}]}{K^{\mathrm{MASP2_{zym}}}_{\mathrm{m\,MASP1^*}}}+\frac{[\mathrm{pCLL1MASP3_{zym}}]}{K^{\mathrm{MASP3_{zym}}}_{\mathrm{m\,MASP1^*}}}\\
&+\frac{[\mathrm{pLFMASP1_{zym}}]}{K^{\mathrm{MASP1_{zym}}}_{\mathrm{m\,MASP1^*}}}+\frac{[\mathrm{pLFMASP2_{zym}}]}{K^{\mathrm{MASP2_{zym}}}_{\mathrm{m\,MASP1^*}}}+\frac{[\mathrm{pLFMASP3_{zym}}]}{K^{\mathrm{MASP3_{zym}}}_{\mathrm{m\,MASP1^*}}}+\frac{[\mathrm{pHFMASP1_{zym}}]}{K^{\mathrm{MASP1_{zym}}}_{\mathrm{m\,MASP1^*}}}+\frac{[\mathrm{pHFMASP2_{zym}}]}{K^{\mathrm{MASP2_{zym}}}_{\mathrm{m\,MASP1^*}}}\\
&+\frac{[\mathrm{pHFMASP3_{zym}}]}{K^{\mathrm{MASP3_{zym}}}_{\mathrm{m\,MASP1^*}}}+\frac{[\mathrm{pMFMASP1_{zym}}]}{K^{\mathrm{MASP1_{zym}}}_{\mathrm{m\,MASP1^*}}}+\frac{[\mathrm{pMFMASP2_{zym}}]}{K^{\mathrm{MASP2_{zym}}}_{\mathrm{m\,MASP1^*}}}+\frac{[\mathrm{pMFMASP3_{zym}}]}{K^{\mathrm{MASP3_{zym}}}_{\mathrm{m\,MASP1^*}}}\\
&+\frac{[\mathrm{pPTX3LFMASP1_{zym}}]}{K^{\mathrm{MASP1_{zym}}}_{\mathrm{m\,MASP1^*}}}+\frac{[\mathrm{pPXT3LFMASP2_{zym}}]}{K^{\mathrm{MASP2_{zym}}}_{\mathrm{m\,MASP1^*}}}+\frac{[\mathrm{pPTX3LFMASP3_{zym}}]}{K^{\mathrm{MASP3_{zym}}}_{\mathrm{m\,MASP1^*}}}
\end{aligned}\right)}
$$

$$
+\frac{k_{\mathrm{cat\,MASP1^*}}^{\mathrm{MASP2_{zym}}}\left[\mathrm{pPTX3LFMASP1^*}\right]\left[\mathrm{pMBL_3MASP2_{zym}}\right]}{K_{\mathrm{m\,MASP1^*}}^{\mathrm{MASP2_{zym}}}\left(\begin{aligned}
&1+\frac{[\mathrm{C4}]}{K_{\mathrm{m\,MASP1^*}}^{\mathrm{C4}}}+\frac{[\mathrm{fC4bC2}]}{K_{\mathrm{m\,MASP1^*}}^{\mathrm{C2}}}+\frac{[\mathrm{fC4bC4bC2}]}{K_{\mathrm{m\,MASP1^*}}^{\mathrm{C2}}}+\frac{[\mathrm{fC3bC4bC2}]}{K_{\mathrm{m\,MASP1^*}}^{\mathrm{C2}}}+\frac{[\mathrm{pC4bC2}]}{K_{\mathrm{m\,MASP1^*}}^{\mathrm{C2}}}+\frac{[\mathrm{pC4bC4bC2}]}{K_{\mathrm{m\,MASP1^*}}^{\mathrm{C2}}}\\
&+\frac{[\mathrm{pC3bC4bC2}]}{K_{\mathrm{m\,MASP1^*}}^{\mathrm{C2}}}+\frac{[\mathrm{pMBL_3MASP1_{zym}}]}{K_{\mathrm{m\,MASP1^*}}^{\mathrm{MASP1_{zym}}}}+\frac{[\mathrm{pMBL_3MASP2_{zym}}]}{K_{\mathrm{m\,MASP1^*}}^{\mathrm{MASP2_{zym}}}}+\frac{[\mathrm{pMBL_3MASP3_{zym}}]}{K_{\mathrm{m\,MASP1^*}}^{\mathrm{MASP3_{zym}}}}\\
&+\frac{[\mathrm{pMBL_4MASP1_{zym}}]}{K_{\mathrm{m\,MASP1^*}}^{\mathrm{MASP1_{zym}}}}+\frac{[\mathrm{pMBL_4MASP2_{zym}}]}{K_{\mathrm{m\,MASP1^*}}^{\mathrm{MASP2_{zym}}}}+\frac{[\mathrm{pMBL_4MASP3_{zym}}]}{K_{\mathrm{m\,MASP1^*}}^{\mathrm{MASP3_{zym}}}}+\frac{[\mathrm{pCLLKMASP1_{zym}}]}{K_{\mathrm{m\,MASP1^*}}^{\mathrm{MASP1_{zym}}}}\\
&+\frac{[\mathrm{pCLLKMASP2_{zym}}]}{K_{\mathrm{m\,MASP1^*}}^{\mathrm{MASP2_{zym}}}}+\frac{[\mathrm{pCLLKMASP3_{zym}}]}{K_{\mathrm{m\,MASP1^*}}^{\mathrm{MASP3_{zym}}}}+\frac{[\mathrm{pCLK1MASP1_{zym}}]}{K_{\mathrm{m\,MASP1^*}}^{\mathrm{MASP1_{zym}}}}+\frac{[\mathrm{pCLK1MASP2_{zym}}]}{K_{\mathrm{m\,MASP1^*}}^{\mathrm{MASP2_{zym}}}}\\
&+\frac{[\mathrm{pCLK1MASP3_{zym}}]}{K_{\mathrm{m\,MASP1^*}}^{\mathrm{MASP3_{zym}}}}+\frac{[\mathrm{pCLL1MASP1_{zym}}]}{K_{\mathrm{m\,MASP1^*}}^{\mathrm{MASP1_{zym}}}}+\frac{[\mathrm{pCLL1MASP2_{zym}}]}{K_{\mathrm{m\,MASP1^*}}^{\mathrm{MASP2_{zym}}}}+\frac{[\mathrm{pCLL1MASP3_{zym}}]}{K_{\mathrm{m\,MASP1^*}}^{\mathrm{MASP3_{zym}}}}\\
&+\frac{[\mathrm{pLFMASP1_{zym}}]}{K_{\mathrm{m\,MASP1^*}}^{\mathrm{MASP1_{zym}}}}+\frac{[\mathrm{pLFMASP2_{zym}}]}{K_{\mathrm{m\,MASP1^*}}^{\mathrm{MASP2_{zym}}}}+\frac{[\mathrm{pLFMASP3_{zym}}]}{K_{\mathrm{m\,MASP1^*}}^{\mathrm{MASP3_{zym}}}}+\frac{[\mathrm{pHFMASP1_{zym}}]}{K_{\mathrm{m\,MASP1^*}}^{\mathrm{MASP1_{zym}}}}+\frac{[\mathrm{pHFMASP2_{zym}}]}{K_{\mathrm{m\,MASP1^*}}^{\mathrm{MASP2_{zym}}}}\\
&+\frac{[\mathrm{pHFMASP3_{zym}}]}{K_{\mathrm{m\,MASP1^*}}^{\mathrm{MASP3_{zym}}}}+\frac{[\mathrm{pMFMASP1_{zym}}]}{K_{\mathrm{m\,MASP1^*}}^{\mathrm{MASP1_{zym}}}}+\frac{[\mathrm{pMFMASP2_{zym}}]}{K_{\mathrm{m\,MASP1^*}}^{\mathrm{MASP2_{zym}}}}+\frac{[\mathrm{pMFMASP3_{zym}}]}{K_{\mathrm{m\,MASP1^*}}^{\mathrm{MASP3_{zym}}}}\\
&+\frac{[\mathrm{pPTX3LFMASP1_{zym}}]}{K_{\mathrm{m\,MASP1^*}}^{\mathrm{MASP1_{zym}}}}+\frac{[\mathrm{pPXT3LFMASP2_{zym}}]}{K_{\mathrm{m\,MASP1^*}}^{\mathrm{MASP2_{zym}}}}+\frac{[\mathrm{pPTX3LFMASP3_{zym}}]}{K_{\mathrm{m\,MASP1^*}}^{\mathrm{MASP3_{zym}}}}
\end{aligned}\right)}
$$

$$
+\frac{k_{\mathrm{cat\,MASP2^*}}^{\mathrm{MASP2_{zym}}}\left[\mathrm{pMBL_3MASP2^*}\right]\left[\mathrm{pMBL_3MASP2_{zym}}\right]}{K_{\mathrm{m\,MASP2^*}}^{\mathrm{MASP2_{zym}}}\left(\begin{aligned}
&1+\frac{[\mathrm{C4}]}{K_{\mathrm{m\,MASP2^*}}^{\mathrm{C4}}}+\frac{[\mathrm{fC4bC2}]}{K_{\mathrm{m\,MASP2^*}}^{\mathrm{C2}}}+\frac{[\mathrm{fC4bC4bC2}]}{K_{\mathrm{m\,MASP2^*}}^{\mathrm{C2}}}+\frac{[\mathrm{fC3bC4bC2}]}{K_{\mathrm{m\,MASP2^*}}^{\mathrm{C2}}}+\frac{[\mathrm{pC4bC2}]}{K_{\mathrm{m\,MASP2^*}}^{\mathrm{C2}}}+\frac{[\mathrm{pC4bC4bC2}]}{K_{\mathrm{m\,MASP2^*}}^{\mathrm{C2}}}\\
&+\frac{[\mathrm{pC3bC4bC2}]}{K_{\mathrm{m\,MASP2^*}}^{\mathrm{C2}}}+\frac{[\mathrm{pMBL_3MASP1_{zym}}]}{K_{\mathrm{m\,MASP2^*}}^{\mathrm{MASP1_{zym}}}}+\frac{[\mathrm{pMBL_3MASP2_{zym}}]}{K_{\mathrm{m\,MASP2^*}}^{\mathrm{MASP2_{zym}}}}+\frac{[\mathrm{pMBL_4MASP1_{zym}}]}{K_{\mathrm{m\,MASP2^*}}^{\mathrm{MASP1_{zym}}}}\\
&+\frac{[\mathrm{pMBL_4MASP2_{zym}}]}{K_{\mathrm{m\,MASP2^*}}^{\mathrm{MASP2_{zym}}}}+\frac{[\mathrm{pCLLKMASP1_{zym}}]}{K_{\mathrm{m\,MASP2^*}}^{\mathrm{MASP1_{zym}}}}+\frac{[\mathrm{pCLLKMASP2_{zym}}]}{K_{\mathrm{m\,MASP2^*}}^{\mathrm{MASP2_{zym}}}}+\frac{[\mathrm{pCLK1MASP1_{zym}}]}{K_{\mathrm{m\,MASP2^*}}^{\mathrm{MASP1_{zym}}}}\\
&+\frac{[\mathrm{pCLK1MASP2_{zym}}]}{K_{\mathrm{m\,MASP2^*}}^{\mathrm{MASP2_{zym}}}}+\frac{[\mathrm{pCLL1MASP1_{zym}}]}{K_{\mathrm{m\,MASP2^*}}^{\mathrm{MASP1_{zym}}}}+\frac{[\mathrm{pCLL1MASP2_{zym}}]}{K_{\mathrm{m\,MASP2^*}}^{\mathrm{MASP2_{zym}}}}+\frac{[\mathrm{pLFMASP1_{zym}}]}{K_{\mathrm{m\,MASP2^*}}^{\mathrm{MASP1_{zym}}}}\\
&+\frac{[\mathrm{pLFMASP2_{zym}}]}{K_{\mathrm{m\,MASP2^*}}^{\mathrm{MASP2_{zym}}}}+\frac{[\mathrm{pHFMASP1_{zym}}]}{K_{\mathrm{m\,MASP2^*}}^{\mathrm{MASP1_{zym}}}}+\frac{[\mathrm{pHFMASP2_{zym}}]}{K_{\mathrm{m\,MASP2^*}}^{\mathrm{MASP2_{zym}}}}+\frac{[\mathrm{pMFMASP1_{zym}}]}{K_{\mathrm{m\,MASP2^*}}^{\mathrm{MASP1_{zym}}}}+\frac{[\mathrm{pMFMASP2_{zym}}]}{K_{\mathrm{m\,MASP2^*}}^{\mathrm{MASP2_{zym}}}}\\
&+\frac{[\mathrm{pPTX3LFMASP1_{zym}}]}{K_{\mathrm{m\,MASP2^*}}^{\mathrm{MASP1_{zym}}}}+\frac{[\mathrm{pPTX3LFMASP2_{zym}}]}{K_{\mathrm{m\,MASP2^*}}^{\mathrm{MASP2_{zym}}}}
\end{aligned}\right)}
$$

$$+\frac{k_{\text{cat MASP2}^{*}}^{\text{MASP2}_{\text{zym}}}\left[\text{pMBL}_{4}\text{MASP2}^{*}\right]\left[\text{pMBL}_{3}\text{MASP2}_{\text{zym}}\right]}{K_{\text{m MASP2}^{*}}^{\text{MASP2}_{\text{zym}}}\left(\begin{array}{l}1+\frac{[\text{C4}]}{K_{\text{m MASP2}^{*}}^{\text{C4}}}+\frac{[\text{fC4bC2}]}{K_{\text{m MASP2}^{*}}^{\text{C2}}}+\frac{[\text{fC4bC4bC2}]}{K_{\text{m MASP2}^{*}}^{\text{C2}}}+\frac{[\text{fC3bC4bC2}]}{K_{\text{m MASP2}^{*}}^{\text{C2}}}+\frac{[\text{pC4bC2}]}{K_{\text{m MASP2}^{*}}^{\text{C2}}}+\frac{[\text{pC4bC4bC2}]}{K_{\text{m MASP2}^{*}}^{\text{C2}}}\\+\frac{[\text{pC3bC4bC2}]}{K_{\text{m MASP2}^{*}}^{\text{C2}}}+\frac{\left[\text{pMBL}_{3}\text{MASP1}_{\text{zym}}\right]}{K_{\text{m MASP2}^{*}}^{\text{MASP1}_{\text{zym}}}}+\frac{\left[\text{pMBL}_{3}\text{MASP2}_{\text{zym}}\right]}{K_{\text{m MASP2}^{*}}^{\text{MASP2}_{\text{zym}}}}+\frac{\left[\text{pMBL}_{4}\text{MASP1}_{\text{zym}}\right]}{K_{\text{m MASP2}^{*}}^{\text{MASP1}_{\text{zym}}}}\\+\frac{\left[\text{pMBL}_{4}\text{MASP2}_{\text{zym}}\right]}{K_{\text{m MASP2}^{*}}^{\text{MASP2}_{\text{zym}}}}+\frac{\left[\text{pCLLKMASP1}_{\text{zym}}\right]}{K_{\text{m MASP2}^{*}}^{\text{MASP1}_{\text{zym}}}}+\frac{\left[\text{pCLLKMASP2}_{\text{zym}}\right]}{K_{\text{m MASP2}^{*}}^{\text{MASP2}_{\text{zym}}}}+\frac{\left[\text{pCLK1MASP1}_{\text{zym}}\right]}{K_{\text{m MASP2}^{*}}^{\text{MASP1}_{\text{zym}}}}\\+\frac{\left[\text{pCLK1MASP2}_{\text{zym}}\right]}{K_{\text{m MASP2}^{*}}^{\text{MASP2}_{\text{zym}}}}+\frac{\left[\text{pCLL1MASP1}_{\text{zym}}\right]}{K_{\text{m MASP2}^{*}}^{\text{MASP1}_{\text{zym}}}}+\frac{\left[\text{pCLL1MASP2}_{\text{zym}}\right]}{K_{\text{m MASP2}^{*}}^{\text{MASP2}_{\text{zym}}}}+\frac{\left[\text{pLFMASP1}_{\text{zym}}\right]}{K_{\text{m MASP2}^{*}}^{\text{MASP1}_{\text{zym}}}}\\+\frac{\left[\text{pLFMASP2}_{\text{zym}}\right]}{K_{\text{m MASP2}^{*}}^{\text{MASP2}_{\text{zym}}}}+\frac{\left[\text{pHFMASP1}_{\text{zym}}\right]}{K_{\text{m MASP2}^{*}}^{\text{MASP1}_{\text{zym}}}}+\frac{\left[\text{pHFMASP2}_{\text{zym}}\right]}{K_{\text{m MASP2}^{*}}^{\text{MASP2}_{\text{zym}}}}+\frac{\left[\text{pMFMASP1}_{\text{zym}}\right]}{K_{\text{m MASP2}^{*}}^{\text{MASP1}_{\text{zym}}}}+\frac{\left[\text{pMFMASP2}_{\text{zym}}\right]}{K_{\text{m MASP2}^{*}}^{\text{MASP2}_{\text{zym}}}}\\+\frac{\left[\text{pPTX3LFMASP1}_{\text{zym}}\right]}{K_{\text{m MASP2}^{*}}^{\text{MASP1}_{\text{zym}}}}+\frac{\left[\text{pPTX3LFMASP2}_{\text{zym}}\right]}{K_{\text{m MASP2}^{*}}^{\text{MASP2}_{\text{zym}}}}\end{array}\right)}$$

$$+\frac{k_{\text{cat MASP2}^{*}}^{\text{MASP2}_{\text{zym}}}\left[\text{pCLLKMASP2}^{*}\right]\left[\text{pMBL}_{3}\text{MASP2}_{\text{zym}}\right]}{K_{\text{m MASP2}^{*}}^{\text{MASP2}_{\text{zym}}}\left(\begin{array}{l}1+\frac{[\text{C4}]}{K_{\text{m MASP2}^{*}}^{\text{C4}}}+\frac{[\text{fC4bC2}]}{K_{\text{m MASP2}^{*}}^{\text{C2}}}+\frac{[\text{fC4bC4bC2}]}{K_{\text{m MASP2}^{*}}^{\text{C2}}}+\frac{[\text{fC3bC4bC2}]}{K_{\text{m MASP2}^{*}}^{\text{C2}}}+\frac{[\text{pC4bC2}]}{K_{\text{m MASP2}^{*}}^{\text{C2}}}+\frac{[\text{pC4bC4bC2}]}{K_{\text{m MASP2}^{*}}^{\text{C2}}}\\+\frac{[\text{pC3bC4bC2}]}{K_{\text{m MASP2}^{*}}^{\text{C2}}}+\frac{\left[\text{pMBL}_{3}\text{MASP1}_{\text{zym}}\right]}{K_{\text{m MASP2}^{*}}^{\text{MASP1}_{\text{zym}}}}+\frac{\left[\text{pMBL}_{3}\text{MASP2}_{\text{zym}}\right]}{K_{\text{m MASP2}^{*}}^{\text{MASP2}_{\text{zym}}}}+\frac{\left[\text{pMBL}_{4}\text{MASP1}_{\text{zym}}\right]}{K_{\text{m MASP2}^{*}}^{\text{MASP1}_{\text{zym}}}}\\+\frac{\left[\text{pMBL}_{4}\text{MASP2}_{\text{zym}}\right]}{K_{\text{m MASP2}^{*}}^{\text{MASP2}_{\text{zym}}}}+\frac{\left[\text{pCLLKMASP1}_{\text{zym}}\right]}{K_{\text{m MASP2}^{*}}^{\text{MASP1}_{\text{zym}}}}+\frac{\left[\text{pCLLKMASP2}_{\text{zym}}\right]}{K_{\text{m MASP2}^{*}}^{\text{MASP2}_{\text{zym}}}}+\frac{\left[\text{pCLK1MASP1}_{\text{zym}}\right]}{K_{\text{m MASP2}^{*}}^{\text{MASP1}_{\text{zym}}}}\\+\frac{\left[\text{pCLK1MASP2}_{\text{zym}}\right]}{K_{\text{m MASP2}^{*}}^{\text{MASP2}_{\text{zym}}}}+\frac{\left[\text{pCLL1MASP1}_{\text{zym}}\right]}{K_{\text{m MASP2}^{*}}^{\text{MASP1}_{\text{zym}}}}+\frac{\left[\text{pCLL1MASP2}_{\text{zym}}\right]}{K_{\text{m MASP2}^{*}}^{\text{MASP2}_{\text{zym}}}}+\frac{\left[\text{pLFMASP1}_{\text{zym}}\right]}{K_{\text{m MASP2}^{*}}^{\text{MASP1}_{\text{zym}}}}\\+\frac{\left[\text{pLFMASP2}_{\text{zym}}\right]}{K_{\text{m MASP2}^{*}}^{\text{MASP2}_{\text{zym}}}}+\frac{\left[\text{pHFMASP1}_{\text{zym}}\right]}{K_{\text{m MASP2}^{*}}^{\text{MASP1}_{\text{zym}}}}+\frac{\left[\text{pHFMASP2}_{\text{zym}}\right]}{K_{\text{m MASP2}^{*}}^{\text{MASP2}_{\text{zym}}}}+\frac{\left[\text{pMFMASP1}_{\text{zym}}\right]}{K_{\text{m MASP2}^{*}}^{\text{MASP1}_{\text{zym}}}}+\frac{\left[\text{pMFMASP2}_{\text{zym}}\right]}{K_{\text{m MASP2}^{*}}^{\text{MASP2}_{\text{zym}}}}\\+\frac{\left[\text{pPTX3LFMASP1}_{\text{zym}}\right]}{K_{\text{m MASP2}^{*}}^{\text{MASP1}_{\text{zym}}}}+\frac{\left[\text{pPTX3LFMASP2}_{\text{zym}}\right]}{K_{\text{m MASP2}^{*}}^{\text{MASP2}_{\text{zym}}}}\end{array}\right)}$$

$$
+\frac{k_{\mathrm{cat\;MASP2^*}}^{\mathrm{MASP2_{zym}}}\left[\mathrm{pCLK1MASP2^*}\right]\left[\mathrm{pMBL_3MASP2_{zym}}\right]}{K_{\mathrm{m\;MASP2^*}}^{\mathrm{MASP2_{zym}}}\left(\begin{array}{l}1+\frac{[\mathrm{C4}]}{K_{\mathrm{m\;MASP2^*}}^{\mathrm{C4}}}+\frac{[\mathrm{fC4bC2}]}{K_{\mathrm{m\;MASP2^*}}^{\mathrm{C2}}}+\frac{[\mathrm{fC4bC4bC2}]}{K_{\mathrm{m\;MASP2^*}}^{\mathrm{C2}}}+\frac{[\mathrm{fC3bC4bC2}]}{K_{\mathrm{m\;MASP2^*}}^{\mathrm{C2}}}+\frac{[\mathrm{pC4bC2}]}{K_{\mathrm{m\;MASP2^*}}^{\mathrm{C2}}}+\frac{[\mathrm{pC4bC4bC2}]}{K_{\mathrm{m\;MASP2^*}}^{\mathrm{C2}}}\\
+\frac{[\mathrm{pC3bC4bC2}]}{K_{\mathrm{m\;MASP2^*}}^{\mathrm{C2}}}+\frac{[\mathrm{pMBL_3MASP1_{zym}}]}{K_{\mathrm{m\;MASP2^*}}^{\mathrm{MASP1_{zym}}}}+\frac{[\mathrm{pMBL_3MASP2_{zym}}]}{K_{\mathrm{m\;MASP2^*}}^{\mathrm{MASP2_{zym}}}}+\frac{[\mathrm{pMBL_4MASP1_{zym}}]}{K_{\mathrm{m\;MASP2^*}}^{\mathrm{MASP1_{zym}}}}\\
+\frac{[\mathrm{pMBL_4MASP2_{zym}}]}{K_{\mathrm{m\;MASP2^*}}^{\mathrm{MASP2_{zym}}}}+\frac{[\mathrm{pCLLKMASP1_{zym}}]}{K_{\mathrm{m\;MASP2^*}}^{\mathrm{MASP1_{zym}}}}+\frac{[\mathrm{pCLLKMASP2_{zym}}]}{K_{\mathrm{m\;MASP2^*}}^{\mathrm{MASP2_{zym}}}}+\frac{[\mathrm{pCLK1MASP1_{zym}}]}{K_{\mathrm{m\;MASP2^*}}^{\mathrm{MASP1_{zym}}}}\\
+\frac{[\mathrm{pCLK1MASP2_{zym}}]}{K_{\mathrm{m\;MASP2^*}}^{\mathrm{MASP2_{zym}}}}+\frac{[\mathrm{pCLL1MASP1_{zym}}]}{K_{\mathrm{m\;MASP2^*}}^{\mathrm{MASP1_{zym}}}}+\frac{[\mathrm{pCLL1MASP2_{zym}}]}{K_{\mathrm{m\;MASP2^*}}^{\mathrm{MASP2_{zym}}}}+\frac{[\mathrm{pLFMASP1_{zym}}]}{K_{\mathrm{m\;MASP2^*}}^{\mathrm{MASP1_{zym}}}}\\
+\frac{[\mathrm{pLFMASP2_{zym}}]}{K_{\mathrm{m\;MASP2^*}}^{\mathrm{MASP2_{zym}}}}+\frac{[\mathrm{pHFMASP1_{zym}}]}{K_{\mathrm{m\;MASP2^*}}^{\mathrm{MASP1_{zym}}}}+\frac{[\mathrm{pHFMASP2_{zym}}]}{K_{\mathrm{m\;MASP2^*}}^{\mathrm{MASP2_{zym}}}}+\frac{[\mathrm{pMFMASP1_{zym}}]}{K_{\mathrm{m\;MASP2^*}}^{\mathrm{MASP1_{zym}}}}+\frac{[\mathrm{pMFMASP2_{zym}}]}{K_{\mathrm{m\;MASP2^*}}^{\mathrm{MASP2_{zym}}}}\\
+\frac{[\mathrm{pPTX3LFMASP1_{zym}}]}{K_{\mathrm{m\;MASP2^*}}^{\mathrm{MASP1_{zym}}}}+\frac{[\mathrm{pPTX3LFMASP2_{zym}}]}{K_{\mathrm{m\;MASP2^*}}^{\mathrm{MASP2_{zym}}}}\end{array}\right)}
$$

$$
+\frac{k_{\mathrm{cat\;MASP2^*}}^{\mathrm{MASP2_{zym}}}\left[\mathrm{pCLL1MASP2^*}\right]\left[\mathrm{pMBL_3MASP2_{zym}}\right]}{K_{\mathrm{m\;MASP2^*}}^{\mathrm{MASP2_{zym}}}\left(\begin{array}{l}1+\frac{[\mathrm{C4}]}{K_{\mathrm{m\;MASP2^*}}^{\mathrm{C4}}}+\frac{[\mathrm{fC4bC2}]}{K_{\mathrm{m\;MASP2^*}}^{\mathrm{C2}}}+\frac{[\mathrm{fC4bC4bC2}]}{K_{\mathrm{m\;MASP2^*}}^{\mathrm{C2}}}+\frac{[\mathrm{fC3bC4bC2}]}{K_{\mathrm{m\;MASP2^*}}^{\mathrm{C2}}}+\frac{[\mathrm{pC4bC2}]}{K_{\mathrm{m\;MASP2^*}}^{\mathrm{C2}}}+\frac{[\mathrm{pC4bC4bC2}]}{K_{\mathrm{m\;MASP2^*}}^{\mathrm{C2}}}\\
+\frac{[\mathrm{pC3bC4bC2}]}{K_{\mathrm{m\;MASP2^*}}^{\mathrm{C2}}}+\frac{[\mathrm{pMBL_3MASP1_{zym}}]}{K_{\mathrm{m\;MASP2^*}}^{\mathrm{MASP1_{zym}}}}+\frac{[\mathrm{pMBL_3MASP2_{zym}}]}{K_{\mathrm{m\;MASP2^*}}^{\mathrm{MASP2_{zym}}}}+\frac{[\mathrm{pMBL_4MASP1_{zym}}]}{K_{\mathrm{m\;MASP2^*}}^{\mathrm{MASP1_{zym}}}}\\
+\frac{[\mathrm{pMBL_4MASP2_{zym}}]}{K_{\mathrm{m\;MASP2^*}}^{\mathrm{MASP2_{zym}}}}+\frac{[\mathrm{pCLLKMASP1_{zym}}]}{K_{\mathrm{m\;MASP2^*}}^{\mathrm{MASP1_{zym}}}}+\frac{[\mathrm{pCLLKMASP2_{zym}}]}{K_{\mathrm{m\;MASP2^*}}^{\mathrm{MASP2_{zym}}}}+\frac{[\mathrm{pCLK1MASP1_{zym}}]}{K_{\mathrm{m\;MASP2^*}}^{\mathrm{MASP1_{zym}}}}\\
+\frac{[\mathrm{pCLK1MASP2_{zym}}]}{K_{\mathrm{m\;MASP2^*}}^{\mathrm{MASP2_{zym}}}}+\frac{[\mathrm{pCLL1MASP1_{zym}}]}{K_{\mathrm{m\;MASP2^*}}^{\mathrm{MASP1_{zym}}}}+\frac{[\mathrm{pCLL1MASP2_{zym}}]}{K_{\mathrm{m\;MASP2^*}}^{\mathrm{MASP2_{zym}}}}+\frac{[\mathrm{pLFMASP1_{zym}}]}{K_{\mathrm{m\;MASP2^*}}^{\mathrm{MASP1_{zym}}}}\\
+\frac{[\mathrm{pLFMASP2_{zym}}]}{K_{\mathrm{m\;MASP2^*}}^{\mathrm{MASP2_{zym}}}}+\frac{[\mathrm{pHFMASP1_{zym}}]}{K_{\mathrm{m\;MASP2^*}}^{\mathrm{MASP1_{zym}}}}+\frac{[\mathrm{pHFMASP2_{zym}}]}{K_{\mathrm{m\;MASP2^*}}^{\mathrm{MASP2_{zym}}}}+\frac{[\mathrm{pMFMASP1_{zym}}]}{K_{\mathrm{m\;MASP2^*}}^{\mathrm{MASP1_{zym}}}}+\frac{[\mathrm{pMFMASP2_{zym}}]}{K_{\mathrm{m\;MASP2^*}}^{\mathrm{MASP2_{zym}}}}\\
+\frac{[\mathrm{pPTX3LFMASP1_{zym}}]}{K_{\mathrm{m\;MASP2^*}}^{\mathrm{MASP1_{zym}}}}+\frac{[\mathrm{pPTX3LFMASP2_{zym}}]}{K_{\mathrm{m\;MASP2^*}}^{\mathrm{MASP2_{zym}}}}\end{array}\right)}
$$

$$
+\frac{k_{\mathrm{cat\ MASP2^*}}^{\mathrm{MASP2_{zym}}}\left[\mathrm{pLFMASP2^*}\right]\left[\mathrm{pMBL_3MASP2_{zym}}\right]}{K_{\mathrm{m\ MASP2^*}}^{\mathrm{MASP2_{zym}}}\begin{pmatrix}1+\frac{[\mathrm{C4}]}{K_{\mathrm{m\ MASP2^*}}^{\mathrm{C4}}}+\frac{[\mathrm{fC4bC2}]}{K_{\mathrm{m\ MASP2^*}}^{\mathrm{C2}}}+\frac{[\mathrm{fC4bC4bC2}]}{K_{\mathrm{m\ MASP2^*}}^{\mathrm{C2}}}+\frac{[\mathrm{fC3bC4bC2}]}{K_{\mathrm{m\ MASP2^*}}^{\mathrm{C2}}}+\frac{[\mathrm{pC4bC2}]}{K_{\mathrm{m\ MASP2^*}}^{\mathrm{C2}}}+\frac{[\mathrm{pC4bC4bC2}]}{K_{\mathrm{m\ MASP2^*}}^{\mathrm{C2}}}\\
+\frac{[\mathrm{pC3bC4bC2}]}{K_{\mathrm{m\ MASP2^*}}^{\mathrm{C2}}}+\frac{[\mathrm{pMBL_3MASP1_{zym}}]}{K_{\mathrm{m\ MASP2^*}}^{\mathrm{MASP1_{zym}}}}+\frac{[\mathrm{pMBL_3MASP2_{zym}}]}{K_{\mathrm{m\ MASP2^*}}^{\mathrm{MASP2_{zym}}}}+\frac{[\mathrm{pMBL_4MASP1_{zym}}]}{K_{\mathrm{m\ MASP2^*}}^{\mathrm{MASP1_{zym}}}}\\
+\frac{[\mathrm{pMBL_4MASP2_{zym}}]}{K_{\mathrm{m\ MASP2^*}}^{\mathrm{MASP2_{zym}}}}+\frac{[\mathrm{pCLLKMASP1_{zym}}]}{K_{\mathrm{m\ MASP2^*}}^{\mathrm{MASP1_{zym}}}}+\frac{[\mathrm{pCLLKMASP2_{zym}}]}{K_{\mathrm{m\ MASP2^*}}^{\mathrm{MASP2_{zym}}}}+\frac{[\mathrm{pCLK1MASP1_{zym}}]}{K_{\mathrm{m\ MASP2^*}}^{\mathrm{MASP1_{zym}}}}\\
+\frac{[\mathrm{pCLK1MASP2_{zym}}]}{K_{\mathrm{m\ MASP2^*}}^{\mathrm{MASP2_{zym}}}}+\frac{[\mathrm{pCLL1MASP1_{zym}}]}{K_{\mathrm{m\ MASP2^*}}^{\mathrm{MASP1_{zym}}}}+\frac{[\mathrm{pCLL1MASP2_{zym}}]}{K_{\mathrm{m\ MASP2^*}}^{\mathrm{MASP2_{zym}}}}+\frac{[\mathrm{pLFMASP1_{zym}}]}{K_{\mathrm{m\ MASP2^*}}^{\mathrm{MASP1_{zym}}}}\\
+\frac{[\mathrm{pLFMASP2_{zym}}]}{K_{\mathrm{m\ MASP2^*}}^{\mathrm{MASP2_{zym}}}}+\frac{[\mathrm{pHFMASP1_{zym}}]}{K_{\mathrm{m\ MASP2^*}}^{\mathrm{MASP1_{zym}}}}+\frac{[\mathrm{pHFMASP2_{zym}}]}{K_{\mathrm{m\ MASP2^*}}^{\mathrm{MASP2_{zym}}}}+\frac{[\mathrm{pMFMASP1_{zym}}]}{K_{\mathrm{m\ MASP2^*}}^{\mathrm{MASP1_{zym}}}}+\frac{[\mathrm{pMFMASP2_{zym}}]}{K_{\mathrm{m\ MASP2^*}}^{\mathrm{MASP2_{zym}}}}\\
+\frac{[\mathrm{pPTX3LFMASP1_{zym}}]}{K_{\mathrm{m\ MASP2^*}}^{\mathrm{MASP1_{zym}}}}+\frac{[\mathrm{pPTX3LFMASP2_{zym}}]}{K_{\mathrm{m\ MASP2^*}}^{\mathrm{MASP2_{zym}}}}\end{pmatrix}}
$$

$$
+\frac{k_{\mathrm{cat\ MASP2^*}}^{\mathrm{MASP2_{zym}}}\left[\mathrm{pHFMASP2^*}\right]\left[\mathrm{pMBL_3MASP2_{zym}}\right]}{K_{\mathrm{m\ MASP2^*}}^{\mathrm{MASP2_{zym}}}\begin{pmatrix}1+\frac{[\mathrm{C4}]}{K_{\mathrm{m\ MASP2^*}}^{\mathrm{C4}}}+\frac{[\mathrm{fC4bC2}]}{K_{\mathrm{m\ MASP2^*}}^{\mathrm{C2}}}+\frac{[\mathrm{fC4bC4bC2}]}{K_{\mathrm{m\ MASP2^*}}^{\mathrm{C2}}}+\frac{[\mathrm{fC3bC4bC2}]}{K_{\mathrm{m\ MASP2^*}}^{\mathrm{C2}}}+\frac{[\mathrm{pC4bC2}]}{K_{\mathrm{m\ MASP2^*}}^{\mathrm{C2}}}+\frac{[\mathrm{pC4bC4bC2}]}{K_{\mathrm{m\ MASP2^*}}^{\mathrm{C2}}}\\
+\frac{[\mathrm{pC3bC4bC2}]}{K_{\mathrm{m\ MASP2^*}}^{\mathrm{C2}}}+\frac{[\mathrm{pMBL_3MASP1_{zym}}]}{K_{\mathrm{m\ MASP2^*}}^{\mathrm{MASP1_{zym}}}}+\frac{[\mathrm{pMBL_3MASP2_{zym}}]}{K_{\mathrm{m\ MASP2^*}}^{\mathrm{MASP2_{zym}}}}+\frac{[\mathrm{pMBL_4MASP1_{zym}}]}{K_{\mathrm{m\ MASP2^*}}^{\mathrm{MASP1_{zym}}}}\\
+\frac{[\mathrm{pMBL_4MASP2_{zym}}]}{K_{\mathrm{m\ MASP2^*}}^{\mathrm{MASP2_{zym}}}}+\frac{[\mathrm{pCLLKMASP1_{zym}}]}{K_{\mathrm{m\ MASP2^*}}^{\mathrm{MASP1_{zym}}}}+\frac{[\mathrm{pCLLKMASP2_{zym}}]}{K_{\mathrm{m\ MASP2^*}}^{\mathrm{MASP2_{zym}}}}+\frac{[\mathrm{pCLK1MASP1_{zym}}]}{K_{\mathrm{m\ MASP2^*}}^{\mathrm{MASP1_{zym}}}}\\
+\frac{[\mathrm{pCLK1MASP2_{zym}}]}{K_{\mathrm{m\ MASP2^*}}^{\mathrm{MASP2_{zym}}}}+\frac{[\mathrm{pCLL1MASP1_{zym}}]}{K_{\mathrm{m\ MASP2^*}}^{\mathrm{MASP1_{zym}}}}+\frac{[\mathrm{pCLL1MASP2_{zym}}]}{K_{\mathrm{m\ MASP2^*}}^{\mathrm{MASP2_{zym}}}}+\frac{[\mathrm{pLFMASP1_{zym}}]}{K_{\mathrm{m\ MASP2^*}}^{\mathrm{MASP1_{zym}}}}\\
+\frac{[\mathrm{pLFMASP2_{zym}}]}{K_{\mathrm{m\ MASP2^*}}^{\mathrm{MASP2_{zym}}}}+\frac{[\mathrm{pHFMASP1_{zym}}]}{K_{\mathrm{m\ MASP2^*}}^{\mathrm{MASP1_{zym}}}}+\frac{[\mathrm{pHFMASP2_{zym}}]}{K_{\mathrm{m\ MASP2^*}}^{\mathrm{MASP2_{zym}}}}+\frac{[\mathrm{pMFMASP1_{zym}}]}{K_{\mathrm{m\ MASP2^*}}^{\mathrm{MASP1_{zym}}}}+\frac{[\mathrm{pMFMASP2_{zym}}]}{K_{\mathrm{m\ MASP2^*}}^{\mathrm{MASP2_{zym}}}}\\
+\frac{[\mathrm{pPTX3LFMASP1_{zym}}]}{K_{\mathrm{m\ MASP2^*}}^{\mathrm{MASP1_{zym}}}}+\frac{[\mathrm{pPTX3LFMASP2_{zym}}]}{K_{\mathrm{m\ MASP2^*}}^{\mathrm{MASP2_{zym}}}}\end{pmatrix}}
$$

$$
\begin{aligned}
&+\frac{k_{\mathrm{cat\ MASP2^*}}^{\mathrm{MASP2_{zym}}}\left[\mathrm{pMFMASP2^*}\right]\left[\mathrm{pMBL_3MASP2_{zym}}\right]}{K_{\mathrm{m\ MASP2^*}}^{\mathrm{MASP2_{zym}}}\left(\begin{aligned}
&1+\frac{[\mathrm{C4}]}{K_{\mathrm{m\ MASP2^*}}^{\mathrm{C4}}}+\frac{[\mathrm{fC4bC2}]}{K_{\mathrm{m\ MASP2^*}}^{\mathrm{C2}}}+\frac{[\mathrm{fC4bC4bC2}]}{K_{\mathrm{m\ MASP2^*}}^{\mathrm{C2}}}+\frac{[\mathrm{fC3bC4bC2}]}{K_{\mathrm{m\ MASP2^*}}^{\mathrm{C2}}}+\frac{[\mathrm{pC4bC2}]}{K_{\mathrm{m\ MASP2^*}}^{\mathrm{C2}}}+\frac{[\mathrm{pC4bC4bC2}]}{K_{\mathrm{m\ MASP2^*}}^{\mathrm{C2}}}\\
&+\frac{[\mathrm{pC3bC4bC2}]}{K_{\mathrm{m\ MASP2^*}}^{\mathrm{C2}}}+\frac{[\mathrm{pMBL_3MASP1_{zym}}]}{K_{\mathrm{m\ MASP2^*}}^{\mathrm{MASP1_{zym}}}}+\frac{[\mathrm{pMBL_3MASP2_{zym}}]}{K_{\mathrm{m\ MASP2^*}}^{\mathrm{MASP2_{zym}}}}+\frac{[\mathrm{pMBL_4MASP1_{zym}}]}{K_{\mathrm{m\ MASP2^*}}^{\mathrm{MASP1_{zym}}}}\\
&+\frac{[\mathrm{pMBL_4MASP2_{zym}}]}{K_{\mathrm{m\ MASP2^*}}^{\mathrm{MASP2_{zym}}}}+\frac{[\mathrm{pCLLKMASP1_{zym}}]}{K_{\mathrm{m\ MASP2^*}}^{\mathrm{MASP1_{zym}}}}+\frac{[\mathrm{pCLLKMASP2_{zym}}]}{K_{\mathrm{m\ MASP2^*}}^{\mathrm{MASP2_{zym}}}}+\frac{[\mathrm{pCLK1MASP1_{zym}}]}{K_{\mathrm{m\ MASP2^*}}^{\mathrm{MASP1_{zym}}}}\\
&+\frac{[\mathrm{pCLK1MASP2_{zym}}]}{K_{\mathrm{m\ MASP2^*}}^{\mathrm{MASP2_{zym}}}}+\frac{[\mathrm{pCLL1MASP1_{zym}}]}{K_{\mathrm{m\ MASP2^*}}^{\mathrm{MASP1_{zym}}}}+\frac{[\mathrm{pCLL1MASP2_{zym}}]}{K_{\mathrm{m\ MASP2^*}}^{\mathrm{MASP2_{zym}}}}+\frac{[\mathrm{pLFMASP1_{zym}}]}{K_{\mathrm{m\ MASP2^*}}^{\mathrm{MASP1_{zym}}}}\\
&+\frac{[\mathrm{pLFMASP2_{zym}}]}{K_{\mathrm{m\ MASP2^*}}^{\mathrm{MASP2_{zym}}}}+\frac{[\mathrm{pHFMASP1_{zym}}]}{K_{\mathrm{m\ MASP2^*}}^{\mathrm{MASP1_{zym}}}}+\frac{[\mathrm{pHFMASP2_{zym}}]}{K_{\mathrm{m\ MASP2^*}}^{\mathrm{MASP2_{zym}}}}+\frac{[\mathrm{pMFMASP1_{zym}}]}{K_{\mathrm{m\ MASP2^*}}^{\mathrm{MASP1_{zym}}}}+\frac{[\mathrm{pMFMASP2_{zym}}]}{K_{\mathrm{m\ MASP2^*}}^{\mathrm{MASP2_{zym}}}}\\
&+\frac{[\mathrm{pPTX3LFMASP1_{zym}}]}{K_{\mathrm{m\ MASP2^*}}^{\mathrm{MASP1_{zym}}}}+\frac{[\mathrm{pPTX3LFMASP2_{zym}}]}{K_{\mathrm{m\ MASP2^*}}^{\mathrm{MASP2_{zym}}}}
\end{aligned}\right)}\\
&+\frac{k_{\mathrm{cat\ MASP2^*}}^{\mathrm{MASP2_{zym}}}\left[\mathrm{pPTX3LFMASP2^*}\right]\left[\mathrm{pMBL_3MASP2_{zym}}\right]}{K_{\mathrm{m\ MASP2^*}}^{\mathrm{MASP2_{zym}}}\left(\begin{aligned}
&1+\frac{[\mathrm{C4}]}{K_{\mathrm{m\ MASP2^*}}^{\mathrm{C4}}}+\frac{[\mathrm{fC4bC2}]}{K_{\mathrm{m\ MASP2^*}}^{\mathrm{C2}}}+\frac{[\mathrm{fC4bC4bC2}]}{K_{\mathrm{m\ MASP2^*}}^{\mathrm{C2}}}+\frac{[\mathrm{fC3bC4bC2}]}{K_{\mathrm{m\ MASP2^*}}^{\mathrm{C2}}}+\frac{[\mathrm{pC4bC2}]}{K_{\mathrm{m\ MASP2^*}}^{\mathrm{C2}}}+\frac{[\mathrm{pC4bC4bC2}]}{K_{\mathrm{m\ MASP2^*}}^{\mathrm{C2}}}\\
&+\frac{[\mathrm{pC3bC4bC2}]}{K_{\mathrm{m\ MASP2^*}}^{\mathrm{C2}}}+\frac{[\mathrm{pMBL_3MASP1_{zym}}]}{K_{\mathrm{m\ MASP2^*}}^{\mathrm{MASP1_{zym}}}}+\frac{[\mathrm{pMBL_3MASP2_{zym}}]}{K_{\mathrm{m\ MASP2^*}}^{\mathrm{MASP2_{zym}}}}+\frac{[\mathrm{pMBL_4MASP1_{zym}}]}{K_{\mathrm{m\ MASP2^*}}^{\mathrm{MASP1_{zym}}}}\\
&+\frac{[\mathrm{pMBL_4MASP2_{zym}}]}{K_{\mathrm{m\ MASP2^*}}^{\mathrm{MASP2_{zym}}}}+\frac{[\mathrm{pCLLKMASP1_{zym}}]}{K_{\mathrm{m\ MASP2^*}}^{\mathrm{MASP1_{zym}}}}+\frac{[\mathrm{pCLLKMASP2_{zym}}]}{K_{\mathrm{m\ MASP2^*}}^{\mathrm{MASP2_{zym}}}}+\frac{[\mathrm{pCLK1MASP1_{zym}}]}{K_{\mathrm{m\ MASP2^*}}^{\mathrm{MASP1_{zym}}}}\\
&+\frac{[\mathrm{pCLK1MASP2_{zym}}]}{K_{\mathrm{m\ MASP2^*}}^{\mathrm{MASP2_{zym}}}}+\frac{[\mathrm{pCLL1MASP1_{zym}}]}{K_{\mathrm{m\ MASP2^*}}^{\mathrm{MASP1_{zym}}}}+\frac{[\mathrm{pCLL1MASP2_{zym}}]}{K_{\mathrm{m\ MASP2^*}}^{\mathrm{MASP2_{zym}}}}+\frac{[\mathrm{pLFMASP1_{zym}}]}{K_{\mathrm{m\ MASP2^*}}^{\mathrm{MASP1_{zym}}}}\\
&+\frac{[\mathrm{pLFMASP2_{zym}}]}{K_{\mathrm{m\ MASP2^*}}^{\mathrm{MASP2_{zym}}}}+\frac{[\mathrm{pHFMASP1_{zym}}]}{K_{\mathrm{m\ MASP2^*}}^{\mathrm{MASP1_{zym}}}}+\frac{[\mathrm{pHFMASP2_{zym}}]}{K_{\mathrm{m\ MASP2^*}}^{\mathrm{MASP2_{zym}}}}+\frac{[\mathrm{pMFMASP1_{zym}}]}{K_{\mathrm{m\ MASP2^*}}^{\mathrm{MASP1_{zym}}}}+\frac{[\mathrm{pMFMASP2_{zym}}]}{K_{\mathrm{m\ MASP2^*}}^{\mathrm{MASP2_{zym}}}}\\
&+\frac{[\mathrm{pPTX3LFMASP1_{zym}}]}{K_{\mathrm{m\ MASP2^*}}^{\mathrm{MASP1_{zym}}}}+\frac{[\mathrm{pPTX3LFMASP2_{zym}}]}{K_{\mathrm{m\ MASP2^*}}^{\mathrm{MASP2_{zym}}}}
\end{aligned}\right)}\\
&-k_{\mathrm{C1INHMASP2}}^{+}\left[\mathrm{C1\text{-}INH}\right]\left[\mathrm{pMBL_3MASP2^*}\right]
\end{aligned}
$$

252

$$
\begin{aligned}
\frac{d\left[\mathrm{fMBL_3MASP3_{zym}}\right]}{dt}&=k_{\mathrm{MBL_3MASP3}}^{+}\left[\mathrm{MBL_3}\right]\left[\mathrm{MASP3}\right]-k_{\mathrm{MBL_3MASP3}}^{-}\left[\mathrm{fMBL_3MASP3_{zym}}\right]\\
&-k_{\mathrm{pMBL_3}}^{+}\left[\mathrm{fMBL_3MASP3_{zym}}\right]\left[\mathrm{Pathogen\ Surface}\right]+k_{\mathrm{pMBL_3}}^{-}\left[\mathrm{pMBL_3MASP3_{zym}}\right]
\end{aligned}
$$

253

$$
\begin{aligned}
\frac{d\left[\mathrm{pMBL_3MASP3_{zym}}\right]}{dt} &= k^{+}_{\mathrm{pMBL_3}}\left[\mathrm{fMBL_3MASP3_{zym}}\right]\left[\mathrm{Pathogen\ Surface}\right] - k^{-}_{\mathrm{pMBL_3}}\left[\mathrm{pMBL_3MASP3_{zym}}\right] \\
&+ k^{+}_{\mathrm{MBL_3MASP3}}\left[\mathrm{pMBL_3}\right]\left[\mathrm{MASP3}\right] - k^{-}_{\mathrm{MBL_3MASP3}}\left[\mathrm{pMBL_3MASP3_{zym}}\right] \\
&- \frac{k^{\mathrm{MASP3_{zym}}}_{\mathrm{cat\ MASP1^*}}\left[\mathrm{pMBL_3MASP1^*}\right]\left[\mathrm{pMBL_3MASP3_{zym}}\right]}{K^{\mathrm{MASP3_{zym}}}_{\mathrm{m\ MASP1^*}}\left(\begin{aligned}
&1 + \frac{\left[\mathrm{C4}\right]}{K^{\mathrm{C4}}_{\mathrm{m\ MASP1^*}}} + \frac{\left[\mathrm{fC4bC2}\right]}{K^{\mathrm{C2}}_{\mathrm{m\ MASP1^*}}} + \frac{\left[\mathrm{fC4bC4bC2}\right]}{K^{\mathrm{C2}}_{\mathrm{m\ MASP1^*}}} + \frac{\left[\mathrm{fC3bC4bC2}\right]}{K^{\mathrm{C2}}_{\mathrm{m\ MASP1^*}}} + \frac{\left[\mathrm{pC4bC2}\right]}{K^{\mathrm{C2}}_{\mathrm{m\ MASP1^*}}} + \frac{\left[\mathrm{pC4bC4bC2}\right]}{K^{\mathrm{C2}}_{\mathrm{m\ MASP1^*}}} \\
&+ \frac{\left[\mathrm{pC3bC4bC2}\right]}{K^{\mathrm{C2}}_{\mathrm{m\ MASP1^*}}} + \frac{\left[\mathrm{pMBL_3MASP1_{zym}}\right]}{K^{\mathrm{MASP1_{zym}}}_{\mathrm{m\ MASP1^*}}} + \frac{\left[\mathrm{pMBL_3MASP2_{zym}}\right]}{K^{\mathrm{MASP2_{zym}}}_{\mathrm{m\ MASP1^*}}} + \frac{\left[\mathrm{pMBL_3MASP3_{zym}}\right]}{K^{\mathrm{MASP3_{zym}}}_{\mathrm{m\ MASP1^*}}} \\
&+ \frac{\left[\mathrm{pMBL_4MASP1_{zym}}\right]}{K^{\mathrm{MASP1_{zym}}}_{\mathrm{m\ MASP1^*}}} + \frac{\left[\mathrm{pMBL_4MASP2_{zym}}\right]}{K^{\mathrm{MASP2_{zym}}}_{\mathrm{m\ MASP1^*}}} + \frac{\left[\mathrm{pMBL_4MASP3_{zym}}\right]}{K^{\mathrm{MASP3_{zym}}}_{\mathrm{m\ MASP1^*}}} + \frac{\left[\mathrm{pCLLKMASP1_{zym}}\right]}{K^{\mathrm{MASP1_{zym}}}_{\mathrm{m\ MASP1^*}}} \\
&+ \frac{\left[\mathrm{pCLLKMASP2_{zym}}\right]}{K^{\mathrm{MASP2_{zym}}}_{\mathrm{m\ MASP1^*}}} + \frac{\left[\mathrm{pCLLKMASP3_{zym}}\right]}{K^{\mathrm{MASP3_{zym}}}_{\mathrm{m\ MASP1^*}}} + \frac{\left[\mathrm{pCLK1MASP1_{zym}}\right]}{K^{\mathrm{MASP1_{zym}}}_{\mathrm{m\ MASP1^*}}} + \frac{\left[\mathrm{pCLK1MASP2_{zym}}\right]}{K^{\mathrm{MASP2_{zym}}}_{\mathrm{m\ MASP1^*}}} \\
&+ \frac{\left[\mathrm{pCLK1MASP3_{zym}}\right]}{K^{\mathrm{MASP3_{zym}}}_{\mathrm{m\ MASP1^*}}} + \frac{\left[\mathrm{pCLL1MASP1_{zym}}\right]}{K^{\mathrm{MASP1_{zym}}}_{\mathrm{m\ MASP1^*}}} + \frac{\left[\mathrm{pCLL1MASP2_{zym}}\right]}{K^{\mathrm{MASP2_{zym}}}_{\mathrm{m\ MASP1^*}}} + \frac{\left[\mathrm{pCLL1MASP3_{zym}}\right]}{K^{\mathrm{MASP3_{zym}}}_{\mathrm{m\ MASP1^*}}} \\
&+ \frac{\left[\mathrm{pLFMASP1_{zym}}\right]}{K^{\mathrm{MASP1_{zym}}}_{\mathrm{m\ MASP1^*}}} + \frac{\left[\mathrm{pLFMASP2_{zym}}\right]}{K^{\mathrm{MASP2_{zym}}}_{\mathrm{m\ MASP1^*}}} + \frac{\left[\mathrm{pLFMASP3_{zym}}\right]}{K^{\mathrm{MASP3_{zym}}}_{\mathrm{m\ MASP1^*}}} + \frac{\left[\mathrm{pHFMASP1_{zym}}\right]}{K^{\mathrm{MASP1_{zym}}}_{\mathrm{m\ MASP1^*}}} + \frac{\left[\mathrm{pHFMASP2_{zym}}\right]}{K^{\mathrm{MASP2_{zym}}}_{\mathrm{m\ MASP1^*}}} \\
&+ \frac{\left[\mathrm{pHFMASP3_{zym}}\right]}{K^{\mathrm{MASP3_{zym}}}_{\mathrm{m\ MASP1^*}}} + \frac{\left[\mathrm{pMFMASP1_{zym}}\right]}{K^{\mathrm{MASP1_{zym}}}_{\mathrm{m\ MASP1^*}}} + \frac{\left[\mathrm{pMFMASP2_{zym}}\right]}{K^{\mathrm{MASP2_{zym}}}_{\mathrm{m\ MASP1^*}}} + \frac{\left[\mathrm{pMFMASP3_{zym}}\right]}{K^{\mathrm{MASP3_{zym}}}_{\mathrm{m\ MASP1^*}}} \\
&+ \frac{\left[\mathrm{pPTX3LFMASP1_{zym}}\right]}{K^{\mathrm{MASP1_{zym}}}_{\mathrm{m\ MASP1^*}}} + \frac{\left[\mathrm{pPXT3LFMASP2_{zym}}\right]}{K^{\mathrm{MASP2_{zym}}}_{\mathrm{m\ MASP1^*}}} + \frac{\left[\mathrm{pPTX3LFMASP3_{zym}}\right]}{K^{\mathrm{MASP3_{zym}}}_{\mathrm{m\ MASP1^*}}}
\end{aligned}\right)}
\end{aligned}
$$

$$
-\frac{k_{\mathrm{cat\,MASP1^*}}^{\mathrm{MASP3_{zym}}}\left[\mathrm{pMBL_4MASP1^*}\right]\left[\mathrm{pMBL_3MASP3_{zym}}\right]}{K_{\mathrm{m\,MASP1^*}}^{\mathrm{MASP3_{zym}}}\left(\begin{aligned}
&1+\frac{[\mathrm{C4}]}{K_{\mathrm{m\,MASP1^*}}^{\mathrm{C4}}}+\frac{[\mathrm{fC4bC2}]}{K_{\mathrm{m\,MASP1^*}}^{\mathrm{C2}}}+\frac{[\mathrm{fC4bC4bC2}]}{K_{\mathrm{m\,MASP1^*}}^{\mathrm{C2}}}+\frac{[\mathrm{fC3bC4bC2}]}{K_{\mathrm{m\,MASP1^*}}^{\mathrm{C2}}}+\frac{[\mathrm{pC4bC2}]}{K_{\mathrm{m\,MASP1^*}}^{\mathrm{C2}}}+\frac{[\mathrm{pC4bC4bC2}]}{K_{\mathrm{m\,MASP1^*}}^{\mathrm{C2}}}\\
&+\frac{[\mathrm{pC3bC4bC2}]}{K_{\mathrm{m\,MASP1^*}}^{\mathrm{C2}}}+\frac{\left[\mathrm{pMBL_3MASP1_{zym}}\right]}{K_{\mathrm{m\,MASP1^*}}^{\mathrm{MASP1_{zym}}}}+\frac{\left[\mathrm{pMBL_3MASP2_{zym}}\right]}{K_{\mathrm{m\,MASP1^*}}^{\mathrm{MASP2_{zym}}}}+\frac{\left[\mathrm{pMBL_3MASP3_{zym}}\right]}{K_{\mathrm{m\,MASP1^*}}^{\mathrm{MASP3_{zym}}}}\\
&+\frac{\left[\mathrm{pMBL_4MASP1_{zym}}\right]}{K_{\mathrm{m\,MASP1^*}}^{\mathrm{MASP1_{zym}}}}+\frac{\left[\mathrm{pMBL_4MASP2_{zym}}\right]}{K_{\mathrm{m\,MASP1^*}}^{\mathrm{MASP2_{zym}}}}+\frac{\left[\mathrm{pMBL_4MASP3_{zym}}\right]}{K_{\mathrm{m\,MASP1^*}}^{\mathrm{MASP3_{zym}}}}+\frac{\left[\mathrm{pCLLKMASP1_{zym}}\right]}{K_{\mathrm{m\,MASP1^*}}^{\mathrm{MASP1_{zym}}}}\\
&+\frac{\left[\mathrm{pCLLKMASP2_{zym}}\right]}{K_{\mathrm{m\,MASP1^*}}^{\mathrm{MASP2_{zym}}}}+\frac{\left[\mathrm{pCLLKMASP3_{zym}}\right]}{K_{\mathrm{m\,MASP1^*}}^{\mathrm{MASP3_{zym}}}}+\frac{\left[\mathrm{pCLK1MASP1_{zym}}\right]}{K_{\mathrm{m\,MASP1^*}}^{\mathrm{MASP1_{zym}}}}+\frac{\left[\mathrm{pCLK1MASP2_{zym}}\right]}{K_{\mathrm{m\,MASP1^*}}^{\mathrm{MASP2_{zym}}}}\\
&+\frac{\left[\mathrm{pCLK1MASP3_{zym}}\right]}{K_{\mathrm{m\,MASP1^*}}^{\mathrm{MASP3_{zym}}}}+\frac{\left[\mathrm{pCLL1MASP1_{zym}}\right]}{K_{\mathrm{m\,MASP1^*}}^{\mathrm{MASP1_{zym}}}}+\frac{\left[\mathrm{pCLL1MASP2_{zym}}\right]}{K_{\mathrm{m\,MASP1^*}}^{\mathrm{MASP2_{zym}}}}+\frac{\left[\mathrm{pCLL1MASP3_{zym}}\right]}{K_{\mathrm{m\,MASP1^*}}^{\mathrm{MASP3_{zym}}}}\\
&+\frac{\left[\mathrm{pLFMASP1_{zym}}\right]}{K_{\mathrm{m\,MASP1^*}}^{\mathrm{MASP1_{zym}}}}+\frac{\left[\mathrm{pLFMASP2_{zym}}\right]}{K_{\mathrm{m\,MASP1^*}}^{\mathrm{MASP2_{zym}}}}+\frac{\left[\mathrm{pLFMASP3_{zym}}\right]}{K_{\mathrm{m\,MASP1^*}}^{\mathrm{MASP3_{zym}}}}+\frac{\left[\mathrm{pHFMASP1_{zym}}\right]}{K_{\mathrm{m\,MASP1^*}}^{\mathrm{MASP1_{zym}}}}+\frac{\left[\mathrm{pHFMASP2_{zym}}\right]}{K_{\mathrm{m\,MASP1^*}}^{\mathrm{MASP2_{zym}}}}\\
&+\frac{\left[\mathrm{pHFMASP3_{zym}}\right]}{K_{\mathrm{m\,MASP1^*}}^{\mathrm{MASP3_{zym}}}}+\frac{\left[\mathrm{pMFMASP1_{zym}}\right]}{K_{\mathrm{m\,MASP1^*}}^{\mathrm{MASP1_{zym}}}}+\frac{\left[\mathrm{pMFMASP2_{zym}}\right]}{K_{\mathrm{m\,MASP1^*}}^{\mathrm{MASP2_{zym}}}}+\frac{\left[\mathrm{pMFMASP3_{zym}}\right]}{K_{\mathrm{m\,MASP1^*}}^{\mathrm{MASP3_{zym}}}}\\
&+\frac{\left[\mathrm{pPTX3LFMASP1_{zym}}\right]}{K_{\mathrm{m\,MASP1^*}}^{\mathrm{MASP1_{zym}}}}+\frac{\left[\mathrm{pPXT3LFMASP2_{zym}}\right]}{K_{\mathrm{m\,MASP1^*}}^{\mathrm{MASP2_{zym}}}}+\frac{\left[\mathrm{pPTX3LFMASP3_{zym}}\right]}{K_{\mathrm{m\,MASP1^*}}^{\mathrm{MASP3_{zym}}}}
\end{aligned}\right)}
$$

$$
-\frac{k_{\text{cat MASP1}^*}^{\text{MASP3}_\text{zym}}\left[\text{pCLLKMASP1}^*\right]\left[\text{pMBL}_3\text{MASP3}_\text{zym}\right]}{K_{\text{m MASP1}^*}^{\text{MASP3}_\text{zym}}\left(\begin{array}{l}1+\frac{[\text{C4}]}{K_{\text{m MASP1}^*}^{\text{C4}}}+\frac{[\text{fC4bC2}]}{K_{\text{m MASP1}^*}^{\text{C2}}}+\frac{[\text{fC4bC4bC2}]}{K_{\text{m MASP1}^*}^{\text{C2}}}+\frac{[\text{fC3bC4bC2}]}{K_{\text{m MASP1}^*}^{\text{C2}}}+\frac{[\text{pC4bC2}]}{K_{\text{m MASP1}^*}^{\text{C2}}}+\frac{[\text{pC4bC4bC2}]}{K_{\text{m MASP1}^*}^{\text{C2}}}\\
+\frac{[\text{pC3bC4bC2}]}{K_{\text{m MASP1}^*}^{\text{C2}}}+\frac{\left[\text{pMBL}_3\text{MASP1}_\text{zym}\right]}{K_{\text{m MASP1}^*}^{\text{MASP1}_\text{zym}}}+\frac{\left[\text{pMBL}_3\text{MASP2}_\text{zym}\right]}{K_{\text{m MASP1}^*}^{\text{MASP2}_\text{zym}}}+\frac{\left[\text{pMBL}_3\text{MASP3}_\text{zym}\right]}{K_{\text{m MASP1}^*}^{\text{MASP3}_\text{zym}}}\\
+\frac{\left[\text{pMBL}_4\text{MASP1}_\text{zym}\right]}{K_{\text{m MASP1}^*}^{\text{MASP1}_\text{zym}}}+\frac{\left[\text{pMBL}_4\text{MASP2}_\text{zym}\right]}{K_{\text{m MASP1}^*}^{\text{MASP2}_\text{zym}}}+\frac{\left[\text{pMBL}_4\text{MASP3}_\text{zym}\right]}{K_{\text{m MASP1}^*}^{\text{MASP3}_\text{zym}}}+\frac{\left[\text{pCLLKMASP1}_\text{zym}\right]}{K_{\text{m MASP1}^*}^{\text{MASP1}_\text{zym}}}\\
+\frac{\left[\text{pCLLKMASP2}_\text{zym}\right]}{K_{\text{m MASP1}^*}^{\text{MASP2}_\text{zym}}}+\frac{\left[\text{pCLLKMASP3}_\text{zym}\right]}{K_{\text{m MASP1}^*}^{\text{MASP3}_\text{zym}}}+\frac{\left[\text{pCLK1MASP1}_\text{zym}\right]}{K_{\text{m MASP1}^*}^{\text{MASP1}_\text{zym}}}+\frac{\left[\text{pCLK1MASP2}_\text{zym}\right]}{K_{\text{m MASP1}^*}^{\text{MASP2}_\text{zym}}}\\
+\frac{\left[\text{pCLK1MASP3}_\text{zym}\right]}{K_{\text{m MASP1}^*}^{\text{MASP3}_\text{zym}}}+\frac{\left[\text{pCLL1MASP1}_\text{zym}\right]}{K_{\text{m MASP1}^*}^{\text{MASP1}_\text{zym}}}+\frac{\left[\text{pCLL1MASP2}_\text{zym}\right]}{K_{\text{m MASP1}^*}^{\text{MASP2}_\text{zym}}}+\frac{\left[\text{pCLL1MASP3}_\text{zym}\right]}{K_{\text{m MASP1}^*}^{\text{MASP3}_\text{zym}}}\\
+\frac{\left[\text{pLFMASP1}_\text{zym}\right]}{K_{\text{m MASP1}^*}^{\text{MASP1}_\text{zym}}}+\frac{\left[\text{pLFMASP2}_\text{zym}\right]}{K_{\text{m MASP1}^*}^{\text{MASP2}_\text{zym}}}+\frac{\left[\text{pLFMASP3}_\text{zym}\right]}{K_{\text{m MASP1}^*}^{\text{MASP3}_\text{zym}}}+\frac{\left[\text{pHFMASP1}_\text{zym}\right]}{K_{\text{m MASP1}^*}^{\text{MASP1}_\text{zym}}}+\frac{\left[\text{pHFMASP2}_\text{zym}\right]}{K_{\text{m MASP1}^*}^{\text{MASP2}_\text{zym}}}\\
+\frac{\left[\text{pHFMASP3}_\text{zym}\right]}{K_{\text{m MASP1}^*}^{\text{MASP3}_\text{zym}}}+\frac{\left[\text{pMFMASP1}_\text{zym}\right]}{K_{\text{m MASP1}^*}^{\text{MASP1}_\text{zym}}}+\frac{\left[\text{pMFMASP2}_\text{zym}\right]}{K_{\text{m MASP1}^*}^{\text{MASP2}_\text{zym}}}+\frac{\left[\text{pMFMASP3}_\text{zym}\right]}{K_{\text{m MASP1}^*}^{\text{MASP3}_\text{zym}}}\\
+\frac{\left[\text{pPTX3LFMASP1}_\text{zym}\right]}{K_{\text{m MASP1}^*}^{\text{MASP1}_\text{zym}}}+\frac{\left[\text{pPXT3LFMASP2}_\text{zym}\right]}{K_{\text{m MASP1}^*}^{\text{MASP2}_\text{zym}}}+\frac{\left[\text{pPTX3LFMASP3}_\text{zym}\right]}{K_{\text{m MASP1}^*}^{\text{MASP3}_\text{zym}}}\end{array}\right)}
$$

$$
-\frac{k_{\mathrm{cat\ MASP1^*}}^{\mathrm{MASP3_{zym}}}\left[\mathrm{pCLK1MASP1^*}\right]\left[\mathrm{pMBL_3MASP3_{zym}}\right]}{K_{\mathrm{m\ MASP1^*}}^{\mathrm{MASP3_{zym}}}\left(\begin{array}{l}
1+\frac{[\mathrm{C4}]}{K_{\mathrm{m\ MASP1^*}}^{\mathrm{C4}}}+\frac{[\mathrm{fC4bC2}]}{K_{\mathrm{m\ MASP1^*}}^{\mathrm{C2}}}+\frac{[\mathrm{fC4bC4bC2}]}{K_{\mathrm{m\ MASP1^*}}^{\mathrm{C2}}}+\frac{[\mathrm{fC3bC4bC2}]}{K_{\mathrm{m\ MASP1^*}}^{\mathrm{C2}}}+\frac{[\mathrm{pC4bC2}]}{K_{\mathrm{m\ MASP1^*}}^{\mathrm{C2}}}+\frac{[\mathrm{pC4bC4bC2}]}{K_{\mathrm{m\ MASP1^*}}^{\mathrm{C2}}}\\
+\frac{[\mathrm{pC3bC4bC2}]}{K_{\mathrm{m\ MASP1^*}}^{\mathrm{C2}}}+\frac{[\mathrm{pMBL_3MASP1_{zym}}]}{K_{\mathrm{m\ MASP1^*}}^{\mathrm{MASP1_{zym}}}}+\frac{[\mathrm{pMBL_3MASP2_{zym}}]}{K_{\mathrm{m\ MASP1^*}}^{\mathrm{MASP2_{zym}}}}+\frac{[\mathrm{pMBL_3MASP3_{zym}}]}{K_{\mathrm{m\ MASP1^*}}^{\mathrm{MASP3_{zym}}}}\\
+\frac{[\mathrm{pMBL_4MASP1_{zym}}]}{K_{\mathrm{m\ MASP1^*}}^{\mathrm{MASP1_{zym}}}}+\frac{[\mathrm{pMBL_4MASP2_{zym}}]}{K_{\mathrm{m\ MASP1^*}}^{\mathrm{MASP2_{zym}}}}+\frac{[\mathrm{pMBL_4MASP3_{zym}}]}{K_{\mathrm{m\ MASP1^*}}^{\mathrm{MASP3_{zym}}}}+\frac{[\mathrm{pCLLKMASP1_{zym}}]}{K_{\mathrm{m\ MASP1^*}}^{\mathrm{MASP1_{zym}}}}\\
+\frac{[\mathrm{pCLLKMASP2_{zym}}]}{K_{\mathrm{m\ MASP1^*}}^{\mathrm{MASP2_{zym}}}}+\frac{[\mathrm{pCLLKMASP3_{zym}}]}{K_{\mathrm{m\ MASP1^*}}^{\mathrm{MASP3_{zym}}}}+\frac{[\mathrm{pCLK1MASP1_{zym}}]}{K_{\mathrm{m\ MASP1^*}}^{\mathrm{MASP1_{zym}}}}+\frac{[\mathrm{pCLK1MASP2_{zym}}]}{K_{\mathrm{m\ MASP1^*}}^{\mathrm{MASP2_{zym}}}}\\
+\frac{[\mathrm{pCLK1MASP3_{zym}}]}{K_{\mathrm{m\ MASP1^*}}^{\mathrm{MASP3_{zym}}}}+\frac{[\mathrm{pCLL1MASP1_{zym}}]}{K_{\mathrm{m\ MASP1^*}}^{\mathrm{MASP1_{zym}}}}+\frac{[\mathrm{pCLL1MASP2_{zym}}]}{K_{\mathrm{m\ MASP1^*}}^{\mathrm{MASP2_{zym}}}}+\frac{[\mathrm{pCLL1MASP3_{zym}}]}{K_{\mathrm{m\ MASP1^*}}^{\mathrm{MASP3_{zym}}}}\\
+\frac{[\mathrm{pLFMASP1_{zym}}]}{K_{\mathrm{m\ MASP1^*}}^{\mathrm{MASP1_{zym}}}}+\frac{[\mathrm{pLFMASP2_{zym}}]}{K_{\mathrm{m\ MASP1^*}}^{\mathrm{MASP2_{zym}}}}+\frac{[\mathrm{pLFMASP3_{zym}}]}{K_{\mathrm{m\ MASP1^*}}^{\mathrm{MASP3_{zym}}}}+\frac{[\mathrm{pHFMASP1_{zym}}]}{K_{\mathrm{m\ MASP1^*}}^{\mathrm{MASP1_{zym}}}}+\frac{[\mathrm{pHFMASP2_{zym}}]}{K_{\mathrm{m\ MASP1^*}}^{\mathrm{MASP2_{zym}}}}\\
+\frac{[\mathrm{pHFMASP3_{zym}}]}{K_{\mathrm{m\ MASP1^*}}^{\mathrm{MASP3_{zym}}}}+\frac{[\mathrm{pMFMASP1_{zym}}]}{K_{\mathrm{m\ MASP1^*}}^{\mathrm{MASP1_{zym}}}}+\frac{[\mathrm{pMFMASP2_{zym}}]}{K_{\mathrm{m\ MASP1^*}}^{\mathrm{MASP2_{zym}}}}+\frac{[\mathrm{pMFMASP3_{zym}}]}{K_{\mathrm{m\ MASP1^*}}^{\mathrm{MASP3_{zym}}}}\\
+\frac{[\mathrm{pPTX3LFMASP1_{zym}}]}{K_{\mathrm{m\ MASP1^*}}^{\mathrm{MASP1_{zym}}}}+\frac{[\mathrm{pPXT3LFMASP2_{zym}}]}{K_{\mathrm{m\ MASP1^*}}^{\mathrm{MASP2_{zym}}}}+\frac{[\mathrm{pPTX3LFMASP3_{zym}}]}{K_{\mathrm{m\ MASP1^*}}^{\mathrm{MASP3_{zym}}}}
\end{array}\right)}
$$

$$-\frac{k_{\text{cat MASP1}^*}^{\text{MASP3}_{\text{zym}}}\left[\text{pCLL1MASP1}^*\right]\left[\text{pMBL}_3\text{MASP3}_{\text{zym}}\right]}{K_{\text{m MASP1}^*}^{\text{MASP3}_{\text{zym}}}\left(\begin{array}{l}1+\frac{[\text{C4}]}{K_{\text{m MASP1}^*}^{\text{C4}}}+\frac{[\text{fC4bC2}]}{K_{\text{m MASP1}^*}^{\text{C2}}}+\frac{[\text{fC4bC4bC2}]}{K_{\text{m MASP1}^*}^{\text{C2}}}+\frac{[\text{fC3bC4bC2}]}{K_{\text{m MASP1}^*}^{\text{C2}}}+\frac{[\text{pC4bC2}]}{K_{\text{m MASP1}^*}^{\text{C2}}}+\frac{[\text{pC4bC4bC2}]}{K_{\text{m MASP1}^*}^{\text{C2}}}\\+\frac{[\text{pC3bC4bC2}]}{K_{\text{m MASP1}^*}^{\text{C2}}}+\frac{\left[\text{pMBL}_3\text{MASP1}_{\text{zym}}\right]}{K_{\text{m MASP1}^*}^{\text{MASP1}_{\text{zym}}}}+\frac{\left[\text{pMBL}_3\text{MASP2}_{\text{zym}}\right]}{K_{\text{m MASP1}^*}^{\text{MASP2}_{\text{zym}}}}+\frac{\left[\text{pMBL}_3\text{MASP3}_{\text{zym}}\right]}{K_{\text{m MASP1}^*}^{\text{MASP3}_{\text{zym}}}}\\+\frac{\left[\text{pMBL}_4\text{MASP1}_{\text{zym}}\right]}{K_{\text{m MASP1}^*}^{\text{MASP1}_{\text{zym}}}}+\frac{\left[\text{pMBL}_4\text{MASP2}_{\text{zym}}\right]}{K_{\text{m MASP1}^*}^{\text{MASP2}_{\text{zym}}}}+\frac{\left[\text{pMBL}_4\text{MASP3}_{\text{zym}}\right]}{K_{\text{m MASP1}^*}^{\text{MASP3}_{\text{zym}}}}+\frac{\left[\text{pCLLKMASP1}_{\text{zym}}\right]}{K_{\text{m MASP1}^*}^{\text{MASP1}_{\text{zym}}}}\\+\frac{\left[\text{pCLLKMASP2}_{\text{zym}}\right]}{K_{\text{m MASP1}^*}^{\text{MASP2}_{\text{zym}}}}+\frac{\left[\text{pCLLKMASP3}_{\text{zym}}\right]}{K_{\text{m MASP1}^*}^{\text{MASP3}_{\text{zym}}}}+\frac{\left[\text{pCLK1MASP1}_{\text{zym}}\right]}{K_{\text{m MASP1}^*}^{\text{MASP1}_{\text{zym}}}}+\frac{\left[\text{pCLK1MASP2}_{\text{zym}}\right]}{K_{\text{m MASP1}^*}^{\text{MASP2}_{\text{zym}}}}\\+\frac{\left[\text{pCLK1MASP3}_{\text{zym}}\right]}{K_{\text{m MASP1}^*}^{\text{MASP3}_{\text{zym}}}}+\frac{\left[\text{pCLL1MASP1}_{\text{zym}}\right]}{K_{\text{m MASP1}^*}^{\text{MASP1}_{\text{zym}}}}+\frac{\left[\text{pCLL1MASP2}_{\text{zym}}\right]}{K_{\text{m MASP1}^*}^{\text{MASP2}_{\text{zym}}}}+\frac{\left[\text{pCLL1MASP3}_{\text{zym}}\right]}{K_{\text{m MASP1}^*}^{\text{MASP3}_{\text{zym}}}}\\+\frac{\left[\text{pLFMASP1}_{\text{zym}}\right]}{K_{\text{m MASP1}^*}^{\text{MASP1}_{\text{zym}}}}+\frac{\left[\text{pLFMASP2}_{\text{zym}}\right]}{K_{\text{m MASP1}^*}^{\text{MASP2}_{\text{zym}}}}+\frac{\left[\text{pLFMASP3}_{\text{zym}}\right]}{K_{\text{m MASP1}^*}^{\text{MASP3}_{\text{zym}}}}+\frac{\left[\text{pHFMASP1}_{\text{zym}}\right]}{K_{\text{m MASP1}^*}^{\text{MASP1}_{\text{zym}}}}+\frac{\left[\text{pHFMASP2}_{\text{zym}}\right]}{K_{\text{m MASP1}^*}^{\text{MASP2}_{\text{zym}}}}\\+\frac{\left[\text{pHFMASP3}_{\text{zym}}\right]}{K_{\text{m MASP1}^*}^{\text{MASP3}_{\text{zym}}}}+\frac{\left[\text{pMFMASP1}_{\text{zym}}\right]}{K_{\text{m MASP1}^*}^{\text{MASP1}_{\text{zym}}}}+\frac{\left[\text{pMFMASP2}_{\text{zym}}\right]}{K_{\text{m MASP1}^*}^{\text{MASP2}_{\text{zym}}}}+\frac{\left[\text{pMFMASP3}_{\text{zym}}\right]}{K_{\text{m MASP1}^*}^{\text{MASP3}_{\text{zym}}}}\\+\frac{\left[\text{pPTX3LFMASP1}_{\text{zym}}\right]}{K_{\text{m MASP1}^*}^{\text{MASP1}_{\text{zym}}}}+\frac{\left[\text{pPXT3LFMASP2}_{\text{zym}}\right]}{K_{\text{m MASP1}^*}^{\text{MASP2}_{\text{zym}}}}+\frac{\left[\text{pPTX3LFMASP3}_{\text{zym}}\right]}{K_{\text{m MASP1}^*}^{\text{MASP3}_{\text{zym}}}}\end{array}\right)}$$

$$-\frac{k_{\mathrm{cat\ MASP1^*}}^{\mathrm{MASP3_{zym}}}\left[\mathrm{pLFMASP1^*}\right]\left[\mathrm{pMBL_3MASP3_{zym}}\right]}{K_{\mathrm{m\ MASP1^*}}^{\mathrm{MASP3_{zym}}}\left(\begin{array}{l}1+\frac{[\mathrm{C4}]}{K_{\mathrm{m\ MASP1^*}}^{\mathrm{C4}}}+\frac{[\mathrm{fC4bC2}]}{K_{\mathrm{m\ MASP1^*}}^{\mathrm{C2}}}+\frac{[\mathrm{fC4bC4bC2}]}{K_{\mathrm{m\ MASP1^*}}^{\mathrm{C2}}}+\frac{[\mathrm{fC3bC4bC2}]}{K_{\mathrm{m\ MASP1^*}}^{\mathrm{C2}}}+\frac{[\mathrm{pC4bC2}]}{K_{\mathrm{m\ MASP1^*}}^{\mathrm{C2}}}+\frac{[\mathrm{pC4bC4bC2}]}{K_{\mathrm{m\ MASP1^*}}^{\mathrm{C2}}}\\
+\frac{[\mathrm{pC3bC4bC2}]}{K_{\mathrm{m\ MASP1^*}}^{\mathrm{C2}}}+\frac{[\mathrm{pMBL_3MASP1_{zym}}]}{K_{\mathrm{m\ MASP1^*}}^{\mathrm{MASP1_{zym}}}}+\frac{[\mathrm{pMBL_3MASP2_{zym}}]}{K_{\mathrm{m\ MASP1^*}}^{\mathrm{MASP2_{zym}}}}+\frac{[\mathrm{pMBL_3MASP3_{zym}}]}{K_{\mathrm{m\ MASP1^*}}^{\mathrm{MASP3_{zym}}}}\\
+\frac{[\mathrm{pMBL_4MASP1_{zym}}]}{K_{\mathrm{m\ MASP1^*}}^{\mathrm{MASP1_{zym}}}}+\frac{[\mathrm{pMBL_4MASP2_{zym}}]}{K_{\mathrm{m\ MASP1^*}}^{\mathrm{MASP2_{zym}}}}+\frac{[\mathrm{pMBL_4MASP3_{zym}}]}{K_{\mathrm{m\ MASP1^*}}^{\mathrm{MASP3_{zym}}}}+\frac{[\mathrm{pCLLKMASP1_{zym}}]}{K_{\mathrm{m\ MASP1^*}}^{\mathrm{MASP1_{zym}}}}\\
+\frac{[\mathrm{pCLLKMASP2_{zym}}]}{K_{\mathrm{m\ MASP1^*}}^{\mathrm{MASP2_{zym}}}}+\frac{[\mathrm{pCLLKMASP3_{zym}}]}{K_{\mathrm{m\ MASP1^*}}^{\mathrm{MASP3_{zym}}}}+\frac{[\mathrm{pCLK1MASP1_{zym}}]}{K_{\mathrm{m\ MASP1^*}}^{\mathrm{MASP1_{zym}}}}+\frac{[\mathrm{pCLK1MASP2_{zym}}]}{K_{\mathrm{m\ MASP1^*}}^{\mathrm{MASP2_{zym}}}}\\
+\frac{[\mathrm{pCLK1MASP3_{zym}}]}{K_{\mathrm{m\ MASP1^*}}^{\mathrm{MASP3_{zym}}}}+\frac{[\mathrm{pCLL1MASP1_{zym}}]}{K_{\mathrm{m\ MASP1^*}}^{\mathrm{MASP1_{zym}}}}+\frac{[\mathrm{pCLL1MASP2_{zym}}]}{K_{\mathrm{m\ MASP1^*}}^{\mathrm{MASP2_{zym}}}}+\frac{[\mathrm{pCLL1MASP3_{zym}}]}{K_{\mathrm{m\ MASP1^*}}^{\mathrm{MASP3_{zym}}}}\\
+\frac{[\mathrm{pLFMASP1_{zym}}]}{K_{\mathrm{m\ MASP1^*}}^{\mathrm{MASP1_{zym}}}}+\frac{[\mathrm{pLFMASP2_{zym}}]}{K_{\mathrm{m\ MASP1^*}}^{\mathrm{MASP2_{zym}}}}+\frac{[\mathrm{pLFMASP3_{zym}}]}{K_{\mathrm{m\ MASP1^*}}^{\mathrm{MASP3_{zym}}}}+\frac{[\mathrm{pHFMASP1_{zym}}]}{K_{\mathrm{m\ MASP1^*}}^{\mathrm{MASP1_{zym}}}}+\frac{[\mathrm{pHFMASP2_{zym}}]}{K_{\mathrm{m\ MASP1^*}}^{\mathrm{MASP2_{zym}}}}\\
+\frac{[\mathrm{pHFMASP3_{zym}}]}{K_{\mathrm{m\ MASP1^*}}^{\mathrm{MASP3_{zym}}}}+\frac{[\mathrm{pMFMASP1_{zym}}]}{K_{\mathrm{m\ MASP1^*}}^{\mathrm{MASP1_{zym}}}}+\frac{[\mathrm{pMFMASP2_{zym}}]}{K_{\mathrm{m\ MASP1^*}}^{\mathrm{MASP2_{zym}}}}+\frac{[\mathrm{pMFMASP3_{zym}}]}{K_{\mathrm{m\ MASP1^*}}^{\mathrm{MASP3_{zym}}}}\\
+\frac{[\mathrm{pPTX3LFMASP1_{zym}}]}{K_{\mathrm{m\ MASP1^*}}^{\mathrm{MASP1_{zym}}}}+\frac{[\mathrm{pPXT3LFMASP2_{zym}}]}{K_{\mathrm{m\ MASP1^*}}^{\mathrm{MASP2_{zym}}}}+\frac{[\mathrm{pPTX3LFMASP3_{zym}}]}{K_{\mathrm{m\ MASP1^*}}^{\mathrm{MASP3_{zym}}}}\end{array}\right)}$$

$$
-\frac{k_{\text{cat MASP1}^{*}}^{\text{MASP3}_{\text{zym}}}\left[\text{pHFMASP1}^{*}\right]\left[\text{pMBL}_{3}\text{MASP3}_{\text{zym}}\right]}{K_{\text{m MASP1}^{*}}^{\text{MASP3}_{\text{zym}}}\left(\begin{array}{l}
1+\frac{[\text{C4}]}{K_{\text{m MASP1}^{*}}^{\text{C4}}}+\frac{[\text{fC4bC2}]}{K_{\text{m MASP1}^{*}}^{\text{C2}}}+\frac{[\text{fC4bC4bC2}]}{K_{\text{m MASP1}^{*}}^{\text{C2}}}+\frac{[\text{fC3bC4bC2}]}{K_{\text{m MASP1}^{*}}^{\text{C2}}}+\frac{[\text{pC4bC2}]}{K_{\text{m MASP1}^{*}}^{\text{C2}}}+\frac{[\text{pC4bC4bC2}]}{K_{\text{m MASP1}^{*}}^{\text{C2}}} \\
+\frac{[\text{pC3bC4bC2}]}{K_{\text{m MASP1}^{*}}^{\text{C2}}}+\frac{\left[\text{pMBL}_{3}\text{MASP1}_{\text{zym}}\right]}{K_{\text{m MASP1}^{*}}^{\text{MASP1}_{\text{zym}}}}+\frac{\left[\text{pMBL}_{3}\text{MASP2}_{\text{zym}}\right]}{K_{\text{m MASP1}^{*}}^{\text{MASP2}_{\text{zym}}}}+\frac{\left[\text{pMBL}_{3}\text{MASP3}_{\text{zym}}\right]}{K_{\text{m MASP1}^{*}}^{\text{MASP3}_{\text{zym}}}} \\
+\frac{\left[\text{pMBL}_{4}\text{MASP1}_{\text{zym}}\right]}{K_{\text{m MASP1}^{*}}^{\text{MASP1}_{\text{zym}}}}+\frac{\left[\text{pMBL}_{4}\text{MASP2}_{\text{zym}}\right]}{K_{\text{m MASP1}^{*}}^{\text{MASP2}_{\text{zym}}}}+\frac{\left[\text{pMBL}_{4}\text{MASP3}_{\text{zym}}\right]}{K_{\text{m MASP1}^{*}}^{\text{MASP3}_{\text{zym}}}}+\frac{\left[\text{pCLLKMASP1}_{\text{zym}}\right]}{K_{\text{m MASP1}^{*}}^{\text{MASP1}_{\text{zym}}}} \\
+\frac{\left[\text{pCLLKMASP2}_{\text{zym}}\right]}{K_{\text{m MASP1}^{*}}^{\text{MASP2}_{\text{zym}}}}+\frac{\left[\text{pCLLKMASP3}_{\text{zym}}\right]}{K_{\text{m MASP1}^{*}}^{\text{MASP3}_{\text{zym}}}}+\frac{\left[\text{pCLK1MASP1}_{\text{zym}}\right]}{K_{\text{m MASP1}^{*}}^{\text{MASP1}_{\text{zym}}}}+\frac{\left[\text{pCLK1MASP2}_{\text{zym}}\right]}{K_{\text{m MASP1}^{*}}^{\text{MASP2}_{\text{zym}}}} \\
+\frac{\left[\text{pCLK1MASP3}_{\text{zym}}\right]}{K_{\text{m MASP1}^{*}}^{\text{MASP3}_{\text{zym}}}}+\frac{\left[\text{pCLL1MASP1}_{\text{zym}}\right]}{K_{\text{m MASP1}^{*}}^{\text{MASP1}_{\text{zym}}}}+\frac{\left[\text{pCLL1MASP2}_{\text{zym}}\right]}{K_{\text{m MASP1}^{*}}^{\text{MASP2}_{\text{zym}}}}+\frac{\left[\text{pCLL1MASP3}_{\text{zym}}\right]}{K_{\text{m MASP1}^{*}}^{\text{MASP3}_{\text{zym}}}} \\
+\frac{\left[\text{pLFMASP1}_{\text{zym}}\right]}{K_{\text{m MASP1}^{*}}^{\text{MASP1}_{\text{zym}}}}+\frac{\left[\text{pLFMASP2}_{\text{zym}}\right]}{K_{\text{m MASP1}^{*}}^{\text{MASP2}_{\text{zym}}}}+\frac{\left[\text{pLFMASP3}_{\text{zym}}\right]}{K_{\text{m MASP1}^{*}}^{\text{MASP3}_{\text{zym}}}}+\frac{\left[\text{pHFMASP1}_{\text{zym}}\right]}{K_{\text{m MASP1}^{*}}^{\text{MASP1}_{\text{zym}}}}+\frac{\left[\text{pHFMASP2}_{\text{zym}}\right]}{K_{\text{m MASP1}^{*}}^{\text{MASP2}_{\text{zym}}}} \\
+\frac{\left[\text{pHFMASP3}_{\text{zym}}\right]}{K_{\text{m MASP1}^{*}}^{\text{MASP3}_{\text{zym}}}}+\frac{\left[\text{pMFMASP1}_{\text{zym}}\right]}{K_{\text{m MASP1}^{*}}^{\text{MASP1}_{\text{zym}}}}+\frac{\left[\text{pMFMASP2}_{\text{zym}}\right]}{K_{\text{m MASP1}^{*}}^{\text{MASP2}_{\text{zym}}}}+\frac{\left[\text{pMFMASP3}_{\text{zym}}\right]}{K_{\text{m MASP1}^{*}}^{\text{MASP3}_{\text{zym}}}} \\
+\frac{\left[\text{pPTX3LFMASP1}_{\text{zym}}\right]}{K_{\text{m MASP1}^{*}}^{\text{MASP1}_{\text{zym}}}}+\frac{\left[\text{pPXT3LFMASP2}_{\text{zym}}\right]}{K_{\text{m MASP1}^{*}}^{\text{MASP2}_{\text{zym}}}}+\frac{\left[\text{pPTX3LFMASP3}_{\text{zym}}\right]}{K_{\text{m MASP1}^{*}}^{\text{MASP3}_{\text{zym}}}}
\end{array}\right)}
$$

$$-\frac{k_{\text{cat MASP1}^*}^{\text{MASP3}_{\text{zym}}}\left[\text{pMFMASP1}^*\right]\left[\text{pMBL}_3\text{MASP3}_{\text{zym}}\right]}{K_{\text{m MASP1}^*}^{\text{MASP3}_{\text{zym}}}\left(\begin{array}{l}1+\frac{[\text{C4}]}{K_{\text{m MASP1}^*}^{\text{C4}}}+\frac{[\text{fC4bC2}]}{K_{\text{m MASP1}^*}^{\text{C2}}}+\frac{[\text{fC4bC4bC2}]}{K_{\text{m MASP1}^*}^{\text{C2}}}+\frac{[\text{fC3bC4bC2}]}{K_{\text{m MASP1}^*}^{\text{C2}}}+\frac{[\text{pC4bC2}]}{K_{\text{m MASP1}^*}^{\text{C2}}}+\frac{[\text{pC4bC4bC2}]}{K_{\text{m MASP1}^*}^{\text{C2}}}\\+\frac{[\text{pC3bC4bC2}]}{K_{\text{m MASP1}^*}^{\text{C2}}}+\frac{[\text{pMBL}_3\text{MASP1}_{\text{zym}}]}{K_{\text{m MASP1}^*}^{\text{MASP1}_{\text{zym}}}}+\frac{[\text{pMBL}_3\text{MASP2}_{\text{zym}}]}{K_{\text{m MASP1}^*}^{\text{MASP2}_{\text{zym}}}}+\frac{[\text{pMBL}_3\text{MASP3}_{\text{zym}}]}{K_{\text{m MASP1}^*}^{\text{MASP3}_{\text{zym}}}}\\+\frac{[\text{pMBL}_4\text{MASP1}_{\text{zym}}]}{K_{\text{m MASP1}^*}^{\text{MASP1}_{\text{zym}}}}+\frac{[\text{pMBL}_4\text{MASP2}_{\text{zym}}]}{K_{\text{m MASP1}^*}^{\text{MASP2}_{\text{zym}}}}+\frac{[\text{pMBL}_4\text{MASP3}_{\text{zym}}]}{K_{\text{m MASP1}^*}^{\text{MASP3}_{\text{zym}}}}+\frac{[\text{pCLLKMASP1}_{\text{zym}}]}{K_{\text{m MASP1}^*}^{\text{MASP1}_{\text{zym}}}}\\+\frac{[\text{pCLLKMASP2}_{\text{zym}}]}{K_{\text{m MASP1}^*}^{\text{MASP2}_{\text{zym}}}}+\frac{[\text{pCLLKMASP3}_{\text{zym}}]}{K_{\text{m MASP1}^*}^{\text{MASP3}_{\text{zym}}}}+\frac{[\text{pCLK1MASP1}_{\text{zym}}]}{K_{\text{m MASP1}^*}^{\text{MASP1}_{\text{zym}}}}+\frac{[\text{pCLK1MASP2}_{\text{zym}}]}{K_{\text{m MASP1}^*}^{\text{MASP2}_{\text{zym}}}}\\+\frac{[\text{pCLK1MASP3}_{\text{zym}}]}{K_{\text{m MASP1}^*}^{\text{MASP3}_{\text{zym}}}}+\frac{[\text{pCLL1MASP1}_{\text{zym}}]}{K_{\text{m MASP1}^*}^{\text{MASP1}_{\text{zym}}}}+\frac{[\text{pCLL1MASP2}_{\text{zym}}]}{K_{\text{m MASP1}^*}^{\text{MASP2}_{\text{zym}}}}+\frac{[\text{pCLL1MASP3}_{\text{zym}}]}{K_{\text{m MASP1}^*}^{\text{MASP3}_{\text{zym}}}}\\+\frac{[\text{pLFMASP1}_{\text{zym}}]}{K_{\text{m MASP1}^*}^{\text{MASP1}_{\text{zym}}}}+\frac{[\text{pLFMASP2}_{\text{zym}}]}{K_{\text{m MASP1}^*}^{\text{MASP2}_{\text{zym}}}}+\frac{[\text{pLFMASP3}_{\text{zym}}]}{K_{\text{m MASP1}^*}^{\text{MASP3}_{\text{zym}}}}+\frac{[\text{pHFMASP1}_{\text{zym}}]}{K_{\text{m MASP1}^*}^{\text{MASP1}_{\text{zym}}}}+\frac{[\text{pHFMASP2}_{\text{zym}}]}{K_{\text{m MASP1}^*}^{\text{MASP2}_{\text{zym}}}}\\+\frac{[\text{pHFMASP3}_{\text{zym}}]}{K_{\text{m MASP1}^*}^{\text{MASP3}_{\text{zym}}}}+\frac{[\text{pMFMASP1}_{\text{zym}}]}{K_{\text{m MASP1}^*}^{\text{MASP1}_{\text{zym}}}}+\frac{[\text{pMFMASP2}_{\text{zym}}]}{K_{\text{m MASP1}^*}^{\text{MASP2}_{\text{zym}}}}+\frac{[\text{pMFMASP3}_{\text{zym}}]}{K_{\text{m MASP1}^*}^{\text{MASP3}_{\text{zym}}}}\\+\frac{[\text{pPTX3LFMASP1}_{\text{zym}}]}{K_{\text{m MASP1}^*}^{\text{MASP1}_{\text{zym}}}}+\frac{[\text{pPXT3LFMASP2}_{\text{zym}}]}{K_{\text{m MASP1}^*}^{\text{MASP2}_{\text{zym}}}}+\frac{[\text{pPTX3LFMASP3}_{\text{zym}}]}{K_{\text{m MASP1}^*}^{\text{MASP3}_{\text{zym}}}}\end{array}\right)}$$

$$-\frac{k^{\mathrm{MASP3_{zym}}}_{\mathrm{cat\,MASP1^*}}\left[\mathrm{pPTX3LFMASP1^*}\right]\left[\mathrm{pMBL_3MASP3_{zym}}\right]}{K^{\mathrm{MASP3_{zym}}}_{\mathrm{m\,MASP1^*}}\left(\begin{aligned}&1+\frac{[\mathrm{C4}]}{K^{\mathrm{C4}}_{\mathrm{m\,MASP1^*}}}+\frac{[\mathrm{fC4bC2}]}{K^{\mathrm{C2}}_{\mathrm{m\,MASP1^*}}}+\frac{[\mathrm{fC4bC4bC2}]}{K^{\mathrm{C2}}_{\mathrm{m\,MASP1^*}}}+\frac{[\mathrm{fC3bC4bC2}]}{K^{\mathrm{C2}}_{\mathrm{m\,MASP1^*}}}+\frac{[\mathrm{pC4bC2}]}{K^{\mathrm{C2}}_{\mathrm{m\,MASP1^*}}}+\frac{[\mathrm{pC4bC4bC2}]}{K^{\mathrm{C2}}_{\mathrm{m\,MASP1^*}}}\\&+\frac{[\mathrm{pC3bC4bC2}]}{K^{\mathrm{C2}}_{\mathrm{m\,MASP1^*}}}+\frac{[\mathrm{pMBL_3MASP1_{zym}}]}{K^{\mathrm{MASP1_{zym}}}_{\mathrm{m\,MASP1^*}}}+\frac{[\mathrm{pMBL_3MASP2_{zym}}]}{K^{\mathrm{MASP2_{zym}}}_{\mathrm{m\,MASP1^*}}}+\frac{[\mathrm{pMBL_3MASP3_{zym}}]}{K^{\mathrm{MASP3_{zym}}}_{\mathrm{m\,MASP1^*}}}\\&+\frac{[\mathrm{pMBL_4MASP1_{zym}}]}{K^{\mathrm{MASP1_{zym}}}_{\mathrm{m\,MASP1^*}}}+\frac{[\mathrm{pMBL_4MASP2_{zym}}]}{K^{\mathrm{MASP2_{zym}}}_{\mathrm{m\,MASP1^*}}}+\frac{[\mathrm{pMBL_4MASP3_{zym}}]}{K^{\mathrm{MASP3_{zym}}}_{\mathrm{m\,MASP1^*}}}+\frac{[\mathrm{pCLLKMASP1_{zym}}]}{K^{\mathrm{MASP1_{zym}}}_{\mathrm{m\,MASP1^*}}}\\&+\frac{[\mathrm{pCLLKMASP2_{zym}}]}{K^{\mathrm{MASP2_{zym}}}_{\mathrm{m\,MASP1^*}}}+\frac{[\mathrm{pCLLKMASP3_{zym}}]}{K^{\mathrm{MASP3_{zym}}}_{\mathrm{m\,MASP1^*}}}+\frac{[\mathrm{pCLK1MASP1_{zym}}]}{K^{\mathrm{MASP1_{zym}}}_{\mathrm{m\,MASP1^*}}}+\frac{[\mathrm{pCLK1MASP2_{zym}}]}{K^{\mathrm{MASP2_{zym}}}_{\mathrm{m\,MASP1^*}}}\\&+\frac{[\mathrm{pCLK1MASP3_{zym}}]}{K^{\mathrm{MASP3_{zym}}}_{\mathrm{m\,MASP1^*}}}+\frac{[\mathrm{pCLL1MASP1_{zym}}]}{K^{\mathrm{MASP1_{zym}}}_{\mathrm{m\,MASP1^*}}}+\frac{[\mathrm{pCLL1MASP2_{zym}}]}{K^{\mathrm{MASP2_{zym}}}_{\mathrm{m\,MASP1^*}}}+\frac{[\mathrm{pCLL1MASP3_{zym}}]}{K^{\mathrm{MASP3_{zym}}}_{\mathrm{m\,MASP1^*}}}\\&+\frac{[\mathrm{pLFMASP1_{zym}}]}{K^{\mathrm{MASP1_{zym}}}_{\mathrm{m\,MASP1^*}}}+\frac{[\mathrm{pLFMASP2_{zym}}]}{K^{\mathrm{MASP2_{zym}}}_{\mathrm{m\,MASP1^*}}}+\frac{[\mathrm{pLFMASP3_{zym}}]}{K^{\mathrm{MASP3_{zym}}}_{\mathrm{m\,MASP1^*}}}+\frac{[\mathrm{pHFMASP1_{zym}}]}{K^{\mathrm{MASP1_{zym}}}_{\mathrm{m\,MASP1^*}}}+\frac{[\mathrm{pHFMASP2_{zym}}]}{K^{\mathrm{MASP2_{zym}}}_{\mathrm{m\,MASP1^*}}}\\&+\frac{[\mathrm{pHFMASP3_{zym}}]}{K^{\mathrm{MASP3_{zym}}}_{\mathrm{m\,MASP1^*}}}+\frac{[\mathrm{pMFMASP1_{zym}}]}{K^{\mathrm{MASP1_{zym}}}_{\mathrm{m\,MASP1^*}}}+\frac{[\mathrm{pMFMASP2_{zym}}]}{K^{\mathrm{MASP2_{zym}}}_{\mathrm{m\,MASP1^*}}}+\frac{[\mathrm{pMFMASP3_{zym}}]}{K^{\mathrm{MASP3_{zym}}}_{\mathrm{m\,MASP1^*}}}\\&+\frac{[\mathrm{pPTX3LFMASP1_{zym}}]}{K^{\mathrm{MASP1_{zym}}}_{\mathrm{m\,MASP1^*}}}+\frac{[\mathrm{pPXT3LFMASP2_{zym}}]}{K^{\mathrm{MASP2_{zym}}}_{\mathrm{m\,MASP1^*}}}+\frac{[\mathrm{pPTX3LFMASP3_{zym}}]}{K^{\mathrm{MASP3_{zym}}}_{\mathrm{m\,MASP1^*}}}\end{aligned}\right)}$$

254

$$\frac{d\left[\mathrm{fMBL_3MASP3^*}\right]}{dt}=-k^{+}_{\mathrm{pMBL_3}}\left[\mathrm{fMBL_3MASP3^*}\right]\left[\mathrm{Pathogen\ Surface}\right]+k^{-}_{\mathrm{pMBL_3}}\left[\mathrm{pMBL_3MASP3^*}\right]$$
$$+k^{+}_{\mathrm{MBL_3MASP3}}\left[\mathrm{MBL_3}\right]\left[\mathrm{MASP3^*}\right]-k^{-}_{\mathrm{MBL_3MASP3}}\left[\mathrm{fMBL_3MASP3^*}\right]$$

255

$$\frac{d\left[\mathrm{pMBL_3MASP3^*}\right]}{dt}=k^{+}_{\mathrm{pMBL_3}}\left[\mathrm{fMBL_3MASP3^*}\right]\left[\mathrm{Pathogen\ Surface}\right]-k^{-}_{\mathrm{pMBL_3}}\left[\mathrm{pMBL_3MASP3^*}\right]$$
$$+k^{+}_{\mathrm{MBL_3MASP3}}\left[\mathrm{pMBL_3}\right]\left[\mathrm{MASP3^*}\right]-k^{-}_{\mathrm{MBL_3MASP3}}\left[\mathrm{pMBL_3MASP3^*}\right]$$

$$+\frac{k_{\text{cat MASP1}^*}^{\text{MASP3}_{\text{zym}}}\left[\text{pMBL}_3\text{MASP1}^*\right]\left[\text{pMBL}_3\text{MASP3}_{\text{zym}}\right]}{K_{\text{m MASP1}^*}^{\text{MASP3}_{\text{zym}}}\left(\begin{array}{l}1+\frac{[\text{C4}]}{K_{\text{m MASP1}^*}^{\text{C4}}}+\frac{[\text{fC4bC2}]}{K_{\text{m MASP1}^*}^{\text{C2}}}+\frac{[\text{fC4bC4bC2}]}{K_{\text{m MASP1}^*}^{\text{C2}}}+\frac{[\text{fC3bC4bC2}]}{K_{\text{m MASP1}^*}^{\text{C2}}}+\frac{[\text{pC4bC2}]}{K_{\text{m MASP1}^*}^{\text{C2}}}+\frac{[\text{pC4bC4bC2}]}{K_{\text{m MASP1}^*}^{\text{C2}}}\\ +\frac{[\text{pC3bC4bC2}]}{K_{\text{m MASP1}^*}^{\text{C2}}}+\frac{\left[\text{pMBL}_3\text{MASP1}_{\text{zym}}\right]}{K_{\text{m MASP1}^*}^{\text{MASP1}_{\text{zym}}}}+\frac{\left[\text{pMBL}_3\text{MASP2}_{\text{zym}}\right]}{K_{\text{m MASP1}^*}^{\text{MASP2}_{\text{zym}}}}+\frac{\left[\text{pMBL}_3\text{MASP3}_{\text{zym}}\right]}{K_{\text{m MASP1}^*}^{\text{MASP3}_{\text{zym}}}}\\ +\frac{\left[\text{pMBL}_4\text{MASP1}_{\text{zym}}\right]}{K_{\text{m MASP1}^*}^{\text{MASP1}_{\text{zym}}}}+\frac{\left[\text{pMBL}_4\text{MASP2}_{\text{zym}}\right]}{K_{\text{m MASP1}^*}^{\text{MASP2}_{\text{zym}}}}+\frac{\left[\text{pMBL}_4\text{MASP3}_{\text{zym}}\right]}{K_{\text{m MASP1}^*}^{\text{MASP3}_{\text{zym}}}}+\frac{\left[\text{pCLLKMASP1}_{\text{zym}}\right]}{K_{\text{m MASP1}^*}^{\text{MASP1}_{\text{zym}}}}\\ +\frac{\left[\text{pCLLKMASP2}_{\text{zym}}\right]}{K_{\text{m MASP1}^*}^{\text{MASP2}_{\text{zym}}}}+\frac{\left[\text{pCLLKMASP3}_{\text{zym}}\right]}{K_{\text{m MASP1}^*}^{\text{MASP3}_{\text{zym}}}}+\frac{\left[\text{pCLK1MASP1}_{\text{zym}}\right]}{K_{\text{m MASP1}^*}^{\text{MASP1}_{\text{zym}}}}+\frac{\left[\text{pCLK1MASP2}_{\text{zym}}\right]}{K_{\text{m MASP1}^*}^{\text{MASP2}_{\text{zym}}}}\\ +\frac{\left[\text{pCLK1MASP3}_{\text{zym}}\right]}{K_{\text{m MASP1}^*}^{\text{MASP3}_{\text{zym}}}}+\frac{\left[\text{pCLL1MASP1}_{\text{zym}}\right]}{K_{\text{m MASP1}^*}^{\text{MASP1}_{\text{zym}}}}+\frac{\left[\text{pCLL1MASP2}_{\text{zym}}\right]}{K_{\text{m MASP1}^*}^{\text{MASP2}_{\text{zym}}}}+\frac{\left[\text{pCLL1MASP3}_{\text{zym}}\right]}{K_{\text{m MASP1}^*}^{\text{MASP3}_{\text{zym}}}}\\ +\frac{\left[\text{pLFMASP1}_{\text{zym}}\right]}{K_{\text{m MASP1}^*}^{\text{MASP1}_{\text{zym}}}}+\frac{\left[\text{pLFMASP2}_{\text{zym}}\right]}{K_{\text{m MASP1}^*}^{\text{MASP2}_{\text{zym}}}}+\frac{\left[\text{pLFMASP3}_{\text{zym}}\right]}{K_{\text{m MASP1}^*}^{\text{MASP3}_{\text{zym}}}}+\frac{\left[\text{pHFMASP1}_{\text{zym}}\right]}{K_{\text{m MASP1}^*}^{\text{MASP1}_{\text{zym}}}}+\frac{\left[\text{pHFMASP2}_{\text{zym}}\right]}{K_{\text{m MASP1}^*}^{\text{MASP2}_{\text{zym}}}}\\ +\frac{\left[\text{pHFMASP3}_{\text{zym}}\right]}{K_{\text{m MASP1}^*}^{\text{MASP3}_{\text{zym}}}}+\frac{\left[\text{pMFMASP1}_{\text{zym}}\right]}{K_{\text{m MASP1}^*}^{\text{MASP1}_{\text{zym}}}}+\frac{\left[\text{pMFMASP2}_{\text{zym}}\right]}{K_{\text{m MASP1}^*}^{\text{MASP2}_{\text{zym}}}}+\frac{\left[\text{pMFMASP3}_{\text{zym}}\right]}{K_{\text{m MASP1}^*}^{\text{MASP3}_{\text{zym}}}}\\ +\frac{\left[\text{pPTX3LFMASP1}_{\text{zym}}\right]}{K_{\text{m MASP1}^*}^{\text{MASP1}_{\text{zym}}}}+\frac{\left[\text{pPXT3LFMASP2}_{\text{zym}}\right]}{K_{\text{m MASP1}^*}^{\text{MASP2}_{\text{zym}}}}+\frac{\left[\text{pPTX3LFMASP3}_{\text{zym}}\right]}{K_{\text{m MASP1}^*}^{\text{MASP3}_{\text{zym}}}}\end{array}\right)}$$

$$
+\frac{k_{\mathrm{cat\ MASP1^*}}^{\mathrm{MASP3_{zym}}}\left[\mathrm{pMBL_4MASP1^*}\right]\left[\mathrm{pMBL_3MASP3_{zym}}\right]}{K_{\mathrm{m\ MASP1^*}}^{\mathrm{MASP3_{zym}}}\left(\begin{array}{l}1+\frac{[\mathrm{C4}]}{K_{\mathrm{m\ MASP1^*}}^{\mathrm{C4}}}+\frac{[\mathrm{fC4bC2}]}{K_{\mathrm{m\ MASP1^*}}^{\mathrm{C2}}}+\frac{[\mathrm{fC4bC4bC2}]}{K_{\mathrm{m\ MASP1^*}}^{\mathrm{C2}}}+\frac{[\mathrm{fC3bC4bC2}]}{K_{\mathrm{m\ MASP1^*}}^{\mathrm{C2}}}+\frac{[\mathrm{pC4bC2}]}{K_{\mathrm{m\ MASP1^*}}^{\mathrm{C2}}}+\frac{[\mathrm{pC4bC4bC2}]}{K_{\mathrm{m\ MASP1^*}}^{\mathrm{C2}}}\\
+\frac{[\mathrm{pC3bC4bC2}]}{K_{\mathrm{m\ MASP1^*}}^{\mathrm{C2}}}+\frac{[\mathrm{pMBL_3MASP1_{zym}}]}{K_{\mathrm{m\ MASP1^*}}^{\mathrm{MASP1_{zym}}}}+\frac{[\mathrm{pMBL_3MASP2_{zym}}]}{K_{\mathrm{m\ MASP1^*}}^{\mathrm{MASP2_{zym}}}}+\frac{[\mathrm{pMBL_3MASP3_{zym}}]}{K_{\mathrm{m\ MASP1^*}}^{\mathrm{MASP3_{zym}}}}\\
+\frac{[\mathrm{pMBL_4MASP1_{zym}}]}{K_{\mathrm{m\ MASP1^*}}^{\mathrm{MASP1_{zym}}}}+\frac{[\mathrm{pMBL_4MASP2_{zym}}]}{K_{\mathrm{m\ MASP1^*}}^{\mathrm{MASP2_{zym}}}}+\frac{[\mathrm{pMBL_4MASP3_{zym}}]}{K_{\mathrm{m\ MASP1^*}}^{\mathrm{MASP3_{zym}}}}+\frac{[\mathrm{pCLLKMASP1_{zym}}]}{K_{\mathrm{m\ MASP1^*}}^{\mathrm{MASP1_{zym}}}}\\
+\frac{[\mathrm{pCLLKMASP2_{zym}}]}{K_{\mathrm{m\ MASP1^*}}^{\mathrm{MASP2_{zym}}}}+\frac{[\mathrm{pCLLKMASP3_{zym}}]}{K_{\mathrm{m\ MASP1^*}}^{\mathrm{MASP3_{zym}}}}+\frac{[\mathrm{pCLK1MASP1_{zym}}]}{K_{\mathrm{m\ MASP1^*}}^{\mathrm{MASP1_{zym}}}}+\frac{[\mathrm{pCLK1MASP2_{zym}}]}{K_{\mathrm{m\ MASP1^*}}^{\mathrm{MASP2_{zym}}}}\\
+\frac{[\mathrm{pCLK1MASP3_{zym}}]}{K_{\mathrm{m\ MASP1^*}}^{\mathrm{MASP3_{zym}}}}+\frac{[\mathrm{pCLL1MASP1_{zym}}]}{K_{\mathrm{m\ MASP1^*}}^{\mathrm{MASP1_{zym}}}}+\frac{[\mathrm{pCLL1MASP2_{zym}}]}{K_{\mathrm{m\ MASP1^*}}^{\mathrm{MASP2_{zym}}}}+\frac{[\mathrm{pCLL1MASP3_{zym}}]}{K_{\mathrm{m\ MASP1^*}}^{\mathrm{MASP3_{zym}}}}\\
+\frac{[\mathrm{pLFMASP1_{zym}}]}{K_{\mathrm{m\ MASP1^*}}^{\mathrm{MASP1_{zym}}}}+\frac{[\mathrm{pLFMASP2_{zym}}]}{K_{\mathrm{m\ MASP1^*}}^{\mathrm{MASP2_{zym}}}}+\frac{[\mathrm{pLFMASP3_{zym}}]}{K_{\mathrm{m\ MASP1^*}}^{\mathrm{MASP3_{zym}}}}+\frac{[\mathrm{pHFMASP1_{zym}}]}{K_{\mathrm{m\ MASP1^*}}^{\mathrm{MASP1_{zym}}}}+\frac{[\mathrm{pHFMASP2_{zym}}]}{K_{\mathrm{m\ MASP1^*}}^{\mathrm{MASP2_{zym}}}}\\
+\frac{[\mathrm{pHFMASP3_{zym}}]}{K_{\mathrm{m\ MASP1^*}}^{\mathrm{MASP3_{zym}}}}+\frac{[\mathrm{pMFMASP1_{zym}}]}{K_{\mathrm{m\ MASP1^*}}^{\mathrm{MASP1_{zym}}}}+\frac{[\mathrm{pMFMASP2_{zym}}]}{K_{\mathrm{m\ MASP1^*}}^{\mathrm{MASP2_{zym}}}}+\frac{[\mathrm{pMFMASP3_{zym}}]}{K_{\mathrm{m\ MASP1^*}}^{\mathrm{MASP3_{zym}}}}\\
+\frac{[\mathrm{pPTX3LFMASP1_{zym}}]}{K_{\mathrm{m\ MASP1^*}}^{\mathrm{MASP1_{zym}}}}+\frac{[\mathrm{pPXT3LFMASP2_{zym}}]}{K_{\mathrm{m\ MASP1^*}}^{\mathrm{MASP2_{zym}}}}+\frac{[\mathrm{pPTX3LFMASP3_{zym}}]}{K_{\mathrm{m\ MASP1^*}}^{\mathrm{MASP3_{zym}}}}\end{array}\right)}
$$

$$
+\frac{k_{\text{cat MASP1}^*}^{\text{MASP3}_{\text{zym}}}\left[\text{pCLLKMASP1}^*\right]\left[\text{pMBL}_3\text{MASP3}_{\text{zym}}\right]}{K_{\text{m MASP1}^*}^{\text{MASP3}_{\text{zym}}}\left(\begin{array}{l}
1+\frac{[\text{C4}]}{K_{\text{m MASP1}^*}^{\text{C4}}}+\frac{[\text{fC4bC2}]}{K_{\text{m MASP1}^*}^{\text{C2}}}+\frac{[\text{fC4bC4bC2}]}{K_{\text{m MASP1}^*}^{\text{C2}}}+\frac{[\text{fC3bC4bC2}]}{K_{\text{m MASP1}^*}^{\text{C2}}}+\frac{[\text{pC4bC2}]}{K_{\text{m MASP1}^*}^{\text{C2}}}+\frac{[\text{pC4bC4bC2}]}{K_{\text{m MASP1}^*}^{\text{C2}}} \\
+\frac{[\text{pC3bC4bC2}]}{K_{\text{m MASP1}^*}^{\text{C2}}}+\frac{[\text{pMBL}_3\text{MASP1}_{\text{zym}}]}{K_{\text{m MASP1}^*}^{\text{MASP1}_{\text{zym}}}}+\frac{[\text{pMBL}_3\text{MASP2}_{\text{zym}}]}{K_{\text{m MASP1}^*}^{\text{MASP2}_{\text{zym}}}}+\frac{[\text{pMBL}_3\text{MASP3}_{\text{zym}}]}{K_{\text{m MASP1}^*}^{\text{MASP3}_{\text{zym}}}} \\
+\frac{[\text{pMBL}_4\text{MASP1}_{\text{zym}}]}{K_{\text{m MASP1}^*}^{\text{MASP1}_{\text{zym}}}}+\frac{[\text{pMBL}_4\text{MASP2}_{\text{zym}}]}{K_{\text{m MASP1}^*}^{\text{MASP2}_{\text{zym}}}}+\frac{[\text{pMBL}_4\text{MASP3}_{\text{zym}}]}{K_{\text{m MASP1}^*}^{\text{MASP3}_{\text{zym}}}}+\frac{[\text{pCLLKMASP1}_{\text{zym}}]}{K_{\text{m MASP1}^*}^{\text{MASP1}_{\text{zym}}}} \\
+\frac{[\text{pCLLKMASP2}_{\text{zym}}]}{K_{\text{m MASP1}^*}^{\text{MASP2}_{\text{zym}}}}+\frac{[\text{pCLLKMASP3}_{\text{zym}}]}{K_{\text{m MASP1}^*}^{\text{MASP3}_{\text{zym}}}}+\frac{[\text{pCLK1MASP1}_{\text{zym}}]}{K_{\text{m MASP1}^*}^{\text{MASP1}_{\text{zym}}}}+\frac{[\text{pCLK1MASP2}_{\text{zym}}]}{K_{\text{m MASP1}^*}^{\text{MASP2}_{\text{zym}}}} \\
+\frac{[\text{pCLK1MASP3}_{\text{zym}}]}{K_{\text{m MASP1}^*}^{\text{MASP3}_{\text{zym}}}}+\frac{[\text{pCLL1MASP1}_{\text{zym}}]}{K_{\text{m MASP1}^*}^{\text{MASP1}_{\text{zym}}}}+\frac{[\text{pCLL1MASP2}_{\text{zym}}]}{K_{\text{m MASP1}^*}^{\text{MASP2}_{\text{zym}}}}+\frac{[\text{pCLL1MASP3}_{\text{zym}}]}{K_{\text{m MASP1}^*}^{\text{MASP3}_{\text{zym}}}} \\
+\frac{[\text{pLFMASP1}_{\text{zym}}]}{K_{\text{m MASP1}^*}^{\text{MASP1}_{\text{zym}}}}+\frac{[\text{pLFMASP2}_{\text{zym}}]}{K_{\text{m MASP1}^*}^{\text{MASP2}_{\text{zym}}}}+\frac{[\text{pLFMASP3}_{\text{zym}}]}{K_{\text{m MASP1}^*}^{\text{MASP3}_{\text{zym}}}}+\frac{[\text{pHFMASP1}_{\text{zym}}]}{K_{\text{m MASP1}^*}^{\text{MASP1}_{\text{zym}}}}+\frac{[\text{pHFMASP2}_{\text{zym}}]}{K_{\text{m MASP1}^*}^{\text{MASP2}_{\text{zym}}}} \\
+\frac{[\text{pHFMASP3}_{\text{zym}}]}{K_{\text{m MASP1}^*}^{\text{MASP3}_{\text{zym}}}}+\frac{[\text{pMFMASP1}_{\text{zym}}]}{K_{\text{m MASP1}^*}^{\text{MASP1}_{\text{zym}}}}+\frac{[\text{pMFMASP2}_{\text{zym}}]}{K_{\text{m MASP1}^*}^{\text{MASP2}_{\text{zym}}}}+\frac{[\text{pMFMASP3}_{\text{zym}}]}{K_{\text{m MASP1}^*}^{\text{MASP3}_{\text{zym}}}} \\
+\frac{[\text{pPTX3LFMASP1}_{\text{zym}}]}{K_{\text{m MASP1}^*}^{\text{MASP1}_{\text{zym}}}}+\frac{[\text{pPXT3LFMASP2}_{\text{zym}}]}{K_{\text{m MASP1}^*}^{\text{MASP2}_{\text{zym}}}}+\frac{[\text{pPTX3LFMASP3}_{\text{zym}}]}{K_{\text{m MASP1}^*}^{\text{MASP3}_{\text{zym}}}}
\end{array}\right)}
$$

$$+\frac{k_{\text{cat MASP1}^*}^{\text{MASP3}_{\text{zym}}}\left[\text{pCLK1MASP1}^*\right]\left[\text{pMBL}_3\text{MASP3}_{\text{zym}}\right]}{K_{\text{m MASP1}^*}^{\text{MASP3}_{\text{zym}}}\left(\begin{array}{l}1+\frac{[\text{C4}]}{K_{\text{m MASP1}^*}^{\text{C4}}}+\frac{[\text{fC4bC2}]}{K_{\text{m MASP1}^*}^{\text{C2}}}+\frac{[\text{fC4bC4bC2}]}{K_{\text{m MASP1}^*}^{\text{C2}}}+\frac{[\text{fC3bC4bC2}]}{K_{\text{m MASP1}^*}^{\text{C2}}}+\frac{[\text{pC4bC2}]}{K_{\text{m MASP1}^*}^{\text{C2}}}+\frac{[\text{pC4bC4bC2}]}{K_{\text{m MASP1}^*}^{\text{C2}}}\\ +\frac{[\text{pC3bC4bC2}]}{K_{\text{m MASP1}^*}^{\text{C2}}}+\frac{[\text{pMBL}_3\text{MASP1}_{\text{zym}}]}{K_{\text{m MASP1}^*}^{\text{MASP1}_{\text{zym}}}}+\frac{[\text{pMBL}_3\text{MASP2}_{\text{zym}}]}{K_{\text{m MASP1}^*}^{\text{MASP2}_{\text{zym}}}}+\frac{[\text{pMBL}_3\text{MASP3}_{\text{zym}}]}{K_{\text{m MASP1}^*}^{\text{MASP3}_{\text{zym}}}}\\ +\frac{[\text{pMBL}_4\text{MASP1}_{\text{zym}}]}{K_{\text{m MASP1}^*}^{\text{MASP1}_{\text{zym}}}}+\frac{[\text{pMBL}_4\text{MASP2}_{\text{zym}}]}{K_{\text{m MASP1}^*}^{\text{MASP2}_{\text{zym}}}}+\frac{[\text{pMBL}_4\text{MASP3}_{\text{zym}}]}{K_{\text{m MASP1}^*}^{\text{MASP3}_{\text{zym}}}}+\frac{[\text{pCLLKMASP1}_{\text{zym}}]}{K_{\text{m MASP1}^*}^{\text{MASP1}_{\text{zym}}}}\\ +\frac{[\text{pCLLKMASP2}_{\text{zym}}]}{K_{\text{m MASP1}^*}^{\text{MASP2}_{\text{zym}}}}+\frac{[\text{pCLLKMASP3}_{\text{zym}}]}{K_{\text{m MASP1}^*}^{\text{MASP3}_{\text{zym}}}}+\frac{[\text{pCLK1MASP1}_{\text{zym}}]}{K_{\text{m MASP1}^*}^{\text{MASP1}_{\text{zym}}}}+\frac{[\text{pCLK1MASP2}_{\text{zym}}]}{K_{\text{m MASP1}^*}^{\text{MASP2}_{\text{zym}}}}\\ +\frac{[\text{pCLK1MASP3}_{\text{zym}}]}{K_{\text{m MASP1}^*}^{\text{MASP3}_{\text{zym}}}}+\frac{[\text{pCLL1MASP1}_{\text{zym}}]}{K_{\text{m MASP1}^*}^{\text{MASP1}_{\text{zym}}}}+\frac{[\text{pCLL1MASP2}_{\text{zym}}]}{K_{\text{m MASP1}^*}^{\text{MASP2}_{\text{zym}}}}+\frac{[\text{pCLL1MASP3}_{\text{zym}}]}{K_{\text{m MASP1}^*}^{\text{MASP3}_{\text{zym}}}}\\ +\frac{[\text{pLFMASP1}_{\text{zym}}]}{K_{\text{m MASP1}^*}^{\text{MASP1}_{\text{zym}}}}+\frac{[\text{pLFMASP2}_{\text{zym}}]}{K_{\text{m MASP1}^*}^{\text{MASP2}_{\text{zym}}}}+\frac{[\text{pLFMASP3}_{\text{zym}}]}{K_{\text{m MASP1}^*}^{\text{MASP3}_{\text{zym}}}}+\frac{[\text{pHFMASP1}_{\text{zym}}]}{K_{\text{m MASP1}^*}^{\text{MASP1}_{\text{zym}}}}+\frac{[\text{pHFMASP2}_{\text{zym}}]}{K_{\text{m MASP1}^*}^{\text{MASP2}_{\text{zym}}}}\\ +\frac{[\text{pHFMASP3}_{\text{zym}}]}{K_{\text{m MASP1}^*}^{\text{MASP3}_{\text{zym}}}}+\frac{[\text{pMFMASP1}_{\text{zym}}]}{K_{\text{m MASP1}^*}^{\text{MASP1}_{\text{zym}}}}+\frac{[\text{pMFMASP2}_{\text{zym}}]}{K_{\text{m MASP1}^*}^{\text{MASP2}_{\text{zym}}}}+\frac{[\text{pMFMASP3}_{\text{zym}}]}{K_{\text{m MASP1}^*}^{\text{MASP3}_{\text{zym}}}}\\ +\frac{[\text{pPTX3LFMASP1}_{\text{zym}}]}{K_{\text{m MASP1}^*}^{\text{MASP1}_{\text{zym}}}}+\frac{[\text{pPXT3LFMASP2}_{\text{zym}}]}{K_{\text{m MASP1}^*}^{\text{MASP2}_{\text{zym}}}}+\frac{[\text{pPTX3LFMASP3}_{\text{zym}}]}{K_{\text{m MASP1}^*}^{\text{MASP3}_{\text{zym}}}}\end{array}\right)}$$

$$+\frac{k_{\text{cat MASP1}^*}^{\text{MASP3}_{\text{zym}}}\left[\text{pCLL1MASP1}^*\right]\left[\text{pMBL}_3\text{MASP3}_{\text{zym}}\right]}{K_{\text{m MASP1}^*}^{\text{MASP3}_{\text{zym}}}\left(\begin{array}{l}1+\frac{[\text{C4}]}{K_{\text{m MASP1}^*}^{\text{C4}}}+\frac{[\text{fC4bC2}]}{K_{\text{m MASP1}^*}^{\text{C2}}}+\frac{[\text{fC4bC4bC2}]}{K_{\text{m MASP1}^*}^{\text{C2}}}+\frac{[\text{fC3bC4bC2}]}{K_{\text{m MASP1}^*}^{\text{C2}}}+\frac{[\text{pC4bC2}]}{K_{\text{m MASP1}^*}^{\text{C2}}}+\frac{[\text{pC4bC4bC2}]}{K_{\text{m MASP1}^*}^{\text{C2}}}\\+\frac{[\text{pC3bC4bC2}]}{K_{\text{m MASP1}^*}^{\text{C2}}}+\frac{[\text{pMBL}_3\text{MASP1}_{\text{zym}}]}{K_{\text{m MASP1}^*}^{\text{MASP1}_{\text{zym}}}}+\frac{[\text{pMBL}_3\text{MASP2}_{\text{zym}}]}{K_{\text{m MASP1}^*}^{\text{MASP2}_{\text{zym}}}}+\frac{[\text{pMBL}_3\text{MASP3}_{\text{zym}}]}{K_{\text{m MASP1}^*}^{\text{MASP3}_{\text{zym}}}}\\+\frac{[\text{pMBL}_4\text{MASP1}_{\text{zym}}]}{K_{\text{m MASP1}^*}^{\text{MASP1}_{\text{zym}}}}+\frac{[\text{pMBL}_4\text{MASP2}_{\text{zym}}]}{K_{\text{m MASP1}^*}^{\text{MASP2}_{\text{zym}}}}+\frac{[\text{pMBL}_4\text{MASP3}_{\text{zym}}]}{K_{\text{m MASP1}^*}^{\text{MASP3}_{\text{zym}}}}+\frac{[\text{pCLLKMASP1}_{\text{zym}}]}{K_{\text{m MASP1}^*}^{\text{MASP1}_{\text{zym}}}}\\+\frac{[\text{pCLLKMASP2}_{\text{zym}}]}{K_{\text{m MASP1}^*}^{\text{MASP2}_{\text{zym}}}}+\frac{[\text{pCLLKMASP3}_{\text{zym}}]}{K_{\text{m MASP1}^*}^{\text{MASP3}_{\text{zym}}}}+\frac{[\text{pCLK1MASP1}_{\text{zym}}]}{K_{\text{m MASP1}^*}^{\text{MASP1}_{\text{zym}}}}+\frac{[\text{pCLK1MASP2}_{\text{zym}}]}{K_{\text{m MASP1}^*}^{\text{MASP2}_{\text{zym}}}}\\+\frac{[\text{pCLK1MASP3}_{\text{zym}}]}{K_{\text{m MASP1}^*}^{\text{MASP3}_{\text{zym}}}}+\frac{[\text{pCLL1MASP1}_{\text{zym}}]}{K_{\text{m MASP1}^*}^{\text{MASP1}_{\text{zym}}}}+\frac{[\text{pCLL1MASP2}_{\text{zym}}]}{K_{\text{m MASP1}^*}^{\text{MASP2}_{\text{zym}}}}+\frac{[\text{pCLL1MASP3}_{\text{zym}}]}{K_{\text{m MASP1}^*}^{\text{MASP3}_{\text{zym}}}}\\+\frac{[\text{pLFMASP1}_{\text{zym}}]}{K_{\text{m MASP1}^*}^{\text{MASP1}_{\text{zym}}}}+\frac{[\text{pLFMASP2}_{\text{zym}}]}{K_{\text{m MASP1}^*}^{\text{MASP2}_{\text{zym}}}}+\frac{[\text{pLFMASP3}_{\text{zym}}]}{K_{\text{m MASP1}^*}^{\text{MASP3}_{\text{zym}}}}+\frac{[\text{pHFMASP1}_{\text{zym}}]}{K_{\text{m MASP1}^*}^{\text{MASP1}_{\text{zym}}}}+\frac{[\text{pHFMASP2}_{\text{zym}}]}{K_{\text{m MASP1}^*}^{\text{MASP2}_{\text{zym}}}}\\+\frac{[\text{pHFMASP3}_{\text{zym}}]}{K_{\text{m MASP1}^*}^{\text{MASP3}_{\text{zym}}}}+\frac{[\text{pMFMASP1}_{\text{zym}}]}{K_{\text{m MASP1}^*}^{\text{MASP1}_{\text{zym}}}}+\frac{[\text{pMFMASP2}_{\text{zym}}]}{K_{\text{m MASP1}^*}^{\text{MASP2}_{\text{zym}}}}+\frac{[\text{pMFMASP3}_{\text{zym}}]}{K_{\text{m MASP1}^*}^{\text{MASP3}_{\text{zym}}}}\\+\frac{[\text{pPTX3LFMASP1}_{\text{zym}}]}{K_{\text{m MASP1}^*}^{\text{MASP1}_{\text{zym}}}}+\frac{[\text{pPXT3LFMASP2}_{\text{zym}}]}{K_{\text{m MASP1}^*}^{\text{MASP2}_{\text{zym}}}}+\frac{[\text{pPTX3LFMASP3}_{\text{zym}}]}{K_{\text{m MASP1}^*}^{\text{MASP3}_{\text{zym}}}}\end{array}\right)}$$

$$+\frac{k_{\text{cat MASP1}^*}^{\text{MASP3}_{\text{zym}}}\left[\text{pLFMASP1}^*\right]\left[\text{pMBL}_3\text{MASP3}_{\text{zym}}\right]}{K_{\text{m MASP1}^*}^{\text{MASP3}_{\text{zym}}}\left(\begin{array}{l}1+\frac{[\text{C4}]}{K_{\text{m MASP1}^*}^{\text{C4}}}+\frac{[\text{fC4bC2}]}{K_{\text{m MASP1}^*}^{\text{C2}}}+\frac{[\text{fC4bC4bC2}]}{K_{\text{m MASP1}^*}^{\text{C2}}}+\frac{[\text{fC3bC4bC2}]}{K_{\text{m MASP1}^*}^{\text{C2}}}+\frac{[\text{pC4bC2}]}{K_{\text{m MASP1}^*}^{\text{C2}}}+\frac{[\text{pC4bC4bC2}]}{K_{\text{m MASP1}^*}^{\text{C2}}}\\+\frac{[\text{pC3bC4bC2}]}{K_{\text{m MASP1}^*}^{\text{C2}}}+\frac{[\text{pMBL}_3\text{MASP1}_{\text{zym}}]}{K_{\text{m MASP1}^*}^{\text{MASP1}_{\text{zym}}}}+\frac{[\text{pMBL}_3\text{MASP2}_{\text{zym}}]}{K_{\text{m MASP1}^*}^{\text{MASP2}_{\text{zym}}}}+\frac{[\text{pMBL}_3\text{MASP3}_{\text{zym}}]}{K_{\text{m MASP1}^*}^{\text{MASP3}_{\text{zym}}}}\\+\frac{[\text{pMBL}_4\text{MASP1}_{\text{zym}}]}{K_{\text{m MASP1}^*}^{\text{MASP1}_{\text{zym}}}}+\frac{[\text{pMBL}_4\text{MASP2}_{\text{zym}}]}{K_{\text{m MASP1}^*}^{\text{MASP2}_{\text{zym}}}}+\frac{[\text{pMBL}_4\text{MASP3}_{\text{zym}}]}{K_{\text{m MASP1}^*}^{\text{MASP3}_{\text{zym}}}}+\frac{[\text{pCLLKMASP1}_{\text{zym}}]}{K_{\text{m MASP1}^*}^{\text{MASP1}_{\text{zym}}}}\\+\frac{[\text{pCLLKMASP2}_{\text{zym}}]}{K_{\text{m MASP1}^*}^{\text{MASP2}_{\text{zym}}}}+\frac{[\text{pCLLKMASP3}_{\text{zym}}]}{K_{\text{m MASP1}^*}^{\text{MASP3}_{\text{zym}}}}+\frac{[\text{pCLK1MASP1}_{\text{zym}}]}{K_{\text{m MASP1}^*}^{\text{MASP1}_{\text{zym}}}}+\frac{[\text{pCLK1MASP2}_{\text{zym}}]}{K_{\text{m MASP1}^*}^{\text{MASP2}_{\text{zym}}}}\\+\frac{[\text{pCLK1MASP3}_{\text{zym}}]}{K_{\text{m MASP1}^*}^{\text{MASP3}_{\text{zym}}}}+\frac{[\text{pCLL1MASP1}_{\text{zym}}]}{K_{\text{m MASP1}^*}^{\text{MASP1}_{\text{zym}}}}+\frac{[\text{pCLL1MASP2}_{\text{zym}}]}{K_{\text{m MASP1}^*}^{\text{MASP2}_{\text{zym}}}}+\frac{[\text{pCLL1MASP3}_{\text{zym}}]}{K_{\text{m MASP1}^*}^{\text{MASP3}_{\text{zym}}}}\\+\frac{[\text{pLFMASP1}_{\text{zym}}]}{K_{\text{m MASP1}^*}^{\text{MASP1}_{\text{zym}}}}+\frac{[\text{pLFMASP2}_{\text{zym}}]}{K_{\text{m MASP1}^*}^{\text{MASP2}_{\text{zym}}}}+\frac{[\text{pLFMASP3}_{\text{zym}}]}{K_{\text{m MASP1}^*}^{\text{MASP3}_{\text{zym}}}}+\frac{[\text{pHFMASP1}_{\text{zym}}]}{K_{\text{m MASP1}^*}^{\text{MASP1}_{\text{zym}}}}+\frac{[\text{pHFMASP2}_{\text{zym}}]}{K_{\text{m MASP1}^*}^{\text{MASP2}_{\text{zym}}}}\\+\frac{[\text{pHFMASP3}_{\text{zym}}]}{K_{\text{m MASP1}^*}^{\text{MASP3}_{\text{zym}}}}+\frac{[\text{pMFMASP1}_{\text{zym}}]}{K_{\text{m MASP1}^*}^{\text{MASP1}_{\text{zym}}}}+\frac{[\text{pMFMASP2}_{\text{zym}}]}{K_{\text{m MASP1}^*}^{\text{MASP2}_{\text{zym}}}}+\frac{[\text{pMFMASP3}_{\text{zym}}]}{K_{\text{m MASP1}^*}^{\text{MASP3}_{\text{zym}}}}\\+\frac{[\text{pPTX3LFMASP1}_{\text{zym}}]}{K_{\text{m MASP1}^*}^{\text{MASP1}_{\text{zym}}}}+\frac{[\text{pPXT3LFMASP2}_{\text{zym}}]}{K_{\text{m MASP1}^*}^{\text{MASP2}_{\text{zym}}}}+\frac{[\text{pPTX3LFMASP3}_{\text{zym}}]}{K_{\text{m MASP1}^*}^{\text{MASP3}_{\text{zym}}}}\end{array}\right)}$$

$$+\frac{k_{\text{cat MASP1}^*}^{\text{MASP3}_{\text{zym}}}\left[\text{pHFMASP1}^*\right]\left[\text{pMBL}_3\text{MASP3}_{\text{zym}}\right]}{K_{\text{m MASP1}^*}^{\text{MASP3}_{\text{zym}}}\left(\begin{array}{l}1+\frac{[\text{C4}]}{K_{\text{m MASP1}^*}^{\text{C4}}}+\frac{[\text{fC4bC2}]}{K_{\text{m MASP1}^*}^{\text{C2}}}+\frac{[\text{fC4bC4bC2}]}{K_{\text{m MASP1}^*}^{\text{C2}}}+\frac{[\text{fC3bC4bC2}]}{K_{\text{m MASP1}^*}^{\text{C2}}}+\frac{[\text{pC4bC2}]}{K_{\text{m MASP1}^*}^{\text{C2}}}+\frac{[\text{pC4bC4bC2}]}{K_{\text{m MASP1}^*}^{\text{C2}}}\\+\frac{[\text{pC3bC4bC2}]}{K_{\text{m MASP1}^*}^{\text{C2}}}+\frac{[\text{pMBL}_3\text{MASP1}_{\text{zym}}]}{K_{\text{m MASP1}^*}^{\text{MASP1}_{\text{zym}}}}+\frac{[\text{pMBL}_3\text{MASP2}_{\text{zym}}]}{K_{\text{m MASP1}^*}^{\text{MASP2}_{\text{zym}}}}+\frac{[\text{pMBL}_3\text{MASP3}_{\text{zym}}]}{K_{\text{m MASP1}^*}^{\text{MASP3}_{\text{zym}}}}\\+\frac{[\text{pMBL}_4\text{MASP1}_{\text{zym}}]}{K_{\text{m MASP1}^*}^{\text{MASP1}_{\text{zym}}}}+\frac{[\text{pMBL}_4\text{MASP2}_{\text{zym}}]}{K_{\text{m MASP1}^*}^{\text{MASP2}_{\text{zym}}}}+\frac{[\text{pMBL}_4\text{MASP3}_{\text{zym}}]}{K_{\text{m MASP1}^*}^{\text{MASP3}_{\text{zym}}}}+\frac{[\text{pCLLKMASP1}_{\text{zym}}]}{K_{\text{m MASP1}^*}^{\text{MASP1}_{\text{zym}}}}\\+\frac{[\text{pCLLKMASP2}_{\text{zym}}]}{K_{\text{m MASP1}^*}^{\text{MASP2}_{\text{zym}}}}+\frac{[\text{pCLLKMASP3}_{\text{zym}}]}{K_{\text{m MASP1}^*}^{\text{MASP3}_{\text{zym}}}}+\frac{[\text{pCLK1MASP1}_{\text{zym}}]}{K_{\text{m MASP1}^*}^{\text{MASP1}_{\text{zym}}}}+\frac{[\text{pCLK1MASP2}_{\text{zym}}]}{K_{\text{m MASP1}^*}^{\text{MASP2}_{\text{zym}}}}\\+\frac{[\text{pCLK1MASP3}_{\text{zym}}]}{K_{\text{m MASP1}^*}^{\text{MASP3}_{\text{zym}}}}+\frac{[\text{pCLL1MASP1}_{\text{zym}}]}{K_{\text{m MASP1}^*}^{\text{MASP1}_{\text{zym}}}}+\frac{[\text{pCLL1MASP2}_{\text{zym}}]}{K_{\text{m MASP1}^*}^{\text{MASP2}_{\text{zym}}}}+\frac{[\text{pCLL1MASP3}_{\text{zym}}]}{K_{\text{m MASP1}^*}^{\text{MASP3}_{\text{zym}}}}\\+\frac{[\text{pLFMASP1}_{\text{zym}}]}{K_{\text{m MASP1}^*}^{\text{MASP1}_{\text{zym}}}}+\frac{[\text{pLFMASP2}_{\text{zym}}]}{K_{\text{m MASP1}^*}^{\text{MASP2}_{\text{zym}}}}+\frac{[\text{pLFMASP3}_{\text{zym}}]}{K_{\text{m MASP1}^*}^{\text{MASP3}_{\text{zym}}}}+\frac{[\text{pHFMASP1}_{\text{zym}}]}{K_{\text{m MASP1}^*}^{\text{MASP1}_{\text{zym}}}}+\frac{[\text{pHFMASP2}_{\text{zym}}]}{K_{\text{m MASP1}^*}^{\text{MASP2}_{\text{zym}}}}\\+\frac{[\text{pHFMASP3}_{\text{zym}}]}{K_{\text{m MASP1}^*}^{\text{MASP3}_{\text{zym}}}}+\frac{[\text{pMFMASP1}_{\text{zym}}]}{K_{\text{m MASP1}^*}^{\text{MASP1}_{\text{zym}}}}+\frac{[\text{pMFMASP2}_{\text{zym}}]}{K_{\text{m MASP1}^*}^{\text{MASP2}_{\text{zym}}}}+\frac{[\text{pMFMASP3}_{\text{zym}}]}{K_{\text{m MASP1}^*}^{\text{MASP3}_{\text{zym}}}}\\+\frac{[\text{pPTX3LFMASP1}_{\text{zym}}]}{K_{\text{m MASP1}^*}^{\text{MASP1}_{\text{zym}}}}+\frac{[\text{pPXT3LFMASP2}_{\text{zym}}]}{K_{\text{m MASP1}^*}^{\text{MASP2}_{\text{zym}}}}+\frac{[\text{pPTX3LFMASP3}_{\text{zym}}]}{K_{\text{m MASP1}^*}^{\text{MASP3}_{\text{zym}}}}\end{array}\right)}$$

$$+\frac{k_{\text{cat MASP1}^*}^{\text{MASP3}_{\text{zym}}}\left[\text{pMFMASP1}^*\right]\left[\text{pMBL}_3\text{MASP3}_{\text{zym}}\right]}{K_{\text{m MASP1}^*}^{\text{MASP3}_{\text{zym}}}\left(\begin{array}{l}1+\frac{[\text{C4}]}{K_{\text{m MASP1}^*}^{\text{C4}}}+\frac{[\text{fC4bC2}]}{K_{\text{m MASP1}^*}^{\text{C2}}}+\frac{[\text{fC4bC4bC2}]}{K_{\text{m MASP1}^*}^{\text{C2}}}+\frac{[\text{fC3bC4bC2}]}{K_{\text{m MASP1}^*}^{\text{C2}}}+\frac{[\text{pC4bC2}]}{K_{\text{m MASP1}^*}^{\text{C2}}}+\frac{[\text{pC4bC4bC2}]}{K_{\text{m MASP1}^*}^{\text{C2}}}\\+\frac{[\text{pC3bC4bC2}]}{K_{\text{m MASP1}^*}^{\text{C2}}}+\frac{\left[\text{pMBL}_3\text{MASP1}_{\text{zym}}\right]}{K_{\text{m MASP1}^*}^{\text{MASP1}_{\text{zym}}}}+\frac{\left[\text{pMBL}_3\text{MASP2}_{\text{zym}}\right]}{K_{\text{m MASP1}^*}^{\text{MASP2}_{\text{zym}}}}+\frac{\left[\text{pMBL}_3\text{MASP3}_{\text{zym}}\right]}{K_{\text{m MASP1}^*}^{\text{MASP3}_{\text{zym}}}}\\+\frac{\left[\text{pMBL}_4\text{MASP1}_{\text{zym}}\right]}{K_{\text{m MASP1}^*}^{\text{MASP1}_{\text{zym}}}}+\frac{\left[\text{pMBL}_4\text{MASP2}_{\text{zym}}\right]}{K_{\text{m MASP1}^*}^{\text{MASP2}_{\text{zym}}}}+\frac{\left[\text{pMBL}_4\text{MASP3}_{\text{zym}}\right]}{K_{\text{m MASP1}^*}^{\text{MASP3}_{\text{zym}}}}+\frac{\left[\text{pCLLKMASP1}_{\text{zym}}\right]}{K_{\text{m MASP1}^*}^{\text{MASP1}_{\text{zym}}}}\\+\frac{\left[\text{pCLLKMASP2}_{\text{zym}}\right]}{K_{\text{m MASP1}^*}^{\text{MASP2}_{\text{zym}}}}+\frac{\left[\text{pCLLKMASP3}_{\text{zym}}\right]}{K_{\text{m MASP1}^*}^{\text{MASP3}_{\text{zym}}}}+\frac{\left[\text{pCLK1MASP1}_{\text{zym}}\right]}{K_{\text{m MASP1}^*}^{\text{MASP1}_{\text{zym}}}}+\frac{\left[\text{pCLK1MASP2}_{\text{zym}}\right]}{K_{\text{m MASP1}^*}^{\text{MASP2}_{\text{zym}}}}\\+\frac{\left[\text{pCLK1MASP3}_{\text{zym}}\right]}{K_{\text{m MASP1}^*}^{\text{MASP3}_{\text{zym}}}}+\frac{\left[\text{pCLL1MASP1}_{\text{zym}}\right]}{K_{\text{m MASP1}^*}^{\text{MASP1}_{\text{zym}}}}+\frac{\left[\text{pCLL1MASP2}_{\text{zym}}\right]}{K_{\text{m MASP1}^*}^{\text{MASP2}_{\text{zym}}}}+\frac{\left[\text{pCLL1MASP3}_{\text{zym}}\right]}{K_{\text{m MASP1}^*}^{\text{MASP3}_{\text{zym}}}}\\+\frac{\left[\text{pLFMASP1}_{\text{zym}}\right]}{K_{\text{m MASP1}^*}^{\text{MASP1}_{\text{zym}}}}+\frac{\left[\text{pLFMASP2}_{\text{zym}}\right]}{K_{\text{m MASP1}^*}^{\text{MASP2}_{\text{zym}}}}+\frac{\left[\text{pLFMASP3}_{\text{zym}}\right]}{K_{\text{m MASP1}^*}^{\text{MASP3}_{\text{zym}}}}+\frac{\left[\text{pHFMASP1}_{\text{zym}}\right]}{K_{\text{m MASP1}^*}^{\text{MASP1}_{\text{zym}}}}+\frac{\left[\text{pHFMASP2}_{\text{zym}}\right]}{K_{\text{m MASP1}^*}^{\text{MASP2}_{\text{zym}}}}\\+\frac{\left[\text{pHFMASP3}_{\text{zym}}\right]}{K_{\text{m MASP1}^*}^{\text{MASP3}_{\text{zym}}}}+\frac{\left[\text{pMFMASP1}_{\text{zym}}\right]}{K_{\text{m MASP1}^*}^{\text{MASP1}_{\text{zym}}}}+\frac{\left[\text{pMFMASP2}_{\text{zym}}\right]}{K_{\text{m MASP1}^*}^{\text{MASP2}_{\text{zym}}}}+\frac{\left[\text{pMFMASP3}_{\text{zym}}\right]}{K_{\text{m MASP1}^*}^{\text{MASP3}_{\text{zym}}}}\\+\frac{\left[\text{pPTX3LFMASP1}_{\text{zym}}\right]}{K_{\text{m MASP1}^*}^{\text{MASP1}_{\text{zym}}}}+\frac{\left[\text{pPXT3LFMASP2}_{\text{zym}}\right]}{K_{\text{m MASP1}^*}^{\text{MASP2}_{\text{zym}}}}+\frac{\left[\text{pPTX3LFMASP3}_{\text{zym}}\right]}{K_{\text{m MASP1}^*}^{\text{MASP3}_{\text{zym}}}}\end{array}\right)}$$

$$+\frac{k_{\text{cat MASP1}^*}^{\text{MASP3}_{\text{zym}}}\left[\text{pPTX3LFMASP1}^*\right]\left[\text{pMBL}_3\text{MASP3}_{\text{zym}}\right]}{K_{\text{m MASP1}^*}^{\text{MASP3}_{\text{zym}}}\left(\begin{array}{l}1+\frac{[\text{C4}]}{K_{\text{m MASP1}^*}^{\text{C4}}}+\frac{[\text{fC4bC2}]}{K_{\text{m MASP1}^*}^{\text{C2}}}+\frac{[\text{fC4bC4bC2}]}{K_{\text{m MASP1}^*}^{\text{C2}}}+\frac{[\text{fC3bC4bC2}]}{K_{\text{m MASP1}^*}^{\text{C2}}}+\frac{[\text{pC4bC2}]}{K_{\text{m MASP1}^*}^{\text{C2}}}+\frac{[\text{pC4bC4bC2}]}{K_{\text{m MASP1}^*}^{\text{C2}}}\\+\frac{[\text{pC3bC4bC2}]}{K_{\text{m MASP1}^*}^{\text{C2}}}+\frac{[\text{pMBL}_3\text{MASP1}_{\text{zym}}]}{K_{\text{m MASP1}^*}^{\text{MASP1}_{\text{zym}}}}+\frac{[\text{pMBL}_3\text{MASP2}_{\text{zym}}]}{K_{\text{m MASP1}^*}^{\text{MASP2}_{\text{zym}}}}+\frac{[\text{pMBL}_3\text{MASP3}_{\text{zym}}]}{K_{\text{m MASP1}^*}^{\text{MASP3}_{\text{zym}}}}\\+\frac{[\text{pMBL}_4\text{MASP1}_{\text{zym}}]}{K_{\text{m MASP1}^*}^{\text{MASP1}_{\text{zym}}}}+\frac{[\text{pMBL}_4\text{MASP2}_{\text{zym}}]}{K_{\text{m MASP1}^*}^{\text{MASP2}_{\text{zym}}}}+\frac{[\text{pMBL}_4\text{MASP3}_{\text{zym}}]}{K_{\text{m MASP1}^*}^{\text{MASP3}_{\text{zym}}}}+\frac{[\text{pCLLKMASP1}_{\text{zym}}]}{K_{\text{m MASP1}^*}^{\text{MASP1}_{\text{zym}}}}\\+\frac{[\text{pCLLKMASP2}_{\text{zym}}]}{K_{\text{m MASP1}^*}^{\text{MASP2}_{\text{zym}}}}+\frac{[\text{pCLLKMASP3}_{\text{zym}}]}{K_{\text{m MASP1}^*}^{\text{MASP3}_{\text{zym}}}}+\frac{[\text{pCLK1MASP1}_{\text{zym}}]}{K_{\text{m MASP1}^*}^{\text{MASP1}_{\text{zym}}}}+\frac{[\text{pCLK1MASP2}_{\text{zym}}]}{K_{\text{m MASP1}^*}^{\text{MASP2}_{\text{zym}}}}\\+\frac{[\text{pCLK1MASP3}_{\text{zym}}]}{K_{\text{m MASP1}^*}^{\text{MASP3}_{\text{zym}}}}+\frac{[\text{pCLL1MASP1}_{\text{zym}}]}{K_{\text{m MASP1}^*}^{\text{MASP1}_{\text{zym}}}}+\frac{[\text{pCLL1MASP2}_{\text{zym}}]}{K_{\text{m MASP1}^*}^{\text{MASP2}_{\text{zym}}}}+\frac{[\text{pCLL1MASP3}_{\text{zym}}]}{K_{\text{m MASP1}^*}^{\text{MASP3}_{\text{zym}}}}\\+\frac{[\text{pLFMASP1}_{\text{zym}}]}{K_{\text{m MASP1}^*}^{\text{MASP1}_{\text{zym}}}}+\frac{[\text{pLFMASP2}_{\text{zym}}]}{K_{\text{m MASP1}^*}^{\text{MASP2}_{\text{zym}}}}+\frac{[\text{pLFMASP3}_{\text{zym}}]}{K_{\text{m MASP1}^*}^{\text{MASP3}_{\text{zym}}}}+\frac{[\text{pHFMASP1}_{\text{zym}}]}{K_{\text{m MASP1}^*}^{\text{MASP1}_{\text{zym}}}}+\frac{[\text{pHFMASP2}_{\text{zym}}]}{K_{\text{m MASP1}^*}^{\text{MASP2}_{\text{zym}}}}\\+\frac{[\text{pHFMASP3}_{\text{zym}}]}{K_{\text{m MASP1}^*}^{\text{MASP3}_{\text{zym}}}}+\frac{[\text{pMFMASP1}_{\text{zym}}]}{K_{\text{m MASP1}^*}^{\text{MASP1}_{\text{zym}}}}+\frac{[\text{pMFMASP2}_{\text{zym}}]}{K_{\text{m MASP1}^*}^{\text{MASP2}_{\text{zym}}}}+\frac{[\text{pMFMASP3}_{\text{zym}}]}{K_{\text{m MASP1}^*}^{\text{MASP3}_{\text{zym}}}}\\+\frac{[\text{pPTX3LFMASP1}_{\text{zym}}]}{K_{\text{m MASP1}^*}^{\text{MASP1}_{\text{zym}}}}+\frac{[\text{pPXT3LFMASP2}_{\text{zym}}]}{K_{\text{m MASP1}^*}^{\text{MASP2}_{\text{zym}}}}+\frac{[\text{pPTX3LFMASP3}_{\text{zym}}]}{K_{\text{m MASP1}^*}^{\text{MASP3}_{\text{zym}}}}\end{array}\right)}$$

256

$$\frac{d\left[\text{fMBL}_3\text{MAp19}\right]}{dt}=k_{\text{MBL}_3\text{MAp19}}^{+}\left[\text{MBL}_3\right]\left[\text{MAp19}\right]-k_{\text{MBL}_3\text{MAp19}}^{-}\left[\text{fMBL}_3\text{MAp19}\right]$$
$$-k_{\text{pMBL}_3}^{+}\left[\text{fMBL}_3\text{MAp19}\right]\left[\text{Pathogen Surface}\right]+k_{\text{pMBL}_3}^{-}\left[\text{pMBL}_3\text{MAp19}\right]$$

257

$$\frac{d\left[\text{pMBL}_3\text{MAp19}\right]}{dt}=k_{\text{pMBL}_3}^{+}\left[\text{fMBL}_3\text{MAp19}\right]\left[\text{Pathogen Surface}\right]-k_{\text{pMBL}_3}^{-}\left[\text{pMBL}_3\text{MAp19}\right]$$
$$+k_{\text{MBL}_3\text{MAp19}}^{+}\left[\text{pMBL}_3\right]\left[\text{MAp19}\right]-k_{\text{MBL}_3\text{MAp19}}^{-}\left[\text{pMBL}_3\text{MAp19}\right]$$

258

$$\frac{d\left[\text{fMBL}_3\text{MAp44}\right]}{dt}=k_{\text{MBL}_3\text{MAp44}}^{+}\left[\text{MBL}_3\right]\left[\text{MAp44}\right]-k_{\text{MBL}_3\text{MAp44}}^{-}\left[\text{fMBL}_3\text{MAp44}\right]$$
$$-k_{\text{pMBL}_3}^{+}\left[\text{fMBL}_3\text{MAp44}\right]\left[\text{Pathogen Surface}\right]+k_{\text{pMBL}_3}^{-}\left[\text{pMBL}_3\text{MAp44}\right]$$

259

$$\frac{d\left[\text{pMBL}_3\text{MAp44}\right]}{dt}=k_{\text{pMBL}_3}^{+}\left[\text{fMBL}_3\text{MAp44}\right]\left[\text{Pathogen Surface}\right]-k_{\text{pMBL}_3}^{-}\left[\text{pMBL}_3\text{MAp44}\right]$$
$$+k_{\text{MBL}_3\text{MAp44}}^{+}\left[\text{pMBL}_3\right]\left[\text{MAp44}\right]-k_{\text{MBL}_3\text{MAp44}}^{-}\left[\text{pMBL}_3\text{MAp44}\right]$$

260

$$\frac{d\left[\text{C1-INHMASP1}^{*}\right]}{dt}=k_{\text{C1INHMASP1}}^{+}\left[\text{C1-INH}\right]\left[\text{fMBL}_{3}\text{MASP1}^{*}\right]+k_{\text{C1INHMASP1}}^{+}\left[\text{C1-INH}\right]\left[\text{pMBL}_{3}\text{MASP1}^{*}\right]$$
$$+k_{\text{C1INHMASP1}}^{+}\left[\text{C1-INH}\right]\left[\text{MASP1}^{*}\right]+k_{\text{C1INHMASP1}}^{+}\left[\text{C1-INH}\right]\left[\text{fMBL}_{4}\text{MASP1}^{*}\right]$$
$$+k_{\text{C1INHMASP1}}^{+}\left[\text{C1-INH}\right]\left[\text{pMBL}_{4}\text{MASP1}^{*}\right]+k_{\text{C1INHMASP1}}^{+}\left[\text{C1-INH}\right]\left[\text{pCLLKMASP1}^{*}\right]$$
$$+k_{\text{C1INHMASP1}}^{+}\left[\text{C1-INH}\right]\left[\text{pCLK1MASP1}^{*}\right]+k_{\text{C1INHMASP1}}^{+}\left[\text{C1-INH}\right]\left[\text{pCLL1MASP1}^{*}\right]$$
$$+k_{\text{C1INHMASP1}}^{+}\left[\text{C1-INH}\right]\left[\text{fCLLKMASP1}^{*}\right]+k_{\text{C1INHMASP1}}^{+}\left[\text{C1-INH}\right]\left[\text{fCLK1MASP1}^{*}\right]$$
$$+k_{\text{C1INHMASP1}}^{+}\left[\text{C1-INH}\right]\left[\text{fCLL1MASP1}^{*}\right]+k_{\text{C1INHMASP1}}^{+}\left[\text{C1-INH}\right]\left[\text{pLFMASP1}^{*}\right]$$
$$+k_{\text{C1INHMASP1}}^{+}\left[\text{C1-INH}\right]\left[\text{pHFMASP1}^{*}\right]+k_{\text{C1INHMASP1}}^{+}\left[\text{C1-INH}\right]\left[\text{pMFMASP1}^{*}\right]+k_{\text{C1INHMASP1}}^{+}\left[\text{C1-INH}\right]\left[\text{fLFMASP1}^{*}\right]$$
$$+k_{\text{C1INHMASP1}}^{+}\left[\text{C1-INH}\right]\left[\text{fHFMASP1}^{*}\right]+k_{\text{C1INHMASP1}}^{+}\left[\text{C1-INH}\right]\left[\text{fMFMASP1}^{*}\right]$$
$$+k_{\text{C1INHMASP1}}^{+}\left[\text{C1-INH}\right]\left[\text{pPTX3LFMASP1}^{*}\right]$$

261

$$\frac{d\left[\text{C1-INHMASP2}^{*}\right]}{dt}=k_{\text{C1INHMASP2}}^{+}\left[\text{C1-INH}\right]\left[\text{pMBL}_{3}\text{MASP2}^{*}\right]+k_{\text{C1INHMASP2}}^{+}\left[\text{C1-INH}\right]\left[\text{fMBL}_{3}\text{MASP2}^{*}\right]$$
$$+k_{\text{C1INHMASP2}}^{+}\left[\text{C1-INH}\right]\left[\text{MASP2}^{*}\right]+k_{\text{C1INHMASP2}}^{+}\left[\text{C1-INH}\right]\left[\text{pMBL}_{4}\text{MASP2}^{*}\right]$$
$$+k_{\text{C1INHMASP2}}^{+}\left[\text{C1-INH}\right]\left[\text{fMBL}_{4}\text{MASP2}^{*}\right]+k_{\text{C1INHMASP2}}^{+}\left[\text{C1-INH}\right]\left[\text{pCLLKMASP2}^{*}\right]$$
$$+k_{\text{C1INHMASP2}}^{+}\left[\text{C1-INH}\right]\left[\text{pCLK1MASP2}^{*}\right]+k_{\text{C1INHMASP2}}^{+}\left[\text{C1-INH}\right]\left[\text{pCLL1MASP2}^{*}\right]$$
$$+k_{\text{C1INHMASP2}}^{+}\left[\text{C1-INH}\right]\left[\text{fCLLKMASP2}^{*}\right]+k_{\text{C1INHMASP2}}^{+}\left[\text{C1-INH}\right]\left[\text{fCLK1MASP2}^{*}\right]$$
$$+k_{\text{C1INHMASP2}}^{+}\left[\text{C1-INH}\right]\left[\text{fCLL1MASP2}^{*}\right]+k_{\text{C1INHMASP2}}^{+}\left[\text{C1-INH}\right]\left[\text{pLFMASP2}^{*}\right]$$
$$+k_{\text{C1INHMASP2}}^{+}\left[\text{C1-INH}\right]\left[\text{pHFMASP2}^{*}\right]+k_{\text{C1INHMASP2}}^{+}\left[\text{C1-INH}\right]\left[\text{pMFMASP2}^{*}\right]+k_{\text{C1INHMASP2}}^{+}\left[\text{C1-INH}\right]\left[\text{fLFMASP2}^{*}\right]$$
$$+k_{\text{C1INHMASP2}}^{+}\left[\text{C1-INH}\right]\left[\text{fHFMASP2}^{*}\right]+k_{\text{C1INHMASP2}}^{+}\left[\text{C1-INH}\right]\left[\text{fMFMASP2}^{*}\right]$$
$$+k_{\text{C1INHMASP2}}^{+}\left[\text{C1-INH}\right]\left[\text{pPTX3LFMASP2}^{*}\right]$$

262

$$\begin{aligned}
\frac{d\left[\mathrm{MASP1}^*\right]}{dt} &= -k^+_{\mathrm{MBL_3MASP1}}\left[\mathrm{MBL_3}\right]\left[\mathrm{MASP1}^*\right] + k^-_{\mathrm{MBL_3MASP1}}\left[\mathrm{fMBL_3MASP1}^*\right] - k^+_{\mathrm{MBL_3MASP1}}\left[\mathrm{pMBL_3}\right]\left[\mathrm{MASP1}^*\right] \\
&+ k^-_{\mathrm{MBL_3MASP1}}\left[\mathrm{pMBL_3MASP1}^*\right] - k^+_{\mathrm{C1INHMASP1}}\left[\mathrm{C1\text{-}INH}\right]\left[\mathrm{MASP1}^*\right] - k^+_{\mathrm{MBL_4MASP1}}\left[\mathrm{MBL_4}\right]\left[\mathrm{MASP1}^*\right] \\
&+ k^-_{\mathrm{MBL_4MASP1}}\left[\mathrm{fMBL_4MASP1}^*\right] - k^+_{\mathrm{MBL_4MASP1}}\left[\mathrm{pMBL_4}\right]\left[\mathrm{MASP1}^*\right] + k^-_{\mathrm{MBL_4MASP1}}\left[\mathrm{pMBL_4MASP1}^*\right] \\
&- k^+_{\mathrm{CLLKMASP1}}\left[\mathrm{CLLK}\right]\left[\mathrm{MASP1}^*\right] + k^-_{\mathrm{CLLKMASP1}}\left[\mathrm{fCLLKMASP1}^*\right] - k^+_{\mathrm{CLLKMASP1}}\left[\mathrm{pCLLK}\right]\left[\mathrm{MASP1}^*\right] \\
&+ k^-_{\mathrm{CLLKMASP1}}\left[\mathrm{pCLLKMASP1}^*\right] - k^+_{\mathrm{CLK1MASP1}}\left[\mathrm{CLK1}\right]\left[\mathrm{MASP1}^*\right] + k^-_{\mathrm{CLK1MASP1}}\left[\mathrm{fCLK1MASP1}^*\right] \\
&- k^+_{\mathrm{CLK1MASP1}}\left[\mathrm{pCLK1}\right]\left[\mathrm{MASP1}^*\right] + k^-_{\mathrm{CLK1MASP1}}\left[\mathrm{pCLK1MASP1}^*\right] - k^+_{\mathrm{CLL1MASP1}}\left[\mathrm{CLL1}\right]\left[\mathrm{MASP1}^*\right] \\
&+ k^-_{\mathrm{CLL1MASP1}}\left[\mathrm{fCLL1MASP1}^*\right] - k^+_{\mathrm{CLL1MASP1}}\left[\mathrm{pCLL1}\right]\left[\mathrm{MASP1}^*\right] + k^-_{\mathrm{CLL1MASP1}}\left[\mathrm{pCLL1MASP1}^*\right] \\
&- k^+_{\mathrm{LFMASP1}}\left[\mathrm{LF}\right]\left[\mathrm{MASP1}^*\right] + k^-_{\mathrm{LFMASP1}}\left[\mathrm{fLFMASP1}^*\right] - k^+_{\mathrm{LFMASP1}}\left[\mathrm{pLF}\right]\left[\mathrm{MASP1}^*\right] + k^-_{\mathrm{LFMASP1}}\left[\mathrm{pLFMASP1}^*\right] \\
&- k^+_{\mathrm{HFMASP1}}\left[\mathrm{HF}\right]\left[\mathrm{MASP1}^*\right] + k^-_{\mathrm{HFMASP1}}\left[\mathrm{fHFMASP1}^*\right] - k^+_{\mathrm{HFMASP1}}\left[\mathrm{pHF}\right]\left[\mathrm{MASP1}^*\right] + k^-_{\mathrm{HFMASP1}}\left[\mathrm{pHFMASP1}^*\right] \\
&- k^+_{\mathrm{MFMASP1}}\left[\mathrm{MF}\right]\left[\mathrm{MASP1}^*\right] + k^-_{\mathrm{MFMASP1}}\left[\mathrm{fMFMASP1}^*\right] - k^+_{\mathrm{MFMASP1}}\left[\mathrm{pMF}\right]\left[\mathrm{MASP1}^*\right] + k^-_{\mathrm{MFMASP1}}\left[\mathrm{pMFMASP1}^*\right] \\
&- k^+_{\mathrm{LFMASP1}}\left[\mathrm{pPTX3LF}\right]\left[\mathrm{MASP1}^*\right] + k^-_{\mathrm{LFMASP1}}\left[\mathrm{pPTX3LFMASP1}^*\right]
\end{aligned}$$

263

$$\begin{aligned}
\frac{d\left[\mathrm{MASP2}^*\right]}{dt} &= -k^+_{\mathrm{MBL_3MASP2}}\left[\mathrm{MBL_3}\right]\left[\mathrm{MASP2}^*\right] + k^-_{\mathrm{MBL_3MASP2}}\left[\mathrm{fMBL_3MASP2}^*\right] - k^+_{\mathrm{MBL_3MASP2}}\left[\mathrm{pMBL_3}\right]\left[\mathrm{MASP2}^*\right] \\
&+ k^-_{\mathrm{MBL_3MASP2}}\left[\mathrm{pMBL_3MASP2}^*\right] - k^+_{\mathrm{C1INHMASP2}}\left[\mathrm{C1\text{-}INH}\right]\left[\mathrm{MASP2}^*\right] - k^+_{\mathrm{MBL_4MASP2}}\left[\mathrm{MBL_4}\right]\left[\mathrm{MASP2}^*\right] \\
&+ k^-_{\mathrm{MBL_4MASP2}}\left[\mathrm{fMBL_4MASP2}^*\right] - k^+_{\mathrm{MBL_4MASP2}}\left[\mathrm{pMBL_4}\right]\left[\mathrm{MASP2}^*\right] + k^-_{\mathrm{MBL_4MASP2}}\left[\mathrm{pMBL_4MASP2}^*\right] \\
&- k^+_{\mathrm{CLLKMASP2}}\left[\mathrm{CLLK}\right]\left[\mathrm{MASP2}^*\right] + k^-_{\mathrm{CLLKMASP2}}\left[\mathrm{fCLLKMASP2}^*\right] - k^+_{\mathrm{CLLKMASP2}}\left[\mathrm{pCLLK}\right]\left[\mathrm{MASP2}^*\right] \\
&+ k^-_{\mathrm{CLLKMASP2}}\left[\mathrm{pCLLKMASP2}^*\right] - k^+_{\mathrm{CLK1MASP2}}\left[\mathrm{CLK1}\right]\left[\mathrm{MASP2}^*\right] + k^-_{\mathrm{CLK1MASP2}}\left[\mathrm{fCLK1MASP2}^*\right] \\
&- k^+_{\mathrm{CLK1MASP2}}\left[\mathrm{pCLK1}\right]\left[\mathrm{MASP2}^*\right] + k^-_{\mathrm{CLK1MASP2}}\left[\mathrm{pCLK1MASP2}^*\right] - k^+_{\mathrm{CLL1MASP2}}\left[\mathrm{CLL1}\right]\left[\mathrm{MASP2}^*\right] \\
&+ k^-_{\mathrm{CLL1MASP2}}\left[\mathrm{fCLL1MASP2}^*\right] - k^+_{\mathrm{CLL1MASP2}}\left[\mathrm{pCLL1}\right]\left[\mathrm{MASP2}^*\right] + k^-_{\mathrm{CLL1MASP2}}\left[\mathrm{pCLL1MASP2}^*\right] \\
&- k^+_{\mathrm{LFMASP2}}\left[\mathrm{LF}\right]\left[\mathrm{MASP2}^*\right] + k^-_{\mathrm{LFMASP2}}\left[\mathrm{fLFMASP2}^*\right] - k^+_{\mathrm{LFMASP2}}\left[\mathrm{pLF}\right]\left[\mathrm{MASP2}^*\right] + k^-_{\mathrm{LFMASP2}}\left[\mathrm{pLFMASP2}^*\right] \\
&- k^+_{\mathrm{HFMASP2}}\left[\mathrm{HF}\right]\left[\mathrm{MASP2}^*\right] + k^-_{\mathrm{HFMASP2}}\left[\mathrm{fHFMASP2}^*\right] - k^+_{\mathrm{HFMASP2}}\left[\mathrm{pHF}\right]\left[\mathrm{MASP2}^*\right] + k^-_{\mathrm{HFMASP2}}\left[\mathrm{pHFMASP2}^*\right] \\
&- k^+_{\mathrm{MFMASP2}}\left[\mathrm{MF}\right]\left[\mathrm{MASP2}^*\right] + k^-_{\mathrm{MFMASP2}}\left[\mathrm{fMFMASP2}^*\right] - k^+_{\mathrm{MFMASP2}}\left[\mathrm{pMF}\right]\left[\mathrm{MASP2}^*\right] + k^-_{\mathrm{MFMASP2}}\left[\mathrm{pMFMASP2}^*\right] \\
&- k^+_{\mathrm{LFMASP2}}\left[\mathrm{pPTX3LF}\right]\left[\mathrm{MASP2}^*\right] + k^-_{\mathrm{LFMASP2}}\left[\mathrm{pPTX3LFMASP2}^*\right]
\end{aligned}$$

264

$$\frac{d\left[\mathrm{MASP3}^*\right]}{dt} = -k^+_{\mathrm{MBL_3MASP3}}\left[\mathrm{MBL_3}\right]\left[\mathrm{MASP3}^*\right] + k^-_{\mathrm{MBL_3MASP3}}\left[\mathrm{fMBL_3MASP3}^*\right] - k^+_{\mathrm{MBL_3MASP3}}\left[\mathrm{pMBL_3}\right]\left[\mathrm{MASP3}^*\right]$$
$$+k^-_{\mathrm{MBL_3MASP3}}\left[\mathrm{pMBL_3MASP3}^*\right] - k^+_{\mathrm{MBL_4MASP3}}\left[\mathrm{MBL_4}\right]\left[\mathrm{MASP3}^*\right] + k^-_{\mathrm{MBL_4MASP3}}\left[\mathrm{fMBL_4MASP3}^*\right]$$
$$-k^+_{\mathrm{MBL_4MASP3}}\left[\mathrm{pMBL_4}\right]\left[\mathrm{MASP3}^*\right] + k^-_{\mathrm{MBL_4MASP3}}\left[\mathrm{pMBL_4MASP3}^*\right] - k^+_{\mathrm{CLLKMASP3}}\left[\mathrm{CLLK}\right]\left[\mathrm{MASP3}^*\right]$$
$$+k^-_{\mathrm{CLLKMASP3}}\left[\mathrm{fCLLKMASP3}^*\right] - k^+_{\mathrm{CLLKMASP3}}\left[\mathrm{pCLLK}\right]\left[\mathrm{MASP3}^*\right] + k^-_{\mathrm{CLLKMASP3}}\left[\mathrm{pCLLKMASP3}^*\right]$$
$$-k^+_{\mathrm{CLK1MASP3}}\left[\mathrm{CLK1}\right]\left[\mathrm{MASP3}^*\right] + k^-_{\mathrm{CLK1MASP3}}\left[\mathrm{fCLK1MASP3}^*\right] - k^+_{\mathrm{CLK1MASP3}}\left[\mathrm{pCLK1}\right]\left[\mathrm{MASP3}^*\right]$$
$$+k^-_{\mathrm{CLK1MASP3}}\left[\mathrm{pCLK1MASP3}^*\right] - k^+_{\mathrm{CLL1MASP3}}\left[\mathrm{CLL1}\right]\left[\mathrm{MASP3}^*\right] + k^-_{\mathrm{CLL1MASP3}}\left[\mathrm{fCLL1MASP3}^*\right]$$
$$-k^+_{\mathrm{CLL1MASP3}}\left[\mathrm{pCLL1}\right]\left[\mathrm{MASP3}^*\right] + k^-_{\mathrm{CLL1MASP3}}\left[\mathrm{pCLL1MASP3}^*\right] - k^+_{\mathrm{LFMASP3}}\left[\mathrm{LF}\right]\left[\mathrm{MASP3}^*\right]$$
$$+k^-_{\mathrm{LFMASP3}}\left[\mathrm{fLFMASP3}^*\right] - k^+_{\mathrm{LFMASP3}}\left[\mathrm{pLF}\right]\left[\mathrm{MASP3}^*\right] + k^-_{\mathrm{LFMASP3}}\left[\mathrm{pLFMASP3}^*\right] - k^+_{\mathrm{HFMASP3}}\left[\mathrm{HF}\right]\left[\mathrm{MASP3}^*\right]$$
$$+k^-_{\mathrm{HFMASP3}}\left[\mathrm{fHFMASP3}^*\right] - k^+_{\mathrm{HFMASP3}}\left[\mathrm{pHF}\right]\left[\mathrm{MASP3}^*\right] + k^-_{\mathrm{HFMASP3}}\left[\mathrm{pHFMASP3}^*\right] - k^+_{\mathrm{MFMASP3}}\left[\mathrm{MF}\right]\left[\mathrm{MASP3}^*\right]$$
$$+k^-_{\mathrm{MFMASP3}}\left[\mathrm{fMFMASP3}^*\right] - k^+_{\mathrm{MFMASP3}}\left[\mathrm{pMF}\right]\left[\mathrm{MASP3}^*\right] + k^-_{\mathrm{MFMASP3}}\left[\mathrm{pMFMASP3}^*\right]$$
$$-k^+_{\mathrm{LFMASP3}}\left[\mathrm{pPTX3LF}\right]\left[\mathrm{MASP3}^*\right] + k^-_{\mathrm{LFMASP3}}\left[\mathrm{pPTX3LFMASP3}^*\right]$$

265

$$\frac{d\left[\mathrm{pMBL_4}\right]}{dt} = k^+_{\mathrm{pMBL_4}}\left[\mathrm{MBL_4}\right]\left[\mathrm{Pathogen\ Surface}\right] - k^-_{\mathrm{pMBL_4}}\left[\mathrm{pMBL_4}\right] - k^+_{\mathrm{MBL_4MASP1}}\left[\mathrm{pMBL_4}\right]\left[\mathrm{MASP1}\right]$$
$$+k^-_{\mathrm{MBL_4MASP1}}\left[\mathrm{pMBL_4MASP1_{zym}}\right] - k^+_{\mathrm{MBL_4MASP2}}\left[\mathrm{pMBL_4}\right]\left[\mathrm{MASP2}\right] + k^-_{\mathrm{MBL_4MASP2}}\left[\mathrm{pMBL_4MASP2_{zym}}\right]$$
$$-k^+_{\mathrm{MBL_4MASP3}}\left[\mathrm{pMBL_4}\right]\left[\mathrm{MASP3}\right] + k^-_{\mathrm{MBL_4MASP3}}\left[\mathrm{pMBL_4MASP3_{zym}}\right] - k^+_{\mathrm{MBL_4MASP1}}\left[\mathrm{pMBL_4}\right]\left[\mathrm{MASP1}^*\right]$$
$$+k^-_{\mathrm{MBL_4MASP1}}\left[\mathrm{pMBL_4MASP1}^*\right] - k^+_{\mathrm{MBL_4MASP2}}\left[\mathrm{pMBL_4}\right]\left[\mathrm{MASP2}^*\right] + k^-_{\mathrm{MBL_4MASP2}}\left[\mathrm{pMBL_4MASP2}^*\right]$$
$$-k^+_{\mathrm{MBL_4MASP3}}\left[\mathrm{pMBL_4}\right]\left[\mathrm{MASP3}^*\right] + k^-_{\mathrm{MBL_4MASP3}}\left[\mathrm{pMBL_4MASP3}^*\right] - k^+_{\mathrm{MBL_4MAp19}}\left[\mathrm{pMBL_4}\right]\left[\mathrm{MAp19}\right]$$
$$+k^-_{\mathrm{MBL_4MAp19}}\left[\mathrm{pMBL_4MAp19}\right] - k^+_{\mathrm{MBL_4MAp44}}\left[\mathrm{pMBL_4}\right]\left[\mathrm{MAp44}\right] + k^-_{\mathrm{MBL_4MAp44}}\left[\mathrm{pMBL_4MAp44}\right]$$
$$+k^+_{\mathrm{C1INHMASP1}}\left[\mathrm{C1\text{-}INH}\right]\left[\mathrm{pMBL_4MASP1}^*\right] + k^+_{\mathrm{C1INHMASP2}}\left[\mathrm{C1\text{-}INH}\right]\left[\mathrm{pMBL_4MASP2}^*\right] - k^+_{\mathrm{MBL_4PTX3}}\left[\mathrm{pMBL_4}\right]\left[\mathrm{PTX3}\right]$$
$$+k^-_{\mathrm{MBL_4PTX3}}\left[\mathrm{pMBL_4PTX3}\right] - k^+_{\mathrm{MBL_4SAP}}\left[\mathrm{pMBL_4}\right]\left[\mathrm{SAP}\right] + k^-_{\mathrm{MBL_4SAP}}\left[\mathrm{pMBL_4SAP}\right] + k^-_{\mathrm{MBL_4PTX3}}\left[\mathrm{pMBL_4PTX3C1q}\right]$$
$$+k^-_{\mathrm{MBL_4PTX3}}\left[\mathrm{pMBL_4PTX3C1}\right] + k^-_{\mathrm{MBL_4PTX3}}\left[\mathrm{pMBL_4PTX3C1}^*\right]$$

266

$$\frac{d\left[\mathrm{pMBL_4MASP1_{zym}}\right]}{dt} = k^+_{\mathrm{pMBL_4}}\left[\mathrm{fMBL_4MASP1_{zym}}\right]\left[\mathrm{Pathogen\ Surface}\right] - k^-_{\mathrm{pMBL_4}}\left[\mathrm{pMBL_4MASP1_{zym}}\right]$$
$$+k^+_{\mathrm{MBL_4MASP1}}\left[\mathrm{pMBL_4}\right]\left[\mathrm{MASP1}\right] - k^-_{\mathrm{MBL_4MASP1}}\left[\mathrm{pMBL_4MASP1_{zym}}\right]$$

$$
-\frac{k_{\mathrm{cat\ MASP1_{zym}}}^{\mathrm{MASP1_{zym}}}\left[\mathrm{pMBL_3MASP1_{zym}}\right]\left[\mathrm{pMBL_4MASP1_{zym}}\right]}{K_{\mathrm{m\ MASP1_{zym}}}^{\mathrm{MASP1_{zym}}}\left(\begin{array}{l}1+\frac{\left[\mathrm{pMBL_3MASP1_{zym}}\right]}{K_{\mathrm{m\ MASP1_{zym}}}^{\mathrm{MASP1_{zym}}}}+\frac{\left[\mathrm{pMBL_3MASP2_{zym}}\right]}{K_{\mathrm{m\ MASP1_{zym}}}^{\mathrm{MASP2_{zym}}}}+\frac{\left[\mathrm{pMBL_4MASP1_{zym}}\right]}{K_{\mathrm{m\ MASP1_{zym}}}^{\mathrm{MASP1_{zym}}}}+\frac{\left[\mathrm{pMBL_4MASP2_{zym}}\right]}{K_{\mathrm{m\ MASP1_{zym}}}^{\mathrm{MASP2_{zym}}}}\\+\frac{\left[\mathrm{pCLLKMASP1_{zym}}\right]}{K_{\mathrm{m\ MASP1_{zym}}}^{\mathrm{MASP1_{zym}}}}+\frac{\left[\mathrm{pCLLKMASP2_{zym}}\right]}{K_{\mathrm{m\ MASP1_{zym}}}^{\mathrm{MASP2_{zym}}}}+\frac{\left[\mathrm{pCLK1MASP1_{zym}}\right]}{K_{\mathrm{m\ MASP1_{zym}}}^{\mathrm{MASP1_{zym}}}}+\frac{\left[\mathrm{pCLK1MASP2_{zym}}\right]}{K_{\mathrm{m\ MASP1_{zym}}}^{\mathrm{MASP2_{zym}}}}\\+\frac{\left[\mathrm{pCLL1MASP1_{zym}}\right]}{K_{\mathrm{m\ MASP1_{zym}}}^{\mathrm{MASP1_{zym}}}}+\frac{\left[\mathrm{pCLL1MASP2_{zym}}\right]}{K_{\mathrm{m\ MASP1_{zym}}}^{\mathrm{MASP2_{zym}}}}+\frac{\left[\mathrm{pLFMASP1_{zym}}\right]}{K_{\mathrm{m\ MASP1_{zym}}}^{\mathrm{MASP1_{zym}}}}+\frac{\left[\mathrm{pLFMASP2_{zym}}\right]}{K_{\mathrm{m\ MASP1_{zym}}}^{\mathrm{MASP2_{zym}}}}\\+\frac{\left[\mathrm{pHFMASP1_{zym}}\right]}{K_{\mathrm{m\ MASP1_{zym}}}^{\mathrm{MASP1_{zym}}}}+\frac{\left[\mathrm{pHFMASP2_{zym}}\right]}{K_{\mathrm{m\ MASP1_{zym}}}^{\mathrm{MASP2_{zym}}}}+\frac{\left[\mathrm{pMFMASP1_{zym}}\right]}{K_{\mathrm{m\ MASP1_{zym}}}^{\mathrm{MASP1_{zym}}}}+\frac{\left[\mathrm{pMFMASP2_{zym}}\right]}{K_{\mathrm{m\ MASP1_{zym}}}^{\mathrm{MASP2_{zym}}}}\end{array}\right)}
$$

$$
-\frac{k_{\mathrm{cat\ MASP1_{zym}}}^{\mathrm{MASP1_{zym}}}\left[\mathrm{pMBL_4MASP1_{zym}}\right]\left[\mathrm{pMBL_4MASP1_{zym}}\right]}{K_{\mathrm{m\ MASP1_{zym}}}^{\mathrm{MASP1_{zym}}}\left(\begin{array}{l}1+\frac{\left[\mathrm{pMBL_3MASP1_{zym}}\right]}{K_{\mathrm{m\ MASP1_{zym}}}^{\mathrm{MASP1_{zym}}}}+\frac{\left[\mathrm{pMBL_3MASP2_{zym}}\right]}{K_{\mathrm{m\ MASP1_{zym}}}^{\mathrm{MASP2_{zym}}}}+\frac{\left[\mathrm{pMBL_4MASP1_{zym}}\right]}{K_{\mathrm{m\ MASP1_{zym}}}^{\mathrm{MASP1_{zym}}}}+\frac{\left[\mathrm{pMBL_4MASP2_{zym}}\right]}{K_{\mathrm{m\ MASP1_{zym}}}^{\mathrm{MASP2_{zym}}}}\\+\frac{\left[\mathrm{pCLLKMASP1_{zym}}\right]}{K_{\mathrm{m\ MASP1_{zym}}}^{\mathrm{MASP1_{zym}}}}+\frac{\left[\mathrm{pCLLKMASP2_{zym}}\right]}{K_{\mathrm{m\ MASP1_{zym}}}^{\mathrm{MASP2_{zym}}}}+\frac{\left[\mathrm{pCLK1MASP1_{zym}}\right]}{K_{\mathrm{m\ MASP1_{zym}}}^{\mathrm{MASP1_{zym}}}}+\frac{\left[\mathrm{pCLK1MASP2_{zym}}\right]}{K_{\mathrm{m\ MASP1_{zym}}}^{\mathrm{MASP2_{zym}}}}\\+\frac{\left[\mathrm{pCLL1MASP1_{zym}}\right]}{K_{\mathrm{m\ MASP1_{zym}}}^{\mathrm{MASP1_{zym}}}}+\frac{\left[\mathrm{pCLL1MASP2_{zym}}\right]}{K_{\mathrm{m\ MASP1_{zym}}}^{\mathrm{MASP2_{zym}}}}+\frac{\left[\mathrm{pLFMASP1_{zym}}\right]}{K_{\mathrm{m\ MASP1_{zym}}}^{\mathrm{MASP1_{zym}}}}+\frac{\left[\mathrm{pLFMASP2_{zym}}\right]}{K_{\mathrm{m\ MASP1_{zym}}}^{\mathrm{MASP2_{zym}}}}\\+\frac{\left[\mathrm{pHFMASP1_{zym}}\right]}{K_{\mathrm{m\ MASP1_{zym}}}^{\mathrm{MASP1_{zym}}}}+\frac{\left[\mathrm{pHFMASP2_{zym}}\right]}{K_{\mathrm{m\ MASP1_{zym}}}^{\mathrm{MASP2_{zym}}}}+\frac{\left[\mathrm{pMFMASP1_{zym}}\right]}{K_{\mathrm{m\ MASP1_{zym}}}^{\mathrm{MASP1_{zym}}}}+\frac{\left[\mathrm{pMFMASP2_{zym}}\right]}{K_{\mathrm{m\ MASP1_{zym}}}^{\mathrm{MASP2_{zym}}}}\end{array}\right)}
$$

$$
-\frac{k_{\mathrm{cat\ MASP1_{zym}}}^{\mathrm{MASP1_{zym}}}\left[\mathrm{pCLLKMASP1_{zym}}\right]\left[\mathrm{pMBL_4MASP1_{zym}}\right]}{K_{\mathrm{m\ MASP1_{zym}}}^{\mathrm{MASP1_{zym}}}\left(\begin{array}{l}1+\frac{\left[\mathrm{pMBL_3MASP1_{zym}}\right]}{K_{\mathrm{m\ MASP1_{zym}}}^{\mathrm{MASP1_{zym}}}}+\frac{\left[\mathrm{pMBL_3MASP2_{zym}}\right]}{K_{\mathrm{m\ MASP1_{zym}}}^{\mathrm{MASP2_{zym}}}}+\frac{\left[\mathrm{pMBL_4MASP1_{zym}}\right]}{K_{\mathrm{m\ MASP1_{zym}}}^{\mathrm{MASP1_{zym}}}}+\frac{\left[\mathrm{pMBL_4MASP2_{zym}}\right]}{K_{\mathrm{m\ MASP1_{zym}}}^{\mathrm{MASP2_{zym}}}}\\+\frac{\left[\mathrm{pCLLKMASP1_{zym}}\right]}{K_{\mathrm{m\ MASP1_{zym}}}^{\mathrm{MASP1_{zym}}}}+\frac{\left[\mathrm{pCLLKMASP2_{zym}}\right]}{K_{\mathrm{m\ MASP1_{zym}}}^{\mathrm{MASP2_{zym}}}}+\frac{\left[\mathrm{pCLK1MASP1_{zym}}\right]}{K_{\mathrm{m\ MASP1_{zym}}}^{\mathrm{MASP1_{zym}}}}+\frac{\left[\mathrm{pCLK1MASP2_{zym}}\right]}{K_{\mathrm{m\ MASP1_{zym}}}^{\mathrm{MASP2_{zym}}}}\\+\frac{\left[\mathrm{pCLL1MASP1_{zym}}\right]}{K_{\mathrm{m\ MASP1_{zym}}}^{\mathrm{MASP1_{zym}}}}+\frac{\left[\mathrm{pCLL1MASP2_{zym}}\right]}{K_{\mathrm{m\ MASP1_{zym}}}^{\mathrm{MASP2_{zym}}}}+\frac{\left[\mathrm{pLFMASP1_{zym}}\right]}{K_{\mathrm{m\ MASP1_{zym}}}^{\mathrm{MASP1_{zym}}}}+\frac{\left[\mathrm{pLFMASP2_{zym}}\right]}{K_{\mathrm{m\ MASP1_{zym}}}^{\mathrm{MASP2_{zym}}}}\\+\frac{\left[\mathrm{pHFMASP1_{zym}}\right]}{K_{\mathrm{m\ MASP1_{zym}}}^{\mathrm{MASP1_{zym}}}}+\frac{\left[\mathrm{pHFMASP2_{zym}}\right]}{K_{\mathrm{m\ MASP1_{zym}}}^{\mathrm{MASP2_{zym}}}}+\frac{\left[\mathrm{pMFMASP1_{zym}}\right]}{K_{\mathrm{m\ MASP1_{zym}}}^{\mathrm{MASP1_{zym}}}}+\frac{\left[\mathrm{pMFMASP2_{zym}}\right]}{K_{\mathrm{m\ MASP1_{zym}}}^{\mathrm{MASP2_{zym}}}}\end{array}\right)}
$$

$$-\frac{k_{\mathrm{cat\,MASP1_{zym}}}^{\mathrm{MASP1_{zym}}}\left[\mathrm{pCLK1MASP1_{zym}}\right]\left[\mathrm{pMBL_4MASP1_{zym}}\right]}{K_{\mathrm{m\,MASP1_{zym}}}^{\mathrm{MASP1_{zym}}}\left(\begin{array}{l}1+\frac{[\mathrm{pMBL_3MASP1_{zym}}]}{K_{\mathrm{m\,MASP1_{zym}}}^{\mathrm{MASP1_{zym}}}}+\frac{[\mathrm{pMBL_3MASP2_{zym}}]}{K_{\mathrm{m\,MASP1_{zym}}}^{\mathrm{MASP2_{zym}}}}+\frac{[\mathrm{pMBL_4MASP1_{zym}}]}{K_{\mathrm{m\,MASP1_{zym}}}^{\mathrm{MASP1_{zym}}}}+\frac{[\mathrm{pMBL_4MASP2_{zym}}]}{K_{\mathrm{m\,MASP1_{zym}}}^{\mathrm{MASP2_{zym}}}}\\+\frac{[\mathrm{pCLLKMASP1_{zym}}]}{K_{\mathrm{m\,MASP1_{zym}}}^{\mathrm{MASP1_{zym}}}}+\frac{[\mathrm{pCLLKMASP2_{zym}}]}{K_{\mathrm{m\,MASP1_{zym}}}^{\mathrm{MASP2_{zym}}}}+\frac{[\mathrm{pCLK1MASP1_{zym}}]}{K_{\mathrm{m\,MASP1_{zym}}}^{\mathrm{MASP1_{zym}}}}+\frac{[\mathrm{pCLK1MASP2_{zym}}]}{K_{\mathrm{m\,MASP1_{zym}}}^{\mathrm{MASP2_{zym}}}}\\+\frac{[\mathrm{pCLL1MASP1_{zym}}]}{K_{\mathrm{m\,MASP1_{zym}}}^{\mathrm{MASP1_{zym}}}}+\frac{[\mathrm{pCLL1MASP2_{zym}}]}{K_{\mathrm{m\,MASP1_{zym}}}^{\mathrm{MASP2_{zym}}}}+\frac{[\mathrm{pLFMASP1_{zym}}]}{K_{\mathrm{m\,MASP1_{zym}}}^{\mathrm{MASP1_{zym}}}}+\frac{[\mathrm{pLFMASP2_{zym}}]}{K_{\mathrm{m\,MASP1_{zym}}}^{\mathrm{MASP2_{zym}}}}\\+\frac{[\mathrm{pHFMASP1_{zym}}]}{K_{\mathrm{m\,MASP1_{zym}}}^{\mathrm{MASP1_{zym}}}}+\frac{[\mathrm{pHFMASP2_{zym}}]}{K_{\mathrm{m\,MASP1_{zym}}}^{\mathrm{MASP2_{zym}}}}+\frac{[\mathrm{pMFMASP1_{zym}}]}{K_{\mathrm{m\,MASP1_{zym}}}^{\mathrm{MASP1_{zym}}}}+\frac{[\mathrm{pMFMASP2_{zym}}]}{K_{\mathrm{m\,MASP1_{zym}}}^{\mathrm{MASP2_{zym}}}}\end{array}\right)}$$

$$-\frac{k_{\mathrm{cat\,MASP1_{zym}}}^{\mathrm{MASP1_{zym}}}\left[\mathrm{pCLL1MASP1_{zym}}\right]\left[\mathrm{pMBL_4MASP1_{zym}}\right]}{K_{\mathrm{m\,MASP1_{zym}}}^{\mathrm{MASP1_{zym}}}\left(\begin{array}{l}1+\frac{[\mathrm{pMBL_3MASP1_{zym}}]}{K_{\mathrm{m\,MASP1_{zym}}}^{\mathrm{MASP1_{zym}}}}+\frac{[\mathrm{pMBL_3MASP2_{zym}}]}{K_{\mathrm{m\,MASP1_{zym}}}^{\mathrm{MASP2_{zym}}}}+\frac{[\mathrm{pMBL_4MASP1_{zym}}]}{K_{\mathrm{m\,MASP1_{zym}}}^{\mathrm{MASP1_{zym}}}}+\frac{[\mathrm{pMBL_4MASP2_{zym}}]}{K_{\mathrm{m\,MASP1_{zym}}}^{\mathrm{MASP2_{zym}}}}\\+\frac{[\mathrm{pCLLKMASP1_{zym}}]}{K_{\mathrm{m\,MASP1_{zym}}}^{\mathrm{MASP1_{zym}}}}+\frac{[\mathrm{pCLLKMASP2_{zym}}]}{K_{\mathrm{m\,MASP1_{zym}}}^{\mathrm{MASP2_{zym}}}}+\frac{[\mathrm{pCLK1MASP1_{zym}}]}{K_{\mathrm{m\,MASP1_{zym}}}^{\mathrm{MASP1_{zym}}}}+\frac{[\mathrm{pCLK1MASP2_{zym}}]}{K_{\mathrm{m\,MASP1_{zym}}}^{\mathrm{MASP2_{zym}}}}\\+\frac{[\mathrm{pCLL1MASP1_{zym}}]}{K_{\mathrm{m\,MASP1_{zym}}}^{\mathrm{MASP1_{zym}}}}+\frac{[\mathrm{pCLL1MASP2_{zym}}]}{K_{\mathrm{m\,MASP1_{zym}}}^{\mathrm{MASP2_{zym}}}}+\frac{[\mathrm{pLFMASP1_{zym}}]}{K_{\mathrm{m\,MASP1_{zym}}}^{\mathrm{MASP1_{zym}}}}+\frac{[\mathrm{pLFMASP2_{zym}}]}{K_{\mathrm{m\,MASP1_{zym}}}^{\mathrm{MASP2_{zym}}}}\\+\frac{[\mathrm{pHFMASP1_{zym}}]}{K_{\mathrm{m\,MASP1_{zym}}}^{\mathrm{MASP1_{zym}}}}+\frac{[\mathrm{pHFMASP2_{zym}}]}{K_{\mathrm{m\,MASP1_{zym}}}^{\mathrm{MASP2_{zym}}}}+\frac{[\mathrm{pMFMASP1_{zym}}]}{K_{\mathrm{m\,MASP1_{zym}}}^{\mathrm{MASP1_{zym}}}}+\frac{[\mathrm{pMFMASP2_{zym}}]}{K_{\mathrm{m\,MASP1_{zym}}}^{\mathrm{MASP2_{zym}}}}\end{array}\right)}$$

$$-\frac{k_{\mathrm{cat\,MASP1_{zym}}}^{\mathrm{MASP1_{zym}}}\left[\mathrm{pLFMASP1_{zym}}\right]\left[\mathrm{pMBL_4MASP1_{zym}}\right]}{K_{\mathrm{m\,MASP1_{zym}}}^{\mathrm{MASP1_{zym}}}\left(\begin{array}{l}1+\frac{[\mathrm{pMBL_3MASP1_{zym}}]}{K_{\mathrm{m\,MASP1_{zym}}}^{\mathrm{MASP1_{zym}}}}+\frac{[\mathrm{pMBL_3MASP2_{zym}}]}{K_{\mathrm{m\,MASP1_{zym}}}^{\mathrm{MASP2_{zym}}}}+\frac{[\mathrm{pMBL_4MASP1_{zym}}]}{K_{\mathrm{m\,MASP1_{zym}}}^{\mathrm{MASP1_{zym}}}}+\frac{[\mathrm{pMBL_4MASP2_{zym}}]}{K_{\mathrm{m\,MASP1_{zym}}}^{\mathrm{MASP2_{zym}}}}\\+\frac{[\mathrm{pCLLKMASP1_{zym}}]}{K_{\mathrm{m\,MASP1_{zym}}}^{\mathrm{MASP1_{zym}}}}+\frac{[\mathrm{pCLLKMASP2_{zym}}]}{K_{\mathrm{m\,MASP1_{zym}}}^{\mathrm{MASP2_{zym}}}}+\frac{[\mathrm{pCLK1MASP1_{zym}}]}{K_{\mathrm{m\,MASP1_{zym}}}^{\mathrm{MASP1_{zym}}}}+\frac{[\mathrm{pCLK1MASP2_{zym}}]}{K_{\mathrm{m\,MASP1_{zym}}}^{\mathrm{MASP2_{zym}}}}\\+\frac{[\mathrm{pCLL1MASP1_{zym}}]}{K_{\mathrm{m\,MASP1_{zym}}}^{\mathrm{MASP1_{zym}}}}+\frac{[\mathrm{pCLL1MASP2_{zym}}]}{K_{\mathrm{m\,MASP1_{zym}}}^{\mathrm{MASP2_{zym}}}}+\frac{[\mathrm{pLFMASP1_{zym}}]}{K_{\mathrm{m\,MASP1_{zym}}}^{\mathrm{MASP1_{zym}}}}+\frac{[\mathrm{pLFMASP2_{zym}}]}{K_{\mathrm{m\,MASP1_{zym}}}^{\mathrm{MASP2_{zym}}}}\\+\frac{[\mathrm{pHFMASP1_{zym}}]}{K_{\mathrm{m\,MASP1_{zym}}}^{\mathrm{MASP1_{zym}}}}+\frac{[\mathrm{pHFMASP2_{zym}}]}{K_{\mathrm{m\,MASP1_{zym}}}^{\mathrm{MASP2_{zym}}}}+\frac{[\mathrm{pMFMASP1_{zym}}]}{K_{\mathrm{m\,MASP1_{zym}}}^{\mathrm{MASP1_{zym}}}}+\frac{[\mathrm{pMFMASP2_{zym}}]}{K_{\mathrm{m\,MASP1_{zym}}}^{\mathrm{MASP2_{zym}}}}\end{array}\right)}$$

$$
-\frac{k^{\mathrm{MASP1_{zym}}}_{\mathrm{cat\ MASP1_{zym}}}\left[\mathrm{pHFMASP1_{zym}}\right]\left[\mathrm{pMBL_4MASP1_{zym}}\right]}{K^{\mathrm{MASP1_{zym}}}_{\mathrm{m\ MASP1_{zym}}}\left(\begin{array}{l}1+\frac{\left[\mathrm{pMBL_3MASP1_{zym}}\right]}{K^{\mathrm{MASP1_{zym}}}_{\mathrm{m\ MASP1_{zym}}}}+\frac{\left[\mathrm{pMBL_3MASP2_{zym}}\right]}{K^{\mathrm{MASP2_{zym}}}_{\mathrm{m\ MASP1_{zym}}}}+\frac{\left[\mathrm{pMBL_4MASP1_{zym}}\right]}{K^{\mathrm{MASP1_{zym}}}_{\mathrm{m\ MASP1_{zym}}}}+\frac{\left[\mathrm{pMBL_4MASP2_{zym}}\right]}{K^{\mathrm{MASP2_{zym}}}_{\mathrm{m\ MASP1_{zym}}}}\\+\frac{\left[\mathrm{pCLLKMASP1_{zym}}\right]}{K^{\mathrm{MASP1_{zym}}}_{\mathrm{m\ MASP1_{zym}}}}+\frac{\left[\mathrm{pCLLKMASP2_{zym}}\right]}{K^{\mathrm{MASP2_{zym}}}_{\mathrm{m\ MASP1_{zym}}}}+\frac{\left[\mathrm{pCLK1MASP1_{zym}}\right]}{K^{\mathrm{MASP1_{zym}}}_{\mathrm{m\ MASP1_{zym}}}}+\frac{\left[\mathrm{pCLK1MASP2_{zym}}\right]}{K^{\mathrm{MASP2_{zym}}}_{\mathrm{m\ MASP1_{zym}}}}\\+\frac{\left[\mathrm{pCLL1MASP1_{zym}}\right]}{K^{\mathrm{MASP1_{zym}}}_{\mathrm{m\ MASP1_{zym}}}}+\frac{\left[\mathrm{pCLL1MASP2_{zym}}\right]}{K^{\mathrm{MASP2_{zym}}}_{\mathrm{m\ MASP1_{zym}}}}+\frac{\left[\mathrm{pLFMASP1_{zym}}\right]}{K^{\mathrm{MASP1_{zym}}}_{\mathrm{m\ MASP1_{zym}}}}+\frac{\left[\mathrm{pLFMASP2_{zym}}\right]}{K^{\mathrm{MASP2_{zym}}}_{\mathrm{m\ MASP1_{zym}}}}\\+\frac{\left[\mathrm{pHFMASP1_{zym}}\right]}{K^{\mathrm{MASP1_{zym}}}_{\mathrm{m\ MASP1_{zym}}}}+\frac{\left[\mathrm{pHFMASP2_{zym}}\right]}{K^{\mathrm{MASP2_{zym}}}_{\mathrm{m\ MASP1_{zym}}}}+\frac{\left[\mathrm{pMFMASP1_{zym}}\right]}{K^{\mathrm{MASP1_{zym}}}_{\mathrm{m\ MASP1_{zym}}}}+\frac{\left[\mathrm{pMFMASP2_{zym}}\right]}{K^{\mathrm{MASP2_{zym}}}_{\mathrm{m\ MASP1_{zym}}}}\end{array}\right)}
$$

$$
-\frac{k^{\mathrm{MASP1_{zym}}}_{\mathrm{cat\ MASP1_{zym}}}\left[\mathrm{pMFMASP1_{zym}}\right]\left[\mathrm{pMBL_4MASP1_{zym}}\right]}{K^{\mathrm{MASP1_{zym}}}_{\mathrm{m\ MASP1_{zym}}}\left(\begin{array}{l}1+\frac{\left[\mathrm{pMBL_3MASP1_{zym}}\right]}{K^{\mathrm{MASP1_{zym}}}_{\mathrm{m\ MASP1_{zym}}}}+\frac{\left[\mathrm{pMBL_3MASP2_{zym}}\right]}{K^{\mathrm{MASP2_{zym}}}_{\mathrm{m\ MASP1_{zym}}}}+\frac{\left[\mathrm{pMBL_4MASP1_{zym}}\right]}{K^{\mathrm{MASP1_{zym}}}_{\mathrm{m\ MASP1_{zym}}}}+\frac{\left[\mathrm{pMBL_4MASP2_{zym}}\right]}{K^{\mathrm{MASP2_{zym}}}_{\mathrm{m\ MASP1_{zym}}}}\\+\frac{\left[\mathrm{pCLLKMASP1_{zym}}\right]}{K^{\mathrm{MASP1_{zym}}}_{\mathrm{m\ MASP1_{zym}}}}+\frac{\left[\mathrm{pCLLKMASP2_{zym}}\right]}{K^{\mathrm{MASP2_{zym}}}_{\mathrm{m\ MASP1_{zym}}}}+\frac{\left[\mathrm{pCLK1MASP1_{zym}}\right]}{K^{\mathrm{MASP1_{zym}}}_{\mathrm{m\ MASP1_{zym}}}}+\frac{\left[\mathrm{pCLK1MASP2_{zym}}\right]}{K^{\mathrm{MASP2_{zym}}}_{\mathrm{m\ MASP1_{zym}}}}\\+\frac{\left[\mathrm{pCLL1MASP1_{zym}}\right]}{K^{\mathrm{MASP1_{zym}}}_{\mathrm{m\ MASP1_{zym}}}}+\frac{\left[\mathrm{pCLL1MASP2_{zym}}\right]}{K^{\mathrm{MASP2_{zym}}}_{\mathrm{m\ MASP1_{zym}}}}+\frac{\left[\mathrm{pLFMASP1_{zym}}\right]}{K^{\mathrm{MASP1_{zym}}}_{\mathrm{m\ MASP1_{zym}}}}+\frac{\left[\mathrm{pLFMASP2_{zym}}\right]}{K^{\mathrm{MASP2_{zym}}}_{\mathrm{m\ MASP1_{zym}}}}\\+\frac{\left[\mathrm{pHFMASP1_{zym}}\right]}{K^{\mathrm{MASP1_{zym}}}_{\mathrm{m\ MASP1_{zym}}}}+\frac{\left[\mathrm{pHFMASP2_{zym}}\right]}{K^{\mathrm{MASP2_{zym}}}_{\mathrm{m\ MASP1_{zym}}}}+\frac{\left[\mathrm{pMFMASP1_{zym}}\right]}{K^{\mathrm{MASP1_{zym}}}_{\mathrm{m\ MASP1_{zym}}}}+\frac{\left[\mathrm{pMFMASP2_{zym}}\right]}{K^{\mathrm{MASP2_{zym}}}_{\mathrm{m\ MASP1_{zym}}}}\end{array}\right)}
$$

$$
-\frac{k^{\mathrm{MASP1_{zym}}}_{\mathrm{cat\ MASP1_{zym}}}\left[\mathrm{pPTX3LFMASP1_{zym}}\right]\left[\mathrm{pMBL_4MASP1_{zym}}\right]}{K^{\mathrm{MASP1_{zym}}}_{\mathrm{m\ MASP1_{zym}}}\left(\begin{array}{l}1+\frac{\left[\mathrm{pMBL_3MASP1_{zym}}\right]}{K^{\mathrm{MASP1_{zym}}}_{\mathrm{m\ MASP1_{zym}}}}+\frac{\left[\mathrm{pMBL_3MASP2_{zym}}\right]}{K^{\mathrm{MASP2_{zym}}}_{\mathrm{m\ MASP1_{zym}}}}+\frac{\left[\mathrm{pMBL_4MASP1_{zym}}\right]}{K^{\mathrm{MASP1_{zym}}}_{\mathrm{m\ MASP1_{zym}}}}+\frac{\left[\mathrm{pMBL_4MASP2_{zym}}\right]}{K^{\mathrm{MASP2_{zym}}}_{\mathrm{m\ MASP1_{zym}}}}\\+\frac{\left[\mathrm{pCLLKMASP1_{zym}}\right]}{K^{\mathrm{MASP1_{zym}}}_{\mathrm{m\ MASP1_{zym}}}}+\frac{\left[\mathrm{pCLLKMASP2_{zym}}\right]}{K^{\mathrm{MASP2_{zym}}}_{\mathrm{m\ MASP1_{zym}}}}+\frac{\left[\mathrm{pCLK1MASP1_{zym}}\right]}{K^{\mathrm{MASP1_{zym}}}_{\mathrm{m\ MASP1_{zym}}}}+\frac{\left[\mathrm{pCLK1MASP2_{zym}}\right]}{K^{\mathrm{MASP2_{zym}}}_{\mathrm{m\ MASP1_{zym}}}}\\+\frac{\left[\mathrm{pCLL1MASP1_{zym}}\right]}{K^{\mathrm{MASP1_{zym}}}_{\mathrm{m\ MASP1_{zym}}}}+\frac{\left[\mathrm{pCLL1MASP2_{zym}}\right]}{K^{\mathrm{MASP2_{zym}}}_{\mathrm{m\ MASP1_{zym}}}}+\frac{\left[\mathrm{pLFMASP1_{zym}}\right]}{K^{\mathrm{MASP1_{zym}}}_{\mathrm{m\ MASP1_{zym}}}}+\frac{\left[\mathrm{pLFMASP2_{zym}}\right]}{K^{\mathrm{MASP2_{zym}}}_{\mathrm{m\ MASP1_{zym}}}}\\+\frac{\left[\mathrm{pHFMASP1_{zym}}\right]}{K^{\mathrm{MASP1_{zym}}}_{\mathrm{m\ MASP1_{zym}}}}+\frac{\left[\mathrm{pHFMASP2_{zym}}\right]}{K^{\mathrm{MASP2_{zym}}}_{\mathrm{m\ MASP1_{zym}}}}+\frac{\left[\mathrm{pMFMASP1_{zym}}\right]}{K^{\mathrm{MASP1_{zym}}}_{\mathrm{m\ MASP1_{zym}}}}+\frac{\left[\mathrm{pMFMASP2_{zym}}\right]}{K^{\mathrm{MASP2_{zym}}}_{\mathrm{m\ MASP1_{zym}}}}\\+\frac{\left[\mathrm{pPTX3LFMASP1_{zym}}\right]}{K^{\mathrm{MASP1_{zym}}}_{\mathrm{m\ MASP1_{zym}}}}+\frac{\left[\mathrm{pPTX3LFMASP2_{zym}}\right]}{K^{\mathrm{MASP2_{zym}}}_{\mathrm{m\ MASP1_{zym}}}}\end{array}\right)}
$$

$$
-\frac{k_{\text{cat MASP1}^*}^{\text{MASP1}_{\text{zym}}}\left[\text{pMBL}_3\text{MASP1}^*\right]\left[\text{pMBL}_4\text{MASP1}_{\text{zym}}\right]}{K_{\text{m MASP1}^*}^{\text{MASP1}_{\text{zym}}}\left(\begin{array}{l}1+\frac{[\text{C4}]}{K_{\text{m MASP1}^*}^{\text{C4}}}+\frac{[\text{fC4bC2}]}{K_{\text{m MASP1}^*}^{\text{C2}}}+\frac{[\text{fC4bC4bC2}]}{K_{\text{m MASP1}^*}^{\text{C2}}}+\frac{[\text{fC3bC4bC2}]}{K_{\text{m MASP1}^*}^{\text{C2}}}+\frac{[\text{pC4bC2}]}{K_{\text{m MASP1}^*}^{\text{C2}}}+\frac{[\text{pC4bC4bC2}]}{K_{\text{m MASP1}^*}^{\text{C2}}}\\
+\frac{[\text{pC3bC4bC2}]}{K_{\text{m MASP1}^*}^{\text{C2}}}+\frac{[\text{pMBL}_3\text{MASP1}_{\text{zym}}]}{K_{\text{m MASP1}^*}^{\text{MASP1}_{\text{zym}}}}+\frac{[\text{pMBL}_3\text{MASP2}_{\text{zym}}]}{K_{\text{m MASP1}^*}^{\text{MASP2}_{\text{zym}}}}+\frac{[\text{pMBL}_3\text{MASP3}_{\text{zym}}]}{K_{\text{m MASP1}^*}^{\text{MASP3}_{\text{zym}}}}\\
+\frac{[\text{pMBL}_4\text{MASP1}_{\text{zym}}]}{K_{\text{m MASP1}^*}^{\text{MASP1}_{\text{zym}}}}+\frac{[\text{pMBL}_4\text{MASP2}_{\text{zym}}]}{K_{\text{m MASP1}^*}^{\text{MASP2}_{\text{zym}}}}+\frac{[\text{pMBL}_4\text{MASP3}_{\text{zym}}]}{K_{\text{m MASP1}^*}^{\text{MASP3}_{\text{zym}}}}+\frac{[\text{pCLLKMASP1}_{\text{zym}}]}{K_{\text{m MASP1}^*}^{\text{MASP1}_{\text{zym}}}}\\
+\frac{[\text{pCLLKMASP2}_{\text{zym}}]}{K_{\text{m MASP1}^*}^{\text{MASP2}_{\text{zym}}}}+\frac{[\text{pCLLKMASP3}_{\text{zym}}]}{K_{\text{m MASP1}^*}^{\text{MASP3}_{\text{zym}}}}+\frac{[\text{pCLK1MASP1}_{\text{zym}}]}{K_{\text{m MASP1}^*}^{\text{MASP1}_{\text{zym}}}}+\frac{[\text{pCLK1MASP2}_{\text{zym}}]}{K_{\text{m MASP1}^*}^{\text{MASP2}_{\text{zym}}}}\\
+\frac{[\text{pCLK1MASP3}_{\text{zym}}]}{K_{\text{m MASP1}^*}^{\text{MASP3}_{\text{zym}}}}+\frac{[\text{pCLL1MASP1}_{\text{zym}}]}{K_{\text{m MASP1}^*}^{\text{MASP1}_{\text{zym}}}}+\frac{[\text{pCLL1MASP2}_{\text{zym}}]}{K_{\text{m MASP1}^*}^{\text{MASP2}_{\text{zym}}}}+\frac{[\text{pCLL1MASP3}_{\text{zym}}]}{K_{\text{m MASP1}^*}^{\text{MASP3}_{\text{zym}}}}\\
+\frac{[\text{pLFMASP1}_{\text{zym}}]}{K_{\text{m MASP1}^*}^{\text{MASP1}_{\text{zym}}}}+\frac{[\text{pLFMASP2}_{\text{zym}}]}{K_{\text{m MASP1}^*}^{\text{MASP2}_{\text{zym}}}}+\frac{[\text{pLFMASP3}_{\text{zym}}]}{K_{\text{m MASP1}^*}^{\text{MASP3}_{\text{zym}}}}+\frac{[\text{pHFMASP1}_{\text{zym}}]}{K_{\text{m MASP1}^*}^{\text{MASP1}_{\text{zym}}}}+\frac{[\text{pHFMASP2}_{\text{zym}}]}{K_{\text{m MASP1}^*}^{\text{MASP2}_{\text{zym}}}}\\
+\frac{[\text{pHFMASP3}_{\text{zym}}]}{K_{\text{m MASP1}^*}^{\text{MASP3}_{\text{zym}}}}+\frac{[\text{pMFMASP1}_{\text{zym}}]}{K_{\text{m MASP1}^*}^{\text{MASP1}_{\text{zym}}}}+\frac{[\text{pMFMASP2}_{\text{zym}}]}{K_{\text{m MASP1}^*}^{\text{MASP2}_{\text{zym}}}}+\frac{[\text{pMFMASP3}_{\text{zym}}]}{K_{\text{m MASP1}^*}^{\text{MASP3}_{\text{zym}}}}\end{array}\right)}
$$

$$
-\frac{k_{\mathrm{cat\,MASP1^*}}^{\mathrm{MASP1_{zym}}}\left[\mathrm{pMBL_4MASP1^*}\right]\left[\mathrm{pMBL_4MASP1_{zym}}\right]}{K_{\mathrm{m\,MASP1^*}}^{\mathrm{MASP1_{zym}}}\left(\begin{array}{l}
1+\frac{[\mathrm{C4}]}{K_{\mathrm{m\,MASP1^*}}^{\mathrm{C4}}}+\frac{[\mathrm{fC4bC2}]}{K_{\mathrm{m\,MASP1^*}}^{\mathrm{C2}}}+\frac{[\mathrm{fC4bC4bC2}]}{K_{\mathrm{m\,MASP1^*}}^{\mathrm{C2}}}+\frac{[\mathrm{fC3bC4bC2}]}{K_{\mathrm{m\,MASP1^*}}^{\mathrm{C2}}}+\frac{[\mathrm{pC4bC2}]}{K_{\mathrm{m\,MASP1^*}}^{\mathrm{C2}}}+\frac{[\mathrm{pC4bC4bC2}]}{K_{\mathrm{m\,MASP1^*}}^{\mathrm{C2}}}\\
+\frac{[\mathrm{pC3bC4bC2}]}{K_{\mathrm{m\,MASP1^*}}^{\mathrm{C2}}}+\frac{[\mathrm{pMBL_3MASP1_{zym}}]}{K_{\mathrm{m\,MASP1^*}}^{\mathrm{MASP1_{zym}}}}+\frac{[\mathrm{pMBL_3MASP2_{zym}}]}{K_{\mathrm{m\,MASP1^*}}^{\mathrm{MASP2_{zym}}}}+\frac{[\mathrm{pMBL_3MASP3_{zym}}]}{K_{\mathrm{m\,MASP1^*}}^{\mathrm{MASP3_{zym}}}}\\
+\frac{[\mathrm{pMBL_4MASP1_{zym}}]}{K_{\mathrm{m\,MASP1^*}}^{\mathrm{MASP1_{zym}}}}+\frac{[\mathrm{pMBL_4MASP2_{zym}}]}{K_{\mathrm{m\,MASP1^*}}^{\mathrm{MASP2_{zym}}}}+\frac{[\mathrm{pMBL_4MASP3_{zym}}]}{K_{\mathrm{m\,MASP1^*}}^{\mathrm{MASP3_{zym}}}}+\frac{[\mathrm{pCLLKMASP1_{zym}}]}{K_{\mathrm{m\,MASP1^*}}^{\mathrm{MASP1_{zym}}}}\\
+\frac{[\mathrm{pCLLKMASP2_{zym}}]}{K_{\mathrm{m\,MASP1^*}}^{\mathrm{MASP2_{zym}}}}+\frac{[\mathrm{pCLLKMASP3_{zym}}]}{K_{\mathrm{m\,MASP1^*}}^{\mathrm{MASP3_{zym}}}}+\frac{[\mathrm{pCLK1MASP1_{zym}}]}{K_{\mathrm{m\,MASP1^*}}^{\mathrm{MASP1_{zym}}}}+\frac{[\mathrm{pCLK1MASP2_{zym}}]}{K_{\mathrm{m\,MASP1^*}}^{\mathrm{MASP2_{zym}}}}\\
+\frac{[\mathrm{pCLK1MASP3_{zym}}]}{K_{\mathrm{m\,MASP1^*}}^{\mathrm{MASP3_{zym}}}}+\frac{[\mathrm{pCLL1MASP1_{zym}}]}{K_{\mathrm{m\,MASP1^*}}^{\mathrm{MASP1_{zym}}}}+\frac{[\mathrm{pCLL1MASP2_{zym}}]}{K_{\mathrm{m\,MASP1^*}}^{\mathrm{MASP2_{zym}}}}+\frac{[\mathrm{pCLL1MASP3_{zym}}]}{K_{\mathrm{m\,MASP1^*}}^{\mathrm{MASP3_{zym}}}}\\
+\frac{[\mathrm{pLFMASP1_{zym}}]}{K_{\mathrm{m\,MASP1^*}}^{\mathrm{MASP1_{zym}}}}+\frac{[\mathrm{pLFMASP2_{zym}}]}{K_{\mathrm{m\,MASP1^*}}^{\mathrm{MASP2_{zym}}}}+\frac{[\mathrm{pLFMASP3_{zym}}]}{K_{\mathrm{m\,MASP1^*}}^{\mathrm{MASP3_{zym}}}}+\frac{[\mathrm{pHFMASP1_{zym}}]}{K_{\mathrm{m\,MASP1^*}}^{\mathrm{MASP1_{zym}}}}+\frac{[\mathrm{pHFMASP2_{zym}}]}{K_{\mathrm{m\,MASP1^*}}^{\mathrm{MASP2_{zym}}}}\\
+\frac{[\mathrm{pHFMASP3_{zym}}]}{K_{\mathrm{m\,MASP1^*}}^{\mathrm{MASP3_{zym}}}}+\frac{[\mathrm{pMFMASP1_{zym}}]}{K_{\mathrm{m\,MASP1^*}}^{\mathrm{MASP1_{zym}}}}+\frac{[\mathrm{pMFMASP2_{zym}}]}{K_{\mathrm{m\,MASP1^*}}^{\mathrm{MASP2_{zym}}}}+\frac{[\mathrm{pMFMASP3_{zym}}]}{K_{\mathrm{m\,MASP1^*}}^{\mathrm{MASP3_{zym}}}}
\end{array}\right)}
$$

$$
-\frac{k_{\mathrm{cat\,MASP1^*}}^{\mathrm{MASP1_{zym}}}\left[\mathrm{pCLLKMASP1^*}\right]\left[\mathrm{pMBL_4MASP1_{zym}}\right]}{K_{\mathrm{m\,MASP1^*}}^{\mathrm{MASP1_{zym}}}\left(\begin{array}{l}
1+\frac{[\mathrm{C4}]}{K_{\mathrm{m\,MASP1^*}}^{\mathrm{C4}}}+\frac{[\mathrm{fC4bC2}]}{K_{\mathrm{m\,MASP1^*}}^{\mathrm{C2}}}+\frac{[\mathrm{fC4bC4bC2}]}{K_{\mathrm{m\,MASP1^*}}^{\mathrm{C2}}}+\frac{[\mathrm{fC3bC4bC2}]}{K_{\mathrm{m\,MASP1^*}}^{\mathrm{C2}}}+\frac{[\mathrm{pC4bC2}]}{K_{\mathrm{m\,MASP1^*}}^{\mathrm{C2}}}+\frac{[\mathrm{pC4bC4bC2}]}{K_{\mathrm{m\,MASP1^*}}^{\mathrm{C2}}}\\
+\frac{[\mathrm{pC3bC4bC2}]}{K_{\mathrm{m\,MASP1^*}}^{\mathrm{C2}}}+\frac{[\mathrm{pMBL_3MASP1_{zym}}]}{K_{\mathrm{m\,MASP1^*}}^{\mathrm{MASP1_{zym}}}}+\frac{[\mathrm{pMBL_3MASP2_{zym}}]}{K_{\mathrm{m\,MASP1^*}}^{\mathrm{MASP2_{zym}}}}+\frac{[\mathrm{pMBL_3MASP3_{zym}}]}{K_{\mathrm{m\,MASP1^*}}^{\mathrm{MASP3_{zym}}}}\\
+\frac{[\mathrm{pMBL_4MASP1_{zym}}]}{K_{\mathrm{m\,MASP1^*}}^{\mathrm{MASP1_{zym}}}}+\frac{[\mathrm{pMBL_4MASP2_{zym}}]}{K_{\mathrm{m\,MASP1^*}}^{\mathrm{MASP2_{zym}}}}+\frac{[\mathrm{pMBL_4MASP3_{zym}}]}{K_{\mathrm{m\,MASP1^*}}^{\mathrm{MASP3_{zym}}}}+\frac{[\mathrm{pCLLKMASP1_{zym}}]}{K_{\mathrm{m\,MASP1^*}}^{\mathrm{MASP1_{zym}}}}\\
+\frac{[\mathrm{pCLLKMASP2_{zym}}]}{K_{\mathrm{m\,MASP1^*}}^{\mathrm{MASP2_{zym}}}}+\frac{[\mathrm{pCLLKMASP3_{zym}}]}{K_{\mathrm{m\,MASP1^*}}^{\mathrm{MASP3_{zym}}}}+\frac{[\mathrm{pCLK1MASP1_{zym}}]}{K_{\mathrm{m\,MASP1^*}}^{\mathrm{MASP1_{zym}}}}+\frac{[\mathrm{pCLK1MASP2_{zym}}]}{K_{\mathrm{m\,MASP1^*}}^{\mathrm{MASP2_{zym}}}}\\
+\frac{[\mathrm{pCLK1MASP3_{zym}}]}{K_{\mathrm{m\,MASP1^*}}^{\mathrm{MASP3_{zym}}}}+\frac{[\mathrm{pCLL1MASP1_{zym}}]}{K_{\mathrm{m\,MASP1^*}}^{\mathrm{MASP1_{zym}}}}+\frac{[\mathrm{pCLL1MASP2_{zym}}]}{K_{\mathrm{m\,MASP1^*}}^{\mathrm{MASP2_{zym}}}}+\frac{[\mathrm{pCLL1MASP3_{zym}}]}{K_{\mathrm{m\,MASP1^*}}^{\mathrm{MASP3_{zym}}}}\\
+\frac{[\mathrm{pLFMASP1_{zym}}]}{K_{\mathrm{m\,MASP1^*}}^{\mathrm{MASP1_{zym}}}}+\frac{[\mathrm{pLFMASP2_{zym}}]}{K_{\mathrm{m\,MASP1^*}}^{\mathrm{MASP2_{zym}}}}+\frac{[\mathrm{pLFMASP3_{zym}}]}{K_{\mathrm{m\,MASP1^*}}^{\mathrm{MASP3_{zym}}}}+\frac{[\mathrm{pHFMASP1_{zym}}]}{K_{\mathrm{m\,MASP1^*}}^{\mathrm{MASP1_{zym}}}}+\frac{[\mathrm{pHFMASP2_{zym}}]}{K_{\mathrm{m\,MASP1^*}}^{\mathrm{MASP2_{zym}}}}\\
+\frac{[\mathrm{pHFMASP3_{zym}}]}{K_{\mathrm{m\,MASP1^*}}^{\mathrm{MASP3_{zym}}}}+\frac{[\mathrm{pMFMASP1_{zym}}]}{K_{\mathrm{m\,MASP1^*}}^{\mathrm{MASP1_{zym}}}}+\frac{[\mathrm{pMFMASP2_{zym}}]}{K_{\mathrm{m\,MASP1^*}}^{\mathrm{MASP2_{zym}}}}+\frac{[\mathrm{pMFMASP3_{zym}}]}{K_{\mathrm{m\,MASP1^*}}^{\mathrm{MASP3_{zym}}}}
\end{array}\right)}
$$

$$
-\frac{k_{\mathrm{cat\,MASP1^*}}^{\mathrm{MASP1_{zym}}}\left[\mathrm{pCLK1MASP1^*}\right]\left[\mathrm{pMBL_4MASP1_{zym}}\right]}{K_{\mathrm{m\,MASP1^*}}^{\mathrm{MASP1_{zym}}}\left(\begin{array}{l}
1+\frac{[\mathrm{C4}]}{K_{\mathrm{m\,MASP1^*}}^{\mathrm{C4}}}+\frac{[\mathrm{fC4bC2}]}{K_{\mathrm{m\,MASP1^*}}^{\mathrm{C2}}}+\frac{[\mathrm{fC4bC4bC2}]}{K_{\mathrm{m\,MASP1^*}}^{\mathrm{C2}}}+\frac{[\mathrm{fC3bC4bC2}]}{K_{\mathrm{m\,MASP1^*}}^{\mathrm{C2}}}+\frac{[\mathrm{pC4bC2}]}{K_{\mathrm{m\,MASP1^*}}^{\mathrm{C2}}}+\frac{[\mathrm{pC4bC4bC2}]}{K_{\mathrm{m\,MASP1^*}}^{\mathrm{C2}}}\\
+\frac{[\mathrm{pC3bC4bC2}]}{K_{\mathrm{m\,MASP1^*}}^{\mathrm{C2}}}+\frac{[\mathrm{pMBL_3MASP1_{zym}}]}{K_{\mathrm{m\,MASP1^*}}^{\mathrm{MASP1_{zym}}}}+\frac{[\mathrm{pMBL_3MASP2_{zym}}]}{K_{\mathrm{m\,MASP1^*}}^{\mathrm{MASP2_{zym}}}}+\frac{[\mathrm{pMBL_3MASP3_{zym}}]}{K_{\mathrm{m\,MASP1^*}}^{\mathrm{MASP3_{zym}}}}\\
+\frac{[\mathrm{pMBL_4MASP1_{zym}}]}{K_{\mathrm{m\,MASP1^*}}^{\mathrm{MASP1_{zym}}}}+\frac{[\mathrm{pMBL_4MASP2_{zym}}]}{K_{\mathrm{m\,MASP1^*}}^{\mathrm{MASP2_{zym}}}}+\frac{[\mathrm{pMBL_4MASP3_{zym}}]}{K_{\mathrm{m\,MASP1^*}}^{\mathrm{MASP3_{zym}}}}+\frac{[\mathrm{pCLLKMASP1_{zym}}]}{K_{\mathrm{m\,MASP1^*}}^{\mathrm{MASP1_{zym}}}}\\
+\frac{[\mathrm{pCLLKMASP2_{zym}}]}{K_{\mathrm{m\,MASP1^*}}^{\mathrm{MASP2_{zym}}}}+\frac{[\mathrm{pCLLKMASP3_{zym}}]}{K_{\mathrm{m\,MASP1^*}}^{\mathrm{MASP3_{zym}}}}+\frac{[\mathrm{pCLK1MASP1_{zym}}]}{K_{\mathrm{m\,MASP1^*}}^{\mathrm{MASP1_{zym}}}}+\frac{[\mathrm{pCLK1MASP2_{zym}}]}{K_{\mathrm{m\,MASP1^*}}^{\mathrm{MASP2_{zym}}}}\\
+\frac{[\mathrm{pCLK1MASP3_{zym}}]}{K_{\mathrm{m\,MASP1^*}}^{\mathrm{MASP3_{zym}}}}+\frac{[\mathrm{pCLL1MASP1_{zym}}]}{K_{\mathrm{m\,MASP1^*}}^{\mathrm{MASP1_{zym}}}}+\frac{[\mathrm{pCLL1MASP2_{zym}}]}{K_{\mathrm{m\,MASP1^*}}^{\mathrm{MASP2_{zym}}}}+\frac{[\mathrm{pCLL1MASP3_{zym}}]}{K_{\mathrm{m\,MASP1^*}}^{\mathrm{MASP3_{zym}}}}\\
+\frac{[\mathrm{pLFMASP1_{zym}}]}{K_{\mathrm{m\,MASP1^*}}^{\mathrm{MASP1_{zym}}}}+\frac{[\mathrm{pLFMASP2_{zym}}]}{K_{\mathrm{m\,MASP1^*}}^{\mathrm{MASP2_{zym}}}}+\frac{[\mathrm{pLFMASP3_{zym}}]}{K_{\mathrm{m\,MASP1^*}}^{\mathrm{MASP3_{zym}}}}+\frac{[\mathrm{pHFMASP1_{zym}}]}{K_{\mathrm{m\,MASP1^*}}^{\mathrm{MASP1_{zym}}}}+\frac{[\mathrm{pHFMASP2_{zym}}]}{K_{\mathrm{m\,MASP1^*}}^{\mathrm{MASP2_{zym}}}}\\
+\frac{[\mathrm{pHFMASP3_{zym}}]}{K_{\mathrm{m\,MASP1^*}}^{\mathrm{MASP3_{zym}}}}+\frac{[\mathrm{pMFMASP1_{zym}}]}{K_{\mathrm{m\,MASP1^*}}^{\mathrm{MASP1_{zym}}}}+\frac{[\mathrm{pMFMASP2_{zym}}]}{K_{\mathrm{m\,MASP1^*}}^{\mathrm{MASP2_{zym}}}}+\frac{[\mathrm{pMFMASP3_{zym}}]}{K_{\mathrm{m\,MASP1^*}}^{\mathrm{MASP3_{zym}}}}
\end{array}\right)}
$$

$$
-\frac{k_{\mathrm{cat\,MASP1^*}}^{\mathrm{MASP1_{zym}}}\left[\mathrm{pCLL1MASP1^*}\right]\left[\mathrm{pMBL_4MASP1_{zym}}\right]}{K_{\mathrm{m\,MASP1^*}}^{\mathrm{MASP1_{zym}}}\left(\begin{array}{l}
1+\frac{[\mathrm{C4}]}{K_{\mathrm{m\,MASP1^*}}^{\mathrm{C4}}}+\frac{[\mathrm{fC4bC2}]}{K_{\mathrm{m\,MASP1^*}}^{\mathrm{C2}}}+\frac{[\mathrm{fC4bC4bC2}]}{K_{\mathrm{m\,MASP1^*}}^{\mathrm{C2}}}+\frac{[\mathrm{fC3bC4bC2}]}{K_{\mathrm{m\,MASP1^*}}^{\mathrm{C2}}}+\frac{[\mathrm{pC4bC2}]}{K_{\mathrm{m\,MASP1^*}}^{\mathrm{C2}}}+\frac{[\mathrm{pC4bC4bC2}]}{K_{\mathrm{m\,MASP1^*}}^{\mathrm{C2}}}\\
+\frac{[\mathrm{pC3bC4bC2}]}{K_{\mathrm{m\,MASP1^*}}^{\mathrm{C2}}}+\frac{[\mathrm{pMBL_3MASP1_{zym}}]}{K_{\mathrm{m\,MASP1^*}}^{\mathrm{MASP1_{zym}}}}+\frac{[\mathrm{pMBL_3MASP2_{zym}}]}{K_{\mathrm{m\,MASP1^*}}^{\mathrm{MASP2_{zym}}}}+\frac{[\mathrm{pMBL_3MASP3_{zym}}]}{K_{\mathrm{m\,MASP1^*}}^{\mathrm{MASP3_{zym}}}}\\
+\frac{[\mathrm{pMBL_4MASP1_{zym}}]}{K_{\mathrm{m\,MASP1^*}}^{\mathrm{MASP1_{zym}}}}+\frac{[\mathrm{pMBL_4MASP2_{zym}}]}{K_{\mathrm{m\,MASP1^*}}^{\mathrm{MASP2_{zym}}}}+\frac{[\mathrm{pMBL_4MASP3_{zym}}]}{K_{\mathrm{m\,MASP1^*}}^{\mathrm{MASP3_{zym}}}}+\frac{[\mathrm{pCLLKMASP1_{zym}}]}{K_{\mathrm{m\,MASP1^*}}^{\mathrm{MASP1_{zym}}}}\\
+\frac{[\mathrm{pCLLKMASP2_{zym}}]}{K_{\mathrm{m\,MASP1^*}}^{\mathrm{MASP2_{zym}}}}+\frac{[\mathrm{pCLLKMASP3_{zym}}]}{K_{\mathrm{m\,MASP1^*}}^{\mathrm{MASP3_{zym}}}}+\frac{[\mathrm{pCLK1MASP1_{zym}}]}{K_{\mathrm{m\,MASP1^*}}^{\mathrm{MASP1_{zym}}}}+\frac{[\mathrm{pCLK1MASP2_{zym}}]}{K_{\mathrm{m\,MASP1^*}}^{\mathrm{MASP2_{zym}}}}\\
+\frac{[\mathrm{pCLK1MASP3_{zym}}]}{K_{\mathrm{m\,MASP1^*}}^{\mathrm{MASP3_{zym}}}}+\frac{[\mathrm{pCLL1MASP1_{zym}}]}{K_{\mathrm{m\,MASP1^*}}^{\mathrm{MASP1_{zym}}}}+\frac{[\mathrm{pCLL1MASP2_{zym}}]}{K_{\mathrm{m\,MASP1^*}}^{\mathrm{MASP2_{zym}}}}+\frac{[\mathrm{pCLL1MASP3_{zym}}]}{K_{\mathrm{m\,MASP1^*}}^{\mathrm{MASP3_{zym}}}}\\
+\frac{[\mathrm{pLFMASP1_{zym}}]}{K_{\mathrm{m\,MASP1^*}}^{\mathrm{MASP1_{zym}}}}+\frac{[\mathrm{pLFMASP2_{zym}}]}{K_{\mathrm{m\,MASP1^*}}^{\mathrm{MASP2_{zym}}}}+\frac{[\mathrm{pLFMASP3_{zym}}]}{K_{\mathrm{m\,MASP1^*}}^{\mathrm{MASP3_{zym}}}}+\frac{[\mathrm{pHFMASP1_{zym}}]}{K_{\mathrm{m\,MASP1^*}}^{\mathrm{MASP1_{zym}}}}+\frac{[\mathrm{pHFMASP2_{zym}}]}{K_{\mathrm{m\,MASP1^*}}^{\mathrm{MASP2_{zym}}}}\\
+\frac{[\mathrm{pHFMASP3_{zym}}]}{K_{\mathrm{m\,MASP1^*}}^{\mathrm{MASP3_{zym}}}}+\frac{[\mathrm{pMFMASP1_{zym}}]}{K_{\mathrm{m\,MASP1^*}}^{\mathrm{MASP1_{zym}}}}+\frac{[\mathrm{pMFMASP2_{zym}}]}{K_{\mathrm{m\,MASP1^*}}^{\mathrm{MASP2_{zym}}}}+\frac{[\mathrm{pMFMASP3_{zym}}]}{K_{\mathrm{m\,MASP1^*}}^{\mathrm{MASP3_{zym}}}}
\end{array}\right)}
$$

$$
-\frac{k_{\mathrm{cat\ MASP1^*}}^{\mathrm{MASP1_{zym}}}\left[\mathrm{pLFMASP1^*}\right]\left[\mathrm{pMBL_4MASP1_{zym}}\right]}{K_{\mathrm{m\ MASP1^*}}^{\mathrm{MASP1_{zym}}}\left(\begin{array}{l}
1+\frac{[\mathrm{C4}]}{K_{\mathrm{m\ MASP1^*}}^{\mathrm{C4}}}+\frac{[\mathrm{fC4bC2}]}{K_{\mathrm{m\ MASP1^*}}^{\mathrm{C2}}}+\frac{[\mathrm{fC4bC4bC2}]}{K_{\mathrm{m\ MASP1^*}}^{\mathrm{C2}}}+\frac{[\mathrm{fC3bC4bC2}]}{K_{\mathrm{m\ MASP1^*}}^{\mathrm{C2}}}+\frac{[\mathrm{pC4bC2}]}{K_{\mathrm{m\ MASP1^*}}^{\mathrm{C2}}}+\frac{[\mathrm{pC4bC4bC2}]}{K_{\mathrm{m\ MASP1^*}}^{\mathrm{C2}}}\\
+\frac{[\mathrm{pC3bC4bC2}]}{K_{\mathrm{m\ MASP1^*}}^{\mathrm{C2}}}+\frac{[\mathrm{pMBL_3MASP1_{zym}}]}{K_{\mathrm{m\ MASP1^*}}^{\mathrm{MASP1_{zym}}}}+\frac{[\mathrm{pMBL_3MASP2_{zym}}]}{K_{\mathrm{m\ MASP1^*}}^{\mathrm{MASP2_{zym}}}}+\frac{[\mathrm{pMBL_3MASP3_{zym}}]}{K_{\mathrm{m\ MASP1^*}}^{\mathrm{MASP3_{zym}}}}\\
+\frac{[\mathrm{pMBL_4MASP1_{zym}}]}{K_{\mathrm{m\ MASP1^*}}^{\mathrm{MASP1_{zym}}}}+\frac{[\mathrm{pMBL_4MASP2_{zym}}]}{K_{\mathrm{m\ MASP1^*}}^{\mathrm{MASP2_{zym}}}}+\frac{[\mathrm{pMBL_4MASP3_{zym}}]}{K_{\mathrm{m\ MASP1^*}}^{\mathrm{MASP3_{zym}}}}+\frac{[\mathrm{pCLLKMASP1_{zym}}]}{K_{\mathrm{m\ MASP1^*}}^{\mathrm{MASP1_{zym}}}}\\
+\frac{[\mathrm{pCLLKMASP2_{zym}}]}{K_{\mathrm{m\ MASP1^*}}^{\mathrm{MASP2_{zym}}}}+\frac{[\mathrm{pCLLKMASP3_{zym}}]}{K_{\mathrm{m\ MASP1^*}}^{\mathrm{MASP3_{zym}}}}+\frac{[\mathrm{pCLK1MASP1_{zym}}]}{K_{\mathrm{m\ MASP1^*}}^{\mathrm{MASP1_{zym}}}}+\frac{[\mathrm{pCLK1MASP2_{zym}}]}{K_{\mathrm{m\ MASP1^*}}^{\mathrm{MASP2_{zym}}}}\\
+\frac{[\mathrm{pCLK1MASP3_{zym}}]}{K_{\mathrm{m\ MASP1^*}}^{\mathrm{MASP3_{zym}}}}+\frac{[\mathrm{pCLL1MASP1_{zym}}]}{K_{\mathrm{m\ MASP1^*}}^{\mathrm{MASP1_{zym}}}}+\frac{[\mathrm{pCLL1MASP2_{zym}}]}{K_{\mathrm{m\ MASP1^*}}^{\mathrm{MASP2_{zym}}}}+\frac{[\mathrm{pCLL1MASP3_{zym}}]}{K_{\mathrm{m\ MASP1^*}}^{\mathrm{MASP3_{zym}}}}\\
+\frac{[\mathrm{pLFMASP1_{zym}}]}{K_{\mathrm{m\ MASP1^*}}^{\mathrm{MASP1_{zym}}}}+\frac{[\mathrm{pLFMASP2_{zym}}]}{K_{\mathrm{m\ MASP1^*}}^{\mathrm{MASP2_{zym}}}}+\frac{[\mathrm{pLFMASP3_{zym}}]}{K_{\mathrm{m\ MASP1^*}}^{\mathrm{MASP3_{zym}}}}+\frac{[\mathrm{pHFMASP1_{zym}}]}{K_{\mathrm{m\ MASP1^*}}^{\mathrm{MASP1_{zym}}}}+\frac{[\mathrm{pHFMASP2_{zym}}]}{K_{\mathrm{m\ MASP1^*}}^{\mathrm{MASP2_{zym}}}}\\
+\frac{[\mathrm{pHFMASP3_{zym}}]}{K_{\mathrm{m\ MASP1^*}}^{\mathrm{MASP3_{zym}}}}+\frac{[\mathrm{pMFMASP1_{zym}}]}{K_{\mathrm{m\ MASP1^*}}^{\mathrm{MASP1_{zym}}}}+\frac{[\mathrm{pMFMASP2_{zym}}]}{K_{\mathrm{m\ MASP1^*}}^{\mathrm{MASP2_{zym}}}}+\frac{[\mathrm{pMFMASP3_{zym}}]}{K_{\mathrm{m\ MASP1^*}}^{\mathrm{MASP3_{zym}}}}
\end{array}\right)}
$$

$$
-\frac{k_{\mathrm{cat\ MASP1^*}}^{\mathrm{MASP1_{zym}}}\left[\mathrm{pHFMASP1^*}\right]\left[\mathrm{pMBL_4MASP1_{zym}}\right]}{K_{\mathrm{m\ MASP1^*}}^{\mathrm{MASP1_{zym}}}\left(\begin{array}{l}
1+\frac{[\mathrm{C4}]}{K_{\mathrm{m\ MASP1^*}}^{\mathrm{C4}}}+\frac{[\mathrm{fC4bC2}]}{K_{\mathrm{m\ MASP1^*}}^{\mathrm{C2}}}+\frac{[\mathrm{fC4bC4bC2}]}{K_{\mathrm{m\ MASP1^*}}^{\mathrm{C2}}}+\frac{[\mathrm{fC3bC4bC2}]}{K_{\mathrm{m\ MASP1^*}}^{\mathrm{C2}}}+\frac{[\mathrm{pC4bC2}]}{K_{\mathrm{m\ MASP1^*}}^{\mathrm{C2}}}+\frac{[\mathrm{pC4bC4bC2}]}{K_{\mathrm{m\ MASP1^*}}^{\mathrm{C2}}}\\
+\frac{[\mathrm{pC3bC4bC2}]}{K_{\mathrm{m\ MASP1^*}}^{\mathrm{C2}}}+\frac{[\mathrm{pMBL_3MASP1_{zym}}]}{K_{\mathrm{m\ MASP1^*}}^{\mathrm{MASP1_{zym}}}}+\frac{[\mathrm{pMBL_3MASP2_{zym}}]}{K_{\mathrm{m\ MASP1^*}}^{\mathrm{MASP2_{zym}}}}+\frac{[\mathrm{pMBL_3MASP3_{zym}}]}{K_{\mathrm{m\ MASP1^*}}^{\mathrm{MASP3_{zym}}}}\\
+\frac{[\mathrm{pMBL_4MASP1_{zym}}]}{K_{\mathrm{m\ MASP1^*}}^{\mathrm{MASP1_{zym}}}}+\frac{[\mathrm{pMBL_4MASP2_{zym}}]}{K_{\mathrm{m\ MASP1^*}}^{\mathrm{MASP2_{zym}}}}+\frac{[\mathrm{pMBL_4MASP3_{zym}}]}{K_{\mathrm{m\ MASP1^*}}^{\mathrm{MASP3_{zym}}}}+\frac{[\mathrm{pCLLKMASP1_{zym}}]}{K_{\mathrm{m\ MASP1^*}}^{\mathrm{MASP1_{zym}}}}\\
+\frac{[\mathrm{pCLLKMASP2_{zym}}]}{K_{\mathrm{m\ MASP1^*}}^{\mathrm{MASP2_{zym}}}}+\frac{[\mathrm{pCLLKMASP3_{zym}}]}{K_{\mathrm{m\ MASP1^*}}^{\mathrm{MASP3_{zym}}}}+\frac{[\mathrm{pCLK1MASP1_{zym}}]}{K_{\mathrm{m\ MASP1^*}}^{\mathrm{MASP1_{zym}}}}+\frac{[\mathrm{pCLK1MASP2_{zym}}]}{K_{\mathrm{m\ MASP1^*}}^{\mathrm{MASP2_{zym}}}}\\
+\frac{[\mathrm{pCLK1MASP3_{zym}}]}{K_{\mathrm{m\ MASP1^*}}^{\mathrm{MASP3_{zym}}}}+\frac{[\mathrm{pCLL1MASP1_{zym}}]}{K_{\mathrm{m\ MASP1^*}}^{\mathrm{MASP1_{zym}}}}+\frac{[\mathrm{pCLL1MASP2_{zym}}]}{K_{\mathrm{m\ MASP1^*}}^{\mathrm{MASP2_{zym}}}}+\frac{[\mathrm{pCLL1MASP3_{zym}}]}{K_{\mathrm{m\ MASP1^*}}^{\mathrm{MASP3_{zym}}}}\\
+\frac{[\mathrm{pLFMASP1_{zym}}]}{K_{\mathrm{m\ MASP1^*}}^{\mathrm{MASP1_{zym}}}}+\frac{[\mathrm{pLFMASP2_{zym}}]}{K_{\mathrm{m\ MASP1^*}}^{\mathrm{MASP2_{zym}}}}+\frac{[\mathrm{pLFMASP3_{zym}}]}{K_{\mathrm{m\ MASP1^*}}^{\mathrm{MASP3_{zym}}}}+\frac{[\mathrm{pHFMASP1_{zym}}]}{K_{\mathrm{m\ MASP1^*}}^{\mathrm{MASP1_{zym}}}}+\frac{[\mathrm{pHFMASP2_{zym}}]}{K_{\mathrm{m\ MASP1^*}}^{\mathrm{MASP2_{zym}}}}\\
+\frac{[\mathrm{pHFMASP3_{zym}}]}{K_{\mathrm{m\ MASP1^*}}^{\mathrm{MASP3_{zym}}}}+\frac{[\mathrm{pMFMASP1_{zym}}]}{K_{\mathrm{m\ MASP1^*}}^{\mathrm{MASP1_{zym}}}}+\frac{[\mathrm{pMFMASP2_{zym}}]}{K_{\mathrm{m\ MASP1^*}}^{\mathrm{MASP2_{zym}}}}+\frac{[\mathrm{pMFMASP3_{zym}}]}{K_{\mathrm{m\ MASP1^*}}^{\mathrm{MASP3_{zym}}}}
\end{array}\right)}
$$

$$
-\frac{k_{\mathrm{cat\ MASP1^*}}^{\mathrm{MASP1_{zym}}}\left[\mathrm{pMFMASP1^*}\right]\left[\mathrm{pMBL_4MASP1_{zym}}\right]}{K_{\mathrm{m\ MASP1^*}}^{\mathrm{MASP1_{zym}}}\left(\begin{array}{l}1+\frac{[\mathrm{C4}]}{K_{\mathrm{m\ MASP1^*}}^{\mathrm{C4}}}+\frac{[\mathrm{fC4bC2}]}{K_{\mathrm{m\ MASP1^*}}^{\mathrm{C2}}}+\frac{[\mathrm{fC4bC4bC2}]}{K_{\mathrm{m\ MASP1^*}}^{\mathrm{C2}}}+\frac{[\mathrm{fC3bC4bC2}]}{K_{\mathrm{m\ MASP1^*}}^{\mathrm{C2}}}+\frac{[\mathrm{pC4bC2}]}{K_{\mathrm{m\ MASP1^*}}^{\mathrm{C2}}}+\frac{[\mathrm{pC4bC4bC2}]}{K_{\mathrm{m\ MASP1^*}}^{\mathrm{C2}}}\\
+\frac{[\mathrm{pC3bC4bC2}]}{K_{\mathrm{m\ MASP1^*}}^{\mathrm{C2}}}+\frac{[\mathrm{pMBL_3MASP1_{zym}}]}{K_{\mathrm{m\ MASP1^*}}^{\mathrm{MASP1_{zym}}}}+\frac{[\mathrm{pMBL_3MASP2_{zym}}]}{K_{\mathrm{m\ MASP1^*}}^{\mathrm{MASP2_{zym}}}}+\frac{[\mathrm{pMBL_3MASP3_{zym}}]}{K_{\mathrm{m\ MASP1^*}}^{\mathrm{MASP3_{zym}}}}\\
+\frac{[\mathrm{pMBL_4MASP1_{zym}}]}{K_{\mathrm{m\ MASP1^*}}^{\mathrm{MASP1_{zym}}}}+\frac{[\mathrm{pMBL_4MASP2_{zym}}]}{K_{\mathrm{m\ MASP1^*}}^{\mathrm{MASP2_{zym}}}}+\frac{[\mathrm{pMBL_4MASP3_{zym}}]}{K_{\mathrm{m\ MASP1^*}}^{\mathrm{MASP3_{zym}}}}+\frac{[\mathrm{pCLLKMASP1_{zym}}]}{K_{\mathrm{m\ MASP1^*}}^{\mathrm{MASP1_{zym}}}}\\
+\frac{[\mathrm{pCLLKMASP2_{zym}}]}{K_{\mathrm{m\ MASP1^*}}^{\mathrm{MASP2_{zym}}}}+\frac{[\mathrm{pCLLKMASP3_{zym}}]}{K_{\mathrm{m\ MASP1^*}}^{\mathrm{MASP3_{zym}}}}+\frac{[\mathrm{pCLK1MASP1_{zym}}]}{K_{\mathrm{m\ MASP1^*}}^{\mathrm{MASP1_{zym}}}}+\frac{[\mathrm{pCLK1MASP2_{zym}}]}{K_{\mathrm{m\ MASP1^*}}^{\mathrm{MASP2_{zym}}}}\\
+\frac{[\mathrm{pCLK1MASP3_{zym}}]}{K_{\mathrm{m\ MASP1^*}}^{\mathrm{MASP3_{zym}}}}+\frac{[\mathrm{pCLL1MASP1_{zym}}]}{K_{\mathrm{m\ MASP1^*}}^{\mathrm{MASP1_{zym}}}}+\frac{[\mathrm{pCLL1MASP2_{zym}}]}{K_{\mathrm{m\ MASP1^*}}^{\mathrm{MASP2_{zym}}}}+\frac{[\mathrm{pCLL1MASP3_{zym}}]}{K_{\mathrm{m\ MASP1^*}}^{\mathrm{MASP3_{zym}}}}\\
+\frac{[\mathrm{pLFMASP1_{zym}}]}{K_{\mathrm{m\ MASP1^*}}^{\mathrm{MASP1_{zym}}}}+\frac{[\mathrm{pLFMASP2_{zym}}]}{K_{\mathrm{m\ MASP1^*}}^{\mathrm{MASP2_{zym}}}}+\frac{[\mathrm{pLFMASP3_{zym}}]}{K_{\mathrm{m\ MASP1^*}}^{\mathrm{MASP3_{zym}}}}+\frac{[\mathrm{pHFMASP1_{zym}}]}{K_{\mathrm{m\ MASP1^*}}^{\mathrm{MASP1_{zym}}}}+\frac{[\mathrm{pHFMASP2_{zym}}]}{K_{\mathrm{m\ MASP1^*}}^{\mathrm{MASP2_{zym}}}}\\
+\frac{[\mathrm{pHFMASP3_{zym}}]}{K_{\mathrm{m\ MASP1^*}}^{\mathrm{MASP3_{zym}}}}+\frac{[\mathrm{pMFMASP1_{zym}}]}{K_{\mathrm{m\ MASP1^*}}^{\mathrm{MASP1_{zym}}}}+\frac{[\mathrm{pMFMASP2_{zym}}]}{K_{\mathrm{m\ MASP1^*}}^{\mathrm{MASP2_{zym}}}}+\frac{[\mathrm{pMFMASP3_{zym}}]}{K_{\mathrm{m\ MASP1^*}}^{\mathrm{MASP3_{zym}}}}\end{array}\right)}
$$

$$
-\frac{k_{\mathrm{cat\ MASP1^*}}^{\mathrm{MASP1_{zym}}}\left[\mathrm{pPTX3LFMASP1^*}\right]\left[\mathrm{pMBL_4MASP1_{zym}}\right]}{K_{\mathrm{m\ MASP1^*}}^{\mathrm{MASP1_{zym}}}\left(\begin{array}{l}1+\frac{[\mathrm{C4}]}{K_{\mathrm{m\ MASP1^*}}^{\mathrm{C4}}}+\frac{[\mathrm{fC4bC2}]}{K_{\mathrm{m\ MASP1^*}}^{\mathrm{C2}}}+\frac{[\mathrm{fC4bC4bC2}]}{K_{\mathrm{m\ MASP1^*}}^{\mathrm{C2}}}+\frac{[\mathrm{fC3bC4bC2}]}{K_{\mathrm{m\ MASP1^*}}^{\mathrm{C2}}}+\frac{[\mathrm{pC4bC2}]}{K_{\mathrm{m\ MASP1^*}}^{\mathrm{C2}}}+\frac{[\mathrm{pC4bC4bC2}]}{K_{\mathrm{m\ MASP1^*}}^{\mathrm{C2}}}\\
+\frac{[\mathrm{pC3bC4bC2}]}{K_{\mathrm{m\ MASP1^*}}^{\mathrm{C2}}}+\frac{[\mathrm{pMBL_3MASP1_{zym}}]}{K_{\mathrm{m\ MASP1^*}}^{\mathrm{MASP1_{zym}}}}+\frac{[\mathrm{pMBL_3MASP2_{zym}}]}{K_{\mathrm{m\ MASP1^*}}^{\mathrm{MASP2_{zym}}}}+\frac{[\mathrm{pMBL_3MASP3_{zym}}]}{K_{\mathrm{m\ MASP1^*}}^{\mathrm{MASP3_{zym}}}}\\
+\frac{[\mathrm{pMBL_4MASP1_{zym}}]}{K_{\mathrm{m\ MASP1^*}}^{\mathrm{MASP1_{zym}}}}+\frac{[\mathrm{pMBL_4MASP2_{zym}}]}{K_{\mathrm{m\ MASP1^*}}^{\mathrm{MASP2_{zym}}}}+\frac{[\mathrm{pMBL_4MASP3_{zym}}]}{K_{\mathrm{m\ MASP1^*}}^{\mathrm{MASP3_{zym}}}}+\frac{[\mathrm{pCLLKMASP1_{zym}}]}{K_{\mathrm{m\ MASP1^*}}^{\mathrm{MASP1_{zym}}}}\\
+\frac{[\mathrm{pCLLKMASP2_{zym}}]}{K_{\mathrm{m\ MASP1^*}}^{\mathrm{MASP2_{zym}}}}+\frac{[\mathrm{pCLLKMASP3_{zym}}]}{K_{\mathrm{m\ MASP1^*}}^{\mathrm{MASP3_{zym}}}}+\frac{[\mathrm{pCLK1MASP1_{zym}}]}{K_{\mathrm{m\ MASP1^*}}^{\mathrm{MASP1_{zym}}}}+\frac{[\mathrm{pCLK1MASP2_{zym}}]}{K_{\mathrm{m\ MASP1^*}}^{\mathrm{MASP2_{zym}}}}\\
+\frac{[\mathrm{pCLK1MASP3_{zym}}]}{K_{\mathrm{m\ MASP1^*}}^{\mathrm{MASP3_{zym}}}}+\frac{[\mathrm{pCLL1MASP1_{zym}}]}{K_{\mathrm{m\ MASP1^*}}^{\mathrm{MASP1_{zym}}}}+\frac{[\mathrm{pCLL1MASP2_{zym}}]}{K_{\mathrm{m\ MASP1^*}}^{\mathrm{MASP2_{zym}}}}+\frac{[\mathrm{pCLL1MASP3_{zym}}]}{K_{\mathrm{m\ MASP1^*}}^{\mathrm{MASP3_{zym}}}}\\
+\frac{[\mathrm{pLFMASP1_{zym}}]}{K_{\mathrm{m\ MASP1^*}}^{\mathrm{MASP1_{zym}}}}+\frac{[\mathrm{pLFMASP2_{zym}}]}{K_{\mathrm{m\ MASP1^*}}^{\mathrm{MASP2_{zym}}}}+\frac{[\mathrm{pLFMASP3_{zym}}]}{K_{\mathrm{m\ MASP1^*}}^{\mathrm{MASP3_{zym}}}}+\frac{[\mathrm{pHFMASP1_{zym}}]}{K_{\mathrm{m\ MASP1^*}}^{\mathrm{MASP1_{zym}}}}+\frac{[\mathrm{pHFMASP2_{zym}}]}{K_{\mathrm{m\ MASP1^*}}^{\mathrm{MASP2_{zym}}}}\\
+\frac{[\mathrm{pHFMASP3_{zym}}]}{K_{\mathrm{m\ MASP1^*}}^{\mathrm{MASP3_{zym}}}}+\frac{[\mathrm{pMFMASP1_{zym}}]}{K_{\mathrm{m\ MASP1^*}}^{\mathrm{MASP1_{zym}}}}+\frac{[\mathrm{pMFMASP2_{zym}}]}{K_{\mathrm{m\ MASP1^*}}^{\mathrm{MASP2_{zym}}}}+\frac{[\mathrm{pMFMASP3_{zym}}]}{K_{\mathrm{m\ MASP1^*}}^{\mathrm{MASP3_{zym}}}}\\
+\frac{[\mathrm{pPTX3LFMASP1_{zym}}]}{K_{\mathrm{m\ MASP1^*}}^{\mathrm{MASP1_{zym}}}}+\frac{[\mathrm{pPXT3LFMASP2_{zym}}]}{K_{\mathrm{m\ MASP1^*}}^{\mathrm{MASP2_{zym}}}}+\frac{[\mathrm{pPTX3LFMASP3_{zym}}]}{K_{\mathrm{m\ MASP1^*}}^{\mathrm{MASP3_{zym}}}}\end{array}\right)}
$$

$$
-\frac{k_{\mathrm{cat\ MASP2^*}}^{\mathrm{MASP1_{zym}}}\left[\mathrm{pMBL_3MASP2^*}\right]\left[\mathrm{pMBL_4MASP1_{zym}}\right]}{K_{\mathrm{m\ MASP2^*}}^{\mathrm{MASP1_{zym}}}\left(\begin{array}{l}1+\frac{[\mathrm{C4}]}{K_{\mathrm{m\ MASP2^*}}^{\mathrm{C4}}}+\frac{[\mathrm{fC4bC2}]}{K_{\mathrm{m\ MASP2^*}}^{\mathrm{C2}}}+\frac{[\mathrm{fC4bC4bC2}]}{K_{\mathrm{m\ MASP2^*}}^{\mathrm{C2}}}+\frac{[\mathrm{fC3bC4bC2}]}{K_{\mathrm{m\ MASP2^*}}^{\mathrm{C2}}}+\frac{[\mathrm{pC4bC2}]}{K_{\mathrm{m\ MASP2^*}}^{\mathrm{C2}}}+\frac{[\mathrm{pC4bC4bC2}]}{K_{\mathrm{m\ MASP2^*}}^{\mathrm{C2}}}\\ +\frac{[\mathrm{pC3bC4bC2}]}{K_{\mathrm{m\ MASP2^*}}^{\mathrm{C2}}}+\frac{[\mathrm{pMBL_3MASP1_{zym}}]}{K_{\mathrm{m\ MASP2^*}}^{\mathrm{MASP1_{zym}}}}+\frac{[\mathrm{pMBL_3MASP2_{zym}}]}{K_{\mathrm{m\ MASP2^*}}^{\mathrm{MASP2_{zym}}}}+\frac{[\mathrm{pMBL_4MASP1_{zym}}]}{K_{\mathrm{m\ MASP2^*}}^{\mathrm{MASP1_{zym}}}}\\ +\frac{[\mathrm{pMBL_4MASP2_{zym}}]}{K_{\mathrm{m\ MASP2^*}}^{\mathrm{MASP2_{zym}}}}+\frac{[\mathrm{pCLLKMASP1_{zym}}]}{K_{\mathrm{m\ MASP2^*}}^{\mathrm{MASP1_{zym}}}}+\frac{[\mathrm{pCLLKMASP2_{zym}}]}{K_{\mathrm{m\ MASP2^*}}^{\mathrm{MASP2_{zym}}}}+\frac{[\mathrm{pCLK1MASP1_{zym}}]}{K_{\mathrm{m\ MASP2^*}}^{\mathrm{MASP1_{zym}}}}\\ +\frac{[\mathrm{pCLK1MASP2_{zym}}]}{K_{\mathrm{m\ MASP2^*}}^{\mathrm{MASP2_{zym}}}}+\frac{[\mathrm{pCLL1MASP1_{zym}}]}{K_{\mathrm{m\ MASP2^*}}^{\mathrm{MASP1_{zym}}}}+\frac{[\mathrm{pCLL1MASP2_{zym}}]}{K_{\mathrm{m\ MASP2^*}}^{\mathrm{MASP2_{zym}}}}+\frac{[\mathrm{pLFMASP1_{zym}}]}{K_{\mathrm{m\ MASP2^*}}^{\mathrm{MASP1_{zym}}}}\\ +\frac{[\mathrm{pLFMASP2_{zym}}]}{K_{\mathrm{m\ MASP2^*}}^{\mathrm{MASP2_{zym}}}}+\frac{[\mathrm{pHFMASP1_{zym}}]}{K_{\mathrm{m\ MASP2^*}}^{\mathrm{MASP1_{zym}}}}+\frac{[\mathrm{pHFMASP2_{zym}}]}{K_{\mathrm{m\ MASP2^*}}^{\mathrm{MASP2_{zym}}}}+\frac{[\mathrm{pMFMASP1_{zym}}]}{K_{\mathrm{m\ MASP2^*}}^{\mathrm{MASP1_{zym}}}}+\frac{[\mathrm{pMFMASP2_{zym}}]}{K_{\mathrm{m\ MASP2^*}}^{\mathrm{MASP2_{zym}}}}\end{array}\right)}
$$

$$
-\frac{k_{\mathrm{cat\ MASP2^*}}^{\mathrm{MASP1_{zym}}}\left[\mathrm{pMBL_4MASP2^*}\right]\left[\mathrm{pMBL_4MASP1_{zym}}\right]}{K_{\mathrm{m\ MASP2^*}}^{\mathrm{MASP1_{zym}}}\left(\begin{array}{l}1+\frac{[\mathrm{C4}]}{K_{\mathrm{m\ MASP2^*}}^{\mathrm{C4}}}+\frac{[\mathrm{fC4bC2}]}{K_{\mathrm{m\ MASP2^*}}^{\mathrm{C2}}}+\frac{[\mathrm{fC4bC4bC2}]}{K_{\mathrm{m\ MASP2^*}}^{\mathrm{C2}}}+\frac{[\mathrm{fC3bC4bC2}]}{K_{\mathrm{m\ MASP2^*}}^{\mathrm{C2}}}+\frac{[\mathrm{pC4bC2}]}{K_{\mathrm{m\ MASP2^*}}^{\mathrm{C2}}}+\frac{[\mathrm{pC4bC4bC2}]}{K_{\mathrm{m\ MASP2^*}}^{\mathrm{C2}}}\\ +\frac{[\mathrm{pC3bC4bC2}]}{K_{\mathrm{m\ MASP2^*}}^{\mathrm{C2}}}+\frac{[\mathrm{pMBL_3MASP1_{zym}}]}{K_{\mathrm{m\ MASP2^*}}^{\mathrm{MASP1_{zym}}}}+\frac{[\mathrm{pMBL_3MASP2_{zym}}]}{K_{\mathrm{m\ MASP2^*}}^{\mathrm{MASP2_{zym}}}}+\frac{[\mathrm{pMBL_4MASP1_{zym}}]}{K_{\mathrm{m\ MASP2^*}}^{\mathrm{MASP1_{zym}}}}\\ +\frac{[\mathrm{pMBL_4MASP2_{zym}}]}{K_{\mathrm{m\ MASP2^*}}^{\mathrm{MASP2_{zym}}}}+\frac{[\mathrm{pCLLKMASP1_{zym}}]}{K_{\mathrm{m\ MASP2^*}}^{\mathrm{MASP1_{zym}}}}+\frac{[\mathrm{pCLLKMASP2_{zym}}]}{K_{\mathrm{m\ MASP2^*}}^{\mathrm{MASP2_{zym}}}}+\frac{[\mathrm{pCLK1MASP1_{zym}}]}{K_{\mathrm{m\ MASP2^*}}^{\mathrm{MASP1_{zym}}}}\\ +\frac{[\mathrm{pCLK1MASP2_{zym}}]}{K_{\mathrm{m\ MASP2^*}}^{\mathrm{MASP2_{zym}}}}+\frac{[\mathrm{pCLL1MASP1_{zym}}]}{K_{\mathrm{m\ MASP2^*}}^{\mathrm{MASP1_{zym}}}}+\frac{[\mathrm{pCLL1MASP2_{zym}}]}{K_{\mathrm{m\ MASP2^*}}^{\mathrm{MASP2_{zym}}}}+\frac{[\mathrm{pLFMASP1_{zym}}]}{K_{\mathrm{m\ MASP2^*}}^{\mathrm{MASP1_{zym}}}}\\ +\frac{[\mathrm{pLFMASP2_{zym}}]}{K_{\mathrm{m\ MASP2^*}}^{\mathrm{MASP2_{zym}}}}+\frac{[\mathrm{pHFMASP1_{zym}}]}{K_{\mathrm{m\ MASP2^*}}^{\mathrm{MASP1_{zym}}}}+\frac{[\mathrm{pHFMASP2_{zym}}]}{K_{\mathrm{m\ MASP2^*}}^{\mathrm{MASP2_{zym}}}}+\frac{[\mathrm{pMFMASP1_{zym}}]}{K_{\mathrm{m\ MASP2^*}}^{\mathrm{MASP1_{zym}}}}+\frac{[\mathrm{pMFMASP2_{zym}}]}{K_{\mathrm{m\ MASP2^*}}^{\mathrm{MASP2_{zym}}}}\end{array}\right)}
$$

$$
-\frac{k_{\mathrm{cat\ MASP2^*}}^{\mathrm{MASP1_{zym}}}\left[\mathrm{pCLLKMASP2^*}\right]\left[\mathrm{pMBL_4MASP1_{zym}}\right]}{K_{\mathrm{m\ MASP2^*}}^{\mathrm{MASP1_{zym}}}\left(\begin{array}{l}1+\frac{[\mathrm{C4}]}{K_{\mathrm{m\ MASP2^*}}^{\mathrm{C4}}}+\frac{[\mathrm{fC4bC2}]}{K_{\mathrm{m\ MASP2^*}}^{\mathrm{C2}}}+\frac{[\mathrm{fC4bC4bC2}]}{K_{\mathrm{m\ MASP2^*}}^{\mathrm{C2}}}+\frac{[\mathrm{fC3bC4bC2}]}{K_{\mathrm{m\ MASP2^*}}^{\mathrm{C2}}}+\frac{[\mathrm{pC4bC2}]}{K_{\mathrm{m\ MASP2^*}}^{\mathrm{C2}}}+\frac{[\mathrm{pC4bC4bC2}]}{K_{\mathrm{m\ MASP2^*}}^{\mathrm{C2}}}\\ +\frac{[\mathrm{pC3bC4bC2}]}{K_{\mathrm{m\ MASP2^*}}^{\mathrm{C2}}}+\frac{[\mathrm{pMBL_3MASP1_{zym}}]}{K_{\mathrm{m\ MASP2^*}}^{\mathrm{MASP1_{zym}}}}+\frac{[\mathrm{pMBL_3MASP2_{zym}}]}{K_{\mathrm{m\ MASP2^*}}^{\mathrm{MASP2_{zym}}}}+\frac{[\mathrm{pMBL_4MASP1_{zym}}]}{K_{\mathrm{m\ MASP2^*}}^{\mathrm{MASP1_{zym}}}}\\ +\frac{[\mathrm{pMBL_4MASP2_{zym}}]}{K_{\mathrm{m\ MASP2^*}}^{\mathrm{MASP2_{zym}}}}+\frac{[\mathrm{pCLLKMASP1_{zym}}]}{K_{\mathrm{m\ MASP2^*}}^{\mathrm{MASP1_{zym}}}}+\frac{[\mathrm{pCLLKMASP2_{zym}}]}{K_{\mathrm{m\ MASP2^*}}^{\mathrm{MASP2_{zym}}}}+\frac{[\mathrm{pCLK1MASP1_{zym}}]}{K_{\mathrm{m\ MASP2^*}}^{\mathrm{MASP1_{zym}}}}\\ +\frac{[\mathrm{pCLK1MASP2_{zym}}]}{K_{\mathrm{m\ MASP2^*}}^{\mathrm{MASP2_{zym}}}}+\frac{[\mathrm{pCLL1MASP1_{zym}}]}{K_{\mathrm{m\ MASP2^*}}^{\mathrm{MASP1_{zym}}}}+\frac{[\mathrm{pCLL1MASP2_{zym}}]}{K_{\mathrm{m\ MASP2^*}}^{\mathrm{MASP2_{zym}}}}+\frac{[\mathrm{pLFMASP1_{zym}}]}{K_{\mathrm{m\ MASP2^*}}^{\mathrm{MASP1_{zym}}}}\\ +\frac{[\mathrm{pLFMASP2_{zym}}]}{K_{\mathrm{m\ MASP2^*}}^{\mathrm{MASP2_{zym}}}}+\frac{[\mathrm{pHFMASP1_{zym}}]}{K_{\mathrm{m\ MASP2^*}}^{\mathrm{MASP1_{zym}}}}+\frac{[\mathrm{pHFMASP2_{zym}}]}{K_{\mathrm{m\ MASP2^*}}^{\mathrm{MASP2_{zym}}}}+\frac{[\mathrm{pMFMASP1_{zym}}]}{K_{\mathrm{m\ MASP2^*}}^{\mathrm{MASP1_{zym}}}}+\frac{[\mathrm{pMFMASP2_{zym}}]}{K_{\mathrm{m\ MASP2^*}}^{\mathrm{MASP2_{zym}}}}\end{array}\right)}
$$

$$-\frac{k_{\mathrm{cat\,MASP2^*}}^{\mathrm{MASP1_{zym}}}\left[\mathrm{pCLK1MASP2^*}\right]\left[\mathrm{pMBL_4MASP1_{zym}}\right]}{K_{\mathrm{m\,MASP2^*}}^{\mathrm{MASP1_{zym}}}\left(1+\frac{[\mathrm{C4}]}{K_{\mathrm{m\,MASP2^*}}^{\mathrm{C4}}}+\frac{[\mathrm{fC4bC2}]}{K_{\mathrm{m\,MASP2^*}}^{\mathrm{C2}}}+\frac{[\mathrm{fC4bC4bC2}]}{K_{\mathrm{m\,MASP2^*}}^{\mathrm{C2}}}+\frac{[\mathrm{fC3bC4bC2}]}{K_{\mathrm{m\,MASP2^*}}^{\mathrm{C2}}}+\frac{[\mathrm{pC4bC2}]}{K_{\mathrm{m\,MASP2^*}}^{\mathrm{C2}}}+\frac{[\mathrm{pC4bC4bC2}]}{K_{\mathrm{m\,MASP2^*}}^{\mathrm{C2}}}+\frac{[\mathrm{pC3bC4bC2}]}{K_{\mathrm{m\,MASP2^*}}^{\mathrm{C2}}}+\frac{[\mathrm{pMBL_3MASP1_{zym}}]}{K_{\mathrm{m\,MASP2^*}}^{\mathrm{MASP1_{zym}}}}+\frac{[\mathrm{pMBL_3MASP2_{zym}}]}{K_{\mathrm{m\,MASP2^*}}^{\mathrm{MASP2_{zym}}}}+\frac{[\mathrm{pMBL_4MASP1_{zym}}]}{K_{\mathrm{m\,MASP2^*}}^{\mathrm{MASP1_{zym}}}}+\frac{[\mathrm{pMBL_4MASP2_{zym}}]}{K_{\mathrm{m\,MASP2^*}}^{\mathrm{MASP2_{zym}}}}+\frac{[\mathrm{pCLLKMASP1_{zym}}]}{K_{\mathrm{m\,MASP2^*}}^{\mathrm{MASP1_{zym}}}}+\frac{[\mathrm{pCLLKMASP2_{zym}}]}{K_{\mathrm{m\,MASP2^*}}^{\mathrm{MASP2_{zym}}}}+\frac{[\mathrm{pCLK1MASP1_{zym}}]}{K_{\mathrm{m\,MASP2^*}}^{\mathrm{MASP1_{zym}}}}+\frac{[\mathrm{pCLK1MASP2_{zym}}]}{K_{\mathrm{m\,MASP2^*}}^{\mathrm{MASP2_{zym}}}}+\frac{[\mathrm{pCLL1MASP1_{zym}}]}{K_{\mathrm{m\,MASP2^*}}^{\mathrm{MASP1_{zym}}}}+\frac{[\mathrm{pCLL1MASP2_{zym}}]}{K_{\mathrm{m\,MASP2^*}}^{\mathrm{MASP2_{zym}}}}+\frac{[\mathrm{pLFMASP1_{zym}}]}{K_{\mathrm{m\,MASP2^*}}^{\mathrm{MASP1_{zym}}}}+\frac{[\mathrm{pLFMASP2_{zym}}]}{K_{\mathrm{m\,MASP2^*}}^{\mathrm{MASP2_{zym}}}}+\frac{[\mathrm{pHFMASP1_{zym}}]}{K_{\mathrm{m\,MASP2^*}}^{\mathrm{MASP1_{zym}}}}+\frac{[\mathrm{pHFMASP2_{zym}}]}{K_{\mathrm{m\,MASP2^*}}^{\mathrm{MASP2_{zym}}}}+\frac{[\mathrm{pMFMASP1_{zym}}]}{K_{\mathrm{m\,MASP2^*}}^{\mathrm{MASP1_{zym}}}}+\frac{[\mathrm{pMFMASP2_{zym}}]}{K_{\mathrm{m\,MASP2^*}}^{\mathrm{MASP2_{zym}}}}\right)}$$

$$-\frac{k_{\mathrm{cat\,MASP2^*}}^{\mathrm{MASP1_{zym}}}\left[\mathrm{pCLL1MASP2^*}\right]\left[\mathrm{pMBL_4MASP1_{zym}}\right]}{K_{\mathrm{m\,MASP2^*}}^{\mathrm{MASP1_{zym}}}\left(1+\frac{[\mathrm{C4}]}{K_{\mathrm{m\,MASP2^*}}^{\mathrm{C4}}}+\frac{[\mathrm{fC4bC2}]}{K_{\mathrm{m\,MASP2^*}}^{\mathrm{C2}}}+\frac{[\mathrm{fC4bC4bC2}]}{K_{\mathrm{m\,MASP2^*}}^{\mathrm{C2}}}+\frac{[\mathrm{fC3bC4bC2}]}{K_{\mathrm{m\,MASP2^*}}^{\mathrm{C2}}}+\frac{[\mathrm{pC4bC2}]}{K_{\mathrm{m\,MASP2^*}}^{\mathrm{C2}}}+\frac{[\mathrm{pC4bC4bC2}]}{K_{\mathrm{m\,MASP2^*}}^{\mathrm{C2}}}+\frac{[\mathrm{pC3bC4bC2}]}{K_{\mathrm{m\,MASP2^*}}^{\mathrm{C2}}}+\frac{[\mathrm{pMBL_3MASP1_{zym}}]}{K_{\mathrm{m\,MASP2^*}}^{\mathrm{MASP1_{zym}}}}+\frac{[\mathrm{pMBL_3MASP2_{zym}}]}{K_{\mathrm{m\,MASP2^*}}^{\mathrm{MASP2_{zym}}}}+\frac{[\mathrm{pMBL_4MASP1_{zym}}]}{K_{\mathrm{m\,MASP2^*}}^{\mathrm{MASP1_{zym}}}}+\frac{[\mathrm{pMBL_4MASP2_{zym}}]}{K_{\mathrm{m\,MASP2^*}}^{\mathrm{MASP2_{zym}}}}+\frac{[\mathrm{pCLLKMASP1_{zym}}]}{K_{\mathrm{m\,MASP2^*}}^{\mathrm{MASP1_{zym}}}}+\frac{[\mathrm{pCLLKMASP2_{zym}}]}{K_{\mathrm{m\,MASP2^*}}^{\mathrm{MASP2_{zym}}}}+\frac{[\mathrm{pCLK1MASP1_{zym}}]}{K_{\mathrm{m\,MASP2^*}}^{\mathrm{MASP1_{zym}}}}+\frac{[\mathrm{pCLK1MASP2_{zym}}]}{K_{\mathrm{m\,MASP2^*}}^{\mathrm{MASP2_{zym}}}}+\frac{[\mathrm{pCLL1MASP1_{zym}}]}{K_{\mathrm{m\,MASP2^*}}^{\mathrm{MASP1_{zym}}}}+\frac{[\mathrm{pCLL1MASP2_{zym}}]}{K_{\mathrm{m\,MASP2^*}}^{\mathrm{MASP2_{zym}}}}+\frac{[\mathrm{pLFMASP1_{zym}}]}{K_{\mathrm{m\,MASP2^*}}^{\mathrm{MASP1_{zym}}}}+\frac{[\mathrm{pLFMASP2_{zym}}]}{K_{\mathrm{m\,MASP2^*}}^{\mathrm{MASP2_{zym}}}}+\frac{[\mathrm{pHFMASP1_{zym}}]}{K_{\mathrm{m\,MASP2^*}}^{\mathrm{MASP1_{zym}}}}+\frac{[\mathrm{pHFMASP2_{zym}}]}{K_{\mathrm{m\,MASP2^*}}^{\mathrm{MASP2_{zym}}}}+\frac{[\mathrm{pMFMASP1_{zym}}]}{K_{\mathrm{m\,MASP2^*}}^{\mathrm{MASP1_{zym}}}}+\frac{[\mathrm{pMFMASP2_{zym}}]}{K_{\mathrm{m\,MASP2^*}}^{\mathrm{MASP2_{zym}}}}\right)}$$

$$-\frac{k_{\mathrm{cat\,MASP2^*}}^{\mathrm{MASP1_{zym}}}\left[\mathrm{pLFMASP2^*}\right]\left[\mathrm{pMBL_4MASP1_{zym}}\right]}{K_{\mathrm{m\,MASP2^*}}^{\mathrm{MASP1_{zym}}}\left(1+\frac{[\mathrm{C4}]}{K_{\mathrm{m\,MASP2^*}}^{\mathrm{C4}}}+\frac{[\mathrm{fC4bC2}]}{K_{\mathrm{m\,MASP2^*}}^{\mathrm{C2}}}+\frac{[\mathrm{fC4bC4bC2}]}{K_{\mathrm{m\,MASP2^*}}^{\mathrm{C2}}}+\frac{[\mathrm{fC3bC4bC2}]}{K_{\mathrm{m\,MASP2^*}}^{\mathrm{C2}}}+\frac{[\mathrm{pC4bC2}]}{K_{\mathrm{m\,MASP2^*}}^{\mathrm{C2}}}+\frac{[\mathrm{pC4bC4bC2}]}{K_{\mathrm{m\,MASP2^*}}^{\mathrm{C2}}}+\frac{[\mathrm{pC3bC4bC2}]}{K_{\mathrm{m\,MASP2^*}}^{\mathrm{C2}}}+\frac{[\mathrm{pMBL_3MASP1_{zym}}]}{K_{\mathrm{m\,MASP2^*}}^{\mathrm{MASP1_{zym}}}}+\frac{[\mathrm{pMBL_3MASP2_{zym}}]}{K_{\mathrm{m\,MASP2^*}}^{\mathrm{MASP2_{zym}}}}+\frac{[\mathrm{pMBL_4MASP1_{zym}}]}{K_{\mathrm{m\,MASP2^*}}^{\mathrm{MASP1_{zym}}}}+\frac{[\mathrm{pMBL_4MASP2_{zym}}]}{K_{\mathrm{m\,MASP2^*}}^{\mathrm{MASP2_{zym}}}}+\frac{[\mathrm{pCLLKMASP1_{zym}}]}{K_{\mathrm{m\,MASP2^*}}^{\mathrm{MASP1_{zym}}}}+\frac{[\mathrm{pCLLKMASP2_{zym}}]}{K_{\mathrm{m\,MASP2^*}}^{\mathrm{MASP2_{zym}}}}+\frac{[\mathrm{pCLK1MASP1_{zym}}]}{K_{\mathrm{m\,MASP2^*}}^{\mathrm{MASP1_{zym}}}}+\frac{[\mathrm{pCLK1MASP2_{zym}}]}{K_{\mathrm{m\,MASP2^*}}^{\mathrm{MASP2_{zym}}}}+\frac{[\mathrm{pCLL1MASP1_{zym}}]}{K_{\mathrm{m\,MASP2^*}}^{\mathrm{MASP1_{zym}}}}+\frac{[\mathrm{pCLL1MASP2_{zym}}]}{K_{\mathrm{m\,MASP2^*}}^{\mathrm{MASP2_{zym}}}}+\frac{[\mathrm{pLFMASP1_{zym}}]}{K_{\mathrm{m\,MASP2^*}}^{\mathrm{MASP1_{zym}}}}+\frac{[\mathrm{pLFMASP2_{zym}}]}{K_{\mathrm{m\,MASP2^*}}^{\mathrm{MASP2_{zym}}}}+\frac{[\mathrm{pHFMASP1_{zym}}]}{K_{\mathrm{m\,MASP2^*}}^{\mathrm{MASP1_{zym}}}}+\frac{[\mathrm{pHFMASP2_{zym}}]}{K_{\mathrm{m\,MASP2^*}}^{\mathrm{MASP2_{zym}}}}+\frac{[\mathrm{pMFMASP1_{zym}}]}{K_{\mathrm{m\,MASP2^*}}^{\mathrm{MASP1_{zym}}}}+\frac{[\mathrm{pMFMASP2_{zym}}]}{K_{\mathrm{m\,MASP2^*}}^{\mathrm{MASP2_{zym}}}}\right)}$$

$$
-\frac{k_{\text{cat MASP2}^*}^{\text{MASP1}_{\text{zym}}}\left[\text{pHFMASP2}^*\right]\left[\text{pMBL}_4\text{MASP1}_{\text{zym}}\right]}{K_{\text{m MASP2}^*}^{\text{MASP1}_{\text{zym}}}\left(\begin{array}{l}1+\frac{[\text{C4}]}{K_{\text{m MASP2}^*}^{\text{C4}}}+\frac{[\text{fC4bC2}]}{K_{\text{m MASP2}^*}^{\text{C2}}}+\frac{[\text{fC4bC4bC2}]}{K_{\text{m MASP2}^*}^{\text{C2}}}+\frac{[\text{fC3bC4bC2}]}{K_{\text{m MASP2}^*}^{\text{C2}}}+\frac{[\text{pC4bC2}]}{K_{\text{m MASP2}^*}^{\text{C2}}}+\frac{[\text{pC4bC4bC2}]}{K_{\text{m MASP2}^*}^{\text{C2}}}\\+\frac{[\text{pC3bC4bC2}]}{K_{\text{m MASP2}^*}^{\text{C2}}}+\frac{[\text{pMBL}_3\text{MASP1}_{\text{zym}}]}{K_{\text{m MASP2}^*}^{\text{MASP1}_{\text{zym}}}}+\frac{[\text{pMBL}_3\text{MASP2}_{\text{zym}}]}{K_{\text{m MASP2}^*}^{\text{MASP2}_{\text{zym}}}}+\frac{[\text{pMBL}_4\text{MASP1}_{\text{zym}}]}{K_{\text{m MASP2}^*}^{\text{MASP1}_{\text{zym}}}}\\+\frac{[\text{pMBL}_4\text{MASP2}_{\text{zym}}]}{K_{\text{m MASP2}^*}^{\text{MASP2}_{\text{zym}}}}+\frac{[\text{pCLLKMASP1}_{\text{zym}}]}{K_{\text{m MASP2}^*}^{\text{MASP1}_{\text{zym}}}}+\frac{[\text{pCLLKMASP2}_{\text{zym}}]}{K_{\text{m MASP2}^*}^{\text{MASP2}_{\text{zym}}}}+\frac{[\text{pCLK1MASP1}_{\text{zym}}]}{K_{\text{m MASP2}^*}^{\text{MASP1}_{\text{zym}}}}\\+\frac{[\text{pCLK1MASP2}_{\text{zym}}]}{K_{\text{m MASP2}^*}^{\text{MASP2}_{\text{zym}}}}+\frac{[\text{pCLL1MASP1}_{\text{zym}}]}{K_{\text{m MASP2}^*}^{\text{MASP1}_{\text{zym}}}}+\frac{[\text{pCLL1MASP2}_{\text{zym}}]}{K_{\text{m MASP2}^*}^{\text{MASP2}_{\text{zym}}}}+\frac{[\text{pLFMASP1}_{\text{zym}}]}{K_{\text{m MASP2}^*}^{\text{MASP1}_{\text{zym}}}}\\+\frac{[\text{pLFMASP2}_{\text{zym}}]}{K_{\text{m MASP2}^*}^{\text{MASP2}_{\text{zym}}}}+\frac{[\text{pHFMASP1}_{\text{zym}}]}{K_{\text{m MASP2}^*}^{\text{MASP1}_{\text{zym}}}}+\frac{[\text{pHFMASP2}_{\text{zym}}]}{K_{\text{m MASP2}^*}^{\text{MASP2}_{\text{zym}}}}+\frac{[\text{pMFMASP1}_{\text{zym}}]}{K_{\text{m MASP2}^*}^{\text{MASP1}_{\text{zym}}}}+\frac{[\text{pMFMASP2}_{\text{zym}}]}{K_{\text{m MASP2}^*}^{\text{MASP2}_{\text{zym}}}}\end{array}\right)}
$$

$$
-\frac{k_{\text{cat MASP2}^*}^{\text{MASP1}_{\text{zym}}}\left[\text{pMFMASP2}^*\right]\left[\text{pMBL}_4\text{MASP1}_{\text{zym}}\right]}{K_{\text{m MASP2}^*}^{\text{MASP1}_{\text{zym}}}\left(\begin{array}{l}1+\frac{[\text{C4}]}{K_{\text{m MASP2}^*}^{\text{C4}}}+\frac{[\text{fC4bC2}]}{K_{\text{m MASP2}^*}^{\text{C2}}}+\frac{[\text{fC4bC4bC2}]}{K_{\text{m MASP2}^*}^{\text{C2}}}+\frac{[\text{fC3bC4bC2}]}{K_{\text{m MASP2}^*}^{\text{C2}}}+\frac{[\text{pC4bC2}]}{K_{\text{m MASP2}^*}^{\text{C2}}}+\frac{[\text{pC4bC4bC2}]}{K_{\text{m MASP2}^*}^{\text{C2}}}\\+\frac{[\text{pC3bC4bC2}]}{K_{\text{m MASP2}^*}^{\text{C2}}}+\frac{[\text{pMBL}_3\text{MASP1}_{\text{zym}}]}{K_{\text{m MASP2}^*}^{\text{MASP1}_{\text{zym}}}}+\frac{[\text{pMBL}_3\text{MASP2}_{\text{zym}}]}{K_{\text{m MASP2}^*}^{\text{MASP2}_{\text{zym}}}}+\frac{[\text{pMBL}_4\text{MASP1}_{\text{zym}}]}{K_{\text{m MASP2}^*}^{\text{MASP1}_{\text{zym}}}}\\+\frac{[\text{pMBL}_4\text{MASP2}_{\text{zym}}]}{K_{\text{m MASP2}^*}^{\text{MASP2}_{\text{zym}}}}+\frac{[\text{pCLLKMASP1}_{\text{zym}}]}{K_{\text{m MASP2}^*}^{\text{MASP1}_{\text{zym}}}}+\frac{[\text{pCLLKMASP2}_{\text{zym}}]}{K_{\text{m MASP2}^*}^{\text{MASP2}_{\text{zym}}}}+\frac{[\text{pCLK1MASP1}_{\text{zym}}]}{K_{\text{m MASP2}^*}^{\text{MASP1}_{\text{zym}}}}\\+\frac{[\text{pCLK1MASP2}_{\text{zym}}]}{K_{\text{m MASP2}^*}^{\text{MASP2}_{\text{zym}}}}+\frac{[\text{pCLL1MASP1}_{\text{zym}}]}{K_{\text{m MASP2}^*}^{\text{MASP1}_{\text{zym}}}}+\frac{[\text{pCLL1MASP2}_{\text{zym}}]}{K_{\text{m MASP2}^*}^{\text{MASP2}_{\text{zym}}}}+\frac{[\text{pLFMASP1}_{\text{zym}}]}{K_{\text{m MASP2}^*}^{\text{MASP1}_{\text{zym}}}}\\+\frac{[\text{pLFMASP2}_{\text{zym}}]}{K_{\text{m MASP2}^*}^{\text{MASP2}_{\text{zym}}}}+\frac{[\text{pHFMASP1}_{\text{zym}}]}{K_{\text{m MASP2}^*}^{\text{MASP1}_{\text{zym}}}}+\frac{[\text{pHFMASP2}_{\text{zym}}]}{K_{\text{m MASP2}^*}^{\text{MASP2}_{\text{zym}}}}+\frac{[\text{pMFMASP1}_{\text{zym}}]}{K_{\text{m MASP2}^*}^{\text{MASP1}_{\text{zym}}}}+\frac{[\text{pMFMASP2}_{\text{zym}}]}{K_{\text{m MASP2}^*}^{\text{MASP2}_{\text{zym}}}}\end{array}\right)}
$$

$$-\frac{k_{\text{cat MASP2}^*}^{\text{MASP1}_{\text{zym}}}\left[\text{pPTX3LFMASP2}^*\right]\left[\text{pMBL}_4\text{MASP1}_{\text{zym}}\right]}{K_{\text{m MASP2}^*}^{\text{MASP1}_{\text{zym}}}\left(\begin{array}{l}1+\frac{[\text{C4}]}{K_{\text{m MASP2}^*}^{\text{C4}}}+\frac{[\text{fC4bC2}]}{K_{\text{m MASP2}^*}^{\text{C2}}}+\frac{[\text{fC4bC4bC2}]}{K_{\text{m MASP2}^*}^{\text{C2}}}+\frac{[\text{fC3bC4bC2}]}{K_{\text{m MASP2}^*}^{\text{C2}}}+\frac{[\text{pC4bC2}]}{K_{\text{m MASP2}^*}^{\text{C2}}}+\frac{[\text{pC4bC4bC2}]}{K_{\text{m MASP2}^*}^{\text{C2}}}\\+\frac{[\text{pC3bC4bC2}]}{K_{\text{m MASP2}^*}^{\text{C2}}}+\frac{\left[\text{pMBL}_3\text{MASP1}_{\text{zym}}\right]}{K_{\text{m MASP2}^*}^{\text{MASP1}_{\text{zym}}}}+\frac{\left[\text{pMBL}_3\text{MASP2}_{\text{zym}}\right]}{K_{\text{m MASP2}^*}^{\text{MASP2}_{\text{zym}}}}+\frac{\left[\text{pMBL}_4\text{MASP1}_{\text{zym}}\right]}{K_{\text{m MASP2}^*}^{\text{MASP1}_{\text{zym}}}}\\+\frac{\left[\text{pMBL}_4\text{MASP2}_{\text{zym}}\right]}{K_{\text{m MASP2}^*}^{\text{MASP2}_{\text{zym}}}}+\frac{\left[\text{pCLLKMASP1}_{\text{zym}}\right]}{K_{\text{m MASP2}^*}^{\text{MASP1}_{\text{zym}}}}+\frac{\left[\text{pCLLKMASP2}_{\text{zym}}\right]}{K_{\text{m MASP2}^*}^{\text{MASP2}_{\text{zym}}}}+\frac{\left[\text{pCLK1MASP1}_{\text{zym}}\right]}{K_{\text{m MASP2}^*}^{\text{MASP1}_{\text{zym}}}}\\+\frac{\left[\text{pCLK1MASP2}_{\text{zym}}\right]}{K_{\text{m MASP2}^*}^{\text{MASP2}_{\text{zym}}}}+\frac{\left[\text{pCLL1MASP1}_{\text{zym}}\right]}{K_{\text{m MASP2}^*}^{\text{MASP1}_{\text{zym}}}}+\frac{\left[\text{pCLL1MASP2}_{\text{zym}}\right]}{K_{\text{m MASP2}^*}^{\text{MASP2}_{\text{zym}}}}+\frac{\left[\text{pLFMASP1}_{\text{zym}}\right]}{K_{\text{m MASP2}^*}^{\text{MASP1}_{\text{zym}}}}\\+\frac{\left[\text{pLFMASP2}_{\text{zym}}\right]}{K_{\text{m MASP2}^*}^{\text{MASP2}_{\text{zym}}}}+\frac{\left[\text{pHFMASP1}_{\text{zym}}\right]}{K_{\text{m MASP2}^*}^{\text{MASP1}_{\text{zym}}}}+\frac{\left[\text{pHFMASP2}_{\text{zym}}\right]}{K_{\text{m MASP2}^*}^{\text{MASP2}_{\text{zym}}}}+\frac{\left[\text{pMFMASP1}_{\text{zym}}\right]}{K_{\text{m MASP2}^*}^{\text{MASP1}_{\text{zym}}}}+\frac{\left[\text{pMFMASP2}_{\text{zym}}\right]}{K_{\text{m MASP2}^*}^{\text{MASP2}_{\text{zym}}}}\\+\frac{\left[\text{pPTX3LFMASP1}_{\text{zym}}\right]}{K_{\text{m MASP2}^*}^{\text{MASP1}_{\text{zym}}}}+\frac{\left[\text{pPTX3LFMASP2}_{\text{zym}}\right]}{K_{\text{m MASP2}^*}^{\text{MASP2}_{\text{zym}}}}\end{array}\right)}$$

267

$$\frac{d\left[\text{fMBL}_4\text{MASP1}_{\text{zym}}\right]}{dt}=k_{\text{MBL}_4\text{MASP1}}^{+}\left[\text{MBL}_4\right]\left[\text{MASP1}\right]-k_{\text{MBL}_4\text{MASP1}}^{-}\left[\text{fMBL}_4\text{MASP1}_{\text{zym}}\right]$$
$$-k_{\text{pMBL}_4}^{+}\left[\text{fMBL}_4\text{MASP1}_{\text{zym}}\right]\left[\text{Pathogen Surface}\right]+k_{\text{pMBL}_4}^{-}\left[\text{pMBL}_4\text{MASP1}_{\text{zym}}\right]$$

268

$$\frac{d\left[\text{fMBL}_4\text{MASP1}^*\right]}{dt}=-k_{\text{pMBL}_4}^{+}\left[\text{fMBL}_4\text{MASP1}^*\right]\left[\text{Pathogen Surface}\right]+k_{\text{pMBL}_4}^{-}\left[\text{pMBL}_4\text{MASP1}^*\right]$$
$$+k_{\text{MBL}_4\text{MASP1}}^{+}\left[\text{MBL}_4\right]\left[\text{MASP1}^*\right]-k_{\text{MBL}_4\text{MASP1}}^{-}\left[\text{fMBL}_4\text{MASP1}^*\right]-k_{\text{C1INHMASP1}}^{+}\left[\text{C1-INH}\right]\left[\text{fMBL}_4\text{MASP1}^*\right]$$

269

$$\frac{d\left[\text{pMBL}_4\text{MASP1}^*\right]}{dt}=k_{\text{pMBL}_4}^{+}\left[\text{fMBL}_4\text{MASP1}^*\right]\left[\text{Pathogen Surface}\right]-k_{\text{pMBL}_4}^{-}\left[\text{pMBL}_4\text{MASP1}^*\right]$$
$$+k_{\text{MBL}_4\text{MASP1}}^{+}\left[\text{pMBL}_4\right]\left[\text{MASP1}^*\right]-k_{\text{MBL}_4\text{MASP1}}^{-}\left[\text{pMBL}_4\text{MASP1}^*\right]$$

$$
+\frac{k_{\mathrm{cat\ MASP1_{zym}}}^{\mathrm{MASP1_{zym}}}\left[\mathrm{pMBL_3MASP1_{zym}}\right]\left[\mathrm{pMBL_4MASP1_{zym}}\right]}{K_{\mathrm{m\ MASP1_{zym}}}^{\mathrm{MASP1_{zym}}}\left(\begin{array}{l}1+\frac{\left[\mathrm{pMBL_3MASP1_{zym}}\right]}{K_{\mathrm{m\ MASP1_{zym}}}^{\mathrm{MASP1_{zym}}}}+\frac{\left[\mathrm{pMBL_3MASP2_{zym}}\right]}{K_{\mathrm{m\ MASP1_{zym}}}^{\mathrm{MASP2_{zym}}}}+\frac{\left[\mathrm{pMBL_4MASP1_{zym}}\right]}{K_{\mathrm{m\ MASP1_{zym}}}^{\mathrm{MASP1_{zym}}}}+\frac{\left[\mathrm{pMBL_4MASP2_{zym}}\right]}{K_{\mathrm{m\ MASP1_{zym}}}^{\mathrm{MASP2_{zym}}}}\\+\frac{\left[\mathrm{pCLLKMASP1_{zym}}\right]}{K_{\mathrm{m\ MASP1_{zym}}}^{\mathrm{MASP1_{zym}}}}+\frac{\left[\mathrm{pCLLKMASP2_{zym}}\right]}{K_{\mathrm{m\ MASP1_{zym}}}^{\mathrm{MASP2_{zym}}}}+\frac{\left[\mathrm{pCLK1MASP1_{zym}}\right]}{K_{\mathrm{m\ MASP1_{zym}}}^{\mathrm{MASP1_{zym}}}}+\frac{\left[\mathrm{pCLK1MASP2_{zym}}\right]}{K_{\mathrm{m\ MASP1_{zym}}}^{\mathrm{MASP2_{zym}}}}\\+\frac{\left[\mathrm{pCLL1MASP1_{zym}}\right]}{K_{\mathrm{m\ MASP1_{zym}}}^{\mathrm{MASP1_{zym}}}}+\frac{\left[\mathrm{pCLL1MASP2_{zym}}\right]}{K_{\mathrm{m\ MASP1_{zym}}}^{\mathrm{MASP2_{zym}}}}+\frac{\left[\mathrm{pLFMASP1_{zym}}\right]}{K_{\mathrm{m\ MASP1_{zym}}}^{\mathrm{MASP1_{zym}}}}+\frac{\left[\mathrm{pLFMASP2_{zym}}\right]}{K_{\mathrm{m\ MASP1_{zym}}}^{\mathrm{MASP2_{zym}}}}\\+\frac{\left[\mathrm{pHFMASP1_{zym}}\right]}{K_{\mathrm{m\ MASP1_{zym}}}^{\mathrm{MASP1_{zym}}}}+\frac{\left[\mathrm{pHFMASP2_{zym}}\right]}{K_{\mathrm{m\ MASP1_{zym}}}^{\mathrm{MASP2_{zym}}}}+\frac{\left[\mathrm{pMFMASP1_{zym}}\right]}{K_{\mathrm{m\ MASP1_{zym}}}^{\mathrm{MASP1_{zym}}}}+\frac{\left[\mathrm{pMFMASP2_{zym}}\right]}{K_{\mathrm{m\ MASP1_{zym}}}^{\mathrm{MASP2_{zym}}}}\end{array}\right)}
$$

$$
+\frac{k_{\mathrm{cat\ MASP1_{zym}}}^{\mathrm{MASP1_{zym}}}\left[\mathrm{pMBL_4MASP1_{zym}}\right]\left[\mathrm{pMBL_4MASP1_{zym}}\right]}{K_{\mathrm{m\ MASP1_{zym}}}^{\mathrm{MASP1_{zym}}}\left(\begin{array}{l}1+\frac{\left[\mathrm{pMBL_3MASP1_{zym}}\right]}{K_{\mathrm{m\ MASP1_{zym}}}^{\mathrm{MASP1_{zym}}}}+\frac{\left[\mathrm{pMBL_3MASP2_{zym}}\right]}{K_{\mathrm{m\ MASP1_{zym}}}^{\mathrm{MASP2_{zym}}}}+\frac{\left[\mathrm{pMBL_4MASP1_{zym}}\right]}{K_{\mathrm{m\ MASP1_{zym}}}^{\mathrm{MASP1_{zym}}}}+\frac{\left[\mathrm{pMBL_4MASP2_{zym}}\right]}{K_{\mathrm{m\ MASP1_{zym}}}^{\mathrm{MASP2_{zym}}}}\\+\frac{\left[\mathrm{pCLLKMASP1_{zym}}\right]}{K_{\mathrm{m\ MASP1_{zym}}}^{\mathrm{MASP1_{zym}}}}+\frac{\left[\mathrm{pCLLKMASP2_{zym}}\right]}{K_{\mathrm{m\ MASP1_{zym}}}^{\mathrm{MASP2_{zym}}}}+\frac{\left[\mathrm{pCLK1MASP1_{zym}}\right]}{K_{\mathrm{m\ MASP1_{zym}}}^{\mathrm{MASP1_{zym}}}}+\frac{\left[\mathrm{pCLK1MASP2_{zym}}\right]}{K_{\mathrm{m\ MASP1_{zym}}}^{\mathrm{MASP2_{zym}}}}\\+\frac{\left[\mathrm{pCLL1MASP1_{zym}}\right]}{K_{\mathrm{m\ MASP1_{zym}}}^{\mathrm{MASP1_{zym}}}}+\frac{\left[\mathrm{pCLL1MASP2_{zym}}\right]}{K_{\mathrm{m\ MASP1_{zym}}}^{\mathrm{MASP2_{zym}}}}+\frac{\left[\mathrm{pLFMASP1_{zym}}\right]}{K_{\mathrm{m\ MASP1_{zym}}}^{\mathrm{MASP1_{zym}}}}+\frac{\left[\mathrm{pLFMASP2_{zym}}\right]}{K_{\mathrm{m\ MASP1_{zym}}}^{\mathrm{MASP2_{zym}}}}\\+\frac{\left[\mathrm{pHFMASP1_{zym}}\right]}{K_{\mathrm{m\ MASP1_{zym}}}^{\mathrm{MASP1_{zym}}}}+\frac{\left[\mathrm{pHFMASP2_{zym}}\right]}{K_{\mathrm{m\ MASP1_{zym}}}^{\mathrm{MASP2_{zym}}}}+\frac{\left[\mathrm{pMFMASP1_{zym}}\right]}{K_{\mathrm{m\ MASP1_{zym}}}^{\mathrm{MASP1_{zym}}}}+\frac{\left[\mathrm{pMFMASP2_{zym}}\right]}{K_{\mathrm{m\ MASP1_{zym}}}^{\mathrm{MASP2_{zym}}}}\end{array}\right)}
$$

$$
+\frac{k_{\mathrm{cat\ MASP1_{zym}}}^{\mathrm{MASP1_{zym}}}\left[\mathrm{pCLLKMASP1_{zym}}\right]\left[\mathrm{pMBL_4MASP1_{zym}}\right]}{K_{\mathrm{m\ MASP1_{zym}}}^{\mathrm{MASP1_{zym}}}\left(\begin{array}{l}1+\frac{\left[\mathrm{pMBL_3MASP1_{zym}}\right]}{K_{\mathrm{m\ MASP1_{zym}}}^{\mathrm{MASP1_{zym}}}}+\frac{\left[\mathrm{pMBL_3MASP2_{zym}}\right]}{K_{\mathrm{m\ MASP1_{zym}}}^{\mathrm{MASP2_{zym}}}}+\frac{\left[\mathrm{pMBL_4MASP1_{zym}}\right]}{K_{\mathrm{m\ MASP1_{zym}}}^{\mathrm{MASP1_{zym}}}}+\frac{\left[\mathrm{pMBL_4MASP2_{zym}}\right]}{K_{\mathrm{m\ MASP1_{zym}}}^{\mathrm{MASP2_{zym}}}}\\+\frac{\left[\mathrm{pCLLKMASP1_{zym}}\right]}{K_{\mathrm{m\ MASP1_{zym}}}^{\mathrm{MASP1_{zym}}}}+\frac{\left[\mathrm{pCLLKMASP2_{zym}}\right]}{K_{\mathrm{m\ MASP1_{zym}}}^{\mathrm{MASP2_{zym}}}}+\frac{\left[\mathrm{pCLK1MASP1_{zym}}\right]}{K_{\mathrm{m\ MASP1_{zym}}}^{\mathrm{MASP1_{zym}}}}+\frac{\left[\mathrm{pCLK1MASP2_{zym}}\right]}{K_{\mathrm{m\ MASP1_{zym}}}^{\mathrm{MASP2_{zym}}}}\\+\frac{\left[\mathrm{pCLL1MASP1_{zym}}\right]}{K_{\mathrm{m\ MASP1_{zym}}}^{\mathrm{MASP1_{zym}}}}+\frac{\left[\mathrm{pCLL1MASP2_{zym}}\right]}{K_{\mathrm{m\ MASP1_{zym}}}^{\mathrm{MASP2_{zym}}}}+\frac{\left[\mathrm{pLFMASP1_{zym}}\right]}{K_{\mathrm{m\ MASP1_{zym}}}^{\mathrm{MASP1_{zym}}}}+\frac{\left[\mathrm{pLFMASP2_{zym}}\right]}{K_{\mathrm{m\ MASP1_{zym}}}^{\mathrm{MASP2_{zym}}}}\\+\frac{\left[\mathrm{pHFMASP1_{zym}}\right]}{K_{\mathrm{m\ MASP1_{zym}}}^{\mathrm{MASP1_{zym}}}}+\frac{\left[\mathrm{pHFMASP2_{zym}}\right]}{K_{\mathrm{m\ MASP1_{zym}}}^{\mathrm{MASP2_{zym}}}}+\frac{\left[\mathrm{pMFMASP1_{zym}}\right]}{K_{\mathrm{m\ MASP1_{zym}}}^{\mathrm{MASP1_{zym}}}}+\frac{\left[\mathrm{pMFMASP2_{zym}}\right]}{K_{\mathrm{m\ MASP1_{zym}}}^{\mathrm{MASP2_{zym}}}}\end{array}\right)}
$$

$$+\frac{k^{\mathrm{MASP1_{zym}}}_{\mathrm{cat\,MASP1_{zym}}}\left[\mathrm{pCLK1MASP1_{zym}}\right]\left[\mathrm{pMBL_4MASP1_{zym}}\right]}{K^{\mathrm{MASP1_{zym}}}_{\mathrm{m\,MASP1_{zym}}}\left(\begin{array}{l}1+\frac{\left[\mathrm{pMBL_3MASP1_{zym}}\right]}{K^{\mathrm{MASP1_{zym}}}_{\mathrm{m\,MASP1_{zym}}}}+\frac{\left[\mathrm{pMBL_3MASP2_{zym}}\right]}{K^{\mathrm{MASP2_{zym}}}_{\mathrm{m\,MASP1_{zym}}}}+\frac{\left[\mathrm{pMBL_4MASP1_{zym}}\right]}{K^{\mathrm{MASP1_{zym}}}_{\mathrm{m\,MASP1_{zym}}}}+\frac{\left[\mathrm{pMBL_4MASP2_{zym}}\right]}{K^{\mathrm{MASP2_{zym}}}_{\mathrm{m\,MASP1_{zym}}}}\\+\frac{\left[\mathrm{pCLLKMASP1_{zym}}\right]}{K^{\mathrm{MASP1_{zym}}}_{\mathrm{m\,MASP1_{zym}}}}+\frac{\left[\mathrm{pCLLKMASP2_{zym}}\right]}{K^{\mathrm{MASP2_{zym}}}_{\mathrm{m\,MASP1_{zym}}}}+\frac{\left[\mathrm{pCLK1MASP1_{zym}}\right]}{K^{\mathrm{MASP1_{zym}}}_{\mathrm{m\,MASP1_{zym}}}}+\frac{\left[\mathrm{pCLK1MASP2_{zym}}\right]}{K^{\mathrm{MASP2_{zym}}}_{\mathrm{m\,MASP1_{zym}}}}\\+\frac{\left[\mathrm{pCLL1MASP1_{zym}}\right]}{K^{\mathrm{MASP1_{zym}}}_{\mathrm{m\,MASP1_{zym}}}}+\frac{\left[\mathrm{pCLL1MASP2_{zym}}\right]}{K^{\mathrm{MASP2_{zym}}}_{\mathrm{m\,MASP1_{zym}}}}+\frac{\left[\mathrm{pLFMASP1_{zym}}\right]}{K^{\mathrm{MASP1_{zym}}}_{\mathrm{m\,MASP1_{zym}}}}+\frac{\left[\mathrm{pLFMASP2_{zym}}\right]}{K^{\mathrm{MASP2_{zym}}}_{\mathrm{m\,MASP1_{zym}}}}\\+\frac{\left[\mathrm{pHFMASP1_{zym}}\right]}{K^{\mathrm{MASP1_{zym}}}_{\mathrm{m\,MASP1_{zym}}}}+\frac{\left[\mathrm{pHFMASP2_{zym}}\right]}{K^{\mathrm{MASP2_{zym}}}_{\mathrm{m\,MASP1_{zym}}}}+\frac{\left[\mathrm{pMFMASP1_{zym}}\right]}{K^{\mathrm{MASP1_{zym}}}_{\mathrm{m\,MASP1_{zym}}}}+\frac{\left[\mathrm{pMFMASP2_{zym}}\right]}{K^{\mathrm{MASP2_{zym}}}_{\mathrm{m\,MASP1_{zym}}}}\end{array}\right)}$$

$$+\frac{k^{\mathrm{MASP1_{zym}}}_{\mathrm{cat\,MASP1_{zym}}}\left[\mathrm{pCLL1MASP1_{zym}}\right]\left[\mathrm{pMBL_4MASP1_{zym}}\right]}{K^{\mathrm{MASP1_{zym}}}_{\mathrm{m\,MASP1_{zym}}}\left(\begin{array}{l}1+\frac{\left[\mathrm{pMBL_3MASP1_{zym}}\right]}{K^{\mathrm{MASP1_{zym}}}_{\mathrm{m\,MASP1_{zym}}}}+\frac{\left[\mathrm{pMBL_3MASP2_{zym}}\right]}{K^{\mathrm{MASP2_{zym}}}_{\mathrm{m\,MASP1_{zym}}}}+\frac{\left[\mathrm{pMBL_4MASP1_{zym}}\right]}{K^{\mathrm{MASP1_{zym}}}_{\mathrm{m\,MASP1_{zym}}}}+\frac{\left[\mathrm{pMBL_4MASP2_{zym}}\right]}{K^{\mathrm{MASP2_{zym}}}_{\mathrm{m\,MASP1_{zym}}}}\\+\frac{\left[\mathrm{pCLLKMASP1_{zym}}\right]}{K^{\mathrm{MASP1_{zym}}}_{\mathrm{m\,MASP1_{zym}}}}+\frac{\left[\mathrm{pCLLKMASP2_{zym}}\right]}{K^{\mathrm{MASP2_{zym}}}_{\mathrm{m\,MASP1_{zym}}}}+\frac{\left[\mathrm{pCLK1MASP1_{zym}}\right]}{K^{\mathrm{MASP1_{zym}}}_{\mathrm{m\,MASP1_{zym}}}}+\frac{\left[\mathrm{pCLK1MASP2_{zym}}\right]}{K^{\mathrm{MASP2_{zym}}}_{\mathrm{m\,MASP1_{zym}}}}\\+\frac{\left[\mathrm{pCLL1MASP1_{zym}}\right]}{K^{\mathrm{MASP1_{zym}}}_{\mathrm{m\,MASP1_{zym}}}}+\frac{\left[\mathrm{pCLL1MASP2_{zym}}\right]}{K^{\mathrm{MASP2_{zym}}}_{\mathrm{m\,MASP1_{zym}}}}+\frac{\left[\mathrm{pLFMASP1_{zym}}\right]}{K^{\mathrm{MASP1_{zym}}}_{\mathrm{m\,MASP1_{zym}}}}+\frac{\left[\mathrm{pLFMASP2_{zym}}\right]}{K^{\mathrm{MASP2_{zym}}}_{\mathrm{m\,MASP1_{zym}}}}\\+\frac{\left[\mathrm{pHFMASP1_{zym}}\right]}{K^{\mathrm{MASP1_{zym}}}_{\mathrm{m\,MASP1_{zym}}}}+\frac{\left[\mathrm{pHFMASP2_{zym}}\right]}{K^{\mathrm{MASP2_{zym}}}_{\mathrm{m\,MASP1_{zym}}}}+\frac{\left[\mathrm{pMFMASP1_{zym}}\right]}{K^{\mathrm{MASP1_{zym}}}_{\mathrm{m\,MASP1_{zym}}}}+\frac{\left[\mathrm{pMFMASP2_{zym}}\right]}{K^{\mathrm{MASP2_{zym}}}_{\mathrm{m\,MASP1_{zym}}}}\end{array}\right)}$$

$$+\frac{k^{\mathrm{MASP1_{zym}}}_{\mathrm{cat\,MASP1_{zym}}}\left[\mathrm{pLFMASP1_{zym}}\right]\left[\mathrm{pMBL_4MASP1_{zym}}\right]}{K^{\mathrm{MASP1_{zym}}}_{\mathrm{m\,MASP1_{zym}}}\left(\begin{array}{l}1+\frac{\left[\mathrm{pMBL_3MASP1_{zym}}\right]}{K^{\mathrm{MASP1_{zym}}}_{\mathrm{m\,MASP1_{zym}}}}+\frac{\left[\mathrm{pMBL_3MASP2_{zym}}\right]}{K^{\mathrm{MASP2_{zym}}}_{\mathrm{m\,MASP1_{zym}}}}+\frac{\left[\mathrm{pMBL_4MASP1_{zym}}\right]}{K^{\mathrm{MASP1_{zym}}}_{\mathrm{m\,MASP1_{zym}}}}+\frac{\left[\mathrm{pMBL_4MASP2_{zym}}\right]}{K^{\mathrm{MASP2_{zym}}}_{\mathrm{m\,MASP1_{zym}}}}\\+\frac{\left[\mathrm{pCLLKMASP1_{zym}}\right]}{K^{\mathrm{MASP1_{zym}}}_{\mathrm{m\,MASP1_{zym}}}}+\frac{\left[\mathrm{pCLLKMASP2_{zym}}\right]}{K^{\mathrm{MASP2_{zym}}}_{\mathrm{m\,MASP1_{zym}}}}+\frac{\left[\mathrm{pCLK1MASP1_{zym}}\right]}{K^{\mathrm{MASP1_{zym}}}_{\mathrm{m\,MASP1_{zym}}}}+\frac{\left[\mathrm{pCLK1MASP2_{zym}}\right]}{K^{\mathrm{MASP2_{zym}}}_{\mathrm{m\,MASP1_{zym}}}}\\+\frac{\left[\mathrm{pCLL1MASP1_{zym}}\right]}{K^{\mathrm{MASP1_{zym}}}_{\mathrm{m\,MASP1_{zym}}}}+\frac{\left[\mathrm{pCLL1MASP2_{zym}}\right]}{K^{\mathrm{MASP2_{zym}}}_{\mathrm{m\,MASP1_{zym}}}}+\frac{\left[\mathrm{pLFMASP1_{zym}}\right]}{K^{\mathrm{MASP1_{zym}}}_{\mathrm{m\,MASP1_{zym}}}}+\frac{\left[\mathrm{pLFMASP2_{zym}}\right]}{K^{\mathrm{MASP2_{zym}}}_{\mathrm{m\,MASP1_{zym}}}}\\+\frac{\left[\mathrm{pHFMASP1_{zym}}\right]}{K^{\mathrm{MASP1_{zym}}}_{\mathrm{m\,MASP1_{zym}}}}+\frac{\left[\mathrm{pHFMASP2_{zym}}\right]}{K^{\mathrm{MASP2_{zym}}}_{\mathrm{m\,MASP1_{zym}}}}+\frac{\left[\mathrm{pMFMASP1_{zym}}\right]}{K^{\mathrm{MASP1_{zym}}}_{\mathrm{m\,MASP1_{zym}}}}+\frac{\left[\mathrm{pMFMASP2_{zym}}\right]}{K^{\mathrm{MASP2_{zym}}}_{\mathrm{m\,MASP1_{zym}}}}\end{array}\right)}$$

$$+\frac{k_{\text{cat MASP1}_{\text{zym}}}^{\text{MASP1}_{\text{zym}}}\left[\text{pHFMASP1}_{\text{zym}}\right]\left[\text{pMBL}_4\text{MASP1}_{\text{zym}}\right]}{K_{\text{m MASP1}_{\text{zym}}}^{\text{MASP1}_{\text{zym}}}\left(\begin{array}{l}1+\frac{\left[\text{pMBL}_3\text{MASP1}_{\text{zym}}\right]}{K_{\text{m MASP1}_{\text{zym}}}^{\text{MASP1}_{\text{zym}}}}+\frac{\left[\text{pMBL}_3\text{MASP2}_{\text{zym}}\right]}{K_{\text{m MASP1}_{\text{zym}}}^{\text{MASP2}_{\text{zym}}}}+\frac{\left[\text{pMBL}_4\text{MASP1}_{\text{zym}}\right]}{K_{\text{m MASP1}_{\text{zym}}}^{\text{MASP1}_{\text{zym}}}}+\frac{\left[\text{pMBL}_4\text{MASP2}_{\text{zym}}\right]}{K_{\text{m MASP1}_{\text{zym}}}^{\text{MASP2}_{\text{zym}}}}\\+\frac{\left[\text{pCLLKMASP1}_{\text{zym}}\right]}{K_{\text{m MASP1}_{\text{zym}}}^{\text{MASP1}_{\text{zym}}}}+\frac{\left[\text{pCLLKMASP2}_{\text{zym}}\right]}{K_{\text{m MASP1}_{\text{zym}}}^{\text{MASP2}_{\text{zym}}}}+\frac{\left[\text{pCLK1MASP1}_{\text{zym}}\right]}{K_{\text{m MASP1}_{\text{zym}}}^{\text{MASP1}_{\text{zym}}}}+\frac{\left[\text{pCLK1MASP2}_{\text{zym}}\right]}{K_{\text{m MASP1}_{\text{zym}}}^{\text{MASP2}_{\text{zym}}}}\\+\frac{\left[\text{pCLL1MASP1}_{\text{zym}}\right]}{K_{\text{m MASP1}_{\text{zym}}}^{\text{MASP1}_{\text{zym}}}}+\frac{\left[\text{pCLL1MASP2}_{\text{zym}}\right]}{K_{\text{m MASP1}_{\text{zym}}}^{\text{MASP2}_{\text{zym}}}}+\frac{\left[\text{pLFMASP1}_{\text{zym}}\right]}{K_{\text{m MASP1}_{\text{zym}}}^{\text{MASP1}_{\text{zym}}}}+\frac{\left[\text{pLFMASP2}_{\text{zym}}\right]}{K_{\text{m MASP1}_{\text{zym}}}^{\text{MASP2}_{\text{zym}}}}\\+\frac{\left[\text{pHFMASP1}_{\text{zym}}\right]}{K_{\text{m MASP1}_{\text{zym}}}^{\text{MASP1}_{\text{zym}}}}+\frac{\left[\text{pHFMASP2}_{\text{zym}}\right]}{K_{\text{m MASP1}_{\text{zym}}}^{\text{MASP2}_{\text{zym}}}}+\frac{\left[\text{pMFMASP1}_{\text{zym}}\right]}{K_{\text{m MASP1}_{\text{zym}}}^{\text{MASP1}_{\text{zym}}}}+\frac{\left[\text{pMFMASP2}_{\text{zym}}\right]}{K_{\text{m MASP1}_{\text{zym}}}^{\text{MASP2}_{\text{zym}}}}\end{array}\right)}$$

$$+\frac{k_{\text{cat MASP1}_{\text{zym}}}^{\text{MASP1}_{\text{zym}}}\left[\text{pMFMASP1}_{\text{zym}}\right]\left[\text{pMBL}_4\text{MASP1}_{\text{zym}}\right]}{K_{\text{m MASP1}_{\text{zym}}}^{\text{MASP1}_{\text{zym}}}\left(\begin{array}{l}1+\frac{\left[\text{pMBL}_3\text{MASP1}_{\text{zym}}\right]}{K_{\text{m MASP1}_{\text{zym}}}^{\text{MASP1}_{\text{zym}}}}+\frac{\left[\text{pMBL}_3\text{MASP2}_{\text{zym}}\right]}{K_{\text{m MASP1}_{\text{zym}}}^{\text{MASP2}_{\text{zym}}}}+\frac{\left[\text{pMBL}_4\text{MASP1}_{\text{zym}}\right]}{K_{\text{m MASP1}_{\text{zym}}}^{\text{MASP1}_{\text{zym}}}}+\frac{\left[\text{pMBL}_4\text{MASP2}_{\text{zym}}\right]}{K_{\text{m MASP1}_{\text{zym}}}^{\text{MASP2}_{\text{zym}}}}\\+\frac{\left[\text{pCLLKMASP1}_{\text{zym}}\right]}{K_{\text{m MASP1}_{\text{zym}}}^{\text{MASP1}_{\text{zym}}}}+\frac{\left[\text{pCLLKMASP2}_{\text{zym}}\right]}{K_{\text{m MASP1}_{\text{zym}}}^{\text{MASP2}_{\text{zym}}}}+\frac{\left[\text{pCLK1MASP1}_{\text{zym}}\right]}{K_{\text{m MASP1}_{\text{zym}}}^{\text{MASP1}_{\text{zym}}}}+\frac{\left[\text{pCLK1MASP2}_{\text{zym}}\right]}{K_{\text{m MASP1}_{\text{zym}}}^{\text{MASP2}_{\text{zym}}}}\\+\frac{\left[\text{pCLL1MASP1}_{\text{zym}}\right]}{K_{\text{m MASP1}_{\text{zym}}}^{\text{MASP1}_{\text{zym}}}}+\frac{\left[\text{pCLL1MASP2}_{\text{zym}}\right]}{K_{\text{m MASP1}_{\text{zym}}}^{\text{MASP2}_{\text{zym}}}}+\frac{\left[\text{pLFMASP1}_{\text{zym}}\right]}{K_{\text{m MASP1}_{\text{zym}}}^{\text{MASP1}_{\text{zym}}}}+\frac{\left[\text{pLFMASP2}_{\text{zym}}\right]}{K_{\text{m MASP1}_{\text{zym}}}^{\text{MASP2}_{\text{zym}}}}\\+\frac{\left[\text{pHFMASP1}_{\text{zym}}\right]}{K_{\text{m MASP1}_{\text{zym}}}^{\text{MASP1}_{\text{zym}}}}+\frac{\left[\text{pHFMASP2}_{\text{zym}}\right]}{K_{\text{m MASP1}_{\text{zym}}}^{\text{MASP2}_{\text{zym}}}}+\frac{\left[\text{pMFMASP1}_{\text{zym}}\right]}{K_{\text{m MASP1}_{\text{zym}}}^{\text{MASP1}_{\text{zym}}}}+\frac{\left[\text{pMFMASP2}_{\text{zym}}\right]}{K_{\text{m MASP1}_{\text{zym}}}^{\text{MASP2}_{\text{zym}}}}\end{array}\right)}$$

$$+\frac{k_{\text{cat MASP1}_{\text{zym}}}^{\text{MASP1}_{\text{zym}}}\left[\text{pPTX3LFMASP1}_{\text{zym}}\right]\left[\text{pMBL}_4\text{MASP1}_{\text{zym}}\right]}{K_{\text{m MASP1}_{\text{zym}}}^{\text{MASP1}_{\text{zym}}}\left(\begin{array}{l}1+\frac{\left[\text{pMBL}_3\text{MASP1}_{\text{zym}}\right]}{K_{\text{m MASP1}_{\text{zym}}}^{\text{MASP1}_{\text{zym}}}}+\frac{\left[\text{pMBL}_3\text{MASP2}_{\text{zym}}\right]}{K_{\text{m MASP1}_{\text{zym}}}^{\text{MASP2}_{\text{zym}}}}+\frac{\left[\text{pMBL}_4\text{MASP1}_{\text{zym}}\right]}{K_{\text{m MASP1}_{\text{zym}}}^{\text{MASP1}_{\text{zym}}}}+\frac{\left[\text{pMBL}_4\text{MASP2}_{\text{zym}}\right]}{K_{\text{m MASP1}_{\text{zym}}}^{\text{MASP2}_{\text{zym}}}}\\+\frac{\left[\text{pCLLKMASP1}_{\text{zym}}\right]}{K_{\text{m MASP1}_{\text{zym}}}^{\text{MASP1}_{\text{zym}}}}+\frac{\left[\text{pCLLKMASP2}_{\text{zym}}\right]}{K_{\text{m MASP1}_{\text{zym}}}^{\text{MASP2}_{\text{zym}}}}+\frac{\left[\text{pCLK1MASP1}_{\text{zym}}\right]}{K_{\text{m MASP1}_{\text{zym}}}^{\text{MASP1}_{\text{zym}}}}+\frac{\left[\text{pCLK1MASP2}_{\text{zym}}\right]}{K_{\text{m MASP1}_{\text{zym}}}^{\text{MASP2}_{\text{zym}}}}\\+\frac{\left[\text{pCLL1MASP1}_{\text{zym}}\right]}{K_{\text{m MASP1}_{\text{zym}}}^{\text{MASP1}_{\text{zym}}}}+\frac{\left[\text{pCLL1MASP2}_{\text{zym}}\right]}{K_{\text{m MASP1}_{\text{zym}}}^{\text{MASP2}_{\text{zym}}}}+\frac{\left[\text{pLFMASP1}_{\text{zym}}\right]}{K_{\text{m MASP1}_{\text{zym}}}^{\text{MASP1}_{\text{zym}}}}+\frac{\left[\text{pLFMASP2}_{\text{zym}}\right]}{K_{\text{m MASP1}_{\text{zym}}}^{\text{MASP2}_{\text{zym}}}}\\+\frac{\left[\text{pHFMASP1}_{\text{zym}}\right]}{K_{\text{m MASP1}_{\text{zym}}}^{\text{MASP1}_{\text{zym}}}}+\frac{\left[\text{pHFMASP2}_{\text{zym}}\right]}{K_{\text{m MASP1}_{\text{zym}}}^{\text{MASP2}_{\text{zym}}}}+\frac{\left[\text{pMFMASP1}_{\text{zym}}\right]}{K_{\text{m MASP1}_{\text{zym}}}^{\text{MASP1}_{\text{zym}}}}+\frac{\left[\text{pMFMASP2}_{\text{zym}}\right]}{K_{\text{m MASP1}_{\text{zym}}}^{\text{MASP2}_{\text{zym}}}}\\+\frac{\left[\text{pPTX3LFMASP1}_{\text{zym}}\right]}{K_{\text{m MASP1}_{\text{zym}}}^{\text{MASP1}_{\text{zym}}}}+\frac{\left[\text{pPTX3LFMASP2}_{\text{zym}}\right]}{K_{\text{m MASP1}_{\text{zym}}}^{\text{MASP2}_{\text{zym}}}}\end{array}\right)}$$

$$
+\frac{k_{\text{cat MASP1}^*}^{\text{MASP1}_{\text{zym}}}\left[\text{pMBL}_3\text{MASP1}^*\right]\left[\text{pMBL}_4\text{MASP1}_{\text{zym}}\right]}{K_{\text{m MASP1}^*}^{\text{MASP1}_{\text{zym}}}\left(\begin{array}{l}1+\frac{[\text{C4}]}{K_{\text{m MASP1}^*}^{\text{C4}}}+\frac{[\text{fC4bC2}]}{K_{\text{m MASP1}^*}^{\text{C2}}}+\frac{[\text{fC4bC4bC2}]}{K_{\text{m MASP1}^*}^{\text{C2}}}+\frac{[\text{fC3bC4bC2}]}{K_{\text{m MASP1}^*}^{\text{C2}}}+\frac{[\text{pC4bC2}]}{K_{\text{m MASP1}^*}^{\text{C2}}}+\frac{[\text{pC4bC4bC2}]}{K_{\text{m MASP1}^*}^{\text{C2}}}\\
+\frac{[\text{pC3bC4bC2}]}{K_{\text{m MASP1}^*}^{\text{C2}}}+\frac{\left[\text{pMBL}_3\text{MASP1}_{\text{zym}}\right]}{K_{\text{m MASP1}^*}^{\text{MASP1}_{\text{zym}}}}+\frac{\left[\text{pMBL}_3\text{MASP2}_{\text{zym}}\right]}{K_{\text{m MASP1}^*}^{\text{MASP2}_{\text{zym}}}}+\frac{\left[\text{pMBL}_3\text{MASP3}_{\text{zym}}\right]}{K_{\text{m MASP1}^*}^{\text{MASP3}_{\text{zym}}}}\\
+\frac{\left[\text{pMBL}_4\text{MASP1}_{\text{zym}}\right]}{K_{\text{m MASP1}^*}^{\text{MASP1}_{\text{zym}}}}+\frac{\left[\text{pMBL}_4\text{MASP2}_{\text{zym}}\right]}{K_{\text{m MASP1}^*}^{\text{MASP2}_{\text{zym}}}}+\frac{\left[\text{pMBL}_4\text{MASP3}_{\text{zym}}\right]}{K_{\text{m MASP1}^*}^{\text{MASP3}_{\text{zym}}}}+\frac{\left[\text{pCLLKMASP1}_{\text{zym}}\right]}{K_{\text{m MASP1}^*}^{\text{MASP1}_{\text{zym}}}}\\
+\frac{\left[\text{pCLLKMASP2}_{\text{zym}}\right]}{K_{\text{m MASP1}^*}^{\text{MASP2}_{\text{zym}}}}+\frac{\left[\text{pCLLKMASP3}_{\text{zym}}\right]}{K_{\text{m MASP1}^*}^{\text{MASP3}_{\text{zym}}}}+\frac{\left[\text{pCLK1MASP1}_{\text{zym}}\right]}{K_{\text{m MASP1}^*}^{\text{MASP1}_{\text{zym}}}}+\frac{\left[\text{pCLK1MASP2}_{\text{zym}}\right]}{K_{\text{m MASP1}^*}^{\text{MASP2}_{\text{zym}}}}\\
+\frac{\left[\text{pCLK1MASP3}_{\text{zym}}\right]}{K_{\text{m MASP1}^*}^{\text{MASP3}_{\text{zym}}}}+\frac{\left[\text{pCLL1MASP1}_{\text{zym}}\right]}{K_{\text{m MASP1}^*}^{\text{MASP1}_{\text{zym}}}}+\frac{\left[\text{pCLL1MASP2}_{\text{zym}}\right]}{K_{\text{m MASP1}^*}^{\text{MASP2}_{\text{zym}}}}+\frac{\left[\text{pCLL1MASP3}_{\text{zym}}\right]}{K_{\text{m MASP1}^*}^{\text{MASP3}_{\text{zym}}}}\\
+\frac{\left[\text{pLFMASP1}_{\text{zym}}\right]}{K_{\text{m MASP1}^*}^{\text{MASP1}_{\text{zym}}}}+\frac{\left[\text{pLFMASP2}_{\text{zym}}\right]}{K_{\text{m MASP1}^*}^{\text{MASP2}_{\text{zym}}}}+\frac{\left[\text{pLFMASP3}_{\text{zym}}\right]}{K_{\text{m MASP1}^*}^{\text{MASP3}_{\text{zym}}}}+\frac{\left[\text{pHFMASP1}_{\text{zym}}\right]}{K_{\text{m MASP1}^*}^{\text{MASP1}_{\text{zym}}}}+\frac{\left[\text{pHFMASP2}_{\text{zym}}\right]}{K_{\text{m MASP1}^*}^{\text{MASP2}_{\text{zym}}}}\\
+\frac{\left[\text{pHFMASP3}_{\text{zym}}\right]}{K_{\text{m MASP1}^*}^{\text{MASP3}_{\text{zym}}}}+\frac{\left[\text{pMFMASP1}_{\text{zym}}\right]}{K_{\text{m MASP1}^*}^{\text{MASP1}_{\text{zym}}}}+\frac{\left[\text{pMFMASP2}_{\text{zym}}\right]}{K_{\text{m MASP1}^*}^{\text{MASP2}_{\text{zym}}}}+\frac{\left[\text{pMFMASP3}_{\text{zym}}\right]}{K_{\text{m MASP1}^*}^{\text{MASP3}_{\text{zym}}}}\end{array}\right)}
$$

$$
+\frac{k_{\mathrm{cat\ MASP1^*}}^{\mathrm{MASP1_{zym}}}\left[\mathrm{pMBL_4MASP1^*}\right]\left[\mathrm{pMBL_4MASP1_{zym}}\right]}{K_{\mathrm{m\ MASP1^*}}^{\mathrm{MASP1_{zym}}}\left(\begin{aligned}&1+\frac{[\mathrm{C4}]}{K_{\mathrm{m\ MASP1^*}}^{\mathrm{C4}}}+\frac{[\mathrm{fC4bC2}]}{K_{\mathrm{m\ MASP1^*}}^{\mathrm{C2}}}+\frac{[\mathrm{fC4bC4bC2}]}{K_{\mathrm{m\ MASP1^*}}^{\mathrm{C2}}}+\frac{[\mathrm{fC3bC4bC2}]}{K_{\mathrm{m\ MASP1^*}}^{\mathrm{C2}}}+\frac{[\mathrm{pC4bC2}]}{K_{\mathrm{m\ MASP1^*}}^{\mathrm{C2}}}+\frac{[\mathrm{pC4bC4bC2}]}{K_{\mathrm{m\ MASP1^*}}^{\mathrm{C2}}}\\
&+\frac{[\mathrm{pC3bC4bC2}]}{K_{\mathrm{m\ MASP1^*}}^{\mathrm{C2}}}+\frac{\left[\mathrm{pMBL_3MASP1_{zym}}\right]}{K_{\mathrm{m\ MASP1^*}}^{\mathrm{MASP1_{zym}}}}+\frac{\left[\mathrm{pMBL_3MASP2_{zym}}\right]}{K_{\mathrm{m\ MASP1^*}}^{\mathrm{MASP2_{zym}}}}+\frac{\left[\mathrm{pMBL_3MASP3_{zym}}\right]}{K_{\mathrm{m\ MASP1^*}}^{\mathrm{MASP3_{zym}}}}\\
&+\frac{\left[\mathrm{pMBL_4MASP1_{zym}}\right]}{K_{\mathrm{m\ MASP1^*}}^{\mathrm{MASP1_{zym}}}}+\frac{\left[\mathrm{pMBL_4MASP2_{zym}}\right]}{K_{\mathrm{m\ MASP1^*}}^{\mathrm{MASP2_{zym}}}}+\frac{\left[\mathrm{pMBL_4MASP3_{zym}}\right]}{K_{\mathrm{m\ MASP1^*}}^{\mathrm{MASP3_{zym}}}}+\frac{\left[\mathrm{pCLLKMASP1_{zym}}\right]}{K_{\mathrm{m\ MASP1^*}}^{\mathrm{MASP1_{zym}}}}\\
&+\frac{\left[\mathrm{pCLLKMASP2_{zym}}\right]}{K_{\mathrm{m\ MASP1^*}}^{\mathrm{MASP2_{zym}}}}+\frac{\left[\mathrm{pCLLKMASP3_{zym}}\right]}{K_{\mathrm{m\ MASP1^*}}^{\mathrm{MASP3_{zym}}}}+\frac{\left[\mathrm{pCLK1MASP1_{zym}}\right]}{K_{\mathrm{m\ MASP1^*}}^{\mathrm{MASP1_{zym}}}}+\frac{\left[\mathrm{pCLK1MASP2_{zym}}\right]}{K_{\mathrm{m\ MASP1^*}}^{\mathrm{MASP2_{zym}}}}\\
&+\frac{\left[\mathrm{pCLK1MASP3_{zym}}\right]}{K_{\mathrm{m\ MASP1^*}}^{\mathrm{MASP3_{zym}}}}+\frac{\left[\mathrm{pCLL1MASP1_{zym}}\right]}{K_{\mathrm{m\ MASP1^*}}^{\mathrm{MASP1_{zym}}}}+\frac{\left[\mathrm{pCLL1MASP2_{zym}}\right]}{K_{\mathrm{m\ MASP1^*}}^{\mathrm{MASP2_{zym}}}}+\frac{\left[\mathrm{pCLL1MASP3_{zym}}\right]}{K_{\mathrm{m\ MASP1^*}}^{\mathrm{MASP3_{zym}}}}\\
&+\frac{\left[\mathrm{pLFMASP1_{zym}}\right]}{K_{\mathrm{m\ MASP1^*}}^{\mathrm{MASP1_{zym}}}}+\frac{\left[\mathrm{pLFMASP2_{zym}}\right]}{K_{\mathrm{m\ MASP1^*}}^{\mathrm{MASP2_{zym}}}}+\frac{\left[\mathrm{pLFMASP3_{zym}}\right]}{K_{\mathrm{m\ MASP1^*}}^{\mathrm{MASP3_{zym}}}}+\frac{\left[\mathrm{pHFMASP1_{zym}}\right]}{K_{\mathrm{m\ MASP1^*}}^{\mathrm{MASP1_{zym}}}}+\frac{\left[\mathrm{pHFMASP2_{zym}}\right]}{K_{\mathrm{m\ MASP1^*}}^{\mathrm{MASP2_{zym}}}}\\
&+\frac{\left[\mathrm{pHFMASP3_{zym}}\right]}{K_{\mathrm{m\ MASP1^*}}^{\mathrm{MASP3_{zym}}}}+\frac{\left[\mathrm{pMFMASP1_{zym}}\right]}{K_{\mathrm{m\ MASP1^*}}^{\mathrm{MASP1_{zym}}}}+\frac{\left[\mathrm{pMFMASP2_{zym}}\right]}{K_{\mathrm{m\ MASP1^*}}^{\mathrm{MASP2_{zym}}}}+\frac{\left[\mathrm{pMFMASP3_{zym}}\right]}{K_{\mathrm{m\ MASP1^*}}^{\mathrm{MASP3_{zym}}}}\end{aligned}\right)}
$$

$$
+\frac{k_{\mathrm{cat\ MASP1^*}}^{\mathrm{MASP1_{zym}}}\left[\mathrm{pCLLKMASP1^*}\right]\left[\mathrm{pMBL_4MASP1_{zym}}\right]}{K_{\mathrm{m\ MASP1^*}}^{\mathrm{MASP1_{zym}}}\left(\begin{aligned}&1+\frac{[\mathrm{C4}]}{K_{\mathrm{m\ MASP1^*}}^{\mathrm{C4}}}+\frac{[\mathrm{fC4bC2}]}{K_{\mathrm{m\ MASP1^*}}^{\mathrm{C2}}}+\frac{[\mathrm{fC4bC4bC2}]}{K_{\mathrm{m\ MASP1^*}}^{\mathrm{C2}}}+\frac{[\mathrm{fC3bC4bC2}]}{K_{\mathrm{m\ MASP1^*}}^{\mathrm{C2}}}+\frac{[\mathrm{pC4bC2}]}{K_{\mathrm{m\ MASP1^*}}^{\mathrm{C2}}}+\frac{[\mathrm{pC4bC4bC2}]}{K_{\mathrm{m\ MASP1^*}}^{\mathrm{C2}}}\\
&+\frac{[\mathrm{pC3bC4bC2}]}{K_{\mathrm{m\ MASP1^*}}^{\mathrm{C2}}}+\frac{\left[\mathrm{pMBL_3MASP1_{zym}}\right]}{K_{\mathrm{m\ MASP1^*}}^{\mathrm{MASP1_{zym}}}}+\frac{\left[\mathrm{pMBL_3MASP2_{zym}}\right]}{K_{\mathrm{m\ MASP1^*}}^{\mathrm{MASP2_{zym}}}}+\frac{\left[\mathrm{pMBL_3MASP3_{zym}}\right]}{K_{\mathrm{m\ MASP1^*}}^{\mathrm{MASP3_{zym}}}}\\
&+\frac{\left[\mathrm{pMBL_4MASP1_{zym}}\right]}{K_{\mathrm{m\ MASP1^*}}^{\mathrm{MASP1_{zym}}}}+\frac{\left[\mathrm{pMBL_4MASP2_{zym}}\right]}{K_{\mathrm{m\ MASP1^*}}^{\mathrm{MASP2_{zym}}}}+\frac{\left[\mathrm{pMBL_4MASP3_{zym}}\right]}{K_{\mathrm{m\ MASP1^*}}^{\mathrm{MASP3_{zym}}}}+\frac{\left[\mathrm{pCLLKMASP1_{zym}}\right]}{K_{\mathrm{m\ MASP1^*}}^{\mathrm{MASP1_{zym}}}}\\
&+\frac{\left[\mathrm{pCLLKMASP2_{zym}}\right]}{K_{\mathrm{m\ MASP1^*}}^{\mathrm{MASP2_{zym}}}}+\frac{\left[\mathrm{pCLLKMASP3_{zym}}\right]}{K_{\mathrm{m\ MASP1^*}}^{\mathrm{MASP3_{zym}}}}+\frac{\left[\mathrm{pCLK1MASP1_{zym}}\right]}{K_{\mathrm{m\ MASP1^*}}^{\mathrm{MASP1_{zym}}}}+\frac{\left[\mathrm{pCLK1MASP2_{zym}}\right]}{K_{\mathrm{m\ MASP1^*}}^{\mathrm{MASP2_{zym}}}}\\
&+\frac{\left[\mathrm{pCLK1MASP3_{zym}}\right]}{K_{\mathrm{m\ MASP1^*}}^{\mathrm{MASP3_{zym}}}}+\frac{\left[\mathrm{pCLL1MASP1_{zym}}\right]}{K_{\mathrm{m\ MASP1^*}}^{\mathrm{MASP1_{zym}}}}+\frac{\left[\mathrm{pCLL1MASP2_{zym}}\right]}{K_{\mathrm{m\ MASP1^*}}^{\mathrm{MASP2_{zym}}}}+\frac{\left[\mathrm{pCLL1MASP3_{zym}}\right]}{K_{\mathrm{m\ MASP1^*}}^{\mathrm{MASP3_{zym}}}}\\
&+\frac{\left[\mathrm{pLFMASP1_{zym}}\right]}{K_{\mathrm{m\ MASP1^*}}^{\mathrm{MASP1_{zym}}}}+\frac{\left[\mathrm{pLFMASP2_{zym}}\right]}{K_{\mathrm{m\ MASP1^*}}^{\mathrm{MASP2_{zym}}}}+\frac{\left[\mathrm{pLFMASP3_{zym}}\right]}{K_{\mathrm{m\ MASP1^*}}^{\mathrm{MASP3_{zym}}}}+\frac{\left[\mathrm{pHFMASP1_{zym}}\right]}{K_{\mathrm{m\ MASP1^*}}^{\mathrm{MASP1_{zym}}}}+\frac{\left[\mathrm{pHFMASP2_{zym}}\right]}{K_{\mathrm{m\ MASP1^*}}^{\mathrm{MASP2_{zym}}}}\\
&+\frac{\left[\mathrm{pHFMASP3_{zym}}\right]}{K_{\mathrm{m\ MASP1^*}}^{\mathrm{MASP3_{zym}}}}+\frac{\left[\mathrm{pMFMASP1_{zym}}\right]}{K_{\mathrm{m\ MASP1^*}}^{\mathrm{MASP1_{zym}}}}+\frac{\left[\mathrm{pMFMASP2_{zym}}\right]}{K_{\mathrm{m\ MASP1^*}}^{\mathrm{MASP2_{zym}}}}+\frac{\left[\mathrm{pMFMASP3_{zym}}\right]}{K_{\mathrm{m\ MASP1^*}}^{\mathrm{MASP3_{zym}}}}\end{aligned}\right)}
$$

$$+\frac{k_{\mathrm{cat\ MASP1^{*}}}^{\mathrm{MASP1_{zym}}}\left[\mathrm{pCLK1MASP1^{*}}\right]\left[\mathrm{pMBL_{4}MASP1_{zym}}\right]}{K_{\mathrm{m\ MASP1^{*}}}^{\mathrm{MASP1_{zym}}}\left(\begin{aligned}&1+\frac{[\mathrm{C4}]}{K_{\mathrm{m\ MASP1^{*}}}^{\mathrm{C4}}}+\frac{[\mathrm{fC4bC2}]}{K_{\mathrm{m\ MASP1^{*}}}^{\mathrm{C2}}}+\frac{[\mathrm{fC4bC4bC2}]}{K_{\mathrm{m\ MASP1^{*}}}^{\mathrm{C2}}}+\frac{[\mathrm{fC3bC4bC2}]}{K_{\mathrm{m\ MASP1^{*}}}^{\mathrm{C2}}}+\frac{[\mathrm{pC4bC2}]}{K_{\mathrm{m\ MASP1^{*}}}^{\mathrm{C2}}}+\frac{[\mathrm{pC4bC4bC2}]}{K_{\mathrm{m\ MASP1^{*}}}^{\mathrm{C2}}}\\&+\frac{[\mathrm{pC3bC4bC2}]}{K_{\mathrm{m\ MASP1^{*}}}^{\mathrm{C2}}}+\frac{[\mathrm{pMBL_{3}MASP1_{zym}}]}{K_{\mathrm{m\ MASP1^{*}}}^{\mathrm{MASP1_{zym}}}}+\frac{[\mathrm{pMBL_{3}MASP2_{zym}}]}{K_{\mathrm{m\ MASP1^{*}}}^{\mathrm{MASP2_{zym}}}}+\frac{[\mathrm{pMBL_{3}MASP3_{zym}}]}{K_{\mathrm{m\ MASP1^{*}}}^{\mathrm{MASP3_{zym}}}}\\&+\frac{[\mathrm{pMBL_{4}MASP1_{zym}}]}{K_{\mathrm{m\ MASP1^{*}}}^{\mathrm{MASP1_{zym}}}}+\frac{[\mathrm{pMBL_{4}MASP2_{zym}}]}{K_{\mathrm{m\ MASP1^{*}}}^{\mathrm{MASP2_{zym}}}}+\frac{[\mathrm{pMBL_{4}MASP3_{zym}}]}{K_{\mathrm{m\ MASP1^{*}}}^{\mathrm{MASP3_{zym}}}}+\frac{[\mathrm{pCLLKMASP1_{zym}}]}{K_{\mathrm{m\ MASP1^{*}}}^{\mathrm{MASP1_{zym}}}}\\&+\frac{[\mathrm{pCLLKMASP2_{zym}}]}{K_{\mathrm{m\ MASP1^{*}}}^{\mathrm{MASP2_{zym}}}}+\frac{[\mathrm{pCLLKMASP3_{zym}}]}{K_{\mathrm{m\ MASP1^{*}}}^{\mathrm{MASP3_{zym}}}}+\frac{[\mathrm{pCLK1MASP1_{zym}}]}{K_{\mathrm{m\ MASP1^{*}}}^{\mathrm{MASP1_{zym}}}}+\frac{[\mathrm{pCLK1MASP2_{zym}}]}{K_{\mathrm{m\ MASP1^{*}}}^{\mathrm{MASP2_{zym}}}}\\&+\frac{[\mathrm{pCLK1MASP3_{zym}}]}{K_{\mathrm{m\ MASP1^{*}}}^{\mathrm{MASP3_{zym}}}}+\frac{[\mathrm{pCLL1MASP1_{zym}}]}{K_{\mathrm{m\ MASP1^{*}}}^{\mathrm{MASP1_{zym}}}}+\frac{[\mathrm{pCLL1MASP2_{zym}}]}{K_{\mathrm{m\ MASP1^{*}}}^{\mathrm{MASP2_{zym}}}}+\frac{[\mathrm{pCLL1MASP3_{zym}}]}{K_{\mathrm{m\ MASP1^{*}}}^{\mathrm{MASP3_{zym}}}}\\&+\frac{[\mathrm{pLFMASP1_{zym}}]}{K_{\mathrm{m\ MASP1^{*}}}^{\mathrm{MASP1_{zym}}}}+\frac{[\mathrm{pLFMASP2_{zym}}]}{K_{\mathrm{m\ MASP1^{*}}}^{\mathrm{MASP2_{zym}}}}+\frac{[\mathrm{pLFMASP3_{zym}}]}{K_{\mathrm{m\ MASP1^{*}}}^{\mathrm{MASP3_{zym}}}}+\frac{[\mathrm{pHFMASP1_{zym}}]}{K_{\mathrm{m\ MASP1^{*}}}^{\mathrm{MASP1_{zym}}}}+\frac{[\mathrm{pHFMASP2_{zym}}]}{K_{\mathrm{m\ MASP1^{*}}}^{\mathrm{MASP2_{zym}}}}\\&+\frac{[\mathrm{pHFMASP3_{zym}}]}{K_{\mathrm{m\ MASP1^{*}}}^{\mathrm{MASP3_{zym}}}}+\frac{[\mathrm{pMFMASP1_{zym}}]}{K_{\mathrm{m\ MASP1^{*}}}^{\mathrm{MASP1_{zym}}}}+\frac{[\mathrm{pMFMASP2_{zym}}]}{K_{\mathrm{m\ MASP1^{*}}}^{\mathrm{MASP2_{zym}}}}+\frac{[\mathrm{pMFMASP3_{zym}}]}{K_{\mathrm{m\ MASP1^{*}}}^{\mathrm{MASP3_{zym}}}}\end{aligned}\right)}$$

$$+\frac{k_{\mathrm{cat\ MASP1^{*}}}^{\mathrm{MASP1_{zym}}}\left[\mathrm{pCLL1MASP1^{*}}\right]\left[\mathrm{pMBL_{4}MASP1_{zym}}\right]}{K_{\mathrm{m\ MASP1^{*}}}^{\mathrm{MASP1_{zym}}}\left(\begin{aligned}&1+\frac{[\mathrm{C4}]}{K_{\mathrm{m\ MASP1^{*}}}^{\mathrm{C4}}}+\frac{[\mathrm{fC4bC2}]}{K_{\mathrm{m\ MASP1^{*}}}^{\mathrm{C2}}}+\frac{[\mathrm{fC4bC4bC2}]}{K_{\mathrm{m\ MASP1^{*}}}^{\mathrm{C2}}}+\frac{[\mathrm{fC3bC4bC2}]}{K_{\mathrm{m\ MASP1^{*}}}^{\mathrm{C2}}}+\frac{[\mathrm{pC4bC2}]}{K_{\mathrm{m\ MASP1^{*}}}^{\mathrm{C2}}}+\frac{[\mathrm{pC4bC4bC2}]}{K_{\mathrm{m\ MASP1^{*}}}^{\mathrm{C2}}}\\&+\frac{[\mathrm{pC3bC4bC2}]}{K_{\mathrm{m\ MASP1^{*}}}^{\mathrm{C2}}}+\frac{[\mathrm{pMBL_{3}MASP1_{zym}}]}{K_{\mathrm{m\ MASP1^{*}}}^{\mathrm{MASP1_{zym}}}}+\frac{[\mathrm{pMBL_{3}MASP2_{zym}}]}{K_{\mathrm{m\ MASP1^{*}}}^{\mathrm{MASP2_{zym}}}}+\frac{[\mathrm{pMBL_{3}MASP3_{zym}}]}{K_{\mathrm{m\ MASP1^{*}}}^{\mathrm{MASP3_{zym}}}}\\&+\frac{[\mathrm{pMBL_{4}MASP1_{zym}}]}{K_{\mathrm{m\ MASP1^{*}}}^{\mathrm{MASP1_{zym}}}}+\frac{[\mathrm{pMBL_{4}MASP2_{zym}}]}{K_{\mathrm{m\ MASP1^{*}}}^{\mathrm{MASP2_{zym}}}}+\frac{[\mathrm{pMBL_{4}MASP3_{zym}}]}{K_{\mathrm{m\ MASP1^{*}}}^{\mathrm{MASP3_{zym}}}}+\frac{[\mathrm{pCLLKMASP1_{zym}}]}{K_{\mathrm{m\ MASP1^{*}}}^{\mathrm{MASP1_{zym}}}}\\&+\frac{[\mathrm{pCLLKMASP2_{zym}}]}{K_{\mathrm{m\ MASP1^{*}}}^{\mathrm{MASP2_{zym}}}}+\frac{[\mathrm{pCLLKMASP3_{zym}}]}{K_{\mathrm{m\ MASP1^{*}}}^{\mathrm{MASP3_{zym}}}}+\frac{[\mathrm{pCLK1MASP1_{zym}}]}{K_{\mathrm{m\ MASP1^{*}}}^{\mathrm{MASP1_{zym}}}}+\frac{[\mathrm{pCLK1MASP2_{zym}}]}{K_{\mathrm{m\ MASP1^{*}}}^{\mathrm{MASP2_{zym}}}}\\&+\frac{[\mathrm{pCLK1MASP3_{zym}}]}{K_{\mathrm{m\ MASP1^{*}}}^{\mathrm{MASP3_{zym}}}}+\frac{[\mathrm{pCLL1MASP1_{zym}}]}{K_{\mathrm{m\ MASP1^{*}}}^{\mathrm{MASP1_{zym}}}}+\frac{[\mathrm{pCLL1MASP2_{zym}}]}{K_{\mathrm{m\ MASP1^{*}}}^{\mathrm{MASP2_{zym}}}}+\frac{[\mathrm{pCLL1MASP3_{zym}}]}{K_{\mathrm{m\ MASP1^{*}}}^{\mathrm{MASP3_{zym}}}}\\&+\frac{[\mathrm{pLFMASP1_{zym}}]}{K_{\mathrm{m\ MASP1^{*}}}^{\mathrm{MASP1_{zym}}}}+\frac{[\mathrm{pLFMASP2_{zym}}]}{K_{\mathrm{m\ MASP1^{*}}}^{\mathrm{MASP2_{zym}}}}+\frac{[\mathrm{pLFMASP3_{zym}}]}{K_{\mathrm{m\ MASP1^{*}}}^{\mathrm{MASP3_{zym}}}}+\frac{[\mathrm{pHFMASP1_{zym}}]}{K_{\mathrm{m\ MASP1^{*}}}^{\mathrm{MASP1_{zym}}}}+\frac{[\mathrm{pHFMASP2_{zym}}]}{K_{\mathrm{m\ MASP1^{*}}}^{\mathrm{MASP2_{zym}}}}\\&+\frac{[\mathrm{pHFMASP3_{zym}}]}{K_{\mathrm{m\ MASP1^{*}}}^{\mathrm{MASP3_{zym}}}}+\frac{[\mathrm{pMFMASP1_{zym}}]}{K_{\mathrm{m\ MASP1^{*}}}^{\mathrm{MASP1_{zym}}}}+\frac{[\mathrm{pMFMASP2_{zym}}]}{K_{\mathrm{m\ MASP1^{*}}}^{\mathrm{MASP2_{zym}}}}+\frac{[\mathrm{pMFMASP3_{zym}}]}{K_{\mathrm{m\ MASP1^{*}}}^{\mathrm{MASP3_{zym}}}}\end{aligned}\right)}$$

$$
+\frac{k_{\mathrm{cat\ MASP1^*}}^{\mathrm{MASP1_{zym}}}\left[\mathrm{pLFMASP1^*}\right]\left[\mathrm{pMBL_4MASP1_{zym}}\right]}{K_{\mathrm{m\ MASP1^*}}^{\mathrm{MASP1_{zym}}}\left(\begin{array}{l}1+\frac{[\mathrm{C4}]}{K_{\mathrm{m\ MASP1^*}}^{\mathrm{C4}}}+\frac{[\mathrm{fC4bC2}]}{K_{\mathrm{m\ MASP1^*}}^{\mathrm{C2}}}+\frac{[\mathrm{fC4bC4bC2}]}{K_{\mathrm{m\ MASP1^*}}^{\mathrm{C2}}}+\frac{[\mathrm{fC3bC4bC2}]}{K_{\mathrm{m\ MASP1^*}}^{\mathrm{C2}}}+\frac{[\mathrm{pC4bC2}]}{K_{\mathrm{m\ MASP1^*}}^{\mathrm{C2}}}+\frac{[\mathrm{pC4bC4bC2}]}{K_{\mathrm{m\ MASP1^*}}^{\mathrm{C2}}}\\
+\frac{[\mathrm{pC3bC4bC2}]}{K_{\mathrm{m\ MASP1^*}}^{\mathrm{C2}}}+\frac{[\mathrm{pMBL_3MASP1_{zym}}]}{K_{\mathrm{m\ MASP1^*}}^{\mathrm{MASP1_{zym}}}}+\frac{[\mathrm{pMBL_3MASP2_{zym}}]}{K_{\mathrm{m\ MASP1^*}}^{\mathrm{MASP2_{zym}}}}+\frac{[\mathrm{pMBL_3MASP3_{zym}}]}{K_{\mathrm{m\ MASP1^*}}^{\mathrm{MASP3_{zym}}}}\\
+\frac{[\mathrm{pMBL_4MASP1_{zym}}]}{K_{\mathrm{m\ MASP1^*}}^{\mathrm{MASP1_{zym}}}}+\frac{[\mathrm{pMBL_4MASP2_{zym}}]}{K_{\mathrm{m\ MASP1^*}}^{\mathrm{MASP2_{zym}}}}+\frac{[\mathrm{pMBL_4MASP3_{zym}}]}{K_{\mathrm{m\ MASP1^*}}^{\mathrm{MASP3_{zym}}}}+\frac{[\mathrm{pCLLKMASP1_{zym}}]}{K_{\mathrm{m\ MASP1^*}}^{\mathrm{MASP1_{zym}}}}\\
+\frac{[\mathrm{pCLLKMASP2_{zym}}]}{K_{\mathrm{m\ MASP1^*}}^{\mathrm{MASP2_{zym}}}}+\frac{[\mathrm{pCLLKMASP3_{zym}}]}{K_{\mathrm{m\ MASP1^*}}^{\mathrm{MASP3_{zym}}}}+\frac{[\mathrm{pCLK1MASP1_{zym}}]}{K_{\mathrm{m\ MASP1^*}}^{\mathrm{MASP1_{zym}}}}+\frac{[\mathrm{pCLK1MASP2_{zym}}]}{K_{\mathrm{m\ MASP1^*}}^{\mathrm{MASP2_{zym}}}}\\
+\frac{[\mathrm{pCLK1MASP3_{zym}}]}{K_{\mathrm{m\ MASP1^*}}^{\mathrm{MASP3_{zym}}}}+\frac{[\mathrm{pCLL1MASP1_{zym}}]}{K_{\mathrm{m\ MASP1^*}}^{\mathrm{MASP1_{zym}}}}+\frac{[\mathrm{pCLL1MASP2_{zym}}]}{K_{\mathrm{m\ MASP1^*}}^{\mathrm{MASP2_{zym}}}}+\frac{[\mathrm{pCLL1MASP3_{zym}}]}{K_{\mathrm{m\ MASP1^*}}^{\mathrm{MASP3_{zym}}}}\\
+\frac{[\mathrm{pLFMASP1_{zym}}]}{K_{\mathrm{m\ MASP1^*}}^{\mathrm{MASP1_{zym}}}}+\frac{[\mathrm{pLFMASP2_{zym}}]}{K_{\mathrm{m\ MASP1^*}}^{\mathrm{MASP2_{zym}}}}+\frac{[\mathrm{pLFMASP3_{zym}}]}{K_{\mathrm{m\ MASP1^*}}^{\mathrm{MASP3_{zym}}}}+\frac{[\mathrm{pHFMASP1_{zym}}]}{K_{\mathrm{m\ MASP1^*}}^{\mathrm{MASP1_{zym}}}}+\frac{[\mathrm{pHFMASP2_{zym}}]}{K_{\mathrm{m\ MASP1^*}}^{\mathrm{MASP2_{zym}}}}\\
+\frac{[\mathrm{pHFMASP3_{zym}}]}{K_{\mathrm{m\ MASP1^*}}^{\mathrm{MASP3_{zym}}}}+\frac{[\mathrm{pMFMASP1_{zym}}]}{K_{\mathrm{m\ MASP1^*}}^{\mathrm{MASP1_{zym}}}}+\frac{[\mathrm{pMFMASP2_{zym}}]}{K_{\mathrm{m\ MASP1^*}}^{\mathrm{MASP2_{zym}}}}+\frac{[\mathrm{pMFMASP3_{zym}}]}{K_{\mathrm{m\ MASP1^*}}^{\mathrm{MASP3_{zym}}}}\end{array}\right)}
$$

$$
+\frac{k_{\mathrm{cat\ MASP1^*}}^{\mathrm{MASP1_{zym}}}\left[\mathrm{pHFMASP1^*}\right]\left[\mathrm{pMBL_4MASP1_{zym}}\right]}{K_{\mathrm{m\ MASP1^*}}^{\mathrm{MASP1_{zym}}}\left(\begin{array}{l}1+\frac{[\mathrm{C4}]}{K_{\mathrm{m\ MASP1^*}}^{\mathrm{C4}}}+\frac{[\mathrm{fC4bC2}]}{K_{\mathrm{m\ MASP1^*}}^{\mathrm{C2}}}+\frac{[\mathrm{fC4bC4bC2}]}{K_{\mathrm{m\ MASP1^*}}^{\mathrm{C2}}}+\frac{[\mathrm{fC3bC4bC2}]}{K_{\mathrm{m\ MASP1^*}}^{\mathrm{C2}}}+\frac{[\mathrm{pC4bC2}]}{K_{\mathrm{m\ MASP1^*}}^{\mathrm{C2}}}+\frac{[\mathrm{pC4bC4bC2}]}{K_{\mathrm{m\ MASP1^*}}^{\mathrm{C2}}}\\
+\frac{[\mathrm{pC3bC4bC2}]}{K_{\mathrm{m\ MASP1^*}}^{\mathrm{C2}}}+\frac{[\mathrm{pMBL_3MASP1_{zym}}]}{K_{\mathrm{m\ MASP1^*}}^{\mathrm{MASP1_{zym}}}}+\frac{[\mathrm{pMBL_3MASP2_{zym}}]}{K_{\mathrm{m\ MASP1^*}}^{\mathrm{MASP2_{zym}}}}+\frac{[\mathrm{pMBL_3MASP3_{zym}}]}{K_{\mathrm{m\ MASP1^*}}^{\mathrm{MASP3_{zym}}}}\\
+\frac{[\mathrm{pMBL_4MASP1_{zym}}]}{K_{\mathrm{m\ MASP1^*}}^{\mathrm{MASP1_{zym}}}}+\frac{[\mathrm{pMBL_4MASP2_{zym}}]}{K_{\mathrm{m\ MASP1^*}}^{\mathrm{MASP2_{zym}}}}+\frac{[\mathrm{pMBL_4MASP3_{zym}}]}{K_{\mathrm{m\ MASP1^*}}^{\mathrm{MASP3_{zym}}}}+\frac{[\mathrm{pCLLKMASP1_{zym}}]}{K_{\mathrm{m\ MASP1^*}}^{\mathrm{MASP1_{zym}}}}\\
+\frac{[\mathrm{pCLLKMASP2_{zym}}]}{K_{\mathrm{m\ MASP1^*}}^{\mathrm{MASP2_{zym}}}}+\frac{[\mathrm{pCLLKMASP3_{zym}}]}{K_{\mathrm{m\ MASP1^*}}^{\mathrm{MASP3_{zym}}}}+\frac{[\mathrm{pCLK1MASP1_{zym}}]}{K_{\mathrm{m\ MASP1^*}}^{\mathrm{MASP1_{zym}}}}+\frac{[\mathrm{pCLK1MASP2_{zym}}]}{K_{\mathrm{m\ MASP1^*}}^{\mathrm{MASP2_{zym}}}}\\
+\frac{[\mathrm{pCLK1MASP3_{zym}}]}{K_{\mathrm{m\ MASP1^*}}^{\mathrm{MASP3_{zym}}}}+\frac{[\mathrm{pCLL1MASP1_{zym}}]}{K_{\mathrm{m\ MASP1^*}}^{\mathrm{MASP1_{zym}}}}+\frac{[\mathrm{pCLL1MASP2_{zym}}]}{K_{\mathrm{m\ MASP1^*}}^{\mathrm{MASP2_{zym}}}}+\frac{[\mathrm{pCLL1MASP3_{zym}}]}{K_{\mathrm{m\ MASP1^*}}^{\mathrm{MASP3_{zym}}}}\\
+\frac{[\mathrm{pLFMASP1_{zym}}]}{K_{\mathrm{m\ MASP1^*}}^{\mathrm{MASP1_{zym}}}}+\frac{[\mathrm{pLFMASP2_{zym}}]}{K_{\mathrm{m\ MASP1^*}}^{\mathrm{MASP2_{zym}}}}+\frac{[\mathrm{pLFMASP3_{zym}}]}{K_{\mathrm{m\ MASP1^*}}^{\mathrm{MASP3_{zym}}}}+\frac{[\mathrm{pHFMASP1_{zym}}]}{K_{\mathrm{m\ MASP1^*}}^{\mathrm{MASP1_{zym}}}}+\frac{[\mathrm{pHFMASP2_{zym}}]}{K_{\mathrm{m\ MASP1^*}}^{\mathrm{MASP2_{zym}}}}\\
+\frac{[\mathrm{pHFMASP3_{zym}}]}{K_{\mathrm{m\ MASP1^*}}^{\mathrm{MASP3_{zym}}}}+\frac{[\mathrm{pMFMASP1_{zym}}]}{K_{\mathrm{m\ MASP1^*}}^{\mathrm{MASP1_{zym}}}}+\frac{[\mathrm{pMFMASP2_{zym}}]}{K_{\mathrm{m\ MASP1^*}}^{\mathrm{MASP2_{zym}}}}+\frac{[\mathrm{pMFMASP3_{zym}}]}{K_{\mathrm{m\ MASP1^*}}^{\mathrm{MASP3_{zym}}}}\end{array}\right)}
$$

$$
+\frac{k^{\mathrm{MASP1_{zym}}}_{\mathrm{cat\,MASP1^{*}}}\left[\mathrm{pMFMASP1^{*}}\right]\left[\mathrm{pMBL_4MASP1_{zym}}\right]}{K^{\mathrm{MASP1_{zym}}}_{\mathrm{m\,MASP1^{*}}}\left(\begin{aligned}&1+\frac{[\mathrm{C4}]}{K^{\mathrm{C4}}_{\mathrm{m\,MASP1^{*}}}}+\frac{[\mathrm{fC4bC2}]}{K^{\mathrm{C2}}_{\mathrm{m\,MASP1^{*}}}}+\frac{[\mathrm{fC4bC4bC2}]}{K^{\mathrm{C2}}_{\mathrm{m\,MASP1^{*}}}}+\frac{[\mathrm{fC3bC4bC2}]}{K^{\mathrm{C2}}_{\mathrm{m\,MASP1^{*}}}}+\frac{[\mathrm{pC4bC2}]}{K^{\mathrm{C2}}_{\mathrm{m\,MASP1^{*}}}}+\frac{[\mathrm{pC4bC4bC2}]}{K^{\mathrm{C2}}_{\mathrm{m\,MASP1^{*}}}}\\
&+\frac{[\mathrm{pC3bC4bC2}]}{K^{\mathrm{C2}}_{\mathrm{m\,MASP1^{*}}}}+\frac{[\mathrm{pMBL_3MASP1_{zym}}]}{K^{\mathrm{MASP1_{zym}}}_{\mathrm{m\,MASP1^{*}}}}+\frac{[\mathrm{pMBL_3MASP2_{zym}}]}{K^{\mathrm{MASP2_{zym}}}_{\mathrm{m\,MASP1^{*}}}}+\frac{[\mathrm{pMBL_3MASP3_{zym}}]}{K^{\mathrm{MASP3_{zym}}}_{\mathrm{m\,MASP1^{*}}}}\\
&+\frac{[\mathrm{pMBL_4MASP1_{zym}}]}{K^{\mathrm{MASP1_{zym}}}_{\mathrm{m\,MASP1^{*}}}}+\frac{[\mathrm{pMBL_4MASP2_{zym}}]}{K^{\mathrm{MASP2_{zym}}}_{\mathrm{m\,MASP1^{*}}}}+\frac{[\mathrm{pMBL_4MASP3_{zym}}]}{K^{\mathrm{MASP3_{zym}}}_{\mathrm{m\,MASP1^{*}}}}+\frac{[\mathrm{pCLLKMASP1_{zym}}]}{K^{\mathrm{MASP1_{zym}}}_{\mathrm{m\,MASP1^{*}}}}\\
&+\frac{[\mathrm{pCLLKMASP2_{zym}}]}{K^{\mathrm{MASP2_{zym}}}_{\mathrm{m\,MASP1^{*}}}}+\frac{[\mathrm{pCLLKMASP3_{zym}}]}{K^{\mathrm{MASP3_{zym}}}_{\mathrm{m\,MASP1^{*}}}}+\frac{[\mathrm{pCLK1MASP1_{zym}}]}{K^{\mathrm{MASP1_{zym}}}_{\mathrm{m\,MASP1^{*}}}}+\frac{[\mathrm{pCLK1MASP2_{zym}}]}{K^{\mathrm{MASP2_{zym}}}_{\mathrm{m\,MASP1^{*}}}}\\
&+\frac{[\mathrm{pCLK1MASP3_{zym}}]}{K^{\mathrm{MASP3_{zym}}}_{\mathrm{m\,MASP1^{*}}}}+\frac{[\mathrm{pCLL1MASP1_{zym}}]}{K^{\mathrm{MASP1_{zym}}}_{\mathrm{m\,MASP1^{*}}}}+\frac{[\mathrm{pCLL1MASP2_{zym}}]}{K^{\mathrm{MASP2_{zym}}}_{\mathrm{m\,MASP1^{*}}}}+\frac{[\mathrm{pCLL1MASP3_{zym}}]}{K^{\mathrm{MASP3_{zym}}}_{\mathrm{m\,MASP1^{*}}}}\\
&+\frac{[\mathrm{pLFMASP1_{zym}}]}{K^{\mathrm{MASP1_{zym}}}_{\mathrm{m\,MASP1^{*}}}}+\frac{[\mathrm{pLFMASP2_{zym}}]}{K^{\mathrm{MASP2_{zym}}}_{\mathrm{m\,MASP1^{*}}}}+\frac{[\mathrm{pLFMASP3_{zym}}]}{K^{\mathrm{MASP3_{zym}}}_{\mathrm{m\,MASP1^{*}}}}+\frac{[\mathrm{pHFMASP1_{zym}}]}{K^{\mathrm{MASP1_{zym}}}_{\mathrm{m\,MASP1^{*}}}}+\frac{[\mathrm{pHFMASP2_{zym}}]}{K^{\mathrm{MASP2_{zym}}}_{\mathrm{m\,MASP1^{*}}}}\\
&+\frac{[\mathrm{pHFMASP3_{zym}}]}{K^{\mathrm{MASP3_{zym}}}_{\mathrm{m\,MASP1^{*}}}}+\frac{[\mathrm{pMFMASP1_{zym}}]}{K^{\mathrm{MASP1_{zym}}}_{\mathrm{m\,MASP1^{*}}}}+\frac{[\mathrm{pMFMASP2_{zym}}]}{K^{\mathrm{MASP2_{zym}}}_{\mathrm{m\,MASP1^{*}}}}+\frac{[\mathrm{pMFMASP3_{zym}}]}{K^{\mathrm{MASP3_{zym}}}_{\mathrm{m\,MASP1^{*}}}}\end{aligned}\right)}
$$

$$
+\frac{k^{\mathrm{MASP1_{zym}}}_{\mathrm{cat\,MASP1^{*}}}\left[\mathrm{pPTX3LFMASP1^{*}}\right]\left[\mathrm{pMBL_4MASP1_{zym}}\right]}{K^{\mathrm{MASP1_{zym}}}_{\mathrm{m\,MASP1^{*}}}\left(\begin{aligned}&1+\frac{[\mathrm{C4}]}{K^{\mathrm{C4}}_{\mathrm{m\,MASP1^{*}}}}+\frac{[\mathrm{fC4bC2}]}{K^{\mathrm{C2}}_{\mathrm{m\,MASP1^{*}}}}+\frac{[\mathrm{fC4bC4bC2}]}{K^{\mathrm{C2}}_{\mathrm{m\,MASP1^{*}}}}+\frac{[\mathrm{fC3bC4bC2}]}{K^{\mathrm{C2}}_{\mathrm{m\,MASP1^{*}}}}+\frac{[\mathrm{pC4bC2}]}{K^{\mathrm{C2}}_{\mathrm{m\,MASP1^{*}}}}+\frac{[\mathrm{pC4bC4bC2}]}{K^{\mathrm{C2}}_{\mathrm{m\,MASP1^{*}}}}\\
&+\frac{[\mathrm{pC3bC4bC2}]}{K^{\mathrm{C2}}_{\mathrm{m\,MASP1^{*}}}}+\frac{[\mathrm{pMBL_3MASP1_{zym}}]}{K^{\mathrm{MASP1_{zym}}}_{\mathrm{m\,MASP1^{*}}}}+\frac{[\mathrm{pMBL_3MASP2_{zym}}]}{K^{\mathrm{MASP2_{zym}}}_{\mathrm{m\,MASP1^{*}}}}+\frac{[\mathrm{pMBL_3MASP3_{zym}}]}{K^{\mathrm{MASP3_{zym}}}_{\mathrm{m\,MASP1^{*}}}}\\
&+\frac{[\mathrm{pMBL_4MASP1_{zym}}]}{K^{\mathrm{MASP1_{zym}}}_{\mathrm{m\,MASP1^{*}}}}+\frac{[\mathrm{pMBL_4MASP2_{zym}}]}{K^{\mathrm{MASP2_{zym}}}_{\mathrm{m\,MASP1^{*}}}}+\frac{[\mathrm{pMBL_4MASP3_{zym}}]}{K^{\mathrm{MASP3_{zym}}}_{\mathrm{m\,MASP1^{*}}}}+\frac{[\mathrm{pCLLKMASP1_{zym}}]}{K^{\mathrm{MASP1_{zym}}}_{\mathrm{m\,MASP1^{*}}}}\\
&+\frac{[\mathrm{pCLLKMASP2_{zym}}]}{K^{\mathrm{MASP2_{zym}}}_{\mathrm{m\,MASP1^{*}}}}+\frac{[\mathrm{pCLLKMASP3_{zym}}]}{K^{\mathrm{MASP3_{zym}}}_{\mathrm{m\,MASP1^{*}}}}+\frac{[\mathrm{pCLK1MASP1_{zym}}]}{K^{\mathrm{MASP1_{zym}}}_{\mathrm{m\,MASP1^{*}}}}+\frac{[\mathrm{pCLK1MASP2_{zym}}]}{K^{\mathrm{MASP2_{zym}}}_{\mathrm{m\,MASP1^{*}}}}\\
&+\frac{[\mathrm{pCLK1MASP3_{zym}}]}{K^{\mathrm{MASP3_{zym}}}_{\mathrm{m\,MASP1^{*}}}}+\frac{[\mathrm{pCLL1MASP1_{zym}}]}{K^{\mathrm{MASP1_{zym}}}_{\mathrm{m\,MASP1^{*}}}}+\frac{[\mathrm{pCLL1MASP2_{zym}}]}{K^{\mathrm{MASP2_{zym}}}_{\mathrm{m\,MASP1^{*}}}}+\frac{[\mathrm{pCLL1MASP3_{zym}}]}{K^{\mathrm{MASP3_{zym}}}_{\mathrm{m\,MASP1^{*}}}}\\
&+\frac{[\mathrm{pLFMASP1_{zym}}]}{K^{\mathrm{MASP1_{zym}}}_{\mathrm{m\,MASP1^{*}}}}+\frac{[\mathrm{pLFMASP2_{zym}}]}{K^{\mathrm{MASP2_{zym}}}_{\mathrm{m\,MASP1^{*}}}}+\frac{[\mathrm{pLFMASP3_{zym}}]}{K^{\mathrm{MASP3_{zym}}}_{\mathrm{m\,MASP1^{*}}}}+\frac{[\mathrm{pHFMASP1_{zym}}]}{K^{\mathrm{MASP1_{zym}}}_{\mathrm{m\,MASP1^{*}}}}+\frac{[\mathrm{pHFMASP2_{zym}}]}{K^{\mathrm{MASP2_{zym}}}_{\mathrm{m\,MASP1^{*}}}}\\
&+\frac{[\mathrm{pHFMASP3_{zym}}]}{K^{\mathrm{MASP3_{zym}}}_{\mathrm{m\,MASP1^{*}}}}+\frac{[\mathrm{pMFMASP1_{zym}}]}{K^{\mathrm{MASP1_{zym}}}_{\mathrm{m\,MASP1^{*}}}}+\frac{[\mathrm{pMFMASP2_{zym}}]}{K^{\mathrm{MASP2_{zym}}}_{\mathrm{m\,MASP1^{*}}}}+\frac{[\mathrm{pMFMASP3_{zym}}]}{K^{\mathrm{MASP3_{zym}}}_{\mathrm{m\,MASP1^{*}}}}\\
&+\frac{[\mathrm{pPTX3LFMASP1_{zym}}]}{K^{\mathrm{MASP1_{zym}}}_{\mathrm{m\,MASP1^{*}}}}+\frac{[\mathrm{pPXT3LFMASP2_{zym}}]}{K^{\mathrm{MASP2_{zym}}}_{\mathrm{m\,MASP1^{*}}}}+\frac{[\mathrm{pPTX3LFMASP3_{zym}}]}{K^{\mathrm{MASP3_{zym}}}_{\mathrm{m\,MASP1^{*}}}}\end{aligned}\right)}
$$

$$
+\frac{k_{\mathrm{cat\ MASP2^{*}}}^{\mathrm{MASP1_{zym}}}\left[\mathrm{pMBL_{3}MASP2^{*}}\right]\left[\mathrm{pMBL_{4}MASP1_{zym}}\right]}{K_{\mathrm{m\ MASP2^{*}}}^{\mathrm{MASP1_{zym}}}\left(\begin{array}{l}
1+\frac{[\mathrm{C4}]}{K_{\mathrm{m\ MASP2^{*}}}^{\mathrm{C4}}}+\frac{[\mathrm{fC4bC2}]}{K_{\mathrm{m\ MASP2^{*}}}^{\mathrm{C2}}}+\frac{[\mathrm{fC4bC4bC2}]}{K_{\mathrm{m\ MASP2^{*}}}^{\mathrm{C2}}}+\frac{[\mathrm{fC3bC4bC2}]}{K_{\mathrm{m\ MASP2^{*}}}^{\mathrm{C2}}}+\frac{[\mathrm{pC4bC2}]}{K_{\mathrm{m\ MASP2^{*}}}^{\mathrm{C2}}}+\frac{[\mathrm{pC4bC4bC2}]}{K_{\mathrm{m\ MASP2^{*}}}^{\mathrm{C2}}}\\
+\frac{[\mathrm{pC3bC4bC2}]}{K_{\mathrm{m\ MASP2^{*}}}^{\mathrm{C2}}}+\frac{\left[\mathrm{pMBL_{3}MASP1_{zym}}\right]}{K_{\mathrm{m\ MASP2^{*}}}^{\mathrm{MASP1_{zym}}}}+\frac{\left[\mathrm{pMBL_{3}MASP2_{zym}}\right]}{K_{\mathrm{m\ MASP2^{*}}}^{\mathrm{MASP2_{zym}}}}+\frac{\left[\mathrm{pMBL_{4}MASP1_{zym}}\right]}{K_{\mathrm{m\ MASP2^{*}}}^{\mathrm{MASP1_{zym}}}}\\
+\frac{\left[\mathrm{pMBL_{4}MASP2_{zym}}\right]}{K_{\mathrm{m\ MASP2^{*}}}^{\mathrm{MASP2_{zym}}}}+\frac{\left[\mathrm{pCLLKMASP1_{zym}}\right]}{K_{\mathrm{m\ MASP2^{*}}}^{\mathrm{MASP1_{zym}}}}+\frac{\left[\mathrm{pCLLKMASP2_{zym}}\right]}{K_{\mathrm{m\ MASP2^{*}}}^{\mathrm{MASP2_{zym}}}}+\frac{\left[\mathrm{pCLK1MASP1_{zym}}\right]}{K_{\mathrm{m\ MASP2^{*}}}^{\mathrm{MASP1_{zym}}}}\\
+\frac{\left[\mathrm{pCLK1MASP2_{zym}}\right]}{K_{\mathrm{m\ MASP2^{*}}}^{\mathrm{MASP2_{zym}}}}+\frac{\left[\mathrm{pCLL1MASP1_{zym}}\right]}{K_{\mathrm{m\ MASP2^{*}}}^{\mathrm{MASP1_{zym}}}}+\frac{\left[\mathrm{pCLL1MASP2_{zym}}\right]}{K_{\mathrm{m\ MASP2^{*}}}^{\mathrm{MASP2_{zym}}}}+\frac{\left[\mathrm{pLFMASP1_{zym}}\right]}{K_{\mathrm{m\ MASP2^{*}}}^{\mathrm{MASP1_{zym}}}}\\
+\frac{\left[\mathrm{pLFMASP2_{zym}}\right]}{K_{\mathrm{m\ MASP2^{*}}}^{\mathrm{MASP2_{zym}}}}+\frac{\left[\mathrm{pHFMASP1_{zym}}\right]}{K_{\mathrm{m\ MASP2^{*}}}^{\mathrm{MASP1_{zym}}}}+\frac{\left[\mathrm{pHFMASP2_{zym}}\right]}{K_{\mathrm{m\ MASP2^{*}}}^{\mathrm{MASP2_{zym}}}}+\frac{\left[\mathrm{pMFMASP1_{zym}}\right]}{K_{\mathrm{m\ MASP2^{*}}}^{\mathrm{MASP1_{zym}}}}+\frac{\left[\mathrm{pMFMASP2_{zym}}\right]}{K_{\mathrm{m\ MASP2^{*}}}^{\mathrm{MASP2_{zym}}}}
\end{array}\right)}
$$

$$
+\frac{k_{\mathrm{cat\ MASP2^{*}}}^{\mathrm{MASP1_{zym}}}\left[\mathrm{pMBL_{4}MASP2^{*}}\right]\left[\mathrm{pMBL_{4}MASP1_{zym}}\right]}{K_{\mathrm{m\ MASP2^{*}}}^{\mathrm{MASP1_{zym}}}\left(\begin{array}{l}
1+\frac{[\mathrm{C4}]}{K_{\mathrm{m\ MASP2^{*}}}^{\mathrm{C4}}}+\frac{[\mathrm{fC4bC2}]}{K_{\mathrm{m\ MASP2^{*}}}^{\mathrm{C2}}}+\frac{[\mathrm{fC4bC4bC2}]}{K_{\mathrm{m\ MASP2^{*}}}^{\mathrm{C2}}}+\frac{[\mathrm{fC3bC4bC2}]}{K_{\mathrm{m\ MASP2^{*}}}^{\mathrm{C2}}}+\frac{[\mathrm{pC4bC2}]}{K_{\mathrm{m\ MASP2^{*}}}^{\mathrm{C2}}}+\frac{[\mathrm{pC4bC4bC2}]}{K_{\mathrm{m\ MASP2^{*}}}^{\mathrm{C2}}}\\
+\frac{[\mathrm{pC3bC4bC2}]}{K_{\mathrm{m\ MASP2^{*}}}^{\mathrm{C2}}}+\frac{\left[\mathrm{pMBL_{3}MASP1_{zym}}\right]}{K_{\mathrm{m\ MASP2^{*}}}^{\mathrm{MASP1_{zym}}}}+\frac{\left[\mathrm{pMBL_{3}MASP2_{zym}}\right]}{K_{\mathrm{m\ MASP2^{*}}}^{\mathrm{MASP2_{zym}}}}+\frac{\left[\mathrm{pMBL_{4}MASP1_{zym}}\right]}{K_{\mathrm{m\ MASP2^{*}}}^{\mathrm{MASP1_{zym}}}}\\
+\frac{\left[\mathrm{pMBL_{4}MASP2_{zym}}\right]}{K_{\mathrm{m\ MASP2^{*}}}^{\mathrm{MASP2_{zym}}}}+\frac{\left[\mathrm{pCLLKMASP1_{zym}}\right]}{K_{\mathrm{m\ MASP2^{*}}}^{\mathrm{MASP1_{zym}}}}+\frac{\left[\mathrm{pCLLKMASP2_{zym}}\right]}{K_{\mathrm{m\ MASP2^{*}}}^{\mathrm{MASP2_{zym}}}}+\frac{\left[\mathrm{pCLK1MASP1_{zym}}\right]}{K_{\mathrm{m\ MASP2^{*}}}^{\mathrm{MASP1_{zym}}}}\\
+\frac{\left[\mathrm{pCLK1MASP2_{zym}}\right]}{K_{\mathrm{m\ MASP2^{*}}}^{\mathrm{MASP2_{zym}}}}+\frac{\left[\mathrm{pCLL1MASP1_{zym}}\right]}{K_{\mathrm{m\ MASP2^{*}}}^{\mathrm{MASP1_{zym}}}}+\frac{\left[\mathrm{pCLL1MASP2_{zym}}\right]}{K_{\mathrm{m\ MASP2^{*}}}^{\mathrm{MASP2_{zym}}}}+\frac{\left[\mathrm{pLFMASP1_{zym}}\right]}{K_{\mathrm{m\ MASP2^{*}}}^{\mathrm{MASP1_{zym}}}}\\
+\frac{\left[\mathrm{pLFMASP2_{zym}}\right]}{K_{\mathrm{m\ MASP2^{*}}}^{\mathrm{MASP2_{zym}}}}+\frac{\left[\mathrm{pHFMASP1_{zym}}\right]}{K_{\mathrm{m\ MASP2^{*}}}^{\mathrm{MASP1_{zym}}}}+\frac{\left[\mathrm{pHFMASP2_{zym}}\right]}{K_{\mathrm{m\ MASP2^{*}}}^{\mathrm{MASP2_{zym}}}}+\frac{\left[\mathrm{pMFMASP1_{zym}}\right]}{K_{\mathrm{m\ MASP2^{*}}}^{\mathrm{MASP1_{zym}}}}+\frac{\left[\mathrm{pMFMASP2_{zym}}\right]}{K_{\mathrm{m\ MASP2^{*}}}^{\mathrm{MASP2_{zym}}}}
\end{array}\right)}
$$

$$
+\frac{k_{\mathrm{cat\ MASP2^{*}}}^{\mathrm{MASP1_{zym}}}\left[\mathrm{pCLLKMASP2^{*}}\right]\left[\mathrm{pMBL_{4}MASP1_{zym}}\right]}{K_{\mathrm{m\ MASP2^{*}}}^{\mathrm{MASP1_{zym}}}\left(\begin{array}{l}
1+\frac{[\mathrm{C4}]}{K_{\mathrm{m\ MASP2^{*}}}^{\mathrm{C4}}}+\frac{[\mathrm{fC4bC2}]}{K_{\mathrm{m\ MASP2^{*}}}^{\mathrm{C2}}}+\frac{[\mathrm{fC4bC4bC2}]}{K_{\mathrm{m\ MASP2^{*}}}^{\mathrm{C2}}}+\frac{[\mathrm{fC3bC4bC2}]}{K_{\mathrm{m\ MASP2^{*}}}^{\mathrm{C2}}}+\frac{[\mathrm{pC4bC2}]}{K_{\mathrm{m\ MASP2^{*}}}^{\mathrm{C2}}}+\frac{[\mathrm{pC4bC4bC2}]}{K_{\mathrm{m\ MASP2^{*}}}^{\mathrm{C2}}}\\
+\frac{[\mathrm{pC3bC4bC2}]}{K_{\mathrm{m\ MASP2^{*}}}^{\mathrm{C2}}}+\frac{\left[\mathrm{pMBL_{3}MASP1_{zym}}\right]}{K_{\mathrm{m\ MASP2^{*}}}^{\mathrm{MASP1_{zym}}}}+\frac{\left[\mathrm{pMBL_{3}MASP2_{zym}}\right]}{K_{\mathrm{m\ MASP2^{*}}}^{\mathrm{MASP2_{zym}}}}+\frac{\left[\mathrm{pMBL_{4}MASP1_{zym}}\right]}{K_{\mathrm{m\ MASP2^{*}}}^{\mathrm{MASP1_{zym}}}}\\
+\frac{\left[\mathrm{pMBL_{4}MASP2_{zym}}\right]}{K_{\mathrm{m\ MASP2^{*}}}^{\mathrm{MASP2_{zym}}}}+\frac{\left[\mathrm{pCLLKMASP1_{zym}}\right]}{K_{\mathrm{m\ MASP2^{*}}}^{\mathrm{MASP1_{zym}}}}+\frac{\left[\mathrm{pCLLKMASP2_{zym}}\right]}{K_{\mathrm{m\ MASP2^{*}}}^{\mathrm{MASP2_{zym}}}}+\frac{\left[\mathrm{pCLK1MASP1_{zym}}\right]}{K_{\mathrm{m\ MASP2^{*}}}^{\mathrm{MASP1_{zym}}}}\\
+\frac{\left[\mathrm{pCLK1MASP2_{zym}}\right]}{K_{\mathrm{m\ MASP2^{*}}}^{\mathrm{MASP2_{zym}}}}+\frac{\left[\mathrm{pCLL1MASP1_{zym}}\right]}{K_{\mathrm{m\ MASP2^{*}}}^{\mathrm{MASP1_{zym}}}}+\frac{\left[\mathrm{pCLL1MASP2_{zym}}\right]}{K_{\mathrm{m\ MASP2^{*}}}^{\mathrm{MASP2_{zym}}}}+\frac{\left[\mathrm{pLFMASP1_{zym}}\right]}{K_{\mathrm{m\ MASP2^{*}}}^{\mathrm{MASP1_{zym}}}}\\
+\frac{\left[\mathrm{pLFMASP2_{zym}}\right]}{K_{\mathrm{m\ MASP2^{*}}}^{\mathrm{MASP2_{zym}}}}+\frac{\left[\mathrm{pHFMASP1_{zym}}\right]}{K_{\mathrm{m\ MASP2^{*}}}^{\mathrm{MASP1_{zym}}}}+\frac{\left[\mathrm{pHFMASP2_{zym}}\right]}{K_{\mathrm{m\ MASP2^{*}}}^{\mathrm{MASP2_{zym}}}}+\frac{\left[\mathrm{pMFMASP1_{zym}}\right]}{K_{\mathrm{m\ MASP2^{*}}}^{\mathrm{MASP1_{zym}}}}+\frac{\left[\mathrm{pMFMASP2_{zym}}\right]}{K_{\mathrm{m\ MASP2^{*}}}^{\mathrm{MASP2_{zym}}}}
\end{array}\right)}
$$

$$+\frac{k_{\text{cat MASP2}^*}^{\text{MASP1}_{\text{zym}}}\left[\text{pCLK1MASP2}^*\right]\left[\text{pMBL}_4\text{MASP1}_{\text{zym}}\right]}{K_{\text{m MASP2}^*}^{\text{MASP1}_{\text{zym}}}\left(\begin{array}{l}1+\frac{[\text{C4}]}{K_{\text{m MASP2}^*}^{\text{C4}}}+\frac{[\text{fC4bC2}]}{K_{\text{m MASP2}^*}^{\text{C2}}}+\frac{[\text{fC4bC4bC2}]}{K_{\text{m MASP2}^*}^{\text{C2}}}+\frac{[\text{fC3bC4bC2}]}{K_{\text{m MASP2}^*}^{\text{C2}}}+\frac{[\text{pC4bC2}]}{K_{\text{m MASP2}^*}^{\text{C2}}}+\frac{[\text{pC4bC4bC2}]}{K_{\text{m MASP2}^*}^{\text{C2}}}\\+\frac{[\text{pC3bC4bC2}]}{K_{\text{m MASP2}^*}^{\text{C2}}}+\frac{\left[\text{pMBL}_3\text{MASP1}_{\text{zym}}\right]}{K_{\text{m MASP2}^*}^{\text{MASP1}_{\text{zym}}}}+\frac{\left[\text{pMBL}_3\text{MASP2}_{\text{zym}}\right]}{K_{\text{m MASP2}^*}^{\text{MASP2}_{\text{zym}}}}+\frac{\left[\text{pMBL}_4\text{MASP1}_{\text{zym}}\right]}{K_{\text{m MASP2}^*}^{\text{MASP1}_{\text{zym}}}}\\+\frac{\left[\text{pMBL}_4\text{MASP2}_{\text{zym}}\right]}{K_{\text{m MASP2}^*}^{\text{MASP2}_{\text{zym}}}}+\frac{\left[\text{pCLLKMASP1}_{\text{zym}}\right]}{K_{\text{m MASP2}^*}^{\text{MASP1}_{\text{zym}}}}+\frac{\left[\text{pCLLKMASP2}_{\text{zym}}\right]}{K_{\text{m MASP2}^*}^{\text{MASP2}_{\text{zym}}}}+\frac{\left[\text{pCLK1MASP1}_{\text{zym}}\right]}{K_{\text{m MASP2}^*}^{\text{MASP1}_{\text{zym}}}}\\+\frac{\left[\text{pCLK1MASP2}_{\text{zym}}\right]}{K_{\text{m MASP2}^*}^{\text{MASP2}_{\text{zym}}}}+\frac{\left[\text{pCLL1MASP1}_{\text{zym}}\right]}{K_{\text{m MASP2}^*}^{\text{MASP1}_{\text{zym}}}}+\frac{\left[\text{pCLL1MASP2}_{\text{zym}}\right]}{K_{\text{m MASP2}^*}^{\text{MASP2}_{\text{zym}}}}+\frac{\left[\text{pLFMASP1}_{\text{zym}}\right]}{K_{\text{m MASP2}^*}^{\text{MASP1}_{\text{zym}}}}\\+\frac{\left[\text{pLFMASP2}_{\text{zym}}\right]}{K_{\text{m MASP2}^*}^{\text{MASP2}_{\text{zym}}}}+\frac{\left[\text{pHFMASP1}_{\text{zym}}\right]}{K_{\text{m MASP2}^*}^{\text{MASP1}_{\text{zym}}}}+\frac{\left[\text{pHFMASP2}_{\text{zym}}\right]}{K_{\text{m MASP2}^*}^{\text{MASP2}_{\text{zym}}}}+\frac{\left[\text{pMFMASP1}_{\text{zym}}\right]}{K_{\text{m MASP2}^*}^{\text{MASP1}_{\text{zym}}}}+\frac{\left[\text{pMFMASP2}_{\text{zym}}\right]}{K_{\text{m MASP2}^*}^{\text{MASP2}_{\text{zym}}}}\end{array}\right)}$$

$$+\frac{k_{\text{cat MASP2}^*}^{\text{MASP1}_{\text{zym}}}\left[\text{pCLL1MASP2}^*\right]\left[\text{pMBL}_4\text{MASP1}_{\text{zym}}\right]}{K_{\text{m MASP2}^*}^{\text{MASP1}_{\text{zym}}}\left(\begin{array}{l}1+\frac{[\text{C4}]}{K_{\text{m MASP2}^*}^{\text{C4}}}+\frac{[\text{fC4bC2}]}{K_{\text{m MASP2}^*}^{\text{C2}}}+\frac{[\text{fC4bC4bC2}]}{K_{\text{m MASP2}^*}^{\text{C2}}}+\frac{[\text{fC3bC4bC2}]}{K_{\text{m MASP2}^*}^{\text{C2}}}+\frac{[\text{pC4bC2}]}{K_{\text{m MASP2}^*}^{\text{C2}}}+\frac{[\text{pC4bC4bC2}]}{K_{\text{m MASP2}^*}^{\text{C2}}}\\+\frac{[\text{pC3bC4bC2}]}{K_{\text{m MASP2}^*}^{\text{C2}}}+\frac{\left[\text{pMBL}_3\text{MASP1}_{\text{zym}}\right]}{K_{\text{m MASP2}^*}^{\text{MASP1}_{\text{zym}}}}+\frac{\left[\text{pMBL}_3\text{MASP2}_{\text{zym}}\right]}{K_{\text{m MASP2}^*}^{\text{MASP2}_{\text{zym}}}}+\frac{\left[\text{pMBL}_4\text{MASP1}_{\text{zym}}\right]}{K_{\text{m MASP2}^*}^{\text{MASP1}_{\text{zym}}}}\\+\frac{\left[\text{pMBL}_4\text{MASP2}_{\text{zym}}\right]}{K_{\text{m MASP2}^*}^{\text{MASP2}_{\text{zym}}}}+\frac{\left[\text{pCLLKMASP1}_{\text{zym}}\right]}{K_{\text{m MASP2}^*}^{\text{MASP1}_{\text{zym}}}}+\frac{\left[\text{pCLLKMASP2}_{\text{zym}}\right]}{K_{\text{m MASP2}^*}^{\text{MASP2}_{\text{zym}}}}+\frac{\left[\text{pCLK1MASP1}_{\text{zym}}\right]}{K_{\text{m MASP2}^*}^{\text{MASP1}_{\text{zym}}}}\\+\frac{\left[\text{pCLK1MASP2}_{\text{zym}}\right]}{K_{\text{m MASP2}^*}^{\text{MASP2}_{\text{zym}}}}+\frac{\left[\text{pCLL1MASP1}_{\text{zym}}\right]}{K_{\text{m MASP2}^*}^{\text{MASP1}_{\text{zym}}}}+\frac{\left[\text{pCLL1MASP2}_{\text{zym}}\right]}{K_{\text{m MASP2}^*}^{\text{MASP2}_{\text{zym}}}}+\frac{\left[\text{pLFMASP1}_{\text{zym}}\right]}{K_{\text{m MASP2}^*}^{\text{MASP1}_{\text{zym}}}}\\+\frac{\left[\text{pLFMASP2}_{\text{zym}}\right]}{K_{\text{m MASP2}^*}^{\text{MASP2}_{\text{zym}}}}+\frac{\left[\text{pHFMASP1}_{\text{zym}}\right]}{K_{\text{m MASP2}^*}^{\text{MASP1}_{\text{zym}}}}+\frac{\left[\text{pHFMASP2}_{\text{zym}}\right]}{K_{\text{m MASP2}^*}^{\text{MASP2}_{\text{zym}}}}+\frac{\left[\text{pMFMASP1}_{\text{zym}}\right]}{K_{\text{m MASP2}^*}^{\text{MASP1}_{\text{zym}}}}+\frac{\left[\text{pMFMASP2}_{\text{zym}}\right]}{K_{\text{m MASP2}^*}^{\text{MASP2}_{\text{zym}}}}\end{array}\right)}$$

$$+\frac{k_{\text{cat MASP2}^*}^{\text{MASP1}_{\text{zym}}}\left[\text{pLFMASP2}^*\right]\left[\text{pMBL}_4\text{MASP1}_{\text{zym}}\right]}{K_{\text{m MASP2}^*}^{\text{MASP1}_{\text{zym}}}\left(\begin{array}{l}1+\frac{[\text{C4}]}{K_{\text{m MASP2}^*}^{\text{C4}}}+\frac{[\text{fC4bC2}]}{K_{\text{m MASP2}^*}^{\text{C2}}}+\frac{[\text{fC4bC4bC2}]}{K_{\text{m MASP2}^*}^{\text{C2}}}+\frac{[\text{fC3bC4bC2}]}{K_{\text{m MASP2}^*}^{\text{C2}}}+\frac{[\text{pC4bC2}]}{K_{\text{m MASP2}^*}^{\text{C2}}}+\frac{[\text{pC4bC4bC2}]}{K_{\text{m MASP2}^*}^{\text{C2}}}\\+\frac{[\text{pC3bC4bC2}]}{K_{\text{m MASP2}^*}^{\text{C2}}}+\frac{\left[\text{pMBL}_3\text{MASP1}_{\text{zym}}\right]}{K_{\text{m MASP2}^*}^{\text{MASP1}_{\text{zym}}}}+\frac{\left[\text{pMBL}_3\text{MASP2}_{\text{zym}}\right]}{K_{\text{m MASP2}^*}^{\text{MASP2}_{\text{zym}}}}+\frac{\left[\text{pMBL}_4\text{MASP1}_{\text{zym}}\right]}{K_{\text{m MASP2}^*}^{\text{MASP1}_{\text{zym}}}}\\+\frac{\left[\text{pMBL}_4\text{MASP2}_{\text{zym}}\right]}{K_{\text{m MASP2}^*}^{\text{MASP2}_{\text{zym}}}}+\frac{\left[\text{pCLLKMASP1}_{\text{zym}}\right]}{K_{\text{m MASP2}^*}^{\text{MASP1}_{\text{zym}}}}+\frac{\left[\text{pCLLKMASP2}_{\text{zym}}\right]}{K_{\text{m MASP2}^*}^{\text{MASP2}_{\text{zym}}}}+\frac{\left[\text{pCLK1MASP1}_{\text{zym}}\right]}{K_{\text{m MASP2}^*}^{\text{MASP1}_{\text{zym}}}}\\+\frac{\left[\text{pCLK1MASP2}_{\text{zym}}\right]}{K_{\text{m MASP2}^*}^{\text{MASP2}_{\text{zym}}}}+\frac{\left[\text{pCLL1MASP1}_{\text{zym}}\right]}{K_{\text{m MASP2}^*}^{\text{MASP1}_{\text{zym}}}}+\frac{\left[\text{pCLL1MASP2}_{\text{zym}}\right]}{K_{\text{m MASP2}^*}^{\text{MASP2}_{\text{zym}}}}+\frac{\left[\text{pLFMASP1}_{\text{zym}}\right]}{K_{\text{m MASP2}^*}^{\text{MASP1}_{\text{zym}}}}\\+\frac{\left[\text{pLFMASP2}_{\text{zym}}\right]}{K_{\text{m MASP2}^*}^{\text{MASP2}_{\text{zym}}}}+\frac{\left[\text{pHFMASP1}_{\text{zym}}\right]}{K_{\text{m MASP2}^*}^{\text{MASP1}_{\text{zym}}}}+\frac{\left[\text{pHFMASP2}_{\text{zym}}\right]}{K_{\text{m MASP2}^*}^{\text{MASP2}_{\text{zym}}}}+\frac{\left[\text{pMFMASP1}_{\text{zym}}\right]}{K_{\text{m MASP2}^*}^{\text{MASP1}_{\text{zym}}}}+\frac{\left[\text{pMFMASP2}_{\text{zym}}\right]}{K_{\text{m MASP2}^*}^{\text{MASP2}_{\text{zym}}}}\end{array}\right)}$$

$$
+\frac{k_{\text{cat MASP2}^*}^{\text{MASP1}_{\text{zym}}}\left[\text{pHFMASP2}^*\right]\left[\text{pMBL}_4\text{MASP1}_{\text{zym}}\right]}{K_{\text{m MASP2}^*}^{\text{MASP1}_{\text{zym}}}\left(\begin{array}{l}1+\frac{[\text{C4}]}{K_{\text{m MASP2}^*}^{\text{C4}}}+\frac{[\text{fC4bC2}]}{K_{\text{m MASP2}^*}^{\text{C2}}}+\frac{[\text{fC4bC4bC2}]}{K_{\text{m MASP2}^*}^{\text{C2}}}+\frac{[\text{fC3bC4bC2}]}{K_{\text{m MASP2}^*}^{\text{C2}}}+\frac{[\text{pC4bC2}]}{K_{\text{m MASP2}^*}^{\text{C2}}}+\frac{[\text{pC4bC4bC2}]}{K_{\text{m MASP2}^*}^{\text{C2}}}\\+\frac{[\text{pC3bC4bC2}]}{K_{\text{m MASP2}^*}^{\text{C2}}}+\frac{\left[\text{pMBL}_3\text{MASP1}_{\text{zym}}\right]}{K_{\text{m MASP2}^*}^{\text{MASP1}_{\text{zym}}}}+\frac{\left[\text{pMBL}_3\text{MASP2}_{\text{zym}}\right]}{K_{\text{m MASP2}^*}^{\text{MASP2}_{\text{zym}}}}+\frac{\left[\text{pMBL}_4\text{MASP1}_{\text{zym}}\right]}{K_{\text{m MASP2}^*}^{\text{MASP1}_{\text{zym}}}}\\+\frac{\left[\text{pMBL}_4\text{MASP2}_{\text{zym}}\right]}{K_{\text{m MASP2}^*}^{\text{MASP2}_{\text{zym}}}}+\frac{\left[\text{pCLLKMASP1}_{\text{zym}}\right]}{K_{\text{m MASP2}^*}^{\text{MASP1}_{\text{zym}}}}+\frac{\left[\text{pCLLKMASP2}_{\text{zym}}\right]}{K_{\text{m MASP2}^*}^{\text{MASP2}_{\text{zym}}}}+\frac{\left[\text{pCLK1MASP1}_{\text{zym}}\right]}{K_{\text{m MASP2}^*}^{\text{MASP1}_{\text{zym}}}}\\+\frac{\left[\text{pCLK1MASP2}_{\text{zym}}\right]}{K_{\text{m MASP2}^*}^{\text{MASP2}_{\text{zym}}}}+\frac{\left[\text{pCLL1MASP1}_{\text{zym}}\right]}{K_{\text{m MASP2}^*}^{\text{MASP1}_{\text{zym}}}}+\frac{\left[\text{pCLL1MASP2}_{\text{zym}}\right]}{K_{\text{m MASP2}^*}^{\text{MASP2}_{\text{zym}}}}+\frac{\left[\text{pLFMASP1}_{\text{zym}}\right]}{K_{\text{m MASP2}^*}^{\text{MASP1}_{\text{zym}}}}\\+\frac{\left[\text{pLFMASP2}_{\text{zym}}\right]}{K_{\text{m MASP2}^*}^{\text{MASP2}_{\text{zym}}}}+\frac{\left[\text{pHFMASP1}_{\text{zym}}\right]}{K_{\text{m MASP2}^*}^{\text{MASP1}_{\text{zym}}}}+\frac{\left[\text{pHFMASP2}_{\text{zym}}\right]}{K_{\text{m MASP2}^*}^{\text{MASP2}_{\text{zym}}}}+\frac{\left[\text{pMFMASP1}_{\text{zym}}\right]}{K_{\text{m MASP2}^*}^{\text{MASP1}_{\text{zym}}}}+\frac{\left[\text{pMFMASP2}_{\text{zym}}\right]}{K_{\text{m MASP2}^*}^{\text{MASP2}_{\text{zym}}}}\end{array}\right)}
$$

$$
+\frac{k_{\text{cat MASP2}^*}^{\text{MASP1}_{\text{zym}}}\left[\text{pMFMASP2}^*\right]\left[\text{pMBL}_4\text{MASP1}_{\text{zym}}\right]}{K_{\text{m MASP2}^*}^{\text{MASP1}_{\text{zym}}}\left(\begin{array}{l}1+\frac{[\text{C4}]}{K_{\text{m MASP2}^*}^{\text{C4}}}+\frac{[\text{fC4bC2}]}{K_{\text{m MASP2}^*}^{\text{C2}}}+\frac{[\text{fC4bC4bC2}]}{K_{\text{m MASP2}^*}^{\text{C2}}}+\frac{[\text{fC3bC4bC2}]}{K_{\text{m MASP2}^*}^{\text{C2}}}+\frac{[\text{pC4bC2}]}{K_{\text{m MASP2}^*}^{\text{C2}}}+\frac{[\text{pC4bC4bC2}]}{K_{\text{m MASP2}^*}^{\text{C2}}}\\+\frac{[\text{pC3bC4bC2}]}{K_{\text{m MASP2}^*}^{\text{C2}}}+\frac{\left[\text{pMBL}_3\text{MASP1}_{\text{zym}}\right]}{K_{\text{m MASP2}^*}^{\text{MASP1}_{\text{zym}}}}+\frac{\left[\text{pMBL}_3\text{MASP2}_{\text{zym}}\right]}{K_{\text{m MASP2}^*}^{\text{MASP2}_{\text{zym}}}}+\frac{\left[\text{pMBL}_4\text{MASP1}_{\text{zym}}\right]}{K_{\text{m MASP2}^*}^{\text{MASP1}_{\text{zym}}}}\\+\frac{\left[\text{pMBL}_4\text{MASP2}_{\text{zym}}\right]}{K_{\text{m MASP2}^*}^{\text{MASP2}_{\text{zym}}}}+\frac{\left[\text{pCLLKMASP1}_{\text{zym}}\right]}{K_{\text{m MASP2}^*}^{\text{MASP1}_{\text{zym}}}}+\frac{\left[\text{pCLLKMASP2}_{\text{zym}}\right]}{K_{\text{m MASP2}^*}^{\text{MASP2}_{\text{zym}}}}+\frac{\left[\text{pCLK1MASP1}_{\text{zym}}\right]}{K_{\text{m MASP2}^*}^{\text{MASP1}_{\text{zym}}}}\\+\frac{\left[\text{pCLK1MASP2}_{\text{zym}}\right]}{K_{\text{m MASP2}^*}^{\text{MASP2}_{\text{zym}}}}+\frac{\left[\text{pCLL1MASP1}_{\text{zym}}\right]}{K_{\text{m MASP2}^*}^{\text{MASP1}_{\text{zym}}}}+\frac{\left[\text{pCLL1MASP2}_{\text{zym}}\right]}{K_{\text{m MASP2}^*}^{\text{MASP2}_{\text{zym}}}}+\frac{\left[\text{pLFMASP1}_{\text{zym}}\right]}{K_{\text{m MASP2}^*}^{\text{MASP1}_{\text{zym}}}}\\+\frac{\left[\text{pLFMASP2}_{\text{zym}}\right]}{K_{\text{m MASP2}^*}^{\text{MASP2}_{\text{zym}}}}+\frac{\left[\text{pHFMASP1}_{\text{zym}}\right]}{K_{\text{m MASP2}^*}^{\text{MASP1}_{\text{zym}}}}+\frac{\left[\text{pHFMASP2}_{\text{zym}}\right]}{K_{\text{m MASP2}^*}^{\text{MASP2}_{\text{zym}}}}+\frac{\left[\text{pMFMASP1}_{\text{zym}}\right]}{K_{\text{m MASP2}^*}^{\text{MASP1}_{\text{zym}}}}+\frac{\left[\text{pMFMASP2}_{\text{zym}}\right]}{K_{\text{m MASP2}^*}^{\text{MASP2}_{\text{zym}}}}\end{array}\right)}
$$

$$+\frac{k_{\text{cat MASP2}^*}^{\text{MASP1}_{\text{zym}}}\left[\text{pPTX3LFMASP2}^*\right]\left[\text{pMBL}_4\text{MASP1}_{\text{zym}}\right]}{K_{\text{m MASP2}^*}^{\text{MASP1}_{\text{zym}}}\left(\begin{array}{l}1+\frac{[\text{C4}]}{K_{\text{m MASP2}^*}^{\text{C4}}}+\frac{[\text{fC4bC2}]}{K_{\text{m MASP2}^*}^{\text{C2}}}+\frac{[\text{fC4bC4bC2}]}{K_{\text{m MASP2}^*}^{\text{C2}}}+\frac{[\text{fC3bC4bC2}]}{K_{\text{m MASP2}^*}^{\text{C2}}}+\frac{[\text{pC4bC2}]}{K_{\text{m MASP2}^*}^{\text{C2}}}+\frac{[\text{pC4bC4bC2}]}{K_{\text{m MASP2}^*}^{\text{C2}}}\\+\frac{[\text{pC3bC4bC2}]}{K_{\text{m MASP2}^*}^{\text{C2}}}+\frac{[\text{pMBL}_3\text{MASP1}_{\text{zym}}]}{K_{\text{m MASP2}^*}^{\text{MASP1}_{\text{zym}}}}+\frac{[\text{pMBL}_3\text{MASP2}_{\text{zym}}]}{K_{\text{m MASP2}^*}^{\text{MASP2}_{\text{zym}}}}+\frac{[\text{pMBL}_4\text{MASP1}_{\text{zym}}]}{K_{\text{m MASP2}^*}^{\text{MASP1}_{\text{zym}}}}\\+\frac{[\text{pMBL}_4\text{MASP2}_{\text{zym}}]}{K_{\text{m MASP2}^*}^{\text{MASP2}_{\text{zym}}}}+\frac{[\text{pCLLKMASP1}_{\text{zym}}]}{K_{\text{m MASP2}^*}^{\text{MASP1}_{\text{zym}}}}+\frac{[\text{pCLLKMASP2}_{\text{zym}}]}{K_{\text{m MASP2}^*}^{\text{MASP2}_{\text{zym}}}}+\frac{[\text{pCLK1MASP1}_{\text{zym}}]}{K_{\text{m MASP2}^*}^{\text{MASP1}_{\text{zym}}}}\\+\frac{[\text{pCLK1MASP2}_{\text{zym}}]}{K_{\text{m MASP2}^*}^{\text{MASP2}_{\text{zym}}}}+\frac{[\text{pCLL1MASP1}_{\text{zym}}]}{K_{\text{m MASP2}^*}^{\text{MASP1}_{\text{zym}}}}+\frac{[\text{pCLL1MASP2}_{\text{zym}}]}{K_{\text{m MASP2}^*}^{\text{MASP2}_{\text{zym}}}}+\frac{[\text{pLFMASP1}_{\text{zym}}]}{K_{\text{m MASP2}^*}^{\text{MASP1}_{\text{zym}}}}\\+\frac{[\text{pLFMASP2}_{\text{zym}}]}{K_{\text{m MASP2}^*}^{\text{MASP2}_{\text{zym}}}}+\frac{[\text{pHFMASP1}_{\text{zym}}]}{K_{\text{m MASP2}^*}^{\text{MASP1}_{\text{zym}}}}+\frac{[\text{pHFMASP2}_{\text{zym}}]}{K_{\text{m MASP2}^*}^{\text{MASP2}_{\text{zym}}}}+\frac{[\text{pMFMASP1}_{\text{zym}}]}{K_{\text{m MASP2}^*}^{\text{MASP1}_{\text{zym}}}}+\frac{[\text{pMFMASP2}_{\text{zym}}]}{K_{\text{m MASP2}^*}^{\text{MASP2}_{\text{zym}}}}\\+\frac{[\text{pPTX3LFMASP1}_{\text{zym}}]}{K_{\text{m MASP2}^*}^{\text{MASP1}_{\text{zym}}}}+\frac{[\text{pPTX3LFMASP2}_{\text{zym}}]}{K_{\text{m MASP2}^*}^{\text{MASP2}_{\text{zym}}}}\end{array}\right)}$$

$$-k_{\text{C1INHMASP1}}^{+}[\text{C1-INH}]\left[\text{pMBL}_4\text{MASP1}^*\right]$$

270

$$\frac{d\left[\text{fMBL}_4\text{MASP2}_{\text{zym}}\right]}{dt}=k_{\text{MBL}_4\text{MASP2}}^{+}[\text{MBL}_4][\text{MASP2}]-k_{\text{MBL}_4\text{MASP2}}^{-}\left[\text{fMBL}_4\text{MASP2}_{\text{zym}}\right]$$

$$-k_{\text{pMBL}_4}^{+}\left[\text{fMBL}_4\text{MASP2}_{\text{zym}}\right][\text{Pathogen Surface}]+k_{\text{pMBL}_4}^{-}\left[\text{pMBL}_4\text{MASP2}_{\text{zym}}\right]$$

271

$$\frac{d\left[\text{pMBL}_4\text{MASP2}_{\text{zym}}\right]}{dt}=k_{\text{pMBL}_4}^{+}\left[\text{fMBL}_4\text{MASP2}_{\text{zym}}\right][\text{Pathogen Surface}]-k_{\text{pMBL}_4}^{-}\left[\text{pMBL}_4\text{MASP2}_{\text{zym}}\right]$$

$$+k_{\text{MBL}_4\text{MASP2}}^{+}[\text{pMBL}_4][\text{MASP2}]-k_{\text{MBL}_4\text{MASP2}}^{-}\left[\text{pMBL}_4\text{MASP2}_{\text{zym}}\right]$$

$$
-\frac{k_{\text{cat MASP1}_{\text{zym}}}^{\text{MASP2}_{\text{zym}}}\left[\text{pMBL}_3\text{MASP1}_{\text{zym}}\right]\left[\text{pMBL}_4\text{MASP2}_{\text{zym}}\right]}{K_{\text{m MASP1}_{\text{zym}}}^{\text{MASP2}_{\text{zym}}}\left(\begin{array}{l}1+\frac{\left[\text{pMBL}_3\text{MASP1}_{\text{zym}}\right]}{K_{\text{m MASP1}_{\text{zym}}}^{\text{MASP1}_{\text{zym}}}}+\frac{\left[\text{pMBL}_3\text{MASP2}_{\text{zym}}\right]}{K_{\text{m MASP1}_{\text{zym}}}^{\text{MASP2}_{\text{zym}}}}+\frac{\left[\text{pMBL}_4\text{MASP1}_{\text{zym}}\right]}{K_{\text{m MASP1}_{\text{zym}}}^{\text{MASP1}_{\text{zym}}}}+\frac{\left[\text{pMBL}_4\text{MASP2}_{\text{zym}}\right]}{K_{\text{m MASP1}_{\text{zym}}}^{\text{MASP2}_{\text{zym}}}}\\+\frac{\left[\text{pCLLKMASP1}_{\text{zym}}\right]}{K_{\text{m MASP1}_{\text{zym}}}^{\text{MASP1}_{\text{zym}}}}+\frac{\left[\text{pCLLKMASP2}_{\text{zym}}\right]}{K_{\text{m MASP1}_{\text{zym}}}^{\text{MASP2}_{\text{zym}}}}+\frac{\left[\text{pCLK1MASP1}_{\text{zym}}\right]}{K_{\text{m MASP1}_{\text{zym}}}^{\text{MASP1}_{\text{zym}}}}+\frac{\left[\text{pCLK1MASP2}_{\text{zym}}\right]}{K_{\text{m MASP1}_{\text{zym}}}^{\text{MASP2}_{\text{zym}}}}\\+\frac{\left[\text{pCLL1MASP1}_{\text{zym}}\right]}{K_{\text{m MASP1}_{\text{zym}}}^{\text{MASP1}_{\text{zym}}}}+\frac{\left[\text{pCLL1MASP2}_{\text{zym}}\right]}{K_{\text{m MASP1}_{\text{zym}}}^{\text{MASP2}_{\text{zym}}}}+\frac{\left[\text{pLFMASP1}_{\text{zym}}\right]}{K_{\text{m MASP1}_{\text{zym}}}^{\text{MASP1}_{\text{zym}}}}+\frac{\left[\text{pLFMASP2}_{\text{zym}}\right]}{K_{\text{m MASP1}_{\text{zym}}}^{\text{MASP2}_{\text{zym}}}}\\+\frac{\left[\text{pHFMASP1}_{\text{zym}}\right]}{K_{\text{m MASP1}_{\text{zym}}}^{\text{MASP1}_{\text{zym}}}}+\frac{\left[\text{pHFMASP2}_{\text{zym}}\right]}{K_{\text{m MASP1}_{\text{zym}}}^{\text{MASP2}_{\text{zym}}}}+\frac{\left[\text{pMFMASP1}_{\text{zym}}\right]}{K_{\text{m MASP1}_{\text{zym}}}^{\text{MASP1}_{\text{zym}}}}+\frac{\left[\text{pMFMASP2}_{\text{zym}}\right]}{K_{\text{m MASP1}_{\text{zym}}}^{\text{MASP2}_{\text{zym}}}}\\+\frac{\left[\text{pPTX3LFMASP1}_{\text{zym}}\right]}{K_{\text{m MASP1}_{\text{zym}}}^{\text{MASP1}_{\text{zym}}}}+\frac{\left[\text{pPTX3LFMASP2}_{\text{zym}}\right]}{K_{\text{m MASP1}_{\text{zym}}}^{\text{MASP2}_{\text{zym}}}}\end{array}\right)}
$$

$$
-\frac{k_{\text{cat MASP1}_{\text{zym}}}^{\text{MASP2}_{\text{zym}}}\left[\text{pMBL}_4\text{MASP1}_{\text{zym}}\right]\left[\text{pMBL}_4\text{MASP2}_{\text{zym}}\right]}{K_{\text{m MASP1}_{\text{zym}}}^{\text{MASP2}_{\text{zym}}}\left(\begin{array}{l}1+\frac{\left[\text{pMBL}_3\text{MASP1}_{\text{zym}}\right]}{K_{\text{m MASP1}_{\text{zym}}}^{\text{MASP1}_{\text{zym}}}}+\frac{\left[\text{pMBL}_3\text{MASP2}_{\text{zym}}\right]}{K_{\text{m MASP1}_{\text{zym}}}^{\text{MASP2}_{\text{zym}}}}+\frac{\left[\text{pMBL}_4\text{MASP1}_{\text{zym}}\right]}{K_{\text{m MASP1}_{\text{zym}}}^{\text{MASP1}_{\text{zym}}}}+\frac{\left[\text{pMBL}_4\text{MASP2}_{\text{zym}}\right]}{K_{\text{m MASP1}_{\text{zym}}}^{\text{MASP2}_{\text{zym}}}}\\+\frac{\left[\text{pCLLKMASP1}_{\text{zym}}\right]}{K_{\text{m MASP1}_{\text{zym}}}^{\text{MASP1}_{\text{zym}}}}+\frac{\left[\text{pCLLKMASP2}_{\text{zym}}\right]}{K_{\text{m MASP1}_{\text{zym}}}^{\text{MASP2}_{\text{zym}}}}+\frac{\left[\text{pCLK1MASP1}_{\text{zym}}\right]}{K_{\text{m MASP1}_{\text{zym}}}^{\text{MASP1}_{\text{zym}}}}+\frac{\left[\text{pCLK1MASP2}_{\text{zym}}\right]}{K_{\text{m MASP1}_{\text{zym}}}^{\text{MASP2}_{\text{zym}}}}\\+\frac{\left[\text{pCLL1MASP1}_{\text{zym}}\right]}{K_{\text{m MASP1}_{\text{zym}}}^{\text{MASP1}_{\text{zym}}}}+\frac{\left[\text{pCLL1MASP2}_{\text{zym}}\right]}{K_{\text{m MASP1}_{\text{zym}}}^{\text{MASP2}_{\text{zym}}}}+\frac{\left[\text{pLFMASP1}_{\text{zym}}\right]}{K_{\text{m MASP1}_{\text{zym}}}^{\text{MASP1}_{\text{zym}}}}+\frac{\left[\text{pLFMASP2}_{\text{zym}}\right]}{K_{\text{m MASP1}_{\text{zym}}}^{\text{MASP2}_{\text{zym}}}}\\+\frac{\left[\text{pHFMASP1}_{\text{zym}}\right]}{K_{\text{m MASP1}_{\text{zym}}}^{\text{MASP1}_{\text{zym}}}}+\frac{\left[\text{pHFMASP2}_{\text{zym}}\right]}{K_{\text{m MASP1}_{\text{zym}}}^{\text{MASP2}_{\text{zym}}}}+\frac{\left[\text{pMFMASP1}_{\text{zym}}\right]}{K_{\text{m MASP1}_{\text{zym}}}^{\text{MASP1}_{\text{zym}}}}+\frac{\left[\text{pMFMASP2}_{\text{zym}}\right]}{K_{\text{m MASP1}_{\text{zym}}}^{\text{MASP2}_{\text{zym}}}}\\+\frac{\left[\text{pPTX3LFMASP1}_{\text{zym}}\right]}{K_{\text{m MASP1}_{\text{zym}}}^{\text{MASP1}_{\text{zym}}}}+\frac{\left[\text{pPTX3LFMASP2}_{\text{zym}}\right]}{K_{\text{m MASP1}_{\text{zym}}}^{\text{MASP2}_{\text{zym}}}}\end{array}\right)}
$$

$$
-\frac{k_{\text{cat MASP1}_{\text{zym}}}^{\text{MASP2}_{\text{zym}}}\left[\text{pCLLKMASP1}_{\text{zym}}\right]\left[\text{pMBL}_4\text{MASP2}_{\text{zym}}\right]}{K_{\text{m MASP1}_{\text{zym}}}^{\text{MASP2}_{\text{zym}}}\left(\begin{array}{l}1+\frac{\left[\text{pMBL}_3\text{MASP1}_{\text{zym}}\right]}{K_{\text{m MASP1}_{\text{zym}}}^{\text{MASP1}_{\text{zym}}}}+\frac{\left[\text{pMBL}_3\text{MASP2}_{\text{zym}}\right]}{K_{\text{m MASP1}_{\text{zym}}}^{\text{MASP2}_{\text{zym}}}}+\frac{\left[\text{pMBL}_4\text{MASP1}_{\text{zym}}\right]}{K_{\text{m MASP1}_{\text{zym}}}^{\text{MASP1}_{\text{zym}}}}+\frac{\left[\text{pMBL}_4\text{MASP2}_{\text{zym}}\right]}{K_{\text{m MASP1}_{\text{zym}}}^{\text{MASP2}_{\text{zym}}}}\\+\frac{\left[\text{pCLLKMASP1}_{\text{zym}}\right]}{K_{\text{m MASP1}_{\text{zym}}}^{\text{MASP1}_{\text{zym}}}}+\frac{\left[\text{pCLLKMASP2}_{\text{zym}}\right]}{K_{\text{m MASP1}_{\text{zym}}}^{\text{MASP2}_{\text{zym}}}}+\frac{\left[\text{pCLK1MASP1}_{\text{zym}}\right]}{K_{\text{m MASP1}_{\text{zym}}}^{\text{MASP1}_{\text{zym}}}}+\frac{\left[\text{pCLK1MASP2}_{\text{zym}}\right]}{K_{\text{m MASP1}_{\text{zym}}}^{\text{MASP2}_{\text{zym}}}}\\+\frac{\left[\text{pCLL1MASP1}_{\text{zym}}\right]}{K_{\text{m MASP1}_{\text{zym}}}^{\text{MASP1}_{\text{zym}}}}+\frac{\left[\text{pCLL1MASP2}_{\text{zym}}\right]}{K_{\text{m MASP1}_{\text{zym}}}^{\text{MASP2}_{\text{zym}}}}+\frac{\left[\text{pLFMASP1}_{\text{zym}}\right]}{K_{\text{m MASP1}_{\text{zym}}}^{\text{MASP1}_{\text{zym}}}}+\frac{\left[\text{pLFMASP2}_{\text{zym}}\right]}{K_{\text{m MASP1}_{\text{zym}}}^{\text{MASP2}_{\text{zym}}}}\\+\frac{\left[\text{pHFMASP1}_{\text{zym}}\right]}{K_{\text{m MASP1}_{\text{zym}}}^{\text{MASP1}_{\text{zym}}}}+\frac{\left[\text{pHFMASP2}_{\text{zym}}\right]}{K_{\text{m MASP1}_{\text{zym}}}^{\text{MASP2}_{\text{zym}}}}+\frac{\left[\text{pMFMASP1}_{\text{zym}}\right]}{K_{\text{m MASP1}_{\text{zym}}}^{\text{MASP1}_{\text{zym}}}}+\frac{\left[\text{pMFMASP2}_{\text{zym}}\right]}{K_{\text{m MASP1}_{\text{zym}}}^{\text{MASP2}_{\text{zym}}}}\\+\frac{\left[\text{pPTX3LFMASP1}_{\text{zym}}\right]}{K_{\text{m MASP1}_{\text{zym}}}^{\text{MASP1}_{\text{zym}}}}+\frac{\left[\text{pPTX3LFMASP2}_{\text{zym}}\right]}{K_{\text{m MASP1}_{\text{zym}}}^{\text{MASP2}_{\text{zym}}}}\end{array}\right)}
$$

$$
-\frac{k_{\text{cat MASP1}_{\text{zym}}}^{\text{MASP2}_{\text{zym}}}\left[\text{pCLK1MASP1}_{\text{zym}}\right]\left[\text{pMBL}_4\text{MASP2}_{\text{zym}}\right]}{K_{\text{m MASP1}_{\text{zym}}}^{\text{MASP2}_{\text{zym}}}\left(\begin{array}{l}1+\frac{\left[\text{pMBL}_3\text{MASP1}_{\text{zym}}\right]}{K_{\text{m MASP1}_{\text{zym}}}^{\text{MASP1}_{\text{zym}}}}+\frac{\left[\text{pMBL}_3\text{MASP2}_{\text{zym}}\right]}{K_{\text{m MASP1}_{\text{zym}}}^{\text{MASP2}_{\text{zym}}}}+\frac{\left[\text{pMBL}_4\text{MASP1}_{\text{zym}}\right]}{K_{\text{m MASP1}_{\text{zym}}}^{\text{MASP1}_{\text{zym}}}}+\frac{\left[\text{pMBL}_4\text{MASP2}_{\text{zym}}\right]}{K_{\text{m MASP1}_{\text{zym}}}^{\text{MASP2}_{\text{zym}}}}\\+\frac{\left[\text{pCLLKMASP1}_{\text{zym}}\right]}{K_{\text{m MASP1}_{\text{zym}}}^{\text{MASP1}_{\text{zym}}}}+\frac{\left[\text{pCLLKMASP2}_{\text{zym}}\right]}{K_{\text{m MASP1}_{\text{zym}}}^{\text{MASP2}_{\text{zym}}}}+\frac{\left[\text{pCLK1MASP1}_{\text{zym}}\right]}{K_{\text{m MASP1}_{\text{zym}}}^{\text{MASP1}_{\text{zym}}}}+\frac{\left[\text{pCLK1MASP2}_{\text{zym}}\right]}{K_{\text{m MASP1}_{\text{zym}}}^{\text{MASP2}_{\text{zym}}}}\\+\frac{\left[\text{pCLL1MASP1}_{\text{zym}}\right]}{K_{\text{m MASP1}_{\text{zym}}}^{\text{MASP1}_{\text{zym}}}}+\frac{\left[\text{pCLL1MASP2}_{\text{zym}}\right]}{K_{\text{m MASP1}_{\text{zym}}}^{\text{MASP2}_{\text{zym}}}}+\frac{\left[\text{pLFMASP1}_{\text{zym}}\right]}{K_{\text{m MASP1}_{\text{zym}}}^{\text{MASP1}_{\text{zym}}}}+\frac{\left[\text{pLFMASP2}_{\text{zym}}\right]}{K_{\text{m MASP1}_{\text{zym}}}^{\text{MASP2}_{\text{zym}}}}\\+\frac{\left[\text{pHFMASP1}_{\text{zym}}\right]}{K_{\text{m MASP1}_{\text{zym}}}^{\text{MASP1}_{\text{zym}}}}+\frac{\left[\text{pHFMASP2}_{\text{zym}}\right]}{K_{\text{m MASP1}_{\text{zym}}}^{\text{MASP2}_{\text{zym}}}}+\frac{\left[\text{pMFMASP1}_{\text{zym}}\right]}{K_{\text{m MASP1}_{\text{zym}}}^{\text{MASP1}_{\text{zym}}}}+\frac{\left[\text{pMFMASP2}_{\text{zym}}\right]}{K_{\text{m MASP1}_{\text{zym}}}^{\text{MASP2}_{\text{zym}}}}\\+\frac{\left[\text{pPTX3LFMASP1}_{\text{zym}}\right]}{K_{\text{m MASP1}_{\text{zym}}}^{\text{MASP1}_{\text{zym}}}}+\frac{\left[\text{pPTX3LFMASP2}_{\text{zym}}\right]}{K_{\text{m MASP1}_{\text{zym}}}^{\text{MASP2}_{\text{zym}}}}\end{array}\right)}
$$

$$-\frac{k_{\text{cat MASP1}_{\text{zym}}}^{\text{MASP2}_{\text{zym}}}\left[\text{pCLL1MASP1}_{\text{zym}}\right]\left[\text{pMBL}_4\text{MASP2}_{\text{zym}}\right]}{K_{\text{m MASP1}_{\text{zym}}}^{\text{MASP2}_{\text{zym}}}\left(\begin{array}{l}1+\frac{\left[\text{pMBL}_3\text{MASP1}_{\text{zym}}\right]}{K_{\text{m MASP1}_{\text{zym}}}^{\text{MASP1}_{\text{zym}}}}+\frac{\left[\text{pMBL}_3\text{MASP2}_{\text{zym}}\right]}{K_{\text{m MASP1}_{\text{zym}}}^{\text{MASP2}_{\text{zym}}}}+\frac{\left[\text{pMBL}_4\text{MASP1}_{\text{zym}}\right]}{K_{\text{m MASP1}_{\text{zym}}}^{\text{MASP1}_{\text{zym}}}}+\frac{\left[\text{pMBL}_4\text{MASP2}_{\text{zym}}\right]}{K_{\text{m MASP1}_{\text{zym}}}^{\text{MASP2}_{\text{zym}}}}\\+\frac{\left[\text{pCLLKMASP1}_{\text{zym}}\right]}{K_{\text{m MASP1}_{\text{zym}}}^{\text{MASP1}_{\text{zym}}}}+\frac{\left[\text{pCLLKMASP2}_{\text{zym}}\right]}{K_{\text{m MASP1}_{\text{zym}}}^{\text{MASP2}_{\text{zym}}}}+\frac{\left[\text{pCLK1MASP1}_{\text{zym}}\right]}{K_{\text{m MASP1}_{\text{zym}}}^{\text{MASP1}_{\text{zym}}}}+\frac{\left[\text{pCLK1MASP2}_{\text{zym}}\right]}{K_{\text{m MASP1}_{\text{zym}}}^{\text{MASP2}_{\text{zym}}}}\\+\frac{\left[\text{pCLL1MASP1}_{\text{zym}}\right]}{K_{\text{m MASP1}_{\text{zym}}}^{\text{MASP1}_{\text{zym}}}}+\frac{\left[\text{pCLL1MASP2}_{\text{zym}}\right]}{K_{\text{m MASP1}_{\text{zym}}}^{\text{MASP2}_{\text{zym}}}}+\frac{\left[\text{pLFMASP1}_{\text{zym}}\right]}{K_{\text{m MASP1}_{\text{zym}}}^{\text{MASP1}_{\text{zym}}}}+\frac{\left[\text{pLFMASP2}_{\text{zym}}\right]}{K_{\text{m MASP1}_{\text{zym}}}^{\text{MASP2}_{\text{zym}}}}\\+\frac{\left[\text{pHFMASP1}_{\text{zym}}\right]}{K_{\text{m MASP1}_{\text{zym}}}^{\text{MASP1}_{\text{zym}}}}+\frac{\left[\text{pHFMASP2}_{\text{zym}}\right]}{K_{\text{m MASP1}_{\text{zym}}}^{\text{MASP2}_{\text{zym}}}}+\frac{\left[\text{pMFMASP1}_{\text{zym}}\right]}{K_{\text{m MASP1}_{\text{zym}}}^{\text{MASP1}_{\text{zym}}}}+\frac{\left[\text{pMFMASP2}_{\text{zym}}\right]}{K_{\text{m MASP1}_{\text{zym}}}^{\text{MASP2}_{\text{zym}}}}\\+\frac{\left[\text{pPTX3LFMASP1}_{\text{zym}}\right]}{K_{\text{m MASP1}_{\text{zym}}}^{\text{MASP1}_{\text{zym}}}}+\frac{\left[\text{pPTX3LFMASP2}_{\text{zym}}\right]}{K_{\text{m MASP1}_{\text{zym}}}^{\text{MASP2}_{\text{zym}}}}\end{array}\right)}$$

$$-\frac{k_{\text{cat MASP1}_{\text{zym}}}^{\text{MASP2}_{\text{zym}}}\left[\text{pLFMASP1}_{\text{zym}}\right]\left[\text{pMBL}_4\text{MASP2}_{\text{zym}}\right]}{K_{\text{m MASP1}_{\text{zym}}}^{\text{MASP2}_{\text{zym}}}\left(\begin{array}{l}1+\frac{\left[\text{pMBL}_3\text{MASP1}_{\text{zym}}\right]}{K_{\text{m MASP1}_{\text{zym}}}^{\text{MASP1}_{\text{zym}}}}+\frac{\left[\text{pMBL}_3\text{MASP2}_{\text{zym}}\right]}{K_{\text{m MASP1}_{\text{zym}}}^{\text{MASP2}_{\text{zym}}}}+\frac{\left[\text{pMBL}_4\text{MASP1}_{\text{zym}}\right]}{K_{\text{m MASP1}_{\text{zym}}}^{\text{MASP1}_{\text{zym}}}}+\frac{\left[\text{pMBL}_4\text{MASP2}_{\text{zym}}\right]}{K_{\text{m MASP1}_{\text{zym}}}^{\text{MASP2}_{\text{zym}}}}\\+\frac{\left[\text{pCLLKMASP1}_{\text{zym}}\right]}{K_{\text{m MASP1}_{\text{zym}}}^{\text{MASP1}_{\text{zym}}}}+\frac{\left[\text{pCLLKMASP2}_{\text{zym}}\right]}{K_{\text{m MASP1}_{\text{zym}}}^{\text{MASP2}_{\text{zym}}}}+\frac{\left[\text{pCLK1MASP1}_{\text{zym}}\right]}{K_{\text{m MASP1}_{\text{zym}}}^{\text{MASP1}_{\text{zym}}}}+\frac{\left[\text{pCLK1MASP2}_{\text{zym}}\right]}{K_{\text{m MASP1}_{\text{zym}}}^{\text{MASP2}_{\text{zym}}}}\\+\frac{\left[\text{pCLL1MASP1}_{\text{zym}}\right]}{K_{\text{m MASP1}_{\text{zym}}}^{\text{MASP1}_{\text{zym}}}}+\frac{\left[\text{pCLL1MASP2}_{\text{zym}}\right]}{K_{\text{m MASP1}_{\text{zym}}}^{\text{MASP2}_{\text{zym}}}}+\frac{\left[\text{pLFMASP1}_{\text{zym}}\right]}{K_{\text{m MASP1}_{\text{zym}}}^{\text{MASP1}_{\text{zym}}}}+\frac{\left[\text{pLFMASP2}_{\text{zym}}\right]}{K_{\text{m MASP1}_{\text{zym}}}^{\text{MASP2}_{\text{zym}}}}\\+\frac{\left[\text{pHFMASP1}_{\text{zym}}\right]}{K_{\text{m MASP1}_{\text{zym}}}^{\text{MASP1}_{\text{zym}}}}+\frac{\left[\text{pHFMASP2}_{\text{zym}}\right]}{K_{\text{m MASP1}_{\text{zym}}}^{\text{MASP2}_{\text{zym}}}}+\frac{\left[\text{pMFMASP1}_{\text{zym}}\right]}{K_{\text{m MASP1}_{\text{zym}}}^{\text{MASP1}_{\text{zym}}}}+\frac{\left[\text{pMFMASP2}_{\text{zym}}\right]}{K_{\text{m MASP1}_{\text{zym}}}^{\text{MASP2}_{\text{zym}}}}\\+\frac{\left[\text{pPTX3LFMASP1}_{\text{zym}}\right]}{K_{\text{m MASP1}_{\text{zym}}}^{\text{MASP1}_{\text{zym}}}}+\frac{\left[\text{pPTX3LFMASP2}_{\text{zym}}\right]}{K_{\text{m MASP1}_{\text{zym}}}^{\text{MASP2}_{\text{zym}}}}\end{array}\right)}$$

$$-\frac{k_{\mathrm{cat\ MASP1_{zym}}}^{\mathrm{MASP2_{zym}}}\left[\mathrm{pHFMASP1_{zym}}\right]\left[\mathrm{pMBL_4MASP2_{zym}}\right]}{K_{\mathrm{m\ MASP1_{zym}}}^{\mathrm{MASP2_{zym}}}\left(\begin{array}{l}1+\frac{\left[\mathrm{pMBL_3MASP1_{zym}}\right]}{K_{\mathrm{m\ MASP1_{zym}}}^{\mathrm{MASP1_{zym}}}}+\frac{\left[\mathrm{pMBL_3MASP2_{zym}}\right]}{K_{\mathrm{m\ MASP1_{zym}}}^{\mathrm{MASP2_{zym}}}}+\frac{\left[\mathrm{pMBL_4MASP1_{zym}}\right]}{K_{\mathrm{m\ MASP1_{zym}}}^{\mathrm{MASP1_{zym}}}}+\frac{\left[\mathrm{pMBL_4MASP2_{zym}}\right]}{K_{\mathrm{m\ MASP1_{zym}}}^{\mathrm{MASP2_{zym}}}}\\+\frac{\left[\mathrm{pCLLKMASP1_{zym}}\right]}{K_{\mathrm{m\ MASP1_{zym}}}^{\mathrm{MASP1_{zym}}}}+\frac{\left[\mathrm{pCLLKMASP2_{zym}}\right]}{K_{\mathrm{m\ MASP1_{zym}}}^{\mathrm{MASP2_{zym}}}}+\frac{\left[\mathrm{pCLK1MASP1_{zym}}\right]}{K_{\mathrm{m\ MASP1_{zym}}}^{\mathrm{MASP1_{zym}}}}+\frac{\left[\mathrm{pCLK1MASP2_{zym}}\right]}{K_{\mathrm{m\ MASP1_{zym}}}^{\mathrm{MASP2_{zym}}}}\\+\frac{\left[\mathrm{pCLL1MASP1_{zym}}\right]}{K_{\mathrm{m\ MASP1_{zym}}}^{\mathrm{MASP1_{zym}}}}+\frac{\left[\mathrm{pCLL1MASP2_{zym}}\right]}{K_{\mathrm{m\ MASP1_{zym}}}^{\mathrm{MASP2_{zym}}}}+\frac{\left[\mathrm{pLFMASP1_{zym}}\right]}{K_{\mathrm{m\ MASP1_{zym}}}^{\mathrm{MASP1_{zym}}}}+\frac{\left[\mathrm{pLFMASP2_{zym}}\right]}{K_{\mathrm{m\ MASP1_{zym}}}^{\mathrm{MASP2_{zym}}}}\\+\frac{\left[\mathrm{pHFMASP1_{zym}}\right]}{K_{\mathrm{m\ MASP1_{zym}}}^{\mathrm{MASP1_{zym}}}}+\frac{\left[\mathrm{pHFMASP2_{zym}}\right]}{K_{\mathrm{m\ MASP1_{zym}}}^{\mathrm{MASP2_{zym}}}}+\frac{\left[\mathrm{pMFMASP1_{zym}}\right]}{K_{\mathrm{m\ MASP1_{zym}}}^{\mathrm{MASP1_{zym}}}}+\frac{\left[\mathrm{pMFMASP2_{zym}}\right]}{K_{\mathrm{m\ MASP1_{zym}}}^{\mathrm{MASP2_{zym}}}}\\+\frac{\left[\mathrm{pPTX3LFMASP1_{zym}}\right]}{K_{\mathrm{m\ MASP1_{zym}}}^{\mathrm{MASP1_{zym}}}}+\frac{\left[\mathrm{pPTX3LFMASP2_{zym}}\right]}{K_{\mathrm{m\ MASP1_{zym}}}^{\mathrm{MASP2_{zym}}}}\end{array}\right)}$$

$$-\frac{k_{\mathrm{cat\ MASP1_{zym}}}^{\mathrm{MASP2_{zym}}}\left[\mathrm{pMFMASP1_{zym}}\right]\left[\mathrm{pMBL_4MASP2_{zym}}\right]}{K_{\mathrm{m\ MASP1_{zym}}}^{\mathrm{MASP2_{zym}}}\left(\begin{array}{l}1+\frac{\left[\mathrm{pMBL_3MASP1_{zym}}\right]}{K_{\mathrm{m\ MASP1_{zym}}}^{\mathrm{MASP1_{zym}}}}+\frac{\left[\mathrm{pMBL_3MASP2_{zym}}\right]}{K_{\mathrm{m\ MASP1_{zym}}}^{\mathrm{MASP2_{zym}}}}+\frac{\left[\mathrm{pMBL_4MASP1_{zym}}\right]}{K_{\mathrm{m\ MASP1_{zym}}}^{\mathrm{MASP1_{zym}}}}+\frac{\left[\mathrm{pMBL_4MASP2_{zym}}\right]}{K_{\mathrm{m\ MASP1_{zym}}}^{\mathrm{MASP2_{zym}}}}\\+\frac{\left[\mathrm{pCLLKMASP1_{zym}}\right]}{K_{\mathrm{m\ MASP1_{zym}}}^{\mathrm{MASP1_{zym}}}}+\frac{\left[\mathrm{pCLLKMASP2_{zym}}\right]}{K_{\mathrm{m\ MASP1_{zym}}}^{\mathrm{MASP2_{zym}}}}+\frac{\left[\mathrm{pCLK1MASP1_{zym}}\right]}{K_{\mathrm{m\ MASP1_{zym}}}^{\mathrm{MASP1_{zym}}}}+\frac{\left[\mathrm{pCLK1MASP2_{zym}}\right]}{K_{\mathrm{m\ MASP1_{zym}}}^{\mathrm{MASP2_{zym}}}}\\+\frac{\left[\mathrm{pCLL1MASP1_{zym}}\right]}{K_{\mathrm{m\ MASP1_{zym}}}^{\mathrm{MASP1_{zym}}}}+\frac{\left[\mathrm{pCLL1MASP2_{zym}}\right]}{K_{\mathrm{m\ MASP1_{zym}}}^{\mathrm{MASP2_{zym}}}}+\frac{\left[\mathrm{pLFMASP1_{zym}}\right]}{K_{\mathrm{m\ MASP1_{zym}}}^{\mathrm{MASP1_{zym}}}}+\frac{\left[\mathrm{pLFMASP2_{zym}}\right]}{K_{\mathrm{m\ MASP1_{zym}}}^{\mathrm{MASP2_{zym}}}}\\+\frac{\left[\mathrm{pHFMASP1_{zym}}\right]}{K_{\mathrm{m\ MASP1_{zym}}}^{\mathrm{MASP1_{zym}}}}+\frac{\left[\mathrm{pHFMASP2_{zym}}\right]}{K_{\mathrm{m\ MASP1_{zym}}}^{\mathrm{MASP2_{zym}}}}+\frac{\left[\mathrm{pMFMASP1_{zym}}\right]}{K_{\mathrm{m\ MASP1_{zym}}}^{\mathrm{MASP1_{zym}}}}+\frac{\left[\mathrm{pMFMASP2_{zym}}\right]}{K_{\mathrm{m\ MASP1_{zym}}}^{\mathrm{MASP2_{zym}}}}\\+\frac{\left[\mathrm{pPTX3LFMASP1_{zym}}\right]}{K_{\mathrm{m\ MASP1_{zym}}}^{\mathrm{MASP1_{zym}}}}+\frac{\left[\mathrm{pPTX3LFMASP2_{zym}}\right]}{K_{\mathrm{m\ MASP1_{zym}}}^{\mathrm{MASP2_{zym}}}}\end{array}\right)}$$

$$-\frac{k_{\text{cat MASP1}_{\text{zym}}}^{\text{MASP2}_{\text{zym}}}\left[\text{pPTX3LFMASP1}_{\text{zym}}\right]\left[\text{pMBL}_4\text{MASP2}_{\text{zym}}\right]}{K_{\text{m MASP1}_{\text{zym}}}^{\text{MASP2}_{\text{zym}}}\left(\begin{array}{l}1+\frac{\left[\text{pMBL}_3\text{MASP1}_{\text{zym}}\right]}{K_{\text{m MASP1}_{\text{zym}}}^{\text{MASP1}_{\text{zym}}}}+\frac{\left[\text{pMBL}_3\text{MASP2}_{\text{zym}}\right]}{K_{\text{m MASP1}_{\text{zym}}}^{\text{MASP2}_{\text{zym}}}}+\frac{\left[\text{pMBL}_4\text{MASP1}_{\text{zym}}\right]}{K_{\text{m MASP1}_{\text{zym}}}^{\text{MASP1}_{\text{zym}}}}+\frac{\left[\text{pMBL}_4\text{MASP2}_{\text{zym}}\right]}{K_{\text{m MASP1}_{\text{zym}}}^{\text{MASP2}_{\text{zym}}}}\\+\frac{\left[\text{pCLLKMASP1}_{\text{zym}}\right]}{K_{\text{m MASP1}_{\text{zym}}}^{\text{MASP1}_{\text{zym}}}}+\frac{\left[\text{pCLLKMASP2}_{\text{zym}}\right]}{K_{\text{m MASP1}_{\text{zym}}}^{\text{MASP2}_{\text{zym}}}}+\frac{\left[\text{pCLK1MASP1}_{\text{zym}}\right]}{K_{\text{m MASP1}_{\text{zym}}}^{\text{MASP1}_{\text{zym}}}}+\frac{\left[\text{pCLK1MASP2}_{\text{zym}}\right]}{K_{\text{m MASP1}_{\text{zym}}}^{\text{MASP2}_{\text{zym}}}}\\+\frac{\left[\text{pCLL1MASP1}_{\text{zym}}\right]}{K_{\text{m MASP1}_{\text{zym}}}^{\text{MASP1}_{\text{zym}}}}+\frac{\left[\text{pCLL1MASP2}_{\text{zym}}\right]}{K_{\text{m MASP1}_{\text{zym}}}^{\text{MASP2}_{\text{zym}}}}+\frac{\left[\text{pLFMASP1}_{\text{zym}}\right]}{K_{\text{m MASP1}_{\text{zym}}}^{\text{MASP1}_{\text{zym}}}}+\frac{\left[\text{pLFMASP2}_{\text{zym}}\right]}{K_{\text{m MASP1}_{\text{zym}}}^{\text{MASP2}_{\text{zym}}}}\\+\frac{\left[\text{pHFMASP1}_{\text{zym}}\right]}{K_{\text{m MASP1}_{\text{zym}}}^{\text{MASP1}_{\text{zym}}}}+\frac{\left[\text{pHFMASP2}_{\text{zym}}\right]}{K_{\text{m MASP1}_{\text{zym}}}^{\text{MASP2}_{\text{zym}}}}+\frac{\left[\text{pMFMASP1}_{\text{zym}}\right]}{K_{\text{m MASP1}_{\text{zym}}}^{\text{MASP1}_{\text{zym}}}}+\frac{\left[\text{pMFMASP2}_{\text{zym}}\right]}{K_{\text{m MASP1}_{\text{zym}}}^{\text{MASP2}_{\text{zym}}}}\\+\frac{\left[\text{pPTX3LFMASP1}_{\text{zym}}\right]}{K_{\text{m MASP1}_{\text{zym}}}^{\text{MASP1}_{\text{zym}}}}+\frac{\left[\text{pPTX3LFMASP2}_{\text{zym}}\right]}{K_{\text{m MASP1}_{\text{zym}}}^{\text{MASP2}_{\text{zym}}}}\end{array}\right)}$$

$$-\frac{k_{\text{cat MASP2}_{\text{zym}}}^{\text{MASP2}_{\text{zym}}}\left[\text{pMBL}_3\text{MASP2}_{\text{zym}}\right]\left[\text{pMBL}_4\text{MASP2}_{\text{zym}}\right]}{K_{\text{m MASP2}_{\text{zym}}}^{\text{MASP2}_{\text{zym}}}\left(\begin{array}{l}1+\frac{\left[\text{pMBL}_3\text{MASP2}_{\text{zym}}\right]}{K_{\text{m MASP2}_{\text{zym}}}^{\text{MASP2}_{\text{zym}}}}+\frac{\left[\text{pMBL}_4\text{MASP2}_{\text{zym}}\right]}{K_{\text{m MASP2}_{\text{zym}}}^{\text{MASP2}_{\text{zym}}}}+\frac{\left[\text{pCLLKMASP2}_{\text{zym}}\right]}{K_{\text{m MASP2}_{\text{zym}}}^{\text{MASP2}_{\text{zym}}}}+\frac{\left[\text{pCLK1MASP2}_{\text{zym}}\right]}{K_{\text{m MASP2}_{\text{zym}}}^{\text{MASP2}_{\text{zym}}}}\\+\frac{\left[\text{pCLL1MASP2}_{\text{zym}}\right]}{K_{\text{m MASP2}_{\text{zym}}}^{\text{MASP2}_{\text{zym}}}}+\frac{\left[\text{pLFMASP2}_{\text{zym}}\right]}{K_{\text{m MASP2}_{\text{zym}}}^{\text{MASP2}_{\text{zym}}}}+\frac{\left[\text{pHFMASP2}_{\text{zym}}\right]}{K_{\text{m MASP2}_{\text{zym}}}^{\text{MASP2}_{\text{zym}}}}+\frac{\left[\text{pMFMASP2}_{\text{zym}}\right]}{K_{\text{m MASP2}_{\text{zym}}}^{\text{MASP2}_{\text{zym}}}}\\+\frac{\left[\text{pPTX3LFMASP2}_{\text{zym}}\right]}{K_{\text{m MASP2}_{\text{zym}}}^{\text{MASP2}_{\text{zym}}}}\end{array}\right)}$$

$$-\frac{k_{\text{cat MASP2}_{\text{zym}}}^{\text{MASP2}_{\text{zym}}}\left[\text{pMBL}_4\text{MASP2}_{\text{zym}}\right]\left[\text{pMBL}_4\text{MASP2}_{\text{zym}}\right]}{K_{\text{m MASP2}_{\text{zym}}}^{\text{MASP2}_{\text{zym}}}\left(\begin{array}{l}1+\frac{\left[\text{pMBL}_3\text{MASP2}_{\text{zym}}\right]}{K_{\text{m MASP2}_{\text{zym}}}^{\text{MASP2}_{\text{zym}}}}+\frac{\left[\text{pMBL}_4\text{MASP2}_{\text{zym}}\right]}{K_{\text{m MASP2}_{\text{zym}}}^{\text{MASP2}_{\text{zym}}}}+\frac{\left[\text{pCLLKMASP2}_{\text{zym}}\right]}{K_{\text{m MASP2}_{\text{zym}}}^{\text{MASP2}_{\text{zym}}}}+\frac{\left[\text{pCLK1MASP2}_{\text{zym}}\right]}{K_{\text{m MASP2}_{\text{zym}}}^{\text{MASP2}_{\text{zym}}}}\\+\frac{\left[\text{pCLL1MASP2}_{\text{zym}}\right]}{K_{\text{m MASP2}_{\text{zym}}}^{\text{MASP2}_{\text{zym}}}}+\frac{\left[\text{pLFMASP2}_{\text{zym}}\right]}{K_{\text{m MASP2}_{\text{zym}}}^{\text{MASP2}_{\text{zym}}}}+\frac{\left[\text{pHFMASP2}_{\text{zym}}\right]}{K_{\text{m MASP2}_{\text{zym}}}^{\text{MASP2}_{\text{zym}}}}+\frac{\left[\text{pMFMASP2}_{\text{zym}}\right]}{K_{\text{m MASP2}_{\text{zym}}}^{\text{MASP2}_{\text{zym}}}}\\+\frac{\left[\text{pPTX3LFMASP2}_{\text{zym}}\right]}{K_{\text{m MASP2}_{\text{zym}}}^{\text{MASP2}_{\text{zym}}}}\end{array}\right)}$$

$$-\frac{k^{\mathrm{MASP2_{zym}}}_{\mathrm{cat\ MASP2_{zym}}}\left[\mathrm{pCLLKMASP2_{zym}}\right]\left[\mathrm{pMBL_4MASP2_{zym}}\right]}{K^{\mathrm{MASP2_{zym}}}_{\mathrm{m\ MASP2_{zym}}}\left(\begin{array}{l}1+\frac{\left[\mathrm{pMBL_3MASP2_{zym}}\right]}{K^{\mathrm{MASP2_{zym}}}_{\mathrm{m\ MASP2_{zym}}}}+\frac{\left[\mathrm{pMBL_4MASP2_{zym}}\right]}{K^{\mathrm{MASP2_{zym}}}_{\mathrm{m\ MASP2_{zym}}}}+\frac{\left[\mathrm{pCLLKMASP2_{zym}}\right]}{K^{\mathrm{MASP2_{zym}}}_{\mathrm{m\ MASP2_{zym}}}}+\frac{\left[\mathrm{pCLK1MASP2_{zym}}\right]}{K^{\mathrm{MASP2_{zym}}}_{\mathrm{m\ MASP2_{zym}}}}\\+\frac{\left[\mathrm{pCLL1MASP2_{zym}}\right]}{K^{\mathrm{MASP2_{zym}}}_{\mathrm{m\ MASP2_{zym}}}}+\frac{\left[\mathrm{pLFMASP2_{zym}}\right]}{K^{\mathrm{MASP2_{zym}}}_{\mathrm{m\ MASP2_{zym}}}}+\frac{\left[\mathrm{pHFMASP2_{zym}}\right]}{K^{\mathrm{MASP2_{zym}}}_{\mathrm{m\ MASP2_{zym}}}}+\frac{\left[\mathrm{pMFMASP2_{zym}}\right]}{K^{\mathrm{MASP2_{zym}}}_{\mathrm{m\ MASP2_{zym}}}}\\+\frac{\left[\mathrm{pPTX3LFMASP2_{zym}}\right]}{K^{\mathrm{MASP2_{zym}}}_{\mathrm{m\ MASP2_{zym}}}}\end{array}\right)}$$

$$-\frac{k^{\mathrm{MASP2_{zym}}}_{\mathrm{cat\ MASP2_{zym}}}\left[\mathrm{pCLK1MASP2_{zym}}\right]\left[\mathrm{pMBL_4MASP2_{zym}}\right]}{K^{\mathrm{MASP2_{zym}}}_{\mathrm{m\ MASP2_{zym}}}\left(\begin{array}{l}1+\frac{\left[\mathrm{pMBL_3MASP2_{zym}}\right]}{K^{\mathrm{MASP2_{zym}}}_{\mathrm{m\ MASP2_{zym}}}}+\frac{\left[\mathrm{pMBL_4MASP2_{zym}}\right]}{K^{\mathrm{MASP2_{zym}}}_{\mathrm{m\ MASP2_{zym}}}}+\frac{\left[\mathrm{pCLLKMASP2_{zym}}\right]}{K^{\mathrm{MASP2_{zym}}}_{\mathrm{m\ MASP2_{zym}}}}+\frac{\left[\mathrm{pCLK1MASP2_{zym}}\right]}{K^{\mathrm{MASP2_{zym}}}_{\mathrm{m\ MASP2_{zym}}}}\\+\frac{\left[\mathrm{pCLL1MASP2_{zym}}\right]}{K^{\mathrm{MASP2_{zym}}}_{\mathrm{m\ MASP2_{zym}}}}+\frac{\left[\mathrm{pLFMASP2_{zym}}\right]}{K^{\mathrm{MASP2_{zym}}}_{\mathrm{m\ MASP2_{zym}}}}+\frac{\left[\mathrm{pHFMASP2_{zym}}\right]}{K^{\mathrm{MASP2_{zym}}}_{\mathrm{m\ MASP2_{zym}}}}+\frac{\left[\mathrm{pMFMASP2_{zym}}\right]}{K^{\mathrm{MASP2_{zym}}}_{\mathrm{m\ MASP2_{zym}}}}\\+\frac{\left[\mathrm{pPTX3LFMASP2_{zym}}\right]}{K^{\mathrm{MASP2_{zym}}}_{\mathrm{m\ MASP2_{zym}}}}\end{array}\right)}$$

$$-\frac{k^{\mathrm{MASP2_{zym}}}_{\mathrm{cat\ MASP2_{zym}}}\left[\mathrm{pCLL1MASP2_{zym}}\right]\left[\mathrm{pMBL_4MASP2_{zym}}\right]}{K^{\mathrm{MASP2_{zym}}}_{\mathrm{m\ MASP2_{zym}}}\left(\begin{array}{l}1+\frac{\left[\mathrm{pMBL_3MASP2_{zym}}\right]}{K^{\mathrm{MASP2_{zym}}}_{\mathrm{m\ MASP2_{zym}}}}+\frac{\left[\mathrm{pMBL_4MASP2_{zym}}\right]}{K^{\mathrm{MASP2_{zym}}}_{\mathrm{m\ MASP2_{zym}}}}+\frac{\left[\mathrm{pCLLKMASP2_{zym}}\right]}{K^{\mathrm{MASP2_{zym}}}_{\mathrm{m\ MASP2_{zym}}}}+\frac{\left[\mathrm{pCLK1MASP2_{zym}}\right]}{K^{\mathrm{MASP2_{zym}}}_{\mathrm{m\ MASP2_{zym}}}}\\+\frac{\left[\mathrm{pCLL1MASP2_{zym}}\right]}{K^{\mathrm{MASP2_{zym}}}_{\mathrm{m\ MASP2_{zym}}}}+\frac{\left[\mathrm{pLFMASP2_{zym}}\right]}{K^{\mathrm{MASP2_{zym}}}_{\mathrm{m\ MASP2_{zym}}}}+\frac{\left[\mathrm{pHFMASP2_{zym}}\right]}{K^{\mathrm{MASP2_{zym}}}_{\mathrm{m\ MASP2_{zym}}}}+\frac{\left[\mathrm{pMFMASP2_{zym}}\right]}{K^{\mathrm{MASP2_{zym}}}_{\mathrm{m\ MASP2_{zym}}}}\\+\frac{\left[\mathrm{pPTX3LFMASP2_{zym}}\right]}{K^{\mathrm{MASP2_{zym}}}_{\mathrm{m\ MASP2_{zym}}}}\end{array}\right)}$$

$$-\frac{k^{\mathrm{MASP2_{zym}}}_{\mathrm{cat\ MASP2_{zym}}}\left[\mathrm{pLFMASP2_{zym}}\right]\left[\mathrm{pMBL_4MASP2_{zym}}\right]}{K^{\mathrm{MASP2_{zym}}}_{\mathrm{m\ MASP2_{zym}}}\left(\begin{array}{l}1+\frac{\left[\mathrm{pMBL_3MASP2_{zym}}\right]}{K^{\mathrm{MASP2_{zym}}}_{\mathrm{m\ MASP2_{zym}}}}+\frac{\left[\mathrm{pMBL_4MASP2_{zym}}\right]}{K^{\mathrm{MASP2_{zym}}}_{\mathrm{m\ MASP2_{zym}}}}+\frac{\left[\mathrm{pCLLKMASP2_{zym}}\right]}{K^{\mathrm{MASP2_{zym}}}_{\mathrm{m\ MASP2_{zym}}}}+\frac{\left[\mathrm{pCLK1MASP2_{zym}}\right]}{K^{\mathrm{MASP2_{zym}}}_{\mathrm{m\ MASP2_{zym}}}}\\+\frac{\left[\mathrm{pCLL1MASP2_{zym}}\right]}{K^{\mathrm{MASP2_{zym}}}_{\mathrm{m\ MASP2_{zym}}}}+\frac{\left[\mathrm{pLFMASP2_{zym}}\right]}{K^{\mathrm{MASP2_{zym}}}_{\mathrm{m\ MASP2_{zym}}}}+\frac{\left[\mathrm{pHFMASP2_{zym}}\right]}{K^{\mathrm{MASP2_{zym}}}_{\mathrm{m\ MASP2_{zym}}}}+\frac{\left[\mathrm{pMFMASP2_{zym}}\right]}{K^{\mathrm{MASP2_{zym}}}_{\mathrm{m\ MASP2_{zym}}}}\\+\frac{\left[\mathrm{pPTX3LFMASP2_{zym}}\right]}{K^{\mathrm{MASP2_{zym}}}_{\mathrm{m\ MASP2_{zym}}}}\end{array}\right)}$$

$$-\frac{k_{\text{cat MASP2}_{\text{zym}}}^{\text{MASP2}_{\text{zym}}}\left[\text{pHFMASP2}_{\text{zym}}\right]\left[\text{pMBL}_4\text{MASP2}_{\text{zym}}\right]}{K_{\text{m MASP2}_{\text{zym}}}^{\text{MASP2}_{\text{zym}}}\left(\begin{array}{l}1+\frac{\left[\text{pMBL}_3\text{MASP2}_{\text{zym}}\right]}{K_{\text{m MASP2}_{\text{zym}}}^{\text{MASP2}_{\text{zym}}}}+\frac{\left[\text{pMBL}_4\text{MASP2}_{\text{zym}}\right]}{K_{\text{m MASP2}_{\text{zym}}}^{\text{MASP2}_{\text{zym}}}}+\frac{\left[\text{pCLLKMASP2}_{\text{zym}}\right]}{K_{\text{m MASP2}_{\text{zym}}}^{\text{MASP2}_{\text{zym}}}}+\frac{\left[\text{pCLK1MASP2}_{\text{zym}}\right]}{K_{\text{m MASP2}_{\text{zym}}}^{\text{MASP2}_{\text{zym}}}}\\+\frac{\left[\text{pCLL1MASP2}_{\text{zym}}\right]}{K_{\text{m MASP2}_{\text{zym}}}^{\text{MASP2}_{\text{zym}}}}+\frac{\left[\text{pLFMASP2}_{\text{zym}}\right]}{K_{\text{m MASP2}_{\text{zym}}}^{\text{MASP2}_{\text{zym}}}}+\frac{\left[\text{pHFMASP2}_{\text{zym}}\right]}{K_{\text{m MASP2}_{\text{zym}}}^{\text{MASP2}_{\text{zym}}}}+\frac{\left[\text{pMFMASP2}_{\text{zym}}\right]}{K_{\text{m MASP2}_{\text{zym}}}^{\text{MASP2}_{\text{zym}}}}\\+\frac{\left[\text{pPTX3LFMASP2}_{\text{zym}}\right]}{K_{\text{m MASP2}_{\text{zym}}}^{\text{MASP2}_{\text{zym}}}}\end{array}\right)}$$

$$-\frac{k_{\text{cat MASP2}_{\text{zym}}}^{\text{MASP2}_{\text{zym}}}\left[\text{pMFMASP2}_{\text{zym}}\right]\left[\text{pMBL}_4\text{MASP2}_{\text{zym}}\right]}{K_{\text{m MASP2}_{\text{zym}}}^{\text{MASP2}_{\text{zym}}}\left(\begin{array}{l}1+\frac{\left[\text{pMBL}_3\text{MASP2}_{\text{zym}}\right]}{K_{\text{m MASP2}_{\text{zym}}}^{\text{MASP2}_{\text{zym}}}}+\frac{\left[\text{pMBL}_4\text{MASP2}_{\text{zym}}\right]}{K_{\text{m MASP2}_{\text{zym}}}^{\text{MASP2}_{\text{zym}}}}+\frac{\left[\text{pCLLKMASP2}_{\text{zym}}\right]}{K_{\text{m MASP2}_{\text{zym}}}^{\text{MASP2}_{\text{zym}}}}+\frac{\left[\text{pCLK1MASP2}_{\text{zym}}\right]}{K_{\text{m MASP2}_{\text{zym}}}^{\text{MASP2}_{\text{zym}}}}\\+\frac{\left[\text{pCLL1MASP2}_{\text{zym}}\right]}{K_{\text{m MASP2}_{\text{zym}}}^{\text{MASP2}_{\text{zym}}}}+\frac{\left[\text{pLFMASP2}_{\text{zym}}\right]}{K_{\text{m MASP2}_{\text{zym}}}^{\text{MASP2}_{\text{zym}}}}+\frac{\left[\text{pHFMASP2}_{\text{zym}}\right]}{K_{\text{m MASP2}_{\text{zym}}}^{\text{MASP2}_{\text{zym}}}}+\frac{\left[\text{pMFMASP2}_{\text{zym}}\right]}{K_{\text{m MASP2}_{\text{zym}}}^{\text{MASP2}_{\text{zym}}}}\\+\frac{\left[\text{pPTX3LFMASP2}_{\text{zym}}\right]}{K_{\text{m MASP2}_{\text{zym}}}^{\text{MASP2}_{\text{zym}}}}\end{array}\right)}$$

$$-\frac{k_{\text{cat MASP2}_{\text{zym}}}^{\text{MASP2}_{\text{zym}}}\left[\text{pPTX3LFMASP2}_{\text{zym}}\right]\left[\text{pMBL}_4\text{MASP2}_{\text{zym}}\right]}{K_{\text{m MASP2}_{\text{zym}}}^{\text{MASP2}_{\text{zym}}}\left(\begin{array}{l}1+\frac{\left[\text{pMBL}_3\text{MASP2}_{\text{zym}}\right]}{K_{\text{m MASP2}_{\text{zym}}}^{\text{MASP2}_{\text{zym}}}}+\frac{\left[\text{pMBL}_4\text{MASP2}_{\text{zym}}\right]}{K_{\text{m MASP2}_{\text{zym}}}^{\text{MASP2}_{\text{zym}}}}+\frac{\left[\text{pCLLKMASP2}_{\text{zym}}\right]}{K_{\text{m MASP2}_{\text{zym}}}^{\text{MASP2}_{\text{zym}}}}+\frac{\left[\text{pCLK1MASP2}_{\text{zym}}\right]}{K_{\text{m MASP2}_{\text{zym}}}^{\text{MASP2}_{\text{zym}}}}\\+\frac{\left[\text{pCLL1MASP2}_{\text{zym}}\right]}{K_{\text{m MASP2}_{\text{zym}}}^{\text{MASP2}_{\text{zym}}}}+\frac{\left[\text{pLFMASP2}_{\text{zym}}\right]}{K_{\text{m MASP2}_{\text{zym}}}^{\text{MASP2}_{\text{zym}}}}+\frac{\left[\text{pHFMASP2}_{\text{zym}}\right]}{K_{\text{m MASP2}_{\text{zym}}}^{\text{MASP2}_{\text{zym}}}}+\frac{\left[\text{pMFMASP2}_{\text{zym}}\right]}{K_{\text{m MASP2}_{\text{zym}}}^{\text{MASP2}_{\text{zym}}}}\\+\frac{\left[\text{pPTX3LFMASP2}_{\text{zym}}\right]}{K_{\text{m MASP2}_{\text{zym}}}^{\text{MASP2}_{\text{zym}}}}\end{array}\right)}$$

$$-\frac{k_{\mathrm{cat\,MASP1^*}}^{\mathrm{MASP2_{zym}}}\left[\mathrm{pMBL_3MASP1^*}\right]\left[\mathrm{pMBL_4MASP2_{zym}}\right]}{K_{\mathrm{m\,MASP1^*}}^{\mathrm{MASP2_{zym}}}\left(\begin{array}{l}1+\frac{[\mathrm{C4}]}{K_{\mathrm{m\,MASP1^*}}^{\mathrm{C4}}}+\frac{[\mathrm{fC4bC2}]}{K_{\mathrm{m\,MASP1^*}}^{\mathrm{C2}}}+\frac{[\mathrm{fC4bC4bC2}]}{K_{\mathrm{m\,MASP1^*}}^{\mathrm{C2}}}+\frac{[\mathrm{fC3bC4bC2}]}{K_{\mathrm{m\,MASP1^*}}^{\mathrm{C2}}}+\frac{[\mathrm{pC4bC2}]}{K_{\mathrm{m\,MASP1^*}}^{\mathrm{C2}}}+\frac{[\mathrm{pC4bC4bC2}]}{K_{\mathrm{m\,MASP1^*}}^{\mathrm{C2}}}\\
+\frac{[\mathrm{pC3bC4bC2}]}{K_{\mathrm{m\,MASP1^*}}^{\mathrm{C2}}}+\frac{[\mathrm{pMBL_3MASP1_{zym}}]}{K_{\mathrm{m\,MASP1^*}}^{\mathrm{MASP1_{zym}}}}+\frac{[\mathrm{pMBL_3MASP2_{zym}}]}{K_{\mathrm{m\,MASP1^*}}^{\mathrm{MASP2_{zym}}}}+\frac{[\mathrm{pMBL_3MASP3_{zym}}]}{K_{\mathrm{m\,MASP1^*}}^{\mathrm{MASP3_{zym}}}}\\
+\frac{[\mathrm{pMBL_4MASP1_{zym}}]}{K_{\mathrm{m\,MASP1^*}}^{\mathrm{MASP1_{zym}}}}+\frac{[\mathrm{pMBL_4MASP2_{zym}}]}{K_{\mathrm{m\,MASP1^*}}^{\mathrm{MASP2_{zym}}}}+\frac{[\mathrm{pMBL_4MASP3_{zym}}]}{K_{\mathrm{m\,MASP1^*}}^{\mathrm{MASP3_{zym}}}}+\frac{[\mathrm{pCLLKMASP1_{zym}}]}{K_{\mathrm{m\,MASP1^*}}^{\mathrm{MASP1_{zym}}}}\\
+\frac{[\mathrm{pCLLKMASP2_{zym}}]}{K_{\mathrm{m\,MASP1^*}}^{\mathrm{MASP2_{zym}}}}+\frac{[\mathrm{pCLLKMASP3_{zym}}]}{K_{\mathrm{m\,MASP1^*}}^{\mathrm{MASP3_{zym}}}}+\frac{[\mathrm{pCLK1MASP1_{zym}}]}{K_{\mathrm{m\,MASP1^*}}^{\mathrm{MASP1_{zym}}}}+\frac{[\mathrm{pCLK1MASP2_{zym}}]}{K_{\mathrm{m\,MASP1^*}}^{\mathrm{MASP2_{zym}}}}\\
+\frac{[\mathrm{pCLK1MASP3_{zym}}]}{K_{\mathrm{m\,MASP1^*}}^{\mathrm{MASP3_{zym}}}}+\frac{[\mathrm{pCLL1MASP1_{zym}}]}{K_{\mathrm{m\,MASP1^*}}^{\mathrm{MASP1_{zym}}}}+\frac{[\mathrm{pCLL1MASP2_{zym}}]}{K_{\mathrm{m\,MASP1^*}}^{\mathrm{MASP2_{zym}}}}+\frac{[\mathrm{pCLL1MASP3_{zym}}]}{K_{\mathrm{m\,MASP1^*}}^{\mathrm{MASP3_{zym}}}}\\
+\frac{[\mathrm{pLFMASP1_{zym}}]}{K_{\mathrm{m\,MASP1^*}}^{\mathrm{MASP1_{zym}}}}+\frac{[\mathrm{pLFMASP2_{zym}}]}{K_{\mathrm{m\,MASP1^*}}^{\mathrm{MASP2_{zym}}}}+\frac{[\mathrm{pLFMASP3_{zym}}]}{K_{\mathrm{m\,MASP1^*}}^{\mathrm{MASP3_{zym}}}}+\frac{[\mathrm{pHFMASP1_{zym}}]}{K_{\mathrm{m\,MASP1^*}}^{\mathrm{MASP1_{zym}}}}+\frac{[\mathrm{pHFMASP2_{zym}}]}{K_{\mathrm{m\,MASP1^*}}^{\mathrm{MASP2_{zym}}}}\\
+\frac{[\mathrm{pHFMASP3_{zym}}]}{K_{\mathrm{m\,MASP1^*}}^{\mathrm{MASP3_{zym}}}}+\frac{[\mathrm{pMFMASP1_{zym}}]}{K_{\mathrm{m\,MASP1^*}}^{\mathrm{MASP1_{zym}}}}+\frac{[\mathrm{pMFMASP2_{zym}}]}{K_{\mathrm{m\,MASP1^*}}^{\mathrm{MASP2_{zym}}}}+\frac{[\mathrm{pMFMASP3_{zym}}]}{K_{\mathrm{m\,MASP1^*}}^{\mathrm{MASP3_{zym}}}}\\
+\frac{[\mathrm{pPTX3LFMASP1_{zym}}]}{K_{\mathrm{m\,MASP1^*}}^{\mathrm{MASP1_{zym}}}}+\frac{[\mathrm{pPXT3LFMASP2_{zym}}]}{K_{\mathrm{m\,MASP1^*}}^{\mathrm{MASP2_{zym}}}}+\frac{[\mathrm{pPTX3LFMASP3_{zym}}]}{K_{\mathrm{m\,MASP1^*}}^{\mathrm{MASP3_{zym}}}}\end{array}\right)}$$

$$
-\frac{k_{\text{cat MASP1}^*}^{\text{MASP2}_{\text{zym}}}\left[\text{pMBL}_4\text{MASP1}^*\right]\left[\text{pMBL}_4\text{MASP2}_{\text{zym}}\right]}{K_{\text{m MASP1}^*}^{\text{MASP2}_{\text{zym}}}\left(\begin{array}{l}1+\frac{[\text{C4}]}{K_{\text{m MASP1}^*}^{\text{C4}}}+\frac{[\text{fC4bC2}]}{K_{\text{m MASP1}^*}^{\text{C2}}}+\frac{[\text{fC4bC4bC2}]}{K_{\text{m MASP1}^*}^{\text{C2}}}+\frac{[\text{fC3bC4bC2}]}{K_{\text{m MASP1}^*}^{\text{C2}}}+\frac{[\text{pC4bC2}]}{K_{\text{m MASP1}^*}^{\text{C2}}}+\frac{[\text{pC4bC4bC2}]}{K_{\text{m MASP1}^*}^{\text{C2}}}\\
+\frac{[\text{pC3bC4bC2}]}{K_{\text{m MASP1}^*}^{\text{C2}}}+\frac{[\text{pMBL}_3\text{MASP1}_{\text{zym}}]}{K_{\text{m MASP1}^*}^{\text{MASP1}_{\text{zym}}}}+\frac{[\text{pMBL}_3\text{MASP2}_{\text{zym}}]}{K_{\text{m MASP1}^*}^{\text{MASP2}_{\text{zym}}}}+\frac{[\text{pMBL}_3\text{MASP3}_{\text{zym}}]}{K_{\text{m MASP1}^*}^{\text{MASP3}_{\text{zym}}}}\\
+\frac{[\text{pMBL}_4\text{MASP1}_{\text{zym}}]}{K_{\text{m MASP1}^*}^{\text{MASP1}_{\text{zym}}}}+\frac{[\text{pMBL}_4\text{MASP2}_{\text{zym}}]}{K_{\text{m MASP1}^*}^{\text{MASP2}_{\text{zym}}}}+\frac{[\text{pMBL}_4\text{MASP3}_{\text{zym}}]}{K_{\text{m MASP1}^*}^{\text{MASP3}_{\text{zym}}}}+\frac{[\text{pCLLKMASP1}_{\text{zym}}]}{K_{\text{m MASP1}^*}^{\text{MASP1}_{\text{zym}}}}\\
+\frac{[\text{pCLLKMASP2}_{\text{zym}}]}{K_{\text{m MASP1}^*}^{\text{MASP2}_{\text{zym}}}}+\frac{[\text{pCLLKMASP3}_{\text{zym}}]}{K_{\text{m MASP1}^*}^{\text{MASP3}_{\text{zym}}}}+\frac{[\text{pCLK1MASP1}_{\text{zym}}]}{K_{\text{m MASP1}^*}^{\text{MASP1}_{\text{zym}}}}+\frac{[\text{pCLK1MASP2}_{\text{zym}}]}{K_{\text{m MASP1}^*}^{\text{MASP2}_{\text{zym}}}}\\
+\frac{[\text{pCLK1MASP3}_{\text{zym}}]}{K_{\text{m MASP1}^*}^{\text{MASP3}_{\text{zym}}}}+\frac{[\text{pCLL1MASP1}_{\text{zym}}]}{K_{\text{m MASP1}^*}^{\text{MASP1}_{\text{zym}}}}+\frac{[\text{pCLL1MASP2}_{\text{zym}}]}{K_{\text{m MASP1}^*}^{\text{MASP2}_{\text{zym}}}}+\frac{[\text{pCLL1MASP3}_{\text{zym}}]}{K_{\text{m MASP1}^*}^{\text{MASP3}_{\text{zym}}}}\\
+\frac{[\text{pLFMASP1}_{\text{zym}}]}{K_{\text{m MASP1}^*}^{\text{MASP1}_{\text{zym}}}}+\frac{[\text{pLFMASP2}_{\text{zym}}]}{K_{\text{m MASP1}^*}^{\text{MASP2}_{\text{zym}}}}+\frac{[\text{pLFMASP3}_{\text{zym}}]}{K_{\text{m MASP1}^*}^{\text{MASP3}_{\text{zym}}}}+\frac{[\text{pHFMASP1}_{\text{zym}}]}{K_{\text{m MASP1}^*}^{\text{MASP1}_{\text{zym}}}}+\frac{[\text{pHFMASP2}_{\text{zym}}]}{K_{\text{m MASP1}^*}^{\text{MASP2}_{\text{zym}}}}\\
+\frac{[\text{pHFMASP3}_{\text{zym}}]}{K_{\text{m MASP1}^*}^{\text{MASP3}_{\text{zym}}}}+\frac{[\text{pMFMASP1}_{\text{zym}}]}{K_{\text{m MASP1}^*}^{\text{MASP1}_{\text{zym}}}}+\frac{[\text{pMFMASP2}_{\text{zym}}]}{K_{\text{m MASP1}^*}^{\text{MASP2}_{\text{zym}}}}+\frac{[\text{pMFMASP3}_{\text{zym}}]}{K_{\text{m MASP1}^*}^{\text{MASP3}_{\text{zym}}}}\\
+\frac{[\text{pPTX3LFMASP1}_{\text{zym}}]}{K_{\text{m MASP1}^*}^{\text{MASP1}_{\text{zym}}}}+\frac{[\text{pPXT3LFMASP2}_{\text{zym}}]}{K_{\text{m MASP1}^*}^{\text{MASP2}_{\text{zym}}}}+\frac{[\text{pPTX3LFMASP3}_{\text{zym}}]}{K_{\text{m MASP1}^*}^{\text{MASP3}_{\text{zym}}}}\end{array}\right)}
$$

$$-\frac{k_{\text{cat MASP1}^*}^{\text{MASP2}_{\text{zym}}}\left[\text{pCLLKMASP1}^*\right]\left[\text{pMBL}_4\text{MASP2}_{\text{zym}}\right]}{K_{\text{m MASP1}^*}^{\text{MASP2}_{\text{zym}}}\left(\begin{array}{l}1+\frac{[\text{C4}]}{K_{\text{m MASP1}^*}^{\text{C4}}}+\frac{[\text{fC4bC2}]}{K_{\text{m MASP1}^*}^{\text{C2}}}+\frac{[\text{fC4bC4bC2}]}{K_{\text{m MASP1}^*}^{\text{C2}}}+\frac{[\text{fC3bC4bC2}]}{K_{\text{m MASP1}^*}^{\text{C2}}}+\frac{[\text{pC4bC2}]}{K_{\text{m MASP1}^*}^{\text{C2}}}+\frac{[\text{pC4bC4bC2}]}{K_{\text{m MASP1}^*}^{\text{C2}}}\\
+\frac{[\text{pC3bC4bC2}]}{K_{\text{m MASP1}^*}^{\text{C2}}}+\frac{[\text{pMBL}_3\text{MASP1}_{\text{zym}}]}{K_{\text{m MASP1}^*}^{\text{MASP1}_{\text{zym}}}}+\frac{[\text{pMBL}_3\text{MASP2}_{\text{zym}}]}{K_{\text{m MASP1}^*}^{\text{MASP2}_{\text{zym}}}}+\frac{[\text{pMBL}_3\text{MASP3}_{\text{zym}}]}{K_{\text{m MASP1}^*}^{\text{MASP3}_{\text{zym}}}}\\
+\frac{[\text{pMBL}_4\text{MASP1}_{\text{zym}}]}{K_{\text{m MASP1}^*}^{\text{MASP1}_{\text{zym}}}}+\frac{[\text{pMBL}_4\text{MASP2}_{\text{zym}}]}{K_{\text{m MASP1}^*}^{\text{MASP2}_{\text{zym}}}}+\frac{[\text{pMBL}_4\text{MASP3}_{\text{zym}}]}{K_{\text{m MASP1}^*}^{\text{MASP3}_{\text{zym}}}}+\frac{[\text{pCLLKMASP1}_{\text{zym}}]}{K_{\text{m MASP1}^*}^{\text{MASP1}_{\text{zym}}}}\\
+\frac{[\text{pCLLKMASP2}_{\text{zym}}]}{K_{\text{m MASP1}^*}^{\text{MASP2}_{\text{zym}}}}+\frac{[\text{pCLLKMASP3}_{\text{zym}}]}{K_{\text{m MASP1}^*}^{\text{MASP3}_{\text{zym}}}}+\frac{[\text{pCLK1MASP1}_{\text{zym}}]}{K_{\text{m MASP1}^*}^{\text{MASP1}_{\text{zym}}}}+\frac{[\text{pCLK1MASP2}_{\text{zym}}]}{K_{\text{m MASP1}^*}^{\text{MASP2}_{\text{zym}}}}\\
+\frac{[\text{pCLK1MASP3}_{\text{zym}}]}{K_{\text{m MASP1}^*}^{\text{MASP3}_{\text{zym}}}}+\frac{[\text{pCLL1MASP1}_{\text{zym}}]}{K_{\text{m MASP1}^*}^{\text{MASP1}_{\text{zym}}}}+\frac{[\text{pCLL1MASP2}_{\text{zym}}]}{K_{\text{m MASP1}^*}^{\text{MASP2}_{\text{zym}}}}+\frac{[\text{pCLL1MASP3}_{\text{zym}}]}{K_{\text{m MASP1}^*}^{\text{MASP3}_{\text{zym}}}}\\
+\frac{[\text{pLFMASP1}_{\text{zym}}]}{K_{\text{m MASP1}^*}^{\text{MASP1}_{\text{zym}}}}+\frac{[\text{pLFMASP2}_{\text{zym}}]}{K_{\text{m MASP1}^*}^{\text{MASP2}_{\text{zym}}}}+\frac{[\text{pLFMASP3}_{\text{zym}}]}{K_{\text{m MASP1}^*}^{\text{MASP3}_{\text{zym}}}}+\frac{[\text{pHFMASP1}_{\text{zym}}]}{K_{\text{m MASP1}^*}^{\text{MASP1}_{\text{zym}}}}+\frac{[\text{pHFMASP2}_{\text{zym}}]}{K_{\text{m MASP1}^*}^{\text{MASP2}_{\text{zym}}}}\\
+\frac{[\text{pHFMASP3}_{\text{zym}}]}{K_{\text{m MASP1}^*}^{\text{MASP3}_{\text{zym}}}}+\frac{[\text{pMFMASP1}_{\text{zym}}]}{K_{\text{m MASP1}^*}^{\text{MASP1}_{\text{zym}}}}+\frac{[\text{pMFMASP2}_{\text{zym}}]}{K_{\text{m MASP1}^*}^{\text{MASP2}_{\text{zym}}}}+\frac{[\text{pMFMASP3}_{\text{zym}}]}{K_{\text{m MASP1}^*}^{\text{MASP3}_{\text{zym}}}}\\
+\frac{[\text{pPTX3LFMASP1}_{\text{zym}}]}{K_{\text{m MASP1}^*}^{\text{MASP1}_{\text{zym}}}}+\frac{[\text{pPXT3LFMASP2}_{\text{zym}}]}{K_{\text{m MASP1}^*}^{\text{MASP2}_{\text{zym}}}}+\frac{[\text{pPTX3LFMASP3}_{\text{zym}}]}{K_{\text{m MASP1}^*}^{\text{MASP3}_{\text{zym}}}}\end{array}\right)}$$

$$-\frac{k_{\text{cat MASP1}^*}^{\text{MASP2}_{\text{zym}}}\left[\text{pCLK1MASP1}^*\right]\left[\text{pMBL}_4\text{MASP2}_{\text{zym}}\right]}{K_{\text{m MASP1}^*}^{\text{MASP2}_{\text{zym}}}\left(\begin{array}{l}1+\frac{[\text{C4}]}{K_{\text{m MASP1}^*}^{\text{C4}}}+\frac{[\text{fC4bC2}]}{K_{\text{m MASP1}^*}^{\text{C2}}}+\frac{[\text{fC4bC4bC2}]}{K_{\text{m MASP1}^*}^{\text{C2}}}+\frac{[\text{fC3bC4bC2}]}{K_{\text{m MASP1}^*}^{\text{C2}}}+\frac{[\text{pC4bC2}]}{K_{\text{m MASP1}^*}^{\text{C2}}}+\frac{[\text{pC4bC4bC2}]}{K_{\text{m MASP1}^*}^{\text{C2}}}\\
+\frac{[\text{pC3bC4bC2}]}{K_{\text{m MASP1}^*}^{\text{C2}}}+\frac{[\text{pMBL}_3\text{MASP1}_{\text{zym}}]}{K_{\text{m MASP1}^*}^{\text{MASP1}_{\text{zym}}}}+\frac{[\text{pMBL}_3\text{MASP2}_{\text{zym}}]}{K_{\text{m MASP1}^*}^{\text{MASP2}_{\text{zym}}}}+\frac{[\text{pMBL}_3\text{MASP3}_{\text{zym}}]}{K_{\text{m MASP1}^*}^{\text{MASP3}_{\text{zym}}}}\\
+\frac{[\text{pMBL}_4\text{MASP1}_{\text{zym}}]}{K_{\text{m MASP1}^*}^{\text{MASP1}_{\text{zym}}}}+\frac{[\text{pMBL}_4\text{MASP2}_{\text{zym}}]}{K_{\text{m MASP1}^*}^{\text{MASP2}_{\text{zym}}}}+\frac{[\text{pMBL}_4\text{MASP3}_{\text{zym}}]}{K_{\text{m MASP1}^*}^{\text{MASP3}_{\text{zym}}}}+\frac{[\text{pCLLKMASP1}_{\text{zym}}]}{K_{\text{m MASP1}^*}^{\text{MASP1}_{\text{zym}}}}\\
+\frac{[\text{pCLLKMASP2}_{\text{zym}}]}{K_{\text{m MASP1}^*}^{\text{MASP2}_{\text{zym}}}}+\frac{[\text{pCLLKMASP3}_{\text{zym}}]}{K_{\text{m MASP1}^*}^{\text{MASP3}_{\text{zym}}}}+\frac{[\text{pCLK1MASP1}_{\text{zym}}]}{K_{\text{m MASP1}^*}^{\text{MASP1}_{\text{zym}}}}+\frac{[\text{pCLK1MASP2}_{\text{zym}}]}{K_{\text{m MASP1}^*}^{\text{MASP2}_{\text{zym}}}}\\
+\frac{[\text{pCLK1MASP3}_{\text{zym}}]}{K_{\text{m MASP1}^*}^{\text{MASP3}_{\text{zym}}}}+\frac{[\text{pCLL1MASP1}_{\text{zym}}]}{K_{\text{m MASP1}^*}^{\text{MASP1}_{\text{zym}}}}+\frac{[\text{pCLL1MASP2}_{\text{zym}}]}{K_{\text{m MASP1}^*}^{\text{MASP2}_{\text{zym}}}}+\frac{[\text{pCLL1MASP3}_{\text{zym}}]}{K_{\text{m MASP1}^*}^{\text{MASP3}_{\text{zym}}}}\\
+\frac{[\text{pLFMASP1}_{\text{zym}}]}{K_{\text{m MASP1}^*}^{\text{MASP1}_{\text{zym}}}}+\frac{[\text{pLFMASP2}_{\text{zym}}]}{K_{\text{m MASP1}^*}^{\text{MASP2}_{\text{zym}}}}+\frac{[\text{pLFMASP3}_{\text{zym}}]}{K_{\text{m MASP1}^*}^{\text{MASP3}_{\text{zym}}}}+\frac{[\text{pHFMASP1}_{\text{zym}}]}{K_{\text{m MASP1}^*}^{\text{MASP1}_{\text{zym}}}}+\frac{[\text{pHFMASP2}_{\text{zym}}]}{K_{\text{m MASP1}^*}^{\text{MASP2}_{\text{zym}}}}\\
+\frac{[\text{pHFMASP3}_{\text{zym}}]}{K_{\text{m MASP1}^*}^{\text{MASP3}_{\text{zym}}}}+\frac{[\text{pMFMASP1}_{\text{zym}}]}{K_{\text{m MASP1}^*}^{\text{MASP1}_{\text{zym}}}}+\frac{[\text{pMFMASP2}_{\text{zym}}]}{K_{\text{m MASP1}^*}^{\text{MASP2}_{\text{zym}}}}+\frac{[\text{pMFMASP3}_{\text{zym}}]}{K_{\text{m MASP1}^*}^{\text{MASP3}_{\text{zym}}}}\\
+\frac{[\text{pPTX3LFMASP1}_{\text{zym}}]}{K_{\text{m MASP1}^*}^{\text{MASP1}_{\text{zym}}}}+\frac{[\text{pPXT3LFMASP2}_{\text{zym}}]}{K_{\text{m MASP1}^*}^{\text{MASP2}_{\text{zym}}}}+\frac{[\text{pPTX3LFMASP3}_{\text{zym}}]}{K_{\text{m MASP1}^*}^{\text{MASP3}_{\text{zym}}}}\end{array}\right)}$$

$$-\frac{k_{\text{cat MASP1}^*}^{\text{MASP2}_{\text{zym}}}\left[\text{pCLL1MASP1}^*\right]\left[\text{pMBL}_4\text{MASP2}_{\text{zym}}\right]}{K_{\text{m MASP1}^*}^{\text{MASP2}_{\text{zym}}}\left(\begin{array}{l}1+\frac{[\text{C4}]}{K_{\text{m MASP1}^*}^{\text{C4}}}+\frac{[\text{fC4bC2}]}{K_{\text{m MASP1}^*}^{\text{C2}}}+\frac{[\text{fC4bC4bC2}]}{K_{\text{m MASP1}^*}^{\text{C2}}}+\frac{[\text{fC3bC4bC2}]}{K_{\text{m MASP1}^*}^{\text{C2}}}+\frac{[\text{pC4bC2}]}{K_{\text{m MASP1}^*}^{\text{C2}}}+\frac{[\text{pC4bC4bC2}]}{K_{\text{m MASP1}^*}^{\text{C2}}}\\+\frac{[\text{pC3bC4bC2}]}{K_{\text{m MASP1}^*}^{\text{C2}}}+\frac{[\text{pMBL}_3\text{MASP1}_{\text{zym}}]}{K_{\text{m MASP1}^*}^{\text{MASP1}_{\text{zym}}}}+\frac{[\text{pMBL}_3\text{MASP2}_{\text{zym}}]}{K_{\text{m MASP1}^*}^{\text{MASP2}_{\text{zym}}}}+\frac{[\text{pMBL}_3\text{MASP3}_{\text{zym}}]}{K_{\text{m MASP1}^*}^{\text{MASP3}_{\text{zym}}}}\\+\frac{[\text{pMBL}_4\text{MASP1}_{\text{zym}}]}{K_{\text{m MASP1}^*}^{\text{MASP1}_{\text{zym}}}}+\frac{[\text{pMBL}_4\text{MASP2}_{\text{zym}}]}{K_{\text{m MASP1}^*}^{\text{MASP2}_{\text{zym}}}}+\frac{[\text{pMBL}_4\text{MASP3}_{\text{zym}}]}{K_{\text{m MASP1}^*}^{\text{MASP3}_{\text{zym}}}}+\frac{[\text{pCLLKMASP1}_{\text{zym}}]}{K_{\text{m MASP1}^*}^{\text{MASP1}_{\text{zym}}}}\\+\frac{[\text{pCLLKMASP2}_{\text{zym}}]}{K_{\text{m MASP1}^*}^{\text{MASP2}_{\text{zym}}}}+\frac{[\text{pCLLKMASP3}_{\text{zym}}]}{K_{\text{m MASP1}^*}^{\text{MASP3}_{\text{zym}}}}+\frac{[\text{pCLK1MASP1}_{\text{zym}}]}{K_{\text{m MASP1}^*}^{\text{MASP1}_{\text{zym}}}}+\frac{[\text{pCLK1MASP2}_{\text{zym}}]}{K_{\text{m MASP1}^*}^{\text{MASP2}_{\text{zym}}}}\\+\frac{[\text{pCLK1MASP3}_{\text{zym}}]}{K_{\text{m MASP1}^*}^{\text{MASP3}_{\text{zym}}}}+\frac{[\text{pCLL1MASP1}_{\text{zym}}]}{K_{\text{m MASP1}^*}^{\text{MASP1}_{\text{zym}}}}+\frac{[\text{pCLL1MASP2}_{\text{zym}}]}{K_{\text{m MASP1}^*}^{\text{MASP2}_{\text{zym}}}}+\frac{[\text{pCLL1MASP3}_{\text{zym}}]}{K_{\text{m MASP1}^*}^{\text{MASP3}_{\text{zym}}}}\\+\frac{[\text{pLFMASP1}_{\text{zym}}]}{K_{\text{m MASP1}^*}^{\text{MASP1}_{\text{zym}}}}+\frac{[\text{pLFMASP2}_{\text{zym}}]}{K_{\text{m MASP1}^*}^{\text{MASP2}_{\text{zym}}}}+\frac{[\text{pLFMASP3}_{\text{zym}}]}{K_{\text{m MASP1}^*}^{\text{MASP3}_{\text{zym}}}}+\frac{[\text{pHFMASP1}_{\text{zym}}]}{K_{\text{m MASP1}^*}^{\text{MASP1}_{\text{zym}}}}+\frac{[\text{pHFMASP2}_{\text{zym}}]}{K_{\text{m MASP1}^*}^{\text{MASP2}_{\text{zym}}}}\\+\frac{[\text{pHFMASP3}_{\text{zym}}]}{K_{\text{m MASP1}^*}^{\text{MASP3}_{\text{zym}}}}+\frac{[\text{pMFMASP1}_{\text{zym}}]}{K_{\text{m MASP1}^*}^{\text{MASP1}_{\text{zym}}}}+\frac{[\text{pMFMASP2}_{\text{zym}}]}{K_{\text{m MASP1}^*}^{\text{MASP2}_{\text{zym}}}}+\frac{[\text{pMFMASP3}_{\text{zym}}]}{K_{\text{m MASP1}^*}^{\text{MASP3}_{\text{zym}}}}\\+\frac{[\text{pPTX3LFMASP1}_{\text{zym}}]}{K_{\text{m MASP1}^*}^{\text{MASP1}_{\text{zym}}}}+\frac{[\text{pPXT3LFMASP2}_{\text{zym}}]}{K_{\text{m MASP1}^*}^{\text{MASP2}_{\text{zym}}}}+\frac{[\text{pPTX3LFMASP3}_{\text{zym}}]}{K_{\text{m MASP1}^*}^{\text{MASP3}_{\text{zym}}}}\end{array}\right)}$$

$$-\frac{k_{\mathrm{cat\ MASP1^*}}^{\mathrm{MASP2_{zym}}}\left[\mathrm{pLFMASP1^*}\right]\left[\mathrm{pMBL_4MASP2_{zym}}\right]}{K_{\mathrm{m\ MASP1^*}}^{\mathrm{MASP2_{zym}}}\left(\begin{array}{l}1+\frac{[\mathrm{C4}]}{K_{\mathrm{m\ MASP1^*}}^{\mathrm{C4}}}+\frac{[\mathrm{fC4bC2}]}{K_{\mathrm{m\ MASP1^*}}^{\mathrm{C2}}}+\frac{[\mathrm{fC4bC4bC2}]}{K_{\mathrm{m\ MASP1^*}}^{\mathrm{C2}}}+\frac{[\mathrm{fC3bC4bC2}]}{K_{\mathrm{m\ MASP1^*}}^{\mathrm{C2}}}+\frac{[\mathrm{pC4bC2}]}{K_{\mathrm{m\ MASP1^*}}^{\mathrm{C2}}}+\frac{[\mathrm{pC4bC4bC2}]}{K_{\mathrm{m\ MASP1^*}}^{\mathrm{C2}}}\\ +\frac{[\mathrm{pC3bC4bC2}]}{K_{\mathrm{m\ MASP1^*}}^{\mathrm{C2}}}+\frac{[\mathrm{pMBL_3MASP1_{zym}}]}{K_{\mathrm{m\ MASP1^*}}^{\mathrm{MASP1_{zym}}}}+\frac{[\mathrm{pMBL_3MASP2_{zym}}]}{K_{\mathrm{m\ MASP1^*}}^{\mathrm{MASP2_{zym}}}}+\frac{[\mathrm{pMBL_3MASP3_{zym}}]}{K_{\mathrm{m\ MASP1^*}}^{\mathrm{MASP3_{zym}}}}\\ +\frac{[\mathrm{pMBL_4MASP1_{zym}}]}{K_{\mathrm{m\ MASP1^*}}^{\mathrm{MASP1_{zym}}}}+\frac{[\mathrm{pMBL_4MASP2_{zym}}]}{K_{\mathrm{m\ MASP1^*}}^{\mathrm{MASP2_{zym}}}}+\frac{[\mathrm{pMBL_4MASP3_{zym}}]}{K_{\mathrm{m\ MASP1^*}}^{\mathrm{MASP3_{zym}}}}+\frac{[\mathrm{pCLLKMASP1_{zym}}]}{K_{\mathrm{m\ MASP1^*}}^{\mathrm{MASP1_{zym}}}}\\ +\frac{[\mathrm{pCLLKMASP2_{zym}}]}{K_{\mathrm{m\ MASP1^*}}^{\mathrm{MASP2_{zym}}}}+\frac{[\mathrm{pCLLKMASP3_{zym}}]}{K_{\mathrm{m\ MASP1^*}}^{\mathrm{MASP3_{zym}}}}+\frac{[\mathrm{pCLK1MASP1_{zym}}]}{K_{\mathrm{m\ MASP1^*}}^{\mathrm{MASP1_{zym}}}}+\frac{[\mathrm{pCLK1MASP2_{zym}}]}{K_{\mathrm{m\ MASP1^*}}^{\mathrm{MASP2_{zym}}}}\\ +\frac{[\mathrm{pCLK1MASP3_{zym}}]}{K_{\mathrm{m\ MASP1^*}}^{\mathrm{MASP3_{zym}}}}+\frac{[\mathrm{pCLL1MASP1_{zym}}]}{K_{\mathrm{m\ MASP1^*}}^{\mathrm{MASP1_{zym}}}}+\frac{[\mathrm{pCLL1MASP2_{zym}}]}{K_{\mathrm{m\ MASP1^*}}^{\mathrm{MASP2_{zym}}}}+\frac{[\mathrm{pCLL1MASP3_{zym}}]}{K_{\mathrm{m\ MASP1^*}}^{\mathrm{MASP3_{zym}}}}\\ +\frac{[\mathrm{pLFMASP1_{zym}}]}{K_{\mathrm{m\ MASP1^*}}^{\mathrm{MASP1_{zym}}}}+\frac{[\mathrm{pLFMASP2_{zym}}]}{K_{\mathrm{m\ MASP1^*}}^{\mathrm{MASP2_{zym}}}}+\frac{[\mathrm{pLFMASP3_{zym}}]}{K_{\mathrm{m\ MASP1^*}}^{\mathrm{MASP3_{zym}}}}+\frac{[\mathrm{pHFMASP1_{zym}}]}{K_{\mathrm{m\ MASP1^*}}^{\mathrm{MASP1_{zym}}}}+\frac{[\mathrm{pHFMASP2_{zym}}]}{K_{\mathrm{m\ MASP1^*}}^{\mathrm{MASP2_{zym}}}}\\ +\frac{[\mathrm{pHFMASP3_{zym}}]}{K_{\mathrm{m\ MASP1^*}}^{\mathrm{MASP3_{zym}}}}+\frac{[\mathrm{pMFMASP1_{zym}}]}{K_{\mathrm{m\ MASP1^*}}^{\mathrm{MASP1_{zym}}}}+\frac{[\mathrm{pMFMASP2_{zym}}]}{K_{\mathrm{m\ MASP1^*}}^{\mathrm{MASP2_{zym}}}}+\frac{[\mathrm{pMFMASP3_{zym}}]}{K_{\mathrm{m\ MASP1^*}}^{\mathrm{MASP3_{zym}}}}\\ +\frac{[\mathrm{pPTX3LFMASP1_{zym}}]}{K_{\mathrm{m\ MASP1^*}}^{\mathrm{MASP1_{zym}}}}+\frac{[\mathrm{pPXT3LFMASP2_{zym}}]}{K_{\mathrm{m\ MASP1^*}}^{\mathrm{MASP2_{zym}}}}+\frac{[\mathrm{pPTX3LFMASP3_{zym}}]}{K_{\mathrm{m\ MASP1^*}}^{\mathrm{MASP3_{zym}}}}\end{array}\right)}$$

$$
-\frac{k_{\mathrm{cat\ MASP1^{*}}}^{\mathrm{MASP2_{zym}}}\left[\mathrm{pHFMASP1^{*}}\right]\left[\mathrm{pMBL_{4}MASP2_{zym}}\right]}{K_{\mathrm{m\ MASP1^{*}}}^{\mathrm{MASP2_{zym}}}\left(\begin{array}{l}
1+\frac{\left[\mathrm{C4}\right]}{K_{\mathrm{m\ MASP1^{*}}}^{\mathrm{C4}}}+\frac{\left[\mathrm{fC4bC2}\right]}{K_{\mathrm{m\ MASP1^{*}}}^{\mathrm{C2}}}+\frac{\left[\mathrm{fC4bC4bC2}\right]}{K_{\mathrm{m\ MASP1^{*}}}^{\mathrm{C2}}}+\frac{\left[\mathrm{fC3bC4bC2}\right]}{K_{\mathrm{m\ MASP1^{*}}}^{\mathrm{C2}}}+\frac{\left[\mathrm{pC4bC2}\right]}{K_{\mathrm{m\ MASP1^{*}}}^{\mathrm{C2}}}+\frac{\left[\mathrm{pC4bC4bC2}\right]}{K_{\mathrm{m\ MASP1^{*}}}^{\mathrm{C2}}}\\
+\frac{\left[\mathrm{pC3bC4bC2}\right]}{K_{\mathrm{m\ MASP1^{*}}}^{\mathrm{C2}}}+\frac{\left[\mathrm{pMBL_{3}MASP1_{zym}}\right]}{K_{\mathrm{m\ MASP1^{*}}}^{\mathrm{MASP1_{zym}}}}+\frac{\left[\mathrm{pMBL_{3}MASP2_{zym}}\right]}{K_{\mathrm{m\ MASP1^{*}}}^{\mathrm{MASP2_{zym}}}}+\frac{\left[\mathrm{pMBL_{3}MASP3_{zym}}\right]}{K_{\mathrm{m\ MASP1^{*}}}^{\mathrm{MASP3_{zym}}}}\\
+\frac{\left[\mathrm{pMBL_{4}MASP1_{zym}}\right]}{K_{\mathrm{m\ MASP1^{*}}}^{\mathrm{MASP1_{zym}}}}+\frac{\left[\mathrm{pMBL_{4}MASP2_{zym}}\right]}{K_{\mathrm{m\ MASP1^{*}}}^{\mathrm{MASP2_{zym}}}}+\frac{\left[\mathrm{pMBL_{4}MASP3_{zym}}\right]}{K_{\mathrm{m\ MASP1^{*}}}^{\mathrm{MASP3_{zym}}}}+\frac{\left[\mathrm{pCLLKMASP1_{zym}}\right]}{K_{\mathrm{m\ MASP1^{*}}}^{\mathrm{MASP1_{zym}}}}\\
+\frac{\left[\mathrm{pCLLKMASP2_{zym}}\right]}{K_{\mathrm{m\ MASP1^{*}}}^{\mathrm{MASP2_{zym}}}}+\frac{\left[\mathrm{pCLLKMASP3_{zym}}\right]}{K_{\mathrm{m\ MASP1^{*}}}^{\mathrm{MASP3_{zym}}}}+\frac{\left[\mathrm{pCLK1MASP1_{zym}}\right]}{K_{\mathrm{m\ MASP1^{*}}}^{\mathrm{MASP1_{zym}}}}+\frac{\left[\mathrm{pCLK1MASP2_{zym}}\right]}{K_{\mathrm{m\ MASP1^{*}}}^{\mathrm{MASP2_{zym}}}}\\
+\frac{\left[\mathrm{pCLK1MASP3_{zym}}\right]}{K_{\mathrm{m\ MASP1^{*}}}^{\mathrm{MASP3_{zym}}}}+\frac{\left[\mathrm{pCLL1MASP1_{zym}}\right]}{K_{\mathrm{m\ MASP1^{*}}}^{\mathrm{MASP1_{zym}}}}+\frac{\left[\mathrm{pCLL1MASP2_{zym}}\right]}{K_{\mathrm{m\ MASP1^{*}}}^{\mathrm{MASP2_{zym}}}}+\frac{\left[\mathrm{pCLL1MASP3_{zym}}\right]}{K_{\mathrm{m\ MASP1^{*}}}^{\mathrm{MASP3_{zym}}}}\\
+\frac{\left[\mathrm{pLFMASP1_{zym}}\right]}{K_{\mathrm{m\ MASP1^{*}}}^{\mathrm{MASP1_{zym}}}}+\frac{\left[\mathrm{pLFMASP2_{zym}}\right]}{K_{\mathrm{m\ MASP1^{*}}}^{\mathrm{MASP2_{zym}}}}+\frac{\left[\mathrm{pLFMASP3_{zym}}\right]}{K_{\mathrm{m\ MASP1^{*}}}^{\mathrm{MASP3_{zym}}}}+\frac{\left[\mathrm{pHFMASP1_{zym}}\right]}{K_{\mathrm{m\ MASP1^{*}}}^{\mathrm{MASP1_{zym}}}}+\frac{\left[\mathrm{pHFMASP2_{zym}}\right]}{K_{\mathrm{m\ MASP1^{*}}}^{\mathrm{MASP2_{zym}}}}\\
+\frac{\left[\mathrm{pHFMASP3_{zym}}\right]}{K_{\mathrm{m\ MASP1^{*}}}^{\mathrm{MASP3_{zym}}}}+\frac{\left[\mathrm{pMFMASP1_{zym}}\right]}{K_{\mathrm{m\ MASP1^{*}}}^{\mathrm{MASP1_{zym}}}}+\frac{\left[\mathrm{pMFMASP2_{zym}}\right]}{K_{\mathrm{m\ MASP1^{*}}}^{\mathrm{MASP2_{zym}}}}+\frac{\left[\mathrm{pMFMASP3_{zym}}\right]}{K_{\mathrm{m\ MASP1^{*}}}^{\mathrm{MASP3_{zym}}}}\\
+\frac{\left[\mathrm{pPTX3LFMASP1_{zym}}\right]}{K_{\mathrm{m\ MASP1^{*}}}^{\mathrm{MASP1_{zym}}}}+\frac{\left[\mathrm{pPXT3LFMASP2_{zym}}\right]}{K_{\mathrm{m\ MASP1^{*}}}^{\mathrm{MASP2_{zym}}}}+\frac{\left[\mathrm{pPTX3LFMASP3_{zym}}\right]}{K_{\mathrm{m\ MASP1^{*}}}^{\mathrm{MASP3_{zym}}}}
\end{array}\right)}
$$

$$-\frac{k_{\text{cat MASP1}^*}^{\text{MASP2}_{\text{zym}}}\left[\text{pMFMASP1}^*\right]\left[\text{pMBL}_4\text{MASP2}_{\text{zym}}\right]}{K_{\text{m MASP1}^*}^{\text{MASP2}_{\text{zym}}}\left(\begin{array}{l}1+\frac{[\text{C4}]}{K_{\text{m MASP1}^*}^{\text{C4}}}+\frac{[\text{fC4bC2}]}{K_{\text{m MASP1}^*}^{\text{C2}}}+\frac{[\text{fC4bC4bC2}]}{K_{\text{m MASP1}^*}^{\text{C2}}}+\frac{[\text{fC3bC4bC2}]}{K_{\text{m MASP1}^*}^{\text{C2}}}+\frac{[\text{pC4bC2}]}{K_{\text{m MASP1}^*}^{\text{C2}}}+\frac{[\text{pC4bC4bC2}]}{K_{\text{m MASP1}^*}^{\text{C2}}}\\+\frac{[\text{pC3bC4bC2}]}{K_{\text{m MASP1}^*}^{\text{C2}}}+\frac{[\text{pMBL}_3\text{MASP1}_{\text{zym}}]}{K_{\text{m MASP1}^*}^{\text{MASP1}_{\text{zym}}}}+\frac{[\text{pMBL}_3\text{MASP2}_{\text{zym}}]}{K_{\text{m MASP1}^*}^{\text{MASP2}_{\text{zym}}}}+\frac{[\text{pMBL}_3\text{MASP3}_{\text{zym}}]}{K_{\text{m MASP1}^*}^{\text{MASP3}_{\text{zym}}}}\\+\frac{[\text{pMBL}_4\text{MASP1}_{\text{zym}}]}{K_{\text{m MASP1}^*}^{\text{MASP1}_{\text{zym}}}}+\frac{[\text{pMBL}_4\text{MASP2}_{\text{zym}}]}{K_{\text{m MASP1}^*}^{\text{MASP2}_{\text{zym}}}}+\frac{[\text{pMBL}_4\text{MASP3}_{\text{zym}}]}{K_{\text{m MASP1}^*}^{\text{MASP3}_{\text{zym}}}}+\frac{[\text{pCLLKMASP1}_{\text{zym}}]}{K_{\text{m MASP1}^*}^{\text{MASP1}_{\text{zym}}}}\\+\frac{[\text{pCLLKMASP2}_{\text{zym}}]}{K_{\text{m MASP1}^*}^{\text{MASP2}_{\text{zym}}}}+\frac{[\text{pCLLKMASP3}_{\text{zym}}]}{K_{\text{m MASP1}^*}^{\text{MASP3}_{\text{zym}}}}+\frac{[\text{pCLK1MASP1}_{\text{zym}}]}{K_{\text{m MASP1}^*}^{\text{MASP1}_{\text{zym}}}}+\frac{[\text{pCLK1MASP2}_{\text{zym}}]}{K_{\text{m MASP1}^*}^{\text{MASP2}_{\text{zym}}}}\\+\frac{[\text{pCLK1MASP3}_{\text{zym}}]}{K_{\text{m MASP1}^*}^{\text{MASP3}_{\text{zym}}}}+\frac{[\text{pCLL1MASP1}_{\text{zym}}]}{K_{\text{m MASP1}^*}^{\text{MASP1}_{\text{zym}}}}+\frac{[\text{pCLL1MASP2}_{\text{zym}}]}{K_{\text{m MASP1}^*}^{\text{MASP2}_{\text{zym}}}}+\frac{[\text{pCLL1MASP3}_{\text{zym}}]}{K_{\text{m MASP1}^*}^{\text{MASP3}_{\text{zym}}}}\\+\frac{[\text{pLFMASP1}_{\text{zym}}]}{K_{\text{m MASP1}^*}^{\text{MASP1}_{\text{zym}}}}+\frac{[\text{pLFMASP2}_{\text{zym}}]}{K_{\text{m MASP1}^*}^{\text{MASP2}_{\text{zym}}}}+\frac{[\text{pLFMASP3}_{\text{zym}}]}{K_{\text{m MASP1}^*}^{\text{MASP3}_{\text{zym}}}}+\frac{[\text{pHFMASP1}_{\text{zym}}]}{K_{\text{m MASP1}^*}^{\text{MASP1}_{\text{zym}}}}+\frac{[\text{pHFMASP2}_{\text{zym}}]}{K_{\text{m MASP1}^*}^{\text{MASP2}_{\text{zym}}}}\\+\frac{[\text{pHFMASP3}_{\text{zym}}]}{K_{\text{m MASP1}^*}^{\text{MASP3}_{\text{zym}}}}+\frac{[\text{pMFMASP1}_{\text{zym}}]}{K_{\text{m MASP1}^*}^{\text{MASP1}_{\text{zym}}}}+\frac{[\text{pMFMASP2}_{\text{zym}}]}{K_{\text{m MASP1}^*}^{\text{MASP2}_{\text{zym}}}}+\frac{[\text{pMFMASP3}_{\text{zym}}]}{K_{\text{m MASP1}^*}^{\text{MASP3}_{\text{zym}}}}\\+\frac{[\text{pPTX3LFMASP1}_{\text{zym}}]}{K_{\text{m MASP1}^*}^{\text{MASP1}_{\text{zym}}}}+\frac{[\text{pPXT3LFMASP2}_{\text{zym}}]}{K_{\text{m MASP1}^*}^{\text{MASP2}_{\text{zym}}}}+\frac{[\text{pPTX3LFMASP3}_{\text{zym}}]}{K_{\text{m MASP1}^*}^{\text{MASP3}_{\text{zym}}}}\end{array}\right)}$$

$$-\frac{k_{\text{cat MASP1}^*}^{\text{MASP2}_{\text{zym}}}\left[\text{pPTX3LFMASP1}^*\right]\left[\text{pMBL}_4\text{MASP2}_{\text{zym}}\right]}{K_{\text{m MASP1}^*}^{\text{MASP2}_{\text{zym}}}\left(\begin{array}{l}1+\frac{[\text{C4}]}{K_{\text{m MASP1}^*}^{\text{C4}}}+\frac{[\text{fC4bC2}]}{K_{\text{m MASP1}^*}^{\text{C2}}}+\frac{[\text{fC4bC4bC2}]}{K_{\text{m MASP1}^*}^{\text{C2}}}+\frac{[\text{fC3bC4bC2}]}{K_{\text{m MASP1}^*}^{\text{C2}}}+\frac{[\text{pC4bC2}]}{K_{\text{m MASP1}^*}^{\text{C2}}}+\frac{[\text{pC4bC4bC2}]}{K_{\text{m MASP1}^*}^{\text{C2}}}\\+\frac{[\text{pC3bC4bC2}]}{K_{\text{m MASP1}^*}^{\text{C2}}}+\frac{[\text{pMBL}_3\text{MASP1}_{\text{zym}}]}{K_{\text{m MASP1}^*}^{\text{MASP1}_{\text{zym}}}}+\frac{[\text{pMBL}_3\text{MASP2}_{\text{zym}}]}{K_{\text{m MASP1}^*}^{\text{MASP2}_{\text{zym}}}}+\frac{[\text{pMBL}_3\text{MASP3}_{\text{zym}}]}{K_{\text{m MASP1}^*}^{\text{MASP3}_{\text{zym}}}}\\+\frac{[\text{pMBL}_4\text{MASP1}_{\text{zym}}]}{K_{\text{m MASP1}^*}^{\text{MASP1}_{\text{zym}}}}+\frac{[\text{pMBL}_4\text{MASP2}_{\text{zym}}]}{K_{\text{m MASP1}^*}^{\text{MASP2}_{\text{zym}}}}+\frac{[\text{pMBL}_4\text{MASP3}_{\text{zym}}]}{K_{\text{m MASP1}^*}^{\text{MASP3}_{\text{zym}}}}+\frac{[\text{pCLLKMASP1}_{\text{zym}}]}{K_{\text{m MASP1}^*}^{\text{MASP1}_{\text{zym}}}}\\+\frac{[\text{pCLLKMASP2}_{\text{zym}}]}{K_{\text{m MASP1}^*}^{\text{MASP2}_{\text{zym}}}}+\frac{[\text{pCLLKMASP3}_{\text{zym}}]}{K_{\text{m MASP1}^*}^{\text{MASP3}_{\text{zym}}}}+\frac{[\text{pCLK1MASP1}_{\text{zym}}]}{K_{\text{m MASP1}^*}^{\text{MASP1}_{\text{zym}}}}+\frac{[\text{pCLK1MASP2}_{\text{zym}}]}{K_{\text{m MASP1}^*}^{\text{MASP2}_{\text{zym}}}}\\+\frac{[\text{pCLK1MASP3}_{\text{zym}}]}{K_{\text{m MASP1}^*}^{\text{MASP3}_{\text{zym}}}}+\frac{[\text{pCLL1MASP1}_{\text{zym}}]}{K_{\text{m MASP1}^*}^{\text{MASP1}_{\text{zym}}}}+\frac{[\text{pCLL1MASP2}_{\text{zym}}]}{K_{\text{m MASP1}^*}^{\text{MASP2}_{\text{zym}}}}+\frac{[\text{pCLL1MASP3}_{\text{zym}}]}{K_{\text{m MASP1}^*}^{\text{MASP3}_{\text{zym}}}}\\+\frac{[\text{pLFMASP1}_{\text{zym}}]}{K_{\text{m MASP1}^*}^{\text{MASP1}_{\text{zym}}}}+\frac{[\text{pLFMASP2}_{\text{zym}}]}{K_{\text{m MASP1}^*}^{\text{MASP2}_{\text{zym}}}}+\frac{[\text{pLFMASP3}_{\text{zym}}]}{K_{\text{m MASP1}^*}^{\text{MASP3}_{\text{zym}}}}+\frac{[\text{pHFMASP1}_{\text{zym}}]}{K_{\text{m MASP1}^*}^{\text{MASP1}_{\text{zym}}}}+\frac{[\text{pHFMASP2}_{\text{zym}}]}{K_{\text{m MASP1}^*}^{\text{MASP2}_{\text{zym}}}}\\+\frac{[\text{pHFMASP3}_{\text{zym}}]}{K_{\text{m MASP1}^*}^{\text{MASP3}_{\text{zym}}}}+\frac{[\text{pMFMASP1}_{\text{zym}}]}{K_{\text{m MASP1}^*}^{\text{MASP1}_{\text{zym}}}}+\frac{[\text{pMFMASP2}_{\text{zym}}]}{K_{\text{m MASP1}^*}^{\text{MASP2}_{\text{zym}}}}+\frac{[\text{pMFMASP3}_{\text{zym}}]}{K_{\text{m MASP1}^*}^{\text{MASP3}_{\text{zym}}}}\\+\frac{[\text{pPTX3LFMASP1}_{\text{zym}}]}{K_{\text{m MASP1}^*}^{\text{MASP1}_{\text{zym}}}}+\frac{[\text{pPXT3LFMASP2}_{\text{zym}}]}{K_{\text{m MASP1}^*}^{\text{MASP2}_{\text{zym}}}}+\frac{[\text{pPTX3LFMASP3}_{\text{zym}}]}{K_{\text{m MASP1}^*}^{\text{MASP3}_{\text{zym}}}}\end{array}\right)}$$

$$-\frac{k_{\text{cat MASP2}^*}^{\text{MASP2}_{\text{zym}}}\left[\text{pMBL}_3\text{MASP2}^*\right]\left[\text{pMBL}_4\text{MASP2}_{\text{zym}}\right]}{K_{\text{m MASP2}^*}^{\text{MASP2}_{\text{zym}}}\left(\begin{array}{l}1+\frac{[\text{C4}]}{K_{\text{m MASP2}^*}^{\text{C4}}}+\frac{[\text{fC4bC2}]}{K_{\text{m MASP2}^*}^{\text{C2}}}+\frac{[\text{fC4bC4bC2}]}{K_{\text{m MASP2}^*}^{\text{C2}}}+\frac{[\text{fC3bC4bC2}]}{K_{\text{m MASP2}^*}^{\text{C2}}}+\frac{[\text{pC4bC2}]}{K_{\text{m MASP2}^*}^{\text{C2}}}+\frac{[\text{pC4bC4bC2}]}{K_{\text{m MASP2}^*}^{\text{C2}}}\\+\frac{[\text{pC3bC4bC2}]}{K_{\text{m MASP2}^*}^{\text{C2}}}+\frac{[\text{pMBL}_3\text{MASP1}_{\text{zym}}]}{K_{\text{m MASP2}^*}^{\text{MASP1}_{\text{zym}}}}+\frac{[\text{pMBL}_3\text{MASP2}_{\text{zym}}]}{K_{\text{m MASP2}^*}^{\text{MASP2}_{\text{zym}}}}+\frac{[\text{pMBL}_4\text{MASP1}_{\text{zym}}]}{K_{\text{m MASP2}^*}^{\text{MASP1}_{\text{zym}}}}\\+\frac{[\text{pMBL}_4\text{MASP2}_{\text{zym}}]}{K_{\text{m MASP2}^*}^{\text{MASP2}_{\text{zym}}}}+\frac{[\text{pCLLKMASP1}_{\text{zym}}]}{K_{\text{m MASP2}^*}^{\text{MASP1}_{\text{zym}}}}+\frac{[\text{pCLLKMASP2}_{\text{zym}}]}{K_{\text{m MASP2}^*}^{\text{MASP2}_{\text{zym}}}}+\frac{[\text{pCLK1MASP1}_{\text{zym}}]}{K_{\text{m MASP2}^*}^{\text{MASP1}_{\text{zym}}}}\\+\frac{[\text{pCLK1MASP2}_{\text{zym}}]}{K_{\text{m MASP2}^*}^{\text{MASP2}_{\text{zym}}}}+\frac{[\text{pCLL1MASP1}_{\text{zym}}]}{K_{\text{m MASP2}^*}^{\text{MASP1}_{\text{zym}}}}+\frac{[\text{pCLL1MASP2}_{\text{zym}}]}{K_{\text{m MASP2}^*}^{\text{MASP2}_{\text{zym}}}}+\frac{[\text{pLFMASP1}_{\text{zym}}]}{K_{\text{m MASP2}^*}^{\text{MASP1}_{\text{zym}}}}\\+\frac{[\text{pLFMASP2}_{\text{zym}}]}{K_{\text{m MASP2}^*}^{\text{MASP2}_{\text{zym}}}}+\frac{[\text{pHFMASP1}_{\text{zym}}]}{K_{\text{m MASP2}^*}^{\text{MASP1}_{\text{zym}}}}+\frac{[\text{pHFMASP2}_{\text{zym}}]}{K_{\text{m MASP2}^*}^{\text{MASP2}_{\text{zym}}}}+\frac{[\text{pMFMASP1}_{\text{zym}}]}{K_{\text{m MASP2}^*}^{\text{MASP1}_{\text{zym}}}}+\frac{[\text{pMFMASP2}_{\text{zym}}]}{K_{\text{m MASP2}^*}^{\text{MASP2}_{\text{zym}}}}\\+\frac{[\text{pPTX3LFMASP1}_{\text{zym}}]}{K_{\text{m MASP2}^*}^{\text{MASP1}_{\text{zym}}}}+\frac{[\text{pPTX3LFMASP2}_{\text{zym}}]}{K_{\text{m MASP2}^*}^{\text{MASP2}_{\text{zym}}}}\end{array}\right)}$$

$$-\frac{k_{\text{cat MASP2}^*}^{\text{MASP2}_{\text{zym}}}\left[\text{pMBL}_4\text{MASP2}^*\right]\left[\text{pMBL}_4\text{MASP2}_{\text{zym}}\right]}{K_{\text{m MASP2}^*}^{\text{MASP2}_{\text{zym}}}\left(\begin{array}{l}1+\frac{[\text{C4}]}{K_{\text{m MASP2}^*}^{\text{C4}}}+\frac{[\text{fC4bC2}]}{K_{\text{m MASP2}^*}^{\text{C2}}}+\frac{[\text{fC4bC4bC2}]}{K_{\text{m MASP2}^*}^{\text{C2}}}+\frac{[\text{fC3bC4bC2}]}{K_{\text{m MASP2}^*}^{\text{C2}}}+\frac{[\text{pC4bC2}]}{K_{\text{m MASP2}^*}^{\text{C2}}}+\frac{[\text{pC4bC4bC2}]}{K_{\text{m MASP2}^*}^{\text{C2}}}\\+\frac{[\text{pC3bC4bC2}]}{K_{\text{m MASP2}^*}^{\text{C2}}}+\frac{[\text{pMBL}_3\text{MASP1}_{\text{zym}}]}{K_{\text{m MASP2}^*}^{\text{MASP1}_{\text{zym}}}}+\frac{[\text{pMBL}_3\text{MASP2}_{\text{zym}}]}{K_{\text{m MASP2}^*}^{\text{MASP2}_{\text{zym}}}}+\frac{[\text{pMBL}_4\text{MASP1}_{\text{zym}}]}{K_{\text{m MASP2}^*}^{\text{MASP1}_{\text{zym}}}}\\+\frac{[\text{pMBL}_4\text{MASP2}_{\text{zym}}]}{K_{\text{m MASP2}^*}^{\text{MASP2}_{\text{zym}}}}+\frac{[\text{pCLLKMASP1}_{\text{zym}}]}{K_{\text{m MASP2}^*}^{\text{MASP1}_{\text{zym}}}}+\frac{[\text{pCLLKMASP2}_{\text{zym}}]}{K_{\text{m MASP2}^*}^{\text{MASP2}_{\text{zym}}}}+\frac{[\text{pCLK1MASP1}_{\text{zym}}]}{K_{\text{m MASP2}^*}^{\text{MASP1}_{\text{zym}}}}\\+\frac{[\text{pCLK1MASP2}_{\text{zym}}]}{K_{\text{m MASP2}^*}^{\text{MASP2}_{\text{zym}}}}+\frac{[\text{pCLL1MASP1}_{\text{zym}}]}{K_{\text{m MASP2}^*}^{\text{MASP1}_{\text{zym}}}}+\frac{[\text{pCLL1MASP2}_{\text{zym}}]}{K_{\text{m MASP2}^*}^{\text{MASP2}_{\text{zym}}}}+\frac{[\text{pLFMASP1}_{\text{zym}}]}{K_{\text{m MASP2}^*}^{\text{MASP1}_{\text{zym}}}}\\+\frac{[\text{pLFMASP2}_{\text{zym}}]}{K_{\text{m MASP2}^*}^{\text{MASP2}_{\text{zym}}}}+\frac{[\text{pHFMASP1}_{\text{zym}}]}{K_{\text{m MASP2}^*}^{\text{MASP1}_{\text{zym}}}}+\frac{[\text{pHFMASP2}_{\text{zym}}]}{K_{\text{m MASP2}^*}^{\text{MASP2}_{\text{zym}}}}+\frac{[\text{pMFMASP1}_{\text{zym}}]}{K_{\text{m MASP2}^*}^{\text{MASP1}_{\text{zym}}}}+\frac{[\text{pMFMASP2}_{\text{zym}}]}{K_{\text{m MASP2}^*}^{\text{MASP2}_{\text{zym}}}}\\+\frac{[\text{pPTX3LFMASP1}_{\text{zym}}]}{K_{\text{m MASP2}^*}^{\text{MASP1}_{\text{zym}}}}+\frac{[\text{pPTX3LFMASP2}_{\text{zym}}]}{K_{\text{m MASP2}^*}^{\text{MASP2}_{\text{zym}}}}\end{array}\right)}$$

$$-\frac{k_{\text{cat MASP2}^*}^{\text{MASP2}_{\text{zym}}}\left[\text{pCLLKMASP2}^*\right]\left[\text{pMBL}_4\text{MASP2}_{\text{zym}}\right]}{K_{\text{m MASP2}^*}^{\text{MASP2}_{\text{zym}}}\left(\begin{array}{l}1+\frac{[\text{C4}]}{K_{\text{m MASP2}^*}^{\text{C4}}}+\frac{[\text{fC4bC2}]}{K_{\text{m MASP2}^*}^{\text{C2}}}+\frac{[\text{fC4bC4bC2}]}{K_{\text{m MASP2}^*}^{\text{C2}}}+\frac{[\text{fC3bC4bC2}]}{K_{\text{m MASP2}^*}^{\text{C2}}}+\frac{[\text{pC4bC2}]}{K_{\text{m MASP2}^*}^{\text{C2}}}+\frac{[\text{pC4bC4bC2}]}{K_{\text{m MASP2}^*}^{\text{C2}}}\\+\frac{[\text{pC3bC4bC2}]}{K_{\text{m MASP2}^*}^{\text{C2}}}+\frac{[\text{pMBL}_3\text{MASP1}_{\text{zym}}]}{K_{\text{m MASP2}^*}^{\text{MASP1}_{\text{zym}}}}+\frac{[\text{pMBL}_3\text{MASP2}_{\text{zym}}]}{K_{\text{m MASP2}^*}^{\text{MASP2}_{\text{zym}}}}+\frac{[\text{pMBL}_4\text{MASP1}_{\text{zym}}]}{K_{\text{m MASP2}^*}^{\text{MASP1}_{\text{zym}}}}\\+\frac{[\text{pMBL}_4\text{MASP2}_{\text{zym}}]}{K_{\text{m MASP2}^*}^{\text{MASP2}_{\text{zym}}}}+\frac{[\text{pCLLKMASP1}_{\text{zym}}]}{K_{\text{m MASP2}^*}^{\text{MASP1}_{\text{zym}}}}+\frac{[\text{pCLLKMASP2}_{\text{zym}}]}{K_{\text{m MASP2}^*}^{\text{MASP2}_{\text{zym}}}}+\frac{[\text{pCLK1MASP1}_{\text{zym}}]}{K_{\text{m MASP2}^*}^{\text{MASP1}_{\text{zym}}}}\\+\frac{[\text{pCLK1MASP2}_{\text{zym}}]}{K_{\text{m MASP2}^*}^{\text{MASP2}_{\text{zym}}}}+\frac{[\text{pCLL1MASP1}_{\text{zym}}]}{K_{\text{m MASP2}^*}^{\text{MASP1}_{\text{zym}}}}+\frac{[\text{pCLL1MASP2}_{\text{zym}}]}{K_{\text{m MASP2}^*}^{\text{MASP2}_{\text{zym}}}}+\frac{[\text{pLFMASP1}_{\text{zym}}]}{K_{\text{m MASP2}^*}^{\text{MASP1}_{\text{zym}}}}\\+\frac{[\text{pLFMASP2}_{\text{zym}}]}{K_{\text{m MASP2}^*}^{\text{MASP2}_{\text{zym}}}}+\frac{[\text{pHFMASP1}_{\text{zym}}]}{K_{\text{m MASP2}^*}^{\text{MASP1}_{\text{zym}}}}+\frac{[\text{pHFMASP2}_{\text{zym}}]}{K_{\text{m MASP2}^*}^{\text{MASP2}_{\text{zym}}}}+\frac{[\text{pMFMASP1}_{\text{zym}}]}{K_{\text{m MASP2}^*}^{\text{MASP1}_{\text{zym}}}}+\frac{[\text{pMFMASP2}_{\text{zym}}]}{K_{\text{m MASP2}^*}^{\text{MASP2}_{\text{zym}}}}\\+\frac{[\text{pPTX3LFMASP1}_{\text{zym}}]}{K_{\text{m MASP2}^*}^{\text{MASP1}_{\text{zym}}}}+\frac{[\text{pPTX3LFMASP2}_{\text{zym}}]}{K_{\text{m MASP2}^*}^{\text{MASP2}_{\text{zym}}}}\end{array}\right)}$$

$$-\frac{k_{\text{cat MASP2}^*}^{\text{MASP2}_{\text{zym}}}\left[\text{pCLK1MASP2}^*\right]\left[\text{pMBL}_4\text{MASP2}_{\text{zym}}\right]}{K_{\text{m MASP2}^*}^{\text{MASP2}_{\text{zym}}}\left(\begin{array}{l}1+\frac{[\text{C4}]}{K_{\text{m MASP2}^*}^{\text{C4}}}+\frac{[\text{fC4bC2}]}{K_{\text{m MASP2}^*}^{\text{C2}}}+\frac{[\text{fC4bC4bC2}]}{K_{\text{m MASP2}^*}^{\text{C2}}}+\frac{[\text{fC3bC4bC2}]}{K_{\text{m MASP2}^*}^{\text{C2}}}+\frac{[\text{pC4bC2}]}{K_{\text{m MASP2}^*}^{\text{C2}}}+\frac{[\text{pC4bC4bC2}]}{K_{\text{m MASP2}^*}^{\text{C2}}}\\+\frac{[\text{pC3bC4bC2}]}{K_{\text{m MASP2}^*}^{\text{C2}}}+\frac{\left[\text{pMBL}_3\text{MASP1}_{\text{zym}}\right]}{K_{\text{m MASP2}^*}^{\text{MASP1}_{\text{zym}}}}+\frac{\left[\text{pMBL}_3\text{MASP2}_{\text{zym}}\right]}{K_{\text{m MASP2}^*}^{\text{MASP2}_{\text{zym}}}}+\frac{\left[\text{pMBL}_4\text{MASP1}_{\text{zym}}\right]}{K_{\text{m MASP2}^*}^{\text{MASP1}_{\text{zym}}}}\\+\frac{\left[\text{pMBL}_4\text{MASP2}_{\text{zym}}\right]}{K_{\text{m MASP2}^*}^{\text{MASP2}_{\text{zym}}}}+\frac{\left[\text{pCLLKMASP1}_{\text{zym}}\right]}{K_{\text{m MASP2}^*}^{\text{MASP1}_{\text{zym}}}}+\frac{\left[\text{pCLLKMASP2}_{\text{zym}}\right]}{K_{\text{m MASP2}^*}^{\text{MASP2}_{\text{zym}}}}+\frac{\left[\text{pCLK1MASP1}_{\text{zym}}\right]}{K_{\text{m MASP2}^*}^{\text{MASP1}_{\text{zym}}}}\\+\frac{\left[\text{pCLK1MASP2}_{\text{zym}}\right]}{K_{\text{m MASP2}^*}^{\text{MASP2}_{\text{zym}}}}+\frac{\left[\text{pCLL1MASP1}_{\text{zym}}\right]}{K_{\text{m MASP2}^*}^{\text{MASP1}_{\text{zym}}}}+\frac{\left[\text{pCLL1MASP2}_{\text{zym}}\right]}{K_{\text{m MASP2}^*}^{\text{MASP2}_{\text{zym}}}}+\frac{\left[\text{pLFMASP1}_{\text{zym}}\right]}{K_{\text{m MASP2}^*}^{\text{MASP1}_{\text{zym}}}}\\+\frac{\left[\text{pLFMASP2}_{\text{zym}}\right]}{K_{\text{m MASP2}^*}^{\text{MASP2}_{\text{zym}}}}+\frac{\left[\text{pHFMASP1}_{\text{zym}}\right]}{K_{\text{m MASP2}^*}^{\text{MASP1}_{\text{zym}}}}+\frac{\left[\text{pHFMASP2}_{\text{zym}}\right]}{K_{\text{m MASP2}^*}^{\text{MASP2}_{\text{zym}}}}+\frac{\left[\text{pMFMASP1}_{\text{zym}}\right]}{K_{\text{m MASP2}^*}^{\text{MASP1}_{\text{zym}}}}+\frac{\left[\text{pMFMASP2}_{\text{zym}}\right]}{K_{\text{m MASP2}^*}^{\text{MASP2}_{\text{zym}}}}\\+\frac{\left[\text{pPTX3LFMASP1}_{\text{zym}}\right]}{K_{\text{m MASP2}^*}^{\text{MASP1}_{\text{zym}}}}+\frac{\left[\text{pPTX3LFMASP2}_{\text{zym}}\right]}{K_{\text{m MASP2}^*}^{\text{MASP2}_{\text{zym}}}}\end{array}\right)}$$

$$-\frac{k_{\text{cat MASP2}^*}^{\text{MASP2}_{\text{zym}}}\left[\text{pCLL1MASP2}^*\right]\left[\text{pMBL}_4\text{MASP2}_{\text{zym}}\right]}{K_{\text{m MASP2}^*}^{\text{MASP2}_{\text{zym}}}\left(\begin{array}{l}1+\frac{[\text{C4}]}{K_{\text{m MASP2}^*}^{\text{C4}}}+\frac{[\text{fC4bC2}]}{K_{\text{m MASP2}^*}^{\text{C2}}}+\frac{[\text{fC4bC4bC2}]}{K_{\text{m MASP2}^*}^{\text{C2}}}+\frac{[\text{fC3bC4bC2}]}{K_{\text{m MASP2}^*}^{\text{C2}}}+\frac{[\text{pC4bC2}]}{K_{\text{m MASP2}^*}^{\text{C2}}}+\frac{[\text{pC4bC4bC2}]}{K_{\text{m MASP2}^*}^{\text{C2}}}\\+\frac{[\text{pC3bC4bC2}]}{K_{\text{m MASP2}^*}^{\text{C2}}}+\frac{\left[\text{pMBL}_3\text{MASP1}_{\text{zym}}\right]}{K_{\text{m MASP2}^*}^{\text{MASP1}_{\text{zym}}}}+\frac{\left[\text{pMBL}_3\text{MASP2}_{\text{zym}}\right]}{K_{\text{m MASP2}^*}^{\text{MASP2}_{\text{zym}}}}+\frac{\left[\text{pMBL}_4\text{MASP1}_{\text{zym}}\right]}{K_{\text{m MASP2}^*}^{\text{MASP1}_{\text{zym}}}}\\+\frac{\left[\text{pMBL}_4\text{MASP2}_{\text{zym}}\right]}{K_{\text{m MASP2}^*}^{\text{MASP2}_{\text{zym}}}}+\frac{\left[\text{pCLLKMASP1}_{\text{zym}}\right]}{K_{\text{m MASP2}^*}^{\text{MASP1}_{\text{zym}}}}+\frac{\left[\text{pCLLKMASP2}_{\text{zym}}\right]}{K_{\text{m MASP2}^*}^{\text{MASP2}_{\text{zym}}}}+\frac{\left[\text{pCLK1MASP1}_{\text{zym}}\right]}{K_{\text{m MASP2}^*}^{\text{MASP1}_{\text{zym}}}}\\+\frac{\left[\text{pCLK1MASP2}_{\text{zym}}\right]}{K_{\text{m MASP2}^*}^{\text{MASP2}_{\text{zym}}}}+\frac{\left[\text{pCLL1MASP1}_{\text{zym}}\right]}{K_{\text{m MASP2}^*}^{\text{MASP1}_{\text{zym}}}}+\frac{\left[\text{pCLL1MASP2}_{\text{zym}}\right]}{K_{\text{m MASP2}^*}^{\text{MASP2}_{\text{zym}}}}+\frac{\left[\text{pLFMASP1}_{\text{zym}}\right]}{K_{\text{m MASP2}^*}^{\text{MASP1}_{\text{zym}}}}\\+\frac{\left[\text{pLFMASP2}_{\text{zym}}\right]}{K_{\text{m MASP2}^*}^{\text{MASP2}_{\text{zym}}}}+\frac{\left[\text{pHFMASP1}_{\text{zym}}\right]}{K_{\text{m MASP2}^*}^{\text{MASP1}_{\text{zym}}}}+\frac{\left[\text{pHFMASP2}_{\text{zym}}\right]}{K_{\text{m MASP2}^*}^{\text{MASP2}_{\text{zym}}}}+\frac{\left[\text{pMFMASP1}_{\text{zym}}\right]}{K_{\text{m MASP2}^*}^{\text{MASP1}_{\text{zym}}}}+\frac{\left[\text{pMFMASP2}_{\text{zym}}\right]}{K_{\text{m MASP2}^*}^{\text{MASP2}_{\text{zym}}}}\\+\frac{\left[\text{pPTX3LFMASP1}_{\text{zym}}\right]}{K_{\text{m MASP2}^*}^{\text{MASP1}_{\text{zym}}}}+\frac{\left[\text{pPTX3LFMASP2}_{\text{zym}}\right]}{K_{\text{m MASP2}^*}^{\text{MASP2}_{\text{zym}}}}\end{array}\right)}$$

$$
-\frac{k_{\text{cat MASP2}^*}^{\text{MASP2}_{\text{zym}}}\left[\text{pLFMASP2}^*\right]\left[\text{pMBL}_4\text{MASP2}_{\text{zym}}\right]}{K_{\text{m MASP2}^*}^{\text{MASP2}_{\text{zym}}}\left(\begin{array}{l}1+\frac{[\text{C4}]}{K_{\text{m MASP2}^*}^{\text{C4}}}+\frac{[\text{fC4bC2}]}{K_{\text{m MASP2}^*}^{\text{C2}}}+\frac{[\text{fC4bC4bC2}]}{K_{\text{m MASP2}^*}^{\text{C2}}}+\frac{[\text{fC3bC4bC2}]}{K_{\text{m MASP2}^*}^{\text{C2}}}+\frac{[\text{pC4bC2}]}{K_{\text{m MASP2}^*}^{\text{C2}}}+\frac{[\text{pC4bC4bC2}]}{K_{\text{m MASP2}^*}^{\text{C2}}}\\+\frac{[\text{pC3bC4bC2}]}{K_{\text{m MASP2}^*}^{\text{C2}}}+\frac{[\text{pMBL}_3\text{MASP1}_{\text{zym}}]}{K_{\text{m MASP2}^*}^{\text{MASP1}_{\text{zym}}}}+\frac{[\text{pMBL}_3\text{MASP2}_{\text{zym}}]}{K_{\text{m MASP2}^*}^{\text{MASP2}_{\text{zym}}}}+\frac{[\text{pMBL}_4\text{MASP1}_{\text{zym}}]}{K_{\text{m MASP2}^*}^{\text{MASP1}_{\text{zym}}}}\\+\frac{[\text{pMBL}_4\text{MASP2}_{\text{zym}}]}{K_{\text{m MASP2}^*}^{\text{MASP2}_{\text{zym}}}}+\frac{[\text{pCLLKMASP1}_{\text{zym}}]}{K_{\text{m MASP2}^*}^{\text{MASP1}_{\text{zym}}}}+\frac{[\text{pCLLKMASP2}_{\text{zym}}]}{K_{\text{m MASP2}^*}^{\text{MASP2}_{\text{zym}}}}+\frac{[\text{pCLK1MASP1}_{\text{zym}}]}{K_{\text{m MASP2}^*}^{\text{MASP1}_{\text{zym}}}}\\+\frac{[\text{pCLK1MASP2}_{\text{zym}}]}{K_{\text{m MASP2}^*}^{\text{MASP2}_{\text{zym}}}}+\frac{[\text{pCLL1MASP1}_{\text{zym}}]}{K_{\text{m MASP2}^*}^{\text{MASP1}_{\text{zym}}}}+\frac{[\text{pCLL1MASP2}_{\text{zym}}]}{K_{\text{m MASP2}^*}^{\text{MASP2}_{\text{zym}}}}+\frac{[\text{pLFMASP1}_{\text{zym}}]}{K_{\text{m MASP2}^*}^{\text{MASP1}_{\text{zym}}}}\\+\frac{[\text{pLFMASP2}_{\text{zym}}]}{K_{\text{m MASP2}^*}^{\text{MASP2}_{\text{zym}}}}+\frac{[\text{pHFMASP1}_{\text{zym}}]}{K_{\text{m MASP2}^*}^{\text{MASP1}_{\text{zym}}}}+\frac{[\text{pHFMASP2}_{\text{zym}}]}{K_{\text{m MASP2}^*}^{\text{MASP2}_{\text{zym}}}}+\frac{[\text{pMFMASP1}_{\text{zym}}]}{K_{\text{m MASP2}^*}^{\text{MASP1}_{\text{zym}}}}+\frac{[\text{pMFMASP2}_{\text{zym}}]}{K_{\text{m MASP2}^*}^{\text{MASP2}_{\text{zym}}}}\\+\frac{[\text{pPTX3LFMASP1}_{\text{zym}}]}{K_{\text{m MASP2}^*}^{\text{MASP1}_{\text{zym}}}}+\frac{[\text{pPTX3LFMASP2}_{\text{zym}}]}{K_{\text{m MASP2}^*}^{\text{MASP2}_{\text{zym}}}}\end{array}\right)}
$$

$$
-\frac{k_{\text{cat MASP2}^*}^{\text{MASP2}_{\text{zym}}}\left[\text{pHFMASP2}^*\right]\left[\text{pMBL}_4\text{MASP2}_{\text{zym}}\right]}{K_{\text{m MASP2}^*}^{\text{MASP2}_{\text{zym}}}\left(\begin{array}{l}1+\frac{[\text{C4}]}{K_{\text{m MASP2}^*}^{\text{C4}}}+\frac{[\text{fC4bC2}]}{K_{\text{m MASP2}^*}^{\text{C2}}}+\frac{[\text{fC4bC4bC2}]}{K_{\text{m MASP2}^*}^{\text{C2}}}+\frac{[\text{fC3bC4bC2}]}{K_{\text{m MASP2}^*}^{\text{C2}}}+\frac{[\text{pC4bC2}]}{K_{\text{m MASP2}^*}^{\text{C2}}}+\frac{[\text{pC4bC4bC2}]}{K_{\text{m MASP2}^*}^{\text{C2}}}\\+\frac{[\text{pC3bC4bC2}]}{K_{\text{m MASP2}^*}^{\text{C2}}}+\frac{[\text{pMBL}_3\text{MASP1}_{\text{zym}}]}{K_{\text{m MASP2}^*}^{\text{MASP1}_{\text{zym}}}}+\frac{[\text{pMBL}_3\text{MASP2}_{\text{zym}}]}{K_{\text{m MASP2}^*}^{\text{MASP2}_{\text{zym}}}}+\frac{[\text{pMBL}_4\text{MASP1}_{\text{zym}}]}{K_{\text{m MASP2}^*}^{\text{MASP1}_{\text{zym}}}}\\+\frac{[\text{pMBL}_4\text{MASP2}_{\text{zym}}]}{K_{\text{m MASP2}^*}^{\text{MASP2}_{\text{zym}}}}+\frac{[\text{pCLLKMASP1}_{\text{zym}}]}{K_{\text{m MASP2}^*}^{\text{MASP1}_{\text{zym}}}}+\frac{[\text{pCLLKMASP2}_{\text{zym}}]}{K_{\text{m MASP2}^*}^{\text{MASP2}_{\text{zym}}}}+\frac{[\text{pCLK1MASP1}_{\text{zym}}]}{K_{\text{m MASP2}^*}^{\text{MASP1}_{\text{zym}}}}\\+\frac{[\text{pCLK1MASP2}_{\text{zym}}]}{K_{\text{m MASP2}^*}^{\text{MASP2}_{\text{zym}}}}+\frac{[\text{pCLL1MASP1}_{\text{zym}}]}{K_{\text{m MASP2}^*}^{\text{MASP1}_{\text{zym}}}}+\frac{[\text{pCLL1MASP2}_{\text{zym}}]}{K_{\text{m MASP2}^*}^{\text{MASP2}_{\text{zym}}}}+\frac{[\text{pLFMASP1}_{\text{zym}}]}{K_{\text{m MASP2}^*}^{\text{MASP1}_{\text{zym}}}}\\+\frac{[\text{pLFMASP2}_{\text{zym}}]}{K_{\text{m MASP2}^*}^{\text{MASP2}_{\text{zym}}}}+\frac{[\text{pHFMASP1}_{\text{zym}}]}{K_{\text{m MASP2}^*}^{\text{MASP1}_{\text{zym}}}}+\frac{[\text{pHFMASP2}_{\text{zym}}]}{K_{\text{m MASP2}^*}^{\text{MASP2}_{\text{zym}}}}+\frac{[\text{pMFMASP1}_{\text{zym}}]}{K_{\text{m MASP2}^*}^{\text{MASP1}_{\text{zym}}}}+\frac{[\text{pMFMASP2}_{\text{zym}}]}{K_{\text{m MASP2}^*}^{\text{MASP2}_{\text{zym}}}}\\+\frac{[\text{pPTX3LFMASP1}_{\text{zym}}]}{K_{\text{m MASP2}^*}^{\text{MASP1}_{\text{zym}}}}+\frac{[\text{pPTX3LFMASP2}_{\text{zym}}]}{K_{\text{m MASP2}^*}^{\text{MASP2}_{\text{zym}}}}\end{array}\right)}
$$

$$
-\frac{k_{\mathrm{cat\,MASP2^*}}^{\mathrm{MASP2_{zym}}}\left[\mathrm{pMFMASP2^*}\right]\left[\mathrm{pMBL_4MASP2_{zym}}\right]}{K_{\mathrm{m\,MASP2^*}}^{\mathrm{MASP2_{zym}}}\left(\begin{array}{l}1+\frac{[\mathrm{C4}]}{K_{\mathrm{m\,MASP2^*}}^{\mathrm{C4}}}+\frac{[\mathrm{fC4bC2}]}{K_{\mathrm{m\,MASP2^*}}^{\mathrm{C2}}}+\frac{[\mathrm{fC4bC4bC2}]}{K_{\mathrm{m\,MASP2^*}}^{\mathrm{C2}}}+\frac{[\mathrm{fC3bC4bC2}]}{K_{\mathrm{m\,MASP2^*}}^{\mathrm{C2}}}+\frac{[\mathrm{pC4bC2}]}{K_{\mathrm{m\,MASP2^*}}^{\mathrm{C2}}}+\frac{[\mathrm{pC4bC4bC2}]}{K_{\mathrm{m\,MASP2^*}}^{\mathrm{C2}}}\\
+\frac{[\mathrm{pC3bC4bC2}]}{K_{\mathrm{m\,MASP2^*}}^{\mathrm{C2}}}+\frac{[\mathrm{pMBL_3MASP1_{zym}}]}{K_{\mathrm{m\,MASP2^*}}^{\mathrm{MASP1_{zym}}}}+\frac{[\mathrm{pMBL_3MASP2_{zym}}]}{K_{\mathrm{m\,MASP2^*}}^{\mathrm{MASP2_{zym}}}}+\frac{[\mathrm{pMBL_4MASP1_{zym}}]}{K_{\mathrm{m\,MASP2^*}}^{\mathrm{MASP1_{zym}}}}\\
+\frac{[\mathrm{pMBL_4MASP2_{zym}}]}{K_{\mathrm{m\,MASP2^*}}^{\mathrm{MASP2_{zym}}}}+\frac{[\mathrm{pCLLKMASP1_{zym}}]}{K_{\mathrm{m\,MASP2^*}}^{\mathrm{MASP1_{zym}}}}+\frac{[\mathrm{pCLLKMASP2_{zym}}]}{K_{\mathrm{m\,MASP2^*}}^{\mathrm{MASP2_{zym}}}}+\frac{[\mathrm{pCLK1MASP1_{zym}}]}{K_{\mathrm{m\,MASP2^*}}^{\mathrm{MASP1_{zym}}}}\\
+\frac{[\mathrm{pCLK1MASP2_{zym}}]}{K_{\mathrm{m\,MASP2^*}}^{\mathrm{MASP2_{zym}}}}+\frac{[\mathrm{pCLL1MASP1_{zym}}]}{K_{\mathrm{m\,MASP2^*}}^{\mathrm{MASP1_{zym}}}}+\frac{[\mathrm{pCLL1MASP2_{zym}}]}{K_{\mathrm{m\,MASP2^*}}^{\mathrm{MASP2_{zym}}}}+\frac{[\mathrm{pLFMASP1_{zym}}]}{K_{\mathrm{m\,MASP2^*}}^{\mathrm{MASP1_{zym}}}}\\
+\frac{[\mathrm{pLFMASP2_{zym}}]}{K_{\mathrm{m\,MASP2^*}}^{\mathrm{MASP2_{zym}}}}+\frac{[\mathrm{pHFMASP1_{zym}}]}{K_{\mathrm{m\,MASP2^*}}^{\mathrm{MASP1_{zym}}}}+\frac{[\mathrm{pHFMASP2_{zym}}]}{K_{\mathrm{m\,MASP2^*}}^{\mathrm{MASP2_{zym}}}}+\frac{[\mathrm{pMFMASP1_{zym}}]}{K_{\mathrm{m\,MASP2^*}}^{\mathrm{MASP1_{zym}}}}+\frac{[\mathrm{pMFMASP2_{zym}}]}{K_{\mathrm{m\,MASP2^*}}^{\mathrm{MASP2_{zym}}}}\\
+\frac{[\mathrm{pPTX3LFMASP1_{zym}}]}{K_{\mathrm{m\,MASP2^*}}^{\mathrm{MASP1_{zym}}}}+\frac{[\mathrm{pPTX3LFMASP2_{zym}}]}{K_{\mathrm{m\,MASP2^*}}^{\mathrm{MASP2_{zym}}}}\end{array}\right)}
$$

$$
-\frac{k_{\mathrm{cat\,MASP2^*}}^{\mathrm{MASP2_{zym}}}\left[\mathrm{pPTX3LFMASP2^*}\right]\left[\mathrm{pMBL_4MASP2_{zym}}\right]}{K_{\mathrm{m\,MASP2^*}}^{\mathrm{MASP2_{zym}}}\left(\begin{array}{l}1+\frac{[\mathrm{C4}]}{K_{\mathrm{m\,MASP2^*}}^{\mathrm{C4}}}+\frac{[\mathrm{fC4bC2}]}{K_{\mathrm{m\,MASP2^*}}^{\mathrm{C2}}}+\frac{[\mathrm{fC4bC4bC2}]}{K_{\mathrm{m\,MASP2^*}}^{\mathrm{C2}}}+\frac{[\mathrm{fC3bC4bC2}]}{K_{\mathrm{m\,MASP2^*}}^{\mathrm{C2}}}+\frac{[\mathrm{pC4bC2}]}{K_{\mathrm{m\,MASP2^*}}^{\mathrm{C2}}}+\frac{[\mathrm{pC4bC4bC2}]}{K_{\mathrm{m\,MASP2^*}}^{\mathrm{C2}}}\\
+\frac{[\mathrm{pC3bC4bC2}]}{K_{\mathrm{m\,MASP2^*}}^{\mathrm{C2}}}+\frac{[\mathrm{pMBL_3MASP1_{zym}}]}{K_{\mathrm{m\,MASP2^*}}^{\mathrm{MASP1_{zym}}}}+\frac{[\mathrm{pMBL_3MASP2_{zym}}]}{K_{\mathrm{m\,MASP2^*}}^{\mathrm{MASP2_{zym}}}}+\frac{[\mathrm{pMBL_4MASP1_{zym}}]}{K_{\mathrm{m\,MASP2^*}}^{\mathrm{MASP1_{zym}}}}\\
+\frac{[\mathrm{pMBL_4MASP2_{zym}}]}{K_{\mathrm{m\,MASP2^*}}^{\mathrm{MASP2_{zym}}}}+\frac{[\mathrm{pCLLKMASP1_{zym}}]}{K_{\mathrm{m\,MASP2^*}}^{\mathrm{MASP1_{zym}}}}+\frac{[\mathrm{pCLLKMASP2_{zym}}]}{K_{\mathrm{m\,MASP2^*}}^{\mathrm{MASP2_{zym}}}}+\frac{[\mathrm{pCLK1MASP1_{zym}}]}{K_{\mathrm{m\,MASP2^*}}^{\mathrm{MASP1_{zym}}}}\\
+\frac{[\mathrm{pCLK1MASP2_{zym}}]}{K_{\mathrm{m\,MASP2^*}}^{\mathrm{MASP2_{zym}}}}+\frac{[\mathrm{pCLL1MASP1_{zym}}]}{K_{\mathrm{m\,MASP2^*}}^{\mathrm{MASP1_{zym}}}}+\frac{[\mathrm{pCLL1MASP2_{zym}}]}{K_{\mathrm{m\,MASP2^*}}^{\mathrm{MASP2_{zym}}}}+\frac{[\mathrm{pLFMASP1_{zym}}]}{K_{\mathrm{m\,MASP2^*}}^{\mathrm{MASP1_{zym}}}}\\
+\frac{[\mathrm{pLFMASP2_{zym}}]}{K_{\mathrm{m\,MASP2^*}}^{\mathrm{MASP2_{zym}}}}+\frac{[\mathrm{pHFMASP1_{zym}}]}{K_{\mathrm{m\,MASP2^*}}^{\mathrm{MASP1_{zym}}}}+\frac{[\mathrm{pHFMASP2_{zym}}]}{K_{\mathrm{m\,MASP2^*}}^{\mathrm{MASP2_{zym}}}}+\frac{[\mathrm{pMFMASP1_{zym}}]}{K_{\mathrm{m\,MASP2^*}}^{\mathrm{MASP1_{zym}}}}+\frac{[\mathrm{pMFMASP2_{zym}}]}{K_{\mathrm{m\,MASP2^*}}^{\mathrm{MASP2_{zym}}}}\\
+\frac{[\mathrm{pPTX3LFMASP1_{zym}}]}{K_{\mathrm{m\,MASP2^*}}^{\mathrm{MASP1_{zym}}}}+\frac{[\mathrm{pPTX3LFMASP2_{zym}}]}{K_{\mathrm{m\,MASP2^*}}^{\mathrm{MASP2_{zym}}}}\end{array}\right)}
$$

272

$$
\frac{d\left[\mathrm{fMBL_4MASP2^*}\right]}{dt}=-k_{\mathrm{pMBL_4}}^{+}\left[\mathrm{fMBL_4MASP2^*}\right]\left[\mathrm{Pathogen\ Surface}\right]+k_{\mathrm{pMBL_4}}^{-}\left[\mathrm{pMBL_4MASP2^*}\right]
$$
$$
+k_{\mathrm{MBL_4MASP2}}^{+}\left[\mathrm{MBL_4}\right]\left[\mathrm{MASP2^*}\right]-k_{\mathrm{MBL_4MASP2}}^{-}\left[\mathrm{fMBL_4MASP2^*}\right]-k_{\mathrm{C1INHMASP2}}^{+}\left[\mathrm{C1\text{-}INH}\right]\left[\mathrm{fMBL_4MASP2^*}\right]
$$

273

$$
\begin{aligned}
\frac{d\left[\mathrm{pMBL_4MASP2^*}\right]}{dt} &= k^{+}_{\mathrm{pMBL_4}}\left[\mathrm{fMBL_4MASP2^*}\right]\left[\mathrm{Pathogen\ Surface}\right]-k^{-}_{\mathrm{pMBL_4}}\left[\mathrm{pMBL_4MASP2^*}\right] \\
&+k^{+}_{\mathrm{MBL_4MASP2}}\left[\mathrm{pMBL_4}\right]\left[\mathrm{MASP2^*}\right]-k^{-}_{\mathrm{MBL_4MASP2}}\left[\mathrm{pMBL_4MASP2^*}\right] \\
&+\frac{k^{\mathrm{MASP2_{zym}}}_{\mathrm{cat\ MASP1_{zym}}}\left[\mathrm{pMBL_3MASP1_{zym}}\right]\left[\mathrm{pMBL_4MASP2_{zym}}\right]}{K^{\mathrm{MASP2_{zym}}}_{\mathrm{m\ MASP1_{zym}}}\left(\begin{aligned}
&1+\frac{\left[\mathrm{pMBL_3MASP1_{zym}}\right]}{K^{\mathrm{MASP1_{zym}}}_{\mathrm{m\ MASP1_{zym}}}}+\frac{\left[\mathrm{pMBL_3MASP2_{zym}}\right]}{K^{\mathrm{MASP2_{zym}}}_{\mathrm{m\ MASP1_{zym}}}}+\frac{\left[\mathrm{pMBL_4MASP1_{zym}}\right]}{K^{\mathrm{MASP1_{zym}}}_{\mathrm{m\ MASP1_{zym}}}}+\frac{\left[\mathrm{pMBL_4MASP2_{zym}}\right]}{K^{\mathrm{MASP2_{zym}}}_{\mathrm{m\ MASP1_{zym}}}} \\
&+\frac{\left[\mathrm{pCLLKMASP1_{zym}}\right]}{K^{\mathrm{MASP1_{zym}}}_{\mathrm{m\ MASP1_{zym}}}}+\frac{\left[\mathrm{pCLLKMASP2_{zym}}\right]}{K^{\mathrm{MASP2_{zym}}}_{\mathrm{m\ MASP1_{zym}}}}+\frac{\left[\mathrm{pCLK1MASP1_{zym}}\right]}{K^{\mathrm{MASP1_{zym}}}_{\mathrm{m\ MASP1_{zym}}}}+\frac{\left[\mathrm{pCLK1MASP2_{zym}}\right]}{K^{\mathrm{MASP2_{zym}}}_{\mathrm{m\ MASP1_{zym}}}} \\
&+\frac{\left[\mathrm{pCLL1MASP1_{zym}}\right]}{K^{\mathrm{MASP1_{zym}}}_{\mathrm{m\ MASP1_{zym}}}}+\frac{\left[\mathrm{pCLL1MASP2_{zym}}\right]}{K^{\mathrm{MASP2_{zym}}}_{\mathrm{m\ MASP1_{zym}}}}+\frac{\left[\mathrm{pLFMASP1_{zym}}\right]}{K^{\mathrm{MASP1_{zym}}}_{\mathrm{m\ MASP1_{zym}}}}+\frac{\left[\mathrm{pLFMASP2_{zym}}\right]}{K^{\mathrm{MASP2_{zym}}}_{\mathrm{m\ MASP1_{zym}}}} \\
&+\frac{\left[\mathrm{pHFMASP1_{zym}}\right]}{K^{\mathrm{MASP1_{zym}}}_{\mathrm{m\ MASP1_{zym}}}}+\frac{\left[\mathrm{pHFMASP2_{zym}}\right]}{K^{\mathrm{MASP2_{zym}}}_{\mathrm{m\ MASP1_{zym}}}}+\frac{\left[\mathrm{pMFMASP1_{zym}}\right]}{K^{\mathrm{MASP1_{zym}}}_{\mathrm{m\ MASP1_{zym}}}}+\frac{\left[\mathrm{pMFMASP2_{zym}}\right]}{K^{\mathrm{MASP2_{zym}}}_{\mathrm{m\ MASP1_{zym}}}} \\
&+\frac{\left[\mathrm{pPTX3LFMASP1_{zym}}\right]}{K^{\mathrm{MASP1_{zym}}}_{\mathrm{m\ MASP1_{zym}}}}+\frac{\left[\mathrm{pPTX3LFMASP2_{zym}}\right]}{K^{\mathrm{MASP2_{zym}}}_{\mathrm{m\ MASP1_{zym}}}}
\end{aligned}\right)} \\
&+\frac{k^{\mathrm{MASP2_{zym}}}_{\mathrm{cat\ MASP1_{zym}}}\left[\mathrm{pMBL_4MASP1_{zym}}\right]\left[\mathrm{pMBL_4MASP2_{zym}}\right]}{K^{\mathrm{MASP2_{zym}}}_{\mathrm{m\ MASP1_{zym}}}\left(\begin{aligned}
&1+\frac{\left[\mathrm{pMBL_3MASP1_{zym}}\right]}{K^{\mathrm{MASP1_{zym}}}_{\mathrm{m\ MASP1_{zym}}}}+\frac{\left[\mathrm{pMBL_3MASP2_{zym}}\right]}{K^{\mathrm{MASP2_{zym}}}_{\mathrm{m\ MASP1_{zym}}}}+\frac{\left[\mathrm{pMBL_4MASP1_{zym}}\right]}{K^{\mathrm{MASP1_{zym}}}_{\mathrm{m\ MASP1_{zym}}}}+\frac{\left[\mathrm{pMBL_4MASP2_{zym}}\right]}{K^{\mathrm{MASP2_{zym}}}_{\mathrm{m\ MASP1_{zym}}}} \\
&+\frac{\left[\mathrm{pCLLKMASP1_{zym}}\right]}{K^{\mathrm{MASP1_{zym}}}_{\mathrm{m\ MASP1_{zym}}}}+\frac{\left[\mathrm{pCLLKMASP2_{zym}}\right]}{K^{\mathrm{MASP2_{zym}}}_{\mathrm{m\ MASP1_{zym}}}}+\frac{\left[\mathrm{pCLK1MASP1_{zym}}\right]}{K^{\mathrm{MASP1_{zym}}}_{\mathrm{m\ MASP1_{zym}}}}+\frac{\left[\mathrm{pCLK1MASP2_{zym}}\right]}{K^{\mathrm{MASP2_{zym}}}_{\mathrm{m\ MASP1_{zym}}}} \\
&+\frac{\left[\mathrm{pCLL1MASP1_{zym}}\right]}{K^{\mathrm{MASP1_{zym}}}_{\mathrm{m\ MASP1_{zym}}}}+\frac{\left[\mathrm{pCLL1MASP2_{zym}}\right]}{K^{\mathrm{MASP2_{zym}}}_{\mathrm{m\ MASP1_{zym}}}}+\frac{\left[\mathrm{pLFMASP1_{zym}}\right]}{K^{\mathrm{MASP1_{zym}}}_{\mathrm{m\ MASP1_{zym}}}}+\frac{\left[\mathrm{pLFMASP2_{zym}}\right]}{K^{\mathrm{MASP2_{zym}}}_{\mathrm{m\ MASP1_{zym}}}} \\
&+\frac{\left[\mathrm{pHFMASP1_{zym}}\right]}{K^{\mathrm{MASP1_{zym}}}_{\mathrm{m\ MASP1_{zym}}}}+\frac{\left[\mathrm{pHFMASP2_{zym}}\right]}{K^{\mathrm{MASP2_{zym}}}_{\mathrm{m\ MASP1_{zym}}}}+\frac{\left[\mathrm{pMFMASP1_{zym}}\right]}{K^{\mathrm{MASP1_{zym}}}_{\mathrm{m\ MASP1_{zym}}}}+\frac{\left[\mathrm{pMFMASP2_{zym}}\right]}{K^{\mathrm{MASP2_{zym}}}_{\mathrm{m\ MASP1_{zym}}}} \\
&+\frac{\left[\mathrm{pPTX3LFMASP1_{zym}}\right]}{K^{\mathrm{MASP1_{zym}}}_{\mathrm{m\ MASP1_{zym}}}}+\frac{\left[\mathrm{pPTX3LFMASP2_{zym}}\right]}{K^{\mathrm{MASP2_{zym}}}_{\mathrm{m\ MASP1_{zym}}}}
\end{aligned}\right)}
\end{aligned}
$$

$$+\frac{k_{\mathrm{cat\ MASP1_{zym}}}^{\mathrm{MASP2_{zym}}}\left[\mathrm{pCLLKMASP1_{zym}}\right]\left[\mathrm{pMBL_4MASP2_{zym}}\right]}{K_{\mathrm{m\ MASP1_{zym}}}^{\mathrm{MASP2_{zym}}}\left(\begin{array}{l}1+\frac{\left[\mathrm{pMBL_3MASP1_{zym}}\right]}{K_{\mathrm{m\ MASP1_{zym}}}^{\mathrm{MASP1_{zym}}}}+\frac{\left[\mathrm{pMBL_3MASP2_{zym}}\right]}{K_{\mathrm{m\ MASP1_{zym}}}^{\mathrm{MASP2_{zym}}}}+\frac{\left[\mathrm{pMBL_4MASP1_{zym}}\right]}{K_{\mathrm{m\ MASP1_{zym}}}^{\mathrm{MASP1_{zym}}}}+\frac{\left[\mathrm{pMBL_4MASP2_{zym}}\right]}{K_{\mathrm{m\ MASP1_{zym}}}^{\mathrm{MASP2_{zym}}}}\\+\frac{\left[\mathrm{pCLLKMASP1_{zym}}\right]}{K_{\mathrm{m\ MASP1_{zym}}}^{\mathrm{MASP1_{zym}}}}+\frac{\left[\mathrm{pCLLKMASP2_{zym}}\right]}{K_{\mathrm{m\ MASP1_{zym}}}^{\mathrm{MASP2_{zym}}}}+\frac{\left[\mathrm{pCLK1MASP1_{zym}}\right]}{K_{\mathrm{m\ MASP1_{zym}}}^{\mathrm{MASP1_{zym}}}}+\frac{\left[\mathrm{pCLK1MASP2_{zym}}\right]}{K_{\mathrm{m\ MASP1_{zym}}}^{\mathrm{MASP2_{zym}}}}\\+\frac{\left[\mathrm{pCLL1MASP1_{zym}}\right]}{K_{\mathrm{m\ MASP1_{zym}}}^{\mathrm{MASP1_{zym}}}}+\frac{\left[\mathrm{pCLL1MASP2_{zym}}\right]}{K_{\mathrm{m\ MASP1_{zym}}}^{\mathrm{MASP2_{zym}}}}+\frac{\left[\mathrm{pLFMASP1_{zym}}\right]}{K_{\mathrm{m\ MASP1_{zym}}}^{\mathrm{MASP1_{zym}}}}+\frac{\left[\mathrm{pLFMASP2_{zym}}\right]}{K_{\mathrm{m\ MASP1_{zym}}}^{\mathrm{MASP2_{zym}}}}\\+\frac{\left[\mathrm{pHFMASP1_{zym}}\right]}{K_{\mathrm{m\ MASP1_{zym}}}^{\mathrm{MASP1_{zym}}}}+\frac{\left[\mathrm{pHFMASP2_{zym}}\right]}{K_{\mathrm{m\ MASP1_{zym}}}^{\mathrm{MASP2_{zym}}}}+\frac{\left[\mathrm{pMFMASP1_{zym}}\right]}{K_{\mathrm{m\ MASP1_{zym}}}^{\mathrm{MASP1_{zym}}}}+\frac{\left[\mathrm{pMFMASP2_{zym}}\right]}{K_{\mathrm{m\ MASP1_{zym}}}^{\mathrm{MASP2_{zym}}}}\\+\frac{\left[\mathrm{pPTX3LFMASP1_{zym}}\right]}{K_{\mathrm{m\ MASP1_{zym}}}^{\mathrm{MASP1_{zym}}}}+\frac{\left[\mathrm{pPTX3LFMASP2_{zym}}\right]}{K_{\mathrm{m\ MASP1_{zym}}}^{\mathrm{MASP2_{zym}}}}\end{array}\right)}$$

$$+\frac{k_{\mathrm{cat\ MASP1_{zym}}}^{\mathrm{MASP2_{zym}}}\left[\mathrm{pCLK1MASP1_{zym}}\right]\left[\mathrm{pMBL_4MASP2_{zym}}\right]}{K_{\mathrm{m\ MASP1_{zym}}}^{\mathrm{MASP2_{zym}}}\left(\begin{array}{l}1+\frac{\left[\mathrm{pMBL_3MASP1_{zym}}\right]}{K_{\mathrm{m\ MASP1_{zym}}}^{\mathrm{MASP1_{zym}}}}+\frac{\left[\mathrm{pMBL_3MASP2_{zym}}\right]}{K_{\mathrm{m\ MASP1_{zym}}}^{\mathrm{MASP2_{zym}}}}+\frac{\left[\mathrm{pMBL_4MASP1_{zym}}\right]}{K_{\mathrm{m\ MASP1_{zym}}}^{\mathrm{MASP1_{zym}}}}+\frac{\left[\mathrm{pMBL_4MASP2_{zym}}\right]}{K_{\mathrm{m\ MASP1_{zym}}}^{\mathrm{MASP2_{zym}}}}\\+\frac{\left[\mathrm{pCLLKMASP1_{zym}}\right]}{K_{\mathrm{m\ MASP1_{zym}}}^{\mathrm{MASP1_{zym}}}}+\frac{\left[\mathrm{pCLLKMASP2_{zym}}\right]}{K_{\mathrm{m\ MASP1_{zym}}}^{\mathrm{MASP2_{zym}}}}+\frac{\left[\mathrm{pCLK1MASP1_{zym}}\right]}{K_{\mathrm{m\ MASP1_{zym}}}^{\mathrm{MASP1_{zym}}}}+\frac{\left[\mathrm{pCLK1MASP2_{zym}}\right]}{K_{\mathrm{m\ MASP1_{zym}}}^{\mathrm{MASP2_{zym}}}}\\+\frac{\left[\mathrm{pCLL1MASP1_{zym}}\right]}{K_{\mathrm{m\ MASP1_{zym}}}^{\mathrm{MASP1_{zym}}}}+\frac{\left[\mathrm{pCLL1MASP2_{zym}}\right]}{K_{\mathrm{m\ MASP1_{zym}}}^{\mathrm{MASP2_{zym}}}}+\frac{\left[\mathrm{pLFMASP1_{zym}}\right]}{K_{\mathrm{m\ MASP1_{zym}}}^{\mathrm{MASP1_{zym}}}}+\frac{\left[\mathrm{pLFMASP2_{zym}}\right]}{K_{\mathrm{m\ MASP1_{zym}}}^{\mathrm{MASP2_{zym}}}}\\+\frac{\left[\mathrm{pHFMASP1_{zym}}\right]}{K_{\mathrm{m\ MASP1_{zym}}}^{\mathrm{MASP1_{zym}}}}+\frac{\left[\mathrm{pHFMASP2_{zym}}\right]}{K_{\mathrm{m\ MASP1_{zym}}}^{\mathrm{MASP2_{zym}}}}+\frac{\left[\mathrm{pMFMASP1_{zym}}\right]}{K_{\mathrm{m\ MASP1_{zym}}}^{\mathrm{MASP1_{zym}}}}+\frac{\left[\mathrm{pMFMASP2_{zym}}\right]}{K_{\mathrm{m\ MASP1_{zym}}}^{\mathrm{MASP2_{zym}}}}\\+\frac{\left[\mathrm{pPTX3LFMASP1_{zym}}\right]}{K_{\mathrm{m\ MASP1_{zym}}}^{\mathrm{MASP1_{zym}}}}+\frac{\left[\mathrm{pPTX3LFMASP2_{zym}}\right]}{K_{\mathrm{m\ MASP1_{zym}}}^{\mathrm{MASP2_{zym}}}}\end{array}\right)}$$

$$
+\frac{k_{\mathrm{cat\ MASP1_{zym}}}^{\mathrm{MASP2_{zym}}}\left[\mathrm{pCLL1MASP1_{zym}}\right]\left[\mathrm{pMBL_4MASP2_{zym}}\right]}{K_{\mathrm{m\ MASP1_{zym}}}^{\mathrm{MASP2_{zym}}}\left(\begin{array}{l}1+\frac{\left[\mathrm{pMBL_3MASP1_{zym}}\right]}{K_{\mathrm{m\ MASP1_{zym}}}^{\mathrm{MASP1_{zym}}}}+\frac{\left[\mathrm{pMBL_3MASP2_{zym}}\right]}{K_{\mathrm{m\ MASP1_{zym}}}^{\mathrm{MASP2_{zym}}}}+\frac{\left[\mathrm{pMBL_4MASP1_{zym}}\right]}{K_{\mathrm{m\ MASP1_{zym}}}^{\mathrm{MASP1_{zym}}}}+\frac{\left[\mathrm{pMBL_4MASP2_{zym}}\right]}{K_{\mathrm{m\ MASP1_{zym}}}^{\mathrm{MASP2_{zym}}}}\\
+\frac{\left[\mathrm{pCLLKMASP1_{zym}}\right]}{K_{\mathrm{m\ MASP1_{zym}}}^{\mathrm{MASP1_{zym}}}}+\frac{\left[\mathrm{pCLLKMASP2_{zym}}\right]}{K_{\mathrm{m\ MASP1_{zym}}}^{\mathrm{MASP2_{zym}}}}+\frac{\left[\mathrm{pCLK1MASP1_{zym}}\right]}{K_{\mathrm{m\ MASP1_{zym}}}^{\mathrm{MASP1_{zym}}}}+\frac{\left[\mathrm{pCLK1MASP2_{zym}}\right]}{K_{\mathrm{m\ MASP1_{zym}}}^{\mathrm{MASP2_{zym}}}}\\
+\frac{\left[\mathrm{pCLL1MASP1_{zym}}\right]}{K_{\mathrm{m\ MASP1_{zym}}}^{\mathrm{MASP1_{zym}}}}+\frac{\left[\mathrm{pCLL1MASP2_{zym}}\right]}{K_{\mathrm{m\ MASP1_{zym}}}^{\mathrm{MASP2_{zym}}}}+\frac{\left[\mathrm{pLFMASP1_{zym}}\right]}{K_{\mathrm{m\ MASP1_{zym}}}^{\mathrm{MASP1_{zym}}}}+\frac{\left[\mathrm{pLFMASP2_{zym}}\right]}{K_{\mathrm{m\ MASP1_{zym}}}^{\mathrm{MASP2_{zym}}}}\\
+\frac{\left[\mathrm{pHFMASP1_{zym}}\right]}{K_{\mathrm{m\ MASP1_{zym}}}^{\mathrm{MASP1_{zym}}}}+\frac{\left[\mathrm{pHFMASP2_{zym}}\right]}{K_{\mathrm{m\ MASP1_{zym}}}^{\mathrm{MASP2_{zym}}}}+\frac{\left[\mathrm{pMFMASP1_{zym}}\right]}{K_{\mathrm{m\ MASP1_{zym}}}^{\mathrm{MASP1_{zym}}}}+\frac{\left[\mathrm{pMFMASP2_{zym}}\right]}{K_{\mathrm{m\ MASP1_{zym}}}^{\mathrm{MASP2_{zym}}}}\\
+\frac{\left[\mathrm{pPTX3LFMASP1_{zym}}\right]}{K_{\mathrm{m\ MASP1_{zym}}}^{\mathrm{MASP1_{zym}}}}+\frac{\left[\mathrm{pPTX3LFMASP2_{zym}}\right]}{K_{\mathrm{m\ MASP1_{zym}}}^{\mathrm{MASP2_{zym}}}}\end{array}\right)}
$$

$$
+\frac{k_{\mathrm{cat\ MASP1_{zym}}}^{\mathrm{MASP2_{zym}}}\left[\mathrm{pLFMASP1_{zym}}\right]\left[\mathrm{pMBL_4MASP2_{zym}}\right]}{K_{\mathrm{m\ MASP1_{zym}}}^{\mathrm{MASP2_{zym}}}\left(\begin{array}{l}1+\frac{\left[\mathrm{pMBL_3MASP1_{zym}}\right]}{K_{\mathrm{m\ MASP1_{zym}}}^{\mathrm{MASP1_{zym}}}}+\frac{\left[\mathrm{pMBL_3MASP2_{zym}}\right]}{K_{\mathrm{m\ MASP1_{zym}}}^{\mathrm{MASP2_{zym}}}}+\frac{\left[\mathrm{pMBL_4MASP1_{zym}}\right]}{K_{\mathrm{m\ MASP1_{zym}}}^{\mathrm{MASP1_{zym}}}}+\frac{\left[\mathrm{pMBL_4MASP2_{zym}}\right]}{K_{\mathrm{m\ MASP1_{zym}}}^{\mathrm{MASP2_{zym}}}}\\
+\frac{\left[\mathrm{pCLLKMASP1_{zym}}\right]}{K_{\mathrm{m\ MASP1_{zym}}}^{\mathrm{MASP1_{zym}}}}+\frac{\left[\mathrm{pCLLKMASP2_{zym}}\right]}{K_{\mathrm{m\ MASP1_{zym}}}^{\mathrm{MASP2_{zym}}}}+\frac{\left[\mathrm{pCLK1MASP1_{zym}}\right]}{K_{\mathrm{m\ MASP1_{zym}}}^{\mathrm{MASP1_{zym}}}}+\frac{\left[\mathrm{pCLK1MASP2_{zym}}\right]}{K_{\mathrm{m\ MASP1_{zym}}}^{\mathrm{MASP2_{zym}}}}\\
+\frac{\left[\mathrm{pCLL1MASP1_{zym}}\right]}{K_{\mathrm{m\ MASP1_{zym}}}^{\mathrm{MASP1_{zym}}}}+\frac{\left[\mathrm{pCLL1MASP2_{zym}}\right]}{K_{\mathrm{m\ MASP1_{zym}}}^{\mathrm{MASP2_{zym}}}}+\frac{\left[\mathrm{pLFMASP1_{zym}}\right]}{K_{\mathrm{m\ MASP1_{zym}}}^{\mathrm{MASP1_{zym}}}}+\frac{\left[\mathrm{pLFMASP2_{zym}}\right]}{K_{\mathrm{m\ MASP1_{zym}}}^{\mathrm{MASP2_{zym}}}}\\
+\frac{\left[\mathrm{pHFMASP1_{zym}}\right]}{K_{\mathrm{m\ MASP1_{zym}}}^{\mathrm{MASP1_{zym}}}}+\frac{\left[\mathrm{pHFMASP2_{zym}}\right]}{K_{\mathrm{m\ MASP1_{zym}}}^{\mathrm{MASP2_{zym}}}}+\frac{\left[\mathrm{pMFMASP1_{zym}}\right]}{K_{\mathrm{m\ MASP1_{zym}}}^{\mathrm{MASP1_{zym}}}}+\frac{\left[\mathrm{pMFMASP2_{zym}}\right]}{K_{\mathrm{m\ MASP1_{zym}}}^{\mathrm{MASP2_{zym}}}}\\
+\frac{\left[\mathrm{pPTX3LFMASP1_{zym}}\right]}{K_{\mathrm{m\ MASP1_{zym}}}^{\mathrm{MASP1_{zym}}}}+\frac{\left[\mathrm{pPTX3LFMASP2_{zym}}\right]}{K_{\mathrm{m\ MASP1_{zym}}}^{\mathrm{MASP2_{zym}}}}\end{array}\right)}
$$

$$
+\frac{k_{\mathrm{cat\ MASP1_{zym}}}^{\mathrm{MASP2_{zym}}}\left[\mathrm{pHFMASP1_{zym}}\right]\left[\mathrm{pMBL_4MASP2_{zym}}\right]}{K_{\mathrm{m\ MASP1_{zym}}}^{\mathrm{MASP2_{zym}}}\left(\begin{array}{l}1+\frac{\left[\mathrm{pMBL_3MASP1_{zym}}\right]}{K_{\mathrm{m\ MASP1_{zym}}}^{\mathrm{MASP1_{zym}}}}+\frac{\left[\mathrm{pMBL_3MASP2_{zym}}\right]}{K_{\mathrm{m\ MASP1_{zym}}}^{\mathrm{MASP2_{zym}}}}+\frac{\left[\mathrm{pMBL_4MASP1_{zym}}\right]}{K_{\mathrm{m\ MASP1_{zym}}}^{\mathrm{MASP1_{zym}}}}+\frac{\left[\mathrm{pMBL_4MASP2_{zym}}\right]}{K_{\mathrm{m\ MASP1_{zym}}}^{\mathrm{MASP2_{zym}}}}\\
+\frac{\left[\mathrm{pCLLKMASP1_{zym}}\right]}{K_{\mathrm{m\ MASP1_{zym}}}^{\mathrm{MASP1_{zym}}}}+\frac{\left[\mathrm{pCLLKMASP2_{zym}}\right]}{K_{\mathrm{m\ MASP1_{zym}}}^{\mathrm{MASP2_{zym}}}}+\frac{\left[\mathrm{pCLK1MASP1_{zym}}\right]}{K_{\mathrm{m\ MASP1_{zym}}}^{\mathrm{MASP1_{zym}}}}+\frac{\left[\mathrm{pCLK1MASP2_{zym}}\right]}{K_{\mathrm{m\ MASP1_{zym}}}^{\mathrm{MASP2_{zym}}}}\\
+\frac{\left[\mathrm{pCLL1MASP1_{zym}}\right]}{K_{\mathrm{m\ MASP1_{zym}}}^{\mathrm{MASP1_{zym}}}}+\frac{\left[\mathrm{pCLL1MASP2_{zym}}\right]}{K_{\mathrm{m\ MASP1_{zym}}}^{\mathrm{MASP2_{zym}}}}+\frac{\left[\mathrm{pLFMASP1_{zym}}\right]}{K_{\mathrm{m\ MASP1_{zym}}}^{\mathrm{MASP1_{zym}}}}+\frac{\left[\mathrm{pLFMASP2_{zym}}\right]}{K_{\mathrm{m\ MASP1_{zym}}}^{\mathrm{MASP2_{zym}}}}\\
+\frac{\left[\mathrm{pHFMASP1_{zym}}\right]}{K_{\mathrm{m\ MASP1_{zym}}}^{\mathrm{MASP1_{zym}}}}+\frac{\left[\mathrm{pHFMASP2_{zym}}\right]}{K_{\mathrm{m\ MASP1_{zym}}}^{\mathrm{MASP2_{zym}}}}+\frac{\left[\mathrm{pMFMASP1_{zym}}\right]}{K_{\mathrm{m\ MASP1_{zym}}}^{\mathrm{MASP1_{zym}}}}+\frac{\left[\mathrm{pMFMASP2_{zym}}\right]}{K_{\mathrm{m\ MASP1_{zym}}}^{\mathrm{MASP2_{zym}}}}\\
+\frac{\left[\mathrm{pPTX3LFMASP1_{zym}}\right]}{K_{\mathrm{m\ MASP1_{zym}}}^{\mathrm{MASP1_{zym}}}}+\frac{\left[\mathrm{pPTX3LFMASP2_{zym}}\right]}{K_{\mathrm{m\ MASP1_{zym}}}^{\mathrm{MASP2_{zym}}}}\end{array}\right)}
$$

$$
+\frac{k_{\mathrm{cat\ MASP1_{zym}}}^{\mathrm{MASP2_{zym}}}\left[\mathrm{pMFMASP1_{zym}}\right]\left[\mathrm{pMBL_4MASP2_{zym}}\right]}{K_{\mathrm{m\ MASP1_{zym}}}^{\mathrm{MASP2_{zym}}}\left(\begin{array}{l}1+\frac{\left[\mathrm{pMBL_3MASP1_{zym}}\right]}{K_{\mathrm{m\ MASP1_{zym}}}^{\mathrm{MASP1_{zym}}}}+\frac{\left[\mathrm{pMBL_3MASP2_{zym}}\right]}{K_{\mathrm{m\ MASP1_{zym}}}^{\mathrm{MASP2_{zym}}}}+\frac{\left[\mathrm{pMBL_4MASP1_{zym}}\right]}{K_{\mathrm{m\ MASP1_{zym}}}^{\mathrm{MASP1_{zym}}}}+\frac{\left[\mathrm{pMBL_4MASP2_{zym}}\right]}{K_{\mathrm{m\ MASP1_{zym}}}^{\mathrm{MASP2_{zym}}}}\\
+\frac{\left[\mathrm{pCLLKMASP1_{zym}}\right]}{K_{\mathrm{m\ MASP1_{zym}}}^{\mathrm{MASP1_{zym}}}}+\frac{\left[\mathrm{pCLLKMASP2_{zym}}\right]}{K_{\mathrm{m\ MASP1_{zym}}}^{\mathrm{MASP2_{zym}}}}+\frac{\left[\mathrm{pCLK1MASP1_{zym}}\right]}{K_{\mathrm{m\ MASP1_{zym}}}^{\mathrm{MASP1_{zym}}}}+\frac{\left[\mathrm{pCLK1MASP2_{zym}}\right]}{K_{\mathrm{m\ MASP1_{zym}}}^{\mathrm{MASP2_{zym}}}}\\
+\frac{\left[\mathrm{pCLL1MASP1_{zym}}\right]}{K_{\mathrm{m\ MASP1_{zym}}}^{\mathrm{MASP1_{zym}}}}+\frac{\left[\mathrm{pCLL1MASP2_{zym}}\right]}{K_{\mathrm{m\ MASP1_{zym}}}^{\mathrm{MASP2_{zym}}}}+\frac{\left[\mathrm{pLFMASP1_{zym}}\right]}{K_{\mathrm{m\ MASP1_{zym}}}^{\mathrm{MASP1_{zym}}}}+\frac{\left[\mathrm{pLFMASP2_{zym}}\right]}{K_{\mathrm{m\ MASP1_{zym}}}^{\mathrm{MASP2_{zym}}}}\\
+\frac{\left[\mathrm{pHFMASP1_{zym}}\right]}{K_{\mathrm{m\ MASP1_{zym}}}^{\mathrm{MASP1_{zym}}}}+\frac{\left[\mathrm{pHFMASP2_{zym}}\right]}{K_{\mathrm{m\ MASP1_{zym}}}^{\mathrm{MASP2_{zym}}}}+\frac{\left[\mathrm{pMFMASP1_{zym}}\right]}{K_{\mathrm{m\ MASP1_{zym}}}^{\mathrm{MASP1_{zym}}}}+\frac{\left[\mathrm{pMFMASP2_{zym}}\right]}{K_{\mathrm{m\ MASP1_{zym}}}^{\mathrm{MASP2_{zym}}}}\\
+\frac{\left[\mathrm{pPTX3LFMASP1_{zym}}\right]}{K_{\mathrm{m\ MASP1_{zym}}}^{\mathrm{MASP1_{zym}}}}+\frac{\left[\mathrm{pPTX3LFMASP2_{zym}}\right]}{K_{\mathrm{m\ MASP1_{zym}}}^{\mathrm{MASP2_{zym}}}}\end{array}\right)}
$$

$$+\frac{k_{\mathrm{cat\ MASP1_{zym}}}^{\mathrm{MASP2_{zym}}}\left[\mathrm{pPTX3LFMASP1_{zym}}\right]\left[\mathrm{pMBL_4MASP2_{zym}}\right]}{K_{\mathrm{m\ MASP1_{zym}}}^{\mathrm{MASP2_{zym}}}\left(\begin{array}{l}1+\frac{\left[\mathrm{pMBL_3MASP1_{zym}}\right]}{K_{\mathrm{m\ MASP1_{zym}}}^{\mathrm{MASP1_{zym}}}}+\frac{\left[\mathrm{pMBL_3MASP2_{zym}}\right]}{K_{\mathrm{m\ MASP1_{zym}}}^{\mathrm{MASP2_{zym}}}}+\frac{\left[\mathrm{pMBL_4MASP1_{zym}}\right]}{K_{\mathrm{m\ MASP1_{zym}}}^{\mathrm{MASP1_{zym}}}}+\frac{\left[\mathrm{pMBL_4MASP2_{zym}}\right]}{K_{\mathrm{m\ MASP1_{zym}}}^{\mathrm{MASP2_{zym}}}}\\+\frac{\left[\mathrm{pCLLKMASP1_{zym}}\right]}{K_{\mathrm{m\ MASP1_{zym}}}^{\mathrm{MASP1_{zym}}}}+\frac{\left[\mathrm{pCLLKMASP2_{zym}}\right]}{K_{\mathrm{m\ MASP1_{zym}}}^{\mathrm{MASP2_{zym}}}}+\frac{\left[\mathrm{pCLK1MASP1_{zym}}\right]}{K_{\mathrm{m\ MASP1_{zym}}}^{\mathrm{MASP1_{zym}}}}+\frac{\left[\mathrm{pCLK1MASP2_{zym}}\right]}{K_{\mathrm{m\ MASP1_{zym}}}^{\mathrm{MASP2_{zym}}}}\\+\frac{\left[\mathrm{pCLL1MASP1_{zym}}\right]}{K_{\mathrm{m\ MASP1_{zym}}}^{\mathrm{MASP1_{zym}}}}+\frac{\left[\mathrm{pCLL1MASP2_{zym}}\right]}{K_{\mathrm{m\ MASP1_{zym}}}^{\mathrm{MASP2_{zym}}}}+\frac{\left[\mathrm{pLFMASP1_{zym}}\right]}{K_{\mathrm{m\ MASP1_{zym}}}^{\mathrm{MASP1_{zym}}}}+\frac{\left[\mathrm{pLFMASP2_{zym}}\right]}{K_{\mathrm{m\ MASP1_{zym}}}^{\mathrm{MASP2_{zym}}}}\\+\frac{\left[\mathrm{pHFMASP1_{zym}}\right]}{K_{\mathrm{m\ MASP1_{zym}}}^{\mathrm{MASP1_{zym}}}}+\frac{\left[\mathrm{pHFMASP2_{zym}}\right]}{K_{\mathrm{m\ MASP1_{zym}}}^{\mathrm{MASP2_{zym}}}}+\frac{\left[\mathrm{pMFMASP1_{zym}}\right]}{K_{\mathrm{m\ MASP1_{zym}}}^{\mathrm{MASP1_{zym}}}}+\frac{\left[\mathrm{pMFMASP2_{zym}}\right]}{K_{\mathrm{m\ MASP1_{zym}}}^{\mathrm{MASP2_{zym}}}}\\+\frac{\left[\mathrm{pPTX3LFMASP1_{zym}}\right]}{K_{\mathrm{m\ MASP1_{zym}}}^{\mathrm{MASP1_{zym}}}}+\frac{\left[\mathrm{pPTX3LFMASP2_{zym}}\right]}{K_{\mathrm{m\ MASP1_{zym}}}^{\mathrm{MASP2_{zym}}}}\end{array}\right)}$$

$$+\frac{k_{\mathrm{cat\ MASP2_{zym}}}^{\mathrm{MASP2_{zym}}}\left[\mathrm{pMBL_3MASP2_{zym}}\right]\left[\mathrm{pMBL_4MASP2_{zym}}\right]}{K_{\mathrm{m\ MASP2_{zym}}}^{\mathrm{MASP2_{zym}}}\left(\begin{array}{l}1+\frac{\left[\mathrm{pMBL_3MASP2_{zym}}\right]}{K_{\mathrm{m\ MASP2_{zym}}}^{\mathrm{MASP2_{zym}}}}+\frac{\left[\mathrm{pMBL_4MASP2_{zym}}\right]}{K_{\mathrm{m\ MASP2_{zym}}}^{\mathrm{MASP2_{zym}}}}+\frac{\left[\mathrm{pCLLKMASP2_{zym}}\right]}{K_{\mathrm{m\ MASP2_{zym}}}^{\mathrm{MASP2_{zym}}}}+\frac{\left[\mathrm{pCLK1MASP2_{zym}}\right]}{K_{\mathrm{m\ MASP2_{zym}}}^{\mathrm{MASP2_{zym}}}}\\+\frac{\left[\mathrm{pCLL1MASP2_{zym}}\right]}{K_{\mathrm{m\ MASP2_{zym}}}^{\mathrm{MASP2_{zym}}}}+\frac{\left[\mathrm{pLFMASP2_{zym}}\right]}{K_{\mathrm{m\ MASP2_{zym}}}^{\mathrm{MASP2_{zym}}}}+\frac{\left[\mathrm{pHFMASP2_{zym}}\right]}{K_{\mathrm{m\ MASP2_{zym}}}^{\mathrm{MASP2_{zym}}}}+\frac{\left[\mathrm{pMFMASP2_{zym}}\right]}{K_{\mathrm{m\ MASP2_{zym}}}^{\mathrm{MASP2_{zym}}}}\\+\frac{\left[\mathrm{pPTX3LFMASP2_{zym}}\right]}{K_{\mathrm{m\ MASP2_{zym}}}^{\mathrm{MASP2_{zym}}}}\end{array}\right)}$$

$$+\frac{k_{\mathrm{cat\ MASP2_{zym}}}^{\mathrm{MASP2_{zym}}}\left[\mathrm{pMBL_4MASP2_{zym}}\right]\left[\mathrm{pMBL_4MASP2_{zym}}\right]}{K_{\mathrm{m\ MASP2_{zym}}}^{\mathrm{MASP2_{zym}}}\left(\begin{array}{l}1+\frac{\left[\mathrm{pMBL_3MASP2_{zym}}\right]}{K_{\mathrm{m\ MASP2_{zym}}}^{\mathrm{MASP2_{zym}}}}+\frac{\left[\mathrm{pMBL_4MASP2_{zym}}\right]}{K_{\mathrm{m\ MASP2_{zym}}}^{\mathrm{MASP2_{zym}}}}+\frac{\left[\mathrm{pCLLKMASP2_{zym}}\right]}{K_{\mathrm{m\ MASP2_{zym}}}^{\mathrm{MASP2_{zym}}}}+\frac{\left[\mathrm{pCLK1MASP2_{zym}}\right]}{K_{\mathrm{m\ MASP2_{zym}}}^{\mathrm{MASP2_{zym}}}}\\+\frac{\left[\mathrm{pCLL1MASP2_{zym}}\right]}{K_{\mathrm{m\ MASP2_{zym}}}^{\mathrm{MASP2_{zym}}}}+\frac{\left[\mathrm{pLFMASP2_{zym}}\right]}{K_{\mathrm{m\ MASP2_{zym}}}^{\mathrm{MASP2_{zym}}}}+\frac{\left[\mathrm{pHFMASP2_{zym}}\right]}{K_{\mathrm{m\ MASP2_{zym}}}^{\mathrm{MASP2_{zym}}}}+\frac{\left[\mathrm{pMFMASP2_{zym}}\right]}{K_{\mathrm{m\ MASP2_{zym}}}^{\mathrm{MASP2_{zym}}}}\\+\frac{\left[\mathrm{pPTX3LFMASP2_{zym}}\right]}{K_{\mathrm{m\ MASP2_{zym}}}^{\mathrm{MASP2_{zym}}}}\end{array}\right)}$$

$$+\frac{k_{\text{cat MASP2}_{\text{zym}}}^{\text{MASP2}_{\text{zym}}}\left[\text{pCLLKMASP2}_{\text{zym}}\right]\left[\text{pMBL}_4\text{MASP2}_{\text{zym}}\right]}{K_{\text{m MASP2}_{\text{zym}}}^{\text{MASP2}_{\text{zym}}}\left(\begin{array}{l}1+\frac{\left[\text{pMBL}_3\text{MASP2}_{\text{zym}}\right]}{K_{\text{m MASP2}_{\text{zym}}}^{\text{MASP2}_{\text{zym}}}}+\frac{\left[\text{pMBL}_4\text{MASP2}_{\text{zym}}\right]}{K_{\text{m MASP2}_{\text{zym}}}^{\text{MASP2}_{\text{zym}}}}+\frac{\left[\text{pCLLKMASP2}_{\text{zym}}\right]}{K_{\text{m MASP2}_{\text{zym}}}^{\text{MASP2}_{\text{zym}}}}+\frac{\left[\text{pCLK1MASP2}_{\text{zym}}\right]}{K_{\text{m MASP2}_{\text{zym}}}^{\text{MASP2}_{\text{zym}}}}\\+\frac{\left[\text{pCLL1MASP2}_{\text{zym}}\right]}{K_{\text{m MASP2}_{\text{zym}}}^{\text{MASP2}_{\text{zym}}}}+\frac{\left[\text{pLFMASP2}_{\text{zym}}\right]}{K_{\text{m MASP2}_{\text{zym}}}^{\text{MASP2}_{\text{zym}}}}+\frac{\left[\text{pHFMASP2}_{\text{zym}}\right]}{K_{\text{m MASP2}_{\text{zym}}}^{\text{MASP2}_{\text{zym}}}}+\frac{\left[\text{pMFMASP2}_{\text{zym}}\right]}{K_{\text{m MASP2}_{\text{zym}}}^{\text{MASP2}_{\text{zym}}}}\\+\frac{\left[\text{pPTX3LFMASP2}_{\text{zym}}\right]}{K_{\text{m MASP2}_{\text{zym}}}^{\text{MASP2}_{\text{zym}}}}\end{array}\right)}$$

$$+\frac{k_{\text{cat MASP2}_{\text{zym}}}^{\text{MASP2}_{\text{zym}}}\left[\text{pCLK1MASP2}_{\text{zym}}\right]\left[\text{pMBL}_4\text{MASP2}_{\text{zym}}\right]}{K_{\text{m MASP2}_{\text{zym}}}^{\text{MASP2}_{\text{zym}}}\left(\begin{array}{l}1+\frac{\left[\text{pMBL}_3\text{MASP2}_{\text{zym}}\right]}{K_{\text{m MASP2}_{\text{zym}}}^{\text{MASP2}_{\text{zym}}}}+\frac{\left[\text{pMBL}_4\text{MASP2}_{\text{zym}}\right]}{K_{\text{m MASP2}_{\text{zym}}}^{\text{MASP2}_{\text{zym}}}}+\frac{\left[\text{pCLLKMASP2}_{\text{zym}}\right]}{K_{\text{m MASP2}_{\text{zym}}}^{\text{MASP2}_{\text{zym}}}}+\frac{\left[\text{pCLK1MASP2}_{\text{zym}}\right]}{K_{\text{m MASP2}_{\text{zym}}}^{\text{MASP2}_{\text{zym}}}}\\+\frac{\left[\text{pCLL1MASP2}_{\text{zym}}\right]}{K_{\text{m MASP2}_{\text{zym}}}^{\text{MASP2}_{\text{zym}}}}+\frac{\left[\text{pLFMASP2}_{\text{zym}}\right]}{K_{\text{m MASP2}_{\text{zym}}}^{\text{MASP2}_{\text{zym}}}}+\frac{\left[\text{pHFMASP2}_{\text{zym}}\right]}{K_{\text{m MASP2}_{\text{zym}}}^{\text{MASP2}_{\text{zym}}}}+\frac{\left[\text{pMFMASP2}_{\text{zym}}\right]}{K_{\text{m MASP2}_{\text{zym}}}^{\text{MASP2}_{\text{zym}}}}\\+\frac{\left[\text{pPTX3LFMASP2}_{\text{zym}}\right]}{K_{\text{m MASP2}_{\text{zym}}}^{\text{MASP2}_{\text{zym}}}}\end{array}\right)}$$

$$+\frac{k_{\text{cat MASP2}_{\text{zym}}}^{\text{MASP2}_{\text{zym}}}\left[\text{pCLL1MASP2}_{\text{zym}}\right]\left[\text{pMBL}_4\text{MASP2}_{\text{zym}}\right]}{K_{\text{m MASP2}_{\text{zym}}}^{\text{MASP2}_{\text{zym}}}\left(\begin{array}{l}1+\frac{\left[\text{pMBL}_3\text{MASP2}_{\text{zym}}\right]}{K_{\text{m MASP2}_{\text{zym}}}^{\text{MASP2}_{\text{zym}}}}+\frac{\left[\text{pMBL}_4\text{MASP2}_{\text{zym}}\right]}{K_{\text{m MASP2}_{\text{zym}}}^{\text{MASP2}_{\text{zym}}}}+\frac{\left[\text{pCLLKMASP2}_{\text{zym}}\right]}{K_{\text{m MASP2}_{\text{zym}}}^{\text{MASP2}_{\text{zym}}}}+\frac{\left[\text{pCLK1MASP2}_{\text{zym}}\right]}{K_{\text{m MASP2}_{\text{zym}}}^{\text{MASP2}_{\text{zym}}}}\\+\frac{\left[\text{pCLL1MASP2}_{\text{zym}}\right]}{K_{\text{m MASP2}_{\text{zym}}}^{\text{MASP2}_{\text{zym}}}}+\frac{\left[\text{pLFMASP2}_{\text{zym}}\right]}{K_{\text{m MASP2}_{\text{zym}}}^{\text{MASP2}_{\text{zym}}}}+\frac{\left[\text{pHFMASP2}_{\text{zym}}\right]}{K_{\text{m MASP2}_{\text{zym}}}^{\text{MASP2}_{\text{zym}}}}+\frac{\left[\text{pMFMASP2}_{\text{zym}}\right]}{K_{\text{m MASP2}_{\text{zym}}}^{\text{MASP2}_{\text{zym}}}}\\+\frac{\left[\text{pPTX3LFMASP2}_{\text{zym}}\right]}{K_{\text{m MASP2}_{\text{zym}}}^{\text{MASP2}_{\text{zym}}}}\end{array}\right)}$$

$$+\frac{k_{\text{cat MASP2}_{\text{zym}}}^{\text{MASP2}_{\text{zym}}}\left[\text{pLFMASP2}_{\text{zym}}\right]\left[\text{pMBL}_4\text{MASP2}_{\text{zym}}\right]}{K_{\text{m MASP2}_{\text{zym}}}^{\text{MASP2}_{\text{zym}}}\left(\begin{array}{l}1+\frac{\left[\text{pMBL}_3\text{MASP2}_{\text{zym}}\right]}{K_{\text{m MASP2}_{\text{zym}}}^{\text{MASP2}_{\text{zym}}}}+\frac{\left[\text{pMBL}_4\text{MASP2}_{\text{zym}}\right]}{K_{\text{m MASP2}_{\text{zym}}}^{\text{MASP2}_{\text{zym}}}}+\frac{\left[\text{pCLLKMASP2}_{\text{zym}}\right]}{K_{\text{m MASP2}_{\text{zym}}}^{\text{MASP2}_{\text{zym}}}}+\frac{\left[\text{pCLK1MASP2}_{\text{zym}}\right]}{K_{\text{m MASP2}_{\text{zym}}}^{\text{MASP2}_{\text{zym}}}}\\+\frac{\left[\text{pCLL1MASP2}_{\text{zym}}\right]}{K_{\text{m MASP2}_{\text{zym}}}^{\text{MASP2}_{\text{zym}}}}+\frac{\left[\text{pLFMASP2}_{\text{zym}}\right]}{K_{\text{m MASP2}_{\text{zym}}}^{\text{MASP2}_{\text{zym}}}}+\frac{\left[\text{pHFMASP2}_{\text{zym}}\right]}{K_{\text{m MASP2}_{\text{zym}}}^{\text{MASP2}_{\text{zym}}}}+\frac{\left[\text{pMFMASP2}_{\text{zym}}\right]}{K_{\text{m MASP2}_{\text{zym}}}^{\text{MASP2}_{\text{zym}}}}\\+\frac{\left[\text{pPTX3LFMASP2}_{\text{zym}}\right]}{K_{\text{m MASP2}_{\text{zym}}}^{\text{MASP2}_{\text{zym}}}}\end{array}\right)}$$

$$+\frac{k_{\text{cat MASP2}_{\text{zym}}}^{\text{MASP2}_{\text{zym}}}\left[\text{pHFMASP2}_{\text{zym}}\right]\left[\text{pMBL}_4\text{MASP2}_{\text{zym}}\right]}{K_{\text{m MASP2}_{\text{zym}}}^{\text{MASP2}_{\text{zym}}}\left(\begin{array}{l}1+\frac{\left[\text{pMBL}_3\text{MASP2}_{\text{zym}}\right]}{K_{\text{m MASP2}_{\text{zym}}}^{\text{MASP2}_{\text{zym}}}}+\frac{\left[\text{pMBL}_4\text{MASP2}_{\text{zym}}\right]}{K_{\text{m MASP2}_{\text{zym}}}^{\text{MASP2}_{\text{zym}}}}+\frac{\left[\text{pCLLKMASP2}_{\text{zym}}\right]}{K_{\text{m MASP2}_{\text{zym}}}^{\text{MASP2}_{\text{zym}}}}+\frac{\left[\text{pCLK1MASP2}_{\text{zym}}\right]}{K_{\text{m MASP2}_{\text{zym}}}^{\text{MASP2}_{\text{zym}}}}\\+\frac{\left[\text{pCLL1MASP2}_{\text{zym}}\right]}{K_{\text{m MASP2}_{\text{zym}}}^{\text{MASP2}_{\text{zym}}}}+\frac{\left[\text{pLFMASP2}_{\text{zym}}\right]}{K_{\text{m MASP2}_{\text{zym}}}^{\text{MASP2}_{\text{zym}}}}+\frac{\left[\text{pHFMASP2}_{\text{zym}}\right]}{K_{\text{m MASP2}_{\text{zym}}}^{\text{MASP2}_{\text{zym}}}}+\frac{\left[\text{pMFMASP2}_{\text{zym}}\right]}{K_{\text{m MASP2}_{\text{zym}}}^{\text{MASP2}_{\text{zym}}}}\\+\frac{\left[\text{pPTX3LFMASP2}_{\text{zym}}\right]}{K_{\text{m MASP2}_{\text{zym}}}^{\text{MASP2}_{\text{zym}}}}\end{array}\right)}$$

$$+\frac{k_{\text{cat MASP2}_{\text{zym}}}^{\text{MASP2}_{\text{zym}}}\left[\text{pMFMASP2}_{\text{zym}}\right]\left[\text{pMBL}_4\text{MASP2}_{\text{zym}}\right]}{K_{\text{m MASP2}_{\text{zym}}}^{\text{MASP2}_{\text{zym}}}\left(\begin{array}{l}1+\frac{\left[\text{pMBL}_3\text{MASP2}_{\text{zym}}\right]}{K_{\text{m MASP2}_{\text{zym}}}^{\text{MASP2}_{\text{zym}}}}+\frac{\left[\text{pMBL}_4\text{MASP2}_{\text{zym}}\right]}{K_{\text{m MASP2}_{\text{zym}}}^{\text{MASP2}_{\text{zym}}}}+\frac{\left[\text{pCLLKMASP2}_{\text{zym}}\right]}{K_{\text{m MASP2}_{\text{zym}}}^{\text{MASP2}_{\text{zym}}}}+\frac{\left[\text{pCLK1MASP2}_{\text{zym}}\right]}{K_{\text{m MASP2}_{\text{zym}}}^{\text{MASP2}_{\text{zym}}}}\\+\frac{\left[\text{pCLL1MASP2}_{\text{zym}}\right]}{K_{\text{m MASP2}_{\text{zym}}}^{\text{MASP2}_{\text{zym}}}}+\frac{\left[\text{pLFMASP2}_{\text{zym}}\right]}{K_{\text{m MASP2}_{\text{zym}}}^{\text{MASP2}_{\text{zym}}}}+\frac{\left[\text{pHFMASP2}_{\text{zym}}\right]}{K_{\text{m MASP2}_{\text{zym}}}^{\text{MASP2}_{\text{zym}}}}+\frac{\left[\text{pMFMASP2}_{\text{zym}}\right]}{K_{\text{m MASP2}_{\text{zym}}}^{\text{MASP2}_{\text{zym}}}}\\+\frac{\left[\text{pPTX3LFMASP2}_{\text{zym}}\right]}{K_{\text{m MASP2}_{\text{zym}}}^{\text{MASP2}_{\text{zym}}}}\end{array}\right)}$$

$$+\frac{k_{\text{cat MASP2}_{\text{zym}}}^{\text{MASP2}_{\text{zym}}}\left[\text{pPTX3LFMASP2}_{\text{zym}}\right]\left[\text{pMBL}_4\text{MASP2}_{\text{zym}}\right]}{K_{\text{m MASP2}_{\text{zym}}}^{\text{MASP2}_{\text{zym}}}\left(\begin{array}{l}1+\frac{\left[\text{pMBL}_3\text{MASP2}_{\text{zym}}\right]}{K_{\text{m MASP2}_{\text{zym}}}^{\text{MASP2}_{\text{zym}}}}+\frac{\left[\text{pMBL}_4\text{MASP2}_{\text{zym}}\right]}{K_{\text{m MASP2}_{\text{zym}}}^{\text{MASP2}_{\text{zym}}}}+\frac{\left[\text{pCLLKMASP2}_{\text{zym}}\right]}{K_{\text{m MASP2}_{\text{zym}}}^{\text{MASP2}_{\text{zym}}}}+\frac{\left[\text{pCLK1MASP2}_{\text{zym}}\right]}{K_{\text{m MASP2}_{\text{zym}}}^{\text{MASP2}_{\text{zym}}}}\\+\frac{\left[\text{pCLL1MASP2}_{\text{zym}}\right]}{K_{\text{m MASP2}_{\text{zym}}}^{\text{MASP2}_{\text{zym}}}}+\frac{\left[\text{pLFMASP2}_{\text{zym}}\right]}{K_{\text{m MASP2}_{\text{zym}}}^{\text{MASP2}_{\text{zym}}}}+\frac{\left[\text{pHFMASP2}_{\text{zym}}\right]}{K_{\text{m MASP2}_{\text{zym}}}^{\text{MASP2}_{\text{zym}}}}+\frac{\left[\text{pMFMASP2}_{\text{zym}}\right]}{K_{\text{m MASP2}_{\text{zym}}}^{\text{MASP2}_{\text{zym}}}}\\+\frac{\left[\text{pPTX3LFMASP2}_{\text{zym}}\right]}{K_{\text{m MASP2}_{\text{zym}}}^{\text{MASP2}_{\text{zym}}}}\end{array}\right)}$$

$$
+\frac{k_{\mathrm{cat\,MASP1^*}}^{\mathrm{MASP2_{zym}}}\left[\mathrm{pMBL_3MASP1^*}\right]\left[\mathrm{pMBL_4MASP2_{zym}}\right]}{K_{\mathrm{m\,MASP1^*}}^{\mathrm{MASP2_{zym}}}\left(\begin{array}{l}1+\frac{[\mathrm{C4}]}{K_{\mathrm{m\,MASP1^*}}^{\mathrm{C4}}}+\frac{[\mathrm{fC4bC2}]}{K_{\mathrm{m\,MASP1^*}}^{\mathrm{C2}}}+\frac{[\mathrm{fC4bC4bC2}]}{K_{\mathrm{m\,MASP1^*}}^{\mathrm{C2}}}+\frac{[\mathrm{fC3bC4bC2}]}{K_{\mathrm{m\,MASP1^*}}^{\mathrm{C2}}}+\frac{[\mathrm{pC4bC2}]}{K_{\mathrm{m\,MASP1^*}}^{\mathrm{C2}}}+\frac{[\mathrm{pC4bC4bC2}]}{K_{\mathrm{m\,MASP1^*}}^{\mathrm{C2}}}\\
+\frac{[\mathrm{pC3bC4bC2}]}{K_{\mathrm{m\,MASP1^*}}^{\mathrm{C2}}}+\frac{[\mathrm{pMBL_3MASP1_{zym}}]}{K_{\mathrm{m\,MASP1^*}}^{\mathrm{MASP1_{zym}}}}+\frac{[\mathrm{pMBL_3MASP2_{zym}}]}{K_{\mathrm{m\,MASP1^*}}^{\mathrm{MASP2_{zym}}}}+\frac{[\mathrm{pMBL_3MASP3_{zym}}]}{K_{\mathrm{m\,MASP1^*}}^{\mathrm{MASP3_{zym}}}}\\
+\frac{[\mathrm{pMBL_4MASP1_{zym}}]}{K_{\mathrm{m\,MASP1^*}}^{\mathrm{MASP1_{zym}}}}+\frac{[\mathrm{pMBL_4MASP2_{zym}}]}{K_{\mathrm{m\,MASP1^*}}^{\mathrm{MASP2_{zym}}}}+\frac{[\mathrm{pMBL_4MASP3_{zym}}]}{K_{\mathrm{m\,MASP1^*}}^{\mathrm{MASP3_{zym}}}}+\frac{[\mathrm{pCLLKMASP1_{zym}}]}{K_{\mathrm{m\,MASP1^*}}^{\mathrm{MASP1_{zym}}}}\\
+\frac{[\mathrm{pCLLKMASP2_{zym}}]}{K_{\mathrm{m\,MASP1^*}}^{\mathrm{MASP2_{zym}}}}+\frac{[\mathrm{pCLLKMASP3_{zym}}]}{K_{\mathrm{m\,MASP1^*}}^{\mathrm{MASP3_{zym}}}}+\frac{[\mathrm{pCLK1MASP1_{zym}}]}{K_{\mathrm{m\,MASP1^*}}^{\mathrm{MASP1_{zym}}}}+\frac{[\mathrm{pCLK1MASP2_{zym}}]}{K_{\mathrm{m\,MASP1^*}}^{\mathrm{MASP2_{zym}}}}\\
+\frac{[\mathrm{pCLK1MASP3_{zym}}]}{K_{\mathrm{m\,MASP1^*}}^{\mathrm{MASP3_{zym}}}}+\frac{[\mathrm{pCLL1MASP1_{zym}}]}{K_{\mathrm{m\,MASP1^*}}^{\mathrm{MASP1_{zym}}}}+\frac{[\mathrm{pCLL1MASP2_{zym}}]}{K_{\mathrm{m\,MASP1^*}}^{\mathrm{MASP2_{zym}}}}+\frac{[\mathrm{pCLL1MASP3_{zym}}]}{K_{\mathrm{m\,MASP1^*}}^{\mathrm{MASP3_{zym}}}}\\
+\frac{[\mathrm{pLFMASP1_{zym}}]}{K_{\mathrm{m\,MASP1^*}}^{\mathrm{MASP1_{zym}}}}+\frac{[\mathrm{pLFMASP2_{zym}}]}{K_{\mathrm{m\,MASP1^*}}^{\mathrm{MASP2_{zym}}}}+\frac{[\mathrm{pLFMASP3_{zym}}]}{K_{\mathrm{m\,MASP1^*}}^{\mathrm{MASP3_{zym}}}}+\frac{[\mathrm{pHFMASP1_{zym}}]}{K_{\mathrm{m\,MASP1^*}}^{\mathrm{MASP1_{zym}}}}+\frac{[\mathrm{pHFMASP2_{zym}}]}{K_{\mathrm{m\,MASP1^*}}^{\mathrm{MASP2_{zym}}}}\\
+\frac{[\mathrm{pHFMASP3_{zym}}]}{K_{\mathrm{m\,MASP1^*}}^{\mathrm{MASP3_{zym}}}}+\frac{[\mathrm{pMFMASP1_{zym}}]}{K_{\mathrm{m\,MASP1^*}}^{\mathrm{MASP1_{zym}}}}+\frac{[\mathrm{pMFMASP2_{zym}}]}{K_{\mathrm{m\,MASP1^*}}^{\mathrm{MASP2_{zym}}}}+\frac{[\mathrm{pMFMASP3_{zym}}]}{K_{\mathrm{m\,MASP1^*}}^{\mathrm{MASP3_{zym}}}}\\
+\frac{[\mathrm{pPTX3LFMASP1_{zym}}]}{K_{\mathrm{m\,MASP1^*}}^{\mathrm{MASP1_{zym}}}}+\frac{[\mathrm{pPXT3LFMASP2_{zym}}]}{K_{\mathrm{m\,MASP1^*}}^{\mathrm{MASP2_{zym}}}}+\frac{[\mathrm{pPTX3LFMASP3_{zym}}]}{K_{\mathrm{m\,MASP1^*}}^{\mathrm{MASP3_{zym}}}}\end{array}\right)}
$$

$$
+\frac{k_{\mathrm{cat\ MASP1^{*}}}^{\mathrm{MASP2_{zym}}}\left[\mathrm{pMBL_{4}MASP1^{*}}\right]\left[\mathrm{pMBL_{4}MASP2_{zym}}\right]}{K_{\mathrm{m\ MASP1^{*}}}^{\mathrm{MASP2_{zym}}}\left(\begin{aligned}
&1+\frac{[\mathrm{C4}]}{K_{\mathrm{m\ MASP1^{*}}}^{\mathrm{C4}}}+\frac{[\mathrm{fC4bC2}]}{K_{\mathrm{m\ MASP1^{*}}}^{\mathrm{C2}}}+\frac{[\mathrm{fC4bC4bC2}]}{K_{\mathrm{m\ MASP1^{*}}}^{\mathrm{C2}}}+\frac{[\mathrm{fC3bC4bC2}]}{K_{\mathrm{m\ MASP1^{*}}}^{\mathrm{C2}}}+\frac{[\mathrm{pC4bC2}]}{K_{\mathrm{m\ MASP1^{*}}}^{\mathrm{C2}}}+\frac{[\mathrm{pC4bC4bC2}]}{K_{\mathrm{m\ MASP1^{*}}}^{\mathrm{C2}}}\\
&+\frac{[\mathrm{pC3bC4bC2}]}{K_{\mathrm{m\ MASP1^{*}}}^{\mathrm{C2}}}+\frac{\left[\mathrm{pMBL_{3}MASP1_{zym}}\right]}{K_{\mathrm{m\ MASP1^{*}}}^{\mathrm{MASP1_{zym}}}}+\frac{\left[\mathrm{pMBL_{3}MASP2_{zym}}\right]}{K_{\mathrm{m\ MASP1^{*}}}^{\mathrm{MASP2_{zym}}}}+\frac{\left[\mathrm{pMBL_{3}MASP3_{zym}}\right]}{K_{\mathrm{m\ MASP1^{*}}}^{\mathrm{MASP3_{zym}}}}\\
&+\frac{\left[\mathrm{pMBL_{4}MASP1_{zym}}\right]}{K_{\mathrm{m\ MASP1^{*}}}^{\mathrm{MASP1_{zym}}}}+\frac{\left[\mathrm{pMBL_{4}MASP2_{zym}}\right]}{K_{\mathrm{m\ MASP1^{*}}}^{\mathrm{MASP2_{zym}}}}+\frac{\left[\mathrm{pMBL_{4}MASP3_{zym}}\right]}{K_{\mathrm{m\ MASP1^{*}}}^{\mathrm{MASP3_{zym}}}}+\frac{\left[\mathrm{pCLLKMASP1_{zym}}\right]}{K_{\mathrm{m\ MASP1^{*}}}^{\mathrm{MASP1_{zym}}}}\\
&+\frac{\left[\mathrm{pCLLKMASP2_{zym}}\right]}{K_{\mathrm{m\ MASP1^{*}}}^{\mathrm{MASP2_{zym}}}}+\frac{\left[\mathrm{pCLLKMASP3_{zym}}\right]}{K_{\mathrm{m\ MASP1^{*}}}^{\mathrm{MASP3_{zym}}}}+\frac{\left[\mathrm{pCLK1MASP1_{zym}}\right]}{K_{\mathrm{m\ MASP1^{*}}}^{\mathrm{MASP1_{zym}}}}+\frac{\left[\mathrm{pCLK1MASP2_{zym}}\right]}{K_{\mathrm{m\ MASP1^{*}}}^{\mathrm{MASP2_{zym}}}}\\
&+\frac{\left[\mathrm{pCLK1MASP3_{zym}}\right]}{K_{\mathrm{m\ MASP1^{*}}}^{\mathrm{MASP3_{zym}}}}+\frac{\left[\mathrm{pCLL1MASP1_{zym}}\right]}{K_{\mathrm{m\ MASP1^{*}}}^{\mathrm{MASP1_{zym}}}}+\frac{\left[\mathrm{pCLL1MASP2_{zym}}\right]}{K_{\mathrm{m\ MASP1^{*}}}^{\mathrm{MASP2_{zym}}}}+\frac{\left[\mathrm{pCLL1MASP3_{zym}}\right]}{K_{\mathrm{m\ MASP1^{*}}}^{\mathrm{MASP3_{zym}}}}\\
&+\frac{\left[\mathrm{pLFMASP1_{zym}}\right]}{K_{\mathrm{m\ MASP1^{*}}}^{\mathrm{MASP1_{zym}}}}+\frac{\left[\mathrm{pLFMASP2_{zym}}\right]}{K_{\mathrm{m\ MASP1^{*}}}^{\mathrm{MASP2_{zym}}}}+\frac{\left[\mathrm{pLFMASP3_{zym}}\right]}{K_{\mathrm{m\ MASP1^{*}}}^{\mathrm{MASP3_{zym}}}}+\frac{\left[\mathrm{pHFMASP1_{zym}}\right]}{K_{\mathrm{m\ MASP1^{*}}}^{\mathrm{MASP1_{zym}}}}+\frac{\left[\mathrm{pHFMASP2_{zym}}\right]}{K_{\mathrm{m\ MASP1^{*}}}^{\mathrm{MASP2_{zym}}}}\\
&+\frac{\left[\mathrm{pHFMASP3_{zym}}\right]}{K_{\mathrm{m\ MASP1^{*}}}^{\mathrm{MASP3_{zym}}}}+\frac{\left[\mathrm{pMFMASP1_{zym}}\right]}{K_{\mathrm{m\ MASP1^{*}}}^{\mathrm{MASP1_{zym}}}}+\frac{\left[\mathrm{pMFMASP2_{zym}}\right]}{K_{\mathrm{m\ MASP1^{*}}}^{\mathrm{MASP2_{zym}}}}+\frac{\left[\mathrm{pMFMASP3_{zym}}\right]}{K_{\mathrm{m\ MASP1^{*}}}^{\mathrm{MASP3_{zym}}}}\\
&+\frac{\left[\mathrm{pPTX3LFMASP1_{zym}}\right]}{K_{\mathrm{m\ MASP1^{*}}}^{\mathrm{MASP1_{zym}}}}+\frac{\left[\mathrm{pPXT3LFMASP2_{zym}}\right]}{K_{\mathrm{m\ MASP1^{*}}}^{\mathrm{MASP2_{zym}}}}+\frac{\left[\mathrm{pPTX3LFMASP3_{zym}}\right]}{K_{\mathrm{m\ MASP1^{*}}}^{\mathrm{MASP3_{zym}}}}
\end{aligned}\right)}
$$

$$
+\frac{k_{\mathrm{cat\,MASP1^*}}^{\mathrm{MASP2_{zym}}}\left[\mathrm{pCLLKMASP1^*}\right]\left[\mathrm{pMBL_4MASP2_{zym}}\right]}{K_{\mathrm{m\,MASP1^*}}^{\mathrm{MASP2_{zym}}}\left(\begin{array}{l}
1+\frac{[\mathrm{C4}]}{K_{\mathrm{m\,MASP1^*}}^{\mathrm{C4}}}+\frac{[\mathrm{fC4bC2}]}{K_{\mathrm{m\,MASP1^*}}^{\mathrm{C2}}}+\frac{[\mathrm{fC4bC4bC2}]}{K_{\mathrm{m\,MASP1^*}}^{\mathrm{C2}}}+\frac{[\mathrm{fC3bC4bC2}]}{K_{\mathrm{m\,MASP1^*}}^{\mathrm{C2}}}+\frac{[\mathrm{pC4bC2}]}{K_{\mathrm{m\,MASP1^*}}^{\mathrm{C2}}}+\frac{[\mathrm{pC4bC4bC2}]}{K_{\mathrm{m\,MASP1^*}}^{\mathrm{C2}}}\\
+\frac{[\mathrm{pC3bC4bC2}]}{K_{\mathrm{m\,MASP1^*}}^{\mathrm{C2}}}+\frac{[\mathrm{pMBL_3MASP1_{zym}}]}{K_{\mathrm{m\,MASP1^*}}^{\mathrm{MASP1_{zym}}}}+\frac{[\mathrm{pMBL_3MASP2_{zym}}]}{K_{\mathrm{m\,MASP1^*}}^{\mathrm{MASP2_{zym}}}}+\frac{[\mathrm{pMBL_3MASP3_{zym}}]}{K_{\mathrm{m\,MASP1^*}}^{\mathrm{MASP3_{zym}}}}\\
+\frac{[\mathrm{pMBL_4MASP1_{zym}}]}{K_{\mathrm{m\,MASP1^*}}^{\mathrm{MASP1_{zym}}}}+\frac{[\mathrm{pMBL_4MASP2_{zym}}]}{K_{\mathrm{m\,MASP1^*}}^{\mathrm{MASP2_{zym}}}}+\frac{[\mathrm{pMBL_4MASP3_{zym}}]}{K_{\mathrm{m\,MASP1^*}}^{\mathrm{MASP3_{zym}}}}+\frac{[\mathrm{pCLLKMASP1_{zym}}]}{K_{\mathrm{m\,MASP1^*}}^{\mathrm{MASP1_{zym}}}}\\
+\frac{[\mathrm{pCLLKMASP2_{zym}}]}{K_{\mathrm{m\,MASP1^*}}^{\mathrm{MASP2_{zym}}}}+\frac{[\mathrm{pCLLKMASP3_{zym}}]}{K_{\mathrm{m\,MASP1^*}}^{\mathrm{MASP3_{zym}}}}+\frac{[\mathrm{pCLK1MASP1_{zym}}]}{K_{\mathrm{m\,MASP1^*}}^{\mathrm{MASP1_{zym}}}}+\frac{[\mathrm{pCLK1MASP2_{zym}}]}{K_{\mathrm{m\,MASP1^*}}^{\mathrm{MASP2_{zym}}}}\\
+\frac{[\mathrm{pCLK1MASP3_{zym}}]}{K_{\mathrm{m\,MASP1^*}}^{\mathrm{MASP3_{zym}}}}+\frac{[\mathrm{pCLL1MASP1_{zym}}]}{K_{\mathrm{m\,MASP1^*}}^{\mathrm{MASP1_{zym}}}}+\frac{[\mathrm{pCLL1MASP2_{zym}}]}{K_{\mathrm{m\,MASP1^*}}^{\mathrm{MASP2_{zym}}}}+\frac{[\mathrm{pCLL1MASP3_{zym}}]}{K_{\mathrm{m\,MASP1^*}}^{\mathrm{MASP3_{zym}}}}\\
+\frac{[\mathrm{pLFMASP1_{zym}}]}{K_{\mathrm{m\,MASP1^*}}^{\mathrm{MASP1_{zym}}}}+\frac{[\mathrm{pLFMASP2_{zym}}]}{K_{\mathrm{m\,MASP1^*}}^{\mathrm{MASP2_{zym}}}}+\frac{[\mathrm{pLFMASP3_{zym}}]}{K_{\mathrm{m\,MASP1^*}}^{\mathrm{MASP3_{zym}}}}+\frac{[\mathrm{pHFMASP1_{zym}}]}{K_{\mathrm{m\,MASP1^*}}^{\mathrm{MASP1_{zym}}}}+\frac{[\mathrm{pHFMASP2_{zym}}]}{K_{\mathrm{m\,MASP1^*}}^{\mathrm{MASP2_{zym}}}}\\
+\frac{[\mathrm{pHFMASP3_{zym}}]}{K_{\mathrm{m\,MASP1^*}}^{\mathrm{MASP3_{zym}}}}+\frac{[\mathrm{pMFMASP1_{zym}}]}{K_{\mathrm{m\,MASP1^*}}^{\mathrm{MASP1_{zym}}}}+\frac{[\mathrm{pMFMASP2_{zym}}]}{K_{\mathrm{m\,MASP1^*}}^{\mathrm{MASP2_{zym}}}}+\frac{[\mathrm{pMFMASP3_{zym}}]}{K_{\mathrm{m\,MASP1^*}}^{\mathrm{MASP3_{zym}}}}\\
+\frac{[\mathrm{pPTX3LFMASP1_{zym}}]}{K_{\mathrm{m\,MASP1^*}}^{\mathrm{MASP1_{zym}}}}+\frac{[\mathrm{pPXT3LFMASP2_{zym}}]}{K_{\mathrm{m\,MASP1^*}}^{\mathrm{MASP2_{zym}}}}+\frac{[\mathrm{pPTX3LFMASP3_{zym}}]}{K_{\mathrm{m\,MASP1^*}}^{\mathrm{MASP3_{zym}}}}
\end{array}\right)}
$$

$$+\frac{k_{\mathrm{cat\ MASP1^*}}^{\mathrm{MASP2_{zym}}}\left[\mathrm{pCLK1MASP1^*}\right]\left[\mathrm{pMBL_4MASP2_{zym}}\right]}{K_{\mathrm{m\ MASP1^*}}^{\mathrm{MASP2_{zym}}}\left(\begin{array}{l}1+\frac{[\mathrm{C4}]}{K_{\mathrm{m\ MASP1^*}}^{\mathrm{C4}}}+\frac{[\mathrm{fC4bC2}]}{K_{\mathrm{m\ MASP1^*}}^{\mathrm{C2}}}+\frac{[\mathrm{fC4bC4bC2}]}{K_{\mathrm{m\ MASP1^*}}^{\mathrm{C2}}}+\frac{[\mathrm{fC3bC4bC2}]}{K_{\mathrm{m\ MASP1^*}}^{\mathrm{C2}}}+\frac{[\mathrm{pC4bC2}]}{K_{\mathrm{m\ MASP1^*}}^{\mathrm{C2}}}+\frac{[\mathrm{pC4bC4bC2}]}{K_{\mathrm{m\ MASP1^*}}^{\mathrm{C2}}}\\+\frac{[\mathrm{pC3bC4bC2}]}{K_{\mathrm{m\ MASP1^*}}^{\mathrm{C2}}}+\frac{[\mathrm{pMBL_3MASP1_{zym}}]}{K_{\mathrm{m\ MASP1^*}}^{\mathrm{MASP1_{zym}}}}+\frac{[\mathrm{pMBL_3MASP2_{zym}}]}{K_{\mathrm{m\ MASP1^*}}^{\mathrm{MASP2_{zym}}}}+\frac{[\mathrm{pMBL_3MASP3_{zym}}]}{K_{\mathrm{m\ MASP1^*}}^{\mathrm{MASP3_{zym}}}}\\+\frac{[\mathrm{pMBL_4MASP1_{zym}}]}{K_{\mathrm{m\ MASP1^*}}^{\mathrm{MASP1_{zym}}}}+\frac{[\mathrm{pMBL_4MASP2_{zym}}]}{K_{\mathrm{m\ MASP1^*}}^{\mathrm{MASP2_{zym}}}}+\frac{[\mathrm{pMBL_4MASP3_{zym}}]}{K_{\mathrm{m\ MASP1^*}}^{\mathrm{MASP3_{zym}}}}+\frac{[\mathrm{pCLLKMASP1_{zym}}]}{K_{\mathrm{m\ MASP1^*}}^{\mathrm{MASP1_{zym}}}}\\+\frac{[\mathrm{pCLLKMASP2_{zym}}]}{K_{\mathrm{m\ MASP1^*}}^{\mathrm{MASP2_{zym}}}}+\frac{[\mathrm{pCLLKMASP3_{zym}}]}{K_{\mathrm{m\ MASP1^*}}^{\mathrm{MASP3_{zym}}}}+\frac{[\mathrm{pCLK1MASP1_{zym}}]}{K_{\mathrm{m\ MASP1^*}}^{\mathrm{MASP1_{zym}}}}+\frac{[\mathrm{pCLK1MASP2_{zym}}]}{K_{\mathrm{m\ MASP1^*}}^{\mathrm{MASP2_{zym}}}}\\+\frac{[\mathrm{pCLK1MASP3_{zym}}]}{K_{\mathrm{m\ MASP1^*}}^{\mathrm{MASP3_{zym}}}}+\frac{[\mathrm{pCLL1MASP1_{zym}}]}{K_{\mathrm{m\ MASP1^*}}^{\mathrm{MASP1_{zym}}}}+\frac{[\mathrm{pCLL1MASP2_{zym}}]}{K_{\mathrm{m\ MASP1^*}}^{\mathrm{MASP2_{zym}}}}+\frac{[\mathrm{pCLL1MASP3_{zym}}]}{K_{\mathrm{m\ MASP1^*}}^{\mathrm{MASP3_{zym}}}}\\+\frac{[\mathrm{pLFMASP1_{zym}}]}{K_{\mathrm{m\ MASP1^*}}^{\mathrm{MASP1_{zym}}}}+\frac{[\mathrm{pLFMASP2_{zym}}]}{K_{\mathrm{m\ MASP1^*}}^{\mathrm{MASP2_{zym}}}}+\frac{[\mathrm{pLFMASP3_{zym}}]}{K_{\mathrm{m\ MASP1^*}}^{\mathrm{MASP3_{zym}}}}+\frac{[\mathrm{pHFMASP1_{zym}}]}{K_{\mathrm{m\ MASP1^*}}^{\mathrm{MASP1_{zym}}}}+\frac{[\mathrm{pHFMASP2_{zym}}]}{K_{\mathrm{m\ MASP1^*}}^{\mathrm{MASP2_{zym}}}}\\+\frac{[\mathrm{pHFMASP3_{zym}}]}{K_{\mathrm{m\ MASP1^*}}^{\mathrm{MASP3_{zym}}}}+\frac{[\mathrm{pMFMASP1_{zym}}]}{K_{\mathrm{m\ MASP1^*}}^{\mathrm{MASP1_{zym}}}}+\frac{[\mathrm{pMFMASP2_{zym}}]}{K_{\mathrm{m\ MASP1^*}}^{\mathrm{MASP2_{zym}}}}+\frac{[\mathrm{pMFMASP3_{zym}}]}{K_{\mathrm{m\ MASP1^*}}^{\mathrm{MASP3_{zym}}}}\\+\frac{[\mathrm{pPTX3LFMASP1_{zym}}]}{K_{\mathrm{m\ MASP1^*}}^{\mathrm{MASP1_{zym}}}}+\frac{[\mathrm{pPXT3LFMASP2_{zym}}]}{K_{\mathrm{m\ MASP1^*}}^{\mathrm{MASP2_{zym}}}}+\frac{[\mathrm{pPTX3LFMASP3_{zym}}]}{K_{\mathrm{m\ MASP1^*}}^{\mathrm{MASP3_{zym}}}}\end{array}\right)}$$

$$+\frac{k_{\text{cat MASP1}^*}^{\text{MASP2}_{\text{zym}}}\left[\text{pCLL1MASP1}^*\right]\left[\text{pMBL}_4\text{MASP2}_{\text{zym}}\right]}{K_{\text{m MASP1}^*}^{\text{MASP2}_{\text{zym}}}\left(\begin{aligned}&1+\frac{[\text{C4}]}{K_{\text{m MASP1}^*}^{\text{C4}}}+\frac{[\text{fC4bC2}]}{K_{\text{m MASP1}^*}^{\text{C2}}}+\frac{[\text{fC4bC4bC2}]}{K_{\text{m MASP1}^*}^{\text{C2}}}+\frac{[\text{fC3bC4bC2}]}{K_{\text{m MASP1}^*}^{\text{C2}}}+\frac{[\text{pC4bC2}]}{K_{\text{m MASP1}^*}^{\text{C2}}}+\frac{[\text{pC4bC4bC2}]}{K_{\text{m MASP1}^*}^{\text{C2}}}\\&+\frac{[\text{pC3bC4bC2}]}{K_{\text{m MASP1}^*}^{\text{C2}}}+\frac{\left[\text{pMBL}_3\text{MASP1}_{\text{zym}}\right]}{K_{\text{m MASP1}^*}^{\text{MASP1}_{\text{zym}}}}+\frac{\left[\text{pMBL}_3\text{MASP2}_{\text{zym}}\right]}{K_{\text{m MASP1}^*}^{\text{MASP2}_{\text{zym}}}}+\frac{\left[\text{pMBL}_3\text{MASP3}_{\text{zym}}\right]}{K_{\text{m MASP1}^*}^{\text{MASP3}_{\text{zym}}}}\\&+\frac{\left[\text{pMBL}_4\text{MASP1}_{\text{zym}}\right]}{K_{\text{m MASP1}^*}^{\text{MASP1}_{\text{zym}}}}+\frac{\left[\text{pMBL}_4\text{MASP2}_{\text{zym}}\right]}{K_{\text{m MASP1}^*}^{\text{MASP2}_{\text{zym}}}}+\frac{\left[\text{pMBL}_4\text{MASP3}_{\text{zym}}\right]}{K_{\text{m MASP1}^*}^{\text{MASP3}_{\text{zym}}}}+\frac{\left[\text{pCLLKMASP1}_{\text{zym}}\right]}{K_{\text{m MASP1}^*}^{\text{MASP1}_{\text{zym}}}}\\&+\frac{\left[\text{pCLLKMASP2}_{\text{zym}}\right]}{K_{\text{m MASP1}^*}^{\text{MASP2}_{\text{zym}}}}+\frac{\left[\text{pCLLKMASP3}_{\text{zym}}\right]}{K_{\text{m MASP1}^*}^{\text{MASP3}_{\text{zym}}}}+\frac{\left[\text{pCLK1MASP1}_{\text{zym}}\right]}{K_{\text{m MASP1}^*}^{\text{MASP1}_{\text{zym}}}}+\frac{\left[\text{pCLK1MASP2}_{\text{zym}}\right]}{K_{\text{m MASP1}^*}^{\text{MASP2}_{\text{zym}}}}\\&+\frac{\left[\text{pCLK1MASP3}_{\text{zym}}\right]}{K_{\text{m MASP1}^*}^{\text{MASP3}_{\text{zym}}}}+\frac{\left[\text{pCLL1MASP1}_{\text{zym}}\right]}{K_{\text{m MASP1}^*}^{\text{MASP1}_{\text{zym}}}}+\frac{\left[\text{pCLL1MASP2}_{\text{zym}}\right]}{K_{\text{m MASP1}^*}^{\text{MASP2}_{\text{zym}}}}+\frac{\left[\text{pCLL1MASP3}_{\text{zym}}\right]}{K_{\text{m MASP1}^*}^{\text{MASP3}_{\text{zym}}}}\\&+\frac{\left[\text{pLFMASP1}_{\text{zym}}\right]}{K_{\text{m MASP1}^*}^{\text{MASP1}_{\text{zym}}}}+\frac{\left[\text{pLFMASP2}_{\text{zym}}\right]}{K_{\text{m MASP1}^*}^{\text{MASP2}_{\text{zym}}}}+\frac{\left[\text{pLFMASP3}_{\text{zym}}\right]}{K_{\text{m MASP1}^*}^{\text{MASP3}_{\text{zym}}}}+\frac{\left[\text{pHFMASP1}_{\text{zym}}\right]}{K_{\text{m MASP1}^*}^{\text{MASP1}_{\text{zym}}}}+\frac{\left[\text{pHFMASP2}_{\text{zym}}\right]}{K_{\text{m MASP1}^*}^{\text{MASP2}_{\text{zym}}}}\\&+\frac{\left[\text{pHFMASP3}_{\text{zym}}\right]}{K_{\text{m MASP1}^*}^{\text{MASP3}_{\text{zym}}}}+\frac{\left[\text{pMFMASP1}_{\text{zym}}\right]}{K_{\text{m MASP1}^*}^{\text{MASP1}_{\text{zym}}}}+\frac{\left[\text{pMFMASP2}_{\text{zym}}\right]}{K_{\text{m MASP1}^*}^{\text{MASP2}_{\text{zym}}}}+\frac{\left[\text{pMFMASP3}_{\text{zym}}\right]}{K_{\text{m MASP1}^*}^{\text{MASP3}_{\text{zym}}}}\\&+\frac{\left[\text{pPTX3LFMASP1}_{\text{zym}}\right]}{K_{\text{m MASP1}^*}^{\text{MASP1}_{\text{zym}}}}+\frac{\left[\text{pPXT3LFMASP2}_{\text{zym}}\right]}{K_{\text{m MASP1}^*}^{\text{MASP2}_{\text{zym}}}}+\frac{\left[\text{pPTX3LFMASP3}_{\text{zym}}\right]}{K_{\text{m MASP1}^*}^{\text{MASP3}_{\text{zym}}}}\end{aligned}\right)}$$

$$
+\frac{k_{\mathrm{cat\,MASP1^*}}^{\mathrm{MASP2_{zym}}}\left[\mathrm{pLFMASP1^*}\right]\left[\mathrm{pMBL_4MASP2_{zym}}\right]}{K_{\mathrm{m\,MASP1^*}}^{\mathrm{MASP2_{zym}}}\left(\begin{array}{l}
1+\frac{[\mathrm{C4}]}{K_{\mathrm{m\,MASP1^*}}^{\mathrm{C4}}}+\frac{[\mathrm{fC4bC2}]}{K_{\mathrm{m\,MASP1^*}}^{\mathrm{C2}}}+\frac{[\mathrm{fC4bC4bC2}]}{K_{\mathrm{m\,MASP1^*}}^{\mathrm{C2}}}+\frac{[\mathrm{fC3bC4bC2}]}{K_{\mathrm{m\,MASP1^*}}^{\mathrm{C2}}}+\frac{[\mathrm{pC4bC2}]}{K_{\mathrm{m\,MASP1^*}}^{\mathrm{C2}}}+\frac{[\mathrm{pC4bC4bC2}]}{K_{\mathrm{m\,MASP1^*}}^{\mathrm{C2}}}\\
+\frac{[\mathrm{pC3bC4bC2}]}{K_{\mathrm{m\,MASP1^*}}^{\mathrm{C2}}}+\frac{\left[\mathrm{pMBL_3MASP1_{zym}}\right]}{K_{\mathrm{m\,MASP1^*}}^{\mathrm{MASP1_{zym}}}}+\frac{\left[\mathrm{pMBL_3MASP2_{zym}}\right]}{K_{\mathrm{m\,MASP1^*}}^{\mathrm{MASP2_{zym}}}}+\frac{\left[\mathrm{pMBL_3MASP3_{zym}}\right]}{K_{\mathrm{m\,MASP1^*}}^{\mathrm{MASP3_{zym}}}}\\
+\frac{\left[\mathrm{pMBL_4MASP1_{zym}}\right]}{K_{\mathrm{m\,MASP1^*}}^{\mathrm{MASP1_{zym}}}}+\frac{\left[\mathrm{pMBL_4MASP2_{zym}}\right]}{K_{\mathrm{m\,MASP1^*}}^{\mathrm{MASP2_{zym}}}}+\frac{\left[\mathrm{pMBL_4MASP3_{zym}}\right]}{K_{\mathrm{m\,MASP1^*}}^{\mathrm{MASP3_{zym}}}}+\frac{\left[\mathrm{pCLLKMASP1_{zym}}\right]}{K_{\mathrm{m\,MASP1^*}}^{\mathrm{MASP1_{zym}}}}\\
+\frac{\left[\mathrm{pCLLKMASP2_{zym}}\right]}{K_{\mathrm{m\,MASP1^*}}^{\mathrm{MASP2_{zym}}}}+\frac{\left[\mathrm{pCLLKMASP3_{zym}}\right]}{K_{\mathrm{m\,MASP1^*}}^{\mathrm{MASP3_{zym}}}}+\frac{\left[\mathrm{pCLK1MASP1_{zym}}\right]}{K_{\mathrm{m\,MASP1^*}}^{\mathrm{MASP1_{zym}}}}+\frac{\left[\mathrm{pCLK1MASP2_{zym}}\right]}{K_{\mathrm{m\,MASP1^*}}^{\mathrm{MASP2_{zym}}}}\\
+\frac{\left[\mathrm{pCLK1MASP3_{zym}}\right]}{K_{\mathrm{m\,MASP1^*}}^{\mathrm{MASP3_{zym}}}}+\frac{\left[\mathrm{pCLL1MASP1_{zym}}\right]}{K_{\mathrm{m\,MASP1^*}}^{\mathrm{MASP1_{zym}}}}+\frac{\left[\mathrm{pCLL1MASP2_{zym}}\right]}{K_{\mathrm{m\,MASP1^*}}^{\mathrm{MASP2_{zym}}}}+\frac{\left[\mathrm{pCLL1MASP3_{zym}}\right]}{K_{\mathrm{m\,MASP1^*}}^{\mathrm{MASP3_{zym}}}}\\
+\frac{\left[\mathrm{pLFMASP1_{zym}}\right]}{K_{\mathrm{m\,MASP1^*}}^{\mathrm{MASP1_{zym}}}}+\frac{\left[\mathrm{pLFMASP2_{zym}}\right]}{K_{\mathrm{m\,MASP1^*}}^{\mathrm{MASP2_{zym}}}}+\frac{\left[\mathrm{pLFMASP3_{zym}}\right]}{K_{\mathrm{m\,MASP1^*}}^{\mathrm{MASP3_{zym}}}}+\frac{\left[\mathrm{pHFMASP1_{zym}}\right]}{K_{\mathrm{m\,MASP1^*}}^{\mathrm{MASP1_{zym}}}}+\frac{\left[\mathrm{pHFMASP2_{zym}}\right]}{K_{\mathrm{m\,MASP1^*}}^{\mathrm{MASP2_{zym}}}}\\
+\frac{\left[\mathrm{pHFMASP3_{zym}}\right]}{K_{\mathrm{m\,MASP1^*}}^{\mathrm{MASP3_{zym}}}}+\frac{\left[\mathrm{pMFMASP1_{zym}}\right]}{K_{\mathrm{m\,MASP1^*}}^{\mathrm{MASP1_{zym}}}}+\frac{\left[\mathrm{pMFMASP2_{zym}}\right]}{K_{\mathrm{m\,MASP1^*}}^{\mathrm{MASP2_{zym}}}}+\frac{\left[\mathrm{pMFMASP3_{zym}}\right]}{K_{\mathrm{m\,MASP1^*}}^{\mathrm{MASP3_{zym}}}}\\
+\frac{\left[\mathrm{pPTX3LFMASP1_{zym}}\right]}{K_{\mathrm{m\,MASP1^*}}^{\mathrm{MASP1_{zym}}}}+\frac{\left[\mathrm{pPXT3LFMASP2_{zym}}\right]}{K_{\mathrm{m\,MASP1^*}}^{\mathrm{MASP2_{zym}}}}+\frac{\left[\mathrm{pPTX3LFMASP3_{zym}}\right]}{K_{\mathrm{m\,MASP1^*}}^{\mathrm{MASP3_{zym}}}}
\end{array}\right)}
$$

$$
+\frac{k_{\mathrm{cat\,MASP1^{*}}}^{\mathrm{MASP2_{zym}}}\left[\mathrm{pHFMASP1^{*}}\right]\left[\mathrm{pMBL_{4}MASP2_{zym}}\right]}{K_{\mathrm{m\,MASP1^{*}}}^{\mathrm{MASP2_{zym}}}\left(\begin{array}{l}
1+\frac{[\mathrm{C4}]}{K_{\mathrm{m\,MASP1^{*}}}^{\mathrm{C4}}}+\frac{[\mathrm{fC4bC2}]}{K_{\mathrm{m\,MASP1^{*}}}^{\mathrm{C2}}}+\frac{[\mathrm{fC4bC4bC2}]}{K_{\mathrm{m\,MASP1^{*}}}^{\mathrm{C2}}}+\frac{[\mathrm{fC3bC4bC2}]}{K_{\mathrm{m\,MASP1^{*}}}^{\mathrm{C2}}}+\frac{[\mathrm{pC4bC2}]}{K_{\mathrm{m\,MASP1^{*}}}^{\mathrm{C2}}}+\frac{[\mathrm{pC4bC4bC2}]}{K_{\mathrm{m\,MASP1^{*}}}^{\mathrm{C2}}}\\
+\frac{[\mathrm{pC3bC4bC2}]}{K_{\mathrm{m\,MASP1^{*}}}^{\mathrm{C2}}}+\frac{\left[\mathrm{pMBL_{3}MASP1_{zym}}\right]}{K_{\mathrm{m\,MASP1^{*}}}^{\mathrm{MASP1_{zym}}}}+\frac{\left[\mathrm{pMBL_{3}MASP2_{zym}}\right]}{K_{\mathrm{m\,MASP1^{*}}}^{\mathrm{MASP2_{zym}}}}+\frac{\left[\mathrm{pMBL_{3}MASP3_{zym}}\right]}{K_{\mathrm{m\,MASP1^{*}}}^{\mathrm{MASP3_{zym}}}}\\
+\frac{\left[\mathrm{pMBL_{4}MASP1_{zym}}\right]}{K_{\mathrm{m\,MASP1^{*}}}^{\mathrm{MASP1_{zym}}}}+\frac{\left[\mathrm{pMBL_{4}MASP2_{zym}}\right]}{K_{\mathrm{m\,MASP1^{*}}}^{\mathrm{MASP2_{zym}}}}+\frac{\left[\mathrm{pMBL_{4}MASP3_{zym}}\right]}{K_{\mathrm{m\,MASP1^{*}}}^{\mathrm{MASP3_{zym}}}}+\frac{\left[\mathrm{pCLLKMASP1_{zym}}\right]}{K_{\mathrm{m\,MASP1^{*}}}^{\mathrm{MASP1_{zym}}}}\\
+\frac{\left[\mathrm{pCLLKMASP2_{zym}}\right]}{K_{\mathrm{m\,MASP1^{*}}}^{\mathrm{MASP2_{zym}}}}+\frac{\left[\mathrm{pCLLKMASP3_{zym}}\right]}{K_{\mathrm{m\,MASP1^{*}}}^{\mathrm{MASP3_{zym}}}}+\frac{\left[\mathrm{pCLK1MASP1_{zym}}\right]}{K_{\mathrm{m\,MASP1^{*}}}^{\mathrm{MASP1_{zym}}}}+\frac{\left[\mathrm{pCLK1MASP2_{zym}}\right]}{K_{\mathrm{m\,MASP1^{*}}}^{\mathrm{MASP2_{zym}}}}\\
+\frac{\left[\mathrm{pCLK1MASP3_{zym}}\right]}{K_{\mathrm{m\,MASP1^{*}}}^{\mathrm{MASP3_{zym}}}}+\frac{\left[\mathrm{pCLL1MASP1_{zym}}\right]}{K_{\mathrm{m\,MASP1^{*}}}^{\mathrm{MASP1_{zym}}}}+\frac{\left[\mathrm{pCLL1MASP2_{zym}}\right]}{K_{\mathrm{m\,MASP1^{*}}}^{\mathrm{MASP2_{zym}}}}+\frac{\left[\mathrm{pCLL1MASP3_{zym}}\right]}{K_{\mathrm{m\,MASP1^{*}}}^{\mathrm{MASP3_{zym}}}}\\
+\frac{\left[\mathrm{pLFMASP1_{zym}}\right]}{K_{\mathrm{m\,MASP1^{*}}}^{\mathrm{MASP1_{zym}}}}+\frac{\left[\mathrm{pLFMASP2_{zym}}\right]}{K_{\mathrm{m\,MASP1^{*}}}^{\mathrm{MASP2_{zym}}}}+\frac{\left[\mathrm{pLFMASP3_{zym}}\right]}{K_{\mathrm{m\,MASP1^{*}}}^{\mathrm{MASP3_{zym}}}}+\frac{\left[\mathrm{pHFMASP1_{zym}}\right]}{K_{\mathrm{m\,MASP1^{*}}}^{\mathrm{MASP1_{zym}}}}+\frac{\left[\mathrm{pHFMASP2_{zym}}\right]}{K_{\mathrm{m\,MASP1^{*}}}^{\mathrm{MASP2_{zym}}}}\\
+\frac{\left[\mathrm{pHFMASP3_{zym}}\right]}{K_{\mathrm{m\,MASP1^{*}}}^{\mathrm{MASP3_{zym}}}}+\frac{\left[\mathrm{pMFMASP1_{zym}}\right]}{K_{\mathrm{m\,MASP1^{*}}}^{\mathrm{MASP1_{zym}}}}+\frac{\left[\mathrm{pMFMASP2_{zym}}\right]}{K_{\mathrm{m\,MASP1^{*}}}^{\mathrm{MASP2_{zym}}}}+\frac{\left[\mathrm{pMFMASP3_{zym}}\right]}{K_{\mathrm{m\,MASP1^{*}}}^{\mathrm{MASP3_{zym}}}}\\
+\frac{\left[\mathrm{pPTX3LFMASP1_{zym}}\right]}{K_{\mathrm{m\,MASP1^{*}}}^{\mathrm{MASP1_{zym}}}}+\frac{\left[\mathrm{pPXT3LFMASP2_{zym}}\right]}{K_{\mathrm{m\,MASP1^{*}}}^{\mathrm{MASP2_{zym}}}}+\frac{\left[\mathrm{pPTX3LFMASP3_{zym}}\right]}{K_{\mathrm{m\,MASP1^{*}}}^{\mathrm{MASP3_{zym}}}}
\end{array}\right)}
$$

$$
+\frac{k_{\mathrm{cat\ MASP1^*}}^{\mathrm{MASP2_{zym}}}\left[\mathrm{pMFMASP1^*}\right]\left[\mathrm{pMBL_4MASP2_{zym}}\right]}{K_{\mathrm{m\ MASP1^*}}^{\mathrm{MASP2_{zym}}}\left(\begin{array}{l}
1+\frac{[\mathrm{C4}]}{K_{\mathrm{m\ MASP1^*}}^{\mathrm{C4}}}+\frac{[\mathrm{fC4bC2}]}{K_{\mathrm{m\ MASP1^*}}^{\mathrm{C2}}}+\frac{[\mathrm{fC4bC4bC2}]}{K_{\mathrm{m\ MASP1^*}}^{\mathrm{C2}}}+\frac{[\mathrm{fC3bC4bC2}]}{K_{\mathrm{m\ MASP1^*}}^{\mathrm{C2}}}+\frac{[\mathrm{pC4bC2}]}{K_{\mathrm{m\ MASP1^*}}^{\mathrm{C2}}}+\frac{[\mathrm{pC4bC4bC2}]}{K_{\mathrm{m\ MASP1^*}}^{\mathrm{C2}}}\\
+\frac{[\mathrm{pC3bC4bC2}]}{K_{\mathrm{m\ MASP1^*}}^{\mathrm{C2}}}+\frac{\left[\mathrm{pMBL_3MASP1_{zym}}\right]}{K_{\mathrm{m\ MASP1^*}}^{\mathrm{MASP1_{zym}}}}+\frac{\left[\mathrm{pMBL_3MASP2_{zym}}\right]}{K_{\mathrm{m\ MASP1^*}}^{\mathrm{MASP2_{zym}}}}+\frac{\left[\mathrm{pMBL_3MASP3_{zym}}\right]}{K_{\mathrm{m\ MASP1^*}}^{\mathrm{MASP3_{zym}}}}\\
+\frac{\left[\mathrm{pMBL_4MASP1_{zym}}\right]}{K_{\mathrm{m\ MASP1^*}}^{\mathrm{MASP1_{zym}}}}+\frac{\left[\mathrm{pMBL_4MASP2_{zym}}\right]}{K_{\mathrm{m\ MASP1^*}}^{\mathrm{MASP2_{zym}}}}+\frac{\left[\mathrm{pMBL_4MASP3_{zym}}\right]}{K_{\mathrm{m\ MASP1^*}}^{\mathrm{MASP3_{zym}}}}+\frac{\left[\mathrm{pCLLKMASP1_{zym}}\right]}{K_{\mathrm{m\ MASP1^*}}^{\mathrm{MASP1_{zym}}}}\\
+\frac{\left[\mathrm{pCLLKMASP2_{zym}}\right]}{K_{\mathrm{m\ MASP1^*}}^{\mathrm{MASP2_{zym}}}}+\frac{\left[\mathrm{pCLLKMASP3_{zym}}\right]}{K_{\mathrm{m\ MASP1^*}}^{\mathrm{MASP3_{zym}}}}+\frac{\left[\mathrm{pCLK1MASP1_{zym}}\right]}{K_{\mathrm{m\ MASP1^*}}^{\mathrm{MASP1_{zym}}}}+\frac{\left[\mathrm{pCLK1MASP2_{zym}}\right]}{K_{\mathrm{m\ MASP1^*}}^{\mathrm{MASP2_{zym}}}}\\
+\frac{\left[\mathrm{pCLK1MASP3_{zym}}\right]}{K_{\mathrm{m\ MASP1^*}}^{\mathrm{MASP3_{zym}}}}+\frac{\left[\mathrm{pCLL1MASP1_{zym}}\right]}{K_{\mathrm{m\ MASP1^*}}^{\mathrm{MASP1_{zym}}}}+\frac{\left[\mathrm{pCLL1MASP2_{zym}}\right]}{K_{\mathrm{m\ MASP1^*}}^{\mathrm{MASP2_{zym}}}}+\frac{\left[\mathrm{pCLL1MASP3_{zym}}\right]}{K_{\mathrm{m\ MASP1^*}}^{\mathrm{MASP3_{zym}}}}\\
+\frac{\left[\mathrm{pLFMASP1_{zym}}\right]}{K_{\mathrm{m\ MASP1^*}}^{\mathrm{MASP1_{zym}}}}+\frac{\left[\mathrm{pLFMASP2_{zym}}\right]}{K_{\mathrm{m\ MASP1^*}}^{\mathrm{MASP2_{zym}}}}+\frac{\left[\mathrm{pLFMASP3_{zym}}\right]}{K_{\mathrm{m\ MASP1^*}}^{\mathrm{MASP3_{zym}}}}+\frac{\left[\mathrm{pHFMASP1_{zym}}\right]}{K_{\mathrm{m\ MASP1^*}}^{\mathrm{MASP1_{zym}}}}+\frac{\left[\mathrm{pHFMASP2_{zym}}\right]}{K_{\mathrm{m\ MASP1^*}}^{\mathrm{MASP2_{zym}}}}\\
+\frac{\left[\mathrm{pHFMASP3_{zym}}\right]}{K_{\mathrm{m\ MASP1^*}}^{\mathrm{MASP3_{zym}}}}+\frac{\left[\mathrm{pMFMASP1_{zym}}\right]}{K_{\mathrm{m\ MASP1^*}}^{\mathrm{MASP1_{zym}}}}+\frac{\left[\mathrm{pMFMASP2_{zym}}\right]}{K_{\mathrm{m\ MASP1^*}}^{\mathrm{MASP2_{zym}}}}+\frac{\left[\mathrm{pMFMASP3_{zym}}\right]}{K_{\mathrm{m\ MASP1^*}}^{\mathrm{MASP3_{zym}}}}\\
+\frac{\left[\mathrm{pPTX3LFMASP1_{zym}}\right]}{K_{\mathrm{m\ MASP1^*}}^{\mathrm{MASP1_{zym}}}}+\frac{\left[\mathrm{pPXT3LFMASP2_{zym}}\right]}{K_{\mathrm{m\ MASP1^*}}^{\mathrm{MASP2_{zym}}}}+\frac{\left[\mathrm{pPTX3LFMASP3_{zym}}\right]}{K_{\mathrm{m\ MASP1^*}}^{\mathrm{MASP3_{zym}}}}
\end{array}\right)}
$$

$$
+\frac{k^{\mathrm{MASP2_{zym}}}_{\mathrm{cat\ MASP1^*}}\left[\mathrm{pPTX3LFMASP1^*}\right]\left[\mathrm{pMBL_4MASP2_{zym}}\right]}{K^{\mathrm{MASP2_{zym}}}_{\mathrm{m\ MASP1^*}}\left(\begin{aligned}
&1+\frac{[\mathrm{C4}]}{K^{\mathrm{C4}}_{\mathrm{m\ MASP1^*}}}+\frac{[\mathrm{fC4bC2}]}{K^{\mathrm{C2}}_{\mathrm{m\ MASP1^*}}}+\frac{[\mathrm{fC4bC4bC2}]}{K^{\mathrm{C2}}_{\mathrm{m\ MASP1^*}}}+\frac{[\mathrm{fC3bC4bC2}]}{K^{\mathrm{C2}}_{\mathrm{m\ MASP1^*}}}+\frac{[\mathrm{pC4bC2}]}{K^{\mathrm{C2}}_{\mathrm{m\ MASP1^*}}}+\frac{[\mathrm{pC4bC4bC2}]}{K^{\mathrm{C2}}_{\mathrm{m\ MASP1^*}}}\\
&+\frac{[\mathrm{pC3bC4bC2}]}{K^{\mathrm{C2}}_{\mathrm{m\ MASP1^*}}}+\frac{\left[\mathrm{pMBL_3MASP1_{zym}}\right]}{K^{\mathrm{MASP1_{zym}}}_{\mathrm{m\ MASP1^*}}}+\frac{\left[\mathrm{pMBL_3MASP2_{zym}}\right]}{K^{\mathrm{MASP2_{zym}}}_{\mathrm{m\ MASP1^*}}}+\frac{\left[\mathrm{pMBL_3MASP3_{zym}}\right]}{K^{\mathrm{MASP3_{zym}}}_{\mathrm{m\ MASP1^*}}}\\
&+\frac{\left[\mathrm{pMBL_4MASP1_{zym}}\right]}{K^{\mathrm{MASP1_{zym}}}_{\mathrm{m\ MASP1^*}}}+\frac{\left[\mathrm{pMBL_4MASP2_{zym}}\right]}{K^{\mathrm{MASP2_{zym}}}_{\mathrm{m\ MASP1^*}}}+\frac{\left[\mathrm{pMBL_4MASP3_{zym}}\right]}{K^{\mathrm{MASP3_{zym}}}_{\mathrm{m\ MASP1^*}}}+\frac{\left[\mathrm{pCLLKMASP1_{zym}}\right]}{K^{\mathrm{MASP1_{zym}}}_{\mathrm{m\ MASP1^*}}}\\
&+\frac{\left[\mathrm{pCLLKMASP2_{zym}}\right]}{K^{\mathrm{MASP2_{zym}}}_{\mathrm{m\ MASP1^*}}}+\frac{\left[\mathrm{pCLLKMASP3_{zym}}\right]}{K^{\mathrm{MASP3_{zym}}}_{\mathrm{m\ MASP1^*}}}+\frac{\left[\mathrm{pCLK1MASP1_{zym}}\right]}{K^{\mathrm{MASP1_{zym}}}_{\mathrm{m\ MASP1^*}}}+\frac{\left[\mathrm{pCLK1MASP2_{zym}}\right]}{K^{\mathrm{MASP2_{zym}}}_{\mathrm{m\ MASP1^*}}}\\
&+\frac{\left[\mathrm{pCLK1MASP3_{zym}}\right]}{K^{\mathrm{MASP3_{zym}}}_{\mathrm{m\ MASP1^*}}}+\frac{\left[\mathrm{pCLL1MASP1_{zym}}\right]}{K^{\mathrm{MASP1_{zym}}}_{\mathrm{m\ MASP1^*}}}+\frac{\left[\mathrm{pCLL1MASP2_{zym}}\right]}{K^{\mathrm{MASP2_{zym}}}_{\mathrm{m\ MASP1^*}}}+\frac{\left[\mathrm{pCLL1MASP3_{zym}}\right]}{K^{\mathrm{MASP3_{zym}}}_{\mathrm{m\ MASP1^*}}}\\
&+\frac{\left[\mathrm{pLFMASP1_{zym}}\right]}{K^{\mathrm{MASP1_{zym}}}_{\mathrm{m\ MASP1^*}}}+\frac{\left[\mathrm{pLFMASP2_{zym}}\right]}{K^{\mathrm{MASP2_{zym}}}_{\mathrm{m\ MASP1^*}}}+\frac{\left[\mathrm{pLFMASP3_{zym}}\right]}{K^{\mathrm{MASP3_{zym}}}_{\mathrm{m\ MASP1^*}}}+\frac{\left[\mathrm{pHFMASP1_{zym}}\right]}{K^{\mathrm{MASP1_{zym}}}_{\mathrm{m\ MASP1^*}}}+\frac{\left[\mathrm{pHFMASP2_{zym}}\right]}{K^{\mathrm{MASP2_{zym}}}_{\mathrm{m\ MASP1^*}}}\\
&+\frac{\left[\mathrm{pHFMASP3_{zym}}\right]}{K^{\mathrm{MASP3_{zym}}}_{\mathrm{m\ MASP1^*}}}+\frac{\left[\mathrm{pMFMASP1_{zym}}\right]}{K^{\mathrm{MASP1_{zym}}}_{\mathrm{m\ MASP1^*}}}+\frac{\left[\mathrm{pMFMASP2_{zym}}\right]}{K^{\mathrm{MASP2_{zym}}}_{\mathrm{m\ MASP1^*}}}+\frac{\left[\mathrm{pMFMASP3_{zym}}\right]}{K^{\mathrm{MASP3_{zym}}}_{\mathrm{m\ MASP1^*}}}\\
&+\frac{\left[\mathrm{pPTX3LFMASP1_{zym}}\right]}{K^{\mathrm{MASP1_{zym}}}_{\mathrm{m\ MASP1^*}}}+\frac{\left[\mathrm{pPXT3LFMASP2_{zym}}\right]}{K^{\mathrm{MASP2_{zym}}}_{\mathrm{m\ MASP1^*}}}+\frac{\left[\mathrm{pPTX3LFMASP3_{zym}}\right]}{K^{\mathrm{MASP3_{zym}}}_{\mathrm{m\ MASP1^*}}}
\end{aligned}\right)}
$$

$$
+\frac{k^{\mathrm{MASP2_{zym}}}_{\mathrm{cat\ MASP2^*}}\left[\mathrm{pMBL_3MASP2^*}\right]\left[\mathrm{pMBL_4MASP2_{zym}}\right]}{K^{\mathrm{MASP2_{zym}}}_{\mathrm{m\ MASP2^*}}\left(\begin{aligned}
&1+\frac{[\mathrm{C4}]}{K^{\mathrm{C4}}_{\mathrm{m\ MASP2^*}}}+\frac{[\mathrm{fC4bC2}]}{K^{\mathrm{C2}}_{\mathrm{m\ MASP2^*}}}+\frac{[\mathrm{fC4bC4bC2}]}{K^{\mathrm{C2}}_{\mathrm{m\ MASP2^*}}}+\frac{[\mathrm{fC3bC4bC2}]}{K^{\mathrm{C2}}_{\mathrm{m\ MASP2^*}}}+\frac{[\mathrm{pC4bC2}]}{K^{\mathrm{C2}}_{\mathrm{m\ MASP2^*}}}+\frac{[\mathrm{pC4bC4bC2}]}{K^{\mathrm{C2}}_{\mathrm{m\ MASP2^*}}}\\
&+\frac{[\mathrm{pC3bC4bC2}]}{K^{\mathrm{C2}}_{\mathrm{m\ MASP2^*}}}+\frac{\left[\mathrm{pMBL_3MASP1_{zym}}\right]}{K^{\mathrm{MASP1_{zym}}}_{\mathrm{m\ MASP2^*}}}+\frac{\left[\mathrm{pMBL_3MASP2_{zym}}\right]}{K^{\mathrm{MASP2_{zym}}}_{\mathrm{m\ MASP2^*}}}+\frac{\left[\mathrm{pMBL_4MASP1_{zym}}\right]}{K^{\mathrm{MASP1_{zym}}}_{\mathrm{m\ MASP2^*}}}\\
&+\frac{\left[\mathrm{pMBL_4MASP2_{zym}}\right]}{K^{\mathrm{MASP2_{zym}}}_{\mathrm{m\ MASP2^*}}}+\frac{\left[\mathrm{pCLLKMASP1_{zym}}\right]}{K^{\mathrm{MASP1_{zym}}}_{\mathrm{m\ MASP2^*}}}+\frac{\left[\mathrm{pCLLKMASP2_{zym}}\right]}{K^{\mathrm{MASP2_{zym}}}_{\mathrm{m\ MASP2^*}}}+\frac{\left[\mathrm{pCLK1MASP1_{zym}}\right]}{K^{\mathrm{MASP1_{zym}}}_{\mathrm{m\ MASP2^*}}}\\
&+\frac{\left[\mathrm{pCLK1MASP2_{zym}}\right]}{K^{\mathrm{MASP2_{zym}}}_{\mathrm{m\ MASP2^*}}}+\frac{\left[\mathrm{pCLL1MASP1_{zym}}\right]}{K^{\mathrm{MASP1_{zym}}}_{\mathrm{m\ MASP2^*}}}+\frac{\left[\mathrm{pCLL1MASP2_{zym}}\right]}{K^{\mathrm{MASP2_{zym}}}_{\mathrm{m\ MASP2^*}}}+\frac{\left[\mathrm{pLFMASP1_{zym}}\right]}{K^{\mathrm{MASP1_{zym}}}_{\mathrm{m\ MASP2^*}}}\\
&+\frac{\left[\mathrm{pLFMASP2_{zym}}\right]}{K^{\mathrm{MASP2_{zym}}}_{\mathrm{m\ MASP2^*}}}+\frac{\left[\mathrm{pHFMASP1_{zym}}\right]}{K^{\mathrm{MASP1_{zym}}}_{\mathrm{m\ MASP2^*}}}+\frac{\left[\mathrm{pHFMASP2_{zym}}\right]}{K^{\mathrm{MASP2_{zym}}}_{\mathrm{m\ MASP2^*}}}+\frac{\left[\mathrm{pMFMASP1_{zym}}\right]}{K^{\mathrm{MASP1_{zym}}}_{\mathrm{m\ MASP2^*}}}+\frac{\left[\mathrm{pMFMASP2_{zym}}\right]}{K^{\mathrm{MASP2_{zym}}}_{\mathrm{m\ MASP2^*}}}\\
&+\frac{\left[\mathrm{pPTX3LFMASP1_{zym}}\right]}{K^{\mathrm{MASP1_{zym}}}_{\mathrm{m\ MASP2^*}}}+\frac{\left[\mathrm{pPTX3LFMASP2_{zym}}\right]}{K^{\mathrm{MASP2_{zym}}}_{\mathrm{m\ MASP2^*}}}
\end{aligned}\right)}
$$

$$
+\frac{k_{\text{cat MASP2}^*}^{\text{MASP2}_{\text{zym}}}\left[\text{pMBL}_4\text{MASP2}^*\right]\left[\text{pMBL}_4\text{MASP2}_{\text{zym}}\right]}{K_{\text{m MASP2}^*}^{\text{MASP2}_{\text{zym}}}\left(\begin{array}{l}1+\frac{[\text{C4}]}{K_{\text{m MASP2}^*}^{\text{C4}}}+\frac{[\text{fC4bC2}]}{K_{\text{m MASP2}^*}^{\text{C2}}}+\frac{[\text{fC4bC4bC2}]}{K_{\text{m MASP2}^*}^{\text{C2}}}+\frac{[\text{fC3bC4bC2}]}{K_{\text{m MASP2}^*}^{\text{C2}}}+\frac{[\text{pC4bC2}]}{K_{\text{m MASP2}^*}^{\text{C2}}}+\frac{[\text{pC4bC4bC2}]}{K_{\text{m MASP2}^*}^{\text{C2}}}\\ +\frac{[\text{pC3bC4bC2}]}{K_{\text{m MASP2}^*}^{\text{C2}}}+\frac{\left[\text{pMBL}_3\text{MASP1}_{\text{zym}}\right]}{K_{\text{m MASP2}^*}^{\text{MASP1}_{\text{zym}}}}+\frac{\left[\text{pMBL}_3\text{MASP2}_{\text{zym}}\right]}{K_{\text{m MASP2}^*}^{\text{MASP2}_{\text{zym}}}}+\frac{\left[\text{pMBL}_4\text{MASP1}_{\text{zym}}\right]}{K_{\text{m MASP2}^*}^{\text{MASP1}_{\text{zym}}}}\\ +\frac{\left[\text{pMBL}_4\text{MASP2}_{\text{zym}}\right]}{K_{\text{m MASP2}^*}^{\text{MASP2}_{\text{zym}}}}+\frac{\left[\text{pCLLKMASP1}_{\text{zym}}\right]}{K_{\text{m MASP2}^*}^{\text{MASP1}_{\text{zym}}}}+\frac{\left[\text{pCLLKMASP2}_{\text{zym}}\right]}{K_{\text{m MASP2}^*}^{\text{MASP2}_{\text{zym}}}}+\frac{\left[\text{pCLK1MASP1}_{\text{zym}}\right]}{K_{\text{m MASP2}^*}^{\text{MASP1}_{\text{zym}}}}\\ +\frac{\left[\text{pCLK1MASP2}_{\text{zym}}\right]}{K_{\text{m MASP2}^*}^{\text{MASP2}_{\text{zym}}}}+\frac{\left[\text{pCLL1MASP1}_{\text{zym}}\right]}{K_{\text{m MASP2}^*}^{\text{MASP1}_{\text{zym}}}}+\frac{\left[\text{pCLL1MASP2}_{\text{zym}}\right]}{K_{\text{m MASP2}^*}^{\text{MASP2}_{\text{zym}}}}+\frac{\left[\text{pLFMASP1}_{\text{zym}}\right]}{K_{\text{m MASP2}^*}^{\text{MASP1}_{\text{zym}}}}\\ +\frac{\left[\text{pLFMASP2}_{\text{zym}}\right]}{K_{\text{m MASP2}^*}^{\text{MASP2}_{\text{zym}}}}+\frac{\left[\text{pHFMASP1}_{\text{zym}}\right]}{K_{\text{m MASP2}^*}^{\text{MASP1}_{\text{zym}}}}+\frac{\left[\text{pHFMASP2}_{\text{zym}}\right]}{K_{\text{m MASP2}^*}^{\text{MASP2}_{\text{zym}}}}+\frac{\left[\text{pMFMASP1}_{\text{zym}}\right]}{K_{\text{m MASP2}^*}^{\text{MASP1}_{\text{zym}}}}+\frac{\left[\text{pMFMASP2}_{\text{zym}}\right]}{K_{\text{m MASP2}^*}^{\text{MASP2}_{\text{zym}}}}\\ +\frac{\left[\text{pPTX3LFMASP1}_{\text{zym}}\right]}{K_{\text{m MASP2}^*}^{\text{MASP1}_{\text{zym}}}}+\frac{\left[\text{pPTX3LFMASP2}_{\text{zym}}\right]}{K_{\text{m MASP2}^*}^{\text{MASP2}_{\text{zym}}}}\end{array}\right)}
$$

$$
+\frac{k_{\text{cat MASP2}^*}^{\text{MASP2}_{\text{zym}}}\left[\text{pCLLKMASP2}^*\right]\left[\text{pMBL}_4\text{MASP2}_{\text{zym}}\right]}{K_{\text{m MASP2}^*}^{\text{MASP2}_{\text{zym}}}\left(\begin{array}{l}1+\frac{[\text{C4}]}{K_{\text{m MASP2}^*}^{\text{C4}}}+\frac{[\text{fC4bC2}]}{K_{\text{m MASP2}^*}^{\text{C2}}}+\frac{[\text{fC4bC4bC2}]}{K_{\text{m MASP2}^*}^{\text{C2}}}+\frac{[\text{fC3bC4bC2}]}{K_{\text{m MASP2}^*}^{\text{C2}}}+\frac{[\text{pC4bC2}]}{K_{\text{m MASP2}^*}^{\text{C2}}}+\frac{[\text{pC4bC4bC2}]}{K_{\text{m MASP2}^*}^{\text{C2}}}\\ +\frac{[\text{pC3bC4bC2}]}{K_{\text{m MASP2}^*}^{\text{C2}}}+\frac{\left[\text{pMBL}_3\text{MASP1}_{\text{zym}}\right]}{K_{\text{m MASP2}^*}^{\text{MASP1}_{\text{zym}}}}+\frac{\left[\text{pMBL}_3\text{MASP2}_{\text{zym}}\right]}{K_{\text{m MASP2}^*}^{\text{MASP2}_{\text{zym}}}}+\frac{\left[\text{pMBL}_4\text{MASP1}_{\text{zym}}\right]}{K_{\text{m MASP2}^*}^{\text{MASP1}_{\text{zym}}}}\\ +\frac{\left[\text{pMBL}_4\text{MASP2}_{\text{zym}}\right]}{K_{\text{m MASP2}^*}^{\text{MASP2}_{\text{zym}}}}+\frac{\left[\text{pCLLKMASP1}_{\text{zym}}\right]}{K_{\text{m MASP2}^*}^{\text{MASP1}_{\text{zym}}}}+\frac{\left[\text{pCLLKMASP2}_{\text{zym}}\right]}{K_{\text{m MASP2}^*}^{\text{MASP2}_{\text{zym}}}}+\frac{\left[\text{pCLK1MASP1}_{\text{zym}}\right]}{K_{\text{m MASP2}^*}^{\text{MASP1}_{\text{zym}}}}\\ +\frac{\left[\text{pCLK1MASP2}_{\text{zym}}\right]}{K_{\text{m MASP2}^*}^{\text{MASP2}_{\text{zym}}}}+\frac{\left[\text{pCLL1MASP1}_{\text{zym}}\right]}{K_{\text{m MASP2}^*}^{\text{MASP1}_{\text{zym}}}}+\frac{\left[\text{pCLL1MASP2}_{\text{zym}}\right]}{K_{\text{m MASP2}^*}^{\text{MASP2}_{\text{zym}}}}+\frac{\left[\text{pLFMASP1}_{\text{zym}}\right]}{K_{\text{m MASP2}^*}^{\text{MASP1}_{\text{zym}}}}\\ +\frac{\left[\text{pLFMASP2}_{\text{zym}}\right]}{K_{\text{m MASP2}^*}^{\text{MASP2}_{\text{zym}}}}+\frac{\left[\text{pHFMASP1}_{\text{zym}}\right]}{K_{\text{m MASP2}^*}^{\text{MASP1}_{\text{zym}}}}+\frac{\left[\text{pHFMASP2}_{\text{zym}}\right]}{K_{\text{m MASP2}^*}^{\text{MASP2}_{\text{zym}}}}+\frac{\left[\text{pMFMASP1}_{\text{zym}}\right]}{K_{\text{m MASP2}^*}^{\text{MASP1}_{\text{zym}}}}+\frac{\left[\text{pMFMASP2}_{\text{zym}}\right]}{K_{\text{m MASP2}^*}^{\text{MASP2}_{\text{zym}}}}\\ +\frac{\left[\text{pPTX3LFMASP1}_{\text{zym}}\right]}{K_{\text{m MASP2}^*}^{\text{MASP1}_{\text{zym}}}}+\frac{\left[\text{pPTX3LFMASP2}_{\text{zym}}\right]}{K_{\text{m MASP2}^*}^{\text{MASP2}_{\text{zym}}}}\end{array}\right)}
$$

$$
+\frac{k_{\mathrm{cat\,MASP2^*}}^{\mathrm{MASP2_{zym}}}\left[\mathrm{pCLK1MASP2^*}\right]\left[\mathrm{pMBL_4MASP2_{zym}}\right]}{K_{\mathrm{m\,MASP2^*}}^{\mathrm{MASP2_{zym}}}\left(\begin{array}{l}1+\frac{[\mathrm{C4}]}{K_{\mathrm{m\,MASP2^*}}^{\mathrm{C4}}}+\frac{[\mathrm{fC4bC2}]}{K_{\mathrm{m\,MASP2^*}}^{\mathrm{C2}}}+\frac{[\mathrm{fC4bC4bC2}]}{K_{\mathrm{m\,MASP2^*}}^{\mathrm{C2}}}+\frac{[\mathrm{fC3bC4bC2}]}{K_{\mathrm{m\,MASP2^*}}^{\mathrm{C2}}}+\frac{[\mathrm{pC4bC2}]}{K_{\mathrm{m\,MASP2^*}}^{\mathrm{C2}}}+\frac{[\mathrm{pC4bC4bC2}]}{K_{\mathrm{m\,MASP2^*}}^{\mathrm{C2}}}\\
+\frac{[\mathrm{pC3bC4bC2}]}{K_{\mathrm{m\,MASP2^*}}^{\mathrm{C2}}}+\frac{[\mathrm{pMBL_3MASP1_{zym}}]}{K_{\mathrm{m\,MASP2^*}}^{\mathrm{MASP1_{zym}}}}+\frac{[\mathrm{pMBL_3MASP2_{zym}}]}{K_{\mathrm{m\,MASP2^*}}^{\mathrm{MASP2_{zym}}}}+\frac{[\mathrm{pMBL_4MASP1_{zym}}]}{K_{\mathrm{m\,MASP2^*}}^{\mathrm{MASP1_{zym}}}}\\
+\frac{[\mathrm{pMBL_4MASP2_{zym}}]}{K_{\mathrm{m\,MASP2^*}}^{\mathrm{MASP2_{zym}}}}+\frac{[\mathrm{pCLLKMASP1_{zym}}]}{K_{\mathrm{m\,MASP2^*}}^{\mathrm{MASP1_{zym}}}}+\frac{[\mathrm{pCLLKMASP2_{zym}}]}{K_{\mathrm{m\,MASP2^*}}^{\mathrm{MASP2_{zym}}}}+\frac{[\mathrm{pCLK1MASP1_{zym}}]}{K_{\mathrm{m\,MASP2^*}}^{\mathrm{MASP1_{zym}}}}\\
+\frac{[\mathrm{pCLK1MASP2_{zym}}]}{K_{\mathrm{m\,MASP2^*}}^{\mathrm{MASP2_{zym}}}}+\frac{[\mathrm{pCLL1MASP1_{zym}}]}{K_{\mathrm{m\,MASP2^*}}^{\mathrm{MASP1_{zym}}}}+\frac{[\mathrm{pCLL1MASP2_{zym}}]}{K_{\mathrm{m\,MASP2^*}}^{\mathrm{MASP2_{zym}}}}+\frac{[\mathrm{pLFMASP1_{zym}}]}{K_{\mathrm{m\,MASP2^*}}^{\mathrm{MASP1_{zym}}}}\\
+\frac{[\mathrm{pLFMASP2_{zym}}]}{K_{\mathrm{m\,MASP2^*}}^{\mathrm{MASP2_{zym}}}}+\frac{[\mathrm{pHFMASP1_{zym}}]}{K_{\mathrm{m\,MASP2^*}}^{\mathrm{MASP1_{zym}}}}+\frac{[\mathrm{pHFMASP2_{zym}}]}{K_{\mathrm{m\,MASP2^*}}^{\mathrm{MASP2_{zym}}}}+\frac{[\mathrm{pMFMASP1_{zym}}]}{K_{\mathrm{m\,MASP2^*}}^{\mathrm{MASP1_{zym}}}}+\frac{[\mathrm{pMFMASP2_{zym}}]}{K_{\mathrm{m\,MASP2^*}}^{\mathrm{MASP2_{zym}}}}\\
+\frac{[\mathrm{pPTX3LFMASP1_{zym}}]}{K_{\mathrm{m\,MASP2^*}}^{\mathrm{MASP1_{zym}}}}+\frac{[\mathrm{pPTX3LFMASP2_{zym}}]}{K_{\mathrm{m\,MASP2^*}}^{\mathrm{MASP2_{zym}}}}\end{array}\right)}
$$

$$
+\frac{k_{\mathrm{cat\,MASP2^*}}^{\mathrm{MASP2_{zym}}}\left[\mathrm{pCLL1MASP2^*}\right]\left[\mathrm{pMBL_4MASP2_{zym}}\right]}{K_{\mathrm{m\,MASP2^*}}^{\mathrm{MASP2_{zym}}}\left(\begin{array}{l}1+\frac{[\mathrm{C4}]}{K_{\mathrm{m\,MASP2^*}}^{\mathrm{C4}}}+\frac{[\mathrm{fC4bC2}]}{K_{\mathrm{m\,MASP2^*}}^{\mathrm{C2}}}+\frac{[\mathrm{fC4bC4bC2}]}{K_{\mathrm{m\,MASP2^*}}^{\mathrm{C2}}}+\frac{[\mathrm{fC3bC4bC2}]}{K_{\mathrm{m\,MASP2^*}}^{\mathrm{C2}}}+\frac{[\mathrm{pC4bC2}]}{K_{\mathrm{m\,MASP2^*}}^{\mathrm{C2}}}+\frac{[\mathrm{pC4bC4bC2}]}{K_{\mathrm{m\,MASP2^*}}^{\mathrm{C2}}}\\
+\frac{[\mathrm{pC3bC4bC2}]}{K_{\mathrm{m\,MASP2^*}}^{\mathrm{C2}}}+\frac{[\mathrm{pMBL_3MASP1_{zym}}]}{K_{\mathrm{m\,MASP2^*}}^{\mathrm{MASP1_{zym}}}}+\frac{[\mathrm{pMBL_3MASP2_{zym}}]}{K_{\mathrm{m\,MASP2^*}}^{\mathrm{MASP2_{zym}}}}+\frac{[\mathrm{pMBL_4MASP1_{zym}}]}{K_{\mathrm{m\,MASP2^*}}^{\mathrm{MASP1_{zym}}}}\\
+\frac{[\mathrm{pMBL_4MASP2_{zym}}]}{K_{\mathrm{m\,MASP2^*}}^{\mathrm{MASP2_{zym}}}}+\frac{[\mathrm{pCLLKMASP1_{zym}}]}{K_{\mathrm{m\,MASP2^*}}^{\mathrm{MASP1_{zym}}}}+\frac{[\mathrm{pCLLKMASP2_{zym}}]}{K_{\mathrm{m\,MASP2^*}}^{\mathrm{MASP2_{zym}}}}+\frac{[\mathrm{pCLK1MASP1_{zym}}]}{K_{\mathrm{m\,MASP2^*}}^{\mathrm{MASP1_{zym}}}}\\
+\frac{[\mathrm{pCLK1MASP2_{zym}}]}{K_{\mathrm{m\,MASP2^*}}^{\mathrm{MASP2_{zym}}}}+\frac{[\mathrm{pCLL1MASP1_{zym}}]}{K_{\mathrm{m\,MASP2^*}}^{\mathrm{MASP1_{zym}}}}+\frac{[\mathrm{pCLL1MASP2_{zym}}]}{K_{\mathrm{m\,MASP2^*}}^{\mathrm{MASP2_{zym}}}}+\frac{[\mathrm{pLFMASP1_{zym}}]}{K_{\mathrm{m\,MASP2^*}}^{\mathrm{MASP1_{zym}}}}\\
+\frac{[\mathrm{pLFMASP2_{zym}}]}{K_{\mathrm{m\,MASP2^*}}^{\mathrm{MASP2_{zym}}}}+\frac{[\mathrm{pHFMASP1_{zym}}]}{K_{\mathrm{m\,MASP2^*}}^{\mathrm{MASP1_{zym}}}}+\frac{[\mathrm{pHFMASP2_{zym}}]}{K_{\mathrm{m\,MASP2^*}}^{\mathrm{MASP2_{zym}}}}+\frac{[\mathrm{pMFMASP1_{zym}}]}{K_{\mathrm{m\,MASP2^*}}^{\mathrm{MASP1_{zym}}}}+\frac{[\mathrm{pMFMASP2_{zym}}]}{K_{\mathrm{m\,MASP2^*}}^{\mathrm{MASP2_{zym}}}}\\
+\frac{[\mathrm{pPTX3LFMASP1_{zym}}]}{K_{\mathrm{m\,MASP2^*}}^{\mathrm{MASP1_{zym}}}}+\frac{[\mathrm{pPTX3LFMASP2_{zym}}]}{K_{\mathrm{m\,MASP2^*}}^{\mathrm{MASP2_{zym}}}}\end{array}\right)}
$$

$$+\frac{k_{\mathrm{cat\,MASP2^*}}^{\mathrm{MASP2_{zym}}}\left[\mathrm{pLFMASP2^*}\right]\left[\mathrm{pMBL_4MASP2_{zym}}\right]}{K_{\mathrm{m\,MASP2^*}}^{\mathrm{MASP2_{zym}}}\left(\begin{array}{l}1+\frac{[\mathrm{C4}]}{K_{\mathrm{m\,MASP2^*}}^{\mathrm{C4}}}+\frac{[\mathrm{fC4bC2}]}{K_{\mathrm{m\,MASP2^*}}^{\mathrm{C2}}}+\frac{[\mathrm{fC4bC4bC2}]}{K_{\mathrm{m\,MASP2^*}}^{\mathrm{C2}}}+\frac{[\mathrm{fC3bC4bC2}]}{K_{\mathrm{m\,MASP2^*}}^{\mathrm{C2}}}+\frac{[\mathrm{pC4bC2}]}{K_{\mathrm{m\,MASP2^*}}^{\mathrm{C2}}}+\frac{[\mathrm{pC4bC4bC2}]}{K_{\mathrm{m\,MASP2^*}}^{\mathrm{C2}}}\\+\frac{[\mathrm{pC3bC4bC2}]}{K_{\mathrm{m\,MASP2^*}}^{\mathrm{C2}}}+\frac{[\mathrm{pMBL_3MASP1_{zym}}]}{K_{\mathrm{m\,MASP2^*}}^{\mathrm{MASP1_{zym}}}}+\frac{[\mathrm{pMBL_3MASP2_{zym}}]}{K_{\mathrm{m\,MASP2^*}}^{\mathrm{MASP2_{zym}}}}+\frac{[\mathrm{pMBL_4MASP1_{zym}}]}{K_{\mathrm{m\,MASP2^*}}^{\mathrm{MASP1_{zym}}}}\\+\frac{[\mathrm{pMBL_4MASP2_{zym}}]}{K_{\mathrm{m\,MASP2^*}}^{\mathrm{MASP2_{zym}}}}+\frac{[\mathrm{pCLLKMASP1_{zym}}]}{K_{\mathrm{m\,MASP2^*}}^{\mathrm{MASP1_{zym}}}}+\frac{[\mathrm{pCLLKMASP2_{zym}}]}{K_{\mathrm{m\,MASP2^*}}^{\mathrm{MASP2_{zym}}}}+\frac{[\mathrm{pCLK1MASP1_{zym}}]}{K_{\mathrm{m\,MASP2^*}}^{\mathrm{MASP1_{zym}}}}\\+\frac{[\mathrm{pCLK1MASP2_{zym}}]}{K_{\mathrm{m\,MASP2^*}}^{\mathrm{MASP2_{zym}}}}+\frac{[\mathrm{pCLL1MASP1_{zym}}]}{K_{\mathrm{m\,MASP2^*}}^{\mathrm{MASP1_{zym}}}}+\frac{[\mathrm{pCLL1MASP2_{zym}}]}{K_{\mathrm{m\,MASP2^*}}^{\mathrm{MASP2_{zym}}}}+\frac{[\mathrm{pLFMASP1_{zym}}]}{K_{\mathrm{m\,MASP2^*}}^{\mathrm{MASP1_{zym}}}}\\+\frac{[\mathrm{pLFMASP2_{zym}}]}{K_{\mathrm{m\,MASP2^*}}^{\mathrm{MASP2_{zym}}}}+\frac{[\mathrm{pHFMASP1_{zym}}]}{K_{\mathrm{m\,MASP2^*}}^{\mathrm{MASP1_{zym}}}}+\frac{[\mathrm{pHFMASP2_{zym}}]}{K_{\mathrm{m\,MASP2^*}}^{\mathrm{MASP2_{zym}}}}+\frac{[\mathrm{pMFMASP1_{zym}}]}{K_{\mathrm{m\,MASP2^*}}^{\mathrm{MASP1_{zym}}}}+\frac{[\mathrm{pMFMASP2_{zym}}]}{K_{\mathrm{m\,MASP2^*}}^{\mathrm{MASP2_{zym}}}}\\+\frac{[\mathrm{pPTX3LFMASP1_{zym}}]}{K_{\mathrm{m\,MASP2^*}}^{\mathrm{MASP1_{zym}}}}+\frac{[\mathrm{pPTX3LFMASP2_{zym}}]}{K_{\mathrm{m\,MASP2^*}}^{\mathrm{MASP2_{zym}}}}\end{array}\right)}$$

$$+\frac{k_{\mathrm{cat\,MASP2^*}}^{\mathrm{MASP2_{zym}}}\left[\mathrm{pHFMASP2^*}\right]\left[\mathrm{pMBL_4MASP2_{zym}}\right]}{K_{\mathrm{m\,MASP2^*}}^{\mathrm{MASP2_{zym}}}\left(\begin{array}{l}1+\frac{[\mathrm{C4}]}{K_{\mathrm{m\,MASP2^*}}^{\mathrm{C4}}}+\frac{[\mathrm{fC4bC2}]}{K_{\mathrm{m\,MASP2^*}}^{\mathrm{C2}}}+\frac{[\mathrm{fC4bC4bC2}]}{K_{\mathrm{m\,MASP2^*}}^{\mathrm{C2}}}+\frac{[\mathrm{fC3bC4bC2}]}{K_{\mathrm{m\,MASP2^*}}^{\mathrm{C2}}}+\frac{[\mathrm{pC4bC2}]}{K_{\mathrm{m\,MASP2^*}}^{\mathrm{C2}}}+\frac{[\mathrm{pC4bC4bC2}]}{K_{\mathrm{m\,MASP2^*}}^{\mathrm{C2}}}\\+\frac{[\mathrm{pC3bC4bC2}]}{K_{\mathrm{m\,MASP2^*}}^{\mathrm{C2}}}+\frac{[\mathrm{pMBL_3MASP1_{zym}}]}{K_{\mathrm{m\,MASP2^*}}^{\mathrm{MASP1_{zym}}}}+\frac{[\mathrm{pMBL_3MASP2_{zym}}]}{K_{\mathrm{m\,MASP2^*}}^{\mathrm{MASP2_{zym}}}}+\frac{[\mathrm{pMBL_4MASP1_{zym}}]}{K_{\mathrm{m\,MASP2^*}}^{\mathrm{MASP1_{zym}}}}\\+\frac{[\mathrm{pMBL_4MASP2_{zym}}]}{K_{\mathrm{m\,MASP2^*}}^{\mathrm{MASP2_{zym}}}}+\frac{[\mathrm{pCLLKMASP1_{zym}}]}{K_{\mathrm{m\,MASP2^*}}^{\mathrm{MASP1_{zym}}}}+\frac{[\mathrm{pCLLKMASP2_{zym}}]}{K_{\mathrm{m\,MASP2^*}}^{\mathrm{MASP2_{zym}}}}+\frac{[\mathrm{pCLK1MASP1_{zym}}]}{K_{\mathrm{m\,MASP2^*}}^{\mathrm{MASP1_{zym}}}}\\+\frac{[\mathrm{pCLK1MASP2_{zym}}]}{K_{\mathrm{m\,MASP2^*}}^{\mathrm{MASP2_{zym}}}}+\frac{[\mathrm{pCLL1MASP1_{zym}}]}{K_{\mathrm{m\,MASP2^*}}^{\mathrm{MASP1_{zym}}}}+\frac{[\mathrm{pCLL1MASP2_{zym}}]}{K_{\mathrm{m\,MASP2^*}}^{\mathrm{MASP2_{zym}}}}+\frac{[\mathrm{pLFMASP1_{zym}}]}{K_{\mathrm{m\,MASP2^*}}^{\mathrm{MASP1_{zym}}}}\\+\frac{[\mathrm{pLFMASP2_{zym}}]}{K_{\mathrm{m\,MASP2^*}}^{\mathrm{MASP2_{zym}}}}+\frac{[\mathrm{pHFMASP1_{zym}}]}{K_{\mathrm{m\,MASP2^*}}^{\mathrm{MASP1_{zym}}}}+\frac{[\mathrm{pHFMASP2_{zym}}]}{K_{\mathrm{m\,MASP2^*}}^{\mathrm{MASP2_{zym}}}}+\frac{[\mathrm{pMFMASP1_{zym}}]}{K_{\mathrm{m\,MASP2^*}}^{\mathrm{MASP1_{zym}}}}+\frac{[\mathrm{pMFMASP2_{zym}}]}{K_{\mathrm{m\,MASP2^*}}^{\mathrm{MASP2_{zym}}}}\\+\frac{[\mathrm{pPTX3LFMASP1_{zym}}]}{K_{\mathrm{m\,MASP2^*}}^{\mathrm{MASP1_{zym}}}}+\frac{[\mathrm{pPTX3LFMASP2_{zym}}]}{K_{\mathrm{m\,MASP2^*}}^{\mathrm{MASP2_{zym}}}}\end{array}\right)}$$

$$
+\frac{k_{\mathrm{cat\ MASP2^*}}^{\mathrm{MASP2_{zym}}}\left[\mathrm{pMFMASP2^*}\right]\left[\mathrm{pMBL_4MASP2_{zym}}\right]}{K_{\mathrm{m\ MASP2^*}}^{\mathrm{MASP2_{zym}}}\left(\begin{array}{l}1+\frac{[\mathrm{C4}]}{K_{\mathrm{m\ MASP2^*}}^{\mathrm{C4}}}+\frac{[\mathrm{fC4bC2}]}{K_{\mathrm{m\ MASP2^*}}^{\mathrm{C2}}}+\frac{[\mathrm{fC4bC4bC2}]}{K_{\mathrm{m\ MASP2^*}}^{\mathrm{C2}}}+\frac{[\mathrm{fC3bC4bC2}]}{K_{\mathrm{m\ MASP2^*}}^{\mathrm{C2}}}+\frac{[\mathrm{pC4bC2}]}{K_{\mathrm{m\ MASP2^*}}^{\mathrm{C2}}}+\frac{[\mathrm{pC4bC4bC2}]}{K_{\mathrm{m\ MASP2^*}}^{\mathrm{C2}}}\\
+\frac{[\mathrm{pC3bC4bC2}]}{K_{\mathrm{m\ MASP2^*}}^{\mathrm{C2}}}+\frac{\left[\mathrm{pMBL_3MASP1_{zym}}\right]}{K_{\mathrm{m\ MASP2^*}}^{\mathrm{MASP1_{zym}}}}+\frac{\left[\mathrm{pMBL_3MASP2_{zym}}\right]}{K_{\mathrm{m\ MASP2^*}}^{\mathrm{MASP2_{zym}}}}+\frac{\left[\mathrm{pMBL_4MASP1_{zym}}\right]}{K_{\mathrm{m\ MASP2^*}}^{\mathrm{MASP1_{zym}}}}\\
+\frac{\left[\mathrm{pMBL_4MASP2_{zym}}\right]}{K_{\mathrm{m\ MASP2^*}}^{\mathrm{MASP2_{zym}}}}+\frac{\left[\mathrm{pCLLKMASP1_{zym}}\right]}{K_{\mathrm{m\ MASP2^*}}^{\mathrm{MASP1_{zym}}}}+\frac{\left[\mathrm{pCLLKMASP2_{zym}}\right]}{K_{\mathrm{m\ MASP2^*}}^{\mathrm{MASP2_{zym}}}}+\frac{\left[\mathrm{pCLK1MASP1_{zym}}\right]}{K_{\mathrm{m\ MASP2^*}}^{\mathrm{MASP1_{zym}}}}\\
+\frac{\left[\mathrm{pCLK1MASP2_{zym}}\right]}{K_{\mathrm{m\ MASP2^*}}^{\mathrm{MASP2_{zym}}}}+\frac{\left[\mathrm{pCLL1MASP1_{zym}}\right]}{K_{\mathrm{m\ MASP2^*}}^{\mathrm{MASP1_{zym}}}}+\frac{\left[\mathrm{pCLL1MASP2_{zym}}\right]}{K_{\mathrm{m\ MASP2^*}}^{\mathrm{MASP2_{zym}}}}+\frac{\left[\mathrm{pLFMASP1_{zym}}\right]}{K_{\mathrm{m\ MASP2^*}}^{\mathrm{MASP1_{zym}}}}\\
+\frac{\left[\mathrm{pLFMASP2_{zym}}\right]}{K_{\mathrm{m\ MASP2^*}}^{\mathrm{MASP2_{zym}}}}+\frac{\left[\mathrm{pHFMASP1_{zym}}\right]}{K_{\mathrm{m\ MASP2^*}}^{\mathrm{MASP1_{zym}}}}+\frac{\left[\mathrm{pHFMASP2_{zym}}\right]}{K_{\mathrm{m\ MASP2^*}}^{\mathrm{MASP2_{zym}}}}+\frac{\left[\mathrm{pMFMASP1_{zym}}\right]}{K_{\mathrm{m\ MASP2^*}}^{\mathrm{MASP1_{zym}}}}+\frac{\left[\mathrm{pMFMASP2_{zym}}\right]}{K_{\mathrm{m\ MASP2^*}}^{\mathrm{MASP2_{zym}}}}\\
+\frac{\left[\mathrm{pPTX3LFMASP1_{zym}}\right]}{K_{\mathrm{m\ MASP2^*}}^{\mathrm{MASP1_{zym}}}}+\frac{\left[\mathrm{pPTX3LFMASP2_{zym}}\right]}{K_{\mathrm{m\ MASP2^*}}^{\mathrm{MASP2_{zym}}}}\end{array}\right)}
$$

$$
+\frac{k_{\mathrm{cat\ MASP2^*}}^{\mathrm{MASP2_{zym}}}\left[\mathrm{pPTX3LFMASP2^*}\right]\left[\mathrm{pMBL_4MASP2_{zym}}\right]}{K_{\mathrm{m\ MASP2^*}}^{\mathrm{MASP2_{zym}}}\left(\begin{array}{l}1+\frac{[\mathrm{C4}]}{K_{\mathrm{m\ MASP2^*}}^{\mathrm{C4}}}+\frac{[\mathrm{fC4bC2}]}{K_{\mathrm{m\ MASP2^*}}^{\mathrm{C2}}}+\frac{[\mathrm{fC4bC4bC2}]}{K_{\mathrm{m\ MASP2^*}}^{\mathrm{C2}}}+\frac{[\mathrm{fC3bC4bC2}]}{K_{\mathrm{m\ MASP2^*}}^{\mathrm{C2}}}+\frac{[\mathrm{pC4bC2}]}{K_{\mathrm{m\ MASP2^*}}^{\mathrm{C2}}}+\frac{[\mathrm{pC4bC4bC2}]}{K_{\mathrm{m\ MASP2^*}}^{\mathrm{C2}}}\\
+\frac{[\mathrm{pC3bC4bC2}]}{K_{\mathrm{m\ MASP2^*}}^{\mathrm{C2}}}+\frac{\left[\mathrm{pMBL_3MASP1_{zym}}\right]}{K_{\mathrm{m\ MASP2^*}}^{\mathrm{MASP1_{zym}}}}+\frac{\left[\mathrm{pMBL_3MASP2_{zym}}\right]}{K_{\mathrm{m\ MASP2^*}}^{\mathrm{MASP2_{zym}}}}+\frac{\left[\mathrm{pMBL_4MASP1_{zym}}\right]}{K_{\mathrm{m\ MASP2^*}}^{\mathrm{MASP1_{zym}}}}\\
+\frac{\left[\mathrm{pMBL_4MASP2_{zym}}\right]}{K_{\mathrm{m\ MASP2^*}}^{\mathrm{MASP2_{zym}}}}+\frac{\left[\mathrm{pCLLKMASP1_{zym}}\right]}{K_{\mathrm{m\ MASP2^*}}^{\mathrm{MASP1_{zym}}}}+\frac{\left[\mathrm{pCLLKMASP2_{zym}}\right]}{K_{\mathrm{m\ MASP2^*}}^{\mathrm{MASP2_{zym}}}}+\frac{\left[\mathrm{pCLK1MASP1_{zym}}\right]}{K_{\mathrm{m\ MASP2^*}}^{\mathrm{MASP1_{zym}}}}\\
+\frac{\left[\mathrm{pCLK1MASP2_{zym}}\right]}{K_{\mathrm{m\ MASP2^*}}^{\mathrm{MASP2_{zym}}}}+\frac{\left[\mathrm{pCLL1MASP1_{zym}}\right]}{K_{\mathrm{m\ MASP2^*}}^{\mathrm{MASP1_{zym}}}}+\frac{\left[\mathrm{pCLL1MASP2_{zym}}\right]}{K_{\mathrm{m\ MASP2^*}}^{\mathrm{MASP2_{zym}}}}+\frac{\left[\mathrm{pLFMASP1_{zym}}\right]}{K_{\mathrm{m\ MASP2^*}}^{\mathrm{MASP1_{zym}}}}\\
+\frac{\left[\mathrm{pLFMASP2_{zym}}\right]}{K_{\mathrm{m\ MASP2^*}}^{\mathrm{MASP2_{zym}}}}+\frac{\left[\mathrm{pHFMASP1_{zym}}\right]}{K_{\mathrm{m\ MASP2^*}}^{\mathrm{MASP1_{zym}}}}+\frac{\left[\mathrm{pHFMASP2_{zym}}\right]}{K_{\mathrm{m\ MASP2^*}}^{\mathrm{MASP2_{zym}}}}+\frac{\left[\mathrm{pMFMASP1_{zym}}\right]}{K_{\mathrm{m\ MASP2^*}}^{\mathrm{MASP1_{zym}}}}+\frac{\left[\mathrm{pMFMASP2_{zym}}\right]}{K_{\mathrm{m\ MASP2^*}}^{\mathrm{MASP2_{zym}}}}\\
+\frac{\left[\mathrm{pPTX3LFMASP1_{zym}}\right]}{K_{\mathrm{m\ MASP2^*}}^{\mathrm{MASP1_{zym}}}}+\frac{\left[\mathrm{pPTX3LFMASP2_{zym}}\right]}{K_{\mathrm{m\ MASP2^*}}^{\mathrm{MASP2_{zym}}}}\end{array}\right)}
$$

$$
-k_{\mathrm{C1INHMASP2}}^{+}\left[\mathrm{C1\text{-}INH}\right]\left[\mathrm{pMBL_4MASP2^*}\right]
$$

274

$$
\frac{d\left[\mathrm{fMBL_4MASP3_{zym}}\right]}{dt}=k_{\mathrm{MBL_4MASP3}}^{+}\left[\mathrm{MBL_4}\right]\left[\mathrm{MASP3}\right]-k_{\mathrm{MBL_4MASP3}}^{-}\left[\mathrm{fMBL_4MASP3_{zym}}\right]
$$

$$
-k_{\mathrm{pMBL_4}}^{+}\left[\mathrm{fMBL_4MASP3_{zym}}\right]\left[\mathrm{Pathogen\ Surface}\right]+k_{\mathrm{pMBL_4}}^{-}\left[\mathrm{pMBL_4MASP3_{zym}}\right]
$$

275

$$\frac{d\left[\mathrm{pMBL_4MASP3_{zym}}\right]}{dt}=k^{+}_{\mathrm{pMBL_4}}\left[\mathrm{fMBL_4MASP3_{zym}}\right]\left[\mathrm{Pathogen\ Surface}\right]-k^{-}_{\mathrm{pMBL_4}}\left[\mathrm{pMBL_4MASP3_{zym}}\right]$$

$$+k^{+}_{\mathrm{MBL_4MASP3}}\left[\mathrm{pMBL_4}\right]\left[\mathrm{MASP3}\right]-k^{-}_{\mathrm{MBL_4MASP3}}\left[\mathrm{pMBL_4MASP3_{zym}}\right]$$

$$-\frac{k^{\mathrm{MASP3_{zym}}}_{\mathrm{cat\ MASP1^*}}\left[\mathrm{pMBL_3MASP1^*}\right]\left[\mathrm{pMBL_4MASP3_{zym}}\right]}{K^{\mathrm{MASP3_{zym}}}_{\mathrm{m\ MASP1^*}}\left(\begin{array}{l}1+\frac{[\mathrm{C4}]}{K^{\mathrm{C4}}_{\mathrm{m\ MASP1^*}}}+\frac{[\mathrm{fC4bC2}]}{K^{\mathrm{C2}}_{\mathrm{m\ MASP1^*}}}+\frac{[\mathrm{fC4bC4bC2}]}{K^{\mathrm{C2}}_{\mathrm{m\ MASP1^*}}}+\frac{[\mathrm{fC3bC4bC2}]}{K^{\mathrm{C2}}_{\mathrm{m\ MASP1^*}}}+\frac{[\mathrm{pC4bC2}]}{K^{\mathrm{C2}}_{\mathrm{m\ MASP1^*}}}+\frac{[\mathrm{pC4bC4bC2}]}{K^{\mathrm{C2}}_{\mathrm{m\ MASP1^*}}}\\ +\frac{[\mathrm{pC3bC4bC2}]}{K^{\mathrm{C2}}_{\mathrm{m\ MASP1^*}}}+\frac{[\mathrm{pMBL_3MASP1_{zym}}]}{K^{\mathrm{MASP1_{zym}}}_{\mathrm{m\ MASP1^*}}}+\frac{[\mathrm{pMBL_3MASP2_{zym}}]}{K^{\mathrm{MASP2_{zym}}}_{\mathrm{m\ MASP1^*}}}+\frac{[\mathrm{pMBL_3MASP3_{zym}}]}{K^{\mathrm{MASP3_{zym}}}_{\mathrm{m\ MASP1^*}}}\\ +\frac{[\mathrm{pMBL_4MASP1_{zym}}]}{K^{\mathrm{MASP1_{zym}}}_{\mathrm{m\ MASP1^*}}}+\frac{[\mathrm{pMBL_4MASP2_{zym}}]}{K^{\mathrm{MASP2_{zym}}}_{\mathrm{m\ MASP1^*}}}+\frac{[\mathrm{pMBL_4MASP3_{zym}}]}{K^{\mathrm{MASP3_{zym}}}_{\mathrm{m\ MASP1^*}}}+\frac{[\mathrm{pCLLKMASP1_{zym}}]}{K^{\mathrm{MASP1_{zym}}}_{\mathrm{m\ MASP1^*}}}\\ +\frac{[\mathrm{pCLLKMASP2_{zym}}]}{K^{\mathrm{MASP2_{zym}}}_{\mathrm{m\ MASP1^*}}}+\frac{[\mathrm{pCLLKMASP3_{zym}}]}{K^{\mathrm{MASP3_{zym}}}_{\mathrm{m\ MASP1^*}}}+\frac{[\mathrm{pCLK1MASP1_{zym}}]}{K^{\mathrm{MASP1_{zym}}}_{\mathrm{m\ MASP1^*}}}+\frac{[\mathrm{pCLK1MASP2_{zym}}]}{K^{\mathrm{MASP2_{zym}}}_{\mathrm{m\ MASP1^*}}}\\ +\frac{[\mathrm{pCLK1MASP3_{zym}}]}{K^{\mathrm{MASP3_{zym}}}_{\mathrm{m\ MASP1^*}}}+\frac{[\mathrm{pCLL1MASP1_{zym}}]}{K^{\mathrm{MASP1_{zym}}}_{\mathrm{m\ MASP1^*}}}+\frac{[\mathrm{pCLL1MASP2_{zym}}]}{K^{\mathrm{MASP2_{zym}}}_{\mathrm{m\ MASP1^*}}}+\frac{[\mathrm{pCLL1MASP3_{zym}}]}{K^{\mathrm{MASP3_{zym}}}_{\mathrm{m\ MASP1^*}}}\\ +\frac{[\mathrm{pLFMASP1_{zym}}]}{K^{\mathrm{MASP1_{zym}}}_{\mathrm{m\ MASP1^*}}}+\frac{[\mathrm{pLFMASP2_{zym}}]}{K^{\mathrm{MASP2_{zym}}}_{\mathrm{m\ MASP1^*}}}+\frac{[\mathrm{pLFMASP3_{zym}}]}{K^{\mathrm{MASP3_{zym}}}_{\mathrm{m\ MASP1^*}}}+\frac{[\mathrm{pHFMASP1_{zym}}]}{K^{\mathrm{MASP1_{zym}}}_{\mathrm{m\ MASP1^*}}}+\frac{[\mathrm{pHFMASP2_{zym}}]}{K^{\mathrm{MASP2_{zym}}}_{\mathrm{m\ MASP1^*}}}\\ +\frac{[\mathrm{pHFMASP3_{zym}}]}{K^{\mathrm{MASP3_{zym}}}_{\mathrm{m\ MASP1^*}}}+\frac{[\mathrm{pMFMASP1_{zym}}]}{K^{\mathrm{MASP1_{zym}}}_{\mathrm{m\ MASP1^*}}}+\frac{[\mathrm{pMFMASP2_{zym}}]}{K^{\mathrm{MASP2_{zym}}}_{\mathrm{m\ MASP1^*}}}+\frac{[\mathrm{pMFMASP3_{zym}}]}{K^{\mathrm{MASP3_{zym}}}_{\mathrm{m\ MASP1^*}}}\\ +\frac{[\mathrm{pPTX3LFMASP1_{zym}}]}{K^{\mathrm{MASP1_{zym}}}_{\mathrm{m\ MASP1^*}}}+\frac{[\mathrm{pPXT3LFMASP2_{zym}}]}{K^{\mathrm{MASP2_{zym}}}_{\mathrm{m\ MASP1^*}}}+\frac{[\mathrm{pPTX3LFMASP3_{zym}}]}{K^{\mathrm{MASP3_{zym}}}_{\mathrm{m\ MASP1^*}}}\end{array}\right)}$$

$$-\frac{k^{\mathrm{MASP3_{zym}}}_{\mathrm{cat\,MASP1^*}}\left[\mathrm{pMBL_4MASP1^*}\right]\left[\mathrm{pMBL_4MASP3_{zym}}\right]}{K^{\mathrm{MASP3_{zym}}}_{\mathrm{m\,MASP1^*}}\left(\begin{array}{l}1+\frac{[\mathrm{C4}]}{K^{\mathrm{C4}}_{\mathrm{m\,MASP1^*}}}+\frac{[\mathrm{fC4bC2}]}{K^{\mathrm{C2}}_{\mathrm{m\,MASP1^*}}}+\frac{[\mathrm{fC4bC4bC2}]}{K^{\mathrm{C2}}_{\mathrm{m\,MASP1^*}}}+\frac{[\mathrm{fC3bC4bC2}]}{K^{\mathrm{C2}}_{\mathrm{m\,MASP1^*}}}+\frac{[\mathrm{pC4bC2}]}{K^{\mathrm{C2}}_{\mathrm{m\,MASP1^*}}}+\frac{[\mathrm{pC4bC4bC2}]}{K^{\mathrm{C2}}_{\mathrm{m\,MASP1^*}}}\\
+\frac{[\mathrm{pC3bC4bC2}]}{K^{\mathrm{C2}}_{\mathrm{m\,MASP1^*}}}+\frac{[\mathrm{pMBL_3MASP1_{zym}}]}{K^{\mathrm{MASP1_{zym}}}_{\mathrm{m\,MASP1^*}}}+\frac{[\mathrm{pMBL_3MASP2_{zym}}]}{K^{\mathrm{MASP2_{zym}}}_{\mathrm{m\,MASP1^*}}}+\frac{[\mathrm{pMBL_3MASP3_{zym}}]}{K^{\mathrm{MASP3_{zym}}}_{\mathrm{m\,MASP1^*}}}\\
+\frac{[\mathrm{pMBL_4MASP1_{zym}}]}{K^{\mathrm{MASP1_{zym}}}_{\mathrm{m\,MASP1^*}}}+\frac{[\mathrm{pMBL_4MASP2_{zym}}]}{K^{\mathrm{MASP2_{zym}}}_{\mathrm{m\,MASP1^*}}}+\frac{[\mathrm{pMBL_4MASP3_{zym}}]}{K^{\mathrm{MASP3_{zym}}}_{\mathrm{m\,MASP1^*}}}+\frac{[\mathrm{pCLLKMASP1_{zym}}]}{K^{\mathrm{MASP1_{zym}}}_{\mathrm{m\,MASP1^*}}}\\
+\frac{[\mathrm{pCLLKMASP2_{zym}}]}{K^{\mathrm{MASP2_{zym}}}_{\mathrm{m\,MASP1^*}}}+\frac{[\mathrm{pCLLKMASP3_{zym}}]}{K^{\mathrm{MASP3_{zym}}}_{\mathrm{m\,MASP1^*}}}+\frac{[\mathrm{pCLK1MASP1_{zym}}]}{K^{\mathrm{MASP1_{zym}}}_{\mathrm{m\,MASP1^*}}}+\frac{[\mathrm{pCLK1MASP2_{zym}}]}{K^{\mathrm{MASP2_{zym}}}_{\mathrm{m\,MASP1^*}}}\\
+\frac{[\mathrm{pCLK1MASP3_{zym}}]}{K^{\mathrm{MASP3_{zym}}}_{\mathrm{m\,MASP1^*}}}+\frac{[\mathrm{pCLL1MASP1_{zym}}]}{K^{\mathrm{MASP1_{zym}}}_{\mathrm{m\,MASP1^*}}}+\frac{[\mathrm{pCLL1MASP2_{zym}}]}{K^{\mathrm{MASP2_{zym}}}_{\mathrm{m\,MASP1^*}}}+\frac{[\mathrm{pCLL1MASP3_{zym}}]}{K^{\mathrm{MASP3_{zym}}}_{\mathrm{m\,MASP1^*}}}\\
+\frac{[\mathrm{pLFMASP1_{zym}}]}{K^{\mathrm{MASP1_{zym}}}_{\mathrm{m\,MASP1^*}}}+\frac{[\mathrm{pLFMASP2_{zym}}]}{K^{\mathrm{MASP2_{zym}}}_{\mathrm{m\,MASP1^*}}}+\frac{[\mathrm{pLFMASP3_{zym}}]}{K^{\mathrm{MASP3_{zym}}}_{\mathrm{m\,MASP1^*}}}+\frac{[\mathrm{pHFMASP1_{zym}}]}{K^{\mathrm{MASP1_{zym}}}_{\mathrm{m\,MASP1^*}}}+\frac{[\mathrm{pHFMASP2_{zym}}]}{K^{\mathrm{MASP2_{zym}}}_{\mathrm{m\,MASP1^*}}}\\
+\frac{[\mathrm{pHFMASP3_{zym}}]}{K^{\mathrm{MASP3_{zym}}}_{\mathrm{m\,MASP1^*}}}+\frac{[\mathrm{pMFMASP1_{zym}}]}{K^{\mathrm{MASP1_{zym}}}_{\mathrm{m\,MASP1^*}}}+\frac{[\mathrm{pMFMASP2_{zym}}]}{K^{\mathrm{MASP2_{zym}}}_{\mathrm{m\,MASP1^*}}}+\frac{[\mathrm{pMFMASP3_{zym}}]}{K^{\mathrm{MASP3_{zym}}}_{\mathrm{m\,MASP1^*}}}\\
+\frac{[\mathrm{pPTX3LFMASP1_{zym}}]}{K^{\mathrm{MASP1_{zym}}}_{\mathrm{m\,MASP1^*}}}+\frac{[\mathrm{pPXT3LFMASP2_{zym}}]}{K^{\mathrm{MASP2_{zym}}}_{\mathrm{m\,MASP1^*}}}+\frac{[\mathrm{pPTX3LFMASP3_{zym}}]}{K^{\mathrm{MASP3_{zym}}}_{\mathrm{m\,MASP1^*}}}\end{array}\right)}$$

$$-\frac{k_{\mathrm{cat\ MASP1^*}}^{\mathrm{MASP3_{zym}}}\left[\mathrm{pCLLKMASP1^*}\right]\left[\mathrm{pMBL_4MASP3_{zym}}\right]}{K_{\mathrm{m\ MASP1^*}}^{\mathrm{MASP3_{zym}}}\left(\begin{aligned}&1+\frac{[\mathrm{C4}]}{K_{\mathrm{m\ MASP1^*}}^{\mathrm{C4}}}+\frac{[\mathrm{fC4bC2}]}{K_{\mathrm{m\ MASP1^*}}^{\mathrm{C2}}}+\frac{[\mathrm{fC4bC4bC2}]}{K_{\mathrm{m\ MASP1^*}}^{\mathrm{C2}}}+\frac{[\mathrm{fC3bC4bC2}]}{K_{\mathrm{m\ MASP1^*}}^{\mathrm{C2}}}+\frac{[\mathrm{pC4bC2}]}{K_{\mathrm{m\ MASP1^*}}^{\mathrm{C2}}}+\frac{[\mathrm{pC4bC4bC2}]}{K_{\mathrm{m\ MASP1^*}}^{\mathrm{C2}}}\\&+\frac{[\mathrm{pC3bC4bC2}]}{K_{\mathrm{m\ MASP1^*}}^{\mathrm{C2}}}+\frac{\left[\mathrm{pMBL_3MASP1_{zym}}\right]}{K_{\mathrm{m\ MASP1^*}}^{\mathrm{MASP1_{zym}}}}+\frac{\left[\mathrm{pMBL_3MASP2_{zym}}\right]}{K_{\mathrm{m\ MASP1^*}}^{\mathrm{MASP2_{zym}}}}+\frac{\left[\mathrm{pMBL_3MASP3_{zym}}\right]}{K_{\mathrm{m\ MASP1^*}}^{\mathrm{MASP3_{zym}}}}\\&+\frac{\left[\mathrm{pMBL_4MASP1_{zym}}\right]}{K_{\mathrm{m\ MASP1^*}}^{\mathrm{MASP1_{zym}}}}+\frac{\left[\mathrm{pMBL_4MASP2_{zym}}\right]}{K_{\mathrm{m\ MASP1^*}}^{\mathrm{MASP2_{zym}}}}+\frac{\left[\mathrm{pMBL_4MASP3_{zym}}\right]}{K_{\mathrm{m\ MASP1^*}}^{\mathrm{MASP3_{zym}}}}+\frac{\left[\mathrm{pCLLKMASP1_{zym}}\right]}{K_{\mathrm{m\ MASP1^*}}^{\mathrm{MASP1_{zym}}}}\\&+\frac{\left[\mathrm{pCLLKMASP2_{zym}}\right]}{K_{\mathrm{m\ MASP1^*}}^{\mathrm{MASP2_{zym}}}}+\frac{\left[\mathrm{pCLLKMASP3_{zym}}\right]}{K_{\mathrm{m\ MASP1^*}}^{\mathrm{MASP3_{zym}}}}+\frac{\left[\mathrm{pCLK1MASP1_{zym}}\right]}{K_{\mathrm{m\ MASP1^*}}^{\mathrm{MASP1_{zym}}}}+\frac{\left[\mathrm{pCLK1MASP2_{zym}}\right]}{K_{\mathrm{m\ MASP1^*}}^{\mathrm{MASP2_{zym}}}}\\&+\frac{\left[\mathrm{pCLK1MASP3_{zym}}\right]}{K_{\mathrm{m\ MASP1^*}}^{\mathrm{MASP3_{zym}}}}+\frac{\left[\mathrm{pCLL1MASP1_{zym}}\right]}{K_{\mathrm{m\ MASP1^*}}^{\mathrm{MASP1_{zym}}}}+\frac{\left[\mathrm{pCLL1MASP2_{zym}}\right]}{K_{\mathrm{m\ MASP1^*}}^{\mathrm{MASP2_{zym}}}}+\frac{\left[\mathrm{pCLL1MASP3_{zym}}\right]}{K_{\mathrm{m\ MASP1^*}}^{\mathrm{MASP3_{zym}}}}\\&+\frac{\left[\mathrm{pLFMASP1_{zym}}\right]}{K_{\mathrm{m\ MASP1^*}}^{\mathrm{MASP1_{zym}}}}+\frac{\left[\mathrm{pLFMASP2_{zym}}\right]}{K_{\mathrm{m\ MASP1^*}}^{\mathrm{MASP2_{zym}}}}+\frac{\left[\mathrm{pLFMASP3_{zym}}\right]}{K_{\mathrm{m\ MASP1^*}}^{\mathrm{MASP3_{zym}}}}+\frac{\left[\mathrm{pHFMASP1_{zym}}\right]}{K_{\mathrm{m\ MASP1^*}}^{\mathrm{MASP1_{zym}}}}+\frac{\left[\mathrm{pHFMASP2_{zym}}\right]}{K_{\mathrm{m\ MASP1^*}}^{\mathrm{MASP2_{zym}}}}\\&+\frac{\left[\mathrm{pHFMASP3_{zym}}\right]}{K_{\mathrm{m\ MASP1^*}}^{\mathrm{MASP3_{zym}}}}+\frac{\left[\mathrm{pMFMASP1_{zym}}\right]}{K_{\mathrm{m\ MASP1^*}}^{\mathrm{MASP1_{zym}}}}+\frac{\left[\mathrm{pMFMASP2_{zym}}\right]}{K_{\mathrm{m\ MASP1^*}}^{\mathrm{MASP2_{zym}}}}+\frac{\left[\mathrm{pMFMASP3_{zym}}\right]}{K_{\mathrm{m\ MASP1^*}}^{\mathrm{MASP3_{zym}}}}\\&+\frac{\left[\mathrm{pPTX3LFMASP1_{zym}}\right]}{K_{\mathrm{m\ MASP1^*}}^{\mathrm{MASP1_{zym}}}}+\frac{\left[\mathrm{pPXT3LFMASP2_{zym}}\right]}{K_{\mathrm{m\ MASP1^*}}^{\mathrm{MASP2_{zym}}}}+\frac{\left[\mathrm{pPTX3LFMASP3_{zym}}\right]}{K_{\mathrm{m\ MASP1^*}}^{\mathrm{MASP3_{zym}}}}\end{aligned}\right)}$$

$$
-\frac{k_{\mathrm{cat\ MASP1^*}}^{\mathrm{MASP3_{zym}}}\left[\mathrm{pCLK1MASP1^*}\right]\left[\mathrm{pMBL_4MASP3_{zym}}\right]}{K_{\mathrm{m\ MASP1^*}}^{\mathrm{MASP3_{zym}}}\left(\begin{array}{l}
1+\frac{[\mathrm{C4}]}{K_{\mathrm{m\ MASP1^*}}^{\mathrm{C4}}}+\frac{[\mathrm{fC4bC2}]}{K_{\mathrm{m\ MASP1^*}}^{\mathrm{C2}}}+\frac{[\mathrm{fC4bC4bC2}]}{K_{\mathrm{m\ MASP1^*}}^{\mathrm{C2}}}+\frac{[\mathrm{fC3bC4bC2}]}{K_{\mathrm{m\ MASP1^*}}^{\mathrm{C2}}}+\frac{[\mathrm{pC4bC2}]}{K_{\mathrm{m\ MASP1^*}}^{\mathrm{C2}}}+\frac{[\mathrm{pC4bC4bC2}]}{K_{\mathrm{m\ MASP1^*}}^{\mathrm{C2}}}\\
+\frac{[\mathrm{pC3bC4bC2}]}{K_{\mathrm{m\ MASP1^*}}^{\mathrm{C2}}}+\frac{[\mathrm{pMBL_3MASP1_{zym}}]}{K_{\mathrm{m\ MASP1^*}}^{\mathrm{MASP1_{zym}}}}+\frac{[\mathrm{pMBL_3MASP2_{zym}}]}{K_{\mathrm{m\ MASP1^*}}^{\mathrm{MASP2_{zym}}}}+\frac{[\mathrm{pMBL_3MASP3_{zym}}]}{K_{\mathrm{m\ MASP1^*}}^{\mathrm{MASP3_{zym}}}}\\
+\frac{[\mathrm{pMBL_4MASP1_{zym}}]}{K_{\mathrm{m\ MASP1^*}}^{\mathrm{MASP1_{zym}}}}+\frac{[\mathrm{pMBL_4MASP2_{zym}}]}{K_{\mathrm{m\ MASP1^*}}^{\mathrm{MASP2_{zym}}}}+\frac{[\mathrm{pMBL_4MASP3_{zym}}]}{K_{\mathrm{m\ MASP1^*}}^{\mathrm{MASP3_{zym}}}}+\frac{[\mathrm{pCLLKMASP1_{zym}}]}{K_{\mathrm{m\ MASP1^*}}^{\mathrm{MASP1_{zym}}}}\\
+\frac{[\mathrm{pCLLKMASP2_{zym}}]}{K_{\mathrm{m\ MASP1^*}}^{\mathrm{MASP2_{zym}}}}+\frac{[\mathrm{pCLLKMASP3_{zym}}]}{K_{\mathrm{m\ MASP1^*}}^{\mathrm{MASP3_{zym}}}}+\frac{[\mathrm{pCLK1MASP1_{zym}}]}{K_{\mathrm{m\ MASP1^*}}^{\mathrm{MASP1_{zym}}}}+\frac{[\mathrm{pCLK1MASP2_{zym}}]}{K_{\mathrm{m\ MASP1^*}}^{\mathrm{MASP2_{zym}}}}\\
+\frac{[\mathrm{pCLK1MASP3_{zym}}]}{K_{\mathrm{m\ MASP1^*}}^{\mathrm{MASP3_{zym}}}}+\frac{[\mathrm{pCLL1MASP1_{zym}}]}{K_{\mathrm{m\ MASP1^*}}^{\mathrm{MASP1_{zym}}}}+\frac{[\mathrm{pCLL1MASP2_{zym}}]}{K_{\mathrm{m\ MASP1^*}}^{\mathrm{MASP2_{zym}}}}+\frac{[\mathrm{pCLL1MASP3_{zym}}]}{K_{\mathrm{m\ MASP1^*}}^{\mathrm{MASP3_{zym}}}}\\
+\frac{[\mathrm{pLFMASP1_{zym}}]}{K_{\mathrm{m\ MASP1^*}}^{\mathrm{MASP1_{zym}}}}+\frac{[\mathrm{pLFMASP2_{zym}}]}{K_{\mathrm{m\ MASP1^*}}^{\mathrm{MASP2_{zym}}}}+\frac{[\mathrm{pLFMASP3_{zym}}]}{K_{\mathrm{m\ MASP1^*}}^{\mathrm{MASP3_{zym}}}}+\frac{[\mathrm{pHFMASP1_{zym}}]}{K_{\mathrm{m\ MASP1^*}}^{\mathrm{MASP1_{zym}}}}+\frac{[\mathrm{pHFMASP2_{zym}}]}{K_{\mathrm{m\ MASP1^*}}^{\mathrm{MASP2_{zym}}}}\\
+\frac{[\mathrm{pHFMASP3_{zym}}]}{K_{\mathrm{m\ MASP1^*}}^{\mathrm{MASP3_{zym}}}}+\frac{[\mathrm{pMFMASP1_{zym}}]}{K_{\mathrm{m\ MASP1^*}}^{\mathrm{MASP1_{zym}}}}+\frac{[\mathrm{pMFMASP2_{zym}}]}{K_{\mathrm{m\ MASP1^*}}^{\mathrm{MASP2_{zym}}}}+\frac{[\mathrm{pMFMASP3_{zym}}]}{K_{\mathrm{m\ MASP1^*}}^{\mathrm{MASP3_{zym}}}}\\
+\frac{[\mathrm{pPTX3LFMASP1_{zym}}]}{K_{\mathrm{m\ MASP1^*}}^{\mathrm{MASP1_{zym}}}}+\frac{[\mathrm{pPXT3LFMASP2_{zym}}]}{K_{\mathrm{m\ MASP1^*}}^{\mathrm{MASP2_{zym}}}}+\frac{[\mathrm{pPTX3LFMASP3_{zym}}]}{K_{\mathrm{m\ MASP1^*}}^{\mathrm{MASP3_{zym}}}}
\end{array}\right)}
$$

$$-\frac{k_{\text{cat MASP1}^*}^{\text{MASP3}_{\text{zym}}}\left[\text{pCLL1MASP1}^*\right]\left[\text{pMBL}_4\text{MASP3}_{\text{zym}}\right]}{K_{\text{m MASP1}^*}^{\text{MASP3}_{\text{zym}}}\left(\begin{array}{l}1+\frac{[\text{C4}]}{K_{\text{m MASP1}^*}^{\text{C4}}}+\frac{[\text{fC4bC2}]}{K_{\text{m MASP1}^*}^{\text{C2}}}+\frac{[\text{fC4bC4bC2}]}{K_{\text{m MASP1}^*}^{\text{C2}}}+\frac{[\text{fC3bC4bC2}]}{K_{\text{m MASP1}^*}^{\text{C2}}}+\frac{[\text{pC4bC2}]}{K_{\text{m MASP1}^*}^{\text{C2}}}+\frac{[\text{pC4bC4bC2}]}{K_{\text{m MASP1}^*}^{\text{C2}}}\\+\frac{[\text{pC3bC4bC2}]}{K_{\text{m MASP1}^*}^{\text{C2}}}+\frac{[\text{pMBL}_3\text{MASP1}_{\text{zym}}]}{K_{\text{m MASP1}^*}^{\text{MASP1}_{\text{zym}}}}+\frac{[\text{pMBL}_3\text{MASP2}_{\text{zym}}]}{K_{\text{m MASP1}^*}^{\text{MASP2}_{\text{zym}}}}+\frac{[\text{pMBL}_3\text{MASP3}_{\text{zym}}]}{K_{\text{m MASP1}^*}^{\text{MASP3}_{\text{zym}}}}\\+\frac{[\text{pMBL}_4\text{MASP1}_{\text{zym}}]}{K_{\text{m MASP1}^*}^{\text{MASP1}_{\text{zym}}}}+\frac{[\text{pMBL}_4\text{MASP2}_{\text{zym}}]}{K_{\text{m MASP1}^*}^{\text{MASP2}_{\text{zym}}}}+\frac{[\text{pMBL}_4\text{MASP3}_{\text{zym}}]}{K_{\text{m MASP1}^*}^{\text{MASP3}_{\text{zym}}}}+\frac{[\text{pCLLKMASP1}_{\text{zym}}]}{K_{\text{m MASP1}^*}^{\text{MASP1}_{\text{zym}}}}\\+\frac{[\text{pCLLKMASP2}_{\text{zym}}]}{K_{\text{m MASP1}^*}^{\text{MASP2}_{\text{zym}}}}+\frac{[\text{pCLLKMASP3}_{\text{zym}}]}{K_{\text{m MASP1}^*}^{\text{MASP3}_{\text{zym}}}}+\frac{[\text{pCLK1MASP1}_{\text{zym}}]}{K_{\text{m MASP1}^*}^{\text{MASP1}_{\text{zym}}}}+\frac{[\text{pCLK1MASP2}_{\text{zym}}]}{K_{\text{m MASP1}^*}^{\text{MASP2}_{\text{zym}}}}\\+\frac{[\text{pCLK1MASP3}_{\text{zym}}]}{K_{\text{m MASP1}^*}^{\text{MASP3}_{\text{zym}}}}+\frac{[\text{pCLL1MASP1}_{\text{zym}}]}{K_{\text{m MASP1}^*}^{\text{MASP1}_{\text{zym}}}}+\frac{[\text{pCLL1MASP2}_{\text{zym}}]}{K_{\text{m MASP1}^*}^{\text{MASP2}_{\text{zym}}}}+\frac{[\text{pCLL1MASP3}_{\text{zym}}]}{K_{\text{m MASP1}^*}^{\text{MASP3}_{\text{zym}}}}\\+\frac{[\text{pLFMASP1}_{\text{zym}}]}{K_{\text{m MASP1}^*}^{\text{MASP1}_{\text{zym}}}}+\frac{[\text{pLFMASP2}_{\text{zym}}]}{K_{\text{m MASP1}^*}^{\text{MASP2}_{\text{zym}}}}+\frac{[\text{pLFMASP3}_{\text{zym}}]}{K_{\text{m MASP1}^*}^{\text{MASP3}_{\text{zym}}}}+\frac{[\text{pHFMASP1}_{\text{zym}}]}{K_{\text{m MASP1}^*}^{\text{MASP1}_{\text{zym}}}}+\frac{[\text{pHFMASP2}_{\text{zym}}]}{K_{\text{m MASP1}^*}^{\text{MASP2}_{\text{zym}}}}\\+\frac{[\text{pHFMASP3}_{\text{zym}}]}{K_{\text{m MASP1}^*}^{\text{MASP3}_{\text{zym}}}}+\frac{[\text{pMFMASP1}_{\text{zym}}]}{K_{\text{m MASP1}^*}^{\text{MASP1}_{\text{zym}}}}+\frac{[\text{pMFMASP2}_{\text{zym}}]}{K_{\text{m MASP1}^*}^{\text{MASP2}_{\text{zym}}}}+\frac{[\text{pMFMASP3}_{\text{zym}}]}{K_{\text{m MASP1}^*}^{\text{MASP3}_{\text{zym}}}}\\+\frac{[\text{pPTX3LFMASP1}_{\text{zym}}]}{K_{\text{m MASP1}^*}^{\text{MASP1}_{\text{zym}}}}+\frac{[\text{pPXT3LFMASP2}_{\text{zym}}]}{K_{\text{m MASP1}^*}^{\text{MASP2}_{\text{zym}}}}+\frac{[\text{pPTX3LFMASP3}_{\text{zym}}]}{K_{\text{m MASP1}^*}^{\text{MASP3}_{\text{zym}}}}\end{array}\right)}$$

$$-\frac{k_{\text{cat MASP1}^*}^{\text{MASP3}_{\text{zym}}}\left[\text{pLFMASP1}^*\right]\left[\text{pMBL}_4\text{MASP3}_{\text{zym}}\right]}{K_{\text{m MASP1}^*}^{\text{MASP3}_{\text{zym}}}\left(\begin{array}{l}1+\frac{[\text{C4}]}{K_{\text{m MASP1}^*}^{\text{C4}}}+\frac{[\text{fC4bC2}]}{K_{\text{m MASP1}^*}^{\text{C2}}}+\frac{[\text{fC4bC4bC2}]}{K_{\text{m MASP1}^*}^{\text{C2}}}+\frac{[\text{fC3bC4bC2}]}{K_{\text{m MASP1}^*}^{\text{C2}}}+\frac{[\text{pC4bC2}]}{K_{\text{m MASP1}^*}^{\text{C2}}}+\frac{[\text{pC4bC4bC2}]}{K_{\text{m MASP1}^*}^{\text{C2}}}\\ +\frac{[\text{pC3bC4bC2}]}{K_{\text{m MASP1}^*}^{\text{C2}}}+\frac{[\text{pMBL}_3\text{MASP1}_{\text{zym}}]}{K_{\text{m MASP1}^*}^{\text{MASP1}_{\text{zym}}}}+\frac{[\text{pMBL}_3\text{MASP2}_{\text{zym}}]}{K_{\text{m MASP1}^*}^{\text{MASP2}_{\text{zym}}}}+\frac{[\text{pMBL}_3\text{MASP3}_{\text{zym}}]}{K_{\text{m MASP1}^*}^{\text{MASP3}_{\text{zym}}}}\\ +\frac{[\text{pMBL}_4\text{MASP1}_{\text{zym}}]}{K_{\text{m MASP1}^*}^{\text{MASP1}_{\text{zym}}}}+\frac{[\text{pMBL}_4\text{MASP2}_{\text{zym}}]}{K_{\text{m MASP1}^*}^{\text{MASP2}_{\text{zym}}}}+\frac{[\text{pMBL}_4\text{MASP3}_{\text{zym}}]}{K_{\text{m MASP1}^*}^{\text{MASP3}_{\text{zym}}}}+\frac{[\text{pCLLKMASP1}_{\text{zym}}]}{K_{\text{m MASP1}^*}^{\text{MASP1}_{\text{zym}}}}\\ +\frac{[\text{pCLLKMASP2}_{\text{zym}}]}{K_{\text{m MASP1}^*}^{\text{MASP2}_{\text{zym}}}}+\frac{[\text{pCLLKMASP3}_{\text{zym}}]}{K_{\text{m MASP1}^*}^{\text{MASP3}_{\text{zym}}}}+\frac{[\text{pCLK1MASP1}_{\text{zym}}]}{K_{\text{m MASP1}^*}^{\text{MASP1}_{\text{zym}}}}+\frac{[\text{pCLK1MASP2}_{\text{zym}}]}{K_{\text{m MASP1}^*}^{\text{MASP2}_{\text{zym}}}}\\ +\frac{[\text{pCLK1MASP3}_{\text{zym}}]}{K_{\text{m MASP1}^*}^{\text{MASP3}_{\text{zym}}}}+\frac{[\text{pCLL1MASP1}_{\text{zym}}]}{K_{\text{m MASP1}^*}^{\text{MASP1}_{\text{zym}}}}+\frac{[\text{pCLL1MASP2}_{\text{zym}}]}{K_{\text{m MASP1}^*}^{\text{MASP2}_{\text{zym}}}}+\frac{[\text{pCLL1MASP3}_{\text{zym}}]}{K_{\text{m MASP1}^*}^{\text{MASP3}_{\text{zym}}}}\\ +\frac{[\text{pLFMASP1}_{\text{zym}}]}{K_{\text{m MASP1}^*}^{\text{MASP1}_{\text{zym}}}}+\frac{[\text{pLFMASP2}_{\text{zym}}]}{K_{\text{m MASP1}^*}^{\text{MASP2}_{\text{zym}}}}+\frac{[\text{pLFMASP3}_{\text{zym}}]}{K_{\text{m MASP1}^*}^{\text{MASP3}_{\text{zym}}}}+\frac{[\text{pHFMASP1}_{\text{zym}}]}{K_{\text{m MASP1}^*}^{\text{MASP1}_{\text{zym}}}}+\frac{[\text{pHFMASP2}_{\text{zym}}]}{K_{\text{m MASP1}^*}^{\text{MASP2}_{\text{zym}}}}\\ +\frac{[\text{pHFMASP3}_{\text{zym}}]}{K_{\text{m MASP1}^*}^{\text{MASP3}_{\text{zym}}}}+\frac{[\text{pMFMASP1}_{\text{zym}}]}{K_{\text{m MASP1}^*}^{\text{MASP1}_{\text{zym}}}}+\frac{[\text{pMFMASP2}_{\text{zym}}]}{K_{\text{m MASP1}^*}^{\text{MASP2}_{\text{zym}}}}+\frac{[\text{pMFMASP3}_{\text{zym}}]}{K_{\text{m MASP1}^*}^{\text{MASP3}_{\text{zym}}}}\\ +\frac{[\text{pPTX3LFMASP1}_{\text{zym}}]}{K_{\text{m MASP1}^*}^{\text{MASP1}_{\text{zym}}}}+\frac{[\text{pPXT3LFMASP2}_{\text{zym}}]}{K_{\text{m MASP1}^*}^{\text{MASP2}_{\text{zym}}}}+\frac{[\text{pPTX3LFMASP3}_{\text{zym}}]}{K_{\text{m MASP1}^*}^{\text{MASP3}_{\text{zym}}}}\end{array}\right)}$$

$$
-\frac{k_{\text{cat MASP1}^*}^{\text{MASP3}_{\text{zym}}}\left[\text{pHFMASP1}^*\right]\left[\text{pMBL}_4\text{MASP3}_{\text{zym}}\right]}{K_{\text{m MASP1}^*}^{\text{MASP3}_{\text{zym}}}\left(\begin{array}{l}
1+\frac{[\text{C4}]}{K_{\text{m MASP1}^*}^{\text{C4}}}+\frac{[\text{fC4bC2}]}{K_{\text{m MASP1}^*}^{\text{C2}}}+\frac{[\text{fC4bC4bC2}]}{K_{\text{m MASP1}^*}^{\text{C2}}}+\frac{[\text{fC3bC4bC2}]}{K_{\text{m MASP1}^*}^{\text{C2}}}+\frac{[\text{pC4bC2}]}{K_{\text{m MASP1}^*}^{\text{C2}}}+\frac{[\text{pC4bC4bC2}]}{K_{\text{m MASP1}^*}^{\text{C2}}}\\
+\frac{[\text{pC3bC4bC2}]}{K_{\text{m MASP1}^*}^{\text{C2}}}+\frac{\left[\text{pMBL}_3\text{MASP1}_{\text{zym}}\right]}{K_{\text{m MASP1}^*}^{\text{MASP1}_{\text{zym}}}}+\frac{\left[\text{pMBL}_3\text{MASP2}_{\text{zym}}\right]}{K_{\text{m MASP1}^*}^{\text{MASP2}_{\text{zym}}}}+\frac{\left[\text{pMBL}_3\text{MASP3}_{\text{zym}}\right]}{K_{\text{m MASP1}^*}^{\text{MASP3}_{\text{zym}}}}\\
+\frac{\left[\text{pMBL}_4\text{MASP1}_{\text{zym}}\right]}{K_{\text{m MASP1}^*}^{\text{MASP1}_{\text{zym}}}}+\frac{\left[\text{pMBL}_4\text{MASP2}_{\text{zym}}\right]}{K_{\text{m MASP1}^*}^{\text{MASP2}_{\text{zym}}}}+\frac{\left[\text{pMBL}_4\text{MASP3}_{\text{zym}}\right]}{K_{\text{m MASP1}^*}^{\text{MASP3}_{\text{zym}}}}+\frac{\left[\text{pCLLKMASP1}_{\text{zym}}\right]}{K_{\text{m MASP1}^*}^{\text{MASP1}_{\text{zym}}}}\\
+\frac{\left[\text{pCLLKMASP2}_{\text{zym}}\right]}{K_{\text{m MASP1}^*}^{\text{MASP2}_{\text{zym}}}}+\frac{\left[\text{pCLLKMASP3}_{\text{zym}}\right]}{K_{\text{m MASP1}^*}^{\text{MASP3}_{\text{zym}}}}+\frac{\left[\text{pCLK1MASP1}_{\text{zym}}\right]}{K_{\text{m MASP1}^*}^{\text{MASP1}_{\text{zym}}}}+\frac{\left[\text{pCLK1MASP2}_{\text{zym}}\right]}{K_{\text{m MASP1}^*}^{\text{MASP2}_{\text{zym}}}}\\
+\frac{\left[\text{pCLK1MASP3}_{\text{zym}}\right]}{K_{\text{m MASP1}^*}^{\text{MASP3}_{\text{zym}}}}+\frac{\left[\text{pCLL1MASP1}_{\text{zym}}\right]}{K_{\text{m MASP1}^*}^{\text{MASP1}_{\text{zym}}}}+\frac{\left[\text{pCLL1MASP2}_{\text{zym}}\right]}{K_{\text{m MASP1}^*}^{\text{MASP2}_{\text{zym}}}}+\frac{\left[\text{pCLL1MASP3}_{\text{zym}}\right]}{K_{\text{m MASP1}^*}^{\text{MASP3}_{\text{zym}}}}\\
+\frac{\left[\text{pLFMASP1}_{\text{zym}}\right]}{K_{\text{m MASP1}^*}^{\text{MASP1}_{\text{zym}}}}+\frac{\left[\text{pLFMASP2}_{\text{zym}}\right]}{K_{\text{m MASP1}^*}^{\text{MASP2}_{\text{zym}}}}+\frac{\left[\text{pLFMASP3}_{\text{zym}}\right]}{K_{\text{m MASP1}^*}^{\text{MASP3}_{\text{zym}}}}+\frac{\left[\text{pHFMASP1}_{\text{zym}}\right]}{K_{\text{m MASP1}^*}^{\text{MASP1}_{\text{zym}}}}+\frac{\left[\text{pHFMASP2}_{\text{zym}}\right]}{K_{\text{m MASP1}^*}^{\text{MASP2}_{\text{zym}}}}\\
+\frac{\left[\text{pHFMASP3}_{\text{zym}}\right]}{K_{\text{m MASP1}^*}^{\text{MASP3}_{\text{zym}}}}+\frac{\left[\text{pMFMASP1}_{\text{zym}}\right]}{K_{\text{m MASP1}^*}^{\text{MASP1}_{\text{zym}}}}+\frac{\left[\text{pMFMASP2}_{\text{zym}}\right]}{K_{\text{m MASP1}^*}^{\text{MASP2}_{\text{zym}}}}+\frac{\left[\text{pMFMASP3}_{\text{zym}}\right]}{K_{\text{m MASP1}^*}^{\text{MASP3}_{\text{zym}}}}\\
+\frac{\left[\text{pPTX3LFMASP1}_{\text{zym}}\right]}{K_{\text{m MASP1}^*}^{\text{MASP1}_{\text{zym}}}}+\frac{\left[\text{pPXT3LFMASP2}_{\text{zym}}\right]}{K_{\text{m MASP1}^*}^{\text{MASP2}_{\text{zym}}}}+\frac{\left[\text{pPTX3LFMASP3}_{\text{zym}}\right]}{K_{\text{m MASP1}^*}^{\text{MASP3}_{\text{zym}}}}
\end{array}\right)}
$$

$$
-\frac{k_{\text{cat MASP1}^*}^{\text{MASP3}_{\text{zym}}}\left[\text{pMFMASP1}^*\right]\left[\text{pMBL}_4\text{MASP3}_{\text{zym}}\right]}{K_{\text{m MASP1}^*}^{\text{MASP3}_{\text{zym}}}\left(\begin{aligned}&1+\frac{[\text{C4}]}{K_{\text{m MASP1}^*}^{\text{C4}}}+\frac{[\text{fC4bC2}]}{K_{\text{m MASP1}^*}^{\text{C2}}}+\frac{[\text{fC4bC4bC2}]}{K_{\text{m MASP1}^*}^{\text{C2}}}+\frac{[\text{fC3bC4bC2}]}{K_{\text{m MASP1}^*}^{\text{C2}}}+\frac{[\text{pC4bC2}]}{K_{\text{m MASP1}^*}^{\text{C2}}}+\frac{[\text{pC4bC4bC2}]}{K_{\text{m MASP1}^*}^{\text{C2}}}\\
&+\frac{[\text{pC3bC4bC2}]}{K_{\text{m MASP1}^*}^{\text{C2}}}+\frac{\left[\text{pMBL}_3\text{MASP1}_{\text{zym}}\right]}{K_{\text{m MASP1}^*}^{\text{MASP1}_{\text{zym}}}}+\frac{\left[\text{pMBL}_3\text{MASP2}_{\text{zym}}\right]}{K_{\text{m MASP1}^*}^{\text{MASP2}_{\text{zym}}}}+\frac{\left[\text{pMBL}_3\text{MASP3}_{\text{zym}}\right]}{K_{\text{m MASP1}^*}^{\text{MASP3}_{\text{zym}}}}\\
&+\frac{\left[\text{pMBL}_4\text{MASP1}_{\text{zym}}\right]}{K_{\text{m MASP1}^*}^{\text{MASP1}_{\text{zym}}}}+\frac{\left[\text{pMBL}_4\text{MASP2}_{\text{zym}}\right]}{K_{\text{m MASP1}^*}^{\text{MASP2}_{\text{zym}}}}+\frac{\left[\text{pMBL}_4\text{MASP3}_{\text{zym}}\right]}{K_{\text{m MASP1}^*}^{\text{MASP3}_{\text{zym}}}}+\frac{\left[\text{pCLLKMASP1}_{\text{zym}}\right]}{K_{\text{m MASP1}^*}^{\text{MASP1}_{\text{zym}}}}\\
&+\frac{\left[\text{pCLLKMASP2}_{\text{zym}}\right]}{K_{\text{m MASP1}^*}^{\text{MASP2}_{\text{zym}}}}+\frac{\left[\text{pCLLKMASP3}_{\text{zym}}\right]}{K_{\text{m MASP1}^*}^{\text{MASP3}_{\text{zym}}}}+\frac{\left[\text{pCLK1MASP1}_{\text{zym}}\right]}{K_{\text{m MASP1}^*}^{\text{MASP1}_{\text{zym}}}}+\frac{\left[\text{pCLK1MASP2}_{\text{zym}}\right]}{K_{\text{m MASP1}^*}^{\text{MASP2}_{\text{zym}}}}\\
&+\frac{\left[\text{pCLK1MASP3}_{\text{zym}}\right]}{K_{\text{m MASP1}^*}^{\text{MASP3}_{\text{zym}}}}+\frac{\left[\text{pCLL1MASP1}_{\text{zym}}\right]}{K_{\text{m MASP1}^*}^{\text{MASP1}_{\text{zym}}}}+\frac{\left[\text{pCLL1MASP2}_{\text{zym}}\right]}{K_{\text{m MASP1}^*}^{\text{MASP2}_{\text{zym}}}}+\frac{\left[\text{pCLL1MASP3}_{\text{zym}}\right]}{K_{\text{m MASP1}^*}^{\text{MASP3}_{\text{zym}}}}\\
&+\frac{\left[\text{pLFMASP1}_{\text{zym}}\right]}{K_{\text{m MASP1}^*}^{\text{MASP1}_{\text{zym}}}}+\frac{\left[\text{pLFMASP2}_{\text{zym}}\right]}{K_{\text{m MASP1}^*}^{\text{MASP2}_{\text{zym}}}}+\frac{\left[\text{pLFMASP3}_{\text{zym}}\right]}{K_{\text{m MASP1}^*}^{\text{MASP3}_{\text{zym}}}}+\frac{\left[\text{pHFMASP1}_{\text{zym}}\right]}{K_{\text{m MASP1}^*}^{\text{MASP1}_{\text{zym}}}}+\frac{\left[\text{pHFMASP2}_{\text{zym}}\right]}{K_{\text{m MASP1}^*}^{\text{MASP2}_{\text{zym}}}}\\
&+\frac{\left[\text{pHFMASP3}_{\text{zym}}\right]}{K_{\text{m MASP1}^*}^{\text{MASP3}_{\text{zym}}}}+\frac{\left[\text{pMFMASP1}_{\text{zym}}\right]}{K_{\text{m MASP1}^*}^{\text{MASP1}_{\text{zym}}}}+\frac{\left[\text{pMFMASP2}_{\text{zym}}\right]}{K_{\text{m MASP1}^*}^{\text{MASP2}_{\text{zym}}}}+\frac{\left[\text{pMFMASP3}_{\text{zym}}\right]}{K_{\text{m MASP1}^*}^{\text{MASP3}_{\text{zym}}}}\\
&+\frac{\left[\text{pPTX3LFMASP1}_{\text{zym}}\right]}{K_{\text{m MASP1}^*}^{\text{MASP1}_{\text{zym}}}}+\frac{\left[\text{pPXT3LFMASP2}_{\text{zym}}\right]}{K_{\text{m MASP1}^*}^{\text{MASP2}_{\text{zym}}}}+\frac{\left[\text{pPTX3LFMASP3}_{\text{zym}}\right]}{K_{\text{m MASP1}^*}^{\text{MASP3}_{\text{zym}}}}\end{aligned}\right)}
$$

$$
-\frac{k_{\text{cat MASP1}^*}^{\text{MASP3}_{\text{zym}}}\left[\text{pPTX3LFMASP1}^*\right]\left[\text{pMBL}_4\text{MASP3}_{\text{zym}}\right]}{K_{\text{m MASP1}^*}^{\text{MASP3}_{\text{zym}}}\left(\begin{array}{l}
1+\frac{[\text{C4}]}{K_{\text{m MASP1}^*}^{\text{C4}}}+\frac{[\text{fC4bC2}]}{K_{\text{m MASP1}^*}^{\text{C2}}}+\frac{[\text{fC4bC4bC2}]}{K_{\text{m MASP1}^*}^{\text{C2}}}+\frac{[\text{fC3bC4bC2}]}{K_{\text{m MASP1}^*}^{\text{C2}}}+\frac{[\text{pC4bC2}]}{K_{\text{m MASP1}^*}^{\text{C2}}}+\frac{[\text{pC4bC4bC2}]}{K_{\text{m MASP1}^*}^{\text{C2}}}\\
+\frac{[\text{pC3bC4bC2}]}{K_{\text{m MASP1}^*}^{\text{C2}}}+\frac{\left[\text{pMBL}_3\text{MASP1}_{\text{zym}}\right]}{K_{\text{m MASP1}^*}^{\text{MASP1}_{\text{zym}}}}+\frac{\left[\text{pMBL}_3\text{MASP2}_{\text{zym}}\right]}{K_{\text{m MASP1}^*}^{\text{MASP2}_{\text{zym}}}}+\frac{\left[\text{pMBL}_3\text{MASP3}_{\text{zym}}\right]}{K_{\text{m MASP1}^*}^{\text{MASP3}_{\text{zym}}}}\\
+\frac{\left[\text{pMBL}_4\text{MASP1}_{\text{zym}}\right]}{K_{\text{m MASP1}^*}^{\text{MASP1}_{\text{zym}}}}+\frac{\left[\text{pMBL}_4\text{MASP2}_{\text{zym}}\right]}{K_{\text{m MASP1}^*}^{\text{MASP2}_{\text{zym}}}}+\frac{\left[\text{pMBL}_4\text{MASP3}_{\text{zym}}\right]}{K_{\text{m MASP1}^*}^{\text{MASP3}_{\text{zym}}}}+\frac{\left[\text{pCLLKMASP1}_{\text{zym}}\right]}{K_{\text{m MASP1}^*}^{\text{MASP1}_{\text{zym}}}}\\
+\frac{\left[\text{pCLLKMASP2}_{\text{zym}}\right]}{K_{\text{m MASP1}^*}^{\text{MASP2}_{\text{zym}}}}+\frac{\left[\text{pCLLKMASP3}_{\text{zym}}\right]}{K_{\text{m MASP1}^*}^{\text{MASP3}_{\text{zym}}}}+\frac{\left[\text{pCLK1MASP1}_{\text{zym}}\right]}{K_{\text{m MASP1}^*}^{\text{MASP1}_{\text{zym}}}}+\frac{\left[\text{pCLK1MASP2}_{\text{zym}}\right]}{K_{\text{m MASP1}^*}^{\text{MASP2}_{\text{zym}}}}\\
+\frac{\left[\text{pCLK1MASP3}_{\text{zym}}\right]}{K_{\text{m MASP1}^*}^{\text{MASP3}_{\text{zym}}}}+\frac{\left[\text{pCLL1MASP1}_{\text{zym}}\right]}{K_{\text{m MASP1}^*}^{\text{MASP1}_{\text{zym}}}}+\frac{\left[\text{pCLL1MASP2}_{\text{zym}}\right]}{K_{\text{m MASP1}^*}^{\text{MASP2}_{\text{zym}}}}+\frac{\left[\text{pCLL1MASP3}_{\text{zym}}\right]}{K_{\text{m MASP1}^*}^{\text{MASP3}_{\text{zym}}}}\\
+\frac{\left[\text{pLFMASP1}_{\text{zym}}\right]}{K_{\text{m MASP1}^*}^{\text{MASP1}_{\text{zym}}}}+\frac{\left[\text{pLFMASP2}_{\text{zym}}\right]}{K_{\text{m MASP1}^*}^{\text{MASP2}_{\text{zym}}}}+\frac{\left[\text{pLFMASP3}_{\text{zym}}\right]}{K_{\text{m MASP1}^*}^{\text{MASP3}_{\text{zym}}}}+\frac{\left[\text{pHFMASP1}_{\text{zym}}\right]}{K_{\text{m MASP1}^*}^{\text{MASP1}_{\text{zym}}}}+\frac{\left[\text{pHFMASP2}_{\text{zym}}\right]}{K_{\text{m MASP1}^*}^{\text{MASP2}_{\text{zym}}}}\\
+\frac{\left[\text{pHFMASP3}_{\text{zym}}\right]}{K_{\text{m MASP1}^*}^{\text{MASP3}_{\text{zym}}}}+\frac{\left[\text{pMFMASP1}_{\text{zym}}\right]}{K_{\text{m MASP1}^*}^{\text{MASP1}_{\text{zym}}}}+\frac{\left[\text{pMFMASP2}_{\text{zym}}\right]}{K_{\text{m MASP1}^*}^{\text{MASP2}_{\text{zym}}}}+\frac{\left[\text{pMFMASP3}_{\text{zym}}\right]}{K_{\text{m MASP1}^*}^{\text{MASP3}_{\text{zym}}}}\\
+\frac{\left[\text{pPTX3LFMASP1}_{\text{zym}}\right]}{K_{\text{m MASP1}^*}^{\text{MASP1}_{\text{zym}}}}+\frac{\left[\text{pPXT3LFMASP2}_{\text{zym}}\right]}{K_{\text{m MASP1}^*}^{\text{MASP2}_{\text{zym}}}}+\frac{\left[\text{pPTX3LFMASP3}_{\text{zym}}\right]}{K_{\text{m MASP1}^*}^{\text{MASP3}_{\text{zym}}}}
\end{array}\right)}
$$

276

$$
\frac{d\left[\text{fMBL}_4\text{MASP3}^*\right]}{dt}=-k_{\text{pMBL}_4}^{+}\left[\text{fMBL}_4\text{MASP3}^*\right]\left[\text{Pathogen Surface}\right]+k_{\text{pMBL}_4}^{-}\left[\text{pMBL}_4\text{MASP3}^*\right]
$$
$$
+k_{\text{MBL}_4\text{MASP3}}^{+}\left[\text{MBL}_4\right]\left[\text{MASP3}^*\right]-k_{\text{MBL}_4\text{MASP3}}^{-}\left[\text{fMBL}_4\text{MASP3}^*\right]
$$

277

$$
\frac{d\left[\text{pMBL}_4\text{MASP3}^*\right]}{dt}=k_{\text{pMBL}_4}^{+}\left[\text{fMBL}_4\text{MASP3}^*\right]\left[\text{Pathogen Surface}\right]-k_{\text{pMBL}_4}^{-}\left[\text{pMBL}_4\text{MASP3}^*\right]
$$
$$
+k_{\text{MBL}_4\text{MASP3}}^{+}\left[\text{pMBL}_4\right]\left[\text{MASP3}^*\right]-k_{\text{MBL}_4\text{MASP3}}^{-}\left[\text{pMBL}_4\text{MASP3}^*\right]
$$

$$+\frac{k_{\mathrm{cat\ MASP1^*}}^{\mathrm{MASP3_{zym}}}\left[\mathrm{pMBL_3MASP1^*}\right]\left[\mathrm{pMBL_4MASP3_{zym}}\right]}{K_{\mathrm{m\ MASP1^*}}^{\mathrm{MASP3_{zym}}}\left(\begin{array}{l}1+\frac{[\mathrm{C4}]}{K_{\mathrm{m\ MASP1^*}}^{\mathrm{C4}}}+\frac{[\mathrm{fC4bC2}]}{K_{\mathrm{m\ MASP1^*}}^{\mathrm{C2}}}+\frac{[\mathrm{fC4bC4bC2}]}{K_{\mathrm{m\ MASP1^*}}^{\mathrm{C2}}}+\frac{[\mathrm{fC3bC4bC2}]}{K_{\mathrm{m\ MASP1^*}}^{\mathrm{C2}}}+\frac{[\mathrm{pC4bC2}]}{K_{\mathrm{m\ MASP1^*}}^{\mathrm{C2}}}+\frac{[\mathrm{pC4bC4bC2}]}{K_{\mathrm{m\ MASP1^*}}^{\mathrm{C2}}}\\
+\frac{[\mathrm{pC3bC4bC2}]}{K_{\mathrm{m\ MASP1^*}}^{\mathrm{C2}}}+\frac{\left[\mathrm{pMBL_3MASP1_{zym}}\right]}{K_{\mathrm{m\ MASP1^*}}^{\mathrm{MASP1_{zym}}}}+\frac{\left[\mathrm{pMBL_3MASP2_{zym}}\right]}{K_{\mathrm{m\ MASP1^*}}^{\mathrm{MASP2_{zym}}}}+\frac{\left[\mathrm{pMBL_3MASP3_{zym}}\right]}{K_{\mathrm{m\ MASP1^*}}^{\mathrm{MASP3_{zym}}}}\\
+\frac{\left[\mathrm{pMBL_4MASP1_{zym}}\right]}{K_{\mathrm{m\ MASP1^*}}^{\mathrm{MASP1_{zym}}}}+\frac{\left[\mathrm{pMBL_4MASP2_{zym}}\right]}{K_{\mathrm{m\ MASP1^*}}^{\mathrm{MASP2_{zym}}}}+\frac{\left[\mathrm{pMBL_4MASP3_{zym}}\right]}{K_{\mathrm{m\ MASP1^*}}^{\mathrm{MASP3_{zym}}}}+\frac{\left[\mathrm{pCLLKMASP1_{zym}}\right]}{K_{\mathrm{m\ MASP1^*}}^{\mathrm{MASP1_{zym}}}}\\
+\frac{\left[\mathrm{pCLLKMASP2_{zym}}\right]}{K_{\mathrm{m\ MASP1^*}}^{\mathrm{MASP2_{zym}}}}+\frac{\left[\mathrm{pCLLKMASP3_{zym}}\right]}{K_{\mathrm{m\ MASP1^*}}^{\mathrm{MASP3_{zym}}}}+\frac{\left[\mathrm{pCLK1MASP1_{zym}}\right]}{K_{\mathrm{m\ MASP1^*}}^{\mathrm{MASP1_{zym}}}}+\frac{\left[\mathrm{pCLK1MASP2_{zym}}\right]}{K_{\mathrm{m\ MASP1^*}}^{\mathrm{MASP2_{zym}}}}\\
+\frac{\left[\mathrm{pCLK1MASP3_{zym}}\right]}{K_{\mathrm{m\ MASP1^*}}^{\mathrm{MASP3_{zym}}}}+\frac{\left[\mathrm{pCLL1MASP1_{zym}}\right]}{K_{\mathrm{m\ MASP1^*}}^{\mathrm{MASP1_{zym}}}}+\frac{\left[\mathrm{pCLL1MASP2_{zym}}\right]}{K_{\mathrm{m\ MASP1^*}}^{\mathrm{MASP2_{zym}}}}+\frac{\left[\mathrm{pCLL1MASP3_{zym}}\right]}{K_{\mathrm{m\ MASP1^*}}^{\mathrm{MASP3_{zym}}}}\\
+\frac{\left[\mathrm{pLFMASP1_{zym}}\right]}{K_{\mathrm{m\ MASP1^*}}^{\mathrm{MASP1_{zym}}}}+\frac{\left[\mathrm{pLFMASP2_{zym}}\right]}{K_{\mathrm{m\ MASP1^*}}^{\mathrm{MASP2_{zym}}}}+\frac{\left[\mathrm{pLFMASP3_{zym}}\right]}{K_{\mathrm{m\ MASP1^*}}^{\mathrm{MASP3_{zym}}}}+\frac{\left[\mathrm{pHFMASP1_{zym}}\right]}{K_{\mathrm{m\ MASP1^*}}^{\mathrm{MASP1_{zym}}}}+\frac{\left[\mathrm{pHFMASP2_{zym}}\right]}{K_{\mathrm{m\ MASP1^*}}^{\mathrm{MASP2_{zym}}}}\\
+\frac{\left[\mathrm{pHFMASP3_{zym}}\right]}{K_{\mathrm{m\ MASP1^*}}^{\mathrm{MASP3_{zym}}}}+\frac{\left[\mathrm{pMFMASP1_{zym}}\right]}{K_{\mathrm{m\ MASP1^*}}^{\mathrm{MASP1_{zym}}}}+\frac{\left[\mathrm{pMFMASP2_{zym}}\right]}{K_{\mathrm{m\ MASP1^*}}^{\mathrm{MASP2_{zym}}}}+\frac{\left[\mathrm{pMFMASP3_{zym}}\right]}{K_{\mathrm{m\ MASP1^*}}^{\mathrm{MASP3_{zym}}}}\\
+\frac{\left[\mathrm{pPTX3LFMASP1_{zym}}\right]}{K_{\mathrm{m\ MASP1^*}}^{\mathrm{MASP1_{zym}}}}+\frac{\left[\mathrm{pPXT3LFMASP2_{zym}}\right]}{K_{\mathrm{m\ MASP1^*}}^{\mathrm{MASP2_{zym}}}}+\frac{\left[\mathrm{pPTX3LFMASP3_{zym}}\right]}{K_{\mathrm{m\ MASP1^*}}^{\mathrm{MASP3_{zym}}}}\end{array}\right)}$$

$$
+\frac{k_{\mathrm{cat\,MASP1^*}}^{\mathrm{MASP3_{zym}}}\left[\mathrm{pMBL_4MASP1^*}\right]\left[\mathrm{pMBL_4MASP3_{zym}}\right]}{K_{\mathrm{m\,MASP1^*}}^{\mathrm{MASP3_{zym}}}\left(\begin{array}{l}
1+\frac{[\mathrm{C4}]}{K_{\mathrm{m\,MASP1^*}}^{\mathrm{C4}}}+\frac{[\mathrm{fC4bC2}]}{K_{\mathrm{m\,MASP1^*}}^{\mathrm{C2}}}+\frac{[\mathrm{fC4bC4bC2}]}{K_{\mathrm{m\,MASP1^*}}^{\mathrm{C2}}}+\frac{[\mathrm{fC3bC4bC2}]}{K_{\mathrm{m\,MASP1^*}}^{\mathrm{C2}}}+\frac{[\mathrm{pC4bC2}]}{K_{\mathrm{m\,MASP1^*}}^{\mathrm{C2}}}+\frac{[\mathrm{pC4bC4bC2}]}{K_{\mathrm{m\,MASP1^*}}^{\mathrm{C2}}}\\
+\frac{[\mathrm{pC3bC4bC2}]}{K_{\mathrm{m\,MASP1^*}}^{\mathrm{C2}}}+\frac{[\mathrm{pMBL_3MASP1_{zym}}]}{K_{\mathrm{m\,MASP1^*}}^{\mathrm{MASP1_{zym}}}}+\frac{[\mathrm{pMBL_3MASP2_{zym}}]}{K_{\mathrm{m\,MASP1^*}}^{\mathrm{MASP2_{zym}}}}+\frac{[\mathrm{pMBL_3MASP3_{zym}}]}{K_{\mathrm{m\,MASP1^*}}^{\mathrm{MASP3_{zym}}}}\\
+\frac{[\mathrm{pMBL_4MASP1_{zym}}]}{K_{\mathrm{m\,MASP1^*}}^{\mathrm{MASP1_{zym}}}}+\frac{[\mathrm{pMBL_4MASP2_{zym}}]}{K_{\mathrm{m\,MASP1^*}}^{\mathrm{MASP2_{zym}}}}+\frac{[\mathrm{pMBL_4MASP3_{zym}}]}{K_{\mathrm{m\,MASP1^*}}^{\mathrm{MASP3_{zym}}}}+\frac{[\mathrm{pCLLKMASP1_{zym}}]}{K_{\mathrm{m\,MASP1^*}}^{\mathrm{MASP1_{zym}}}}\\
+\frac{[\mathrm{pCLLKMASP2_{zym}}]}{K_{\mathrm{m\,MASP1^*}}^{\mathrm{MASP2_{zym}}}}+\frac{[\mathrm{pCLLKMASP3_{zym}}]}{K_{\mathrm{m\,MASP1^*}}^{\mathrm{MASP3_{zym}}}}+\frac{[\mathrm{pCLK1MASP1_{zym}}]}{K_{\mathrm{m\,MASP1^*}}^{\mathrm{MASP1_{zym}}}}+\frac{[\mathrm{pCLK1MASP2_{zym}}]}{K_{\mathrm{m\,MASP1^*}}^{\mathrm{MASP2_{zym}}}}\\
+\frac{[\mathrm{pCLK1MASP3_{zym}}]}{K_{\mathrm{m\,MASP1^*}}^{\mathrm{MASP3_{zym}}}}+\frac{[\mathrm{pCLL1MASP1_{zym}}]}{K_{\mathrm{m\,MASP1^*}}^{\mathrm{MASP1_{zym}}}}+\frac{[\mathrm{pCLL1MASP2_{zym}}]}{K_{\mathrm{m\,MASP1^*}}^{\mathrm{MASP2_{zym}}}}+\frac{[\mathrm{pCLL1MASP3_{zym}}]}{K_{\mathrm{m\,MASP1^*}}^{\mathrm{MASP3_{zym}}}}\\
+\frac{[\mathrm{pLFMASP1_{zym}}]}{K_{\mathrm{m\,MASP1^*}}^{\mathrm{MASP1_{zym}}}}+\frac{[\mathrm{pLFMASP2_{zym}}]}{K_{\mathrm{m\,MASP1^*}}^{\mathrm{MASP2_{zym}}}}+\frac{[\mathrm{pLFMASP3_{zym}}]}{K_{\mathrm{m\,MASP1^*}}^{\mathrm{MASP3_{zym}}}}+\frac{[\mathrm{pHFMASP1_{zym}}]}{K_{\mathrm{m\,MASP1^*}}^{\mathrm{MASP1_{zym}}}}+\frac{[\mathrm{pHFMASP2_{zym}}]}{K_{\mathrm{m\,MASP1^*}}^{\mathrm{MASP2_{zym}}}}\\
+\frac{[\mathrm{pHFMASP3_{zym}}]}{K_{\mathrm{m\,MASP1^*}}^{\mathrm{MASP3_{zym}}}}+\frac{[\mathrm{pMFMASP1_{zym}}]}{K_{\mathrm{m\,MASP1^*}}^{\mathrm{MASP1_{zym}}}}+\frac{[\mathrm{pMFMASP2_{zym}}]}{K_{\mathrm{m\,MASP1^*}}^{\mathrm{MASP2_{zym}}}}+\frac{[\mathrm{pMFMASP3_{zym}}]}{K_{\mathrm{m\,MASP1^*}}^{\mathrm{MASP3_{zym}}}}\\
+\frac{[\mathrm{pPTX3LFMASP1_{zym}}]}{K_{\mathrm{m\,MASP1^*}}^{\mathrm{MASP1_{zym}}}}+\frac{[\mathrm{pPXT3LFMASP2_{zym}}]}{K_{\mathrm{m\,MASP1^*}}^{\mathrm{MASP2_{zym}}}}+\frac{[\mathrm{pPTX3LFMASP3_{zym}}]}{K_{\mathrm{m\,MASP1^*}}^{\mathrm{MASP3_{zym}}}}
\end{array}\right)}
$$

$$
+\frac{k_{\mathrm{cat\ MASP1^{*}}}^{\mathrm{MASP3_{zym}}}\left[\mathrm{pCLLKMASP1^{*}}\right]\left[\mathrm{pMBL_{4}MASP3_{zym}}\right]}{K_{\mathrm{m\ MASP1^{*}}}^{\mathrm{MASP3_{zym}}}\left(\begin{aligned}&1+\frac{[\mathrm{C4}]}{K_{\mathrm{m\ MASP1^{*}}}^{\mathrm{C4}}}+\frac{[\mathrm{fC4bC2}]}{K_{\mathrm{m\ MASP1^{*}}}^{\mathrm{C2}}}+\frac{[\mathrm{fC4bC4bC2}]}{K_{\mathrm{m\ MASP1^{*}}}^{\mathrm{C2}}}+\frac{[\mathrm{fC3bC4bC2}]}{K_{\mathrm{m\ MASP1^{*}}}^{\mathrm{C2}}}+\frac{[\mathrm{pC4bC2}]}{K_{\mathrm{m\ MASP1^{*}}}^{\mathrm{C2}}}+\frac{[\mathrm{pC4bC4bC2}]}{K_{\mathrm{m\ MASP1^{*}}}^{\mathrm{C2}}}\\
&+\frac{[\mathrm{pC3bC4bC2}]}{K_{\mathrm{m\ MASP1^{*}}}^{\mathrm{C2}}}+\frac{\left[\mathrm{pMBL_{3}MASP1_{zym}}\right]}{K_{\mathrm{m\ MASP1^{*}}}^{\mathrm{MASP1_{zym}}}}+\frac{\left[\mathrm{pMBL_{3}MASP2_{zym}}\right]}{K_{\mathrm{m\ MASP1^{*}}}^{\mathrm{MASP2_{zym}}}}+\frac{\left[\mathrm{pMBL_{3}MASP3_{zym}}\right]}{K_{\mathrm{m\ MASP1^{*}}}^{\mathrm{MASP3_{zym}}}}\\
&+\frac{\left[\mathrm{pMBL_{4}MASP1_{zym}}\right]}{K_{\mathrm{m\ MASP1^{*}}}^{\mathrm{MASP1_{zym}}}}+\frac{\left[\mathrm{pMBL_{4}MASP2_{zym}}\right]}{K_{\mathrm{m\ MASP1^{*}}}^{\mathrm{MASP2_{zym}}}}+\frac{\left[\mathrm{pMBL_{4}MASP3_{zym}}\right]}{K_{\mathrm{m\ MASP1^{*}}}^{\mathrm{MASP3_{zym}}}}+\frac{\left[\mathrm{pCLLKMASP1_{zym}}\right]}{K_{\mathrm{m\ MASP1^{*}}}^{\mathrm{MASP1_{zym}}}}\\
&+\frac{\left[\mathrm{pCLLKMASP2_{zym}}\right]}{K_{\mathrm{m\ MASP1^{*}}}^{\mathrm{MASP2_{zym}}}}+\frac{\left[\mathrm{pCLLKMASP3_{zym}}\right]}{K_{\mathrm{m\ MASP1^{*}}}^{\mathrm{MASP3_{zym}}}}+\frac{\left[\mathrm{pCLK1MASP1_{zym}}\right]}{K_{\mathrm{m\ MASP1^{*}}}^{\mathrm{MASP1_{zym}}}}+\frac{\left[\mathrm{pCLK1MASP2_{zym}}\right]}{K_{\mathrm{m\ MASP1^{*}}}^{\mathrm{MASP2_{zym}}}}\\
&+\frac{\left[\mathrm{pCLK1MASP3_{zym}}\right]}{K_{\mathrm{m\ MASP1^{*}}}^{\mathrm{MASP3_{zym}}}}+\frac{\left[\mathrm{pCLL1MASP1_{zym}}\right]}{K_{\mathrm{m\ MASP1^{*}}}^{\mathrm{MASP1_{zym}}}}+\frac{\left[\mathrm{pCLL1MASP2_{zym}}\right]}{K_{\mathrm{m\ MASP1^{*}}}^{\mathrm{MASP2_{zym}}}}+\frac{\left[\mathrm{pCLL1MASP3_{zym}}\right]}{K_{\mathrm{m\ MASP1^{*}}}^{\mathrm{MASP3_{zym}}}}\\
&+\frac{\left[\mathrm{pLFMASP1_{zym}}\right]}{K_{\mathrm{m\ MASP1^{*}}}^{\mathrm{MASP1_{zym}}}}+\frac{\left[\mathrm{pLFMASP2_{zym}}\right]}{K_{\mathrm{m\ MASP1^{*}}}^{\mathrm{MASP2_{zym}}}}+\frac{\left[\mathrm{pLFMASP3_{zym}}\right]}{K_{\mathrm{m\ MASP1^{*}}}^{\mathrm{MASP3_{zym}}}}+\frac{\left[\mathrm{pHFMASP1_{zym}}\right]}{K_{\mathrm{m\ MASP1^{*}}}^{\mathrm{MASP1_{zym}}}}+\frac{\left[\mathrm{pHFMASP2_{zym}}\right]}{K_{\mathrm{m\ MASP1^{*}}}^{\mathrm{MASP2_{zym}}}}\\
&+\frac{\left[\mathrm{pHFMASP3_{zym}}\right]}{K_{\mathrm{m\ MASP1^{*}}}^{\mathrm{MASP3_{zym}}}}+\frac{\left[\mathrm{pMFMASP1_{zym}}\right]}{K_{\mathrm{m\ MASP1^{*}}}^{\mathrm{MASP1_{zym}}}}+\frac{\left[\mathrm{pMFMASP2_{zym}}\right]}{K_{\mathrm{m\ MASP1^{*}}}^{\mathrm{MASP2_{zym}}}}+\frac{\left[\mathrm{pMFMASP3_{zym}}\right]}{K_{\mathrm{m\ MASP1^{*}}}^{\mathrm{MASP3_{zym}}}}\\
&+\frac{\left[\mathrm{pPTX3LFMASP1_{zym}}\right]}{K_{\mathrm{m\ MASP1^{*}}}^{\mathrm{MASP1_{zym}}}}+\frac{\left[\mathrm{pPXT3LFMASP2_{zym}}\right]}{K_{\mathrm{m\ MASP1^{*}}}^{\mathrm{MASP2_{zym}}}}+\frac{\left[\mathrm{pPTX3LFMASP3_{zym}}\right]}{K_{\mathrm{m\ MASP1^{*}}}^{\mathrm{MASP3_{zym}}}}\end{aligned}\right)}
$$

$$+\frac{k_{\text{cat MASP1}^*}^{\text{MASP3}_{\text{zym}}}\left[\text{pCLK1MASP1}^*\right]\left[\text{pMBL}_4\text{MASP3}_{\text{zym}}\right]}{K_{\text{m MASP1}^*}^{\text{MASP3}_{\text{zym}}}\left(\begin{array}{l}1+\frac{[\text{C4}]}{K_{\text{m MASP1}^*}^{\text{C4}}}+\frac{[\text{fC4bC2}]}{K_{\text{m MASP1}^*}^{\text{C2}}}+\frac{[\text{fC4bC4bC2}]}{K_{\text{m MASP1}^*}^{\text{C2}}}+\frac{[\text{fC3bC4bC2}]}{K_{\text{m MASP1}^*}^{\text{C2}}}+\frac{[\text{pC4bC2}]}{K_{\text{m MASP1}^*}^{\text{C2}}}+\frac{[\text{pC4bC4bC2}]}{K_{\text{m MASP1}^*}^{\text{C2}}}\\ +\frac{[\text{pC3bC4bC2}]}{K_{\text{m MASP1}^*}^{\text{C2}}}+\frac{\left[\text{pMBL}_3\text{MASP1}_{\text{zym}}\right]}{K_{\text{m MASP1}^*}^{\text{MASP1}_{\text{zym}}}}+\frac{\left[\text{pMBL}_3\text{MASP2}_{\text{zym}}\right]}{K_{\text{m MASP1}^*}^{\text{MASP2}_{\text{zym}}}}+\frac{\left[\text{pMBL}_3\text{MASP3}_{\text{zym}}\right]}{K_{\text{m MASP1}^*}^{\text{MASP3}_{\text{zym}}}}\\ +\frac{\left[\text{pMBL}_4\text{MASP1}_{\text{zym}}\right]}{K_{\text{m MASP1}^*}^{\text{MASP1}_{\text{zym}}}}+\frac{\left[\text{pMBL}_4\text{MASP2}_{\text{zym}}\right]}{K_{\text{m MASP1}^*}^{\text{MASP2}_{\text{zym}}}}+\frac{\left[\text{pMBL}_4\text{MASP3}_{\text{zym}}\right]}{K_{\text{m MASP1}^*}^{\text{MASP3}_{\text{zym}}}}+\frac{\left[\text{pCLLKMASP1}_{\text{zym}}\right]}{K_{\text{m MASP1}^*}^{\text{MASP1}_{\text{zym}}}}\\ +\frac{\left[\text{pCLLKMASP2}_{\text{zym}}\right]}{K_{\text{m MASP1}^*}^{\text{MASP2}_{\text{zym}}}}+\frac{\left[\text{pCLLKMASP3}_{\text{zym}}\right]}{K_{\text{m MASP1}^*}^{\text{MASP3}_{\text{zym}}}}+\frac{\left[\text{pCLK1MASP1}_{\text{zym}}\right]}{K_{\text{m MASP1}^*}^{\text{MASP1}_{\text{zym}}}}+\frac{\left[\text{pCLK1MASP2}_{\text{zym}}\right]}{K_{\text{m MASP1}^*}^{\text{MASP2}_{\text{zym}}}}\\ +\frac{\left[\text{pCLK1MASP3}_{\text{zym}}\right]}{K_{\text{m MASP1}^*}^{\text{MASP3}_{\text{zym}}}}+\frac{\left[\text{pCLL1MASP1}_{\text{zym}}\right]}{K_{\text{m MASP1}^*}^{\text{MASP1}_{\text{zym}}}}+\frac{\left[\text{pCLL1MASP2}_{\text{zym}}\right]}{K_{\text{m MASP1}^*}^{\text{MASP2}_{\text{zym}}}}+\frac{\left[\text{pCLL1MASP3}_{\text{zym}}\right]}{K_{\text{m MASP1}^*}^{\text{MASP3}_{\text{zym}}}}\\ +\frac{\left[\text{pLFMASP1}_{\text{zym}}\right]}{K_{\text{m MASP1}^*}^{\text{MASP1}_{\text{zym}}}}+\frac{\left[\text{pLFMASP2}_{\text{zym}}\right]}{K_{\text{m MASP1}^*}^{\text{MASP2}_{\text{zym}}}}+\frac{\left[\text{pLFMASP3}_{\text{zym}}\right]}{K_{\text{m MASP1}^*}^{\text{MASP3}_{\text{zym}}}}+\frac{\left[\text{pHFMASP1}_{\text{zym}}\right]}{K_{\text{m MASP1}^*}^{\text{MASP1}_{\text{zym}}}}+\frac{\left[\text{pHFMASP2}_{\text{zym}}\right]}{K_{\text{m MASP1}^*}^{\text{MASP2}_{\text{zym}}}}\\ +\frac{\left[\text{pHFMASP3}_{\text{zym}}\right]}{K_{\text{m MASP1}^*}^{\text{MASP3}_{\text{zym}}}}+\frac{\left[\text{pMFMASP1}_{\text{zym}}\right]}{K_{\text{m MASP1}^*}^{\text{MASP1}_{\text{zym}}}}+\frac{\left[\text{pMFMASP2}_{\text{zym}}\right]}{K_{\text{m MASP1}^*}^{\text{MASP2}_{\text{zym}}}}+\frac{\left[\text{pMFMASP3}_{\text{zym}}\right]}{K_{\text{m MASP1}^*}^{\text{MASP3}_{\text{zym}}}}\\ +\frac{\left[\text{pPTX3LFMASP1}_{\text{zym}}\right]}{K_{\text{m MASP1}^*}^{\text{MASP1}_{\text{zym}}}}+\frac{\left[\text{pPXT3LFMASP2}_{\text{zym}}\right]}{K_{\text{m MASP1}^*}^{\text{MASP2}_{\text{zym}}}}+\frac{\left[\text{pPTX3LFMASP3}_{\text{zym}}\right]}{K_{\text{m MASP1}^*}^{\text{MASP3}_{\text{zym}}}}\end{array}\right)}$$

$$+\frac{k_{\mathrm{cat\ MASP1^*}}^{\mathrm{MASP3_{zym}}}\left[\mathrm{pCLL1MASP1^*}\right]\left[\mathrm{pMBL_4MASP3_{zym}}\right]}{K_{\mathrm{m\ MASP1^*}}^{\mathrm{MASP3_{zym}}}\left(\begin{aligned}&1+\frac{[\mathrm{C4}]}{K_{\mathrm{m\ MASP1^*}}^{\mathrm{C4}}}+\frac{[\mathrm{fC4bC2}]}{K_{\mathrm{m\ MASP1^*}}^{\mathrm{C2}}}+\frac{[\mathrm{fC4bC4bC2}]}{K_{\mathrm{m\ MASP1^*}}^{\mathrm{C2}}}+\frac{[\mathrm{fC3bC4bC2}]}{K_{\mathrm{m\ MASP1^*}}^{\mathrm{C2}}}+\frac{[\mathrm{pC4bC2}]}{K_{\mathrm{m\ MASP1^*}}^{\mathrm{C2}}}+\frac{[\mathrm{pC4bC4bC2}]}{K_{\mathrm{m\ MASP1^*}}^{\mathrm{C2}}}\\&+\frac{[\mathrm{pC3bC4bC2}]}{K_{\mathrm{m\ MASP1^*}}^{\mathrm{C2}}}+\frac{[\mathrm{pMBL_3MASP1_{zym}}]}{K_{\mathrm{m\ MASP1^*}}^{\mathrm{MASP1_{zym}}}}+\frac{[\mathrm{pMBL_3MASP2_{zym}}]}{K_{\mathrm{m\ MASP1^*}}^{\mathrm{MASP2_{zym}}}}+\frac{[\mathrm{pMBL_3MASP3_{zym}}]}{K_{\mathrm{m\ MASP1^*}}^{\mathrm{MASP3_{zym}}}}\\&+\frac{[\mathrm{pMBL_4MASP1_{zym}}]}{K_{\mathrm{m\ MASP1^*}}^{\mathrm{MASP1_{zym}}}}+\frac{[\mathrm{pMBL_4MASP2_{zym}}]}{K_{\mathrm{m\ MASP1^*}}^{\mathrm{MASP2_{zym}}}}+\frac{[\mathrm{pMBL_4MASP3_{zym}}]}{K_{\mathrm{m\ MASP1^*}}^{\mathrm{MASP3_{zym}}}}+\frac{[\mathrm{pCLLKMASP1_{zym}}]}{K_{\mathrm{m\ MASP1^*}}^{\mathrm{MASP1_{zym}}}}\\&+\frac{[\mathrm{pCLLKMASP2_{zym}}]}{K_{\mathrm{m\ MASP1^*}}^{\mathrm{MASP2_{zym}}}}+\frac{[\mathrm{pCLLKMASP3_{zym}}]}{K_{\mathrm{m\ MASP1^*}}^{\mathrm{MASP3_{zym}}}}+\frac{[\mathrm{pCLK1MASP1_{zym}}]}{K_{\mathrm{m\ MASP1^*}}^{\mathrm{MASP1_{zym}}}}+\frac{[\mathrm{pCLK1MASP2_{zym}}]}{K_{\mathrm{m\ MASP1^*}}^{\mathrm{MASP2_{zym}}}}\\&+\frac{[\mathrm{pCLK1MASP3_{zym}}]}{K_{\mathrm{m\ MASP1^*}}^{\mathrm{MASP3_{zym}}}}+\frac{[\mathrm{pCLL1MASP1_{zym}}]}{K_{\mathrm{m\ MASP1^*}}^{\mathrm{MASP1_{zym}}}}+\frac{[\mathrm{pCLL1MASP2_{zym}}]}{K_{\mathrm{m\ MASP1^*}}^{\mathrm{MASP2_{zym}}}}+\frac{[\mathrm{pCLL1MASP3_{zym}}]}{K_{\mathrm{m\ MASP1^*}}^{\mathrm{MASP3_{zym}}}}\\&+\frac{[\mathrm{pLFMASP1_{zym}}]}{K_{\mathrm{m\ MASP1^*}}^{\mathrm{MASP1_{zym}}}}+\frac{[\mathrm{pLFMASP2_{zym}}]}{K_{\mathrm{m\ MASP1^*}}^{\mathrm{MASP2_{zym}}}}+\frac{[\mathrm{pLFMASP3_{zym}}]}{K_{\mathrm{m\ MASP1^*}}^{\mathrm{MASP3_{zym}}}}+\frac{[\mathrm{pHFMASP1_{zym}}]}{K_{\mathrm{m\ MASP1^*}}^{\mathrm{MASP1_{zym}}}}+\frac{[\mathrm{pHFMASP2_{zym}}]}{K_{\mathrm{m\ MASP1^*}}^{\mathrm{MASP2_{zym}}}}\\&+\frac{[\mathrm{pHFMASP3_{zym}}]}{K_{\mathrm{m\ MASP1^*}}^{\mathrm{MASP3_{zym}}}}+\frac{[\mathrm{pMFMASP1_{zym}}]}{K_{\mathrm{m\ MASP1^*}}^{\mathrm{MASP1_{zym}}}}+\frac{[\mathrm{pMFMASP2_{zym}}]}{K_{\mathrm{m\ MASP1^*}}^{\mathrm{MASP2_{zym}}}}+\frac{[\mathrm{pMFMASP3_{zym}}]}{K_{\mathrm{m\ MASP1^*}}^{\mathrm{MASP3_{zym}}}}\\&+\frac{[\mathrm{pPTX3LFMASP1_{zym}}]}{K_{\mathrm{m\ MASP1^*}}^{\mathrm{MASP1_{zym}}}}+\frac{[\mathrm{pPXT3LFMASP2_{zym}}]}{K_{\mathrm{m\ MASP1^*}}^{\mathrm{MASP2_{zym}}}}+\frac{[\mathrm{pPTX3LFMASP3_{zym}}]}{K_{\mathrm{m\ MASP1^*}}^{\mathrm{MASP3_{zym}}}}\end{aligned}\right)}$$

$$+\frac{k_{\text{cat MASP1}^*}^{\text{MASP3}_{\text{zym}}}\left[\text{pLFMASP1}^*\right]\left[\text{pMBL}_4\text{MASP3}_{\text{zym}}\right]}{K_{\text{m MASP1}^*}^{\text{MASP3}_{\text{zym}}}\left(\begin{array}{l} 1+\frac{[\text{C4}]}{K_{\text{m MASP1}^*}^{\text{C4}}}+\frac{[\text{fC4bC2}]}{K_{\text{m MASP1}^*}^{\text{C2}}}+\frac{[\text{fC4bC4bC2}]}{K_{\text{m MASP1}^*}^{\text{C2}}}+\frac{[\text{fC3bC4bC2}]}{K_{\text{m MASP1}^*}^{\text{C2}}}+\frac{[\text{pC4bC2}]}{K_{\text{m MASP1}^*}^{\text{C2}}}+\frac{[\text{pC4bC4bC2}]}{K_{\text{m MASP1}^*}^{\text{C2}}} \\ +\frac{[\text{pC3bC4bC2}]}{K_{\text{m MASP1}^*}^{\text{C2}}}+\frac{\left[\text{pMBL}_3\text{MASP1}_{\text{zym}}\right]}{K_{\text{m MASP1}^*}^{\text{MASP1}_{\text{zym}}}}+\frac{\left[\text{pMBL}_3\text{MASP2}_{\text{zym}}\right]}{K_{\text{m MASP1}^*}^{\text{MASP2}_{\text{zym}}}}+\frac{\left[\text{pMBL}_3\text{MASP3}_{\text{zym}}\right]}{K_{\text{m MASP1}^*}^{\text{MASP3}_{\text{zym}}}} \\ +\frac{\left[\text{pMBL}_4\text{MASP1}_{\text{zym}}\right]}{K_{\text{m MASP1}^*}^{\text{MASP1}_{\text{zym}}}}+\frac{\left[\text{pMBL}_4\text{MASP2}_{\text{zym}}\right]}{K_{\text{m MASP1}^*}^{\text{MASP2}_{\text{zym}}}}+\frac{\left[\text{pMBL}_4\text{MASP3}_{\text{zym}}\right]}{K_{\text{m MASP1}^*}^{\text{MASP3}_{\text{zym}}}}+\frac{\left[\text{pCLLKMASP1}_{\text{zym}}\right]}{K_{\text{m MASP1}^*}^{\text{MASP1}_{\text{zym}}}} \\ +\frac{\left[\text{pCLLKMASP2}_{\text{zym}}\right]}{K_{\text{m MASP1}^*}^{\text{MASP2}_{\text{zym}}}}+\frac{\left[\text{pCLLKMASP3}_{\text{zym}}\right]}{K_{\text{m MASP1}^*}^{\text{MASP3}_{\text{zym}}}}+\frac{\left[\text{pCLK1MASP1}_{\text{zym}}\right]}{K_{\text{m MASP1}^*}^{\text{MASP1}_{\text{zym}}}}+\frac{\left[\text{pCLK1MASP2}_{\text{zym}}\right]}{K_{\text{m MASP1}^*}^{\text{MASP2}_{\text{zym}}}} \\ +\frac{\left[\text{pCLK1MASP3}_{\text{zym}}\right]}{K_{\text{m MASP1}^*}^{\text{MASP3}_{\text{zym}}}}+\frac{\left[\text{pCLL1MASP1}_{\text{zym}}\right]}{K_{\text{m MASP1}^*}^{\text{MASP1}_{\text{zym}}}}+\frac{\left[\text{pCLL1MASP2}_{\text{zym}}\right]}{K_{\text{m MASP1}^*}^{\text{MASP2}_{\text{zym}}}}+\frac{\left[\text{pCLL1MASP3}_{\text{zym}}\right]}{K_{\text{m MASP1}^*}^{\text{MASP3}_{\text{zym}}}} \\ +\frac{\left[\text{pLFMASP1}_{\text{zym}}\right]}{K_{\text{m MASP1}^*}^{\text{MASP1}_{\text{zym}}}}+\frac{\left[\text{pLFMASP2}_{\text{zym}}\right]}{K_{\text{m MASP1}^*}^{\text{MASP2}_{\text{zym}}}}+\frac{\left[\text{pLFMASP3}_{\text{zym}}\right]}{K_{\text{m MASP1}^*}^{\text{MASP3}_{\text{zym}}}}+\frac{\left[\text{pHFMASP1}_{\text{zym}}\right]}{K_{\text{m MASP1}^*}^{\text{MASP1}_{\text{zym}}}}+\frac{\left[\text{pHFMASP2}_{\text{zym}}\right]}{K_{\text{m MASP1}^*}^{\text{MASP2}_{\text{zym}}}} \\ +\frac{\left[\text{pHFMASP3}_{\text{zym}}\right]}{K_{\text{m MASP1}^*}^{\text{MASP3}_{\text{zym}}}}+\frac{\left[\text{pMFMASP1}_{\text{zym}}\right]}{K_{\text{m MASP1}^*}^{\text{MASP1}_{\text{zym}}}}+\frac{\left[\text{pMFMASP2}_{\text{zym}}\right]}{K_{\text{m MASP1}^*}^{\text{MASP2}_{\text{zym}}}}+\frac{\left[\text{pMFMASP3}_{\text{zym}}\right]}{K_{\text{m MASP1}^*}^{\text{MASP3}_{\text{zym}}}} \\ +\frac{\left[\text{pPTX3LFMASP1}_{\text{zym}}\right]}{K_{\text{m MASP1}^*}^{\text{MASP1}_{\text{zym}}}}+\frac{\left[\text{pPXT3LFMASP2}_{\text{zym}}\right]}{K_{\text{m MASP1}^*}^{\text{MASP2}_{\text{zym}}}}+\frac{\left[\text{pPTX3LFMASP3}_{\text{zym}}\right]}{K_{\text{m MASP1}^*}^{\text{MASP3}_{\text{zym}}}} \end{array}\right)}$$

$$
+\frac{k_{\mathrm{cat\ MASP1^*}}^{\mathrm{MASP3_{zym}}}\left[\mathrm{pHFMASP1^*}\right]\left[\mathrm{pMBL_4MASP3_{zym}}\right]}{K_{\mathrm{m\ MASP1^*}}^{\mathrm{MASP3_{zym}}}\left(\begin{aligned}&1+\frac{[\mathrm{C4}]}{K_{\mathrm{m\ MASP1^*}}^{\mathrm{C4}}}+\frac{[\mathrm{fC4bC2}]}{K_{\mathrm{m\ MASP1^*}}^{\mathrm{C2}}}+\frac{[\mathrm{fC4bC4bC2}]}{K_{\mathrm{m\ MASP1^*}}^{\mathrm{C2}}}+\frac{[\mathrm{fC3bC4bC2}]}{K_{\mathrm{m\ MASP1^*}}^{\mathrm{C2}}}+\frac{[\mathrm{pC4bC2}]}{K_{\mathrm{m\ MASP1^*}}^{\mathrm{C2}}}+\frac{[\mathrm{pC4bC4bC2}]}{K_{\mathrm{m\ MASP1^*}}^{\mathrm{C2}}}\\
&+\frac{[\mathrm{pC3bC4bC2}]}{K_{\mathrm{m\ MASP1^*}}^{\mathrm{C2}}}+\frac{[\mathrm{pMBL_3MASP1_{zym}}]}{K_{\mathrm{m\ MASP1^*}}^{\mathrm{MASP1_{zym}}}}+\frac{[\mathrm{pMBL_3MASP2_{zym}}]}{K_{\mathrm{m\ MASP1^*}}^{\mathrm{MASP2_{zym}}}}+\frac{[\mathrm{pMBL_3MASP3_{zym}}]}{K_{\mathrm{m\ MASP1^*}}^{\mathrm{MASP3_{zym}}}}\\
&+\frac{[\mathrm{pMBL_4MASP1_{zym}}]}{K_{\mathrm{m\ MASP1^*}}^{\mathrm{MASP1_{zym}}}}+\frac{[\mathrm{pMBL_4MASP2_{zym}}]}{K_{\mathrm{m\ MASP1^*}}^{\mathrm{MASP2_{zym}}}}+\frac{[\mathrm{pMBL_4MASP3_{zym}}]}{K_{\mathrm{m\ MASP1^*}}^{\mathrm{MASP3_{zym}}}}+\frac{[\mathrm{pCLLKMASP1_{zym}}]}{K_{\mathrm{m\ MASP1^*}}^{\mathrm{MASP1_{zym}}}}\\
&+\frac{[\mathrm{pCLLKMASP2_{zym}}]}{K_{\mathrm{m\ MASP1^*}}^{\mathrm{MASP2_{zym}}}}+\frac{[\mathrm{pCLLKMASP3_{zym}}]}{K_{\mathrm{m\ MASP1^*}}^{\mathrm{MASP3_{zym}}}}+\frac{[\mathrm{pCLK1MASP1_{zym}}]}{K_{\mathrm{m\ MASP1^*}}^{\mathrm{MASP1_{zym}}}}+\frac{[\mathrm{pCLK1MASP2_{zym}}]}{K_{\mathrm{m\ MASP1^*}}^{\mathrm{MASP2_{zym}}}}\\
&+\frac{[\mathrm{pCLK1MASP3_{zym}}]}{K_{\mathrm{m\ MASP1^*}}^{\mathrm{MASP3_{zym}}}}+\frac{[\mathrm{pCLL1MASP1_{zym}}]}{K_{\mathrm{m\ MASP1^*}}^{\mathrm{MASP1_{zym}}}}+\frac{[\mathrm{pCLL1MASP2_{zym}}]}{K_{\mathrm{m\ MASP1^*}}^{\mathrm{MASP2_{zym}}}}+\frac{[\mathrm{pCLL1MASP3_{zym}}]}{K_{\mathrm{m\ MASP1^*}}^{\mathrm{MASP3_{zym}}}}\\
&+\frac{[\mathrm{pLFMASP1_{zym}}]}{K_{\mathrm{m\ MASP1^*}}^{\mathrm{MASP1_{zym}}}}+\frac{[\mathrm{pLFMASP2_{zym}}]}{K_{\mathrm{m\ MASP1^*}}^{\mathrm{MASP2_{zym}}}}+\frac{[\mathrm{pLFMASP3_{zym}}]}{K_{\mathrm{m\ MASP1^*}}^{\mathrm{MASP3_{zym}}}}+\frac{[\mathrm{pHFMASP1_{zym}}]}{K_{\mathrm{m\ MASP1^*}}^{\mathrm{MASP1_{zym}}}}+\frac{[\mathrm{pHFMASP2_{zym}}]}{K_{\mathrm{m\ MASP1^*}}^{\mathrm{MASP2_{zym}}}}\\
&+\frac{[\mathrm{pHFMASP3_{zym}}]}{K_{\mathrm{m\ MASP1^*}}^{\mathrm{MASP3_{zym}}}}+\frac{[\mathrm{pMFMASP1_{zym}}]}{K_{\mathrm{m\ MASP1^*}}^{\mathrm{MASP1_{zym}}}}+\frac{[\mathrm{pMFMASP2_{zym}}]}{K_{\mathrm{m\ MASP1^*}}^{\mathrm{MASP2_{zym}}}}+\frac{[\mathrm{pMFMASP3_{zym}}]}{K_{\mathrm{m\ MASP1^*}}^{\mathrm{MASP3_{zym}}}}\\
&+\frac{[\mathrm{pPTX3LFMASP1_{zym}}]}{K_{\mathrm{m\ MASP1^*}}^{\mathrm{MASP1_{zym}}}}+\frac{[\mathrm{pPXT3LFMASP2_{zym}}]}{K_{\mathrm{m\ MASP1^*}}^{\mathrm{MASP2_{zym}}}}+\frac{[\mathrm{pPTX3LFMASP3_{zym}}]}{K_{\mathrm{m\ MASP1^*}}^{\mathrm{MASP3_{zym}}}}\end{aligned}\right)}
$$

$$
+\frac{k_{\text{cat MASP1}^*}^{\text{MASP3}_{\text{zym}}}\left[\text{pMFMASP1}^*\right]\left[\text{pMBL}_4\text{MASP3}_{\text{zym}}\right]}{K_{\text{m MASP1}^*}^{\text{MASP3}_{\text{zym}}}\left(\begin{array}{l}1+\frac{[\text{C4}]}{K_{\text{m MASP1}^*}^{\text{C4}}}+\frac{[\text{fC4bC2}]}{K_{\text{m MASP1}^*}^{\text{C2}}}+\frac{[\text{fC4bC4bC2}]}{K_{\text{m MASP1}^*}^{\text{C2}}}+\frac{[\text{fC3bC4bC2}]}{K_{\text{m MASP1}^*}^{\text{C2}}}+\frac{[\text{pC4bC2}]}{K_{\text{m MASP1}^*}^{\text{C2}}}+\frac{[\text{pC4bC4bC2}]}{K_{\text{m MASP1}^*}^{\text{C2}}}\\
+\frac{[\text{pC3bC4bC2}]}{K_{\text{m MASP1}^*}^{\text{C2}}}+\frac{\left[\text{pMBL}_3\text{MASP1}_{\text{zym}}\right]}{K_{\text{m MASP1}^*}^{\text{MASP1}_{\text{zym}}}}+\frac{\left[\text{pMBL}_3\text{MASP2}_{\text{zym}}\right]}{K_{\text{m MASP1}^*}^{\text{MASP2}_{\text{zym}}}}+\frac{\left[\text{pMBL}_3\text{MASP3}_{\text{zym}}\right]}{K_{\text{m MASP1}^*}^{\text{MASP3}_{\text{zym}}}}\\
+\frac{\left[\text{pMBL}_4\text{MASP1}_{\text{zym}}\right]}{K_{\text{m MASP1}^*}^{\text{MASP1}_{\text{zym}}}}+\frac{\left[\text{pMBL}_4\text{MASP2}_{\text{zym}}\right]}{K_{\text{m MASP1}^*}^{\text{MASP2}_{\text{zym}}}}+\frac{\left[\text{pMBL}_4\text{MASP3}_{\text{zym}}\right]}{K_{\text{m MASP1}^*}^{\text{MASP3}_{\text{zym}}}}+\frac{\left[\text{pCLLKMASP1}_{\text{zym}}\right]}{K_{\text{m MASP1}^*}^{\text{MASP1}_{\text{zym}}}}\\
+\frac{\left[\text{pCLLKMASP2}_{\text{zym}}\right]}{K_{\text{m MASP1}^*}^{\text{MASP2}_{\text{zym}}}}+\frac{\left[\text{pCLLKMASP3}_{\text{zym}}\right]}{K_{\text{m MASP1}^*}^{\text{MASP3}_{\text{zym}}}}+\frac{\left[\text{pCLK1MASP1}_{\text{zym}}\right]}{K_{\text{m MASP1}^*}^{\text{MASP1}_{\text{zym}}}}+\frac{\left[\text{pCLK1MASP2}_{\text{zym}}\right]}{K_{\text{m MASP1}^*}^{\text{MASP2}_{\text{zym}}}}\\
+\frac{\left[\text{pCLK1MASP3}_{\text{zym}}\right]}{K_{\text{m MASP1}^*}^{\text{MASP3}_{\text{zym}}}}+\frac{\left[\text{pCLL1MASP1}_{\text{zym}}\right]}{K_{\text{m MASP1}^*}^{\text{MASP1}_{\text{zym}}}}+\frac{\left[\text{pCLL1MASP2}_{\text{zym}}\right]}{K_{\text{m MASP1}^*}^{\text{MASP2}_{\text{zym}}}}+\frac{\left[\text{pCLL1MASP3}_{\text{zym}}\right]}{K_{\text{m MASP1}^*}^{\text{MASP3}_{\text{zym}}}}\\
+\frac{\left[\text{pLFMASP1}_{\text{zym}}\right]}{K_{\text{m MASP1}^*}^{\text{MASP1}_{\text{zym}}}}+\frac{\left[\text{pLFMASP2}_{\text{zym}}\right]}{K_{\text{m MASP1}^*}^{\text{MASP2}_{\text{zym}}}}+\frac{\left[\text{pLFMASP3}_{\text{zym}}\right]}{K_{\text{m MASP1}^*}^{\text{MASP3}_{\text{zym}}}}+\frac{\left[\text{pHFMASP1}_{\text{zym}}\right]}{K_{\text{m MASP1}^*}^{\text{MASP1}_{\text{zym}}}}+\frac{\left[\text{pHFMASP2}_{\text{zym}}\right]}{K_{\text{m MASP1}^*}^{\text{MASP2}_{\text{zym}}}}\\
+\frac{\left[\text{pHFMASP3}_{\text{zym}}\right]}{K_{\text{m MASP1}^*}^{\text{MASP3}_{\text{zym}}}}+\frac{\left[\text{pMFMASP1}_{\text{zym}}\right]}{K_{\text{m MASP1}^*}^{\text{MASP1}_{\text{zym}}}}+\frac{\left[\text{pMFMASP2}_{\text{zym}}\right]}{K_{\text{m MASP1}^*}^{\text{MASP2}_{\text{zym}}}}+\frac{\left[\text{pMFMASP3}_{\text{zym}}\right]}{K_{\text{m MASP1}^*}^{\text{MASP3}_{\text{zym}}}}\\
+\frac{\left[\text{pPTX3LFMASP1}_{\text{zym}}\right]}{K_{\text{m MASP1}^*}^{\text{MASP1}_{\text{zym}}}}+\frac{\left[\text{pPXT3LFMASP2}_{\text{zym}}\right]}{K_{\text{m MASP1}^*}^{\text{MASP2}_{\text{zym}}}}+\frac{\left[\text{pPTX3LFMASP3}_{\text{zym}}\right]}{K_{\text{m MASP1}^*}^{\text{MASP3}_{\text{zym}}}}\end{array}\right)}
$$

$$+\frac{k_{\text{cat MASP1}^*}^{\text{MASP3}_{\text{zym}}}\left[\text{pPTX3LFMASP1}^*\right]\left[\text{pMBL}_4\text{MASP3}_{\text{zym}}\right]}{K_{\text{m MASP1}^*}^{\text{MASP3}_{\text{zym}}}\left(\begin{array}{l}1+\frac{[\text{C4}]}{K_{\text{m MASP1}^*}^{\text{C4}}}+\frac{[\text{fC4bC2}]}{K_{\text{m MASP1}^*}^{\text{C2}}}+\frac{[\text{fC4bC4bC2}]}{K_{\text{m MASP1}^*}^{\text{C2}}}+\frac{[\text{fC3bC4bC2}]}{K_{\text{m MASP1}^*}^{\text{C2}}}+\frac{[\text{pC4bC2}]}{K_{\text{m MASP1}^*}^{\text{C2}}}+\frac{[\text{pC4bC4bC2}]}{K_{\text{m MASP1}^*}^{\text{C2}}}\\
+\frac{[\text{pC3bC4bC2}]}{K_{\text{m MASP1}^*}^{\text{C2}}}+\frac{\left[\text{pMBL}_3\text{MASP1}_{\text{zym}}\right]}{K_{\text{m MASP1}^*}^{\text{MASP1}_{\text{zym}}}}+\frac{\left[\text{pMBL}_3\text{MASP2}_{\text{zym}}\right]}{K_{\text{m MASP1}^*}^{\text{MASP2}_{\text{zym}}}}+\frac{\left[\text{pMBL}_3\text{MASP3}_{\text{zym}}\right]}{K_{\text{m MASP1}^*}^{\text{MASP3}_{\text{zym}}}}\\
+\frac{\left[\text{pMBL}_4\text{MASP1}_{\text{zym}}\right]}{K_{\text{m MASP1}^*}^{\text{MASP1}_{\text{zym}}}}+\frac{\left[\text{pMBL}_4\text{MASP2}_{\text{zym}}\right]}{K_{\text{m MASP1}^*}^{\text{MASP2}_{\text{zym}}}}+\frac{\left[\text{pMBL}_4\text{MASP3}_{\text{zym}}\right]}{K_{\text{m MASP1}^*}^{\text{MASP3}_{\text{zym}}}}+\frac{\left[\text{pCLLKMASP1}_{\text{zym}}\right]}{K_{\text{m MASP1}^*}^{\text{MASP1}_{\text{zym}}}}\\
+\frac{\left[\text{pCLLKMASP2}_{\text{zym}}\right]}{K_{\text{m MASP1}^*}^{\text{MASP2}_{\text{zym}}}}+\frac{\left[\text{pCLLKMASP3}_{\text{zym}}\right]}{K_{\text{m MASP1}^*}^{\text{MASP3}_{\text{zym}}}}+\frac{\left[\text{pCLK1MASP1}_{\text{zym}}\right]}{K_{\text{m MASP1}^*}^{\text{MASP1}_{\text{zym}}}}+\frac{\left[\text{pCLK1MASP2}_{\text{zym}}\right]}{K_{\text{m MASP1}^*}^{\text{MASP2}_{\text{zym}}}}\\
+\frac{\left[\text{pCLK1MASP3}_{\text{zym}}\right]}{K_{\text{m MASP1}^*}^{\text{MASP3}_{\text{zym}}}}+\frac{\left[\text{pCLL1MASP1}_{\text{zym}}\right]}{K_{\text{m MASP1}^*}^{\text{MASP1}_{\text{zym}}}}+\frac{\left[\text{pCLL1MASP2}_{\text{zym}}\right]}{K_{\text{m MASP1}^*}^{\text{MASP2}_{\text{zym}}}}+\frac{\left[\text{pCLL1MASP3}_{\text{zym}}\right]}{K_{\text{m MASP1}^*}^{\text{MASP3}_{\text{zym}}}}\\
+\frac{\left[\text{pLFMASP1}_{\text{zym}}\right]}{K_{\text{m MASP1}^*}^{\text{MASP1}_{\text{zym}}}}+\frac{\left[\text{pLFMASP2}_{\text{zym}}\right]}{K_{\text{m MASP1}^*}^{\text{MASP2}_{\text{zym}}}}+\frac{\left[\text{pLFMASP3}_{\text{zym}}\right]}{K_{\text{m MASP1}^*}^{\text{MASP3}_{\text{zym}}}}+\frac{\left[\text{pHFMASP1}_{\text{zym}}\right]}{K_{\text{m MASP1}^*}^{\text{MASP1}_{\text{zym}}}}+\frac{\left[\text{pHFMASP2}_{\text{zym}}\right]}{K_{\text{m MASP1}^*}^{\text{MASP2}_{\text{zym}}}}\\
+\frac{\left[\text{pHFMASP3}_{\text{zym}}\right]}{K_{\text{m MASP1}^*}^{\text{MASP3}_{\text{zym}}}}+\frac{\left[\text{pMFMASP1}_{\text{zym}}\right]}{K_{\text{m MASP1}^*}^{\text{MASP1}_{\text{zym}}}}+\frac{\left[\text{pMFMASP2}_{\text{zym}}\right]}{K_{\text{m MASP1}^*}^{\text{MASP2}_{\text{zym}}}}+\frac{\left[\text{pMFMASP3}_{\text{zym}}\right]}{K_{\text{m MASP1}^*}^{\text{MASP3}_{\text{zym}}}}\\
+\frac{\left[\text{pPTX3LFMASP1}_{\text{zym}}\right]}{K_{\text{m MASP1}^*}^{\text{MASP1}_{\text{zym}}}}+\frac{\left[\text{pPXT3LFMASP2}_{\text{zym}}\right]}{K_{\text{m MASP1}^*}^{\text{MASP2}_{\text{zym}}}}+\frac{\left[\text{pPTX3LFMASP3}_{\text{zym}}\right]}{K_{\text{m MASP1}^*}^{\text{MASP3}_{\text{zym}}}}\end{array}\right)}$$

278

$$\frac{d\left[\text{fMBL}_4\text{MAp19}\right]}{dt}=k_{\text{MBL}_4\text{MAp19}}^{+}\left[\text{MBL}_4\right][\text{MAp19}]-k_{\text{MBL}_4\text{MAp19}}^{-}\left[\text{fMBL}_4\text{MAp19}\right]$$
$$-k_{\text{pMBL}_4}^{+}\left[\text{fMBL}_4\text{MAp19}\right][\text{Pathogen Surface}]+k_{\text{pMBL}_4}^{-}\left[\text{pMBL}_4\text{MAp19}\right]$$

279

$$\frac{d\left[\text{pMBL}_4\text{MAp19}\right]}{dt}=k_{\text{pMBL}_4}^{+}\left[\text{fMBL}_4\text{MAp19}\right][\text{Pathogen Surface}]-k_{\text{pMBL}_4}^{-}\left[\text{pMBL}_4\text{MAp19}\right]$$
$$+k_{\text{MBL}_4\text{MAp19}}^{+}\left[\text{pMBL}_4\right][\text{MAp19}]-k_{\text{MBL}_4\text{MAp19}}^{-}\left[\text{pMBL}_4\text{MAp19}\right]$$

280

$$\frac{d\left[\text{fMBL}_4\text{MAp44}\right]}{dt}=k_{\text{MBL}_4\text{MAp44}}^{+}\left[\text{MBL}_4\right][\text{MAp44}]-k_{\text{MBL}_4\text{MAp44}}^{-}\left[\text{fMBL}_4\text{MAp44}\right]$$
$$-k_{\text{pMBL}_4}^{+}\left[\text{fMBL}_4\text{MAp44}\right][\text{Pathogen Surface}]+k_{\text{pMBL}_4}^{-}\left[\text{pMBL}_4\text{MAp44}\right]$$

281

$$\frac{d\left[\text{pMBL}_4\text{MAp44}\right]}{dt}=k^{+}_{\text{pMBL}_4}\left[\text{fMBL}_4\text{MAp44}\right]\left[\text{Pathogen Surface}\right]-k^{-}_{\text{pMBL}_4}\left[\text{pMBL}_4\text{MAp44}\right]$$
$$+k^{+}_{\text{MBL}_4\text{MAp44}}\left[\text{pMBL}_4\right]\left[\text{MAp44}\right]-k^{-}_{\text{MBL}_4\text{MAp44}}\left[\text{pMBL}_4\text{MAp44}\right]$$

282

$$\frac{d\left[\text{CLLK}\right]}{dt}=-k^{+}_{\text{CLLKMASP1}}\left[\text{CLLK}\right]\left[\text{MASP1}\right]+k^{-}_{\text{CLLKMASP1}}\left[\text{fCLLKMASP1}_{\text{zym}}\right]-k^{+}_{\text{CLLKMASP2}}\left[\text{CLLK}\right]\left[\text{MASP2}\right]$$
$$+k^{-}_{\text{CLLKMASP2}}\left[\text{fCLLKMASP2}_{\text{zym}}\right]-k^{+}_{\text{CLLKMASP3}}\left[\text{CLLK}\right]\left[\text{MASP3}\right]+k^{-}_{\text{CLLKMASP3}}\left[\text{fCLLKMASP3}_{\text{zym}}\right]$$
$$-k^{+}_{\text{CLLKMAp19}}\left[\text{CLLK}\right]\left[\text{MAp19}\right]+k^{-}_{\text{CLLKMAp19}}\left[\text{fCLLKMAp19}\right]-k^{+}_{\text{CLLKMAp44}}\left[\text{CLLK}\right]\left[\text{MAp44}\right]$$
$$+k^{-}_{\text{CLLKMAp44}}\left[\text{fCLLKMAp44}\right]-k^{+}_{\text{pCLLK}}\left[\text{CLLK}\right]\left[\text{Pathogen Surface}\right]+k^{-}_{\text{pCLLK}}\left[\text{pCLLK}\right]-k^{+}_{\text{CLLKMASP1}}\left[\text{CLLK}\right]\left[\text{MASP1}^{*}\right]$$
$$+k^{-}_{\text{CLLKMASP1}}\left[\text{fCLLKMASP1}^{*}\right]-k^{+}_{\text{CLLKMASP2}}\left[\text{CLLK}\right]\left[\text{MASP2}^{*}\right]+k^{-}_{\text{CLLKMASP2}}\left[\text{fCLLKMASP2}^{*}\right]$$
$$-k^{+}_{\text{CLLKMASP3}}\left[\text{CLLK}\right]\left[\text{MASP3}^{*}\right]+k^{-}_{\text{CLLKMASP3}}\left[\text{fCLLKMASP3}^{*}\right]+k^{+}_{\text{C1INHMASP1}}\left[\text{C1-INH}\right]\left[\text{fCLLKMASP1}^{*}\right]$$
$$+k^{+}_{\text{C1INHMASP2}}\left[\text{C1-INH}\right]\left[\text{fCLLKMASP2}^{*}\right]$$

283

$$\frac{d\left[\text{pCLLK}\right]}{dt}=k^{+}_{\text{pCLLK}}\left[\text{CLLK}\right]\left[\text{Pathogen Surface}\right]-k^{-}_{\text{pCLLK}}\left[\text{pCLLK}\right]-k^{+}_{\text{CLLKMASP1}}\left[\text{pCLLK}\right]\left[\text{MASP1}\right]$$
$$+k^{-}_{\text{CLLKMASP1}}\left[\text{pCLLKMASP1}_{\text{zym}}\right]-k^{+}_{\text{CLLKMASP2}}\left[\text{pCLLK}\right]\left[\text{MASP2}\right]+k^{-}_{\text{CLLKMASP2}}\left[\text{pCLLKMASP2}_{\text{zym}}\right]$$
$$-k^{+}_{\text{CLLKMASP3}}\left[\text{pCLLK}\right]\left[\text{MASP3}\right]+k^{-}_{\text{CLLKMASP3}}\left[\text{pCLLKMASP3}_{\text{zym}}\right]-k^{+}_{\text{CLLKMASP1}}\left[\text{pCLLK}\right]\left[\text{MASP1}^{*}\right]$$
$$+k^{-}_{\text{CLLKMASP1}}\left[\text{pCLLKMASP1}^{*}\right]-k^{+}_{\text{CLLKMASP2}}\left[\text{pCLLK}\right]\left[\text{MASP2}^{*}\right]+k^{-}_{\text{CLLKMASP2}}\left[\text{pCLLKMASP2}^{*}\right]$$
$$-k^{+}_{\text{CLLKMASP3}}\left[\text{pCLLK}\right]\left[\text{MASP3}^{*}\right]+k^{-}_{\text{CLLKMASP3}}\left[\text{pCLLKMASP3}^{*}\right]-k^{+}_{\text{CLLKMAp19}}\left[\text{pCLLK}\right]\left[\text{MAp19}\right]$$
$$+k^{-}_{\text{CLLKMAp19}}\left[\text{pCLLKMAp19}\right]-k^{+}_{\text{CLLKMAp44}}\left[\text{pCLLK}\right]\left[\text{MAp44}\right]+k^{-}_{\text{CLLKMAp44}}\left[\text{pCLLKMAp44}\right]$$
$$+k^{+}_{\text{C1INHMASP1}}\left[\text{C1-INH}\right]\left[\text{pCLLKMASP1}^{*}\right]+k^{+}_{\text{C1INHMASP2}}\left[\text{C1-INH}\right]\left[\text{pCLLKMASP2}^{*}\right]$$

284

$$\frac{d\left[\text{fCLLKMASP1}_{\text{zym}}\right]}{dt}=k^{+}_{\text{CLLKMASP1}}\left[\text{CLLK}\right]\left[\text{MASP1}\right]-k^{-}_{\text{CLLKMASP1}}\left[\text{fCLLKMASP1}_{\text{zym}}\right]$$
$$-k^{+}_{\text{pCLLK}}\left[\text{fCLLKMASP1}_{\text{zym}}\right]\left[\text{Pathogen Surface}\right]+k^{-}_{\text{pCLLK}}\left[\text{pCLLKMASP1}_{\text{zym}}\right]$$

285

$$\frac{d\left[\text{pCLLKMASP1}_{\text{zym}}\right]}{dt}=k^{+}_{\text{pCLLK}}\left[\text{fCLLKMASP1}_{\text{zym}}\right]\left[\text{Pathogen Surface}\right]-k^{-}_{\text{pCLLK}}\left[\text{pCLLKMASP1}_{\text{zym}}\right]$$
$$+k^{+}_{\text{CLLKMASP1}}\left[\text{pCLLK}\right]\left[\text{MASP1}\right]-k^{-}_{\text{CLLKMASP1}}\left[\text{pCLLKMASP1}_{\text{zym}}\right]$$

$$
-\frac{k_{\mathrm{cat\ MASP1_{zym}}}^{\mathrm{MASP1_{zym}}}\left[\mathrm{pMBL_3MASP1_{zym}}\right]\left[\mathrm{pCLLKMASP1_{zym}}\right]}{K_{\mathrm{m\ MASP1_{zym}}}^{\mathrm{MASP1_{zym}}}\left(\begin{array}{l}1+\frac{\left[\mathrm{pMBL_3MASP1_{zym}}\right]}{K_{\mathrm{m\ MASP1_{zym}}}^{\mathrm{MASP1_{zym}}}}+\frac{\left[\mathrm{pMBL_3MASP2_{zym}}\right]}{K_{\mathrm{m\ MASP1_{zym}}}^{\mathrm{MASP2_{zym}}}}+\frac{\left[\mathrm{pMBL_4MASP1_{zym}}\right]}{K_{\mathrm{m\ MASP1_{zym}}}^{\mathrm{MASP1_{zym}}}}+\frac{\left[\mathrm{pMBL_4MASP2_{zym}}\right]}{K_{\mathrm{m\ MASP1_{zym}}}^{\mathrm{MASP2_{zym}}}}\\+\frac{\left[\mathrm{pCLLKMASP1_{zym}}\right]}{K_{\mathrm{m\ MASP1_{zym}}}^{\mathrm{MASP1_{zym}}}}+\frac{\left[\mathrm{pCLLKMASP2_{zym}}\right]}{K_{\mathrm{m\ MASP1_{zym}}}^{\mathrm{MASP2_{zym}}}}+\frac{\left[\mathrm{pCLK1MASP1_{zym}}\right]}{K_{\mathrm{m\ MASP1_{zym}}}^{\mathrm{MASP1_{zym}}}}+\frac{\left[\mathrm{pCLK1MASP2_{zym}}\right]}{K_{\mathrm{m\ MASP1_{zym}}}^{\mathrm{MASP2_{zym}}}}\\+\frac{\left[\mathrm{pCLL1MASP1_{zym}}\right]}{K_{\mathrm{m\ MASP1_{zym}}}^{\mathrm{MASP1_{zym}}}}+\frac{\left[\mathrm{pCLL1MASP2_{zym}}\right]}{K_{\mathrm{m\ MASP1_{zym}}}^{\mathrm{MASP2_{zym}}}}+\frac{\left[\mathrm{pLFMASP1_{zym}}\right]}{K_{\mathrm{m\ MASP1_{zym}}}^{\mathrm{MASP1_{zym}}}}+\frac{\left[\mathrm{pLFMASP2_{zym}}\right]}{K_{\mathrm{m\ MASP1_{zym}}}^{\mathrm{MASP2_{zym}}}}\\+\frac{\left[\mathrm{pHFMASP1_{zym}}\right]}{K_{\mathrm{m\ MASP1_{zym}}}^{\mathrm{MASP1_{zym}}}}+\frac{\left[\mathrm{pHFMASP2_{zym}}\right]}{K_{\mathrm{m\ MASP1_{zym}}}^{\mathrm{MASP2_{zym}}}}+\frac{\left[\mathrm{pMFMASP1_{zym}}\right]}{K_{\mathrm{m\ MASP1_{zym}}}^{\mathrm{MASP1_{zym}}}}+\frac{\left[\mathrm{pMFMASP2_{zym}}\right]}{K_{\mathrm{m\ MASP1_{zym}}}^{\mathrm{MASP2_{zym}}}}\end{array}\right)}
$$

$$
-\frac{k_{\mathrm{cat\ MASP1_{zym}}}^{\mathrm{MASP1_{zym}}}\left[\mathrm{pMBL_4MASP1_{zym}}\right]\left[\mathrm{pCLLKMASP1_{zym}}\right]}{K_{\mathrm{m\ MASP1_{zym}}}^{\mathrm{MASP1_{zym}}}\left(\begin{array}{l}1+\frac{\left[\mathrm{pMBL_3MASP1_{zym}}\right]}{K_{\mathrm{m\ MASP1_{zym}}}^{\mathrm{MASP1_{zym}}}}+\frac{\left[\mathrm{pMBL_3MASP2_{zym}}\right]}{K_{\mathrm{m\ MASP1_{zym}}}^{\mathrm{MASP2_{zym}}}}+\frac{\left[\mathrm{pMBL_4MASP1_{zym}}\right]}{K_{\mathrm{m\ MASP1_{zym}}}^{\mathrm{MASP1_{zym}}}}+\frac{\left[\mathrm{pMBL_4MASP2_{zym}}\right]}{K_{\mathrm{m\ MASP1_{zym}}}^{\mathrm{MASP2_{zym}}}}\\+\frac{\left[\mathrm{pCLLKMASP1_{zym}}\right]}{K_{\mathrm{m\ MASP1_{zym}}}^{\mathrm{MASP1_{zym}}}}+\frac{\left[\mathrm{pCLLKMASP2_{zym}}\right]}{K_{\mathrm{m\ MASP1_{zym}}}^{\mathrm{MASP2_{zym}}}}+\frac{\left[\mathrm{pCLK1MASP1_{zym}}\right]}{K_{\mathrm{m\ MASP1_{zym}}}^{\mathrm{MASP1_{zym}}}}+\frac{\left[\mathrm{pCLK1MASP2_{zym}}\right]}{K_{\mathrm{m\ MASP1_{zym}}}^{\mathrm{MASP2_{zym}}}}\\+\frac{\left[\mathrm{pCLL1MASP1_{zym}}\right]}{K_{\mathrm{m\ MASP1_{zym}}}^{\mathrm{MASP1_{zym}}}}+\frac{\left[\mathrm{pCLL1MASP2_{zym}}\right]}{K_{\mathrm{m\ MASP1_{zym}}}^{\mathrm{MASP2_{zym}}}}+\frac{\left[\mathrm{pLFMASP1_{zym}}\right]}{K_{\mathrm{m\ MASP1_{zym}}}^{\mathrm{MASP1_{zym}}}}+\frac{\left[\mathrm{pLFMASP2_{zym}}\right]}{K_{\mathrm{m\ MASP1_{zym}}}^{\mathrm{MASP2_{zym}}}}\\+\frac{\left[\mathrm{pHFMASP1_{zym}}\right]}{K_{\mathrm{m\ MASP1_{zym}}}^{\mathrm{MASP1_{zym}}}}+\frac{\left[\mathrm{pHFMASP2_{zym}}\right]}{K_{\mathrm{m\ MASP1_{zym}}}^{\mathrm{MASP2_{zym}}}}+\frac{\left[\mathrm{pMFMASP1_{zym}}\right]}{K_{\mathrm{m\ MASP1_{zym}}}^{\mathrm{MASP1_{zym}}}}+\frac{\left[\mathrm{pMFMASP2_{zym}}\right]}{K_{\mathrm{m\ MASP1_{zym}}}^{\mathrm{MASP2_{zym}}}}\end{array}\right)}
$$

$$
-\frac{k_{\mathrm{cat\ MASP1_{zym}}}^{\mathrm{MASP1_{zym}}}\left[\mathrm{pCLLKMASP1_{zym}}\right]\left[\mathrm{pCLLKMASP1_{zym}}\right]}{K_{\mathrm{m\ MASP1_{zym}}}^{\mathrm{MASP1_{zym}}}\left(\begin{array}{l}1+\frac{\left[\mathrm{pMBL_3MASP1_{zym}}\right]}{K_{\mathrm{m\ MASP1_{zym}}}^{\mathrm{MASP1_{zym}}}}+\frac{\left[\mathrm{pMBL_3MASP2_{zym}}\right]}{K_{\mathrm{m\ MASP1_{zym}}}^{\mathrm{MASP2_{zym}}}}+\frac{\left[\mathrm{pMBL_4MASP1_{zym}}\right]}{K_{\mathrm{m\ MASP1_{zym}}}^{\mathrm{MASP1_{zym}}}}+\frac{\left[\mathrm{pMBL_4MASP2_{zym}}\right]}{K_{\mathrm{m\ MASP1_{zym}}}^{\mathrm{MASP2_{zym}}}}\\+\frac{\left[\mathrm{pCLLKMASP1_{zym}}\right]}{K_{\mathrm{m\ MASP1_{zym}}}^{\mathrm{MASP1_{zym}}}}+\frac{\left[\mathrm{pCLLKMASP2_{zym}}\right]}{K_{\mathrm{m\ MASP1_{zym}}}^{\mathrm{MASP2_{zym}}}}+\frac{\left[\mathrm{pCLK1MASP1_{zym}}\right]}{K_{\mathrm{m\ MASP1_{zym}}}^{\mathrm{MASP1_{zym}}}}+\frac{\left[\mathrm{pCLK1MASP2_{zym}}\right]}{K_{\mathrm{m\ MASP1_{zym}}}^{\mathrm{MASP2_{zym}}}}\\+\frac{\left[\mathrm{pCLL1MASP1_{zym}}\right]}{K_{\mathrm{m\ MASP1_{zym}}}^{\mathrm{MASP1_{zym}}}}+\frac{\left[\mathrm{pCLL1MASP2_{zym}}\right]}{K_{\mathrm{m\ MASP1_{zym}}}^{\mathrm{MASP2_{zym}}}}+\frac{\left[\mathrm{pLFMASP1_{zym}}\right]}{K_{\mathrm{m\ MASP1_{zym}}}^{\mathrm{MASP1_{zym}}}}+\frac{\left[\mathrm{pLFMASP2_{zym}}\right]}{K_{\mathrm{m\ MASP1_{zym}}}^{\mathrm{MASP2_{zym}}}}\\+\frac{\left[\mathrm{pHFMASP1_{zym}}\right]}{K_{\mathrm{m\ MASP1_{zym}}}^{\mathrm{MASP1_{zym}}}}+\frac{\left[\mathrm{pHFMASP2_{zym}}\right]}{K_{\mathrm{m\ MASP1_{zym}}}^{\mathrm{MASP2_{zym}}}}+\frac{\left[\mathrm{pMFMASP1_{zym}}\right]}{K_{\mathrm{m\ MASP1_{zym}}}^{\mathrm{MASP1_{zym}}}}+\frac{\left[\mathrm{pMFMASP2_{zym}}\right]}{K_{\mathrm{m\ MASP1_{zym}}}^{\mathrm{MASP2_{zym}}}}\end{array}\right)}
$$

$$
-\frac{k_{\mathrm{cat\ MASP1_{zym}}}^{\mathrm{MASP1_{zym}}}\left[\mathrm{pCLK1MASP1_{zym}}\right]\left[\mathrm{pCLLKMASP1_{zym}}\right]}{K_{\mathrm{m\ MASP1_{zym}}}^{\mathrm{MASP1_{zym}}}\left(\begin{array}{l}1+\frac{\left[\mathrm{pMBL_3MASP1_{zym}}\right]}{K_{\mathrm{m\ MASP1_{zym}}}^{\mathrm{MASP1_{zym}}}}+\frac{\left[\mathrm{pMBL_3MASP2_{zym}}\right]}{K_{\mathrm{m\ MASP1_{zym}}}^{\mathrm{MASP2_{zym}}}}+\frac{\left[\mathrm{pMBL_4MASP1_{zym}}\right]}{K_{\mathrm{m\ MASP1_{zym}}}^{\mathrm{MASP1_{zym}}}}+\frac{\left[\mathrm{pMBL_4MASP2_{zym}}\right]}{K_{\mathrm{m\ MASP1_{zym}}}^{\mathrm{MASP2_{zym}}}}\\+\frac{\left[\mathrm{pCLLKMASP1_{zym}}\right]}{K_{\mathrm{m\ MASP1_{zym}}}^{\mathrm{MASP1_{zym}}}}+\frac{\left[\mathrm{pCLLKMASP2_{zym}}\right]}{K_{\mathrm{m\ MASP1_{zym}}}^{\mathrm{MASP2_{zym}}}}+\frac{\left[\mathrm{pCLK1MASP1_{zym}}\right]}{K_{\mathrm{m\ MASP1_{zym}}}^{\mathrm{MASP1_{zym}}}}+\frac{\left[\mathrm{pCLK1MASP2_{zym}}\right]}{K_{\mathrm{m\ MASP1_{zym}}}^{\mathrm{MASP2_{zym}}}}\\+\frac{\left[\mathrm{pCLL1MASP1_{zym}}\right]}{K_{\mathrm{m\ MASP1_{zym}}}^{\mathrm{MASP1_{zym}}}}+\frac{\left[\mathrm{pCLL1MASP2_{zym}}\right]}{K_{\mathrm{m\ MASP1_{zym}}}^{\mathrm{MASP2_{zym}}}}+\frac{\left[\mathrm{pLFMASP1_{zym}}\right]}{K_{\mathrm{m\ MASP1_{zym}}}^{\mathrm{MASP1_{zym}}}}+\frac{\left[\mathrm{pLFMASP2_{zym}}\right]}{K_{\mathrm{m\ MASP1_{zym}}}^{\mathrm{MASP2_{zym}}}}\\+\frac{\left[\mathrm{pHFMASP1_{zym}}\right]}{K_{\mathrm{m\ MASP1_{zym}}}^{\mathrm{MASP1_{zym}}}}+\frac{\left[\mathrm{pHFMASP2_{zym}}\right]}{K_{\mathrm{m\ MASP1_{zym}}}^{\mathrm{MASP2_{zym}}}}+\frac{\left[\mathrm{pMFMASP1_{zym}}\right]}{K_{\mathrm{m\ MASP1_{zym}}}^{\mathrm{MASP1_{zym}}}}+\frac{\left[\mathrm{pMFMASP2_{zym}}\right]}{K_{\mathrm{m\ MASP1_{zym}}}^{\mathrm{MASP2_{zym}}}}\end{array}\right)}
$$

$$
-\frac{k_{\mathrm{cat\ MASP1_{zym}}}^{\mathrm{MASP1_{zym}}}\left[\mathrm{pCLL1MASP1_{zym}}\right]\left[\mathrm{pCLLKMASP1_{zym}}\right]}{K_{\mathrm{m\ MASP1_{zym}}}^{\mathrm{MASP1_{zym}}}\left(\begin{array}{l}1+\frac{\left[\mathrm{pMBL_3MASP1_{zym}}\right]}{K_{\mathrm{m\ MASP1_{zym}}}^{\mathrm{MASP1_{zym}}}}+\frac{\left[\mathrm{pMBL_3MASP2_{zym}}\right]}{K_{\mathrm{m\ MASP1_{zym}}}^{\mathrm{MASP2_{zym}}}}+\frac{\left[\mathrm{pMBL_4MASP1_{zym}}\right]}{K_{\mathrm{m\ MASP1_{zym}}}^{\mathrm{MASP1_{zym}}}}+\frac{\left[\mathrm{pMBL_4MASP2_{zym}}\right]}{K_{\mathrm{m\ MASP1_{zym}}}^{\mathrm{MASP2_{zym}}}}\\+\frac{\left[\mathrm{pCLLKMASP1_{zym}}\right]}{K_{\mathrm{m\ MASP1_{zym}}}^{\mathrm{MASP1_{zym}}}}+\frac{\left[\mathrm{pCLLKMASP2_{zym}}\right]}{K_{\mathrm{m\ MASP1_{zym}}}^{\mathrm{MASP2_{zym}}}}+\frac{\left[\mathrm{pCLK1MASP1_{zym}}\right]}{K_{\mathrm{m\ MASP1_{zym}}}^{\mathrm{MASP1_{zym}}}}+\frac{\left[\mathrm{pCLK1MASP2_{zym}}\right]}{K_{\mathrm{m\ MASP1_{zym}}}^{\mathrm{MASP2_{zym}}}}\\+\frac{\left[\mathrm{pCLL1MASP1_{zym}}\right]}{K_{\mathrm{m\ MASP1_{zym}}}^{\mathrm{MASP1_{zym}}}}+\frac{\left[\mathrm{pCLL1MASP2_{zym}}\right]}{K_{\mathrm{m\ MASP1_{zym}}}^{\mathrm{MASP2_{zym}}}}+\frac{\left[\mathrm{pLFMASP1_{zym}}\right]}{K_{\mathrm{m\ MASP1_{zym}}}^{\mathrm{MASP1_{zym}}}}+\frac{\left[\mathrm{pLFMASP2_{zym}}\right]}{K_{\mathrm{m\ MASP1_{zym}}}^{\mathrm{MASP2_{zym}}}}\\+\frac{\left[\mathrm{pHFMASP1_{zym}}\right]}{K_{\mathrm{m\ MASP1_{zym}}}^{\mathrm{MASP1_{zym}}}}+\frac{\left[\mathrm{pHFMASP2_{zym}}\right]}{K_{\mathrm{m\ MASP1_{zym}}}^{\mathrm{MASP2_{zym}}}}+\frac{\left[\mathrm{pMFMASP1_{zym}}\right]}{K_{\mathrm{m\ MASP1_{zym}}}^{\mathrm{MASP1_{zym}}}}+\frac{\left[\mathrm{pMFMASP2_{zym}}\right]}{K_{\mathrm{m\ MASP1_{zym}}}^{\mathrm{MASP2_{zym}}}}\end{array}\right)}
$$

$$
-\frac{k_{\mathrm{cat\ MASP1_{zym}}}^{\mathrm{MASP1_{zym}}}\left[\mathrm{pLFMASP1_{zym}}\right]\left[\mathrm{pCLLKMASP1_{zym}}\right]}{K_{\mathrm{m\ MASP1_{zym}}}^{\mathrm{MASP1_{zym}}}\left(\begin{array}{l}1+\frac{\left[\mathrm{pMBL_3MASP1_{zym}}\right]}{K_{\mathrm{m\ MASP1_{zym}}}^{\mathrm{MASP1_{zym}}}}+\frac{\left[\mathrm{pMBL_3MASP2_{zym}}\right]}{K_{\mathrm{m\ MASP1_{zym}}}^{\mathrm{MASP2_{zym}}}}+\frac{\left[\mathrm{pMBL_4MASP1_{zym}}\right]}{K_{\mathrm{m\ MASP1_{zym}}}^{\mathrm{MASP1_{zym}}}}+\frac{\left[\mathrm{pMBL_4MASP2_{zym}}\right]}{K_{\mathrm{m\ MASP1_{zym}}}^{\mathrm{MASP2_{zym}}}}\\+\frac{\left[\mathrm{pCLLKMASP1_{zym}}\right]}{K_{\mathrm{m\ MASP1_{zym}}}^{\mathrm{MASP1_{zym}}}}+\frac{\left[\mathrm{pCLLKMASP2_{zym}}\right]}{K_{\mathrm{m\ MASP1_{zym}}}^{\mathrm{MASP2_{zym}}}}+\frac{\left[\mathrm{pCLK1MASP1_{zym}}\right]}{K_{\mathrm{m\ MASP1_{zym}}}^{\mathrm{MASP1_{zym}}}}+\frac{\left[\mathrm{pCLK1MASP2_{zym}}\right]}{K_{\mathrm{m\ MASP1_{zym}}}^{\mathrm{MASP2_{zym}}}}\\+\frac{\left[\mathrm{pCLL1MASP1_{zym}}\right]}{K_{\mathrm{m\ MASP1_{zym}}}^{\mathrm{MASP1_{zym}}}}+\frac{\left[\mathrm{pCLL1MASP2_{zym}}\right]}{K_{\mathrm{m\ MASP1_{zym}}}^{\mathrm{MASP2_{zym}}}}+\frac{\left[\mathrm{pLFMASP1_{zym}}\right]}{K_{\mathrm{m\ MASP1_{zym}}}^{\mathrm{MASP1_{zym}}}}+\frac{\left[\mathrm{pLFMASP2_{zym}}\right]}{K_{\mathrm{m\ MASP1_{zym}}}^{\mathrm{MASP2_{zym}}}}\\+\frac{\left[\mathrm{pHFMASP1_{zym}}\right]}{K_{\mathrm{m\ MASP1_{zym}}}^{\mathrm{MASP1_{zym}}}}+\frac{\left[\mathrm{pHFMASP2_{zym}}\right]}{K_{\mathrm{m\ MASP1_{zym}}}^{\mathrm{MASP2_{zym}}}}+\frac{\left[\mathrm{pMFMASP1_{zym}}\right]}{K_{\mathrm{m\ MASP1_{zym}}}^{\mathrm{MASP1_{zym}}}}+\frac{\left[\mathrm{pMFMASP2_{zym}}\right]}{K_{\mathrm{m\ MASP1_{zym}}}^{\mathrm{MASP2_{zym}}}}\end{array}\right)}
$$

$$
-\frac{k_{\text{cat MASP1}_{\text{zym}}}^{\text{MASP1}_{\text{zym}}}\left[\text{pHFMASP1}_{\text{zym}}\right]\left[\text{pCLLKMASP1}_{\text{zym}}\right]}{K_{\text{m MASP1}_{\text{zym}}}^{\text{MASP1}_{\text{zym}}}\left(\begin{array}{l}1+\frac{\left[\text{pMBL}_3\text{MASP1}_{\text{zym}}\right]}{K_{\text{m MASP1}_{\text{zym}}}^{\text{MASP1}_{\text{zym}}}}+\frac{\left[\text{pMBL}_3\text{MASP2}_{\text{zym}}\right]}{K_{\text{m MASP1}_{\text{zym}}}^{\text{MASP2}_{\text{zym}}}}+\frac{\left[\text{pMBL}_4\text{MASP1}_{\text{zym}}\right]}{K_{\text{m MASP1}_{\text{zym}}}^{\text{MASP1}_{\text{zym}}}}+\frac{\left[\text{pMBL}_4\text{MASP2}_{\text{zym}}\right]}{K_{\text{m MASP1}_{\text{zym}}}^{\text{MASP2}_{\text{zym}}}}\\+\frac{\left[\text{pCLLKMASP1}_{\text{zym}}\right]}{K_{\text{m MASP1}_{\text{zym}}}^{\text{MASP1}_{\text{zym}}}}+\frac{\left[\text{pCLLKMASP2}_{\text{zym}}\right]}{K_{\text{m MASP1}_{\text{zym}}}^{\text{MASP2}_{\text{zym}}}}+\frac{\left[\text{pCLK1MASP1}_{\text{zym}}\right]}{K_{\text{m MASP1}_{\text{zym}}}^{\text{MASP1}_{\text{zym}}}}+\frac{\left[\text{pCLK1MASP2}_{\text{zym}}\right]}{K_{\text{m MASP1}_{\text{zym}}}^{\text{MASP2}_{\text{zym}}}}\\+\frac{\left[\text{pCLL1MASP1}_{\text{zym}}\right]}{K_{\text{m MASP1}_{\text{zym}}}^{\text{MASP1}_{\text{zym}}}}+\frac{\left[\text{pCLL1MASP2}_{\text{zym}}\right]}{K_{\text{m MASP1}_{\text{zym}}}^{\text{MASP2}_{\text{zym}}}}+\frac{\left[\text{pLFMASP1}_{\text{zym}}\right]}{K_{\text{m MASP1}_{\text{zym}}}^{\text{MASP1}_{\text{zym}}}}+\frac{\left[\text{pLFMASP2}_{\text{zym}}\right]}{K_{\text{m MASP1}_{\text{zym}}}^{\text{MASP2}_{\text{zym}}}}\\+\frac{\left[\text{pHFMASP1}_{\text{zym}}\right]}{K_{\text{m MASP1}_{\text{zym}}}^{\text{MASP1}_{\text{zym}}}}+\frac{\left[\text{pHFMASP2}_{\text{zym}}\right]}{K_{\text{m MASP1}_{\text{zym}}}^{\text{MASP2}_{\text{zym}}}}+\frac{\left[\text{pMFMASP1}_{\text{zym}}\right]}{K_{\text{m MASP1}_{\text{zym}}}^{\text{MASP1}_{\text{zym}}}}+\frac{\left[\text{pMFMASP2}_{\text{zym}}\right]}{K_{\text{m MASP1}_{\text{zym}}}^{\text{MASP2}_{\text{zym}}}}\end{array}\right)}
$$

$$
-\frac{k_{\text{cat MASP1}_{\text{zym}}}^{\text{MASP1}_{\text{zym}}}\left[\text{pMFMASP1}_{\text{zym}}\right]\left[\text{pCLLKMASP1}_{\text{zym}}\right]}{K_{\text{m MASP1}_{\text{zym}}}^{\text{MASP1}_{\text{zym}}}\left(\begin{array}{l}1+\frac{\left[\text{pMBL}_3\text{MASP1}_{\text{zym}}\right]}{K_{\text{m MASP1}_{\text{zym}}}^{\text{MASP1}_{\text{zym}}}}+\frac{\left[\text{pMBL}_3\text{MASP2}_{\text{zym}}\right]}{K_{\text{m MASP1}_{\text{zym}}}^{\text{MASP2}_{\text{zym}}}}+\frac{\left[\text{pMBL}_4\text{MASP1}_{\text{zym}}\right]}{K_{\text{m MASP1}_{\text{zym}}}^{\text{MASP1}_{\text{zym}}}}+\frac{\left[\text{pMBL}_4\text{MASP2}_{\text{zym}}\right]}{K_{\text{m MASP1}_{\text{zym}}}^{\text{MASP2}_{\text{zym}}}}\\+\frac{\left[\text{pCLLKMASP1}_{\text{zym}}\right]}{K_{\text{m MASP1}_{\text{zym}}}^{\text{MASP1}_{\text{zym}}}}+\frac{\left[\text{pCLLKMASP2}_{\text{zym}}\right]}{K_{\text{m MASP1}_{\text{zym}}}^{\text{MASP2}_{\text{zym}}}}+\frac{\left[\text{pCLK1MASP1}_{\text{zym}}\right]}{K_{\text{m MASP1}_{\text{zym}}}^{\text{MASP1}_{\text{zym}}}}+\frac{\left[\text{pCLK1MASP2}_{\text{zym}}\right]}{K_{\text{m MASP1}_{\text{zym}}}^{\text{MASP2}_{\text{zym}}}}\\+\frac{\left[\text{pCLL1MASP1}_{\text{zym}}\right]}{K_{\text{m MASP1}_{\text{zym}}}^{\text{MASP1}_{\text{zym}}}}+\frac{\left[\text{pCLL1MASP2}_{\text{zym}}\right]}{K_{\text{m MASP1}_{\text{zym}}}^{\text{MASP2}_{\text{zym}}}}+\frac{\left[\text{pLFMASP1}_{\text{zym}}\right]}{K_{\text{m MASP1}_{\text{zym}}}^{\text{MASP1}_{\text{zym}}}}+\frac{\left[\text{pLFMASP2}_{\text{zym}}\right]}{K_{\text{m MASP1}_{\text{zym}}}^{\text{MASP2}_{\text{zym}}}}\\+\frac{\left[\text{pHFMASP1}_{\text{zym}}\right]}{K_{\text{m MASP1}_{\text{zym}}}^{\text{MASP1}_{\text{zym}}}}+\frac{\left[\text{pHFMASP2}_{\text{zym}}\right]}{K_{\text{m MASP1}_{\text{zym}}}^{\text{MASP2}_{\text{zym}}}}+\frac{\left[\text{pMFMASP1}_{\text{zym}}\right]}{K_{\text{m MASP1}_{\text{zym}}}^{\text{MASP1}_{\text{zym}}}}+\frac{\left[\text{pMFMASP2}_{\text{zym}}\right]}{K_{\text{m MASP1}_{\text{zym}}}^{\text{MASP2}_{\text{zym}}}}\end{array}\right)}
$$

$$
-\frac{k_{\text{cat MASP1}_{\text{zym}}}^{\text{MASP1}_{\text{zym}}}\left[\text{pPTX3LFMASP1}_{\text{zym}}\right]\left[\text{pCLLKMASP1}_{\text{zym}}\right]}{K_{\text{m MASP1}_{\text{zym}}}^{\text{MASP1}_{\text{zym}}}\left(\begin{array}{l}1+\frac{\left[\text{pMBL}_3\text{MASP1}_{\text{zym}}\right]}{K_{\text{m MASP1}_{\text{zym}}}^{\text{MASP1}_{\text{zym}}}}+\frac{\left[\text{pMBL}_3\text{MASP2}_{\text{zym}}\right]}{K_{\text{m MASP1}_{\text{zym}}}^{\text{MASP2}_{\text{zym}}}}+\frac{\left[\text{pMBL}_4\text{MASP1}_{\text{zym}}\right]}{K_{\text{m MASP1}_{\text{zym}}}^{\text{MASP1}_{\text{zym}}}}+\frac{\left[\text{pMBL}_4\text{MASP2}_{\text{zym}}\right]}{K_{\text{m MASP1}_{\text{zym}}}^{\text{MASP2}_{\text{zym}}}}\\+\frac{\left[\text{pCLLKMASP1}_{\text{zym}}\right]}{K_{\text{m MASP1}_{\text{zym}}}^{\text{MASP1}_{\text{zym}}}}+\frac{\left[\text{pCLLKMASP2}_{\text{zym}}\right]}{K_{\text{m MASP1}_{\text{zym}}}^{\text{MASP2}_{\text{zym}}}}+\frac{\left[\text{pCLK1MASP1}_{\text{zym}}\right]}{K_{\text{m MASP1}_{\text{zym}}}^{\text{MASP1}_{\text{zym}}}}+\frac{\left[\text{pCLK1MASP2}_{\text{zym}}\right]}{K_{\text{m MASP1}_{\text{zym}}}^{\text{MASP2}_{\text{zym}}}}\\+\frac{\left[\text{pCLL1MASP1}_{\text{zym}}\right]}{K_{\text{m MASP1}_{\text{zym}}}^{\text{MASP1}_{\text{zym}}}}+\frac{\left[\text{pCLL1MASP2}_{\text{zym}}\right]}{K_{\text{m MASP1}_{\text{zym}}}^{\text{MASP2}_{\text{zym}}}}+\frac{\left[\text{pLFMASP1}_{\text{zym}}\right]}{K_{\text{m MASP1}_{\text{zym}}}^{\text{MASP1}_{\text{zym}}}}+\frac{\left[\text{pLFMASP2}_{\text{zym}}\right]}{K_{\text{m MASP1}_{\text{zym}}}^{\text{MASP2}_{\text{zym}}}}\\+\frac{\left[\text{pHFMASP1}_{\text{zym}}\right]}{K_{\text{m MASP1}_{\text{zym}}}^{\text{MASP1}_{\text{zym}}}}+\frac{\left[\text{pHFMASP2}_{\text{zym}}\right]}{K_{\text{m MASP1}_{\text{zym}}}^{\text{MASP2}_{\text{zym}}}}+\frac{\left[\text{pMFMASP1}_{\text{zym}}\right]}{K_{\text{m MASP1}_{\text{zym}}}^{\text{MASP1}_{\text{zym}}}}+\frac{\left[\text{pMFMASP2}_{\text{zym}}\right]}{K_{\text{m MASP1}_{\text{zym}}}^{\text{MASP2}_{\text{zym}}}}\\+\frac{\left[\text{pPTX3LFMASP1}_{\text{zym}}\right]}{K_{\text{m MASP1}_{\text{zym}}}^{\text{MASP1}_{\text{zym}}}}+\frac{\left[\text{pPTX3LFMASP2}_{\text{zym}}\right]}{K_{\text{m MASP1}_{\text{zym}}}^{\text{MASP2}_{\text{zym}}}}\end{array}\right)}
$$

$$
-\frac{k_{\mathrm{cat\,MASP1^*}}^{\mathrm{MASP1_{zym}}}\left[\mathrm{pMBL_3MASP1^*}\right]\left[\mathrm{pCLLKMASP1_{zym}}\right]}{K_{\mathrm{m\,MASP1^*}}^{\mathrm{MASP1_{zym}}}\left(\begin{aligned}&1+\frac{[\mathrm{C4}]}{K_{\mathrm{m\,MASP1^*}}^{\mathrm{C4}}}+\frac{[\mathrm{fC4bC2}]}{K_{\mathrm{m\,MASP1^*}}^{\mathrm{C2}}}+\frac{[\mathrm{fC4bC4bC2}]}{K_{\mathrm{m\,MASP1^*}}^{\mathrm{C2}}}+\frac{[\mathrm{fC3bC4bC2}]}{K_{\mathrm{m\,MASP1^*}}^{\mathrm{C2}}}+\frac{[\mathrm{pC4bC2}]}{K_{\mathrm{m\,MASP1^*}}^{\mathrm{C2}}}+\frac{[\mathrm{pC4bC4bC2}]}{K_{\mathrm{m\,MASP1^*}}^{\mathrm{C2}}}\\
&+\frac{[\mathrm{pC3bC4bC2}]}{K_{\mathrm{m\,MASP1^*}}^{\mathrm{C2}}}+\frac{[\mathrm{pMBL_3MASP1_{zym}}]}{K_{\mathrm{m\,MASP1^*}}^{\mathrm{MASP1_{zym}}}}+\frac{[\mathrm{pMBL_3MASP2_{zym}}]}{K_{\mathrm{m\,MASP1^*}}^{\mathrm{MASP2_{zym}}}}+\frac{[\mathrm{pMBL_3MASP3_{zym}}]}{K_{\mathrm{m\,MASP1^*}}^{\mathrm{MASP3_{zym}}}}\\
&+\frac{[\mathrm{pMBL_4MASP1_{zym}}]}{K_{\mathrm{m\,MASP1^*}}^{\mathrm{MASP1_{zym}}}}+\frac{[\mathrm{pMBL_4MASP2_{zym}}]}{K_{\mathrm{m\,MASP1^*}}^{\mathrm{MASP2_{zym}}}}+\frac{[\mathrm{pMBL_4MASP3_{zym}}]}{K_{\mathrm{m\,MASP1^*}}^{\mathrm{MASP3_{zym}}}}+\frac{[\mathrm{pCLLKMASP1_{zym}}]}{K_{\mathrm{m\,MASP1^*}}^{\mathrm{MASP1_{zym}}}}\\
&+\frac{[\mathrm{pCLLKMASP2_{zym}}]}{K_{\mathrm{m\,MASP1^*}}^{\mathrm{MASP2_{zym}}}}+\frac{[\mathrm{pCLLKMASP3_{zym}}]}{K_{\mathrm{m\,MASP1^*}}^{\mathrm{MASP3_{zym}}}}+\frac{[\mathrm{pCLK1MASP1_{zym}}]}{K_{\mathrm{m\,MASP1^*}}^{\mathrm{MASP1_{zym}}}}+\frac{[\mathrm{pCLK1MASP2_{zym}}]}{K_{\mathrm{m\,MASP1^*}}^{\mathrm{MASP2_{zym}}}}\\
&+\frac{[\mathrm{pCLK1MASP3_{zym}}]}{K_{\mathrm{m\,MASP1^*}}^{\mathrm{MASP3_{zym}}}}+\frac{[\mathrm{pCLL1MASP1_{zym}}]}{K_{\mathrm{m\,MASP1^*}}^{\mathrm{MASP1_{zym}}}}+\frac{[\mathrm{pCLL1MASP2_{zym}}]}{K_{\mathrm{m\,MASP1^*}}^{\mathrm{MASP2_{zym}}}}+\frac{[\mathrm{pCLL1MASP3_{zym}}]}{K_{\mathrm{m\,MASP1^*}}^{\mathrm{MASP3_{zym}}}}\\
&+\frac{[\mathrm{pLFMASP1_{zym}}]}{K_{\mathrm{m\,MASP1^*}}^{\mathrm{MASP1_{zym}}}}+\frac{[\mathrm{pLFMASP2_{zym}}]}{K_{\mathrm{m\,MASP1^*}}^{\mathrm{MASP2_{zym}}}}+\frac{[\mathrm{pLFMASP3_{zym}}]}{K_{\mathrm{m\,MASP1^*}}^{\mathrm{MASP3_{zym}}}}+\frac{[\mathrm{pHFMASP1_{zym}}]}{K_{\mathrm{m\,MASP1^*}}^{\mathrm{MASP1_{zym}}}}+\frac{[\mathrm{pHFMASP2_{zym}}]}{K_{\mathrm{m\,MASP1^*}}^{\mathrm{MASP2_{zym}}}}\\
&+\frac{[\mathrm{pHFMASP3_{zym}}]}{K_{\mathrm{m\,MASP1^*}}^{\mathrm{MASP3_{zym}}}}+\frac{[\mathrm{pMFMASP1_{zym}}]}{K_{\mathrm{m\,MASP1^*}}^{\mathrm{MASP1_{zym}}}}+\frac{[\mathrm{pMFMASP2_{zym}}]}{K_{\mathrm{m\,MASP1^*}}^{\mathrm{MASP2_{zym}}}}+\frac{[\mathrm{pMFMASP3_{zym}}]}{K_{\mathrm{m\,MASP1^*}}^{\mathrm{MASP3_{zym}}}}\end{aligned}\right)}
$$

$$
-\frac{k_{\mathrm{cat\,MASP1^*}}^{\mathrm{MASP1_{zym}}}\left[\mathrm{pMBL_4MASP1^*}\right]\left[\mathrm{pCLLKMASP1_{zym}}\right]}{K_{\mathrm{m\,MASP1^*}}^{\mathrm{MASP1_{zym}}}\left(\begin{aligned}&1+\frac{[\mathrm{C4}]}{K_{\mathrm{m\,MASP1^*}}^{\mathrm{C4}}}+\frac{[\mathrm{fC4bC2}]}{K_{\mathrm{m\,MASP1^*}}^{\mathrm{C2}}}+\frac{[\mathrm{fC4bC4bC2}]}{K_{\mathrm{m\,MASP1^*}}^{\mathrm{C2}}}+\frac{[\mathrm{fC3bC4bC2}]}{K_{\mathrm{m\,MASP1^*}}^{\mathrm{C2}}}+\frac{[\mathrm{pC4bC2}]}{K_{\mathrm{m\,MASP1^*}}^{\mathrm{C2}}}+\frac{[\mathrm{pC4bC4bC2}]}{K_{\mathrm{m\,MASP1^*}}^{\mathrm{C2}}}\\
&+\frac{[\mathrm{pC3bC4bC2}]}{K_{\mathrm{m\,MASP1^*}}^{\mathrm{C2}}}+\frac{[\mathrm{pMBL_3MASP1_{zym}}]}{K_{\mathrm{m\,MASP1^*}}^{\mathrm{MASP1_{zym}}}}+\frac{[\mathrm{pMBL_3MASP2_{zym}}]}{K_{\mathrm{m\,MASP1^*}}^{\mathrm{MASP2_{zym}}}}+\frac{[\mathrm{pMBL_3MASP3_{zym}}]}{K_{\mathrm{m\,MASP1^*}}^{\mathrm{MASP3_{zym}}}}\\
&+\frac{[\mathrm{pMBL_4MASP1_{zym}}]}{K_{\mathrm{m\,MASP1^*}}^{\mathrm{MASP1_{zym}}}}+\frac{[\mathrm{pMBL_4MASP2_{zym}}]}{K_{\mathrm{m\,MASP1^*}}^{\mathrm{MASP2_{zym}}}}+\frac{[\mathrm{pMBL_4MASP3_{zym}}]}{K_{\mathrm{m\,MASP1^*}}^{\mathrm{MASP3_{zym}}}}+\frac{[\mathrm{pCLLKMASP1_{zym}}]}{K_{\mathrm{m\,MASP1^*}}^{\mathrm{MASP1_{zym}}}}\\
&+\frac{[\mathrm{pCLLKMASP2_{zym}}]}{K_{\mathrm{m\,MASP1^*}}^{\mathrm{MASP2_{zym}}}}+\frac{[\mathrm{pCLLKMASP3_{zym}}]}{K_{\mathrm{m\,MASP1^*}}^{\mathrm{MASP3_{zym}}}}+\frac{[\mathrm{pCLK1MASP1_{zym}}]}{K_{\mathrm{m\,MASP1^*}}^{\mathrm{MASP1_{zym}}}}+\frac{[\mathrm{pCLK1MASP2_{zym}}]}{K_{\mathrm{m\,MASP1^*}}^{\mathrm{MASP2_{zym}}}}\\
&+\frac{[\mathrm{pCLK1MASP3_{zym}}]}{K_{\mathrm{m\,MASP1^*}}^{\mathrm{MASP3_{zym}}}}+\frac{[\mathrm{pCLL1MASP1_{zym}}]}{K_{\mathrm{m\,MASP1^*}}^{\mathrm{MASP1_{zym}}}}+\frac{[\mathrm{pCLL1MASP2_{zym}}]}{K_{\mathrm{m\,MASP1^*}}^{\mathrm{MASP2_{zym}}}}+\frac{[\mathrm{pCLL1MASP3_{zym}}]}{K_{\mathrm{m\,MASP1^*}}^{\mathrm{MASP3_{zym}}}}\\
&+\frac{[\mathrm{pLFMASP1_{zym}}]}{K_{\mathrm{m\,MASP1^*}}^{\mathrm{MASP1_{zym}}}}+\frac{[\mathrm{pLFMASP2_{zym}}]}{K_{\mathrm{m\,MASP1^*}}^{\mathrm{MASP2_{zym}}}}+\frac{[\mathrm{pLFMASP3_{zym}}]}{K_{\mathrm{m\,MASP1^*}}^{\mathrm{MASP3_{zym}}}}+\frac{[\mathrm{pHFMASP1_{zym}}]}{K_{\mathrm{m\,MASP1^*}}^{\mathrm{MASP1_{zym}}}}+\frac{[\mathrm{pHFMASP2_{zym}}]}{K_{\mathrm{m\,MASP1^*}}^{\mathrm{MASP2_{zym}}}}\\
&+\frac{[\mathrm{pHFMASP3_{zym}}]}{K_{\mathrm{m\,MASP1^*}}^{\mathrm{MASP3_{zym}}}}+\frac{[\mathrm{pMFMASP1_{zym}}]}{K_{\mathrm{m\,MASP1^*}}^{\mathrm{MASP1_{zym}}}}+\frac{[\mathrm{pMFMASP2_{zym}}]}{K_{\mathrm{m\,MASP1^*}}^{\mathrm{MASP2_{zym}}}}+\frac{[\mathrm{pMFMASP3_{zym}}]}{K_{\mathrm{m\,MASP1^*}}^{\mathrm{MASP3_{zym}}}}\end{aligned}\right)}
$$

$$-\frac{k_{\mathrm{cat\ MASP1^*}}^{\mathrm{MASP1_{zym}}}\left[\mathrm{pCLLKMASP1^*}\right]\left[\mathrm{pCLLKMASP1_{zym}}\right]}{K_{\mathrm{m\ MASP1^*}}^{\mathrm{MASP1_{zym}}}\left(\begin{array}{l}1+\frac{[\mathrm{C4}]}{K_{\mathrm{m\ MASP1^*}}^{\mathrm{C4}}}+\frac{[\mathrm{fC4bC2}]}{K_{\mathrm{m\ MASP1^*}}^{\mathrm{C2}}}+\frac{[\mathrm{fC4bC4bC2}]}{K_{\mathrm{m\ MASP1^*}}^{\mathrm{C2}}}+\frac{[\mathrm{fC3bC4bC2}]}{K_{\mathrm{m\ MASP1^*}}^{\mathrm{C2}}}+\frac{[\mathrm{pC4bC2}]}{K_{\mathrm{m\ MASP1^*}}^{\mathrm{C2}}}+\frac{[\mathrm{pC4bC4bC2}]}{K_{\mathrm{m\ MASP1^*}}^{\mathrm{C2}}}\\+\frac{[\mathrm{pC3bC4bC2}]}{K_{\mathrm{m\ MASP1^*}}^{\mathrm{C2}}}+\frac{[\mathrm{pMBL_3MASP1_{zym}}]}{K_{\mathrm{m\ MASP1^*}}^{\mathrm{MASP1_{zym}}}}+\frac{[\mathrm{pMBL_3MASP2_{zym}}]}{K_{\mathrm{m\ MASP1^*}}^{\mathrm{MASP2_{zym}}}}+\frac{[\mathrm{pMBL_3MASP3_{zym}}]}{K_{\mathrm{m\ MASP1^*}}^{\mathrm{MASP3_{zym}}}}\\+\frac{[\mathrm{pMBL_4MASP1_{zym}}]}{K_{\mathrm{m\ MASP1^*}}^{\mathrm{MASP1_{zym}}}}+\frac{[\mathrm{pMBL_4MASP2_{zym}}]}{K_{\mathrm{m\ MASP1^*}}^{\mathrm{MASP2_{zym}}}}+\frac{[\mathrm{pMBL_4MASP3_{zym}}]}{K_{\mathrm{m\ MASP1^*}}^{\mathrm{MASP3_{zym}}}}+\frac{[\mathrm{pCLLKMASP1_{zym}}]}{K_{\mathrm{m\ MASP1^*}}^{\mathrm{MASP1_{zym}}}}\\+\frac{[\mathrm{pCLLKMASP2_{zym}}]}{K_{\mathrm{m\ MASP1^*}}^{\mathrm{MASP2_{zym}}}}+\frac{[\mathrm{pCLLKMASP3_{zym}}]}{K_{\mathrm{m\ MASP1^*}}^{\mathrm{MASP3_{zym}}}}+\frac{[\mathrm{pCLK1MASP1_{zym}}]}{K_{\mathrm{m\ MASP1^*}}^{\mathrm{MASP1_{zym}}}}+\frac{[\mathrm{pCLK1MASP2_{zym}}]}{K_{\mathrm{m\ MASP1^*}}^{\mathrm{MASP2_{zym}}}}\\+\frac{[\mathrm{pCLK1MASP3_{zym}}]}{K_{\mathrm{m\ MASP1^*}}^{\mathrm{MASP3_{zym}}}}+\frac{[\mathrm{pCLL1MASP1_{zym}}]}{K_{\mathrm{m\ MASP1^*}}^{\mathrm{MASP1_{zym}}}}+\frac{[\mathrm{pCLL1MASP2_{zym}}]}{K_{\mathrm{m\ MASP1^*}}^{\mathrm{MASP2_{zym}}}}+\frac{[\mathrm{pCLL1MASP3_{zym}}]}{K_{\mathrm{m\ MASP1^*}}^{\mathrm{MASP3_{zym}}}}\\+\frac{[\mathrm{pLFMASP1_{zym}}]}{K_{\mathrm{m\ MASP1^*}}^{\mathrm{MASP1_{zym}}}}+\frac{[\mathrm{pLFMASP2_{zym}}]}{K_{\mathrm{m\ MASP1^*}}^{\mathrm{MASP2_{zym}}}}+\frac{[\mathrm{pLFMASP3_{zym}}]}{K_{\mathrm{m\ MASP1^*}}^{\mathrm{MASP3_{zym}}}}+\frac{[\mathrm{pHFMASP1_{zym}}]}{K_{\mathrm{m\ MASP1^*}}^{\mathrm{MASP1_{zym}}}}+\frac{[\mathrm{pHFMASP2_{zym}}]}{K_{\mathrm{m\ MASP1^*}}^{\mathrm{MASP2_{zym}}}}\\+\frac{[\mathrm{pHFMASP3_{zym}}]}{K_{\mathrm{m\ MASP1^*}}^{\mathrm{MASP3_{zym}}}}+\frac{[\mathrm{pMFMASP1_{zym}}]}{K_{\mathrm{m\ MASP1^*}}^{\mathrm{MASP1_{zym}}}}+\frac{[\mathrm{pMFMASP2_{zym}}]}{K_{\mathrm{m\ MASP1^*}}^{\mathrm{MASP2_{zym}}}}+\frac{[\mathrm{pMFMASP3_{zym}}]}{K_{\mathrm{m\ MASP1^*}}^{\mathrm{MASP3_{zym}}}}\end{array}\right)}$$

$$-\frac{k_{\mathrm{cat\ MASP1^*}}^{\mathrm{MASP1_{zym}}}\left[\mathrm{pCLK1MASP1^*}\right]\left[\mathrm{pCLLKMASP1_{zym}}\right]}{K_{\mathrm{m\ MASP1^*}}^{\mathrm{MASP1_{zym}}}\left(\begin{array}{l}1+\frac{[\mathrm{C4}]}{K_{\mathrm{m\ MASP1^*}}^{\mathrm{C4}}}+\frac{[\mathrm{fC4bC2}]}{K_{\mathrm{m\ MASP1^*}}^{\mathrm{C2}}}+\frac{[\mathrm{fC4bC4bC2}]}{K_{\mathrm{m\ MASP1^*}}^{\mathrm{C2}}}+\frac{[\mathrm{fC3bC4bC2}]}{K_{\mathrm{m\ MASP1^*}}^{\mathrm{C2}}}+\frac{[\mathrm{pC4bC2}]}{K_{\mathrm{m\ MASP1^*}}^{\mathrm{C2}}}+\frac{[\mathrm{pC4bC4bC2}]}{K_{\mathrm{m\ MASP1^*}}^{\mathrm{C2}}}\\+\frac{[\mathrm{pC3bC4bC2}]}{K_{\mathrm{m\ MASP1^*}}^{\mathrm{C2}}}+\frac{[\mathrm{pMBL_3MASP1_{zym}}]}{K_{\mathrm{m\ MASP1^*}}^{\mathrm{MASP1_{zym}}}}+\frac{[\mathrm{pMBL_3MASP2_{zym}}]}{K_{\mathrm{m\ MASP1^*}}^{\mathrm{MASP2_{zym}}}}+\frac{[\mathrm{pMBL_3MASP3_{zym}}]}{K_{\mathrm{m\ MASP1^*}}^{\mathrm{MASP3_{zym}}}}\\+\frac{[\mathrm{pMBL_4MASP1_{zym}}]}{K_{\mathrm{m\ MASP1^*}}^{\mathrm{MASP1_{zym}}}}+\frac{[\mathrm{pMBL_4MASP2_{zym}}]}{K_{\mathrm{m\ MASP1^*}}^{\mathrm{MASP2_{zym}}}}+\frac{[\mathrm{pMBL_4MASP3_{zym}}]}{K_{\mathrm{m\ MASP1^*}}^{\mathrm{MASP3_{zym}}}}+\frac{[\mathrm{pCLLKMASP1_{zym}}]}{K_{\mathrm{m\ MASP1^*}}^{\mathrm{MASP1_{zym}}}}\\+\frac{[\mathrm{pCLLKMASP2_{zym}}]}{K_{\mathrm{m\ MASP1^*}}^{\mathrm{MASP2_{zym}}}}+\frac{[\mathrm{pCLLKMASP3_{zym}}]}{K_{\mathrm{m\ MASP1^*}}^{\mathrm{MASP3_{zym}}}}+\frac{[\mathrm{pCLK1MASP1_{zym}}]}{K_{\mathrm{m\ MASP1^*}}^{\mathrm{MASP1_{zym}}}}+\frac{[\mathrm{pCLK1MASP2_{zym}}]}{K_{\mathrm{m\ MASP1^*}}^{\mathrm{MASP2_{zym}}}}\\+\frac{[\mathrm{pCLK1MASP3_{zym}}]}{K_{\mathrm{m\ MASP1^*}}^{\mathrm{MASP3_{zym}}}}+\frac{[\mathrm{pCLL1MASP1_{zym}}]}{K_{\mathrm{m\ MASP1^*}}^{\mathrm{MASP1_{zym}}}}+\frac{[\mathrm{pCLL1MASP2_{zym}}]}{K_{\mathrm{m\ MASP1^*}}^{\mathrm{MASP2_{zym}}}}+\frac{[\mathrm{pCLL1MASP3_{zym}}]}{K_{\mathrm{m\ MASP1^*}}^{\mathrm{MASP3_{zym}}}}\\+\frac{[\mathrm{pLFMASP1_{zym}}]}{K_{\mathrm{m\ MASP1^*}}^{\mathrm{MASP1_{zym}}}}+\frac{[\mathrm{pLFMASP2_{zym}}]}{K_{\mathrm{m\ MASP1^*}}^{\mathrm{MASP2_{zym}}}}+\frac{[\mathrm{pLFMASP3_{zym}}]}{K_{\mathrm{m\ MASP1^*}}^{\mathrm{MASP3_{zym}}}}+\frac{[\mathrm{pHFMASP1_{zym}}]}{K_{\mathrm{m\ MASP1^*}}^{\mathrm{MASP1_{zym}}}}+\frac{[\mathrm{pHFMASP2_{zym}}]}{K_{\mathrm{m\ MASP1^*}}^{\mathrm{MASP2_{zym}}}}\\+\frac{[\mathrm{pHFMASP3_{zym}}]}{K_{\mathrm{m\ MASP1^*}}^{\mathrm{MASP3_{zym}}}}+\frac{[\mathrm{pMFMASP1_{zym}}]}{K_{\mathrm{m\ MASP1^*}}^{\mathrm{MASP1_{zym}}}}+\frac{[\mathrm{pMFMASP2_{zym}}]}{K_{\mathrm{m\ MASP1^*}}^{\mathrm{MASP2_{zym}}}}+\frac{[\mathrm{pMFMASP3_{zym}}]}{K_{\mathrm{m\ MASP1^*}}^{\mathrm{MASP3_{zym}}}}\end{array}\right)}$$

$$
-\frac{k_{\text{cat MASP1}^*}^{\text{MASP1}_{\text{zym}}}\left[\text{pCLL1MASP1}^*\right]\left[\text{pCLLKMASP1}_{\text{zym}}\right]}{K_{\text{m MASP1}^*}^{\text{MASP1}_{\text{zym}}}\left(\begin{array}{l}
1+\frac{[\text{C4}]}{K_{\text{m MASP1}^*}^{\text{C4}}}+\frac{[\text{fC4bC2}]}{K_{\text{m MASP1}^*}^{\text{C2}}}+\frac{[\text{fC4bC4bC2}]}{K_{\text{m MASP1}^*}^{\text{C2}}}+\frac{[\text{fC3bC4bC2}]}{K_{\text{m MASP1}^*}^{\text{C2}}}+\frac{[\text{pC4bC2}]}{K_{\text{m MASP1}^*}^{\text{C2}}}+\frac{[\text{pC4bC4bC2}]}{K_{\text{m MASP1}^*}^{\text{C2}}}\\
+\frac{[\text{pC3bC4bC2}]}{K_{\text{m MASP1}^*}^{\text{C2}}}+\frac{[\text{pMBL}_3\text{MASP1}_{\text{zym}}]}{K_{\text{m MASP1}^*}^{\text{MASP1}_{\text{zym}}}}+\frac{[\text{pMBL}_3\text{MASP2}_{\text{zym}}]}{K_{\text{m MASP1}^*}^{\text{MASP2}_{\text{zym}}}}+\frac{[\text{pMBL}_3\text{MASP3}_{\text{zym}}]}{K_{\text{m MASP1}^*}^{\text{MASP3}_{\text{zym}}}}\\
+\frac{[\text{pMBL}_4\text{MASP1}_{\text{zym}}]}{K_{\text{m MASP1}^*}^{\text{MASP1}_{\text{zym}}}}+\frac{[\text{pMBL}_4\text{MASP2}_{\text{zym}}]}{K_{\text{m MASP1}^*}^{\text{MASP2}_{\text{zym}}}}+\frac{[\text{pMBL}_4\text{MASP3}_{\text{zym}}]}{K_{\text{m MASP1}^*}^{\text{MASP3}_{\text{zym}}}}+\frac{[\text{pCLLKMASP1}_{\text{zym}}]}{K_{\text{m MASP1}^*}^{\text{MASP1}_{\text{zym}}}}\\
+\frac{[\text{pCLLKMASP2}_{\text{zym}}]}{K_{\text{m MASP1}^*}^{\text{MASP2}_{\text{zym}}}}+\frac{[\text{pCLLKMASP3}_{\text{zym}}]}{K_{\text{m MASP1}^*}^{\text{MASP3}_{\text{zym}}}}+\frac{[\text{pCLK1MASP1}_{\text{zym}}]}{K_{\text{m MASP1}^*}^{\text{MASP1}_{\text{zym}}}}+\frac{[\text{pCLK1MASP2}_{\text{zym}}]}{K_{\text{m MASP1}^*}^{\text{MASP2}_{\text{zym}}}}\\
+\frac{[\text{pCLK1MASP3}_{\text{zym}}]}{K_{\text{m MASP1}^*}^{\text{MASP3}_{\text{zym}}}}+\frac{[\text{pCLL1MASP1}_{\text{zym}}]}{K_{\text{m MASP1}^*}^{\text{MASP1}_{\text{zym}}}}+\frac{[\text{pCLL1MASP2}_{\text{zym}}]}{K_{\text{m MASP1}^*}^{\text{MASP2}_{\text{zym}}}}+\frac{[\text{pCLL1MASP3}_{\text{zym}}]}{K_{\text{m MASP1}^*}^{\text{MASP3}_{\text{zym}}}}\\
+\frac{[\text{pLFMASP1}_{\text{zym}}]}{K_{\text{m MASP1}^*}^{\text{MASP1}_{\text{zym}}}}+\frac{[\text{pLFMASP2}_{\text{zym}}]}{K_{\text{m MASP1}^*}^{\text{MASP2}_{\text{zym}}}}+\frac{[\text{pLFMASP3}_{\text{zym}}]}{K_{\text{m MASP1}^*}^{\text{MASP3}_{\text{zym}}}}+\frac{[\text{pHFMASP1}_{\text{zym}}]}{K_{\text{m MASP1}^*}^{\text{MASP1}_{\text{zym}}}}+\frac{[\text{pHFMASP2}_{\text{zym}}]}{K_{\text{m MASP1}^*}^{\text{MASP2}_{\text{zym}}}}\\
+\frac{[\text{pHFMASP3}_{\text{zym}}]}{K_{\text{m MASP1}^*}^{\text{MASP3}_{\text{zym}}}}+\frac{[\text{pMFMASP1}_{\text{zym}}]}{K_{\text{m MASP1}^*}^{\text{MASP1}_{\text{zym}}}}+\frac{[\text{pMFMASP2}_{\text{zym}}]}{K_{\text{m MASP1}^*}^{\text{MASP2}_{\text{zym}}}}+\frac{[\text{pMFMASP3}_{\text{zym}}]}{K_{\text{m MASP1}^*}^{\text{MASP3}_{\text{zym}}}}
\end{array}\right)}
$$

$$
-\frac{k_{\text{cat MASP1}^*}^{\text{MASP1}_{\text{zym}}}\left[\text{pLFMASP1}^*\right]\left[\text{pCLLKMASP1}_{\text{zym}}\right]}{K_{\text{m MASP1}^*}^{\text{MASP1}_{\text{zym}}}\left(\begin{array}{l}
1+\frac{[\text{C4}]}{K_{\text{m MASP1}^*}^{\text{C4}}}+\frac{[\text{fC4bC2}]}{K_{\text{m MASP1}^*}^{\text{C2}}}+\frac{[\text{fC4bC4bC2}]}{K_{\text{m MASP1}^*}^{\text{C2}}}+\frac{[\text{fC3bC4bC2}]}{K_{\text{m MASP1}^*}^{\text{C2}}}+\frac{[\text{pC4bC2}]}{K_{\text{m MASP1}^*}^{\text{C2}}}+\frac{[\text{pC4bC4bC2}]}{K_{\text{m MASP1}^*}^{\text{C2}}}\\
+\frac{[\text{pC3bC4bC2}]}{K_{\text{m MASP1}^*}^{\text{C2}}}+\frac{[\text{pMBL}_3\text{MASP1}_{\text{zym}}]}{K_{\text{m MASP1}^*}^{\text{MASP1}_{\text{zym}}}}+\frac{[\text{pMBL}_3\text{MASP2}_{\text{zym}}]}{K_{\text{m MASP1}^*}^{\text{MASP2}_{\text{zym}}}}+\frac{[\text{pMBL}_3\text{MASP3}_{\text{zym}}]}{K_{\text{m MASP1}^*}^{\text{MASP3}_{\text{zym}}}}\\
+\frac{[\text{pMBL}_4\text{MASP1}_{\text{zym}}]}{K_{\text{m MASP1}^*}^{\text{MASP1}_{\text{zym}}}}+\frac{[\text{pMBL}_4\text{MASP2}_{\text{zym}}]}{K_{\text{m MASP1}^*}^{\text{MASP2}_{\text{zym}}}}+\frac{[\text{pMBL}_4\text{MASP3}_{\text{zym}}]}{K_{\text{m MASP1}^*}^{\text{MASP3}_{\text{zym}}}}+\frac{[\text{pCLLKMASP1}_{\text{zym}}]}{K_{\text{m MASP1}^*}^{\text{MASP1}_{\text{zym}}}}\\
+\frac{[\text{pCLLKMASP2}_{\text{zym}}]}{K_{\text{m MASP1}^*}^{\text{MASP2}_{\text{zym}}}}+\frac{[\text{pCLLKMASP3}_{\text{zym}}]}{K_{\text{m MASP1}^*}^{\text{MASP3}_{\text{zym}}}}+\frac{[\text{pCLK1MASP1}_{\text{zym}}]}{K_{\text{m MASP1}^*}^{\text{MASP1}_{\text{zym}}}}+\frac{[\text{pCLK1MASP2}_{\text{zym}}]}{K_{\text{m MASP1}^*}^{\text{MASP2}_{\text{zym}}}}\\
+\frac{[\text{pCLK1MASP3}_{\text{zym}}]}{K_{\text{m MASP1}^*}^{\text{MASP3}_{\text{zym}}}}+\frac{[\text{pCLL1MASP1}_{\text{zym}}]}{K_{\text{m MASP1}^*}^{\text{MASP1}_{\text{zym}}}}+\frac{[\text{pCLL1MASP2}_{\text{zym}}]}{K_{\text{m MASP1}^*}^{\text{MASP2}_{\text{zym}}}}+\frac{[\text{pCLL1MASP3}_{\text{zym}}]}{K_{\text{m MASP1}^*}^{\text{MASP3}_{\text{zym}}}}\\
+\frac{[\text{pLFMASP1}_{\text{zym}}]}{K_{\text{m MASP1}^*}^{\text{MASP1}_{\text{zym}}}}+\frac{[\text{pLFMASP2}_{\text{zym}}]}{K_{\text{m MASP1}^*}^{\text{MASP2}_{\text{zym}}}}+\frac{[\text{pLFMASP3}_{\text{zym}}]}{K_{\text{m MASP1}^*}^{\text{MASP3}_{\text{zym}}}}+\frac{[\text{pHFMASP1}_{\text{zym}}]}{K_{\text{m MASP1}^*}^{\text{MASP1}_{\text{zym}}}}+\frac{[\text{pHFMASP2}_{\text{zym}}]}{K_{\text{m MASP1}^*}^{\text{MASP2}_{\text{zym}}}}\\
+\frac{[\text{pHFMASP3}_{\text{zym}}]}{K_{\text{m MASP1}^*}^{\text{MASP3}_{\text{zym}}}}+\frac{[\text{pMFMASP1}_{\text{zym}}]}{K_{\text{m MASP1}^*}^{\text{MASP1}_{\text{zym}}}}+\frac{[\text{pMFMASP2}_{\text{zym}}]}{K_{\text{m MASP1}^*}^{\text{MASP2}_{\text{zym}}}}+\frac{[\text{pMFMASP3}_{\text{zym}}]}{K_{\text{m MASP1}^*}^{\text{MASP3}_{\text{zym}}}}
\end{array}\right)}
$$

$$-\frac{k_{\mathrm{cat\ MASP1^*}}^{\mathrm{MASP1_{zym}}}\left[\mathrm{pHFMASP1^*}\right]\left[\mathrm{pCLLKMASP1_{zym}}\right]}{K_{\mathrm{m\ MASP1^*}}^{\mathrm{MASP1_{zym}}}\left(\begin{aligned}&1+\frac{[\mathrm{C4}]}{K_{\mathrm{m\ MASP1^*}}^{\mathrm{C4}}}+\frac{[\mathrm{fC4bC2}]}{K_{\mathrm{m\ MASP1^*}}^{\mathrm{C2}}}+\frac{[\mathrm{fC4bC4bC2}]}{K_{\mathrm{m\ MASP1^*}}^{\mathrm{C2}}}+\frac{[\mathrm{fC3bC4bC2}]}{K_{\mathrm{m\ MASP1^*}}^{\mathrm{C2}}}+\frac{[\mathrm{pC4bC2}]}{K_{\mathrm{m\ MASP1^*}}^{\mathrm{C2}}}+\frac{[\mathrm{pC4bC4bC2}]}{K_{\mathrm{m\ MASP1^*}}^{\mathrm{C2}}}\\&+\frac{[\mathrm{pC3bC4bC2}]}{K_{\mathrm{m\ MASP1^*}}^{\mathrm{C2}}}+\frac{\left[\mathrm{pMBL_3MASP1_{zym}}\right]}{K_{\mathrm{m\ MASP1^*}}^{\mathrm{MASP1_{zym}}}}+\frac{\left[\mathrm{pMBL_3MASP2_{zym}}\right]}{K_{\mathrm{m\ MASP1^*}}^{\mathrm{MASP2_{zym}}}}+\frac{\left[\mathrm{pMBL_3MASP3_{zym}}\right]}{K_{\mathrm{m\ MASP1^*}}^{\mathrm{MASP3_{zym}}}}\\&+\frac{\left[\mathrm{pMBL_4MASP1_{zym}}\right]}{K_{\mathrm{m\ MASP1^*}}^{\mathrm{MASP1_{zym}}}}+\frac{\left[\mathrm{pMBL_4MASP2_{zym}}\right]}{K_{\mathrm{m\ MASP1^*}}^{\mathrm{MASP2_{zym}}}}+\frac{\left[\mathrm{pMBL_4MASP3_{zym}}\right]}{K_{\mathrm{m\ MASP1^*}}^{\mathrm{MASP3_{zym}}}}+\frac{\left[\mathrm{pCLLKMASP1_{zym}}\right]}{K_{\mathrm{m\ MASP1^*}}^{\mathrm{MASP1_{zym}}}}\\&+\frac{\left[\mathrm{pCLLKMASP2_{zym}}\right]}{K_{\mathrm{m\ MASP1^*}}^{\mathrm{MASP2_{zym}}}}+\frac{\left[\mathrm{pCLLKMASP3_{zym}}\right]}{K_{\mathrm{m\ MASP1^*}}^{\mathrm{MASP3_{zym}}}}+\frac{\left[\mathrm{pCLK1MASP1_{zym}}\right]}{K_{\mathrm{m\ MASP1^*}}^{\mathrm{MASP1_{zym}}}}+\frac{\left[\mathrm{pCLK1MASP2_{zym}}\right]}{K_{\mathrm{m\ MASP1^*}}^{\mathrm{MASP2_{zym}}}}\\&+\frac{\left[\mathrm{pCLK1MASP3_{zym}}\right]}{K_{\mathrm{m\ MASP1^*}}^{\mathrm{MASP3_{zym}}}}+\frac{\left[\mathrm{pCLL1MASP1_{zym}}\right]}{K_{\mathrm{m\ MASP1^*}}^{\mathrm{MASP1_{zym}}}}+\frac{\left[\mathrm{pCLL1MASP2_{zym}}\right]}{K_{\mathrm{m\ MASP1^*}}^{\mathrm{MASP2_{zym}}}}+\frac{\left[\mathrm{pCLL1MASP3_{zym}}\right]}{K_{\mathrm{m\ MASP1^*}}^{\mathrm{MASP3_{zym}}}}\\&+\frac{\left[\mathrm{pLFMASP1_{zym}}\right]}{K_{\mathrm{m\ MASP1^*}}^{\mathrm{MASP1_{zym}}}}+\frac{\left[\mathrm{pLFMASP2_{zym}}\right]}{K_{\mathrm{m\ MASP1^*}}^{\mathrm{MASP2_{zym}}}}+\frac{\left[\mathrm{pLFMASP3_{zym}}\right]}{K_{\mathrm{m\ MASP1^*}}^{\mathrm{MASP3_{zym}}}}+\frac{\left[\mathrm{pHFMASP1_{zym}}\right]}{K_{\mathrm{m\ MASP1^*}}^{\mathrm{MASP1_{zym}}}}+\frac{\left[\mathrm{pHFMASP2_{zym}}\right]}{K_{\mathrm{m\ MASP1^*}}^{\mathrm{MASP2_{zym}}}}\\&+\frac{\left[\mathrm{pHFMASP3_{zym}}\right]}{K_{\mathrm{m\ MASP1^*}}^{\mathrm{MASP3_{zym}}}}+\frac{\left[\mathrm{pMFMASP1_{zym}}\right]}{K_{\mathrm{m\ MASP1^*}}^{\mathrm{MASP1_{zym}}}}+\frac{\left[\mathrm{pMFMASP2_{zym}}\right]}{K_{\mathrm{m\ MASP1^*}}^{\mathrm{MASP2_{zym}}}}+\frac{\left[\mathrm{pMFMASP3_{zym}}\right]}{K_{\mathrm{m\ MASP1^*}}^{\mathrm{MASP3_{zym}}}}\end{aligned}\right)}$$

$$-\frac{k_{\mathrm{cat\ MASP1^*}}^{\mathrm{MASP1_{zym}}}\left[\mathrm{pMFMASP1^*}\right]\left[\mathrm{pCLLKMASP1_{zym}}\right]}{K_{\mathrm{m\ MASP1^*}}^{\mathrm{MASP1_{zym}}}\left(\begin{aligned}&1+\frac{[\mathrm{C4}]}{K_{\mathrm{m\ MASP1^*}}^{\mathrm{C4}}}+\frac{[\mathrm{fC4bC2}]}{K_{\mathrm{m\ MASP1^*}}^{\mathrm{C2}}}+\frac{[\mathrm{fC4bC4bC2}]}{K_{\mathrm{m\ MASP1^*}}^{\mathrm{C2}}}+\frac{[\mathrm{fC3bC4bC2}]}{K_{\mathrm{m\ MASP1^*}}^{\mathrm{C2}}}+\frac{[\mathrm{pC4bC2}]}{K_{\mathrm{m\ MASP1^*}}^{\mathrm{C2}}}+\frac{[\mathrm{pC4bC4bC2}]}{K_{\mathrm{m\ MASP1^*}}^{\mathrm{C2}}}\\&+\frac{[\mathrm{pC3bC4bC2}]}{K_{\mathrm{m\ MASP1^*}}^{\mathrm{C2}}}+\frac{\left[\mathrm{pMBL_3MASP1_{zym}}\right]}{K_{\mathrm{m\ MASP1^*}}^{\mathrm{MASP1_{zym}}}}+\frac{\left[\mathrm{pMBL_3MASP2_{zym}}\right]}{K_{\mathrm{m\ MASP1^*}}^{\mathrm{MASP2_{zym}}}}+\frac{\left[\mathrm{pMBL_3MASP3_{zym}}\right]}{K_{\mathrm{m\ MASP1^*}}^{\mathrm{MASP3_{zym}}}}\\&+\frac{\left[\mathrm{pMBL_4MASP1_{zym}}\right]}{K_{\mathrm{m\ MASP1^*}}^{\mathrm{MASP1_{zym}}}}+\frac{\left[\mathrm{pMBL_4MASP2_{zym}}\right]}{K_{\mathrm{m\ MASP1^*}}^{\mathrm{MASP2_{zym}}}}+\frac{\left[\mathrm{pMBL_4MASP3_{zym}}\right]}{K_{\mathrm{m\ MASP1^*}}^{\mathrm{MASP3_{zym}}}}+\frac{\left[\mathrm{pCLLKMASP1_{zym}}\right]}{K_{\mathrm{m\ MASP1^*}}^{\mathrm{MASP1_{zym}}}}\\&+\frac{\left[\mathrm{pCLLKMASP2_{zym}}\right]}{K_{\mathrm{m\ MASP1^*}}^{\mathrm{MASP2_{zym}}}}+\frac{\left[\mathrm{pCLLKMASP3_{zym}}\right]}{K_{\mathrm{m\ MASP1^*}}^{\mathrm{MASP3_{zym}}}}+\frac{\left[\mathrm{pCLK1MASP1_{zym}}\right]}{K_{\mathrm{m\ MASP1^*}}^{\mathrm{MASP1_{zym}}}}+\frac{\left[\mathrm{pCLK1MASP2_{zym}}\right]}{K_{\mathrm{m\ MASP1^*}}^{\mathrm{MASP2_{zym}}}}\\&+\frac{\left[\mathrm{pCLK1MASP3_{zym}}\right]}{K_{\mathrm{m\ MASP1^*}}^{\mathrm{MASP3_{zym}}}}+\frac{\left[\mathrm{pCLL1MASP1_{zym}}\right]}{K_{\mathrm{m\ MASP1^*}}^{\mathrm{MASP1_{zym}}}}+\frac{\left[\mathrm{pCLL1MASP2_{zym}}\right]}{K_{\mathrm{m\ MASP1^*}}^{\mathrm{MASP2_{zym}}}}+\frac{\left[\mathrm{pCLL1MASP3_{zym}}\right]}{K_{\mathrm{m\ MASP1^*}}^{\mathrm{MASP3_{zym}}}}\\&+\frac{\left[\mathrm{pLFMASP1_{zym}}\right]}{K_{\mathrm{m\ MASP1^*}}^{\mathrm{MASP1_{zym}}}}+\frac{\left[\mathrm{pLFMASP2_{zym}}\right]}{K_{\mathrm{m\ MASP1^*}}^{\mathrm{MASP2_{zym}}}}+\frac{\left[\mathrm{pLFMASP3_{zym}}\right]}{K_{\mathrm{m\ MASP1^*}}^{\mathrm{MASP3_{zym}}}}+\frac{\left[\mathrm{pHFMASP1_{zym}}\right]}{K_{\mathrm{m\ MASP1^*}}^{\mathrm{MASP1_{zym}}}}+\frac{\left[\mathrm{pHFMASP2_{zym}}\right]}{K_{\mathrm{m\ MASP1^*}}^{\mathrm{MASP2_{zym}}}}\\&+\frac{\left[\mathrm{pHFMASP3_{zym}}\right]}{K_{\mathrm{m\ MASP1^*}}^{\mathrm{MASP3_{zym}}}}+\frac{\left[\mathrm{pMFMASP1_{zym}}\right]}{K_{\mathrm{m\ MASP1^*}}^{\mathrm{MASP1_{zym}}}}+\frac{\left[\mathrm{pMFMASP2_{zym}}\right]}{K_{\mathrm{m\ MASP1^*}}^{\mathrm{MASP2_{zym}}}}+\frac{\left[\mathrm{pMFMASP3_{zym}}\right]}{K_{\mathrm{m\ MASP1^*}}^{\mathrm{MASP3_{zym}}}}\end{aligned}\right)}$$

$$
-\frac{k^{\mathrm{MASP1_{zym}}}_{\mathrm{cat\,MASP1^*}}\left[\mathrm{pPTX3LFMASP1^*}\right]\left[\mathrm{pCLLKMASP1_{zym}}\right]}{K^{\mathrm{MASP1_{zym}}}_{\mathrm{m\,MASP1^*}}\left(\begin{array}{l}
1+\frac{[\mathrm{C4}]}{K^{\mathrm{C4}}_{\mathrm{m\,MASP1^*}}}+\frac{[\mathrm{fC4bC2}]}{K^{\mathrm{C2}}_{\mathrm{m\,MASP1^*}}}+\frac{[\mathrm{fC4bC4bC2}]}{K^{\mathrm{C2}}_{\mathrm{m\,MASP1^*}}}+\frac{[\mathrm{fC3bC4bC2}]}{K^{\mathrm{C2}}_{\mathrm{m\,MASP1^*}}}+\frac{[\mathrm{pC4bC2}]}{K^{\mathrm{C2}}_{\mathrm{m\,MASP1^*}}}+\frac{[\mathrm{pC4bC4bC2}]}{K^{\mathrm{C2}}_{\mathrm{m\,MASP1^*}}}\\
+\frac{[\mathrm{pC3bC4bC2}]}{K^{\mathrm{C2}}_{\mathrm{m\,MASP1^*}}}+\frac{[\mathrm{pMBL_3MASP1_{zym}}]}{K^{\mathrm{MASP1_{zym}}}_{\mathrm{m\,MASP1^*}}}+\frac{[\mathrm{pMBL_3MASP2_{zym}}]}{K^{\mathrm{MASP2_{zym}}}_{\mathrm{m\,MASP1^*}}}+\frac{[\mathrm{pMBL_3MASP3_{zym}}]}{K^{\mathrm{MASP3_{zym}}}_{\mathrm{m\,MASP1^*}}}\\
+\frac{[\mathrm{pMBL_4MASP1_{zym}}]}{K^{\mathrm{MASP1_{zym}}}_{\mathrm{m\,MASP1^*}}}+\frac{[\mathrm{pMBL_4MASP2_{zym}}]}{K^{\mathrm{MASP2_{zym}}}_{\mathrm{m\,MASP1^*}}}+\frac{[\mathrm{pMBL_4MASP3_{zym}}]}{K^{\mathrm{MASP3_{zym}}}_{\mathrm{m\,MASP1^*}}}+\frac{[\mathrm{pCLLKMASP1_{zym}}]}{K^{\mathrm{MASP1_{zym}}}_{\mathrm{m\,MASP1^*}}}\\
+\frac{[\mathrm{pCLLKMASP2_{zym}}]}{K^{\mathrm{MASP2_{zym}}}_{\mathrm{m\,MASP1^*}}}+\frac{[\mathrm{pCLLKMASP3_{zym}}]}{K^{\mathrm{MASP3_{zym}}}_{\mathrm{m\,MASP1^*}}}+\frac{[\mathrm{pCLK1MASP1_{zym}}]}{K^{\mathrm{MASP1_{zym}}}_{\mathrm{m\,MASP1^*}}}+\frac{[\mathrm{pCLK1MASP2_{zym}}]}{K^{\mathrm{MASP2_{zym}}}_{\mathrm{m\,MASP1^*}}}\\
+\frac{[\mathrm{pCLK1MASP3_{zym}}]}{K^{\mathrm{MASP3_{zym}}}_{\mathrm{m\,MASP1^*}}}+\frac{[\mathrm{pCLL1MASP1_{zym}}]}{K^{\mathrm{MASP1_{zym}}}_{\mathrm{m\,MASP1^*}}}+\frac{[\mathrm{pCLL1MASP2_{zym}}]}{K^{\mathrm{MASP2_{zym}}}_{\mathrm{m\,MASP1^*}}}+\frac{[\mathrm{pCLL1MASP3_{zym}}]}{K^{\mathrm{MASP3_{zym}}}_{\mathrm{m\,MASP1^*}}}\\
+\frac{[\mathrm{pLFMASP1_{zym}}]}{K^{\mathrm{MASP1_{zym}}}_{\mathrm{m\,MASP1^*}}}+\frac{[\mathrm{pLFMASP2_{zym}}]}{K^{\mathrm{MASP2_{zym}}}_{\mathrm{m\,MASP1^*}}}+\frac{[\mathrm{pLFMASP3_{zym}}]}{K^{\mathrm{MASP3_{zym}}}_{\mathrm{m\,MASP1^*}}}+\frac{[\mathrm{pHFMASP1_{zym}}]}{K^{\mathrm{MASP1_{zym}}}_{\mathrm{m\,MASP1^*}}}+\frac{[\mathrm{pHFMASP2_{zym}}]}{K^{\mathrm{MASP2_{zym}}}_{\mathrm{m\,MASP1^*}}}\\
+\frac{[\mathrm{pHFMASP3_{zym}}]}{K^{\mathrm{MASP3_{zym}}}_{\mathrm{m\,MASP1^*}}}+\frac{[\mathrm{pMFMASP1_{zym}}]}{K^{\mathrm{MASP1_{zym}}}_{\mathrm{m\,MASP1^*}}}+\frac{[\mathrm{pMFMASP2_{zym}}]}{K^{\mathrm{MASP2_{zym}}}_{\mathrm{m\,MASP1^*}}}+\frac{[\mathrm{pMFMASP3_{zym}}]}{K^{\mathrm{MASP3_{zym}}}_{\mathrm{m\,MASP1^*}}}\\
+\frac{[\mathrm{pPTX3LFMASP1_{zym}}]}{K^{\mathrm{MASP1_{zym}}}_{\mathrm{m\,MASP1^*}}}+\frac{[\mathrm{pPXT3LFMASP2_{zym}}]}{K^{\mathrm{MASP2_{zym}}}_{\mathrm{m\,MASP1^*}}}+\frac{[\mathrm{pPTX3LFMASP3_{zym}}]}{K^{\mathrm{MASP3_{zym}}}_{\mathrm{m\,MASP1^*}}}
\end{array}\right)}
$$

$$
-\frac{k^{\mathrm{MASP1_{zym}}}_{\mathrm{cat\,MASP2^*}}\left[\mathrm{pMBL_3MASP2^*}\right]\left[\mathrm{pCLLKMASP1_{zym}}\right]}{K^{\mathrm{MASP1_{zym}}}_{\mathrm{m\,MASP2^*}}\left(\begin{array}{l}
1+\frac{[\mathrm{C4}]}{K^{\mathrm{C4}}_{\mathrm{m\,MASP2^*}}}+\frac{[\mathrm{fC4bC2}]}{K^{\mathrm{C2}}_{\mathrm{m\,MASP2^*}}}+\frac{[\mathrm{fC4bC4bC2}]}{K^{\mathrm{C2}}_{\mathrm{m\,MASP2^*}}}+\frac{[\mathrm{fC3bC4bC2}]}{K^{\mathrm{C2}}_{\mathrm{m\,MASP2^*}}}+\frac{[\mathrm{pC4bC2}]}{K^{\mathrm{C2}}_{\mathrm{m\,MASP2^*}}}+\frac{[\mathrm{pC4bC4bC2}]}{K^{\mathrm{C2}}_{\mathrm{m\,MASP2^*}}}\\
+\frac{[\mathrm{pC3bC4bC2}]}{K^{\mathrm{C2}}_{\mathrm{m\,MASP2^*}}}+\frac{[\mathrm{pMBL_3MASP1_{zym}}]}{K^{\mathrm{MASP1_{zym}}}_{\mathrm{m\,MASP2^*}}}+\frac{[\mathrm{pMBL_3MASP2_{zym}}]}{K^{\mathrm{MASP2_{zym}}}_{\mathrm{m\,MASP2^*}}}+\frac{[\mathrm{pMBL_4MASP1_{zym}}]}{K^{\mathrm{MASP1_{zym}}}_{\mathrm{m\,MASP2^*}}}\\
+\frac{[\mathrm{pMBL_4MASP2_{zym}}]}{K^{\mathrm{MASP2_{zym}}}_{\mathrm{m\,MASP2^*}}}+\frac{[\mathrm{pCLLKMASP1_{zym}}]}{K^{\mathrm{MASP1_{zym}}}_{\mathrm{m\,MASP2^*}}}+\frac{[\mathrm{pCLLKMASP2_{zym}}]}{K^{\mathrm{MASP2_{zym}}}_{\mathrm{m\,MASP2^*}}}+\frac{[\mathrm{pCLK1MASP1_{zym}}]}{K^{\mathrm{MASP1_{zym}}}_{\mathrm{m\,MASP2^*}}}\\
+\frac{[\mathrm{pCLK1MASP2_{zym}}]}{K^{\mathrm{MASP2_{zym}}}_{\mathrm{m\,MASP2^*}}}+\frac{[\mathrm{pCLL1MASP1_{zym}}]}{K^{\mathrm{MASP1_{zym}}}_{\mathrm{m\,MASP2^*}}}+\frac{[\mathrm{pCLL1MASP2_{zym}}]}{K^{\mathrm{MASP2_{zym}}}_{\mathrm{m\,MASP2^*}}}+\frac{[\mathrm{pLFMASP1_{zym}}]}{K^{\mathrm{MASP1_{zym}}}_{\mathrm{m\,MASP2^*}}}\\
+\frac{[\mathrm{pLFMASP2_{zym}}]}{K^{\mathrm{MASP2_{zym}}}_{\mathrm{m\,MASP2^*}}}+\frac{[\mathrm{pHFMASP1_{zym}}]}{K^{\mathrm{MASP1_{zym}}}_{\mathrm{m\,MASP2^*}}}+\frac{[\mathrm{pHFMASP2_{zym}}]}{K^{\mathrm{MASP2_{zym}}}_{\mathrm{m\,MASP2^*}}}+\frac{[\mathrm{pMFMASP1_{zym}}]}{K^{\mathrm{MASP1_{zym}}}_{\mathrm{m\,MASP2^*}}}+\frac{[\mathrm{pMFMASP2_{zym}}]}{K^{\mathrm{MASP2_{zym}}}_{\mathrm{m\,MASP2^*}}}
\end{array}\right)}
$$

$$-\frac{k_{\mathrm{cat\,MASP2^*}}^{\mathrm{MASP1_{zym}}}\left[\mathrm{pMBL_4MASP2^*}\right]\left[\mathrm{pCLLKMASP1_{zym}}\right]}{K_{\mathrm{m\,MASP2^*}}^{\mathrm{MASP1_{zym}}}\left(\begin{array}{l}1+\frac{[\mathrm{C4}]}{K_{\mathrm{m\,MASP2^*}}^{\mathrm{C4}}}+\frac{[\mathrm{fC4bC2}]}{K_{\mathrm{m\,MASP2^*}}^{\mathrm{C2}}}+\frac{[\mathrm{fC4bC4bC2}]}{K_{\mathrm{m\,MASP2^*}}^{\mathrm{C2}}}+\frac{[\mathrm{fC3bC4bC2}]}{K_{\mathrm{m\,MASP2^*}}^{\mathrm{C2}}}+\frac{[\mathrm{pC4bC2}]}{K_{\mathrm{m\,MASP2^*}}^{\mathrm{C2}}}+\frac{[\mathrm{pC4bC4bC2}]}{K_{\mathrm{m\,MASP2^*}}^{\mathrm{C2}}}\\+\frac{[\mathrm{pC3bC4bC2}]}{K_{\mathrm{m\,MASP2^*}}^{\mathrm{C2}}}+\frac{[\mathrm{pMBL_3MASP1_{zym}}]}{K_{\mathrm{m\,MASP2^*}}^{\mathrm{MASP1_{zym}}}}+\frac{[\mathrm{pMBL_3MASP2_{zym}}]}{K_{\mathrm{m\,MASP2^*}}^{\mathrm{MASP2_{zym}}}}+\frac{[\mathrm{pMBL_4MASP1_{zym}}]}{K_{\mathrm{m\,MASP2^*}}^{\mathrm{MASP1_{zym}}}}\\+\frac{[\mathrm{pMBL_4MASP2_{zym}}]}{K_{\mathrm{m\,MASP2^*}}^{\mathrm{MASP2_{zym}}}}+\frac{[\mathrm{pCLLKMASP1_{zym}}]}{K_{\mathrm{m\,MASP2^*}}^{\mathrm{MASP1_{zym}}}}+\frac{[\mathrm{pCLLKMASP2_{zym}}]}{K_{\mathrm{m\,MASP2^*}}^{\mathrm{MASP2_{zym}}}}+\frac{[\mathrm{pCLK1MASP1_{zym}}]}{K_{\mathrm{m\,MASP2^*}}^{\mathrm{MASP1_{zym}}}}\\+\frac{[\mathrm{pCLK1MASP2_{zym}}]}{K_{\mathrm{m\,MASP2^*}}^{\mathrm{MASP2_{zym}}}}+\frac{[\mathrm{pCLL1MASP1_{zym}}]}{K_{\mathrm{m\,MASP2^*}}^{\mathrm{MASP1_{zym}}}}+\frac{[\mathrm{pCLL1MASP2_{zym}}]}{K_{\mathrm{m\,MASP2^*}}^{\mathrm{MASP2_{zym}}}}+\frac{[\mathrm{pLFMASP1_{zym}}]}{K_{\mathrm{m\,MASP2^*}}^{\mathrm{MASP1_{zym}}}}\\+\frac{[\mathrm{pLFMASP2_{zym}}]}{K_{\mathrm{m\,MASP2^*}}^{\mathrm{MASP2_{zym}}}}+\frac{[\mathrm{pHFMASP1_{zym}}]}{K_{\mathrm{m\,MASP2^*}}^{\mathrm{MASP1_{zym}}}}+\frac{[\mathrm{pHFMASP2_{zym}}]}{K_{\mathrm{m\,MASP2^*}}^{\mathrm{MASP2_{zym}}}}+\frac{[\mathrm{pMFMASP1_{zym}}]}{K_{\mathrm{m\,MASP2^*}}^{\mathrm{MASP1_{zym}}}}+\frac{[\mathrm{pMFMASP2_{zym}}]}{K_{\mathrm{m\,MASP2^*}}^{\mathrm{MASP2_{zym}}}}\end{array}\right)}$$

$$-\frac{k_{\mathrm{cat\,MASP2^*}}^{\mathrm{MASP1_{zym}}}\left[\mathrm{pCLLKMASP2^*}\right]\left[\mathrm{pCLLKMASP1_{zym}}\right]}{K_{\mathrm{m\,MASP2^*}}^{\mathrm{MASP1_{zym}}}\left(\begin{array}{l}1+\frac{[\mathrm{C4}]}{K_{\mathrm{m\,MASP2^*}}^{\mathrm{C4}}}+\frac{[\mathrm{fC4bC2}]}{K_{\mathrm{m\,MASP2^*}}^{\mathrm{C2}}}+\frac{[\mathrm{fC4bC4bC2}]}{K_{\mathrm{m\,MASP2^*}}^{\mathrm{C2}}}+\frac{[\mathrm{fC3bC4bC2}]}{K_{\mathrm{m\,MASP2^*}}^{\mathrm{C2}}}+\frac{[\mathrm{pC4bC2}]}{K_{\mathrm{m\,MASP2^*}}^{\mathrm{C2}}}+\frac{[\mathrm{pC4bC4bC2}]}{K_{\mathrm{m\,MASP2^*}}^{\mathrm{C2}}}\\+\frac{[\mathrm{pC3bC4bC2}]}{K_{\mathrm{m\,MASP2^*}}^{\mathrm{C2}}}+\frac{[\mathrm{pMBL_3MASP1_{zym}}]}{K_{\mathrm{m\,MASP2^*}}^{\mathrm{MASP1_{zym}}}}+\frac{[\mathrm{pMBL_3MASP2_{zym}}]}{K_{\mathrm{m\,MASP2^*}}^{\mathrm{MASP2_{zym}}}}+\frac{[\mathrm{pMBL_4MASP1_{zym}}]}{K_{\mathrm{m\,MASP2^*}}^{\mathrm{MASP1_{zym}}}}\\+\frac{[\mathrm{pMBL_4MASP2_{zym}}]}{K_{\mathrm{m\,MASP2^*}}^{\mathrm{MASP2_{zym}}}}+\frac{[\mathrm{pCLLKMASP1_{zym}}]}{K_{\mathrm{m\,MASP2^*}}^{\mathrm{MASP1_{zym}}}}+\frac{[\mathrm{pCLLKMASP2_{zym}}]}{K_{\mathrm{m\,MASP2^*}}^{\mathrm{MASP2_{zym}}}}+\frac{[\mathrm{pCLK1MASP1_{zym}}]}{K_{\mathrm{m\,MASP2^*}}^{\mathrm{MASP1_{zym}}}}\\+\frac{[\mathrm{pCLK1MASP2_{zym}}]}{K_{\mathrm{m\,MASP2^*}}^{\mathrm{MASP2_{zym}}}}+\frac{[\mathrm{pCLL1MASP1_{zym}}]}{K_{\mathrm{m\,MASP2^*}}^{\mathrm{MASP1_{zym}}}}+\frac{[\mathrm{pCLL1MASP2_{zym}}]}{K_{\mathrm{m\,MASP2^*}}^{\mathrm{MASP2_{zym}}}}+\frac{[\mathrm{pLFMASP1_{zym}}]}{K_{\mathrm{m\,MASP2^*}}^{\mathrm{MASP1_{zym}}}}\\+\frac{[\mathrm{pLFMASP2_{zym}}]}{K_{\mathrm{m\,MASP2^*}}^{\mathrm{MASP2_{zym}}}}+\frac{[\mathrm{pHFMASP1_{zym}}]}{K_{\mathrm{m\,MASP2^*}}^{\mathrm{MASP1_{zym}}}}+\frac{[\mathrm{pHFMASP2_{zym}}]}{K_{\mathrm{m\,MASP2^*}}^{\mathrm{MASP2_{zym}}}}+\frac{[\mathrm{pMFMASP1_{zym}}]}{K_{\mathrm{m\,MASP2^*}}^{\mathrm{MASP1_{zym}}}}+\frac{[\mathrm{pMFMASP2_{zym}}]}{K_{\mathrm{m\,MASP2^*}}^{\mathrm{MASP2_{zym}}}}\end{array}\right)}$$

$$-\frac{k_{\mathrm{cat\,MASP2^*}}^{\mathrm{MASP1_{zym}}}\left[\mathrm{pCLK1MASP2^*}\right]\left[\mathrm{pCLLKMASP1_{zym}}\right]}{K_{\mathrm{m\,MASP2^*}}^{\mathrm{MASP1_{zym}}}\left(\begin{array}{l}1+\frac{[\mathrm{C4}]}{K_{\mathrm{m\,MASP2^*}}^{\mathrm{C4}}}+\frac{[\mathrm{fC4bC2}]}{K_{\mathrm{m\,MASP2^*}}^{\mathrm{C2}}}+\frac{[\mathrm{fC4bC4bC2}]}{K_{\mathrm{m\,MASP2^*}}^{\mathrm{C2}}}+\frac{[\mathrm{fC3bC4bC2}]}{K_{\mathrm{m\,MASP2^*}}^{\mathrm{C2}}}+\frac{[\mathrm{pC4bC2}]}{K_{\mathrm{m\,MASP2^*}}^{\mathrm{C2}}}+\frac{[\mathrm{pC4bC4bC2}]}{K_{\mathrm{m\,MASP2^*}}^{\mathrm{C2}}}\\+\frac{[\mathrm{pC3bC4bC2}]}{K_{\mathrm{m\,MASP2^*}}^{\mathrm{C2}}}+\frac{[\mathrm{pMBL_3MASP1_{zym}}]}{K_{\mathrm{m\,MASP2^*}}^{\mathrm{MASP1_{zym}}}}+\frac{[\mathrm{pMBL_3MASP2_{zym}}]}{K_{\mathrm{m\,MASP2^*}}^{\mathrm{MASP2_{zym}}}}+\frac{[\mathrm{pMBL_4MASP1_{zym}}]}{K_{\mathrm{m\,MASP2^*}}^{\mathrm{MASP1_{zym}}}}\\+\frac{[\mathrm{pMBL_4MASP2_{zym}}]}{K_{\mathrm{m\,MASP2^*}}^{\mathrm{MASP2_{zym}}}}+\frac{[\mathrm{pCLLKMASP1_{zym}}]}{K_{\mathrm{m\,MASP2^*}}^{\mathrm{MASP1_{zym}}}}+\frac{[\mathrm{pCLLKMASP2_{zym}}]}{K_{\mathrm{m\,MASP2^*}}^{\mathrm{MASP2_{zym}}}}+\frac{[\mathrm{pCLK1MASP1_{zym}}]}{K_{\mathrm{m\,MASP2^*}}^{\mathrm{MASP1_{zym}}}}\\+\frac{[\mathrm{pCLK1MASP2_{zym}}]}{K_{\mathrm{m\,MASP2^*}}^{\mathrm{MASP2_{zym}}}}+\frac{[\mathrm{pCLL1MASP1_{zym}}]}{K_{\mathrm{m\,MASP2^*}}^{\mathrm{MASP1_{zym}}}}+\frac{[\mathrm{pCLL1MASP2_{zym}}]}{K_{\mathrm{m\,MASP2^*}}^{\mathrm{MASP2_{zym}}}}+\frac{[\mathrm{pLFMASP1_{zym}}]}{K_{\mathrm{m\,MASP2^*}}^{\mathrm{MASP1_{zym}}}}\\+\frac{[\mathrm{pLFMASP2_{zym}}]}{K_{\mathrm{m\,MASP2^*}}^{\mathrm{MASP2_{zym}}}}+\frac{[\mathrm{pHFMASP1_{zym}}]}{K_{\mathrm{m\,MASP2^*}}^{\mathrm{MASP1_{zym}}}}+\frac{[\mathrm{pHFMASP2_{zym}}]}{K_{\mathrm{m\,MASP2^*}}^{\mathrm{MASP2_{zym}}}}+\frac{[\mathrm{pMFMASP1_{zym}}]}{K_{\mathrm{m\,MASP2^*}}^{\mathrm{MASP1_{zym}}}}+\frac{[\mathrm{pMFMASP2_{zym}}]}{K_{\mathrm{m\,MASP2^*}}^{\mathrm{MASP2_{zym}}}}\end{array}\right)}$$

$$
-\frac{k_{\mathrm{cat\ MASP2^*}}^{\mathrm{MASP1_{zym}}}\left[\mathrm{pCLL1MASP2^*}\right]\left[\mathrm{pCLLKMASP1_{zym}}\right]}{K_{\mathrm{m\ MASP2^*}}^{\mathrm{MASP1_{zym}}}\left(\begin{array}{l}1+\frac{\left[\mathrm{C4}\right]}{K_{\mathrm{m\ MASP2^*}}^{\mathrm{C4}}}+\frac{\left[\mathrm{fC4bC2}\right]}{K_{\mathrm{m\ MASP2^*}}^{\mathrm{C2}}}+\frac{\left[\mathrm{fC4bC4bC2}\right]}{K_{\mathrm{m\ MASP2^*}}^{\mathrm{C2}}}+\frac{\left[\mathrm{fC3bC4bC2}\right]}{K_{\mathrm{m\ MASP2^*}}^{\mathrm{C2}}}+\frac{\left[\mathrm{pC4bC2}\right]}{K_{\mathrm{m\ MASP2^*}}^{\mathrm{C2}}}+\frac{\left[\mathrm{pC4bC4bC2}\right]}{K_{\mathrm{m\ MASP2^*}}^{\mathrm{C2}}}\\+\frac{\left[\mathrm{pC3bC4bC2}\right]}{K_{\mathrm{m\ MASP2^*}}^{\mathrm{C2}}}+\frac{\left[\mathrm{pMBL_3MASP1_{zym}}\right]}{K_{\mathrm{m\ MASP2^*}}^{\mathrm{MASP1_{zym}}}}+\frac{\left[\mathrm{pMBL_3MASP2_{zym}}\right]}{K_{\mathrm{m\ MASP2^*}}^{\mathrm{MASP2_{zym}}}}+\frac{\left[\mathrm{pMBL_4MASP1_{zym}}\right]}{K_{\mathrm{m\ MASP2^*}}^{\mathrm{MASP1_{zym}}}}\\+\frac{\left[\mathrm{pMBL_4MASP2_{zym}}\right]}{K_{\mathrm{m\ MASP2^*}}^{\mathrm{MASP2_{zym}}}}+\frac{\left[\mathrm{pCLLKMASP1_{zym}}\right]}{K_{\mathrm{m\ MASP2^*}}^{\mathrm{MASP1_{zym}}}}+\frac{\left[\mathrm{pCLLKMASP2_{zym}}\right]}{K_{\mathrm{m\ MASP2^*}}^{\mathrm{MASP2_{zym}}}}+\frac{\left[\mathrm{pCLK1MASP1_{zym}}\right]}{K_{\mathrm{m\ MASP2^*}}^{\mathrm{MASP1_{zym}}}}\\+\frac{\left[\mathrm{pCLK1MASP2_{zym}}\right]}{K_{\mathrm{m\ MASP2^*}}^{\mathrm{MASP2_{zym}}}}+\frac{\left[\mathrm{pCLL1MASP1_{zym}}\right]}{K_{\mathrm{m\ MASP2^*}}^{\mathrm{MASP1_{zym}}}}+\frac{\left[\mathrm{pCLL1MASP2_{zym}}\right]}{K_{\mathrm{m\ MASP2^*}}^{\mathrm{MASP2_{zym}}}}+\frac{\left[\mathrm{pLFMASP1_{zym}}\right]}{K_{\mathrm{m\ MASP2^*}}^{\mathrm{MASP1_{zym}}}}\\+\frac{\left[\mathrm{pLFMASP2_{zym}}\right]}{K_{\mathrm{m\ MASP2^*}}^{\mathrm{MASP2_{zym}}}}+\frac{\left[\mathrm{pHFMASP1_{zym}}\right]}{K_{\mathrm{m\ MASP2^*}}^{\mathrm{MASP1_{zym}}}}+\frac{\left[\mathrm{pHFMASP2_{zym}}\right]}{K_{\mathrm{m\ MASP2^*}}^{\mathrm{MASP2_{zym}}}}+\frac{\left[\mathrm{pMFMASP1_{zym}}\right]}{K_{\mathrm{m\ MASP2^*}}^{\mathrm{MASP1_{zym}}}}+\frac{\left[\mathrm{pMFMASP2_{zym}}\right]}{K_{\mathrm{m\ MASP2^*}}^{\mathrm{MASP2_{zym}}}}\end{array}\right)}
$$

$$
-\frac{k_{\mathrm{cat\ MASP2^*}}^{\mathrm{MASP1_{zym}}}\left[\mathrm{pLFMASP2^*}\right]\left[\mathrm{pCLLKMASP1_{zym}}\right]}{K_{\mathrm{m\ MASP2^*}}^{\mathrm{MASP1_{zym}}}\left(\begin{array}{l}1+\frac{\left[\mathrm{C4}\right]}{K_{\mathrm{m\ MASP2^*}}^{\mathrm{C4}}}+\frac{\left[\mathrm{fC4bC2}\right]}{K_{\mathrm{m\ MASP2^*}}^{\mathrm{C2}}}+\frac{\left[\mathrm{fC4bC4bC2}\right]}{K_{\mathrm{m\ MASP2^*}}^{\mathrm{C2}}}+\frac{\left[\mathrm{fC3bC4bC2}\right]}{K_{\mathrm{m\ MASP2^*}}^{\mathrm{C2}}}+\frac{\left[\mathrm{pC4bC2}\right]}{K_{\mathrm{m\ MASP2^*}}^{\mathrm{C2}}}+\frac{\left[\mathrm{pC4bC4bC2}\right]}{K_{\mathrm{m\ MASP2^*}}^{\mathrm{C2}}}\\+\frac{\left[\mathrm{pC3bC4bC2}\right]}{K_{\mathrm{m\ MASP2^*}}^{\mathrm{C2}}}+\frac{\left[\mathrm{pMBL_3MASP1_{zym}}\right]}{K_{\mathrm{m\ MASP2^*}}^{\mathrm{MASP1_{zym}}}}+\frac{\left[\mathrm{pMBL_3MASP2_{zym}}\right]}{K_{\mathrm{m\ MASP2^*}}^{\mathrm{MASP2_{zym}}}}+\frac{\left[\mathrm{pMBL_4MASP1_{zym}}\right]}{K_{\mathrm{m\ MASP2^*}}^{\mathrm{MASP1_{zym}}}}\\+\frac{\left[\mathrm{pMBL_4MASP2_{zym}}\right]}{K_{\mathrm{m\ MASP2^*}}^{\mathrm{MASP2_{zym}}}}+\frac{\left[\mathrm{pCLLKMASP1_{zym}}\right]}{K_{\mathrm{m\ MASP2^*}}^{\mathrm{MASP1_{zym}}}}+\frac{\left[\mathrm{pCLLKMASP2_{zym}}\right]}{K_{\mathrm{m\ MASP2^*}}^{\mathrm{MASP2_{zym}}}}+\frac{\left[\mathrm{pCLK1MASP1_{zym}}\right]}{K_{\mathrm{m\ MASP2^*}}^{\mathrm{MASP1_{zym}}}}\\+\frac{\left[\mathrm{pCLK1MASP2_{zym}}\right]}{K_{\mathrm{m\ MASP2^*}}^{\mathrm{MASP2_{zym}}}}+\frac{\left[\mathrm{pCLL1MASP1_{zym}}\right]}{K_{\mathrm{m\ MASP2^*}}^{\mathrm{MASP1_{zym}}}}+\frac{\left[\mathrm{pCLL1MASP2_{zym}}\right]}{K_{\mathrm{m\ MASP2^*}}^{\mathrm{MASP2_{zym}}}}+\frac{\left[\mathrm{pLFMASP1_{zym}}\right]}{K_{\mathrm{m\ MASP2^*}}^{\mathrm{MASP1_{zym}}}}\\+\frac{\left[\mathrm{pLFMASP2_{zym}}\right]}{K_{\mathrm{m\ MASP2^*}}^{\mathrm{MASP2_{zym}}}}+\frac{\left[\mathrm{pHFMASP1_{zym}}\right]}{K_{\mathrm{m\ MASP2^*}}^{\mathrm{MASP1_{zym}}}}+\frac{\left[\mathrm{pHFMASP2_{zym}}\right]}{K_{\mathrm{m\ MASP2^*}}^{\mathrm{MASP2_{zym}}}}+\frac{\left[\mathrm{pMFMASP1_{zym}}\right]}{K_{\mathrm{m\ MASP2^*}}^{\mathrm{MASP1_{zym}}}}+\frac{\left[\mathrm{pMFMASP2_{zym}}\right]}{K_{\mathrm{m\ MASP2^*}}^{\mathrm{MASP2_{zym}}}}\end{array}\right)}
$$

$$
-\frac{k_{\mathrm{cat\ MASP2^*}}^{\mathrm{MASP1_{zym}}}\left[\mathrm{pHFMASP2^*}\right]\left[\mathrm{pCLLKMASP1_{zym}}\right]}{K_{\mathrm{m\ MASP2^*}}^{\mathrm{MASP1_{zym}}}\left(\begin{array}{l}1+\frac{\left[\mathrm{C4}\right]}{K_{\mathrm{m\ MASP2^*}}^{\mathrm{C4}}}+\frac{\left[\mathrm{fC4bC2}\right]}{K_{\mathrm{m\ MASP2^*}}^{\mathrm{C2}}}+\frac{\left[\mathrm{fC4bC4bC2}\right]}{K_{\mathrm{m\ MASP2^*}}^{\mathrm{C2}}}+\frac{\left[\mathrm{fC3bC4bC2}\right]}{K_{\mathrm{m\ MASP2^*}}^{\mathrm{C2}}}+\frac{\left[\mathrm{pC4bC2}\right]}{K_{\mathrm{m\ MASP2^*}}^{\mathrm{C2}}}+\frac{\left[\mathrm{pC4bC4bC2}\right]}{K_{\mathrm{m\ MASP2^*}}^{\mathrm{C2}}}\\+\frac{\left[\mathrm{pC3bC4bC2}\right]}{K_{\mathrm{m\ MASP2^*}}^{\mathrm{C2}}}+\frac{\left[\mathrm{pMBL_3MASP1_{zym}}\right]}{K_{\mathrm{m\ MASP2^*}}^{\mathrm{MASP1_{zym}}}}+\frac{\left[\mathrm{pMBL_3MASP2_{zym}}\right]}{K_{\mathrm{m\ MASP2^*}}^{\mathrm{MASP2_{zym}}}}+\frac{\left[\mathrm{pMBL_4MASP1_{zym}}\right]}{K_{\mathrm{m\ MASP2^*}}^{\mathrm{MASP1_{zym}}}}\\+\frac{\left[\mathrm{pMBL_4MASP2_{zym}}\right]}{K_{\mathrm{m\ MASP2^*}}^{\mathrm{MASP2_{zym}}}}+\frac{\left[\mathrm{pCLLKMASP1_{zym}}\right]}{K_{\mathrm{m\ MASP2^*}}^{\mathrm{MASP1_{zym}}}}+\frac{\left[\mathrm{pCLLKMASP2_{zym}}\right]}{K_{\mathrm{m\ MASP2^*}}^{\mathrm{MASP2_{zym}}}}+\frac{\left[\mathrm{pCLK1MASP1_{zym}}\right]}{K_{\mathrm{m\ MASP2^*}}^{\mathrm{MASP1_{zym}}}}\\+\frac{\left[\mathrm{pCLK1MASP2_{zym}}\right]}{K_{\mathrm{m\ MASP2^*}}^{\mathrm{MASP2_{zym}}}}+\frac{\left[\mathrm{pCLL1MASP1_{zym}}\right]}{K_{\mathrm{m\ MASP2^*}}^{\mathrm{MASP1_{zym}}}}+\frac{\left[\mathrm{pCLL1MASP2_{zym}}\right]}{K_{\mathrm{m\ MASP2^*}}^{\mathrm{MASP2_{zym}}}}+\frac{\left[\mathrm{pLFMASP1_{zym}}\right]}{K_{\mathrm{m\ MASP2^*}}^{\mathrm{MASP1_{zym}}}}\\+\frac{\left[\mathrm{pLFMASP2_{zym}}\right]}{K_{\mathrm{m\ MASP2^*}}^{\mathrm{MASP2_{zym}}}}+\frac{\left[\mathrm{pHFMASP1_{zym}}\right]}{K_{\mathrm{m\ MASP2^*}}^{\mathrm{MASP1_{zym}}}}+\frac{\left[\mathrm{pHFMASP2_{zym}}\right]}{K_{\mathrm{m\ MASP2^*}}^{\mathrm{MASP2_{zym}}}}+\frac{\left[\mathrm{pMFMASP1_{zym}}\right]}{K_{\mathrm{m\ MASP2^*}}^{\mathrm{MASP1_{zym}}}}+\frac{\left[\mathrm{pMFMASP2_{zym}}\right]}{K_{\mathrm{m\ MASP2^*}}^{\mathrm{MASP2_{zym}}}}\end{array}\right)}
$$

$$-\frac{k_{\text{cat MASP2}^*}^{\text{MASP1}_{\text{zym}}}\left[\text{pMFMASP2}^*\right]\left[\text{pCLLKMASP1}_{\text{zym}}\right]}{K_{\text{m MASP2}^*}^{\text{MASP1}_{\text{zym}}}\left(\begin{array}{l}1+\frac{[\text{C4}]}{K_{\text{m MASP2}^*}^{\text{C4}}}+\frac{[\text{fC4bC2}]}{K_{\text{m MASP2}^*}^{\text{C2}}}+\frac{[\text{fC4bC4bC2}]}{K_{\text{m MASP2}^*}^{\text{C2}}}+\frac{[\text{fC3bC4bC2}]}{K_{\text{m MASP2}^*}^{\text{C2}}}+\frac{[\text{pC4bC2}]}{K_{\text{m MASP2}^*}^{\text{C2}}}+\frac{[\text{pC4bC4bC2}]}{K_{\text{m MASP2}^*}^{\text{C2}}}\\+\frac{[\text{pC3bC4bC2}]}{K_{\text{m MASP2}^*}^{\text{C2}}}+\frac{\left[\text{pMBL}_3\text{MASP1}_{\text{zym}}\right]}{K_{\text{m MASP2}^*}^{\text{MASP1}_{\text{zym}}}}+\frac{\left[\text{pMBL}_3\text{MASP2}_{\text{zym}}\right]}{K_{\text{m MASP2}^*}^{\text{MASP2}_{\text{zym}}}}+\frac{\left[\text{pMBL}_4\text{MASP1}_{\text{zym}}\right]}{K_{\text{m MASP2}^*}^{\text{MASP1}_{\text{zym}}}}\\+\frac{\left[\text{pMBL}_4\text{MASP2}_{\text{zym}}\right]}{K_{\text{m MASP2}^*}^{\text{MASP2}_{\text{zym}}}}+\frac{\left[\text{pCLLKMASP1}_{\text{zym}}\right]}{K_{\text{m MASP2}^*}^{\text{MASP1}_{\text{zym}}}}+\frac{\left[\text{pCLLKMASP2}_{\text{zym}}\right]}{K_{\text{m MASP2}^*}^{\text{MASP2}_{\text{zym}}}}+\frac{\left[\text{pCLK1MASP1}_{\text{zym}}\right]}{K_{\text{m MASP2}^*}^{\text{MASP1}_{\text{zym}}}}\\+\frac{\left[\text{pCLK1MASP2}_{\text{zym}}\right]}{K_{\text{m MASP2}^*}^{\text{MASP2}_{\text{zym}}}}+\frac{\left[\text{pCLL1MASP1}_{\text{zym}}\right]}{K_{\text{m MASP2}^*}^{\text{MASP1}_{\text{zym}}}}+\frac{\left[\text{pCLL1MASP2}_{\text{zym}}\right]}{K_{\text{m MASP2}^*}^{\text{MASP2}_{\text{zym}}}}+\frac{\left[\text{pLFMASP1}_{\text{zym}}\right]}{K_{\text{m MASP2}^*}^{\text{MASP1}_{\text{zym}}}}\\+\frac{\left[\text{pLFMASP2}_{\text{zym}}\right]}{K_{\text{m MASP2}^*}^{\text{MASP2}_{\text{zym}}}}+\frac{\left[\text{pHFMASP1}_{\text{zym}}\right]}{K_{\text{m MASP2}^*}^{\text{MASP1}_{\text{zym}}}}+\frac{\left[\text{pHFMASP2}_{\text{zym}}\right]}{K_{\text{m MASP2}^*}^{\text{MASP2}_{\text{zym}}}}+\frac{\left[\text{pMFMASP1}_{\text{zym}}\right]}{K_{\text{m MASP2}^*}^{\text{MASP1}_{\text{zym}}}}+\frac{\left[\text{pMFMASP2}_{\text{zym}}\right]}{K_{\text{m MASP2}^*}^{\text{MASP2}_{\text{zym}}}}\end{array}\right)}$$

$$-\frac{k_{\text{cat MASP2}^*}^{\text{MASP1}_{\text{zym}}}\left[\text{pPTX3LFMASP2}^*\right]\left[\text{pCLLKMASP1}_{\text{zym}}\right]}{K_{\text{m MASP2}^*}^{\text{MASP1}_{\text{zym}}}\left(\begin{array}{l}1+\frac{[\text{C4}]}{K_{\text{m MASP2}^*}^{\text{C4}}}+\frac{[\text{fC4bC2}]}{K_{\text{m MASP2}^*}^{\text{C2}}}+\frac{[\text{fC4bC4bC2}]}{K_{\text{m MASP2}^*}^{\text{C2}}}+\frac{[\text{fC3bC4bC2}]}{K_{\text{m MASP2}^*}^{\text{C2}}}+\frac{[\text{pC4bC2}]}{K_{\text{m MASP2}^*}^{\text{C2}}}+\frac{[\text{pC4bC4bC2}]}{K_{\text{m MASP2}^*}^{\text{C2}}}\\+\frac{[\text{pC3bC4bC2}]}{K_{\text{m MASP2}^*}^{\text{C2}}}+\frac{\left[\text{pMBL}_3\text{MASP1}_{\text{zym}}\right]}{K_{\text{m MASP2}^*}^{\text{MASP1}_{\text{zym}}}}+\frac{\left[\text{pMBL}_3\text{MASP2}_{\text{zym}}\right]}{K_{\text{m MASP2}^*}^{\text{MASP2}_{\text{zym}}}}+\frac{\left[\text{pMBL}_4\text{MASP1}_{\text{zym}}\right]}{K_{\text{m MASP2}^*}^{\text{MASP1}_{\text{zym}}}}\\+\frac{\left[\text{pMBL}_4\text{MASP2}_{\text{zym}}\right]}{K_{\text{m MASP2}^*}^{\text{MASP2}_{\text{zym}}}}+\frac{\left[\text{pCLLKMASP1}_{\text{zym}}\right]}{K_{\text{m MASP2}^*}^{\text{MASP1}_{\text{zym}}}}+\frac{\left[\text{pCLLKMASP2}_{\text{zym}}\right]}{K_{\text{m MASP2}^*}^{\text{MASP2}_{\text{zym}}}}+\frac{\left[\text{pCLK1MASP1}_{\text{zym}}\right]}{K_{\text{m MASP2}^*}^{\text{MASP1}_{\text{zym}}}}\\+\frac{\left[\text{pCLK1MASP2}_{\text{zym}}\right]}{K_{\text{m MASP2}^*}^{\text{MASP2}_{\text{zym}}}}+\frac{\left[\text{pCLL1MASP1}_{\text{zym}}\right]}{K_{\text{m MASP2}^*}^{\text{MASP1}_{\text{zym}}}}+\frac{\left[\text{pCLL1MASP2}_{\text{zym}}\right]}{K_{\text{m MASP2}^*}^{\text{MASP2}_{\text{zym}}}}+\frac{\left[\text{pLFMASP1}_{\text{zym}}\right]}{K_{\text{m MASP2}^*}^{\text{MASP1}_{\text{zym}}}}\\+\frac{\left[\text{pLFMASP2}_{\text{zym}}\right]}{K_{\text{m MASP2}^*}^{\text{MASP2}_{\text{zym}}}}+\frac{\left[\text{pHFMASP1}_{\text{zym}}\right]}{K_{\text{m MASP2}^*}^{\text{MASP1}_{\text{zym}}}}+\frac{\left[\text{pHFMASP2}_{\text{zym}}\right]}{K_{\text{m MASP2}^*}^{\text{MASP2}_{\text{zym}}}}+\frac{\left[\text{pMFMASP1}_{\text{zym}}\right]}{K_{\text{m MASP2}^*}^{\text{MASP1}_{\text{zym}}}}+\frac{\left[\text{pMFMASP2}_{\text{zym}}\right]}{K_{\text{m MASP2}^*}^{\text{MASP2}_{\text{zym}}}}\\+\frac{\left[\text{pPTX3LFMASP1}_{\text{zym}}\right]}{K_{\text{m MASP2}^*}^{\text{MASP1}_{\text{zym}}}}+\frac{\left[\text{pPTX3LFMASP2}_{\text{zym}}\right]}{K_{\text{m MASP2}^*}^{\text{MASP2}_{\text{zym}}}}\end{array}\right)}$$

286

$$\frac{d\left[\text{fCLLKMASP1}^*\right]}{dt}=-k_{\text{pCLLK}}^{+}\left[\text{fCLLKMASP1}^*\right]\left[\text{Pathogen Surface}\right]+k_{\text{pCLLK}}^{-}\left[\text{pCLLKMASP1}^*\right]$$
$$+k_{\text{CLLKMASP1}}^{+}\left[\text{CLLK}\right]\left[\text{MASP1}^*\right]-k_{\text{CLLKMASP1}}^{-}\left[\text{fCLLKMASP1}^*\right]-k_{\text{C1INHMASP1}}^{+}\left[\text{C1-INH}\right]\left[\text{fCLLKMASP1}^*\right]$$

287

$$\frac{d\left[\mathrm{pCLLKMASP1}^{*}\right]}{dt}=k_{\mathrm{pCLLK}}^{+}\left[\mathrm{fCLLKMASP1}^{*}\right]\left[\mathrm{Pathogen\ Surface}\right]-k_{\mathrm{pCLLK}}^{-}\left[\mathrm{pCLLKMASP1}^{*}\right]$$

$$+k_{\mathrm{CLLKMASP1}}^{+}\left[\mathrm{pCLLK}\right]\left[\mathrm{MASP1}^{*}\right]-k_{\mathrm{CLLKMASP1}}^{-}\left[\mathrm{pCLLKMASP1}^{*}\right]$$

$$+\frac{k_{\mathrm{cat\ MASP1_{zym}}}^{\mathrm{MASP1_{zym}}}\left[\mathrm{pMBL_3MASP1_{zym}}\right]\left[\mathrm{pCLLKMASP1_{zym}}\right]}{K_{\mathrm{m\ MASP1_{zym}}}^{\mathrm{MASP1_{zym}}}\left(\begin{array}{l}1+\frac{\left[\mathrm{pMBL_3MASP1_{zym}}\right]}{K_{\mathrm{m\ MASP1_{zym}}}^{\mathrm{MASP1_{zym}}}}+\frac{\left[\mathrm{pMBL_3MASP2_{zym}}\right]}{K_{\mathrm{m\ MASP1_{zym}}}^{\mathrm{MASP2_{zym}}}}+\frac{\left[\mathrm{pMBL_4MASP1_{zym}}\right]}{K_{\mathrm{m\ MASP1_{zym}}}^{\mathrm{MASP1_{zym}}}}+\frac{\left[\mathrm{pMBL_4MASP2_{zym}}\right]}{K_{\mathrm{m\ MASP1_{zym}}}^{\mathrm{MASP2_{zym}}}}\\+\frac{\left[\mathrm{pCLLKMASP1_{zym}}\right]}{K_{\mathrm{m\ MASP1_{zym}}}^{\mathrm{MASP1_{zym}}}}+\frac{\left[\mathrm{pCLLKMASP2_{zym}}\right]}{K_{\mathrm{m\ MASP1_{zym}}}^{\mathrm{MASP2_{zym}}}}+\frac{\left[\mathrm{pCLK1MASP1_{zym}}\right]}{K_{\mathrm{m\ MASP1_{zym}}}^{\mathrm{MASP1_{zym}}}}+\frac{\left[\mathrm{pCLK1MASP2_{zym}}\right]}{K_{\mathrm{m\ MASP1_{zym}}}^{\mathrm{MASP2_{zym}}}}\\+\frac{\left[\mathrm{pCLL1MASP1_{zym}}\right]}{K_{\mathrm{m\ MASP1_{zym}}}^{\mathrm{MASP1_{zym}}}}+\frac{\left[\mathrm{pCLL1MASP2_{zym}}\right]}{K_{\mathrm{m\ MASP1_{zym}}}^{\mathrm{MASP2_{zym}}}}+\frac{\left[\mathrm{pLFMASP1_{zym}}\right]}{K_{\mathrm{m\ MASP1_{zym}}}^{\mathrm{MASP1_{zym}}}}+\frac{\left[\mathrm{pLFMASP2_{zym}}\right]}{K_{\mathrm{m\ MASP1_{zym}}}^{\mathrm{MASP2_{zym}}}}\\+\frac{\left[\mathrm{pHFMASP1_{zym}}\right]}{K_{\mathrm{m\ MASP1_{zym}}}^{\mathrm{MASP1_{zym}}}}+\frac{\left[\mathrm{pHFMASP2_{zym}}\right]}{K_{\mathrm{m\ MASP1_{zym}}}^{\mathrm{MASP2_{zym}}}}+\frac{\left[\mathrm{pMFMASP1_{zym}}\right]}{K_{\mathrm{m\ MASP1_{zym}}}^{\mathrm{MASP1_{zym}}}}+\frac{\left[\mathrm{pMFMASP2_{zym}}\right]}{K_{\mathrm{m\ MASP1_{zym}}}^{\mathrm{MASP2_{zym}}}}\end{array}\right)}$$

$$+\frac{k_{\mathrm{cat\ MASP1_{zym}}}^{\mathrm{MASP1_{zym}}}\left[\mathrm{pMBL_4MASP1_{zym}}\right]\left[\mathrm{pCLLKMASP1_{zym}}\right]}{K_{\mathrm{m\ MASP1_{zym}}}^{\mathrm{MASP1_{zym}}}\left(\begin{array}{l}1+\frac{\left[\mathrm{pMBL_3MASP1_{zym}}\right]}{K_{\mathrm{m\ MASP1_{zym}}}^{\mathrm{MASP1_{zym}}}}+\frac{\left[\mathrm{pMBL_3MASP2_{zym}}\right]}{K_{\mathrm{m\ MASP1_{zym}}}^{\mathrm{MASP2_{zym}}}}+\frac{\left[\mathrm{pMBL_4MASP1_{zym}}\right]}{K_{\mathrm{m\ MASP1_{zym}}}^{\mathrm{MASP1_{zym}}}}+\frac{\left[\mathrm{pMBL_4MASP2_{zym}}\right]}{K_{\mathrm{m\ MASP1_{zym}}}^{\mathrm{MASP2_{zym}}}}\\+\frac{\left[\mathrm{pCLLKMASP1_{zym}}\right]}{K_{\mathrm{m\ MASP1_{zym}}}^{\mathrm{MASP1_{zym}}}}+\frac{\left[\mathrm{pCLLKMASP2_{zym}}\right]}{K_{\mathrm{m\ MASP1_{zym}}}^{\mathrm{MASP2_{zym}}}}+\frac{\left[\mathrm{pCLK1MASP1_{zym}}\right]}{K_{\mathrm{m\ MASP1_{zym}}}^{\mathrm{MASP1_{zym}}}}+\frac{\left[\mathrm{pCLK1MASP2_{zym}}\right]}{K_{\mathrm{m\ MASP1_{zym}}}^{\mathrm{MASP2_{zym}}}}\\+\frac{\left[\mathrm{pCLL1MASP1_{zym}}\right]}{K_{\mathrm{m\ MASP1_{zym}}}^{\mathrm{MASP1_{zym}}}}+\frac{\left[\mathrm{pCLL1MASP2_{zym}}\right]}{K_{\mathrm{m\ MASP1_{zym}}}^{\mathrm{MASP2_{zym}}}}+\frac{\left[\mathrm{pLFMASP1_{zym}}\right]}{K_{\mathrm{m\ MASP1_{zym}}}^{\mathrm{MASP1_{zym}}}}+\frac{\left[\mathrm{pLFMASP2_{zym}}\right]}{K_{\mathrm{m\ MASP1_{zym}}}^{\mathrm{MASP2_{zym}}}}\\+\frac{\left[\mathrm{pHFMASP1_{zym}}\right]}{K_{\mathrm{m\ MASP1_{zym}}}^{\mathrm{MASP1_{zym}}}}+\frac{\left[\mathrm{pHFMASP2_{zym}}\right]}{K_{\mathrm{m\ MASP1_{zym}}}^{\mathrm{MASP2_{zym}}}}+\frac{\left[\mathrm{pMFMASP1_{zym}}\right]}{K_{\mathrm{m\ MASP1_{zym}}}^{\mathrm{MASP1_{zym}}}}+\frac{\left[\mathrm{pMFMASP2_{zym}}\right]}{K_{\mathrm{m\ MASP1_{zym}}}^{\mathrm{MASP2_{zym}}}}\end{array}\right)}$$

$$+\frac{k_{\mathrm{cat\ MASP1_{zym}}}^{\mathrm{MASP1_{zym}}}\left[\mathrm{pCLLKMASP1_{zym}}\right]\left[\mathrm{pCLLKMASP1_{zym}}\right]}{K_{\mathrm{m\ MASP1_{zym}}}^{\mathrm{MASP1_{zym}}}\left(\begin{array}{l}1+\frac{\left[\mathrm{pMBL_3MASP1_{zym}}\right]}{K_{\mathrm{m\ MASP1_{zym}}}^{\mathrm{MASP1_{zym}}}}+\frac{\left[\mathrm{pMBL_3MASP2_{zym}}\right]}{K_{\mathrm{m\ MASP1_{zym}}}^{\mathrm{MASP2_{zym}}}}+\frac{\left[\mathrm{pMBL_4MASP1_{zym}}\right]}{K_{\mathrm{m\ MASP1_{zym}}}^{\mathrm{MASP1_{zym}}}}+\frac{\left[\mathrm{pMBL_4MASP2_{zym}}\right]}{K_{\mathrm{m\ MASP1_{zym}}}^{\mathrm{MASP2_{zym}}}}\\+\frac{\left[\mathrm{pCLLKMASP1_{zym}}\right]}{K_{\mathrm{m\ MASP1_{zym}}}^{\mathrm{MASP1_{zym}}}}+\frac{\left[\mathrm{pCLLKMASP2_{zym}}\right]}{K_{\mathrm{m\ MASP1_{zym}}}^{\mathrm{MASP2_{zym}}}}+\frac{\left[\mathrm{pCLK1MASP1_{zym}}\right]}{K_{\mathrm{m\ MASP1_{zym}}}^{\mathrm{MASP1_{zym}}}}+\frac{\left[\mathrm{pCLK1MASP2_{zym}}\right]}{K_{\mathrm{m\ MASP1_{zym}}}^{\mathrm{MASP2_{zym}}}}\\+\frac{\left[\mathrm{pCLL1MASP1_{zym}}\right]}{K_{\mathrm{m\ MASP1_{zym}}}^{\mathrm{MASP1_{zym}}}}+\frac{\left[\mathrm{pCLL1MASP2_{zym}}\right]}{K_{\mathrm{m\ MASP1_{zym}}}^{\mathrm{MASP2_{zym}}}}+\frac{\left[\mathrm{pLFMASP1_{zym}}\right]}{K_{\mathrm{m\ MASP1_{zym}}}^{\mathrm{MASP1_{zym}}}}+\frac{\left[\mathrm{pLFMASP2_{zym}}\right]}{K_{\mathrm{m\ MASP1_{zym}}}^{\mathrm{MASP2_{zym}}}}\\+\frac{\left[\mathrm{pHFMASP1_{zym}}\right]}{K_{\mathrm{m\ MASP1_{zym}}}^{\mathrm{MASP1_{zym}}}}+\frac{\left[\mathrm{pHFMASP2_{zym}}\right]}{K_{\mathrm{m\ MASP1_{zym}}}^{\mathrm{MASP2_{zym}}}}+\frac{\left[\mathrm{pMFMASP1_{zym}}\right]}{K_{\mathrm{m\ MASP1_{zym}}}^{\mathrm{MASP1_{zym}}}}+\frac{\left[\mathrm{pMFMASP2_{zym}}\right]}{K_{\mathrm{m\ MASP1_{zym}}}^{\mathrm{MASP2_{zym}}}}\end{array}\right)}$$

$$+\frac{k_{\mathrm{cat\ MASP1_{zym}}}^{\mathrm{MASP1_{zym}}}\left[\mathrm{pCLK1MASP1_{zym}}\right]\left[\mathrm{pCLLKMASP1_{zym}}\right]}{K_{\mathrm{m\ MASP1_{zym}}}^{\mathrm{MASP1_{zym}}}\left(\begin{array}{l}1+\frac{\left[\mathrm{pMBL_3MASP1_{zym}}\right]}{K_{\mathrm{m\ MASP1_{zym}}}^{\mathrm{MASP1_{zym}}}}+\frac{\left[\mathrm{pMBL_3MASP2_{zym}}\right]}{K_{\mathrm{m\ MASP1_{zym}}}^{\mathrm{MASP2_{zym}}}}+\frac{\left[\mathrm{pMBL_4MASP1_{zym}}\right]}{K_{\mathrm{m\ MASP1_{zym}}}^{\mathrm{MASP1_{zym}}}}+\frac{\left[\mathrm{pMBL_4MASP2_{zym}}\right]}{K_{\mathrm{m\ MASP1_{zym}}}^{\mathrm{MASP2_{zym}}}}\\+\frac{\left[\mathrm{pCLLKMASP1_{zym}}\right]}{K_{\mathrm{m\ MASP1_{zym}}}^{\mathrm{MASP1_{zym}}}}+\frac{\left[\mathrm{pCLLKMASP2_{zym}}\right]}{K_{\mathrm{m\ MASP1_{zym}}}^{\mathrm{MASP2_{zym}}}}+\frac{\left[\mathrm{pCLK1MASP1_{zym}}\right]}{K_{\mathrm{m\ MASP1_{zym}}}^{\mathrm{MASP1_{zym}}}}+\frac{\left[\mathrm{pCLK1MASP2_{zym}}\right]}{K_{\mathrm{m\ MASP1_{zym}}}^{\mathrm{MASP2_{zym}}}}\\+\frac{\left[\mathrm{pCLL1MASP1_{zym}}\right]}{K_{\mathrm{m\ MASP1_{zym}}}^{\mathrm{MASP1_{zym}}}}+\frac{\left[\mathrm{pCLL1MASP2_{zym}}\right]}{K_{\mathrm{m\ MASP1_{zym}}}^{\mathrm{MASP2_{zym}}}}+\frac{\left[\mathrm{pLFMASP1_{zym}}\right]}{K_{\mathrm{m\ MASP1_{zym}}}^{\mathrm{MASP1_{zym}}}}+\frac{\left[\mathrm{pLFMASP2_{zym}}\right]}{K_{\mathrm{m\ MASP1_{zym}}}^{\mathrm{MASP2_{zym}}}}\\+\frac{\left[\mathrm{pHFMASP1_{zym}}\right]}{K_{\mathrm{m\ MASP1_{zym}}}^{\mathrm{MASP1_{zym}}}}+\frac{\left[\mathrm{pHFMASP2_{zym}}\right]}{K_{\mathrm{m\ MASP1_{zym}}}^{\mathrm{MASP2_{zym}}}}+\frac{\left[\mathrm{pMFMASP1_{zym}}\right]}{K_{\mathrm{m\ MASP1_{zym}}}^{\mathrm{MASP1_{zym}}}}+\frac{\left[\mathrm{pMFMASP2_{zym}}\right]}{K_{\mathrm{m\ MASP1_{zym}}}^{\mathrm{MASP2_{zym}}}}\end{array}\right)}$$

$$+\frac{k_{\mathrm{cat\ MASP1_{zym}}}^{\mathrm{MASP1_{zym}}}\left[\mathrm{pCLL1MASP1_{zym}}\right]\left[\mathrm{pCLLKMASP1_{zym}}\right]}{K_{\mathrm{m\ MASP1_{zym}}}^{\mathrm{MASP1_{zym}}}\left(\begin{array}{l}1+\frac{\left[\mathrm{pMBL_3MASP1_{zym}}\right]}{K_{\mathrm{m\ MASP1_{zym}}}^{\mathrm{MASP1_{zym}}}}+\frac{\left[\mathrm{pMBL_3MASP2_{zym}}\right]}{K_{\mathrm{m\ MASP1_{zym}}}^{\mathrm{MASP2_{zym}}}}+\frac{\left[\mathrm{pMBL_4MASP1_{zym}}\right]}{K_{\mathrm{m\ MASP1_{zym}}}^{\mathrm{MASP1_{zym}}}}+\frac{\left[\mathrm{pMBL_4MASP2_{zym}}\right]}{K_{\mathrm{m\ MASP1_{zym}}}^{\mathrm{MASP2_{zym}}}}\\+\frac{\left[\mathrm{pCLLKMASP1_{zym}}\right]}{K_{\mathrm{m\ MASP1_{zym}}}^{\mathrm{MASP1_{zym}}}}+\frac{\left[\mathrm{pCLLKMASP2_{zym}}\right]}{K_{\mathrm{m\ MASP1_{zym}}}^{\mathrm{MASP2_{zym}}}}+\frac{\left[\mathrm{pCLK1MASP1_{zym}}\right]}{K_{\mathrm{m\ MASP1_{zym}}}^{\mathrm{MASP1_{zym}}}}+\frac{\left[\mathrm{pCLK1MASP2_{zym}}\right]}{K_{\mathrm{m\ MASP1_{zym}}}^{\mathrm{MASP2_{zym}}}}\\+\frac{\left[\mathrm{pCLL1MASP1_{zym}}\right]}{K_{\mathrm{m\ MASP1_{zym}}}^{\mathrm{MASP1_{zym}}}}+\frac{\left[\mathrm{pCLL1MASP2_{zym}}\right]}{K_{\mathrm{m\ MASP1_{zym}}}^{\mathrm{MASP2_{zym}}}}+\frac{\left[\mathrm{pLFMASP1_{zym}}\right]}{K_{\mathrm{m\ MASP1_{zym}}}^{\mathrm{MASP1_{zym}}}}+\frac{\left[\mathrm{pLFMASP2_{zym}}\right]}{K_{\mathrm{m\ MASP1_{zym}}}^{\mathrm{MASP2_{zym}}}}\\+\frac{\left[\mathrm{pHFMASP1_{zym}}\right]}{K_{\mathrm{m\ MASP1_{zym}}}^{\mathrm{MASP1_{zym}}}}+\frac{\left[\mathrm{pHFMASP2_{zym}}\right]}{K_{\mathrm{m\ MASP1_{zym}}}^{\mathrm{MASP2_{zym}}}}+\frac{\left[\mathrm{pMFMASP1_{zym}}\right]}{K_{\mathrm{m\ MASP1_{zym}}}^{\mathrm{MASP1_{zym}}}}+\frac{\left[\mathrm{pMFMASP2_{zym}}\right]}{K_{\mathrm{m\ MASP1_{zym}}}^{\mathrm{MASP2_{zym}}}}\end{array}\right)}$$

$$+\frac{k_{\mathrm{cat\ MASP1_{zym}}}^{\mathrm{MASP1_{zym}}}\left[\mathrm{pLFMASP1_{zym}}\right]\left[\mathrm{pCLLKMASP1_{zym}}\right]}{K_{\mathrm{m\ MASP1_{zym}}}^{\mathrm{MASP1_{zym}}}\left(\begin{array}{l}1+\frac{\left[\mathrm{pMBL_3MASP1_{zym}}\right]}{K_{\mathrm{m\ MASP1_{zym}}}^{\mathrm{MASP1_{zym}}}}+\frac{\left[\mathrm{pMBL_3MASP2_{zym}}\right]}{K_{\mathrm{m\ MASP1_{zym}}}^{\mathrm{MASP2_{zym}}}}+\frac{\left[\mathrm{pMBL_4MASP1_{zym}}\right]}{K_{\mathrm{m\ MASP1_{zym}}}^{\mathrm{MASP1_{zym}}}}+\frac{\left[\mathrm{pMBL_4MASP2_{zym}}\right]}{K_{\mathrm{m\ MASP1_{zym}}}^{\mathrm{MASP2_{zym}}}}\\+\frac{\left[\mathrm{pCLLKMASP1_{zym}}\right]}{K_{\mathrm{m\ MASP1_{zym}}}^{\mathrm{MASP1_{zym}}}}+\frac{\left[\mathrm{pCLLKMASP2_{zym}}\right]}{K_{\mathrm{m\ MASP1_{zym}}}^{\mathrm{MASP2_{zym}}}}+\frac{\left[\mathrm{pCLK1MASP1_{zym}}\right]}{K_{\mathrm{m\ MASP1_{zym}}}^{\mathrm{MASP1_{zym}}}}+\frac{\left[\mathrm{pCLK1MASP2_{zym}}\right]}{K_{\mathrm{m\ MASP1_{zym}}}^{\mathrm{MASP2_{zym}}}}\\+\frac{\left[\mathrm{pCLL1MASP1_{zym}}\right]}{K_{\mathrm{m\ MASP1_{zym}}}^{\mathrm{MASP1_{zym}}}}+\frac{\left[\mathrm{pCLL1MASP2_{zym}}\right]}{K_{\mathrm{m\ MASP1_{zym}}}^{\mathrm{MASP2_{zym}}}}+\frac{\left[\mathrm{pLFMASP1_{zym}}\right]}{K_{\mathrm{m\ MASP1_{zym}}}^{\mathrm{MASP1_{zym}}}}+\frac{\left[\mathrm{pLFMASP2_{zym}}\right]}{K_{\mathrm{m\ MASP1_{zym}}}^{\mathrm{MASP2_{zym}}}}\\+\frac{\left[\mathrm{pHFMASP1_{zym}}\right]}{K_{\mathrm{m\ MASP1_{zym}}}^{\mathrm{MASP1_{zym}}}}+\frac{\left[\mathrm{pHFMASP2_{zym}}\right]}{K_{\mathrm{m\ MASP1_{zym}}}^{\mathrm{MASP2_{zym}}}}+\frac{\left[\mathrm{pMFMASP1_{zym}}\right]}{K_{\mathrm{m\ MASP1_{zym}}}^{\mathrm{MASP1_{zym}}}}+\frac{\left[\mathrm{pMFMASP2_{zym}}\right]}{K_{\mathrm{m\ MASP1_{zym}}}^{\mathrm{MASP2_{zym}}}}\end{array}\right)}$$

$$
+\frac{k_{\mathrm{cat\ MASP1_{zym}}}^{\mathrm{MASP1_{zym}}}\left[\mathrm{pHFMASP1_{zym}}\right]\left[\mathrm{pCLLKMASP1_{zym}}\right]}{K_{\mathrm{m\ MASP1_{zym}}}^{\mathrm{MASP1_{zym}}}\left(\begin{aligned}&1+\frac{\left[\mathrm{pMBL_3MASP1_{zym}}\right]}{K_{\mathrm{m\ MASP1_{zym}}}^{\mathrm{MASP1_{zym}}}}+\frac{\left[\mathrm{pMBL_3MASP2_{zym}}\right]}{K_{\mathrm{m\ MASP1_{zym}}}^{\mathrm{MASP2_{zym}}}}+\frac{\left[\mathrm{pMBL_4MASP1_{zym}}\right]}{K_{\mathrm{m\ MASP1_{zym}}}^{\mathrm{MASP1_{zym}}}}+\frac{\left[\mathrm{pMBL_4MASP2_{zym}}\right]}{K_{\mathrm{m\ MASP1_{zym}}}^{\mathrm{MASP2_{zym}}}}\\&+\frac{\left[\mathrm{pCLLKMASP1_{zym}}\right]}{K_{\mathrm{m\ MASP1_{zym}}}^{\mathrm{MASP1_{zym}}}}+\frac{\left[\mathrm{pCLLKMASP2_{zym}}\right]}{K_{\mathrm{m\ MASP1_{zym}}}^{\mathrm{MASP2_{zym}}}}+\frac{\left[\mathrm{pCLK1MASP1_{zym}}\right]}{K_{\mathrm{m\ MASP1_{zym}}}^{\mathrm{MASP1_{zym}}}}+\frac{\left[\mathrm{pCLK1MASP2_{zym}}\right]}{K_{\mathrm{m\ MASP1_{zym}}}^{\mathrm{MASP2_{zym}}}}\\&+\frac{\left[\mathrm{pCLL1MASP1_{zym}}\right]}{K_{\mathrm{m\ MASP1_{zym}}}^{\mathrm{MASP1_{zym}}}}+\frac{\left[\mathrm{pCLL1MASP2_{zym}}\right]}{K_{\mathrm{m\ MASP1_{zym}}}^{\mathrm{MASP2_{zym}}}}+\frac{\left[\mathrm{pLFMASP1_{zym}}\right]}{K_{\mathrm{m\ MASP1_{zym}}}^{\mathrm{MASP1_{zym}}}}+\frac{\left[\mathrm{pLFMASP2_{zym}}\right]}{K_{\mathrm{m\ MASP1_{zym}}}^{\mathrm{MASP2_{zym}}}}\\&+\frac{\left[\mathrm{pHFMASP1_{zym}}\right]}{K_{\mathrm{m\ MASP1_{zym}}}^{\mathrm{MASP1_{zym}}}}+\frac{\left[\mathrm{pHFMASP2_{zym}}\right]}{K_{\mathrm{m\ MASP1_{zym}}}^{\mathrm{MASP2_{zym}}}}+\frac{\left[\mathrm{pMFMASP1_{zym}}\right]}{K_{\mathrm{m\ MASP1_{zym}}}^{\mathrm{MASP1_{zym}}}}+\frac{\left[\mathrm{pMFMASP2_{zym}}\right]}{K_{\mathrm{m\ MASP1_{zym}}}^{\mathrm{MASP2_{zym}}}}\end{aligned}\right)}
$$

$$
+\frac{k_{\mathrm{cat\ MASP1_{zym}}}^{\mathrm{MASP1_{zym}}}\left[\mathrm{pMFMASP1_{zym}}\right]\left[\mathrm{pCLLKMASP1_{zym}}\right]}{K_{\mathrm{m\ MASP1_{zym}}}^{\mathrm{MASP1_{zym}}}\left(\begin{aligned}&1+\frac{\left[\mathrm{pMBL_3MASP1_{zym}}\right]}{K_{\mathrm{m\ MASP1_{zym}}}^{\mathrm{MASP1_{zym}}}}+\frac{\left[\mathrm{pMBL_3MASP2_{zym}}\right]}{K_{\mathrm{m\ MASP1_{zym}}}^{\mathrm{MASP2_{zym}}}}+\frac{\left[\mathrm{pMBL_4MASP1_{zym}}\right]}{K_{\mathrm{m\ MASP1_{zym}}}^{\mathrm{MASP1_{zym}}}}+\frac{\left[\mathrm{pMBL_4MASP2_{zym}}\right]}{K_{\mathrm{m\ MASP1_{zym}}}^{\mathrm{MASP2_{zym}}}}\\&+\frac{\left[\mathrm{pCLLKMASP1_{zym}}\right]}{K_{\mathrm{m\ MASP1_{zym}}}^{\mathrm{MASP1_{zym}}}}+\frac{\left[\mathrm{pCLLKMASP2_{zym}}\right]}{K_{\mathrm{m\ MASP1_{zym}}}^{\mathrm{MASP2_{zym}}}}+\frac{\left[\mathrm{pCLK1MASP1_{zym}}\right]}{K_{\mathrm{m\ MASP1_{zym}}}^{\mathrm{MASP1_{zym}}}}+\frac{\left[\mathrm{pCLK1MASP2_{zym}}\right]}{K_{\mathrm{m\ MASP1_{zym}}}^{\mathrm{MASP2_{zym}}}}\\&+\frac{\left[\mathrm{pCLL1MASP1_{zym}}\right]}{K_{\mathrm{m\ MASP1_{zym}}}^{\mathrm{MASP1_{zym}}}}+\frac{\left[\mathrm{pCLL1MASP2_{zym}}\right]}{K_{\mathrm{m\ MASP1_{zym}}}^{\mathrm{MASP2_{zym}}}}+\frac{\left[\mathrm{pLFMASP1_{zym}}\right]}{K_{\mathrm{m\ MASP1_{zym}}}^{\mathrm{MASP1_{zym}}}}+\frac{\left[\mathrm{pLFMASP2_{zym}}\right]}{K_{\mathrm{m\ MASP1_{zym}}}^{\mathrm{MASP2_{zym}}}}\\&+\frac{\left[\mathrm{pHFMASP1_{zym}}\right]}{K_{\mathrm{m\ MASP1_{zym}}}^{\mathrm{MASP1_{zym}}}}+\frac{\left[\mathrm{pHFMASP2_{zym}}\right]}{K_{\mathrm{m\ MASP1_{zym}}}^{\mathrm{MASP2_{zym}}}}+\frac{\left[\mathrm{pMFMASP1_{zym}}\right]}{K_{\mathrm{m\ MASP1_{zym}}}^{\mathrm{MASP1_{zym}}}}+\frac{\left[\mathrm{pMFMASP2_{zym}}\right]}{K_{\mathrm{m\ MASP1_{zym}}}^{\mathrm{MASP2_{zym}}}}\end{aligned}\right)}
$$

$$
+\frac{k_{\mathrm{cat\ MASP1_{zym}}}^{\mathrm{MASP1_{zym}}}\left[\mathrm{pPTX3LFMASP1_{zym}}\right]\left[\mathrm{pCLLKMASP1_{zym}}\right]}{K_{\mathrm{m\ MASP1_{zym}}}^{\mathrm{MASP1_{zym}}}\left(\begin{aligned}&1+\frac{\left[\mathrm{pMBL_3MASP1_{zym}}\right]}{K_{\mathrm{m\ MASP1_{zym}}}^{\mathrm{MASP1_{zym}}}}+\frac{\left[\mathrm{pMBL_3MASP2_{zym}}\right]}{K_{\mathrm{m\ MASP1_{zym}}}^{\mathrm{MASP2_{zym}}}}+\frac{\left[\mathrm{pMBL_4MASP1_{zym}}\right]}{K_{\mathrm{m\ MASP1_{zym}}}^{\mathrm{MASP1_{zym}}}}+\frac{\left[\mathrm{pMBL_4MASP2_{zym}}\right]}{K_{\mathrm{m\ MASP1_{zym}}}^{\mathrm{MASP2_{zym}}}}\\&+\frac{\left[\mathrm{pCLLKMASP1_{zym}}\right]}{K_{\mathrm{m\ MASP1_{zym}}}^{\mathrm{MASP1_{zym}}}}+\frac{\left[\mathrm{pCLLKMASP2_{zym}}\right]}{K_{\mathrm{m\ MASP1_{zym}}}^{\mathrm{MASP2_{zym}}}}+\frac{\left[\mathrm{pCLK1MASP1_{zym}}\right]}{K_{\mathrm{m\ MASP1_{zym}}}^{\mathrm{MASP1_{zym}}}}+\frac{\left[\mathrm{pCLK1MASP2_{zym}}\right]}{K_{\mathrm{m\ MASP1_{zym}}}^{\mathrm{MASP2_{zym}}}}\\&+\frac{\left[\mathrm{pCLL1MASP1_{zym}}\right]}{K_{\mathrm{m\ MASP1_{zym}}}^{\mathrm{MASP1_{zym}}}}+\frac{\left[\mathrm{pCLL1MASP2_{zym}}\right]}{K_{\mathrm{m\ MASP1_{zym}}}^{\mathrm{MASP2_{zym}}}}+\frac{\left[\mathrm{pLFMASP1_{zym}}\right]}{K_{\mathrm{m\ MASP1_{zym}}}^{\mathrm{MASP1_{zym}}}}+\frac{\left[\mathrm{pLFMASP2_{zym}}\right]}{K_{\mathrm{m\ MASP1_{zym}}}^{\mathrm{MASP2_{zym}}}}\\&+\frac{\left[\mathrm{pHFMASP1_{zym}}\right]}{K_{\mathrm{m\ MASP1_{zym}}}^{\mathrm{MASP1_{zym}}}}+\frac{\left[\mathrm{pHFMASP2_{zym}}\right]}{K_{\mathrm{m\ MASP1_{zym}}}^{\mathrm{MASP2_{zym}}}}+\frac{\left[\mathrm{pMFMASP1_{zym}}\right]}{K_{\mathrm{m\ MASP1_{zym}}}^{\mathrm{MASP1_{zym}}}}+\frac{\left[\mathrm{pMFMASP2_{zym}}\right]}{K_{\mathrm{m\ MASP1_{zym}}}^{\mathrm{MASP2_{zym}}}}\\&+\frac{\left[\mathrm{pPTX3LFMASP1_{zym}}\right]}{K_{\mathrm{m\ MASP1_{zym}}}^{\mathrm{MASP1_{zym}}}}+\frac{\left[\mathrm{pPTX3LFMASP2_{zym}}\right]}{K_{\mathrm{m\ MASP1_{zym}}}^{\mathrm{MASP2_{zym}}}}\end{aligned}\right)}
$$

$$+\frac{k^{\mathrm{MASP1_{zym}}}_{\mathrm{cat\ MASP1^*}}\left[\mathrm{pMBL_3MASP1^*}\right]\left[\mathrm{pCLLKMASP1_{zym}}\right]}{K^{\mathrm{MASP1_{zym}}}_{\mathrm{m\ MASP1^*}}\left(\begin{aligned}&1+\frac{[\mathrm{C4}]}{K^{\mathrm{C4}}_{\mathrm{m\ MASP1^*}}}+\frac{[\mathrm{fC4bC2}]}{K^{\mathrm{C2}}_{\mathrm{m\ MASP1^*}}}+\frac{[\mathrm{fC4bC4bC2}]}{K^{\mathrm{C2}}_{\mathrm{m\ MASP1^*}}}+\frac{[\mathrm{fC3bC4bC2}]}{K^{\mathrm{C2}}_{\mathrm{m\ MASP1^*}}}+\frac{[\mathrm{pC4bC2}]}{K^{\mathrm{C2}}_{\mathrm{m\ MASP1^*}}}+\frac{[\mathrm{pC4bC4bC2}]}{K^{\mathrm{C2}}_{\mathrm{m\ MASP1^*}}}\\&+\frac{[\mathrm{pC3bC4bC2}]}{K^{\mathrm{C2}}_{\mathrm{m\ MASP1^*}}}+\frac{[\mathrm{pMBL_3MASP1_{zym}}]}{K^{\mathrm{MASP1_{zym}}}_{\mathrm{m\ MASP1^*}}}+\frac{[\mathrm{pMBL_3MASP2_{zym}}]}{K^{\mathrm{MASP2_{zym}}}_{\mathrm{m\ MASP1^*}}}+\frac{[\mathrm{pMBL_3MASP3_{zym}}]}{K^{\mathrm{MASP3_{zym}}}_{\mathrm{m\ MASP1^*}}}\\&+\frac{[\mathrm{pMBL_4MASP1_{zym}}]}{K^{\mathrm{MASP1_{zym}}}_{\mathrm{m\ MASP1^*}}}+\frac{[\mathrm{pMBL_4MASP2_{zym}}]}{K^{\mathrm{MASP2_{zym}}}_{\mathrm{m\ MASP1^*}}}+\frac{[\mathrm{pMBL_4MASP3_{zym}}]}{K^{\mathrm{MASP3_{zym}}}_{\mathrm{m\ MASP1^*}}}+\frac{[\mathrm{pCLLKMASP1_{zym}}]}{K^{\mathrm{MASP1_{zym}}}_{\mathrm{m\ MASP1^*}}}\\&+\frac{[\mathrm{pCLLKMASP2_{zym}}]}{K^{\mathrm{MASP2_{zym}}}_{\mathrm{m\ MASP1^*}}}+\frac{[\mathrm{pCLLKMASP3_{zym}}]}{K^{\mathrm{MASP3_{zym}}}_{\mathrm{m\ MASP1^*}}}+\frac{[\mathrm{pCLK1MASP1_{zym}}]}{K^{\mathrm{MASP1_{zym}}}_{\mathrm{m\ MASP1^*}}}+\frac{[\mathrm{pCLK1MASP2_{zym}}]}{K^{\mathrm{MASP2_{zym}}}_{\mathrm{m\ MASP1^*}}}\\&+\frac{[\mathrm{pCLK1MASP3_{zym}}]}{K^{\mathrm{MASP3_{zym}}}_{\mathrm{m\ MASP1^*}}}+\frac{[\mathrm{pCLL1MASP1_{zym}}]}{K^{\mathrm{MASP1_{zym}}}_{\mathrm{m\ MASP1^*}}}+\frac{[\mathrm{pCLL1MASP2_{zym}}]}{K^{\mathrm{MASP2_{zym}}}_{\mathrm{m\ MASP1^*}}}+\frac{[\mathrm{pCLL1MASP3_{zym}}]}{K^{\mathrm{MASP3_{zym}}}_{\mathrm{m\ MASP1^*}}}\\&+\frac{[\mathrm{pLFMASP1_{zym}}]}{K^{\mathrm{MASP1_{zym}}}_{\mathrm{m\ MASP1^*}}}+\frac{[\mathrm{pLFMASP2_{zym}}]}{K^{\mathrm{MASP2_{zym}}}_{\mathrm{m\ MASP1^*}}}+\frac{[\mathrm{pLFMASP3_{zym}}]}{K^{\mathrm{MASP3_{zym}}}_{\mathrm{m\ MASP1^*}}}+\frac{[\mathrm{pHFMASP1_{zym}}]}{K^{\mathrm{MASP1_{zym}}}_{\mathrm{m\ MASP1^*}}}+\frac{[\mathrm{pHFMASP2_{zym}}]}{K^{\mathrm{MASP2_{zym}}}_{\mathrm{m\ MASP1^*}}}\\&+\frac{[\mathrm{pHFMASP3_{zym}}]}{K^{\mathrm{MASP3_{zym}}}_{\mathrm{m\ MASP1^*}}}+\frac{[\mathrm{pMFMASP1_{zym}}]}{K^{\mathrm{MASP1_{zym}}}_{\mathrm{m\ MASP1^*}}}+\frac{[\mathrm{pMFMASP2_{zym}}]}{K^{\mathrm{MASP2_{zym}}}_{\mathrm{m\ MASP1^*}}}+\frac{[\mathrm{pMFMASP3_{zym}}]}{K^{\mathrm{MASP3_{zym}}}_{\mathrm{m\ MASP1^*}}}\end{aligned}\right)}$$

$$+\frac{k^{\mathrm{MASP1_{zym}}}_{\mathrm{cat\ MASP1^*}}\left[\mathrm{pMBL_4MASP1^*}\right]\left[\mathrm{pCLLKMASP1_{zym}}\right]}{K^{\mathrm{MASP1_{zym}}}_{\mathrm{m\ MASP1^*}}\left(\begin{aligned}&1+\frac{[\mathrm{C4}]}{K^{\mathrm{C4}}_{\mathrm{m\ MASP1^*}}}+\frac{[\mathrm{fC4bC2}]}{K^{\mathrm{C2}}_{\mathrm{m\ MASP1^*}}}+\frac{[\mathrm{fC4bC4bC2}]}{K^{\mathrm{C2}}_{\mathrm{m\ MASP1^*}}}+\frac{[\mathrm{fC3bC4bC2}]}{K^{\mathrm{C2}}_{\mathrm{m\ MASP1^*}}}+\frac{[\mathrm{pC4bC2}]}{K^{\mathrm{C2}}_{\mathrm{m\ MASP1^*}}}+\frac{[\mathrm{pC4bC4bC2}]}{K^{\mathrm{C2}}_{\mathrm{m\ MASP1^*}}}\\&+\frac{[\mathrm{pC3bC4bC2}]}{K^{\mathrm{C2}}_{\mathrm{m\ MASP1^*}}}+\frac{[\mathrm{pMBL_3MASP1_{zym}}]}{K^{\mathrm{MASP1_{zym}}}_{\mathrm{m\ MASP1^*}}}+\frac{[\mathrm{pMBL_3MASP2_{zym}}]}{K^{\mathrm{MASP2_{zym}}}_{\mathrm{m\ MASP1^*}}}+\frac{[\mathrm{pMBL_3MASP3_{zym}}]}{K^{\mathrm{MASP3_{zym}}}_{\mathrm{m\ MASP1^*}}}\\&+\frac{[\mathrm{pMBL_4MASP1_{zym}}]}{K^{\mathrm{MASP1_{zym}}}_{\mathrm{m\ MASP1^*}}}+\frac{[\mathrm{pMBL_4MASP2_{zym}}]}{K^{\mathrm{MASP2_{zym}}}_{\mathrm{m\ MASP1^*}}}+\frac{[\mathrm{pMBL_4MASP3_{zym}}]}{K^{\mathrm{MASP3_{zym}}}_{\mathrm{m\ MASP1^*}}}+\frac{[\mathrm{pCLLKMASP1_{zym}}]}{K^{\mathrm{MASP1_{zym}}}_{\mathrm{m\ MASP1^*}}}\\&+\frac{[\mathrm{pCLLKMASP2_{zym}}]}{K^{\mathrm{MASP2_{zym}}}_{\mathrm{m\ MASP1^*}}}+\frac{[\mathrm{pCLLKMASP3_{zym}}]}{K^{\mathrm{MASP3_{zym}}}_{\mathrm{m\ MASP1^*}}}+\frac{[\mathrm{pCLK1MASP1_{zym}}]}{K^{\mathrm{MASP1_{zym}}}_{\mathrm{m\ MASP1^*}}}+\frac{[\mathrm{pCLK1MASP2_{zym}}]}{K^{\mathrm{MASP2_{zym}}}_{\mathrm{m\ MASP1^*}}}\\&+\frac{[\mathrm{pCLK1MASP3_{zym}}]}{K^{\mathrm{MASP3_{zym}}}_{\mathrm{m\ MASP1^*}}}+\frac{[\mathrm{pCLL1MASP1_{zym}}]}{K^{\mathrm{MASP1_{zym}}}_{\mathrm{m\ MASP1^*}}}+\frac{[\mathrm{pCLL1MASP2_{zym}}]}{K^{\mathrm{MASP2_{zym}}}_{\mathrm{m\ MASP1^*}}}+\frac{[\mathrm{pCLL1MASP3_{zym}}]}{K^{\mathrm{MASP3_{zym}}}_{\mathrm{m\ MASP1^*}}}\\&+\frac{[\mathrm{pLFMASP1_{zym}}]}{K^{\mathrm{MASP1_{zym}}}_{\mathrm{m\ MASP1^*}}}+\frac{[\mathrm{pLFMASP2_{zym}}]}{K^{\mathrm{MASP2_{zym}}}_{\mathrm{m\ MASP1^*}}}+\frac{[\mathrm{pLFMASP3_{zym}}]}{K^{\mathrm{MASP3_{zym}}}_{\mathrm{m\ MASP1^*}}}+\frac{[\mathrm{pHFMASP1_{zym}}]}{K^{\mathrm{MASP1_{zym}}}_{\mathrm{m\ MASP1^*}}}+\frac{[\mathrm{pHFMASP2_{zym}}]}{K^{\mathrm{MASP2_{zym}}}_{\mathrm{m\ MASP1^*}}}\\&+\frac{[\mathrm{pHFMASP3_{zym}}]}{K^{\mathrm{MASP3_{zym}}}_{\mathrm{m\ MASP1^*}}}+\frac{[\mathrm{pMFMASP1_{zym}}]}{K^{\mathrm{MASP1_{zym}}}_{\mathrm{m\ MASP1^*}}}+\frac{[\mathrm{pMFMASP2_{zym}}]}{K^{\mathrm{MASP2_{zym}}}_{\mathrm{m\ MASP1^*}}}+\frac{[\mathrm{pMFMASP3_{zym}}]}{K^{\mathrm{MASP3_{zym}}}_{\mathrm{m\ MASP1^*}}}\end{aligned}\right)}$$

$$+\frac{k_{\mathrm{cat\,MASP1^*}}^{\mathrm{MASP1_{zym}}}\left[\mathrm{pCLLKMASP1^*}\right]\left[\mathrm{pCLLKMASP1_{zym}}\right]}{K_{\mathrm{m\,MASP1^*}}^{\mathrm{MASP1_{zym}}}\left(\begin{array}{l}1+\frac{[\mathrm{C4}]}{K_{\mathrm{m\,MASP1^*}}^{\mathrm{C4}}}+\frac{[\mathrm{fC4bC2}]}{K_{\mathrm{m\,MASP1^*}}^{\mathrm{C2}}}+\frac{[\mathrm{fC4bC4bC2}]}{K_{\mathrm{m\,MASP1^*}}^{\mathrm{C2}}}+\frac{[\mathrm{fC3bC4bC2}]}{K_{\mathrm{m\,MASP1^*}}^{\mathrm{C2}}}+\frac{[\mathrm{pC4bC2}]}{K_{\mathrm{m\,MASP1^*}}^{\mathrm{C2}}}+\frac{[\mathrm{pC4bC4bC2}]}{K_{\mathrm{m\,MASP1^*}}^{\mathrm{C2}}}\\
+\frac{[\mathrm{pC3bC4bC2}]}{K_{\mathrm{m\,MASP1^*}}^{\mathrm{C2}}}+\frac{[\mathrm{pMBL_3MASP1_{zym}}]}{K_{\mathrm{m\,MASP1^*}}^{\mathrm{MASP1_{zym}}}}+\frac{[\mathrm{pMBL_3MASP2_{zym}}]}{K_{\mathrm{m\,MASP1^*}}^{\mathrm{MASP2_{zym}}}}+\frac{[\mathrm{pMBL_3MASP3_{zym}}]}{K_{\mathrm{m\,MASP1^*}}^{\mathrm{MASP3_{zym}}}}\\
+\frac{[\mathrm{pMBL_4MASP1_{zym}}]}{K_{\mathrm{m\,MASP1^*}}^{\mathrm{MASP1_{zym}}}}+\frac{[\mathrm{pMBL_4MASP2_{zym}}]}{K_{\mathrm{m\,MASP1^*}}^{\mathrm{MASP2_{zym}}}}+\frac{[\mathrm{pMBL_4MASP3_{zym}}]}{K_{\mathrm{m\,MASP1^*}}^{\mathrm{MASP3_{zym}}}}+\frac{[\mathrm{pCLLKMASP1_{zym}}]}{K_{\mathrm{m\,MASP1^*}}^{\mathrm{MASP1_{zym}}}}\\
+\frac{[\mathrm{pCLLKMASP2_{zym}}]}{K_{\mathrm{m\,MASP1^*}}^{\mathrm{MASP2_{zym}}}}+\frac{[\mathrm{pCLLKMASP3_{zym}}]}{K_{\mathrm{m\,MASP1^*}}^{\mathrm{MASP3_{zym}}}}+\frac{[\mathrm{pCLK1MASP1_{zym}}]}{K_{\mathrm{m\,MASP1^*}}^{\mathrm{MASP1_{zym}}}}+\frac{[\mathrm{pCLK1MASP2_{zym}}]}{K_{\mathrm{m\,MASP1^*}}^{\mathrm{MASP2_{zym}}}}\\
+\frac{[\mathrm{pCLK1MASP3_{zym}}]}{K_{\mathrm{m\,MASP1^*}}^{\mathrm{MASP3_{zym}}}}+\frac{[\mathrm{pCLL1MASP1_{zym}}]}{K_{\mathrm{m\,MASP1^*}}^{\mathrm{MASP1_{zym}}}}+\frac{[\mathrm{pCLL1MASP2_{zym}}]}{K_{\mathrm{m\,MASP1^*}}^{\mathrm{MASP2_{zym}}}}+\frac{[\mathrm{pCLL1MASP3_{zym}}]}{K_{\mathrm{m\,MASP1^*}}^{\mathrm{MASP3_{zym}}}}\\
+\frac{[\mathrm{pLFMASP1_{zym}}]}{K_{\mathrm{m\,MASP1^*}}^{\mathrm{MASP1_{zym}}}}+\frac{[\mathrm{pLFMASP2_{zym}}]}{K_{\mathrm{m\,MASP1^*}}^{\mathrm{MASP2_{zym}}}}+\frac{[\mathrm{pLFMASP3_{zym}}]}{K_{\mathrm{m\,MASP1^*}}^{\mathrm{MASP3_{zym}}}}+\frac{[\mathrm{pHFMASP1_{zym}}]}{K_{\mathrm{m\,MASP1^*}}^{\mathrm{MASP1_{zym}}}}+\frac{[\mathrm{pHFMASP2_{zym}}]}{K_{\mathrm{m\,MASP1^*}}^{\mathrm{MASP2_{zym}}}}\\
+\frac{[\mathrm{pHFMASP3_{zym}}]}{K_{\mathrm{m\,MASP1^*}}^{\mathrm{MASP3_{zym}}}}+\frac{[\mathrm{pMFMASP1_{zym}}]}{K_{\mathrm{m\,MASP1^*}}^{\mathrm{MASP1_{zym}}}}+\frac{[\mathrm{pMFMASP2_{zym}}]}{K_{\mathrm{m\,MASP1^*}}^{\mathrm{MASP2_{zym}}}}+\frac{[\mathrm{pMFMASP3_{zym}}]}{K_{\mathrm{m\,MASP1^*}}^{\mathrm{MASP3_{zym}}}}\end{array}\right)}$$

$$+\frac{k_{\mathrm{cat\,MASP1^*}}^{\mathrm{MASP1_{zym}}}\left[\mathrm{pCLK1MASP1^*}\right]\left[\mathrm{pCLLKMASP1_{zym}}\right]}{K_{\mathrm{m\,MASP1^*}}^{\mathrm{MASP1_{zym}}}\left(\begin{array}{l}1+\frac{[\mathrm{C4}]}{K_{\mathrm{m\,MASP1^*}}^{\mathrm{C4}}}+\frac{[\mathrm{fC4bC2}]}{K_{\mathrm{m\,MASP1^*}}^{\mathrm{C2}}}+\frac{[\mathrm{fC4bC4bC2}]}{K_{\mathrm{m\,MASP1^*}}^{\mathrm{C2}}}+\frac{[\mathrm{fC3bC4bC2}]}{K_{\mathrm{m\,MASP1^*}}^{\mathrm{C2}}}+\frac{[\mathrm{pC4bC2}]}{K_{\mathrm{m\,MASP1^*}}^{\mathrm{C2}}}+\frac{[\mathrm{pC4bC4bC2}]}{K_{\mathrm{m\,MASP1^*}}^{\mathrm{C2}}}\\
+\frac{[\mathrm{pC3bC4bC2}]}{K_{\mathrm{m\,MASP1^*}}^{\mathrm{C2}}}+\frac{[\mathrm{pMBL_3MASP1_{zym}}]}{K_{\mathrm{m\,MASP1^*}}^{\mathrm{MASP1_{zym}}}}+\frac{[\mathrm{pMBL_3MASP2_{zym}}]}{K_{\mathrm{m\,MASP1^*}}^{\mathrm{MASP2_{zym}}}}+\frac{[\mathrm{pMBL_3MASP3_{zym}}]}{K_{\mathrm{m\,MASP1^*}}^{\mathrm{MASP3_{zym}}}}\\
+\frac{[\mathrm{pMBL_4MASP1_{zym}}]}{K_{\mathrm{m\,MASP1^*}}^{\mathrm{MASP1_{zym}}}}+\frac{[\mathrm{pMBL_4MASP2_{zym}}]}{K_{\mathrm{m\,MASP1^*}}^{\mathrm{MASP2_{zym}}}}+\frac{[\mathrm{pMBL_4MASP3_{zym}}]}{K_{\mathrm{m\,MASP1^*}}^{\mathrm{MASP3_{zym}}}}+\frac{[\mathrm{pCLLKMASP1_{zym}}]}{K_{\mathrm{m\,MASP1^*}}^{\mathrm{MASP1_{zym}}}}\\
+\frac{[\mathrm{pCLLKMASP2_{zym}}]}{K_{\mathrm{m\,MASP1^*}}^{\mathrm{MASP2_{zym}}}}+\frac{[\mathrm{pCLLKMASP3_{zym}}]}{K_{\mathrm{m\,MASP1^*}}^{\mathrm{MASP3_{zym}}}}+\frac{[\mathrm{pCLK1MASP1_{zym}}]}{K_{\mathrm{m\,MASP1^*}}^{\mathrm{MASP1_{zym}}}}+\frac{[\mathrm{pCLK1MASP2_{zym}}]}{K_{\mathrm{m\,MASP1^*}}^{\mathrm{MASP2_{zym}}}}\\
+\frac{[\mathrm{pCLK1MASP3_{zym}}]}{K_{\mathrm{m\,MASP1^*}}^{\mathrm{MASP3_{zym}}}}+\frac{[\mathrm{pCLL1MASP1_{zym}}]}{K_{\mathrm{m\,MASP1^*}}^{\mathrm{MASP1_{zym}}}}+\frac{[\mathrm{pCLL1MASP2_{zym}}]}{K_{\mathrm{m\,MASP1^*}}^{\mathrm{MASP2_{zym}}}}+\frac{[\mathrm{pCLL1MASP3_{zym}}]}{K_{\mathrm{m\,MASP1^*}}^{\mathrm{MASP3_{zym}}}}\\
+\frac{[\mathrm{pLFMASP1_{zym}}]}{K_{\mathrm{m\,MASP1^*}}^{\mathrm{MASP1_{zym}}}}+\frac{[\mathrm{pLFMASP2_{zym}}]}{K_{\mathrm{m\,MASP1^*}}^{\mathrm{MASP2_{zym}}}}+\frac{[\mathrm{pLFMASP3_{zym}}]}{K_{\mathrm{m\,MASP1^*}}^{\mathrm{MASP3_{zym}}}}+\frac{[\mathrm{pHFMASP1_{zym}}]}{K_{\mathrm{m\,MASP1^*}}^{\mathrm{MASP1_{zym}}}}+\frac{[\mathrm{pHFMASP2_{zym}}]}{K_{\mathrm{m\,MASP1^*}}^{\mathrm{MASP2_{zym}}}}\\
+\frac{[\mathrm{pHFMASP3_{zym}}]}{K_{\mathrm{m\,MASP1^*}}^{\mathrm{MASP3_{zym}}}}+\frac{[\mathrm{pMFMASP1_{zym}}]}{K_{\mathrm{m\,MASP1^*}}^{\mathrm{MASP1_{zym}}}}+\frac{[\mathrm{pMFMASP2_{zym}}]}{K_{\mathrm{m\,MASP1^*}}^{\mathrm{MASP2_{zym}}}}+\frac{[\mathrm{pMFMASP3_{zym}}]}{K_{\mathrm{m\,MASP1^*}}^{\mathrm{MASP3_{zym}}}}\end{array}\right)}$$

$$
+\frac{k_{\mathrm{cat\ MASP1^*}}^{\mathrm{MASP1_{zym}}}\left[\mathrm{pCLL1MASP1^*}\right]\left[\mathrm{pCLLKMASP1_{zym}}\right]}{K_{\mathrm{m\ MASP1^*}}^{\mathrm{MASP1_{zym}}}\left(\begin{array}{l}1+\frac{[\mathrm{C4}]}{K_{\mathrm{m\ MASP1^*}}^{\mathrm{C4}}}+\frac{[\mathrm{fC4bC2}]}{K_{\mathrm{m\ MASP1^*}}^{\mathrm{C2}}}+\frac{[\mathrm{fC4bC4bC2}]}{K_{\mathrm{m\ MASP1^*}}^{\mathrm{C2}}}+\frac{[\mathrm{fC3bC4bC2}]}{K_{\mathrm{m\ MASP1^*}}^{\mathrm{C2}}}+\frac{[\mathrm{pC4bC2}]}{K_{\mathrm{m\ MASP1^*}}^{\mathrm{C2}}}+\frac{[\mathrm{pC4bC4bC2}]}{K_{\mathrm{m\ MASP1^*}}^{\mathrm{C2}}}\\
+\frac{[\mathrm{pC3bC4bC2}]}{K_{\mathrm{m\ MASP1^*}}^{\mathrm{C2}}}+\frac{[\mathrm{pMBL_3MASP1_{zym}}]}{K_{\mathrm{m\ MASP1^*}}^{\mathrm{MASP1_{zym}}}}+\frac{[\mathrm{pMBL_3MASP2_{zym}}]}{K_{\mathrm{m\ MASP1^*}}^{\mathrm{MASP2_{zym}}}}+\frac{[\mathrm{pMBL_3MASP3_{zym}}]}{K_{\mathrm{m\ MASP1^*}}^{\mathrm{MASP3_{zym}}}}\\
+\frac{[\mathrm{pMBL_4MASP1_{zym}}]}{K_{\mathrm{m\ MASP1^*}}^{\mathrm{MASP1_{zym}}}}+\frac{[\mathrm{pMBL_4MASP2_{zym}}]}{K_{\mathrm{m\ MASP1^*}}^{\mathrm{MASP2_{zym}}}}+\frac{[\mathrm{pMBL_4MASP3_{zym}}]}{K_{\mathrm{m\ MASP1^*}}^{\mathrm{MASP3_{zym}}}}+\frac{[\mathrm{pCLLKMASP1_{zym}}]}{K_{\mathrm{m\ MASP1^*}}^{\mathrm{MASP1_{zym}}}}\\
+\frac{[\mathrm{pCLLKMASP2_{zym}}]}{K_{\mathrm{m\ MASP1^*}}^{\mathrm{MASP2_{zym}}}}+\frac{[\mathrm{pCLLKMASP3_{zym}}]}{K_{\mathrm{m\ MASP1^*}}^{\mathrm{MASP3_{zym}}}}+\frac{[\mathrm{pCLK1MASP1_{zym}}]}{K_{\mathrm{m\ MASP1^*}}^{\mathrm{MASP1_{zym}}}}+\frac{[\mathrm{pCLK1MASP2_{zym}}]}{K_{\mathrm{m\ MASP1^*}}^{\mathrm{MASP2_{zym}}}}\\
+\frac{[\mathrm{pCLK1MASP3_{zym}}]}{K_{\mathrm{m\ MASP1^*}}^{\mathrm{MASP3_{zym}}}}+\frac{[\mathrm{pCLL1MASP1_{zym}}]}{K_{\mathrm{m\ MASP1^*}}^{\mathrm{MASP1_{zym}}}}+\frac{[\mathrm{pCLL1MASP2_{zym}}]}{K_{\mathrm{m\ MASP1^*}}^{\mathrm{MASP2_{zym}}}}+\frac{[\mathrm{pCLL1MASP3_{zym}}]}{K_{\mathrm{m\ MASP1^*}}^{\mathrm{MASP3_{zym}}}}\\
+\frac{[\mathrm{pLFMASP1_{zym}}]}{K_{\mathrm{m\ MASP1^*}}^{\mathrm{MASP1_{zym}}}}+\frac{[\mathrm{pLFMASP2_{zym}}]}{K_{\mathrm{m\ MASP1^*}}^{\mathrm{MASP2_{zym}}}}+\frac{[\mathrm{pLFMASP3_{zym}}]}{K_{\mathrm{m\ MASP1^*}}^{\mathrm{MASP3_{zym}}}}+\frac{[\mathrm{pHFMASP1_{zym}}]}{K_{\mathrm{m\ MASP1^*}}^{\mathrm{MASP1_{zym}}}}+\frac{[\mathrm{pHFMASP2_{zym}}]}{K_{\mathrm{m\ MASP1^*}}^{\mathrm{MASP2_{zym}}}}\\
+\frac{[\mathrm{pHFMASP3_{zym}}]}{K_{\mathrm{m\ MASP1^*}}^{\mathrm{MASP3_{zym}}}}+\frac{[\mathrm{pMFMASP1_{zym}}]}{K_{\mathrm{m\ MASP1^*}}^{\mathrm{MASP1_{zym}}}}+\frac{[\mathrm{pMFMASP2_{zym}}]}{K_{\mathrm{m\ MASP1^*}}^{\mathrm{MASP2_{zym}}}}+\frac{[\mathrm{pMFMASP3_{zym}}]}{K_{\mathrm{m\ MASP1^*}}^{\mathrm{MASP3_{zym}}}}\end{array}\right)}
$$

$$
+\frac{k_{\mathrm{cat\ MASP1^*}}^{\mathrm{MASP1_{zym}}}\left[\mathrm{pLFMASP1^*}\right]\left[\mathrm{pCLLKMASP1_{zym}}\right]}{K_{\mathrm{m\ MASP1^*}}^{\mathrm{MASP1_{zym}}}\left(\begin{array}{l}1+\frac{[\mathrm{C4}]}{K_{\mathrm{m\ MASP1^*}}^{\mathrm{C4}}}+\frac{[\mathrm{fC4bC2}]}{K_{\mathrm{m\ MASP1^*}}^{\mathrm{C2}}}+\frac{[\mathrm{fC4bC4bC2}]}{K_{\mathrm{m\ MASP1^*}}^{\mathrm{C2}}}+\frac{[\mathrm{fC3bC4bC2}]}{K_{\mathrm{m\ MASP1^*}}^{\mathrm{C2}}}+\frac{[\mathrm{pC4bC2}]}{K_{\mathrm{m\ MASP1^*}}^{\mathrm{C2}}}+\frac{[\mathrm{pC4bC4bC2}]}{K_{\mathrm{m\ MASP1^*}}^{\mathrm{C2}}}\\
+\frac{[\mathrm{pC3bC4bC2}]}{K_{\mathrm{m\ MASP1^*}}^{\mathrm{C2}}}+\frac{[\mathrm{pMBL_3MASP1_{zym}}]}{K_{\mathrm{m\ MASP1^*}}^{\mathrm{MASP1_{zym}}}}+\frac{[\mathrm{pMBL_3MASP2_{zym}}]}{K_{\mathrm{m\ MASP1^*}}^{\mathrm{MASP2_{zym}}}}+\frac{[\mathrm{pMBL_3MASP3_{zym}}]}{K_{\mathrm{m\ MASP1^*}}^{\mathrm{MASP3_{zym}}}}\\
+\frac{[\mathrm{pMBL_4MASP1_{zym}}]}{K_{\mathrm{m\ MASP1^*}}^{\mathrm{MASP1_{zym}}}}+\frac{[\mathrm{pMBL_4MASP2_{zym}}]}{K_{\mathrm{m\ MASP1^*}}^{\mathrm{MASP2_{zym}}}}+\frac{[\mathrm{pMBL_4MASP3_{zym}}]}{K_{\mathrm{m\ MASP1^*}}^{\mathrm{MASP3_{zym}}}}+\frac{[\mathrm{pCLLKMASP1_{zym}}]}{K_{\mathrm{m\ MASP1^*}}^{\mathrm{MASP1_{zym}}}}\\
+\frac{[\mathrm{pCLLKMASP2_{zym}}]}{K_{\mathrm{m\ MASP1^*}}^{\mathrm{MASP2_{zym}}}}+\frac{[\mathrm{pCLLKMASP3_{zym}}]}{K_{\mathrm{m\ MASP1^*}}^{\mathrm{MASP3_{zym}}}}+\frac{[\mathrm{pCLK1MASP1_{zym}}]}{K_{\mathrm{m\ MASP1^*}}^{\mathrm{MASP1_{zym}}}}+\frac{[\mathrm{pCLK1MASP2_{zym}}]}{K_{\mathrm{m\ MASP1^*}}^{\mathrm{MASP2_{zym}}}}\\
+\frac{[\mathrm{pCLK1MASP3_{zym}}]}{K_{\mathrm{m\ MASP1^*}}^{\mathrm{MASP3_{zym}}}}+\frac{[\mathrm{pCLL1MASP1_{zym}}]}{K_{\mathrm{m\ MASP1^*}}^{\mathrm{MASP1_{zym}}}}+\frac{[\mathrm{pCLL1MASP2_{zym}}]}{K_{\mathrm{m\ MASP1^*}}^{\mathrm{MASP2_{zym}}}}+\frac{[\mathrm{pCLL1MASP3_{zym}}]}{K_{\mathrm{m\ MASP1^*}}^{\mathrm{MASP3_{zym}}}}\\
+\frac{[\mathrm{pLFMASP1_{zym}}]}{K_{\mathrm{m\ MASP1^*}}^{\mathrm{MASP1_{zym}}}}+\frac{[\mathrm{pLFMASP2_{zym}}]}{K_{\mathrm{m\ MASP1^*}}^{\mathrm{MASP2_{zym}}}}+\frac{[\mathrm{pLFMASP3_{zym}}]}{K_{\mathrm{m\ MASP1^*}}^{\mathrm{MASP3_{zym}}}}+\frac{[\mathrm{pHFMASP1_{zym}}]}{K_{\mathrm{m\ MASP1^*}}^{\mathrm{MASP1_{zym}}}}+\frac{[\mathrm{pHFMASP2_{zym}}]}{K_{\mathrm{m\ MASP1^*}}^{\mathrm{MASP2_{zym}}}}\\
+\frac{[\mathrm{pHFMASP3_{zym}}]}{K_{\mathrm{m\ MASP1^*}}^{\mathrm{MASP3_{zym}}}}+\frac{[\mathrm{pMFMASP1_{zym}}]}{K_{\mathrm{m\ MASP1^*}}^{\mathrm{MASP1_{zym}}}}+\frac{[\mathrm{pMFMASP2_{zym}}]}{K_{\mathrm{m\ MASP1^*}}^{\mathrm{MASP2_{zym}}}}+\frac{[\mathrm{pMFMASP3_{zym}}]}{K_{\mathrm{m\ MASP1^*}}^{\mathrm{MASP3_{zym}}}}\end{array}\right)}
$$

$$
+\frac{k_{\mathrm{cat\ MASP1^{*}}}^{\mathrm{MASP1_{zym}}}\left[\mathrm{pHFMASP1^{*}}\right]\left[\mathrm{pCLLKMASP1_{zym}}\right]}{K_{\mathrm{m\ MASP1^{*}}}^{\mathrm{MASP1_{zym}}}\left(\begin{array}{l}
1+\frac{\left[\mathrm{C4}\right]}{K_{\mathrm{m\ MASP1^{*}}}^{\mathrm{C4}}}+\frac{\left[\mathrm{fC4bC2}\right]}{K_{\mathrm{m\ MASP1^{*}}}^{\mathrm{C2}}}+\frac{\left[\mathrm{fC4bC4bC2}\right]}{K_{\mathrm{m\ MASP1^{*}}}^{\mathrm{C2}}}+\frac{\left[\mathrm{fC3bC4bC2}\right]}{K_{\mathrm{m\ MASP1^{*}}}^{\mathrm{C2}}}+\frac{\left[\mathrm{pC4bC2}\right]}{K_{\mathrm{m\ MASP1^{*}}}^{\mathrm{C2}}}+\frac{\left[\mathrm{pC4bC4bC2}\right]}{K_{\mathrm{m\ MASP1^{*}}}^{\mathrm{C2}}}\\
+\frac{\left[\mathrm{pC3bC4bC2}\right]}{K_{\mathrm{m\ MASP1^{*}}}^{\mathrm{C2}}}+\frac{\left[\mathrm{pMBL_{3}MASP1_{zym}}\right]}{K_{\mathrm{m\ MASP1^{*}}}^{\mathrm{MASP1_{zym}}}}+\frac{\left[\mathrm{pMBL_{3}MASP2_{zym}}\right]}{K_{\mathrm{m\ MASP1^{*}}}^{\mathrm{MASP2_{zym}}}}+\frac{\left[\mathrm{pMBL_{3}MASP3_{zym}}\right]}{K_{\mathrm{m\ MASP1^{*}}}^{\mathrm{MASP3_{zym}}}}\\
+\frac{\left[\mathrm{pMBL_{4}MASP1_{zym}}\right]}{K_{\mathrm{m\ MASP1^{*}}}^{\mathrm{MASP1_{zym}}}}+\frac{\left[\mathrm{pMBL_{4}MASP2_{zym}}\right]}{K_{\mathrm{m\ MASP1^{*}}}^{\mathrm{MASP2_{zym}}}}+\frac{\left[\mathrm{pMBL_{4}MASP3_{zym}}\right]}{K_{\mathrm{m\ MASP1^{*}}}^{\mathrm{MASP3_{zym}}}}+\frac{\left[\mathrm{pCLLKMASP1_{zym}}\right]}{K_{\mathrm{m\ MASP1^{*}}}^{\mathrm{MASP1_{zym}}}}\\
+\frac{\left[\mathrm{pCLLKMASP2_{zym}}\right]}{K_{\mathrm{m\ MASP1^{*}}}^{\mathrm{MASP2_{zym}}}}+\frac{\left[\mathrm{pCLLKMASP3_{zym}}\right]}{K_{\mathrm{m\ MASP1^{*}}}^{\mathrm{MASP3_{zym}}}}+\frac{\left[\mathrm{pCLK1MASP1_{zym}}\right]}{K_{\mathrm{m\ MASP1^{*}}}^{\mathrm{MASP1_{zym}}}}+\frac{\left[\mathrm{pCLK1MASP2_{zym}}\right]}{K_{\mathrm{m\ MASP1^{*}}}^{\mathrm{MASP2_{zym}}}}\\
+\frac{\left[\mathrm{pCLK1MASP3_{zym}}\right]}{K_{\mathrm{m\ MASP1^{*}}}^{\mathrm{MASP3_{zym}}}}+\frac{\left[\mathrm{pCLL1MASP1_{zym}}\right]}{K_{\mathrm{m\ MASP1^{*}}}^{\mathrm{MASP1_{zym}}}}+\frac{\left[\mathrm{pCLL1MASP2_{zym}}\right]}{K_{\mathrm{m\ MASP1^{*}}}^{\mathrm{MASP2_{zym}}}}+\frac{\left[\mathrm{pCLL1MASP3_{zym}}\right]}{K_{\mathrm{m\ MASP1^{*}}}^{\mathrm{MASP3_{zym}}}}\\
+\frac{\left[\mathrm{pLFMASP1_{zym}}\right]}{K_{\mathrm{m\ MASP1^{*}}}^{\mathrm{MASP1_{zym}}}}+\frac{\left[\mathrm{pLFMASP2_{zym}}\right]}{K_{\mathrm{m\ MASP1^{*}}}^{\mathrm{MASP2_{zym}}}}+\frac{\left[\mathrm{pLFMASP3_{zym}}\right]}{K_{\mathrm{m\ MASP1^{*}}}^{\mathrm{MASP3_{zym}}}}+\frac{\left[\mathrm{pHFMASP1_{zym}}\right]}{K_{\mathrm{m\ MASP1^{*}}}^{\mathrm{MASP1_{zym}}}}+\frac{\left[\mathrm{pHFMASP2_{zym}}\right]}{K_{\mathrm{m\ MASP1^{*}}}^{\mathrm{MASP2_{zym}}}}\\
+\frac{\left[\mathrm{pHFMASP3_{zym}}\right]}{K_{\mathrm{m\ MASP1^{*}}}^{\mathrm{MASP3_{zym}}}}+\frac{\left[\mathrm{pMFMASP1_{zym}}\right]}{K_{\mathrm{m\ MASP1^{*}}}^{\mathrm{MASP1_{zym}}}}+\frac{\left[\mathrm{pMFMASP2_{zym}}\right]}{K_{\mathrm{m\ MASP1^{*}}}^{\mathrm{MASP2_{zym}}}}+\frac{\left[\mathrm{pMFMASP3_{zym}}\right]}{K_{\mathrm{m\ MASP1^{*}}}^{\mathrm{MASP3_{zym}}}}
\end{array}\right)}
$$

$$
+\frac{k_{\mathrm{cat\ MASP1^{*}}}^{\mathrm{MASP1_{zym}}}\left[\mathrm{pMFMASP1^{*}}\right]\left[\mathrm{pCLLKMASP1_{zym}}\right]}{K_{\mathrm{m\ MASP1^{*}}}^{\mathrm{MASP1_{zym}}}\left(\begin{array}{l}
1+\frac{\left[\mathrm{C4}\right]}{K_{\mathrm{m\ MASP1^{*}}}^{\mathrm{C4}}}+\frac{\left[\mathrm{fC4bC2}\right]}{K_{\mathrm{m\ MASP1^{*}}}^{\mathrm{C2}}}+\frac{\left[\mathrm{fC4bC4bC2}\right]}{K_{\mathrm{m\ MASP1^{*}}}^{\mathrm{C2}}}+\frac{\left[\mathrm{fC3bC4bC2}\right]}{K_{\mathrm{m\ MASP1^{*}}}^{\mathrm{C2}}}+\frac{\left[\mathrm{pC4bC2}\right]}{K_{\mathrm{m\ MASP1^{*}}}^{\mathrm{C2}}}+\frac{\left[\mathrm{pC4bC4bC2}\right]}{K_{\mathrm{m\ MASP1^{*}}}^{\mathrm{C2}}}\\
+\frac{\left[\mathrm{pC3bC4bC2}\right]}{K_{\mathrm{m\ MASP1^{*}}}^{\mathrm{C2}}}+\frac{\left[\mathrm{pMBL_{3}MASP1_{zym}}\right]}{K_{\mathrm{m\ MASP1^{*}}}^{\mathrm{MASP1_{zym}}}}+\frac{\left[\mathrm{pMBL_{3}MASP2_{zym}}\right]}{K_{\mathrm{m\ MASP1^{*}}}^{\mathrm{MASP2_{zym}}}}+\frac{\left[\mathrm{pMBL_{3}MASP3_{zym}}\right]}{K_{\mathrm{m\ MASP1^{*}}}^{\mathrm{MASP3_{zym}}}}\\
+\frac{\left[\mathrm{pMBL_{4}MASP1_{zym}}\right]}{K_{\mathrm{m\ MASP1^{*}}}^{\mathrm{MASP1_{zym}}}}+\frac{\left[\mathrm{pMBL_{4}MASP2_{zym}}\right]}{K_{\mathrm{m\ MASP1^{*}}}^{\mathrm{MASP2_{zym}}}}+\frac{\left[\mathrm{pMBL_{4}MASP3_{zym}}\right]}{K_{\mathrm{m\ MASP1^{*}}}^{\mathrm{MASP3_{zym}}}}+\frac{\left[\mathrm{pCLLKMASP1_{zym}}\right]}{K_{\mathrm{m\ MASP1^{*}}}^{\mathrm{MASP1_{zym}}}}\\
+\frac{\left[\mathrm{pCLLKMASP2_{zym}}\right]}{K_{\mathrm{m\ MASP1^{*}}}^{\mathrm{MASP2_{zym}}}}+\frac{\left[\mathrm{pCLLKMASP3_{zym}}\right]}{K_{\mathrm{m\ MASP1^{*}}}^{\mathrm{MASP3_{zym}}}}+\frac{\left[\mathrm{pCLK1MASP1_{zym}}\right]}{K_{\mathrm{m\ MASP1^{*}}}^{\mathrm{MASP1_{zym}}}}+\frac{\left[\mathrm{pCLK1MASP2_{zym}}\right]}{K_{\mathrm{m\ MASP1^{*}}}^{\mathrm{MASP2_{zym}}}}\\
+\frac{\left[\mathrm{pCLK1MASP3_{zym}}\right]}{K_{\mathrm{m\ MASP1^{*}}}^{\mathrm{MASP3_{zym}}}}+\frac{\left[\mathrm{pCLL1MASP1_{zym}}\right]}{K_{\mathrm{m\ MASP1^{*}}}^{\mathrm{MASP1_{zym}}}}+\frac{\left[\mathrm{pCLL1MASP2_{zym}}\right]}{K_{\mathrm{m\ MASP1^{*}}}^{\mathrm{MASP2_{zym}}}}+\frac{\left[\mathrm{pCLL1MASP3_{zym}}\right]}{K_{\mathrm{m\ MASP1^{*}}}^{\mathrm{MASP3_{zym}}}}\\
+\frac{\left[\mathrm{pLFMASP1_{zym}}\right]}{K_{\mathrm{m\ MASP1^{*}}}^{\mathrm{MASP1_{zym}}}}+\frac{\left[\mathrm{pLFMASP2_{zym}}\right]}{K_{\mathrm{m\ MASP1^{*}}}^{\mathrm{MASP2_{zym}}}}+\frac{\left[\mathrm{pLFMASP3_{zym}}\right]}{K_{\mathrm{m\ MASP1^{*}}}^{\mathrm{MASP3_{zym}}}}+\frac{\left[\mathrm{pHFMASP1_{zym}}\right]}{K_{\mathrm{m\ MASP1^{*}}}^{\mathrm{MASP1_{zym}}}}+\frac{\left[\mathrm{pHFMASP2_{zym}}\right]}{K_{\mathrm{m\ MASP1^{*}}}^{\mathrm{MASP2_{zym}}}}\\
+\frac{\left[\mathrm{pHFMASP3_{zym}}\right]}{K_{\mathrm{m\ MASP1^{*}}}^{\mathrm{MASP3_{zym}}}}+\frac{\left[\mathrm{pMFMASP1_{zym}}\right]}{K_{\mathrm{m\ MASP1^{*}}}^{\mathrm{MASP1_{zym}}}}+\frac{\left[\mathrm{pMFMASP2_{zym}}\right]}{K_{\mathrm{m\ MASP1^{*}}}^{\mathrm{MASP2_{zym}}}}+\frac{\left[\mathrm{pMFMASP3_{zym}}\right]}{K_{\mathrm{m\ MASP1^{*}}}^{\mathrm{MASP3_{zym}}}}
\end{array}\right)}
$$

$$+\frac{k_{\text{cat MASP1}^*}^{\text{MASP1}_{\text{zym}}}\left[\text{pPTX3LFMASP1}^*\right]\left[\text{pCLLKMASP1}_{\text{zym}}\right]}{K_{\text{m MASP1}^*}^{\text{MASP1}_{\text{zym}}}\left(\begin{array}{l}1+\frac{[\text{C4}]}{K_{\text{m MASP1}^*}^{\text{C4}}}+\frac{[\text{fC4bC2}]}{K_{\text{m MASP1}^*}^{\text{C2}}}+\frac{[\text{fC4bC4bC2}]}{K_{\text{m MASP1}^*}^{\text{C2}}}+\frac{[\text{fC3bC4bC2}]}{K_{\text{m MASP1}^*}^{\text{C2}}}+\frac{[\text{pC4bC2}]}{K_{\text{m MASP1}^*}^{\text{C2}}}+\frac{[\text{pC4bC4bC2}]}{K_{\text{m MASP1}^*}^{\text{C2}}}\\ +\frac{[\text{pC3bC4bC2}]}{K_{\text{m MASP1}^*}^{\text{C2}}}+\frac{\left[\text{pMBL}_3\text{MASP1}_{\text{zym}}\right]}{K_{\text{m MASP1}^*}^{\text{MASP1}_{\text{zym}}}}+\frac{\left[\text{pMBL}_3\text{MASP2}_{\text{zym}}\right]}{K_{\text{m MASP1}^*}^{\text{MASP2}_{\text{zym}}}}+\frac{\left[\text{pMBL}_3\text{MASP3}_{\text{zym}}\right]}{K_{\text{m MASP1}^*}^{\text{MASP3}_{\text{zym}}}}\\ +\frac{\left[\text{pMBL}_4\text{MASP1}_{\text{zym}}\right]}{K_{\text{m MASP1}^*}^{\text{MASP1}_{\text{zym}}}}+\frac{\left[\text{pMBL}_4\text{MASP2}_{\text{zym}}\right]}{K_{\text{m MASP1}^*}^{\text{MASP2}_{\text{zym}}}}+\frac{\left[\text{pMBL}_4\text{MASP3}_{\text{zym}}\right]}{K_{\text{m MASP1}^*}^{\text{MASP3}_{\text{zym}}}}+\frac{\left[\text{pCLLKMASP1}_{\text{zym}}\right]}{K_{\text{m MASP1}^*}^{\text{MASP1}_{\text{zym}}}}\\ +\frac{\left[\text{pCLLKMASP2}_{\text{zym}}\right]}{K_{\text{m MASP1}^*}^{\text{MASP2}_{\text{zym}}}}+\frac{\left[\text{pCLLKMASP3}_{\text{zym}}\right]}{K_{\text{m MASP1}^*}^{\text{MASP3}_{\text{zym}}}}+\frac{\left[\text{pCLK1MASP1}_{\text{zym}}\right]}{K_{\text{m MASP1}^*}^{\text{MASP1}_{\text{zym}}}}+\frac{\left[\text{pCLK1MASP2}_{\text{zym}}\right]}{K_{\text{m MASP1}^*}^{\text{MASP2}_{\text{zym}}}}\\ +\frac{\left[\text{pCLK1MASP3}_{\text{zym}}\right]}{K_{\text{m MASP1}^*}^{\text{MASP3}_{\text{zym}}}}+\frac{\left[\text{pCLL1MASP1}_{\text{zym}}\right]}{K_{\text{m MASP1}^*}^{\text{MASP1}_{\text{zym}}}}+\frac{\left[\text{pCLL1MASP2}_{\text{zym}}\right]}{K_{\text{m MASP1}^*}^{\text{MASP2}_{\text{zym}}}}+\frac{\left[\text{pCLL1MASP3}_{\text{zym}}\right]}{K_{\text{m MASP1}^*}^{\text{MASP3}_{\text{zym}}}}\\ +\frac{\left[\text{pLFMASP1}_{\text{zym}}\right]}{K_{\text{m MASP1}^*}^{\text{MASP1}_{\text{zym}}}}+\frac{\left[\text{pLFMASP2}_{\text{zym}}\right]}{K_{\text{m MASP1}^*}^{\text{MASP2}_{\text{zym}}}}+\frac{\left[\text{pLFMASP3}_{\text{zym}}\right]}{K_{\text{m MASP1}^*}^{\text{MASP3}_{\text{zym}}}}+\frac{\left[\text{pHFMASP1}_{\text{zym}}\right]}{K_{\text{m MASP1}^*}^{\text{MASP1}_{\text{zym}}}}+\frac{\left[\text{pHFMASP2}_{\text{zym}}\right]}{K_{\text{m MASP1}^*}^{\text{MASP2}_{\text{zym}}}}\\ +\frac{\left[\text{pHFMASP3}_{\text{zym}}\right]}{K_{\text{m MASP1}^*}^{\text{MASP3}_{\text{zym}}}}+\frac{\left[\text{pMFMASP1}_{\text{zym}}\right]}{K_{\text{m MASP1}^*}^{\text{MASP1}_{\text{zym}}}}+\frac{\left[\text{pMFMASP2}_{\text{zym}}\right]}{K_{\text{m MASP1}^*}^{\text{MASP2}_{\text{zym}}}}+\frac{\left[\text{pMFMASP3}_{\text{zym}}\right]}{K_{\text{m MASP1}^*}^{\text{MASP3}_{\text{zym}}}}\\ +\frac{\left[\text{pPTX3LFMASP1}_{\text{zym}}\right]}{K_{\text{m MASP1}^*}^{\text{MASP1}_{\text{zym}}}}+\frac{\left[\text{pPXT3LFMASP2}_{\text{zym}}\right]}{K_{\text{m MASP1}^*}^{\text{MASP2}_{\text{zym}}}}+\frac{\left[\text{pPTX3LFMASP3}_{\text{zym}}\right]}{K_{\text{m MASP1}^*}^{\text{MASP3}_{\text{zym}}}}\end{array}\right)}$$

$$+\frac{k_{\text{cat MASP2}^*}^{\text{MASP1}_{\text{zym}}}\left[\text{pMBL}_3\text{MASP2}^*\right]\left[\text{pCLLKMASP1}_{\text{zym}}\right]}{K_{\text{m MASP2}^*}^{\text{MASP1}_{\text{zym}}}\left(\begin{array}{l}1+\frac{[\text{C4}]}{K_{\text{m MASP2}^*}^{\text{C4}}}+\frac{[\text{fC4bC2}]}{K_{\text{m MASP2}^*}^{\text{C2}}}+\frac{[\text{fC4bC4bC2}]}{K_{\text{m MASP2}^*}^{\text{C2}}}+\frac{[\text{fC3bC4bC2}]}{K_{\text{m MASP2}^*}^{\text{C2}}}+\frac{[\text{pC4bC2}]}{K_{\text{m MASP2}^*}^{\text{C2}}}+\frac{[\text{pC4bC4bC2}]}{K_{\text{m MASP2}^*}^{\text{C2}}}\\ +\frac{[\text{pC3bC4bC2}]}{K_{\text{m MASP2}^*}^{\text{C2}}}+\frac{\left[\text{pMBL}_3\text{MASP1}_{\text{zym}}\right]}{K_{\text{m MASP2}^*}^{\text{MASP1}_{\text{zym}}}}+\frac{\left[\text{pMBL}_3\text{MASP2}_{\text{zym}}\right]}{K_{\text{m MASP2}^*}^{\text{MASP2}_{\text{zym}}}}+\frac{\left[\text{pMBL}_4\text{MASP1}_{\text{zym}}\right]}{K_{\text{m MASP2}^*}^{\text{MASP1}_{\text{zym}}}}\\ +\frac{\left[\text{pMBL}_4\text{MASP2}_{\text{zym}}\right]}{K_{\text{m MASP2}^*}^{\text{MASP2}_{\text{zym}}}}+\frac{\left[\text{pCLLKMASP1}_{\text{zym}}\right]}{K_{\text{m MASP2}^*}^{\text{MASP1}_{\text{zym}}}}+\frac{\left[\text{pCLLKMASP2}_{\text{zym}}\right]}{K_{\text{m MASP2}^*}^{\text{MASP2}_{\text{zym}}}}+\frac{\left[\text{pCLK1MASP1}_{\text{zym}}\right]}{K_{\text{m MASP2}^*}^{\text{MASP1}_{\text{zym}}}}\\ +\frac{\left[\text{pCLK1MASP2}_{\text{zym}}\right]}{K_{\text{m MASP2}^*}^{\text{MASP2}_{\text{zym}}}}+\frac{\left[\text{pCLL1MASP1}_{\text{zym}}\right]}{K_{\text{m MASP2}^*}^{\text{MASP1}_{\text{zym}}}}+\frac{\left[\text{pCLL1MASP2}_{\text{zym}}\right]}{K_{\text{m MASP2}^*}^{\text{MASP2}_{\text{zym}}}}+\frac{\left[\text{pLFMASP1}_{\text{zym}}\right]}{K_{\text{m MASP2}^*}^{\text{MASP1}_{\text{zym}}}}\\ +\frac{\left[\text{pLFMASP2}_{\text{zym}}\right]}{K_{\text{m MASP2}^*}^{\text{MASP2}_{\text{zym}}}}+\frac{\left[\text{pHFMASP1}_{\text{zym}}\right]}{K_{\text{m MASP2}^*}^{\text{MASP1}_{\text{zym}}}}+\frac{\left[\text{pHFMASP2}_{\text{zym}}\right]}{K_{\text{m MASP2}^*}^{\text{MASP2}_{\text{zym}}}}+\frac{\left[\text{pMFMASP1}_{\text{zym}}\right]}{K_{\text{m MASP2}^*}^{\text{MASP1}_{\text{zym}}}}+\frac{\left[\text{pMFMASP2}_{\text{zym}}\right]}{K_{\text{m MASP2}^*}^{\text{MASP2}_{\text{zym}}}}\end{array}\right)}$$

$$+\frac{k_{\mathrm{cat\,MASP2^*}}^{\mathrm{MASP1_{zym}}}\left[\mathrm{pMBL_4MASP2^*}\right]\left[\mathrm{pCLLKMASP1_{zym}}\right]}{K_{\mathrm{m\,MASP2^*}}^{\mathrm{MASP1_{zym}}}\left(\begin{aligned}&1+\frac{[\mathrm{C4}]}{K_{\mathrm{m\,MASP2^*}}^{\mathrm{C4}}}+\frac{[\mathrm{fC4bC2}]}{K_{\mathrm{m\,MASP2^*}}^{\mathrm{C2}}}+\frac{[\mathrm{fC4bC4bC2}]}{K_{\mathrm{m\,MASP2^*}}^{\mathrm{C2}}}+\frac{[\mathrm{fC3bC4bC2}]}{K_{\mathrm{m\,MASP2^*}}^{\mathrm{C2}}}+\frac{[\mathrm{pC4bC2}]}{K_{\mathrm{m\,MASP2^*}}^{\mathrm{C2}}}+\frac{[\mathrm{pC4bC4bC2}]}{K_{\mathrm{m\,MASP2^*}}^{\mathrm{C2}}}\\&+\frac{[\mathrm{pC3bC4bC2}]}{K_{\mathrm{m\,MASP2^*}}^{\mathrm{C2}}}+\frac{[\mathrm{pMBL_3MASP1_{zym}}]}{K_{\mathrm{m\,MASP2^*}}^{\mathrm{MASP1_{zym}}}}+\frac{[\mathrm{pMBL_3MASP2_{zym}}]}{K_{\mathrm{m\,MASP2^*}}^{\mathrm{MASP2_{zym}}}}+\frac{[\mathrm{pMBL_4MASP1_{zym}}]}{K_{\mathrm{m\,MASP2^*}}^{\mathrm{MASP1_{zym}}}}\\&+\frac{[\mathrm{pMBL_4MASP2_{zym}}]}{K_{\mathrm{m\,MASP2^*}}^{\mathrm{MASP2_{zym}}}}+\frac{[\mathrm{pCLLKMASP1_{zym}}]}{K_{\mathrm{m\,MASP2^*}}^{\mathrm{MASP1_{zym}}}}+\frac{[\mathrm{pCLLKMASP2_{zym}}]}{K_{\mathrm{m\,MASP2^*}}^{\mathrm{MASP2_{zym}}}}+\frac{[\mathrm{pCLK1MASP1_{zym}}]}{K_{\mathrm{m\,MASP2^*}}^{\mathrm{MASP1_{zym}}}}\\&+\frac{[\mathrm{pCLK1MASP2_{zym}}]}{K_{\mathrm{m\,MASP2^*}}^{\mathrm{MASP2_{zym}}}}+\frac{[\mathrm{pCLL1MASP1_{zym}}]}{K_{\mathrm{m\,MASP2^*}}^{\mathrm{MASP1_{zym}}}}+\frac{[\mathrm{pCLL1MASP2_{zym}}]}{K_{\mathrm{m\,MASP2^*}}^{\mathrm{MASP2_{zym}}}}+\frac{[\mathrm{pLFMASP1_{zym}}]}{K_{\mathrm{m\,MASP2^*}}^{\mathrm{MASP1_{zym}}}}\\&+\frac{[\mathrm{pLFMASP2_{zym}}]}{K_{\mathrm{m\,MASP2^*}}^{\mathrm{MASP2_{zym}}}}+\frac{[\mathrm{pHFMASP1_{zym}}]}{K_{\mathrm{m\,MASP2^*}}^{\mathrm{MASP1_{zym}}}}+\frac{[\mathrm{pHFMASP2_{zym}}]}{K_{\mathrm{m\,MASP2^*}}^{\mathrm{MASP2_{zym}}}}+\frac{[\mathrm{pMFMASP1_{zym}}]}{K_{\mathrm{m\,MASP2^*}}^{\mathrm{MASP1_{zym}}}}+\frac{[\mathrm{pMFMASP2_{zym}}]}{K_{\mathrm{m\,MASP2^*}}^{\mathrm{MASP2_{zym}}}}\end{aligned}\right)}$$

$$+\frac{k_{\mathrm{cat\,MASP2^*}}^{\mathrm{MASP1_{zym}}}\left[\mathrm{pCLLKMASP2^*}\right]\left[\mathrm{pCLLKMASP1_{zym}}\right]}{K_{\mathrm{m\,MASP2^*}}^{\mathrm{MASP1_{zym}}}\left(\begin{aligned}&1+\frac{[\mathrm{C4}]}{K_{\mathrm{m\,MASP2^*}}^{\mathrm{C4}}}+\frac{[\mathrm{fC4bC2}]}{K_{\mathrm{m\,MASP2^*}}^{\mathrm{C2}}}+\frac{[\mathrm{fC4bC4bC2}]}{K_{\mathrm{m\,MASP2^*}}^{\mathrm{C2}}}+\frac{[\mathrm{fC3bC4bC2}]}{K_{\mathrm{m\,MASP2^*}}^{\mathrm{C2}}}+\frac{[\mathrm{pC4bC2}]}{K_{\mathrm{m\,MASP2^*}}^{\mathrm{C2}}}+\frac{[\mathrm{pC4bC4bC2}]}{K_{\mathrm{m\,MASP2^*}}^{\mathrm{C2}}}\\&+\frac{[\mathrm{pC3bC4bC2}]}{K_{\mathrm{m\,MASP2^*}}^{\mathrm{C2}}}+\frac{[\mathrm{pMBL_3MASP1_{zym}}]}{K_{\mathrm{m\,MASP2^*}}^{\mathrm{MASP1_{zym}}}}+\frac{[\mathrm{pMBL_3MASP2_{zym}}]}{K_{\mathrm{m\,MASP2^*}}^{\mathrm{MASP2_{zym}}}}+\frac{[\mathrm{pMBL_4MASP1_{zym}}]}{K_{\mathrm{m\,MASP2^*}}^{\mathrm{MASP1_{zym}}}}\\&+\frac{[\mathrm{pMBL_4MASP2_{zym}}]}{K_{\mathrm{m\,MASP2^*}}^{\mathrm{MASP2_{zym}}}}+\frac{[\mathrm{pCLLKMASP1_{zym}}]}{K_{\mathrm{m\,MASP2^*}}^{\mathrm{MASP1_{zym}}}}+\frac{[\mathrm{pCLLKMASP2_{zym}}]}{K_{\mathrm{m\,MASP2^*}}^{\mathrm{MASP2_{zym}}}}+\frac{[\mathrm{pCLK1MASP1_{zym}}]}{K_{\mathrm{m\,MASP2^*}}^{\mathrm{MASP1_{zym}}}}\\&+\frac{[\mathrm{pCLK1MASP2_{zym}}]}{K_{\mathrm{m\,MASP2^*}}^{\mathrm{MASP2_{zym}}}}+\frac{[\mathrm{pCLL1MASP1_{zym}}]}{K_{\mathrm{m\,MASP2^*}}^{\mathrm{MASP1_{zym}}}}+\frac{[\mathrm{pCLL1MASP2_{zym}}]}{K_{\mathrm{m\,MASP2^*}}^{\mathrm{MASP2_{zym}}}}+\frac{[\mathrm{pLFMASP1_{zym}}]}{K_{\mathrm{m\,MASP2^*}}^{\mathrm{MASP1_{zym}}}}\\&+\frac{[\mathrm{pLFMASP2_{zym}}]}{K_{\mathrm{m\,MASP2^*}}^{\mathrm{MASP2_{zym}}}}+\frac{[\mathrm{pHFMASP1_{zym}}]}{K_{\mathrm{m\,MASP2^*}}^{\mathrm{MASP1_{zym}}}}+\frac{[\mathrm{pHFMASP2_{zym}}]}{K_{\mathrm{m\,MASP2^*}}^{\mathrm{MASP2_{zym}}}}+\frac{[\mathrm{pMFMASP1_{zym}}]}{K_{\mathrm{m\,MASP2^*}}^{\mathrm{MASP1_{zym}}}}+\frac{[\mathrm{pMFMASP2_{zym}}]}{K_{\mathrm{m\,MASP2^*}}^{\mathrm{MASP2_{zym}}}}\end{aligned}\right)}$$

$$+\frac{k_{\mathrm{cat\,MASP2^*}}^{\mathrm{MASP1_{zym}}}\left[\mathrm{pCLK1MASP2^*}\right]\left[\mathrm{pCLLKMASP1_{zym}}\right]}{K_{\mathrm{m\,MASP2^*}}^{\mathrm{MASP1_{zym}}}\left(\begin{aligned}&1+\frac{[\mathrm{C4}]}{K_{\mathrm{m\,MASP2^*}}^{\mathrm{C4}}}+\frac{[\mathrm{fC4bC2}]}{K_{\mathrm{m\,MASP2^*}}^{\mathrm{C2}}}+\frac{[\mathrm{fC4bC4bC2}]}{K_{\mathrm{m\,MASP2^*}}^{\mathrm{C2}}}+\frac{[\mathrm{fC3bC4bC2}]}{K_{\mathrm{m\,MASP2^*}}^{\mathrm{C2}}}+\frac{[\mathrm{pC4bC2}]}{K_{\mathrm{m\,MASP2^*}}^{\mathrm{C2}}}+\frac{[\mathrm{pC4bC4bC2}]}{K_{\mathrm{m\,MASP2^*}}^{\mathrm{C2}}}\\&+\frac{[\mathrm{pC3bC4bC2}]}{K_{\mathrm{m\,MASP2^*}}^{\mathrm{C2}}}+\frac{[\mathrm{pMBL_3MASP1_{zym}}]}{K_{\mathrm{m\,MASP2^*}}^{\mathrm{MASP1_{zym}}}}+\frac{[\mathrm{pMBL_3MASP2_{zym}}]}{K_{\mathrm{m\,MASP2^*}}^{\mathrm{MASP2_{zym}}}}+\frac{[\mathrm{pMBL_4MASP1_{zym}}]}{K_{\mathrm{m\,MASP2^*}}^{\mathrm{MASP1_{zym}}}}\\&+\frac{[\mathrm{pMBL_4MASP2_{zym}}]}{K_{\mathrm{m\,MASP2^*}}^{\mathrm{MASP2_{zym}}}}+\frac{[\mathrm{pCLLKMASP1_{zym}}]}{K_{\mathrm{m\,MASP2^*}}^{\mathrm{MASP1_{zym}}}}+\frac{[\mathrm{pCLLKMASP2_{zym}}]}{K_{\mathrm{m\,MASP2^*}}^{\mathrm{MASP2_{zym}}}}+\frac{[\mathrm{pCLK1MASP1_{zym}}]}{K_{\mathrm{m\,MASP2^*}}^{\mathrm{MASP1_{zym}}}}\\&+\frac{[\mathrm{pCLK1MASP2_{zym}}]}{K_{\mathrm{m\,MASP2^*}}^{\mathrm{MASP2_{zym}}}}+\frac{[\mathrm{pCLL1MASP1_{zym}}]}{K_{\mathrm{m\,MASP2^*}}^{\mathrm{MASP1_{zym}}}}+\frac{[\mathrm{pCLL1MASP2_{zym}}]}{K_{\mathrm{m\,MASP2^*}}^{\mathrm{MASP2_{zym}}}}+\frac{[\mathrm{pLFMASP1_{zym}}]}{K_{\mathrm{m\,MASP2^*}}^{\mathrm{MASP1_{zym}}}}\\&+\frac{[\mathrm{pLFMASP2_{zym}}]}{K_{\mathrm{m\,MASP2^*}}^{\mathrm{MASP2_{zym}}}}+\frac{[\mathrm{pHFMASP1_{zym}}]}{K_{\mathrm{m\,MASP2^*}}^{\mathrm{MASP1_{zym}}}}+\frac{[\mathrm{pHFMASP2_{zym}}]}{K_{\mathrm{m\,MASP2^*}}^{\mathrm{MASP2_{zym}}}}+\frac{[\mathrm{pMFMASP1_{zym}}]}{K_{\mathrm{m\,MASP2^*}}^{\mathrm{MASP1_{zym}}}}+\frac{[\mathrm{pMFMASP2_{zym}}]}{K_{\mathrm{m\,MASP2^*}}^{\mathrm{MASP2_{zym}}}}\end{aligned}\right)}$$

$$
+\frac{k_{\mathrm{cat\,MASP2^*}}^{\mathrm{MASP1_{zym}}}\left[\mathrm{pCLL1MASP2^*}\right]\left[\mathrm{pCLLKMASP1_{zym}}\right]}{K_{\mathrm{m\,MASP2^*}}^{\mathrm{MASP1_{zym}}}\left(\begin{array}{l}1+\frac{[\mathrm{C4}]}{K_{\mathrm{m\,MASP2^*}}^{\mathrm{C4}}}+\frac{[\mathrm{fC4bC2}]}{K_{\mathrm{m\,MASP2^*}}^{\mathrm{C2}}}+\frac{[\mathrm{fC4bC4bC2}]}{K_{\mathrm{m\,MASP2^*}}^{\mathrm{C2}}}+\frac{[\mathrm{fC3bC4bC2}]}{K_{\mathrm{m\,MASP2^*}}^{\mathrm{C2}}}+\frac{[\mathrm{pC4bC2}]}{K_{\mathrm{m\,MASP2^*}}^{\mathrm{C2}}}+\frac{[\mathrm{pC4bC4bC2}]}{K_{\mathrm{m\,MASP2^*}}^{\mathrm{C2}}}\\+\frac{[\mathrm{pC3bC4bC2}]}{K_{\mathrm{m\,MASP2^*}}^{\mathrm{C2}}}+\frac{[\mathrm{pMBL_3MASP1_{zym}}]}{K_{\mathrm{m\,MASP2^*}}^{\mathrm{MASP1_{zym}}}}+\frac{[\mathrm{pMBL_3MASP2_{zym}}]}{K_{\mathrm{m\,MASP2^*}}^{\mathrm{MASP2_{zym}}}}+\frac{[\mathrm{pMBL_4MASP1_{zym}}]}{K_{\mathrm{m\,MASP2^*}}^{\mathrm{MASP1_{zym}}}}\\+\frac{[\mathrm{pMBL_4MASP2_{zym}}]}{K_{\mathrm{m\,MASP2^*}}^{\mathrm{MASP2_{zym}}}}+\frac{[\mathrm{pCLLKMASP1_{zym}}]}{K_{\mathrm{m\,MASP2^*}}^{\mathrm{MASP1_{zym}}}}+\frac{[\mathrm{pCLLKMASP2_{zym}}]}{K_{\mathrm{m\,MASP2^*}}^{\mathrm{MASP2_{zym}}}}+\frac{[\mathrm{pCLK1MASP1_{zym}}]}{K_{\mathrm{m\,MASP2^*}}^{\mathrm{MASP1_{zym}}}}\\+\frac{[\mathrm{pCLK1MASP2_{zym}}]}{K_{\mathrm{m\,MASP2^*}}^{\mathrm{MASP2_{zym}}}}+\frac{[\mathrm{pCLL1MASP1_{zym}}]}{K_{\mathrm{m\,MASP2^*}}^{\mathrm{MASP1_{zym}}}}+\frac{[\mathrm{pCLL1MASP2_{zym}}]}{K_{\mathrm{m\,MASP2^*}}^{\mathrm{MASP2_{zym}}}}+\frac{[\mathrm{pLFMASP1_{zym}}]}{K_{\mathrm{m\,MASP2^*}}^{\mathrm{MASP1_{zym}}}}\\+\frac{[\mathrm{pLFMASP2_{zym}}]}{K_{\mathrm{m\,MASP2^*}}^{\mathrm{MASP2_{zym}}}}+\frac{[\mathrm{pHFMASP1_{zym}}]}{K_{\mathrm{m\,MASP2^*}}^{\mathrm{MASP1_{zym}}}}+\frac{[\mathrm{pHFMASP2_{zym}}]}{K_{\mathrm{m\,MASP2^*}}^{\mathrm{MASP2_{zym}}}}+\frac{[\mathrm{pMFMASP1_{zym}}]}{K_{\mathrm{m\,MASP2^*}}^{\mathrm{MASP1_{zym}}}}+\frac{[\mathrm{pMFMASP2_{zym}}]}{K_{\mathrm{m\,MASP2^*}}^{\mathrm{MASP2_{zym}}}}\end{array}\right)}
$$

$$
+\frac{k_{\mathrm{cat\,MASP2^*}}^{\mathrm{MASP1_{zym}}}\left[\mathrm{pLFMASP2^*}\right]\left[\mathrm{pCLLKMASP1_{zym}}\right]}{K_{\mathrm{m\,MASP2^*}}^{\mathrm{MASP1_{zym}}}\left(\begin{array}{l}1+\frac{[\mathrm{C4}]}{K_{\mathrm{m\,MASP2^*}}^{\mathrm{C4}}}+\frac{[\mathrm{fC4bC2}]}{K_{\mathrm{m\,MASP2^*}}^{\mathrm{C2}}}+\frac{[\mathrm{fC4bC4bC2}]}{K_{\mathrm{m\,MASP2^*}}^{\mathrm{C2}}}+\frac{[\mathrm{fC3bC4bC2}]}{K_{\mathrm{m\,MASP2^*}}^{\mathrm{C2}}}+\frac{[\mathrm{pC4bC2}]}{K_{\mathrm{m\,MASP2^*}}^{\mathrm{C2}}}+\frac{[\mathrm{pC4bC4bC2}]}{K_{\mathrm{m\,MASP2^*}}^{\mathrm{C2}}}\\+\frac{[\mathrm{pC3bC4bC2}]}{K_{\mathrm{m\,MASP2^*}}^{\mathrm{C2}}}+\frac{[\mathrm{pMBL_3MASP1_{zym}}]}{K_{\mathrm{m\,MASP2^*}}^{\mathrm{MASP1_{zym}}}}+\frac{[\mathrm{pMBL_3MASP2_{zym}}]}{K_{\mathrm{m\,MASP2^*}}^{\mathrm{MASP2_{zym}}}}+\frac{[\mathrm{pMBL_4MASP1_{zym}}]}{K_{\mathrm{m\,MASP2^*}}^{\mathrm{MASP1_{zym}}}}\\+\frac{[\mathrm{pMBL_4MASP2_{zym}}]}{K_{\mathrm{m\,MASP2^*}}^{\mathrm{MASP2_{zym}}}}+\frac{[\mathrm{pCLLKMASP1_{zym}}]}{K_{\mathrm{m\,MASP2^*}}^{\mathrm{MASP1_{zym}}}}+\frac{[\mathrm{pCLLKMASP2_{zym}}]}{K_{\mathrm{m\,MASP2^*}}^{\mathrm{MASP2_{zym}}}}+\frac{[\mathrm{pCLK1MASP1_{zym}}]}{K_{\mathrm{m\,MASP2^*}}^{\mathrm{MASP1_{zym}}}}\\+\frac{[\mathrm{pCLK1MASP2_{zym}}]}{K_{\mathrm{m\,MASP2^*}}^{\mathrm{MASP2_{zym}}}}+\frac{[\mathrm{pCLL1MASP1_{zym}}]}{K_{\mathrm{m\,MASP2^*}}^{\mathrm{MASP1_{zym}}}}+\frac{[\mathrm{pCLL1MASP2_{zym}}]}{K_{\mathrm{m\,MASP2^*}}^{\mathrm{MASP2_{zym}}}}+\frac{[\mathrm{pLFMASP1_{zym}}]}{K_{\mathrm{m\,MASP2^*}}^{\mathrm{MASP1_{zym}}}}\\+\frac{[\mathrm{pLFMASP2_{zym}}]}{K_{\mathrm{m\,MASP2^*}}^{\mathrm{MASP2_{zym}}}}+\frac{[\mathrm{pHFMASP1_{zym}}]}{K_{\mathrm{m\,MASP2^*}}^{\mathrm{MASP1_{zym}}}}+\frac{[\mathrm{pHFMASP2_{zym}}]}{K_{\mathrm{m\,MASP2^*}}^{\mathrm{MASP2_{zym}}}}+\frac{[\mathrm{pMFMASP1_{zym}}]}{K_{\mathrm{m\,MASP2^*}}^{\mathrm{MASP1_{zym}}}}+\frac{[\mathrm{pMFMASP2_{zym}}]}{K_{\mathrm{m\,MASP2^*}}^{\mathrm{MASP2_{zym}}}}\end{array}\right)}
$$

$$
+\frac{k_{\mathrm{cat\,MASP2^*}}^{\mathrm{MASP1_{zym}}}\left[\mathrm{pHFMASP2^*}\right]\left[\mathrm{pCLLKMASP1_{zym}}\right]}{K_{\mathrm{m\,MASP2^*}}^{\mathrm{MASP1_{zym}}}\left(\begin{array}{l}1+\frac{[\mathrm{C4}]}{K_{\mathrm{m\,MASP2^*}}^{\mathrm{C4}}}+\frac{[\mathrm{fC4bC2}]}{K_{\mathrm{m\,MASP2^*}}^{\mathrm{C2}}}+\frac{[\mathrm{fC4bC4bC2}]}{K_{\mathrm{m\,MASP2^*}}^{\mathrm{C2}}}+\frac{[\mathrm{fC3bC4bC2}]}{K_{\mathrm{m\,MASP2^*}}^{\mathrm{C2}}}+\frac{[\mathrm{pC4bC2}]}{K_{\mathrm{m\,MASP2^*}}^{\mathrm{C2}}}+\frac{[\mathrm{pC4bC4bC2}]}{K_{\mathrm{m\,MASP2^*}}^{\mathrm{C2}}}\\+\frac{[\mathrm{pC3bC4bC2}]}{K_{\mathrm{m\,MASP2^*}}^{\mathrm{C2}}}+\frac{[\mathrm{pMBL_3MASP1_{zym}}]}{K_{\mathrm{m\,MASP2^*}}^{\mathrm{MASP1_{zym}}}}+\frac{[\mathrm{pMBL_3MASP2_{zym}}]}{K_{\mathrm{m\,MASP2^*}}^{\mathrm{MASP2_{zym}}}}+\frac{[\mathrm{pMBL_4MASP1_{zym}}]}{K_{\mathrm{m\,MASP2^*}}^{\mathrm{MASP1_{zym}}}}\\+\frac{[\mathrm{pMBL_4MASP2_{zym}}]}{K_{\mathrm{m\,MASP2^*}}^{\mathrm{MASP2_{zym}}}}+\frac{[\mathrm{pCLLKMASP1_{zym}}]}{K_{\mathrm{m\,MASP2^*}}^{\mathrm{MASP1_{zym}}}}+\frac{[\mathrm{pCLLKMASP2_{zym}}]}{K_{\mathrm{m\,MASP2^*}}^{\mathrm{MASP2_{zym}}}}+\frac{[\mathrm{pCLK1MASP1_{zym}}]}{K_{\mathrm{m\,MASP2^*}}^{\mathrm{MASP1_{zym}}}}\\+\frac{[\mathrm{pCLK1MASP2_{zym}}]}{K_{\mathrm{m\,MASP2^*}}^{\mathrm{MASP2_{zym}}}}+\frac{[\mathrm{pCLL1MASP1_{zym}}]}{K_{\mathrm{m\,MASP2^*}}^{\mathrm{MASP1_{zym}}}}+\frac{[\mathrm{pCLL1MASP2_{zym}}]}{K_{\mathrm{m\,MASP2^*}}^{\mathrm{MASP2_{zym}}}}+\frac{[\mathrm{pLFMASP1_{zym}}]}{K_{\mathrm{m\,MASP2^*}}^{\mathrm{MASP1_{zym}}}}\\+\frac{[\mathrm{pLFMASP2_{zym}}]}{K_{\mathrm{m\,MASP2^*}}^{\mathrm{MASP2_{zym}}}}+\frac{[\mathrm{pHFMASP1_{zym}}]}{K_{\mathrm{m\,MASP2^*}}^{\mathrm{MASP1_{zym}}}}+\frac{[\mathrm{pHFMASP2_{zym}}]}{K_{\mathrm{m\,MASP2^*}}^{\mathrm{MASP2_{zym}}}}+\frac{[\mathrm{pMFMASP1_{zym}}]}{K_{\mathrm{m\,MASP2^*}}^{\mathrm{MASP1_{zym}}}}+\frac{[\mathrm{pMFMASP2_{zym}}]}{K_{\mathrm{m\,MASP2^*}}^{\mathrm{MASP2_{zym}}}}\end{array}\right)}
$$

$$
+\frac{k_{\mathrm{cat\ MASP2^*}}^{\mathrm{MASP1_{zym}}}\left[\mathrm{pMFMASP2^*}\right]\left[\mathrm{pCLLKMASP1_{zym}}\right]}{K_{\mathrm{m\ MASP2^*}}^{\mathrm{MASP1_{zym}}}\left(\begin{aligned}&1+\frac{[\mathrm{C4}]}{K_{\mathrm{m\ MASP2^*}}^{\mathrm{C4}}}+\frac{[\mathrm{fC4bC2}]}{K_{\mathrm{m\ MASP2^*}}^{\mathrm{C2}}}+\frac{[\mathrm{fC4bC4bC2}]}{K_{\mathrm{m\ MASP2^*}}^{\mathrm{C2}}}+\frac{[\mathrm{fC3bC4bC2}]}{K_{\mathrm{m\ MASP2^*}}^{\mathrm{C2}}}+\frac{[\mathrm{pC4bC2}]}{K_{\mathrm{m\ MASP2^*}}^{\mathrm{C2}}}+\frac{[\mathrm{pC4bC4bC2}]}{K_{\mathrm{m\ MASP2^*}}^{\mathrm{C2}}}\\&+\frac{[\mathrm{pC3bC4bC2}]}{K_{\mathrm{m\ MASP2^*}}^{\mathrm{C2}}}+\frac{\left[\mathrm{pMBL_3MASP1_{zym}}\right]}{K_{\mathrm{m\ MASP2^*}}^{\mathrm{MASP1_{zym}}}}+\frac{\left[\mathrm{pMBL_3MASP2_{zym}}\right]}{K_{\mathrm{m\ MASP2^*}}^{\mathrm{MASP2_{zym}}}}+\frac{\left[\mathrm{pMBL_4MASP1_{zym}}\right]}{K_{\mathrm{m\ MASP2^*}}^{\mathrm{MASP1_{zym}}}}\\&+\frac{\left[\mathrm{pMBL_4MASP2_{zym}}\right]}{K_{\mathrm{m\ MASP2^*}}^{\mathrm{MASP2_{zym}}}}+\frac{\left[\mathrm{pCLLKMASP1_{zym}}\right]}{K_{\mathrm{m\ MASP2^*}}^{\mathrm{MASP1_{zym}}}}+\frac{\left[\mathrm{pCLLKMASP2_{zym}}\right]}{K_{\mathrm{m\ MASP2^*}}^{\mathrm{MASP2_{zym}}}}+\frac{\left[\mathrm{pCLK1MASP1_{zym}}\right]}{K_{\mathrm{m\ MASP2^*}}^{\mathrm{MASP1_{zym}}}}\\&+\frac{\left[\mathrm{pCLK1MASP2_{zym}}\right]}{K_{\mathrm{m\ MASP2^*}}^{\mathrm{MASP2_{zym}}}}+\frac{\left[\mathrm{pCLL1MASP1_{zym}}\right]}{K_{\mathrm{m\ MASP2^*}}^{\mathrm{MASP1_{zym}}}}+\frac{\left[\mathrm{pCLL1MASP2_{zym}}\right]}{K_{\mathrm{m\ MASP2^*}}^{\mathrm{MASP2_{zym}}}}+\frac{\left[\mathrm{pLFMASP1_{zym}}\right]}{K_{\mathrm{m\ MASP2^*}}^{\mathrm{MASP1_{zym}}}}\\&+\frac{\left[\mathrm{pLFMASP2_{zym}}\right]}{K_{\mathrm{m\ MASP2^*}}^{\mathrm{MASP2_{zym}}}}+\frac{\left[\mathrm{pHFMASP1_{zym}}\right]}{K_{\mathrm{m\ MASP2^*}}^{\mathrm{MASP1_{zym}}}}+\frac{\left[\mathrm{pHFMASP2_{zym}}\right]}{K_{\mathrm{m\ MASP2^*}}^{\mathrm{MASP2_{zym}}}}+\frac{\left[\mathrm{pMFMASP1_{zym}}\right]}{K_{\mathrm{m\ MASP2^*}}^{\mathrm{MASP1_{zym}}}}+\frac{\left[\mathrm{pMFMASP2_{zym}}\right]}{K_{\mathrm{m\ MASP2^*}}^{\mathrm{MASP2_{zym}}}}\end{aligned}\right)}
$$

$$
+\frac{k_{\mathrm{cat\ MASP2^*}}^{\mathrm{MASP1_{zym}}}\left[\mathrm{pPTX3LFMASP2^*}\right]\left[\mathrm{pCLLKMASP1_{zym}}\right]}{K_{\mathrm{m\ MASP2^*}}^{\mathrm{MASP1_{zym}}}\left(\begin{aligned}&1+\frac{[\mathrm{C4}]}{K_{\mathrm{m\ MASP2^*}}^{\mathrm{C4}}}+\frac{[\mathrm{fC4bC2}]}{K_{\mathrm{m\ MASP2^*}}^{\mathrm{C2}}}+\frac{[\mathrm{fC4bC4bC2}]}{K_{\mathrm{m\ MASP2^*}}^{\mathrm{C2}}}+\frac{[\mathrm{fC3bC4bC2}]}{K_{\mathrm{m\ MASP2^*}}^{\mathrm{C2}}}+\frac{[\mathrm{pC4bC2}]}{K_{\mathrm{m\ MASP2^*}}^{\mathrm{C2}}}+\frac{[\mathrm{pC4bC4bC2}]}{K_{\mathrm{m\ MASP2^*}}^{\mathrm{C2}}}\\&+\frac{[\mathrm{pC3bC4bC2}]}{K_{\mathrm{m\ MASP2^*}}^{\mathrm{C2}}}+\frac{\left[\mathrm{pMBL_3MASP1_{zym}}\right]}{K_{\mathrm{m\ MASP2^*}}^{\mathrm{MASP1_{zym}}}}+\frac{\left[\mathrm{pMBL_3MASP2_{zym}}\right]}{K_{\mathrm{m\ MASP2^*}}^{\mathrm{MASP2_{zym}}}}+\frac{\left[\mathrm{pMBL_4MASP1_{zym}}\right]}{K_{\mathrm{m\ MASP2^*}}^{\mathrm{MASP1_{zym}}}}\\&+\frac{\left[\mathrm{pMBL_4MASP2_{zym}}\right]}{K_{\mathrm{m\ MASP2^*}}^{\mathrm{MASP2_{zym}}}}+\frac{\left[\mathrm{pCLLKMASP1_{zym}}\right]}{K_{\mathrm{m\ MASP2^*}}^{\mathrm{MASP1_{zym}}}}+\frac{\left[\mathrm{pCLLKMASP2_{zym}}\right]}{K_{\mathrm{m\ MASP2^*}}^{\mathrm{MASP2_{zym}}}}+\frac{\left[\mathrm{pCLK1MASP1_{zym}}\right]}{K_{\mathrm{m\ MASP2^*}}^{\mathrm{MASP1_{zym}}}}\\&+\frac{\left[\mathrm{pCLK1MASP2_{zym}}\right]}{K_{\mathrm{m\ MASP2^*}}^{\mathrm{MASP2_{zym}}}}+\frac{\left[\mathrm{pCLL1MASP1_{zym}}\right]}{K_{\mathrm{m\ MASP2^*}}^{\mathrm{MASP1_{zym}}}}+\frac{\left[\mathrm{pCLL1MASP2_{zym}}\right]}{K_{\mathrm{m\ MASP2^*}}^{\mathrm{MASP2_{zym}}}}+\frac{\left[\mathrm{pLFMASP1_{zym}}\right]}{K_{\mathrm{m\ MASP2^*}}^{\mathrm{MASP1_{zym}}}}\\&+\frac{\left[\mathrm{pLFMASP2_{zym}}\right]}{K_{\mathrm{m\ MASP2^*}}^{\mathrm{MASP2_{zym}}}}+\frac{\left[\mathrm{pHFMASP1_{zym}}\right]}{K_{\mathrm{m\ MASP2^*}}^{\mathrm{MASP1_{zym}}}}+\frac{\left[\mathrm{pHFMASP2_{zym}}\right]}{K_{\mathrm{m\ MASP2^*}}^{\mathrm{MASP2_{zym}}}}+\frac{\left[\mathrm{pMFMASP1_{zym}}\right]}{K_{\mathrm{m\ MASP2^*}}^{\mathrm{MASP1_{zym}}}}+\frac{\left[\mathrm{pMFMASP2_{zym}}\right]}{K_{\mathrm{m\ MASP2^*}}^{\mathrm{MASP2_{zym}}}}\\&+\frac{\left[\mathrm{pPTX3LFMASP1_{zym}}\right]}{K_{\mathrm{m\ MASP2^*}}^{\mathrm{MASP1_{zym}}}}+\frac{\left[\mathrm{pPTX3LFMASP2_{zym}}\right]}{K_{\mathrm{m\ MASP2^*}}^{\mathrm{MASP2_{zym}}}}\end{aligned}\right)}
$$

$$
-k_{\mathrm{C1INHMASP1}}^{+}\left[\mathrm{C1\text{-}INH}\right]\left[\mathrm{pCLLKMASP1^*}\right]
$$

288

$$
\frac{d\left[\mathrm{fCLLKMASP2_{zym}}\right]}{dt}=k_{\mathrm{CLLKMASP2}}^{+}\left[\mathrm{CLLK}\right]\left[\mathrm{MASP2}\right]-k_{\mathrm{CLLKMASP2}}^{-}\left[\mathrm{fCLLKMASP2_{zym}}\right]
$$

$$
-k_{\mathrm{pCLLK}}^{+}\left[\mathrm{fCLLKMASP2_{zym}}\right]\left[\mathrm{Pathogen\ Surface}\right]+k_{\mathrm{pCLLK}}^{-}\left[\mathrm{pCLLKMASP2_{zym}}\right]
$$

289

$$
\begin{aligned}
\frac{d\left[\mathrm{pCLLKMASP2_{zym}}\right]}{dt} &= k^{+}_{\mathrm{pCLLK}}\left[\mathrm{fCLLKMASP2_{zym}}\right]\left[\mathrm{Pathogen\ Surface}\right]-k^{-}_{\mathrm{pCLLK}}\left[\mathrm{pCLLKMASP2_{zym}}\right] \\
&+k^{+}_{\mathrm{CLLKMASP2}}\left[\mathrm{pCLLK}\right]\left[\mathrm{MASP2}\right]-k^{-}_{\mathrm{CLLKMASP2}}\left[\mathrm{pCLLKMASP2_{zym}}\right] \\
&-\frac{k^{\mathrm{MASP2_{zym}}}_{\mathrm{cat\ MASP1_{zym}}}\left[\mathrm{pMBL_3MASP1_{zym}}\right]\left[\mathrm{pCLLKMASP2_{zym}}\right]}{K^{\mathrm{MASP2_{zym}}}_{\mathrm{m\ MASP1_{zym}}}\left(\begin{aligned}&1+\frac{\left[\mathrm{pMBL_3MASP1_{zym}}\right]}{K^{\mathrm{MASP1_{zym}}}_{\mathrm{m\ MASP1_{zym}}}}+\frac{\left[\mathrm{pMBL_3MASP2_{zym}}\right]}{K^{\mathrm{MASP2_{zym}}}_{\mathrm{m\ MASP1_{zym}}}}+\frac{\left[\mathrm{pMBL_4MASP1_{zym}}\right]}{K^{\mathrm{MASP1_{zym}}}_{\mathrm{m\ MASP1_{zym}}}}+\frac{\left[\mathrm{pMBL_4MASP2_{zym}}\right]}{K^{\mathrm{MASP2_{zym}}}_{\mathrm{m\ MASP1_{zym}}}}\\
&+\frac{\left[\mathrm{pCLLKMASP1_{zym}}\right]}{K^{\mathrm{MASP1_{zym}}}_{\mathrm{m\ MASP1_{zym}}}}+\frac{\left[\mathrm{pCLLKMASP2_{zym}}\right]}{K^{\mathrm{MASP2_{zym}}}_{\mathrm{m\ MASP1_{zym}}}}+\frac{\left[\mathrm{pCLK1MASP1_{zym}}\right]}{K^{\mathrm{MASP1_{zym}}}_{\mathrm{m\ MASP1_{zym}}}}+\frac{\left[\mathrm{pCLK1MASP2_{zym}}\right]}{K^{\mathrm{MASP2_{zym}}}_{\mathrm{m\ MASP1_{zym}}}}\\
&+\frac{\left[\mathrm{pCLL1MASP1_{zym}}\right]}{K^{\mathrm{MASP1_{zym}}}_{\mathrm{m\ MASP1_{zym}}}}+\frac{\left[\mathrm{pCLL1MASP2_{zym}}\right]}{K^{\mathrm{MASP2_{zym}}}_{\mathrm{m\ MASP1_{zym}}}}+\frac{\left[\mathrm{pLFMASP1_{zym}}\right]}{K^{\mathrm{MASP1_{zym}}}_{\mathrm{m\ MASP1_{zym}}}}+\frac{\left[\mathrm{pLFMASP2_{zym}}\right]}{K^{\mathrm{MASP2_{zym}}}_{\mathrm{m\ MASP1_{zym}}}}\\
&+\frac{\left[\mathrm{pHFMASP1_{zym}}\right]}{K^{\mathrm{MASP1_{zym}}}_{\mathrm{m\ MASP1_{zym}}}}+\frac{\left[\mathrm{pHFMASP2_{zym}}\right]}{K^{\mathrm{MASP2_{zym}}}_{\mathrm{m\ MASP1_{zym}}}}+\frac{\left[\mathrm{pMFMASP1_{zym}}\right]}{K^{\mathrm{MASP1_{zym}}}_{\mathrm{m\ MASP1_{zym}}}}+\frac{\left[\mathrm{pMFMASP2_{zym}}\right]}{K^{\mathrm{MASP2_{zym}}}_{\mathrm{m\ MASP1_{zym}}}}\\
&+\frac{\left[\mathrm{pPTX3LFMASP1_{zym}}\right]}{K^{\mathrm{MASP1_{zym}}}_{\mathrm{m\ MASP1_{zym}}}}+\frac{\left[\mathrm{pPTX3LFMASP2_{zym}}\right]}{K^{\mathrm{MASP2_{zym}}}_{\mathrm{m\ MASP1_{zym}}}}\end{aligned}\right)} \\
&-\frac{k^{\mathrm{MASP2_{zym}}}_{\mathrm{cat\ MASP1_{zym}}}\left[\mathrm{pMBL_4MASP1_{zym}}\right]\left[\mathrm{pCLLKMASP2_{zym}}\right]}{K^{\mathrm{MASP2_{zym}}}_{\mathrm{m\ MASP1_{zym}}}\left(\begin{aligned}&1+\frac{\left[\mathrm{pMBL_3MASP1_{zym}}\right]}{K^{\mathrm{MASP1_{zym}}}_{\mathrm{m\ MASP1_{zym}}}}+\frac{\left[\mathrm{pMBL_3MASP2_{zym}}\right]}{K^{\mathrm{MASP2_{zym}}}_{\mathrm{m\ MASP1_{zym}}}}+\frac{\left[\mathrm{pMBL_4MASP1_{zym}}\right]}{K^{\mathrm{MASP1_{zym}}}_{\mathrm{m\ MASP1_{zym}}}}+\frac{\left[\mathrm{pMBL_4MASP2_{zym}}\right]}{K^{\mathrm{MASP2_{zym}}}_{\mathrm{m\ MASP1_{zym}}}}\\
&+\frac{\left[\mathrm{pCLLKMASP1_{zym}}\right]}{K^{\mathrm{MASP1_{zym}}}_{\mathrm{m\ MASP1_{zym}}}}+\frac{\left[\mathrm{pCLLKMASP2_{zym}}\right]}{K^{\mathrm{MASP2_{zym}}}_{\mathrm{m\ MASP1_{zym}}}}+\frac{\left[\mathrm{pCLK1MASP1_{zym}}\right]}{K^{\mathrm{MASP1_{zym}}}_{\mathrm{m\ MASP1_{zym}}}}+\frac{\left[\mathrm{pCLK1MASP2_{zym}}\right]}{K^{\mathrm{MASP2_{zym}}}_{\mathrm{m\ MASP1_{zym}}}}\\
&+\frac{\left[\mathrm{pCLL1MASP1_{zym}}\right]}{K^{\mathrm{MASP1_{zym}}}_{\mathrm{m\ MASP1_{zym}}}}+\frac{\left[\mathrm{pCLL1MASP2_{zym}}\right]}{K^{\mathrm{MASP2_{zym}}}_{\mathrm{m\ MASP1_{zym}}}}+\frac{\left[\mathrm{pLFMASP1_{zym}}\right]}{K^{\mathrm{MASP1_{zym}}}_{\mathrm{m\ MASP1_{zym}}}}+\frac{\left[\mathrm{pLFMASP2_{zym}}\right]}{K^{\mathrm{MASP2_{zym}}}_{\mathrm{m\ MASP1_{zym}}}}\\
&+\frac{\left[\mathrm{pHFMASP1_{zym}}\right]}{K^{\mathrm{MASP1_{zym}}}_{\mathrm{m\ MASP1_{zym}}}}+\frac{\left[\mathrm{pHFMASP2_{zym}}\right]}{K^{\mathrm{MASP2_{zym}}}_{\mathrm{m\ MASP1_{zym}}}}+\frac{\left[\mathrm{pMFMASP1_{zym}}\right]}{K^{\mathrm{MASP1_{zym}}}_{\mathrm{m\ MASP1_{zym}}}}+\frac{\left[\mathrm{pMFMASP2_{zym}}\right]}{K^{\mathrm{MASP2_{zym}}}_{\mathrm{m\ MASP1_{zym}}}}\\
&+\frac{\left[\mathrm{pPTX3LFMASP1_{zym}}\right]}{K^{\mathrm{MASP1_{zym}}}_{\mathrm{m\ MASP1_{zym}}}}+\frac{\left[\mathrm{pPTX3LFMASP2_{zym}}\right]}{K^{\mathrm{MASP2_{zym}}}_{\mathrm{m\ MASP1_{zym}}}}\end{aligned}\right)}
\end{aligned}
$$

$$-\frac{k_{\text{cat MASP1}_{\text{zym}}}^{\text{MASP2}_{\text{zym}}}\left[\text{pCLLKMASP1}_{\text{zym}}\right]\left[\text{pCLLKMASP2}_{\text{zym}}\right]}{K_{\text{m MASP1}_{\text{zym}}}^{\text{MASP2}_{\text{zym}}}\left(\begin{array}{l}1+\frac{\left[\text{pMBL}_3\text{MASP1}_{\text{zym}}\right]}{K_{\text{m MASP1}_{\text{zym}}}^{\text{MASP1}_{\text{zym}}}}+\frac{\left[\text{pMBL}_3\text{MASP2}_{\text{zym}}\right]}{K_{\text{m MASP1}_{\text{zym}}}^{\text{MASP2}_{\text{zym}}}}+\frac{\left[\text{pMBL}_4\text{MASP1}_{\text{zym}}\right]}{K_{\text{m MASP1}_{\text{zym}}}^{\text{MASP1}_{\text{zym}}}}+\frac{\left[\text{pMBL}_4\text{MASP2}_{\text{zym}}\right]}{K_{\text{m MASP1}_{\text{zym}}}^{\text{MASP2}_{\text{zym}}}}\\+\frac{\left[\text{pCLLKMASP1}_{\text{zym}}\right]}{K_{\text{m MASP1}_{\text{zym}}}^{\text{MASP1}_{\text{zym}}}}+\frac{\left[\text{pCLLKMASP2}_{\text{zym}}\right]}{K_{\text{m MASP1}_{\text{zym}}}^{\text{MASP2}_{\text{zym}}}}+\frac{\left[\text{pCLK1MASP1}_{\text{zym}}\right]}{K_{\text{m MASP1}_{\text{zym}}}^{\text{MASP1}_{\text{zym}}}}+\frac{\left[\text{pCLK1MASP2}_{\text{zym}}\right]}{K_{\text{m MASP1}_{\text{zym}}}^{\text{MASP2}_{\text{zym}}}}\\+\frac{\left[\text{pCLL1MASP1}_{\text{zym}}\right]}{K_{\text{m MASP1}_{\text{zym}}}^{\text{MASP1}_{\text{zym}}}}+\frac{\left[\text{pCLL1MASP2}_{\text{zym}}\right]}{K_{\text{m MASP1}_{\text{zym}}}^{\text{MASP2}_{\text{zym}}}}+\frac{\left[\text{pLFMASP1}_{\text{zym}}\right]}{K_{\text{m MASP1}_{\text{zym}}}^{\text{MASP1}_{\text{zym}}}}+\frac{\left[\text{pLFMASP2}_{\text{zym}}\right]}{K_{\text{m MASP1}_{\text{zym}}}^{\text{MASP2}_{\text{zym}}}}\\+\frac{\left[\text{pHFMASP1}_{\text{zym}}\right]}{K_{\text{m MASP1}_{\text{zym}}}^{\text{MASP1}_{\text{zym}}}}+\frac{\left[\text{pHFMASP2}_{\text{zym}}\right]}{K_{\text{m MASP1}_{\text{zym}}}^{\text{MASP2}_{\text{zym}}}}+\frac{\left[\text{pMFMASP1}_{\text{zym}}\right]}{K_{\text{m MASP1}_{\text{zym}}}^{\text{MASP1}_{\text{zym}}}}+\frac{\left[\text{pMFMASP2}_{\text{zym}}\right]}{K_{\text{m MASP1}_{\text{zym}}}^{\text{MASP2}_{\text{zym}}}}\\+\frac{\left[\text{pPTX3LFMASP1}_{\text{zym}}\right]}{K_{\text{m MASP1}_{\text{zym}}}^{\text{MASP1}_{\text{zym}}}}+\frac{\left[\text{pPTX3LFMASP2}_{\text{zym}}\right]}{K_{\text{m MASP1}_{\text{zym}}}^{\text{MASP2}_{\text{zym}}}}\end{array}\right)}$$

$$-\frac{k_{\text{cat MASP1}_{\text{zym}}}^{\text{MASP2}_{\text{zym}}}\left[\text{pCLK1MASP1}_{\text{zym}}\right]\left[\text{pCLLKMASP2}_{\text{zym}}\right]}{K_{\text{m MASP1}_{\text{zym}}}^{\text{MASP2}_{\text{zym}}}\left(\begin{array}{l}1+\frac{\left[\text{pMBL}_3\text{MASP1}_{\text{zym}}\right]}{K_{\text{m MASP1}_{\text{zym}}}^{\text{MASP1}_{\text{zym}}}}+\frac{\left[\text{pMBL}_3\text{MASP2}_{\text{zym}}\right]}{K_{\text{m MASP1}_{\text{zym}}}^{\text{MASP2}_{\text{zym}}}}+\frac{\left[\text{pMBL}_4\text{MASP1}_{\text{zym}}\right]}{K_{\text{m MASP1}_{\text{zym}}}^{\text{MASP1}_{\text{zym}}}}+\frac{\left[\text{pMBL}_4\text{MASP2}_{\text{zym}}\right]}{K_{\text{m MASP1}_{\text{zym}}}^{\text{MASP2}_{\text{zym}}}}\\+\frac{\left[\text{pCLLKMASP1}_{\text{zym}}\right]}{K_{\text{m MASP1}_{\text{zym}}}^{\text{MASP1}_{\text{zym}}}}+\frac{\left[\text{pCLLKMASP2}_{\text{zym}}\right]}{K_{\text{m MASP1}_{\text{zym}}}^{\text{MASP2}_{\text{zym}}}}+\frac{\left[\text{pCLK1MASP1}_{\text{zym}}\right]}{K_{\text{m MASP1}_{\text{zym}}}^{\text{MASP1}_{\text{zym}}}}+\frac{\left[\text{pCLK1MASP2}_{\text{zym}}\right]}{K_{\text{m MASP1}_{\text{zym}}}^{\text{MASP2}_{\text{zym}}}}\\+\frac{\left[\text{pCLL1MASP1}_{\text{zym}}\right]}{K_{\text{m MASP1}_{\text{zym}}}^{\text{MASP1}_{\text{zym}}}}+\frac{\left[\text{pCLL1MASP2}_{\text{zym}}\right]}{K_{\text{m MASP1}_{\text{zym}}}^{\text{MASP2}_{\text{zym}}}}+\frac{\left[\text{pLFMASP1}_{\text{zym}}\right]}{K_{\text{m MASP1}_{\text{zym}}}^{\text{MASP1}_{\text{zym}}}}+\frac{\left[\text{pLFMASP2}_{\text{zym}}\right]}{K_{\text{m MASP1}_{\text{zym}}}^{\text{MASP2}_{\text{zym}}}}\\+\frac{\left[\text{pHFMASP1}_{\text{zym}}\right]}{K_{\text{m MASP1}_{\text{zym}}}^{\text{MASP1}_{\text{zym}}}}+\frac{\left[\text{pHFMASP2}_{\text{zym}}\right]}{K_{\text{m MASP1}_{\text{zym}}}^{\text{MASP2}_{\text{zym}}}}+\frac{\left[\text{pMFMASP1}_{\text{zym}}\right]}{K_{\text{m MASP1}_{\text{zym}}}^{\text{MASP1}_{\text{zym}}}}+\frac{\left[\text{pMFMASP2}_{\text{zym}}\right]}{K_{\text{m MASP1}_{\text{zym}}}^{\text{MASP2}_{\text{zym}}}}\\+\frac{\left[\text{pPTX3LFMASP1}_{\text{zym}}\right]}{K_{\text{m MASP1}_{\text{zym}}}^{\text{MASP1}_{\text{zym}}}}+\frac{\left[\text{pPTX3LFMASP2}_{\text{zym}}\right]}{K_{\text{m MASP1}_{\text{zym}}}^{\text{MASP2}_{\text{zym}}}}\end{array}\right)}$$

$$-\frac{k_{\text{cat MASP1}_{\text{zym}}}^{\text{MASP2}_{\text{zym}}}\left[\text{pCLL1MASP1}_{\text{zym}}\right]\left[\text{pCLLKMASP2}_{\text{zym}}\right]}{K_{\text{m MASP1}_{\text{zym}}}^{\text{MASP2}_{\text{zym}}}\left(\begin{array}{l}1+\frac{\left[\text{pMBL}_3\text{MASP1}_{\text{zym}}\right]}{K_{\text{m MASP1}_{\text{zym}}}^{\text{MASP1}_{\text{zym}}}}+\frac{\left[\text{pMBL}_3\text{MASP2}_{\text{zym}}\right]}{K_{\text{m MASP1}_{\text{zym}}}^{\text{MASP2}_{\text{zym}}}}+\frac{\left[\text{pMBL}_4\text{MASP1}_{\text{zym}}\right]}{K_{\text{m MASP1}_{\text{zym}}}^{\text{MASP1}_{\text{zym}}}}+\frac{\left[\text{pMBL}_4\text{MASP2}_{\text{zym}}\right]}{K_{\text{m MASP1}_{\text{zym}}}^{\text{MASP2}_{\text{zym}}}}\\+\frac{\left[\text{pCLLKMASP1}_{\text{zym}}\right]}{K_{\text{m MASP1}_{\text{zym}}}^{\text{MASP1}_{\text{zym}}}}+\frac{\left[\text{pCLLKMASP2}_{\text{zym}}\right]}{K_{\text{m MASP1}_{\text{zym}}}^{\text{MASP2}_{\text{zym}}}}+\frac{\left[\text{pCLK1MASP1}_{\text{zym}}\right]}{K_{\text{m MASP1}_{\text{zym}}}^{\text{MASP1}_{\text{zym}}}}+\frac{\left[\text{pCLK1MASP2}_{\text{zym}}\right]}{K_{\text{m MASP1}_{\text{zym}}}^{\text{MASP2}_{\text{zym}}}}\\+\frac{\left[\text{pCLL1MASP1}_{\text{zym}}\right]}{K_{\text{m MASP1}_{\text{zym}}}^{\text{MASP1}_{\text{zym}}}}+\frac{\left[\text{pCLL1MASP2}_{\text{zym}}\right]}{K_{\text{m MASP1}_{\text{zym}}}^{\text{MASP2}_{\text{zym}}}}+\frac{\left[\text{pLFMASP1}_{\text{zym}}\right]}{K_{\text{m MASP1}_{\text{zym}}}^{\text{MASP1}_{\text{zym}}}}+\frac{\left[\text{pLFMASP2}_{\text{zym}}\right]}{K_{\text{m MASP1}_{\text{zym}}}^{\text{MASP2}_{\text{zym}}}}\\+\frac{\left[\text{pHFMASP1}_{\text{zym}}\right]}{K_{\text{m MASP1}_{\text{zym}}}^{\text{MASP1}_{\text{zym}}}}+\frac{\left[\text{pHFMASP2}_{\text{zym}}\right]}{K_{\text{m MASP1}_{\text{zym}}}^{\text{MASP2}_{\text{zym}}}}+\frac{\left[\text{pMFMASP1}_{\text{zym}}\right]}{K_{\text{m MASP1}_{\text{zym}}}^{\text{MASP1}_{\text{zym}}}}+\frac{\left[\text{pMFMASP2}_{\text{zym}}\right]}{K_{\text{m MASP1}_{\text{zym}}}^{\text{MASP2}_{\text{zym}}}}\\+\frac{\left[\text{pPTX3LFMASP1}_{\text{zym}}\right]}{K_{\text{m MASP1}_{\text{zym}}}^{\text{MASP1}_{\text{zym}}}}+\frac{\left[\text{pPTX3LFMASP2}_{\text{zym}}\right]}{K_{\text{m MASP1}_{\text{zym}}}^{\text{MASP2}_{\text{zym}}}}\end{array}\right)}$$

$$-\frac{k_{\text{cat MASP1}_{\text{zym}}}^{\text{MASP2}_{\text{zym}}}\left[\text{pLFMASP1}_{\text{zym}}\right]\left[\text{pCLLKMASP2}_{\text{zym}}\right]}{K_{\text{m MASP1}_{\text{zym}}}^{\text{MASP2}_{\text{zym}}}\left(\begin{array}{l}1+\frac{\left[\text{pMBL}_3\text{MASP1}_{\text{zym}}\right]}{K_{\text{m MASP1}_{\text{zym}}}^{\text{MASP1}_{\text{zym}}}}+\frac{\left[\text{pMBL}_3\text{MASP2}_{\text{zym}}\right]}{K_{\text{m MASP1}_{\text{zym}}}^{\text{MASP2}_{\text{zym}}}}+\frac{\left[\text{pMBL}_4\text{MASP1}_{\text{zym}}\right]}{K_{\text{m MASP1}_{\text{zym}}}^{\text{MASP1}_{\text{zym}}}}+\frac{\left[\text{pMBL}_4\text{MASP2}_{\text{zym}}\right]}{K_{\text{m MASP1}_{\text{zym}}}^{\text{MASP2}_{\text{zym}}}}\\+\frac{\left[\text{pCLLKMASP1}_{\text{zym}}\right]}{K_{\text{m MASP1}_{\text{zym}}}^{\text{MASP1}_{\text{zym}}}}+\frac{\left[\text{pCLLKMASP2}_{\text{zym}}\right]}{K_{\text{m MASP1}_{\text{zym}}}^{\text{MASP2}_{\text{zym}}}}+\frac{\left[\text{pCLK1MASP1}_{\text{zym}}\right]}{K_{\text{m MASP1}_{\text{zym}}}^{\text{MASP1}_{\text{zym}}}}+\frac{\left[\text{pCLK1MASP2}_{\text{zym}}\right]}{K_{\text{m MASP1}_{\text{zym}}}^{\text{MASP2}_{\text{zym}}}}\\+\frac{\left[\text{pCLL1MASP1}_{\text{zym}}\right]}{K_{\text{m MASP1}_{\text{zym}}}^{\text{MASP1}_{\text{zym}}}}+\frac{\left[\text{pCLL1MASP2}_{\text{zym}}\right]}{K_{\text{m MASP1}_{\text{zym}}}^{\text{MASP2}_{\text{zym}}}}+\frac{\left[\text{pLFMASP1}_{\text{zym}}\right]}{K_{\text{m MASP1}_{\text{zym}}}^{\text{MASP1}_{\text{zym}}}}+\frac{\left[\text{pLFMASP2}_{\text{zym}}\right]}{K_{\text{m MASP1}_{\text{zym}}}^{\text{MASP2}_{\text{zym}}}}\\+\frac{\left[\text{pHFMASP1}_{\text{zym}}\right]}{K_{\text{m MASP1}_{\text{zym}}}^{\text{MASP1}_{\text{zym}}}}+\frac{\left[\text{pHFMASP2}_{\text{zym}}\right]}{K_{\text{m MASP1}_{\text{zym}}}^{\text{MASP2}_{\text{zym}}}}+\frac{\left[\text{pMFMASP1}_{\text{zym}}\right]}{K_{\text{m MASP1}_{\text{zym}}}^{\text{MASP1}_{\text{zym}}}}+\frac{\left[\text{pMFMASP2}_{\text{zym}}\right]}{K_{\text{m MASP1}_{\text{zym}}}^{\text{MASP2}_{\text{zym}}}}\\+\frac{\left[\text{pPTX3LFMASP1}_{\text{zym}}\right]}{K_{\text{m MASP1}_{\text{zym}}}^{\text{MASP1}_{\text{zym}}}}+\frac{\left[\text{pPTX3LFMASP2}_{\text{zym}}\right]}{K_{\text{m MASP1}_{\text{zym}}}^{\text{MASP2}_{\text{zym}}}}\end{array}\right)}$$

$$-\frac{k_{\text{cat MASP1}_{\text{zym}}}^{\text{MASP2}_{\text{zym}}}\left[\text{pHFMASP1}_{\text{zym}}\right]\left[\text{pCLLKMASP2}_{\text{zym}}\right]}{K_{\text{m MASP1}_{\text{zym}}}^{\text{MASP2}_{\text{zym}}}\left(\begin{array}{l}1+\frac{\left[\text{pMBL}_3\text{MASP1}_{\text{zym}}\right]}{K_{\text{m MASP1}_{\text{zym}}}^{\text{MASP1}_{\text{zym}}}}+\frac{\left[\text{pMBL}_3\text{MASP2}_{\text{zym}}\right]}{K_{\text{m MASP1}_{\text{zym}}}^{\text{MASP2}_{\text{zym}}}}+\frac{\left[\text{pMBL}_4\text{MASP1}_{\text{zym}}\right]}{K_{\text{m MASP1}_{\text{zym}}}^{\text{MASP1}_{\text{zym}}}}+\frac{\left[\text{pMBL}_4\text{MASP2}_{\text{zym}}\right]}{K_{\text{m MASP1}_{\text{zym}}}^{\text{MASP2}_{\text{zym}}}}\\+\frac{\left[\text{pCLLKMASP1}_{\text{zym}}\right]}{K_{\text{m MASP1}_{\text{zym}}}^{\text{MASP1}_{\text{zym}}}}+\frac{\left[\text{pCLLKMASP2}_{\text{zym}}\right]}{K_{\text{m MASP1}_{\text{zym}}}^{\text{MASP2}_{\text{zym}}}}+\frac{\left[\text{pCLK1MASP1}_{\text{zym}}\right]}{K_{\text{m MASP1}_{\text{zym}}}^{\text{MASP1}_{\text{zym}}}}+\frac{\left[\text{pCLK1MASP2}_{\text{zym}}\right]}{K_{\text{m MASP1}_{\text{zym}}}^{\text{MASP2}_{\text{zym}}}}\\+\frac{\left[\text{pCLL1MASP1}_{\text{zym}}\right]}{K_{\text{m MASP1}_{\text{zym}}}^{\text{MASP1}_{\text{zym}}}}+\frac{\left[\text{pCLL1MASP2}_{\text{zym}}\right]}{K_{\text{m MASP1}_{\text{zym}}}^{\text{MASP2}_{\text{zym}}}}+\frac{\left[\text{pLFMASP1}_{\text{zym}}\right]}{K_{\text{m MASP1}_{\text{zym}}}^{\text{MASP1}_{\text{zym}}}}+\frac{\left[\text{pLFMASP2}_{\text{zym}}\right]}{K_{\text{m MASP1}_{\text{zym}}}^{\text{MASP2}_{\text{zym}}}}\\+\frac{\left[\text{pHFMASP1}_{\text{zym}}\right]}{K_{\text{m MASP1}_{\text{zym}}}^{\text{MASP1}_{\text{zym}}}}+\frac{\left[\text{pHFMASP2}_{\text{zym}}\right]}{K_{\text{m MASP1}_{\text{zym}}}^{\text{MASP2}_{\text{zym}}}}+\frac{\left[\text{pMFMASP1}_{\text{zym}}\right]}{K_{\text{m MASP1}_{\text{zym}}}^{\text{MASP1}_{\text{zym}}}}+\frac{\left[\text{pMFMASP2}_{\text{zym}}\right]}{K_{\text{m MASP1}_{\text{zym}}}^{\text{MASP2}_{\text{zym}}}}\\+\frac{\left[\text{pPTX3LFMASP1}_{\text{zym}}\right]}{K_{\text{m MASP1}_{\text{zym}}}^{\text{MASP1}_{\text{zym}}}}+\frac{\left[\text{pPTX3LFMASP2}_{\text{zym}}\right]}{K_{\text{m MASP1}_{\text{zym}}}^{\text{MASP2}_{\text{zym}}}}\end{array}\right)}$$

$$-\frac{k_{\text{cat MASP1}_{\text{zym}}}^{\text{MASP2}_{\text{zym}}}\left[\text{pMFMASP1}_{\text{zym}}\right]\left[\text{pCLLKMASP2}_{\text{zym}}\right]}{K_{\text{m MASP1}_{\text{zym}}}^{\text{MASP2}_{\text{zym}}}\left(\begin{array}{l}1+\frac{\left[\text{pMBL}_3\text{MASP1}_{\text{zym}}\right]}{K_{\text{m MASP1}_{\text{zym}}}^{\text{MASP1}_{\text{zym}}}}+\frac{\left[\text{pMBL}_3\text{MASP2}_{\text{zym}}\right]}{K_{\text{m MASP1}_{\text{zym}}}^{\text{MASP2}_{\text{zym}}}}+\frac{\left[\text{pMBL}_4\text{MASP1}_{\text{zym}}\right]}{K_{\text{m MASP1}_{\text{zym}}}^{\text{MASP1}_{\text{zym}}}}+\frac{\left[\text{pMBL}_4\text{MASP2}_{\text{zym}}\right]}{K_{\text{m MASP1}_{\text{zym}}}^{\text{MASP2}_{\text{zym}}}}\\+\frac{\left[\text{pCLLKMASP1}_{\text{zym}}\right]}{K_{\text{m MASP1}_{\text{zym}}}^{\text{MASP1}_{\text{zym}}}}+\frac{\left[\text{pCLLKMASP2}_{\text{zym}}\right]}{K_{\text{m MASP1}_{\text{zym}}}^{\text{MASP2}_{\text{zym}}}}+\frac{\left[\text{pCLK1MASP1}_{\text{zym}}\right]}{K_{\text{m MASP1}_{\text{zym}}}^{\text{MASP1}_{\text{zym}}}}+\frac{\left[\text{pCLK1MASP2}_{\text{zym}}\right]}{K_{\text{m MASP1}_{\text{zym}}}^{\text{MASP2}_{\text{zym}}}}\\+\frac{\left[\text{pCLL1MASP1}_{\text{zym}}\right]}{K_{\text{m MASP1}_{\text{zym}}}^{\text{MASP1}_{\text{zym}}}}+\frac{\left[\text{pCLL1MASP2}_{\text{zym}}\right]}{K_{\text{m MASP1}_{\text{zym}}}^{\text{MASP2}_{\text{zym}}}}+\frac{\left[\text{pLFMASP1}_{\text{zym}}\right]}{K_{\text{m MASP1}_{\text{zym}}}^{\text{MASP1}_{\text{zym}}}}+\frac{\left[\text{pLFMASP2}_{\text{zym}}\right]}{K_{\text{m MASP1}_{\text{zym}}}^{\text{MASP2}_{\text{zym}}}}\\+\frac{\left[\text{pHFMASP1}_{\text{zym}}\right]}{K_{\text{m MASP1}_{\text{zym}}}^{\text{MASP1}_{\text{zym}}}}+\frac{\left[\text{pHFMASP2}_{\text{zym}}\right]}{K_{\text{m MASP1}_{\text{zym}}}^{\text{MASP2}_{\text{zym}}}}+\frac{\left[\text{pMFMASP1}_{\text{zym}}\right]}{K_{\text{m MASP1}_{\text{zym}}}^{\text{MASP1}_{\text{zym}}}}+\frac{\left[\text{pMFMASP2}_{\text{zym}}\right]}{K_{\text{m MASP1}_{\text{zym}}}^{\text{MASP2}_{\text{zym}}}}\\+\frac{\left[\text{pPTX3LFMASP1}_{\text{zym}}\right]}{K_{\text{m MASP1}_{\text{zym}}}^{\text{MASP1}_{\text{zym}}}}+\frac{\left[\text{pPTX3LFMASP2}_{\text{zym}}\right]}{K_{\text{m MASP1}_{\text{zym}}}^{\text{MASP2}_{\text{zym}}}}\end{array}\right)}$$

$$-\frac{k_{\mathrm{cat\ MASP1_{zym}}}^{\mathrm{MASP2_{zym}}}\left[\mathrm{pPTX3LFMASP1_{zym}}\right]\left[\mathrm{pCLLKMASP2_{zym}}\right]}{K_{\mathrm{m\ MASP1_{zym}}}^{\mathrm{MASP2_{zym}}}\left(\begin{array}{l}1+\frac{\left[\mathrm{pMBL_3MASP1_{zym}}\right]}{K_{\mathrm{m\ MASP1_{zym}}}^{\mathrm{MASP1_{zym}}}}+\frac{\left[\mathrm{pMBL_3MASP2_{zym}}\right]}{K_{\mathrm{m\ MASP1_{zym}}}^{\mathrm{MASP2_{zym}}}}+\frac{\left[\mathrm{pMBL_4MASP1_{zym}}\right]}{K_{\mathrm{m\ MASP1_{zym}}}^{\mathrm{MASP1_{zym}}}}+\frac{\left[\mathrm{pMBL_4MASP2_{zym}}\right]}{K_{\mathrm{m\ MASP1_{zym}}}^{\mathrm{MASP2_{zym}}}}\\ +\frac{\left[\mathrm{pCLLKMASP1_{zym}}\right]}{K_{\mathrm{m\ MASP1_{zym}}}^{\mathrm{MASP1_{zym}}}}+\frac{\left[\mathrm{pCLLKMASP2_{zym}}\right]}{K_{\mathrm{m\ MASP1_{zym}}}^{\mathrm{MASP2_{zym}}}}+\frac{\left[\mathrm{pCLK1MASP1_{zym}}\right]}{K_{\mathrm{m\ MASP1_{zym}}}^{\mathrm{MASP1_{zym}}}}+\frac{\left[\mathrm{pCLK1MASP2_{zym}}\right]}{K_{\mathrm{m\ MASP1_{zym}}}^{\mathrm{MASP2_{zym}}}}\\ +\frac{\left[\mathrm{pCLL1MASP1_{zym}}\right]}{K_{\mathrm{m\ MASP1_{zym}}}^{\mathrm{MASP1_{zym}}}}+\frac{\left[\mathrm{pCLL1MASP2_{zym}}\right]}{K_{\mathrm{m\ MASP1_{zym}}}^{\mathrm{MASP2_{zym}}}}+\frac{\left[\mathrm{pLFMASP1_{zym}}\right]}{K_{\mathrm{m\ MASP1_{zym}}}^{\mathrm{MASP1_{zym}}}}+\frac{\left[\mathrm{pLFMASP2_{zym}}\right]}{K_{\mathrm{m\ MASP1_{zym}}}^{\mathrm{MASP2_{zym}}}}\\ +\frac{\left[\mathrm{pHFMASP1_{zym}}\right]}{K_{\mathrm{m\ MASP1_{zym}}}^{\mathrm{MASP1_{zym}}}}+\frac{\left[\mathrm{pHFMASP2_{zym}}\right]}{K_{\mathrm{m\ MASP1_{zym}}}^{\mathrm{MASP2_{zym}}}}+\frac{\left[\mathrm{pMFMASP1_{zym}}\right]}{K_{\mathrm{m\ MASP1_{zym}}}^{\mathrm{MASP1_{zym}}}}+\frac{\left[\mathrm{pMFMASP2_{zym}}\right]}{K_{\mathrm{m\ MASP1_{zym}}}^{\mathrm{MASP2_{zym}}}}\\ +\frac{\left[\mathrm{pPTX3LFMASP1_{zym}}\right]}{K_{\mathrm{m\ MASP1_{zym}}}^{\mathrm{MASP1_{zym}}}}+\frac{\left[\mathrm{pPTX3LFMASP2_{zym}}\right]}{K_{\mathrm{m\ MASP1_{zym}}}^{\mathrm{MASP2_{zym}}}}\end{array}\right)}$$

$$-\frac{k_{\mathrm{cat\ MASP2_{zym}}}^{\mathrm{MASP2_{zym}}}\left[\mathrm{pMBL_3MASP2_{zym}}\right]\left[\mathrm{pCLLKMASP2_{zym}}\right]}{K_{\mathrm{m\ MASP2_{zym}}}^{\mathrm{MASP2_{zym}}}\left(\begin{array}{l}1+\frac{\left[\mathrm{pMBL_3MASP2_{zym}}\right]}{K_{\mathrm{m\ MASP2_{zym}}}^{\mathrm{MASP2_{zym}}}}+\frac{\left[\mathrm{pMBL_4MASP2_{zym}}\right]}{K_{\mathrm{m\ MASP2_{zym}}}^{\mathrm{MASP2_{zym}}}}+\frac{\left[\mathrm{pCLLKMASP2_{zym}}\right]}{K_{\mathrm{m\ MASP2_{zym}}}^{\mathrm{MASP2_{zym}}}}+\frac{\left[\mathrm{pCLK1MASP2_{zym}}\right]}{K_{\mathrm{m\ MASP2_{zym}}}^{\mathrm{MASP2_{zym}}}}\\ +\frac{\left[\mathrm{pCLL1MASP2_{zym}}\right]}{K_{\mathrm{m\ MASP2_{zym}}}^{\mathrm{MASP2_{zym}}}}+\frac{\left[\mathrm{pLFMASP2_{zym}}\right]}{K_{\mathrm{m\ MASP2_{zym}}}^{\mathrm{MASP2_{zym}}}}+\frac{\left[\mathrm{pHFMASP2_{zym}}\right]}{K_{\mathrm{m\ MASP2_{zym}}}^{\mathrm{MASP2_{zym}}}}+\frac{\left[\mathrm{pMFMASP2_{zym}}\right]}{K_{\mathrm{m\ MASP2_{zym}}}^{\mathrm{MASP2_{zym}}}}\\ +\frac{\left[\mathrm{pPTX3LFMASP2_{zym}}\right]}{K_{\mathrm{m\ MASP2_{zym}}}^{\mathrm{MASP2_{zym}}}}\end{array}\right)}$$

$$-\frac{k_{\mathrm{cat\ MASP2_{zym}}}^{\mathrm{MASP2_{zym}}}\left[\mathrm{pMBL_4MASP2_{zym}}\right]\left[\mathrm{pCLLKMASP2_{zym}}\right]}{K_{\mathrm{m\ MASP2_{zym}}}^{\mathrm{MASP2_{zym}}}\left(\begin{array}{l}1+\frac{\left[\mathrm{pMBL_3MASP2_{zym}}\right]}{K_{\mathrm{m\ MASP2_{zym}}}^{\mathrm{MASP2_{zym}}}}+\frac{\left[\mathrm{pMBL_4MASP2_{zym}}\right]}{K_{\mathrm{m\ MASP2_{zym}}}^{\mathrm{MASP2_{zym}}}}+\frac{\left[\mathrm{pCLLKMASP2_{zym}}\right]}{K_{\mathrm{m\ MASP2_{zym}}}^{\mathrm{MASP2_{zym}}}}+\frac{\left[\mathrm{pCLK1MASP2_{zym}}\right]}{K_{\mathrm{m\ MASP2_{zym}}}^{\mathrm{MASP2_{zym}}}}\\ +\frac{\left[\mathrm{pCLL1MASP2_{zym}}\right]}{K_{\mathrm{m\ MASP2_{zym}}}^{\mathrm{MASP2_{zym}}}}+\frac{\left[\mathrm{pLFMASP2_{zym}}\right]}{K_{\mathrm{m\ MASP2_{zym}}}^{\mathrm{MASP2_{zym}}}}+\frac{\left[\mathrm{pHFMASP2_{zym}}\right]}{K_{\mathrm{m\ MASP2_{zym}}}^{\mathrm{MASP2_{zym}}}}+\frac{\left[\mathrm{pMFMASP2_{zym}}\right]}{K_{\mathrm{m\ MASP2_{zym}}}^{\mathrm{MASP2_{zym}}}}\\ +\frac{\left[\mathrm{pPTX3LFMASP2_{zym}}\right]}{K_{\mathrm{m\ MASP2_{zym}}}^{\mathrm{MASP2_{zym}}}}\end{array}\right)}$$

$$
-\frac{k_{\mathrm{cat\ MASP2_{zym}}}^{\mathrm{MASP2_{zym}}}\left[\mathrm{pCLLKMASP2_{zym}}\right]\left[\mathrm{pCLLKMASP2_{zym}}\right]}{K_{\mathrm{m\ MASP2_{zym}}}^{\mathrm{MASP2_{zym}}}\left(\begin{array}{l}1+\frac{\left[\mathrm{pMBL_3MASP2_{zym}}\right]}{K_{\mathrm{m\ MASP2_{zym}}}^{\mathrm{MASP2_{zym}}}}+\frac{\left[\mathrm{pMBL_4MASP2_{zym}}\right]}{K_{\mathrm{m\ MASP2_{zym}}}^{\mathrm{MASP2_{zym}}}}+\frac{\left[\mathrm{pCLLKMASP2_{zym}}\right]}{K_{\mathrm{m\ MASP2_{zym}}}^{\mathrm{MASP2_{zym}}}}+\frac{\left[\mathrm{pCLK1MASP2_{zym}}\right]}{K_{\mathrm{m\ MASP2_{zym}}}^{\mathrm{MASP2_{zym}}}}\\+\frac{\left[\mathrm{pCLL1MASP2_{zym}}\right]}{K_{\mathrm{m\ MASP2_{zym}}}^{\mathrm{MASP2_{zym}}}}+\frac{\left[\mathrm{pLFMASP2_{zym}}\right]}{K_{\mathrm{m\ MASP2_{zym}}}^{\mathrm{MASP2_{zym}}}}+\frac{\left[\mathrm{pHFMASP2_{zym}}\right]}{K_{\mathrm{m\ MASP2_{zym}}}^{\mathrm{MASP2_{zym}}}}+\frac{\left[\mathrm{pMFMASP2_{zym}}\right]}{K_{\mathrm{m\ MASP2_{zym}}}^{\mathrm{MASP2_{zym}}}}\\+\frac{\left[\mathrm{pPTX3LFMASP2_{zym}}\right]}{K_{\mathrm{m\ MASP2_{zym}}}^{\mathrm{MASP2_{zym}}}}\end{array}\right)}
$$

$$
-\frac{k_{\mathrm{cat\ MASP2_{zym}}}^{\mathrm{MASP2_{zym}}}\left[\mathrm{pCLK1MASP2_{zym}}\right]\left[\mathrm{pCLLKMASP2_{zym}}\right]}{K_{\mathrm{m\ MASP2_{zym}}}^{\mathrm{MASP2_{zym}}}\left(\begin{array}{l}1+\frac{\left[\mathrm{pMBL_3MASP2_{zym}}\right]}{K_{\mathrm{m\ MASP2_{zym}}}^{\mathrm{MASP2_{zym}}}}+\frac{\left[\mathrm{pMBL_4MASP2_{zym}}\right]}{K_{\mathrm{m\ MASP2_{zym}}}^{\mathrm{MASP2_{zym}}}}+\frac{\left[\mathrm{pCLLKMASP2_{zym}}\right]}{K_{\mathrm{m\ MASP2_{zym}}}^{\mathrm{MASP2_{zym}}}}+\frac{\left[\mathrm{pCLK1MASP2_{zym}}\right]}{K_{\mathrm{m\ MASP2_{zym}}}^{\mathrm{MASP2_{zym}}}}\\+\frac{\left[\mathrm{pCLL1MASP2_{zym}}\right]}{K_{\mathrm{m\ MASP2_{zym}}}^{\mathrm{MASP2_{zym}}}}+\frac{\left[\mathrm{pLFMASP2_{zym}}\right]}{K_{\mathrm{m\ MASP2_{zym}}}^{\mathrm{MASP2_{zym}}}}+\frac{\left[\mathrm{pHFMASP2_{zym}}\right]}{K_{\mathrm{m\ MASP2_{zym}}}^{\mathrm{MASP2_{zym}}}}+\frac{\left[\mathrm{pMFMASP2_{zym}}\right]}{K_{\mathrm{m\ MASP2_{zym}}}^{\mathrm{MASP2_{zym}}}}\\+\frac{\left[\mathrm{pPTX3LFMASP2_{zym}}\right]}{K_{\mathrm{m\ MASP2_{zym}}}^{\mathrm{MASP2_{zym}}}}\end{array}\right)}
$$

$$
-\frac{k_{\mathrm{cat\ MASP2_{zym}}}^{\mathrm{MASP2_{zym}}}\left[\mathrm{pCLL1MASP2_{zym}}\right]\left[\mathrm{pCLLKMASP2_{zym}}\right]}{K_{\mathrm{m\ MASP2_{zym}}}^{\mathrm{MASP2_{zym}}}\left(\begin{array}{l}1+\frac{\left[\mathrm{pMBL_3MASP2_{zym}}\right]}{K_{\mathrm{m\ MASP2_{zym}}}^{\mathrm{MASP2_{zym}}}}+\frac{\left[\mathrm{pMBL_4MASP2_{zym}}\right]}{K_{\mathrm{m\ MASP2_{zym}}}^{\mathrm{MASP2_{zym}}}}+\frac{\left[\mathrm{pCLLKMASP2_{zym}}\right]}{K_{\mathrm{m\ MASP2_{zym}}}^{\mathrm{MASP2_{zym}}}}+\frac{\left[\mathrm{pCLK1MASP2_{zym}}\right]}{K_{\mathrm{m\ MASP2_{zym}}}^{\mathrm{MASP2_{zym}}}}\\+\frac{\left[\mathrm{pCLL1MASP2_{zym}}\right]}{K_{\mathrm{m\ MASP2_{zym}}}^{\mathrm{MASP2_{zym}}}}+\frac{\left[\mathrm{pLFMASP2_{zym}}\right]}{K_{\mathrm{m\ MASP2_{zym}}}^{\mathrm{MASP2_{zym}}}}+\frac{\left[\mathrm{pHFMASP2_{zym}}\right]}{K_{\mathrm{m\ MASP2_{zym}}}^{\mathrm{MASP2_{zym}}}}+\frac{\left[\mathrm{pMFMASP2_{zym}}\right]}{K_{\mathrm{m\ MASP2_{zym}}}^{\mathrm{MASP2_{zym}}}}\\+\frac{\left[\mathrm{pPTX3LFMASP2_{zym}}\right]}{K_{\mathrm{m\ MASP2_{zym}}}^{\mathrm{MASP2_{zym}}}}\end{array}\right)}
$$

$$
-\frac{k_{\mathrm{cat\ MASP2_{zym}}}^{\mathrm{MASP2_{zym}}}\left[\mathrm{pLFMASP2_{zym}}\right]\left[\mathrm{pCLLKMASP2_{zym}}\right]}{K_{\mathrm{m\ MASP2_{zym}}}^{\mathrm{MASP2_{zym}}}\left(\begin{array}{l}1+\frac{\left[\mathrm{pMBL_3MASP2_{zym}}\right]}{K_{\mathrm{m\ MASP2_{zym}}}^{\mathrm{MASP2_{zym}}}}+\frac{\left[\mathrm{pMBL_4MASP2_{zym}}\right]}{K_{\mathrm{m\ MASP2_{zym}}}^{\mathrm{MASP2_{zym}}}}+\frac{\left[\mathrm{pCLLKMASP2_{zym}}\right]}{K_{\mathrm{m\ MASP2_{zym}}}^{\mathrm{MASP2_{zym}}}}+\frac{\left[\mathrm{pCLK1MASP2_{zym}}\right]}{K_{\mathrm{m\ MASP2_{zym}}}^{\mathrm{MASP2_{zym}}}}\\+\frac{\left[\mathrm{pCLL1MASP2_{zym}}\right]}{K_{\mathrm{m\ MASP2_{zym}}}^{\mathrm{MASP2_{zym}}}}+\frac{\left[\mathrm{pLFMASP2_{zym}}\right]}{K_{\mathrm{m\ MASP2_{zym}}}^{\mathrm{MASP2_{zym}}}}+\frac{\left[\mathrm{pHFMASP2_{zym}}\right]}{K_{\mathrm{m\ MASP2_{zym}}}^{\mathrm{MASP2_{zym}}}}+\frac{\left[\mathrm{pMFMASP2_{zym}}\right]}{K_{\mathrm{m\ MASP2_{zym}}}^{\mathrm{MASP2_{zym}}}}\\+\frac{\left[\mathrm{pPTX3LFMASP2_{zym}}\right]}{K_{\mathrm{m\ MASP2_{zym}}}^{\mathrm{MASP2_{zym}}}}\end{array}\right)}
$$

$$
-\frac{k_{\text{cat MASP2}_{\text{zym}}}^{\text{MASP2}_{\text{zym}}}\left[\text{pHFMASP2}_{\text{zym}}\right]\left[\text{pCLLKMASP2}_{\text{zym}}\right]}{K_{\text{m MASP2}_{\text{zym}}}^{\text{MASP2}_{\text{zym}}}\left(\begin{array}{l}1+\frac{\left[\text{pMBL}_3\text{MASP2}_{\text{zym}}\right]}{K_{\text{m MASP2}_{\text{zym}}}^{\text{MASP2}_{\text{zym}}}}+\frac{\left[\text{pMBL}_4\text{MASP2}_{\text{zym}}\right]}{K_{\text{m MASP2}_{\text{zym}}}^{\text{MASP2}_{\text{zym}}}}+\frac{\left[\text{pCLLKMASP2}_{\text{zym}}\right]}{K_{\text{m MASP2}_{\text{zym}}}^{\text{MASP2}_{\text{zym}}}}+\frac{\left[\text{pCLK1MASP2}_{\text{zym}}\right]}{K_{\text{m MASP2}_{\text{zym}}}^{\text{MASP2}_{\text{zym}}}}\\+\frac{\left[\text{pCLL1MASP2}_{\text{zym}}\right]}{K_{\text{m MASP2}_{\text{zym}}}^{\text{MASP2}_{\text{zym}}}}+\frac{\left[\text{pLFMASP2}_{\text{zym}}\right]}{K_{\text{m MASP2}_{\text{zym}}}^{\text{MASP2}_{\text{zym}}}}+\frac{\left[\text{pHFMASP2}_{\text{zym}}\right]}{K_{\text{m MASP2}_{\text{zym}}}^{\text{MASP2}_{\text{zym}}}}+\frac{\left[\text{pMFMASP2}_{\text{zym}}\right]}{K_{\text{m MASP2}_{\text{zym}}}^{\text{MASP2}_{\text{zym}}}}\\+\frac{\left[\text{pPTX3LFMASP2}_{\text{zym}}\right]}{K_{\text{m MASP2}_{\text{zym}}}^{\text{MASP2}_{\text{zym}}}}\end{array}\right)}
$$

$$
-\frac{k_{\text{cat MASP2}_{\text{zym}}}^{\text{MASP2}_{\text{zym}}}\left[\text{pMFMASP2}_{\text{zym}}\right]\left[\text{pCLLKMASP2}_{\text{zym}}\right]}{K_{\text{m MASP2}_{\text{zym}}}^{\text{MASP2}_{\text{zym}}}\left(\begin{array}{l}1+\frac{\left[\text{pMBL}_3\text{MASP2}_{\text{zym}}\right]}{K_{\text{m MASP2}_{\text{zym}}}^{\text{MASP2}_{\text{zym}}}}+\frac{\left[\text{pMBL}_4\text{MASP2}_{\text{zym}}\right]}{K_{\text{m MASP2}_{\text{zym}}}^{\text{MASP2}_{\text{zym}}}}+\frac{\left[\text{pCLLKMASP2}_{\text{zym}}\right]}{K_{\text{m MASP2}_{\text{zym}}}^{\text{MASP2}_{\text{zym}}}}+\frac{\left[\text{pCLK1MASP2}_{\text{zym}}\right]}{K_{\text{m MASP2}_{\text{zym}}}^{\text{MASP2}_{\text{zym}}}}\\+\frac{\left[\text{pCLL1MASP2}_{\text{zym}}\right]}{K_{\text{m MASP2}_{\text{zym}}}^{\text{MASP2}_{\text{zym}}}}+\frac{\left[\text{pLFMASP2}_{\text{zym}}\right]}{K_{\text{m MASP2}_{\text{zym}}}^{\text{MASP2}_{\text{zym}}}}+\frac{\left[\text{pHFMASP2}_{\text{zym}}\right]}{K_{\text{m MASP2}_{\text{zym}}}^{\text{MASP2}_{\text{zym}}}}+\frac{\left[\text{pMFMASP2}_{\text{zym}}\right]}{K_{\text{m MASP2}_{\text{zym}}}^{\text{MASP2}_{\text{zym}}}}\\+\frac{\left[\text{pPTX3LFMASP2}_{\text{zym}}\right]}{K_{\text{m MASP2}_{\text{zym}}}^{\text{MASP2}_{\text{zym}}}}\end{array}\right)}
$$

$$
-\frac{k_{\text{cat MASP2}_{\text{zym}}}^{\text{MASP2}_{\text{zym}}}\left[\text{pPTX3LFMASP2}_{\text{zym}}\right]\left[\text{pCLLKMASP2}_{\text{zym}}\right]}{K_{\text{m MASP2}_{\text{zym}}}^{\text{MASP2}_{\text{zym}}}\left(\begin{array}{l}1+\frac{\left[\text{pMBL}_3\text{MASP2}_{\text{zym}}\right]}{K_{\text{m MASP2}_{\text{zym}}}^{\text{MASP2}_{\text{zym}}}}+\frac{\left[\text{pMBL}_4\text{MASP2}_{\text{zym}}\right]}{K_{\text{m MASP2}_{\text{zym}}}^{\text{MASP2}_{\text{zym}}}}+\frac{\left[\text{pCLLKMASP2}_{\text{zym}}\right]}{K_{\text{m MASP2}_{\text{zym}}}^{\text{MASP2}_{\text{zym}}}}+\frac{\left[\text{pCLK1MASP2}_{\text{zym}}\right]}{K_{\text{m MASP2}_{\text{zym}}}^{\text{MASP2}_{\text{zym}}}}\\+\frac{\left[\text{pCLL1MASP2}_{\text{zym}}\right]}{K_{\text{m MASP2}_{\text{zym}}}^{\text{MASP2}_{\text{zym}}}}+\frac{\left[\text{pLFMASP2}_{\text{zym}}\right]}{K_{\text{m MASP2}_{\text{zym}}}^{\text{MASP2}_{\text{zym}}}}+\frac{\left[\text{pHFMASP2}_{\text{zym}}\right]}{K_{\text{m MASP2}_{\text{zym}}}^{\text{MASP2}_{\text{zym}}}}+\frac{\left[\text{pMFMASP2}_{\text{zym}}\right]}{K_{\text{m MASP2}_{\text{zym}}}^{\text{MASP2}_{\text{zym}}}}\\+\frac{\left[\text{pPTX3LFMASP2}_{\text{zym}}\right]}{K_{\text{m MASP2}_{\text{zym}}}^{\text{MASP2}_{\text{zym}}}}\end{array}\right)}
$$

$$-\frac{k_{\mathrm{cat\ MASP1^*}}^{\mathrm{MASP2_{zym}}}\left[\mathrm{pMBL_3MASP1^*}\right]\left[\mathrm{pCLLKMASP2_{zym}}\right]}{K_{\mathrm{m\ MASP1^*}}^{\mathrm{MASP2_{zym}}}\left(\begin{array}{l}1+\frac{[\mathrm{C4}]}{K_{\mathrm{m\ MASP1^*}}^{\mathrm{C4}}}+\frac{[\mathrm{fC4bC2}]}{K_{\mathrm{m\ MASP1^*}}^{\mathrm{C2}}}+\frac{[\mathrm{fC4bC4bC2}]}{K_{\mathrm{m\ MASP1^*}}^{\mathrm{C2}}}+\frac{[\mathrm{fC3bC4bC2}]}{K_{\mathrm{m\ MASP1^*}}^{\mathrm{C2}}}+\frac{[\mathrm{pC4bC2}]}{K_{\mathrm{m\ MASP1^*}}^{\mathrm{C2}}}+\frac{[\mathrm{pC4bC4bC2}]}{K_{\mathrm{m\ MASP1^*}}^{\mathrm{C2}}}\\
+\frac{[\mathrm{pC3bC4bC2}]}{K_{\mathrm{m\ MASP1^*}}^{\mathrm{C2}}}+\frac{[\mathrm{pMBL_3MASP1_{zym}}]}{K_{\mathrm{m\ MASP1^*}}^{\mathrm{MASP1_{zym}}}}+\frac{[\mathrm{pMBL_3MASP2_{zym}}]}{K_{\mathrm{m\ MASP1^*}}^{\mathrm{MASP2_{zym}}}}+\frac{[\mathrm{pMBL_3MASP3_{zym}}]}{K_{\mathrm{m\ MASP1^*}}^{\mathrm{MASP3_{zym}}}}\\
+\frac{[\mathrm{pMBL_4MASP1_{zym}}]}{K_{\mathrm{m\ MASP1^*}}^{\mathrm{MASP1_{zym}}}}+\frac{[\mathrm{pMBL_4MASP2_{zym}}]}{K_{\mathrm{m\ MASP1^*}}^{\mathrm{MASP2_{zym}}}}+\frac{[\mathrm{pMBL_4MASP3_{zym}}]}{K_{\mathrm{m\ MASP1^*}}^{\mathrm{MASP3_{zym}}}}+\frac{[\mathrm{pCLLKMASP1_{zym}}]}{K_{\mathrm{m\ MASP1^*}}^{\mathrm{MASP1_{zym}}}}\\
+\frac{[\mathrm{pCLLKMASP2_{zym}}]}{K_{\mathrm{m\ MASP1^*}}^{\mathrm{MASP2_{zym}}}}+\frac{[\mathrm{pCLLKMASP3_{zym}}]}{K_{\mathrm{m\ MASP1^*}}^{\mathrm{MASP3_{zym}}}}+\frac{[\mathrm{pCLK1MASP1_{zym}}]}{K_{\mathrm{m\ MASP1^*}}^{\mathrm{MASP1_{zym}}}}+\frac{[\mathrm{pCLK1MASP2_{zym}}]}{K_{\mathrm{m\ MASP1^*}}^{\mathrm{MASP2_{zym}}}}\\
+\frac{[\mathrm{pCLK1MASP3_{zym}}]}{K_{\mathrm{m\ MASP1^*}}^{\mathrm{MASP3_{zym}}}}+\frac{[\mathrm{pCLL1MASP1_{zym}}]}{K_{\mathrm{m\ MASP1^*}}^{\mathrm{MASP1_{zym}}}}+\frac{[\mathrm{pCLL1MASP2_{zym}}]}{K_{\mathrm{m\ MASP1^*}}^{\mathrm{MASP2_{zym}}}}+\frac{[\mathrm{pCLL1MASP3_{zym}}]}{K_{\mathrm{m\ MASP1^*}}^{\mathrm{MASP3_{zym}}}}\\
+\frac{[\mathrm{pLFMASP1_{zym}}]}{K_{\mathrm{m\ MASP1^*}}^{\mathrm{MASP1_{zym}}}}+\frac{[\mathrm{pLFMASP2_{zym}}]}{K_{\mathrm{m\ MASP1^*}}^{\mathrm{MASP2_{zym}}}}+\frac{[\mathrm{pLFMASP3_{zym}}]}{K_{\mathrm{m\ MASP1^*}}^{\mathrm{MASP3_{zym}}}}+\frac{[\mathrm{pHFMASP1_{zym}}]}{K_{\mathrm{m\ MASP1^*}}^{\mathrm{MASP1_{zym}}}}+\frac{[\mathrm{pHFMASP2_{zym}}]}{K_{\mathrm{m\ MASP1^*}}^{\mathrm{MASP2_{zym}}}}\\
+\frac{[\mathrm{pHFMASP3_{zym}}]}{K_{\mathrm{m\ MASP1^*}}^{\mathrm{MASP3_{zym}}}}+\frac{[\mathrm{pMFMASP1_{zym}}]}{K_{\mathrm{m\ MASP1^*}}^{\mathrm{MASP1_{zym}}}}+\frac{[\mathrm{pMFMASP2_{zym}}]}{K_{\mathrm{m\ MASP1^*}}^{\mathrm{MASP2_{zym}}}}+\frac{[\mathrm{pMFMASP3_{zym}}]}{K_{\mathrm{m\ MASP1^*}}^{\mathrm{MASP3_{zym}}}}\\
+\frac{[\mathrm{pPTX3LFMASP1_{zym}}]}{K_{\mathrm{m\ MASP1^*}}^{\mathrm{MASP1_{zym}}}}+\frac{[\mathrm{pPXT3LFMASP2_{zym}}]}{K_{\mathrm{m\ MASP1^*}}^{\mathrm{MASP2_{zym}}}}+\frac{[\mathrm{pPTX3LFMASP3_{zym}}]}{K_{\mathrm{m\ MASP1^*}}^{\mathrm{MASP3_{zym}}}}\end{array}\right)}$$

$$-\frac{k_{\text{cat MASP1}^*}^{\text{MASP2}_{\text{zym}}}\left[\text{pMBL}_4\text{MASP1}^*\right]\left[\text{pCLLKMASP2}_{\text{zym}}\right]}{K_{\text{m MASP1}^*}^{\text{MASP2}_{\text{zym}}}\left(\begin{array}{l}1+\frac{[\text{C4}]}{K_{\text{m MASP1}^*}^{\text{C4}}}+\frac{[\text{fC4bC2}]}{K_{\text{m MASP1}^*}^{\text{C2}}}+\frac{[\text{fC4bC4bC2}]}{K_{\text{m MASP1}^*}^{\text{C2}}}+\frac{[\text{fC3bC4bC2}]}{K_{\text{m MASP1}^*}^{\text{C2}}}+\frac{[\text{pC4bC2}]}{K_{\text{m MASP1}^*}^{\text{C2}}}+\frac{[\text{pC4bC4bC2}]}{K_{\text{m MASP1}^*}^{\text{C2}}}\\+\frac{[\text{pC3bC4bC2}]}{K_{\text{m MASP1}^*}^{\text{C2}}}+\frac{\left[\text{pMBL}_3\text{MASP1}_{\text{zym}}\right]}{K_{\text{m MASP1}^*}^{\text{MASP1}_{\text{zym}}}}+\frac{\left[\text{pMBL}_3\text{MASP2}_{\text{zym}}\right]}{K_{\text{m MASP1}^*}^{\text{MASP2}_{\text{zym}}}}+\frac{\left[\text{pMBL}_3\text{MASP3}_{\text{zym}}\right]}{K_{\text{m MASP1}^*}^{\text{MASP3}_{\text{zym}}}}\\+\frac{\left[\text{pMBL}_4\text{MASP1}_{\text{zym}}\right]}{K_{\text{m MASP1}^*}^{\text{MASP1}_{\text{zym}}}}+\frac{\left[\text{pMBL}_4\text{MASP2}_{\text{zym}}\right]}{K_{\text{m MASP1}^*}^{\text{MASP2}_{\text{zym}}}}+\frac{\left[\text{pMBL}_4\text{MASP3}_{\text{zym}}\right]}{K_{\text{m MASP1}^*}^{\text{MASP3}_{\text{zym}}}}+\frac{\left[\text{pCLLKMASP1}_{\text{zym}}\right]}{K_{\text{m MASP1}^*}^{\text{MASP1}_{\text{zym}}}}\\+\frac{\left[\text{pCLLKMASP2}_{\text{zym}}\right]}{K_{\text{m MASP1}^*}^{\text{MASP2}_{\text{zym}}}}+\frac{\left[\text{pCLLKMASP3}_{\text{zym}}\right]}{K_{\text{m MASP1}^*}^{\text{MASP3}_{\text{zym}}}}+\frac{\left[\text{pCLK1MASP1}_{\text{zym}}\right]}{K_{\text{m MASP1}^*}^{\text{MASP1}_{\text{zym}}}}+\frac{\left[\text{pCLK1MASP2}_{\text{zym}}\right]}{K_{\text{m MASP1}^*}^{\text{MASP2}_{\text{zym}}}}\\+\frac{\left[\text{pCLK1MASP3}_{\text{zym}}\right]}{K_{\text{m MASP1}^*}^{\text{MASP3}_{\text{zym}}}}+\frac{\left[\text{pCLL1MASP1}_{\text{zym}}\right]}{K_{\text{m MASP1}^*}^{\text{MASP1}_{\text{zym}}}}+\frac{\left[\text{pCLL1MASP2}_{\text{zym}}\right]}{K_{\text{m MASP1}^*}^{\text{MASP2}_{\text{zym}}}}+\frac{\left[\text{pCLL1MASP3}_{\text{zym}}\right]}{K_{\text{m MASP1}^*}^{\text{MASP3}_{\text{zym}}}}\\+\frac{\left[\text{pLFMASP1}_{\text{zym}}\right]}{K_{\text{m MASP1}^*}^{\text{MASP1}_{\text{zym}}}}+\frac{\left[\text{pLFMASP2}_{\text{zym}}\right]}{K_{\text{m MASP1}^*}^{\text{MASP2}_{\text{zym}}}}+\frac{\left[\text{pLFMASP3}_{\text{zym}}\right]}{K_{\text{m MASP1}^*}^{\text{MASP3}_{\text{zym}}}}+\frac{\left[\text{pHFMASP1}_{\text{zym}}\right]}{K_{\text{m MASP1}^*}^{\text{MASP1}_{\text{zym}}}}+\frac{\left[\text{pHFMASP2}_{\text{zym}}\right]}{K_{\text{m MASP1}^*}^{\text{MASP2}_{\text{zym}}}}\\+\frac{\left[\text{pHFMASP3}_{\text{zym}}\right]}{K_{\text{m MASP1}^*}^{\text{MASP3}_{\text{zym}}}}+\frac{\left[\text{pMFMASP1}_{\text{zym}}\right]}{K_{\text{m MASP1}^*}^{\text{MASP1}_{\text{zym}}}}+\frac{\left[\text{pMFMASP2}_{\text{zym}}\right]}{K_{\text{m MASP1}^*}^{\text{MASP2}_{\text{zym}}}}+\frac{\left[\text{pMFMASP3}_{\text{zym}}\right]}{K_{\text{m MASP1}^*}^{\text{MASP3}_{\text{zym}}}}\\+\frac{\left[\text{pPTX3LFMASP1}_{\text{zym}}\right]}{K_{\text{m MASP1}^*}^{\text{MASP1}_{\text{zym}}}}+\frac{\left[\text{pPXT3LFMASP2}_{\text{zym}}\right]}{K_{\text{m MASP1}^*}^{\text{MASP2}_{\text{zym}}}}+\frac{\left[\text{pPTX3LFMASP3}_{\text{zym}}\right]}{K_{\text{m MASP1}^*}^{\text{MASP3}_{\text{zym}}}}\end{array}\right)}$$

$$
-\frac{k^{\mathrm{MASP2_{zym}}}_{\mathrm{cat\ MASP1^*}}\left[\mathrm{pCLLKMASP1^*}\right]\left[\mathrm{pCLLKMASP2_{zym}}\right]}{K^{\mathrm{MASP2_{zym}}}_{\mathrm{m\ MASP1^*}}\left(\begin{aligned}&1+\frac{[\mathrm{C4}]}{K^{\mathrm{C4}}_{\mathrm{m\ MASP1^*}}}+\frac{[\mathrm{fC4bC2}]}{K^{\mathrm{C2}}_{\mathrm{m\ MASP1^*}}}+\frac{[\mathrm{fC4bC4bC2}]}{K^{\mathrm{C2}}_{\mathrm{m\ MASP1^*}}}+\frac{[\mathrm{fC3bC4bC2}]}{K^{\mathrm{C2}}_{\mathrm{m\ MASP1^*}}}+\frac{[\mathrm{pC4bC2}]}{K^{\mathrm{C2}}_{\mathrm{m\ MASP1^*}}}+\frac{[\mathrm{pC4bC4bC2}]}{K^{\mathrm{C2}}_{\mathrm{m\ MASP1^*}}}\\
&+\frac{[\mathrm{pC3bC4bC2}]}{K^{\mathrm{C2}}_{\mathrm{m\ MASP1^*}}}+\frac{[\mathrm{pMBL_3MASP1_{zym}}]}{K^{\mathrm{MASP1_{zym}}}_{\mathrm{m\ MASP1^*}}}+\frac{[\mathrm{pMBL_3MASP2_{zym}}]}{K^{\mathrm{MASP2_{zym}}}_{\mathrm{m\ MASP1^*}}}+\frac{[\mathrm{pMBL_3MASP3_{zym}}]}{K^{\mathrm{MASP3_{zym}}}_{\mathrm{m\ MASP1^*}}}\\
&+\frac{[\mathrm{pMBL_4MASP1_{zym}}]}{K^{\mathrm{MASP1_{zym}}}_{\mathrm{m\ MASP1^*}}}+\frac{[\mathrm{pMBL_4MASP2_{zym}}]}{K^{\mathrm{MASP2_{zym}}}_{\mathrm{m\ MASP1^*}}}+\frac{[\mathrm{pMBL_4MASP3_{zym}}]}{K^{\mathrm{MASP3_{zym}}}_{\mathrm{m\ MASP1^*}}}+\frac{[\mathrm{pCLLKMASP1_{zym}}]}{K^{\mathrm{MASP1_{zym}}}_{\mathrm{m\ MASP1^*}}}\\
&+\frac{[\mathrm{pCLLKMASP2_{zym}}]}{K^{\mathrm{MASP2_{zym}}}_{\mathrm{m\ MASP1^*}}}+\frac{[\mathrm{pCLLKMASP3_{zym}}]}{K^{\mathrm{MASP3_{zym}}}_{\mathrm{m\ MASP1^*}}}+\frac{[\mathrm{pCLK1MASP1_{zym}}]}{K^{\mathrm{MASP1_{zym}}}_{\mathrm{m\ MASP1^*}}}+\frac{[\mathrm{pCLK1MASP2_{zym}}]}{K^{\mathrm{MASP2_{zym}}}_{\mathrm{m\ MASP1^*}}}\\
&+\frac{[\mathrm{pCLK1MASP3_{zym}}]}{K^{\mathrm{MASP3_{zym}}}_{\mathrm{m\ MASP1^*}}}+\frac{[\mathrm{pCLL1MASP1_{zym}}]}{K^{\mathrm{MASP1_{zym}}}_{\mathrm{m\ MASP1^*}}}+\frac{[\mathrm{pCLL1MASP2_{zym}}]}{K^{\mathrm{MASP2_{zym}}}_{\mathrm{m\ MASP1^*}}}+\frac{[\mathrm{pCLL1MASP3_{zym}}]}{K^{\mathrm{MASP3_{zym}}}_{\mathrm{m\ MASP1^*}}}\\
&+\frac{[\mathrm{pLFMASP1_{zym}}]}{K^{\mathrm{MASP1_{zym}}}_{\mathrm{m\ MASP1^*}}}+\frac{[\mathrm{pLFMASP2_{zym}}]}{K^{\mathrm{MASP2_{zym}}}_{\mathrm{m\ MASP1^*}}}+\frac{[\mathrm{pLFMASP3_{zym}}]}{K^{\mathrm{MASP3_{zym}}}_{\mathrm{m\ MASP1^*}}}+\frac{[\mathrm{pHFMASP1_{zym}}]}{K^{\mathrm{MASP1_{zym}}}_{\mathrm{m\ MASP1^*}}}+\frac{[\mathrm{pHFMASP2_{zym}}]}{K^{\mathrm{MASP2_{zym}}}_{\mathrm{m\ MASP1^*}}}\\
&+\frac{[\mathrm{pHFMASP3_{zym}}]}{K^{\mathrm{MASP3_{zym}}}_{\mathrm{m\ MASP1^*}}}+\frac{[\mathrm{pMFMASP1_{zym}}]}{K^{\mathrm{MASP1_{zym}}}_{\mathrm{m\ MASP1^*}}}+\frac{[\mathrm{pMFMASP2_{zym}}]}{K^{\mathrm{MASP2_{zym}}}_{\mathrm{m\ MASP1^*}}}+\frac{[\mathrm{pMFMASP3_{zym}}]}{K^{\mathrm{MASP3_{zym}}}_{\mathrm{m\ MASP1^*}}}\\
&+\frac{[\mathrm{pPTX3LFMASP1_{zym}}]}{K^{\mathrm{MASP1_{zym}}}_{\mathrm{m\ MASP1^*}}}+\frac{[\mathrm{pPXT3LFMASP2_{zym}}]}{K^{\mathrm{MASP2_{zym}}}_{\mathrm{m\ MASP1^*}}}+\frac{[\mathrm{pPTX3LFMASP3_{zym}}]}{K^{\mathrm{MASP3_{zym}}}_{\mathrm{m\ MASP1^*}}}\end{aligned}\right)}
$$

$$-\frac{k_{\text{cat MASP1}^*}^{\text{MASP2}_{\text{zym}}}\left[\text{pCLK1MASP1}^*\right]\left[\text{pCLLKMASP2}_{\text{zym}}\right]}{K_{\text{m MASP1}^*}^{\text{MASP2}_{\text{zym}}}\left(\begin{array}{l}1+\frac{[\text{C4}]}{K_{\text{m MASP1}^*}^{\text{C4}}}+\frac{[\text{fC4bC2}]}{K_{\text{m MASP1}^*}^{\text{C2}}}+\frac{[\text{fC4bC4bC2}]}{K_{\text{m MASP1}^*}^{\text{C2}}}+\frac{[\text{fC3bC4bC2}]}{K_{\text{m MASP1}^*}^{\text{C2}}}+\frac{[\text{pC4bC2}]}{K_{\text{m MASP1}^*}^{\text{C2}}}+\frac{[\text{pC4bC4bC2}]}{K_{\text{m MASP1}^*}^{\text{C2}}}\\ +\frac{[\text{pC3bC4bC2}]}{K_{\text{m MASP1}^*}^{\text{C2}}}+\frac{[\text{pMBL}_3\text{MASP1}_{\text{zym}}]}{K_{\text{m MASP1}^*}^{\text{MASP1}_{\text{zym}}}}+\frac{[\text{pMBL}_3\text{MASP2}_{\text{zym}}]}{K_{\text{m MASP1}^*}^{\text{MASP2}_{\text{zym}}}}+\frac{[\text{pMBL}_3\text{MASP3}_{\text{zym}}]}{K_{\text{m MASP1}^*}^{\text{MASP3}_{\text{zym}}}}\\ +\frac{[\text{pMBL}_4\text{MASP1}_{\text{zym}}]}{K_{\text{m MASP1}^*}^{\text{MASP1}_{\text{zym}}}}+\frac{[\text{pMBL}_4\text{MASP2}_{\text{zym}}]}{K_{\text{m MASP1}^*}^{\text{MASP2}_{\text{zym}}}}+\frac{[\text{pMBL}_4\text{MASP3}_{\text{zym}}]}{K_{\text{m MASP1}^*}^{\text{MASP3}_{\text{zym}}}}+\frac{[\text{pCLLKMASP1}_{\text{zym}}]}{K_{\text{m MASP1}^*}^{\text{MASP1}_{\text{zym}}}}\\ +\frac{[\text{pCLLKMASP2}_{\text{zym}}]}{K_{\text{m MASP1}^*}^{\text{MASP2}_{\text{zym}}}}+\frac{[\text{pCLLKMASP3}_{\text{zym}}]}{K_{\text{m MASP1}^*}^{\text{MASP3}_{\text{zym}}}}+\frac{[\text{pCLK1MASP1}_{\text{zym}}]}{K_{\text{m MASP1}^*}^{\text{MASP1}_{\text{zym}}}}+\frac{[\text{pCLK1MASP2}_{\text{zym}}]}{K_{\text{m MASP1}^*}^{\text{MASP2}_{\text{zym}}}}\\ +\frac{[\text{pCLK1MASP3}_{\text{zym}}]}{K_{\text{m MASP1}^*}^{\text{MASP3}_{\text{zym}}}}+\frac{[\text{pCLL1MASP1}_{\text{zym}}]}{K_{\text{m MASP1}^*}^{\text{MASP1}_{\text{zym}}}}+\frac{[\text{pCLL1MASP2}_{\text{zym}}]}{K_{\text{m MASP1}^*}^{\text{MASP2}_{\text{zym}}}}+\frac{[\text{pCLL1MASP3}_{\text{zym}}]}{K_{\text{m MASP1}^*}^{\text{MASP3}_{\text{zym}}}}\\ +\frac{[\text{pLFMASP1}_{\text{zym}}]}{K_{\text{m MASP1}^*}^{\text{MASP1}_{\text{zym}}}}+\frac{[\text{pLFMASP2}_{\text{zym}}]}{K_{\text{m MASP1}^*}^{\text{MASP2}_{\text{zym}}}}+\frac{[\text{pLFMASP3}_{\text{zym}}]}{K_{\text{m MASP1}^*}^{\text{MASP3}_{\text{zym}}}}+\frac{[\text{pHFMASP1}_{\text{zym}}]}{K_{\text{m MASP1}^*}^{\text{MASP1}_{\text{zym}}}}+\frac{[\text{pHFMASP2}_{\text{zym}}]}{K_{\text{m MASP1}^*}^{\text{MASP2}_{\text{zym}}}}\\ +\frac{[\text{pHFMASP3}_{\text{zym}}]}{K_{\text{m MASP1}^*}^{\text{MASP3}_{\text{zym}}}}+\frac{[\text{pMFMASP1}_{\text{zym}}]}{K_{\text{m MASP1}^*}^{\text{MASP1}_{\text{zym}}}}+\frac{[\text{pMFMASP2}_{\text{zym}}]}{K_{\text{m MASP1}^*}^{\text{MASP2}_{\text{zym}}}}+\frac{[\text{pMFMASP3}_{\text{zym}}]}{K_{\text{m MASP1}^*}^{\text{MASP3}_{\text{zym}}}}\\ +\frac{[\text{pPTX3LFMASP1}_{\text{zym}}]}{K_{\text{m MASP1}^*}^{\text{MASP1}_{\text{zym}}}}+\frac{[\text{pPXT3LFMASP2}_{\text{zym}}]}{K_{\text{m MASP1}^*}^{\text{MASP2}_{\text{zym}}}}+\frac{[\text{pPTX3LFMASP3}_{\text{zym}}]}{K_{\text{m MASP1}^*}^{\text{MASP3}_{\text{zym}}}}\end{array}\right)}$$

$$
-\frac{k_{\mathrm{cat\ MASP1^*}}^{\mathrm{MASP2_{zym}}}\left[\mathrm{pCLL1MASP1^*}\right]\left[\mathrm{pCLLKMASP2_{zym}}\right]}{K_{\mathrm{m\ MASP1^*}}^{\mathrm{MASP2_{zym}}}\left(\begin{array}{l}
1+\frac{[\mathrm{C4}]}{K_{\mathrm{m\ MASP1^*}}^{\mathrm{C4}}}+\frac{[\mathrm{fC4bC2}]}{K_{\mathrm{m\ MASP1^*}}^{\mathrm{C2}}}+\frac{[\mathrm{fC4bC4bC2}]}{K_{\mathrm{m\ MASP1^*}}^{\mathrm{C2}}}+\frac{[\mathrm{fC3bC4bC2}]}{K_{\mathrm{m\ MASP1^*}}^{\mathrm{C2}}}+\frac{[\mathrm{pC4bC2}]}{K_{\mathrm{m\ MASP1^*}}^{\mathrm{C2}}}+\frac{[\mathrm{pC4bC4bC2}]}{K_{\mathrm{m\ MASP1^*}}^{\mathrm{C2}}}\\
+\frac{[\mathrm{pC3bC4bC2}]}{K_{\mathrm{m\ MASP1^*}}^{\mathrm{C2}}}+\frac{[\mathrm{pMBL_3MASP1_{zym}}]}{K_{\mathrm{m\ MASP1^*}}^{\mathrm{MASP1_{zym}}}}+\frac{[\mathrm{pMBL_3MASP2_{zym}}]}{K_{\mathrm{m\ MASP1^*}}^{\mathrm{MASP2_{zym}}}}+\frac{[\mathrm{pMBL_3MASP3_{zym}}]}{K_{\mathrm{m\ MASP1^*}}^{\mathrm{MASP3_{zym}}}}\\
+\frac{[\mathrm{pMBL_4MASP1_{zym}}]}{K_{\mathrm{m\ MASP1^*}}^{\mathrm{MASP1_{zym}}}}+\frac{[\mathrm{pMBL_4MASP2_{zym}}]}{K_{\mathrm{m\ MASP1^*}}^{\mathrm{MASP2_{zym}}}}+\frac{[\mathrm{pMBL_4MASP3_{zym}}]}{K_{\mathrm{m\ MASP1^*}}^{\mathrm{MASP3_{zym}}}}+\frac{[\mathrm{pCLLKMASP1_{zym}}]}{K_{\mathrm{m\ MASP1^*}}^{\mathrm{MASP1_{zym}}}}\\
+\frac{[\mathrm{pCLLKMASP2_{zym}}]}{K_{\mathrm{m\ MASP1^*}}^{\mathrm{MASP2_{zym}}}}+\frac{[\mathrm{pCLLKMASP3_{zym}}]}{K_{\mathrm{m\ MASP1^*}}^{\mathrm{MASP3_{zym}}}}+\frac{[\mathrm{pCLK1MASP1_{zym}}]}{K_{\mathrm{m\ MASP1^*}}^{\mathrm{MASP1_{zym}}}}+\frac{[\mathrm{pCLK1MASP2_{zym}}]}{K_{\mathrm{m\ MASP1^*}}^{\mathrm{MASP2_{zym}}}}\\
+\frac{[\mathrm{pCLK1MASP3_{zym}}]}{K_{\mathrm{m\ MASP1^*}}^{\mathrm{MASP3_{zym}}}}+\frac{[\mathrm{pCLL1MASP1_{zym}}]}{K_{\mathrm{m\ MASP1^*}}^{\mathrm{MASP1_{zym}}}}+\frac{[\mathrm{pCLL1MASP2_{zym}}]}{K_{\mathrm{m\ MASP1^*}}^{\mathrm{MASP2_{zym}}}}+\frac{[\mathrm{pCLL1MASP3_{zym}}]}{K_{\mathrm{m\ MASP1^*}}^{\mathrm{MASP3_{zym}}}}\\
+\frac{[\mathrm{pLFMASP1_{zym}}]}{K_{\mathrm{m\ MASP1^*}}^{\mathrm{MASP1_{zym}}}}+\frac{[\mathrm{pLFMASP2_{zym}}]}{K_{\mathrm{m\ MASP1^*}}^{\mathrm{MASP2_{zym}}}}+\frac{[\mathrm{pLFMASP3_{zym}}]}{K_{\mathrm{m\ MASP1^*}}^{\mathrm{MASP3_{zym}}}}+\frac{[\mathrm{pHFMASP1_{zym}}]}{K_{\mathrm{m\ MASP1^*}}^{\mathrm{MASP1_{zym}}}}+\frac{[\mathrm{pHFMASP2_{zym}}]}{K_{\mathrm{m\ MASP1^*}}^{\mathrm{MASP2_{zym}}}}\\
+\frac{[\mathrm{pHFMASP3_{zym}}]}{K_{\mathrm{m\ MASP1^*}}^{\mathrm{MASP3_{zym}}}}+\frac{[\mathrm{pMFMASP1_{zym}}]}{K_{\mathrm{m\ MASP1^*}}^{\mathrm{MASP1_{zym}}}}+\frac{[\mathrm{pMFMASP2_{zym}}]}{K_{\mathrm{m\ MASP1^*}}^{\mathrm{MASP2_{zym}}}}+\frac{[\mathrm{pMFMASP3_{zym}}]}{K_{\mathrm{m\ MASP1^*}}^{\mathrm{MASP3_{zym}}}}\\
+\frac{[\mathrm{pPTX3LFMASP1_{zym}}]}{K_{\mathrm{m\ MASP1^*}}^{\mathrm{MASP1_{zym}}}}+\frac{[\mathrm{pPXT3LFMASP2_{zym}}]}{K_{\mathrm{m\ MASP1^*}}^{\mathrm{MASP2_{zym}}}}+\frac{[\mathrm{pPTX3LFMASP3_{zym}}]}{K_{\mathrm{m\ MASP1^*}}^{\mathrm{MASP3_{zym}}}}
\end{array}\right)}
$$

$$-\frac{k_{\mathrm{cat\ MASP1^*}}^{\mathrm{MASP2_{zym}}}\left[\mathrm{pLFMASP1^*}\right]\left[\mathrm{pCLLKMASP2_{zym}}\right]}{K_{\mathrm{m\ MASP1^*}}^{\mathrm{MASP2_{zym}}}\left(\begin{array}{l}1+\frac{[\mathrm{C4}]}{K_{\mathrm{m\ MASP1^*}}^{\mathrm{C4}}}+\frac{[\mathrm{fC4bC2}]}{K_{\mathrm{m\ MASP1^*}}^{\mathrm{C2}}}+\frac{[\mathrm{fC4bC4bC2}]}{K_{\mathrm{m\ MASP1^*}}^{\mathrm{C2}}}+\frac{[\mathrm{fC3bC4bC2}]}{K_{\mathrm{m\ MASP1^*}}^{\mathrm{C2}}}+\frac{[\mathrm{pC4bC2}]}{K_{\mathrm{m\ MASP1^*}}^{\mathrm{C2}}}+\frac{[\mathrm{pC4bC4bC2}]}{K_{\mathrm{m\ MASP1^*}}^{\mathrm{C2}}}\\+\frac{[\mathrm{pC3bC4bC2}]}{K_{\mathrm{m\ MASP1^*}}^{\mathrm{C2}}}+\frac{[\mathrm{pMBL_3MASP1_{zym}}]}{K_{\mathrm{m\ MASP1^*}}^{\mathrm{MASP1_{zym}}}}+\frac{[\mathrm{pMBL_3MASP2_{zym}}]}{K_{\mathrm{m\ MASP1^*}}^{\mathrm{MASP2_{zym}}}}+\frac{[\mathrm{pMBL_3MASP3_{zym}}]}{K_{\mathrm{m\ MASP1^*}}^{\mathrm{MASP3_{zym}}}}\\+\frac{[\mathrm{pMBL_4MASP1_{zym}}]}{K_{\mathrm{m\ MASP1^*}}^{\mathrm{MASP1_{zym}}}}+\frac{[\mathrm{pMBL_4MASP2_{zym}}]}{K_{\mathrm{m\ MASP1^*}}^{\mathrm{MASP2_{zym}}}}+\frac{[\mathrm{pMBL_4MASP3_{zym}}]}{K_{\mathrm{m\ MASP1^*}}^{\mathrm{MASP3_{zym}}}}+\frac{[\mathrm{pCLLKMASP1_{zym}}]}{K_{\mathrm{m\ MASP1^*}}^{\mathrm{MASP1_{zym}}}}\\+\frac{[\mathrm{pCLLKMASP2_{zym}}]}{K_{\mathrm{m\ MASP1^*}}^{\mathrm{MASP2_{zym}}}}+\frac{[\mathrm{pCLLKMASP3_{zym}}]}{K_{\mathrm{m\ MASP1^*}}^{\mathrm{MASP3_{zym}}}}+\frac{[\mathrm{pCLK1MASP1_{zym}}]}{K_{\mathrm{m\ MASP1^*}}^{\mathrm{MASP1_{zym}}}}+\frac{[\mathrm{pCLK1MASP2_{zym}}]}{K_{\mathrm{m\ MASP1^*}}^{\mathrm{MASP2_{zym}}}}\\+\frac{[\mathrm{pCLK1MASP3_{zym}}]}{K_{\mathrm{m\ MASP1^*}}^{\mathrm{MASP3_{zym}}}}+\frac{[\mathrm{pCLL1MASP1_{zym}}]}{K_{\mathrm{m\ MASP1^*}}^{\mathrm{MASP1_{zym}}}}+\frac{[\mathrm{pCLL1MASP2_{zym}}]}{K_{\mathrm{m\ MASP1^*}}^{\mathrm{MASP2_{zym}}}}+\frac{[\mathrm{pCLL1MASP3_{zym}}]}{K_{\mathrm{m\ MASP1^*}}^{\mathrm{MASP3_{zym}}}}\\+\frac{[\mathrm{pLFMASP1_{zym}}]}{K_{\mathrm{m\ MASP1^*}}^{\mathrm{MASP1_{zym}}}}+\frac{[\mathrm{pLFMASP2_{zym}}]}{K_{\mathrm{m\ MASP1^*}}^{\mathrm{MASP2_{zym}}}}+\frac{[\mathrm{pLFMASP3_{zym}}]}{K_{\mathrm{m\ MASP1^*}}^{\mathrm{MASP3_{zym}}}}+\frac{[\mathrm{pHFMASP1_{zym}}]}{K_{\mathrm{m\ MASP1^*}}^{\mathrm{MASP1_{zym}}}}+\frac{[\mathrm{pHFMASP2_{zym}}]}{K_{\mathrm{m\ MASP1^*}}^{\mathrm{MASP2_{zym}}}}\\+\frac{[\mathrm{pHFMASP3_{zym}}]}{K_{\mathrm{m\ MASP1^*}}^{\mathrm{MASP3_{zym}}}}+\frac{[\mathrm{pMFMASP1_{zym}}]}{K_{\mathrm{m\ MASP1^*}}^{\mathrm{MASP1_{zym}}}}+\frac{[\mathrm{pMFMASP2_{zym}}]}{K_{\mathrm{m\ MASP1^*}}^{\mathrm{MASP2_{zym}}}}+\frac{[\mathrm{pMFMASP3_{zym}}]}{K_{\mathrm{m\ MASP1^*}}^{\mathrm{MASP3_{zym}}}}\\+\frac{[\mathrm{pPTX3LFMASP1_{zym}}]}{K_{\mathrm{m\ MASP1^*}}^{\mathrm{MASP1_{zym}}}}+\frac{[\mathrm{pPXT3LFMASP2_{zym}}]}{K_{\mathrm{m\ MASP1^*}}^{\mathrm{MASP2_{zym}}}}+\frac{[\mathrm{pPTX3LFMASP3_{zym}}]}{K_{\mathrm{m\ MASP1^*}}^{\mathrm{MASP3_{zym}}}}\end{array}\right)}$$

$$
-\frac{k_{\mathrm{cat\ MASP1^{*}}}^{\mathrm{MASP2_{zym}}}\left[\mathrm{pHFMASP1^{*}}\right]\left[\mathrm{pCLLKMASP2_{zym}}\right]}{K_{\mathrm{m\ MASP1^{*}}}^{\mathrm{MASP2_{zym}}}\left(\begin{aligned}
&1+\frac{[\mathrm{C4}]}{K_{\mathrm{m\ MASP1^{*}}}^{\mathrm{C4}}}+\frac{[\mathrm{fC4bC2}]}{K_{\mathrm{m\ MASP1^{*}}}^{\mathrm{C2}}}+\frac{[\mathrm{fC4bC4bC2}]}{K_{\mathrm{m\ MASP1^{*}}}^{\mathrm{C2}}}+\frac{[\mathrm{fC3bC4bC2}]}{K_{\mathrm{m\ MASP1^{*}}}^{\mathrm{C2}}}+\frac{[\mathrm{pC4bC2}]}{K_{\mathrm{m\ MASP1^{*}}}^{\mathrm{C2}}}+\frac{[\mathrm{pC4bC4bC2}]}{K_{\mathrm{m\ MASP1^{*}}}^{\mathrm{C2}}}\\
&+\frac{[\mathrm{pC3bC4bC2}]}{K_{\mathrm{m\ MASP1^{*}}}^{\mathrm{C2}}}+\frac{\left[\mathrm{pMBL_{3}MASP1_{zym}}\right]}{K_{\mathrm{m\ MASP1^{*}}}^{\mathrm{MASP1_{zym}}}}+\frac{\left[\mathrm{pMBL_{3}MASP2_{zym}}\right]}{K_{\mathrm{m\ MASP1^{*}}}^{\mathrm{MASP2_{zym}}}}+\frac{\left[\mathrm{pMBL_{3}MASP3_{zym}}\right]}{K_{\mathrm{m\ MASP1^{*}}}^{\mathrm{MASP3_{zym}}}}\\
&+\frac{\left[\mathrm{pMBL_{4}MASP1_{zym}}\right]}{K_{\mathrm{m\ MASP1^{*}}}^{\mathrm{MASP1_{zym}}}}+\frac{\left[\mathrm{pMBL_{4}MASP2_{zym}}\right]}{K_{\mathrm{m\ MASP1^{*}}}^{\mathrm{MASP2_{zym}}}}+\frac{\left[\mathrm{pMBL_{4}MASP3_{zym}}\right]}{K_{\mathrm{m\ MASP1^{*}}}^{\mathrm{MASP3_{zym}}}}+\frac{\left[\mathrm{pCLLKMASP1_{zym}}\right]}{K_{\mathrm{m\ MASP1^{*}}}^{\mathrm{MASP1_{zym}}}}\\
&+\frac{\left[\mathrm{pCLLKMASP2_{zym}}\right]}{K_{\mathrm{m\ MASP1^{*}}}^{\mathrm{MASP2_{zym}}}}+\frac{\left[\mathrm{pCLLKMASP3_{zym}}\right]}{K_{\mathrm{m\ MASP1^{*}}}^{\mathrm{MASP3_{zym}}}}+\frac{\left[\mathrm{pCLK1MASP1_{zym}}\right]}{K_{\mathrm{m\ MASP1^{*}}}^{\mathrm{MASP1_{zym}}}}+\frac{\left[\mathrm{pCLK1MASP2_{zym}}\right]}{K_{\mathrm{m\ MASP1^{*}}}^{\mathrm{MASP2_{zym}}}}\\
&+\frac{\left[\mathrm{pCLK1MASP3_{zym}}\right]}{K_{\mathrm{m\ MASP1^{*}}}^{\mathrm{MASP3_{zym}}}}+\frac{\left[\mathrm{pCLL1MASP1_{zym}}\right]}{K_{\mathrm{m\ MASP1^{*}}}^{\mathrm{MASP1_{zym}}}}+\frac{\left[\mathrm{pCLL1MASP2_{zym}}\right]}{K_{\mathrm{m\ MASP1^{*}}}^{\mathrm{MASP2_{zym}}}}+\frac{\left[\mathrm{pCLL1MASP3_{zym}}\right]}{K_{\mathrm{m\ MASP1^{*}}}^{\mathrm{MASP3_{zym}}}}\\
&+\frac{\left[\mathrm{pLFMASP1_{zym}}\right]}{K_{\mathrm{m\ MASP1^{*}}}^{\mathrm{MASP1_{zym}}}}+\frac{\left[\mathrm{pLFMASP2_{zym}}\right]}{K_{\mathrm{m\ MASP1^{*}}}^{\mathrm{MASP2_{zym}}}}+\frac{\left[\mathrm{pLFMASP3_{zym}}\right]}{K_{\mathrm{m\ MASP1^{*}}}^{\mathrm{MASP3_{zym}}}}+\frac{\left[\mathrm{pHFMASP1_{zym}}\right]}{K_{\mathrm{m\ MASP1^{*}}}^{\mathrm{MASP1_{zym}}}}+\frac{\left[\mathrm{pHFMASP2_{zym}}\right]}{K_{\mathrm{m\ MASP1^{*}}}^{\mathrm{MASP2_{zym}}}}\\
&+\frac{\left[\mathrm{pHFMASP3_{zym}}\right]}{K_{\mathrm{m\ MASP1^{*}}}^{\mathrm{MASP3_{zym}}}}+\frac{\left[\mathrm{pMFMASP1_{zym}}\right]}{K_{\mathrm{m\ MASP1^{*}}}^{\mathrm{MASP1_{zym}}}}+\frac{\left[\mathrm{pMFMASP2_{zym}}\right]}{K_{\mathrm{m\ MASP1^{*}}}^{\mathrm{MASP2_{zym}}}}+\frac{\left[\mathrm{pMFMASP3_{zym}}\right]}{K_{\mathrm{m\ MASP1^{*}}}^{\mathrm{MASP3_{zym}}}}\\
&+\frac{\left[\mathrm{pPTX3LFMASP1_{zym}}\right]}{K_{\mathrm{m\ MASP1^{*}}}^{\mathrm{MASP1_{zym}}}}+\frac{\left[\mathrm{pPXT3LFMASP2_{zym}}\right]}{K_{\mathrm{m\ MASP1^{*}}}^{\mathrm{MASP2_{zym}}}}+\frac{\left[\mathrm{pPTX3LFMASP3_{zym}}\right]}{K_{\mathrm{m\ MASP1^{*}}}^{\mathrm{MASP3_{zym}}}}
\end{aligned}\right)}
$$

$$
-\frac{k_{\mathrm{cat\ MASP1^{*}}}^{\mathrm{MASP2_{zym}}}\left[\mathrm{pMFMASP1^{*}}\right]\left[\mathrm{pCLLKMASP2_{zym}}\right]}{K_{\mathrm{m\ MASP1^{*}}}^{\mathrm{MASP2_{zym}}}\left(\begin{array}{l}
1+\frac{[\mathrm{C4}]}{K_{\mathrm{m\ MASP1^{*}}}^{\mathrm{C4}}}+\frac{[\mathrm{fC4bC2}]}{K_{\mathrm{m\ MASP1^{*}}}^{\mathrm{C2}}}+\frac{[\mathrm{fC4bC4bC2}]}{K_{\mathrm{m\ MASP1^{*}}}^{\mathrm{C2}}}+\frac{[\mathrm{fC3bC4bC2}]}{K_{\mathrm{m\ MASP1^{*}}}^{\mathrm{C2}}}+\frac{[\mathrm{pC4bC2}]}{K_{\mathrm{m\ MASP1^{*}}}^{\mathrm{C2}}}+\frac{[\mathrm{pC4bC4bC2}]}{K_{\mathrm{m\ MASP1^{*}}}^{\mathrm{C2}}}\\
+\frac{[\mathrm{pC3bC4bC2}]}{K_{\mathrm{m\ MASP1^{*}}}^{\mathrm{C2}}}+\frac{\left[\mathrm{pMBL_{3}MASP1_{zym}}\right]}{K_{\mathrm{m\ MASP1^{*}}}^{\mathrm{MASP1_{zym}}}}+\frac{\left[\mathrm{pMBL_{3}MASP2_{zym}}\right]}{K_{\mathrm{m\ MASP1^{*}}}^{\mathrm{MASP2_{zym}}}}+\frac{\left[\mathrm{pMBL_{3}MASP3_{zym}}\right]}{K_{\mathrm{m\ MASP1^{*}}}^{\mathrm{MASP3_{zym}}}}\\
+\frac{\left[\mathrm{pMBL_{4}MASP1_{zym}}\right]}{K_{\mathrm{m\ MASP1^{*}}}^{\mathrm{MASP1_{zym}}}}+\frac{\left[\mathrm{pMBL_{4}MASP2_{zym}}\right]}{K_{\mathrm{m\ MASP1^{*}}}^{\mathrm{MASP2_{zym}}}}+\frac{\left[\mathrm{pMBL_{4}MASP3_{zym}}\right]}{K_{\mathrm{m\ MASP1^{*}}}^{\mathrm{MASP3_{zym}}}}+\frac{\left[\mathrm{pCLLKMASP1_{zym}}\right]}{K_{\mathrm{m\ MASP1^{*}}}^{\mathrm{MASP1_{zym}}}}\\
+\frac{\left[\mathrm{pCLLKMASP2_{zym}}\right]}{K_{\mathrm{m\ MASP1^{*}}}^{\mathrm{MASP2_{zym}}}}+\frac{\left[\mathrm{pCLLKMASP3_{zym}}\right]}{K_{\mathrm{m\ MASP1^{*}}}^{\mathrm{MASP3_{zym}}}}+\frac{\left[\mathrm{pCLK1MASP1_{zym}}\right]}{K_{\mathrm{m\ MASP1^{*}}}^{\mathrm{MASP1_{zym}}}}+\frac{\left[\mathrm{pCLK1MASP2_{zym}}\right]}{K_{\mathrm{m\ MASP1^{*}}}^{\mathrm{MASP2_{zym}}}}\\
+\frac{\left[\mathrm{pCLK1MASP3_{zym}}\right]}{K_{\mathrm{m\ MASP1^{*}}}^{\mathrm{MASP3_{zym}}}}+\frac{\left[\mathrm{pCLL1MASP1_{zym}}\right]}{K_{\mathrm{m\ MASP1^{*}}}^{\mathrm{MASP1_{zym}}}}+\frac{\left[\mathrm{pCLL1MASP2_{zym}}\right]}{K_{\mathrm{m\ MASP1^{*}}}^{\mathrm{MASP2_{zym}}}}+\frac{\left[\mathrm{pCLL1MASP3_{zym}}\right]}{K_{\mathrm{m\ MASP1^{*}}}^{\mathrm{MASP3_{zym}}}}\\
+\frac{\left[\mathrm{pLFMASP1_{zym}}\right]}{K_{\mathrm{m\ MASP1^{*}}}^{\mathrm{MASP1_{zym}}}}+\frac{\left[\mathrm{pLFMASP2_{zym}}\right]}{K_{\mathrm{m\ MASP1^{*}}}^{\mathrm{MASP2_{zym}}}}+\frac{\left[\mathrm{pLFMASP3_{zym}}\right]}{K_{\mathrm{m\ MASP1^{*}}}^{\mathrm{MASP3_{zym}}}}+\frac{\left[\mathrm{pHFMASP1_{zym}}\right]}{K_{\mathrm{m\ MASP1^{*}}}^{\mathrm{MASP1_{zym}}}}+\frac{\left[\mathrm{pHFMASP2_{zym}}\right]}{K_{\mathrm{m\ MASP1^{*}}}^{\mathrm{MASP2_{zym}}}}\\
+\frac{\left[\mathrm{pHFMASP3_{zym}}\right]}{K_{\mathrm{m\ MASP1^{*}}}^{\mathrm{MASP3_{zym}}}}+\frac{\left[\mathrm{pMFMASP1_{zym}}\right]}{K_{\mathrm{m\ MASP1^{*}}}^{\mathrm{MASP1_{zym}}}}+\frac{\left[\mathrm{pMFMASP2_{zym}}\right]}{K_{\mathrm{m\ MASP1^{*}}}^{\mathrm{MASP2_{zym}}}}+\frac{\left[\mathrm{pMFMASP3_{zym}}\right]}{K_{\mathrm{m\ MASP1^{*}}}^{\mathrm{MASP3_{zym}}}}\\
+\frac{\left[\mathrm{pPTX3LFMASP1_{zym}}\right]}{K_{\mathrm{m\ MASP1^{*}}}^{\mathrm{MASP1_{zym}}}}+\frac{\left[\mathrm{pPXT3LFMASP2_{zym}}\right]}{K_{\mathrm{m\ MASP1^{*}}}^{\mathrm{MASP2_{zym}}}}+\frac{\left[\mathrm{pPTX3LFMASP3_{zym}}\right]}{K_{\mathrm{m\ MASP1^{*}}}^{\mathrm{MASP3_{zym}}}}
\end{array}\right)}
$$

$$-\frac{k_{\mathrm{cat\;MASP1}^*}^{\mathrm{MASP2_{zym}}}\left[\mathrm{pPTX3LFMASP1}^*\right]\left[\mathrm{pCLLKMASP2_{zym}}\right]}{K_{\mathrm{m\;MASP1}^*}^{\mathrm{MASP2_{zym}}}\left(\begin{aligned}&1+\frac{[\mathrm{C4}]}{K_{\mathrm{m\;MASP1}^*}^{\mathrm{C4}}}+\frac{[\mathrm{fC4bC2}]}{K_{\mathrm{m\;MASP1}^*}^{\mathrm{C2}}}+\frac{[\mathrm{fC4bC4bC2}]}{K_{\mathrm{m\;MASP1}^*}^{\mathrm{C2}}}+\frac{[\mathrm{fC3bC4bC2}]}{K_{\mathrm{m\;MASP1}^*}^{\mathrm{C2}}}+\frac{[\mathrm{pC4bC2}]}{K_{\mathrm{m\;MASP1}^*}^{\mathrm{C2}}}+\frac{[\mathrm{pC4bC4bC2}]}{K_{\mathrm{m\;MASP1}^*}^{\mathrm{C2}}}\\&+\frac{[\mathrm{pC3bC4bC2}]}{K_{\mathrm{m\;MASP1}^*}^{\mathrm{C2}}}+\frac{\left[\mathrm{pMBL_3MASP1_{zym}}\right]}{K_{\mathrm{m\;MASP1}^*}^{\mathrm{MASP1_{zym}}}}+\frac{\left[\mathrm{pMBL_3MASP2_{zym}}\right]}{K_{\mathrm{m\;MASP1}^*}^{\mathrm{MASP2_{zym}}}}+\frac{\left[\mathrm{pMBL_3MASP3_{zym}}\right]}{K_{\mathrm{m\;MASP1}^*}^{\mathrm{MASP3_{zym}}}}\\&+\frac{\left[\mathrm{pMBL_4MASP1_{zym}}\right]}{K_{\mathrm{m\;MASP1}^*}^{\mathrm{MASP1_{zym}}}}+\frac{\left[\mathrm{pMBL_4MASP2_{zym}}\right]}{K_{\mathrm{m\;MASP1}^*}^{\mathrm{MASP2_{zym}}}}+\frac{\left[\mathrm{pMBL_4MASP3_{zym}}\right]}{K_{\mathrm{m\;MASP1}^*}^{\mathrm{MASP3_{zym}}}}+\frac{\left[\mathrm{pCLLKMASP1_{zym}}\right]}{K_{\mathrm{m\;MASP1}^*}^{\mathrm{MASP1_{zym}}}}\\&+\frac{\left[\mathrm{pCLLKMASP2_{zym}}\right]}{K_{\mathrm{m\;MASP1}^*}^{\mathrm{MASP2_{zym}}}}+\frac{\left[\mathrm{pCLLKMASP3_{zym}}\right]}{K_{\mathrm{m\;MASP1}^*}^{\mathrm{MASP3_{zym}}}}+\frac{\left[\mathrm{pCLK1MASP1_{zym}}\right]}{K_{\mathrm{m\;MASP1}^*}^{\mathrm{MASP1_{zym}}}}+\frac{\left[\mathrm{pCLK1MASP2_{zym}}\right]}{K_{\mathrm{m\;MASP1}^*}^{\mathrm{MASP2_{zym}}}}\\&+\frac{\left[\mathrm{pCLK1MASP3_{zym}}\right]}{K_{\mathrm{m\;MASP1}^*}^{\mathrm{MASP3_{zym}}}}+\frac{\left[\mathrm{pCLL1MASP1_{zym}}\right]}{K_{\mathrm{m\;MASP1}^*}^{\mathrm{MASP1_{zym}}}}+\frac{\left[\mathrm{pCLL1MASP2_{zym}}\right]}{K_{\mathrm{m\;MASP1}^*}^{\mathrm{MASP2_{zym}}}}+\frac{\left[\mathrm{pCLL1MASP3_{zym}}\right]}{K_{\mathrm{m\;MASP1}^*}^{\mathrm{MASP3_{zym}}}}\\&+\frac{\left[\mathrm{pLFMASP1_{zym}}\right]}{K_{\mathrm{m\;MASP1}^*}^{\mathrm{MASP1_{zym}}}}+\frac{\left[\mathrm{pLFMASP2_{zym}}\right]}{K_{\mathrm{m\;MASP1}^*}^{\mathrm{MASP2_{zym}}}}+\frac{\left[\mathrm{pLFMASP3_{zym}}\right]}{K_{\mathrm{m\;MASP1}^*}^{\mathrm{MASP3_{zym}}}}+\frac{\left[\mathrm{pHFMASP1_{zym}}\right]}{K_{\mathrm{m\;MASP1}^*}^{\mathrm{MASP1_{zym}}}}+\frac{\left[\mathrm{pHFMASP2_{zym}}\right]}{K_{\mathrm{m\;MASP1}^*}^{\mathrm{MASP2_{zym}}}}\\&+\frac{\left[\mathrm{pHFMASP3_{zym}}\right]}{K_{\mathrm{m\;MASP1}^*}^{\mathrm{MASP3_{zym}}}}+\frac{\left[\mathrm{pMFMASP1_{zym}}\right]}{K_{\mathrm{m\;MASP1}^*}^{\mathrm{MASP1_{zym}}}}+\frac{\left[\mathrm{pMFMASP2_{zym}}\right]}{K_{\mathrm{m\;MASP1}^*}^{\mathrm{MASP2_{zym}}}}+\frac{\left[\mathrm{pMFMASP3_{zym}}\right]}{K_{\mathrm{m\;MASP1}^*}^{\mathrm{MASP3_{zym}}}}\\&+\frac{\left[\mathrm{pPTX3LFMASP1_{zym}}\right]}{K_{\mathrm{m\;MASP1}^*}^{\mathrm{MASP1_{zym}}}}+\frac{\left[\mathrm{pPXT3LFMASP2_{zym}}\right]}{K_{\mathrm{m\;MASP1}^*}^{\mathrm{MASP2_{zym}}}}+\frac{\left[\mathrm{pPTX3LFMASP3_{zym}}\right]}{K_{\mathrm{m\;MASP1}^*}^{\mathrm{MASP3_{zym}}}}\end{aligned}\right)}$$

$$-\frac{k_{\mathrm{cat\;MASP2}^*}^{\mathrm{MASP2_{zym}}}\left[\mathrm{pMBL_3MASP2}^*\right]\left[\mathrm{pCLLKMASP2_{zym}}\right]}{K_{\mathrm{m\;MASP2}^*}^{\mathrm{MASP2_{zym}}}\left(\begin{aligned}&1+\frac{[\mathrm{C4}]}{K_{\mathrm{m\;MASP2}^*}^{\mathrm{C4}}}+\frac{[\mathrm{fC4bC2}]}{K_{\mathrm{m\;MASP2}^*}^{\mathrm{C2}}}+\frac{[\mathrm{fC4bC4bC2}]}{K_{\mathrm{m\;MASP2}^*}^{\mathrm{C2}}}+\frac{[\mathrm{fC3bC4bC2}]}{K_{\mathrm{m\;MASP2}^*}^{\mathrm{C2}}}+\frac{[\mathrm{pC4bC2}]}{K_{\mathrm{m\;MASP2}^*}^{\mathrm{C2}}}+\frac{[\mathrm{pC4bC4bC2}]}{K_{\mathrm{m\;MASP2}^*}^{\mathrm{C2}}}\\&+\frac{[\mathrm{pC3bC4bC2}]}{K_{\mathrm{m\;MASP2}^*}^{\mathrm{C2}}}+\frac{\left[\mathrm{pMBL_3MASP1_{zym}}\right]}{K_{\mathrm{m\;MASP2}^*}^{\mathrm{MASP1_{zym}}}}+\frac{\left[\mathrm{pMBL_3MASP2_{zym}}\right]}{K_{\mathrm{m\;MASP2}^*}^{\mathrm{MASP2_{zym}}}}+\frac{\left[\mathrm{pMBL_4MASP1_{zym}}\right]}{K_{\mathrm{m\;MASP2}^*}^{\mathrm{MASP1_{zym}}}}\\&+\frac{\left[\mathrm{pMBL_4MASP2_{zym}}\right]}{K_{\mathrm{m\;MASP2}^*}^{\mathrm{MASP2_{zym}}}}+\frac{\left[\mathrm{pCLLKMASP1_{zym}}\right]}{K_{\mathrm{m\;MASP2}^*}^{\mathrm{MASP1_{zym}}}}+\frac{\left[\mathrm{pCLLKMASP2_{zym}}\right]}{K_{\mathrm{m\;MASP2}^*}^{\mathrm{MASP2_{zym}}}}+\frac{\left[\mathrm{pCLK1MASP1_{zym}}\right]}{K_{\mathrm{m\;MASP2}^*}^{\mathrm{MASP1_{zym}}}}\\&+\frac{\left[\mathrm{pCLK1MASP2_{zym}}\right]}{K_{\mathrm{m\;MASP2}^*}^{\mathrm{MASP2_{zym}}}}+\frac{\left[\mathrm{pCLL1MASP1_{zym}}\right]}{K_{\mathrm{m\;MASP2}^*}^{\mathrm{MASP1_{zym}}}}+\frac{\left[\mathrm{pCLL1MASP2_{zym}}\right]}{K_{\mathrm{m\;MASP2}^*}^{\mathrm{MASP2_{zym}}}}+\frac{\left[\mathrm{pLFMASP1_{zym}}\right]}{K_{\mathrm{m\;MASP2}^*}^{\mathrm{MASP1_{zym}}}}\\&+\frac{\left[\mathrm{pLFMASP2_{zym}}\right]}{K_{\mathrm{m\;MASP2}^*}^{\mathrm{MASP2_{zym}}}}+\frac{\left[\mathrm{pHFMASP1_{zym}}\right]}{K_{\mathrm{m\;MASP2}^*}^{\mathrm{MASP1_{zym}}}}+\frac{\left[\mathrm{pHFMASP2_{zym}}\right]}{K_{\mathrm{m\;MASP2}^*}^{\mathrm{MASP2_{zym}}}}+\frac{\left[\mathrm{pMFMASP1_{zym}}\right]}{K_{\mathrm{m\;MASP2}^*}^{\mathrm{MASP1_{zym}}}}+\frac{\left[\mathrm{pMFMASP2_{zym}}\right]}{K_{\mathrm{m\;MASP2}^*}^{\mathrm{MASP2_{zym}}}}\\&+\frac{\left[\mathrm{pPTX3LFMASP1_{zym}}\right]}{K_{\mathrm{m\;MASP2}^*}^{\mathrm{MASP1_{zym}}}}+\frac{\left[\mathrm{pPTX3LFMASP2_{zym}}\right]}{K_{\mathrm{m\;MASP2}^*}^{\mathrm{MASP2_{zym}}}}\end{aligned}\right)}$$

$$-\frac{k_{\mathrm{cat\ MASP2^*}}^{\mathrm{MASP2_{zym}}}\left[\mathrm{pMBL_4MASP2^*}\right]\left[\mathrm{pCLLKMASP2_{zym}}\right]}{K_{\mathrm{m\ MASP2^*}}^{\mathrm{MASP2_{zym}}}\left(\begin{array}{l}1+\frac{[\mathrm{C4}]}{K_{\mathrm{m\ MASP2^*}}^{\mathrm{C4}}}+\frac{[\mathrm{fC4bC2}]}{K_{\mathrm{m\ MASP2^*}}^{\mathrm{C2}}}+\frac{[\mathrm{fC4bC4bC2}]}{K_{\mathrm{m\ MASP2^*}}^{\mathrm{C2}}}+\frac{[\mathrm{fC3bC4bC2}]}{K_{\mathrm{m\ MASP2^*}}^{\mathrm{C2}}}+\frac{[\mathrm{pC4bC2}]}{K_{\mathrm{m\ MASP2^*}}^{\mathrm{C2}}}+\frac{[\mathrm{pC4bC4bC2}]}{K_{\mathrm{m\ MASP2^*}}^{\mathrm{C2}}}\\+\frac{[\mathrm{pC3bC4bC2}]}{K_{\mathrm{m\ MASP2^*}}^{\mathrm{C2}}}+\frac{[\mathrm{pMBL_3MASP1_{zym}}]}{K_{\mathrm{m\ MASP2^*}}^{\mathrm{MASP1_{zym}}}}+\frac{[\mathrm{pMBL_3MASP2_{zym}}]}{K_{\mathrm{m\ MASP2^*}}^{\mathrm{MASP2_{zym}}}}+\frac{[\mathrm{pMBL_4MASP1_{zym}}]}{K_{\mathrm{m\ MASP2^*}}^{\mathrm{MASP1_{zym}}}}\\+\frac{[\mathrm{pMBL_4MASP2_{zym}}]}{K_{\mathrm{m\ MASP2^*}}^{\mathrm{MASP2_{zym}}}}+\frac{[\mathrm{pCLLKMASP1_{zym}}]}{K_{\mathrm{m\ MASP2^*}}^{\mathrm{MASP1_{zym}}}}+\frac{[\mathrm{pCLLKMASP2_{zym}}]}{K_{\mathrm{m\ MASP2^*}}^{\mathrm{MASP2_{zym}}}}+\frac{[\mathrm{pCLK1MASP1_{zym}}]}{K_{\mathrm{m\ MASP2^*}}^{\mathrm{MASP1_{zym}}}}\\+\frac{[\mathrm{pCLK1MASP2_{zym}}]}{K_{\mathrm{m\ MASP2^*}}^{\mathrm{MASP2_{zym}}}}+\frac{[\mathrm{pCLL1MASP1_{zym}}]}{K_{\mathrm{m\ MASP2^*}}^{\mathrm{MASP1_{zym}}}}+\frac{[\mathrm{pCLL1MASP2_{zym}}]}{K_{\mathrm{m\ MASP2^*}}^{\mathrm{MASP2_{zym}}}}+\frac{[\mathrm{pLFMASP1_{zym}}]}{K_{\mathrm{m\ MASP2^*}}^{\mathrm{MASP1_{zym}}}}\\+\frac{[\mathrm{pLFMASP2_{zym}}]}{K_{\mathrm{m\ MASP2^*}}^{\mathrm{MASP2_{zym}}}}+\frac{[\mathrm{pHFMASP1_{zym}}]}{K_{\mathrm{m\ MASP2^*}}^{\mathrm{MASP1_{zym}}}}+\frac{[\mathrm{pHFMASP2_{zym}}]}{K_{\mathrm{m\ MASP2^*}}^{\mathrm{MASP2_{zym}}}}+\frac{[\mathrm{pMFMASP1_{zym}}]}{K_{\mathrm{m\ MASP2^*}}^{\mathrm{MASP1_{zym}}}}+\frac{[\mathrm{pMFMASP2_{zym}}]}{K_{\mathrm{m\ MASP2^*}}^{\mathrm{MASP2_{zym}}}}\\+\frac{[\mathrm{pPTX3LFMASP1_{zym}}]}{K_{\mathrm{m\ MASP2^*}}^{\mathrm{MASP1_{zym}}}}+\frac{[\mathrm{pPTX3LFMASP2_{zym}}]}{K_{\mathrm{m\ MASP2^*}}^{\mathrm{MASP2_{zym}}}}\end{array}\right)}$$

$$-\frac{k_{\mathrm{cat\ MASP2^*}}^{\mathrm{MASP2_{zym}}}\left[\mathrm{pCLLKMASP2^*}\right]\left[\mathrm{pCLLKMASP2_{zym}}\right]}{K_{\mathrm{m\ MASP2^*}}^{\mathrm{MASP2_{zym}}}\left(\begin{array}{l}1+\frac{[\mathrm{C4}]}{K_{\mathrm{m\ MASP2^*}}^{\mathrm{C4}}}+\frac{[\mathrm{fC4bC2}]}{K_{\mathrm{m\ MASP2^*}}^{\mathrm{C2}}}+\frac{[\mathrm{fC4bC4bC2}]}{K_{\mathrm{m\ MASP2^*}}^{\mathrm{C2}}}+\frac{[\mathrm{fC3bC4bC2}]}{K_{\mathrm{m\ MASP2^*}}^{\mathrm{C2}}}+\frac{[\mathrm{pC4bC2}]}{K_{\mathrm{m\ MASP2^*}}^{\mathrm{C2}}}+\frac{[\mathrm{pC4bC4bC2}]}{K_{\mathrm{m\ MASP2^*}}^{\mathrm{C2}}}\\+\frac{[\mathrm{pC3bC4bC2}]}{K_{\mathrm{m\ MASP2^*}}^{\mathrm{C2}}}+\frac{[\mathrm{pMBL_3MASP1_{zym}}]}{K_{\mathrm{m\ MASP2^*}}^{\mathrm{MASP1_{zym}}}}+\frac{[\mathrm{pMBL_3MASP2_{zym}}]}{K_{\mathrm{m\ MASP2^*}}^{\mathrm{MASP2_{zym}}}}+\frac{[\mathrm{pMBL_4MASP1_{zym}}]}{K_{\mathrm{m\ MASP2^*}}^{\mathrm{MASP1_{zym}}}}\\+\frac{[\mathrm{pMBL_4MASP2_{zym}}]}{K_{\mathrm{m\ MASP2^*}}^{\mathrm{MASP2_{zym}}}}+\frac{[\mathrm{pCLLKMASP1_{zym}}]}{K_{\mathrm{m\ MASP2^*}}^{\mathrm{MASP1_{zym}}}}+\frac{[\mathrm{pCLLKMASP2_{zym}}]}{K_{\mathrm{m\ MASP2^*}}^{\mathrm{MASP2_{zym}}}}+\frac{[\mathrm{pCLK1MASP1_{zym}}]}{K_{\mathrm{m\ MASP2^*}}^{\mathrm{MASP1_{zym}}}}\\+\frac{[\mathrm{pCLK1MASP2_{zym}}]}{K_{\mathrm{m\ MASP2^*}}^{\mathrm{MASP2_{zym}}}}+\frac{[\mathrm{pCLL1MASP1_{zym}}]}{K_{\mathrm{m\ MASP2^*}}^{\mathrm{MASP1_{zym}}}}+\frac{[\mathrm{pCLL1MASP2_{zym}}]}{K_{\mathrm{m\ MASP2^*}}^{\mathrm{MASP2_{zym}}}}+\frac{[\mathrm{pLFMASP1_{zym}}]}{K_{\mathrm{m\ MASP2^*}}^{\mathrm{MASP1_{zym}}}}\\+\frac{[\mathrm{pLFMASP2_{zym}}]}{K_{\mathrm{m\ MASP2^*}}^{\mathrm{MASP2_{zym}}}}+\frac{[\mathrm{pHFMASP1_{zym}}]}{K_{\mathrm{m\ MASP2^*}}^{\mathrm{MASP1_{zym}}}}+\frac{[\mathrm{pHFMASP2_{zym}}]}{K_{\mathrm{m\ MASP2^*}}^{\mathrm{MASP2_{zym}}}}+\frac{[\mathrm{pMFMASP1_{zym}}]}{K_{\mathrm{m\ MASP2^*}}^{\mathrm{MASP1_{zym}}}}+\frac{[\mathrm{pMFMASP2_{zym}}]}{K_{\mathrm{m\ MASP2^*}}^{\mathrm{MASP2_{zym}}}}\\+\frac{[\mathrm{pPTX3LFMASP1_{zym}}]}{K_{\mathrm{m\ MASP2^*}}^{\mathrm{MASP1_{zym}}}}+\frac{[\mathrm{pPTX3LFMASP2_{zym}}]}{K_{\mathrm{m\ MASP2^*}}^{\mathrm{MASP2_{zym}}}}\end{array}\right)}$$

$$
-\frac{k_{\mathrm{cat\ MASP2^*}}^{\mathrm{MASP2_{zym}}}\left[\mathrm{pCLK1MASP2^*}\right]\left[\mathrm{pCLLKMASP2_{zym}}\right]}{K_{\mathrm{m\ MASP2^*}}^{\mathrm{MASP2_{zym}}}\left(\begin{aligned}&1+\frac{[\mathrm{C4}]}{K_{\mathrm{m\ MASP2^*}}^{\mathrm{C4}}}+\frac{[\mathrm{fC4bC2}]}{K_{\mathrm{m\ MASP2^*}}^{\mathrm{C2}}}+\frac{[\mathrm{fC4bC4bC2}]}{K_{\mathrm{m\ MASP2^*}}^{\mathrm{C2}}}+\frac{[\mathrm{fC3bC4bC2}]}{K_{\mathrm{m\ MASP2^*}}^{\mathrm{C2}}}+\frac{[\mathrm{pC4bC2}]}{K_{\mathrm{m\ MASP2^*}}^{\mathrm{C2}}}+\frac{[\mathrm{pC4bC4bC2}]}{K_{\mathrm{m\ MASP2^*}}^{\mathrm{C2}}}\\&+\frac{[\mathrm{pC3bC4bC2}]}{K_{\mathrm{m\ MASP2^*}}^{\mathrm{C2}}}+\frac{[\mathrm{pMBL_3MASP1_{zym}}]}{K_{\mathrm{m\ MASP2^*}}^{\mathrm{MASP1_{zym}}}}+\frac{[\mathrm{pMBL_3MASP2_{zym}}]}{K_{\mathrm{m\ MASP2^*}}^{\mathrm{MASP2_{zym}}}}+\frac{[\mathrm{pMBL_4MASP1_{zym}}]}{K_{\mathrm{m\ MASP2^*}}^{\mathrm{MASP1_{zym}}}}\\&+\frac{[\mathrm{pMBL_4MASP2_{zym}}]}{K_{\mathrm{m\ MASP2^*}}^{\mathrm{MASP2_{zym}}}}+\frac{[\mathrm{pCLLKMASP1_{zym}}]}{K_{\mathrm{m\ MASP2^*}}^{\mathrm{MASP1_{zym}}}}+\frac{[\mathrm{pCLLKMASP2_{zym}}]}{K_{\mathrm{m\ MASP2^*}}^{\mathrm{MASP2_{zym}}}}+\frac{[\mathrm{pCLK1MASP1_{zym}}]}{K_{\mathrm{m\ MASP2^*}}^{\mathrm{MASP1_{zym}}}}\\&+\frac{[\mathrm{pCLK1MASP2_{zym}}]}{K_{\mathrm{m\ MASP2^*}}^{\mathrm{MASP2_{zym}}}}+\frac{[\mathrm{pCLL1MASP1_{zym}}]}{K_{\mathrm{m\ MASP2^*}}^{\mathrm{MASP1_{zym}}}}+\frac{[\mathrm{pCLL1MASP2_{zym}}]}{K_{\mathrm{m\ MASP2^*}}^{\mathrm{MASP2_{zym}}}}+\frac{[\mathrm{pLFMASP1_{zym}}]}{K_{\mathrm{m\ MASP2^*}}^{\mathrm{MASP1_{zym}}}}\\&+\frac{[\mathrm{pLFMASP2_{zym}}]}{K_{\mathrm{m\ MASP2^*}}^{\mathrm{MASP2_{zym}}}}+\frac{[\mathrm{pHFMASP1_{zym}}]}{K_{\mathrm{m\ MASP2^*}}^{\mathrm{MASP1_{zym}}}}+\frac{[\mathrm{pHFMASP2_{zym}}]}{K_{\mathrm{m\ MASP2^*}}^{\mathrm{MASP2_{zym}}}}+\frac{[\mathrm{pMFMASP1_{zym}}]}{K_{\mathrm{m\ MASP2^*}}^{\mathrm{MASP1_{zym}}}}+\frac{[\mathrm{pMFMASP2_{zym}}]}{K_{\mathrm{m\ MASP2^*}}^{\mathrm{MASP2_{zym}}}}\\&+\frac{[\mathrm{pPTX3LFMASP1_{zym}}]}{K_{\mathrm{m\ MASP2^*}}^{\mathrm{MASP1_{zym}}}}+\frac{[\mathrm{pPTX3LFMASP2_{zym}}]}{K_{\mathrm{m\ MASP2^*}}^{\mathrm{MASP2_{zym}}}}\end{aligned}\right)}
$$

$$
-\frac{k_{\mathrm{cat\ MASP2^*}}^{\mathrm{MASP2_{zym}}}\left[\mathrm{pCLL1MASP2^*}\right]\left[\mathrm{pCLLKMASP2_{zym}}\right]}{K_{\mathrm{m\ MASP2^*}}^{\mathrm{MASP2_{zym}}}\left(\begin{aligned}&1+\frac{[\mathrm{C4}]}{K_{\mathrm{m\ MASP2^*}}^{\mathrm{C4}}}+\frac{[\mathrm{fC4bC2}]}{K_{\mathrm{m\ MASP2^*}}^{\mathrm{C2}}}+\frac{[\mathrm{fC4bC4bC2}]}{K_{\mathrm{m\ MASP2^*}}^{\mathrm{C2}}}+\frac{[\mathrm{fC3bC4bC2}]}{K_{\mathrm{m\ MASP2^*}}^{\mathrm{C2}}}+\frac{[\mathrm{pC4bC2}]}{K_{\mathrm{m\ MASP2^*}}^{\mathrm{C2}}}+\frac{[\mathrm{pC4bC4bC2}]}{K_{\mathrm{m\ MASP2^*}}^{\mathrm{C2}}}\\&+\frac{[\mathrm{pC3bC4bC2}]}{K_{\mathrm{m\ MASP2^*}}^{\mathrm{C2}}}+\frac{[\mathrm{pMBL_3MASP1_{zym}}]}{K_{\mathrm{m\ MASP2^*}}^{\mathrm{MASP1_{zym}}}}+\frac{[\mathrm{pMBL_3MASP2_{zym}}]}{K_{\mathrm{m\ MASP2^*}}^{\mathrm{MASP2_{zym}}}}+\frac{[\mathrm{pMBL_4MASP1_{zym}}]}{K_{\mathrm{m\ MASP2^*}}^{\mathrm{MASP1_{zym}}}}\\&+\frac{[\mathrm{pMBL_4MASP2_{zym}}]}{K_{\mathrm{m\ MASP2^*}}^{\mathrm{MASP2_{zym}}}}+\frac{[\mathrm{pCLLKMASP1_{zym}}]}{K_{\mathrm{m\ MASP2^*}}^{\mathrm{MASP1_{zym}}}}+\frac{[\mathrm{pCLLKMASP2_{zym}}]}{K_{\mathrm{m\ MASP2^*}}^{\mathrm{MASP2_{zym}}}}+\frac{[\mathrm{pCLK1MASP1_{zym}}]}{K_{\mathrm{m\ MASP2^*}}^{\mathrm{MASP1_{zym}}}}\\&+\frac{[\mathrm{pCLK1MASP2_{zym}}]}{K_{\mathrm{m\ MASP2^*}}^{\mathrm{MASP2_{zym}}}}+\frac{[\mathrm{pCLL1MASP1_{zym}}]}{K_{\mathrm{m\ MASP2^*}}^{\mathrm{MASP1_{zym}}}}+\frac{[\mathrm{pCLL1MASP2_{zym}}]}{K_{\mathrm{m\ MASP2^*}}^{\mathrm{MASP2_{zym}}}}+\frac{[\mathrm{pLFMASP1_{zym}}]}{K_{\mathrm{m\ MASP2^*}}^{\mathrm{MASP1_{zym}}}}\\&+\frac{[\mathrm{pLFMASP2_{zym}}]}{K_{\mathrm{m\ MASP2^*}}^{\mathrm{MASP2_{zym}}}}+\frac{[\mathrm{pHFMASP1_{zym}}]}{K_{\mathrm{m\ MASP2^*}}^{\mathrm{MASP1_{zym}}}}+\frac{[\mathrm{pHFMASP2_{zym}}]}{K_{\mathrm{m\ MASP2^*}}^{\mathrm{MASP2_{zym}}}}+\frac{[\mathrm{pMFMASP1_{zym}}]}{K_{\mathrm{m\ MASP2^*}}^{\mathrm{MASP1_{zym}}}}+\frac{[\mathrm{pMFMASP2_{zym}}]}{K_{\mathrm{m\ MASP2^*}}^{\mathrm{MASP2_{zym}}}}\\&+\frac{[\mathrm{pPTX3LFMASP1_{zym}}]}{K_{\mathrm{m\ MASP2^*}}^{\mathrm{MASP1_{zym}}}}+\frac{[\mathrm{pPTX3LFMASP2_{zym}}]}{K_{\mathrm{m\ MASP2^*}}^{\mathrm{MASP2_{zym}}}}\end{aligned}\right)}
$$

$$-\frac{k_{\text{cat MASP2}^*}^{\text{MASP2}_{\text{zym}}}\left[\text{pLFMASP2}^*\right]\left[\text{pCLLKMASP2}_{\text{zym}}\right]}{K_{\text{m MASP2}^*}^{\text{MASP2}_{\text{zym}}}\left(\begin{array}{l}1+\frac{[\text{C4}]}{K_{\text{m MASP2}^*}^{\text{C4}}}+\frac{[\text{fC4bC2}]}{K_{\text{m MASP2}^*}^{\text{C2}}}+\frac{[\text{fC4bC4bC2}]}{K_{\text{m MASP2}^*}^{\text{C2}}}+\frac{[\text{fC3bC4bC2}]}{K_{\text{m MASP2}^*}^{\text{C2}}}+\frac{[\text{pC4bC2}]}{K_{\text{m MASP2}^*}^{\text{C2}}}+\frac{[\text{pC4bC4bC2}]}{K_{\text{m MASP2}^*}^{\text{C2}}}\\+\frac{[\text{pC3bC4bC2}]}{K_{\text{m MASP2}^*}^{\text{C2}}}+\frac{[\text{pMBL}_3\text{MASP1}_{\text{zym}}]}{K_{\text{m MASP2}^*}^{\text{MASP1}_{\text{zym}}}}+\frac{[\text{pMBL}_3\text{MASP2}_{\text{zym}}]}{K_{\text{m MASP2}^*}^{\text{MASP2}_{\text{zym}}}}+\frac{[\text{pMBL}_4\text{MASP1}_{\text{zym}}]}{K_{\text{m MASP2}^*}^{\text{MASP1}_{\text{zym}}}}\\+\frac{[\text{pMBL}_4\text{MASP2}_{\text{zym}}]}{K_{\text{m MASP2}^*}^{\text{MASP2}_{\text{zym}}}}+\frac{[\text{pCLLKMASP1}_{\text{zym}}]}{K_{\text{m MASP2}^*}^{\text{MASP1}_{\text{zym}}}}+\frac{[\text{pCLLKMASP2}_{\text{zym}}]}{K_{\text{m MASP2}^*}^{\text{MASP2}_{\text{zym}}}}+\frac{[\text{pCLK1MASP1}_{\text{zym}}]}{K_{\text{m MASP2}^*}^{\text{MASP1}_{\text{zym}}}}\\+\frac{[\text{pCLK1MASP2}_{\text{zym}}]}{K_{\text{m MASP2}^*}^{\text{MASP2}_{\text{zym}}}}+\frac{[\text{pCLL1MASP1}_{\text{zym}}]}{K_{\text{m MASP2}^*}^{\text{MASP1}_{\text{zym}}}}+\frac{[\text{pCLL1MASP2}_{\text{zym}}]}{K_{\text{m MASP2}^*}^{\text{MASP2}_{\text{zym}}}}+\frac{[\text{pLFMASP1}_{\text{zym}}]}{K_{\text{m MASP2}^*}^{\text{MASP1}_{\text{zym}}}}\\+\frac{[\text{pLFMASP2}_{\text{zym}}]}{K_{\text{m MASP2}^*}^{\text{MASP2}_{\text{zym}}}}+\frac{[\text{pHFMASP1}_{\text{zym}}]}{K_{\text{m MASP2}^*}^{\text{MASP1}_{\text{zym}}}}+\frac{[\text{pHFMASP2}_{\text{zym}}]}{K_{\text{m MASP2}^*}^{\text{MASP2}_{\text{zym}}}}+\frac{[\text{pMFMASP1}_{\text{zym}}]}{K_{\text{m MASP2}^*}^{\text{MASP1}_{\text{zym}}}}+\frac{[\text{pMFMASP2}_{\text{zym}}]}{K_{\text{m MASP2}^*}^{\text{MASP2}_{\text{zym}}}}\\+\frac{[\text{pPTX3LFMASP1}_{\text{zym}}]}{K_{\text{m MASP2}^*}^{\text{MASP1}_{\text{zym}}}}+\frac{[\text{pPTX3LFMASP2}_{\text{zym}}]}{K_{\text{m MASP2}^*}^{\text{MASP2}_{\text{zym}}}}\end{array}\right)}$$

$$-\frac{k_{\text{cat MASP2}^*}^{\text{MASP2}_{\text{zym}}}\left[\text{pHFMASP2}^*\right]\left[\text{pCLLKMASP2}_{\text{zym}}\right]}{K_{\text{m MASP2}^*}^{\text{MASP2}_{\text{zym}}}\left(\begin{array}{l}1+\frac{[\text{C4}]}{K_{\text{m MASP2}^*}^{\text{C4}}}+\frac{[\text{fC4bC2}]}{K_{\text{m MASP2}^*}^{\text{C2}}}+\frac{[\text{fC4bC4bC2}]}{K_{\text{m MASP2}^*}^{\text{C2}}}+\frac{[\text{fC3bC4bC2}]}{K_{\text{m MASP2}^*}^{\text{C2}}}+\frac{[\text{pC4bC2}]}{K_{\text{m MASP2}^*}^{\text{C2}}}+\frac{[\text{pC4bC4bC2}]}{K_{\text{m MASP2}^*}^{\text{C2}}}\\+\frac{[\text{pC3bC4bC2}]}{K_{\text{m MASP2}^*}^{\text{C2}}}+\frac{[\text{pMBL}_3\text{MASP1}_{\text{zym}}]}{K_{\text{m MASP2}^*}^{\text{MASP1}_{\text{zym}}}}+\frac{[\text{pMBL}_3\text{MASP2}_{\text{zym}}]}{K_{\text{m MASP2}^*}^{\text{MASP2}_{\text{zym}}}}+\frac{[\text{pMBL}_4\text{MASP1}_{\text{zym}}]}{K_{\text{m MASP2}^*}^{\text{MASP1}_{\text{zym}}}}\\+\frac{[\text{pMBL}_4\text{MASP2}_{\text{zym}}]}{K_{\text{m MASP2}^*}^{\text{MASP2}_{\text{zym}}}}+\frac{[\text{pCLLKMASP1}_{\text{zym}}]}{K_{\text{m MASP2}^*}^{\text{MASP1}_{\text{zym}}}}+\frac{[\text{pCLLKMASP2}_{\text{zym}}]}{K_{\text{m MASP2}^*}^{\text{MASP2}_{\text{zym}}}}+\frac{[\text{pCLK1MASP1}_{\text{zym}}]}{K_{\text{m MASP2}^*}^{\text{MASP1}_{\text{zym}}}}\\+\frac{[\text{pCLK1MASP2}_{\text{zym}}]}{K_{\text{m MASP2}^*}^{\text{MASP2}_{\text{zym}}}}+\frac{[\text{pCLL1MASP1}_{\text{zym}}]}{K_{\text{m MASP2}^*}^{\text{MASP1}_{\text{zym}}}}+\frac{[\text{pCLL1MASP2}_{\text{zym}}]}{K_{\text{m MASP2}^*}^{\text{MASP2}_{\text{zym}}}}+\frac{[\text{pLFMASP1}_{\text{zym}}]}{K_{\text{m MASP2}^*}^{\text{MASP1}_{\text{zym}}}}\\+\frac{[\text{pLFMASP2}_{\text{zym}}]}{K_{\text{m MASP2}^*}^{\text{MASP2}_{\text{zym}}}}+\frac{[\text{pHFMASP1}_{\text{zym}}]}{K_{\text{m MASP2}^*}^{\text{MASP1}_{\text{zym}}}}+\frac{[\text{pHFMASP2}_{\text{zym}}]}{K_{\text{m MASP2}^*}^{\text{MASP2}_{\text{zym}}}}+\frac{[\text{pMFMASP1}_{\text{zym}}]}{K_{\text{m MASP2}^*}^{\text{MASP1}_{\text{zym}}}}+\frac{[\text{pMFMASP2}_{\text{zym}}]}{K_{\text{m MASP2}^*}^{\text{MASP2}_{\text{zym}}}}\\+\frac{[\text{pPTX3LFMASP1}_{\text{zym}}]}{K_{\text{m MASP2}^*}^{\text{MASP1}_{\text{zym}}}}+\frac{[\text{pPTX3LFMASP2}_{\text{zym}}]}{K_{\text{m MASP2}^*}^{\text{MASP2}_{\text{zym}}}}\end{array}\right)}$$

$$
-\frac{k_{\mathrm{cat\,MASP2^*}}^{\mathrm{MASP2_{zym}}}\left[\mathrm{pMFMASP2^*}\right]\left[\mathrm{pCLLKMASP2_{zym}}\right]}{K_{\mathrm{m\,MASP2^*}}^{\mathrm{MASP2_{zym}}}\left(\begin{aligned}&1+\frac{[\mathrm{C4}]}{K_{\mathrm{m\,MASP2^*}}^{\mathrm{C4}}}+\frac{[\mathrm{fC4bC2}]}{K_{\mathrm{m\,MASP2^*}}^{\mathrm{C2}}}+\frac{[\mathrm{fC4bC4bC2}]}{K_{\mathrm{m\,MASP2^*}}^{\mathrm{C2}}}+\frac{[\mathrm{fC3bC4bC2}]}{K_{\mathrm{m\,MASP2^*}}^{\mathrm{C2}}}+\frac{[\mathrm{pC4bC2}]}{K_{\mathrm{m\,MASP2^*}}^{\mathrm{C2}}}+\frac{[\mathrm{pC4bC4bC2}]}{K_{\mathrm{m\,MASP2^*}}^{\mathrm{C2}}}\\
&+\frac{[\mathrm{pC3bC4bC2}]}{K_{\mathrm{m\,MASP2^*}}^{\mathrm{C2}}}+\frac{[\mathrm{pMBL_3MASP1_{zym}}]}{K_{\mathrm{m\,MASP2^*}}^{\mathrm{MASP1_{zym}}}}+\frac{[\mathrm{pMBL_3MASP2_{zym}}]}{K_{\mathrm{m\,MASP2^*}}^{\mathrm{MASP2_{zym}}}}+\frac{[\mathrm{pMBL_4MASP1_{zym}}]}{K_{\mathrm{m\,MASP2^*}}^{\mathrm{MASP1_{zym}}}}\\
&+\frac{[\mathrm{pMBL_4MASP2_{zym}}]}{K_{\mathrm{m\,MASP2^*}}^{\mathrm{MASP2_{zym}}}}+\frac{[\mathrm{pCLLKMASP1_{zym}}]}{K_{\mathrm{m\,MASP2^*}}^{\mathrm{MASP1_{zym}}}}+\frac{[\mathrm{pCLLKMASP2_{zym}}]}{K_{\mathrm{m\,MASP2^*}}^{\mathrm{MASP2_{zym}}}}+\frac{[\mathrm{pCLK1MASP1_{zym}}]}{K_{\mathrm{m\,MASP2^*}}^{\mathrm{MASP1_{zym}}}}\\
&+\frac{[\mathrm{pCLK1MASP2_{zym}}]}{K_{\mathrm{m\,MASP2^*}}^{\mathrm{MASP2_{zym}}}}+\frac{[\mathrm{pCLL1MASP1_{zym}}]}{K_{\mathrm{m\,MASP2^*}}^{\mathrm{MASP1_{zym}}}}+\frac{[\mathrm{pCLL1MASP2_{zym}}]}{K_{\mathrm{m\,MASP2^*}}^{\mathrm{MASP2_{zym}}}}+\frac{[\mathrm{pLFMASP1_{zym}}]}{K_{\mathrm{m\,MASP2^*}}^{\mathrm{MASP1_{zym}}}}\\
&+\frac{[\mathrm{pLFMASP2_{zym}}]}{K_{\mathrm{m\,MASP2^*}}^{\mathrm{MASP2_{zym}}}}+\frac{[\mathrm{pHFMASP1_{zym}}]}{K_{\mathrm{m\,MASP2^*}}^{\mathrm{MASP1_{zym}}}}+\frac{[\mathrm{pHFMASP2_{zym}}]}{K_{\mathrm{m\,MASP2^*}}^{\mathrm{MASP2_{zym}}}}+\frac{[\mathrm{pMFMASP1_{zym}}]}{K_{\mathrm{m\,MASP2^*}}^{\mathrm{MASP1_{zym}}}}+\frac{[\mathrm{pMFMASP2_{zym}}]}{K_{\mathrm{m\,MASP2^*}}^{\mathrm{MASP2_{zym}}}}\\
&+\frac{[\mathrm{pPTX3LFMASP1_{zym}}]}{K_{\mathrm{m\,MASP2^*}}^{\mathrm{MASP1_{zym}}}}+\frac{[\mathrm{pPTX3LFMASP2_{zym}}]}{K_{\mathrm{m\,MASP2^*}}^{\mathrm{MASP2_{zym}}}}\end{aligned}\right)}
$$

$$
-\frac{k_{\mathrm{cat\,MASP2^*}}^{\mathrm{MASP2_{zym}}}\left[\mathrm{pPTX3LFMASP2^*}\right]\left[\mathrm{pCLLKMASP2_{zym}}\right]}{K_{\mathrm{m\,MASP2^*}}^{\mathrm{MASP2_{zym}}}\left(\begin{aligned}&1+\frac{[\mathrm{C4}]}{K_{\mathrm{m\,MASP2^*}}^{\mathrm{C4}}}+\frac{[\mathrm{fC4bC2}]}{K_{\mathrm{m\,MASP2^*}}^{\mathrm{C2}}}+\frac{[\mathrm{fC4bC4bC2}]}{K_{\mathrm{m\,MASP2^*}}^{\mathrm{C2}}}+\frac{[\mathrm{fC3bC4bC2}]}{K_{\mathrm{m\,MASP2^*}}^{\mathrm{C2}}}+\frac{[\mathrm{pC4bC2}]}{K_{\mathrm{m\,MASP2^*}}^{\mathrm{C2}}}+\frac{[\mathrm{pC4bC4bC2}]}{K_{\mathrm{m\,MASP2^*}}^{\mathrm{C2}}}\\
&+\frac{[\mathrm{pC3bC4bC2}]}{K_{\mathrm{m\,MASP2^*}}^{\mathrm{C2}}}+\frac{[\mathrm{pMBL_3MASP1_{zym}}]}{K_{\mathrm{m\,MASP2^*}}^{\mathrm{MASP1_{zym}}}}+\frac{[\mathrm{pMBL_3MASP2_{zym}}]}{K_{\mathrm{m\,MASP2^*}}^{\mathrm{MASP2_{zym}}}}+\frac{[\mathrm{pMBL_4MASP1_{zym}}]}{K_{\mathrm{m\,MASP2^*}}^{\mathrm{MASP1_{zym}}}}\\
&+\frac{[\mathrm{pMBL_4MASP2_{zym}}]}{K_{\mathrm{m\,MASP2^*}}^{\mathrm{MASP2_{zym}}}}+\frac{[\mathrm{pCLLKMASP1_{zym}}]}{K_{\mathrm{m\,MASP2^*}}^{\mathrm{MASP1_{zym}}}}+\frac{[\mathrm{pCLLKMASP2_{zym}}]}{K_{\mathrm{m\,MASP2^*}}^{\mathrm{MASP2_{zym}}}}+\frac{[\mathrm{pCLK1MASP1_{zym}}]}{K_{\mathrm{m\,MASP2^*}}^{\mathrm{MASP1_{zym}}}}\\
&+\frac{[\mathrm{pCLK1MASP2_{zym}}]}{K_{\mathrm{m\,MASP2^*}}^{\mathrm{MASP2_{zym}}}}+\frac{[\mathrm{pCLL1MASP1_{zym}}]}{K_{\mathrm{m\,MASP2^*}}^{\mathrm{MASP1_{zym}}}}+\frac{[\mathrm{pCLL1MASP2_{zym}}]}{K_{\mathrm{m\,MASP2^*}}^{\mathrm{MASP2_{zym}}}}+\frac{[\mathrm{pLFMASP1_{zym}}]}{K_{\mathrm{m\,MASP2^*}}^{\mathrm{MASP1_{zym}}}}\\
&+\frac{[\mathrm{pLFMASP2_{zym}}]}{K_{\mathrm{m\,MASP2^*}}^{\mathrm{MASP2_{zym}}}}+\frac{[\mathrm{pHFMASP1_{zym}}]}{K_{\mathrm{m\,MASP2^*}}^{\mathrm{MASP1_{zym}}}}+\frac{[\mathrm{pHFMASP2_{zym}}]}{K_{\mathrm{m\,MASP2^*}}^{\mathrm{MASP2_{zym}}}}+\frac{[\mathrm{pMFMASP1_{zym}}]}{K_{\mathrm{m\,MASP2^*}}^{\mathrm{MASP1_{zym}}}}+\frac{[\mathrm{pMFMASP2_{zym}}]}{K_{\mathrm{m\,MASP2^*}}^{\mathrm{MASP2_{zym}}}}\\
&+\frac{[\mathrm{pPTX3LFMASP1_{zym}}]}{K_{\mathrm{m\,MASP2^*}}^{\mathrm{MASP1_{zym}}}}+\frac{[\mathrm{pPTX3LFMASP2_{zym}}]}{K_{\mathrm{m\,MASP2^*}}^{\mathrm{MASP2_{zym}}}}\end{aligned}\right)}
$$

290

$$
\frac{d\left[\mathrm{fCLLKMASP2^*}\right]}{dt}=-k_{\mathrm{pCLLK}}^{+}\left[\mathrm{fCLLKMASP2^*}\right]\left[\mathrm{Pathogen\ Surface}\right]+k_{\mathrm{pCLLK}}^{-}\left[\mathrm{pCLLKMASP2^*}\right]
$$

$$
+k_{\mathrm{CLLKMASP2}}^{+}\left[\mathrm{CLLK}\right]\left[\mathrm{MASP2^*}\right]-k_{\mathrm{CLLKMASP2}}^{-}\left[\mathrm{fCLLKMASP2^*}\right]-k_{\mathrm{C1INHMASP2}}^{+}\left[\mathrm{C1\text{-}INH}\right]\left[\mathrm{fCLLKMASP2^*}\right]
$$

291

$$
\begin{aligned}
\frac{d\left[\text{pCLLKMASP2}^{*}\right]}{dt} &= k^{+}_{\text{pCLLK}}\left[\text{fCLLKMASP2}^{*}\right]\left[\text{Pathogen Surface}\right] - k^{-}_{\text{pCLLK}}\left[\text{pCLLKMASP2}^{*}\right] \\
&+ k^{+}_{\text{CLLKMASP2}}\left[\text{pCLLK}\right]\left[\text{MASP2}^{*}\right] - k^{-}_{\text{CLLKMASP2}}\left[\text{pCLLKMASP2}^{*}\right] \\
&+ \frac{k^{\text{MASP2}_{\text{zym}}}_{\text{cat MASP1}_{\text{zym}}}\left[\text{pMBL}_3\text{MASP1}_{\text{zym}}\right]\left[\text{pCLLKMASP2}_{\text{zym}}\right]}{K^{\text{MASP2}_{\text{zym}}}_{\text{m MASP1}_{\text{zym}}}\left(\begin{aligned}
&1+\frac{\left[\text{pMBL}_3\text{MASP1}_{\text{zym}}\right]}{K^{\text{MASP1}_{\text{zym}}}_{\text{m MASP1}_{\text{zym}}}}+\frac{\left[\text{pMBL}_3\text{MASP2}_{\text{zym}}\right]}{K^{\text{MASP2}_{\text{zym}}}_{\text{m MASP1}_{\text{zym}}}}+\frac{\left[\text{pMBL}_4\text{MASP1}_{\text{zym}}\right]}{K^{\text{MASP1}_{\text{zym}}}_{\text{m MASP1}_{\text{zym}}}}+\frac{\left[\text{pMBL}_4\text{MASP2}_{\text{zym}}\right]}{K^{\text{MASP2}_{\text{zym}}}_{\text{m MASP1}_{\text{zym}}}} \\
&+\frac{\left[\text{pCLLKMASP1}_{\text{zym}}\right]}{K^{\text{MASP1}_{\text{zym}}}_{\text{m MASP1}_{\text{zym}}}}+\frac{\left[\text{pCLLKMASP2}_{\text{zym}}\right]}{K^{\text{MASP2}_{\text{zym}}}_{\text{m MASP1}_{\text{zym}}}}+\frac{\left[\text{pCLK1MASP1}_{\text{zym}}\right]}{K^{\text{MASP1}_{\text{zym}}}_{\text{m MASP1}_{\text{zym}}}}+\frac{\left[\text{pCLK1MASP2}_{\text{zym}}\right]}{K^{\text{MASP2}_{\text{zym}}}_{\text{m MASP1}_{\text{zym}}}} \\
&+\frac{\left[\text{pCLL1MASP1}_{\text{zym}}\right]}{K^{\text{MASP1}_{\text{zym}}}_{\text{m MASP1}_{\text{zym}}}}+\frac{\left[\text{pCLL1MASP2}_{\text{zym}}\right]}{K^{\text{MASP2}_{\text{zym}}}_{\text{m MASP1}_{\text{zym}}}}+\frac{\left[\text{pLFMASP1}_{\text{zym}}\right]}{K^{\text{MASP1}_{\text{zym}}}_{\text{m MASP1}_{\text{zym}}}}+\frac{\left[\text{pLFMASP2}_{\text{zym}}\right]}{K^{\text{MASP2}_{\text{zym}}}_{\text{m MASP1}_{\text{zym}}}} \\
&+\frac{\left[\text{pHFMASP1}_{\text{zym}}\right]}{K^{\text{MASP1}_{\text{zym}}}_{\text{m MASP1}_{\text{zym}}}}+\frac{\left[\text{pHFMASP2}_{\text{zym}}\right]}{K^{\text{MASP2}_{\text{zym}}}_{\text{m MASP1}_{\text{zym}}}}+\frac{\left[\text{pMFMASP1}_{\text{zym}}\right]}{K^{\text{MASP1}_{\text{zym}}}_{\text{m MASP1}_{\text{zym}}}}+\frac{\left[\text{pMFMASP2}_{\text{zym}}\right]}{K^{\text{MASP2}_{\text{zym}}}_{\text{m MASP1}_{\text{zym}}}} \\
&+\frac{\left[\text{pPTX3LFMASP1}_{\text{zym}}\right]}{K^{\text{MASP1}_{\text{zym}}}_{\text{m MASP1}_{\text{zym}}}}+\frac{\left[\text{pPTX3LFMASP2}_{\text{zym}}\right]}{K^{\text{MASP2}_{\text{zym}}}_{\text{m MASP1}_{\text{zym}}}}
\end{aligned}\right)} \\
&+ \frac{k^{\text{MASP2}_{\text{zym}}}_{\text{cat MASP1}_{\text{zym}}}\left[\text{pMBL}_4\text{MASP1}_{\text{zym}}\right]\left[\text{pCLLKMASP2}_{\text{zym}}\right]}{K^{\text{MASP2}_{\text{zym}}}_{\text{m MASP1}_{\text{zym}}}\left(\begin{aligned}
&1+\frac{\left[\text{pMBL}_3\text{MASP1}_{\text{zym}}\right]}{K^{\text{MASP1}_{\text{zym}}}_{\text{m MASP1}_{\text{zym}}}}+\frac{\left[\text{pMBL}_3\text{MASP2}_{\text{zym}}\right]}{K^{\text{MASP2}_{\text{zym}}}_{\text{m MASP1}_{\text{zym}}}}+\frac{\left[\text{pMBL}_4\text{MASP1}_{\text{zym}}\right]}{K^{\text{MASP1}_{\text{zym}}}_{\text{m MASP1}_{\text{zym}}}}+\frac{\left[\text{pMBL}_4\text{MASP2}_{\text{zym}}\right]}{K^{\text{MASP2}_{\text{zym}}}_{\text{m MASP1}_{\text{zym}}}} \\
&+\frac{\left[\text{pCLLKMASP1}_{\text{zym}}\right]}{K^{\text{MASP1}_{\text{zym}}}_{\text{m MASP1}_{\text{zym}}}}+\frac{\left[\text{pCLLKMASP2}_{\text{zym}}\right]}{K^{\text{MASP2}_{\text{zym}}}_{\text{m MASP1}_{\text{zym}}}}+\frac{\left[\text{pCLK1MASP1}_{\text{zym}}\right]}{K^{\text{MASP1}_{\text{zym}}}_{\text{m MASP1}_{\text{zym}}}}+\frac{\left[\text{pCLK1MASP2}_{\text{zym}}\right]}{K^{\text{MASP2}_{\text{zym}}}_{\text{m MASP1}_{\text{zym}}}} \\
&+\frac{\left[\text{pCLL1MASP1}_{\text{zym}}\right]}{K^{\text{MASP1}_{\text{zym}}}_{\text{m MASP1}_{\text{zym}}}}+\frac{\left[\text{pCLL1MASP2}_{\text{zym}}\right]}{K^{\text{MASP2}_{\text{zym}}}_{\text{m MASP1}_{\text{zym}}}}+\frac{\left[\text{pLFMASP1}_{\text{zym}}\right]}{K^{\text{MASP1}_{\text{zym}}}_{\text{m MASP1}_{\text{zym}}}}+\frac{\left[\text{pLFMASP2}_{\text{zym}}\right]}{K^{\text{MASP2}_{\text{zym}}}_{\text{m MASP1}_{\text{zym}}}} \\
&+\frac{\left[\text{pHFMASP1}_{\text{zym}}\right]}{K^{\text{MASP1}_{\text{zym}}}_{\text{m MASP1}_{\text{zym}}}}+\frac{\left[\text{pHFMASP2}_{\text{zym}}\right]}{K^{\text{MASP2}_{\text{zym}}}_{\text{m MASP1}_{\text{zym}}}}+\frac{\left[\text{pMFMASP1}_{\text{zym}}\right]}{K^{\text{MASP1}_{\text{zym}}}_{\text{m MASP1}_{\text{zym}}}}+\frac{\left[\text{pMFMASP2}_{\text{zym}}\right]}{K^{\text{MASP2}_{\text{zym}}}_{\text{m MASP1}_{\text{zym}}}} \\
&+\frac{\left[\text{pPTX3LFMASP1}_{\text{zym}}\right]}{K^{\text{MASP1}_{\text{zym}}}_{\text{m MASP1}_{\text{zym}}}}+\frac{\left[\text{pPTX3LFMASP2}_{\text{zym}}\right]}{K^{\text{MASP2}_{\text{zym}}}_{\text{m MASP1}_{\text{zym}}}}
\end{aligned}\right)}
\end{aligned}
$$

$$+\frac{k_{\mathrm{cat\ MASP1_{zym}}}^{\mathrm{MASP2_{zym}}}\left[\mathrm{pCLLKMASP1_{zym}}\right]\left[\mathrm{pCLLKMASP2_{zym}}\right]}{K_{\mathrm{m\ MASP1_{zym}}}^{\mathrm{MASP2_{zym}}}\left(\begin{array}{l}1+\frac{\left[\mathrm{pMBL_3MASP1_{zym}}\right]}{K_{\mathrm{m\ MASP1_{zym}}}^{\mathrm{MASP1_{zym}}}}+\frac{\left[\mathrm{pMBL_3MASP2_{zym}}\right]}{K_{\mathrm{m\ MASP1_{zym}}}^{\mathrm{MASP2_{zym}}}}+\frac{\left[\mathrm{pMBL_4MASP1_{zym}}\right]}{K_{\mathrm{m\ MASP1_{zym}}}^{\mathrm{MASP1_{zym}}}}+\frac{\left[\mathrm{pMBL_4MASP2_{zym}}\right]}{K_{\mathrm{m\ MASP1_{zym}}}^{\mathrm{MASP2_{zym}}}}\\+\frac{\left[\mathrm{pCLLKMASP1_{zym}}\right]}{K_{\mathrm{m\ MASP1_{zym}}}^{\mathrm{MASP1_{zym}}}}+\frac{\left[\mathrm{pCLLKMASP2_{zym}}\right]}{K_{\mathrm{m\ MASP1_{zym}}}^{\mathrm{MASP2_{zym}}}}+\frac{\left[\mathrm{pCLK1MASP1_{zym}}\right]}{K_{\mathrm{m\ MASP1_{zym}}}^{\mathrm{MASP1_{zym}}}}+\frac{\left[\mathrm{pCLK1MASP2_{zym}}\right]}{K_{\mathrm{m\ MASP1_{zym}}}^{\mathrm{MASP2_{zym}}}}\\+\frac{\left[\mathrm{pCLL1MASP1_{zym}}\right]}{K_{\mathrm{m\ MASP1_{zym}}}^{\mathrm{MASP1_{zym}}}}+\frac{\left[\mathrm{pCLL1MASP2_{zym}}\right]}{K_{\mathrm{m\ MASP1_{zym}}}^{\mathrm{MASP2_{zym}}}}+\frac{\left[\mathrm{pLFMASP1_{zym}}\right]}{K_{\mathrm{m\ MASP1_{zym}}}^{\mathrm{MASP1_{zym}}}}+\frac{\left[\mathrm{pLFMASP2_{zym}}\right]}{K_{\mathrm{m\ MASP1_{zym}}}^{\mathrm{MASP2_{zym}}}}\\+\frac{\left[\mathrm{pHFMASP1_{zym}}\right]}{K_{\mathrm{m\ MASP1_{zym}}}^{\mathrm{MASP1_{zym}}}}+\frac{\left[\mathrm{pHFMASP2_{zym}}\right]}{K_{\mathrm{m\ MASP1_{zym}}}^{\mathrm{MASP2_{zym}}}}+\frac{\left[\mathrm{pMFMASP1_{zym}}\right]}{K_{\mathrm{m\ MASP1_{zym}}}^{\mathrm{MASP1_{zym}}}}+\frac{\left[\mathrm{pMFMASP2_{zym}}\right]}{K_{\mathrm{m\ MASP1_{zym}}}^{\mathrm{MASP2_{zym}}}}\\+\frac{\left[\mathrm{pPTX3LFMASP1_{zym}}\right]}{K_{\mathrm{m\ MASP1_{zym}}}^{\mathrm{MASP1_{zym}}}}+\frac{\left[\mathrm{pPTX3LFMASP2_{zym}}\right]}{K_{\mathrm{m\ MASP1_{zym}}}^{\mathrm{MASP2_{zym}}}}\end{array}\right)}$$

$$+\frac{k_{\mathrm{cat\ MASP1_{zym}}}^{\mathrm{MASP2_{zym}}}\left[\mathrm{pCLK1MASP1_{zym}}\right]\left[\mathrm{pCLLKMASP2_{zym}}\right]}{K_{\mathrm{m\ MASP1_{zym}}}^{\mathrm{MASP2_{zym}}}\left(\begin{array}{l}1+\frac{\left[\mathrm{pMBL_3MASP1_{zym}}\right]}{K_{\mathrm{m\ MASP1_{zym}}}^{\mathrm{MASP1_{zym}}}}+\frac{\left[\mathrm{pMBL_3MASP2_{zym}}\right]}{K_{\mathrm{m\ MASP1_{zym}}}^{\mathrm{MASP2_{zym}}}}+\frac{\left[\mathrm{pMBL_4MASP1_{zym}}\right]}{K_{\mathrm{m\ MASP1_{zym}}}^{\mathrm{MASP1_{zym}}}}+\frac{\left[\mathrm{pMBL_4MASP2_{zym}}\right]}{K_{\mathrm{m\ MASP1_{zym}}}^{\mathrm{MASP2_{zym}}}}\\+\frac{\left[\mathrm{pCLLKMASP1_{zym}}\right]}{K_{\mathrm{m\ MASP1_{zym}}}^{\mathrm{MASP1_{zym}}}}+\frac{\left[\mathrm{pCLLKMASP2_{zym}}\right]}{K_{\mathrm{m\ MASP1_{zym}}}^{\mathrm{MASP2_{zym}}}}+\frac{\left[\mathrm{pCLK1MASP1_{zym}}\right]}{K_{\mathrm{m\ MASP1_{zym}}}^{\mathrm{MASP1_{zym}}}}+\frac{\left[\mathrm{pCLK1MASP2_{zym}}\right]}{K_{\mathrm{m\ MASP1_{zym}}}^{\mathrm{MASP2_{zym}}}}\\+\frac{\left[\mathrm{pCLL1MASP1_{zym}}\right]}{K_{\mathrm{m\ MASP1_{zym}}}^{\mathrm{MASP1_{zym}}}}+\frac{\left[\mathrm{pCLL1MASP2_{zym}}\right]}{K_{\mathrm{m\ MASP1_{zym}}}^{\mathrm{MASP2_{zym}}}}+\frac{\left[\mathrm{pLFMASP1_{zym}}\right]}{K_{\mathrm{m\ MASP1_{zym}}}^{\mathrm{MASP1_{zym}}}}+\frac{\left[\mathrm{pLFMASP2_{zym}}\right]}{K_{\mathrm{m\ MASP1_{zym}}}^{\mathrm{MASP2_{zym}}}}\\+\frac{\left[\mathrm{pHFMASP1_{zym}}\right]}{K_{\mathrm{m\ MASP1_{zym}}}^{\mathrm{MASP1_{zym}}}}+\frac{\left[\mathrm{pHFMASP2_{zym}}\right]}{K_{\mathrm{m\ MASP1_{zym}}}^{\mathrm{MASP2_{zym}}}}+\frac{\left[\mathrm{pMFMASP1_{zym}}\right]}{K_{\mathrm{m\ MASP1_{zym}}}^{\mathrm{MASP1_{zym}}}}+\frac{\left[\mathrm{pMFMASP2_{zym}}\right]}{K_{\mathrm{m\ MASP1_{zym}}}^{\mathrm{MASP2_{zym}}}}\\+\frac{\left[\mathrm{pPTX3LFMASP1_{zym}}\right]}{K_{\mathrm{m\ MASP1_{zym}}}^{\mathrm{MASP1_{zym}}}}+\frac{\left[\mathrm{pPTX3LFMASP2_{zym}}\right]}{K_{\mathrm{m\ MASP1_{zym}}}^{\mathrm{MASP2_{zym}}}}\end{array}\right)}$$

$$+\frac{k_{\mathrm{cat\ MASP1_{zym}}}^{\mathrm{MASP2_{zym}}}\left[\mathrm{pCLL1MASP1_{zym}}\right]\left[\mathrm{pCLLKMASP2_{zym}}\right]}{K_{\mathrm{m\ MASP1_{zym}}}^{\mathrm{MASP2_{zym}}}\left(\begin{array}{l}1+\frac{\left[\mathrm{pMBL_3MASP1_{zym}}\right]}{K_{\mathrm{m\ MASP1_{zym}}}^{\mathrm{MASP1_{zym}}}}+\frac{\left[\mathrm{pMBL_3MASP2_{zym}}\right]}{K_{\mathrm{m\ MASP1_{zym}}}^{\mathrm{MASP2_{zym}}}}+\frac{\left[\mathrm{pMBL_4MASP1_{zym}}\right]}{K_{\mathrm{m\ MASP1_{zym}}}^{\mathrm{MASP1_{zym}}}}+\frac{\left[\mathrm{pMBL_4MASP2_{zym}}\right]}{K_{\mathrm{m\ MASP1_{zym}}}^{\mathrm{MASP2_{zym}}}}\\+\frac{\left[\mathrm{pCLLKMASP1_{zym}}\right]}{K_{\mathrm{m\ MASP1_{zym}}}^{\mathrm{MASP1_{zym}}}}+\frac{\left[\mathrm{pCLLKMASP2_{zym}}\right]}{K_{\mathrm{m\ MASP1_{zym}}}^{\mathrm{MASP2_{zym}}}}+\frac{\left[\mathrm{pCLK1MASP1_{zym}}\right]}{K_{\mathrm{m\ MASP1_{zym}}}^{\mathrm{MASP1_{zym}}}}+\frac{\left[\mathrm{pCLK1MASP2_{zym}}\right]}{K_{\mathrm{m\ MASP1_{zym}}}^{\mathrm{MASP2_{zym}}}}\\+\frac{\left[\mathrm{pCLL1MASP1_{zym}}\right]}{K_{\mathrm{m\ MASP1_{zym}}}^{\mathrm{MASP1_{zym}}}}+\frac{\left[\mathrm{pCLL1MASP2_{zym}}\right]}{K_{\mathrm{m\ MASP1_{zym}}}^{\mathrm{MASP2_{zym}}}}+\frac{\left[\mathrm{pLFMASP1_{zym}}\right]}{K_{\mathrm{m\ MASP1_{zym}}}^{\mathrm{MASP1_{zym}}}}+\frac{\left[\mathrm{pLFMASP2_{zym}}\right]}{K_{\mathrm{m\ MASP1_{zym}}}^{\mathrm{MASP2_{zym}}}}\\+\frac{\left[\mathrm{pHFMASP1_{zym}}\right]}{K_{\mathrm{m\ MASP1_{zym}}}^{\mathrm{MASP1_{zym}}}}+\frac{\left[\mathrm{pHFMASP2_{zym}}\right]}{K_{\mathrm{m\ MASP1_{zym}}}^{\mathrm{MASP2_{zym}}}}+\frac{\left[\mathrm{pMFMASP1_{zym}}\right]}{K_{\mathrm{m\ MASP1_{zym}}}^{\mathrm{MASP1_{zym}}}}+\frac{\left[\mathrm{pMFMASP2_{zym}}\right]}{K_{\mathrm{m\ MASP1_{zym}}}^{\mathrm{MASP2_{zym}}}}\\+\frac{\left[\mathrm{pPTX3LFMASP1_{zym}}\right]}{K_{\mathrm{m\ MASP1_{zym}}}^{\mathrm{MASP1_{zym}}}}+\frac{\left[\mathrm{pPTX3LFMASP2_{zym}}\right]}{K_{\mathrm{m\ MASP1_{zym}}}^{\mathrm{MASP2_{zym}}}}\end{array}\right)}$$

$$+\frac{k_{\mathrm{cat\ MASP1_{zym}}}^{\mathrm{MASP2_{zym}}}\left[\mathrm{pLFMASP1_{zym}}\right]\left[\mathrm{pCLLKMASP2_{zym}}\right]}{K_{\mathrm{m\ MASP1_{zym}}}^{\mathrm{MASP2_{zym}}}\left(\begin{array}{l}1+\frac{\left[\mathrm{pMBL_3MASP1_{zym}}\right]}{K_{\mathrm{m\ MASP1_{zym}}}^{\mathrm{MASP1_{zym}}}}+\frac{\left[\mathrm{pMBL_3MASP2_{zym}}\right]}{K_{\mathrm{m\ MASP1_{zym}}}^{\mathrm{MASP2_{zym}}}}+\frac{\left[\mathrm{pMBL_4MASP1_{zym}}\right]}{K_{\mathrm{m\ MASP1_{zym}}}^{\mathrm{MASP1_{zym}}}}+\frac{\left[\mathrm{pMBL_4MASP2_{zym}}\right]}{K_{\mathrm{m\ MASP1_{zym}}}^{\mathrm{MASP2_{zym}}}}\\+\frac{\left[\mathrm{pCLLKMASP1_{zym}}\right]}{K_{\mathrm{m\ MASP1_{zym}}}^{\mathrm{MASP1_{zym}}}}+\frac{\left[\mathrm{pCLLKMASP2_{zym}}\right]}{K_{\mathrm{m\ MASP1_{zym}}}^{\mathrm{MASP2_{zym}}}}+\frac{\left[\mathrm{pCLK1MASP1_{zym}}\right]}{K_{\mathrm{m\ MASP1_{zym}}}^{\mathrm{MASP1_{zym}}}}+\frac{\left[\mathrm{pCLK1MASP2_{zym}}\right]}{K_{\mathrm{m\ MASP1_{zym}}}^{\mathrm{MASP2_{zym}}}}\\+\frac{\left[\mathrm{pCLL1MASP1_{zym}}\right]}{K_{\mathrm{m\ MASP1_{zym}}}^{\mathrm{MASP1_{zym}}}}+\frac{\left[\mathrm{pCLL1MASP2_{zym}}\right]}{K_{\mathrm{m\ MASP1_{zym}}}^{\mathrm{MASP2_{zym}}}}+\frac{\left[\mathrm{pLFMASP1_{zym}}\right]}{K_{\mathrm{m\ MASP1_{zym}}}^{\mathrm{MASP1_{zym}}}}+\frac{\left[\mathrm{pLFMASP2_{zym}}\right]}{K_{\mathrm{m\ MASP1_{zym}}}^{\mathrm{MASP2_{zym}}}}\\+\frac{\left[\mathrm{pHFMASP1_{zym}}\right]}{K_{\mathrm{m\ MASP1_{zym}}}^{\mathrm{MASP1_{zym}}}}+\frac{\left[\mathrm{pHFMASP2_{zym}}\right]}{K_{\mathrm{m\ MASP1_{zym}}}^{\mathrm{MASP2_{zym}}}}+\frac{\left[\mathrm{pMFMASP1_{zym}}\right]}{K_{\mathrm{m\ MASP1_{zym}}}^{\mathrm{MASP1_{zym}}}}+\frac{\left[\mathrm{pMFMASP2_{zym}}\right]}{K_{\mathrm{m\ MASP1_{zym}}}^{\mathrm{MASP2_{zym}}}}\\+\frac{\left[\mathrm{pPTX3LFMASP1_{zym}}\right]}{K_{\mathrm{m\ MASP1_{zym}}}^{\mathrm{MASP1_{zym}}}}+\frac{\left[\mathrm{pPTX3LFMASP2_{zym}}\right]}{K_{\mathrm{m\ MASP1_{zym}}}^{\mathrm{MASP2_{zym}}}}\end{array}\right)}$$

$$+\frac{k_{\text{cat MASP1}_{\text{zym}}}^{\text{MASP2}_{\text{zym}}}\left[\text{pHFMASP1}_{\text{zym}}\right]\left[\text{pCLLKMASP2}_{\text{zym}}\right]}{K_{\text{m MASP1}_{\text{zym}}}^{\text{MASP2}_{\text{zym}}}\left(\begin{array}{l}1+\frac{\left[\text{pMBL}_3\text{MASP1}_{\text{zym}}\right]}{K_{\text{m MASP1}_{\text{zym}}}^{\text{MASP1}_{\text{zym}}}}+\frac{\left[\text{pMBL}_3\text{MASP2}_{\text{zym}}\right]}{K_{\text{m MASP1}_{\text{zym}}}^{\text{MASP2}_{\text{zym}}}}+\frac{\left[\text{pMBL}_4\text{MASP1}_{\text{zym}}\right]}{K_{\text{m MASP1}_{\text{zym}}}^{\text{MASP1}_{\text{zym}}}}+\frac{\left[\text{pMBL}_4\text{MASP2}_{\text{zym}}\right]}{K_{\text{m MASP1}_{\text{zym}}}^{\text{MASP2}_{\text{zym}}}}\\+\frac{\left[\text{pCLLKMASP1}_{\text{zym}}\right]}{K_{\text{m MASP1}_{\text{zym}}}^{\text{MASP1}_{\text{zym}}}}+\frac{\left[\text{pCLLKMASP2}_{\text{zym}}\right]}{K_{\text{m MASP1}_{\text{zym}}}^{\text{MASP2}_{\text{zym}}}}+\frac{\left[\text{pCLK1MASP1}_{\text{zym}}\right]}{K_{\text{m MASP1}_{\text{zym}}}^{\text{MASP1}_{\text{zym}}}}+\frac{\left[\text{pCLK1MASP2}_{\text{zym}}\right]}{K_{\text{m MASP1}_{\text{zym}}}^{\text{MASP2}_{\text{zym}}}}\\+\frac{\left[\text{pCLL1MASP1}_{\text{zym}}\right]}{K_{\text{m MASP1}_{\text{zym}}}^{\text{MASP1}_{\text{zym}}}}+\frac{\left[\text{pCLL1MASP2}_{\text{zym}}\right]}{K_{\text{m MASP1}_{\text{zym}}}^{\text{MASP2}_{\text{zym}}}}+\frac{\left[\text{pLFMASP1}_{\text{zym}}\right]}{K_{\text{m MASP1}_{\text{zym}}}^{\text{MASP1}_{\text{zym}}}}+\frac{\left[\text{pLFMASP2}_{\text{zym}}\right]}{K_{\text{m MASP1}_{\text{zym}}}^{\text{MASP2}_{\text{zym}}}}\\+\frac{\left[\text{pHFMASP1}_{\text{zym}}\right]}{K_{\text{m MASP1}_{\text{zym}}}^{\text{MASP1}_{\text{zym}}}}+\frac{\left[\text{pHFMASP2}_{\text{zym}}\right]}{K_{\text{m MASP1}_{\text{zym}}}^{\text{MASP2}_{\text{zym}}}}+\frac{\left[\text{pMFMASP1}_{\text{zym}}\right]}{K_{\text{m MASP1}_{\text{zym}}}^{\text{MASP1}_{\text{zym}}}}+\frac{\left[\text{pMFMASP2}_{\text{zym}}\right]}{K_{\text{m MASP1}_{\text{zym}}}^{\text{MASP2}_{\text{zym}}}}\\+\frac{\left[\text{pPTX3LFMASP1}_{\text{zym}}\right]}{K_{\text{m MASP1}_{\text{zym}}}^{\text{MASP1}_{\text{zym}}}}+\frac{\left[\text{pPTX3LFMASP2}_{\text{zym}}\right]}{K_{\text{m MASP1}_{\text{zym}}}^{\text{MASP2}_{\text{zym}}}}\end{array}\right)}$$

$$+\frac{k_{\text{cat MASP1}_{\text{zym}}}^{\text{MASP2}_{\text{zym}}}\left[\text{pMFMASP1}_{\text{zym}}\right]\left[\text{pCLLKMASP2}_{\text{zym}}\right]}{K_{\text{m MASP1}_{\text{zym}}}^{\text{MASP2}_{\text{zym}}}\left(\begin{array}{l}1+\frac{\left[\text{pMBL}_3\text{MASP1}_{\text{zym}}\right]}{K_{\text{m MASP1}_{\text{zym}}}^{\text{MASP1}_{\text{zym}}}}+\frac{\left[\text{pMBL}_3\text{MASP2}_{\text{zym}}\right]}{K_{\text{m MASP1}_{\text{zym}}}^{\text{MASP2}_{\text{zym}}}}+\frac{\left[\text{pMBL}_4\text{MASP1}_{\text{zym}}\right]}{K_{\text{m MASP1}_{\text{zym}}}^{\text{MASP1}_{\text{zym}}}}+\frac{\left[\text{pMBL}_4\text{MASP2}_{\text{zym}}\right]}{K_{\text{m MASP1}_{\text{zym}}}^{\text{MASP2}_{\text{zym}}}}\\+\frac{\left[\text{pCLLKMASP1}_{\text{zym}}\right]}{K_{\text{m MASP1}_{\text{zym}}}^{\text{MASP1}_{\text{zym}}}}+\frac{\left[\text{pCLLKMASP2}_{\text{zym}}\right]}{K_{\text{m MASP1}_{\text{zym}}}^{\text{MASP2}_{\text{zym}}}}+\frac{\left[\text{pCLK1MASP1}_{\text{zym}}\right]}{K_{\text{m MASP1}_{\text{zym}}}^{\text{MASP1}_{\text{zym}}}}+\frac{\left[\text{pCLK1MASP2}_{\text{zym}}\right]}{K_{\text{m MASP1}_{\text{zym}}}^{\text{MASP2}_{\text{zym}}}}\\+\frac{\left[\text{pCLL1MASP1}_{\text{zym}}\right]}{K_{\text{m MASP1}_{\text{zym}}}^{\text{MASP1}_{\text{zym}}}}+\frac{\left[\text{pCLL1MASP2}_{\text{zym}}\right]}{K_{\text{m MASP1}_{\text{zym}}}^{\text{MASP2}_{\text{zym}}}}+\frac{\left[\text{pLFMASP1}_{\text{zym}}\right]}{K_{\text{m MASP1}_{\text{zym}}}^{\text{MASP1}_{\text{zym}}}}+\frac{\left[\text{pLFMASP2}_{\text{zym}}\right]}{K_{\text{m MASP1}_{\text{zym}}}^{\text{MASP2}_{\text{zym}}}}\\+\frac{\left[\text{pHFMASP1}_{\text{zym}}\right]}{K_{\text{m MASP1}_{\text{zym}}}^{\text{MASP1}_{\text{zym}}}}+\frac{\left[\text{pHFMASP2}_{\text{zym}}\right]}{K_{\text{m MASP1}_{\text{zym}}}^{\text{MASP2}_{\text{zym}}}}+\frac{\left[\text{pMFMASP1}_{\text{zym}}\right]}{K_{\text{m MASP1}_{\text{zym}}}^{\text{MASP1}_{\text{zym}}}}+\frac{\left[\text{pMFMASP2}_{\text{zym}}\right]}{K_{\text{m MASP1}_{\text{zym}}}^{\text{MASP2}_{\text{zym}}}}\\+\frac{\left[\text{pPTX3LFMASP1}_{\text{zym}}\right]}{K_{\text{m MASP1}_{\text{zym}}}^{\text{MASP1}_{\text{zym}}}}+\frac{\left[\text{pPTX3LFMASP2}_{\text{zym}}\right]}{K_{\text{m MASP1}_{\text{zym}}}^{\text{MASP2}_{\text{zym}}}}\end{array}\right)}$$

$$+\frac{k_{\text{cat MASP1}_{\text{zym}}}^{\text{MASP2}_{\text{zym}}}\left[\text{pPTX3LFMASP1}_{\text{zym}}\right]\left[\text{pCLLKMASP2}_{\text{zym}}\right]}{K_{\text{m MASP1}_{\text{zym}}}^{\text{MASP2}_{\text{zym}}}\left(\begin{array}{l}1+\frac{\left[\text{pMBL}_3\text{MASP1}_{\text{zym}}\right]}{K_{\text{m MASP1}_{\text{zym}}}^{\text{MASP1}_{\text{zym}}}}+\frac{\left[\text{pMBL}_3\text{MASP2}_{\text{zym}}\right]}{K_{\text{m MASP1}_{\text{zym}}}^{\text{MASP2}_{\text{zym}}}}+\frac{\left[\text{pMBL}_4\text{MASP1}_{\text{zym}}\right]}{K_{\text{m MASP1}_{\text{zym}}}^{\text{MASP1}_{\text{zym}}}}+\frac{\left[\text{pMBL}_4\text{MASP2}_{\text{zym}}\right]}{K_{\text{m MASP1}_{\text{zym}}}^{\text{MASP2}_{\text{zym}}}}\\+\frac{\left[\text{pCLLKMASP1}_{\text{zym}}\right]}{K_{\text{m MASP1}_{\text{zym}}}^{\text{MASP1}_{\text{zym}}}}+\frac{\left[\text{pCLLKMASP2}_{\text{zym}}\right]}{K_{\text{m MASP1}_{\text{zym}}}^{\text{MASP2}_{\text{zym}}}}+\frac{\left[\text{pCLK1MASP1}_{\text{zym}}\right]}{K_{\text{m MASP1}_{\text{zym}}}^{\text{MASP1}_{\text{zym}}}}+\frac{\left[\text{pCLK1MASP2}_{\text{zym}}\right]}{K_{\text{m MASP1}_{\text{zym}}}^{\text{MASP2}_{\text{zym}}}}\\+\frac{\left[\text{pCLL1MASP1}_{\text{zym}}\right]}{K_{\text{m MASP1}_{\text{zym}}}^{\text{MASP1}_{\text{zym}}}}+\frac{\left[\text{pCLL1MASP2}_{\text{zym}}\right]}{K_{\text{m MASP1}_{\text{zym}}}^{\text{MASP2}_{\text{zym}}}}+\frac{\left[\text{pLFMASP1}_{\text{zym}}\right]}{K_{\text{m MASP1}_{\text{zym}}}^{\text{MASP1}_{\text{zym}}}}+\frac{\left[\text{pLFMASP2}_{\text{zym}}\right]}{K_{\text{m MASP1}_{\text{zym}}}^{\text{MASP2}_{\text{zym}}}}\\+\frac{\left[\text{pHFMASP1}_{\text{zym}}\right]}{K_{\text{m MASP1}_{\text{zym}}}^{\text{MASP1}_{\text{zym}}}}+\frac{\left[\text{pHFMASP2}_{\text{zym}}\right]}{K_{\text{m MASP1}_{\text{zym}}}^{\text{MASP2}_{\text{zym}}}}+\frac{\left[\text{pMFMASP1}_{\text{zym}}\right]}{K_{\text{m MASP1}_{\text{zym}}}^{\text{MASP1}_{\text{zym}}}}+\frac{\left[\text{pMFMASP2}_{\text{zym}}\right]}{K_{\text{m MASP1}_{\text{zym}}}^{\text{MASP2}_{\text{zym}}}}\\+\frac{\left[\text{pPTX3LFMASP1}_{\text{zym}}\right]}{K_{\text{m MASP1}_{\text{zym}}}^{\text{MASP1}_{\text{zym}}}}+\frac{\left[\text{pPTX3LFMASP2}_{\text{zym}}\right]}{K_{\text{m MASP1}_{\text{zym}}}^{\text{MASP2}_{\text{zym}}}}\end{array}\right)}$$

$$+\frac{k_{\text{cat MASP2}_{\text{zym}}}^{\text{MASP2}_{\text{zym}}}\left[\text{pMBL}_3\text{MASP2}_{\text{zym}}\right]\left[\text{pCLLKMASP2}_{\text{zym}}\right]}{K_{\text{m MASP2}_{\text{zym}}}^{\text{MASP2}_{\text{zym}}}\left(\begin{array}{l}1+\frac{\left[\text{pMBL}_3\text{MASP2}_{\text{zym}}\right]}{K_{\text{m MASP2}_{\text{zym}}}^{\text{MASP2}_{\text{zym}}}}+\frac{\left[\text{pMBL}_4\text{MASP2}_{\text{zym}}\right]}{K_{\text{m MASP2}_{\text{zym}}}^{\text{MASP2}_{\text{zym}}}}+\frac{\left[\text{pCLLKMASP2}_{\text{zym}}\right]}{K_{\text{m MASP2}_{\text{zym}}}^{\text{MASP2}_{\text{zym}}}}+\frac{\left[\text{pCLK1MASP2}_{\text{zym}}\right]}{K_{\text{m MASP2}_{\text{zym}}}^{\text{MASP2}_{\text{zym}}}}\\+\frac{\left[\text{pCLL1MASP2}_{\text{zym}}\right]}{K_{\text{m MASP2}_{\text{zym}}}^{\text{MASP2}_{\text{zym}}}}+\frac{\left[\text{pLFMASP2}_{\text{zym}}\right]}{K_{\text{m MASP2}_{\text{zym}}}^{\text{MASP2}_{\text{zym}}}}+\frac{\left[\text{pHFMASP2}_{\text{zym}}\right]}{K_{\text{m MASP2}_{\text{zym}}}^{\text{MASP2}_{\text{zym}}}}+\frac{\left[\text{pMFMASP2}_{\text{zym}}\right]}{K_{\text{m MASP2}_{\text{zym}}}^{\text{MASP2}_{\text{zym}}}}\\+\frac{\left[\text{pPTX3LFMASP2}_{\text{zym}}\right]}{K_{\text{m MASP2}_{\text{zym}}}^{\text{MASP2}_{\text{zym}}}}\end{array}\right)}$$

$$+\frac{k_{\text{cat MASP2}_{\text{zym}}}^{\text{MASP2}_{\text{zym}}}\left[\text{pMBL}_4\text{MASP2}_{\text{zym}}\right]\left[\text{pCLLKMASP2}_{\text{zym}}\right]}{K_{\text{m MASP2}_{\text{zym}}}^{\text{MASP2}_{\text{zym}}}\left(\begin{array}{l}1+\frac{\left[\text{pMBL}_3\text{MASP2}_{\text{zym}}\right]}{K_{\text{m MASP2}_{\text{zym}}}^{\text{MASP2}_{\text{zym}}}}+\frac{\left[\text{pMBL}_4\text{MASP2}_{\text{zym}}\right]}{K_{\text{m MASP2}_{\text{zym}}}^{\text{MASP2}_{\text{zym}}}}+\frac{\left[\text{pCLLKMASP2}_{\text{zym}}\right]}{K_{\text{m MASP2}_{\text{zym}}}^{\text{MASP2}_{\text{zym}}}}+\frac{\left[\text{pCLK1MASP2}_{\text{zym}}\right]}{K_{\text{m MASP2}_{\text{zym}}}^{\text{MASP2}_{\text{zym}}}}\\+\frac{\left[\text{pCLL1MASP2}_{\text{zym}}\right]}{K_{\text{m MASP2}_{\text{zym}}}^{\text{MASP2}_{\text{zym}}}}+\frac{\left[\text{pLFMASP2}_{\text{zym}}\right]}{K_{\text{m MASP2}_{\text{zym}}}^{\text{MASP2}_{\text{zym}}}}+\frac{\left[\text{pHFMASP2}_{\text{zym}}\right]}{K_{\text{m MASP2}_{\text{zym}}}^{\text{MASP2}_{\text{zym}}}}+\frac{\left[\text{pMFMASP2}_{\text{zym}}\right]}{K_{\text{m MASP2}_{\text{zym}}}^{\text{MASP2}_{\text{zym}}}}\\+\frac{\left[\text{pPTX3LFMASP2}_{\text{zym}}\right]}{K_{\text{m MASP2}_{\text{zym}}}^{\text{MASP2}_{\text{zym}}}}\end{array}\right)}$$

$$+\frac{k_{\mathrm{cat\ MASP2_{zym}}}^{\mathrm{MASP2_{zym}}}\left[\mathrm{pCLLKMASP2_{zym}}\right]\left[\mathrm{pCLLKMASP2_{zym}}\right]}{K_{\mathrm{m\ MASP2_{zym}}}^{\mathrm{MASP2_{zym}}}\left(\begin{array}{l}1+\frac{\left[\mathrm{pMBL_3MASP2_{zym}}\right]}{K_{\mathrm{m\ MASP2_{zym}}}^{\mathrm{MASP2_{zym}}}}+\frac{\left[\mathrm{pMBL_4MASP2_{zym}}\right]}{K_{\mathrm{m\ MASP2_{zym}}}^{\mathrm{MASP2_{zym}}}}+\frac{\left[\mathrm{pCLLKMASP2_{zym}}\right]}{K_{\mathrm{m\ MASP2_{zym}}}^{\mathrm{MASP2_{zym}}}}+\frac{\left[\mathrm{pCLK1MASP2_{zym}}\right]}{K_{\mathrm{m\ MASP2_{zym}}}^{\mathrm{MASP2_{zym}}}}\\+\frac{\left[\mathrm{pCLL1MASP2_{zym}}\right]}{K_{\mathrm{m\ MASP2_{zym}}}^{\mathrm{MASP2_{zym}}}}+\frac{\left[\mathrm{pLFMASP2_{zym}}\right]}{K_{\mathrm{m\ MASP2_{zym}}}^{\mathrm{MASP2_{zym}}}}+\frac{\left[\mathrm{pHFMASP2_{zym}}\right]}{K_{\mathrm{m\ MASP2_{zym}}}^{\mathrm{MASP2_{zym}}}}+\frac{\left[\mathrm{pMFMASP2_{zym}}\right]}{K_{\mathrm{m\ MASP2_{zym}}}^{\mathrm{MASP2_{zym}}}}\\+\frac{\left[\mathrm{pPTX3LFMASP2_{zym}}\right]}{K_{\mathrm{m\ MASP2_{zym}}}^{\mathrm{MASP2_{zym}}}}\end{array}\right)}$$

$$+\frac{k_{\mathrm{cat\ MASP2_{zym}}}^{\mathrm{MASP2_{zym}}}\left[\mathrm{pCLK1MASP2_{zym}}\right]\left[\mathrm{pCLLKMASP2_{zym}}\right]}{K_{\mathrm{m\ MASP2_{zym}}}^{\mathrm{MASP2_{zym}}}\left(\begin{array}{l}1+\frac{\left[\mathrm{pMBL_3MASP2_{zym}}\right]}{K_{\mathrm{m\ MASP2_{zym}}}^{\mathrm{MASP2_{zym}}}}+\frac{\left[\mathrm{pMBL_4MASP2_{zym}}\right]}{K_{\mathrm{m\ MASP2_{zym}}}^{\mathrm{MASP2_{zym}}}}+\frac{\left[\mathrm{pCLLKMASP2_{zym}}\right]}{K_{\mathrm{m\ MASP2_{zym}}}^{\mathrm{MASP2_{zym}}}}+\frac{\left[\mathrm{pCLK1MASP2_{zym}}\right]}{K_{\mathrm{m\ MASP2_{zym}}}^{\mathrm{MASP2_{zym}}}}\\+\frac{\left[\mathrm{pCLL1MASP2_{zym}}\right]}{K_{\mathrm{m\ MASP2_{zym}}}^{\mathrm{MASP2_{zym}}}}+\frac{\left[\mathrm{pLFMASP2_{zym}}\right]}{K_{\mathrm{m\ MASP2_{zym}}}^{\mathrm{MASP2_{zym}}}}+\frac{\left[\mathrm{pHFMASP2_{zym}}\right]}{K_{\mathrm{m\ MASP2_{zym}}}^{\mathrm{MASP2_{zym}}}}+\frac{\left[\mathrm{pMFMASP2_{zym}}\right]}{K_{\mathrm{m\ MASP2_{zym}}}^{\mathrm{MASP2_{zym}}}}\\+\frac{\left[\mathrm{pPTX3LFMASP2_{zym}}\right]}{K_{\mathrm{m\ MASP2_{zym}}}^{\mathrm{MASP2_{zym}}}}\end{array}\right)}$$

$$+\frac{k_{\mathrm{cat\ MASP2_{zym}}}^{\mathrm{MASP2_{zym}}}\left[\mathrm{pCLL1MASP2_{zym}}\right]\left[\mathrm{pCLLKMASP2_{zym}}\right]}{K_{\mathrm{m\ MASP2_{zym}}}^{\mathrm{MASP2_{zym}}}\left(\begin{array}{l}1+\frac{\left[\mathrm{pMBL_3MASP2_{zym}}\right]}{K_{\mathrm{m\ MASP2_{zym}}}^{\mathrm{MASP2_{zym}}}}+\frac{\left[\mathrm{pMBL_4MASP2_{zym}}\right]}{K_{\mathrm{m\ MASP2_{zym}}}^{\mathrm{MASP2_{zym}}}}+\frac{\left[\mathrm{pCLLKMASP2_{zym}}\right]}{K_{\mathrm{m\ MASP2_{zym}}}^{\mathrm{MASP2_{zym}}}}+\frac{\left[\mathrm{pCLK1MASP2_{zym}}\right]}{K_{\mathrm{m\ MASP2_{zym}}}^{\mathrm{MASP2_{zym}}}}\\+\frac{\left[\mathrm{pCLL1MASP2_{zym}}\right]}{K_{\mathrm{m\ MASP2_{zym}}}^{\mathrm{MASP2_{zym}}}}+\frac{\left[\mathrm{pLFMASP2_{zym}}\right]}{K_{\mathrm{m\ MASP2_{zym}}}^{\mathrm{MASP2_{zym}}}}+\frac{\left[\mathrm{pHFMASP2_{zym}}\right]}{K_{\mathrm{m\ MASP2_{zym}}}^{\mathrm{MASP2_{zym}}}}+\frac{\left[\mathrm{pMFMASP2_{zym}}\right]}{K_{\mathrm{m\ MASP2_{zym}}}^{\mathrm{MASP2_{zym}}}}\\+\frac{\left[\mathrm{pPTX3LFMASP2_{zym}}\right]}{K_{\mathrm{m\ MASP2_{zym}}}^{\mathrm{MASP2_{zym}}}}\end{array}\right)}$$

$$+\frac{k_{\mathrm{cat\ MASP2_{zym}}}^{\mathrm{MASP2_{zym}}}\left[\mathrm{pLFMASP2_{zym}}\right]\left[\mathrm{pCLLKMASP2_{zym}}\right]}{K_{\mathrm{m\ MASP2_{zym}}}^{\mathrm{MASP2_{zym}}}\left(\begin{array}{l}1+\frac{\left[\mathrm{pMBL_3MASP2_{zym}}\right]}{K_{\mathrm{m\ MASP2_{zym}}}^{\mathrm{MASP2_{zym}}}}+\frac{\left[\mathrm{pMBL_4MASP2_{zym}}\right]}{K_{\mathrm{m\ MASP2_{zym}}}^{\mathrm{MASP2_{zym}}}}+\frac{\left[\mathrm{pCLLKMASP2_{zym}}\right]}{K_{\mathrm{m\ MASP2_{zym}}}^{\mathrm{MASP2_{zym}}}}+\frac{\left[\mathrm{pCLK1MASP2_{zym}}\right]}{K_{\mathrm{m\ MASP2_{zym}}}^{\mathrm{MASP2_{zym}}}}\\+\frac{\left[\mathrm{pCLL1MASP2_{zym}}\right]}{K_{\mathrm{m\ MASP2_{zym}}}^{\mathrm{MASP2_{zym}}}}+\frac{\left[\mathrm{pLFMASP2_{zym}}\right]}{K_{\mathrm{m\ MASP2_{zym}}}^{\mathrm{MASP2_{zym}}}}+\frac{\left[\mathrm{pHFMASP2_{zym}}\right]}{K_{\mathrm{m\ MASP2_{zym}}}^{\mathrm{MASP2_{zym}}}}+\frac{\left[\mathrm{pMFMASP2_{zym}}\right]}{K_{\mathrm{m\ MASP2_{zym}}}^{\mathrm{MASP2_{zym}}}}\\+\frac{\left[\mathrm{pPTX3LFMASP2_{zym}}\right]}{K_{\mathrm{m\ MASP2_{zym}}}^{\mathrm{MASP2_{zym}}}}\end{array}\right)}$$

$$+\frac{k_{\text{cat MASP2}_{\text{zym}}}^{\text{MASP2}_{\text{zym}}}\left[\text{pHFMASP2}_{\text{zym}}\right]\left[\text{pCLLKMASP2}_{\text{zym}}\right]}{K_{\text{m MASP2}_{\text{zym}}}^{\text{MASP2}_{\text{zym}}}\left(\begin{array}{l}1+\frac{\left[\text{pMBL}_3\text{MASP2}_{\text{zym}}\right]}{K_{\text{m MASP2}_{\text{zym}}}^{\text{MASP2}_{\text{zym}}}}+\frac{\left[\text{pMBL}_4\text{MASP2}_{\text{zym}}\right]}{K_{\text{m MASP2}_{\text{zym}}}^{\text{MASP2}_{\text{zym}}}}+\frac{\left[\text{pCLLKMASP2}_{\text{zym}}\right]}{K_{\text{m MASP2}_{\text{zym}}}^{\text{MASP2}_{\text{zym}}}}+\frac{\left[\text{pCLK1MASP2}_{\text{zym}}\right]}{K_{\text{m MASP2}_{\text{zym}}}^{\text{MASP2}_{\text{zym}}}}\\+\frac{\left[\text{pCLL1MASP2}_{\text{zym}}\right]}{K_{\text{m MASP2}_{\text{zym}}}^{\text{MASP2}_{\text{zym}}}}+\frac{\left[\text{pLFMASP2}_{\text{zym}}\right]}{K_{\text{m MASP2}_{\text{zym}}}^{\text{MASP2}_{\text{zym}}}}+\frac{\left[\text{pHFMASP2}_{\text{zym}}\right]}{K_{\text{m MASP2}_{\text{zym}}}^{\text{MASP2}_{\text{zym}}}}+\frac{\left[\text{pMFMASP2}_{\text{zym}}\right]}{K_{\text{m MASP2}_{\text{zym}}}^{\text{MASP2}_{\text{zym}}}}\\+\frac{\left[\text{pPTX3LFMASP2}_{\text{zym}}\right]}{K_{\text{m MASP2}_{\text{zym}}}^{\text{MASP2}_{\text{zym}}}}\end{array}\right)}$$

$$+\frac{k_{\text{cat MASP2}_{\text{zym}}}^{\text{MASP2}_{\text{zym}}}\left[\text{pMFMASP2}_{\text{zym}}\right]\left[\text{pCLLKMASP2}_{\text{zym}}\right]}{K_{\text{m MASP2}_{\text{zym}}}^{\text{MASP2}_{\text{zym}}}\left(\begin{array}{l}1+\frac{\left[\text{pMBL}_3\text{MASP2}_{\text{zym}}\right]}{K_{\text{m MASP2}_{\text{zym}}}^{\text{MASP2}_{\text{zym}}}}+\frac{\left[\text{pMBL}_4\text{MASP2}_{\text{zym}}\right]}{K_{\text{m MASP2}_{\text{zym}}}^{\text{MASP2}_{\text{zym}}}}+\frac{\left[\text{pCLLKMASP2}_{\text{zym}}\right]}{K_{\text{m MASP2}_{\text{zym}}}^{\text{MASP2}_{\text{zym}}}}+\frac{\left[\text{pCLK1MASP2}_{\text{zym}}\right]}{K_{\text{m MASP2}_{\text{zym}}}^{\text{MASP2}_{\text{zym}}}}\\+\frac{\left[\text{pCLL1MASP2}_{\text{zym}}\right]}{K_{\text{m MASP2}_{\text{zym}}}^{\text{MASP2}_{\text{zym}}}}+\frac{\left[\text{pLFMASP2}_{\text{zym}}\right]}{K_{\text{m MASP2}_{\text{zym}}}^{\text{MASP2}_{\text{zym}}}}+\frac{\left[\text{pHFMASP2}_{\text{zym}}\right]}{K_{\text{m MASP2}_{\text{zym}}}^{\text{MASP2}_{\text{zym}}}}+\frac{\left[\text{pMFMASP2}_{\text{zym}}\right]}{K_{\text{m MASP2}_{\text{zym}}}^{\text{MASP2}_{\text{zym}}}}\\+\frac{\left[\text{pPTX3LFMASP2}_{\text{zym}}\right]}{K_{\text{m MASP2}_{\text{zym}}}^{\text{MASP2}_{\text{zym}}}}\end{array}\right)}$$

$$+\frac{k_{\text{cat MASP2}_{\text{zym}}}^{\text{MASP2}_{\text{zym}}}\left[\text{pPTX3LFMASP2}_{\text{zym}}\right]\left[\text{pCLLKMASP2}_{\text{zym}}\right]}{K_{\text{m MASP2}_{\text{zym}}}^{\text{MASP2}_{\text{zym}}}\left(\begin{array}{l}1+\frac{\left[\text{pMBL}_3\text{MASP2}_{\text{zym}}\right]}{K_{\text{m MASP2}_{\text{zym}}}^{\text{MASP2}_{\text{zym}}}}+\frac{\left[\text{pMBL}_4\text{MASP2}_{\text{zym}}\right]}{K_{\text{m MASP2}_{\text{zym}}}^{\text{MASP2}_{\text{zym}}}}+\frac{\left[\text{pCLLKMASP2}_{\text{zym}}\right]}{K_{\text{m MASP2}_{\text{zym}}}^{\text{MASP2}_{\text{zym}}}}+\frac{\left[\text{pCLK1MASP2}_{\text{zym}}\right]}{K_{\text{m MASP2}_{\text{zym}}}^{\text{MASP2}_{\text{zym}}}}\\+\frac{\left[\text{pCLL1MASP2}_{\text{zym}}\right]}{K_{\text{m MASP2}_{\text{zym}}}^{\text{MASP2}_{\text{zym}}}}+\frac{\left[\text{pLFMASP2}_{\text{zym}}\right]}{K_{\text{m MASP2}_{\text{zym}}}^{\text{MASP2}_{\text{zym}}}}+\frac{\left[\text{pHFMASP2}_{\text{zym}}\right]}{K_{\text{m MASP2}_{\text{zym}}}^{\text{MASP2}_{\text{zym}}}}+\frac{\left[\text{pMFMASP2}_{\text{zym}}\right]}{K_{\text{m MASP2}_{\text{zym}}}^{\text{MASP2}_{\text{zym}}}}\\+\frac{\left[\text{pPTX3LFMASP2}_{\text{zym}}\right]}{K_{\text{m MASP2}_{\text{zym}}}^{\text{MASP2}_{\text{zym}}}}\end{array}\right)}$$

$$
+\frac{k_{\mathrm{cat\ MASP1}^*}^{\mathrm{MASP2_{zym}}}\left[\mathrm{pMBL_3MASP1}^*\right]\left[\mathrm{pCLLKMASP2_{zym}}\right]}{K_{\mathrm{m\ MASP1}^*}^{\mathrm{MASP2_{zym}}}\left(\begin{array}{l}
1+\frac{[\mathrm{C4}]}{K_{\mathrm{m\ MASP1}^*}^{\mathrm{C4}}}+\frac{[\mathrm{fC4bC2}]}{K_{\mathrm{m\ MASP1}^*}^{\mathrm{C2}}}+\frac{[\mathrm{fC4bC4bC2}]}{K_{\mathrm{m\ MASP1}^*}^{\mathrm{C2}}}+\frac{[\mathrm{fC3bC4bC2}]}{K_{\mathrm{m\ MASP1}^*}^{\mathrm{C2}}}+\frac{[\mathrm{pC4bC2}]}{K_{\mathrm{m\ MASP1}^*}^{\mathrm{C2}}}+\frac{[\mathrm{pC4bC4bC2}]}{K_{\mathrm{m\ MASP1}^*}^{\mathrm{C2}}}\\
+\frac{[\mathrm{pC3bC4bC2}]}{K_{\mathrm{m\ MASP1}^*}^{\mathrm{C2}}}+\frac{\left[\mathrm{pMBL_3MASP1_{zym}}\right]}{K_{\mathrm{m\ MASP1}^*}^{\mathrm{MASP1_{zym}}}}+\frac{\left[\mathrm{pMBL_3MASP2_{zym}}\right]}{K_{\mathrm{m\ MASP1}^*}^{\mathrm{MASP2_{zym}}}}+\frac{\left[\mathrm{pMBL_3MASP3_{zym}}\right]}{K_{\mathrm{m\ MASP1}^*}^{\mathrm{MASP3_{zym}}}}\\
+\frac{\left[\mathrm{pMBL_4MASP1_{zym}}\right]}{K_{\mathrm{m\ MASP1}^*}^{\mathrm{MASP1_{zym}}}}+\frac{\left[\mathrm{pMBL_4MASP2_{zym}}\right]}{K_{\mathrm{m\ MASP1}^*}^{\mathrm{MASP2_{zym}}}}+\frac{\left[\mathrm{pMBL_4MASP3_{zym}}\right]}{K_{\mathrm{m\ MASP1}^*}^{\mathrm{MASP3_{zym}}}}+\frac{\left[\mathrm{pCLLKMASP1_{zym}}\right]}{K_{\mathrm{m\ MASP1}^*}^{\mathrm{MASP1_{zym}}}}\\
+\frac{\left[\mathrm{pCLLKMASP2_{zym}}\right]}{K_{\mathrm{m\ MASP1}^*}^{\mathrm{MASP2_{zym}}}}+\frac{\left[\mathrm{pCLLKMASP3_{zym}}\right]}{K_{\mathrm{m\ MASP1}^*}^{\mathrm{MASP3_{zym}}}}+\frac{\left[\mathrm{pCLK1MASP1_{zym}}\right]}{K_{\mathrm{m\ MASP1}^*}^{\mathrm{MASP1_{zym}}}}+\frac{\left[\mathrm{pCLK1MASP2_{zym}}\right]}{K_{\mathrm{m\ MASP1}^*}^{\mathrm{MASP2_{zym}}}}\\
+\frac{\left[\mathrm{pCLK1MASP3_{zym}}\right]}{K_{\mathrm{m\ MASP1}^*}^{\mathrm{MASP3_{zym}}}}+\frac{\left[\mathrm{pCLL1MASP1_{zym}}\right]}{K_{\mathrm{m\ MASP1}^*}^{\mathrm{MASP1_{zym}}}}+\frac{\left[\mathrm{pCLL1MASP2_{zym}}\right]}{K_{\mathrm{m\ MASP1}^*}^{\mathrm{MASP2_{zym}}}}+\frac{\left[\mathrm{pCLL1MASP3_{zym}}\right]}{K_{\mathrm{m\ MASP1}^*}^{\mathrm{MASP3_{zym}}}}\\
+\frac{\left[\mathrm{pLFMASP1_{zym}}\right]}{K_{\mathrm{m\ MASP1}^*}^{\mathrm{MASP1_{zym}}}}+\frac{\left[\mathrm{pLFMASP2_{zym}}\right]}{K_{\mathrm{m\ MASP1}^*}^{\mathrm{MASP2_{zym}}}}+\frac{\left[\mathrm{pLFMASP3_{zym}}\right]}{K_{\mathrm{m\ MASP1}^*}^{\mathrm{MASP3_{zym}}}}+\frac{\left[\mathrm{pHFMASP1_{zym}}\right]}{K_{\mathrm{m\ MASP1}^*}^{\mathrm{MASP1_{zym}}}}+\frac{\left[\mathrm{pHFMASP2_{zym}}\right]}{K_{\mathrm{m\ MASP1}^*}^{\mathrm{MASP2_{zym}}}}\\
+\frac{\left[\mathrm{pHFMASP3_{zym}}\right]}{K_{\mathrm{m\ MASP1}^*}^{\mathrm{MASP3_{zym}}}}+\frac{\left[\mathrm{pMFMASP1_{zym}}\right]}{K_{\mathrm{m\ MASP1}^*}^{\mathrm{MASP1_{zym}}}}+\frac{\left[\mathrm{pMFMASP2_{zym}}\right]}{K_{\mathrm{m\ MASP1}^*}^{\mathrm{MASP2_{zym}}}}+\frac{\left[\mathrm{pMFMASP3_{zym}}\right]}{K_{\mathrm{m\ MASP1}^*}^{\mathrm{MASP3_{zym}}}}\\
+\frac{\left[\mathrm{pPTX3LFMASP1_{zym}}\right]}{K_{\mathrm{m\ MASP1}^*}^{\mathrm{MASP1_{zym}}}}+\frac{\left[\mathrm{pPXT3LFMASP2_{zym}}\right]}{K_{\mathrm{m\ MASP1}^*}^{\mathrm{MASP2_{zym}}}}+\frac{\left[\mathrm{pPTX3LFMASP3_{zym}}\right]}{K_{\mathrm{m\ MASP1}^*}^{\mathrm{MASP3_{zym}}}}
\end{array}\right)}
$$

$$+\frac{k_{\text{cat MASP1}^*}^{\text{MASP2}_{\text{zym}}}\left[\text{pMBL}_4\text{MASP1}^*\right]\left[\text{pCLLKMASP2}_{\text{zym}}\right]}{K_{\text{m MASP1}^*}^{\text{MASP2}_{\text{zym}}}\left(\begin{array}{l}1+\frac{[\text{C4}]}{K_{\text{m MASP1}^*}^{\text{C4}}}+\frac{[\text{fC4bC2}]}{K_{\text{m MASP1}^*}^{\text{C2}}}+\frac{[\text{fC4bC4bC2}]}{K_{\text{m MASP1}^*}^{\text{C2}}}+\frac{[\text{fC3bC4bC2}]}{K_{\text{m MASP1}^*}^{\text{C2}}}+\frac{[\text{pC4bC2}]}{K_{\text{m MASP1}^*}^{\text{C2}}}+\frac{[\text{pC4bC4bC2}]}{K_{\text{m MASP1}^*}^{\text{C2}}}\\ +\frac{[\text{pC3bC4bC2}]}{K_{\text{m MASP1}^*}^{\text{C2}}}+\frac{[\text{pMBL}_3\text{MASP1}_{\text{zym}}]}{K_{\text{m MASP1}^*}^{\text{MASP1}_{\text{zym}}}}+\frac{[\text{pMBL}_3\text{MASP2}_{\text{zym}}]}{K_{\text{m MASP1}^*}^{\text{MASP2}_{\text{zym}}}}+\frac{[\text{pMBL}_3\text{MASP3}_{\text{zym}}]}{K_{\text{m MASP1}^*}^{\text{MASP3}_{\text{zym}}}}\\ +\frac{[\text{pMBL}_4\text{MASP1}_{\text{zym}}]}{K_{\text{m MASP1}^*}^{\text{MASP1}_{\text{zym}}}}+\frac{[\text{pMBL}_4\text{MASP2}_{\text{zym}}]}{K_{\text{m MASP1}^*}^{\text{MASP2}_{\text{zym}}}}+\frac{[\text{pMBL}_4\text{MASP3}_{\text{zym}}]}{K_{\text{m MASP1}^*}^{\text{MASP3}_{\text{zym}}}}+\frac{[\text{pCLLKMASP1}_{\text{zym}}]}{K_{\text{m MASP1}^*}^{\text{MASP1}_{\text{zym}}}}\\ +\frac{[\text{pCLLKMASP2}_{\text{zym}}]}{K_{\text{m MASP1}^*}^{\text{MASP2}_{\text{zym}}}}+\frac{[\text{pCLLKMASP3}_{\text{zym}}]}{K_{\text{m MASP1}^*}^{\text{MASP3}_{\text{zym}}}}+\frac{[\text{pCLK1MASP1}_{\text{zym}}]}{K_{\text{m MASP1}^*}^{\text{MASP1}_{\text{zym}}}}+\frac{[\text{pCLK1MASP2}_{\text{zym}}]}{K_{\text{m MASP1}^*}^{\text{MASP2}_{\text{zym}}}}\\ +\frac{[\text{pCLK1MASP3}_{\text{zym}}]}{K_{\text{m MASP1}^*}^{\text{MASP3}_{\text{zym}}}}+\frac{[\text{pCLL1MASP1}_{\text{zym}}]}{K_{\text{m MASP1}^*}^{\text{MASP1}_{\text{zym}}}}+\frac{[\text{pCLL1MASP2}_{\text{zym}}]}{K_{\text{m MASP1}^*}^{\text{MASP2}_{\text{zym}}}}+\frac{[\text{pCLL1MASP3}_{\text{zym}}]}{K_{\text{m MASP1}^*}^{\text{MASP3}_{\text{zym}}}}\\ +\frac{[\text{pLFMASP1}_{\text{zym}}]}{K_{\text{m MASP1}^*}^{\text{MASP1}_{\text{zym}}}}+\frac{[\text{pLFMASP2}_{\text{zym}}]}{K_{\text{m MASP1}^*}^{\text{MASP2}_{\text{zym}}}}+\frac{[\text{pLFMASP3}_{\text{zym}}]}{K_{\text{m MASP1}^*}^{\text{MASP3}_{\text{zym}}}}+\frac{[\text{pHFMASP1}_{\text{zym}}]}{K_{\text{m MASP1}^*}^{\text{MASP1}_{\text{zym}}}}+\frac{[\text{pHFMASP2}_{\text{zym}}]}{K_{\text{m MASP1}^*}^{\text{MASP2}_{\text{zym}}}}\\ +\frac{[\text{pHFMASP3}_{\text{zym}}]}{K_{\text{m MASP1}^*}^{\text{MASP3}_{\text{zym}}}}+\frac{[\text{pMFMASP1}_{\text{zym}}]}{K_{\text{m MASP1}^*}^{\text{MASP1}_{\text{zym}}}}+\frac{[\text{pMFMASP2}_{\text{zym}}]}{K_{\text{m MASP1}^*}^{\text{MASP2}_{\text{zym}}}}+\frac{[\text{pMFMASP3}_{\text{zym}}]}{K_{\text{m MASP1}^*}^{\text{MASP3}_{\text{zym}}}}\\ +\frac{[\text{pPTX3LFMASP1}_{\text{zym}}]}{K_{\text{m MASP1}^*}^{\text{MASP1}_{\text{zym}}}}+\frac{[\text{pPXT3LFMASP2}_{\text{zym}}]}{K_{\text{m MASP1}^*}^{\text{MASP2}_{\text{zym}}}}+\frac{[\text{pPTX3LFMASP3}_{\text{zym}}]}{K_{\text{m MASP1}^*}^{\text{MASP3}_{\text{zym}}}}\end{array}\right)}$$

$$+\frac{k_{\mathrm{cat\,MASP1^*}}^{\mathrm{MASP2_{zym}}}\left[\mathrm{pCLLKMASP1^*}\right]\left[\mathrm{pCLLKMASP2_{zym}}\right]}{K_{\mathrm{m\,MASP1^*}}^{\mathrm{MASP2_{zym}}}\left(\begin{array}{l}1+\frac{[\mathrm{C4}]}{K_{\mathrm{m\,MASP1^*}}^{\mathrm{C4}}}+\frac{[\mathrm{fC4bC2}]}{K_{\mathrm{m\,MASP1^*}}^{\mathrm{C2}}}+\frac{[\mathrm{fC4bC4bC2}]}{K_{\mathrm{m\,MASP1^*}}^{\mathrm{C2}}}+\frac{[\mathrm{fC3bC4bC2}]}{K_{\mathrm{m\,MASP1^*}}^{\mathrm{C2}}}+\frac{[\mathrm{pC4bC2}]}{K_{\mathrm{m\,MASP1^*}}^{\mathrm{C2}}}+\frac{[\mathrm{pC4bC4bC2}]}{K_{\mathrm{m\,MASP1^*}}^{\mathrm{C2}}}\\+\frac{[\mathrm{pC3bC4bC2}]}{K_{\mathrm{m\,MASP1^*}}^{\mathrm{C2}}}+\frac{[\mathrm{pMBL_3MASP1_{zym}}]}{K_{\mathrm{m\,MASP1^*}}^{\mathrm{MASP1_{zym}}}}+\frac{[\mathrm{pMBL_3MASP2_{zym}}]}{K_{\mathrm{m\,MASP1^*}}^{\mathrm{MASP2_{zym}}}}+\frac{[\mathrm{pMBL_3MASP3_{zym}}]}{K_{\mathrm{m\,MASP1^*}}^{\mathrm{MASP3_{zym}}}}\\+\frac{[\mathrm{pMBL_4MASP1_{zym}}]}{K_{\mathrm{m\,MASP1^*}}^{\mathrm{MASP1_{zym}}}}+\frac{[\mathrm{pMBL_4MASP2_{zym}}]}{K_{\mathrm{m\,MASP1^*}}^{\mathrm{MASP2_{zym}}}}+\frac{[\mathrm{pMBL_4MASP3_{zym}}]}{K_{\mathrm{m\,MASP1^*}}^{\mathrm{MASP3_{zym}}}}+\frac{[\mathrm{pCLLKMASP1_{zym}}]}{K_{\mathrm{m\,MASP1^*}}^{\mathrm{MASP1_{zym}}}}\\+\frac{[\mathrm{pCLLKMASP2_{zym}}]}{K_{\mathrm{m\,MASP1^*}}^{\mathrm{MASP2_{zym}}}}+\frac{[\mathrm{pCLLKMASP3_{zym}}]}{K_{\mathrm{m\,MASP1^*}}^{\mathrm{MASP3_{zym}}}}+\frac{[\mathrm{pCLK1MASP1_{zym}}]}{K_{\mathrm{m\,MASP1^*}}^{\mathrm{MASP1_{zym}}}}+\frac{[\mathrm{pCLK1MASP2_{zym}}]}{K_{\mathrm{m\,MASP1^*}}^{\mathrm{MASP2_{zym}}}}\\+\frac{[\mathrm{pCLK1MASP3_{zym}}]}{K_{\mathrm{m\,MASP1^*}}^{\mathrm{MASP3_{zym}}}}+\frac{[\mathrm{pCLL1MASP1_{zym}}]}{K_{\mathrm{m\,MASP1^*}}^{\mathrm{MASP1_{zym}}}}+\frac{[\mathrm{pCLL1MASP2_{zym}}]}{K_{\mathrm{m\,MASP1^*}}^{\mathrm{MASP2_{zym}}}}+\frac{[\mathrm{pCLL1MASP3_{zym}}]}{K_{\mathrm{m\,MASP1^*}}^{\mathrm{MASP3_{zym}}}}\\+\frac{[\mathrm{pLFMASP1_{zym}}]}{K_{\mathrm{m\,MASP1^*}}^{\mathrm{MASP1_{zym}}}}+\frac{[\mathrm{pLFMASP2_{zym}}]}{K_{\mathrm{m\,MASP1^*}}^{\mathrm{MASP2_{zym}}}}+\frac{[\mathrm{pLFMASP3_{zym}}]}{K_{\mathrm{m\,MASP1^*}}^{\mathrm{MASP3_{zym}}}}+\frac{[\mathrm{pHFMASP1_{zym}}]}{K_{\mathrm{m\,MASP1^*}}^{\mathrm{MASP1_{zym}}}}+\frac{[\mathrm{pHFMASP2_{zym}}]}{K_{\mathrm{m\,MASP1^*}}^{\mathrm{MASP2_{zym}}}}\\+\frac{[\mathrm{pHFMASP3_{zym}}]}{K_{\mathrm{m\,MASP1^*}}^{\mathrm{MASP3_{zym}}}}+\frac{[\mathrm{pMFMASP1_{zym}}]}{K_{\mathrm{m\,MASP1^*}}^{\mathrm{MASP1_{zym}}}}+\frac{[\mathrm{pMFMASP2_{zym}}]}{K_{\mathrm{m\,MASP1^*}}^{\mathrm{MASP2_{zym}}}}+\frac{[\mathrm{pMFMASP3_{zym}}]}{K_{\mathrm{m\,MASP1^*}}^{\mathrm{MASP3_{zym}}}}\\+\frac{[\mathrm{pPTX3LFMASP1_{zym}}]}{K_{\mathrm{m\,MASP1^*}}^{\mathrm{MASP1_{zym}}}}+\frac{[\mathrm{pPXT3LFMASP2_{zym}}]}{K_{\mathrm{m\,MASP1^*}}^{\mathrm{MASP2_{zym}}}}+\frac{[\mathrm{pPTX3LFMASP3_{zym}}]}{K_{\mathrm{m\,MASP1^*}}^{\mathrm{MASP3_{zym}}}}\end{array}\right)}$$

$$+\frac{k_{\mathrm{cat\,MASP1^*}}^{\mathrm{MASP2_{zym}}}\left[\mathrm{pCLK1MASP1^*}\right]\left[\mathrm{pCLLKMASP2_{zym}}\right]}{K_{\mathrm{m\,MASP1^*}}^{\mathrm{MASP2_{zym}}}\left(\begin{array}{l}1+\frac{[\mathrm{C4}]}{K_{\mathrm{m\,MASP1^*}}^{\mathrm{C4}}}+\frac{[\mathrm{fC4bC2}]}{K_{\mathrm{m\,MASP1^*}}^{\mathrm{C2}}}+\frac{[\mathrm{fC4bC4bC2}]}{K_{\mathrm{m\,MASP1^*}}^{\mathrm{C2}}}+\frac{[\mathrm{fC3bC4bC2}]}{K_{\mathrm{m\,MASP1^*}}^{\mathrm{C2}}}+\frac{[\mathrm{pC4bC2}]}{K_{\mathrm{m\,MASP1^*}}^{\mathrm{C2}}}+\frac{[\mathrm{pC4bC4bC2}]}{K_{\mathrm{m\,MASP1^*}}^{\mathrm{C2}}}\\
+\frac{[\mathrm{pC3bC4bC2}]}{K_{\mathrm{m\,MASP1^*}}^{\mathrm{C2}}}+\frac{\left[\mathrm{pMBL_3MASP1_{zym}}\right]}{K_{\mathrm{m\,MASP1^*}}^{\mathrm{MASP1_{zym}}}}+\frac{\left[\mathrm{pMBL_3MASP2_{zym}}\right]}{K_{\mathrm{m\,MASP1^*}}^{\mathrm{MASP2_{zym}}}}+\frac{\left[\mathrm{pMBL_3MASP3_{zym}}\right]}{K_{\mathrm{m\,MASP1^*}}^{\mathrm{MASP3_{zym}}}}\\
+\frac{\left[\mathrm{pMBL_4MASP1_{zym}}\right]}{K_{\mathrm{m\,MASP1^*}}^{\mathrm{MASP1_{zym}}}}+\frac{\left[\mathrm{pMBL_4MASP2_{zym}}\right]}{K_{\mathrm{m\,MASP1^*}}^{\mathrm{MASP2_{zym}}}}+\frac{\left[\mathrm{pMBL_4MASP3_{zym}}\right]}{K_{\mathrm{m\,MASP1^*}}^{\mathrm{MASP3_{zym}}}}+\frac{\left[\mathrm{pCLLKMASP1_{zym}}\right]}{K_{\mathrm{m\,MASP1^*}}^{\mathrm{MASP1_{zym}}}}\\
+\frac{\left[\mathrm{pCLLKMASP2_{zym}}\right]}{K_{\mathrm{m\,MASP1^*}}^{\mathrm{MASP2_{zym}}}}+\frac{\left[\mathrm{pCLLKMASP3_{zym}}\right]}{K_{\mathrm{m\,MASP1^*}}^{\mathrm{MASP3_{zym}}}}+\frac{\left[\mathrm{pCLK1MASP1_{zym}}\right]}{K_{\mathrm{m\,MASP1^*}}^{\mathrm{MASP1_{zym}}}}+\frac{\left[\mathrm{pCLK1MASP2_{zym}}\right]}{K_{\mathrm{m\,MASP1^*}}^{\mathrm{MASP2_{zym}}}}\\
+\frac{\left[\mathrm{pCLK1MASP3_{zym}}\right]}{K_{\mathrm{m\,MASP1^*}}^{\mathrm{MASP3_{zym}}}}+\frac{\left[\mathrm{pCLL1MASP1_{zym}}\right]}{K_{\mathrm{m\,MASP1^*}}^{\mathrm{MASP1_{zym}}}}+\frac{\left[\mathrm{pCLL1MASP2_{zym}}\right]}{K_{\mathrm{m\,MASP1^*}}^{\mathrm{MASP2_{zym}}}}+\frac{\left[\mathrm{pCLL1MASP3_{zym}}\right]}{K_{\mathrm{m\,MASP1^*}}^{\mathrm{MASP3_{zym}}}}\\
+\frac{\left[\mathrm{pLFMASP1_{zym}}\right]}{K_{\mathrm{m\,MASP1^*}}^{\mathrm{MASP1_{zym}}}}+\frac{\left[\mathrm{pLFMASP2_{zym}}\right]}{K_{\mathrm{m\,MASP1^*}}^{\mathrm{MASP2_{zym}}}}+\frac{\left[\mathrm{pLFMASP3_{zym}}\right]}{K_{\mathrm{m\,MASP1^*}}^{\mathrm{MASP3_{zym}}}}+\frac{\left[\mathrm{pHFMASP1_{zym}}\right]}{K_{\mathrm{m\,MASP1^*}}^{\mathrm{MASP1_{zym}}}}+\frac{\left[\mathrm{pHFMASP2_{zym}}\right]}{K_{\mathrm{m\,MASP1^*}}^{\mathrm{MASP2_{zym}}}}\\
+\frac{\left[\mathrm{pHFMASP3_{zym}}\right]}{K_{\mathrm{m\,MASP1^*}}^{\mathrm{MASP3_{zym}}}}+\frac{\left[\mathrm{pMFMASP1_{zym}}\right]}{K_{\mathrm{m\,MASP1^*}}^{\mathrm{MASP1_{zym}}}}+\frac{\left[\mathrm{pMFMASP2_{zym}}\right]}{K_{\mathrm{m\,MASP1^*}}^{\mathrm{MASP2_{zym}}}}+\frac{\left[\mathrm{pMFMASP3_{zym}}\right]}{K_{\mathrm{m\,MASP1^*}}^{\mathrm{MASP3_{zym}}}}\\
+\frac{\left[\mathrm{pPTX3LFMASP1_{zym}}\right]}{K_{\mathrm{m\,MASP1^*}}^{\mathrm{MASP1_{zym}}}}+\frac{\left[\mathrm{pPXT3LFMASP2_{zym}}\right]}{K_{\mathrm{m\,MASP1^*}}^{\mathrm{MASP2_{zym}}}}+\frac{\left[\mathrm{pPTX3LFMASP3_{zym}}\right]}{K_{\mathrm{m\,MASP1^*}}^{\mathrm{MASP3_{zym}}}}\end{array}\right)}$$

$$
+\frac{k_{\mathrm{cat\,MASP1^*}}^{\mathrm{MASP2_{zym}}}\left[\mathrm{pCLL1MASP1^*}\right]\left[\mathrm{pCLLKMASP2_{zym}}\right]}{K_{\mathrm{m\,MASP1^*}}^{\mathrm{MASP2_{zym}}}\left(\begin{array}{l}1+\frac{[\mathrm{C4}]}{K_{\mathrm{m\,MASP1^*}}^{\mathrm{C4}}}+\frac{[\mathrm{fC4bC2}]}{K_{\mathrm{m\,MASP1^*}}^{\mathrm{C2}}}+\frac{[\mathrm{fC4bC4bC2}]}{K_{\mathrm{m\,MASP1^*}}^{\mathrm{C2}}}+\frac{[\mathrm{fC3bC4bC2}]}{K_{\mathrm{m\,MASP1^*}}^{\mathrm{C2}}}+\frac{[\mathrm{pC4bC2}]}{K_{\mathrm{m\,MASP1^*}}^{\mathrm{C2}}}+\frac{[\mathrm{pC4bC4bC2}]}{K_{\mathrm{m\,MASP1^*}}^{\mathrm{C2}}}\\
+\frac{[\mathrm{pC3bC4bC2}]}{K_{\mathrm{m\,MASP1^*}}^{\mathrm{C2}}}+\frac{[\mathrm{pMBL_3MASP1_{zym}}]}{K_{\mathrm{m\,MASP1^*}}^{\mathrm{MASP1_{zym}}}}+\frac{[\mathrm{pMBL_3MASP2_{zym}}]}{K_{\mathrm{m\,MASP1^*}}^{\mathrm{MASP2_{zym}}}}+\frac{[\mathrm{pMBL_3MASP3_{zym}}]}{K_{\mathrm{m\,MASP1^*}}^{\mathrm{MASP3_{zym}}}}\\
+\frac{[\mathrm{pMBL_4MASP1_{zym}}]}{K_{\mathrm{m\,MASP1^*}}^{\mathrm{MASP1_{zym}}}}+\frac{[\mathrm{pMBL_4MASP2_{zym}}]}{K_{\mathrm{m\,MASP1^*}}^{\mathrm{MASP2_{zym}}}}+\frac{[\mathrm{pMBL_4MASP3_{zym}}]}{K_{\mathrm{m\,MASP1^*}}^{\mathrm{MASP3_{zym}}}}+\frac{[\mathrm{pCLLKMASP1_{zym}}]}{K_{\mathrm{m\,MASP1^*}}^{\mathrm{MASP1_{zym}}}}\\
+\frac{[\mathrm{pCLLKMASP2_{zym}}]}{K_{\mathrm{m\,MASP1^*}}^{\mathrm{MASP2_{zym}}}}+\frac{[\mathrm{pCLLKMASP3_{zym}}]}{K_{\mathrm{m\,MASP1^*}}^{\mathrm{MASP3_{zym}}}}+\frac{[\mathrm{pCLK1MASP1_{zym}}]}{K_{\mathrm{m\,MASP1^*}}^{\mathrm{MASP1_{zym}}}}+\frac{[\mathrm{pCLK1MASP2_{zym}}]}{K_{\mathrm{m\,MASP1^*}}^{\mathrm{MASP2_{zym}}}}\\
+\frac{[\mathrm{pCLK1MASP3_{zym}}]}{K_{\mathrm{m\,MASP1^*}}^{\mathrm{MASP3_{zym}}}}+\frac{[\mathrm{pCLL1MASP1_{zym}}]}{K_{\mathrm{m\,MASP1^*}}^{\mathrm{MASP1_{zym}}}}+\frac{[\mathrm{pCLL1MASP2_{zym}}]}{K_{\mathrm{m\,MASP1^*}}^{\mathrm{MASP2_{zym}}}}+\frac{[\mathrm{pCLL1MASP3_{zym}}]}{K_{\mathrm{m\,MASP1^*}}^{\mathrm{MASP3_{zym}}}}\\
+\frac{[\mathrm{pLFMASP1_{zym}}]}{K_{\mathrm{m\,MASP1^*}}^{\mathrm{MASP1_{zym}}}}+\frac{[\mathrm{pLFMASP2_{zym}}]}{K_{\mathrm{m\,MASP1^*}}^{\mathrm{MASP2_{zym}}}}+\frac{[\mathrm{pLFMASP3_{zym}}]}{K_{\mathrm{m\,MASP1^*}}^{\mathrm{MASP3_{zym}}}}+\frac{[\mathrm{pHFMASP1_{zym}}]}{K_{\mathrm{m\,MASP1^*}}^{\mathrm{MASP1_{zym}}}}+\frac{[\mathrm{pHFMASP2_{zym}}]}{K_{\mathrm{m\,MASP1^*}}^{\mathrm{MASP2_{zym}}}}\\
+\frac{[\mathrm{pHFMASP3_{zym}}]}{K_{\mathrm{m\,MASP1^*}}^{\mathrm{MASP3_{zym}}}}+\frac{[\mathrm{pMFMASP1_{zym}}]}{K_{\mathrm{m\,MASP1^*}}^{\mathrm{MASP1_{zym}}}}+\frac{[\mathrm{pMFMASP2_{zym}}]}{K_{\mathrm{m\,MASP1^*}}^{\mathrm{MASP2_{zym}}}}+\frac{[\mathrm{pMFMASP3_{zym}}]}{K_{\mathrm{m\,MASP1^*}}^{\mathrm{MASP3_{zym}}}}\\
+\frac{[\mathrm{pPTX3LFMASP1_{zym}}]}{K_{\mathrm{m\,MASP1^*}}^{\mathrm{MASP1_{zym}}}}+\frac{[\mathrm{pPXT3LFMASP2_{zym}}]}{K_{\mathrm{m\,MASP1^*}}^{\mathrm{MASP2_{zym}}}}+\frac{[\mathrm{pPTX3LFMASP3_{zym}}]}{K_{\mathrm{m\,MASP1^*}}^{\mathrm{MASP3_{zym}}}}\end{array}\right)}
$$

$$
+\frac{k_{\text{cat MASP1}^*}^{\text{MASP2}_{\text{zym}}}\left[\text{pLFMASP1}^*\right]\left[\text{pCLLKMASP2}_{\text{zym}}\right]}{K_{\text{m MASP1}^*}^{\text{MASP2}_{\text{zym}}}\left(\begin{array}{l}1+\frac{[\text{C4}]}{K_{\text{m MASP1}^*}^{\text{C4}}}+\frac{[\text{fC4bC2}]}{K_{\text{m MASP1}^*}^{\text{C2}}}+\frac{[\text{fC4bC4bC2}]}{K_{\text{m MASP1}^*}^{\text{C2}}}+\frac{[\text{fC3bC4bC2}]}{K_{\text{m MASP1}^*}^{\text{C2}}}+\frac{[\text{pC4bC2}]}{K_{\text{m MASP1}^*}^{\text{C2}}}+\frac{[\text{pC4bC4bC2}]}{K_{\text{m MASP1}^*}^{\text{C2}}}\\
+\frac{[\text{pC3bC4bC2}]}{K_{\text{m MASP1}^*}^{\text{C2}}}+\frac{\left[\text{pMBL}_3\text{MASP1}_{\text{zym}}\right]}{K_{\text{m MASP1}^*}^{\text{MASP1}_{\text{zym}}}}+\frac{\left[\text{pMBL}_3\text{MASP2}_{\text{zym}}\right]}{K_{\text{m MASP1}^*}^{\text{MASP2}_{\text{zym}}}}+\frac{\left[\text{pMBL}_3\text{MASP3}_{\text{zym}}\right]}{K_{\text{m MASP1}^*}^{\text{MASP3}_{\text{zym}}}}\\
+\frac{\left[\text{pMBL}_4\text{MASP1}_{\text{zym}}\right]}{K_{\text{m MASP1}^*}^{\text{MASP1}_{\text{zym}}}}+\frac{\left[\text{pMBL}_4\text{MASP2}_{\text{zym}}\right]}{K_{\text{m MASP1}^*}^{\text{MASP2}_{\text{zym}}}}+\frac{\left[\text{pMBL}_4\text{MASP3}_{\text{zym}}\right]}{K_{\text{m MASP1}^*}^{\text{MASP3}_{\text{zym}}}}+\frac{\left[\text{pCLLKMASP1}_{\text{zym}}\right]}{K_{\text{m MASP1}^*}^{\text{MASP1}_{\text{zym}}}}\\
+\frac{\left[\text{pCLLKMASP2}_{\text{zym}}\right]}{K_{\text{m MASP1}^*}^{\text{MASP2}_{\text{zym}}}}+\frac{\left[\text{pCLLKMASP3}_{\text{zym}}\right]}{K_{\text{m MASP1}^*}^{\text{MASP3}_{\text{zym}}}}+\frac{\left[\text{pCLK1MASP1}_{\text{zym}}\right]}{K_{\text{m MASP1}^*}^{\text{MASP1}_{\text{zym}}}}+\frac{\left[\text{pCLK1MASP2}_{\text{zym}}\right]}{K_{\text{m MASP1}^*}^{\text{MASP2}_{\text{zym}}}}\\
+\frac{\left[\text{pCLK1MASP3}_{\text{zym}}\right]}{K_{\text{m MASP1}^*}^{\text{MASP3}_{\text{zym}}}}+\frac{\left[\text{pCLL1MASP1}_{\text{zym}}\right]}{K_{\text{m MASP1}^*}^{\text{MASP1}_{\text{zym}}}}+\frac{\left[\text{pCLL1MASP2}_{\text{zym}}\right]}{K_{\text{m MASP1}^*}^{\text{MASP2}_{\text{zym}}}}+\frac{\left[\text{pCLL1MASP3}_{\text{zym}}\right]}{K_{\text{m MASP1}^*}^{\text{MASP3}_{\text{zym}}}}\\
+\frac{\left[\text{pLFMASP1}_{\text{zym}}\right]}{K_{\text{m MASP1}^*}^{\text{MASP1}_{\text{zym}}}}+\frac{\left[\text{pLFMASP2}_{\text{zym}}\right]}{K_{\text{m MASP1}^*}^{\text{MASP2}_{\text{zym}}}}+\frac{\left[\text{pLFMASP3}_{\text{zym}}\right]}{K_{\text{m MASP1}^*}^{\text{MASP3}_{\text{zym}}}}+\frac{\left[\text{pHFMASP1}_{\text{zym}}\right]}{K_{\text{m MASP1}^*}^{\text{MASP1}_{\text{zym}}}}+\frac{\left[\text{pHFMASP2}_{\text{zym}}\right]}{K_{\text{m MASP1}^*}^{\text{MASP2}_{\text{zym}}}}\\
+\frac{\left[\text{pHFMASP3}_{\text{zym}}\right]}{K_{\text{m MASP1}^*}^{\text{MASP3}_{\text{zym}}}}+\frac{\left[\text{pMFMASP1}_{\text{zym}}\right]}{K_{\text{m MASP1}^*}^{\text{MASP1}_{\text{zym}}}}+\frac{\left[\text{pMFMASP2}_{\text{zym}}\right]}{K_{\text{m MASP1}^*}^{\text{MASP2}_{\text{zym}}}}+\frac{\left[\text{pMFMASP3}_{\text{zym}}\right]}{K_{\text{m MASP1}^*}^{\text{MASP3}_{\text{zym}}}}\\
+\frac{\left[\text{pPTX3LFMASP1}_{\text{zym}}\right]}{K_{\text{m MASP1}^*}^{\text{MASP1}_{\text{zym}}}}+\frac{\left[\text{pPXT3LFMASP2}_{\text{zym}}\right]}{K_{\text{m MASP1}^*}^{\text{MASP2}_{\text{zym}}}}+\frac{\left[\text{pPTX3LFMASP3}_{\text{zym}}\right]}{K_{\text{m MASP1}^*}^{\text{MASP3}_{\text{zym}}}}\end{array}\right)}
$$

$$+\frac{k_{\text{cat MASP1}^*}^{\text{MASP2}_{\text{zym}}}\left[\text{pHFMASP1}^*\right]\left[\text{pCLLKMASP2}_{\text{zym}}\right]}{K_{\text{m MASP1}^*}^{\text{MASP2}_{\text{zym}}}\left(\begin{array}{l}1+\frac{[\text{C4}]}{K_{\text{m MASP1}^*}^{\text{C4}}}+\frac{[\text{fC4bC2}]}{K_{\text{m MASP1}^*}^{\text{C2}}}+\frac{[\text{fC4bC4bC2}]}{K_{\text{m MASP1}^*}^{\text{C2}}}+\frac{[\text{fC3bC4bC2}]}{K_{\text{m MASP1}^*}^{\text{C2}}}+\frac{[\text{pC4bC2}]}{K_{\text{m MASP1}^*}^{\text{C2}}}+\frac{[\text{pC4bC4bC2}]}{K_{\text{m MASP1}^*}^{\text{C2}}}\\+\frac{[\text{pC3bC4bC2}]}{K_{\text{m MASP1}^*}^{\text{C2}}}+\frac{[\text{pMBL}_3\text{MASP1}_{\text{zym}}]}{K_{\text{m MASP1}^*}^{\text{MASP1}_{\text{zym}}}}+\frac{[\text{pMBL}_3\text{MASP2}_{\text{zym}}]}{K_{\text{m MASP1}^*}^{\text{MASP2}_{\text{zym}}}}+\frac{[\text{pMBL}_3\text{MASP3}_{\text{zym}}]}{K_{\text{m MASP1}^*}^{\text{MASP3}_{\text{zym}}}}\\+\frac{[\text{pMBL}_4\text{MASP1}_{\text{zym}}]}{K_{\text{m MASP1}^*}^{\text{MASP1}_{\text{zym}}}}+\frac{[\text{pMBL}_4\text{MASP2}_{\text{zym}}]}{K_{\text{m MASP1}^*}^{\text{MASP2}_{\text{zym}}}}+\frac{[\text{pMBL}_4\text{MASP3}_{\text{zym}}]}{K_{\text{m MASP1}^*}^{\text{MASP3}_{\text{zym}}}}+\frac{[\text{pCLLKMASP1}_{\text{zym}}]}{K_{\text{m MASP1}^*}^{\text{MASP1}_{\text{zym}}}}\\+\frac{[\text{pCLLKMASP2}_{\text{zym}}]}{K_{\text{m MASP1}^*}^{\text{MASP2}_{\text{zym}}}}+\frac{[\text{pCLLKMASP3}_{\text{zym}}]}{K_{\text{m MASP1}^*}^{\text{MASP3}_{\text{zym}}}}+\frac{[\text{pCLK1MASP1}_{\text{zym}}]}{K_{\text{m MASP1}^*}^{\text{MASP1}_{\text{zym}}}}+\frac{[\text{pCLK1MASP2}_{\text{zym}}]}{K_{\text{m MASP1}^*}^{\text{MASP2}_{\text{zym}}}}\\+\frac{[\text{pCLK1MASP3}_{\text{zym}}]}{K_{\text{m MASP1}^*}^{\text{MASP3}_{\text{zym}}}}+\frac{[\text{pCLL1MASP1}_{\text{zym}}]}{K_{\text{m MASP1}^*}^{\text{MASP1}_{\text{zym}}}}+\frac{[\text{pCLL1MASP2}_{\text{zym}}]}{K_{\text{m MASP1}^*}^{\text{MASP2}_{\text{zym}}}}+\frac{[\text{pCLL1MASP3}_{\text{zym}}]}{K_{\text{m MASP1}^*}^{\text{MASP3}_{\text{zym}}}}\\+\frac{[\text{pLFMASP1}_{\text{zym}}]}{K_{\text{m MASP1}^*}^{\text{MASP1}_{\text{zym}}}}+\frac{[\text{pLFMASP2}_{\text{zym}}]}{K_{\text{m MASP1}^*}^{\text{MASP2}_{\text{zym}}}}+\frac{[\text{pLFMASP3}_{\text{zym}}]}{K_{\text{m MASP1}^*}^{\text{MASP3}_{\text{zym}}}}+\frac{[\text{pHFMASP1}_{\text{zym}}]}{K_{\text{m MASP1}^*}^{\text{MASP1}_{\text{zym}}}}+\frac{[\text{pHFMASP2}_{\text{zym}}]}{K_{\text{m MASP1}^*}^{\text{MASP2}_{\text{zym}}}}\\+\frac{[\text{pHFMASP3}_{\text{zym}}]}{K_{\text{m MASP1}^*}^{\text{MASP3}_{\text{zym}}}}+\frac{[\text{pMFMASP1}_{\text{zym}}]}{K_{\text{m MASP1}^*}^{\text{MASP1}_{\text{zym}}}}+\frac{[\text{pMFMASP2}_{\text{zym}}]}{K_{\text{m MASP1}^*}^{\text{MASP2}_{\text{zym}}}}+\frac{[\text{pMFMASP3}_{\text{zym}}]}{K_{\text{m MASP1}^*}^{\text{MASP3}_{\text{zym}}}}\\+\frac{[\text{pPTX3LFMASP1}_{\text{zym}}]}{K_{\text{m MASP1}^*}^{\text{MASP1}_{\text{zym}}}}+\frac{[\text{pPXT3LFMASP2}_{\text{zym}}]}{K_{\text{m MASP1}^*}^{\text{MASP2}_{\text{zym}}}}+\frac{[\text{pPTX3LFMASP3}_{\text{zym}}]}{K_{\text{m MASP1}^*}^{\text{MASP3}_{\text{zym}}}}\end{array}\right)}$$

$$
+\frac{k_{\mathrm{cat\,MASP1^*}}^{\mathrm{MASP2_{zym}}}\left[\mathrm{pMFMASP1^*}\right]\left[\mathrm{pCLLKMASP2_{zym}}\right]}{K_{\mathrm{m\,MASP1^*}}^{\mathrm{MASP2_{zym}}}\left(\begin{array}{l}
1+\frac{[\mathrm{C4}]}{K_{\mathrm{m\,MASP1^*}}^{\mathrm{C4}}}+\frac{[\mathrm{fC4bC2}]}{K_{\mathrm{m\,MASP1^*}}^{\mathrm{C2}}}+\frac{[\mathrm{fC4bC4bC2}]}{K_{\mathrm{m\,MASP1^*}}^{\mathrm{C2}}}+\frac{[\mathrm{fC3bC4bC2}]}{K_{\mathrm{m\,MASP1^*}}^{\mathrm{C2}}}+\frac{[\mathrm{pC4bC2}]}{K_{\mathrm{m\,MASP1^*}}^{\mathrm{C2}}}+\frac{[\mathrm{pC4bC4bC2}]}{K_{\mathrm{m\,MASP1^*}}^{\mathrm{C2}}}\\
+\frac{[\mathrm{pC3bC4bC2}]}{K_{\mathrm{m\,MASP1^*}}^{\mathrm{C2}}}+\frac{\left[\mathrm{pMBL_3MASP1_{zym}}\right]}{K_{\mathrm{m\,MASP1^*}}^{\mathrm{MASP1_{zym}}}}+\frac{\left[\mathrm{pMBL_3MASP2_{zym}}\right]}{K_{\mathrm{m\,MASP1^*}}^{\mathrm{MASP2_{zym}}}}+\frac{\left[\mathrm{pMBL_3MASP3_{zym}}\right]}{K_{\mathrm{m\,MASP1^*}}^{\mathrm{MASP3_{zym}}}}\\
+\frac{\left[\mathrm{pMBL_4MASP1_{zym}}\right]}{K_{\mathrm{m\,MASP1^*}}^{\mathrm{MASP1_{zym}}}}+\frac{\left[\mathrm{pMBL_4MASP2_{zym}}\right]}{K_{\mathrm{m\,MASP1^*}}^{\mathrm{MASP2_{zym}}}}+\frac{\left[\mathrm{pMBL_4MASP3_{zym}}\right]}{K_{\mathrm{m\,MASP1^*}}^{\mathrm{MASP3_{zym}}}}+\frac{\left[\mathrm{pCLLKMASP1_{zym}}\right]}{K_{\mathrm{m\,MASP1^*}}^{\mathrm{MASP1_{zym}}}}\\
+\frac{\left[\mathrm{pCLLKMASP2_{zym}}\right]}{K_{\mathrm{m\,MASP1^*}}^{\mathrm{MASP2_{zym}}}}+\frac{\left[\mathrm{pCLLKMASP3_{zym}}\right]}{K_{\mathrm{m\,MASP1^*}}^{\mathrm{MASP3_{zym}}}}+\frac{\left[\mathrm{pCLK1MASP1_{zym}}\right]}{K_{\mathrm{m\,MASP1^*}}^{\mathrm{MASP1_{zym}}}}+\frac{\left[\mathrm{pCLK1MASP2_{zym}}\right]}{K_{\mathrm{m\,MASP1^*}}^{\mathrm{MASP2_{zym}}}}\\
+\frac{\left[\mathrm{pCLK1MASP3_{zym}}\right]}{K_{\mathrm{m\,MASP1^*}}^{\mathrm{MASP3_{zym}}}}+\frac{\left[\mathrm{pCLL1MASP1_{zym}}\right]}{K_{\mathrm{m\,MASP1^*}}^{\mathrm{MASP1_{zym}}}}+\frac{\left[\mathrm{pCLL1MASP2_{zym}}\right]}{K_{\mathrm{m\,MASP1^*}}^{\mathrm{MASP2_{zym}}}}+\frac{\left[\mathrm{pCLL1MASP3_{zym}}\right]}{K_{\mathrm{m\,MASP1^*}}^{\mathrm{MASP3_{zym}}}}\\
+\frac{\left[\mathrm{pLFMASP1_{zym}}\right]}{K_{\mathrm{m\,MASP1^*}}^{\mathrm{MASP1_{zym}}}}+\frac{\left[\mathrm{pLFMASP2_{zym}}\right]}{K_{\mathrm{m\,MASP1^*}}^{\mathrm{MASP2_{zym}}}}+\frac{\left[\mathrm{pLFMASP3_{zym}}\right]}{K_{\mathrm{m\,MASP1^*}}^{\mathrm{MASP3_{zym}}}}+\frac{\left[\mathrm{pHFMASP1_{zym}}\right]}{K_{\mathrm{m\,MASP1^*}}^{\mathrm{MASP1_{zym}}}}+\frac{\left[\mathrm{pHFMASP2_{zym}}\right]}{K_{\mathrm{m\,MASP1^*}}^{\mathrm{MASP2_{zym}}}}\\
+\frac{\left[\mathrm{pHFMASP3_{zym}}\right]}{K_{\mathrm{m\,MASP1^*}}^{\mathrm{MASP3_{zym}}}}+\frac{\left[\mathrm{pMFMASP1_{zym}}\right]}{K_{\mathrm{m\,MASP1^*}}^{\mathrm{MASP1_{zym}}}}+\frac{\left[\mathrm{pMFMASP2_{zym}}\right]}{K_{\mathrm{m\,MASP1^*}}^{\mathrm{MASP2_{zym}}}}+\frac{\left[\mathrm{pMFMASP3_{zym}}\right]}{K_{\mathrm{m\,MASP1^*}}^{\mathrm{MASP3_{zym}}}}\\
+\frac{\left[\mathrm{pPTX3LFMASP1_{zym}}\right]}{K_{\mathrm{m\,MASP1^*}}^{\mathrm{MASP1_{zym}}}}+\frac{\left[\mathrm{pPXT3LFMASP2_{zym}}\right]}{K_{\mathrm{m\,MASP1^*}}^{\mathrm{MASP2_{zym}}}}+\frac{\left[\mathrm{pPTX3LFMASP3_{zym}}\right]}{K_{\mathrm{m\,MASP1^*}}^{\mathrm{MASP3_{zym}}}}
\end{array}\right)}
$$

$$+\frac{k_{\mathrm{cat\ MASP1^*}}^{\mathrm{MASP2_{zym}}}\left[\mathrm{pPTX3LFMASP1^*}\right]\left[\mathrm{pCLLKMASP2_{zym}}\right]}{K_{\mathrm{m\ MASP1^*}}^{\mathrm{MASP2_{zym}}}\left(\begin{array}{l}1+\frac{[\mathrm{C4}]}{K_{\mathrm{m\ MASP1^*}}^{\mathrm{C4}}}+\frac{[\mathrm{fC4bC2}]}{K_{\mathrm{m\ MASP1^*}}^{\mathrm{C2}}}+\frac{[\mathrm{fC4bC4bC2}]}{K_{\mathrm{m\ MASP1^*}}^{\mathrm{C2}}}+\frac{[\mathrm{fC3bC4bC2}]}{K_{\mathrm{m\ MASP1^*}}^{\mathrm{C2}}}+\frac{[\mathrm{pC4bC2}]}{K_{\mathrm{m\ MASP1^*}}^{\mathrm{C2}}}+\frac{[\mathrm{pC4bC4bC2}]}{K_{\mathrm{m\ MASP1^*}}^{\mathrm{C2}}}\\ +\frac{[\mathrm{pC3bC4bC2}]}{K_{\mathrm{m\ MASP1^*}}^{\mathrm{C2}}}+\frac{[\mathrm{pMBL_3MASP1_{zym}}]}{K_{\mathrm{m\ MASP1^*}}^{\mathrm{MASP1_{zym}}}}+\frac{[\mathrm{pMBL_3MASP2_{zym}}]}{K_{\mathrm{m\ MASP1^*}}^{\mathrm{MASP2_{zym}}}}+\frac{[\mathrm{pMBL_3MASP3_{zym}}]}{K_{\mathrm{m\ MASP1^*}}^{\mathrm{MASP3_{zym}}}}\\ +\frac{[\mathrm{pMBL_4MASP1_{zym}}]}{K_{\mathrm{m\ MASP1^*}}^{\mathrm{MASP1_{zym}}}}+\frac{[\mathrm{pMBL_4MASP2_{zym}}]}{K_{\mathrm{m\ MASP1^*}}^{\mathrm{MASP2_{zym}}}}+\frac{[\mathrm{pMBL_4MASP3_{zym}}]}{K_{\mathrm{m\ MASP1^*}}^{\mathrm{MASP3_{zym}}}}+\frac{[\mathrm{pCLLKMASP1_{zym}}]}{K_{\mathrm{m\ MASP1^*}}^{\mathrm{MASP1_{zym}}}}\\ +\frac{[\mathrm{pCLLKMASP2_{zym}}]}{K_{\mathrm{m\ MASP1^*}}^{\mathrm{MASP2_{zym}}}}+\frac{[\mathrm{pCLLKMASP3_{zym}}]}{K_{\mathrm{m\ MASP1^*}}^{\mathrm{MASP3_{zym}}}}+\frac{[\mathrm{pCLK1MASP1_{zym}}]}{K_{\mathrm{m\ MASP1^*}}^{\mathrm{MASP1_{zym}}}}+\frac{[\mathrm{pCLK1MASP2_{zym}}]}{K_{\mathrm{m\ MASP1^*}}^{\mathrm{MASP2_{zym}}}}\\ +\frac{[\mathrm{pCLK1MASP3_{zym}}]}{K_{\mathrm{m\ MASP1^*}}^{\mathrm{MASP3_{zym}}}}+\frac{[\mathrm{pCLL1MASP1_{zym}}]}{K_{\mathrm{m\ MASP1^*}}^{\mathrm{MASP1_{zym}}}}+\frac{[\mathrm{pCLL1MASP2_{zym}}]}{K_{\mathrm{m\ MASP1^*}}^{\mathrm{MASP2_{zym}}}}+\frac{[\mathrm{pCLL1MASP3_{zym}}]}{K_{\mathrm{m\ MASP1^*}}^{\mathrm{MASP3_{zym}}}}\\ +\frac{[\mathrm{pLFMASP1_{zym}}]}{K_{\mathrm{m\ MASP1^*}}^{\mathrm{MASP1_{zym}}}}+\frac{[\mathrm{pLFMASP2_{zym}}]}{K_{\mathrm{m\ MASP1^*}}^{\mathrm{MASP2_{zym}}}}+\frac{[\mathrm{pLFMASP3_{zym}}]}{K_{\mathrm{m\ MASP1^*}}^{\mathrm{MASP3_{zym}}}}+\frac{[\mathrm{pHFMASP1_{zym}}]}{K_{\mathrm{m\ MASP1^*}}^{\mathrm{MASP1_{zym}}}}+\frac{[\mathrm{pHFMASP2_{zym}}]}{K_{\mathrm{m\ MASP1^*}}^{\mathrm{MASP2_{zym}}}}\\ +\frac{[\mathrm{pHFMASP3_{zym}}]}{K_{\mathrm{m\ MASP1^*}}^{\mathrm{MASP3_{zym}}}}+\frac{[\mathrm{pMFMASP1_{zym}}]}{K_{\mathrm{m\ MASP1^*}}^{\mathrm{MASP1_{zym}}}}+\frac{[\mathrm{pMFMASP2_{zym}}]}{K_{\mathrm{m\ MASP1^*}}^{\mathrm{MASP2_{zym}}}}+\frac{[\mathrm{pMFMASP3_{zym}}]}{K_{\mathrm{m\ MASP1^*}}^{\mathrm{MASP3_{zym}}}}\\ +\frac{[\mathrm{pPTX3LFMASP1_{zym}}]}{K_{\mathrm{m\ MASP1^*}}^{\mathrm{MASP1_{zym}}}}+\frac{[\mathrm{pPXT3LFMASP2_{zym}}]}{K_{\mathrm{m\ MASP1^*}}^{\mathrm{MASP2_{zym}}}}+\frac{[\mathrm{pPTX3LFMASP3_{zym}}]}{K_{\mathrm{m\ MASP1^*}}^{\mathrm{MASP3_{zym}}}}\end{array}\right)}$$

$$+\frac{k_{\mathrm{cat\ MASP2^*}}^{\mathrm{MASP2_{zym}}}\left[\mathrm{pMBL_3MASP2^*}\right]\left[\mathrm{pCLLKMASP2_{zym}}\right]}{K_{\mathrm{m\ MASP2^*}}^{\mathrm{MASP2_{zym}}}\left(\begin{array}{l}1+\frac{[\mathrm{C4}]}{K_{\mathrm{m\ MASP2^*}}^{\mathrm{C4}}}+\frac{[\mathrm{fC4bC2}]}{K_{\mathrm{m\ MASP2^*}}^{\mathrm{C2}}}+\frac{[\mathrm{fC4bC4bC2}]}{K_{\mathrm{m\ MASP2^*}}^{\mathrm{C2}}}+\frac{[\mathrm{fC3bC4bC2}]}{K_{\mathrm{m\ MASP2^*}}^{\mathrm{C2}}}+\frac{[\mathrm{pC4bC2}]}{K_{\mathrm{m\ MASP2^*}}^{\mathrm{C2}}}+\frac{[\mathrm{pC4bC4bC2}]}{K_{\mathrm{m\ MASP2^*}}^{\mathrm{C2}}}\\ +\frac{[\mathrm{pC3bC4bC2}]}{K_{\mathrm{m\ MASP2^*}}^{\mathrm{C2}}}+\frac{[\mathrm{pMBL_3MASP1_{zym}}]}{K_{\mathrm{m\ MASP2^*}}^{\mathrm{MASP1_{zym}}}}+\frac{[\mathrm{pMBL_3MASP2_{zym}}]}{K_{\mathrm{m\ MASP2^*}}^{\mathrm{MASP2_{zym}}}}+\frac{[\mathrm{pMBL_4MASP1_{zym}}]}{K_{\mathrm{m\ MASP2^*}}^{\mathrm{MASP1_{zym}}}}\\ +\frac{[\mathrm{pMBL_4MASP2_{zym}}]}{K_{\mathrm{m\ MASP2^*}}^{\mathrm{MASP2_{zym}}}}+\frac{[\mathrm{pCLLKMASP1_{zym}}]}{K_{\mathrm{m\ MASP2^*}}^{\mathrm{MASP1_{zym}}}}+\frac{[\mathrm{pCLLKMASP2_{zym}}]}{K_{\mathrm{m\ MASP2^*}}^{\mathrm{MASP2_{zym}}}}+\frac{[\mathrm{pCLK1MASP1_{zym}}]}{K_{\mathrm{m\ MASP2^*}}^{\mathrm{MASP1_{zym}}}}\\ +\frac{[\mathrm{pCLK1MASP2_{zym}}]}{K_{\mathrm{m\ MASP2^*}}^{\mathrm{MASP2_{zym}}}}+\frac{[\mathrm{pCLL1MASP1_{zym}}]}{K_{\mathrm{m\ MASP2^*}}^{\mathrm{MASP1_{zym}}}}+\frac{[\mathrm{pCLL1MASP2_{zym}}]}{K_{\mathrm{m\ MASP2^*}}^{\mathrm{MASP2_{zym}}}}+\frac{[\mathrm{pLFMASP1_{zym}}]}{K_{\mathrm{m\ MASP2^*}}^{\mathrm{MASP1_{zym}}}}\\ +\frac{[\mathrm{pLFMASP2_{zym}}]}{K_{\mathrm{m\ MASP2^*}}^{\mathrm{MASP2_{zym}}}}+\frac{[\mathrm{pHFMASP1_{zym}}]}{K_{\mathrm{m\ MASP2^*}}^{\mathrm{MASP1_{zym}}}}+\frac{[\mathrm{pHFMASP2_{zym}}]}{K_{\mathrm{m\ MASP2^*}}^{\mathrm{MASP2_{zym}}}}+\frac{[\mathrm{pMFMASP1_{zym}}]}{K_{\mathrm{m\ MASP2^*}}^{\mathrm{MASP1_{zym}}}}+\frac{[\mathrm{pMFMASP2_{zym}}]}{K_{\mathrm{m\ MASP2^*}}^{\mathrm{MASP2_{zym}}}}\\ +\frac{[\mathrm{pPTX3LFMASP1_{zym}}]}{K_{\mathrm{m\ MASP2^*}}^{\mathrm{MASP1_{zym}}}}+\frac{[\mathrm{pPTX3LFMASP2_{zym}}]}{K_{\mathrm{m\ MASP2^*}}^{\mathrm{MASP2_{zym}}}}\end{array}\right)}$$

$$+\frac{k_{\text{cat MASP2}^*}^{\text{MASP2}_{\text{zym}}}\left[\text{pMBL}_4\text{MASP2}^*\right]\left[\text{pCLLKMASP2}_{\text{zym}}\right]}{K_{\text{m MASP2}^*}^{\text{MASP2}_{\text{zym}}}\left(\begin{array}{l}1+\frac{[\text{C4}]}{K_{\text{m MASP2}^*}^{\text{C4}}}+\frac{[\text{fC4bC2}]}{K_{\text{m MASP2}^*}^{\text{C2}}}+\frac{[\text{fC4bC4bC2}]}{K_{\text{m MASP2}^*}^{\text{C2}}}+\frac{[\text{fC3bC4bC2}]}{K_{\text{m MASP2}^*}^{\text{C2}}}+\frac{[\text{pC4bC2}]}{K_{\text{m MASP2}^*}^{\text{C2}}}+\frac{[\text{pC4bC4bC2}]}{K_{\text{m MASP2}^*}^{\text{C2}}}\\+\frac{[\text{pC3bC4bC2}]}{K_{\text{m MASP2}^*}^{\text{C2}}}+\frac{[\text{pMBL}_3\text{MASP1}_{\text{zym}}]}{K_{\text{m MASP2}^*}^{\text{MASP1}_{\text{zym}}}}+\frac{[\text{pMBL}_3\text{MASP2}_{\text{zym}}]}{K_{\text{m MASP2}^*}^{\text{MASP2}_{\text{zym}}}}+\frac{[\text{pMBL}_4\text{MASP1}_{\text{zym}}]}{K_{\text{m MASP2}^*}^{\text{MASP1}_{\text{zym}}}}\\+\frac{[\text{pMBL}_4\text{MASP2}_{\text{zym}}]}{K_{\text{m MASP2}^*}^{\text{MASP2}_{\text{zym}}}}+\frac{[\text{pCLLKMASP1}_{\text{zym}}]}{K_{\text{m MASP2}^*}^{\text{MASP1}_{\text{zym}}}}+\frac{[\text{pCLLKMASP2}_{\text{zym}}]}{K_{\text{m MASP2}^*}^{\text{MASP2}_{\text{zym}}}}+\frac{[\text{pCLK1MASP1}_{\text{zym}}]}{K_{\text{m MASP2}^*}^{\text{MASP1}_{\text{zym}}}}\\+\frac{[\text{pCLK1MASP2}_{\text{zym}}]}{K_{\text{m MASP2}^*}^{\text{MASP2}_{\text{zym}}}}+\frac{[\text{pCLL1MASP1}_{\text{zym}}]}{K_{\text{m MASP2}^*}^{\text{MASP1}_{\text{zym}}}}+\frac{[\text{pCLL1MASP2}_{\text{zym}}]}{K_{\text{m MASP2}^*}^{\text{MASP2}_{\text{zym}}}}+\frac{[\text{pLFMASP1}_{\text{zym}}]}{K_{\text{m MASP2}^*}^{\text{MASP1}_{\text{zym}}}}\\+\frac{[\text{pLFMASP2}_{\text{zym}}]}{K_{\text{m MASP2}^*}^{\text{MASP2}_{\text{zym}}}}+\frac{[\text{pHFMASP1}_{\text{zym}}]}{K_{\text{m MASP2}^*}^{\text{MASP1}_{\text{zym}}}}+\frac{[\text{pHFMASP2}_{\text{zym}}]}{K_{\text{m MASP2}^*}^{\text{MASP2}_{\text{zym}}}}+\frac{[\text{pMFMASP1}_{\text{zym}}]}{K_{\text{m MASP2}^*}^{\text{MASP1}_{\text{zym}}}}+\frac{[\text{pMFMASP2}_{\text{zym}}]}{K_{\text{m MASP2}^*}^{\text{MASP2}_{\text{zym}}}}\\+\frac{[\text{pPTX3LFMASP1}_{\text{zym}}]}{K_{\text{m MASP2}^*}^{\text{MASP1}_{\text{zym}}}}+\frac{[\text{pPTX3LFMASP2}_{\text{zym}}]}{K_{\text{m MASP2}^*}^{\text{MASP2}_{\text{zym}}}}\end{array}\right)}$$

$$+\frac{k_{\text{cat MASP2}^*}^{\text{MASP2}_{\text{zym}}}\left[\text{pCLLKMASP2}^*\right]\left[\text{pCLLKMASP2}_{\text{zym}}\right]}{K_{\text{m MASP2}^*}^{\text{MASP2}_{\text{zym}}}\left(\begin{array}{l}1+\frac{[\text{C4}]}{K_{\text{m MASP2}^*}^{\text{C4}}}+\frac{[\text{fC4bC2}]}{K_{\text{m MASP2}^*}^{\text{C2}}}+\frac{[\text{fC4bC4bC2}]}{K_{\text{m MASP2}^*}^{\text{C2}}}+\frac{[\text{fC3bC4bC2}]}{K_{\text{m MASP2}^*}^{\text{C2}}}+\frac{[\text{pC4bC2}]}{K_{\text{m MASP2}^*}^{\text{C2}}}+\frac{[\text{pC4bC4bC2}]}{K_{\text{m MASP2}^*}^{\text{C2}}}\\+\frac{[\text{pC3bC4bC2}]}{K_{\text{m MASP2}^*}^{\text{C2}}}+\frac{[\text{pMBL}_3\text{MASP1}_{\text{zym}}]}{K_{\text{m MASP2}^*}^{\text{MASP1}_{\text{zym}}}}+\frac{[\text{pMBL}_3\text{MASP2}_{\text{zym}}]}{K_{\text{m MASP2}^*}^{\text{MASP2}_{\text{zym}}}}+\frac{[\text{pMBL}_4\text{MASP1}_{\text{zym}}]}{K_{\text{m MASP2}^*}^{\text{MASP1}_{\text{zym}}}}\\+\frac{[\text{pMBL}_4\text{MASP2}_{\text{zym}}]}{K_{\text{m MASP2}^*}^{\text{MASP2}_{\text{zym}}}}+\frac{[\text{pCLLKMASP1}_{\text{zym}}]}{K_{\text{m MASP2}^*}^{\text{MASP1}_{\text{zym}}}}+\frac{[\text{pCLLKMASP2}_{\text{zym}}]}{K_{\text{m MASP2}^*}^{\text{MASP2}_{\text{zym}}}}+\frac{[\text{pCLK1MASP1}_{\text{zym}}]}{K_{\text{m MASP2}^*}^{\text{MASP1}_{\text{zym}}}}\\+\frac{[\text{pCLK1MASP2}_{\text{zym}}]}{K_{\text{m MASP2}^*}^{\text{MASP2}_{\text{zym}}}}+\frac{[\text{pCLL1MASP1}_{\text{zym}}]}{K_{\text{m MASP2}^*}^{\text{MASP1}_{\text{zym}}}}+\frac{[\text{pCLL1MASP2}_{\text{zym}}]}{K_{\text{m MASP2}^*}^{\text{MASP2}_{\text{zym}}}}+\frac{[\text{pLFMASP1}_{\text{zym}}]}{K_{\text{m MASP2}^*}^{\text{MASP1}_{\text{zym}}}}\\+\frac{[\text{pLFMASP2}_{\text{zym}}]}{K_{\text{m MASP2}^*}^{\text{MASP2}_{\text{zym}}}}+\frac{[\text{pHFMASP1}_{\text{zym}}]}{K_{\text{m MASP2}^*}^{\text{MASP1}_{\text{zym}}}}+\frac{[\text{pHFMASP2}_{\text{zym}}]}{K_{\text{m MASP2}^*}^{\text{MASP2}_{\text{zym}}}}+\frac{[\text{pMFMASP1}_{\text{zym}}]}{K_{\text{m MASP2}^*}^{\text{MASP1}_{\text{zym}}}}+\frac{[\text{pMFMASP2}_{\text{zym}}]}{K_{\text{m MASP2}^*}^{\text{MASP2}_{\text{zym}}}}\\+\frac{[\text{pPTX3LFMASP1}_{\text{zym}}]}{K_{\text{m MASP2}^*}^{\text{MASP1}_{\text{zym}}}}+\frac{[\text{pPTX3LFMASP2}_{\text{zym}}]}{K_{\text{m MASP2}^*}^{\text{MASP2}_{\text{zym}}}}\end{array}\right)}$$

$$
+\frac{k_{\mathrm{cat\ MASP2^*}}^{\mathrm{MASP2_{zym}}}\left[\mathrm{pCLK1MASP2^*}\right]\left[\mathrm{pCLLKMASP2_{zym}}\right]}{K_{\mathrm{m\ MASP2^*}}^{\mathrm{MASP2_{zym}}}\left(\begin{array}{l}1+\frac{[\mathrm{C4}]}{K_{\mathrm{m\ MASP2^*}}^{\mathrm{C4}}}+\frac{[\mathrm{fC4bC2}]}{K_{\mathrm{m\ MASP2^*}}^{\mathrm{C2}}}+\frac{[\mathrm{fC4bC4bC2}]}{K_{\mathrm{m\ MASP2^*}}^{\mathrm{C2}}}+\frac{[\mathrm{fC3bC4bC2}]}{K_{\mathrm{m\ MASP2^*}}^{\mathrm{C2}}}+\frac{[\mathrm{pC4bC2}]}{K_{\mathrm{m\ MASP2^*}}^{\mathrm{C2}}}+\frac{[\mathrm{pC4bC4bC2}]}{K_{\mathrm{m\ MASP2^*}}^{\mathrm{C2}}}\\
+\frac{[\mathrm{pC3bC4bC2}]}{K_{\mathrm{m\ MASP2^*}}^{\mathrm{C2}}}+\frac{[\mathrm{pMBL_3MASP1_{zym}}]}{K_{\mathrm{m\ MASP2^*}}^{\mathrm{MASP1_{zym}}}}+\frac{[\mathrm{pMBL_3MASP2_{zym}}]}{K_{\mathrm{m\ MASP2^*}}^{\mathrm{MASP2_{zym}}}}+\frac{[\mathrm{pMBL_4MASP1_{zym}}]}{K_{\mathrm{m\ MASP2^*}}^{\mathrm{MASP1_{zym}}}}\\
+\frac{[\mathrm{pMBL_4MASP2_{zym}}]}{K_{\mathrm{m\ MASP2^*}}^{\mathrm{MASP2_{zym}}}}+\frac{[\mathrm{pCLLKMASP1_{zym}}]}{K_{\mathrm{m\ MASP2^*}}^{\mathrm{MASP1_{zym}}}}+\frac{[\mathrm{pCLLKMASP2_{zym}}]}{K_{\mathrm{m\ MASP2^*}}^{\mathrm{MASP2_{zym}}}}+\frac{[\mathrm{pCLK1MASP1_{zym}}]}{K_{\mathrm{m\ MASP2^*}}^{\mathrm{MASP1_{zym}}}}\\
+\frac{[\mathrm{pCLK1MASP2_{zym}}]}{K_{\mathrm{m\ MASP2^*}}^{\mathrm{MASP2_{zym}}}}+\frac{[\mathrm{pCLL1MASP1_{zym}}]}{K_{\mathrm{m\ MASP2^*}}^{\mathrm{MASP1_{zym}}}}+\frac{[\mathrm{pCLL1MASP2_{zym}}]}{K_{\mathrm{m\ MASP2^*}}^{\mathrm{MASP2_{zym}}}}+\frac{[\mathrm{pLFMASP1_{zym}}]}{K_{\mathrm{m\ MASP2^*}}^{\mathrm{MASP1_{zym}}}}\\
+\frac{[\mathrm{pLFMASP2_{zym}}]}{K_{\mathrm{m\ MASP2^*}}^{\mathrm{MASP2_{zym}}}}+\frac{[\mathrm{pHFMASP1_{zym}}]}{K_{\mathrm{m\ MASP2^*}}^{\mathrm{MASP1_{zym}}}}+\frac{[\mathrm{pHFMASP2_{zym}}]}{K_{\mathrm{m\ MASP2^*}}^{\mathrm{MASP2_{zym}}}}+\frac{[\mathrm{pMFMASP1_{zym}}]}{K_{\mathrm{m\ MASP2^*}}^{\mathrm{MASP1_{zym}}}}+\frac{[\mathrm{pMFMASP2_{zym}}]}{K_{\mathrm{m\ MASP2^*}}^{\mathrm{MASP2_{zym}}}}\\
+\frac{[\mathrm{pPTX3LFMASP1_{zym}}]}{K_{\mathrm{m\ MASP2^*}}^{\mathrm{MASP1_{zym}}}}+\frac{[\mathrm{pPTX3LFMASP2_{zym}}]}{K_{\mathrm{m\ MASP2^*}}^{\mathrm{MASP2_{zym}}}}\end{array}\right)}
$$

$$
+\frac{k_{\mathrm{cat\ MASP2^*}}^{\mathrm{MASP2_{zym}}}\left[\mathrm{pCLL1MASP2^*}\right]\left[\mathrm{pCLLKMASP2_{zym}}\right]}{K_{\mathrm{m\ MASP2^*}}^{\mathrm{MASP2_{zym}}}\left(\begin{array}{l}1+\frac{[\mathrm{C4}]}{K_{\mathrm{m\ MASP2^*}}^{\mathrm{C4}}}+\frac{[\mathrm{fC4bC2}]}{K_{\mathrm{m\ MASP2^*}}^{\mathrm{C2}}}+\frac{[\mathrm{fC4bC4bC2}]}{K_{\mathrm{m\ MASP2^*}}^{\mathrm{C2}}}+\frac{[\mathrm{fC3bC4bC2}]}{K_{\mathrm{m\ MASP2^*}}^{\mathrm{C2}}}+\frac{[\mathrm{pC4bC2}]}{K_{\mathrm{m\ MASP2^*}}^{\mathrm{C2}}}+\frac{[\mathrm{pC4bC4bC2}]}{K_{\mathrm{m\ MASP2^*}}^{\mathrm{C2}}}\\
+\frac{[\mathrm{pC3bC4bC2}]}{K_{\mathrm{m\ MASP2^*}}^{\mathrm{C2}}}+\frac{[\mathrm{pMBL_3MASP1_{zym}}]}{K_{\mathrm{m\ MASP2^*}}^{\mathrm{MASP1_{zym}}}}+\frac{[\mathrm{pMBL_3MASP2_{zym}}]}{K_{\mathrm{m\ MASP2^*}}^{\mathrm{MASP2_{zym}}}}+\frac{[\mathrm{pMBL_4MASP1_{zym}}]}{K_{\mathrm{m\ MASP2^*}}^{\mathrm{MASP1_{zym}}}}\\
+\frac{[\mathrm{pMBL_4MASP2_{zym}}]}{K_{\mathrm{m\ MASP2^*}}^{\mathrm{MASP2_{zym}}}}+\frac{[\mathrm{pCLLKMASP1_{zym}}]}{K_{\mathrm{m\ MASP2^*}}^{\mathrm{MASP1_{zym}}}}+\frac{[\mathrm{pCLLKMASP2_{zym}}]}{K_{\mathrm{m\ MASP2^*}}^{\mathrm{MASP2_{zym}}}}+\frac{[\mathrm{pCLK1MASP1_{zym}}]}{K_{\mathrm{m\ MASP2^*}}^{\mathrm{MASP1_{zym}}}}\\
+\frac{[\mathrm{pCLK1MASP2_{zym}}]}{K_{\mathrm{m\ MASP2^*}}^{\mathrm{MASP2_{zym}}}}+\frac{[\mathrm{pCLL1MASP1_{zym}}]}{K_{\mathrm{m\ MASP2^*}}^{\mathrm{MASP1_{zym}}}}+\frac{[\mathrm{pCLL1MASP2_{zym}}]}{K_{\mathrm{m\ MASP2^*}}^{\mathrm{MASP2_{zym}}}}+\frac{[\mathrm{pLFMASP1_{zym}}]}{K_{\mathrm{m\ MASP2^*}}^{\mathrm{MASP1_{zym}}}}\\
+\frac{[\mathrm{pLFMASP2_{zym}}]}{K_{\mathrm{m\ MASP2^*}}^{\mathrm{MASP2_{zym}}}}+\frac{[\mathrm{pHFMASP1_{zym}}]}{K_{\mathrm{m\ MASP2^*}}^{\mathrm{MASP1_{zym}}}}+\frac{[\mathrm{pHFMASP2_{zym}}]}{K_{\mathrm{m\ MASP2^*}}^{\mathrm{MASP2_{zym}}}}+\frac{[\mathrm{pMFMASP1_{zym}}]}{K_{\mathrm{m\ MASP2^*}}^{\mathrm{MASP1_{zym}}}}+\frac{[\mathrm{pMFMASP2_{zym}}]}{K_{\mathrm{m\ MASP2^*}}^{\mathrm{MASP2_{zym}}}}\\
+\frac{[\mathrm{pPTX3LFMASP1_{zym}}]}{K_{\mathrm{m\ MASP2^*}}^{\mathrm{MASP1_{zym}}}}+\frac{[\mathrm{pPTX3LFMASP2_{zym}}]}{K_{\mathrm{m\ MASP2^*}}^{\mathrm{MASP2_{zym}}}}\end{array}\right)}
$$

$$+\frac{k_{\text{cat MASP2}^*}^{\text{MASP2}_{\text{zym}}}\left[\text{pLFMASP2}^*\right]\left[\text{pCLLKMASP2}_{\text{zym}}\right]}{K_{\text{m MASP2}^*}^{\text{MASP2}_{\text{zym}}}\left(\begin{array}{l}1+\frac{[\text{C4}]}{K_{\text{m MASP2}^*}^{\text{C4}}}+\frac{[\text{fC4bC2}]}{K_{\text{m MASP2}^*}^{\text{C2}}}+\frac{[\text{fC4bC4bC2}]}{K_{\text{m MASP2}^*}^{\text{C2}}}+\frac{[\text{fC3bC4bC2}]}{K_{\text{m MASP2}^*}^{\text{C2}}}+\frac{[\text{pC4bC2}]}{K_{\text{m MASP2}^*}^{\text{C2}}}+\frac{[\text{pC4bC4bC2}]}{K_{\text{m MASP2}^*}^{\text{C2}}}\\+\frac{[\text{pC3bC4bC2}]}{K_{\text{m MASP2}^*}^{\text{C2}}}+\frac{[\text{pMBL}_3\text{MASP1}_{\text{zym}}]}{K_{\text{m MASP2}^*}^{\text{MASP1}_{\text{zym}}}}+\frac{[\text{pMBL}_3\text{MASP2}_{\text{zym}}]}{K_{\text{m MASP2}^*}^{\text{MASP2}_{\text{zym}}}}+\frac{[\text{pMBL}_4\text{MASP1}_{\text{zym}}]}{K_{\text{m MASP2}^*}^{\text{MASP1}_{\text{zym}}}}\\+\frac{[\text{pMBL}_4\text{MASP2}_{\text{zym}}]}{K_{\text{m MASP2}^*}^{\text{MASP2}_{\text{zym}}}}+\frac{[\text{pCLLKMASP1}_{\text{zym}}]}{K_{\text{m MASP2}^*}^{\text{MASP1}_{\text{zym}}}}+\frac{[\text{pCLLKMASP2}_{\text{zym}}]}{K_{\text{m MASP2}^*}^{\text{MASP2}_{\text{zym}}}}+\frac{[\text{pCLK1MASP1}_{\text{zym}}]}{K_{\text{m MASP2}^*}^{\text{MASP1}_{\text{zym}}}}\\+\frac{[\text{pCLK1MASP2}_{\text{zym}}]}{K_{\text{m MASP2}^*}^{\text{MASP2}_{\text{zym}}}}+\frac{[\text{pCLL1MASP1}_{\text{zym}}]}{K_{\text{m MASP2}^*}^{\text{MASP1}_{\text{zym}}}}+\frac{[\text{pCLL1MASP2}_{\text{zym}}]}{K_{\text{m MASP2}^*}^{\text{MASP2}_{\text{zym}}}}+\frac{[\text{pLFMASP1}_{\text{zym}}]}{K_{\text{m MASP2}^*}^{\text{MASP1}_{\text{zym}}}}\\+\frac{[\text{pLFMASP2}_{\text{zym}}]}{K_{\text{m MASP2}^*}^{\text{MASP2}_{\text{zym}}}}+\frac{[\text{pHFMASP1}_{\text{zym}}]}{K_{\text{m MASP2}^*}^{\text{MASP1}_{\text{zym}}}}+\frac{[\text{pHFMASP2}_{\text{zym}}]}{K_{\text{m MASP2}^*}^{\text{MASP2}_{\text{zym}}}}+\frac{[\text{pMFMASP1}_{\text{zym}}]}{K_{\text{m MASP2}^*}^{\text{MASP1}_{\text{zym}}}}+\frac{[\text{pMFMASP2}_{\text{zym}}]}{K_{\text{m MASP2}^*}^{\text{MASP2}_{\text{zym}}}}\\+\frac{[\text{pPTX3LFMASP1}_{\text{zym}}]}{K_{\text{m MASP2}^*}^{\text{MASP1}_{\text{zym}}}}+\frac{[\text{pPTX3LFMASP2}_{\text{zym}}]}{K_{\text{m MASP2}^*}^{\text{MASP2}_{\text{zym}}}}\end{array}\right)}$$

$$+\frac{k_{\text{cat MASP2}^*}^{\text{MASP2}_{\text{zym}}}\left[\text{pHFMASP2}^*\right]\left[\text{pCLLKMASP2}_{\text{zym}}\right]}{K_{\text{m MASP2}^*}^{\text{MASP2}_{\text{zym}}}\left(\begin{array}{l}1+\frac{[\text{C4}]}{K_{\text{m MASP2}^*}^{\text{C4}}}+\frac{[\text{fC4bC2}]}{K_{\text{m MASP2}^*}^{\text{C2}}}+\frac{[\text{fC4bC4bC2}]}{K_{\text{m MASP2}^*}^{\text{C2}}}+\frac{[\text{fC3bC4bC2}]}{K_{\text{m MASP2}^*}^{\text{C2}}}+\frac{[\text{pC4bC2}]}{K_{\text{m MASP2}^*}^{\text{C2}}}+\frac{[\text{pC4bC4bC2}]}{K_{\text{m MASP2}^*}^{\text{C2}}}\\+\frac{[\text{pC3bC4bC2}]}{K_{\text{m MASP2}^*}^{\text{C2}}}+\frac{[\text{pMBL}_3\text{MASP1}_{\text{zym}}]}{K_{\text{m MASP2}^*}^{\text{MASP1}_{\text{zym}}}}+\frac{[\text{pMBL}_3\text{MASP2}_{\text{zym}}]}{K_{\text{m MASP2}^*}^{\text{MASP2}_{\text{zym}}}}+\frac{[\text{pMBL}_4\text{MASP1}_{\text{zym}}]}{K_{\text{m MASP2}^*}^{\text{MASP1}_{\text{zym}}}}\\+\frac{[\text{pMBL}_4\text{MASP2}_{\text{zym}}]}{K_{\text{m MASP2}^*}^{\text{MASP2}_{\text{zym}}}}+\frac{[\text{pCLLKMASP1}_{\text{zym}}]}{K_{\text{m MASP2}^*}^{\text{MASP1}_{\text{zym}}}}+\frac{[\text{pCLLKMASP2}_{\text{zym}}]}{K_{\text{m MASP2}^*}^{\text{MASP2}_{\text{zym}}}}+\frac{[\text{pCLK1MASP1}_{\text{zym}}]}{K_{\text{m MASP2}^*}^{\text{MASP1}_{\text{zym}}}}\\+\frac{[\text{pCLK1MASP2}_{\text{zym}}]}{K_{\text{m MASP2}^*}^{\text{MASP2}_{\text{zym}}}}+\frac{[\text{pCLL1MASP1}_{\text{zym}}]}{K_{\text{m MASP2}^*}^{\text{MASP1}_{\text{zym}}}}+\frac{[\text{pCLL1MASP2}_{\text{zym}}]}{K_{\text{m MASP2}^*}^{\text{MASP2}_{\text{zym}}}}+\frac{[\text{pLFMASP1}_{\text{zym}}]}{K_{\text{m MASP2}^*}^{\text{MASP1}_{\text{zym}}}}\\+\frac{[\text{pLFMASP2}_{\text{zym}}]}{K_{\text{m MASP2}^*}^{\text{MASP2}_{\text{zym}}}}+\frac{[\text{pHFMASP1}_{\text{zym}}]}{K_{\text{m MASP2}^*}^{\text{MASP1}_{\text{zym}}}}+\frac{[\text{pHFMASP2}_{\text{zym}}]}{K_{\text{m MASP2}^*}^{\text{MASP2}_{\text{zym}}}}+\frac{[\text{pMFMASP1}_{\text{zym}}]}{K_{\text{m MASP2}^*}^{\text{MASP1}_{\text{zym}}}}+\frac{[\text{pMFMASP2}_{\text{zym}}]}{K_{\text{m MASP2}^*}^{\text{MASP2}_{\text{zym}}}}\\+\frac{[\text{pPTX3LFMASP1}_{\text{zym}}]}{K_{\text{m MASP2}^*}^{\text{MASP1}_{\text{zym}}}}+\frac{[\text{pPTX3LFMASP2}_{\text{zym}}]}{K_{\text{m MASP2}^*}^{\text{MASP2}_{\text{zym}}}}\end{array}\right)}$$

$$
+\frac{k_{\text{cat MASP2}^*}^{\text{MASP2}_{\text{zym}}}\left[\text{pMFMASP2}^*\right]\left[\text{pCLLKMASP2}_{\text{zym}}\right]}{K_{\text{m MASP2}^*}^{\text{MASP2}_{\text{zym}}}\left(\begin{array}{l}1+\frac{[\text{C4}]}{K_{\text{m MASP2}^*}^{\text{C4}}}+\frac{[\text{fC4bC2}]}{K_{\text{m MASP2}^*}^{\text{C2}}}+\frac{[\text{fC4bC4bC2}]}{K_{\text{m MASP2}^*}^{\text{C2}}}+\frac{[\text{fC3bC4bC2}]}{K_{\text{m MASP2}^*}^{\text{C2}}}+\frac{[\text{pC4bC2}]}{K_{\text{m MASP2}^*}^{\text{C2}}}+\frac{[\text{pC4bC4bC2}]}{K_{\text{m MASP2}^*}^{\text{C2}}}\\
+\frac{[\text{pC3bC4bC2}]}{K_{\text{m MASP2}^*}^{\text{C2}}}+\frac{\left[\text{pMBL}_3\text{MASP1}_{\text{zym}}\right]}{K_{\text{m MASP2}^*}^{\text{MASP1}_{\text{zym}}}}+\frac{\left[\text{pMBL}_3\text{MASP2}_{\text{zym}}\right]}{K_{\text{m MASP2}^*}^{\text{MASP2}_{\text{zym}}}}+\frac{\left[\text{pMBL}_4\text{MASP1}_{\text{zym}}\right]}{K_{\text{m MASP2}^*}^{\text{MASP1}_{\text{zym}}}}\\
+\frac{\left[\text{pMBL}_4\text{MASP2}_{\text{zym}}\right]}{K_{\text{m MASP2}^*}^{\text{MASP2}_{\text{zym}}}}+\frac{\left[\text{pCLLKMASP1}_{\text{zym}}\right]}{K_{\text{m MASP2}^*}^{\text{MASP1}_{\text{zym}}}}+\frac{\left[\text{pCLLKMASP2}_{\text{zym}}\right]}{K_{\text{m MASP2}^*}^{\text{MASP2}_{\text{zym}}}}+\frac{\left[\text{pCLK1MASP1}_{\text{zym}}\right]}{K_{\text{m MASP2}^*}^{\text{MASP1}_{\text{zym}}}}\\
+\frac{\left[\text{pCLK1MASP2}_{\text{zym}}\right]}{K_{\text{m MASP2}^*}^{\text{MASP2}_{\text{zym}}}}+\frac{\left[\text{pCLL1MASP1}_{\text{zym}}\right]}{K_{\text{m MASP2}^*}^{\text{MASP1}_{\text{zym}}}}+\frac{\left[\text{pCLL1MASP2}_{\text{zym}}\right]}{K_{\text{m MASP2}^*}^{\text{MASP2}_{\text{zym}}}}+\frac{\left[\text{pLFMASP1}_{\text{zym}}\right]}{K_{\text{m MASP2}^*}^{\text{MASP1}_{\text{zym}}}}\\
+\frac{\left[\text{pLFMASP2}_{\text{zym}}\right]}{K_{\text{m MASP2}^*}^{\text{MASP2}_{\text{zym}}}}+\frac{\left[\text{pHFMASP1}_{\text{zym}}\right]}{K_{\text{m MASP2}^*}^{\text{MASP1}_{\text{zym}}}}+\frac{\left[\text{pHFMASP2}_{\text{zym}}\right]}{K_{\text{m MASP2}^*}^{\text{MASP2}_{\text{zym}}}}+\frac{\left[\text{pMFMASP1}_{\text{zym}}\right]}{K_{\text{m MASP2}^*}^{\text{MASP1}_{\text{zym}}}}+\frac{\left[\text{pMFMASP2}_{\text{zym}}\right]}{K_{\text{m MASP2}^*}^{\text{MASP2}_{\text{zym}}}}\\
+\frac{\left[\text{pPTX3LFMASP1}_{\text{zym}}\right]}{K_{\text{m MASP2}^*}^{\text{MASP1}_{\text{zym}}}}+\frac{\left[\text{pPTX3LFMASP2}_{\text{zym}}\right]}{K_{\text{m MASP2}^*}^{\text{MASP2}_{\text{zym}}}}\end{array}\right)}
$$

$$
+\frac{k_{\text{cat MASP2}^*}^{\text{MASP2}_{\text{zym}}}\left[\text{pPTX3LFMASP2}^*\right]\left[\text{pCLLKMASP2}_{\text{zym}}\right]}{K_{\text{m MASP2}^*}^{\text{MASP2}_{\text{zym}}}\left(\begin{array}{l}1+\frac{[\text{C4}]}{K_{\text{m MASP2}^*}^{\text{C4}}}+\frac{[\text{fC4bC2}]}{K_{\text{m MASP2}^*}^{\text{C2}}}+\frac{[\text{fC4bC4bC2}]}{K_{\text{m MASP2}^*}^{\text{C2}}}+\frac{[\text{fC3bC4bC2}]}{K_{\text{m MASP2}^*}^{\text{C2}}}+\frac{[\text{pC4bC2}]}{K_{\text{m MASP2}^*}^{\text{C2}}}+\frac{[\text{pC4bC4bC2}]}{K_{\text{m MASP2}^*}^{\text{C2}}}\\
+\frac{[\text{pC3bC4bC2}]}{K_{\text{m MASP2}^*}^{\text{C2}}}+\frac{\left[\text{pMBL}_3\text{MASP1}_{\text{zym}}\right]}{K_{\text{m MASP2}^*}^{\text{MASP1}_{\text{zym}}}}+\frac{\left[\text{pMBL}_3\text{MASP2}_{\text{zym}}\right]}{K_{\text{m MASP2}^*}^{\text{MASP2}_{\text{zym}}}}+\frac{\left[\text{pMBL}_4\text{MASP1}_{\text{zym}}\right]}{K_{\text{m MASP2}^*}^{\text{MASP1}_{\text{zym}}}}\\
+\frac{\left[\text{pMBL}_4\text{MASP2}_{\text{zym}}\right]}{K_{\text{m MASP2}^*}^{\text{MASP2}_{\text{zym}}}}+\frac{\left[\text{pCLLKMASP1}_{\text{zym}}\right]}{K_{\text{m MASP2}^*}^{\text{MASP1}_{\text{zym}}}}+\frac{\left[\text{pCLLKMASP2}_{\text{zym}}\right]}{K_{\text{m MASP2}^*}^{\text{MASP2}_{\text{zym}}}}+\frac{\left[\text{pCLK1MASP1}_{\text{zym}}\right]}{K_{\text{m MASP2}^*}^{\text{MASP1}_{\text{zym}}}}\\
+\frac{\left[\text{pCLK1MASP2}_{\text{zym}}\right]}{K_{\text{m MASP2}^*}^{\text{MASP2}_{\text{zym}}}}+\frac{\left[\text{pCLL1MASP1}_{\text{zym}}\right]}{K_{\text{m MASP2}^*}^{\text{MASP1}_{\text{zym}}}}+\frac{\left[\text{pCLL1MASP2}_{\text{zym}}\right]}{K_{\text{m MASP2}^*}^{\text{MASP2}_{\text{zym}}}}+\frac{\left[\text{pLFMASP1}_{\text{zym}}\right]}{K_{\text{m MASP2}^*}^{\text{MASP1}_{\text{zym}}}}\\
+\frac{\left[\text{pLFMASP2}_{\text{zym}}\right]}{K_{\text{m MASP2}^*}^{\text{MASP2}_{\text{zym}}}}+\frac{\left[\text{pHFMASP1}_{\text{zym}}\right]}{K_{\text{m MASP2}^*}^{\text{MASP1}_{\text{zym}}}}+\frac{\left[\text{pHFMASP2}_{\text{zym}}\right]}{K_{\text{m MASP2}^*}^{\text{MASP2}_{\text{zym}}}}+\frac{\left[\text{pMFMASP1}_{\text{zym}}\right]}{K_{\text{m MASP2}^*}^{\text{MASP1}_{\text{zym}}}}+\frac{\left[\text{pMFMASP2}_{\text{zym}}\right]}{K_{\text{m MASP2}^*}^{\text{MASP2}_{\text{zym}}}}\\
+\frac{\left[\text{pPTX3LFMASP1}_{\text{zym}}\right]}{K_{\text{m MASP2}^*}^{\text{MASP1}_{\text{zym}}}}+\frac{\left[\text{pPTX3LFMASP2}_{\text{zym}}\right]}{K_{\text{m MASP2}^*}^{\text{MASP2}_{\text{zym}}}}\end{array}\right)}
$$

$$
-k_{\text{C1INHMASP2}}^{+}\left[\text{C1-INH}\right]\left[\text{pCLLKMASP2}^*\right]
$$

292

$$
\frac{d\left[\text{fCLLKMASP3}_{\text{zym}}\right]}{dt}=k_{\text{CLLKMASP3}}^{+}\left[\text{CLLK}\right]\left[\text{MASP3}\right]-k_{\text{CLLKMASP3}}^{-}\left[\text{fCLLKMASP3}_{\text{zym}}\right]
$$

$$
-k_{\text{pCLLK}}^{+}\left[\text{fCLLKMASP3}_{\text{zym}}\right]\left[\text{Pathogen Surface}\right]+k_{\text{pCLLK}}^{-}\left[\text{pCLLKMASP3}_{\text{zym}}\right]
$$

293

$$\frac{d\left[\text{pCLLKMASP3}_{\text{zym}}\right]}{dt}=k_{\text{pCLLK}}^{+}\left[\text{fCLLKMASP3}_{\text{zym}}\right]\left[\text{Pathogen Surface}\right]-k_{\text{pCLLK}}^{-}\left[\text{pCLLKMASP3}_{\text{zym}}\right]$$

$$+k_{\text{CLLKMASP3}}^{+}\left[\text{pCLLK}\right]\left[\text{MASP3}\right]-k_{\text{CLLKMASP3}}^{-}\left[\text{pCLLKMASP3}_{\text{zym}}\right]$$

$$-\frac{k_{\text{cat MASP1}^*}^{\text{MASP3}_{\text{zym}}}\left[\text{pMBL}_3\text{MASP1}^*\right]\left[\text{pCLLKMASP3}_{\text{zym}}\right]}{K_{\text{m MASP1}^*}^{\text{MASP3}_{\text{zym}}}\left(\begin{array}{l}1+\frac{\left[\text{C4}\right]}{K_{\text{m MASP1}^*}^{\text{C4}}}+\frac{\left[\text{fC4bC2}\right]}{K_{\text{m MASP1}^*}^{\text{C2}}}+\frac{\left[\text{fC4bC4bC2}\right]}{K_{\text{m MASP1}^*}^{\text{C2}}}+\frac{\left[\text{fC3bC4bC2}\right]}{K_{\text{m MASP1}^*}^{\text{C2}}}+\frac{\left[\text{pC4bC2}\right]}{K_{\text{m MASP1}^*}^{\text{C2}}}+\frac{\left[\text{pC4bC4bC2}\right]}{K_{\text{m MASP1}^*}^{\text{C2}}}\\+\frac{\left[\text{pC3bC4bC2}\right]}{K_{\text{m MASP1}^*}^{\text{C2}}}+\frac{\left[\text{pMBL}_3\text{MASP1}_{\text{zym}}\right]}{K_{\text{m MASP1}^*}^{\text{MASP1}_{\text{zym}}}}+\frac{\left[\text{pMBL}_3\text{MASP2}_{\text{zym}}\right]}{K_{\text{m MASP1}^*}^{\text{MASP2}_{\text{zym}}}}+\frac{\left[\text{pMBL}_3\text{MASP3}_{\text{zym}}\right]}{K_{\text{m MASP1}^*}^{\text{MASP3}_{\text{zym}}}}\\+\frac{\left[\text{pMBL}_4\text{MASP1}_{\text{zym}}\right]}{K_{\text{m MASP1}^*}^{\text{MASP1}_{\text{zym}}}}+\frac{\left[\text{pMBL}_4\text{MASP2}_{\text{zym}}\right]}{K_{\text{m MASP1}^*}^{\text{MASP2}_{\text{zym}}}}+\frac{\left[\text{pMBL}_4\text{MASP3}_{\text{zym}}\right]}{K_{\text{m MASP1}^*}^{\text{MASP3}_{\text{zym}}}}+\frac{\left[\text{pCLLKMASP1}_{\text{zym}}\right]}{K_{\text{m MASP1}^*}^{\text{MASP1}_{\text{zym}}}}\\+\frac{\left[\text{pCLLKMASP2}_{\text{zym}}\right]}{K_{\text{m MASP1}^*}^{\text{MASP2}_{\text{zym}}}}+\frac{\left[\text{pCLLKMASP3}_{\text{zym}}\right]}{K_{\text{m MASP1}^*}^{\text{MASP3}_{\text{zym}}}}+\frac{\left[\text{pCLK1MASP1}_{\text{zym}}\right]}{K_{\text{m MASP1}^*}^{\text{MASP1}_{\text{zym}}}}+\frac{\left[\text{pCLK1MASP2}_{\text{zym}}\right]}{K_{\text{m MASP1}^*}^{\text{MASP2}_{\text{zym}}}}\\+\frac{\left[\text{pCLK1MASP3}_{\text{zym}}\right]}{K_{\text{m MASP1}^*}^{\text{MASP3}_{\text{zym}}}}+\frac{\left[\text{pCLL1MASP1}_{\text{zym}}\right]}{K_{\text{m MASP1}^*}^{\text{MASP1}_{\text{zym}}}}+\frac{\left[\text{pCLL1MASP2}_{\text{zym}}\right]}{K_{\text{m MASP1}^*}^{\text{MASP2}_{\text{zym}}}}+\frac{\left[\text{pCLL1MASP3}_{\text{zym}}\right]}{K_{\text{m MASP1}^*}^{\text{MASP3}_{\text{zym}}}}\\+\frac{\left[\text{pLFMASP1}_{\text{zym}}\right]}{K_{\text{m MASP1}^*}^{\text{MASP1}_{\text{zym}}}}+\frac{\left[\text{pLFMASP2}_{\text{zym}}\right]}{K_{\text{m MASP1}^*}^{\text{MASP2}_{\text{zym}}}}+\frac{\left[\text{pLFMASP3}_{\text{zym}}\right]}{K_{\text{m MASP1}^*}^{\text{MASP3}_{\text{zym}}}}+\frac{\left[\text{pHFMASP1}_{\text{zym}}\right]}{K_{\text{m MASP1}^*}^{\text{MASP1}_{\text{zym}}}}+\frac{\left[\text{pHFMASP2}_{\text{zym}}\right]}{K_{\text{m MASP1}^*}^{\text{MASP2}_{\text{zym}}}}\\+\frac{\left[\text{pHFMASP3}_{\text{zym}}\right]}{K_{\text{m MASP1}^*}^{\text{MASP3}_{\text{zym}}}}+\frac{\left[\text{pMFMASP1}_{\text{zym}}\right]}{K_{\text{m MASP1}^*}^{\text{MASP1}_{\text{zym}}}}+\frac{\left[\text{pMFMASP2}_{\text{zym}}\right]}{K_{\text{m MASP1}^*}^{\text{MASP2}_{\text{zym}}}}+\frac{\left[\text{pMFMASP3}_{\text{zym}}\right]}{K_{\text{m MASP1}^*}^{\text{MASP3}_{\text{zym}}}}\\+\frac{\left[\text{pPTX3LFMASP1}_{\text{zym}}\right]}{K_{\text{m MASP1}^*}^{\text{MASP1}_{\text{zym}}}}+\frac{\left[\text{pPXT3LFMASP2}_{\text{zym}}\right]}{K_{\text{m MASP1}^*}^{\text{MASP2}_{\text{zym}}}}+\frac{\left[\text{pPTX3LFMASP3}_{\text{zym}}\right]}{K_{\text{m MASP1}^*}^{\text{MASP3}_{\text{zym}}}}\end{array}\right)}$$

$$-\frac{k_{\mathrm{cat\,MASP1^*}}^{\mathrm{MASP3_{zym}}}\left[\mathrm{pMBL_4MASP1^*}\right]\left[\mathrm{pCLLKMASP3_{zym}}\right]}{K_{\mathrm{m\,MASP1^*}}^{\mathrm{MASP3_{zym}}}\left(\begin{array}{l}1+\frac{[\mathrm{C4}]}{K_{\mathrm{m\,MASP1^*}}^{\mathrm{C4}}}+\frac{[\mathrm{fC4bC2}]}{K_{\mathrm{m\,MASP1^*}}^{\mathrm{C2}}}+\frac{[\mathrm{fC4bC4bC2}]}{K_{\mathrm{m\,MASP1^*}}^{\mathrm{C2}}}+\frac{[\mathrm{fC3bC4bC2}]}{K_{\mathrm{m\,MASP1^*}}^{\mathrm{C2}}}+\frac{[\mathrm{pC4bC2}]}{K_{\mathrm{m\,MASP1^*}}^{\mathrm{C2}}}+\frac{[\mathrm{pC4bC4bC2}]}{K_{\mathrm{m\,MASP1^*}}^{\mathrm{C2}}}\\ +\frac{[\mathrm{pC3bC4bC2}]}{K_{\mathrm{m\,MASP1^*}}^{\mathrm{C2}}}+\frac{[\mathrm{pMBL_3MASP1_{zym}}]}{K_{\mathrm{m\,MASP1^*}}^{\mathrm{MASP1_{zym}}}}+\frac{[\mathrm{pMBL_3MASP2_{zym}}]}{K_{\mathrm{m\,MASP1^*}}^{\mathrm{MASP2_{zym}}}}+\frac{[\mathrm{pMBL_3MASP3_{zym}}]}{K_{\mathrm{m\,MASP1^*}}^{\mathrm{MASP3_{zym}}}}\\ +\frac{[\mathrm{pMBL_4MASP1_{zym}}]}{K_{\mathrm{m\,MASP1^*}}^{\mathrm{MASP1_{zym}}}}+\frac{[\mathrm{pMBL_4MASP2_{zym}}]}{K_{\mathrm{m\,MASP1^*}}^{\mathrm{MASP2_{zym}}}}+\frac{[\mathrm{pMBL_4MASP3_{zym}}]}{K_{\mathrm{m\,MASP1^*}}^{\mathrm{MASP3_{zym}}}}+\frac{[\mathrm{pCLLKMASP1_{zym}}]}{K_{\mathrm{m\,MASP1^*}}^{\mathrm{MASP1_{zym}}}}\\ +\frac{[\mathrm{pCLLKMASP2_{zym}}]}{K_{\mathrm{m\,MASP1^*}}^{\mathrm{MASP2_{zym}}}}+\frac{[\mathrm{pCLLKMASP3_{zym}}]}{K_{\mathrm{m\,MASP1^*}}^{\mathrm{MASP3_{zym}}}}+\frac{[\mathrm{pCLK1MASP1_{zym}}]}{K_{\mathrm{m\,MASP1^*}}^{\mathrm{MASP1_{zym}}}}+\frac{[\mathrm{pCLK1MASP2_{zym}}]}{K_{\mathrm{m\,MASP1^*}}^{\mathrm{MASP2_{zym}}}}\\ +\frac{[\mathrm{pCLK1MASP3_{zym}}]}{K_{\mathrm{m\,MASP1^*}}^{\mathrm{MASP3_{zym}}}}+\frac{[\mathrm{pCLL1MASP1_{zym}}]}{K_{\mathrm{m\,MASP1^*}}^{\mathrm{MASP1_{zym}}}}+\frac{[\mathrm{pCLL1MASP2_{zym}}]}{K_{\mathrm{m\,MASP1^*}}^{\mathrm{MASP2_{zym}}}}+\frac{[\mathrm{pCLL1MASP3_{zym}}]}{K_{\mathrm{m\,MASP1^*}}^{\mathrm{MASP3_{zym}}}}\\ +\frac{[\mathrm{pLFMASP1_{zym}}]}{K_{\mathrm{m\,MASP1^*}}^{\mathrm{MASP1_{zym}}}}+\frac{[\mathrm{pLFMASP2_{zym}}]}{K_{\mathrm{m\,MASP1^*}}^{\mathrm{MASP2_{zym}}}}+\frac{[\mathrm{pLFMASP3_{zym}}]}{K_{\mathrm{m\,MASP1^*}}^{\mathrm{MASP3_{zym}}}}+\frac{[\mathrm{pHFMASP1_{zym}}]}{K_{\mathrm{m\,MASP1^*}}^{\mathrm{MASP1_{zym}}}}+\frac{[\mathrm{pHFMASP2_{zym}}]}{K_{\mathrm{m\,MASP1^*}}^{\mathrm{MASP2_{zym}}}}\\ +\frac{[\mathrm{pHFMASP3_{zym}}]}{K_{\mathrm{m\,MASP1^*}}^{\mathrm{MASP3_{zym}}}}+\frac{[\mathrm{pMFMASP1_{zym}}]}{K_{\mathrm{m\,MASP1^*}}^{\mathrm{MASP1_{zym}}}}+\frac{[\mathrm{pMFMASP2_{zym}}]}{K_{\mathrm{m\,MASP1^*}}^{\mathrm{MASP2_{zym}}}}+\frac{[\mathrm{pMFMASP3_{zym}}]}{K_{\mathrm{m\,MASP1^*}}^{\mathrm{MASP3_{zym}}}}\\ +\frac{[\mathrm{pPTX3LFMASP1_{zym}}]}{K_{\mathrm{m\,MASP1^*}}^{\mathrm{MASP1_{zym}}}}+\frac{[\mathrm{pPXT3LFMASP2_{zym}}]}{K_{\mathrm{m\,MASP1^*}}^{\mathrm{MASP2_{zym}}}}+\frac{[\mathrm{pPTX3LFMASP3_{zym}}]}{K_{\mathrm{m\,MASP1^*}}^{\mathrm{MASP3_{zym}}}}\end{array}\right)}$$

$$-\frac{k_{\text{cat MASP1}^*}^{\text{MASP3}_{\text{zym}}}\left[\text{pCLLKMASP1}^*\right]\left[\text{pCLLKMASP3}_{\text{zym}}\right]}{K_{\text{m MASP1}^*}^{\text{MASP3}_{\text{zym}}}\left(\begin{array}{l}1+\frac{[\text{C4}]}{K_{\text{m MASP1}^*}^{\text{C4}}}+\frac{[\text{fC4bC2}]}{K_{\text{m MASP1}^*}^{\text{C2}}}+\frac{[\text{fC4bC4bC2}]}{K_{\text{m MASP1}^*}^{\text{C2}}}+\frac{[\text{fC3bC4bC2}]}{K_{\text{m MASP1}^*}^{\text{C2}}}+\frac{[\text{pC4bC2}]}{K_{\text{m MASP1}^*}^{\text{C2}}}+\frac{[\text{pC4bC4bC2}]}{K_{\text{m MASP1}^*}^{\text{C2}}}\\
+\frac{[\text{pC3bC4bC2}]}{K_{\text{m MASP1}^*}^{\text{C2}}}+\frac{[\text{pMBL}_3\text{MASP1}_{\text{zym}}]}{K_{\text{m MASP1}^*}^{\text{MASP1}_{\text{zym}}}}+\frac{[\text{pMBL}_3\text{MASP2}_{\text{zym}}]}{K_{\text{m MASP1}^*}^{\text{MASP2}_{\text{zym}}}}+\frac{[\text{pMBL}_3\text{MASP3}_{\text{zym}}]}{K_{\text{m MASP1}^*}^{\text{MASP3}_{\text{zym}}}}\\
+\frac{[\text{pMBL}_4\text{MASP1}_{\text{zym}}]}{K_{\text{m MASP1}^*}^{\text{MASP1}_{\text{zym}}}}+\frac{[\text{pMBL}_4\text{MASP2}_{\text{zym}}]}{K_{\text{m MASP1}^*}^{\text{MASP2}_{\text{zym}}}}+\frac{[\text{pMBL}_4\text{MASP3}_{\text{zym}}]}{K_{\text{m MASP1}^*}^{\text{MASP3}_{\text{zym}}}}+\frac{[\text{pCLLKMASP1}_{\text{zym}}]}{K_{\text{m MASP1}^*}^{\text{MASP1}_{\text{zym}}}}\\
+\frac{[\text{pCLLKMASP2}_{\text{zym}}]}{K_{\text{m MASP1}^*}^{\text{MASP2}_{\text{zym}}}}+\frac{[\text{pCLLKMASP3}_{\text{zym}}]}{K_{\text{m MASP1}^*}^{\text{MASP3}_{\text{zym}}}}+\frac{[\text{pCLK1MASP1}_{\text{zym}}]}{K_{\text{m MASP1}^*}^{\text{MASP1}_{\text{zym}}}}+\frac{[\text{pCLK1MASP2}_{\text{zym}}]}{K_{\text{m MASP1}^*}^{\text{MASP2}_{\text{zym}}}}\\
+\frac{[\text{pCLK1MASP3}_{\text{zym}}]}{K_{\text{m MASP1}^*}^{\text{MASP3}_{\text{zym}}}}+\frac{[\text{pCLL1MASP1}_{\text{zym}}]}{K_{\text{m MASP1}^*}^{\text{MASP1}_{\text{zym}}}}+\frac{[\text{pCLL1MASP2}_{\text{zym}}]}{K_{\text{m MASP1}^*}^{\text{MASP2}_{\text{zym}}}}+\frac{[\text{pCLL1MASP3}_{\text{zym}}]}{K_{\text{m MASP1}^*}^{\text{MASP3}_{\text{zym}}}}\\
+\frac{[\text{pLFMASP1}_{\text{zym}}]}{K_{\text{m MASP1}^*}^{\text{MASP1}_{\text{zym}}}}+\frac{[\text{pLFMASP2}_{\text{zym}}]}{K_{\text{m MASP1}^*}^{\text{MASP2}_{\text{zym}}}}+\frac{[\text{pLFMASP3}_{\text{zym}}]}{K_{\text{m MASP1}^*}^{\text{MASP3}_{\text{zym}}}}+\frac{[\text{pHFMASP1}_{\text{zym}}]}{K_{\text{m MASP1}^*}^{\text{MASP1}_{\text{zym}}}}+\frac{[\text{pHFMASP2}_{\text{zym}}]}{K_{\text{m MASP1}^*}^{\text{MASP2}_{\text{zym}}}}\\
+\frac{[\text{pHFMASP3}_{\text{zym}}]}{K_{\text{m MASP1}^*}^{\text{MASP3}_{\text{zym}}}}+\frac{[\text{pMFMASP1}_{\text{zym}}]}{K_{\text{m MASP1}^*}^{\text{MASP1}_{\text{zym}}}}+\frac{[\text{pMFMASP2}_{\text{zym}}]}{K_{\text{m MASP1}^*}^{\text{MASP2}_{\text{zym}}}}+\frac{[\text{pMFMASP3}_{\text{zym}}]}{K_{\text{m MASP1}^*}^{\text{MASP3}_{\text{zym}}}}\\
+\frac{[\text{pPTX3LFMASP1}_{\text{zym}}]}{K_{\text{m MASP1}^*}^{\text{MASP1}_{\text{zym}}}}+\frac{[\text{pPXT3LFMASP2}_{\text{zym}}]}{K_{\text{m MASP1}^*}^{\text{MASP2}_{\text{zym}}}}+\frac{[\text{pPTX3LFMASP3}_{\text{zym}}]}{K_{\text{m MASP1}^*}^{\text{MASP3}_{\text{zym}}}}\end{array}\right)}$$

$$-\frac{k_{\text{cat MASP1}^*}^{\text{MASP3}_{\text{zym}}}\left[\text{pCLK1MASP1}^*\right]\left[\text{pCLLKMASP3}_{\text{zym}}\right]}{K_{\text{m MASP1}^*}^{\text{MASP3}_{\text{zym}}}\left(\begin{array}{l}1+\frac{[\text{C4}]}{K_{\text{m MASP1}^*}^{\text{C4}}}+\frac{[\text{fC4bC2}]}{K_{\text{m MASP1}^*}^{\text{C2}}}+\frac{[\text{fC4bC4bC2}]}{K_{\text{m MASP1}^*}^{\text{C2}}}+\frac{[\text{fC3bC4bC2}]}{K_{\text{m MASP1}^*}^{\text{C2}}}+\frac{[\text{pC4bC2}]}{K_{\text{m MASP1}^*}^{\text{C2}}}+\frac{[\text{pC4bC4bC2}]}{K_{\text{m MASP1}^*}^{\text{C2}}}\\+\frac{[\text{pC3bC4bC2}]}{K_{\text{m MASP1}^*}^{\text{C2}}}+\frac{[\text{pMBL}_3\text{MASP1}_{\text{zym}}]}{K_{\text{m MASP1}^*}^{\text{MASP1}_{\text{zym}}}}+\frac{[\text{pMBL}_3\text{MASP2}_{\text{zym}}]}{K_{\text{m MASP1}^*}^{\text{MASP2}_{\text{zym}}}}+\frac{[\text{pMBL}_3\text{MASP3}_{\text{zym}}]}{K_{\text{m MASP1}^*}^{\text{MASP3}_{\text{zym}}}}\\+\frac{[\text{pMBL}_4\text{MASP1}_{\text{zym}}]}{K_{\text{m MASP1}^*}^{\text{MASP1}_{\text{zym}}}}+\frac{[\text{pMBL}_4\text{MASP2}_{\text{zym}}]}{K_{\text{m MASP1}^*}^{\text{MASP2}_{\text{zym}}}}+\frac{[\text{pMBL}_4\text{MASP3}_{\text{zym}}]}{K_{\text{m MASP1}^*}^{\text{MASP3}_{\text{zym}}}}+\frac{[\text{pCLLKMASP1}_{\text{zym}}]}{K_{\text{m MASP1}^*}^{\text{MASP1}_{\text{zym}}}}\\+\frac{[\text{pCLLKMASP2}_{\text{zym}}]}{K_{\text{m MASP1}^*}^{\text{MASP2}_{\text{zym}}}}+\frac{[\text{pCLLKMASP3}_{\text{zym}}]}{K_{\text{m MASP1}^*}^{\text{MASP3}_{\text{zym}}}}+\frac{[\text{pCLK1MASP1}_{\text{zym}}]}{K_{\text{m MASP1}^*}^{\text{MASP1}_{\text{zym}}}}+\frac{[\text{pCLK1MASP2}_{\text{zym}}]}{K_{\text{m MASP1}^*}^{\text{MASP2}_{\text{zym}}}}\\+\frac{[\text{pCLK1MASP3}_{\text{zym}}]}{K_{\text{m MASP1}^*}^{\text{MASP3}_{\text{zym}}}}+\frac{[\text{pCLL1MASP1}_{\text{zym}}]}{K_{\text{m MASP1}^*}^{\text{MASP1}_{\text{zym}}}}+\frac{[\text{pCLL1MASP2}_{\text{zym}}]}{K_{\text{m MASP1}^*}^{\text{MASP2}_{\text{zym}}}}+\frac{[\text{pCLL1MASP3}_{\text{zym}}]}{K_{\text{m MASP1}^*}^{\text{MASP3}_{\text{zym}}}}\\+\frac{[\text{pLFMASP1}_{\text{zym}}]}{K_{\text{m MASP1}^*}^{\text{MASP1}_{\text{zym}}}}+\frac{[\text{pLFMASP2}_{\text{zym}}]}{K_{\text{m MASP1}^*}^{\text{MASP2}_{\text{zym}}}}+\frac{[\text{pLFMASP3}_{\text{zym}}]}{K_{\text{m MASP1}^*}^{\text{MASP3}_{\text{zym}}}}+\frac{[\text{pHFMASP1}_{\text{zym}}]}{K_{\text{m MASP1}^*}^{\text{MASP1}_{\text{zym}}}}+\frac{[\text{pHFMASP2}_{\text{zym}}]}{K_{\text{m MASP1}^*}^{\text{MASP2}_{\text{zym}}}}\\+\frac{[\text{pHFMASP3}_{\text{zym}}]}{K_{\text{m MASP1}^*}^{\text{MASP3}_{\text{zym}}}}+\frac{[\text{pMFMASP1}_{\text{zym}}]}{K_{\text{m MASP1}^*}^{\text{MASP1}_{\text{zym}}}}+\frac{[\text{pMFMASP2}_{\text{zym}}]}{K_{\text{m MASP1}^*}^{\text{MASP2}_{\text{zym}}}}+\frac{[\text{pMFMASP3}_{\text{zym}}]}{K_{\text{m MASP1}^*}^{\text{MASP3}_{\text{zym}}}}\\+\frac{[\text{pPTX3LFMASP1}_{\text{zym}}]}{K_{\text{m MASP1}^*}^{\text{MASP1}_{\text{zym}}}}+\frac{[\text{pPXT3LFMASP2}_{\text{zym}}]}{K_{\text{m MASP1}^*}^{\text{MASP2}_{\text{zym}}}}+\frac{[\text{pPTX3LFMASP3}_{\text{zym}}]}{K_{\text{m MASP1}^*}^{\text{MASP3}_{\text{zym}}}}\end{array}\right)}$$

$$-\frac{k_{\text{cat MASP1}^*}^{\text{MASP3}_{\text{zym}}}\left[\text{pCLL1MASP1}^*\right]\left[\text{pCLLKMASP3}_{\text{zym}}\right]}{K_{\text{m MASP1}^*}^{\text{MASP3}_{\text{zym}}}\left(\begin{array}{l}1+\frac{[\text{C4}]}{K_{\text{m MASP1}^*}^{\text{C4}}}+\frac{[\text{fC4bC2}]}{K_{\text{m MASP1}^*}^{\text{C2}}}+\frac{[\text{fC4bC4bC2}]}{K_{\text{m MASP1}^*}^{\text{C2}}}+\frac{[\text{fC3bC4bC2}]}{K_{\text{m MASP1}^*}^{\text{C2}}}+\frac{[\text{pC4bC2}]}{K_{\text{m MASP1}^*}^{\text{C2}}}+\frac{[\text{pC4bC4bC2}]}{K_{\text{m MASP1}^*}^{\text{C2}}}\\+\frac{[\text{pC3bC4bC2}]}{K_{\text{m MASP1}^*}^{\text{C2}}}+\frac{\left[\text{pMBL}_3\text{MASP1}_{\text{zym}}\right]}{K_{\text{m MASP1}^*}^{\text{MASP1}_{\text{zym}}}}+\frac{\left[\text{pMBL}_3\text{MASP2}_{\text{zym}}\right]}{K_{\text{m MASP1}^*}^{\text{MASP2}_{\text{zym}}}}+\frac{\left[\text{pMBL}_3\text{MASP3}_{\text{zym}}\right]}{K_{\text{m MASP1}^*}^{\text{MASP3}_{\text{zym}}}}\\+\frac{\left[\text{pMBL}_4\text{MASP1}_{\text{zym}}\right]}{K_{\text{m MASP1}^*}^{\text{MASP1}_{\text{zym}}}}+\frac{\left[\text{pMBL}_4\text{MASP2}_{\text{zym}}\right]}{K_{\text{m MASP1}^*}^{\text{MASP2}_{\text{zym}}}}+\frac{\left[\text{pMBL}_4\text{MASP3}_{\text{zym}}\right]}{K_{\text{m MASP1}^*}^{\text{MASP3}_{\text{zym}}}}+\frac{\left[\text{pCLLKMASP1}_{\text{zym}}\right]}{K_{\text{m MASP1}^*}^{\text{MASP1}_{\text{zym}}}}\\+\frac{\left[\text{pCLLKMASP2}_{\text{zym}}\right]}{K_{\text{m MASP1}^*}^{\text{MASP2}_{\text{zym}}}}+\frac{\left[\text{pCLLKMASP3}_{\text{zym}}\right]}{K_{\text{m MASP1}^*}^{\text{MASP3}_{\text{zym}}}}+\frac{\left[\text{pCLK1MASP1}_{\text{zym}}\right]}{K_{\text{m MASP1}^*}^{\text{MASP1}_{\text{zym}}}}+\frac{\left[\text{pCLK1MASP2}_{\text{zym}}\right]}{K_{\text{m MASP1}^*}^{\text{MASP2}_{\text{zym}}}}\\+\frac{\left[\text{pCLK1MASP3}_{\text{zym}}\right]}{K_{\text{m MASP1}^*}^{\text{MASP3}_{\text{zym}}}}+\frac{\left[\text{pCLL1MASP1}_{\text{zym}}\right]}{K_{\text{m MASP1}^*}^{\text{MASP1}_{\text{zym}}}}+\frac{\left[\text{pCLL1MASP2}_{\text{zym}}\right]}{K_{\text{m MASP1}^*}^{\text{MASP2}_{\text{zym}}}}+\frac{\left[\text{pCLL1MASP3}_{\text{zym}}\right]}{K_{\text{m MASP1}^*}^{\text{MASP3}_{\text{zym}}}}\\+\frac{\left[\text{pLFMASP1}_{\text{zym}}\right]}{K_{\text{m MASP1}^*}^{\text{MASP1}_{\text{zym}}}}+\frac{\left[\text{pLFMASP2}_{\text{zym}}\right]}{K_{\text{m MASP1}^*}^{\text{MASP2}_{\text{zym}}}}+\frac{\left[\text{pLFMASP3}_{\text{zym}}\right]}{K_{\text{m MASP1}^*}^{\text{MASP3}_{\text{zym}}}}+\frac{\left[\text{pHFMASP1}_{\text{zym}}\right]}{K_{\text{m MASP1}^*}^{\text{MASP1}_{\text{zym}}}}+\frac{\left[\text{pHFMASP2}_{\text{zym}}\right]}{K_{\text{m MASP1}^*}^{\text{MASP2}_{\text{zym}}}}\\+\frac{\left[\text{pHFMASP3}_{\text{zym}}\right]}{K_{\text{m MASP1}^*}^{\text{MASP3}_{\text{zym}}}}+\frac{\left[\text{pMFMASP1}_{\text{zym}}\right]}{K_{\text{m MASP1}^*}^{\text{MASP1}_{\text{zym}}}}+\frac{\left[\text{pMFMASP2}_{\text{zym}}\right]}{K_{\text{m MASP1}^*}^{\text{MASP2}_{\text{zym}}}}+\frac{\left[\text{pMFMASP3}_{\text{zym}}\right]}{K_{\text{m MASP1}^*}^{\text{MASP3}_{\text{zym}}}}\\+\frac{\left[\text{pPTX3LFMASP1}_{\text{zym}}\right]}{K_{\text{m MASP1}^*}^{\text{MASP1}_{\text{zym}}}}+\frac{\left[\text{pPXT3LFMASP2}_{\text{zym}}\right]}{K_{\text{m MASP1}^*}^{\text{MASP2}_{\text{zym}}}}+\frac{\left[\text{pPTX3LFMASP3}_{\text{zym}}\right]}{K_{\text{m MASP1}^*}^{\text{MASP3}_{\text{zym}}}}\end{array}\right)}$$

$$
-\frac{k_{\mathrm{cat\ MASP1^*}}^{\mathrm{MASP3_{zym}}}\left[\mathrm{pLFMASP1^*}\right]\left[\mathrm{pCLLKMASP3_{zym}}\right]}{K_{\mathrm{m\ MASP1^*}}^{\mathrm{MASP3_{zym}}}\left(\begin{array}{l}
1+\frac{[\mathrm{C4}]}{K_{\mathrm{m\ MASP1^*}}^{\mathrm{C4}}}+\frac{[\mathrm{fC4bC2}]}{K_{\mathrm{m\ MASP1^*}}^{\mathrm{C2}}}+\frac{[\mathrm{fC4bC4bC2}]}{K_{\mathrm{m\ MASP1^*}}^{\mathrm{C2}}}+\frac{[\mathrm{fC3bC4bC2}]}{K_{\mathrm{m\ MASP1^*}}^{\mathrm{C2}}}+\frac{[\mathrm{pC4bC2}]}{K_{\mathrm{m\ MASP1^*}}^{\mathrm{C2}}}+\frac{[\mathrm{pC4bC4bC2}]}{K_{\mathrm{m\ MASP1^*}}^{\mathrm{C2}}}\\
+\frac{[\mathrm{pC3bC4bC2}]}{K_{\mathrm{m\ MASP1^*}}^{\mathrm{C2}}}+\frac{\left[\mathrm{pMBL_3MASP1_{zym}}\right]}{K_{\mathrm{m\ MASP1^*}}^{\mathrm{MASP1_{zym}}}}+\frac{\left[\mathrm{pMBL_3MASP2_{zym}}\right]}{K_{\mathrm{m\ MASP1^*}}^{\mathrm{MASP2_{zym}}}}+\frac{\left[\mathrm{pMBL_3MASP3_{zym}}\right]}{K_{\mathrm{m\ MASP1^*}}^{\mathrm{MASP3_{zym}}}}\\
+\frac{\left[\mathrm{pMBL_4MASP1_{zym}}\right]}{K_{\mathrm{m\ MASP1^*}}^{\mathrm{MASP1_{zym}}}}+\frac{\left[\mathrm{pMBL_4MASP2_{zym}}\right]}{K_{\mathrm{m\ MASP1^*}}^{\mathrm{MASP2_{zym}}}}+\frac{\left[\mathrm{pMBL_4MASP3_{zym}}\right]}{K_{\mathrm{m\ MASP1^*}}^{\mathrm{MASP3_{zym}}}}+\frac{\left[\mathrm{pCLLKMASP1_{zym}}\right]}{K_{\mathrm{m\ MASP1^*}}^{\mathrm{MASP1_{zym}}}}\\
+\frac{\left[\mathrm{pCLLKMASP2_{zym}}\right]}{K_{\mathrm{m\ MASP1^*}}^{\mathrm{MASP2_{zym}}}}+\frac{\left[\mathrm{pCLLKMASP3_{zym}}\right]}{K_{\mathrm{m\ MASP1^*}}^{\mathrm{MASP3_{zym}}}}+\frac{\left[\mathrm{pCLK1MASP1_{zym}}\right]}{K_{\mathrm{m\ MASP1^*}}^{\mathrm{MASP1_{zym}}}}+\frac{\left[\mathrm{pCLK1MASP2_{zym}}\right]}{K_{\mathrm{m\ MASP1^*}}^{\mathrm{MASP2_{zym}}}}\\
+\frac{\left[\mathrm{pCLK1MASP3_{zym}}\right]}{K_{\mathrm{m\ MASP1^*}}^{\mathrm{MASP3_{zym}}}}+\frac{\left[\mathrm{pCLL1MASP1_{zym}}\right]}{K_{\mathrm{m\ MASP1^*}}^{\mathrm{MASP1_{zym}}}}+\frac{\left[\mathrm{pCLL1MASP2_{zym}}\right]}{K_{\mathrm{m\ MASP1^*}}^{\mathrm{MASP2_{zym}}}}+\frac{\left[\mathrm{pCLL1MASP3_{zym}}\right]}{K_{\mathrm{m\ MASP1^*}}^{\mathrm{MASP3_{zym}}}}\\
+\frac{\left[\mathrm{pLFMASP1_{zym}}\right]}{K_{\mathrm{m\ MASP1^*}}^{\mathrm{MASP1_{zym}}}}+\frac{\left[\mathrm{pLFMASP2_{zym}}\right]}{K_{\mathrm{m\ MASP1^*}}^{\mathrm{MASP2_{zym}}}}+\frac{\left[\mathrm{pLFMASP3_{zym}}\right]}{K_{\mathrm{m\ MASP1^*}}^{\mathrm{MASP3_{zym}}}}+\frac{\left[\mathrm{pHFMASP1_{zym}}\right]}{K_{\mathrm{m\ MASP1^*}}^{\mathrm{MASP1_{zym}}}}+\frac{\left[\mathrm{pHFMASP2_{zym}}\right]}{K_{\mathrm{m\ MASP1^*}}^{\mathrm{MASP2_{zym}}}}\\
+\frac{\left[\mathrm{pHFMASP3_{zym}}\right]}{K_{\mathrm{m\ MASP1^*}}^{\mathrm{MASP3_{zym}}}}+\frac{\left[\mathrm{pMFMASP1_{zym}}\right]}{K_{\mathrm{m\ MASP1^*}}^{\mathrm{MASP1_{zym}}}}+\frac{\left[\mathrm{pMFMASP2_{zym}}\right]}{K_{\mathrm{m\ MASP1^*}}^{\mathrm{MASP2_{zym}}}}+\frac{\left[\mathrm{pMFMASP3_{zym}}\right]}{K_{\mathrm{m\ MASP1^*}}^{\mathrm{MASP3_{zym}}}}\\
+\frac{\left[\mathrm{pPTX3LFMASP1_{zym}}\right]}{K_{\mathrm{m\ MASP1^*}}^{\mathrm{MASP1_{zym}}}}+\frac{\left[\mathrm{pPXT3LFMASP2_{zym}}\right]}{K_{\mathrm{m\ MASP1^*}}^{\mathrm{MASP2_{zym}}}}+\frac{\left[\mathrm{pPTX3LFMASP3_{zym}}\right]}{K_{\mathrm{m\ MASP1^*}}^{\mathrm{MASP3_{zym}}}}
\end{array}\right)}
$$

$$-\frac{k_{\text{cat MASP1}^*}^{\text{MASP3}_{\text{zym}}}\left[\text{pHFMASP1}^*\right]\left[\text{pCLLKMASP3}_{\text{zym}}\right]}{K_{\text{m MASP1}^*}^{\text{MASP3}_{\text{zym}}}\left(\begin{aligned}&1+\frac{[\text{C4}]}{K_{\text{m MASP1}^*}^{\text{C4}}}+\frac{[\text{fC4bC2}]}{K_{\text{m MASP1}^*}^{\text{C2}}}+\frac{[\text{fC4bC4bC2}]}{K_{\text{m MASP1}^*}^{\text{C2}}}+\frac{[\text{fC3bC4bC2}]}{K_{\text{m MASP1}^*}^{\text{C2}}}+\frac{[\text{pC4bC2}]}{K_{\text{m MASP1}^*}^{\text{C2}}}+\frac{[\text{pC4bC4bC2}]}{K_{\text{m MASP1}^*}^{\text{C2}}}\\&+\frac{[\text{pC3bC4bC2}]}{K_{\text{m MASP1}^*}^{\text{C2}}}+\frac{\left[\text{pMBL}_3\text{MASP1}_{\text{zym}}\right]}{K_{\text{m MASP1}^*}^{\text{MASP1}_{\text{zym}}}}+\frac{\left[\text{pMBL}_3\text{MASP2}_{\text{zym}}\right]}{K_{\text{m MASP1}^*}^{\text{MASP2}_{\text{zym}}}}+\frac{\left[\text{pMBL}_3\text{MASP3}_{\text{zym}}\right]}{K_{\text{m MASP1}^*}^{\text{MASP3}_{\text{zym}}}}\\&+\frac{\left[\text{pMBL}_4\text{MASP1}_{\text{zym}}\right]}{K_{\text{m MASP1}^*}^{\text{MASP1}_{\text{zym}}}}+\frac{\left[\text{pMBL}_4\text{MASP2}_{\text{zym}}\right]}{K_{\text{m MASP1}^*}^{\text{MASP2}_{\text{zym}}}}+\frac{\left[\text{pMBL}_4\text{MASP3}_{\text{zym}}\right]}{K_{\text{m MASP1}^*}^{\text{MASP3}_{\text{zym}}}}+\frac{\left[\text{pCLLKMASP1}_{\text{zym}}\right]}{K_{\text{m MASP1}^*}^{\text{MASP1}_{\text{zym}}}}\\&+\frac{\left[\text{pCLLKMASP2}_{\text{zym}}\right]}{K_{\text{m MASP1}^*}^{\text{MASP2}_{\text{zym}}}}+\frac{\left[\text{pCLLKMASP3}_{\text{zym}}\right]}{K_{\text{m MASP1}^*}^{\text{MASP3}_{\text{zym}}}}+\frac{\left[\text{pCLK1MASP1}_{\text{zym}}\right]}{K_{\text{m MASP1}^*}^{\text{MASP1}_{\text{zym}}}}+\frac{\left[\text{pCLK1MASP2}_{\text{zym}}\right]}{K_{\text{m MASP1}^*}^{\text{MASP2}_{\text{zym}}}}\\&+\frac{\left[\text{pCLK1MASP3}_{\text{zym}}\right]}{K_{\text{m MASP1}^*}^{\text{MASP3}_{\text{zym}}}}+\frac{\left[\text{pCLL1MASP1}_{\text{zym}}\right]}{K_{\text{m MASP1}^*}^{\text{MASP1}_{\text{zym}}}}+\frac{\left[\text{pCLL1MASP2}_{\text{zym}}\right]}{K_{\text{m MASP1}^*}^{\text{MASP2}_{\text{zym}}}}+\frac{\left[\text{pCLL1MASP3}_{\text{zym}}\right]}{K_{\text{m MASP1}^*}^{\text{MASP3}_{\text{zym}}}}\\&+\frac{\left[\text{pLFMASP1}_{\text{zym}}\right]}{K_{\text{m MASP1}^*}^{\text{MASP1}_{\text{zym}}}}+\frac{\left[\text{pLFMASP2}_{\text{zym}}\right]}{K_{\text{m MASP1}^*}^{\text{MASP2}_{\text{zym}}}}+\frac{\left[\text{pLFMASP3}_{\text{zym}}\right]}{K_{\text{m MASP1}^*}^{\text{MASP3}_{\text{zym}}}}+\frac{\left[\text{pHFMASP1}_{\text{zym}}\right]}{K_{\text{m MASP1}^*}^{\text{MASP1}_{\text{zym}}}}+\frac{\left[\text{pHFMASP2}_{\text{zym}}\right]}{K_{\text{m MASP1}^*}^{\text{MASP2}_{\text{zym}}}}\\&+\frac{\left[\text{pHFMASP3}_{\text{zym}}\right]}{K_{\text{m MASP1}^*}^{\text{MASP3}_{\text{zym}}}}+\frac{\left[\text{pMFMASP1}_{\text{zym}}\right]}{K_{\text{m MASP1}^*}^{\text{MASP1}_{\text{zym}}}}+\frac{\left[\text{pMFMASP2}_{\text{zym}}\right]}{K_{\text{m MASP1}^*}^{\text{MASP2}_{\text{zym}}}}+\frac{\left[\text{pMFMASP3}_{\text{zym}}\right]}{K_{\text{m MASP1}^*}^{\text{MASP3}_{\text{zym}}}}\\&+\frac{\left[\text{pPTX3LFMASP1}_{\text{zym}}\right]}{K_{\text{m MASP1}^*}^{\text{MASP1}_{\text{zym}}}}+\frac{\left[\text{pPXT3LFMASP2}_{\text{zym}}\right]}{K_{\text{m MASP1}^*}^{\text{MASP2}_{\text{zym}}}}+\frac{\left[\text{pPTX3LFMASP3}_{\text{zym}}\right]}{K_{\text{m MASP1}^*}^{\text{MASP3}_{\text{zym}}}}\end{aligned}\right)}$$

$$-\frac{k_{\mathrm{cat\ MASP1^*}}^{\mathrm{MASP3_{zym}}}\left[\mathrm{pMFMASP1^*}\right]\left[\mathrm{pCLLKMASP3_{zym}}\right]}{K_{\mathrm{m\ MASP1^*}}^{\mathrm{MASP3_{zym}}}\left(\begin{aligned}&1+\frac{[\mathrm{C4}]}{K_{\mathrm{m\ MASP1^*}}^{\mathrm{C4}}}+\frac{[\mathrm{fC4bC2}]}{K_{\mathrm{m\ MASP1^*}}^{\mathrm{C2}}}+\frac{[\mathrm{fC4bC4bC2}]}{K_{\mathrm{m\ MASP1^*}}^{\mathrm{C2}}}+\frac{[\mathrm{fC3bC4bC2}]}{K_{\mathrm{m\ MASP1^*}}^{\mathrm{C2}}}+\frac{[\mathrm{pC4bC2}]}{K_{\mathrm{m\ MASP1^*}}^{\mathrm{C2}}}+\frac{[\mathrm{pC4bC4bC2}]}{K_{\mathrm{m\ MASP1^*}}^{\mathrm{C2}}}\\&+\frac{[\mathrm{pC3bC4bC2}]}{K_{\mathrm{m\ MASP1^*}}^{\mathrm{C2}}}+\frac{[\mathrm{pMBL_3MASP1_{zym}}]}{K_{\mathrm{m\ MASP1^*}}^{\mathrm{MASP1_{zym}}}}+\frac{[\mathrm{pMBL_3MASP2_{zym}}]}{K_{\mathrm{m\ MASP1^*}}^{\mathrm{MASP2_{zym}}}}+\frac{[\mathrm{pMBL_3MASP3_{zym}}]}{K_{\mathrm{m\ MASP1^*}}^{\mathrm{MASP3_{zym}}}}\\&+\frac{[\mathrm{pMBL_4MASP1_{zym}}]}{K_{\mathrm{m\ MASP1^*}}^{\mathrm{MASP1_{zym}}}}+\frac{[\mathrm{pMBL_4MASP2_{zym}}]}{K_{\mathrm{m\ MASP1^*}}^{\mathrm{MASP2_{zym}}}}+\frac{[\mathrm{pMBL_4MASP3_{zym}}]}{K_{\mathrm{m\ MASP1^*}}^{\mathrm{MASP3_{zym}}}}+\frac{[\mathrm{pCLLKMASP1_{zym}}]}{K_{\mathrm{m\ MASP1^*}}^{\mathrm{MASP1_{zym}}}}\\&+\frac{[\mathrm{pCLLKMASP2_{zym}}]}{K_{\mathrm{m\ MASP1^*}}^{\mathrm{MASP2_{zym}}}}+\frac{[\mathrm{pCLLKMASP3_{zym}}]}{K_{\mathrm{m\ MASP1^*}}^{\mathrm{MASP3_{zym}}}}+\frac{[\mathrm{pCLK1MASP1_{zym}}]}{K_{\mathrm{m\ MASP1^*}}^{\mathrm{MASP1_{zym}}}}+\frac{[\mathrm{pCLK1MASP2_{zym}}]}{K_{\mathrm{m\ MASP1^*}}^{\mathrm{MASP2_{zym}}}}\\&+\frac{[\mathrm{pCLK1MASP3_{zym}}]}{K_{\mathrm{m\ MASP1^*}}^{\mathrm{MASP3_{zym}}}}+\frac{[\mathrm{pCLL1MASP1_{zym}}]}{K_{\mathrm{m\ MASP1^*}}^{\mathrm{MASP1_{zym}}}}+\frac{[\mathrm{pCLL1MASP2_{zym}}]}{K_{\mathrm{m\ MASP1^*}}^{\mathrm{MASP2_{zym}}}}+\frac{[\mathrm{pCLL1MASP3_{zym}}]}{K_{\mathrm{m\ MASP1^*}}^{\mathrm{MASP3_{zym}}}}\\&+\frac{[\mathrm{pLFMASP1_{zym}}]}{K_{\mathrm{m\ MASP1^*}}^{\mathrm{MASP1_{zym}}}}+\frac{[\mathrm{pLFMASP2_{zym}}]}{K_{\mathrm{m\ MASP1^*}}^{\mathrm{MASP2_{zym}}}}+\frac{[\mathrm{pLFMASP3_{zym}}]}{K_{\mathrm{m\ MASP1^*}}^{\mathrm{MASP3_{zym}}}}+\frac{[\mathrm{pHFMASP1_{zym}}]}{K_{\mathrm{m\ MASP1^*}}^{\mathrm{MASP1_{zym}}}}+\frac{[\mathrm{pHFMASP2_{zym}}]}{K_{\mathrm{m\ MASP1^*}}^{\mathrm{MASP2_{zym}}}}\\&+\frac{[\mathrm{pHFMASP3_{zym}}]}{K_{\mathrm{m\ MASP1^*}}^{\mathrm{MASP3_{zym}}}}+\frac{[\mathrm{pMFMASP1_{zym}}]}{K_{\mathrm{m\ MASP1^*}}^{\mathrm{MASP1_{zym}}}}+\frac{[\mathrm{pMFMASP2_{zym}}]}{K_{\mathrm{m\ MASP1^*}}^{\mathrm{MASP2_{zym}}}}+\frac{[\mathrm{pMFMASP3_{zym}}]}{K_{\mathrm{m\ MASP1^*}}^{\mathrm{MASP3_{zym}}}}\\&+\frac{[\mathrm{pPTX3LFMASP1_{zym}}]}{K_{\mathrm{m\ MASP1^*}}^{\mathrm{MASP1_{zym}}}}+\frac{[\mathrm{pPXT3LFMASP2_{zym}}]}{K_{\mathrm{m\ MASP1^*}}^{\mathrm{MASP2_{zym}}}}+\frac{[\mathrm{pPTX3LFMASP3_{zym}}]}{K_{\mathrm{m\ MASP1^*}}^{\mathrm{MASP3_{zym}}}}\end{aligned}\right)}$$

$$-\frac{k_{\text{cat MASP1}^*}^{\text{MASP3}_{\text{zym}}}\left[\text{pPTX3LFMASP1}^*\right]\left[\text{pCLLKMASP3}_{\text{zym}}\right]}{K_{\text{m MASP1}^*}^{\text{MASP3}_{\text{zym}}}\left(\begin{array}{l}1+\frac{[\text{C4}]}{K_{\text{m MASP1}^*}^{\text{C4}}}+\frac{[\text{fC4bC2}]}{K_{\text{m MASP1}^*}^{\text{C2}}}+\frac{[\text{fC4bC4bC2}]}{K_{\text{m MASP1}^*}^{\text{C2}}}+\frac{[\text{fC3bC4bC2}]}{K_{\text{m MASP1}^*}^{\text{C2}}}+\frac{[\text{pC4bC2}]}{K_{\text{m MASP1}^*}^{\text{C2}}}+\frac{[\text{pC4bC4bC2}]}{K_{\text{m MASP1}^*}^{\text{C2}}}\\+\frac{[\text{pC3bC4bC2}]}{K_{\text{m MASP1}^*}^{\text{C2}}}+\frac{[\text{pMBL}_3\text{MASP1}_{\text{zym}}]}{K_{\text{m MASP1}^*}^{\text{MASP1}_{\text{zym}}}}+\frac{[\text{pMBL}_3\text{MASP2}_{\text{zym}}]}{K_{\text{m MASP1}^*}^{\text{MASP2}_{\text{zym}}}}+\frac{[\text{pMBL}_3\text{MASP3}_{\text{zym}}]}{K_{\text{m MASP1}^*}^{\text{MASP3}_{\text{zym}}}}\\+\frac{[\text{pMBL}_4\text{MASP1}_{\text{zym}}]}{K_{\text{m MASP1}^*}^{\text{MASP1}_{\text{zym}}}}+\frac{[\text{pMBL}_4\text{MASP2}_{\text{zym}}]}{K_{\text{m MASP1}^*}^{\text{MASP2}_{\text{zym}}}}+\frac{[\text{pMBL}_4\text{MASP3}_{\text{zym}}]}{K_{\text{m MASP1}^*}^{\text{MASP3}_{\text{zym}}}}+\frac{[\text{pCLLKMASP1}_{\text{zym}}]}{K_{\text{m MASP1}^*}^{\text{MASP1}_{\text{zym}}}}\\+\frac{[\text{pCLLKMASP2}_{\text{zym}}]}{K_{\text{m MASP1}^*}^{\text{MASP2}_{\text{zym}}}}+\frac{[\text{pCLLKMASP3}_{\text{zym}}]}{K_{\text{m MASP1}^*}^{\text{MASP3}_{\text{zym}}}}+\frac{[\text{pCLK1MASP1}_{\text{zym}}]}{K_{\text{m MASP1}^*}^{\text{MASP1}_{\text{zym}}}}+\frac{[\text{pCLK1MASP2}_{\text{zym}}]}{K_{\text{m MASP1}^*}^{\text{MASP2}_{\text{zym}}}}\\+\frac{[\text{pCLK1MASP3}_{\text{zym}}]}{K_{\text{m MASP1}^*}^{\text{MASP3}_{\text{zym}}}}+\frac{[\text{pCLL1MASP1}_{\text{zym}}]}{K_{\text{m MASP1}^*}^{\text{MASP1}_{\text{zym}}}}+\frac{[\text{pCLL1MASP2}_{\text{zym}}]}{K_{\text{m MASP1}^*}^{\text{MASP2}_{\text{zym}}}}+\frac{[\text{pCLL1MASP3}_{\text{zym}}]}{K_{\text{m MASP1}^*}^{\text{MASP3}_{\text{zym}}}}\\+\frac{[\text{pLFMASP1}_{\text{zym}}]}{K_{\text{m MASP1}^*}^{\text{MASP1}_{\text{zym}}}}+\frac{[\text{pLFMASP2}_{\text{zym}}]}{K_{\text{m MASP1}^*}^{\text{MASP2}_{\text{zym}}}}+\frac{[\text{pLFMASP3}_{\text{zym}}]}{K_{\text{m MASP1}^*}^{\text{MASP3}_{\text{zym}}}}+\frac{[\text{pHFMASP1}_{\text{zym}}]}{K_{\text{m MASP1}^*}^{\text{MASP1}_{\text{zym}}}}+\frac{[\text{pHFMASP2}_{\text{zym}}]}{K_{\text{m MASP1}^*}^{\text{MASP2}_{\text{zym}}}}\\+\frac{[\text{pHFMASP3}_{\text{zym}}]}{K_{\text{m MASP1}^*}^{\text{MASP3}_{\text{zym}}}}+\frac{[\text{pMFMASP1}_{\text{zym}}]}{K_{\text{m MASP1}^*}^{\text{MASP1}_{\text{zym}}}}+\frac{[\text{pMFMASP2}_{\text{zym}}]}{K_{\text{m MASP1}^*}^{\text{MASP2}_{\text{zym}}}}+\frac{[\text{pMFMASP3}_{\text{zym}}]}{K_{\text{m MASP1}^*}^{\text{MASP3}_{\text{zym}}}}\\+\frac{[\text{pPTX3LFMASP1}_{\text{zym}}]}{K_{\text{m MASP1}^*}^{\text{MASP1}_{\text{zym}}}}+\frac{[\text{pPXT3LFMASP2}_{\text{zym}}]}{K_{\text{m MASP1}^*}^{\text{MASP2}_{\text{zym}}}}+\frac{[\text{pPTX3LFMASP3}_{\text{zym}}]}{K_{\text{m MASP1}^*}^{\text{MASP3}_{\text{zym}}}}\end{array}\right)}$$

294

$$\frac{d\left[\text{fCLLKMASP3}^*\right]}{dt}=-k_{\text{pCLLK}}^{+}\left[\text{fCLLKMASP3}^*\right]\left[\text{Pathogen Surface}\right]+k_{\text{pCLLK}}^{-}\left[\text{pCLLKMASP3}^*\right]$$
$$+k_{\text{CLLKMASP3}}^{+}\left[\text{CLLK}\right]\left[\text{MASP3}^*\right]-k_{\text{CLLKMASP3}}^{-}\left[\text{fCLLKMASP3}^*\right]$$

295

$$\frac{d\left[\text{pCLLKMASP3}^*\right]}{dt}=k_{\text{pCLLK}}^{+}\left[\text{fCLLKMASP3}^*\right]\left[\text{Pathogen Surface}\right]-k_{\text{pCLLK}}^{-}\left[\text{pCLLKMASP3}^*\right]$$
$$+k_{\text{CLLKMASP3}}^{+}\left[\text{pCLLK}\right]\left[\text{MASP3}^*\right]-k_{\text{CLLKMASP3}}^{-}\left[\text{pCLLKMASP3}^*\right]$$

$$
+\frac{k_{\mathrm{cat\,MASP1^*}}^{\mathrm{MASP3_{zym}}}\left[\mathrm{pMBL_3MASP1^*}\right]\left[\mathrm{pCLLKMASP3_{zym}}\right]}{K_{\mathrm{m\,MASP1^*}}^{\mathrm{MASP3_{zym}}}\left(\begin{array}{l}
1+\frac{[\mathrm{C4}]}{K_{\mathrm{m\,MASP1^*}}^{\mathrm{C4}}}+\frac{[\mathrm{fC4bC2}]}{K_{\mathrm{m\,MASP1^*}}^{\mathrm{C2}}}+\frac{[\mathrm{fC4bC4bC2}]}{K_{\mathrm{m\,MASP1^*}}^{\mathrm{C2}}}+\frac{[\mathrm{fC3bC4bC2}]}{K_{\mathrm{m\,MASP1^*}}^{\mathrm{C2}}}+\frac{[\mathrm{pC4bC2}]}{K_{\mathrm{m\,MASP1^*}}^{\mathrm{C2}}}+\frac{[\mathrm{pC4bC4bC2}]}{K_{\mathrm{m\,MASP1^*}}^{\mathrm{C2}}}\\
+\frac{[\mathrm{pC3bC4bC2}]}{K_{\mathrm{m\,MASP1^*}}^{\mathrm{C2}}}+\frac{\left[\mathrm{pMBL_3MASP1_{zym}}\right]}{K_{\mathrm{m\,MASP1^*}}^{\mathrm{MASP1_{zym}}}}+\frac{\left[\mathrm{pMBL_3MASP2_{zym}}\right]}{K_{\mathrm{m\,MASP1^*}}^{\mathrm{MASP2_{zym}}}}+\frac{\left[\mathrm{pMBL_3MASP3_{zym}}\right]}{K_{\mathrm{m\,MASP1^*}}^{\mathrm{MASP3_{zym}}}}\\
+\frac{\left[\mathrm{pMBL_4MASP1_{zym}}\right]}{K_{\mathrm{m\,MASP1^*}}^{\mathrm{MASP1_{zym}}}}+\frac{\left[\mathrm{pMBL_4MASP2_{zym}}\right]}{K_{\mathrm{m\,MASP1^*}}^{\mathrm{MASP2_{zym}}}}+\frac{\left[\mathrm{pMBL_4MASP3_{zym}}\right]}{K_{\mathrm{m\,MASP1^*}}^{\mathrm{MASP3_{zym}}}}+\frac{\left[\mathrm{pCLLKMASP1_{zym}}\right]}{K_{\mathrm{m\,MASP1^*}}^{\mathrm{MASP1_{zym}}}}\\
+\frac{\left[\mathrm{pCLLKMASP2_{zym}}\right]}{K_{\mathrm{m\,MASP1^*}}^{\mathrm{MASP2_{zym}}}}+\frac{\left[\mathrm{pCLLKMASP3_{zym}}\right]}{K_{\mathrm{m\,MASP1^*}}^{\mathrm{MASP3_{zym}}}}+\frac{\left[\mathrm{pCLK1MASP1_{zym}}\right]}{K_{\mathrm{m\,MASP1^*}}^{\mathrm{MASP1_{zym}}}}+\frac{\left[\mathrm{pCLK1MASP2_{zym}}\right]}{K_{\mathrm{m\,MASP1^*}}^{\mathrm{MASP2_{zym}}}}\\
+\frac{\left[\mathrm{pCLK1MASP3_{zym}}\right]}{K_{\mathrm{m\,MASP1^*}}^{\mathrm{MASP3_{zym}}}}+\frac{\left[\mathrm{pCLL1MASP1_{zym}}\right]}{K_{\mathrm{m\,MASP1^*}}^{\mathrm{MASP1_{zym}}}}+\frac{\left[\mathrm{pCLL1MASP2_{zym}}\right]}{K_{\mathrm{m\,MASP1^*}}^{\mathrm{MASP2_{zym}}}}+\frac{\left[\mathrm{pCLL1MASP3_{zym}}\right]}{K_{\mathrm{m\,MASP1^*}}^{\mathrm{MASP3_{zym}}}}\\
+\frac{\left[\mathrm{pLFMASP1_{zym}}\right]}{K_{\mathrm{m\,MASP1^*}}^{\mathrm{MASP1_{zym}}}}+\frac{\left[\mathrm{pLFMASP2_{zym}}\right]}{K_{\mathrm{m\,MASP1^*}}^{\mathrm{MASP2_{zym}}}}+\frac{\left[\mathrm{pLFMASP3_{zym}}\right]}{K_{\mathrm{m\,MASP1^*}}^{\mathrm{MASP3_{zym}}}}+\frac{\left[\mathrm{pHFMASP1_{zym}}\right]}{K_{\mathrm{m\,MASP1^*}}^{\mathrm{MASP1_{zym}}}}+\frac{\left[\mathrm{pHFMASP2_{zym}}\right]}{K_{\mathrm{m\,MASP1^*}}^{\mathrm{MASP2_{zym}}}}\\
+\frac{\left[\mathrm{pHFMASP3_{zym}}\right]}{K_{\mathrm{m\,MASP1^*}}^{\mathrm{MASP3_{zym}}}}+\frac{\left[\mathrm{pMFMASP1_{zym}}\right]}{K_{\mathrm{m\,MASP1^*}}^{\mathrm{MASP1_{zym}}}}+\frac{\left[\mathrm{pMFMASP2_{zym}}\right]}{K_{\mathrm{m\,MASP1^*}}^{\mathrm{MASP2_{zym}}}}+\frac{\left[\mathrm{pMFMASP3_{zym}}\right]}{K_{\mathrm{m\,MASP1^*}}^{\mathrm{MASP3_{zym}}}}\\
+\frac{\left[\mathrm{pPTX3LFMASP1_{zym}}\right]}{K_{\mathrm{m\,MASP1^*}}^{\mathrm{MASP1_{zym}}}}+\frac{\left[\mathrm{pPXT3LFMASP2_{zym}}\right]}{K_{\mathrm{m\,MASP1^*}}^{\mathrm{MASP2_{zym}}}}+\frac{\left[\mathrm{pPTX3LFMASP3_{zym}}\right]}{K_{\mathrm{m\,MASP1^*}}^{\mathrm{MASP3_{zym}}}}
\end{array}\right)}
$$

$$+\frac{k_{\text{cat MASP1}^*}^{\text{MASP3}_{\text{zym}}}\left[\text{pMBL}_4\text{MASP1}^*\right]\left[\text{pCLLKMASP3}_{\text{zym}}\right]}{K_{\text{m MASP1}^*}^{\text{MASP3}_{\text{zym}}}\left(\begin{array}{l}1+\frac{[\text{C4}]}{K_{\text{m MASP1}^*}^{\text{C4}}}+\frac{[\text{fC4bC2}]}{K_{\text{m MASP1}^*}^{\text{C2}}}+\frac{[\text{fC4bC4bC2}]}{K_{\text{m MASP1}^*}^{\text{C2}}}+\frac{[\text{fC3bC4bC2}]}{K_{\text{m MASP1}^*}^{\text{C2}}}+\frac{[\text{pC4bC2}]}{K_{\text{m MASP1}^*}^{\text{C2}}}+\frac{[\text{pC4bC4bC2}]}{K_{\text{m MASP1}^*}^{\text{C2}}}\\
+\frac{[\text{pC3bC4bC2}]}{K_{\text{m MASP1}^*}^{\text{C2}}}+\frac{[\text{pMBL}_3\text{MASP1}_{\text{zym}}]}{K_{\text{m MASP1}^*}^{\text{MASP1}_{\text{zym}}}}+\frac{[\text{pMBL}_3\text{MASP2}_{\text{zym}}]}{K_{\text{m MASP1}^*}^{\text{MASP2}_{\text{zym}}}}+\frac{[\text{pMBL}_3\text{MASP3}_{\text{zym}}]}{K_{\text{m MASP1}^*}^{\text{MASP3}_{\text{zym}}}}\\
+\frac{[\text{pMBL}_4\text{MASP1}_{\text{zym}}]}{K_{\text{m MASP1}^*}^{\text{MASP1}_{\text{zym}}}}+\frac{[\text{pMBL}_4\text{MASP2}_{\text{zym}}]}{K_{\text{m MASP1}^*}^{\text{MASP2}_{\text{zym}}}}+\frac{[\text{pMBL}_4\text{MASP3}_{\text{zym}}]}{K_{\text{m MASP1}^*}^{\text{MASP3}_{\text{zym}}}}+\frac{[\text{pCLLKMASP1}_{\text{zym}}]}{K_{\text{m MASP1}^*}^{\text{MASP1}_{\text{zym}}}}\\
+\frac{[\text{pCLLKMASP2}_{\text{zym}}]}{K_{\text{m MASP1}^*}^{\text{MASP2}_{\text{zym}}}}+\frac{[\text{pCLLKMASP3}_{\text{zym}}]}{K_{\text{m MASP1}^*}^{\text{MASP3}_{\text{zym}}}}+\frac{[\text{pCLK1MASP1}_{\text{zym}}]}{K_{\text{m MASP1}^*}^{\text{MASP1}_{\text{zym}}}}+\frac{[\text{pCLK1MASP2}_{\text{zym}}]}{K_{\text{m MASP1}^*}^{\text{MASP2}_{\text{zym}}}}\\
+\frac{[\text{pCLK1MASP3}_{\text{zym}}]}{K_{\text{m MASP1}^*}^{\text{MASP3}_{\text{zym}}}}+\frac{[\text{pCLL1MASP1}_{\text{zym}}]}{K_{\text{m MASP1}^*}^{\text{MASP1}_{\text{zym}}}}+\frac{[\text{pCLL1MASP2}_{\text{zym}}]}{K_{\text{m MASP1}^*}^{\text{MASP2}_{\text{zym}}}}+\frac{[\text{pCLL1MASP3}_{\text{zym}}]}{K_{\text{m MASP1}^*}^{\text{MASP3}_{\text{zym}}}}\\
+\frac{[\text{pLFMASP1}_{\text{zym}}]}{K_{\text{m MASP1}^*}^{\text{MASP1}_{\text{zym}}}}+\frac{[\text{pLFMASP2}_{\text{zym}}]}{K_{\text{m MASP1}^*}^{\text{MASP2}_{\text{zym}}}}+\frac{[\text{pLFMASP3}_{\text{zym}}]}{K_{\text{m MASP1}^*}^{\text{MASP3}_{\text{zym}}}}+\frac{[\text{pHFMASP1}_{\text{zym}}]}{K_{\text{m MASP1}^*}^{\text{MASP1}_{\text{zym}}}}+\frac{[\text{pHFMASP2}_{\text{zym}}]}{K_{\text{m MASP1}^*}^{\text{MASP2}_{\text{zym}}}}\\
+\frac{[\text{pHFMASP3}_{\text{zym}}]}{K_{\text{m MASP1}^*}^{\text{MASP3}_{\text{zym}}}}+\frac{[\text{pMFMASP1}_{\text{zym}}]}{K_{\text{m MASP1}^*}^{\text{MASP1}_{\text{zym}}}}+\frac{[\text{pMFMASP2}_{\text{zym}}]}{K_{\text{m MASP1}^*}^{\text{MASP2}_{\text{zym}}}}+\frac{[\text{pMFMASP3}_{\text{zym}}]}{K_{\text{m MASP1}^*}^{\text{MASP3}_{\text{zym}}}}\\
+\frac{[\text{pPTX3LFMASP1}_{\text{zym}}]}{K_{\text{m MASP1}^*}^{\text{MASP1}_{\text{zym}}}}+\frac{[\text{pPXT3LFMASP2}_{\text{zym}}]}{K_{\text{m MASP1}^*}^{\text{MASP2}_{\text{zym}}}}+\frac{[\text{pPTX3LFMASP3}_{\text{zym}}]}{K_{\text{m MASP1}^*}^{\text{MASP3}_{\text{zym}}}}\end{array}\right)}$$

$$+\frac{k_{\text{cat MASP1}^*}^{\text{MASP3}_{\text{zym}}}\left[\text{pCLLKMASP1}^*\right]\left[\text{pCLLKMASP3}_{\text{zym}}\right]}{K_{\text{m MASP1}^*}^{\text{MASP3}_{\text{zym}}}\left(\begin{array}{l}1+\frac{[\text{C4}]}{K_{\text{m MASP1}^*}^{\text{C4}}}+\frac{[\text{fC4bC2}]}{K_{\text{m MASP1}^*}^{\text{C2}}}+\frac{[\text{fC4bC4bC2}]}{K_{\text{m MASP1}^*}^{\text{C2}}}+\frac{[\text{fC3bC4bC2}]}{K_{\text{m MASP1}^*}^{\text{C2}}}+\frac{[\text{pC4bC2}]}{K_{\text{m MASP1}^*}^{\text{C2}}}+\frac{[\text{pC4bC4bC2}]}{K_{\text{m MASP1}^*}^{\text{C2}}}\\
+\frac{[\text{pC3bC4bC2}]}{K_{\text{m MASP1}^*}^{\text{C2}}}+\frac{[\text{pMBL}_3\text{MASP1}_{\text{zym}}]}{K_{\text{m MASP1}^*}^{\text{MASP1}_{\text{zym}}}}+\frac{[\text{pMBL}_3\text{MASP2}_{\text{zym}}]}{K_{\text{m MASP1}^*}^{\text{MASP2}_{\text{zym}}}}+\frac{[\text{pMBL}_3\text{MASP3}_{\text{zym}}]}{K_{\text{m MASP1}^*}^{\text{MASP3}_{\text{zym}}}}\\
+\frac{[\text{pMBL}_4\text{MASP1}_{\text{zym}}]}{K_{\text{m MASP1}^*}^{\text{MASP1}_{\text{zym}}}}+\frac{[\text{pMBL}_4\text{MASP2}_{\text{zym}}]}{K_{\text{m MASP1}^*}^{\text{MASP2}_{\text{zym}}}}+\frac{[\text{pMBL}_4\text{MASP3}_{\text{zym}}]}{K_{\text{m MASP1}^*}^{\text{MASP3}_{\text{zym}}}}+\frac{[\text{pCLLKMASP1}_{\text{zym}}]}{K_{\text{m MASP1}^*}^{\text{MASP1}_{\text{zym}}}}\\
+\frac{[\text{pCLLKMASP2}_{\text{zym}}]}{K_{\text{m MASP1}^*}^{\text{MASP2}_{\text{zym}}}}+\frac{[\text{pCLLKMASP3}_{\text{zym}}]}{K_{\text{m MASP1}^*}^{\text{MASP3}_{\text{zym}}}}+\frac{[\text{pCLK1MASP1}_{\text{zym}}]}{K_{\text{m MASP1}^*}^{\text{MASP1}_{\text{zym}}}}+\frac{[\text{pCLK1MASP2}_{\text{zym}}]}{K_{\text{m MASP1}^*}^{\text{MASP2}_{\text{zym}}}}\\
+\frac{[\text{pCLK1MASP3}_{\text{zym}}]}{K_{\text{m MASP1}^*}^{\text{MASP3}_{\text{zym}}}}+\frac{[\text{pCLL1MASP1}_{\text{zym}}]}{K_{\text{m MASP1}^*}^{\text{MASP1}_{\text{zym}}}}+\frac{[\text{pCLL1MASP2}_{\text{zym}}]}{K_{\text{m MASP1}^*}^{\text{MASP2}_{\text{zym}}}}+\frac{[\text{pCLL1MASP3}_{\text{zym}}]}{K_{\text{m MASP1}^*}^{\text{MASP3}_{\text{zym}}}}\\
+\frac{[\text{pLFMASP1}_{\text{zym}}]}{K_{\text{m MASP1}^*}^{\text{MASP1}_{\text{zym}}}}+\frac{[\text{pLFMASP2}_{\text{zym}}]}{K_{\text{m MASP1}^*}^{\text{MASP2}_{\text{zym}}}}+\frac{[\text{pLFMASP3}_{\text{zym}}]}{K_{\text{m MASP1}^*}^{\text{MASP3}_{\text{zym}}}}+\frac{[\text{pHFMASP1}_{\text{zym}}]}{K_{\text{m MASP1}^*}^{\text{MASP1}_{\text{zym}}}}+\frac{[\text{pHFMASP2}_{\text{zym}}]}{K_{\text{m MASP1}^*}^{\text{MASP2}_{\text{zym}}}}\\
+\frac{[\text{pHFMASP3}_{\text{zym}}]}{K_{\text{m MASP1}^*}^{\text{MASP3}_{\text{zym}}}}+\frac{[\text{pMFMASP1}_{\text{zym}}]}{K_{\text{m MASP1}^*}^{\text{MASP1}_{\text{zym}}}}+\frac{[\text{pMFMASP2}_{\text{zym}}]}{K_{\text{m MASP1}^*}^{\text{MASP2}_{\text{zym}}}}+\frac{[\text{pMFMASP3}_{\text{zym}}]}{K_{\text{m MASP1}^*}^{\text{MASP3}_{\text{zym}}}}\\
+\frac{[\text{pPTX3LFMASP1}_{\text{zym}}]}{K_{\text{m MASP1}^*}^{\text{MASP1}_{\text{zym}}}}+\frac{[\text{pPXT3LFMASP2}_{\text{zym}}]}{K_{\text{m MASP1}^*}^{\text{MASP2}_{\text{zym}}}}+\frac{[\text{pPTX3LFMASP3}_{\text{zym}}]}{K_{\text{m MASP1}^*}^{\text{MASP3}_{\text{zym}}}}\end{array}\right)}$$

$$
+\frac{k_{\text{cat MASP1}^*}^{\text{MASP3}_{\text{zym}}}\left[\text{pCLK1MASP1}^*\right]\left[\text{pCLLKMASP3}_{\text{zym}}\right]}{K_{\text{m MASP1}^*}^{\text{MASP3}_{\text{zym}}}\left(\begin{array}{l}1+\frac{[\text{C4}]}{K_{\text{m MASP1}^*}^{\text{C4}}}+\frac{[\text{fC4bC2}]}{K_{\text{m MASP1}^*}^{\text{C2}}}+\frac{[\text{fC4bC4bC2}]}{K_{\text{m MASP1}^*}^{\text{C2}}}+\frac{[\text{fC3bC4bC2}]}{K_{\text{m MASP1}^*}^{\text{C2}}}+\frac{[\text{pC4bC2}]}{K_{\text{m MASP1}^*}^{\text{C2}}}+\frac{[\text{pC4bC4bC2}]}{K_{\text{m MASP1}^*}^{\text{C2}}}\\
+\frac{[\text{pC3bC4bC2}]}{K_{\text{m MASP1}^*}^{\text{C2}}}+\frac{[\text{pMBL}_3\text{MASP1}_{\text{zym}}]}{K_{\text{m MASP1}^*}^{\text{MASP1}_{\text{zym}}}}+\frac{[\text{pMBL}_3\text{MASP2}_{\text{zym}}]}{K_{\text{m MASP1}^*}^{\text{MASP2}_{\text{zym}}}}+\frac{[\text{pMBL}_3\text{MASP3}_{\text{zym}}]}{K_{\text{m MASP1}^*}^{\text{MASP3}_{\text{zym}}}}\\
+\frac{[\text{pMBL}_4\text{MASP1}_{\text{zym}}]}{K_{\text{m MASP1}^*}^{\text{MASP1}_{\text{zym}}}}+\frac{[\text{pMBL}_4\text{MASP2}_{\text{zym}}]}{K_{\text{m MASP1}^*}^{\text{MASP2}_{\text{zym}}}}+\frac{[\text{pMBL}_4\text{MASP3}_{\text{zym}}]}{K_{\text{m MASP1}^*}^{\text{MASP3}_{\text{zym}}}}+\frac{[\text{pCLLKMASP1}_{\text{zym}}]}{K_{\text{m MASP1}^*}^{\text{MASP1}_{\text{zym}}}}\\
+\frac{[\text{pCLLKMASP2}_{\text{zym}}]}{K_{\text{m MASP1}^*}^{\text{MASP2}_{\text{zym}}}}+\frac{[\text{pCLLKMASP3}_{\text{zym}}]}{K_{\text{m MASP1}^*}^{\text{MASP3}_{\text{zym}}}}+\frac{[\text{pCLK1MASP1}_{\text{zym}}]}{K_{\text{m MASP1}^*}^{\text{MASP1}_{\text{zym}}}}+\frac{[\text{pCLK1MASP2}_{\text{zym}}]}{K_{\text{m MASP1}^*}^{\text{MASP2}_{\text{zym}}}}\\
+\frac{[\text{pCLK1MASP3}_{\text{zym}}]}{K_{\text{m MASP1}^*}^{\text{MASP3}_{\text{zym}}}}+\frac{[\text{pCLL1MASP1}_{\text{zym}}]}{K_{\text{m MASP1}^*}^{\text{MASP1}_{\text{zym}}}}+\frac{[\text{pCLL1MASP2}_{\text{zym}}]}{K_{\text{m MASP1}^*}^{\text{MASP2}_{\text{zym}}}}+\frac{[\text{pCLL1MASP3}_{\text{zym}}]}{K_{\text{m MASP1}^*}^{\text{MASP3}_{\text{zym}}}}\\
+\frac{[\text{pLFMASP1}_{\text{zym}}]}{K_{\text{m MASP1}^*}^{\text{MASP1}_{\text{zym}}}}+\frac{[\text{pLFMASP2}_{\text{zym}}]}{K_{\text{m MASP1}^*}^{\text{MASP2}_{\text{zym}}}}+\frac{[\text{pLFMASP3}_{\text{zym}}]}{K_{\text{m MASP1}^*}^{\text{MASP3}_{\text{zym}}}}+\frac{[\text{pHFMASP1}_{\text{zym}}]}{K_{\text{m MASP1}^*}^{\text{MASP1}_{\text{zym}}}}+\frac{[\text{pHFMASP2}_{\text{zym}}]}{K_{\text{m MASP1}^*}^{\text{MASP2}_{\text{zym}}}}\\
+\frac{[\text{pHFMASP3}_{\text{zym}}]}{K_{\text{m MASP1}^*}^{\text{MASP3}_{\text{zym}}}}+\frac{[\text{pMFMASP1}_{\text{zym}}]}{K_{\text{m MASP1}^*}^{\text{MASP1}_{\text{zym}}}}+\frac{[\text{pMFMASP2}_{\text{zym}}]}{K_{\text{m MASP1}^*}^{\text{MASP2}_{\text{zym}}}}+\frac{[\text{pMFMASP3}_{\text{zym}}]}{K_{\text{m MASP1}^*}^{\text{MASP3}_{\text{zym}}}}\\
+\frac{[\text{pPTX3LFMASP1}_{\text{zym}}]}{K_{\text{m MASP1}^*}^{\text{MASP1}_{\text{zym}}}}+\frac{[\text{pPXT3LFMASP2}_{\text{zym}}]}{K_{\text{m MASP1}^*}^{\text{MASP2}_{\text{zym}}}}+\frac{[\text{pPTX3LFMASP3}_{\text{zym}}]}{K_{\text{m MASP1}^*}^{\text{MASP3}_{\text{zym}}}}\end{array}\right)}
$$

$$
+\frac{k_{\mathrm{cat\ MASP1^{*}}}^{\mathrm{MASP3_{zym}}}\left[\mathrm{pCLL1MASP1^{*}}\right]\left[\mathrm{pCLLKMASP3_{zym}}\right]}{K_{\mathrm{m\ MASP1^{*}}}^{\mathrm{MASP3_{zym}}}\left(\begin{array}{l}
1+\frac{[\mathrm{C4}]}{K_{\mathrm{m\ MASP1^{*}}}^{\mathrm{C4}}}+\frac{[\mathrm{fC4bC2}]}{K_{\mathrm{m\ MASP1^{*}}}^{\mathrm{C2}}}+\frac{[\mathrm{fC4bC4bC2}]}{K_{\mathrm{m\ MASP1^{*}}}^{\mathrm{C2}}}+\frac{[\mathrm{fC3bC4bC2}]}{K_{\mathrm{m\ MASP1^{*}}}^{\mathrm{C2}}}+\frac{[\mathrm{pC4bC2}]}{K_{\mathrm{m\ MASP1^{*}}}^{\mathrm{C2}}}+\frac{[\mathrm{pC4bC4bC2}]}{K_{\mathrm{m\ MASP1^{*}}}^{\mathrm{C2}}}\\
+\frac{[\mathrm{pC3bC4bC2}]}{K_{\mathrm{m\ MASP1^{*}}}^{\mathrm{C2}}}+\frac{\left[\mathrm{pMBL_{3}MASP1_{zym}}\right]}{K_{\mathrm{m\ MASP1^{*}}}^{\mathrm{MASP1_{zym}}}}+\frac{\left[\mathrm{pMBL_{3}MASP2_{zym}}\right]}{K_{\mathrm{m\ MASP1^{*}}}^{\mathrm{MASP2_{zym}}}}+\frac{\left[\mathrm{pMBL_{3}MASP3_{zym}}\right]}{K_{\mathrm{m\ MASP1^{*}}}^{\mathrm{MASP3_{zym}}}}\\
+\frac{\left[\mathrm{pMBL_{4}MASP1_{zym}}\right]}{K_{\mathrm{m\ MASP1^{*}}}^{\mathrm{MASP1_{zym}}}}+\frac{\left[\mathrm{pMBL_{4}MASP2_{zym}}\right]}{K_{\mathrm{m\ MASP1^{*}}}^{\mathrm{MASP2_{zym}}}}+\frac{\left[\mathrm{pMBL_{4}MASP3_{zym}}\right]}{K_{\mathrm{m\ MASP1^{*}}}^{\mathrm{MASP3_{zym}}}}+\frac{\left[\mathrm{pCLLKMASP1_{zym}}\right]}{K_{\mathrm{m\ MASP1^{*}}}^{\mathrm{MASP1_{zym}}}}\\
+\frac{\left[\mathrm{pCLLKMASP2_{zym}}\right]}{K_{\mathrm{m\ MASP1^{*}}}^{\mathrm{MASP2_{zym}}}}+\frac{\left[\mathrm{pCLLKMASP3_{zym}}\right]}{K_{\mathrm{m\ MASP1^{*}}}^{\mathrm{MASP3_{zym}}}}+\frac{\left[\mathrm{pCLK1MASP1_{zym}}\right]}{K_{\mathrm{m\ MASP1^{*}}}^{\mathrm{MASP1_{zym}}}}+\frac{\left[\mathrm{pCLK1MASP2_{zym}}\right]}{K_{\mathrm{m\ MASP1^{*}}}^{\mathrm{MASP2_{zym}}}}\\
+\frac{\left[\mathrm{pCLK1MASP3_{zym}}\right]}{K_{\mathrm{m\ MASP1^{*}}}^{\mathrm{MASP3_{zym}}}}+\frac{\left[\mathrm{pCLL1MASP1_{zym}}\right]}{K_{\mathrm{m\ MASP1^{*}}}^{\mathrm{MASP1_{zym}}}}+\frac{\left[\mathrm{pCLL1MASP2_{zym}}\right]}{K_{\mathrm{m\ MASP1^{*}}}^{\mathrm{MASP2_{zym}}}}+\frac{\left[\mathrm{pCLL1MASP3_{zym}}\right]}{K_{\mathrm{m\ MASP1^{*}}}^{\mathrm{MASP3_{zym}}}}\\
+\frac{\left[\mathrm{pLFMASP1_{zym}}\right]}{K_{\mathrm{m\ MASP1^{*}}}^{\mathrm{MASP1_{zym}}}}+\frac{\left[\mathrm{pLFMASP2_{zym}}\right]}{K_{\mathrm{m\ MASP1^{*}}}^{\mathrm{MASP2_{zym}}}}+\frac{\left[\mathrm{pLFMASP3_{zym}}\right]}{K_{\mathrm{m\ MASP1^{*}}}^{\mathrm{MASP3_{zym}}}}+\frac{\left[\mathrm{pHFMASP1_{zym}}\right]}{K_{\mathrm{m\ MASP1^{*}}}^{\mathrm{MASP1_{zym}}}}+\frac{\left[\mathrm{pHFMASP2_{zym}}\right]}{K_{\mathrm{m\ MASP1^{*}}}^{\mathrm{MASP2_{zym}}}}\\
+\frac{\left[\mathrm{pHFMASP3_{zym}}\right]}{K_{\mathrm{m\ MASP1^{*}}}^{\mathrm{MASP3_{zym}}}}+\frac{\left[\mathrm{pMFMASP1_{zym}}\right]}{K_{\mathrm{m\ MASP1^{*}}}^{\mathrm{MASP1_{zym}}}}+\frac{\left[\mathrm{pMFMASP2_{zym}}\right]}{K_{\mathrm{m\ MASP1^{*}}}^{\mathrm{MASP2_{zym}}}}+\frac{\left[\mathrm{pMFMASP3_{zym}}\right]}{K_{\mathrm{m\ MASP1^{*}}}^{\mathrm{MASP3_{zym}}}}\\
+\frac{\left[\mathrm{pPTX3LFMASP1_{zym}}\right]}{K_{\mathrm{m\ MASP1^{*}}}^{\mathrm{MASP1_{zym}}}}+\frac{\left[\mathrm{pPXT3LFMASP2_{zym}}\right]}{K_{\mathrm{m\ MASP1^{*}}}^{\mathrm{MASP2_{zym}}}}+\frac{\left[\mathrm{pPTX3LFMASP3_{zym}}\right]}{K_{\mathrm{m\ MASP1^{*}}}^{\mathrm{MASP3_{zym}}}}
\end{array}\right)}
$$

$$+\frac{k_{\text{cat MASP1}^*}^{\text{MASP3}_{\text{zym}}}\left[\text{pLFMASP1}^*\right]\left[\text{pCLLKMASP3}_{\text{zym}}\right]}{K_{\text{m MASP1}^*}^{\text{MASP3}_{\text{zym}}}\left(\begin{array}{l}1+\frac{[\text{C4}]}{K_{\text{m MASP1}^*}^{\text{C4}}}+\frac{[\text{fC4bC2}]}{K_{\text{m MASP1}^*}^{\text{C2}}}+\frac{[\text{fC4bC4bC2}]}{K_{\text{m MASP1}^*}^{\text{C2}}}+\frac{[\text{fC3bC4bC2}]}{K_{\text{m MASP1}^*}^{\text{C2}}}+\frac{[\text{pC4bC2}]}{K_{\text{m MASP1}^*}^{\text{C2}}}+\frac{[\text{pC4bC4bC2}]}{K_{\text{m MASP1}^*}^{\text{C2}}}\\
+\frac{[\text{pC3bC4bC2}]}{K_{\text{m MASP1}^*}^{\text{C2}}}+\frac{[\text{pMBL}_3\text{MASP1}_{\text{zym}}]}{K_{\text{m MASP1}^*}^{\text{MASP1}_{\text{zym}}}}+\frac{[\text{pMBL}_3\text{MASP2}_{\text{zym}}]}{K_{\text{m MASP1}^*}^{\text{MASP2}_{\text{zym}}}}+\frac{[\text{pMBL}_3\text{MASP3}_{\text{zym}}]}{K_{\text{m MASP1}^*}^{\text{MASP3}_{\text{zym}}}}\\
+\frac{[\text{pMBL}_4\text{MASP1}_{\text{zym}}]}{K_{\text{m MASP1}^*}^{\text{MASP1}_{\text{zym}}}}+\frac{[\text{pMBL}_4\text{MASP2}_{\text{zym}}]}{K_{\text{m MASP1}^*}^{\text{MASP2}_{\text{zym}}}}+\frac{[\text{pMBL}_4\text{MASP3}_{\text{zym}}]}{K_{\text{m MASP1}^*}^{\text{MASP3}_{\text{zym}}}}+\frac{[\text{pCLLKMASP1}_{\text{zym}}]}{K_{\text{m MASP1}^*}^{\text{MASP1}_{\text{zym}}}}\\
+\frac{[\text{pCLLKMASP2}_{\text{zym}}]}{K_{\text{m MASP1}^*}^{\text{MASP2}_{\text{zym}}}}+\frac{[\text{pCLLKMASP3}_{\text{zym}}]}{K_{\text{m MASP1}^*}^{\text{MASP3}_{\text{zym}}}}+\frac{[\text{pCLK1MASP1}_{\text{zym}}]}{K_{\text{m MASP1}^*}^{\text{MASP1}_{\text{zym}}}}+\frac{[\text{pCLK1MASP2}_{\text{zym}}]}{K_{\text{m MASP1}^*}^{\text{MASP2}_{\text{zym}}}}\\
+\frac{[\text{pCLK1MASP3}_{\text{zym}}]}{K_{\text{m MASP1}^*}^{\text{MASP3}_{\text{zym}}}}+\frac{[\text{pCLL1MASP1}_{\text{zym}}]}{K_{\text{m MASP1}^*}^{\text{MASP1}_{\text{zym}}}}+\frac{[\text{pCLL1MASP2}_{\text{zym}}]}{K_{\text{m MASP1}^*}^{\text{MASP2}_{\text{zym}}}}+\frac{[\text{pCLL1MASP3}_{\text{zym}}]}{K_{\text{m MASP1}^*}^{\text{MASP3}_{\text{zym}}}}\\
+\frac{[\text{pLFMASP1}_{\text{zym}}]}{K_{\text{m MASP1}^*}^{\text{MASP1}_{\text{zym}}}}+\frac{[\text{pLFMASP2}_{\text{zym}}]}{K_{\text{m MASP1}^*}^{\text{MASP2}_{\text{zym}}}}+\frac{[\text{pLFMASP3}_{\text{zym}}]}{K_{\text{m MASP1}^*}^{\text{MASP3}_{\text{zym}}}}+\frac{[\text{pHFMASP1}_{\text{zym}}]}{K_{\text{m MASP1}^*}^{\text{MASP1}_{\text{zym}}}}+\frac{[\text{pHFMASP2}_{\text{zym}}]}{K_{\text{m MASP1}^*}^{\text{MASP2}_{\text{zym}}}}\\
+\frac{[\text{pHFMASP3}_{\text{zym}}]}{K_{\text{m MASP1}^*}^{\text{MASP3}_{\text{zym}}}}+\frac{[\text{pMFMASP1}_{\text{zym}}]}{K_{\text{m MASP1}^*}^{\text{MASP1}_{\text{zym}}}}+\frac{[\text{pMFMASP2}_{\text{zym}}]}{K_{\text{m MASP1}^*}^{\text{MASP2}_{\text{zym}}}}+\frac{[\text{pMFMASP3}_{\text{zym}}]}{K_{\text{m MASP1}^*}^{\text{MASP3}_{\text{zym}}}}\\
+\frac{[\text{pPTX3LFMASP1}_{\text{zym}}]}{K_{\text{m MASP1}^*}^{\text{MASP1}_{\text{zym}}}}+\frac{[\text{pPXT3LFMASP2}_{\text{zym}}]}{K_{\text{m MASP1}^*}^{\text{MASP2}_{\text{zym}}}}+\frac{[\text{pPTX3LFMASP3}_{\text{zym}}]}{K_{\text{m MASP1}^*}^{\text{MASP3}_{\text{zym}}}}\end{array}\right)}$$

$$+\frac{k_{\text{cat MASP1}^*}^{\text{MASP3}_{\text{zym}}}\left[\text{pHFMASP1}^*\right]\left[\text{pCLLKMASP3}_{\text{zym}}\right]}{K_{\text{m MASP1}^*}^{\text{MASP3}_{\text{zym}}}\left(\begin{array}{l}1+\frac{[\text{C4}]}{K_{\text{m MASP1}^*}^{\text{C4}}}+\frac{[\text{fC4bC2}]}{K_{\text{m MASP1}^*}^{\text{C2}}}+\frac{[\text{fC4bC4bC2}]}{K_{\text{m MASP1}^*}^{\text{C2}}}+\frac{[\text{fC3bC4bC2}]}{K_{\text{m MASP1}^*}^{\text{C2}}}+\frac{[\text{pC4bC2}]}{K_{\text{m MASP1}^*}^{\text{C2}}}+\frac{[\text{pC4bC4bC2}]}{K_{\text{m MASP1}^*}^{\text{C2}}}\\ +\frac{[\text{pC3bC4bC2}]}{K_{\text{m MASP1}^*}^{\text{C2}}}+\frac{[\text{pMBL}_3\text{MASP1}_{\text{zym}}]}{K_{\text{m MASP1}^*}^{\text{MASP1}_{\text{zym}}}}+\frac{[\text{pMBL}_3\text{MASP2}_{\text{zym}}]}{K_{\text{m MASP1}^*}^{\text{MASP2}_{\text{zym}}}}+\frac{[\text{pMBL}_3\text{MASP3}_{\text{zym}}]}{K_{\text{m MASP1}^*}^{\text{MASP3}_{\text{zym}}}}\\ +\frac{[\text{pMBL}_4\text{MASP1}_{\text{zym}}]}{K_{\text{m MASP1}^*}^{\text{MASP1}_{\text{zym}}}}+\frac{[\text{pMBL}_4\text{MASP2}_{\text{zym}}]}{K_{\text{m MASP1}^*}^{\text{MASP2}_{\text{zym}}}}+\frac{[\text{pMBL}_4\text{MASP3}_{\text{zym}}]}{K_{\text{m MASP1}^*}^{\text{MASP3}_{\text{zym}}}}+\frac{[\text{pCLLKMASP1}_{\text{zym}}]}{K_{\text{m MASP1}^*}^{\text{MASP1}_{\text{zym}}}}\\ +\frac{[\text{pCLLKMASP2}_{\text{zym}}]}{K_{\text{m MASP1}^*}^{\text{MASP2}_{\text{zym}}}}+\frac{[\text{pCLLKMASP3}_{\text{zym}}]}{K_{\text{m MASP1}^*}^{\text{MASP3}_{\text{zym}}}}+\frac{[\text{pCLK1MASP1}_{\text{zym}}]}{K_{\text{m MASP1}^*}^{\text{MASP1}_{\text{zym}}}}+\frac{[\text{pCLK1MASP2}_{\text{zym}}]}{K_{\text{m MASP1}^*}^{\text{MASP2}_{\text{zym}}}}\\ +\frac{[\text{pCLK1MASP3}_{\text{zym}}]}{K_{\text{m MASP1}^*}^{\text{MASP3}_{\text{zym}}}}+\frac{[\text{pCLL1MASP1}_{\text{zym}}]}{K_{\text{m MASP1}^*}^{\text{MASP1}_{\text{zym}}}}+\frac{[\text{pCLL1MASP2}_{\text{zym}}]}{K_{\text{m MASP1}^*}^{\text{MASP2}_{\text{zym}}}}+\frac{[\text{pCLL1MASP3}_{\text{zym}}]}{K_{\text{m MASP1}^*}^{\text{MASP3}_{\text{zym}}}}\\ +\frac{[\text{pLFMASP1}_{\text{zym}}]}{K_{\text{m MASP1}^*}^{\text{MASP1}_{\text{zym}}}}+\frac{[\text{pLFMASP2}_{\text{zym}}]}{K_{\text{m MASP1}^*}^{\text{MASP2}_{\text{zym}}}}+\frac{[\text{pLFMASP3}_{\text{zym}}]}{K_{\text{m MASP1}^*}^{\text{MASP3}_{\text{zym}}}}+\frac{[\text{pHFMASP1}_{\text{zym}}]}{K_{\text{m MASP1}^*}^{\text{MASP1}_{\text{zym}}}}+\frac{[\text{pHFMASP2}_{\text{zym}}]}{K_{\text{m MASP1}^*}^{\text{MASP2}_{\text{zym}}}}\\ +\frac{[\text{pHFMASP3}_{\text{zym}}]}{K_{\text{m MASP1}^*}^{\text{MASP3}_{\text{zym}}}}+\frac{[\text{pMFMASP1}_{\text{zym}}]}{K_{\text{m MASP1}^*}^{\text{MASP1}_{\text{zym}}}}+\frac{[\text{pMFMASP2}_{\text{zym}}]}{K_{\text{m MASP1}^*}^{\text{MASP2}_{\text{zym}}}}+\frac{[\text{pMFMASP3}_{\text{zym}}]}{K_{\text{m MASP1}^*}^{\text{MASP3}_{\text{zym}}}}\\ +\frac{[\text{pPTX3LFMASP1}_{\text{zym}}]}{K_{\text{m MASP1}^*}^{\text{MASP1}_{\text{zym}}}}+\frac{[\text{pPXT3LFMASP2}_{\text{zym}}]}{K_{\text{m MASP1}^*}^{\text{MASP2}_{\text{zym}}}}+\frac{[\text{pPTX3LFMASP3}_{\text{zym}}]}{K_{\text{m MASP1}^*}^{\text{MASP3}_{\text{zym}}}}\end{array}\right)}$$

$$+\frac{k_{\text{cat MASP1}^*}^{\text{MASP3}_{\text{zym}}}\left[\text{pMFMASP1}^*\right]\left[\text{pCLLKMASP3}_{\text{zym}}\right]}{K_{\text{m MASP1}^*}^{\text{MASP3}_{\text{zym}}}\left(\begin{array}{l}1+\frac{[\text{C4}]}{K_{\text{m MASP1}^*}^{\text{C4}}}+\frac{[\text{fC4bC2}]}{K_{\text{m MASP1}^*}^{\text{C2}}}+\frac{[\text{fC4bC4bC2}]}{K_{\text{m MASP1}^*}^{\text{C2}}}+\frac{[\text{fC3bC4bC2}]}{K_{\text{m MASP1}^*}^{\text{C2}}}+\frac{[\text{pC4bC2}]}{K_{\text{m MASP1}^*}^{\text{C2}}}+\frac{[\text{pC4bC4bC2}]}{K_{\text{m MASP1}^*}^{\text{C2}}}\\
+\frac{[\text{pC3bC4bC2}]}{K_{\text{m MASP1}^*}^{\text{C2}}}+\frac{\left[\text{pMBL}_3\text{MASP1}_{\text{zym}}\right]}{K_{\text{m MASP1}^*}^{\text{MASP1}_{\text{zym}}}}+\frac{\left[\text{pMBL}_3\text{MASP2}_{\text{zym}}\right]}{K_{\text{m MASP1}^*}^{\text{MASP2}_{\text{zym}}}}+\frac{\left[\text{pMBL}_3\text{MASP3}_{\text{zym}}\right]}{K_{\text{m MASP1}^*}^{\text{MASP3}_{\text{zym}}}}\\
+\frac{\left[\text{pMBL}_4\text{MASP1}_{\text{zym}}\right]}{K_{\text{m MASP1}^*}^{\text{MASP1}_{\text{zym}}}}+\frac{\left[\text{pMBL}_4\text{MASP2}_{\text{zym}}\right]}{K_{\text{m MASP1}^*}^{\text{MASP2}_{\text{zym}}}}+\frac{\left[\text{pMBL}_4\text{MASP3}_{\text{zym}}\right]}{K_{\text{m MASP1}^*}^{\text{MASP3}_{\text{zym}}}}+\frac{\left[\text{pCLLKMASP1}_{\text{zym}}\right]}{K_{\text{m MASP1}^*}^{\text{MASP1}_{\text{zym}}}}\\
+\frac{\left[\text{pCLLKMASP2}_{\text{zym}}\right]}{K_{\text{m MASP1}^*}^{\text{MASP2}_{\text{zym}}}}+\frac{\left[\text{pCLLKMASP3}_{\text{zym}}\right]}{K_{\text{m MASP1}^*}^{\text{MASP3}_{\text{zym}}}}+\frac{\left[\text{pCLK1MASP1}_{\text{zym}}\right]}{K_{\text{m MASP1}^*}^{\text{MASP1}_{\text{zym}}}}+\frac{\left[\text{pCLK1MASP2}_{\text{zym}}\right]}{K_{\text{m MASP1}^*}^{\text{MASP2}_{\text{zym}}}}\\
+\frac{\left[\text{pCLK1MASP3}_{\text{zym}}\right]}{K_{\text{m MASP1}^*}^{\text{MASP3}_{\text{zym}}}}+\frac{\left[\text{pCLL1MASP1}_{\text{zym}}\right]}{K_{\text{m MASP1}^*}^{\text{MASP1}_{\text{zym}}}}+\frac{\left[\text{pCLL1MASP2}_{\text{zym}}\right]}{K_{\text{m MASP1}^*}^{\text{MASP2}_{\text{zym}}}}+\frac{\left[\text{pCLL1MASP3}_{\text{zym}}\right]}{K_{\text{m MASP1}^*}^{\text{MASP3}_{\text{zym}}}}\\
+\frac{\left[\text{pLFMASP1}_{\text{zym}}\right]}{K_{\text{m MASP1}^*}^{\text{MASP1}_{\text{zym}}}}+\frac{\left[\text{pLFMASP2}_{\text{zym}}\right]}{K_{\text{m MASP1}^*}^{\text{MASP2}_{\text{zym}}}}+\frac{\left[\text{pLFMASP3}_{\text{zym}}\right]}{K_{\text{m MASP1}^*}^{\text{MASP3}_{\text{zym}}}}+\frac{\left[\text{pHFMASP1}_{\text{zym}}\right]}{K_{\text{m MASP1}^*}^{\text{MASP1}_{\text{zym}}}}+\frac{\left[\text{pHFMASP2}_{\text{zym}}\right]}{K_{\text{m MASP1}^*}^{\text{MASP2}_{\text{zym}}}}\\
+\frac{\left[\text{pHFMASP3}_{\text{zym}}\right]}{K_{\text{m MASP1}^*}^{\text{MASP3}_{\text{zym}}}}+\frac{\left[\text{pMFMASP1}_{\text{zym}}\right]}{K_{\text{m MASP1}^*}^{\text{MASP1}_{\text{zym}}}}+\frac{\left[\text{pMFMASP2}_{\text{zym}}\right]}{K_{\text{m MASP1}^*}^{\text{MASP2}_{\text{zym}}}}+\frac{\left[\text{pMFMASP3}_{\text{zym}}\right]}{K_{\text{m MASP1}^*}^{\text{MASP3}_{\text{zym}}}}\\
+\frac{\left[\text{pPTX3LFMASP1}_{\text{zym}}\right]}{K_{\text{m MASP1}^*}^{\text{MASP1}_{\text{zym}}}}+\frac{\left[\text{pPXT3LFMASP2}_{\text{zym}}\right]}{K_{\text{m MASP1}^*}^{\text{MASP2}_{\text{zym}}}}+\frac{\left[\text{pPTX3LFMASP3}_{\text{zym}}\right]}{K_{\text{m MASP1}^*}^{\text{MASP3}_{\text{zym}}}}\end{array}\right)}$$

$$
+\frac{k_{\mathrm{cat\ MASP1^*}}^{\mathrm{MASP3_{zym}}}\left[\mathrm{pPTX3LFMASP1^*}\right]\left[\mathrm{pCLLKMASP3_{zym}}\right]}{K_{\mathrm{m\ MASP1^*}}^{\mathrm{MASP3_{zym}}}\left(\begin{array}{l}
1+\frac{[\mathrm{C4}]}{K_{\mathrm{m\ MASP1^*}}^{\mathrm{C4}}}+\frac{[\mathrm{fC4bC2}]}{K_{\mathrm{m\ MASP1^*}}^{\mathrm{C2}}}+\frac{[\mathrm{fC4bC4bC2}]}{K_{\mathrm{m\ MASP1^*}}^{\mathrm{C2}}}+\frac{[\mathrm{fC3bC4bC2}]}{K_{\mathrm{m\ MASP1^*}}^{\mathrm{C2}}}+\frac{[\mathrm{pC4bC2}]}{K_{\mathrm{m\ MASP1^*}}^{\mathrm{C2}}}+\frac{[\mathrm{pC4bC4bC2}]}{K_{\mathrm{m\ MASP1^*}}^{\mathrm{C2}}}\\
+\frac{[\mathrm{pC3bC4bC2}]}{K_{\mathrm{m\ MASP1^*}}^{\mathrm{C2}}}+\frac{\left[\mathrm{pMBL_3MASP1_{zym}}\right]}{K_{\mathrm{m\ MASP1^*}}^{\mathrm{MASP1_{zym}}}}+\frac{\left[\mathrm{pMBL_3MASP2_{zym}}\right]}{K_{\mathrm{m\ MASP1^*}}^{\mathrm{MASP2_{zym}}}}+\frac{\left[\mathrm{pMBL_3MASP3_{zym}}\right]}{K_{\mathrm{m\ MASP1^*}}^{\mathrm{MASP3_{zym}}}}\\
+\frac{\left[\mathrm{pMBL_4MASP1_{zym}}\right]}{K_{\mathrm{m\ MASP1^*}}^{\mathrm{MASP1_{zym}}}}+\frac{\left[\mathrm{pMBL_4MASP2_{zym}}\right]}{K_{\mathrm{m\ MASP1^*}}^{\mathrm{MASP2_{zym}}}}+\frac{\left[\mathrm{pMBL_4MASP3_{zym}}\right]}{K_{\mathrm{m\ MASP1^*}}^{\mathrm{MASP3_{zym}}}}+\frac{\left[\mathrm{pCLLKMASP1_{zym}}\right]}{K_{\mathrm{m\ MASP1^*}}^{\mathrm{MASP1_{zym}}}}\\
+\frac{\left[\mathrm{pCLLKMASP2_{zym}}\right]}{K_{\mathrm{m\ MASP1^*}}^{\mathrm{MASP2_{zym}}}}+\frac{\left[\mathrm{pCLLKMASP3_{zym}}\right]}{K_{\mathrm{m\ MASP1^*}}^{\mathrm{MASP3_{zym}}}}+\frac{\left[\mathrm{pCLK1MASP1_{zym}}\right]}{K_{\mathrm{m\ MASP1^*}}^{\mathrm{MASP1_{zym}}}}+\frac{\left[\mathrm{pCLK1MASP2_{zym}}\right]}{K_{\mathrm{m\ MASP1^*}}^{\mathrm{MASP2_{zym}}}}\\
+\frac{\left[\mathrm{pCLK1MASP3_{zym}}\right]}{K_{\mathrm{m\ MASP1^*}}^{\mathrm{MASP3_{zym}}}}+\frac{\left[\mathrm{pCLL1MASP1_{zym}}\right]}{K_{\mathrm{m\ MASP1^*}}^{\mathrm{MASP1_{zym}}}}+\frac{\left[\mathrm{pCLL1MASP2_{zym}}\right]}{K_{\mathrm{m\ MASP1^*}}^{\mathrm{MASP2_{zym}}}}+\frac{\left[\mathrm{pCLL1MASP3_{zym}}\right]}{K_{\mathrm{m\ MASP1^*}}^{\mathrm{MASP3_{zym}}}}\\
+\frac{\left[\mathrm{pLFMASP1_{zym}}\right]}{K_{\mathrm{m\ MASP1^*}}^{\mathrm{MASP1_{zym}}}}+\frac{\left[\mathrm{pLFMASP2_{zym}}\right]}{K_{\mathrm{m\ MASP1^*}}^{\mathrm{MASP2_{zym}}}}+\frac{\left[\mathrm{pLFMASP3_{zym}}\right]}{K_{\mathrm{m\ MASP1^*}}^{\mathrm{MASP3_{zym}}}}+\frac{\left[\mathrm{pHFMASP1_{zym}}\right]}{K_{\mathrm{m\ MASP1^*}}^{\mathrm{MASP1_{zym}}}}+\frac{\left[\mathrm{pHFMASP2_{zym}}\right]}{K_{\mathrm{m\ MASP1^*}}^{\mathrm{MASP2_{zym}}}}\\
+\frac{\left[\mathrm{pHFMASP3_{zym}}\right]}{K_{\mathrm{m\ MASP1^*}}^{\mathrm{MASP3_{zym}}}}+\frac{\left[\mathrm{pMFMASP1_{zym}}\right]}{K_{\mathrm{m\ MASP1^*}}^{\mathrm{MASP1_{zym}}}}+\frac{\left[\mathrm{pMFMASP2_{zym}}\right]}{K_{\mathrm{m\ MASP1^*}}^{\mathrm{MASP2_{zym}}}}+\frac{\left[\mathrm{pMFMASP3_{zym}}\right]}{K_{\mathrm{m\ MASP1^*}}^{\mathrm{MASP3_{zym}}}}\\
+\frac{\left[\mathrm{pPTX3LFMASP1_{zym}}\right]}{K_{\mathrm{m\ MASP1^*}}^{\mathrm{MASP1_{zym}}}}+\frac{\left[\mathrm{pPXT3LFMASP2_{zym}}\right]}{K_{\mathrm{m\ MASP1^*}}^{\mathrm{MASP2_{zym}}}}+\frac{\left[\mathrm{pPTX3LFMASP3_{zym}}\right]}{K_{\mathrm{m\ MASP1^*}}^{\mathrm{MASP3_{zym}}}}
\end{array}\right)}
$$

296

$$
\frac{d[\mathrm{fCLLKMAp19}]}{dt}=k_{\mathrm{CLLKMAp19}}^{+}[\mathrm{CLLK}][\mathrm{MAp19}]-k_{\mathrm{CLLKMAp19}}^{-}[\mathrm{fCLLKMAp19}]
$$

$$
-k_{\mathrm{pCLLK}}^{+}[\mathrm{fCLLKMAp19}][\mathrm{Pathogen\ Surface}]+k_{\mathrm{pCLLK}}^{-}[\mathrm{pCLLKMAp19}]
$$

297

$$
\frac{d[\mathrm{pCLLKMAp19}]}{dt}=k_{\mathrm{pCLLK}}^{+}[\mathrm{fCLLKMAp19}][\mathrm{Pathogen\ Surface}]-k_{\mathrm{pCLLK}}^{-}[\mathrm{pCLLKMAp19}]
$$

$$
+k_{\mathrm{CLLKMAp19}}^{+}[\mathrm{pCLLK}][\mathrm{MAp19}]-k_{\mathrm{CLLKMAp19}}^{-}[\mathrm{pCLLKMAp19}]
$$

298

$$
\frac{d[\mathrm{fCLLKMAp44}]}{dt}=k_{\mathrm{CLLKMAp44}}^{+}[\mathrm{CLLK}][\mathrm{MAp44}]-k_{\mathrm{CLLKMAp44}}^{-}[\mathrm{fCLLKMAp44}]
$$

$$
-k_{\mathrm{pCLLK}}^{+}[\mathrm{fCLLKMAp44}][\mathrm{Pathogen\ Surface}]+k_{\mathrm{pCLLK}}^{-}[\mathrm{pCLLKMAp44}]
$$

299

$$\frac{d[\text{pCLLKMAp44}]}{dt} = k^{+}_{\text{pCLLK}}[\text{fCLLKMAp44}][\text{Pathogen Surface}] - k^{-}_{\text{pCLLK}}[\text{pCLLKMAp44}] + k^{+}_{\text{CLLKMAp44}}[\text{pCLLK}][\text{MAp44}] - k^{-}_{\text{CLLKMAp44}}[\text{pCLLKMAp44}]$$

300

$$\frac{d[\text{CLK1}]}{dt} = -k^{+}_{\text{CLK1MASP1}}[\text{CLK1}][\text{MASP1}] + k^{-}_{\text{CLK1MASP1}}[\text{fCLK1MASP1}_{\text{zym}}] - k^{+}_{\text{CLK1MASP2}}[\text{CLK1}][\text{MASP2}] + k^{-}_{\text{CLK1MASP2}}[\text{fCLK1MASP2}_{\text{zym}}] - k^{+}_{\text{CLK1MASP3}}[\text{CLK1}][\text{MASP3}] + k^{-}_{\text{CLK1MASP3}}[\text{fCLK1MASP3}_{\text{zym}}] - k^{+}_{\text{CLK1MAp19}}[\text{CLK1}][\text{MAp19}] + k^{-}_{\text{CLK1MAp19}}[\text{fCLK1MAp19}] - k^{+}_{\text{CLK1MAp44}}[\text{CLK1}][\text{MAp44}] + k^{-}_{\text{CLK1MAp44}}[\text{fCLK1MAp44}] - k^{+}_{\text{pCLK1}}[\text{CLK1}][\text{Pathogen Surface}] + k^{-}_{\text{pCLK1}}[\text{pCLK1}] - k^{+}_{\text{CLK1MASP1}}[\text{CLK1}][\text{MASP1}^{*}] + k^{-}_{\text{CLK1MASP1}}[\text{fCLK1MASP1}^{*}] - k^{+}_{\text{CLK1MASP2}}[\text{CLK1}][\text{MASP2}^{*}] + k^{-}_{\text{CLK1MASP2}}[\text{fCLK1MASP2}^{*}] - k^{+}_{\text{CLK1MASP3}}[\text{CLK1}][\text{MASP3}^{*}] + k^{-}_{\text{CLK1MASP3}}[\text{fCLK1MASP3}^{*}] + k^{+}_{\text{C1INHMASP1}}[\text{C1-INH}][\text{fCLK1MASP1}^{*}] + k^{+}_{\text{C1INHMASP2}}[\text{C1-INH}][\text{fCLK1MASP2}^{*}]$$

301

$$\frac{d[\text{pCLK1}]}{dt} = k^{+}_{\text{pCLK1}}[\text{CLK1}][\text{Pathogen Surface}] - k^{-}_{\text{pCLK1}}[\text{pCLK1}] - k^{+}_{\text{CLK1MASP1}}[\text{pCLK1}][\text{MASP1}] + k^{-}_{\text{CLK1MASP1}}[\text{pCLK1MASP1}_{\text{zym}}] - k^{+}_{\text{CLK1MASP2}}[\text{pCLK1}][\text{MASP2}] + k^{-}_{\text{CLK1MASP2}}[\text{pCLK1MASP2}_{\text{zym}}] - k^{+}_{\text{CLK1MASP3}}[\text{pCLK1}][\text{MASP3}] + k^{-}_{\text{CLK1MASP3}}[\text{pCLK1MASP3}_{\text{zym}}] - k^{+}_{\text{CLK1MASP1}}[\text{pCLK1}][\text{MASP1}^{*}] + k^{-}_{\text{CLK1MASP1}}[\text{pCLK1MASP1}^{*}] - k^{+}_{\text{CLK1MASP2}}[\text{pCLK1}][\text{MASP2}^{*}] + k^{-}_{\text{CLK1MASP2}}[\text{pCLK1MASP2}^{*}] - k^{+}_{\text{CLK1MASP3}}[\text{pCLK1}][\text{MASP3}^{*}] + k^{-}_{\text{CLK1MASP3}}[\text{pCLK1MASP3}^{*}] - k^{+}_{\text{CLK1MAp19}}[\text{pCLK1}][\text{MAp19}] + k^{-}_{\text{CLK1MAp19}}[\text{pCLK1MAp19}] - k^{+}_{\text{CLK1MAp44}}[\text{pCLK1}][\text{MAp44}] + k^{-}_{\text{CLK1MAp44}}[\text{pCLK1MAp44}] + k^{+}_{\text{C1INHMASP1}}[\text{C1-INH}][\text{pCLK1MASP1}^{*}] + k^{+}_{\text{C1INHMASP2}}[\text{C1-INH}][\text{pCLK1MASP2}^{*}]$$

302

$$\frac{d[\text{fCLK1MASP1}_{\text{zym}}]}{dt} = k^{+}_{\text{CLK1MASP1}}[\text{CLK1}][\text{MASP1}] - k^{-}_{\text{CLK1MASP1}}[\text{fCLK1MASP1}_{\text{zym}}] - k^{+}_{\text{pCLK1}}[\text{fCLK1MASP1}_{\text{zym}}][\text{Pathogen Surface}] + k^{-}_{\text{pCLK1}}[\text{pCLK1MASP1}_{\text{zym}}]$$

303

$$\frac{d[\text{pCLK1MASP1}_{\text{zym}}]}{dt} = k^{+}_{\text{pCLK1}}[\text{fCLK1MASP1}_{\text{zym}}][\text{Pathogen Surface}] - k^{-}_{\text{pCLK1}}[\text{pCLK1MASP1}_{\text{zym}}] + k^{+}_{\text{CLK1MASP1}}[\text{pCLK1}][\text{MASP1}] - k^{-}_{\text{CLK1MASP1}}[\text{pCLK1MASP1}_{\text{zym}}]$$

$$
-\frac{k_{\mathrm{cat\,MASP1_{zym}}}^{\mathrm{MASP1_{zym}}}\left[\mathrm{pMBL_3MASP1_{zym}}\right]\left[\mathrm{pCLK1MASP1_{zym}}\right]}{K_{\mathrm{m\,MASP1_{zym}}}^{\mathrm{MASP1_{zym}}}\left(\begin{array}{l}1+\frac{\left[\mathrm{pMBL_3MASP1_{zym}}\right]}{K_{\mathrm{m\,MASP1_{zym}}}^{\mathrm{MASP1_{zym}}}}+\frac{\left[\mathrm{pMBL_3MASP2_{zym}}\right]}{K_{\mathrm{m\,MASP1_{zym}}}^{\mathrm{MASP2_{zym}}}}+\frac{\left[\mathrm{pMBL_4MASP1_{zym}}\right]}{K_{\mathrm{m\,MASP1_{zym}}}^{\mathrm{MASP1_{zym}}}}+\frac{\left[\mathrm{pMBL_4MASP2_{zym}}\right]}{K_{\mathrm{m\,MASP1_{zym}}}^{\mathrm{MASP2_{zym}}}}\\+\frac{\left[\mathrm{pCLLKMASP1_{zym}}\right]}{K_{\mathrm{m\,MASP1_{zym}}}^{\mathrm{MASP1_{zym}}}}+\frac{\left[\mathrm{pCLLKMASP2_{zym}}\right]}{K_{\mathrm{m\,MASP1_{zym}}}^{\mathrm{MASP2_{zym}}}}+\frac{\left[\mathrm{pCLK1MASP1_{zym}}\right]}{K_{\mathrm{m\,MASP1_{zym}}}^{\mathrm{MASP1_{zym}}}}+\frac{\left[\mathrm{pCLK1MASP2_{zym}}\right]}{K_{\mathrm{m\,MASP1_{zym}}}^{\mathrm{MASP2_{zym}}}}\\+\frac{\left[\mathrm{pCLL1MASP1_{zym}}\right]}{K_{\mathrm{m\,MASP1_{zym}}}^{\mathrm{MASP1_{zym}}}}+\frac{\left[\mathrm{pCLL1MASP2_{zym}}\right]}{K_{\mathrm{m\,MASP1_{zym}}}^{\mathrm{MASP2_{zym}}}}+\frac{\left[\mathrm{pLFMASP1_{zym}}\right]}{K_{\mathrm{m\,MASP1_{zym}}}^{\mathrm{MASP1_{zym}}}}+\frac{\left[\mathrm{pLFMASP2_{zym}}\right]}{K_{\mathrm{m\,MASP1_{zym}}}^{\mathrm{MASP2_{zym}}}}\\+\frac{\left[\mathrm{pHFMASP1_{zym}}\right]}{K_{\mathrm{m\,MASP1_{zym}}}^{\mathrm{MASP1_{zym}}}}+\frac{\left[\mathrm{pHFMASP2_{zym}}\right]}{K_{\mathrm{m\,MASP1_{zym}}}^{\mathrm{MASP2_{zym}}}}+\frac{\left[\mathrm{pMFMASP1_{zym}}\right]}{K_{\mathrm{m\,MASP1_{zym}}}^{\mathrm{MASP1_{zym}}}}+\frac{\left[\mathrm{pMFMASP2_{zym}}\right]}{K_{\mathrm{m\,MASP1_{zym}}}^{\mathrm{MASP2_{zym}}}}\end{array}\right)}
$$

$$
-\frac{k_{\mathrm{cat\,MASP1_{zym}}}^{\mathrm{MASP1_{zym}}}\left[\mathrm{pMBL_4MASP1_{zym}}\right]\left[\mathrm{pCLK1MASP1_{zym}}\right]}{K_{\mathrm{m\,MASP1_{zym}}}^{\mathrm{MASP1_{zym}}}\left(\begin{array}{l}1+\frac{\left[\mathrm{pMBL_3MASP1_{zym}}\right]}{K_{\mathrm{m\,MASP1_{zym}}}^{\mathrm{MASP1_{zym}}}}+\frac{\left[\mathrm{pMBL_3MASP2_{zym}}\right]}{K_{\mathrm{m\,MASP1_{zym}}}^{\mathrm{MASP2_{zym}}}}+\frac{\left[\mathrm{pMBL_4MASP1_{zym}}\right]}{K_{\mathrm{m\,MASP1_{zym}}}^{\mathrm{MASP1_{zym}}}}+\frac{\left[\mathrm{pMBL_4MASP2_{zym}}\right]}{K_{\mathrm{m\,MASP1_{zym}}}^{\mathrm{MASP2_{zym}}}}\\+\frac{\left[\mathrm{pCLLKMASP1_{zym}}\right]}{K_{\mathrm{m\,MASP1_{zym}}}^{\mathrm{MASP1_{zym}}}}+\frac{\left[\mathrm{pCLLKMASP2_{zym}}\right]}{K_{\mathrm{m\,MASP1_{zym}}}^{\mathrm{MASP2_{zym}}}}+\frac{\left[\mathrm{pCLK1MASP1_{zym}}\right]}{K_{\mathrm{m\,MASP1_{zym}}}^{\mathrm{MASP1_{zym}}}}+\frac{\left[\mathrm{pCLK1MASP2_{zym}}\right]}{K_{\mathrm{m\,MASP1_{zym}}}^{\mathrm{MASP2_{zym}}}}\\+\frac{\left[\mathrm{pCLL1MASP1_{zym}}\right]}{K_{\mathrm{m\,MASP1_{zym}}}^{\mathrm{MASP1_{zym}}}}+\frac{\left[\mathrm{pCLL1MASP2_{zym}}\right]}{K_{\mathrm{m\,MASP1_{zym}}}^{\mathrm{MASP2_{zym}}}}+\frac{\left[\mathrm{pLFMASP1_{zym}}\right]}{K_{\mathrm{m\,MASP1_{zym}}}^{\mathrm{MASP1_{zym}}}}+\frac{\left[\mathrm{pLFMASP2_{zym}}\right]}{K_{\mathrm{m\,MASP1_{zym}}}^{\mathrm{MASP2_{zym}}}}\\+\frac{\left[\mathrm{pHFMASP1_{zym}}\right]}{K_{\mathrm{m\,MASP1_{zym}}}^{\mathrm{MASP1_{zym}}}}+\frac{\left[\mathrm{pHFMASP2_{zym}}\right]}{K_{\mathrm{m\,MASP1_{zym}}}^{\mathrm{MASP2_{zym}}}}+\frac{\left[\mathrm{pMFMASP1_{zym}}\right]}{K_{\mathrm{m\,MASP1_{zym}}}^{\mathrm{MASP1_{zym}}}}+\frac{\left[\mathrm{pMFMASP2_{zym}}\right]}{K_{\mathrm{m\,MASP1_{zym}}}^{\mathrm{MASP2_{zym}}}}\end{array}\right)}
$$

$$
-\frac{k_{\mathrm{cat\,MASP1_{zym}}}^{\mathrm{MASP1_{zym}}}\left[\mathrm{pCLLKMASP1_{zym}}\right]\left[\mathrm{pCLK1MASP1_{zym}}\right]}{K_{\mathrm{m\,MASP1_{zym}}}^{\mathrm{MASP1_{zym}}}\left(\begin{array}{l}1+\frac{\left[\mathrm{pMBL_3MASP1_{zym}}\right]}{K_{\mathrm{m\,MASP1_{zym}}}^{\mathrm{MASP1_{zym}}}}+\frac{\left[\mathrm{pMBL_3MASP2_{zym}}\right]}{K_{\mathrm{m\,MASP1_{zym}}}^{\mathrm{MASP2_{zym}}}}+\frac{\left[\mathrm{pMBL_4MASP1_{zym}}\right]}{K_{\mathrm{m\,MASP1_{zym}}}^{\mathrm{MASP1_{zym}}}}+\frac{\left[\mathrm{pMBL_4MASP2_{zym}}\right]}{K_{\mathrm{m\,MASP1_{zym}}}^{\mathrm{MASP2_{zym}}}}\\+\frac{\left[\mathrm{pCLLKMASP1_{zym}}\right]}{K_{\mathrm{m\,MASP1_{zym}}}^{\mathrm{MASP1_{zym}}}}+\frac{\left[\mathrm{pCLLKMASP2_{zym}}\right]}{K_{\mathrm{m\,MASP1_{zym}}}^{\mathrm{MASP2_{zym}}}}+\frac{\left[\mathrm{pCLK1MASP1_{zym}}\right]}{K_{\mathrm{m\,MASP1_{zym}}}^{\mathrm{MASP1_{zym}}}}+\frac{\left[\mathrm{pCLK1MASP2_{zym}}\right]}{K_{\mathrm{m\,MASP1_{zym}}}^{\mathrm{MASP2_{zym}}}}\\+\frac{\left[\mathrm{pCLL1MASP1_{zym}}\right]}{K_{\mathrm{m\,MASP1_{zym}}}^{\mathrm{MASP1_{zym}}}}+\frac{\left[\mathrm{pCLL1MASP2_{zym}}\right]}{K_{\mathrm{m\,MASP1_{zym}}}^{\mathrm{MASP2_{zym}}}}+\frac{\left[\mathrm{pLFMASP1_{zym}}\right]}{K_{\mathrm{m\,MASP1_{zym}}}^{\mathrm{MASP1_{zym}}}}+\frac{\left[\mathrm{pLFMASP2_{zym}}\right]}{K_{\mathrm{m\,MASP1_{zym}}}^{\mathrm{MASP2_{zym}}}}\\+\frac{\left[\mathrm{pHFMASP1_{zym}}\right]}{K_{\mathrm{m\,MASP1_{zym}}}^{\mathrm{MASP1_{zym}}}}+\frac{\left[\mathrm{pHFMASP2_{zym}}\right]}{K_{\mathrm{m\,MASP1_{zym}}}^{\mathrm{MASP2_{zym}}}}+\frac{\left[\mathrm{pMFMASP1_{zym}}\right]}{K_{\mathrm{m\,MASP1_{zym}}}^{\mathrm{MASP1_{zym}}}}+\frac{\left[\mathrm{pMFMASP2_{zym}}\right]}{K_{\mathrm{m\,MASP1_{zym}}}^{\mathrm{MASP2_{zym}}}}\end{array}\right)}
$$

$$-\frac{k_{\text{cat MASP1}_{\text{zym}}}^{\text{MASP1}_{\text{zym}}}\left[\text{pCLK1MASP1}_{\text{zym}}\right]\left[\text{pCLK1MASP1}_{\text{zym}}\right]}{K_{\text{m MASP1}_{\text{zym}}}^{\text{MASP1}_{\text{zym}}}\left(\begin{array}{l}1+\frac{\left[\text{pMBL}_3\text{MASP1}_{\text{zym}}\right]}{K_{\text{m MASP1}_{\text{zym}}}^{\text{MASP1}_{\text{zym}}}}+\frac{\left[\text{pMBL}_3\text{MASP2}_{\text{zym}}\right]}{K_{\text{m MASP1}_{\text{zym}}}^{\text{MASP2}_{\text{zym}}}}+\frac{\left[\text{pMBL}_4\text{MASP1}_{\text{zym}}\right]}{K_{\text{m MASP1}_{\text{zym}}}^{\text{MASP1}_{\text{zym}}}}+\frac{\left[\text{pMBL}_4\text{MASP2}_{\text{zym}}\right]}{K_{\text{m MASP1}_{\text{zym}}}^{\text{MASP2}_{\text{zym}}}}\\+\frac{\left[\text{pCLLKMASP1}_{\text{zym}}\right]}{K_{\text{m MASP1}_{\text{zym}}}^{\text{MASP1}_{\text{zym}}}}+\frac{\left[\text{pCLLKMASP2}_{\text{zym}}\right]}{K_{\text{m MASP1}_{\text{zym}}}^{\text{MASP2}_{\text{zym}}}}+\frac{\left[\text{pCLK1MASP1}_{\text{zym}}\right]}{K_{\text{m MASP1}_{\text{zym}}}^{\text{MASP1}_{\text{zym}}}}+\frac{\left[\text{pCLK1MASP2}_{\text{zym}}\right]}{K_{\text{m MASP1}_{\text{zym}}}^{\text{MASP2}_{\text{zym}}}}\\+\frac{\left[\text{pCLL1MASP1}_{\text{zym}}\right]}{K_{\text{m MASP1}_{\text{zym}}}^{\text{MASP1}_{\text{zym}}}}+\frac{\left[\text{pCLL1MASP2}_{\text{zym}}\right]}{K_{\text{m MASP1}_{\text{zym}}}^{\text{MASP2}_{\text{zym}}}}+\frac{\left[\text{pLFMASP1}_{\text{zym}}\right]}{K_{\text{m MASP1}_{\text{zym}}}^{\text{MASP1}_{\text{zym}}}}+\frac{\left[\text{pLFMASP2}_{\text{zym}}\right]}{K_{\text{m MASP1}_{\text{zym}}}^{\text{MASP2}_{\text{zym}}}}\\+\frac{\left[\text{pHFMASP1}_{\text{zym}}\right]}{K_{\text{m MASP1}_{\text{zym}}}^{\text{MASP1}_{\text{zym}}}}+\frac{\left[\text{pHFMASP2}_{\text{zym}}\right]}{K_{\text{m MASP1}_{\text{zym}}}^{\text{MASP2}_{\text{zym}}}}+\frac{\left[\text{pMFMASP1}_{\text{zym}}\right]}{K_{\text{m MASP1}_{\text{zym}}}^{\text{MASP1}_{\text{zym}}}}+\frac{\left[\text{pMFMASP2}_{\text{zym}}\right]}{K_{\text{m MASP1}_{\text{zym}}}^{\text{MASP2}_{\text{zym}}}}\end{array}\right)}$$

$$-\frac{k_{\text{cat MASP1}_{\text{zym}}}^{\text{MASP1}_{\text{zym}}}\left[\text{pCLL1MASP1}_{\text{zym}}\right]\left[\text{pCLK1MASP1}_{\text{zym}}\right]}{K_{\text{m MASP1}_{\text{zym}}}^{\text{MASP1}_{\text{zym}}}\left(\begin{array}{l}1+\frac{\left[\text{pMBL}_3\text{MASP1}_{\text{zym}}\right]}{K_{\text{m MASP1}_{\text{zym}}}^{\text{MASP1}_{\text{zym}}}}+\frac{\left[\text{pMBL}_3\text{MASP2}_{\text{zym}}\right]}{K_{\text{m MASP1}_{\text{zym}}}^{\text{MASP2}_{\text{zym}}}}+\frac{\left[\text{pMBL}_4\text{MASP1}_{\text{zym}}\right]}{K_{\text{m MASP1}_{\text{zym}}}^{\text{MASP1}_{\text{zym}}}}+\frac{\left[\text{pMBL}_4\text{MASP2}_{\text{zym}}\right]}{K_{\text{m MASP1}_{\text{zym}}}^{\text{MASP2}_{\text{zym}}}}\\+\frac{\left[\text{pCLLKMASP1}_{\text{zym}}\right]}{K_{\text{m MASP1}_{\text{zym}}}^{\text{MASP1}_{\text{zym}}}}+\frac{\left[\text{pCLLKMASP2}_{\text{zym}}\right]}{K_{\text{m MASP1}_{\text{zym}}}^{\text{MASP2}_{\text{zym}}}}+\frac{\left[\text{pCLK1MASP1}_{\text{zym}}\right]}{K_{\text{m MASP1}_{\text{zym}}}^{\text{MASP1}_{\text{zym}}}}+\frac{\left[\text{pCLK1MASP2}_{\text{zym}}\right]}{K_{\text{m MASP1}_{\text{zym}}}^{\text{MASP2}_{\text{zym}}}}\\+\frac{\left[\text{pCLL1MASP1}_{\text{zym}}\right]}{K_{\text{m MASP1}_{\text{zym}}}^{\text{MASP1}_{\text{zym}}}}+\frac{\left[\text{pCLL1MASP2}_{\text{zym}}\right]}{K_{\text{m MASP1}_{\text{zym}}}^{\text{MASP2}_{\text{zym}}}}+\frac{\left[\text{pLFMASP1}_{\text{zym}}\right]}{K_{\text{m MASP1}_{\text{zym}}}^{\text{MASP1}_{\text{zym}}}}+\frac{\left[\text{pLFMASP2}_{\text{zym}}\right]}{K_{\text{m MASP1}_{\text{zym}}}^{\text{MASP2}_{\text{zym}}}}\\+\frac{\left[\text{pHFMASP1}_{\text{zym}}\right]}{K_{\text{m MASP1}_{\text{zym}}}^{\text{MASP1}_{\text{zym}}}}+\frac{\left[\text{pHFMASP2}_{\text{zym}}\right]}{K_{\text{m MASP1}_{\text{zym}}}^{\text{MASP2}_{\text{zym}}}}+\frac{\left[\text{pMFMASP1}_{\text{zym}}\right]}{K_{\text{m MASP1}_{\text{zym}}}^{\text{MASP1}_{\text{zym}}}}+\frac{\left[\text{pMFMASP2}_{\text{zym}}\right]}{K_{\text{m MASP1}_{\text{zym}}}^{\text{MASP2}_{\text{zym}}}}\end{array}\right)}$$

$$-\frac{k_{\text{cat MASP1}_{\text{zym}}}^{\text{MASP1}_{\text{zym}}}\left[\text{pLFMASP1}_{\text{zym}}\right]\left[\text{pCLK1MASP1}_{\text{zym}}\right]}{K_{\text{m MASP1}_{\text{zym}}}^{\text{MASP1}_{\text{zym}}}\left(\begin{array}{l}1+\frac{\left[\text{pMBL}_3\text{MASP1}_{\text{zym}}\right]}{K_{\text{m MASP1}_{\text{zym}}}^{\text{MASP1}_{\text{zym}}}}+\frac{\left[\text{pMBL}_3\text{MASP2}_{\text{zym}}\right]}{K_{\text{m MASP1}_{\text{zym}}}^{\text{MASP2}_{\text{zym}}}}+\frac{\left[\text{pMBL}_4\text{MASP1}_{\text{zym}}\right]}{K_{\text{m MASP1}_{\text{zym}}}^{\text{MASP1}_{\text{zym}}}}+\frac{\left[\text{pMBL}_4\text{MASP2}_{\text{zym}}\right]}{K_{\text{m MASP1}_{\text{zym}}}^{\text{MASP2}_{\text{zym}}}}\\+\frac{\left[\text{pCLLKMASP1}_{\text{zym}}\right]}{K_{\text{m MASP1}_{\text{zym}}}^{\text{MASP1}_{\text{zym}}}}+\frac{\left[\text{pCLLKMASP2}_{\text{zym}}\right]}{K_{\text{m MASP1}_{\text{zym}}}^{\text{MASP2}_{\text{zym}}}}+\frac{\left[\text{pCLK1MASP1}_{\text{zym}}\right]}{K_{\text{m MASP1}_{\text{zym}}}^{\text{MASP1}_{\text{zym}}}}+\frac{\left[\text{pCLK1MASP2}_{\text{zym}}\right]}{K_{\text{m MASP1}_{\text{zym}}}^{\text{MASP2}_{\text{zym}}}}\\+\frac{\left[\text{pCLL1MASP1}_{\text{zym}}\right]}{K_{\text{m MASP1}_{\text{zym}}}^{\text{MASP1}_{\text{zym}}}}+\frac{\left[\text{pCLL1MASP2}_{\text{zym}}\right]}{K_{\text{m MASP1}_{\text{zym}}}^{\text{MASP2}_{\text{zym}}}}+\frac{\left[\text{pLFMASP1}_{\text{zym}}\right]}{K_{\text{m MASP1}_{\text{zym}}}^{\text{MASP1}_{\text{zym}}}}+\frac{\left[\text{pLFMASP2}_{\text{zym}}\right]}{K_{\text{m MASP1}_{\text{zym}}}^{\text{MASP2}_{\text{zym}}}}\\+\frac{\left[\text{pHFMASP1}_{\text{zym}}\right]}{K_{\text{m MASP1}_{\text{zym}}}^{\text{MASP1}_{\text{zym}}}}+\frac{\left[\text{pHFMASP2}_{\text{zym}}\right]}{K_{\text{m MASP1}_{\text{zym}}}^{\text{MASP2}_{\text{zym}}}}+\frac{\left[\text{pMFMASP1}_{\text{zym}}\right]}{K_{\text{m MASP1}_{\text{zym}}}^{\text{MASP1}_{\text{zym}}}}+\frac{\left[\text{pMFMASP2}_{\text{zym}}\right]}{K_{\text{m MASP1}_{\text{zym}}}^{\text{MASP2}_{\text{zym}}}}\end{array}\right)}$$

$$-\frac{k_{\mathrm{cat\ MASP1_{zym}}}^{\mathrm{MASP1_{zym}}}\left[\mathrm{pHFMASP1_{zym}}\right]\left[\mathrm{pCLK1MASP1_{zym}}\right]}{K_{\mathrm{m\ MASP1_{zym}}}^{\mathrm{MASP1_{zym}}}\left(\begin{array}{l}1+\frac{\left[\mathrm{pMBL_3MASP1_{zym}}\right]}{K_{\mathrm{m\ MASP1_{zym}}}^{\mathrm{MASP1_{zym}}}}+\frac{\left[\mathrm{pMBL_3MASP2_{zym}}\right]}{K_{\mathrm{m\ MASP1_{zym}}}^{\mathrm{MASP2_{zym}}}}+\frac{\left[\mathrm{pMBL_4MASP1_{zym}}\right]}{K_{\mathrm{m\ MASP1_{zym}}}^{\mathrm{MASP1_{zym}}}}+\frac{\left[\mathrm{pMBL_4MASP2_{zym}}\right]}{K_{\mathrm{m\ MASP1_{zym}}}^{\mathrm{MASP2_{zym}}}}\\+\frac{\left[\mathrm{pCLLKMASP1_{zym}}\right]}{K_{\mathrm{m\ MASP1_{zym}}}^{\mathrm{MASP1_{zym}}}}+\frac{\left[\mathrm{pCLLKMASP2_{zym}}\right]}{K_{\mathrm{m\ MASP1_{zym}}}^{\mathrm{MASP2_{zym}}}}+\frac{\left[\mathrm{pCLK1MASP1_{zym}}\right]}{K_{\mathrm{m\ MASP1_{zym}}}^{\mathrm{MASP1_{zym}}}}+\frac{\left[\mathrm{pCLK1MASP2_{zym}}\right]}{K_{\mathrm{m\ MASP1_{zym}}}^{\mathrm{MASP2_{zym}}}}\\+\frac{\left[\mathrm{pCLL1MASP1_{zym}}\right]}{K_{\mathrm{m\ MASP1_{zym}}}^{\mathrm{MASP1_{zym}}}}+\frac{\left[\mathrm{pCLL1MASP2_{zym}}\right]}{K_{\mathrm{m\ MASP1_{zym}}}^{\mathrm{MASP2_{zym}}}}+\frac{\left[\mathrm{pLFMASP1_{zym}}\right]}{K_{\mathrm{m\ MASP1_{zym}}}^{\mathrm{MASP1_{zym}}}}+\frac{\left[\mathrm{pLFMASP2_{zym}}\right]}{K_{\mathrm{m\ MASP1_{zym}}}^{\mathrm{MASP2_{zym}}}}\\+\frac{\left[\mathrm{pHFMASP1_{zym}}\right]}{K_{\mathrm{m\ MASP1_{zym}}}^{\mathrm{MASP1_{zym}}}}+\frac{\left[\mathrm{pHFMASP2_{zym}}\right]}{K_{\mathrm{m\ MASP1_{zym}}}^{\mathrm{MASP2_{zym}}}}+\frac{\left[\mathrm{pMFMASP1_{zym}}\right]}{K_{\mathrm{m\ MASP1_{zym}}}^{\mathrm{MASP1_{zym}}}}+\frac{\left[\mathrm{pMFMASP2_{zym}}\right]}{K_{\mathrm{m\ MASP1_{zym}}}^{\mathrm{MASP2_{zym}}}}\end{array}\right)}$$

$$-\frac{k_{\mathrm{cat\ MASP1_{zym}}}^{\mathrm{MASP1_{zym}}}\left[\mathrm{pMFMASP1_{zym}}\right]\left[\mathrm{pCLK1MASP1_{zym}}\right]}{K_{\mathrm{m\ MASP1_{zym}}}^{\mathrm{MASP1_{zym}}}\left(\begin{array}{l}1+\frac{\left[\mathrm{pMBL_3MASP1_{zym}}\right]}{K_{\mathrm{m\ MASP1_{zym}}}^{\mathrm{MASP1_{zym}}}}+\frac{\left[\mathrm{pMBL_3MASP2_{zym}}\right]}{K_{\mathrm{m\ MASP1_{zym}}}^{\mathrm{MASP2_{zym}}}}+\frac{\left[\mathrm{pMBL_4MASP1_{zym}}\right]}{K_{\mathrm{m\ MASP1_{zym}}}^{\mathrm{MASP1_{zym}}}}+\frac{\left[\mathrm{pMBL_4MASP2_{zym}}\right]}{K_{\mathrm{m\ MASP1_{zym}}}^{\mathrm{MASP2_{zym}}}}\\+\frac{\left[\mathrm{pCLLKMASP1_{zym}}\right]}{K_{\mathrm{m\ MASP1_{zym}}}^{\mathrm{MASP1_{zym}}}}+\frac{\left[\mathrm{pCLLKMASP2_{zym}}\right]}{K_{\mathrm{m\ MASP1_{zym}}}^{\mathrm{MASP2_{zym}}}}+\frac{\left[\mathrm{pCLK1MASP1_{zym}}\right]}{K_{\mathrm{m\ MASP1_{zym}}}^{\mathrm{MASP1_{zym}}}}+\frac{\left[\mathrm{pCLK1MASP2_{zym}}\right]}{K_{\mathrm{m\ MASP1_{zym}}}^{\mathrm{MASP2_{zym}}}}\\+\frac{\left[\mathrm{pCLL1MASP1_{zym}}\right]}{K_{\mathrm{m\ MASP1_{zym}}}^{\mathrm{MASP1_{zym}}}}+\frac{\left[\mathrm{pCLL1MASP2_{zym}}\right]}{K_{\mathrm{m\ MASP1_{zym}}}^{\mathrm{MASP2_{zym}}}}+\frac{\left[\mathrm{pLFMASP1_{zym}}\right]}{K_{\mathrm{m\ MASP1_{zym}}}^{\mathrm{MASP1_{zym}}}}+\frac{\left[\mathrm{pLFMASP2_{zym}}\right]}{K_{\mathrm{m\ MASP1_{zym}}}^{\mathrm{MASP2_{zym}}}}\\+\frac{\left[\mathrm{pHFMASP1_{zym}}\right]}{K_{\mathrm{m\ MASP1_{zym}}}^{\mathrm{MASP1_{zym}}}}+\frac{\left[\mathrm{pHFMASP2_{zym}}\right]}{K_{\mathrm{m\ MASP1_{zym}}}^{\mathrm{MASP2_{zym}}}}+\frac{\left[\mathrm{pMFMASP1_{zym}}\right]}{K_{\mathrm{m\ MASP1_{zym}}}^{\mathrm{MASP1_{zym}}}}+\frac{\left[\mathrm{pMFMASP2_{zym}}\right]}{K_{\mathrm{m\ MASP1_{zym}}}^{\mathrm{MASP2_{zym}}}}\end{array}\right)}$$

$$-\frac{k_{\mathrm{cat\ MASP1_{zym}}}^{\mathrm{MASP1_{zym}}}\left[\mathrm{pPTX3LFMASP1_{zym}}\right]\left[\mathrm{pCLK1MASP1_{zym}}\right]}{K_{\mathrm{m\ MASP1_{zym}}}^{\mathrm{MASP1_{zym}}}\left(\begin{array}{l}1+\frac{\left[\mathrm{pMBL_3MASP1_{zym}}\right]}{K_{\mathrm{m\ MASP1_{zym}}}^{\mathrm{MASP1_{zym}}}}+\frac{\left[\mathrm{pMBL_3MASP2_{zym}}\right]}{K_{\mathrm{m\ MASP1_{zym}}}^{\mathrm{MASP2_{zym}}}}+\frac{\left[\mathrm{pMBL_4MASP1_{zym}}\right]}{K_{\mathrm{m\ MASP1_{zym}}}^{\mathrm{MASP1_{zym}}}}+\frac{\left[\mathrm{pMBL_4MASP2_{zym}}\right]}{K_{\mathrm{m\ MASP1_{zym}}}^{\mathrm{MASP2_{zym}}}}\\+\frac{\left[\mathrm{pCLLKMASP1_{zym}}\right]}{K_{\mathrm{m\ MASP1_{zym}}}^{\mathrm{MASP1_{zym}}}}+\frac{\left[\mathrm{pCLLKMASP2_{zym}}\right]}{K_{\mathrm{m\ MASP1_{zym}}}^{\mathrm{MASP2_{zym}}}}+\frac{\left[\mathrm{pCLK1MASP1_{zym}}\right]}{K_{\mathrm{m\ MASP1_{zym}}}^{\mathrm{MASP1_{zym}}}}+\frac{\left[\mathrm{pCLK1MASP2_{zym}}\right]}{K_{\mathrm{m\ MASP1_{zym}}}^{\mathrm{MASP2_{zym}}}}\\+\frac{\left[\mathrm{pCLL1MASP1_{zym}}\right]}{K_{\mathrm{m\ MASP1_{zym}}}^{\mathrm{MASP1_{zym}}}}+\frac{\left[\mathrm{pCLL1MASP2_{zym}}\right]}{K_{\mathrm{m\ MASP1_{zym}}}^{\mathrm{MASP2_{zym}}}}+\frac{\left[\mathrm{pLFMASP1_{zym}}\right]}{K_{\mathrm{m\ MASP1_{zym}}}^{\mathrm{MASP1_{zym}}}}+\frac{\left[\mathrm{pLFMASP2_{zym}}\right]}{K_{\mathrm{m\ MASP1_{zym}}}^{\mathrm{MASP2_{zym}}}}\\+\frac{\left[\mathrm{pHFMASP1_{zym}}\right]}{K_{\mathrm{m\ MASP1_{zym}}}^{\mathrm{MASP1_{zym}}}}+\frac{\left[\mathrm{pHFMASP2_{zym}}\right]}{K_{\mathrm{m\ MASP1_{zym}}}^{\mathrm{MASP2_{zym}}}}+\frac{\left[\mathrm{pMFMASP1_{zym}}\right]}{K_{\mathrm{m\ MASP1_{zym}}}^{\mathrm{MASP1_{zym}}}}+\frac{\left[\mathrm{pMFMASP2_{zym}}\right]}{K_{\mathrm{m\ MASP1_{zym}}}^{\mathrm{MASP2_{zym}}}}\\+\frac{\left[\mathrm{pPTX3LFMASP1_{zym}}\right]}{K_{\mathrm{m\ MASP1_{zym}}}^{\mathrm{MASP1_{zym}}}}+\frac{\left[\mathrm{pPTX3LFMASP2_{zym}}\right]}{K_{\mathrm{m\ MASP1_{zym}}}^{\mathrm{MASP2_{zym}}}}\end{array}\right)}$$

$$
-\frac{k_{\mathrm{cat\,MASP1^*}}^{\mathrm{MASP1_{zym}}}\left[\mathrm{pMBL_3MASP1^*}\right]\left[\mathrm{pCLK1MASP1_{zym}}\right]}{K_{\mathrm{m\,MASP1^*}}^{\mathrm{MASP1_{zym}}}\left(\begin{array}{l}1+\frac{[\mathrm{C4}]}{K_{\mathrm{m\,MASP1^*}}^{\mathrm{C4}}}+\frac{[\mathrm{fC4bC2}]}{K_{\mathrm{m\,MASP1^*}}^{\mathrm{C2}}}+\frac{[\mathrm{fC4bC4bC2}]}{K_{\mathrm{m\,MASP1^*}}^{\mathrm{C2}}}+\frac{[\mathrm{fC3bC4bC2}]}{K_{\mathrm{m\,MASP1^*}}^{\mathrm{C2}}}+\frac{[\mathrm{pC4bC2}]}{K_{\mathrm{m\,MASP1^*}}^{\mathrm{C2}}}+\frac{[\mathrm{pC4bC4bC2}]}{K_{\mathrm{m\,MASP1^*}}^{\mathrm{C2}}}\\
+\frac{[\mathrm{pC3bC4bC2}]}{K_{\mathrm{m\,MASP1^*}}^{\mathrm{C2}}}+\frac{\left[\mathrm{pMBL_3MASP1_{zym}}\right]}{K_{\mathrm{m\,MASP1^*}}^{\mathrm{MASP1_{zym}}}}+\frac{\left[\mathrm{pMBL_3MASP2_{zym}}\right]}{K_{\mathrm{m\,MASP1^*}}^{\mathrm{MASP2_{zym}}}}+\frac{\left[\mathrm{pMBL_3MASP3_{zym}}\right]}{K_{\mathrm{m\,MASP1^*}}^{\mathrm{MASP3_{zym}}}}\\
+\frac{\left[\mathrm{pMBL_4MASP1_{zym}}\right]}{K_{\mathrm{m\,MASP1^*}}^{\mathrm{MASP1_{zym}}}}+\frac{\left[\mathrm{pMBL_4MASP2_{zym}}\right]}{K_{\mathrm{m\,MASP1^*}}^{\mathrm{MASP2_{zym}}}}+\frac{\left[\mathrm{pMBL_4MASP3_{zym}}\right]}{K_{\mathrm{m\,MASP1^*}}^{\mathrm{MASP3_{zym}}}}+\frac{\left[\mathrm{pCLLKMASP1_{zym}}\right]}{K_{\mathrm{m\,MASP1^*}}^{\mathrm{MASP1_{zym}}}}\\
+\frac{\left[\mathrm{pCLLKMASP2_{zym}}\right]}{K_{\mathrm{m\,MASP1^*}}^{\mathrm{MASP2_{zym}}}}+\frac{\left[\mathrm{pCLLKMASP3_{zym}}\right]}{K_{\mathrm{m\,MASP1^*}}^{\mathrm{MASP3_{zym}}}}+\frac{\left[\mathrm{pCLK1MASP1_{zym}}\right]}{K_{\mathrm{m\,MASP1^*}}^{\mathrm{MASP1_{zym}}}}+\frac{\left[\mathrm{pCLK1MASP2_{zym}}\right]}{K_{\mathrm{m\,MASP1^*}}^{\mathrm{MASP2_{zym}}}}\\
+\frac{\left[\mathrm{pCLK1MASP3_{zym}}\right]}{K_{\mathrm{m\,MASP1^*}}^{\mathrm{MASP3_{zym}}}}+\frac{\left[\mathrm{pCLL1MASP1_{zym}}\right]}{K_{\mathrm{m\,MASP1^*}}^{\mathrm{MASP1_{zym}}}}+\frac{\left[\mathrm{pCLL1MASP2_{zym}}\right]}{K_{\mathrm{m\,MASP1^*}}^{\mathrm{MASP2_{zym}}}}+\frac{\left[\mathrm{pCLL1MASP3_{zym}}\right]}{K_{\mathrm{m\,MASP1^*}}^{\mathrm{MASP3_{zym}}}}\\
+\frac{\left[\mathrm{pLFMASP1_{zym}}\right]}{K_{\mathrm{m\,MASP1^*}}^{\mathrm{MASP1_{zym}}}}+\frac{\left[\mathrm{pLFMASP2_{zym}}\right]}{K_{\mathrm{m\,MASP1^*}}^{\mathrm{MASP2_{zym}}}}+\frac{\left[\mathrm{pLFMASP3_{zym}}\right]}{K_{\mathrm{m\,MASP1^*}}^{\mathrm{MASP3_{zym}}}}+\frac{\left[\mathrm{pHFMASP1_{zym}}\right]}{K_{\mathrm{m\,MASP1^*}}^{\mathrm{MASP1_{zym}}}}+\frac{\left[\mathrm{pHFMASP2_{zym}}\right]}{K_{\mathrm{m\,MASP1^*}}^{\mathrm{MASP2_{zym}}}}\\
+\frac{\left[\mathrm{pHFMASP3_{zym}}\right]}{K_{\mathrm{m\,MASP1^*}}^{\mathrm{MASP3_{zym}}}}+\frac{\left[\mathrm{pMFMASP1_{zym}}\right]}{K_{\mathrm{m\,MASP1^*}}^{\mathrm{MASP1_{zym}}}}+\frac{\left[\mathrm{pMFMASP2_{zym}}\right]}{K_{\mathrm{m\,MASP1^*}}^{\mathrm{MASP2_{zym}}}}+\frac{\left[\mathrm{pMFMASP3_{zym}}\right]}{K_{\mathrm{m\,MASP1^*}}^{\mathrm{MASP3_{zym}}}}\end{array}\right)}
$$

$$
-\frac{k_{\mathrm{cat\,MASP1^*}}^{\mathrm{MASP1_{zym}}}\left[\mathrm{pMBL_4MASP1^*}\right]\left[\mathrm{pCLK1MASP1_{zym}}\right]}{K_{\mathrm{m\,MASP1^*}}^{\mathrm{MASP1_{zym}}}\left(\begin{array}{l}1+\frac{[\mathrm{C4}]}{K_{\mathrm{m\,MASP1^*}}^{\mathrm{C4}}}+\frac{[\mathrm{fC4bC2}]}{K_{\mathrm{m\,MASP1^*}}^{\mathrm{C2}}}+\frac{[\mathrm{fC4bC4bC2}]}{K_{\mathrm{m\,MASP1^*}}^{\mathrm{C2}}}+\frac{[\mathrm{fC3bC4bC2}]}{K_{\mathrm{m\,MASP1^*}}^{\mathrm{C2}}}+\frac{[\mathrm{pC4bC2}]}{K_{\mathrm{m\,MASP1^*}}^{\mathrm{C2}}}+\frac{[\mathrm{pC4bC4bC2}]}{K_{\mathrm{m\,MASP1^*}}^{\mathrm{C2}}}\\
+\frac{[\mathrm{pC3bC4bC2}]}{K_{\mathrm{m\,MASP1^*}}^{\mathrm{C2}}}+\frac{\left[\mathrm{pMBL_3MASP1_{zym}}\right]}{K_{\mathrm{m\,MASP1^*}}^{\mathrm{MASP1_{zym}}}}+\frac{\left[\mathrm{pMBL_3MASP2_{zym}}\right]}{K_{\mathrm{m\,MASP1^*}}^{\mathrm{MASP2_{zym}}}}+\frac{\left[\mathrm{pMBL_3MASP3_{zym}}\right]}{K_{\mathrm{m\,MASP1^*}}^{\mathrm{MASP3_{zym}}}}\\
+\frac{\left[\mathrm{pMBL_4MASP1_{zym}}\right]}{K_{\mathrm{m\,MASP1^*}}^{\mathrm{MASP1_{zym}}}}+\frac{\left[\mathrm{pMBL_4MASP2_{zym}}\right]}{K_{\mathrm{m\,MASP1^*}}^{\mathrm{MASP2_{zym}}}}+\frac{\left[\mathrm{pMBL_4MASP3_{zym}}\right]}{K_{\mathrm{m\,MASP1^*}}^{\mathrm{MASP3_{zym}}}}+\frac{\left[\mathrm{pCLLKMASP1_{zym}}\right]}{K_{\mathrm{m\,MASP1^*}}^{\mathrm{MASP1_{zym}}}}\\
+\frac{\left[\mathrm{pCLLKMASP2_{zym}}\right]}{K_{\mathrm{m\,MASP1^*}}^{\mathrm{MASP2_{zym}}}}+\frac{\left[\mathrm{pCLLKMASP3_{zym}}\right]}{K_{\mathrm{m\,MASP1^*}}^{\mathrm{MASP3_{zym}}}}+\frac{\left[\mathrm{pCLK1MASP1_{zym}}\right]}{K_{\mathrm{m\,MASP1^*}}^{\mathrm{MASP1_{zym}}}}+\frac{\left[\mathrm{pCLK1MASP2_{zym}}\right]}{K_{\mathrm{m\,MASP1^*}}^{\mathrm{MASP2_{zym}}}}\\
+\frac{\left[\mathrm{pCLK1MASP3_{zym}}\right]}{K_{\mathrm{m\,MASP1^*}}^{\mathrm{MASP3_{zym}}}}+\frac{\left[\mathrm{pCLL1MASP1_{zym}}\right]}{K_{\mathrm{m\,MASP1^*}}^{\mathrm{MASP1_{zym}}}}+\frac{\left[\mathrm{pCLL1MASP2_{zym}}\right]}{K_{\mathrm{m\,MASP1^*}}^{\mathrm{MASP2_{zym}}}}+\frac{\left[\mathrm{pCLL1MASP3_{zym}}\right]}{K_{\mathrm{m\,MASP1^*}}^{\mathrm{MASP3_{zym}}}}\\
+\frac{\left[\mathrm{pLFMASP1_{zym}}\right]}{K_{\mathrm{m\,MASP1^*}}^{\mathrm{MASP1_{zym}}}}+\frac{\left[\mathrm{pLFMASP2_{zym}}\right]}{K_{\mathrm{m\,MASP1^*}}^{\mathrm{MASP2_{zym}}}}+\frac{\left[\mathrm{pLFMASP3_{zym}}\right]}{K_{\mathrm{m\,MASP1^*}}^{\mathrm{MASP3_{zym}}}}+\frac{\left[\mathrm{pHFMASP1_{zym}}\right]}{K_{\mathrm{m\,MASP1^*}}^{\mathrm{MASP1_{zym}}}}+\frac{\left[\mathrm{pHFMASP2_{zym}}\right]}{K_{\mathrm{m\,MASP1^*}}^{\mathrm{MASP2_{zym}}}}\\
+\frac{\left[\mathrm{pHFMASP3_{zym}}\right]}{K_{\mathrm{m\,MASP1^*}}^{\mathrm{MASP3_{zym}}}}+\frac{\left[\mathrm{pMFMASP1_{zym}}\right]}{K_{\mathrm{m\,MASP1^*}}^{\mathrm{MASP1_{zym}}}}+\frac{\left[\mathrm{pMFMASP2_{zym}}\right]}{K_{\mathrm{m\,MASP1^*}}^{\mathrm{MASP2_{zym}}}}+\frac{\left[\mathrm{pMFMASP3_{zym}}\right]}{K_{\mathrm{m\,MASP1^*}}^{\mathrm{MASP3_{zym}}}}\end{array}\right)}
$$

$$
-\frac{k_{\text{cat MASP1}^*}^{\text{MASP1}_{\text{zym}}}\left[\text{pCLLKMASP1}^*\right]\left[\text{pCLK1MASP1}_{\text{zym}}\right]}{K_{\text{m MASP1}^*}^{\text{MASP1}_{\text{zym}}}\left(\begin{array}{l}1+\frac{[\text{C4}]}{K_{\text{m MASP1}^*}^{\text{C4}}}+\frac{[\text{fC4bC2}]}{K_{\text{m MASP1}^*}^{\text{C2}}}+\frac{[\text{fC4bC4bC2}]}{K_{\text{m MASP1}^*}^{\text{C2}}}+\frac{[\text{fC3bC4bC2}]}{K_{\text{m MASP1}^*}^{\text{C2}}}+\frac{[\text{pC4bC2}]}{K_{\text{m MASP1}^*}^{\text{C2}}}+\frac{[\text{pC4bC4bC2}]}{K_{\text{m MASP1}^*}^{\text{C2}}}\\
+\frac{[\text{pC3bC4bC2}]}{K_{\text{m MASP1}^*}^{\text{C2}}}+\frac{[\text{pMBL}_3\text{MASP1}_{\text{zym}}]}{K_{\text{m MASP1}^*}^{\text{MASP1}_{\text{zym}}}}+\frac{[\text{pMBL}_3\text{MASP2}_{\text{zym}}]}{K_{\text{m MASP1}^*}^{\text{MASP2}_{\text{zym}}}}+\frac{[\text{pMBL}_3\text{MASP3}_{\text{zym}}]}{K_{\text{m MASP1}^*}^{\text{MASP3}_{\text{zym}}}}\\
+\frac{[\text{pMBL}_4\text{MASP1}_{\text{zym}}]}{K_{\text{m MASP1}^*}^{\text{MASP1}_{\text{zym}}}}+\frac{[\text{pMBL}_4\text{MASP2}_{\text{zym}}]}{K_{\text{m MASP1}^*}^{\text{MASP2}_{\text{zym}}}}+\frac{[\text{pMBL}_4\text{MASP3}_{\text{zym}}]}{K_{\text{m MASP1}^*}^{\text{MASP3}_{\text{zym}}}}+\frac{[\text{pCLLKMASP1}_{\text{zym}}]}{K_{\text{m MASP1}^*}^{\text{MASP1}_{\text{zym}}}}\\
+\frac{[\text{pCLLKMASP2}_{\text{zym}}]}{K_{\text{m MASP1}^*}^{\text{MASP2}_{\text{zym}}}}+\frac{[\text{pCLLKMASP3}_{\text{zym}}]}{K_{\text{m MASP1}^*}^{\text{MASP3}_{\text{zym}}}}+\frac{[\text{pCLK1MASP1}_{\text{zym}}]}{K_{\text{m MASP1}^*}^{\text{MASP1}_{\text{zym}}}}+\frac{[\text{pCLK1MASP2}_{\text{zym}}]}{K_{\text{m MASP1}^*}^{\text{MASP2}_{\text{zym}}}}\\
+\frac{[\text{pCLK1MASP3}_{\text{zym}}]}{K_{\text{m MASP1}^*}^{\text{MASP3}_{\text{zym}}}}+\frac{[\text{pCLL1MASP1}_{\text{zym}}]}{K_{\text{m MASP1}^*}^{\text{MASP1}_{\text{zym}}}}+\frac{[\text{pCLL1MASP2}_{\text{zym}}]}{K_{\text{m MASP1}^*}^{\text{MASP2}_{\text{zym}}}}+\frac{[\text{pCLL1MASP3}_{\text{zym}}]}{K_{\text{m MASP1}^*}^{\text{MASP3}_{\text{zym}}}}\\
+\frac{[\text{pLFMASP1}_{\text{zym}}]}{K_{\text{m MASP1}^*}^{\text{MASP1}_{\text{zym}}}}+\frac{[\text{pLFMASP2}_{\text{zym}}]}{K_{\text{m MASP1}^*}^{\text{MASP2}_{\text{zym}}}}+\frac{[\text{pLFMASP3}_{\text{zym}}]}{K_{\text{m MASP1}^*}^{\text{MASP3}_{\text{zym}}}}+\frac{[\text{pHFMASP1}_{\text{zym}}]}{K_{\text{m MASP1}^*}^{\text{MASP1}_{\text{zym}}}}+\frac{[\text{pHFMASP2}_{\text{zym}}]}{K_{\text{m MASP1}^*}^{\text{MASP2}_{\text{zym}}}}\\
+\frac{[\text{pHFMASP3}_{\text{zym}}]}{K_{\text{m MASP1}^*}^{\text{MASP3}_{\text{zym}}}}+\frac{[\text{pMFMASP1}_{\text{zym}}]}{K_{\text{m MASP1}^*}^{\text{MASP1}_{\text{zym}}}}+\frac{[\text{pMFMASP2}_{\text{zym}}]}{K_{\text{m MASP1}^*}^{\text{MASP2}_{\text{zym}}}}+\frac{[\text{pMFMASP3}_{\text{zym}}]}{K_{\text{m MASP1}^*}^{\text{MASP3}_{\text{zym}}}}\end{array}\right)}
$$

$$
-\frac{k_{\text{cat MASP1}^*}^{\text{MASP1}_{\text{zym}}}\left[\text{pCLK1MASP1}^*\right]\left[\text{pCLK1MASP1}_{\text{zym}}\right]}{K_{\text{m MASP1}^*}^{\text{MASP1}_{\text{zym}}}\left(\begin{array}{l}1+\frac{[\text{C4}]}{K_{\text{m MASP1}^*}^{\text{C4}}}+\frac{[\text{fC4bC2}]}{K_{\text{m MASP1}^*}^{\text{C2}}}+\frac{[\text{fC4bC4bC2}]}{K_{\text{m MASP1}^*}^{\text{C2}}}+\frac{[\text{fC3bC4bC2}]}{K_{\text{m MASP1}^*}^{\text{C2}}}+\frac{[\text{pC4bC2}]}{K_{\text{m MASP1}^*}^{\text{C2}}}+\frac{[\text{pC4bC4bC2}]}{K_{\text{m MASP1}^*}^{\text{C2}}}\\
+\frac{[\text{pC3bC4bC2}]}{K_{\text{m MASP1}^*}^{\text{C2}}}+\frac{[\text{pMBL}_3\text{MASP1}_{\text{zym}}]}{K_{\text{m MASP1}^*}^{\text{MASP1}_{\text{zym}}}}+\frac{[\text{pMBL}_3\text{MASP2}_{\text{zym}}]}{K_{\text{m MASP1}^*}^{\text{MASP2}_{\text{zym}}}}+\frac{[\text{pMBL}_3\text{MASP3}_{\text{zym}}]}{K_{\text{m MASP1}^*}^{\text{MASP3}_{\text{zym}}}}\\
+\frac{[\text{pMBL}_4\text{MASP1}_{\text{zym}}]}{K_{\text{m MASP1}^*}^{\text{MASP1}_{\text{zym}}}}+\frac{[\text{pMBL}_4\text{MASP2}_{\text{zym}}]}{K_{\text{m MASP1}^*}^{\text{MASP2}_{\text{zym}}}}+\frac{[\text{pMBL}_4\text{MASP3}_{\text{zym}}]}{K_{\text{m MASP1}^*}^{\text{MASP3}_{\text{zym}}}}+\frac{[\text{pCLLKMASP1}_{\text{zym}}]}{K_{\text{m MASP1}^*}^{\text{MASP1}_{\text{zym}}}}\\
+\frac{[\text{pCLLKMASP2}_{\text{zym}}]}{K_{\text{m MASP1}^*}^{\text{MASP2}_{\text{zym}}}}+\frac{[\text{pCLLKMASP3}_{\text{zym}}]}{K_{\text{m MASP1}^*}^{\text{MASP3}_{\text{zym}}}}+\frac{[\text{pCLK1MASP1}_{\text{zym}}]}{K_{\text{m MASP1}^*}^{\text{MASP1}_{\text{zym}}}}+\frac{[\text{pCLK1MASP2}_{\text{zym}}]}{K_{\text{m MASP1}^*}^{\text{MASP2}_{\text{zym}}}}\\
+\frac{[\text{pCLK1MASP3}_{\text{zym}}]}{K_{\text{m MASP1}^*}^{\text{MASP3}_{\text{zym}}}}+\frac{[\text{pCLL1MASP1}_{\text{zym}}]}{K_{\text{m MASP1}^*}^{\text{MASP1}_{\text{zym}}}}+\frac{[\text{pCLL1MASP2}_{\text{zym}}]}{K_{\text{m MASP1}^*}^{\text{MASP2}_{\text{zym}}}}+\frac{[\text{pCLL1MASP3}_{\text{zym}}]}{K_{\text{m MASP1}^*}^{\text{MASP3}_{\text{zym}}}}\\
+\frac{[\text{pLFMASP1}_{\text{zym}}]}{K_{\text{m MASP1}^*}^{\text{MASP1}_{\text{zym}}}}+\frac{[\text{pLFMASP2}_{\text{zym}}]}{K_{\text{m MASP1}^*}^{\text{MASP2}_{\text{zym}}}}+\frac{[\text{pLFMASP3}_{\text{zym}}]}{K_{\text{m MASP1}^*}^{\text{MASP3}_{\text{zym}}}}+\frac{[\text{pHFMASP1}_{\text{zym}}]}{K_{\text{m MASP1}^*}^{\text{MASP1}_{\text{zym}}}}+\frac{[\text{pHFMASP2}_{\text{zym}}]}{K_{\text{m MASP1}^*}^{\text{MASP2}_{\text{zym}}}}\\
+\frac{[\text{pHFMASP3}_{\text{zym}}]}{K_{\text{m MASP1}^*}^{\text{MASP3}_{\text{zym}}}}+\frac{[\text{pMFMASP1}_{\text{zym}}]}{K_{\text{m MASP1}^*}^{\text{MASP1}_{\text{zym}}}}+\frac{[\text{pMFMASP2}_{\text{zym}}]}{K_{\text{m MASP1}^*}^{\text{MASP2}_{\text{zym}}}}+\frac{[\text{pMFMASP3}_{\text{zym}}]}{K_{\text{m MASP1}^*}^{\text{MASP3}_{\text{zym}}}}\end{array}\right)}
$$

$$-\frac{k_{\mathrm{cat\,MASP1^*}}^{\mathrm{MASP1_{zym}}}\left[\mathrm{pCLL1MASP1^*}\right]\left[\mathrm{pCLK1MASP1_{zym}}\right]}{K_{\mathrm{m\,MASP1^*}}^{\mathrm{MASP1_{zym}}}\left(\begin{array}{l}1+\frac{[\mathrm{C4}]}{K_{\mathrm{m\,MASP1^*}}^{\mathrm{C4}}}+\frac{[\mathrm{fC4bC2}]}{K_{\mathrm{m\,MASP1^*}}^{\mathrm{C2}}}+\frac{[\mathrm{fC4bC4bC2}]}{K_{\mathrm{m\,MASP1^*}}^{\mathrm{C2}}}+\frac{[\mathrm{fC3bC4bC2}]}{K_{\mathrm{m\,MASP1^*}}^{\mathrm{C2}}}+\frac{[\mathrm{pC4bC2}]}{K_{\mathrm{m\,MASP1^*}}^{\mathrm{C2}}}+\frac{[\mathrm{pC4bC4bC2}]}{K_{\mathrm{m\,MASP1^*}}^{\mathrm{C2}}}\\
+\frac{[\mathrm{pC3bC4bC2}]}{K_{\mathrm{m\,MASP1^*}}^{\mathrm{C2}}}+\frac{\left[\mathrm{pMBL_3MASP1_{zym}}\right]}{K_{\mathrm{m\,MASP1^*}}^{\mathrm{MASP1_{zym}}}}+\frac{\left[\mathrm{pMBL_3MASP2_{zym}}\right]}{K_{\mathrm{m\,MASP1^*}}^{\mathrm{MASP2_{zym}}}}+\frac{\left[\mathrm{pMBL_3MASP3_{zym}}\right]}{K_{\mathrm{m\,MASP1^*}}^{\mathrm{MASP3_{zym}}}}\\
+\frac{\left[\mathrm{pMBL_4MASP1_{zym}}\right]}{K_{\mathrm{m\,MASP1^*}}^{\mathrm{MASP1_{zym}}}}+\frac{\left[\mathrm{pMBL_4MASP2_{zym}}\right]}{K_{\mathrm{m\,MASP1^*}}^{\mathrm{MASP2_{zym}}}}+\frac{\left[\mathrm{pMBL_4MASP3_{zym}}\right]}{K_{\mathrm{m\,MASP1^*}}^{\mathrm{MASP3_{zym}}}}+\frac{\left[\mathrm{pCLLKMASP1_{zym}}\right]}{K_{\mathrm{m\,MASP1^*}}^{\mathrm{MASP1_{zym}}}}\\
+\frac{\left[\mathrm{pCLLKMASP2_{zym}}\right]}{K_{\mathrm{m\,MASP1^*}}^{\mathrm{MASP2_{zym}}}}+\frac{\left[\mathrm{pCLLKMASP3_{zym}}\right]}{K_{\mathrm{m\,MASP1^*}}^{\mathrm{MASP3_{zym}}}}+\frac{\left[\mathrm{pCLK1MASP1_{zym}}\right]}{K_{\mathrm{m\,MASP1^*}}^{\mathrm{MASP1_{zym}}}}+\frac{\left[\mathrm{pCLK1MASP2_{zym}}\right]}{K_{\mathrm{m\,MASP1^*}}^{\mathrm{MASP2_{zym}}}}\\
+\frac{\left[\mathrm{pCLK1MASP3_{zym}}\right]}{K_{\mathrm{m\,MASP1^*}}^{\mathrm{MASP3_{zym}}}}+\frac{\left[\mathrm{pCLL1MASP1_{zym}}\right]}{K_{\mathrm{m\,MASP1^*}}^{\mathrm{MASP1_{zym}}}}+\frac{\left[\mathrm{pCLL1MASP2_{zym}}\right]}{K_{\mathrm{m\,MASP1^*}}^{\mathrm{MASP2_{zym}}}}+\frac{\left[\mathrm{pCLL1MASP3_{zym}}\right]}{K_{\mathrm{m\,MASP1^*}}^{\mathrm{MASP3_{zym}}}}\\
+\frac{\left[\mathrm{pLFMASP1_{zym}}\right]}{K_{\mathrm{m\,MASP1^*}}^{\mathrm{MASP1_{zym}}}}+\frac{\left[\mathrm{pLFMASP2_{zym}}\right]}{K_{\mathrm{m\,MASP1^*}}^{\mathrm{MASP2_{zym}}}}+\frac{\left[\mathrm{pLFMASP3_{zym}}\right]}{K_{\mathrm{m\,MASP1^*}}^{\mathrm{MASP3_{zym}}}}+\frac{\left[\mathrm{pHFMASP1_{zym}}\right]}{K_{\mathrm{m\,MASP1^*}}^{\mathrm{MASP1_{zym}}}}+\frac{\left[\mathrm{pHFMASP2_{zym}}\right]}{K_{\mathrm{m\,MASP1^*}}^{\mathrm{MASP2_{zym}}}}\\
+\frac{\left[\mathrm{pHFMASP3_{zym}}\right]}{K_{\mathrm{m\,MASP1^*}}^{\mathrm{MASP3_{zym}}}}+\frac{\left[\mathrm{pMFMASP1_{zym}}\right]}{K_{\mathrm{m\,MASP1^*}}^{\mathrm{MASP1_{zym}}}}+\frac{\left[\mathrm{pMFMASP2_{zym}}\right]}{K_{\mathrm{m\,MASP1^*}}^{\mathrm{MASP2_{zym}}}}+\frac{\left[\mathrm{pMFMASP3_{zym}}\right]}{K_{\mathrm{m\,MASP1^*}}^{\mathrm{MASP3_{zym}}}}\end{array}\right)}$$

$$-\frac{k_{\mathrm{cat\,MASP1^*}}^{\mathrm{MASP1_{zym}}}\left[\mathrm{pLFMASP1^*}\right]\left[\mathrm{pCLK1MASP1_{zym}}\right]}{K_{\mathrm{m\,MASP1^*}}^{\mathrm{MASP1_{zym}}}\left(\begin{array}{l}1+\frac{[\mathrm{C4}]}{K_{\mathrm{m\,MASP1^*}}^{\mathrm{C4}}}+\frac{[\mathrm{fC4bC2}]}{K_{\mathrm{m\,MASP1^*}}^{\mathrm{C2}}}+\frac{[\mathrm{fC4bC4bC2}]}{K_{\mathrm{m\,MASP1^*}}^{\mathrm{C2}}}+\frac{[\mathrm{fC3bC4bC2}]}{K_{\mathrm{m\,MASP1^*}}^{\mathrm{C2}}}+\frac{[\mathrm{pC4bC2}]}{K_{\mathrm{m\,MASP1^*}}^{\mathrm{C2}}}+\frac{[\mathrm{pC4bC4bC2}]}{K_{\mathrm{m\,MASP1^*}}^{\mathrm{C2}}}\\
+\frac{[\mathrm{pC3bC4bC2}]}{K_{\mathrm{m\,MASP1^*}}^{\mathrm{C2}}}+\frac{\left[\mathrm{pMBL_3MASP1_{zym}}\right]}{K_{\mathrm{m\,MASP1^*}}^{\mathrm{MASP1_{zym}}}}+\frac{\left[\mathrm{pMBL_3MASP2_{zym}}\right]}{K_{\mathrm{m\,MASP1^*}}^{\mathrm{MASP2_{zym}}}}+\frac{\left[\mathrm{pMBL_3MASP3_{zym}}\right]}{K_{\mathrm{m\,MASP1^*}}^{\mathrm{MASP3_{zym}}}}\\
+\frac{\left[\mathrm{pMBL_4MASP1_{zym}}\right]}{K_{\mathrm{m\,MASP1^*}}^{\mathrm{MASP1_{zym}}}}+\frac{\left[\mathrm{pMBL_4MASP2_{zym}}\right]}{K_{\mathrm{m\,MASP1^*}}^{\mathrm{MASP2_{zym}}}}+\frac{\left[\mathrm{pMBL_4MASP3_{zym}}\right]}{K_{\mathrm{m\,MASP1^*}}^{\mathrm{MASP3_{zym}}}}+\frac{\left[\mathrm{pCLLKMASP1_{zym}}\right]}{K_{\mathrm{m\,MASP1^*}}^{\mathrm{MASP1_{zym}}}}\\
$$
[truncated: 531,879 more chars]
